# Supplementary material for: Profile of the in silico secretome of the palm dieback pathogen, Fusarium oxysporum f. sp. albedinis, a fungus that puts natural oases at risk
Source: PLoS One. 2022 May 26;17(5):e0260830. doi: 10.1371/journal.pone.0260830 (PMC9135196; doi:10.1371/journal.pone.0260830)
Supplement: S2 File — (PDF) [file pone.0260830.s002.pdf]

>FUN_000151-T1 | Effector probability: 0.765
MLTSTFFLSLLGLAAASPVQPHINVNPQYPSRSTSKGFKLVVNVTDHSKDFDPPIHNTELTSIHTGAGLALVGVIEKDGRIFY
QNGTKEERQNGEATVISDGGTPLTPNGIALVADKKNKNLFGATLNFEPGTPGVQLNQPEDPYTYLLPETFVACNKSLEYYQGK
HFIVIEQAHLTIDKNNKIQRNIPKGCAPIRLVPQCAELETLPKGSYSSHKYALESKCYKDVSKIQWEKYSP
>FUN_000354-T1 | Effector probability: 0.586
MFIKTVIILLASLSHGKEIMQRRHDVSSPIIDQFPWSSFLNKTGLDPLQYLKPPSDDIPETLACTTEPGPDRLACPVAIAAWS
NLADAEQYIYIPFDQCLSATEGTCRTVACALTGDLTIKNDEITGRMWNPVSTNCVFGGVGGIWQNDGSTLVIEMGYASQK
>FUN_000387-T1 | Effector probability: 0.615
MLLLPLLSAITLAVASPVALDDYVNSLEERAVGVTTTDFSNFKFYIQHGAAAYCNSEAAAGSKITCSNNGCPTVQGNGATIVT
SFVGSKTGIGGYVATDSARKEIVVSFRGSINIRNWLTNLDFGQEDCSLVSGCGVHSGFQRAWNEISSQATAAVASARKANPSF
KVISTGHSLGGAVAVLAAANLRVGGTPVDIYTYGSPRVGNAQLSAFVSNQAGGEYRVTHADDPVPRLPPLIFGYRHTTPEFWL
SGGGGDTVDYTISDVKVCEGAANLGCNGGTLGLDIAAHLHYFQATDACNAGGFSWRRYRSAESVDKRATMTDAELEKKLNSYV
QMDKEYVKNNQARS
>FUN_000536-T1 | Effector probability: 0.68
MLVSTALLALSSVAFAAATPAPAQNPETDFSFWLGGPILEKCLAEKNCEDRLLDNPYYVEAKKTNHAKRYSCHGAGMHTVVTS
GKNFVKFGSFNPYDLFHHVYDLCHEGGCDSGSDWARSTKYVTATGIYSKDIKVSAQGVYSGWDMRNALAQSLADASGRNQVWS
EEVACPSGGRACSRVKAWQGYAPNFHQSAIFNNCNQYGWMNAKTDGLTGITSGGDCKSIVEKLGAIIGETVPFGAGTIPLLGA
LCG
>FUN_000554-T1 | Effector probability: 0.583
MKCLFSTLCLALAHFCQVAETKSGSWSGTNNYFLQGISDSAQDAYIQTLSSYDTKVVRLWVNQASKGCQKGSQIVRDIPQLET
TIGQYNKVTLDEIDKLLVKLSDKNIKAIISPHDANSLIGDYRKDAYWARWGSGYFYEKQDAFDAYDARLSYILNYKGTYSGKV
WKNWPNAIFSFNLQNEPMTPGPSNCKNGDPSGWACGRATFMRNAGLDSHILISTGGLGGDFSHGCTFLPAVTVPGKWSANLPN
WLNQANGKKVYLEEWGVYSQQYDQKSAFVSEVNDMNSAGLPNMYWQLLPPSSGGCSYNPKNDAGNPFGIYTNSGVNLAGPING
ATSSGAAQDWTGSLY
>FUN_000555-T1 | Effector probability: 0.648
MPSLITLSMMVGTIATSVVADVYNVNVPSGFFPGPQSGIGSWYRASTGQDSTNGNSWCGYKYYNSDPLFAPSLKAMGGATYNS
NPDAWRQQTRKYCGLEAKVTDPTTGKSQLMYIGDSFDDQWVRTPASIDIMIDAFSNIHGNPNGDKNNVIKGVQWELTGRVNTK
YAAPGASWPPASGGSGTGGLGAKCDGQTKLCNSGLTCLSPDGVCSDKACAKDGLAAQVCRAKSPGTVSAIFATFRKGNGDPDY
S
>FUN_000557-T1 | Effector probability: 0.877
MSVKYLLFSVLLVGPVQAEDSKDLSKHNCCLHIKDASDAWWMTNPDITKEVCKSYYKDVAQFDNKTGTCNEHKNGTINDVDWK
SKCKIYGTSLGWTDDRVGAGYDATCYPIDA
>FUN_000617-T1 | Effector probability: 0.654
MHFPILPILITLAGSALAAPHLPKRQNPCFVTGSEALPDEVSTQATNLASVITCDNSKTTIDGVPDVSSGGVTFSSINFAESG
QSPLAFALDKFATTSPLANNNLDTFQNELNVYLATEAGIRSTGGNLAIKVPKFFLQFQMARIQQAQGAVSDIPGQTVDHQLEK
VLKNAAGEDQALLDQVNELAVNLN
>FUN_000671-T1 | Effector probability: 0.556
MAPIKNIILALFVAADLAAVSAHSVITNAVGDAGGSGMALGVDTSTPRDGTRRRPFQQDATRFRGNAADTVGETLSNGDNDIE
QGTLDIMEETGSQLPQVNPGGSLEMTVHQVNSDGAGPYTCMINADGTGTSWDNIPVTTNVEGNERGRNRDGEMGDFPLVASIP
AGQNCTGTVAGEDNVCLVRCQNPARAGPFGGVIPVQMAQANGGNAGNA
>FUN_000750-T1 | Effector probability: 0.557
MLFNVAFFCSAALAVSASNEWRAPTASDRRSPCPMVNAVANHGYLPRDGLHISLEDLIVAFTDAINLDPAATALVGQKALTTG
NNGTFNLDDLNKHGVIEHDGSLSRADIFFGDNHSFNETIWASTASHFTEPTISIATAAKARKERLKAAEAANPEFSLPADLQQ
FSFIETALYLSVFGNLNDGNAKTEWVKTLFQEERLPIEEGFKRSDDVITAAGILGLVAKVAVASI
>FUN_000782-T1 | Effector probability: 0.606
MKLASRSILAGLATALPTNPPDQYEYIIIGSGPGGGTLAANLARAGHSVFLIEAGGDQGNTPSQRIPAMADQVAEHPSMSWSF
YVNHYQNETQARRDSKYAYRLSNGTLWSGLNPPEGAEPLGILYPRGATLGGSAQLNAMNFALPPDNDWDAIAELTGDESWRFH
KMRELFIEIENCTYAPEGTPGHGFDGFIQSNRNNISYITDRPGVVQSLKHAFEQTEGIQVEDAAEVGRLMQ
>FUN_000784-T1 | Effector probability: 0.74
MNQLASLLLLASALSSNAAASPKGPPLNTGLLQMVTYIKRNPNMTLDEFWQYWDTQHAPKVIPLATHFGITRYEQVRVAGKIV

PTDAGATEPVSNNLVDFDGIAMFLYESADVLIDMLSHSYYVNVVEPDEHVFIDKSAHGNGMVATYIGEHIDAVDDAKSVWKGD
KKTLSKYQKLFKKY
>FUN_000801-T1 | Effector probability: 0.595
MRINLPQIAFSNKFLLHGTLAIAALHLSRFKKNASEANSYMMKALHHYGTALRTATSLMASINAQNGPALYLFSMLCFSFTLG
LGPKPGDFLLFGKQGIAQWLGQLQGMRSLLETKPELFQDDTLAPMFQLSVRCFAQSISRTDHFPQLREQIQQAASGDPEL
>FUN_000811-T1 | Effector probability: 0.966
MQFLSITSLLALATLASAKLHSQAVCVTNRHYDPIGGTPWSPSYNWKVNYEILPGATNCACNYYRNRNTGNKQWDKCPDCRFD
GLVCESKDWHIGGDEFTYYCEKKCGAQGAEAN
>FUN_000848-T1 | Effector probability: 0.653
MAVLSVSHLLSFLALFAIAAAQIPGNLPIDEAEKQYLESLPPDKLAEILKIYEQGIAGQYPASPDVIEAQELIWSYGQSPPVY
PTREFNPSHLLRRAAPGKGLGDWSSVYQQASALVAQMSIKEKVAVTSGQTTVTNGCAGMIPGVQRLGFPGMCLSDGPNRLREV
EAVNGYASAITVGAAWNKELALARGYHMGLEAKSKGGNSTHLIKCFSRDS
>FUN_000901-T1 | Effector probability: 0.607
MHSSLFTLLVVAASAQASCARATRKFSLKTTYDSSNFFDQFYFRDAAYYDSIDPSYGGDPTHGSVNYLSKSKAQSAGLAKTTN
GQVYVGVDSVNKAKLLGSSKTRHGRDSVRLESKATYGNGLLIADIEHMPGTACGVWPSFWSYNFDEDPVGEIDIIEGINDQSQ
NVVSLHTCGACKFTKIGGLDERPNCNNGGTESEQCEDGTNYDGCGSTHASGSYGSAFNKGKGGVYAVWLESDALKIYWFNRQS
IPADIKSGNPDPSKWGTPASQFLSGSGCNVGKYFKGQTIIINTAFCGDNIDQGTWNEECKASTGSKTCDDYVTNHPEAFKESY
WLFNSIKYYQ
>FUN_000927-T1 | Effector probability: 0.66
MHFATILITAVATLASTATAAPKTETFATASVYAFNRDKNTHKFVDVPFGKLTHIDYQITDLELRGVQFLLPDGEKIDPDKVT
CQMYKDSLGTQPGSAAFTKQTPAHISLFNPVQFGWVLCYVNVGSA
>FUN_000939-T1 | Effector probability: 0.934
MKFALIASIFIAGCVASPLVEAQAESAEHINEIKYVDIAPAELEPKNANLVSRDTLPVTSCPAGQTYDRSVCFKSDRIRSFCV
ANPRQNREQITDTPCKPQEVCVQRNLSTGKSYAKCIPIHNLVSWKTSPDGGEGCTTSKVTPSGYYSLGTILYDVNKNPIQVNK
IKYLGEPGDKDEGIGGSTSSFSSSIFNFVSGQYMKSKDIRKLLNIKLIRTQPVLPAAALET
>FUN_000950-T1 | Effector probability: 0.885
MQFKTLFAASFLSGFSAAAPAASEDANNCGKPFQLMALRSGSKVHFAPFSAAHAGLLLNLPDGAQGADCGPGGGYPNNRATFR
INDEGELLLNSVGLIQQRFYTDRSGMGQGILQYNNGTSLPRNAETKGWKIDKAGNLNFADTDGFTVCPNGVDGSWNVWIPTGN
AHPGNTNVNCTSVHARTVEVENPPSCLYTTS
>FUN_001126-T1 | Effector probability: 0.925
MHFPSAIALLTALPSVLACKGYTGGLPKHTGTKTLSAPQYIKKGQTFDAGWVKYDRGVKCTGQDEGGEKDTVFVLEDGAKLRN
VIIGANQREGVYCLGSCTLEFVWFEDVCEDAISIKGGGTANIIGGGAYKAADKIIQHNGCGHVNIINFYANDYGKVYRSCGNC
KGNCRRSVHMEGTTAVNGGELMGINTNLGDKATYSNNCYPKVQCQGYNGCDKGNGACEPTKAGLC
>FUN_001131-T1 | Effector probability: 0.667
MKLSLSLAALAAGAFQVVSGQTVYFAGDSTMAKNGANDGVTDGWGNYIGKYLTVTAVNKAIGGRSARSYTNEGRFTEIVNLVK
KGDIVVIEFGHNDGGSPEKNDNNRSDCPGAGTEVCISDKTGEKVYTFVFYVSQAAKALVAKGATVILSSQTPNNQWETGTFEP
GAPRFVGYVPTAAKALNSPSVTYVDHFAAVTKMYQKLGNAKVNSLYPKDHTHTSPEAADLIAKAFVQAIDEELSGKTSLKPYI
KTPVTKVY
>FUN_001167-T1 | Effector probability: 0.747
MKLAHVLPILTLVVPIACGKYSEPPWEAKVPTAEEKKAREAWLDTQRDITAACEDLILETTRPRAQHQEQLNNRVVVVQEVTE
EASNRLLTFEGPMVRDSCYDLVHYFPKVVEKCKKALGALDAPKLACSNALIKPEKSNLGKKLRGLRFLFTVYDYNFSKRCVKD
TRIAAPPLPMVDDIRWYLMNTANAYSDIDEYCKSKGLNSSEIAD
>FUN_001186-T1 | Effector probability: 0.921
MKLSGVLSALALTSAVSATTVSYDTGYDDKSRPMTAVSCSDGKNGLITKYGWKTQGNVPTKYVGGVNIIEGWNSANCGGCYRL
EYKGKKINVLAIDHAASGFNIGLDAMNALTNGQAVKLGRINAQVYHANPSDCGLKK
>FUN_001252-T1 | Effector probability: 0.838
MKSFTLVTLLCTVAIAAVIPRDTVFDGHCCFTLQDVATSATVQQNSDGNLLLNAGKTNGFYCIDLSNSQDILRDNAFNACFLS
PSGALKCVDPTPGFQSWTLQKSGSNTLLQHDGGSTFNSCSKTSTSAKGTVLYGDKHTDATACKKVTLKAKNFKGTCKSLQG
>FUN_001330-T1 | Effector probability: 0.655
MSSRLLAAALALGGLSMGQTTGNFNFLTYNVAGLPAIINNNEVPGDKATNANLIGTVLATQKYDIVHMQEDFNYHAYIYATDN

HPYRTPTSGGVPFGDGLNTVANYDWTGLVRKKWNKCNLNSGDCLTPKGFSFMRMKIQGIEVDLYNLHADAGSDQGDVDARSAG
IDQILAYINANSQGRAVIVAGDTNDRWTNAGRSINKLTDAGFSDSWVQLIQGGKFPTAGATANPCKVPAADNTCEIVDKVFYR
SGSSVKLTAKSFNYVPKVFVQPDGNILSDHNPVLVEFSWST
>FUN_001364-T1 | Effector probability: 0.656
MTFIKALIPLALYLAGAQAKSCSAGGKPTAEFCSKDLPLSCHNTTAVEDTCCFIPAGQLLQTQFWDSDPVAGPHDSWTIHGLW
PDYCDGTYPQFCDKSREYTNIKDLVTKFLGKKTVSYMDKYWVSQDGNDESLWEHEFNKHGTCISTLEPSCYTNYETGAEAADY
VKKTISLFKTLPTYKWLAEAGIKPSKTKTYTADEIVDALAEHHGARVTIGCSNGSLSEVWYHFNVKGSLQDGQFVSSEPDGSK
SSCPDSGIKYAPKK
>FUN_001376-T1 | Effector probability: 0.556
AHAAFGLLPETLFLTVNLLDRYCSKRVVYKQHYQLVGCAALLIAAKYCDKKDRVPQIHELNNMCCGLYEAGMFTEMEMHVVNT
LDWAIGPTTVGFSQLMVAEEGDDQEVAHVATYLCEIALYHRDFVSTKPSVMARSSLSLARAILGRPEINNRGWGQTENVTLNT
LAYYLHQTSPTLAYKYSTTGFSRVSQKLARFMAEQAAMTAGAANPATPLAEPVNKHTSNIFRAPQKGHSTMGFNGYSTPPITS
DSNSLMRNHNMAKESYDLPPQCQVTPIPPNHAALYGQAQYVVYSNQHGMNQ
>FUN_001418-T1 | Effector probability: 0.607
MLNLTNKVALIIGLGQTGTEGWGIGAACAVTLARQGAIIFGGNRTLASTTKTKETIEAEGGTCDIIATDATDSASVKALVDAC
MKKHGRIDILLTSVGQSQPGDPASMSEEVWDSQMDINLKSVYLACHHVLPIMESQKSGSVICISSIAGLRYIGKPQVAYNTAK
AAILQFVKATAIIYAAKGVRLNAVVPGLMNTPYTRSLAERFGKGYEEFCRTREEQVPMERMGDAWDVASAVAFLAADEARYVT
GQKIVVDGGITSSTGRA
>FUN_001454-T1 | Effector probability: 0.564
MKLSIVAFLAAVPATIAAPAAARVATPIAYPYPATTDPAAVPDSGWSKDHKYYPAPKGGYKYPEYSPPKKDYGSHGGHHDEGK
KGNKGNKGNKGDEYRHGQKGNKGDNYGHGQKGNKGDGYRHGQKGNKGDGYRHGQKGNKGDDYGHGQKGNKGDSYGYGQKGNKG
DDYGYGQEGNKGSKYGHNNGH
>FUN_001455-T1 | Effector probability: 0.821
MKLSILTLAAAVPALAAAVPTSVDTAEVKRANCKLTLQWISNWSEAALRRYRVQLITSPRNDAHLGQYCDLFKQSASGVQNVQ
CFWTDGMFVIDDSQGEGSPGHTLYLKDFNNAAHYFELITGCDTVRNL
>FUN_001460-T1 | Effector probability: 0.583
MKAYLLAISLFVSNCFAVTPEAVYGGGFDHNKNDTIKLNIANGGAGQSGLIKELANAYIKKRVADGEKPFQVAWIKSDTFYSI
QYLKTGDADIGITYNPAAEEIAIKQGIAKSPSYYAFRDHFLLVGPKENPANISKSDDIMTTFANLHEAAEGPATEPPVRFLSR
YDKSATNIKETLLWAGIGQVPWATAYSTWYHQYIAFPIQALTAAIILQEYTITDRGTILSLDAELRNQTVVYKAGSDKADDPL
LNPAHALVGEKAPNGKEAAEFIKWLVSDKGQDVIAGFKKDGQVLYSKAPKGQD
>FUN_001469-T1 | Effector probability: 0.713
MFKHTIVFILTSLLLEISCTTYQPNQHILIPAPEEDASQMLTEGGWDGDFGVTSFPRCDFGSRVGGVVHGWPAKGGFYAHLCS
VAELEFLGLDRFKPSNKSDDPEKEEAHCSKMRQLGAKWFRNPDHQLRAGEKLRNGEPDAPLLFVGWPAGGGVWVIHTTLSQSA
RKGLGRIGNAFTMEERCKMVEQLGGRFYADPKDCPHLDLDGSREGAR
>FUN_001473-T1 | Effector probability: 0.901
MKFAAFTALAAVFGSAAAANKANVINDCTNTIYVQSFPYGGGAPGPLTTVKPGQRFSEDLRASGSTIKIATTRTLTKPLFFGY
SSESQPNNVYYEFSTEFGNPFADKHNILSPGEGCEKFDCQPNDAKCYSTPSMKKVYGCPSPVNLYAKVCAK
>FUN_001488-T1 | Effector probability: 0.829
MKSLLSLALVSLTAIASGVDAIAPKCRCLPGDTCWPSDTGNVWSRFKKTVNGLLIKTVPIGSPCHDPNYDEEACTALQQAWRL
PQTHIDSSSSVMQPYFANQSCDPFLAQSRRPAYIDIHRNSGPHIDIQISGRNYMDIDIP
>FUN_001504-T1 | Effector probability: 0.684
MRASLDSTLLILANVLAVKAQSCPDIHIFGARETSVAPGFGSAGQLVDMIKADHPGATSEAIDYPACGGQASCGGVQYGDSAK
QGTQAVTTAVNGLNQRCPQTKIVLVGYSQGGQIMDNNICGGPDSGAGISDSSVPLSASAVQQVKAVIMLGDPRFVSGLSYGVG
TCNAGGFDARPAGFSCPNADKVQIYCDSQDPFCCDGNDSNHHQQYVNIYGKEALAFVNSKL
>FUN_001516-T1 | Effector probability: 0.944
MKFTAALLLLIPLAAAGPAISTAEGLPDLATSGEDGYSLSHGEALQLAAKVCPAKFPRKCSIGNFCCRTKKCCKKECCKNTAR
YCSAGRCYR
>FUN_001517-T1 | Effector probability: 0.823
MKATLLTLLSSATVAASAAALPDGVQAVDNPLEGYTLIPMEWTGSIKEGADPITLSGTAEEVVAQIQKLNPDYVFPEDNTSEP
EIEKRSQGHIICKVGGFGAMDVRAAHRERNYLRSLGNNVCHVGAGPRTCTKIACATGDAIILCNDNGHAISPRCSYLADYIDH

IIRACSWTVNSPPCTVRPCGPSWSVDMVRGQQFDSDNYNVIVAKDTC
>FUN_001525-T1 | Effector probability: 0.586
MKFTSTSLALLASIPLVFSLPTEVKPSEQLEHPQGFDKIQKADPEHWLQAVKEEAARQEAAAASTFNETEALEARASWIYQVH
GMYTDNLVNVGDVDTFYMLWTRMYDSSDDKGGLSDITRNAWSKFCNSPYGNNGGVTTRFILDGQWGAVGRLSGWSMRDALIHS
MWQTADGIGKKNGYTVYNGCYGFTWQESKPGKANSACGGRSGKACPYNDDCPLAGMECTGLKWGTWMPSIIRMNVYNRDGSLR
ADAYQARISSQAVGSGGCSKAQTISAYVADFIPIVGPYFATGIRINCLYQS
>FUN_001537-T1 | Effector probability: 0.835
MQSAWTLVTLLLASSAPAVLGGECNAPREAGENNNCLGGSYNDCVTRNNQLCTGECFGQPAGGAGAPCYTGCTTRNQQYCAGY
CMKISNCDDCIESLKQMGAAGSDEQHKETCSQEGDSYYCNCDS
>FUN_001540-T1 | Effector probability: 0.575
MASDSIVIVGAGIIGLDVALVLSQQGYGKNITVIAEYLPGDTSPSYTSPWAGCNFSAISGTDSNAIKWDRHGYAHLKKLAAED
SDKSFVKRTPSIEFWDDNVPHDKIKAMADYLDDFKALSAQELPEGVKFGCAFTTLTVNAPAHCLYLYKRLRKDFGVRFIRRKL
GNIYEAYNNPATKVVFNCTGNAAKTLAGVQDEKCYPTRGQVLLVRASHVSTNVMRHGKDYETYVIPRPGSNGNVILGGYMQKG
NDDSATYSSESESILQRTTELSTELQQREPEVLAAFAGMRPSREGGARIERDEILVNGERRVIVHNYGAGGTGFQAGYGMALD
AVKSIEDILSTLPTRSLL
>FUN_001554-T1 | Effector probability: 0.864
MGPYLLMKLSLLGLALINPVQSNDGCQPATWKASVMDVGGINWRLSTLTGSQIDDNTCALILKKYHITIDTFYDLNHRLNNDC
KTIQPNIRYCVEGFPEPLRAYNGLCGPDNGNATCVGTDKQYCNKNTWTCGDTLYVPEYSG
>FUN_001559-T1 | Effector probability: 0.896
MKLIYTALFALASTAVNACDSTLCEGASVLRVVPCTKECMGRPCHLYECPDSPYTYICGKDRTKCDEIW
>FUN_001689-T1 | Effector probability: 0.841
MRLSNILSLTLAFIAPATVLAAPANTLHRRDCPSVDTIRQWIRDNASVGENTIFYTAGAKQEQAKAFAEQKVTDGNYWGKVFD
NNKYLDWIEECGEGPEQDKLFPRMGEALARESSGTAYVIMIKGNAIANFWKDNEYPYLDENGVKIIAVNAENFDDQKDYNGQP
FKRAIEF
>FUN_001809-T1 | Effector probability: 0.57
MKFSAIIAAGVLSVSALAKSGYKYERLDKNDAVLLVVDIQEGLYQLTRDWDPTLYHHQSMAHAALGQAFDMPVILTTSADQGP
NGPLMREIREMYPKAPLVQRQGQVNAWDSEEFRDAVKATNKSQVILAGITTDVCTTFLALSLRDAGYSVWANMEASGTTTPLI
RDISNDRMRDAGVQVVSLFSIVCELMRDWRNKPGAKEIYPWLGQYYPAAGYMARGHAAAITNGTIQPGEDGYTHYP
>FUN_001813-T1 | Effector probability: 0.849
MKFSVSLLTLIPAVFALPTGEDAAVSKRQSANTVTDQLLFSVTLPTFTARRNARDPPTLDWTSDGCTSSPDNPFGFPFVPACN
RHDFGYNNYRKQSRFTVSAKARIDSNFKTDLYYQCTSSSAAGACRALADVYYAAVRAFGGGDATPGKRDEDLVKEYEEKVAIY
NKAVEEAQAKGELPRLD
>FUN_001859-T1 | Effector probability: 0.816
MQFYTIVSLFLAGTAYALPATSANGYEACPSGGLFGNPQCCSLNLVGVLSGDCRAPTKTPNSAKEFQAICAESGQKARCCGLS
EILELGAFCQKPVGVSA
>FUN_001945-T1 | Effector probability: 0.704
MKFSAFFVIASAAFAVAAPGVATFSQERAALNESAPSRDREEGRADLREPEREEGREGEREDDREDRAGRGRQDGRLNRGGLA
FSQVDLNYLLRVNQLNLGKLQVLSQRNNFNVVVFQDLFASRDFSLQSLLQLQQLSTMLAIAETGIFDQFELSRLDLGNLNLGL
INGIAGLNLAQFIDAALKPQITIISKEVNNNIIIAK
>FUN_001967-T1 | Effector probability: 0.57
MAELLGIVAGGAGLASLALQLVDGGQKLRHRYKNAKGMEVNVLWLSEDIELIGKQLIQLEASADDIMQEQLGPIMMGRCRDRS
AKVADRLANLAGDLPVNSSRIQMIRTTFRSGQWKGELDELQALVTGLKQDISQYTTPPLILMEQD
>FUN_002029-T1 | Effector probability: 0.88
MRFSTVIATALAAFSNASPCLAGAPEPVICGGNGYVGATNTPWYKKSQAEGTIEGCFNSCTGGCKAISFDTQYKTCYFYTAEV
SKMRLYQVTNSPMTFHYFDRACVADAVGKTCGATGYVGATNTPWYKKSQAEGNMKNCLDACRGGCKAVSFDVKYKTCYFYTAE
VSKMQLYTVSSPMSFYYFDRACGHEVTETANV
>FUN_002057-T1 | Effector probability: 0.737
MKFLPLAAGILTLFGGVEAKKSPFFILTGDSTVATGGGWGDALLNSTKKPAGGINIAKNGATTVSFRSQGLWDTALDNVKSHK
KAHEAIVTIQFGHNDQKTLTLEQYSDNLATMIGEVKEAGGTAIIITSLTRRTFKDGKVVENLSNERDAAIAVANKAGVKYLDL
NTASTKYVNAIGQENADKYNEIEGDRTHLNFSGKLVFGRIVMDLLVEKRRDLARYIATNKKLSQLIKDGIYATGAE

>FUN_002136-T1 | Effector probability: 0.665
MRFSIAAAATALLSLAEARIIGIRVPSEIKLGEPFNAIIVRENYIQAVTDIGIVFGYAPVDQYPETIGQVANVFFLGPDESNK
IGPLSKEVTIPAPNAPDAPHGEGLVTAALASIYGASGSPTFSYYNVTVKYGNITTDTYKSSSEWVDLSG
>FUN_002151-T1 | Effector probability: 0.869
MVNLSKTWVACLAFGFVEPSLGFYDKSSQRVGYNPLVKQWNGVDSNLTATLDLVDPGYVSVFEKRRHIDVTAWATVALAAGTT
ILAAEAGINIYKQIADIIKSKSNHNSCSMTTGTDSNGYMIEGYAYLATTSGHDCKTTAETKTILAAVKDCADWQHKHGAIMGC
CVLSHGGTWEGHLRLTSQPNKYPAHAVNCG
>FUN_002176-T1 | Effector probability: 0.925
MHASSLLTLLTTLPAAMACLGYTGGVPKATGTKSLSAPQYLKKGQVFDAKWVRYDRGVKCSGQSEGGEKDAVFVLEDGATLRN
VVIGANQKEGVHCLGACNLEFVWFEDVCEDAISIKGSGTANIIGGGAYKAADKIIQHNGCGHVNIVNFYANDYGKVYRSCGNC
KGNSKCKRSVHMEGVTAVNGGELIGINTNLGDKATYSNNCYPKTQCQGYNGCDKSNGECEPSKAGKC
>FUN_002201-T1 | Effector probability: 0.651
MRIINPSLCLLSAAMLGSVGADRDCCYVWQGDEGIVQQLHVSWTASSQTATCKIAGWGTDTKCSVETSVVDNAWLKANATVSF
KEPNVFHFSFGMNNNDCDFLDDDKVARFWDSYLFASFTYLTQKDVCPDGGVF
>FUN_002205-T1 | Effector probability: 0.553
MISLFPLLSLLTLSWASPAVLTPLSIFNDNSIKTVNQPDSGLLNFTLHSRDTLEKRALTLAYLLCDVTFNGLNNANWQPFQVK
GELMLVQGIPSSGTTNGANPYDVVISIGTPISNPVAGSISYVTNRYLNPFISGRRDLTRLDFARVSATSNTVTVSVDTSLAAA
NQISVFNARSGFTANIYNPATGGFDLVFGNNGAISGRIVITGRAPVSGGQAPYQAIISGKVKQKGTFTL
>FUN_002258-T1 | Effector probability: 0.94
MHASTILYGLVFLQGAMASPVGNVAAADVEPRATEPEGLETRDIERRAPALGDMPFPSFPSPKKKGQGSGSKDCSSSEQTTLC
SSGSSYCCSPDGNGGTKCAISDSCNSDSKVICCNNNNGYQMCIGEIDFNMPVTININIYKGGKGKNGGK
>FUN_002259-T1 | Effector probability: 0.926
MQFSIVALLVAATGALAAPGNRGGDRGDRGGDRGDRGGDRGDRNTNTNTQSISCNGGSAYCCSPEYDGGYFNNYKCDKNINSC
NQGSAIVCCNQNINGNNNHQSQYCSAFGNQKVIFA
>FUN_002323-T1 | Effector probability: 0.903
MKNFLAIILALPAVFAAPAAKAGRHVKACACANDAGETQIGGYCPYIAGSNINVDGQDYCFPAATWSEYMDTRFTAEFCPGYF
PGYPNPVCKTVTVCPLIGDYQQIC
>FUN_002346-T1 | Effector probability: 0.855
MALRWLQSLLSCARAQAITLLNVCLAIGGWGDNSGFDEGVKTSSSRERFAKNIASTVDRLGFDCTLTGNILVAMARTICKSRT
AKKKNEIKAFPLLLKEIKKYIGRKELSIAVPGLERDMIAYTSAETPRIKESVDFINVVYIAQ
>FUN_002499-T1 | Effector probability: 0.787
MKFFTTALLSAAAVSGYSVYKVKDFTASCVPHSTFCNYEFKVIQSGSMETWKTPVHCSAHVQSANYLLPDVKDAKCKDSSRTF
SVKRSKKGLTFSVSQPVSPISNTVGKHFIPNKQLWQSKEPNAEIQAYKGPKDFKLNAVE
>FUN_002520-T1 | Effector probability: 0.638
MPSINALLTASLAFASIALGAPAAQDKKFTVEQVKNPKFIKNGPLALAHVYAKYGVPLPKGLEKAVKASDRNGLVWS
>FUN_002731-T1 | Effector probability: 0.784
MVKIQLLYSLAVAASALAAPSGVFSSLFSSLDKRACTNPSNLLKNAGFESKSIKPWVYSPYYPKLTSQKLVASGYKSDQALQI
SGLAKTQNDYGFSRLEQNFTNCKFGKYELSWSMYLPKGAAQGLDPRNPAMTIWVYDRAGDGQVGTLSFGPNSFNSSLGYPVQR
GTHKVNQWVNLSMKLPYNVNVGKCTLVINWIVPGQETGNGDGKLTLKLDNFVLKPAK
>FUN_002733-T1 | Effector probability: 0.583
MKSFLKAAVSTFLLVGTVASAAIEDPVELFNRQAAGVVIRQCNRPGVLALAYDDGPGQYTSQLVDILNNAGAKATLFLTGTLY
GCIYNQQAAIKKAYNSGHQIASHTWTHPQNFGSLSTDQLKQEMQKVEQALVNIIGKKPAYMRPPYLATGGNVLPTMQQLGYKV
ITDDVDSGDWNGQSAQQSLQKFQQAGAGGNGHIPLMHETYASTVQTLTPALINWAKQNNLKLVTVADCLGNAGGAYQSGTFNG
NGQNFC
>FUN_002735-T1 | Effector probability: 0.647
MKILALLASAAAVSLAAPTVDTHEIDARAVDTVLYPAGTYRYWIQSGKIIWDPQDQLLIVKNGKAADETTTIVTFEFDESTRG
KTCELLFFELWDRDVSTGTKTLDVFTYSDPPTGPRAFSAADAANWASTKSRGNHVGRIRVPKPGNATWEQSYQEWPKIPCPAGQ
LIGVEYVGVGDRVQVRWDIGVTGPRFKVVG
>FUN_002900-T1 | Effector probability: 0.584
MKASLILALPALAVAAATPQVEERQLGSLFDPACLLRITRITECLPDLSLDSIVGIFDIIGCPVEIACDITRCARIPGLPLPG

LPCTN
>FUN_002922-T1 | Effector probability: 0.729
MLPSFLLSSLLCLSTVSALPNPIIAERAACDCTGTRDGGSSSKDYICRDARLGPTKLPKKLPLSATVESYNRFGGLTPIQFLQ
TWTDEKGNYKYPPQNGFQLDANGNAINGSMVLQVGTLVDRFGSEYGSYVSAASAPYSQRALPPSNLATNPDTPDFPYNYHVYR
VIKPLTVVGGPIAPWFGQPGLGAQFFTGETGNVKFLIEQNYLQKEDPSALVYKSDGCA
>FUN_002923-T1 | Effector probability: 0.721
MHCNLSLGVVAALLAGANAHGHVAKVIAGGKEYTGGIPHGAPSDAVGWAAGNQDNGFVSPDAFKSADIICHKSAKPVSNAVVV
AAGDVVTLKWDTWPESHHGPVTEYLAPVSGDFASINKQSLRWVKVAQKGLKSGSNPGDWASDDLIRDGFSWKFTVPKNLKAGK
YVLRHEIIGLHSAGQANGAQAYPQCINLEVTGSGGQAISGGGDFTTFYTPTDPGILFNLYQSFSSYPIPGPAVRTI
>FUN_003019-T1 | Effector probability: 0.891
MKFTFSVLALVASANAWTMQFDCPRGSHFEGSYSGSKNKGCTSIPACVPGDHMVWGNTKKSNCVLRLYAAAPCAADQEIGHSK
ENWDHKFGQAVFAWGVTNC
>FUN_003068-T1 | Effector probability: 0.733
MLFLSTLVLSFVAVGYATPLAIRLDNTEPPENATFVQCVEGTTDPICLKIRGPSSDVITEIVHPDGTVEVHRNKYTREQLKDI
RHKNKVKSVKHKPGHTVRDGSAAFISDLTKRESGPRICKSETQRWWDQNDWGYWYQAWHQVGNCFYCNQCTEAIAVGFSVSQT
WTVGLSAKFGEVIEASFEFSWGETHTLTDTRTCQWNNVQSGCHSIWYQPLMSYHNGYANYQTHTHCGAGQGQGASDSYYDHNY
AYANVNQIGNNNGVNQGNLGCDSGCQGNDHRQCQYGNNGGSLWPNAN
>FUN_003091-T1 | Effector probability: 0.794
MVRLNHLLLLAGTLLPVFGAATKSSNSAKAPEPQQPGIVSNCKSYYLVEKGETCSEVAAKNKISLSDFLEWNPKTGTDCNALL
ANAYACVSVTETKGSSSAKPPAKKYSPTQAGIAKNCAKYALVGKTTTCKSIETQNKLSFANFYKWNPAVGKHCQGLKKGYYVC
VGVEKTATPTPTTPEQCVHQSSYLAWLVVIG
>FUN_003099-T1 | Effector probability: 0.638
AGVFFDLIQCLIQRALLYATQDVRECYILVKNALLDDHKKKVILILHSQCGIEGGMIIDWLLDKMPLDKLQKLEVYTFGNLAN
HFSNPYRGNSTRSSVIPHIEHHHQLNQHYLDDMFPLEKTLQRARAATEGDFMHRKVRVRTDGTMKVTTKAELPPGFVVTGDEG
SKNASMRPDRVPMMGDLSRLWKYRNGQLPDSFKSVLTDGM
>FUN_003251-T1 | Effector probability: 0.622
MHFLSILLLAGSALAAPRAAQSSPHQPRGDSIDDMLPPIVPINTRSGDRDLISKIITAPTQAERVKLLNQPGDYVFDFKAGTG
AGEASGKGGKSVSATALTMPALIGNGASMTVAFLGPCGMNTAHVHNRATELNIIVKGRLVTNFVVENGAKPIANTMDTFQMSV
FPQGAIHQEFNPDCEDAVFVAAFDNADPGVNQIAQNFFSLNGDVVKATLGGVQTIDGKDIESFRAHIPANIALGIDACLNKCG
IKRNSKRDISELLN
>FUN_003325-T1 | Effector probability: 0.597
MKDARAETICQPKASKKCSLFSFFANLLFALKPFPLKAQTLDFCGEPDKSDYTLIQDPFLGDIDHGQPGITFETAYGEGGPRW
FMVYQNGEDAKPYRAMKVSSNTTVFVDLTTSCSTFSGRLVRGNDIDFDGGAHNLGTWAEMNWQSYPLVYGGVSVIEGNDGPIL
LQSEDPNTPSMGFTEDIIPRAPKECRVKKDSGGMALKPTDKDGYDEATREFTKRQLDNQKVSIDKSYTATVMSHNGRFKIVFL
HGNH
>FUN_003382-T1 | Effector probability: 0.781
MKSIVASALLFLGFTSAQYGGQIKVKDDGCPQFTAGEKSQPLSWVKGNNICADLSDICPDGRCFMAFQALVTGTDSRTPAKMG
ACSTDDCSSDCQTWDVDSQSNSISVDCAEFTGQHYFYLGD
>FUN_003505-T1 | Effector probability: 0.761
MCIQRIIIALTLSGAAVSQNLNIPTPVGSIVSLSAPSVISGSKDMGNKEYDRGRPCNTDEDTGSENAVFILENGATLSNVIIG
SNQLEGIHCKGACTLNNVWFRDVCEGEAMENGRSSLGLARANICIDAISALGNGDVLIQGGGAQEAKDKVVQHNGRGTVTIKD
FTVVNAGKLYRGCGDCTNNGGPRNVVIQNVRAKGVSELVGINSNYGDTADVSESCGSNVKKVCQEYKGVEKGSGDSKEVKTTA
NCKGQQSFSAC
>FUN_003512-T1 | Effector probability: 0.743
MVNLLYVVYALSAVAGVQACETTCSATGDSGSTCSYICNQACPDVPAHEARNNFLAALQSGGHSCSAVGASGVRCQKTDGFGS
CYDHYWLCGDC
>FUN_003596-T1 | Effector probability: 0.789
MLRNTVLVLLIAAEATHGQAAFTLRKTYDSSNFLDSFNFRDRAYFDSIDPGYEGDPTGGSVNYLSRSQAVASGIVNTNNGKVH
LGVNSVDKAALLTPGGSRHGRGSVRLESKESYSSGILFADIEHMPGTACGVWPAYWSYNFDEDPVGEIDIIEGINGNQNGNYV
SLHTCGACIFNRPGGADPRNNCNIGGSDTRYCTDGNNYSGCGNTMPSGSYGKTFNANKGGVYATWLTTEAVKVWWFPRNNIPA

DIKNGKPEPNTWGQPATSQFVNANGNCDVGRYFKKQTIIFNTAFCGSNIDQGIWNQECRASTGYATCDDYVTNQPGAFKEAYW
TINSLKLYQ
>FUN_003601-T1 | Effector probability: 0.674
MNIPRLYGIRLVLCFPLTSGADKLQIYENLKKGLAHTVTSIPWISGVIGPEEGQDPKTRRVQIVDSPSGFKFPYKDLSDTLPS
YAALKEKSFALSEFSTAPLGPIDVTPQGPD
>FUN_003622-T1 | Effector probability: 0.694
MFTLQALQLASMCTLVYGHGYLSKPMSRTGLNAEAGPDTCPECTILEPVTAWPDLDSAKVGRSGPCGYNARVSVDYNQPGANW
GKEPIATYKPGQVIDVQWCVDNNGDHGGMYAYRICQDQDIVDKFLDPNYIPTEAEKQAAEDCFEEGLLPCTDVNGQECGYSPD
CSADQPCHRNDWFTCKSFDGNSDGKGCRGVDNAPINSCYTSIAGGYTVSGKIKIPDYVSNHTLLSFKWNAFQTPQVYLTCADI
AISA
>FUN_003865-T1 | Effector probability: 0.925
MQLINLYALTVFATSALAADCFGNGNKDVGKFITAYWDARERMCSNSGCTYQEACTTSGSYTVKGLGVDTILNVEIKRKNTGG
KKGFKDCWDATEDIINQCPKGGSKQLSGSWEANGQLYQVNGYFSFN
>FUN_003868-T1 | Effector probability: 0.758
MKVSSFLLSLLPLTAAATSIPQPQAVSAPEPATSAECCPFTYKPTCTKNLERVFHIKLFYNRKEGITPEQFNAYWANNHTKTA
GDFHLRFGVYKYSQYHSTPELRDLLRVPGAAPVLEFDGAAEFWVPTMETFQAMGSDPFYRDVITPDENNFIDHSSMRMIVGFD
YIMVDNQNAVTEHGRTFQ
>FUN_003875-T1 | Effector probability: 0.657
MKFFTVTILAMVSGALAMPVAAPNGGTINYEGLKGPSNTNPQPYKPSRPCLPSQQCRGKN
>FUN_003909-T1 | Effector probability: 0.854
MRSSGYIASLVAAGAMAVLHNGKLQNDNKHYEIVTKFEYVTHYVIGGGSDAPQTTCVSQPKMSNFEKQPDISREYITTAIYAQ
SINVQPVKRPKQAPNRDSVSGFGLNYGLSTDQQEAVNLHNDGRKAVGNGPLSWDDSLAAGAQQWANHLASIGSLQHSKGNHGE
NLYMGTTDSPYSVSAKTFLAENSQYNGEAISASNYLHFGHYTQCVWKHTNKIGLAVSKDSHGVSWVVARYQRPGNIIGEKPY
>FUN_003933-T1 | Effector probability: 0.847
MVSFKSLLLAASALTGALARPFDFLDEQDDGNSTSVLEARQVTGNSEGYHNGYFYSWWSDGGGYANYRMGEGSHYQVDWRNTG
NFVGGKGWNPGTGRTINYGGSFSPQGNGYLCVYGWTRSPLVEYYVIESYGTYNPGSAGQHKGTVYNDGDTYDLYQTTRVQQPS
IDGIQTFNQYWAIRRNKRTSGAVNMQTIFNAWNSAGMRLGNHYYQILATEGYQSSGSSSIYVQTK
>FUN_004006-T1 | Effector probability: 0.847
MQFSIFSILAIATVVIAAPQSSCNNGGLFYSDPKTLEPCKSECAGGTCAFQGKCETDPNIPLLCLAKCTC
>FUN_004107-T1 | Effector probability: 0.664
MRILLVALLSLLANSANAYKTSLIGYGQSWYDPPCAYAYRAVIGNAPLNYPSMAHGSMGTSKHSHGGSALAPCIATNNDFLHT
LAYYLSTRCADVSPSKLESYWAGQATGDKFVSAKWTYVAVLADVTAPPKRTYIAGDTLNYTALIADTDYKYQYDFNVFFDWEE
AVQSTYV
>FUN_004316-T1 | Effector probability: 0.806
MLAQTIFSILAIATTGLAAPVEPRSNDYFTPSEIHTYNINNGAIYDTDEGRVTKAPTNGGNDITTLLTFKYPEAARGKQCQFA
FYLAPGENVIGSKKLDLYSSLAPAPGPRAGWGPGNQRNVHLGRMDVKVGGFATWEATYGPYMTQKTDCKKPGTVEGLELVGVY
DLDAIYWNPTKSGPRIVIS
>FUN_004319-T1 | Effector probability: 0.595
MVSWNNIFTLALGLIGSARAYTNPIRNPGGGDPQITYTGGYYYLISTEWTNLQLSRATTIEGLKTATPKVIYTDSDPSRSSNV
WAPELHYLGGKWYIYYTAGKAEDLTGQRSHVIKGGASPWDSWSYGAKLSDDWGIDGTILRTNQFGNYFVYSCMTGVQYQSTCI
RKLGSDFLSVGALSIISQPDQSWEKSGTPVQEGPNALYFGGKTYISYSANYCWTPDYCVALLEWDGKTDPAKASAWKKSNGCV
LKSANGSYGTGHNSFFQSPDGKQTFITFHATSNKNGACDDTRYAMTQPLTANADGTPNFGSVQPFSHQFAEPSK
>FUN_004335-T1 | Effector probability: 0.629
MKPTQALIALATIVYAAPIVEQTKALTPPLAELDTHFEHHIKRCGKDEPPFLGRFPFANETYNQLTDGTPCRNVTMIYARGSR
QAGNVGKANNTGPALFNSLADRIGLENLAVQGVTYKARRRDFIFKGGCNEGSKTMAKLINQAASQCPDTKIVIAGYSQGAQLL
HKAAKNVTAGVTQHIAAAVTLGYPKKPMGKIAASRSLSVCRPGDSFCDKTIPWKPFVPIVGAPLWWFITMLDTHGDYNENATA
VANWIADRVTNIG
>FUN_004374-T1 | Effector probability: 0.778
MLHLSSVLTAGLALITAANAACGDGSPQGVVSGSGTFTATVNGANVYTGTDYRLAIQTALDRISSGQRVTVRASGSIGANTIS
ITSGKTFEVCGTMNVGNKSGRGAIEAINQNDVKIPYLKMTGNPYFGLRFSGTRNLALGDITLNLSGGIGIRFDRDANWNYDVS

MGNIVVTGAGSHAVETFKIDGLTITSVKAKDVGECGLLIQESRNVKVGYVEGINAGAGTGYATLRFANNNGQLANGEYTTTNV
FIDKVYSRGGGRGIFCVSMSGATEIKNIDLADNGNNAILIENCYNFAIRDGLINGGGEVRVSARSEFPNTSGIYVKAQVNSNT
VRESPCAENIYWGITGNAQKNIC
>FUN_004382-T1 | Effector probability: 0.639
MKILILIAPLLSLPLAFANSGGDYGYGYGEKISTVTATVTHTVTKPIYKAPITKTKTETVTNFKPTVTKYKTKTKTVTKKPYP
TYHKPGYGDGKHKDGEYNG
>FUN_004399-T1 | Effector probability: 0.713
MQLSSLLTVISLVSASLAATGDRGSYTVSGLGARKKAILNAGGNTLDLAIAMLEDEHMSTDYKYGDGKTLDAANFGLFKVNWG
MLRVCAKRAGFVGQSESQWENGAKLNSDIYADVASRWDCQEYYGYDKWFAGHRNGATGLSNPNTEDIRFYRESVEWIQAQIDS
KSTYKTDDTRFWVDVNPI
>FUN_004423-T1 | Effector probability: 0.87
MRLYYALFAAAPLFCNATEIDAHIEGYGVAVPEWEVEITPRGPTTVLNGTIEEVHEELLQLNPDWDEEYTVNSTESELAERDS
SVELFARTDFSDAEYHCGGRWPKCRTTIINQGISYLRRIKGKPRNGPGPGNCGRVSCSFNSAIWWCNDNAKSKTLNSFSSIAD
GGAFIVKKCYIPDRGPPVPGKWNMLSGQAFHNSKWNVIVRKDKC
>FUN_004543-T1 | Effector probability: 0.588
MRFALALSWLPLSLALHLPESLDRRTKHEPITDCSAEQVVDLLKLEPNTEKGYFVQTFVDPTTVPGTNRSISTAIYYLLEGSA
GQSLWHKLDAAEVWHYYAGAPLVLSLSKNDGSCTRDHVMGNDLFGGQRPQVVVAAEEWQSARSLGDWTLVGTTVAPGFDPTGQ
VLKPEGWKPKSCKRPH
>FUN_004609-T1 | Effector probability: 0.604
MKLSQILSTAALCSFALASPLPGNAIAEREANPVTPPGYGAKYILEDDLDKREPQRNAYGKAYTLKMKDDGFEKRDPQNTYGK
AYTLKMKDDGFEKREPQRNTYGKAYTLKMKDDGFEKRDPQNTYGKAYTLKMKDDGFEKREPESGA
>FUN_004612-T1 | Effector probability: 0.795
MKFNLIIIGTLAIGQGLAADYNLHATYTDELVNVGNLDSFWSVWNRMYDVSDDKGGLSDHTTWQYTGNCNSGYDKPKTSVRVQ
LDGQWGTVGKASGWQMRDALIQAMWATVQAAGQQNQYPVYTDCSGFTWQESTPNNKNAACGRATTKKVYCPCEYMVECQKFSW
GRKLPSQVRINVYNRDGSLRADMYQIKIGAQTLRGESGCGKAGAIAEVLASFVPGVGQYFDKGIKIACRQGSLW
>FUN_004711-T1 | Effector probability: 0.81
MHFINLVAVFIGLAAAAPTATPTKDNMIAVSTTAPSAYEDPYTYWAAGNHSFSTCDKKTYNAHQAPKSTKRANYRDCAALLST
FGTRNGTFSIPAASDDKREYGVGDGLVNIVKSGSCAFAVRADKGLKVGDDDVVWIMQKAVLEYSAGTEMAARGSVKCAADEAG
KKGGLYWQVHGIESAGY
>FUN_004806-T1 | Effector probability: 0.833
MVATKFLLMVLAAYVTATPVASAPKPATTQWKPLDIKATIDWDGIDKKAYQDPANWNTTDHVIPAAKGKSANPHFHILSGPCE
QGSCPDYSAAFDLVYTFTAVPVDGDPPLTIFESQSSIRINDCNECLIHKVGSNLGNSVPGGCWDFRSCGRDQTICVDPGNQRA
HRIWKGYVKKCYHMRVEYLGDCGFIKSRIVLHPDAEVPCNW
>FUN_004812-T1 | Effector probability: 0.96
MKIHIFAVHFLAVASNAAFLITDEGVRCRSGPTTCNAIQRQSAKGTDVAITSQTEGTHIKGNVPWDKTTFGCYISDYYVAKGS
SGYVTSKCRSCKAPKSNAATVNLIASFEGFRPDVLNSKKLLADGMKEFEICITAMLNSKANLNRNQYGALISSAFNMGCGNAD
SSTLVGRLNNGEDPNTVISQELPQWVNSNDQRLPGLVRRHNAEIELAQKPTRRRALPKRC
>FUN_004846-T1 | Effector probability: 0.702
MKTFSFYILLGALFSRTDAAEQKHIDEACSDVWGVINCDSSITYPNYPRPVNDINGIQIDTEIVPKEVNICDAVRDALGSDDF
CLSNNDYTIITDRASVVKNQLAATNGWTFVRAPEVDVATCKRLSKAVMSCYTGTCDGPKVRAIFASYVENADSIIDSDFVRML
NKWVTLFESLKKRTTEVQTYSKLVQAHLKTVSSKVNSVKANVCKNNACKASTPANAFKKFATVKSLQGVPIAAGKALANIPKM
TQITRNAIKYTTTPSDEACYLNLMNE
>FUN_004867-T1 | Effector probability: 0.952
MHLSSLIPIITLSLGAANAMAIEEEGTLEARADQCNPGINLIKLKIKPGKYFTGVGKPGKCYNLPANIKYFDVYSDDTKSLVS
CFDCKVYTEANCKGSYVSIEGADNFAFKTTKKPHYKSWRCGPP
>FUN_004956-T1 | Effector probability: 0.688
MKFFALLASAAALSFAAPTVDTHEIDARAVDTVLYPAGTYRYWIQSGKIIWDPQDQLLIVKNGNAADETTTIVTFEFDESTRG
KTCELLFELWDRDVSSGTKTLDIFTYSDPPTGPRAFSAADAAKWASTKSRDNHVGRIRVPKPGNATWEQSYQGWPKIPCPAGQ
LVGVEYVGVGDRVQVRWDIGVTGPRFKVMG
>FUN_004994-T1 | Effector probability: 0.67

MKGSLVFLAGLFAPFALAQSLCDQYSYYANGGYEFNNNRWGQSSGSGSQCTYIDWSNSNGAGWHVDWSWSGGQDNVKAYPNSA
LQIGTKRIVSSISNMQSAAAWSYSGTNVRANVAYDLFTAADPNHVTYSGDYELMIWLARYGNVQPIGSKQTTVNIEGRSWELW
VGMNGSMKVFSFVASSPVNSFNSDVKQFFNYLANSQGYPASKQYLLTFQFGTEPFTGSNAKLTVTNFNAHVN
>FUN_004995-T1 | Effector probability: 0.793
MRSLLTLTLALFASAGLGDAASAGCGKQPPSSGVKTMQVNGKNREYTLQLPNNYQNNKPHRLVFGYHWLSGNMGNVVQGGYYG
LRNLAGDSTIFIAPNGLNAGWANQGGEDITFTDQMLAFAKQNLCIDEKQVFATGFSYGGAMSHSVACSRPNDFAAVAVISGAL
LSGCNGGNTPVSYLHIHGSADNVLSIQQGRQLRDKWIGTNGCQQKQVNDPAPGAQNYVKTSYTCSRKPVTWIGHGGGHVADPT
ANGQKFAPGETWSFFNAAAGKSAKLRC
>FUN_005007-T1 | Effector probability: 0.958
MRAVSVIGILATIISLAAANPHGACGCQINTDGALDDDTTDTCCIRFGGKTSFLTTSRKVRFSGKYCLRGKIDGNSWYNCCRQ
FRPEGDGACPW
>FUN_005041-T1 | Effector probability: 0.706
MKLQLSLVLFAAVALCNAKVFDRQALSYAPIPEEIYQPPTKSGETTLLDFIKSREDLSELSKLVEQTPGFLQAFSTSADWQYT
FIAPSNEAFNNTGEYYKTFASTPKGRWWSGQMLQHHYIPNSRLYTSNFTAEKTRFQLGSYLYASAEIKGGDLVLNNVATIVEA
NIPVTNGLVHISDRILAGDAMIYEADIGTTKQGFIPGSCSNPNLPYC
>FUN_005045-T1 | Effector probability: 0.721
MRFSSSIVSLMATGAVAAPHDDNWQNGNKHSKLVTKFGFVTRYLVGEGGSPQTTCVPQPQKSDFKQHIPAPVYAQPATAQFVN
EPKHAPNQDSSSDFGPSYGLSTDQEKAIHLHNEARKAVGNDPLSWDDTLAYGAQEWADHLASKGLLQHSQGGDGENLYMGTTD
SPYSAAVKAFLAEGSQYNGEAISGSNYMSFGHYT
>FUN_005118-T1 | Effector probability: 0.585
MRITTFLSTICAAVLVAAEQRTAQVYVQPILSSPSKPQILAEVAYDAASLVSTEIVSYEAPELPETASLVRVGIYDPKSSRWI
AGTTAASTENFDRGYAPTILLSVDESGDVRSVVLKGVRVDAGQTRDFGPKAVVIAEKKGAQPELNKPVVLSPNGRKAAEEEPK
SLLQKYWWLIAIVVVMSMAGGNEK
>FUN_005229-T1 | Effector probability: 0.748
MQTFNILFITLATAVAGVTSSSSCPPFPASMTPFSYGFKEPKPPAIKPEYQAHFVQHKWNGELSHITAGFIQNSPSKGFVRAD
EAFDGVLASSTFDYSNVTKLGLVDNTLITYDPKKAKPSVWRGYVNSNYPILNKTVLIDNGAVFEGFVKRDFTPTPVAAWSIMY
QNAIPVTVFVNECGVIVGYDYFAPELRTRVIMEFFNIQAK
>FUN_005233-T1 | Effector probability: 0.8
MHLTSPLSIGLSLCIAAATGSCHPSSAKLTRCIEKPSINATADNYPGSASSAHSTTYPYNVTCLNDVPVAASACKSNDPDEAL
ACARKYIDAIDEQISFLYARRLGYAAVAGAAKFKNGTALNDPTRNQAVAAGMAARVLKYGGSGETGRIMGGEGCQIYASLKYE
AQQIQNCGGKLNETFERVCK
>FUN_005310-T1 | Effector probability: 0.556
MTPKKHRIRCIGHIVNLSLQAFLLGPSNEALIAALEAASEATSEDLLIRFSQALTTRRRRGRRRLSRSDVDEDYLGIEGIPAL
RKLHGLAVWARCSSLNSQLWDDEVGLRLGIDNETRWSSWYHVLDKLFKKKAQIVQFMHENEQILGENRLTAADWDLLQKAYLF
FQPFDGATLCAEGNKASLSQSLFLMDCLLSHYENEK
>FUN_005326-T1 | Effector probability: 0.652
MLVKSLLSSSLLLAIAQAGPTISGQGLAKRDGFPVCDDTSRTYEGPYTDGEGTYVTSDRTTHPYKFPKVRKCWYDYFVLETSK
EYTPWQKASGNIYCTGTQVCTATKLVGNEVCQERSDSVSVSVGGEIEGFSLGLDFTTTDTESKCVQAQDLTACTWDDGACHTV
WTQQEVMRQKGYRRQRCNWGNGDETQCMDNWEQTTPSDFIDYGCGSKCTDTNECGNTDGKPCSK
>FUN_005477-T1 | Effector probability: 0.611
MKFTTALVSAVLAFTSFAEAAPRNTRRTDSSKPLSLTAQLRLADTAADRFALLPEDDDFVFDFNKKQDNPGKGGELIAANRAN
FPALVGTGAGMAFGRIDPCGMNTLHVHPRSAELQVVTSGRLITEMVPENGVLDKDGKRRVIRTELTANMMTPFYQGSIHTQFN
PDCEPATFIATFATEDFGTGQIADELVSLSDDVIAATFGQSIAGEDIDKVRQAVPKSIALGVDKCLETCGLKKRSI
>FUN_005525-T1 | Effector probability: 0.687
MISLTVGVLALQGGFAEHIDLVRKAAEYLSSTEGISKTKFHCIEVRTKEELDQCNALIIPGGESTTISFVAAQSGLLEPLRDF
VKVQKRPVWGTCAGLILLSDEANATKKGGQELIGGLAVRVHRNHFGRQMESFESGMNLPFLNDDKPFPGVFIRAPVVEEVIGS
SDDGRPPVEVLAKLPGRVDKMKSGVSQANTKDDSGDIVAVRQGNVLGTSFHPELTKDERIHVWWLKEILNQQ
>FUN_005559-T1 | Effector probability: 0.899
MKSSLFLASSLLALFPSCLQALAIQGYPENVRFGEPTFVEGGNTTTAKRQVATPPDNCWVQGLGQSSYWFLPVYSGDDVYACI
MARKRMTGSSWVCPRDDWWPDSYVDEIVSAGQEQIFKEGGGKTTEKGVFKARFEGTTRKIEDKEGMSAMLDMLFRYSLPNCDT

SYSLCVTKSRHYYYKYKGDTIGIVHRESDCRGWFGIGGDTYAYCPGGSHCQKD
>FUN_005579-T1 | Effector probability: 0.963
MKLVVVLSIISSVLAAAYSDPAARLDNGLFTRQDEGEAVCANCQNSVTCNGVKVNCSGKRAAAPHFFKRQQEQEIRNVCNNCK
NNVICNGKRI
>FUN_005597-T1 | Effector probability: 0.838
MKGLYTVFLAFTAVQATPTINSESKALNARAVDLKVQYYKDGGCTDYAVSIWPGTSNKCYGYSYSNTNSANIVSCGGRGVACT
CYMFEQDNCKGNSVRTVALSGSGSACVSNWGKGYKSMQCYTTQIA
>FUN_005612-T1 | Effector probability: 0.563
MSYPSDYTVGWICALSIEAVAATVFLDHEIEGPATQAKDDTNNYIYGRIQKHNVVIATLPVGEYGTATAASVAKDLTRSFPNV
RIGLMVGIGGGAPSLNKDIRLGDVVVSTPNGEHGGIFQYDYGKSLQDQVFQHTRILDQPPQLLRSAVSSLQVQHRIHGNGLQE
SIATILEQYPRLRDEYGRPSDSADILFKPAVVHAGGESCDICDKDETDIVNRKPREHRKSWIHYGIIASANSLMKNAMLRDKL
ATQKGVLCFETEAAGLMNGFSCLVIRGICDYSDSHKNKVWQGYAAMAAAAYAKQIITGIPRGRFLILGPSIHADAFQALPN
>FUN_005622-T1 | Effector probability: 0.579
MVSKLALALFFGNALAGLAGRAIPPRCAIADPTPEQVQHAQNLKKIESVSKVAATSITVDTYFHVVSSSSSKYITKAKLQAQL
KALNDAYGPSDVTFNLIDTTFTTNSNWAAGNGEVAMKRQLRQGDYKTLNLYFTDVAKLDGLDALGYCFFPEPNVSTGSSTFIR
DGCVIVAETVPGGSMAPYNLGGTAVHEVGHWFNLFHTFQDGCTGGDLVSDTPAQASQTSGCPARRDSCPNQAGDDPIHNFMDY
SDDVCYEEFTPGQKTRMHSAWTTYRK
>FUN_005653-T1 | Effector probability: 0.748
MVSLRDIGLLLLAAPIAMAAPKAPSEVTIKTLPFSKPEDISKAPSTSAVASPASDVSQKAACQLPLRFTNFALYSDTGCKNVI
FDPFMLTWDPCKGYQWTNALANGVIFQSMRWTGGSNAQDFYACQQGFSCNANVARISQSTNVCSSGGGLHFDKLAVNP
>FUN_005744-T1 | Effector probability: 0.817
MRFSSYVVSLMAAGAVAAPHNDNWQNGNKHSKVVTKFEYVTHYVVGEGGSPQTTCVPQPRRSDFKQYIPAPVYAQPATAQFVK
>FUN_005760-T1 | Effector probability: 0.653
MKFFTIALLSSVSTVMAIDCFCVTTDDDVAAGRLPQRTFRLSGRCEELGGKPDIESRTCELLPDAAAFTEADCNADSGDRVLV
PNCFASVL
>FUN_005816-T1 | Effector probability: 0.615
MASMSFKTMAILAFTVSQPVLGAVFPSNIFSRSEIEAMPLEKRGSMDAYQLWDAAEIPYILEGLPHDLSGSIRSAMREWEQST
CIRFLPKTTQSAWANFKK
>FUN_005855-T1 | Effector probability: 0.881
MKFTATLFGLFGLFAAQAFAVNMYSAPNAAAECGALGVAEWDLDTLPAGMDVSELRKCKEHPLSVKIHSRNPDSTKVKVLEKR
KCTSSKWKPKAGCDKHWCWQNCGSAKEVEWGFWCWSAHKDGKGAWMSCSNDSDCPTDDKFCGKGDCQQCGCGC
>FUN_005883-T1 | Effector probability: 0.628
MYFSNFLVTLASMASMASAIPAPDALLDTTSNISPSDFTIRSDSNNLEARTTFSCPGAMSYCPWTKACSCPPGQSWDGKAKKC
AGKKASGCWPKPSASVYAGAGVDVKLGTYCAASPYKIVKYDTKHAYCQASLKNTVFLAPLSIGAEVALYGGAAINVKGQLSAS
LKTVCGGLAGLYLESSADAVALFNTNKYGYAVRPGSVTGGLVYAVVDFVKSITCFLGLGNCQVYDCVSYCSKGCKNYVDVRGS
IGGYVNGLVGFCIVKDTVLFVNKVGACASVKIQGLIRIVASIHVSLKGIFGCNC
>FUN_006171-T1 | Effector probability: 0.643
MHFLYSLLATAGLAAAHGYVESGTIGGQTYQFYNPYTDPYMNPVPKRISRPIPGNGPVEDVTSIDMQCNGYTAGGIKGSSPAA
LHADAKAGSTVNLKWTLWPDSHVGPVITYMARCPDSGCDTWEPGTQKVWFKIQEAGRDGTSNNWATSPLMKSGNSGVNYTIPE
CIKPGYYLVRHELIALHSASGYPGAQFYPGCHQLKVSGSGSTTPSNLVAFPGAYASTDPGVTYNPYQATTYKIPGPAVFKC
>FUN_006195-T1 | Effector probability: 0.679
MRFSIAAASAALLALAEARITGIKVPKEIAVGEPFEAIIVRENYIQSVFDSSIVFGYAPESQYPGTIGQVANSFALGKKESNK
VEPLKKKLTIPEGATKGKGLVTAALLSIYGASGSPTISEYNVTVTFGDKTSKEYASSS
>FUN_006251-T1 | Effector probability: 0.722
MQFKTLFTASLLGLAAAAPAEECTKTGPAKSGNSPAPKTFGLVALRSASPIHFTHFSASENGFLLGLPKDKQNATCTGKSDGS
AVFRLSEGELFLYNTGKKQQRAYTDRSGMGQGVLQYATVTKNPLPEAFETKGWKIDKSGNLNFDNASGFTACPNGPDGSWTVW
VATGNSRPGNSDKECLGFNARVAEIEHPTSCFYSEYSG
>FUN_006256-T1 | Effector probability: 0.939
MQLSKFVVLSVTALLSATGEACKCYGTKGNLNNGATHKCCNDYHGVYSGNDCKASSISEHLSGFDRCCKGQGSDCDFPGRAAA
LGQEMRAVKHVEIKTALAK

>FUN_006403-T1 | Effector probability: 0.654
MHLSSILVLAAAFMPFAATDDCVQRTTPCSPIAVCDSMSGINVCGTTENPSQCSTMVVPDDDSGSVSCQCCKA
>FUN_006410-T1 | Effector probability: 0.585
MHHLSLVYALLISATGAIPLEDSPFPGAIKWTPNHQIQPGDVIVPVKSVSYVVKEEAYLAKLKAEGIKIGAPELDPSWISYNG
TDLPKPKYPKASKGQLGKRDDCDGTFYITTDKTETFVDWDVQMSPVVCAVGDMDISVSSGYSVANTVGGSAGVDLTFVKDKLA
GSLGINYSRTWTTQTSVITKATIKDGQCGVMITKPLTTRRSGRQFRGCIGSATEVGTWYADSRGEGSYNGITWIEGAISACAK
PGSNPPLSRCNGQGNFR
>FUN_006411-T1 | Effector probability: 0.864
MQISLFTVALMAISGAHAALHKAAACVSNRVSSPVGGTPFSPSYNWQTTYEVLADATKCACDMYKQRNTGGEMWDTCPDCTFD
GTICNSAEGHMGGDEFTYYCEEKCGAQGAEAD
>FUN_006582-T1 | Effector probability: 0.935
MLFQNILIAASIGLAAASQSSGRECGRKIAPCPEDTTCKPILANCKDLNKCSGMCHFKNEYQTCGGFRAKPPPPCKKGTHCVN
DPRTPGDCGMTCDKPRICAPKNPHTCGGFMGKKCPKGLYCYDDPSDKCDPKKGGADCSGICL
>FUN_006626-T1 | Effector probability: 0.874
MKFSILAQAILALPALATAIPEPVDTTEVDKRANCKLTLQWKSNWYENALRRYRVQLITSPRNDDHLGLYCNQFKVNAVQLEN
VQCFWTDGMYVLDVSEVQGPAGYAQYKNQHNLASKTFNRGTGCDVIQNL
>FUN_006627-T1 | Effector probability: 0.895
MKFTLFTQAAMAIPFITGALATPTPDASEVKRADCSMTVRYRKDWVESGLDRYRMQLITNPRNDRHLDVYCDAFKAPVGFTIN
PQCFWGDAYYVDVSAARGPAGRRAVQDAHNKACNDFEVYTGCRTIREF
>FUN_006652-T1 | Effector probability: 0.675
LNAPMWKRLALAAELLDLARQQQGGQGNQGTTLASTSTSTSTIGDETKSFLLRIANRIFTSRELSQPEVLGYLLGFGTDFTNV
PAWTWVHINSLYWACAKQWPGLKEALLALGRDPQPDNIYFQTDGFKLPYLEAYKHRGPILQELCFYDYMSFVVLKKERYHWRG
TTPIPFPPTATVCKGWVQCLRTPGKMAVPVFDGRLTDEFDEQDEVFIKK
>FUN_006655-T1 | Effector probability: 0.733
MKLSSLLTFTTIAIAGTDAWSFTVWTGKDQHGKKRHYHSSVGDNDCYNFDKAITSKGVGSFEFCSFIWTRCSVSIHSDIGCRG
KKLGSATAADKGDLWPTKWSKNTSKEAKKMKSFRIQGCKDIPVTGGNLDIASCK
>FUN_006656-T1 | Effector probability: 0.908
MKFSAVIALLLPLSGVLASPVADSGPVGIGRDEHATGTGPLSLDQSEADLVGRDLFARSTRKCKIVNVSNRAFCRSGPGTNYP
IKYSVYKGQEYKFDCYKKGTCHEGNCTWDRLPHAEGYCYVSGAYTDGRCTKAALGKC
>FUN_006677-T1 | Effector probability: 0.725
MLGKFLVAALAVVGGVNPVEAQQQNIFVTGVPVTGGGAVPARKNINDMHKAGGPAWDLYMRAVRSMHDAKETDWKSYFQVAGI
HGKPFIQWNGGGGRAGNGWPGYCPH
>FUN_006684-T1 | Effector probability: 0.564
MKFSLLLALTASSVAQAAQLAFPGAEGFGRYAVGGRQGEVYKVTNLNDSGTGSLRDAVSKPNRIVVFDVGGVIKISERIVVSK
NIYIAGQTAPGGGIVVYGNGWSLSNANDSIVRYITIRMGKGGTSGKDAIGIADGKNIIFDHVSVSWGRDETFSINGDVTNVTI
QNTIIAQGLVSHSCGGLMQTDGGVSLFRNLYIDNKTRNPKVKGVNDFQNNVVYNWGGGGGYIAGDSQADSYANIINNYFISGP
DTTVTAFTRGNSFFHAYVKDNFYDSNRNGKLDGAALCEKASCYSDIDFVKTPYNYPAPTALTPQAAVELVLKGVGNSLHRDTV
DTALIDQVKSYGTKGGQISDEKEFGGVGEIANGAALKDSDGDGIPDEWETKNGLNPNDASDGMKVASNGYANLENYVNSLV
>FUN_006748-T1 | Effector probability: 0.719
MKASIIYGLAALFGASRVIADCVEGHCETITPDYIVEHKCNMYRTGTTHERVASAKDCAALCQAAGVEVCPYHPPSKKCIVSK
PDGEDRTRADVMYITKVEIDDPFVEPDLFAETDAEAKEACLAREAALESELCSTDLAACKAGCAAVGDGTTCRSYAHDGGSPG
TCLLYYKHIRELQPAGNFKVEGTWRSWDKEC
>FUN_006937-T1 | Effector probability: 0.636
MRAFLFSSAFFAIVASALPDPKGHEFRAAGPYDSRSPCPGLNALANHGYLPRDGANINYDMINHAAQAAYHFEDGFYIDAVNM
VFQLDISTTSRPNETFHLRDLAQHDQIEVDGSLTRNDIFFGDDLHFDATVFDPVARDLGLDKISRKDKFVTIETAAKATKNRL
DLAKRANPEFNVSVHQHETEYGTTALYLLTLWDEHQKAAPKHWVKALLGEDRIAYREGYNKGKSVKTNKQVGAMTQAVRAVVG
WKP
>FUN_006953-T1 | Effector probability: 0.886
MQISALITLFSLSTGTNAWAQAGNGEWIANNKIYDVTNSGFAKATIEACTYRNTETRVPIGQPCKYWLDGNGRIASGVCREDQ
YMYYCA

>FUN_007055-T1 | Effector probability: 0.855
MRFSSYVVSLMATGAVAAPHNDNWQNGNKHSKVVTKFEYVTHYVVGEGGSPQTTCDPKPQKSDFKQYIPAPVYAQPATAQFVK
EPKHAPNQDSSSDLGPSYGLSTDQEKAIHLHNEARKAVGNDP
>FUN_007216-T1 | Effector probability: 0.893
MKFFTVIATIISAVSISAVPILKERAVGGVLICTGANSTGTCTHEVYTLNKCHQLSEPFLRNVTTFAPDGEYFNCYPRTTGCN
DTCTSPTGCTIGPIDFDYKFKFNFTRVGWPKLFNSFDCSLKKTTKGENMFD
>FUN_007375-T1 | Effector probability: 0.648
MASLIASAAYTLLVKFGTGLAGKAGGWVFQEGLAAVTGGTDTEKLRKDGLRTYITDIETYYSTIVDIMQEAFQLPDRNLTDAD
RVRQAQGLQKRLDNRLRACSNDVPGYLDQINDFLNEQGSNAFLQQAAQQALD
>FUN_007376-T1 | Effector probability: 0.771
MNSLSLIVLFFGAAMAGIHHNCGCTVRGSYRQDLAQAACALWGSNQPHTHWDGYSCVDKGTGRGIDGAPWETTCKQAWDINFG
GNKNDAWGKCWH
>FUN_007377-T1 | Effector probability: 0.842
MARFILILAALATAAFASTTANKALEKTPVKVTEVNYKVGEVSKCITLSHLQALGQTFTNLNATGDYKLPEIPTTYINDPTAC
IPESLFDQYFEPIVAISKEQNLLESFDAEKVHGRDLEVRARNCPQIKDAALGRVHSWSCDSAPHPSSCRSCANFSSINFVAAC
VACAAKANQESIFCCAAAATTFATYYSQVCLEK
>FUN_007380-T1 | Effector probability: 0.636
MKLSSFLYTASLVAAIPTAIEPRQASDSINKLIKNKGKLYYGTITDPNLLGVAKDTAIIKADFGAVTPENSGKWDATEPSQGK
FNFGSFDQVVNFAQQNGLKVRGHTLVWHSQLPQWVKNINDKATLTKVIENHVTNVVGRYKGKIYAWDVVNEIFDWDGTLRKDS
HFNNVFGNDDYVGIAFRAARKADPNAKLYINDYSLDSGSASKVTKGMVPSVKKWLSQGVPVDGIGSQTHLDPGAAGQIQGALT
ALANSGVKEVAITELDIRTAPANDYATVTKACLNVPKCIGITVWGVSDKNSWRKEHDSLLFDANYNPKAAYTAVVNALR
>FUN_007478-T1 | Effector probability: 0.637
MQFGLLLFSALPATWAAHLYAVPQSLSLLETSAEDNGCTLPDTYRIRNFKAESKDSGKTLSAFDFVFFDEDTKLTTPCHKNAS
SKALTGLGGRDRFACDNDVVEFIWTDDTKKLWMMEKVCQQQDGSVPYEASGSIILNVKCARTGSCSSNSTDQKSAFTSLQPVR
EAPPS
>FUN_007542-T1 | Effector probability: 0.914
MKFLPLAVALFATLTVAAPSSPSDIQARSCVCKKVGDDWICTGTKCYDKVKRDLVPRQCSCHKIGDEWLCGGPKCPRDLPEEN
KLAKRQCSCHKVGDEWLCGGPKCPRSLPAEESGLEKRQCSCKKVAGEWICSGRKCPRDLSHLMGEE
>FUN_007609-T1 | Effector probability: 0.602
MVVIKLTPAIVLAFLAGANAKDIFVSPTGTGSGTLAAPYGSIQSAIDAAKAGDFIYLRKGTYAPSKNIQVKTSGAKGSPITVK
PYQNEKVVIDGENMPGTPKAVGESLPNAERGVFHIQNAEWWSFYNLEIINGPYGIYARDASNNYYEGLSTHDNYETGFQLEGA
SSNNQVINLDSYRNRDPRKNGESADGFACKDGQGEGNVLRNARLWDNVDDGLDLYMFGSPVTLDEVYAWGNGFNRWSFTPFEG
DGNGFKLGITNNPPANHIVKNCIAFQNAKKGFIDNGNPGSLTFDRNTAWKNGDNGFNLRSSSSKVTKNVAASNTGDQVSLNSK
VSASGNSWDSKDTWNDASFKSTDASTLKGARGTDGRVKASDFLIPASGAAIGATTKAKV
>FUN_007667-T1 | Effector probability: 0.888
MRFSTMFTALVACASTTSAAINWSLEKVSNPSADQADAYSRIENAMRLAAARYNRLGRATKTIRVSYVPGVPTADANFNGSLR
FGSNRSYMSERTALHEISHTLGIGQTAAFDRKCAANDWRTATPLLQSWDGAGVRINCGGGHIWPYGLNYDNEWSETNANRHVQ
LVNAMIADGLQG
>FUN_007671-T1 | Effector probability: 0.702
MSSLANTFTALTCLLAVAGAVPTALPRASGNCPSTGKTTRQEPSALYSVFPGSPDVAKKSVGFNVATYNNASQIEQLLVFTGI
PAEAKKCTLGWAQGEQPERLFIVKGGDALTEFKQLSGFPGKAVTYNTAKEFDTAGESVGAADFTNWDDLPAQTHIVGNIDCKS
TVYLKAALRNPNGNTKVFLEQSDKNGVYIEYSC
>FUN_007688-T1 | Effector probability: 0.555
MYSILRIASLMALIGYAQADFAVGTSASQMGIGEIVPDTGGDIYNTDAVAVSYPGGCLDNVQQFECVSERCLIEGFFEIRGLK
TEDQYGFDWNGEGWEFSQNSEILGFCTKNDDEGCTKTGVNYSLGFFSHMLCTGSINSGF
>FUN_007980-T1 | Effector probability: 0.797
MLLKMLVSITAAVAVTATGIDKPVGVAGCKWSGTAPYCAGECEPGWHERGRSQCGDGSCCWTGSKALCCEDEDAVQDLMREKE
EVGVDSCYWSGTAPYCAGECGPGEREIGRSKCGDGACCWTGEKAYCCNDEESDNRDL
>FUN_008054-T1 | Effector probability: 0.641
MKSSDACLPAALLSVSQVQATCVPGTRETISPDYIVEYQCNWLRIGKSHTGINSPTECAALARDAGATASAYHPPTKKCVVGR

EGGTEKANADTYYMVKEDEDEEDPFAMTCAEEKEACLERETVLKAELVSSKDQLAASQAECDSSRADSALLKDILQSNCTSQH
TKYGMVAGTRYRFWCGRFHEPAGQRESHSTATMEACVKLCTSKPWCTMVLHGIFRETCQLYDRKVKIEATPPQSSVLWNSAVN
DQA
>FUN_008138-T1 | Effector probability: 0.842
MHFTSVFTALSLAVIAVAAPGDRGHYTVSGLGARKQAVLNAGGNTLDLAIAMLEDEHMQTNYKYGDGKTRDAANFGIFKVNWG
MLRVCASRAGFKGQSEDQWNNGDKLNSDIYADVASRWDCQKHYGYEKWFAGHRNGATGLDNPNTEDIKFYRGSVEWIKSQIDS
DPKYKSDDTRFWVDVTPI
>FUN_008152-T1 | Effector probability: 0.672
MHFTTLLTSAIALASTATSQPTARQAPTANAVIMIVNSADHSRQPVRIPLAQLTTLDYQVTELRLESLNVNIPNIESPELSDV
VCQRYKDKYGVQLGSAEFSHEKPALISTNPVEFGWVLCYHQDRA
>FUN_008163-T1 | Effector probability: 0.66
MPSLKAISASLVGLLSLGSASAIPPVNSEGYLVASTYFAGFHANRGFPVSAMPWNKYTDVKYAFAETSPDGTLDVSKSQPENI
PCFVKDAKKHNVKPLISIGGWTGSRHFSTNIGSAKNRTAFAKKVINFVDKYGFEGFDIDWEYPNRQGLGCNDINENDTANLLE
FFKEIRKDPKGKKFYITAAGSVFPWNDKTGVASKDVSGFAEVINYIMLMNYDYYGAWSAVAGPNSPLYRKCDARNDQGAAENS
IAQWTGAGMPASQLVLGAPNYGHGFKVNSTSAFGKSKNHKLQLYPAQNSTDRFQGSSWDNDPLVDACGNPNPPGGTYPFWSLI
KEAKFLDTKGNPAPGIASAWDNCSQTPVLYDAKREIYVSYDNAASFYDKGKFVLKNKLAGFGTYEAGGDYNNILIDSIRSAVG
LH
>FUN_008319-T1 | Effector probability: 0.574
MKLTLITALIAPVLAINIPKELQERSGCGDNCARAVVPGFRGPAVVASYKAECEAYLQVTTTPDASTVYVTKSIPTYASACSG
EPRYLTACSCASASVVTVAAPTPTVTKVVFV
>FUN_008323-T1 | Effector probability: 0.689
MYPLTTLASVLAVAGAVTATLEPAQSNTKGKYPKSPSCSPSKTSNAIQAAECAYNTRVSGQQTFAIFKVDHQYDANNGAPYGT
CEAYECDAPTSDELTADADYWTFFWNDNGESSGVGTTCIKDPNDGTCGCENSDGTFVHGGTNCK
>FUN_008401-T1 | Effector probability: 0.963
MKFSSIFITAAVFVGAISAAAVPDTNIPGIDLNKRSCNEEQFRQCTRNCPTGTGGGPIGCPIGKQQFGIPV
>FUN_008409-T1 | Effector probability: 0.609
MKPLIALTALFSLTTASQSPLRDNQAPLPKPPIPLKGHLPLMSSNPPSVQPSVALGDILGSNRGLTSFSSFARMQPSTDTRLS
DLSTNTTVLAPLNSAVDALPRKPWEQPADYNNFGADAYEGDGGQDRARENMKRFVEAHLVPISPWEKGEKSKTLGGKEVWWEV
KEGRRVIMPDEVEVERVASQVGNGELWILKGVLNYA
>FUN_008417-T1 | Effector probability: 0.66
MHSTIFALVAVAGYVSAGPTARSQTECSGVGQFYTCANNGFRGYCSVDPCAIKWCTDFVEGTCDPVFITKPVVATETEEEEEE
HWEPETTPASLPEGQCAPGTGFFQVCSNGFKGCCKSDACAGKDAICPDDKKVKRTDDPTVCAPGTGFFQSCSNGFRGCCKSDA
CTGTAGVCPDNKPEAPKPKPETWEHKPETTTETAPASLPDGQCAPGTGFFQVCSNGFRGCCKSDACTGNAGVCPDKKTKRSDD
PTVCPPGTGFFQSCSNGFRGCCKSDACSQSQPICPDAIAKRSDDPTVCAPGTGFFQSCSNGFRGCCKSDACSGAAPICPDTVA
KRSDDPTVCPPGTGFFQSCSNGFRGCCKGDACGNTWCPDYKTGTYEPAQTLKVKARSDGTCRPGTGFFQVCANGFKGCCKKDA
CSQKKPVCPK
>FUN_008476-T1 | Effector probability: 0.555
MKFLGLLNLAALVSAVPTPTFQEAGKTLGKRAAITDAANIGYATQNGGTTGGAGGATVTVSSLAEFSKAAESEGKQVIYVKGQ
ISGNNKIRVKSDKTIVGAAGASLDNIGLYINKQKNVIVRNLKIKNVVAANGDAIGIQKSTNVWVDHCELSSDLSKDKDFFDGL
LDVTHASDFVTVSNTHLHDHHKASLVGHSDNNGSEDKGTLHVTYANNHWSSIGSRAPSVRFGFVHVFNNYYEDISVTGVNSRM
GAQVLVESTTFTSAKKALTSKDSKETGSISVNDVNLGGSTNDAPKGSISKSNIPYQYSLVGSSKVKSAVVGVAGATLKL
>FUN_008564-T1 | Effector probability: 0.562
MIFKSLLLSSLLATGLALYIPHEDITSKECDCSGEHVRENNKLKHIYICGDERLGPTDLPTNLPLSTYVAGYDRFGGLTPNEF
LEKWWNNTARPDGKKPVGWKYPLKNGFELDDDERPIRANVNLAPGTLVDRFGEPTGRYLSPATAPFSQRALHPGNLITGENKE
FPNNYHVYKVTKSFTVQAGPIRPWFGQPGYGVQFFLGVGITVKDYLDNGSLQWVNASALVREAKHCALSGGDTDLESEEL
>FUN_008604-T1 | Effector probability: 0.666
MQFTKIASVLAMAAAAIAAPAPGNYEIEPRTGGGNKNQPACSAQSQQVCCTGLSCLVQVLGGCSTSSFCCETDAPDGALVNVA
LLNCVNLL
>FUN_008640-T1 | Effector probability: 0.924
MKFSAILLVLAPLVSAAASKEAAAGLPDLASAGEKGHDLSSHGEGLQLAAKVCPAKFPRKCSLGNFCCRTLKCCKKECCQNSA

RYCSNGRCYK
>FUN_008688-T1 | Effector probability: 0.909
MLAKAVVLTTFACNAWASPYVVTNPKDLKNDWHSNDYTGTRGKDQMIHWKFTIEGHNAVPDPGDFSTVCKGVQNGELCGEPCT
KPGAFVPCKSETYEAGQWFNEYGTTVEVRRTTFPDAGKKVITSAMANITEQDVVGTVHYKLKFGDFQKQFISTFNPPTA
>FUN_008812-T1 | Effector probability: 0.742
MLRQVALAALSVLPVALGQVAEDFESGWDKVAWPTYAPDCDQGGSVELDKTTAHSGKNSIKVTGGPNGFCGHKFFGTDAVPEG
HVYVRTWMKTAKAFDDSHVTFITMPDAAQGKGKHLRIGGQSKIMMYNHESDDATLPDLSPDGIAASKNIPANTWQCLEYHLSP
DGTIATWLNQ
>FUN_008868-T1 | Effector probability: 0.84
MHFTTTALSALLASAVSAVPLNSTPYDNPDSNIFPSFHRYSDWAICKGKITKDRFPNLQAPNREGGCIRYYQGIDMTGVVTEQ
HFFFKDGFKTACDCAAKCLEEPNKCTNWVWKHTFMPEDGGKRSCTLYSSPNLPTDVTLKYDLANSKGFNLLQATNNPQAGAPA
PLTFLDAAGTIPDKFGVSGFMVQDQNGRQFC
>FUN_009230-T1 | Effector probability: 0.891
MKFSIASTLLLANGIAAMPWSSLNAKQSNSEEITLRIQVSQPSSNHAFSPNHKGVVNVHNYEVCLKVCWPEEPKCPEGWGDGD
YPCWTCCKKTGDDDDL
>FUN_009470-T1 | Effector probability: 0.671
MRSLRTTVLAVAASAIAVVNADYVIDPESVPLSQRRVWCQNEIETCPMICGQTSKGDTKVNECDPKTLTYGCICGDGKQPNVS
EYSLTLPFYVCQEWGNQCVEDCKGSASCASDCRQNHPCGASNPKKYNTTSTATDTAAVKATASATDDPNVVYTGTPGGDGGDN
SDSSTTTKTNGAAVIEAGRTWGLTIVLSTIFAGFAMF
>FUN_009533-T1 | Effector probability: 0.624
MHIASYVSVAFTVLGLAAASPKGCTTKRSPEPKLEDGHYIVDRATKFANKKVWTFNGKSLPEGLYSSDYPVGKTHVFTPSGVK
VRNGYLELTVPGGQKSKPYKAAEVATEIENIKYASVRTTAILSEPAGVCNGMFFYQSDSQETDIEWLSDPKSESNYDGIRRLW
FTNQDNDGDGEPSHKPVLPPSNPTTTEHEYRIDWTKGLVQFYVDGVKQWSTKKDVPNVPGPWIWNNWSNGDKGWSAGPPKQNA
VFKIKKIEMYYNTA
>FUN_009556-T1 | Effector probability: 0.618
MLVNTSILTLLMAAFAAASPITESSPTLTKRNSVNDCGDSTFENHSSGGSPQVSDCQQIARNIAGGGTWTVGAGGEHHQLVQY
GTCAFGAQGAGSNMNAAHIGNQDIIDLINSSIEKFQWEGKVGAAGVMGCQSLTGLVGGVDMRWGIYHT
>FUN_009563-T1 | Effector probability: 0.616
MWKLLVSGFVAVASLSGVNAAYPNPGPVTGDTRVHDPTVVKTPSGGYLLAHTGDNVSLKTSSDRTAWKDAGAVFPNGAPWTTQ
YTKGDKNLWAPDISYHNGQYYLYYSASSFGQRTSAIFLATSKTGASGSWTNQGVVVESNNNNDYNAIDGNLFVDSDGKWWLSF
GSFWSGIKLIQLDPKTGKRTGSSMYSLAKRDASVEGAVEAPFITKRGSTYYLWVSFDKCCQGAASTYRVMVGRSNSITGPYVD
KAGKQMMSGGGTEIMASHGSIHGPGHNAVFTDNDADVLAYHYYNNAGTALLGINLLRYDNGWPVAY
>FUN_009592-T1 | Effector probability: 0.639
MFGSISTYFVTLLAAASTVANAAATSKNPVYTGLGTRYGDSDGCTEEDCWQKGACSFVDYKLPAGIDGTTCVSEDIWKDGANC
GGCIQVSYKGKSLKIMVTNKTGGDKNHLDMTPATWSKLTNGMTGGGVDGIKWKWIACPLKSPLQVHMHGGASKYWFAATIENI
THRVKAVEVSSDSGKTWKATTLKDPNMWILKGTLPNDTAWVRVTSVNNKKVIVKNVALKSGVVTKGTSNF
>FUN_009609-T1 | Effector probability: 0.905
MPFHTLLLAGIMAMIGHAKKPTKNLNSIYFHGNFTTISGGTGNKFGHNTGLTITDSARKEPYSEPYSGGASPCANPNMELKIK
SSCWEDELAIKD
>FUN_009621-T1 | Effector probability: 0.751
MELSWLQNLNLLLNRTVGACHQTSDRRNKVVDEVKHVLLCLANRRKLSRERLVVTNEHLNRALHYFGILILITDVTFDKEEET
SIPQNDHFRNPSIPDINGTPNTHQPPSNTQPGSSS
>FUN_009680-T1 | Effector probability: 0.59
MTRSLQGSVVLVTAGTSGLGLCVAEKLIQAGASVVINYASNEERAHDAFVHLNQLAAKSFSQDTGSRCLKIKADVTKKEGIQD
LVNQTVSAMGRIDAVVSNAGWTKFANFHDIDDNVDEEVWDRCYAANVKSHMFLCHAAKKYLEEAKGAFVMTASVAGVKPSGSS
IAYSVTKAAQIHLSKTLATVMAPSIRVNSVSPGFMETNWIVNMPQSKIDDAREKTLLKQITKVEDVAEQIILLIKSESVTGSN
VVIDSGFSI
>FUN_009791-T1 | Effector probability: 0.899
MLVNTFCLIAFATLTVAAPQRPDLNRDPDNNGDRIAQTKRVEDLRNKGLTCNEGAQEAFICSDGLGGDCFINILGEGNCLI
>FUN_009792-T1 | Effector probability: 0.882

MFAKIFSAFLLITAVTAKLHDNCACHNGDSYNWRLTAAACTEYNDEGYEWGGATYNETSGRCAQATTEDKIAGDQWEDACRKI
AEEGYDCADGEGKCYANPKKVRGRC
>FUN_009845-T1 | Effector probability: 0.751
MKFTTLSLIASLASLSAASLSAVPIIEAIAPKSKICPTGNKECRTATQAAPFIISSFQSQQIYSPAEIAAVLALMAFESGDFQ
YKRNHYPGRPGQGTANMQMPNYNLLYAKSIPELAKGWQGIESVEGLSDQELGDLLDDVTVDKYNFGSGPWFLKTQCKEDVRQA
FKTDVDTGFQKYIEECVGTDLQPRLEYFQRAKTAFGL
>FUN_009890-T1 | Effector probability: 0.879
MKLIAFLLFLLGLVNAGVVRTPAEAATLTTETTAPVATPDAFVAKGEEMASCNIQCAFWYQKCYYAPWSYSCGNDGRFRRHEW
NPICEKNCWCNCDSK
>FUN_009902-T1 | Effector probability: 0.626
MKPRCPSFWITLGILYFNIGQNNDSLDALTRAVELNAHIWEPWYNLGVLYDSCNGQHSDAADAFYKCLQRKPELSNVRARLEA
QQSYAEGLNEELLGGSLIHEMVDSPLDGKHGW
>FUN_009956-T1 | Effector probability: 0.913
MKATLFVSLLLSLGVTATPATDTVTIEDGGYTYTGIDKPLLALRGLEARCSCFPCYSPHADCQPGKCQCAGDYGCWSCGGGRM
QCQPGPGSGQCWT
>FUN_010001-T1 | Effector probability: 0.612
MRFTDFLLASAGASLALAAPHTTRAKGKFLFTGSNESGGEFGEGNLPGTLNKDYIWPTTSSIDTLASTGMNTFRVGFRMERVT
PSGITGAIDEAYFKGLQDTVNHITSKGHYAVIDPHNYGRFNGNIIKSSDFSVWWQKVAKRFANNKLVIFDTNNEYHDMDNSVV
AALNQAAVDAIRKAGATSQYIFVEGNAYTGAWSWVSSGNGAAMKDLKDPQNKIIYEMHQYLDSDSSGTSETCVSSTIGAERLK
AATQWLKANKKKGILGETAGGANAQCIAALKGELQHLLDNSDVWTGWLYWAAGPWWGDYMFSMEPTTGTAYKKVLPQIKQFIG
A
>FUN_010040-T1 | Effector probability: 0.71
MKAYLALSILACAISTTAKNIHRRADGFLPAAGIPKGGYNAYGGNYDHPETNKYQPVGQKQASSSGATNTGGKKATTQSTKCI
SKSKLESIVNKYVSTFSGITDGGALAKTIFEEDVKFYSQSIWWTSSSSKISKYAKNDDFPPIYKNRKELIEGNTEKTNDPSAF
IKGPIAYGCNSFTFYWKGDFEVPKGTRRGRGNGIDMVFLNPETGKVKKAYSEYNTLNQVYNWGAHITWSKDDVCCDCPVVFDP
KCKCKK
>FUN_010192-T1 | Effector probability: 0.84
MQFFTLLCLATSALALPQTLTKRETCMDKGSKVTEWTVKDFKYEAVYTQNTPTKQTNSATVTFTLQNRGVGYEGKCSAKSTDA
KKDFFTGNTDYNCDVPFEGDSASFKYNRKSGVIAIFQHWSCVKEGGWYEAKGNTTFTPKCTEKTWKNAHYKAGGDKAYSNRRV
TCQQKQLKVPVLEMQAVL
>FUN_010220-T1 | Effector probability: 0.835
MHSPTLSIFFTLLLSSHSLCATNEPCYGPSGRAGVCITEASCTSAGGTAISGACPADAANIKCCSKPTCSSGNCRWSSDCAGT
SASNQCPGPAQMKCCSSAATGFGGYSAPTIPAVGACKQVSVNAAKAVVNQFPGRIREIGCKRDCSCPGSSDHCCGLATDFMCS
DAGGSATLSGKEIAEWCMRNRNTLNLKYVIWGQKIWTTSVDKTEKNWENWRTMEDRGDLTQNHWDHVHVSYNG
>FUN_010325-T1 | Effector probability: 0.788
MRFFAVAALLSVAVAAPAYDKADGTCCCCDISKPATVCKKDVKPEDCFCAAVVCPAGAPTIWATSTTSPATTTTAAPVKREEK
TPAPAGPPCCCCDPSKNAIVCTVRAEGEDNSCICPMVMCPADASTLTVYSTPKETGK
>FUN_010455-T1 | Effector probability: 0.559
MRLLNILFLSGLATAAPGANLQARVAANDPCNIGYCTQNGGTTGGGSAAQVTVKTLAELTAAAAADGPSVILVQGSISGAAKV
QVTSNKSIIGKTGSSLTGIGLTINGQKNVIVRNMKISKVEADYGDAITIQKSTNVWVDHCDLSAVRGDDKDFYDGLVDLSHAA
DWVTISYTYFHDHSKGSLVGHSDKNAAEDVGTLRVTYANNHFNNVRSRGPLLRFGTAHIFNQYYDTMDTGLNSRMGAQALIQS
SVFTNVGKKAIFSESSSEVGYVVAEDVVLGGESQNTAPKGTLSSSKIPYQFTLLGSGKVASTVPGQAGQKLSF
>FUN_010462-T1 | Effector probability: 0.828
MLLNKAFLGALLAMGTVTALPNPDAEPADLEDRSILHHCGKHASWDHAKSECVCHDSGKVYTKKHHKCKCPKGEKWHHIEKKC
KK
>FUN_010591-T1 | Effector probability: 0.575
MHLSTLYLHSSLLLASSFLSTVIAAPSNNLLDADISVDGFGLQDERGDSDGVSAKAKVSAENDYKVPSDYNKPRVLEANIDVT
RLGLDPVIFYPDNDDEDTEVDDTDEEKRSIFARLFTRALSSDKQEALRLHNVARSNVKVKAIVWDSKLESAATAYAKKLAKAG
KMQHSAGKDRPNQGENLAYAWASNGFKNPITAGAQGWLNEKKYYKGETIPKGNFSKYGHYTQCVWKSSTKIGIGAAKDSKGAW
YTVARYSGPGNVVGQKPY

>FUN_010692-T1 | Effector probability: 0.668
MKFFQPIAVFLSLLTSIAPSGVMAAEESAANQHQKGETVKYQGLNLYVSKPGRFNLGHKKPGKRTGVLFLTDVYGIQLKENRE
LVDNFSKEGFVVAAPDLFQGNPARETPDFNITEFLAKHPPSVTDPIVAKAINYLRNELKVNSIAATGYCYGGRYVFRSLGQNG
KVDVGFTAHPSLLATEEIEAVRKPVSIAGAAEDQIFPQPRQAETEAILTKIGKPFTSVLYSGTTHGFAVRANASNPQQVFAKD
EAFYQAVRFFNAWD
>FUN_010742-T1 | Effector probability: 0.681
MFAQAFLLIAFVCNVSGTPYVVTDPKDLHSTWSSDDYKATHDTHSIRWEFTVKGRNGVPDPGNFAASCHGLQVISDDLDLTGK
PDFERCNDTAYEARQYFSGEYTTVEVRRTDYPDLGKEVQISATANYTSTNDNIVNGHLKFGDFQTKFIPTPIEG
>FUN_010801-T1 | Effector probability: 0.654
MQFSLAIVTLLATAVSALPTEEKRQAYIPCSGLYGTSQCCATDVLGVADLDCGNPPSTPANATDFSAICSAIGQRARCCVLPI
LDQGILCNTPTGVQD
>FUN_011068-T1 | Effector probability: 0.67
MHSFLFLAIASFAAAHVPSVSVPSCPRSNRITFSKSVPDQDPFPRTQIDLCYTSTALSLTFTALDEKSFYFNSSQGTNDDIWE
YEVVEAFIYKGTNDPQTYLEYEINPNNVTYQAFVVYNPTKDREEGAPFDHFFIVDPEADGFEAETKLNRRAGTWVSKVKIPLGL
FNVDPGCAKGTKWRMNFFRTVTNPKIFPDQELGGWSSPDKASFHITKFFGHVDFV
>FUN_011113-T1 | Effector probability: 0.848
MVATKFLIMAMAAIVTATPVASAPKAESTAWSPLNIEDKIDWDGVDRKAFKDPANWNNTASAGNTKRPADSMVILAGPCDQGT
CPDYNAAFDLVYTFTAVPVPPSNPNDPPLTIFSSNSDIRVNDCNNCQRTKVGSSLGGTVAGGCLDFTSCGRPQTICVDPGKSR
AHRIWKDKNVKTCYNMRVENLGSCGFVKSRIVLHPTGETACNW
>FUN_011150-T1 | Effector probability: 0.919
MKFSLFAVALSTLAAASPTPDMEHEKRVLGLLKPKGGIPFTFTSIWEVLATPDQVVDADNKYTGGLKGSKGLFKFGINSNEDV
ICYNITLYGFRGDYQSPANTATHIHEAVKGKSGPPRIAFPNPVGDEKSRNAVGCLQGPFRTGVIQNGQDTGVGFTLKQIEKNP
EKFFADSHSSLAVPGAFRGQLSSGKVC
>FUN_011267-T1 | Effector probability: 0.59
MKLDIAKLTAFLVANSTGTGVLVCPGGGYSHVSIVKEGYTPAAYLNKLGIDAWVLDYTTTSNATAPIYPKPEDEVFAALKKIR
HDNPKIEKLGIWGFSAGGHLASTTLTNPKAGLDFGILAYPVITLEGNYTHIGSRDNLVGPNATAEELHDLSAQNLVSDTTPPT
FLFHTFDDQAVPVQNTLMFAEAMAAHKRKAQVLILPDGPHGLGLALDDPVRSWTSELTRFLTYSI
>FUN_011341-T1 | Effector probability: 0.933
MRFEIAAVIMALAMNVSADRMEIFTKCTLGCDSSDAFFYTDYGAYKVNANEGCRSTSVPGMVEFCVDWGKRRSHFRFSGQNKR
CLIQDSESAYGCAYNSCWKTTWKEISCHWREAPNPDIEKEPVSSAAAAKATASAFTA
>FUN_011483-T1 | Effector probability: 0.761
MAYGVSAGALIVLAMFANGWSVERCGVEFQKLAKFAFRPPTTAAVPGMNWIRAILSDSLYSETDIEIALKSVFGEKAFTEAAY
AQRIGAKIGIPAATIKYPSSLCLFTNYNSSKQETRGYRVMTEAENIKTWEV
>FUN_011497-T1 | Effector probability: 0.761
MDSLNIPGPRALLHAGAVVLAFLPASWAITLPERLTGEPCVSVNGAWQITEDCVDPKYSQPVIDSETDETSPVPHRKVSGHFE
GTTIDFNVYLPKEGFKGRFFQILYPLQTSTSLPQEIGFGAESGGYTVRAKGFPSYRGDAAAAKVGMMIARDYYKPKQEDPRLL
LRWKRWLPPDGWSYGEHH
>FUN_011499-T1 | Effector probability: 0.591
MLGIFKLVIGGMLAIASIAPGVNAAYPPPGPVSGNVGAHDPTVVKRPNGGYLMAVTAPNVLLKTSSDRTKWSDAGAVFPSGAP
WTDVYRKDDPNLWAPDLSYHNGKYYLYYSASSFGSRHSAIFLATSTTGASGSWKNEGIVIETKANSAYNAIDPNLVVDKDGKW
WMSFGSFGDGIKVISINPSTGKRSGSNMVSIARRSNEAVEAPVIVRHGNYYYLWVSFDRCCQGAASTYRIMVGRSSNITGPYL
DKSGKDMMAGGGTEVLAGQGSVHGPGHNAVFTDSDADVLVYHWYNNDGRSTIGINLLRYDNGWPVAY
>FUN_011609-T1 | Effector probability: 0.708
MKILILIAPLLSLPLAFANYGGDYGYGYGEKISTVTATVTHTVTKPIYKAPITKTKTETVTNFKPTVTKYKTKTKTVTEKPYP
TYHKPGYGDGKHKDGEYKG
>FUN_011758-T1 | Effector probability: 0.691
MLFNVAFFYSAALAVSASNEWRAPTASDPNNHRPKPLSHGDGYLPRDGLNISLDDLIVAFTDAINLDPAATTLIGKKALTTGN
NGTFNLDDLNKHGEEERVPFEEGFKRSDDVINAAGILGLVAKLAVASIWDSAMVVH
>FUN_011792-T1 | Effector probability: 0.659
MSLTQAVVLPLAITTASVSLYHPNHLALPLPQRSPIETGKTLFIWGGASSVGSTAIQLARASGLRVVSTASSHNHELVRSLGA

SVVLDYKSPTIVNDAVAALADCDLVGCYDAISESASLEPLGAILDRIGPHKVCLIVTPRQALSSNMKWTLSLAFEIMDDKGKH
LVDHIWHNFVPEALADGQLQPKPDHLVIGRGLETLQSGLDRLRKGVSARKLIIEV
>FUN_011799-T1 | Effector probability: 0.802
MQLLNIVLALVPLVAVSEAACHQSSEASVLSGSYGQGQKKTHCIFVPGSGKHTDLEVKRLTGGSTTLSEYGCYYYLNREITGC
YKGGRRAYDNWEFSADPNAGAY
>FUN_011832-T1 | Effector probability: 0.647
MKILILIAPLLSLPLAFANSGGDYGYGYGEKISTVTATVTHTVTKPIYKAPITKTKTETVTNFKPTVTKYKTKTKTVTKKPYP
TYHKPGYGDGKHKDGEYKG
>FUN_011866-T1 | Effector probability: 0.773
MFSKIVSATLLLAATVSAAPASKTVRSTPDKTVTLTGVTHSVNAGLGGLRFDPDNVVAEVGDVVEWHFLPKNHTVAQSSFGEP
CEPLADGSGFFAGFNFPTQEGQAPDVFQIVVEDSKPIWYYCAQQMGDHCQNGMVGVINQNFDNQDFSLRRHKELAAETVKSVI
PPVQQGGQVIPNPNPNGGF
>FUN_011926-T1 | Effector probability: 0.967
MKTVFFNIAALALTISGVFANPAPNGMAVDISERDGVLEVREVPRAEAGIQCRECVHLGGRCTIGDGSCYASEHASCRWCGNN
CKSRCIGDGQTCEQWCL
>FUN_011991-T1 | Effector probability: 0.824
MKCTPTAFALLAAAAAGVQAANIQLVGSVLNSAAKEPFEKSKTLVYAGGKTKRDAPKVYEPDPNRDYNLEDGYIFVLQCTTVG
FRPECISFGSEPGKCVSYFDFDPKKGDDPTSISDAFNHNVTSISTNTGGVCQFYHYTGCDDKLDDRGLSTSYNYNLNVSLPED
SRTVEYFRNITSWRC
>FUN_012210-T1 | Effector probability: 0.786
MKLAATIIAVMATFAAAQAASDGNLVGCQPGTYRCAGTTGWEVCNTRAVFVNGGTCPPDTVCKFFKPSKSPYCVPPNFEFPKM
>FUN_012301-T1 | Effector probability: 0.85
MKFVPTLLFPLSYGVLALQGVLQRSDPQFIHLTFHGGLASYSVAFPVDGSTRKTDHDLSVDVIHNSDYNALEQCFFAIEGQAK
LTSKVSTKDGSQHILDNSPRVIIAVDCTGSCVPMYGECYDTRGQPVGPCCNGMCMANRCQPWNSTLS
>FUN_012370-T1 | Effector probability: 0.579
MRTAAFITLLASVVSATPFGQRSQVLARASETCPVVFDGRVPANASLTDFDTVNGGGWNPYNPGFVKGNNISWSEILQLPKTK
TKSRFDTEAGTIPLEVTISDKSIFMKQLGFRRAGLQFNKDSNEGSPGSKGVKTLHFSIMQDDKRPLNLSHEYLNVWHEKADFS
GNQFQFQAGQLIGQNGTAATWKLLDQDFKLLWETPMLKKVWQNFAITLNYEKNTIQAYYSKGCKPLKVATQPIARNLTGQGQF
QIGILKKPTGTDDVANSGFQEANLNEGLIYGGIFLEDSADGCVSL
>FUN_012505-T1 | Effector probability: 0.881
MHFSQLLITAAAFFTQGTLAVGIKTYSGRDCTGTEQTLTVDHNAACNPKVQRFQSYKENGFGPSNGQRIAFYAQPSCSQESFI
YDTYSYNGDYFHSKQCYNIDGHSPVKYAQGAKLY
>FUN_012557-T1 | Effector probability: 0.697
MKFTSVLLAGPALVAGRYLSDEVTSGTSTHYGGNVSGGNCGFVSYTIPSGIYGTAFSGPNWDNAGVCGSCIEVTGPTGKKIKA
MIVDKCNECNKGHLDLFENAFTAVGGTNGLVQTSWRLISCDITTPLVLRNKEGTSAYWFSMQVRNSNLPVKSLEVSTNSGKTW
TGTTRRDYNFFENPSGFLVDTVDVRITSSTGSTIIVKNVGAKPSTEYPASGNFA
>FUN_012600-T1 | Effector probability: 0.716
MYTSTLLLTLVASASAHIASWNKGMYCKGGNDSSVDNANTNLAVNPLYDLPKSKWWMQADRGCDAVPPPKGEFLELPAGKSFM
TELANNRAFTTLSYKGELTTEWQDGKNRSMPWRGPEGGCLMDGGDGSGGELHTKNIESTGGTAWAISYESDISKVTMDNLVVF
SVRYYSPFFRETWYDVPADMPACPEEGCYCAWLWIPDGCGQPNMYMQNHRCKVTGSTSTKKLGKPKPPVYCRDNPTKCVPGPK
QMMAWNQAEGNNVNPPNGKTPTYNQRMGFMDGAQDDIFVDE
>FUN_012721-T1 | Effector probability: 0.844
MFVPLLLLNLLLPSCLAAPYLNVRDCQINEITASLSGAYIDRDNKNNVSFRLYSEGIDTRCPPLNKDPEKNTAKPFTSKNVYE
CANPAVTFSYNGDDGQLRIWISNEKGSFGGFVTISNPSSEVISTTLKCLDN
>FUN_012726-T1 | Effector probability: 0.932
MKFTTLAATLLTLGLGADIAAAACCDVKVCDDFNLKGNCKNDCYPYLKTVNINKSGLRSSIASGKTDKDCFCTFGKDSQSCML
VDDKSKNAPNHCLTGINKLYCSRN
>FUN_012739-T1 | Effector probability: 0.818
MHLALLLSLVATTVANAVKSRNILYFDQWHTTDFPPPDLTRAVTHVMMSFADSSLFAAQPAGKYEPFQSHEQVREPFDHKLNV
CLAIGGWGDNSGFDEGVKTSSSRQRFAKDIASTVDRLGFDCVDIDWEYPGGNGQDYNQVPNCKKKNEIKGFPLLLKEIKKYIG

RKELSIAVSGLERDMTAYTLM
>FUN_012745-T1 | Effector probability: 0.965
MHFATIIATAALVMGVAAEPASKDLPFNVISARIWSSKDCGGDNNNGNLGELTTHRDELNECFKFTDKVKSVSQYEHAKGCKL
LLFTDSNCKRGQKTVKDDQCRETSSHFSSYKTVCN
>FUN_012823-T1 | Effector probability: 0.84
MFSPLLLLTLTLSAIATSPSSSLNTTNISQHPSRVAPRFISGGFYDKCKDVRFYLAKVDDTHPRKNSFNGYKSSPWLVAKCPD
KNGKYLCTWLALSKCLLNSEGELYRGSNGNFHGSCTAYVAVGAVDGYLTCHGNWGILDYCSGRQSHDPFLDSK
>FUN_012842-T1 | Effector probability: 0.812
MKFTAVTLLTLAAGAMAAPVAEAAPEAAPGYTTYGDYKGAGENLPSYPSYGSYGAKPKPKPKPAPAPKKYTNYGSYNYKKYSS
YGHYKREAEPEAAPEAAPEAAPEAEAAPGYTTYGDYKGAGENLPSYPSYGSYGAKPKPKPKPAPAPKKYTNYGSYNYKKYSSY
GNYKREAEPEAAPEAEAEPETYSKYGSYPKKYTNYGSYNYKKYSSYGTYKRAKEFINSLF
>FUN_012858-T1 | Effector probability: 0.796
MFSKPTNTILAAAATLISAGALPRSEAGSVSITPHDQYSSSIGVLGCKIDTNRVAYWPGSVDCNNICVKVSYESRSVYLLKID
SSGGAYDISYDAWNYLGFGKSASDSPQQGGGIAMIYEFVHASQCTDILDNGKLPLAAANSMNYVASCLSEPNSWVAQNYELYN
INDPVCKRGVNEKCYLNLAISNQPECPSGLGAVGELNLTVENILYPSGKIVPAQ
>FUN_012886-T1 | Effector probability: 0.586
MLSKLLFAAMGSILAQSAAVHASGSIGYLFTFGDSYSQTGFDPNGAKPSAANPLGNPPFPGWTAAGGANWVGDIVKEQNNSLV
LSYNFAYGGATVDANIVKPYASTVLSFVDQVNQFSNSVGKHPAGTSWTAQNTIAGVWIGVNDVGNSFYLQDADAVVEKATTRY
FELLQVLYKAGLRKFVLLSVPPTELTPLMIQQGADSNALLVKAIKLYNSKLASKLSAFKKANSGVKTLLVDTSVSFKKAINNP
KAYGAPDATCYNSDGKSCLWFNDYHPGIAINQLVAEQVANELEANGFGW
>FUN_012913-T1 | Effector probability: 0.807
MKFSIVFAGLFASATMASPQGNITPNTMGALERRASFPIPASKGSVTYKKVQTISGVFDGGMKTYGRGVKCTGQAEGGDADAV
FLLKNGATLKNVIIGADQIEGVHCEGSCTIENVWWKKVCEDALSLKGDGNALIKGGGATGADDKVIQHNGLGTVTIDGFTVVD
FGKLYRSCGNCKKMGTRNVVVKNVKAFNGKVLTGINSNKGDVSTITGTCASSVKEICVEYQGTVPGKEPKKLGSGPSKACKYS
SVKSC
>FUN_013086-T1 | Effector probability: 0.601
MKFSILTALTAIVGSAAAANQAVVTNDCSGTIYVQSWPYNGGAPGPLVTLKPGQKFSENLRSTGSTVKIATTKTLTNPLFFGY
SSTSKPNYVYYEFSTQWGNPFANKHNILTTGKGCKQFDCKAGDASCYSTPSMKKVYGCPSPATISATICAK
>FUN_013106-T1 | Effector probability: 0.73
MVSFTSLLAAVSAVTGVMALPSAQPVDGMSVVERDPPTNVLDKRTQPTTGTSGGYYFSFWTDTPNSVTYTNGNGGQFSMQWSG
NGNHVGGKGWMPGTSRTIKYSGSYNPNGNSYLAVYGWTRNPLIEYYIVENFGTYNPSSGGQKKGEVNVDGSVYDIYVSTRVNA
PSIDGNKTFQQYWSVRRNKRSSGSVNTGAHFQAWKNVGLNLGTHDYQILAVEGYYSSGSASMTVSQ
>FUN_013121-T1 | Effector probability: 0.777
MKYSAVAILSLAQGIVAAPSFLEKIKGSKPKALSTDQDISLSIKFSQVPMQREEHQAVKSEICWLLCADKDINCPEGLYRSKK
VRSPPWPKFDNN
>FUN_013147-T1 | Effector probability: 0.978
MKFPIANSLAILAAVPKVSAWGITFYNNVDNCDVNDETEYQILEGNQGDCYTFGSSMPGVSCGHYIRGGVENKGCSGMFKATS
VWTKENSNCKFYAYTDCRDYGTQRENRECLNTRELLVFNPDVDSWKYIASFRCESQVQRLGTHILLTDIAITAKH
>FUN_013383-T1 | Effector probability: 0.723
MHSYTIITTLTLSTAASAYQLPQNLKSIYDNHKAGSCSNRLSDRFPEGARYCGDIPGAVFLKGSDGNYDNMDIDCDGANNHAG
ACSNDPTGQGETAFKDTVNQYGISDLDANVHPYVVFGNEGASPSFDPQQHGIKPLSVMAVHYGIWGDTNGGTSTGEASISLAE
LCFPNQGLNGDMGHGEKDVLYLAFKGDEAVPGKNGADWKTTSRANFSKSIRALGDKLVAKL
>FUN_013393-T1 | Effector probability: 0.627
MKSLSLILSALAVQVAVAQTPDKAKEQHPKLETYRCTKASGCKKQTNYIVADAGIHGIHQKNGAGCGDWGQKPNATACPDEAS
CAKNCILSGMDSNAYKNAGITTSGNKLRLQQLINNQLVSPRVYLLEENKKKYEMLHLTGTEFSFDVEMEKLPCGMNGALYLSE
MPQDGGKSTSRNSKAGAYYGAGYCDAQCYVTPFINGVGNIKGQGVCCNELDIWEANSRATHIAPHPCNKPGLYGCTGDECGSS
GICDKAGCGWNHNRINVTDFYGRGKQYKVDSTRKFTVTSQFVANKQGDLIELHRHYIQDNKVIESAVVNISGPPKINFINDKY
CAATGANEYMRLGGTKQMGDAMSRGMVLAMSVWWSEGDFMAWLDQGVAGPCDATEGDPKNIVKVQPNPEVTFSNIRIGEIGST
SSVKAPAYPGPHRL
>FUN_013400-T1 | Effector probability: 0.576

MQFSLACLAAILATSVSAAPAPAVNMMAASPQWTIENMQRSCAKDDSSCTWNFKIDTHKGAATGCKYVVKGSKASQRNGGPVK
CGDFTITSGWSGQFGPGNGFTTFSVVSSKRQIVWPAYTDKQVSSGKVVKPDQSYTPANLPN
>FUN_013404-T1 | Effector probability: 0.77
MHIKYLLINLFALTVSVKALPDADNDFGLEARDDSDALVQRWDCGHDAKEVYGKCVCNDKQLTWDGKKCVCPKGTTWEYGKCV
PNKPSCHKPQVYNPHSKKCECPHGTEWKYGKCIPKCRHGQYFDKDQWKCVCPKGTEYQYGKCIPKCPHGQSYDKDQGKCVCPK
GTEWKYGKCIPKCPDGQYFDKHQWKCVCPAGTSWKYGKCVKNCPKGQEYNKWSHKCECPKGTEWKYGK
>FUN_013453-T1 | Effector probability: 0.599
MRFSTILGSVLLAAPIQAAKLLICSDSTTANYATGNALQGWGFYIQDYTTLTVSNLAKNGRSTRSFINEGLWSDLLSKTSSGD
FVVIEMGHNDDGDPTTSDRATLPGTGEETVTVTTTTGSKEVVHTFGWYLRKMIADVKAKGATPIISGMVNRNYWTGNTLQSKW
PFADYAEAVAKAAGVEYINHTKYSVALFQAMGPTKAKTYYPNDNTHTNWDGAKLNTQTFVQAVKYKCGGTSVLKKYLNAAGNA
IKSPAQQSC
>FUN_013691-T1 | Effector probability: 0.857
MFTKFTTILAASSAALVSAGPLPRGDSGSASITPHDQYSSSIGVLGCKINTNRVAYWPGSVDCNNICVKVSNEGRSVYLLKID
SSGGAHDISYDAWNYLGFGESATKDPQQGGGIAMNYEYVHASKCKDILDNGKLPLAAANSMNYVASCLSEPKSWVAQNYELYN
INDPVCKHGVDEKCHLNLAVSNQPECPSGLGSVKDLNLKVENIAYGSGKKVAAQ
>FUN_013712-T1 | Effector probability: 0.858
MVNFITSFAILLTAASSALAAPQPLEARDDTSCMDNLPGNTLANVNEAVECINYLASLGDQACVAGVSGQSFCRRGNTQITGL
AVGLNSDQTSSSPCRDVARGAGLVMDRCTRADGKVRGQNPAWGNGHLMVDIRNVPQ
>FUN_013733-T1 | Effector probability: 0.959
MKISFVPLAFVAALAGFGEARNCTPKLDHCGHFLLDIGKYYSTISDELTRATKATCFKKNQINDALFSCGPNGTIKFKKYCKN
GCQVGAMGKHDYCWT
>FUN_013735-T1 | Effector probability: 0.853
MVAFNTLLTLAATLVPLVTGAALPTHEDSSLTRRQYQDKSWDAYHRQNGAFARQGLCLFYLDPSPRDGAWACGVYCKNDRTKI
CTTVHPSKFTNDMLGANPDGERYAIGQCQCDTSAADFLATATVDFTARGLDKGFREIGSVTCEIMVNVMKEAVFASTYAIPGV
GPVATAARTVAKGVKLASKTQGGKDMWTDAVKESCNFRRDEELLNKGFGIFEGAPDEF
>FUN_013805-T1 | Effector probability: 0.844
MQIKALLITPLVAAGVVSAAPKASSTPKSTFFQGLALRSASPIHFNYIQANKESFELKLKKQEASCDDGKHHNDVTFHLYNDE
LWLYSVGNPGQQAYVDLSGMGQGKFGYTTGAQPMPRNGQRKGWKIDKDGMLTFDGSSFVACPNGDNLEKTSWSVWVYNSIDNP
GGNKNCLPFSVKAAKVKKPVGCLYSQVQPDE
>FUN_013815-T1 | Effector probability: 0.841
MRFNITALLASLALAASADRMEVFTSCGGWTCSSFDGWFYTDYGTYKVNVNTGCRGTSVPAMVEFCVDWDNRRAHFRFSGQGK
RCMIQDSESAYGCAGTCYKTTWREIPCNWRMVSEEDPATEIASSAFVTTTKAAGN
>FUN_013888-T1 | Effector probability: 0.94
MQIQHVITVLWLSSTSTGQTLNIPSRVGSIVSLPSASVISGSKDMGNKEYDRGRRCNTDADTGSNNAVFILENGAALSNVIIG
ANQLEGVHCKGACTLNNVWFRDVCEDAISALGNGDVLIEGGGAQEAKDKVVQHNGRGTVTIKDFTVVNAGKLYRGCGDCTNNG
GPRNVVVQNVKAKGVSELVGINSNYGDTANISGSCGSNVNKVCQEYKGVQKGNGDSEKVKTTANCKGQQSFPAC
>FUN_013969-T1 | Effector probability: 0.63
MESASLVIGLAGLAGLFNACLEAVDKVQSYQTFGTDSHVLDTRFKAAKTRFERWGPGVGIKQGKLLPNYHPALDDHDTSAVVT
DLLHIIIKTICDASNSPQRRTRATGPDVDDSSALHEPGLPFAAASRSRRGKMSWALWGKRGRIEQVELFEKLVQQLHYLVPPT
TGASPRPTHKPDPGRSDTLARGMDSRS
>FUN_014065-T1 | Effector probability: 0.772
MKIAAFLTSALAGAAAARDSVYLVNSYKGNEISSGIAYYADGHSATGGSRPDDYVDVVHGSNIIWEGQTVKGTFGSGVSFTSN
IFADAGGKQGNAWAGTGTNGFHTYNCYKGTNPNGKPWVLYTVDGWTVNVIYFCNPFN
>FUN_014090-T1 | Effector probability: 0.618
MKFAITLAALASLAAATPLDASTSSCKPGTYSCTPDKTGWQVCDVNHKYVAAGVCPPGTSCVFYKKSASPYCVPPGFKFPQA
>FUN_014164-T1 | Effector probability: 0.597
MLSLWSLGLLLLSAPAAIAAPGSSLDVSPTNLPFTESANTAEASTATAVATDTAKTVGHVLKGCQENLRYTNWELYSDPNCQN
AIFNPLMVIWDPCKKFQETAPLSSGVIFQGIRWIGGGNAQHFYACQQGFSCREQVSEIPQNPGVCLSSGGQHWDKIATVP
>FUN_014277-T1 | Effector probability: 0.724
MRFPFAGLVLTVIGSVQSSPVAQAAKPPQFFLVGDSTVAVDGGWGNGLLSYLNAPAKGDNRGVSGSTTVSWKSNGRWDILIKD

ISAAKAEFEPVVTIQFGHNDQKVMQLNEFHANLVDIGNQIKAAGGTPIFITSLTRRTFQNGEVVQNLKEWAAETIAAAGDVGA
QYLELNKASTDYVNAIGNENAQRYNWGEGDRTHLNPAGEIVFGRMVVDLLLEKRDDFSSYFTPNKALSDKIAKGEFATGDE
>FUN_014286-T1 | Effector probability: 0.815
MKFSIITLSLITLASAAPAAKPQSGEISYGALNRDHIPCSVKGASAANCRPGAEANPYNRGCNAIEKCRGGVGDNRGSIKFGY
WNGLVTGS
>FUN_014305-T1 | Effector probability: 0.734
MKLNPKQLTAFLAANSTGVGVLVCPGGGYSVMSISYEGFGPAEYLNTLGIDAWVLNYTTASIKTPPLYPTPMDEALAAVELIR
KQSPGTKKLGVMGFSAGGHLAGTTLTTPKAKLDFGILSYPVITMEDDYTHENSRYNLLGNNPTRKQIESLSVQNRVSDKTPPT
FLFHTSNDELVPVQNTYLYANAMAKHGRKVQVVVLPDGKHGIGLGVDDPVRDWRLELERFLKYSI
>FUN_014381-T1 | Effector probability: 0.674
MKFLLPLLSLSAAANALHFFIDGTTPRCFYEELPKDTLVVGHYTAEEWDDRVSAWVKHDGISIYINVDEIFDNDHRVVSQRGA
ASGRFTFSAADAGDHKICFTPSSSSGRTSWLSAKNPNGGIKLTLDLVIGETNQIESSDKGKIQDITSRVKDLNARLADIRREQ
VFQREREAEFRDQSESTNARVIRWIIIQLIVLGITCTWQLSHLRSFFIKQKLT
>FUN_014425-T1 | Effector probability: 0.853
MVNPILVITTILATTAHAYKWDRCSGVQKCVASGPPPIYGDYTDFKYDSSNGYWYSKQIDGLYINPDGYYEPNGNGHVLQVWN
KPGNTLSRWRAPGKTACCLPDDVGTNIQGVSAQGYQ
>FUN_014469-T1 | Effector probability: 0.568
MRSLQIISVLGALFLTTVQAVTLPAGVPRDISEFRDKYPYAPPKHEHRRIIRIRASTNNADDVSDEFRRGVRKANGGGTLHLA
KGKTYVIGKALDLTGLEDIHINLEGEIRFTDDVEYWQENAWYHPFQKSIMFWKWGGKDIKIYGNGVIEGQGQRWWNEFESGTG
SILNPDNKYYRPILFYAENTTNLDVSGIHLKDSPCWNNFIVSSNNVKYTDVVATALSNNGSIIPKNTDFMNTMNTSAVRIERT
WVNIDDDCFSPKPNSSDLYVNTMYCNGTHGQSMGSLGQYKGEVSNVYDVHIENVWMMNGDYSAARIKVWAGEETGTGFVNNVT
FK
>FUN_014476-T1 | Effector probability: 0.656
MRFTTSLHLLGAALLASIASAQIAPAPDGWPNFWYKGHVTNKATFEYNPTNEFIFPSIFHAGEYLDDPLGEWYLYYAPHENPG
GISLVYSDSLEGPWKEYENNPIIANKWDSYYSVPHVSSPDASWNSDAGRMFLYFHGDNTQTRWAESSNGVDFRYGGVAVNNQM
SGSNTTESSYARVFAHPNSASKYNYAMFYMANEKDNRRKIRLAESVDGRKWTVDSDYVVQPGGPEGTDVSGANYWTWNGQTYV
IYHGSTGKIYARTIDQTLRDVGAEPILLYQSRGKGEDVGRVAAPDIASSGGNTYLFYESGDRLGATIAWAKMQKQ
>FUN_014477-T1 | Effector probability: 0.67
MQLTNLFYFAAALTSVSAATVSYDPGYGESGRALTAVACSDGKNGLITKYKWKTQGQIPKFPYIGGAQAVAGWNSPNCGTCWK
LTYKGKSINVLAIDHTAAGFNISPAAMNALTNNQAVKLGRVDATATQVAISNCGLK
>FUN_014485-T1 | Effector probability: 0.844
MKFSTITTLLSTSAGVLAAGPSATAKKATAIESIKGDNGITTPLPIQPGMVDDCDAFYYVKPGDNCLIISAQFGISFDQFKEW
NPTVGKDCLSLWADANVCVRTIGFEYPETAACYVNEDILPWGSNKVAAAKAATEWCSNGAQGVYNIGEKRAKCVDAPSGDGKF
IFEIYNEWGIRQGLPATECRKKLLLPISKCTDGGQGRVKSWHTETYLEKGKC
>FUN_014516-T1 | Effector probability: 0.61
MLFFKSIASLAALVSLAVAGPIESRQSATTCGSTNYSASQVRAAANAACQYYQNDDTAGSSTYPHTYNNREGFDFIVDGPYQE
FPIKSGGVYTGGSPGADRVVINTNCEYAGAITHTGASGNNFVGCSGTN
>FUN_014517-T1 | Effector probability: 0.588
MRLTSFFESAALVVASLSELSQAHAFTPRGFTTPKRKELWQPEVGTPWQIILSEVVKVPKAGVSSMTPDVPIWDMDLFENSKS
TITAMKKGGKKVICYFSAGSWENWRKDKDSFPKKDLGKVMDGWPDERWVNISSVAVRAIMTQRIKLAAEKGCDAVDPDNMDGY
QNDNGLGLTEGDTISYVKFLSAEAAKYNMVMGMKNGGDVTEEVLPYVAFCINESCIQYSECDLYQPYIDAGKPVFNIEYPKGA
PKVKAKDQKKICSTSGAAEGSDGFSKVIKKMNLDKWVLYC
>FUN_014538-T1 | Effector probability: 0.728
MKTSFATLVIPLVATVSAVKLSVSDDGFLHLNGKQLVASDGVIDFQASGSDFTVGKDGTLSLSNGQQVNINRNGDAIYGSGAG
AKDFGIKDGKMTWARGDIYACLTEENYQKIRFAADKARGTAIGDDCSQITVSVL
>FUN_014564-T1 | Effector probability: 0.712
MRFLPFVAAALAVVPGALAVDQKKSAIVWFEDESTPDSIVDECKNALIKAGGKITHVYSIIKGFSVIAPEKALEIVQVNHENH
QIRVEKDEVVTTD
>FUN_014633-T1 | Effector probability: 0.603
MHFFQKPALLLLPAAALAGAIPYEVRKDFANPKSGITLKKAFDIHAQEENGFLSYKGTQPGKVVQDGVKLRILSVGDSITVGY

GKGTDGNGYRKRLRKDLSGNEIVWAGTEKTKGNMKDGHFAAWSGKTVQYINDHVDPSLEQRPNLILIHAGTNDMNSNHRVSTD
GNHPQETTNRLKSMVEKMISKCPDATIIIGMITDVCDNKSYHFQRERTKIYRGHIAKLAAELSKDGSHVLAADFGPFDDTLLS
DCVHPTQKGYEILGDWWYDFIHQIPEGWIKDPVGPDPVRD
>FUN_014872-T1 | Effector probability: 0.874
MQLINLSALALFATSALAADCFGNRQDGIERFEQAYWDARQKMCSNSDCTYQQGCTTRGSKTIKGLASITVNVELSRKNTGGK
KGFKDCWDATENIINQCVKGSHQLSGTWEYNGQLYQVNGYYS
>FUN_014876-T1 | Effector probability: 0.687
MRSLLLYPLLAASAMAEKLLYRNTFNSTEAIADWVAEGPVKATVSNNTLELAAPGDFVYWVPEVFPERIRITWEFSPIEEPGL
AIIFFGAAAAKDGGSIFDKDLKPRNGSYPQYHSSDIRTLHASYFRRRWPEERAFHLANLRKSPGFHLVAQGADPLPNVEDTQG
AYYKVEVIHDKRDVKFSINGLELFSWEDVDRSTGPVIRGGRIGFRQMNPLVARYRNLEVWKI
>FUN_014921-T1 | Effector probability: 0.915
MRFNTITLSILLAQSTCLAAPADGIDSSPGFSLAPRSKLETRDSYDCNGSGLCGTISVKNCDDAVNNRLIRNNDVNYGAPGSG
RPQTGTCQGACGIFIQGRSTCARTGNQI
>FUN_014929-T1 | Effector probability: 0.67
MKFFALLSSAAAVSFAAPTVDTHEIDARAVDTILYPAGTYRYWVQSGKIIWDPQDQLLIVKNGKAADDTTTIVTFEFDESTRG
KTCELLFELWDRDVSTGTKTLDVFTYSDPPTGPRAFSAADAANWASTKSRDNHVGRIRVPKPGNATWEQSYQGWPKIPCPAGQ
LIGVEYVGVGDRVQVRWDIGVTGPRFKVMG
>FUN_014934-T1 | Effector probability: 0.88
MKLLTLISAFATATLVSADQRAQLSAPDGSVHHLSARDSTCPRPMCKTPASQGPNDPPACGDSYAACKFDQFPCDEHFSPKVT
DTHHCYCILANKKAMDTYCQERGFKSGTNPWKYYYAVECHGAVSNQVCNKDCHDQGRGNGRIDKAHPNGACACDKPNPPYDTC
KA
>FUN_014952-T1 | Effector probability: 0.964
MLFNNAIVAIALSVAVNALPQTYPNKCGDQVCPADKAKCCEVIVNGVAEIGCFAECPPIQALQRRQTYPNKCGDQVCPSGKPK
CCEVIVNGVSEIGCFEECPAPPSAQEKLQRRDQPTATAAAFPPIPTFGPKCGDSFFCPVGQVCCPNALYHCADPDKVSQQCPQ
>FUN_015146-T1 | Effector probability: 0.629
MKFTLFALSTLTASLAAAYPITGNDVKCRSGPGTSYAVKKVLKKGTDVKITCQTEGTNISGNTIWDKISDGCYVSDYYVKTGS
SGYIKPKCGGGCSAPSSNQATVDLIGEFEGFVPHIYKDAAGYPTVGYGHLCSNSKCTDVKYPIPLSKANGKKLLADDMRKFEK
CIAKMVSSKVTLNKNQFGALVSWSFNLGCGAAEGSQLLKRLNKGEKPNTVISQELPKWVYAGGRKLPGLVRRRNAEVALAKKA
TSEKALPVKC
>FUN_015147-T1 | Effector probability: 0.788
MKFTTLCAYALAFFSTGVHSYPVTSDNLNCRSGPGTAFAIKKSYKKGQDVTITCQTQGDNVEGNSIWDKTSDGCYVADKYVKT
GKDGYVKGKCTNVPKPPKNKKIPGPRVNDYPYKNSCGPADKWLYFKCQCTSFVAWRVNERLGIKFHNKYKGKAWGNGNQWDEA
ARASGVRVDNKPVPGCIAQTNAGKSGHVAWVSAVDGDMVTVEEYNWNNYRAYGTRKVHKSKFNYIHLKV
>FUN_015242-T1 | Effector probability: 0.629
MAPSVEHFKFRILIVGAGPVGLTAAAALSQDGHSVTVLERHPDLQTQGGTPTVQPAAARAYTHIGLGAALAAISITGHGLHSW
PYKDSFGPIGVLSYGNVKAFMTERPAVQLMAYDVSIAAGATVLFNCQVTGLDQNALPVRLWMADGQEYTADLIIAADGIKSKI
RQIIYPDRAVEPVPTPECIFQSQVPRRILKSDDRVAPYLEPNTTHGTLGPSKFSICRATVEGNFAMTSIVMDYGLPPA
>FUN_015322-T1 | Effector probability: 0.778
MKFSAAVFTLLAATGVTAAPAAESVNMMAATPQWTIRDAKRYCRSDDSICNWKFGIDTGNGKPYECRYDVKGPGASKKRSAGP
STCGDFTITSGWSDQFGANNGFTTLSVVSKSKRQIIWPAYTDKQLAGAKIVKPDQSYAPASLPK
>FUN_015389-T1 | Effector probability: 0.559
MTTLAALLALLCLSGQAISTPAPHHKPPVAITTNGSYYGTYNAEYDQDIFFGMPYAQPPVGDLRFARPQSLNTSWSGLRNATT
PGFSCIGYGPESYMASPNREDCLNLEVIRPAREEKPLPVLVWIHGGGFTEGSSTTPYYNTSFIVQRSQETGQPIMTVSINYRL
AAWGFIWSDQIVEEGLTNLGLRDQRMALQWVQENIAAFGGDPSRVTIMGESA
>FUN_015411-T1 | Effector probability: 0.961
MLFNNIIVAIALSGAATALPQKYPIKCGDSVCPADKPKCCEVLVNGDLEIGCFEECPALPPVQAKLRHREQPTITIAASPPIQ
TFGPKCGDSYFCPVGQVCCPNALYHCADPDKVAQECPL
>FUN_015415-T1 | Effector probability: 0.607
MELSLLYFLCILSLASASFPFNFGLSSSPVLLPRAKNPTSKDGNCGSNSETNATCLTSTFGNCCSEKGFCGKTSAYCSEGCQE
AFGSCSSSADGQLVSTSGSCGATSTSNITCEGSTYGDCCSEKGYCGKNATYCGAGCQSMFGTCSSGDESSTTTATSDKTSTST

AASSTSLGAISIDGNCGSNSDINATCKDSTFGDCCSAKGYCGGTSALDAKLNTVPAIRHPRLRPHLRPPRHPRLNRPRQPLRQ
PRQQAP
>FUN_015427-T1 | Effector probability: 0.61
MRLSSFITGAALLSSGANALNILLNNDDGFGSGNLREMYRIFKEKGHNVWLVAPATKQSGKGGTSDFTTEGNLTAPSQYDLIP
KGAPSVGHDPKDSQIWYYNGTPAACTFVALDYVLPKFANFSVPDLVVTGPNYGTNLGGFVWTLSGTAGAAYAATNRGIPAIAI
SASNQEVPYFEVKNRTNPATWAAQASVKFVENFIATSPKNGPLLPLGYGVSVNLPVLTKKNQNPDFVQTRFTGNAHVNEAVLD
KEKGTFTWANIKPYAAGVNACINGDCSLPGETYVVENGKASVSFYTVDYSAPSTEYTKSLIQRVASFVSSDK
>FUN_015451-T1 | Effector probability: 0.844
MLVSQSLLSLGSIFNSVTTLPGCGEVNVFYTRRLACRHTYVTQQGYDAALVEAQIFNHTRQLREAGYNVRAVWRGPEIPGNEM
SRYMKDVHWNVAGIGFGVRGSQISDVITLFEGQ
>FUN_015685-T1 | Effector probability: 0.553
MLALKTLLGLATVQAVSAHFGLVFPPWRADTLSEEGEERYNQWEQPCARVDFNKKNLTDWPLDGGALTLDLHHEWSYIFVNLG
LGENSTNFNISLTPEFLNATNSGTLFIDKLELPSDVKPKDGDIASIQVVTVGDTGNALYNCADIRFKENAKGPSNKTGDVDYV
KIKQQKGNGTEEDSSSNSTSSGDSKDNAAGTMGVNTMVMTSVVGLAAAFVMGLGL
>FUN_015834-T1 | Effector probability: 0.816
MRYLFAVLSLLTLVNAQFGGFFDQMFNQQGGHEHHQAQQQNNPSDANHYKQNYENSVCDKYLCPDTLACVHFPHHCPCAWDAQ
QEKFELADGQRLCVSRGGFRPAETARKIELARKGLL
>FUN_015892-T1 | Effector probability: 0.715
MKAVTILLSICLAVRAMATDMCSTYSRTPCICPAGTEYAQSASWAVVGANAKDVEELMNDYFECGWRGSVPYETLGPNNHPGK
SVRVTTFKTLQGIFNFSEILTDQRVQSDGSFIQKFEYLSTIPGSNKNGTQTPWGGYWITITADHIFGNETLIRWSTYLCSRGY
VNGM
>FUN_015983-T1 | Effector probability: 0.937
MKFSIVLAVAALSAETFACANIGQVCQKGDPDVCQCDAPLTLSCRGDLATRPGGARRFHYQLGDICPLLKRNGRCVNGKCVPG
PRLTAVTPTPTPE
>FUN_015993-T1 | Effector probability: 0.662
MRLSLLPLIALAGSAFASGDSISTAIDNISNATLALNKTVATWPQTLLGALPITTKSTLLLTEIHKGFVIARESEPLSLEETL
QVAKATSELSADVELTINTIIAAKPNFDRLQVSPVILLNLNLQRALSQDFSEAVISKVPKDLQGNAKALVQGIDDSFARAISK
YSKLRG
>FUN_016007-T1 | Effector probability: 0.799
MKYTAILALAGVSSAAVTKTLPKSAGATSFPTAVPVKGSYDGGMKRFERSPSVCQGQSETGEKDAMFILENGATLSNVIIGAS
QAEGVHCKGTCTLNNVWWADVCEDAVTLKQTSGTSYINGGGAFHASDKIVQFNGRGTVQIKDFYAEDYGKLVRSCGNCKDNGG
PRNVVISGSVAVDGGVLCGINTNYGDTCKITSSCQNKGKYCDRYEGNSSGAEPSKIGSGPDGKYCTTSGVTTSC
>FUN_016040-T1 | Effector probability: 0.564
MLARFFFVLPSAAFALASPPGHKLCPSQSGMPTKPTPVLPSNGGGSELPEPSEDVSLKHIALGFGIQNYTCADTAASPTPVGA
LAVLYDVTHLYPGQGHSSLTQDEWASLPGDILDTLKVPLNLNEKGTGASLVKPFPKKQDLKIRSLSKKIPYLGHHYFNAAGVP
TFDLDKARQLLVAKKMGDIKAPASSPAGPEGTGAVNWLFLGDAGGSHGISYAYRVLTAGGASHGCKAKGADSTSYTAMYWFYN
>FUN_016044-T1 | Effector probability: 0.882
MHFITAALSAHLASAALAVPLNSTPHDNPDSNIFPDFDRYSDWAICKGKITKGRFPNLQAPNRDGGCVRYYRGIDMTGVVTEQ
HFFFKDGFKTACDCAARCLEEPNKCTNWAWKHTFMPEDGGKRSCTLYSSPNLPTDVTLKYDLANSKGFNLLQAANNPQAGAPA
PLTFLDAAGTIPDKFGVSGFMVQDQNGSQFC
>FUN_016093-T1 | Effector probability: 0.842
MKTSFALAVLSLFYTHCLAASVDMWSSPLKGRAALKYEAIEPEVIKARLGTTPDENSEGGRESGMVYFCREENWGAPCFAYRP
ELEYTCNELGPELKGHIGSVFIDPGMICRLAGFNNDRCAPIKIFAWPETQHGWPDLFHQNAPGGAMNLGAATTHFTCAECTNC
VEKSG
>FUN_016145-T1 | Effector probability: 0.686
MSSLLPLISEILPMIMPASANITRAKQLEPTHPTVEGPVIERPAVVGKCDNMCVTVLTTRPHSKSTVRHNSEQDTIIYAVSGN
GVLIVNEAVNSELKHHDLAPGDFAFVPAWIEHQVKNDTDEDVQWLMIQSGSTPIRADLTEWGGDVIQSKN
>FUN_016177-T1 | Effector probability: 0.769
MYSLTKLFVAVMALSTFASTNPIADKGDTQNLEARGGCLGYANYNACLAGRHRACPIGAGQATCFATASKVCQQNC
>FUN_016237-T1 | Effector probability: 0.921

MQFALVSVLFAVGVFAKNGCGFPDGPDCVSLGKGSDGLEVFRDNDGCCLLPARCGNEAGVNCRRENEGPDGLPINTKRVAHRR
RAQKDYRA
>FUN_016248-T1 | Effector probability: 0.666
MSMLRLVLIESIWSGSSINIGHSEILQKLNAIEGGISSDNNLAGDGRSRLTAYAQQKIVQIPRTILALAPRFLNAQDINPRFP
KMARRMILHLAFIQSCPCCPSDIRQEATDILMRLEKGIELSQAQFAANLLYQ
>FUN_016264-T1 | Effector probability: 0.958
MKFTALATAIFALGFGAEMATAAECCQMKVCDKMNLGGNCKHGCYPIRKTAKLNLSGLKSSVSSAKTENRCFCTIGKEVESCM
EVTSNAKGTNVPNHCLTGAKYAYCQFK
>FUN_016315-T1 | Effector probability: 0.921
MKFSVVTFLALSMVASAEADKRAAKQMQINYFDDNNCNRFLGQIDVTWASKHLSSGKKNCYNYNYGNS
>FUN_016318-T1 | Effector probability: 0.895
MKTSTILTALGGMLAFNAQVVNAGCYTTGDPWPNKDQAAQFVWDACYGSQGMFSGQFRPKQTKSMCPRSGQLGLVFEVENQWD
QTLDLNNDDCYTRLKNEIYGCDRGGESTVSKWRFRADPGNC
>FUN_016658-T1 | Effector probability: 0.643
MLYPRFQSATVAVIAAVLTPLVLGAATPTPQPSIQWETTTKDILLSEIGPFDLESQLTSSGSTSGTMNKRSSYSEGVCGAIPF
KPQTGGGWHFKQAWCDRTGTDVNTFKVDCFGGRNYVEELPKRKGACGKGEWCVDFHGYNTKGDAADDVTCVNRKNIHTWVANT
KTRPVEDRVTCSSGWRNDYKRSAKATFEVDVMDSAGKDRISPENVYYILNQKRIGVSRSNDAEVGSGNIIIPPGGAIQACVTA
KVAQLQILNILGAVTSFKLL
>FUN_016830-T1 | Effector probability: 0.612
MTGTHSIITPAIMYWGTPVALITTSNDDGTVNIGPISSVWWLGHRCLLGLASGSQTTLNLFRTKHCVINLPSNDMAHYINPIA
KTTGTPTVPPGKKDRGYKHCKDKFAASGLTQQASDLVQPPRISECPVQMEAEVTNSMDLMQDVPDRKGLLVAVEVKILRTHVL
NDLRMEGYENRIDPDRWRPLIMSFQEFYGLMPAKVAESSLGTIDEEKYRFITRSDVVKQGGDMDALDCEAGAGR
>FUN_016872-T1 | Effector probability: 0.715
MRLVSLPSTLLLAANAAATYNALFHFGPTAQVETRSIDQIYKAALEEGGVVTTWFGGDEKNQEDAVKKAFETAFPVMKLNLTV
DLSKYLDGNIDQQLANNNVYVDTVAL
>FUN_016873-T1 | Effector probability: 0.72
MTPFRCAATWAFILMVVMGSPSEATPITSEGAGAHRSINPRDSVPWQAKHRMDSSNFQDTFDKMVHNKYHLTYVSGYPINGNP
RFAAIWDQSPTSDWVARHGMTSTEYQGQFDALVSKGFRLRLVDGYPVAGNTRFAAIWDKSGGSAWVARHDLTSSEYQNAFNTF
FSQGYRLKHVSGYAQGDQARYAALWEKSATNITWAAHHGMTSSDYQKLSDKYVAMGYRTVHVNGYVVNNIDFYAAIWDKSPSG
PWVSRHRMSSANYQNEFDEWTGKGYRLRLVSGYTMGSDEDMYAAIWVRG
>FUN_016923-T1 | Effector probability: 0.575
MVKIATVIATVIALFNNLASADPAIRITGLGCGLYDGNGNSVFTLKSRTVITHSENGNVVCQATVTPSTAGKARTFNFKNTNV
FCCTLAGCTPNWHETISASGQATLTCHV
>FUN_016939-T1 | Effector probability: 0.592
MLLKHFLLGALLALGSVTALPNPEADLHDVEARDNKHHRPRCGKDADFDRMKNKCICKDEDKELMMGVCKCKGDLKPNRHGEC
RCPDDQRPDRHDPHKCVCKDKDKDKDLMTG
>FUN_016969-T1 | Effector probability: 0.845
MIINSLVLSTLLTFANKATATNTQRVVEIAGINTSNVTIWDCPSTTSPDLSQSMIQTAMNDFAYTFYTEKDVKRAFERYVASN
YVQHNPSIPDGRDAAVKILSPLFGSKDNTFEIARVMVGPEYTTIHIKAGGANDSLTNVFDVYRTKGSCIVEHWDCLQAMEKNT
TSHHPYF
>FUN_016990-T1 | Effector probability: 0.8
INWDPKRHRIRCILHVINLSLQAFLFASLREALQAALDAASDITEDELYERFNLALNDASGGDTLNQLDQIGAQTRHPGGVAF
KKASVQKGNPSRSDNRSGNNARWKGWIMIPAPRKLHRIGLWLPYLFERHKGTKRSRQGYQAN
>FUN_016997-T1 | Effector probability: 0.818
MHLALLLGLVATAVANAAKSRNILYFDQWHTADFPPPDLTGAVTHVMMSFANSSLFAAQPAGEYKPFQSLEQVRQLFDHELNV
CLSIGGWNDNSGFDEGVKTSSSRERFAKNIASTVDRLGFDCIDIDWEYPGGNGQDYKQVPNCKKRNEIKAFPLLLKEIKKYIR
TKELSIAVPGLERDMIAYTSAETPRINESVDFINVHSPFKLNLRES
>FUN_017069-T1 | Effector probability: 0.939
MQFSITAIVLGLAAFASAGIVDTEGVRNAPRSAVLVTRQNGQNGGRPVPSGECCVANTSLKQDACTASNGQAGRCVPGGNNCG
GRLSCVAQANLQCDANVIERGKDLCRAKAANGLFDGGNIIQNLSQ
