# Supplementary material for: Profile of the in silico secretome of the palm dieback pathogen, Fusarium oxysporum f. sp. albedinis, a fungus that puts natural oases at risk
Source: PLoS One. 2022 May 26;17(5):e0260830. doi: 10.1371/journal.pone.0260830 (PMC9135196; doi:10.1371/journal.pone.0260830)
Supplement: S3 File — (PDF) [file pone.0260830.s003.pdf]

>Foa133 SIX1-1
ATGGCGCCCTATAGCATGGTACTCCTTGGCGCCCTCTCAATCCTTGGGTTTGGGGCTTATGCTCAAGAGGCTGCGGTTCAGGA
ACCCCAGATATTTTTCAACTTGACCTACACGGAATATCTGGATAAGGTGGCAGGATCCCACGGGAGTTCCCAGACGAGTGATT
TGCCCTGGAATGACACTATGAGCGGCTTTCCTGGGAACGAGACGGGCAGCAGCCTATCGAGGCGTGGTCGCATTGTCAACCTT
GGAAAGCGTGACCCTGTCGGCGGGGAGTCTTTCAACGATCGTGTCACCAATGACATGCTCCAGGCGCTTCATGGTTTATGCGT
TGAAAATTTTGGCACAGGCTTTCGAGCGACTAGTGGGTACTGTCGCGCTAACCGTCGGGCGTCTAGGGAACTCCAATGT
>Foa133 SIX1-2
TCAATGACCAGAGCTTGCCCAACGAACCAGGAGTGCACCACTTTCGTGGCGCACAACTTTCGTAGCCAGTCGCCTACCAGCGA
CAAGTCTACTTTCCCTGTCTGTGGGCCCCGAATTGAGGTGAAGGAAAGACATGATATAGGGATCCACACGGAGTGGGATGGAA
TTTGGTACCCCGAATCGCCTAAATCGCCTGGGACCTACGATTCTTTCGCCCAGATGGCGGGCAGTCTCAATGGGTACTTTGAC
TTTAATGGTGTTTATTCGAATGGAGAGGGGATGAGCTCTCGGGGAAATGGACACTCATGGTCATGCATTGGTTGCCCGGGCGG
CAAGCTAACTATTACTAGCACAGTTCGCCCAACTTGGGCGATTGGATATACCAGCCCCCACT
>Foa133 SIX9
ATGAAGCTTCTAGCAGGCGCAGCCACGGTCTTGGCCGCCTTCTCTACCGCCGCGGCTTAGACAATTCAAGTTGGTTGCCGCGC
TCCTGACACCAAGAACGACGGTCTTCTGACCGAGCTTCTTCATAACCCATCTGCGCGTGGGGTCGCCGACCCAGACCTGCGTT
ATGGATTTTGGGATGCAAGGTGGCGAAAGTGCTGCAACGAGCATAACATATGCGACAGATGCTACACGTTTAGCTACAACCAC
CCATACCCTTGGGCTTACAGACAGCGCAGGGGAACTATCCGCGGCCAACAGTTCGACTTTGCTTGCGTCAACTGGCACACGGG
AGCTTGTAAATGA
>Foa133 SIX11
ATGATGTTCTCCAAAGCCATCTCAATTATCTCAATCCTTATGAGCACCAGCCACGCCATCAATATATGTTGCTCCTCCTTTGC
TGGCCACACCTGCACGAAAGATCAATACAATAACCACCGCCAGAATGTTATCTTGAACCAGATCATCGACAAAGACGGGATGA
ATTGCGTACGAAAGGGCGCTGGGCCGGGACGATGGACCAGGAAAGGTGACTGGTCAGAGTGGTATGACTGTCAGCAGTGGAAC
GGGCCTGAGCAGCATCAGATCGAGGTTGGAGAGTGTACTCTGTTTTGTGTCACACCGAGTGGCATTCTCAATAGACCCTGCAT
TTGA

Query: Foa133 SIX1-1 Query ID: lcl|Query_6543 Length: 411
>Fusarium oxysporum f. cubense strain BRIP62895 secreted in xylem 1 (SIX1) gene, complete cds
Sequence ID: KX434994.1 Length: 834
Range 1: 1 to 411
Score:715 bits(387), Expect:0.0,
Identities:403/411(98%), Gaps:0/411(0%), Strand: Plus/Plus

```
Query  1    ATGGCGCCCTATAGCATGGTACTCCTTGGCGCCCTCTCAATCCTTGGGTTTGGGGCTTAT  60
            ||||||||||||||||||||||||||||||||||||||||||||||||||||||||||||
Sbjct  1    ATGGCGCCCTATAGCATGGTACTCCTTGGCGCCCTCTCAATCCTTGGGTTTGGGGCTTAT  60

Query  61   GCTCAAGAGGCTGCGGTTCAGGAACCCCAGATATTTTTCAACTTGACCTACACGGAATAT  120
            |||||||||||||||||| |||||||||||||||||||||||||||||||||||||||||
Sbjct  61   GCTCAAGAGGCTGCGGTTGAGGAACCCCAGATATTTTTCAACTTGACCTACACGGAATAT  120

Query  121  CTGGATAAGGTGGCAGGATCCCACGGGAGTTCCCAGACGAGTGATTTGCCCTGGAATGAC  180
            |||||||||||||||| ||||||||||||||||||||||||||||||||| |||||||||
Sbjct  121  CTGGATAAGGTGGCAGCATCCCACGGGAGTTCCCAGACGAGTGATTTGCCGTGGAATGAC  180

Query  181  ACTATGAGCGGCTTTCCTGGGAACGAGACGGGCAGCAGCCTATCGAGGCGTGGTCGCATT  240
            ||||||||||||||||||||||||||||||||||||||||||||||||||||||||||||
Sbjct  181  ACTATGAGCGGCTTTCCTGGGAACGAGACGGGCAGCAGCCTATCGAGGCGTGGTCGCATT  240
```

```
Query  241  GTCAACCTTGGAAAGCGTGACCCTGTCGGCGGGGAGTCTTTCAACGATCGTGTCACCAAT  300
            ||||||||||||||||||||||||||||||||||||||||||||||||||||||||||||
Sbjct  241  GTCAACCTTGGAAAGCGTGACCCTGTCGGCGGGGAGTCTTTCAACGATCGTGTCACCAAT  300

Query  301  GACATGCTCCAGGCGCTTCATGGTTTATGCGTTGAAAATTTTGGCACAGGCTTTCGAGCG  360
            |||||||||||||||||||||||| | |||||||||||||||||||||||||||||||||
Sbjct  301  GACATGCTCCAGGCGCTTCATGGTCTCTGCGTTGAAAATTTTGGCACAGGCTTTCGAGCG  360

Query  361  ACTAGTGGGTACTGTCGCGCTAACCGTCGGGCGTCTAGGGAACTCCAATGT  411
            |||| ||||||||||||| |||||||||||||| |||||||||||||||||
Sbjct  361  ACTAATGGGTACTGTCGCCCTAACCGTCGGGCGACTAGGGAACTCCAATGT  411
```

Query: Foa133 SIX1-2 Query ID: lcl|Query_39917 Length: 394
>Fusarium oxysporum f. cubense strain BRIP44012 secreted in xylem 1 (SIX1) gene, complete cds
Sequence ID: KX434997.1 Length: 834
Range 1: 439 to 832
Score:612 bits(331), Expect:9e-171,
Identities:373/394(95%), Gaps:0/394(0%), Strand: Plus/Plus

```
Query  1    TCAATGACCAGAGCTTGCCCAACGAACCAGGAGTGCACCACTTTCGTGGCGCACAACTTT  60
            ||| |||||||||||||||||  ||||||||| |||||||||||| ||||||||||||||
Sbjct  439  TCAGTGACCAGAGCTTGCCCACGGAACCAGGAATGCACCACTTTCATGGCGCACAACTTT  498

Query  61   CGTAGCCAGTCGCCTACCAGCGACAAGTCTACTTTCCCTGTCTGTGGGCCCCGAATTGAG  120
            ||||| | |||||||||||| |||||||||||||||||||||||||||||||||||||||
Sbjct  499  CGTAGTCCGTCGCCTACCAGTGACAAGTCTACTTTCCCTGTCTGTGGGCCCCGAATTGAG  558

Query  121  GTGAAGGAAAGACATGATATAGGGATCCACACGGAGTGGGATGGAATTTGGTACCCCGAA  180
            |||| ||| |||||||||||||||||||||||||||||||||||||| ||||||||||||
Sbjct  559  GTGACGGAGAGACATGATATAGGGATCCACACGGAGTGGGATGGAATCTGGTACCCCGAA  618

Query  181  TCGCCTAAATCGCCTGGGACCTACGATTCTTTCGCCCAGATGGCGGGCAGTCTCAATGGG  240
            |||||||||||||||||||||||||||| ||||||||||||| |||||| ||||||||||
Sbjct  619  TCGCCTAAATCGCCTGGGACCTACGATTTTTTCGCCCAGATGACGGGCACTCTCAATGGG  678

Query  241  TACTTTGACTTTAATGGTGTTTATTCGAATGGAGAGGGGATGAGCTCTCGGGGAAATGGA  300
            ||||||||||||||||||||||||||||||||||||||||||||||||||||||||||||
Sbjct  679  TACTTTGACTTTAATGGTGTTTATTCGAATGGAGAGGGGATGAGCTCTCGGGGAAATGGA  738

Query  301  CACTCATGGTCATGCATTGGTTGCCCGGGCGGCAAGCTAACTATTACTAGCACAGTTCGC  360
            ||||||||||||||||||  |||||||||||||||||| ||||||||||||||   ||||
Sbjct  739  CACTCATGGTCATGCATTTATTGCCCGGGCGGCAAGCTGACTATTACTAGCACCTATCGC  798

Query  361  CCAACTTGGGCGATTGGATATACCAGCCCCCACT  394
            |||||||||||||||||||||||||||||| |||
Sbjct  799  CCAACTTGGGCGATTGGATATACCAGCCCCTACT  832
```

Query: Foa133 SIX9 Query ID: lcl|Query_496725 Length: 345
>Fusarium oxysporum f. sp. passiflorae ICMP:21871 secreted in xylem 9 (SIX9) gene, complete cds
Sequence ID: MH230159.1 Length: 377
Range 1: 16 to 360
Score:499 bits(270), Expect:7e-137,
Identities:320/345(93%), Gaps:0/345(0%), Strand: Plus/Plus

```
Query  1    ATGAAGCTTCTAGCAGGCGCAGCCACGGTCTTGGCCGCCTTCTCTACCGCCGCGGCTTAG  60
            ||||||||||||||||  | |||||||| |||||||| |||||||||||||| |||  ||
Sbjct  16   ATGAAGCTTCTAGCAGTTGTAGCCACGGCCTTGGCCGTCTTCTCTACCGCCGAGGCCCAG  75

Query  61   ACAATTCAAGTTGGTTGCCGCGCTCCTGACACCAAGAACGACGGTCTTCTGACCGAGCTT  120
            |||| |||||||||||| ||||||| ||||||||||||||||||||||||||| ||||||
Sbjct  76   ACAACTCAAGTTGGTTGTCGCGCTCTTGACACCAAGAACGACGGTCTTCTGACTGAGCTT  135

Query  121  CTTCATAACCCATCTGCGCGTGGGGTCGCCGACCCAGACCTGCGTTATGGATTTTGGGAT  180
            |||| ||||||||| |||||||||| ||||||||||||||||||||||||||||||||||
Sbjct  136  CTTCTTAACCCATCAGCGCGTGGGGCCGCCGACCCAGACCTGCGTTATGGATTTTGGGAT  195

Query  181  GCAAGGTGGCGAAAGTGCTGCAACGAGCATAACATATGCGACAGATGCTACACGTTTAGC  240
            |||| |||||||| ||||||||||||||| |||||||||||||||| |||||||||||||
Sbjct  196  GCAAAGTGGCGAAGGTGCTGCAACGAGCAAAACATATGCGACAGATACTACACGTTTAGC  255

Query  241  TACAACCACCCATACCCTTGGGCTTACAGACAGCGCAGGGGAACTATCCGCGGCCAACAG  300
            ||||||||||||||||||||| ||||||||||||||| ||||||||||||| ||||||||
Sbjct  256  TACAACCACCCATACCCTTGGTCTTACAGACAGCGCACGGGAACTATCCGCCGCCAACAG  315

Query  301  TTCGACTTTGCTTGCGTCAACTGGCACACGGGAGCTTGTAAATGA  345
            |||||||||||||||||||||||||| || ||||| |||||||||
Sbjct  316  TTCGACTTTGCTTGCGTCAACTGGCATACTGGAGCCTGTAAATGA  360
```

Query: Foa133 SIX11 Query ID: lcl|Query_9109 Length: 336
>Fusarium oxysporum f. sp. sesami isolate FS08027 secreted in xylem 11 gene, complete cds
Sequence ID: MN417218.1 Length: 336
Range 1: 1 to 336
Score:599 bits(324), Expect:6e-167,
Identities:332/336(99%), Gaps:0/336(0%), Strand: Plus/Plus

```
Query  1    ATGATGTTCTCCAAAGCCATCTCAATTATCTCAATCCTTATGAGCACCAGCCACGCCATC  60
            ||||||||||| || |||||||||||||||||| ||||||||||||||||||||||||||
Sbjct  1    ATGATGTTCTCAAAGGCCATCTCAATTATCTCACTCCTTATGAGCACCAGCCACGCCATC  60
```

```
Query  61   AATATATGTTGCTCCTCCTTTGCTGGCCACACCTGCACGAAAGATCAATACAATAACCAC  120
            ||||||||||||||||||||||||||||||||||||||||||||||||||||||||||||
Sbjct  61   AATATATGTTGCTCCTCCTTTGCTGGCCACACCTGCACGAAAGATCAATACAATAACCAC  120

Query  121  CGCCAGAATGTTATCTTGAACCAGATCATCGACAAAGACGGGATGAATTGCGTACGAAAG  180
            ||||||||||||||||||||||||||||||||||||||||||||||||||||||||||||
Sbjct  121  CGCCAGAATGTTATCTTGAACCAGATCATCGACAAAGACGGGATGAATTGCGTACGAAAG  180

Query  181  GGCGCTGGGCCGGGACGATGGACCAGGAAAGGTGACTGGTCAGAGTGGTATGACTGTCAG  240
            |||||||||||||| |||||||||||||||||||||||||||||||||||||||||||||
Sbjct  181  GGCGCTGGGCCGGGGCGATGGACCAGGAAAGGTGACTGGTCAGAGTGGTATGACTGTCAG  240

Query  241  CAGTGGAACGGGCCTGAGCAGCATCAGATCGAGGTTGGAGAGTGTACTCTGTTTTGTGTC  300
            ||||||||||||||||||||||||||||||||||||||||||||||||||||||||||||
Sbjct  241  CAGTGGAACGGGCCTGAGCAGCATCAGATCGAGGTTGGAGAGTGTACTCTGTTTTGTGTC  300

Query  301  ACACCGAGTGGCATTCTCAATAGACCCTGCATTTGA  336
            ||||||||||||||||||||||||||||||||||||
Sbjct  301  ACACCGAGTGGCATTCTCAATAGACCCTGCATTTGA  336
```
