# Supplementary material for: Profile of the in silico secretome of the palm dieback pathogen, Fusarium oxysporum f. sp. albedinis, a fungus that puts natural oases at risk
Source: PLoS One. 2022 May 26;17(5):e0260830. doi: 10.1371/journal.pone.0260830 (PMC9135196; doi:10.1371/journal.pone.0260830)
Supplement: S4 File — (PDF) [file pone.0260830.s004.pdf]

>FUN_001102-T1
MKSIITFAGLAFASFAAAGPCRPRTTTTAAAAVSSSTEIASSTATETAASTSTDYSVPVV
SESSTLATVIVSETSATETSAAETSTVGTTTAETSAAGTTTAETSAAGTTTAETTTAEGT
TTAEGTTTAETTTAEGTTTAEGTTTADTTTAEATTTEATTTEATTTDAETTSGTATETTT
SAEPTADQSCDNAGLEYAIYEHTFYNSDPPHFSSFDPTFFHTATPTFQGETTRIGIPPGT
ASDTSFAIYDDSPVQRFQYKAVNHQAFLYAPDTGDYKVTIPNSDEITLIWFGTKAISGWT
RQNADLEQDYPGGTSKSFTIHLTAGTYTPFRLLWANAQGDLNFIAEVQAPDGTVIVNGDG
SDNRYFVRFACDESTPSFPAFGGSG
>FUN_001507-T1
MRVLALQMLAICQLACASPCKPSSSASVTLSTTSAESTSAPAVSSSTIADETETSTTFFT
GSTAVSETETATTETETETVSGTTTEVASDLATSTILITTSSAEPTTLATSTAEPTTTTT
AAAEPTNVLTNPGFDSGTITPWYTIGQRGTLSLSDEGTHASEFSGHFYADYNGPVVLGVD
HPVDQSLIKVDTEYHYSIWIKTTAAVDCQTRKITCGAGGGYILGTDWAGPYNEWTQFTMS
CIWNQNFLDLVPSIQIRAECQSLEFYVDDAILIEAN
>FUN_002662-T1
MRLYASFSLAFGLVAASPCKPLTSGSTGVSATFSSSATEIYTTFADSATSTVETLATETS
TVIESETSTAIDTTATEASTFVGSTSTETTDATISASETSSQATSGTALFTSVTSADATT
TVTTETSTAETSAATTTTVETSTAVTTTTTSEEPGPTDHLLIAGQGSAQGLPLKTTKQNG
DFVIFGERSSSWTVGRFVVDPTTGYLTRDGIPVCAKYHYMDRRASLTLCSTEYNLYGTAR
LTCARNPAAGTALQCSAVKLDCVSSGPGDSQRCSVSTEANWTKFFISVSSDYTVYLGADD
LAVSSFAPVDIFVDRAPTAAAPTVP
>FUN_003643-T1
MRSIIHLRLAFGLLAIGSAVASPCKPLSATTTSGAISVAVTAATSTETSLATSVASTTED
GEATSSTVVGSSTETSASSAETSGSSITVETSTASATTEFTTETTTAEPTTTVLTTTAAE
TTSSAAIPTFTMFASGSNAVAGRSLQSYNRGNTVAVFDPATDDDNPSTRPYSIDSQGRIV
NDLGWFLCGYYGATNEELNKPATVVTCHSETPLQTAFLTCELSGTFGIECSVPAISCVSS
GSTDMPPTCVAATGTWSLNSVGVRAFGHTWQIGSSDTPANYERMTMSIREV
>FUN_002171-T1
MISRNLLTGTVVLLAASMANAGPCRPSSIASSSTVITSVSETATSSFESESATSTASVDA
TQTATTATESESESETGTTFIVETTPTVSSVETTTTALVDTTTAAPVITSADTTTEATTT
TAAATTTSEEPEALQTIYLYAGGSNDPALAPLGGTGFTALGDTSLPDVEYIDFTTDTTSA
LFFTLGERTGKVKIGNGANVGKLAGYSTSGDFSLVIVAETTLAEENGISPLDCEIVDGNG
FQMLQCQYGDEGVADLWTCAGHLTLVKPGVDFTSMCARAATAYKLGYIQVVDY
>FUN_007104-T1
MPSSKVFAAILAALAMADASPCKPKPVTTSGSTAAVVTTSGSTDILSSTATTETAATTTT
GAACSHYTPYTDIVPADCGKTGVAPSCADKVIGSPITVTDYAQCGNTCGMTVGCKSFSIK
GSSCTFYKAPVSSLGYVFNEGSDASHFYDLDLCFGCGEGTTTSGTETITGSQTTGTETET
SATGSETGTTTATGSETETSGTAHATGTTDTETSATGSQTETTTAPGATTETSATETLTA
NTDTTTAAATTTTSAGCTAYTPVANPPAADCAVRGAPNGFAAFMAQLIGIPNSNTEECAR
QCGMYRSSTVVDDKCLSFGLDASNTCYLYKVGLSSLSVDAAGTPVLEFHDFEACYSCDEG
GATTTTEAGSGTTTTAAGVDTTTTETGSET
>FUN_007541-T1
MHSKGISFLAFGLLVAQSVVASPCKPLSFSTSSSALTTALESIISISVPSSTGSETIVLE
TTSTESEAVTTAETNVAGTGTTTAITIDTAISSTKLPTESETSPSVIETSTLATQTTTVA
KGSTSVADDRTTTTIAVSTAAESATTTETTKAETITGTSTAIDTTATTAKTTTGTETTTS
AVGATTTSAKTSTVSTDNTTVTFEESRASTSEDVTTTAKDSTTSVSMGSTTAEGSSTTSD
SANSSSEPITTSATDYGATLVESSTVTSGDGTNTIATSSVAITTTTTSSEYSTTTTDASTT
TETDGAALSTTASSEDISTTAKDSTTDMTTSSEVVTETSGLSATTSGESTTTVITSSEDS
STTIVASQGESTAATDGTTTTAAAAEYSTTTSAESITTAKDSTTVAEQSTTTTDELTTTA
AESTTTAAATTTGCIPNTVLASPTPLFTSGDVHIDEYDTVQLPFQVGIYGSLSNTVYVGV

TGMLSILEAPKFEYSGSYYLPFPYFPPVSIFPYWGDQFTSTETCGSGIAYAVHETSRGQT
FTVEYNTIAVGGNGVDLDHFTVSLYKDHPGLVRFVYYHTENNGRGATVGIQAGNSYSQYS
YDMRSILDNSYVEIDTSSGDALTTSGQL
>FUN_008700-T1
MRFSLQPLAICLVSAQARFALASPCKPLTTTALAATTTTTAVEEASTTTATTDDSTTTVA
GETTIVTLSEATTTTALSDLSTTLTTLTLSEGSTTTAFGEESTTTTLAEDTTTTAVTDEP
TTTTFEATTTTAAEATTTTEALEGTQMAAVFADNTEKDTYIDESGGTYSTPQEGGSTSKA
RFELEPETNRLFTHLVDGTKVYLFTVIPDGPNYAFQFDTAANIDNVDVYHYVLCTADADN
VLSCASESGPTPIVWYWVDSAERYYGNSNPSFDSYPIVHFKLG
>FUN_008841-T1
MRFYASFSLAIGLVAASPCKPPSSTVVSDVISSSVATETSSALTESASSTVDSTATTAET
DVAETSTSAGSTVIETSTAIGSTSTDFTEVSISTTESLVEPTSTATTSEAATSVDTTTAL
TTEASSVETSTAAATTTSEEAGPTGYFLVAGEGPALGGKVKSNGDTFTPMVFGDRGSTFN
PVRFLVDETTGELQQDGVPVCAYQQVGLPYALITRCENANLYYGSVPLNCAQFEGPGTSI
RCSAVKLDCWQTGPGSSQACTRSMDPDWDTFFITNSGDYIWYLSSGTLQNDFMTTVKVFV
DHVEVSAPVQT
>FUN_009673-T1
MVSNKLIVAAFAALASVANASPCKPSSTKAITTSATETASAGFTSKTSTSVETSTAITES
GTVIENETAATATDATTEALSTEASTTVSESATTTATVDEPLLTNTGFEDGTTAPWQLLT
QHEDSLVLGSGYQSPASGKVQFGDEDGSQYSNLIIQKINKKALQAGSYVLEGRTRVDEYS
QSGDGCSTIIAACLTGGTGSWVPVPASLSRASAETSVGHWSQIDTTCTFTEEMLSADADI
SVVFGFYCANSGAYLDSVELKPAAVIPNTDTTTVATNSETSMIITTTQATTMATSIEGST
IATETATTTTATDADPTPLLINGSFDLETTEPWLSTRSESVDRDTNSPFEGPASGRLVFG
VDGGQAYNNYFYQKIDTKDLKAASYRLSGFVRVDYYTNSINGDGCNSMAVGCTLGDPNNL
DRVPGSTVMGTPSVAVDNWFPLDTTCTLTEEMLSQHDYVSVTFGFSCAEVGANLDAVTFQ
EVV
>FUN_009795-T1
MYTRSFLSLAVGLIGVAASPCKPLTSASVVSSATLTSSVAEPTVSTSVESVATETSNSVE
SLVTDALTSTTALTATSTDSTTLFMTETSAATTDFSSTETSADTTTADATTTTSDAPAEI
TTFSIIAGSGPAADNNLRSDNNFGTPLYFGSRVTLPAQHYNIDAATGQIEAGTKSICAWF
QNSDTTKAYITACTSDNENPNEGIVFLNCQVPTEGSALQCSVPVLSCIQQGGGEQTCAAT
TDTLSHFSMEGDDRMVYIVGDGFTGINQTPVDLVIDQAAVAAPTA
>FUN_013109-T1
MRSKVLLLAIGLLVADSALASPCKPRSPTSETGISTSVDSTTQPTSTVEETPTVASSETS
TETGAITTTAPDTTATSEAETTTAVQVTTTTAEAETTTAVISTTTTAEAESTTTTAVATP
TFTIVGGGGAIQGAPLTGIDQDGSVLLFNPQTGTSRTRTIILDPDTGRLRDKDTGIQICA
YYGSANTIFDPANFAFCQNGNTGPNTSFDYMTCQIVSGKLSCTAPQAACPTDDDGVSLGC
TTVDGQLDNEFYYKTQPGLGTYLYISSGSPNGYTPVDVIAQEA
>FUN_015362-T1
MRFTHPALLLALCQIASSSPCKPSSSVTSSAPTSTDNESASSATETSTAISVETGSSTIS
HDTTLAASETASASDLSISTTLVSTSSAEPTTTLEATTDLSTTLDETTTTFESETTSGSA
TLISTTTAEASTTTSATPEIPSNRVLNPGFEDASVFPWQSMSGTLSPSSSEVHSGSQAGF
FSGTTPMSAMLGSRQFINPTWITPGKSYKFSAWIKITNTVNCGSRTIVCGHGAGQGTTSS
VGSITETGDFSLASVTCSWTQAQWEAGPSVQIRSYCTGLSFFVDDVTLEEVETLG
FUN_002171-T1
MISRNLLTGTVVLLAASMANAGPCRPSSIASSSTVITSVSETATSSFESESATSTASVDA
TQTATTATESESESETGTTFIVETTPTVSSVETTTTALVDTTTAAPVITSADTTTEATTT
TAAATTTSEEPEALQTIYLYAGGSNDPALAPLGGTGFTALGDTSLPDVEYIDFTTDTTSA
LFFTLGERTGKVKIGNGANVGKLAGYSTSGDFSLVIVAETTLAEENGISPLDCEIVDGNG
FQMLQCQYGDEGVADLWTCAGHLTLVKPGVDFTSMCARAATAYKLGYIQVVDY

>FUN_004837-T1
MISSSFLSGALAVLAIHSVNAGPCRPSSVSTTKTLTAIKSTTTESSVSSSPETTLPLSTT
AELSVVATQTTTISDVVSSSETFADGTTTTQAVEDTTTTAFETTSATGLSVTTTEATTVS
GDISSSVTLSDDTTTLLAITTTTQAVQDTTTTAPATTTTTAAAEPGECTFNVECEALNGG
ANPVCDAGTCVPDNSQPDPCTDDSDCTTPGETCSISGFCHVASEEPDFCVDENDCLLSVK
PICVLGLCECSESTHQCAPRSDLQLCQSNAGCSAGAACQQGICVDQVACSGPDSCLANLD
LCVLPGVCVCRNGVCALDG
>FUN_006504-T1
MLSSNVLRALALLVATVNAGPCRPTTTLAGNTIEATSTATVIESTADLTSTIVESAAETE
TTDIPTTTTAIVEESTTTDAASTTTSAAVDNTSCSISDDCVANTEFCLVNGLNMCVCLDA
VCVTVD
>FUN_012629-T1
MVRYSILNLAAIATLLLGAEAGPCRPATTVATSIAETTSTLAADTSATSIDTTTVTTLAD
TTTTDGEESTTTTLADTTITTQAESTTTTAAAACAETQLFINPGFDDSPSGIAPWTSNAN
LIQSQAQSGTNALSAVFSNGQPDYYFKQTLQNLNGDYEFSYYYRVVSVSQNADYVCNIEL
KVGDTSKFGAMYDSAGGWRSDSVSFSIAGEAVAQADVQLILTCYGEFVRIEVNIDTLAFT
RVCSA
>FUN_016404-T1
MPSVNTFITALVAGFAIGAQAGPCRPHAPSSSVIQYPISTTQQTAAGTSAAYTDVVSKSE
TKTSLSTAAVQVTSTTKDESKPATAEETTTGYPAAETTSASAGTLTNGSVYPTSEAETTE
PAAETTPTAKEDTTSAPHTTQDSSAAPYTTTSVPESTSKPKTTLDTTTFAPTTTSEPTTT
TSEAYTTTSAAATFSCPPRSELTCAKTGFFNNADSNLIQVYYDYDLNQCQEECDKTDNCK
TIGITTSNQCELYDAAVSALGFEFRDGWYYSVYDACCFNGE
>FUN_001681-T1
MLALVGLALANLAAAGPCKPSGPTLVSSLAVTSSIPTDISETSEVPSLTVPQSTTTTQEV
DIIITNAIEGGSFAARNANNPPSGLTNFGASGNAEFHEGGCYKVDGSPDDGCAALTASGN
PAGKRDTGSFASIWQTLNSLSTVSQRKYTVQFYYLVASAGSQDCTVTAALGNKQFYSQSL
SSVGTSVSWTHVLEQVDAESASATFAISMTCSGNGISVILVDSIFISNQVTPANIGNFVL
DFGVPTTNPVTTSESLPTSTERTIEPETTSASSSSEVFTERTMPETTTTSPPNTDTTRPA
QPTETACKPTCELRAGFDNTPEWNCRVYGLYSGLTYQLPNQDDDETRPWYDGAEDCAEIC
KTLPGCKSAGYMFAPRKCFFSNNVVTHSEVRNMMDDGVDVDWYGMDCFACSACEHGAAKT
TVAEVSSTPVNTDTTIAPEPTTFTTRTSDSPATTTSPADVCLYNRGQECEFNRFKDHSDT
LCIWAAIFTGTTWKESREDYPYQDGPYQCAAICQTLKNCESSGYYSTENRCLFTSKKLQR
SDMISHEDRPFDHSVWSHNSCWTCPTCSETAALPATRYCSYDQGDSCRAVSGKPGALCNY
QGFWGAYNQWDLARFPDQSSPEKCAAICMALDYCVASGYKDDRCMFSFRELKVADFTDWP
DHSRDGTWSDNSCFECPGCTA
>FUN_002166-T1
MRFNSILSLAFGLPAAIAGPCKPGSSSQSSDAVLTTATQATASGVASTETASTTVPGTIE
TSTADVSFASTSIESDTTTPITSLDTTETTTLAPTTTTSEEPAVTTFNIIAEGGPADGIV
MGQRENYYNLEFSSNPDWEPVALNLEEGTGYLRRADPYRPNFPLVCIRWGSGTVPNPGWF
IDCSASDTYYGAPVKCDLKAGGELSCYIPAGHCRFYTVPPRDDDAWDCQADDGVFRNFYT
EESTDEDDVTYYDPWMGFGGYEGKGFYNPLEPVTFRWRSAD
>FUN_002344-T1
MMLLKYSAAVAAFALCGQVSAGPCKPITRVTSSSLGTSSTAATEASLTATSSLSTETSAT
IDTSSAATSVESSATTVSSEASSTTGDSSTLFTSTTETATLDATTTEFVSTSTEASDVST
TATATTSAAPVCEFTGDYTNYVQNPSFDDLDGNGQPTVEPWIMLGVSSLTTNNPRTGARS
MAYSYPDAAIGGSSAILQQLTNTVAGHEYIFKYHWVLIEGQPLQSEECRIGTFAGSEGAN
SQFFSVDGDQEIVQGQYYEQEYRITAENDNQRLSIGFFCSSQPSAGTVKIQIDDVSVYDY
YEGCESP
>FUN_003526-T1

MIRNTLVLAALAATAVAGPCKPGSSSTKLLSTTIGSTETSYFETSITEATSSAAVTETIP
SIASSDATTTAEVTTTSGSGSTTTSADVCVQSLAAPNGEPRFTDRLADCQEFNIVTVSSY
EVTLTAYKRGNVITIPTNAIVRRAEGEAATTILPTGTPAYATYCDSPAAYYEACSELGVT
AFTTTIPEPTTTEVIITN
>FUN_003619-T1
MVHHQGRNVHYKSLFVALAAVSTVAAGPCKPSTTSAGLSTTATVLEEPTSTQTSAIEETT
TTVDETTAAATATAPIEAALTTTTVAADTTTTAAPSGVCGITGYFLPNQELTYLNSPGKK
DSVRQCLEACAAYAGCEVIAFYTDRSFVTDIGRCEFFSGELVDDRQDTSYEWSEVGCLDN
LQD
>FUN_003904-T1
MHLSILRLLAVGLAGLDVTFAGPCKPRSSASESSIATTATTAGTSSFTLSSDTSATFAST
TTEAVTTADTTTEATTEVTSAVTETDTASTEATTSGDTTTEATTFATSTGTTEATSTTGA
DATTETMTTSGTVTASSEITQAPTTTEETTTAAATTTAVPMFRLLAENGGYANQPLLATR
YPFTPLMFSSAQGYTEAYFTVDATTGHLLLDGHLPVCGFQPGDGTSSFTVCSNNMGAQEQ
FLTCEAPTSSQLECTVPEVQCSMFSQTCTNTGALWGTTYIGASGGTIGDKAILGPQSMQG
YTPVPFLITFDSD
>FUN_007311-T1
MIAKSLVFAVLATHAAAGPCKPGGLSSNTLSASESASEPTTSETSVLDSVTTTTSEQATT
TTEAATTTTSAAVPAGEAIIFQVNPRRRLSKRDTTFVGSNNPTECTFASVFRLDEGKLLE
NGVPIYYAGSGFQELAAQGEPPADSVTTTFSIAGGRLSWTDSSFGEAGFCQTESDGQVYI
TFGSEPVGCEPVTLTAYKEEQCQNGQIVGIETSSAETTTAAETTASSDAVQPTTSSNACV
VGIDGLNGEPPRESRLADCSRLNTVTVSPYPETSTVFKREVAFRIPTAFPTWRPAMNTLA
VRAEGEPTATTIQPTEVPVYATYCDSPEEYYAACSEAGITAFTTTLPTTTTSTSTTVSDC
PAKRLVRRAGEHMGYEFEDNWDAHIMPGYKLF
>FUN_007611-T1
MARQLVAILAAAVAFSGVSAGPCKPVSSLALSSAILLETTTTATSHETASSTVTVDVIET
TGAFDTTVTDATTDATGTTDAVDPTITESATDAATAGSITTFLTTTTTEQSPEQSTTTTA
GPVGPGPCLEEQVLYNPSFDDSISSWPWDLNSGVQVSSVKPRSPSYCLLNTLISSHPSTT
FSQALPALGDYEYELVYYISMQNFNGFDLLCYANAFVNGKKMWDSGNFDGLGPFTYQRVS
EVFTPQNSDDAGELRFEIQCEGEFQYAEMAVDDVSLTRRCGA
>FUN_007889-T1
MRSFIAIAALAISGAVAGPCKPRTTETSASVASETSSAAIEATTLPAPPVDSIITNTCTG
GGLTSLDPFVTDGDVTLNTNDGYNPGGGSSDKSCAALSAQSSGTRKRQTLGDIAALKQLL
TGLNVRTQYTVQFFYLVFTPPQATTSCVLEAFIGSQKFFTTGIFSNGASVSYNEVLVSTS
APATQGFLNIQTACSAGGSATVLVDSIFISNQVTPENINDFRLDFGGGDIREPGNTPATT
QVAPTTQRGPATQTLPATQNGPATQPEPATQNGPATQSEPATQNGPATQSEPATQSEPAT
QSEPATQNGPSTATNAPNQGTTTAPQNQGTESTQGTVIPTETAVNGATTAGGESSQSASA
AQNNQQTTETAQGNSQSTGTVQVNAQSTTFPGTGSSESTSAPVNAASQATTSSANGDAST
DGNFASTTTRPDTQPATPTPVGFQTVTAFDPDSTVGPLNNAEPAQDNCVSTNLVPNGGFE
SASSGWQISGNAGITYNGEFGPQARANSGNLAMALHWPQKDGVKAGFKRTISGLVPGQTY
YLGYGHHTGNGAHLPYYECDILVKFDGVNFDDFDPFYDPNAWWQYRNRMNYVTPTHSTVE
LSFVLTCQNNPASFTLLMDDVFLSACQK
>FUN_008367-T1
MRSAFALTGLAILGNINVALAGPCKPHGATSHTEATVTTAAPTTSETKGPLVVKNVIGNG
NFALRDPTNPSYIPNYTVEGQGQIVENQGYTGDGSKEKGCVELQAANSPPGRKRAIGNIV
SISQQLDSLDIKKKYTVRFFYAVVTASQINVCTLSASIAGHQFYTSTILSIGTAIDWNTV
LEQTDVPNTQGAFSVSVNCPIGGIAAIYVDSIFMSNQVTPQTINDVSIDFGDGNDVATTS
GLPVGSTTSNAPNSKESTKSPSTQTLTSTGADAVTSEIAHPTTGLSMGSDTETFHPSIKS
VIGSDTVTHGSDGAQTKEPTSATGESTASEPTTQKPATASESDIPTLTLNPTTSALHPST
ASDSSPSTASSLPTGSRVCPVGAPPPGYCKPVQPEVTQTVSLPGIPQLSDNDRPTAPRAC

WAFGKAKQGTWGRTKSSNPRQNSIADCALLCKQEGSACKAFALYNLGGETSCWMLGDRLG
VVGIDLNQQYSLMWNDFDCFECQDCDIKNPADIETSTLAPSTTSLPAIMTTSVSPSTPTL
CPNCHLRSSPSSDLVCEKIGNLGAVDLQPYANNDFDQTTMQTNSEQCAAICYQLDGCAAS
AHDAARGRCIFSNVAMTSSAFQEAQALNAHDYILPWSSQGCWSCSNDCSVQQDTTSEAPS
PTEHTVEHTTITPSRTEPLTTFISSYVPTTTESAMPQCTLALSDGCTFDQNNYNYQDCSK
YGTMKNTFTLGDNEYPWQDGINTYQNCVAICNQMPHRCKSSAWDQNQSACVFSSNSIFTS
AFTSDSGDDSDMNWSDQSCFKCFCHDQDRDAYSASLRTALPTATCTPSITSEDAVCSIKQ
VDAGDLVCQHQGYFPWQWDEAPSKFPNQDSEERCAALCNSNPDCQASGWSEEYGKCAIGG
FQLKDITWQQFGDTKLSWSDKGCWDCSDCIKSQKWRIYN
>FUN_009826-T1
MLFHKVFLAAAAVATTYAGPCKPVSSRASVSSSADASIPSSTETVEISDATRTATSESSG
TEEATSSVTIGSETVTSGGSTTLATLLSNSGSVSEGRSTSETGSRSSDVLVSTSGTETLG
PSTDTAVTGITTGTTDVNPTTQPTTAPTTDDTTDATATGTTTTELTGQATIDPATQSSTE
PTTEPTDGPTTGTATTDSPTTLPTTDDTTGSATQPTTDTIEPTSGATTDPSTTQHSTTEG
TTTEATTAAPTSTSEFCYDNAYVISMLAFDPVASPFCVSYLSMTTHTITDYATTDPTNVY
QVDVATITADALTRIETNYDGITAIVTEYKTNTEWSEATVTSTRSIETITCLDTAYSYTA
PVPTFDRGGPYREKRGQDEIEIPEAIPSDWTEAQTSDICSCLDLEEPTAHTTITETVEPT
TFITASTDVVTPIEEYTKVITTTSVSVFTSTVTEKKTSTVTAWVVETTFADNNDIAYRRF
NCPFDANIYDDGFTTNYFKGKTPLSSGNTQTLQFSTCSTLSLSGSGTFDPSQVALLYNAY
FYAKETGMYTFSVPDTIDNWGYLWVKDAAYTWNVNAWAIQGTRTGQNSQLWKGGSYQIQL
KKGDAIPFTYLWANGGGCAGNDLSVRTPSGKSIPGMAGSTVKACHLNKFT
>FUN_010739-T1
MIPQTYAAAFITACLVAGSQAGPCKPSSRITEGFSTSDVSTTLGSTTASSFVETTTVAAS
STYFSNEESSVIVTESTSSTDTSVDPKETSTTALSTAEGTTTEIATTTDAATTTEAATDT
ATTTTSEASSAVPFISNAGFDDDDSSVAPWETYYGEYDVSIASDVKHDGRNSALMAATLG
GKLLGDYIKQPLRGSITAGVTYTISAWVNANYFCPAATLICSYQNNAWTDPTTFDLTAKA
GQWTYISSTCTYTQEQIDSGGLYFMIGLTCIGFPGQAYIDTADFSA
>FUN_013434-T1
MTVKTIMTAFIAALLLAGSEAGPCKPRPRTTNILTTVEITSSSTKATSTTEAGLSTTDIS
STGYLIFTTDQAESTSETSTESKDTTTTPAPVISTTKTSSSVALTSSVIESSVEPTTTTE
DAMTTAVSITSTAELSSTIIESRADTSTISEDTTATIIAVLTTEVSLTRELTSETIESCT
EESTTTEASFITTLSTSAPELSPTVIESSTGVSTVTEDATTTTISALETSSAVDISSTIV
ESSTEITTSSQEPTATVSISTTVLIPTTTESTTTSEVTTTTTTEAAGPPTLVNLGFDDTT
EPWSVTQPNLVSLSLDNNIKHDGRSSARMSFSTAGGSTSYISQAYSSPAQAGVGYPASAW
VRPGPGCTIAVLGCAYGNAGLFSGRRTLVVTATTTQPGTINQWQEISYTCTYTQSQIDQG
GLALNIGFMCVSGSEAWIDSVTFYGQ
>FUN_013812-T1
MNSKTLISVLACLSVGVSAGPCKPVTSQATQVTSATGELSTSIDLTTTVSASDAASTTEL
SSKTTEESNTEIATTTTAADTTTEISTTAAVDTTSEAPTTTTEEATTTTGAAQCPTPSAC
NNLGFDWAYYSNPNQNTDTTYSSFVPESFKQDNPLYVGTTRQIGGLFQPSNGDTTGPIYG
STQNFPLDYFALNHHGYLYSCDAGTYKFDIPYANDALYLWVGDKAYGGWSSGDADAKALY
NQPDHIAGSAHFEIDLRAGEYIPIRFVYGQAQYGGGFTFTVTAPNGQVLVSNDDTASPYI
VRYSCDGVDAPVYPPFGSEI
>FUN_013948-T1
MQSKIALVSLALVGFCSAGPCKPSSRTTLISHSITSSIATIDVSASSTDSHTNFDSLTTT
SSAETTEVPSITLPQSSTTTEEEVIIITNAILGGSFASRDPNSPSGLTNFDASGNAEFHQ
GGCYRADGSLDDGCAALSASGDVTKRSFFGSFASIFQTVRSVPRKKYTIQFFYLINSAGS
QGCVASAKFGNAEFYSQPASNLRASWIRVLGQVEAASDLPTFAISLTCSGAGASSILTID
DFKLDLGGSPPPVETTTAIRAEESPSLTTEPVTPKRTGGLSNTSTSMPVSTDSQGDTSSA
VTNTKRTSSDPKTSSAAHESIETMTTSAVRHTSEAMIDTTPITPSSKTLALDETSKAETT

GPVSQAIGSTSQPVTTAGPSQPTSTACKPKCEMINDFYAHLDDFGCDLNGVFVKSDAIYT
LPGEEVGTTQTHWYNSNDECAEICKTLPGCLSAGFQRLSGRCFFSNTLVTREDIRDGRDS
QMVHWFDMECFICGCSSGDTSSTSFPTTTLVPEPTSVTTTTKATQPTGVCHNNRGQECEI
NPSNVENNDYVCIGGGVFTGETWTVPRSLYPMQENGEQCAAICDTLENCESSGFFGMENH
CLFTTTKIKTSDFADPDPNYDDPGLDPKNSVWSHRSCWTCPTCVLSNTPLPKSPTCNYKP
GDACTRVSADGVICNYSGMLPGTFGIDGSYPDQSSSGKCAAICRKMTGCLGSGYRNGQCM
FTRTKLTAGDFLDRHTYDLVWDDPSCFECPGCST
>FUN_015255-T1
MVFNKLIAAAFANLALLASVNAGPCKPQSIATSATEIGSTSIVSDTTTALSTTIETSTAI
SETETATESETTTTLTEDGTTMSASMSETSTTIAVSESTTVVSSSDTTIVTSSEATTAPT
TTESTTTMVESATTTTTAEAEPTELLINGNFDLGTVDPWLSRNDAIQLGSSSPYEGPAYA
VLQFGISDGESYNNGVYQKIDKSLLKTGVYRLSAQLRVDFATNTQFGDGCNAMAVGCYYG
DPNNVVPVQGGSVTVSADDAVGQWTPLDTTCSITEERLSQYGYLSVSIGFSCANAEANVD
AVTFEEVV
>FUN_015297-T1
MRASQTLIALSGVLGLAQAGPCKPHPPASVISSTVVIESASSTASIPQPTTTAAETSTVE
TATEVLSTDEPTSTEIKATTTTAAIETSATSATTDVPVPTPCAIEPGCQAAGFNVDYYKN
VFERGYGNDEMSVPPSYYITDNLTPLDSSVTSETYFAQDYMQDLAGYPQIFPDSAYAGKA
WYVGYHRTLAGGIKVDANNFTLVYTGYYQAPETGTYELCVTADNANTLYFGQGNAFECGT
GETDPNAPALVLTATGYHFNNPTQCGHVDLIAGRHYPVRNVMGNKNAVSAFEFSVTTPSG
SKTHDFEGQAFPVACGVKN
