# Supplementary material for: Profile of the in silico secretome of the palm dieback pathogen, Fusarium oxysporum f. sp. albedinis, a fungus that puts natural oases at risk
Source: PLoS One. 2022 May 26;17(5):e0260830. doi: 10.1371/journal.pone.0260830 (PMC9135196; doi:10.1371/journal.pone.0260830)
Supplement: S2 Table — (PDF) [file pone.0260830.s006.pdf]

Gene ID	HMMER	Hotpep	DIAMOND	Signalp	#ofTools
FUN_002139-T1	AA1_2(43-353)	AA1	AA1_2	Y(1-22)	3
FUN_004641-T1	AA1_3(51-363)	AA1	AA1_3	Y(1-21)	3
FUN_005360-T1	AA1_3(97-416)	AA1	AA1_3	N	3
FUN_000196-T1	AA11(18-208)	AA11	AA11	Y(1-23)	3
FUN_002231-T1	AA11(19-212)	AA11	AA11	N	3
FUN_004567-T1	AA11(20-212)	AA11	AA11	Y(1-20)	3
FUN_015233-T1	AA11(21-227)	AA11	AA11	Y(1-15)	3
FUN_008496-T1	AA12(24-429)	AA12	AA12	N	3
FUN_004999-T1	AA12(25-424)	AA12	AA12	Y(1-22)	3
FUN_008686-T1	AA12(27-429)	AA12	AA12	Y(1-20)	3
FUN_003622-T1	AA13(19-251)	AA13+CBM	AA13	N	3
FUN_012600-T1	AA14(17-289)	N	AA14	Y(1-19)	2
FUN_001410-T1	AA16(17-183)	AA16	AA16	Y(1-34)	3
FUN_006201-T1	AA2(184-432)	AA2	AA2	Y(1-22)	3
FUN_013647-T1	AA2(60-222)	N	AA0	N	2
FUN_014533-T1	AA3(20-602)	AA3	AA3	N	3
FUN_000302-T1	AA3_1(16-539)	AA3+AA8	AA3_1	N	3
FUN_002454-T1	AA3_1(18-545)	AA3+AA8	AA3_1	Y(1-22)	3
FUN_007060-T1	AA3_2(23-596)	AA3	AA3_2	Y(1-18)	3
FUN_003518-T1	AA3_2(23-636)	AA3	AA3	N	3
FUN_004870-T1	AA3_2(28-635)	AA3	AA3	Y(1-23)	3
FUN_000884-T1	AA3_2(33-607)	AA3	AA3_2	Y(1-23)	3
FUN_005029-T1	AA3_2(40-600)	AA3	AA3_2	N	3
FUN_005765-T1	AA3_2(43-668)	AA3	AA3	Y(1-22)	3
FUN_009743-T1	AA3_2(65-639)	AA3	AA3_2	Y(1-22)	3
FUN_002187-T1	AA5(390-895)	AA5	AA5_1	Y(1-23)	3
FUN_004605-T1	AA5_2(154-708)	AA5+CBM3	AA5_2	Y(1-21)	3
FUN_014079-T1	AA5_2(457-1069)	AA5+CBM3	AA5_2+CB	Y(1-31)	3
FUN_013182-T1	AA5_2(60-677)	AA5+CBM1	AA5_2+CB	Y(1-21)	3
FUN_010257-T1	AA5_2(62-677)	AA5+CBM1	AA5_2+CB	Y(1-29)	3
FUN_007549-T1	AA5_2(64-678)	AA5+CBM3	AA5_2+CB	Y(1-27)	3
FUN_001774-T1	AA7(114-306)	N	N	Y(1-27)	1
FUN_015263-T1	AA7(117-572)	N	N	N	1
FUN_000911-T1	AA7(118-568)	N	GH43_11	Y(1-15)	2
FUN_014851-T1	AA7(119-566)	N	N	N	1
FUN_008843-T1	AA7(121-328)	N	N	Y(1-21)	1
FUN_011865-T1	AA7(124-322)	N	N	Y(1-22)	1
FUN_016735-T1	AA7(131-576)	N	N	Y(1-25)	1
FUN_002498-T1	AA7(173-386)	N	N	Y(1-26)	1
FUN_006754-T1	AA7(191-405)	N	N	N	1
FUN_011465-T1	AA7(52-249)	N	N	N	1
FUN_010690-T1	AA7(52-263)	N	AA7	Y(1-22)	2
FUN_010463-T1	AA7(54-471)	N	N	Y(1-20)	1
FUN_000807-T1	AA7(54-491)	AA7+CBM1	AA7	Y(1-23)	3
FUN_013670-T1	AA7(54-491)	AA7	AA7	Y(1-18)	3
FUN_014227-T1	AA7(55-264)	N	N	Y(1-27)	1

FUN_003412-T1	AA7(59-258)	N	N	Y(1-23)	1
FUN_013947-T1	AA7(59-484)	N	N	N	1
FUN_000550-T1	AA7(61-266)	N	N	Y(1-19)	1
FUN_006750-T1	AA7(62-497)	N	N	Y(1-27)	1
FUN_004066-T1	AA7(66-526)	N	N	Y(1-27)	1
FUN_011841-T1	AA7(720-917)	N	N	Y(1-20)	1
FUN_014350-T1	AA7(75-278)	N	N	Y(1-28)	1
FUN_001132-T1	AA8(11-758)	AA3+AA8	AA3_1+AA	Y(1-25)	3
FUN_000878-T1	AA8(4-767)	AA3+AA8	AA3_1+AA	Y(1-20)	3
FUN_002458-T1	AA8(4-833)	AA3+AA8+	AA3_1+AA	Y(1-22)	3
FUN_004439-T1	AA9(10-234)	AA9+CBM1	AA9	Y(1-22)	3
FUN_013069-T1	AA9(10-234)	AA9+CBM1	AA9+CBM1	Y(1-24)	3
FUN_008784-T1	AA9(13-238)	AA9+CBM1	AA9	Y(1-22)	3
FUN_000172-T1	AA9(17-239)	AA9+CBM1	AA9	Y(1-28)	3
FUN_010535-T1	AA9(4-226)	AA9+CBM1	AA9+CBM1	Y(1-17)	3
FUN_002496-T1	AA9(6-227)	AA9	AA9	N	3
FUN_006171-T1	AA9(6-233)	AA9	AA9	N	3
FUN_003018-T1	AA9(7-210)	AA9+CBM1	AA9+CBM1	Y(1-22)	3
FUN_015179-T1	AA9(7-222)	AA9	AA9	Y(1-21)	3
FUN_007381-T1	AA9(8-228)	AA9	AA9	Y(1-20)	3
FUN_013416-T1	AA9(9-223)	AA9	AA9	Y(1-23)	3
FUN_013405-T1	AA9(9-225)	AA9	AA9	Y(1-21)	3
FUN_002923-T1	AA9(9-227)	AA9+CBM1	AA9	N	3
FUN_007566-T1	AA9(9-229)	AA9+CBM1	AA9	Y(1-20)	3
FUN_012557-T1	CBM63(131-201)	CBM63	CBM63	N	3
FUN_009893-T1	CBM63(232-302)	CBM63	CBM63	Y(1-32)	3
FUN_008826-T1	CBM87(43-259)+C	N	CBM87+CE	Y(1-34)	2
FUN_016380-T1	CBM87(43-259)+C	N	CBM87+CE	Y(1-23)	2
FUN_011844-T1	CE1(35-201)	N	CE1	Y(1-18)	2
FUN_004995-T1	CE1(39-211)	CE1+CBM1	CE1	Y(1-21)	3
FUN_009814-T1	CE1(42-233)	CE1+CBM2	CE1	N	3
FUN_013944-T1	CE1(42-235)	CE1+CBM1	CBM1+CE1	Y(1-21)	3
FUN_013453-T1	CE12(21-228)	CE12	CE12	Y(1-33)	3
FUN_001131-T1	CE12(22-235)	CE12	CE12	Y(1-21)	3
FUN_002057-T1	CE12(26-210)	CE12	CE12	N	3
FUN_014277-T1	CE12(30-215)	CE12	CE12	Y(1-25)	3
FUN_012886-T1	CE16(29-287)	CE16+CBM	CE16	Y(1-23)	3
FUN_006721-T1	CE16(29-290)	N	CE0	Y(1-18)	2
FUN_017110-T1	CE16(32-319)	CE0	GH3	N	3
FUN_013111-T1	CE16(33-282)	N	CE16	Y(1-27)	2
FUN_003628-T1	CE16(38-307)	N	N	Y(1-20)	1
FUN_013787-T1	CE3(19-209)	CE3	CE3	Y(1-16)	3
FUN_003793-T1	CE3(57-244)	CE3	CE3	Y(1-21)	3
FUN_007495-T1	CE3(57-254)	CE3	N	N	2
FUN_014633-T1	CE3(70-270)	CE3	CE3	N	3
FUN_005618-T1	CE3(90-280)	CE3	CE3	Y(1-27)	3
FUN_007035-T1	CE4(111-237)	CE4+CBM1	CBM18+CE	Y(1-18)	3

FUN_015746-T1	CE4(115-248)	CE4+CBM1	CBM18+CE	Y(1-27)	3
FUN_009278-T1	CE4(124-255)	CE4+CBM1	CBM18+CE	Y(1-19)	3
FUN_002733-T1	CE4(45-167)	N	CE4	Y(1-22)	2
FUN_008114-T1	CE5(170-339)	CE5	N	N	2
FUN_001504-T1	CE5(22-227)	CE5+CBM1	CE5	Y(1-21)	3
FUN_002001-T1	CE5(24-228)	CE5	CE5	N	3
FUN_006786-T1	CE5(30-222)	N	CE5	N	2
FUN_015673-T1	CE5(32-221)+CE5	CE5	CE5	N	3
FUN_000775-T1	CE5(47-223)	CE5	CE5	Y(1-26)	3
FUN_017065-T1	CE5(48-252)	N	CE5	Y(1-22)	2
FUN_007245-T1	CE5(52-228)	CE5	CE5	Y(1-23)	3
FUN_014468-T1	CE5(53-228)	CE5	CE5	Y(1-29)	3
FUN_004335-T1	CE5(70-257)	CE5	CE5	N	3
FUN_008398-T1	CE8(27-306)	CE8	CE8	Y(1-43)	3
FUN_013225-T1	CE8(33-165)	N	N	Y(1-48)	1
FUN_013642-T1	CE8(383-648)+CE	CE8	CE8	Y(1-20)	3
FUN_008559-T1	CE8(47-312)	CE8	CE8	Y(1-27)	3
FUN_007109-T1	CE8(51-381)	CE8	CE8	Y(1-22)	3
FUN_013224-T1	CE8(54-378)	CE8	CE8	Y(1-18)	3
FUN_012839-T1	GH1(161-619)	N	GH1	N	2
FUN_002082-T1	GH10(16-311)	GH10	GH10	Y(1-17)	3
FUN_007984-T1	GH10(20-330)	GH10+CBM	CBM1+GH1	Y(1-18)	3
FUN_007380-T1	GH10(29-326)	GH10+CBM	GH10	Y(1-26)	3
FUN_009722-T1	GH10(33-324)	GH10	GH10	N	3
FUN_017120-T1	GH10(34-351)	GH10+CBM	GH10	Y(1-21)	3
FUN_004762-T1	GH105(32-378)	GH105	GH105	N	3
FUN_005603-T1	GH105(36-377)	GH105	GH105	N	3
FUN_008147-T1	GH106(309-823)	N	GH106	N	2
FUN_003885-T1	GH106(32-996)	GH106	GH106	N	3
FUN_008648-T1	GH106(33-963)	GH106	GH106	Y(1-24)	3
FUN_016900-T1	GH11(44-219)	GH11+CBM	GH11	Y(1-20)	3
FUN_003933-T1	GH11(51-226)	GH11+CBM	GH11	Y(1-20)	3
FUN_013106-T1	GH11(54-228)	GH11+CBM	GH11	Y(1-20)	3
FUN_001732-T1	GH114(43-261)	N	GH114	Y(1-27)	2
FUN_014517-T1	GH114(48-245)	GH114	GH114	Y(1-26)	3
FUN_011288-T1	GH115(35-706)	GH115	GH115	Y(1-19)	3
FUN_003419-T1	GH115(45-674)	GH115	GH115	N	3
FUN_007040-T1	GH12(100-248)	GH12	GH12	Y(1-25)	3
FUN_005772-T1	GH12(135-280)	N	GH12	N	2
FUN_002648-T1	GH12(209-358)	GH12	GH12	N	3
FUN_010440-T1	GH12(214-363)	N	GH12	N	2
FUN_004994-T1	GH12(90-237)	GH12+CBM	GH12	Y(1-19)	3
FUN_013233-T1	GH125(70-497)	GH125	GH125	N	3
FUN_003866-T1	GH125(86-495)	GH125	GH125	Y(1-20)	3
FUN_010240-T1	GH125(87-523)	GH125	GH125	Y(1-21)	3
FUN_000418-T1	GH128(172-385)	N	GH128	N	2
FUN_010225-T1	GH128(42-280)	GH128	GH128	N	3

FUN_008178-T1	GH128(48-275)	GH128	GH128	Y(1-22)	3
FUN_003013-T1	GH13_1(58-345)	CBM20	GH13_1	Y(1-20)	3
FUN_012370-T1	GH131(35-290)	GH131	GH131	Y(1-28)	3
FUN_001730-T1	GH132(137-438)	GH132	GH132	N	3
FUN_005476-T1	GH132(46-307)	GH132	GH132	Y(1-22)	3
FUN_004399-T1	GH134(24-184)	GH134	GH134	Y(1-19)	3
FUN_008138-T1	GH134(24-184)	GH134	N	Y(1-19)	2
FUN_008839-T1	GH139(11-775)	GH139	GH139	N	3
FUN_014265-T1	GH145(40-337)	N	GH145	Y(1-20)	2
FUN_002507-T1	GH145(69-278)	N	GH145	Y(1-39)	2
FUN_010770-T1	GH146(36-553)	GH146+CB	GH146	Y(1-25)	3
FUN_011660-T1	GH16_1(67-299)	N	GH16_1	Y(1-20)	2
FUN_003596-T1	GH16_1(73-320)	N	GH16_1	Y(1-21)	2
FUN_000901-T1	GH16_1(77-321)	N	GH16_1	Y(1-23)	2
FUN_006685-T1	GH16_10(41-227)	N	GH16_10	N	2
FUN_009413-T1	GH16_18(67-223)	N	GH16_18	Y(1-19)	2
FUN_011000-T1	GH16_18(68-224)	N	GH16_18	Y(1-22)	2
FUN_008661-T1	GH16_18(70-223)	N	GH16_18	Y(1-42)	2
FUN_008478-T1	GH16_18(70-226)	N	GH16_18	Y(1-22)	2
FUN_012671-T1	GH16_18(70-226)	N	GH16_18	Y(1-19)	2
FUN_002103-T1	GH16_19(105-268	GH16+CBM	CBM18+GH	Y(1-20)	3
FUN_007194-T1	GH16_22(80-296)	N	GH16_22	N	2
FUN_016214-T1	GH16_22(83-291)	N	GH16_22	Y(1-21)	2
FUN_000558-T1	GH16_22(93-307)	N	GH16_22	N	2
FUN_009533-T1	GH16_23(83-259)	N	GH16	Y(1-20)	2
FUN_004584-T1	GH16_3(29-284)	N	GH16_3	N	2
FUN_001993-T1	GH16_3(30-277)	N	GH16_3	Y(1-18)	2
FUN_004822-T1	GH16_3(31-293)	CBM56+CB	N	Y(1-20)	2
FUN_012622-T1	GH16_3(32-283)	N	GH16_3	Y(1-18)	2
FUN_009618-T1	GH16_3(32-284)	N	GH16_3	Y(1-20)	2
FUN_010730-T1	GH16_3(43-299)	N	GH16_3	Y(1-22)	2
FUN_010454-T1	GH162(32-523)	GH162	GH162	Y(1-21)	3
FUN_004659-T1	GH17(104-310)	GH17	GH17	Y(1-19)	3
FUN_011031-T1	GH17(112-356)	GH17	GH17	Y(1-51)	3
FUN_002055-T1	GH17(49-302)	GH17	GH17	Y(1-24)	3
FUN_006687-T1	GH17(64-291)	GH17	GH17	N	3
FUN_002046-T1	GH18(115-462)	GH18+CBM	CBM18+GH	Y(1-21)	3
FUN_012739-T1	GH18(20-176)	CBM18	N	Y(1-18)	2
FUN_016997-T1	GH18(20-201)	CBM18	N	Y(1-18)	2
FUN_014144-T1	GH18(21-423)	GH18+CBM	CBM18+GH	Y(1-18)	3
FUN_015472-T1	GH18(21-423)	CBM18	CBM18+GH	N	3
FUN_016129-T1	GH18(28-369)	GH18+CBM	GH18	Y(1-22)	3
FUN_007727-T1	GH18(324-673)	GH18	GH18	Y(1-21)	3
FUN_014570-T1	GH18(41-406)	GH18	GH18	Y(1-23)	3
FUN_008163-T1	GH18(49-404)	N	GH18	Y(1-23)	2
FUN_003651-T1	GH18(58-421)	N	GH18	Y(1-23)	2
FUN_015414-T1	GH18(85-412)	CBM18	CBM18+GH	Y(1-26)	3

FUN_005436-T1	GH2(53-371)	GH2+CBM4	GH2	Y(1-23)	3
FUN_007019-T1	GH2(69-806)	GH2	GH2	N	3
FUN_009858-T1	GH20(169-509)	GH20	GH20	N	3
FUN_007433-T1	GH20(213-558)	GH20	GH20	Y(1-22)	3
FUN_015146-T1	GH24(102-242)	N	GH24	Y(1-19)	2
FUN_004812-T1	GH24(121-209)	N	N	Y(1-18)	1
FUN_005040-T1	GH27(113-338)	GH27+CBM	GH27	Y(1-22)	3
FUN_008806-T1	GH28(149-396)	N	GH28	Y(1-18)	2
FUN_000587-T1	GH28(192-450)	GH28	GH28	Y(1-24)	3
FUN_008788-T1	GH28(37-362)	GH28	GH28	Y(1-17)	3
FUN_003536-T1	GH28(39-353)	GH28	GH28	Y(1-18)	3
FUN_010091-T1	GH28(54-376)	GH28	GH28	Y(1-24)	3
FUN_013226-T1	GH28(57-392)	GH28	N	Y(1-21)	2
FUN_008558-T1	GH28(59-328)	GH28	GH28	Y(1-32)	3
FUN_009561-T1	GH28(66-375)	GH28	GH28	Y(1-37)	3
FUN_014469-T1	GH28(71-334)	GH28	GH28	Y(1-21)	3
FUN_017109-T1	GH28(73-428)	GH28	GH28	N	3
FUN_005719-T1	GH28(74-425)	GH28	GH28	N	3
FUN_003337-T1	GH28(83-437)	GH28	GH28	Y(1-29)	3
FUN_014262-T1	GH29(22-398)	GH29+CBM	GH29	Y(1-24)	3
FUN_007341-T1	GH3(100-321)	GH3+CBM1	GH3	N	3
FUN_017148-T1	GH3(107-331)	GH3	GH3	N	3
FUN_014860-T1	GH3(107-346)	GH3	GH3	N	3
FUN_005414-T1	GH3(111-334)	GH3	GH3	Y(1-22)	3
FUN_005852-T1	GH3(163-248)	CBM1	GH3	Y(1-22)	3
FUN_001872-T1	GH3(68-284)	GH3+CBM1	GH3	Y(1-19)	3
FUN_015538-T1	GH3(72-290)	GH3+CBM1	GH3	N	3
FUN_002283-T1	GH3(75-324)	GH3	GH3	Y(1-24)	3
FUN_013181-T1	GH3(76-290)	GH3+CBM1	GH3	Y(1-21)	3
FUN_008653-T1	GH3(84-301)	GH3+CBM1	GH3	N	3
FUN_004816-T1	GH3(88-300)	GH3+CBM1	GH3	N	3
FUN_009586-T1	GH3(93-308)	GH3+CBM1	GH3	Y(1-22)	3
FUN_011906-T1	GH3(98-321)	GH3+CBM1	GH3	Y(1-22)	3
FUN_008182-T1	GH30_7(21-447)	N	GH30_7	Y(1-20)	2
FUN_004983-T1	GH30_7(36-481)	GH30	GH30_7	Y(1-24)	3
FUN_005025-T1	GH31(292-842)	N	GH31	Y(1-18)	2
FUN_011767-T1	GH31(327-908)	GH31	GH31	Y(1-25)	3
FUN_008134-T1	GH32(30-323)+CB	GH32+CBM	CBM38+GH	Y(1-23)	3
FUN_005204-T1	GH32(45-354)	GH32+CBM	GH32	Y(1-35)	3
FUN_009236-T1	GH35(45-248)	N	GH35	N	2
FUN_005425-T1	GH35(45-390)	GH35	GH35	N	3
FUN_015261-T1	GH36(37-727)	GH36	GH36	Y(1-25)	3
FUN_014691-T1	GH37(51-626)	GH37	GH37	Y(1-30)	3
FUN_005630-T1	GH43_1(28-343)	GH43	GH43_1	Y(1-21)	3
FUN_005035-T1	GH43_13(23-315)	N	GH43_13	Y(1-22)	2
FUN_015231-T1	GH43_14(27-303)	GH43	GH43_14	N	3
FUN_015482-T1	GH43_21(76-303)	N	GH43_21	Y(1-20)	2

FUN_001804-T1	GH43_22(153-367	GH43	GH43_22	Y(1-22)	3
FUN_017061-T1	GH43_22(185-425	N	GH43_22	Y(1-31)	2
FUN_008181-T1	GH43_22(91-306)	N	GH43_22	Y(1-31)	2
FUN_002724-T1	GH43_24(42-257)	CBM32	GH43_24	N	3
FUN_004464-T1	GH43_24(44-285)	CBM35	CBM35+GH	Y(1-25)	3
FUN_010607-T1	GH43_24(46-279)	GH43+CBM	CBM35+GH	Y(1-20)	3
FUN_004319-T1	GH43_26(21-306)	GH43	GH43_26	N	3
FUN_006500-T1	GH43_26(21-316)	N	GH43_26	Y(1-21)	2
FUN_005599-T1	GH43_29(24-304)	GH43+CBM	CBM6+GH4	Y(1-23)	3
FUN_007891-T1	GH43_30(45-315)	N	GH43_30	N	2
FUN_008321-T1	GH43_33(30-458)	N	GH43_33	Y(1-26)	2
FUN_009564-T1	GH43_34(31-314)	GH43	GH43_34	N	3
FUN_005598-T1	GH43_36(20-286)	GH43	GH43_36	N	3
FUN_013116-T1	GH43_36(30-300)	GH43	GH43_36	Y(1-19)	3
FUN_009563-T1	GH43_5(34-312)	GH43	GH43_5	Y(1-21)	3
FUN_011499-T1	GH43_5(38-313)	N	GH43_5	Y(1-25)	2
FUN_010441-T1	GH43_5(38-316)	GH43	GH43_5	Y(1-35)	3
FUN_001994-T1	GH45(22-220)	GH45+CBM	CBM1+GH4	Y(1-18)	3
FUN_008124-T1	GH47(59-536)	GH47	GH47	Y(1-17)	3
FUN_015443-T1	GH49(24-598)	GH49	GH49	N	3
FUN_008156-T1	GH5_15(82-384)	GH5	GH5_15	Y(1-24)	3
FUN_013382-T1	GH5_15(98-401)	GH5	GH5_15	N	3
FUN_014512-T1	GH5_16(34-373)	GH5	GH5_16	Y(1-21)	3
FUN_002723-T1	GH5_16(42-378)	GH5	GH5_16	Y(1-20)	3
FUN_004118-T1	GH5_23(131-410)	N	GH5_23	Y(1-19)	2
FUN_004972-T1	GH5_23(83-364)	N	GH5_23	Y(1-21)	2
FUN_003025-T1	GH5_27(58-389)	N	GH5_27	N	2
FUN_000554-T1	GH5_31(47-222)	N	GH5	Y(1-22)	2
FUN_011239-T1	GH5_31(52-329)	N	GH5	N	2
FUN_010001-T1	GH5_5(31-309)	GH5+CBM1	GH5_5	Y(1-26)	3
FUN_010079-T1	GH5_5(84-360)	GH5+CBM1	CBM1+GH5	Y(1-23)	3
FUN_001438-T1	GH5_7(55-331)	N	GH5_7	Y(1-21)	2
FUN_002167-T1	GH5_7(60-348)	GH5+CBM1	CBM1+GH5	Y(1-19)	3
FUN_008140-T1	GH5_7(64-278)	GH5+CBM1	N	N	2
FUN_009601-T1	GH51(206-653)	GH51	GH51	N	3
FUN_009818-T1	GH53(21-345)	GH53	GH53	Y(1-19)	3
FUN_009235-T1	GH53(36-358)	GH53	N	Y(1-23)	2
FUN_003033-T1	GH54(22-336)+CB	GH54+CBM	CBM42+GH	N	3
FUN_009508-T1	GH55(17-767)	GH55	GH55	N	3
FUN_013514-T1	GH6(130-422)	GH6+CBM1	CBM1+GH6	Y(1-17)	3
FUN_015435-T1	GH62(28-296)	GH62+CBM	GH62	Y(1-44)	3
FUN_000031-T1	GH63(587-798)	GH63	GH63	N	3
FUN_012932-T1	GH64(69-441)	GH64+CBM	GH64	Y(1-25)	3
FUN_016734-T1	GH64(75-444)	GH64	GH64	Y(1-19)	3
FUN_009189-T1	GH64(75-447)	GH64+CBM	GH64	N	3
FUN_011086-T1	GH64(75-447)	GH64+CBM	GH64	Y(1-31)	3
FUN_014333-T1	GH64(77-449)	GH64+CBM	GH64	Y(1-19)	3

FUN_013515-T1	GH67(18-697)	GH67	GH67	N	3
FUN_017060-T1	GH67(22-692)	GH67	GH67	N	3
FUN_013393-T1	GH7(22-416)	GH7	GH7	Y(1-19)	3
FUN_007818-T1	GH7(23-429)	N	GH7	N	2
FUN_004977-T1	GH71(21-411)+CB	GH71+CBM	CBM24+GH	Y(1-17)	3
FUN_002450-T1	GH71(42-431)+CB	GH71+CBM	CBM24+GH	Y(1-18)	3
FUN_000252-T1	GH72(17-332)	GH72	GH72	Y(1-26)	3
FUN_016792-T1	GH72(28-328)	GH72	GH72	Y(1-28)	3
FUN_014467-T1	GH74(82-179)	GH74+CBM	GH74	Y(1-17)	3
FUN_010210-T1	GH75(12-253)	GH75	GH75	N	3
FUN_013383-T1	GH75(7-225)	GH75	GH75	N	3
FUN_004652-T1	GH76(29-396)	GH76	GH76	Y(1-24)	3
FUN_000915-T1	GH76(31-373)	N	GH76	N	2
FUN_013542-T1	GH76(85-498)	GH0+GH76	GH0	Y(1-23)	3
FUN_005668-T1	GH78(359-762)	GH78	GH78	Y(1-19)	3
FUN_012197-T1	GH78(404-810)	GH78	GH78	Y(1-27)	3
FUN_007315-T1	GH79(41-418)	GH79	GH79	Y(1-35)	3
FUN_012051-T1	GH81(173-847)	GH81	GH81	Y(1-22)	3
FUN_004327-T1	GH93(40-354)	GH93	GH93	Y(1-22)	3
FUN_016968-T1	GH93(48-354)	GH93	GH93	Y(1-19)	3
FUN_001686-T1	GH93(49-351)	GH93	GH93	Y(1-31)	3
FUN_009788-T1	GH93(49-351)	N	GH93	N	2
FUN_013136-T1	GH93(57-361)	GH93	GH93	Y(1-20)	3
FUN_003524-T1	GH95(26-768)	GH95	GH95	N	3
FUN_005570-T1	GT20(173-628)	GT20	GT20	Y(1-45)	3
FUN_011036-T1	GT24(1167-1414)	GT24	GT24	Y(1-31)	3
FUN_009630-T1	N	CE3	CE3	Y(1-20)	2
FUN_001493-T1	N	GH17	N	N	1
FUN_006531-T1	N	GH27	GH27	N	2
FUN_016977-T1	N	GH27+CBM	CBM35+GH	Y(1-30)	2
FUN_010794-T1	N	GH39	GH39	Y(1-22)	2
FUN_006232-T1	N	GH39	GH39	Y(1-18)	2
FUN_007997-T1	N	AA1	AA1	Y(1-25)	2
FUN_009624-T1	N	AA1	AA1	Y(1-24)	2
FUN_009071-T1	N	GT31	GT31	N	2
FUN_010781-T1	N	CBM1	CBM1	Y(1-24)	2
FUN_013925-T1	N	CBM1	CBM1	N	2
FUN_015415-T1	N	CBM18	N	Y(1-18)	1
FUN_002346-T1	N	CBM18	N	Y(1-18)	1
FUN_015337-T1	N	CBM2	N	N	1
FUN_010457-T1	N	CBM5+CBM	N	Y(1-18)	1
FUN_013275-T1	N	CBM5	N	Y(1-28)	1
FUN_005695-T1	N	CBM5	N	Y(1-21)	1
FUN_016008-T1	N	CBM5	N	Y(1-22)	1
FUN_009406-T1	N	CBM5	N	Y(1-32)	1
FUN_004489-T1	N	CBM5	N	Y(1-21)	1
FUN_015702-T1	N	CBM5	N	Y(1-28)	1

FUN_007797-T1	N	CBM5	N	N	1
FUN_009592-T1	N	CBM63	CBM63	Y(1-23)	2
FUN_000084-T1	N	N	CBM12	Y(1-29)	1
FUN_000782-T1	N	N	AA3	N	1
FUN_000798-T1	N	N	AA0	Y(1-27)	1
FUN_000848-T1	N	N	GH3	Y(1-20)	1
FUN_002047-T1	N	N	CBM50+GH	N	1
FUN_002048-T1	N	N	CBM50	N	1
FUN_002142-T1	N	N	GH0	Y(1-26)	1
FUN_002451-T1	N	N	GH39	Y(1-24)	1
FUN_003071-T1	N	N	CBM50	Y(1-21)	1
FUN_004374-T1	N	N	CBM1	Y(1-21)	1
FUN_004799-T1	N	N	AA0	Y(1-27)	1
FUN_006251-T1	N	N	GT90	Y(1-21)	1
FUN_006936-T1	N	N	GT1	N	1
FUN_007535-T1	N	N	GH78	Y(1-36)	1
FUN_008190-T1	N	N	GH146	N	1
FUN_009765-T1	N	N	GH31	N	1
FUN_010613-T1	N	N	CBM18+CB	Y(1-24)	1
FUN_011848-T1	N	N	CBM18+GH	N	1
FUN_013584-T1	N	N	GH0	N	1
FUN_013687-T1	N	N	CBM20+GH	Y(1-24)	1
FUN_013919-T1	N	N	CBM20+GH	Y(1-22)	1
FUN_013932-T1	N	N	GH51	Y(1-19)	1
FUN_014485-T1	N	N	CBM50	Y(1-19)	1
FUN_014499-T1	N	N	GH0	Y(1-21)	1
FUN_014600-T1	N	N	GH146	N	1
FUN_016114-T1	N	N	AA0	Y(1-27)	1
FUN_016128-T1	N	N	GH18	Y(1-20)	1
FUN_016803-T1	N	N	GH0	Y(1-21)	1
FUN_017013-T1	N	N	GH18	Y(1-22)	1
FUN_002508-T1	PL1_10(80-253)	PL1	PL1_10	Y(1-35)	3
FUN_006684-T1	PL1_2(70-245)	PL1+CBM6	PL1_2	Y(1-18)	3
FUN_006940-T1	PL1_4(109-294)	PL1	CBM1+PL1	Y(1-46)	3
FUN_008802-T1	PL1_4(110-293)	PL1	PL1_4	Y(1-25)	3
FUN_004117-T1	PL1_4(111-295)	PL1	PL1_4	N	3
FUN_002649-T1	PL1_4(111-296)	PL1	PL1_4	Y(1-20)	3
FUN_009983-T1	PL1_4(112-295)	PL1	PL1_4	Y(1-27)	3
FUN_004350-T1	PL1_4(114-297)	PL1	PL1_4	Y(1-21)	3
FUN_010455-T1	PL1_7(73-252)	PL1	PL1_7	Y(1-16)	3
FUN_001807-T1	PL1_7(76-257)	PL1	PL1_7	Y(1-32)	3
FUN_008476-T1	PL1_7(80-260)	PL1	PL1_7	Y(1-70)	3
FUN_013637-T1	PL1_9(83-263)	PL1	PL1_9	Y(1-24)	3
FUN_005018-T1	PL11_2(22-593)	PL11+CBM	PL11_2	N	3
FUN_004325-T1	PL26(31-917)	PL26	PL26	Y(1-21)	3
FUN_003505-T1	PL3(31-235)	PL3	N	N	2
FUN_009771-T1	PL3_2(168-345)	PL3	CBM1+PL3	Y(1-18)	3

FUN_016007-T1	PL3_2(26-212)	PL3	PL3_2	Y(1-28)	3
FUN_001126-T1	PL3_2(30-215)	PL3	PL3_2	N	3
FUN_002176-T1	PL3_2(30-217)	PL3	PL3_2	Y(1-19)	3
FUN_013888-T1	PL3_2(33-214)	PL3	N	N	2
FUN_012913-T1	PL3_2(43-228)	PL3+CBM1	PL3_2	Y(1-18)	3
FUN_000916-T1	PL3_2(44-233)	PL3+CBM1	PL3_2	Y(1-18)	3
FUN_001808-T1	PL3_2(59-246)	N	PL3_2	Y(1-16)	2
FUN_012149-T1	PL4_1(17-529)	PL4	PL4_1	Y(1-19)	3
FUN_007064-T1	PL4_3(21-647)	PL4	PL4_3	Y(1-29)	3
FUN_000164-T1	PL4_3(23-655)	PL4	PL4_3	Y(1-23)	3
FUN_007609-T1	PL9_3(20-377)	PL9	PL9_3	Y(1-21)	3
