# Supplementary material for: Profile of the in silico secretome of the palm dieback pathogen, Fusarium oxysporum f. sp. albedinis, a fungus that puts natural oases at risk
Source: PLoS One. 2022 May 26;17(5):e0260830. doi: 10.1371/journal.pone.0260830 (PMC9135196; doi:10.1371/journal.pone.0260830)
Supplement: S3 Table — (PDF) [file pone.0260830.s007.pdf]

#id	product	contains disulfidebonds	confidence	bondcount
FUN_003798-T1	hypothetical protein	y	1.0	1
FUN_015451-T1	hypothetical protein	y	1.0	1
FUN_001167-T1	hypothetical protein	y	1.0	1
FUN_001702-T1	hypothetical protein	y	1.0	1
FUN_016968-T1	hypothetical protein	y	1.0	1
FUN_000347-T1	hypothetical protein	y	1.0	1
FUN_000512-T1	hypothetical protein	y	1.0	1
FUN_000572-T1	hypothetical protein	y	1.0	1
FUN_000617-T1	hypothetical protein	y	1.0	1
FUN_000661-T1	hypothetical protein	y	1.0	1
FUN_000927-T1	hypothetical protein	y	1.0	1
FUN_001330-T1	hypothetical protein	y	1.0	1
FUN_001519-T1	hypothetical protein	y	1.0	1
FUN_001689-T1	hypothetical protein	y	1.0	1
FUN_002293-T1	hypothetical protein	y	1.0	1
FUN_002451-T1	hypothetical protein	y	1.0	1
FUN_003380-T1	hypothetical protein	y	1.0	1
FUN_003875-T1	hypothetical protein	y	1.0	1
FUN_003997-T1	hypothetical protein	y	1.0	1
FUN_004316-T1	hypothetical protein	y	1.0	1
FUN_004399-T1	hypothetical protein	y	1.0	1
FUN_004611-T1	hypothetical protein	y	1.0	1

FUN_005039-T1	hypothetical protein	y	1.0	1
FUN_005229-T1	hypothetical protein	y	1.0	1
FUN_005315-T1	hypothetical protein	y	1.0	1
FUN_005337-T1	hypothetical protein	y	1.0	1
FUN_005416-T1	hypothetical protein	y	1.0	1
FUN_005430-T1	hypothetical protein	y	1.0	1
FUN_005614-T1	hypothetical protein	y	1.0	1
FUN_005706-T1	hypothetical protein	y	1.0	1
FUN_005818-T1	hypothetical protein	y	1.0	1
FUN_005840-T1	hypothetical protein	y	1.0	1
FUN_005999-T1	hypothetical protein	y	1.0	1
FUN_006251-T1	hypothetical protein	y	1.0	1
FUN_006537-T1	hypothetical protein	y	1.0	1
FUN_006687-T1	hypothetical protein	y	1.0	1
FUN_007671-T1	hypothetical protein	y	1.0	1
FUN_008152-T1	hypothetical protein	y	1.0	1
FUN_008201-T1	hypothetical protein	y	1.0	1
FUN_009132-T1	hypothetical protein	y	1.0	1
FUN_009609-T1	hypothetical protein	y	1.0	1
FUN_009621-T1	hypothetical protein	y	1.0	1
FUN_009917-T1	hypothetical protein	y	1.0	1
FUN_010742-T1	hypothetical protein	y	1.0	1
FUN_010780-T1	hypothetical protein	y	1.0	1

FUN_011238-T1	hypothetical protein	y	1.0	1
FUN_011259-T1	hypothetical protein	y	1.0	1
FUN_011483-T1	hypothetical protein	y	1.0	1
FUN_011497-T1	hypothetical protein	y	1.0	1
FUN_011868-T1	hypothetical protein	y	1.0	1
FUN_013089-T1	hypothetical protein	y	1.0	1
FUN_013127-T1	hypothetical protein	y	1.0	1
FUN_013584-T1	hypothetical protein	y	1.0	1
FUN_013694-T1	hypothetical protein	y	1.0	1
FUN_014065-T1	hypothetical protein	y	1.0	1
FUN_014538-T1	hypothetical protein	y	1.0	1
FUN_014721-T1	hypothetical protein	y	1.0	1
FUN_015685-T1	hypothetical protein	y	1.0	1
FUN_016315-T1	hypothetical protein	y	1.0	1
FUN_013142-T1	hypothetical protein	y	1.0	1
FUN_002507-T1	hypothetical protein	y	1.0	1
FUN_010412-T1	hypothetical protein	y	1.0	1
FUN_012541-T1	hypothetical protein	y	1.0	1
FUN_004028-T1	hypothetical protein	y	1.0	1
FUN_012222-T1	hypothetical protein	y	1.0	1
FUN_006945-T1	hypothetical protein	y	1.0	1
FUN_013160-T1	hypothetical protein	y	1.0	1
FUN_015020-T1	hypothetical protein	y	1.0	1

FUN_015389-T1	hypothetical protein	y	1.0	1
FUN_010001-T1	hypothetical protein	y	1.0	1
FUN_011150-T1	hypothetical protein	y	1.0	1
FUN_016145-T1	hypothetical protein	y	1.0	1
FUN_010903-T1	hypothetical protein	y	1.0	1
FUN_013153-T1	hypothetical protein	y	1.0	1
FUN_002650-T1	hypothetical protein	y	1.0	1
FUN_001403-T1	hypothetical protein	y	1.0	1
FUN_003206-T1	hypothetical protein	y	1.0	1
FUN_012886-T1	hypothetical protein	y	1.0	1
FUN_006721-T1	hypothetical protein	y	1.0	1
FUN_009630-T1	hypothetical protein	y	1.0	1
FUN_004994-T1	hypothetical protein	y	1.0	1
FUN_005772-T1	hypothetical protein	y	1.0	1
FUN_013416-T1	hypothetical protein	y	1.0	1
FUN_015179-T1	hypothetical protein	y	1.0	1
FUN_015435-T1	hypothetical protein	y	1.0	1
FUN_011000-T1	hypothetical protein	y	1.0	1
FUN_001804-T1	hypothetical protein	y	1.0	1
FUN_005424-T1	hypothetical protein	y	1.0	1
FUN_016665-T1	hypothetical protein	y	1.0	1
FUN_007837-T1	hypothetical protein	y	1.0	1
FUN_009212-T1	hypothetical protein	y	1.0	1

FUN_013233-T1	hypothetical protein	y	1.0	1
FUN_000270-T1	hypothetical protein	y	1.0	1
FUN_002309-T1	hypothetical protein	y	1.0	1
FUN_007535-T1	hypothetical protein	y	1.0	1
FUN_007819-T1	hypothetical protein	y	1.0	1
FUN_008148-T1	hypothetical protein	y	1.0	1
FUN_016973-T1	hypothetical protein	y	1.0	1
FUN_007281-T1	hypothetical protein	y	1.0	1
FUN_016563-T1	hypothetical protein	y	1.0	1
FUN_002531-T1	hypothetical protein	y	1.0	1
FUN_003305-T1	hypothetical protein	y	1.0	1
FUN_003006-T1	Trichothecene C-3 esterase	y	1.0	1
FUN_001399-T1	hypothetical protein	y	1.0	1
FUN_015257-T1	hypothetical protein	y	1.0	1
FUN_005967-T1	hypothetical protein	y	1.0	1
FUN_008690-T1	hypothetical protein	y	1.0	1
FUN_015388-T1	hypothetical protein	y	1.0	1
FUN_002735-T1	hypothetical protein	y	1.0	1
FUN_004956-T1	hypothetical protein	y	1.0	1
FUN_014929-T1	hypothetical protein	y	1.0	1
FUN_011036-T1	hypothetical protein	y	1.0	1
FUN_015916-T1	hypothetical protein	y	1.0	1
FUN_003730-T1	hypothetical protein	y	0.913881	2

FUN_007028-T1	hypothetical protein	y	0.866295	2
FUN_007912-T1	hypothetical protein	y	0.867879	2
FUN_000078-T1	hypothetical protein	y	0.826494	2
FUN_000151-T1	hypothetical protein	y	0.747651	2
FUN_000209-T1	hypothetical protein	y	0.843	2
FUN_000264-T1	hypothetical protein	y	0.478461	2
FUN_000363-T1	hypothetical protein	y	0.717306	2
FUN_000585-T1	hypothetical protein	y	0.672377	2
FUN_000763-T1	hypothetical protein	y	0.871214	2
FUN_000812-T1	hypothetical protein	y	0.80746	2
FUN_001455-T1	hypothetical protein	y	0.659624	2
FUN_002171-T1	hypothetical protein	y	0.800416	2
FUN_002260-T1	hypothetical protein	y	0.852986	2
FUN_002499-T1	hypothetical protein	y	0.72796	2
FUN_003046-T1	hypothetical protein	y	0.440022	2
FUN_003320-T1	hypothetical protein	y	0.783263	2
FUN_003459-T1	hypothetical protein	y	0.797699	2
FUN_003526-T1	hypothetical protein	y	0.789706	2
FUN_003583-T1	hypothetical protein	y	0.784587	2
FUN_003904-T1	hypothetical protein	y	0.819327	2
FUN_004320-T1	hypothetical protein	y	0.820984	2
FUN_004321-T1	hypothetical protein	y	0.818091	2
FUN_004374-T1	hypothetical protein	y	0.844385	2

FUN_004655-T1	hypothetical protein	y	0.81571	2
FUN_004861-T1	hypothetical protein	y	0.956766	2
FUN_005310-T1	hypothetical protein	y	0.808016	2
FUN_005339-T1	hypothetical protein	y	0.704632	2
FUN_005723-T1	hypothetical protein	y	0.84208	2
FUN_006626-T1	hypothetical protein	y	0.660496	2
FUN_006627-T1	hypothetical protein	y	0.571978	2
FUN_006652-T1	hypothetical protein	y	0.757141	2
FUN_006953-T1	hypothetical protein	y	0.411642	2
FUN_006991-T1	hypothetical protein	y	0.782312	2
FUN_007034-T1	hypothetical protein	y	0.797202	2
FUN_007070-T1	hypothetical protein	y	0.875053	2
FUN_007208-T1	hypothetical protein	y	0.868941	2
FUN_007889-T1	hypothetical protein	y	0.885004	2
FUN_008401-T1	hypothetical protein	y	0.393191	2
FUN_008463-T1	hypothetical protein	y	0.823559	2
FUN_008688-T1	hypothetical protein	y	0.692261	2
FUN_008700-T1	hypothetical protein	y	0.864888	2
FUN_008839-T1	hypothetical protein	y	0.869219	2
FUN_009213-T1	hypothetical protein	y	0.870271	2
FUN_009596-T1	hypothetical protein	y	0.815603	2
FUN_009738-T1	hypothetical protein	y	0.740854	2
FUN_009791-T1	hypothetical protein	y	0.366953	2

FUN_009793-T1	hypothetical protein	y	0.782977	2
FUN_009845-T1	hypothetical protein	y	0.819244	2
FUN_010074-T1	hypothetical protein	y	0.816359	2
FUN_011799-T1	hypothetical protein	y	0.636473	2
FUN_012263-T1	hypothetical protein	y	0.931762	2
FUN_012505-T1	hypothetical protein	y	0.65702	2
FUN_012629-T1	hypothetical protein	y	0.800405	2
FUN_012721-T1	hypothetical protein	y	0.695649	2
FUN_012742-T1	hypothetical protein	y	0.821245	2
FUN_013111-T1	hypothetical protein	y	0.709089	2
FUN_013136-T1	hypothetical protein	y	0.868754	2
FUN_013400-T1	hypothetical protein	y	0.706228	2
FUN_013543-T1	hypothetical protein	y	0.771124	2
FUN_013671-T1	hypothetical protein	y	0.874442	2
FUN_013805-T1	hypothetical protein	y	0.662669	2
FUN_013967-T1	hypothetical protein	y	0.755519	2
FUN_014323-T1	hypothetical protein	y	0.86682	2
FUN_014425-T1	hypothetical protein	y	0.706691	2
FUN_015255-T1	hypothetical protein	y	0.823286	2
FUN_015322-T1	hypothetical protein	y	0.711509	2
FUN_015428-T1	hypothetical protein	y	0.817661	2
FUN_015858-T1	hypothetical protein	y	0.725615	2
FUN_016093-T1	hypothetical protein	y	0.565555	2

FUN_016803-T1	hypothetical protein	y	0.931836	2
FUN_016939-T1	hypothetical protein	y	0.435219	2
FUN_004596-T1	hypothetical protein	y	0.876257	2
FUN_002174-T1	hypothetical protein	y	0.878081	2
FUN_010192-T1	hypothetical protein	y	0.697054	2
FUN_008737-T1	hypothetical protein	y	0.801591	2
FUN_005476-T1	hypothetical protein	y	0.802149	2
FUN_002925-T1	hypothetical protein	y	0.844631	2
FUN_009975-T1	hypothetical protein	y	0.814615	2
FUN_014892-T1	hypothetical protein	y	0.835623	2
FUN_003295-T1	hypothetical protein	y	0.856852	2
FUN_008654-T1	hypothetical protein	y	0.802008	2
FUN_001186-T1	hypothetical protein	y	0.697352	2
FUN_014477-T1	hypothetical protein	y	0.661393	2
FUN_005909-T1	hypothetical protein	y	0.959116	2
FUN_000775-T1	securin, sister chromatid separation inhibitor	y	0.305091	2
FUN_007245-T1	hypothetical protein	y	0.176101	2
FUN_008114-T1	hypothetical protein	y	0.315647	2
FUN_014468-T1	hypothetical protein	y	0.523933	2
FUN_006839-T1	hypothetical protein	y	0.81073	2
FUN_013049-T1	hypothetical protein	y	0.132846	2
FUN_004600-T1	hypothetical protein	y	0.782695	2
FUN_015012-T1	hypothetical protein	y	0.924517	2

FUN_013602-T1	hypothetical protein	y	0.919667	2
FUN_016266-T1	saccharopepsin	y	0.925117	2
FUN_001817-T1	hypothetical protein	y	0.794165	2
FUN_013383-T1	hypothetical protein	y	0.141203	2
FUN_001857-T1	hypothetical protein	y	0.880025	2
FUN_008178-T1	hypothetical protein	y	0.816316	2
FUN_000172-T1	hypothetical protein	y	0.866509	2
FUN_007566-T1	hypothetical protein	y	0.425555	2
FUN_008784-T1	hypothetical protein	y	0.113793	2
FUN_000915-T1	hypothetical protein	y	0.879186	2
FUN_002346-T1	hypothetical protein	y	0.760487	2
FUN_000587-T1	hypothetical protein	y	0.875032	2
FUN_008806-T1	hypothetical protein	y	0.923078	2
FUN_002724-T1	hypothetical protein	y	0.861022	2
FUN_010607-T1	hypothetical protein	y	0.87861	2
FUN_000387-T1	hypothetical protein	y	0.0925445	2
FUN_004711-T1	hypothetical protein	y	0.7647	2
FUN_009556-T1	hypothetical protein	y	0.730268	2
FUN_000916-T1	hypothetical protein	y	0.950471	2
FUN_002904-T1	hypothetical protein	y	0.77461	2
FUN_000164-T1	hypothetical protein	y	0.148865	2
FUN_007064-T1	hypothetical protein	y	0.092785	2
FUN_005622-T1	hypothetical protein	y	0.931203	2

FUN_009534-T1	hypothetical protein	y	0.740884	2
FUN_001813-T1	hypothetical protein	y	0.914819	2
FUN_009053-T1	hypothetical protein	y	0.937839	2
FUN_003475-T1	hypothetical protein	y	0.844286	2
FUN_014286-T1	hypothetical protein	y	0.959309	2
FUN_012149-T1	hypothetical protein	y	0.957906	2
FUN_014516-T1	hypothetical protein	y	0.910875	2
FUN_007911-T1	hypothetical protein	y	0.72417	2
FUN_011715-T1	hypothetical protein	y	0.873585	2
FUN_001395-T1	hypothetical protein	y	0.902328	2
FUN_006972-T1	Protein disulfide-isomerase erp38	y	0.851899	2
FUN_008564-T1	hypothetical protein	y	0.843073	2
FUN_002576-T1	hypothetical protein	y	0.836236	3
FUN_004450-T1	hypothetical protein	y	0.546417	3
FUN_000354-T1	hypothetical protein	y	0.561882	3
FUN_000557-T1	hypothetical protein	y	0.566339	3
FUN_001280-T1	hypothetical protein	y	0.807175	3
FUN_001469-T1	hypothetical protein	y	0.574753	3
FUN_001473-T1	hypothetical protein	y	0.507995	3
FUN_001488-T1	hypothetical protein	y	0.812093	3
FUN_001507-T1	hypothetical protein	y	0.564936	3
FUN_001687-T1	hypothetical protein	y	0.645191	3
FUN_002151-T1	hypothetical protein	y	0.616884	3

FUN_002166-T1	hypothetical protein	y	0.559157	3
FUN_002201-T1	hypothetical protein	y	0.564011	3
FUN_002344-T1	hypothetical protein	y	0.582389	3
FUN_002557-T1	hypothetical protein	y	0.527604	3
FUN_002662-T1	hypothetical protein	y	0.511567	3
FUN_002853-T1	hypothetical protein	y	0.597087	3
FUN_002900-T1	hypothetical protein	y	0.542558	3
FUN_003019-T1	hypothetical protein	y	0.345672	3
FUN_003029-T1	hypothetical protein	y	0.334083	3
FUN_003296-T1	hypothetical protein	y	0.577084	3
FUN_003619-T1	hypothetical protein	y	0.518187	3
FUN_003643-T1	hypothetical protein	y	0.52028	3
FUN_003648-T1	hypothetical protein	y	0.581478	3
FUN_003865-T1	hypothetical protein	y	0.591671	3
FUN_004392-T1	hypothetical protein	y	0.475182	3
FUN_004654-T1	hypothetical protein	y	0.581286	3
FUN_004867-T1	hypothetical protein	y	0.518769	3
FUN_005222-T1	hypothetical protein	y	0.563999	3
FUN_005579-T1	hypothetical protein	y	0.576176	3
FUN_005612-T1	hypothetical protein	y	0.53454	3
FUN_005653-T1	hypothetical protein	y	0.571369	3
FUN_005760-T1	hypothetical protein	y	0.576794	3
FUN_005811-T1	hypothetical protein	y	0.527803	3

FUN_006410-T1	hypothetical protein	y	0.550661	3
FUN_006504-T1	hypothetical protein	y	0.508196	3
FUN_006655-T1	hypothetical protein	y	0.651909	3
FUN_006671-T1	hypothetical protein	y	0.544097	3
FUN_007376-T1	hypothetical protein	y	0.56687	3
FUN_007478-T1	hypothetical protein	y	0.575355	3
FUN_007564-T1	hypothetical protein	y	0.316414	3
FUN_007611-T1	hypothetical protein	y	0.53551	3
FUN_007688-T1	hypothetical protein	y	0.573322	3
FUN_007930-T1	hypothetical protein	y	0.531711	3
FUN_008319-T1	hypothetical protein	y	0.616907	3
FUN_008809-T1	hypothetical protein	y	0.606915	3
FUN_008841-T1	hypothetical protein	y	0.514957	3
FUN_009230-T1	hypothetical protein	y	0.571364	3
FUN_009351-T1	hypothetical protein	y	0.646071	3
FUN_009483-T1	hypothetical protein	y	0.52436	3
FUN_009673-T1	hypothetical protein	y	0.528706	3
FUN_009795-T1	hypothetical protein	y	0.556964	3
FUN_010169-T1	hypothetical protein	y	0.524644	3
FUN_010413-T1	hypothetical protein	y	0.626516	3
FUN_010454-T1	hypothetical protein	y	0.528819	3
FUN_010462-T1	hypothetical protein	y	0.68173	3
FUN_010613-T1	hypothetical protein	y	0.542231	3

FUN_010880-T1	hypothetical protein	y	0.650071	3
FUN_011419-T1	hypothetical protein	y	0.563122	3
FUN_011934-T1	hypothetical protein	y	0.515148	3
FUN_011991-T1	hypothetical protein	y	0.355187	3
FUN_012210-T1	hypothetical protein	y	0.57561	3
FUN_012557-T1	hypothetical protein	y	0.535813	3
FUN_012745-T1	hypothetical protein	y	0.641295	3
FUN_013086-T1	hypothetical protein	y	0.885521	3
FUN_013109-T1	hypothetical protein	y	0.515217	3
FUN_013120-T1	hypothetical protein	y	0.558233	3
FUN_013434-T1	hypothetical protein	y	0.519643	3
FUN_013595-T1	hypothetical protein	y	0.582376	3
FUN_013733-T1	hypothetical protein	y	0.532275	3
FUN_014090-T1	hypothetical protein	y	0.576529	3
FUN_014164-T1	hypothetical protein	y	0.570739	3
FUN_014630-T1	hypothetical protein	y	0.618883	3
FUN_014706-T1	hypothetical protein	y	0.613575	3
FUN_014872-T1	hypothetical protein	y	0.588171	3
FUN_014887-T1	hypothetical protein	y	0.558238	3
FUN_015017-T1	hypothetical protein	y	0.551219	3
FUN_015362-T1	hypothetical protein	y	0.5648	3
FUN_015477-T1	hypothetical protein	y	0.531091	3
FUN_015834-T1	Long chronological lifespan protein 2	y	0.496606	3

FUN_015892-T1	hypothetical protein	y	0.496142	3
FUN_016013-T1	hypothetical protein	y	0.563114	3
FUN_016177-T1	hypothetical protein	y	0.369779	3
FUN_016237-T1	hypothetical protein	y	0.604464	3
FUN_016318-T1	hypothetical protein	y	0.368152	3
FUN_016923-T1	hypothetical protein	y	0.518501	3
FUN_006531-T1	hypothetical protein	y	0.840585	3
FUN_010739-T1	hypothetical protein	y	0.562734	3
FUN_013337-T1	hypothetical protein	y	0.361129	3
FUN_015861-T1	hypothetical protein	y	0.551628	3
FUN_007668-T1	hypothetical protein	y	0.79495	3
FUN_002931-T1	hypothetical protein	y	0.487316	3
FUN_008154-T1	hypothetical protein	y	0.553938	3
FUN_010079-T1	Endoglucanase gh5-1	y	0.639763	3
FUN_002167-T1	hypothetical protein	y	0.556351	3
FUN_012113-T1	hypothetical protein	y	0.655175	3
FUN_005233-T1	hypothetical protein	y	0.678523	3
FUN_004335-T1	hypothetical protein	y	0.814758	3
FUN_006786-T1	hypothetical protein	y	0.672163	3
FUN_012823-T1	hypothetical protein	y	0.567817	3
FUN_013944-T1	hypothetical protein	y	0.679635	3
FUN_000911-T1	hypothetical protein	y	0.876624	3
FUN_005356-T1	hypothetical protein	y	0.579774	3

FUN_013812-T1	hypothetical protein	y	0.530801	3
FUN_000252-T1	hypothetical protein	y	0.624355	3
FUN_016792-T1	hypothetical protein	y	0.568965	3
FUN_001732-T1	hypothetical protein	y	0.533561	3
FUN_006171-T1	hypothetical protein	y	0.864569	3
FUN_013069-T1	glycoside hydrolase 61	y	0.826476	3
FUN_002450-T1	hypothetical protein	y	0.36456	3
FUN_007194-T1	hypothetical protein	y	0.79217	3
FUN_011660-T1	hypothetical protein	y	0.444955	3
FUN_003536-T1	Polygalacturonase 1	y	0.769514	3
FUN_008181-T1	hypothetical protein	y	0.594483	3
FUN_001873-T1	hypothetical protein	y	0.607254	3
FUN_016078-T1	Phosphatidylglycerol/phosphatidylinositol transfer protein	y	0.833661	3
FUN_007374-T1	hypothetical protein	y	0.537192	3
FUN_001358-T1	hypothetical protein	y	0.589983	3
FUN_012946-T1	hypothetical protein	y	0.472316	3
FUN_004117-T1	hypothetical protein	y	0.607007	3
FUN_004350-T1	hypothetical protein	y	0.563609	3
FUN_006486-T1	hypothetical protein	y	0.745604	3
FUN_009765-T1	hypothetical protein	y	0.536677	3
FUN_016153-T1	hypothetical protein	y	0.499872	3
FUN_013787-T1	hypothetical protein	y	0.449237	3

FUN_001474-T1	hypothetical protein	y	0.834083	3
FUN_003983-T1	hypothetical protein	y	0.354319	3
FUN_016730-T1	hypothetical protein	y	0.693173	3
FUN_005717-T1	hypothetical protein	y	0.789623	3
FUN_013902-T1	hypothetical protein	y	0.751725	3
FUN_010457-T1	hypothetical protein	y	0.4429	3
FUN_001012-T1	hypothetical protein	y	0.880063	3
FUN_011341-T1	hypothetical protein	y	0.209258	4
FUN_005507-T1	hypothetical protein	y	0.91779	4
FUN_002067-T1	hypothetical protein	y	0.209804	4
FUN_008451-T1	hypothetical protein	y	0.85216	4
FUN_000038-T1	hypothetical protein	y	0.193086	4
FUN_000554-T1	hypothetical protein	y	0.83729	4
FUN_000555-T1	hypothetical protein	y	0.194779	4
FUN_000811-T1	hypothetical protein	y	0.197139	4
FUN_000939-T1	hypothetical protein	y	0.226475	4
FUN_001041-T1	hypothetical protein	y	0.819493	4
FUN_001252-T1	hypothetical protein	y	0.198934	4
FUN_001337-T1	hypothetical protein	y	0.215298	4
FUN_001502-T1	hypothetical protein	y	0.238106	4
FUN_001525-T1	hypothetical protein	y	0.187995	4
FUN_001554-T1	hypothetical protein	y	0.829878	4
FUN_001559-T1	hypothetical protein	y	0.197165	4

FUN_001617-T1	hypothetical protein	y	0.188708	4
FUN_001773-T1	hypothetical protein	y	0.208163	4
FUN_002258-T1	hypothetical protein	y	0.832931	4
FUN_002259-T1	hypothetical protein	y	0.194782	4
FUN_002323-T1	hypothetical protein	y	0.285294	4
FUN_003382-T1	hypothetical protein	y	0.206023	4
FUN_004006-T1	hypothetical protein	y	0.276398	4
FUN_004331-T1	hypothetical protein	y	0.261726	4
FUN_004343-T1	hypothetical protein	y	0.191722	4
FUN_004368-T1	hypothetical protein	y	0.242072	4
FUN_004423-T1	hypothetical protein	y	0.282022	4
FUN_004612-T1	hypothetical protein	y	0.190215	4
FUN_004728-T1	hypothetical protein	y	0.224785	4
FUN_005007-T1	hypothetical protein	y	0.198768	4
FUN_005354-T1	hypothetical protein	y	0.20627	4
FUN_005559-T1	hypothetical protein	y	0.197208	4
FUN_005597-T1	hypothetical protein	y	0.190107	4
FUN_005703-T1	hypothetical protein	y	0.198086	4
FUN_005994-T1	hypothetical protein	y	0.257585	4
FUN_006114-T1	hypothetical protein	y	0.23239	4
FUN_006256-T1	hypothetical protein	y	0.209346	4
FUN_006403-T1	hypothetical protein	y	0.190214	4
FUN_006411-T1	hypothetical protein	y	0.197139	4

FUN_006656-T1	hypothetical protein	y	0.263222	4
FUN_006814-T1	hypothetical protein	y	0.203822	4
FUN_007216-T1	hypothetical protein	y	0.214263	4
FUN_007803-T1	hypothetical protein	y	0.210114	4
FUN_008323-T1	hypothetical protein	y	0.273511	4
FUN_008604-T1	hypothetical protein	y	0.195383	4
FUN_008745-T1	hypothetical protein	y	0.281011	4
FUN_008822-T1	hypothetical protein	y	0.201541	4
FUN_008868-T1	hypothetical protein	y	0.296675	4
FUN_009349-T1	hypothetical protein	y	0.222137	4
FUN_009421-T1	hypothetical protein	y	0.270118	4
FUN_009792-T1	hypothetical protein	y	0.284637	4
FUN_009890-T1	hypothetical protein	y	0.18237	4
FUN_009905-T1	hypothetical protein	y	0.263455	4
FUN_009959-T1	hypothetical protein	y	0.262534	4
FUN_010040-T1	hypothetical protein	y	0.211031	4
FUN_010211-T1	hypothetical protein	y	0.283649	4
FUN_011266-T1	hypothetical protein	y	0.208792	4
FUN_011935-T1	hypothetical protein	y	0.207534	4
FUN_012301-T1	hypothetical protein	y	0.184719	4
FUN_012694-T1	hypothetical protein	y	0.249774	4
FUN_012858-T1	hypothetical protein	y	0.206213	4
FUN_013147-T1	hypothetical protein	y	0.210038	4

FUN_013691-T1	hypothetical protein	y	0.206213	4
FUN_013735-T1	hypothetical protein	y	0.211624	4
FUN_013815-T1	hypothetical protein	y	0.208796	4
FUN_014418-T1	hypothetical protein	y	0.247188	4
FUN_014498-T1	hypothetical protein	y	0.27178	4
FUN_014499-T1	hypothetical protein	y	0.289439	4
FUN_015273-T1	hypothetical protein	y	0.209151	4
FUN_015983-T1	hypothetical protein	y	0.204006	4
FUN_016044-T1	hypothetical protein	y	0.296675	4
FUN_016404-T1	hypothetical protein	y	0.186117	4
FUN_016658-T1	hypothetical protein	y	0.211484	4
FUN_016703-T1	hypothetical protein	y	0.205963	4
FUN_016828-T1	hypothetical protein	y	0.201517	4
FUN_017069-T1	hypothetical protein	y	0.207206	4
FUN_003033-T1	hypothetical protein	y	0.430435	4
FUN_000355-T1	hypothetical protein	y	0.626558	4
FUN_000075-T1	hypothetical protein	y	0.926188	4
FUN_006548-T1	hypothetical protein	y	0.58553	4
FUN_006583-T1	hypothetical protein	y	0.281502	4
FUN_006663-T1	hypothetical protein	y	0.239331	4
FUN_012447-T1	hypothetical protein	y	0.269824	4
FUN_013174-T1	hypothetical protein	y	0.872343	4
FUN_017065-T1	hypothetical protein	y	0.784606	4

FUN_013354-T1	hypothetical protein	y	0.271453	4
FUN_012785-T1	hypothetical protein	y	0.198499	4
FUN_010716-T1	hypothetical protein	y	0.6015	4
FUN_015263-T1	FAD-linked oxidoreductase hmp9	y	0.648365	4
FUN_001774-T1	hypothetical protein	y	0.706576	4
FUN_006754-T1	hypothetical protein	y	0.687394	4
FUN_008843-T1	hypothetical protein	y	0.494932	4
FUN_011865-T1	hypothetical protein	y	0.440176	4
FUN_010781-T1	hypothetical protein	y	0.523763	4
FUN_013925-T1	hypothetical protein	y	0.602067	4
FUN_010210-T1	hypothetical protein	y	0.80436	4
FUN_001859-T1	hypothetical protein	y	0.877304	4
FUN_010801-T1	beta ketoadipyl CoA thiolase, th1	y	0.882378	4
FUN_013673-T1	Protein transport protein yos1	y	0.74477	4
FUN_000418-T1	hypothetical protein	y	0.446734	4
FUN_013542-T1	hypothetical protein	y	0.846394	4
FUN_010091-T1	hypothetical protein	y	0.500786	4
FUN_000084-T1	hypothetical protein	y	0.235127	4
FUN_001374-T1	hypothetical protein	y	0.412973	4
FUN_002513-T1	hypothetical protein	y	0.610104	4
FUN_003091-T1	hypothetical protein	y	0.935059	4
FUN_011272-T1	hypothetical protein	y	0.380959	4
FUN_016031-T1	hypothetical protein	y	0.216162	4

FUN_008867-T1	hypothetical protein	y	0.660927	4
FUN_004812-T1	hypothetical protein	y	0.748569	4
FUN_008322-T1	hypothetical protein	y	0.631714	4
FUN_013172-T1	hypothetical protein	y	0.411376	4
FUN_015764-T1	hypothetical protein	y	0.278708	4
FUN_013071-T1	hypothetical protein	y	0.739889	5
FUN_000315-T1	hypothetical protein	y	0.588221	5
FUN_000536-T1	hypothetical protein	y	0.391672	5
FUN_001118-T1	hypothetical protein	y	0.609073	5
FUN_001516-T1	hypothetical protein	y	0.258053	5
FUN_001517-T1	hypothetical protein	y	0.346726	5
FUN_001977-T1	hypothetical protein	y	0.938328	5
FUN_002155-T1	hypothetical protein	y	0.39905	5
FUN_002891-T1	hypothetical protein	y	0.506152	5
FUN_003069-T1	hypothetical protein	y	0.663889	5
FUN_003512-T1	hypothetical protein	y	0.24465	5
FUN_004567-T1	hypothetical protein	y	0.541542	5
FUN_004764-T1	hypothetical protein	y	0.319324	5
FUN_004806-T1	hypothetical protein	y	0.353617	5
FUN_004846-T1	hypothetical protein	y	0.396565	5
FUN_006508-T1	hypothetical protein	y	0.737698	5
FUN_007310-T1	hypothetical protein	y	0.691356	5
FUN_007311-T1	hypothetical protein	y	0.557094	5

FUN_007322-T1	hypothetical protein	y	0.360038	5
FUN_007334-T1	hypothetical protein	y	0.313009	5
FUN_007377-T1	hypothetical protein	y	0.361298	5
FUN_007622-T1	hypothetical protein	y	0.448406	5
FUN_007879-T1	hypothetical protein	y	0.382056	5
FUN_008640-T1	hypothetical protein	y	0.258901	5
FUN_008798-T1	hypothetical protein	y	0.462169	5
FUN_009956-T1	hypothetical protein	y	0.256743	5
FUN_011113-T1	hypothetical protein	y	0.355579	5
FUN_011926-T1	hypothetical protein	y	0.261685	5
FUN_012600-T1	hypothetical protein	y	0.474452	5
FUN_013782-T1	hypothetical protein	y	0.561557	5
FUN_015036-T1	hypothetical protein	y	0.342263	5
FUN_015046-T1	hypothetical protein	y	0.48835	5
FUN_015354-T1	hypothetical protein	y	0.777903	5
FUN_015679-T1	hypothetical protein	y	0.80873	5
FUN_016009-T1	hypothetical protein	y	0.89786	5
FUN_016264-T1	hypothetical protein	y	0.262644	5
FUN_006581-T1	hypothetical protein	y	0.952217	5
FUN_005896-T1	hypothetical protein	y	0.748307	5
FUN_013162-T1	hypothetical protein	y	0.263765	5
FUN_002001-T1	hypothetical protein	y	0.685468	5
FUN_001504-T1	hypothetical protein	y	0.799303	5

FUN_009243-T1	hypothetical protein	y	0.897382	5
FUN_000306-T1	hypothetical protein	y	0.706837	5
FUN_001776-T1	hypothetical protein	y	0.927781	5
FUN_009826-T1	hypothetical protein	y	0.814999	5
FUN_000196-T1	hypothetical protein	y	0.604279	5
FUN_003018-T1	hypothetical protein	y	0.760614	5
FUN_003596-T1	hypothetical protein	y	0.672501	5
FUN_003337-T1	hypothetical protein	y	0.942933	5
FUN_005719-T1	hypothetical protein	y	0.924195	5
FUN_017109-T1	hypothetical protein	y	0.938333	5
FUN_008788-T1	hypothetical protein	y	0.939321	5
FUN_013514-T1	hypothetical protein	y	0.876415	5
FUN_014485-T1	hypothetical protein	y	0.587514	5
FUN_001410-T1	hypothetical protein	y	0.562932	5
FUN_013128-T1	hypothetical protein	y	0.879433	5
FUN_012913-T1	hypothetical protein	y	0.857789	5
FUN_013888-T1	hypothetical protein	y	0.838844	5
FUN_015630-T1	hypothetical protein	y	0.927537	5
FUN_000500-T1	hypothetical protein	y	0.952494	5
FUN_001364-T1	hypothetical protein	y	0.86552	5
FUN_010054-T1	hypothetical protein	y	0.838524	5
FUN_010425-T1	hypothetical protein	y	0.780527	5
FUN_014890-T1	hypothetical protein	y	0.83542	5

[RK]C..C.{
12}H position

KCPDCRF
DGLVCES
KDWH 75-93

Cluster (6)	Cluster (4)	Cluster (2)	Cluster (1.4)
215	211	201	178
500	487	463	424
110	113	115	110
249	239	225	198
25	35	41	44
93	95	98	96
140	139	138	181
142	141	140	130
354	340	317	278
100	5	2	1
227	219	208	186
233	225	214	113
241	233	221	194
362	348	325	286
18	4	7	2
182	180	173	156
161	159	155	140
10	14	8	15
386	373	349	310
81	83	85	82
298	286	265	230
202	199	191	169

150	149	146	132
400	387	363	324
195	193	128	121
286	274	254	221
177	175	168	19
160	6	13	10
407	394	370	331
410	397	373	334
80	82	84	81
209	206	198	175
413	400	376	337
61	62	66	65
209	206	198	175
38	40	44	48
327	313	291	253
227	219	208	186
65	115	117	10
286	274	254	221
338	324	302	263
441	428	404	365
334	320	298	259
211	208	199	176
457	444	420	381

333	319	297	258
195	193	128	121
459	446	422	383
186	184	177	158
93	95	98	96
473	460	436	397
80	82	84	81
121	122	123	118
478	465	441	402
51	55	61	61
25	35	41	44
16	23	25	31
342	328	305	266
511	498	474	435
150	149	146	132
271	260	244	213
41	45	4	6
41	45	4	6
1	1	1	4
1	1	1	4
1	1	1	4
94	96	1	4
1	1	1	4

1	1	1	4
259	249	235	207
14	21	4	6
76	78	80	76
10	14	8	15
475	462	438	399
11	15	15	7
11	15	15	7
11	15	15	7
84	86	89	86
84	86	89	86
55	59	22	28
125	125	53	53
125	125	53	53
13	20	3	5
13	20	3	5
499	486	462	423
39	41	45	30
72	74	77	74
403	390	366	327
513	500	476	437
33	32	38	32
33	32	38	32

198	196	188	167
136	136	135	127
179	177	170	153
45	49	55	55
45	49	55	55
45	49	55	55
56	60	64	63
324	310	288	250
197	195	187	166
373	359	336	297
110	113	115	110
174	36	42	46
113	117	118	114
8	12	16	16
203	200	192	30
24	16	20	29
24	16	20	29
81	83	85	82
81	83	85	82
81	83	85	82
112	116	8	15
62	63	67	66
15	22	15	7

56	60	64	63
97	99	101	99
215	211	201	178
90	91	94	92
349	335	312	273
49	53	60	60
351	337	314	275
143	142	141	131
148	147	33	38
149	148	145	39
165	163	159	144
78	80	82	77
178	176	169	152
372	358	335	296
276	265	248	216
379	366	342	303
282	270	129	122
285	273	37	2
283	271	129	122
30	18	21	14
69	70	74	71
69	70	74	71
14	21	4	6

101	102	104	1
293	281	260	113
205	202	194	171
103	104	106	103
307	295	273	238
165	163	159	144
165	163	159	144
126	126	126	3
276	265	248	216
283	271	129	122
282	270	129	122
322	309	287	249
16	23	25	31
18	4	7	2
156	155	151	137
270	259	243	212
211	208	199	176
333	319	297	258
13	20	3	5
294	282	261	226
34	33	49	50
223	109	111	106
202	199	191	169

149	148	145	39
132	132	56	56
19	25	27	17
184	182	175	35
342	328	305	266
465	452	428	389
34	33	49	50
202	199	191	169
467	454	430	391
84	86	89	86
25	35	41	44
103	104	106	103
477	464	440	401
17	24	26	33
61	62	66	65
181	179	172	155
183	181	174	157
486	473	449	410
240	232	71	42
103	104	106	103
196	194	186	165
503	490	466	427
507	494	470	431

316	304	282	80
339	325	92	90
150	149	146	132
113	117	118	114
451	438	414	375
107	110	112	107
171	168	164	149
1	1	1	4
1	1	1	4
1	1	1	4
1	1	1	4
1	1	1	4
157	156	152	138
157	156	152	138
7	11	9	11
57	42	46	24
57	42	46	24
57	42	46	24
57	42	46	24
22	10	19	27
22	10	19	27
82	84	86	83
15	22	29	7

11	15	15	7
11	15	15	7
121	122	123	118
237	229	218	191
173	170	4	6
53	57	63	62
12	19	3	5
12	19	3	5
12	19	3	5
152	151	148	134
35	34	40	12
54	58	32	19
177	175	168	19
119	120	121	116
119	120	121	116
139	138	137	129
87	88	91	89
440	427	403	364
108	43	24	25
105	106	108	37
91	92	95	93
91	92	95	93
207	204	196	173

207	204	196	173
363	349	326	287
437	424	400	361
197	195	187	166
82	84	86	83
91	92	95	93
30	18	21	14
292	280	259	225
174	36	42	46
8	12	16	16
67	68	72	70
274	263	247	215
74	76	23	12
48	52	59	59
350	336	313	274
353	339	316	277
159	158	154	139
4	7	11	20
239	231	220	193
29	5	2	1
229	221	210	42
112	116	8	15
370	356	333	294

216	18	21	14
2	2	5	9
34	33	49	50
37	39	18	8
30	18	21	14
135	135	134	126
143	142	141	131
374	361	337	298
375	362	338	299
195	193	128	121
247	360	7	2
77	79	81	14
16	23	25	31
290	278	257	223
154	64	37	2
395	382	358	319
45	49	55	55
183	181	174	157
310	298	276	240
52	56	62	3
311	299	277	241
411	398	374	335
412	399	375	336

149	148	145	39
88	89	39	43
7	11	9	11
26	17	10	18
115	118	119	5
423	410	386	347
16	23	25	31
34	33	49	50
427	414	390	351
208	205	197	174
78	80	82	77
258	248	234	206
30	18	21	14
137	137	136	128
270	259	243	212
233	225	214	113
240	232	71	42
30	18	21	14
10	14	8	15
46	50	57	57
38	40	44	48
339	325	92	90
456	443	419	380

159	158	154	139
77	79	81	14
52	56	62	3
319	109	111	106
341	327	304	265
158	157	153	111
468	455	431	392
239	231	220	193
77	79	81	14
327	313	291	253
242	234	71	42
49	53	60	60
77	79	81	14
341	327	304	265
311	299	277	241
490	477	453	414
492	479	455	416
290	278	257	223
216	18	21	14
77	79	81	14
229	221	210	42
208	205	197	174
502	489	465	426

87	88	91	89
338	324	302	263
16	23	25	31
509	496	472	433
184	182	175	35
3	3	6	13
106	107	109	105
242	234	71	42
1	1	1	4
1	1	1	4
1	1	1	4
94	96	1	4
1	1	1	4
259	249	235	207
164	162	158	143
157	156	152	138
22	10	128	121
57	42	46	24
58	108	110	24
471	458	434	395
95	97	99	97
29	5	2	1
401	388	364	325

41	45	4	6
135	135	134	126
135	135	134	126
251	241	227	200
12	19	3	5
12	19	3	5
97	99	101	99
102	103	105	102
151	150	147	133
144	143	142	19
72	74	77	74
258	248	234	206
506	493	469	430
136	136	135	127
136	136	135	127
256	246	232	204
19	25	27	17
19	25	27	17
218	213	203	180
52	56	62	3
28	29	2	1
55	59	22	28

359	345	322	283
292	280	259	225
118	36	42	46
8	12	16	16
126	126	126	3
11	15	15	7
61	62	66	65
297	285	264	229
404	391	367	328
368	354	331	292
31	30	34	40
347	333	310	271
220	214	204	182
352	338	315	276
224	217	206	184
2	2	5	9
153	153	149	135
23	27	36	3
9	13	17	26
360	346	323	284
99	101	103	101
68	69	73	35
169	166	162	147

35	34	40	12
117	119	120	115
267	256	240	211
267	256	240	211
171	168	164	149
380	367	343	304
293	281	260	113
131	131	10	18
170	167	163	148
16	23	25	31
166	164	160	145
99	101	103	101
14	21	4	6
115	118	119	5
170	167	163	148
405	392	368	329
406	393	369	330
312	300	278	242
145	144	9	11
168	64	37	2
55	59	22	28
309	297	275	87
224	217	206	184

85	87	90	88
223	109	111	106
419	406	382	343
78	80	82	77
81	83	85	82
312	300	278	242
127	127	127	54
335	321	299	260
331	317	295	257
269	258	242	2
226	111	113	39
115	118	119	5
445	432	408	369
153	153	149	135
236	228	217	2
448	435	411	372
153	153	149	135
78	80	82	77
9	13	17	26
130	130	130	22
249	239	225	198
117	119	120	115
319	109	111	106

117	119	120	115
480	467	443	404
297	285	264	229
485	472	448	409
226	111	113	39
488	475	451	412
34	33	71	42
505	492	468	429
331	317	295	257
170	167	163	148
15	22	29	7
8	12	16	16
335	321	299	260
518	505	481	442
119	120	121	116
1	1	1	4
26	17	10	18
26	17	10	18
131	131	10	18
26	17	10	18
214	17	10	18
26	17	10	18
58	42	46	24

212	209	200	177
10	14	8	15
15	22	29	7
29	5	2	1
29	5	2	1
100	5	2	1
29	5	2	1
29	5	2	1
98	100	102	100
98	100	102	100
237	229	218	191
173	170	4	6
173	170	4	6
76	78	80	76
53	57	63	62
152	151	148	134
144	143	142	19
21	6	13	10
234	226	215	189
234	226	215	189
68	69	73	35
138	94	97	95
43	47	51	3

218	213	203	180
85	87	90	88
64	66	69	68
112	116	8	15
65	6	13	10
18	4	7	2
51	55	61	61
99	101	103	101
155	154	150	136
243	235	222	195
166	164	160	145
110	113	115	110
69	70	74	71
114	4	7	2
376	363	339	300
183	181	174	157
92	93	96	94
131	131	10	18
301	289	268	233
40	44	48	49
97	99	101	99
421	408	384	345
285	273	37	2

422	409	385	346
63	65	68	67
131	131	10	18
236	228	217	2
269	258	242	2
243	235	222	195
334	320	298	259
447	434	410	371
301	289	268	233
462	449	425	386
343	329	306	267
209	206	198	175
336	322	300	261
15	22	29	7
154	64	37	2
117	119	120	115
65	115	117	10
344	330	307	268
317	305	283	246
214	17	10	18
26	17	10	18
58	108	110	24
58	108	110	24

212	209	200	177
10	14	8	15
15	22	29	7
41	45	4	6
92	93	96	94
13	20	3	5
151	150	147	133
54	58	32	19
246	237	32	19
54	58	32	19
144	143	142	19
98	100	102	100
68	69	73	35
140	139	138	181
33	32	38	32
60	43	24	25
60	43	24	25
1	1	1	4
53	57	63	62
64	66	69	68
24	16	20	29
24	16	20	29
317	305	283	246
