# Supplementary figures and images for: Profile of the in silico secretome of the palm dieback pathogen, Fusarium oxysporum f. sp. albedinis, a fungus that puts natural oases at risk
Source: PLoS One. 2022 May 26;17(5):e0260830. doi: 10.1371/journal.pone.0260830 (PMC9135196; doi:10.1371/journal.pone.0260830)

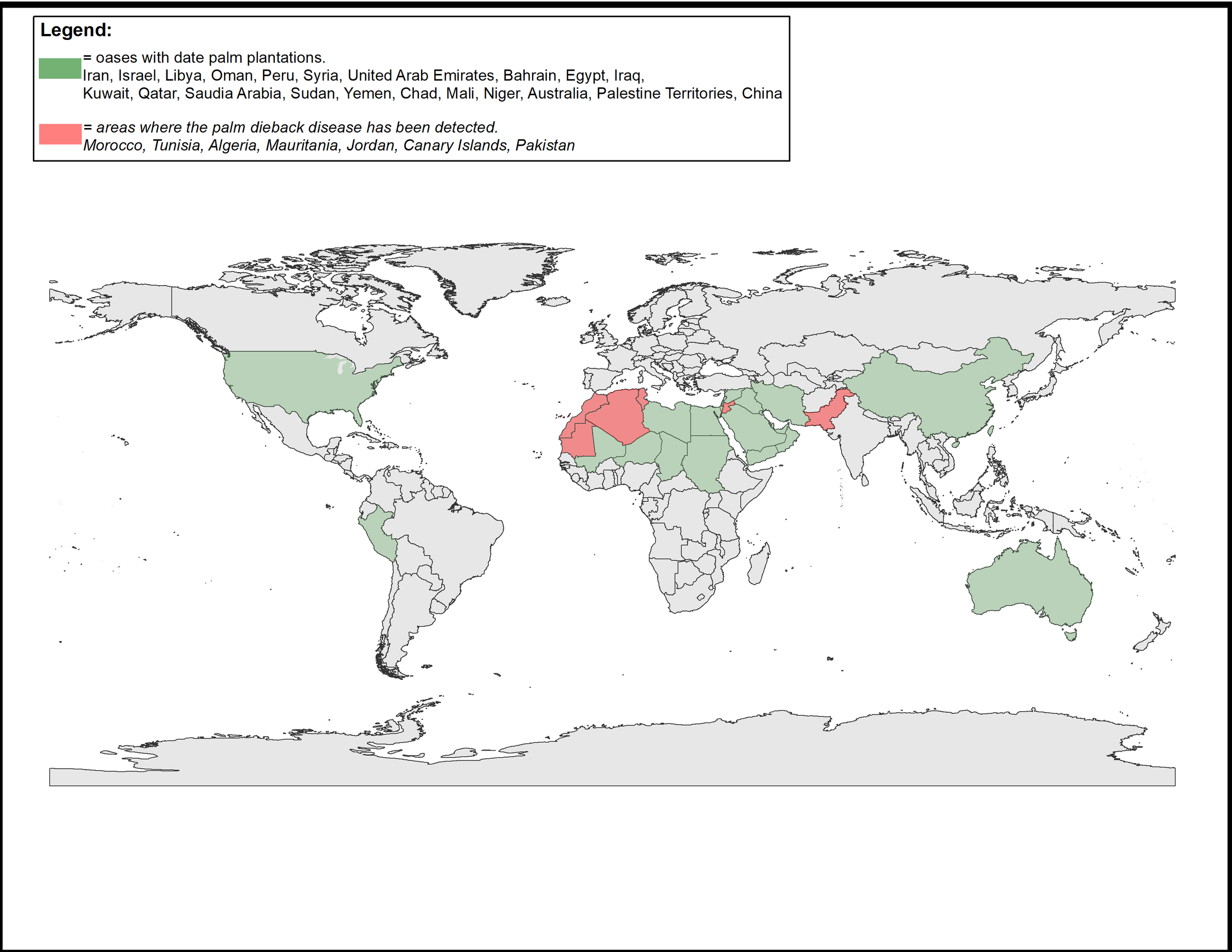

Supplement: S1 Fig — Map was developed using ArcGIS 10.8 with layer from natural earth dataset. http://www.naturalearthdata.com/. (TIF) [file pone.0260830.s009.tif]
