# Supplementary material for: Profile of the in silico secretome of the palm dieback pathogen, Fusarium oxysporum f. sp. albedinis, a fungus that puts natural oases at risk
Source: PLoS One. 2022 May 26;17(5):e0260830. doi: 10.1371/journal.pone.0260830 (PMC9135196; doi:10.1371/journal.pone.0260830)
Supplement: S1 File — (PDF) [file pone.0260830.s001.pdf]

```
GeneID  Feature Contig  Start   Stop    Strand  Name    Product BUSCO   PFAM
InterPro        EggNog  COG     GO Terms        Secreted        Membrane
Protease        CAZyme  Notes   Translation
FUN_000001      CDS     scaffold_1      555     3150    +
hypothetical protein            PF00241

DRLGRPVVQIKVNNVGPGAAAAPDDKAGNLAKQILICIGARLMPTSNLWQPVGSATALAFDKYNGPVFLTTPDGKKIVPILSV
ERDFLIGATLCARTQFPLIVCYAITVHKSQSITEDMIVTDLSCRDFQTGLSYVAVSRVETLEGLMLEAPFDRNHLVYGSPPDG
MKMKMRDQELRKRQVLTQNPATVSQDCITAFNDLKLNKKYKYIVYKLSDDYKEIVVEHASDNSDWEDFREKLVNATSKSRTGA
VGKGPRYAVYDFEYSLASGDGIRNKITFIAWSPDDAGIQPKMIYASSKEALKRSLTGIATELQANDTDDIEYDSILKTVSKGL
AG
FUN_000002      CDS     scaffold_1      3523    4185    -
hypothetical protein            PF12754;PF17183         ENOG41

MSETAFAQTFLASLESRPVRLSADHVEDPKTYPARPPYIIPRMPKAMSKPNNLAPGSERSITVSLKSLRNPPLSIKLTSQPLD
TSILDIKANIEKQTRIPAAKTKLLHNKKPIPDSKILKDLLGETDMSIEFTVMVIGGAAAIPPEEPEATPEAQPVGAQALQTEA
FWSDLKGFLMQRLKDEAEAERLSELFKSSWESSQANP
FUN_000003      CDS     scaffold_1      4636    6676    +
hypothetical protein            PF01189;PF17125

MGVGRRMKKQGPPQPLSEEHFAKLKRKAGLPVDPVVEAPEKKKRKTNTKERTVIEPKRNGVATKKDVKKTKVTKPMKAAAPAP
EPKANGKVKKSKKAPVPALEDVSDEEMDDEFDLEDLEDDSDLGGGAGLGDDFLGSDDDDSVFDSDEDAGGKNTKAMFSEDEDE
SDAEEKLTAANIAGLSRKLDAQMAEEAAEAEAEMAQEALQTNIHGDKPHILSDEDSDDELSKKTNALLAPDLQLLRTRITENI
RVLDDFANLSEEGRSRVEYTSQLIKDICSYYGYSEYLAEKLFNLFTPREAFAFFEANESARPVVIRTNTLRTHRRDLAQALIN
RGVTLEPVGKWSKVGLQVFESSVPLGATPEYLAGHYILQAASSFLPCMALDPQENERVLDMAAAPGGKTTYMAAMMKNTGIVV
ANDPNKARAKGLIGNIHRLGTRNVIVSNYDAREFPKPMGGFDRVLLDAPCSGTGVIAKDPSVKTNKTERDFMQLPHIQKQLVL
AAIDSVNHASKTGGYIVYSTCSVTVEENEQVVQYALSRRPNVRLVDAGLAFGKEGFTSYMGKKFDPSMRHTRRYYPHTYNVDG
FFVAKFHKIGPTPANASNARDRSTGPADEDEVIDRTPIATDDEETGKAKPDDDFGGWDEEEDKEYMEKGRRNAMRRRGLDPKS
NSKKAKKEKEIKKQK
FUN_000004      CDS     scaffold_1      7341    10415   -
hypothetical protein                                    ENOG41

MEPLRLKPRQPVEADIENWDDDDFVVEGDDLSFRSPSTATNNPSRPSSSRRRDSGSSHFSFRSELEGEEEQHVQIPGDDEKST
LDAIAAAQSAGIPLPSNVPSSALMGGTIKRLGGRKIRKIIQDDWENDLEIPDSSQGLKMKKLEQPKSPETSRHVSFGSTHTSP
MSWGNSPPTSPFDKVNRRESTQSIQSIQSMQSIQSATSSLSAAINLDRFKDADDDDDFFGDGGDTIRASKNRQLPKPVSFITP
PTPQREAKPSNPEDDFEMDLELPSDGKLKLSTRKEIPKTPSNQSEDLDWGEGSLGTRYGGTRRDARSARSSAASALSPSVSSS
ITAESEDETFDGLVLPPGPVNWTERLQQRRKSRSPNRISEEPIVPAKKIPAAEADKPDFLDGLDLGEGDVFDSSKLTLHKNVR
VKETRPASPARPKAAVSLTFTHKPLNSTRIPRLNHHERTHSTSLEPVSESGGPIPQRTRRSHSRLGHSSQSSIVSLPTPTTTS
PSHGLPPTPRRREVNLRTSTNSLRTEPTTTSAQLLKQKRSLPAIRGLNTPAKQPSYRHERPPSRSENNRPSSGVRPKTPTERQ
RHGATDSPATVRKSHLPFLPAGASQSQSQHVATKQTRGFRRHDSDNSISSIDLRPNSRTLSRSTMRSPSPNHRHRVTADTWER
LSKPKNKKNFGDGHELDGFDDLPTSKETETKYLKQPTSSGPKVALRNKLYQNVLPDRNTTTSPSIPPTPRPSFTPHFARDTAA
SRIARETALAQRVPSSGPLTPLNSQRVAHLSSRGNLTPTIPQSNIRSRKHKRPQQTKPHLISNLNSGKESKMVNGMFYNADTY
KWEGNENALNVFEPPVQTPTQAVVSSNTREKETSTPRPALITNISATKGVQVVGGMVFDPQNMCWLKLDNPAKPTSETSDTMD
GFDALDDEDVFKDIPDLEDNAADDEGAQGRVSDIKDEWLVGEEFDVGPEFIRRQREEEDRWRKKCEKWIGRGSRDREAWRWTI
RELVSQFDDLAM
FUN_000005      CDS     scaffold_1      12901   14148   +
hypothetical protein                                    ENOG41

MASQARVLKFSRSDDKSSFVLLQATPEGSKQLDLKLVGTEGEEPYVASLKHDRVVSLRVKNCPASESEWQRILEDLFQQEPLP
DIQATATVQSEKSISITIRKDIQGITQRLGSITLNHDPDEAIELFDWCGAAVESSSSSKQTAANLSAKSSESEALVAQLQLQL
```

EELIKAKDEDETALLRKFRDLLNEKKLKIREQQQVLATLSTNPSMAGQSQHSQVVEASVQQPKPRKPTRQAGKSRASKRKAPA
SRILEESEDDDAVDTMSVDLKQEAVDTDPGNTTEATASVDSDDEDGDGLDSGPPQQNKAPDAVSRGKTPSKESERPPPPRALP
FATKKSTKATPAPAPAGSDTESDDEL
FUN_000006 CDS scaffold_1 14999 16030 +
hypothetical protein PF00248

MGSVATKDVPTYKLNDGNEIPVLAFGLGTAQYKKDPESGLDQHVVDITTKAIKTGYRHLDGAEVYGNEEELGQAIKAGGVPRE
QLYVTTKIPAEKKGSAIDSFNVSLQKLGLEYVDLYLIHGPWFAETDEDLQQRWAELEQIKESGKAKSIGVSNFLQSHLEAVLK
TAKIKPAINQIEYHPYLQHGDLVDFHKKHNIAVSAYGPLTPIVTAKGGPVDPFYTKLAEKYNVTESEVGLRWVIDQDIIALTT
SNKVERLEGYLAKLPKFKLTQDEIAEISRLGAKKHFRGFWRNKFDENDRS
FUN_000007 CDS scaffold_1 16951 19006 +
hypothetical protein EOG092618J9 PF02696 ENOG41

MLCRNALRRAVPRSPPASHLPRLKQMAAHISNGNGALSPASSYEGATLADLPKSWHFTESLPADSIFPTPADSHKTPRDQITP
RQVRNAAYTWVRPAEQKDPELLAISPAALRDLGIKSGEESTDDFRQLVAGNKLYGWDEEKLEGGYPWAQCYGGFQFGQWAGQL
GDGRAISLFETTNPASGERYELQLKGAGMTPYSRFADGKAVLRSSIREFIVSEALNALKIPTTRALSLTLLPDSKVRRETIEP
GAIVLRFAQSWLRLGNFDILRARGDRKLIRQLATYIAEDVFGGWDKLPGRLEDPDEPVKSLDPKRGVSSETIEGDNGSEENRF
TRFYREVVRRNAKVVANWQAYGFMNGVLNTDNTSIYGLSIDFGPFAFMDNFDPAYTPNHDDYTLRYSYRNQPTIIWWNLVRFG
EAIGELMGAGANVDDSTFVNEGVTKEQEAEVVARAEKLITQAGEEYKAVFLAEYKRLMTARLGLKTHKDSDFDILFSEALDTL
EALELDFHHFFRRLSKIKLQDIATGEARNEKASVFFHKEGPPTTISEEDARARLAIWLESWRERIIEDWKDEGEDVSESTDSD
RIEAMKRMNPNFVPRGWILDELIQRVEKDGERDVLNRVMHMSLNPFEDRWDGETFDGKTYNGDKDEELRWTDDPPKTERAMQC
SCSS
FUN_000008 CDS scaffold_1 19529 22029 + NUO78 NADH
dehydrogenase (ubiquinone) 78K chain precursor, 5-prime end
PF00111;PF00384;PF09326;PF10588;PF13510

MLRNTLARSAWRTSRRAVNVSRTFATTSQRPAEVELTIDGKKVSIEAGSALIQACEKAGSTVPRYCYHEKLMIAGNCRMCLVE
VERAPKPVASCAWPVQPGMVVKTNSPLTHKAREGVMEFLLANHPLDCPVCDQGGECDLQDQSMRYGADRGRFHEIGGKRAVED
KNIGPLIKTSMNRCIHCTRCIRFSNDIAGAPEMGSTGRGNDIQIGTYLEKNLDSEMSGNVIDLCPVGALTSKPYAFRARPWEL
KHTESIDVLDGLGSNIRVDSRGLEVMRILPRLNDDVNEEWINDKTRFACDGLKTQRLTMPLVRREGRFEPADWEEALSEIGRA
YQIKNPQGNEFKVIAGALTEVESLVVAKDMANKLGSENLALDTPTGSQPIAHGVDVRSNFLFNSQIWGIEEADAILIVGSNPR
HEAAVLNARIRKQWLRSDLEIGVVGETWDSTFEFEHFGTDHAALKSALSGPFGETLKNAKRPMIIVGSGVTDHADAKAYYETI
GAFVDKHASNFRTEEWQGYNVLQREASRAGAFEVGFTTPSAEVAQTKPKFVWLLGADEVNEADIPKDAFVVYQGHHGDKGAQI
ADIVLPGAAYTEKAGTYINTEGRVQMTRAATSLPGASRTDWKILRAASEFLGAPLPYDDVAMVRDRMVEISPALAAYDVVEPV
ALPALSKVQLVDQNKGAQVTGTPLKKVIDNFYFTDVISRSSPTMARCSAAKATGDPRTNFMAPGMEEDKPMGQVEYGV
FUN_000009 CDS scaffold_1 22323 22944 -
hypothetical protein ENOG41

MAQPPRPATTVDPLMGVLDPEGHPIIPRLMSCAPAIFCLIGENDKLKDSLSSSSFTKLDRLQKELDTLNTHAVRDRETFLALI
VRERERIFQEGRRFQALEELHGGQVKADVPLGLISQELGLMVANMEAPAKSGIPNTENIPRPTLDSSNARSLRERQAYEQMRN
VLNFSQIAANREAEINHRRELLEADIKKEQLSQDQERRVN
FUN_000010 CDS scaffold_1 23667 24683 - ATG37 Autophagy
protein 37 PF00887 ENOG41

MADSVDRVFVHALNTVKKIPKTGASRPPPTDRLRLYGLYKQAMEGDVDGVMERPTAASGMASDDLQREKDKWDAWNLQKGLSR
TESKRRYIEALIDTMHRYATTPDAEELVSELEFVWNQIKNNSPSSSLSSPRANRSAGASQPYEDAAQASDAEGPMKEIRPMSE
YDEAELRSQKQLELEEDDIDGSQNHDRVSGRWQRKVERALTTMSAEVAALREQITTGREWRAKKERSLPALVKWFAWVVVKHL
FADFVILTVVLLWLRKRKDRRLEDLVRAAVKLVREYVRNVLPSRG
FUN_000011 CDS scaffold_1 25554 26592 -
hypothetical protein PF00636;PF14622

MSKRPNQDAHSEEDEPRKRVQLNSEKAPLSGTIATVAPGVSRWTVADIPPSLPPLPRISPELEEQVFRHPGLGEPNYERLEWY
GDAALEMISTELVFETFEYLPTGRSSQLREQLVRNLTLSDYYRQYGMQHKTKIPADMGSMVDMIRRSGRDKETIKIQGDVFEA
YVGAVVKSDPQHGAANAVAWLKALWARTIKDQIRQAEQHRKNDLKGQRFLARSSATTPPTEQTVTTQAIPTAKPKAPSTNKDR
LSRAIVVRGIKLRYEDMPCNKKDKNLKLPLFTVGIYLDGWGEAGKLLGFGTALGKKEAGERAAKSVLENKKQLKVYEAKKEAW
MKEAHEKEPAAQA
FUN_000012 CDS scaffold_1 27391 27863 +
hypothetical protein PF08293

MSVPRARILDLVKAQCQVFATSYNPEGVRMGNKVLRQRLRGPAMAAYYPRKTATIKDLKREFGPTLATWDEGEEDRFEYIEEL
KLRGKSAPKKKKGPPAPTGKKR
FUN_000013 CDS scaffold_1 29089 31633 +
hypothetical protein PF00806 ENOG41

MSSAGSTGVSPERTVPIFAASVQAENSNPLPLAPGSKEAGKSIPAKKNIAALSTSKLANQGLPKKMKLSHDSSTADQVYPVDK
FLAKLSEQQSALNQQNDANKLRDETNQYARAFDHASSSNSLPVTPATDAFPSTAPTTRPASATLDEARSESEEVMRLRLQLAQ
AQNEISKLDQELAETRVVKTTADVPPFGTRGPVYPPRENAWGPPDDSHSDTSDAMSASTFNRTREIWGNQPIFANTVQTPVIE
PTPGKWLGGRLFSQPCSEPTGTPFPVMEGYRSERLTPDTEMMRSNYGRRGNRFEGRFNSPQPFGPGCGGGFNSPMNQSDYMGS
PAPGAPSNAPQGLGPMGVYPPYPNPAGTPLSPHASEFTTGSGWKNEVGLPALYSHRTLNPRQVLAPDDQTYLAPTEPLNYRRL
LDRNVNCNWKYIVDKIVCNNDQQASIFLQQKLKVGTADQKFEIVDAIVAQAYPLMINRFGNFLVQRCFEHGTPDQVINIAEAI
RGNTLNLSMDPFGCHVVQKAFDSVPENFKAIMVHELLRRIPETVIHRYACHVWQKLFELRWTESPPQIMKFVNDALRGMWHEV
ALGETGSLVVQNIFENCLEEDKRPCIEEVLANIDIVAHGQFGNWCIQHICEHGAPPDRSRAIDHVIRYAAEYSTDQFASKVVE
KCLKIGGAEFLGRYLDRVCEGRRDRTRIPLIDIASDQYGNYLIQWILNNASPQHREIVAAHIRKHMVSLRGSKFGSRVGMLCT
NHAVATRPGPGAGPGMGGRMGPGPRYNNGYR
FUN_000014 CDS scaffold_1 32411 33247 -
hypothetical protein PF17733 ENOG41

MDQQQEASSAPRSLTGADRIRALKDETAIFTAFDTYPWTKDKNFMSGLYAILGEPGQQNPQASLADMAIHARIFYYAQRIGVS
IEFSAYKAWLASNSDHQPPDVLPEEYRQRDEANASPAPVLDWQKAAPKTDLYVDRKTAAAQSGSQDQPSYPMGFAEMIKLIQE
GKPVPGIRQIPNTVVRDPAVKPVGARAVPRKPWEKDTTVDMPVLPDLPKALDTEFPPVQDDGLVSSAAGAS
FUN_000015 CDS scaffold_1 34036 35938 +
hypothetical protein PF00481

MNRAAVRALRTTSLSTVRTLSRTPRRGFAVSFFALAQARSKASAHARSARRNPAGAIPLRSSMYFRRLPVALVSSIVVGYGAW
YSLNSSDIPPGSGVGSRTSLASTQTADVLPTRTVLVVGADELRQGTIVGEGPISKATSDDGRRIVEMLTPDQATQKLRRLEQS
FSVSRGQGVTRYDLVQLPSNDPIEDDHAEKIVQVPSRSSVETDNSDWMFWGVFDGHSGWTTSATLRESLINYVARELNETYKK
ASGDLPSEDAVNLAIKTGFNNLDNEIVHKSVEKVFKGGSKTVAAELLQPALSGSCALLSFYDSRSNLLRVACTGDSRAVLGRR
AGNGKWTATALSEDQTGSNPQEVERMRKEHPGEDNVIRNGRVLGGLEPSRAFGDAVYKWSRDVAWKLRENFFGRSPSPLLKTP
PYVTAEPIVTTTKVHPENGDFLVLATDGLWEMLTNEEVVGLVGKWIETQGQSGTNSQFDAAWNKIFGSSKTPLPVEGSKTAGP
DGNKTPIRVQQWGIDPDAKDRFTVKDKNVATHLVRNALGGNNDEQVCALLTLPSPFSRRYRDDLTVQVIFFGHGEKTGEVTVN
LEATAGEGVKAKL
FUN_000016 CDS scaffold_1 36450 38349 -
hypothetical protein PF03595 ENOG41

MAPASDPMVAGAELQPPSPSPVPPSGSTSALATGYESPSEENPPFFSVVASAPAAPGTAHSRHHVMPRPALSQRPSYITAIAP
EHTNGHARPGRLLNNESTLTSNSLNHVIATAEKGPGNQKVSIRDRIACYQWTYFTMTMSTGGVANIIYSLKWEAPWLRGIGLF
FFFLNIVLFAMNCILICTRFHLRPGSFVNSFTDQVESLFISASLVSIAVILINTCQYGIPYTGVWLVHTMECVFWVYAALSVS
LSAFLYLILWSTLIFPVHTMTPTWVFPAYPLLLNAPFAANLIAAADSAGHKLSTNTVAMALGATAIQGTGCLIAFMISSAFIY
RLMTQKLPRDMQRPGIFMSIGPYGFTAAGIAQLGSQADLVIPPNFLDNPQFAAIIKVISILVSLWLWGLAMWFFIVCVGALWK
YSLSGHHLPFQMTWWSFVFPNTALVTATSVMGKIFDSDGLHIFASVMTAAIIIVWALIFIRMCWSLKSRKLLWPKDGK

FUN_000017 CDS scaffold_1 40470 41344 +
hypothetical protein EOG09264W1U PF08551 ENOG41

MSFTNTPVTRLGVVGLVAGSIIASVLDVKHYFYILIDTHIWRYRQFWRILAYQLCYVNSTEVLFAAMSLYNLRIVERMWGSRK
FASFLTVTFFITSIIPPVVSMVLRPLAAGWFNYIPAGPTPIIFAILAQYHAMVPHMYKYRVATSEAPPTDRPFVGLTFSDKSY
KYAIALHLALLQWPGSVLGAVTGWIVGYSWREGLLPAALVRWRVPGWVVGLSSHRRSAEFEGLRRRLEDENSTTSVATGAQAS
TEGQAERRRTMGQQIMDQVREVL
FUN_000018 CDS scaffold_1 42580 45984 +
hypothetical protein PF15456 ENOG41

MSAYAREGSPYGYGSNRMSPPAPSPRGKIFVEGYRSDIINGFEKDNQLYNPMNPDRPQSSALVDLRDPIQVHLLTETALSDSK
EYTILSQEEVDDLKKQSQSLSQRVESTRQNLTIQSKYRDAAISMARLYNPGKLDGKRRSLLGRNSGDHARAKEAEAERQASER
KCEELAAELFNLEKRLMEPQRRLLEHTAGILQLTHKASNKKKDLTPPLQPLNGMPGSPESLYTYTRNSMDRAGDEHYFNDNDY
YDQANGVSTRGQPRQQPIEIPLKSPIREHNQLRDEMEKMKDENLHLQGQANSLVKSITEMEVRLENLNGSLRDAIVRFNPSKN
EDYYDPPFGVSGMKPGELLNNQLDYLEMGLNAIEVEQEESSGETIKTDGAFEKRIESLNQQVRDLLLTVDPSYPAVPHSPQDN
FETKLDYLEDALRAADSELERAVQAAQAGPPEKQDSDQVERILMNLWNMMQTGFAKIKKEKEERRRARMERGYEDEEVSDDEF
DIEEAFSLPGFSNRVQWLYSQATTLKDQKSVLKRQIKQQRELNNKSDAEKDEELARKQEELDETQVLLARAERDAMTAQTMLS
ETLEELESARSVAHNVGPSKAEAEGSSRKAAELEADVKRLQADLAGAAGAAGAAHAQLEERDEKIVKLERDLDQLKSDLASAA
ASAAAAQSQIEDRESKITALEAELDYLETAQSEIEERNKRIAALQSELEQANAAHVQIEERDAKIATLHTEVQEANTLRDQVE
VHSATIATLKAELEKTNDLEARLEERNARVTHLESDMELLQADLAGAAGAAGAAQAQLEERNSKIAALESDIKQLEERLSAAE
SSSNNTRNQLTGVDSVIDALNAQLDEVNRSKQMAEDNARSLQQRVDSQKEELATKKKMLKDKEDELELLNMNLVEMKTELTIA
QAELDGAYGSRAERAADVAAIKTSGEVMKLQNQLTRLKNELAGTVQELEEVTRETLSAEREKIELENKLQDVQSAKKTLEGEV
MTLRQRLEADMEKAREHIAKLQEDLDGERLRAIPPNGGVGRGASMLSEQFRATMREERKKFQESLKEERARARQLEEELAKFK
RDQGSARASLNPQ
FUN_000019 CDS scaffold_1 47780 49697 -
hypothetical protein PF05794 ENOG41

MTADQGFGGPVERSRRSLSTSGPGPNGEGETRPEKETARKPSTDEKDDAITTSQVLPRRYDSPSDTPAPVAAPLSRNPSEMST
TSSPGPESRRSPAPSQPLEPPVTKSSLGELEVSKIINNPKLRHDINFDPELHFRPNVEGDKGRRKHDRASIFWTTLKEELVEF
IVDRDSFYAKHGTGDDWCLPKLLKSVKEIIQTLVPQRDRQFLDEGLNVELLMQQFHRGIADLEKLASWLSRVLKSHCAPMRDE
WVDSMYKQLSNGNSNNDVEELVNGMRSLLSVLEAMKLDVANHQIRCLRPALIEETTAFEQKFLMKKLRNRKMDIGDAKPWYQE
AARTYPSGAGKMYSCFGDMGVFFEGISRAMLPSAGEKPLPGTFFFDEDRLGRVRSDILDAINLDVCMRMYEDLERIAKLNSPP
AFASAMSDDHEFNFNTPPSSSRPSSVTLSSADSASSSPRSSMVLPLYLNSETNEAKSRARNLRDSLVALLQQAPHDQHYHEKW
RSMAPSMALQIFRFTNAPMDMLVPFEEKLLENVCHISSPIYKEMEEAYRIRLLNELAMRVRYFKALSGVCLFSIATGNRVPTS
SRSLDAGRDRDLDAAIRMGQQEGGIEDIATRIAHVGVVHWRIWARMAYVDDDDMSMD
FUN_000020 CDS scaffold_1 57454 57999 +
hypothetical protein PF02269

MEPRARAGKNVGKMNFSHAELAQLLYAHGDVQNPLPETVRVLDEILTDFMQSIAFEATRAANYSGRQKIKYEDFEFAFRKNPA
FLGKVQEVFEKQKEIKKAREILRDGEDEIMKDAADEEKKREKSDTRTGGAVGAATSRVEEELGEADDDAEAELDALGKKR
FUN_000021 CDS scaffold_1 60609 61977 +
hypothetical protein PF00892 ENOG41

MNPTPPPYEEVPQSKLLPGDTHESHQHLLPPSPSPNLLSSKVDSSSFDDFRRMSSSPSPCPSPIPLRHFSQQGFDLPAETPPS
KWRQFWIKNKPAAYVALSQFFGALMNLSARLVELEGDGMHPVQALLMRHSITALFCSAYMWWNKTPDFPFGKKEIRWLLWLRG
ASGFWGIFGVWYSMMYISLADATVITFLAPGVAGFICWFALREPFTRIEQLATLVALLGVVLIARPASLFSGSDDDNSSTEPP
ESSGIPGIDHEATPEERLIAVGIALLGVLGAAGAFTTLRTIGKRAHPLISVNYFGIISTFIALSMLIFAPILDIQQPGLRWVT
PTTVKQWVLLLPLGVLGFIMQYLLTAGLGADKSNRANSMVYTHMLFAASFDRWVFGHRMDFISGAGCTLILGSAIGVVMLKKP
PAAKQPLDDVERHGNLEGEMEGSPMLVPDSGASEELRLSR
FUN_000022 CDS scaffold_1 62280 65074 -

hypothetical protein PF00172 ENOG41

MAEDSRAQLDDGPDEGSDGPQNHSNLAIRRACDACRTRKIRCDRNSPCSHCIHAKIPCTHHDTRPKEKRTRILLSAQYERKID
IIDRRLEALTELMRDMKMNMSSTSTQPNQNHMPGSDVPRQSPAQASSSTPYGHLAQQTADSPVIEGESSLAAHGEFANEFLKN
AVGTESLQGASLELRETLDSLHHIVSSLKQQTAATEMSYPHARQLPRPSLKNNELPPIQTAVALIRECETENSEVSAWIREFF
SMESFSGMCLHVYFSEDVSEADFILVNVGLMYMFMDRQQRTTDAKEQEECEGYMKTLRENTETALANLPFHLPATSNMISALL
LGAFYAIEISKPSLSWTLSCKASELCQTLGYHRISTMKNDKPREAQRKQFLFWNTYFIDKSLSLRLGRASTIQDWDVTVPLLT
SNPPNATPLSAFICLWVATARCQGHIYELLYSPDSMTQSDDVRRFRAQKLVNDLKDISRRSKELSKSREKEVKTNFGEYFFDF
ILISDDVLRLSLLTLIYRATPAPPNSKVTFIPECIEAARATLHRHQDCMDLLGRDNTVYFPSYVHWTLLFAPFIPFIVVFCQV
IETQDQTDLARLHSFCTSLETTIQLSDAAAKTYRLFQVLYTVALRYIEFRTSTPPADQTQASAELNNYLAALGFPPAGLNNGV
QQSTHMDPSQADAFSQPLGDMSMLDGADGQRGANTMMWMGNTAQLGEWFNSSQQMMELLEEPSFTFPQ
FUN_000023 CDS scaffold_1 66268 70264 +
hypothetical protein PF00005;PF00664

MAPSINEKENEKVAGGSDATSTPEDAVVNAEGGDAPSAGEEGEIEGSWGDYKRILTYAGPTEYLMQGIALVAAIGSGAGIALQ
NLIFGQFITTITDFTSGDSQPSKFRKEVADLALYFVYLGIGRFVLSYIWNVFLTYSSYRIVRNIRKEYLRAALRQEVAYYDFG
SGGSIATQATSNGRLIQTGIAEKLGLFFQGIAAFLTAFIVAFVVQWKLTLICLCIAPATIIVMTVVATMEAVHEVKILEIHGQ
ANSLAEGILASVRTVHAFEMRARLVDKFDKHLEEAHTVGKKISILFGLMFSADYTIIYLGFGLAFWQGVRMLSTGEIKSSGVI
FTVLLSVVIAAVQLTQLTPYTIDFSRAMSGAAQLFTLIDRASAIDPLSKDGETPSETVGHVELENVSFAYPTRPGVTVLEDFS
LNVPAGKVTALVGQSGSGKSTIVGLIERWYNPKSGTIKLDGRPIDKLNLNWLRRNVRLVQQEPVLFEGSVFDNIKHGLVGTEW
ENSPAEQQMERIQEAAKMAFAHNFIMELPEGYNTQIGQRGGLLSGGQKQRVAIARSVVSQPKVLLLDEATSALDPHAESVVQQ
ALDKASEGRTTIVIAHKLATIRKADNIVVMSKGSIIEQGSHESLIAQDGAYAKLVKIQNLAVENDPNNVSTDEEEEVAPEGDK
EELGLSKTVTQYETHVQERLEAGKELGNYDNHKQMGIFYVVYRLIRESPEVAWSYFFVLAGCLGAVAAFPGQAILLAHVVEVF
TLTGDRMRDRGDFYASMFIVLAAGLLVSYFALGYATNTIAQFLSHKLRKQSLRDMLRQDLQFFDRKENSTGALASRVDSNPQS
ILELMGFNVALILIAILNLAACSLLAIIHSWKLGLVVVCAGLPPLVSSGYFKIRLDAKLDRDTSKRYSTSASIASEAITAIRT
VSSLAIEESVLDKYVDELNQAVTGSKNDLFRIMIFFALTQSIEYWFQALGFWYGCRLLSYGDISMYNFFVAFLGVFYSGQASA
QLFQFSTSITKGVNAANYIFWLNQLQPSVQETPENRDNGPKSGGPVVLDDVRFSYPLRPHAPVLRGIDLDVKPGQFVALVGAS
GCGKSTMIAMLERFYDPSSGTISIANSALPTLNPRLYRRIIGLVQQEPTLFQGTIRENIALGVDDPETEGKDPATSVPDKRIE
AALRAANAWDFVSSLPEGLDTQAGSNGAQLSGGQRQRIAIARALIRNPKVLLLDEATSALDTESEKIVQGALAEAAKEGDRIT
IAVAHRLSTIKDADKICVFLGGKITEAGTHQELLAQGGLYRKMCEAQALDS
FUN_000024 CDS scaffold_1 71342 77176 +
hypothetical protein PF12931;PF12932;PF12935

MASESPKATDLATMPNHPDPTSSTEAPAHATSSTTPDAVAQADVPTSPDVSSQKDQNSSEQNGAANAWPSHEDDDDDSNEWLA
SDGAVPNQTQEETAFEETELEVASQPKAPVSQHSSSMSFARTVSHEISFGDEEEGEWNLSRTDTDPFKFMPPTDRTNSFPPVP
PAHTNSEVEDQQPLPSNQALDVLEETEKEADAEEELYQREETTDGLDAPRRGHSSSISIGGDLKSPEGHDSDERYEEGMPLIP
PPEKENHEVPSEQPNHTLSDSFDDETTEGDDFFSQVQGSEPASDEKPAPVLERKSTMQVLDSMNAGAISNKSALEGTPEEEEE
DEEEEEEEEEEEEEEEEEEEDSSDDDEDSDEDSEEDKKAAQPAPTPASSDPAPAAPDSSPAKKEDLASKWEQAFADDEDDDFL
LEENVGDDKQVDPAAFFGSDDEGFLEDEEPAPAPASAPAPTSRQPSGSNPYMPQVPATHPAPSPYAPSMQVPAAAAPQTSAYN
TPAPVVTPFGTAAQYGQPPPARPDMSKAQSFADKSKGGYHSPYDLPTDLVTNLVKPRKRASMQQLSQANQLHAPVQPPPRSAS
VGMPAPPTRLTPPSSSHGLPSHQQSTAPPKPTTPGAQHKENFFEELPMTSRPRPSSRTSQRALSPARNAPPMAHAPHIPSPLA
PAAPNHMAPPPLPTTAVTPGESYSEPPSQPALVPGAGLVAPERVSPYAALTTSANHMPPPASTSASRYSPAPGTSHTAPPAPA
SSRYSPAPPVPKSHGSYGAVATSGPGPVLPHQPRTSSPLTHSESAHFEPRMSRVSSLPPTREVDEEDEQQTPTRSASATHAIP
HHDSRYNPASPPKSTQTPPPPSYAGQLTLSPPKQSGPYAPHAPPTAQAGFVPPPRAQTQSPSASFGHKQAQRSSDSARRPSSA
HSHSYPSPVGAKPAPSPYAPVAPTAPMAPAPGVSTVRTRGQSITMNMVAPTDGREHDELQRWKGVPIISWGVGGTVVTSFPKS
VPRYGINQTVPMVVRTPGEVKVQSVKDIEPLHEHVAKFPGPLRGKSKKKETIGWLSAGIELLEKDLPDVSFHSQLSLEAKRAV
ERLLLWKIMRIFIENDGILEGNPTVEAAVRDVLSPGENTSSARDDALFPAAASFDTGVPSITSMQADGVDVSSMDQIRHHLLR
GDRDKAVWALVDKRLWGHAMLISHTVEGDLYKRVAQEFVRKEVNYPGHANESMAALYKILSGNLEDCVDELVPVHARAGLQLM
STGGGSGPTKDTLDGLDKWRETLSLVLSNRSTDDIQGLNALGNLLSSYGRAEAAHICFIFGRHVSIFGGLDDPNANFVLIGAD
HRQQSDQFAKETEALQLSEVYEYGLSLAGGALAAAGAPHLAAYKLEHAMTLAEYGHRDKAMQYCDAILTAMSSQTKRSPYHHH

VLETAVEDFMLRLKQAPKEESGSWISKPSMNKVSDSMWNRFNKFVAGDDNEENNGGTGEAGPFNRPSGEFSRSPSVSNFDVYG
QQSPSFGMTPAQPSASIAASKYAPNISTPAASANLYSPTSQYTPGRSSTEYAPNSYEPSYPGAASADRGEGYTPSVPQSEPTS
AGLGGPAGLAPAAHITQGYSPAGYQPYGVPASTSMGNDEKPADSSSQGFQPLSYGYEPPQMSSNPPDQASEHNTGESGNGGYE
PPSFQPYGYEPPSYEPQSTAEDDDEQPKPKKKSFMDDDEDDFPSMKPTEKSKAEKDRENDEMFRKAAEEDAKRAAAQQSSKKG
WGFGGWFGGSKKAPLEQTSSGDSSPGKPIRAKLGEASSFVYDPELKRWVNKKPGAENTPAKTATPPPPKAGPRSVSGTPPPPT
GTPPPPLVTSNSAPPPLMPPKVRAGTPELSKQASTESLGLAPPAMARSVSNTSNASAPPSRPTTSMSNASSIDDLLSVAPRKA
SDKKKPRKGGRYVDVMAK
FUN_000025 CDS scaffold_1 77637 78853 -
hypothetical protein PF04005

MRFRTELKNIRTFSKLTAALGSLEKIAWLRLSDDTARFTVIPDMGSQVWASLAMDFIFDGYHIQSAEAGNTINLELPLQPLQR
ALKSALNSISASLRLTKKDGLPVLSMTITTTTSATNPAGAAKPSGTATGDDPFDDDEMFQAEHLETSLRREHEKIITQDIPVR
VLHPETVETIMQPRVREPDVHIQLPPLLQLKAISDRFTKLAMASNSGSSAHTKSPKLELSANMHGGLRLRIATETTDICSVWS
NLENPELDPAQLDCPVEEHPSTKFRAGGPNHWATVRVDGKDWSRVLSVGRLEGRVIACFADNHALILYVYVPQYDDATADDSV
VTYYVQSYSM
FUN_000026 CDS scaffold_1 79471 81043 +
hypothetical protein ENOG41

MDIAEGRSVTESSEQQIKNIQSRHEDTPPVSQVPEVQNPVEASSSVPAEGNTGKTAKKPFFKRTWSKATERLFRRNTGNEARR
FFIHNVRQLPPAHQRGPSDPSGFVNLGRQDPRARDPKVKHPDSGPDHCVMCNKQLHDSTSDYHSWKRCETRRAWHEWKFLRVS
RLLDDEWLASRTKQFQACEARRLAWLNIVIEPPANRSPEEANRYRRAIDAPEIAILPSQREKSNLPPGPSTEMREDRNQAGTK
KEKTVEFPSAVGNVDGHDHITPAEASTSWKLPEVAKQPPFDVPTPGEAGGSATGAASINGTAGSKNKTNSTVTDVTVPQPPMQ
KTASESEPGFIDKKTNVGPRGDMAEEEPEKLVTLPDPPKDVPKKSAKETESKAEGKRKVAQPTAS
FUN_000027 CDS scaffold_1 81607 82189 -
hypothetical protein ENOG41

MGATMSVIKTLIVPAVISLILFILLTYVLIPLWRRYARYSQYLPLDTLSSQTSSLRQRITSRMATWRSNRGVAFASEDVSDDG
LEDDDGEELGNVDEDTWRQMEADTRAARPDNTRRLSRDLEEGFRDDSDDEPDRLARNNFRS
FUN_000028 CDS scaffold_1 84595 89906 +
hypothetical protein PF02181;PF06367;PF06371

MSDKTRQSSGGRSLFSRSKHKDKRLTEESRYPADDAASFRSSRHKRESSAISLDRPESSDGGINQMAGVITSIPYDAVGGGSR
SPIPVEYLPKGEQMPVRREPLPHHLNKNGLDFHQYPSWDGTSAQSGAHSPGRQPLGYGYGNVTMASTGRQTQYQQWGPPRGSS
SHSNNPPNPRYDSYMSSNARGSADNLSIQSAMPSASSQSSYAASQHSNRDSHRFTKFPSGPPPGQSDPQGGFYFPKPDDDNVV
EQMFLQLMQKRGWHNLPEQARRQMMAYPAQKKWTLLYQDRLTEWQGEQKRRQTARPNQYTATPDITTYSDEEGTPEWYVRRVM
EDRLDTKGMGSLEVNLRTQQIGWVKRFVECQGQVALMTLLLKINRKTAQGPAQDNTRIDKNLDREYDIIKCVKALMNNKFGAD
DALIHQKVMVALASSLISPRLTTRKLVSEIITFLCTWGENAEGHLKVIQALDEVKTASGENGRFDAWMRLVEVTIDGRGKMGS
LVGASEELRTGGIGMENLLMEYAVATLMLVNMIIDSPERDLELRIHIRAQFTACGIKRILTKMEEFQYELLDKQIERFRTNEA
IDYEDMLERENSSIKDDVEGEVKDLTDPVQIADAIQQRLHGTKTNDYFISALQHLMLIRANDGEERLRMFQLVDSMLSYVAMD
RRLPNMDLKQSLNFTVQSLLDKLHTDSEARQAQDEALESRQIAEAAMAERDEMKAQLELGADGLVAKLQKQLDEQSRFIDAQR
RQADGLKAELDSMQTMRAKEAQRYELETRELYLMLRDAQDVAASKAIKSAAASASASSKTAGPEDPARMQGILDRERLMERLQ
MQIERQKTQYKLEGRVWGDAVGPSDRLRALREEMDDRPGTPPGGGTPPRDFTNSVLGSIHRNTKIPRKPLKRRSDGEVIDEDD
ETEGEDGVIFEKPRIVEMKRPTIDPKQQAGLLGEIGSKVKKFDASDSEDDTTGPSHPSMETSSPITPPGDSETPKIEVTGAAP
PPPPPPPPPPPMPGQIPGAPPPPPPPPPPPPMPGQIPGAPPPPPPLPGMLMPGGGPPPPPPPPPLPGAGGMPPPPPPPLPGAMS
GHYLSRQPAFGAAPSIGLPVVRPKKKLKALHWEKVDAPETSHWAAHTPSAEAREEKYQELSKKGILDEVEKLFMAKEIKKIGI
GSSSKKDDKKQIISSDLRKAYEIAFAKFSQYSVEKIVQMIIHCDPEILDNTVVMDFLQKDDLCNIPDNTVKQMAPYSKDWTGP
DAKSQDRELDPSELTRQDQLYLYTAFELHHYWKSRMRALALTRSFEQEYEEINEKIRQVVTVSESLRDSVSLMNVLGLILDIG
NYMNDANKQARGFKLSSLARLGMVKDDKNESTLADLVERIVRNQYPEWETFADDINGVMTAQKINIEQLQADAKKYIDNIRNI
QMSLDSGNLSDPKQFHPQDRVSQIAQRIMKEARRKSEQMELYLEEMMKTYKDIMVFYGEDPADDGARRDFFAKLALFVGEWKK
SREKNVQVEETRKRNEASMKRKHTAQLKLTNANAEGPTSPSNTGAMDSLLEKLRAAAPQARDQRDRRRRARLKDRHQVRVAS

GQKIPDLDEIPEVEAGLKNKEEPTEDDGKVLSPGLSSPREGEDDVADRAAALLQGMRGGDGADDNDPERRETLRKARRQTAEE
ERRLRRRRRERATTNQSEENPDEQKEELREESKVEEGEAAKEPPKEEEAPIEDDVPTPRAATTSDGAPEEEQGEKA
FUN_000029 CDS scaffold_1 90460 91084 -
hypothetical protein

MAIPAPCSIFCSFEGVVLSITAIHIVLTTLWNWICGPATPTPEELIRSRRGNHAGRSAVNRVAEAAPRPAADKHPYHRPCHSW
WWYAMALWPARDDLLRKPRRKWWWYILALWPARDDPGRRFILTTAWSISILTFIFFLREISPDIYQGHVRFFTILSFLALEVG
RAAYFYHLLKDCSVLVQEVCIGLMGHVTGGFCRLGSLFAVP
FUN_000030 CDS scaffold_1 91438 92232 -
hypothetical protein PF08648

MGDSRRNRRPDSRQMWDESDRRDRRGGHNRDNDRDRRGYRSRSRERRGYRDRSRSPDRRHRDRDGDRNRPRDRGPRHHDDRDR
RDRRGEDAEGAPPRLRGDERRDDDRDKRSKSRRSASPQSRSPTHEALPTRPRPDAKRPAPNMSFNVGSRASRSPPPPSRAQRE
EESNRSEEGQASEVEEDPMDAEDDDMAAMQAMMGFGGFGTTKNKKVSGNNAGGVHKEKKTEYRQYMNRNGGFNRPLSPSR
FUN_000031 CDS scaffold_1 92625 95179 +
hypothetical protein PF03200;PF16923

MVKIAHWVSAATLALGVSAADDASSVLTTEIGRQNNQSLFWGPYKSNLYFGVRPRTPDGLWTGLMWGKVDNYHDIQNGFRYTC
EQGEDMHGYGWDEYDARIGGVQSIHDKGNNIDLTTTFVKIPGGNHGGSWAARIRGELRDGAPKNTRTMLFYYIAQDGDGELAV
EGEGTKYGFKDDVTLKGSSKALGDYKLLITKGTGEHPSTDHKFLEDRHGDTTIVSSTEVPADLTWQGKPVVFKQLQDAVSPVQ
QNADQADPPPPWQVYRIAHAPGKGNVHIVEKTFEGRFEFDVIFSSESGGKEFTSEEVTEQIEKTSEIFNERFTNTFKLKEPFT
QEKYKKFGKSMLSNLIGGIGYFHGHQLIDRSYAPEYEEQNDGFWEEAAQAKARTQPELEGPYELFTSVPSRPFFPRGFLWDEG
FHLIPIADWDIDLTLEIVKSWYNTMDEDGWIAREQILGHEARSKVPPEFQVQFPHYANPPTLFLIIEGFMERLRASNGTKSEA
KERILGADPLQTAHLDNIELGENYLRKLYPLLRQQYDWFRKTQRGDIKNYDREAYSTKEAYRWRGRTETHILTSGLDDYPRPQ
PPSPGELHVDLMSWVGLMTKSLMNIADALGMAEDVNEYRKNLDGIEHNLNDLHWSDEDGCYCDATIDDYEEHALVCHKGYISL
FPFLVGLMKADDPKLGKILDLVGDEEHLWSPHGIRSLSKKDEFYATGENYWRSPVWMPINYLALSQLQNVASQEGPYRGKAKD
LFTRLRKNLVDTVYKSWEETGFAWEQYNPDTGAGQRTQHFTGWTSLVVKIMAMEDPSQEAHAKDEL
FUN_000032 CDS scaffold_1 98568 98811 +
hypothetical protein ENOG41

MAAFTLTFAPTTRSFGRSGYNKDGDDSDSKPTNNRGRSGYNEPEEGDSGRSGYNGPEDDDSSKGNKGRSGYNGPSDNDEE
FUN_000033 CDS scaffold_1 99357 101139 -
hypothetical protein PF01926;PF10396;PF12631

MLRRSLLRRLNCKANRSRVFLSPFYISKCNDQLFTQPLRYSIASVSKPYSHQSWHTQNPEFVSGLPIIDDTIYALSTAQGRAG
IAVIRISGPSCLEIYEALCPSKPLPKPRYAKVRSLYDPQHANNEQIVLDSEALVLYFPSPKTVTGDDVLELHVHGGSATVKAV
LAAIPKCSATHRIRYAEPGEFTKRAFFNDRLDLAQIESLSDTLAAETEQQRRAAVRGNSGVLGRQYETWREQLLLARGEIEAL
IDFSEDQHFDESQAELLQNVTAQVARMLHSIELHEQGSQRSELLRNGIRIALLGPPNVGKSSLMNLIVGREASIVSGEAGTTR
DIVEASLDIRGYLCSFADTAGFRSKGSQVINGADSGAIGAVEEEGIRRAKQRALDSDLVIVLASVEDGQNGPFVQYDQETLDL
AAGAEDCVVVVNKQDAVGKEEFEKLVQDFRKIVRIRAPKLAAAEFVSVSCKEAQAGTWESKDPGGIQAVITKLVASFEKMTSM
PVDLQDLLGVTERQRQLLIKCRRHLEDFMMEAAPEEGLDADAVLAAEYLRYAANCLARITGRDEFGDVEDVLGVIFEK
FUN_000034 CDS scaffold_1 101614 102993 -
hypothetical protein EOG09262B1U

MPSRSEITYFGAGPALLPTDVLEKAAQALIDYEHTGLGIAEHSHRSELATNIINEAKADLASYIDIPEDYEVLFMQGGGSGEF
SATMYNLVGAWVTKKKAQIVANLKAPEDDPRVEQELRNAVEKELKTDYIVTGGWSQKASEEAKRLLGPEHVNIVADARQINNG
KYGKIPEESTWNLSKEAALVYYCDNETVDGVEFPAFPQSLTPGPDGEGPIVVADMSSNILSRRIPVRNFSVIFFGAQKNLGCT
GVTVVIIKKSLLPPKTPQPPPALLRRLGLPIPPIIFSYETIAKNNSLYNTLSIFDVYIAGQVLKKSLNTYNKVEGQEAVSAKK
AELIYGALDAHPDVYRVVPDKSVRSRMNICFRVTKNGDTDGTEKAFLKEATAQGLTGLKGHRSVGGIRASSYNSISLEGAEKL
AKFIETFATS

FUN_000035 CDS scaffold_1 104442 105378 +
hypothetical protein EOG09264441 PF00227;PF10584
T01A
MFRNNYDNDSVTFSPQGRIFQIEYAAEAVKQGSVVVGIASKTHAVLCAVKRNAEELSSYQKKLFTVDEHAGIAIAGLTSDARV
LSNFMKQQCLGHRLTYGRAIPLRSLVDMIGEKAQMNTQMYGKRPYGVGLLVAGVDERGPHLFEFQPSGMTEEMLAFAIGARSQ
MARTYLERNIDEFADCSREELVKHGLKALKESLVQDKELTVENTSVGVVGINTKDGKKKVEPFKLYDGFEVQPWIESVGEGQG
GAEEGDGEVMDVDS
FUN_000036 CDS scaffold_1 105664 106479 -
hypothetical protein PF03641 ENOG41

MTADSTIPSQAAVVSQNGHPRTKICVYCGAAPGASPAHIEAARALGRAMAENNIDLVYGGGTVGLMGEVAKSLCAINGPESVH
GIIPEALVKFERDGTYGTVNEHNHIVPEESVYGRTTVVKDMHTRKKLMAEEVFAGGPGSGFIGLSGGFGTMEEIFETTTWNQL
GIHNRGIVLLNIEGYWDGIVQWMDRASEEGFVKPANKNILVNADTPEGAIKALREYKVSDAIYQLQWGSQ
FUN_000037 CDS scaffold_1 107521 109605 +
hypothetical protein PF05602 ENOG41

MAEAQVLPPRQGEQQQGGGGGGGFSFNKLLLGGAIYFGINAALNLALKKDQRGVTVTNPETGEAVTVPGNVEGIPPFQLRPKE
LNEGATYRNIPKNIAPIWPQDSHVDIIVTLSQSFNPAPISQVPDEYLVLQEKEFHLSNKSDKRTIETKFTVPPAVQHNGTLWG
HFYIGLPGSNLDPKQPGYDTSKAYYFTWPLTQYLPKKKVAKTRNLLEDIPKHEEEPEEEEPTGPIIANYYHPNASLSFVPDLG
VKDFAAMAPQMRQYLRLEATGARDGSGQHAWYYPVLFVNTFWQLTNQMTLLNDTVKELPLRIDLGNLASWQFQLMSTLETNSK
ESARQAAFGGSLPGGGDGTEIEMIKEVFLDSNPILLGVTIVVSILHMILETLAFGSDIAHYRKKKDNVGISVRSILANVFMQT
VIFLYLLDNSQNTSWMILGSQVVGIVIEFWKITTVVNVRIRPGGPESFLPYTIAFEDKQKLTETEEKTKEYDEIAFKYMYIAG
IPLLIAYGIYSLYYDSHKSWYSYIITTLVGSVYAYGFLMMVPSLYINYRLKSVAHMPAKAMMYKFLNTFIDDLFAFTIKMPFL
HRLATLRDDVIFFVYLYQRWAYRIDYSRVNEFGQGGEDEAVDDKEAKEKAKDKLVEDVKAEAKTSSADTGKAKKRK
FUN_000038 CDS scaffold_1 110811 111903 +
hypothetical protein ENOG41

MSSLDSVGSLLAAIATLLSQGLRILSLADKSHWGPDEHEQVHALASALDEAKKDFQELAPLVNGQIYYETDRKHESIEELRAL
KAQFTAHIEQIRDWSRSGGPINPIWVRETHALQRKLHRAQCRAARRIYSSEKEGSARCLGAFLVYRQQRQWTLNKTVPDEAEY
SRRYREELRLCNTIGSFKRFGDRDIAFVCDYCDGHIVWEDIENMPSIRTFQEAAASPILTLSPTIDNPHWQATAFTLSGHQEK
QVVFAPVAIANHMAPQPRDWQAGLLCPYCEVESTEPQEQYDDEDAYRPDLGYEDMEAFQEHLEWQHTVTAPTSQAQAASDNCI
VM
FUN_000039 CDS scaffold_1 112286 113043 -
hypothetical protein EOG09264IG5 PF00957;PF13774

MIYSTQISRLDGLMLCASVDDEQAESTLAETKQNVRQVLRKLTRNSECQASIETPNHTLHYLIDSDIVFLAICAPSYPRKLAF
TYLADLAREFTTTYPASQVHSPALRPYAFMEFDTFISKTKTTYADSRASANLDKLNDELRDVTKVMTKNIEDLLYRGDSLERM
GEISSRLRDDSKKYRRAAVRINWELLLKQYGPFAALGFIIIFFLYWRFF
FUN_000040 CDS scaffold_1 113669 114812 -
hypothetical protein PF10483

MAPTSNVHARSHSLLLLQKLLNLRDGASPLTLVIDNLEQPARPVLSEFVSRAKIAKTQVIFLSLVTLKKPQDADVFIKAAGRD
LQTVRKDLLNHYPAFNPLLDKGKPTQRAIVIVDSLNALASAAPQSLASFLSSIITPAVSIVATYHDDVPIVLPRSFSEYEPHP
FTVLCHLATAILRLSSLYQEIERQKARNRSIQEPEWGLNEDREGVLVGLGEKGRDRNEDSNGVVINMELRRRSGRTVSEKFIL
SSKGLVTAPQPGKVCLLTDHPMFAAPTDNGETGEGEEEPESTFNLGLTEKQRKDREGIVLPYFDAQTDIGAGEGGRILYEMGR
EDDFDDEEDEI
FUN_000041 CDS scaffold_1 115206 116173 -
hypothetical protein PF13919 ENOG41

MADKHEETITVGINGGESNEARKSALPSSPLSSAPNHEDYGTLTMADQPPDTPSTSLNGRENTTATTPMKMAKGKRRVLVKKV

PKKSKWNVDNILTDPKSPLASADLRSILSNPMAWDTLDKEEKAEILALFPDSQHILSPGTEDACPDFASLMNDDSFRYDCAAY
TENIAQGRHDPEWLAHAWTAHERRKMGDFDEFLDNKFKDEWDVELPPELKTKRGPAVSKEASDVTMEDTEPVKNEGDKKEDID
MENSTSEKKDNPDIDSEVDELQRCDPAHEGPIVVAISQRKSSMMEIDGDDTRDELA
FUN_000042 CDS scaffold_1 116900 119191 +
hypothetical protein PF10022 ENOG41

MAPNQPLCLGYNGIHPFSKVEIKDRTSVQELLRTVLDPLEPFFSPQKARVKCPGATAVRFDQTASEVEGICRPLWGLAALLAG
GGDYEGKEWWIQGIKSGTDPENPEYWGYPQDNDQRMVEMCPLGWALAVVPEFWSSLSDKEKSNVETWLGNSINEKNMPNTNWL
WFRVFANLGLKKNGGKFSQERLDSDIEHLETFYRGGGWSNDGPEGIHQMDYYSSSFAIQLLQLLYAKLAGEEDPKRAEEFKRR
AQQVALDLIHYFDDEGRAIPYGRSVGYRFAMVSFWGALAHADVELPAPLTWGMVKGVVFRHLRWWQTQSDIWTSSGTLSIGYS
YPNMYMAENYNSPGSPYWACLAFMCLATPEDHPFWTSDEESTSGVIPKTKALSQPGHIMSYIGGHCMLLSSGQACSYPMKGTH
AKYGGFAYSSAYGYSVPPGLFSLEQYALASQLGLSDDGGEYWKTRRLCEYAGIENREGTPVLVSIWKPFPDVTIRTVLIPSQE
ETPNWHLRVHHIKSGREVMTADGSFAISNVNSTNGRYLEPYDETKHEGTSPKIIGNYDLKTPDGWASGKSGAFAVSKGAVGIK
ALEPAEQRQAMLVNADPNSNLVESRSTIPTLQHTIKAGESVWYVSAIYAKPSGVGVNKESYLDGWAKTPPIPGWLEKEMNA
FUN_000043 CDS scaffold_1 120663 122576 +
hypothetical protein PF00083;PF07690 ENOG41

MGLAGILKKVIRNDAMKTDPEEIYNWRVFAIVAGSCFGGMLFGWDTGAIGGVLAMKATQERFNYTPEAKVTLDQNIVSTLQAG
CFAACFFTSYFTEKYGRRWCLVGTGIVTTIGVILQAASTANGSLPLMYVGRFVGGLGVGAASSLVPLYVSECAPRAIRGGLTS
FYQLFIVTGVMLSFWVNYGALLHLKAPTVYALPLALQALPAVLLITCMLLAPESPRWCARKDDWERAKSILIDLRMLPEDHEY
VQAEIQEMSAQLEAEKRLTGDASASTLWKELVTIPGNRKRAIISVLLMICQQMTGVNAVNYYAPQIFQSLGMTGTTVSLFATG
VYGIVKVLGCAAFLIFCADSLGRRRSLLWTSAAQALTMYIIGIYGRVEPPVAGQGISAFGYVAIVCIYLWAALFQFGWGPCCW
ILVSEIPTARLRALNVAIGAATQWLFNFIIARTVLTMQKTMGPAGYGMFFMFGSFDLLMGIFVWFFVPETKGLSLEQMDELFG
VGDLHSKLDAEGPEGARTPSIREEVADIKNTKH
FUN_000044 CDS scaffold_1 126665 127893 +
hypothetical protein PF00106;PF13561 ENOG41

MADADSNPSTATHNSPRTWLLTSALSPLSVRLIRLLLSHGDYVVACLPPYEIDHEDRSAEFRELVNECKSSRKDREGWKDRIR
GIRCDGAAMGSCGAAVAEAVQVFGRIDILLCCKSDAVVGTVEELSTTPFTQNLVRDQFESVFFSQVNFIRAALPQLRSQHTGH
IIVLTSTGGHIGTPGMSIYTAATWALEGFCDSLAYEIAPFNIKVTVVQPNKEILSLTNRLTFAPQMPAYDQYSETAPSIRDIL
ANVLNTHPDTALPYPTSPADYGPSPMSPASTSLEPDAAPGEILHRYPKLPPGAADKLVMETVHALSAIGGHENPPARHIVGHE
AAVAVKEKLKTVTEELEDFVEASLSVDTFESELQAEARERKPSEQSPQGPPSSVG
FUN_000045 CDS scaffold_1 128507 132835 +
hypothetical protein EOG0926077L PF08626

MSLDPLAPVAPARVKTLLLPLGQIKADRFASFVERLNKEHVVHLRDISADGRPNRNMFSPLAYPDGAIIYNLVTHVPPPSHLA
LTPFDLYREPLAIIALADGTELHQKSFNKRQSINGTGPTTTEKNIRALYQELEDLRDMYPKALVHQVLLFDYANPHDTEIPMP
EGLVDIPPLENCTVTTLRTVMCDISSLLLAEMTTLAKSFEAMTNIESPVPYSSTSKHTNGGSWGGEGGLNGLLRRGSSISSRH
SVRSNSAGGIMDKTQARMSMPANARPGLHSSSSTPGLSSTPPKSSLSNPPITTDSQSPPASDRSTPDPQPAPQETTESSRSAS
RDRISTQGFGSGGVNDRWRLRTKGRAAVVVGSMFLQAGRWTDSIKELSDGAMAARSVNDHLWHGKALELILVNLFLLGWSNIE
FHVPTVLIQFPDKPAHKQTPDPTPEDPDQPKHLRNLQWLLPELLERIIALYSRVSSDSLPPLPMSEAFIRFSKIYSALHLCNG
YLNRESLAMIATGIVPEQPYVAPPRFGVTPSRQQIGAMLFKAFPAGASELLTTVDRASILSGIATVLGPLGLHRKKAMVIREL
VSVLIGGLVEARTRGAADMGIHPAAGLMSLTSGGGGSSSGSMALEIGECDVEQGIEALMTLLCKSYGIVGFDLTRKAGAKNNE
DADDSDEAVIARIRGQSAARFFGFPDVKLNILRACINFSEALPDFDGVLRFSSDLLRTAGSGVAPGPRREDASPMITRDEQVR
LATGISRTANLVQRLGYNDIAAEYWDEFFVRGIKLEPSLNSKTPVAHSKSILPGATASRASQDLDPFIYNPFLKKPDEMVNQT
LVADEYATFKITLQNPYDIEVDVESVHLATEGVQFDAVKEATVVGPYRTQVIRLRGRAKEAGTVKVTGAIIKVRGCRERRFPV
FSMPWTPKQEAKIKAKGLAALEEAVTDVKPFAPKLEAEILSLTAIQAQPLVIVKSTTLAQSSIMILEGERQVFSVTLQNLAST
PVDFMLFSFKDSTQEPLQTAINNRDATPSELYEYELALMKKQALRLPNSQQTRHIAPNGEATFDFEILGKPGLTAATIQVDYT
HLGCPQEEMPDQFYTRQVSLDLTVTVNASVELARIDALPVHGEIPQPLWDRLGSSFTAKPDEYCLLSVDLRNVWPSQMVVQLE
SNDGISVQEDILPGNTSRVILPVKRVYLEDPHATIPTLNPSRNRQFVVSASKISPEMERANREGFWYRERVLSCLRATWKTTS

LPKRSGSIDLRSIRLTSRMIEAIKVDEVDIDISVEDKDGNAAEKDVAYVDEFMQLRVRLTNRTAQKIHPLVRILPALRHRPAN
VGLEFTRKLAWNGTLQQLIPVLEGHGSAEISVGMTVLCRGEFEISASVEEVQVWDEPRSEPTRARSESQTMMDAALGVKERRI
WHARQPCLLMTRDR
FUN_000046 CDS scaffold_1 134169 138221 +
hypothetical protein

MFDEFSNAIEAMAEGLDNNSPDEGTAFVDAPPQEAIDIEEVPNGVPRKMEAGDLRTLTKAIMVASKCTLESNTIPNDLPDHIV
QSIKRHDSWYMAIVDQVFDTVLKYTTRPKEQSPFSPEMSRWLKDISDLNTRASLYEFLLHPAVRSHRCRAAIGREPVSRKQES
RLFNDLEFFPVTECGSAVPNVNGTYLLHGVRASDKHPSLDPIYVGQAAAVKLNSTGAVGVRKRAMQHHDHISPGGEKFLHVHQ
RLSEPDIERVDVTVLSMFPYPTPDMGEEVLRHCTALLTLVETIDVLLIDYLSTSGFSNIAVCIGSWEGLEDLRPPCLPRRSYK
GLNRALPCNQGRPRLDGLSINTNKMFTSQSLNGIIKVDFEIIVSQLELRGVRKTKAEVLKGYRMLASNPNSGLTTCKTVRWRR
IWDQVYRVKRHLEEEGLVLPPQDYTDPFFHVPALENGHDTSWHFKTLLKKSGYTDFDHPIMDVQGGFWATYLPRLLRREVWER
LTETAPERIIVGHQAMPKRLFNQALMVVLHFTQRRCTVTSNTVIVPRFEWSDLIEIWHQAFAQLIADGVPRSEILVGGACQIA
RLLNAHRRRLRDPSYSSTLPPWKYDDSKKASEKPAKLQPRFVDEQSLEGHDESHNIDISEETDGIYHWPSEQDRYEAVGATHQ
MVALAAKHRLREPTETVKLNTRRSGPCTMAQHTSSTLRILTMIMIMAGHHRSVFDKYSESYAGDDTFWEIIFVELGKKWNIET
QAQLQHRFGWVLKYLRSGGLPEEAKTASQILQQCLSNSVLSSEKHPYNYDVKTWTGDLLNPDLSSNWVKKLLVFNGVWDTLKD
CVQERMGGCGIVGFCFMYGLLIPHVWEESMTTQGHRYPALEQIRHGETMASSKDSETSEAIDVHNTTLSESSGPSSRTSASQS
KSLSSTLPLSTVPGAVRHKATTSPRGVGPARVNDRRSLAPETSVPSPSSLTLQSTDLSPSLSPPPSPPPYPPSQEFLTNKKRW
QPDEDDYVKYLMRLPISRDEQLEKLRQRFQSNHRSKAALAKRIERLRNDPGKSITDKPQLERNKRYEAAEEEYLRQLIETHPC
SSWDEVVAKMEQRFSKGRSKAAYQLHAAKLGLNTSRISNVWTAEHDECLRNFIRNETPPKRYSDLITEKFGTYRSRAARHRRL
TELGFVGGYGTWTEEETKFIEENRDLNPRELCENFWARFGHGRSSDSIKNKRVGVLNSKGIQWTADQLRFFSEWPHSSTTVVE
AYRERFKEDTRADPKIIKMYREMKGRRAQMNLEEQDLEEEDEEDPDDEHSTCT
FUN_000047 CDS scaffold_1 138842 141335 +
hypothetical protein PF00117;PF00425;PF04715;PF07722
C26
MTTGSEPSVGAPKRILFIDAYDSFTNNIVSLLRTIVGADIFVIRIDLSVVDRASDDDAPTKWTEQEFINNLAQFDAVVCGPGP
GSPLNSEDVGAFSLLWDLPEHLQLPVLGICLGFQSLLAAHGGSVRRLKRGLHGMVREIEHRGEDIFCGVPPFKATLYHSLCVD
IGQYSDGWAEENRWRSTSEFSPLAWATEFREDGRREQILQGVRHNKKPFWGLQYHPESVCTEKNAQGVLINWFQAALQWNKYR
GRRVQGPLLEIQTLSPPNHLESAAAHKEHLGNWWSTSNSSEISLRDFAKGSEYTYRTITLPQGAGMPELVEMLGLEKGEAVIL
DSSSSKNGDALALNSIVALEVDDALRFEYNVCNDYVTVRHPSTDGEDKTEMISLENGTVNVWEVISDFWETRSHPPGYDCSNS
AFKGGFMGFITYEMGLHGLEKKMVPEDRGHKRSDICLAWVTKSIVLDHRAGVAYVQNLKARGSNDSWLDRMTERIQQSDCWNA
IKMSNGVNGHVIENRAQNKEINITTPQPDRYEEQVRVCQDFIAAGESYELCLTSQTTMARPRSRNNERSPWTIYQTLRKRQPA
PFGSFIRLGGATMLSCSPERFLRYDTNGLCSMRPMKGTVRKSEAVSTLAQAEKILHVPKEVAENLMIVDLVRHDLHGVCGVGH
VTVPDLMKVEEYATVFQMITVVNGQLPGRNGNKPHGARRSSFDSHCPYTGLDALAAALPPGSMTGAPKKRSCELLQIIEGQHE
RSLYSGVVGYMDVAGAGDWSVTIRTMFRWDDEVAPAEEGETEPREVWRIGAGGAVTILSTPEGERDEMFTKLAGPMGVFRDAA
FUN_000048 CDS scaffold_1 141780 142255 -
hypothetical protein PF04133 ENOG41

MPTAGLKTIIALSFVLAVGFLLVILSCALWKVYYPLLVVATYVIAPIPNWICGHCANPDDFVESSGAAVLDLGRFFTGFFVVM
GIALPVVLAHSGLIEVQAMAMSIIGGLLIYGTIVSFGMFFHEEQDF
FUN_000049 CDS scaffold_1 143111 144181 +
hypothetical protein EOG09264L3W PF01778;PF04874

MSSDEIVWQIIGQQFCAFKIKTNKAQTFCRNEYNVTGLCNRQSCPLANSRYATVREDAGKLFLLVKTPERSHLPSKLWQRYKL
PNNYSKALSTIDEKLIYWPNFLIHKCKQRLTRLTQVQTRMRRIAAEEERLGEKLVPKMAPKIRHREQARERKAEAAAKLERTI
ERELVERLRQGAYGDQPLNVSESIWKKVLNAMERDGEGQRDKDMDKGLDENGEEADWSEEEDDNLENQVEYVSDIEESDEELG
DLEDWLDSENEEEDEDEDDDDSEESETEKSGNKRKRGRAIKMKNKKPRQEEKEKLMLTNDLTW
FUN_000050 CDS scaffold_1 144956 145709 +
hypothetical protein PF14475 ENOG41

MASWYSNLVQKTSSQISSLRQNLLSGEQDGDTEDDTHVCRVLRGYYTEKGRPFPSWLPPDPKAPAQQPIQPVMTPQAGQRLGA
QGQQHGGLSSLWDNGPQQPQQQAPQSLRAGGGRTPASLQPGGGGGDINRRPLPSQQRAGSYQNTGGQFGRPETASVPPGGASG
GGGGGSAQDRLRQRLWGGGSRTTSPQAGSQGPFNPPGGNNGGGGGGGGGYEDRFAPGGMYDQASGGGNAGLRRGGLPSGPRGY
R
FUN_000051 CDS scaffold_1 146033 148700 -
hypothetical protein EOG09260N53 PF00566;PF12068

MSQQDSGAADAPPQQPSRPSASPSNSFYALSDDEEGEYNTIRNAETGRGVKLLFSKSKVYVHPTPSAKDNIPGYVALLQQRGH
HEERPSSSASNESTTIASSDLLLAWIPESALGDSASIYVKVDLCDGDSPPKQSYLVPPPPTVTSHVGSVGGYAFAIPVSAVYS
LLVRPPSLGWWYGSVIINSRAGDSFPALFFHDNECQSTILQKKKIARDTFDPFGESGQMFWGGDEVVKWLRRYVKIERSGAEP
NIYLIEPSKEDSEAFGHKLTSSPSQIGRQDSNVGAQRGAAGPSNRDAEMDPFVKLIKETGWNIMEKFSKVTTFTRRAAQDIVE
NPNLPPQVRRLLRNPEVQTLQDEFDSARIYLARWAMGIQEQSDRDRRQRIWSANDVMELEDTDVGEFELLEGASNLSLEERRK
TVTMKEWNTFFDPQTGRLSVTIDEVKERVFHGGLDSEDGVRKEAWLFLLGVYEWYSTADERKAQIASLRDQYYKLKLSWWERL
EGDGGEGETGEWWREQKGRIEKDVHRTDRNVPIFMGEDIPHPDPSSPFAEVGTNVHLEQMKEMLLTYNEYNKDLGYVQGMSDL
LAPIYAVIQDDAVAFWGFQKFMERMERNFLRDQSGMRNQLLTLDQLVQFMDPALWNHLQKADSTNFFFFFRMILVWYKREFAW
LDVLRLWEGLWTDYMSANFHLFIALAILERHRDVIMEHLQHFDEVLKYVNELSNTIDLEATLIRAETLFKRFQRLVDAVDKKQ
NFPAPRFSQKEPSKSSEQAKSGPSEGGKASGKGKAAEPAAPPPQPKTITPELRKLLSKEVEVLPRKTVAQKGDGMPNK
FUN_000052 CDS scaffold_1 150007 151327 -
hypothetical protein ENOG41

MGVGSFLEPLIVVTLLFGGAYFNRSKDYNFWTNKSGIASIKSYKRSDDLPKRDSTDSLMSGWSGSRSPSLDSSSQPALRRRRL
QVLGYKRIVSTPNTHIFEDRLLSRLLKKLPFLVECWYWFLIYFVYQVGRAITALTLDEGTVDVARRHALQIIHLEQRLHIFWE
IDFQKWFLARPTLLHWINRIYSFIHIPGTITFLVVLYYFTTTRHRRYAAGRVRSDTVAAGPALYEARRRTMAMCNLIAFVVFT
SWPCMPPRLLSDPEYKGPDAEEAKSFGFVDSVHSGTGESSVWTTNRFCNQYAAMPSLHFGYSLLVGLTVATIPITGLRSTSWK
RIIIVAAGMSYPVLILTAIVATANHFILDAVAGAMVCSIAWNCNDFLLNFCILEDYFLSMLRLHKPVNWTDPETAVEPEFQTG
LLSEDV
FUN_000053 CDS scaffold_1 153842 157646 -
hypothetical protein PF00072;PF00512;PF02518 ENOG41

MASCSPKQVSEPARERESFKYGASLLNDITDSPTEGAPPGQPSANGTVPSDEIKRQETPRDTVLASLAQLGLWKTGTDRALIS
LFDRNHQYIVAETTQRSSLAKSPIRPKDRNGDFWLYPGTFPRRLSVCGRALMAQSHDSSSSDKELPLTLIPDLKADARLCKIQ
HPLSEFPARFYAAVPVRSPTGVDIGVYCVIDSEPREAESWTTNDTEVLREISSNIMDYLRSKSVGDAYRRSVRMTRGIGSFVE
KESSMASWRARTNSPAFHDDATREGNLNSEQQALQQFRDISLQKTLPVHVPMQPNTTGETTAISSGGPGEANMTSTSSKSMSV
RESDANAKEKSIAKIFSRAANLIREGIEIEGVVFLDANFETSATDSMLHEGQSSTESSSNSDESSDGIEQGKSCCGILGFATS
TSSSINLDVGSDFHTTMPMKLLAALLRRYPAGKIFNFDEDGSLLSSGSSEADLCTPMPKQEYFGEESTAALSGKSQRPWSRSL
EGPSVSKIFPGARSVAFVPVWDPKRDRWLAGGFAYTRTPTRTFTIEGELSYLRGFGMLAMSEAQRLEALLVNKAQSDVLGSLS
HELRTPLHGVMLSAELLSDTQLDVFQGNLLRTLETCGRTLSETIDHLLYFSKVNQVAAHRKKERRARKRSIVSDSATTVPASV
STSEVRLDALIEEVMDSVFAGHVFKGNEAQKAWQTANRSKRVNTEDENDLTGGQSEDPVKYEVPLGHDSQVAIFLDIDPYVPY
RFHVAAGALRRVVMNLFGNALKYTHKGTITVALSQKPLSSNNPSSYRRLVRISVTDTGQGISPDFLRNDLFKPFSQENHLSHG
TGLGLSLVKKIVASMGGKISIESEVNVGTTATVTLPLLRSKDSDSSNLGQEKNEVDEETKRLKGLRLRLVGFPSEEEIKSNTG
RGIPMSAMKVMCHRWLEMDVINEAKCQEVAPDLVLCNEIAIKDPVLSSEELVRCPVVVVCANAITAHKRSSESKASENQRVYE
FVSQPIGPHKLARILHIALCRWGDLQALSPAVPVGEDEEHLDYASDDAKRCSTGSNTEGDAPDDKSTPGPGAASLKRPGPPSR
VPSKRPPQATQQQGSTAASEPKSFLLVDDNPINLSVLCAYMKKLKTKYTTAADGLEAVERFQENPDEFSCILMDISMPRMDGI
EATKQIRAFENKEDREPVVILALTGLASASAQQEAYASGVDVFLSKPVKLKELSSILRERSLA
FUN_000054 CDS scaffold_1 158874 162463 -
hypothetical protein PF00533;PF16589

MLAPAGSNETQDSQPIFEAYKAEFGVGTLSSSPPKYVKSPKISTTSPKQIPSPMSEVVVPSSDPTAIDLAAPATTVPRDALSD
KARPGGPTTENEDPSHWDTTRVALTHSKKQCATLEADVVNGVAQGLKDRVNSNSNVNSSVWVPNTFVKSKHLANLSARHDSPQ
PQSVQDNAPKLLIKCLEPPASAVTAALRKMDYSQQTPTQVNDDRDYSEYCDIVPSSPVNGDTQPASHEPRTLQDDDTGAVNFA

NMSELGRPSSQISEDAGFDNTRGDWRHPDGTSQLNNGQTPHRPHAPTFETPAVPKNPFAARPSIAAPLAGSQLFGQTQFSSAI
KHISPTSSRPSPKLFNSISPNIIETSPLKNRANVSSPSDIRTSSPQRRLHDIPETSLQDLDEGPVRASTPVNDQSTAGEMIPE
SPPSTDPTPRAKAPRSSNATEPMAHYEPMKKSQERKSMENFVITPWDSDDDSDDAIRRLERRKKIERKKAQAAEEMGRVSFTP
KIRRNSAEQPSRKKRRVLPLEEPETTRKPSPKTDRGKGQELPPLVDDSQKGLVASNETLPVTSTKATPGNSASDNPQIDHDGD
IVLVDADKVVLDDDMIPATSPAPSLPPTAPVEQPPPSEPELPQLRVEYEGLPQGPNDHSEPSSLPPARKRTTRTYGRAARQKR
RNPFLSSSFSDGLMSDLDPKPTPVSSPLQIVAAVSIIEEEPEEELSVSRTTEEKSEKKSAPRSRKPARDVYADLPPPMTTRSR
RSDRAETAPVTPLASRSVGQMPPTSSSLSILSTTPLPSAKTTPGTQDSPGSERPESVTLPSPRGSRDFHKEEARKGSKTESPQ
PAKRAARVSKRFSYLDSDSTDELHHSPAASVLERTMVHSKSSRSFKQSFAQSHRVGRLFEGMVFAISFSDQFKAQERKKLATK
ITQSGGIILAEGFQDMFEHSSIMNATDPVMDEQDSLQLTKLISESGFTALIADKHSRKVKYMQALALGLPCLAPQWITTCVNK
GVIVDWEPYVLCAGASTVLGNAIRSRILAPYSATEARLAEVIQQRPRLLDGQRVLVVIDSKKSRNEAKEPYIFLASVLGPSIS
RVFSTQQARELLLEHQKAGTPFDWLYLDKGTGTVDAVLAPTETTGNKKRRRSTATQPRIENIRVLSDELVIQSLILGRMVEED
EMYT
FUN_000055 CDS scaffold_1 163364 163926 +
hypothetical protein

MQSVQERKNIIVEAANALMLDVNCSSYPLITSSNTTLVSIISGLTLNPKNIIETIGIVKACTARVGQGAFKAEDTGDIGTKLQ
EMAGKGNSNRQKTQITSINYCNFLNLTKLDALDTFETIEVAVAYKFDGVELEHYPADLDMLARAEVVYHELPGWQKPTTGANT
FYGLPKQAR
FUN_000056 CDS scaffold_1 177033 177495 +
hypothetical protein PF12855 ENOG41

MDSDSWAHSFCLACDKQVQSSTDAYCSESCRLADFEKTSTTSSQASSPGLASPPCQWSSKSSSSGFYLSPAYDFSNAKPYGSR
HMSQPTFKPYGTEQSTIRSLTPSSSHSSLCSMQSTSTTGESSQLSDKARKELRAYAVSFDNVRTQRRRSY
FUN_000057 CDS scaffold_1 186872 188094 +
hypothetical protein ENOG41

MNSKHVTYPMRFGSDGKYLKPIFGEEGLSILHSVTHIECSPYWYRFFVKSFPSRPWPLTINERPFTIASKTTEDLLLTPIEGK
RHERTRGRISLFQHIEPQVPNTDIFAGNQNLHICVNIDARQDRFPETSLRAMVQDVTEIFFQIVVSLGWEIDLKSKANQLPGY
IACCEVVDTGVDDTSHERWSHWGSRQLTDHPWPGHVSELPLFPGTKLRGSKARIVHPGDVVGMREFFLQNRIGIAVDVVPGTD
VALVKLDEAINYKNQHGTMSFARLLGEDPDDALPWDTIVSLATRWLPFTKGIIVAKSVRVVRNPATTHYRVYHWAWTGQPEDE
KARTLRQETKWLELLRRTRLEL
FUN_000058 CDS scaffold_1 188455 189736 -
hypothetical protein PF00155 ENOG41

MALSNRAHEAEEACKGIALWEVITNLYDQENNPDGIVSLGVAENTLMHNVLRKHIHDNLALTNPAFTYGDGTTGTKRAKQAVS
RFLNKHLKPFRDIEPAHISMTNGCSAAIEHLSWAVANPGDAILLGQPYYGTFVPDLTYRFGAKLLPVAFGDVDPLCEEAVTKY
EEVILDTQAKGTKVAGLVISHPHNPLGRCYSRKALIGFMKLCQKYKVHFISDEIYALSVWPNTVDQHPPPIPFESALAIDTTG
IIDPERLHVLWGMSKDFGANGIRVGAIVSQANSSLHAAIVAVGLYSSVSSISDHITANVLEDDAFVDAYLAENQRKLSAQYAR
VVSWAVKNNIDYAPGVNAAFFLWVDLGKVYEALHPKVETDDITDFVMDKLMERRVFLASGKAFGSEKPGWFRIVFSHPDEYLD
LGLQRVMEALQ
FUN_000059 CDS scaffold_1 190292 191862 - GTR1 GTP-binding
protein gtr1 EOG09262H1X PF00070;PF02852;PF07992;PF13738

MQALAHSLRSSTLSSSSRTTPLRIASITRHLSTSARNMAPITKETDYLVIGGGSGGLASARMASSKFGVKATIVENKRLGGTC
VNVGCVPKKVTYNAAALAEAIHDAKAYGFSVEQTAPFDWSSFKTKRDAYIKRLNGIYERNLNNDKVDYLHGWARLVSKNQAEV
TLDDNSKVLVNAKKILVAVGGKPTIPPEIPGAEYGTNSDGFFDISTQPKKVAIVGAGYIAVEFAGMFNALGTETHLFIRHDTF
LRNFDPMIQESVTKEYERLGVKLHKRSQASKVEKDSNGKLTITYKDDQGNESVVSDVDNLIWAIGRTPETKDIGLEEAGVKLG
EKGHILVDEYQNTAVDNIYALGDVTGEVELTPVAIAAGRRLAHRLFGGPEFANLKLDYNNVPSVVFSHPEVGSIGLTEPQAIE
KYGKDNIKVVYKTSFTAMYYAMMEPEQKGPTNYKLIVAGPEEKVIGLHIMGLGSGEMLQGFGVAVKMGATKADFDSCVAIHPTS
AEELVTLK

FUN_000060	CDS	scaffold_1	193698	197615	+
hypothetical protein		PF12763

MAADEAPNLNLSPEEKRTYGQLFRQADSDSVGVVVGEIAVRFFHKTGLDSRILGEIWQIADKENRGFLTPAGFGIALRLIGHA
QAGREPTPEIALQQAPLPRFDGIVPQVTGTGGIPPPPPVPVSSPPPPAALQAQSTGGPIRIPPLTPEKVAQYTGLFERQPLQG
GQLPGDQAKSIFEKSGLPNEALGRIWQLADTEQRGALVLTEFIIAMHLLTSMKTGALRSLPSVLPAGLYEAASRRGPASRQSS
TGPSISAIPRQVSGTAQVRTNSPLGRPPMSPQQSGASDWAVTPADKARFDQIYADLDKGNKGYITGEEAVTFFSQSNLPEDSL
AQIWDLADTKSQGQLSRDEFAVAMYLIRQQRSGRSVPLPTTLPPNLVPPSMRTQIRPQTATSAFDPPPPLVVQAPPPAPKSAL
DDLFGLESGTSSPAPPPPAQAPMSTGGSNANDPFAGGSALTPTSPVKPSLTGTGGSSFKPFVPSSSFGRGLTQQPTGGSAGSG
QKLPAPSASEDLLSDNDEASKNISGETTELANLSNQISSLSKQTQDVQAKRNTTQNELNQATSQKQNFEQRLAQLRTLYEKEA
QDTRALEEQLSNARKETAKLQSECMALEGTYRDTQTQHQQVLAALQADQQENTNLRERIRVVNGEIAQLKPQIEKLKSDARQQ
KGLVAINKKQLSTTEGERDKLKGEAEELTKSIEDLRLANTGSPASASAQIASPAASTSSANNPFFRRTASTDIMGAFASPPPT
KNVSDKSFDDLFGPSFPPSASATPPPPPQTSFKPQHTGASTASAGSYSTPPAASSPTMSRQATLAADPPAPPESRQISSSFLP
FPDHTESLSSSRQVSPPASRADDPLHGSATPVPGDLKASDSPAGVPGAFPGDELDKSEVKDVASAPDDTTRDAVKDEAPKATE
SADPFGGNDEAKAKADFENAFAAFTTAKNHSKPSPEANKSSAFDSEFPPISELERDDEEESDSSSDNGGFDDNFTPASPPAKP
FGEKANAGESPGATHAAPASTSPQPAQGTTPPAEPKAPSTEQPSSPATVTESTAQKSSVDDIFGAAATGTAPASQQPAPSNPP
QTKGGFEDLDSDFEGLEDAKEGSADEDFANISRSGLDDFNPVFDSSPPGSQTKTESTAFGNESFDFISHSSTGAAQPAVGTNQ
QKAPEAHDWDAIFSGLDSPSATAAQPASAEKPAGAESHPGQQALNRTLSTESEHDDPILKSLTSMGYKRPEALDALEKYDYDL
DKVS
FUN_000061	CDS	scaffold_1	198162	200421	-
hypothetical protein	EOG09260K5F	PF06046

MDDTPAPKLSELLRHPDDLDKIPALKLEFSRKKGAVDGQLRSGLREQLETTQSGMTGLSDGQKTVQMIKEEMIKIDKLCSESQ
NMIKDFASINLVSQAHRNFGAVETMRRNLETFNERISRVELMLREDDEDSDNMPNLLPCHYELTQLRNIRDDAMEQIQRAEDE
SLEATLVDYFSRLDDTIDWFDEHVGIIALNLINLVVQDNNGLVVRFAVVMEAEESSDQRVLALQEALKDHKEMATRFQSITDG
AKKVRGYKEKFIQAIRLSAEQQFEGAKEEFLEDPSKLDKIMKWYFNDLNVVKVGMSHLMPKKWNIVKTYAGVYHQLMHDFLVG
MVDGNEASSAHTLEIVGFPEKYYRKMVKIGLKQEELIPHVIDNREAELVRDFRELIIKFLDEWIDRIFAQEKRDLAERNVEGS
NLDQDEYGYFRTKNFVALWRMLREQVDTAANSQRADVVEGVIDAMFARLRTRQQSWQAMLEDEAIPYEEGKIPELEGFQALQD
WLVATANDQIACIDDNEDENRLAYLSSFRRLVEQHVTPAYLERIESEVNTLRDGYVDFSTWCINRFAQLIFSVDFGTVMPDFF
TPRWYTTTAMKQMVVTFEEYVNDYRHVLHHSLVDIFVEIFAEELLVRYLSAVRNKGAKFRRTDPFQDKLFNDISTAFECFSTL
PSPDIGASIKETWRVTEHFLRLLTADRDAIVDAYVTFKMAYWDLNVQWVEAVLKSRDDFERSWIGAVKKEAANIDSGRGPETI
MSRVK
FUN_000062	CDS	scaffold_1	200822	201950	-
hypothetical protein			ENOG41

MAPSKLRTPAGVDHDYNFLHGIERSVERAEKLLVEERGIVQEEELRPMTVQEVKWKPGKDGRKRKVLVTRVLREAKGRTFERF
LAARLRKLNITIVCAPLGMARQKENHTTLNRRTNRINWQVEWMVLENHPDAEPKKVRMLSKVMDDVPLYEAYHTSLEEQQKAK
GQQAKKTPRAGTDGQVQDLVNYTWSSGSFALQDPSTVTWTNHNDTEPGLWPSEKIEAQKKQFQFFLAKFRTPSDKPSVVTRLD
PEDCLREVLRNTRVLEFPTIWVLDQGQSLPETFTLGPKDKAPQESNKRKNPPGKKGPAKPNKRRNKGQDVEEGEVRSDEEGNE
SDDGINTGFKGVSLEEGDVIAEQSLGEEDDDDDETSSSGSDSD
FUN_000063	CDS	scaffold_1	202696	205462	-
hypothetical protein		PF02141;PF03456		ENOG41

MPPSLNTHPSFTRPRTSDRDGRPSTRDQGADQNLLIPSRTSSLHSRITQPIPSTLNMKPQQRTPKTLTHAYMVCGVGREPSQW
VKAPAPAQGKIGHMKGAVGQFWLPEILGSSPRLEQDNEIARALHSAMRACFPHDVEICTGRSQPHCVHHAFVLQQDSSHTLYG
ICLRVWSRADEKRAETIRDLRKRTESDYYDNPDETYWIPYCLSFLSRYPLYNLLGDYLRGMWIHWNKATNLFHAEEVSRILSF
PAPRLNDLVRIDMKDYALCYQFPSSPTGFQNFAMWPLFNCLSIPNIVGVIEAAISPTRRIIFVSHYPAMLTMAAETVRYCVRV
YEWSGLYVPVVHARHAKELVQEPGPYILGITVECRSLFTAPTDALVVDLDRNFVLTSSPPTALTPGQRNKFVTRLTQALNGDV
TPSGVPQHLRSAYGGGKLVPAGQIIVMRGEVESIQDPEWWNQDAVMAVMDHVCEKLGRNTGIKAVFGGSVKKPLMTKVSMRHL
NEIVRERNQYSRDALEAWQDFINLKGRMDTELNKVTKRNNYLVEELESWKQQFLKFQAFAETLTKETQDLKVKIDTHKRENRR

LAGLIDQQKDDNARLSVRLTGTEKQRDDALEALVLQQEIAEELERERKRNKKELSQLQHTNMTIMRQRDEARRVVLHLRSLIG
GQSHHMEHLIQSLTKPDDLTHEIEEGYDEAEEDVQQAGEPGRLTPSPNPRNKRYSSSSFTDVADRHLKDKTDAIAHIVRNIAE
QCQAAVEGLQLAHDAELGSSSRRNSSLSTTQSDDGHSAATSETGDDSLLAPRSGRASSIPPTPDLIPNRSSTAMSFASTATTP
ERASQQYSLRDEIPTKIVEDDEEDFEENRSDNGGVTHETSVVSKHQLQHQQSLMHRPSGARISALGGTR
FUN_000064 CDS scaffold_1 210345 210987 -
hypothetical protein ENOG41

MENNQEPLLTADMEVGAVTVEAPFDEPKKGRVLILNGFPGTGKTSVVKNLKERLPGQKIQIVDSPIMAGPVCPHAYQRGCRSA
YLHEIRRLADQGYTILVTASLANNISSQQVIEDILRIICGKDISLFWININRQEVVQDQQISNSTRLTNGESKTENSDVLPAT
AERGQLVLPSSLGHKLDGISLVTRVMNLGNDIEDAVKKVSEVMAREE
FUN_000065 CDS scaffold_1 212193 212971 -
hypothetical protein ENOG41

MEHTASEYERRRLLIGPVSAQGLDEKMALVTRQIPSPDERNQEYWARTFFQVAKRDGIKAEIKTIISTDLSKFETRDYSGYFH
PGRPTRKPRDIDERKIKKIILSKAHQAFFFIGMMASNLAVKVMPGEELTARTLSSKDLFELTIPMTEGEKQQVQQERGPKYYW
QRARLIGANATVEAGISNHEYEFATHPKMGMNNRPVVACCSTNALRPLALIQVVMNASDNGDSTDVISSMTLSRTSRPARGWR
LC
FUN_000066 CDS scaffold_1 213950 214502 +
hypothetical protein

MPTISSPSLIFASCGSSPHGSSQDQAIELDTWRPRPSVRDSKSFTPDIKLEEIPPNVDEHAPPDAQGRYLIKDEEGNFPCTQV
IANTILNEWRRQNGRAEVILSYLAGANEPAHKVRFLGDEHPPEAGPSNFKVDDLPPLIGRSCFSLGTRWKFLNLEGRTYWQPS
DP
FUN_000067 CDS scaffold_1 215670 216537 -
hypothetical protein ENOG41

MDHLEPNLERIRVRLRGIQSLTTVEIDLIAQHHEWLTALDERERLALSEEDSPRTWTEDLATFARGCLKALSGETADVDDDMD
GSRSGTEHEEDTCMSPDLPEIITLSPEFARHMRDQIRDLPCRAVDWDYPLEILKEGAPAKEDVLYAIRAIMCGWVNELINARA
VLEHRIRGVTSENRAIFMLQVPTHLPRMVGVLDIEKDGKIGSYAEWKSLDKGFPTRYRYLWDYIDYMWPILADVRAWNVVSRN
LDNIYQSDACTNQMELLLKFSVDLHTIRFDHHPNGRPRF
FUN_000068 CDS scaffold_1 218146 219495 -
hypothetical protein ENOG41

MDEEPDRWRTSAAGKRLAQTLKRARSPTDSTNKLNNIDLAIVSAYPAYEPTSTEADKIDKARERLDRGSQDPEVREDSFFSVT
TALTTMAEDAKSYAEDIQVLEHLDEDDKHTHNNVAEAALLSGETDATGHGTRAPVPSTGLSENLTSQTASDGDNEGTVLKTLK
SRKATSAWVQPNRTVIAAYHHEDMLDSNDEPHQVPVHGTNFDNVWTPGNSGSTVTNGDMRIVRKYVRASRLFIPRDGAKHMPD
RLPVEIPGPESATPAVYVGISRVAIQHVFMRNKAKVNKVSFADNTESFDLRAEPSNVVAGINTIFHEGCTVPGNISRLKRFVK
DKTTVFKEQPAPMERLVRQNNRVLQYLPVEPVQNRKASDDAGHQPSYRIVNRRELETILIGFILPRTWDV
FUN_000069 CDS scaffold_1 222103 224327 +
hypothetical protein PF00155 ENOG41

MSFSGRKFSINRHTGAPKTLTRRFSTTEPSMNEFQSKVHRQFRSAHEGHMPHAGLDASRSSTGVIWCTERASEYGYLENPDAW
ANLGQGAPEVEDDIEGCFPRPETINISMAGREYGPTAGIKPLREAVAKLYNEAHRQGKESQYTWENVAIVPGGRAGLIRIAAV
LGNSYLSFFLPDYTAYNEMLSLFKNFAAIPVPLAEEDGYHIHPDKIAEEIARGTSVILTSNPRNPTGRVIANPELAEIQDICR
DRATFISDEFYSGYNYTSNCDGSTISAAENVDDVDEDDVLIIDGLTKRFRLPGWRIAWILGPKEYINAIGSCGSYLDGGACHP
FQEAAIPMLEPSVVKNEMIHLQSHFRDKRDFVVRRLREMGFIIKYVPDSTFYLWLNLEGLPESISDGLNFFQACLEEKVIVVP
GIFFDLNPSRRRDLFDSPCHHFVRLSYGPRMDVLKMGMDAIERVVQKHRKLN
FUN_000070 CDS scaffold_1 225551 225968 +
hypothetical protein ENOG41

MLKRLPHLTITRLYNTTFAPRFSSTMSSNDQQTQTGSSEAKDTNKTPLPLPAPEHAAADQGDDVTSLRVGESVKLDAMGPLVV
NTDGTMGRIGNWAGMTEHERAQTLRLLGKRNKERMDVLKAKKAEEEQKSGTNAEK
FUN_000071 CDS scaffold_1 226481 228047 -
hypothetical protein PF07716 ENOG41

MSNFNGRRGPNVSQYLRDLNAINRQETAHEEPFNMEEDLALFTNTQFFDFETGQNTDYQAQPLKVDIEAPQSTSPSDGMTPAP
SVVGDINPNSFDFIQGDFSFPDFTSTYPSTPLNGFADGAQNFPSMQPNAPAGYQPVQQQQQAPHYGQQATPQPSTEKRGSESS
PGAGRGSLNFEEASRHAAEEDKRRRNTAASARFRIKKKQREQALEKSAKEMSEKVSVLESKVQQLEMENKWLKNLLVEKNEGN
DEIVTLWKEFAASKSTDKSESKAKSAVKEETR
FUN_000072 CDS scaffold_1 231319 232837 +
hypothetical protein PF05238

MARYSIPTKARLPSSLRVDASNPAVVKSLNRLSRESLISLALDWLDDESLPNSIPYIERRDEDDDEENDDLYPPCQTIDELQQ
LYFDMQQQKGSKRDVVSRIVEGDWRLGLTLFQLAMADMAYFEQNPTSQKWSAYQILPLKQPSQDAGEDQVLRVDQESLTIPRF
HPSTFLQNLQTHVLPDVKAHYHFHRPTAIPVLLLRIFVIDSPYNTDFALSSRNPNGTATNFDSSRTVYIAFPDGSPALYITKS
QATGNSGSGESKSLHSLIVDGVPKALSRPRERYTLKSGNLQSKNLDALLEKKGSGRTNSAGGGWSIYSNEATKISPLDTVIPT
PPLSRESSSSNLSLKRGVSFTESERVAKKSRLVAKARFGDSGFVTDGKGVEKVDIIIQDPFPSTSEDTGDTEENEDDATAGSK
NRRKSKITAVVREANITAEEDNDDTISSDRWTPTIKVTFSGTHVWAGVRQLVEAGIIDGERMPGWMTGEEGVTTGLVRHGRIR
GYKGSGM
FUN_000073 CDS scaffold_1 232925 234226 -
hypothetical protein PF01026

MASSPTPQPVEGSSYKPRYIDIGINLTDPIFRGKYHGKERHPDDLDAIIGRAREVGCTKLIVTGSDLGNSRDALTLARDYAGT
IFGTAGIHPCSSSVFSEAGPSHESEHTTPCDPDPSAPVSEEHPPCPTKTGKLIRDLTSLVKEAQASGQKSLVAMGEFGLDYDR
LHYCSKSIQLHSFAAQLQVAASISPQLPLFLHSRAAHNDFVRLLKEAFGEKLEKLEKGSVVHSFTGTAEEMRELMDLGLYIGI
NGCSFKTVENCAVVKEVHLDRLMIETDGPWCEVRPSHEGYKYLIEKKETPNTENQQNGTAATAESAQKPQKQSKKNQKKEPEV
PERYKTVKKEKWEEGAMVKGRNEPCNIERVAKIIAGIKGVSIEEVCEAAWKNTVTVFGLEES
FUN_000074 CDS scaffold_1 235161 238014 + RNR1
ribonucleotide-diphosphate reductase subunit rnr1
PF00317;PF02867;PF03477

MFVRKRDGRQERVQFDKITARVSRLCYGLDMDHVDPVAITQKVISGVYGGVTTVQLDDLAAETAAYMTVTHPDYAILAARIAV
SNLHKQTKKQWSAVVSDLYHYVNPKNGRPSPMISKETYECVMRHKEELDSAIVYDRDFQYQYFGFKTLERSYLLKIDGKIVER
PQHMIMRVSVGIWGDDIERVLETYNLMSSKFFTHASPTLFNAGTPQPQLSSCFLVDMKDDSIEGIYDTLKTCAMISKMAGGIG
LNVHRIRATGSYIAGTNGTSNGVVPMLRVFNNTARYVDQGGNKRPGAFAIYLEPWHSDVFEFLDLRKNHGKEEVRARDLFLAL
WIPDLFMKRVEKNGDWTLMCPNECPGLADCYGEEFEALYEKYEKEGKGRKTIKAQKLWYAILEAQTETGNPFMLYKDACNRKS
NQKNLGTIRSSNLCTEIIEYCAPDEVAVCNLASLALPSFINYDEACYDFKKLHKVSQVVIRNLNKIIDVNHYPVQEARNSNMR
HRPIGLGVQGLADAFLALRMPFESPEARELNKQIFETIYHAALTASVQLAKEEGPYSTFKGSPASEGILQFDMWNVKPSDLWD
WETLREEVKTHGIRNSLLLAPMPTASTSQILGNNECFEPYTSNIYQRRVLAGEFQVVNPWLLKDLVDMGLWSDAMKNRIIAEN
GSIQNIPNIPADVKALYKTVWEISQRQVVQMAADRGAFIDQSQSLNIHMKDPTMGKITSMHFAGWKLGLKTGMYYLRTQAAAA
PIQFTVDQQALKLADSNSAQIKPLKKRAPPAGSSYLMSSPSTMGRTELNGSRTGSQDSGLNGNAAKSLNGTATVPGRAPTIKA
DVEEGDSPRALPTEPAEKVQEEELGVKKNPQSEDQDKDNEDRERDIYSEAVLQCSIEDPESCIMCSG
FUN_000075 CDS scaffold_1 242358 243456 +
hypothetical protein PF05730 ENOG41

MKSSFLTIFGLAAAAVAQSSDDLPQCGRTCAGNMVSAEKSQELGCDAGDIGCLCTNQNFIYGLRDCSAAICNGEQAAQVLNYG
LEICRRAGVQITTGASGEVSATATGSGAVRTVLSTLTSGDSTITSALSTISGTATGASDDVSVSTYTSVLTNSEGDEFTTTGK
AILGGAVVTTFTSGGSTIVSTITSGSETETSGAESAEVTTFTSDGTEIVRTLVTETASTDSAASAEVTTFTTDGTEVVRTLTT
VTSGSQSESVSETVTDASTATEGATSATGTDASATTTGTDNAAAAQMTGAPAGVIAAAGIAMLLL
FUN_000076 CDS scaffold_1 244277 245470 -

hypothetical protein PF02678;PF05726;PF07883

MTLFRSIFIPVFIALAAIALFQHRIKDTATSTFEFLGTLTPLTFHKANTQHKEHHLFATEMSVIRAVRKVFLAVEQSEGAGAR
VRRSIGTPQLRNFSPFLMLDHFSVKPGAGFPDHPHRGQETITYLLEGGMDHEDFAGNRGTLSAGDLQFMTAGKGIVHAEMPRQ
NEDGSANVGLQLWVDLPKDLKACEPRYRDLRGSEIPIAKVDDDKVTVKVISGQSHGIDSVKDLAYTPVWFLDIEIRPGGKITQ
PLPANWNAFAYTLEGDVIVGKDDQRRVVEQYHNIVFEPQGDVVHFEVDAGASKPARLALIAGIPLDQPVIQYGPFVLTSKEDV
AKALFDYQTHSNGFERAENWQSEIGKSMMD
FUN_000077 CDS scaffold_1 246052 247732 +
hypothetical protein ENOG41

MPRQTITQSSWYDNARRLEDPKPRLEWDEVTEWEEANLCNKITIFDLPLECPFPLTNRRNACALIENFLDAVRYHRELFPYWR
RRAIKYFIWREWFVIRNNDEWKDYRNHPLFKNRDALRALAKKQFQNAMGLASGLLFVLHGLYTNDWELEFLLKGQQKLIDTYN
MCDVNMTLWNREKMLDILFIECQWLKDTLLEAHMKEKKEIDEEWEHRQTKTLEEMEVFIDETPELMDEDEDDDNAQGNAQGNA
QGNAQGNAQNNAQGITQNNTQNNTRGIDQGNAQDIDPWDVPITMCMNQWDDQSFAQQFEQMLRRQEKDLRQWDAQCYAQGLNE
SLVHSMAQILKFEVEDAQYFAQRRAQDVNQRKARSIAQERARQHRNRRQAQIRAQIRAQRRAQGRTQGRTQDKARGIAQNNTQ
RIVQYFDRDTSQNIIQSLSRGNTKKSVKAFMEGVYQELARGIAQDKAQQRRTQRRAHRTVQENIRETTQNTAKENGQEGTQNN
LQDSTATAHHHQRTDSQAPEEPVNTAVPPIDPRPALPARPVDDDEDIYGYSGGEDGNTKKK
FUN_000078 CDS scaffold_1 248221 248941 -
hypothetical protein ENOG41

MIFYNLLSFALFVTSSLSRATDKEIHLQWSLCDRDGEAVLEKLGEEVRPPYKRNPITYFDTMPPTHAQQGVMFRTKTNKGNPF
SIIKVRFDEKPERIPPGAHCVYDRYGDNVPFTCGQRYLLGDKTKEIWSDDQVRFAEKYDDIDWDGLVPYGPFPDGKWKLKILG
YKAKLDDVVAGELHLMEIELSTPKAGSEKVYQDVTEYLREHDVLLCDPQASKTLRLFHDMGYIDDGDTWIEEL
FUN_000079 CDS scaffold_1 252884 253484 +
hypothetical protein ENOG41

MSHQVNGDVSAAQYSAFVQHLLNYPVISDGVHSFKSNEFGQRSIKLTDAAYQTFAAPVVPYFAKPYQYVSPYVQKVDSLGDKT
LDRIDEKFPVVKKPTDELYQDTRALILFPIQKGLEGKDHVFQVYNSEIKKVEQGGLVAHGKAAVTTVLVVSNETLSWLSSFLH
QKKAETTTAVNEKINQ
FUN_000080 CDS scaffold_1 255328 257419 - FET3 ferroxidase
fet3 PF00394;PF07731;PF07732
AA1_2;AA1_1;AA1_3
MHFNSLSVEAALLLALPHLASAATRKFDFDIGWVRANPDNAFERPVIGINGKWPIPTIEVDIGDRVIINAHNNLGNQSTSLHF
HGLYMNGTTHMDGPAGVSQCPIVPGTSFTYNFTVDQPGTYWYHSHTAAQYPDGLRAPFIVHDKDFPYKDKYDEEVIFTLSDWY
HDEMQSMIPKFMSKANPSGAEPVPNNALMNETTNFTMSVKPETTYLFRTINIGAFAGQYLWFEGHKMRIVEVDGIYTEEAEAE
MIYISAAQRVSFLLTTKKDTSKNFPIVASMDTTLFDVLPPDLNYNATGWLVYDKKADKPDAATVETLDPHDDMKLVPYDQMGI
LGKPDQEFTLDVKMENLKDGANYAWFNNITYVEAKVPTLYTALSAGKDAEDPSVYGTYTHSMVLKKNEIVQIVVNNLDSGRHP
FHLHGHAFQSIYRSEEEAGIWADANVTEADLPKTPMRRDTLVIYPNGNIVMRFKADNPGVWLFHCHIEWHVVSGLIATFVEDP
LTLQETIQIPKNHLDACAAAGMPTKGNAAANTENFLDLTGENKPAKALPAGFTPRGIVALVFSCICGILGVAVVAWYGFSAPI
DPTSAGALSAGIVDGSDSGDGNPAHKGPQETVVSPSGDARSH
FUN_000081 CDS scaffold_1 260226 261450 +
hypothetical protein PF03239

MAVDVFAVPVFLVVFRETLETVIIVSVLLAFLKQTLDGPNGDIKVYKQLRRQVWLGTGIGFFICLVVAAAVIGVFYTVGRNSW
EKNEYYYEGAFCLFASLIISVMGAALLRIGKMQAKWRVKLAKALESPIKAGSKGWFKQFIEKYAMFVLPFVTVLREGIEAVVF
VAGVSFSASAKSIPLPTVVGLFAGCCVGYLLYKGGASTKLQLFLVLSTCLLYLVGAGLFSRSVWSFEMAKWNEYIGGEADEFG
NGPGSYDIDQSVWHVNCCASTEAIQNGWGIFNAILGWTNSATYGSVISYNLYWICVMIGFIVMRFKETHGRLPFGKANAPANA
VDDAESHASSTPKNAATEKTTTA
FUN_000082 CDS scaffold_1 261587 263065 -
hypothetical protein ENOG41

MGESGTTTSAEADGPEASKKDLPPLVEIDPAGDIVLDVTFETSISTLNKTRKAELAASRKAGTQPPDRSSLKSKVRVAYRVNL
GALKKHSKYFSNLLSNSQFREAKLISDIHEKLAQLKIKVNEANVEDLPWIPITDDDDATKAAGRENAMEDILNIIHQRPTKTS
RGTMSYVTTLAIVADRFDCVAAVARVLNTDLKFKWPLTSNKPLRSGDGRPTETEQVLRQKVLVAWLLGQPMRLQQSTRELIMR
GSSLWSEFYDTDTDMTAAWWNLPDGLEEELRHRRECILNTVSSVQRHFLALFSSRERQCKLGYDSSAACDSYQLGQMLKFLVN
KNLLFLVDYSPASLNMVPDTAMIEIEDLLSTLQQCPSYQIDKHHTNCGLRVRLEPILSYMRSMLSAGVIAIPYADWKKRPIEI
SWLALREHAVDDKDNPPKKFQFTRAIANDQRLRYEGALYVDKMAKAMFMADEWDWTPEN
FUN_000083 CDS scaffold_1 264017 266006 +
hypothetical protein EOG09261KRX PF00481

MAQFTRAVDSTATPAIQLLKISARPLTTPNRFAHTDALQRARRNSRHIDYTRLTTFYSTCATWTGSSRQARLTGPGSQLGLFS
PLAVETYGTRGFHTYFVTHLPSSSVHPDSRPRGPGHKLPRDAATPHTPSSGPVPAVAPPYIPSTRDLTVVRIPMRRAKHHLGV
ATDRGSRPYNEDREQAGTISLPAFAKRAPRSVQQKPGEATAADSAWGDPQIFYFGVFDGHGGTECADFVRDELPGYIEKASEE
FGLQSSLRKRKPGREGIHNHHHDHKHKQGRPSQPIQSTKSPSTKREALDAVPMKGPEEVKSEMNVPSASNGGLSDKPVHGERI
HSAASPAAPVSDLAKAIRMEKNLVKEYRETIGGYFRRFHPEHFVLSRDDNPGESHKITIESVLTYAFLRADLDFVSAQARKID
PNDTKGADTPVNNDEVFGHPSTPSGHGIGGPERFKGGSTASVVLISTPTPAPFWHPAAQSTLLCAHVGDSRILLCDTATGLAQ
PLTSDHHPSTPTESRRLRRYAPAGSMVSGDSFGEERIAGLANSRAFGDMGSKRIGVSAEPELTRVEMGPAQYSFLVLMTDGIS
GTLSDQEIVDVVKEARTPEDGARNIVKYATEVSSDGDNATCQVVRLGGWERRSEGGLGSLGTKEIRDARIAEAQDPRRGKR
FUN_000084 CDS scaffold_1 266523 269058 -
hypothetical protein PF07217 ENOG41

MPSFSMPRGSWLLLIAVCLILLPGKAEAFGAGNIPSIAQVEGHNWRHGDIEDMLETIAFLHGKKWTSMLIKRTYFGNWLRDYS
QAVDIGSLKGVNAETIRILVWVLSFMAHGYATGEFEVTADRLGVYRPEEHIDNPLGYGDGVDARTFDKRLRGPVEKIETEIDS
RTGMKNYIANENGNWATSAAYLRYSFARSIHYGRLYTSGSKKGNEGDLCEALRCLGQALHTMEDFSAHSNYCELALRELGYHS
VFPHCGAHTEIHLHGKRVYPLVTGTFGAVDFLHSVIGEASDHFTQSEVDEVDVALKNAEGASNRSGGGGGGERSLLGGFLGIK
SSPSDFIGLVSKLPGVGDGFAAQARDLKASSEAQEQRNMSYQQTRGDLSRDNANQVPGMSPDFDPVETARKIYPILEFRDKIV
KAISRGIAKVPGLEKLLEKISETLTAFILGLLAPFIRPIINQVSKVLKDGSSGVITASAKSQYEPWDNPQCSDPTHSMLSKDH
FTNILNSCAGRIAATILQYTVPRVLYAWENTGVSVDEVVNDVLRAFHHPAARDDHIEIQRDMFKTVKKWADEHPRRHELAHLL
SSDSVKNHKNHILNQVQGNSRSIGGGGCNHGTFGDMANAGHGKVAGSLWSQVKTRDLDSLEGKDGNPAAGYLSSSPAPPSQSS
FEYGQNPNYAASNSGHSQQGGYGAPPPQPPYGGGYGGPPQPQYGAPPGQYGGPPPGQYGAPPPQQYGGPGYGAPQYPPYGQQG
YGGPPPGQQPPGWGQYPGSRHGY
FUN_000085 CDS scaffold_1 271252 272452 +
hypothetical protein EOG09263KDI PF00173;PF04116

MPGRTLPTFTRAEVEAHNSGDSCYVTIGNKVYDITDFVEDHPGGPEYILEYAGRDIEEILKDSDSHTHSDSAYEILDENLVGF
LVSEKAVNGHANGKANGNGNAKLPNGEANGDANGSVHPRTGMSCAEDLSKDTDYNLDYKKNKFLDLNRPLFPQIWFGGFSKEF
YLDQVHRPRHYKGGQSAPLFGNFLEPLSKTAWWVVPMVWLPCVAYGTWVASQGFDNQLFTVGYWLFGVFFWTIIEYVLHRFLF
HLDYYLPDNRVGITLHFILHGIHHYLPMDKYRLVMPPTLFAALAAPFWKFAHAVLFHNWYAATAAYCGGIFGYVCYDLTHYFL
HHQDLPLWYKELKKYHLAHHFLDYELGFGVTSKFWDSVFGTELIYNTKKTK
FUN_000086 CDS scaffold_1 273008 274852 +
hypothetical protein PF00076;PF15519

MATLDVEALLDATAKDTSEQKTKSPVEDRTRDEPERRDRERDRGRDRDGSRHRDRDHGRRRDRRDSPNRSRRGTPDVGTPRSD
AGSHKSRRRSRSRDSDRRHSRRNRDGDYYRGGRRSRSRSRSPYRHYRPRDDRDHRDRRDRDRRNRDYGRGRDDERRETKREEG
NPQLTEDERDRRTVFVQQLAARLRTRELKEFFEKVGPVNEAQIVKDRISQRSKGVGYVEFKNEESVTQALQLTGQKLLGIPVI
VQVTEAEKNRQARNPEASGPHPNSIPFHRLYVGNIHFNVTEQDLQAVFEPFGELEFVQLQKDENGRSRGYGFVQFRDAGQARE
ALEKMNGFDLAGRPIRVGLGNDKFTPESTANMLQRFSGQNQNQNFQGSAFSGSGGRGPQSSTFDRAGGRDNEKTGGASALDDT
DVAGVNFNNYSRDALMRKLARTDDSATNGHDERQVLKPKTETKPLPVNVNMASRCVVLHNMFDPEEEEGTDWVKELEDDVRQE
AESKYGHVVHISADPNSKGDIYLKFDKVQGGENAIKGLNGRYFGGRMIDASPVVDAVYSSLFSRTRAI
FUN_000087 CDS scaffold_1 276562 278806 +

hypothetical protein ENOG41

MLSINFSTPLAGAARSERIKQMTNNHPAFEKARMSQEILVKIQTSLRRMPGVSSNESNEIITKLQEVGQIISSETSQLVDALM
NLTLDQEDTDKAIARMSIEANLAKAEADDQSHNLGNLRTELADEKGKRQEAEAEAVKLMGHLEKLMDELKSLKAKVRDGGSEG
TRSSADHDEMVKQLEISIKHIENQINARRSMWPMSNSSPDSVARALETIAEACPDKNAAQKSIMSLRPNGTDSPKKAVDINDG
ALQPQRPSSALQNSRPPNLPTRFGSHIVPPPSRPGSAAPGERRFNQWAPPTPAQSQPRYAVPPPNGAGPQSMFGRNAPRRGFS
NGTYRPNAPEFYPQSYGQPEKGPSAQSNGGFGQQRREFYPPTPSHNRNKYSRFRDPVQAPDFAGPPGALTTRPSFPGPPIHIT
EMTIGAWHDQLTELYTTVRLFVDVYADKPTFVNPMDLSSTRLWPVLLATYHPLSETEAISYLDFHLKDRSSKSCLVTRVIIDY
IVNRVWVPRAWMGAETDATYGLAEVEKDLERTQGQTAVRRQHILDRQAAIVDSVLKLDSFEEFSKVRCQEISDVILHMVEPLL
NPTADPETTYKELEKVSSAAWALSSRILSSRLTFDFRFPEIGSRFSHQSMFPIWPDSDPHELQAEHWRIALVTTPVVTCRNDT
GSNISAHSVCMADVVCMR
FUN_000088 CDS scaffold_1 279643 280621 -
hypothetical protein PF00092 ENOG41

MPHAASPSPPKMFSRIKDKFSTKKSSAESHTHFDMGNSNSTPKNNPFLDVPEEAPPAYEPTQRTAPLLNVPQRGASPAPSISS
ITSAEDRYAFLSTFDTIFVIDDSGSMAGRSWREVREALSTIAPICTSHDPDGIDVYFLNHRSPAVGTGVQAPGGYFQIRDANQ
VQHLFESVRPCGATPTGSRLHSILKPYVAHLSRRAANVDSTKPVNIIVITDGCPTDDPESIIVHHAKKLDQIEAPPHQVGVQF
FQVGNELGAAKALRELDDDLADQGIRDMVDTATWNSTTSDNSKALTADGILKVVLGAVVRRLDRKSTRGSPQGRRH
FUN_000089 CDS scaffold_1 283859 284896 -
hypothetical protein EOG09264CP8 PF01066

MSPQTRRQAAKAQNPSSDSEPEVRSNGNGSANGVADASDDAPRENIFLFIPNIIGYARIVLAIASLYYMPLHPRTCSFLYSVS
CLLDALDGYAARIYEQSTRFGAVLDMVTDRCTTACLIVFLSSAFPRWAIVFQGLIALDLASHYMHMYATLVVSGTNASHKSID
ESKNWLLRLYYTNKNVLFALCALNELFFIALYLLCFSSPLVSPYLIKPIQTVGAELQAGAQVNTSILAQIFPDPFSPAALELA
RANKMDSTVPWIIAGVSFPFMFLKQFINCVQLYNASLGLAEIDLKTRREQGLPRPKPKEA
FUN_000090 CDS scaffold_1 289690 290791 + URA3 orotidine
5'-phosphate decarboxylase EOG09263Q7N PF00215

MSPHPTLKATFASRAETATHPLSRHLYKLMDLKASNLCLSADVATARELLYFADKIGPSIVVLKTHYDMVAGWDFDPRTGTGA
KLASLARKHGFLIFEDRKFGDIGNTVELQYTSGAARIIEWAHITNVNMVPGKASVTSLAHAANRWLERYHYEVKTSISIGTPT
ASQLDEDSERSDNENQKDTPEPARADSGRKGSIVSTTTVTQQYESADSPRLVKTIPEGDETVFPGIDEAPIERGLLILAQMSS
QGNFMNKEYTQACVEAAREHKNFVMGFVSQECLNTEPEDDFIHMTPGCQLPPEGADENEAIKGDGKGQQYNTPQKIIGIAGAD
IAIVGRGIIKASDPEEEAERYRSAAWKAYTERVR
FUN_000091 CDS scaffold_1 291474 293785 +
hypothetical protein PF00172;PF04082 ENOG41

MTAAAENADGPRLRKRRRIVISCTECHRRKQRCDREQPCGNCKSRNKESACVYETGAPTAKQSKQTTKASPTYSDTFVKQAEP
SPAQSVDSSNERLGEPLSSKVADWGYAHNGASTMGILKRIETLTDGEDGPPDLTSRDSSGGSPGKELALREKYRAIIRQLPAR
NYIEKLVEMYMRGFNWQYYPIDPDIFYAQLDEWNSLPFSVFSNVGPRGLNPELRAFPAVVFQIIATALLLLPEKPDSTFDSLK
FAGGMRFEDLAVEYSESGQAVLDLLGKKNLSLSTVQAQFLRASFHKFTAKVTDAWHTIAIAIRDAQDLGMHRDSLDPRPKDAS
VESILENQWLIQRRRRIYILLAIWDLNCAMILGRPGTVDWNQTLPTPPVDAPIPQNRTKMPVVPRSEDDHPTPITRLLWNYEL
CGPLRAIQNLEVDGSYPMDPAKIDEIHQCILNLDKKMPASFRMENPDTRWDHLPEAHWYPANRFYFASLHEFSKMALHRPFIF
NRLESRVEAIHASLKTLEIQKMTFEGLPPDSWRNFMLFFASFDAIVLLASVYILFPREHTEYTDKTIEHFQWTIERFSAIQER
NPLAKSAQGVLRAIVARFKRAMNKTQTGSLPPLAPSSSAGSIKANADSTPGSSSLCSSDFSGLDSTWIVPSVDNLNAMAPFFP
TGDLVYNDLTAGPGMTMLPLPLQGGQGDIGNDSMWQFGGGWGDDTVWQMLNQFPAATDGSIPDII
FUN_000092 CDS scaffold_1 296062 297826 +
hypothetical protein ENOG41

MASNTQMNDLVSRFNALQAREMPEQKYIKDLVERLHALQETNHALQEKLADASLGLNHMSQLRKLYEGTRLQCETLTADLNSI
RSANNYVFIIMDGNGLIFDNRFIKQGREGGKQAAQALLLAVARLCPSLPSSFEIFCDVLVNIAEMGRALLNDGSIDDPSLFHE

FAIGFTEAKSSFNFIDIGLGKGTLVRKIQETAMWHLGCYNCRHVMLGVGHSAQYAPFLRRVNEAGDARMCVSILLGEPIVREI
EACGNTVYALDGDIFRTTKLVDKTLIEKPSVQHTEATKPQSSPVARAATQAVLTPATSTSSMSPPQTSWAKITKSASPPPKLT
MPLPPKQDKTKPASKTPPQPAWSPGPRGRDPPITVGIPAMEDIKRREDTDKLCNNHFLRGPCTRIGACTFNHTFKPSKEQLKA
LAMLARQNPCINGQDCDVNDCIYGHNCPNVTNGVCTRSHCRFKVDGHPPNTKFTNSKIHEN
FUN_000093 CDS scaffold_1 298760 299588 +
hypothetical protein EOG092647CM PF01922

MSHPRIEEVSDSDFDSDPAEADIDDFADSDIMRRVEPSEAAPKTVPAAAAPPRQMPAIVPADAPAGGTMPASADRSAYADFQC
LYPVVYFDASRSRAEGRRVGAELAVKDPLAREIANACSRLRLPTLFEPEKVHPKDWANPGRVKVGLKKTPGHPVKNKHHLYRLV
AEHLRENPTTEDSPGLRVRIGGQLQPEAGKPYPKPAVPRGWKMGELVPYLSAAMTGGGVSENLFKDMMKEMQGGGDPMSALMG
AQGGGGEESSSGGSKKKKDKKGKGKA
FUN_000094 CDS scaffold_1 299847 303894 -
hypothetical protein PF01302;PF12455

MPDFKPGQTVQLNDGSKAIVRFVGTTHFQVGEWIGVELENKTGKNDGSVQGERYFDCPMGYGMFVKPVMAKIIAQAPTPKPAA
RKPAARPSSFAPTSGRASSAAGDAGLGRRKSLNAPSPSPVPRVSRPTSIARSPTKSPTKQLSAASSTTVSRTGTPSNARAPSV
GAKSRPSIGGPRTSMGPPPPTARTRQASTSSGTARAASVAPRTTTSRMSLAGPPRPASRPASRPSSAARRTSVDSQARRGSSD
RDDIASPIKDNGDILSPQPRSPVQARTKALEKLTGGPSSRTTTPPAARKPSSTTTGPRPAASTAASREIEDLKAKLKVLERKR
AEDRDKLKQLDKVQGERDKFESVIQMLQQKYQPQQQENAELKKLLKEAEMRLDSVEELQAEHDSILELATLDREMAEETAEVL
KAELEALKEKAEEMELEVEILREENEEYSKGMAPEERASSGWLQMERANERLKEALMRLRDLTQETEEELKGQIKGLEEDVKE
FNIMKEEFSKCRGKLEQSESAVEDLRQQLDNALGAEDMIEDLTERNMSMSEQIEELKAVIDDLESLKEINDELEINHVQNEKE
MQEELDFKDSVIAEQARRAAQQEESLEDMEYTLSRFRELVTSLQSDLDDMKASQAVTEGESEKLNDRSRAMMDLNMKLQISAS
KAQVKTIDLELRRLDAQEAEQHLEIVKLFLPDTYKEDQDSVLSLLRFRRVAFKANLLNSFIRERLNGQPHPGHEDDVFAGCDA
SDKLVWVSTMCDRFVSDMTHCTIEQFSKYQNALHELEPVERALNVWIDGLRRDDLKEKTCADELHRTIALLSHLGEVHISNSL
ANYADDLHMKAMVMQSHLDSAAVSFTTVRGLVQRVVPAEGEEDELAQHFAKKSEAVVTHTRGTKVIAAKAVRALQDLRTRSLS
LLPDTNEAFEQCCEATQELADLARQIGLGIHSLFTADEGRTEPFSYAEVQTAVYKTVLSVSTSSESDLFSTYLSKLRAVTTQI
SDLVSLATDLDQVQEFDVTPAPWRLRSQELKALKTIPVDAEEELRRLKEEHSEARRTIAQRDEHLSTAVLKIETLESRMRDAQ
ANIDRIAKLQEELEASGQQVGSLKEDIEKQDRELKNLESERDKWKKIASDSRAYADGADAAGAKAGQERAVATAREMDALKKD
IESLQSAVRYLREDNRRARTSEQHKYEWLAEPLKKPTSVAEKRRNMIAAEGKDVLGELVKMASSASLYDFASLPQDKLAWKPV
RSTPQYHAAKQMEDYASWESWQESVLKKAHVLQGQTANAERRKKTPTAARLRIKLPGVDGKVVPGSGRGVQIVGSQEWEALQG
RLAV
FUN_000095 CDS scaffold_1 304607 306777 -
hypothetical protein PF00505;PF09011

MTQVISLVTPSQAMTPAHSPMDSDDYDRPRAFRALPDTPDSPSPRTAGLGRKRAAPINTSDASYGRLERLKLSTPSSMASNEA
TRDICLCTPAPKIPRPRNAFILYRQHHQAQVVARNPNLSNPDISKIIGEQWKDEPQEVKDNWKALADEEKQRHQQQYPDYRYQ
PRRGNKSQGLRSGSVSADESGRCLKCNLRCIATPSTPSTPYPTPSASEGRAPPYPYTPALRDPEAARRGSFGNLAMRQQQPSK
RSPYREVEEYGPESPEMKRRRYNTTVSYHPISSPGTVGRALSLGAAPQRPYPVTPLPEPYFTRSKSGPMPPPPRPAAGGPWPH
QEHRGRHPSYDDESLRLPPLQTQTAMSPTNAQEVDIRQLQTPVTGLGISNARDPQARSVEAMVMSIPFTRKLSVLSKINHTPI
SSLGPGTLSLEKVENRGAVIAVEGPKPRILKQVGLLVERALSTSNEIALKTWGQMTGDDIKSNQTGSQDNDEPSESNASLVSC
FETMIQWHQKSREMVKHITSQPESLRGSQRERDLDSPISRRGSVEEDNASRSSWDDNGSSKIPVALIKEGFSLTLSDSFACKI
PIADAYAPIDHWQWMATLWRGTINPDLVVYVKPSLEDEIAKCGTVDSYKGLMVVRVAVGKDLDDATERRIVFEVIEWVRGGSF
REKADKTR
FUN_000096 CDS scaffold_1 308712 310287 - RVB1 RuvB
ATP-dependent DNA helicase pontin PF06068;PF17856

MVQISEVKGNKRDNRTAAHTHIKGLGLKPDGYAEKQAAGFVGQVGARESCGVVVDLIRAQKMAGRGVLLAGGPGTGKTALALA
ISQELGTKIPFCPIVGSEIYSTEVKKTEMLMENFRRAIGLKVRETKEVYEGEVTELTPEEAENPLGGYGKTISTLLIGLKSAK
GQKKLRLDPSIYEAIQKERVTVGDVIYIEANTGACKRVGRSDAYATEFDLEAEEYVPIPKGEVHKKKEIVQDVTLHDLDVANA
RPQGGQDIMSMMGQLMKPKMTEITDKLRGEINKVVSKYIDQGVAELVPGVLFIDEAHMLDVECFTYLNRALESPISPIVVLAS

NRGMCTIRGTDDIVAAHGIPADFLTRLLIIPTTPYEAEEIKRIVRIRSSTEGVSVSDAAIDKISEHGVRISLRYCLQLLTPAS
ILAKANGRSQIDVQDVAECEDLFLDARRSAALLSSEAGRGYLA
FUN_000097 CDS scaffold_1 312004 316012 +
hypothetical protein EOG09260AZM PF00702;PF16209;PF16212

MPPSPRYRASDPPDSPANDSDSDLDLDIQELDPISTTPAPRDNERPSTEPQTSQIALRNLRMGGVRRAGKRNRGYGDLGQDRD
GNDEHSQALLGEHNSSASRWSEGSGYNEDDQPLLGGQPSQSRKSMNSDRVSSRLRLPSFMSGSKKPEPSDAEDQEDDDPSSSR
HVAVGSTQPVRFPNNIISNAKYTALTFLPITLYNEFSFFFNMYFLLVALSQAIPALRIGYLLTYIAPLAFVLCITMGKEAFDD
IARRRRDTEANSEEYNILVFQDADSNLAPVRQRKLLKSEVLQKHSRKKSKGARENLSDILEEDDVEAPPPSSRVVEVSRKSKD
LKVGDVLKLTKGQRVPADVVILKCFTSEAPAPAPIPEEPAEEDTLLAFDGEEPQAKGKQPAEAAPEQETESGPGGETFIRTDQ
LDGETDWKLRLASPLTQNLPTEEFVRLRVTGGKPDRKVNEFLGTIELVESRKDALAHHATVQDSDASHTAALSIDNTAWANTV
IASQATTLAVIMYTGPQTRSALSTAPSRSKTGLLEYEINSLTKILCALTLALSIILVALEGFGNTKDNVWYVKIMRFLVLFST
IVPISLRVNLDMGKSAYSWFIQRDPGMPGAVVRTSTIPEDLGRIEYLLSDKTGTLTQNEMEMKKIHVGTVSYANEAMDEVSTY
VKQGFYIPPSADSAAQNMLITPSSTYSSTTTMGTTRTRREIGTRVRDVVLALALCHNVTPTVDIEDGKEVTGYQASSPDEIAI
VKWTESVGLRLVYRDRKSMVLEYTNSKRPVVRVRILDVFPFTSEGKRMGIIVHFHEDVKVKNPSLSTGEIWFFQKGADTVMSS
IVAANDWLDEETANMAREGLRTLVVGRKKLSYDQYKEFSARYQEASLSISGRDAGMQRVVSHYLEHDLELIGVTGVEDKLQKD
VKPSLELLRNAGIKIWMLTGDKVETARCVAVSSKLVARGQYIYTVSKLKKKDNAQEHLDFLRSKTDACLLVDGESLALFLTHF
RLEFISIAVQLPTVVACRCSPNQKAEVAKLIREYTKKRVCCIGDGGNDVSMIQAADVGVGIVGKEGRQASLAADFSIEQFCHL
VKLLVWHGRNSYKRSAKLAQFVIHRGLIIAVCQTMYSIAIKFEPEGLYKDWLLVGYATVYTAAPVLSLVLDKDVDENLANLYP
ELYKELTSGRSLSYRTFFVWVFVSIYQGGMIQGLSQILTEVDGPKMVAVSYTVLVLNELLMVAIEITTWHPIMIISIIGTFLL
YIGSIPFLGGYFDLRFLITWGFVWRVLAIGAVSLIPPYAGKLIRRTMKPPSYRKVQSH
FUN_000098 CDS scaffold_1 316182 317517 -
hypothetical protein PF00970

MPSPPTDMMRATSRIITCSSRSITQNRRILTKCGQFQSRLSIAKTQPPRISSRSLSTSSPPRQHASEKPDFSTPLQSRIFPSL
IVVGIILVSGGYYLLTPPSRPNTLNEITFVPYGITAREAISPTSFVITAVPRTPNPSLPYLNPSDNHWRYPLWSVEFKQPEVQ
ISRHYTPLPPLSTEDPIDGSLRFYIRTVGDGEMSNYLGRRQVGEDVFLRGPHVGFELAQRLGEHSRLVFLAGGTGVVPGIQAA
KAVLEANEKSSVDLLWAVRKREEVQKSAPPRQSSWKFWQEKKPTTLGTEVESPSPVTKRLQDLKMAYGDRLRIQVVVDEEGTR
FQDRDISNAIAASPGTVASFDAGCRFHDQAMHVHASEFALAEGPGCVCKPSEGTTPGKNLFIVSGPDGFIEYYAGPKIWLGGQ
QTQGPVSGIAGYLQKQNPLLARDWLVLKM
FUN_000099 CDS scaffold_1 317895 319013 -
hypothetical protein PF06999 ENOG41

MSLRPSINLTRRCLFSLSSTSRRTFAQQTRQTRPPPPPPFQTVATCPEPTCGCAATPAMPEDLPLDREGPLKGAIAGYAEHVL
VCTGNADWPSRIEDDNGGDCLAADLKELFGRGGTYSDPFHNISVLNSSFPSSVSPRPEVQTTSVYLVPSFKYVPFLPRVSFDS
VEALAKGFLLPKKLHPAHEGLSPIHRDRLTRKEGYQGLLPGVQDVRDVLVLICGHTGRDARCGIMAPVLQTEFEDKLEMEGFD
VLHGPVQVNLGDKQRIQGETGEGKTTARVGLISHIGGHKFAGNVIIYLPPDLKMGDEPHPLAGCGIWYGRVDPKNVEGIVKET
ILRGNVVADMFRGGIDAEHKMLRM
FUN_000100 CDS scaffold_1 319517 321868 -
hypothetical protein PF01490

MASPSRIPSEQPPRSASPSSFRPRSISASVPRADLTARLASPIPSQLGTTPPRGGSPRPDAAPRTENLEDLTQLPGSGTSAQG
PGVSALAAALSNSLGQSPPRHGTPAARVATPPIRSQSPLLGGRNSGTPTNYGSFNSRSQNALGTSAPYEDPEIVKRHLVQPSD
DNPGSEESSIQGNSKGKQPAEIGGAGLGEDEFSSLKLQGGDVTRGIYKWTEQAEARAKYNRSKSFDLGRPEPETEVLDINSIK
VPGGFRRNHLRRNVQSPGPHGHPEDGHASPAPGQQRLFTSSFLEFLTIYGHFAGEELEEDDEDLGPNEYFSSGEDTDEYNSDD
EREPMEDSALLTPSRRRRKRKVRGGTGNNSPMNAALLLLKSFVGTGVLFLPRAYLNGGMLFSNLILFGVAALSYYCFVLLVQT
QLKVGGSFGDLGGALYGKHMRTLILASIVISQIGFVAAYTVFTAANLQAFVRAVSDCKSSISIQWLILIQMLIFLPFALLRDI
GKLAFTALVADAFILIGLAYLLYYDILTLNANGISDIIMFNKKDWTLFIGTAIFTFEGIGLIIPVQESMRHPQKFPRVLLIVM
IIITVLFIGMGAISYAAYGSHTETVVLLNLPQDNKMVNGVQFLYSVAILLSTPLQIFPAIRIAETELFTRSGKYNPWVKWQKN
VFRFFVVMLCAAIAWLGADHLDKFVALVGNFACIPLVFIYPPMLHYKAIARTKFWKVADILLCIFGFIAMAYATTLTAMSWAT

AEPKHPGYCDEKGTMVGF
FUN_000101 CDS scaffold_1 324180 327282 +
hypothetical protein PF00324;PF13520

MTTPQQSDIKGVDNGGERQRNSITESHGGRGSVSVTMEPRHNKLGSISGVYIPVFLNIMSILMFLRFGQIIGKIGFVGILGLL
VTAYSIDLLTTLSLSAIASNGEVKGGGAYYLISRSLGPEFGGSIGILFYLAQVLNASMNVVGLIDCVRLNLGAAFPSGYWTGY
GLQTAALLLCTGLCFLGSATFSRASNALLAILSLAIVSIPVSAVFKAPFHDRDLGIHFTGPSLDTLTDNFLPHSGPQFKGLET
FRDLFGILFPATSGIFAGASMSGDLKDPSRSIPHGTLWAMLTTFIIYFVVILSLAASTTHSSFLANDNVIPLVNLSQPVILAG
ECAVTFFSALMGLIGASKLFQAFARDKLLPGLGFFSKGTKHGDEPIYALLLTYVIAQLALFADLNQIATFISMGYQMTFFVMN
LACFLLKVGSAPNFRPSFKFFTWQTAFLAGILSGFAMFFIDETYAAIAITVLVLLFLLIHYLSPPKHWGDVSQNLIYHQVRKY
LLRLRPEHIKFWRPHIILLINNPRRQTRLIQFCNSLKKGSLYILGHVIVTDDFNSGVHEARLQQHAWTKYINEFSKIKAFVQL
TMSPTITWGIRNLILSAGLGGMRPNIAVLGFYNMEELRKSNPKLRVPDVPVSLSSQMRRPPRSNGKAPERPRRRRGDTSARLM
EGILPTDVIRTENMMSPKEYLTILEDLALRYRLNIAVGCGFESLETPRKDGTNTKKYIDLWPIQMSAEVTSDGKSLLTTNFDT
YTLILQLGHILHSVQLWKHVYTLRVMVFVEYESEVNEEHARVLALLEKLRIDAEVKVFCLASGSLNTYDLIVNGIGNDIDWEI
VVNDTLRNEEWWDDVQMFRGRSDNMTSTQELDQLEHIYDTISGRPGLYNPHEEIYDRRRASTTDVPELPRRPAIRMLSKMGVS
MGIHTHHLPDGALDETDNEDDEAEDSQAELLDDEEMGYSSTLDHESIDERNIGPSDAARQPLLGGWRFS
FUN_000102 CDS scaffold_1 327385 328354 +
hypothetical protein

MSSSATVKGVGVGVGGVQTSQSASASASQVAPESSATQTNERPHGGSTDSAPAEFPPLVSRDSLGPSAAFSRVRPPSPTREDT
VRPYRSGASTPGRPNLSRQSSAVKFSSRPVPETKIVASEAEGSTISFAPPSSTESETPKAGPYRPSYSRQSSLGKFPNRSRPS
GQVLAGEESRKVSFAEQSDCEPYSTHHSRFHSRRNSRGSTQGGGESYDQIPEMLERYRLSSHLEEEGQNSGYTPQGLELSFNE
LPSRAQHLIVNELMRQHSKETAVLLSTLPIPSEGTCLDEASTIQYLSDVEVLCNELPPTLLVLSNNMTVTVNL
FUN_000103 CDS scaffold_1 329700 331128 +
hypothetical protein ENOG41

MLHYPDRPARRITEADIPSDEECKKSLINMRKASFVEQIHRRHEIQHQKQLSPVREPTQEELPALRPPQHELPRIVQPQPQPP
TEQPRAIQPQIKQPRAQQPLRLTFGPFNGQFRSAFGPLTPAEEEPCPLEKVDTIGSTATATPQDSLQVPEPSVYIRKSLLVVD
DLKIAREPSPSAVISEAKTIKLERVDNHATPGVSPISSPRPSDASASGWPRRKQSVQMVAIRRSSQDSQKRKQSAHTVVVRRP
SKPTADALNSHPVHAPSTQVHVRQDSVSDPMCRVCHNGIAETSGTCSSCENDAITATPVEISPNFSRLHHKPTIKKYNRPPPL
IPQSLDGIAKLHRPSASLTSDPEANSLSPPASPTSHCSSPAETVKTVATGPSVPPTPMSALSTPEVFKRFQEEVESRAKADDS
PAFDDPWMIKSITPEADVYEEDWADYYFDEQNFNDDKVTNGPAPGANFVPSPVNPGPGWI
FUN_000104 CDS scaffold_1 331336 332452 -
hypothetical protein PF02492 ENOG41

MAPIPITIVTGFLGSGKTTLILNLIPQLRAQNPDYKLALLKNEFGDLAVDSQLAANSAISGVQELLNGCICCNLVGQLGPALE
ELEKTVTPDRIVIETSGSAFPATLALEVNRLARESGKYILDGVISVIDVENWKGYEDTSYTARIQARYTDLIVFNKWENVSML
RYDECLDRVGDLEVDVAKVKSDKGKVSMDLIFGVDGGLAKELTEVDLEKTNGHSHDHSNGTTNGHSHDHQSEVEVLSVVLKAD
PSARVSTAKLIAFLSSAPKDEAYRIKALVSLSSTPTNSDPDIPPPEPSASGRYILNWAFGRWTFTPVAESLQEHDSSSDVTLR
MTIIFARYESNKWKKRIEAGGLIELEGGDSSGLAITKIL
FUN_000105 CDS scaffold_1 332898 333263 +
hypothetical protein ENOG41

MASQESGQNNNVQDESFKEQLDKAADSTRELENQKPNPVIEKITEYVPAAAKILPARQSPPKEEDKPPGPPERPHHDDKIEDF
VRDQHRSQGVDGILGDSPNDE
FUN_000106 CDS scaffold_1 336782 338554 -
hypothetical protein PF00085;PF05903;PF08324 ENOG41
C97
MDVQLFVYDLSRGMARQMSMGLLGFQLDAIYHTSIELNGKEYVVYDGGIIAIRPGSSHLGQPLEKIRLGTTNLPMDVIEEFLDS
LRPIFTLEAYDLFHHNCNNFSDSFANFLLGKGIPEHIVKMPQAVLDSPMGRMLLPQLTQGINAGRQNGSILGLQQSAQTPSAP

KHGVKIVSNSAEFDRLMNGAKNSCAVVFFTSATCPPCKVLYPIYDELAEEVGEKATLIKVDIAQPQAHEIGSRYSIRATPTIV
TFLRGDEENRWSGADPAALRGNVQLLVQMAHPVHPHERLRLPTFANPNAKPVLYAKVPPLDKLLVKMGDEVARKPEVQALKKY
LEDRAKDGPSSAVIPEMNHLSSLVRDSVTALPLDILFTIVDLFRCALSDPRVSGYFAEEKNHETVRTVLDFVNQQPGCPYALR
LVTLQMACNFFSTPLFSDEIMRDNSLRSAVILLVSSSFLDESHNNVRVAGSSLLFNLSVANRRARQESKPTLLGDDEIELAAS
VVEAIALEEKSAEALHGMLLALGHLVYGTPLDGDLPDLLQTVGAGDNILGKKSKFPNEKLITEVGKELMGKGFRKP
FUN_000107 CDS scaffold_1 339514 341272 + GDA1
Guanosine-diphosphatase EOG09261EAB PF01150

MRTPTSPPSSKFPVFDPHEKPDRYKFKPKRQGIIGRMKESWMTQSQRTRWIKTAAIVFAIVTLFYFISPKGVEVYHEVTHPAQ
GNNGQSPSDSSYGTERCTKSYSKDKPIVQYVLMIDAGSTGSRIHVYKFNNCGDTPELEHEEFKMTEKDVGGLSKYNGDPVAAA
KSLDPLMKVAMDTVPNALKGCTPVAVKATAGLRMIGKEASDAILAEVRRHLEQDYPFPVVDKENEGVVIMDGSLEGVYAWITT
NYLLGKIGGPDKSETAAVFDLGGGSTQIVFEPTFKGAAHGGMPEKLAEGDHKYDLDFGGRHFELYQHSHLGYGLMAARKAIHG
ALIKDIESKNTDKAWVKQPIVNPCIAPGMNKTVEVTLDGSEEPVEVTFVGPSQPAPAQCRNLAEKILNKDEECKLAPCSFNGI
HQPLLSKTFTKEDVYIFSYFFDRTKPLGMPDSFTLREMHDLTQTVCMGKSGWDVFTTIPDAMAELDGRPEWCLDLNFMMALLH
SGYDMPIDREVKIAKKIKGNELGWCLGASLPLLAGGSGWSCKVNQIA
FUN_000108 CDS scaffold_1 341517 342666 -
hypothetical protein PF17863 ENOG41

MADDGLLDKVHALSDLELALLLCLISREHCLVSTPSDAIDDLIQELQLVATKTFGLSWVVIDCTPSTTLEDFASALLLGNAAS
PSSHSPTASRHPDSYFTSRPSTSHPRTPLSPLTPSGAFTSRIANVVFAKNLDRAPQAVQIQALELLRTRRIFTRTSVQTAPKQ
FVFIPVLEAASGGEARVTAHLNDFLYIAYWHNPEDGFVNLEEADGGNDADTASTGSVVKRGPGEETSSDPPLIVESDISYLGT
LSKEAQVDVEIIRYQMNIVSFLRMHRAVAGGISPTATKHFGQLMRCLAALHRLDFVTPALVGLAARKVYLHRIQLTPPEKERS
MQWGSRLEAVEALLEDVGPEDVIEDVLGMVTAPV
FUN_000109 CDS scaffold_1 343190 344142 +
hypothetical protein PF00025;PF00071;PF01926;PF04670;PF08477

MSSRKKVLLKVIILGDSGVGKTSLMNQYVNKKFSASYKATIGADFLTREVLVDDRQVTMQLWDTAGQERFQSLGVAFYRGADC
CVLVYDVNNAKSFEALDSWRDEFLIQASPRDPPNFPFVVLGNKIDVEESKRVISNKRAMTFCQSKGDIPYFETSAKEAINIDQ
AFEVIARNALAQEESEEFSGDFDDPINIHIENDRDGCAC
FUN_000110 CDS scaffold_1 344931 346452 +
hypothetical protein PF00441;PF02770;PF02771;PF08028

MSARIPLIAANRVSDAAKKQLDLVAKFVEEECIPADPVVEAQAGEGDARWEGHPSVIEDLKAKARKLGLWNMFLPKGHYKESP
GWTNLEYGLMAEWLGRSHVASEACNCSAPDTGNMEVLAKYGNDAQKEQWLRPLMDGKIRSAFLMTEPQIASSDATNIELDIRR
EGNEYVLNGQKWWSSGAGDPRCKIYIVMGKTDANNKDPYRQQSVVLVPAGTPGITIDRMLKVYGFDDAPHGHGHITFKNVRVP
VSNLVLGEGRGFEIIQGRLGPGRIHHAMRSIGAAERALDWMLLRVNDESKKPFGKLLREHGVILEWIAKSRIEIDAARLIVLN
AAIKMDDLGPKKALKEIAEAKVLIPQTALNVIDRAVQAYGGAGVSQDTPLAYMWAGIRTLRLADGPDEVHLQQLGRNENKRSA
EATATIKRQRAKTEELLKQYGVERSQPGARIKHNAKL
FUN_000111 CDS scaffold_1 347447 347875 +
hypothetical protein PF05032 ENOG41

MSAKVLADKDVNAPMIEQPVTKDVKSMEYHRQVLQSKMAEEETANKYVSPSDNIMSPCSAKINALRNKHASKAKPKSLFAQAS
AKKLQGDNPLGAKPAPAKAGFQL
FUN_000112 CDS scaffold_1 348240 349761 - RBG2
Ribosome-interacting GTPase 2 PF01926;PF02421;PF02824;PF16897

MVNITEKIKEIEEEMKKTQKNKATEYHLGLLKGKLARLRAQLLEPGPGAGGGGGSGFDVSKSGDARIALVGFPSVGKSTFLSK
VTKTRSEVASYAFTTLTAIPGVLEYGGAEIQLLDLPGIIEGASEGKGRGRQVISAAKTSDLILMVLDATKKAEQRALLEAELE
AVGIRLNREPPNIYLKVKTAGGMKITFQSPPKNLDEKMLYNILRDYKILNCEVLVRDENATVDDFIDVIMKDHRKYIKCLYVY
NKIDSVSLDFLDKLAREDHTVVMSCELDLGIQDVVDRCWKELKLIRIYTKRKGAEPDFGEALIVRSNSTIEDVCDRIHRTLKD

TFKYALVWGASARHVPQRVGLAHPVADEDVVYIVSAWKA
FUN_000113 CDS scaffold_1 350084 351348 + CWC2
Pre-mRNA-splicing factor PF00076;PF16131 ENOG41

MADTAEQIEAAPAVEQEGQDMAVATTNTEVAAPTGKKVVKKIIRKKKRPARAQVDPDFVTSEPPPQTGTIFNIWYNKWSGGDR
EDKYLSKTKAKGRCNVAKDSGYTNADAMPGSYFCLRFARGVCPKGQDCEFLHRLPGPYDHFNPNVDCFGRDKFSDYRDDMGGV
GSFMRQNRTVYVGRIHVTDDIEEIVARHFAEWGPVERIRVLNTRGVAFITYVNQANAEFAKEAMAHQSLDHEEVLNVRWATAD
PNPMAQAREARKIEEQAAEAIRRALPAEFVAEIEGRDPEARKRRKIESSYGLEGYEAPDEIHFARGTQAVNPLGREGFEVEYQ
ERPMIENGESGEPPAQVEEQQPQPQQETGGIFSGSTLAALNKAKVSVASKPKAAATTGPLVAYGSDSDSDDE
FUN_000114 CDS scaffold_1 351428 352333 -
hypothetical protein PF01730

MPSQILTPDTANVIQDEIFYLEKRLQDAKARLDKVLPSPPLSMATEPHLASTTHFLLLLSDSALPLGSFAFSSGLESYLAHEP
RASASFASFLPSSLSSFAATTLPFVLAAHRDPDSLPQLDDQLDAAIICTVGRRASVAQGRALLGIWERSFRASCPDVDGRPLR
EFAAFLRRESQNEVPLVSAHLAPLFGAICALVGLGLRQTAYVFMLSHVKALISAAVRASVFGPYQAQKVLAGQQVQKMIDDMI
DREWNTPVEEAGQTVPIMDLWIGRHETLYSRIFNS
FUN_000115 CDS scaffold_1 352615 354715 -
hypothetical protein PF11500 ENOG41

MLGWMLRRGENAPEPVNDGDTTQIDQPDTPAPVFAARAFKSALFGTPARPTNQTTRAVAKDRNDKTDQMSRTPPRPQGILLTP
GTGTTRRKRVSFGQDVKKNNNLSKEPETRQRTRLNDALEKASKSVNQKNTTQQDQGDSSDEWEEADDEDYCTHDITVDLNEPH
SQSGRYWKEEFEKYHEDAKAEMEKLLKYKQLAKSYAKQKDAEAIELAVKLKEEQQKVIEMERQIAEGASKIASKRVDRSDEVS
SELLSTLTKQTALAVQYRTRVQELEDQLEEFLREREDDADPKDRKRRQATSPRTQKTLIETQRELRRARMQVKEVGELREQIS
SLKSQLRAAEKRATRAEAMNETKSTQEKPENESVREGSRAQELRAQLREAREENRKKDEELRQLKQEFEAYRNETQAHNADTN
GVLERAHTKIAELKKEIRILKTGDNYALEQNDNAARPKSWHVQTDAGRLADEASKPRNVDVDDSTKNRRHNMERRSFDLTELG
NDTMELKAADPNVPSLRQKFHEDAVPKVTKSDIGTSKPISSSLGDKPDIGRPRWQPFVPRSPRNRAYLGEEITKRIENGGATP
GRTGLDDIAAPDLPALAKSIARSKRITSGEKPEERIDLLHDHYARVGGPDPNNSTFMANAPKSVLPPERRAAAIARIEQRMAE
KKRARGRKAYDKENVRP
FUN_000116 CDS scaffold_1 355878 360963 +
hypothetical protein PF00069;PF05773;PF07714;PF12745;PF13393

MAGKQSGVWNTPSGLKNNNKNESSFPGLLPAASPEVTNKIEYEELQQNELLALEAIYSDDFVMHTETQNAWKKSEPHFDIRIK
ASSDEDFACTLGFVMTATYPKSPPLITLKNYDLKEVTTFKIQKYLETKPKLFAQDAQEMIDQIVEGVRDILEDAAQAKANGKH
LPSLEEERERHEASMAKLAEEKKEEEARKKIEAMQEEERIMAEMLQQQIDRQKQKAKESKRRNGGNTPQQSSTSLETEEIIEF
EQLCNATDQSGNTLVFKSVTSKRHLRQGPVSTVYEVRPVLINGQGNLPMALKQTVIRTTNKDTKEFKLQLQSLESRLQDLKSV
KRVHHRHLVEVLGFKVQAGLSTDPAVSNVCTVNVLSPLAEKNSLEEFLELAGRIENIGRVRSWTRDLLDALNFLHNKNIAHQD
IHPGNILLFREPNGQTVPKISDAWYQREIHSITSNKPGLPGLNTAKSAYWLPPEIAGASKPSYTFKTDIWDFGVVFIQMIFGL
DALRTYSSPKNLMESVTLSHSLHELVNRFFKEDKQKRPRAFELGSSEFLATDAPVLFQDSPAVPSTSSSVSTQNLPAKPRHEF
TTHRGPALSRYTEDFIEEARLGKGGFGEVVKARMKLDGQIYAIKKIKPRSQANLTEILKEVRLLSQLNHPAVVRYNTAWVEEA
PDQAETEDDTSTGYFTEENTQGTGSAGIDIEFATSTGGLDFISSNANVDYGFEDSEDSEDDSDEDETEDEDDTEDEMSIHRVS
SPDKERNAMFQRRARYQRSYRTMLYISMEYCEKRTLRDLIARNLYKNTPEIWRLFSQILEGLAHIHGLSIVHRDLKPENIFIT
SDSDGIDNVKIGDFGLATSGQFSLEKANGNTLETDDMTRSIGTAYYSAPEVRSTVNGIYSTKVDMYSLGIIFFEMCYLPMMGM
QKADVLGQLRRPKPVLPSDFKPTDKAQADIILSLVNHNPKERPSSTDLLNNEELPIQIESVRGRQTLAILANPSSPYYHKTLS
WLFSKRMDPVEDYAWDMSARSLSPQELLNQVLVKQELVSIFRRHGALEPPRSSIYPRTSHYGDNAVQLLTPRGKVVQLPYDLT
LGNARMMAYFDPVVERSFTFGNVFRDKNDGGHPLMFGEADFDIVTTDALDFALNEAEVLKVMDEIVHTFPSLSTTPMCFHLGH
SDLLQLVFEYCGISPASRRAAADVLSKLNIHNFNWQKIRVELRSPTVGVSAISVDELQKFDFRDTPNKTFSKLKTLFEGSNMY
QRASPTIAHLKEVIEYCKRLGVGTKIYINPLNSLKEAFYTGGIIFSCIYDKKVKDVFAAGGRYDQLIREFRPRAGGQVREKHA
VGFSLAWERLAKIPKAGGRSFLKKSEDESSGIFNSRRCDCLIASFNAAVSRSLGAEILQTLWAHSISAELAKDARSPEDLLSK
HREEEYSWLIIIKQDAMLKIKSLGRKDVPDADIPTTQLLSWLRNEIRERDSKTVVKLRGNSSADTNGSGEKEEQEVRVLVAQT
RSKKFNRRTVVEQAQSSASSLVQSFLDGPILAIETTDQVMDLIRGTCLSEVEGWRQVEQSVTNTERKYIREIHDELDNLRFKY

QKKNDGSRHAFLYNFRSGNCVYYDLGA
FUN_000117 CDS scaffold_1 362415 363143 +
hypothetical protein PF00179

MAQKRLMQELQPLQKEKWVNIETDDSNLLLWNIGLWVVNPDSVWHGAYLKAEMKFPNDYPYQPPSFKFLTKNICHPNVYTDGN
LCISILHKPGEDEQSGELASERWNVLHGVESVLRSVLLLLDDPEINSPANVDASVLYRDNKTEYNKRAKDIVDKSQKDIPPGS
RMPTPSELAPAPQKPIDDDADFWNMTDEEEDFGGSDSDEDMEDFDDDDEDDDDVRDQK
FUN_000118 CDS scaffold_1 363805 366784 -
hypothetical protein ENOG41

MEASQQEAIGQVIEFTGLDPVDDRELVIQALKQSNWKPDSVVSQYYENQDGFRAKYKQLWDDSMFTADRDGSDNTTGISFHVE
SSDHNIIQGVTPPSESHAYGAPSRPPSRSNNRSPLGTMVDWTAAHVPGNSLGHHHEYALTRTHTGVPESQAQEDDDMQRAIRA
SAQEAGLPVSHHESGIIGTSEPPPPHFGPANRETYVAADWAIVQSDPMKESSVNEFAPSKRKRAPGAPAMLVISSAQGGEHRL
GGLLTILHEIPLARNILLGIGSPATTYGCNNDWWRGQIILSPEVLAKMAEEGSSDPSVHKSGSAFEDEVHRLMAFLDSTERGY
GSISVLADILADSDKQGMEKQFYEMLGKRYPDVAQPMMQVAGVSMFYGDDEEEVASFGLLEIEHFQHEYSYIKTLYEALDHVM
WSDTVGLDKIHDGSKMAFFKKMGDVLVLDTTGDGPSDSFEIPLEFYPERYLMNRKDEARRIQHGWCQTKEQTKRILDKKEQLV
SLAGTWGDATNDKGAVLKKAIDQWEGYRSYLESFLRFKTLEKSGFDIDKYPDYRTAPPDVDDNVYLESQKVTDVIDYSESLLV
DLETKMKGLDVELEQITAKQRALGRLLTVPDKPSRPKPMSCKRYLLRGLVASPSVLYVCQREEADLIDLSDVETKPRDQWWRL
AYTPYGDQAVRAEKTTIDIVLVDIWKDTKKPLLVYATEDALGTPKDHLSSQLERFIKVENKAFRQELSKEESTANESKQTGNF
ESSSSLSPHSSFSPSKRKHRSDSGGSMDSNRASIGSDDDRNGFDNPFLPDDDERVGTEMTDYVHSFTDMEGHIPDSLTGRTQD
STAPTEPTSATMTPSTVTADYLDTNTAFAAEEPRSPEMQEKARPPPFMSLSRNSSMRAEPTNLMDMDIPDEKS
FUN_000119 CDS scaffold_1 368782 370152 +
hypothetical protein EOG092641A6 PF00226;PF01556

MVKETKLYDTLNVKPEATQDEIKKGYKKAALKWHPDKNKDSPDAAEKFKECSQAYEILSDPEKRKIYDQYGLEFLLRGGTAQP
EGGAGGNPFAAGGMPGGFEGFNFQGGMPGGGGTRTFHFNTSSGAGGFGFSNPEDIFAEFMRNGSAGGMHGGDEDDIAGMFGGF
GGAGPRSRSSRTRSGFEPRPREATPEVTTVERPLPLTLEELFNGVTKKMKIKRKTYDETGKRVQTDQILEVPIKPGLKKGSKI
KFNGVGDQVEGGRQDLHFIVEEKEHPLYKREDNDLVHVVTLDLKEALTGWRRTVTTIDGRQLNLEKGGPTQPNSEERYPGLGM
PISKKPGQRGDFVIKYKINFPASLTADQKQKLREIL
FUN_000120 CDS scaffold_1 371678 372470 +
hypothetical protein PF00702;PF13242;PF13419 ENOG41

MPEINTLLFDCDNTLVLSEELAFEACAGLINEICEARQVEMRFTGETLIKEFVGQNFRGMLTTLQKNYNIEISPEDMETYVRK
EEDAVIAKLKESLRPCVGVDEQLEKLAASGKYTMSVVSSSALRRVRASIEKVGQDKYFQGDVVFSAATSLEKPTSKPDPAIYL
HALEKLGKKAEEAVAIEDSKSGTLSGTRAGIKVIGYVGPYAEDKQPEMENTLRDAGAVVIMRHWSEFPAALQKIEAGEV
FUN_000121 CDS scaffold_1 372722 374197 -
hypothetical protein EOG09261N2L PF04179;PF17184

MPEPRFSDLILPQNEQLSMSQLMASFKRTTLTIHNRLTSIQTDANFVDLVSQAYSPRPLVANERCGSWYIPPERKGASAYFKS
TDGHERAWKFSTRRLNLHLVELIENNDGIIIVDSTRRGKRMPDALSSTIPIWCTVLNLTLLPSNPSSANLYLPAHLPATTHSQ
ICALIPGFVASFKALNLNLPTCLTKPLRPFWVTPDSSLPSVEDTSSIFDDFRPVICCTASRRVIGSEMDEGGYIQGAADDTEM
WAHDLTAPLFWENIDELLKTPEAELPDLISRLVSDHAANKKDEGTHIKLTPHLSVCPLPLPANPSECRIVVTQDTTPKDNWIK
SKTYMQAGLGKNKLASRNLRTSLPEICDFAAKYLASATEPNQQQIIVACESGRDLSVGVALAISCYLFDDGGNIRKSTEDVSF
TKTFIKSRLGVIMTAYPGANPSRATLQSVNSFLMDWRK
FUN_000122 CDS scaffold_1 374528 375895 -
hypothetical protein PF01408

MEPLNVLMIGTGEYTTGFVGGGASGSDKKVGVVGLSLFDLRRREKVNKLGMVGVNGKKFPAIREHLNKNIQQVYNNLDTSFDS
FPENDKVDPDAYKTAIDALKPGDAITIFTPDPTHFPIAKYAIERGIHVMITKPAVKVLEEHQELVDLAQKKGVYVYVEHHKRY
DPAYADARAKAQKLGDFNYFYSYMSQPKSQLETFKAWAGKESDISYYLNSHHIDICDSMVQERGYVPVSVNASSSKGVAVDLG

CDPITEDTISLLVTWNKNGEPSKRAVGVYTASWTAPQKAGVHSNQYFHYLAANGEIRVDQAKRGYDVADDSIGQLMWYNPFYM
KYAPDEDGNFAGQTGYGYISIEKFVDGCRAVNSGKLKPEDLDAKPLPTLKNTIATTAILEAGRRSIDENREVKIVVENGKWRL
E
FUN_000123 CDS scaffold_1 378268 379261 + LGA1
L-threo-3-deoxy-hexylosonate aldolase PF00701

MTTSNGVTNGQNGHAAAPKSLPTGIYAPVMTFFDPETEELDIPTIKKHAVRLGQAGLAGLVAMGSNGEAVHCTREEKIAVTKA
TREALDEAGFQSVPVFLGATEGSVKGTIELSKLAAEVGAAATLVLPPSYYKAQMSEDAIYNYFTDLADASPIPIVLYNYPGAV
AGLDMDSDLLIKLGQHPNIVGTKFTCGSTGKLTRVALATDAKTPFQEGSGYLAFGGIADFTIQTLISGGSGIIAGGANVMPKT
CVKVWDLYVKGKKDEAQALQKKLSKADWVLTKAAIAGTKSAIQSYYGYGGYPRRPLPRLTPEQAKGIAEGIKELMEIENSL
FUN_000124 CDS scaffold_1 380186 381953 +
hypothetical protein PF07690 ENOG41

MREPASGSVASAVAENGTNALSDERTPLLKSTSQPQNQDAEPLSEAQGDDPDRGLPAGQEEETTVVAEEVSTGRLVLIFGTAW
IGVFLGAIDSTIIATLSGPISSEFQSLSLLSWLATAYLISNAACQPISGRLTDIFGRGPGLVFSNIFFAAGNLICGLARDQTT
MIIGRVVAGIGGGGLMSISTFLGSDLVPLRKRGIIQGLGNICYGSGAMLGGVFGGLINDHTSQGWRLAFLIQVPPVLVSAVAV
HFLVRVPPKQSDKSFLARIDFMGAFLTSSFLVFLLLGLNSGGNIVPWTHPLPLTTIPLAFITFGLFLFWESKARQPIIPVKLL
LDRTVLNACLCNLACTMAVMGALFYVPLYLQVLGSSATQSGIQILPSPIGISVGSLLSGFIMKRTGKYTKLGVVSLLFLIAGV
VVLTIQNEHSPKWLMGVSFFLIGSGYGATLTTTLLACIAAVDHSQQAVITSATYLARSLGSTVGVTIGSAVYQNILKARLWDR
FGAEPGAADEIRRIRDDLDEIKHLPEGWYDGVISSFVEAFRGVWLTLLAFAIIGLLCISLMRQHTLHSTLERR
FUN_000125 CDS scaffold_1 385317 386160 +
hypothetical protein ENOG41

MKHLFILLSALWSTQAAAQRYTYTTRTMTECFSATQTSFGQAEPTQGADKYWTIEMPECQDCLCTDCAYTNTYTTTLDVFSPS
GIAKFPYQVKEVYRGMSAMPADATPTAVPYGFTSGVETCSICGDEPVTATMTYPRGGSPYQESVPTATLAAQTAFERPSTLRT
QVADTEATDSPSNENNTRPLENTVLEHKAIQYSGQGPDMADFVKFSLASKSGPNVDDAESKTPGKPSRDINSYAKMRFDAAEA
APSKDVDGAAASKHPPAGVAIAVLVPIALLL
FUN_000126 CDS scaffold_1 389539 390496 +
hypothetical protein

MRFEIDPDIVPNLKLGLHSFQIIISLVAWCLQIGVFNAKDAEVTGRNGWTFAVCFLSVPAWIFLIMTPRWGRTRRFAQPHAML
AVDAAFTIIWLSAFATQAAYNAAGQCGKACSISKGIVGCGVLVTLLFAGSTFVSAFTLNYYNNHNILPGYDNRQMGSDNIDPD
KAAFSMAPHGDEAYERVDMDDHEPSGSAYGGGYNNNSYNSHSRYGDANPYSADDEDPDRFGALPPRNNTLFDNDTEYHSGGAP
PSMPASYANPTGGHPYEDVPAQFPAGNYDRGH
FUN_000127 CDS scaffold_1 390991 391691 -
hypothetical protein PF09420

MGRELQKKKARSGRQPIRQLNRSKKILNPRGNDAIAKNWNKKETLSQNYRRLGLVARLKAPTGGTEKKLGATTTRAYPNDPFS
IATVENAIVSEARVERDADGKIIRILGEAKPNPLNDPLNDLDNDSDAEPAEEWGGIEDDADATDVVKTLLEQSKQPDLPKKRH
QSTREKEWLDKLVAKYGDDTAAMARDRKLNPMQQTAADIAKRIRKMNKE
FUN_000128 CDS scaffold_1 392080 393467 -
hypothetical protein PF01266;PF13450 ENOG41

MDAKRNIVVVGGGIIGSTTAYYLTRHSKFNPALHTITLLEAAPTIAQGASGKAGGLLALWAYPECLVPLSYRLHAELAAEHNG
PQRWGYRQLGCGSFDAVVTRDKIKSLQANGNGSANGSENGKDWEKLPKQNGAAKGLLEEGILPKDLDWVDHSIINSWSEMGAP
GKTETSQVHPLHFTTAIAELAQQGGAQIHTNAKVTKINSTKNGVESIEYLDRNTGETKTIASVTDIVVAAGPWTNKVLPRARI
EGLRAHSVVFDANVSPYAVFTDIQLPPDFIPEHRAKMGQKRRHRGNVDPEIYARPFNEAYACGEPDTNVPLPETADQVECDEA
QCDDIISYISTFSPVLAAAPIKAKQACYLPRHIRFGQESGPLIGRTTVPGLFVAAGHTCWGIQNGPGTGKLMSEFVFDGGAKS
ADVDKLDPRKFKV
FUN_000129 CDS scaffold_1 394501 395101 + HYR1

peroxiredoxin hyr1 PF00255

MASATSFYDFKPLDKRGQEVPLADYKGKVVLIVNTASKCGFTPQYAGLEKLWTKLKEKYPEDFVILGFPCNQFGGQEPGTDDD
IQEFCQLNYGVTFPIMQKTEVNGDNTNPLWAWLKDQQSGLLGLKRIKWNFEKFLVGRDGKVKGRWASTTKPESLEEPILKALA
EKAEA
FUN_000130 CDS scaffold_1 395547 397408 -
hypothetical protein PF00225

MDQFFLDHAQRYQELVNRFKPTPVKSKTSNEESNPDITIATRLRPILDDEKEGGQLIGAFPRNNAPGVIDLHELRRPVRGPPP
LVSTNYRVDRVFNSEATTEGIYQELVKPLVPWAWGGGVSTMFAYGQTGSGKTYTVSGLERLIAETLFSCDIKGTRNIFISIIE
LAGNSASDLLNARKPISILEDSFGSTHLAGASEYHVTDADTLIKHINNAASFRRTASTQKNDSSSRSHAICKIRLENPELPQS
DDGLLYLIDLAGSEAARDVTEHSAQRIKESREINASLSVLKDCIRGRASVDAAGLATGKKVYVPFRQSALTKVLKHVFDPAAE
RSCKTVVVACVNPSFLDVGATKNTLRYGEMLKITVPKAAVPNYDASKPSTWPQNLLHDWILKNSGTPPVSPTALAPKETGIQL
LRLPVPEFITRCLKTPGITLEQATAFQAKFWRLHIDSQRAANNKPEATQKDAKSEKEEAGKQGLSSQDPRPEMADVPFKERLR
PGMVVSWNPSDDTPGFYRLPGRNLVMILSPEGEGIFRCAVTVPAIMPGAFEIYLWQQVVAQVDTMEAEVWLEYDPGTRYYYEN
V
FUN_000131 CDS scaffold_1 409705 412305 +
hypothetical protein PF00172;PF11951 ENOG41

MSQPTVTVKDPSAVRKRRRRAPAGGASDDCFSCSKKNIKCDRRRPYCSQCLEVGSECSGYKTQLTWGVGVASRGKLRGLSLPI
AKAPPVTREPRKHSTASSRSSAIARSGTEDEVSRMRQGPIDIPAVTQVASAPTTPFTMAGYDYLSIPHPEHATPIHQGNWGAV
NYSPAQETAQNLHRFPVPLVTENLSSSIDPLTPDVGYVSPMSQSYTRDDVSYVHSPSYMYDSYTTGTSSPGPQSPPASLYAEH
ARTFAPTSCPSLAHAPSEMSSSLHSHTDTFDSQMGNRMVRDCDSFGVPEVDTYGASYSSMPISWQSPSMQDEDVKSHHSDFAP
SGWPTASENHSVHVSQDLISKMPFFMDYYENIMCPSMVFIDGPHNPFRDHILRLAVNSQSLSHAICALASCNLRMKRKLSLGR
DTRDLSEKLMADKHAAEAMADTQPDDLSLAEEYQHRNLAVHLLNEQLNDRTKAIHDSVLATILLLCHYRMVESGIAKFHTQFA
GVSKILALRTQHLATSSNSVWMEAIFNYFDAISASINERESQVYPGFSGVSDSQLLPVGAENLVGCDRELFKTISRLGRLNLL
SQNRSVRKPAAAYGTQEVPERSPSLSYISPLGHARRESLTGFNVNDQIGDMSSVTSHGLGDGRSNTGIHEDRAAPTSAPATSY
EEGRSLFWHEWKEARLALQNWNFDASRVSASLPEPCTQSQLRDLESLSEAFRYAALLYTERLVSPSLPSGHQSIQSLVSQVVY
YATNLDTGSSAEKFLLWPLFVAGCECVNELQQNMVRSKCRDIMFRSGYMNNLAALEVLEKLWAQDWAGEEPLMLGTRTGDQRG
PFNWTKCIGGPGVEVEWIMF
FUN_000132 CDS scaffold_1 414319 415199 -
hypothetical protein ENOG41

MTEQRGTLSDDMPCGVAIIRYEQCLHKLLFKVGCTQACKGLCPPSRQRVLVVTSYLWLCEDCHKREFNTELVERYHKWTNLQR
GVPLDYPPEQQRMLSIVLQNRECLDNAFRESVRAMKLEEIQWVAEWTYEYGLMLYDVTVARKWNRSKAMRRIDQLRSLRLWDL
VVIKDGLRCSKELFEDQPEETYWTISEQFAEQHYDRACEVQPPLFSEIENTPETKESVESPDMMDWVHDGEDDDDEDPITEQP
PTSQPLLDEQLHGTATTTSTHVSLNR
FUN_000133 CDS scaffold_1 415987 417647 -
hypothetical protein PF13230;PF13522

MCRFLVYKGSDEILLSKLILDPTHSILKQSFDSRLRLDTRRGQNNADGFGIGFYTDPKLGSAPCLFTSTIPAWNCTNLQRIAS
KTASRLIFGHVRATTEGSLSEDNCHPFTHGSLMWMHNGGLGGWKYIKRRLGERLADKWYLGVKGGTDSEWAFALFLDTLERLG
HDPSACPETGFGPTILREAMKRTIAQINEMTDAIPESILQNEDVDTRSLLNFAVTDGHSVICTRYISSSKDEAASLYYSSGTQ
WTTRTQSASDKQYQMERKDRGADIVLVASEPLTFERENWVNVPTNSILTIHRQTVMVHPIIDKYYERDPHHVRSSAFVQAKGL
TSNEKNRSDVASPAGVDRQQQELDGYRKMMTGTLNFGPPRSLSPDMCRQTRKVAPVCESIPVPQPQAQPPPAQTTPPSEQGNK
PAPAQGNIKVKRRSVQLLDGNQGQSSNDYDQISDTEEPTRADVHNPRKLSRYFPELTLASHAGS
FUN_000134 CDS scaffold_1 418950 421207 +
hypothetical protein PF00400

MSSDSPDTLPTPASSSRPKGTPSITPRRFNRFWQPRQFQTQPLPKFRTALGSLDESDTNQQPISPQSLLHSDPILPSSPSDRF

QDESRKRKLQAEASGDEVVFKRPTLNLDDMPPLRFSTTTTSSNTLPGNDNEIQMSESQISLESLDSYRKATLSNFFRTSRGVA
RTLKKSPFLVNTPSITEEPQPDNELANYAPRPIRKFGNRGVASQLLNREHGLGYSTGQTYLHTPACDPRAETARFHSRCIDSY
ECLTPSGSRTIPFCLTSTHNAPVTAVGDEEGCVRFFDTSPNDDPNEEKDLGVHSVHDNAIWDMDFSQDDMRLATTAGDAALIM
DVTTKSIAARLADGHYNSTRQIAWQKGQSIGNVLTTSDKAGRIRLWDLRCPHSHVRSFSTISSSETGRRTVTVRDETIRPFQA
STVNTLDNTHERTAHGKTSPASVTAIQWLPAGREHLLLSASEAEAVVKLWDLRYINRRHQDKSTPLSATTVPSNHAWRSYGIT
SLSLSSDAARMYAVCKDNTIYAYSMSHLMLGHAPELKDLATKRRPIAAQGLGPLYGFKHDSFAARTFFVKCRMRPKGLSHSSD
LLAVGSSESSVVLFPTDERYLRSACAQRAHLLDPPGSAPTPSRSFSSAAGTEPTSSTIPIFRNGTALVHGHQREVSNVSWSYD
GKLVSASDDSIVRQWQEDEGRARYFRQIGDFGGERYMAGWADVGSDWDDEDDE
FUN_000135 CDS scaffold_1 421686 423213 -
hypothetical protein PF01126 ENOG41

MASLRADVSQHPQPQPASDPLPQPQPQHRQHDLSLAESVAIATRSVHAKLNKLIIARLPLALPPLATDSSAYITGLLHVTPIY
VAFETLWRDILESGLPVDLPGEADETCELETPPNSAETAFLASDLHELSSCSRMQSILQQMYLPGLTRSDRLKADIANLAGWS
NDTVEEQLRLVEENGRLGEFIQHIKRTVQHRPHVLMAYSYILFMALFAGGRFIRATLESAGDDFWDRLPSPVQPNRLPCEERS
RPTKRASGLSDEEIPIDDFHRHATHTMPLRFFHFQTPEDGEDLKREFKRRLASMEGQLTAREKQDIVQESICIFDNMTLLVQQ
LDMVCDAPARKESGGSTTSSLLDLVDQLHPFRSRLRDSVSIARDRIQRSIKLAPSGTKLVWKIWNYNATASTVEPESGIKLPI
GHPGLSKDDAAAIELCPAFSKSMRFDKTLPRPMRLPAKTNTTEHELKDCLQVASKKFDSMSLFNWLIVLTVGVILLGALCSAR
RGRGVMIIEA
FUN_000136 CDS scaffold_1 424733 426192 -
hypothetical protein EOG09263M8W PF06027;PF13127

MAPAMSTLSDEGSLSRERQHVQDENDVAVPSSPLLESRSRSPAGLRARLGLGGVARRTLGFALLLLTVFLWTLYNFIASYMFS
DQTYNKPFFIVYLNTSIFAISLIPKFFKYLRKHGISGMRHDATQLWSDYKHGRINTKSLYQSEDDQATERLISQGYGSTETAA
SEEKLTFKETAVLSFEFCMLWFSANYFASACLEYTSVASVTILTSTSSVWTLIFCSLFGIERFSTAKILGVGASLAGVILIST
VDLSGKSDEDRGSFPHKSPTQIAVGDSMALLSAVIYGLYVTVMKRKVPNEDKVDMQLFFGLVGVFNVVLLWPIFFILHWTGLE
PFELPPSSTLWGIIIFNAVSSFISDISWALALLMTTPLVVTVGLSLTIPLSLIGEILQYQQYSSFTYWVGAAVVFVSFIVVSR
ESHDGEVPKKDDDLERSVGV
FUN_000137 CDS scaffold_1 427669 428625 +
hypothetical protein ENOG41

MNRICPPWRQFELLGGRKRMADTNGMDFRNERAKDALRIVLEIVHGKEVVDYENSSPRLLFYILEVHAWLGHPKATYSSGMDP
KMVGTQGAQFSPFFHKDAISGCIFDMAKKAKYLYRVKDWLLLALVAEKLRLHDVMQLVLDNLCLFCRAGKRELPEEARDCIKD
KDWARIQDLRLIDKNLLNKREFYVDKIFKGLRLLSHQVLYFDGGILPNEKIFNTYQDYKVAACSYCRSLSSDEFHRELISAHL
WPLCAETYRERVIDLLHVIRDMEERTLVGRNCNQLTHLYDHLRRMCTESER
FUN_000138 CDS scaffold_1 428773 429714 -
hypothetical protein ENOG41

MSSPLRIGYVPEHFSTPLYFAQKHFGLDAELIPFPSGTGHMVTAIRAGEIDVAVGLTEGWIAGLGKEGIEGDGGYRLVGTYVE
TPLCWAISTGAKRPEITSVDSLKSGKIGVSRIGSGSYVMGFVLADQQGWLTPGAAEKPFSYTVVLNNFENLRNAVNSGEADFF
MWEHFTSKKFYDSGEIRRVGEIYTPWSSWKIVASTKLTQNGLDARVKDLFGKLDEGIKHFNDNQEEAVAYISSSLGYTEPDAR
EWLKTVRFPTKTEGVRAEVVQNTVSILRKAGVLAEGKGMEPQAMVFSS
FUN_000139 CDS scaffold_1 430442 432168 +
hypothetical protein PF07690 ENOG41

MVPQYHPVSSEDVELESHLDVPLLDLEPLEDDADTSTDIIDEMKCKVDEVQSQLGYLYGWNSLITSIVGLVVAFPYGMMSDKI
GRKPTVMLAWSGIAVCFLFAPFSMKAFQGELREKPYLLIVGGFFQIFGGGVPVMFSTLYSIAADVSSEEDKAKHFLWLTFGAT
LGGISGPIIAGILMNKYGPWVPVYLVFATVPIIVGILCLLPETLTINLKHQKAQAGKSAPTTFKGHVSHATKDLKHSVGMLKD
PSVLMIMVTFFVTNARFTAYTTTLGQYISKHFKWKMAEVSLILSPLGILNLIILAGLPKVSEILVSPRFRMSAFGKDLFLTRI
STGILAFAAFWQGISHNAVMFLFGLFIGTFGAADSPLARATVTHYVQPEYTARLYALIGMIEVIGSFIGGPVLAWFFDQGLKR
KGIWIGLPWFYVSFLCSLSWIALFFVKPPTKRSREDPADSDGYETDDYMPDDPLRLQ

FUN_000140 CDS scaffold_1 432889 437940 +
hypothetical protein PF00005;PF00664 ENOG41

MDGYNSTACGDSLFGPSVWTEGCRGGFDFTLSFQESILSIVPSAIFLAIAGPRAFYLFRSPDKTKRHATYTPKLITSAIYSIL
QVVLIALVPSAKKLNTRFSLAAAILNLLAAFAILLLAHVEHVKSVRPSFILTAYLFVSLLFDAARLRTEWLMSLNIAYAATLS
TSTIFKLTLLVLETIEKRKILISSEKPISKESTSGPFSRGFFVWLNSLLISGWATVLTNNDLPTIYEKLSSEKLAVRFGKSWK
KATSKSKKPSLFLATVTVLRWELLGIIPPRIGMIALSISQPFLVSNALRFLTMPASDSTMNLGYGLIGAFAFVFIGSALLTAW
YEHLTFRAGAMVRGGLMTMIYSKMMKLPTDDLGESSAVTLMGNDVETLIEKLHMLLVESWANTLTVGIAMYLLAAQLGAVCVA
PIVTAIICLLLTGSIGKMMVARQIKYQKATQDRINLTSEVLGSMKPVKMLGLSERFSSLISQKRDEEIKTGKHYRLINVYLNC
ITNCNVGMTEAITFGAYALAAKFGNGEPFSAAQAITALSILGVMMDPLSHLLGSIPGSFSVFGCFRRMEDFLNLEERIDRRQV
NGRVKNTSRSSSESSQPVQETIEATPLKDIPVGADGSRIVIEDGNFLWGEKAVLQDINTTFPSHKNGSLTMLVGPVGSGKSSL
LKAILGETTSSMGNVSLDASEVAFCDQTPWVMNATIRANIIAESKGYDSAWFETVINACDLTIDLGRLPEGDLTQVGEQGVKL
SGGQKQRIAIARALYSRKPVAIFDDVFSGLDKVTEQIIFTRVFGKDGLLRKNGTTVILATHAVNRLPESDFVVVLEKGGRLVE
QGTYHELRSGNGYIHDLDISSHDDHDDQPSAETKPVEESDKTDNKDAVEDEPTEVPSDRSVFMYYFKANGIHNMLAQILLIAS
AGVITSFRYVWVTWWGDGKGRDSRDIGYWLSIYTVLSAFEGILITLAIAMFLLVCGPVSGKLLHSRLLNAAMGAPLSFLHNSA
AGSLINRFSQDLRHVDIILPLAFSIFLFEVAACFGAAGLAMAAVSWFAISIPFVIVVLALIQRFYVRTSKQLRLLEIEHKAPL
FSHFLETIDGLATIRAFGWIQPYTDKALSRIDDAQKPAYLLNCIQRWLTLVLDMVVAFLTIILVVFAVTLREKIDPSLLGIAL
VNMMRLGTNMKGIILNWSILETSLGAIARIRMFCSSAPNEQKTNEDNEPGELWPRQGSLEVKNLDVQYEQTTEPVLRGLSFSI
KPEQKLGLCGRSGSGKSSFVQALLRMAEIVNGQITLDGQDISTVPRYLIRQRLSCLTQDPFVFGDTIRANLDPCNTASDDEIR
SALERVGIWSVLEAKIDDNKDPLDGKMDENVLSHGQRQLFCLARALLKRSSLLILDEPTSSVDTQTDARMQEVIRTEFSDCTI
IMIAHRMDTLLDFDKVAVLDKGSLVEFGAPQELLKDEAGAFARLYRANEGRKTEEQ
FUN_000141 CDS scaffold_1 443029 445786 -
hypothetical protein ENOG41

MEDPWGSPWTNDEPPKIDLPAPPPHAHFTADHSGSSQRVSPAHTPWNEDDDAWGGWAEAGKDSSPRWGRSPGLRPMGGSPAGS
RLPSPTPDPWGRSPTFDTARLRKEDNGDSAISLGEGLRPIVGRVATRTPSPARSLKENIADIWQQADPALSNRSLSPLPIEDE
GRPESPSGVPRPALQRGIRPPALRQPSNKVSELVEMYDDMAKSSHSTSPIDPSMRKVSDNASVQDVTIDMKVDEPDGIEEELQ
AKVEAEEELKEESNEKLEVGLEEEMEAEVEEKSKLQPEPDAKVEIVSEETLDQDENVMENGIAESPKDEDQGQTDSDSWSDFE
SPMKEAQQDEAKQPEVEVSKEQPTVTTTDTASHEETSKEPAPAPPKPPSEPFAIDMTKLDDIFPSIETSFPSPEPIPDVIIDD
SFTSISERKAWYLMSRPGSMRKHNLGDDENYVRVGWGNSQVREQAIRIVRRWMEEDSITGRVVLGRRGGAAGAKIFNWDSSAP
STEISISELLARKNHSRQASAASKGTAASPTAAAFGWGSNPIPSPTVAAPSSAPRTSTTSEPRSSVETSPVEATKTSPVSKTK
APPLASPPRPRITEEPPSPIKPTPLKPTNRPISITQPLHFPASPTISEPQSPLNPPSFLSTNRPVSVSQAPTSPLAQPPTNAG
WGEDDDDDDWGDMVSSPTAETNGGFTSMDAIVDSSSNDIKDHTSQPSITAQSPLDDIVQSAPQSEIVTNGRPSSDTLPLPSQS
SAPNHAQSPTDPWNISALQGIASSHSRSTTVDLTASPVKSLIPNHSRSTTIDLGTPRPPLASLASANLISNKAKSPIEQANKV
STWDSWGLGLLDDSNKPVRPKEDGMFASQQLQAPTPLDTFKDMASPKKPRPVSLPVPPRPKPAIESFKDDEIVANILRDLPDL
SYMLR
FUN_000142 CDS scaffold_1 446543 448512 -
hypothetical protein PF12253

MPLYELSPNIQESEATGRKRSHDEFMGNNDGIDTAEDIKLPSLASDRPDGEFALSSASSGERAPSPASSELSDPGTSTPPRRS
PSPQTPAKTQITQTTINSTSKPATTLVKRKKLTPAEKAEKEKENARQKAEKEKENARLKEEREQKAAIRAVEKAKIEAEKAAK
AKERDEKRRQKEEEKKRKDDEKKRKEEEEEKKARQQRTLGSFFKVPSTPKKTVDIPQPKNATSGDTSPAKPASEPPKNEYEKI
FKPFFIKDNTRIAHIGPEMDTETREVKSKILDECVGGQRAGELGPSRFDPTRLFSLPSKPRKRGVLHHPVKHIMEQVYRETEK
AENAGPEHVDKIMRDTRQKLSKVPMKVISFSQDVRPPYYGTVTFKPFVLGKLNMSQLARTPTARRLPLDYDYDSEAEWQEEEE
GEDLDVDDDEEEMDDEDDMDEFLDDSEDAGLSRRIFANTIEPDSTGICFENGSTVANQVIYDHRMEFMHDGLQQTWGIDPFST
EYWEPEVKNKILKPTKTTAASDGNSKMPPPPTPANAFSALTGSNGNTGGDAPKLVKSELLDDVKRAILSNKALSKVGIIDFLY
HQFRDDVSRTEVKNTVELVAEKIGKGRSKEWALKSGHEIAS
FUN_000143 CDS scaffold_1 449728 450772 -
hypothetical protein ENOG41

MLEPQAMRGPIDQTVGYGHSRTRTTSSTAFPMQFQTSHLAQPGPNLSQNRRSPSVNTFSTASSIPPPAAYRSSSQSELRRSTS
SRSGGASSQPSSYVALLRKQKGTVWCDRSQYEDPRLLAQQRAAKMRATLEVVGGSRSASGGAGARTSTGLSTTNKVAAKIPKI
RHHGKNPVVGYAPGENHVGVGGVPMRLSATEVEAESSEDENAGNRPHHRRTGSSGRSSTTSSRRNLAYRTSGGMGSQHSRRWS
PGDTPERTGSLVEATPEDIVDDAASGKARSMASGSSGERADNVGELSNAPRLASNSLMHSALTREKSVKSADELRRRGSVDER
TSTLTSGRLFIANPD
FUN_000144 CDS scaffold_1 458502 461651 +
hypothetical protein PF03105;PF03124 ENOG41

MKFAKELEREAVPEWRIKYLNYKQGKKHIKAVGRALARANGTPRGLGQLRTGTFASPATLRQPFTRENTALNSATIPSVAAVA
PPRSIPTPRSRTAENESLTDSGDNRHYGSFVNTPPEPSPLAGETKHAEFQLPAPALKVHDHESDLPASPATVGPTEVMRRNIQ
RIDPRRTSSASVVSTIASPDVNNSTKKPSESGAQDSPSHLRRLFTHQSTAKSDRRVDINMQNFDLVKEREREFFDFLDSELEK
VESFYKMKEEQAGQRLDILRVQLHEMRNRRIQEMADERAREENPQKKGTHDSANGKLNGIMDPIKSKIFPIGPNTKALQKMNT
TPNINGGVVGDAERDYIRRPTQHEVSYRTAKRKLKLALQEFYRGLELLKSYAMLNRTAFRKLNKKYDKAVNARPPYRYLNEKV
NQSWFVNSDVLEGHIRTVEDLYARYFERGNHKLAAGKLRSLNKKPEDQSIGMFQNGLLIGTGAVFAIQGLIYGAQLLYDDDVE
VRVRTSYLMQIYGGYFLMLYLFSLFCIDCLIWNQNKVNYPFIFEFDQRHHLDWRELAQFPSFFFLVLGLFMWVNFSRYGDPDM
YIYYPVILIFFTIVILFFPAPTLLYKSRRWFAYSHWRLLLAGLYPVEFRDFFLGDIYCSLTYATANIELFFCLYANYWQNPVQ
CNSSHSRALGFLTALPPIWRFLQCLRRYKDTRNVFPHLVNGGKYTATIIAAVMLSFYRIHDSKMHLALFITFSTINSIYCSFW
DLFMDFSLLQPNSRHWCLRDILALKRRWLYYFIMVVDPILRFAWIFYAIFTHNTQHSTIVSFMVAFMEVTRRGMWTLFRVENE
HCGNVSQYKASRDVPLPYRIEPLIARASMDSTVASRQPDDTEGVASLETPSRHGTAESTAVSAGQAEEGLSRRRTGGVAPPPP
PKRTFSRMLAEAHKQDFEKKRRPLEQTSEDPAAELGQSDDEDDADEAVSLLEMSEAEDLARASSSSRYR
FUN_000145 CDS scaffold_1 462180 462996 +
hypothetical protein ENOG41

MAHQSIEMALIPIKGVELEDFHGDSSITTSRAPDAIITHLCHLTYLERHELRDLLIAWLEVSHNTTDLLSLCPITQSQRESFF
SSIFSDWESSKPLYSPEYIPCVFTDGKTEVGVWSQDLPVSREERKRALENGEQEPIRQHTTARQAGQKVFMIPSMYKDGKKIV
IVFLYMDADDEFGDPEHVTFKPPGSTREDAYMRSIKDYDKSQASRVHKYNLEAIIHTARKRIVKYANDGVMEGRLPAYTGHRV
PTRFGTDPILPKIESGDACWET
FUN_000146 CDS scaffold_1 463865 465542 +
hypothetical protein PF02127
M18
MTQVTPAMLTARASSLSLRQQALSSSATLNNNATAPQKTEPKQSSKNFIISDMKGRDTKDNRVCASCITKLAPDEVGQVNWHL
EAGQACKLCQVEKMEPECFTKPFCDFLRENPTIFHTVDYFENKLKALGYEQLSPRDNWSGKIQPGGKYWVTRNGSSLIAFKVG
KAYKPGDGVAMIGGHIDALAAKLKPVSTKPVKAGYIQLGAAPYAGALNATWWDRDLSIGGRVILDDKETGKKTVKLVKLDWPI
ARIPTLAPHFGVGMMGNNNAETQAVPIIGLESSQRASTEALGPAGSFVNTQPPRLVELIAKELNIQSYSSIVNWELELYDSQP
AQTGGLDREFIFAGRIDDKLCSWAALTALLASSENSEEGGIKLVALFDDEEIGSLLRQGARGNFLPSVVERTVESLSPDVYGP
GLQGQTWSASFLASADVTHAGNPNFLEKYLPEHVPELNVGVAITYDSNAHMTTDSVSAAIMERAGELGGCRTQRFQIRSDSRS
GGTIGPALSSMIGVRAADVGLPQLSMHSIRATTGALDPGLGVKFFKSFLDNWEKIDAEWH
FUN_000147 CDS scaffold_1 466170 467664 -
hypothetical protein ENOG41

MSSNVTTSESPSTTGPQVHNKATHAQQVSTTHQNTQVAQAHQSQEGLQHPPAPLELIDHVDELVADIPNQQQNVQNLIADQAD
MQSVAGLPFNQHSPYQNIVQQSNVFPQQPSNPNFAQHTYGYPSQQAFQYPSHAGQQQYPQMGQRFPSQSAFHFNQQFGNSYLS
QQNFQQSSQLQAHPHHYAVFQDPRDGAPPQHQSTHSMPLNLAGGGNALAMQHPQQLGNPAQQVAPAASTHPICHEVGFKNLLE
DQDFVESRGADECVSEHSQLVIKEKMSLHDLIKLYEYVQRKGDVYLPADDTYKLFWSSLFKNWNSKMPVSESHPLPARSKSDI
HIQIGYYSTDASSNQLANGRRSSKARKGNAVPIYLSLDLDAEGINWLYKDNNGQRVHRSQVKLLVGLSDTQAKHNVLTHYDTY
EKNRVCGHNLQLIIEAARRRVQKWAIAGSGANVELGVKCRPPTNLLRLVLASEHFRNQHSKNCAVADLEALRLKSGYGDRKN
FUN_000148 CDS scaffold_1 474697 475318 +
hypothetical protein ENOG41

MRCVDLAEQDQEVQQGLGVPYQVEDLRRFWRELSRDWNAFTSNADNRHMPAITRTDARVQVGYLKYDHPQRLHYPDAPGAARK

HLASVYMSISTATRDQMFTTTWKGGDRKFINPSHAELNGGTIARCIDTVIQRVDSNTKERTKAYNIRLIINAARRGVMRFAQT
GTGLGAYLPPDTERPALLETAFVRDRARQMVSNSGAGPVP
FUN_000149 CDS scaffold_1 476997 477876 -
hypothetical protein ENOG41

MMEDHPGNVGHANMAPSSTIKEEPDSRPLPVTGTRYEDFESDGGCPTSRPGGYFMNRPTDLNVHEKFDVGNNLRKWVKTEHPE
LYHKVRPEEINISANQRADFFCTLYKDWTSAADLEEVPSSPRPKTKDDTRIHVGKWNQYLPPSDNKRRRDEGDDNKSPTPSPK
KPKKREEARLRDKPAYLAVGYSAGKGVDKVTSDFKDGSGQMATSQYIDWKSKDNIDQLKRRCLTKWERNERTRIRQFNQGRIL
ISSRNYIIEAAAAGFVEGSRPSVNLPGVIELVEHERMRRSSRS
FUN_000150 CDS scaffold_1 480493 480980 +
hypothetical protein ENOG41

MSQFDDINTILCLAGAAGYRHRTQKLFDVTWYQVTEDGNTQWRTEDPVALLNIENAWARDRLEHFNGYFHDNIYIAEGQYISR
CIGATCFRVTNFLDQLILEAERERLREVHGFYVFLRRARILAQELGIIDDSESVSTD
FUN_000151 CDS scaffold_1 482334 483048 -
hypothetical protein ENOG41

MLTSTFFLSLLGLAAASPVQPHINVNPQYPSRSTSKGFKLVVNVTDHSKDFDPPIHNTELTSIHTGAGLALVGVIEKDGRIFY
QNGTKEERQNGEATVISDGGTPLTPNGIALVADKKNKNLFGATLNFEPGTPGVQLNQPEDPYTYLLPETFVACNKSLEYYQGK
HFIVIEQAHLTIDKNNKIQRNIPKGCAPIRLVPQCAELETLPKGSYSSHKYALESKCYKDVSKIQWEKYSP
FUN_000152 CDS scaffold_1 485301 485822 +
hypothetical protein ENOG41

MSDTQSLPQIQDEPTELSFVGSDNSLSLSPTDFEALFTVTYSQDVDQSLMGSWPQDVVSLGPLMDQENVLGLAMDSAPPAVDD
CSEVDFAAPIIGMDSHLSNVEAAQFNLSSSPVSLLDLPESAHEVLPGYVSLSGSPDPTSTL
FUN_000153 CDS scaffold_1 486528 488070 -
hypothetical protein PF14441 ENOG41

MGVGKPRLDKYEKILSRLFENLALFHILKGIDGPHMVTAHAPTDVQGIRRRFLKNLSFICDYRKGGDTTTSIALESQTNEVIL
WVAANCTPHDKVIRFLNDVLQQLQRDPKGTEEERERLREKLTKKCVGFAAPRLKKECKLLLRAASYCEKYLKEDGTTVQGAER
RSLLEWLPRFSATNTDTLALCKTAYDSRHDLQMSSLGSLSRELGVAPRETAMNIKAVRHLIGRLAERIRIPALLIGDFLMLGP
LFNSYQIKKVQAPIPAIVPPADGLRNLNSILKRILQPRDPRLEDMQNYLARLDGPLKLEEAIRSMYDEDKGQARVHSEIQVLE
EFHRNERSFVGNDRYIACSKLSCLCCKFYFKYHPGRLEEPESHQKAYLNWRPIELPGGRENEHWAHQRRVLGMLSSELSTAVE
KQIETRQRPTPWQPDSVTNITEAMNSASLGEVEADEVLGNGDGWSDGTDSASLAHMNEDEFSDESEDSDDESDGGAGLDA
FUN_000154 CDS scaffold_1 489191 492032 +
hypothetical protein PF00023;PF12796;PF12937;PF13606

MPSLDRLPNEVLCNIAGFLVKKYDIASLSRANRHCYDIASPILWKREARSDRPGAIHWAVEHGDINVLRRALEAGVDASRVAF
THRPKPRIDRAHKWYNYHSNYYDDPVWRVHDDDHSVDLDASYVLHDMCDCFESGCDDGIFSDCRWEPIHVAASKGRIDMIEIL
LDAGAPIDAPSWGVCLCRPHLPMEAFTESPAVDEGELEDLHGGNWTPLHVAICHNQYETAKFLLKRGASPVIYQGIDFELEPL
AWRQDDTPVDSSRFKLTALHHAAKENMMDLVEFLIDEKYQEDIDVEGPFMGTPLFQAIWYGHWDTVVPYLLQHGADLDKRLIE
TGLTPLMMACFSRRFDDAARLVDLGSNVTTLSVHQFSILHLILGPRHLRPEDFFDPLGSPPKQSSKVSEAEIIQKFITKGFDV
DARETLLGMTPLMVASASCNTDAMQALLEGGANVNAVDNEGLSALARVGETAEGSTILGLYDSAKMLLDAGATLEDSRDEITP
LNIICTRQCEPYFNREWDNQHATLAKLLIEHGADPNEKGVATSRPFTEAIIHGNLNIARAIIENGGRPEKGDLERLLSIFVHE
SYDDGKTDFILHIDYAKYGISGPSEGFLVDLLKTALNNQLWTRAADLMRSVSTPSVFRKGLIYRCLEKPSPIADEPSSLIQVL
LDRGQDPNELFEGEPPLYYSFKSGSCWRTTPVLVNGGADIHMPTNAMPDGALMYTITHGYQPQTCQLLAKHPHALRGKPERLH
RECWSSLIHWEPHPSRVLVGRSDDRVPQLNWTFTSRLISAGLRTDVTTLDDEDVKELVEQAIPKTEVLKVEGRKVLEALDIAY
VEPSVSDMLNATIGMDVEGEDAEEEEEGEDFEYGSVSDEIDDHSDYYDVDSDGVPFELFPPYMGGGDGDEDEEDENDQGEQDG
EDGWGEGNSSDFDDYDDGAPMPIPAPLLFGFLM
FUN_000155 CDS scaffold_1 494754 496339 +

hypothetical protein PF00389;PF02826

MVKVLAVLYDGGQHAKDQPLLLGTTENELGIRKWLEDQGHTLITTSDKDDEGSTFDKELEDAEIIITTPFHPGYLTAERLARA
KKLKLAVTAGIGSDHVDLNAANKTNGGITVAEVTGSNVVSVAEHVLMTILVLIRNFVPAHEQIEAGDWNVAHAAKQEFDLEGK
VVGTVAVGRIGERVLRRLKPFDCKELLYFDYQPLSPEKEKEIGCRRVDTLEEMLAQCDIVTINCPLHEKTKGLFNKDLIAKMK
KGSYLVNTARGAIVVKEDVAAALKSGHLAGYGGDVWDHQPAPKDHPLRTAKNNWGGGNAMVPHMSGTSLDAQIRYANGTKAII
DSYLSGRHDYKPEDLIVHQGDYATKAYGQREKK
FUN_000156 CDS scaffold_1 497002 498885 -
hypothetical protein PF00324;PF13520 ENOG41

MVPPYGLPSNMRDSLELASLASSSQLNEEGATEISSRPSISSSRRLSLEQDDPLDSANPANRMNRSYSVSSAFDFSANMFPLS
STAGAEGYAPIGAPTSRSRPSGGLGGGSLEKNKTLTYLNGLSLIVGLIIGSGIFSSPGSVSTRVGSPGAAILVWIIAGILAWT
GAASYAELGGAIPLNGGAQVYLAKTFGEVIGFLFTWVAMLVLKPGSAAIIAIIMGEYFVRAFIGPDAEHVSPWINKAVALVGL
FIVTFLNCVSTKLGTRVNDTLMFMKFIALLGVTVTGVVVAITGKTLKGKSEVEWKPHQLFENTSTDMSAWALALYAGLWAYDG
WDNTNYVVGEFKNPTKDLPRVIHTAMPLVILCYVLANIAYFLVLPLESMDGSNTVAVMFGNRVFGSVGSLILALIVSASCFGA
LNSSTFTSSRLAYVAGKEGYIPSIFGTIGIGGSPHEHELSTQRTRSWFTRKMRRMFGDEDAGLFYTPVYALILNAVLTVGYVI
VGEFSTLVTFYGVAGYTFYFLTVLGLIILRVREPGLVRPYKTWITTPIIFCCVSLFLLSRAVFAQPFQTIAVVFFVVAGVPVY
FWRIRGRDEQVLRRRGQPSEEKKWWQFWR
FUN_000157 CDS scaffold_1 499393 499999 +
hypothetical protein PF08555

MDQKHITSLAQPLALFSGAQPLAPPHIAYLSHSFMINVKSMIIAFPTTLLKHHNTMGSDDYAAVGGGGALKLKGAKIHKKKKK
RDKTDLEKNLDTGNKDEEKRKKKDREEQEPNDEDDEDKPEVQKTEAQKRHEEIKKKRLLKLAESSSSRPELLKTHKERVEELN
TYLSRLSEHHDMPRIGPG
FUN_000158 CDS scaffold_1 500617 502436 -
hypothetical protein ENOG41

MASVDIKNDYSMEAGLLSTDREERTMLSKEALLQDSESAQAIPRTKTRRISPAGKALIALAACCLLGVGVASIGGATRFCPGH
LRHGDVVGKHGMNAHRPNLSDSMAQMVRRQNTPAPTDTEQGTGPTDAASDGASNAPSGGSSGGASGGASNTVQQPTATNEASE
TPAQPSATAEESTDQASATDEASNTQTQPSVTQPSATSEPSSPSRPASSEPEEPTSNPDTGPPSATARPTEDSATEASPSATE
SQGEPTEPASTAASTQRTTRRTTENRPESTEDSSPESTTDDSEATATGTSVSEEEPSETAPSSRASTSNPSAPSAATSTAAST
STRRTSLPDTSSFSELPSETASSGETLQSTALDDTSTVIETATVPPSTVAEATTVLSDLTSESASGPTQESETASQASEETET
TTGDQETATQTDEASQTSDNAESATETKSDRSTVTGTGTRRTSSAVPKTFTSTLEDGGVTTMTSTSWVEVVPSAEATAGSNPD
LQNAAPRSVGSVLAAVLGMVFGGFILL
FUN_000159 CDS scaffold_1 506711 509428 +
hypothetical protein EOG09260LVD

MTEHLSPDSLNTLDPDEQDSLRVRSRSPHPYHHQNIDLPHLSDRFTLRNRQAQSAKTGDDAPHEPSPTAWPSFLKDSPQTSDS
GSEADDEHYLKGLPAPKANLHKGLRGLNEPLSGFSTPLPSPALLDSHAIRTVDKPPMPTETPETKRLLEALKKRRRVVVRRIT
EAVIVLALGLMVASNSQVLQLIKIWGKDILLCGLVYAGLLALYPVRVVAWAYRNRTPSHPIPLELPAHFDPAPILYPSAITLY
VSLLISSSNPAAILPNLILSLASIPQSVIPKVDPYATYDVLAWGLTCIPLIWSPKQREYFESKHPESFISGETAILLYPLHQT
LCVTLHYLTTTSLLTAELQLLSIALINVLLLASSPQVLILKALLWGGGLSLLFFCGRVISWGIALARVPKWRFRRVSVSQRPP
LWKSFKQIFSLRRLRHELLRPEYEDAVYDTIGSSEDEAGPLFKKPTRVRTLGPSSPNPAEPDSDPSSPTAADGFGNWSFARRH
TLPHLDHGNRRNATHTPSGRKKRATSMSVRPFFKLTQEQATLRKWLYAGYVYLSIIAIILGGIRTYVQWYALDGNEPIGWALG
YLFGDWSWFRYQVVSLNLERWICLPPRIDPTDDKQCHSGWVQHVRHNDFGEANTRLLLSTYWMGILILGLIIVFRLKDIYEVD
TRRKVFHFMMVGMFLPATFVDPTYAALALSLILAVFLILDLLRASQLPPLSKPIASFLAPYVDGRDFRGPVVISHIFLLIGCA
IPLWLGLASLPRSGSGYLSGWEVTTRDVSLVSGVICVGLGDAAASLIGRRYGRRKWFWGGGKSLEGSVAFAVAVMLGLGAASM
WLRIGGWPAAGEQPGVIAGTRNAAMCASMASLTEAVLTGGNDNVIVPVVLWTCVKSLGV
FUN_000160 CDS scaffold_1 509711 511495 -
hypothetical protein PF04084

MPPKKAVPEPDTQQDLPRSLRKRGHQDLEPIPESDLETSPVKRQRSAAHHSPKATTGTNGHTEGIHRDVIGDSDASSSESLSR
VDPDETPKPAPKRRGRPPKKAVNGETPTPKANRTALFETPNKKTPIALNGATPGGADRSAKRKSTRALIEHVVGDDLTDEEEY
DGLAQQIYESSEDEEALEGDIAISTEASGVDEAATPSKSTPRRKAQRKAPARSPTPPRDLPPHELYFAHNKPGRAKTSNNTLG
SLALLTHDEYFTIMRETQDHHEADIEFLESLHAESFPQWAFELSQGFSLCLYGYGSKRRLLHKLAGHLYSSIRKEKGDKIVII
NGYAHNTTMREVLSTIGAAVDPSHRIPLTQPAVMVPAILSHLATTSSTLTLVVNSIDAAPLRKSGSQSALAQLAAHPQIRLVC
SADTPDFALLWDIGVRSAFNMVFHDCTTFAPYGAELDVVDEVHELLGRNAHRVNGREGVAFVLRSLPENAKNLFRLLVGEVLI
AIEEEGDSGDEPTGVEYRMVYNKAVEEFICSSEMAFRTLLKEFHDHQIITSMKDALGTELLSLPFRKDELEAILEDLMS
FUN_000161 CDS scaffold_1 512910 514071 +
hypothetical protein ENOG41

MGGKTWSVDEERMLWEVVVPRSVAAANPADRGMSWEQCAQYMNDNAGDIAQRNYTKNMLYEHHYQNIVKGGSSPNAGPFVERH
LRLIEWYKNHRSPPPASSPPVPRPPQNPELTALLSEQSKPKRRRGKNAAKTRVAENRNALPTIDEDGPGPGLSGSGTSSVPYV
RSFAPYDWAQQQSFEENQSPPDDPTAKYALDFRPLPRTVRAARPGGNFLSRPMEKLEGGLWRLVPETRENENFGESMSMVPRN
SNVQARHSRAQEESSLTRPYTGPPQRSPQGPQGGSLPSIRQTFPFINFRQPPNSAPLRETSHQTGKRGYSGGQDQNQAPKRRR
LPDRPVPRQSNQQPSSQRK
FUN_000162 CDS scaffold_1 514639 515289 +
hypothetical protein PF02466

MNFPGMTPSGGAAAPPGIGGPQDPNVKAVQSAMESCFGKSVMSGVMGFGMGGLFGMFMASMSYDTPYHTAAPGSPQSTITSLP
LKQQLKIGFKDMGTRSWSMAKNFGKVGALYSGVECGIEGLRAKNDLTNSVAAGCLTGGILAKNAGPQAAAGGCLAFAAFSAAI
DAYMRSPPKDD
FUN_000163 CDS scaffold_1 515710 517067 -
hypothetical protein ENOG41
GH16
MSRYSSGVKPGEIPPIYDEIMLARKGQEVRHASSTMPWWNPRYWRKRVWAAIIIVIIVIVIIIVAVAVGKARKNIYPDYTPLS
YSLKDTYGGESFFDQFNYFTGYDPTEGFVHYVPKAQAELLNLTYASPSTAVIKVDTSVGPNDKPNASTGRFSVRIESKKTYEN
GLFIFDVKHTPYGCGTWPALWLTDPFNWPNHGEIDVMESTNKAEDGNQMTLHTTGDCSMDVRREDTGKTLQKNCNHEKDDNAG
CGVLSEPAGYGTVFNKNGGGVMAVEWRHEGIRMWQFGRDSIPSDIKGNNPKPDTWGTAVADFPNTHCNIGTHFKNQSIIANID
LCGSLVYHVWDNSGCSGKCTDLVANNPEAFENAYWEFGTFQVVYQAS
FUN_000164 CDS scaffold_1 519287 521497 -
hypothetical protein PF14683;PF14686
PL4_3;PL4;PL4_4;PL4_5
MKFRKLSAAFLVSLLQAPQLVAAVLNATETDTQLVISNDRLYAAVQKKGGAIVKLTLDGTNLLGSPSGSTGIGPYLDCYCTPK
GFWTPGSVAPEYKLFKGKDGKGKDYGGIVMSDTYTETGQVLEQYWFLRDGETGLHTFSRVAYHNEEQPFLRNLQELRTLFRPN
NDMWTHLLTNTKQYAPLPGKEAKEKQVVVQDATWYLGNTPNDPYVKQEADYFTKYTFQDSWRDIDAYGLFADGSKTEDGDAYG
AWLVMNTKDTYFGGPLHSDLVVDGILYNYISSNHHGDQTPNITNGFDRTFGPQYFHFNRFPGETDILKAQADAAQYADPEWNA
DFYDSIAKHVPNYVPTKSRGSFEVKVDLPKGAKNAIAVLAQSGVDFQDNVFDTKAYQYWANLDESGRATIPRVKSGTYRLTVY
ADNIFGQYTQDKVKIKAGKTEKKHVRWREESAGKELWRIGTPDKTSGEYRHGFEPDTSKPLQPEQYRIYWANWDFVKDFPEGV
NFKVGESDVGKDLNYVHWSVFGGKGNSVRPEQYVGDGNVNNWTIAFDLKESQVKQKKHATFTVQLAGAKTAAGNTDIYNASEP
HSNLKYTVNINGKDLEPWVIPYDHSSSCAVRSSVSCYNIAHKFEFDAKLLKKGENEIILSLPYNATNYESAVLPTSVYVQYDA
LRLEVE
FUN_000165 CDS scaffold_1 522168 523983 - PRP45 mRNA
splicing protein EOG09264DT4 PF02731

MTSIAGSLQAALPKPKYTGEDEEPPARAQQRGVRIVGPGQLDETQLVLKRSGPPAYGQRSGWRPRSQEDFGDGGAFPEIPIAQ
YPLEMGKKGGSSSNALAIQVDSEGKVKYDAIARQGHSENRIVHTSFKELIPLRQRADAGEIDLSRPDKESVEATTERTKNALA
ALVSGAVAAQKPKNVNIGQRKDATFVRYTPANQMGDNSKKQDRIMKIVERQRDPMEPPKFKHKKIPRGPPSPPPPVMHSPPRK
LTAEDQEMWRIPPPISNWKNPKGFTVPLDKRLAADGRGLQDVVISDKHAQLAEAVKMAERHARDEVQKRAMMQQRLAEKEKSQ
KEDTLRELAQKARAERAAAGRGRRDSRDSGDSRDSRSRSRSYSYSESDRSDSEDEEVRERVKARQEKQREEERKLRQNRMGAE

RRAQVMAREQGRDISEKIALGLAKPTQSKETMYDSRLFNQTSGFDSGINEDNPYDKPLFAAQDAISSIYRPRANVDDDDDGEA
GDREMAKIQRSSRFGEALGKGTFKGAADAEAREGPVQFEKDAGDPFNVDKFLSEVDQNSSSKRGYGLQDEDSRQSKRPRVDPD
DDED
FUN_000166 CDS scaffold_1 525137 525885 -
hypothetical protein PF01105

MRLSTLVAGLFAGLVTNVAATALTYKLDANEKACFHTTTKKEGEKIAFYFAVQSGGSFDVDYVVEGPNGKIIMDGQKERQGDF
VFSANVVGDYSFCFDNEMSTFAEKFVDFEIAVENESRAQLPSKQGTTPEQTSILEDSIFKVSGQLSTISRNQKYFRTRENRNF
ATVNSTEGRIINFSMIQIGLIICMGALQVFVVRFFFQGARKGYV
FUN_000167 CDS scaffold_1 526334 527825 - LEU1_1
3-isopropylmalate dehydratase PF00180

MAEHNIVVFAGDHCGPEVIAEAIKVIKAVEDISPTAGKFNLEHLLLGGCSIDKTGTPLTDEALAAAKASDAVLLGAIGGPEWG
TGAVRPEQGLLKLRSEMCAYGNLRPCFFASDALVETSPLKASVCRGVDMMLVRELTSGLYFGERKEYDGVNAFDTTVYTKPEI
ERIARLGGYLARTRGDSRVISLDKANVLATSRLWRSVVDEVYKNEFPDLKVEHQLIDSAAMIMVKNPTQLNGVMIAPNLAGDI
LSDEASAITGSIGLLPSASLCGVPEVGSKVLSIYEPIHGSAPDISGKGIVNPIGTILSVAMMFRYSLNLAHEAKLVEDAVRAA
IDGGLRTKDMGGSTGTAEAGDAIVAELVKTLKA
FUN_000168 CDS scaffold_1 528477 530516 +
hypothetical protein PF09423

MAVQTKAAKLSSVALRISALIFFRLFPAHFPSIAFALFAIYVPSYISSYFAQPEIGVLEDEVDVTVKESTITSETPLLTEDGV
AVAPEAEVVAEVVDIEEKLTVGEKVPSIANILLSGAPSVRRPIASILTFLINAILIGLTADALFRARWYYPSDDLSFVRLGYV
SPSEARFVIREPDQLQMPITFEYHIKDDEPTYDTKIWLTAGYVTSTDNSTDFTTTLTVPLDSAKQNIYEWRTSNNHTGEFRSA
PKIGEMPNTKDGLFTFLSTSCILPRFPYNPMEHALAIPGLRNLAGLLPKLDPQFMLFLGDFIYVDVPERFGKTTEEYRMQYRQ
IYASPDWAPVAQNLSWIHVLDDHEISNDWSSNTTGIYSAAVDPWHIYQANVNPPISEAAGPLNLRRNATWYEFTQGPASFFMM
DTRSYRSSNNAPFNATDKTMLGKEQLADLLAWLERPASQGVRWKFIASSVPFTKNWPINVKDTWGGFLAERKQVLEAMWDSGA
RGASVVILSGDRHEFAATRFPPPVDSRWPQSAAAHEFSTSPLNQFASPFPTYKQTDDEDIMMNYIPGGKSKFGSFSIEKLENG
GSSLHYTLYIDGKETWKTTVEAPDAPEAVPVETVVSPSLWDKLSKFF
FUN_000169 CDS scaffold_1 530893 531796 +
hypothetical protein EOG092653LT PF07890

MAGPGNKKKRQHDGRLQRPSKKQKRKQLRDYNSDSEPEEAQEFDAVNLLDSDDDIHNVKVDDVGDSENEVSSSEDEDAVTNKP
LKKTKTNAKNARDSKPEAESDEGEDDDDDEGSDDDDEDGASTNKRKKSKRNDPNAFATSLSKILSTKLSSSKRSDPVLARSAA
AHEASKAAVDSALESKAKKQMREQKKRAFEKGRVKDVLIATTNDATGELETSTSEIMETERRLRKVAQRGVVKLFNAVRAAQV
KAVEAEKGVKKEGVIGMKKREEKVNEMSKKGFLELIASGGGGLKKGALEEA
FUN_000170 CDS scaffold_1 532012 532634 -
hypothetical protein PF00111

MSASRSFATCLAKLPATAPRTIQSMRLGQNVLSRTSKGRSPTISRFTRFSPVSHRAFTTTTQRRHGHIDPPKPGEELYVTFIE
KDGTENKFAVSEGDNLLDIAQANDLEMEGACGGSCACSTCHVIVADDAYFDKMPEPEDDENDMLDLAFGLTETSRLGCQVKMT
KELDGLVVKLPSMTRNLQASDFS
FUN_000171 CDS scaffold_1 533077 534649 +
hypothetical protein PF00153

MSTHREGVNPLRPYYIPPTIGEAAETVNKSSPSPNPFPDGRHVTAAGERYASKARDILSDLEYNDYLGDTSPSMVQNVKDLID
ELLWKYTSVLMAQPFEVAKTLLQVRNQDENAVYETPVEPETPKRQPSFHGSSMYGGFGDSDSEGEEPAYFTSHVPSTPSPSTP
RSSHGRRAPSPVKKPNVPEHYLTLRRPDSILEVIGQLWGKEGAWGVWKGSNATFLYTVLQSLLENWGRSFLSAIFNVPDMGVR
EDIDRMIDIASPYPWASLFVAATAAVATGLALSPLDLVRTRLIVTPSSNGQRRTLATLRNLPSYLCSSMLVLPTVLNSLIHPL
ITLSTPLVLRTQFMIDSQVSPMTFSIAKFVASSSAILLKLPIETVLRRGQVSILSSHEYVQALSGPDQQFTTIVPPGRYEGTF
GTMFHIVNEEGTHEVASTKPAVAKKGKAKTKGVAATVYKGQGREGLWRGWKVNWWGLVGLWTASVVGHGGEGEF

FUN_000172 CDS scaffold_1 535698 536529 +
hypothetical protein PF03443
AA9
MSLLSKMTRFKAACWLALLLPAITVSAHGHVDEIIINGVSYQGYGSTDFPYMENPPVVAGWTISQRDNGFVSPEAYGDPDIIC
HRDATPAEGHIEVTAGDVITLRWSGWPENHIGPVLNYLANCKGPCERVDKTELEFFKIDGLGQLEQGTPGRYADSVLQENGDR
WNVRIPESIASGNYVLRHEIIALHNALERGGAQNYPQCFNLRITGDGSDSPSGYLGTELYDVEDPGILVNVYSSSVDYEVPGP
TIVEGGVSSVEQSPSRATTTAKCTTRY
FUN_000173 CDS scaffold_1 537254 539764 +
hypothetical protein PF05205 ENOG41

MAQSGVEQPPLNGEKAPAPEPRKFKASDLPLPSATRAAIESLAHSFKKEGAYDSIRKQVWDKFEASDYEAQVTKSILEVAEQE
IERNPQQLLTLDRRKAAALIDGALERSGVYHKAEDVISQLIDVGAIEERIRETRRAEVGVEAAEAEKLRGAKTDEEYEAETEA
RRAEREKVREELRQKEAAIEEEKRRILKEERKKEEREREKAEIKRQEERDERRRKREQEREERERLRDIEREKRHKERERDRD
RDRDTRDRDRDRDRDRDRDRGRDRDRDRDRDRHRDHDRHRDDRHKEKPAVSTELKEKLSKEEHDQLEQEALADLLRESSKPAQ
KQELEIDSSLAPPPRKTGPVSAINPIRRERSSIAEGRKTSDAGLGAERDGPGTPAQAKRPDADDRKSSRSASRIVDRDGDRER
DRERDRLRERDSRRDRRSNSPRRGISGSRVRDRRDRSRSRARRDRTRSRTGREDRAVRSRSRPKYDHYERSRSRRRDRSRSRP
RVVERRERSQSRPRPERRDRSRDRRDRSRLRSDRRERSRSRTRRDQSRSHDTRLGGADHYDPRERDRERDRDRPRDRERGRER
SPLRRRSRERPGNDRDQAKPREKEQEKAAPEKEREKEKPNERRSRSPSRATATAARPSSSKAQDGLEEWKMEEVRKREKEAKA
YLAAQKDARNKGLPIPGLDDKKSSGEISPDLRRRRIQDDIDRYTPGGREERRRSRSRSRSRQRDATKDRDRERERDRDRDRGD
RDKLRGGDSYRERDRDRDRDRDRDRRDRDRDRDTDRRDRDRDRERERSRDRRNRRERSLSPRRERRGSRSRR
FUN_000174 CDS scaffold_1 540027 541473 -
hypothetical protein PF03151 ENOG41

MAGSNDLEANNRMSRGSEAVDNGFSEKPLNQGTGSRIHASVYIITWIFFSNTTILFNKWLIDTAGFRYPIILTTWHLVFATIA
TQLLARTTTLLDSRHSLPITRRLYIRTILPIGVLYSASLVFSNIVYLYLSVAFIQMLKSTGPVCTLVASWVWGVAQPDSKTFG
NIMLIVAGVAISSFGEIEFSWWGFIFQMCGTIAEAVRVVMIQVMLSAEGLRMDPLVGLYYYAPVCTLMNMVVVLFSEGPRFKW
EDAAQAGYGVLLANACLAFFLNVISVFLIGKTSGLVMTLSGILKSILLVAASVVLWGTHISLTQTLGYAVALMGLVLYSIGYE
QLLNMWEEAVAWGTGTLNREGEMSPTLRKAIMIGCLGFITVIIAGALWHYHGLSSEHVTRVTSSWFAH
FUN_000175 CDS scaffold_1 543638 545762 -
hypothetical protein PF00443;PF13423
C19
MTSQHPDFDEFYDDQVAAHRQLFPQQNPWDRITQPSVLISCLIVVVTITYNLYASTTTFQESIQLLGTSLWDGLVFIAPESLL
NALDTPDNSTTIATSESMSLVERSHHAVKSDKMRRVLGLTRPGGVMSSVFEVRNRALSMTGSVLGLKTESDRPPGLGNMDNSC
YQNSILQGLSSLKPLPEYLSTYLEASNKSGDQDVAQTLRTLIADLNDVSNYGRTLWTPSLLKSMSTWTQQDAQEYYSKLLDDI
DKSVAKAIKNAQPQHPGLAFKIDKDDAAVSEHSDDSGYQSVITAPEIKSGHNPLEGMLAQRVACVQCEHSEGLSMIPFTCLTL
SLGLNKNQHDLYERLDAYSKVEAIENVECAKCTLLKHEDLLSKLVDRMRHNGTPDEQLQKALRRLEAVKLALEEDDFEEKTLT
EKCSIPTNNKVSSTKTKQIVISRAPKSLAIHMQRSVFDPSTFNMMKNSAPVNFPMTLDLGPWCLGSAPNSSTQASSAEEKWDG
DPRESMIAGDQGSSKFTGPIYELRAVVTHYGRHENGHYICYRRYPRHSPPVKTVDDEEPDSKVDIRPELESKNQENDIDEPEM
DWWRLSDHNVSKVSEDVVLSLSPGVFMLFYDCVDSTMAMNNQDESMQDANEVADLGAATGLASATDNTAQITQGHETQASNGK
PEEASNLEEDATSCIANDQAAVETTTRGSNLITNTAPLVHVAP
FUN_000176 CDS scaffold_1 546534 547242 -
hypothetical protein ENOG41

MNGARRCCPFGYSCKDDKECVLDKDQDKSYAFLLPVPEYTSATTSAPTSTTTSSSTSDILSVETSEVPDRVFQPPSASADPTT
STTESTTPGDNEEEEKGSGISPTGAVTAGTVAGVCCLVGVGIFVWMKWFRKKGDRDTPEMSLARESWGYFSTRSAPSTLHLHL
ARGPDDKFIVTPTTAGFTPSYPPLAPIVDENQSPVELPATPVSLCMWSNFENAAVEEPKLAYIIPAKRQ
FUN_000177 CDS scaffold_1 549254 551010 +
hypothetical protein PF02668;PF06155

MGMQIEKSAHKTTIQRTETSQIRMERQALDGKSTTEYRTFPNMWLRDNCQCSQCVNQDTKQRNVDTFQLKEEDLKVVDFKETE

DKLHVNWSDGHESQHPLSYLYCWFKGAYAKEFGKDHMIRLWDSSIGTHPPSVPYNQFFVDHGWEAIGELTKNIRIHGFCFVTD
APVYPESTEDLLKRLGPIRNTHYGGFYDFTPDLAMADTAYTNLALPAHTDTTYFTEPAGLQAFHCLSHEPAPGEALEEGESLG
GESLLVDGFKAAVTLRKENPWAFKTLAQVELPWHASGNVGISISPDKVYPVIEQEQGGHLKRIRWNNDDRGAVNPYEAKNWYA
AARAWNEILRRKENEYWFKLEPGKIVIFNNWRVLHGRSAFKGIRRICGAYIPYDDFVSCYRVTNTPGGRVDGRSYNNRPRVIG
HTRSKNYKDVTKAPEQEAPVIQES
FUN_000178 CDS scaffold_1 551816 552850 + LXR3 L-xylulose reductase PF00106;PF08659;PF13561

MDPKTGAFHHDNMAVPDTSRVLPLFSLKGRTAIVSGAGAGIGLAIAQGLAEAGANVAIWYNSNKQALKEAEKIEKDYGVKCRA
YQVNVISPEAVDKAINDIISDFNGRLDVFVANSGIAWEDGTFIDGPTERARRVMEVNVDGVMWCAKSAGAHFRRQKKEGTTLD
GKKLTNFVAGSFIATASMSGSIVNIPQLQAVYNASKAAVIHFCKSLAVEWTGFARVNTVSPGYIKTEITDFISEDVKNIFKEK
TVAGRQGETGELKGAYLYLASDASSFTTGHDLVVDGGYCVP
FUN_000179 CDS scaffold_1 554113 556030 + CNA1 3',5'-cyclic-nucleotide phosphodiesterase (PDEase) (3':5'-CNP) PF00149

MDSEGEANTAPSTEERRNVQVDNAIRAIQEKKPVPEIDFTIHTMEDNTQVSTLERVCKDVQAPAMYKPTDEQFFEDDTHSKPN
LQFLKQHFYREGRLTEEQALWIIRKGTELLRAEPNLLEMDAPITVCGDVHGQYYDLMKLFEVGGDPAETRYLFLGDYVDRGYF
SIECVLYLWCLKIHYPKTLWLLRGNHECRHLTDYFTFKLECKHKYSETIYEACMESFCALPLAAVMNKQFLCIHGGLSPELHT
LDDLKSIDRFREPPTQGLMCDILWADPLEDFGQEKTSDYFLHNHVRGCSYFFSYPAACAFLEKNNLLSIIRAHEAQDAGYRMY
RKTRTTGFPSVMTIFSAPNYLDVYNNKAAVLKYENNVMNIRQFNCTPHPYWLPNFMDVFTWSLPFVGEKITDMLIAILSTCSE
EELKEETPSSTSPGPASPALSNDPESIEVRRRAIKNKILAIGRLSRVFQVLREESEKVTELKTVSGGRLPAGTLMLGAEGLKN
AINGFEDARKVDLQNERLPPTHEEVVKHQEEERNTALQKAADDANNDKKLQQLSRRLST
FUN_000180 CDS scaffold_1 556621 557117 - hypothetical protein

MAAVDVRQDSFPSRSREVSGLVNGIATEITSTSFADKILITISQEGRLSQWIQVPLTGSSSGVVEMNLPSSNKGLLPSTHLTP
TTLFGGGGEERETLGQLYAAQIASQICLRSPDDRRILVLGLGLSKTDLEREAFFDLLELAQKVL
FUN_000181 CDS scaffold_1 557399 561041 + hypothetical protein EOG092610VI PF00270;PF00271;PF04408;PF07717

MAKYVPRQRKHKVLARERAKENAQHEVQDNPNQEILLPTAKADREAKKAQMKAELRQEGAKMSGKKAKRLEKYIEGKLRKDES
RELMAKLAEQQIDTTLFSSSRVLGQGRETKKEALRRAMREEKAGLEGGEEERNEILLEKRKELNADEEISSDESEESEPDEQK
SSKPTPTPAPAPTPTPTPKESDQPKTEISAPTIGSGLKRPLEVDDEGRPVIKKRQKRGGVKSKFSLAPIETPLEPESEEADSV
ASDSDSDEWNGFSDDSAEKDDIAEDDSESEEEDDEESSEGSEESDEDMEDADENKAQRSSSFKAWAHQQRNEALGYQSIEGTT
ANLEIPKPENFVPRAPEQDPLPMELQPTQNINRKVYSVTVTRIPEVQEARLKLPVVSEEQRIMEAIHNHDIVVVCGSTGSGKT
TQIPQFLYESGYGSSDSPTPGMIGITQPRRVAAVSMSKRVGEELGDKSEVVAYQIRFEGTVDPKTAIKFMTDGVLLREVAQDI
TLKKYSAIVIDEAHERSVNTDILIGMLSRVIKLRAELAAEDPTVKPLKLIIMSATLRIEDLTMNPTLFATPPPVLEVEGRQHP
VTIHFSRRTQHDYVEEAFRKISRGHKKLPPGDILVFLTGRNEILELSKKLKATFGGPKTADGPKVQISASEAPIEVEDIEFGD
VDDRNGDDFDEIPTDDEDEDDEDEFQIEEDQEVAPLKMRVLPLYSLLPTREQMRVFEPAPEGTRNIILATNVAETSLTIPGIR
YVFDCGRSKERQYDRLSGVQSYDIGWISKASANQRSGRAGRTGPGHCYRLYSSAVYERDFPPFTDPELLRMPIEGIVLQLKAM
NLQHVVNFPFPTPPDRRALAKSEKLLTYLSAISSTGQVTQIGQTMSVFPLSPRFARILLVGHLHDCIHYTIALVAGLSAAEIF
IPENQAIPALAAKDETAIRTTSDVIAEDRQANVRKMFNEVHRNFCYLDDKSDAIKLLQVVGEFAHEPTEEWCESHFVRYKVLK
EIQQLRRQITELLRTNVSAFTNLKYQDKLDPPSPKQVAALKQMVAAGFVDQVAIRADLTPNPPEQFRKPRRSIDVPYIPLIPI
HAGKEVESDRAVYIHPTSPLAHISIQECPEYIVYSYLQRAAQSVDAEKRPKTRMHALTDVTGGQLAGLAKGTPLITYGKPIKE
LKPTTGTDGAAVRECFVVPYLRAESTGGQGWPLPAKKVKQRKVPGKGWVVE
FUN_000182 CDS scaffold_1 561322 562382 + hypothetical protein PF00106;PF08659;PF13561 ENOG41

MSERSNALFPGVALVTGAASGIGRQVAISFAIEGCRRLALFDKDSTGLNDTKATIKTTSGDANPDVFIRHVDNLDTYEVSRSM
ELAIKHFGRIDYAVNCAGIYGPTKQSHEVTPEEFDHVINTNFRGLWLFAREELKYMTSQDVGVTHDGRPGFRGSIVNVGSNLG

LVGKPKTPVYSASKAAIISLTRSDAIDYAKDQIRVNCVCPGVIETPMTANVPEDDPAVQTAVMKRKGTPQEVADAVLFLSSPK
ASFIQGAALSVDGGYTIS
FUN_000183 CDS scaffold_1 562502 563509 -
hypothetical protein EOG09264S40 PF04934

MSNPNDPPLDEIQWRSPMAIQQMGGLHNNTILFYFAESPFFERTSNNAVVYNQAMNVPSMYPVIQTREAFETHLNTMSGLEFR
VVEEPAETGPGAGTGVWVIRKQTRRKSAYDDDEITVHATYFVVGENIYMAPTLSAILSARILTISSQVTNAVTTAESVRKWGP
SMGNFYQLPSAKTSTKPRIHTSAAATPMPVSDEASKAPAAPTAAAQKDDEKSLERLAEESFMIHMKYGGEYIDENPITGRPGE
FHLTTTGRKPPQIAKTDGPIRSINTPTINTKVDDKKESKERTPKSATAPKPRRKKSKMVNSTSTPAAS
FUN_000184 CDS scaffold_1 563851 564610 +
hypothetical protein PF10159 ENOG41

MDLLSSIRKTGSRGGVNFSWDEVASSSHRENYLGHSLKAPVGRWQKGRDLNWYAKAEDDSGEPNEDGETEEERRERERKEELR
KIKEAEEDAIAKALGLPPPVRNSSGANAVEVDPSKRKVGSESGPPEEAGNEKRESSRRHDDTERRERRRHRSHDRGHDRERRH
RRHRRSRSRDRDGGRERDEDGPRHRHRDRDGDRESSHGHRRHREAHQRGGPKEDERRHRDRSRERRRSRSRDRRRRSASPRPH
RRD
FUN_000185 CDS scaffold_1 564833 566440 -
hypothetical protein EOG09262A8G PF00764

MSKGRVCLAYSGGLDTSTILKWLILEGYEVVCFLADVGQVEDFDAVEKKALALGAERMIIENLQKEFVEQIVFRAIQCNAIYE
DRYLLGTALARPVIARAQVRVAEKYTCNLLSHGCTGKGNEYDTFHYEKILIHRSLTEPLSQVRFELAFKACNPSIKVIAPWRL
PEFCEKFQGRQDLLKFAAENNIPVSSTPKAPWSQDENLVHCSYEAGILEDPDHTPPKDLWTQTVDPLEAPDKPLDFTVYFEKG
LPVKVATPDQEATDSVELFKLLNKIGHDHGVGRIDIVENRWIGLKSRGCYDTPGLTIARLAHVDLEGLVLDSKVRKLRDQFVT
IEWSQCLYNGMYFSPEREWLDEAIVSAQKGVNGQVRLRAYKGNVYVLGRSSDTSSLYSQEDASMDSLDTFSPMDSTGFIAIQS
IRLEKYGAQKAKDGQPLSQS
FUN_000186 CDS scaffold_1 567841 573198 +
hypothetical protein PF12449 ENOG41

MDYSRLRAAALRDGEDEEAVTVDTRALIDKVLARYSGEWTTLRELIQNAADAQAKTVSVKWETLPSTQVPLPNTTSRSEILKH
TLSNHTLRRLVVQNDGQPFTKTDWGRLKRIAEGNPDETKIGAFGVGFYSVFADCEEPFVSSGNEAMAFYWKGNALFTKKSQIP
ADQATHHTTFVLDYRNTTTPLPNLLSVSQFLATSLTFVALEHIEFWIDDYQILSLKKKSSPSVELPLPRDLEARTKDGLMKVT
SVDRTSTQIDASYMAAIGWKPQATTSTTKNESYGAPETPSLRSFFARLTSSASQAGIRTKAQHEENAAQVTISQDVTKLSSST
IFLRATSASIRTSVASSFASELERATKKPPPKTTKIAILTSSYDETQASQEAATSGALGKATDVFASVLPNKKPGGRIFIGFP
TMQTTGAGLHISAPSVIPTVEREAIDLNARWVRTWNLEILRAAGIITRLAFANEMSDLSSRIKQVMAKETKFSPAVIAKFMPE
ALHIHKTFTFEDSTPSSQVGQIIEEAFWMCFKKASIETYSTRGVLQTVDVRIASDEMSKFVNSIPVVPEEMKGVPFVKKLIDF
GLISHITVTDVKKELETKAMNKVQLMNFISWAGKKSLSGELDPGSRSALLEVAVGTMSDDGEQGEIIALGSIRNHLIANRIPA
NLPIPPTTIPHALTANSQQAELRALGWEPLEILPWLRFLLDTNSSRSEEQNLTQSSKFAIQVLTVLSKNFENSSPNARTTIVS
LLQANTVVPTKQGMRKPTESFFPIVKLFDDLPVIQGCEKIKEKFLVIIGVRKTVDLETIFTRLLNASSEGHQKWSHMELIKYL
ASVREDIPGEDLSKLKQTRFCPAEAGPKGMESTKGTTTLYKVSELFEPKDSLRALRLPILQWPGPPGSYRPGSPEARFINSLG
LRPYPSVPELVELMSGKDESLRVSSMTYFLANHQINGYGSFQLGGSPKAIMPLQGSTRLVPPSQCFYNPRASILGFNILRRDL
HDHAIKFGVARDPPMVDCVNRLLASPPQDHQNAVALYSYFASRITELGENNLVKLRNASIVPITRTTRSSEKSTSSMTHVSPS
RVYLGASTTYGDIFDFVDFGPEANAFLFQCGAKSEPTKLEVAQMACSEPARLLGVLQSSEKYLDLLKSLAEASSTLHRDKELW
RKMRTAPFLLAYKELAPPKGKSDDLEEEDEPIRQYQLASAGQIVILDDIISYRLFKDRLICAPEEDALEEFYLALGAQKLSSV
VQEEVRIGPHSDKQKAALSLRKHVVERSKIFLFEYTNYRRDAIKHDVKWLDKNLTVHIVRSVALRRTLKGQYQTHTEKRSAAS
EKTSNGWVLYVSDESKPDMYQIGQAICQMLLNRPNQQAYLFFEPFLTLDLYGLRNRGYNVDRILRAKAAEARIAEEERRKALE
EEQRQIKEREEQWGAEAKAAEAAREATRIPEPLGPTMPGSWDSPEETKRSPEQQARKSRGLFSNISRRLGFESDQHEDESHKE
LDKFLDRPREIEASGGSSSNTGKKPQQDEGRVTSPALVQQNLLNAIKSTRAYGSDSVFSEPKVNEVKEQATYCDKTPAQNITF
IAETSNGMKIFFSKDVKDPGSFLSKHLNNLNVFSSMLIEVGNTYSMSPKVLHIFYDEAGGTIAFNTGGSIFCNFRFFLQLHAA
QIEGRSGQAKAEAATWWWVVLAHELAHNLVSLHNADHSYYTESFIQQYFPKIVAKAVQWTANAGSRSETPTIAPPPSGAPPPY
PGQLGRSLLD

FUN_000187	CDS	scaffold_1	573833	574207	+
hypothetical protein				ENOG41

MVHRIAFWSLFGLGARFWQMGIEMRPFFNKSSLWVYPVYAAGGASFGYWLQGVDDSQTSTLQERKALLLEKRARKAERDAKAE
A
FUN_000188	CDS	scaffold_1	574624	576033	-
hypothetical protein	EOG09262JZW	PF01008

MSTEAEGSVPAPQTTENNDVKTEKKEQAQPQKPKEQQQAALVGEKKLSGAELKKKAKEEKAARRAQAKASQASQGPSQGGQQA
SGDGKGGKAKPKQDGQHQHQQHGGAKLPIRPAAATAVVKDDKPSIPDCFSHLSMARRIEMTNADKDVHPAVLVLGEHMSAFAI
SDSVTRLEATLYAFQKVIDSYTTPPGFTFSRHFTPHVLNPQIEYLTACRPMCFSMGNAIRWLKLQISKVDVDLHDNDVKKLLK
ESIDNYIRERITLADYVIVETAANMIGHGDVVLTYAHHHLVERTLLQARKNKTDFRVILVDDPYERVGIDLAKRLSAAGIQVT
YASDLGALRTHLYEATKAFLAAEAIFSNGAMYARAGSCDIATAATDLNVRVVALCETVNFTERVSIDSLTYNEIDPERSTDVG
FRLLFDTTRDKYISVVVTELGNSSATSVPAILRKLEEL
FUN_000189	CDS	scaffold_1	578435	579849	+
hypothetical protein	EOG09262528	PF01960
T05
MMKKFARAYSSASATLGSNPVPPSKIQYIPTSGKYPKGFIASGVLAGVKPGNTSKPDIALVTSDRPCAAAGVFTKNKFQAAPV
TYSRKLLQQKKNAGLRSVVVNSGNANAVTGTGGLEDATSMARTTDQRVGDEDSTIVMSTGVIGQRLPIQKILDKIPTAVSKAG
GSHENWLECAKAICTTDTFPKLMSRSFELPSSPGVEYRIAGMTKGAGMIAPNMATLLTILATDAPISPDVMPNVLRHAVDRSF
NSITIDGDTSTNDTVALLANGAAGGKEVTSEQSPDYQAFQKLLTEFSVDLAKYVVRDGEGATKFVTIRVVDSASEEAAREIGR
SIAKSPLVKTALYGKDANWGRVLCATGYALISPPGQPVNDVPEITPERTNVSFVPTDGSAELKLLVDGEPEQVDEKRASEILA
MEDLEIVVKLGTGDKSAVHYTCDFSHDYVTINGDYRT
FUN_000190	CDS	scaffold_1	580153	580895	-
hypothetical protein	EOG09264TJN	PF00475

MASSQPTRWAALARDTNETKIQLALNLDGGDFPPDTDSRLLNDGDSHASQASKSQKIAVNTGIGFLDHMLHALAKHAGWSLAL
NCKGDLHIDDHHTAEDVCIALGYAFAQALGTPAGLARFGYAYAPLDEALSRAVIDLSNRPYSVVDLGLKREWLGQLSTEMVPH
CLQSFAQGARVTLHVHCLHGDNDHHRAESAFKALAVAIKAATTRIPGKEGEVPSTKGTLSA
FUN_000191	CDS	scaffold_1	581349	583260	+
hypothetical protein	EOG09260S5R	PF12895;PF13181;PF13432

MENFLREWRQDALNKAQYESAIFIGDKLLALTNDDQDAFWLAQVHFATGNYTRAQAFLSSQDLIARNPSCRYLAGHCLIKQSR
FDEALSILGDRNPTHLIANGTSNKRKTQPRHGRRRDPAAEEEAANRRYEAAMCFLRGICYAKQNAFDRAKECYKDAVRIDVQC
FEAFQQLMNNSLLSPDEEWQFLESLDFDSIHVSGDVSSSQQAADFTKMLYTTRLSKYRNPAAFETAYDSLSTHYHLASNPDLQ
LARADLLYTQCRYRDALNITNGILQEDKYNFSVYPVHLACLFELKEKNLLFLIAHDLADSHPEEPCSWLAVGIYYFSIGKIPE
ARRYFSKASMMDAHFGPAWIGFAHTFAAEGEHDQAISAYSTAARLFMGTHLPQVFLGMQNHALNNMTLAEEFLKTAYGLCKTD
PLLLNEMGIVKYHQDKPKEAAQYFTAALKIADDMDSDPSAWLAARTNLGHAFRRLRHFNRALAEFDEVLRQGGKDAAIFSAKG
LILMEQSKPEEAVVVLHEALAINPQDSIATELLNKALEETALIDGAAESEAEDLLDFERTLGQRKHDAAVKVNGGRKTAGRTE
KGKGRLRRTRRMTVLEDEDGSPEKEGSMMEMSDDE
FUN_000192	CDS	scaffold_1	583813	584323	+
hypothetical protein		PF08560		ENOG41

MSRFFPHTPYAEDQPLARTILTTHVIARAITLSSIIATGITTTRHFVPFLRPKTPTLTFFPRLIRSASTGAVAAVGVGALMTI
SRMRGREEVEWQDRSWRLLENRGQLETDDWTAIGAGAGALIGANALKGWRGAVGGLGLGSMGGALAWMAWRFGVNGGKFPKKE
EDL
FUN_000193	CDS	scaffold_1	584363	586660	-
hypothetical protein		PF00501;PF16177

MDLTKTPRKVWEHPNPKSTAMWAFMQEANKLHGLNLKTFNDLYQWSVSKRSDFYAQLWASQNWIHEGSYSYVVDESIPITKLP

AWFPGIRINYAENLLWTGAIGGAPGERSTLHKEDDTIAITEVREGNSSVKNVTWGELRRRVGELASALKQRGVQKGDRVVMVG
AHAVETLVVFLAATWLGGLFSSSSTDMGVGGLLQRTVQIDPKFVFFDDGALYNGKVIDLRDKIEGVVEGMKECPSFQGAIIVQ
RFDSPHDTSQIPKTQRLESFLSSAPSTPPPIERVDFQDPMVVYYSPGTTGTPKAIVHGVGPILVSVAKEAILHREITPKDVAL
QYTTTGWIMYLASVTRLAFGGRTVFYDGSPFIPDRSVLLRIVEEQRVTNLGISPRWLGELIKEGIVPQKEADLSSLTAVQSTG
MVLKEQVFDWFYDGAFPTNIKLANFSGGTDIAACFVMENPLSPIYAGGCPGRVIGTPMAIYPSSPDLDRPISPVPDGEPGDLV
GTAAFPNVPLYLWNDTTPAPGKKYTSAYFDRFPNVWSQGDFAAVHPQTGHIHILGRSDGVLNPSGIRFGSADIYAVLEGSFAK
EVAESLCVGQRRPQDHDESVVLFLLMKPGVKFNAELASRIRERIAKELTKRHVPKYIFEVPDIPTTVNGKKVELPVKQIISGS
NIKPSGTLLNPQSLDFFYQFQKIEEIDQVKAKL
FUN_000194	CDS	scaffold_1	587320	598852	-
hypothetical protein	EOG092605T6	PF00454;PF02259;PF02260

MPIFFTILRGPCIFQSNSPDQKLRMAILEVIHRLPTQPAPPEPFEPHAEETVDLLMQLVRTDNEENATICVKIISDIMRHQHK
VLQGKVQLFLTLIQELFEQMEKVVREQLDNAPHNAPSQPGAPSTPGNSQTNFQSPRPGSPVASVNDLGTDTQQQNRPLLKGMH
SFKVLSECPIIVVSIFQVYRNTVQQNVRKFVPLIQNVLCLQASAQQQAHADAAAKGTIHTGVSPAIKNRVAFADFITAQVKTM
SFLAYLLRQYASHLNDFLPRLPDIVLRLLKDCPREKSGTRKELIVAIRHIINYNFRKIFIPKIDELLDERTLTGDGLTVHETM
RPLAYSMLADLIHHVRDSLKPEQIRKTVEVYTRNLQDNFPGTSFQTMSAKLLLNMAECIAKLPNKVDARHYLIMILNAIADKF
AAMNRQYPNAVKLSALYREQMKAGTPETYLADMDSPPDWDETDIFSAVPIKTSNPRDRGADPVVDNKFLFRNLMTGLKNTFYQ
LRTCNVGSPIDAQNAPTHWQDVSYGFTAEEVKVIIKLFREGAYVFRYYEIEKPAAESQYMSPVEYMANFYMVSCSKEEKDLLE
TFATVFHAIDPATFHEVFQQEIPHLYDMIFEHTALLHIPQFFLASEATSPSFCGMLLQFLMERIDQVGSADVKKSSILLRLFK
LAFMAVTLFANQNEQVLLPHVVNIVTKSIELSTKAEEPMNYFLLLRSLFRSIGGGKFEQLYKQILPLLEMLLDVLNNLLMAAR
KPSERDLYVELCLTVPARLSHLLPHLSFLMRPLVVALRAGTDLVGQGLRTLELCVDNLTADYLDPIMAPVIDELMTALFDHLK
PHPYSHFHAHTTMRILGKLGGRNRKFMTSAVPLTYREYADDPSSFDLRLLGSKKDRAFPADMGIDFAIQKLMEFPKATKNNHN
KQYDGYYKKQALHLIKAQLKLRIGYDQLPDDLPRLLRLQAEDLVARRYEINTANFEISDRERSIPKKESEDLVVKRLLKAIMF
AHSFPEFKDEASTFLLNVCKHFAIIEIGRALVDLKRTFSPFDPNAGEGPLHIDTRNLSDAIVESLASDHPDVREAGKSAIREM
YDATAIIFGSESDVGKLPFFSHLSSTFCHSCYEEEWFTKTGGSLGINYLLTELDLGDTWITSKQTEFIRALTYVVKDMPQDLP
EKTRCLAQTSLEVLLKRITKDITKEDTLPITQQPGQPQNPQLKQPRLSQICQQFGNDLYHMNKHVRETAKHSLELIATAAKCE
VWELLEPCKDKILQPIFAKPLRALPFSIQIGYINAMTYHMSLKNDWVPFDENLNRLLMESLALADASDESLANKPAEFRTHEH
IVNLRVSCIKLLTTAMTFEEFSNQPTKTKILSVFFKCLYSESKPTIIAANDALKSVLSVDRRLPKELLQGGLRPVLQSLSDPK
RLSTAGLDNLSRLLKLLTSYFKVEIGARLLEQIDSIVEPSALQQISFTFFDQHPQMKIITAILTIFHLLPAPAEAFKERLIDC
FLGLEEKLRRTQLSPFRLPIYRYMNRYPKEIWAYLFGKVEDLKYGRLLAQVLAHADSSALREVAIENLDGLITRCNELISQNN
EAKFIAMVNAIHIFESLSHFPSAEKWMDKKEHLDWLKSIGKELETHLRQHTLPAHLRLPAYQAAEELMTILVKSLERAPKDLD
PLFNLIECVTSDELRITQELFSFIYQKIICSDAVDFWKTIVLRCLDTYAGKSASNKMKHFLLHYIVNPIVAMDVMRNWKQLDQ
NRTPRLMDRTVIDAVSSKIWKVQPESTTDDQAQPGIDHTRYEVLQLSAMLVKYYHTPLQDARKDIIKFGWTFIRLEDVINKHA
AYVVIGYFIAHYETPPKIVTQVYLSLLKANQNEGRALVTQALELIAPMLPKRCGTGQNERVAAWAIAPRRVLVDEGQNAQQMT
SIYHFLVRHPDLFYEARDRYITLMISSLRKLATPQNASHESKRLCLNMMWLIWTWEERRVEGKSAAFGARSLSQSPNTKKRKL
ESDQANTSSQPVGRAEYQIPPLFRLKMIKYLVEFIAQLNERYELPSAKPRDHVSSSIPPVPLALTDLCKKAMKLLYNLVQPQY
WGDLDLDLFPNVTQVILASERTQTILTADPTDKEKFDDKFLTNIINTLQVVRIVLNSKSDEWIQKSMPAIQKVLEKCLKSENP
EIQDCLHLSDKKFDDNRELRSIVKRILDSVPDDTPMEDADADGETETQTSEIVAYLSQVATEAMNSSNFTSGVNILWSLGQRK
PNVIDQHINSLMKALQSKHAREHVQHYNAIANQAAGVNRNQDPNTPSGEMSAYDLEIQTKIMIKEIQVVALRMEVLGDNRRPF
LSVLATLVEKSMSIELCEEILNMVEGWVFRSEGTWPTLKEKTAVLHKMLSFEHRQDPTMLSKFLDLVIRIYEDPKITRTELTV
RMEHAFLIGTRAADVEMRNRFMAIFDKSLSKSASARLSYVLTSQNWDTLSDSYWLAQATQLLLGAVETNANIQLYQNDFRTLP
ISRLLTIFTKEMEAREPTVIIDDKFESFMATHRRFMSELGDIKVKDVIEPLVQLQHVDSQLAHNLWVTLFPIYWSSVAKDDRV
DLERGIVALLTKDYHSRQIDKRPNVIQSILDGAAKTWPECKIPPHVLKFEAKTYDAWYTALVQLENSAIKPQVDSATVRESNL
DALVELYASLQEDDLFYGTWRRRCQFVETNAALSYEQNGMWDKAQKLYENAQIKARTGVIPFSQAEYMLWEDHWVLCAQKLQQ
WEILQDFAKHENFQDLLLECAWRNTEMWQDEQHREALDNIIKGVMDAPTPRRAFFQSFMSLLKFHNQKEASTDFARVCDEAIQ
LSIRKWHQLPERLTNAHVPLLQNFQQLVELHDASVICQSLANTNQTNLDVKSGELKLLLGAWRDRLPNVWDDITAWQDLVTWR
QHIFSLINQTYLQLLPQQGQQNAGGASSFAYRGYHETAWIINRFAHVARKHNLPDVCINQLSRIYTLPNIEIQEAFLKLREQA
KCHYENPEELSSGLDVINNTNLNYFSPSQKAEFYTLKGMFLEKLKQKDEADSAYGTALYFDIGAAKAWAEWGYFNDRKFKEDP
TDLNAARQALTSYLQAAGSYKNAKSRKLLARILWLLSLDDAKGTIALGFDDFKGETPVWYWITFIQQLITGLGHKEAPRVFQL
LLKIAKSYPQALYFQLRTNREDLLVIKKNQEARDRTARQRAQSVASNGKASASPSMTKQDPPRPGTASSRPGTANATDGTPVK

AEGEANGSNAAGTPAPTNGQTPDPSKAAQKATPQPGQPGQPQKKPPWDYTEEVMSVLKTAFPLLALSMETMVDQIQKNFKCPP
DEDAYRLIVALLNDALAYVSRTPPSFAKNVKLPAATETNITRFAETILPSHIKKSFEADFVTVKPTMYEYIYKLRRWRNKFEE
KLDHRIPRAFLENFSPHLSEFRYGKFDEVEVPGQYLQHKDKNQDFIRIDRFLPNIDLVRSISASYRRIKMRGHDGSIHKWAIQ
HPAARHCRREERILQLFRSLNQTLGRKKESRRRDLQFTIPIMVPFAPHIRLVQEDTSYTTLQGVYEDHCRNMGMSKDDPVLFT
MEKLRGALESKSSNKPEQAATARLEVFNAIQEKWVPSTVAIEYFQKVFPQFAEFWLFRRQFSYQLAALTFITYIMYMHNRYPQ
KINISRATGKVWGSEMMSYMSANKPFFHNPEPVPFRLTPNLQTLMGPLATEGIFACSLMAIARCLTEPEYELEHALTLLVRDE
MLFWFTGSHRNGVITEGQLRDSVQVNSESIVKRALSLAHSPVGNLPANQTVIDAIAKAVNPMNLAQCDALWMPYL
FUN_000195 CDS scaffold_1 600090 600756 +
hypothetical protein PF06094 ENOG41

MSGDFTAFFYGTLMAPEVFFTVCYGNRDPPKAIKDMHTFTPAILDGYCRHRVESADYPAVVPEKGHTVLGIYATGLTDGNVDK
LDMFEGSEYFKEKVKVKLLNKDGTAPEKGEAKETFVYVFNNPDHLEKGEWDFEEFRKSKMKNWTRAGLGFGEDIPEGTANNEW
DEWK
FUN_000196 CDS scaffold_1 604435 605665 +
hypothetical protein ENOG41
AA11
MFAKAFTIATLASFASAHMLMANPKPYGNPNNSPLEADGSDFPCKGKVNDGSGDNVYKQGSTQELSFTGQAVHGGGSCQVSIT
TDKNPTKDSVWKVIKSIEGGCPSRTATGNLGGNADAPNPDKYDFTIPKELAAGEYTLAWTWFNHVGNREMYMNCAAITVEGSG
GSKDLLNSLPDMFVANIGNKCETLPDTDLAFPNPGKDVSKLKSKLDGPSGTGCQKAGSGSGDGGSDGGSGGGDSQPSTPVAAP
TTNNGAQPTQPAATPAPSAGNGSGSGSGSGDGSNGTPEIPGGAFITVSQPSASQPTATQAPATPETPQDGSGSGEGSGSGDGN
QSGGSGSGSSGFAAGTACTNEGEWNCIGGSTFQRCASGAWTATQQLSSGVSCTPGQGADIAMTTKRGKRNMRRAQRA
FUN_000197 CDS scaffold_1 606138 607491 -
hypothetical protein ENOG41

MSPAFAPRFKNESVFDLSRDLNRNLITTSLYQDHLYVAMANLTSGTILPPWISQEYFFQKYELQDYSMNRTGDIYTVSTRGYG
AAANCTTVSASKLTTEYEIPEDWPTEMMNLSQCTTDAQFVAAAVPVIRSAANNRSTGISSLEYSLTVDRTFTRSPCGRTLPLG
WARTQEAKDVNSTVDASFVICRPIFETAIFNVTIDPLGHVISYERTSNLTTTLDYDESELHTDILFQTYNSRWDQDPQWHNHS
LSMNWMNHLIMVVNGSRSAFDPNDPVPDPKELLPAVSDIYRRVYAILLGLNDHIFETSNRGEPISAIRHTKETRIFMEDASLI
ITMTILALNTIVAGLFYIRAAAFVLPRMPTTLGAVVAYFAPSRLATPAYKDAPGQSSRTLSFGRYIGMDGNVHVGIEADPHVV
PIDPSSLGPQADYLKFLRRRKKGDTNQPNNSETWI
FUN_000198 CDS scaffold_1 607867 609946 -
hypothetical protein PF11915 ENOG41

MSSWETTALRLQGNVAADSTPSSLDKSLPLLPKTNVKARTTTGVKKVSPEEETCKAKGWKPLSLSTPMLLAMIAFTILLAVAV
ETLAQRSAAQGGLALSPSLDDMPGYAKFGYLYVPTIIAVLYSMLWSWIDLDVKRMQPWFELSKREGATAENSLFLDYQYEFVA
LVPFKAAKRKHWPVFFGGTAMVIVFWALTPLQSALLGTGIIKQTDMTSLVNRSQLLPVAEHVEVLDPEFLNTGYAIGWLGQQF
PAFTTAGYALLPFYPDTSPKLASFRKSTTVSPNITAETTKLWTELNCWPAEIARIGVRHQEQFSFLNGQGCNATAGFGARNET
RMFYIGYFTSPYSDFQIANPNCGRTPDSIHQFLAIWGKAIPVDWDPSPTFNISAMFCQPQYFKQRVLATVNANTFEPDSKSIK
DLGPRETLSDNEFNRTAFEYLLANGMAETPIVKDYPFNAVVEQHPRLNHTNITFPVSNMVGFALAGKDLDKDQYVHHDVLHKA
YSDAHKYLFSVATTTILINSTKFSNNTVSVEYFMTGIIVSRAFATAVECFLLVVTIFTGFVLWFSRAAPSNLPVNPSSIRRYI
DFFSNSPDALSAFKPMDHADDEGLLEDFKMDGFQLISKNDGADVEILLLPRLRGSESYDKSIQRGYYEPVKPLALKRWVGLLF
VLVLIGAMAFLSYLKHQESSLNGSPRSS
FUN_000199 CDS scaffold_1 612966 617116 +
hypothetical protein EOG092604S1 PF00443;PF13423;PF13446
C19
MSWDERHSQESCNPQQEQWPPSDARFPWHHLVWVGQESQSSPLVKIDDRQRNKYYPLVAREPFACSAGPCTFSVTLEISEPRM
TREWIDLLLDHDTIRKNVRRAKEQDPDRYAAATDDWALQAPLNLNTYLKNLLEATSQENARSISKRNKRFFVLFGTRCFEMFR
QLEFEETVDVAEDGVDGGTFTPPIPPRSDNPSGATDLGTYRAYIEDVRSEVQCIIHRGGQPAELCTPGLHADLGCAEVPNVDS
VLVNITRYEIMGVLPNQPKEVVVNAYKRQWEMIPDKRKALIDALMSIGNDLNDEQLNEYAVTQSSIFESQVQSQTNGDDGGLT
NQALLFFELQPLNDYKAQSIVQAFRRKVAQDPSCANTARNMLMLISQASTDDNYTAELVMEYGEGFSLPTAKEILGLSDASGF

GPDTLDDVKEKIRNAKDKDAKTTYLEAMEKIAEHTNSTGLKSTVSDLREMHGIAVPDGSSATGASAQAANFDLPVGLENIGNT
CYLNSLLQYLFTVKPVRDVVINYDKFKLELTDEKIKERLLGGNKMQLDRGEAVVAQAFVQELSDLFNNLEQSDKVATRPSQRL
ANAVLLSTGTLLKETKSSAEATTESQPPPLPTRPPPGPPAKENNDVEMITVTANDSRDPVDTASTVSSQTLVEEDSDRSYDKV
ETSPTTTEDQVIDTVMEVGQDEDVVKISRTSDRPMEISEAVEKPKNGTSQDIESTDVNMRDVEEPETVDQKVLTALEHQKRSS
GTDQQDVEEVMGSIINRLQAAIRPSSVDESTGIQFEEIMKTFFVTTINYTKKFDDKTYQKEISFDRSITAFPAPKGSCSLYEA
LGRNFDQEIIEEDKLSRYTAIKTLPPVLHVLIQRSQSMGSKNGNPVLIPEILYLDRYMDAPHESLTFRKRVESWATASRISDI
KASKAAVGSTIPTGPILESIGQEEDAKGSIAAVTEDSEMTDGVKIVEEDWSFDGTVDDDYLLVGRPSVNSVTSAGLPSWPKDI
KDTETAIREKMDSELRDKEEALENYYSKLKSNPYRLHAVICHRGQLMSGHYWVWIYDFEQDVWRKYNDSNVEVKRSTDEVLNT
LSTSGEPYFLCYVRDEDKETYVDVPRRRRPAPPSDTSFENLDADGDITVTNSEPLKTSESPQELPPAYSEQNNEDDASASTEA
DVYEAPTSPTKRPMLVKNDDRTSEPPKLPHVSEKQSLDDPETEPVIHVKFTPEDCDTIAQECKRSELENPDRAIRAVKKRIKL
KVKPRTNPPADARRHGPRGMAHHIIEAAKLREKLKKATEDEETKKL
FUN_000200 CDS scaffold_1 617429 618439 -
hypothetical protein PF00378

MAPRLSQPVVTLRLLRRYYSSATEPLIRVTNLPAPNTGHIRILELNRPSARNAISKALLAKLRAEIDAVHIQYGPNGEELPLQ
KRFGGAAGVDEKGPTRAVVLASAVDTSFCAGADLKERKGMSQGETAEFLTNLRNTLTSLSNLPIPTISAISSVALGGGLELAL
STHFRVLSSNATVGLPETRLGIIPGAGGTHRLPALIGLSRARDLILTGRRVGAPEAYFLGIADRLVEIVPEDERDGSDVLGEA
RKAALSEAVRLAQEICEGGPIGVRAALQAVAWAREEVENKMYERVVNTEDRNEALKAFQEKRKPIFTGR
FUN_000201 CDS scaffold_1 619220 621330 +
hypothetical protein ENOG41

MASASVLQSLLTSTTLASSVPSSTASSTPLTILIRDSIEGTSDFFPFLDIAGLGSGSDDVDSGLTFKELFATSLDGNQEITLF
SSGCQVNGRTDCTRACNNSSLFFSSLETFYNCAALAAAAHWTQDAKSYYVSQETERNASAIMGSGTLSEFDGRPVLRSLIACA
QDSCDNDGLSKPCNRTIDRLSYDTSTPEEIFEALDEFCPELPAEVNPDIFGPGVLISYVLQVCFAASLYLLLKGFTLWVTITQ
DTSRRKSEPKPYRLKRSLTRIESMIWRDSGGLSRTSIAIATTLVEFQEAQCWFVFAVQIASILAIVVNSQEGTFWGEIVVNAA
IAFHVSQNGILPMFLVQVCLHHEGIRNWHTFLGFFMEYLLAIVATSQKIYFKDAFALFKNQNQIEACGFNPSPRTYCAATQGI
DGQSFSFFPHPLLYKMTFLILDTIAIIALVADQLAWTLRKHHLTKHVQIGSYRLGRWPRWGKQKRGLFIKIWFWRILEVAYLV
INILYMISLTKVINAESFAANRWSYGQIIAVTVWGPVIVKLFDLLLSGPPKNGMRLNSGPPRLRIDNVINGRVGTSAVEVIED
DNETDEKCDEPAPRARDEGFMIRQAATMQLGDSNHDGRDIEGEAIETGPKAASPEGISAERDVKGTTK
FUN_000202 CDS scaffold_1 622205 622643 +
hypothetical protein ENOG41

MSFLTENVIRRVALAPRVIAFQAPRTFTTSFALQRTATDAVKDTAKKVDRVVSDKIVDGIEAGENVAAKAKDVAQDVTGGSAT
GKAQELRGEAAGKAQELKGEAKGAAEQAKGKVKGAAEEVKGKL
FUN_000203 CDS scaffold_1 623086 623960 -
hypothetical protein PF01184

MSSGTPPSHGATTPTQYTDKEEGHYHCNHPNHYYEERNRNVQKPHQSMVSQVYNPSFFKVANPGPLGLISFALTTFVLGLYQC
GAGLPGSNPFGGVGPDQAIFGLAVFFGGIAQLFAGLMEFRVGNTFGTTVHCSYGAFWLAFAMFFVPDLGIKDAYKGDDRAYSF
ALGIFLILWCFLTLIFFVAALKTNIAILIVLGFLFLAFLFLSLAQFLQTTHPTAAIRINKTGGAFSCICAFAAFYAGAAGIMT
EDTTWVRFPLGEIDVQPKKTNIA
FUN_000204 CDS scaffold_1 626233 626902 +
hypothetical protein PF00226 ENOG41

MKSDAPYTKHRFYQLVKVYHPDRHSHAPDIDNIPHKVRLERYRLIVAANDLLSDPSKRQLYDVHGVGWTGGRPQTLNETVRSA
DRAWRHRAGSAAHNATWEDWERWYDARDGRVKDPLYMSNGLFATLVVVMCMVGAFAQMSRADQYGADLVEMKNQSNLAIGQQV
ARRNTIAAGRSKDERVDMFLKDRENLNYAFQPGKYDNEVLQAAHGHGPSHEHNGKV
FUN_000205 CDS scaffold_1 627527 628379 -
hypothetical protein ENOG41

MATMIPADVTVPAHEDRDVPRTWQSSHGSTFKTVVHGDRDSESGVMTETVWETMNPYSSDPDATPVATTNNYTTYSTATPTSS
QSSGSLASTLHHSGGLSDGAIAAIVIGVLFGLAIAALATWLLVRKSRRRSRVTQVTPLQTPIYSPSPPSSPLPTPPPLTESGN
DRISSLEDSWVSYGDRVRSPTPQPAVAEVRSFTRAWQKPRPYTVSRGRMTEVHGSEGSESPPSPDDDMSPISPLTRAGSSQAD
RRVVSPVSLVSRFSNVKQD
FUN_000206 CDS scaffold_1 628743 630597 -
hypothetical protein EOG09262MFL PF02637;PF02934

MSRISTSILSRYLLKGQISRQGCISARHVSRHTSLPSAAITIANTTRRLHAAPEVVTPPPDQPVPLRKQLKDEAKKAKKQGKK
KPKGDSQTVDGWELTVGIEIHAQLNTARKLFSPAITSFNDEPNSHVALFDVAMPGSQPLFQKETLIPALRAALALNCEVQEVS
RFDRKHYFWWDQPSGYQITQYYEPFAKNGYITLLARDGISPQDGERVTIGIKQVQLEQDTAKTLAQAGKVQWLDFNRVGVPLI
EIITEPELHHPRTAAVLVRKIQMLLNTVDACVSGMETGGLRADVNVSVRRTDGSNPSLGTRTEIKNLSTIKAVEDAIIAERDR
QIRELEAGRSIPSETRGWSIGSTETRRLRGKEGEVDYRYMPDPDIAPLVIGNDLVGHLRQSLAVTPDSELDTLIARFGLTAKD
ALSLINLENGSRVQYYYKVLRSIQDKLSEDLSEAEMPEFKSYSALVGNWIIHELGRLTTYKAGPLAARDLTFTPEGDCEQVPD
SDLSQLLYHLIRKQITGKVAKELLFAIYLKEIEGGVTQAIEENDLWFKEIPEEEYEQLADEAMEGEHKILQEFASSETFPQGK
LMFLVGKMMRLGPTERIVPANAERVMRAKVEGLRNP
FUN_000207 CDS scaffold_1 631888 633061 +
hypothetical protein ENOG41

MSDRAQTRPRPSWSSSEGAIQEAVSQQSKVEDYEETNSQSPSEGATHYPTPPSSSSKMEHTRSNVQRTVSGSAVTQLTSTDDT
AVTKPTSNKRKMLREARNMKNKGAHRPASISSSGPDPHFVPQEAFASSPESSYFKNTPSGRQAAPSAYAPTTVAVANYPQSPV
SPMSYAGWGPNHTPAGYPPSQDPFSPQQSGFPLIEQPYHPGFTYVNQKASTLEPPFVDQNPSYDTSYPETQDEMAAPNGELPI
EALHGYASIAARISGRAEPQLIPIYRRFDWLLHRNLLRYQDQLGMLEEELLRMDSITTRLGGHIPVSAREERRFGRQIHKDRV
DLMERVDIILSKYSKCIKDSGTTSSNWVRNGDVDTGRHAKTTVANERRNTGLQNLSEP
FUN_000208 tRNA scaffold_1 633679 633769 + tRNA-Ala

FUN_000209 CDS scaffold_1 635543 636451 +
hypothetical protein ENOG41

MAVLKLKAIAIILALCAASVVATGGETATCLSDHTTTETAVTDAGYTISDGTLVSTMNICTVTHETKCDHVASVPYSVEMHPS
SAAGMPSAPTGATAPADSSATSVGAQGVSDTTEAGPPAEQTGAQSTGAPVVPVVPVPPSSGPSYVPGPGTASGEQGTNPSPTN
SASVENPSPSAVVSATDVPGVQTTTAAYGSSVTDTAVVVTDSETGYETAISSTATDTYPSSAEETDESTPTGTPGATASAAAK
MLAPGAALSIAGMLIAVIF
FUN_000210 CDS scaffold_1 636823 641046 -
hypothetical protein EOG09260EQD PF00637

MTEQSPQPSHELAHDTDAQQPEHEPAVDANETFPPVENKAPDDVAHEKDGKSKDATGKEGGAEARDVKNGDSNDGKSGHDENN
GEEEEEDDDDDDEADDDDEDDEDDEDDDEDEEDEEPRLKYARLTQHLNGVYRNGDATSAFLVAGDKMIVGTHNGNIHIVQLPI
FQSMRVYHAHSASVTSISISPYPPPLPTEKKPEAAPRHTTSSNATVSSGRQADASPAAASRRPREASQIPRTPSNDIYVATSS
LDGNICVQSLVDMKDVQLRNFARPVQAVALSPEFKTDRTYLSGGLAGQLILTAGGGPGRSTSTTTGTAAATASGWLGSMGLGG
NSGKDTILHSGEGTISTIKWSLSGKYVVWLNEHGIKIMRSKLHLESADIEDAWKRVGHIDRPQTDEWETMASVWKGRVEWIDE
QAVEVDESDQSTVDKGISPAAHKLREQAVMGKKSIERLLVGWGGTIWIIHVHPGGVGVGRHAGEKTVGRAEIVKILRMDCIIS
GISLYTHNLLLVLAYCLPEDEDEDEDSGSVSNSPDKKHKSNPSTGSEPSGGVRRRQNNQPPELRLIDLNSQAEADKDSLSVSR
YERLSSGDYHLGVLPARNAASAIASSRGALEAIAGIGTDMWNAAINPRSLFSSGASIRSRGSGDDSSLIGSTAGTIRPGTNQR
LSPTVHSGLVKPGVKIFIHSPYDCILATRRDLSDHLGWLLERQQYQRAWELLDEHPEIMAPLNERANDGTPSTPTLNQEPSDE
FNDDESVVDSQLRDFYSSAEREKRRIGELWIQELIEENNWASAGKVCGQVLKTPDRWEKWVWTFAGAKKFDAITDYIPTEPMH
PPLPSTIYEVVLGHYIQNDKPRFRELLDRWSPELFDIKTITTALENQLKYRDVREDSIDEGERGRDWRIVIESLARLHEANGR
HREALKCYIKLHDADSAFRLIRDNHLAEAVEDDIPSFIGLRVPSGKVDEMTAEELDEATSEAITLLVDEAQHGLLRPDIVVEQ
LLSKKLNLYIYFYFRGLWKGDGIQEHSGENVDRLVMDSQSLVDNFADLAVHLFATFDRALLMQYLKTSVSYTFEKAVQECETF
SYYDELVFLYSKTGQMKRALYLIIDRLKNVHKAIEFAKEQDDPDLWEDLLKYSMDKPSFIRGLLEQVGTAINPITVVQRIPEG

LEIEGLREGLTHMMKEHEIQYSISSGVARVLRSEVATAQNELRAGQRKGIKFEVVIQEQEHVDIQVKDVSTDPDNPEPNISHP
STAHDHRVPKPGHCARCHEPFTEFEMETLVGYACGHVFHVSHLLEMLHGNKKVDVDLGNGSEEGSRYSVGVKVMRARLLKDEM
KGGCPVCHVKE
FUN_000211 CDS scaffold_1 641537 647464 +
hypothetical protein EOG0926049S PF12859

MATVKSLGLHQPSGLQHAINEQLLPPNPPSTSYTWDISTEDRFDGDLEDEILSTEHCVIWCRGGIFRKTFRFELEKEPVIQAL
LAYFPASKDDKSTQEEEAQSDQRPALSKALVVFLKTQAHIYFLSGTSHIVHMPFEVETAFAGPVGVIIQRKQKAESVAPISLK
FPRVPPNSFVSSQLTAFNSSQLTAFSVEGLGKPKALPLRLSSTLDNLWEAPLEQPESRWPRLVSLTDPLLELGLVVTNQEAQN
GKSLRQTAAKKLAFLDSAEEVLHVEEIKIPGALTQDLSQPLIIAVTINRETSEYTVWRLTYLQHEDRFLARQKDAKSKTARRR
SSMPPAFASTPGTPVQPNLRESFGAPLPGKRPRKSERLEKPMIDLVSSLEQQDKEGSGVARRSSRRVSSMLARADLSASHERA
ILPDQPLLSSHVGARRHESQGSHARLSANYAHQIHPSLSSLLAAPFYEGLDEGFHNMGLDDHEFDGLQHEILFTKLHSVPMNN
SNVRYSDQPARTQTKVFILVAPPFAIDGHDQSQLLIGIQDATERRLQLLTLELGIQRGTNMGAKPGKKNIPDGTTVVATVVDR
RHAQNVVDSCKLMDGDHSAILILSESMDGRHELSTQAPWSVLTKISLSLLFVDNTRSLQYRGRVVDRDVTQRKSEVIDFSNGS
IVGVRHPRQGGIVDVVDAEGRLHELRIQLEPRSPHVRRVLDVCRSILPHALGEHIFAGWLHCMQWIGGHDETSTDIEWSAMTI
LLLSLFLSLGRTDTKPFPKTRQLPRRRRHPSGSFGSIKESEDWGSLEKAETSNSLGCPTWMMNGGWQWALDEDGDDSGDENLS
TTFISKHISMAKEYMVSILGETAFGAAGYMPTALGKSMESRRKVAVDVFMGLHLLLEEEKLDIMTPEFRSPGRADLRVIMCQI
ARWLKWPSFIAIYEAGIQEDVDQRHDSDLNLRPAIPQPPVRPDVVNWIQSRLIGERRVPYITPADVYYAGSQLSEAEKSQDRR
WESILPRTLMFKQFFKFMKPSTSAVQMVEAMRDAGITPLILDTLPEAILAPLRDAISLCQPHPPTSWSKDLLELVDRRDISLI
LAPGKQPKPSASKILTPTHNATWDYKLLCQSVDESNSIGYDEGEGTERQAIIRSLFKEDRRLNEAQDLLSTHKARLVRLDPHP
GWPESEYLEKQKELVTRIATGTLAIPAGRALLYYSLRYPLITQKFHIGGFNLNCIVKPTNTTVGVDKSQFSEEKVCWGFFHQG
VAAGLAISPQAQGIDTSWILYNKPGQDLNNRHAGFLLALGLNGHLKDVAKWVAFKYLTPKHTMTSIGLLLGLAASYMGTMDSL
ITRLLSVHATRMLPRGAAELNLSPLTQTSGIMGIGLLYANSQHRRMSEIMLSEIEHIDEEDEEEPLRSECYRLAAGFALGFIN
LGKGNDLKGLHDMRLTEKLISHATTTKNIEIVHILDRAAAGAVMALALIYMKSEDQIVARKIDIPNSVLQFDYIRPDILLLRT
LTKNLIMWSKIEPTFAWIRKNLPRPYRSQFKLQSTTKLQSTDMAFHSIVAGLCFSIALRFSGSASPKVRDLLLYYLDQFMRIA
QIPSTASMHPNGTPPYDEELTRTNARMCQDIVAISASIVMAGTGDIPVLRRLRALHGRDDPETPYGSHLAAHLAIGALFLGCG
TATFGTSNLAIASLLVAFYPIFPTSVMDNRSHLQALRHFWVLATEQRCLVTKDVLTGQPITVPVQIRMRKNTSTEPVLNRTAP
CILPPIDQIASLSTTCGPQFWDVELDFSNPEVRAAFQDTQSLYLRRRPPREGAFASTLRALGRDEKGKDPLEWVFGLEGLRGI
SYAERAVVLERGDDTQHSGSAVDARLEMAKGIAEGTDRERLEGARLLFEWASVRDRLRGTNMFDSQETITESHVRREVEQEDD
QDATVEDGGVWWMRDSVIENLKGMVWLASREGEH
FUN_000212 CDS scaffold_1 648077 649459 - INT6 eukaryotic
translation initiation factor 3 subunit E PF01399;PF09440

MEDVLNAVSADGSSIMPRLAPHLSRHLLFPLIQFESERAEEQGQDAKAKEILSGKIKLLEDTNMTDYVATLYCELHGVSEAPE
QYNKKRQEVLSQLENYEQATAKIADLLTQDEVVNGLRSDKVANLEFLKNQHGVTMEMVNALYDFGQFQFRCGQYGPAADMLYQ
FRVLSTDNDKVSAATWGKLASEILQTNWESAVDELKNVRENIDSKLFNNPRAQLDHRTMLLHWSLFPLFNWEGAREPILDMFF
SAPYINTIQTSCPWILRYLIAAVITGRGRARNSSIQQKQIKDIIRYVRQEAYEYTDPITQFVNALYIAHDFEAAREALSMAAE
VCRSDFFLASSADAFVDAARHLICESYCKIFSRMNIRDLSAKLGLNPDDGEKWIVNLIRETRLDAKIDSQDGTVVMNHPPNNV
YQQVIEKTKGGFFRTQVLNAAVSK
FUN_000213 CDS scaffold_1 649918 650486 +
hypothetical protein PF05046

MHFLTSRVLSIGRQAIAQKPAIFTQRASYSAAATSSTNTFKTHVSYKPNPLRQPVNPNRKNKIPYKDRPLKQPFVPPPVSKNP
EKLGATLRYVIRRTPSSQLPVYRKWMSGGNRMIVIIKKIDGDRSKFIQDLARDLEIKPVDIRLNPTTQHVEIKGHVYDKAVEW
ILKTGF
FUN_000214 CDS scaffold_1 650897 654080 -
hypothetical protein PF01388

MNDAVPNHNGNGFPHMNDSNAAGNMMDPSAFMANPGQFNPAQFNQQMGGAMANGPGSMRNASPSFQNPVYQTNSVIPSKRPRP
REDSLAGSPSQNPGMLPTSRSETPQQQPFAGGFQPGAVQQNPGQFSHLQPNGSANASPSPIMSNQMRPGSVPQRVATASPHPF

SPSGQQFNPQTSPIPSEHGTPQPNPYMQNMAQGFNPNFAPSPSNARPSPNPNAMTGNQMMAQQMSQMPQHMGQMQGNMFPPQM
QQAQQQQGTPQQQGQQQQGQQTPQRPGMMDAQKMAAYQMRLQQQLQGNTQIQAQMQAQNMGRGMMPNKQVPGMPNGQMQQGGM
RPQQRMMANVNPEQFMKNLTTLMNSKGLQLDANPMVMDRPVNLMVLFQAVQNKGGYKQATTTPNGWPHVAQMLGLPPQNPIVP
QTLKTIYERNLYKFEEVWIAQQQKQRMMQQQQQQSQQQSQQQPQQPQTPGTLMGQATPQKQMQPGQQMTPGAMGQSGPPAQMQ
QTPMKQMQPGQPPVNGFSTPLPQMQPQPPVMPGQNRTLSQTIDASAVPEFPGAPSPATRRAGSISQNEGRQASAAPVVVEKMP
RLAPQTDEYSPCARELSTFGGVDLNAFSKLGAELERWKPDVPPLQELGNIDIGALTKSLQSGIHGETRLALDVLAAASCSMNQ
LHFIQLRYCDELIEALIECAEDQVEMLAEHTAEVSDEIQISPYEDVLRACRLERFNIRDLPVFGTQEYELDRAVDRLICVTTI
LRNLSFPGEQNDNHSVLADEIVIKFVCAVIRYLGTRTMLLRSHNNTLDFMKDVVILLSNIAGSVEIPGREQALCLLQFLLAFA
PAPNPTVSDGTLFFTQYEPSLHAYLPHAVDALAKLLARDEPNRGHYKAVFALDSNSSPPYELLTRAFGLAIAPIPDKARTQTR
QPNLPSLVEVRKPFLMQGLLSAEILASLAPGHDSGVAQSWLSSGNDFAQNLFRLIRELSQLYEQPQVQGPRTAPRKDPELVYI
VVVAVALLRRLAEKARDPNDPTSSIPAKAMPAPHALLEALSMQSAEWSKDEVLQKLSTFFALGR
FUN_000215 CDS scaffold_1 659283 660528 -
hypothetical protein PF08640

MAGVADKARFYLERAVPQLREWEEKEIFSKDEIRTIVQKRNDYEHRVLSPGNKPSDWSSYAQWEQSLESLRSKRCKRLKIRHL
QSAHAGQGRTLAIYERGVNRHPGSSALWREYLSYTSSVKASKRWRKTMTNALRMMPTDPELWAMAGRRSAKNGDMAAARGFFM
RGCRFCTTNEKLWVEYARTEMEWLEKVDKRKAVAKPGQDVLRPDREEDGDELRLVDSDDDEDDDDLPEPSNAQAKVIDKQTAQ
HLKSNPAMDGAIPMAIFDISKKQAFFSANTAEAFFDLFVSFTHVPAQPRISQHVLDALDQEYPNHPATCNVHIRQPIMGVNPQ
TAEFPKNLREVLSRLNRYLEATTDRTELQKKTVAWIDGYLALDVLDEGIQAVLEHTKKKMESI
FUN_000216 CDS scaffold_1 661015 664328 +
hypothetical protein EOG092605KN PF10277 ENOG41

MASKYKDKDTGVVLSFNGSLVSWAHTAVAYTAFFSALVIGVYLHYHKIVQNEFYGYPQEWFPSVSATIGDRYPERSFFMIFIA
ITSGPRFALVGLWYLLTRKPGQKLPTFVAVMGLLRTLTCGGWTYITSTDDHDWHDILMISYIVATLPWTTGCIALSPANSQAI
KYRKYLASAFFGTLVPLIYFFIQHKVHRVAGAYTTYAFFEWSLIILDVAFDAVTALDFSTFAIEVRDIKGASKGENLSSIPSA
VLEKEKEKATGGVFAVRFSWPEALDIAAEVYHGYVFWTILTSLGLVVWYFPLWYMGISGYEVLVMTTISPFILASRSARCLVV
NNLRAVHLLSLAGIAAYLVEEPSYRLFTVGFGVFMSCLGWAGTLFAESVHEGRLESKVMGWMIGLILSSTAKFAWWTNNPIWP
IMHAANGGWNNTGLVLGVLAALRFTRRAPLAAGLAPRPAKSSSSFLSSLGLAGLFFGLHSLLSDTSTMILWGWEGYPIRGPYF
STHGWLTVLAMSLGLFGGVWQPRLASSWGVYIIGTVGAMFLTFFSHWSGYYGALTLATYLMAYSVPILTHAAKTNPTTTFGNG
FLIYNFLVLFHVWVVAYAFVPGGQLVREHTDWIMYTMMTCIGAGVYGINASGYQRQPSKRSVPTQQRKYFGVATILINVFFLI
SAFQRFPSNNYQPYHADDRILTAGIWTIHFSFDNDMWASEYRMRDLIKELEIDVIGLLESDNQRIIMGNRDATQFLAEDLGMY
VDYGPGPNKHTWGAALLSKFPILNSTHHLLPSPVGELAPAIHATLDVYGQLVDVFVFHSGQEEDPEDRRLQSLYLADLMGSTP
RPAFLLSYLVTKPKEGNYNTYVSEKSGMKDVDPSDWDRWCEYILFKRLKRVGYARVSRSSITDTELQVAKFKIPESKEEIEKL
DAQPDKERNRRVKEEEVPEGWRFPAMFRGDGVREHRYHVFNEPRYFN
FUN_000217 CDS scaffold_1 666032 667783 +
hypothetical protein PF00067 ENOG41

MTFNLTHLLVYLGLLVGGLALYGFGIVVYRVFFHPLAKYPGPFLAKITDGYQLYHAWKGDRQLEFWRMHQKYGPVVRFGPNSL
CFNSNKALKEIYGFRTNVRKAEFYNAFVHPTANTHNTRDKEVHARKRRVMSQAFSESAMKEMQRYILGNVRTFCEQIGMLEGS
GEETKGWTKPRKMSDWCNYLAMDILGDLCFGKAFHMLESPTNRFALELVEAATTRHLLCGTMPIVNKLNLDKILFPGLAAGRA
RYMGYSKGQLAERTKLGEETDRRDFFYYLLKARDPETGQGFSTPELWSESNLLIIAGSDTTSTAMAATLFYLVRCPRALERVT
EEIRSKFNDVEEICQGATLASCTYLRACIDEAMRLSPSVGGILPREVLSGGITVEGRAIPEGTVIGVPHYTIHHNESYYPSPY
EYVPERWLVGALNPLTGEKTTEDEVALAASAFCPFSIGPRGCIGKGLAYVEMTNTLARTMYLYDMRKAIGIVDPAEGNPKNEW
GRHRPGEMQLVDTFTSAKNGPMIEFRKAAHTNA
FUN_000218 CDS scaffold_1 669213 670728 +
hypothetical protein PF13634

MQTALRDVKGAIQFIVGEENNHPNRHDICKQGTQGAPFGEFLVGKRPKSTIADTGAQPTPFRSNNNNPSSPFGGGASTGGSAF
GQPSTLGAKPSAFGAPSFGQPSQPAQGGGSVFGQPSQPSAFGQPSQLGQTASAFGKPAQTSTFGQPSQPGSAFGQTSALGAKP
NPFGTPAFGQPSQPTAQGSVFGQPSQPNTQGSAFGQPSQLNVQGSAFGQASQLGQKPSPFGAPSGTNNSSSPFGAVANNNNAP

AANPFGAPSGSATTSQPANPFGSNNNNQNNAPANPFGQPSQPAQGSSPFGQPSNAVAPATSTPFGASNATSNQPTNSPFGQPS
QTQSNGFASQNNQPPASNPFGQPSQQKPANPFGQPSNAAPAAANPFATTQAQKPPAAAAATSTGPYPPNSSRHHPPIESYSSK
GMDGRLLMFKGKSVIYKDGKPGIREFDGTWRRIWFPDGPPAFSADTELPPEMYDDKSKAQWMAFAQTGTFEGGLMPELPPPRE
CTLWDF
FUN_000219 CDS scaffold_1 671445 673459 +
hypothetical protein PF02845

MVSLPPLAPFPQSSWRQHLSSSEWNTLLQSWTSICRAISSLSEKDIKTALARDDSIATFLVSFAEETAEAGFTILDSHSTTLP
KAVFQLVSRVFTVSPPPQLFDYAFLANIARIFPRKATAPLIARLFQRHAAALEAALTALKKQLIPQLEAGIKGDLKSVEARLI
RLNYLLHASSDTCTLFLAGSDFLDGLIVCFRVMNPSLRKVILTTTYLCLVGLIDTEPAKWSMLSDQLYALKEAADAHKAGPLN
VNDSLVPELITNTPLLKVLLRRAEDTGAATENFKKRITALEGYRKGTMVRPKRLIRRRLDKGKGKITQQEVNADIHVHKMSQI
TQVQDLFPDLGSGFVSKCLDEYGEDVEQVVANLLSETLPPHLANADRSESLSSHPEAGHTELAPRSTPPQVPTRHNVFDDDDF
DRLAMDVSKVSFGKKPGKNADEMLKDKATAPNKSAILSALAAFDSDDDERDDTYDADDVGGTVDASNQEADAASDSNEEALFR
AYQMDSKLFDRDAGTRRGNPRAKLREETGMTDEAIEGWALILVRNPQQKRRLEAKYAFSGQQAQLERTSWRASPAGSGEEGSE
PDGGSSRGGRGGGRGRGRGRGRGRGGNVAGPTGEKETESARKNKEANKGSRANHNRRDARAKKMARGGFAG
FUN_000220 CDS scaffold_1 673459 675420 -
hypothetical protein ENOG41

MAHPEDGRDDAKEWELPAKINTSMETGNRSSSSGTPTTTLASLKRKNRDHDTEMSMDVVKTKHQCQDRLQAVVEKRARKNAGT
PRSAESPTDLIRGRASRCIEITSFPLVFEHAILPFLSQYLTIWVGPRHVVTVTRGKSPQMKRICIMAPKSISRARKIIIVSHV
QDLIPSNFSKLVTFAFTQGEINRTVTWARGLGKDHKDDICAARNPYFFRDPCMGDSIGVTGNGVFEDSTATLGPCITVGGGSY
WLGNFHPFMEAYQQLAQVEIEHPSSQDRKRCIDEGHDAMAQETNFRLGKLEVTSGLNLKTTRISHDPYWDECDMDKPLVVTDW
ALIGARTSQANILRKFPSETQPPTQEPTVATTTAIVPGADVLSSGRTSGYQRGQICEIPAYVSGVENQTQKATREWFIEEPWP
QEDEDAWIRGGIGVNGDSGAGVVDANTHGLIGQVWGRNNYWGPGQRVTYFTPIADIFDDIQEKCGQQSRPQLPQHRDEANCFP
LHPSCRQCFDLRVYLNSSRRSSRMSLQSMIMGTGDGDQDLTSIEAVSELATPKDYHRYSGIEETLASLSGIVSPAGPSGYPGT
PMIADMKSPYATELDLGDLYGPEATAPTQARKRRTSCLAAAPGSDRWKKQRTGD
FUN_000221 CDS scaffold_1 679092 685960 - POL2 DNA
polymerase epsilon catalytic subunit EOG092600W1
PF00136;PF03104;PF08490;PF10108;PF13482

MPNTSLRRPQKGFRRGGKVAYHGNRTRTFAASSRNEATSADEKWERTRLAHSIDENMGFARYESGKKREGWLVNIQPTSIEDE
RIPGGRAAVDCYFIEEDGSTFKATVEFDPYFLIAVKKGRESEVEEWVKRVAGGGVVKSIRRVEKDDLNMPNHLLGYRRTFLEL
RFANVNDLMAARKDIMPIAEKNKKNMNAMDAYAEVATSNGDFDLFDDSRDDDRNMNTALSDASDFIVDIREYDVPYHVRVMID
LDIRAGKWYFVEAKHGVTKIYPNEERSLPAEPVVMAYDIETSKLPLKFPDAATDQIIMISYMIDGQGFLITNRQILSEDIGDF
DYTPKPEYPGPFMIFNEPDEKAVIERFFLHIKEARPTVIATYNGDFFDWPFVEARASINGIDMYQEIGWKKDNEDQYKCNYSV
HMDCFHWVNRDSYLPQGSRGLKAVTVAKLGYDPDELDPELMTPYATERPQTLAEYSVSDAVATYYLYMKYVHPFIFSLCTILP
LSGDEVLRKGTGTLCEMLLMVQAYQREIVLPNKYITPKEAFWDGHLLDSETYVGGHVESIEAGVFRADIPVDFAVDPGAIDEL
LGDLDAALKFVITVEEKKNFDDVENYEEVKTQIVERLNKLKETPNRNEKPLIYHLDVASMYPNIMTTNRLQPDSMIQESDCAA
CDFNRPGKTCDRRLPWAWRGEYLPPKRDEYNMIKNALQNEKFPGKYPSSPMRTFQELSADEQANLVRKRLQLYSQKVYHKIHD
STTIVREAIICQRENPFYINTVRDFRDRRYDYKGKAKVWKGKTSSLQSAGAAQSEVDAAKKMIILYDSLQLAHKVILNSFYGY
VMRKGSRWYSLEMAGVTCLTGAHIIQMARQLVERLGRPLELDTDGIWCMLPATFPENFSFKLKGGKKLNISYPCVMLNHHVHA
KFTNHQYQTLVDKKTLKYETHSDNSIFFEVDGPYKAMVLPTSKEEDKNLKKRYAVFNDDGSLAELKGFEVKRRGELKLIKIFQ
QQIFKFFLEGETLAECYGAVAKVANRWLDVLHSKGTTLADEELMELISENRSMSKTLEEYGNQKSTSITTAKRLADFLGEQMV
KDKGLNCKFIICARPKNAPVTERAVPVAIFSAEESVKRTYLRKWLKEEPTDTDPRALLDWDYYLERLGSVIQKLITIPAALQK
VRNPVPRVPHPDWLQRRINIKDDKLKQKKLTDLFKKSPLEDITNINGPRLDDVEDFGSKLLKPKQVNAVIASSQAATTVQKRK
SPEPAENPDPMSALPEVMPSASENYEAFLMYQKKKWKLQKQARIRRRQLFGDRRGGAVNNLQQTFMKQAHTTYMSNWQVLHLK
ATETPGIVMAYVLIDVKIHTLKVNVPRQVFLNLKSKDLPDVEVEGCEVEQVNYTLPNGHPSSHLFKLTVSEDVYFNESEKFSL
LFNHPSVEGVYEKQVPLNIRAVLRLGNQCTIDAAQHAVLGKGLEQGFDLAGLKRPAKTRTYLETSPLAYIYVSHIATGERQIF
GIFSTTNDQAHVVILQKNKDSGQDLPNISKMYSEMLARRHAEAAGTNWQDCFTYQEKLHIKITQVTTRRKAHLEIGDVVKKMR
KDEPRPQMMVIQSSQRKLLIHDVPILGEFPVLPLKYDVADSSLPPLGWQSVIAKRLVGHYLGLGSWILHLTALARYGDVPLCN

LERDDPRFLIDVAYARRLQANGVVLWWSPDARPDHAGYEKDDLLGPLDTVKMPNVNNPGTFSSVCIDIDVRNLAINTILTSSL
INELEGADSVSFNPAADDSETLASENAFANAGVQVLREMVKAWWTEACRGSTMADVLVQHLVRWVENPDSFMYDRALHYYVQM
MSRKAFQQLMADFRRVGSQVIFANANRLILQTTKAEVGNAYAYSEYIIKSIKSKPLFHFIDLEIKEYWDYLVWYDEFNYGGKA
CQEVIEAEEQNLETVMHWQMATFLPVRLQSTFQDWVIEFIQLMHQLKRPHNGDPDATPRLTQLPSKGLEDHDGQILLGKAFEK
PLKKDIIGLLNKQKRELLHPELAQDYTFPVLPGSHLNPRNPILELVKSLMQVLSLDKNITLEARLLRKELLALFEVREFSKDG
TFTNPSESLRLPQISCDSCTMMRDLDLCRDEDLFGEGAAWRCGFCGTEFDRVAIEERLLGIVESWIVEWTTQDLKCARCGALR
LNDFMEHCTCSGEWKEIVSRQDVSKKLGVMKRVAKFYGLRMLSDVVEELYKGL
FUN_000222 CDS scaffold_1 686428 686993 +
hypothetical protein PF12597

MASNPQDGQSMPGDKTLHVWSKPIEQNEATTVNGEHGATPTNNRPTISEAVSMIKKDDFTNVANTPCARQGFITGIASGAGIG
GLRFVLKGNPGSAANWAVGMFVLGSMASYEWCQYLRREERRQMKRHIEVVSENRREQARKIQEARREHDKLEAEKKAAVKPWY
KVW
FUN_000223 CDS scaffold_1 687424 688336 -
hypothetical protein ENOG41

MATASANISSTPKRKRGEETWASPIQFTFVHEPLSSSFHPSSVPTPADTEDGSDSSRSRVLAHKFRGLALESGGGATVSNEDS
DNLMDEDKSMRKRQKPDEIMSGADAAPTVQEVVDLDGKSVLPSKEPLQPFKGRVHEEPQQQKQNHGSLHHAYPSINRLSESKS
RVKKRTGSPPLRMKRPSQRPFEDPDEEVQIVDPVRAALTWHEDEITIYDPDDEDDDGIGINGIGFKPTPALAHARAMRRRQQM
AEYRKREESEARAQRSQRRGRGSGVKFGRSEEQSPPRKVRFTDTEASNIAITTG
FUN_000224 CDS scaffold_1 689288 689865 -
hypothetical protein PF00536;PF07647 ENOG41

MSSYSSSLSSSYSGSPTSSSASLSFHHHVSSSRRDTGKIGDEMGIIRTWFSVLDDSERSSALHSIAELPSLREIEPMIQALKD
RKRELWRQQEELIIQPPVKPKWIMPVFPHNSQDPRWAAKWLRALRLHKYERCLLGMSPAQVSCLEDEDLQQLGIDTVGARAKM
LRAIHNV
FUN_000225 CDS scaffold_1 691363 691963 -
hypothetical protein PF04145

MDHGGMGGSGAACKVEMLWNWNTIDACFLASSWQIKNQGMMAATCIGVILLVILVEFFRRMGKEYDALLQRGFQRQATTHSVS
LAAAGCTGAVVPTSQTLTYRASPLQQFIRAFIHAATFAGAYIIMLLAMYFNGYVIISIFIGAGLGKFFCDWLVVRIDIEDLTA
PEDKAKGIEETTVCCG
FUN_000226 CDS scaffold_1 692938 694894 +
hypothetical protein PF00175;PF01794;PF08022;PF08030 ENOG41

MVSLSQLDARHIQNMSEAKTLEPHWGYADRALPCTNDKGSCEYLDLVYYAHDLGMLYSGILWATILAVFFLWAILRHVGKPSS
PAITLGVNPKAGLAKVRSSLSSLTRQYLLPDACRTIFGRTTRFQVAVMLSLVGYLTVWSFVGMTYQKWVTPVKDMPGVYNTRT
TLGPWSDRVGILAYALTPLSVLLASRESVLSLITGVPYQSFIFLHRWLGYIIFLQGSLHTIAWTVVEVRLYQPQPKVAAEWIV
QEYMIWGVVAMILLLLMFIGSTPWGIRATGYEFFRKSHYVLAMVYIGACWGHWKQLKCFLLPSLLFWFADRGFRLLRTAYLHY
HHLPSGKMGFQAAQAVITRFPDAEHGDILRFDLENDQDPWKIGQHYFLCFTESNIWQSHPFTPINAPVVEKGVVKHSYIMRAK
GGETKKMAELAAKKLTISERATTSVILTGAYGETTMDHVAPDTNIVCVAGGTGIAYVLPVLLELARSPISRDRKIELIWAIRH
AADAEWVQEEMDLLQQARKALNLKIRVYATRDSSSSSKLQVSDEKDVTDATRQLGSSSSSDVIEEGCACGTDVPVAKIGNGIV
DEDRHPNLSKLISDFVSNTITGPTTVFASGPGGMISDLRTIVAGCNSGNKVWNGNERFDVKLVCDDRLEW
FUN_000227 CDS scaffold_1 695831 696854 +
hypothetical protein PF01263

MADAPISFLPLGAIIQSLVVNGVNIVQGFPTQEDYEKHNSPYFGVTVGRVANRIKGARIDSLNGKEVTLAANDGQNHLHGGKI
GWSSRVWDGPKPVGTRQVPGVEGLEGGESVAFILTSEDGDEGYPGTVEVTVTYTTGTQNVDGKEVIILAMEYEAKLVGGADET
VINMTNHSYFNPSGKETIAGTDITLPTANYLAVGSDLIPTGKIEPFPGVTANKTFTLGPQEPNIDHCFVLNTDPSSVPLDTRN
QPLNLNVKAHHSGSGVNLEVYSTEPTFQIYTGAGINVPAVNGLPARGARAGFCCEPARYVNAANVPEWKNQVLLKKGDTYGAR

TVYKAWAD
FUN_000228 CDS scaffold_1 697066 698411 -
hypothetical protein ENOG41

MAPSEDEIEWALLDATYQVYQATPDETTVNKVRKQTEENLSLDEGFLSSAEWKQKSKSLIKERVEKLMDGWIPDSNKNADSDG
EDKTGVKRSSPDAESPEPKPKRQKRVTMPKSKPKSKKANKKEESDIESDLESKPSVSSELSELSDLSEEEKPQKKRKRAAPKK
TAPKHKSKAVKPEGGDEEDEEDAIKSEPEVPGDNDDEVENGELKSDKATTSTKPQNPEDREEGSKGGENKPVVDEEEEYSDVI
DEPVKPKRKKKEKKEAASKPAKDTKVAAKKVTTSDDSNTEEIKRLQGQLTKCGVRKLWHNELKKYGDDAKAKIRHLRKMLTDI
GMDGRFSEAKAREIKERRELLAEVESAQEMSALWGVEGRGRASRSKSKSAKVVESGDSDAEENDGDENNDDEEETYAARRKRA
RADLAFLGDDDSDSD
FUN_000229 CDS scaffold_1 698942 699393 +
hypothetical protein PF05254

MSASLAPECNEVKERYDTCFLKWYSEKYLRGQEKDNKECADMFKEYQNCLKVALKDRGVDKLVEEAREENKENDLKHLGPRK
FUN_000230 CDS scaffold_1 699949 702666 +
hypothetical protein PF05024

MHPFSILTLGIFVAGYITARWDLVTRLYELAIFAWENGVVTRAAKAFAALSVLFLSICAPTVLLARKETNLHPRGTGTGVSAR
EQLRRREHDGLMRVFWPTDIRKSDRPGVVVGWRNSILDVFVVTILEDVDARNTEFHLKAGTLFRSELHPTSQITDMCGHSSMH
VLGLSNAIDAAAADPSWFCATTNSDPREPKITCAKAISVQLILYDRPQPQGMQYVSLNPIALALGHDGSLLGDTTVSEGEQET
RERQKKEEKRKLAEKLKQHTIFKRVPSARDRALPKIINQINWSWELEKTLHKNVGRLGSRPRRSLSVSERVVETASTMRNYVI
LQLWTWFTLYLFPTIRKTFVLVLMGHRVVAEMLLLLLEFRAKPGYAALKDISATAQQVEIRLQQFCYWPMQYMTLRQRKNNWA
SVTTSHPDYIRFYNSLWLVANDVIIGIALGSYIIENADWVAEQIGDLLQTYTVDALQSSISWLMGWPAGLKLNGELAAFLGDL
FLWVIDYWSSCIETLTPALPQMVWFIGFSSFAGASMPIAMFSDMLSTLTIHIYSFYLASGRIYHWQLTILQSLFHLFRGKKHN
VLRNRIDSCDYDLDQLLVGTILFTLLFFLLPTVAVFYLNFAIARMAIISLKAGFDTLLSCLNHFPLFALMLRIKDPRRLPGGI
RFELRDSHDYRHDKANNADGLPPTSVIYLKSIPLTFRIMFNQYFQMANRIRKHYLSPKVFFCLLTGKFVPPINRKNLYSLQYS
MLPARRATIWEMWRALNTETKPAKRMPLPYIPPLSNAGRRTSANNGRAR
FUN_000231 CDS scaffold_1 704617 705875 +
hypothetical protein PF07986;PF16752

MDPKQRFYNHFLESVTVLQDLIDELPSIASVGGERQEAIDHILASIAKLQNEVSDAADYTPSYDRKQYSETIKTLQDKLNETL
TKITPKSKFQFRRKTDHVDMGAPENDPRLSPGSHSRAKHDTAIASTIASPPSVRKEDTLSELPSKDTYKNYNEEMARPSASSL
RKPSFSAAKNISISNHSGLHIILPSSASRATSSGSLTDLKNCIIDMSIPTSSGSAFPGLAIKNVSKSLIVGGRVNGAVHITGV
SDSTIVVVARQVRIHECSNVDIYLHCGSHPIIEDCSGMRFSPLPKAYMTDAEEPEENQWDQVDDFKWLKAGHSPNWTTLSENE
RLSDDLWRSVVPGQPGVSVEESLKKLGIPRR
FUN_000232 CDS scaffold_1 706089 706536 -
hypothetical protein PF07544 ENOG41

MSPSSASAPASASGPHPLALPPTFSPDTLDALSELSLVLARVRAGIQSSAGITTEPAPGTTGHNASGPTLSFKDVPGATDGLK
HKLQRARAQVRELPDMDRSIAEQNDEIRDLENRIEKQRALLQRLRDEATGRDRKEEVSAGDKMES
FUN_000233 CDS scaffold_1 707211 709138 +
hypothetical protein PF01676;PF06415

MSKTDQKACLIVIDGWGIPSADSPKDGDAITNAKTPVMDALYKDSKGYTELEASSLAVGLPEGLMGNSEVGHLNIGAGRVVWQ
DVVRIDQTIKNGQLGQNDVIKKTFQSVAAGNGRLHLCGLVSHGGVHAKQTHLYALLKAAKEYGVPKVFIHFFGDGRDTDPKSG
AGYMQELVDTAKEIGIGEIGTVVGRYFAMDRDKRWDRLEVALKGLVLGEGEASEDPVATVKARYERGGDFDRDEFLTPIIVGG
DERRIKDDDTVFFFNYRSDRVRQITQLLGDVDRSPLPNFKYPKVKPLVTMTQYKGDYPFEVAFKPQHMGNVLAEWLGKQNVEQ
VHVAETEKYAHVTFFFNGGVEKVFPLETRDQSQDLVPSNKSVATYDLAPEMSADGVADQVVKRLGEQKFPFVMNNFAPPDMVG
HTGVYDAAVIGCEATDKAIGKILEACKKEGYVLFITADHGNAEEMKFPDGKPKTSHTTNKVPLLMANYPEGWSLKKTDEGVLG
DVAPTVLAAMGLPQPEEMTGKSLLQKA

FUN_000234 CDS scaffold_1 711293 711656 -
hypothetical protein

MRFPDDGDLNNLHVDHPFIVGIAAGRVSPFDLEWMNVLITGGFVRDHRQGRNCKRSVVALRYEGRIARCNANLDCSTWDLVLS
QIDHFIASYNRKITKPLLYTTMARHKHPSPPGNFQDL
FUN_000235 CDS scaffold_1 715348 718380 +
hypothetical protein PF09421 ENOG41

MPLHETTYHGTQPPPSSSKTGHPLPRRTSPANSVTLRHHRLAQDASIKAGQGSGPAVKTTKTGSPRRNSSGDSHETGQSDPNT
WFDQSNQNPTATFDSNVMEVDPPFFQKESDSSNEEKIHYNHQLAPPKLTAAHSSSADDYRSVIDDLTVEIQKLREELKRYKNT
GPDMLRKEKLFEIKVHGLPKKKKRELEATLRDFAASLEGSPNASSTQNIKKSRHATREKMYSESGSKHASSSSGSNFRPADSA
YASMSTGANSGTSLSRPSMGSQAKSSEAVESYLRDIPEGLYPRHMIMTDKERKKLVVRRLEQLFTGKIRGRHVVKKQPVLPSG
SSAALAPVVAETQPTMSNTSNSIHEPPALQTSGKEPTREARILPLEQQSGHSGKKSRSADNASASNSNGDNTESGGNGNSTGS
GTNTSPPMTTLPEQRPTRPRDLDPDRAQVPAENMDYIRHLGLVPPELLAEQHSEDVHPDAEGWVYLNLLCNLAQLHMINVTPD
FVRSAVSGISTKFQLSPDGRKIRWRGGTEGTRFSSDSSGYNSQKSPSTDDTEDGMEKKRKRQKTGRSTGDEFQSGSSSKNGLM
FDPQLCAPSESFHYKPLFAQQDSSGGQTSLDETVSSFGPPEESNIGESRWGLSGSGASSRRKRRHDGAIIYYSGAPFCTDLSG
DPGDMSSTTQMRASGQTQTDQDNFKIPPSPLRRTDSGSFINYRPLTDRGPLSSQQSTAMDVDSEDPPSLTDDADDVSEVDLDF
TWSDNAQYMQHFPLEPCGLGGVLPEDHFMVVVSTKRPKQDDLSALPQVGGRRFNENTDGIIRRLATMSTSSPDLGASKSLPAT
HSLPIEIEYMSGRIKRLTPVSLPPPAIFFPPFSPTESTADDDYDDSDDVDMSSSLGELIGQRTVQRHSDGYPDGVDLSSGDEE
GDEPDDSPNQNTYDINRDPKALPNRPRQAARRTSSAAAAAGTARGASKSNSANPALSHESSVDTAGAIESGYSSSEESA
FUN_000236 CDS scaffold_1 719145 721004 +
hypothetical protein PF01593;PF07156;PF13450

MLHLALLGALASVLCRAEDVHNVAIIGAGAAGSSAAFHLWQYAIEEDIAINITVFEKTDRIGGRTLTVPAYNDPSLPIELGAS
IFVGANHILANASERYHLPVSEPHRLARDDMTVIWDGYEFVFQTTEGSWAWWDLAKMFWRYGLAPYRSKQIVDAMIEHFLKLY
EAPYFPFKSLTQRAEQLGWNEITSITGEQMLKQNEIDPRFAREILQVGTRVNYASNLAYIHGLETLVSLATDNAMSVESGNWR
IFEHMVIESGASISRNTTVASIEKVQKKTRSSSPVYVITTKDASSKDDTLKEYDVEFDNVIIANPWQFSNIKAGEGVLDHKID
EIPYTKLHVTLFASPLELSPEFFGLKPGSKAPATVYTTLGEDEEPGKGADGVGRTGFYSISTLKYVVNPKTGQKERVYKIFSP
TPVTADFLTRLLGTDIPDSVISGKLQDDASTSNPISWYHPHWFNSYPVELPRVTFEDPLIGEGVYYTSGIESFVSCMETSALM
GKNVARLAVDKFAGISRPEPPNEWEGYEAGDEEEGGIRVQAESEL
FUN_000237 CDS scaffold_1 722054 724664 +
hypothetical protein EOG09260WG2 PF08553;PF17747;PF17748

MFALRGIGKLIFGSSTQEALIELPQGQLYLVRPLSPKGYSELIFRDSAIRIRRTNQEFQYQLVVQRVFEEGEAELLAEEEGED
AEIDALASEKDEKTFLLDEALSFRSEIRESGDKVLAWKDLSGDTGDLFQFVCDPSVTSSQVQQFVQVAQQCQYERKYRKSHST
ATASDLQQFEFEEEDPIPPASPLHSPTLARSIESVDDMLSKTQANSAARREPAFVPEQEIEAPVLTGPDASNPPEALEIYAAV
VAELHVFDPQPGHFALVDDSVIAQVSEVGKWEYWLQIESNDKVYLGTPVVADFNPVFDFEYLSFVFNHFSSDGTARSWLLRFK
DQPTLEKFQEAIMQAIWEKLNETKWTKMQDKEREYVLDAFGDLTMEDAPSTEEEEEEEEEEEEELEDDRAQEEEYDTDEENDS
SFPKNADGEVNSQLAVGYKHDRSFVVRGSKIGVFKHTPNNHLEFSTNISKVTTPSGKLMNPKKVMLHSEDRDLILQNEIDPNK
LYRMDLEYGKVVDEWNVHDDIPVVTFAPENKFAQMTSEQTFLGVSNNALYRVDPRLSGNKLVDADMKQYASKNDFSALATTEK
GYIAVASNKGDIRLFDRLGIRAKTQLPALGDPITGIDVSADGRWILGTTKNYILLVDAQQKSGKNEGKLGFEKPFAANDKPHP
RRLALTPEHVAQFYHETGKPVDFTPAKFNTGEGAEETSIITATGPYIIEWNLKRILRGMKAAYKIKRYEEEVKADDFKYGSDK
NVIVALPNEVNMVAKQSLRKPTRESIIGNVRLSDSRRGSGRIGTRDSGRYKLGKDDIVNAPY
FUN_000238 CDS scaffold_1 725187 726845 -
hypothetical protein PF00982
GT20
MPGAVDENGQAAPGRLLLLSNRLPITIKRSEDGSYSFSMSSGGLVTGLSGLSKTTSFQWYGWPGLEVPDNEVEGMKQRLKDEY
GAHPVFIDDELADRHYNGFSNSILWPLFHYHPGEITFDESAWAAYQEVNRLFAKTVIKDVQDGDLIWVHDYHLMLLPQMLREE
IGESKKNVKIGFFLHTPFPSSEIYRILPVREALLTGLLDCDLIGFHTYDYARHFLSSCSRILECPTTPNGVDWNGRFVTVGAF
PIGIDPDNFVEGLKKPKVQERIAALSRKFEGVKLVVGVDRLDYIKGVPQKLHALEVFLTEHPEWIGKIVLVQVAVPSRQDVEE

YQNLRAVVNELVGRINGKFGTIEFMPIHFLHQSVTFDELTALYAVSDVCLVSSTRDGMNLVSYEYIATQQKNHGVMILSEFTG
AAQSLNGSLIVNPWNTEELANALHDAVTMSPEQREANYKKLERYVFKYTSAWWGASFVSEMTRLSTEGSQPKTLRNVSGAVIG
LEQKAQQAVADLEKKAEELLTIDEKKEADSQTEPSQ
FUN_000239 CDS scaffold_1 729370 732005 +
hypothetical protein EOG0926158Y PF00570;PF01612;PF08066

MEQSQDFKSLQESVQKSLISTIKTVNRIAAEDLSFQRTVNPSVGQLLEDRTSRILELSTQLLLTAGKACGVKAPKLEDVEDIE
MKWRGVVDVVDSSLEKADTALDEYTGLVKRKEPPLPDPANKSKKPKSTNKVIRNANMTKPQLLFERKPDNFAPAPWKPMLTSK
PNAKLSLEESLNVVPNEAGTPQYQHPYEHEIVRMSYPKRVFKESEPIMYQPVETTEATFVDTYEGVLEMLEELKGAKEIAIDL
EHHDFRSYVGLVSLMQISTREKDWIVDTLQPWRHKLEVLNQVFTDPSVVKVFHGAYMDMVWLQRDLGLYVNGLFDTFFACEQL
HYPAKSLAYLLSKFVDFDADKQYQLADWRIRPLPEEMMYYARSDTHYLLYIYDRVRNELVAASDRGDVDKDYIGRAVEKSKEQ
SLSRYEHPGYDEETGEGSRGWYGYIFKNSHLAFDSEQFAVFRALWKWRDNTARKEDESTNYVLSTRDITEIARINPPDAKALH
SLLPLNASLARPRFNEIWEQIKETKAKGGLSLLHFFTSMAPDSVRKNGMPIAARKTTKLPDLDGEVTVSRLTRSQLFGDMPIS
SKWDPSTRPTETEEELIPFPWQRFIQQGSIKGDVQHDTAAEEVNVSEQTDTVESAVGNAEVEEADEEFTLRRGQKRKSQAIEE
SEPSEEDTDSSQYDSDEEMQEGGGVIAVEDEPSQKASRSARRKLRKAEEKKAEEERLRRQEAKKVRKAQKKQKKQDDKTKKFD
AVPFDYSKATSVLHSNRESNGGQADGKGKKKVFDPYSKSADADIKGARKAPPVRGERSATFKK
FUN_000240 CDS scaffold_1 735694 736415 -
hypothetical protein PF00137

MASSRVFASRLASQMAVKAARPAVRAPVAAASKRTISGASPLQAMKRQTLLQATSRNAFQVQRRAYSSEIAQAMVEVSKNLGM
GAAAIGLTGAGIGIGLVFAALLNGVARNPALRGQLFSYAILGFAFVEAIGLFDLMVALMAKFVSQFPILALLFPFEHELTCST
LDLSYFPANWVGSCRMIESTRQRRLLSAVHCAMS
FUN_000241 CDS scaffold_1 739402 741417 -
hypothetical protein PF01853

MPQKRKRPDQDPSEQELLPASALKSTSSDSQSKSASARRATRQTPVIPPTPPTPVTQLQPQPRTETAVPPPIIPTLSSASSKP
TPSPEQKRSRASNGRPQLPPSQPKAQSQHPPSVLRWAPLEQPGPAVRPPPHHLHPTTMAAMPPPRPIAAGRPPSDLVAPEITY
HVPQSHTNSFQRTSALVRPHGPTAQPPRILERPPPPTVTPVHPPKPPVPPSVRPPLRADRNIDKVVLGNMCFSTWYPSYYGKE
VLGESSGNLGKTGSKDLGAKDQATGGKSSSRRDREHHPVVDRLYVCPTCFKYSKELVAWCHHVHVCERKTHIPGRKVYVHPRG
RRKILVPHDNKTSGPKRRKGEGNIRLVEEIVQDEGEWSIWEVDGEKDGLFCQNLSLFAKLFLDNKSVFFDVTGFNYFLLVYTP
PTRSLQTATEPEPPSPRITGFFSKEKMSWDNNNLACILIFPPWQRKGLGALLMGASYEISRREGILGGPEKPISDLGKKGYKR
YWAGEIARWLLTINMDSKTPQNEVLVDLSDCSKATWILPEDCLQVLRDMGVVEEAGLGPSKPEEKPVAEAEAEAEEKKGEETT
RTSPTADGGVKEETVPVVKTVPRVRMDKQAVRRYVAENRISLERMCDPAGFVEGYAIKAPEVVEEEVVEAEE
FUN_000242 CDS scaffold_1 742192 744244 -
hypothetical protein PF13516 ENOG41

MASATELRPTQPPSFSTHQHATPSLPSVPSTKYHDEVEVDLAIHLATLCRKSLVELDSHLPGFKPSHPFTADEIVSLPLDKHR
VDDTLHELPRRRSARHNLAVAISSCLYPVHQGSLTAIIRYEEDRVRLRAWAALQRRYDMLKATIRNGHTARGLSSGAAPGVQA
PVWASRITSQGVWDPAKHPISLEGVPAIPMPVEIAHEADLVPFLNHLRGGGTSKLNGDEKGFELDDGRGEPYYGVKGAEFRKG
VVYEDGRMDLCKMVVGPDHIGKLMDSLRPNEFVRHFLLGNNIIGPVGAHEIANFIHDLPGRIDTWYLAGNCIDRPSFNILVDA
MIESDAITNIWLKRNPLGSHASEDVYRLITGVRNLRTLDLDQTQLGDRGVSDLFNRLAGHEMPEGAKLPLKHIYLNGNGISNE
GAQAIGKFLVSPHCGLTSIYMSSNPMGDSGANALAQALPKASYLTRLLLQSIGVSTKGAVALCKAATGHPALEVLDLGQAYAT
HDLGQAYNYIEDGAVTAIQDLVSAESHLSYLNFGHMAVTPPAINLISEAVLKSPSLVYFAAYPILPDPALKAATFRPAVDTIF
ANPAEPTRPQILMSKAVQEHLSANVKTRYGEETSYSQFMEEDRRWLVSDKTDVRKIDSVYRNRDAGLARRRLLTLVKNWEEGD
ETLDRVMAAHAPTCSLRRH
FUN_000243 tRNA scaffold_1 745335 745424 - tRNA-Trp

FUN_000244 CDS scaffold_1 745882 747732 -
hypothetical protein EOG09262CMP PF01399;PF08375

MPSKTPNNNGKKPAGNNVQKNKDVEMTDSTKPKGKKSAKDGDEEMTVVVPPSKSKRSDKTAADAEGDVSMGEEEEEAKVDPVT
QTIADIKSNFALIDRAVSLFDARFSLRALRSISIIRKRLTPDIIAQVISEAFPATANSSAIVKPLLSAIEREDVILGRQSGSE
MEIDEAKTSGKNGAKKEAKEPIPEIDIFLGILVQVYLFDSKQFQRGADFSKYLSDRVQSLNRRTLDSLSAKVYFYYSIFCEHI
APLPPSPQSPVVAIRPTLLAALRTAVLRKDVDTQASVIVLLLRNYLSTSHINQADLLVSHTQFPENAVNNQVARFLYYLGRIR
AIQLRYTEAHEHLTAATRKAPSSSCALGFAQTATKLLYVVELLMGDIPDRSMFRQPTMEVALQPYFLLVKSVRVGNLEDFETA
IADHADTFRRDGTYSLILRLRQNVIKTGIRMMSLSYSRISLRDICIRLHLGSEESAEYIVAKAIRDGVIEATLDRERGFMKSK
EVGDVYATREPGEAFHDRIRACLALHDESVKAMRFPMNQHRLELKNAQEAREREREMAKEIQEGDLDEDDLGGDFDGI
FUN_000245 CDS scaffold_1 748789 750904 -
hypothetical protein PF03847

MNPQMAQQGQGQQAGAGQAQQRPPPMYQPNQIRNLPTLSDEEKSKYEAGLQGLWNKANNSPQNSPDHIAARQKIIEFSKMLIT
KIQQRRSQQMQGQNAQNQQARPQQMQQNAAGQANQAQPQQQAAGAAAGNQSAAAAAVRRNPVPDHIQQHVNKINFRAPQSVID
KSAVEATKWIEEIKERYTRALMTMDASKQKVAQIDKIINDRAAAGQPFKEDELRQLQLKKDQQLKAHTDAHKWVDSVRKQQGS
LQGTGQAQAQAQTQPQAQNSVRLSQQGNQPVQQAAATQQNQPQAQPRPQQQQQQQQQPQATQNQPQAAQQQNNAQTNTAPINA
AVEVAKQQLAAVSRASPVNGTPGATPAQVRPAQGTPQPGRQQLPAAQVQVKQEPGAAPINPVMASTAAQNQAGTPTQQNAPRI
PTPQQAAAASAPVTAAGPQQALSHSAAMDLANQKAINTPGSASVPGRPAATGTPTPGSAVNQGVMGPNVPQQPTPQGHPHAHP
PQPQQTPMQSKMPIPKVLPEKATAVPQGVSVGGGVNTGRPTMSQGSGTLGGVMNQPAMARIPAYNHEAEGDHVLSKKKLDELV
RQVCGGPAEGQDGNLLTPEVEENVLNMADSFVDSVLHAACRNSKERGSKVLEIRDIQLVLERTYNIRVPGYSSDELRTVRKIQ
PSTGWIAKMSAVQAAKVMPGKGE
FUN_000246 CDS scaffold_1 751989 752921 +
hypothetical protein

MRFSSSTVLAALPALAAAQDSPIDQYKAQFQNVIGQFASYIPNPGHHDPVAAAEAKAGSMRLSVLTLENWKQTLYEPVSAGAT
TPEEWWLLISGGNKTCYGRCGTVEQAFNETAAKFALLDNTPHMAYLNCDNQPILCNAWSAGPASLYIIEMLPEPAPVEIYSKR
LNLTTTTSDTLVAFHESGSKDQFHRIDSSWFHPFNGKLAEYGVAVPFGYLMWAMGLIPNWLFMLVVSFGSRAMMSRRMQGVNG
APQAGAQRAQ
FUN_000247 CDS scaffold_1 753084 753697 -
hypothetical protein PF09447

MAPTEAELLANYLIQPSPLTAIVTLEQFKALFPRPLQSSPQVRSLFRDLQAQRTDLLDQVAENIAHEAKRGITMRREVVRAKR
EAEREDIDAEIEMERALFGDASGAASAKHTLNSVIPELEGAAGALHAELAHLKEEEATLLDSVQQTIGALSDLRYGKFANGRI
SEGVIDGLKNVEAACENKS
FUN_000248 CDS scaffold_1 755328 757416 +
hypothetical protein PF05712;PF11717

MAPARQQPAPPPFSKDEKVLCFHMDMLYEAKIMDVQPGEKPSDGYKYKVHYKGWKNTWDDWVLVDRIRPFDDEHKELAAQLHA
QLKHNIQRSTKQPKKGLRSGAESARVSEERSGSATVQGGRGGRRGKDWELEQPGKSMDSFKSSKKVKLTDLKEPKDHLTLLPS
SGHRYVTESGKLIHDDRYVMPLTVEDEPKQPRQKPLKPSAKPKPRSCDQLETEDAFHNKPMIKLPVPDHIQAMLVDDWENITK
NNQLVPLPHNKPVTKIFEDYLAHERPHREEGSSSMDILEEVVAGFREYFEKALSRILLYRFERHQYMDLKKLWENTEANPEIT
NVCDVYGAEHLARLIVSLPELLAQTNMDQQSVSRLREEIGKFNVWLGRNCETYFVNEYETPSQEYIDKARSF
FUN_000249 CDS scaffold_1 757877 758141 -
hypothetical protein PF00327

MSFFRITLHRSAIGLPERTRGVLAALGLRRRMQTVFHPVHPQFAGMILKVKELVRVEEVDRALSKREVKAARTPDPGFYIEKA
VPRM
FUN_000250 CDS scaffold_1 758944 760979 -
hypothetical protein PF00285;PF00549;PF02629

MAPASSSGLSANDNIQRFPAPSRPLSPLPEHALFTDKTRCFVYGLQPRAVQGMLDFDFICKRSKPSVAGIIYTFGGQFVSKMY

WGTSETLLPVYQQVDKAMSKHPDVDVVVNFASSRSVYSSTMELMENPQVKTIAIIAEGVPERRAREIAHVAKKKGVTIIGPAT
VGGIKPGSFKIGNTGGMMDNIVASKLYRKGSVGYVSKSGGMSNELNNIISQNTDGVYEGIAIGGDRYPGTTFIDHLLRYQADP
ECKILVLLGEVGGVEEYKVIDAVKQGIITKPIVAWAIGTCASMFKTEVQFGHAGSFANSQLETAKMKNEKMKEAGFYVPATFE
DLPATLKEVYDKLVSQGTIVPQPEPVVPKIPLDYSWAQELGLIRKPAAFISTISDDRGQELLYAGMPISDVFKEDIGIGGVMS
LLWFRRRLPPYASKFLEMVLMLTADHGPAVSGAMNTIITTRAGKDLISALVSGLLTIGSRFGGALDGAAEEFTRAFDKGLSPR
DFVDSMRKANKLIPGIGHRIKSRNNPDLRVELVKEYVLNNFPSHKLLDYALAVETVTTSKKDNLILNVDGCIAVCFVDLVRNC
GAFSAEEAEDYLKMGVLNGLFVLGRSIGLIAHFLDQKRLRTGLYRHPWDDITYLLPNLREAGAPGAEGRVEVSL
FUN_000251 CDS scaffold_1 764429 766276 +
hypothetical protein PF08442;PF16114

MSAKSILEADGKAILNYHLTRAPVIKPSPLPAPTTHNPPSRLASLHFPEDANVEDILNQAEVTYPWLLQPDAKFVAKPDQLIK
RRGKSGLLALNKTWPEAKAWVAERAGKEQQVEHTTGVLRQFLVEPFVPHPQDTEYYININSVRDGDWILFTHEGGVDVGDVDA
KAEKLLIPVDLAEYPSNEEIAATLLKKVPQGVHNVLVDFITRLYAVYVDCQFTYLEINPLVVIPNEDKTSAAVHFLDLAAKLD
QTADFECGVKWAIARSPAALGLTNIAPSADGKINIDAGPPMEFPAPFGRELTKEEAYIADLDAKTGASLKLTVLNAKGRIWTL
VAGGGASVVYADAIASAGFADELANYGEYSGAPTESQTYHYARTVLDLLLRAPQSDEGKVLFIGGGIANFTNVASTFKGVIRA
LRDFAPKLIEHNVSIWVRRAGPNYQEGLKNMKAATQELGLNAKIFGPEMHVSGIVPLALVPGKWEESKAQEFQA
FUN_000252 CDS scaffold_1 767372 768963 -
hypothetical protein PF03198 ENOG41
GH72
MLIKAALMALGAAVVGAVPPLEIKGADFINPETGDKFQIVGMAYQPGGSAGYDPSSGRDPLSHKDECMRDAALMQILGINAIR
VYNLDPNINHDECASIFNAAGMYMMIDVNSPLPGEALHSEKPWESYYTAYLNRTFAITEAFGNYPNTLLFFSANEVINNQATA
EFAPQYIRAVTRDLKNYIKNNLKRQIPVGYSAADVRDVLWDTWNYLQCADSEDKGDMSRADLFALNSYSWCGPEATFETSSYD
DLVAGFNSTSIPIFFSEYGCNKPQPRYWNETQAMYGDEMTPVFSGGVVYEYTEEDNNYGLVKITGDKLRILGDFNRLKNQFAR
INWKEVQSQPASKSSPKAPACKASIIEDSGFDNNFTLPTIPDDAQKLVDNGIKNKPSGKIIDISDWNVKLDVSNADGSAMKNL
KVIPLKDDESNASGKNDADTGSETTDDNNSTNTDNSTNSNGGDAKDDEDAAILNRPLAWAMALPLAAMVFAL
FUN_000253 CDS scaffold_1 771912 773102 - SPN2 Septin Spn2
PF00735;PF01926

MAAAPPNTIYPQSHVGFDSITSQIEKKLLKRGFQFNVICVGQTGMGKSTLINTIFASHLIDSKGRFQPDEPIRQTTEIQAVSH
NIEENGVRLRLNIVDTPGYGDLVNNDRCWDPIVKYIKDQHSAYLRKELTAQRERYIQDTRIHCCLFFIQPSGHSLKPIDIVVL
KKLSDVVNVVPVIAKADTLTVEERQEFKERIKEEFAFHNLKMYPYDNEEFDDEERALNGQIKNLVPFAVVGSEKSIIVNGKQV
RGRQNRWGVINVEDETHCEFVYLRDFLLRTHLQDLIETTSQIHYETFRAKQLLALKESSAHGGASSRPISPAADRELSRNSQR
MTMNGY
FUN_000254 CDS scaffold_1 774658 775799 +
hypothetical protein PF00400

MSKQYLTTHTVDNAHITDIFSIATTSKAVISGSGSSTLHIHDTTDPSFPLKQSISDAHKLGCHHVCTSRNGNVAASAGFGGEV
KIWKVDSDTGEWSLNGEITGSSSKPGEAWAMALSENGSYLATTTNDGRVNVWDVMDEKKPKIREYETGSAGSGSFGMSVDLSR
DGKYTASGHQNGSVYIFNNDTGRVLYSLSGLAKPVRSVAFSPGNTRLAAAGDAGIIALYDMKHGEHVGNLTGHSSWITSLDWS
DTGEYLLSGSMDGKVKVWSIERSACVATHSETDKALWSVKWLPKTVRSEMFCTAGANRSLSFYREATGG
FUN_000255 CDS scaffold_1 776457 777117 +
hypothetical protein PF04081

MPTTRRSAASTRSRGLPAKGQSTLSFSNKVTKPVPKSAKKSAISASITKLDPSQHATQREVEDIVVNDHESIEVDKEEEVEAA
TETEVVPEPAKSELELQAEKVTDAQIKKYWKSIEDQWTTPRLHQQGVSLSEKVLRYFDVSSQYGPCIGMPRIKRWKRAERLGL
NPPVEVLAVLLKEERKGNDKVETAHMDEILNSTAVGA
FUN_000256 CDS scaffold_1 777326 778590 -
hypothetical protein PF10343 ENOG41

MSDDEVDTELLELLRQHLQGKIDIQEEPETGVLESAEYVYDSCIDVAVDMRASKKAAESIYEQMQQKSYSTATWSEHELHPKA

KDETTVNFIFTMDLLNFSFWSELPDDERFAIEYRGKRWTGYWSLVAALQRAIDEGIPITDPFYWKNEEECTLESLTHVFRSCT
EEEIPLLEERLDCLREAGQVLFKHYDGSVAELIYAADGSAARLVNLLAQDFDCFRDEHRFEDGKMIRLMKRAQILVADLWACF
NGASYGEFRDIDKITMFADYRIPQILMTMGALYCSPTVAAAIKDRKMIESGCSWELQIRACSIWCVELIRREILRQHPDAHVN
AILIDFFLYDSMKELEAAGKEPIPHHRTRSIWY
FUN_000257 CDS scaffold_1 779564 782054 +
hypothetical protein PF00899;PF14732

MNATSADQAPAQTQNAAAAPAVAPASTQQPQIQTQVQRQQEQQQKKTAPSENASPRRTLAMTRDRHNQQSLGASLNTSVKQAR
VLMVGAGGIGCELLKNLVLTGFGEIHIVDLDTIDLSNLNRQFLFRHEHIKKSKALVAKEAAERFNPNVKIVAHHANIKDDEFT
VAWFQQFRIAFNALDNLEARRHVNKMCLAADVPLIESGTTGFNGQVQVIKKGVTACYDCTPKEAPKSFPVCTIRSTPSQPIHC
IVWGKSYLLNEIFGTSEDQAAFDHSTDADNAKEIEELKKESEALKMIRDATGTSKFPQMLFDKVFNADIERLRSVEGMWTSRR
APEPLQYQTILAQACEAIANKDKILNDDQRVWSLEESLVVFNDSLDRLSKRILELKKNKKPEDPDPTITFDKDDIDTLDFVTA
SANIRSTIFGIDKKSRFDTKQMAGNIIPAIATTNAIVAGLCVLQSFKVLKGEYAQSKEVFLTPFAPARLLAPDRSREPNPECP
VCSVYFTSIVADLSRATLKDLVDDIVMSKLGFEGKEFVVNNDIGTLVECFEDGDDENLPKKLTDLGIKKDSFLTVIDQDDEDT
LVNVVINVQEGTLKEDEKPVKATFSEIPEIPRRPKKLQPVAANGNGELNDEQAVSAEPKGIKRPHGEDGEPPLKKLKITESGT
DIVDVDEVQSRAAGGAIVIDD
FUN_000258 CDS scaffold_1 782110 783115 -
hypothetical protein PF00106;PF01370;PF08659;PF13561

MSFSLRGKHAIVVGGTGTLGFRIASALADRGSVVTIMSRNAVDDRQVLEPRLKPFEPLPYREDFDGGRRPTEHQFKRLDVQRG
DIFKGLFPTLGPVDVLVNCAGTSQASALKRTGEEEIQRILNLNLRATILASKYAKLTNHGCIINVSSLMATKMGAGATVYAAS
KAGVIAFTRALALEYKSRSIRVNTLLPGWIQSPMWNELTETAREAYLKQCPLGRLGTPDEVADAAMFLITNRFANNCVLNLDG
GLSAN
FUN_000259 CDS scaffold_1 784274 786219 +
hypothetical protein PF01569

MSDPQRDAPPLSANIPAATTTTTVLPTTNGSAKGDDVPDAGLRSLDHYKRALPKWRYNLRQQMLPLIRWETPYLAWMQEKLRT
PALDSYFAITANLGTHTFFMIGLPICFWCGYAAFGKGLVHILALGVFWTGFIKDFYSLPRPLSPPLHRITMSGSAALEYGFPS
THSANAVSVAVYALLILRSPDNTLAPTTKFALECLSYFYAASIIFGRLYCGMHGFLDVIVGSIMGAAISLLEFYYGPPLDEYM
HSSSWAAPLVAALIILVLVRIHPEPADDCPCYDDSVAFAGVVIGLEFGTWTYGKIALDPWETHAHGGGSVDITHLGLAANVAR
IVFGVLVVFAWRETMKPLLLKLLPHLFRIFEQVGVNMPRRFFTPASKYKTVPTGSRIDTLFPSPSDFPRMVESIRNPTTRGRS
VSIGPQSAADAYETLAYRERKRRESVSSNHSLKSKSSSLELQTTHEDYSGKGAQASGAQANRIVEFEQMMGTGEVVTTPAADD
DRVEIFVTGTEDGLGEREMFSQLIKPRVRYDVEVVTKLVVYTGIAWFAVAIIPIMFEIVGLGTNQLRER
FUN_000260 CDS scaffold_1 786784 788065 +
hypothetical protein PF00004;PF03215;PF08542;PF13177

MSDIGDEMDVDVPVVSKDVIFSSEAKQGKRSAANLPVEAEDSLPWVEKYRPNTLDDVSGHQDILATINKFIDQNRLPHLLLYG
PPGTGKTSTILALARRIYGAANMRQMVLELNASDDRGIDVVREQIKTFASTKQIFSMGGASARSGNSMAGFKLIVLDEADAMT
STAQMALRRIMEKYTTNTRFCIIANYSHKLSPALLSRCTRFRFSPLKEGDIRVLVDKVVEEEHVRIGGEAVDALVKLSKGDMR
RALNVLQACHASSTPLRAKDAPKVPDSEIERENITTETIYNCIAAPPPDAIKEIVSTLLKTSDVTSCLNTINALKVSRGLALA
DIITALSEELVKLEVGPEVMITWLDGLANIEHRVAGGGSELIQTGAVVGVVRNGVELMSR
FUN_000261 CDS scaffold_1 788515 790074 +
hypothetical protein PF02214 ENOG41

MTSIASSDAGNLTVEPLSERNVSRIPLILPHERVFPIQIGSELFKLSGASLSSDAPSYFSQYFVCQLESAKERNDESSSAIRT
LYIDRDPVIFRDISLHLQGYHVQPRDGEHFVRLFSDAQFYSLPKLISQLYEESIFISIGHREFQIPREIFNDPGNSPNYFSLG
FAAFFSRPDDLFPGLDREGLIRPPSILPPSVPKRSADTFAELLHFLRGYPIRIRDEAHRQDLLSDARYFHFKGLEQRLIPHSL
SYNQATRRDEIVLRLENIQKSGVSVFISNTDPLTGFVQYARPYVDEKPAELVLEIGGETTKLHFSGSNARAEFFRDTKARVAK
LFELVSSKLNFSQAIKTEHLTNHGQLTNLGSALLKEDLVRIALEPESAIILDGKDYLEDFINAAASESSTNEYPRKRKRADGD
SGDEEWVVTTGQWRLRIQNAPNGRGVECILVAAKLDAMSSQLSRNMSRSFLGS

FUN_000262 CDS scaffold_1 790702 792724 -
hypothetical protein ENOG41

MEETNSPPGSPGGPLQAVTADRVNQQRERESVFSHLRGGSRDNSVHDKISQFNNLSVTMQSKQLERKTADAALKRAMLGREEA
ETELRRYREENKNLRKAIDEGKDRERKVGERLESLMENYGRAKETHAHTQALWEKEIRRARKETFKTQSSIVKLQEELKSART
NAKMIDENLKREKARSKVREQEAFEARYQIVGVQEQLDQALERIKLVEQERDAYKTAAKNEEVARIAAEGRIPLPPQDADDEF
GSPKKDKRRVSRDPRVSLSTMDIVSSEASEVEIEELSIQLQWERQRADRAHEMIEFLQAECHLRCCACSKSTRRASAEAPRQK
RRSSFGLEGPNDKVLKLDNEAVDGEKRATTPPRIEEPQARTEPETGAEPDVQPEPAQEQHPQEPPQRIIKSRKEPRRSTIFCP
KEGIFRTVSEQEAEIIEAQRKIEETEAAAKAQRESHAESEFQDVDEVVIEEQVEAETTIGDGTEMEPRYSGEYDNYRRYARTP
SVEPPAFAMLAQERTSLASLLNAPHSHSAPVPSVPTMPDVAEDILVSPDTRPHTTNAFYTVTTTTTVPVHDDKVRNSASFNER
LRTPSHNSTGTTFDMSNPALTPTMTREQALAKIRERRGRARSMAQGAMTPSRRMVEGIERRDMSAPTGKVAGKGR
FUN_000263 CDS scaffold_1 793361 794223 +
hypothetical protein PF00173 ENOG41

MADESTLRQRKPQPKNETESEVSQPSTPTKKGKKRSSAQVDEEDPWDGYSPYLDVVRVISFIIVASMGLSYVISGGESFWWGH
KNKPNWMTQRFYKDLILGPPPPVYMTLEELSLHDGTDPDRPLLLAINGTIYDVSNGRRMYGPGGSYSYFAATDAARGFVTGCF
AEDQTADLRGYEETFLPLDDPEVDSHWTPEELAELKIKEREEAKKKADAALQHWVDFFANSKKYTKVGYVYREPDWLENEKPK
KLCDQAQRSRKTRKIPKKD
FUN_000264 CDS scaffold_1 794570 795516 -
hypothetical protein ENOG41

MYFSQIAIVALAAVAEAVDVQVVSVGRNSATNATGLKFWPEKITAEPGTMVQFQFWAGNHTVTQSTFDDPCVPIGNVMSNVTG
IYSGYQPVEASMSKGMIPTYTIMVKDKKPMWLFCSKAKHCQGGMSMVINENTSANATRSLNNYKSLCSSATVSEVVPVQGGGS
PQGGNGGNGTNTGGSGGGSSGGDGSGNDSSDGGSSSGGDSSDGGSSSGDDSSDDGSSDDGSEDGSGTPTGVAAPGATGTPTAG
SSGDPVVTAAAAELKAPISMLLAVGAAAMYVL
FUN_000265 CDS scaffold_1 797734 798139 -
hypothetical protein PF09415 ENOG41

MPPKQSSGTGRGRPKATTKKGAKASEPESEPQSSNPFELSDDDNNRHGISTREAQAVEEEEPDKSIPPELLTRLLHEFFAKDA
TRISRDANAAAGKYFDVFVREAIARAAVEKDGGFLEVEDLEKVSPQLLLDL
FUN_000266 CDS scaffold_1 798660 800008 -
hypothetical protein PF00153

MSAEQPQKVLGMPPFMADFLMGGVSAAVSKTAAAPIERVKLLIQNQDEMLKTGRLDRKYAGIGDCFKRTMADEGVMSLWRGNT
ANVIRYFPTQALNFAFRDKFKKMFGYKKDKDGYALWMAGNLASGGAAGATSLLFVYSLDYARTRLANDAKNAKKGGDRQFNGL
VDVYKKTLASDGIAGLYRGFMPSVAGIVVYRGLYFGMYDSIKPVVLVGNLANNFLASFALGWIVTTGAGIASYPLDTIRRRMM
MTSGEAVKYKNTMDAARQIVAKEGVKSLFKGAGANILRGVAGAGVLSIYDQLQVLLFGKAFK
FUN_000267 CDS scaffold_1 804069 804574 - RPS25 40S
ribosomal protein S25 EOG09265SHL PF03297

MAPAAGAKKQKKKWSKGKVKDKAQHAVVLDKTTSEKLYKDVQSYRLVTIATLVDRMKINGSLARQCLADLEEKGIIKPVVTHS
KMKIYTRAVGGSD
FUN_000268 CDS scaffold_1 804918 805726 + RPS5 ribosomal
protein S5 PF00177

MSDAGEIEVENSALYEVLPKDVVKEVGNVKLFNKWDYDVEVRDISLTDYISLRNPVYVTHTAGRYATKRFRKANCPIIERLTN
SLMHHGRNNGKKLMAVRIVAHAFEIIHLMTDQNPIQVAVDAIVNCGPREDSTRIGSAGTVRRQAVDVSPLRRVNQAISLLTTG
AREASFRNVKSIAECLAEELINAAKGSSNSYAIKKKDELERVAKSNR
FUN_000269 CDS scaffold_1 806057 806903 -
hypothetical protein ENOG41

MGTPFISLLEPSKLDGYKRGAPLEEQPSSISQTFLDAMEVREQVFVKEQNVPAENEVDDDDPRSCHWVVYASVNKVEELEVRN
EEGNVIQPRKSSTRSTPIGTIRLVPFPHDPHPEDGGKYWNGILQDDNGSQNGHKNGHKKMKASSGIKPYIKDRKTTFHNGQEP
YVKLGRLAVIKEFRGHGISGLLVNTVLSWLKAHPSYFNPNIKEVGLEHLNPVGDVMVIPQWSGLVCVHAQKQVVQLWKKWGFE
IDEEMGTWWEEGMPHVGMFQRLEISEKISRLD
FUN_000270 CDS scaffold_1 807883 808639 -
hypothetical protein PF05630 ENOG41

MVLITQLLSGLALASGILASPIERRAVINHDAVVGFPQTVPSNTAGSLYLKYKPYLKVFNGCVPFPAVDSNGNTGGGLATSGS
SNGGCSSSPGQVYVRGGTYNGRYGIMYSWYMPKDSPSPGLGHRHDWENAVIWLSGESTSATVVGMAVSQHGGYDKRTSGTFSG
NSPLVGYTAIWPTNHQMIFTNDQGGQQPLIAWESLTAAARTALTNTDFGSANVPFKDGSFESNLDKAAV
FUN_000271 CDS scaffold_1 812042 818185 +
hypothetical protein PF00018;PF06920;PF14429;PF16172

MPWQPLPRIAFAVATYPFPASSPADLPLEIGDELYIIEETPDGNWLRGYLVAPPSLLAGLTSVKGQTLEARVFSGIFPRSCVE
VREMLGEPEEGDENEDAASDGPALDGDMPRASDSAKSGVTNISARKSRKDGNRSTISSKGRTPFKELPNGTLGSLSIPVDRDP
NAPRPAAPVPMLKIGDETPTSLSEPLIDEIASCLREWHSTNLHELLLSRQYSRLDQLSQLITGLNLHRQQFLYNVLTAHEYDR
LRERTVWDLVRVNKLCGGEVIVRDPEARGRVLTADDCIVKVTKLQSVMSLLDEPPQSLVELTALHHLMVDIKGFAGASIEETS
LVFYLVSKPQYGQPTTLSEGFTVKIPAGGTLVNLAKHAQTKTLFTDLSSQDVGDVPSAETELFLVVKVLASQQIIPSQPLSRS
GTGPTAPYSRDNMKPTSSGGMKSTRRSLMWGGKSSRSGISRGSPVTRMDAVAEQNDERLSTSGAESREGTGPPSTAGSKTGSP
PDGVQIADRTIGLGVLRLNPIMKQDDEVEHLVNIWSTSSRHAGEKDAGDDWDPLVKELLDSPSGHYEKSRRGERLQLQLKSFN
HSDADILIKSTPTMLSGVTKTSKMGFSGAPTRPRSDIYLTLTDAALGKQNLLSRFGGSATAMPSSVHGNNLQITLEVRKTSGE
RIENCIFASSNTEALSTFKSIATERGEPWNQTLRLALAPNEVPQAHVVMFVSDMPNPPFAIGHMPLWDQGAFIRDGLHGLLLY
KIDEHTSTAQPGPTGKGGYLSLSWSPQGKDDHPAEVTGPLAVLRIDTYLCSTRFSQDRVILGLLKWRDLQREEVPGVLRQLIF
VPEIEVVKLLSDVLDGLFGILVEYSGSHEFEDLVFTALVRVLGIVHDRRFNLGPLVDQYAENQFNYPFATPCLMRSFTRLLQN
PTEPETARKLRATFKVVRHILKFITHARTQQKEKEAGIGITSNTTGFTRELRAIFKALDAMMRNSAPALVGTQTLAVQHFHTW
LPELAGLLTTEEILHIAIDFMDSCIDVKGKLVLYKLVLIINYGKLDLFAHPDQKLALSTNTVRWITPHWGHSEEVTDQWRDQI
RLCCSILASQIDYLGSEIPDHIPKIIDSYLAILAAPRTPKDRLSLLFPSSYPFPTKPVNEDLQFDEALVELSAILSAISNSPS
GMQLELAEDDLTTLLENLLRVHMSILKGEAYPLGWLSVHIHHHKSTMRTLEYLAGTMLEAFLPDPEEAESFNTELWKLFFTTL
LKLVGSTSLALETFPEQKRRAVWKIAGDVREHGAELLRRTWEAIGWETTPDERSRYNLSKMGGYQVQYVPALVGPIVELCLSV
HEGLRRMAVEVLQTMIVSEWTLSEDLSIIQTETIDRLDLFFKEKPLTESTLQKLFIPELLERFEPLAEIPDEPLYTALREFVG
TIDEFLDLLVAVHSSEDTGEATHLINRLRLMEFLRDMQKEEIFVRYVHQLADLQAESRNHTEAGLALRLHADLYEWDPASQTS
ALPDPEFPAQTQFERKERLYFDMIKYFEEGESWSHALAAYKELQTQYETNIFDFSKLARTERAIATIYETISKSDKLVPKYFK
VVYKGLGFHANLRDKEYIYEGSPSERASAFTDRMQEQYPAAQIVTGGDVDDVEGQFLVISAVTPHRDLSHHVFQRARVPQVIR
DFLLSSHPQTFSISTRRNTNGPVKEHSAEKLVFTTADAFPTILRRSEVVDTQEIRLSAHETALERIVRKTQEMTALEHRVADG
NGDHAQLLLDAVSVSVNPTSESSVACYRQLLPEPKEVNDDADEDEDQEASEVELTPQDSAIKMALVDHAIMIKRCLAMFARSP
NEILNSRYEELHRFFESTFASEIAHFTPPQPQREMVATPSPTWRRSSRSSEASPKHKNIGVSVNGVTTEEASVIRPVSKRGTR
LSFLGGSKKKEPELPTEPEEPAVDIETASNLSRSLSRSQSKEHNRRHSFFRTQSHDEKHPESPGGFSSRGSFDQQFGGGNEKA
VENKIPSKKGSVRKRFSMLKLGRKGSKGNGMMGSLDEE
FUN_000272 CDS scaffold_1 819245 820805 -
hypothetical protein ENOG41

MPGSWDAESPSTTKTHEYYERDIGTRKGTSQHPLKEFHHSREIFNFLSSPGEAQPQSLRKAEPSLTTEEIADEKADRLTKKVM
SMEHRLSSHMTLASKAKELAEENRVLREQFNDAQSHIFSLQPYRKDLTPEEVGQDYDALVEQIQDWVQKLVNPWLEDCDDDAH
AFLTHAKRRKADANRFKSAWRYVCVKFETVNTSNGADHFTVRTWTAEAYNTLLSCPRFKPVRQRQAKEMAVQLSDILGIFPQE
ENLHSCYQDLTGLVVFIDSFMRLDCKNVLQNRKGFNLQKLDPPPSEEDLYHKLHSVYTVVPAFYMRQVGQGDALRSPIVVRKQ
QILVAWG
FUN_000273 CDS scaffold_1 822683 824572 +
hypothetical protein ENOG41

MEVGKPQTADRKIIVGVDFGTTYSGVAWAENRNPSRRTCITQWPVSSANKEGQSSDKVPTRIRYNGDKIEWGFSVPVNAPQHE
VVDWFKLDLDPSTQAVGYNPSTEGARSGRNVDELVTDFISQLVDHLMYTLREKLGSGLVDSTPLQFLITVPAIWSDLAKEKTK
KACEQAMISVSGHHVHLVSEPEAAAIYALHDLDMNGLDAGDTIVVVDAGGGTVDLISYTILSTKPILEVQEAAPGSGALCGST
FLNMRFSKFLQSKLGKEEGFDDEIMAEALDVFENKVKRQFALNVEPDETYNIPVAGLANNKSLGISRGRFALKASDVNKIFEP
VVLEVIRLVKDQITASNVPVQAILLVGGFGSSNYLKERLRAAIDSSVRIMQPPNAWQAIVQGAVLKGLAQSSPDCAVVKVENR
KARKHYGTEWSVRWDEKSHGDLKSERYWCGLDGCYKVRVMEWFITRGSTVSENDPYIAAFVWTNPVSKGRIRKVKMTIYNDHL
SRDAPLSRNDNVKMLSIVEADVNHIPENQLRRRKGCDDQWYYELNCKIEAIYTSASTSYTLIYQNQRFSTVTCEYV
FUN_000274 CDS scaffold_1 825360 827226 +
hypothetical protein PF01485 ENOG41

MLCFKGSKGYKVVPKKKKVKAATSTSTSTNQTSAPRTRNGTGTGTAAVTQPTNRVAAKSTRPSPDRSRNPPYSNSRSSPSRRH
NPEQGPPIQPPRQQSKVARTNNMASQNGIFYGRVDSAITSGFEDDESATAVQNPTSKSQGSSSSNTAQSQAYDTIPASSTQAP
FRYQRPSFLDDFDFTAESRRDIVVAHGVTSALSPRQGYHPTPASILNFADFDLENTDTKISDIRHDRIPFHERPIINTAAAAS
SSSSSSASPYVDLPESLDSDATTTRAIDRRECIVCSDIKATDSFPDFVTSKCTHTPSICLDCMERSIQVGMKSKRWTDIRCDE
CQENLEYDDIQRFADEQTIAKYERQALRAAVEEDENFFWCTSDCGSGQIHDSGSAQPIVVCIKCSHRSCFRHGVNWHEDLSCE
EYDRLQEDPDFRGHLELENERWSKAQAAQAAQEEADRDLARRFVAKERAKIKRQEAQDQRERERERERQKSQRERERERQKYQ
ERRRELEREKQERQRVRVRERQKNQERQRERARERQKNQERQRELERERQERQRALREKASAQKAAEERRQIAARRKIEEAKS
IATLRATTKPCGGCGWAIEKNSGW
FUN_000275 CDS scaffold_1 828192 830475 -
hypothetical protein PF00005;PF06472

MASQSKLALPGDRTLRGIISQLTTLYVSNRTRISRAVWITLFAALANRIRLAISEQKAASAREAAQRAARRGTTSSGSEETPR
KKVELNREFFRSLLRLLKIVVPGWRSKESRLLMSHSFFLVLRTLISLRVAEMDGAIVKALVKGNGKEFLKRIVWWMLIAVPAT
FTNSMLSYHQAELSLKYRSRLTQHIHDKYLSNLTFYGISALDDRIKNPDQLIAVDVSKFSNSLAELYSNLAKPLLDMTIYTWS
LSKSVGGEGVVFMSLLVQLSANVMRALTPPFGKYVADEARLEGEFRFQHSRLIDYSEEVALYGGHNAEKDTLDKGYFTLIKHV
NYILRRRFYHGFMEDFVIKYFWGALGLMLCSVPVFVKMPGHIAMNMGDRTEAFVTNRRMLLSASDAFGRIMFSYREVMELAGY
TSRVATLLDVMDDVQAGHFEKKLVSSSGVEGNEAVLKGRGTVHESNDITFIDVPIISPNGDVLIKALSFTLKHGDHLLVIGPN
GCGKSSLFRILGGLWPVYGGTVYKPPFHAIFYIPQRPYLSRGSLRQQIIYPDSLRQMRARGVTDVDLLEYLKILGLEHLPELY
EEGWDAEAEWRDVLSGGLQQRVAMARLFYHRPKYAILDECTSSVTLETEKAMYDTAKALGVTLMTVSHRRSLWKYHSHILQFD
GQGNYVFTKLDADRRLKLEDEKEELEVRLRQVPELERRIAELTAA
FUN_000276 CDS scaffold_1 831105 831416 + DPH3 Diphthamide
biosynthesis protein 3 PF05207

MSDDEGLSIYDEVEIEDMTFDEAMGVYQFPCPCGDKFQITLEDLLDEQDIAVCPSCSLMIRVIFDLDDLPKPPTSGTSGGQVP
IAA
FUN_000277 CDS scaffold_1 832144 832442 -
hypothetical protein

MHASAAVLAFVAVAAAAQTPVAPVATPVATPGAPTNGTKPGSGSGSGSGSGSGSGSTPANGAAALGLSGLAGALAVALAL
FUN_000278 CDS scaffold_1 835324 839023 - LEU6
2-isopropylmalate synthase EOG092601KZ PF08264

MASTDSVPKAMEALNISKTKELKGTEKRDTLVALEKKYQQKWQDDKVFEANAPTTDEVPLHSIPAAELREQQPKFFGCMAYPY
MNGTLHAGHSFSVSKVEFAAGVARMQGKRALFPMGFHCTGMPIKACADKLVDEVKMFGQDFSGYKDDEPVVEEKAPVVKLIKE
DITKFKATKGKAAAKSVKMKYQFQIMQAIGIPTEEIHKFADPQHWLQHFPPLCREDLTNFGCRIDWRRSFVTTDANPYYDAFV
RWQMNRLKELNKIKFGKRYTIYSIKDGQPCMDHDRSEGEGVGPQEYTALKIKVLEWAPKAAEALKGKLPEGANVYKIAATLRP
ETMYGQTCCFVGPKITYGVFKANDNDFYVMTERAARNMAHQGIFATEGVVEKVADVLGSDMVGTLVDAPLSLHKEGVRVLPME
TVLPTKGTGVVTSVPSDSPDDFATVTDLAKKADYYGIQKEWAELEIFPIIDTPSYGGLCAPFLVKKLKIASPKDTKQLEEAKE
LAYKEGFYQGVLKVGEFKGEKVEVAKPKVRQQMIDAGQAFAYSEPERKVVSRSGDDCIVALMDQWYLDYGEESWKKTALDWVE
NGLNTYTTETKNSFEGVLNWLNQWACARTYGLGSKLPWDPQFLVESLSDSTVYMAYYTIAHLLHNDIFGKTKGSANVGPEQMV

DEVWDYVFCRQELSDEILTNSKIPKETLESMRREFEYFYPLDVRVSGKDLIANHLTFFLYSHLALFSQEYWPQSIRANGHLML
NGEKMSKSTGNFMTLRDLTEKYGADASRIALADAGDGVNDANFEEDVADTNILRLYTLKEWCEDMVRDQDQLRTGELNSFQDA
LFNNDMNAVIKEAVEQYVNTNYKLALKAGLYELTSARDFYREACAAAGIKLHRDLALRYIEVQALLLAVVAPHWSEYIWLEVL
KNEGTIHNARFPEIKEVDASLSAKRDYVRNTASNVNSAEGLQLKKKAKGKETSFDPKKPKQLTIFVTDKFPAWQAKYIDLLKE
MWNVEAKSVNDKELNGKIGKMGEMKKAMPFVQNLKRRLQAGEPASVVLEQKLAFNEKETLLQMVPGLKRTSGLVACHIISVEE
GSKKGTNLGDGKEVELSVPTAEHAVPGQPTFHFENVEA
FUN_000279 CDS scaffold_1 839361 839895 +
hypothetical protein ENOG41

MFPTLIRRMASSSKDSLLKKAPLTVATNPYKARKVWPPNFKQLTVQQQLRFEKKYKRRIILASRAPRWVKFVKTAQLLTIVAA
LIWICFYSEFEWWGQKYKPSEEIRKKTQYLFGTVDPEKRYERRPDAPPFHPPKDEPESK
FUN_000280 CDS scaffold_1 840041 840665 -
hypothetical protein EOG09265FGA PF01217

MINAFLVFNGQGQPRLTKFYTQLETSIQQRLISEIFTLVSNRPAGSCNFLPLPPLLAASGTSHTSSEEQNDVPTLVTYRNYAT
LYFIVISTSTESPLALIDLIQVYVEALDKLFENVCELDLIFNFETLHSTLSEMIIGGVVIETNLDRIVSGVKAQGTVAKRPVN
EGRGPTGLGSGLGMGANFAWTGR
FUN_000281 CDS scaffold_1 841076 841743 +
hypothetical protein EOG092659DX PF01491

MSRQIAFQASRLASRTIRSSTPICRSSFRIAPAITHLSIRSTLSQKCFSTTVSIKRGIMPDTENPAKPLNEAEEIHQNAVVAD
ITEQEYHDLADEYLDNVVGKFEELQDQREDIDVDFSAGVLSISWPDKGTYIINKQPPNKQIWLSSPISGPKRYDWCLFGEGQN
EKEGTAVGDWIYVRDGSSLNQVFSEELGVDIDVPSEE
FUN_000282 CDS scaffold_1 842257 842698 -
hypothetical protein ENOG41

MATPTEIRHAYRHLYRNLLKAVQYAIPARFVARDQLRRAFREPGATYDGRGIKRTIWFLQAAAKEKGLEHKILKNLLRVRQMR
YRKKDYSAYDPLKYAEQEMKEANATAYNHYDMTVAMLNKTMGICLR
FUN_000283 CDS scaffold_1 843201 845717 -
hypothetical protein PF00083;PF07690 ENOG41

MTSLSLGLSPSLSPHLPKIPPSKSSRLPSNIPLRTLRPDNQQYQDPDSQATSLLASRNDHDRDYDDDSDDNRQHRSDVAKSHR
RSSYSGSESSWTDTGDIGEQLADEHDPVRLQLSSDVEDELLANVHRRHPKHGNQKRVRIQDSHHHRHSQSRSALINKEAIEVP
NFNPPGPSRAERVLGAIMAGRTGSIHGLTGKALIYFTSIFVSLGVFLFGYDQGVMSGIITGPYFMDYFDHPSKAYVGTMVAIL
EIGAFITSLMVGRIGDIIGRRRTIFYGSCIFFVGGALQTLATSMPMMMAGRFVAGFGVGMLSTIVPVYQSEISPPHNRGKLAC
IEFTGNIVGYSTSVWVDYGCGFIESDYSWRIPLGLQCFMGALLGLGSLVIVESPRWLLDNDHDEEGMVVIANLYGGGDIHDPK
ARDEYREIKMNVLLQRQEGERTYKDMFRRYKTRVFIAMSAQALAQLNGINVISYYAPYVFESAGWVGHDAVLMTGFNSLTYLM
STIPPWYLVDRWGRRKILLSGATAMAISLSLISYFLFLDIKWTPRLVVLFVMIYNAAFGYSWGPIPWLYPPEILPLSIRSKGA
SLSTATNWAANWLVGEMTPILQEWIKWRMYLVHAFFCVASVVIVYFIYPETCGVRLEDMDSLFGDASTVAGTPSLHGTETGSL
MRAGSPVGSSRGLIGPSSAIPGLSLDPPAIDDDSKSQMQTGGDERSIGGWLSRVVGRNRSDSGSSAQGRYAPLGQQEEGREGR
RDDY
FUN_000284 CDS scaffold_1 852967 854066 +
hypothetical protein PF12271 ENOG41

MGSTEFGNFHDFCRDSTLPVCNLLSQNHDQNGNWGGCELTGISLSGGRHLGNLGSILLAGAAIVTAVFLLLRSEKKRAAVGRR
EMQMFLIGYIIISICEIFSVGEFPLNSTVRIAFSAIHIGMIIATCWILMLNAVVGYQIIDDGTPLSMALIAISALLLLIGTGY
IALDTGFSWTGYWDDSYEAPRNRNIALYVLYQLVPLILLVAFLVLEAILVIRILGETRPMIYLAAAALLFAIGQVFNYAISKY
ICDGTSGKIDGALFQTLFTLLSVIMVWVFWSSITEDDWPMPVTNTYP
FUN_000285 CDS scaffold_1 854918 856378 +
hypothetical protein EOG09262ILV PF04056;PF07975;PF13519

MADSDGEYVADMSDDDLMDHRVTDDDPQAAKGKTAKSRKGDSARAWEVSKRTWETNLPEEEDGILSLTALEAEKRKRLLRDTT
PLQRGIIRHMVLVLDMSFAMTEKDLLPTRYRLMLSYAAAFVREFFEQNPISQLGIIGMRDGVAVRISDVGGNPTEHLEKLKGL
ENEDPQGNPSLQNALEMCRGALFHAPSHGTREVLIIYGALLSSDPGDIHETIGNLITDRIRVSIVGLSAQVAICADLCSRTNA
GDESQYNIAMDEVHFRELFLAATTPPVTRTAEQSTASLLMMGFPSRTLVPNGTTSYCACHNRPFREGYLCTRCGARVCRIPAE
CPACDLTLILSTHLARSYHHLFPLRNWVEVPWTKASRSAACFSCLAPFPEPPKGKAPDKSREDSGAPKTAKGVSESGRYACEV
CGQHFCIDCDVFAHEVVHNCPGCQSLLSKTDAAASSGEPNGTAAYTNGDVVMT
FUN_000286 CDS scaffold_1 856792 858067 -
hypothetical protein PF00903

MSPSAITNSPPQQATQPTTVPITSALDRADNTFAVQPPPNFTGYDHITWWVGNAKQAASYYTNLFGFKPIAYKGLETGSRYFA
SYVIENNDVRFVFTSPLRSEAHFPDDEPISKSDRKLLKEMYAHLERHGDAVKDVAFEVENVDGVYHKAVQEGAIAVQDPKVSK
DKEHGSVSTAVICTYGDTTHTLISRQNYTGPFLPGYRTWKKHSATVSLPDVPLARIDHCVGNQSWNEMDSACAFYEQCLSFHR
FWSVDDSQICTEFSALSSVVMASPNNIVKMPINEPALGKKKSQIEEYVIFNSGPGVQHIALLTPDIITAVSALRARGVEFIDV
PPSYYTDMRHRLKTERRNWELKEEFETLERLNILIDYDEGGYLLQLFTKPLMDRPTVFIEIIQRNDFDGFGAGNFKSLFEAIE
REQAERGNL
FUN_000287 CDS scaffold_1 859139 859937 +
hypothetical protein ENOG41

MASVCLSKHDINILEKIKDPESNPYAGIVLDSSLPRDPNITDATIYERVVERERDIIRSIQSLETQLKSLGSEEGKDIAVKGY
QQSLSAVESMIAEHPNYASARNNRVQVLRRLYGDAMMLSDTKDNSAPLIESPDQEERKKAVVTALSDIEASITLLTPSSPTMP
ISPQVARTLSMAHTQRAALYLKTARLMLSRSLDIDGTLEESKWEKLDFEGAASRDLAFGGRYGNPIAKGLAVSVNPTAKLCGQ
IVREAMKKEYGPSFGD
FUN_000288 CDS scaffold_1 860610 861317 +
hypothetical protein PF01777

MKFLKVGRVAIITRGRYAGKKVVIIQPVDNGNKPHPFGHALVAGIERYPSKITRRMSKTRQEKRNKIKPFIKVINYNHLMPTR
YTLELEGLKGAVSNDTFKEVSQREDAKKTVKKVLEERYTSGKNRWFFTPLSMLYHRCAIPPEQSCIKTRLVLLTCI
FUN_000289 CDS scaffold_1 862127 863529 -
hypothetical protein PF03151 ENOG41

MSADEKIRVSGETPRATTPVLPTVNPGLEKSQSARASIHPTFYVIAWIGFSSSVILFNKWLLDTLNFRYPVILTTYHLTFSTV
VTQIMARWTPYLDGRKTVKMTARVYIRAVVPIGIFFSLSLICGNLTYLYLSVAFIQMLKATTPVAVLISGWILGVSAPNLKQF
LNVSAIVVGVIIASMGEIHFVTVGVLFQMGGIIFEALRLTMVQRLLSSADYKMDPLVSLYYFAPICAVMNGVVALIWEIPRCS
MAEVYHVGLFTFFLNGLCAFMLNVSVVFLIGKTSAVVLTLCGVLKDILLVIASMMIWGTQVTGLQFFGYSIALGGMVYYKLGY
EQIKGHIADANRQWAEFGARKPILRKVTIIIATAFLIFTFFGGLAPGYSSEVDPTRLANEVSNRFGINDAQHV
FUN_000290 CDS scaffold_1 868146 869964 +
hypothetical protein PF01593;PF13450 ENOG41

MADVNVDVLVIGMGPTGLGAAKRLNHINGPSWLIVDSSDKAGGLAGTDTTPEGFLYDVGGHVIFSHYKYFDDCIDEALPKDDD
WYTHQRISYVRYKGLWVPYPFQNNISMLPKEDQVSCIEGIIDSALECRVANTKPKDFDEWIVRMMGEGIANIFMRPYNYKVWA
VPTTKMQCQWLGERVAAPDAKLVTKNVILNKVAGNWGPNATFRFPARDGTGGIWIAVAETIPKEKKRFGKQGEVTKIDAEKKI
AYFADGSTIGYNKLINTMAVDHLAEKLGNQELVSLTKQLYYSTTHVIGVGIRGERPERIGDKCWLYFPEDNCPFYRATIFSNY
SPYNQPQKDAKLPTLYLANGEKPASSEAKEGPYWSIMLEVSQSTMKPVDVENLLKDSIQGLINTEMIKPEDEIVSTYHRAFDH
GYPTPTLEREGVLKQVLPKLEAMDILSRGRFGSWRYEVGNQDHSFMLGVEAVDAIHSGAVELTLNYPDFVNSRQNTERRLTQN
FIVAPPQTNGTKDIPSHSKAQ
FUN_000291 CDS scaffold_1 873333 880214 +
hypothetical protein PF00072;PF00512;PF01590;PF02518;PF13185;PF13191

MPAEPDSRTDIYSLGVLFWTLLTQRPVFEGDSPLDIVQGVLGRRIPNVSTVRLDIPDVVGRIIQRCTARNVQDRYHSASGLRH

DLVKVQEFLGDGDWLALKEWRIGTKDVSSFFMLPAIMIGRQHEQAELNKVIDRVAKSHAMNLKGNTNRFSDGSTLSNDMAAFD
DISSEGASSVDGTNYRSGSFTHTASSDPKLPKTNFNPLFSDTQTVSGDTISSSPSGTLPRIARPWERHQSISIETRSLVDSSG
PDSQNRHSVVESTASSLSRQLGSAKFRRRDHCEVVTIEGAGGLGKSCLVQSVLADARRRGYCATAKFDTARRTAFGPLLKLLS
SLFKQVWGERNTETPFHQGLKQFVRPVWPMLHRLLGLPEFLLGPPEASIGRSISSSLQFASIRGNGKSGLKRRGSSPGSSPKP
VARNPSVASQSSSDFLRTGTSTKTMRLMNIFLDVLRVFTAHKFICFCLDDLHFADDESMELISQIIASRMKMVIIMTYRTEEI
TWDKIQSMIHLPEGEAEYPRSGGPLVTRITLSPLTEDDIVQYVSTTLCRPKEEVLPLALVLQSKTAGNPFYMREMLSACHRKK
CIWYDYRDSQWHYDLDRLFEQFQGEKDYDILDTGFITHRLSELPAAARTLLAWAALIGNSFSFELICHLMGGEFDYHEDDKGS
CCATFTHKTYSQGEAIAGLQAAIQAYIIVPSETDDRFRFAHDRYIHASADLQECNSRKMHFIIAQTLQKYYGGENQQLDNTAS
HICAAVDIIKRRISNRYQYRKLLTSCAQAAAESGAKPTAAKYYNTALTLLQDDPWTDGKEDVSYDETMQLYLRAAECYLYMGQ
YTAANEILNTIFKNGKSPFDKAPAYVLQSRIYAQSGDSASALVSLKDCLVGLGVTVDDEPSFQKCDEKFERLSIQIQTMGRGE
LLNYKQAEDPSVAPVGAVLAETISAAWWSDGLRYYHLTLMMVEMHLERGAYPQSGMAFLYLAMIALSRFNMTQFAVELGNTSQ
ELLYQSRDPFFLARGLMLYSMCIGHVHMTMPMIMAQMEDAVEYASVAGDRISTIMSYGVSSVFKFFSGETCSDLEGFCQYGCE
EIPNWYADSRGGSILIAVRQLCRALQGKTRTSNALEVMNDETHNAENYKKWLISCSRKSNRSVTLYESFEIIPLFLYGHYERA
VEIGSHAVDQLSMLWSAHNSRLILLFHGLARAGYLLQKLQLPCSPTEDFSSQAEEVIKELRRYVKMMDEWCTVSDINYLSWIK
FLQAQIEELSLNHGNAIQHYEQALDHAAEHNSVFDEALGNYLMAGMFIRRSARRSARAALLEAVGLFRQMGALGVSKAIEEEH
SLLLHGPTRNYRTCDTGVQTDFVADATSVQYRTGDAEDGGDLARIPSNTMNMADLNKGERIGAWRGSMNIQAEPGAGLPALDM
IDLHAILVSSQVISSMLQVDELLKTMCDVILQTCGGSATLAAIVVQENDMDTWCVAASGDPERGASAHIPGIPISGTSLIAEN
VVLYSTRFLESVFVPDLISDERFGNVNESWLQRNPVGKSIIAIPICHGNKPLLGVLYLEGEPGAFTDRNVTVLQLLVNQIGIS
YSNALAMKNVEKISAENRSMVAVQKRALAKALEAETKAKNAEAEAKRNVKLAEEAAKAKSIFLANVSHELRTPLNGVIGNSEL
LRDSDLNKDQLEMADSIRVSADLLLTVINDILDFSKMEADKMKLYIIAFNPEDMVREVVRAVSYSNREKTSKKNVKIVQNINL
PPMLIYGDPIRLHQVLGNLIGNSLKFTEDGSITIGARLDKETKEHATLTFWVRDTGIGIPPQQLAKLFQPFSQADASTARKYG
GSGLGLSICKSLIETMMKGKIRLESEENVGTTAWFTVTFEKAKSDVAAGDAQTKATPSVDRYYAATSPVERAESPNPYLDLSK
VPKDEIRICVAEDNPINQKIAIQYVQRLGYKGVVAYDNGLKAVEGLRQKAKEGKPYHVVLMDVQMPVLDGYEATKLIRKDSLD
AVRTILVIAMTASAIQGDREKCLAAGMNDYLAKPVRSEILKKKLDAYLSVEHPPPKPESSPPLQRDSSSTPSPHTQSGVATQR
SNSSLVLPSPMVKPSAGTKGLANVSLNPMQEEAVFNQPVDQTALQAQDSPVYNREQEPSPLAIRGSSPHSMNASGLGRVSSDD
RSIDTASIADSSTSQSQKRLPRKLVKNRRNSESNSERPSLSNISNDKSRGVLKKPQRQSTTNLVEEGQKAENNSYSFGKDSSK
RSSLTSSASSLKDRIFPH
FUN_000292 CDS scaffold_1 890361 891964 +
hypothetical protein ENOG41

MLTVKQPPTYGYKSVHDLPTPPSTSRPSPPLIYREPANNSLPVAYRGHSPPSQPMSAPHRGLPPPAAMTLPPQQPPAAGAPPS
THLPPHPPPPPPPPQPSLGPPGHQQRDSWGQLPAPPQQWQGAEESMRHWLQARAEEDKRKQEEERTKQEEARTRQESLRLEQRK
VEMDMLRTSLQAGIPPPMVPLVFAGMGGGGVAPQAALEWAQQFMPPGQAPPRAQIMPAQWPVSPEHQREPQTLSHTHAQHPGI
PSASTPAAGYVYPPSPSRPRSQTVSGAIGRPMGISTLPSLNTNVPQPAHAPPMHSHQHPHMQQAQQQQQEPQSSPSIYFHHWQ
PPTSQASGSSNRPGSPSESAKQQGIMPLPPPAVSSDIDHLRLLVKAALRAQLFVDRDVVTRDKEVTCHPSGSSGRDGLAERVL
ADLYAECHRYAAQRLYPRKKDPAARTNSRYQLYCQRSRRLAHGIQGPVARAKSEKTATSSHHLEIQSRIGVSRKPQNREKIQG
KDKALDGSAP
FUN_000293 CDS scaffold_1 894589 897989 -
hypothetical protein PF00566

MLPSKFGSVRQKQPERRTGLRLDPSIVALRYEKTIPAEPPILIVSRDPTRLSRRPSSSSASSHSSRHRPIVIVPPGIVAPPAE
EHPALRGSSSSSSSSSSSTPTLRTSPVSVEEWKRDSGVARNKSTSTIHEEEDEDGHSESECKNKIPAVESNASSLVSSVMPQP
STTTQRDEDPFVDENSIFEPLTSIPTDPFCVVYNARCTTPPPPPQVSHSDTPSPLPAPATPTRPSRTTASTVPFTIPYSECLA
GKVPSSPTYPPLSGSRSHKSPCSSLPSSPRTSCSSSPPTISKGISRSGSSRFGTFSQMFNTPIQPLWRGTASTANSSASSTPT
VSAQSTITTALGATANATVNAPATDADTAIDLAESGSLPSDKEQGSSRFWNRSRSASTTSNNSTSTATLAGTTTISSNNDLPF
VPLDVSIPCESLLDDAFMNTVSFSNRGSIMLGAQDAAAIDGAIKQHTMSIDSSSSTSTSTNADTFATRNNDESDSGRATPTPT
PSTTAPRSITPTPTTPTTPKSQTLPRPSDESNDMNNSVKGAGDGSPQGLMSTPDIRVLAADVEKESQKVRSLYTVGDGSHGEP
GKRHSYCERLEPTPEVPSEENELDPVPNHLSPAWQMPASGSSSSLRSRGGGLEDWDDLEGAHVDRYGFITLPPPPGSRMGTPT
ETRSASGSPRKRSLLQKRDPYSLTSTLSGSGRRTPTRKVSARSLNTQTSELSVSTSLRSSRSVIRQASNLLPHNRDRRWMDEA
GDMLNAAPSLQDIVDEIQAEKLTEVMKRKEWERSEKWRKMAKVVRRGGEGEGMEFEFDMRNPKLIERTWKGIPDRWRGAAWWS

FMATSAREHEGSPSEERIVAEFHRLQRRSSPDDVQIDLDVPRTISRHIMFRRRYRGGQRLLFRVLHAISIYYPETGYVQGMAS
LAATLLCYFDEEKCFVMLVRMWHLRGLARLYRPGFEELMAAMGDFSKHWLNKEVASKLDELCIDTTAYGTRWYLTLFNLSVPF
PAQLRIWDVFLLLGNDQPSMSESRKSESNTPQTGEYDVLHATSAALAQALREVLLDAEFENAMKALTSSIPVKDEDLLMKVVK
AEYKQHHGKKKT
FUN_000294 CDS scaffold_1 902209 904831 -
hypothetical protein PF00023;PF01843;PF12796;PF13637;PF13857
ENOG41
MDLGDSINPEGQPEEKPRTLPADLPRSLDDRRHVPNEHLVTETEMYDGWQGQSQFLTTPAIAKPLNFGNLSLNDPEYEDDITK
GPKDSDTRLMEMLAAQAAHQAAPAFENEDEVVNSESLSEAEKKEKLQKALNMAASNGDVDRIRKLLNGKAKDYLDLDAEDEDG
TPPLIYASCFGHEPVVQALIDAGADVNKQDRNQWTALMWAMTNRHKGIAKLLLDNNASSDQKTSSGRTAFDFVPPDSEMSFYL
HDNGYNIGSAGTDDFYRPGFSQDRFEEEMAENEMRRRLMMESARDLEVDLGNVGMDDQPEPVDEFEEEQQEFDWNRCLHDQMF
VFQEHELERILDIVITNMTPQRSPTQKPVPANMIFLSARYAHYHSSPELLERLLVSAMDYINDVVERCQWDMTILAFWISNAT
LLLHYLKKDPGLFEATGEFQAQLAELINEIFILIVRDAERRLDKVLDVAMLEHETIPGFEDITFQNEWKLFKRKKEVKEEPLE
KRFRPPSPKQRAKPAPRNVTSLLSSTLFVLDLYDIHSVITAQIISQLIYWIGAELFNRIMSNRKYLARTKAMQIRMNISILED
WARTNNRQAEHFEGGEMRSSGESTMDAARRHLAPVIQLLQWLQCFSSLNGDDMEALVITLQQLKRLSPQQLIHSANHYRPEVG
EKGLPKSAMKYLINLQKEAALKRERRRSGVPSPQKSGQDSTPVTPVKGRSNGNNLATPGSTDSPPEDDWDEDDDMPEHLLLDP
ALMLPFVLPSVTDMLVTYGAGLGGVNRERERKYIPTVPPEFLEKLEVSGGTRKDPMFGEADWENEEV
FUN_000295 CDS scaffold_1 905429 906569 - ALC1
Allantoicase PF03561

MTSIADEIEYKLDNVEVSRVRPDDINKTFRSTCIDLISSGLGGKVLGYSDQWFCEASNLLNPRAPIAQPGKMVFTGAWYDGWE
TRRHNQESFDYAIIKLGVASGTIEGVEIDTAFFNGNHAPAISVEGVFSQDDKVVSWGGGRGEWETILGIQECGASQRFAWKLK
SPSQKAYTHVRLNMYPDGGIARFRLYGHAVPVFPEDKDVIFDLAAAQNGGIAVSCSDEHFGTKDNLIIPGRGKDMGDGWETKR
SRGKDHTDFAIIKLGAPGYIENWVVDTAHFRGNYPQKVSIEGCEWTGHGDPVADATAWRVFVPPSKTGPDQEHEFESEEKEKK
VLVTHVKLIMIPDGGVKRLRAFGKRAV
FUN_000296 CDS scaffold_1 907554 908361 +
hypothetical protein PF03188 ENOG41

MASAAGVPERFPPIEDLSARETEPLLGRPGDAAQEDGVPLIKNLVLGTGIVAQLGVVLLTILIWASVLSKPLILFSAHPLLQS
LAVLTLAQSVLSLQPTHTAEQKRVGQRVHASLNLVAFLILVAGVTIIEYNKIANNGPHFHSVHGYLGVITSIVLLLQYGVGFT
MWATPALYGGVDNAKAVWKYHRWSGYIIFVLLLTTVVSAVETDYNHNVLKIKLWAVLLLAIITLVGVVPRIQKQKLGFPGPGS
T
FUN_000297 CDS scaffold_1 914753 920815 +
hypothetical protein PF00072;PF00512;PF02518;PF08447;PF08448;PF13426
ENOG41
MTTDRHPSHPPSSPLDAQSKEPRVHNNMIGDPTDESTVSVIKGSGNTAQPLAARAGTDALSPRRVGFGGAPSPTQASSQKQLN
EYREKLARDLERREMSARGLMASPNEKNRVSPIPEEGLSSSPLQTSALTTASTESNATVRGAPTPGPIAGTPSYPFPRMAPAG
FVPSYLHRPFTTLSPTGAPRSFSYGSFDALGMAAQDKVLSSTSTPASNFTFNPSAMSPQPENLDFPNPNLYDLSLMLAAEPGL
DAWWSNVVQIMRDVYKAERVTLAIPADTTDIENVPWGQKATFNLHKEDDLSLGYMAAKQSSSIVLSTSDYDRSWPGSNATPSI
SDDNLHAKSASRPDLFSRHSFTTYEDKRERTRTTPNAQARRPAFLARSQSNYPAGPASKESYVDYAKLNKTTLDEHDAIEQQI
PSWEAPLMGEKESQGRVLPVLQALDYEADPLIDHSGIMRVLERGRAIALTRSYPYLADEAPAESSKKPKPAAPEPSNAPRNFA
KPRSDSFSKLSSMFNSASSSRVPTRSKTHSAADRAKSIASRLEGRLEDDMPRPPTPKYEEFEQAPPSPWSQSPAPSPAVRADP
KENPFFTDAMVDEDSFNPDTGTEDYTGMRPPEAIGVDNSWTVLHIPLSHVLLSKPTRTFKLDSTLMEQRSHARTKSEGVPAPS
DEGGRNTPDSLKRDKPAPIAILSILSPIIPYPSNLKHSLEHLAPHMATTFSLCRHYSNLETELAGLQRKRPQTAGFGAVAHDG
RPLESMAYLTADDIVPHQSLAGSMTSPSDYSGVSKSATGSPLGTPGWDQGSGGFHADKRPPPTSSPAATPQGEGYFSSRQRPV
PARHETFGGIGTPRARTSSKDTSPAEKRSIRVSGSFLTQESPEQNLVADNKATRTSSDESTGGLAKKSTQVDAPDVSIDTLDP
TNNDNTAEDLADSTKTGRGTRQGHSMLHSYGADFASTFQSLPPSSTLSARPTPVSAPSRSGSLTTTAVDMPPPSDRLKGLILD
SLPAHVFVALPQTGEIVWVNSRYLSYRGQTATDLAADPWGSLHPDDRDDYLKAWGHSIRTGEQFSRTVRIKRFDGAYRWFYAR
AVASKDKRGVIMQFLGSYMDIHDQHIAELKAARQEEIEASEAKHRLLANLIPQIIFTATEDEGITFANEQWLSYTGQSFSDSL
GLGFMDFVHPDDLAKCRIPSTQPSTPGGIRREGHSKIYRQGSAQSEAASTTTIQPPRSRTPQDSPLRNVQQALSRNNSSSSS

AYEWPAADLTELARKGIIKVATDSAGRVSYTTEVRLLSKTGEYRWHLIRCVEIDTIDFGSGASSYFGSATDINDHKLLETKLK
EAMETKGRFLSNMSHEIRTPLIGISGMVSFLQDTTLNEEQLDYTNTIQTSANSLIMIINDILDLSKVDAGMMKLKYEWFHTRS
IIEDVNELVSTMAIAKRLELNYLVEADVPAWVKGDKVRIRQVLLNVTGNAIKFTSEGEVFSRCRVYVAEGSHVDENEIMLEFS
VTDTGRGFSKEEAELIFKPFSQIDGSSTRQHGGSGLGLVISRQLAELHGGSMTGRATLGKGSTFTFTARFGLPTADDHPNLPD
SPPATGAFTPRPEMVDNVPSTIKPLPPPKHLRNAEGASPGGTTSEFTSPGGLSVGSSNPSVRSAKSQVTDRSSATSVTGGLAR
FSEAAKASGQDLSQMKLEMPSGRSSPGTHLLPGSGSPEDFRPPMYSILIICPQFHSREATTQHIEMTLPKDVPHQITALASVN
EAQTLIGGEDPIKFTHIVINLPSAEAIIGLMDDITKSQTIDKTTILVLSDSVQRQAVIKLAANTKYEQLLSDNMVTFIYKPVK
PSRFAVIFDPNKMRDLSIDRNRSTAQQMVENQKASYQEIERRMGNKGYKVLLVEDNPVNQKVLMKYLKKIGVDVEIAVDGAEC
TNMVLSKPHKHYSLILCDLHMPRKDGYQACREIRQWEVANNFPQMPIIALSANVMSDVQDKCVAAGFSDYVTKPVDFIDLSRA
MSKFF
FUN_000298 CDS scaffold_1 921961 923541 -
hypothetical protein PF00300 ENOG41

MTKPRLIILVRHGQSEGNKNREIHQTVPDHRVKLTPEGWNQAHDAGRRLRSLLRPDDTLQFFTSPYRRTRETTEGILESLTSD
EGSPSPFRRANIKVYEEPRLREQDFGNFQPCSAEMERMWQERADYGHFFYRIPNGESAADAYDRVSGFNESLWRQFGEDDFPS
VCVLVTHGLMSRVFLMKWYHFTVEYFEDLRNINHCEFLIMRKQENNKYLLENKLRTWSDLRRERKDKEDKDKDKDKDGSKLPR
TKTFVVTRRWGGCPNGCDHSRHYAKREDLEIMRKKDHENGGPEPYGKITNTDEIIITIVSRKQKAQLDCIGTNSESATLGAHDE
DGPEIDISSNHEGKSVSDLNDTPSVISASEYIQSPYLHIGRDGGGSYSGHTSAAETDNDSSEDENISLHRIASLNAQLNRTVG
ERSEKPSYNQKPLLKAGPEHLAYTSDKDSPDEYARTNRLGDASTDASCDGDAELVEDLDRAEKEDRSIRGSVY
FUN_000299 CDS scaffold_1 925789 926428 +
hypothetical protein PF06658 ENOG41

MSAPGPESVPTSADPRSHRPTKKRALTPVSAQAASVEALFSKPDQIIRIPESSTSGSGSSALRSAPPEIVTNVQGSSAGAGSG
EFHVYKASRRREYERLRSMDEDLRREKDIEEFNKDKIERDRKDEERTRKNREKREKMKARKSKKGKGPATTDKTPNATQSKDD
SSLAENDAEKDTADVDSKTKTEHGKDDTESTPSMQPAGLVIHDEDD
FUN_000300 CDS scaffold_1 926852 928832 - CCT3 T-complex
protein 1 subunit gamma PF00118

MQAPVVVMNTNSGERQTGRKAQLSNIAAAKTVADIIRSCLGPKAMLKMLLDPMGGIVLTNDGHAILREIEVSHPAAKSMIELS
RTQDEEVGDGTTTVIILAGEILAQALPQLDRNIHPVVIISAFKRALKDALEIIEEISLPIDVNDDKAMYQLISSSIGTKFVSR
WMDQMCDLALKAVRTVTWEAGNGKTEVDIKRYARVEKVPGGEIEDSRVLDGLMLNKDITHPKMRRRIENPRIVLLDCPLEYKK
GESQTNIEITKEDDWNRILQIEEEQVKALCEAIVAVKPDLVITEKGVSDLAQHYFMKANITALRRVRKTDNNRIARATGATIV
NRVEDLQDSDVGTRCGLFEIEKIGDEYFTFLTKCQDPKACTVLLRGPSKDVLNEIERNLQDAMGVARNVMFSPRLSPGGGATE
MAVSVRLGQLAKSIEGVQQWPYKAVADALEVIPRTLVQNAGKSPVRVLTDLRAKQAEGKSTWGVNGDTGAIADMKEYGVWEPQ
AIKLQSIKTAIEAACLLLRVDDICSAKKAAQIGGGGGGEE
FUN_000301 CDS scaffold_1 930241 931968 -
hypothetical protein ENOG41

MSATQRKSNEAKVPKIVEPDPGPTTGSFAASYPLRPAASRQRTYPQQQEEERDEDQATLYRYTNHDLSQQHLSTLSVREKLRK
GIAMGFANNLARTNATQEVLQQGPQGNPGPQPMDTMTISGMMMKMDVYHHMMPELSRLHSKGVSNRKGVEMVNHELINNAAGA
TGRAEFLKKRSQHIRAREVPVRVASERLHVITGSANNNRYAPFPILDLPSERLMRVEPFPNKNDSSLIVPLNSEGLMAWLEWA
CRRFGYWKLELDSLHFKHDMVRNKMLKRSVSLGTHSPVSAEIADSLDALKLDARKGSITLYEANVTCGSPLQQQRWTWFKIVK
PLLDIKGPVPEKYHTTDATMVFAIPTSKIKKIKEEWDPPLVVPQAKSQGSSQQDAFAHLGDFGRLNLESYTQEATRQLTLEWR
YSEKGIPTFAVTPHPWFMKKFPPDFLWIEDAPSYSADESWWDRLGEKAISDRDHWHYVQALMSGDICLVEAKSKKGGKWTKVA
PQVSKGNAGRTSMSNPGVGNHQSKTAWAASGLRNEVQRD
FUN_000302 CDS scaffold_1 934161 935842 +
hypothetical protein PF00732;PF05199
AA3_1;AA8;AA3;AA3_2;AA3_3
MKFATIIGSALLGFASAGNTTTRKTTYDYIVAGAGASGLIVAERLANAGHKVLLVERGGPSFYSTGNRDDLMKWNKTVTAYDV
PGMAYYTDISPTIQWCTDIAASAGCLLGGSTMLNALMFVKPRAADFEGWPRGWQWEDGVSDAAKSVFERMPGSILPSADGKRY

DDGAFEVMSKFLESNGWDEVNALENVEAKDKIFTHPPWLISNGLRGGPVRDILPAAQALDNFSLQLHAKVIRAVRKGPIITGI
EMEHDDGSREIIKLSKGGSVVLSSGTHSTPRILFNSGIGPKKQIRIVQSGSTNVKLPPASQWIDLPVGQRLKDHPIVVVDFQT
KNKLQALPREAFTEPNDETVELFAEGSGLLSQSAQRLNFWKSVEGKDGVTRYVQGTVNAPENNTISAKVYLTHGLTSHGTLEI
TPGGNTNITQKPFLNTEGDRDALKTFVAELLSYARKPGSVLSVPNNVTADTILEVSYSGNHHLGSANMGAKNDGKSVVGPDTR
VWGTKNLFVVDGSMHPEVPTGNTQASIMVAAAHAANKILAVSSKRH
FUN_000303 CDS scaffold_1 936099 936683 -
hypothetical protein ENOG41

MHDNKRSHPLLEQVPLTVSPFVSLPTATTLSYNYKTMPSNIPPSSLGIEAASDNPAGQTKPKYVVSNSGHAAHPEDIIASCRA
LQAYVTKMQEDAERELREFDEKIKARELAEKRRVAPGWLDSEMHMLEPERSTPVQQQGAGNVPEHQNTQSGTNTNATEDQGAE
LDRVFGGMALK
FUN_000304 CDS scaffold_1 937012 937953 +
hypothetical protein PF03517

MLPTTIRTPPKAEDYTPLAEYQSTTPESFVSGKPVLHYHLEGAKAWIPKTECGSLALFPADSNEKPSAPEGDSINGDVEELVE
QAVDVFVNSEALTIFSPKVEAGVSIPYPSISIHAIKQLGPETDAPRTQAVWMQLEFSDGGAEDDDFSTIDLTIIPPKSDSEPS
AAKQLYDAMANCSDLHPDPNDGEEDEDEYDRIVFEGSEEHQAIEGFTGVLRGASNGGLPPPMPGSGGWITADNVNEYFDEDGN
WIGPGAEGQEEELGEGAGRVRDRDEAEGADAVETAAADDPENKRPRVE
FUN_000305 CDS scaffold_1 938322 940929 -
hypothetical protein ENOG41

MMDLSNISRRAIHGAGGLGLGWILTFSIGGAILLFAAIGFLLTWRVKSRDKEDATTVYQVSENVEDHRRSIFTKRLVKRKYAM
SRSVSRLSLSLPPVLPPLPTYNSFTFFGGNGKRGRSSSWVEEDRFHGPRVSRSRRDSLFSRDGWLGRAPTIPTLMTDDDLEKA
QGSQELEQGIKVQKRQSAETLKPSKTAPELPMPEDEAQTSPVRAPSRARVRASVTDTDLRDILRSTEQRLRDGTSRSPSKNSR
SSPTKRSPQKISSVRSTPRKNSPTKTPHSHKTISSEDSTNTTGTVRISRIPPSPTKRATIHVVPTDAQSRQVSTSSIGSAANS
LIAEATQELVLPGGLSSPSRMRGRQWDAPEDPSPEKQNPQKKTSHKQSAKEDSPDRRASQDSQASSSLSTLYSANDPEEKDQP
DPFVERKTPGFMSSESRGSLFGNRASHKRGRNLSISIPGGQSIFNTTGQLRPSFVSTQAVGGPPISSYLQPPPERFQRNDTYS
RDQDGIALAFPVSNSYTSILTPSVTTTEGDSTLSLGNRMSESPKPEVPERVRPLLQENRHSVATVPSPSTMTSSPFDEQDMLS
LLMGTAPKRALPEPPKHIAQVDQIVMPKPVSPMPRREFSQHLRQMSSTANTIYHDEPPVDSPSAISTGSPLRRSISRSKKDMP
NLAPPTVPSMGSLGNSIMELRRMNSMVSSYSGNSLGSSVNEPDSPTLPMLNLTSSFSRRTINPVTPRASMSGRQNYLNLGSPN
KNSSGSVVPPMPTRPLPQSRSNRSNLGVEIREDDIDEGKENQEPSSTSSGLRETRRSANMGRDTRDLSRAPSKTKLGTVSELI
AESKRDSKRKSVDSIGLYDADGFLKSSPVDAKGRCLRM
FUN_000306 CDS scaffold_1 941680 943549 -
hypothetical protein PF09362 ENOG41

MLKSLITLSAGLVGGAHAFWRMECPGRVGLARIDPIVDPGTVSKHAHSIHGSSNFAETVTTEQLLDADCTSCRVTQDKSSYWH
PAMYFQDGDSGEFELVPQVGGMLAYYLLFGDNVTAFPPDFRMLSGSNDRRSYTIGDPSKPDPEKSMWQALGQTTQSDLAQRAL
GFNCLNYQKAPEGTLYRHYMPDKAYLDANCADGIRLEIMFPSCWKGGDAVDSQNHKDHVAFPDLVMTGTCPEEYPVRLPSLMY
EVIWNTAAFSDRNGRFVFANGDTTGYGLHADFIMGWEEEFLQDAINTCTSETGRIEDCPLFDVVSEQKATSCEMKLPKALANE
DVKGPMKELPGGDGVPNGDGSDVEQPDPTDGAHAPTLTYSPGDRPENSASPLPGQVFKVSSAYGAPAPGPSSKTTTKDVPSSV
EQKPSLSTDEVLIPTIDAGDVGVAAIKESTIAEPLPASAPTTTPAPELQPVTDTKSYFSTQFVTNGNLVSKILWDEEVVYVTD
LQEEVVVVTVTSTAVVTPSAAPAAAPPRRRRRGAHLHGHGHGHRYF
FUN_000307 CDS scaffold_1 947701 950451 +
hypothetical protein ENOG41

MDDLSGLDWSSKPAGGQPKPPIMNSAFAPMRPTPSPLGSGRNTPLSTQGSGSVAPKPAPTKPSQDSFSNLMNFGPAKLKQNLS
LAERQAQLEAEKRQKEEERRKQQEAQFGNGQFWDALGSRGASTSPALQPPPISNTPKIDDDDLFAAFNKDTKVDNSSYYPPPE
SHRSTSANGAPAAAPINLSDPNAWNPNGKGANGGAFGVEDDDPFGLNQMKPAAAPAASTQLADDDDFLGDLARPVEEVRRKQE
ASRPNAEPGKPIEDEDSSSSEEEATPAREQYIERTPRVNKDPFDRAVAQLVDYGFSADDARRALVESGDGYNAQAAVNWLLDE
AHRKSKEQAQAKPSSASSSRPGSRPPRGDSRSPAAGDQDLAKTAAAMGNSLFKTANSLWKTGQKKMQQAVADFQQEGDPNQPK

WMREAQQQSRSQSAGKARADATDEAMMLEAGGRPQPRRTNSRPTEQRATPQTSRGQSPAHSAGSRASPAPRWQQQAQPPTSAF
DSKSRLNRLAADDEISTYRSPNRRNKTSSQASSPAPTSKPAEPESDLLFNSQEVRPSQSMPSRPAQPSPRPTPQAKRPSPPVS
RPSPRPAREIPSISPVALQTSTQHRLQGTAHFKRGDYAAAHSSYSSSLSAVPPTHPLAILLLCNRALTALKTGEPKQAVEDAD
SALKLIGPGNGQGEVVAVKNESGVDENRDLKDLYGKALSRKAEALEQMEKWGDASAVWQLCVESGVGGSNAIKGRQRCQNTLA
PKPKPAPKPAAAKPRPRPSATASLGPQKSSEAVTRLREANEAAAKEDDEKFVLSEKVDAKVSAWRDGKRDNIRALLASLDSVL
WENSGWKKVGLHELVMNNKVKISYMKAIAKTHPDKLPQNASTEVRLIAGLVFSTLNESWDKFKADNGL
FUN_000308 CDS scaffold_1 950636 951182 - UBC7
Ubiquitin-conjugating enzyme E2 7 PF00179

MTSTAHRRLLQEYRALTNNPPEGITAGPISEDDLLHWECLIQGPEGTPFEGGVFPAELKFPKDYPLAPPSMKFLADVWHPNVY
PSGLVCISILHPPGDDPNHYEHASERWSPIQSVEKILISVMSMLAEPNDESPANVEAAKMWRERRTEYENKVREGVRKMLGL
FUN_000309 tRNA scaffold_1 951996 952087 - tRNA-Glu

FUN_000310 CDS scaffold_1 956529 961419 -
hypothetical protein ENOG41

MSGLPEGWESDYDGRRWFYKYKPTGHIQYHFPKEGDEFPDFIDTFSPAPVLAPEERLESQQQVRRYASTTTPAKLSPKKEDGG
YGMSATARPVSMTWDGGFEDEENGVFQPENFMYLGPGTYNDVSPLAEEEEEAARRVVAGGIKGRVEKSSSKGVSPLNSERTTP
AQGTTIAGPVTGEPVLVPPTVVEEVHEMPVVEPPPPHDPVGIIAEMPTDDTAQAHIEKHPPPVEMADNTVLAPIETAVSMMAE
LPERTSPKTKKKEEPQLAPGSLRDYGAQMQPLRISKKPAEEMPPGFQSYKPADISVDIPPNPAPLRRATFQPGGSMMDASPVR
PPDRGHSGTPNVLSPPQVPPKRPLDEPSQPQIPPKHPEQPPPLSIANDGSLVPDQQTELSHMPSVLKPARGKVPGQQTVPGPA
RPPQGHGYVPPTQGKHPPSMTPAPLAQQQRLEEPRMGVQRVNTVPELLPSQRPQSTVQTGHQVLAPSTMNQQHVPKRPASVMP
EMVAGQSREQIQGRYNAGPQQGGNLPYRGPVENMSPAGLPRSETAGPITPFPPYPVDNPPYPDDGPVYHRENPPYPEEPFVQK
QISGQGSSDPSPPISSNRRHSSFSSAVISPDSRHGSMSFPTQTPSPMEHSRRASTNSSINPNYTPSPVSQTSSSMQSFSPTPP
SVPTNQYQQGSYFTMQDVEGQGHNIAARDVLRKKSLSRSTDARRSSVGADPVAAQRGSPLAQQTGQGYNIAPQGVSRSQSLRQ
LPQGNVAPQNQPPTAPTPPPPQGQQGLGRIEEYEEAPGATVSRSSTVSVTTDMRKGSVGSSVQPSPMGSRRTSWQAESPKNDS
PIQFQGQQGPIPQGHAGQPMHQMQAPQGAPMAAQNSKQLQRKPSQGHAPQPQNQQLSARDSRAPQGVPPQQGPGSQGQPMQGQ
VIPPQVQGKAPHPGQVSTPVQNVLHKPPPAPAPTPPALAPKPTPTRLQKRSSYPASPAGQLPPQLQFPQGPVPPGQMPQGPTP
GPMPPQGQQQMAYHGQGSNGRMQPQVQIPPNQQQWQPGMQPMTPQGSRPVSMQMPTQASQSAGAKEGKEGKKWLKWLKGGSKS
VSHSPTTPVISSPISPAVGRPSWGGGEYAQQAVWQPGQPMSSAKPGFQGNTVPPQSQPGQMHQPAQPPTQPVQAPPQHGQMGF
RPGQPLPRTGPLPPQAAQFPQTSQASQQPSQIPPQSGQMLYQGGPPAQPGQIIPQTMQLPSQQRQPPQNRQMPLQQGQINTQP
GPAPTPSSQIPPQAMQLPPQNRQSPPQQERIPTQQVQTQPQFNQMTFKPGQVPPPGPAPRDRQPEPRGYSTGGQSVGVSATQP
VTIPPKVPDPSMAPPVRVEPSNISQRQAPPQRQPSPAQPKALEQAPMPQSKAPKPPQPPQIATPNTLQPQPNSQRGISPPPSA
VSPGLSDAAASSISHMSSYRRDSFSDAGSITTIEVAQAQPQPVLKPSIVQVHRRSTDMISKSQVHLNNDSQSSSDVTPRISAE
TARVGTEAAPPPSNLQYQQATSRPEPIPQVNTSKHDITTAPLPSKPDEPKFVQPHTRDQHMNQEPAPHKADHRSAAYANSSIQ
NQHSNNNTKPISNIPQPAPAKPAAPAAEDKWAKKPVVDYSGGDWGDDDDWDY
FUN_000311 CDS scaffold_1 962484 965067 -
hypothetical protein PF00924

MSSDPPPHGFEEKELKDFNLKQPPPTRNPSQFSASQQQVPARTDSPNPAPEQKQSAPSLDTFPTLQAPHETNDSTSIRSPIGQ
RADASRLDDNIELLRIERAVSADDASQMRKRAHNVEPEDAFNAPIPAETYHNEKKVDPDASLLKLWMFLRKFPRFVRYVVYLI
PGAAILLIPVLLGAFALKSDGKRRDVNGVDLMWFGIWLEIVWGVLWVSRMITSLLPPTFKLVAKLFGSTNAKKWKDIGYQLDL
HTAVFLWFLAILISFEPTMLNHNFRDRKPNWVNIMNKVIIALFTLATLNFVEKILIQWIAFTFHQRTYATRIDNNKADIGQLV
HLYEHAKAHNEKTDYFFQRGSNSASGAQTPLQTLQDNARNAWNKVGYVAGRVGNDLIGRKVDSNHPRRVVNELLKTMSTAHTL
ARLIYRCAVKEGEDLVYLEDMEKIFETEEEAEAAFMMFDKDMNGDISLDEFEAVCNEIHLEKKAIAASLKDLDSVIKKLDKVF
VFIIIVITIIVFISILSGSAAAALGSAGTVVLGLAWVLQATAQEFLQSIIFVFVKHPFDVGDRVTVYGSTGDTMMGDDYYVTE
ISLLYTEFKKMQGHIVQAPNSLLNNLFILNQRRSNGLADVLPLVMRFGTPQHMIDDLKARMTDFCLANKRDYAPRIITEMTKV
DEVRSCSMNMIFFHKTNFQNELLRLNRHNKFVTELMTQMVNVGIQSPFRIEPGGSREHPMYWSGLQPPPAYGKEPDHGGVELH
DKPAHDRPLHSEGPPLSHVSSTYSRRGVDRMGSIDNNVNDFQDVFENRRDNIHAQRLASIREKERGSRIEDEKERRSLASSSG

VDRRTSTDSRSRVFGRVRTGSKSRRPGDIV
FUN_000312	CDS	scaffold_1	968693	971636	-
hypothetical protein	EOG09260B3X	PF01055;PF13802;PF17137
GH31
MRLNTASSFRWTALLLLLGTLTCLFQSAIAVKDKDFKKCDQSGFCKRNRAYADNAVAQGSNWKAPYEISADSTVFEDGKLQAV
VVKTINSHGETVRLPLTISFLKSGAARVTMDEEKRRNKDVVLRDDSPVRKERYDEADDWVLVGGLDLDKEAQLAFQDKSQINI
KYGPEAKQEAIIKFSPFEIDFRRDGNSHVKFNDRGLLNMEHWRPKIENKEGEEAAEDESTWWDESFGGNTDTKPRGPESVALD
ITFHGYEHVFGIPEHTGPLSLKQTRVGDGNYDEPYRMYNADVFEYILDSPMTLYGSIPFMQAHRKGSSVGVFWFNVAETWVDI
TKAKNQANPLSLGVGGKTSTHTHWISEAGLLDVFVLLGPTPQDLTKTYGELTGYTAMPQEFAIAYHQCRWNYISSDDVRNVDR
NMDKHKIPYDVIWLDLEYTDDRKYFTWEPHSFPDPIDMGEHLDAHGRQLVVLLDPHIKKTDNYAASEELVAQDLGVHDKEQKL
YEGWCWPGASNWIDCFNPKAIEWWKTMLKFDKFKGTMSNTWMWNDMSEPSVFNGPEVTMPKDNIHHGGWEHRDVHNLNGLTFQ
NATFQALLHREKGELRRPFILTRAFYAGSQKLGAMWTGDNQADWGHLATSIPMTINQGISGFPFAGADVGGFFGNPEKDLLVR
WYQTGIWYPFFRAHAHIDARRREPYLLGEHYTPIATAAIRLRYSLLPAWYTAFYNAAQDGSPIIRPMFWTHPSEEAGFALEDQ
FFVGSTGLLVKPVTEQNKESVDIWIPDDEVYYDYFTYDVQKTSKGKYLTVSAPLDKIPVLLRGGHIIPRRDIPRRSSALMRFD
DYTLVVSASKSGAAEGELYVDDGDSFDYEQGQYIHRKFTLSGNTLTSIEAEGRDTKSIKSGPWLKAMEKVHVDKIIIVGAPKT
WDQKEVQVESEGRTWTAKVQYHAAKGSRAAFAVVGRVGAKVGEDWSIKLA
FUN_000313	CDS	scaffold_1	972281	973339	+	NDK1	nucleoside
diphosphate kinase	EOG09264YKY	PF00334

MAAEEPRQREDPAASGKNWKQQQVRRSAENLAPRFFALLFALLALYILFSPPSSSLSPPVDLSSTSASYSVPTSQVIPDKNIA
KMSSSEQTFIAIKPDGVQRGLVGPIISRFENRGFKLAAIKLVSPGKEHLEKHYADLAGKPFFPGLIEYMSSGPICAMVWEGRD
AVKTGRAILGATNPLASSPGTIRGDYAIDVGRNVCHGSDSVENAQKEIALWFKEGEVLSWKSAQFNWVYEKA
FUN_000314	CDS	scaffold_1	973473	974085	-
hypothetical protein		PF10306		ENOG41

MRRNLISFNWRPSLRARAPPRINTSYPRAESTQASRIDRITSKLPRRLQRYTSGLRNAPVSHVVSFLILHEITAIVPVLGLFG
LFHYTNYVPVEYVTGHFGSYVEDGVGRFERYFTRKGWFGFGQNEEGEASNAATTHPDSKSEDAVERWHSGDGRYKVVVEVALA
YAITKALLPVRIIGSVWATPWFAGVLMRAKGIFTSKP
FUN_000315	CDS	scaffold_1	975128	976295	+
hypothetical protein			ENOG41

MISVKLAAIGALLATAQAQIRHPNEDLILADCGIGDNKAHPGWSTSRQVNWYQDIQWPDDVSKFPPPSDLEVEVPYADGTYPW
NPKGTTVKLSNGVVWTAYINPRIADGQPAGSAVTTKEGGQELTCYAYRGRPVSAAINTTVNADAVCLTAFVCNRDNRAPPRPS
GMENPTSTLETSPAPPATTTFVSQPPATPSPTGGEPNPPADPNAGKLFVSILLSPRFINWPNTWQAFIEKFSWDRDSGKCVTE
PVKGDGYTINIDCAGIQIDSDSHMTLIMIKILHDIGMSSTFFNQNPTVPGKRNSTADHWVVMPESFSLQAIDVSHQKTIGYLA
YNTTYDNFLAGPCSSCQFERFNKDFFDPALEAVKGTYPLYNSYNIQAQCSPWMACW
FUN_000316	CDS	scaffold_1	976550	978782	-
hypothetical protein		PF06985		ENOG41

MRVGLRSSTQLLAQIQPFHLAAGATIIALIVNAQHLRSLLVTTSQYIYDSLSNALFNVSQTLFGFSYDDTIFDQHIRLIHLSD
SASNSSPNLTLHTFKLDECPPYIALSYTWGPVTQNTIFDQSSILLNARPFPVLPNLHHAIKQLRISRSGQYLWVDGICINQRS
LPERSAQVAIMDLIYNGAAETIIWLGLATDQTARAIELVQKIAKGAESKIIEWGRVQSYGDAYIMNDPELLKRNGLPNLTEND
WLTLRDIYTRPWFGRVWMLQEVALSRNPRVLIGHHETAWDSVGDTAGLINMSGALIGLFTVGSGSETAPLIYSLVHAAGLHVT
RQWAQDKNSRYKEILRSIDYSVGIRQDHPKKLLLELLLSSIGFKATIPRDRVYSLLGIVNFMERAKGRPRLDVIVDYKSPNND
ILIDLGISFFRETKSLHLLSLAGITSRAVPADLPTWIPSFETVHVPVLGPNYTRMLPFDAAAREEADFTIDRSLLHLNIKIFS
PHLGTIEELGESWSDVIQGKFNDTMKMLLHCGTTYIPTGQPIVEAFWRTLIMDCDLSQRPAPSHLAMSFSKWMLLVTIQALIA
YQLKNPSMYDQFDALEPLWTLANTRDSTNTLPKSGDMIPLLFELGLRHDPSVRVDTESEKTAKFASWAEISAPFGALLRVNLA
ASRRIARTIRGYLCLVPLQAKAGDKIMIVAGCAAPLVLRRSGPSESRFQLVGDAYVHGAMFGEHITGTGESSWRDVHLV
FUN_000317	CDS	scaffold_1	979207	980242	-
hypothetical protein		PF00704

GH18
MPPLPPSANPLPRLITYYQTHHDSSGNLISPLSLITQPGIAITHVIVAAIHINDDPQKITLNDYHPSHPIFQTMWAELRVLQA
SGVKVMGMLGGACKGSYARLDADQETFDRYYGPVKDMIRERGLDGLDLDVEEEMTLGGIVRLIDRLRSDFGPAFIITLAPVAM
SLLDFTKNLSGFDYEALEVMRGRDIAWYNTQFYCGWGDCSNPLMYDLMVRKGWPPEKIVIGLVTNPENGSGYIPMNPLNMVLT
TLRGRYGSFGGIMGWEYFNSLPGGKERPWEWAREMTKLLRGHLLSAPAQATQPVLPAAVESGSKAKNEVVDPDGPGGQDAPVP
KQFEYYSDGLDD
FUN_000318 CDS scaffold_1 980503 981458 -
hypothetical protein PF04479 ENOG41

MSSSGERPINGYVDPNFPNPNGEWDTPVIIYGYTPAFSLAVFAAAWFALFLVVHLVQTIRFRSWYFITFPIGLLFEIVGYVAR
SLSAKKDPYHLLYFILNYFFIVTAPVFLAAGVYTIFSALIPRLGRRYSFLPPKFILWFFITSDVIATVVQITGAALIGVRQSN
RDDPTDANNILLGGLAYQVFSLGVFVVLTASFLFRARREVSSRGKKLSLFCVAFGVATIMIYLRTCFRLAETAEGLGGHLYSN
EIFFACLEFAPVALAVLLFAIWHPGRCVGKKVRVQDLEKEQTRVQDF
FUN_000319 CDS scaffold_1 982335 983412 -
hypothetical protein ENOG41

MAAVMEPNPPPSFNSHIQMPDIKDAIRAAFPRTAELLSLLEQTKHIRTDLSLQQKIVSDLESQLSESNRELDGLDRKRLADLE
SHKKYRDGHVMKFLYKASGKENSFTGQAEREEQNYHNTLQQAHLASEHNSSVKAKLDEELRKKNDLDQSMQGYLDLQKQLDDI
YDDIFSGPTPEFPEEDAKEKQSNNALSAYVTTKTAFELHQKALGLLEQATATMTAGLQQVDKALQSGDMNHLRALNKGRELVQ
QSKMTVDQLVQLGADVIELPPEANPRTMEVTSNLGDVWGKVDITGGRQEVARCTAALNNCLSQAKERKYYLSKELKRKGEEMD
EARTELQKVRKGIFEEVMGDDLVKGS
FUN_000320 CDS scaffold_1 984353 985385 -
hypothetical protein EOG09263720 PF00398

MPKSSDSRRGGARKGPYDKPEARKSIFKYNTNIGQHILKNPGIADMIVAKANLKPTDTVLEIGPGTGVLTTRILEQAKAVKAV
ELDIRMGAELTKRVQGKPEQQKLEIIMGDFAKLDLPEALPPFDVCISNTPYQISSIIISKLISLPRPPRVSILMVQREFGLRL
CARPGDSLYSRLSVNTQFTSKVALIAKVGKNNFSPPPEVESVVVRLEPRPEVPAANIQELDGMLRICFSRKNKTLRASFIDSE
ELCQRNWITWAAMDPEKVSEQDLQFFRDSEDTIDAHQSVCGIPVSKATLKSFIRSKIEHVLEKTELSEARSAKCDENDFLRLL
LAFRENNIYFT
FUN_000321 CDS scaffold_1 986005 987916 +
hypothetical protein ENOG41

MASPRARLPRAGKLGVLIPRSKPHGVSICPFCCITSRPRIRPRRAILGKRFYTVSTNPRAELEQHLLDIQKLAPSLVNLSRLQ
LALQGLRQIPGQEAVRVAILGLAPGSNAGQTAKNVLRVLVTDPLQDEQEWERQLKDHDPKNPLVVRIRSTAEQQSTLTVTRTS
LLSEVNVSSAEWNNLNLELLFMETHLPEFARDEQTRAIEVEESVLVPKVGIPSAEDRVTPVTTPVHRAILVGDGFEGAVNVTS
VPVAKEEESVLKVVDLRGVDSKQLDTPFALVDTSLAERGVSLFRQSSSNAIEYERLWFNSNFPALTTWLKSGISSPDGTTKPA
VRGLITSLLNNILARCEEENRREIRETYQAAPTLNGQEPDFSPVLIQAAGAWSQQAHAELQDELDLAFTGRRWRKLGWWQLFW
RVDDVAMLTNDMLNQRFLPTAERELVYLTGKIAQAANADQSYPQPHSVNDSDANIELKRLGPGELEPLALVNNRPALPKWPGH
IAFTRRYLQNETVPALQALAQRLVVQSLGTSGLTTSLAALLYISSFSSTVYEAGAVAALGIVWSCSRLQKKWDAARTFWEGEV
REEGRKALRGVEDSVAEVLSHEKQQEKPVVDQKGDEINEVRELVVKAQEALGRMK
FUN_000322 CDS scaffold_1 988186 988665 - SMD3 small
nuclear ribonucleoprotein Sm D3 PF01423

MTSTIGIPIKLLNEAQGHIVTLEITSGQTYRGKLLDAEDNMNVQLKDITVTARDGRVSHLDQVYIRGSHVRFFIVPDMLRNAP
MFRSRNVRGRGVGLARGRATVSRARAGGRGGR
FUN_000323 CDS scaffold_1 989006 989609 -
hypothetical protein ENOG41

MVNKRHTAHESQPSTPASKAIEPLPGRSRWSEWALHNSGLYYYRARYISFEDISSIPPTGRNSIVPNVQGGYIHYQSMSVNEA
TSQGSSQAPELSTCSTVTTPTTSDPPVSTQEVYGQQNDQLVVSTKTTEDSMLMSGALQAEADTTETVVVISNSNAPKRKSTTS

KKQKEKKKHGKKLDVDKRKQVSNKAKVNKWLDTL
FUN_000324 CDS scaffold_1 990300 991809 -
hypothetical protein EOG09262H50 PF08704

MVQPSPFLEPGTTTSTGDLAIVLLSRDNLQPITLEQSTGAVDGYLEGATLNTRFGSFPHSTLLGIPWGSQVRASNVDTGSRGR
KRRREATDDDTPTTTGPDDDVADVTSKPAKKAVAAASGFIHILRPTPELWTSSLPHRTQVVYTPDYSYILQRIRAVPGTRIIE
AGAGSGSFTHASARAVYNGYPKDENDKKGKVFSFEYHEPRYIKMQEEIHEHKLDGVVQLTHRDVYGEGFNVDGKSPQATSVFL
DLPAPWEALHHLSRQKPDKLEKESENADTPEWVSPLDPKRSVHICTFSPCIEQVTRTIEELRLLGWKDIDMVEISARRINTIR
ERVGANLPAERGNVQTPANVKEAMQRLKEINRKTKEFHKVHFKSTNGDDDSSAMDVDTPASNSPKTDANGKAAEDSKPWMHGN
LYHRPENDLKTHTSYLTFAVLPREWTEEDEAAAEAKWPCGKEGGVIGSLNRQARKQQTREKLAAKKKQKQENDTQKEGVVEET
AEKQ
FUN_000325 CDS scaffold_1 992387 994941 +
hypothetical protein EOG092611G7 PF01406

MESLKLYNSLKPGAPVPFVPIEKGKVTWYACGPTVYDKSHLGHARNYVSTDIIRRILMHYFGFDVKFVMNITDIDDKIIIKAR
RQRLLELEKNKNYTKDELRDLALAAFRAYAKSSLPLLLKDGEDIDATNYAQRREAGYGHVLAGGTISGEGKPGDDEAKVKMHL
SNMTAAAEAIASGEIFPGTDEILLPFLDSLYKETIDTRDQTMFTDLTQSMEKLFMDDMDALNVLRPDVITRVTEYVPQIADFV
KRIVDKGFAYESEGSVYFDISAFEQAGNTYARLRPDNRNDKSLQEEGEGSLSKNLGGKKNPGDFALWKKSKAGEPFWPSPWGD
GRPGWHIECSVMCSDVLGATIDIHSGGIDLAFPHHDNELAQSEAYFCEHGKGEHTWVNYFIHMGHLSISGSKMSKSLKNFQTI
QDALATNYSSRGMRIVFLMGRWNDGVEISPDMRLQADNWESTISNFFINVKALLAEAGISHDVKSLSLSADGKASEGLLAELE
QAKKDFEAALVNSIDTPKAMSVILKLVNTANVHLRDNKDADLVALESIARWITKIVGIFGLDSNASPPYEGLGWATVIASDVE
PKTAVQPYAEVFTKVKSDVSGLSLESAEISSLLEQDPTAEFESIASGGSRDPEQLALPYLRAVSKLRDELRRIVSNQAPETKK
AILSLTDRIRDEDLTNLGVYLDDRPDGQASLIKFIPAAELIAAREEKAAQAAEKARKKEEARLAREKADQEAREKAKVRPEDL
FKGDERYSAWDEQGLPTKMKDGSDVPKSQLKGLKKQWDRQKKAHDDLKAKGLL
FUN_000326 CDS scaffold_1 995757 997748 +
hypothetical protein PF07690 ENOG41

MEKNFDETVVGSQDGSSSDLEHIEVDWTEEEEAKLIRKVDFTIMPLLVLGFFALQLDRGNIGNALTDFFMADVGITQSQFNVG
QQLFSAGIVLLEIPSNFILYRVGPAKWIGSQIIAWGLVATFQAFQKGLGAFLSTRLLLGLCEAGFIPAGLYTITRWYKRDETS
KRFSIYFLGNMIASGASGLIAYGILHMRGVGGLGGWQWLFILEGIFTILVGILFILFFPDSPFNPVSLFKFGIFTERERILT
KRILKDDPSKIHAKPNVTKEEFRNVMTNWRLIPHVLLTISALAPATTMGSYSPSLVKSFGYERLKCNALVSIGSWALVVTNLL
WGYLGDKLQMRGPLVAFGIFMFWAFTIADRVMVFSPDGHTRFALLTLTHAFGWQWHPLNGSWMSLNAGSAGERSITMAILIMS
ANTSGIIGSQLFQERDAPLYKTGWTAIMALASVGLVMAVVANLQYFFLNGRRIGRKGLKYSP
FUN_000327 CDS scaffold_1 997888 999115 -
hypothetical protein ENOG41

MLPARPLIRSTVPRAARASRASRQVRFQSTASSSSSSAHLASGIAGGFVGSAIFYGIYSYTPAGRAASGINKAAKEAQQKYEA
AAKKLQKNTPDADQAVNYIKEFAYSYVGWIPGGRAYVDAAFKDWEKVRENNKDEADKLVNDAYKQFQDLSKSGLSLETASKAY
DVLADLAKKVANLAGDAISDIIDNHPQVKEKLGGNVDQLKELGDKYGPEAKKQVDETWKQVKDIFAGGFSAASISKARKLIEE
KVEQIKKLGDEAWKKGLEEAKPYLDKNPKVKELIEKNADALKEGNTAELFKRAKSAVDSGDLGDLEKYVKDATEKVKSKGNEL
TGGWVDIEKYIKEIPNGGEVMEKLQQLSEVADKHKEEGEKLFKETVEELRQVLEKKSEKAQEIAGEAKKDAKKETK
FUN_000328 CDS scaffold_1 1000615 1001176 -
hypothetical protein

MSQSSNGHPSEGSASSPAMCMSSSPSVVSSSPTPANSESVPNRISPMASETPSANSQYKDYEESHDDRLPAAPSQMGMTDHGS
RTYLSLGDVRHHPVFISRRPLTCPDQLARQTKLLATHYDKLSGNIAIFGDRLHNLHDAINLVLERQNQEVNNPRSFVTDPVHW
PDEAASAQHSAHSQSTPDHN
FUN_000329 CDS scaffold_1 1002843 1003484 +
hypothetical protein PF03928 ENOG41

MAPKALTRETTTGAALEKVQGAIQSPTTEVIPKPPSDLEELKADCDSFTFTSFTTEDAFVLGNLLYARLYPYAVKGKPTVISI
ALANTSQVVFQTVTGPGTAPDNEQWVRRKRNTVLRFGNSTWFMHNKFKGDEVAFAAKYAIADSNKGDYAIHGGAIPIRVQGVE
GIVAVVVVSGLKQDEDHGVIADVIKSNWSC
FUN_000330 CDS scaffold_1 1003752 1006032 +
hypothetical protein PF00172 ENOG41

MNTTKWRISKACQECRAKKIKCNGETPCQNCSMRNLSCVYREKARNRTRKVKPRTAAYETIMSHDAMESTSLEPSDEGTVPPT
PNADDAASVGPTGSAMDSERSLTHNSVAATHRASPSCFLQLYYGPSSNFALLNSIYHQIAGTCPNDPPSRSGIVEEVGPGLDL
FSHRRLFFGDLADNQRPSSVPDDCSAMLIDPDTAHRLLERYLLTYWHGLPIMSKDHYRRRLSALYQPPGIFDYDAPETIIIML
AVGLGASMTGEEAIADFLFQKTKQGVAKLDEVVNMQMLTHSHAHFQSERARPNSGFLHVGTACRKAVAAGLHKDGCSRPGYTE
DDTNQRRITFWSLFFWETWQCFVLGRPSSLPDPGSIIPLPKGQKLLKSLVTLSRIMDKCVKKIYSPRHDSLLPVWNAAIEIRR
ELHQFAEQQLKDMKFGLVGDPSTGELGVCQAMVSTMYHHTLLLTFRPFLILRAKLNHDRAAATSAENLQMPPPWLDSACEYCL
EAARNSVGFLTGSCATNILCRDIKYHGFFMEGACYALAFDMLQEKKTAHRNLPWIHSGLRCLRSMMGSAGVNAGQLPITIASI
EQMVRSAGFELKDPVDTNQQKQPSQSTLPGSPAPVSVAGGMRSEPAFTFPSMPFGFDVTGQMNGSSSSPAGAASEDMADFTAA
DVGWDIDFGTMNMEAFLSLDSAEAFNFAP
FUN_000331 CDS scaffold_1 1006362 1006836 -
hypothetical protein PF08592 ENOG41

MDSTVRTFQVFGLTSSLVLAGINLGSSLLTVPLLYNQPTSINTPFFKDFYTRGAVTLVPLALFSGASSGIVAYLVPAQRTLWA
VAAVATVSQLPWTVLGMMATNNRLNDIAASSVEQEKVGRDEVVALLKKWRWMNIVRGLLAFTGGLSAILALQNE
FUN_000332 CDS scaffold_1 1007500 1009199 +
hypothetical protein ENOG41

MELSSLYQTASATFGSLPVAYKFLCAVLVIFAFSKAFSGAERKGYKKNLPRYAPIELAIASYILSGDGIGRRIFSALSRYGGS
LFGLTSGHQIFVDLPGVERLLAQSHHTFNSDPAQYSLCTLVFGSTDTPELRAKFDTSLKDLVPPLERTFLNDAASTANVEKSR
VAERGASLVTFSSNPKDMARWELSAGIKVLQPETLDSPGKVEANLQSLTRDFGACMSVPLIYGQDILDRNPRLLDDFWTFDND
LFPLLMVGVPKWAPFRIMQDGLKARARIIDSLEAVYRRVDQYEKGEPVDLGADMSDVGMMLRERNKIYNRDGWTFPERAAADL
GTFWGLNANAQPVLFWFLLYVYSTPGLLKRIRDEISPYVTLSDGAKPEIKSMDFPSLFRKCQLFKACIFETYRLVNEPTSIRY
VTQPVSLNDGNIKHDLQEGTYISVPLALKNKNPSIYEDPDTFIPDRFLDTDPETGKAMARYGKLKPWGLGAAMCKGRTFAEKE
IISLGSAVVSLWDISPAEGEWKLPAMIPGTGVKRPVSDIRVVISRRSPLKS
FUN_000333 CDS scaffold_1 1011435 1012218 -
hypothetical protein PF00625

MRWFLGYAKRNSNLLIPSTKPFSQVPKRSFAMASPSDRRPLVISGPSGVGKGTLIKMLFSRHPDIFTLSVSHTTRNPREGETD
GVEYHFVTKDAFRDLIAKDGFVEHAQFGSNLYGTSKATIEEQTAKGKVVVLDIEMEGVKQVKASSIDARYVFISPPDTEELEK
RLRGRGTETEESIQQRLTRAQDELAWAKNAEFDKILVNDDLEKTYQELDAFVYSEKTN
FUN_000334 CDS scaffold_1 1012617 1014249 - GCS1_1 Zn
finger-containing GTPase- Activating Protein for ARF
GT21
MWSLKEVIAGVFLAVAGLVATVIAIGVRAIFRNFASRPAPSPNLGDNTPHVTIIRPIKGVEPRLYDCIAASFRQNYPQDKISI
RLCLENDTDPAYPILQRVLEDFPGIDARILFEVDDLALGTTPNMGPNPKIRNLSRAYREARGDIVWIVDCNVWMAKGVLGRMV
AKLKGHNVGGDNIPYKFVHQLPIVVDLIDYSTPVAADGQPLLAASSVEDDDSDHLGEGGSHDNPKVWAQGGGRLDEMFMATSH
AKFYSAINTSRLAPCAVGKSTMFCKTQLDHATDPALNPKIPRGKSLPTGVDYFSHNICEDHMIGDVLWNAEFSGYRNHGLVWG
DIAVQPMSDMSVKAYAARRSRWLRARKFTVLPATIFEPFTESFVFATYFTFAITTLRIYGIPPTWPARVITWLTTITVWMLVD
FLGYRHLHSGVTMEVHEDTPRFARGSMNRDGIKSRKFSEFLAAWIVREALALPIWAYAVVFGNTVNWRGRRFRIRRDTTVEAL
DPEEEERSRQVPTPELERASSQNKQRVD
FUN_000335 CDS scaffold_1 1015316 1015912 -
hypothetical protein PF04828 ENOG41

MTSFDDHGFPVVHLSSDGWSTEDEATATCFCGAVQLVLPLKKPGLLNRHVCHCIDCRKIGSAFYQSNITVDDTYLKHVRGEDN

LSTFSEEKTIRANATMTNYFCKTCGTLMYRRGARFPGLTILRTGTVDDLSLADTKLRPQVEQFIERRASWSKPIDGAAQVVGM
HQPSDIEGIP
FUN_000336 CDS scaffold_1 1018008 1019826 +
hypothetical protein EOG09262WXK PF01974

MAEIQSSNTVSVGTPPTTDSADKRQSQQRGPPLHQIYALPAPIRTFPLPAFYPNNPISFFHVAYAWLGQLWSPPKAEPSVVHN
GVWSAATSSVHITDDASTRALWEQGFYGKGSLSRSEPAWLKREQVRQGLADAHVSEIMTVQRREERMRAKWERARLEQEAIRE
TRRKEAEETKAREIKAREAKAFEAKERELRARESKDIAEPRSIKPISLSAALPTYESPVSPAALLALPNSLVDIFKPTPIKPV
NLPVSTPTHASPVCPATILGMPNSAAEIVEESAIETAEEIDGLGISGVHPTAMGLSVDTLLNGHVMEPVANGILHHDANGTSV
VASSSDEEDNTKPKKRRKSVRFSPTVESTTFHFGDPPSPKRSTPNGTPNGLADIRAASPVALNGQNEENFLERTSKPVLKNME
HLQLTPEEAFFLSFGLGVLTVTDPSSGAQLSSSDLLRLFRQHSYFPPRVEPADPALQPDDNFLIHYAVYHYFRSLGWVPRAGI
KFGVDWLLYAKGPVFDHAEFGLIVVPSYSHALWKEAGKQDPQKTWQWLHGTVRVLSHVTKSLVLVYYVDVPPPSKFEKALEQGV
AEALKLYKVREVMVKRWSANRNRG
FUN_000337 CDS scaffold_1 1020324 1021851 +
hypothetical protein PF02668;PF06155 ENOG41

MFSCRLAAYSCRALPRGLSSTRASAQRISLNPTNSQCRTHRRTLTTKNFGIPPRFEPTTTLSAKDNNLTISSGDRFIQIQDEK
HGGWTRLDATILRDSCTCTTCVDPASGQKSFATTAIPSDIAIDNIHTTSTGVGISFKNDMYKDHEMILPWSSVDKALGHAPVE
RMPYPRQDAVYPKTGRTFWDKATIQQRVRKIDYHEFMKGDDAFWHTLLDLSNLGLVFLKNVPHDENSIVNITTRIANIKETFY
GRTFDVRAKPDAENVAYTSGYLGLHQDLLYLESPPAIQLLHCMENSCNGGESLFSDGLFAGKLLWLQNSPTVKNLARVKIPYH
YEKHGYFYRQQRSLFDVGPDGNLAAVYWSPPFQNQFQVPAVDARAWLEPAQLFDGLINDPDAMFEMKMKPGECVLFDNMRVMH
GRNQFDVGGGSRWLRGAYIAREDLVSRVLHVPEALAAEYRSGKEWSRTLEDEELKASKWFDKVGGQVESVVDGLKRKGAAELG
ERDYEVLNLG
FUN_000338 CDS scaffold_1 1022034 1023493 -
hypothetical protein EOG09264LH2 PF04969;PF05002

MSHITLAQQGLAAAEARNWDEAIEKLSTALKASQNPAWLVARSKALINKKRFQEALDDANLAWHTAYQRNKRPLLIDAHYRRA
VAYFRLGQYANADACCVYAMRLVKGFPAVEKEDPAKKNTDENGFYKVTLKDAQEESKTDDINKSQGGAAGALGAQGNDVANAK
EWRIASTLRMQILFAMDKLPEDDPARKLTTANKPELKELSDVGSKKAETKESAAATQTAPQPTVPADTPVRLQEFQNDTTMTV
SIFSKGVNKDKLQVQFSPKSAHLDSIIWPSGDEKPFTLDLWGEIDTEASTYKVTPNKVELALKKKTPGKWAQLKGEAGDSAPD
AAAAEEAEKLKVLKDARKKAMDNAAAEAPAQEKPTDSTEAKEKTPAAQDSSKPAYPSSSRTGPKNWDTIGDDIDSDEEKDVNV
FFKKLFKDASPEQQRAMMKSFTESNGTSLSTDWDDVKNRTVETVPPEGVEAKKW
FUN_000339 CDS scaffold_1 1024918 1027407 -
hypothetical protein EOG09260SIZ PF00432;PF13243;PF13249

MVANSTGREGSALKSRKRAADSESEPLLKQGQPFPKQPRISSQLDTTRWRLKDDDSRHTWHYLENDDDAKEWPQSYAEKWYLN
LPMDLPDLPTPDNPLAAAENGLDFFEKLQLPSGHWGCEYGGPMFLLPGIVMTWYVTRTPISSAKATAIYNYVSARAHPEDGGW
GLHIEGESSVFGTLMNYVALRLVGVDPEDPVLVKARGTLHKMGGALYAPHWAKFWMAILGVMSWDIVNPVPPEFWLLPDWVPF
APWRWWIHIRMVFLPMGWLYSRRWSCEETDVIRSLKKEVFLEDYAKINWSSHRNSIGVVDNYHPKSWLLNAANWVLANIWMPY
MRPNFLKEKAEAWASKQVDMEDANTGYACLAPVNASMNTVLCYARDGPDDYGVKRHIERLEEYLWVKDEGMLANGTNGVQCWD
TAFLIQAVFEAGLHKNEKYRPMLMKSLHYLERQQIREDCVDQEVCYRQPRKGGWPFSNRDQGYGVSDCISEALKAIILLQKVG
GLPEVLEDRRLFDAVDTLLLYQNDNGGMSSYEKRRGGEWLEMLNAAEVFGRIMIEYDYPECTTACVTALSMFNKHWPDYRTDE
VRTLIRTAAEWIKSNQGPDGSWYGSWGICFTYAGMFALESMKHIGQTYATGENSRRGCDFFISKQRADGGWSESYKACETMEY
VEHPSGSLVVQTAWALIGLMEAEYPHVEPLRRGIQFIMDRQKPNGEWLQEAIEGVFNKSCMISYPNYKFTFTIKALGMFAKRF
PEEKLVPTWAVAQNGTNGVKTNGVKTVEVKVNGVKPNGVKTNGVKGKGI
FUN_000340 CDS scaffold_1 1027806 1029340 +
hypothetical protein PF04406

MDEEAEIAQSIAIPELTQNPQANRTSSTTANENTDTNTIINNPSAGTVVTRIEDILAQIISALAAGQELSIPFSARRSTRRAA
NVQPEQVHFPGRNQQEAVKFARILLILQLSHDALVSGTVLTKRHIFYQHQHLFEKQGQVDDLVDDIAFSLGISRGDLNIVAAS

KGALAGPLLIRFRDGSTLNPCSGDLGVAIPTVQSISSVDVQNIKWILVVEKDAVFRPLCSSQFWRTCIFGPGVLVTAKGYPDL
TTRSFLNFVHTQYPQLPLLGLFDYDPDGIKILRCYRHGSERLSHEADLGIESLRWLGIRSVHLSRGHTNTSLSNYGQPSHVSI
TSIQCRDPVTYLNGRERAAAISTLKKISLYSPNELEGSELRLELQLMIMLGVKAEIEWLDESGDLCSWLDGEIGEALISERI
FUN_000341 CDS scaffold_1 1029613 1030269 +
hypothetical protein PF00023;PF12796;PF13606;PF13637;PF13857

MAPKLTEEEIDDLIYFARAGEQEDLTETIKSLAERENVSPAEIVAAAQDASNKSTCLHMATGNGHLEIVRELIQYFDTQPKEQ
KQAFLDEANEAGNTGLHWAALGGHLDVIKLLLEQGASPALANEQNYVPLDLAYFNHKNDVAEYFLSTAKKLEEKNQEEGGLSA
AVGSVEIEEGDEKAEETKETS
FUN_000342 CDS scaffold_1 1032251 1034844 -
hypothetical protein PF00096;PF04082 ENOG41

MKGRPRLPVPLCRFCNKEFKRQEHLERHIRTHTRERPYGCPCGRTFTRQDLLNRHRRLSQCSTHGSGKQAESNLAISPDGAVT
TSPSISQASDENNHHSHQLQSQGNPSNLMPMEVSPHTMSSQQEMMGRFSIHDRPLGYQEQTPSHIFEPPKDPIRNSNVTPAGA
LDSLDFMQFESFNADMFPDEMDMINSYLGEVLPPEHSHQGFTPDTTNFFTQSFVPEPVLSPAPSSHLQPEAPGHIELSNAPKS
TLPFCQKQQAQPTPVRNKVDRLLDTTEDIVATNPWAVSAAAHERLSLEFSKHRSDIPQSFTLPSRHALSRYVASWIRGYHPHL
PFVHLPTTNLESMSPMLLLTLAATGSFYGFEHPQGYAMYFVAKAMINHELEERRRVSNRHILSGFPRFAALPTSSTEVYDAPT
NLPADLTKFDIELLQSLLIAVMTMSWLDGPFAEEALSMSSKLTALTRDILKRLPEENANDTWEDWGRDEERRRTLLSAYFTLN
IQTICFNVPPQMTATEFSFELPCSEAEFNAPDSETWNRVRRKVDPRKLNFQSCFKQLLSGEPLAKEVSATEFGNYMLIQSLLI
QIYFERQVSSALLTPSPSLSESTIATYAAALGAWQSCWDSAIESAPDPSSRNSPLPFNSTAMLRLAHIHLGFGLYSQCELLSR
DPIAKAQVFEPYQNPLPLRAPHLDQAVLHAIYALRIPVRVGIAFVARGRTGHWSVQHAISHFGCALLLTHWLENIYQLVLSDG
TSALREEEKRLLSMVDRLVEETHLEASLGPKSDFPDRIRRLAVAAVKLWAETCKGIQVYEIVHVVGETLSLVAESLEKQI
FUN_000343 CDS scaffold_1 1035290 1039062 -
hypothetical protein
PF00289;PF00364;PF02222;PF02626;PF02682;PF02785;PF02786;PF07478

MGSLDLHTLQHIRKVLIANRGEIAVRCINACRKLGLHVVSVFTNADATSLHVKLADEAILLPGEDSSAYTDGDAILDICKRTG
ANVVFPGYGFLSENAEFADTISSAGIIFAGPSTASIRAMGLKHEARGIAQSANVPVIPGTQLLASADEATKAATELGFPVMLK
ATGGGGGMGLQICNSADEIAKAFAMVESRAGTLFKNKGVFLEKYYPRSRHIEVQVAGNGEIVVAFGERECSLQRRHQKVVEES
PSPFLERHAGLRERMLDAAINYALQLRYKSVGTVEFLVDDDTAQFFFLEMNTRLQVEHGISELCYGVDLVELMLRQADYELRG
QLGIPSETLKGLGRPQPLGSAIEVRVYAEIPLCNFAPSPGVLQHVHWPQGDGVRVDTWVQSGQRITPLYDPLIGKVMVHSPDG
REAARRKMLAALADTELQGTQSNLEYLSAILDSELFASGNTLTNSLSTFEFKSCSMQVLEPGVSTTIQDYPGRVSVGHGVPPG
GPMDDLSARIANILVGNDEGVELLEVTLSGPKLLFHEAAIVSVCGAELPIAVDGSAQPLWSRLVVKQGQILSLGKVTGSGARA
YLAVRGGFPGVPAFLGSKSTAPELGFGGYQGRKLQTHDILALSPDSKVQAAEATTFTFPSHHVPKFGSTTICCIDGPYGSDDI
LTEEGSKTLYEGEWSVSHNSGRSGIRLQGPRLKWARTSGGGGGSHPSNVFEYGYPNGGVNFTGESPIIFANDRPDLGGFVCPT
TICSGEMWKLGQLKPGDTVRLQSVSYEDALKITHEKNAYLEALAAFANGKSGDVSAPQTAFAAKPPSSILHQSPARGVHPQVI
YRQGGDTSIIVQYGNQTSDLRNTVCVQLLTQQLAAAKLPSVRGDPNIATLTVRFDPVHIDRSELLQQLLTFDTNIGDTSGVKI
PARQIRLPVCLDHSSLEESAQRYMENIRPTAAYLPDNVEYLRKNNALATRRDVLDSLLKTPWLTVAVGFFVGTPILFPLDPWR
VLTGQKYNPSRGYTPSGSVGLGGSTVAIYPVAAPGGYQLMGRTLGGWDSTGNRPGFSPEKPWLFNHLDLVSFYEVTEQEYDKM
ERDFGAGQYAFDISETAIDMDQYIAKFDTAEKDPEYQAWRERQSEAADKLAALEQKLFDEWTISKQSGGGADEDGDIDSDEIA
VIESPVNANVWKVLVKPGDVLEEGQTVAVLEAMKMEINLVVGEDQVGAEVVKVVQPPGSVVSPGTVVVKARRSQE
FUN_000344 CDS scaffold_1 1039791 1041498 -
hypothetical protein PF07690 ENOG41

MTSGVAIPGTVQLVDVQGIFNVKHGQQSRDIVLVPQPTDDPDDPLRWSIPRKTRNIVCAMVWCFFVAAMISGLSPAYILIEED
TGISIADLSTGNGILFLFLGWGTMITQCVALAYGRRLTLIVSVILTTGITLWTAFIKSRGEFFTNRVLLGICASPQETLIEVI
IGDIFFAHDRGFYMGAYSWTLWCGAFLAPVASGYVAQELGWRWIQYILTIIGGSVAIITFFLFEETMFYRHHDVGNLSDTAAL
DPATIKASATVGKDVEAPSEKQDPSVQDEKSTSRSMEAAAYTPGESEKTYLGKLKVWGYHDSRQPSTFKLFFLPIRLLFSFPG
IGFGGLLVGGILAWYNVVGGSLALILGNPPYNFSANNIGLTYLASVVGVTVGCFLSGWMSDALALFLARRNGGVMEPEQRLWT
CLVALVMHPAGCLLYGVGASYHIHWFGVVFGLGLICVTLPMGSNLAFTYILDTYKEVAGEGLVSTILIRNMMGFAFGYAVVPM

IENLGLRNAFILIAVLGEAVWCTAIMMIYVGKPLRRWTAHSYWNLIEHYGATAH
FUN_000345 CDS scaffold_1 1042123 1042969 -
hypothetical protein PF03746

MTGLAQRYEINADMGEGFGRWKMGPDEELMPLIDAANIACGFHAGDPSIMLKTVRLCKQHGVKAGAHPGLQDLFGFGRRKMEI
DPKDMYAMVLYQVGALKAMLDAEGVELSHIKPHGELFFYMQRDKGIMRAVLEACATFKVPVYACQNPEQEAMCNEMGVPFQGE
VYVDIDYSPEGRLVPFAQSHPVTPELCHERAFGAAMKDTGKDINGDPFNFGFKGKPFSICLHSDSPTVLENAKLVRQAVDEAN
RKKFPSQNTNGHRL
FUN_000346 CDS scaffold_1 1044132 1044950 +
hypothetical protein PF08241;PF08242;PF13489;PF13649;PF13847
ENOG41
MGEQNKAHYNNAPPALFRWPWIIELCNQISKELRSNIDWIGIRPPSSRPTKLLDYACGDGVASRALAPFMSTVRGMDIASGMV
EQYNKMALKAGYTSIKMRAVQGDLIDPELTPSPETNAPGFFDFDVVVMCMALHHIEDPDNMILQLSKRLRPGGILLIIDWVAS
SAGSSAQTGNRTLGAISRMGFQENEVKSSFDKAGLEDWAWKLTSAPSQVPREIGGEQQLFLARGKKPFSQHVL
FUN_000347 CDS scaffold_1 1046052 1046322 +
hypothetical protein ENOG41

MKPQVLLAAGLATVAIADQWPMAKEKCGRLGPMEWDPNDLPPDVDVMQIRMCADHPMGAGNYWGWGEYLPDWFPRNPFADMEI
DWLWWP
FUN_000348 CDS scaffold_1 1046590 1047594 -
hypothetical protein PF03435;PF05368;PF13460 ENOG41

MTDFQPKSILIFGATGNIGRYITNAIANAQPAFDHVAIFTSEDTVTRKPELIKELKSKAVKIITGDVNNPEDVKRAYQGVDTV
ISAVGRNVIETQIELFKLAAESGSVKWFFPSEYGTDIEYGPQSASEKPHQLKLKVRKYIRENANGLKYTFVVTGPYIDMYFTL
SPDVIEAGGFDHKNKKAVLIDNGEGKIGFTTMPDVGKAVVAALRHPAESFNRALIVQSFVVNSKQILKELEKQTGGEPWEVSS
YTLDELREAEKKGWAAGNPKAVSYTLRRIWSEGGTLYEKTDNERIGLKEEDLETLEDAVKRELTTGY
FUN_000349 CDS scaffold_1 1048889 1049525 +
hypothetical protein ENOG41

MVKLDELARFYRTQLYMDIELGLHNLLIKKRDALSPPHSSPAQHYYAAFSRPPNCCWDEDSDRYTEEGNDCETPYPILGKDMK
FKICQRDHPDGEGCADRVCFIPNASARKYMLDFMAKRSWQTPSLDRLEPVAYCLVRKYCSNIPSKDIETFSRIVRMLFEDLRY
PDPRNWDPEVHGVLNWKGKPIQTCVDDFMSEIHGVKWKRDMREYF
FUN_000350 CDS scaffold_1 1049769 1051893 -
hypothetical protein PF00004 ENOG41

MTTDTMDSTKSSGDESTVILTPADTEPKEETTETKTEQKTADSKTEDSKTEETPNEDEEKKEKVMVGLLTESKDLYAKFDKNG
DRSWTDKYPDDLEEAAENEETQKYAVIIRKRKPKEADSNKPLDIDSLVIQSPYLKRVLGKVFEDYPGIVTDVSRLKFHAPFEC
FVHRWGQFTAAKDDPNYDQITREHVSLLHKIMRDELGEIIQLREDYFKNRAVAFEHVWTLFPPGCTVWGSVKGRPVAVRFDSG
YLAKTQCGGLAYMMNCKGIDWDGRSMGWCDITMQINDFPGTAPFSSLSCYPLEYHPNHDQARNMLLERGRKFESLAGYHYKAY
SGTAIWWVNATKSRKETVNSRIVIDGANWEKQNPDHTVWLNYLTGDGPVASSDRDSDAGNNDDDEDCYSDCYNDSGVKKPEFD
DDQRAPLSEEQLIMTSPMVRGYALKNKRWMEFFIDDVTEVKFNEKAFDSLVLPADQKELILAFAQSQVKFKNVFDDIISGKGK
GIIMLLSGGPGIGKTLTAESVAEEMKVPLYIMSAGDLGSDAYDIEENLQRILEMVANWNAVLLLDECDVFLEARSAHDIERNR
IVSIFLRMLEYYEGILFLTTNRVKDMDQAFQSRIHMSLEYPPLDNTARESVWRGFLSRAISVDAKIEGESAHEITEGEIKALA
GLELNGRQIKNVLKTANLLACHKGEKLAFTHLRTVLRVEGHSL
FUN_000351 CDS scaffold_1 1052581 1053391 -
hypothetical protein PF13714 ENOG41

MSNDLATQLKALHKPSSPVVFPNIWDVASFQTVTSLNNGTSKPVKALATASWAVAATYGIQDEELTLEQNMEAISKVAPLAKA
AGIPLSADLQDGYGSQIESTVKRAVELGVVGANIEDVSSETGEFYPIDEQVQRLKTALKAASDAGCPDFVINARCDTFHVDTG
LSEAEAMKEAIKRGKAYLEAGATTVFYWGGSGRGLRNAWVETLVKELDGRVAVKLAHRTKDALSTKELGEIGVARISVGPSLF

LLAQAAIKEAAESILNGGNL
FUN_000352 CDS scaffold_1 1053916 1054573 +
hypothetical protein PF00583;PF13673 ENOG41

MPLQLRPAVPEDIPEMCQVYYSAFGDTSIGSRIFTSDIEASNRFFQKTFTDDMADPQCELLVVTHKSSPDSKDEKVVSLAKWT
LPGAPIQDPPPAEAWPANGDLAVEFFGAMTRGHRKFMGDRPHYYLEVICTHETWQRKGAGTLILRWGIERADTDGLPCFLEAT
PKGKLVYEKLGFKVKAEEEFKWSFGTFVETYMERDAKIEKRTMTRPKSKEFA
FUN_000353 CDS scaffold_1 1055901 1057633 +
hypothetical protein PF03403

MNASKMSYLSRLSPIPSFPEYTGPYKVGTVDVEIPISELEAPSAAPEGVDKIHTVQYRMFYPAVPESHEKHISWLPNPQRQHL
VAYTKFLGIGPMLAEFLSFLPRHLHYTTIPAHKNAKLLESSTENKRWPTMIFSHGLGGCRNSYSYIAGSLASHGIVVICPEHR
DGSAVASFIRVPEKQNGTITSNGRIHVPYEKISHDVSPEVYQAREAQLRIRCWELGMIHQAMLAVDNGTQLTNLNRSTPSLDQ
FIGRCNIHEPGSIIFAGHSFGAATMTQFLKSTYYADVPEVAAMEKPIFVPAEGSDIRKQVTEKTLVMLLDMWCMPLMAPNSAP
LFNLPLPAYADKASAPGGKAILAVESEHFFKWTDHLHMKARVLSPDPTVKVVTTQLFERPSGYKMSEPNFFYVVNSAHLNQSD
FGILFPWLTQKIFKAEQPERALRLNLRAQLQMLRENNVPVGRTFAGDLVDGTSFDKLDKFNEANGDPCKDGINNDQAIFDKSG
NNPVEFWRWIDIIGLGDSVGKKTTEKKVEEGEEEMKGELDPSEELPGAPPSMTGAVSAAAA
FUN_000354 CDS scaffold_1 1057797 1058336 -
hypothetical protein ENOG41

MFIKTVIILLASLSHGKEIMQRRHDVSSPIIDQFPWSSFLNKTGLDPLQYLKPPSDDIPETLACTTEPGPDRLACPVAIAAWS
NLADAEQYIYIPFDQCLSATEGTCRTVACALTGDLTIKNDEITGRMWNPVSTNCVFGGVGGIWQNDGSTLVIEMGYASQK
FUN_000355 CDS scaffold_1 1059671 1061338 -
hypothetical protein PF00135
S09X
MVSCKFLTAALCAGATLALPQSKTPSNPTATIDNGIIIGTSTSIPDSKTKVNQFLGIPFAEKPIRFSPPTPAKSWDTPYNATV
YKPACFMKFNYPEERRNRTIEIFATPEPPAGTSEDCLNLNIYAPAGAKAGSKPVAFWIHGGSFSHGSGSLPYYEGSKMAGYED
LVVVTVNYRTNILGFPETYDLPKGQWNLGFLDQRLALTWVQDNIAAFGGDPKKVTIFGESAGAGSVDDLITAPPDPLPFRAAI
LQSGSASTNVTSTGSWTNATKLAGCDKGDFDKVLKCMREVPAAKLKDIIERGMLDFAPLSDDGVTLSNYPRDIRLESKDNPKL
MARVPVMLGTTADEARIPDFMNITVKDALKTLAPGISDFQVSVLKFFYPIGSPGINNEFDQVTRMATEIGMQCPIRYVAEDFV
ETGIKTWRYIYNASFANTEIFMGSGAYHSAEIPTLFGTFPEKGATDFQEKLSREMQKAWGKFIRDPANGPGWAQVSKIGVFGD
GVRPDSGNKPKKAMKVVDANIIEPRCLMFKGIWGKGQTKA
FUN_000356 CDS scaffold_1 1061939 1063574 -
hypothetical protein PF06985 ENOG41

MALEPDLKEELRDQIHDCINKRGVHQECDVGWFRQDLKPNPPTRLIDVDTNDPSIVRLIVTAEDLQKDFIPKYLTLSYCWGST
NGHAKTTRATIAARREGIAVHSLPKTIQDAIQLTRLLKFRYLWIDAICIIQSDLDDVYLDDWNTEAPRIGSYYLHSKCLISAS
AASDSSQGLFVKQNARKYPLRTCALAFKNEKQEYICLSVPRPSPSEDWSAEPLRSRGWCLQEAVLSPRILHWSKHALIWQCHG
TTKSPTYGNDLNTARDIRTGQSHISFAQEPDHAMAIAWTELISRYSKMHFTFETDRLVAIQGLANRLADLHGGEYFAGVFRSH
LADGLLWKNSYDKAHNALAGVPTWSWATRCLNIWFLPVSHSFIRSTKPNVFPYNRSPINLNTPEKRALRFEAPLLNINLGRPF
TETDIVSTVQRPVFSCHVSFTEDSEDEYVVNFEYDAERLMPERFDMLEVLFLGLHVLHKLRGYISPSEFEESTVIDPDTIVSC
EGILLRKAGQYYERIGRLDFDMPKNYKRRISLKKLMDSNRKNVCLI
FUN_000357 CDS scaffold_1 1064301 1065633 -
hypothetical protein

MRTSTIIASLAAAVSAYEPWLNEPDTGLNTYLASTNWTEGSRPLLKDIRGVPDFDFAARQVLTDQQYAFYRTAAAGEWAYRNN
LKVWEKARLRPHQLASVKGLNETLGVTILGHNFSAPIFISPAARAGYGDSDRGELNFVDAAADEDILYVAALYATKTIEEIGT
QRKKRGNTIFQQIYSNANLSVTWDAMKRAEDQGVKAFVWTIDAPATSTRHRAARYDTTNANGATSVLSWDLFKEIKSHTKLPI
ILKGITTTEDALRAVEAGADGIWLSNHGGRQVDYSPSPLEIAYELRRNAPEIFKKTEVIADSGVRYGSDVIKLLALGVKAVGL
GRPFMYSNVYGVEGPKKLIQILKTEILADAAQIGITDLHNIPSKVLNTRALERDVYLMDEN

FUN_000358 CDS scaffold_1 1067200 1068574 -
hypothetical protein PF04666 ENOG41

MLSLLVKATTCIALLLCLTWYGQTHFYRDPGSVFFDKARAYETRYSEHRKAEVEKLINSYPELKKPALGKARNGNKLLCVALS
SVKRETQYLPTTIGSMVHGLVKEERDDLHISVLIAETDPRRHPGWNHQWLNRAADDIFTYDLNDTQTKHLNDLEQNGRYQEKG
VFDYTYALERCYATGALYVGMFEDDIILAEGWFMRFLQGLSQISDSGNWLFMRLFNQERSTGWSSREIGGNNEFLIILGIDIG
IAASVWFVRRQWRGSRKYLDLETLAVTAFILVPGLIVLLYQSGKASLFPPPPGVFKEPFGCCSQAMVFPRHKVPLLIDSLKGR
REGQIDLMLDEIASSNGLDRYALYPVQAQHIGIDSARKTTKDEAQAIWSMAFENQNPIILKKEHSKLLEKYELWREKVEQDAL
DSMYLDELS
FUN_000359 CDS scaffold_1 1073212 1074904 +
hypothetical protein PF00083;PF07690

MVKQPSAGEPAGPVVKGVIPTAKQALSDLFIWKQRVVITNEHGEETTEWRDPDPIKNPISLMAQLSGKDWIFFLVGFCAWTAD
AFDFHALSIQTKKLSVYYDTSKTSITTAITLTLLLRSVGAAMFGLAGDKWGRKWPMVFNMIILGVLQIATIYSTTFQQFLAVR
SLFGLFMGGVYGNAIAMALESCPSNARGLMSGILQQGYSFGYVLAACANLGVGGSTESWKTVFWIAAGISVAVGIVRIFFPES
QQFLEARAKGKKSSTPGAFWADCKKMLIAEWKMCVYCCFLMTWFNYYSHTSQDSYTTFMLEQKELNNSAASRASILMKTGACV
GGTIIGYLSQFVGRRRAIIVSAFMSALMIPAWILPTTERGLSASGFFIQFFVQGAWGVIPIHLNELSPPAFRSSFPGVTYQIG
NMISSPSAQIVNAIAEKTFVTLKNGDKVEAYGPVMGVATAIIALGIMFTTMFGPEKRGRAFEHAVAGVHDEDLPQHQKKDIET
ASVEQVEMDERNKNKEEA
FUN_000360 CDS scaffold_1 1075027 1077920 -
hypothetical protein PF00023;PF12796;PF13637

MKHLLTTRGPTVEIFAVYLLFVALRVEGSKGMEFLRFLLESGVSPDSIDPNDYRYSALHRAVRLENRAAVQLLLHFGADPDAK
MQGNREERLGSPLSFAVDLRYSRTIAEMLIGAGADTNLGPDKPLIRAARRWDTVLVIRLLEVGADPMLLPAGGLSMIYFAIAS
HNLDLVNMMIETGVNPNLSIEELQITDLELLRDEFRFYRVGQILTPIQFAVLVGPANIVKRLIEGGAVMDQLIEPDVQENRNQ
PPRYEGILTPLQMSIQQNWGDITKILLDAGATIDYRHPTTATALQMACGLSSPDRMKTELIEVLLAKGADINALPGENGGRTT
IQAAVECGDHELLKLLLSKGGDLFASSSKNNGLTVFQAALRSGSAELMTYVFLELGSQGGSANCVDGTNYLLEAISTGNKQIL
GTLMMSWNRLGLHWHKEYILSAAKVAIRKDLTHLVETHIVEFLDHVPPSITQEDVNSMICECIWSGDHRTFDLLMHGSIEHNL
DCAQSEYPTPLWLAIHEGKHYMAQCLINAGANPNQESLAICRKGLCCDIRLEMPLNQAILQLDSKFMEILLDKGADPNKVGLL
DERTALGLALHQDVSLSKYGGHHLNKSHEEPFIHLNRSHKKPFIHLNRSHEKPFIALMLCRYVNYFCQLAPTLMLQLEALRFN
VELAKFLVEVGANVNTSSIRTSALQLAVSNNNLDLVNFFLDEGASVDASVAGQTALQTAANLENLELVKNLIDRGADTDAKFP
DSLTTALQYAAMRGNIEIVKYLVDNRASVNEEASPYYNATALGLAVVNNHTRVAIFLIEKGASIDANPALYGGATLLQIASRH
GNHEIVTCLVGNGAAVNAAPATERGATALQFAAVNGNIKMAVLLLENGAHVSAKGAEVDGRTALEGAAEHGRLDMVYLLLEND
EEPETIEERCHDAAKFAEAEYHDVIARILRDYKRP
FUN_000361 CDS scaffold_1 1080517 1082374 -
hypothetical protein PF00144 ENOG41
S12
MKIQVQPLPLLLLLTGDVLAAKNYHCPPIGPVLPAPTSPSSHQNVKNAIEAITETIKAYSSLLHSSAVSVGVTSIYEDKPLLD
FHHTPENLDPRGVSKIDADSVYRIGSISKAYTTLAALKLKRVGMHDPVTKYVPELRKLNKQQSENNAITTVDWDKVSLQALAS
HMGGMPADLVTDLTSFRNWTDLGLPAAHDVLGCAGLVGIPPCDVQDFWDNFGKRPPTFAPWGNPVYSNIAFFVMSLVIERVSG
QSYEEFVQKNVLDVAGMNSTTYAKPDDSVGAIGPDDIFWNTSIGILSPAGSFYSSTKDLLAFGSSILKHEFLDLPETNKWLKP
VTFTSGRGQFIGAPWEIVRSNKLTSDERVVNVYTKGGDIGTYHSIFAMIPDYGIVISVLVGGPEIAGGLPLVFFSLITKALVP
ALEAAGKDQAKKTFAGTYINEDTNSTLVLDVDDDGPGLSITKWIVRGTDVSTHWLHYLSVLNPNVPKIPLSGRLYPTDLTAGD
KTEWRAMFRIGTANQIGMQEGLLFWEDASCMSWAMIDRAAYEFLGLDEMVFESEDGRTEEVELVGFKTTLKKAK
FUN_000362 CDS scaffold_1 1083182 1087395 -
hypothetical protein PF08623

MASGQINATPQAVMGHVTKLSDNDPDFRFMALNDLLQLLNIAKPDFLHHDYNIAARTVDSLIKVLDDQNGEVQNLAVKCLGPF
VGKIPTPVIAPLIEKLSSLKLKNSVDNAVPSLALRNVIIALPRPVPGIPPASDVQEAYSAISRVLIPRLIGPGPKTQVPKNPR
VPLPPVPEGLLQNEGDLNAEAVDVLIEVVRCFGPMLVQVEVEAMQEVVIQLLESEKGTSVVKKRAVVAISMLAVYLSDEHLEE

VVRRITLGLTKKISPVTRRLYISILGSMARSIPARFGPHLAGTAPLIIKALSEEELQEHLDALSDGDDLGQDFNEVREAALVA
LDAFLASCPQEMRPFTDETIEACLRYLKYDPNYNVDDEDEDMEDEEEDEEMDEDDEFDADDGFEDDDDDASWKVRRCAAKTIY
TVISTRGSGDLLENGVLYNQTAPHLIKRISEREENVRLEIISALSLLVRKTGEGLPTTDLSLDDLEPDSETRIPISRKRRRQS
SGGGASASQFMSGSGLVSPVLEKIPPSGPRADLARLTPSIVKAITKQLKGKTIPTKQAVIKLLNDIVSVQHGGLAEYFDQVIG
PIIEAIQPTGAVSASTHVASHSGSSSATPSTLRITALSVISDIAKTHSSTILQPYLTKIVDGVASAANNRFYKISSEAIRTVE
ELVKTITPPRSRNAASKYKAELEKLYTVIIDRSSAQDADAEVRQRAIHALGVLISRTSTSEGSSLLSEDKVKTALEVLQERLK
NETTRLAAVRAVENVARYARTPEQLDKAWVQDVTLELSAQLRKANRSLRGSSIIALRNLVLSPATKGQLEPDTIQGIVTDLMP
IIVNSDAHLLGPTLIILANLVPEHPELVVTDEMIAAISQLLKEYHAGIVLDQLLVLVSNIGESGAGQGLMQGLLKNVSVAGDP
PVVGKVIGTLLVTGGDSVGVKLDSFVTELYTSAKTNDEARVSLALAVLGEAGKRLGTSSTLKPDLFLDQFHKEPDKVSLSAAV
ALGQAGSGNVPEFLPVILKTMQKGGNTQYLLIQSIKEILQSISAQSTDLRNYAPAIWDELLKASDNADNKVVSAECVGRLVTL
DPAVFIPRLQALLKDQSLGIRGMAVQAVRYTLPESDEILDVMLRDVLIQMLLTMLQDSDMDIRRLAMTTLTTAARSKPDLIHP
HLGELMPFVLQESVIKKELIKEVMMGPFKHTVDDGLEVRKSAYETLYALMETAFSRINNIDFYDRVVAGLKDDNDIRQLCNLM
VTKLIAIDPDETTRRLNSIAEAYRTVLSVKLKDNAVKQDVEKQEEANKSILRVTLLLGEKMKAMTGNAGAATSNAGAASTWTG
YWDWVNKEFEKQLKALREENNQLQTRMV
FUN_000363 CDS scaffold_1 1088401 1088878 +
hypothetical protein ENOG41

MRLLLAFVSLLSLTTAGPVSRSPPDKDGEYLWGCGKTAPFSISDCNELLDWFKTVSKTGWFGIGKVNGLTKYWGSCQLYIQPG
PLGHFALEIQQDEFVDVVHRGMVYKCPDKWARTTVKPDNNTWIAFVTEKGWEPHDEF
FUN_000364 CDS scaffold_1 1090685 1092438 +
hypothetical protein ENOG41

MSDPSKPGSSMESSNDIDMASDTSDNKMSPVEKIPPEVLSYALSFIPDRASIRAAMISGPTLYNAFKQRQAYIASCILFNSMD
EPVYREAIVTFNMKIAEWKGVKAGIKAINRVYSSEQQRIYSQFLTFDRVKEMWRLHKAVEYFAERLPSSLIRKHPVVKYKNAY
SINTNVRVRFQRALYRLDACLKITELMIASQLHDNHGKNRAPTEEEHKEAHWVHCLRDLEEHRILKAFHSQYSAVEIEQLTSI
CGMLVTEIAPSFNTFLERDIELGTQLPYYITHALCPGSMSLISQGLPFLKDFLTAGSRPLRAKVMRRINPPIDWNPNLPTRPP
FPGNDEQLTCIVHDYEGTPGKWARMDTPDFMMRTPFIKDPDPGPECAWHVLSRYAIFLNESDEEVTPYQSLPWGYVFWDIDMF
EKAEFTHIRIEHHNEFGFTLEANHPIHDSWQPLAKWDSNMATDRLLMSSQEKVYLKNRGETGYFNFDSWILETEAHLFQELNN
MPREVLANAMRDMATPELLQQFFQTMEAQHPEIVQMAIDLSTAGVGNAWVAGTLTETPVPEDEDEDDAN
FUN_000365 CDS scaffold_1 1092875 1094411 -
hypothetical protein ENOG41

MATSGEPQPATVPQPFITPRLPLDILYHIGSLLASESITEHEHQIDSNSSCDCCMDKSSASLYKLTYTSKATRDVLEPLLYRN
LILTTPQDVTNIFINLIQRPELRQHVQYILCYAPLAGPRVRKLEMPECKRIWSKRCPSDKPALMRLLDGAGLHKLAWSACMLE
RTKQRFMFSPDFKHDGILELLFASILFLSPNVTSFTWRDCNNNPKAFILDHIMCKAVHDGLPLMPKLQKLSTEKAEFEESKQA
QFFTPHINLWENLYSLQLNDIDLDMEFVLMLFNGSFKENRPVRELCVRCVAGSERPESLTSFPPGFELASTMLLGDKNLDNYK
DQFKAFPNLNLLDVTFVFHRRRMEEGSLTLKAFLHAVGSPERLCLTGHPLPTSALDTGVTHSRLKYLKVRELQPNAPAKTTSK
DNLIAGLNQFWNKKSIIVPNLEEIDWDHYTFKREDMEGEDKAVWELVGEEEWEDDSGSDEDEDDDDAFWYSMERGDIDAFIEG
AIGVEDMWDAWGP
FUN_000366 CDS scaffold_1 1094909 1095269 +
hypothetical protein PF05839 ENOG41

MNKDSSFTFVHMHRSRDADLFEDFCKDKLPDDEIYVPPVHQPINPEDEDDVVPDQHAAFGIQRATQKSKEPAWKDLGLSELMN
KGPSIGTGGKPKGSGSALPR
FUN_000367 CDS scaffold_1 1095446 1096332 -
hypothetical protein EOG09263ZW6 PF04051

MSPPSTSPSRQAHVYTCDNEPRSTTTTLDFPRLRAYTNPAKVVLKLRNAPATVSQPIMSNLQPPNTVAPTPTPSGNKDPGLRY
PSNGKNIYSRPLNRTKTAELSQASFAYLFGEMVTYAQRRVKGIQELEQRLNLQGHSIGLKLLDLLLFREPVRTQTRPLSIIAL
LHFIKQNIWQHLFGRQADRLEKSANPETPDEYMIIDNEPLVNQYISVPKEMSQLNCAAYVAGIVEGVCDGADFPARVTAHTVA

EGDMWPGKTVFLVKFRPEVLEREGFLGKN
FUN_000368 CDS scaffold_1 1096601 1098602 -
hypothetical protein PF00004;PF08740

MDITKMSKMGWPPFLQAMNSTAQPLNSSSSFPGIMDTFLVSAGQASPLLQLFLFVYRFVGAQLGLDPSLLLTLLGCLWGLSKL
FDQVYATIDNFLHTYFRCTIYVSENDQIYSTLMQFLSEQQDIATNRHLTAQTVFKSAWDEEEDQADVLATNTVEDGEDSPKYL
NFASDAARCNPRYVPAMGTTGFWHDRTYFRVNRKRESLQSTNGWGGTKDVEELKISCFGRSIDPIKQLLADAKTAYFLDTRHK
TTIYRPRIKESRRDAWSMWQQVARRPIRPMRTVILEHEEKHDVLRDINEYLHPGTPKWYASRGIPLRRGYLFHGPPGTGKTSF
SFALAGVFGIDIYVISLQDPTVSEEDLAVLFTRLPRRCVVLLEDIDTAGLRRPNDEEDEEENEDGTGEKAGEAKGKKTEKAEK
KEKKKSKKAKDSSDSDTDSSEEDRKRRRKRRRNRRDRENTSNRGTNNILTVESISLSGLLNAIDGVASHEGRILIMTTNKPES
LDEALIRPGRVDVQVGFKNATSAQASELFYRMYEMSRNKPVPMSKTKPTAPKPQNGSIHSLVDNKGKETTLSIDDLKTISQEF
GQLIPEGMFSPAEIQGFLLKRKKSPRKALEDASGWIEATVKQKELKSKVVTVQ
FUN_000369 CDS scaffold_1 1100438 1101537 -
hypothetical protein PF00650;PF03765 ENOG41

MASSTENTAGPRKTPIASPGPNSKPLPRPELTDEQKTKYEALLEQVKGITEIQCEKKADEEDKSGPITDHERSWLTRECLLRY
LRATKWSVDDSAKRLKATLAWRREYGLEGFTPEYISPEQETGKQMIIGYDRQGRPCQYLNPARQNTDTSPRQLHHLFYMVERV
TDLMPPGVEMLSLMINFKPSKERKNTSVPVSVAREVLHILQNHYPERLGKALIINVPWIVWGFFKIITPFIDPVTREKLKFNE
DMKQYVPPEQLWSLDWGGDMDFEYDHETYWPALNELCRQKRDEKYRRWEAAGKEIGESEDYLAGGTDVSVKGFKFGEDAGVKD
VQEKLAETKLEEQSVAA
FUN_000370 CDS scaffold_1 1102760 1106533 -
hypothetical protein PF03372 ENOG41

MEPTNGEGPDGSSLKPVSSLRARFENMGNSTNSNVSKPVDVPPTTAPAATGPPSRPISPAPKPNRLRDFKPGQEVPPASPSTP
AIPARPKPKPAALSVNHDAPPPPTSARPAPTPKPQTVQLQQPPASATLPAVTVLPPQSPPKGRISALAVGDQPSFVNPDAVAT
PPTPTGGSRSFKIPSRSLNTSPSFNAPKSPRSGGSRPPSPPPPRRSGELIRTREPKVPPPPPAPRNEKALIPAPPKIASLSGP
TVIGKGGISQENSPFNSPPSSLGSGEETPPKLPTRPRPQSHIEPSRPKSVVMGFDPPPVHHSITTMRKDREEANGFSRGHITP
QVTGEPRPALPARPANFDIPPRPPPVNNNSNSMPPPPRPQRPAATTAVQAVDPAPTSSPPKRVVSTPTTQQPQQQLAAPSRGH
GRSMTIDRASDRTPAEFRIAPTTVAAPIAAAAPPKSVQSTPVVAAKIAPKAEPIHHVTTYPEASNTNRRPPFSKHGTYEIPTK
YDARSFDVCGEFICASGYMTRVWSLIDGEQIMSLQHTEGVKATAVSFKPAADPREEGKRIWIGTNTGDLMEVEVATHAITPSK
PNVHGRNEVVRIFRHWNEIWTMDESGTLLVWGPDEKAVANITGHPTQSYKLPKGHTHSMVIGDYLWHATGKEIRVFAPTVDGR
TQFQVLVRPLIADSAGEVTSGTLVKSHPDKVFFGHVDGKVTVYNTNDYSCVMMMNISTWKINALASAGNYIWAAYNTGKICVY
DVEEAPWVILKEWQGHENPIVKMAADPSCSYFLDRQQIISLGADNKIKAWDGLLEDDFLEDSMKSKDTQYCDFDEIRALFFTW
NAGASTPSSLRHSGGDSTFFQDLVQSSGSPDILVFGFQELVDLEDKKLTAKRFLKPKKKEAGTDQERMSHQYRDWRDFLMKTL
DDYMPADDLYHLLHTAPMVGLFTCVFVKATLRDRIRGLQGAEVKRGMGGLHGNKGCVAVRFRVDDTSICLVNCHLAAGQSQAN
ARHNDIAAILEANLFAVERDSASRIDSFVGGGDGSLIMDHELCILNGDLNYRIDTMSRDTVVTAVKQNNLGKLLERDQLLVAR
RRNPAFKLRAFEEMPITFAPTYKYDVGTDNYDTSEKRRSPAWCDRLLYRGRGRVEQFDYRRHEVRVSDHRPVTGTFNLWVKKV
DPRGRATAWMESQQSFEDMREKETSEDKLLFLRDTCGFDEETSKRLIREKSARRDNRSPVRA
FUN_000371 CDS scaffold_1 1107925 1108444 +
hypothetical protein PF15932 ENOG41

MNTVKSFWLGWGSLCVAGGGAYYFAKQQINADRQAKMEAHRKKRQMIENLETTEGNGSPARTDSTGSPSQEASSDPAPTRHAP
ETESQRIGEKSKYESANVYRSPKGDRFS
FUN_000372 CDS scaffold_1 1109059 1110723 +
hypothetical protein PF04818 ENOG41

MATPELAIAKATLSATLFRADPTSLSRPEVDAFFPILTDALAKCSRPNVQKCKVWIIDKIAPSAARTTALAKYFNALSKSLQD
DGSRPSLKRRRLHLLYVLNDVLYHAVTRNDDFKFATAWEPVLPGMIASTAAFDNCPKHKQKIANLIRLWENQQYVKSSFAKKL
EDALASGVAPQTAQEAQTSNVTLKLAKDAPFALPSFHGDSTVPWYDLPATTWLPHLTPNSTKPMFPDQIRPIQLAAGPADKVL
VNAVQDLLGEVERMFSKEQKWNDEPGNDLNELGERVVLDEITGEVIAGDTYYGWSRQFCEKMKERKKKGRKGSDRGRSRSRSR

SYSRSRSRSSSRPTHKRRRDSKDSRGRSLSRSRSRSYSRDNDRSTKRPRSPSYSRSRSRSRSRKARFERPGYSRSPSRSPPPF
QGKDPQDRSYRPPPFPQQAPPAPFPPIPPPGDFPVPPPPPAGYQGPWPPPPPPPHMAPGWFPPNPAMMGQMMGGWNVPPPPPP
PHNGYQGRGRGNGYRGRGRGGYDRGRGW
FUN_000373 CDS scaffold_1 1111590 1113777 - PPP2R5D
Serine/threonine-protein phosphatase 2A 56 kDa regulatory subunit delta isoform
EOG09261T98 PF01603

MKRFSQRVLSRGKDPNKSSKKNKDSKDGTASPSSRDSNQSPVLTPSSSTSTLNDPRNKPLPQNNAGHGGEHGSGSQPSNLSNV
TQAGSTPDRFATGGASSPNGGSANSRLPPTVVISPTPGHIPPPGAAETMPHDLAPPKAGQKSLLIHRGIDNRDAIPEGLRTPK
RQHSSRFDISAHRELEKLPGFHEVPPNRRQELFMQKIDQCNVIFDFNDASGDMKSKEIKRLALHELLDYVANNRQVITEPMYP
KVVDMFAKNLFRPIPPPMNPQGEAFDPEEDEPVLEVAWPHIQVVYEFFLRFIESQDFNTNIAKAYIDHSFVLQLLELFDSEDP
RERDFLKTTLHRIYGKFLNLRSFIRRSINNVFFQFTYETERFNGIAELLEILGSIINGFALPLKEEHKIFLTRVLLPLHKPKS
LSMYHPQLAYCIVQFLEKDASLTEDVVLGLLRYWPKVNSTKEVMFLNEVEDIFEVMDPAEFAKVQEPLFHQLAKSVASPHFQV
AERALYFWNNEYFCNLVSDNVEIILPIMFAPLYENSKGHWNRTIHGMVYNAMKLFMEINPQLFDDCSHDYTEQQNSAAAREAL
RERKWAALSEKANQQRESNGNGTADAPSAQNYVNPMPRVDEVDPVTEDNSKRLDSLKLQDGRPNLHERQNSVGSTRSR
FUN_000374 CDS scaffold_1 1115973 1117882 +
hypothetical protein EOG09263GSR PF09734;PF17682

MEISSDENGYESLDDTQLENDNGERANDSVHPDSAPRYTVPSRAIGAVEIPAVVENIDRTIKAFGRVPNLQHVMDAGRNSIPL
YLTPEMPFCKPIMSHNARSHNVVLKITVPKRTGRKRKRGTDGPWEGDVEINDAENQPQENDRVSSHARLDDPKVLRRTMADNV
DKYQVEAVGVIRNTHRFRGLADFYWDMSKSSFAQQYVDKVLPGDVDKIKEFKFQPGTDKGPNVDILPPPMFTHMSLPFNYQYS
QNPYVRATEDGGTVNTTAVKQVGYFIGAEDPAPAGPQLEPDMTDPRMVEIMAELEAAFEERPVWTRRSLLNHLGGKLKNWNEL
KKYLNYAAYQFKGGPWRDGVVPYGIDPRTDPKYRTYQTLMFKLPKQKRAQRGQTWKSLRKVQMGPLKEFLEELSESHVFDGDT
FHTDGKVWQVCDITDPLLKELLENAAIRPEWDPSSGWYHGGLWAKVKAIMKTKLVAIQFDRHLTREDFAMTLQAGDETPIRSN
QATFHLPLPNLRLTDEELTQLRGRQPTKKNKHKGYSVRVRDPNAGPKRAPAEETRAVERGEEEARILEEMGSGNEDSDDDDSG
DDEDGDITAVRDEDIDREMMYG
FUN_000375 CDS scaffold_1 1118634 1119863 +
hypothetical protein

MAAVKRQSNGATAIVVSEYKTGSGTLTSDRFWCDPCKRIFSTWQQLHDHKEKMSAQGKAKHIHCQFCSRDFHTESAKITHIQQ
DHPQEQSLYCTGCGKGPFVRVGGLVSHVEKECLILNTNTIETMREEKMEFSQALTALTKEPLKNNYGGYMPENANKGLPDATW
ESTGSIPAPFTIEQSQFPSLGESSSTTLQPTKEKKENSWNKGKNLFPDAPAAQRPTQEQLKQVTAPSARATFDLMSDLDPKHP
NFNVGRFYSEYTQKFNCRMGRCGKAFKTAKGFIAHLTSEVHSASKYRCPYCLNTFGSLASITQHVESNSNKCKIRDTEQYNSY
MDQLLASMVNVKDRHADGTVRYETSKTFGKGPSPGSDLVEQKENPKVNGGDPYTGMQIHW
FUN_000376 CDS scaffold_1 1120582 1122273 + PFA3
palmitoyltransferase for Vac8p PF01529

MATARRWARKIERCCCTFATYFPLAFVYGLTSWAVWVVVCIGSASRKSSWIGTGSSIIGVVLYIMLNWCYTTAVFTPPGSTTN
DMGYGLLPTQNTPQGTSFTVKSNGEFRFCKKCQARKPDRAHHCSTCRRCVLKMDHHCPWLATCIGLRNHKAFLLFLIYTTVFC
FWSFAVSGSWVWYEALDDQEYIDTFLPVNFIMLSVISGIIGLVVGAFTGWHIHLARCGQTTIECLEKTRYLSPLRKTYNSAHN
PANNVPEAARQFVDFHTNALPGITRPEEGEERRSYPPDGSHPVQLSYAQREREQRQRRYEEYLDEQDSEKLPNVFDLGWKRNL
LHLFGPTPALWFFPVSNTTGDGWTWEASSKWLEARDRLKAEREQQRAREVNAGWGSPDDIPDVPERPTGAGRHFTPGPKLAGP
KTMSKADRVLGRDPNLYADATQNVPMSRLSPRGRTVDDELADLDSDDDDGFIDTNNPEPENGKLSPSFTSEAQRRDDAEARAL
EVVTNGNWGRGGASGMLRKGSSQSSPLSRTPSNLSRSGTPKFQDEGVD
FUN_000377 CDS scaffold_1 1122774 1123449 -
hypothetical protein PF01966 ENOG41

MMSDINPLVLEKIPQADTCLSALELARDALPIPILNHSLRVYLLARYIAEKEDSPFKSEDQSPLLFVAAIHHDIGASHLCNGE
QRFEICSADCAKAHLAKSGYSEAASHQVWTAIAVHTSPGIAERIDPLSRLIRLGVLSDFGSKDYRTSLGVDEYYTEIEKLLPR
LDAEKCLGDAVVSQAKEIPHVDSLTWPNDAKFPAASWPGILLRAHAENPGHDGVNPAF

FUN_000378 CDS scaffold_1 1125011 1128353 -
hypothetical protein EOG09260GIX PF00168;PF00387;PF00388

MSFSFDSDSKSTLTTHNLSEMSSLTRYQLGQVQTAFPSQTQPVPIPMSAISASSGASNSQEGSPLMSPDTAVLTTYSSVQPSP
EPMRGREEEVFPSSFQLPESVLSRKTSTNSLGYNAMSEVANNNKGGLIRRFSNRAQRFAGRRRPSSGAPASRDGSVGPSILRR
RSDSNATAPQENSALTDTDEEPEIIPDDCISMVGLDGATNTSSANSTHGSISASAGAMAGPVLPAELRNGCPVRKVSKKSRPK
RINLVYETESNKLSWDATRPQKSLHVDEIREIRTGSDIQQYIIDYGLGEEAAKYSNKWFTIIYTVPENSKTRFLHIVTDNTKS
LSLWTEFLDAMLRYRQELMTSLMAFNDRAVAQYWQTEMAKEFGDQVRSPEQEEMNIAGVKRVCQNLHIYSSQATLEVNFHLSD
SRRRQKLNFREFKDFVRRMKQRTDVQRIIRGFAANPEFGLTSTEFLTFLRDVQGEDVDSNRVAWEKLFSRFCRQYRNDEVDLQ
ENAEIMSEAAFVGFLTSEHNDVIQPEPQNIVLDRPMNEYFISSSHNTYLLGRQVAGQSSVEGYISALVRGCRCVEVDCWDGYN
GQPEVNHGRTMTTSISFKEVMTTINKYAFVKSKYPLWISLEVHCSPAQQATMTEIIKECFGSRLVTETLEAFPDKLPTPSELM
ERVLIKVKKPQIKEEPVSVGNDFRGRRRGNSLNSPMMRPSAPDGTPLMPSQSLPQSPMLTPSHSSRRLVSKTRVNTITEGRVQ
DMMSSSTSDNESAGEQPAKKAPNKTVKVLGELGVYCAGVKFSGFDTVDAKQYNHIFSFMESSFAKHSRAKDQKMALDIHNMRY
MMRVYPDRTRITSNNFDPLIYWRRGVQMAALNWQTFDLGMQLNRAMFDGKDASGYVLKPAELRDIQVLPYNSDIAEGKKERSV
VSFSIDVISAQQLMRPANLPANKSMDPYVEIEIFHANDKRDKKEADSNLVMEHDSPLKVQTEVIRENGFNPMFDKQFKFKVTT
KHPDLIFVRWSVKLSNDGESYNERPAVATYTAKLTNLKQGYRTLPLLNHAGDQYLFSKLFCKIKVDSVQKMLIDAPRRTQDGN
KLNRLGGKVFSRINTSPKSTIEKSSSEKTSFDSY
FUN_000379 CDS scaffold_1 1144471 1147236 -
hypothetical protein

MAATTEQAPQQLSDALVAFSLEGRFPDNISVLPPVSETDLQPAIQALAKAKRDLEAELHTINQETKQDVDSWIRNSKTLQEDI
FRSKAMANEIERQSEAPDASGEAIQDAEEKAEFLNREVQYSQQLHGALQGIKRVNQLLSEVEAAKNERRILDSLRLLEKSWEA
IDQIGVSKTCRVMKLLNLRSFELKSSVHEVFDHIWKTLIHADIETRQFAVYNAVEDEQMNLSDAVVGLQAYKEVDERMEQLWR
NVDAAIISPRMDIKNNTLPTIQADGEVLKLSGEASRSVDALLTDLETSFTFLAQKLPSDLLPPLGNFMMADVIPKLIGEWLEV
AVPSSLKEMDNFQAMIKRAREFCQSLTQHGYTGFTDLQDWVDNAPSIWLGKCRETTLDSVRSKLLGGIGESKQVEKVEKQMVS
LAEGKEITKTPGGAAANTEAADWGADWGDAWEEDNEQPEVQNKFASKGPGSAKADEDDGADAWGWDEEDTTTEAKDTKEAPEK
DEEDDSAAWGWGEEDTTQEPVQAPKPKGKAPNAQNETRELVLKETYSVSSMPEPVLELIYAILEDGAALTRDDVEYAPVAATA
PGLFGLPTFALALFRAISPHYYSRSEGGNMFLYNDAMYLAEKLSEFADGWKKRDDLTPRARNMLRLDNDIKSLQAFANRSYAN
EMNIQKTILRDFLGGAQSLMQQDEMEACVELASARIRAMASVWKPILARSVWTQALGSLADALATKLITDVLEMSSIGQDEAY
NIAKTIATATELDDLFLPSTLTGTAKSEDEVPQTAQYAPSWLRLKYLSEVLQSNLNEVRYLWCESELSLYFSVSEVIDLIEAS
FEANSRTRDTIREIQSKPTPLAGR
FUN_000380 CDS scaffold_1 1148866 1152114 +
hypothetical protein EOG09260DFH PF00675;PF05193 ENOG41
M16C
MSPESSKSHFRKIQSFKTDYAPCTITQYVSDRSGMQVVVADRKGPKLNGYFTLATEIFDDSGAPHTLEHLVFMGSKSYQYKGL
LDKLSSRAYSHTNAWTATDHTAYTLETAGWDGFAQVLPVYLEHVILPTITDESIVTEVWHIDGEGNDAGVVYSEMQAVQFRSS
ELMDIKARRLLYPENVGFRYETGGMTDALRVLAPERIRQFHRDMYQPRNLCLVLVGEVNQDELIHILEDFEESIKDDIPSLDT
PFKRPWIDSAQPPKINETQIVTVEFPEEDESVGEVLVGFFGPNCNDLIQSSALNVLLTYLCGSSVSILENVMVEKEELASSVS
YWTDPRPDMVIWLQPTGVATEKLEFVEKRLFELLKEVASKPIDMDYMRECIRRERRQVKYHAETSESFYATNIITDYLFGKRD
GSTLKDLKDLEEYDVLEKWTDQQWRDFLSKWMADAPHISILGKPSHELATKMKKDEEARIAKRKEDLGAEGLEKLAKRLEDAK
KKNDEPIPASVVDRWSVPSTDSIHFIESDTARSGHARAVGLGSGSAQKLIDGTTQGKLPLFIQFEDVPTNFVHITIHIGTSQV
PNELKPLMPIFSDNFFNTHIMRDGKQVGFEQVVMELERDTISYSLHSARYISDPDGIMIQFQVEPQKYAAAVEWIRTMMFDSI
FDPQRLKASVTKALADIPESKRDGKSMASEVNAAIHMEKSSLAVAKRVLVRAVYLKRLKKLLEKEPEKVVGWFNTVRDSLFTF
QNLRFLVTANLETLPDPLTTWDTLSKSLTVAENMVEIPKPVSLLNDEGRNPGSVGAIIVPMTTLESSYSVSTAQGLASFDDPR
LASIMVAIGYLEAAEGPLWNAVRGAGYAYGVYFSRDINSGIISYHVYRSPDAYKAIKASKEAISKIAKGEVDIDRHLLEGTIS
QIVVMFADEQSTMPSAAQQNFVQGVVRGLPKDWGKEVLRRVRAVTEDEIKTALLETVLPCFEPGKSNIVVTAAKIMQEGMETA
FKDMGYKVQTHELSYFHDDYGLKPEEGEEEESSEEDEGLEGSEGSYDSDESYDD
FUN_000381 CDS scaffold_1 1152384 1153899 -
hypothetical protein PF01435;PF16491
M48A

MDFLQRLARFLDRPLFPWKKLIMGFSVGQYLFESFLTLRQYRVLQKTSPPAVLSKEVSQEVFDKSQAYGRAKAKFEIINGLYS
QVQNLAFMHFDVLPKLWSWTGDLLLKWAPARFTGEISHTIVFVLTFAVISQLLRLPSSIYQTFVLEEKFGFNKQTPKLFVTDL
IKTQALTFVLAPPFLAGFLKIIQKTGNQFFYYLWLFVIALQVFMITIYPVAILPLFNKLSPLEDGELKTKVEALAASLKFPLH
ELYVIDGSKRSAHSNAYFFGLPWKKHIVIYDTLIEKSEPDEVVAVLAHELGHWKLGHTTSLFGISQAHTFYIFLLFSVFINNH
SLYSSFGFLKQHPIIIGFILFSDALAPMDLVINLLMHIVSRKFEFQADAFAKQLGYPEQLARSLLKLQIQNLSTMDADWMYAS
YHFSHPHLSERLKALGWKGSEGVTDGVTDKSIGNEKEGVVKASGRDEL
FUN_000382 CDS scaffold_1 1154558 1156118 -
hypothetical protein PF09728 ENOG41

MPASQVDAPVANGHDGHAHSPTKKGKGKKGMDNNEASRLLHARISQLEQDAAGDKEQELEIEREVKRANRDLLQQVSKMDNMQ
KIDHLTKRSSELLADMRRLERENQKNKRRGDTLQKERDASRTELNKTVGLKEKLEKLCRELQRDNNKMKNENKELQTTQKRNN
AHWDEKYATLLTKLDGYQEEKDTPKKQVVDMEVDELFRIRFKSFIEQYELRELHFHSLMRTKELEVQYHMARYEREKKNAEGE
STKARHLQAQVQAFTKTETELRNQLNVYVDKFKQVEDTLNNSNDLFLSFRKEMEDMSKKGKRLEKENEALKRQKEATAANIIR
MAEERQEWKKKTETAEKKTEKLMSIIQQMQQQGRRVPPGMSNTVESGYSDSHGGNEGDESDYSDEEGEEEELSEFDDDTEEEP
QGNEQGTPVAYGPERPPQPVPSAATNGH
FUN_000383 CDS scaffold_1 1157026 1160094 + CYM1
Mitochondrial presequence protease PF00675;PF05193;PF08367
M16C
MLRNAAAGARKAVTELSQFPKPGEKLHGFTLVRSKHVPELELTALHLQHDKTGADYLHIARDDSNNVFSIGFKTNPPDDTGIP
HILEHTTLCGSEKYPIRDPFFKMLPRTLSNFMNAFTASDHTFYPFATTNAQDFKNLMSVYLDSTLHPLLKKSDFTQEGWRIGP
ENPLAEDEASKKLVFKGVVYNEMKGQMSDAGYLYYIRFHDHIFPDINNSGGDPQKITDLTYGQLRKFHAEHYHPSNAKVFTYG
DMPLVDHLQQVDAQLQAFEKIQGDKQIHEPVTLSGPKEVTLYGPLDPLVDPDRQYKTSVSWIMGDTTDVLESFSLALLSTLLM
DGYGSPLYRGLVEAGMGADWSPNAGYDSSAKKGIFSIGLTGVQEADVPKLKEKVQQILRDVREKGFDKTKIDGSLHQLELSLK
HKTANFGFSMLNRLKPKWFNGVDPFDSLAWNDTINGFQTKMAEGNYLEGLIDKYLLNDNTLTFTMAPSTTYGEDLVKEEQERL
STRIQAAIKEAGSEENARKHFEKQEQELLVEQNKTNTEDLGCLPTVHVKDIPRSKEPVVVRDENANGTKIQWHEAPTNGLTYF
RAINTLENLPDELRELVPLFTDSIMRLGTKDLNMEQLEDLIKLKTGGVSVGYHCTPSPTDFHAASEGLIFTGMALDRNVPVMF
DIIQKLVLGTDFDSPEAALRIRQLLQASADGVVNDIASTGHRFAMGSAESGLTRSSWLRQQVSGLSQVQLVTSLASRPETDKL
EDVISKLKQIQSIALAGGNLRTAITCGPESVAENGASLQKFVGNLSRDPLDLKNPSPRQLPKDSKTFYPLPYQVYYGGLSVPT
TSYTAAEGAPLQILSQLLTHKHLHHEIREKGGAYGGGAYSRPLDGLFGFYSYRDPNPQNTLSIMRGAGQWAVDKKWSDRDLEE
AKISVFQGVDAPKSVNQEGMGRFLSGITEEMKQKKREELLDVTKDQVREVAQRYLVDGLAKGEGRVAFLGEKQSWVDGEWKIR
EMDVKGAE
FUN_000384 CDS scaffold_1 1162268 1163665 + SPE1 Ornithine
decarboxylase EOG09261V03 PF00278;PF02784

MVMATAVLDTYNHNVNHPHIALKKPLLQEPLEQYGVITPKQLIGQALHQRVEAIDHEMCEPGDEDTFFVADLGEVYRQHLRWK
KNLPRVRPFYAVKCNPDPQIIKLLSELGTGFDCASKTEIEQVLSAGLSPDRIIYAQPCKTNSYVRYVKSVGVKQMTFDNADEL
YKIAKLYPGAELFLRIMTDDSESLCRFSMKFGAAMDTTEGLLALAKDLGLNVVGVSFHVGSGASDPLAFYKAVRDAHTVFQLA
HEFGFNMRTLDVGGGFSGDTFETMAAVLGDALDEFFPPHSNIEIIAEPGRYYVATAFTIACNIIARRTVEDPTLDGKGYMLYV
NDGVYGNFSNIMFDHQQPIAKVLRASGKTLFETTAAHPTSEGEGFEYSVWGPTCDGIDRITESTRFDSILDVGDWLYFEDMGA
YTKCSATQFNGFSNAHDVIYVCSEPGAKALLGL
FUN_000385 CDS scaffold_1 1164197 1167522 -
hypothetical protein EOG09260NNR PF00097;PF00176;PF00271;PF04851

MPRGRPSTRGTPITAADTSRDTSTASNRMTRSARTRSQAASSIPTPTPAVDIQQDAPSGSRPRGRPRRSIPEVEASPLSQFPT
SDIALPTKRRGRPSKAPIIESESVSSVPTPLDPDTGSDYSTPASSKVPTPAAVDHGKSTIFEVQLPAPSTSRPVDRERGLHKS
AYSMNSRAKGRMVIEDSDDEDFDNYRNAQVARRLQDEELKQASSAASSSLPLRRSTRPSLSGSFNTPAQASVKRERASTLTDS
TAKRGRPAKKPKVIADSDDEEIDMDAEIAQGLDLDSGSSLSSYEDESFADDDASDKEAARYISSDDEDVPMAMSRKAAGKRPA
RPMARTVPRVKAPAATFAHSARKAAQADGPSGDELDALESALGDAESTDFATDNNDGYIDLSSSDADADDADNRDHLATIANN
RRAFRNQGVSRRAIRERERLEKNHPEIIGMWDRIKKEPVIKAGKATQPERISRQLKPFQLEGLAWMMEMEKAKYEGGLLGDEM
GLGKTIQAVSLIMSDYPAKQPSLVLVPPVALMQWQQEIKSYTDGALNTFVFHGTNQKTKGITVKELKKFDVIMMSYNSLESVY

RKQEKGFKRKDGIYKEKSAIHAIDFHRVILDEAHCIKTRTTMTAKACFALKTTYRWCLTGTPLQNRIGEFFSLVRFLQVDTFA
SYLCKQCPCSTLEWSMDEHSRCTGCKHPGVQHVSLFNQELLNPIQKYGNAGAGATAFERLRLMTDRIMLRRLKKDHTNSMELP
VKEVYVDRQFFGEEENDFANSIMTNGQRKFDTYVAQGVLLNNYANIFGLIMQMRQVADHPDLILKKNAEGGQNVLVCCICDEP
AEDTIRSRCKHDFCRACVGSYVRSTDEPDCPRCHIPLSIDLEQPEIEQDENLVKKNSIINRIKMENWTSSSKIELLVHELHKL
RSDNASHKSIIFSQFTTMLQLIEWRLRRAGITTVMLDGSMTPAQRQASIEHFMNNVDVECFLVSLKAGGVALNLTEASRVFIV
DPWWNPAAEWQSADRCHRIGQTRPCTITRLCIEDSVESRMVLIQEKKTNMIHSTVNSDTKAMESLTPQDMQFLFRGS
FUN_000386 CDS scaffold_1 1168413 1170659 +
hypothetical protein EOG092621X3

MRKRERHCTALHLSELLLTLLFCANSFSSLILPSLSFTGWLMARATRSNNQNAQDQNAQAGQNGQNGQNGQTNQSNRQGRAVY
GPQSALTDFLASHNISAAQIRSDAEARRRQAAREDAAQDENGNGESSAAGARSGRRSSRNDNDEASLKRKREEEKKLAKIKES
KAFKKRKQRSDLSDDEIARALMESNSGPLPGQIEHCEICEKRFTVTPYSVAGPNGGLLCPPCGREIAKEREGGQKKKPKKPVN
TGPVGRRRAIQSRILDGDVGTKSLATLCVQTLAKNVDLADSLGDLPDHLIDKIARMFSKRRLLKPETLPLFVQPGTEDLHIYD
GAKLGEQDYISIFQTASKLKNLKIRCGIQFKDEVMDYLLSRDINLETFYLHGANLLSDEKWHKFIQEKGEKLKGIQVYYTDNH
FGDETIATLRDHCPNLKRLKVENNQKLTNDGVKTIASLSALEHLGLQLQHKTESDAYIEVVSKIGANLKTLSLKIVPEVDDGL
LQAIHEHCRSLSKFRITDSEFMTDQGFVDLFTKWANPPLHFVDLQKCRQIDASKPRENPDSVGLCSEGFKALMAHSGEKLQYL
NIHACRHISREAFEEVFHEDARYPELKELEISFCEEVTDFILGSIFRSCPKIREVNVFGCMKVKEVRVPRCVILVGVPNAQGM
VVEGSHD
FUN_000387 CDS scaffold_1 1170863 1171999 -
hypothetical protein PF01764 ENOG41

MLLLPLLSAITLAVASPVALDDYVNSLEERAVGVTTTDFSNFKFYIQHGAAAYCNSEAAAGSKITCSNNGCPTVQGNGATIVT
SFVGSKTGIGGYVATDSARKEIVVSFRGSINIRNWLTNLDFGQEDCSLVSGCGVHSGFQRAWNEISSQATAAVASARKANPSF
KVISTGHSLGGAVAVLAAANLRVGGTPVDIYTYGSPRVGNAQLSAFVSNQAGGEYRVTHADDPVPRLPPLIFGYRHTTPEFWL
SGGGGDTVDYTISDVKVCEGAANLGCNGGTLGLDIAAHLHYFQATDACNAGGFSWRRYRSAESVDKRATMTDAELEKKLNSYV
QMDKEYVKNNQARS
FUN_000388 CDS scaffold_1 1174229 1174654 -
hypothetical protein PF00468

MFSSITRLARPVLAKPLTQSPRTFTTLVPLRPSLTPMNGAIRRTALPSSFTPLTAASADTVPSSAISAHPALGDMQIRCGPRN
TMNGHTRLVQKRRHGFLCRKRSKTGRKILLRRKIKGRRHIAQ
FUN_005867 CDS scaffold_2 678 3883 -
hypothetical protein PF11635

MTSEKMPLILDNPMPVELNDVDDLFGDGVGLSLPARAQSKQLQQRMDDVRIRGCCQTVAWSRTGTIANITPDGQNLELRFLRR
SPEDGSWDLSEPTTCPFVKGLPTIPIVHLVWAGTSSPDLAVIDAVGRVSIVSFSISLNHPFPQRKWDADPTDDVHTVVGAYWL
NVSPPNQQASYNVMYGPATKHGNGYTYESSFVHAGGPTHPSPAKSALLTITTHGVIRMFWSQNTNRLEETTMELESISASDEL
ITHASFASEKKHLLLAVATTSKQLKLIKIEIQWGQASQADKATGRPAGNLSPSLVEKHLATTNWLQGGPGDSSLDISMIELSH
LEVLPSVVDSTGKNTTPPMVVTARSRTPTESSYQGSQSVVDRWEAIEQKQNLPSAYEQLGSRRNSISSELPAVTQLQKVAPVT
ANKVVVAFQTTSFGKILVLAFADGTVEYRDRLTFEELYTTQELNKVQNLRQIGWTFTDEGPCQQVAFSPTFCSMVQMGEDGKI
KWNKLHYPMGDIGNSMHDAQYCGSIAALTVTAAPSMFYQNNYDDLLAIVRPYTTKKRFVQDLVTELIRILKIQIDYSEEIHHD
SLVRNGSLQYCLSIMNALGFRGDFHPRSFQGKFSMLFLNVRNVVVLITIASNTPVTVREKLSPLDDPEVIETLVGCARWALDL
IAWLMDCLFELMNDNHFQELLTRERFHELAPYLHEKNNVAFHFLMSSSSRGFLSAICRRLAHLEALSGRAIEFYRKQSAVVEG
VAGGRAAPQLQQAYQAMQQVTSSALVKVSEVETLLTGLSNEIRQAYQIFLPSLAKSQNNQSQGKQLDMTMKAARVQMELSILL
SAAPPAPFLQIIKKFFNTDLPAFRNTVDPGRLFFANYDLLEVEDDEHSLAAKKARGMVYVDVFKRMQIRPSPNKQWRRCSRCT
AVMEDVFGSRSGFTFVLGQQRKCSCGGQWTLLPKGHVA
FUN_005868 CDS scaffold_2 8143 11413 +
hypothetical protein ENOG41

MSQGGGIAALDSTLGWNEDQWPDRAEAVVAIDSNSMNARDSGSVNSQGPGRPDDIMSFFDNETLSSGSASLSQPLYPRPLHHY

TNQTSHINKSRIHAQPSHVYAPIFETMHSLQRPKPPLQPQRQRRVPTRLDTDDSTDTSAIDQSQGLGDHSFLLPSRPMSIATT
DVMTTKDDPTCSNSFYRAWAEEEQIELRIHSETNPDTDNLRHNSQYHDLLSEIDTECLFPDDSLTTDHRPSSSRSHLLQWRRS
PNAAASTSPKHQRVSSDVQPPTTENRPSATITRQEFEALPPTIQRKYFSTIERSQLAQSPDLGHCQRAGSVGQDQLLSPWSSL
PKPRGSYEHQERPITSDDSVLRTDRPRIAKTDQKFYANLPEKIKRRHLTEEELQLAYKSQNTFSNAPEAVSNKVDIPSQEVKM
PSPLPSPGLSEGTSPFSGMLSRQNDRAMTSSDVKRSDSFYDSFRWLDDEEGLDLRLYLDDYHINLREEVPVPNKSRRPSFRRH
LSINKLPFGKPSVNGLGEGPASPTALPPSASHSSSVGSSGHVRKKSRARALSLISANRQTPLASPSPLTPPLDPSAAHYRDPE
ARKKLRAYLASPQKFDEAIEFGFCSKDGPRPHTGFNKASACEPDRLRSFLEDDRSSRYSDDASAAEPDSPKTPQLFDKTPHIR
SLRSSIEHSSYSRGEKSIYDPAASREMTLRMTLTRPDLRQSEDQIYGWKQAGSRVSSSRGEHTPSPALYAREGNPKQSIERQL
AAQDQWEDPVDHDNRTVKRFWNRVKRS
FUN_005869 CDS scaffold_2 12545 17147 - VPS10 vacuolar
protein sorting/targeting protein PEP1 PF15901;PF15902 ENOG41

MMRSSAWAALSWRALIFSLLWTAVAAKQDKPTVDASVAKHPPLNLNYFEDSDVVVFQDIEERNIWRSEDAGKTWAQVPDIPDR
SATFLYLHPFDSNSAFVLTKDRKHYKTEDRGKSWSEFNSGTMPSAFQPDTLVFHAGDPKRIIFNGMNCDGIFCDEETTYTIDG
FKTVQQLRPSTSGCWWAKANREFTTGDAELDKTRILCIVTDPLSLFKTSQKLCVSDNFFAKGSGGKFDEFEPSLDGQRDVTGV
VNIAAVKSFILLASSSAGSDEMTLFVTSDAQFWHRAMFPTDDSHDHSHKINQEAYTVLESTNYSIQVDVMTSHPSTPMGVIFT
SNSNGTYFTENIPYTNRNVKGHVDFEKISGIQGIFLVNTVENGKDVDAKNARKVVVTQITFDDGRTFEPVKAGQDRIHLHSMT
DIDNIGRIFSSPAPGLVMGNGNTGAALGEFESSNLYVSDNAGVSWKKALEGPHKYEFGDTGGILVAVRDSLKEDVDKISFSLD
YGENWESAPLPDGLKVRPVILTTTQDSTSLKFLLVGEKDRTFHMIAINFEGMEKRTCESKDMESWYARVDDKGAATCIMGHKQ
TYNRRKKSADCFLKADFRDPEPVIENCECTDADFECDYNFQRDPDDNKVCKKVGPVPIPEGSCKGKDETFKGSSGWRLIPGNT
CTRKSGAQKDDPVERKCSDGGSSGGGSTPGTPASGEISLKINEFTDIKGQDMQKFYLMGVESESPTSEVVIARPIGEKLPDGR
VEVENKLWITADHGKTWKRILEKENILALIPHTYFREVVFFHTDSEKVIYTIDRGHSFHSFKTPFSDPSAAMSFHPDKKDWMI
WIGKRCGDVAGSKDCYPEASISTDRGDNWKTLQRYATKCEFTGNSAFKFRAQNQIVCLVHKDENIDNDKTIVTIEDFSADDKI
IHNGTVAAFATMNEFILATDEVIEDGKESAGLQAIASMDGKHFEAAQFPRNFHDSHSSLYTVLDSSNHAVNLFVATDLSEGRR
RGSIIKSNSNGTTYVLSASNVNSDELGYVDFEKVAGLEGVTLINSVSNPVDKNGKKVIQTKISHNDGAEWGFLPPPSKGADGK
SYSCSSSGDSKCALHLHHYTERENKGRTFSASTAVGLIFGYGNVGPSLGDVKDADTFMSADGGINWKSVKKGVWTWQYGDQGS
IIVLAQRATHVNKVKSNIVSYSTDEGNTWTDYKFTDKEVTIQDLTSVHSGTSRNFLVWYQTDDKKLFAANLDFTGLTNQPCKY
SDDSSSDYDLWSPKHPLQKDDCLFGHKAKYLRKKTDRKCYNQASMSRLRGYENCECTRRDFECAYNFELDNHGQCTLVPDHDA
ISGEEWCKQHPNETSYFDPTGYRRIPLTTCEGGQELDKTSTEHACAGHEEDFARKRGASGWAIFFAIIIPIGLAAAFGWYAWR
NWSGKFGQIRLGDNSATFDSEQPWIKYPVIAISALAAVVAALPLVLTSIWRSATGVYERVSNRSRGGNWSRRYTTRDSFARGR
GDYSMVDDDEGELLGEESDEEV
FUN_005870 CDS scaffold_2 19464 21634 +
hypothetical protein ENOG41

MAATYKQFLAAPSSSLLADTAALHYVTTTTTFSGPTEIIKHLNALQKQVAKKKEEVFNIVDGQNVIVLEVDTGLGFQTGGGPY
LPGLDDNFLSARDVYLPITHFVTFDEAGKILQIRLQWDQGSLLKQVEVIGKSGRNWPIRDSREQIAFIQSCIRTAGTGAPVAP
SHNETVARNRTNSTNALRDPHASLRLFGPRDEIEAEPASVVSPYAGTSRPRQRSFTEILGDEPTSPSSGRRNMSPSKRGQGKN
FQPSRIFDGQEHVEQPEEQPQPKNKRYIRPNPKKFSHFDFADGSDPQDAPQPGQSFDERPKSKHDSQWSFQDFVTPQKVKPSR
TMRTQDVRHWDTDKDVVDETPGHPAAKPRRDADAHFELLDDGERVPQQPRPGSHPPGYAHNEGLGLYKNQLFDKEDVGQNERA
LGNITNVKDRGKDFDHHFDISDESPAHPQDRVPEGHQKAVQMMQHNWDTYDRSPAAQKENRPIENTDSHKINIAGDGMGGRKG
TNRDWLYGETQDVPQSIPTRKQPNVVASGVPLTKSAKIHIAGDGMGGKKGTERDWLYNETSETAGDSKTTKPIPTRTPGSNAA
PASEIKENTDKHKINIAGDGMGGKKGTERDWLYNETSETSGGQATTTKSIPTRTPGSSTAPTNEVRENTDKHKINIAGDGMGG
RKGTNRDWLYGELDEQPLPTRQNASSKKDFWDF
FUN_005871 CDS scaffold_2 22337 24317 -
hypothetical protein PF00324;PF13520

MAPHDVERGSISKMKEESLAPGSSFSMREPEYGDISPPSYWDRFVDGFRRDQRSSLFTNDPLGQHEGSGRVHDGAHYYDIQSA
MLETANSGLARELKGRHLQMIAIGGSIGTGLFVASGRALADGGPASILLAFTIVGAMLFCTCQALGELAVIFPIAGSFSSWAT
RFIDPSWGFAMGWNYAMQWLIVLPLEIIAASLTLSYWDESLTRAIFVSVFLVVIIVINMFGVKGYGEAEFIFSIIKVIAVIGF
ILLGIVLNCGGTPDSGYIGGRYWQNPGAFNNGFKGMCNVFVTAAFSFAGTELIGLAAAETANPRKSLPTALKQVFWRITLFYI

VALTLVGLLVPHNDPRLVGGNSDADASASPFVIAIEEAGIQVLPSVMNAVILTAVLSVGNSAVFGSSRTLAALANLRQAPKIV
GYVDRKGRPLVAIAIASAFGLIAFLADLPEQGAVLDWLMAISGLSTIFTWGSICVCHIRFRRAWAARGRSVSELPFQSQVGVV
GSWVGITLNVLVIIAQFWVGAFPIGWQELTSAQVAQNFFHKWVGAPCVLAFYIFHKLYFRTTFVRIRDMDVDTGRRDFNVPIL
VAQEREEREGWPRWKRYYKFMC
FUN_005872 CDS scaffold_2 26057 28243 -
hypothetical protein PF01532
GH47
MIRDPFNIHRTTSTFGRPQSRNIPAPSQAEADEVAMAFSMPRTVPSFDSRQRELEDRFWGSGQRSNKSDRLPMYKDKPYASPY
DDEYRPFWKRKKWMAIIAFVVLTLIYWTGSSKKPSPKRTVTTDWSYTGLSKDDRKANWDHRRDRVVEAFELSWDAYKRYAWGF
DEYHPISKTGENMAPKGLGWIIIDSLDTMILMNLTSRVQDARHWISDSLTWDQDQDVNTFETTIRMLGGLLSAHYLSTEFPGL
APLAEDDEGAAGEDLYLEKAKDLADRLMSAFDSPSGIPYASVNLAKFEGIPSHADMGASSTAEATTLQLEFKYLAKLTGEKDF
WDRVENVMKLVDNQGAQDGLVPIFIYATSGEFRGENIRLGSRGDSYYEYLIKQYLQTNKQEPIYQDMWYEALQGVRKHLVTYT
SNSKFTIIGERPNGLEMPLSPKMDHLVCFMPGTIALAATEGLTEAQARKLPTWSKKNEEDMQLAREVMETCWGMYKYMATGLS
AEITYFEIDNPPTAYGNPRNGPVNFDTSPDAEWRKDFTVKSGDVHNLQRPETVESLFYMWRITNDNRYREWGWEMFKSFMNHT
AVRNGGGFTSLRNANVVPPKVRDNMESFWLAETLKYFYLLFSPPDLLPLDKVVINTEGHPLPRFDMGQLFSTGWKRKPRDANG
KIIATAVKVESKEKEAVMQDAKKVN
FUN_005873 CDS scaffold_2 29102 29958 + ATG10 E2-like
conjugating enzyme atg10 PF03987

MAIENFPSLTAEEFSEACHHLDRQYCQASLGPQRARWKLRLCNALCTDFAYGGGFTTYIQIRRPLEFDLDHGDLSLDLDGFSF
SDDKTHVPIGGDKDMLDAEEADEAALIRQRTRPEVAMVEYEIHLHPTYRVPCLWFNLRNLPADEPAFNIDTVFRRLVPDEYKA
GLRALGNVGGISADHHPITGVPSFFVHPCLLGDAISKFECDRTNYLMIWLGLVGGCVGLWVPKEMAM
FUN_005874 CDS scaffold_2 30018 30739 -
hypothetical protein PF00076;PF13893 ENOG41

MAAVTTAPRGLQPNASLPAKVQPIPPNQTLYVTNLPSSKIQKADLRTALYLLFSTYGPVLDVVALKTMKMRGQAHIVFRDVQS
ATQAMRSLEGQAFLGRDLKIQYAKSKSNFVAKLDGTFKIPNVAGATNVEQTELQQSIFNAPPPGSAPAPTPGSGLPAKPAANA
DHVMEDADTPGGRGQKRTRDDEDSDSDVAMEEDSDDD
FUN_005875 CDS scaffold_2 31152 32355 +
hypothetical protein EOG09262VPD PF00400

MAAPEVHHLFHHPIADHSFSVDHSVLAVARDTAVELYGKVGNAFKLKDELKGHDKTVTSVDIAPNSGRIVTCSQDRNALVWEP
SPTGYKPTLVLLRISRAATFVRWSPSENKFAVGSGDRVIAVCYFEEENDWWVSKHLKKPIRSTITNVAWHPNSVLLAAGSTDA
HARVFSAFIKGMDSRPPPGVWGERLPFNTVCGEFLNNSAGWVHSVSFSPSGDSLAFAAHDSSITVVYPSGPEQPPRAVVTVST
QQLPFKSLIWTSEDEIIAAGYDCEAFRFQGGEAGWQLTGAIEQKGRPGLGEHREESALNMFRQMDLKGKAKDDTQLKTIHQNT
ISTVRPFETSGDRVTKFSTSGVDGRVVVWST
FUN_005876 CDS scaffold_2 32921 33456 -
hypothetical protein PF01042 ENOG41

MSSDQQIVFTKDAPAALGPYSQAIKTPNMIYCSGQIPLTPEGEMVQGITEQTRQACKNVQAVVEAAGSSISKVVKTTVFISDM
SYFAEINTEYEKWFSHKPARSCVAVKTLPKNVDVEIEVIALP
FUN_005877 CDS scaffold_2 35111 37532 +
hypothetical protein EOG09260J53 PF02366;PF02815;PF16192
GT39
MARSSTPQGSLRQRGAPSKKPFEEDSFDPNIELDKLAKAGAQRAAAQSETEYKIGLFLITILSFVTRFWGISHPNEVVFDEVH
FGKFASYYLERTYFFDVHPPFGKLLFAFVGWLVGYDGNFHFENIGDSYIANKVPYVAYRALPATLGALTVSVTYLIMWESGYS
LPACILAAGLVLLDNAHIGQTRLILLDATLVLAMACSLLFYIKWYKLRHEPFSRKWWKWLILTGFALSCDISVKYVGVFAFVT
IGSAVVIDLWDLLNINRPGGAISLQEFTKHFAARAFGLIIMPFLFYLFWFQVHFAVLYRSGPGDDFMTPEFQETLSDNVMLAN
SIDIQYYDQITIRHKETKTYLHSHEDRYPLRYDDGRVSSQGQQITGYPYNDTNNYWEILPANNDKQIGRIVKNHELVRLRHVG
TDKILLSHDVASPYYPTNQEFTAVTPEEAFGKREKDTLFEVRIEHGKKNQNFKTVAGHFKLIHNPSKVAMWTHTKPLPEWGYK

QQEINGNKQIAPSSNVWIAEDIPSLPADHPRRQKPERKVKSLPFLQKWFELQRAMFYHNSKLTSSHPYASHPYQWPFLLRGVS
FWTQSETRQQIYFLGNPIGWWLASSLLAVYAGILLADQVSLRRGVDALDRRTRSRLYNSTGFFFLAWATHYFPFFLMGRQLFL
HHYLPAHLASCLVAGALLEFIFNSEAPEEVTIKDKKGPVSPRHHVTARERFAGQSMLGAWIACGAILSLIIAGWYFFLPLTYG
YPGLSVDAILRRKWLGYDLHFAK
FUN_005878 CDS scaffold_2 38843 40166 +
hypothetical protein PF11735 ENOG41

MVSHTNRLYLRRLLRSRFPKIVFILVVIINVLDVLRIQRNLLDADRTPAPKLSQPPGRIYIASMHFNNERVIRDHWGPAVIEL
AKLFGRENVFVSVYESGSWDKTKRELHHMDQELERLGVPHRVEMSDVTHKDKIENPNKGEGWIDTPRGKRELRRIPFLAKLRN
RTLQDLIDLSKKGEHFDKVLFLNDVVFTTDDVLKLLGTNGGDYAAACSLDFSKPPQYYDTFALRDTNGQAHAMPTWPYFKGSV
SRNALVNHLDAVPVASCWNGIVAMPVEPFTSSSKLRFRGIPDSLAEHHLEGCECCLIHADNPLSKTRGVYLNPHVRVGYNLRA
YQALHPEQGAWVSTWQIFSGLWTNRIMRWVSSPFDAWVVRRRVAEWEKLGGREPGEFCLINEMQVLVERGWAHV
FUN_005879 CDS scaffold_2 42419 42866 +
hypothetical protein ENOG41

MASQPTPQSASDRLYTSPPGVYARNFSPAPSQGAKQPRRQSNDHYANFSKRERTVRGQRVGNKVIIDKGVPLPPQGQYKAADP
SRSRSVRHSPRRAGSVQDRIDRSYTVSPLTEDERNAPHVDIPSHDEYTRWI
FUN_005880 CDS scaffold_2 46855 49186 + ACE1
copper-binding transcription factor ENOG41

MSFSHPRRRTPVTRPDSDTDNALSLKNSSTLRKGATFHSPTSSSSTLDNTFVPPTLPRAQSHLDDVVDANRRRVALALNDIDE
ALSLDQLSLSPKSKIKTLRDTSLPIPRGFLEGPIVDPKMTKEEERRVLRPRSVRHSRHHESDSGLGTSVASTNEKRGAVTSAK
KETKVQTRSAITRSVAAASEKLPSLGPKAINRIHEHTLRPLLAKPTLKEFEPIVLDIPRRIRSKEIICLRDLEKTLIFMAPVS
QLLTGFGVWENTYRNLCLKEKTKSATLYLDFCLTSVQCIQATVEYLSDREQIRPADRPYTNGYFLDLKDQILQYGKQLAAKNS
GDDMDIDAYVPSFVPLDYPTNSCRRSDEIKLIGGLHEGRPVELVRVRKDGTYISLDTGKPVEVDSDSPMQVKRSLSQQLEDEE
EIQRSMARRKKNASPEELAPKKCREPGCNKEFKRPCDLTKHEKTHSRPWKCPVPTCKYHTYGWPTEKEMDRHHNDKHSAAPAM
YECMFKPCPYKSKRESNCKQHMEKAHGWTYVRTKTNGKKLPSVAGSVQQQTPPLGNMSTPSSTEYNGVPTPPQNDVTQFVGDF
PLYPNDSDWMSINNIPTETLHLDLAMDSTSPASASSYEQYAPYQNGSAFILDNEDLYAAHMQLPAQLPTPEQPVMYNPNLKMM
QQQLPMYQQPQQQIPIQTVAPHFSPTGQETAMLYTPNSLRDVDEGFDDSFAGDGMDFPLFPNDNGNGMTKTNDYQSLFGEIPS
ANLGFSQNSQDIFQMMDWSNVDLQQNLGE
FUN_005881 CDS scaffold_2 50142 51799 +
hypothetical protein PF13537 ENOG41

MQLDDLFLTFTSTVLSLRGDHVAKQPLVDHVTESILCWNGEAWGIRGESVQGNDGEAILALLAEASRGAGDVLDILRAIEGPF
AFIYLDKPAKRLYYGRDRLGRRSLLVRDGSPFVLSSIAETPVDGWTEVEADGCYTLDLSKGDSPEGLVPGRHDWTADTTLISS
IGSFNEDLPRKNFALGQDSRSVQELRSRLVESLRLRVLDVPLPPKAKPTDARVAVLFSGGLDCTVLARLSHDMIPADQCIDLI
NVAFENPRIAGQFKDLSREELYEKCPDRMTGRNASAELSRVCPGRAWRFVAVNVPYAENLEHRAEVIRLIYPHNTEMDLSIAC
ALYFAARGQGLGETTADSNPQPYSTTARVLLSGLGADELFGGYGRHGVAYTHRGYGGVVQELKLDVSRLGKRNLGRDDRVMAH
WGREVRFPYLDERFVKWAIESPVWEKCDFETPGGEGNLDAEKRVLRLVAQSLGMSSVSKEKKRAIQFGARTAKMESGKVKGTT
VLST
FUN_005882 CDS scaffold_2 52767 53704 +
hypothetical protein ENOG41

MLNKFIDVRDRDSYSTFTSATGVSILTSFLTEETDDMRKGTRPPPLNLSIVTRPRSLPRLSTTPIARELTPLLTPLPEEVEEP
TGAAIRAVRVAVDFMQIVSCLLVILMLAIFFISYSGAAVSSHGIKAFILIAALTCDVGLGMWSIVHHDRPWTGPAVLLRTLTA
SVLLGSLVSFLAVERVFPSDYTYWGLPLSQTGAPVLGLVSAILGWDLVHVVLSRRTVECWARMCSWGHRQMLRRRWLSSRGHR
RGRSGPWWFNSRQSALEKRGWTCVSVS
FUN_005883 CDS scaffold_2 56461 57373 +
hypothetical protein ENOG41

MYFSNFLVTLASMASMASAIPAPDALLDTTSNISPSDFTIRSDSNNLEARTTFSCPGAMSYCPWTKACSCPPGQSWDGKAKKC
AGKKASGCWPKPSASVYAGAGVDVKLGTYCAASPYKIVKYDTKHAYCQASLKNTVFLAPLSIGAEVALYGGAAINVKGQLSAS
LKTVCGGLAGLYLESSADAVALFNTNKYGYAVRPGSVTGGLVYAVVDFVKSITCFLGLGNCQVYDCVSYCSKGCKNYVDVRGS
IGGYVNGLVGFCIVKDTVLFVNKVGACASVKIQGLIRIVASIHVSLKGIFGCNC
FUN_005884 CDS scaffold_2 57752 59043 -
hypothetical protein ENOG41

MMDQDTQQLRVESPKTIELDVTMEDMSAKDVISITPAPKLSYTPQFPSESSVILSRLKNDSQTEGNENVREKHFADLKDTLTM
PTPTKAKGQSSLSYSLNAGVKRKREEPSEKIDLTQSTVPFPWKVRSQIQQATIEAQHTREETRCSMCDGSAQVSPLVTCIRCL
VSWHQKCHPPLMRGEDMTSASFTCADCIAGREQTVRLRGRVGQQRQHEIERLRQKRLSALPKGVVPAKPHLVGFGPGRASDVS
RAQYFESMPKTDLLNILSLCDQLKPNLLVDVLVSISKRHPEMPIFDSPDWDSQLLNTSRPPKTKHDEKPRHGHVLVNRAAKPK
QKTTKKILKRTRVIEVITSAPEEDNVDILPPTWAKAGQGLYSQLLPDTEDRSLLMDENDEESFSHFLVDGAGRQIMEPVGA
FUN_005885 CDS scaffold_2 59465 60982 -
hypothetical protein PF02515

MNLFLAARSLRSKPVNGCAHGVRGYSSAVKTGLPLEGIRVLDMTRVLAGPYCTQILGDLGAEVIKIEHPVRGDDTRAWGPPYA
KYKDGSSAKGPGESAYFLGANRNKKSLGLSFQHKEGVEVLHRLAAKCDVLVENYLPGTLKKYSMDYETLRKINPGLIYASITG
YGQTGPYSNRAGYDVMVEAEFGLMHITGERDGPPVKVGVAVTDLTTGLYTSNSVMAALLGRAKSGKGQHIDVALSDCQTATLA
NIASSCLVSGKKDSGRWGTAHRKFDCLNKNFNANSEASIVPYKSFETKDGGILFGGGNDRLFGVLCDGLGQPQWKDDARYKTN
ADRVEHRNDLEAKIEAITKLKTTQEWLEIFEGKGMPYAAINDVQGTLNHEHTKARNMVVEMEHDECGPLKMVNTPIKLSETPP
TIRSAPPMLGQHTNEVLQEHLGLNETDITALREKGIVG
FUN_005886 CDS scaffold_2 63384 64676 -
hypothetical protein EOG09262N10 PF00591;PF02885

MASQSQDDGQNALPPVNIKPLLTKLWPNESAVSPQEIAEAISHFFTNQVTEAQTASLLMALHFTKLDFRADVLAECARVMREA
AASIPVEELKEVIERRGRKEGDYNGGLCDIVGTGGDGHDTFNISTTSSILASALLMVSKHGNKASTSKSGSADLVACMKPQPP
IISAIRPGTLVKAYSETNYTFLFAPVFHTGMRYVAPIRKQLPWRTVFNNLGPLANPVEDVLEARVIGVGRRDLGPAFAEALCM
AGFKKALIICGEEELDEVSCAGNTLCWRVNETSAGRLEVEHFTVHPSDFGLSTHPLDTVSSGKEPSENAEILSRILHNELPDD
DPILEFVLLNTAALLVTSGICESDTSNMGEGDDGKVITERGPGGQRWKEGIRRARWALKSGEAWRQWEKFVKVTNEIGA
FUN_005887 CDS scaffold_2 65930 66968 +
hypothetical protein EOG09262PAY PF00382;PF08271

MPPNGSDAPAFEEDLSNILVCPECNISPPHLVEEFSSGDTVCEDCGMVVGSRIIDTRSEWRTFANDDQGGDDPSRVGGPQDEF
VEGQQLATTVAFSETKAHKALSRTQNNTNQDKSQKGLMQAYKEIVSYCEAINMGTNVSNAAKHIFKLVDKHKFLKGKPQEAVI
AGCIFIACRQNNVPRTFREIFNLTSVSKKEVGRVFKQLQSFLQKLQDQDGEATGLNTVTNYENTSVGAEDLCGRYVSQLGFTK
QNKISKISRSLAERANNISALAGRSPLSVAAACIYMACQIVGESRSSLPIAKQAGVSDGTVKTAYKHLYAAREQLITKEWFEG
GASMEKLTPATAA
FUN_005888 CDS scaffold_2 69529 70843 +
hypothetical protein PF00710;PF17763

MMPSFKRLHGRALVATAPVYCSGPAASSTIKMAPSSASWTTCLWRIILSVLAPSTALLQFGSWAASVFGPPVHELYLQPHFSA
LQKAPIQTGIPFEISTTPEFNCFSPNLPNITIYATGGTIAGSASSADQTTGYQSAALSVQSLIDAVPQLCNVANVRGVQFANT
DSIDMSSDMLQALAEQIQADLNSPSTQGAVVTHGTDTLDESAFFLDLTIQSDKPVVVTGSMRPATAISADGPMNLLSSVTLAA
SQNARGRGVMIAINDRIGSARFTTKVNANHLDAFQAPDSGLLGTFVNIQPVFFYPPSRPLGHHHFKLQSRPPSSALPQVDILY
AYQELSVGMFKAAVDLGARGIVLAGLGAGFWTSKGTEEIRRIVRETKIPVIVSRRPEGGFVGPCQAGIGAGFLNPQKARIQLQ
LALEAKMDNDAIRALFEHAGVH
FUN_005889 CDS scaffold_2 72719 77130 +
hypothetical protein PF00122;PF13246;PF16209;PF16212

MFQDRSGAEVPSRPESPKASSKVNVRRRNSGAGRSAPLVNRIKDVAADVYQKTVVELILRRKHVTVSTDGRRIPLELEHETPL

IDTRRGLAYVSNSIRTSRYTVWDFIPKQLFFQFSRVGNFYFLCVGIPQMVPGLSTTGSYTTILPLLFFVMLTIVKEGYDDYRR
YRLDKIENAGFATVLGREDKYTGKLQPVNKWTKLNPFLTHSTAEPYPVPEEEFNGLRWVPVRWSEIKVGDVIRLCRDEPIPAD
LVLLHSDGENKLAYIETMALDGETNLKSKQVTPALEGCDTIEGISKCKADFVVENPNPDLYNFDGRVTVSGKTVPLTSNEVIY
RGSVVRNTNTAIGLVINTGEDCKIRMNANKHPKAKKPALERVVSKIVVTLATYVVVLSVGVSMGYVKWQKSTERHSWYLEQAK
VPFYQIIIAFIIMFNNVVPLSLYISLEIVKIGQLLMLNSDVEMYDEETDTPARCNTNTILENLGQVGYVFSDKTGTLTDNIMK
FRKISVAGTVWLHEMDLEPKVDEAEAFKLDEESEPSEPSVYKTDPVTVVIREEQAEASNEPSTPLALASPSRPSMSHRPSMAP
SRPSMAPSRNSFGSRRSSSQWRSTGRPDHIQPDVTTSDLIEYLRLRPNSGFARKAKQYILAVALCHTCLPEYKENGELVFQAA
SPDELALVRAAQELGYLVVNRTTQTITLRVTQPDGHEEELKYEVLDVIEFTSARKKMSIVVRFPDGRVSVICKGADSAILPRL
KMSQIAKQKASEVRKSAELEREMHRRSEQQEPRNSFGGRPSLTIRRNPGVSRDRSTSRRPNVDRSKSFEFGRLSRRSEDKPRL
SIATRGVSIDLPRGQYLSTPVHYQQPVPEHLAFLEDPALLDDSETFAKCFKHLDDFATEGLRTLLFAQKFITEQEYQAWKKTW
DEATTSLSDRQQRIEDAGDMIEQSFDLVGATAIEDKLQKGVPETIERLRKANIKVWMLTGDKRETAINIAHSARICRPGSDLY
ILDISKGSLDSQLIALQEDLSTGSVHSVVVIDGQTLAAVEKSPELSATFFKVMLQVNSVICCRASPAQKALLVTTVRSRLKKY
HGRKRRGLTLAIGDGANDLAMISASHVGIGISGKEGLQAARVADYAIAQFRFLQRMLLVHGRWNYVRTAKFILYTFWKEMFFY
LPTAQYQRYTGYSGTSLYEATSLTVFNTLFTSLCVICMGIWEQDLSAETLLAVPELYVYGQRNQGLNIWKFGRWMLLGAIEGV
VCWYGVWAGYGWITPAARDQGLYALGTLAFSVGVLWINWKLFIFETHYKSTIVMASFFITTVGWFAWLCFLDAAFAGRPSGPY
AIRNSFTEAFGADAVWWATLFIVLGLLGLFEIVLKCVKRLLLIAGLWDWPPWGKSRRGENIEEWDVELWQELEQDPALRERLK
RLARDEQVEEEDEFENGIEEGIWGR
FUN_005890 CDS scaffold_2 77263 78655 -
hypothetical protein PF03435 ENOG41

MIDIALLGATGYTGRLCASYMAQVLPGNVSWAIAGRNKSKLQVLHRELQLEEPKCTVYALDLASDEAISELVKTSRVVINTIG
PYATTCGTAVIRACAENGTDYVDCSGEPAWMRDIIAQYDTTACNSGSRIIMTAGWAAVPADLSVYLAALKLQRQFSLPTREVL
VCLEDVRGSFSGGSLNSLCSLEPVNIAPFGLSPVSRTEQQVAKNGVLPPPNALDVQVVDELGVLVESTHAQIEKTLVGRSWGL
YAGHGSDFPSPEGYGEDFFFSSRMRCSNALSALMFRTSLSLLTNAITKIPPIRYLAARMFPPGTGPSEEARRSHFFKYRTLAI
ADNKGAEPVPKVEVKFAYGGDPYVFTGVALTQAALVLLQGIIPAHRRGGILTPATLGEEFVARLKQPEAGVEIEVEVLSE
FUN_005891 CDS scaffold_2 78950 80855 -
hypothetical protein PF07690 ENOG41

MADHSNEKRPDFVIDSASQNTALTSSGQEKIVPGDVIDNEESSHPLNSDNRPEENISSNDDGSGDAESVDSKDELRLSKARCI
ALVCTVTGASFLNTLASQSVVIILPQIGRALDVPDTRLQWVVSSYVLTFGCFLLLWGRIADIYGKRLIFILGSFWVTICCVVN
AFIPTEIAFDLFRGLHGLGAAANVPTAIGILGTTFPPGKAKNYAFSAYAAGAPLGSVCGNLVSGLIAEWASWKWVFGALAIMA
GMIAVAGVFCIPPPPPELRPEHNNLRSKTAAVDWIGATLITCGLLALMFALTEGNVVGWTAPWIPVLIVVSFGVIVAFFFWQR
YLEQKTNRPPLVKVSIFSNWRFTAVMIIMGFFFGGFNNYLIYATYYFQDYQGLSPLQTMLRFIPTGVSGFVVAFFTAYFLSRV
PTFFILAFGHVAVCIAAVLYAVPIPPTTSYFAWGFWAMILSVVGADTAWPCLTLFTSHSLPKEDQAVGGALINSSGMIGRAIA
LAIATSIQSSVMAKERGVSVQDVGSVKEWDAPSLKGLRAGAWTNFGILFIALALVVYTFRSMEIIGKIPDRPERSEGVVNQEE
TDVERRG
FUN_005892 CDS scaffold_2 82709 85693 -
hypothetical protein PF02845 ENOG41

MSEVTSRTSASRGRGSGRGGRGGFAGRGGRRTNGDKPDHSTADSQGAFEDEGDFGELRKQYGDKTSVIREMFPDWSEADVLFA
LQETNGDENEAVTRIAEGTISQWGEVSKPKKASRAKVKDQAPASSNDATSTGPRATRGGRAVSEGGRGRGRATDRGGRSTRGR
SSVVPTNGAGTKDSSALSVPTEESSAWGTKEPKKDTSEENQPIADQPAPSATTEAPKPAAPAVKTWASMLRQSAPPKAPPKPK
EAPATPQPAAEHAETESAPEPVEPEAEVVPEPESENVPAPAVDEADETTPVVDTATPTEVPHVNVEPEVALPPSKDELTESNL
EQVVDESKPPPTGTVASTAADSWDPRQNPASVTATPISAAQQQHAAPPSGFAATAAKATAERTARTPSHHHHQRRVLDQEEAV
RMPGNREVDRAAVQFGAFSLNGDEDIDGDREEPETRAQPPADSPITHPRTSLPPATQAAVPDSFAQKPTSTVAPIAAPTAPAA
QAQAQIPGQGPASAQQYGRFGQAGVQETVGTPQKPLDPFNQQSTPSTQPPFDNFAAQTSQAQQPGGAFSSAPSDYSSYYTANQ
SDRNPYNYYGQQFGQQGGQGQQDGIASQQRPFGGYGASQVDNLSQYPQSGLHNQPRFGGSAVDAQNSGNSTPNPTTQAQQQQP
QQPGQQQQQQPGQGSQPQSHGQQYPGYNHPYYSNPYYHQYYSGYGQGGFGPYGKGGMYGQPYGISPNAPYDHSSSPAGFAQSS
LHRDSGLGSGLGDYGRAATTQAGSQPGLGGSTFNSVHDTFNRGGSAFPSQGQSFNSPAQPGNAAADDLKPFGEPKAASGPSPS
LGGARPGSATNAPPAQSGLPPPQNVGAYGGYPSHLQGHGLHGSNAYGMGGNAGTNQHGNSPYGSYGQGFSASGYYSGNQQQQQ

QQQRGGWGGNYH
FUN_005893 CDS scaffold_2 87573 88782 +
hypothetical protein ENOG41

MFTTKSVQSLKNLFASYHEPLPLSKQQSQKLLDGLKTSFRKQLDREYGESPNSLSPAAAKPVDVDPARLSATNRHLKDILSNP
LFSYNKDVTSTFPSSLASPSLVAPTRDPIEVFDHAVARGLMTPKAAIGVMVAKAKQLQANEPGLGSLATSGMSGRIIRWLRAC
GAEQDLRFMDNYTFIRALSPFLVAEGMEEIAWEWLARTINNTSGDLSNEKRLRRASNLLGELVKTKCQPQYGNLDSGISTMLQ
AQQLFRTSPLLSDLLVSSWRSVSWLSTVESYSRPAPSEELFDAHIATADRLPQPFLLERAHLHLYHPTHPDHLPALNFFKDKQ
RLRKLVNTVEPKKSKPDKLSTVSWLAFLGHDTVNHLTQSGRTQEAQGVTELLQAEVASLFNHKSEPALGS
FUN_005894 CDS scaffold_2 89068 91056 -
hypothetical protein EOG09260ZG2 PF10637;PF13640;PF13661 ENOG41

MKRKAEAAVGTSKTDKKKSKPSLSAEEAQKRFRAGLFDKKVLNSYTDQYAQSEPYKHAVINGLVDDSLLRSVRSEIKANVEFT
PKETDIYKIHQSGDLANLDGLDDESLAKLPSLLKLRDAIYSEAFRNYVSHITNCGPLSGRKTDMAINIYTPGCYLLCHDDVIG
SRRVSYILYLVDPDTPWKPEWGGALRLFPVQELKDKEGEIAKTPLPDVTKVIPPAWNQLSFFAVQPGESFHDVEEVYRAETKE
QLERDGGRIRMAISGWFHIPQIGEEGYIKGEEERNAKNSGLMQLQGNPAQYDMPQPRVMKVDKASQEGFDEADLEFLLKYISP
AYLVPDALEEISETFNEMFEITLPDILGKKFAQRLRDYVEAEEKKPAQEDTATIEKTTSWRVAKPPHKARYLYQQPSGPDQLR
TSQEESPITELLDIFLPSRQFRQWLQMATKTTIESADLLARRFRRGLDYTLATGHEGKARVEINLGFTPTSGWGDDEEEDPEE
EAENQNGEDVKSKGKGKAKAVEKQKKVEEETPEVGGQEIFMSGDDDADEDAAVYKTGDDDDNILFFQAASWNKMTIVMRDSGA
LKFVKYVSKSAKGDRWDISGAFEIEEEDDDEDAGEGAAPDDDDEEEEEEFQGFSPDAAESDSE
FUN_005895 CDS scaffold_2 92024 92933 -
hypothetical protein EOG092644Z6 PF00834

MAPKTIIAPSILSADFAQLGHDCARTMEQGADWLHVDIMDGHFVPNITFGPPVVASIRGHVDQPTEAHGRGTFDCHMMIAEPK
KWVKEFKKAGCNLYCFHYEAAFSSAAESPEQQTDEKTNPKALIRYIHDQGLLAGIAIKPDTSVDVLWEILENSDEKERPDMVL
VMTVYPGFGGQKFMASELPKVQALREKYPELNIEVDGGLGPKTIDEAADAGANVIVAGSAVFGAKDPSEVIAQLRQAVDARSA
K
FUN_005896 CDS scaffold_2 93601 94165 -
hypothetical protein PF05730

MKLSVFTSVLLAGLVAAQQEKLPKCAQPCVDQYTTGGGVAGCGQLDVKCICSNKNFLSGIACCLEDKCDAAGRDTAVKYAKQI
CSTAGVTDLPDQVTCDKNAASGSATGATTPTSSAGSSQTSPATGSAGGDSGNAAAREGAAGVLGVVMAMLFAL
FUN_005897 CDS scaffold_2 95307 96626 - PPE1 Protein
phosphatase methylesterase 1 EOG09264E5J PF00561;PF12697
S33
MSDLQRAWAKAKYSVSNDTFDEVDEEHENDVLPELPEPVDDDSSSASSASSVSSTGTIIPSPNQKLFARPQGVARGRTLEQIP
WTTYFERELSLKSEQEPEVVYHAYLTSPVGKGPLFVMHHGAGSSGLSFAVVASEIKKRLSTAGILAIDCRGHGSTNAPGDKAL
DMRLDTLSSDLFSMVQLTKNEMAWSEMPPIVLVGHSLGGAVVTDLAKSGRLGTSVLGYAVLDVVEGSAIDALQSMHTYLSTRP
LGFATLQAGIEWHIRSRTIRNSISARTSVPALLVFNENDDPTRPWRWRTNLGATQPYWEGWFIGLSKKFLGAKGGKLLLLAGT
DRLDTELTIGQMQGKYALQVFPEAGHFIHEDLPEKTAVSLVDFFRRNDRSALVLPPKVSDLLKQGKRV
FUN_005898 CDS scaffold_2 104102 105383 +
hypothetical protein PF00172 ENOG41

MVDPHTPSLGQDHGLDMKGSYSAPNNAALLASEAEEQQQNGNLGEKKRNKLGYHRTSVACGHCRRRKIRCITSPNDTQGRCIN
CIRLKKDCSFFPVDQASIDDSRSKQTSKSSTGPKGSSATSSPATPISNPVEQPKKTKPPFVPSSKRPGPITVPKITKAEGVKG
FPPQTKASGTSPTSMTPPEAGNQHPTSWIITAPEHSPTSSSNLSTPWHTYATGSPMSAQFSPFTSVNHSPSGWPPVNSEPVPQ
GDMDMAWGQFAPPTRSMSYGGEPLSSGHPSQYSLMAPGRQFERRPSALSDAYTTSMSGMVPGFDGSSMNTAVPFPPNAVPATN
YAAWNQTQAYPGYTYVKNQETYGDDWSHERRGQDRQLQGNSGQQVINNATMNAYQTQR
FUN_005899 CDS scaffold_2 107458 107876 -
hypothetical protein ENOG41

MSYEDQDMLLLVEVRAAQRTFEGAYVRTALGQFSFALIILKVFTEEFYAIGALFAVYGVAVMLVAIYRRYEGQNQFFTSESAD
GSLKRKFRTSGNSVLLLTILSLSAYITLMVLTWRLVS
FUN_005900 CDS scaffold_2 111017 114485 +
hypothetical protein ENOG41

MASTFDVPPASLHDKDNQPFPTSNQQAPTSAFDSNQLPTTLSRVDSDSLSPINYLETPDSTSSLSHYQASDFSDLDDDPFFGA
DFNADGGAPNFLEQDLNGLDQSFTPGLMPDKDVDSGTYPLTPEQTASVHATPPQGDLSYAIKGSAPSELPNCISPQEIQKRFN
PHPINTQTSELTPSHSSSCRSSEDGLAPGVATMTSPRVTVSMWGNHDETPVHTSVERSFEDSPTTVRGGFESAGDLISSGDNS
SARDSTGRWQQNPLTGQSGLDPNNRPSNEVPSINELATRRDDDERKELVGDWLADKMNDLSVRPDDNSRDQINELDSQRDDPH
EHMIPFGRETENKFIPGQTYYRDQGEMSQQDRDIIAANRNWADAPMFPSISRGVPGRNQPESSAAAMERFERMYRDTDSILSR
AATWGTRRRSLPSVFDLDMADGTSGGILKKLSSSRESRSSKPGSLLRDLRGLVRRPSASRLKRTLSSGNDEESLPSSEGRGSQ
DSKRDSISKRGDSVSHLAPPNRIGSWGKKPMPSINTAIASMGSNIASIGTTHARSGSVSGSGTPGGSITSPKSPFSGLSVKKS
IRRPRSKSELPKPTPAGMQTESHPTLVSMWKRETGGPPVAALAKTKHADLDDDDDDDDDFSDEADMRTNPNVIDNITPNLEGF
QQHIIDLNPGLATTNTFLVDRIAYHQVQRYKYLLKNKVAHIGLGAACNSGIMCVSRGGSATLLDQNKNVRELDPLSSPNDEDD
GSPVEGAINTESFPQDIPMPPTSHLPAEFECRLCFAAKKFMKPSDWTKHVHEDVQPFTCTWDKCPGPKMFKRKADWVRHENEG
HRQLEWWTCDVEACHHQCYRRDNFLQHLVREHKYPEPKVKSKAAVKKAGGSEPTWQKVEQCHHETTKKPHDEPCRFCGKTFPT
WKKLTVHLAKHMEQISLPVLRLVDAKASELSADTVISPVQDPPPRNILTTPIQTNMPMQYPVGGQHQQIPQATPQIYQNHNQN
QMAYPMMPTEPYHQQQQQQQQQQGFYTDQYTNASNATHNFPSAHAEMGMHPMNHQSFNAAPMQEMTASSAGYQQNANAFMMPS
NGLETYNQMNPLGLQSHGMPMGQMGPMVYDGITDPSSGNGSPFSGQGSMSPYSHSPNLNANTGNGMWGDRRMAGYQ
FUN_005901 CDS scaffold_2 117678 120034 +
hypothetical protein PF06011;PF14558 ENOG41

MTRVHSNWSAWKVAVTAATLVTGVLGDNILKTSGVADCGSEPGISIDKLDISYDNDKQTVTFDVAGTSDKQRNVSAILEVNAY
GRKVYENSINPCSKDTPIPQLCPLPNKHFAAAGNQKIDSEWANKVPSIAFQVPDISAQATLRLVSVDNPEEQVACFQAQVGNG
KTASVPAVTYLSVGVVGAALLVSGASAASSALSGTAGAGHGGAPSPSFAETLGWFQGMAMNGMLSVDYPPVYRSFAKNFGFSA
GIIPWESLQKSIDNFRNMTGGDLTNDSFEVLRNATLVFSDNSTSSPEDHSYKVKRAFNTFESLANLAIRGVETSVGDASESEG
GQSLKNQTLSVSGISAYIQQYSVPKSNTFMMVLLVVAIIIATIIVSILLVKAILEFWALFGSFPQSLAGFREHYWGSIARTIT
HLILVLYGIWVLYCVFQFTVGDSWAAQLLAGVTLAIFTAILGWFTWKIFSTVRKLKSTEGNADALYNDKETWVKYSLFYESYK
KDYWWIFIPTIVYLFAKGCTLAVGDGNGMAQTIAQLVVESIFLCLLVWTRPFERKSSNIIGIAIQTVRVLSVALILVFVHELK
IPQDSKTIAGVVLIAVQSALTGLLVILIIWNAINVLVKENPHRKRRKEMEKAKRDMDTLTPLDARNSLLLDRKDNNNISMFAM
PAPQEKGSGRSMSPDQYRDAESGYQQPPPNYLSSSPPMQRNLTNMEHNNDSQYLMDNAAPVAWTNRDPSPVGMGRYDSGDTGY
SPLNKQQTYGYGNGGGYRGF
FUN_005902 CDS scaffold_2 120377 121490 -
hypothetical protein PF09414 ENOG41

MPRKLVTVRHVSAITAIPRADRIAAATVGGWTCVVPVNVFEVGDRAVYFEIDSLLPTTDPRFAPLAPKIIGPDGPTSAPDIRV
QTIQIRGVLSQGLLLPLADFPEIVAQLDGIPSDKLRDVGFEDILNVGKFEKPAMPLQHTSTSDAPLPEYPDFIPRTNQERVQN
LTDVFAEHGTEMFEESTKMDGSSMTVFYLNDANPLASTVPSETRHNGVAVCSRNRILVENHPRSPPLFYATARALNLHETLPK
IGRNIALQGELCGSSIQSNFEGFAKGTHCFFLFAVYDIDEQRYLPPREVYETWAPLLGVKHVPVHGYRPLNEMGSQVTDLVNR
AEGRGINGRKREGIVFKREDGQFSFKAISNSYLLTHGE
FUN_005903 CDS scaffold_2 122237 124785 -
hypothetical protein EOG0926499W PF02204;PF02845;PF18151

MSPPEQSTTSSSQPVLDETLSNKPNVELMSAQDPAAGTPETTDHHSHGSDSRDNTGKEDNLIDFGDQAADVAAHEHKADAAEQ
GPSEMVKEKQAADDYQAPSGYLNTHASTAAKIKPIALPSSNDATTQYLTAEPSTYVDPTPATPITSQPPSRTPSNAARSHVSG
DSTPPKSDAEFDERRYISEDEHEGGSQSEIQSIMEQFSEFGGGPGEEEVMSPRLEIASPMLGHPVQHPPRKSSLEPLVPTLSG
QMQGLHISTSSPPAETPNNTGVEDLGPPVVPPKDGVHGTPPRPKIERNMSVASPSSPAQSHRPPPPEPEPEPEPTQPFDFHRFLEQ
LRNKKADPVARYLKSFLSEFGKRQWMVHEQVKIIGDFLAFIANKMAQCEVWRDVSDAEFDNAREGMEKLVMNRLYTQTFSPAI
QAPKPIPGAKPKRKGGDIPLGPGRRGQHQEDVERDDIVRQKMSIYGWVREEHLDIPPVGDSGRRFLKLAQQELLKIKSYRAPR

DKIICVLNCCKVIFGLLKHNKSDSSADSFMPLLIYVVLQSNPEHLVSNVQYILRFRNQEKLGGEAGYYLSSLMGAVQFIENMD
RTTLTITDEEFEKHVEEAVSAIAEKHAQSPPVTQQPVFNEKSGPLPGETSARPSLDGPRTSTSNDEYTGEEKAAITGLLKTIQ
KPLSSIGRMFSDDPGPSMDPGPSSAPRTPAPPERHSCEEPREHQQAPSKHALSAEEAAARQASAEAAEAQRLSRAEHANVVET
LAGMFPDLDKDVISDVVYEKEGRVGLAVDACLALST
FUN_005904 tRNA scaffold_2 125484 125579 - tRNA-Leu

FUN_005905 CDS scaffold_2 125826 126462 +
hypothetical protein PF14124 ENOG41

MPTALSTAPYRQVRACYNDTTIIVYQAYKESIAKAAVDAQKLNASPDFKSGRMTWIKPSWAWMMYRAGYSYKDPGQSRILALR
MKHEHFIALLERGVLSSHAHKLNPGEKRETTVDVRIQWDPERTAKLEVLPYRSIQIGIPGALSEQWANEWIVEIEDVTDKARE
LKRVIDERPGITDEELLSMGLIFEERPFEVPEAVHTKLEMTDVPV
FUN_005906 CDS scaffold_2 126816 128260 -
hypothetical protein PF06985 ENOG41

MRLINTHTFQLEFYPGGSRRYAILSHVWGDDEVTFQDVASGDPEEALHLERYSKLRESCRMARSLKLDYLWIDTCCIDKSSSA
ELSEAINSMFRWYAESTICIAFLEDVPSSESEEEQRKTFATSRWFTRGWTLQELIAPGKVIFYGRGWQRLGSRSELKEDIKSV
TGIDYELLDATHHMAEIRQSQLGEFSVAQKMFWAAGRETTRPEDIAYCLLGIFDINMPLLYGEGQVKAFKRLQEEILKSTDDE
SIFAWRQPRYRVEGKTYWSLLANSPSAFDLGQTSKDLNGLVPKRSKYLSLRSGSSMSMTNRGLDLELPLTPFPIDTSGTIFLA
FLNCEFRRGQTSINPAILLQRAAWDRNSHFVRIRPDILALSMMNSIILPDELLNMIRSGQKDVLQEAIPRQIFVPHNTPDLRY
LKGVIFRPEMKGLAKESRMVVRHLVLHLDHYKHP
FUN_005907 CDS scaffold_2 128653 129712 -
hypothetical protein PF00106;PF08659;PF13561 ENOG41

MTTQNFKNEKDIPVSHQDFPGTEREMPNPKATRDELPEAGGNSRTYQGSGKLKGKKALITGGDSGIGAASALLFGREGVSDIV
IAYLPEEEKDAQDTKKQVENEGAKVHLISLDLSKQENCKKLVDFAVEKMNGIDILFNNAAYQMMVQDIKDLPEEQWIHTFNIN
IHSFFYISKYALPHMKTGATIINNASINAYIGRPDLLDYTSTKGAIVSFTRGLSNQYVGKGIRVNAVAPGPVWTPLIPATMDD
EAQKQFTSPMGRPAQPSEIATCVVFLASSDSSCVSGQTIHCNGGTIVNG
FUN_005908 CDS scaffold_2 130785 131186 -
hypothetical protein

MKPVVSAFNAWSCTVLSVFAIVILSALAGLYRSNHEEFTGGVDDPEDGKAVSDTIFIAVLVYVAFFVFCGFQGFLHTRESRRG
AIAL
FUN_005909 CDS scaffold_2 133215 134056 +
hypothetical protein PF00080

MRTQAIVAVLFSALAGQVIGQDDPHDAPRVNDNPIGVKYKATLPKEPFFKNAAIDGNVKGFIQAEAPSDGHGVQFKVQFSNLP
KEGGPFTYHIHVEPVPDNGNCTATLAHLDPFARGEEPPCDPEKPASCQVGDNSGKHGKITSDPFTADYIDYYASTKEGIGAYF
GNRSFVLHYANKTRLTCANFVNLNPGVIDSNFTAPAYLPTPTETVNPETNTPTPSGTGTGGKPISTETSVVSPNGASSATLPI
NLALAGVFALILAI
FUN_005910 CDS scaffold_2 135490 137386 +
hypothetical protein

MSPSVMVDTNTALEQRSERDIRSPDMEYNLSVEDFREYEYPNMVQGAYLDHGGATIYARSLITGFSQAMIGNLWGNPHSENLP
AKLSGDMIDNIRAKALDFLGADPKYFDLVFVANATAAIKLVADAFRDIGEKTPTKGFWYGCHKEAHTSIIGVRALTSGDYHCF
EDDESVEEWISRPFSCQSRRGKSTSLGLFAYPGQSNLSGRRLPQDWSKRIRQHPQLRNIYTLFDAAALAMTSSLSSLFHDPMD
APDFTCLSFYKIFGFPDLGALVVRRASGHILNLRRYFGGGTIAQLSPSKDSRVMKKVPGLGDLHKIWDIHEGVEDGTLPFHSI
LALGVAIDTHLRLYGSMDMISRHCCYLARYLYERLVDLKHRNGSPLIELYVDSPFMYGDPSLQGPTFAFNIMKEDGSYIPWTE
VERLANSAGVYIRAGGVCCPGGVAKALDYEDWEWDRIFSSGHACGSSEMAVIHNKPTGIVRASLGPMTTKRDIQAFISFLSNE

FSTKSAPIPRLLGTAKDLPLREKFPYLTSEKEFSERDLVYEH
FUN_005911 CDS scaffold_2 138366 140724 +
hypothetical protein ENOG41

MAKKKGNQTPGGEGQNATLDNASQTHPQTSNTMDTNGGSQKKKPKTSKEGPSSSSSSFQTLNICRNKHWRYISSFHGPWLQMP
IEILETIANINYNTPRPCPIDPAVLYDLLKIRRLVDEATNLAVRAASDIASPVLTNVHGGLPGASSMSVLGMTGPGHGTRLSR
ERKFRMREQASQKLSRAYHLDEIACSVATMQGASPIDEIGALVLLRNPQDQDAKYVHFFHEKIPSRQLAESTSLQPLTDIISE
RPNESEALRTRAIVKTFKEDFEGAAHDLTVALTVCRIHQQPHRLSGNELESQYAHRGRRWRQDITPSEKDQPSSLESQLLFLR
GAAYLSLACQYVEGGIPTSQSQSGHADSHSPDEAFDPEVSSRKSPEIEEKSLREQAEARKLVKKYAKWALRDLLTFMSHFEYA
PNLPSSIAKDFNDRVNSSAKGTRHPRPSEATYSLEPHTTYAISELFAAVPPSDLPPYPNQEVKNSNGKTQPSDMPQTCEWSTY
HPLMADALHSLLLCHCLTQTSAKELQRHTYMAARLIRLADGYPVFQPSRSAARSDWVEILRRADDNWLQLSASWETLCIPAPL
PFLYDTLPQDVSKMHIGASRKEAAAAAASLINGGSDNARPAVLSPADKRRQRKREHDQRVYEALNDERVCDDATFRASIEARE
KRDEKERAATAAAANGSSSNATSTNNSGLKGWSDDDSKDYPLCSVRASLIAQWVQKAPIVTGTTRRKKRAKKTVSRAASLAGL
PEKRNLEDGK
FUN_005912 CDS scaffold_2 141090 144065 -
hypothetical protein PF00172;PF04082 ENOG41

MDPADIFRQGFGQVAPRQNPVFMGHPQHHMVQFQQQPPPPPAFRKPVQQTDETSDGDGSSHRIAHTLTACCRCRQRKTRCDPT
LPRCLPCERSGSTCEYLDAAKGRKINRHYVIRLQDKVRQLEAELSQYTDDENEYPTSNEDMVRPGGMVRLNGGDETPRYLGPS
SGIAMTRLLMEQAKRYTDSKRISDLIPSVRARQARMQSIQMTGPSARRKSYPMISQHPAESLPTRAVADKLLEIFNQKAQLFW
PVLHEKDLLQDLDAVYNGDTDPYRLFIVRMLVAISLQKLDTQYAGLADSYYLAAMQLFEDVVRPKDLKTLQCLVLLGEYSLLT
PTRTPIYYVIGLAARICQQEGLASEKTISTGYNLDAKTIDMRRRIIWTVAAMEYGLAHSMGRPNSFATGDDRLDVEFFAAVDD
DNITDQGIQPGPPSERKLVAIHFYRMRMCQAEIRRALYEKKKEEPKNDSHPWFARIEKMNKEWLDASPTSPDWCKPWFTGRYH
QMRIFMYRPSPQIPKPSPRAAAICYESAAYIIQLNAKQMGSGADLTWVFLLTVNMSLNTLLWTVSYPEVRRAHPRHEVETLVQ
TSMEVLDRCTERWPGTASASQLYSIFSKACLQGYDERPMTGQAAGFFTTPPSFADPNSPEAFQNLQQVQAPYQNPPQFNHVFN
SPPEAMNAYAFDPNFPPPQPSFRSNSIFFNPASNEPTGRRFSYFPPDFMQPGENMMDDPTPPATTTPEQHITSPPDHMSEQLP
TPPDSIPTGNMATPTPSNTLSPSNTLTAHPTPIMQHASPAGVPLVPVQTNMSPPIKIEQSQRGPTFVVPQNPQAAPTQRPLPT
PSGIGDWFSPPAPFISPYAFNNMSNSFFNDSMANPGNYGDMSGSGLGLQNIQGGNAPPPQFDYGFARQGSLTQSQQLELMNVL
ETEGMGDIDAFLNGGNISNNRWY
FUN_005913 CDS scaffold_2 152042 153667 +
hypothetical protein PF00083;PF07690 ENOG41

MPSKTRVYNWFIAMVAASCMTLYGFDSSVFNALQASENWLNWFNLDLKNDSYTVGLINTCYTIGAIVSGFFIGGPLADWLGRR
AGMGIGCFITIIAAIIQAFAPKGKLGVFILGRVLIGIGQGTALTAGPVYIGEVSPPEIRGQVMTFWQLFYSVGAFIAYWINFA
CGKNREKLGEWDWRMVVIFQVLVPILVIILLPFQPESPRFLIKKGKIDDARSALRSIRDTEEEVEDEVLAIQAAIEYEKEAIS
PGYRALFKDPSIRKRFGIAVVLNVGQQLTGQGTLNTYSTSIYKQVWTSTEKINLINALWATMGILFTLNAVWTADRFGRRWLF
MVGAAGMALCMLIVPVIGLATPTPKTEPTAIGIVVMLYLFIFFYKPSWGAGVWMWTSEVFSANVRTQAVGMASQCQNVANTIF
QQFFPTFLAKTGLKCLFFFFAVNILLCVFVFFFVPETKGISLEHMDTVFGGADHTEKGAQMLGVDAAQHQTQEFKDGAEIQQS
EKKRESV
FUN_005914 CDS scaffold_2 154431 156103 -
hypothetical protein PF00734;PF00840
GH7
MYRIVATASALIAAARAQQVCSLNTETKPALTWSKCTSSGCSDVKGSVVIDANWRWTHQTSGSTNCYTGNKWDTSICPDGKTC
ADKCCLDGADYAGTYGITSSGNQLSLGFVTNGPYSKNIGSRTYLMENENTYQMFQLLGNEFTFDVDVSGIGCGLNGALYFVSM
DEDGGKAKYSGNKAGAKYGTGYCDAQCPRDVKFINGVANSEGWKPSASDVNAGVGNMGTCCPEMDIWEANSISTAFTPHPCTK
LTQHSCTGDSCGGTYSSDRYGGTCDADGCDFNAYRQGNKTFYGPGSNFNIDTTKKMTVVTQFHKGSNGRLSEITRLYVQNGKV
IANSESKIAGNPGSSLTSDFCSKQKSVFGDIDDFTKKGGWNGMSDALSAPMVLVMSLWHDHHSNMLWLDSTYPTDSTKVGSQR
GSCAITSGKPSDLERDVPNSKVSFSNIKFGPIGSTYKSDGTTPNPPASSSTTGSSTPTNPPAGSVDQWGQCGGQNYSGPTTCK
SPFTCKKINDFYSQCQ
FUN_005915 CDS scaffold_2 159140 160931 -

hypothetical protein PF04855

MERSKPQAFLTSYAPRLRAYNNSLLTPVLPSSAPAGPASRTTKRGTTIINYAEDGYDDLEDDSDDPRRRPTGLRSLRKEDSVS
RQDLADKIGKDTKEPVEVQGVWRDWIAKHRLLRSDQQNAVQATLPLTLIPIRIDVDVPAFIPPAPYPMPHNSSTVDTSLPQYR
QQEATVPYKLRDIFCWNLHETLISTDQFAQTLVQDLDLPNRQVVIADISKQIRTQLEEYSGVALHPLFHSDPQALSVPQPTTA
ALTPRPSFALKSRDDSPVSAIRGASRPDTPAQTGGAATPSQSQVPDVTAEATPILPESDDYNPDDTYRCIINLNLNLASMLYT
DKFEWSLLHPPGTAEAFAKVTCTDLGLTGEWIPAMTHAIYEAVLRLKKEACEAGGLVAGWGGMQQELPNDAAHGSEAGWRYDP
EHLGDDWEPKVEFLSKEEIEKREGDREREVRRLRRETARFSSTTGMLGGTPFGAPMEVEEERMGRGERSKKKRRFRSLSPLGR
GGTPGGRGTPDVGGYGGGGALTDQERYQWRCMHCRIWGTSVWAVRDGPAGPRTLCANCGYLYERDQRLPRQSKNIHISDIRG
FUN_005916 CDS scaffold_2 161663 162029 -
hypothetical protein PF00188 ENOG41

MRLTTLGASLCCMDFRPGDSVIESQDIVHGLLRLNSARNANGYRPLIWDPALASNAQDYAIQLGCGAVAPGKPERTAVYKEIS
ATCAGQPHRRTFETAANFWLTANYRPNRVPEDYYNYRK
FUN_005917 CDS scaffold_2 162999 163542 +
hypothetical protein EOG092658X5 PF05180

MASRAPSFASKVLRQLTKQPQRSLPFLRASFIRPATTNLQSFRAAHNIPRPPTQTYAKPKPEQAQTGETPKSEGERPEIKASH
YQLSFTCIPCGHRSHHNVSKQGYHYGSTLITCPECRNRHVISDHLNIFGDRKVTIEDLMREKGRLVKRGSLGEDGDIEFWPEE
SLPGDIQAGNNESS
FUN_005918 CDS scaffold_2 164272 165733 -
hypothetical protein PF03972

MSEPKPKSSSPTVQLSQWISKLKLDDIPDRVCTRAKYLILDGLACAFVGSHLPWSETAAQAILNLEPTQGDASLIGWEGRKVT
ALTAALLNGTFIQGFELDDWHSEAPLHSNSIILPALLAAAEQANAQASHFTTSGKDFLLATIAGYETGPRVGRSLWGTHVLSS
GWHSGAVFGPAAAAAAASVSKLYGLDADTIEDAFGIACTQSCGLMSAQFESDVKRMHHGFAARNGLLAAVLAQGGYVGIKQVF
EREYGGFLKQFSSGNGKQPQYLPQELTSELGIRWQTNGIVIKPYAAMAGTHPSIDCIRHLQEQNPDRMKRFDGIKKIDILLGE
AAFHHGGWRAAKPLTATGAQMSNSFTAATQIVHGQVLMPQFTPDTLVDEDVWRLVDLTECKLHITDGDSIGCQEVRIRFEDGT
VLHHSVPSAFGVEPPLSNDDIVAKWRQLTRDIVENEVVEKIEEIVLSLEEQDDLVTLFELIRQTSKNPLTR
FUN_005919 CDS scaffold_2 166585 167638 +
hypothetical protein PF07690 ENOG41

MPLYLSFFYPRDKVGFRQGIFLSGSALANAYGGVLGYAILGVRGSVAPWRCLFLIEGLPVWIVAILAWFYLPDDLLSAKFLTD
REKEGAGGVSWAEWRSAFLDWRSYVPGLMYFSCNVCFGSLPLFIPTIISEMGTFNHQESNGLSAPPYALCFVTILACAFASDR
AKLRGPFVAAAAIVSTVGYCLLGVSEGVAARYAGVFLSVQIFVSISILLPWVSSLHHTESKRAGGWAIFATLGQFGPLVGTNI
FPKDQGPYYHKGAWISCGFTSLVVLLSIAYSLLLRWENKRLDRVMIEDAGVGVGMEGDDHPRGFRYVV
FUN_005920 CDS scaffold_2 168120 169930 +
hypothetical protein ENOG41

MHIECNNIVEAPEATVDASPARSDRTAVGTSSSALIYTAAEPSLEVDPSYAVQSDRSSSLSEILGREGIAVKQIDDCFARFFN
HYSKLLPGISNENEVNCESYLEESSLLFWTTIATGSRRLEHAMKLHRIAVQHIRGGTFQPLLHITNPIPIIKSPLILCLWPLP
VDTIWKDPSHVYAGTAHSLAVQNGLFVNGHEQDFARTHTSLSKEIRDIRAHLWLNCCLVFQALCDGLPYLSISESKPYQRKTR
LEEIVDTSFHYRIKCHDILIDATRAFGDIIDEQGEGTTEVLDAVPRLDAMQDFALYASNYYTRMALLAAFCILRLVRSQLKEH
IDLRDAQQSLFKAISFVKRRSMQHGALDERYGTILSQLWSNSDSMDTTTANGLDLRIRSRLFMSVVFDSLWWTAPESVQTGAT
DASTYWNSTAAPVGDSFGSSKFPEVYMDWNFPLDLDYLG
FUN_005921 CDS scaffold_2 170026 171060 -
hypothetical protein PF13714

MSSQGEDVCTTITAGKLLRQRIEAGGFILAPGVHDGFSARIALEVGFDVLYMTGAGVTASVHGCADLGIATPNDMRRSAEMIA
SLSPFTPVIADADTGYGGPIMVARTVEQYSRSGVGVLHIEDQVQTKRCGHLAGKVLVDLKEYLARIRAAVQARRRIGSDIVII

ARTDSIQKHGYEEALARLRAARDAGADAGADAAFPEGFRSVEEARRMIADLAPWPVLLNMVENSVTPKISAKEAKGLGFSMMI
VPLATLAPAYTAIKAGLQKLKETGLADTELTPQDLFRVCGLDESMKIDEEAGASNFEGGVD
FUN_005922	CDS	scaffold_2	171681	179666	-
hypothetical protein	EOG092600S9	PF07539

MPSNSSGRITKARKNKNTTPHQKNHRWESFTTKISKFNSLQPLRKVRRHDLDTEDLSTTTSYFQTGLQKWGELNVSKPFSAFK
TKVWPLCESLPQLLHFEQRIMDLLADFIAGQDKEALEPLLDLLTAFSHDLGVRFEKHYGRSLDLIVAIAAKPQDVDVIEWTFG
ALAFLFKYLSKLLVPDLRPTFNVMAPLLGKSRHPPHIARFAAEALSFLVKKAAAPSHRETALKRLVECARDDLLSIKDDRQFT
LYKDGLMNMFAEAIKGTDNIIHSTGPAVFAALVDAIPEEERTLTEDTTWTDVVCGVLTSVIHHATADTFGDFATGVHETIRAN
MERVSPADRQWQAVPLFRIYGTMAGVRKGNRLTDWAPLIKNFVELLSEVTKAGLEVPHDAASAVWKFVIVNIAIVWHHAPIDA
LIPHIVPLTQSLSREPFMKWFIPFCSYFCGLDAQRFGSLFRNDFQRFIANHWSEEANEDMICILLPKMIETRAFPPAGEKDSC
RMPQGWQDQIVSKFERLEISPFPERGPYNKDPQVWRDRCLPKYSALLQILELTTVHPSTNARIAELLLRKLKLALRPSSTLAS
DEVHFIVGQGFHAYLRMSKAAGSVDITLSPLLRAAVPRFSQSVSFLHAYLAYEEILQSKEIARRQDSTSSDSSSSEEDPVTSS
LIENLSSPSHDLRLASLNLLKALSPTPDNWNAVETMIETEETPLSLAHTRTIAMHLRKLGQNYASFEEGSWLQRAVPAFLFGM
LTVKLSPVWDDSVETMKQITQTKAGEEAICEVAFRWLKIPSARWGPSQPETQSSRPAFVSDFECTNVRRLEEVAAEVENIIDR
PDELMLQNFDDKQQTAETAAGNSRSRALKVFNAIPSIAERRSRLLVPHFLAWASEDRTPDSANDQDVSEGATWSLADRKAMLS
VFSQFINPRVLYQHEEVYTALMQLMENGDVEVQKVTLKAILAWKQEAIKTYQENLEFLLDEARFKNELTVFLQDENAIKPEHR
ADLMPVLLRLLYGRTISKKGAASGRHGLQATRLAVLRNLSVEDMGSFLDIATGKLKDVKVVGASKTIFEEPILPIRKQVGFLN
MISSVISELGTNATPYLDTLLNAVLYCLVFACRQLSAQGMGPESEEEEEKASTQSLLRVIRSTGLKCLIALFQNAQSFQWAPY
QDIILEDVVAPRLENLPSETTQGVSGMLQLFATWSVLPRIALFLAPHSKVPEGILPKVIECLSIEKGKDEVKIHVLTIIRNLI
KLATAPAQESEFNEVIKADLLDTNSKTILSQISTVLDMSGISNDLMEACVETILTMAPIVQDTEDIQAVIEISIFLLNQPPRR
VSPKLKGRILLIVEQYVTRSTAFENQALLDNVYDTISSLFSYFKDRENRQSLSRAMLAIAEKDNSLQEVAGLCAALNSFKEGR
IDEPDYDRRLAAYSAISGDRETPLTPKQWLPLLHNLTYHIRDDAEFGILSSNSADGLRRFIMDAATCSSEISRGRFDIHLRGV
ILPALYAGAREPSDTARREYLRVMGHLLSTMPEWEPVADLSALLNDRTEDSAELTFFFNILSPATARQLEALHILEAANKQKE
MGSQNLAQFFIPLLEHFIFGRADGVDDHGLGAQAIITIGNLASSLHWKHYRTTLQRYIGYVEAKPEQQKLTIRLLSKVADALV
DAAENEPQERMEIDEVDASSSATTRLPLTIPTAEKLSIEVTNSFLPTLVKHLHEKDESEVSYRVPVGVIIVRLLKLLPSDQMD
QKLAGVLTDISHILRSKSVEARDMARDTLVKIAVILGSSYFGFILKELRGALTRGYQLHVLSYTMHSILVAIIPICSPGDLDY
CLTFVVTVIMDDIFGVIGQEKDAEGYTTQTKEIKSSKSQDSMELIAKNASISRLIELVKPLRALLMQKVDLKMVRKLDTLMAR
ITAGLLQNPAAESRDTLVFCYEVIQDVYNSQKPEVEEKLDPRVKKYLVQKGAKKSGDRGKTTKHTYKLIRFAVDILRAILKKH
DSLRTPTNISGFIPILGDAMVGGEDEVKISAFRLLAVIVKVPFLDDEGSKLYKVAVKEATKSISMAVSTTTDLSQAALKMLAV
VLRERRDVVVKDAAVDMLLAKLKDDLTEPLYRHVTFNFLRSVLDRHLETAAVYDTLDYVGTVMITNDDKDTRDLARGAFFQFI
REYPQKKARWAKQLNFIVANLKYEREGGRLSVMEIVHLLLMKSSDDFVQEISSTCFLPLFFVLANDDSEKCRVAAAGLLKEIF
RKADKERTQTFLGLVRTWLDQDGNEAVLKLAFQVFGFYLESDENASKNKKDFKLVLGKISSVIEAEDIREADGELLDAALEVI
RVSLSVFPDKILSSSSEKMWSSILRCLSHQSTSVKLSSIGLTSSYLADFAQQASDVAVGEAVVGSHGLTLDLPKVQNLVRLGL
GVLATRDIDEKLGAEAVQVLAFLAPRLPLRPVTEGERSDDDVEQEQQNEEEEDEDEEEEEEQAEGTQRKTDLQHLFWKISHII
RREIPPRAAAITPKVAAMELLETICRRSSADRLRPSFKTILVPLHNLTDPSIAPPFSNDELFKTKHEALKTRAQILMDSLQKK
FGTAEYSKQLLAIREEVRAKREQRSSKRKIEALAQPEKYGRDKRKKFEKNKERKKTRSKEQKVMRQSFKRW
FUN_005923	CDS	scaffold_2	179975	181693	+
hypothetical protein	EOG09262HKC	PF04676;PF04677	ENOG41

MAAPKIIVLGSLNGQLEPAFKKLATLHTKNSFSMAILTGDVFSATQDDDSVNALLDGAIEVPLPTYFTIGTHPLPPRIAAKVE
AEEDICENLHFLGKRSITKTSDGVRIVVLGGQLDANLIAGLSKEQHLPFHTADDAKSLRGANNADILLTSIWPASVWTGSQVA
LGPTSQASLTVSDNIAELCAALKPRYHLTASPEAFFYEREAFVHPTEKETDNTCITRFISMAPFGNDAKAKSLYAFSLNKGDA
SVPPGATASPFNPKTKKRAPKDDSYSRYGGDDNSRGHRGRRQKQRHRSPPPGPDRCYFCLSNPNLSAHMCCSIGDDAYISTAK
GPLPTSNTFAEQGLAFPGHLIIIPLPHNPTIPSIGPVTDPAGEAAKTYNEMTRFREAVQAMIASKSSHKLGVVTWEISRERNV
HLIWQLMPLAADLVRKGVAEAAFKVEAENQSLPAFTARELTLEQQAESGGDFFRVWLWADDGEDRIKGKALVMPLPSDMRFDL
QFGRRVLAKLLGLESRLVWKDCEQTVEEETKDVEAFRAAFKEWDFTLQEGEEAAT
FUN_005924	CDS	scaffold_2	183168	185032	-
hypothetical protein	EOG09262FE3	PF00581

MEASSPLAALHRPSMLPSWGSRDVFRGHPHYSTSSVTSASMSLREQMHKHSGDYFNVKDVRGSSPAASLAADLSQNFRLDSDS
SPQFPTPRRALFTAGMMGSLEGRDSVTTPPLPSSSPAPLAELKELMEVSPLPHKTPFFTQFEITSPTPGTTPAADDEMVLDSP
APISRQSSLEPPKPIMAENRRIAVPRRPSLTRMKGFSTTAVPSRQADTELPPFRFGAGSRLNHTSSNLSLSECFESTSPPQER
RPASANSPCPGLSIGRTRPQFLNFNACSRSNGSPSMNSHSRRQSNPFLRNRKQFRRSLSMFEHPADVMKSNSEGEETTSSALQ
SVMDVEEAQEPVLPHFLLEDPTDTIPRITRETLVDVLDGKYSSHFDQKIVIDCRFEYEYEGGHIDGAVNYNDKDLLTNQLFQT
PMDGRTLLIFHCEYSAHRAPLMARHVRSEDRTVNAEFYPKLTYPEVYILDGGYSGFFTEHRGRCYPQEYVEMSDEAHQRTCER
EMGRLKSRKGLSRAATFAFGQREPCVDESPTAPSRPQSRNLHAPMSLLGASPMIGDRSHARRMASY
FUN_005925 CDS scaffold_2 197319 200393 +
hypothetical protein EOG09260M44 PF04931

MGSKRKRGAKEGTNGAQKAPKRTKSDNESVRPSSKPASDKSPFVEAPIGDERKREAGLYDLLGSEDPDERIEAADCIVPSLLA
EGVSETVLQRHLDRRLFRGLASGRNASRLGFSLVLTEVLGQLFGEKNLAESRYQGLTFEKILEIFIENTQAIGNLPGQEERDF
VFGRLFGIFAFVRSGILFSDISRWNAVLDLILDLSSRKVWLRPQCGWVIVQSIEQLNKKQAETTLEKVAEANLAKTPEGVAIW
IAAQTRFPGLRIKPWPVPLHKNTFGDLAAILRESFKDSFADQSEPGQQINFPGWSPQLHYVWSILLEHFLEKREAGLEDFKQF
WTRVVDDSLFSKRATEGQKFRGFMVFRKMLEGLFELPAHIKVLFSKNLTLCLMNQASKEDRYLHRAANKALKAIEATTSAHPA
TLVPILKGLLGENGAYNFDQRTNTKTVDQILQNISGETGEEILKIIRTPLSTIAKQDTKKASSTLRVYVDYLSKILNASASPT
SGEFQQTVFSAALQELSQLAYSQPKNIPVDTLTEGVRELCRTRLESSFAKVSRRTEDYGSLCLAVSSIDPNSVAMTEEIKTAV
QEALSRMQKLLRRKAGDDSEKNLFQSIAMLHAVSIFRLYNEDPDAMEVLNDLAQCSDRLEKGKSAESEAGTSEIILEILLSMV
VRPSSLMRQVTQQVFGAFTPQISAAGLELLTGPLTSGENTKGQKELFSTGEDEMEVDEDEDEEGTDADEEDNSDIEIDSDVEF
IDIKETNDESGEDEEDDEDEDGEKSKGEFDRPEDLDNELEKILNSHRLDKDVNAESSDSEGDMSDSEMFAIDEQLAAAIKPRI
QDRSNDSKKQKKEAKQSVLNFKNRILDFIDIYVRNESRSPLSFTLLLPLLDLMRTTSTKPLAARASKIISNYQKGLKKARSNR
QEVQILEPDDMLDLLREIHKAAGQDDSHAYAKAASTASLIVVSALVAADKSKMEDILAVYSKTQASCFSGEVKLQSSFIGDWQ
NWYRNAMQQVRN
FUN_005926 CDS scaffold_2 200546 201575 -
hypothetical protein EOG092644WX PF00227;PF10584
T01A
MTSIGTGYDLLNSIFSPDGRNFQVEYAVKAVENGGTSIGIRAKDGVVLAIEKVVSSKLLKPGANKRIATIDSHVGAVSSGMVP
DGRHFVDRARDEAQSWRQNFKTPIPTSDLASRMGGYLQAYTMYGSVRPFGITAIVGGYDTPEETPVDGEVGSGPKVGAGGKIE
GKHGGPFLYMIEPSGMYWGYYGAATGKGRQAAKAELEKLDLPAGNITLEEAVKQAARIIYIAQKDNKDKDFELEMTWISGPDG
PTKGRHVEVPKELREEAERLAKAEDEDDDDDDDDEDEDAKDDKMED
FUN_005927 CDS scaffold_2 203684 204461 +
hypothetical protein ENOG41

MSFSPSAMQQDELAALLARNLSFNPIPEPAPAQQQEPKADHAEPIIYTSVHYTHSAHIAQAAQHQDVPRRASEPPQTQGPRVE
AILQHHGVNPATLTPSQLQLFKVAEPSQQERLIELWNICPPGNGGDIPALAWSSTTVEHEEQLARLRYERLNQQQQVMSLDGT
QVQAVDGRWVRNSEPEVEPYMTSGYEELMRREREREARDSQPRSTYGLYSHATDPVYMGPDYAHEQQLLEMATQYGAYQGFRA
PEVDTMDVM
FUN_005928 CDS scaffold_2 205181 206496 -
hypothetical protein EOG09260KNI PF13516

MASSDKIFSLEGKGLKLDTAEDLEPHIAPLRSADVEEVRILGNTLGVGACKLLGEVLATKKNLRVANFADIFTGRLLSEIPDA
ISSLLTSVLNLPKLNTINLNDNAFGLNVQAPLVAFLAAHVPLQHLYLNNNGMGPHAGILIADALSELHSKKEAARKEGKEVPD
LETVICGRNRLENGSMTAWAKAYKLHNKIKVIKMVQNGIRQEGISHLLAEGLSHASKLEVLDLQDNTFTVTGARALSKVVANW
TSLQELGVGDSLLGAKGGVLVAAALAKGKNAKLETLRLQYNEITSKGIKAFATAAKDGLPALKRIEINGNILTEDDESIPVLQ
QLLEERKEKFGGDIVNEDDWGVDELEDLEEPDSDAEEEEEEEEEIEPEDRAEKLIKEAEEAQEEPVIPVKDKEVDELAKKLEK
TGI
FUN_005929 CDS scaffold_2 207281 208586 + CYT1 cytochrome
c1 PF02167

MLARSCLRSTRTFNGLRNGPSAVTKVWHDPTTSASELPVCREIITTNLDTPQPRQSQQNAPQLHRAASSSSSAASDASATRLN

LAAAASTTLALGSMAWYYHLYGPVAFAMTPAEEGLHATKYPWVHNKWFKTFDHQALRRGFQVYQEVCQSCHSLSRIPYRTLVG
SVLTVDEAKALAEENEYPGEPDEQGEIQMRPGKLADYMLPPYKNEEAARFANNGALPPDLSLIIKARHGGCDYIFSLLTGYPE
EPPAGVQVAPGMNFNPYFPGTGIAMARVLYDGLVEYEDGTPATTSQMAKDVVEFLNWAAEPEMDDRKRMGMKVLVVSASLWAV
SVWVKRYKWAWLKSRKIAYDPPKEVKVRR
FUN_005930 CDS scaffold_2 209657 210344 +
hypothetical protein ENOG41

MPFVGEDAHAGRKRRWSDDNEQPRLSLYPSEEAKDYYILDQRPELALRKVLPVSKRTRITGDDIHVTDGLNASSFSFNTPHRR
RRSSQLKMLPVQPAFAKSRVTGALLVPCNICHRKPTKKSDLDSFADCQGCGERTCFICIRECHGWNLDGGSGVSEQEVLSRSF
HMDDVDDRQQQEQNDTKNGTRQADKGWNAVGHRAVVCSLCSIERGAEGDVACLGCFSRMEGS
FUN_005931 CDS scaffold_2 210800 212291 - RPT1 26S
proteasome regulatory subunit 7 PF00004;PF17862

MPSATGSNWEKYQKNFADDEIEEKKITPLTDEDIQVLKTYGAAPYGTSIKKLEKQIKEKQQSVDEKIGVKESDTGLAPPHLWD
VAADRQRMSEEQPFQVARCTKIIADEKGDEAKSKYVINVKQIAKFVVQLGERVSPTDIEEGMRVGVDRNKYQIMLPLPPKIDA
SVTMMTVEEKPDVTYGDVGGCKEQVEKLREVVEMPLLSPERFVNLGIDPPKGALLYGPPGTGKTLCARAVANRTDATFIRVIG
SELVQKYVGEGARMVRELFEMARTKKACIIFFDEIDAIGGARFDDGAGGDNEVQRTMLELITQLDGFDARGNIKVMFATNRPS
TLDPALMRPGRIDRKIEFSLPDLEGRANILRIHAKSMSVERDIRWELISRLCPNATGAELRSVCTEAGMYAIRARRKVASEKD
FLSAVDKVIKGNLKFNSTATYMQYN
FUN_005932 CDS scaffold_2 212745 214515 + CCT4 T-complex
protein 1 subunit delta EOG09261XNJ PF00118

MSAPVAAPAPAAGGSSNATFRDKEKPMAVRSSNIVAARAVADAIRTSLGPRGMDKMIRGGKGETIITNDGNTMLKSMSVMHPT
AKMLVNLSGAQDVEAGDGTTSVVVICGSLLGAADRLLSKGIHPSVISESFQRAAAAAVEVLHDMSLPITLSDTSSLLQAANTS
LSSKIVSQYSNLLGPMAVNSVTKTIDLKTAENVDLKNIRIVKRVGGTIEDSELVDGLVLTQPVLKNAGGPARMEKARIGLIQF
QLSPPKPDMENTIQVNDYRQMDKIVKEERLYLLNMAKKIKKAKCNVLFIQKSILRDAVNDLSLHFLAKLGILAVKDIERDEVE
FICKSTGCKPIADIDSFTEDKLGYADLVEEVQSSGSRMVKVTGTKSVGKTVSVVVRGANSLILEEAERSLHDALCVVRCLVKK
KALIAGGGAPEIEIAAQLSKQARSLTGTEAICWKAFADALEVIPTTLAENAGLNSIKVVTELRHRHEMGEKNAGVSIKSGGVN
TNISKENVLQPLLVSTSAIELAAETVKMILRIDDIALTR
FUN_005933 CDS scaffold_2 214895 215387 +
hypothetical protein ENOG41

MASSNKSDYSSNEDDNPDFAAKAQGRLLQLKKGRDERMNAIVQESATGMDKLRTSIARFQQDRRTKEVRAVAISVTRIIEAVE
RRKGIEQQMETLVSRLINKTQEVESMMKTGLRGREDDVKKTK
FUN_005934 CDS scaffold_2 215749 216097 -
hypothetical protein PF05051

MDAAQTTISSPVGAAANVASPSTTAASKPKPCCVCKDEKAKRDECMLFSNAKDPAADCKSLIDQYRSCMSGFGYQV
FUN_005935 CDS scaffold_2 216884 218036 +
hypothetical protein PF07859 ENOG41
S09X CE10
MLNIEEIRALGNVHPEFEPIIRAHNPMLNGWDMNTDLETFREMMAQVKQYRPKSDAAALSYQTKDFKIPLRDGFEVDARSYMP
DGDVPTDGLSGLVVFHGGGFITGDLDTEAGLCAEFTKLGGIAVNIDYRHAPEHVFPQAINDAFDATIWVSQNVDKLGINPSKG
FIIGGTSSGADISLTISHLYREAETLHPLTGVYAPITSGVNDQTVPEKYKEYFISYEQNAKVPVFNAESMKFVHSKYRPDPKS
PLAFPVAFPSHANLPKTYFQACGMDPVRDCSIVLEQVYKDEGVPTKIDIYPGLPHAFWAIFPELEISQKRERDAVEGLKWLLA
G
FUN_005936 CDS scaffold_2 219209 220262 +
hypothetical protein PF00011

MSRQPNPFNNANFWDFIQHFDPNQATGRGVDHQTETPFPSFMAGFPFGSPAGPHPPHHPHPHGHGHGPRGPPPPPEADGFVWG

PWFSGEGAPDSRIRNEQRQQANETQQATSSESHESDETMNVPDPEEVAPEENQCGARPEWAGRGRGRGRGGPRCGRGRGRGGF
PHGPHHGSHHGPHHGPHPPPPPYGGPFDLSGLFRGWGNHPFFRNMREQFQENQNQEQDTNDSSDSSFNPPIDIFNTEKSYVIH
VALPGAKKEDIGVNWDADRNLLKIAGVVHRPGDEAFLSTLSSGERKVGMFERNVALPPTGADERDEVDGLGITAKMEDGVLAI
TVPKAEKEWTEIHKIDIE
FUN_005937 CDS scaffold_2 221014 222622 -
hypothetical protein ENOG41

MMAATNDQTPSFRCDSPPPDALPTPAIGACPELSRCLSTTSSWSHGSNDANPRDALGIEKFNRHSTGSLDTSTLKNRHSDISA
ISRHSSYNFDGRSRRRGYMRPQGTDFAASARSRESVMSLGSIAHLQYYFARTGLLDGKGGQLARKKQQRATLDLSSLDTNAEA
LPKIVGSDFDSSYASTDSSPDFSSQGFGTDLVESPIEELDDYYEDNFSDLGGDMLPPTVSTYHHREKVVPKPPTILELKSELE
QTLGDAKKALNEVEARRTGTYEAPSSAPDYPNMPAENTQGWFELQGMHILDVVTLAIRAAKIYYTAHELPDRLDSIKPEKQVR
ADLLAVMETLRQMATRNFKEGMRQEEIKTMNDWVESVFDILKKEQEIEDAERAERVGWTWLKGDWSGRELERERAFLMSMDTA
SEPLPEWKSMSPADAKPTPFLEAMQNGVRLVKLHNAVVHKSRRRFGAIGSFHTDTQKPYRCADNLRYWVKAAELRFEVMLKVD
VLGVQYNTSPQVWVDFETAILKWSRHVREEITRELEL
FUN_005938 CDS scaffold_2 226363 229621 +
hypothetical protein PF08580 ENOG41

MNTGDLNATTSAVKDNNDSDWMPKADPFLSTPAASPETNRPTDVGELTSGNCYSDQDHDVPTDSLLNFADVPGQLSHLPFNPG
SANPSSTYTPDPNSSLLTFSSSIIDTTPTSSASSVTDTPTTSTIVGASAATTPATITKITSTSTNALLPFPTPTSPLPSSALR
KTSPGLAARLKALGFGAQKFSTPPITPQNDRIGRLNEDQLRQLDEKHQAGSVKSVIARRGRPWKGAGASSSPKPVAHDPNLEL
PPFKVEIPESTVALLPEIQTPEPLDMDTQKYRLPDHTNGNGTKVTLDTRREHIERTTRPPSPDSAGLPPPPPPPKDTPPIATS
TPATPPTDYVPSLNSYFTPGHNRPGSIYTLSRASFASQLAQLTSLQLPDAESLASQVTAIPTAQVAAKALINAAEQIRSWISK
ASEVVEGLDSEDDVEWAAAGGREGLAEVEMAITRFEELVKAYVGAIEQLQNRKDVANVSVQDLTQAVSQMESVIHEWEKIRRT
LHVVRAQVEIAMEWEELWNNVLGDVQGEMDELSRLVFEMEERRHKSLMAANADSVDIGDLETIVEENPIQAARLQAPSRYSLP
TFPVTPNSPGTPTLSQDDSSLLALFARMQPLRASLDFLPMRLSMFEARAQPSFPTACEELNMRREGLDASYKKLEKDAESLRK
ELGEDRWVIVFRGAGRQAQKMQESVERSLLKLREAADSGMHLTNQPAMMKKLESYEAKRMHYGPAIERVLSIIDKGVSERLTV
NGEILRLHNDMQNRWQALKQQMKDAESLIEEIHADKGQQLRDSISSMLSNDRSTSGSGRETPGSSPPSSVIMSSLGLDPHTPL
SKNPKVRATPGAANRQIPSNRRHSSLPAPTSQTSRKSGFSRLSTITGSSSTASLPPSQIKRPDSKLAHRPRWNASTNTSDVDT
GHNFKPLTLTTPSPYAKKSPVAHKSAGSTTPGSGTSKLPTLRSPLARASSASPRVDETPTRGSHSNLSFRERLASPGANSQPL
PKPRLASHQSTTALSGRRSSLQASRSIGPDGDRHSRPASSLAMSTRRISLLPQPTETGRASPASVSSIRSTMRKPSPAEPKDS
KPRWRH
FUN_005939 CDS scaffold_2 236949 242854 +
hypothetical protein PF00023;PF12796 ENOG41

MAGLPKLPAPHSELAKYIFDHKDINITEIMEPYREYEANLRSIYAQDRQNPILGDPYLNVVPLFTENTKFITTRARNLDAESD
EEKSKYIMALPDDKRRAHDSPATVANLEEFQKNFNIFSESSLADLDWSNVVAAGSSVVNCLLPVPKEFNTTKRKLREYYHEKF
CPASDVDLFLYGLNHDQAIDKIKQIEQAIKDALLNEVTVVRTKYAITIASQYPVRHIQIVLRVYKSVSEILTGFDIDAAGGAY
DGTQVYVTPRALGSFITQINHIDLTRRSPSYENRLSKYSHRNFEVYWPELDRNQIDPTIFERSFRRTLGLARLLVLERLPTTT
ARDTYLDKRREERGRPRVYRSHQVLHGNIKDYHEDEIADWLSEEDVSNYHTFTVPYGQHFNAKRIEKLCYTRDLLLNAEWNQP
DDREVYLHRHPAFFGRVEDVIEDCCGCCPEAVTDQEKEVAEKEAEIYIKGKVSFLIDDPGRQQIGSFNPLTEQDWTDMAYVGN
VTRLCQSIVDGDVDDVSNWLSQEDADPNKRDYTGRTPLHLAVMTSTPEVIKCLVERGARLTARLADGKNALHLAASRGDVEII
KILMEKSIENEEAEEERQDKKRRAAKKPSSDNNDGVRNEGAASDPDGESDGEIIDDETTDADAASMTTGSFVKIKQVEAENAE
DLVPEESSDEPDFFNVDALAWDLQCSPLHLAIAEGNEDAVRVLCDYGTDSILPVKSLAEHGDSRVILTLVLALTLPNDKAKSM
ANLLMRLGATSAQADSKGCTAFHRYVESGEVELIDTLLDNDKTGIKAAINHMVCDGSYWNAETIAPINTAVQSGDVALVLKLL
TAGAAAHIDFDNWLKSAKVSDMSGRLGNLETNQKMYKETVEQPLITAIRSGRTDVAIKLLEGGADPNSLNTETHRLIFNEYQR
NWNKGQSALDLIQKSLKSLRGFTVETNGPVKPRETPGMDEFLSHFTPGSYSHWVVSEQVGSEKRSFASRLKRYEEDIKKNPEP
KGVPEKLEAIKELSAGFKALEEELISRGGKTFDELHPDIKTNLRNNASSTSSRIKDEKKAIKPFEFHFSFNNDRDMTEKRRDG
YIELMEAAWAGDIEKVKELSLQAWGAEKDQPPLKIAVSDNMGNTPFSVAFLRGHHDLARALLEIVKAQWSPPKKDKVRFRMEA
GNEDEEYESDSDDSDNSSENEPRIVSEKVQDKFTIDDIGHVSMEVESHTKPLQIICESVPSWLIEKKVVYRYQKRSLFVHTFD
TNDTTGLKLLLDLAQHYSGQKFEGDDADQEDEGSTCSFTFPQSDFKWAVEHGKTQLLALVIKRTGAGIPLDHLVKKSGVELKR

KPRYYQGLTVYGRKRKDWANAGRGMVIKSSGLRTPPLLHAALGGSVESVELFLGDAPHRLYGEFAKSKASREDSRFKHLKESP
GGFDRAISKWLGADNDLVIHCAVLAYPGDNANELLDYLVETCPDFIEKKNSEGDTPLMVACRLGRIDAVKILLAANADQSARN
QRGENILHAAIERSPKAYQLRELLDLLDSGLRSHLFLQRKNLNENGTTPVHAWISQVSGMTLGADNRNNYRRYNGSGYAKPYI
GQEKELVLMLKLLLEYSKGEELEMLNGAGDTCLHTAIMTGMIAAVRVLVEFKPSLVYRENAVGRTPAELAHDKLTSQKFTMPD
KPSIKDRNRVHDTLRKNSEEFIQEAAMDAHSTEQRRSLESLGLSDTYKPQEVPLILASMGVEKNRSSKRLESRSIDLVLWDLC
RTTMKQHPGKRRLVSLNEANDVAKRLGEKYSASRYFSVQARVDDEEVDPDEKESGTTIDFAVQEMKSRLSQAWETKRRHGSKN
SSSGSDYGRSSSSDESSDIYESLY
FUN_005940 CDS scaffold_2 243152 243967 - ATG33 Autophagy-protein 33

MTATGTSVSLLKFVGTVSLGLLTGVSYSITSLALPVLLRLPSSNSASHGLTSLNAALKTPILALTSLASAPFLISFALAPRSS
RHPYLLYTGLLATLSFVAPLLIPAPAPRHVASSAPRQSPRAKMEASYEVLGDAHSEPASEEDIDDINGEGVRVEVEGLVHSYI
ARTAFSALGFTMAVIGIWGDGAPQAVVYVS
FUN_005941 CDS scaffold_2 245153 248429 - MPH1 3'-5' DNA helicase EOG092614O9 PF00270;PF00271;PF04851

MDSDEFDDDIADEDFITALDQVSSSMNGQGNRKALHAVVSQPSNRPNSAAVAALELEDLPSDAFSSPEPQSRSNVSVAAPAGP
APRTGFNRTSSGNWRQTTLFGGSLSSDGPRPSQPAATRVFRADLPREEPTHHEVDTEAMQTWVYPTNLGAIRNYQFSIVKNSL
FNNTLVALPTGLGKTFIAATVMLNFYRWTKKAKLVFVAPTKPLVAQQVDACYNIAGIPRSETTLLTGDIPPALRVDEWESRRV
FFMTPQTLLNDISHGYADPKSIALLVIDEAHRAVGEYAYAKVTKLIRRFSKSFRVLALTATPGSKIETVQEVIDNLGISHCEI
RTEDSIDIRQYVHQRNIEQVVLDPSDEMVLVSELFTKALKPMTDKLSSQNIWFGRSPMAITAYGLMQSQREWFASRGRHANQG
VQHMMRAVFSVLTSIAHSIKLLNFHGIKPFYDNLVDLRSEQEGRGEKGSKYKRQLIQDSNFQEMMDKISKWLRTEGFVGHPKL
TALADTVLNHFMDQSDNSATRVIVFSEYRDSAEDIVRMLNKHQPLIKASVFVGQADGKRGEGMKQAQQIEAINRFKKGDFNVL
VATSIGEEGLDIGQVDLIVCYDSSASPIRMLQRMGRTGRKRAGNIVLLLMRGKEEDQFAKSKDNYEKMQTLICEGSRFNFRFD
LSTRIVPRGVVPEVDKRHVDIPVENTQDQSLPEPKKRRAPAGKKKPPKKFHMPDGVETGFQSVASMLKIGGRAKQQQNKRNPE
LGDLATVPELSKVLLDEEELKELNRAYRNLPFNHSIIEETDMPDMTAHPELQRQLRPVFKLKHGTRTKRFVKMWHKMAHDPES
LVLPCRYQDRSNYLEIPVHAFAGSESETEPTDETPTTKAKGIGATASVKKKQNRKPPSKVQPRAKRRRLSSDLALQTFAVGSM
VDEGLSEENDEEDDDDEEDEVIPRGRGRPKRGSKPRSKVKRKPRQKNGGLNSDEVGDDCERDSDMIETDGSDNGEDLLDFIVS
DNHPMSSGREPVSLRTSFSSASSTSTPECAHDKTTKPFYVPTQFPGTQESDDIPDLGEVLKMTGKQRGQEFSDNDVIVRRTAR
GRRVVVDESDSD
FUN_005942 CDS scaffold_2 249692 251880 + hypothetical protein EOG09263MNN PF02136;PF03943

MAPRGRQNTSGRETRNARKPATTSRGGIQKRKAGRVDGDGDLDMDSAGRRAKKATTADIKPTRSSTRTSTRGGPSKNAQNVLK
HLSNGTAASLASRISSASSGKGPKTRSQDMTGLTVLRVGGLKESKAASNAGGGVNDLLGFMERKAGSFRTGSKRKVAIKKHHT
VGDYVVYIGASGEDASELIKLNTFTFAGAPLEVIEVDEVPDRGTAIESKSTLELRTKLQEILSSRYLGTNKLLNLDALASDANL
VTLGMFESRERALKTFRGLMAVCESLFKTDKEKEDAIESISLANNNIDDVGQVEVVATTFPRLKNLDMSGNQVASMQALQPWK
GKFKYLETLFMTGNPIETTDPSYPSTLLEWFPRLQNINGTQLRTLEQIAEQEAALRPKPIPQNGPDFRDVGGIGENFLLEFFA
AYDSDRPGLASRLYDEDSRFSISVDTRAAPDPNAAAPMSWASYIKLSRNLTKITMPNPRIQRLFRGTASIQDAWKTLPLTRHP
DIKVEINKYIMDCHPLQGLVDPSGQSSGGVDGLIIAVHGEFEEHDPNTNATGKRSFSRTFVLGPAKPGQGVIRVVSDMLSLRT
YSLLPNVFAAPASIPTTAPADQHQAMITELCKQTGMTPQYSEMCLTQVNWEFDQALVIFNEKKSQLPAEAFATMIGS
FUN_005943 CDS scaffold_2 255770 258008 + hypothetical protein ENOG41

MSLPEPPSSLDNGCSVVHDNTLYVFTPTAFLSLPLEEGAEWKKLKMGEQVTGGACVGTDAALFIIGGSGGQADYNGLQKYVFA
DKKWETLPIDDTAFRNLQHHGAAYIKNTDQIVVSGGSQDGRETPSDTSRYFAASGPYILQSAAGSAASVNPILLAWSDAEVCL
LGGSDWNTQVLLFNAATSWRTTGITLSTPVAASSKAMLVQGDDGSKSLYVFDLSVSPNKVHRYVVWGNNNAPGQNAADQNVKR
DLSLNDWPEYNSTLAPTTARSDYSMAQDSNGRVVFVGGNKDEPLAFFDMKENSWLDASGIVGDESQKILSAESTTGTKTRTST
TKTSSTESSTKTKSVSSDLSSTLVTSTTSATATDDSFSSATISDSSSATNSDIAPVGGASSDDKSGLSSNAILGITLGTILGF
IAVLIVILLLLRRRKKTRQPSPENTQPRHDFPSDEKDPMGLGGGPVSPFRGHHSNMSQESFSSMAILMGRAGKNKPGISRNLS

NGTNRSSVSTEHKQLKSTIGKPVLQEMQHPVLQGQDTRGVAFDPTVAEPRPRNGPLETQDGLRRSSGWNRYWSGGSALQILGF
GSSKRNTVGSDSDSHYSDAIPHRNPRVTQDSATVPPLNFEFRPEMNRVNSGSPVVAEYNKNIPFRDGVAGKIERPISKASSGY
SSGIPESVNETWDPDHKDKPWGTDRATSSIYNPSFYFGAPLSPRVPPPQTPPSGVSTQPQLAMASQSSDMSWLNLGDRSRQ
FUN_005944 CDS scaffold_2 262695 264728 +
hypothetical protein PF00010 ENOG41

MSSTAWNGQDQTMASTGEDDFHQFLDMSSMGSLGDGMHFDFQSFQDGSAQGLMSQTRDAPDTIMTDSDNPGLMSTPNTMPMST
STVQSTIPAHMMTPASDPISNIDAQIQYLQQQKFQQQQRQMQEQQVAFFHNHNHSVPPTPQSLEMPNSGQFYSQAEQIPTSGA
YDRNYHQRMKEQDMAFTPLVSPAVTPLDPNFSMDSGYTIPSAYFSPLTSPALHAQNDPSTVYDPRLTNTNNSPIDIDLDRSAA
PVTSVFDLPKKATRKKTTTKTRAKASIRSSPIVKPQRRKTGPSPAIVSHVLSEVEENNGAFLPMPATSTETSAEENSSVSPEA
LSEMPPPPIPNRRSTSKSPYIQAQSSNQQTPVSGPVDSRPAPATPASLMKLPASRASKQANGPQEPVVSEHIESLELPESVSG
KTMTPVISRSSVQSPSVEPISRRGSSFQPLPSPVFPKASGPASASASPQLAPGSAGPSARKTPQLAPRSNRKRSTGSVHVSPA
LLPRISPSIKPLLPGTPGMTPAESAASQLLMSKSNYQNILEGNKVPGISYPSELSTNLTSKRTSHKIAEQGRRNRINSALQVM
AGLLPGGDKTNLADEGDKKDGKQANAQNSKASVVENAIVHMKSLEKENGDLKKEVEELKRRLEGLQGSTDDKEKA
FUN_005945 CDS scaffold_2 265208 265669 -
hypothetical protein PF09341

MTAIPDPEFPCSMQATPLSCDDDDPNVLNVPFPTARLATTALKAIQVDPELSPLVRRQLTVVPAPTSTQGSPSGGDADVRLLE
VQYRATTNRMLRVAVNGFMESLKLVVEVMEQLDTDVLDHQQ
FUN_005946 CDS scaffold_2 267333 269263 +
hypothetical protein EOG09262E7I PF00782;PF14671

MLLPTSWLNYISDRLYLASYIQPPTPDTPFPYPEEPQPASPKKRSQRAAAGATPLAAKSNRPQPCYFTVDDTLLYNAFHHDFG
PLHIGHLYRFAIQFHDILGAKQNKDRPIVFWSAADPRSRANAACMLACYMVLIQNWPPHLALAPIAQVDPPLMPFRDAGYSQA
DYGISVQDVVYGVWKAKEEKCCDLDNFDLDEYERFERVEHGDFNWITPHFLAFASPQHAPVQKITEGSDLYPLLPRTLAAVDA
HPKLPKPFKNVLKHFSEKNIGLVVRLNSQLYSPSYFEALGIQHLDMIFDDGTCPSLSTVRKFIRLAHETITVRKQGIAVHCKA
GLGRTGCLIGAYLIYRHGFTANEVISFMRFMRPGMVVGPQQHWLHLNQGTFREWWVEERIERRLRREMAAANPIPSTPIRAMQ
KTTLRNGQASTPPNRSPSNRTPLSEVDHDRNNIGVQEDYLPAPTPGQPRKTARDRHHPYQRSTSNGLAVEEQRTIEQEAEYIA
THSQAHGGESDEELHLRMRRHRKATSQSPARSEKTRSVSQTTAIYTIDNDASHDAENIGSVRTKHVERVASTPGVLTKVRGSK
RQGESPLRAKESAGIRKTSGRVGSANHGASVTAASTARKVSGA
FUN_005947 CDS scaffold_2 269952 271773 -
hypothetical protein PF14479 ENOG41

MAEAVGTALAVVGVLGQLFDGCVRAYSLFTAAANLDTDSQRLLCKVRIEEMRLVVWGRDWGVAEGRLEAHLESTRNPQLRSLA
LQILEELHSAVTDFKKLKNRYGLVDEGRASLEVTGSKPRKSPSPSRKRPKDDESRDLNGASRVTSERNWGKEMGLRARWVIAD
KDKFINLLRDLRDFNDGLERLFPTSHLPSFQRAWTNQLLESAQRDVTQLTLLEIASDGVYPKLTTSANLKKLRINLDAKPQAS
FKPTFALKVPRAALDLSGDGGDGGRSTGRHESAGDVVIEWVDYDRDDVDERVAHVRRLDDLARMMHSASDCHPDLHSIDCVGY
VDDTSRCRYGLVYKAPSPSFSTLHELIASSDLKTPNLDDRVRLAHTLAVALWSLHSLDWLHKSLCSSNILFFPSAFSASAHSS
TAAGALVPDIQCPYLTGFDASRPDLDTALSVAPRNPSILTLHRHPASLRGFPHCKPMDIYSLGLVLLEIGLWKVLQTYHKPHY
STGRWRDKVLRAALVPGLGSKVGSRYRDVVDKCLAVSEEMTSAEAGKVIEDVVTALEAIRT
FUN_005948 CDS scaffold_2 272185 276290 -
hypothetical protein PF00005;PF00664 ENOG41

MAVTLDQSGTEKPKRSFLSSLKTCFIYLELLFAAGPTWVDYFLIALGTLCAVGAGVPFPLMGVLFGQLIDNFNGATCAAGGSG
SGASDAASDPLDYENAINDKVIKTAWIGAIALVLIYGHLTCWNIISQRLAQRLRTRYVSALLRQPPEFFDTRGASGQVSSRLQ
GDITAVQAGTSEKVGNIITTMSFFVTVFIIAFTKQPRLAGILICMLPAFLLSGILGGRYLGKFVVKQTEASAAASSIASEALS
HVAVVQAFGAAPRLEEKFAEHMARSRKYAIAKSSIAAIQTGLLYFIAYSGNALAFWQGSKKIVESVARNDGSTTVGEIYAIVY
LLVDACVMLGGMAPTLPFLGAAVGAFQRLKEDIDAPSSIDGTSREGVVLPPSAAKTLTFRDVSFEYASRRGQPVLENVNLDFP
AGKYTAIVGLSGSGKSTIASLIARLHDPTKGTVELEGHDLRELNVKSLRSFISFVQQEPSLLDRSILENIALGLVNSPKESHQ
HLKPLLKGPELAKMAAKGKDALDSAASLGPEFVEIANLIRHATEQADAASFIERLELGYGTTAGPKGSLVSGGQRQRIALARA

LIRDPEILVLDEATAALDSASEKRIQLAVERAAAEKRTIISIAHRLSTIRNADNIVVMKAGRVVEQGTYDDLMAKEDGEFAHM
AKLQTVGNKSDTGSVDGESIGASTLRNDTQPNEKTEVSGAKQSLDSQILGREKEETKKKEEIASDDHELDDVKPFSSVIKGLV
WLIRPSLGWFTLAMIAAVFVGATFSGSGIIFGFTVGALNPCANTLDHIRSMGNMFAGLFFMLAGVELIANFLAWLGFGIVAER
LLYNLRVLSFRSLLEQGIHWHQSEGRTPTSLLGIITKDSMSVGAFSGSTFGTVFAILINVLIAVIISHIFAWKIALVCLVTLP
ILLGSGFMQLRMLARYEERHREAFSTATALATEAIQSIRTVAVLSLENEYMEGFARLLKPPREQVVRASVTTNIWLAISYTTG
TFINALAYWWGSQLIMKGEYTQKDFLIILVAMLTSAQLWSGMFSLAPEFSRARLALSRVMTVVNMGSSTHAGKPGQDPSKTGR
DIEASGGEKSLSPADNHSRGGAKVTFKGVSFAYPSRPDATILDNVSFTLSPNQFCGLVGPSGAGKSTIMNLVQRLYEPTSGSV
LIDDNDISKLPASFRDTIALVPQDPALFDGSVRFNVGLGAPPGHEATDDEIKEACRLANIHDVIAALPDGYDTECGPSASRLS
GGQRQRLAIARALVRRPRLLLLDESTSALDAASEAALQEGLERASRGTTVLAITHRLHTVQKADIIFIVENGQIIDSGRHSQL
MERRESYRLNAMQQMLQ
FUN_005949 CDS scaffold_2 278207 278792 +
hypothetical protein PF00583;PF13302

MSQATLRTSRLELRPLGHEHGDHIYQLDSNPEVMKYIGYGKPLTADESAIVHKLLLETASFGSGLGCWAGFAGDDFVGWWVLA
PSQSNDDPPQVNKKRVEFGLRILPKFWGQGFAKEGSRALVKHGLEDLGVEEVFGETMAVNQGSRAIMAKIGLKHVRTFHNKYD
NPPPGIEEGEVEYRVTRDEWSSQQACAA
FUN_005950 CDS scaffold_2 280431 282366 + MIR1_1
mitochondrial phosphate carrier protein ENOG41

MSALAKIREGLAREDNTAVAHTLAPHEKEIEETPETITETGPGHDKEATAGALPPDDKGNDSDVPSEDVQTGVKEIQAITLTW
GKGSLAALLCLIWTLFLISGFRGSFYLVLVPYVTSEWQAHSLMTTIPIVSDAMTAACYIPMAKALDVWGRAEGFLLMSGFATL
GLILMAVSQNLATFCAAQVFYSVGWGGMIYAVGVLAADASNLRNRGLAFAFTSSPYMITAFAGSKAAAAFVLDVKNWRWGFGW
IAIVLPCVTIPLFLVLKVNLHKAFKKGTVTKTHRSRGFFGSIWWVFNEFDVIGIFLFGGGLVVFLLPFNLAAHAPDGWSTSYI
IAMIVVGFCTLVFFGVWEYWLAPVPFLQGRFLLDRSVVAACMIDLTYQISYYTWNYFFTSFLQVVVNLGPAEAGYVNSTFQVV
SGVLLFIVGFLIRKTGFYKWTFYFAVPIYIFALGLMIHFRAPNQYVGYIIMCEIFISIGGAVFILVMQLAVLAAVAHQYVAAA
LATLYVAGGVGGAVGGAISGAIWTNTFIPQLMKNLPESELANAPLIAGNIVNQLAYPVNSPARLAIQESYGFAQVRMLAAGVG
IASLFFIWVPMLRNINVKKLKQTKGLVL
FUN_005951 CDS scaffold_2 283981 285077 -
hypothetical protein PF02077

MSQRFNSGYGLGGPAPYGGSQAADPQQGEGFLEQIRPYTSKIEDALDTISEPIKPYLPAIGRFLIVVTFFEDALRIVTQWSDQ
LLYLKDYRHIPSGITHLFLLVNIIAMFSCSTLVIIRKHSDYAVAGLMGVVVTQALGYGLIFDLNFFLRNLSVIGGLVMVLSDS
WVRKTQVFAGLPQIDEKDRKMYFQLAGRVLLIFLFIGFVFSGEWSLWRVIVSSLGGISCVMVVVGFKAKYSATLLVVILSIFN
LLVNNFWTLHEHHPHKDFAKYDFFQILSIVGGLLLLVNSGPGQFSIDEKKKVY
FUN_005952 CDS scaffold_2 286073 287660 -
hypothetical protein

MSGSTTPRFGYDVLSVFDVPPNARDFLADVIESNRAPEETEELFQESLLARAADWHYLIECAKSMRRARPEDRNSLQAQDVLA
DVWYTLSGQDPPPGEEPWELTDRLIARAKQLDVDPKIRPPHITEQIENHRQQWLGSKVFPKTTTCLTTSPYWSDQPHGRRPEG
SNNLAKWKDRACLPLASSQARVSAILHSQTPDNETYQTLSVDKPAGGQGSPQEVALCDMTLILKPQSSISPYFVATSASDEAG
QAIALARKRPLRGTVPSVPFAPLTSQEFGLIQEKFAHEPFWLLTVVTFLIRTKGTQAIPTFYKVKERFPTPTHIASKGNTEPL
VEMIRHLGLAEHRVSLMKKYARGFLDQPPVAGIYHKVKKYDHRDIDPLSLYQQKGSSALLVDKDSYGQDLLEAWEIGHLTQGK
YALDSWRIFCRDELLGRATGWNGEDRDPKLQPEWMRVRPDDKELRAYLRWMWMKEGWEWNPDTGERTVLCEELRNAVNEGRVV
YDEKGGLKIHNLAYSANALILGEEELMAES
FUN_005953 CDS scaffold_2 289118 289887 +
hypothetical protein PF00076

MSSRGGKLAPEVNRALFVKNLSYNVTPEELFDLFGKFGPIRQVRQGIANNTKGTAFVVYEDVADAKQACDKLNGFNFQNRYLV
VLYHQPDKMAKSKEDLEARRESLAQLKQQHGID
FUN_005954 CDS scaffold_2 292332 293694 +

hypothetical protein PF04434 ENOG41

MSPETRSRTRAQSLGRKTEQSHHDESSSDSDTSSDSSGYVDYSSEFDDDDEPSAIRSPTKLTYLVGHLPEETQSIVRDTFKEP
PKIALEKCRRIDDTYAFQMTELVTRSVRIRAPQDGTSNLSCSCRDDGQPCQHLLWLLDQIVKQTLYETDLNKPLRMNPVGYAE
EMGEVFQNISNFRLDVLAEGLHCHLVTPDSEYNNEPDAFRAQEAREMLSSVYKMDPGDFRPDIFEKVSHRRKIIKRNDLEQTI
SRMLLDNHHFFHYFRSLSRPQDPIKDVFYKLTQRINRVVRDLDTPGQTSDPDPPRDVIWAASHILGCVRRMRHDIYSRESPIS
AREALSAARSLVHILDLVVSRNTDHGPIDAPRIDRNLYLRLIGDRDQDFILNVLNLIPEAASQFLHNLEEVLGKIEIYGAPAG
YVQRFRAMLGRLRTSSTGSGLKRSVQGQTGGRKTRRTR
FUN_005955 CDS scaffold_2 293947 295675 -
hypothetical protein ENOG41

MELSQESIHDVIHPTAAFSGPPSTTQLGSAVQDSHVADWQNSSLNPKNRIDSLSPLDRPLWRIDGCTAFGSQFYAVPLFIDSM
SPIRMDVFIPEPSKLSPELRLALDVDVAFHTTSAMRISRLGITQHVLRILQHWTARQQEPLQIFSNIPFGSRIVLRNMPMKVA
DAEVTIAPTHYLERQLLSVASLENAWGSDVELPPTVDLGDVVYVSQLHDSVCLVRIGGKIWIFKALTSYTKYLYHELRQLLII
PPHPNIVSRPMHLVTKKCSFGSKVAVVGFTLEYHIHGSLRDLIPFLKLHNMVSLADETKWAIQLASALVHLRTTSSIFYPDLR
LDNIVLSASRDAVMVDFEQRGVWCEFAAPEVNALEYVRLLAIDEEIPTEVSEKYSKLLTEMLPHWEAMGESEEYKWPCKGYNV
PWACLTPKEQEACEVYMLGRVLWCIFEGNSAPQRAAVWLSYRWEPLVEFPGYTKTPGAMQKLIDRCTRGRQNGLSRLIVRERN
QLVLRELEKTGLSTPDGVQRTAKEWWSREIDASERWLRRRIEGMKRGDWKENYYDRPSLKDVLADLEAFRDERGFKF
FUN_005956 CDS scaffold_2 301394 303707 +
hypothetical protein ENOG41

MPSSTSPLARRMADLTLFFCPCCGWDPRHQDGYQQLPSSAPAVSGPDAPYMAEKQPSMTMPSSEPYDNTTLSSGQQPDYSFQQ
TNLNVAPLVNFPLISDQTALAEPLMSGPGPTHQPNLSARDFYVAQAFKEEPQLENQVMMPSQQLVTKALPQVGYSSDPNLIDT
NTITHQSLAPPDSVSQYGMLINTQLGEDTVQNGWSLMDQQANFEIVLPNQRGGKRGPFKDPNLREQTAQTRKIGSCIRCRMQR
IRCENNPEEEGGACLTCKKVSNSRAGRFPCLRYKITDIRLFKPGQVPGYEWTRRWTNNISDPIQSWATTEEPRTIYLSEGLSN
KFVKVKVQRFIPQAGDKLERTWDYKGVKKSVKIPPYAMVNLEEAKKEYLDHIENSMQDAFTKLLGPPEGLLFRTYLRAWKIFK
DPSTTPECVELIHHTLLLWMSIRLTTRSSFIVGEETLGMKQNILDETNPNHGKIPLPPVLGAQMDLILIHHIQTKLRRELLDK
LQKMMSKNKQSTWLVTYLVIFILLHNTALITAHDAGYAKKHGMKRRFAREEKVKEYHLGANILLAHFHYCNKGIYPFSEDCKD
QDLRTLAGLDEEKIKFVHHTSNLARRYALQWEEIRNKAVYEHDYFFVSQLFETNWQPRTTI
FUN_005957 CDS scaffold_2 306853 308288 +
hypothetical protein PF02668 ENOG41

MVSSLAGPIIGPSPAVELAPAAAGCVSRVADGPAGVPEGWPKFLDEPMAWVGPQFTDESEYIHTLSESDLQEAENALLVFKAL
GLDGDCVSRDNFPLPTLGPRLDRIRRDVHQGKGFGVIRGLDPQKYSVEDLTVLYLGIQSYIANRHGRQDRKGNMLVHIIADNS
SKLKAGHHRHSTSPITFHNEEAGDIVSWLTRSTAVSGGKCIIASAYTVVYNVLATSRPDMVRTLARSDWPFALPRFQCRPVLFH
HEGRVLINFGRVALTGNAIHPRPTNLPAVTPRQMEALDSIENIAKATQLEISTHAGDMHFINNLTILHRREGFVNGEAARERR
HLVRMRLRDDELGWKLPADLRQEWSAAFNKEAPKIWHIEPMPDGFFPLRSQAN
FUN_005958 CDS scaffold_2 310830 312866 +
hypothetical protein PF00328

MAPASGILKSKAAPGASGYQPVHQDEGDGHDDGPREPPAQPIIPRGPIRSYRNTIMLLSGLVVILMAINLYLSLPLAFSGSGS
GSCPGDPPRVPQYFQTSPQLWPGVTETGKPAFMAQTHVFEPTATFVPNDPLQTSIPIEGMKEGNRSIFQMMGYLSPYSPSTGF
GVDEYPIPPGAEIVQVQMLSRHGARYPTPGSNVATLGERLANASAGLKTSGALEFLMDWKYELGKAILVPRGRQELFESGVLH
SYMYGSLYNPQSKLIVRTTTQDRMLKSAENWMAGFFGLEWTNNATIEVIIEAPGFNNSLAGGLNCPNSDKADYVTPVAAWYKK
YLKDATARFNNVTKGFTWTPADVWAAQNMCPYETVAYGFSRFCDLFTYEEWEHFGYSIDLGFSSGAGFQSPVGRATGLGYQQE
VMARLKNHTLGYSGSQINTTLDSMKETFPLNQSLYFDFSHDTNIISILTAFGLRQFSEELPTDKYPGEHEFVVSKLTPFGARL
DIEIIKAPSPVSPKRDGYLDGKETKYIHFVLNQRTVPLGRSFPECDVNRKDGWCDLDTFIEVQDKMAEKAQFDYSCFGDYSSL
PYGKVTDGVPPS
FUN_005959 CDS scaffold_2 313399 315067 -
hypothetical protein PF00909

MSYVIPGPPTAFNGTNEELGGDSTTENLNQWYQSGDQAFIILSACLVLLMVPGIAFLYSGLARRKSALSMLWVVMMSFSVIVF
QWYFWGFSLAFSTTANNPFIGDLKHFGLMKVWGAPSVGSPLVPALLYSFYQMMFCAVTGALTVGAVAERGRVVPSMVFIFFWA
TLVYCPLAYWVWNPNGWGFVNGVLDYAGGVPVEIGSGMSAFAYSWVLGRRNEKMMMNFRPHNISLITLGTMFLWFGWLGFNGG
SSFGANLRAVMACWNSCLTAMFAAMTWCILDFRLARKWSMVGWCSGTISGLVAATPASGFLDPWASIILGVVAGVACNFATKI
KFLVRIDDSLDVFAEHGVGGIVGLIFNAFFARGSIIGLDGVNTGATGGFMDGNYVIIGWQIAAIVAASAYAFVMSAIIAKIID
FIPGLKLRASEEAELLGMDDDQHGEFSYDYVEVRRDFLAWSPHRGEPAEDGDVLEPTHGIQEHQSMMNPSESDDVRKTPSLNT
PDVVAPDAVITEKHA
FUN_005960 CDS scaffold_2 319148 321552 -
hypothetical protein ENOG41

MESQQPYKRNFQPISVGKCYAQTATDRFGDGAKAAVRDRERVTLGMHPQSAELPFTWPGNDSDDERNAGPSIYRLIPSMQKFR
SNLTALSQVYNLYFSAYQGRIFVYRPRAAPSQALPSQPDLQLKPKASTAAQLVGGQLDESRPHTVNHMITGFLGDEEIVLACY
DDGDVVAYYTKEIEKQAKECRATSDRSHKSVPKQFFHENVGISAWGLAVHRKSRLIAVSSNRHEVVVFALALTQDPLSRRAGL
SRTTRLDIPTRTRNWRIVIRMAPSADNIPNVCFVDDCQGHAQRICAIDIKGTAWLADIWQGNRGLIVIPPLCSNLLKSEEFHP
APSRGWGIFALDERDFLKVKTTEELFGVPTNRLEVIPAGSGCQQPLVNVRNAVANIPDNPHNMLRPLSQPGPMIPLAWEPDMP
QLGFVVQAEDLLGSDVGEEEFEDTEDETDEEHDEGGDSEDGSEVDSEEDAIVAGTDWSSFYFQAHELAQGLSTYKSPGSSPDN
LYAWDATFKQQDSEGQGENKEDLGHAMNGNATAAHKLDMTYFPHSNTVYRTPRKAEMMMAYWRRPPEYNRHPQLPEAYLADLG
KRLFLLRTYEKDIELHSFSPIPNRALPTEFGVICSDALKFGRFRDPGLRRHFHATSRLNMIALAPELSLMAIGSPTGRVVLLT
LTRKAVPAERDDGTWEHGFRVEWVLPTGSDETEHRKTLRPMHGMAMGPVQVGDEIGGKVGGVGPAMPRRYRLMLHYRNHDILS
YELTREEQSGKLCIF
FUN_005961 CDS scaffold_2 322616 324164 -
hypothetical protein PF00150
GH5_24;GH5_51;GH5_11;GH5_1;GH5;GH5_20
MWSGLRVPHFLGGTRPRSPTPTPTVDDPQQRNHLLGDWTDIEQNRIMKEGNERQNEDEELDQDLGPTKKTRRRVRFADFEKPT
TQRRSWIRTRTRRCSCLRSPSSAILFAALLLCLGVGYVYRWKVETALHRSWIPDAASERFSQNPLPPPPEHLQITNYTLPLRT
RGRDIVDVNGRRFKLASVNWYGASDEFFVAGGLDIQHRDDIAKTIKKLGFNSVRLPYADELVIKNPVVEEKHLRANHDLVGLR
ALDIFHIIVQTLTKSGIAVIVNNHITSATWCCGADPCDAGWSNDHLGPICRVRQTEDEWIQHWETIMLPHINNPLVIGVDLRN
EIRGLWGTMPWSKWAPAAERCGNRLLQMNRDWLVIVEGTESSNDLSKVCKRPILLDVTHRLVYSAHVYAWSGWGSWQGRFLQR
DYDSFAKTMHHNWGYIVDKQIAPVWVGEIGAPVQPSVGDANYWQHLVRFLQEKDSDFGYWALNARKPKGNATERYGLLQDDWK
TPVLDYRMKDMLELMSA
FUN_005962 CDS scaffold_2 325568 327210 -
hypothetical protein PF00002 ENOG41

MANNTSGVNLCPPPFWDAALFPSEGGFIDGRLCQTVATIQCCLPCPMTDWVYPDRFDTLTAIANWVSLFSQICCIFLLLSWAF
LPVEKTNRHYLSICLTVAILLMNMGFVIPLAAQPEQCYNTITPHSMQTSKVCGASGTFLLLGGWAGIMWVFLRSVSLHLQICW
QVLVGKNFMIFAQAAGWGVPILGIILALVFSGVSFRFGATCHINHENSLADFWIPLLLFAGLTVIITFATFGYCIKVYLASLS
DNGASSEGSSLPTYTQSIKTMNPRQVYRRVRRVIALQWRGIAIVLIIIADVIFFSVVFVFQDNVVQSVADDPTKAGAFVKCLF
QEKGKKEPCLDKASSLVINEATVTAVLLLLALNGLWLILFLGRWSMVQGWIDLIKKPFTRNKKEFVSVDARVDVKNHPRSYEM
LSRDSSAAVTPITPVKSPESGRRTPDYFGSTARYHAPVRSFSSPRPPPTGRETGDSYPMPTYSKEMNPLAMNRT
FUN_005963 CDS scaffold_2 335098 336091 +
hypothetical protein PF02152 ENOG41

MGKETPLVSSRRLSNAEGDPVAVVRVRNLQTTIQGPKDAWGRGSKQQPLLISAEVSLTHAFPSSSSDDKVASDTVHYGLLSKS
ILSTLEKLPRSALSLSDVLNRLWVDLTGFQYTGVKEPSAQNRKAFLDMSLIRRLSVSLVLPKASLMGSAIRLSGSCLFADSAI
TARSLELGLEGIRVPTLIGVNSNERNAKQVVITNIRIDEYQTAHDDYAVIESTIVDAMSESSFETLEALAASLAFHIARELRT
KRDDFYGQIIRIGLEKPTAVPLAEAACVELTVRTEDVKIEVSKTKDVRFDDTRNQIAHSNDRKS
FUN_005964 CDS scaffold_2 336347 337075 -
hypothetical protein PF12239 ENOG41

MGSTETVPFWNMNIPKDQRTEECPDFLQGLHKKDQGILSTPDQEYHIFSWAEVRDIIQTNRLEKFKRVPSELRRYKAFTFYLK
QKYGSIANFIHEHRLGWSTPLTPRGAPFEFEDDYKFLWNDAPYGVDPRIAHLVVWTKFDLVEDPATGDLTDKARKEIDDFVTK
TFRAHIPEENVLWFRNWHSLQSVNTVQHFHVMLFNPDPDFVRKITKGDVPRAGMEVYK
FUN_005965 CDS scaffold_2 339813 341904 + FYU1 putative UTP--glucose-1-phosphate uridylyltransferase PF01704

MASAIKSALPTHLKPNSGDEQGNERRHGKTRSHMAFENTSTNVAAAQMRNALTNLAETVKDPEQKKLFETEMDNFFALFRRYL
NDKAKGNAVDWDRIAPPAQGQVVDYEDLANTESVQFLNKLAVLKLNGGLGTSMGCVGPKSVIEVRDGMSFLDLSVRQIEYLNR
TYDVNVPFILMNSFNTNDDTAAIIKKYEGHNVDILTFNQSRYPRVYKDSLLPVPKHNDSPINEWYPPGHGDVFESLYNSGILD
KLLERGIEIIFLSNVDNLGAVVDLRILQHMMETNAEYIMELTNKTKADVKGGTIIDYEGSVRLLEIAQVPKEHVNEFKSIKKF
KYFNTNNIWLNLKAIKRVVENDELEMEIIPNGKTIPGDKKGESDISIIQLETAVGAAIRHFNNAHGVNVPRRRFLPVKTCSDL
MLVKSDLYTLKHGQLQMSAARFGDAPLIKLGGDFKKVSDFQKRIPSIPKVLELDHLTITGAVNLGRGVTLKGTVIIVATEGST
IDIPPGSILENVVVQGSLRLLEH
FUN_005966 CDS scaffold_2 344568 344751 - hypothetical protein

MSAAAVVPPSSAVWMLAAIGNLVSLAWSPYRIGKYFNDFAPRLVNSPNDELQLKTFPAST
FUN_005967 CDS scaffold_2 346673 347361 + hypothetical protein PF10342 ENOG41

MKYTLATIAALASVALAKPAFLNTEFDLTEGKPYTIRYSGCDDGCTIVLQNGPSDDLSDYKTLTTSATGDSFTFTPSELPSDT
YNFKITDKAGEVNYSAQFPYKGSYAAPSVTKSATATAETTAAVSTKEATTLASVTKSAEETTTVAKPIIPTHAPSKNATTPTA
AHPTPSKTGSAGGETSVPSVPESGAARMTSSLALIAGAVMAMVYLN
FUN_005968 CDS scaffold_2 348929 349502 - hypothetical protein ENOG41

MASMTLSDKHESNSDSSISLSSSILLEDRSSSIFDFYGQVMDMDDYQHPSPPTIDMSTSMVLERTTAIRSSPSEADDFSMSGW
TIVGTSSAASETTCHAPASVSASVADLTASELLRLELQDTRPSGSLASPGSHVVRDRASEMSVGSDSLFSYSYTSVQDDQGLC
GIGAWADLMTSSFVAKKRRITPRQ
FUN_005969 CDS scaffold_2 351284 353085 - hypothetical protein PF01734;PF11815

MSLQKLRSWYARKSPVELWLDLLRTAEAFEDWEEAALHLDNLLGLDLWRNNPASKYYDWRLIAERLDSLATAREDGNFQQLVN
LLRSGLVRNLGNITSPKLYNRSFAGTKYLIEEYITQIAEAVEDIRALPTTPSAIHGTGPSLTTQMKLDCIHDTRQAFGRSTLV
LQGGAIFGMCHLGVVKALFLRGLLPRIITGTATGALIAALVAIHTEDELPAVLSGDGIDLSAFASKNGHENGETSTAQTFRSR
WETLLRRIRRFSKEGYFLDVTVLEECVRANVGDLTFEEAYNRSKRVLNITVATEGQGGVPTLLNYLTAPNVLIWTAAVASNAS
SPSLYGHRKTTMLCKDAHGSIVPWAPANTIDFRHWTHTSYSDRDSPLRRIAELFNVNHFIVSQARPYLIPFIQSDMHGPSLVE
SRSKTTQISAFLVRMVGLEIRHRLSQLDTLNLLPTGIRRFLVDEQVPAASMVLVPEVTAGDFVRLLETPTRETLNYWVLRGER
SVWPAVAALRIRCAVENELDRSYQVVRKFKAPGLRRKGSMAATLEAERRERNGQFGSAVTDISAT
FUN_005970 CDS scaffold_2 353876 355156 - hypothetical protein PF04004

MSDIEDPIESVDEGGDDLFGDDGDDDVVPTKEPVNEDDELASDPEGDSYARYRNDDDDQAQVQTKERAIQNVETYRHRIPKPK
DGALRILRIPKFMKIMPEVYDPETFEPTEFDLANAKAEHPKHVARVRKDHSTGEFKSNTNIFHWSDGSVTISVGGEHYEIQRK
ALAPPPDKPYNEVNDGHYYAAAAELHSNILMTVGHITEQYNVRPNKAVGDDALTLLAERMAVASKPANATDMIIQTTVDPELQ
KKQAELAEKERMKAQRRRENAAAKMDGGYGRSRGGLSIGDLEGGRRGAGGSRKRGAPGSGKPRRRRDEESDEELEHAVGRHEG
YSLDDGFIVNSDDEEVESGGDDDDEDDFPDDDEEEKPRSKRQRTRDRSEDEDDLSEVEQVAGRSRRRNVIDDDDD
FUN_005971 CDS scaffold_2 355956 357917 + hypothetical protein PF03637 ENOG41

MSNLFSGINARFRGGSAKPSSGPQKSPTGSTASQPPPPELPTQGSQSSSASLAPKVPPLPNSPSLAQTIGMDDSSGVMSGDEL
ISSYHLPRPLPLWLNAQYAKHIVKGNFMTLSARPKTVEQGEWIAHQVVEHYRNLWNFVRVLHEKEDDGTSICNSTSCPRMSAG
ANHSFTWLNRNREPVELPAYEYMTLMQRWISGKIDDTNIFPTDPSGVSYAHNPSITTTPLSQLSNPGEPEYIGKRSGFPDKFV
DICQMIFRQMFRVYAHLYWAHFTEPFYHLNLEKQLNSCFSHFVLTATALDMLKPAELEPMQPLIDLWAANGTFPPESKAYEYA
NIRAGERLLQLSNVPQ
FUN_005972 CDS scaffold_2 358375 359177 -
hypothetical protein PF11578 ENOG41

MKLTNFPTLIPAFTAQIAINDPLVITSNLLNIPFLPKAGTLISEPGYEPPLEATFIHGSDFIRRDPDGQWVKLEVTSVARDTS
GSLLRFSYNGIVNMAGDEGKVIRGDTNATTTGFGNAFVQIRFETDAPGLKLLQDKLYVGSGRFVIEEDRPVIVEYKISEVSAQ
CNKGSKND
FUN_005973 CDS scaffold_2 367442 369349 +
hypothetical protein PF00170;PF03131 ENOG41

MTSTFQMQQPFYRPASLTVDTQHAQKYFEDEDNSVLDDNILEHNTIDSGLELSPPMADSRRESFAVGPPLFSPKQEDWQSVEM
QSVSSNNPFFDQHSNNPFMRPDHTQNNGFAATANWFGNTSGTCTPLNPFDGVPAEYDANASLFHRPMAGPTPFTNPGNMFPSL
PVGSQSIPTSPQKEWMTPQQPINKNKMRPSSPGLRSHNEMRRGDGIRKKNARFDIPAERNLSNIDHLISQSTDEQEIKELKQQ
KRLLRNRQAALDSRQRKKQHTERLEDEKKQYTALMTDMEEELKMLHARVEQLALEKQECIKFIETQRLDKEEMIRVHTNETGE
LRKKISVLTDHVQRLDSAVPATGDNGFSNGFDAMDAMDGLGMPGAWDASNFLHDYPEPEVKQEMAIVPAKKNDNPFAESEKPA
AQGGLLFMLFLVGAFVLSSRSTPAIPRVSEDVRAASATILDNVLKDAGVNSQSASLQAAPQPSGAANWGNTISMNDMAMDGVA
PSMLGELGDQLTQPTQEQTNEQIFSLSAAQYNGVNSQDFLQNAPERSSPSQGRRNLAEALATMRIPNKQNDAAEVYTRSLLWD
QIPNDVVRNFAKMVSECNSAQNEQQCNELRT
FUN_005974 CDS scaffold_2 371658 373494 -
hypothetical protein PF08238 ENOG41

MGIRDILKKKDDLSTGQPPEKGTIDHLAAPEFTIIRSDTSTQERIYPPSMKSVDNLHLAVQEPKEKDKPRRSLDVFRSGRSRS
ASESSQTKKESGARRLSQRLHLSRAPPSSENVPENLPDIVTSDEAQWEKRATMLAGQNNRARSNSRPGTPSDSSRGEERAVSS
QQIDQDIQEAIRLHEEGKLELSTRMFGRLADPQGANNPLSQVLYGLALRHGWGCAPDPEGAVKYLSAAASNSASIEQMALQAG
MKKGGAAKGELVLAIFELANCFRHGWGIEKDAYAAKQYYETAANLGDTDAMNEIAWCYVEGFGCKKDKFAAARYYRLAEKAGN
KTLGNSWIWKEKYDPPGDGNRK
FUN_005975 CDS scaffold_2 374606 376150 -
hypothetical protein ENOG41

MSEEERELLARISQVAGQINRHKNQQAGSRGSPSHHPAHHRQNSYRHASSPYPARHNRIGRHPTAHHHRTLHLNGDNSTASRS
ASSGAETPPGWVSRTDRHRQLINANVYEKETQNRAKAIEQTRQRKISGRRQREKAQFNEFLKHQATASSAQTNPADRNVLTIE
GAQFRVMDGGKKLVKIPGACKLRCDSDACALPAADALNSSSRTPKFATVAGVKFYRTKTGNLVANRFVNDQRYEVLFSETKLL
TGLRRTGAVKKINQPCKIFSTTGNFSSPGWSATHLLYLDRPSGHGAKLTSTLHIGSCTKGPRCRYIHDPNKVALCKDILKDGQ
CVNGESCDLSHDMTPERTPNCLHYAKGHCAKADCPYTHSKASPAAPVCRNFGFNGYCEMGAECTDRHVFECPDFSNTGRCKVK
GCKLPHRERASVLRNKTNAAGEPLGDISSDDEAADSDDVDSDEVAEFIDADSDLSDFEEQKDFLSI
FUN_005976 CDS scaffold_2 376574 377636 +
hypothetical protein PF05615

MASTWDLLDPEEEAELHKTRLLNVEEKPFKRLTKRINTISQLLLPFDPAINGANTSEDIRSKLKQLKEDMNLDFDAFGSSITR
MQFLHNANSQERERYRVDQDRILADCQSVRQNNIQLREQLEAAQATLAQRKQFDELAEKITSNRLLRPREDQIANLAKLEEEC
RELERESETYSETWRERREQFNRIMEEGMLLRRQIRDEKEEVDRREGMNEDAEDDAEVDKVGQTPRNEPSGNATPHPDGDSQM
KADGDAGTPNPEALNGGTTPMRDTPAPDTLKPPSGHTESQSRGDSQAPSRDVSPMPPHDERKAESGEDVDMEEPEFSRAHDDG
GMTPLVTVEPPESNDKMEVDN
FUN_005977 CDS scaffold_2 377785 378202 -
hypothetical protein PF02970

MPPPSQLAIATGSVNRLLKEEASYHKEVEEEEAKVKALKEKIDSGANDDENASFMLKQQQTALEQTKAVFEPLRQKIAVAVEK
LAEQLTVSEELNAPEDQVVQAKEALVKAKATQADA
FUN_005978 CDS scaffold_2 378791 380696 -
hypothetical protein EOG0926115V PF04824;PF04825

MFYSETLLNKSGPLARVWLSANLERKLSKNHILQSNVTDSVEAIITPNQAPMALRLSSQLLLGVVRIYQRKTRYLLDDCNEAM
MKIKMAFRSSGNNDMAVNLQIPNREALTLPDRITPYDNFELPPPPDANWLLSQVEDVTAAPIGRKGRASQRDINLQEDYDNSQ
FLNDGMGMEDDMIAPLGNIDLELDFGLDDLGLEGTGLEPEGGRREDRTTAGPQIDDSELDFPQKDDDRAMTLDLPDERVQIHD
GEDPDLMAIDFNPDDVSQVPDIAAYPPPRISESPLSDIDEDRAAQLEQDHSRYQMADLYEPDHEEDQIVRRPAQRAKKQKILT
PDEEIALSSNHIKQQQANRDNIIKEGGFLPRDPFLLALMDMQKSGGFVSSIMGEGRSSAWAPELRNMLTLDTARGMNELKRKR
DSGIADVESDHGASKSPRMLDVGSDTGLGFEGAFGEMSIPPEESRHEIPADDEMEDEEAGTMMPGFEDTTVALVHPAESGPVS
QVTKHVVHQLRERFGDDAADSPSKRTQRAVLLQNLCPRQQTTKADATKMFFECLVLATKDAIKVEQGPGLGDDIRIRAKRGLW
GAWAEREAGGEMSQDNDAPNEPERAAVASTAVAVSA
FUN_005979 CDS scaffold_2 382259 385646 +
hypothetical protein PF00122;PF00689;PF00690;PF00702;PF13246

MQLPWGGRDGPGNDTLLPTSNIPSITGHAADGIDSKDSDRPQRPSRHVRTTSSDARNVADHFSYMTPSEAAANLRTSLTLGLT
PTEALTRLGEYGPNEIPHDPPEPLWLRFVKQFQEPLILLLLVSAGTSLLLGNMDDAISITVAVTIVVSVGFIQEYRSEKSIEA
LNHLVPNHAHLVRGQSKISPLGKTTTWPPRTDDADSALTPGSVTPVSDILDATSSKVMASQLVPGDLVLFTTGDRIPADIRVT
KAADLTIDASNLTGETEPVRVTAEARSRGFGSYGLDKSQLPRPNSLAPSEHGDSHGDGIHNIAYMGTLVKSGHGQGIVFATGG
HTHFGTIAVSVSGTESPRSPLQLSMDDLGSQLSKASFVVIGLISVVGWLQGKKLLEIFTISISLAVAAIPEGLPIIVTVTLAL
GVHRMARHNAIVRRMPKVETLGSVNVVCTDKTGTLTTNHMTTTRMWYFGAQEPVPVESDHDDAETNPDLNQATLRVLRIGNIA
NDARLAQKYTEHGQAATAVLSSTLGRGQVSTYTRWVGQPTDVAMLDLLDRFKEHDVRESIGPRVSETPFSSERKWMGVTIGSE
TKGDKEFAYMKGSIEKVLAACDTYLEKDGREIVLDSARRQEALQAAEAMAVQGLRVLAFASGSVTRPLRSKSAARSTPGFDRS
ESPFSHSPEDIYKNLTFAGLVGMRDPPRPGVGRSIRRLMRGGVKVIMITGDAETTALAIGKQLGMNIAVPSGHAGGQNTVRPV
LRGDEVDRMSEEDLAQAMQHTTIFARTNPDHKLKIIRALQSRGDIVAMTGDGVNDAPALKKADIGISMGRHGTDVAKEAADMI
LTDDDFSTILRAIEEGKGIFNNIQNFLTFQLSTSAASLALVFICTCFGFKSPLNAMQILWINIIMDGPPAQSLGVERVDPDVM
TKPPRRRGDPVLTKSLVTRVLTSAAIITIGTMLIYRREMMADAQVTRRDTTMTFTCFVFFDMFNALSCRSESKSVLQGEVGLF
SNNLFNWAVSLSIAGQLLVIYFPWLQETFQTEALGFFDLVRLVLLCSTVFWVDELRKYLKYKKRRFGNGYSQAV
FUN_005980 CDS scaffold_2 386100 388559 +
hypothetical protein PF08318

MLSLQNGISHSEHPVVETPAAVSTSSLDNANTESEIREALASLHAHEVSITSRLDALVASQADLSRDLGRLDLLRAGLGAQVI
AARSIGNDMLATAADTAGRLSDRVKELDLEKSRVEETLGVVEQVAELKACVNGVVGSMGAPQDWEAAAGYISRASKIPEEITK
GSFAVGIVPSVEVPDPPWVTLEEAKESLCGLFLREFEKAAEESDGTKVTRFFKLFPLIGRAEVGLDVYGRYVCQGVAGTARAT
LKDALNGQRREGFFYANALTKLFEHIAQIVEGHGGLVERHYGSGKMVRVIERLQMEADVQGGIIVDTWSDERGIDRKLTDVRS
YPFSFLVQSFLPQPPRGGTPRVNSPAIGVGNNPRESEDEGVNMREVDGLLSEIAVMLGRWSLYTRFLAGKCKNPDTPDEAPLV
IPDVLVKSNLYRKVSTKLTSPYNVMTTFFFRRSVEKAFQLDEYPTGLSLSLSRQIEGNAPYIILAVDDVMYIVNAVIQKSIST
SQRDVIASVVPTVGRVLGSDFVGMIQRKMRDESYPRPVVQGGFPPEDKIIQFIVLINSLDMANEYLTRIITGRIGESSHLPNG
DAQNGPLKDSFPPFERDVIFVANALHTLQTSFIGKTTELLNEGIQVLFNQVVKLRLRPVLTDTFRDADYTLSEDDIADIAQQND
EDEDELLEQVPRLFEHGWDQLMKPIARLMTPGTYTTLLDTTARYLSKIWEKKIMGYAGRTNALGAIRLERDFIALVDIVSRGD
YAVREVFAKVLQILMVANMEDDEWDEVMAQDGEDDAIEWVLTEEERRRARSLVRG
FUN_005981 CDS scaffold_2 389339 390635 +
hypothetical protein EOG09262FW1 PF01300;PF03481

METRIVKVPSKGSLGQFRPGDGLAKLNHWEVQCENKLALEALQDAAHLLRTKDTPVAFPTETVVYGLGADATRTPSVKGIYSAK
GRPSDNPLISHVCDLEMLRQYIGHTDETDPIPQRYRALIERFWPGPLTILLPNPVPSKLAPEVTAGLKTFGVRMPSSTLALSL
IKLAGVPLAAPSANASTKPSPTAAQHVMDDLNGKIELILDGGHCQVGVESTVVDGLCDPPVVLRPGGIGIDELRSCPGWENVS
IAYQDKSEEGKAAPRAPGMKYKHYSPKATVVLYESSFGEGGNGIASLDLKAANGTVNGHASNGERKGRVVGVIRTQRWKSGAG
LRCASFQHLTDVQGPDEDDSPFQVYEGDLLDEKEQPIGKILDIDLGRGTEGIARGLFAALREFDRRGADTIFVDGIEDRSDIA

AAVMNRLRKAASETRA
FUN_005982 CDS scaffold_2 390828 391561 -
hypothetical protein EOG09264OBA PF01217

MAPGMSLFSVQAILILSTEDGSRIFAKYFQPPHSAPNAPTSTSANPYSDAKSQKAFEKGLIDKTAKQNGDIILYDNRIVLYKM
ESDVMMYVVGSVDENEILLYNTILALRDSLHILFKQSVDKRTIVENYDLVSLAIDEIVDDGIILETDPTIITQRVSRAPTQDM
PVGRIDLSEQGVNNLAQLGKSKLADWLRQGL
FUN_005983 CDS scaffold_2 391928 395076 +
hypothetical protein PF00493;PF07728;PF14551;PF17207;PF17855

MSSPTKRSTRSSATPRRAAGNSETPRQGNSQLDAPSSPLHYQSSSPGPSNQNNLPGDVSSPFGHMTNSQSDVPSPPLRQMTDT
QSDLDPERTPRANPTTPAYHGGSSPIHYEPSSSPGRSLRQQSELRSESSGLFLGSQLGTPAPHRRGDINSDATRTPRASRRVI
LDDAGRVVREGPADGSDAASFANRDPNTSEADVLGGQGQSLIWGTTVSIDDTFASFKEFLRNFTQKYRMYRDGASDEDVQNAP
DAESKPYWEALENMLLLGTTRLYLDISDLNLYPPTRKLWHQIQAYPQEIVPVMDQSVHDMMVEIARAETLRNRQSQSSAGHQA
SQQSTQSSEPVFPSSDRPEEAPTPRTQPDQQQQASLEDQVASSIYVVRPFGLDKTTNLRDLNPSDMDRLISIKGLVIRTTPVI
PDMKDAFFRCNVCNHSVNVGLDRGKIREPTECPREICKSKNSMLIVHNRCSFEDKQVIKLQETPDAVPAGQTPHSVSVCVYNE
LVDFCKAGDRVELTGIFRVSPVRVNPRQRALKSVHKTYVDVLHIQKVDKKRMGADPSTLGVAGEEEAEAGENGIEETRKITIE
DEEKIRETAARDDIYELLARSLAPSIYEMDDVKKGILLQLFGGTNKTFQKGGSPKYRGDINVLLCGDPSTAKSQMLSYVHKIA
PRGVYTSGKGSSAVGLTAYVTRDPETRQLVLESGALVLSDGGVCCIDEFDKMSDATRSVLHEVMEQQTVSVAKAGIITTLNAR
TSILASANPIGSRYNPDLPVPQNIDLPPTLLSRFDLVYLILDNADEKNDRRLAKHLLSLYLEDKPQSAPNKNDILPVEFLTLY
ISYARSKIQPTISQEAAQELVDCYVAMRSLGQDVRAADKRITATTRQLESMIRLSEAHAKMRLSETVTRDDVREANRLIQSAL
KTAATDAQGRIDMSLLTEGTSAADRKRRDELRTALLRLLDDMTAGGNTVRWTDVARRLSEGASIPVEQSEFNDVMRALEAESA
IMITGEGARRTIRRVTSVA
FUN_005984 CDS scaffold_2 395283 397054 -
hypothetical protein PF01053

MVVLKTQSEVGHAPPPQAPYSVITNLPGWDVARAIREGDPAPMKKLVHIYPRFMPTHYSAQLGHEIAKRLGHTDKAALVYLNP
AVWPYTLHHVTHENRGDKRLTVQDATLKCVDIAGHRVYLILVSPTAMPAVVLTWQNPGLGISIRGAEDLLKSIDTIKEVPFEA
EKGPIPSPTWTPESEAHGDLRTRIVELLHRAPVDADKVKCTPSDVFLYPTGMAAIFHSSVLLTKHRPGTIVILGIIFHNTYHH
LIEECPQGMKHFGKVDKEGLSVFEDWLKHEKEEGRPVSHAFVEVPGNPTLETVDTKRLKELSEEYGFFLIIDDTISGFANVDV
LSNSDVLLSSLTKSFSGYANVMGGSIVLNPTSPHYHNLRSLFKESHHNELFASDAKVLLSNSKDFLERTKILNRNALAMANFL
HKAISIPDSPVVNVQYPSQLGSKSNYDAILRRATPELPEPGYGCLLTVEFSTVEAAIAFYDRAGFYPSPHLGGHVTIMFAYNM
VVFGKKPDEKAYMHELGVREASVRISAGLENEEDLIDTLRDALEVATKVKKGLEAGQ
FUN_005985 CDS scaffold_2 397932 400461 -
hypothetical protein EOG09260KCW PF02656;PF09359 ENOG41

MLIVQDLKTRSRTWLSTRDGKNLSVRPGVVVMKFGEQLRSSVIQEYQWYYIDYDGLKNELKGPTGPLKAGKGPEWTEDDETRF
VERLESELDKVHTKQKVKAMEISRRIAVSEREVKDVVNRLNERGLGENGPSEEEFMLLEEDLSDIIADVHDLAKFVQLNYTGF
YKIIKKHDKTTGWHLKPVFDSRLKAKPFYKENYDAAVIKLSKLYDLVRTRGNPVKGDSAAGGGQANFIRQTTKYWVHPDNVTE
LKLIILKHLPVLVFNANKDFDPEDSAITSIYYDNPDTWDLYEGRLKKTEGAEAIRLRWYGGMKTETIFVERKTHREDWTGEKS
VKARFAMKEKNVNAYMKGELLPAAIFEKARKEGKKSEKAIAEDERLASEIQYSVIKKGYKPVCRSFYNRTAFQLPADARVRIS
LDTELTMVREDNLDGRVRSGDNWRRMDIGVDYPFSQLPAEDIERFPYAVLEVKLQTQSGQKPPEWVRQLISSHLVEAVPKFSK
FIHGTATLFPDRINLLPFWMPQMDVDIRKPVTHDFGIRRPGLSGTTNTSDDDEELDSDDEELQASGRNGTNGESSAQGARGLR
ELPPIDMEGQMTDLPAADEDYAIYNSDDEYDERYELAQAKRSGGWRYYSTLLSAKTRHLRQHFGKHVISALKLAIPAPRSTQV
ERSDRLQALFGHDPIQNKKFKAPPGKKIYVPVRVEPKVYFAAERTFLGWLEFSIYIGTIAVTLLNFGSHPTPTHFWISGLFTA
LAIASLAYSVFTYLHRSKGIRERKAIGFYDRWGPSILCIALFVGVAANFGFEGKERGIW
FUN_005986 CDS scaffold_2 401952 403289 + RMT2 Arginine
N-methyltransferase 2 EOG09262N47

MAPKIDDSMPARISSDCPVEIREVLYHAWGHDRSGLQKLLKTTGKANAQDPKTGETPLHAAIRACGPASGDDDDQEEDGSVEE

AKEIVHDLFLRGAIWNDVDSNNETPGCLALRLGRKPLYNLCVEAGVRAELLFALMGDYEALSSGSDDGDEEMEAEQDDEEAPQ
LVSAEDTAPPPEEPKFIPPDAQEKQVTSEEYLNSKLVYDDAKLVDSDLNGVMMAWETDIMRRSVAALIPDSAPGKRILNIGFG
MGIVDGMFADLKPSRHHIIEAHPSVLEHLSKDESKFGPSWENSGPEEGAFKVHKGKWQDIVPKLLEDGEVYDAIYFDTFGEDY
SQLRHFFSECIIGIMDQDGKFSFFNGLGADRKICYDVYTKVVEMQCADAGLDVEWEESDVDMSGLEKAGEGEWEGVRRRYWTL
DKYRLPICTFMG
FUN_005987 CDS scaffold_2 403475 405794 -
hypothetical protein PF00349;PF03727

MVGLGPRRPPSRKGSMADVPKDLADEIHKLEKLFTVEPAKLKEITDHFVSELAKGLSVEGGSIPMNPTWVMSYPDGYETGTYL
ALDMGGTNLRVCQITLTEEKSEFDILQSKYRMPEELKSGTSEELWEYIAECLYQFLETHHGDCSKLEKIPLGFTFSYPATQNY
IDEGILQRWTKGFDIDGVEGKNIVPMFEEALKARGVPIKLAAIINDTTGTLIASAYTDTAMKIGCIFGTGCNAAYMEDCGSIP
KIADLNLPADLPMAINCEWGAFDNEHKVLPRTAYDVTIDKESPRPGQQAFEKMIAGLYLGEIFRLILVDLHDNKAIHIFENQD
IALLRKPYSLDASFLSAIEEDPWENLTETYDLFVKKLNLKPTRPELELVRRTAELIGTRAARLSACGVAAICKKKGYESCHVG
ADGSVFNKYPYFKERGAQALREILDWPAKTDKKAEDPIEVLTAEDGSGVGAALIAALTLKRINEGNMAGILHPENFK
FUN_005988 CDS scaffold_2 407276 408882 +
hypothetical protein EOG092628A0 PF04597

MKPFAITTALFSFVSFALAGATTDSKTSKVILPVDFKPPQVFKNANLVHVISLEKTYVKEQINVLVENVAKEPQTEYYVPFTA
EQLPRIGGFEVKDRKDANAGPFVAETVEYDSLSDIQYYRIRLPTPLKPGAQQTLGITYYYLKAYTPLPAAVSQDDDQYLSFNF
SVYAPSAYITKKQKTELKAASADVPDYTKLPGSGEVKEFPVKQGTKLIYGPFDEKPAGAVSPANVRFQFTKPVIHVKELDRLI
EVSHWGGNIAFEEHYEMYHGGANLSDNFDRIKYSQHSLYRQHGVAGVRPSHYLDQLRIPLPGGSVDAYYTDVIGNVTTSTWRT
DNRDALLVLKPRYPLFGGWRYPFTIGWNSDASNFLRKTTTGSFVLRIPFIEGPKQPEGVEYEHINVNVLLPEGAENVKFHTNI
PESSIVSTSVDLTRTYLDTVGRTAVSIKARNLVDEFRDRQLIISYDAPLSSALRKPLVIFASAMVVFVTTWALGQVQVGFKPK
K
FUN_005989 CDS scaffold_2 410930 412009 +
hypothetical protein PF13805 ENOG41

MSRFSFDASTSRHEPLCLESPIDVKPFSRNRALSIRSNKSKGNSGAGAGAKRGFSFNSLRGQVQPELSRKLFRLIKSENNLIG
AHETAGRERVSIATQLSEWGEHTGDDSISDISDKVGVVLSEMGEQEDAYAHALDDSRAYLKAIRNTEKSVQPSRENKDKIADE
IQKLKLKEPGSTRLPVLEQELVRAEAENLVAEAQLTNITRQKLKEAYAAEFAATIERAEKQIILAKHGRRLLELLDDSPVVPG
DTRVPYQHSSQARQILNDAEDDLRDWRPEAEGFSTPVQSRSPTLEGKGKEPLAAGDNLSPVQSEAATVESETSPLEQRGTKSS
VYAEVAG
FUN_005990 CDS scaffold_2 413438 414628 + IPP1 Inorganic
pyrophosphatase PF00719

MASNYTVRKVAAPNTLEHRVYIEKDGQPVSPFHDIPLYANQEQTILNMVVEIPRWTNAKLEISKEELLNPIKQDIKKGKLRYV
RNCFPHKGYLWNYGAFPQTWEDPNSVHPETKAKGDNDPLDVCEIGELVGYTGQVKQVKVLGVMALLDEEETDWKVIVIDINDP
LASKLNDVEDVERHLPGLLRATNEWFRIYKIPDGKPENQFAFTGECKNKSYALDVVRECAEAWERLITGKTPAGGVSTTNVTV
QNSPSRVSPDQLPPLPPNEDVPAEKIDASIDKWFFISGASA
FUN_005991 CDS scaffold_2 414866 415218 -
hypothetical protein PF10780

MITKFMTEVSAKFNPFSACAKPARLFLTFLPPNARANGTTITSTILPRTSKEPSSLRVKFKDGKELNFDCQKINIKGLVEEVD
RHSRQLQKAADLTD
FUN_005992 CDS scaffold_2 415509 416718 +
hypothetical protein PF03908

MSFEGLQERLTALQETTTQLKELIDRLATLKFQPGSVPLTTDEESSESGELSAEITSTLRDGEEEHELLQEEVEFLRGAEHDK
DRLKESVEKIGKELESCRLSFRKARLSAKHSLAQAQRREREILLTSFSQPTSETNSLYPDDEKTARPTRHQQHVQKQQSSLTE
EDQQNVGASANVTNALRRTHDLIQAELARSEFAHETLTESSAALKQLNESYGSLDTMLASSKDLLGTLLRSQKSDTWYLQTTF

YMLACTLGWLLFRRLLYGPMWWIVWLPLRLMFGLGTSAGSAVMQAGSGQGQVKEAGQASKGVPVEGLPDDELPTVKVGTEQQA
EVLEEVDKIVNVVREADELGNIPEGDEDNIGNPKKRMWEEPDVVEQQRPRDEL
FUN_005993 CDS scaffold_2 417128 418169 - MDE1
Methylthioribulose-1-phosphate dehydratase PF00596

MSTEQRPTGDALITSEDPNHPANLIPSLCAKFWTLGWVTGTGGGCSIRDDDLVYIAPSGVQKELMKNTDIYVMALSEQDPNHN
KLNQRTYLRSPPCYKPSQCTPLFLAAFTRRGAGCCIHTHSQWAVLVTLLLESQGPGKDRVFEINNIEQIKGFGRGMNKTGNLG
YHDTLRIPVIENTPHEEDLTEYLEEAMDKYPDTYAVLVRRHGVYVWGDNVHKAKTQCESLDYLFQLAVEMKKLGLPWISEVEQ
IAPQRT
FUN_005994 CDS scaffold_2 421571 422221 +
hypothetical protein ENOG41

MRFTSIFVAGAFATMAAAQSKTASVSPAQQSQLDCLDACDAGDVKCQSYCITVPSPDEKNINATTECVAACPKGKGSASDTEK
YSVCLQDCIADNYWKTIDGTPQETGAAGSKDKASSAAESAASKATGTASDEGSTATASASESDATATDASETASGSASGTASG
SAAQSSETGNAATALAGGVSLLGLVAAVFAL
FUN_005995 CDS scaffold_2 423836 425683 +
hypothetical protein PF00262

MKLNAVAAAMSAAMLTGNVHAEDIKEASPSVPDKLPTFTPTVVKADFLEQFTDDWDQRWKPSHAKKDTSGSEEEWAYVGEWAV
EEPVQYKGIEGDKGLVVKNPAAHHAISAKFPKKIDNKDNTLVVQYEVKLQNGLECGGAYMKLLRDNKALHQEEFANTTPYVIM
FGPDKCGHTNKVHFIFNHKNPKTGEYEEKHLDSPPTAKITKTTELYTLIVHPNNTYVIKQNNEEVKTGSLLEDFTPAVNPPAE
IDDANDKKPEDWVDQARIPDPEAKKPEDWDEDAPYEIVDEEAEKPEDWLENEPVTIPDPEAEKPDDWDDEEDGDWIAPTVPNP
KCADASGCGPWTKPMKRNPDYKGKWTAPYIENPAYKGTWAPRKIKNPDYFEDKNPANFEPMGAIGFEIWTMQNDILFDNIYIG
HSIEDANKLAEETFGVKHPIEKALFDADKPKQEDKPKSPSDLKFLDDPVHYITEKVELFKAIAAQDPIEAIKFVPEVAGAFAA
IIIGAAGLIAVLFNLGKSPAVQQTAEKASDKAKQVKDKAAEATATGAEQVKGAVNKRTTRSQS
FUN_005996 CDS scaffold_2 426737 428501 + ALDH6A1
Methylmalonate-semialdehyde dehydrogenase [acylating] mitochondrial
PF00171
MRRAISRGLGASSRSTPALSRSSLLASSNITSGSSHVAARRIHATSKQLQPVTAALASTASSYPTTHAKVEAVDTPYFIDNKF
VSSSTDKYIDLHDPATNELVTRVPQMTDAEMKAAVASAEKAFKSWKNTTVISRQQIMFRFVQLIRENWDRLAASITLEQGKTF
ADAKGDVLRGLQVAEAAIAAPELLKGEVLEVSKDMETRTYREPLGVTAAICPFNFPAMIPLWCIPIATITGNTLILKPSERDP
GAAMIIAELVQKAGFPEGVVNIIHGAHRTVDFILDEPAIKAISFVGGNKAGEYIFSRGSANGKRVQANLGAKNHAVVSPDANK
NQFINSITGAAFGAAGQRCMALSTLVMVGETKEWLNEVAEQAKHLNVNGGFEEGADLGPVISPQSKERIEKLIESAEKEGATI
LLDGRGYKPSKYPNGNWVGPTIITNVTPDMTCYKEEVFGPVLVCLNAESIDDAIDLVNKNEYGNGTAIFTRSGATAETFRKNI
EAGQVGINVPIPVPLPMFSFTGNKKSIAGGGASTFYGKPGINFYTQLKTVTALWQSADAVAKKADVSMPTQQ
FUN_005997 CDS scaffold_2 429540 431687 -
hypothetical protein PF01494;PF07976 ENOG41

MVPSIVQSDDMDQMEIPFPAHRERQEGSPPPFQDPLALLRSLPEPPLECQVCVVGAGPAGLMLAANLTRFGINVEVIDDRADQ
TPVGRADGLQPKTIETFRQMRLSDTLLKQGVRVFDIAFWRSTADEPLRRLGREVHYPPIIDVLDPYILLVHQGMVEGLFLEDM
KKRGKEVRRNMAFESYSVPDNKTGPLQVNCRANVNQDKRSVLTQYLIGCDGAHSKVRKSIPDVKAVGMSQAAIWGVLDGELIT
DFPDIWSKTLVYSQEHGSILIIPRERNMTRFYIELKAGAKFDRRDLGQEFMMKRAKKIMAPFRLDWKYVEWFGRYQVGQRVAS
RFTDGHLRAFLAGDASHTHSPKSAQGMNTSMHDSWNLSWKLNLAVRGLAKPNLLESYEEERRKIALDLVNFDYEHANQIAGGD
AIALAENFRTNVRFISGIGAEYGENAINRPGIGNNHFVMGDAKPGCLLPPAKVTRYIDSNPVDIQLDIPMLGQFRIYLLMWDV
QQSAPFLQTFCHAIAGTDSFISRLSAAASASYASQPRAPAPEDVYSRPERYTVVSHLFTFGLITTMPKTEIEISDLPPLLQDS
RWTFYLDDIPDQDTRGSLCTNKWLGSLGPGEVAIVNVRPDGYVGSIGRWDSSLDESGVQAARWLDEYYDGFMKIPTQ
FUN_005998 CDS scaffold_2 434970 436181 -
hypothetical protein PF00106;PF08659;PF13561 ENOG41

MPMHQGLIPREGFCADVVLRLIRQTALNPALLLPLVLFARLTKKGQDLSILHPSASGHLKTLFYVALARWASGWWSDKTRNNW

VDDKYDWKREVVLVTGGAGGIGGRIVKLFEEMGVKVVVLDVQPMSFEVSANVHHFRCDLRSPENVEAVAEKIRAEVGHPTVVI
NVAGVVRGKTILESQPSDIRFTFDVNTFAPFWIAKTFVPDMVAKNHGMVVTLTSYASWLTIPNLVDYGASKAAALAFHEGLTA
ELTTRYNAPKVRTVIVHPGPTNTALFKGYYQNTDFLMPPLAPESVADAVVRQVLTGRSGHVVIPGTGTILAALRMQPDWYAIP
VRAKAEAYMKNFSGRQVIEDVDTAIENDGEEKEADGNATASTVLVS
FUN_005999 CDS scaffold_2 436908 437756 -
hypothetical protein ENOG41

MLSSKQQLTAVLLLAGRALGLNFTISNGQIFTPGFVVLDAPQPNTPLGGDTLHVAIDVTANGKLPLPPHDEGDDNQIFSIEMF
LYSYMTGRNLTISNGTATANNASLGEIMAQEPGSTVKHINWVWPDCLVGDGNPGGDSDRGVYNISIRQNFRLNGDDHYTIFDV
PINVTNSIPEDDDRPSCDELSNEILSPEEIDAEAANKVGVLFAPGDSTELDASGEDSKGSALDSKLAVYAGLVSLFMAI
FUN_006000 CDS scaffold_2 438910 441380 +
hypothetical protein PF00294;PF04227

MSSSLAGVKDLLKISDEITDAVATNKPIVALESTIYTHGALGNELALQHSDLVRSNGGIPAIIAIVDGVPTVGATASDIIRMI
ESGNAVKASRRDIAYLAGMGLTGQKIHGGTTISGTMLLARLAGIRVFGTGGLGGVHRGGHDSMDVSADLTELGRTRVAVISAG
CKGFLDIPRTLEFLETQGAHVSTFSDGRSGKIDFPAFWARDSGIKSPSVVHTEKEAAAIILAQEKLGIESGLLFANPIPEEFG
IPAPEMQAYIEQAVREANEKGFTGSANTPYILGRIKELTGEKAVIANKALVTSNITRATNIAVELSKLLSSGSEPVKTFNSYY
AASSASAPASPAPTELKGPEVTSDSKADILVAGSVAVDLSCDYAPKSGDASPVLHTSNPSSISQSIGGVGHNVALAAHSVSKH
ARVRLCSMVGDDVAGKTILNGLKASGLDTTYIRQLGHEYHGSNRTAQYVAVNDANKNLVMAMADMGIFTNHSFPEYWKSAVRG
TKPKWLVVDGNWSEKDIRAWLKAGQDQDCKIAFEPVSTAKSMNLFCPQKGHPKLRVFPKPTVDIASPNSYELAAMYSAARDNG
YFEAPEWFDIIDAFGMRGARDRFVKMTSAELTDAGVPVQSVQLLPYIPTIVTKLGPQGVLLTAILGKDDPRLKDPDSEEYILA
RSMSDHPSVGGLYMRLFSAAEKVQNIVSVNGVGDTFFGVLISGLAQGGKVENLIDVAQAGSCLTLKSPESVSPDLHRLESVLA
QAAAE
FUN_006001 CDS scaffold_2 441871 443617 +
hypothetical protein

MAPPQDEEMLDPSSSSSSENESQFDSEDEVPQKKPTIALYDKDSDEEELERLVLGNKAGFRAQLFKDDGIFDSSWGAEGRDLQ
LVEQDPGLEEIDDADLFMFDTGTGPGAAEVTKAAKPTDGNAPVWEDSDDDRLAISLAGAARLRKLRKTEAEDLVSGTEYSRRL
RQQYLRLNPLPSWAREADGRPGKRRRSSAASDSSADSDSDSEISAQPLEKFLRDVNSLAGAGSTKKRRLRPEVIDIQRTREIP
DKHKAPVANLSFHPEFPVLLSSSTASVLYLHHIAPAAHPTPNPQLTSVQVKQVDVRKAEFLYPRGDKVFFAGRRKYFHHWDLK
SGTVQKTTQILGHRLEHKTMERFKLSPCGRYMAIVASTRKGGGIINVLNTTSMQWISAARLASQNGIADFAWWSTGNGMTILG
RDGQVGEYSMESRSFLGIWHDEGCVGGIVLGLGGHQGPTLLGDDRWVAVGSNSGITNIYDRNELVILKSEDVSIKERPTPTRV
FEQLVTPITHIAFSPDGQLMAFGSQHKKDALRLVHLPTCTVYRNWPTEQTPLGRITAVAFGNQSDLLAVGNDTGKIRLWEIRS
FUN_006002 CDS scaffold_2 443738 444198 -
hypothetical protein ENOG41

MARPLDEDSDSDRVDRSGVIVLAPSNSSESSNKHVTLMDQWNDYFKKGTLQDFQRLCADLGLPSDLPSKTKCRDALKSINVNI
KQFLQCETKPDDVELFKNRKELIVWTRKRKAFFPQRQLPKGSPLRTLLKHMFN
FUN_006003 tRNA scaffold_2 444956 445051 + tRNA-Ile

FUN_006004 CDS scaffold_2 448644 452397 +
hypothetical protein ENOG41

MPLRRSSNSADGHSVTSSPSFSLSSTKRRFPLLSRRRNRHSDVSQSSIPPLPSPSPPASPSPSQKVAKPGLRVHVQVRFESPH
GSEHVRDYEASPQLKATDRICQALLSRLQHCSSELITRHDSNAIDPLRKPHRDVKPLRYRITYRVERDGLILVEKSLRSFQEY
ELTHDDAREVVAATDRIIGLFLVRHDPGFQWSESVESEATSPESETFRPCTGRPQSMACIPRSHFVSDTQKFEFVSGYSIELF
LRSRCATRYPENRNASIKIDSRQPSPLPLLLGEELTTRVSNLIIDPVDAWKRKFDKRHKSCAGLEGSGGCTHIEDGAVDIMVK
VRNHIGPDYTYFSHRIQTSKVLFNDHDGRDFDEFANQVKAKLEKARDMTDKSVRSLDDFTLTIRELRGKNWSVHEPLIVRLDP
TVTYCRQTMEAVMERLQTGISNVLEHHEDALATMTVHKRGHLIFDGFLDGAGGDDSVHHEKFATPDLERKALEAHLKERIRSD

ITMLCRDTCAIDCPDTLPNLRLKTSGTAASSIGATQLMRPPLSTAGSSSAAPTTPSLAASPLPRQPSETSFHGSMASTRSDKS
LAKKIAKQNLRDSVECPSTPATPSPDLRRVPSGVEMAKKHGYMSDFMADDENQSEPPSTPSLVDTDSLSPRDSVVATPSRAMT
HDQSNEGLRIIDDGRRIIYQEQSDMDAIASGMMQFHRPITDDDEPIQLAKEKPAENSDKKAARRSLDTRMPDLSGNTDQLSEA
KKHELAITQQESQPHATTTPELQKAKVAEEPSTESVAEQAPRSEVSVPGQTKAEAIAEMGTGETHRASQEEQWETAETRDTPR
VNPTGIEVNSKSETSFGTTPEASIRGDAKIKDTSIPDVAVTATAEEQPSKEQQIEAQLSNDSKVQTPKAEPVVAEKLHVPPAD
AAQKTLEASVLGEFPKPPSIHSYTDEASHDDQQEEHGQEIPIQTSPSASRQAEEDPEVSTSLAPKGAHTSSDSGISMEGDAKD
REAQSPAVTKLIENTSLPSKKTDDQSSVKADVVDKEEQHTPHSDVTDEHFPEMVTHFLETVPEDTLLEPTEVVAEDVEIVTSD
SPKTPEESTPLEERPLMPTIASAPPESLLTLENFFSSSSEENEKGKESPRKPFDTSDFADALARPRTRPRTATLPERRYSTAL
TLEININAHRDPDQEHSPIEDAPKSASGAWRPDGLHRRQPSAGFIGLPDQRRLHSVGLRSAVLPYRWQQMRREQALWEGRPGT
SHSEA
FUN_006005 CDS scaffold_2 452771 453599 -
hypothetical protein PF00106;PF13561 ENOG41

MFCSDHHHTQRDMTITTHIIEKGTIMSLPTITRNTTIALVTGANQGIGFEIAKKLASENNDYHVIVSGRRLQAVQEANSLLQA
QGLNVSPLILDITSDDSISAAVEAIGDKFGRLDILVNNSAISGYGMAVSPRELMMAVYNTNCIGTALVTDSFIPLLERSTKIR
RIVFVSTALSSLTLKADPTNPMHSFDFGVYTHSKSALNMIALHYATRYENDSQWKINIACPGHNSRTALNGFAGTEPVDLGAI
SSVKAATLGPDGETGTFKDDKRIIPW
FUN_006006 CDS scaffold_2 454058 454826 - SLD5 GINS
complex subunit EOG09264VZ7 PF05916;PF16922

MDIDDILRQVDPTSHRIPSETRDLQALTRLWVAERSAPELLEWPTDGLFERVNARIKSQIEKVEDMTGDMDPKTNFALIVIQT
ELERYKFLVRSFLRARMAKIDKHTLHYLSTQELRDRMSPTEAAYATRHQALLHNHYLSSFLASFPQQLQNLNDTAGNISMIDS
PDLDTAVFVRMLRDKDVYGKGTDDDITLPAKNGDVLILRWSSAKHMVDVGDAELV
FUN_006007 CDS scaffold_2 455275 456034 -
hypothetical protein PF04750 ENOG41

MAPRHPLQRLTSPSRDVSLLLHIIGIASFSYNFHFLTVWDTPIARSYGWHMQFLTIIGLSASLIAFVLGALADITLSPTLFQA
KNAVAVLATPLEVVISILYWGLRLIDPKLLMPDDFYLHIIPDMGFHLAPAVLLSLDLVLLSPPWAIPAYGVMASSTVIAFAYW
YWVELCFSHNGWYPYPLFELLSTTQRVMLFTFSAGLVTVSSSFLKWVYGRVNGYEKAEREAHKPLKKVQ
FUN_006008 CDS scaffold_2 456461 458428 +
hypothetical protein PF00160;PF00400

MASDDDNRSQKRNHSEFRDDASDSSSDDDMGPQLPSQAPKKKRRVLPHEKLYVAALPKSPRYSKSLMHKEQILFATWTPLTEF
LITSSIDGVVKFWKKIAQGIEFVKEFKAHNGEIKSVSVSKDGRSFATAGVDETVKIFDVITFDLLSMISLNYAPNCVCWVHSK
GASLPLLAVSEETKPLIHIYDGRGEKEEAIHTIKGLHRKPVHLMAFNEAYDCVVSADEGGMLEYWRPSGDYEKPDGVFEYKSS
TNLFDFKKAKSVPSCLSVSPNGKSLVAFSLPDRKIRIFDFATAKLHREYDESLQVAEEMHRSGAGAAKLDNVEFGRRAAHERD
IESQTLKYKSNVIFDESRNFIIYGSMLGIKVLNTYTNQVVKVYGKDENFRAVNIAIYQGAPQKKGVTTVEMGASSNPLLQEAE
TRDPILVATGVGKVRFYMFSNEEDISKSTRDVQNEKPMMLGNKKDAQTKKTETGTGAVIHTTYGDIHIRLFPDAAPKTVENFV
THSKNGYYNNTIFHRVIRKFMIQGGDPLGDGTGGESIWGREFEDEFSSLKHDKPYTVSMANAGPNTNASQFFITTEKTPWLDG
KHTIFGRATQGFDIIHKIENVRTHKEKPEEDIKVLNIDII
FUN_006009 CDS scaffold_2 462259 463566 + AMT16
Phospho-2-dehydro-3-deoxyheptonate aldolase amt16 PF00793

MSSFFIPNKNVGNQADSEDWRIRGYNPLTPPDLLQHEISQTPESKKTVLQGRNETVAIVNGTDEKNRLLVVIGPCSIHDPAAA
LAYCDLLLKEKEKHKDELLIVMRSYLEKPRTTVGWKGLINDPDIDGSFQINKGLRISRQLFVDLTAKGMPIASEMLDTISPQF
LADLLSVGAIGARTTESQLHRELASGLSFPVGFKNGTDGSLNVAIDAIGAAQHPHHFLSVTKPGVVAIVGTEGNNDCFVILRG
GSKGTNFDAESIAAAKEALKKKGVRQRVMVDCSHGNSSKDHKNQPKVAADIAGQISKGEEGIMGVMIESNINEGNQKVPPEGK
SGLKYGVSITDACISWEDTVSTLDTLAQAVKARRELSNGA
FUN_006010 CDS scaffold_2 464170 465327 +
hypothetical protein PF00096 ENOG41

MVRDYSPPESPLSSVDESDQYEEDGHEYDESTLRPSKRQRLEAGSTTSSAVVPDVEAEAVPEPDPLEGMSDVSSDTSGDIPSS
PINARLDEEDFQEQVTKCDWDGCPAGDQGNMDKLVEHIHNSHIENRQKKYTCEWMSCTRKGLPHASGYALKAHMRSHTREKPF
YCYLPECDRSFTRSDALAKHMRTVHETEALRPSDPVPKSMQAGPQGKGGKIKIIMKTNQQSQGPHDDMDDAANGDESNAEYFT
PLTPDLFTPDELELPVDKLYRKCHWESKWAEEIGDSLTKECKEWEEIYYKEWLEKEVLLSQVIKSEVNWHERRQAILTGVVDV
HVPGAVESVEGERTNGANAESEGVANGTKVAELTA
FUN_006011 CDS scaffold_2 465580 466243 -
hypothetical protein ENOG41

MRFHYHALNSPTDNFQAIASYTYGNFLWLSTQALPLIVWPSFVGSLLRPGNETSTSWALLQLLAIVSANNSLALETYFGRSLG
LALLALGLTVVVLSGVLPLDSSSKEAPEGAPSPYASAAVLISTLHHASSAFYCYGRYSWTGETGFLLGCVGSAVFATFGLYCV
LFAGDTAMTSRYHKFDQSTSGFPFKNSQSYRAKKKAL
FUN_006012 CDS scaffold_2 467026 468757 + NAR1 Cytosolic
Fe-S cluster assembly factor nar1 EOG09261P7G PF02906 ENOG41

MSAILSADDLNDFISPGVACIKPVETLPAAPPPQPQSLETEVILDGQQPATNSNAPAQISLTDCLACSGCVTSAEAVLVSLQS
HAEVLTTLDSAPALKLVTDDSGKFRVEGLEEENAKLFVVSVSPQTRANLAAACGGGVSEGDAGHMLDNLFRGPSGIASGGKYH
NEFTWVVDTNTAREATLVLGADEVLGTSQTETATPAKPILASSCPGWVCYAEKTHPHVLPHISKVKSPQALMGTLLKTTLSRT
LGIAPGRIWHLAVMPCFDKKLEASREELTEEVWAEGETRGRGVRDVDCVITSKEILMLAESRGLSFFNIPKTPLQSKSLTPFP
DPKLHEFLFPRIRTGNAPREAGTSGGLLYHILKSRAAQTPGAEIVHTRGRNVDVAEYSILVNGEPVFRAARYYGFRNIQNLVR
RLKPARPSRMPGGKPFGSARKPAGKAASLEYSYVEVMACPGGCTNGGGQIKVDDPVVMERKNYSGNPGPQEQKLWLSEVDEAY
FSGDEAGTEVSVVGTAESIGGISHSYINDTLAHWAEVTGIGLDRLAFTTYREVISNVGKDTTTDETVVQLAGKIGGGW
FUN_006013 CDS scaffold_2 468782 469693 -
hypothetical protein PF12752 ENOG41

MGKNTTVIPDAWEDDWETQADKMASEPEQPTQPVPLSKSERLAQHAEQNRKLWESADAPQTFHYVEAGSNSGVPLTTPFKPQV
KVLSRKPIIAKRDATAGMSGLSLDDDNETKKEVPMSPEEVRAKQKRDREEKQRRYEEARAKIFGESNPSSGASSPGTVTPPKV
DGYAGRGRGRGRGGYRNNDTRQIDNRQPENRAFDAPRRMQNMSGSGRELFDPNFSPKPEQPPQRRQPDYSSPGRSVTPRDEQQ
QQLQPRAPIRSPKGPDGSGRGGFGFAHRGNRGS
FUN_006014 CDS scaffold_2 470444 470748 +
hypothetical protein ENOG41

MAGGLRSSPIPQQEMADRAANTSSRRNAEETETLRSKLVDQHISMLNYPDPLKPRAPLQVYPQGITPEMEARWLQMLKKNGV
FUN_006015 CDS scaffold_2 476019 478756 +
hypothetical protein PF00069;PF00433;PF07714;PF14531

MHGVINKNVRSAADEDGADHIGLETPRSGVATPQPDLQDKRLPGIMSYFGQVRKYPSTPSSDPNSSQSDTTLDRISVVPTPPA
GLQLQAHTEGSPRGCESSALDRSLLQHERPGSMPSTTERDEHNLSDPYPTPPTSQPSSSGGSISQDMISADSGAHGRATVEQK
SLTQQSQFKKPTASPFTTTHFQTSNDPSLPELDSSKNAAPTKDSIIPSHPEPSTSSGACLAAAPGKWHFLNGLKELTRMTFKS
GNSTPTRAMSAARPSGSDRAPSSGRTSHDGAEVSGTQTPRSSAGAQAPAAKGKLTIKINEARGLRKSRDPYVVVVFQRSELIS
GGPHHIDEDDNLSVEPPPAAGGIPIQRSGSDSGRPLAIPMRSRQSSNTSINDYGSFRNRSGGQLTFTSPKWDAEAEFDVVDYD
MLVDVSVYDHGATGDEFLGHVDFQASKDPGATVQGWFQLQGHADTMAENAPTGEIFLEAIYHRAERKQFGPTDFEILKLIGKG
TFGQVYQVRKKDTQRIYAMKVLQKKVIVQKKEVAHTVGERNILVRTAMSDSPFIVGLKFSFQTPSELYLVTDYMSGGELFWHL
QKEGRFDEKRAKFYIAELILAIQHLHNNDIVYRDLKPENILLDANGHIALCDFGLSKANLTKNDTTNTFCGTTEYLAPEVLLD
ESGYTKMVDFWSLGVLVFEMCCGWSPFYAEDTQQMYKNIAFGKVRFPRDTLSQEGRNFVKGLLNRNPKHRLGATDDAEELKRH
PFFADVDWTLLSKKLITPPFKPKLKSETDVSYFDPEFTTALDQNGSLNERAAALARGYAASTPLSPSVQANFQGFTFVDESAL
DDHMRDRAGLVDEDMDDGQNHRNCDNDDWDNLDDIDLRKANRMSGIVKTGHDEHMVGGSHFDV
FUN_006016 CDS scaffold_2 479891 480626 -
hypothetical protein PF10775

MLIQLSRASRARSAVAAVSRVARPNAVQVRGFIAPTVSRKADFVQELYLKELKAYKIPAVKESDAEGNVQTFNVPKTPASPEE
TDLAGNLKEYESMAVEIEGQDSSAQSGTPEVADWLEAEEEDEAHH
FUN_006017 CDS scaffold_2 481389 482689 -
hypothetical protein PF10348;PF16010 ENOG41

MKSTFSASLAVLSAASSALAVTSSFCPDNGNVCFRWGIPEASASAGSGSFYFQIKAPTSAQWAGLGTGSQMAGSNMFIIYQDG
NGNVTLSPRRGVGEVMPQYTKRSGLELLDGSGIKDNQMIANIRCNNCVDLSLKGTSSWIAAWKNGNSLDSTSMEERISEHDTH
ADFSVDFSKATMSSDSNPFTGSNEDTNSNSGSSSGGAVTQKGSGSPSKTVLRAHGIIMSIVFIAGYPLGAILMPMLGKWLIHA
GWQVVMLLLMWAGFGLGYVYARDGGYWGKQAHTRMGTAVCALMTLQPVLGYMHHRYFVSHRKRGVVSHVHVWFGRALIIIGIV
NGGLGLQLANSSTAYIIAYSVIAGIAAILYLAAAFIGERRRNASRAKQISPQMSQEEAR
FUN_006018 CDS scaffold_2 485034 486582 -
hypothetical protein PF00069;PF07714

MISQAPAAVTTVRGFQTTPATSGDEDSDHGHSEAPTPRRKTPGRNLIINDVVSANCSPTFRAHPSPAMSGIAKLRMQMEPLSL
DGGSPCHSMSRAGSQQGLALEFRKQMISRTQSREDVRSEAGSEISDSSISYEVNLEHDFASESVRERSGYLETLEGGLAPNRK
MTSEDFEQLRCLGKGTYGTVLLVKQRATGRLYAQKQFKKASLVVHKKLVEQTKTERQILESVNRHPFVVKLFYAFQDQEQLYL
ILEYGQGGELFTHLNTEKMFSEDVAAFYMAEMLLAISHLHNDLGVVYRDLKPENCLLDAEGHLLLTDFGLSKVSVDQNESCNS
MLGTVEYMAPEVVLGKKYGKAVDWWSFGALGYDLMTGNPPFRGQNHAKIQDNIVKQKLVLPYFLSPDAKDLLTRLLRKDPSKR
LGAVMPKDLATMKKHRFFRKIDWKKLAERELEPPIQPMITDPELAENFAPEFTELSISPVLPNKEAWPRSLPKDELFGGFSFV
ASSSLLNEDRFAAFAQS
FUN_006019 CDS scaffold_2 488274 489834 +
hypothetical protein EOG09262CXO PF00400;PF08799

MMHPSRQAYVEDAEPQGIALEDVPDDHDYDIPMGGTGVPSEKASAILSQFNRKRLAATIAVPTDDTRVRAKLREMGEPVTLFG
EALVDRRDRLRELLTIQAEMTGLENGDITMEDAGDQDQEEEQEEEFYSRGGPELLQARLKLAEYSLPRAKKRVQFQKVESTIP
LRTQVKFRKQVKERLQAFELQGSQTVGERHVSMTRISPNGELVAVGNWGGQVKLVEIPSLNQKTTFRGHTNKISGLSWFPGST
LPEHNVSPDTVNLASGGAEGSIHLWSLNQDTPLSTLEGHSQRVCRIEFHPSGRYLASASEDTSWRLWDVDTTAEVLLQEGHSR
GVYAVSFNTDGSLLASAGLDSIGRIWDLRSGRTVMILDGHLDGHIKPIHALDWSSDGHRVLSGSADGWIKCWDVRKVQRTGGI
GAHTSAVSDMRWFKGLDDPLTGVPPGVDDKGAQLPKKSGTFFVSSGFDRNVKIFSADDWTLVQTLSGHTGPVASVDYSRDGKW
IVSGGHDRTVKLWGRNDGEGV
FUN_006020 CDS scaffold_2 490337 491781 +
hypothetical protein PF00134

MASIDRYRPPREGYQPPTLPANPRPERDSRSRSPRRRDAVPPAVPTPPQHSTRTSPPRPQARQPQESPARSGAQTPVASLNQW
NFTSDEVRSTPSIVEGLSPAEERMRRAKGVNFIYQAGVMLDLPQITLWVAGVFFHRFFMRCHMVQEKGGIHHYNIAATALFLA
NKTEENCRKTKDIIIAVAKVAQKNAKLIIDEQSKEYWRWRDSILTYEEVMLEQLTFDLMVDNPYHHLFKLLDQLGIVHNKNLR
QAAWAFCNDACLTSIPLLIGPRDVAISAIFFASIYANQQIEDINGEPWWKLLKGDEVLCSQAIEVMRQFYTENPLRKQNPSLP
SPAFHLENTRKRGDTLLSQPDTLSSTTGTPFEVDRASRSPGVSSRVNGAAQDGEGPYPERDEGSQPRHDENGRPPRSPLKRRE
PDDGEEQSDRAEKRAKLSEDEEGELVED
FUN_006021 CDS scaffold_2 493789 494203 +
hypothetical protein PF01423

MATLGGYLNKKVLIVTADSRILVGTLAACDQTTNLVLNNAVERIIRTPDDLEPSAQVPLGLYLVRGDNVCSIGLVDEALDDSI
NWTEVKGSAIGGIKHV
FUN_006022 CDS scaffold_2 494453 495920 -
hypothetical protein PF01156 ENOG41

MAPKQKIIIDTDPGVDDILALLLALSAKPEELEVLMVSVTYGNVPLQSCLRNVVSLFHVLGKELEWRKSTGKPEGFGAMKANK
PIVAVGPDHPLCDEELMADYFHGIDGLHNVHEAHPDLSPAETWKGIFSDGANEAGDYSPFFTPSKAPSHHEILRILKENPMDT
VSILAVGPLTNVALAAAEDPETFLRVKELVVMGGAVNVEGNCTPVAEFNCYADAVAAARVYALTSPKPSSTMPTIPQELSTLK

AYPTKLSRQLKLTLCPLDITTPHLIGKNYFKENIQAHVEAGSPLARWVSHFVTKSFSKIEDMEGDENEPGLSLHDPLTVWYML
TRDDPKWKTPEKLEDIRVETSGQWTHGMHVVDRRLRKKPAEAAVALSENPNEDPHILTLDEVPGDDHGWLSVVKGNRLNRVID
SPGHDIFKEVLMQRIFG
FUN_006023 CDS scaffold_2 497978 499181 +
hypothetical protein ENOG41

MGYLYTIAGYALLFSVAYALYRLSQGQKAGNQPRAQIKPAKSSQAETRKEDRKKKQRQEAFASEAQEASKKPKAESETSAWTS
SAKDKDDNVDNREFARQLAKAKEGTKFAAKSDAGKQREKSVKQSRANKVASAAEEKESAPSSTTGADADDDQSPVTSPEVGPA
VAVAGDVSDMLETAPARPTVLRLTDTEPKKQNKKTPKAEAPVETKKQRQNRKKAEAAKAAREESEKERKVLEEKQRRAARIAE
GRAAKDGSQFTAAQSKSSAWKEGAPKAPEAAQTNGFHQPLDTYEEAPASAVNAPKSTDRKWIESLPSEEEQIQLLQNDDDWST
VKTKSKKGAKNATSAGSGDEAAARPAVQPKQPTGPNKASQPSQSFGSFSALTTKGDAAEEEEEEEWDV
FUN_006024 CDS scaffold_2 503244 504471 +
hypothetical protein ENOG41

MSLPVAIQSAVFYILACTPCAQVRHRQKVREQSRREREEKAKVVGEQPDVYQHPSPFNTNPYWQEEISMGPSLPQKKSASKNS
SQRGLTSQGGESSAFSVSEQTHQGGSRINFGASNSIIAEDDNLSDDWNRRHGYQREDEELWGQWGGQKFKDAISKARGSAGRL
IESTLGLEKEVTEQQRHDFYFPKNPPINEYHPPVVSSKIPSRNAHQWMLQPPPPAKVMEGKVPVSRAGSSGSKSSGRTLVGDD
MQLGRLVHEKLVMEKLKKENSNPTETELIESLFLNRTSQSLSVHRTRSLSFDTSDDSLDSGFAKRKTRLRPVAAPPGYDSSDD
SDSDTPIPFSQSAMNLGTRRTPSSTGHAAQRPKLETIMSTRSATAIRMSSKRSKRQKSTRSKRLSGAASPVGDDTD
FUN_006025 CDS scaffold_2 505113 505770 -
hypothetical protein ENOG41

MSYLQLHLFTRFSSYLIMSSTSTSADTTPIAPARFAAALKDLSVGMLHLKVLEIRNSIAHLQYSNDQLKPFAEGTETTPSGEA
SGPDQDCIDAIRENEQVIDRMAERIALIRAEVEERGLSWTEFQNRDETTSKNDEDAAAVNGDTQASTAASDNENRHNAWNDGT
FQTGTIRNGEVQVDRQAGRPEGGSLSDEQLRRALEERMRDLGTDDDEGGMHL
FUN_006026 CDS scaffold_2 506034 507720 +
hypothetical protein PF01694
S54
MSLFACPNSSRVLLRSALQRAVSKASASAAQPFAPTRWISSCVSRNRSGLLPSSTNCRSTIFRNNNSSYETRRTIFFDKTIRN
YEDLPRDYRDQVGLRFRSKDLTDAEVVEAFGKGINPKRANQLLRILHGRRVAGTLDDPAFAVHTSSYTKSQIAKGLEYLRKSV
KVDEVLNAGLRAEDELAQLEAEKEAAKKPKQASKKNKNEAETKPEEQPAPVYKPDPVYGHSKLDELRAQNVAKRKAKEALEAE
ARKAAEAKGEVNSGTLANLDHKAERQIANPKIAEYYKKAQSDLEAPVELKTWERVLPSATLVALVIGFLAAVSTVYEEPAPRY
RVFPDISTAHATLGAIVAVNVLVWAAWKAPPLWRLLNRYMIIAVGAVKPVSMFTAPFSHQYFSHLLMNMVPLFLVGSALHEDI
GRANLLTLYVGCGAVGFVGSVATYAMRGMLGVTTLGASAATLGVCAAYFWEHRLDGYRFFGLPQDGVPGIIFLALICVPQLAA
FGKTVKLKIDIASHLCGILSGIFGMELINRTRSEREKKTIDVAGTPVRAARLPRPDESVEGSN
FUN_006027 CDS scaffold_2 510115 511990 +
hypothetical protein PF02146

MSPSSPLSFVPDGETSTLSSPLSNLSRTPSLPSSPDMPDPTKRYPSPSTTASGPHSPDKTCDLPSEDEIVAKPLDDSTSGPPP
AKKRRISPPKERTTEYLDLMKPEDELTAEDRGQLKRLIDTLRKKKKIVVIAGAGISVSAGIPDFRSSTGLFATVKTQHNLKGS
GKHLFDASVYKHDSTTQSFHAMVREMATMTKAAKPTPFHELLASLAQEGRLLRLYSQNIDCLDTQLKPLATEVPLNPKGPWPV
TIQLHGGLQKMVCTKCNNIREFNGEVFDGPEAPLCPTCKDLDEVRTAHAGKRSHGIGRLRPRFVLYNEYNPDEDAIGNVSSAD
LKARPDAVLVVGTTLKVPGTRRLVREMCQVTRGRKNGTAIWINVDDEPKGVDLKNCWDIVVRSKCDDVARSAALPPWNITIGQ
DYEVSEEDFSKSQEDRKHFNFFVEIPVNSVSTHSHTEGQGAAKSKQVKQVQGIPTPHASPKISAIKNPAKTMQTTISFDGKVT
TDQPSQLSKRGSSKQSGRKRKAPGGHKEAQLKSFKATKNIIGQEKAGGSASATQTKRNVTGRGKNRHPLPSQRPTNAPHKETL
TDTKDLDEKVRQRETGYPSTPTTKMNGTISPRSYPNNTRHLID
FUN_006028 CDS scaffold_2 512517 514026 -
hypothetical protein PF11976 ENOG41

MKKLPFKPTALRKAAPKPKPTESEDSKDSDDDGLSLFRRSKEMAPIMAADQQRRWRRHREAEQEAERKRLEAIGEKRPREDSP

DSDGLTNKDPGPKVSDGLPSLPGRAEGTPVTDQPLTQDGADRTSELVTPPSSKRSRLDSSSAQKPTLSSQLDIDEDPFPDASP
TRRLGPIRNPPTPSRNLKTEGFKPQAKPSTDPITIDSDSESDSEPPKPKTDLSSRPRSSSIELAERTLKPSKEPSPPPVEEDE
FAEYIRKAEEKRARQQALQANKSEEQSKEAFNIFITSTIPHTVPLQVRFSFNKPLRIVRDAWVKHQMNKGVSIQADDVTLTWN
NKKVYNTSTLIGLGIRPIGNGRAVSDSQGSAGFRENMTSMNVQAWNPELFQKMEQEQELQRKRDAGELSDEEEEKPAERIRFV
IMLKGRDVEPLACKILPETTVETLISYFRQQRNVGSDKQVSLWWDGERLEEHVEMKDAEIEAQDTIEVHID
FUN_006029 CDS scaffold_2 514524 514920 + LSM6 U4/U6-U5
snRNP complex subunit lsm6 PF01423

MENGAISQGEGKDPSSFLSDIIGNPVTVKLNSGVVYKGELQSVDGYMNIALEKTAEYVNGQKRREYGDAFVRGNNVMYISADS
FUN_006030 CDS scaffold_2 514993 516010 -
hypothetical protein PF08241;PF13649 ENOG41

MASQTIHDIATKGFDDALSYDAHRPSYPPAAVASLLGHLGLEGQSGRHILDLAAGTGKFTELLSARPEGYEIIAVEPLDSMRS
TLAAKKLSKVDVRPGTAAEMKDVGDGWADGCIVAQAFHWFAREESLKEIHRVLKPGAKLGLIWNAEEYNRPESWPASTKWEQE
LSELNFNEKADNEPRFRHLLWKKVLERQAEAEKPFFSTPIETEKITWSIWLTPDALWDRFDTLSWNKLRQGEERRLFREKFDK
IIKEGDATFNENGEVELHGCTFFVWTSRLDGPE
FUN_006031 CDS scaffold_2 516381 517307 -
hypothetical protein ENOG41

MTAFFVDWDLWQEMTFVLGCCIVLVFAMGLVKLWWSNRAIRRLEIIDEEKRARISLMSRCGIENMRAPGIPFGVRAILSGVEV
EGIWISRPNSPGPCQLTPVATQVGRRIRIAKGKGKMIDIGSSECLSDTLDSETTPSYPPSTKTSQQDGIISTEGIQSQQEHVG
SASSLTKTQVDSSVRRNAQINCNYTGSRSSSSCNDDFITPEQTPPGSTISQTEAFDKGKGHSVSDTIFSTEQLNALPRVRKPS
AGSVAMPSGHPRVLRSEKRRLISESSSS
FUN_006032 CDS scaffold_2 520346 521366 +
hypothetical protein ENOG41

MPIRNPFARRPGSVIVQDENQRPDSTPGFEKVDTVGSKSTPVLSIRSQGRDNGEYKMSGMSTGPGRFPSLMASPLNLRIVNDS
GVYLPPSPTEEKGQWPRKYLSTRNSTDTRSSSGDIEHFSISRESFDSYRRSFDISARSPMPNSDFPTRQSLDSARLPRLPRSA
VERSFEQPPTAEERFEDVGLDDHKQQQQQLAPQAQPQKRGFFSKFSDKDSSSNPTVSRFLMHGRKRAQSGQGSELTPMDNASP
KVTVSTDGQEMQ
FUN_006033 CDS scaffold_2 522254 523700 +
hypothetical protein PF01053

MASDEVPTNLKDVSSAPDGTAGARTGLDSSLAGLSLASRAVHADEGIQSHRAVAPAMHVSTTFRYSNDPDALKAWDNTDPHNP
LDSHVYSRATGPNTTRLEAILTSVIGAPTITYSSGLSAFHAMLVHLNPKRIAIGDGYHGCHGVISILSRLTGLKQLPLDCAVE
DLEPGDVIHVETPLNPTGEARDLAAYAEKAKKAGAYLTVDATFAPPPLQDPFKYDVDVVMHSGTKYFGGHSDMLCGTLSVNRK
HRDWESALLNDRLLLGSVMGSLEGWLGIRSLRTLELRVQRQSATATALVAWLAEQQRDPSSAIGALVERVQHASLQPEASVEG
SWLQKQMPNGYGPVFSVYLRDGDVAKRLPSKLELFHHATSLGGVESLIEWRAMTDPKIDKRLLRVSIGVEGLEDLKNDLLQGL
EALRKEKEKGGSK
FUN_006034 CDS scaffold_2 524252 524943 -
hypothetical protein ENOG41

MSRRQDHHQRHYGQTYNTESSRNWSEEAVRGSWDSNYQRTTSANTSPRDQSQAWRRPSPQAYDRRQTMVNQWLGQQYSHDPSN
VMDAVSDLTTPTTSPAFSDSPTFPSRTQEAPPYQTHTVANDFLAPPLRTSIPHRPHDGHHHNAPPASSRGSTSYRLDNRSLDY
RHMEPRSAEQDLRGVGSFADNDRSRREVKYKPLSNWFGLWLPPQE
FUN_006035 CDS scaffold_2 526616 527435 +
hypothetical protein PF01522 ENOG41
CE4
MARFSLFRLPTKLRRRVRRNRMATLIALVVLVGLLIFPFYSAYCVYKPPRYLISWLRRKYPDVLFEEPTDQKIIALSIDDAPS
AHTDDIMQVLEENEAHATFFVIGSQVEGRKDKLVKLVAKGHELGNHAMHDEPSKSLSNEKLLEEVHLVKDMLTEAYAANGQIL

PNNYFRPGSGIFNRRMRDVLGNQGFRITLGSVYPHDPQIPYPDTNAKHILSMAHPGAIIICHDRRSWTVPMLRTVLPELKRQG
YRVVTITDLVKAVSSQNQRQQHL
FUN_006036 CDS scaffold_2 527538 528663 -
hypothetical protein PF19086 ENOG41

MKYSHLNQAAIVDALRGQTLSIPNLHNFFHVWPNPQGQLNYHHEHVTPIVNRAVERMAMVYPLIACRKKDNIASLACSLYPQA
RRRQIEALTLYTTWLVCWDDAVDANEGDLAGDFMRAERWRGQTMRMVREALAVDERNTSEADDDPINGVFREFGARFCQTAPR
DQRRLLHDEIQFFINSCAAEQELRLASRIPDYEPYMKMRIGTVGGRMLCSLVPYATDERLPGALSSAPEIVHLWTQVSILQSL
MNDMLSLKKELRTDCVINAVAAIMEPGMSLDVVVAKLEERMKMAVNNFDDAANMLLRKTEFQEQTHAIVKRYVDGCRSIVAGT
LRFTLTSPRYNIHKLIEEDGSLKITL
FUN_006037 CDS scaffold_2 530079 530982 -
hypothetical protein ENOG41

MSLSRAFTTRKAKMAGQTEMGEQQVQVPHRSNSARDRQHPILRHQISAPLQLVHTTNMLSYNAPDIPHAALRSKSSLRSVGEE
SEAPSTTESTPPTSPDVSPVDECPGPKPNHLSTYFSVSDKAAVLPKISEAPAIPKRSPSHTKQNSYEALARQRSTSQMSRDSE
HSVSTKASFTFSRSSSTSTRASSASYVSASQAQKAPPMPVPAVPAVAPVPPPSFQQRALKDSHPFGHELAQVTELAEEYSTKP
TTKEDEEDHRYMKSRGLNKFSPEEYLSDVQSLILSFFPEVSHAKPTTPQWI
FUN_006038 CDS scaffold_2 534336 536016 -
hypothetical protein EOG09261DW8 PF00400

MSSMITAAQWVPRGFAAPFPQKYTLDEAEFERIAELAKLQLDDAQEDLEEAEAAQKGAKANETEDNEEDSEMKIDEDKPTEKT
EINLNDDDLKEYDLENYDNDDGEDEPGNGEGMGMFGNIKSLAYYESNKDDPYITIDADKEQEDEDREELQILATDNLIVAAKV
EDEMAHLEVFVYEDEADNLYVHHDIMLPAIPLCVEWIDMPVNKPGAEKDSTGNFVAVGTMDPDIEVWDLDTIDCMYPNAILGQ
GGNEEEKKSKKKKKKSKKSNDEYHVDAVLSLAANRKHRNLMASASADKTVKLWDLNTAKCAKSYTYHTDKVCSLAWHANEPTV
LLSGSYDRTVVAADMRAPDAKPPRWGVESDVENVRWDPHDPNFFFVSTENGVIHYHDVRNAPSDPTGTKAVWTLQAHDESVSS
FDINPVVPGYMVTGSTDKTVKLWNIQPTGPSVVVSRNLDVGKVFSTTFAPDPEVAFRLAVAGSKGTMHVWDTSTNASVRKAFA
QRVPEQRGKGEDRLVGVNNDDSSDDDDEEDDKQDDDDSMDED
FUN_006039 CDS scaffold_2 536529 539454 - PRP16 DEAH-box
RNA helicase prp16 PF00270;PF00271;PF04408;PF07717

MAEDREYDGYNPNPKRRRLDTPGQRFQNDSRASTPHRAQSRYASTPPPRNIGPTEEDSKALDRDWYGGDEFGGHSFGDEAHNP
FASYDTWEGQQQETARHEKMTSRFDARRDQRNRENDAWETNRMLQSGVAQRRDMASDFVDDDESTRVHLLVHDLRPPFLDGRT
IFTKQLEPIPAVKDPQSHMAVFSRKGSKVVKEARQQRERQRQAKEATSMTGTTLGNIMGAKEEDGDSALPVPAEDNAEPSERK
GNKFSAHMKKADGASNFSQSKTLREQREYLPAFAVREDLLSVIRENQVVICVGETGSGKTTQLTQFLQEDGYGKTGMIGCTQP
RRVAAMSVAKRVAEEMEVELGSTVGYAIRFEDCTSKETVIKYMTDGVLLRESLNEPDLDRYSCVIMDEAHERALNTDILMGLF
KKILQRRRDLKLIVTSATMNSKRFSDFFGGAPEFIIPGRTFPVDVMFHRSPVEDYVDQAVHQVLSIHVSMGPGDILVFMTGQE
DIEITCELIQKRLDALNDPPKLSILPIYSQMPADLQAKIFDKAAPGVRKCIVATNIAETSLTVDGIKYVVDAGYSKMKVYNPK
IGMDTLQITPISQANASQRSGRAGRTGPGKAFRLYSEKEFKEDLYLQTIPEIQRTNLANTVLMLKSLGVKDLLDFDFMDPPPQ
DTITTSMFDLWALGALDNLGELTDLGRKMSAFPMDPSLAKLLITAEEYGCSEEMITIVSMLSVPNVFYRPKERQEEADAAREK
FWVHESDHLTYLQVYTNWKANGYSDGWCVKHFLHPKSLRRAKEIREQLLDIVRMQKMELTSCGMDWDIVRKCICSGYYHQAAK
YKGSGEYINLRTNLGVQLHPTSALYAGHPPDYIVYHELILTSKVYVSTVTAVDPHWLADLGGVFYSVKEKGYSVRDKRITETE
FNRKMEIEAKMADDKRKDEERKQMEEERTQKKSKKIGADGKKFVTQGAVKKPVIKRRGRGF
FUN_006040 CDS scaffold_2 539921 540406 -
hypothetical protein PF00708

MSKRVYFAVEGRVQGVGFRYFAQKKAQEYGVTGWCRNTKDEKVEGEAQASEEILSKFFKDVDNGPRSARVVRVSQEDRQIIED
ENDFVVTR
FUN_006041 CDS scaffold_2 540814 541974 +
hypothetical protein PF07052 ENOG41

MEAHSSEPATEPVRFRAGKKRKAYRQRVQEDDAAVAETIQSPPTASASEPSDEFARKTSSDRAQDEGESVAAALKLRNARRAR
LGGVAFRNTNRPDDDMNNERALIPHDADYTSNNEPIMKGVADRFTHQTGRLTDLNEKHMMDYIESRLYNRAGGNSSQNTLPGT
TSDPARQPSATTTNHESGRAVMQGQLMEINLDDHSARNENAVQADSATDGGPRAKKPRLRRDGKPWRPRNRRDSDALKRDQIV
DEILHETRLDLYEPTPEQNSRTVVADQDGAADARLAEEFRQQFLEDVAERQLRKKKATNQTTRAGTEEVLKGPKLGGSRNARA
QMRDLLLKKEKEGKK
FUN_006042 CDS scaffold_2 542614 544323 -
hypothetical protein ENOG41

MPPRSSLTSSFSITDSNNEVVCPLRNQDGSSCRKRCIGEKRYRSMQEHIRRAHPEHYISKLPATEESFLLMINTPPSERRPLD
QTSAPNAQGLSHSHSSLPPPIDHLQGFHERHNYHRDEPSNPGTPRLLDDFSNGGPVSGPMLGTASAAAALAELHGVKSERDMD
LDGDYYSDMDVRRRPRTSIELPPLNLSNHDITSDPYSSAKSNRQRDFLPSILANSPPGRSSTLPPLQRSVGPNRPRKQSVTKR
GREPHHKKKNSKGSATDWLRRIQNEERYRPGNDRKALSAEPSADFGKRWEDLIDAADQAASAAGDIDEDRTPVPQSPVSMHRS
SLPPFSHQPQFQPASYQASPLQQALTPPSYGQDAVEPFPSVESGESGDNFHMGTRGLSDSSPTYSAQNIQIYCAACQGFSLLK
DSYACTECICGLCPTCVEVLMTEHGARRKCPRCATIGGRFKPFQLDIR
FUN_006043 CDS scaffold_2 549178 550326 -
hypothetical protein PF00036;PF13202;PF13499;PF13833 ENOG41

MAYNRPYDEDALPRFAEPEPKPGQSRTPAPQQHPPPQQQHQQQQYAPQQQYHHQQPRYDKPLPTQRDARSHSLGQGAYGHMSP
PPNPGGARPQAHNRPAPNSRPPPSPGIDGSGSDPSLLPLFRAVDKDGTGHLSERELSAALVNGDWTAFDPHTVRMMIRMFDSD
RSGTIGFEEFCGLWSFLASWRTLFDRFDADRSGNISLSEFNNALVAFRYRLSPQFVELLFNTYDKRNEGVMSFDLFVQSCISL
KRMTDVFKKYDDDRDGYITLSFEDFLTEILKQLK
FUN_006044 CDS scaffold_2 551735 552657 +
hypothetical protein PF04752

MPDQDSSQPSEFWLYGYGSLIWKPPPHFDRRIPGWVDGYVRRFWQARYVFKQPNEDHRGTPEAPGRVVTLIERSYWEQLTDSH
DSAPERVWGVAYRIIPEKVAEVKEYLDIREINGYTIHYAPFHPADGSPPIRTLVYIGTPDNEQFVGPQEPQELAEHIFRSQGP
SGLNKDYLLSLDTALSELAPDSGDHHIHDLARRVRELEKNCEDETKLLNPTTSVHQQKHSTEEAEEIEEPHH
FUN_006045 CDS scaffold_2 553221 554034 -
hypothetical protein PF00076 ENOG41

MSKLFIGGLAWHTEEATLRQKFEEFGPVEEAVVVKDRDTGRSRGFGFVRYTQEGDAQKAIATMNNVEFDGRTIRVDKASDNGP
RGGFGRGGGYGRGFGGPAPYGMAPPGHGYQVPAPNMYAPMPYGRGYPPPQQAYGAPPQGFMPQQQFGYPDPSQMPPQQQHPQG
GRGY
FUN_006046 CDS scaffold_2 557431 559176 +
hypothetical protein ENOG41

MNNFASGAIPNTRVTMRPNLTAGLFLNGRSSTYERGADITFNDMTLIETCGEKGKADKIPQHSTSENSSGQDDVSNSGHISRS
ATELPRTTTNDEEGEVQSLADDGSQENQQNNIQKEGSEAHSGSVSSTETTQASDYETPEAMLKRLIETGIFDGTGIFKTTSHR
AMEPNIEYHDCLGMNGTEQSSKNPTYQDKGVMADRSRQSTLGPNIQAVTNNTQFDLNSGAKDAPVPTKASRSQYEQELPVPIC
STSTQVQPPNLPTSVIENNTHNIPEEPCVKQPQGTKSTSTTNSRGHPLSRISERGHRYTEQNDRPGLMFPNLIAEDFNVEHLY
HQDSTENNESQSKGLFVRRGLQSHDNMIPISGSPGFQHACRPLNGHISSAGLYSAVFPTVHSETYSDHREIQHDNRQLTQPGY
CEDGETIHEFIKRIEGEVSMKWDNSQQSVGVGCINDIEECAKSESFEMDSNEDLSQCSDIEHDAAIQSSLHGIFSSSDQGSVS
ANPAMAEDWEHSGYEQSRYWTGKYYDEHGVSDAKMVGFWRPNHF
FUN_006047 CDS scaffold_2 559235 560251 -
hypothetical protein PF03328

MAARSPSIIRRSLLYVPGSSQKMLTKSLGLKSDNVTYDLEDSVTPSLKDTARNQLREHISNLKARPSGISELAVRINAVSTPF
ALSDLTTLAPLPHVDAVVVPKVNSAADLTFVTDVLRHVAPERHTVEADNPIKIIALIESARSIMDLAQISRASPYLSGFIFAA
EDFALDLSLTRTPSLTEFLYARSAIVTAARAAGLPSAIDLVCTSYKGEQGLKTLEEECAGGKSIGFNGKQCIHPSQVEVVQRM
FAPDQNEVEWAIRVSIADEKASASGRGAWTLDGKMIDAPVVGKASAVIVKAEQCGIDVQSLRNKWKDQEPE

FUN_006048 CDS scaffold_2 560714 561502 + TVP23 Golgi apparatus membrane protein tvp23 PF05832 ENOG41

MDATQPQPAPGSLSWRLSAHPITLLTFLGFRISSVLIYFLGLWIIKSMIMIFIITILLLAADFYYIKNIAGRRLVGLRWWNEV
DPQTGESQWVFESSEPGTKTVNPTDSRFFWLALYIQPMLWVLMAILALVRLQFLWLPLVVIALVLTIMNTLAFSRCDKFSNAS
SIAGSAFGTGNLAGSLASNMVSNWFSRS

FUN_006049 CDS scaffold_2 562223 563461 -
hypothetical protein ENOG41

MEQIANQIDRDDTGDIVKDMQNAPSSTNINFASPSNKGDSEGVVDGSGNGNTGVGGSSSQTKVIVFNSTNFADGHVFQLKDSD
QNKKIIGFSKTDSKTTLDVVQWQVISGDNIEPLGDAEFSDAKPDEQLIDVDNNSVTLNFEGKTSKYYGKEMYIQIDWKKDGAS
GYTKTELFTVTNTDNTTEITELNNQLKRDQSQQETLESTTSSSSSATATLSPSSTSGAEESSGGGGGGGLSTGATAGIAVGAV
IGGLLLIGALAWFFLRRRRRSKQAGDEYVTQQTYAVDKETHGRATDSPNSPYSDENHMQPVALGSLDRDRGVAPTPPPGGVPR
SSIGSHDRAAHSGAQTPQGMSSNVAHLVEDGMTVDEIRRLEEEERQLDDEIERAARR

FUN_006050 CDS scaffold_2 566158 569714 -
hypothetical protein EOG0926146A PF05277

MTTPAEVDDFGLPIRRFPTPKNDEPTETPKDEKPAASPVADALSGEQPRASETKNDKSSDQTTPPAVEPVKDDDKSETFEDAR
SEQSNPSKDASNPPVVTHETPPPTFIKTPAKIEPAEPESINDPEPKDSRKTRSETPEAPKEPTDIDVAIKKQETPKPDDPTNK
GPVETQPEAADPVKKSTGDEVAVSKEEPPQPDKVTPTPVSPTQANRKDVSEFSHQKISTKPEEPQKGVDDDDWQEMPSYARYD
MYNDDDKLVAREYDPEQDESYEYGGLGGAGKGYTRVTLDDDAESATSMDDNTKYLFKSAAGTSMVDDDEGRDAIGQMQATKDL
LTEGQRVAYVGLVRLEIVRLVKEAEKMKSFKKTKKEVGVSVESMLMWGQKMMLRLYAHMEINEAEQIMIEQLAEHGVMPQDLA
PALNTNARVSNPMADSESSMPSTPSLDEKPAEAPPPYEALDGEELSDVKTPSQLPTTAKIDIDLRWTVLCDLFLLLIADSIYD
SRSRVLLERVGESLDITWVDICRFEKKVTEALEMQQAAEKENWNEEEHTETRRKNALTRRYVMMGLATVGGGLVIGLSAGLLA
PVIGAGLATGLTAIGVTGTGSFLGGVGGAAIITSGAAASGSIIGGRAADRRTGAVRTFEYRPLHNNKRVNLIVTISGWLTGKV
DDVRLPFSTVDPVMGDLYSVLFEPEMLRSMGDTINILATEALTQSIQQILGTTILAALMSALQLPIILTKLAYLIDNPWAVSL
DRATSAGKILADSLLERNLGTRPITLVGFSIGARVVFSCLQELSKKGAVGIVQNVYMFGSPIVVKKEEYIKAKTVVSGRFLNA
YNRNDWILGYLFRLTSGGIRRVAGLAPVEDCPFVENMDVTDLVNGHMDYRQKMPVLLIRCGWSVESEEFVEIEDPDPDNHNER
QRELINEIEEARKNLEKEGKAKKSGRFSFFGRRKNAEKQDWEIYEDSKNKGEGKEKAKEGEQADNNTGVLFDIDAIRAEIAKD
ARDHYASPEDLQVKEIKSTLPPMKLDVSSLPASPSAVANGSRAGSRTDLTGARDSHDTRVSQERSHSYTPSYTRQASPPGYSS
PYGGGFGSGHGDAYGSGRHDEDDIQMTFDTSFDDHPRSATVAPTETAATRPQVKTAQTLPTMTLHEPWGDSDDDDFGKEKEIS
MTFA

FUN_006051 CDS scaffold_2 570702 571668 -
hypothetical protein PF04900

MRGKRSKQYRKLMEQFSQTFGFREPYQVLVDAEMVRDSSRFKMDLEPALSRTVHGKVKPMITQCEIRKLYAARNEPGVHEAID
LAKTLERRRCGHHPDDYPEPLSTQECLRSVVDPKATLQNKHRYVVASQDQEVRRMLRGIKGVPLIYIKRSVMILEPMADESVQ
VRAKEERSKFRAEIKNQLGKRKRDDADDDEKADKTDGNSEEQKKKKKKGHGPKGPNPLAVQKPKKAKTEGQQPRKQESTDAKD
APQEGAGKRKRRRRNKATAGTADDGETDTAAAEVTMAET

FUN_006052 CDS scaffold_2 572101 573357 -
hypothetical protein

MPSQPSSILAAVPTSFLPSLPVRRLTCTTWRPCRKRRFASTTTRLLHDVPILPFRFETGIGLFAKRPPRPFPPPFLSPPSTSF
TDPLSTHHQSRDRRAFVNGQLIRGKTNGDDAVYASDYFICANDGVGAWATRPRGHAGLWSRLIGHFWSSAIEEELVRLPKSQE
PNPIASLQSAYEQTLEATMSHDCLGTTTACGAQLHYKTCTENKTQASPVLYVTNVGDCQVMVLRPSTEKVIYKTVEQWHWFDC
PRQLGTNSPDTPTDNAVMDKVDLEVGDIVLAMSDGVIDNLWEHEIVARILKSIKEWESGTHAEAHKGDRTGGRNGGMRVAAQD
LMEAAREIAVDPFAESPFMEHAIEEGLASEGGKLDDISVVAALCVKNEG

FUN_006053 CDS scaffold_2 578723 583086 +
hypothetical protein PF00169

MSQFSAGTKTPPPSTSPPSNSPHRPGHRKTNSVTEPFPDHRVSADISRPNTPPSRPDYGTPINGGSPIEPSARRRRSSSAASS
RPPSMVLAHQPPIMDITEDTIPELQPIFSFLNSHSNKLYQEGYFLKLDDQNTQGKPNPDRTWTECFAQLVGTVLSLWDAAELD
AAGEDGEVLPKFINLTDASIKMIESLPTRSSDEQPLQNILSISTAGRNRYLLHFNSRHSLLQWTAGIRLAIFEHSSLQEAYTG
ALIAGKGKSLNNIGVIMERARFPTEEWVRVRFGAGVPWRRCWCVITPPDEKQYAKLQKELKKRSPYDRSPVPTLKGDIKFYDQ
RKDGKKQKKMQPIATITDAYSAYAIYPQSKSLIDASTLLKVEGNIAIHTDPPSSTEGFVFIMPETHPAVSGFEMLLRFLFPTW
DTFGLYGRPGRLVASTLDSRSLMFAMPKSRRYGYLENLDVSNLILEDGSSGWGERDWRKKLKECTGTRMNAVDDAPTTRSRSN
SRKSVRLSFGDGPPKPKAGFSDEQVPTRSSRSFSLTERTRTDSAPADPARVAFGGHGRNSSDPNQLGGPPFRPGTNGNSPYSG
SPQRGQTPVGAPRHNQASRDESPAYQNERAPSGSPPLKDLDGMRQMHTPEPVSRPPAFSHGSQARPVSRAYHSPELRRANSRL
SVTTLAQLATAGGLGEPNDSNPSPRGDEVDAARSGPSVLPPHANSVGISANDNRSREALSPLNQDPSSNSLPPPANLSQQRSR
SPLAQPPLGPPNTYGRGPNSRPGTSEGRRSPMPPGMPPQQRPPHPQNTNRRPDVGPDGRRPSDPRRPGPDGRRPSDPRGPGFD
GRRPSDPRGRGNGPPPGHMPPPPGPHGNYRPGPGRPGPPGPPGYRPNGPPHGPPNGPPGPMGHRKPVPGGPPPPAPFENSKGN
TMSGSSGITTNEIIDHYAFDQGRQTPRQGTPNSERRPGPPGRQESHTSNQSSHYEDNSPTSSLKNFVDTYQYSEPKPRAGVLK
TVGGAEEPDPRDPGFDIPDINFGPTINYAATKDRSKTPGALTPGGSSQRPHTPGGQGPTPPPHKSSGSQGSLGRKESPDPKRT
MAWQPGASTPPSNAHGLSPEEFVQQRAAHARSASSNTLGGSPSPGMKRTSSSDMLQAIQNGRHSRSSSADLLARPGSRGASST
LNFASSGETSSHLSAREQEHVARLTGQPLISMPGHQRQGSQGSFVPGHQRQASSGSGLVGAIEAREREKQQMKQGVNSQAVQH
AISQRQQQQAAMAYQQQMAQQQMAQQQMMQQQMAQQQQIAYQQQAQQQQNYQQGYQQPMMGAGNIPPYSPMGQPAGQYTPGGS
PYGPAPQQMGGHGQPNSFSRPLRAAQQVDPRFVPPQGQYGPSPGAQQTTRNVHPQYQGQAF
FUN_006054 CDS scaffold_2 584540 588233 +
hypothetical protein PF00069;PF00498;PF07714

MDGDEPTQATQNVLDPRRVGKQNSGFSDEDISDIICVLYPHSDSARVELERLVGENSPHIIGKDEADGVEPDYALEDQAARFL
ANPTGTGTHAIILRLSSHLKNPKAGFVFGRNPVRCDVVFVNDPLKRISNIHFRIYVNEYGTVMVEDQSTNGTIVDDHLLTSHP
QAKGRSDPPISKWVLSSGSIIKIYLHKEARDLTFRVRIPRRDNEYDQAYTDKVDDFFTRHGLPRQNETITPGPGGHVDLFKQP
AQTNLRREETNEITQRTPQTLSKRRDAPREWTGSGKYNRTGSIGKGAFAVVHRVTSKYDGLPYAAKEIEKRRFIKNGVLDQKV
ENEMKIMQRVKHPHIVRYMEHFEWDDRLLIIIMEFVPGGDLGKLISDQKALREDVVRTMSGQLLGALGYLHANNITHRDVKPD
NILINSLNPIDLKLTDFGLSKMVDSEQTFLRTFCGTLLYCAPEVYTEYAEYDDDGVRSRGKKMRRPPGQRYNHAVDIWSLGGV
LFYALTASPPYPVKSGISYSELLHKIMTTRLNIAPLQRNDVSEQAIDFLCRMLQRRPENRATVAELESHPWSSGEDSIINASQ
SFDEISDDDMFDNFSQLQPRHYEEDRVSDSMGEESEKETPTVPLESNQPRLFGEVGVSAIGSSGVIPEDHLNLENSSNPSADE
TEILRKDEDEAYQSDELVTPRNRNRRTYRQNNISMAQNQSEDQLQSLVEDVASQSLGGNEPSLQGPGQSRYSLQSAEFNTSKR
KPPSYDTSDEFDNTPQGKPTMKRLKSEGNIEDLANDVLEEYKLLVQVPSAEKQASGRQIDKPYNKTCFWEQDKTTWHFDYPEL
THLQHKAFERGAAARNEKFEPGKSPLWDLAMKYFSPVSKAGSQINQARRSSRTPQDTAADEPLEFPPTAAPLENSPIPDTLPT
HTQIIVPVQEDEPNRRAIGVLESHHDSCVPGISIPIKDTLVSFGRGPANTVVFRDIQESRVPKYAFKLLLWKEDGTFDPSKDP
DKVPPPWLRDAGDPNEYGFYISTKASVGISINGYQLASSDAKNHSGPSHHWARIWNGDTVMIWGDRKSTQTKLVFQCFWGASS
QAREENQGLQLASPSLSQKLDAACQKAEKRIKEVIEKKKIKDAINADLRERTENIEREQERSRAFEKKRKEAIAYLDSRQTPL
SRMASPASAPGTSNYREPLMFRMSSDNNLSIQ
FUN_006055 CDS scaffold_2 589603 592434 -
hypothetical protein ENOG41

MDIFNKAIRRRRHRSGYDVDLLSAAFGLPFLHVNGPGNNSARRVALDYVSESEPDSECGDGSEDGSEGAFSSSSEDDRGRQQR
GYDTPERVSTSRPLPHSSPHPSKRSPKRKKHHRRQLAKSSPRSSPRKSSRRQGSRSSSRRSTVEIMNSQQQPVSHVRPSATFP
PQFPIPLSKQHCNIVQSSSFPIPSLSQPLRVPVCQPFPQQPVCYHHQINYVPHRTSTLMQAHPAQYIAPVTQPVAARTPASDQ
APTVSMPPAAFKNTLNSAGERGDRHKKSGNQGDAKLSGPLTNEVQRLQKHIEAKMADLAEEPNSRILRRDLRRLQDRLNFALN
KAITASKKPHARQVSNSTFSGFSQPAHDKKTAQEKDFQQSRKREQCGQRDVSPQRIERHHICTECGNMRSALYHKKFPVGPGP
STKINLCESCREMEYKRGVVRQYHFCFNCGSARSKTFHAQHPVFPGEPIFTNYCDACVLEFHYNGSLPDDSVIGLAPRLKDHI
ARRAAIQKEDDMDSLTVNRHRNRNKGEPKASLDQDEAIEYKKQAPTIGKVRGRRPEPREPRDTVVSPETTVVSPESSYCPSRN
TGSSNRRAERKSLGDTNTDPPPPPPPHKESEKRPRNYQAPYVEDSVSTAHSKGDTHIDSHEGEMTGQKFANKVYEPWKTLPRD
FVLKSAMASDRSSSDGSGKAQDATEKAPHRPREKSLDSERSDPSSSKSSGSKTVRFKKSIDVRTPLAHEGGTEFSEPESPIRM
RLPGSPLISRKKPSYYNSYSPERKATDKNLHPHYGQSSRNSSVPNRENLRTPDDFRPGAPSKGYSQGAFSKDFDNGGDWDSFR
SPAYSYVEQYTDDGPLHERHETDEGFQAPIPKFHDYSYHDQYMESTASLPSRMSSGSVFSSFFKKKKNKSSSVPPGDRAGSYP
PSHNFNETARDN

FUN_006056 CDS scaffold_2 593590 596126 - STT3
oligosaccharyl transferase stt3 subunit EOG09260TVA PF02516
GT66
MAVKTAKTEDVLAGVNGQNTRTLLRVIILLLIAGAAVSSRLFSVIRFESIIHEFDPWFNFRATKYLVANGFYKFWDWFDDRTW
HPLGRVTGGTLYPGLMVTSGVIYHALRALTVPVDIRNICVLLAPAFSGLTAYAAYLLTNEMTTSSSAGLLAAVFMGIAPGYIS
RSVAGSYDNEAIAIFLLVLTFYLWVKALKLGSMLWGALCALSYAYMVASWGGYAFITCLLPVHAFVLICMGRYSDRLYVSYTT
WYAIGTMASFQVPFVGFLCVKTNEHMPALGVFGLLQVIAFIVYVKSVIPGRQITTIVVAAGVATFFVGLAGLILLTSFGYIAP
WGGRFYSLFDTGYAKIHIPIIASVSEHQPTAWPSFFFDLNFLIWLFPAGVYMCFQNLADEHVFIVVYALFASYFAGVMVRLML
TLTPVVCVAAAMAASQLLDTYLRVNQPEPTQAAQGEAVDGSKKAKGALRATAKPDVGIYTTLSKITVVAGMVIYLVMFVTHCT
WVTSNAYSAPSVVLSSRRMDGSPVIIDDYREAYQWLRQNTGKDAKIMSWWDYGYQIGGMADRPTLVDNNTWNNTHIATVGKAM
SSREEVSYPIMRQHEVDYVLVVFGGVLGYSGDDINKFLWMVRIAEGIWPDEVKERNFFTERGEYRVDAGATETMKNSLMYKMS
YHNFNKTNSPGKIVDRVRGVKMPDTSPTLDVLEEAFTSENWLIRIYKVKDLDNVGRDHFEAGAFSRGQKKKKAVQKRGARVLR
VD
FUN_006057 CDS scaffold_2 596950 597467 -
hypothetical protein PF05680

MASTGVNVLRWSALGLGIFYGFTHQRAITASQKAEHAQHEYEKKEKLIQQAKAEFAKKNNPTSGDSVITDPSDPKFDLEKLLL
KVQKESP
FUN_006058 CDS scaffold_2 598149 600286 +
hypothetical protein

MQQRRDEILAKKAKLAELKRQRELRASQATASRSNIGTSSDLISPTPGRADNRREIETLINSLVGDSRPGSTVTGGPGSPAPR
GSRPNSVLSAGEFSNEPSEVTTTSHVQVPAQPPPSLSTAPLLTVFECPPSPVKEVFSYSKGVQTTEEWTTPTRTRAQSLISEA
EDVPAVTSTPSKRLSRRERDREEELRQKIREEVEEELRATKEFLSDGVLPSGSGLNYPSRNLTAEELDAVAHSDEFVDFLDKS
TKVIERALDQETYDILTDYALQGQDNDDEDEDSGNTGGKGRHRIKEVVQFFDERWSKKRMISGIDFSPKFSELVLASYTKNPT
APHEPDGLVQVWNMHMQDRPEYVFTAQSDVLTAKFSPYHNNLIIGGSYSGQVLLWDTRAKAAPVQKTPLTGYGHAHPIYSVDI
VGTQNANNIISCSTDGVVCGWTMDVFAQPQELLELKNPSQAKVAVEDVSPTCLSFPATDPTFFLVGSEEGTIFPCHRYDRAGA
KAGVDKKISYKGHTAPVMSVDFHPARGPVDLGDLVISSSLDWSVKLWKVRAPAATSTIGSGDGTISPLIDFVREDVVYDAKWS
PVKPNIFALVDGAGWLELWDIAVETEEPVSKISPSSRKDGRTMLSKSLNKVAWEPNDGKRLATGGIDGSLTVFEVASGLGGKD
GPKNEDWANVKKLVNRLEAVGSNGALVV
FUN_006059 CDS scaffold_2 600894 601982 -
hypothetical protein PF00248

MPEILGKHVGALGYGLMGFTWRPKPTPLDQALEAMHTAFANGCNLWSGAEFYGPPEYNSMTLIKAYFTKFPEDAEKITIAMKG
TYNPDTFQLDGSPENVRRSIDNVLNQLGGVKKLDIFAPSRRDHNVPFEETLNVIQKEYVDSGKVGGIALSECSAETIEEAVKH
VKVALVEVECSLFSPDILKNGVAAACAKHNIPIMAYAPIGRGMLTGRFADSSQFQGQGIASGFPRFQPESFQHNLKLVDQVKA
VAERKGITPAQLAINWVRGLNGRKGMPTIIPTPGATTTARVKENAQYFPLSEEEMETLEDIAYAFEVSGGRYPEGVPMEA
FUN_006060 CDS scaffold_2 602525 603473 -
hypothetical protein ENOG41

MGIKPQFFLVRPGAEQITPNGQVLSQPATAVPLIPADLLPEWIEVIGAPRSLSPDETRDMGNLGVIHAELDTYKLRFAPITGD
GASTSDESDTSEERAPGFRPGPCAEGASSSHNYYEKPKPTTTTTQLNTPKSKLKPAQGLSSSRHNPANQQQPAHLSPTSHKET
PQIPTNTTAPSSQLPSLCRHWCHHGVCKWGRDCHYEHIMPITAEGLGEVGLTGFPDWWPQARGLMPMSPQALRAADAPNGRRT
ARKIGNLLRRKQRARILAQRRDVHFRNLEEGSEAEDEYEDEEDVEEEVVKPEPKRGGEEGILIDLD
FUN_006061 CDS scaffold_2 603853 605702 -
hypothetical protein EOG09261DJC PF01302

MTTTHHVGQRISYDGALCTVRFVGEVSGTTGTWLGVEWDDSARGKHDGCHKGIRYFTCKSKFPTAASFVRPSRPADAPRHFLA
AVNHKYASEYTLPDGRRAAEEIVFFGKRAEEVGFEKIRRKQANIGELTVVILEDLQVSSARADDESEGIIAKTCPKITQLDLS
RNLFERLDPVFEICRELPNLQHLTLNGNRFQNVLGDQANDAVNNVKELSLEETLLSWEEVCHIATKSPSLAALDAGSNQFQSL

PALDYGNLSSTLTSINLEINEFTTFVDISTLATLRSLRNIHLKGNNISTIAPEGIEGPIFSDTVQYLDVSYNNIQSWEFVNQL
PKHFPGLVGLRIGHNPFYDAADADAKASSSEESHMFTVARLGSLKSLNFSQITPADRSNAEMFYLSRIAKQLAAAPEDAEQEV
LANHPRYADLCKFYGEPNIVRRLEVNPAFLEARLVTVGFRLQDGEEKVKKIPKSYDIYAVKGIAGKLFGLSPLKLGLIWETGE
WDPVAGFDDQEGDSSDEEDAEEERERKGIDTHAAEEDTAGKPGRWVKREVELKDGPRQLGYCVDGVDVKIRVEAR
FUN_006062 CDS scaffold_2 606808 608260 -
hypothetical protein ENOG41

MLWRIITAIKDLLIPPELNALYFSLRTSLSNTVGLETTQNFTMASLEALLGPMARRLLKVAVAKTTNVVRTKLVFMTRPLQSQ
LTLRTARVARQPIHPVAVLRQQKRHTRWFSSSAAQNVNRVIRRFVSSEPKAARFDRSKIPTSNTSRRVAQLSGRAPFANALRP
NLTGGAFPRSAGGYSLGGSARYFSHTPAAPAQVVQNVSQAMRAFFLSGQKLRYDGLGPRGERQYRAVSPVEDQAMHKLSAFSH
SAPGSFIDFQLSPTVTALSPLAAAIPFASETSGFKAEAAAAGASLNTEGFLDVLSSDFGRALQDLTAVNADLRRLAVLGDLPV
LLEKHNVLRVRFPGVDAETIERLCDDIGIQRGVVGQDADFENTVGVPVALRFPFAPDGMKSAFTPSVSVRSLEGYETDEISTL
DEEYFVQQSYLDEMINSPWLAETEGYETMSPPDSSSIAGPSEDYEGLEGIYRFLEECDQAKGRIGRAN
FUN_006063 CDS scaffold_2 609855 611271 -
hypothetical protein PF00388

MAHLTIRNLTINPIELIEIERFEGQTVRTGNVLSNMTSHISGFIKATDFSSRQTQAKGDPIHKEKASVKVEPFKTKDSGIRAA
DKNKEVVRLTFKYEDHKYQVDIPSPSNKSSVMKNLDGGKHDLTVVYTPNGAFLAIFSSAQLHRWMHELHDEWPLTLLSIPGTH
NSPTCYTALPSVRCQAVDVMEQLKNGVRFLDVRVSVSPDDDELALVHSVFPISLTGTKYFKDMLEDIYKFLDENSSETVIMSI
KREGTGKGNDEQLGKYLKHSYVDKRRSRWWTEPKIPTLGGARGRIVIVRRFNLDGEMKKTCWDGRGWGIDAGAWPDNCEDGKV
PGGIIRVQDFYEITESQNIEKKIEYCRRQLERAAEQTFALAGMDGHKEGASLPPLFVNFLTASNFFNATCWPERIAAKVNPSV
IEYLCMCHGEDGKGPNKLKIGSAGTGVVVTDWVGHKDDWDLIRCIVGMNARLQLKK
FUN_006064 CDS scaffold_2 613160 613781 -
hypothetical protein ENOG41

MDAPNNTSQSQDPQVDVPKENSPPPAYTPRRVPVTDFTAMMARIIQERRDALSNMGGNNPRPQPQQTTCTSLLSDTVDDDQDE
TSEGSSPISLKIDTSVNVSRNNNIVCLTVTPAEQANAIAKAVVQALHEGSSGRCGIPMIDESGCPRPLSIQVDAGLNVEGTGN
VIGSRDVIGGLIQKRAGSSLRLDLDDEEDMAPTKRQRTSH
FUN_006065 CDS scaffold_2 619740 621131 - ETH1
S-adenosylmethionine synthase EOG09262QJW PF00438;PF02772;PF02773

MSATNGTNGVHAELSSWKHYNEGSFLFTSESVGEGHPDKIADQVSDAILDACLREDPLSKVACETATKTGMIMVFGEITTQAK
LDYQKVVRDAIKDIGYDDSAKGFDYKTCNLLVAIEQQSPDIAQGLHYEKALEQLGAGDQGIMFGYATDETPELFPLTLLFAHK
LNAAMSAARRDGSLPWLRPDTKTQVTIEYKHDNGAVVPQRVHTVVVSAQHSPDISTEELRKEIKEKIIKKVIPAKYLSDETIY
HIQPSGLFIIGGPQGDAGLTGRKIIVDTYGGWGAHGGGAFSGKDFSKVDRSAAYVGRWIAKSLVNAKLCRRALVQLSYAIGVA
EPLSIYVDSYGTSEKTSEELVQIIRDNFDLRPGVIVRELNLDHPIYLQTAKNGHFGTNQSFSWEQPKELKF
FUN_006066 CDS scaffold_2 622970 624030 +
hypothetical protein PF03029

MSKFGAMIMGPAGAGKSTFCAALITHLNLNRRSAFYINLDPAAESFEHEPDLDIKELISLKDAMEEVGLGPNGGLIYCFEFLM
ENLDWLTEALDSLTEEYLIIFDMPGQIELYTHVPILPALVKFLSQPGSLDIRMAAVYLLEATFVVDRAKFFSGTLSAMSAMLM
LEVPHINILSKMDLVKGQVKKKDLKRFLTPDVGLLDDDPIERARRVTEGPEAEDDESKAPDEKEQVMKGASFRRLNRAVAGLI
ESFSMINYLRLDVTNEDSVGAILSYIDDCIQFHEAQDPKEPHDEEFEDHDD
FUN_006067 CDS scaffold_2 624395 624935 -
hypothetical protein PF12716 ENOG41

MPSVEPPTLATHLLTALLPPELADSIQKHILHPRSPVQIIARNVLDQAQNLLDTAAPLLEPLFDRVMIVLAENQGLVGVIASL
VVLATVVIILNWIRRLLMWWTRMAMRIATWAFLLALAAWIWERGVFESARDMAVMGGKIVGYLAVVKDVWLQEYDRYEAQQGM
AGTRGTASGRRSR
FUN_006068 CDS scaffold_2 625844 628600 -

hypothetical protein ENOG41

MAAQTFPNYSRDVRHGKSGSGSSLNYGPVPGSPTLTNPDMILPDYDCDVSLDERSQSPIRMWNNAQTSDMRFHLPSSQNQFIA
GPIAPSTPIIYGNGTMLSDIGEVTEVESVVGKGGRRGSAHLSVYSNDDHVLGSSPTTGVTRIKSRSQILQRERKSSIDSNSTI
TSQGRAAPFGDFDDSVSVDDVNFQGDDEESMASSYVEGTAVREPRVPRIARTQEATEQRYSTSSLSDRAEQILANAKQRLTTM
EGNLSRARSSLFTPMSDGSTPSPPIVRPATSLRESTIPLSSSQGHSRNLSETSSQNGLASPGFPQRSASALGAAGGYRQPLTS
SRSADALGIGYGATSHRSPTTLDPMLETLSEDEGGANGDSRRSSHLYNQLSPTFGSMTDTDMPRSASVAQVRDLKHQMKDLRG
KISSLKEQARADSLKRRSLQSLRTPSPFTHARWDQGLPESRTSQETTPETPAWQAPWNQGSPKDNVNDQMSPKEGSKEPSEES
AKTLDAGAEHGPRTSDAPGVVVAQEQSGSPQFEAEPDLGDDIQVQTQVQENDDMNDDGETWEGDLEQEEEDVKQEGNGYGLHN
GEYNDDVEDDDDDEFVDFESESGESSYHDAFQTPVSHEDREDAFDYEHFFLHSAMGTMSQHRRESWSSVSSGDSVETTRGPIV
APASAPDSDSDHERRPSVDTMSSFNSFATATEGRMSRNSEFEDATETIVIMPGDSDAPLSSKRATFGNFKFPRSGAPSRNGSV
DYHQSHPRRNTVVRRPSSSAATYARHRPSMSSFGSTGTNRSFPLVDKSKGTNGILTPTGTPGGSPDSELRQISESLLNEAAGL
GGIEGGQKNGNTSAVQGLDKESQILVERLVASLGKCVLGLTESSRAGTEARMYRRRIDAARRILEGLEE
FUN_006069 CDS scaffold_2 630714 631913 -
hypothetical protein EOG09263RW3 PF00763

MRSQIITCFRSLRTFAMATSTVPKTCKVITAETIAKGYLSEVKDTLAKIQGENPCTPTLAAFLANGDPAAVKYAEWSKKTCEE
NGFNFDLRTVDKELLEEEIMKANEDDNVDGMIVYYPIFPNNPSHDKYIQESVDLGKDVEGLRHKHIHNMYHNIRFIDPPENRK
KSILPCTPLAVVKILEYLQIYNPILASGNRLFGKTITVINRSEVNGRPLAALLANDGATVYSVDVTGVQIFTRGEGIKKARHQ
VEDKEGWKLEDCLPLSDVVIGGVPVESFKVPTELIRDGAVCINFSSYRNFDGPAIKEKASIYVPSVGKVTIAILLRNLVRLIA
NRPSKDESQKSVEARAEAFADD
FUN_006070 CDS scaffold_2 634608 636285 +
hypothetical protein PF07690

MGFQIAHLWKAPEVNPISRKARSVPMLNPIDIYGRVFSFSWLGFMLAFWAWYTFPPLLTVTIKEDLHLTPAQIANSNIVSLSA
TFFLRFLTGPLCDLYGPRRVFAYLILLGCFPIGLAPLVNSATGLYISRFFIGILGATFVPCQVWCTGFFDKNVVGTANALAGG
WGNAGGGITYFIMPAVFDSLVAHQGMSPSKAWRVTFIVPLVCLIVCGLGMLFLCPDSPMGSWEDQAMLVRKNMEAHGMSSSGD
VTPVGTPDRCGSDEEKNPGEERDVKVGDHEHPISRNEAMEFARGEVIVKPTLRDALHVCYSPQTIFHVATYACSFGGELAINA
ILSSYFKKNFPHLDQTKASNYAAIFGFLNFLNRPLGGVIADIIYNKCGRNLWLKKVWIVACGVLTGALLIVIGKVNPSEANGG
DIGTLVGLVVVMAIFIEAGNGANFALVPHVHPSANGILSGLTGGGGNIGGIMFAVVFRFMHGGNDYAMGLWVIGIITIALNLA
VCWIPPLPKGQIGGH
FUN_006071 CDS scaffold_2 638052 638898 -
hypothetical protein PF10342 ENOG41

MQFTISAAALMAFAAKALAQVADFDPVLTPTNWEEVSAGKTLEITWQAKPKYSGEKISISLIGGATQNTQVPIKTIATGIDND
AASYSWAIDSTLGTENVYGLVLKLESNPEVFQYSFPFKIEGAKKSEDKPSQTKNDYEVPSSVAPKPTKPAYPVPETTVVAVSE
TVTVPCNNTAGSPTTFVPVVKTHYPAPPAPPANNGTAPNPPVYTHPAQPPVVPTQPAGQPPVYGQPTPTPVPVSGAARFGAPI
AVVAGLVMAAFAL
FUN_006072 CDS scaffold_2 644052 645575 +
hypothetical protein PF00400;PF08662;PF12894

MASEDDRPSDSPRNKRRKTSGDSSPPYAPQRDAEDNDRSPRQSRQLSIRDNRRNSRTISPSPGEDGAGNKYYESRRTSRSRSR
LRSRSLSSIDSRRHSRSRSRLTTRSQSPRSSRSRSRSQLSFRRSRTDSLSARGAEPTPPPEPFKPNYRARLVLHGHTKPVSQV
RISPNGKFIASASADATIKIWDATTGEHMDTLVGHMAGVSCLAWTPDSNTIASGSDDKAIRLWDRVTGRPKTTTRKSVAGQEM
GPLKGHHNYIHCLAFSPKGNILASGSYDEAVFLWDVRAGRQMRSLPAHSDPVSGIDFSRDGTLVVSCSTDGLIRIWDTSTGQC
LRTLVHEDNPAVANVCFSPNGRFVLAFNLDNCIRLWDYVSGTVKKTYQGHINEKFAVGGCFGVLDGAPFIASASEDGSIVMWD
VVSKTVLQRVEGHKGVCFWVDVHGETMVTGGQDNTVKVYRHFRDANKVNGDTEKVGKEPQAEGSLAQQDVAMEGI
FUN_006073 CDS scaffold_2 645830 648039 -
hypothetical protein PF00012 ENOG41

MTANEPVDDFIIIGLDFGTTYSGIAWAYSREPEEIELVTSWDSEFNNCSDVEKAPTQLLYDDKKGTSWGYSIPASKDALKWFK
LLLLDSDDVPIMVSKSSQMRCAQKLLDKMRKDPVEVIACYLRKIWNHAIDSIQRAVGAELLQKSPFHVVITLPAIWPPYAQQR
MKQAAKTSGILDARSCGETKLRFISEPEAAALATIKDLSKRSTIKVGDAMVICDAGGGTVDLISYQIESISPFVVKECVKGDG
GLCGGVFLDEQFLELIKRKLVPGSWDNVTSAEEKKFLNECWEHGIKPQFSNQNRDWLVDLPDSCDVASSGRKLKRRKTLELSS
SDILSACTPIIDKIEALLRRQTQAVKSKLGKSAKYIILVGGFGRSSYLYSKLQSAFFESTVLQSRGNKPEKRWSAICRGAVVH
CITNHGLSATLGVTVGARVARYSYGVRFHTDFDPQKHQLSDKYWSEERQKWRARNQMQWFLREGDNLLTKKPVRHDYERLYSQ
RIGHVSETIYICSEFPPPKSSGQAVEKLCEIRWTRNISLESLPTWTNPLGKVYQKLSYAIEMTCEYGIVDFTVYYKDKRVGAH
NVDVQFR
FUN_006074 CDS scaffold_2 648736 650222 +
hypothetical protein ENOG41

MEAQGPDQKPGFPLNLETEVSGPLNTIMDQSTPLTKLEEDYNDLLGRIPKFPAFPDLPIGSEFQDKPQNHPVPMQSNTTSEDW
TQEDMFGAQQENYTAFLDNYRVVSSEERSNLAQVIQTEVQNGMDRVFNYVKSFIPQPAQASTTSTTTELRAQLKKERNYVQKL
SGDYNNLLQHSHKQKQDLEKVNARLDDVLRERDQLRQLLEGGSLANSDKATDDSIRGKWKELAYNIRCLARHLAHNPPRQQLD
DIAKERLRFIAGDYHTLLEDEDYRELIIMSYLWVTVQDDVFDARQTVWGGPELKSFKAVRDRIISRVGDGINIPNCDDPIAHA
ARWLAQGSVMMCNLWERDDRGIRRLVVAEAKRLRPFHSIHQSTADRCDRKVVDQLREILDSATELDQMMMRSKAIFQIHWRDQ
SQSLSTAEMWNEDVMDSEASTKKLSPKSRVIFFISPIVYKAGTADGQRYNSRMVLAKASVVCN
FUN_006075 CDS scaffold_2 650850 652541 -
hypothetical protein ENOG41

MRLPAIVGAVGLLGTVVEAAPRGSWFSSFQQRRVARSPNGNAYDPPSYTPVNTYTPANTYTATKNTTTPTTHSTTLEPSIQCP
SNYVFVSTKTVDVTVTVTASSNGSYYTTAVCHDCTKTLTISNTIYVPPSKYASTMPYGEQNTGSPYPANSTYATPSQTKNDYP
YMNSTIGYSASTHTGYQTPGQSAVYSTADGYVSPPVYTKPAYTKPVYSDPTYGNSTGTQSGYVPPVYTKPAYSSVASGNGSVP
VYTKPVDTPTYSAPVYTKPVLPGNSSVVDPPVYTKPVETPSYSAPVYTKPILPGNSSTYDTPTYTKPVDSPVYTPPAYTKPAY
SQPIIPGNSSTVDPPVYTKPAETPTYTPPVYTKPVYFQPSNSSTIVPPVISTTSDEAVYTPPVYTKPAYSVPGNSSTVNPIIP
TSASSDDSPVYTPPVYTDPAVSSESSEYSTVSIPTNSTIPPVISTTYQDTTVLSTAESTISSSEVPPVIPTYPASEDITSTLD
TTIYETETETPTTSATETPYPTPTGADYEQPRQGGLGAAVKIGLGLSA
FUN_006076 CDS scaffold_2 653656 654709 -
hypothetical protein ENOG41

MEPVPETERMEEFRPTPTPAPAPVSTSASASASASSSLSASPSLSPGYPSLRLQNPSAFSNLSIELPRKPPNLRRSTDPSPTS
PASPSWSATPFIPYRPRTTSPLSGAHSRSRSTTSLAPPMSRTQSMPGVNGSGHILFSPQHRPGSPSGSPSRVRIPRKPADEAF
PPISPVRTSVLEPEQMHERRSTSPIMGVSTSTPARLRRPSSPLRTFTSSSTGSLGTFPSTPSSSISSTSSYRSYDALSGSYGT
TYSSVPSTPTSTRSRSPSISSLETIPDTPDAEEAALEAERIAQLKADADAAEGNESSDLKGKDGQTRGRTMGFGSRDKRKRWS
VCGAERRGDLDLETIWED
FUN_006077 CDS scaffold_2 658351 658993 -
hypothetical protein EOG09264RJL PF00383;PF08211

METPETISRGDTAKAAEVCSAHGITPKEFSELRERAVAAKATAYCPYSQFRVGATVLSSEGELTSGANVENASYPVGTCAERV
ALGTAVTSGHRGFRAIAVATDIAPPASPCGMCRQFIREFCTLDTPIIMFDKNEDFVIAKLRQLLPMSFGPDQLPPPGAPGTR
FUN_006078 CDS scaffold_2 659620 663695 -
hypothetical protein EOG092605FC PF00069;PF07714

MTESSPRAVRFSGTEEDGRIDKNRSHRHETPIETNGDPHATERQDELGNLSRYETSGHTGSLTSLLPGTVINGAPKSQRVAAG
DDGAAYTPNGSWPQRPLGPARTPSNTYNPATSRKPAHPPVTPSFTESIRSSSKTRPRQSESRFRAQERAYVQKLRQDYSGGYF
SSYSNLNGNDSDSEGETPSSEGPYDDRFDQETIMFYGNDNLQPTDEDLKDPENRERLEWHGMLEAVLTGDVVRQEKKRLIGSA
DQQAGKSAHKTELWLGIRSKVCGRHIPVQRRMLEESRSTLDRTLDEIINFQVKGESEAGKPPYEQVKDMVAKIEMCENLYPSW
ASLAAEHKAADSATFKEAHDAIISWYNTNEMINTELNILRKWVGNDELDFTRTKQRSPAVNGISSDETSFLDRLMKEDGLQSL
YNDDNGNLEKGMLAPISTVISKAKDTLIRNSEPFRKRHLPPYLEELLTLISFPSRLIEEITRTRLAYARRVKESAQQNPLMQD
QMISQFQLLLKFAIRIKIECTSIAHPEPGWDLPPCIDESFDQVVLEALKYYFKMLNWKLSGNKNTFKEAELLFQEWDFANEIG

SHLQRGHIEVAEQFSSLTFKALNRLSQTFEKELQAKPKETASEMSKRYKACLDSVRVRQRMLQRFTRMLNDNYEHASDYSISF
PPETIQKFYDQLVASGHFQVDTRLLEQDNTYVIASPELRERLEDIPVMMAITSKDQFPDELGEQYVLILRPEDPINWFGEVIE
VPLRDLNLDLKRGHIRLCASSPQTLHNARRAFLDAVDMHVDLVQESRSNIHKVNSRLTETRRVAYKLSNTFMDSVEIIRKQAR
GKDCQELIQTCFVFATEFGQRSLLYMDSNRRQMNNLKLTKLALDWVSFICDDCVASDRKTFRWAVLALELAMGMTRGRHILAL
GEDEYAKLRSKVAGCMSLLISHFDIMGARSNLAAQAEKERIEALVGQFRRLDKNRMLNDEEASRFITEQRMEELERVDEYRRE
KVAERQGVGRVLEGNNEVDRSLAYLSSSATNVTMRWQQGQFVGGGTFGNVYAAMNLDTGHLMAVKEIRLQDPKLIPQIAEQIR
EEMGVLEVLDHPNVVSYYGIEVHRDRVYIFMEFCSGGSLASLLEHGRIEDEQVIMVYALQLLEGLVYLHESGIAHRDIKPENI
LLDHNGIIKYVDFGAAKVIARQGRTLAGEKQATMPNRSMTGTPMYMSPEVIKGENPGKPGAVDIWSLGCVILEMATGRRPWAQ
LDNEWAIMYNIAQGNPPQLPPTDQLSPQGIDFLKKCFTRDPRNRSSAVELLQHEWITSIRSQVVEPATPSDTSSAQSTPTVSS
TSSRLGGSDGFY
FUN_006079	CDS	scaffold_2	671962	673061	+
hypothetical protein		PF00106;PF08659;PF13561

MPVPESEYLSPVWRDGIFNGRVVFVTGGAGSICSMQTRALVRLGANACIIGRSVEKTELAAKDIAGVREGAKVIGIGGCDVRK
IDSLQAAAERCVKELGGIDFVIAGAAGNFVAPIEGLSSNAFKSVMDIDVLGTFNTIKATMPYLLKSSTPRIIYVSATFHYTGM
PLQAHVSAAKASIDSLMASVALEYGPRGVTSNVIAPGGIDGTEGLARLGSDAESEKKRYAKGIPLGRAGTVRDIADATVFLFG
EAGSYVSGQVLAVDGAAWRRQGAISVGTEAGMEYPDYILNGEFSKNLRDPRKNGKAKL
FUN_006080	CDS	scaffold_2	674429	675893	+
hypothetical protein			ENOG41

MHAKPLSLLFLSSLPSLTAAEDVLGLYVFHRHGDRTAKAWKPVNFTALGADEVHSSGSWYRDTYVSKDASRKITGLSPESAVL
SQLDVTSPADAVLQNSALVFLQGLYPPTKQFETLANGSKVEAPFSGYQYIPIAAVSTAASDKNSENSAWLQGNSGCTNAEASS
NDYFSSPEYAGVYKDSESFYQGLLPVINGTYGKDEANFQNAYTIFDLINVARIHNSSISSDDLLDDSTLEKLYNLASIHEWNL
AYNSSEPVRAIAGSVLAGQIIEALEPLAEGKKGPKVNIQFGAYAAFMSFFGLAGLDKVSSDFKGVVDYASSMAFELVTNATNP
TADDVSVRFYFANGTAAEHTPKMFPLFGKDETTISWKDFKTGMSDFAIEDTKHWCKLCGNHDGNCASNSDDDSTSSQLSSGGS
GNGVSKPVAGVIGALVTLVVILGVQAAFILLGGLRLVKKSTLAAAGASQAGAVKA
FUN_006081	CDS	scaffold_2	676221	678208	-
hypothetical protein		PF07690		ENOG41

MPLGILEAKGIEHVPGTTQYFDDPSRPQVAGEHHGNLKRVQVGTETIILIPQPSDDPNDPLNWSLLRRDLITFLLCFAGILAT
ALGPILAANTITISLLFSKDFTKVALLTGYFLLGCGAGAIFFVPSGRIWGKRHLFLIGILILIASSAWAGSVGTNYGSFIGAR
IVQGVGCAPYESLLNAAVGDLYFVHQRGVRMAFTNLAVFGGAFFTPILVGKITDSMGWKWTFYFVAIFLAATLPAIFFFCPET
AYRREASLNTDTTGELGIELTTKDKRQAPSPQTESGSSRSPEPTQHSTGFTFLPKSNALQPIGHSNTPKKTFLQSISLFDGRK
TDERYWVLLLRPFPLLTHPAFIWGCLIQGAMIGWTVFIGVIVAAIFIGSPYYWDEVDAGYTYTGPFIGAVLGFVLAGLLADTS
VKYMTKLNKGIYEPEFRILLVIPMMIIGGIGLYGFALTAPGVVKKEYSYVVPLIFFGFEVAGMVIGAVASSLYIVDAYRDLTI
EGFTIMIIFKNFFSFILTFFAYNWINDGGIERTMLAIASIQVVVCLLSIPMYIYGKRVRAFYYRHDLLDMTGLR
FUN_006082	CDS	scaffold_2	682543	685238	+
hypothetical protein		PF00172;PF04082		ENOG41

MDPGLSSSSNVSPGPGPQSNRKERGAIAAQACETCRNRKQRCDEQRPKCGTCQRFKLECRYREPQPTKKDKTLVEILDRLKTV
ESKIDNLGQRENTTPPLFTTPQPPAVYSTNTPLLVDPDSHDPLPGTSLPNHPTSPTPNRDTGGYRYDSSVSKMSEWPVVRQMF
ESLGQKAPSALGEIPALPRGLRASNVTLPSDGLQPVGIPSNSTLQIPLHLSGSTSTLNLSPPSVDWETMQRLSKAYFDVINML
HPVLDRQWFNSNVLSSIINNGFQEGALSSLVLLVFALGEVALTTSEVPISAYKQRPSGIKGGTIDRPPGLALFNEARKRMGFA
MSEVSLENVQMLALASLYYSSCGQALECWRMNVYASLACQGLITSKPDELHGPRTDLVKRVFWHCSIMETCFHMEFGLPLTGL
EKLEETIGLPDFSGPMTDEDYIVNQQTHFHEHFASQIVLRRLSANFHSVLNKTFGTDASLPVAGFAPFNISSSPGTAAVMKQL
DAQLDQWRSMLPSHLRWQDNQDVAFADPSHNAFGDVYTEESLQSNYMFTPDLDTPPATYPFAADIQTALLRTRYSYNKYVIHR
PCIFKALHHPESMTREDAEGAAECLKASVKWPIALSPPCTNKRLVPITFFWSQNLLGVLILLHLSQQHPMLGRIRSSLCGQRF
DVEASQTVTVYLDWLRDMKKIDSTANWCWNIARLVYQLDD
FUN_006083	CDS	scaffold_2	687028	690209	-
hypothetical protein			ENOG41

MDIVEFYVSCNPPLLPKGVALIFASVEERKDDLGILEILYEPEIPWFKLIAPRASQREVSDWMHSQLEELIESEADAVIRDED
EDDLSTSAIQLDIEVLVEEPHIIAFPSLQTIQKDILNQYDTFRYPDTIKTLSYKTVWRVPESSGVTIMQILSNYESLGSVSPL
GPSIDSLQALTKCVITYNFQGSLLYIGSSESEQALSVAKRKLDTLANFMDSPLPPTAHMVLTETSSLVTLCYRYMTHTGLSRL
TYVDSAGTYQSEDQEYASIASAVSLRIESVSRYRQPVPDSVKYPIPEPGNLATWQPEFHPFSGYSYGNKRSGTIAVSENKRPR
QKVQHQESDSIDKITSPEGNRNLKHISTTDLPQVRKCLEAPRYHARNHKKITNWLYSIESSGDGDHLKPPSFLIEGNGEQGQG
ISTESPCDSCPETSRSIDEPREPRLVNTKDVPLLEAKIKYQMPESDYQASSSQLPLGLDLPENSLKLLNFPNPSDPQSNTTKD
LMDINDGPITTPALMDSIPLHDRGNRLQTASEDQGVHSEVTFGKRNHQEKRLFDTMRQKAASGLSWANVASKKKEEKRDDKPT
FGLNVPVTFVPGNRSATPTSATPTSEVIASFSHVPAKQKEEHAKDKSNSTEKPKPDPIPQTFQIVPGMDMPSTDQVDYSEILS
QAENKLSKLVEVLQIVPGKVSIQAQFGRLCRKDTFPALVYDGQAPSWAVNKTVEALNTENPHMGFYPILTTSGAEANTIPQMF
GGRSPWLLTEKRVYYEFHCVAEKTLEAVVEVDADTFQHQYFPPATEVSKAFVHCTRRAWDVKFSVSRMYMEGMADDMEEFAAS
LVRSMSIETNEMGELVISTETKSTSNLGVDKVRICHEARYRSGGKGPSCLKVTMIRRVEKLPGIPKGTYRGQSVPVTPPGNGR
PGQWFETSISSTRAEEIFQENMGLEFGDKTHWTPETLQHQGVFRAIAEPALQMVSQMDHVGDSNSNGQGPRTDRQSYSAAQDS
KDRQKGKYFW
FUN_006084 CDS scaffold_2 691621 693212 -
hypothetical protein ENOG41

MRNRLHKKDFSIHKDTVNAAATVASKSTEPHAPPLDEEKTLTPASTGAINSMKARSPTSYKPAVARSSTIRSVRSFEGSESSP
ESKYSNFSSSMNSTTSALSNSNSDSNFHNQTHLKSKTEHDTHTATPSRWSTGAARVVEPTPDPDNSAMSENVEAPDSQWDSTI
GKAGLGKTGRVINKLVSDNEALKRDIKIERLKADEAKQAAKLVEDKLERMVNDYESRLLEANVTKTLLARKERQVETLQTTVE
LEKKKAIDALQREKSWREEMEKTRKDATTQVEEATSYAQLMEGRYNAISSHWQDQGEEVKRAVSKMKSEIDQLNEERRADDDK
IQTLRDLCDQQDSNIKQLRHEKEEIARMFEEYKQSQEQGLKDIKTNARKREDEQEALLEASRETLNKLRWALNVKENVKGAQ
FUN_006085 CDS scaffold_2 694512 696877 -
hypothetical protein EOG09260C2V PF11935

MAANLSVSDQIRQLDDARKLVLGDVKYYPSVVKGILPIIGPSAALELRRWGAEFLAEAFSTPALPNGEKETMQPYVLPTLESM
LENEREDPQVLRSVIQCVASIYPLAMRWIINNGYDTITWERMVAVKQKVLRIWDNASPTVRICCIKFAQRVVLAQTAATGSEL
RYGGTLDVSLDKVPPNHQSLDPRNLEAEASGLLDRMLATLQESSDALLVDATLNCMSILVRTRPGTSNRVINALLNFNPLKLA
NSPLTPKTRVMIRSMEKTTRMLLIHLVKRDPNNPMTPRIQQHVERMMRTMAEFFDDSGRKRPFEPAPQDAYDAKRQRLSPAQI
QIPPLGPGPHSLADVFTLIDNTALKNFDISQVPAPLVARIAVTTLSRLDPQVLSKAVDGIRGRLDALANAPAPELNPNTAPLG
VEEDDDDYEPDFYQAEDTEQILNKLDSSPMHDAPVLDDGLALKSFHLHQPSALTPETALTAGNGTVTRVIEMMKSLEDPAKKS
KAGFSRLAASSGNRDSWMTILARLATRSVAGLEETSIKEEGSSSAPQSLSNNIRDVLYSYVMEDFRKHIDVAVSWLCEEWYND
KLQQKKGGDRPLYYEKCCLRLIDGFLPYLHPQDKVLTRFLSEIPELNKNLLSRVKHMCRDPSVVQLALTSLLYLVMMRPPVKE
IALDTVQDIWTEFEESRPMAGKYLTKYRPTFMESARNAASESGQSASPAITA
FUN_006086 CDS scaffold_2 697485 697823 +
hypothetical protein EOG09265KSE

MPTSPHSTYYDRRLRQGPALVRARRPYLVKNAVTGLGLLAVVGSIYWYTLNAVGQDNFEDVKVPDAPAKPSASK
FUN_006087 CDS scaffold_2 698315 700375 -
hypothetical protein PF08598 ENOG41

MAATASAPPPLSSPALGVADHEDSNISSPLSEVDTKDDNDEDIEHMNLDHDDEDSVRRSPRKKPPPTSDSDSVLSDAHSDVPS
DGNVTEAETERLYDTPKHQRQRDVVVDQFNDGQVYEHTPSKLRRTANLHNDDEDESAIDDDDASTGSPTLGEDSPTKHAIAHN
AGEEDEHKNDAQERKRKRSPAADQSETEQPLRKRASSEHATDVNTPQQPEETILQEDGPTPAHESTGTHTPAEDTDASPQKRS
TNRETDLAERLSRAVKKNTRGSKRKAAAAVADLDPDTDSGSHDGARDSARGTDVDHHDEADADADEEVDSATAHDEELERKQA
AFKDWTVIEEMFGVFRDRLYKDRLERLEEEEQSLLASEPTHPEYLNMKQCLDDRLEQKLREINNEHEYRLKAHERRSVAQRAQ
IWGQYYQAVREKRERTLEALNQEWYDIQTARRSAHSLQDCGLLYPKDPAQRVRNAIAYNTEVSALATIAKYEGFPAGPEMTGA
TPSELEDDIAAIERAKRSRHKPVNQRREEYYAQPFDRLGPAGEQFLRETPWANPNHLLHKMHPTAAPAEAPADDMTSRGAHQA
SVPPNAQITQSPSLSARLSESPELTRTLLSPAHQAKRVSNSLPGVNRGTKTAAT
FUN_006088 CDS scaffold_2 701047 702890 + NOP12 Nucleolar

protein 12	EOG09264IQ7	PF00076

MAKRSKGLTASSKAVDPTLDALFASSAGPVQAPAKSRYSTLVEQKVREPPKPRVQLEEQDADDEVLSEISEELSFEEDGPSDE
EEDGSDASEPEGESEDEQEQEEESSDEPMKDAPVELDDIIDATEDKSNKDRKRKRKNENDDLEGRYLDKLVAEEEAERAGKRQ
KNDALNKTEKAVAEDEDAGNDSDIPVHETLAKDSKSSDLEKAARTVFLANVSTEAINSKAAKKTLMAHLSSILDKDATPPQTI
ESLRFRSVAFAGGSLPKRAAYITKSLMDATTKSANAYVVYSTSAAARKAAAELNGTQVLERHLRVDSVAHPSPTDHRRCVFVG
NLGFVDDETVLNTNADGDTTEKKKNKTPSDVEEGLWRTFSTQGKVENVRVVRDSKTRVGKGFAYVQFYDGNDVEAALLLDGKK
FPPMLPRKLRVTRAKDPRKTALAQERARGKNVVPNGAAKSTKYKHKATPEEQSMAGRTSKLLGRSAAVQQRHKKRPSTQGASE
DAQNIPSNIKGPEQFVFEGRRASAKDGTPKDLKLGKKGKGKGRSSRPQNRGARRAAEWKKKS
FUN_006089	CDS	scaffold_2	703674	705387	-	TUB1_2
alpha-tubulin		PF00091;PF03953

MKGEILHLHLGQAGTQLGNSAWELYLLEHGLGPDGRPDPNAPDIGDPGSFETFFTETSSGKHVPRSLFVDLDPSPIDEIRTGE
YRNLFHPELLISGKEDAANNYARGHYTIGKEMVDNVIDRIRRVADNCQSLQGFLIFHSFGGGTGSGFGALLLERLSTEYGKKS
KLEFAVYPAPRTSTAVVEPYNAVLSTHSTIENSDCTFLVDNEAVYDICRRNLDIPRPSYEHLNRLIAQVVSSITSSLRFDGAL
NVDLNEFQTNLVPYPRIHYPLISYAPVISSAKSEHESFKVQELTFQCFEPNNQMVVCDPRNGKYMAVALLYRGDVVPRDCNAA
IAALKAKASFNLVEWCPTGFKLGINYQKPMAVPAAPEAGGLAPVKRSVSMLSNTTAIAEAWSRLDYKFDLMHSKRAFVHWYVG
EGMEEGEFTEAREDLAALEKDYEEVAADSFEPEEELEY
FUN_006090	CDS	scaffold_2	707293	709248	-
hypothetical protein				ENOG41

MASILDRDPDADKKMDPRNSIASDFAEDAVVHDFGHSPSYARVFRSLGAMSLIMAMASPMCGIFIAVSYQINYGGYWGLTWYE
YLLFNGANYWWTAALAPPSISRPLSFIAGMANIMNALTSIASFAWASSSSICTIIGLFTGWIPTNPIIFAVAIGTCAAWVFTT
SIRMDKASLVFISSATLVLVSSACFVIGLPVAQKTQGIPFTSAATVFGDYTNYSDWGRPVSVPFTFFCAAWVITGWNAPSAVA
EETHNARIVAPRSIINKYCLQAGMAASDPAGYAAFTLLIDRWGLKVAGAFLLIIFLNTSIGGAAVLVMMSCQTAAFARDGGIL
FNDKPSYVSPRINMPIYTTMFLTIGGMLILCLGFSPIAPETIYSLAVIAIMVLYALHMIFRVFDNGRWVPGPWNMGRFGKPVH
VLGFLTVVYMMIMECFPPQGNWTGATLNYNWAVFLGAVLLSTLLWFSHGSRSYKGVNQEMLRVWREQEHDRGLNQEGSGESRS
ETYIEGVKP
FUN_006091	CDS	scaffold_2	709866	711178	+
hypothetical protein		PF01266	ENOG41

MQSSWRPATQRVVLLEEMAQCEFEAVNYQETAKFIQENNLAAEVDLVQYRSADVYLTPESWKTGLASYNGFKESGGDVSDINV
LSKAEVEESLRVKGCYGAITFPAASLWPHKLAIAVLRKALDLGLSLHTNTPVTSVSPDTSGPWKVSTPRGVVMANKVIHATNG
YASYLLPELDGRIIPLKGHVSAITPPPAYMDQPLTTSFAFISNDNYDYLIQRPGSEKYLIWGGGEVAHPKGLSGGFGDCDDRH
VVSEVESYILNAPVEIFRDWEESRESSGTSPEGAVAFSWSGIMGLSKDLLPFLGELPGKPGQYLAGGYHGHGMARIFLSCKAF
CEKFLGEPIDPRVPTTYFNLTSRLKDPIDLEIMDNIA
FUN_006092	CDS	scaffold_2	711822	712583	-
hypothetical protein

MEGGQGPYLYSVDKREYLDFVSDYSAAFYGHSNPAITEAISSALYTGFSLGSVTRKECHLGERIKRRFLSMERVRFCNSGNEA
NTYALVTATEFTGRTKILVFDIGYHGDTLNFGSGDNKLNLPHDFIFGTYNNIEANQKHTSSDLAAIIVEPMLSAGGQIPVTRE
FLSFLRKTADVTGAVLIFDEVVTSRLHINGLQGFHKIMPDMTTLGKYIGGGAPTSWLSMRRELGN
FUN_006093	CDS	scaffold_2	713967	714317	-
hypothetical protein

MKYWLYESISNILDWTCATIPVGHVDFLKDPKPSNGGDFKPLSSLDRDNWNLYSPELYSDAPTCLQVLGQKFTEEKVLACLRV
IEA
FUN_006094	CDS	scaffold_2	714414	715617	-
hypothetical protein		PF01425

MPHLTVTNVVEWPYKSGQLSARQIEITDSEPSQLLKMLASGAWTAQEVLLAFIARCIIAHHLTNPLTDPMFDQGFRRAVELDD
YHRRTGKTAGPFHGLPISLIDVFNMQGMPTTLGFVARANAHPLYSGELVNRLSAAGAIFYCKTNIPQSLMSGECHNFLFGRTA
TPYNTTLSAGGSSGGEGSLIALGGSPLGIGSDIAGSIRTRANFNGIYGLCPTNGRLPLHDAEQSNAGYLINGVAGPLSKSIDR
LEVYARTLLSLKPWEWDSACERLPWDEQIYQETLLIGLSGKRQLCLGFVANDRITTPHPPIQRGMRETRATLEKAGVQVVDVQ
LFDGTEGMWEMITRIFSADGGNAFREEIAKSGEPISEEIELANPQDAMNVRQLLDHGKKILKNLATYT
FUN_006095 CDS scaffold_2 717108 718278 -
hypothetical protein ENOG41

MSSSAEDDESTSSGLSSIASAAPSSTISLSDPTPAPTTLVIAPKPTNEDDLDDDGIFPPMTTPWTGPVDCTWTYDANKVPKSG
SDGLAAMLDLQPIAGAKSLSCYPDAMFDKGLTGVYSPGTCPHGWTTVSLRVDTSENRDDETTTAICCSSEYTLDGNLCKRSVS
SVLAVPYTYNQTAQTYDAVSDTPTTLYGATVAVYTIRALFKDSDKDALGLKDEDDIPGTEHHGRSLSLGARIGIGVGVAVFVL
LALGALAFWLIRRERQKTEKRRAHELGAVGSMRHTSPGPGDNFYAAADAIHRRDRSRQTAEPPPAYEAATDSNSMTENDSRLS
EDTATRDEEIRALQVQKEAIQRRIEELERSETQSQEDNRRD
FUN_006096 CDS scaffold_2 720454 721255 + RPS15 ribosomal
protein S15 EOG092658SK PF00203

MADEYDAEQAAELKRKRAFRKFSYRGIDLDQLLDLSSDQLRDVVHARARRRINRGLKRRPMGLIKKLRKAKQEAQPNEKPDLV
KTHLRDMIVVPEMIGSVIGIYSGKEFNQVEIKPEMVGHYLAEFSISYKPVKHGRPGIGATHSSRFIPLK
FUN_006097 CDS scaffold_2 721616 721988 -
hypothetical protein ENOG41

MRRYNMNATESVCLYKFKRDCAFQDPSGTRVLIYSLVSNINHACPDCAQATFLVAEAYDITVTLVKDVRASEEIFIDFGEARS
HFDCSLCNTREPIWKRRKRKKDGRDTKRRGDTDGEEPQNP
FUN_006098 CDS scaffold_2 722791 723492 -
hypothetical protein ENOG41

MFVARQRVAGAARQLQRTARTYASDAHAHHKAPEVNESFSTGSLLAVGGFFGSVLVYQFVPQEGEQSSILSLIDKYTSRGKDW
EDINALHTKAMEQAGYDRNLFENGGNKHRFVDVAYPEAMTSYAPRNNVAGHLANMDYVVEHYRQKHIQEEERKAAKLAQKEE
FUN_006099 CDS scaffold_2 727104 727431 +
hypothetical protein ENOG41

MCHTVIAQRICKDCRHSLGETVIDFTRCARKCSSPFYCLTPEPQMELCNLCSATATLSTPILAQHALEDTTTAAAAAAASAGA
RLYDHSIAKAAAYPGPGVARHTAAH
FUN_006100 CDS scaffold_2 729013 730150 +
hypothetical protein ENOG41

MSTLYGVIERANKAGLLDEYARLWKLFIEGIDEVVGYVREEDCMKIQWDVKWFHCETVNKKGDEVRRITIVRNNNYLAAEGFS
HIPSPEEACKIAFQKLLAANADKFPAFGVWKMTKEADQEYCPIHLADPGVNLLPLRIPLPLRGVLGVLTVGVHLNVYRVKEED
GKETIDQIWVSHRSKDPNYSYPGMLDQIVAGGADTNDTIEGQLAPCKTLAREAREEAGLTIDHLTRQVFFEEMGGDDGDKKKR
RILVGTVERASSITFFDLKDESAGDLYNNHLEPGLRFVYDLKITNPSFRPKKMESAIERFEPMGVSQVTESLSSNRWKPNCGL
VMLDFMLRHGLVTKHDESRFERIRNDLRRPLCFAFLTELHRITKGW
FUN_006101 CDS scaffold_2 730330 730882 -
hypothetical protein PF07883 ENOG41

MFSEAKPLPPVKRFITTHDSNGNAIWDETIPIQVEGNRSGGFTTHVSYVNEDDTNTLTGDNDLKSYRENPTEKDIAPASGSAL
RVVDFWPGFPAIMHRTASVDYGIIIEGEIDCILDNGATKTFRRGDIIIQRGTNHAWKNTGTEVCRICVTLLPAPPVKIRGKEL
EVHGFADFPRLAEESTV
FUN_006102 CDS scaffold_2 735726 737151 +
hypothetical protein ENOG41

MHRSATLLALGALSSTATAAWLNSNQERSWEPPRQTGVFGEEAELGQVAMGWSPVPTGAPKLAGAMDMGFALGKRAVTDNTCG
YDTVGNSFTCTNTLSATCTLSDGYVGCCDTARSCNSVKTTCIPYSRSRDECTSFSDLHTLCCGQRDRPNCFEWVATTGVDEFT
LFDCAAGNGTSVLLLSDPLALSSTSGDDSSTTDNPSTTTDDGSSTTTSAAATSTSADNGGGGSSTNVGAIAGGVVGGVAALAL
VGLAGFLLFRRRNKTTPAAATTPGDGTQNQPSMAQGPQSPGPSFVPSSPSNAAYPSGVPSNYQGGYQQAYDPSLAAPYGQPQG
YQQPGYSGYSPQPQNFQGQYPQQTQYPSQYGVGGYGAPSTSPPPHFTPSPGPNKDGPEGAPQQHVQELPAVQPLGNEGNRAEL
S
FUN_006103 CDS scaffold_2 737472 739181 -
hypothetical protein PF01408 ENOG41

MSQLKNLLTKLTKTQDGDAQPQPETLVSKPPPKPEMSVTPPRFLIIGAGSRGQNYAAAIDSVSNGVVAAVAEPLKFKRESLGR
AHIWGDGSPGEGQSFHDWREFFAYEQDRRRRAASGEENVPEGVDGVFVCVLDEMHREVIVGLAPLGLHIMCEKPLACSLQDCI
DMYKAMRPSQSTKIFSIGHVLRYSPHNILLRKLLIEDRVIGEISSAVHTEPVGWWHFTHSYVRGNWRNENTTAPSLLTKSCHD
IDLLLWLLCSPEKPGQGEPHLPETVSSVGNLHLFKKSRKPKAVGSATNCMKCPLGDEGCKFSAKNIYLGPKLKGLQAGNTRWP
VSIVVHDIEDYPSHQKKEEMLIKALEEDYNDSTPKSEIQARNWFGRCVFEADNNVCDDQFVTITWPESTKPSKTATLHMVAQT
TKICERYSNFYGEHGEVHANSRRIVVEDFNTGEVKTYHPRVEDLGHGGGDLGLTRQFVMACDRVKNHDWEAPRAQDEFIGCTL
DEVLRSHAMVFAAEEARLGRKVLDWEDWWNKAVGKQMETNRHA
FUN_006104 CDS scaffold_2 739685 740379 +
hypothetical protein EOG09264V2U PF00929

MSTVAEVQADNQPKSASDDPLVWIDCEMTGLDQDNDEIIEIYCIITTGNLEILDEEGFHAIIHFPQSRLDQMDEWCTKTHADS
GLTAAVLESTTTPEQAADALYEYITRFIPERKRGLLAGNSVHCDRAFLRREPYKRVMRHLHHRILDVSSIKEAARRWAAKRIV
NKVPNKKGLHKARDDILESIEEARYYREVIFRPTFDAVPAKNKPNKGK
FUN_006105 CDS scaffold_2 740993 741555 +
hypothetical protein PF02878

MGVQTVEFKPFQDQKPGTSGLRKKVTVFQQPHYSEAFITSILLSIPEGVEGSFLVIGGDGRYWNPEVIQLIAKIGAAYGVKKL
VIGQNGILSTPAASHVIRLRKATGGILLTASHNPG
FUN_008869 CDS scaffold_3 426 1287 -
hypothetical protein EOG09263X4B PF00005

MTPPKPPRVEVNNLSYTFPDYSTGVNNITLDLPPRSRTLLIGANGAGKTTLLRLLAGKRLAPSDTISICGVDPFKEGLEGVTY
LGLEWVLNPIVRTDIGVNELLRSVGGDAYPDRRDELVAMLDVDTNWRMHAVSDGERRRVQLAMGLLRPWTVLLLDEITVDLDV
LSRAEFLAWLKRETEIRECTIVYATHILDNLAGWPTHLAHMHLGTVKEWDEADKMLATIDGTVGASGNSRLGELVLSWLREDL
KERGPRSNMKRGPEGKTYGFGGIQIGGYGDESKQREA
FUN_008870 CDS scaffold_3 1946 3623 +
hypothetical protein EOG09263CUU PF04042;PF18018

MVTLEDVEGSLLRKPSETSHQVPSRSASVYKPLQSFVLDKQRSYQQQYGDMYFLRLTKIKPAVDKVAAEAWTGTKIGEEEAQR
VERVLDVRQGELCWVTGTVYMEMPLKPNILEDVSKDRWISAPILTPKYFSKDDQVMLEDESGRIRLVGDVLKTVNLVTGCIIG
VMGTENANGELEVIDIKFPDLPPQPDRWSLSKPNSEKAKTKDEDEDMADASEAKKGNSKIAIVSGLSFSGTDASHTLELELLS
EYLLGEAMTPLSQIDVSHISRLVIAGNSISTTDRKPPAADEALPEKKAQKKYGYDASAYNPLPSQLFDAFIAELLPSIPITML
PGAQDPANASYPQQPVHPAMFPSARAYARDPAAPATQPGWFDTVTNPWEAELEGWRFLGTGGQNVDDVFKYVGSDDRLGMMEA
MCRWRCCAPTAPDTLWSYPFQDDDPFVMQTCPHLYFVGNQPHFTTKVIHGPEGQSVRLVTVPSFAETKELVLIDTETLEVERV
KISAT
FUN_008871 CDS scaffold_3 3842 5528 -
hypothetical protein ENOG41

MAPTCNVKQKTMTRAQVGQVIRSTSCSSSPSCSSSSSSPSAPGSPSSWSESAVSIGNKRSPMTANNEPPKKRYKPQPDKDSQI
VKASNNNKMIIEAPLLKDFTKEFGIPASQFVWGPRETTPEEESFDEDETAPPPSIPNAYQRDPGSRRGRALVVLTKNVNPTPA
RYQNRTQRGRRGVLRNSPAPEPRPRLPIRIKVKSPRRLSFMGLPAEVREQIYRGLLVSSKPIPVYSSWRRVYQREKPGLDISI

LMVSKKVFVEARGVMYGENIFLYLLRDAPTQYHEVANIQDLVNDDIYVPEPGMRDQENIANRDTDPLALPIHEPGTIDTAKYA
QYFRFITVKADSSRSTPSTKEFMVDAIKMFAKTPYNANIHTLKIIISPSFKHGKFTFVDFFEPASELMVALKSLPCEIIHVQI
LNKLLNNGAWPSSTDLILRAHQLRFYRQLALQEREDKRKDEGDDRDVKGQSSDLFRTDAKMKNFRFNKLCWIHQKMAQLSKFI
LQVCEKCAQDDDDKQGNTGGAPPGATDDDEDDWYIYEHDELEESEEEDQDFVQPSDNESDYEA
FUN_008872 CDS scaffold_3 7642 8717 +
hypothetical protein PF00291

MGSLGSDAKLPWIRTPCLLNPQLSRVAGCNVYLKLENLQPSGSFKSRGIGNLMTQAAAAATGPVHFYCSSGGNAGLACATSAL
SLGQKATIVIPTIITELMKGKLLDLGVEVHQKGRNWAECDKYMREELLANDPMGIYVPPFDDPRVWDGAATMVDEIRDQLEEP
IEGIVCSVGGGGLINGLMQGVEGRPWQDKKPAVVAVETKGADSLNASVLAKEHVTLPEMTSIATSLGATRVSEQTWKWSQHSS
ETMKSLVVSDADAAISAVRFADDGRHLVEVACGAALAPAYRGDLRRVLGQGMSDEEWGKKNVVIVLCGGSNVTLGMLNEYRDK
YRGESSIKI
FUN_008873 CDS scaffold_3 8882 9344 -
hypothetical protein PF15630 ENOG41

MADTTSDEDRERLKAALWYAVGQIVDEESLRRNRNATPQFIGALTELVWTQVENVATDLESFSNHAGRSTVITDDVLLLARKN
PDLHQIMKEFVDQAKAEKGTAKSRGGASKR
FUN_008874 CDS scaffold_3 10638 11596 +
hypothetical protein ENOG41

MGAKSGTALKFLQWFIRGVQFACAALILAIYSYFLATLHNHNLHISDTIRAVEGISGVAVLYTAAALVLVCCLAGRTLSSLLA
IILDVAFIGAFIYVAVANKGGASSCTGYLDTPFGKGKAGNTAEGSDGFTALPSYRTACKLQSACLAVSIIAIIFFIFSIVIEV
ALARHHRKEKRFGPSPANDYTSGYGAKGGFLGRLFRRRKTAAAEEGHDLPEHTHPDQLDTYRQSHTTDRTAVNNYPDNTYNKY
DNTGFSYQDTKYGRQDGYGQIHYPEPEYGVTQPPARQTENPNYRYEDGVYDRN
FUN_008875 CDS scaffold_3 12139 15509 -
hypothetical protein EOG09260RVQ PF07923;PF11882 ENOG41

MSNVWPPRASQDDAQPVTPIEDVTDTGEIADLAGEAEQPDLPPVPAPPTAARPPLERNTLQGAPPAAPMAPPPSNDQPSGTDS
LSLQQLRHIVAEFNRADPVAYDFEYTDMGPHAEEIDEWFNYQVPQWVRLSAAQRAFKWHWQHESRSQDSWDDADNDTRARFVQ
TAIAGVQSNDAALRSASIGKLVYLVLGRWNDTAMPNATPGDSRSVASIPQLQAIKAGVECLSSLEGLPVIWEALRNSFEVQWS
GEPLGQHNSAQEAQDELMNLMTIMYVAVQETLNDPEDMSSTYGKLLELNPSMVDYMFTATSKLRWDEQNAMPQTQILLLFWKS
ILLVFGGTKELEAIKDATNEMGDKAADKKTITASPLDYHVFRQEITSKYPAYVPPRPAIPLEADNTSLLPPLSSQVSRNNGAN
GIIPAPPQVHSGGTSILDQPVHIATPAPSPPPSPAVNGKGGKKQNYQTNQNFPFMYPPLDASSNFAGGKGPSGLQHALVGRKW
EGSDIPASILEAGELFSKRVRMTRATRQLWEERERFLKFERGYNSDHNDDDDEIEGLDLNELSAEEQEILKAVKAQTKAEEKR
KHPIAPEPEIDYGPRPELLSERDKQRLAAVEKFYRDALPHLQSLVIVLLRPILLNVTAIVAQQPQQAPPGMGRGNNPVMNGGP
SANRQQQELPGQNLPKPPTPELSLEEVDAARTREITSKAMTGILILLLKWLRVSHVLKFEYMTQLLLDSNYVPLVLKLFAHQD
VQQVVDSKMDRAENSFFHFCNLLSQSRDKAAADSVQEEDEGDDEEIEESEDEAAPPPIRRRRSPTAQNDDDTDSNSDDHATST
RPEVDELGYPLNQQPTEPITDFSRRNFFSLINYLRVMQKICKNKAHRNLLLVQYKSSTILRKSLKVPQPELRLYTLKLFKNQV
PYCGRKWRQSNMRVITAIYLHCRPELKDEWLAGSDVDAEVDAALPLEQALRSLTHWLNLRRYPDKIAPDIRAAMRDEQDFFSR
ELEKLELNWTDGGGDDGMSELEPSEVW
FUN_008876 CDS scaffold_3 16185 17064 -
hypothetical protein PF09428 ENOG41

MFEVPEAKRVRREDLDKSDDGAENWSDDGICDAELRATLNAQIARSLGLEILAESTPEVAMKDCSLTGGKESRDDDIGDSEAI
PAAEDDQEEEFVFRLFSKAPPSQKVVLEEDPGPTGDGTFVSGRPLTYYVVKNISAERKHEYTVAAVSGDQVLARSHGRAWGLE
VPWKVTKLAITRKARANEKPKDEVAQRKRRPGKKQRISLRKRAKEREERKAAEVKKMAEKEEHVKDKKKRMNRLKKLRKRAKA
KGQKQALREGDGDNGSHADSSGSE
FUN_008877 CDS scaffold_3 18713 20814 -
hypothetical protein PF05224 ENOG41

MTTATAYPATMADLRGHPGHSSMWSSYGGSVHIPGRVADPMPSTSSPLSGRTSSNHTEMDQPSYPYSRYPQDDQEAYDRPAHP
EIVSHHGYPNLKRSFSEAEPPAYQEIVQDLRDDNSKMTASHEHKLLAFKRTQDKHTIVDQQGRMQQLELSAQLHGMFFLSEMP
ANTSDGSLRQPELTCYRRNLFQISGTLVTPRGQLSVITESGETVAVSNMEVTISAIESVDGNPVRLIVIPWKTPPPNSPETNQ
TPDQEPQSLPLIPFQDDGTESDGEYAVYPIGWRRLQFRIATANNGRRKELQQHFVLHLKVVGTLANGTKTVLTESTTAPIVVR
GRSPRNFQARKEIPLLGSSAGSRGQALVETGVGVVAGPLSAKHQEAKPRGIDAQLPRTAFTFNAPKIPGTQLGPIRSNSYPTW
GTPPAQVPLGHIPTSGAESYPAASMGPLAGTTSFPAESQGMPIQSSMEPPVPISMVTNDSEHPPVRSQYTYNMQTTTGPPQLS
IHTTPLGSHDQAMNIPRYVDNPRPLKSPRHMSHPSIRSAGSVANTDASPEYRYAPYAPVNSSSSEVAQPSYNPETSGPPSVPA
RDYYAPAHTWTSAPGEHSTNLAYASTAESRPYPFPQDEYKSTPAGTSPTKTESSQPQASSVYNGATRGSFDAMHQYSWNGN
FUN_008878 CDS scaffold_3 29398 31495 -
hypothetical protein PF01485;PF05773

MDYSDDPRLIELGSIEAIYPEIRRPDAKDPFTFEIDLPVEPAAPVTVTFPAASAPAHPGLTTLGQATENAQPEVDSLEVSYLP
PLRLRVRLPEGYPADAPPDIVVSTIPQWLSRETIKRLEDDGPRLWDEIGRDMVAFTYIDHIQRAAEDVFGTISPGGTLQVDPE
HKLAVLDHDIKAKKAAFEKETFDCGVCLDPKKGSKCHRMLDCRHIFCIQCLQDFYNDAINEGNLSTVRCLSPNCAKKRAASKD
TKKHKVAISPSELLQIGLSEEMVKRYVTLKYKTELESDKNTIYCPRQWCNGAARSKRHKKPEGLEFAETSDAESGKEEEENEK
EKEQEQGGVSAKSGKRKNKETFSKADLLSICEDCGFAFCGQCYQSWHGEFVRCAPRRDKGELSEEEKASLEYLQLHTSPCPTC
NAPAQKTHGCNHMICSRCDTHFCYLCSSWLDPVNPYQHYNQLAGGKVTSCYMRLWELEGGDGDDVGLGFVGGRGPVADAPAAL
DDIELMPLPEVIESDDSDGEDEANVQGNNRPLGGQGQGIEIAREGPLVIRLVDNQARDGRRAIPPAAPDAPQQLAGRGHNGPP
RGPAGRGRGARGAGRGGGAVARGRGGGGGARGGGAVANRQRVNQRQPQLPQQAQGQDENNQDGLDPAQEAWVRRFVQMALNDE
EDLVDGDSDEDDGNWIIR
FUN_008879 CDS scaffold_3 32427 33191 +
hypothetical protein PF01569

MDDSSTPLASLSVTHVYYDPNDHVSLACAYLALLPQALCVVYATLVLFTREVEVALMFLGQLACEVLNFALKRLIKEERPRRI
HGKGYGMPSSHAQFVAFWSVSLALFLLVRHKPPRVHQVRAESGLHRPWSVLERVAVSVAGAAIAAATAWSRVYLNYHTPKQVV
VGSVAGVISALGWFLIVAAVRQTGWLNWALETPLARAFRVRDLVTEEDMCQAGWEKWEKRRAAARTIKKDR
FUN_008880 CDS scaffold_3 33674 35637 -
hypothetical protein ENOG41

MDNDPTMDSTGFQFQANSTSKWDNELKFAAAGSVRTSIIVLSVFNVIVAFAVTISILLRSWRTLKQVEPIWDFKSSWLRLVKG
RDVYPFVLSLGIVVQGIVYATAQAKGLESLMILGCATISRMMLLAFFIVPFIQMLFGLELTIRITRPSIFPFRGKFNTLACLV
TIGTLLLVVFSITFAVLPSEFCFASLISFLHRYDAGCFGVLLTVILIVLIECGVICFKLHTGARMRIAERDEASRMVYHMIIA
VISYTLQITFFYNTAFRDTGAADETSMTLNMIGTVVMNMSGLLLGCLYLFLRSSRSPTGCSDDDIEASQGFRKWGDDQDQEPP
TPGSAMLQPLDLPKLVRKAESKEDLTLDDRVEDKVFGARKDFLNRGLKPLRLGSMRNSGGLPTVPKPVRTSSKESIKNFKRSM
YSIFPKAEPPKSPILLPTTTYKDSAPKKTPALQNLADELLAPPALRLPDKRFRSSGASMLSTATVQIGLRLSNIGDMRPAKPE
LKLDTNTKQVHNLDCPNSTVRFFPRKTSPLAIAIVDVPGEGTRYKIQDNFDERLSEKELPPVPLSAGMNTPPETTLSSAGWIS
QFCKSS
FUN_008881 CDS scaffold_3 39287 41175 +
hypothetical protein PF01513

MGDHTEKHIHFEDGRNREYDGSSDEDCKAVEVVFDPGNPYRRKSSMVASEIKAPVMRHPSRRDECLVHQFLDSQRRAKDAASS
ASEPESEHSIGNNPPSYLSNNPNPSHATFHKALNGDHLEAVRDDSLDSIVDPKYQKKCRAVVEPGGMEHSGQADQQAWTQQMT
KSMSEVDLSGYQDDVRSRLLTKQQLSDMAWGVRELSRRLSSMRLRFKVKSIFILTKIYDQDLIPKTRELVKWLLQHNHEVAYI
VYVQDKLKTNRKFDVSGIIDEVSEGYLDTGDMNEQTVKETLNRRLRYWDENMCRTRPHTFDFVISLGGDGTVLYASWLFQRIV
PPVLSFALGSLGFLTKFDFEDYQQTLLTAFTKGVTVSLRLRFEGTVMRSQPRKRAQLSKGSEEDEEPSRDLVEELIGEEREDE
HTHRPDGTFEILNEVVVDRGPNPSENPRVNVTPAGDVLMYILAMSTTEIFGDDEHFTSVLADGICVSTPTGSTAYNLAAGGSL
CHPENPVMLVTSICAHTLSFRPIILPDTIVLRVGVPYSARTASWASFDGRERVELKPGDYVTISASRFPFASVQAQGRRSEDW
VNSISGKLGWNTRQKQKSYKEWEK
FUN_008882 CDS scaffold_3 41784 42563 -
hypothetical protein PF05241 ENOG41

MAIEPSSSLPPDLFDQTTIISLLSTLAIVFAAYAASLRFLPSSSSGVLRFLFIWHLADALCHFLLEGSFLYHCFFSHQPLVDD
AKTDLFPTPPGFLGYSDRVYGAQSGGSNPFAQLWMVYARADKRWAGVDLGVVSLELLTVFFDGPLAVYVCYCLAKKDPKVSIW
MIVLATCELYGGFMTFCPEWLIGSANLDTSNFMYLWVYLFFFNTLWVWIPLWVIWYSVKDISNALNLRQSKKTL
FUN_008883 CDS scaffold_3 43285 43670 -
hypothetical protein PF11034 ENOG41

MDTIKNTVNQATEKVQEGISGTSKEANKEVAKDSNAGVGTRASAAKDAVSDKIDESTHGAKGEAYKQNP
FUN_008884 CDS scaffold_3 52440 53202 +
hypothetical protein

MKHLIAVGACYLDTILTNVTSTAVPFFPSEDSKLRATSLNIRRGGNCPNSLQVLEQLLSEHDTLKLHLVSPLPAAASSATQRV
ISSFGAQSNIDFNHCVYREESTEAANSYIIRSEASGSRTLVNYNDLLEMTEDEFGNIARSFNPDQETWWHFEGRIPDTIQSCI
RLLRNVLPKATISVEIEKPGREGLPELAAEADVVFYSRSWAEVRMDSWISAVTIVVT
FUN_008885 CDS scaffold_3 64451 65492 +
hypothetical protein ENOG41

MMHPLFGVQPAGSLPITYPYDPMLDDHTQQMACSQASRRFSRGSSGQRSGGAMRVTKPSSANNSPRSSGFMARRKTLMNDGNS
QRRQQQIMEQLSTFCDVESQQPAPRSSRPVSWHPGSYGQQPYVQAQQPAYALTTSHLPTDHQDLHGNYAHYSPMMGSTSCGTS
PLAFSHLPLPYQSTDNMTCNPYHGTNMSHHSPAASMVLPGRQLPVISAPAEATDSNGWDWNTFIMHGIGSTTPPTPETLPQTQ
LSQPAVSEDPHYQALEDAAEEDGEILVGMGLYDTPEKYNEDPQLNNYRSTVSSLLGSSFRTHEPQGKGLKLEETWEPPKSDEE
DEDDDEDEDDDDEQ
FUN_008886 CDS scaffold_3 67171 68147 +
hypothetical protein PF04031

MVQYVFTPWRDRYELLLVREQMYTGIDTNATEAKGHSGHSEGSINIQNQQILDDQETQKRQHKAVARVSMWMQRGNCPHMVES
TALLMAAMLSDKEAAAGENAASSAYAIRAAYSAAFSRFVTGLLDGHQDKQRKQSMYSIAKTIGLPATFVELRHQSTHEQLPSL
AKLRTAAKKALLWIWDYYWQQLGEDNSNACRKAVLRYLSEGDESKLAALFQEFERWPPERVLKTVQEVKGSLPGNQAFLKCAE
LMQRLRESEEDKKSSKSGTADTTMKDTEPDTGEADEEDDFGWSQFKGTWKPKPIGIV
FUN_008887 CDS scaffold_3 68530 69732 +
hypothetical protein PF16123

MHIQSIPMWEGSSNNYAYLVVDDKSKDAVIVDPANPPEVAPILKDAIQAGKINLTAIVNTHQRQQEADIIGGKDCEGVTKTPG
HGETFKLGDITFKGVHTPCHTQDSICFFVEDGKDKAVFTGDTLFIGGCGRFFEGNAEEMHEALNKRLAALPDDTVVYPGHEYT
KANVKFAASVSWRDAVQKLHSFAENNKVTTGKFTIGDEKEHNVFMRVEDPEIQKQTGETEPVAVMAKLREMKNNFK
FUN_008888 CDS scaffold_3 70500 71185 +
hypothetical protein ENOG41

MPPKRKAPATSATAAPKARQSKLAMEHNVTAQEEGEIREAFSLFAEPMDGEKHGVLPIDDVKSALIALGVPPSSHAELKEFIS
ILDPENDGYATFEPFFAICALKFHTREHDSDAHRAEVEEAFRLFTNGQDGPITLAHLRRVAAVLKEDVDEELLKDMILEANGG
VGVARGVGVEEFDGVMKSAGVWR
FUN_008889 CDS scaffold_3 71770 72593 -
hypothetical protein PF01652 ENOG41

MSSRLPLFTRGSADSQSQENDPSSPAEQRDDAKRNFLKTMRALPTQHYWNVYFDRQAKEGESGDQYHSGLEQLGTQIESVQDF
WRYANNTPVGNIGVRESLYLFKSGFRPVWEDRRNILGGSWTFRFPKSIGPDVWTRVQLLAIGEKLQSVLDDDDQLCGVGLSVR
FNSHLITVWHRDASKKNSIDNIVKCIMEELPPEMHPKPDAYYYKRHSDHAGFNPPPELKVVLDSRRQEEEKLAAAAKGGDAKP
AVTVESPQ
FUN_008890 CDS scaffold_3 74713 76625 -
hypothetical protein PF00225;PF16796 ENOG41

MWVILRHRLHDSIDNVNSILMTMALDSRLMPSPSSPSASFYEGRVHSSASTPSLRSRDAALSMPARMEGGGNVKVVVRVRAFL
KRELERQAKCLIQMDPITQLTTLSAPDDPDPEAAKLKAKKVIEEKKFTFDNSFWSHDTNDKHYATQEDVYNSLGEDFLDHNFE
GYHTCIFAYGQTGSGKSYTMMGTPDQPGLIPRTCEDLFERIDAAHCENSNVAYNVRVSYFEVYNEHVRDLLVPPQTHKAPNYL
KIRESPTEGPYVKDLTEVPVRNINEILRYMKLGDTSRTVASTKMNDTSSRSHAVFTIMLKQIHHDMETDETTERSSRIRLVDL
AGSERAKATEATGARLREGSNINKSLTTLGRVIAALADPKAGRGGKRKEVVPYRDSILTWLLKDSLGGNSKTAMIACIAPSDY
DETLSTLRYADQAKRIRTRAVVNQDHISTAERDAQIAAMAEEIRILQLSVSDSRQREKNAKEAEDKLEEYQTHVNSMQRMMEE
RSMVAESKIKKLQTENEALKLHLKLAIESLKNPIPPVVVKPEREVDEQDKENASEYSEYDDDQYDSESTAYGEDELQEEMQQY
LQDMGNLRKLMQGDLTRFKNENSARMPLGAKETL
FUN_008891 CDS scaffold_3 77199 77784 +
hypothetical protein

MARTKEDVASKGDASPRIPKSSAGVTKNTAEAPVKRGRGRPPKGAAPMPKKVYVPTGRPRGRPPGSGKKTTAGPKKVAAVNDD
GTPKQGRGRPRRAASGTESAATTPKDGKKRGRKPKAADVSEDEETKHDEPADEDDAANESPAKDEAHDEDGEASE
FUN_008892 CDS scaffold_3 78041 78927 -
hypothetical protein PF01230;PF11969;PF16278

MSHQDSEPEEAITREEIRGEEPTKKSTRNAFAELMAPKRKTPPETNTARPSRSGNPFKDRMGLGAYLEDPASYPSSRVIYHNE
DFVAINDRYPKATIHTLLLPRSPKYNLLHPFDALDDPEVLAKVKTETQRLKALVAKELQRRLGTHSQSDAHRQAVLDGDAEPD
QSGELPEGRDWAAEVKAGVHAVPSMRHLHVHVFSRDMFSEALRHRKHYNSFNTPFLVDLDDFPLAADDPRRNTKEEAYLRWDM
KCWRCGLNFGNQFQKLKRHLEEEYDTWKKL
FUN_008893 CDS scaffold_3 79279 83388 -
hypothetical protein PF00270;PF00271;PF04408;PF05773;PF07717

MVKKGKGKSSGGIDLSDPMPSAPPKDQGKGKKGQKGGTSTPEAVPSGPPKPTVKQLIGGSSWTGKLPVNLLSEYCQKQKWERP
DYDTKKTPDGFSVWVTMSAKDPKTQQITKLEPFKIPATHKHLIMRPTAIEAKHAAATYALFRVCSMQNKHTVLPPEHKSLWKD
FQALKTQDVKEGKAWMYEPDPFKSLQERQEAKAAAEKKRKEIEAAKAKAAAMPGASGLVLMSNAGKGDSRSSNIMKGWSTAPK
VEMGKKTRAQLETLLRSGVKWNPNGVHMSKPQKDAIISELSKLSFRKSHVEEAVEYCKDREETLEWLLIHVPEDDLPRWALPE
SYAAGVSVGATNLRREGIIKRLAQAGYSLELATRVLDEQGVDEDKAAETLQNLLFPRESQDSADADFLDLGSPEEQWEEEVGS
LEAIYGEGFEREGDNVIRIKLDSVKNGQQTVDASFQVRRPATYPANLILSIVANIPSYIKLSIIRKALKYVDESLRDEPMKIY
LAMDWIQQNINGIIEEPGKLVDISAVSSAAAEYKPVAAIAQNKRSTRAPKMIKWIKDEKSREQWLRRQESSSLKNMVSKRQGL
PAWQMREAIIGTVRSNHVTIISGETGSGKSTQSMQFILDDLYAQGLGGCANMIVTQPRRISALGLADRVAEERCTRVGEEVGY
AIRGESRRSKDTKITFVTTGVLLRRLQTSGGRVEDVVASLADVSHVVIDEVHERSLDTDFLLNLIRDVMKAKKDMLKLILMSA
TLDAATFKRYFASEGLSVGTVEIAGRTFPVDEYYLDDVIRMTAYGVESSDSEFISGEALGKVIQKLGHRINYNLLVETVKAVD
FELSYEKKPGGILIFLPGVGEINQACRALKAINSLHVLPLHASLETREQKRVFSGAPPGKRKVVVATNVAETSITIDDIVVVV
DSGKVKETSFDVQNNMRKLEETWASRAACKQRRGRAGRVQEGRCYKLFTQNLEQQMPERPEPEIRRVPLEQLCLSVRAMGMKD
VAGFLGRSPTPPDATAIDGAMKLLQRMGALDGDELTAMGQQLAMLPADLRCGKLMVFGAIFGCLDDCVTIAAILSTRSPFVSP
QEKRDEAKEARMKFYRGDGDLLTDLQAFQEWDFMMQDHIPHRQIRSWCEENFLNFQTLSDISNTRAQYYTALGEIGIVAPSEA
TIEAHARGASSDGSQLLRALVAAAFTPQIARIQYPDKKFASSMSGAVELDPEARSIKYFNQENGRVFVHPSSTLFGSQGFSGN
AAYMAYFSLISTSKIFIRDLTPFNAYTLLLFSGPIELDTLGRGLLVDGWLRLRGWARIGVLLARLRGMVDELIAKKVESPEVN
VKDDEVIMLVRKMIELDGMDA
FUN_008894 CDS scaffold_3 84581 86597 -
hypothetical protein EOG09261OSU
PF00514;PF01749;PF02985;PF13513;PF13646;PF16186 ENOG41

MAERYIPEHRRTQFKAKNTFKPEELRRRREEQQVEIRKAKREENLAKRRGIGTGENRPGASLGAAPDSDDESAPTESQLNEDL
PQMVQGVFSDQIDSQIQATTKFRKLLSKERNPPIEEVIKTGVVSRFVEFLRSPHTLVQFEAAWALTNIASGSATQTQVVIEAG
AVPIFVELLASPEPDVREQAVWALGNIAGDSPHCRDYVLSCGALKPLLNLLGDSRKLSMLRNATWTLSNFCRGKTPQPDWTTI
APALPVLSKLVYSLDDEVLIDACWAISYLSDGSNDKIQAVIEAGIPRRLVELLMHASTSVQTPALRSVGNIVTGDDVQTQVII
NCGALPCLLSLLSSTKDGIRKEACWTISNITAGNSSQIQAVIDANIVPPLIHLLQNGDLKTRKEACWAISNATSGGLQKPEQI

RYLVSQGCIKPLCDLLGCPDNKIIQVALDGLENILKIGDLDKQAAGESGDSINRYALFIEECGGMEKIHDCQNNANEEIYMKA
YNIIEKYFSDDEENADEGMAQQPAGPNGTFGFGTNGAAPSGGFDFAKGSDAMDM
FUN_008895 CDS scaffold_3 87682 89855 +
hypothetical protein PF02765;PF16686 ENOG41

MPPTSTSTLPGYVTIRDILDGKIANGALVNVLGLVSDFRDAIQTRKTDCKCQVRLFDSSVDDDGSIAFDIFRPKENLPNPSSG
DVMLIRQARVQSYASGGPSLLSNYNTRISQYKASKIPRNPGEASCALCPPSGPKDTPPGDKENAFVSAMYHSIDKSRLPNEEM
FETMVVASRNVKDKFKLLQDVQEGCFCDIVAQVVRPPHDGGDKMTLWVSDYTENPFFHNFSIGFDESSIGRDGDPFGYTDKFT
MAAKPSGWPGPFGKRCIQITCWEPHVTAIRDQDIGTLAWVLIKNLQIKLGHSGANLEGFLREDRESHGPKIGIRMVDSNPDSE
NFDPRAKEALQRKREYERLRQGQAREIKEALRAGQKRKHGVESKSEPKKGNSRSRRSEKRSKSKANKQGEGREPTEQGVVPVV
DLNIRVKCENEDKPASLIAEITKLVQHETTIDDGLVKLPLPFVNLNYRANVRVVDFSPSNLADFAYPKKESEYDDLSDDGEES
LSNSETEAEDGQPIQRTLDDFSKVRNWEWRFFLELEDAAVAEKHKKQRLWVAVDNQSAQLLTGLDACNLRRDKQALVTLRDKM
FTLWGNLEEKKVAAKEAARKGKPPVDSDDEDQRLPEPKNFGKAQLTNRAFPCCVKQYGIKVAEPEPSQANAGNGKRWQRMFQL
FGARIVGE
FUN_008896 CDS scaffold_3 90285 93890 -
hypothetical protein EOG09260R84 PF08514

MDNNASPSPDPDATTRRRSGRVVRAPTKFTPDTATQSTNPKRKRDDQDDEEEDEEEDAEIEAPDSDEEMSNEPDNDSDEDHPA
PRSRKKSSQTSRAKKPSAKKPKINGSKPLRPARVTHAAKSLPSRPKKSVRIEDAAVKGHGLYSHLFGSGDSSKSVAEEWLASY
KTNAVAATADLVNCILQCAGCDQLITEDDVLDLDNVAGRLADLQSIYQEQQITDYPLISRTKHSRSFRDVLISFFECLIVDMH
QGRLMYRDETFLDNLHTWLASMSSSSLRPFRHTATTISLAVQSALVTVANDLDYRLKKSTEKRDKEQTNKDRKACADAIQSFF
DTVFVHRYRDVDPKIRTECVEALGNWIISLPVIFLEPGYLRYLGWMLSDTNAPTRLEVLKQLTKVFRRDAGQLGHFIDRFRPR
LVEMACKDSDVAVRVAAIGVIDVLRDQDLLEGEEVDAIGKLIFDNELRIRKAVVGFFVACTKDAISEKTKDLRDSEATEDIPR
GKKASVDAPRMEWIDLKCLAETLAIYDNQIEEEHQSGQILGLDVAVDLLESAVPETRISLAAQVLFEKVNAIKQWTLLAGYLL
FDHTTSSKSRSKGNNPDLAFKKAVAPTTEEESILLDVLSSAVKSSLTHAPDHDRSRKKPQTGKEAEDVRISPEDTAAELVIVI
PPLLSKFGAEPDTAAIVLRLAHFLDLEIFKQLRQNTNRYEKLLDEIVTQFNRHDDKRVLSEATAALLHSRQYGELEEITDNKL
SYLWETVITSLHNFDKTYELSSRGNLGEAPLRELSTVLLKISKLASISDCIDVLEADGDSSESTTNPAIQILANIVHRGKYEP
LDDQDIDDLEDEVVSFAIKTCQFYFMWKTLAYSRLLSTTGSIADAELDRLSALRQTFRRHLIETLSSRAAIDQLRLFATGSLC
DLHLTFATLRPTIDQLRPSSSSESQSSNGEKYKTLIQDIESGLIPELIAIFDGTEKQYAKKIKKDKTLNEPAEDEDPMADDED
EEDEDEDEGLSEEERFTAELKAEKALCELTAKYVLVITARLVDHKGSHGGKLRRRLLRNQNKLGNNFKEVVTYLDEEKLAERT
RKKAAHAQAPTSAKPQKAQLSEAIVIGEDDEDDIFDDTPPPEEGSREDLRAKGLLEDDPIEDDDENEDEEAERPAHESDDDVI
GD
FUN_008897 CDS scaffold_3 94619 95609 +
hypothetical protein PF01774

MDSPFPPSSSSPGDGRIVVGLLPAGESGLETMTYQYPLKLISPAPSSNQPSTLVFLLSYGGGLVAGDGVNLTIHVKPQAKLSI
ATQGHTKIFKSPTADVITTQNLDVTIDDDASMCLLPDPVQPFEDSVYAQKQIFKLRSQASLCLLDWVTQGRVARGENWSFRKW
TGRNEIWLQDSETTSADRLLVRDSIILSRDASKSVGRTLPEMMQHMAISGTLILRGSKTKSLGEFFQDEFAALPRIGARDFRS
PEAMARSITDVSDFERWRLQRLKEEKEGGVLWSAANVRGCMVVKFGAKTVEAGREWIGSMIIQEGSIASEFGDKALMCLR
FUN_008898 CDS scaffold_3 96974 98667 +
hypothetical protein PF05132

MNRGRAAARGSRRGAAAASSRAAGESNAGSSTPAVADTPGSATPVPQSIAATSTRGGATTRATRAPAAGRFRPKNIRRDEAAR
DSLARQEEQKASERAADERKARGRSRFRSKRSRGDAMGRGGFGRVISGASGPFSSGAAGSGGRGSGGWFGGGGSGGGGFGGGG
GKGFKSDSKPGFAQASRSREARINADKLHVHTPEEDLDSEDEAMIEALNSRAVSVLPMGIYRREHKDEGVIVATTAELEAAEN
AQAIPPTPSKEEESLWVDGDADEPLSTDESAEDGGIWKNNSKTPVIKKEPGLDDSMDLDIEAKADAGDKDKQASETPAPQTKK
PKQDPEEAMIQTDLEILANELGAVEISEDEDGKTEGPSNKDGRMYLFQFPPVLPPLKPITQPSSINKVKPEPGEGEDNVLEST
PAAADTEPVDLTNDKNTTGFNVPGFEDPEQKAGFMSSLLSTGGMVGQLKVRKSGRVELDWGGSTMELNPATGMNFLTTAVIIE
ENDEKPRPGVIGGESIGMGKIMGRFVLAPKWDDEEDWEVSSEDLRIE
FUN_008899 CDS scaffold_3 99272 100772 -

hypothetical protein ENOG41

MATNKDIDLHEENGSVLVATAITLLTLSWFSVGLRTYTRAVLMKSLQLDDWLMLVAQIIFTISCAFILEGVQQGIGKHNDAIK
PDEAKVQALMWQALATATYILDMMFIKLSIGVFLLRLCVKRVYVWIIWISLAIISIWSIVLFFWNLFQCKPVEMQWDFRIKDG
TCVSADQIVSAAYAISVMTVVSDWLYALLPIPMLWSVKMTKQAKATVIAILGLGIFASVATLVRLRFLADLTDTEDILFAGTD
AMVWTLIEPGVAIVASSLATIRPLLRAMRIRGFESTENTYGTGHSAAVRSNANRSKPGILIMPGFGPDDVSLTNMVSQNNTNS
LSHPNDIEILPPRHVADGPISPGQASIVKSEILVIQGNSSPPSSWQSRNDRSASSSFDQIHDLEAYSQELERDNIRGRGR
FUN_008900 CDS scaffold_3 103434 104342 +
hypothetical protein PF07859
CE10
MADIAEDKSLGLTYASHQYGKHNRQKLGVWRFADRPAQTTGYWVVFIHGGAWRDPRNDENDFTESIKRIVISGAVAILDIAGF
VSIDYRLSPHPDFPQESATVKHPDHLEDIWSALNYLQGKYELSNNYILVGHSAGATLAFQLLMSGDILASHPKGPLPIAIVGV
SGIFDLVGLNDRHNGGYAGFIEAAFGDDKSAWEKASPARFGSSFKDNWASGKLAMLAWSAEDTLIDEPEIDNMASLLTEQGLK
VEVNKHLRGEHDFVWQDGSQIARLVITTLHHLHRI
FUN_008901 CDS scaffold_3 104477 105943 -
hypothetical protein PF07690 ENOG41

MFGKDVEKTPTRRQSSPCPSEGNPEDPQNLHHTISNTNAPPPVFDPESFDPPDGGFTAWSQVLAGVFINCLAWGYPASFGVYQ
LHYEESLGLPSSQISWIGSCQTFLTFAFCGPAGRLADAGYTRETVAFGSFLAILGTFMTSLCKEYWQIFLSQGVCTGIGLALL
FMPAVTVVGSYFKAKKAFALALAATGTGLGSIIFPATLQYLIPQIGFPWAVRCAGFVALVLIVAANLILKPYLAPRKSGPLIE
WEAFRELPYLLFVLGAFLNFYALYFGFFYINVYARNVIGFTAVEAVSLLLIVNAMSIPARPIMGYLADNILGPINAFLIGMTA
LSVMVYCWIGVKSVAGMYAFCVFFGFVNGACQGTFVGANASLTKDPRKMGTRFGMVQTISAFASLAGPPTAGAILDKSNGNFV
YAQIWGGTVFLFGALMIASSRVASTGFVLMKKK
FUN_008902 CDS scaffold_3 108436 110412 -
hypothetical protein ENOG41

MASMSPLNTAVVPSGASSNGGSVSPQQTSTPKNVAFELLFLESPHCRARLPMRVQIYPHDTTDSIVTTVKNFYGLYSGPTGSK
GVSFEDDLGNTLIARYENFRNNMVVYVRVIEEPPANATTYSPHPYHNSSVGTHPYYSDNGYAATQAQRFPQELSRPSSRTSRR
RSPSPNTARGRRSASASTNGKKGRSRSSKTRASASRSHTELYSDSINGYSSNDEHDSTSGKNKDQLPTTDISVENIVEGGRRK
RAKFESSELPLFAPPQMPAATSNPSVSPARRAEPHRPAMPFVQPHQNPFTNPRPMQSPQGYSHGITHPNLYSTPGPDRRNRNS
GGYTSSAMGILPTPDPTIGSCVSEEDKDVAIQLMRLGDMSNVSHGRTSASTLDDTFSGRADAASSTGATSDGESESEAELPPA
RRQKLDISGTVGNVYQTTESHFMPPPESAEVSGDDADYEDGADESLLSTGKSKPGKTPKASTKPKASASVSKAPSAKSSKTAR
PSNGAKGKKLPPVPSGPMSPASMPPSRKQSVASTGQTPLAPGEDDQPDLSTKPRCQRCRKSKKGCDRQRPCGRCRDAGLSADQ
CISEDEGNGRKGRYGRHMGVPIKKDEVTAVVQPALLPAAPIAAAAMTLDKSKKRKR
FUN_008903 CDS scaffold_3 117344 119498 +
hypothetical protein PF00134;PF02984

MDARPFRPRGAENATHLEQSNKSSGNLAMANTNTAAGFRGPAKRAAFGDMTNVSKQAVYTRDEGKSMKIYASKGVNNNHGGAP
LNKENTVYNKDSFSRPAQRLPNISSTRTVLENKSSDANKKTSRNNVQETYVRKDTHNATKAPALSTQNAPALQPRHYKSQPQL
KPQQQQASLRRTQSKQLEKIVPKADVELGSKSSNPAVMSLQEADYPYDQGAYLESMYLPIETGVDLDMLQKEEYPESHIKLPE
ICEEEPIVPIPYDPSVPAMSEPEECWEEADDEDFDDQDQAYTTAHSVRSRDMTAGGATEIVPLPRMTARVQRELEDAREEVER
TRTQDEVEEELWDVSMVAEYGDEIFEYMRELEIKMLPNAHYMDNQTEIQWSMRSVLMDWLVQVHNRFGLLPETLFLTVNYIDR
FLSQKIVSIGKLQLVGATAILVASKYEEINCPSLGEIVYMVDNGYTADEVLKAERFMLSMLSFELGWPGPMSFLRRVSKADDY
DLETRTLAKYFLELTIMDERFVASPPSFLAAGAHCLSRLILKKGDWTKQHVFYSGYTWSQLKNLVTMMIECCDRPDQHHAAVF
EKYTDRRYKGASLLVQEALDTGFTLPHQLSPVRRELQGYREGSAELSIAQPQLIPIEG
FUN_008904 CDS scaffold_3 123387 125615 +
hypothetical protein PF00036;PF00153;PF13202;PF13405;PF13499;PF13833

MSKLAVAKEAVKEAVIGTHEPEQLAAHTKARFTKHAIKDPETGELYLGPDEFINAVAPEGEDYHKISRDQYSILFSVADVNGK
GKVSLADWGIFEHLLNKPDAEYQIAFRLFDVERTGSVKYDDFRTLYELNKGPDNLTFDWDSDWAKIYIGNKKHRHPLDYPQFC

QMLRGLQGERIRQAFHQLDKDGDGFISGEEFERIIVETARHKLSEHILDNLHTLVNLSIGSKISYANVRAFQNVIGGMDLVEL
ILRRAIGQSKDGKITRPEFLNEAAKLTRFSLFTPMEADILFHFAGLDEPSGRLGLSDFAKVLDPSWRNPIYDAVEATKAKVAA
GGILMGVLTSGYNFALGSVAGAFGAFMVYPIDLVKTRLQNQRGAQPGQRLYKNSIDCFQKVIRNEGFRGLYSGVLPQLVGVAP
EKAIKLTVNDLARKYFTDKNGNITVLSEMISGGSAGACQVVFTNPLEIVKIRLQVQGEVAKTVEGTPKRSAMWIVRNLGLVGL
YKGASACLLRDVPFSAIYFPTYSHLKKDFFGESPTNKLGVLQLLTAGAIAGMPAAYLTTPCDVIKTRLQVEARKGEATYNGLR
HAAKTIWKEEGFTAFFKGGPARIFRSSPQFGFTLAAYEVLQTLLPMPGTQKEKIPTGVSDAVSTAKGSLDTSPYGRSRNALKV
ILDLDEDFGKVKLPNEKGWRSLPKIMGGGGQ
FUN_008905 CDS scaffold_3 127243 127873 -
hypothetical protein ENOG41

MATTIQQQEPHKTGFLDLPVEIRLEIYDQLLLTAPYTRCGRPCPEQKVHASLLRTNRQIHDEATDLLYGCNTFLSHPTLLTSF
PRLRNWYGPVKESSIIPSIRRFHTQVRLDVDLPYDADAVTKAYSGLDELTIDVVQSMFLGVGYRNLRMFEGVRGVKKVNVTGS
TTGFEDYVEWLKMSMMAEPGAKVPEYEPQQQAGWIQRLATVHY
FUN_008906 CDS scaffold_3 130488 131556 +
hypothetical protein PF04193

MAVGFLTFLSGLFGWIYFLAWSASFYPQPLLNWRRRSTSGTTIDFPFINVLGFAAYFSSNVAFYYSPEIRAQYAARHKGLEST
VQFNDITFALHALFLSIITTSQYFAPSLWGFTPNSGNRPSRFILGVAAGCITGVLLTCVIVASSPGNDAVYDWVALDIVYAVG
YVKLIVTLIKYTPQIVTNYNNQSTDGWSISQILLDLTGGVLSVSQQAIDSYQQRDWSGITGNPVKFALGNISMVYDTVFIIQH
YVLYHDSEKPQSSGRENQRLLDEERRAERSE
FUN_008907 tRNA scaffold_3 132720 132818 - tRNA-Ser

FUN_008908 CDS scaffold_3 133138 136063 - LEU5 coenzyme A
transporter PF08264;PF13603

MPLLHQRLMSCATARRALSSLYSSSIRHPIIAHTKTATLGAISRRFYAIDLTSIDEKWRQRWRESNETFKVSEKGTNGQDKHY
VLPMFPYPSGTLHLGHLRVYSIADVVARFRTLRGEKVMLPMGWDAFGLPAENAAIERGVAPEKWTRSNMAKMKEQLELMNGSW
DWDRELSTCDPGFYKHTQKLFLMLHERGLAYQAEAEVNWDPVDKTVLANEQVDANGNSWRSGVKVEKRKLKQWFFRISEFREA
LLEELEALAKENAWPERVLAQQKNWLGKSTGASVKFPVLALGHDIHAAIEVFTTRPDTLFGVQYVALAASHPIVAKLAASDPE
LQAFIDIIPGLPADSKVGYLLPHLRAINPLAYHEETPDATKVSLPVYVAPYVLGDYGEGAVMGVPGHDLRDNAFWKEHHYDVP
VRQVLSASEDESTTAMKNEPFVEHGVMTEHSGSFKGKNSVAAGEEIVKLLEKADLAKPVEKWRLRDWLISRQRYWGTPIPIIH
CDSCGPVPVPDEQLPVKLPDVDWAETRNGSPLESSPGFVNTTCPKCNSPARRDTDTMDTFVDSSWYYARFADPHNQEQLFSPE
AGRTLPVDTYIGGIEHAILHLLYSRFIFKFLASTPLLPQYNEETHKSAEPFKRLITQGMVHGRTYINPDNGRFLKPDEVDLTD
PNQPKVVSSGKTANVSFEKMSKSKYNGVDPSEFISKYGADATRAHMLFQAPVGDVLNWDEAKISGVTRWLQRLHDQIVAIAAE
TADQTTTAREYLVEKHKGLGSASTEELKQWDAEGAIWRHLQRTITSITTSYEEVYSLNTVVSDLMILTNTLAENDAASPIVKH
EAARALISMMAPITPAFAEECWALLCPSASSIFSSTSFPVPDNSLAELVRPRIQPCAVQVNGKVRGVVDIPAPPAELSGDELR
DWMVREILATKEGAARFSEGPYDLRAAKRAIPVRGGTTINFVLK
FUN_008909 CDS scaffold_3 136715 138737 +
hypothetical protein PF02219

MDKITDRIAALPADGTYFSLEFFPPKTAMGFSNLRDRLHRMERALRPLFVNVTWGAGGSTSQKSLELAELCQREVGLTTCLHL
TCTNMSKKLIDEALSDAKALGIRNILALRGDPPRREEYRDPEDSEDDGGQDFNWAVDLVRYIRKTHGDYFCIGVAAYPEGHAE
ESHPLGQSLEHDLPYLVQKVQAGADFIQTQLFFDIEAYEHFETTLREHPSGAFKGIPIIPGLMPIQSYQMIKRTTKLSHAKIP
DHLLARLDAVKGDDERVKMVGVDIVSELIDRIKAIKSKTPGPKGFHFYTLNLEKAVSFILERANLIPDPPENEEAIAVDELAI
PSLQINGSANMRSSSHSRSRQSRRHSSVASDPHNRVIVGDRPAIYPEWEATGQEAGVRAEAINTRANTLAISEGEGVLGREAT
WDDFPNGRFGDARSPAYGEIDGYGVSLHMSVTQAVKLWGYPKTRQDINDLFVKHIRGELSAIPWSEEELRAESSTIQPNLLQL
NRKGWWTVASQPAVNGLRSSDGTFGWGPPNGFVFQKSFVEFFIPANEWDTLKAKLASPELQDSVCFYASNARGDYLSSDNSDH
VNGSTEAGPSTNAVTWGVFPGKEIITPTIIEEVSFRAWSEEAFGIWGEWAKVYGRGSESEKLLSGIKDDYWLVNVIHHDFVEK
DALWQLLLS

FUN_008910 CDS scaffold_3 139471 140679 -
hypothetical protein ENOG41

MAPANAKANYKTYEAQARMVRAIVAAHPDVKWNYKEIAACYGSDMTDHALNHRFRKVRATVSIIHAAREQGLDVKDLTTDENL
LPTTQGAIDKNNIAKYFGQSTADGIQFQFRTIKKDAEALRYAANNGDDVANCLNLGTASGLPNTPSKPTPSRQPGSRSAKGSK
RKTSYTPLNPIKRSGSEDEDDDELNFDEMEHTPSKKVKTTGRTPRGPTPSRTAAQKAAATIAGASAQYNDSESPYEDAPTPTP
GPTSAPVGLPAATLPPSATVAPTSIFGNVERKPQPSVVGNSGLDMINSVGLGYPDIFSVAGTFRAPPPDDSSYMEWGDGEI
FUN_008911 CDS scaffold_3 142442 143441 -
hypothetical protein PF04193 ENOG41

MEPGLVGWVMELTAWVAPFFIVMSPILSYADQIISMYRNKTSAGFSLDIPLIMLVASFLRIFYWPQAQYDTSLLLQSLLMVIV
QIALLKVALDYRPPPPTKGGEAGLPFAGADDGLMGFQRPYNFWQWRSPRRYWHAIMYFAAGLVFLELLLSQMPGLYAVYANTI
GCIGLGVEATLPIPQILVNMRSKSSKGLRLSVLAAWIGGDTMKLFWFFTSKSEIPWSFKISGMFQASCDFFLGFQYLLYNSPE
EPAQIKEHPMVEWEAPKSTTRSHSRSLTPTRRPAPFVGAEEAK
FUN_008912 CDS scaffold_3 145508 147039 +
hypothetical protein ENOG41

MEFTTPERRERPSRAAAPKSFKDPESDSDEPIVPRFCSMSSTGSRVIQVKDETAHSSSDPAIKTSPLATINQSTVLKKRSSSD
EEANSPNKKVRKIVLKTNKSATASNNAATPQPAPPALSHIEVSPSGNLSEIQSLHEGLTFLQERISATLHTTRFAVELDAAKK
KITSLERQLLEASSPDDSTSKLKQCEKKLAMSLDRNGRQLDDNVELTRRFKETRAYADELRVEMAAMLAEKDPNFNDAFKVTD
DEVERIWGGIRYQIFAFVAQVLTVKPYRKAAPREAEHSVVEALKKEQKKDIHLADFYFEQYIWKCLVRYIFQGEAAIFGGPAG
RVFQLFCLDISKIDCESMPELSRVKAHTANLLNEQFDQDNNREVEGIVQKLKNDLAIFMGSDEEDKAEEKLGNIVSKAVKLNS
YFLKSRAFFVTDWDTDDESNNFDDLIVRYKSGKQGGEPVVGVQISPRLSKIGNADGEFFKSANAMVIYKPMVTLNYE
FUN_008913 CDS scaffold_3 147725 148391 + URA6
bifunctional uridylate/adenylate kinase EOG0926406J PF00406;PF13207;PF13238

MPAIEQDAPATIAPQKSTPTFDPKDVTVIFVLGGPGAGKGTQCSKLVSDHGFCHLSAGDLLRAEQERPGSQYGDLIKDYIRNG
LIVPMEVTIALLENAMAADLKAKASQKGRFLIDGFPRKMDQAVKFEETVCPAKLVLFFDCPEDVMESRLLERGKTSGRDDDNA
ESIRKRFRTFIETSMPVVNRFEEVGKVLKLDATPPPDEVYANTEKALAERLGPDF
FUN_008914 CDS scaffold_3 148781 149964 -
hypothetical protein PF06839 ENOG41

MVSTPRPKSRRDPQTPRSQKKRMPKGRYSDGHWWCNCEPRQKATLREVKKSGPNNGKLFWKCDDCNFFLWRDEAKVRESGLRA
SRRGSESSKSEPPPMTQQSLVSYGYQVTPSRRQSDDDSASSTESGEDSEADTPMPKSVRNRTQIADHSKLPSPPTAAVETPSR
GPSKRRRDVFEEDEDEFSDIASDEERQMAAIADKSAEKAAQSRFTTLTTTRSTDAISGIPTPSVSRTLFPTSESKRQKQVSFE
YTPSMSSTTLSANTTPSKTPCRMQEPPASSPPETSYDVTDEVMNLLRGQNIDKSVLSSVQSILETAARRTKGIVLGRDSARSS
LKTKDDNIATMQERITALENRERMHRSQMTNIKAGLMKMYDDN
FUN_008915 CDS scaffold_3 151634 153120 -
hypothetical protein PF01399;PF18005

MAETKTAGQPTLVFVDGSFEDLAAEMADYLKAEDAKQLLSQEKPPSQEEVISKLVSASGALNTVPEKEYTAASNLMTHLVLMS
SDPKKFLPTLCTTFAKPLLNSPVHGAGLSLNALTTVFNLLDPADPVRARVFMEILKFLRAHGMFESLRTYLDKLPEWLATWGT
DADFQRKIYEEVAEVAIEAGEEQQAYEYVVKALRTFDGDAISSEEAQRLSLRAVKKALTSNNHFLFQDLRGIPSVQALSDSHP
VYSQLLDIFAEQDLEDYNDFNDEHQGWVEKEKLDHEKLHRKMRLLTFSSLAASTPSREIEYSNITKALQIPENEIEMWAIDVI
RAGLVEGKLSQQRQKFLVHKVTYRVFGQKQYQELANRVDHWRTTLQNVLGVLRQEQANAKAQKEREIQELERKVANAGVSGNQ
DGGRRRQNQNREPREPREPREPREPRERTEDDD
FUN_008916 CDS scaffold_3 154168 156518 -
hypothetical protein PF00069;PF00498;PF07714;PF16697

MAPQPEKSQLKRPRGSLPDDETETKKPRRAERLEADPDKTPIVNKQHLPSPLTHLTDDSNELNKEPTATPPGQKQHEITPKKT

EDTYSQGQALSSPPQDTQPLSQFVDRHPAISDDIEDEIREGVWGYLVPLDPKYGDKPIVLKKRSACPLPDTVADSAKQDNKHH
TNENGKSAAMKDEEAFEKKKKEKGVSSGGYLIGRHPECDIVVNDGIVSNRHCLIFTENKGNDTVAIVEDLSSNGTYVNEAIVG
RNQRRELEEQDEIAVHGKARFVFRYPQSRQTSAFLQQYTLLEKLGKGHFAEVYLCVEKATGQRFAVKIFTKHPGVEDRSKTEG
LQQEIGVLMGVSHPNVLCLKDTFNERDRVYLVLELAPEGELFNYIVMKQKLSEDESRKLFVQLFQGIKYLHERNIVHRDIKPE
NILLVDKNLHVKLADFGLAKIIGEESFTTTLCGTPSYVAPEILADSKQRKYTKAVDVWSLGVVLYICLCGFPPFSDELYSRDF
PFTLSQQIKSGRFDYPSPYWDSVGDPALDLIDSMLIVDPEKRFTIDQCLQHPWLTQSSPGVNDSTGGLVGGIAGLEVNRRAPA
RERTLLASLNTVQVTAQLEVGKDKNPVKVFSKNKGRVTNIAKESDPAAQRAPDEFIEMGGKGDQELFADDDSSIYPIGDNGEK
IKANKAKR
FUN_008917 CDS scaffold_3 158273 161880 -
hypothetical protein PF00169;PF01412;PF16746

MGIVSSKPEEGPALYLRDQNRLSISSLVVTNPRKRTSVNIVPNAFPATRVSAQRPLGDTSPVEFVQDPDISNPTAPPNFLVKL
SNDDELIFSFTFVMRQAQLAQPATSSEPASAPIDTSISGLTYVYASNLKEVENLVTREFHADPNLHRNANVALVGDYTTGGSP
SVSFEWTWKWKPPKPTEDKGGGWRNSCSFVEYDPRAHRLHTLASFSYFVAGTNPRDALYLSSAHAFSGTPASLSHPNSPSPPF
LLASPPKIRVASSQSVDSRITAPDIIDEPVSPLIPTAEMPLTPGFPQAKETVKVDVACPKPNEESMVSDDGPVFRATLKALEQ
KTGNMRTQMKKVLKKAEHAHSCQIEANDAFTAFVDALREAAATNANAVQPALTHYFDKIAREILSYERQNTYNLQKIIIEPVT
KLYQVDIKQAESKKRDFEEESKDYYAYVSRYLGQRQDSVKAKKLAEADSKYQNKRRNFELKRFDYSSFMQDLHGGRKEQEVLS
HLTRFADAQTRGFMATAKKIEVLLPQLEALSNEVQEADKEYQYQRREREEKRRLLEKSNTPYKEPEQNSTASNMPVGSATNGN
TGNTSDSDLGRADSTGSQLNAGSVNVPSGVELSRSPGSLGQTGVGSPAAASKFKGIRDLEERDNMPSAVTHRKEGLLWALNRP
GGHVDPRNLNKQGWHKFWIVLDQGKLSEYSNWKQKLDLHIDPIDLRMASVREARNAERRFCFEVITPSFKRVYQATSEEDMNS
WITAINNALQSAMEGRSYQGKPTSSNGDSSLRRDIGSVLTGKTQSLSHGNHHSTANTGIPSRRITVGARPSAVRTSSSGYDEN
PDRVLQMVRDNDQGNLWCADCGSGSKVEWVSINLGIILCIECSGIHRSLGTHISKVRSLTLDIKSFTIDIVELLLLIGNRVSN
MIWEAKLDQSLKPTAQASREQRLRFITSKYVDRLFVEPISPTLSRYGTADETLLAAIKKNEIQQVLYALALKANPNTVDKMRG
THAVWLALAAADPAAPSPGTPHTVTDAEAKAVPFPVAELLVQNGAEIPATMPAFPLGRFAQIYVEQKRGRTVIEGSMDAVPSL
PSNMSPAERLQREKEARLQKRVSTGGRLARSPIPER
FUN_008918 CDS scaffold_3 165667 169856 +
hypothetical protein EOG092608WU PF00168;PF10540 ENOG41

MSSESRRSYNPGRRMGSSRDLGRARAASRAQAIVVEHDEARSFALRSAYLHYLLQPKAKRKQYVPAPKQPVRTHTSVGALVQE
FTSGSSSSFKLPHNFTSALLERCGGVLRGSERLPGYNDAAVKRSFAEAYTTFSERNFRKTIDKERKLEPLILMFYTSTTKAAQ
KGAAPDDDSWKLIPDRHLALFVRLIATILRDQGHDRDKTELMSKLSVLEKKLLTNDQNLVGGGSDGAGTTIEVVVPLSYDVKD
MPLVQQVVRIFGLSLSEAQAEINENKGVWTEEAALKDLKSYQHRLNSDMPGALRSDDFDLDEAFQEWKKAESPHLSQMMLDIL
TAKPELAKTSSSNIDKGFSSRPQSTYSDPAFDASMGFDPAINLASLNIGGDSSIRTVEENVYTFVPPDPRAFFKYILKHAMTF
DQINSDPDLDYQPLSKQSMDLLTELCVRWRVPQCSRLITLLEVASRNYLDRELLPEELDAVFDFVKTPLPEVKKPPHISNYST
PLSEIDPSRWTVHDFAVYQHTLNTLHETLLRDLYDRMEQCYDTKPPQIGVVQYILDNHIFSDPCFSPRPEATEEFSQHLSQRL
HDQAQEAYRQYMEKEISADRNDWDFANVVKLGKTVIKLCERINKRYNKNPEIMGVNPFQILVEEVFPRFEEDASAIIESILEM
AQETGTELDIQDGFELYRELVAIRRIHNDSLPGRPFAFNVEDLLVDFVWRWVRAAESRVEEYVEQAVKQDQFQVRTENPEDIP
RDSERHSVSIIDIFMLFNQTLDTVFNLNWGNDEHHARFMTSLSKAFAAGIGKYCELVDQKFAKEMDRPSAEELAAQTQTTQEK
WMRYAKDAWNNKDRVEPFQFYSESFVKLNNIEYAMQELDKLERTMDPDACAALLERIDGPKKKARRPNKYTFTIKVVEAEDLK
ACDANGYSDPYVVFGDEYQKRLHKTRIIQRSLNPRWDETFDITVQGPVNMIATIWDYDTFGDHDYVGRTSLKLDPVHFSDYLP
REFWLDLDSQGRLLIRVSMEGERDDIQFHFGKAFRHLKRTERDMVRKVTDKLTSQINHTLSLETLRHLLGTGGIGASVTNLWK
KRASTVQTATPAQVEAALAPLFAYFDENFAIMKQTLTKATMDAVMARLWKEVLMVIENLLVPPLSEKPSQQRPLTRKELDIVY
HWLDLLFQFFNARDDSGEQLGVPAETLKSPKWHELASLNFFYFEDTNSLIRESERMAAATTERARLALQQQNSQSNRLSAPAA
LGASLGGAGSFASMGTIRRGKSIMMSRNLGTMRKAKEAKRREAQADPSDDMILRILRMRPEAAHYLKDRHRQKERQAATAAAA
LIVKNSVTQGWNSSAGPAFSGNFGRNNLPRR
FUN_008919 CDS scaffold_3 176216 180396 +
hypothetical protein PF00072;PF00512;PF00672;PF02518;PF18947

MVDDAALAAAASIVASIAPDPRLPNSIPVGVASQVQLPGPDTPAKRKLELELQNLALRVGKLESQASATSPFPETPNEVIDTL
FGEEAQAVAVRPKPKVFHAQGSLHSPHLPSYQLTEEALEGLREHVDDQSKLLDSQRQELAGVNAQLLEQKQLQERALEILEQE

RIATLERELWKHQKANEAFQKALREIGEIVTAVARGDLTMKVRMNTVEMDPEITTFKRTINAMMDQLQIFASEVSRVAREVGT
EGLLGGQARIGGVDGTWKELTDNVNVMAQNLTDQVREIASVTTAVAHGDLTKKIERPARGEILQLQQTINTMVDQLRTFASEV
TRVARDVGTEGMLGGQADVGGVQGMWNDLTVNVNAMANNLTTQVRDIIKVTTAVAKGDLTQKVQADCRGEIFELKSTINSMVD
QLQQFAREVTKIAREVGTEGRLGGQATVHDVEGTWRDLTENVNGMAMNLTTQVREIAKVTTAVAKGDLTKKIGVEVKGEIAEL
KNTINQMVDRLGTFAVEVSKVAREVGTDGTLGGQAQVANVEGKWKDLTENVNTMASNLTVQVRSISTVTQAIANGDMSQKIKV
EANGEIQVLKETINNMVDRLSSFCYEVQRVAKDVGVDGKMGAQADVGGLDGRWKEITTDVNTMASNLTTQVRAFSDITNLATD
GDFTKLVDVEASGEMDELKRKINQMISNLRDSIQRNTQAREAAELANKTKSEFLANMSHEIRTPMNGIIGMTQLTLDTDLTQY
QREMLNIVNNLANSLLTIIDDILDLSKIEARRMVIEEIPYTLRGTVFNALKTLAVKANEKFLDLTYKVDSSVPDYVIGDSFRL
RQIILNLVGNAIKFTEHGEVSLTIKESMGQNNVRPGEYAVEFVVEDTGIGIAQDKLDLIFDTFQQADGSMTRKFGGTGLGLSI
SKRLVNLMGGDLWVNSEHGKGSEFHFTCLVKLAPDDAALIEQQIRPYRGHQVLFVDKAQSQNATSIKPMLEKIGLKPVVVDSE
KSPALTRLQSGGSLPYDAILVDSIDTARRLRAVDDFKYLPIVLLAPVVHVSLKSCLDLGITSYMTTPCKLIDLGNGMIPALEN
RATPSLADNTKSFEILLAEDNTVNQRLAVKILEKYHHVVTVVGNGWEAVKAVQSKKFDVILMDVQMPIMGGFEATGKIREYER
GIGSHRTPIIALTAHAMMGDREKCIQAQMDEYLSKPLQQNHLIQTILKCATLGGPLLEKNRERELALHAETKSKHKEGGQGLL
RPTLESRSFTSREPLLGNGKESPAILATDEDPLARARLDLSDMRSLTN
FUN_008920 CDS scaffold_3 181336 183068 -
hypothetical protein ENOG41

MAPSDTNNRATKPPASGSSSGDADLAQAYRDLARGEQAATALENNLSNLESRLDAILAALEARETPQMPATAAKANSSPGAAD
KDQKGRSLDGANGDAEKIEKDAT
FUN_008921 CDS scaffold_3 190434 192414 +
hypothetical protein PF00505;PF00536;PF07647;PF09011 ENOG41

MSPDLESIFAELGLSQYLGAFVEQGFDEWDIILDIQESDLDALGVKLGHRRKLQRRIANARGISPSVSLVTSVRPTSEDAKSD
SVQPEPTRTELPPEGQGVAKRKYRRHPKPDENAPERPPSAYVLFSNKMREDLKSQNLTFTEIAKLVGENWQNLNASEKEAYES
QANADKEKYHRDLMEYKKTADYRKYMQYLHEFKEKQAKRTQVDVSKRTKLDPVRLRHSSSSSSTMTPGGNTSSGSGSERLQGS
EPPPCRRERLNSIASIAESQHSSTTPTLLSQANSNDETMSSPQAAHFDAGSPREPHYQASKRQQSWREGGRGVEVSHQHLPSL
SNMLEDGRKGMQVPSGSEGNPYSSGFVAANHPRSVPEVPNVLPSAPPKPPLLRHEPSSSSSIGSVSPAAGFARTPGEGPLPIH
ALLSHHQTLPTPVVAANAAMFERGSPVSGLGTGTTTPSPTDPVPLVRPYFGHGVVPRGYGSQPLPPTGPPARNDEYMADSDVP
MTPAPDLIAPIEDRSFKTRLDGMSVLLRAGELVEKHERDERR
FUN_008922 CDS scaffold_3 194349 197023 -
hypothetical protein EOG09260WU6 PF05786

MPRVAQVPRTGSARNNTGSSTASPFITQSPVKIPLNDDVSEKGQRMSSRRALHERQFNEIKKAATPRKVGLRLDDMENGDPQT
PRGGRASYIEDDEGFVVGGSAVTPMKRVPLLANFEEWMKMATDNKINATNSWNFALIDYFHDMSLLKEGDGVNFQKASCTLDG
CVKIYTNRVDSVATETGKLLSGLADSNNAKKKDKDGEDADESDEEELDEDGNVKKKPKKKTQRSSEATLAPSFNSLQLKKFEL
EFAVDPLFKKASADFDEGGAKGLLLNHLMIDSQGRIVFDSSDDTGDAATLGKTKSDDEEGDGEDEEAENDPEEGLEDEEEADD
VEIDVGVLGAKFIPDLHRLDELDVCPSLKTFDLGDPSGSMDIPFLKAPEDWRQDQDKEKTPGALGDFSGLVIDGDEAAGFDDD
DLALGAFDAADNVAFGEGGEAWAREAALEPQMRVYDAGLGEEGGDGDEIDGNGEYVVSMTSAQKADKMHEDILGFFDQALQKN
WASAEHWRIRKIKDVNKPATETKKRKEKEPFEIDFAAPLDSHTSDIIHTQATNNSAISMPKKDWKSKSRNLLPDDKHFNSKSL
LSLFLKPKARMSKRRTGFSSRGGGGFGNTGADNQPDGEMDEAFWANQKAPQHTDETALPEGDYDANFFQDDVMPFPGGGDLDD
DDDLEFADAREHFSPGLDGQAGLTDGGGLTALLNGETVTNTGAFGTTLVTQTRRVRPEYVQYARVAKKVDVRRLKEELWKGMD
VDILQKQPEPQAVDVSEPKQEETLKFTEIMNNLQTVYPKPVMDDISTSFCFICLLHLANEKGLVIDNTPGLSELEIRRDWSAE
IVEGE
FUN_008923 CDS scaffold_3 198119 201277 +
hypothetical protein PF01699

MTNTTPRGLLCPISETTKGPNATPKRFPRGSRLQSRPFYTTILLLSVLTAYSFLSHTTFAQSVSNRATDPDLLFKRSFSTVET
PECNQVHDAEDKCAFVRKYCSDDDAGLIHYIELYYCSFGNVRPIAFTALVLWLGLLFTTIGIAASDFFSVNLSTIATVLGLSE
SLAGVTFLAFGNGSPDVFSTFAAMGSNSASMAVGELIGAASFITGVVAGSMALVREFRVDRKSYTRDICFFILAVVFTMIFLA
DGHLHLWECWVMIGYYGVYVVTVVTWHWYSTRRKARQRREGEARSHVYAALGHSGDELAGEPYRDDPDDVGSGPGTAHATPPD

ISLLEAGPRIEVDGTPQRAESDTSSEDHDRMVAAEVASSMRVLRGRRVRRNTMTPIRPSLVGALEFRSALAQLQREGNLQLST
IPGRSYSDYHVHRRRQTTATIAESARSDGQLDPNTGEHAPIRNRALSSGDVPVGVPAHPDLHIPRRNGSEPTLSVPGSTASGT
RASSPSPSYTVGGNLAAPPVGPSGTTHDAPSEGQKGQLLTPELRLQIPSSRRSSYSDRSSPSTPFPVYSDSPALLTPNYQNDP
IEFVSPGGVSPSTVAPGPASRETPFADLQLTVDTPSRPVRWWPYAVLPAPHVLWATLFPTLQGWKEKTAWDKFVSAISVPSIF
LLVVTLPVVDSETTDGESSILEALNDHTDHYHHDHQVVGHMAPPISIEHSAIEPEGESEWERYRRHTITSRGNSHVGLATTPS
TAHAADGETLAASQFSLGPPAIVAKPASDFHSSSSVKNDCTGWNRWLVALQLFTGPQFAVLVLWANTLEDWESPHKALIRMVL
YTLLASLILLGVLVVFTSEDRPPRYHYMLCFMGFIISIAWISTIAGEVVGVLKTVGVILNISEALLGLTIFAAGNSVGDLVAD
ITVARLGYPVMALSACFGGPMLNILLGIGIGGAMMTIQKANKKHRKNPSHPIKYKPYRIQVGGTLMISAITLLVTLVGLLIVV
PMNKWILSRKIGWGLITLWAVSTIVNVIVELTGAWGEMA
FUN_008924 CDS scaffold_3 202279 203205 + PMM1
Phosphomannomutase 1 EOG09263YFH PF03332

MALASPPSYTPLQERPLKDTICLFDVDGTLTPARLDASPEILDILQKLRSKCAIGYVGGSDFAKQQEQLGKPAGQPVTALFDF
CFSENGLTAFKLGEPLESNSFIKFIGEEQYKELANFVLHYVADLDIPVKRGTFIEFRNGMINISPVGRNASTQERNDFEAFDK
DAKVREKFVAVLKERFGHLGLTFSIGGQISFDVFPTGWDKTYCLQHLENEAKKPDGIAYKTIHFFGDKTFEGGNDYEIYTDSR
TTGHSVTGPEDTMRILKELFDL
FUN_008925 CDS scaffold_3 203484 207178 -
hypothetical protein PF00397;PF19050 ENOG41

MERSRGVPDEEMFLSKPAPEDCDSPTVEPLRIFKPQSPQPAKDGRSSAFRYPAPPSSTSPASKHSFPLPPGASSSAAPLPFPD
DDDIRKPTPAKPFGSAYNDTSPRLETSPPGKKPGLAERRGAAPKPISSPTSPDADTDLFQRPLADSHRPGSSNNNYPSYQKTY
YPPPGVASGSSSNAPAQPAAVQQLKMNDQGVNRFASTASTSTTRASRGSPPPPETPIVEPGQAPADAIEARYAAAGISGTATL
NSLGAPSAAASQRLAQYGNQPPQRPWTPTETPDQAPSGPPTVYQGMNAISSPAQSHQSFSPPPQPEQQQQQKPQPQGQGQGQA
QVSVLEQDFQRMNTNSPPPAYSSLNPGGNSSYPNEKQRPQQAGSSSAAAQPQQQATTSTAPTKAAAVAAASTVAGGLASPALQ
HPGHPAFANDPHPAFVNEPHPEQNGQPSQQQVVAPAQTFETQNPASPPPLPEGWIAHLDQNSGQYYYIHLASQATQWEFPKGP
NPISHEQAPLSPTASTYGNPLASPMFGKQAMASPMFPPHTPGYAESIMSVAASATPTAAGFTGPPPSAGVDMYRIQPTNGVYF
GPYLRYVNMDIEKGLWLGSILIVTDAPQPPTIHIHLSHDLAPNPRQAEPRPIFTHQRWKFYKYDLELPMSEAGTERWTYAVTS
HLGCTRYEFVVAGRQETSWRFIAHSGNDFAAGTSQNERAKLGGVGFMWKDVLQKNVDCGGFHVQLGLGDQIYGDRLWKEVPLL
KQWLAMSGRDNKKNVQWTARHEEDVAHAYFHYYTSHFDQPFLREAFAQIPHVLQIDDHDIFDGYGSYPDYMQSSPMFKNIGRI
ATEMYLLFQHHTTVEMLRNVSTDHDIFTITGTGWHFVKYLGPAVVVVGPDCRSERTQAQVMAGPTYQGIFPKVATLPPSVQHC
IWMISVPLVYPRLDTVESLANTMAAGKKAVNTTYNILGKVTSSVAGVVGGKDVVAQGFSQVKKAVGKTGLMGNVLNQFGELDI
QEVLKDMWTHDTKDLERTYLIRTLQGIAQQKGIRMTFLSGDVNASGAGLVHDPTHPGDHKTMYQIISSPIVAAPQSNYVLKML
HNQKTLYVPQNGKKSTHEVSDTKEDMMEIFHSDASGAARELKKLMGRRNYVAFVSYDQDAAAAVPQTPFSPNPSLNGSQQGLS
KVSLAVDFVVQGDGAFTAPTKYGPVIIPHLEYGR
FUN_008926 CDS scaffold_3 209302 212678 + MSH3 Mismatch
repair protein msh3 EOG09260LI6 PF00488;PF01624;PF05188;PF05190;PF05192

MAPTPKSSEKKQQSLTSFFTPKTVNGLAAVFEKQSRAQAAKSQPQSQPQSSSKDNESPSRKRALDDAGKENEKPGKTLKRARV
NDTPANEDTSTFFSRSKAGDQETIGTRTERYRYDGSSAPTTQDGDKNTESIQNEDEDAEERRQHQELHKRFVKKLGHPDALVW
RRNLQDAAPAGEDEDADADEEDAPAPAKTKKKGSKTGKLTPMEIQFLDIKRKHMDTILIMEVGYKFRFFGEDARIAAKELSIV
CIPGKMRYDEHPSEAHLDRFASASIPVHRLPVHAKRLVAAGHKVGVVRQIETAALKKAGDNRNTPFVRKLTNLYTKGTYIDEN
GELDQSGGSGAPSGGYLLCLTESKAKGWGTDEKVDVGIIAVQPATGDIIYDNFEDGFMRSEIETRLLHISPCEFLIVGDLTKG
TDKLVQHLSGTSTNVFGDRSRVERVPKSKTIAAEAYSHVTQFYADKLKDNAQDETAAALLDKVLKLPESVTICLSAMINHLQE
YGLEHIFDLTKYFHSFSTRSHMLINGTTLESLEVYRNSTDHSEKGSLFWALNKTLTRPGHRLLRKWVGRPLLDQQLLEARLNA
VEELLEKQSTVPVSQLESLLANTKTDLERSLIRIYYGKCTRPELFSVLQALQRVASYYSTVKSPSDAPFSSSLLNDAVCALPQ
ILDTVVSYLERINLVAAQKDDKYGFFRDEFQTEDMQDHQLGIAHVEHELDGHRAVAAEKIKKKTVDYVTVAGIEYLIEVPNTD
IKNVPASWAKISGTKKLSRFHTPEVLRLITERDQHREALAAACDKAFTDLLASISADYQPLRDAVSALATLDCILSLSKVAAQ
PGYSRPSFLPSTADPTISITNGRHAIAEHTLDGGYIPFSTSLMHPSPLAHLITGPNMGGKSSFVRALALIVLLSQVGSYVPAD
ALSLTLCDAIHTRTGARDNLFAGESTFMVEVSETARILRSAGPRSLVILDELGRGTSTHDGAAIAQAVLQHVVTETRCLTLFI
THYQNLARVAEGLDGVKNVHMKFKAEKGEDGEEQVTFLYEVGEGVAHRSYGLNVARLARIPKKVIDVAALKSGQMEQEMKIRR

FRGVCRALSDVIHNGPDQLDPLVSGIEQL
FUN_008927 CDS scaffold_3 213320 215007 -
hypothetical protein ENOG41

MRAKQLKEEARSRRNQGNHFASAKQNKRNIKQGTSMSPAIKLEGDELLDDFPIFPGFFDHDQDHDVQEEFSLGADSMSLKGQV
WPGMGKMDLADDEMRRTRNQKKPKSVIDRMKRASERIEPTQFIMTSELEVERTKDVYDDASSPAPGQEESTPPRKAPKTKRKK
ATPLAEISANAPKQRRSTARGPKSGTGKETNPTKKKSREKEPKTYPPPEKPQDIHDVFRDEKDRPASISEPPLPATRNDRRFD
LRTKHGARSIDNFLHPNLVSPTPHPRDLAPRQLLGRETSNTLRPESFPPGTFSHVEASYALKDATIYNASSRLPFVPPNFDQY
RGLGPDQFRLAADYGFQLKHEDYTGSITGDTTQGTNNSPFMGVPGTNPLFSHDRPFLNPYSQATPNATFSPLSFSSINRQQDH
LHNDAGMKPPQAQICEPNENNGGLGEEQELSMNVPWNLHGTENDLDFSHGLAVDDSQM
FUN_008928 CDS scaffold_3 217316 218352 -
hypothetical protein EOG09263G3M PF00432

MSQDSTGQKGPELAVDAHVKYVQSLDTRKDELDYWLTEHLRLNGLYWGLNALFLLGRPEALPRQDVIDFILSCQHENGGFGAA
PGHDAHMLSTVSAVQILAMTDALDQLETKGKGKNQVGKFIAGLQNQESGTFAGDEWGEEDTRFLYGAFNALSLLGLMSLVNVD
KAVAHIIACANFDGGYGTGPGAESHSGQIFTCVAALAIVGRLDLVDKEKLGRWLSERQVPCGGLNGRPEKDEDVCYSWWVLSS
LAMIERTHWIDRDALIAFILKCQDTEIGGISDRPGNMVDVWHTQFGLCGLSLLGYPGLEAVDPVYCMPKSITKRLLG
FUN_008929 CDS scaffold_3 218972 219887 +
hypothetical protein PF00561;PF12697 ENOG41

MHKEYFTTNDGCKITFQSSLPLKEVAGVAAEKCVLLMHGFSGSSDYFIRNFTSLSKSLWVVAPDMRGHGDSSQTRGGYHVARL
AADLSGLVAHIRKTASVSIVPVGCSIGAAVLWTYVELFGCNDFAGFVFVDQAPLQDRSAFDEWDETRAHTGCYDEKTMLAAQY
NWINDSKKAHIDLAIGCLGYRHAPKEEDGISAEQQAKDEAFFTDISAKCDQTWLARLLADHTRYDHRESIETITVPVLVMAGR
RSSCFPLEGMLESVRRVEKSRPGLARSSVFDSGHWLFYEEPERFNKEIAEFVNSC
FUN_008930 CDS scaffold_3 223398 225765 +
hypothetical protein PF00620 ENOG41

MGRKAAPQPLALSSQTSPQAQAQAQTQSESGAALRSRDDSPTAETVSPGVSPVESKSPRSPRSSPFHSRFSPLRNQGGKHRKV
SHTTAALADATAEGKPSSSTPPPQPSAPVPIPVQRPSTATASTEWSREESVPRQPLRHTREDPSTYPSISSTLEEHLPDPPAT
TAATTQSEAVQPIPSDSKKGAKNGFFHFNKQSKSSNQSQPHAHHRKHTETRAQVMSRGSDGSNRPRHGDKHDPSPSASYTDHT
LHKPVSADPLRSDLSLSSAGDYDTASPQFSTKKGRSKPFGLLNRSRSNRDKEDASPELKPASSPRQGNEPELHLNSVPYKTAN
QGGDRSIRDMMNSAVRNRSEDRAPRDSSSTRRAQRDKESKTQPSSLNENGGSFFNGLKSSGTRAAGMLSDRFFNKSGRGGRED
KDALDDEHYQIKVINLPLVEQTRLTRISKRLEDSRDKTEFWMPAFPWRAIDYLNYKGCEVEGLYRVPGSGPQIKKWQRKFDEQ
YDVNLFNERDLYDINIIGSMLKAWLRELPDELFPKAAQDRIARECAGAEEVPELLIEELSNLSPFNYYLLFAITCHLSLLLAH
SDKNKMDFRNLCICFQPCMKIDAFCFKFLVCDWRDCWRGCKNEAKYIEEEYMIFDQPPPRGLAEPAKPRPQDESEDDRQQLSS
SGSSKQSGRLSPSDQKPKLKKKQLQISPSNGSVVSNESTAPTTLTINSDRELGRTSSELRPLSPIMPLSPLGF
FUN_008931 CDS scaffold_3 226986 227568 -
hypothetical protein PF04939

MALPSSKPKLPVAVEKPTPYTFDLGHLLAEDPNPVTLDRDNLEQSLAELARDGAQSLINQFLSTCPLNSTAEGVLLTLPAPST
RLPREKPVPQAKPPTKWERFAAKKGIKPKTREQRRNLAFDEQTGEWQRKWGYKALNKKGEDWPIVEVDMEAEKKRKEGTSIRG
DGRRERKERIKRNERKMRKNQAQGTGK
FUN_008932 CDS scaffold_3 228030 229469 +
hypothetical protein EOG092625U6 PF10236

MASANSLRCLMRPTIPRAPRIQPVLLAPFSTSNAALAVSAPPAIKSRRDLPQKVKKSYKKRANVTPVRKPQPGERKAFRKRIQ
LSNNSALPVAGLENLDASNMTKAESAGKMFAIPDQVVDHLRALEAFKTTQTWNLFRKPHVLQRRETVELMKKLELSAKDKKAL
KCVLTGSKLSGKSMAMLQAMAYALLNNWVVFHVPEGQDLTNGNTEYSPIPETEPMQFSQPIYCLKMIQSLYRANRVVLEKLNI
EKDWSKFTNLKEGATLADLALSAKEAEYAWPTLLALWTELTLPGRPPVLFALDGLAHINKISDYRDPSFNEVHAHELTLVRLF
IDALSGKTPLPNGGAVIAATSENNSHHHPSQELVLSQLEAGQAGREIPKPDPYERKYDERVYEALKNSWVLRLEGITKDEARS

LMEYWGASGLFRHVLNSRTVSEKWTVGGHGNVGEMERAALMQMRL
FUN_008933 CDS scaffold_3 229734 232400 -
hypothetical protein PF00493;PF07728;PF14551;PF17207;PF17855

MALREFKAPVSYEKQQHAFQEFLTGFKTSPEQTITTALGNITIGEDDLDDEYDLMEEDGQDANRQQAKERRAPQYKYKNMLQQ
LSDRTIDEATIDLDDLATWENQAFDGEESMRLVDSIEMNTKHYVEIFSRAVDEVMPPMSADVNFKDDVLDVLMARRQIRNREL
DEAAERDPTAADDKFPAELTRRYTLVFKPRTSTSSQSSKALAVRQVRGEHLGHLITIRAIATRVSDVKPIVQVSAYTCDRCGC
EIFQPVTDKQYGPLTMCPSEDCRQNQAKGQLNPSSRASKFLPFQEVKVQEMAEQVPIGQIPRSLTVLCHGTLVRQINPGDVVD
ISGIFLPTPYTGFKAMKAGLLTDTYLEAHHVLQHKKAYSEMIVDPTLVRRIEKYRQTGQVYELLAKSIAPEIFGHLDVKKALL
LLLIGGVTKEMGDGMKIRGDINICLMGDPGVAKSQLLKYISKVAPRGVYTSGRGSSGVGLTAAVMRDPVTDEMVLEGGALVLA
DNGICCIDEFDKMDETDRTAIHEVMEQQTISISKAGISTTLNARTSILAAANPVYGRYNPRISPVENINLPAALLSRFDILFL
LLDTPTRETDEQLAKHVAFVHMNSRHPDIGTGNVVFSPHEVRSYIAQARTYRPVVPETVSEYMIKTYVRMRDQQQRAEKKGKQ
FTHTTPRTLLGVVRLAQALARLRFSNEVTQDDVDEALRLVEASKESLNNDLGTGRARMDPSSRIYNFVKQLADAGQCRPEDAA
GEEELGIELSMTQLKARVIAQGFTADQWSNAVQEYTSVDIWQTTRNGARLVFVNSDPDNDQEDDMDF
FUN_008934 CDS scaffold_3 232923 236607 +
hypothetical protein PF00004;PF09262;PF17862

MSPRRNAQSVSAEISLSHLKSCLVNLPTSLVSLLVNVNTPAQNVIIELSYREASPTGAGSQQRSIFVGWTGMPSKRRTAPPGT
RDGPNGSRSSRDQEVQLVELDATLAKSLGISEGQKIMATIHLDPPLAHTINIEPLTPEDWEIIELHATFLELNLISQIRALPN
PLYKIGDNPVEPHALTLHLSPTSTANIKVISLDPSPPADLPFAKISPDAEVIVAPKTRQKSSHSSGDHRSVASTSKSKRSATS
TVRRRSAKEERRPTMFLRGLDRSSCEEWFDEGPLVQDLSVWVDHDLLSGEAFKGVNYIAVDVIRPAGLQQPQPEEGGVPAGTT
AATKVIAHLRSWIDPPNSQSVALSSPLCASLGCSGMVGGIVKLQAAPQQLPKTAVQSIKVFPFATSAKTVEGLRFGGESKAEK
EESAKRFRQIYAATRGTDGLLQGPLTDGSIMGLYEGLDPPQGWEGGIIKFTFGGDFQENKDAANWLLGLDTKLPIDFQPPTPR
PSRLTETDGFERQPTHDSLLVGIDSLLDNLQSNLVHLSSVLLTGGMGSGKSSIARYLAQKLRQDSLFHTLYYPCRKLVNDETR
ISTIKETLNRLFATASWGARLNGKAVVVLDDLDKLCPVETELQVGNDNGRSRQVGEILCSIVRQYCTRDSGVVLLATAEGKES
INGVVVSGHVVREIVELKAPDKESRRRVMESIVMQDAMTADEAQTQFSDESRPQTADGSMTEDSGAWMDGASHASKESRPAKT
SGFILDPDLDFLDISGSTDGYMPGDINVLVSRARNEAIIRAIAESPDSTGAIHLARADFDKALKGFTPASLRNVSLQSSSTSF
KSIGGLQETRQVLLETLQYPTKYAPIFAQCPLRLRSGLLLYGYPGCGKTLLASAVAGECGLNFISVKGPEILNKYIGASEKSV
RDLFDRAQAAKPCVLFFDEFDSIAPKRGHDSTGVTDRVVNQLLTQMDGAEGLSGVYVLAATSRPDLIDPALLRPGRLDKSLLC
DMPSLEDRVDIIKALFQKVRLSDELTESDGPLTDIARRTEGFSGADLQALVSNAQLEAIHDVLDVDGPTVTSKRTNGTDGKSN
TTPSFVQFRYGKDAKPLTEATPKSKSAALVENASILAKLEEIKIARKKSKQIHGAPAANTDAKAESSDQREVVIERGHLIKAL
DSTRASISSEEKARLQRIYTEFVVGRSGQMKDGQGSMEIGGRSSLM
FUN_008935 CDS scaffold_3 236718 237777 -
hypothetical protein PF00459

MSSYTLERQIAELAVLRASILTKRVQSTVSGISKADDSPVTAADFAAQAVLISALRKAFPGDHFVGEEDSSALREDPALKQRV
WELASGAHLENADDDALLASPKDVDELLEVIDLGGRGQGGRKGRFWVMDPIDGTATFLKGEQYAVSLALVEDGKEVVGVLGCA
NLKPVDDTVAESTIDKDGLGLMLTAVRGQGTTIRKMDFSGLQPAQPLDSVAKASSPAEAQIINYSSGSTSRHDLIRKLASSFG
AKFPNIELYSSHIRYAALLVGGGDFQLRIPSSDDVVMHIWDHAGAQLILTEAGGKVTDLDGKDMDFGAGRDLSRNNGLLAARQ
GIHAAVLEGMKKILTEDAST
FUN_008936 CDS scaffold_3 238607 240769 -
hypothetical protein PF14441 ENOG41

MPVTNTEIECARNIALLYYLGRIGCKPQKNSIQDCEPIRKADINRALSLQDEKLLTSTLAFLSSIRDDAMSVTAVCVEEKKSS
VVVMVAANAKDSHNSSPYLDAVKEGFDKIFRSLNRETPDAFQIKLAQGPSNTSALDKKLELIINTFADISRISSLSTMLLEDI
GPRMEPDLCSGLLNTIHKLAHYKTCAWTLVKLSRRYSILGRTSTIAVRLDDTAFGKPPAETVGFKLEEHLKKLKKEYNTNWDL
DNFGQRLATNTKKFWEDFLRVTNEPKIHAEIQLMWHLERHPSSKPPRVLASNKDACFLCNAFISFHGKYMIPKTHGRIYPGWR
LPSTGLNETRRLFVRELERIAVERLNVVNQQGFEKNIYPLESTISLVATSTTSLNRSRETKDMQPAMTLTNTGINNEVLDTID
EKRPAICLLPPTKPGQSKAGVKTKTKNLDVESTEVSLIPRNSPISSQETMVLTQKPANETEKIQEQAPISVGEPNDIASEETE
YEPNKITSPETYQTRQSSNYFPEPKRSDHKSSDSPTAWEQVKKDSTKRIRLNSFNLYIEYTSSDPNTSKVLKIKTKRLSVEEA

ETATDGSDHVYDLDSLSTLGCDLGNCQQVKMKVKGDVYKVDLGAFVCHDE
FUN_008937 CDS scaffold_3 241722 246844 -
hypothetical protein EOG092607ZS PF00176;PF00271;PF00385;PF13907

MMSTPTLGGPSNGHLSPVDGISMSFDTGDHATDSDSHDAPDLTLDQPSPASSDEANNDSNIAHDNTHMSASEQSSEDNASDDG
DFDMEESLPSQNEDAMEDRDSSTDSNRASKRKAPVEEDEYIKANPELYGLRRSTRPREQRKIVESDDSDSEPPVNRRTVKRRR
VESSRPSSKIGTPTLRASTADSDSDSDTYGGARAKSLQKKARMQREAQPDLALAEKRWSSRRAAQVQQGAYEESDVDEDDDDD
ELAPMAYTVDYIDDSPYIEKVVRHRLKDGFEISYGLTKNDFEYFIKWQGKSHLHDTWETFQDIRDYRGYRKVENYFRKVIEYE
VDIRVGDDIPPETKEQFFLDRERDEEAFEDYTKVERVVAVRDGEDDTEYLVKWKGLTYEECTWEVASEISDAFQDQIDQYLDR
ASRSWQSDRKETNLDTRSRMVKLEEQPDFIKGGELRNFQLRGLNFLCLNWTKGNNVILADEMGLGKTVQTVSFLSWLRNARRQ
EGPSLVVAPLSVIPAWCDTFNHWSPDINYVVYLGPEDARKIIREHELLVDGNPKKPKFNVLVTSYEFILQDWQFLQSIKWQTL
AVDEAHRLKNRESQLYNRLVSFGIPCKILITGTPIQNNLAELSALLDFLNPGKVDIDEDLDSLSASDAQEKLQQLHKAIAPFI
LRRTKETVESDLPPKTEKIIRVELSDVQLEYYKNILTRNYTALCDATNGHKNSLLNIMMELKKISNHPYMFPGAEEKVLAGSV
RREDQIKGLIASSGKMMLLDQLLSKLNKDGHRVLIFSQMVKMLDILGDYCSLRGYKFQRLDGTIAAGPRRMAINHFNADDSDD
FCFLLSTRAGGLGINLMTADTVIIFDSDWNPQADLQAMARAHRIGQKRPVNIYRLVSKETVEEEVLERARNKLLLEYLTIQAG
VTDDGKAAFKEELNKKGLRVEGPSSSEDIQMVLKMRSSKMFEQSGNQERLEQLDIDSILENAEVTKTKVDDKINLSSGGIDWD
NFMQITDVKVDDINLDWDQIIPADKIAEIKAEEEKKQHEAYVAKVAAESAPRRAAIKSRHRESERDVRLKKRQKEQQDKEEDD
RRPVPLDPKRPLNDKEQRSLIKAYFRYGSMDDRGDEIIKEAKLKERDPDYVKSVLDEFIKAAKEAVDDNYAQMVEEEKRLGKT
LTKKDRKAVLIDFGDLKKVNAETAIERPKQLQLLRQVIRSHNDWHTFRLPDATKAASYSCAWGAKEDAMLLVGIDRHGFGAWP
QIRDDPDLDMADKLFLEEHRVEKKEERSKGNDKMKAPGAVHLVRRSEYLLSVLQAKHSNDRVVQRAVENHHRNNKKSLANGHR
GSATASPAPHNGKKPRDRDRDHLHGDLHRSRSHAEERGTPRPDFKRKHLSHEDSRSPKHRRIEEHRRSSKHGEDRERSEKRRH
RDDEDRRDERRDERRDDRRHDKHRLSHSHRDDRREDRRDDRRDDRRDDRRHDRHREDRERDRERDRERERERAHDSHPQDERR
AKALRRLDELRRIGDNKDKRAEDNDAMIWFLLKPVRENFERILSTTKDNVKSSKERASIFGVELVVIGTFLDEKLAATAADEG
LKSNFWDFLAALWPVDDTSKSVTGKRLSNMYRTLHSRSKGSGSTKTNGA
FUN_008938 CDS scaffold_3 254056 256141 + RLI1 Fe-S cluster-binding ribosome biosynthesis protein EOG09261127
PF00005;PF00037;PF04068

MSDKLTRIAIVNSDKCRPRKCRQECKKSCPVVRSGKLCIEVQPDSKLAFISESLCIGCGICPKKCPFDAITIINLPTNLESQV
THRYGPNSFKLHRLPMPRPGQVLGLVGTNGIGKSTALKILSGKLKPNLGRHDNPPDWEDVIKHFRGSELQNYFTKLLEDDLKA
VVKPQYVDQIPRAVRGPNKSVRFLIESRASLGNMEQVANVLELNHIMDRDINHLSGGELQRFAIGTVAVQNSDIYMFDEPSSY
LDVKQRLSAALIIRSLLRDNDYVIVVEHDLSVLDYLSDYICVLYGKPAVYGVVTLPHSVREGINIFLDGHIPTENLRFRDESL
TFRIAETTDDFAIDKSRAFTYPKMEKTLGSFKLNVEAGDFTDSEIIVMMGENGTGKTTFCRLLAGALKPDSQKSVPEMRISMK
PQTITPKFDGTVRQLFFKKIKQSFLSPQFQTDVVKPLKLDDFIDQEVKNLSGGELQRVAIVLALGIPADIYLIDEPSAYLDSE
QRIIASRVIKRFIMHSKKTAFIVEHDFIMATYLADRVIVFDGQPGIDAHANKPESLLTGCNTFLKNLDVTFRRDPTNYRPRIN
KSGSQLDQEQKMSGNFFFLEETPDKS
FUN_008939 CDS scaffold_3 256920 259495 -
hypothetical protein EOG09261K9J PF00397;PF01846

MNGFGSPYGQPVTAWQEHHTPDGRAYYYNPTTKATQWTKPEEMMSSAERALANQPWKEYTAEGGRKYWYNTETKQSSWEMPDV
YKTALGTTSKPATPASATPYTPPASAGGYNQAPYDQYRDQRDTYPESRQITYGNDPKVQAFVPATNDPEYATTEEAEAAFAKL
LRRSGVQPDWTWEQTIRATARDPQFRAIKDPKDRKAAFDKYCQDVVVQDKERAKERLAKLRADFETMLKRHPEITHYTRWKTA
RPIIEGETIFRSTNNESERRQLFEEYIISLKKAHAEQQTTLRKNAMDGLIDLLPKLNLEPYTRWADAQGIISSTPPFQNDEKY
QALTKFDILTAFQNHMKALERRFNDTKQEEKNRKFRKERKARDAFKSLLTELRRNGKINAGTKWSQIVPLIENDNRYTDAVGQ
SGSTPQELFWDVIEEEERGLRGPRNDVLDVLEDKRFDLTPTSDFEEFLSIMTDDRRTANIEPDILKLIFDRLREKRSSKRGDD
DRQSERQQRRAIDDLRAYMKRMEPPITLSDTYDKVRSRLLKSDEFQAVASEDARRNAFDKHIRRLREKEDEADRSYRRRDRMS
SERDMHRRERERSRGERSHRSGGRGSRRSRSPEPDAYEADRRKAIAERERNHRKSTMAEGLLGADRSRLSPPPRRERERDRDR
ERERDYDRPSRSRRDDDSYYDRERRDREDERERLYRRRTDRGSHDELNYGDERPSGSRRRRPDDEDEYTRHDSRDAKRTKRER
SRERTPPRAEERPRKKTPPPTTKDVHSGSEEGEIEED
FUN_008940 CDS scaffold_3 259900 261896 +

hypothetical protein PF12735 ENOG41

MSGSGNTTDRESIPTEATQHEPESLTQLKRSLFITDPTTEFAEQSHLSYLIPENTDLDLEAAFKDVEPGKSILDSIKRRDTLF
FDETVNVLLLLKSPWKDEASLRTHLNRLVISVEAQIVNSKTHGKENSTTDTIFSGTVPDIDDPFIIVDGGEEEEEEEEEEEEE
EEEEEEEEEEEEEEDSNDSEESGDDANLDQSVFAVWKLPVFLSRPRARISAPAIIFTASASMKPEVSTDLIGKGSGYLQSGVP
SGFNLLESFGSDAALGGVKPRLSALRVSRVTPVTRSQDVTKHIRALPQVQHKIFPVVHTRIRFSRPTTAPTSSAVIALLEVDF
TSHFECEVSLDQIELSILDATVENLNNDAGMQLPLTCVSHDHITFLYRIAPRQHDVTVKNPMRELDIQISATAQVIPGRCTPS
MNMSWSTQLDFTLPVNPGYGSATAGTGIVRAHRPSQLSITGQAVNPLVSPSIIRPDALPTLEAATSNTDASITELGITMTFTG
PSEPVYPGQVFSWTVYIVNRTTEKNSAPPRKLALVAIPKRRRSEVRMTRPPSVGGRRHGEKDVADAVTDENVLHALQKNSSVE
STDVVCLSADTRVGPLAPGACHVVELQFLALQEGVVGLEAMRVVDLGSQEHVDIRDLPTMLVEPAAA
FUN_008941 CDS scaffold_3 262087 265039 -
hypothetical protein PF08639

MSSSAVAGIDATRPARSRILTPSSEGSSNHKDELRSPEGHKRKRADSVAMEHLLKPSIAIKPHPPKLNVQPRILYPLMVLPRE
RLPLSCIDFHATNVDLASYRFFESHVKILDLESRMGSGPVVLLARKEASRAVYALERQPNGVYVVCRLGPWTDLEALAEDASA
VCRERLRPTARTEPQSQYGPSVITTPHIHKEEKIKRAAIEAIQTLVRKRPRSQSVSTLAESVKIEAAPEAAIIADSKLPSPTF
KPEEVAQTLGEQQTQASRPTTAISTSGNIDEPSQQLTTESIFETIRTQYYEMLYKSMGSLAYFAKGPLSRARSAFHLDLEANL
DIGDLIDFLKGLVLTTVQIDKKYRETIPALIAQMKTVVDSSDEGPKKKRRAKKMKIGKDGLYPYEEAHIRKWWATNKPELKED
ETNVPTQQIQSLTSMLRTRETQLQMIIILEILALTLLKPADDAEDSQLPLLPGAAESQGDAVVPSAKKRSKHNLPVLVDVHAD
RLTIWQSTASDEQLLLEDSQITQAPDGQSQQKASSEPLKDFCVDIIVPFFSHRLPELCDSINRKLGGPVIVKPTRPKVLKRPS
SKRSPKPGAVAKRSISGQPTRTLQRALSTEQQSRRSVSRGPSNMIALMRSATSTSLPGIKREASDPSLVKSVLNAEPDLMNRK
SGPLSRSSSVSNLQDAKSNKKAQVEAELKEAISSLRKPNREVVGKALAEAAERRATVGSSAKKVRKPGRSSLGVSVVKATPAN
TRFRDVFAKPHVTDTPIMSTEDVIPPSSLLSMVPSTGLRGAQRDGFRQSPTPDIERIGSTPTKAGSSFIRRPRNDEDVLPFPP
SSPSLERLTMSAADLFNPGGVTNRKRKVSFTFSRNDELLATPVKGMKTKNIDMENQAAKPMPAKSVSIYEKLGWDDDLDDLL
FUN_008942 CDS scaffold_3 267722 269190 -
hypothetical protein PF00046 ENOG41

MSRTTETKPRLSKEEVEILEAEFQKNHKPNSTTKKALAESMRVDNSRINNWFQNRRAREKKEKNIREYAAKQRMDKDTTANES
GVHSDDDHLSDRVVSSAPFPAPRPLEIRSTDVSSPEHESENDASHSDFGAGSSPNLSSQPTPEPVSASLNAPLPSYSQYPQLI
VPNEDGEPTPSLSQQCFPRLSTSPIQEQYVYSSNNLAVDQTSQSSMGLKPSPSMDIASRRNRRPPQLAINASRSYSASGPRTG
LDMGRRADVGHSMRRVASATGVGRISKPCGGPRSPYFERNPEALMQLNRSPNFQSAATIAPPTPNTPVVANQQGLCEATPAST
VAYEEKYPMDLAIHDPTLRTPPTTPGAMDQLYSNESVYQVAVADEPLVTPGLAPYPNEFEVPGTSSQVPNYVSQGCSSQPQTP
SYGAPMGPTYFGFAGGNAEYNWSDDASLSAHSSPGQSQQSVNFMNMTPSSFTYSDK
FUN_008943 CDS scaffold_3 270230 270646 -
hypothetical protein ENOG41

MDEQRWKGNVNDNENTNSWPNETRSRHRNWSAAALGPGPTNLHQPLELSARSSADSTVAALASSFGNGDFKPLRGRLRRDTEL
WINERGQGAATTAAQAGAERSLLCSAHYSM
FUN_008944 CDS scaffold_3 279229 280217 -
hypothetical protein PF13419

MGSTPEYNLPPQELTFDGFLFDMDGTIIDSTEAVVKHWETIGNEIGVAPEVILETSHGRRSIDILKILAPEKANWDYVRDMEG
RLPKYHGHEAVEIPGARSMLEALIARSSPWAIVTSGTVPLVTGWLRARDLPTPPSDHLVTAESVENGKPDPACYRLGRERLSL
QAEDAQVLVLEDSPAGIRAGKAAGCKVLGLVTSHTVEQVIAAEPDWVVRDLSSVKVLRSEGGKVTIEISNALRI
FUN_008945 CDS scaffold_3 281211 282167 +
hypothetical protein PF01470
C15
MGSQIGDPDGITVLVTGFGPFREQYPINPSWEIANSLPSHLPPLRAKDPNSRHAAVVLPKVRIVVHPEAIRVNYRVVRGLVPS
FHDTPQKFDIVIHIGMAGPRPFYSIERRGHRDGYKHPDVDGEYIDGEEERERNDWPWRGLPEEIETELNLNEILPLWQGHSSE
ADLRISEDAGHFMCDFIYYSSLSELWKLQRPRKALFLHVPADASPTSIAKGRELTLNLIRSVVESEMIDKDKLLVSKTAGEEL

FUN_008946 CDS scaffold_3 282632 282852 +
hypothetical protein ENOG41
MCKKATCGTCKKTSWWGCGSHIQSVIDNVPEAERCECEPKVDVGGASYPPMAASPN
FUN_008947 CDS scaffold_3 283280 285570 -
hypothetical protein PF13415;PF13418;PF13422;PF13854 ENOG41

MAKDKKKNADAKKAKKAEKAAKQANKGEKKAKNKAAKIEGSDAEDVDLDEVLEEYRRQQEQFLKITETVIDAPPRARAASTLM
ASPHDSNTLLLFGGEYFNGSLAQFFNDLNIYNINRDEWRCVTSPNAPLPRSGHAWTRAGNPNHVYLFGGEFSSPKQGTFHHYS
DFWRLEPATREWTKIETKGKDKSPSARSGHRMTYWKQYIILFGGFQDTSNQTKYLSDLWIFDTVNFVWHSPQLPPAQLKPDAR
SSFTLLPHEQGAVLYGGYSRVKATVNVKQKGNKGPSQAQRNVLIPKVHEDCFFLRISQPATDANPNTPPVVRWEKRKKPANAP
NPTRAGATMAWHKGRGILFGGVHDVEASEEGMDSEFFNQLFAWNIERNRFMPLGLRKPRQQKKAAAEPRGGRRGRAQANEDEL
LRQLAALETGASLDDADEIELAKKEEERDDDKPAREMPVTMEPPHVRFNAQLAIQDDVLYIYGGTFEKGDREFTFDDLYAIDL
GKLDGCKEIFSRPVEDWIESDDEDEDDEDEDEEDEEDEDEEETGQQLYTPSKRKKKQDEVSEASSEPSSEPSTPSEDDETETA
ATSVDDGLPHPRPFESRREFFVRTSAEWQEILMTSLRWKNIQPETLAIKEIKAKAFELSEEKWWDCREEIVALEEEQEAAGIQ
EVVSLAERGDAASGGGARRR
FUN_008948 CDS scaffold_3 285992 286895 -
hypothetical protein EOG09263060 PF09445

MSSSGVEMSLDDSEYSMKPAEELPLTDDCHHYQGKHEVPWDIQKYFAQRYSIFSLYDYGVYMTDDAWFGVTPEPVANQVAHDM
YGTDQKKHILIDVFGGAGGNSIAFALSERWSRVISIERDPSTLACAQNNAKVYGIAPGLITWVLGDSFEYLDKLFNHTEELHP
NLRVSLDETVLFASPPWGGPGYRTDEVFNLYNMQPYNLDDLHTAYNRLDHALFLPRTSDIRQIARLAPPDRKVEVVQYCMEGA
SKALVAYLPGKYSKARQ
FUN_008949 CDS scaffold_3 287238 288355 +
hypothetical protein EOG09262V9N PF04495 ENOG41

MFNALNRFISRLDGDVQQQRQQERGSFGFQVLRNTNLELAIEPWFDFIVGINGRPIEDPEPALFSQEVRNCAGSTVTLGLWNA
KGQRTREIHAPVALDTASLGLSLQYAPLALAANIWHVLDVPANSPADAAGLLPYSDYILGSPEGALYGEGGLGELVEDFIGRP
MRIWVYNNEYNVTREVTIQPSRDWGGQGALGCVLGYGALHRIPPPLSEPVDAPGETMFDGATNEKSEDAFIPAVGGSEPAPPA
GDFLVPAQMIGDAPVSAPPRGAGKKKERHGHNPSRLMDDYFKEEEEKSRAVDNAPSARNSPAPPPPKGGPPRDSPKPETQASG
GEAE
FUN_008950 CDS scaffold_3 288537 289167 -
hypothetical protein EOG09265JA7 PF02109

MAAKKNVRDITPSAAATTQASTTSAGSVPPQPQPKPVAIAKNEIKGEVQWDKVVQNLYNHYVNNTPQRIKLVDAFMVFLVVVG
VLQFINCALAGTFPPFNAFLSGFCVTVGQFVLTASLRLQTNEAVKNDCPSVSPERAFADYIVCSLILHFFAINFIN
FUN_008951 CDS scaffold_3 289560 290012 +
hypothetical protein PF03647 ENOG41

MATVNYHDLELPAYILGALTAGGGIMGYARTRSVPSIVAGCAVGFLYALGGYRIQNEQPYGVELGLLASVILGGSAFPRALRL
RKPVPILLSVISAFGLFTYGSALRRRA
FUN_008952 CDS scaffold_3 291190 294302 +
hypothetical protein ENOG41

MGFHLSRLVALAALLRASYAFSVTANTDGNALASAIFGNGISVISASFTGASDSSGTFTDGPFGMGDAAILTSGAAVGALPNG
DHYVNNGAAGSATYCGGLSNNAAILTADVMLIPGFGGMRFEVVMASEESGGAADAIGIFVNNQNYALDGNGNRLNAVSPWLSQ
PLVIVPPNSVTSYAGSSPPYWIDVPSLPGGMQTVVVAICDAGDNMWDSALMIKAEACVDCKEPFRLAYVTTTTTLTAGDTPYT
STITASGTLSGTIEIGVTAEETSTTTEEPTTTIVVDETTTTSEEPPTPTTQDESTTTSQETTSTAEDTIMATTSKETATITTP
EETTTTTTAKETTFTASGTTTDTTTDVMETSTEASSPVTSEKLPNSTMITADTTSVERSDTVTESSTIVVESTTAPMSDTATKP
APNSPNESTTSLETTSLSDSPIDSTTTSWEEVQVPSTILAVPTSTDEVTLSLSSDLSSESATTPIPDMTATESLETSAAEETE
SSTDTISNPAEETSFSDVITSTAQDTPSPATLAVPTSSTVPSNLAVIGNFKLFGCLGSAAGYPSFDLIGEGPDMTTAECARLG

RGRAYIGIYQRSCYAADTLDSAEIVTNGRCDLPCPGDPGLFCGGVILTDSSLDSRLRRRDAPPGILLTLYAQIEATSSSLTTS
DAPSTAEIPSLTVSTRITLQPILTSESSGFPTDNPTISNSPSSTTETFVDSVISLEPSVPVTRSQGAKPITPPFPTTVSYNAG
NFTRTEAVAPIITTVTYTIVDPNDPSYLTITEYCTTLRPPPCHRCQYQKPPTVEMTTIEVGCNRCGHNGENTITLNIPAGAVV
AAPTREYAAHETHSVQHYEPKPDTQGTRPKNSSPAAKGSHGQAEPSAVPLGGNGYPDAPKPTFHRRPAPAHTPVIVPDAPIVI
VSGAVVKAIEGMLMTMLLVVGLAFLL
FUN_008953 CDS scaffold_3 294730 295017 -
hypothetical protein PF08583

MHPHLHTKNALACEEVIAALEQCHSQGFMHKAVGSCNDAKEKVNECLKIERSKMQAENRNAARAKRDKIREQQRELGL
FUN_008954 CDS scaffold_3 295259 295874 +
hypothetical protein EOG09265BAK PF01920

MSSAAQVPAKKQQDLQVTYSNYKNTLQQIAQRIGDIEQEAEEHKLVLETLEPLSEDRKCFRLINGVLVERTVKDVVPALKTNQ
EGLRKVLDDLVKQYKTKQDDLEKWKKKNNVQVVQQ
FUN_008955 CDS scaffold_3 300196 301249 +
hypothetical protein PF00125;PF00808

MSDSPQSAAPKEVDHGAPSPDDEAQMNEQQDPQAANSAGYEYEGVKEQDRWLPIANVARIMKNALPENAKIAKEAKECMQECV
SEFISFITSEASEKCQQEKRKTVNGEDILFAMTSLGFENYAEALKVYLSKYREQQNQSNRDRVGMDSNPQWLNDAKPEPSAVG
EFGQDGNGVEAGGDPNYIYASQPGHNGNAAEGY
FUN_008956 CDS scaffold_3 302583 304650 +
hypothetical protein EOG09261660 PF05327

MRPFTPATRNFSGPVPRSSTPVRSILKPASVLGRRKAEYAGLSDPVDAPESPTKRRKIFIDETRNVTYEVGRRTMDEVKLEVR
TALEEHLRGNDSQYDTLKELFASDKQRYLPPVVGEEDDTLKPHELQVYVVALTGCVPILKNRLCNGLVHTVLNCSWLGRDDAF
VKVFTHFLAALVSAQGSYLSPVLMMMVGKFSDSRSSVWSVPDFPEVSRETMRNRLHSTLHYLLQMFPSAISVLEELLVSKLPF
HNESMRVHMAYVDNLLRIKDYVPDSRDEVFDIILKRVVQIDSQMQTDLEDLDDDVTAAVMYALREHSEGDEWENDDDSDDESD
AESVHSDDPDFDENVARIKSIKGSVEKMDAMMDTLFSVYTPHFTNPGSDTAFDMFTVLLREFEQLVLPTYKSRHTQFLIFHFA
QMHERLTDAFCGQLIATAFQGNTPNVLKQSAAAYLASFVARGAHLPSGLIRSIFGLLLHHLDQYRKKYEPLCRGPDLMRFTPY
YSLVQATLYIFCFRWQDLIVSTPEFVDPEDPASYIGQEMEWMGTCRKDLSVQIFSKLNPLKVCAPVIVEEFAKISHRLNFMYV
YPLVESNKRVRLTQYLTATYSTGGALRDAGYETQDESFHQLDPYFPFDPYQLPVSKRWLENDYVHWKAVQGLNAEDEDDSDDD
IDDDLDEDEIEEGTATDSDGEDYE
FUN_008957 CDS scaffold_3 305652 307244 +
hypothetical protein ENOG41

MANTATADERPSFTEFLKKTKQKVAGTPCEGPDCATPQRRGRKSRGSVGVSSESPENSSETSPQKSRKSLTATEKAKLRRTQV
RRAQIQHRQRKAEYQKQLELDITHFRELIALTEFESEQLKKDNDSIKELLTNKGITIPRCKSGTCSIRPRLTKDVVTGDNDAW
VDDTLGVKLRAEDPSFQEYDQDGGEIFADINVDDIIVTLKKDESMETPAFSIRSNESSSNATSSPPPLPDLNLTPDEEQKAVN
FILSLEHICWDHFFVGDFPSHSHLSNDEPKGHCLMASSLCMANAPLDVFGDRKLVSSASSCHNRRSLGLDPYVPPVHLEWPSP
RISLSSLYGLAQSLNPGDLEITPVQAWFELASRFDKSLLLERLDLLGTELVGVSKCLEFGAVMEKDAFESVVARVYGGTLEEA
MAAAAIIDPRMGSVCDISRMRNFQANLLTNQLGFVGYAQS
FUN_008958 CDS scaffold_3 307583 313450 -
hypothetical protein EOG092605QM PF04118 ENOG41

MALEPGSGRRSVSPESSGRESPILRQWRNQLGEEDYAIKDKSYRKYAHGVDRALVLFETALEEWADYISFLNKLLKALQARPK
SINAIPSKATVAKRLSQCLNPSLPSGVHQKALELYNLVFSVIGKDGLSRDLPLYLPGLATVLSFASLSVRAPYLDLLERHFLG
IDPRSLRPAMKSIILALLPGLEEETSEDFDRTLRLVESFKHAIRPSYSEEITNQHSSGDAFFWQCFFLASITSQSRRSGALAY
LVRSLPPLGPRAAPDPNKSATSRSDGDEDTKSKLAEVVTSPEPGLLLRCFASGLSDEQLLIQRGFLDLLVSHLPLNAHVIQSR
VKPDDLELLLKAAVGVVTRRDMSLNRRLWAWLLGPEPAAEHEASGDNSSSPPADQHAFLASKTNYFEEFGLQPLTKAILQMIE
NSSHNTASERAKPYRICLSLMDRWEIGGLVVPEVFLPIIESVRRFKDHASNKAEFNEVLRSASVFFDGIESGLIYGEIAALLA

QALGPGALSPPERNDKLSLVNFILSNFNVREEEMVTIHAPLCCLTALAMLQDFKERVSARNHLDPQMFTLSTQALSVAMSLLE
LVPERVFPVISKDSENRSKSLPTNVEILKKSQSFYVNEQGNIEASASPFDALTIGRAILEQAAQFVCQDLGSSETDLNACIRL
LVLAIYKTPRTYEAKEEELLQLMLELLEHPDIMAFSSFSAILQLSTQLYYAERIETAQLSSLVPLLVRHAWTYLSASEPKYHV
EAVRGLWQLQSALSTSNRDIEAALSSLIISQETARLGAIEAVDKGRALSVLWSHTLQDNVSPERRGSKTPTQDMKSLPRLAGA
DNYEVMLTQPVFLILDALQDDRTPLYMTVKAWLNTMLGIDRLFLIFISRLSDIPFLQTLTQNDEKPNQDSLSFSEDEDLDLSL
YYLRTLSNILTCAGDISWTVLSSKHVSFHGQHMQISSGGEENEMTLQEFFIRVCMRCITANTPDNATKEFEDRVNQLYRYALS
LLHRFLLSPFAASLSAQHLENVLIDRLEKSLRGSDGYVQVLLLDVVYDTLKLRDIAPADVPTSPISERRPSSFDPRGSRPSLS
VPEARIVSPPPPQLLQCLQDGLSSPSSRPVLDSWVAFVSECLPLYSDSIFQVLIPLVETLCREIGTTFTNLQETFSSNALSPG
SEKSSPESTLIFLLNGLEQVLARAHQQLLSEEARTQVAKSPDQPQSLFGSMVSNVWQSDAPQSRSATANDRLTVHLAFQDAVR
ICYKIWTWGQGNDSSKQDQGSFGSFNYTSLRMRNRARRLLEHLFAAENLQCLETVIALWKQSIDGAESIQVFNMLSALEACRP
KHCMPALFNAIYRRTGAGAVDTASKSTMTISLQDTDLVWFLVEYTRTLDDDAMDEIWQDCMVFLKDLLTNPFPHRQTLPNLLG
FAALLGEKVDNTNFGEQRRMRKELGDTFIRLLAALFTTRPVAFTDSTTSGSGIPKSDSENSLNGAREGPDDIISILASIVPKL
TKILVEPDRVLSAAATISTNVIGPTLRAKGFPDIVSKNTMCLLHELSRVHNNQKNWKKDVGDAFNDPKFFGMDLDLAKEDWLP
LLRQWTLTDKEKMPEIVGRISAPSTAGIVFGVGATSARLEADRKTQLNLRRIATLILAGSEDAFVSDLSEIFSKLAELLGASS
TSSPSSTTRADIYMVFRALALRTSAIHLGMLWPVVNAEIHAAISSVAAPDNSTASDTYTNTSVLQACKLLDLLICVAPDDFQL
HEWLFITDTIDAVYRPSTYQPVALVDELSDELGASVNSSGFQSSSVVNPVTRGSSRRPLLGPGGINDDVSLDRKDELVAKVLR
PFFGQLSIFAFESTYAMGRVDRDACISSLLKDIFDERSIVRAL
FUN_008959 CDS scaffold_3 314143 315949 + MON1 Vacuolar fusion protein mon1 EOG092624JL PF19036;PF19037;PF19038

MESDEMTPKPASPTNEDYRPPLPPRPSTSATDTASLQAHATTAITPVEIQTLSFPDGSRGTFSTPGPDALPSPAEGGHASPNR
QESSGSGEADETASIMSFAPTMRPAGDIASLLVGELHKKSPAWKMLRTQSATVLPFEKGRIGANDRLAEFHHEFNSLPEDYDE
DVESDILHLWKSKLKHYLILSSAGKPIWSRYGDLSLINSSMGVVQTIISFYEGAKNPLQAFSAGDTRFVILTKGPLYFVAISK
LGESDSQIQAQLDALYMQILSTLTLPRLTHIFANRPSTDLRKPLEGTEGLLSSLADTFTKGSASALLGSLECLRLRKSQRHAI
NNTFLKLRTDKLLYGLIVAGGKLVSVIRPRRHSLHPSDLQLIFNMLFESDGIKGGGGENWIPICLPAFNNKGYLYMYVSFFDG
TSGDQASSPAAAQGRASEEEIAMILVSTDRESFFELKQMRDNVAQQLAKNGTLAIIQNATRLGRPKIHEIAPGSQVSHFLYKS
RANVQFCMASLEPHFTGLVERRRLMTVYHELHASIHAKHSHLKVLHAIGDDATSLAWTTPVFEFYCVAGPNVSRATITQGANK
IIQWAKREEERLFIIGGGVF
FUN_008960 CDS scaffold_3 316135 316672 - hypothetical protein ENOG41

MTDRRPILTLPLPARDPFESQRPGSSLQSPSIRSPRFHEDFDAPFSLDIMNASRTTLATDTMSYPSTGTTSRNSFGCDGDRPP
PLQYRNASWESETKRRSKVNDRILEWAKRSWGAVRTRSNSKDDYFEGHSAQSPTSDMTSPSSDNITPSEPDGGRTAHDNQVFA
VTRLPSSKAGSK
FUN_008961 CDS scaffold_3 321074 322037 + hypothetical protein ENOG41

MYASSPANNAIRLESKVGVDAMFSKPVSRTNDSTKPYYHPTQTNPSRFKVRLRSSRSGLQSKWSLESPGIPERSSSNPIKPRL
RLKVSRSRMSNKLMGPDGTIVRNSPLKQYSSLLELKNFPHRDVFTDRSSFREALEEQLAQFGVDKRLSNIDEGTIRGPSRQLS
DQFDIPYPPSTKGIVTAKLVSQSKFDSGLGIFDRWDFDKTLYSGKNFVRKNFDRTLYSKPIRKKSSFLRHRASPTNIRKRPRG
PANLSDPTPLRSEITQGSSFEDSMLTPSVMLPQRIRNRTRRVKRWAAEAKRAVQALMRRTLSRSQDSDSEG
FUN_008962 CDS scaffold_3 322404 324485 - hypothetical protein PF00135 ENOG41
S09X
MTAAPKQSDPASEEDSVHAPNNANNIHDEAAIKKQPGRKKWFIIGATVFLIVLILSLTLGLYYGLRKEHNVDETAAKDNTNQT
PSPQPSDNPNNNSDDNDNNKDKGKSSDDDKEEPAKKREGPYVDLGYSKYEGNVLESNIHEYLGIRYAKAPTGDLRWRAPEEPE
STTGTLKAQEYAPYCPGVNDGLSSRIDEDCLFANVWTPANATSDSKLPVMVFFQSGGYIRNASPYVNGTQLISASNNNIIFVN
FNYRVGMFGFLASKEVKEDGHLNAGLMDQRFLLNWIQKHIEEFGGDPEHVILHGESAGAGSVALQLVAYGGKDEGLFAGAIAE
STFMPGLPNPDDLQYQFDRVVNATDCADADDTLKCLRGIKSTDLQAHNAKAPFDGRAYRSYFYWAPTTDGDMFPDLPSKLYEK
GEFVKVPLLSGSCTNEGSNYAVNAGSSAQFIRYMQNEYPYLTTEDTETILDLYPQEPKIVKHDTWFPSASRAYGEATFICPTN

NILNAFAEHADPKTLWSYRYNVAIEEFTNDGYGVPHVSNAPAVFGPDMTAAKAGPSYRTYNAPMVPIVQNYWISFVRSFDPNT
HRDEDAPRWENWGDDQRRLVFELNNNTMETVDKGQRERCEAWLDMSGSTKH
FUN_008963 CDS scaffold_3 326917 329142 +
hypothetical protein PF00172;PF04082 ENOG41

MEETPMGESIKISCERCRRRKIKCDRKRPCSRCIKAGTECILSGSGEKQRPISKGYVQALEGQVASLELLLRKLAVADSAERD
EMLSEIALSPPASLDITQKSQIDHAKSNTDPKVVAAQVRSGQLRRPRSSHATQFFGGTSAFHIHLSQEVSLSPDEITTHLESN
STPLMMNIPEVSASSSPRDPSFVYAPHDETSQTCMAAYFKYQYQFHMLIYREYFLRDYDVGSGRYYSDALLFAICSLGAMQLD
DSRSVSDIFAHQAQQLIYAALDSPELTTLQALALLGYREIGVGHTSKGWLFAGMAFRLAHEMGLHLDPNNWDASTEGASNRDT
EILRRVYWAIFIADKQLSLYFGRPPALHPSESDVRNTIRLQYPPDWEGLLDTYICKGASATEFEDGVALVGSFIYQAELCKIA
HLMITDLFENRRGNVDATVAAARSRQIHVSLTKWLSSLPGTLHWNQWTVGVVQPSVLRMHMLFHTIMIILHRPPSHMYEKQGI
AESEDVEICYESLQAILRLMRTYSRHYRFRSLPLDFVQTLSTAAGTIMMKRFFQGASWEDSDIARSLSTVIDAMEEVQHTWPC
MGEIKDYVVRARNAQVVMPPEDPLIGPDLMNGLELGHNVTAELMAQWGEELGTLVTDEFLSMQLQGSEQGVMAPFDFNQPPLG
PQ
FUN_008964 CDS scaffold_3 330983 331901 +
hypothetical protein

MKAAEQLGPSTIPSTTATNALLVIGVKAPGALQELVTTRENGGDGTAVVNSADQGLEHIETWLKERTLCGQYPKSYGLLQSSQ
LAVVVGVIEDKLTQLYFATKGMFERPEEYNGDKDVEHFIRLLKEAWFPTEKGDSIDRLKITVAKQLINGLKHAALDGHSTPAV
MWRSFRCSDLLQLTRETGG
FUN_008965 CDS scaffold_3 332136 332304 -
hypothetical protein
MEDLRVHFEGDQMEGAGQDSAENHSPQTKVAEGKGIDVKMTDAEVDEARYMSQAE
FUN_008966 CDS scaffold_3 332897 334274 -
hypothetical protein PF03959;PF12146
S09X
MSSSNSPTSQTSNSYVEQTVSVLSSVASYMRLPALASTGLAAVLTSLLYFKQKSVSQHHHRLRLPFLATTNLLFLCLTRIIIK
PSPANPACIRALIYPSNIPANSRSDVPKPSDFGISNFEELYIPTDDGEKLSAFYIRGPRGHKNSNITILMFHGNAGNIGHRLP
IARMIINYIGCNVFMLEYRGYGSSTGQPDESGLNVDAQTGLNYLRQRAETRDHKLMVYGQSLGGAVAIKLVSKNQEAGDIAGL
ILENTFLSIRKLIPSVVPPAKYLTLLCHQVWPSESVLPNITKVPTLFISGSQDEIVPPKHMKQLYDLSAAPTKRWKPLPGGDH
NSSVLEEGYFEAMSDFIAEVTGDTPQEKTRL
FUN_008967 CDS scaffold_3 339600 341407 +
hypothetical protein PF08790

MDSYAAIQRLPPLKPYSYPILLRFHISNKCPFTPVNASPNANTNAHPPKQTSYTPDITPTTPFLSRTASFKGHPFFPMPISWP
TTTVLPCSRLPLYSSNIPACGDVLTKKKLDPHRNRCRGATFTCIDCMVYFPGVQYRSHTSCMTEDQKYQGALYKDKKNKKQKH
SHPNDNQQHYTQDQAMAEVQTLPNNTGNMSHYAYVEDVPEDQFGWAEYEESSDDESPNGPPPEAPTPPSAAPEPHVDVFEFLV
GTGQTPNASNMNLDAEPGNSLVRYEPKDKSDEYGYMEDDDEPAVEYGSGPVPAAEFVTPASKSERRKSRTSTEKKDKKRKRLH
IDVPSDQVMTDAPPVLHSGLTGGINRMMRPVFPPSPDYSGGDAPEISPASPLKKSKHSKHSKHSKHASAGHSLFGMIAGSKPK
SKSKTKTKSSSKTKKSSSSSKKSHKEKKPQQLIEYRPQSKDGKTDPSAGQVVLYKPRADVFLSFVDKGPESEKGVSLNRVLKR
FHREREATGSELGKGKEEKELWRSLRLRQNEQGEIVLFSVE
FUN_008968 CDS scaffold_3 342046 343177 -
hypothetical protein EOG092635ST
PF00097;PF04757;PF13445;PF13639;PF13920;PF13923;PF14634

MRMRALFCRSQNIMSSSSASSSPYPFATAPDIIRSHQKDAYFTGHLTQILSDLHRRLRGARLTHAHAPEIQTLATLAYFALTT
IPGNRTLGEEYCDLVQIDARDGQLPAIGRRAGYVAASILLPYIAARILPGLRARLRKLLQRRLESLRKRDDGSATGREARIWS
YLDQHLGSFTSGAPFKAVILALFYFSGTYYQLSKRLLSLRYVFTRTVPDTPDRAGYELLGVLLVIQLAVQGYTHIRSTITESA
ARERAAFGASDDISLNHDGAYNGDNNLLLSTGASSSKAKVDIFVATHTPAAAVPRVQLTDDKAMGYIKGGQQRKCTLCLEEMR
DPSATQCGHVFCWECIGDWVREKPECPLCRREAMAQHILPLRVM

FUN_008969 CDS scaffold_3 344084 347544 -
hypothetical protein EOG09261727 PF01422;PF01424

MAEQETSHTSHQARNNAPNRSQNEGQGSASRSRGGSGGGRRRGRGGRNRGQRQDDSAQVSEGRGNAPQTGPADVTIAAQTIAA
PESPGASRGRRNRRGNRGGSRGQGNQGRGVFSMGPQRTFGGRLTTTEQPTETAQDASLSADAPEFVPGQPVSQRSAQQQSSSA
SQPTAQGRSRNRTRNRKQDRPRQEVPKSTASELWQRIQEDIANWNYECRICTEEVTRKTEVWSCTTCWTVVHLECAHQWWDTS
MKVNEESGDRSWRCPGCNSTLTDEPGDSSCWCGKETQPSRNSILPPHSCGQTCSKPRSTCSHPCTLQCHPGPCPPCTVLSPPE
PCYCGKHSQRKNCRDTDYYNGWSCRERCGDFLPCGQHECSQICHPGVCGTCEVPVEVKCYCGRVQKEMQCSKQDDICDSFDLA
NNSWFEGSFSCESACGRTFDCGVHRCQISCHPQEEISAHCPFSPDVVTHCPCGKTPLKELMEQPRQTCEDHVPHCERICEKKL
ECGHLCQSLCHTGECDPCTQTMEIDCRCGRVTGETICHEGNVQQPLCFKICQATRNCGRHRCGEHCCPGEKKASQRIAQQKKQ
RLGPDSLPVEAEHICVAVCGRPLKCGSHFCEQLCHRGACQSCPEAIWEEVSCNCGQTVLHPPQPCGTRQPSCTNQCQRQTACG
HPSVDHQCHPDDVSCPPCTYLVQKRCICGKTTFHNKPCHLQEAHCGEQCGQKLSCGLHTCKKLCHRPGECGDAQNGCEQICGK
PKLLCNHPCQSVCHGQTPCKESTACQVKMSTSCACGNHKQSFKCLASTSNPTPSRPEIRCDEECERLDRNRRLAAALNIDPAS
HTNDHIPFSDNTLKLYKQMQSWGDAQESQFRVFAANKDEVRLRYEPMKNQSRQFLHLLAEDFGFESKSEDYDIHRSVLVWKSD
KFVSAPTKTLAQCVKIRASQAAEAAAAAAIRPPSPPILETEPFNALVLTEPRFGLTIEEVNTALEPDLTSLQGFSFKVDFLNE
EVLIKATVSYSAFLTPAPMEKSLEILKPRIEQTIRREKLAESVLLCHSDANGAISRREVPLRAGTGGWSAVAGRAASKPMSLS
STPAPEEAKPGRKLLLGLKKKRPQQPEAGKVWAALDGDVEC
FUN_008970 CDS scaffold_3 350443 351196 + MPC1 pyruvate
transporter mpc1 PF03650

MAALIKAANAKIRSNPVTDYVCSTHFWGPVSNFGIPLAAIMDTQKSPELISGQMTGALIIYAGTFMRYSLAVTPKNYLLFACH
FVNAGAQLTQGYRYLNYHYWGGKENMPKEQLAQAAEAAKGKVEKATEKVQNAVSK
FUN_008971 CDS scaffold_3 356100 360283 +
hypothetical protein
PF00289;PF00364;PF00682;PF02222;PF02436;PF02655;PF02785;PF02786;PF07478;PF08443;PF1
3533
MAGIAPAAPQQPLTFNDVFEEEDIDEPKEANTVHHIRANSSIMHVKKILVANRGEIPIRIFRTAHELSLHTVAVFSYEDRLSM
HRQKADEAYVIGKRGQYTPVGAYLAGDEIIKIAVEHGVQMIHPGYGFLSENAEFARSVEKAGLIFVGPSPDVIDSLGDKVSAR
KLANAANVPVVPGTQGAVERYEEVKDFTDKYGFPIIIKAAFGGGGRGMRVVREQESLKESFERATSEAKTAFGNGTVFVERFL
DKPKHIEVQLLGDNHGNIVHLYERDCSVQRRHQKVVEIAPAKDLPAEVRDNILADAVRLAKTAGYRNAGTAEFLVDQQNRYYF
IEINPRIQVEHTITEEITGIDIVAAQIQIAAGATLNQLGLTQDRISTRGFAIQCRITTEDPAKGFSPDTGKIEVYRSSGGNGV
RLDTGNGFAGAVITPHYDSMLTKCTCHGSTYEIARRKVLRALIEFRIRGVRTNIPFLASLLTHPTFIDGNCWTTFIDDTPQLF
DLIGSQNRAQKLLAYLGDVAVNGSSIKGQIGEPKFKGEIQVPELINSAGEKIDTSTPCTKGWRNIILEQGPKAFAKAVREYKG
TLLMDTTWRDAHQSLLATRVRTVDLLGIAKETSHALSNLYSLECWGGATFDVAMRFLYEDPWDRLRKMRKLVPNIPFQMLLRG
ANGVAYSSLPDNAIDHFVDQAKKNGVDIFRVFDALNDINQLEVGIKAVQKAGGVAEGTISYSGDMLRPGKKYNLEYYLDLADK
LVALDIDILGIKDMAGVLKPHAATLLIGSIRKKYPDLPIHVHTHDSAGTGVASMVACAKAGADAVDAATDSLSGMTSQPSINA
IIASLEGSECDPGLDPKLVRSLDVYWQQLRLLYSPFEAHLAGPDPEVYEHEIPGGQLTNMMFQASQLGLGSQWLETKKAYEHA
NELLGDIVKVTPTSKVVGDLAQFMVSNGLSPEDVKAKASQLDFPGSVLEFLEGLMGQPYGGFPEPLRSDALRGRRKLDKRPGL
FLDPVDFVKTKRELGKKYGAPVTECDVASYVMYPKVFEDYKKFVQQYGDLSVLPTRYFLSRPEIGEEFNVELEKGKVLILKLL
AVGPLSENTGQREVFFEMNGEVRQVTVVDKKAAVENISRPKADANDSSQVGAPMSGVLVELRVHEGSEVKKGDPIAILSAMKM
EMSISAPHSGKVSNLQVREGDSVDGSDLVCRIEKA
FUN_008972 CDS scaffold_3 363438 364123 +
hypothetical protein ENOG41

MESLQLSQVLADLSNIGAADPGAAEAIVSANNPSNSSYTRVEVTAAATAPTSSPTTNQQRRPTGLQRHWSSEKFDKFGRRILS
PGSHSGSAVNSSPGTPRRGESDFEEDLERAGTLMQLYDIRSKIKQQDNSSLLRAREKVNAIAARQQAQQAAERNMKAADELRR
QRYSFPRAGL
FUN_008973 CDS scaffold_3 365789 366348 +
hypothetical protein PF12273 ENOG41

MAPTEDVFARDYYRCSYGWNWNGRRCVRNNWSYWGRWVLAGIVIFFFVLFLFCCLFSRRRKRRGVKPVYGTGWMAAKPWGNNN
NNHQMYNYGNQGGYNQGYQQNNGGYGAPPPPPAYGQQQQPQYTGTTFNTNDGYYGQGQYSGVQQPQGTYQRDGAYSPPAGPPP
GK
FUN_008974 CDS scaffold_3 368470 369879 +
hypothetical protein ENOG41

MPSIDEAMARSLRGSAWEVVKDKFVRTIVADLSRRDMLADAAQTTDEVAQKSEDFATAFSSWDNCMNVAWCKWPVIGVMIIGG
LIIFSIVWCIIRCCCWGLSCCCSCCQCLKCCGNCCGACDPPRSATKHLDDPYAPQSQHHGYRSEPPMNSSAPQYGVTKPMRSE
PPQYAEFETSKGRDDDALPEMPSWEGASSKKVEVHTEAVELDTLPSKPPHSQQSNMMGGVAPNNLTPYSQAQSNSSGYLGANQ
APDAYSPIDQQGYGYQAPGMNPMYSNAAAVAPMPHERRSPALNTNGYGNQGYGHDQGFEQPQGYGQMQGYGQQGYGQATSPND
YAATYSGYRGTPPPRPASFAKRTSMHQDEFGTYGQFPPRRSPAIPEDYGYGSPVRQSPGPQNDFGYAQPPRQSPAPMNDYRTP
PPGPEASYGANPVQQRQHMPESHSPQEPYPGFKPYQP
FUN_008975 CDS scaffold_3 370548 371777 -
hypothetical protein PF12400 ENOG41

MQPPSNLEDIPVAVDDASSLILAQSSTVLEMATSVIASAASMASSTAASATFIASGAPQSYDEDDSNGECSLLGSFALLVQGA
LGALALLSLVYKRWRERPQRPLKIWFFDVSKQVFGSVLVHIANIFMSMLTSGRFSVQVDPSATSMTRRSDDDNYAPNPCSFYL
LNLAIDTTLGIPILIILLKILTGLVRFTPVGQPPESVQSGNYGTPPNAWWWLKQSVIYFCGLFGMKICVLIIFIMMPWISKAG
DWALGWTEGNERLQIAFVMMIFPLIMNALQYYIIDSFIKLKETNHESHGNESDDEGRRRRYDDSEDETSRVRLVDSEHETDSE
SDDDDDKHVTRPTHSTQPKGRDSGEYDPERDGDDRTIIGSSASARGPQDKVPQELYPKE
FUN_008976 CDS scaffold_3 372469 373414 +
hypothetical protein PF02777

MLRPILRVPRSAFGLGLRSTVNARRSIHHVPQLPHDYSEGVPNLMSPGGFSIAWTEYMKLTVEKLNALTVGTELEDKDTKTIA
LMTAREPNQAPIFNYASMAHNNHFFFQGIAPEGTPMPDALRSELEASFSSIETLRREFIITASAMFGPGFLWLVKAGPGDYRL
LPTYLAGSPYPGAHWRAQTTDMNAVGKDGSARTFMHNQAFGANKRNSDLPPGGVELEPLLCLNTWEHAWLLDWGVGAGGQGGK
AAFAESWWKLIDWEKVAQKSGVLRPGFKSA
FUN_008977 CDS scaffold_3 374735 377337 -
hypothetical protein PF07647 ENOG41

MEYTSFRGGDRLTYSQMRQQPLPRDTFFSLKNDSQLLDRPISVATEFVDTDWEEEDEIISDEEDNSPRISLQSAGQPSFTTVS
SYDEVPTPRSSRSQEVPLDYSPKQVEGPRGPHLFRTSTDQYSATEEDIILTLSPITPKGPRNIADFLMTSQPPPPRASPFQYT
DVELDTTTLASWSTEMVAQAMLNAGVELSVADRFMENDISGAILITLKFEDLKELNIQSFGIRTKVWHQIQALRDSRPASPRP
ETPIEDVPSKEVVREARRGEEGGLKRRQSSKRRHRARTNGDDITPMESVSIIGIEQVIPKPHHCSKGENCSKFRKQQQLIRAF
KKEYPHVDINATGTVMVGDAGNPETAKALDPNKVRPVSDAVPSVVASSDVMGPGMPPLQYLYEASLRGVQSRDPQDNVRQFLS
FQHQQQGDCSAEVPPTPPFDGMPTAQAQLPHQGLRRLPKLSIPGKPQLPPSRLGASSVPPPQKKPTQSFAQHQQQLQQQHQPA
FVPYQMEKTSPRSPDLEMTPRNSFRHGTPFSELDVPITAVPIGPVARDFSQSVPPDMNYRTSPTNNVSPPRCQSRTSARRPSF
PVMPALEENKPVLVSRPSQGSSSPQSPRGSRSQQPLQPPPRDNLPFSRGQLNGSEKVLAPTITPLGAKGKSPSADPANGISHQ
GPMKKRKTKMLRHEWQDGYFTLKGTRLNMHKDAEELGRTLEYVDIDDYAIACSSVASSSKLTAAFKTMHLSRGSHSREKSDPV
GAFAFQLIPQEKNTARLRKRESHLQPAGAIPAEGVNGTGKTHHFAVKNRDDRIDWMRELMLAKALRQKGEGFEISVNGNMI
FUN_008978 CDS scaffold_3 388122 390239 + ADV1
Transcriptional regulatory protein pro1 PF00172;PF11951

MSTLPPDHRKGVSRATGNTVNMTVSQSSKAKPASKASNGKPKTKTQMHRRSRTGCYTCRLRRKKCDEGTPMCTACKHLGLQCE
YKRPMWWSNNDMRRKHKEDIKMIIKRKKLSEKSSHNIQNSVTSSPPGLTHSLPTSATFTDPLDRTRSASIDSQFPAAFNFNSP
PSGNEYGFGAPMHPEFMFGSYSPYEIDVKTERQMFVNDVPTVRESHISTFSTYHTPPPAGTVLPNGPLDGEWAEQVFQERKES
LSEETLNCNFFDFSHGPSTESRQVQIELDENDQRLLDHFIQFVLPTIFPILESNQHCSVSSDLILPALQSNSAYLHCCLSVAA
QHLKSYTNGSTSTEDIDNDIMRHRYATIWALCEALKKDENHQQILEATLGLIFFQSVVGRYDDGLLDIPWHQHFQAAISLVQK
LDLPGIVSDPTRASMQTPFNMSLSSWIDILGATMKGRSPTFAHTYREKHLSQLNPSLGLRELMGCDDRVMYLISEIACLESLK
KDGMDDFTLCQHVSALGEQISLTEMGDAGPKMPFNANGSLSPKQLSKNMTRAFRIAARIFLCSLVPGFNPRQPSPMGLVEKLT

TVLQHIPSGPNGFDRNLAWVYLIGGSISVPGSSFRAFFEDRLAQLGDSARFGTMGRVATLLHEVWVQNDSLSGVSTPGSTTSE
AAQLHIHWRDVMESKGWDFLLI
FUN_008979 CDS scaffold_3 393291 394554 +
hypothetical protein ENOG41

MLLGRLPTPLRVLFITALYASTSSALPANSVFAREDTCDADQTSCNSDIANFCCPKGNSCRLLAGKTTAVCCPRGQTCNVIQP
ITCDIRGQDQREFPNAPVKTIVFNLDLQKCGQKCCPFGYSCEDEQCVIDKDQTNVPEGAELPNETSTPVPSAITTATGAIETI
SISAAPSTSAEPTAAVGSDNSSDKDGDSKSDNSGPATTSIVGGVVGGSIILLIIAVVIFLYVRRQNKRDAKGSEKGSHIYGYG
RRSTSDASFGNIISEPISQPNSYRADFILKTPSTQRSSIVPARQLSNASTRLNAPPPRIRISIPNPFDSPNPSPNAQPSPLDN
EESLRHGNVRLPPIRAMKGSSYCSRRPSTPELQREPSSENINVFADPSTIVKPRPLTRATTFTDLMDEADLGAVRRGKPYVPG
TTPRI
FUN_008980 CDS scaffold_3 395237 398996 -
hypothetical protein ENOG41

MDASVTDVRRSSIEKMPMSPTPPFDRLPDEIIEQILRQTDPNGFASLVLLNRKWRGVSQRAHLYRHQLSRCPSYSASHPTPPQ
WDDDKLPELRRLFAREVKRNLFQAYLRPRETTFKLISTSISSSSCPGGEGMQYSASPKGHHLLAYNSGRIYVIDLKGLNLEVK
REFKILRRPVAACINDKGTLLVVLSTEMQVDLYDLQRSPPRRSRSIILDNTPRAIALSPCGSVLAAAYEGGIEVSSLRPDALA
TDRRAVKCDAVDALSFSFDGTQILGTTIHSAPPSTVIITAPYYDPGPHMSEDNLSPLWTTSILFPNTSRDCSHAVLLQDGSEE
EASWTFTYDRSFETFRAVRIDDLRNGTTYFTGPVPSASPQASLIPSTLPSTTYRGELVSAGFQGREVWIYGIPEDLDAVPDNV
SSSNDGSASGLGRQNSDRSGTSRRASTRINNPEGARVPKWQILCDQLRNTFVSGFKVAEHGEVKTVKWVAGHDSSSTQERLVI
AARGVGAPRLVTEEEDMDFVDGGRVTLLDFDYGISNGTKSEIIIEVGTDNPEVLEEEQRDLATEVAIVRRRTVAQRRGGRGGS
SLLRAATSASRPRAAPDASAIPAVPRLPLNEDHNNDDDDPLLPRRIGQLPSQPANRRAVVEPVSPSEDGDAPSIEEMEALDAP
YSHASPRSQTTLRRAATAAAVDRSLHPRTADGRRIEYRRADGRREHPHESDADNWVPPPPPYQKEDPGPDLPAFMRGPSVAPI
GLGGASVPAPAPVPALPDHATSASDRPGPENHRASGTDSKSEQPGNNSSQRASGQHPLRGYTTRDQLQVGDAAQNSVSPLPQT
DEDADIYDVTPPRSPRQQAAQHTGGTSETRARQTSISSAVSALTSRAVTVPINSREGRSPSPVQADISAYDHFGPGTRIPAVN
VESVPSTVSTAQARSDPAPRARRLSNAQTWPRVAGHEAEQNVASADGYPFPMSAPAERQAADESISGLPPLPSSSQLASLSKR
VSQGNPRRLSGGFQVPRRPVGSFDGSSYVSSSLPDSLLAGPSNLSRPEPDQPLIISTPKGVSGAFDSPSRQVSGRRNETPLLA
PIPRHPRPVPGSNQRPTVERLETIYSIASSHGGNPPTTVPLPNQQVPAWLHAPPMPVTRTSPTVNRRPSRAERSAAKNIKDAK
KRGWSAKPKKKKKKAADAASSAGWTDISTGSGEREKEKEKDKKCIVM
FUN_008981 CDS scaffold_3 404142 405110 -
hypothetical protein ENOG41

MSSSQSPSFTAEFIKEEPGKPVPQKPVRRRGLNDQIKWVKAWMSKLPQGDEDWDNNRPSTLEDILRLRDRLTISHVESRRDMD
WLTLLETYAAASKDFEGRETQLHCMVMVAACHVAHDQGLTINDVMDAMAKCVTGGSDTLRSKRFALPKCVQIGDELAKVLGPR
AYELPLRVNSYFTFGQHFTVECFPILRRESAFAHRPNNKLPSELLRIPSLVYELCDGKVSLQQIEKALEPGGAKAKARSKSQT
KSKARSTGSPDSTWSVVSSADTEHDMIHIMK
FUN_008982 CDS scaffold_3 406860 408129 +
hypothetical protein PF03124 ENOG41

MDGDPAVDPGLDSFSLAFPLPYRVGFIATLAVWGWGLNLHCLYLFNVDVPQLIRYPGRASPQHIPHHGSTYRLAIALSALFAL
SVSLFWVCTWGIPERVVAFGWMPLTYLTALIAMFVVPLRNLPSGGRRRFLATLRRVSLGGLAEPQDGKFGDILLADVLTSYAK
VFGDLFITLCMFFSAEGSSTQRPDRNCGGTVLVPFIMGVPSLIRFRQCLIEYFRVRRAPYKESTGWGGQHLANALKYSTAFPV
LITSAWQRSTEDPDSKAALHKAWLVAVLINSLYSFYWDVAKDWDMTLFSPKQDRESPTHPFGLRDRLVFRYPNLYYLVIGMDL
MLRCTWSMKLNGRLDRFSDLEGGIFLIEFLEVFRRWVWIFFRVETEWLRNNSSGLGVDDILLGDYQGKDNEDD
FUN_008983 CDS scaffold_3 409489 410818 +
hypothetical protein ENOG41

MTSCTRPVSSIELVLPPQNAYLYRNRPLPLPPTTLSRTSSKAKPPPRFSAFPQTLGPGNDPKALPQQPPVIVPKALTRQRASR
MSISGTTSLRERRSSQKIQQVTGHDVGPGIDWTSAISRPQYSPSISPKSMRDDSSSGYGISLDEPIFDDEPTPLADYQPRSSD
SSMPPLEPDCDSVLSTHSYTSPESPKAQRGSVSLHIAKTKRLSSATSTMAALEPVSNLEDPFDEDVNWQARSCYNYFSDLETA

DEYHRIATRLANNDKRQSIYGTSSLTRSRTSLSRRLSVGARSLFTRRKDSALSASSSRASLTAASYPTKGPYSPSIPPSSETG
SVVSPPRSTFEIDDEDELYADDGSMREVVKDFLARRSEERNSMELGIPRSIPQFSEKSPEHKSLSRTGVQVKDLISNARDGAR
LIQARGERRRTQLRTQIKMIPEEEVGR
FUN_008984 CDS scaffold_3 411751 413028 +
hypothetical protein PF00293 ENOG41

MIYDTGASINNETSIETQTGSHSITDADVVEALQKGIREATREELINHGEASLYYDSLTMSPLNAMSAAAIARFRAYKPPPFP
LWDRLPVRRRAAVLVLLYADRRGDLRVVITMRAASLRNFSGHAAFPGGKADDLEETPFQIARREAWEEIGLPMDDSKLPKPFR
VEHLCYLPPSLARTHLVVRPCVAFLHADQRTAGEPAPTVEETMIPRLDAREVAAVFSAPFYNFLHAQDLPPAPGETLPEGQWY
DGFWNHWKDHPWRVHNFYVPVNNQSISKPRRDSEQSRLVDKLEKEEEPDGRFKVWGMTARILVDAARIAYAQEPGFEHNDSFG
DEEIIAHADSLGDLGEKIPEKRTEPGDASKM
FUN_008985 CDS scaffold_3 413241 414019 -
hypothetical protein PF04145

MDHSGMSHSGMDHGDMDHGHGGGMKDMCSMNMLFTWDTTNLCIVFRQWHVRSTTSLFFSLVAVIFLAVGYEALRSLSRQYEEA
LDNRVRAAPSLDVPVTESTPILAGQSQGQADQRAHLIKAVLYGLQNFYAFMLMLIFMTYNGWVMIAVSLGAFLGYLFFGKRTS
ATKDNACH
FUN_008986 CDS scaffold_3 414725 415414 + BNA1
3-hydroxyanthranilic acid dioxygenase PF06052

MLGPPVNLPKWLEENSHLLQPPINNYCVYNEDFSVMIVGGPNARTDYHINQTPEWFYQYKGAMMLKVVDEGEFRDIIIREGDM
FLLPGNTPHNPVRFANTVGVVLEQRRPKDSLDRMRWYCGDCGEIVHEAAFHCTDLGTQIKAAVQDFKSSEDKRTCSKCGTMAD
AAPKPGTIQDPNLA
FUN_008987 CDS scaffold_3 415586 416430 -
hypothetical protein PF01557

MFALRRTMATMAPQATSSLKQAGKVVCIGRNFAEHIAELNNTKPKQPFFFLKPTSSIVLPGEGPVLRPKGIDMHYEVELALVI
GKLTRDLQASDTQGALDAIKAYAIAIDFTARNAQNEAKKKGLPWDIAKGFDTFLPLSNIIPKTAIKDPHNVELFLQLNGETKQ
QGSTNLMIYQIPRILSDISKVMTLHPGDIVLTGTPAGVGPAVPGDVVRAGVRIDGKELEEGTIEIPVEESPSSYSFAET
FUN_008988 CDS scaffold_3 417579 420513 -
hypothetical protein EOG09260EE7 PF00400;PF04003

MVKSYLKYEHGKFFGVVASPTSNIVWSSKDRTGTSAGQAIVAANEEVLCWDIKKGEITNRWRDERCTIPVTAIAQSKSDKDVF
AVGYEDGSIRLWDSKISSVILNFNGHKSAITKLVFDKSGVRLASGSKDTDIIIWDLVAEVGQFKLRGHKDQVTGLHFVEPEPQ
VQDEDGAHAMVLDGEGTHDGFLLTTGKDSLIKLWDLSSRHCIETHISQTNGECWALGLAPDYSGCVTAGNDGEIKVWSLDADG
LALSARQVDTPAAVQYLQDRGTLHRQSKDRTTEVIFHPKKDYFAVHGSEKGVDVWRIRSEAEIKKALARKRRRRREKAKDGRV
VEEETNAEEPVDVSKADISDVFVQYVIVRTGGKVRSVDWALPSGQKDLHLLVGTTNNLLEYYSLPSKEKSEKSKKDDVPDYTR
ALSVELPGHRTDIRALSLSSDDKMLASASNGSLKIWNIKTQNCIRTFECGYALCCSFLPGDKVVVVGTKSGELQLFDVASAAL
LDSVDAHEGAIWSLNVHPDGQSVVSGSADKTAKFWDFKIVQEEVLGTRRTTPKLKLQQSRTLKVSDDILSLKFSPDARLIAVA
LLDNTVKVFFVDSLKLYLNLYGHKLPVLSMDISFDSKLIVTSSADKNIRIWGLDFGDCHKALFGHQDSILQVAFVPHNNDGNG
HHFFSSSKDRTIRYWDGDKFEQIQRLDGHHGEVWAIAVAHSGDLLVSAGHDKSIRVWDETEEQIFLEEEREKEIEELYESTLT
TSLERDPDAQDENNEVAAASKQTTETLMAGERIAEALEMGMADLNLLREYEEAKLTNPHAQPPQRNPVFLALGNITAEAYVLS
VLQKIKASALHDALLVLPFASVPILFTFLNIFAMRSMNMPLTCRILFFMLKTHHKQIVASRTMKAMLDGIRVNLRASLKRQKD
EMGVNIAALKVVGMQIRDKSIKDYVDENWEEESDERNAKKRTFVHVA
FUN_008989 CDS scaffold_3 420780 421951 +
hypothetical protein EOG09263EQZ PF04045

MLLLDYQNVLIQSVLTERFSGAPPASIDQTVSDFDGVTFHISTPESKTQILLSIQIRCFPDLVKYGAEEVLQREYGDYMTSVE
PGFDFSVLVDLENLPESKEERNELALKFALLKRNAMAAPFEQAYKEHYALKEEASKFTSEEAPQGVREGGEVKAIHYREEEAI
YVKASHDRVTVIFSTVFREETDRVFGKVFIQEFVDARRRAIQNAPQVLFRNDAPLELQGVPGVQDTGSGDIGYVTFVLFPRHL

TPQRMTEVISHIQTFRDYFHYHIKASKAYIHSRMRKRTADFLQVLRRARPENEEKERKTASGRTFKVQGN
FUN_008990 CDS scaffold_3 422332 423191 + LCL3 putative endonuclease lcl3 EOG092644K0 PF00565

MIWPFGSRDADKEKEQKLKEEAKRQHVSWTDSINRIRSAPVDAAREWAPVVLFPLAGMAALQIYANYVRRIPGSAYVRPNFFR
NRSIFGRVTSVGDGDNFHLFHTPGGRLLGWGWLRKVPEARKELKDRTISIRIAGVDAPECAHFGRPAQPFSAEALAWLRNYIS
KRNVRAYVYRRDQYDRIVATVYTRRWLFRKDVGLEMIKAGMATTYEAKQGAEFGGRQKIYERAEAKAKQKRKGMWSGKSKDFE
SPRAYKTKWAGVNQEKTGT
FUN_008991 CDS scaffold_3 425871 429626 +
hypothetical protein EOG092605VU PF12768 ENOG41

MRLPFQRRSPSARQSHSLGPVLGPSLAVLAALAPSLTHAITFNPVPSPNLDFSNLGRIGIVGDYDGISLYEYEEQKESARSSN
GSESLLAPMPNGAFASILSTDASIRAMCSFQLSNGDMQGVVIGGNFTSLDGTQSKAVALFNPNTTEITPLDGIEGEVNALYCD
QERDTVYVGGNFKGANSTNAIAWVGTDGWTNLPFAGFNGPVKAITKASNGHIIFGGTFTGLGNSSTPNGSSSQVINLSTANIT
DENGSSGTDPKDIVCPGSSSTPWLLQDDTPGYWEADFSFGFAPTKLRLRNTRQEGRGTKTFRFLAYPINGIMNLTYVDPESGN
NVTCTSECPLRDDEDYQDFTFVNRVGMNKFRIAISDWYGSGGGLESIELFQSDIFAYAITDFNEPKCGGVKNPASATHTGPWK
VSPSLESSSEYLTAELNGDDDPEKISVTFFPNIMESGNYSVNIYTPGCQADDTCATRGRVNITGVMSGDNDKDTNFTSTLYQT
NDFDKYDQIYFGYIEKSSGSFTPSVTLRPLAGQDVNTLTIVAQRVEFTLTNSTGGLNGLFDFDPEQAVVNATSIVNSPINKLG
ASFDRASGVGVLVTDSNTTYVGGNFTSKDHDNILAIIENDDVKEPGGGLNGEVFAMYLNATQLYVGGDFSNTASGPVTGLDNV
AVYDTKEDKWGALGAGLNGPVMHAVPLMLNITADTPELVIAFTGEFSSCNSFDNYDAISVDGFAIWVASQGNWLQNIDGNYPA
FDGSLTAAVLDIPMDGGSLYAGALSSAQLGTNGAATLSDDGLGQFPLKIRAATPSSNSSALSRRDVTSFNKNTTGVVTGIFYD
ENDYNITILAGHFTTESTKGDKVQNIVLLEGDNVTGLGSDISNGSTFVALAVRNGILFAGGDISGTIDGTDIRGLVSYDLESK
SFNSQPASVSGANSTVAAIAVRPGDSGDVYVGGSFDTAGALDCPGLCTYNVNSGQWSRPGSELEGTVYSLLWLSKNQLVVGGD
LRGNGSDQRFLASWDANSETWSNFPGSEDIPGPVAVIAPGSIDYDQLWVAGTSLKDDSVFVMKYDGEKWHTAGPSLSSDTIIR
GLQVFSVTEDHEAADLLDKNQVLMLTGSIDITDFGVASAALFNGTTYQPYAMTIKAGDGPGTIAGIFTQRDSFFKEEKHMALG
FVVLIGLAIALGLMLLVVLCGIILDRIRKKREGYVPAPTSMYDRGSGMRHIPPHELLESLGHGRGGAPPRV
FUN_008992 CDS scaffold_3 440097 441308 +
hypothetical protein PF00096;PF12874 ENOG41

MTMTLDTTQQRFGPSLNFDYSAPAQPPAFSNPWSSSSPSQHPPAGSSLFVGSQPALNPSIMAGKPPQSRASTSSTSSMASYGG
AMPVPNTSADLLSINRMQTTSAAYGDPTYTTSASPVNHQFAPTSAAPYDTLGYAPAPVRPPPFGLAPADDHARRYSQHSIQDE
RRSFADALDASHGMLAMSQETPRPIYGARSDRSSVDSYGFPSTHSTSSSISSSGNFSSYYGDSVSDYSTAGSDIESVSSRTLP
RPQGLMGSQIPPAPQSMMGQFSSKVSSSTQKKHKCKVCDKRFTRPSSLQTHMYSHTGEKPFACEVEGCGRHFSVVSNLRRHRK
VHRGDARSEAGSEDHHSD
FUN_008993 CDS scaffold_3 449692 453325 -
hypothetical protein EOG092608T8 PF08920

MSDADFETIKKLQQERNAASAGNKGSRTFDTANQRVDDTKQKLTDSADSTLYDRDGADRFSGYHTSLPMGDDDEEMGDGDNTR
RLIGQYTASREMIDEFARGGGVEEDDILAGKGEKSGRIVDRETDYQKRRFNRALTPTRADPFAANRQAGASENGTSYREIMEA
RELEREEQRVLQAIKAKQEGKTGDDGDAQPMLTDGNAEAEATEASSTARKRKKRWDVSSAPAEDDTAEAGEAAKPKRSRWDQA
PAPGAEVSKKRSRWDQAPSATPMGNTGLATPAHPSSAPTLPTTFGTDIGRNMPLSDEELDLLLPGESDGYKILEPPPGYEPVR
APAHKLMATPAPQSGFMMQDPEQVRFSRKPMPAEIPGVGDLQFFKAEDMAYFGKLTDGSDENALTVEELKERKIMRLLLKIKN
GTPPMRKTALRQITDNARQFGAGPLFDQILPLLMEKTLEDQERHLLVKVIDRILYKLDDLVRPYVHKILVVIEPLLIDQDYYA
RVEGREIISNLSKAAGLATMISTMRPDIDHVDEYVRNTTARAFAVVASALGIPALLPFLRAVCRSKKSWQARHTGVKIVQQIP
ILMGCAVLPHLKGLVECIGPNLNDEQTKVRTVTSLAIAALAEASNPYGIESFDDILNPLWTGARKQRGKGLAGFLKAVGYIIP
LMDEEYANYYTSQIMEILLREFSSPDEEMKKVVLKVVSQCAGTDGVTAGYLKEHVLDEFFKSFWVRRMALDKRNYRQVVETTV
DIGQKVGVSEIVERIVNNLKDESEPYRKMTVETVEKIVASLGAADIGERLEERLVDGILHAFQEQSVEDIIMLNGFGSVVNAL
GTRCKPYIPQIVSTILWRLNNKSATVRQQAADLISRIAMVMKQCGEDALMGKLGVVLYEYLGEEYPEVLGSILGALRSIVTVV
GIAQMQPPIKELLPRLTPILRNRHEKVQENTIDLVGRIADRGPESVNAREWMRICFELLDMLKAHKKGIRRAANNTFGFIAKA
IGPQDVLATLLNNLRVQERQSRVNTAVAIGIVAETCAPFTVLPALMNEYRVPELNVQNGVLKSLSFLFEYIGEMAKDYVVYAVT

PLLEDALIDRDQVHRQTAASVVKHIALGVVGLGCEDAMVHLLNLLYPNLFETSPHVIDRIVEAIEAIRMAVGPGLVLNYVWAG
LFHPARKVRTPYWRLYNDAYVQGADAMVPYYPNLDEDKMDRPELAIVL
FUN_008994 CDS scaffold_3 453993 455286 -
hypothetical protein EOG092628HC PF00730;PF07934

MSVVGKTDWHKLPVTLGELCINTTLRCGQSFRWQKINHEWTCTLHGRLLHLKQDSTHLHYRVTFPAPKPLTPAPNNTEALLRH
YFNLDTSLEPLYKQWSDADANFKKRAPQFKGVRILSQDAWETLICFICSSNNNITRISQMVYKLCQNYGPLIAYMGDEPFHDF
PSPQDLTGDDVESHLRELGFGYRAKYIAETARMVANEKPETWLESLRNPDQPGFNTTPVPKEKHASYKEALAQLLTLKGVGPK
VADCVCLMGLGWGEAVPVDTHVWQIAQRDYKFGKSKAKTLNKATYEAVGDHFRSLWGPFAGWAHSVLFTADLREFAAQAAKEE
DEPTVIKLTAYGSTEHTRSKTRKTITITDSDTVVKEETPVIEPTDEQGLEEVKQMRRSKRVRTS
FUN_008995 CDS scaffold_3 455899 457840 +
hypothetical protein PF00076

MNGDSYSSRDGRRGRDYPPRGERGERGDRDDRRDRHRDRDRRRSRSPEHRSHRRGEGDVDAYSSSRNHRDREREDRYSGRERR
GGDREWDRDRGSSRRDARRDDDERPNRRDRDPYEDRRRGGRDRREDRFPAQQERRSASPPPKKREPTPDLTNIVPVLDRKRRL
TQWDIKPPGYENVTAEQAKLSGMFPLPGAPRQQPMDPSKLQAFMNQPGGQVTSAGLKANNSRQSKRLLVSRIPSGTSEEALMS
FFNLQLNGLNVIDTTDPCVLCQFSNDRSFAVIEFKDAPEATVALAMDGISMEASDASNGTDGGHRGLEIRRPRDYVVPAVTEE
VSYDSEVVSNIVPDTVNKLSITNIPTFLTEEQIIELLASFGKPKAFVLVKDRGTEESRGIAFAEYQDPAASNPTALDTLNGME
IGGKKLKVSKASIGPTQVANFDVGITAISGLASQTANEVESSRVLQLLNMVTAEELLDNDDYEEICEDVKEECSKFGKIIDVK
VPRPTGGSRQSAGVGKIFVKYEKAEDTAKALQALAGRKFADRTVVTTYFPEENFDVGAW
FUN_008996 CDS scaffold_3 459226 459913 + RPS13 ribosomal
40S subunit protein S13 EOG0926514P PF00312;PF08069

MGRLHSKGKGISASALPYSRSAPAWLKTTPEQVVEQIAKLARKGATPSQIGVVLRDSHGIAQVKLVTGNRILRILKSSGLAPE
LPEDLYMLIKKAVAVRKHLERNRKDKDSKFRLILIESRIHRLARYYKTVGVLPPTWKYESATASTIVA
FUN_008997 CDS scaffold_3 460215 460836 -
hypothetical protein PF05186

MADEAPRTDPAAETPQVVAPAESAVNGHNSDGPQKDVTMSDAPVDHPASSPAPATHAPSPVPARTGTPAQGSRAASAHPDSGL
SLPAEAPPHGDSTRRYLNTKVTGVLLEGVKQLAKDKPSDPLRVLGEYLIQKSKELEGTG
FUN_008998 CDS scaffold_3 461132 463748 +
hypothetical protein PF08774

MDAFVTRLSKPPSQLREQVRGDSPEKERPSKRAKRQGSPDSEGGLCPDHPQDISPREDFPDNEAAKTKYQTEEHPRITGIESA
LPPAAEGQEAVEQYETMKSSQASAHDEDDTTETTQPLWVKGRSSIYVDAFNLALDTVLEEESHLFDGKETEVFKQWRGLNYEA
QYLYVRLFLRKTASWHRHSRLGYHNDISDLEAAITTLQSSRVLPASTATPIQSPVHGLELEESRLDETFSFADRSQDHFDSVE
EAASLLSLDELKELAKEAKVQGRNKSELIRSLVKMSKQQSGLMSVGLSRHNSRGSVSEDQENLDAKAKRKDTAKLRREDSNRE
QHFLTKIIAILGPCIRLSPPAFKLFERVHLVFYRSTEWTEKSLTTIILAKIARRHFPEYIVCRTSTIFMSRLHLLEYETAIRM
EAEVDAWEFSSPPGDEGLRLMVNIFEKVYPRWKILVEEEQHKEHTVYEMGEGAYLRRFTPGHSYTRIVHKAAPVFGKLKQHLR
EHELLTELLDQRLFHPARRGSWYQRKALLEEHYMPALDPNPKFTDPEQQKKHWKKIAVATCEAGLQDPDCHLIFHYDLQKRLV
KLEKKLRIPRRLQHDFGHVDLQKPIEHTIKGIQLKKDIIAKGGRQVSTKTIWLDELDTQEECSVEAMCLSQYRSEGWKGYHAE
GGIIRTLFAYLFYDILFIYIPNVFQTAYQTCPLDLHTDAFYPSRASEINHRLVEIANGEAPRLIRKVWESEHERRTSVVGLNW
DFEIDDLVELAGCFEGSALAAVCKVMAQEYRQRGGGIPDLILWRTNNLPEDSNKVPGRPKGEVMFSEVKSANDRLSDTQRLWI
HVLTGAGVKVALCNAIAKEVREVD
FUN_008999 CDS scaffold_3 466602 467295 -
hypothetical protein ENOG41

MPTAKPKLGKLTTPVTATFPSEITSATPISAVSFTFKPDPDFLKTPISPPMAYTDFLTKAMALNSPATSSGQTTPDSLPDTAT
SEETDTSSKSEEGTPPSSASSTTSKKFARPTPPATAPMVPPSSFTCSTPMSAPPTGPVCFPSLKVPPSPSVSNIDSPLSASTA
RSPFSARPVHSVFDWDAALKARLLEKKQKTSRTSVRHIREVVTRTVTYTPRMAPAPKGKRRKVE

FUN_009000 CDS scaffold_3 472027 473665 +
hypothetical protein EOG092627XA PF01485

MDSEEEYMSALSSDDEIMQDESGDEISAGEGVFPNILRDPPYSTTLVTNDVDITDFDDEEFDEPDPDFGLIKDAEKKKKSAHV
VSYKVYEPSDIQRQQDDMISEVNMILDMQKEDAAILLRHFRWNKERLLEDYMDRPEKVLEAAGLSSNTSSPPKLEVIPGFTCD
ICCEDEEGLESFAMKCGHRYCVDCYRHYLTQKIREEGEAARIQCPSDGCGRILDSASLDVLVTPALADRYQELLNRTYVEDKD
NFKWCPAPDCPNALECGVKKKDLGKIVPTVECRCGYRFCFGCPNPDHQPAPCELVKKWLKKCADDSETANWISANTKECPKCN
STIEKNGGCNHMTCRKCKYEFCWMCMGLWSEHGTSWYNCNRYEEKSGSEARDAQAKSRTSLERYLHYYNRYANHEQSAKLDKD
IAQKTEKKMVQLQTASGMSWIEVQYLNSASQALQTCRQTLKWTYAFAFYLARNNLTEIFEDNQKDLEMAVENLSEMFEKPTAE
LSDPKLKVDIMDKTSYCNKRRVILLEDTAENLAIGITTLTPFSIPFI
FUN_009001 CDS scaffold_3 474754 477102 -
hypothetical protein EOG09262A65 PF00752;PF00867

MGVSGLLPLLKSIQKPTQLKKYDGQVLGVDAYGWLHRAAYCCALELGQGKPTQKYLHAAMNRVRMLRHFGVTPYMVFDGDFLP
SKAATEESRDTKRNEKKKAAMELLRAGKPAQATQEFQKCIDITPEMASALIQLLKKLDIPYVVAPYEADAQLVYLERQGLING
IISDDSDLLVFGAKKLLTKLDQYGNCIEINRKDFCACREVSLTGWSDTEFRRMAIFSGCDYLRGLPGIGLKTAYRMLRKTKAP
ERIVRMVQMQGRKVSENYLTQFYQAELTFLHQWVFCPTKRELVHLTDLDGERTAEEMPFIGAFVEPEIARSIARGDMNPITKT
LIVTSTTPSKRRHSQLAHGAIDQPPPTMKPISSYFKGSSRIPMGEMDPNCFEVDPQRVSQITGGGLVPRVFPLPRPYLDETIQ
TAPSHSRGSDVTRTRTSPRLHRRRTEPIANMLNSFVNSPTTTRRTSSAGTEPCRTTASASTDPANRPPKKARLCEENEAGPEG
GLPQKSRFFPAPKLRRSPRRAKSDAYLLSDDSLDEALLALPDFEGWKSADKTKKSVSVFEEKQSQETSTTADSESHISFVKDD
ETLMSSCDASMPPPSSAASASRVSSTPSRPDIRQFSYTKPNETPASTASRRSSVFSAASTPSTAPSTAASRMTPLQRLGARAM
NGPMSPKLPKPRTNNTDKQFLSGVPVNPAFIPLPKVNLDEVADLNKCGSEDQIIPASDEEDEDEDEVDLPPKKLNLSRFAFA
FUN_009002 CDS scaffold_3 478262 478980 +
hypothetical protein PF11176

MPTTLHKTRKQISKKRNGVVNALHEKSRDSMRLHKAGVRDQRIEKLAAARSKKEQPLVDRVAFFQQALRLKDRDNKGAPEIDE
VQHMIHSFVHQYDEEYNETKKARRPGRPASVKEDLLKAKINILEEEYKSGFVMPDLLDNVNVNALHLWEGSWSYLTQLKWVKV
NSEGQVRPTSFPSGGTN
FUN_009003 CDS scaffold_3 479905 480833 +
hypothetical protein EOG0926577T PF02330

MFSARNVVRSAPRAVSRLSGAALRQTARPRAFTKAASALRPAQAAFSTTSFRKATENDGELSAKLESEIKIEEDIKASEQDPA
SIKDFLNNSAFELVDTPGQEIVKLVRDFGDEKITVSFSIADITNYDPYGEESALEEEGFEDEGAQGSQRNARANEEIDEELDE
GAEEESNPPINLSIVVEKPGKASGALNIDASAQEGHIVVENLFYYTDASVAKVESPDAAQKRADVYPGPPFGSLDDDLQVLME
RFLEERGITQALAVFVPDYVDVKEQREYTRWLNNVKAFIDA
FUN_009004 CDS scaffold_3 481425 483147 -
hypothetical protein PF01237

MSSSTPPPTVDAPDETQSDTSKLRTFLGILKKFIGVSDLAAVRFSLPSQLLEPTPNLEYWTYIDAPNAFVAMGTSDDPLDRML
EVVRFWLTKDLKYAKGRPCKPYNSCLGEFFRCNWETEDNAPKIDTSSLQQTNGTSKGGLSALKVAAKGDKNASNVSLSVPQHG
AAKDDKLIRVSYLTEQTSHHPPVSAFNVTCPEKGITARGFDQITAKFTGTSVKVLPGEHNMGIFITLEKRDGETYQLTHPAAH
LGGLLRGALSVSVSEFAYITCPKTKIKVILHYVEEGWLGRTTNKIDGVVFKYDPENDDKSRVQDVPDEDVLARLSGPWKEKVV
FTLGPRPVKSVPVEEQYTIIDIAPLSVAPKIIPPKEKQLPNESLTLWGGVTDAIHAKQYGKATQVKVELEEAQREKARQREAN
KETWQPVFFKHVTGNDGKPDLTEKGREVLERAQKGDWSLEGIM
FUN_009005 CDS scaffold_3 484768 485931 -
hypothetical protein ENOG41

MPKRSLDAANMAGPIKPAPTRKTERSHEENQERAYIAASRRADRSLEARVQSARMASEIHKKRTGKAFRITEEIVMKEEMYEE
EDDEFPRSYRILKQNMQTDNPAFNARVDAYLINRVAMSQMISATESDWRNNEINMEFARMFPRAEQQAQSLSHRYSAPGYSAL
RTQNNTISTPMEQHFEPNFQPINYGPQCNNSHGDRSLSMSGPISESRNDTAMSPSALTPGSGSHPETPQPRCTSSFANVQPPP

IMEYNSDSSVFTAELPPEAKLLMGGGMNDTFSSALYPEPHNWGVPQSYPYSGDSKYIKEEDTGLGVAGESYPELSETLPWDRF
DPGTPTKTFGDQEPAWDYFLNENWGNDQQQQ
FUN_009006 CDS scaffold_3 493571 495860 - NOP9 Nucleolar protein 9 EOG09261BPJ

MPKPRTRRGAGREEKKRKRIEEAEQQHETYESENKRQRTYEDNDNTDGFDYQDGQDPQQNGLGEKEFFGMLAEEEQEYFRRAD
ELLELNQFPSTEDRDIFLENVWKEAEGKELKLASSQSCSRLMERLIQLSNTAQKKRLFEAFGGHFLSLVQHRFASHCCEALFL
RSAGVVSQELSGYVLDTKGTDVDMQKPEASMENLFLATLDELEGALSYLITDRFASHTLRVLLLVLAGRPLEDASVRSLIKSK
KKEKISVAGGSATDEANQGLRAVPESFSMAAKKIIQDSTAGMDATALRVLARHPIGNPTLQLLLELDLTLNKTEQKAESEQPT
LLFQLLPGAPKSLSDGSSEASEFINGMIYDQIGSRLIETLITHCPGKVFKALNQNIFLPRIEGYVRNDVSCYAAIRLLNRLSK
DDLVQAVEKITPTVPQLVAKSRLNVLKTLFERCNARGANDEIKKLNKGLKEGCGKTPADLVIFLCGLKDEEKKKKDVQQLSKN
EYAIQSHGAQLLTTLLSIPGPTKGVQESLLALEPQTLVRLATTSMPTVTVLTTALATSSSNPAFHKSIASAILPHTHELAISQ
FGHNLINAIVEVPSKGKERSIPFHMKEAFMARLGDHEAELRDSWMGRSVWRNWKGDMWKTRRGDWKLWMREVDANVASSLPQR
GPKVAEREKPPAKPVPEVVEEHPDQEPAAQEHITEDNMKMVVDDVVEAAVNGEEEESKKDKKVKTKKSKKDKKEKKEKKDKKK
KDKSAEAKADEETEE
FUN_009007 CDS scaffold_3 505372 507342 + hypothetical protein PF07690 ENOG41

MADNGPSNGSPSDLKPDDRTGDSSRQQDKFSPADSIPATRALDVIGAESGSCTQIKSENAIGNLSPSSGPSSLQLKELNELPA
SLSRGWRFYGAFGTLCLVTFIIALDSTIICVALPTIAEDIGATAIEAFWCGTSFLLASTVVQPPVASLSHIFGRRPALLASLT
VFAGGSIMAALAKNIAVLLGGRTLQGLGSGGILALTYVIATDLVSLRERGKWFGLISLNWAIGSILGPLIGGALVEVSWPWLF
WINLPFCGIAYIAIPLTMRIKRKGDTSVNTKIKEFDWIGTIIFVGSLTSFLIPLSCGGIMYDWSSPRTVIPLVFGILGLISFG
IHIKFCRKYCKCDPLLRPSLFTSLTALSAYFATVIHGITVWCLLYYVPLYHEVRGSSPITAGIAVMPFTGTVAPAAVIVGLLI
AKTGTYRPFIWAGWVLVPVGMGLMLLLDNHTETFRWVLIYLTGGLGLGILYSAQAFAAQASASNSDLPFAASFYAFCRSLGQG
IGVAVGGVTFQNEFRKQVEKSPEFASKANEWAKDASALVQILKKSPSSMQLMKDTIIDGYINGLRTVWLVLTLLACIALLVSL
VCVKAKSLDRKFETEHALDQAGPKEKKDQRVPEVNV
FUN_009008 tRNA scaffold_3 509668 509772 + tRNA-Leu

FUN_009009 CDS scaffold_3 510262 512018 + hypothetical protein ENOG41

MATASSSSNLSSPSSFVTAHDATLDKSTAASSPVHENPCPYRDARQLPRDLKAHCQILLEEQLYGSAINLLNSIAASGISKHT
PSKKHVLVPPPSHLALLNTLVIHPLHTTRAEKKDQLDVASQALDYLRNILLVAGPINADLRTAFQFSSTPRSGRRWDQYGQGN
DSDISDTESNGDDERLRGKLANDGSIWNRGQDFWSTIGWAFNCSTLYPKRWQYWKAWLELMLDVLEADWNERERCDKEAQLAN
GPESELPRTSRNESIIVMYMEQQNGRQNGAKGFVKAIFADGGEISSSAFREVFDKEPRGPSKMSNKRKREQVLDLENDKFGDY
FDDESISSGVSEPPTPQKPKDTRKLGAASVHSQGLVESVDIRLRLFRLLSAVTWSLRKPSELHRLYEEYTASLKLLPLPMFTL
FACQRPNPLLSEAHITITKELFDLLLPSSYKDPSKVDPEGDAEGSLTLSMLEQCFVPHPANTVALEDNAKLSLVVEDAIQLLW
ACDLMVYSEEFEEAVEKGIKAREAKAKKRRTGKVKGDEADALAQEILTNSGERIRMLLEVLKASQEAES
FUN_009010 CDS scaffold_3 512248 512876 - hypothetical protein PF05071

MATNQIGPVARAWYKWKALRLPWRKRFLVGYDLQGNTYWEFRLTRGAESNERWRRIVKYPRSTHYSSVKVSPQWHQWLRHTRE
EPPSIEEQHSDVLRRVRMQKLAAEADARWEAKPRVMEAPQAAPAPLLSPAKASPLQQDARSGEQTDTGAKKTKEEAEKKDDPW
ATAKARAPGETWQPTSWSPTAAKKR
FUN_009011 CDS scaffold_3 513440 515464 + hypothetical protein PF00307

MASVSSLDKDLRKMRLEKYTPAAANEARSWIEDILGESLPSSDLLEGLKDGVALCKLANLALPPPGVRFKQSAMPFVQMENIS
LFLRACQSPPLNLHQHDTFLTVDLFEQKDPAQVLQCIGAFSRAAHAANPQNFPTSIGPKNRPETVTPQGTGSRTPTGTRDRGA

SVTSNKSPAFGRSAALPHRTGDSGSGRWSPTKSPTPKNGPESPGAVSSWSKKEHEGTTAPAWNIAQYGYLGGASQGNLGISFG
GRRQITNAGPRVPTLAEKERRRKEEEERAHREADEGERQRAEEEDRARVEEELRWEQETEQLREKERQAAEEEKRRWEEEERQ
WKLTEEKRRKEEEDAEARLAEERKSVKNRGDSKLTGQFLSQYRADQGASQQDGASEDDYKNRVKQLEQELELARQREAEYERE
RQERAQRQGSIETKPTPKPKPKLVSSPPTRSDSWSKDEREVLRTRSPFNREPLQTNNAPVLPSRSPRPLPDPTALPQAKSQNT
SSRPLPDPAAYSSRPTPAAPSRTDRFLSSNPAPSPPAAQATYSRELGATAERDAEDQRRAQSQNKTKAGGWASKSLLEREMEM
ERQRQREWEEAQKETAAAVRSNEGVDGIGGGIGGRWDVGQWAGYTGGDSQNKGSVGIGAGRRQIVGPRPLPEQPR
FUN_009012 CDS scaffold_3 515906 517628 -
hypothetical protein PF00856 ENOG41

MKRLALDLTSHHLDDLDPDMDPTARNEQLLDRRSQLTEGLSSLPYDLILYLNRAAIHSDLGYPDLAAGDAYRALLLADEVLNE
GFEYHEQALESLQMHTAVPLPDVLAHGNLPQDELQSPETDLEVEDEAVKRLAILAQVRAYQILSLGLLLCGSLQSAASFCQRG
LQLSPSNQELLDTKNNIVTVARRRLRRDDIDIDYPNLPDQGLVRREVYPWNDHEPDRFAPESLAELNERLSSMAPKCVVEVAT
LPVLLEGASSTDDYEIIPTCKQLGVFAKEDIAPGEVVLKEYSLLTANNRLKDSICDACSSDLPPLGSENEPVSCPECYDTVFC
TQYCFDQAMERYHPAVCEKDVDAIAKDPDAFEADQTLYLLLLSRVLAIAAHEEVNPLDVREVKYIWGDFVPTRTNDINVSPNA
GPPPEWTLPFSFKYNIETPLHVLEKMDIDIYSSLAENDLWVLNTLYAKFRGTASARKNPRDGRPDVAAVHPYWCLANHDCDPN
VTWEWGGRMVLEARKERVVGGRPGGIKKGEEILNHYCDVNLPVQQRREWARGSLGGWCMCKRCRDEAAMGEANHV
FUN_009013 CDS scaffold_3 521157 521940 +
hypothetical protein PF01182

MGKTQPNLFAFRNVDALAPALRSYVIQSQAAGIARHGAFKVGVSGGSLPKTLAQALLAPSSGPNDVVDFKRWEIFFADERAVP
LDHEDSNYALLKKELLDKIPEDQKPTVHPIDTEHLNDLQELADQYEKTLVASFASRDSVRLPIFDLLLLGCGPDGHTCSLFPG
HELLREVDAWVAPIEDSPKPPPKRITLTLPVVNHSVRVAFVATGGGKKDIMKQIFDTDGGLPCTLVNEGTGERCSWFVDEPAV
EGVSFPRRAFL
FUN_009014 CDS scaffold_3 522696 524058 -
hypothetical protein ENOG41

MSTLQRSRSLRKPASSTTTTATANANAPDGSPSRLPVKPLTRSATTSSNARTLRNGTTTGLSRATSVRQPAKPVTSEPAKRET
RYPPSTTTTRPRPAVRPTSADGPTQATRKAPAPTHTRAKSTATGLRNAPVLRPPSSGSSASSTTTTTTTRPTRSSTTSTNEKL
SGAAPRLRPAFSTLQQHYSPAKSSAPKPLTSTFLAPPSPSKLPANVAASAETSRLQTELLQLHLLHRDAPTVDATWRASAQRK
LGERFSELASASKQIDDQEKAEVERENILALRKWAIGGGLEERIQILDAAIAGLWALEEPGGRYARVVRRFERWVDRMCEIEE
ARRAGGALIQENDVLFIGELDSAWKDECAGLVRRLDTIKRQLDQLGDFARDDQEGESKDQSSLQRMVEGSRELVQGMLTELAV
MDDIERDALAREDEWIERMNRDDEMEDTPRAGAVWRAV
FUN_009015 CDS scaffold_3 525060 527025 -
hypothetical protein ENOG41

MATMLNTNSLQPPKTGSSRWSKALPDVPGPDDPSFYDDYYEEPSPRLPPAKELPPPPPRSTSIANAKLLPPSLPPLHLLAVDS
PPSGPPKMAIPRRPIGKLPANPPPLPQKEPQQPNSSQIPPSPSESLSSILSAYSRSSGESLVRSPCEANDSVRQSEAISSPSH
QAPPANTNTGTFISPSAQQQQHHYQQQQPQQYVAQKLPANHTRPPPLPSKDTKYHLPATPAPVRKPVPENKLSELNSASSPSS
QQQPQPQLWRRRSSKAERSLELPDLKLVASHGSTAATQPVVAPPHVQQQLSSNGPKISTIPNWQPGSTNPIAPAGQDSQTSPA
AEQVPDTPTMGINSSKLNRLKGKLHLHRRGKSSGDTSKSASTPRPGANRPPTPEYQKEDIKTPVVDSFVSPLSPASSPEPAAQ
VSPEIPTDATSIFPQAQTQEHLRSTGSITRKAVQVPRLPSPSEDKPVSVSPEAASLPPSVTDSRGSRGSSGSDTAVHQVQPPT
EVSNRFPPRHSSVRSNAPPRQSAVEHDPRLVPSESHGPLYRGRDGTLYPEMKVLQEPDPRAAYFPKQSESPIPEGTIFKAPPL
KKSHFNCYHGHKTMNRRTNRYYPLTCQTCDKSDVEDRWTCSFCHLRICEHCVKKLNNSGNDLRRLVGHPQLTS
FUN_009016 CDS scaffold_3 529905 531749 +
hypothetical protein PF07690 ENOG41

MNSSLSSESPSEGSPLLEDRNHYEASRNIENEQASDGYTPKALYSDKKLHLLIAAVGIGVYLAAADQLLTVATYAKIGNELKA
LNNTSWIATAYFLTLTSSQPLYGKLSDIFGRKPCLLFAYIVFSLGCLGCGLAQGMAQLCAARAVAGIGGGGMNSVVAILLSDL
VPLKDRGVWQGKISILFFAGTATGAPLGGILADSVGWRWSFLGQVPLCFLAFIAVYIVLDLPPVEHDHWLAKVRKIDFLGAFT
LVAAVVALLLGLDSGSNLGWSHIMTVVALSSTPVLFALFLLIEINVASHPFAPGHIIFDRSLFACYGVGFFSGAGQTSTIFFL

PLIFQAVQGLGATQSGSLLVPGMIAGVAGSILGGWIIKRTEKFYAVTLSAYALVLVSVIPMGMFVWLRSTTGETIGLTIMSFG
AGCAFVTTLIGLLANAAAEDTAVVVACSYLFRSLGASIGISTGSAVLQQVLRIQLAARLPDGEEARKIEEKVRQSLDYIKELP
PHLADQVRSSYQIAGISSLALISIYLVVSFLLAFWIKEKPLRR
FUN_009017 CDS scaffold_3 534019 535176 -
hypothetical protein ENOG41

MLINGNHRAQAQFDRAKWRPMVMIPTWALQLGLSMSMMGLFAYRLGDSMKVNKDNDKKNDDPTIEIVWEATNVALWFVASLCS
FVEIAKYFAEALTPWTMLFTHVIKLTCAIATLALDIVVYTEKHDKHYSLVALGLDCAFIITSLVLVFYSVRRYRRLSAYDDYT
HPVNVKPYGFNDDLERDTSYARQSTDKRFSSTSSRASIISKRESVEMGTVQRTPSVYSHKRDTQFDDYVARRGSATKERIGSG
DFSYAGAQGEAQDSGATLTPTRPRGASSGRAASWTSDRGLVAVPEEDDDITAGKESKTEKEKKDREALLGNTPRESIDGLVVP
QEADLSEPRWQREQ
FUN_009018 CDS scaffold_3 537588 537894 -
hypothetical protein ENOG41

MRFSIFTLVALVSASSASLVRRVDVNVPAMTNADGVVVPFDTAGVVQPAKKRDLEQKKRDLAQRKRHISRKRRAVSQEKQKQQ
FUN_009019 CDS scaffold_3 539071 539821 +
hypothetical protein EOG09263ZSC PF01871 ENOG41

MATRDHCLLCFEALEAHLTHRKALTLEEIQEYSASASDSEPVAPKTLDDPSLRHLAIPDASASSSSSSTSLGASTPATSTSSL
PIPPTSAPLFVTWNTMEDGEPVLRGCIGTFEAQDLAEGIPEYALISALQDTRFSPIRKSELPTLQVAVTLLTDFEEVDDIFNW
EIGVHGIRLSFYDRGRRYGSTYLPDVASEQGWTKEETLFSLIRKAGWTGSRGRWKDLDLKVTRYQGKKTTLEYADYKKWIDEA
FUN_009020 CDS scaffold_3 540585 541368 -
hypothetical protein PF00071;PF08477 ENOG41

MTAPTQTPVPISITICGDGGCGKSSITLRLVRSQWTSDYDPTIEDSYSVTRRIDGVTYHLSLTDTAGQEEYRGMWASSNLGAD
AFLLVYDITSKDSLDALQHFNELIDMEAETRLDNADRARRAGLRSGHDTRSRTIPPVKLVAGNKCDLQESRQVPAAQGLEWAR
KRGCGFMETSARLEVNIEETFALVVRRVVEARRLADTGPDAAEFNARGMTKPLTPLPTDSDTEKRAVGVRGPNLQGDRVGGKK
DNGFWKSLRCW
FUN_009021 CDS scaffold_3 544565 546257 +
hypothetical protein PF00282;PF01212

MTVTNGRNGNPSITTLHRAEEVNDLIDAVKGLIVPYIKAADDAAAERATGDIPPSSAGVKNNVLVDFQKPQELAQRLKFSLPN
SGQGKEGLLEIIQQVLQNSVNTWDQGFLDKLYASTNAVGVVSDMVLSVLNTNLHVFQVSPALTIIEKTTAKTLAHLFGFTGSR
AGGISCQGGSASNLTSLVVARNTLFPECRASGNGNHDFVVFTSAHGHYSVEKSAMICGLGSNSVWPVPVDEFGCMKPGALREL
VVRARNEGKTPFYVNSTAGTTVMGSYEPFEEISKICKEFGLWMHIDASWGGPAIFSSKQKHKLNGAHLADSLTVNPHKMMNVP
VTCSFLLGPDMNIFNKANSTAAGYLFHTSDSGDIWDLADLTLQCGRRGDSLKLALAWIYYGAAGFEKQIDHAFEQAEYLANFI
KQSDNFVLVSQDPPPCLQVCFYYSPGRNLSDNKEENTLRTKTMVEKMILRGYMVDYAPGPKGSFFRVVVNCQTLPGTVEGLVK
GLEEVGRQ
FUN_009022 CDS scaffold_3 546845 548246 +
hypothetical protein PF06991

MPPKRMTANPVKPARYRAGKPTGAESDSDSDASENEESNEPALPPPPKATSAGKISSGSLGKVDLNARRKEAEAAEERRLAKE
KAERLAAEEGFVTEEEEESEEEEDDDEEEESESEEESSEDEAPRRLMIRPKFVPKSQRGNAKTPAQEEEEARQAEEEARKKAA
DELVEEQIKKDLAARAAGKKHWDDDENEDSDVDTTDGLDPEAEEAAWRVRELKRLKRARAIIEEREKELAEVERRRNLTEEER
QAEDEAFLAQQKEEKEGKGKMSFMQRYLHKGAFYQDEMKAAGLDKRDIMGSRIQDDVRNREALPEYLQRRDMAKLGRKGATKY
RDMRTEDTGRWGDIGDGRKRDGGRFEEDERFRPDDDRFRRDERSAGGANAIPLGDRKRDDRNERDNGHRDRRDRDRDSYRSRD
DGQSRRRSRSRSRSRSPRRENDRDDYRRRKRSTSRDDERYESDKRRRVDAR
FUN_009023 CDS scaffold_3 548373 550442 -
hypothetical protein EOG09261V87 PF04042;PF08418

MADEIESRFSPNKPLEPDVLSELESIMRLHGLSAEDLFFKWESYCIKLDLDAQALSLEVLRNLKQSIQDELENSHRKVQVKTE
RKVANTPRGTAKGGDVFGMLDGLVPSTPASGAKLNRGAASGSAVKRKTETPKGLMSSPATGMNEQLKSLNGLPPTSFSDRANP
GETVEILNDRLNAAEPPIAPFAEPRVKLTAASDQKKLGYKPLAMKLSEASEILDDRIDNFMTLIQQHHKIDENEFGSAAAQST
TEVVAVGRIASDSSEGKLNAASLVLETSRRTGMGLRVPLKVDNIRSWSFFPGQIVAFRGTNASGNEFVVKEVLEVPLLPSAAS
LPSALEGHRERFRGGPDAMDEDSEPAPLNILYASGPYTADDNLDFEPLHALCSQAGDTYADALVLTGPFLDIDHPLITTGDFD
LPEEANFDPDTATMATVFKYLVAPAFNRLAASNPQITIILVPSVRDVLSKHVSWPQDSIPRKELGLPKMVRIVTNPMTLSINE
LVLGVSSQDILSQLRSEEVVSRGGGQPGTDLMSRLCRYLVEQRHYFPLFPPTDRSKLPKTGTEDGLATGAALDLSYLELGEMV
NVRPDVMIVPSLLPPFAKVVESVLLINPGYLSKRRGAGTYARMTLYPPPVSNGSGDDVMGHQIYDRARVEIVRI
FUN_009024 CDS scaffold_3 551045 554965 -
hypothetical protein ENOG41

MASQMPNPIQGGWNHNGQTMQQNVETNEAGVQVRPMGLKVHYTFDKEAKVNCLARSTQTLFVQTIPLDEQNSIGIVDLRACLQ
AITECSPELGNQDSEGDYTIYALDYSEPDTPLVGQGMLSWALDSMRGGEWLSQSPKMVTGRVTKNLLAVFGGGNRETLEVKLK
FTVGSRLQRREPNVDIPPVEMPPHPMQPTPLRPASLQPASLPLSRSAEMAVASGAAEWSSFIQANPHIGHTAHVSRVASPALS
QGAPPPATIERRDSIDPAISQANSGSDVQRIAPMPVPPSTTAASTSRPSSRSSRNRKPPTGRPRGRPRKKPAEGNTSGYEDGT
EGEDGGSVPVKKRAKPMKVEKATPNPFTSGPESLRVTASTAGSLRNFRPVNANNEAVAGNHLQEVPRAPTPVPEGGPLNGPAQ
GGPKGVKPRRESSMRKELGATLSSQIPPRPLRALSPSQEDGRSPESPVETPAFSEDSPQDIRSSPPMPRTASFMRSSPPPSSP
VLPPMPSLPRQISSFAENETDDLLDEPVLPTAKEGQTYSQPKTQKVMEDVDNTGIPTQVFRIEDGPEGQGLVHIQNFNTPYPT
STPAGPPPSEPVTAKKVAIPRLVRNSSQKRPPGSKQQPRSYAPTPPPTTDAAEKDSPVPTTASSLPDSTQMQGNQAMEAMEED
ALFELVRAVSSSSEPNTSISTNQSTEGSLPTSKSSSRVIAPKPRPSRPLGRSQSAGILALPSVPASEPAGPSSLSQSVLAEPP
RASTDTTTCLRRSKSVSVPKPIAASDPLGPPETYLITNTLPTLSEAIFPQSDFPPMPSSPPCKSNKNQIKKDSIKQRLESAIA
AGEMPPFCTNCGAIETPTWRKIWVQEHDGVPENVEYSEKPGRVTAIEILKRDKDKNPLSHRLIKKALGFDDDRSDWSEKLLCN
PCGIWMSKYHNHRPQDKWDNGLLGQSRKKRGSAGPESQAKRARTKSSSAPHPPSEAFPVTDSIGPVERSSPKQPDVVSMGSRV
DEDAAMQNVFHLLAADGRHTKNMSQSVPGSSHSRASRGTGTADSPIEMDLDDELGSTRRLLFPSPRKEGTPKTLGEIDANVTK
TADCRQNKEICGKENAVCDAQNEDLIMDDDIEALFNSPVRPSTPPPKSKADADSALFKTPTRPTPSHRPITRSVSRSLRSVRD
LLSPSQQALLQRTPTRSPASVRRSPRLNLESKLESVLDTPLSRTITQLLSEPNFDLASSNLDFSTLPLLDTDPASLGDFGSFL
STDGIAPSSSPKDVHVNFAYGGSDNVWAEWGGIEHKSTVQEKEK
FUN_009025 CDS scaffold_3 556547 558116 -
hypothetical protein EOG0926213Q PF00378;PF16113

MTFRTLTVRAGASANVASRSSPRIFNMVQQSKILSSSLGARQLSSEATNIREKRNDDPNDVVFESKYGLRTVMLNRPKKLNSL
NASMIRKIVPRLIEWEKSDLANVVVMKGAGEKALCAGGDVAALAKLNSESEDGWQKSAQYFALEYKLDHYIATYKKPYIAFMD
GITMGGGVGLSTHAPFRIATEKTVFAMPETTIGFFPDVGASFFLPRMNGSVGTYLALTSERLTGPNVFYSGIATHYLHSTSLP
DLEARLAELRFRDSDGLPERLALINQTLEEFCTGLPYDQPITLSGEIRQAIDRCFNKHTISEIIAALQAERGPTEEWAQKQLK
TLHKRSPTAVHVALRQMRIGGEWDIAETFKREHQIATKFMQHPDFTEGVSALLIRKEAPKWQPESLEAIGGTNVAKPFFEYDS
NNELALFTDRTFKEYPHRELGVPSEKEIEQVLSSGTYTQEQLANKIVSSRNGRQGIAEVAREIIARKTAVDDQGKAVWMADES
LPGSRL
FUN_009026 CDS scaffold_3 558471 559030 + SMD2 mRNA
splicing protein EOG09265IT6 PF01423

MSTDPKIQELLSKSRNELTEYEIAQLEEHEFSAGPLSILQTAVRSHVQVLISIRNNRKLLARVKAFDRHCNMVLENVKEMWTE
TPRLANGKKGRPVNKDRFISKMFLRGDSVILVLLS
FUN_009027 CDS scaffold_3 559269 561057 -
hypothetical protein PF02733;PF02734

MSAKHFVNDPTHLVSSALHSLTLTNPSLALDPDFKVIYRRPDSNAKAQVSIISGGGSGHEPSFAGMVGQGMLSAAVAGTIFAS
PSAEQIRTAITSRVDTSKGVLVTVMNYTGDVLNFGMAVEKAKAAGLEVEMVVVGDDVGVGRAKAGKVGRRGIAGTVLVHKISG
ALAALGKPLDQVAKYAQLTADNLVSVGASLEHVHVPGRKVDTEGSLAADEVELGMGIHNEPGSGREKAEVPDLVSKMLKQLLD
TADKDRAFVDVNSKEVVLMINNLGGVSVLELGGITAEVASQLESNYSIRPVRILSGTFMTSLNGLGFSISLLNVVSPDFEAPS
MIELLDAPSEVVGWSAPVQAGTWKTKNTNTRTGRAGATGDIKPSGLKTDPSAAQAVLKKGLQKVIDAEPEVTHYDTIVGDGDC

GIGLKRGAEAILKHIDEQPLTGDVVVDLSSIVTIVETAMDGTSGALYAIFLNALVHALRELSPGTASPEVWAKALKMSSDALA
KYTPARPGDRTLVDALHPFVEALNQTGDVKNAADAALEGANKTKGMQASLGRTVYIGGSGYQEVPDPGAWGLASFFLGLSS
FUN_009028 CDS scaffold_3 563355 564847 +
hypothetical protein PF03663
GH76
MHSLLRATTAILGWAAAVTAYDVTWDDNKSIEKAAGQAAFGLVSYYTGNNTGDTPGNLPDPYYWWEAGAMFGTLVDYWWLTGD
DSYNTITKQAMIHQAGTDGDYMPDNQTMTEGNDDQGFWAMAAMSAAEHQFPDPPEDTPGWLAQVQAVFNEYVSRWDADYCGGG
MRWQIFKWNTGFDYKNAISNGCFFNVASRLALYTGNDTYADWAEKLWKWHEDSGIITDKFAVQDGVHISDAKGKKCTDIDKTQ
WSYNTGIFLHGAAVMYNLTSSDSWKKRADGLLDDVFNKFVKDEIIYEQFCEPHKQCSQDQQSFKGYLARWLAATTQLYSSTND
KIMKLIKTSAQAAAKVCTGSPTEGYKGPAGTACGFSWTTGSFDGSVGVGPQMNALSILMYTLVESAKGPVTSKTGGTSKGNPG
GGNTDINDNDGTPPLKDITTADKAGAGILTFLFLAGVIGGVSFLVIDF
FUN_009029 CDS scaffold_3 565330 566236 -
hypothetical protein PF13826 ENOG41

MKKSEFRSKMEPTSRPLTFASTAGMLFMLKDCFKPQTTVVVGSFIQLALCAVLPLRWAVVPPLAVFFNSIVTTTIQLYSTNPN
EYNKNVVPGRATAQLPFSTGSFGSTPGASPVVVFHLGIQANHPLGLAAPGMEQISKYFMAMHNDLASKRDEYGMLSSSTWRGD
EKSSNNTLLLIYYFRDIESLHRFAHDELHRKAWDYVNKSKLKHVGIFHETFNVPAHEYENVYVNCHPVLMGHATVKTTEGGEE
EERWINSLVSADTPLLKTQYARMSRDEHGRLKDTE
FUN_009030 CDS scaffold_3 567553 569263 -
hypothetical protein PF13257 ENOG41

MASQQQAIRRRYSVADRLPADLVLPTTPSNSNFETAPSSTISATASPTVGVAASVNPRYAPVRDLAQGSNPRHLNSSSHNRHG
SMTSTAAMPSRPASKDESVPFRRGHARAKSSVSSMNRQVNRLSLTLPIAPPTSDPSRPTPTSASMSSVPPTPIESGIATPADA
NEFIIAIAAQERRVLELREELSRAEADLTLLKKKWTAQEKKGDTIPLEAFRSPILSSEDDAASIRRSVDSDRRKLLQQTQNPA
TPNRRRVLRGGHTRTLSLLSPAKPDAGFSLYQDRDHEHEQVKLPSIERRTAQLTNPHLSKRASWQPRTQQNGPVVPQIVEDFK
LGLRAFVEDIRQITVGDEPINGQPIRSNSVNERGEANRNQDTIRPNRPLRPKVSTVFEPPHNNSDTTETKSSTSTSTASKRPE
LPARSKTKSKNKSFSWQPLGFDSMDDNDWSNWESPASSSKTSRWSGSTIGSGGLDDMSATSDEGEPADSPSKKNSTGYETPLL
SPKLEELLPNIVNQFSPSNIKRTATTLMDEWEKSLTDPNYPHQTQNKENTA
FUN_009031 CDS scaffold_3 572509 574968 +
hypothetical protein PF01068

MGTVKPQLGVKIGRQPWVKGRSIKHCLDMGHGRMSVEEKIDGEYCQIHVDPSKGDKCIQIFSKSGKDSTEDRVALHGIIQESL
KIGQPDTRVKKACILEGELVVYDDSQEKILPFHRIRKHVSRRGRFLNTELDSLPGPQEHLMIIYYDVMLLEDQSLINLRHSER
FKILSNLVCCRKGWAGLVPRQVIDFGQPLGASTLRKAFAMVILAWKEGLVLKPDEPYFDFTDQRRRFSSCCIKLKKEYIGNFG
DVGDFSVVGARYDSTKALSYRIPGLKWTHFYIGCLDNREAVKSWGAKPEFTIVNVVELNETILREVVIYSNPEPVSPDDNGAL
ALKLAPGVEQGSPPTFIFTKPLVFDLKCFSFDRVGNTGFWSLRFPSVTKVHFDREFTDTISFEQLQALAKEATTAGKLEDSQE
NLQWIAKLEAADPRGIAVDAASQLTVTTMPTPSPRKSTQNTTSTWSPKSPLATKSPVQVSPCRGRRERVHRAPSLDLPSITAT
TSLRTEPLSPTKLCAKHKRGLPSTGPTSQHKRLKSSSETSISQNDYPHIDIASQPRAPLAARDNNPRPQPVKSSCAPCCPSSP
SESFFDESSNSDGINAEVVKSIINPGTGELQTETNPERLKPSTTKVTTALKRKCVYAGEDCMLANAVVLFLPGLLAMKEAKKL
LAQHGVDAPVTDIDTWLKAEKEARQKGKITIPSKAYVVCDSDNKLVFKHLAEKFKELRKDNLFNKGTHIEIHDWRFLSYLTTQ
EDESIEEKYFHGFSDWRMRWKLHTI
FUN_009032 CDS scaffold_3 575091 576423 -
hypothetical protein PF03171;PF14226 ENOG41

MNYPMRDKAAHFRRPLGQDQNTANIPIIDISAPDADQKEVAKQLVDAAEEHGFIYIRNLGHDIQAKDIDGAFDLVRKSFECPL
EEKQRCTIQTNNRGWSGMHSETLDPKHQKVGDFKEAFNFGEFADGKAQQPLPSDLVPDEPQISAFANSCHDLCQKLLYLLGLG
LGVDDFFSSAHNTEKGASSSILRFLRYPPPESTAHSEDDIRAGAHSDYGSITLLFRLKGQAGLEVLRKDNVWAPVPVCPAGTE
EDPSPPILINIGDLLSYWTNGLFRSTLHRVVFPSEGSSGVQGETSNGPRYSIAYFCHPVGAAPLEPVPSERVKNFTPSEGVSS
ENPYATRKVMTADEHLFLRLKESYGDLYEKKS
FUN_009033 CDS scaffold_3 577228 579969 +

hypothetical protein   EOG09260BFE   PF00752;PF12246;PF12247   ENOG41

MPFLVEDSWINDQVSTHDISELEGCAIAVDATYYLSQLLETPPAHEPLLSALGGLTGVEAHINQNLDLWAKSEIVPFFVFDGQ
PVTGQDDITLDRGLKANKKTDEAWNLYSQGAAEEAVTTFGTSPGAFRIQNLYPLLQTVLKNRGLHFLVAPYTACAQLAYFEMI
DSDQCSGVMGSQELLLYPVKDSVIRAFDWEAKTVSAISKKKVMRSLTPTASEPRFIDSFLMAGTSFLPPFPALLESSIYSDYN
ISTAANLLRTAENSVATACASFNDILQSKDSGWLDKYRKARMVVHHFVYIAESGEIRVNDYEHLTSDNHEYLGLQLPAELFHY
LNTGLIGPRLLGNITHGQVLIQPTLDGVASDGYKKLITDRIVPIKEHALSLLIPRLHRGIQHKNIKVRVWFDPKYSYTINHRS
VNPPPSQRVASWSVKDEDLRAFFPDDFAGPVSLEVLSLVNSDFVAKTFPKGRPIKGIDSTDMVTSVAIWRFLHLRGYANDEHK
LTPWGNALANTLLILQDAKENHPEVTGLPEAALVAFELIRNGLLTGKHTEGQAGLPRKGSYEEKATLVLISECASLLKLRHQV
YGYTGPLNKNLLSFWSLASAVREADRDLVEAIVASMFLYGQSKRERDDQLEISRRLPFHQEPDIGLGIAVRTFFDDDEAGGDQ
EARLQRLEEFPKTFVPYAESLTKDFRVVRDFIDALVKGVKMLGTDELRAEDKDAWTKAQAYLEARPF
FUN_009034   CDS   scaffold_3   580504 581522 -
hypothetical protein   PF13668   ENOG41

MASLKTILFGLFATTALAMPSGPRFSKRQLQYHDLSKRQNAAAAAAGLSDLDILQFALTLEHLEDTFYREAFLTLSDEAFAPL
GLSTQTLDDIKAIGKTEAAHVVLLQSALAGNGITPVQECKYDFKGATADPAAMVATAAILESVGVSAYLGAAPLLSDPAILGT
AGAILTVEARHQTAIRIFSQAKAVPQPLDTALGPRAVFSLAAPFITECPEGSNLKIEAFPTLAMAEGQDVKAVAIGTKVKLAS
EAAAGATHCGFTSGGQLPGGTKFTTFTEGEGCEVPQGAAGVVYVTLTSAGPLEGVLSDDITVAGPMVLTLS
FUN_009035   CDS   scaffold_3   582725 587053 -
hypothetical protein   PF00773;PF17849;PF17877

MEQQQQQPPQQPQQQQPQQQQSSQQQASQQQPQVGGVPGPAGRRLHIAHRRSPSELTPLMSMFANPGMEQLAIQQQIELLQQQ
QQQIQATHQQYVNMGMVPPGQPLGPGGAFNPLQPMPNMSQAAFQFPNQVPQQNMAPPTQPLSHRRNQSALPNMGMGPPPAPSS
GASGSGFGNFEAPQAQGRENTGGRGGRGGGAGGGHQRRHSLALADAKKAAEIAQQKRTTTGFSFPASPAPDEENKPASQSTLE
VPVAQGSTRGGRGGHGRSQSMAVNGRGGGRGGLNAESNEFQRRGSGHARTGSRNFEGNWRTQGQGQEQSGNAGQSQGFQPGHR
PRGSMNQSVSGIGGFQYNPNQPQVMQLPNQMMMPQMYGQQLNPMQLNQLQALQAAQMNGQPFMGLQGSQHAGQLGGQQQQQQR
KTLFTPYLPQASLPALLADGQLVSGILRVNKKNRSDAYVTTQDGLLDADIFICGSKDRNRALEGDLVAIELLDVDEVWGQKRE
KEEKKKRKDITDTRSGSTNQGNQNSGNNNDGNAPEGGIRRRGSLRQRPTQKKNDDVEVEGQSLLLVEEEEINDEQKPLYAGHV
VAVVERVAGQMFSGTLGLLRPSSQATKEKQEAERAAREGNNSRHPEPRQQEKPKIVWFKPTDKRVPLIAIPTEQAPRDFVEKH
QDYADRIFVACIKRWPITSLHPFGTLVEQLGRMGDLKVETDALLRDNNFASDEFSDAVLRSVGLQDWSLDKEDEAALAERRDY
REENVFTIDYNGGAELGNAVHVKSRPDGKIEIGIHVADIAHFVKPNSLVDREAKKRGTSVQLKNRFCALLPPKLSAEVCSLTP
EQDRLAVSVVFNVNPHTGSVAEGDAWIGRSIIKSAGKVSLDDIDDALTNPSEYKNPAVPVNTIQILNAVSQKFREARLGAGGE
PIAPLRLLQQLDDENDPVQDNLFDSTQALELVEELIHKANAHVAQRLVEGLPEKAFLRRQSAPNPRRLQTFVERMTALGYDID
SSGSGALQNSLFKVDDPDLRKGMETLVVKSMQRAKYFISGKTGKQLWPHYALNLPLYTHFTNPTRRYADIIVHRQLEAVLSEG
KIEFTEDLENLVKTAESCNTKKDSAQNAQEQSVHIESCRTMDKKRQEANGDLIAEGIVLCVYESAFDVLIPEWGFEKRVHCDQ
LPLKKAEFRKEKRVLELYWEKGVPSSAFVPEDERPKAAASIRHSNALAAQRQAEEAERVRKEREEATRKQTDTGTISTDDVDA
LFDDDDDNTSDVTEAMAGASLAERPTQSVPGSPARSSSNAGNLHRTRSDSKVPVAEAVETRLTNKEKYLSFFSLREEGGDYIQ
DVTEMTRVPVILKTDLTKSPPCLTIRSLNPYAL
FUN_009036   CDS   scaffold_3   590620 592274 +
hypothetical protein   PF07817

MTFSSPVRRNQLLSSPDRNIAATFLLDSRNNELNHRDALAAAQLEHDRVRQAAIRVYELHELQEEHKRIVAEERREEERLKAE
AAVVAEEKRLRELKAKTVPRLPPEPAPQPPSPAKPEPTKTNGATQIKDVTPALDAKKPIVSPATGPASASATTASFTQTKPSN
QFGTTQPASESLPQKSNGATIQSTALAATTIPQPAVKSAAPTVQPVQSKSAPSIDRYSQIHQELKKLRKELQAQSKVAGSPLK
GKLGAARREIRVAIGQLTGGKGANAQPINKITAALKEALEGRIPSPPIDVSLFVVDKREPVEGSPNNEATLPSLFIYLINICA
KGIVSQFINEGGANPKAADPVGVFAAHIFSTKEFQWRGQSLVDILMAKYRIVCPVLFGHRGSDKTERGRMAIGWKKDGPSWIT
EQSHNDRMTGLGAGFASLSLRDFGKSSKKNPYPPTNYWRALAYIVNSPPNETSNTQYVVLRSMIQGHEQRFLNFYGNAALAAL
RLALIEFPKKAPQNATAAGSLAALADVLKTESGLILT
FUN_009037   CDS   scaffold_3   592649 593411 -
hypothetical protein   EOG09265B1X   PF09177

MMSSTNDEDPFLQVQQDVLNQMANARPLFASYLRIRSLSTDANSPELASARSDLESALSSLADDLADLVASVRAVESEPSQFG
LSPHEVTRRKRLVQEVGGEIDDMHDELAKKVDAGDLPDPNAFAIDGDAEDNYAEFEQQQQMEMMHEQDQHLDGVFQTVGNLRR
QADDMGRELEEQREMLEEVDTVADRVGGRLATGVQKLQYVMRQNEDRYSSCCIAVLIFVLILLLILLLIL
FUN_009038 CDS scaffold_3 594044 595838 +
hypothetical protein PF13679 ENOG41

MIPENPLPFNGNFSSPEEYIDELLKFVRTSETFQILCGGVHILDFFTIEPGLFHYAIPKEWHAFILSRSLMEFLDLLMRDDLD
NLKLEGEQPPASLIDYIRAVRSLSLGRSFTPPSEKLPVLPRSVAVGMNVKKTHEVTNFADYLARLSKDISSQCGYEISHYVDF
GSGQNYLGRAMASEPYNRHVVAVEGRENNVTAARGLDVSSGLAVKPKVMRNKKLWTKILEARGPDGQEDPEALAKAIREVAGD
EAFEFRPVKELEAEYTVEKGKGSVQYISGRLETGDLADVIAQINPGSQTEDEKKDLSLMAMSIHSCGNLSHFGIRSLVLNPDI
RAVAIVGCCYNLMTEKLGPPTYKHAFLRPTLQAVNGRLVRESEKHDPQGFPMSQKFSAYQGDGVRLNITARMMACQAPQNWTE
KESAGFFSRHFYRAVLQKMFLDRGVVKKVRHTGSQGESQADTAASTQDDSESPFDISTNPVIIGSLRKSCYGSFKSYVRGAVE
KLTTNNEYKQYADVMQEKMGDISDEEIERYEALYMPRKKELCAVWSLMAFSAMAVESLIVSDRWTFLKEHDDLVRHAWVETVF
DYEQSPRNLVVVGVKR
FUN_009039 CDS scaffold_3 599404 600688 +
hypothetical protein PF13840 ENOG41

MNAQVSFLDGTYTLIHIPLSLYSTLLQPILRVLLPQSQGTGGLRDSPEYELQGLTPDGQHGFLNISITPLECSVVCHTSWAQN
VFEPVLKAMPRDIANTVSVSKESYMVLSVISAGLDAGGRVMELTSPLALAGIPIFFITTYYSDFILVPTKERENVVKSLIAKG
FELSDNESNFITSAANHKQGTGLSATSPPHEGLPLNVAELQTRIFGTLKKRHVAPQIEEALELVQCSGREASQLANFTHQRPS
ISRHATGNGRRPSWADNVDTKLYTCIISALVSQPRFMSVTLAQDDPPSLLLDKNLLDIFGDSLVGDTDASLIPIFLDLKTLPL
EATGIVCGVAGILVQDTQIAASSELSYLSTAQAGAVILSEEQSVRAMGILKPLLSEEL
FUN_009040 CDS scaffold_3 601307 602117 -
hypothetical protein PF01205;PF05773 ENOG41

MSEELQDEVEAINSIYGDDSLVPTEDDSSVFILKLPGDASSLRLKFPSDYPSKPPSALSTHHSSGGVKGAGARDLALFRDALG
EVFQEGLVCLFDAVEEFTRRAEEQKPEPESEPKAPSTPEEEDYEQPDFPPPEWVLSDLVTESKSTFLAHVARVTSPDQARYYV
QLLLSSDKRIRSATHNMTAWRIRGPGATSFQDCDDDGETAAGGRMLHLMQVMDIWDAMVVVTRWYGGVQLGPRRFALINAVAR
DGFVKSGLVKEERQEKKRGK
FUN_009041 CDS scaffold_3 602587 605373 - VMA1
H(+)-transporting V1 sector ATPase subunit A EOG09261225
PF00006;PF02874;PF16886

MLGIRPTLRARRSLLDLIRTRGEDDDNNSQREQGSDSDDDSLSSGSGARGSRYRGSFSDKAPAPPVNVATVRDPTRKASSSSW
LHSRAGKARAAALALVDVARQVPLPNSPPPSPSPSLSPYSPSSRPQPSSQPPPSSTLSPGSTASSIRPQSPHSPTSPHLQLDN
APVLSLKMPPKTSKAANNSSKTNEEDGYQTGKIYSISGPVVVAEDMIGVAMYELVRVGHDNLVGEVIRISGDQASIQVYEETS
GVMVGDPVTRTGKPLSVELGPGLLNGIYDGIQRPLEAISKMAKSIYIPRGIAVPALDREKKWEFTPSVKVGDHLSGGDVWGSV
FENSFLADHKILFPPRARGTVTKIAAKGEYTVVENILEVEFDGKKTEYPMMQTWPVRVPRPSNDKKSADQPFIVGQRVLDALF
PSVQGGTVAIPGAFGCGKTVISQSVSKFSNSDVIVYVGCGERGNEMAEVLKDFPELTIEVDGRKEPIMKRTTLIANTSNMPVA
AREASIYTGITVAEYFRDQGLNVAMMADSSSRWAEALRELSGRLGEMPADQGFPAYLGAKLASFYERAGRVQTLGSPEREGSV
SIVGAVSPPGGDFSDPVTTSTLGIVQVFWGLDKKLAQRKHFPSINTSLSYSKYNTILDKYYEKNYPDFPRLRDRIKQLLSDSE
ELDQVVQLVGKSALSDPDKITLDIAGLIKEDFLQQNGYSDYDQFCPIWKTEWMMKLMVGFHDEAQKVIAQGQSWAKVREATSD
LQANLRQLKFEVPTDGQDVISKRYEEIQQKMTDKFAAVMDE
FUN_009042 CDS scaffold_3 605923 607761 -
hypothetical protein PF00743;PF07992;PF13450;PF13738

MDAQNQELNFDVCVVGAGPLGLLALKNLREQGLNAKAFERQEYVGGTWHASNNIEQTTALEYTTANTSKQCCSITDFPMPDDF
PVHPPQKDLERYFESYAEQFGLYPHIRFSVSVDHIERDEPRKAWRVFLKDVKTGVEEVRTFGRVVVATGMLNTRHLPHVKGLE
RFSGDAIHSRQFKDASKYQGKNVVVVGIGATGVDSTSFLVKAGTNKVYLSHRGTVFVLPRRVKGKAFEHTLSRRVSICVRTIG

NIMPNIFAGLMTKMMINMRDKEWPHLKEVFGTRPVDGVLHRVPLFSEHLADNLKDGTVKSVQGIKEVTGPKTITLTDGTVLED
VDAIIFCSGYGYDFSIVEGPGDPTDPAIAPDHHKRIEATKFYGEENKFARLFHGFISEQFPDSLAFLGHMIIMRPPFVLYDLT
TMALASLWSGDYPMPSAEERRRDIDDHYDYVVKTLQRGPAPHPGFRWRAKPTYEFLNQAAGTGVTNRLGCFTWEAWKLWWNDR
KFYNLLMDGTDVPAVYRLFETGRGRKPWPGARESIEKTNQEVKELGEQWERENKDKKTK
FUN_009043 CDS scaffold_3 609011 611119 -
hypothetical protein PF00149

MRLSTTLLPCLGAITAVIGTDGQAVVPQGLNENQKTLYTNDWADRIWKQIQGVSSCSGCQGLLLTFKSLANLGDRYFVNTLQD
VCKKSKVEDADVCDGTIELQGPIIAEALRNVAIGSKTAQHFCVTFLGLCHYPAIDEWEVPVPPDHLRRKRPVSSGRDPIKVVH
YSDIHVDQLYTEGSNSECNKPICCRPFTENDEPGRAVSPAGPYGEHTCDSPASLEHSMYEAIKKLIPDAAFTIFTGDIVDHSI
WNTSWDYNEHQIIQSYEHMDRHLGIVYGTAGNHESHPTNAYQPSSIGDASSWIYDLLAGTWSRWIGHESASTAARIGAYSTKY
PHGNLRVISLNTNLFYRGNFWLFQRKMLRDPSGQIDWLVEELHAAEKAGERVYIIGHMPLGDRNAFHDQSNYLNQVVNRYSAT
IAAMFFGHTHRDHFQITYSDAPGKTFNNALITSYVGPSLTPMSGMPSFRVYDVDPVTFAVLDATTYSADMTSPIYQTQGPVWK
KYYSAKETYGPLTNPPLIDHAAELTAAFWHNVTHVFEKNQTAFDEYMLRLSRGWVQPECTGECRSSQICMLRAARSQDGCDVP
TLGSSYHTRMENVAERDECGVSVLQATFSALVAKEGALKILQEILAGLDVKLHG
FUN_009044 CDS scaffold_3 611725 614905 -
hypothetical protein EOG09260JDM PF08634 ENOG41

MLRLRRGIAPAATFAHICRPHLQCLRQCSPLRLFATETSPDDTSIPTPKPVPDSEDALNLMKAASEKIEKGAKAAERRKEDKK
RRAARFEVKGSTEGTTETDISDALAVAKRVYGIDKSKKKKKKKGSKPSSPNEQQTTGKADSSEASSGVVGQQAGAWASLQEKL
QQELGAEPSNDPAAPQEDHTSPSPDLDQHIIASSHETEPSVAAPPSKKKTKAAKTQKALPPVYTIMPHHLKLKAVEEESRRSV
PTLAHQLDKVLFNGGPYQLQDQRTRVYNFDPYLASIMPVDEFDFDALKEYVTSSKDSRLTDLCAKYKKKYCGSTSSMTAMLSQ
FHFLLSAWRPLSFRHLSRSFKVEYDTFTALTRGPAAAFARYKDGVYAIDADKEFDTANVLSMLGKSMEKLLTLKKTHFEKYRR
SRSHELSEEEKNADEAFHFSTLGDFMMRSQLDAYDPRLPGTGMFDLKTRAVVSIRMDVGGYQKGVGYEIRSRFGTWESFEREY
YDMIRAAFLKYSLQVRMGRMDGIFVAFHNTQRIFGFQYISLEEMDEALHGTANRRVGNEEFKVSVKLLNNLLDRATKRFPEKS
LRLHIETRPTNPPLTYFFAEPVSDEEMQETQEKGSEAVAMFKNNMLRISQDEEKGRMSDEAEPQTKEDTQKLSSTERQKSWDE
MMAKVDQTVEDDAAGLDSVREAIEQALEQSGLLAGKPEPERETYINELVEALTEELKENKEAASELEDTTQKDSAEDDALDAS
ESSSETEDALFPSIDASEGGSSNNDVDSKDVEYAGKETESGLTEAVETRAEDEGHSPILSDAVDGTDTSLNSDDSADATLKDL
IIKFAQGVDNNPSNLGTFERVLSELVRDQKEPSSEVDESNTASIGVSPAESVKTSGADKSSSTEEPRSDKESADIPKKEILGM
YVTVRNMVDGEVVERISCSEPEEPTNWKVEYTVVELPSERAHRILRELKKRRKKALVSTPEDREREWHKMWGGTLPQRTEAGR
KYRDSVMQREEQTGVKLAWETDARIKTNKNKNKEPHSRKKGSEKKVRSKKKTKKEEKTSPIKE
FUN_009045 CDS scaffold_3 615234 618008 +
hypothetical protein PF09742;PF12722 ENOG41

MGASDSKLVFKRGIFRLSEERHIPADDAYWTSFWELPESSEDVFSLFTPADIRRTRDRALENLETLILAITSRLFILRHHPSF
PDPELAPEREALNCIRVLTRILPYLYEKESLSDWEQQFFWAQRRKRTRHGAIANEILFDEAQEAERKTQTAEFEEVKPLAEEL
VDTLVDLLFFSDLTLPRQPNGRPKVSYAIWQSGVGCNTAVATTKEYESNRSEILRLLLTLSGQSLYMTAGVLPQNGVRTLTHL
CACPDKQVVLSVLCSLLNTTLKYNPATWRVPYNTLVFKDGKQMLVTYSLQFLLVLLLYPIPENPENPSPKHSYRHFLGRLHRP
QDFQFIVDGMTRILNQPLQEKTSYLPGSQSSGHFAPEILMLFWEILQCNKRFRSFIVDTERAHDFVVLALFYALEYKNDATKQ
GVVRMCAFLLQTLSVETNFGVNLNKYFEGQESLPMSIRIPGFRGTYADFLIHSIYNLITTSQGKLSAIYPALLSVINNIAPYL
EGLSSTSSSKLMQLFSSMSSPSFLLANETNHALLHSLLESISSILENKYQKNPELVFAILKNKKRVEALRSFTLESGQEEIER
RNRRRKDSGHHFDPFDAASVRSSVDSLRSPNSAQPRHSSLEEVPEDGAFAIGDDDDDDDDDDDDDSDDEERQETPARSTSSEA
PSRRSSTPNVEDAVPTQLRGMSEKARGKMPAGVPTFSRQNSTTSLGGRSISGQSQAGAFEPTTHWIDSWLLELPLHTILAVIQ
QVSALLPRQELAADIPTPETLRRISGVHVVGIEPSAPRVHSFEWSQLALGWYESLLWGVIFTNEMQVSRGTMGIWNGTAIKLF
RVQETAPEGPTLTSPRGAVDAVGSNIVSRIGQINLRGGPQGNSQPPQRNN
FUN_009046 CDS scaffold_3 619053 620709 +
hypothetical protein PF06441 ENOG41

MSQQQPGDSGDEVQPYRIRVSSKYLDITRQKLELTRLPHEPHSKDWWEPKPQVESLVDFWQESFSWQNHEEELNQTLPQFRTS
FQTSTSSTPVRLHFIHARSPHANAVPLLLIPPFPLTNLSLGHLVQPLSDPEDAGTNQPFHVVIPALPGLGFSDAVPSDVPPIS

TTAQLLDSLMKRLAYEHYVGSNAGSASISPAAIDWRLARHLSIHYSESCLGFHFIAPPLTCPTLSDSASQWLKWKLARLLGSS
VLGYSKDDLSAIQKQKTPQLSKKKSAVNLGQHSRSDFEPNTPSYALCDSPTGLLLYVLKLLRTLGPKKELTPKDIITLTSLIW
LPGPEAALRFWAQCASKIEPVEEKKTNKKPKVAITVFTGDEDQVEQQRTLPRPAKTMYSCPGWANREYQIVHLNRAPGTPGFL
AWDRQDVIVEGVRGLVKAILAKDKRMQVAGQPGAVLQDQLEVQDGRIAQADLSGTTVQDPNASPGGEGPPKTGVQKPAGGDHQ
HLVPPQAAIHGGTH
FUN_009047 CDS scaffold_3 621453 624720 + SEC8 exocyst subunit PF04048

MSNRYDRPHREGNGYGNFGRSNDDYRSGYQDGYGSDRYATPPATQQPRPPPSLRQMSSRSRIPQVQAPPQPADTHAARQITEV
IDHIKKEWPAMCQDDCVPVQLALQLLDRSSVGRAHEYAQFQQSHNYLQESLKGIVHEHHQGFNSSIGTFHKIQSSIQQSQKRV
RALKESLASSKTSLCATDPELKKLSKSSQEFDALVQTLNELDDLRAVPDQLEARISEKRFLTAVEVLQNALRKLRKPELDDIG
ALTDLRSYLANQETALMDILVEELHEHLYLKSPYCQERWQSLAKTQGAVTEGFGDIKPIAPFHVILDAIDVDKTVREDPMKNP
EADTFYYVGLLVESLNRLGRLQNAVETLKQRLPVELFTIVNETINEVDQRHPSSLRGGSARGDGLHVYGTRETQMRADVIYDL
LWSLYGKFEAIGEGHRVFHESIKVLIRREGAGNNSALLGSFKELWNLYQNEIRSLLHNYVTTDADVYQFDSPNPGDTLHGKKD
IREHLFKFSEANTKSVEMTSEYDALDNIIQDAVPGLGSRKQDRKGRNVQDGGRRGAGAGAGSDGPFGSSHQTTGSYKSLVEPS
VFNMSLLLPPTLVFLQRLKNIVPPGSDLAASTLTTFLDNFLVNVFQPQLDETLAKLSDTVFGEADAFTQDPAWSQVARRPVFR
GTTAFFEVVTAFCRMLGTIPPDQALSSLIVTQMMRYYDRCFSWFKTLVVKAQDPATEISNDNLRSSARFSLDQGEIQETMKKL
FMSEEMDWELAEKEIHLLMELTDESPLDSGDIIQDRDSISSLCLLYTSMKWLAVKIAGLRQITTHDTDTSRQNLPRHSSRRWT
LMNDTGKTAGEEGPVSLPLTQETVQAFDSIVSSFEELAGTALLTLHMEIRCRIVHSLSIALSPKVAPYLLDQEVSEPDPQILS
LNSELVFFDETIVRYLREKEIAFIRTGLGLLINCYLVSNACKASPMNDKGCGRMQLNILVLQQNLKNVEEGVDLARAANYFSL
YEQGADAIVEKAKEDKEQQRGTSAQDPDNFSYDELKALVELCYSEAMANPERGIATAAKRQMQDKLLGLSEHMWQT
FUN_009048 CDS scaffold_3 627273 628963 + hypothetical protein PF09346 ENOG41

MANTFGATIRSFWHTMTSYDRHSSFDSPYRTGRHVPLQNGRGGIMTGVATASDSRNDVSSPYSDETGRASPLQPDGATFAPTS
APYSPGLRSMSARNASQGDGFEVQSPGDVPMQNFADGLPPAPPVAHSWRRIDAWADENYPELYDQLCEGATNNDLNDLEHQLD
CSLPQDVRDSLMVHDGQERGGMPTGIIFSHMLLDCEEIVQEWDNWRTVNHQYLLETSINKPSTPSKAFGGSNEASSSKQGAAA
GGSPGPWRQDLLSRQDCVPPNAVQKAYAHSSWIPLVRDWGGNNLAVDLAPGPGGQWGQIILFGRDYDTKYVVARSWAAFLAVV
ADDLNSGKWYVDEETNELKLREFKETRVEPSYFSILRWRMDQKYGRRAASNRRSMGPKPNSPLGSRSSSPYGSGGENGEPRGR
SMQRLSGSSPLASPRPGPGYGKATPLSKVTEETTIPELADAYIQPEKLVEVESPRQSQDTKTPKVTTSIPRVESDLLKVEEEP
SPKANGKKPEVVDESMKTIEI
FUN_009049 CDS scaffold_3 629868 632904 + hypothetical protein PF01968;PF05378;PF06032 ENOG41

MSKTIASRRLRIGIDVGGTNTDGVLIDPLMSSGPDRGIIAWHKEPTTVNPSVGINNALTTMLSSSSIRPHEVASVTIGTTHFV
NAVVERDAARLSRVAVIRLCGPFSKHNLPCVDWPDDMRELILGHHALVHGGLEVDGRLISDIDPDEIKTQCSIIKQKGIKCVV
VVGIFSPIDTVERQEERAADIIKAEIPGCDVVCSKEVANLGFLERENAAMLNASILPFARKTIRSFHEPVKRLGLNCAVFITQ
NDGTVLSGELAARLPIRTFSSGPTNSMRGAAFLVHGDLDEAMMVVDIGGTTSDVGILLENGFPRQQAAYSDLSGVRMNFSCPD
IKSIGLGGGSIVRIDSSVSVGPDSVGYKLPEEAVVFGGKVLTATDCTVLANPELKIGDPALMKDALSEDQLTKVSLTIKQKLE
KVIDTMKTSPKDIPVILVGGGAVIAPDELKGASKVIKPQWSQVANAIGAAIARVSAVVDTVQSTVSKSTNECLDEVSRAAVEK
TVEAGALRSTVKVVEKEGFPLQYIKNKTRFVVRATGDFDFSKEVVAPEFQAEHGDHQNMGHYEKNTKNTGPKHDTQQDEDFDI
LSYRPDVRNRTWYVSERDVSWIATGCYILGTGGGGSPYGLMIRLRTQLRNGSIIRVVNPEDLPDDARVGCGGGAGSPTVAIEK
LAGDELLEAQQELYKMCNTSATHMISVEVGGANGLSGLLLGSSDQMDIPTVDGDWMGRAYPTKWQTTPVVFKERDTIWSPIAV
SDGNGNVLVMPKASSDEAVERIIRAALSEMGSQVGAADAPVTGEETKRWAVEHTLSQSWRIGRAVARARKENRVDNVAETIIE
ECGGPGAGKVLWKGKIIGVDRTLRNGHIYGECLIEGADVRDEHVASGDISEQFKGVVKIPFKNENIAALRVYNDRKEELQEDV
LAIVPDLVCVIDAQNGEAVGTPEYRYGLLVVVLGIAASDRWTGTERGIKIGGPQAFGLGHLKFEPLGKYFKPRSVIDEFDEC
FUN_009050 CDS scaffold_3 633297 633782 + TXNL4A Thioredoxin-like 4A EOG0926506U PF02966

MGSVVLPHLNTGWHVDQAILSEEDRLVVIRFGRDWDPDCMRQDEVLYKIADKVRNFAVIYLCDIDQVPDFNQMYELYDPCSLM

FFFRNKHMMIDLGTGDNNKIKWVLEDKQELIDIFETVYRGAKKGRGLVVSPKDYSTRHRY
FUN_009051 CDS scaffold_3 634284 635704 -
hypothetical protein PF05139 ENOG41

MEVSSAEQLKSNVKPFKSIAQDVETVTKYFDSFGDCKVLLIGDASHGTSEFYSVRAEITKYMIQNHGFNIVAIEADWPDAESV
DRYVRHRPGPGQATVETTQMAKRKGREPAFLRFPTWMWRNIEVRDFVEWMRSYNSDLKEEEAAGFYGLDLYSMGTSMKAVVDY
LDTVDKDMAQVAKGRYSKLMHWADDPHEYGLESLAASFQGYEKDVIAMLKDILIKRIEYSAALDDGTEFHSGEQNARVVKDAE
QYYKAMYRGRDETWNLRDTHMFETLTRLLEHRGRDSKAIVWAHNSHVGDARATSMGWSREELNLGQLCKERFGVQALSIGTGT
NTGTVAAAQNWDGDMNGMELQQGLPGSYEELMHAAGIDNFVLDLRKGKCDERLREILNEKRLERFIGVLYRPETEKASHYSYA
VLPEQFDGFIWFDKSKHVGTLEVHQPKSPREYHETWPFGL
FUN_009052 CDS scaffold_3 640664 642260 +
hypothetical protein ENOG41

MSAPLVCRPNPSQGLVNTRAVKTFRIGELLLLIVEELGERTEQKPGYQNLGSLGNLILTNKSLFISLQAYLYTQDLEDDCHKG
LQHAVFNCSDQVGYRIITKYPTELLRSNINKIYKHNTANTRATFTLLHIAAARQQHRVIRKLGRLGAKYLDVKNLHIVLPQEF
RDRLEQETALFNHLPRLRWKPALAPLVLHQVETFDLLDEYWKAAYVGTFNFRAIAVAFAVEGVATPEPPFWPMTVHHLAATLT
DHNYSVQLLRHAVELHPSHNEAPGGLERYSVLHFAIQAYNFRALRYLLESGVGLGRYMVDALGNNPLHCVFRLALESANKASS
RLACRKMVDDLMDNHGFHHRMVRTCYPYESPILIVAQSARIDWSGRHHMIKYLLGKCLKSEMDMRFHRNYNPALHPNILNVPD
ACGNTALAFIAQAIVVHRGNEGLEAVFRNMIRHGAQINLDINPRYRPRRYAHSIKYIVQFAEGCTKFKKLVDRLGGRLHQAEV
DDTAIGYDGVEHGDHEPHVRSLPPQHLFAQEHP
FUN_009053 CDS scaffold_3 643674 644500 +
hypothetical protein PF07510 ENOG41

MKFSIALLVPVAGVLAAPTPPGIPSDSTARSLLSGLTVAASTNTGTYDRDLFPHWETYEGACNTREYVLKRDGTNVVTNSACA
ATSGTWKSPYDGATWTQASDIDIDHMVPLKNAWISGAASWTTAKRTQFANDVTRPQLWAVTDNVNQAKSDKSPDSWKPPLTSF
YCTYAKSWIQVKSYWQLTITSAEKTALGSMLDYC
FUN_009054 CDS scaffold_3 645018 649699 -
hypothetical protein PF01369;PF15410

MPSNSTSSSNDNARRKTRRRPHTDLNINTNTSSLASPLSRERDAIPHPQTPPPQDSEYRVSQSKSKSKSRSLANTNSDNNSSS
NNRAPTRLPASNTHAHESFLENPTPVEDDMADRLPPSSTQRDSHDLSLSPRNVTRDSLVANMLMSLDQFSMAQLSDKYGPSST
PASRTHTFDETSPQFGAAEQTRSWVASNSVAGPPRSNGHQYSYSSDLEGADDSSRISSRGRRSNSSSNFQSSFPRLNSVREGH
RAGSSRAMHSRGGKPGSKSSSTASVDAGYVQVLGSQRYGRGFGRSASFDYGPQHQAAQPPVVEPSPFRVEFPNSYIDDEYDAA
PTPTVPGGPRREPSIPAMSAMPSSIPPPMPPPPLEPPTLDHKRSSRSLKSVSGRKPATSREPTPAVPALDLDSAPAPNVGYGK
SKDPTVHSGPTPATHAPAPVKERPGFFRRVFGSSRNNSHNNETTNPPAPQSNPSADKMAGSASAPPSRDTSSSHSHHPVLQKK
PSSFFRRRKKSVTEDTPPLPPMPAAPVPTAAPNLAPQDRDGNYPPQPSPVSSLRKVMNPYLRSGQSAPGTSASQGPSPLADIS
NMTPERDDRAAEREGYQREFSPDYSPSPNARIRTVRSESDEQLNKRNNNSTPSRPPPERPTKAIESRNNSFLNLDGASDNEDE
RGFSNRKPASVSQTKSKQPDNTPARDIPRPGKDHVGSRKPSHRPQQSDSEDEPDRSNLALPIEGARASSPGSASTGTDYKSAL
SAPPSVRIESSADPSPKVLGTFDSIKNKGIDEPEFVIGDPTEDDRQKAQKIYDGNEDFIQKEKAASWMGEEGPIRQRTLQAYM
ELYDFTNQSIVQALRQVCGRLVFRAETQQVDRILVAFSKRWCDCNPNHGFKATDVIHTICYSIMLLNTDLHLADIESKMTRSQ
FIKNTMTTILQAVDESAPDAFHRPSILPDKNGLAAGENQDPPSEQDRRSFRNSFRPPARADSGTLPGEGIEDCGPLVKSRFNG
TRKAWEEQIETVLKGIYTSIRDERLPLFGAESEKNASLQPSHSGGLSVMGMLKRTPSVLSKAPSESQLSVRGRINENGRANTA
RWTSKSRSRAGMSRNGFSSSRTSFDDGNSLWSPTPSSATWSRHSLGRTQTSVSQDSFGSHLPRGDYQQSIGFANALSQAIIRD
EEMYGDGAPSIMSSEIPATQLLEDESLELAGPPWVKEGMVTHKHHLDGVDKKAKARNWSEVFAVIQKGQLNLFSFQPNKSVRS
RNRGRNAGGAPAIVGGGNWQDNATNLGCYNLRQTLASALPPPGYSRARPHVWALSLPNGAVHLFQVGTPEICKEFVTTANYWS
ARLSTHPLTGGISNIEYGWSEAIVNNALVSAINETTTGKTSRPGSSAAHGRRSSVNSGSFRGSSFDHVAGAFTNNSGRGKLPG
DRIHIAEWAPPTQSMRPSNQPEAEQLQTLDTYVKGIEAELQTHNRLRSPMLLAFTPRGSNAVKAMANWERKSAYLLREIVKFT
TYVDCLRQADSRKQEIYHERDLAQRAARGELSEGDMDVSSDDEGDVTLRP
FUN_009055 CDS scaffold_3 652476 653913 +
hypothetical protein PF11951 ENOG41

MASFMADNSSSPSNSWPLDQFVQDPGYLAYQEELRCLIFNTAQTAAPSREASPTLGSYGPTDEELAAQAQTKTALASGRRLEY
LKNYVAQVAPWVLTSRLDMFDSNGAFTVQVPVLARSCPALLYAVLALSARQMERKEARQHSFDSLELYQEAIRLLNPLLQARD
LTIIPICVILCCLEMMSASPQDWRRHLEGCAALFDSFDVNGFSGGLLQAVFWCYARMDLCGALISDGTQGTLVPLDKWLPPGS
DPNSAADIFRDHRSPDMHANYSVYLCSKVCELVADRTHYVELGENNGCDSATFERRWLRLWHDLQSWLEDRPAEVVPVQFIES
RPFPQILFVHWAGISSNQLYHTACTLLLGSKPREVKLDVGPTGSSIWHAKCICGISLTNPHQGCLNNAIQPLWIAGRLLSHKS
EHLLLSKLILSIEAMTGWATNWRIADLEHAWGYKVR
FUN_009056 CDS scaffold_3 654093 655173 - XYL1_1
NAD(P)H-dependent D-xylose reductase (XR) PF00248

MAPSTIKLASGHEMPLVGFGLWKVPADQAAETVYNAIKSGYRLFDGAYDYQNEKEAGQGIQRAIKEGLVKREDIFITTKLWNN
YHAKEHALAFAKSQNEAWGLGYIDLYLIHFPVALEYIEPEKRRYPAWWMDDQGTVKPANVPIRETWEALEEVVDQGIAKSIGV
SNFQAQSLYDVQRYTRHPISSLQVEHHPYLVQPDLIAMAQENNIAVTAYSSFGPQSFKELPGIFSKRAHGAEPLLESSLIKGI
AGKYGKTPAQVLLRWATQRNVAVIPKSNNPSRLAQNLDVVSFDLSREEIESISALDRGLRFNDPGFYLPNFPLRIFA
FUN_009057 CDS scaffold_3 655555 657533 +
hypothetical protein PF00172 ENOG41

MGDAENEAKTRLKRTRTGCFKCRVRRRKCDEGKPSCQRCVDGGFECQYGTKLSFLQKNAKTSSGSRNAKQSYAKLRFVVPETA
TKSGTHVQHNEFASSEPEKTEGSSIEVDATAAAQENTSQHQEPSQQTKPPVQPPPPAIISPSVETYVTSHQAPSHSLQDSVDF
NIVISPSNAAYETALDGLLALGNDHYVSPSAPTVSQTGTDGGLFGQLPQNAQFEVSDKPRGDVFPNCLHMPQDRAVELLRHYR
YNIAPWLDINDPDQTFGLFVPRVAMDSIYVLDSLLSLSLETLGNRTQLDPNEQSLLPDTSTSSIEILCRVLVFTFTTLRRQFA
TQPTSWLSPSKSTGYETINSTPFQLHHPTAALAVSWMVLRLGVSVGLMNGSSVPIPSIVSCEVSSPIGMLSREPLRCGREPIL
LCAEVLNYCSGGDIQTDPDTIRLPAAHRWKAFSEALHKWYANRPTDFKPMLETEDGEQLFPLVLFTNGAAILANQLYHTSMLL
LLQNRPRTLPKEHGRNIYLSPLWHAQRICGISLNNDTRTSWDFSLLASFYFAAKRMTYEPQQHAILRGIDRIGSLTGWNVNSL
SAQLMHEWQPD
FUN_009058 CDS scaffold_3 658310 659722 +
hypothetical protein PF00107;PF08240

MCSLAASQPLTHDATPVVDELKVKLNGYPSDPQTLKAPLPNPSLQVTADHNLKRVDAPVYAPKSGEVLLHIKATGICGSDVHF
WKTGCIGSLVFEGDCIIGHEAAGVVIRVGEDVTDLKPGDRVAVEPGVPCGHCFLCKDGRYNLCEDVQFSGVYPYAGTLQRYKV
HPAKWLHKLPDSVSFAEGALLEPLSVVLHGINSAPITLGTPAVICGAGPIGLLALAAARASGAHPLVITDVEPKRLAFAKDFV
PTAITYQVDISKSNEQNGKAVRKLFGETEYAQPRVVFECTGIESSVCSAAYMVRRGGVLMVVGVGRSIMNNLPFMHLSLAEIQ
LRFINRYKDTWPAGIACIEGGLIDAKKLVSHVFPLEQALEGLTLSADPKNGCIKVQIVDETETTYF
FUN_009059 CDS scaffold_3 660740 662319 +
hypothetical protein ENOG41

MGATQYYRSQIGEESVIDYSHTDFPFKLLAKDVYYFFVYVWALPWVIWPVYPFKCKVFDELYPSPQNVFCVVVHLVLFFLQLG
FIIALPFSIVFPVWMAATGIAGFMFTNWLICKLLNGSKETYTSDEKYAKARPEHAHEQWVFLNGVAVGDHWMQSNLNRLALTF
GRPILGIHNQTRGIIFDVVECLIQRNFGYATGDVRICFRLLKDILYDESKSKVVFVLHSQGGIEGGLVLDWLLQELPQDLMSK
LEVYTFGNAANHFNNPHRHRSSQMLARLKPLSAMSTVLTESSFDSPSSSPLEAKDDLALKILNPVRKESSGVSTRSISAAKDR
CILHVEHYANSTDFVAIWGILHFATNRMESRELPRFQGRLFARADGHHGGHQMNQHYLNAMFPLKKDAETGEYVGADEDNEFM
NEAIKVGEEGSASANAREAFEISWAGTQGFGSGDASTPIEVHGISNRIKEASDDHGIRVKDVSRLWSYRNGRSPVELPPMLVS
ENGVPRTATL
FUN_009060 CDS scaffold_3 662940 666819 -
hypothetical protein PF00931;PF05057 ENOG41

MGSENRSIARYETTAVYTHPEAKVDIVLVHGLNGTPDKTWTAKNGTFWPLDLLPVALRGAQANVLVYGYNADVYSKKNNQTAS
DSFIHQHAQNLITNLTLHRQSEGTFKNPIIWVCHSLGGILVKRALLYSSDVREPHLEHFRSIFVSTFGLVFLGTPHVGSDAAT
WGLILQAMSDAVIPKRFFDSESVLLRTLKKDNETLANINSHFLDIYQRFEIHMVRENQKTDIKGHKIFIVDANSAGPQLPGVT
YYGIEASHSNMCKFDGTSSPGFRNIAITIQQWIQRAPSATQERWLAEEDERRARAMIRANEIMSPYTSQTQTPVLSQGISSFK

GKRPVLEGSSLALLNRDESIFVHPERFRPNSLFRGRQQELQDLHTMLMDRQRRDQGTSAVLIWSVPGGGKTHLAREYAFKHRH
DYSCGVFWVRTKTPEDLEDGFLRIAKHAMSRAEIDIQDETTLQNPSKVIPLVRNWLDRSENWLLILDGALYDTPNLKNCIPDA
RNSSLILTSTDTSIAGDHHFDNPQKLELGPLADNDARTLLLEDMDKKRPWTQDDLDRALEVVQLVEGLPLAIHTAAGQMKATK
EPLAKYIRSYKKRPRTGGLGAYKAVREKLKERGETAALNLMYLLAFWGGRVPVEMLSLGLKALDKRTPVRTHDLIGKRSLNKT
FTVLIAFALIERDEIEDVPSVSTPGETIPSSRPMEPLDVLKIHGIVQSFFLEALKKDGQYNFWLERAAAVFLESFDRGDSRAK
NNKEMGIPDDYRRYATQCRRILKHLQEVDRPSLELMAVEQALQGRFEDMQVKIRTLARSMTTDSEGRWVRGPYVSVFEKSNSA
SLSSSFTTPDDDNIEEEKQAPWDLRTSFPSLEADNDVPYPYDSTMPVPVFYEDDDTLMGSVVSQQRPAERRSSGNSPRVSERD
LNESWTLIREPSVSRSRPIFEPAERVVRPTVRGQSVGAIPRPDSHPREITRERVEENPFMGSGDGIIPTMEIANADWLTHDRI
TDRPRASHSDTSLDADIDQWAPGLPITSHSVSSLRPVSHRQQPASYHRRTLSTGEVLSSSLPNSLASSTLRPPSSGPESHSSQ
PMSRNSSSRSDQPVSSNPPVPMWARRVPAATATEPSPRRSTNSPDNISLGYQSWQLRQSGPWNSEGASRREITPSPRPEVEAD
GADMVRSGSGGIQFHGRIIEFGRLQPATAQPVYPLEISESTHALESQTTATRRRRNTNSQPPGYPRPDTSR
FUN_009061	CDS	scaffold_3	669190	670075	-
hypothetical protein		PF00498

MASDEERESRPSRRRYEGEDNESRDDRRSKYTERDSGRRERRRSRDRRERRRSRTPESSRRRSRSRDRQRDTNREHRRRRSRD
DKSSRRRRDDDDGGGDRRVAKRSPVRRTGPLPSQADSFAVTTGEAPEKPKEKPNFGSTGVLAAASNSVAQADGTTITLKYHEP
SEARKPSPRDVWKLFVFKGQDIVDTIELSTRSCWLIGREMSVVDLPAEHPSISKQHAVIQFRYVEKRNEFGDKIGKVKPYLID
LESANGTMLNDSKIPDSRYLELRDKDMIQFGHSTREYVIMLAPRD
FUN_009062	CDS	scaffold_3	670556	671228	-
hypothetical protein			ENOG41

MHPSKLLTNQPSKAIYIDSIATLSDPLSSMSSTFSLVSLLTMAYHIRTPLPSNQERLLTLLNTMSPALRTETESRYSREEILN
SFVHFLSPVLNGMAGMLELLTNVDAFSRPEDFRQFWSLERSNLIRDFVESGGPLRGMQGYDVPFLETFVKDPSCRIALGSNNG
MLLLVPGDAEVGDDVWWDEVEQHLTVSRKGAEDLNTNAEAYLDNGSRITVMAISSAT
FUN_009063	CDS	scaffold_3	674258	675041	+
hypothetical protein			ENOG41

MSTTNNNPENYALFRDCLSTTLLQPASSAPDPPKRRRRGKNTGAPHVTSTPAPDPEKDAEELAEFVDYLATEIFDNLPEELQD
LEYRTWREDEALQEQYSLPLTKDSLLLFNLPPSVIETLTTYNLISADPYVESHLPSSPESFLLPILTSYITPLIEPPPATRST
RADACELCSRSWVPLSYHHLIPRFVHEKAVKRGWHRKEDLQNVAWLCGACHRFVHHFKTHEELARDYYTVELLLDEDEVQRWA
SWVGRLRWKGR
FUN_009064	CDS	scaffold_3	675713	676715	-
hypothetical protein		PF01585;PF13821		ENOG41

MSTEHKNGVGAQDEEEDDYMNMSFDDPTPVKETSIQRTQRLKRESRARGIIKSKEQIAEEGAAAREKALSTSMLDDAKAKKSK
GLAMMAKMGFTGGGLGKKTEDGAAPGRTEPIKVSVKDDRGGIGLDNEKKRKVREAAEERDMKAVKMDPDEYRERVRKEREDAR
LEKQFFAAQRTAERMDDEKTEVYGPGASLPDSKDEKEKSRLISSRPLKSIPIVYRGLVRHREEVERDRRMRYDLEQSLSRLPT
YEDDQEDADDKRALGKGHTVYATAEDLDEEDKELDQFNELEIGERLKSVLEYLREKHQYCFWCKMAYPDAEMEGCPGLTEEDH
D
FUN_009065	CDS	scaffold_3	677400	680254	-
hypothetical protein	EOG092620IA	PF04147

MAGSQLKRLKASLKEQGIVGPQQSKKQKRRNAQDERSRNDKRLQRGVVLEGIREQFNPFDLKHAKGPKFEVTSNRPTLTAGSI
KGRPGQAKAASEERRRQTLLVEMQRRNKVGGILDRRFGENDPTMAPEDKMLERFAREKQRSHKKNSMFDLEDDDASEGLTHMG
KSLSFDDMDDFKEDDLEEDYDSDGSVREQQRLKRIRAIAAQGSDGEDDEPERKKTKKEVMEEVIAKSKHYKYERQAAKEEDEE
LREKLDKELQNIQYMLHHSRPGAKPQNEGAAKPTIAGVDRDAFEKNFDLQVKKLAQDKRAQPADRTKTEEEKAEEEESTRLKEL
EDKRQKRMRGESVSDSEDEEVGNKGKKPELDAMDLDDEEDFGLGSGIRARPTATELGFDDEDDFVIDDDLVASGSDLALDSED
ESDMEDGSDEEAEDEEDDFTKGLLNEEESRNPVFDADSTTKASAIQKGDEQGLPYTFECPQTCDELQVTLAPYPAKTFPTIVQ
RIRALYHPKLSSMNKEKLGNFATALVDYIGLPWDPATSAPFTVLESIVRHIHSLAKMFPIEISKQFRKNLEEMGESRPLALGT
GDLVFLSAVGTIFPTSDHFHQVVTPAVLTITRYLGQRVPQNLAHYAIGTYLCILAVSYQKLAKRYVPEVINFSLNTLLALAPV

ATSKKVGNFPLHNALEGVRTEAAAESELRRLNFSDCIEQEAKKTETIARKIAILDTTAQVIEAAADTWTGKSAFLETFGQAAS
VLKHLGSKTCRSQLPTALNERIEKLQVKFDRMLKVAQLSRRNVELHHHRPLAIKTYVPKFEETFDPDKHYDPDRERAELAKLK
KEHKKERKGALRELRKDANFMAREKLRIKKAKDEAYEKKYKRLVAEIQSEEGREANAYEREKSARKRAKNR
FUN_009066 CDS scaffold_3 680516 681559 +
hypothetical protein ENOG41

MGWADSLLSFARDERVVLVTLGFATFGVVSTVFTALTIIRDDNEILPPQPKTQYITQDTEDSLELETLEKLLDHPNYSIREVA
IKILCDRAVNDSETTHILLFGITRPDYDERMRCLRALALLTGQTIGLDGLSKLNNPRAYSALVRSLELSLDDTERPSLTDSHW
DEYYLRDMAERFCLMFILELANKYGASMLVKARFVEKWLAKQEWGDTPEERRQSFRDYMALKTNRIVEIVDRIKESRRGLRAL
ERAGLMDKENSRRRIRELPDLIMEIEEEAGNLPLVEQQVTRTREHSAEEQRLRRQHREAMVLNDGTRPLAREDIIEREHTSPA
FUN_009067 CDS scaffold_3 682035 683138 -
hypothetical protein ENOG41

MVPVDDIDPRYQTWNIGTDVSLRRMSEWAGYKQLNGLEIVSSVLRDTPECWEEVLDMVSILRNNFRLTVGKSCGFHIHVSKVT
GPILLHLKEVKIRTESSIITGTYIYRQWPERNVPTDFEQYIPVDKVVDCRTLGIMKKLWTAETLQRLKLFITPSMGSKSCLGL
SKCYSFDEFDEGSEDKCRGTVEFRYLEGALDPELILRWSQLMVSLFQFADLASPQAWQNFVPAILQCPVLGKMDPNVLRVFLV
SLGKGDDHGFWINRIEIMSNLPLDAQQLARRPADNNEILPPLDNGYIDALRDELCTREMKLPCTIQKAATLNESESSTDPDDF
AKLLSEKACFFF
FUN_009068 CDS scaffold_3 683735 684452 -
hypothetical protein ENOG41

MALHASNLTTANPQRGLMVKNELESWTVEYDGLINEHQCKEYEWVGLRSKSPPFRPETFIPGTDSKTAIDIMTKGFLTVPNAE
TRLVVKVFIPKHLVSLDSMKAIASMLWLVDPLLSDIHLAHCGPNSLYSLGLQYTNLARDDANDLQAEMLSAVEVEDPWNNRLS
PERKLLELLPHPGDLGETKYRHRIYRILGATSVQDLIHLMDIAIYGEEGRYPHASPAYCFLENKTTIAIEFN
FUN_009069 CDS scaffold_3 685148 687587 -
hypothetical protein PF00566

MRPLQESLSRWRETQSQGAHLGDLRRAVRYNGPASPCISGCRSLCWKTFLLSTAAEGLSWSQVLDDERKLYAEKRDHFLKYIK
HPEALAELNIDPLTEDPSSPWNTIRQDEVVRAEIQQDVQRLPDEASYHEDQTQSIILDILFMYCKLNPERGGYRQGMHELLAP
ILHVIERDAVDPTTLPEEIPLDDALVKTLDHSFIEHDAFVLFSKLMERAQSFYEVKDVIPAPGSSLRPPKFAEQSSAIVERSR
FIHEVCLQKVDPELATHLTNIEILPQIFLIRWIRLLFSREFPFEQFLVLWDTIFAVDPTLELIDLICVAMLIRIRWDLLEADY
SVCLQLLLKYPPPSGAHGPHTFVDDALYLRDHLSPSGGSSLIMKYTGKMPTLSSPPQTSRAGTPSFRGFNSFKHRGPGPRPQI
SSPSRFLQQQGGMEALFQGAAKGAKGVYERGEKLGINQAVRDAMGEIKRNLNEARSAPRSPQPLVNDQESARSLVALERRNQQ
LAAMLDETVDNLKAISASNLDDKAKSLELIEIAAAKVQFVKIYLDDSSMEVPALQSPPPEDSPQEVAVAETTIVQPEPVSVAS
TEVDISALQISESKKSPESESARPPSSDRMDIVAHDDGKSANSLAPSRPAPVPTRSTLAQSSFSWMLEPDESGSSKASPASNK
SPPPQHKKKMSNSVSREKNAFLFGEVTESRNPLNSDEIFGLEPLKKLKNAAQEEKTKE
FUN_009070 CDS scaffold_3 688098 690066 +
hypothetical protein ENOG41

MSADLFAEFNSLSNNSAPPPGQQQQQQATPFQTQAQQQPEPLQRAATQDLFGLLGNTSNVSSVKTHPWPAFESQSSAHSAWGS
ASVPSKPAETNDDDDGWGDFEVAEPSQPATVNATPVQTLSPAPFSASGWGSAANAPPNSANSSSIPWPKPDPSSAAAVAQRVP
GVKQQPPTRVVRASTMDLLSNSLVDIGGASKPASAPKAQPSGTQSRFTQEWRQPTVDQEPQQRMKAKLKPSAQQDPSVIFDAE
DFELQVAEGEDDDDGDDEFGDFESVPPPVAMKMAPAITAQEPPPAPPSMDLLSLDEPAPAPSQTTKKPPPTKLLGPLAFGATA
TNYPQAPKSPSFQDRNPFPELAIKTPTFEEPKPVKKPKEETPTTAWPSFESPKGANLAETKYQDDEWDAWDDFTASDNKKANA
STGKAPEGWDWDAVDNVQPSHKESQDDAPPPINVPPPSVILSAFPDLLNSGTAFFKPMSGHSTSIKQRILSDPKATEFLRGYI
LLATTAARVIAGRKQRWHRDKILAKSMSISAAGSKGMKLAGVDKTQAAREDREAADVVAVWREQVGRLRSAVAAAKSVDKNLS
LQVPEISENMQVHTAKMVPTAPRACIICGLKRDERVAKVDYEVEDSFGEWWLDHWGHKACKNFWIEHEQKLRQR
FUN_010805 CDS scaffold_4 324 6139 -
hypothetical protein PF00176;PF00271;PF04851 ENOG41

MDDIEDDPYSWDIDVVVNRLCAPGVPWSRDPAFLATRIQEEEFDGKTLLTFEHVCSRQELMDCLGIRIARHKAALAEAIVTLR
SRSKGYWEWQADFRRKQSGFPGEETEASTHATEQRRSSNLPNGDHHKEKETPVSVSDTNGTIPHAAGNPLQQELDSQNHTHTH
QAHDQLQLATHSRQPPIHEPPDQIPEVDERSSKRRRVAPMLLTDIPRNADPAFLPTEADVLDYATTKAGMKFADDNSAGFPWD
NAPSYAYLGKGKISREDMLTPVGMLSSLLNESDDSFTSFSIHTIPPGRRLAANNILKRLLTGRYRIHMHSSRSPSHLSNESDE
ILELTDLDNELDAETLREIKEEEAENERLDALDDNPQPYPFVTPERIAEILNEAITAIEENWAEKKLPKYERKAYSLWQKSRR
LGIKGIQIQNAYKTAKHLIERLQKLCLEIQKTDSASEAHYREAAKSLEQSLEDKKYQEWLIKLLESSRPPPKPQTVARPKPRA
VRQLDTLALMEDEVLTSSEEEDFVVPDDHMDVIENGLTTDRDSTPPPMKQEWGDFNTDFMDLTQDDMDEPRGLGYDMTNPIDL
TSPAKQANSPLNGDRGNAVPPRKNLSEREALDAGPSDAAITNGESLTTDDPANVENKPLEPDVVCCQAPINHVSKKGHDENQS
REIQPEKTQSPRHDGGFHPPPIESFGDLQQLASQPLKKYVKRNDRWRLLIGEMWHMEHARRKSAVDLILSDHPNNVWEEHIQP
YLFEPLEDPEQLPQGNVKVVLFDVVRLCRCFFVCQHTTEEQMLSYKMGKLRKTLNQARVKFFEPLCLFVAALTPHFPQDSQIY
RQETDILDDLFPEEQDEMDDFDDVVEEGRAGRRRPKEKEIIRDKAAVDLRERENQRLREQEARRKKLRETLALDGSVSRDGTR
LIINESKEEDQGLIYIHEDIGRRIKDHQIDGVRFIWNQIVRDPSVRQGCLLAHTMGLGKTMQVITVLVALAEAAQSKDPSVVA
QIPEDLREPRILVLCPAALVDNWIDELLKWAPADLLGELRKVSSNTPVEERAAVVSSWASGRGVLTLGYEMFKKIMNMSEELA
DLLTNRPDVVIADEAHKMKNRESQTNLASSRFRTKSRIALTGSPLSNSVLEYFAMIDWVAPNFLGPFSEFSHIYASPVERGLY
NDSTPAEKRRAQMRLKALEQLVAPKINRHTIAALKSDLPPKQEFIIFVPPTTPQKQLYQLYIRGVAREGTDSQAETFAAINHL
GLICSHPRCFEAKVKAIQKGIRSNSDNEDKSFPKSMIPEFLETLHSFRDLETPTLSLKTELLTIILDEARQVKDKVLIFSQSL
HTLDYIENMCRMQRRTVSRLDGSTPVPSRQRQTKDFNEGSKEVFLISTTAGGVGLNIQGANRVVIFDVRYNPSDEQQAVGRAY
RIGQQKPVFVYRFMVAGTFEDNLHNRQVFKMQLASRVVDKKNPISWSKRKGDVVSVIRHCPPSDLTPYLGKDRILDKLINHRE
NGESIRSIVTTDTFEEEDLGAALTEDEKKQVAQMIELNRLRETNPEEYLRAKDRVDLKEQARLHGEQGELLRASSLSQQFVPR
LIDDTSDMLHHARFLPPATAPALGQDMLGAGKLSHETSSPTNRLVSQQPTASSHGPAPMPMAGANTFFGEHNHSGPLIEPHST
PIISAQPRASPIFKQGGVFNVTENPAMVEFEGCLRESLQKMQQRNVLQTGGDPGEMAKSTTMRVNEVRRKGQYGLLPDTKHWR
VLIRLLSHQKWVIAIATGFIAPEYLAQAEEKDLEKRLEAINALTETEIATRALEKTSSPDPNNLQNIRRRSSHRGEKRSRATD
DMKIMREAADNRRNRAFRLPPWANEALFEEKTRTSPAMGTREGQFGFRDVTPGL
FUN_010806 CDS scaffold_4 7622 9325 +
hypothetical protein PF03199;PF03917

MTSLTDGIYPPTLKAEEKDALIETVKDWSIGNGLAVRPPPTVIAAEADPKSIAAINVPVTLFPSPFPKQCFAQGKAVQKTYNE
LYASVSRDEEFLAQVVKEVSDGDEFIRNLWDVHTKVKAEGYTQPLSLGLFRSDYMVHQGTETTKSSLQVKQVEFNTIASSFGG
LSTHTSALHKYLATTEYPILENPITQGSLDLPENTSTRGLAAGIVEAYNIYPDSELGHQKCVIFLVQDGERNIFDQRHLEYQI
ASSSSSIPMFRLPYSQILQHSKIADTSKRQLLYILPRNRSKIYEVAVIYMRSGYGPSDYPDQQAWQARYHLERSNAIKCPSVL
TQLAGTKKVQQILATPRPSKESSALSRFIRDDTDDATELWRTFTNIYPMDSSDAGLEARKKALDPKICQNYVLKPQREGGGNN
IYRGAIPDFLKSVPESHWGSYILMELITPPPVTNIILRNGNLEQGGVICELGIYGTCIWDQSTGKVHHNEEAGYLLRTKGDKS
EEGGVAAGFGCMDSCALV
FUN_010807 CDS scaffold_4 9799 10332 -
hypothetical protein EOG09265JNA PF00436 ENOG41

MSFSSVFRRAAVAPASAARAFSTTTPRPLAKITIIGNLADTPEVHSTSTGREILRYAVASNSGPRDNRKTSWFRVTSFAEGPQ
RDYLMNLPKGASVYVEGDASLSNYTDSNGQNRSSFNIVQRNLEVLRRPQAPEQGE
FUN_010808 CDS scaffold_4 11625 13418 +
hypothetical protein PF02076

MIDSAHLFGRDDLSIIMAPGPGTITTPSLTTNLVCRVLLGLLANIACIVPLKNLYRNGEFAAVVFIANIEWSNLDTIINSLIW
RNDDTSKWWNGEGFCDLNAYYVNFTNALFGTCLLAIMRNLAQQVGLLRANALTVRERRRRNLVQALIMFPLPIVQVAWVWPLT
TQRYAVATLAGCSWIAWPSWPYMVFFVLAPITVALITSGYAILVYIRFRDIAETTRSAVNSSRSANQRAQRTKRRLYLMVLAI
LVPYLPLVVTLAVLNMLKAFPLQPFDYDLIHNRVIPYPWSAVIFVPSNSVNFGYLNNCYIHILSAIPVVIFFGMTKDAMNSYR
RGLLCLGFGCLFPKLHEEYDPDRTIGGSSSGHSHLMGSTTSTASSISSKIRSLLSQRNMSTASSASLNPISLQSTDAAPVAHE
YELGPRLGGLQVSQTPLREISYPPPTVSAFSEPLNPPLRNPFLFRMRFDMPAIPFPSLSSFRFNKKDKRPPLDRGLPLDSLPS
VFNNQLWEDSSATQPCSQTLVWADREITPTPGTFTTQPSLLVPMSSTYSVTTEPLQETYRR
FUN_010809 CDS scaffold_4 14002 17402 -
hypothetical protein PF13886 ENOG41

MHSRWLRLWVLFCLLFIVQHVSGEVFRIAPRADDNPEKTTAIDSSTTSTRAHSSATKTSEVSARQTELSGASESQSQTESISV
AATETEKTQSSDPSSTSTDGSLESGLDDSSYYNATIPAGQLPMEPRLTPGWGVAGTILLITGLAYGLVGIKNRMIHTFFSTAF
AASLGVAVLIVYVMKSEVSDALQGGYVVAAVISGCVLGGASMFFREITEGLGCALGGFCISMWLLCLVPGGLLGPVVAKAIFI
ACFTVGGFAFYFSHYTRDWALILMISFSGATVTVLGIDCFSRAGLKEFWAWVWELNDDLFPLGANTYPVTKGIRVETAAIIII
FLFGIISQIKLWKIVREKREKKAEEAAEGERNLREEEEQVGRNIEEANARERRQWERIYGDGDVGSLTASRDSDDAEPTNEKR
NRSSQTDSSKTRSTSIVEVIEMAEMPDAVQTKKQPTLTLMSSDQDEDGRVTVRVATDDTVQPVMGRGDQQLDTGTTMQNLPLT
GEEVDRRISTQSSVAKSAVPPVIPLPFTIPVADDNDDALTNGDRSSVATFADEEDDTGIHASGHRHLLAKRLSRLSRGSMELL
GNISHRSSRVFGDHQENEHGESTDELVIPRSRPRDDDGSVAATIDDDSMSGDGRQSRPISELPKSIEINAQLSEKDDKGKPSP
SPIDQETVMHADGNDAIKAEEPPSADVEKTRSATSGSSARVSLTKDRLPRSLSRVALSYRTNEWAKHLSNAEAPEVDEIHIEA
SRNPVVPTIEAPAPVHVDELRKSSAEGTPAPMIPRPDSQASNYSQTASRRNVKQHVPAALALLTGESQSRSPGTTPTSSLMAR
TSSGGLRIASGGIAPIAEEQIVPSLTPPIAEEETMGMQNLNAPAANESQRTSTPGVVSYSSPQTLLGQREMYLRNKSAANLVP
SSSDVNLPTRHRASSDAGSLINYPMYAAAIGVDVDDLPLNQRKELMRQSSLSPSVSTPSLQRLSGGSNSNGFNSSEALLNTHQ
PKRVSTLYTSSAREAALATFRQSVQHELRAGTPVISNSGRETPFTPSSLLASREVEVQRSVDMSRNILLSQKQAEAQRRETQQ
REKEWADKAFDERMRNGDLLDVHREAMRKLQRHAKDM
FUN_010810 CDS scaffold_4 22302 23818 -
hypothetical protein ENOG41

MTDDNTASSAAGRPATGTSSSFSAPPPGHGLRRAMTVSVAEDAASRRQHMSPPGFDTIPPRRSSNFSEYSLSEARDLLNPQLR
DPSSTESSPGETSSLASLSLAFALLPAISGVLFKNGNAVVTDVMLLGLAGVFLHWSVTQPWAWYHSAQEVRIQHESTTDSVID
DDSDLDSSTHMPAPHSPLDHVPEYQEVQTEPTEELQDTKPSNQQQEALAELYYYEIIALASCFILPLLGAYLLHAIRSQLSRP
SEGLVSNYNLTIFLLVAELRVLSHMIKLVQSRTLHLQRVVQGGPSRFQKSSENSGQLESVLARLERLESRFAAEETMSVQETK
QEISKAKQKDSVVARDVRNAIQPELDALNRAVRRYEKKATLLQFQTESRFSGVDARLDDAIALAASAAKNSASHKNMFMWTVE
SLTVIILLPFKTLLRIMLLPLSTLLALTSKSKKKSPPSSKMSRSSRNGKASVQPRYNGDRVPSRVAKR
FUN_010811 CDS scaffold_4 26434 28484 +
hypothetical protein PF10256 ENOG41

MLASRASSSPSSTKPPVNLSPAPFRSRHWPTVSEFPLVDVQSLYCFFSCSLSPLPVNDSSNSLARCRSTIHHYEIEADTVHDD
DFTNATVFSPPFSLSAPDLSLATAATCSCRIPSFSFSAFVFRQHRLRAQAISAQPNSPGRSQQREKDLAISSERGRPGGRADN
LFPTDPQLSRPFPSNPIPAAVDAHGNPLILSLEPPRSAYPGTGSSSGQSAVAAAASNSQRQPARPFDGYQATALHPSPFRPQK
AAGVANRPRRLSAVRLWNPTNSTPRPHTKPESSRKRRPSTPPPPSIPLKHPTFDTRAPPDPIGTGPSDYPLLTLSEQHQIRHS
LTPRASLQVDRAGSSDRRISLPNSVRASYDEKGSRRGAVSAEEPRLSRDLFAQETVVEHPIVKIDKGKGKAVMMPETDDPMPS
YGKDLERGPDIMDPRISNVSAGDGIGSALSTTNSSIMGEDVEPDAAGEWGPQHPCYPHLNPHVPVDSPEYVNTRIIRIRRDWL
IKGDLAPTFSNLYPEILDPAGLSEQEFRRIIEKLNGELIPAFDPYGLRNIIDALLGLVTGWIWDDVGLTGIKSRLNSLEKWIE
QWNLEMEKTIGTEDGAIPPKILPLRQTGYMTLDIQIPDPEIAPAPSTTNPNDSRTALPLEPPSAVV
FUN_010812 CDS scaffold_4 29679 30720 +
hypothetical protein PF01758;PF13593

MLAIVHISIAAGALRSQTPSPPILIGLLTTACLPTTIASNVVMTRASGGDEAAAIISVVIGNVAGSFLSPLLIYGFFPAGHPE
AFDSWRPADPSTLGHMYADVAKQLCLSVVLPLIVGQAVRWRWEDRTVKVLNTLKLAKVSTLCLVLLVWTTFSGAFGTGALKDL
STSNVLFLVFMNVALYALFTVICFFLARPPGRFVRTVSSSWLRQVFKRMSKEQTIAVCFCGAAKTTSLGIPLASSMWARADDL
TRAYIQIPVLLYTIEQVFMAQGLVYVFRYYMRRGNKEHGSEEAEQGTSQEQCSIEVQMQPDDLSRQVIDEDVEQKGQRGQ
FUN_010813 CDS scaffold_4 31035 32563 -
hypothetical protein PF00232
GH1;GH5_19
MSLPKDFQWGFATASYQIEGAIDKDGRGPANWDTFCAKPGKIADGSSGVTACDSYNRTAEDIALLKSVGAKAYRFSLCWSRII
PLGGRNDPINQAGIDHYRKFVDDLLEAGITPFITLFHWDVPDELDRRYGGLLNREEFPLDYERYARVVFESIPRCKNWITHNE
PWCSAILGYSTGSNAPGRCSDRKKSDVGDSSTEPWIVGHNLLVAHGRAVKIYREEFKPKNGGEIGITLNGDATYPWNPKDPRD
VEAAERKIEFAISWFADPIYFGDYPASMRAQLGDRLPTFTPEEKALVLGSNDFYGMNHYTANYVKHREGEAAPEDYVGNLELH
FWNHRGDCIGEETQSTWLRPCALGFRDLLVWISKRYGFPRIYVTENGTSIKGENDMPREKILQDDFRVKYYDDYVRAMADASR

LDGVDVHGYFAWSLLDNFEWAEGYETRFGVTYVDYENDQKRYPKKSAQHLKPLFDSLIKQEEHAVNGNGVKAGQT
FUN_010814 CDS scaffold_4 34222 35028 -
hypothetical protein ENOG41

MSTLRPLRGGCQCGRNRYIIAVPQNGINEAQVLFNTESAHQIPLATPLAAYIRVPLSWYHSTTYSFFPDETHALIRRVYTHPS
QQYSKRHFCGYCGTPLSYWSENPHTEAEFINLTLGSLLREDLRDLEDMGLIPEEEDDKAPAGQSPAVVGRNTALRQSYGVPWF
DGMVEGTRLGNMRRSQGVKQSHDGRVKVEWEIVEYSNGEESTAPMEEDNDAEMSNPGKRKLQDRDDSEAGAVGN
FUN_010815 CDS scaffold_4 36192 37145 +
hypothetical protein PF00248 ENOG41

MTQLNHRATSATTTTLGAQDCLTLQNTTATIPLLGFGTYKIRGQTCTTAVLAALLAGYSHIDSAALYRNEVCVYEAIEQSDVP
REAIFLTTKVGSPRRMAGQGDVYQDVVETVDRIAGVDGYVDLLLMHVPGPSRDYRQRLWAAMEMVKRKGRAKSIGVSNFRVRH
LEELKEYATEWPPSANQIELHPWCQQRDVVTYCQDHGIVIEAYSPLATGTRLNDPEVQRVASKHNKTPAKILIRYSLQKGWIP
LPKSSNPGRIQENIMVFDFALDLDDMMALDALDEGPAGAVFRMNVD
FUN_010816 CDS scaffold_4 37335 39266 -
hypothetical protein PF00501;PF13193

MSDPPSRIRSILNHLRPHPTAPVNFHTLSPTTFLERAASIEPDAEAIFHITVNGAVLRRSYAEFADRARGLAYYFLKHGYRRV
GILAPNTPAFLESIYGIVAAGAAIVPVNYRLKQDDIAYIFDFAEVDCIIVDTEFVGLLDAYKEKHQSVPLIVDMVDFLPRTHV
HSCLEVVANLARQDTDATEGQLSGPFDEAVLEGLKHDIDQGSHGWAGLHAQARSEDDMLAIPFTSGTTSRPKGVVYTHRGAYL
AAMANIIESGLNIGRCKYLWTLPMFHAIGWTFPWSVVAVRGTNVCLRKIDYPLIWKLLKEEGITHFNAAPTVNTLLVAAREAE
RLPQEVKVTVAASPPTGYLFEQMTKLNLMPVHVYGMTETYGPITKCYILPEWDNLPPQEKFAKMSRQGHGFITSQPIRVIKPD
QPQGVLIDVAKDGKEVGEIVFIGNICAKGYYKDPEATRQLFAGGVLHSGDLAVWHPDGSAQIQDRAKDIIISGGENISSVALE
SMLAQHPDILEAGAVAVPDSHWGERPKVYITVKDGKSVTGEEVISWAKHQSDISKFMVPREVEVVDELPKTSTGKIKKNVLRE
WAKHGRE
FUN_010817 CDS scaffold_4 41093 43335 +
hypothetical protein PF02204 ENOG41

MASRDPSEGPSARPSTTRTASHSHTPKTTAPSSDDRLKVPKRAHTFANATSAKDGTASPDAFDTAEHSDSDDGIETTRASIEL
DILPIELITLTDSFIESLNAKVHPTPPNIERLSQKFQEFYTQASANINTHINALASRQSRDVSPAPSTSSISSAASRLRSKAS
ALTKAEPEQQMITSEELASRKRTRKALEAKRSLLEEAVERRLCEGIYDKIYRHRSTQDEAQDDKLRSKTAALALVGIGPADLG
IDLTGETDESEGKAGPTVEDIKEKLEPARRDIAMMTQKRYPLGKLNHLKAAHRSIVDTLAHFHPSASADEIMPMLIYTLITLP
PENLHVISDVHFIQYFRWEPKLTGEAAYCLTNLEAAISFLQTVDLTTLRADEQLSGPAKSTSPRTETFPPAYQQGEAPSSANV
PETSKTKAEGAKLSAPDVKASVALRNRRLSDLVNGPAQAFGAASDAVFNTADQSLKNISNSLGDSYKFLVGKLREHQDSPRES
ITVPRTLDDARKLVSTPSPDEEDSSGLNRLLAPDDNDLLKRPASREERVLNLIGGRRDRSADSTRSGRSASSSKKVLFASDEP
KTASQIPGQNPAVLDQMRNLGNSFNPMARLPSISMIRGFGRNTPSAPATPATKEPVKPADGGDLGTAFPDIAPALPPKEIPRI
APPNKRFMEIQTPGELKLGEVLDLLRDYRRLAGALKDIGAFETK
FUN_010818 CDS scaffold_4 43937 46939 -
hypothetical protein PF02386 ENOG41

MLDETRASVWARLKAVKPPFMSKKPHFNFISVHYTWIIGATLLASVIIYGAGRGQTSYVDSLMFASGANTQAGLNPIDVNLLN
TFQQIVVYLFTITSNPITLHTSVVFLRLYWFEKRFQGWVTDARQRRATLSKAKTRPRNEREAEEGVNGRHITVVPYQGRPPRI
TNDGILLDGNDVIQSQAIADDDDDENLPHKPPPASSDESDTVSAGRSNSIELSPLEKPDQGFGDGREANEGQSRSQNAPTGIK
FAETVKRSDGVDDDLTKFPQRRSHAEHIAILERQRNQDNEILRIPGPRETERGMGPRRLDNDDTQDGDDDNNTIAMTRTVESR
PDHATLDSRAPTGRRPTIVIAEPKRPLKHELTDDAKAVGGTLNALRVRKPRVFNRGQKEVHDDNESTMGRAFRTRTIDTIKSA
LSNDHTKDMPYLSYTPTMGRNSNFVGLTLEQREELGGIEYRSLRTLALILLSYFWGFQLIAVTFLLPFILHNEKYGIIVEGNA
ISRTWWGFWTANSAFNDLGLTLTPDSMNSFNTSEYVMMIMWFFIIIGNTGFPVMLRFVIWVLAQIVPKGTGLWEELRFLLDHP
RRCFTLLFPSSANWWLFWILAALNAIDLLFFVVLDLNSGPVAELPVHTRIADGLFQAASTRTAGFSCFSLSDLHPAIPVLYMI
MMYISIFPIAISIRRTNVYEEKSLGVYHNKKDEDEEESASALNYVGTHLRRQLSFDLWYVFVGFFLLAISEGESLKAKDFNLF
DVLFEVISAYGTVGLSMGVPNVNASLCSQFTVVGKLIIVAMQIRGRHRGLPYGLDRAVLLPSEARFQKEAEENQPILVRTRTN

ASMGTTSGMEPPISPGLPGRRGSMGRIRERANSRIISQFLYPGPVIHSNEIHGHKRATSNNSNNLRDFPRFNTEPVFEEEKDD
VPPLRTIESHSGGPRRAETNPMP
FUN_010819 CDS scaffold_4 50589 53067 - PTR8_1
transcription factor TFIIH complex ERCC-3 subunit EOG09260HSP
PF00271;PF04851;PF13625;PF16203

MPPKRKAGAAVQGGAKVGRSSRMSTPGAATPRTIDSNEDMPDEEEGPVDEALERDLNKNIDIFSLDRYQKRHAIRDPLPHIFG
DRDFSYLVLKKDHQNRPLWIDPQKGRIILESFNPLAEQAQDFLITISEPLSRPTFMHEYALTTHSLYAAVSVGLSPEDIINTL
DRFLKTPLPDEIRNFITSCTQSYGKVKLVLKNTKYYVESPDPNMLQTLLKNPKIGPLRVQGTEEITTSVAPKIGGLVIPGTQN
AAGVKQANGLSQAGEQGKDGQPVQEGEVYATLNEEDDEDQEVTHSFEIADKDVETVQKECLNLGYPVLEEYDFRRDEANANLD
IDLKPGTQIRPYQEKSLSKMFGNGRAKSGLIVLPCGAGKTLVGITAACTIKKGVIVLCTSSMSVVQWRNEFLKWSNINPDDIV
AFTSDSKNNVFTGSTGIIVTTYAMVTQSRARSYDAEKMMKFLTGREWGLMLLDEVHVVPANIFRKVTSSIKTHSKLGLTATLL
REDDKISDLNFLIGPKLFEANWMELSKQGHIARVQCAEVWCPMPTEFYDQYLKAPSRKKGLLYIMNPRKFQACQYLINYHESR
GDKIIVFSDNVYALKAYALKLQKAFIYGGTGQAERLQVLENFQHNPNVNTLFLSKIGDTSLDLPEATCLIQISSHYGSRRQEA
QRLGRILRAKRRNDEGFNAFFYSLVSKDTQEMYFSSKRQAFLVDQGYAFKVITQLANIEKTPGLAFADVSERRELLQKVLVEN
ESMEEDDPNDDMFHQGTMGRKKKKGARRTAGTLGELSGGQDMAYIEQNKKLSAKRKKGDSNAFFKKIGRENARRAAAQ
FUN_010820 CDS scaffold_4 53463 53964 +
hypothetical protein PF05495 ENOG41

MCKHILNAQVSVRSPCCRKWFDCVECHSEQESHPLKESFEMIFACKKCRKCFRKDVKEFEEADEFCPHCDNHFVIDAKTPQAA
LSIESDDVRMDNKMLKDDRQKTKGLRSIFDPTEEADKLG
FUN_010821 CDS scaffold_4 54148 55180 - PRE6 Proteasome
subunit alpha type-4 PF00227;PF10584
T01A
MASGYDRALSVFSPDGHVFQVEYAGEAVKRGTCAVGVKGADVVVLGCEKRSAMKLQDTRITPSKIQLLDHHVALAFAGLNADA
RILVDKARLEAQSHRLSVEDPVTIDYITKYVAGVQQRYTQAGGVRPFGISTLIVGFDNGSDVPRLYQTEPSGIYSAWKANAIG
RSSKTVREFLERNYKEDMDREATIRLAIKSLLEVVQTGAKNIEISLMAPGATIETLPTSEIEGYVKEIEQEKQEEAAKKKTGR
TPGTGSAAILTRSQDDSAAE
FUN_010822 CDS scaffold_4 55778 57380 -
hypothetical protein PF00172 ENOG41

MVGVPGKYKGCETCRRRRVKCSNERPYCQKCISSGRQCEGYERERVFITGTPQNKGRVASHPKKSSSSKKDSSPSYEEPPRTL
DITPVHPLTSAWDDHTLVSSQGVEYSVLVSALHTRLPYTLPDHSADGSTPFRITFPPYTPTDLQSLLGEGDFGVRAQCLARLP
SVYEQDDSAQSYCIFFFEHETTYAFGESGSQQMRRMGPAYFSHFPNHHFFVRVYRPLATGFALLRRQDTFLSSPDWKTVPWQR
HPKSLLDHLLDLVLLLPAIFSQVDQIVPSEPTLHRRHSAQQLLRDCLSLERHLDAWFQMANRPSFEHPVAYWTEELISPGGLI
PFTNSYAFRDANTGLAFLYYWMTQILFHQCIESLHRAIYQPVIDAYPNMWPDLPFDLQIDLNRYQHGRMFAADICRGLDSVLH
DTVQPDMLIMPMAVAMDLYRDINSVSQDGLMEIMWIDNFRSRLIEKGQHVAGVLQSQTWSEVATF
FUN_010823 CDS scaffold_4 58725 60540 -
hypothetical protein PF01565;PF02913

MSCRSQLFRALRAPASCSGSRRTVSLRTNILERSLAHSRTPARCIATTAPRLISQTRAQQSKLTSETYPQLERDARFAQVTPE
HVARFREILGNNPSAIIDGITGGGAGVDAADFETYNEDWMHKYKGQSKLVLRPGTTDEVSGILKYCNEQRLAVVPQGGNTGLV
GGSIPVFDEIVISMARMNEIRSFDEVSGSLVIDAGCVLETVDSYLAQKGYIFPLDLGAKGSCHVGGNVATNAGGLRLLRYGSL
HGTVLGVEAVLPNGTVINDLCTLRKNNTGYDVKQLFIGAEGTLGIITKIAIQCPQRSPAVNVAVFGIESYDKAQLAFREAKKQ
LSEILSAFELMDGRSQRIVSEVKGQEHPLEGEYPFYCLIETSGSNGEHDYAKLETFLEDVMTREVIADGVVAQDETQLRNLWG
WREGITECLGHWGGTYKYDISIPLDEMYTIVEDTKARLIDLGLLGDTSDHPVVDVLGYGHMGDSNLHLNIPVRRYDPAVEKAL
EPWVYEWIQKRSGSISAEHGLGIAKKKFIGYSRDDTTIGLMKQIKNLFDPNGIMNPYKYI
FUN_010824 CDS scaffold_4 61687 64531 -
hypothetical protein PF00307;PF08726;PF11971

MDATKDSAQAFQGMLPLQPGNVEGNEKFPNFCLHADAQSSDDCDCNQQQRYQENKEDYYVYQTDLSINNNSNQHHQHFHNSPS
ASISASSRLSYISTVSASASSSSSRISTASFESLPITPISVFEASPHSPRSLSNRLSSPVTGFIARRRTFTQDSCSLSYSPPS
SPTPRTRRDKSQLHAARVQRSGSRTTNTTFLLGLQIGKQSTNSRRFPPSASTPADALAAVEPPHDQKLTRMAFAEQQRWVTVQ
QKTFTKWLNTKIEARNLEVKDLVKDLSDGVMLIHLLECLSHESLGRYASKPKLRVQKFENANLALDFVKSRGIQMTNIGAEDV
VDGNQKIVLGLIWTLILRFTISDINEEGMSAKEGLLLWCQRKTACYDEVEVRDFSASWNDGLAFCALLDIHRPDLIDYDALDK
KDHRGNMQLAFDIAHKEIGIPKLLDVEDVCDVAKPDERSLMTYIAYWFHAFSQMEKVENAGRRVEKFVNNMQGAWEMQSAYER
RMRALLQAIQEQIEVWKQATFEGTYADAKAQSNQFFEYKKGKKRQWVAEKSDLATLLGNIKTKLGTYRLRPYDPPAELSLDAL
ERKWAELASNEMTRAQLINETIRDIKNALRKSFADKANDFAMALNTMQLAISGLDGDVEDQLHHVRKLSESLSPLDQYLDKIS
ELDQKCQEANIEENDFTTYTYDELSYELGLVKTSVQKKLAFLENQMVARSMTNLTPIQLEEFESVFRHFDRDDTNSLQELEFS
AALASLGLVFSEDEMHDYFLETSGGRDYVTFEQFIRFMVDVTEDQNTAEQVFQSFREVADGKPYVTEMDLRHSLVPDEVIDQL
IEIMPPHSGPDMSEDRGMPQYDYISFMEKLINDQNNQQENPEATQQNRSDAEPQSPHTNGV
FUN_010825 CDS scaffold_4 66006 66754 -
hypothetical protein EOG09264I6B PF01245

MNVASVGRPVGCLKSALRRTRLLRTFERSVSSTAVEPVPKPIPNAFSAEQRADLTKVSKFHIYPRVPSIRTTHPDPMPALLQK
QLAKLDPTGARTRLFSREHADSAKVGDVLMVTTKGGEPFAGAFLQIRRRGQDTAIQLRGQMMKVGVEMWFKIYSPTVTGIDII
WRRPKRARRARLTYMRKPKHDMGSVDQMVFAWKKERYTLRSRANQSGKPSGRQHAKILGQKKK
FUN_010826 CDS scaffold_4 66988 68271 +
hypothetical protein ENOG41

MLNRSLISGLRPVANLRASLALPAIQYRRFSISPARSFSAVFAETKNPQLNEILIEIQEKIILPAHLPEKARKLVFNPDKSDY
IQRNPVTIEVDGLEHTFKTIDRFHDVPNSMKAFAEATKLMKTKEEWDNISTLLAGYKKAGIKLKRWHADAVTRRAGHHGQPYV
VIECAQQVEKTGLSLQRDCTVILLLASINRKIISPKGEESETQQALKWNKMVWDIIQRPEHLAKNTPPRRQPHMNPVIRGMIL
FSQASAVKEQQAAGAPVDDLIRELRDNVEFITSAWAERESDDLTSSPYLNPINSRVKPNSDNYVPERQHVSPFNYILTLTENI
RAIELAQEIIGEDAKDLQPIHDLMEKHIEDFVMMSPDKNSFGWDMAYERVTGRQPSWKQHLPAAPRPRNDVEDN
FUN_010827 CDS scaffold_4 68394 69566 - CYS17 Cysteine
synthase 1 PF00291

MFRPSVRRLAAAAAKAAAAPSSHTLNVSRAQGVSRGLTGAIGNTPLIRLNHLSEETGCEILGKAEFMNPGGSVKDRAALYVVK
DAEERGLLKPGGTVVEGTAGNTGIGLAHVCRSRGYKLVIYMPNTQSQGKIDLLRLLGAEVYPVPAVAFDNPENYNHQARRHAE
RLDNAVWTNQFDNTANRRAHIETTGPEIWAQTDGKIDAFTCATGTAGTLAGITRYLKDVSDGRVKSFLADPPGSVLHSYVSSG
GKLTERTGSSITEGIGQGRITDNLQPDIGLVDGSLHISDEKSIEMVYRCLDEEGLYLGASSSLNVVAAKEVAEKLGKGHTVVT
VLCDGAYRYADRLFSRKWLTDKKLLGAIPKHLEKYIVLP
FUN_010828 CDS scaffold_4 69870 72750 -
hypothetical protein PF00018;PF00097;PF13445;PF13923

MEPTNAGLDLEKELTCSICTELLYQPLTLLDCLHTFCGACLKEWFTFQAATAERAPNPPAPGTNIFTCPSCRAAVRTTQHNAT
VVTLLDMFIAASPGKDRSAAEKEEMARKYKPGDQVLPTINTHRTADERRIDAEDRRLVEEVRELSLQEAGVSSGPPRTRRRRE
SRSADDRRRTSRDRERDRGRDRSVDSRHQRRGSRRPRADEGGRHSSDQLHVEGERRRRRSESRQRQIEHQSSIRSLISSGDMS
ERDIEREIESFARQIQEEGLLDGLDLDNIDLSRDDELSRRITEAYRRRQRDRTRQEARRNNGNNYPPLTRYAENAAAGDPRML
APEGGRPRERSRPHSRSASAASATSAASQTEERSRPPTSTNLANLEVQEPIRRPRRRTASGGRSSTTPVFPTTAEPRPAARSS
TDLGSRPQASDITQSRPSFSEGRSTSTPIHTVPFPNPTEAASFSNNTGNMSFASRQGTSQSASVPPLFSHHDAAAGRANRPRP
VDLAIVHQTVTSPISSPTVSGHQRTRSQLYPEPSVSCSRCNKQHIEYELHYNCSICANGQWNVCLNCYRAGKGCHHWFGFGHG
AFSKWEKIRQQKGDASLPQPHTLTALRYRPPRSSPGGADGRKTLTADDPRHRLESGTFCAKCSAWTNECYWRCDICNEGDWGF
CNDCVNQGRSCTHPLLPMAHEATQSNQHRPGSPPRSPGRPTAATIFKGPNASSIKPFEYLSFTTRCDLCQEPIQPNQARYHCY
QCTSSLVSDAAPGDYDICSSCYGNLIVQRQISPENGHSGWRRCLNGHRMVVIAFTDGKSGRWRYIAQDLVGGQGLKPEAAEKT
EHREKGLQKWVWHENGQDLARLVTKDVSETAPSESGFAKSLPPDGGVGLKATARFGWYPRPGADDELLFPKGAEIKEIEDVNG
EWFFGAYMGTRGLFPAPYVRLEQDA
FUN_010829 CDS scaffold_4 73511 74452 - ECHS1 putative
enoyl-CoA hydratase, mitochondrial PF00378;PF16113

MNTFRCMRPVAARVAFQRSTLPRVARAYSSTTYEYIQVSRPKPGVGQVTLNRPKALNALCTPLIKELNQALLEFNVADDTSVI
ILTGSQKAFAAGADIKEMAPLTFAEAYTKSFIESWSDLTTQVKKPIIAAVSGHALGGGCELAMMCDLIYCTENANFGQPEIKL
GTVPGAGGSQRLTRAIGKSKAMELILTGKSLSGVDAEKWGLAARVFPTYEALMEETLKTAETIAGYSKVAVQAAKEVVNKSQD
LALRDGVEYERRVFHSLFGSQDQKIGMKAFAEKKKAEWSHS
FUN_010830 CDS scaffold_4 75564 76298 + RER1 retention in endoplasmic reticulum protein 1 EOG09265ANI PF03248 ENOG41

MNAPEPEQTPFEAVSVHTSRLQRKYQALLDQSTPFVLYRWVGTVVCLLLFFLRILLAQGWYIVAYALGIYLLNLFLAFLQPKF
DPSNEEADNEMEDGSVGTLPTKQDEEFKPFIRRLPEFKFWYWATRAIIISFFCSWFEIFNVPVFWPVLVMYWFILFFLTMRKQ
IQHMIKYRYVPFTVGKKNYAKNSS
FUN_010831 CDS scaffold_4 77011 78500 +
hypothetical protein EOG09264331 PF03985

MSSSAPRTGERMVHQDFIARIRFSNALPPPPHPPKLLDIPNTGLASGQYTTPGFASRLAREQPLNIEADAELGMPLDLVGMPG
VFDGDERSIQAPPHPPALHPHDRPLLRPIAALGKHKVAEANVSFLRRTEYISSTVTKRQDGAAPRALLTKANKNRRIPEPAAD
TPHVIKRKIDKGFDIAEHEFKDPKRAKHPSKRNLKVVDVSPFIPDLEAFPDSGAYVTIKFHQNPVVSTKDYDRRLLSGLFRPI
DRTAEEEEAYEAAVAAHEQDPENNPKPQNWMNFDYYLAATPDVGDRFRRKFDVEDADHNEDELYTHDSVNGGCFQYNRLRAYE
TAQETNYDHETKFDAEVLLAYNEDTFYPNQKAVYYYPVMQKSVIRPQRTKNIARNIGITDEEQVVDQLDVTVEDPREDIREMI
KQHRDHPLGTGQADEEEGNEEEHHEGQDAEGEEEGEHHRVSSPAGRRSRSASEEHDAEGEDEE
FUN_010832 CDS scaffold_4 78876 80576 -
hypothetical protein PF05686 ENOG41

MHPIGYLTRNAQEEFGKIKARQSKTLEEAVQEYRRRYDIPPPPLFDEWFRFAKDNDVKLIDEFDTIHDLITPFWGLKPKTIRA
RAREALGFDNGLIGVSIRDHKITYIQNGVEWQQNATKRMMGKFLKYLPDMDLAFNFHDESRVILSHEDLTRLVKMAKEQNMPA
ALAKSQLANDFTQTNSELYDGKSFDEISLTRFNSFAHQSTWSHSRLSCPPDTPARCLEEEEAVDYRNRYGMSDLGFVYNTTAM
SDICLSPSLKSNFGFFGGPNTYRIVQDLFPIFSQSKISSYSDLVYPSPWDWAGMVEYDEEMDMEWVKKESKLYWRGSTTGGYS
RNGRWRHQHRQRLVQKLNARDQAHILTKQDDPSWATSEVPRGDYSEMIDVHFSHIGQCDQGDCEAQRAFFNVTEAVDQQDAWS
YKYLLDMDGNAFSGRFTAFLRSRSLTFKLAVFREWHAEWLKPWAHYVPLSIQGDDWLETVRFFESEEAGREEGERIAAAGREW
ANQALRQVDMEAWFFRLMLEYARVIDDKREVIGYDRSSANLKLPKVES
FUN_010833 CDS scaffold_4 81505 82200 - CRP27 40S ribosomal protein S22 PF00410

MVRTSVLHDALNAINNAEKAGKRQVLIRPSSKVIIKFLQVMQRHGYIGEFEEVDDHRSGKIVVQLNGRLNKTGVISPRYNVRL
SELEKWVVKLLPARQFGYVILTTSAGIMDHEEARRKHVSGKIIGFFY
FUN_010834 CDS scaffold_4 82780 83424 + RPS12 40S ribosomal protein S12 EOG09265CN0 PF01248

MSDVEDNTPEVADVVEVSGDAPKGQMSILDALKGVLKLSLMHDGLARGLREASKALDRRQAHMCVLNENCEEEAYKKLVVALC
NEHNIPLIQIPDGKQLGEWAGLCVLDREGNARKVVNCSCVVVKDWGEESQERSILLNYFQTEQ
FUN_010835 CDS scaffold_4 83686 84176 - PIN4
Peptidyl-prolyl cis-trans isomerase pin4 PF00639;PF13616

MGKNDKKGADKGGKGKADKGKDAKDSGGKSKGAQSINVRHILCEKHAKKEEALAKLNDGLKFDEVAREYSEDKARQGGSLGWK
TKGSLDPKFEEVAFALEPSTTASPKFVECKTEFGYHIIMVEGRK
FUN_010836 CDS scaffold_4 84592 86103 -
hypothetical protein ENOG41

MPVIRSGSTRDIYKAPLSAVLRRHGRERRERWSQGEDPGDFMPPHQDEEPSLEPSESSKGKQPEPQRLPSRAGYDTAVLLDLC
DTIRSSLHEGRPEDRWEEAMGFLAALLRDEEHYNRAIEFETIRDTHLDRLISDLMDPKYRHPRVPIRFSKDVERAETLERKWV

ERFRGPYFNMEQNRYRDLPKTGRLRDVALNPNADNSEERWQAKEGKTLSELEGNLEIEPGHWWLNLACAHRDGIVGSAREKPT
KGKYGVAALPLLTGQEKIDEGEGTLKYVRQGRITDIHVSLISQVGATFRILRGHHLRSPYAPKVGIRYDGLYTIRRYSQWFNE
VSDRHHMMLTLERVEDQKPIEDILHIPRPSEMDDWELYEKYEGEAIKKHKGNKALLD
FUN_010837 CDS scaffold_4 87742 90076 +
hypothetical protein EOG09261EY9 PF01142

MAEQSHHAVRGTVSYTRSIGISQRVTPLATPWTGEMRTRFSDFQVNEIKEDGTVLHLQQVGLADEEAPAIEVKKEFNGESTAQ
ETKTDESAKDALKQTYSETATKPESAPQPETVNEVSPEDATILEGLTDQRFAQELIGVYRSGHGADHEKKKSVTSEPMDDRAK
RGQVHQEIRRIFKSRIDTNTTDEGAIVATLIPPRKANSKKRGRGGRGGGREEKPAGEYLHFTLFKENRDTMDAVNQIARFLKV
KPQVIGYAGTKDRRASTVQRCSVRYMRPRNLAGINGRIWGVSTGDYDYKDKPIYLGQLLGNEFVIAIKSCQIVGESKDQPIAQ
RVEVLKKNVDLALNHMTEHGWINYFGHQRFGTHEVGTHQVGQLILGDKYEEAVMSLLHYDDKIAQRAEAGDIPDEPSKRDEYL
RNQACMLFLTDKDVDRAIKLMPRRFSAENSIFRHLNRQGAQSRRDFIGSLVHITRGLRSMYLHAYQSYIWNHAASRRWELHGE
NVIAGDLIIAPTESTPLVSGQDQDGDDIINPVEDDEDAPVRARPLTAEEAASGNYTIFDIVLPTPGYDVVYPENDIGEFYKDF
MGRDENGNLDPYKMRRMRREFSLPGRYRKIMNRFLATPSAEVRAYSDNAEQMHPTDLDNIKASKGPSRKRSLEDVDSDSTVKK
TKVENGTPASTEVEMTYVQAEIADDGSAPEATPAPQEPTKVAVVVKFQLGRSAYATIALRELMGDPPEDTETVARDT
FUN_010838 CDS scaffold_4 90642 92379 -
hypothetical protein PF00069;PF07714

MADQQPQSSDSTPLPQQPAAPTPISLPTHNSGPSPVPLLRPAIPGARSGGARTPRLGLAIPPSPNAKAVGNQGAAAAAPSRPP
LPTLHLATPMGSQVTPHEQLPRSQCATQASAGGGSESSAAHSRSGSFGPLDGRASNPTSAGSQYSALSFASQYGIGVSRPQGT
PDPVSAVGSMYSERSEGGVSMERDGSLQGLEAFDKLSLEKARTLDVDELDEEGWRIASLEKRIVEIGNLGEGAGGAVTRCKLK
GGNTVFALKVITTNPDPDVKKQILRELGFNKECASDHICKYYGAFVDPATATISIAMEFCEGGSLDSIYKEVKRLGGRTGEKV
LGKIAEGVLGGLTYLHTRRIIHRDIKPSNILLCRDGAVKLCDFGVSGDFGTKGEANTFIGTSYYMAPERITGQSYTITSDVWS
TGVTLLEVAQHRFPFPADGTEMQPRAGLIDLLTYIVRQNVPKLKDEPEMDVYWSDNFKYFIECCLEKQPNRRASPWKMMEHPW
MVEMRSKRVNMVKYLSFVWGWDEQPKDS
FUN_010839 CDS scaffold_4 94857 96477 + DBP8 Putative
RNA helicase PF00270;PF00271;PF04851

MVPPADPERATAASDEEILSGAASSSDESDYLDSATSRKRRRTAEVPQTGGDEVKDEDEEDNELKRVLSTIKVPSRIKRAAQV
DQREAQTKPLIAPKNSIQAPTDPNTTFSSLNVRPWLVQSLANMAIKRPTGIQKGCIPEILKGRDCIGGSRTGSGKTVAFAVPI
LQKWSEDPTAIFAIVLTPTRELALQIFEQFKAISSPQSLKAILVTGGSDMRTQAIEIGKRPHVIIATPGRLADHIRTSGEDTI
CGLRRVRYVVLDEADRLLNANGPGSMLPDVEECLSVLPPATERQTLLFTATITPEVRALKDMPIKPGKQPVFVCEVDTQTLAI
PTTLKQMYIKTPVTHKEHYLHVFLLTEANVDKTVILFCNRTSTADYLHHLLRMLDHRVTSLHSKLPQRQRIDNLARFRASAAR
ILVATDVAARGLDIPEVSLVINYDLPRDPDDYIHRVGRTARAGRKGEAVSFVGQRDVELALTIEKRVGRDMESWEEEGVNLET
RVVRDALKIVSEKKREALLEVEEGREVGGKRKRTKQKLRVE
FUN_010840 tRNA scaffold_4 96721 96816 - tRNA-Ile

FUN_010841 CDS scaffold_4 102499 103249 +
hypothetical protein ENOG41

MSTTTTTTSIHRPTASKTSSLRQSTLKKNMSITQTYYLAHKARAKLSREAAQPDHDLRLLVGHANLLDSLMLELADAEREQER
WFNQSVRGATNAQQERRVQWADNIVEEEDEDDYDSDSSDSDDYDSEDDDVEMTSTSLPVKPMSPRITHIELDEDMEEDDLEED
YAQLELVRTPSHSNSPPELVDHDSDSSDDDSMPPSPPQSILTFDEKDVKEQSQQEQESNSLYDEEYYLPPRNPARLVSAISVY
FUN_010842 CDS scaffold_4 106493 107681 +
hypothetical protein EOG092620V3

MRLLPRRALAHDLSPGLICRTYRLQRRQFATSLSPPPPPLSGYTSLTSRRLISVTGPDSAKFLQGIVTANVTTEDGKPRSDGF
YAAFLTATGRVLYDVFIYPNHNTSGASAEEPGYLIEVDADQASTLAKHIKRYKLRAKLAVRLLENDEATVWHAWDDSNSANSS
SIVKSANFSLQDPRAPGLGYRLLQLDQHPPQIDLEPSTQEAYTIRRYLNGIPEGQDEISREHALPQETNMDIMKGIDFHKGCY

VGQELTIRTRHRGVVRKRILPCVVYEKEHAPPTTLQYHADDTTSSLESVTADMIPRDTSIGRFEKRGRSAGKWLKGVGNIGLG
LCRLEIMTDVTLPGDAASGAFNPEDEFVLDWGEEESRNRVKVKAFVPDWLREGLDAENQRNEK
FUN_010843 CDS scaffold_4 108921 109686 +
hypothetical protein PF09994 ENOG41

MATETTPKTIKRLIACCDGTWMDSDNGYEEAGLLRKEGSLQIPSNVTRISRCFEKRCSDGKLQVVNYESGVGTGSNVLDSITG
GAFGMGLAERMRETYSFLCSNYMDGDEIILVGFSRGAFTVRSVAGMIGNLGLLTREGVEFFYPIFKDMQHWMDDDYEDPFPNI
PFPNKPKGKDAADVYRARLEQLGYTRVRRSEGEGDIITIKAVGVWDTVGSLGIPRVLWLEKLGIRADNDE
FUN_010844 CDS scaffold_4 111164 112013 +
hypothetical protein PF00226

MSDTKAADLLQFAQEYASKDVDLYELLGIDALTPKEDIHRAWRKRSLKYHPDKAGDNFDAEKWQLFERARDILSEPGARGAYD
GAIKAALLRKQERETMDKQRKAFVDDLEARENAWKVQREEKEQREKQEIEKERSRLVEQRRMREEEERRQAAAQEVEDLAEA
RRRLKEKKEKKKQDEAREKFLRKSRRAAEAADGKPASGPINGVMDVPGDFPVDFGTEQKFYWELVCDKLRAVQAVRNLRQKEA
TPEEYQQAEQGLLDAKTRIHQAEVRFAEHTSVS
FUN_010845 CDS scaffold_4 112110 113247 -
hypothetical protein PF04909 ENOG41

MSSSSSLVVDIHTHMYPPSYIRILESRSKIPLVRKFSQAPDPRLILLEAEVKALEEATQNPEAKPPGRPLTSHYASLAQKLHF
MDTHKIDISVISLANPWLDFLSPSESGAIAQSVNEEFSRMCSDNPGRLFFFGTLPLTAALETVLASIEHLRILRYCRGVILGT
SGLGKGLDDPDLLPIFEALATTGLTVFLHPHYGLPNDVWGPRASAEYGHVLPLALGFPMETTIAVARMYLAGVFDKVPELRMI
LAHSGGTLPFLAGRIESCIAHDGQLLREGKLTKDRRTVWEVLREQVYLDAVIYSEVGLKAAVQASGADRLMFGTDHPFFPPLT
TDEQGEWESVSLNAEAVALAVGQDTGDFKNVMGMNAVKILRLDAES
FUN_010846 CDS scaffold_4 113793 114959 +
hypothetical protein PF03171;PF14226 ENOG41

MASGLAVSKEGLSIPLIDFSRFLSGTPVERNETANAILDGFKTAGFIYLKNHPILPKDLQHAFNLSARFFNEPLEKKMEVVWT
TPEANRGYSAPGREKVSQLKDIQEVEKVRAALPDLKESLEIGREDEPGYPNRWLEETGDLVGFRKDMLGFFEQCRQLHVEVMK
AIAVGMGLDDRFFDSFVDVGDNTLRLLHYPAVQSNIFKTNPGTVRAGAHSDYGSITLLFQDARGGLQVKSPTGQFVDATPIEG
TVVVNAGDLLARWSNDTIKSTIHRVVEPPQKQAESYPPRYSIAYFCNPNFRSYIEAIPGTFGTEKDKKYEGINSGDYLVQRLT
ATY
FUN_010847 CDS scaffold_4 115407 116185 +
hypothetical protein PF04387

MASNTAAAKRPLKQASTANKAYLIFYNFISAVLWSVVLGRTAMLVSAYGPEYVVYPNTGQYTKWTQTLAGLEVLHSLLGVVRAP
LMTTLMQVASRFLLVWAIVDVFPFLALSPFYSSMLIAWSVTEIIRYSFFALSLSGYQPNFLTWLRYNTFFVLYPIGIFSECTL
IWFATEPAGHSNELYKWALYAILAIYVPGSYVLYTYMMSQRRKVMRNLKAQGQKAQ
FUN_010848 CDS scaffold_4 116343 117767 -
hypothetical protein PF05057 ENOG41

MKKTLLLLCFIHGFKGGEDTFGVDYQFTKDLRDLVGEALPKVNVEVVVYPKYETRGDLGSCVSRFRDWLQDKVIDMEVAAGTPS
PTIEPSVRTILVGHSMGGIVAAEMLIGLASEKPIYTEDGIQKSEKPTFNSLMFPYIQGVLAFDTPYLGISPGVVAHGAEDQYQ
NAAQAVGAISQLSGLGASLWGAKTAGSPSTSPAPPKIAGALPAPSASGGNSWQKWGKMAMYAGAAGAVAAGGAAAWMKRDQLG
EGWSWASSHLEFVGCLARGEELRKRVACLLQLREELDVGFANLYTRLGKAAPSKQINVVGTVLGQDRTFCNLPKKQPGGVWME
AINNKATDEAGAHMNMFTPRENPGYNKLAQDATEVIRGWLQNGWYETSAEEPLLLQEAPLTEDGLSPETPDVEKTV
FUN_010849 CDS scaffold_4 118358 122758 -
hypothetical protein
PF00176;PF00271;PF00439;PF04851;PF07529;PF08880;PF14619

MASVHAPHAVQHPGAPMPAGATKQQAEEVFRKLKQMKEQGVPPSDPEYIKASQFLMSFQQQHNMRRNQQQFLQQQQKQQMQNA

TNGTPANGVMSGRPMQQGSPQSTQPPSNPLGASAMPNQTTSTPVSTGTSPSTAPSSNHFTQQQLGLLRQQIHAFKLLGKNAGV
SLQLQQAIFNQRHRRQAAVADAAQVTQATKSNQADSEASKEGQNGPGAATEDEGSEIPKAHTFKTVKSPYGTSMIRPEIKYFD
HSQRKNRWFIPGVFPTGIDFDHLRYEREVVVSNRMRQRYAELKNLPGDLAHWDSSKETLEADDSLKRKAIIEMKSIALYAKQR
ALRDKIGRQMMHYDNLAMTTNRSGFRRMKKQNVREARITEKLEKQQRDARENREKKKHTDFLRAIYTHRQEIQESASSQRTKS
HKLSRLMYQQHFNIEKEEQKRIERTAKQRLQALKANDEEAYLKLLDQAKDTRITHLLKQTDGFLHQLASSVKAQQRQAAERYG
DGDDPQMDDASDYDEDDESSKKIDYYAVAHRIREEVTEQANMLVGGKLKEYQVKGLQWMISLYNNNLNGILADEMGLGKTIQT
ISLITYLIERKQQAGPYLVIVPLSTLTNWNLEFERWAPSVSRIVYKGPPNARKQQQDKIRQGGFQVLLTTYEYIIKDRPILSK
IKWFHMIIDEGHRMKNSNSKLSFTIQQYYHTRFRLILTGTPLQNNLSELWAMLNFVLPNIFKSATTFDEWFNTPFANTGGQDK
MELTEEEQILVIRRLHKVLRPFLLRRLKKDVEKDLPDKTEKVIKCKFSALQSKLYKQMVTHNRLVVSDGKGGKTGARGLSNMI
MQLRKLCNHPFVFDVVENVMNPLSISNDLLWRTAGKFELLDRILPKYQATGHRVLMFFQMTAIMDIMEDYLRYKRFEYLRLDG
TTKSDERSDLLREFNAPDSKYFMFLLSTRAGGLGLNLQTADTVIIYDSDWNPHQDLQAQDRAHRIGQKNEVRILRLISSNSVE
EKILERARFKLDMDGKVIQAGRFDNKSSETDRDAMLRTLLETADMAESGEQDEMEDEELNMLLARSDDEIAVFQKIDEERQRN
SPYGNGPGSKPRLMGEDELPDIYLNEGNPISDETEDVVLGRGARERTKVKYDDGLTEEQWLMAVDDDDDSPEAAAARKQARKD
RRENNRLKKSGISNSVDESPSGSRASTEEIETPKKRGRKPGSKNEKRKAEDGNDEPPPKKRRGPQGRPSKVSLESRLPPHQRE
VLQKSLRNLYDALMTLEVDDIEPPEDDESDPGKRLIIGPFVKLPPKRDYADYYLIIQNPICMNQIQTRIKKEEYTSLSGLRKD
IELMIRNCQTYNEDGSILYQDAKVMNEFFNSKYQEELAAHPELQELEEGGKDSSVAPSGGGGTPQPSGTRIKLISSGSKEANG
GSTAAQSDEE
FUN_010850 CDS scaffold_4 123807 125514 -
hypothetical protein PF00400

MSSDDDFEIEVSELAADDPMRAFLPTSFGKKSKEADVAAQIERSRRKVAEVSGGKKKQDSSDDSDSDNSDDSDQDEFPVSHEL
VLKTHDRAVTTVSLDPAGGRLATASTDCTVKLHDFASMTPSTLRSFRSVDPYETKASDVNSQSHPIHRIEFNPLAGGVFLCVS
AHPQAKILSRDGDIITEFVKGDMYLRDMHNTKGHISEITTGTWHPTDKNLCITAGTDSTLRIWDINNKRTQRDVVVFKSKAAG
SAGRTKMTAVAWGSSPQGSSNVLVAAALDGSLVMYSGNSPFSRPAGEIRDAHKPQTWTSGIDISSDGRMVVTRGGDDLIKLWD
TRKFTKPLVTAAHTSTSDHFPTSNIKYAPNSTSIITGSATGHLHILNPGNLRAEHVTEITPGSPLITVDWHPKINQIVTGSAN
AETHVLYNPQMSSRGAVDVMSRAPKKRHIDDNPELTMDQSVGVSGDAIVVPGATRSSKPSGVTVSGRSKDPRRPHIPQQTPFQ
KNQPDEKLIAENIPLARMLHEDPREALLKYADKAQSDPMFTKAWSKTQPQTQYAELSDEEDGPDKKRLKR
FUN_010851 CDS scaffold_4 126418 127435 + SWC5 swr complex
subunit PF07572 ENOG41

MPPDSLLDEDESYVSSEDSDFALDDSPDQTSDHSDLEDHSDKTANKRSRPQDEADAGYDNSGDEAIIKRGEKRRKKTQAKGVR
EHDEGEGGLIKTRRQRAAEKEERQYATNNEPVTIDVDALWAQMISKPPTPESKDDRPNDADTSTNFIDKPNDSAKKKSNSTIS
DQSSTMIRIKRTYNFAGRIHIEEKLVPRDSAEAKLYLASQGQEAPKDSPEKRVTRKAFRSAFEPQVDLLPHRADLNLGMAARI
KAGKEAQAKKLNVVEKSRMDWAGYVDKEGIKDELELAGKSKDSYMAREDFLAKSEALREEDSRRARLAGKA
FUN_010852 CDS scaffold_4 127843 128797 +
hypothetical protein PF15375 ENOG41

MAILGKRKAEPEPLISEEDAAAIFRRHFEAEFAPLPEAEAAKSKFKKTKHEGNSNDIESEEEEENNDSNGDDGEWAGLSDEDS
VTEEEENPTIQVVDHSSKQPPKPAAMSKRELKAFMSSRPPEQTTPKTEQPLSATSSSSNTLPEDAPSLLAQDLELRRLLAESH
LLAPTISASGTTIAPKAFATGRTRQKATDLRVQALGSKVSIHKQEKMPMNMRKGIVAAADAREAKRRREAKENGVILERETGK
KKGRRDRGRDVAVDRPGVGRLKGAELRLSDKDIKSIEGGRDTFGRRSRR
FUN_010853 CDS scaffold_4 129273 131571 -
hypothetical protein PF00096;PF02200;PF13894

MYPQHSTMAPQKPETFMLSTEAQQALPHDAQVALQQVDNLKYFLISAPVDWQPDQYIRRFLLPTGEYVSCILWNNLFHISGTD
IVRCLSFRFQAFGRPVKNSKKFEEGIFSDLRNLKSGTDASLEEPKSAFLDFLYKNNCIRTQKKQKVFYWYSVPHDRLFLDALE
RDLKREKMGQEATTVAVSEPALSFQYDSSQSLYEQLTKAQQANSSSFSAQQSAFSQSQSTSPVMRAMDSMPPPPTMMPQSMPP
LAEGMDAMVPYGAMAVPHPMAQAVPVKREPDFTRVQYNHNGVPITQGHQRHASMPAYGLEYSPAPSFVSSQYEDYSNRGISFE
PITPPQQALGISAEPAYIANEETGLYSAIPDHMASMNGLNGMVQLPPSNLAGPQFSRPYGTNNVYSVIEGSPTYKQRRRRSSI
PPSMSAITTPAATAQAASHTHTHRPSELRRSISASVGPVAEGDESADNSPPGLSYSTSTVSVNTQHHQNMSRHGTPLSTVEGS

PAAHSMGMHQQEFPHLASEEFSGDVNDQRRSVPAPNGAVRRARSATVMEVGPYPQKSHSCPIPTCGRLFKRLEHLKRHVRTHT
QERPYICPHCSKAFSRSDNLAQHKRTHGREDGGDGSLNLSGEEDEDFSGDDHLGSLEEASPHSDSAYVTGSLNAAAHGSTPPS
SMAPTQSYNSLETLSMPMTMSQPAAINASGMM
FUN_010854 CDS scaffold_4 139399 141639 - CRN1
Coronin-like protein crn1 EOG092627F1 PF00400;PF08953;PF16300

MAGRFVRASKYRHVFGKSTRKEFCYDNLHISRNAWDTNLVKVNPEYLSVNWDASGGGAFAVIPLNERGKLPDQIPLFRGHTAA
VLDTDWNPFHDNIIASASDDGKVFIWEVPEGFTLHTDAEEITDVAPVSKLAGHPRKVGQVLFNPAAENILASASGDFTVKLWD
VSTGQSPLALKHNDIVQSLSWNASGSMLVTTSRDKKIRVWDVRQEKPVHEAPGHGGAKNSRAVWLGEHNRFATTGFSRMSERQ
IALWEPGRTEPIGGFTMLDSISGVCMPFWDDGSNCLYLAGKGDGNIRYFEYENDKFEFLSEYKSGDPQRGIAFMPRRGINTHE
NEVMRAYKTVNDAYIEPISFTVPRRAETFQADIFPPATGTKPAATAQEWFDGKTAIPNKIDLESVYDGTAPKEIASDYKAPAA
PSPVTEKPAPKMEEPKKEEPKPAPALRSPPPTMSEQKGSISAMASRYQDKQEEEEDDDDASSFEEVSRPAQRQAVPAKFTPP
AQPQTASTPSPSTVAKPSSPVKTPAAAASAAPPPTSSAPRAVSSAPSGSDSSLAEIKQLIEGQTKLINSQSQQITLLTAEVEA
LKRKVSSGSQDQSERIRQLELELEEARS
FUN_010855 CDS scaffold_4 142381 146760 -
hypothetical protein PF00169;PF08174 ENOG41

MASGIPRPLSELSPTEMRRNSPSWHRQSTGSPKKSPGNPDSSPFQPSPVESVTSPRLFWQNRNSENLYGSGSPSPNRRSSIER
LQKASRVRNSNILALEQKQEYDPTRIPQIERPLAKVQGNAFGSTGSFRSSTIERPIFGHSRSDSAMTIPAVSPKSSTQTAGSP
NRPLTPSRDQVSPTKSSLSHSRFKNSFDMEKNTWSFGSPEEDDDHPMGRRHAKSVTFDAAPPQINEYEMATPDLSSIGTNSRE
GSYESIEYDEDEDDVLYDPGRVDLEGDSFDASLEDTDKTPVVGPDDWRGDSPMVNRNGPREYSDSPMPEGTPSAATAGRPLHI
RSDSTTSNGEHRPLPPLPGFMSHSRNNSAPSSPASPSLSATAERMLGSNRNLPSPPQASTTSKSDIHSIGNAKMSLEERLRLM
MLSDDGDGKTAAEQQRERRLRRANGRERLSSPISEPESTTSFNDALQEQDDVVGDMSALDDYQLPPRISRDSIMRKVNGNNKT
SDQEKDCIFSSPPASRSPSRSPERQVPADPDVPIPSTEDSLHDMSDEEEEEEEEGSVIIRRMPESEIEFYEDSDVDFDEEESR
LELPADSDSESHYSSQEPTPAQAKPEEVQEEARKESPELDDLREEVMTPRPTHTQNESVSSLPDFASGDKDASFSRDFESYML
PEPEEPKVVEEPVKEAKEPEAPKMADAHAYLQRPFTPEHLSKPEYDGTGWGDPEDEYDDDCPSTPDSVIHHPVPVRVEEPEQA
TIPERSATIKAPGSKLKTRLSNTPSDISAMREARRQVSMEVPDVPPIPDKHSKRLSKDNNGLLLTGDEFINRHPSFKNRSLTL
DLDLGLSLDQDFERVIEAQKVAYNDLFTQPPAHNSPSRQVSAAKAQQQIAEGLNANVTSRRQRGYLMRQNTKVVTASDKDTED
FRVARSVGNSPVKEDRPMSCIVEPWNGKPRQRSVRKRVGGSTGPVPPMPGQESNATAVSHAPVDEDLTLEMATPESGERGRLF
VKVMGVKDLDLPLPRNERTWFSLTLDNGVHCVTTAWLELARNAPIGQEFELVVPNDLEFQLTLNVKLEKPVERVKPPPPTVKT
HKHKTSTFSRVFASPKKRKELELRQREEEERAVQQQKDAQAKQRTGPPTAYELLSPLAAEDGSFGRAYVCLREHESRCFGRPY
MAEVACFNEWATEEAGFASSVKSKRGNTAVVRRAPYKVGKLELQLLFVPRPKNATDDDMPKSMNSCIRELKAAEERMSRNWEG
HLSQQGGDCPYWRRRYFKLVGTKLTAYHEATRQPRATINLANAKRLIDDRRALTEKETTGRGGRRRRSAFAEDEEGYMFVEEG
FRIRFNNGELIDFYADTAADKEGWMKVLTDVVGRDNPDDDSSNQRAKAKWCQLVLRREEQLRRRASGRRVNSRPKSMFL
FUN_010856 CDS scaffold_4 148656 149531 - HIS7 Histidine
biosynthesis bifunctional protein hisB EOG092640IZ PF00977

MTKFRPCIDLHAGQVKQIVGGTLDSTSSALQTNYVSQHPPAHFAQLYRDNDLTGAHVIMLGPGNEGPAKEALEAWPGGLQVGG
GINDKNAKEWLNAGAEKVIITSYLFPEGRFSQVRLDAVLQALGGDKNKLVIDLSCRRRGEDSWFVAMNKWQTITDMEVNQESI
KSLEPYCAEFLIHAADNEGLQRGVDEKLVQRLSEWCSIPVTYAGGGRHLEDLENVKRLSNGKVDLTIGSALDCFGGSGVKFDD
CVAWNKKQSI
FUN_010857 CDS scaffold_4 150090 151110 -
hypothetical protein PF12706;PF13483 ENOG41

MSDKGSIGLGLSKRQTLLKDKSTRGDDQSLLPKAIKTHPRGSEGDENANVYFIGTATTIIEWEGFRLLTDPNFLHAGDHVHLG
PGVTAQRQTNPAVDLHELPSLDAILLSHYHEDHFDRLVEDSLNRDFLIVTTPHAHKCLTSKNEPFRNVKPLDFFDHLELKNED
SKAGEPLPVIKVTGMPGKHVPPGPLAAVNDLLGAVPPTNGWMLELGYHKVENGKLQTGYRIYISGDTLLVDELKEIPKWLREE
RIDLMLIHLGGTTIPGPSAPLIMVTLDAKQGVELMKMMDPEVTIPIHYDDYDVFLSPRKDFETAVKEAGMENRVVILDRGDRY
GFTVKKI
FUN_010858 tRNA scaffold_4 151542 151643 + tRNA-Val

FUN_010859 CDS scaffold_4 152136 154596 +
hypothetical protein PF06985 ENOG41

MPDRSPSHDSRDESSPEPERDRDRPRDRDRDRDRERDRDRDRERDRERDRERDRERDHDRDLDRDLDIDGVVDRDLDRDKDRD
RDRDYDRDKDRERDRDRDRDRDRDRDRPPDDARNRDRDNGRDRDDDHGRDRDYANGKDRDRLKDRDIDRDRRRESRVPPRVPR
EPRDRDHPPNSSKRNSITDRLLGSFRAVRGEKEKEIPIPAVASSIQRRASSAVTKGGPVLRIREWLDACNADHHHHCAVSSDI
DITTWRPTWLIDVVERRLVKATPKDRYLALSYVNGTQRNGAPMFTHLLQSNVEAFQDRLPNLDMPQTYLDSMWLAKKLGIRHI
WIDRICITQDNREEMDDHIKHMAYVFSNSYLTIVAATGDVYTGLPSLDPKRSTRGLRTTSRTHQELVAASPWYTRGWTLVERI
YSRRSVFFFEDSITWECHCETWQGSPNSVMKKLRGGRQECTGAVPDAAFAFQHPPWPDLDEYARYVMEYSARKLTLVDDTLPA
FAGITHVLSRSFPGGFIYGMPIAFLDIALLWRPHASIRRRALSRPPFLPSWSWMGWWFDNIPVDLTLWRAAADYVEEARTGKR
GQETKRYRSAHPFRIKPTVTWSLTDRAATAPVMNTGLQYRDLRGRRSPSSELPPGWSRSGSYYVHDSDDSALFKYPIPVEDPP
ETGGYEPPVGEQAYPGPLLSFKTTTGFFEVEFHTTLAHRDRTNPPLAVGTIWNKANRWAGQFRAHDAWLGIQSSNYDGEERLE
FISISTATERRGSHVFDNEKFEEHMDADEMIDFVNVLWIERIGDIAYRRGVGQILLKTWEAQAKDEVGILLG
FUN_010860 CDS scaffold_4 157756 159190 +
hypothetical protein PF01733

MERVRQALGRQHSSTSTSEYEPLTGTEEATPLEGSTVLEGQHELPFSWIEYSIFALLGVAMLWAWNMFLAAAPYFTARFAGDA
WIQSNFQSAILTVSTVTNLGAMLVLTSIQYSASYPFRINLALVINVFTFSLLTASTVIGLSASPTLYLVFLLATVAAAAWAAG
LIQNGAFAFAASFGRPEYMQAIMAGQGIAGVLPPVAQVFTVLVFPPEKDQNTSIKEPSSEDGQTSAFVYFLTAVVVSVAALLS
FVPLVRRHNHIIENRMVEQMNESMHSIEEAERAARKVTSLWRLFTKLHWLAIGVALTFIATMFMPVFTAKIHSVKENSGALYQ
PSAFIPLGFFFWNLGDLGGRVATILPFSLRHRPFALFVLSIIRFGILPLYLLCNIDGRGAIVSSDFFYLFIVQLVFGLTNGWL
GSSFMMASGEWVDEGEREAAGGFMGLCLVAGLSIGSLLSFTVAGV
FUN_010861 CDS scaffold_4 159549 161150 -
hypothetical protein

MSTPSLSDGQLTPTDTDPDLCFSSSEDDHVWPTPQDVVEMSIMLNGSAVPLSWTRESLKRLPMGTRPIKMVGEGAANVVFELG
IPEGNMWANDFKGWLLRVAKAPASGQPARFNYLKQQEFYAKQITPFLKTHAIQQRLVVLRHTNIIPQLNAFLRSIDHQRKEKF
RGSFISESNWGLLVEDMRVSEPKNSILIEFKPKWLNQSPSAPQGAIRCRQCAMELFNFLRDPSPSRHRPEQKPCPLTLANPDA
PADISSPFRFAPKLATQANNPMVRELLAKTADHQVIRDLRWLQNLSDTRGPLRAEKGDPMFSLAMTVRDCTCFVQMNLGHDVP
PEQRLRVRLGDFDLKDTDIKFKRWTSAEKDLIDSGCYTADWIMCNGQYYHPPTKCLLEWTRRRDRFVKIIGIKDKDPYLHPLP
NKTAHFPTEAMTKQALVYWMTTHPIKLTGLLEPGRKDKPKAEVCPFRNEPPNLMKWLSARP
FUN_010862 CDS scaffold_4 166526 168526 + MDM34 ERMES
complex subunit EOG09261S0S

MAFNFNWSPLTADADFYRRARDLLTKALNKSPKPPIIVDDILVSEFNLGTVPPDLEILEIGDLAEDRFRGIFKMTYSGDAFLT
LKTRVQANPLNTYLYSKPTFTSPQPLAAASGLTIPLSITLSDFKLSAFIILVFSKQKGLTLVFRNDPLESLKVSSTFDSIQFV
RDYLQRTIEQQLRNLMMDELPAIIHRLSLQLWCPDQANKGTETPAEKEDEAGVDPLASPPLDPVDANGNLLDPNAISELTLNG
SGEVQSLFSQKNLLRLAALTDSHRTLSLFTPGIRDVVFRAWTGYGERSETSTPLLSTPTLTKTMSFHAGNSTTYTFSDTASNA
HGHLPSRPSLISMNSATNGRSLGAGRSRTGRKKKTRVVNLRRTKSEVTTPETGSEASDTASVDVPLSEPMIDESIPEEAESSI
IAEASTPNKVRFRSAGEAGRPSVMATLEEARRMGSPLKNAVEPSESQNYPSIAAITSELKSEKGKSPGLENQSISSETSSVIL
EQAWIMKMAGEIAKRVYDEKNRQRSPWDEREDAPPPAYEAATQ
FUN_010863 CDS scaffold_4 169474 170331 - YAF9 NuA4
histone H4 acetyltransferase complex and the SWR1 complex subunit EOG09262UTQ
PF03366
MAPQNQLGKRVKLTQVRRPFIVGSTAKPFSDTNPRPAGVPDTHTHSWQVFVKGLDDTDITYWLRRVQFKLHESIPNYVRMVEG
EPGKPFVVEETGWGEFDITIKLYYVNDSGEKPQTLYHYLRLHPYGRNEEEKQAMIASNGEIRAWSYEEQLFNEPYEVFYNLLT
QGAVPKGWKPSGGKGKGKTRAPPPFPTDSNEVWEHSAMIPAHNRPGQPFSLETEAAEIRKLADAQTQAEDMAKRILAELKAKE
ELLAKLKGENQAAAAAAKS

FUN_010864 CDS scaffold_4 170775 171698 +
hypothetical protein EOG09262ZZ8 ENOG41

MSASKKLVVCGGTGFLGSRICKYAVARGWDVTSISRSGDPRWDTISASATPPSWAHKVSWERGDILRPATYAPLLNGADFVVH
SMGILLEADYKGAISGKESPIAGLQKAFAPVRDRGVDPLAKGQGEDIKPPNPKDQFTYEIMNRDSAVTLAKHAVAAKVNAFCY
ISAAAGAPVLPQRYISTKREAEITIASKFPELRGLFIRPSFMFDSSRPVTMPLAAMVGLGTAFNGLTGNYFKTFIGAAGVKPL
KVETVAEAVVEALGDETIKGAIDVPEIEELASKAWRKTML
FUN_010865 CDS scaffold_4 171786 172587 - SYF2
pre-mRNA-splicing factor syf2 PF08231

MAPSPTGKKDEAQQMNVDNMSKAQDRLARFKALQARAKTSSEKNLKEATLESQRLATDPSQITALHRKHAIASHKLLKADVEE
AGGDFERKRAWDWTVDESEKWDKRLKKKTTRRDNNAFADHTQEANKIYKRQLKNIDMNMEQYEKDKKALIEKAAASGGLDIVE
TEDGELIAVDKDGSFYSTADSTTFAENKPDKAAVDRLVADLQRAEEQRLKKRKERMAQNGDDGDVTYINEKNKQFNQKLSRFY
NKYTAEIRDSFERGTMI
FUN_010866 CDS scaffold_4 173656 175144 +
hypothetical protein ENOG41

MQRRAASTALDAGKTKSKMFLKNETRWGETYYVVNRWPETPKLPEEHEYFRINDLLLDLDDMFSKHVRPSSEEDEERALKRWS
RDNKRDDVRRKLHRTETSLTWSRIKSFEMYSEPSSSWRLGSHDIFSAALRAPSPAPLIDSSKVQQGSANNATAEREPNKLHLI
SSNNGIPKQALEDDSSLLRWFQSRTQLSWSGQYPAVSPSHSGQLSAALRKQDSLTSLRRLVSQYLSVETSAISFHQLKSPGQE
KSTENLSSDLRNACENFLKQDTLQNKHDVLVFLGNLGQRLTARGDHLGGPLCGLGLRLSAEICKPTASKRYFQIGLQTGYWTG
SNQGLIDILHCLETYQCHFNSVSQPNGLDLYGRQELLELLLGRSRTHNHSALSLINACLHGCKQETAFEAYRASILLAGHLGM
VEWLNQEQRTSAAIIQRLARSSNSKDKKRQLEAILSEAFESAKSIADTSVVAQSHDAVLTDPSLAGDVCEEKDRNQYCPP
FUN_010867 CDS scaffold_4 176772 178557 +
hypothetical protein PF13854 ENOG41

MPQPYANLSKNSTVPDVSGGIFWPDTINKKIYLFGGEFNGVTPWDFDLYAYDIINDEWDNMGVSRTDDVVGLSYGAGLSIPDR
GEAYYYGGWMNNATDANWGDAPQIPTSYLLRYEMDTNNWSNDTGPDDIGRAEGVMVHIPAGDGGMLVYFGGIRAANDGGWEGQ
PMDQIILYDVLSGKSYFQNATGDVPESRRRFCAGATWTKDQSSYNIYLYGGAGEEQGSSGFDDIYILTLPTFTWIKMYPEGNG
TGDYPHHSFTCNVVNEAQMLIHGGFFPLTNDCDVPDQWGLHDMSLGRQNKDKSPWMLYDPELTKYAVPTDIISVVGGNPTGGA
TKTAPADGFDHQDLKALMTRTASAGSRTATRDVSGPTETGDHESGATLSTGAIAGIAAGGAVALIAVLVTCFCLIRKHRRRRE
RVGSQQPMTQSYDYHHPQHPSFASNQPSPSPWSPQSSSFTPSSPVGYTGPQLAQSYSGPPVELPSGHPEDSSYVSPRTESASA
VEPKYDANGNLWVPQVSMMQTPDQGHSPGSPPYYDGTMVSNSSKNGPEYFPPGTPQELAAERRTSYVPGQPVHQTYYHP
FUN_010868 CDS scaffold_4 186170 187229 +
hypothetical protein PF12855 ENOG41

MHHSRKKSGHGLPNSSATDVRKAVTATDPAKYKRPVMTRRHTPQKLGRSQREREREWTESWEDERESFPQFCMTCEKQFIPHD
DMFLYCSDNCRRVDQTASSQPSSSVRHYASSNYPFHSAGNPEPKDIIPRASPSRPSSMILSSPPATPGTGAPNFQHTSAISAL
RSLNVRPPSPPSPTGSSSNLWPFSRSAATSPYSSYSRPSAPYLSSTYDGGYYGAGGSGYNYEATSGGMDRPLPSRHPSTYSRP
RSIELVTPMVSR
FUN_010869 CDS scaffold_4 190147 194349 - DRS2
aminophospholipid translocase PF00122;PF13246;PF16209;PF16212

MAGRPPGGGFDTGHSNNRDDLLLDLDNDQPVYSGGQRSNLNDDDLMRFHEQNQDPPPGRTSVSYDDFVGARDANHASTAQPAG
GLGVPPRSGPGSNPYLTRQYSQTSELGNYQRYADDFDDYPVDGDSYYHQDGGARVDETTPGLGRNNARNRNSVLGLGGGFLGK
VKNRLGLGQGYSEMDLPLTEPGHERGDSVGGNSHQPPQQAQKGRFDMGNFKFGFGRSKPDPSTLGPRLIYLNNPPANAANKYV
DNHISTAKYNVATFLPKFLYEQFSKFANIFFLFTAALQQIPNLSPTNPYTTIAPLIVVLIISAVKELVEDYRRKQADNALNTS
KARVLRGSTFQETKWINVAVGDIIRVESEEPFPSDLVLLASSEPEGLCYIETANLDGETNLKIKQGLPETSSMVSPNELSRLG
GRIKSEQPNSSLYTYEATLTMQTGGGEKEFALNPEQLLLRGATLRNTPWVHGVVVFTGHETKLMRNATAAPIKRTKVERKLNM
LVLLLVGILLVLSIVCTVGDLIQRKVEGDALSYLFLDPTNTAGQITQTFLKDMVTYWVLFSALVPISLFVTVELVKYWHGILI

NDDLDMYYDKNDTPATCRTSSLVEELGMVEYVFSDKTGTLTCNQMEFKQCSIAGIQYSEDVPEDRRPTMVDGVEVGLFDYKAL
KANLANGHETAPAIDHFLSLLATCHTVIPEMDEKGGIKYQAASPDEGALVAGALDLGFKFTARKPKSVIIDANGRELEYELLA
VCEFNSTRKRMSTIYRCPDGKIRCYCKGADTVILERLNDQNPHVEVTLRHLEEYASEGLRTLCLAMREIPENEFQEWYKIYDT
AQMTVGGNRAEEVDKASEIIEKDFFLLGATAIEDRLQDGVPETIHTLQQANIKVWVLTGDRQETAINIGMSCKLLSEDMMLLI
VNEETAAATRDNIQKKMDAIRTQGDGTIETETLALIIDGKSLTFALEKDLEKLFLDLAIMCKAVICCRVSPLQKALVVKLVKK
YQKESILLAIGDGANDVSMIQAAHIGIGISGEEGLQAARSADVAIAQFRFLRKLLLVHGAWSYQRVTKTILFSFYKNIALYMT
QFWYTFQNVFSGQVIYESWTLSFYNVFYTVLPPLALGILDQFISARLLDRYPQLYMMGQQNYFFRFKVFTQWIGNAIYHSIVL
YIWAQLFWYGDLIQGDGKIAGHWVWGTALYGATLLTVLGKAALVTNNWTKYHVLAIPGSMAIWYVLTAVYGIVAPMAGVSMEY
FGTIPRIYESPVFWLQTVCLAIMCLLRDFVWKYAKRMYRPQTYHHIQEIQKYNIQDYRPRMEQFQKAIRKVRQVQRMRKQRGY
AFSQADESQTRVLQAYDTTKHRGRYGEMASSRPAGR
FUN_010870 CDS scaffold_4 196044 197737 +
hypothetical protein PF12330

MTRSTAVKKYGKPAKRSKAERLFAELPQSPVRPQQEPNRKKDSISLITNKVSSLHLDEKSAPKTKAKLPKKTIESVQEKISEV
INIKEDTTLKVPEKRGTPEAKEPSSDPPVDENDSVDFSRLLEAQIASEAAAQQRQLRTLTWEDVCPPGDRIDKIAEASYAEVY
RVTNERGTSIIKVIRLPSPIKPQTKAQIRSGLVDEEPHPEEDVQGELQISEWLADIPGFVVYKERYVVQGKTTRQLLETHQSF
QKKMKRQDPDRAQFYPSPSRYLDDTRFLVVELGDAGTSLEDWKLTSESQLWDIFFLQAIALARAEDLVMFEHRDLHEGNLCIR
QVKPPRDMGPPSAGFFGFSGLDITILDYGLSRGEDLSIEDAKPVAFDLERDLSLFTSTHAAQCKVYRQMRSFLLRADRTCLPP
EAHDTPYAEGIDGQLSWDAYAPYSNVLWLAYLYEYLISHFKGDKKELARFRKETREMWNYLDPDAGDELPCFSCAADVVCFAV
EAGWVRQEQLNGVDESLIEREDSIIGVRDDIKDVKQESAPARRSTRRQ
FUN_010871 CDS scaffold_4 199333 200729 +
hypothetical protein ENOG41

MSSSTGPSGLPNSLLSTSISSLASSTSSRHPSQISKTYRQASTLFLTRRLPEALTTLLPLITPPEHVDDGEPAPVARASRTTR
IKVWSLYLTILNGIVELEPEEGKDAFGNQEWRALCTKVREGEIWDEVVTNGYHGVEGDVDSDVVINLATILLAHARDQKLNQH
KLEAYLAASNTPNLDVAGRLAESTSSPMSNRYRSPAKGASGANTPRDLNARVKILELYTLHVLLRNNEWDYAREFISVSSVLD
EERREAFLQALQSLHDEQQEQERLENEERQRQENELKREIEEAKRLRAENEEKERKRIEEERLKREGSEVDYGIDDTASHTGS
TRPRKARAPSSSSSRQSRPRGTNQAGPKANGKAVSQAPTLTSRASMVIAQLRTIIEHLGSSLNSNPMFLFKFLAFVMGLILML
SNANFRERVKRILAVFWSKVKGTAGMGVKVSYI
FUN_010872 CDS scaffold_4 201010 201464 - PHF5A PHD
finger-like domain-containing protein 5A EOG09265DWT PF03660

MSRHHPDLVMCRKQAGIAIGRLCDKCDGKCPVCDSYVRPTTLVRICDECSFGNYQNKCVVCGGEGISDAFYCFECTRLEKDRD
GCPKIINLGSSRTDLFYQKKTNRTTQY
FUN_010873 CDS scaffold_4 201838 202880 +
hypothetical protein PF04696 ENOG41

METRNELRPLDDTKADHEQHNDTLSEDAPNKDSMKRKASINADELSSKRTKHDDYFREAPSEPRRRNSSPTRHNSYGDSPDID
VDRRKNATQEEKRRGKRLFGGLLSTLSQTGGSVQQKRRLEIERRQQDRLQKQNIEEDRLREEKRLRLTEVRRGEQLTFDEEVM
HSKHTKMLAMARYLRTKSRPHIYYLPWKLTAEQEDTIDDQIQQTKVTIEREVESLNARKGRQAKGGRQPSETTAPSRQELDHD
SKDTNGPEKSPHGQEKPLQASNHDHHHDESADVLEEADEDMVIY
FUN_010874 CDS scaffold_4 203714 204749 +
hypothetical protein PF00561;PF12146;PF12697
S33
MAPDKLVPNDSRVKTETAQIRGKNYKYIVGEPEGTPLETIVLIHGFPDLGFGWRYQVPYLMSLGFRVIVPDMVGYGGTDAPES
LEEYTYKSLSADINELARKYVGEDGQIILGGHDWGGAIVWKAACWYPQLIKGVFSVCTPYMQPRETFVPLEAIIQSGHLLNFS
YQLQFKGPEVQSRLQGEEKIRQFLNALYGGRTPSGDLGFSAKDGVYFDKLPELGPSPLVSKEEMDYYAKEYASKEPPELRGPL
NYYRMQELNHRDDAEVAKKGGHKFEMPALFITATDDSALPPSMSKGMDSAFTNLTREEVKASHWALWQASEAVNQHITKWLDV
VLDGALKAKASL
FUN_010875 CDS scaffold_4 205606 207002 +

hypothetical protein PF07946 ENOG41

MASFLKNAFGGAKAPEPASPDSDFADFAEAPNPAPEAATQDATAGSAAAAATAVPYTKWYNVHERHSLSEFRLEGIILLISSF
IFLLHMIGARRNRSRAKAWIRAHAPVLQKEYALVGFGGVPTLDNQDVNPDTLIKEKSLFEFATYATGRQNTAFTDFKLTLTKK
FNPIVNGFEHVASFFVETVDAPKDAVEVLTYPFDGKESLTVPSAPGASEIRAKDAKSTYDGFVWGVVHKDVMRRVRDDRYDVS
LTFTKDNPKLPAWLTVMSESAEITDTLLTPELIAAVEAAGDLFEYLIISDQPVDKPATLEETTPRKRLFLKYALPSDGNYDNL
LPLFSHFLQLPDVLVKVAHFRPEVSKKVRSTREHAISELKKTAESQRQEELLLEKEKARKAKRDAELKGLDAKAQKRYLEKER
EKEMRRSQKRQTQRA
FUN_010876 CDS scaffold_4 207949 210719 -
hypothetical protein PF00172;PF04082 ENOG41

MDKQQQTVQKSRQRREAERERKRALRACDGCRRQKEKCDGGVPCRRCTRLHRQCEFLGPTARDAEGGDGASRNRSGPGNAELL
QRASYMEKIITHYAGSVSLDTESLKAMAESIDNKNFDANQVQRQAPIMDSPGSDFLGAEDENYTVQPLDNNTTHYSGEFSHWN
FSMRIKQWIEQCVPERHDAPGPLSFKEYYRAEELQSANNTQASLSALPPRYVAEFLVQAFYKHAETNYFYVERGWLAEQIDLI
YQNPGALSRREVGTLCMIWIIFAIGTQYAYLDSISGRDAHGKSNDSSPFSEDTIGVMFYQQACKLVPDVITVASLESVQAILL
IGIYTLPLDASGLSYIYLNLAVKLAIQNGMHRKWPAGGLDPYVRETRNRVWWTAYTTEKRVGIYHGRPLSILSKDVDAEMPTD
RPDIMPSSPTNVAHMLATLRLNQALGKIAHEISVLRTAQKHEMNEGLQRVIDMKEELRRWWDALPETAFHEEVNPQNPITRAD
THLKLEYCLLRMYAGRPFILPREVVRGNASTSSSPADSNNQRPNTPHKSNPRSILVADCVEAALTIIDTCHLLQSTIGLARAS
YTEFSSCRAALLVIITQCLQKKTDRLRDALRTGMAMIKEMSAGGESARSEASLIEVFERAISRLDATADPSGRETDYSRFKKW
ELLWKNDVPLQDFRQEESPETSLPLPQNTNAFWRGSTSATARPGMPTMQAPSPFVGMDTNFPSVPQVMDEFSTLFGYGFGPSP
DSMAGTANGGMWMGP
FUN_010877 CDS scaffold_4 215114 216547 -
hypothetical protein PF00202

MPSFVESPVRTEIPGPVSKASSKRLDAIFDARAVHFVVDYDKSHDNYIVDVDGNKYLDVYAQIASIPVGYNNDTLIEAAKSPE
MISALVNRPAIGNFPSDQWVDILRNGLMKVAPKGLSYIFTAQSGSEANELAYKAAFMLYSRRKRGDADWNEEEINSCLENSKP
GSPELAIMSFKNSFHGRGFGSLSTTRSKAVHKLDIPSFNWPQAPFPALKYPLEEHVEENAAEEKRCLEEVERIMTTWHCPVAG
LIVEPIQSEGGDNHASPAFFQGLRDITKKHGSVLIADEVQTGFGATGSFWGHDHWNLTSPPDMVTFSKKAQTAGYFFGNEMLI
PDKAYRQFNTWIGDPARVIMCKAVIQEILDKKLVEQTARVGTHLYAELARLAAKYPEHIQNLRGKDQGTFIAFDTKDPAGLVR
SMRHIGVNIGTCGKNTVRLRPMLIFQEEHIPILINAFDKVIGAL
FUN_010878 CDS scaffold_4 217962 219660 +
hypothetical protein PF00171

MTAPKLTDPTLIKNACYVNGEWVAAKSGKDFAVENPSTLEKIGNCPEFDASDTEAAIAAADAAYKTYRKTPARQRARYLRRWY
DLMMENSEDIARLITLENGKPLADGRTEANYAAAFFEWFSEEAPRIYGETIEATNPSCRLSTIKQPVGVCGLIAPWNFPAAMI
TRKAGPALAAGCTVVIKAPAEAPLTALALAELAHRAGIPAGVVNVITALDNTAEVGKVLTTHPKVKKVSFTGSTGVGRLLMNQ
SSSTIKKLSFELGGNAPFIVFEDADLDKAVKGVIACKFRNSGQTCVCANRILVHRSIYDKFVQMVLDVVKTFVVGDGFGEKTT
HGPLIHGRAVAKAAEHVEDALSKGAKLIHGGERIPHLGPNYFDLTMLTGMKPGMKICSEETFGPVAAFFAFDNEDEAIELAND
SDVGLGGYFFSNNVNRCYRVAEALEVGMVGVNCGVLSDPAAPFGGIKQSGFGREGSKYGIDEFTITKMVMTNIDP
FUN_010879 CDS scaffold_4 221918 222511 -
hypothetical protein PF01910 ENOG41

MDYESIATPESCYVDFCLLPIGTGTVSVAEDIAEVQKVLKASGLKYTLHSAGTTVEGSWNEVMAAVGKAHAVVHRRGVVRIQS
SMRVGSRTDKKQTAEDKVKRVENLLAKDESK
FUN_010880 CDS scaffold_4 223081 224170 -
hypothetical protein ENOG41

MLQSSLLSVVAGVLAATSTVYALPQSNSGSGSGSSTACNNSPDLCSRSYNNITHMGAHGSSFLRDGNNGLAAAGNQNFNATDA
LDAGLRLLQAQVHKENNTLRLCHTSCGILDAGPLEDWLTKINVWMKANKNEVVTLLLVNSDDAKPDEFGQAINGSGIAELAYA
PATQEPTSEWPTLKSMIDNSTRLVTFVTNIDASTQYPYLMPEFDYVFETAFEVPSLTGFNCTVDRPSKIKDGATAMASNYMGL

VNHFKYQSLSDNSDLFVPDTENIDTVNSDGTSEDGQLGKHLQECRQEWGAVPNFVLVDFFEKGQVLAATDKMNGISDATGREE
VSDESIGSTTDRQVGMVALTAFVAAAVLLV
FUN_010881 CDS scaffold_4 225660 227213 -
hypothetical protein EOG09262SMG PF03234;PF08564;PF08565

MPVDYSKWDALELSDDSDIEVHPNVDKRSFIRAKQNQIHQERQHRKLQIETYKYERVVNDGLLKRISGLLASLRAHASEAATR
NPAEVAFQAVMESAGDPKDDKPPSPPEGVHTQEKEQPSYTKMMATLLDQVNKALDEKKPENRYEAMISEIQSHEDKVMDLQKQ
LVAKLEELQKEEGRKITSEGIHTGFDSSHVAKSKPEEKKDSTQVELLNPSFAETSSSSASGNQDVKVDDDSDIESSPAGKKFG
TIRANDYAASLQFLSQNPQLLVERETDGLLVQAFDAALENKDELSRQLVHQALLLQYCRALGKDGVGLFFKRITTKGHQAQDV
FYKDVQDTYMKIRTRSREILAERAKEGQAEGVEQIQLHAVEPGTVINIKVPPADSEDPEEQEARKIFESFKPEMRKALETGNL
DEVNKVLGEMKIDEAEELVGLFGEANILSLEEEIIDATTEEGKQQLKEIEAAASVDKKEEYGDPE
FUN_010882 CDS scaffold_4 227713 233576 - MOT1
TATA-binding protein-associated factor mot1 EOG092602I6
PF00176;PF00271;PF12054

MASRLDRLVTILETGSTRLIRDTAVNQLADWQKQHPDELFNLLSRVVPYLRHKEWETRRTAASAIGKIVEHAPVYDPNSEDAP
EEGKKEEPVPENGHIKKEEEDDAKKLPSDDGLFKLESLNVEMILKYGKELLRGGGIEYGLAALDPQARLTHQKKTLNGRLGLL
GRKYEDDEIAYTGGDNLTAPMTPMDTTNGHGHARPDGSGAQSQAPEESQLSSRQLNVLKRKRKREAMKASQGKGGFGDLSVRR
SMTSGSDNLGDDTAMPDGDTKKNSKVNEYFNLDRPADVDEDTKVVSEFKGPVVPIKSELEAEETMEGAEWPYDRLCDFLKVDL
FDPSWETRHGAAMGLREVIRVHGGGAGKLRDKTRKENDVLNQKWLDDMACRLCCVLMLDRFTDYSSDTSVAPIRETIGQSLGS
VLKHLPSPSVYSTFKILYRMVMQEDLKLDRPVWSVCHGGMIGLRYVVAVRKDLLLQDSDMIDGIIKTVMKGLGDMDDDVRSVS
AATLIPMAKEFVTLRPTQLEGLVNIIWESLSNLGDDLSASTGRIMDLLATLCGFPEVLEAMKASAAQDEERSFTLLVPRLYPF
LRHTITSVRVAVLKALSTFAKLDAETSQGWLNGRILRLIFQNILVERDRDALNMSLDLWTSLVESLASKPAVLADEFTAHIDP
MMQLTLHPIGVSRNPIPMNASLFQKPSGGTYTMPGVTQHTPRKPSSPDGSDRAPKRRRKSTKVDDTPTASLTHDVDGHMMQGD
VDLVGMDVLIRSRVSAAKAMGFIMSRVPVASLDDYDALLIPGLGSAFSSSQMTACVIIDEFAMNSQHLGDSPRYLENLQRIID
SERPAAYRDLVNFIQRVRTQCQQLIHLFRDHGKVSHSKLPTLPVVVQGEAEAGPSAFSIATAEKCIGDDYEKLKKAMPPGQRL
IASQQLSDARDMTILAIEEAKTVKAARDVRVKAAAACAMVGMKVLPKKPSPLIKGIMDSVKTEENQKLQIRSADTIARLVQLF
TEKGRKGPADKVVSNLVKFSCVEVAETPEFPVHASKTDCVLSMQKEEDRVDHPDAAKWAREAKAARITRRGAKEALEILSRTY
GASLLETVPSLRTFMEDPLVRAFSLDTLPGEAKDPEQTFGQEIVDAMSVIRTMTPTLDKALQPFIMQMMPLVIKALHSELSVF
RYMAAKCMATICSVMTVEGMTALVEKVLPSINNPVDLNFRQGAIEAIYHLIAVMGDAILPYVIFLIVPVLGRMSDSDNEIRLI
ATTSFATLVKLVPLEAGIPDPPGLSEELLKGRDRERTFIAQLLDPKKVEQFQIPVAIKAELRSYQQEGVNWLNFLNKYHLHGI
LCDDMGLGKTLQTLCIVASDHHQRAEEFAKTQAPDVRKLPSLIVCPPTLSGHWQQEIKTYAPFLSVTAYVGPPAERKAMKDRL
GETDIIVTSYDVCRNDAEVLDKHSWNYVVLDEGHLIKNPKAKITQAVKKLASNHRLILTGTPIQNNVLELWSLFDFLMPGFLG
AEKVFLDRFAKPIAASRFSKASSKEQEAGALAIEALHKQVLPFLLRRLKEEVLNDLPPKILQNYYCDLSDLQQKLFEDFTKKQ
GKKIQAEAGREDKEAKQHIFQALQYMRKLCNSPAMVMKPGSTLYDETQKILAKQGTSIEDAQHAPKLTALRDLLVDCGIGVEG
SDSNDPLYQPIKPHRALIFCQMKEMLDMVQNKVLKELLPSVSHLRLDGSVEANKRQDIVNKFNSDPSYDVLLLTTSVGGLGLN
LTGADTVIFVEHDWNPQKDLQAMDRAHRIGQKKVVNVYRLITRGTLEEKILNLQRFKIDVASTVVNQQNAGLSTMDTDQILDL
FNVGDAAPNLLADKEKNNIDGREEDMVDIETGDVRVPGKKAWLDDLGDLWDNKQYEESFDLDDFMKTMS
FUN_010883 CDS scaffold_4 236000 239768 +
hypothetical protein PF02463;PF06470;PF13175;PF13304;PF13476

MYIKQIIIQGFKSYKDQTVIEPFSPKTNVIVGRNGSGKSNFFAAIRFVLSDAYTQMSREERQGLLHEGSGSAVMSAYVEIIFD
NSDDRFPTGNKDVILRRTIGLKKDEYSVDRKVVTKADVMNLLEAAGFSRSNPYYIVPQGRVTALTNMKESDRLNLLKEVAGTQ
VYETRRAESLKIMHETNNKREKIDELLEYIKERLSELEEEKEELRAFQDKDRERRCLEYAYYHNIQLGIQANLDELDNVRQDG
IDSSDTNRAEYTEGEKAISRLDSEIHKLQREMELLQIERRQVEEDRRDGAKALAKAEMKVKNLREGQSAQEQARAQHAAELES
VQNEIASKEQQLSTINPAYNQKKQEEDEIRRQLDHAEATRNRLFAKQSRGSQFRNKSERDTWLRKEIQELELNISTQKANKID
ADEEVERVRESIAQAEQDVADLRNRLANFSGEKTALEEEVAKARDIIDKLNDERKLVRREDDKLNSVIANARQEKETAERELA
HAMDGSTARGLATIRRLKQERDIPGAYGTLAELLEVSDAYRLPVEQIAGASLFHYVVNNADTATYLADTLYRQQGGRVTFMPL
AQLRPRQIKLPRSNDAVPLLSKINYNEEYEKAFQQVFGKAVVCPNLTVASQYARSHGVDGITPEGDTTNKRGAMTGGYIDPRK
SRLHAVQAVNKWRDEYERLLAQSRDIRKQTELKDQEITAAMSDLQKANERLRQAVDGFEPLKHELINKSKHLEKELSHLDAAI

KRRDAVEKNMNSFLEDLAAHEAELRSDFKKTLTAAEERQLEELGTSTQELQKQWNELSRARRDLERQKQLLEVDLRQNLQMKL
DQLNSQAFEDSTGSSGGGLKDAQRELKKAQKVQKAVEASLQELETKMDNTQARLEELANEKAQLEQAQSEISARIERQQKKMD
KSLRKKAVLSTQAAECAQTIRDLGVLPEEAFDKYENMDPNQVSTKIKKVNEALKKYKHVNKKAFEQYNNFTTQQDQLMKRRKE
LDDSQESIEVLVEHLDRRKDEAIERTFKQVSKEFTTIFGKLVPAGHGRLLIQRRADRRQEPVDESDGEARGVENYTGVGISVS
FNSKHLDEQQKIQQLSGGQKSLCALCLIFALQATESSPMVIFDEVDANLDAQYRTAVAALLESISKEIGTQFICTTFRPEIVH
VADRCYGVTFRNKTSSIDCVSTEQALEFVEGQAKPT
FUN_010884 CDS scaffold_4 240198 241370 -
hypothetical protein EOG09263FAK PF11788

MTASSRGSKAAQAVLSTSPNLCTRCASSSSVSRYPARLYSSLAAAVAKTPTDTSTPAPARSPPPYDVRSGVILTRPPLVTRKL
HPFENAFFFYQKRLEERLNTPFITSIYFKPDTARRLDWNIKVQERKGTVAKELGVYNGKSSKAWDDELKVGDELSNQETIVKS
LLRDAEARVSDDAEIIAPEDVIPVEPPADRVTEADRKGDVQRLDRQLDRTLYLVVKGKDGWGFPADVIPKDENLHESAKRVLD
QAAGVNMNTWIVSRVPVAHVVSRPKMTADGVVEKKGEKTFFIKGRIMAGQADLKNNPFGYTEFKWLTREELEKELPKDYWKGV
RNMMTDR
FUN_010885 CDS scaffold_4 242240 244326 -
hypothetical protein PF00097;PF00628;PF12678;PF13639;PF13831
ENOG41
MSEAEQCIICLDPLPRPSAALQAPAALTVAASDAAGGVSGGEPASAPESAPVPTTTSTASDVLENDSNYLNIVASLDGCNHII
HDACIRSWAQKTNTCPICRNPFHSVRVYNGVDGTAISKYDVQDKKQVAEFDVRQWLGENPEDEEEEEQGNPCPICNSSEREDV
LLLCDSCDAAYHTHCIGLDAIPDGDWYCMECSHLFQLVEEPRTTESGEQSPRPSYVRRPNPRNVRGYHVRTRARLRRARRQAR
NVEWQGAWGQFSGRFYEMSDLDLDNHDDEDEELEQYRRFQELGRRELERWQQRMDIARRLGARDVFINNIPSAISERLQSAPE
PVQETADERRAWGALERARETDEPSTTSRKRKARSVTASPREPVQEPERKLKRPRTRRLPTQNGEGSSSVSSPAPAGPSSTRA
VPNGSSSRNGTVDHSSIDPPLVSSLLKELEPSIMSEDETMTTNGWRHAPEASSPALSPAISPSPSNYSSPRALSLTPPPMPNF
NGRPTSPTLSLSSTIQPRYAPANYSPTRSNRDHSDSETRSTRPHANYSPTHTSNDQSDSETRSVKVPESRPLELRQPRPRRPS
EVPAQKEEQPSADSNITQEDKISINEIVKGALRPHWRSRKLTTEQYSVINRDISRKLYDEVKGAASLSDEARRQWENRATKEV
AQAVAELSA
FUN_010886 CDS scaffold_4 246531 247193 - RPL43 60S
ribosomal protein L43 PF01780

MTKRTKKVGVTGKYGTRYGASLRKQVKKMEVTQHAKYTCTFCGKVTVKRQATGIWDCKSCKRTVAGGAYTVATPAAAAMRSTL
RRLREIAEV
FUN_010887 CDS scaffold_4 247988 249495 +
hypothetical protein PF03435 ENOG41

MPLVKNHGRQYDIVVLGATGYTGKLTAEYISMHLATDLKWAVSGRNESKLKAVVEECRELNPDRLPPAIEVVNLNDADLSVLA
KKTCIIVTTIGPYSLYGEHAYKACAEAGTHYVDVTGEAAWVHKMIKTYEATAKQTGAILIPQAGIESAPADLITWTMAKAIRT
DLGSQTRDVVVSLHKINSAPSGGTLATALSIWDVFSLQEIKEASSPYAQSPIPRNNEPTRPRSSIFQMIFGVRTIPNLGLQTT
SVTNSTDVAVVERTWGLLSSTPSRKDEFYGPNFVWVEHMKARNWLHGIFIHWLLIVGGILLVSVAPLRSFLKKRVYQPGEGAK
REDTAKDELEYRGIAYPDSEKAAGKAAFCRMWYHGGMYFLTGMLLAEIAATILEDDIELDGGSYTPACLGQGLVDRLDKSGFR
TDVKIIDA
FUN_010888 CDS scaffold_4 250418 252112 +
hypothetical protein PF02582 ENOG41

MDPHTTSSDAVLNESSPLLPTTENRPKGTRSVTFSDNPVTKTFEPTRQQFQHRSPGHHAVGSVSGAAGGPPMLTALNNKLRRR
NSQGSVPAVANLSFGPKIGPQRSTKKTEKLKLLPNTDLDDPEDEESGRDVYSQYTRIKDPAARRDAAKLGKADRDRLPRVTAY
CTANRYEMEALMRFLKGRGKTRGANPKLIDECIYTPYNYTAKNARSRDPVQNYERRHSTGEEAGSEAHGHNGHAHNDANGNGN
GSHIDSADLLGSAIEDHDSISETNPDFDTQVHTPEVFLFDYGVVVIWGMTEAQEARFLKEIAKFETEKLAPDDVETELFNFYY
TKDYQARIYNDFITLRDKNNYMTKLAISHALAQSVKTSLFEELIASTVDTCKDIPTQIATTGKIALRRSQINMQIGELFILRI
NIHLNGSVLDTPELFWVEPQLEPVYQAVRSYLEMDQRVGLLTERLDVIADLLAVLKGELSHGHDEKLEWIVIVLIAAEILVAA
VNIVVDLYVGE

FUN_010889 CDS scaffold_4 252476 253455 -
hypothetical protein PF00505 ENOG41

MPITLKRKASEADLPPRTPAIPSTLPPSVEEAYRRKCVQLKNRTNEVEDANDAARLRLARIKRQVEKLRIERAFLLEQLAKRT
SANVEDSDGSPSPPPTDYQFTIPSELANHQKPKDKPLRTKRGHRKSMMPDSDSKAPVAASFTNTAGSPTSETFSHPPESQPKP
AHANGISASDSKKPTSAFELFCLETRPGLENKEKDNEIPVNIEDELTRIWKGLSDGEKDEYEAKFELAMAKSDESKKSPASDK
KDDKPAESKQLVQDEDIEMGDDTEDQDTQAGEKVEE
FUN_010890 CDS scaffold_4 254669 256552 + CCT8 T-complex
protein 1 subunit theta PF00118

MSLNIPNAPNAGLFKQGYNNYDSEDGAVLRNIDACRAIASTVQTSLGPYGRNKIVINHLQKMILTSDAATILRELDVVHPAAK
LLVMASQQQEAEMGDATNLVIVLAGELLRKAEDLLRMGLKTSDIVIGYEKAQKFALETLEELAVDKVEDMRDQEELSKAISTV
IASKQNGNETFLADLVAEAVLNVLPKNPANFNVDNIRVVKIMGGSLEQSRVVKGMVFPKEPDGSVKKASRAKVGVFTCPIDAG
QTETKGTVLLHNAKEMMDFTKGEESQLETSIKELYDSGLRVVVCGERVGDLAMHYLNRFGILCIRILSKFELRRVCRVVGATP
LARLGAPMPDEMGSIDVVETLEIGGDRVTVFRQEDEVTRTATLVLRGATQNHLDDIERAVDDGVNVVKAITRDPRLVPGAGAT
EVELVERIQAHGEKTPGLSQYAIKKYGEAFEVVPRTIAESAGLDATEVLSRLYAAHANSDAWDIGVDIENDDNTGTIDAKDEG
ILDLLISKQWAIKLATEAARTVLSVDQIIVARQAGGPKPPGPNPNWDED
FUN_010891 CDS scaffold_4 257532 261062 -
hypothetical protein PF00092;PF13768;PF15411 ENOG41

MFRRLRKNQDHNTTSSQGRDDDKAIATNLRELGLVGSRASSRRPRTGTDSKPFTEGHSIKAKSLRGQDSVSSAGSVRSGVSNA
SRRVGSIDNNVMATNADRSRSRRERTFVGSECAVCEEPLEHTLRGERILQFSCSHVSHEACFYEFIREFESQYCPTCDAPLHL
DTSRGGNVIDIEKISNMVRSVGTDSRSAMTPTPTATPWDNQPARAPSVESNQRRPVQQQHVGGRDSVARGSMRDSRDAPLSDR
YGPSRHARSDSEATGVASSGGYPETTQSGPARRHDYDVQAMETTPASPRPMTRNPIPAPIVTVRSEFPTLNRSRQQQTLTCLV
TVEVPDNNWRPDPEDLGAAAPLPPPPTTNRAEEAYGRPPSPARSAPRFYPYESTEVLEEMTDSLRNRVDNWHGLDFSRFGKLR
LYGTLRVGKDKVSWQELECFLFAEMLICVKEKKIPQAQQWDENGAPRKTTRCTLKGSILIKKHLNEVSETGNIDENILTLSLS
VAELPQFHLRFENRNQLKLWQQALLDLNAVETSPVRSPEYDRGESETDEELEWQRSRPQRVSSVASSWGGARSTTTAPTDYTN
FPRSPAMAKSVHVPIDVVVVVPISSSMQGVKINLVRDALKFMVNTLGERDRMGLVTFGSGGGGVPIVGMTTKAWPGWSNILTS
IKPVGQKSHRADVVEGANVAMDLLMQRKYNNPIASIMLISDASTSDADSVDFVVSRAEAAKITIHSFGLGMTHKPDTMIELST
RTKASYTYVKDWMMLRECLAGCLGSMQTLSHQNVKLKLKLPEGSPAKFHKISGALQITKRATGRDAEASLGDLRFGDKRDVLV
QLVILPDNTSQEQLPQDPWDNIVSGLEALGGPMDNDNERTLSVEEVPLIQADLSWGDILREGTVTHMPRPSLLAITMLPVSGG
SKKAWQNSPPIPPHPSIVQRRMELLTSDMLTRALTLVSRGQHDRAHTLLNETRSILKGLGKGGLPPIPPPSKSTPSAPSTPHP
ANDASPGQSTTPDRKRSPSPPASAISGVNGLPSSQLGRSRSNDGLGLGAGIDANTVAALDAELESSLEWINHPAVFSRDSRKA
VLQAIGVISSQRAFTYRTPIEGLWACRVSGVKKLTEKSREWREDGGGEGGITEES
FUN_010892 CDS scaffold_4 268633 269516 -
hypothetical protein PF08297

MAPQTRKRNYNQSTTKDTSVEKPTSEEKRKLPVRAKDGDSSDGATPISTESKGTKVVFGDDDNDNAAPPPVVVPKPAAPVEEE
DEEDNSDDEAPEAVSTSKVASDIKKSNQAAQKAAQEEAAAQKRKRRERDDFFKQQAEERKKFEEQEKAGGDEKASVEEEPAQS
LIQRSKAEKAVNLLPAEFLTDSSEDEADGIDDQPEERPRKRRVATVERSLARQERGPRDERVGSTVYRVAKKTDERMAPKLKK
HAKSSKDILLKRNRSVTKQRSGFFVR
FUN_010893 CDS scaffold_4 269935 271066 +
hypothetical protein PF12895;PF14559 ENOG41

MSTAKLHVKPRNVYQLLRGDFTLPLAPVAQSSPHRAALSTSRISSSIGYQTRSYATPKGARKPLRPPPRAPKLVKTQPSSSLQ
TDFDLSQISPMDFELAMRASENQVGTLRPDEYYEYASKFSNAIRKGAYPSRVNLSENQIPVEVAHEMACLLWLFRRMKAHSSF
SMALWASASELNYNPAVVSLVSQLFASGSWRKITAFADVENRFMKLVAEAKNCNALTVYGEYLFQDGKYDQAVAILNQALDVD
DGVFEWKRKCLTCLAKSYAKLGKVHEAKETLELLGDPEADAELDQLLRSSDAEMTRQQLYTDAVKGKHDLYSQLAEVEFERET
KEMDVELKKNHHLWGLEWSRLADPGAKF
FUN_010894 CDS scaffold_4 271235 273208 -

hypothetical protein PF00875;PF03441

MAKPRVIYWFRTDLRLHDSPALKAALDLDPAVLWPIFTWDPHYVYRARGGLNRWQFLLDCQNDLSRSISQVNPKSKLFVLREA
PQTLFPKLFKAWKVTHLVFEKDTDSYGRERDSVVVQAAKDAGVEVLVRSGRTLWDSDQIVEKHGGKPTMSITQLQTAGSKLGE
IRKPIPAPKHLPDPGDMPVNFEQDEPSTKPDFNAGFRTEGDKAYTRIAGPNDDFAIETMEELGFPPATTPHRGGETRALKELN
KLIADKKYTATFQKPKTNPAQFEPQATTLLSPFLHFGALSVRLFYWRVREIVDSYGKGASTPPESLIGQLLFRDMYFAAQAAL
GYVFSQTANNPYCRFIPWHLPSKRDSETGLITGEYHIDSEEAEIWFRRWRVGMTGFPWIDALMRQLKDEGWIHHLGRHAVACF
LTRGGCYIDWERGCEVFEEWLIDHEPACNAGNWQWLSCTAFFSQYFRCYSPIAFGQKWDKEGNFIRRYVPELKNMDSKYIYEP
WKAPLPDQKKAGVRIKGDGLNSTEEGTYPKPMFDFAKRRDVCISAMKTAYQVGLHGNDGQALDGTWRKLFPTSRGEIQGDIES
DNGEHADYADDEGDREDNEAKEKGEGIKSIENGDRAMSKRSGRRHSNENTTKKQKT
FUN_010895 CDS scaffold_4 279834 281193 +
hypothetical protein ENOG41

MAVIAVLIVLTIHTPKESSGRTLSKVSVALEVVTLVILCWFTSTYVSTSFRRWSTRCLGVDFGACLLAILFATVATVATLIQF
SKSKFENEDGFTGSSKSSLLVGLSASLGVCFFLQTGFLSVHFFLTRQSDQKALSLHTLEEDRGLPSFRSKSSLKSIPYSHTAS
DHDTAPRVMNSMDSQTPTPSIRGRSRSGTATSIKVQLSNAIRSATVRSHVSSSEFRRPISTDSTIYQSSEDAFDTWDTSSVDT
NNRQVVMEATTPPQQTQGHFLETIPGSPSPSRTSTPNNDMTLEPPRIRMRSRSFSPVTITREQMSPQTSTTDLPEPSLSESHI
HPLFRSDSPTPPPMATPGTVVLASPNAGQVITHRQSVRSLRSASITVGPSPLSHRDEHESIREEQESETDSLTTIDRPLTPPI
PEWVMGAGARSSWSDYNNRKSRIDNAGDGESKKQEKN
FUN_010896 CDS scaffold_4 282482 284040 +
hypothetical protein EOG09264CST PF00240;PF00627;PF09280;PF11976

MKVTFKDLKQQKFTLDVEPSELISAVKEKISAEKGWEPKLQKLIYSGKILKDEETVGSYNIEEKGFVVCMVNKPKPKPEPKAA
ESSAPPATPAQAVANTPAAPAAPAHSSSHQTAVPATPTPQRSVDAGTGAQAEEPSGLAMGSQRTEAIANMEAMGFERSQIEAA
MRAAFNNPDRAVEYLLNGIPDNIRQEQQQREAAPAAPASQPAQPAASAQGASDEGGVNLFDLAAQRGGSGGRGGNGGNQAAAA
AAAAAAAGQGGDLGNLDFLRHNAQFQQLRQVVQQQPQMLEPILQQLGAGNPQLAELIATNPDQFLQLLGEYADDDTPLPPGAQ
AISVTEEERDAIERLCRLGFDRDQAIQAYFACDKNEELAANFLFDQPEDDEPAPNNNN
FUN_010897 CDS scaffold_4 285534 287268 -
hypothetical protein EOG09265C25 PF00249;PF15963

MSSIFKKKGGPAFKPKIPAARRPAAPVPSQPPKAPPAASPPHDSRDSEAQEVSHRSLETPSAKDPVSSPIESQIQDSRELGRQ
SPTPPSTIQEPAKPDEHAATEGFAQERPPEEKQSELANQNTERNPEKTGVPTTAAQATTSSEQEQSQQPPAQYSEIHSSSTDD
APSPVLQTPTPDDSEGPSGTDLSNTPSLPAADLNDTHEETSGEPQNVPKSTRRPRTRKQINQVNQDEVEREGGSKTKKRQRKT
DEQSAAPRQPRKRRTTQSGTSTPKNRRARSLTPEDSESQLVDLQKLKMADLTKDLHIGKKFSRHDELRERERRARMKIKLGVD
GERDSSATPEDGGQGGKIGSPAASSPAPSVSAPVAPSGPQFRIVDGQIVIDQSSLAVDRHARAAAAAGDMETVEENDFTRLIT
SNSFMNTSKLKGPNIWTDAETELFYRGLRMLGTDFEMISKMFPGKQRRHVKLKFNREERHCPQRVNAALIGEKTVKIDLEEYK
AFTGSQFEPVADIEAEQRKIQEEYEAEEKRRADEQAEIMRKRREELFKDDDEGDAKKKKRKKKQAVAYGLNGEPIAQEA
FUN_010898 CDS scaffold_4 287804 290388 + BRE1 E3
ubiquitin-protein ligase bre1 PF00097;PF08647;PF13445;PF13920;PF14447

MEDRKRPAISSADDIAPPSKRVAVNGSKAKDDSSDMKEESWIEAYTKGAIYRQMQEYSRKASTYESRLEELHKRSVHHDDHLR
LIDAWWRQVLEEMELLSDSEITSTTPSDPPYLSGVTFKDLHEFQKHLSDKGKTIKSRAEALLGRLASSRGKIDPDAAALENKV
ASLLATQKEYLFKLDRLKSEKDQLSEQLNAATLRYFKAEKKLDRAKSFQVQKLEQQAFANATRPSASGDGSAEAGETNGNAGE
LLLKYEEATAAATKQKEQLDVILAEIKTLQDENSTLKAKRETLTDEDFIRTDVFKQFKNQNEDLIKRINTLEATNKQLREEAE
KLQAERSMFRTQLEADANQVTQELEAEIISRDQDLARVRSARDELLAETTQHKARLEQDRASIDQVKALASAKEDRITALEAK
LSRLQPNEDQQAIRPDIEALTIDELRLHYTKLERDFNSINTEFPAMEKAYKKIIQIAQKKAMDTSAAEERMAILIAEKSKADQ
KYFAARKDADTRNNEIRSLRHQNSKSSEIIAQLKDLESQNRTLLGNLDKQLTDLKQANAALMTENKKMETASLDALRRTESLN
KQVADLTNLVKSKDAASAVVRERNTMQETEVEKMKVRLEHTQKDRDNWKNKALSNASEEEEMLRTFALCTICRINFKNTALKT
CGHLFCNQCVDDRISNRMRKCPTCSRAFDKMDVMPVHH
FUN_010899 CDS scaffold_4 290844 291744 -

hypothetical protein PF06682 ENOG41

MIPGSLAWLLALPAFALAARPKDAIKLSDVRSLTLRGNGAMTNHRRVGAIPQLRCVSKKALCELYDIDVMRCTNEGSGWGDED
IQWSCTASLPEELKLVTTDVICEGYNSPDDPYVLKGSCGVEYRVALTRKGERRYPNIANGGWFSDGRGGTDWGALLFTIIFVA
ILGWIIYSACYRAQEAGGNSNRPRRRRDGWSSGGWGPGWGPDGDDPPPPYPGSKPSSQSDTWRPGFWSGAAGGAAAGYWAGSR
NQQNHHNHHNDGYGSIGRGGSGWGGRSGSSSGSNNSNRHESTGFGSTSRR
FUN_010900 CDS scaffold_4 295558 300119 +
hypothetical protein PF00005;PF00664

MTEISEKTAAATSSSDPSRPSSSSHSGSGSTDKDAVRPHMKSSSDPNNEKLDLTKADSAVIVPPKAENIEDHYRHLPPDEAEV
LRRQVVSPEVKQGVAVLYRYASRNDILIILISGLCAIAGGAALPLMTVVFGNLQGVFQDFFVNRTLTSSAFNDKLVEFVLYFV
YLGIGEFIVVYISTVGFIWTGEHIAGKIRSHYLESCLRQNIGFFDQIGAGEVVTRITSDTNLIQDGISEKVSLTLAAVATFVS
AFIIGFIKYWKLTLILFSTVIALLINMGGGSSFILKYNRQSLEAYAHGGSLADEVISSIRNAVAFGTQERLARQYDAHLKNAE
YFGFRVKGAVACMIAGMMLVLYLNYGLAFWQGSKMLVDGETSLSNILTILMAVMIGAFNLGNVAPNIQAFTNAVAAAAKIFNT
IDRVSPLDSSSNEGEKLENIQGSIRLSKIKHIYPSRPEVTVMDDVSLEIPAGKVTALVGASGSGKSTIVGLVERFYDPVQGTV
YLDGHDISKLNLRWLRQQMALVSQEPTLFGTTIFNNIRHGLIGTKHEEASEEKQRELVIEAAKKANAHDFVSSLPEKYETNVG
ERGFLLSGGQKQRIAIARAIVSDPKILLLDEATSALDTKSEGVVQAALENASEGRTTITIAHRLSTIRDAHNIVVMSNGRIVE
QGTHNELLENKGPYSKLVSAQKIAAAETMTPEEQAAIDEKEVSLMRKMTSEKQAAIIADPNDDIAARLDRTSTTKSASSLALQ
GRKAEAEQKYGLWILIKLIASFNKREWGFMITGLIFSAICGGGNPTQAVFFAKQITTLSVPVTDQNRHQIKKDSDFWSAMYLM
LAFVQLFAFIIQGVLFAKCSERLVHRVRDRAFRAMLRQDVAFFDRDENTAGALTSFLSTETTHVAGLSGVTLGTLLMVGTTLI
AAIVLSLAIQWKLSLVCISLIPVLLGCGFFRFWILAKFQHRAKAAYDSSAGFASEAISAIRTVASLTREEDVLKTYRDSLAVQ
QRKSLISVLKSSTLYAASQSLLFACFAVGFYYGGTLIAKFELSMFQFFLCFMAIIFGAQSAGTIFSFAPDMGKAHHAAGELKK
LFDRQPVVDTWSDTGERLSQVEGTLEFRDVHFRYPTRPEQPVLRGLNLVVRPGQYIALVGASGCGKSTTIALLERFYDPLSGG
VFIDGHEISTLNINDYRSHIALVSQEPTLYQGTIKENILLGTAREDVSDKDVEFACREANIYDFIISLPDGFNTIVGSKGALL
SGGQKQRIAIARALIRDPKILLLDEATSALDSESEHVVQAALDKAAKGRTTIAVAHRLSTIQKADVIYVFDQGRIVEQGTHTE
LMKKKGRYAELVNLQSLEKQS
FUN_010901 CDS scaffold_4 303438 304563 +
hypothetical protein ENOG41

MASSSWSASTTTCSRPNSTSYRAPRLRIALSFLLLLACLTSTVLAGTETSSSSLRHRRHHALTINRYELHRRKDEDEVKEIVN
KETPSKTEGKSKATTVTIQRAAKTAAATNTPLPEAFDGNLSAELSTTSDSNCPAFLNGILSDPSYETCYPLSMMLQTSRGFFE
AQKQLLSIVRVLDATCAANVTYCTDFFDAAARNLTASENCKQEWESGNTIVKQFLYGMKAYEMMYKATCLQTPEDDMYCYANA
VTNTTTAANAYFYYLPYNLTLPGSTNPSCSWCIQETMNIFHAAAEDRSQPVALTYESAARQVNTLCGPDFVNSTLPPEETNLA
HIFAPSWVGLVLALGSAALVNAVL
FUN_010902 CDS scaffold_4 310446 311295 +
hypothetical protein ENOG41

MCAVPPFTEYHFNPAATTNGGPPHSNSPMVSPTEPRPDHMKIPDILTTGEGSGGRMSANNCSGDVRFRLQIRQQPIAARSCGF
GERDRRVIDPPPIVQLLIEGANLTKEETVKHLRYPHYVMSCSIYDESGSRDASFMPEEYRQQRRLMGLLVSAPFVGKDEHGEE
GCFFCFPDLSCRTPGSFRLHFALVKIDPIRAKEVKRFPTLVDDKSDVFTVYTAKDFPGMQASTKLTKRLKEQGCIISIKKGND
RSKNARSHDDSSDGEQDEGEASLQGKRRRRSAR
FUN_010903 CDS scaffold_4 318420 320082 +
hypothetical protein PF09362 ENOG41

MKSAFATAAAVLAGAASAAHETGTFAVLRFTNDQLTRGRMDPILFPGQTSTHVHHIMGGSGFSTKATGEDLQKSKCSNALVKG
DNSNYWFPTLYFHDPKSGEFEDVEFDYFNAYYFFEKTHDDIKPFPVGLQIVAGDAMTRTMPKAGSKPNLDPSKGPVNPARMTC
PRLNNDYTLPSWDAKSDGKMAGVGDPVNKGEGVGFPDRTCDGKYSPLRADVHFPSCYNPEAGLTDFKNNMAYPEDNDGYLDCP
KGWIHVPHLFYEAYWRTDKFAGRWTEGEGKQPFVFSNGDVTGYSSHADFMAGWDEDLLQHIIDTCNAGTEGMDHCPGLTYGLN
KGDCTIESEVDEKITGTLSKLPGNNPLSGFSYGDKAPSMPSGGDKESSPSKPSVQPSASKSASGEKTTAVELPGYTKPTSDST
GAHEEPVVPKPTLPSHPAVVSEAAAQATDVLSSAPKPTDIFDKPEKPKKCKTRVHTVWNTVTVTQTASVPVQTEAGTPSYKRD

HVRRHAHHHGSGHNHLRRRSHLRR
FUN_010904 CDS scaffold_4 320759 322623 +
hypothetical protein PF01189

MSLYHEAAEILAGSSFEGGSLKSRVFKKKNLKSAPNQVYALVLESCKWSLILKEVIERSELLKLERKLTPTLSLLLVHDLLLA
KKGIALPQGHGLRASIERHKGRISSEFKLARLRRKMPTLEALREQVERQCAGEEANYPRWVRVNAVKSTLEEQLETTFSKYTR
ATSIKEVVTNTGRLIYIDPHVPNLLAITAGIDLTKTEAYTTGKIILQDKASCFPAYLLDPRAEDGDLIDACSAPGNKTTHLAA
ILKEHMPESNAPEQTIYAFEKDSRRAQTLEKMVKIAGSKPVTKIGFGQDFLQVDPMAEKYKSVGALLLDPSCSGSGIVGRDSM
PDLHLPEGISSTGKGPAAKQNGRKRKHEQTETPEDRIMIDDDGNETAIKSEKDLETRLEALSSFQLTLLLHAFKFPSAKRITY
STCSIHIQENERVVTKALESDIAKQRGWRILLRKDQVSGLREWPVRGLPEACGGDEDIAEACIRSYKDDGQGVMGFFVAGFVR
PDVQNFGTTGNEGPYLRDDSGAIIRDMLGMPVLKTTGEHVSLTAKDDNSGDRKEEEEDEEEAEDSESESTSSTAAHDLNARQI
AVDARQGTDTDEDEWEGLGD
FUN_010905 CDS scaffold_4 324024 325973 + FUB11_2 Efflux pump
fub11 PF07690 ENOG41

MTVATQLQPPTSSASSTIDNATDVESQQINAKASHFNLILDQVGITDAVLNYDYTGHGTADSPYLVDFLPNDNRNALNFSRSK
KWLITILQAIATLAVTFASTAYSGGLSSILMHFHVSTEVAILGISMFVLGFAIGPLLWAPLSELYGRQIPFFVSFMALTAFNA
GAAGAPTMAALIVLRFFAGSFGSSPLTNAGGVIADMFHANERGLATALFAMAPFLGPTIGPIAGGFLGEHEGFRWVAGMSAIF
TGIVWIVNSLVIPETYAPYLLRRRAAVLSKRTGKVYISKIDVGRPQATIATQFKTAILRPWILLFKEPIVLLTSLYMAIIYGT
LYLCFAAFPIVFQQGRGWSPGIGGLAFTGIAVGMLFAVTGTILDNKRYARAAEAAGGHASPEARLPPSLVGSILIPVGLFWFA
WTNGKDVHWIVSIIGSAFFACGLVTVFLSLLNYLIDSYVIFAASVLAANSILRSLFGAAFPLFTTYMYNDLGIHWASSIPAFL
AVACVPFPFLFYKYGETIRMKCEFAAEAANVLQRMRTKHEVITEDQAAEEAHEAELERRASNALRRSLTKTRTSKSIH
FUN_010906 CDS scaffold_4 326782 329107 - MCM5
minichromosome maintenance protein 5
PF00493;PF07728;PF14551;PF17207;PF17855

MDRGSIYSAHVYEPSYAENGDTRMQLQTQLETFILDFRLDNNFVYRDQLRENALLKRYFCDVNINDLISFNEELAHRLTSEPA
EIIPLFENALKKCTHRIVFPHEPKAEIPDHQLLLHSNADDVSIRNLDSVTIARLVRVPGIVIGASVMSSKATGLHIQCRNCGH
TQNIPVLGGFTGVTLPRQCARSRVPNDPTPKCPLDPYFVVHEKSGFVDQQIIKLQEAPDQVPVGELPRHVLISADRYLTNRVV
PGSRCTVMGIFSIYQNKASKNSSNGGAVAIRTPYLRAVGIQTDIDQAAKGNATFSEEEEQEFLELSRRPDIYNVMADCIAPSI
YGNRDIKKAILCLLLGGSKKILPDGMKLRGDINVLLLGDPGTAKSQLLKFVEKAAPISIYTSGKGSSAAGLTASVQRDQSTRE
FYLEGGAMVLADGGVVCIDEFDKMRDEDRVAIHEAMEQQTISIAKAGITTILNARTSVLAAANPIFGRYDDMKTPGENIDFQT
TILSRFDMIFIVKDDHSREKDETMAKHVLSIQMNGRGAEDMTETEIPIDKMRRYITYCKTRCAPRLSSEAAEKLSSHFVSIRR
QVHAAEMEANTRSSIPITVRQLEAIVRITESLAKLTLSPIATEVHVDEAIRLFLCSTMDAVNQGSNQGSRELNDEVNRLETEL
KRRLPIGWSTSLSTLRREMVEGKGYSEQALNRALMVLQRRDTIMFRNQGAQVYRNGA
FUN_010907 CDS scaffold_4 329663 333133 +
hypothetical protein EOG09260WCZ PF07928

MFSPSSPNATKSADNLAALSPTGRGELPFHSNNHRHTQMHPHPSSRRGSTASSIHSVGGILDSAPSNLAASVYESSQNAISTL
LQPPIVRTGLQPHTSAPASTAHKPPTARDIPPVTLTNITKVDAEEFKPYLSQIGSLYEQLQRLKESDEEANKEQRPGRQDTPT
ESSGDGYLRPGPKPRPTRKGSIASLSSMNSNDGHSPVRRPSAGFTRRSTHGPPPLSTIPTVYFDDEFHLENPRTFDIVSERSE
VIRPANADDGQSSLNGSAATPRKALATNAILQEKLSWYMDTIEVHLINSISTASTTFFTALGSLKELHSEAAESVGRIKTLRK
ELKALDEEIATRGLQIVYQRRRQENLRQLNDAVLQLRQIADGLASCETLVDEGEVDKALANIDSLEKLIAGERDATAANEPGI
QLRDLRSASALQGVHADLATLRSRIGKAYETNFQNILIRDLRRHAESVSSQEILLRWSNAASRSKGSHARSPSVFPTYLTNTD
ELKTELLPNMIGLHRAKHIAEATVAYRESVLREIRNLIRRPLPSSTDDDNESMMSVSTASGGRHLSNQEKSSILARNLRSLDP
EDGEDLLIKIYIGVTETLRRLTTQVKVLLEITSSLTENPQGEGLMSPTVRSPISSPRPDRFPSNPMNSATFEVQEELHKSLDV
TNLLGQAVDVAIEKIVKVLKVRAEQTAQLPLTLFLRYFTLNLYFATECESISGRSGTSLKNVVNGHIKDFVQRNRDAEMQKLA
QGMESDQWNAKDFTEKNTELLNTILGSSTHDPHEWTDSTKIWIPSSELEPVDEDEAIETNGTGKEKTRTATIDSETFMLPKSA
ILCLDGIAKFSHLISGIPSMTVDIATSLIAYLQFFNSRCTQLILGAGATRSAGLKNITTKHLALASQALSFTATLIPHVREFV
RRHAGSGASVSNLMGEFDKIRRLLQEHQDSIYQKLVEIMGGRAAIHSRTMKTIDWEADGDKGVHSYMETLAKETTTLHRVLTK

HLPETSIQMIMLPVFASYRDQLGSSFREADPKTETGLKSMNNDIEFLTTKLGKLDGFGETGEYLQKIVKAKQVKVVEPVIEKP
ADERKDDESKESEAVPASESTAEKAEK
FUN_010908 CDS scaffold_4 335250 335751 -
hypothetical protein ENOG41

MKFATALVGLVASATYAAAASVTFVTLDDKERTIIFTPDPGYEGPESVTVSSAKEVTVDFPDKYIGNFYAVQKGKPDQPGMLG
EVTFGGWNGKTYFDVSAIVDPNDKNNVKQMWPKSAATPMSGCETFPCDNCYWLPDDVQTKVTDEVDLITTLGDGPSPYKAKSN
FUN_010909 CDS scaffold_4 341499 342449 +
hypothetical protein

MKIFQTFSAVVLIRGVAAGSFLMMSPVPKPPFKNETEHTTAKTIVTTITSDYSFYCPEPSTFHHKNVTYTATEPTYLTITNCP
CTVTYTQNIKSPGTAVYPTAPPGQPMPPTPPVMSSSAPAPPLPPDKPAPPPSEYSAPISGPPPPPPKATAPYSEPSLPPAIPK
STVPIPEAPPPPPPPPHATAPAPAPEPSPPKVTVPAAGTLPPPPPPEVSVPAPELPPLPPPSNLERPAVPPSQAASPPVNPTG
TIEASVPSAAANKVSGSFGAFVLAGVAALAM
FUN_010910 CDS scaffold_4 343081 345240 -
hypothetical protein EOG092613S3 PF04049;PF13181;PF13414;PF13432

MTLSVHEISQLRAALQDAVVKCSERCLYQSSKWAAELLNALPEIEEDEENINPADPGHASPIFAANSDPEEAILEAKELSKYL
LAKSLFDCREYDRCAAVFLPDSLLSSVLASRVDSTSASTTTGKGKGKASSASAVTPLPKLSQKSLFLALYAKFMSGEKRRNED
SEMVMGPQDLGTVANKQLLVIGRFLATWFEERTTEDDEVLGSQGWLEYLYGMVLAKEKNDDKALEYFIRSVHKYTMNWGCWLE
MTSLISRVEDLNRISRHCPQNIVSYMFHLHTSLELYQQGPGLANSLEQLLSIFPTSSFLLTCNALLAYHAKDLMAAEQHFTRL
LALHPHRLDSLDHYSNILYVLNLRPKLAFLAHLCSSVDKFRPESCVVIGNYYSLLSMHEKAVQYFRRALTLDRSCLSAWTLMG
HEYVELKNTHAAIESYRRAVDVNRRDYRAWYGLGQTYEMLEMHTYSLWYYKKAAGLRPWDGKMWMAVGSCLQKMGRERDGIKA
LKRALLADAYYDVGSSFGSGDLLGSRSATGHMDPDILLQIAVMYDQLGEEEEARSYMELCVAQEDGGGAAEADPAESIAIHND
SPPGSDNGAEGNENPGNEGTGVTAATSKARMWLAKFSMRTGDYITASRLAGELCQDGVEVEEAKALVREVRSRMEATGMLDPL
S
FUN_010911 CDS scaffold_4 348761 349876 +
hypothetical protein EOG092633US PF00348

MAQQTTLQEFNSVYPKLEEALLDHARSYKLPQEQLDWYKRSLEVNPLGGKCNRGMSVPDSVSLLLEKPLTEEQYFQAATLGWM
TELLQAFFLVSDDIMDSSITRRGQPCWYRQEGVGMIAINDAFMLESAIYTLLKKYFRSHPAYVDLIELFHETTFQTELGQLCD
LLTAPEDNVNLDNFSLEKYSFIVIYKTAYYSFYLPVALALHQLNLATPSNLKQAEDILIPLGEYFQIQDDYLDNFGKPEHIGK
IGTDIKDNKCSWLVNQALAVATPEQRKILEENYGRKDDEKEKVVKKLYDDLNLEQRYLDYEEKVVGQIRERIANIDENEGLKK
TVFEAFLAKIYKRSK
FUN_010912 CDS scaffold_4 350481 353362 -
hypothetical protein ENOG41

MTMGPIGTVRIRCVLCLRSFAPRFPSAESQTAPSPAVPPGPSSPLASPLYVLTPKTTNIPPFLSNLASKPLPLRSPNLNQEFS
RSTAHLTGRDHSRSRSRSRSRSRSRSRSRPRGPDLFKPLPPTYAFDLPAPESPILRTVPSASDIARFKRRNLHALRINTETAE
RAAKIEREDLEREEDEAERLADEVARLEAETDRILAEQKKRDLARRRAQLPTPSPKFPRFLVLDKFSFLYRSRRSNALISQAG
TPSPGAPTIFSLDLSRPSSPEESSSPGKMSFIEQGGKGIVPGTDAPTSASNGGERRVCVRCLSSVINLPVNTDTSPVDILYST
ANLTSHDIDPKASVLIECYVELGLERRLRRYERIRDVMNSWDRDQQNSLLVVTNDISQSDDPDLDMKSVPRTPTPPPGFTLQM
YHSSRPGKWNKRWITLLESGQMFASKKPDSKASDKDSTVLCHLSDFDIYTPRESEARRHLKAPRRFCYAVKSQQKTFVFPNGE
NFVHFFCAEDGKLARRFLELVQRWRSWYLVNKQVDFEKKDKPPQLAMHLENDTAPKAISRSKSSAAYKGHSTQVSVDETPYTI
GAFQPLLDMDRFDKPLEEFGKDLEQDKTKYGDPDTEFKSGLVRKATCKQPNVLTKSKSIHHKHRSPPTPRDEAQFSSTGLLGQ
GYEEKRKIAERAPIISPPPTNAAFTKGASLLGQGYEQKRKISERAPVNNIVPSTNAGFTEGPSLLNGGVVSPTSPNENMADSK
SWFPLGADQSSQPRYRSRSVRDPNRRPTTSEKVPDIPQPLVNLTNSFPEPPRWRENKGHGVKAPAGGPLISLATGGSTNNSFN
TRSASGLIGQSRPLPSSSSMTVGSRPRSRSITGTQQSPSHAPPVPPLPIRSLRRENTAPLEQSRGRDPRPREPLINRAGTMGF
SSRN
FUN_010913 CDS scaffold_4 355149 356348 -

hypothetical protein PF01370;PF02719;PF04321;PF07993

MSERTVLVTGATGLLGREVATAFGLKNWNVKGTGYSRADGINTFKVNLSNETEVGEFLDETKFAVPSLYGCCASRLTLLPTFR
PQVIVHCAAQRFPDKVDKDPEGARELNVAASKALAKLAADRDIFVIYISTDYVFPGTPGDAPYEADAKPQPTNLYGQTKLDGE
RAVLDTLKQAGKEGLGVVLRVPVLYGNAETPAESAVNVLMDALWKAQTQGAEISMDHWAIRYPTNTEDIGRVCHDISVKYLDT
PSKERSCLPQVLQFSSEDRMTKYEIVTLFGEIMGLSTEGIKPNTEGNDPNASVQRPYDCHLSTKGLKDLGIDVSTCDFKGWWR
REVRAFRK
FUN_010914 CDS scaffold_4 357904 359646 -
hypothetical protein ENOG41

MGMQGPLTAQALGRSPYDDVVEWDDDHDEPPNTNDSGQQYDHRGRPINPETKRINRDIIRSHNEVMLVIGVAEQENPTTNPEA
ESDKRHAMYEDDVGINLAFSALRCVDAAGAFGLDGFRQRVLIYKRYSHIPFWDLYAQSRNTFSISQDLLPGASVNLFSNYIDR
QIALLWRDRPDRVFARRLAHEVWSYVRVHLELYIALQRLGLASSTNWLPSLSFFVPFTQDSPIPAPPPVKDLSFQSLLQWVGG
LCICSTPFLVWVMSQRLIRDWRPQIWARIFRWLPNTGSLPGVTNSWLPADRPRDRSEHQRARSTVDDVSPIRPIDGHMPNDTG
PVEAVRRPSTFSARGDDYATDEEDNEGLSATLISFDVEATAETSEAPPGLWSAELRPSVEARGALMPTTTYCDTMLTQLPPLI
ASHIFADAVLRLVTAPWEATALRLMAHSFRQRLNLPTHDVCEINVFSHLNLTFAINFLGTQVLHLALCGEIWAAFTTVATVLH
TTPDEWREAEAEEQEKRQDWTDEST
FUN_010915 CDS scaffold_4 360023 362848 +
hypothetical protein PF00400;PF08625

MANKQPLKTTFDVNHVIRPIFTGGSVAIDNSARILATVLGEDAILTDPTNGKHLAQIESDGEFISTLTLTPSGSHLIICSRSL
SMRIYTLRVSQEGGTIDATLTRTVKAHTTPVVVLAVDRTSTLLATGGTDGAVKVWDIIGGFVTHTFRGSSVLVSALLFFEAAA
RPSDSQSGKGKKTKRQEEPDEEEEDSSTVNFRLSCGQQDGKVRVWDLHKRSCVANLDSHVSDVQGLDYSPEQNALVTAGRDKT
ITWWDARSWKIRKVVPCLELVEAAGFIDDGSLTYSAGANGSLRIWDTDMGKEITPQQPAKSEEEGIVSGVYRPGLPFILLVQV
DHTLALYRPPRKADTASLPTPEPFRRISGTHDDIIDLGYLLPDRSMVALATNSEDIRIVSVTEAQNGEADWDSNSNPYFGQDV
ALLKGHNEIVISLDIDWSGHWIATGAKDNTARLWRIDPTNNSYICWAVFSGHAESLGAVALPKGVPPESSPARSDPLNHPPAF
LITGSQDQTVKKWDIPRTAQQKGQKGGSRAIFTRKAHEKDINAINVHHSGQLFASASQDKTVKIWSVEEGEVQGILRGHRRGV
WTVQFSPVNMPAIQGEDGPITGKGAVLTGSGDKTIKLWNLASYTCVRTFEGHSNSVLKVAWLNMPASQEQSKKRVQFTSAGGD
GLVKVWDANSGEAHCTLDNHEDRVWAVAVHPEDNTIVSGSGDSTVTFWKDTSSETQAAASQAALKLIEQEQELENHIHAGSFR
EAIILALQLNHPGRLLNLFTSVITTNNPDKGSLSGLSAVDEVLGSLSDEQIFMLLLRLRDWNTNARTAPVAQRILWTLIRSYP
ASKFSNLSVKGAKGQKSLKDVLHAIRVYTERHYRRTEELVDESYLVEYTLQEMDDLAPLLEDGDGLIKDAGNDVVMAG
FUN_010916 CDS scaffold_4 363263 364880 +
hypothetical protein EOG09262QL6 PF00443;PF02148
C19
MAKRQASEALDDLVGVASPASKRSRLDDIDSPEPNGASNELIDESNTHGNAQEDDADEEDDQDPTATAPIRQSAPTDGYDDLY
LDTIDRNVLDFDFEKLCSVSLSNINVYACLVCGKYFQGRGPKSHAYFHSLDEDHHVYINLETQRVYVLPEGYEVKSRALDDIK
YVSDPRYSKKEVIEMDRTPRTSYTLDRKEYIPGFVGMNNIKDNDYLNVIVQALAHVAPLRNYLLLEDFSNKTELVKRCSILVR
KIWNPRAFKAHVSPHELLQEISLRSNKRFTLTTQSDPVDFLSWFLNNLHLGLGGSKTKPGSSMIQRTFQGKMKVESQAITARA
DATDRLRFEDADVKVDIVRFLLLTLDLPSAPLFQDELEKNIIPQVPLTTILTKYDGQRAQEHHAQRKRYRLMHPLPPFLAFHV
KRFSQNKFVSERNPTIVTFDARNLDMSPYVEPNPKEWPPGEPIWYDLVANVIHEAVRVREDVVDSGEERKTWKVQLKNKATGE
WVVCQDLYVDKVQSELLYLGEAYLQIWERRRDPRGKGKAS
FUN_010917 CDS scaffold_4 365345 366478 -
hypothetical protein PF01652

MDTSNLWSRRTNTGKLSLSTPGQGSSGDSFNRNASFPKRQGGDTPSSTKPNPFNTTPGGLVSPTSGASSAFGLGSGAFASFGS
AKTPKNSANPFDTAMASAVVKSGTKDPSKNAGKGAGMTAISENNQSGVSTANPSHALVDSWTFWYRPPISKAHGFIEYEKTLH
EIATVRTGEEFWEIYRHLKRPSTLPVVSDFHLFKKGIRPIWEDDVNKKGGKWVVRMKKGVADRYWEDLILSLIGDQFADAGDD
VCGAVLSVRNGEDILSIWTRTDGGRVIKIRETMKHVLNFPASTRIEFKSHDSSIQQRTAIEEQRREKAGQHHHHDKRHTGGPS
KQAAEQSHP
FUN_010918 CDS scaffold_4 368353 368997 + LOC1 60S

ribosomal subunit assembly/export protein

MGVTRTKTIKNKHAAAPGAAKTSKRSATDGIAKSKKPKGPTLSKQVKDKGREALLQKFKNPKKKKYTDQQLGIPELNTVTPVG
VIKPRGKKKGKVFADDQESMSTIMALVQAEKDGQIESKMIKQRQMEEIREARKAEAEKKEQEKQSKLEDTKDSLRKRRKRKST
GDAEDNIKEFTTSGSKGTKSKGKKRVSFA
FUN_010919 CDS scaffold_4 369359 370938 -
hypothetical protein PF12146
S33
MEWLGHAELNFTHAPSPRKLQEKDGNVTDLLSICEKATPPCRLNPLLFNGHLQTMWTATKPAGPKIYYKRKIFDADHKIYHGT
FAVDFAVEPFEAPEDPSLSRRTAYFTPEEFEHIGSDDHKPMLVVLHGLSGGSHEIYLRHTIAPLIGEGGWEVCVVNSRGCARS
KITSGVLYNARATWDIRQTIKWLQKTFPNRPLFGLGFSLGANMLTNYIKVSSKILQNSYIGKEVYLRVMGSALKELAATHRTE
LEKYSKIDVEAVMNITYLYEFDRLIQCPSWGYPTESAYYRDASSVDSILSIEIPFLAVHSTDDPIAVKEAIPYEEFKQNPNTV
LLTSSLGGHLCWFETSGDRWFTKPVANFLNHLAFKTDLNDLRPLVNPNNNDHLADGHGYIPMRRKLVIVED
FUN_010920 CDS scaffold_4 374635 376667 +
hypothetical protein PF00069;PF07714

MEWSYPPQYPQGSSHGPPSGPSSHYHSNTTRSPPYAAPTGPMQQFPQGGSYRGGYRGGGFRGRGRGGFKNSHWNSGPPSRGHH
DDLGNERFSNVDDHGNTGSEPMDVQEEVFQSHDELGEDTSQSVQDRAGPLTTPNRPPPSGPGGSSGSKFSFAFKPSSKPAVTA
PKPEISQKLNAAPRREPQPENRDRDRARDQEREPPRSAPTEPASSRPRHERRYPPEGPRVAPRMRKVMKIMKRPKPRPTLPAD
LVDSESVFFRKPGNESVVGSGTYGKVFKGLNVYTKGMVALKRIRMEGERDGFPVTAIREIKLLQSLRHVNIVNLQEVMVEKND
CFMVFEYLSHDLTGLLNHPTFKLETAQKKDLAKQMFEGLDYLHTRGVLHRDIKAANILVSNEGVLKIADFGLARFYAKRHQLD
YTNRVITIWYRSPELLLGETKYTAAVDVWSAACVMVEIFDRNAIFPGDGTELSQLEKIYNVMGTPNLKDWPNLIDMAWFELLR
PTVKRRNVFAEKYRDKMSPAAFDLLSAMFHYDPAKRPSAAQVLQHAYFTEEAPAARQATELATHNDWHEFESKALRKENDRRE
REAKKLAKENAGRDKEKDKKRINEGTDQRDTKRLQVDKNGGNKPTPAVAPPNSTSEA
FUN_010921 CDS scaffold_4 377576 379091 -
hypothetical protein ENOG41

MRSPSPEEPPQPPQIHQHKPSRHHASTSTSSTPALSKPRPHSLQFSFPPLFQTNASSTSLVPSTAEGKPIPVDNSTAEHRTSA
LRELNSNFPTRHRYAQSTGAQSSTYSQPVIVRSYYAPVPAHPADRGVIVVNGRGHRSALSESSGPSAFVRRVLPFGTGSASVR
NGIMGTMARNRTKKRLENEPEEPKLPSVEAFRFKSFMANLEDQSGGTDINADLDRIAEICAKSRYSLSNQYEVHYAPHGSGSS
FMAPAVESQEAQGPTLQAVSSDDERNINRSRKRPMGVRRNSRAMGTLETIMSSSRSSDEDKSKKKSAAEIAEDVRGRAAQKGS
SKHGSSVSSEGGASENVFLEEEDVRQRSPRQKRSGSLALIDGNRLSMHLNDLESSRPTAAGLVSEPALPQTSSSQLEIRTAPE
VTNKDHLPGLPKKCLAATEGLDQPVVHGSISAIETSNQRSNLMSTLSGWMSWRSSDTLPTTKGRAEGSLRELLKTRDIKGKGV
EAAAYQ
FUN_010922 CDS scaffold_4 381077 381471 -
hypothetical protein PF01679 ENOG41

MGAISVVFLILITIFFPPIGVWAVAGCGMDLFINICLTLLGFLPGHIHAFYLEYIYYDRREQAREGRFATGPAPGIYSDNVQT
GGQGYGTMGRAA
FUN_010923 CDS scaffold_4 385305 387582 +
hypothetical protein PF00076;PF00641;PF00929 ENOG41

MASQQLPQLNIDRYVVIHVATTCDEHGVYVTKDSAEVIELGWILVDANSLEEITHESVLVKPVNTPITPLCTSLTTLTWEHVR
NAGTFRDAITRFDTFATEYLTSKNLDFVFVTLDAWDLRVQLPREARDKAVVLPPYLQHSRTFDLRTEYQRWQQHHPESLPFGP
SMLSNICAALEVEPVQSSAPIKHNLPFHLQALAPASPRRAMEEAVTLARVLRGLIRKSQPPQEHPDVLTRPMDARADVRAFLS
ERSKVLHMSGLPHDTTQSELESWFTQFGGRPIAFWTLRTPEQHKPTGTGFAVFSSHEEAAESLCMNGRALNEKAIEVSPSSSR
VLDRAQDILTPFPPSKNRPRPGDWTCPSCGFSNFQRRTACFRCSFPAVGAGPSNDMGGGNNNYGGGYGYGPPAMMPPPPHGGH
HGPMGHGGGRMGGSGVVPFRAGDWKCGNEVCGYHNFAKNVCCLRCGASRAGAAVVADSGYPSPMDNASQYGMNQGSMGGAPGP
GPFGSGSSFGGSGGGYNQQHFGGPPSHYLPSGLGGGAAAYPSSLNTHGSFGSGPASHAAGPFDSRAAEAAFQSATNGPASGGP
SNNFYNNNANNNGGNTENDPFAFLSSGIGGLSVSGGDGRQNGGSAPPNKSPA

FUN_010924 CDS scaffold_4 389053 391153 -
hypothetical protein ENOG41

MVSNPSHPDAGPSAPHNVPRNTFNMQPATSNTAQSGVNSQTTPQDQGQGQVQSPDELLTHPERQFELLTDGMRRRLEEAVARE
SRTNEELAAMGVDPEDYDEHGNKYMDTNIDLGPDVEIKEPDSQLSIAKRVKFSKAYAHGLFEGENPYEIENEVDHLTLSLKHT
SLDLITALCCNLELAIEIGKHLSPRDIVGLYIASPAFRKAITGHMLSCIRMWIDTNAPEAGKVFHWKLYGKTLIKDPADRNSS
THDARFLEGRWETTKTNPSQIRLIPGFKYLELVIGRDRYCREIIAMMARMGFRMPHTMHSTLLRLWVLLDIPTTRQRQLFLRS
RKYWTDIHLYNAQFFMVKLSMAFTHPFFSPINIDMVKLMMGQKGLYPLWQCLMRQKYRTMSECMELKARYDLLLPRSVWGLIQ
REKYPSAYGVPVHDVGLGHLEGWGKGVLHLGRPDQILPIEAVSRGLHLDDHITQMMVWGYIDFETGENLVPNEEEMYISDEDE
KLNKADNTHHWQRKHALKKRWNELTPEQQEIKDDDADEHLRAMAWSSVDHEGLDWDRESDDADDESDGEYDINAEINRGYRM
PATPNESSSPEDNMSDIPAGGEATRVGNQEISADEVAETMGGMDVPVRREEIGAWNTTLSELFVNSPPEVNDADYKMARTWDI
WFTNGSSGQPPFATAPNTMGIQNQAAGNNLNNNGQ
FUN_010925 CDS scaffold_4 391723 393342 - HAT2 Histone
acetyltransferase type B subunit 2 PF00400;PF12265

MAPAQSAERDVDIEMTHEDDDDQGERMINEEYKTWKKNSPFLYDMILGTALTWPTLTVQWFPDVKEPEGKNYSVHRLLLGTHT
SDESPNFLQIANVQIPKAVAPNPKDYDEERGEIGGYGKPGDVAAIKCEIVQKIEHPGEVNKARYQPQNPDIIATLCVDGKILI
FDRTKHPLQPTSLGKVNAQIELVGHKAEGFGLNWNPHEEGCLASGSEDTTMCLWDLKTLKGDSRILNPSRKYTHHTQIVNDVQ
YHPISKNFIGSVSDDQTLQIVDVRHSETAKAAVVAKRGHLDAINALAFNPNSEVLVATASADKTIGIWDLRNVKEKVHTLEGH
NDAVTSLAWHPTEAGILGSASYDRRIIFWDLSRVGEEVLPDDQDDGPPELLFMHGGHTNHLADFSWNLNEPWLVASAAEDNLL
QIWKVAESIVGKDDGDLPVDELDR
FUN_010926 CDS scaffold_4 394112 398574 +
hypothetical protein PF03159;PF17846;PF18129;PF18194;PF18332;PF18334

MGVPKFFRWLSERYPAISQLIAENRIPEFDCLYLDMNGIIHNCTHKDAGEDVSFRLSEEEMFIRIFNYIEHLFGKIKPKQLFF
MAIDGVAPRAKMNQQRSRRFRTALDAEKAREKAIKEGVEIPKEEPFDSNCITPGTEFMAKLSQQLRYFVNKKVSEDTDWQGCE
IVLSGHEVPGEGEHKIMEYIRNAKAQPNYNQNVRHCLYGLDADLIMLGLLSHDPHFCLLREEVTFGRASKTKSKELEHQNFYL
LHLCIVREYLEMEFQELQEDGALSFPFDLERVIDDFILMAFFVGNDFLPNLPSLHINEGALAKMFRIYKSILPKCDGYMNENG
VINMERLQRLLGELSKTEIDAFENDVSDEKWFASKQMEKKLENKNAEAKAPKGGQLVITSAQRDLWKQKIRPYISKRSEQPLD
LGTSLKAADRKFVQDLADSMHLQWSTKEDDEGHRHLIVSFPPKVKDDADDDEEDDDDDEEGNLAAYRVMKTYDKALVIDLTPE
DAQKHYEKLYQDKYQGWKTKYYLQKFEEWAPDNYDKELTALCENYVQGLQWVLYYYYQGIASWPWFYGYHYAPLISDVVKGVG
ADLNFKKGQPFLPYEQLMGVLPDRSKKIVPKVYHDLMTNPDSPIIDFYPRDFELDMNGKKMDWEAVIKIPFIDEKRLLAAMAP
KNELLEPDEKARNGFGVPLKFTYSPEVDFVYPSPLPGVFPEIQNCRCIENIFDLPDMEGLEYLSGLTNGALLSVDALAGFPSL
HTLPYTAQLVEGYGVNVFQADSRNPSICVTLTETENRTKIETWKAKIGHRCFVGYPFLQEAKVIKVQDELFSYEMAENGKDIV
TKDHNNREAADWAKEADYLENWHAKRLAITIGQVECLVYVHMLKGLIRTEEGALIKEYAENPSLRSTYAAQTIVDEVVNEDER
FIEKEALPIEEEFPRGTRAFFLGEYAYGRPLEVAAHANNKAEIVVSMLKNKEPDFAKQIIHQAERSNPYTPSFAVAKQLGIHP
LVLSKITSSYQIINSAGLRLNLGLNLKFEGRKLKVLGYSRKSHTGWEFSGMAIKLIADYMVAFPDFFAAIQREPQKSEVLETD
LWNDPSVASQRVKEIVAWLKKQETSKFERVPLEAEQLDSEVVKALANVGEQIHAASQDIDIKKLRGVPRSALLKPSDAEMVLG
SQNFALGDRVTYVSAAGKVPIATRGTVAGISRTATALLLDVVWDTSFMSGTTLNERAPMFRGQTVASSSVLNTTNKHVISTTS
KSQQRRPVIAAQGGYANVGVTQYKDAPAPGPLRGGWRGALNGSNSPGRNSPGRGRGNGARGGAPNLMHSTLVYRNGPPDGATQ
GQDANGHGTRGRGGRGRARGTHHSSPGPDGHRGVEPPQEQRYGNVPPPASLDSPRGRGRGRGRGGRGRGGASRGRGNGGAAAD
GTQG
FUN_010927 CDS scaffold_4 398883 399667 -
hypothetical protein EOG09264VC6 PF04573

MYNSLTRIQNTFGFFTTVAFVVAAFIAASDLLAPRAPDAGIFQKTNAQVVKGRPHYYSSKKEEYAIIRFNLDADLRSLFTWNT
KQVFVYVTAEWPGPNNSTNEAVIWDKIITNPSADHLQNIGPVAMKKLKRSAEGKSIDPERGFIGLKNQKPKYQITHPSGKVAE
IDDVKLKLHYNVQPWVGFLTWDQSRDIGHWRALKWGESPKFQLPALKTKKKDAPKKSY
FUN_010928 CDS scaffold_4 400801 404017 +
hypothetical protein PF02902

C48
MGGGQSTEARSYYSEPRDDRPNKRVKPSGPELSNTNLSRFFPTGSLHGPLEAIEDEEPQTSQQKCHDLTREDSMVETIDGSSI
ISFGRTAGATSNQQEYRNVHRQNSVKRTRRRRRHESKISSSPIEIDDTSAVAHRNLAPQTSHPPTSPDQLVRENEDGPPPANI
TSDVVSQPKRPAAEYSQSAPKRFKVHPQQSDDELAPRAPGGRETAKKPSFPKLISQPRTQRGDIQPTTFKTTKAMPGRETLAN
KGSALKEVPLEAAICGKYSFSPADNIEPVTLRLGPDAAEPMHENSGDLSWLQVLRSSVKGVKHNNTNSPIVLISRSARTSFSC
GKLALKFVSDADASFFIHWLHGLPCLVQHETDKLDATFHCAKKEADGYRTNSAKASGIFLPDDSHRANSARPQQHHRDPFLGD
PVSPGLPNSKQKKLKDGMQSIFQPKEDVQIEPLFTDDLFTGLARNQRLGTRHLTRRESPQSQHERTPKPWTACNPGWEKKWHR
SLVWPPTGKNRATVDKDDIQRLDEGEFLNDNIISFYLRYLQDQLEKERPEVLKKVYIFSTFFFEKLRSSRGKINYDGVKAWTA
RVELLSYEFIVVPVNENAHWYLAIIYNAPRLLQPEVKAETNIKKESSSPQDAIIVEDNDPVLNLAESPPAATKSSIVSVDVEV
TRSTRSRAINDSNSSLLGDKHTPNIVTPGKTSKRQSTGGNQKFSLDDSKIITLDSLGSSHAPTCRCLKDYLLEEAKHKKGLEI
ASVPSGMTARGIPMQDNFCDCGVYVLGYMENFLKDPDEAVRRLLHKEDTEWEINAPEIRAKVRDLLFECQEKQHERLEKEKEI
KRQRRATKGPVSSPQIAASSPQVPRREPETPQVSRYLTSPSVNGASGSPFEAARQTGTTSSYFAVASAERPSQSPTSKRNDEP
SLVQPLREDSGNESKASSSGDVYHSARSSPINSVPPVPSGLTTEGSRDERTPKQPSTPEFVHRLPDSPDDMGPTATSSTVKKT
SSPLLSLVTRQHPSPGSQGSQRAVQLEQTVVRSIEDDMSPPRGPQYDGVDQSIDLTV
FUN_010929 CDS scaffold_4 404412 406161 -
hypothetical protein PF00400

MSSFFTVPGAQKKRKRTETSEQPKKRVAPSKSAGKTNSKNAGPTKKRVERDDESISGSDSDDDESYDSASVASDVEMEDNESD
DEEGETAAERRLRLAERYLENVKEHVDEAGFDAADIDRDLIAERLQEDVAEGKGRVYRQLASELGFDNASRTFFRSNTNTVTA
IAICAPYIYTTTKDLYLQKWKTQDLPQHQYPQTTKRKPKKPPAPPRKRPELEAWLKGNTTKSKDKDYQRHTDRILAVAASPDG
KYVATGGADRKLIIYDAHALKPIKVFTHHRDAVTGLVFRRGTNQLYSCSKDRTVKVWSLDEMAYVETLFGHQDEILDVDALAQ
ERCVSVGARDRTARLWKVAEETQLVFRGGAVDKKSRPTGVHPRSLLHEGSMDRVAMIDDDLFVTGSDNGALALWSVQKKKPVY
IEPIAHGIDPPLEPEEASAEVNPDPKIVPPPTPRWITAIRTIPYSDVILTGSWDGHVRIWQLSEDKRKIEFIGVLAKPEDGEK
ASHEENVHGPLHGVVNDIDIFERGDRGKDGLCVVVAIGNEHRLGRWNPPAKAGRNGGFIFEIPRIQRPVVNGTNESSNGDEQD
E
FUN_010930 CDS scaffold_4 406659 407350 + PCB4 putative proteasome subunit beta type-4 PF00227
T01A
MRGATILKASDDKTRALNKHTLLAFSGEAGDTVQFAEYIQRNAQLYSMRNESDLSPSGLAHFVRGELATSLRSRKPYNVNLLM
GGVDPITGKPSLYWLDYLASLADVPYAAHGYAQYYCLSILDKHHHPDITLHQGIKILTMCTDELKRRLPIDFKGMVVKAVKAD
GIVDIEFDDDKVVKSA
FUN_010931 CDS scaffold_4 410462 412532 + DED1 DEAD-box ATP-dependent RNA helicase PF00270;PF00271

MADQINMGGLSLNDGAQQGPPQGQRSYIPPHMRRQGGAPQGGPPAMNGGPPPAGPGPNGLGNSAWANQNYGARQSNWGNDVPT
YQNNNNNRRGGWGGRGGYSGGGGNLDGHSGGGGGYAARGSGDGQWRDGKHVPGPANPRVERELFGTPDDPSKQHTGINFEKYD
DIPVEASGHEVPEPVHQFTTPPLDEHLCRNIELAHYKVPTPVQKYSIPIVMGGRDLMACAQTGSGKTGGFLFPILSQAFINGP
SPVPANAAGQFGRQRKAYPTSLILAPTRELVSQIFDESRKFAYRSWVRPCVVYGGADIGSQLRQIERGCDLLVATPGRLVDLI
ERGRISLQNIKYLVLDEADRMLDMGFEPQIRRIVEGEDMPQVQDRQTLMFSATFPRDIQMLARDFLKDYIFLSVGRVGSTSEN
ITQKVEYVEDVDKRSVLLDILHSNANGLTLIFVETKRMADSLSDFLINQNFPATSIHGDRTQRERERALEFFRNGRCPILVAT
AVAARGLDIPNVLHVINYDLPTDVDDYVHRIGRTGRAGNTGIATAFFNRGNRGIVRELMDLLKEANQEVPAFLETIARESSFG
GRGGRSRGGGGRGGATRDFRKFGGGGGGGFGGNNGGGGGFNAPSQGSGYGGGGFGGSGGYGGGGYSGGGGYGGGGYGNPSGPG
AQSSWW
FUN_010932 CDS scaffold_4 414475 416263 +
hypothetical protein ENOG41

MGVFDSLDAGVTNLFGQWNVYSTGLVTLLLAVVSYRVISTRDPDVHPMLLARQAVPSSVRNEGESPIYRSQAAPHGMPLNTGL
NVKDTGASKWSRGRDGDLRDIWKRAAEGGENGAKGRILTVLGSQNVVDHKLDDITRQINLIGQHIAESGGIRVAVYLPNSVEL
LVTLFACSFYQNLTTVLIPFDVSNDELVSMLRRSAADTLITAPGAFPFDAVIQAYPSLRQLIWVTDEGSSHMDWNEIPEGTGG
NINVATWQDILRDSPAHAGTELPTIDPGKTPSDVVTFWQTEPGEVEEMVRFSQANLVSAISAQLAAIPTKERINPSDLFLPAD

SLANVYTLVLTLGALYSNASIALNSVAGKSPDLVLATQGVAPTVLVASPETLLKTHEESTSRLRSSLANASHVMATRSLALEG
VHATSNLLSGFSSGAAPALGTTPGKLRLVLVAERAGSDTPLLPANVLSDLRIFTGARVVYALTAAKVAGAICQTALYDYRLPV
DAKTHFGPPLTSVELYLKDAGAHKTTDEKIEGHIVVRGPSVAGGEVNVGIPGKIRHDNTLAYV
FUN_010933 CDS scaffold_4 418539 420867 +
hypothetical protein PF06985 ENOG41

MSQNGTAAAKPSAGQSTAARVGMKKISRLFAFTRLGRGRPESANGHHQTEDSASTRESSPDVHQLTSRRRRGAAPEHRKPARN
GLSRLIADEENQPCAICATIDFPSLLNWRPGQPRPWIPLAHTLAELSACPYCIFFQALVGAEPDITRKFTPYLRIRQAFERLG
VGEKHELGGAVLFEVTTKSKVLPWGYIVRIVDGDDDMAGYSETTPVIRGRVIPPKLDIALPRTWLDFCRSNHSHTKCASSGPP
IRGLKLVDCQDNQVVFVDDLNVEALEYVTLSYVRGSPTDEEWDHNGLPEVLPPLLTDAISVTKSLGFRYLWIDRFCFPQYNAH
ERRRQLEKMGEIYARSALTIIVAAGQGVQDGIPGISMAREEQLSLQTEAGCFTTSLTRPDVEVANSKWASRGWTYQEGLLSRR
RLVFTPSQIYFQCQTFHCHESISFPLQTKPALNLGRVFPDNGAGGRTGDYRNIVSGYVSREFSFPEDRLDAFRGILDKYAQMD
NALETVLGLPLFHEKDFKNMPSPTRTDRLAVGLGWLYDPPGNADISTHPLQLQGPFPSWSWLGWKLRPEYTGHGPNLSFYQVG
EEAPVVDNISAVPNMTISIGFKDDKILCWETEGDEIAARSTPITFLRLTTYCFEIRLTVPSPTTSSASASSSAPAITLTSPDL
LDAFHSPIAALLLRAAQSPPESPSELPPGEHELIGAFISGRDWKVDESYDSANVLICGRRNWNAEDVLVRLGILELGAGGLLG
VDEDTAVVKGAMNNERRLDVQLRELDIY
FUN_010934 CDS scaffold_4 423053 425466 +
hypothetical protein PF05057 ENOG41

MDPSSPPPPAYSERPPIPPRKPVNSSTSAIYQTPPSAASSSTSTPGPYMHTLSSGPWLGPDPRSSSTQSLLPDTSRDSQAATP
ADYLGRRKLLIIYIHGFLGNDQSFRSFPAHVHALLANLLAESHVIHSKIYPRYKTYRSVGVARDNFSEWLRPHESPNTDVVLV
GHSMGGILAADVVLMPNPSPYGLYPFKHRIIGHVSLDCPFIGLHPGIVVSGITSLFKPAPTPPQDSNQKGTSGTTPNSPEAST
PGDPSVSSLYLTNSQSTDPGSSTSLPSIASSSQPQRDPFANQPYFNQPFFNDSGLQERPFVQRMFHFANKYRSEGLIQAAAKH
IFSYLEFGSCLADPTGLESRYRKIRALEDIDDLQTGEMNQGSDRPLTRVRFVNYYTLCTGRPKSPKASPDVSSPQLSTANITC
PKTRYAQSNSSASSSSVELDFLPNKISIDERIQPHTRQESPLPIITLEHRDTLGNSRDHQTLLDSDTKARASDTTIKDNPVDD
YEALASDINRLSMQSIDPTPIDEESLAHPLPDTATLTFQDEAPIDAKGTNNPLPGKEEADLPPLPDPPTTPDIPDPSLYTEKD
ERKQVEREAKRIRRAHENALKDHAKAVREREKLIEKRQKRSQKEAEKALKEAARQEKEALKESQRKEKESQKEEQRLIKEASE
HENEAQKMQEHEDTQRTKNLENQYELEAEAEAITKARGLKSSKPRKLRKFCNLPTKGGRIDDPTWVDIYMADVDEVGAHCGLF
FAGQHYEKLVGDVGNRIMGWVQDDMTKRTIQDMEKGTFE
FUN_010935 CDS scaffold_4 429817 432048 +
hypothetical protein ENOG41

MTYSSSGPSLAQALDMGKESNNGNMDTDVDPLHSNLDPSKSHSLPTSDLSVLSESDPGSIQQSRSASHPRLLRSSESEKDISP
NHIHHAHLQLHQLENVDDRAVDEPAEQSHLQLHKTGHHSHHSHHHLHRSKSPANLHLRAAQPKHVHAIADADTLKPRAEQDLV
TQVVQTVSVIQVVDSVGSPIEVQTHYAPPATVVVDSASGITVAAISDPEPKISVAAVASDPAASYNVPSPPSAPAATAVETPG
LTDPSPSAPVPDATVSDSIPEPVNDTTLLPTADLPSNTVPPTNETNGPVTSTDPDTASHETTSPTAPTTVVDTTASDVSGSET
ALVTTDDLTATTTDTPTLIETTSDLTSEATQDVSSEVTSDETLIATTDVTQSESKPSTTTIPPTTNTASRTRSSSTVLVVSST
FVTSSINPSDTSTYSYSDSVYSSISAQETWSSGSWGDEDNEGGSGAATADNQPPAATTTSNSNNTDTGTLSPQQKQVIGGVVG
SIAGVAFLAFFVLFALRYKKKHPGGGILGGSQSGTRSIGGPTSGPDGGSGASMAERAGASAAITAAFAGLMGKKQRQTSELEP
ASERGFYRVSGKKLPSVLQVGGDGYSDPRTEPTSTDRANRESVMSGNSDYWRGSMAFDPSDRDSPHLALGSPMRPISGVPVIR
SGPARTPVTESNPFTDPSRPRPDSDALGGSTGGGSIASRDGSFGSPSRFQERI
FUN_010936 CDS scaffold_4 435435 438400 +
hypothetical protein PF00172;PF04082 ENOG41

MDHMGFQVPGMAPPIMNHPPQVFGGYDGIPQLPPEIAAQMFNDSAMLLEDANDPKRRRIARACDMCRKKKIKCDGKMPSCTHC
INYKTECVFTQVEKKRAPPKGAKYIEGLENRLNRMEHLLRLSGLLDEDDDDLGALEKRLLERQEKSRQVSMPAASNPNSPSQS
TSGPSAAADGGEMTPQSSLTSPKDDKRKSATPAPSTAPSTAPAPAANVEEIKQEDQEEVEALSELMCSLVTNNFGETRYIGSS
SGFSIFSPKGVSWVNSKTGDDSFQRTISDISVDDHKWTNWKPEVFGDLFQRPIFRPLPPKPEALSLLQDFFDNFNCIFPLFHR
PTFMHLVHLQYSSDPYQGSGWWASLNCALAIAHRLRVMSNLVPQDEDEKAWGHLKNAIGVFPELTMRNTDLLSVQALLGMALF
LQGTPNPQPTLLLVSAAMRLAHSIGLHKRGTGFNLNPIEIEQRKRVFWIAYMLDKDLCLRAGRPPAQDDDDMNVELPDADPED

NIGNIPLADGKGKMNLFRAMCEFATIESEVYKRLYSVQATKQSDGELLHTIGELDHKLEEWKDRIPIDYRPEHDINASHTPLI
LHVVMLHLTYYNCLTTIHRMSVAHGLWTSRLANYAIQGLNARPLNPRVFSSAALCTAAARASVSLLKYVPQGDFACVWMILYF
PVSALVTLFGNILQNPLDPRAKSDTRLMNIVVTFLSMLGQEAEQGGVHRMLGICAEFERIAKAVIDKAEKEQSSRRKRKNQDT
NKSSANATTATARQSSTAESATTTSVSSNSQRRSSQAQLSPPQNGASMGQFSMGSPMNDPSPSAMSAGWPQEFPVPQSGDFDS
MNYGDTNMNSSPQIPVAAFQQPLLPQDLFSLPMTLDWSWAEMTTGAYPTVENGNFGEDCNNC
FUN_010937 CDS scaffold_4 440854 441889 -
hypothetical protein PF08633 ENOG41

MSFHPQTPQSPSQFSSGTSEPVLSVTTSMTSTATPTTLPTPAHSVTGSAHHDLAMTDDSPHKRKRSVDDSGDREQKKVHTEES
KLGIEDLHLDVGKKYLLCQTPHPESLPRVSEDLYDMFNLSGLAAEVAREKPNGEKNALRSSYTGHMKNLGIAGNWKVRVTDKD
SNTEKPDFFDILHMPDEDWDNTIVKGQNIKHGLSQASLSALGRAVTMAKGPIGKKDWDISRLGLQSPGLDSKQTSSARPTAPN
TPLNVPGAVGRLKAQGPSANDPNRPKRNVKKRTYGDSSFEGYGEGYPDDDTAVEGGYSTGEGEGGQKRRKKNPGNTSPYPSAM
RQQSYGPGMVGA
FUN_010938 CDS scaffold_4 444963 446584 -
hypothetical protein PF00046 ENOG41

MLVTRQYETEHSRWPFSRYEASLTSTSPRMSNPYDVNVELPAPTSWQAHNPTTSHSGSGPAQQHQSRNEASRLSLMHHPAENR
GNALGIHMRPGATQMYSPHLQTIDRGGMSLPGPRDSVLQNDSYGMKPSTADMEDVISSGLISTSKTATNSTFVPPSRQTNDVS
STQSSQGARIGSPQDEEEDDLDDDDMLDGEGEHSAQTPAERAAARRKMKRFRLTHQQTRFLMSEFAKQPHPDAAHRERLSREI
PGLSSRQVQVWFQNRRAKIKRLNADDRDRMIKMRAVPEGFDNVQALHSPYGAVHGMNAPMPSPGHFNTQSYAQHMIRPLIVDG
RRHDTSEHASPTAMTPGFGGMGYSPAGSLTSSGLASPLSPAPSDRYPYGGHFTAPLTGSPRTSHPFAQHHGPGTPVEIHRSSP
QPIPPPLLRDPLCRARSESSQSPLRPNMPWKGDAGDYSYRGTNTSPNLSDRHPSIYHPAPLSNPSIAMSGYDANSVPGSRAPP
ENQVHRTEI
FUN_010939 CDS scaffold_4 453925 454495 -
hypothetical protein

MIANSTFRQGIPCDAVGLGKSSTSLLAALEIKEVRQIWASHLIITEPFWAPGREEQVIERAHRLPQKREVHVCRAVSHPSMVD
ELVNDSRSSQLGQIHKDLHGVLIGKDDETFHRELLSTRAAPEQEEEEEEEVEVRVELLDLEDANDRIMKRINAN
FUN_010940 CDS scaffold_4 455996 457012 -
hypothetical protein PF00725;PF02737

MAASIFRMPSMAAARHARCFSSSPRQYAAAEVKKLGVIGAGQMGLGIALVAAQKAQVPVTLIDTSDKALDKGIAFAEKLLAKD
VSKSRITQEQADSARSLLSTSTKMEDLSSADMVIEAVPELPNLKFDIFGKLAQICPKHAILATNTSSISITRIAAATTKDPTD
TSASSRVVSTHFMNPVPVQKGVEIISGLQTSQDTLDTAIEFCKRMGKITSVSADSPGFLANRILMPYINEAIICLETGVGDRD
SIDAIMKNGTNVPMGPLQLADFIGLDTCLSIMKVLYEETADSKYRPSVLLKNMVNAGWYGKKSGKGFYDY
FUN_010941 CDS scaffold_4 457396 460932 +
hypothetical protein EOG09260H81 PF04821

MELADGSTDIVHPEVRAHINSLVSALGGISADSDGGYQLGDDALEVLRDLKRWIRFYDEKTNRMDVARCIHEANLVEGDLLPI
LSTWPENATDSKFKSRMALACFELMVPLTWPMARDRERMTVNHHRHMPVLELAQVGYKRAVVNYDGARVLHTAIRVALPSMAI
PIGDRTQRDQGIIKLVLFFLRNIAMIEPPPDVKYEGDESQISRSATIDAFSYQDIFLVLLTLASNMGEDFRAEDTSVMEIIYH
LVKQVDIEKLFMNEQQLSKAKAGELADMMSKESSMLKAYNRKGPTRHNRFGTMIWVKRDDGKMSSLSGQDALADASTRNQKMD
NSKTFRPPRRARKGDKDEKDLGLPTKLNSRAYDQLRSFVEDFLDSGFNPLFQHVRKTIDREAPHVMQYHRRQFFYLVAWFLEA
ERMRRKARKQSSQNPSEDISSFNLVAGVLNQEMFITLNKALHESLEMKDWHELTAVMRCFTQILLTVQEMSESGNEDNEEIAE
NVLSRLFYEEATHDAIATIIRTYKDQSFNYLDAATELVHHFLRILEGYSKQNIDMQVRSRKRTRRKKKAAQDAAGIENDNDEN
DGSDNDAASAERTTKERKFDFHRFATRFTPQSVVDTFVAFTKYYRDLTDSQLKRAHRYFYRVAFKAEASVMLFRVDIIHLFYN
MIKGPAALEKSSRVFKEWEELVKQVLRKCFKKLEQRPELLVEMLFSKGSSTAFFLEYGFERQTVTTASKAKPAAELVFKNTEE
LDRQIAIVVGAMLDKNQANHVAWIKKILGDAESERKAFMAAEEVMASVEPVQDEAEEQPTESGPKAPPIFYVRPDDKDRRNAM
FKNSHMRLLMKLVGLQLLDPASEETPESAWIIPSDVSADVLKDAIHYINQAEFSPPTFEEGVLAEHQLKRKSAPRKKADFDDD
EDGEPDDPMFGPLGPTVRRPIDEEDDPKKSPRKRRPRKELTEEERQKKRKERRRKEQDRLMSFRSEEFVRPEDDETDSEYDEA

FFAREREIKAKAAAIRHVAERLPVPRKPTRKRGIAATESASSSEEHQEGSGDSESGHDSDPGDDFILRAMNTTLEEENEGQPE
GTLPDGSDGESRKRRRISVEKTQPDEDEEMGGVNKNGDEDEDEAPVVTRRPRVRGGFVVDSDDDE
FUN_010942 CDS scaffold_4 461248 463381 -
hypothetical protein ENOG41

MVSASPGHERDHDGMASPRQKQEAWWRIHLFRGMVSDIRRRIPYYVSDWTDAWDYRVVPATVYMYFANILPALAFSLDMFQKT
GSNYGVNEVLLSSVLGAVIFSLFAAQPLVIVGVTGPITVFNYTVYDIMKPTGVNYIGFMCWIGIWSLILHWILAVTNSCNWLR
WVTRFPCDIFGFYVAFIYLQKGIQVLEHLGRGQEFYLSIVAALLVFMVAYVCGELGGSSLFRHPLRVFLKDYGTPLTIIFFTG
FVHFGRMQEVDLEVLPTGIAFEPTSGRDWLVRFWDLSVGDIFLALPFAVLLTILFWFDHNVSSLIAQGSEFPLRKPAGFHWDI
FLLGLTTGVAGILGLPFPNGLIPQAPFHTESLCVTKAVKEDDEDGNPVGGGYSFEATHVVEQRFSNFAQGLLTLGTMTGPLLV
VLHLIPHGVLAGLFFVMGVQALEGNGITGKLIFLARDRALTPSNSPLLQIRRRSAVWYFVLIELVGFGATFAITQTVAAVGFP
VIIFALIPVRALLLPYIFSPEELSLLDQPTASEFIMEGIGGSWGGKDDEKSALQQNDALRRPEHRDSVLAHPGAPRKSQEDLA
ELGQIQSGQSTATRRRSLERRPQRKSTP
FUN_010943 CDS scaffold_4 465406 468139 -
hypothetical protein PF00004;PF01434;PF06480;PF17862
M41
MSRFLRQSSQLARATRLSTQPALRLCARPSLVTRSIPAKTSAAFAIRARTYSSQPPPPGKDPRSNPDKDDEHGSKPLNQHSAK
DGPDGSKSEAPKPPPLPEGWIHLNKEELAHLEEFTSRLPEAQRPVAKDILEKLQIVGAPAETRDLLQKQRQNGNLSILDKGRL
MRCVYMVTERIIELEDQQDNRDGQFSSFRMDQEPNTQDGKGGADKSKQQQQQQQQQKGGKPPKNERSGWLEAVQTGIAIGVTV
WIAELFANPFSEKEITWQEMRKAFLDKGLVQKLIVVNGSHVRVELHPSATGATGEGGQPARKSYVFSIGSVESFERKLEQAQD
ELGIPPSERIPVSYEAGGSTLGNLLLAFGPTLLFIGLILWTQRSMGGRGGAGGGMFNFGKSKAKKFNAESAVKVKFSDVAGLE
EAKTEIMEFVSFLKQPEKFEKLGAKIPRGAILAGPPGTGKTLLAKATAGESGVPFFSVSGSEFVEMFVGVGPSRVRDLFAEGR
KNAPCIIFIDEIDAIGRARQESGRGFGGNDEREATLNQILTEMDGFNTREQVVVLAGTNRADMLDKALMRPGRFDRHIFIDRP
TMKGRQEIFKVYLNKIVTNEDHEYLVGRLATLTPGFSGADIANVVNEAALIAARGNADEVKMDHFERAIERVIGGLERKSLVL
KPEEKKTVAYHEAGHAICGWFLEHADPLLKVSIIPRGQGALGYAQYLPQDAYLMNTNQLMDRMAMTMGGRVSEELHFPTVTTG
ASDDFKKVSQMARNMVTQWGMSEKVGPVHFENDPNRMQKPFAESTAQQIDQEVSRIVEAAYQRCRDLLTSKKKEVGLIAQELL
KKEVLVRDDMVRILGKRPFGDNEDFEKYFGGGKEESIPPPFPEETDTPKDPPTPAPAFKKLE
FUN_010944 CDS scaffold_4 469060 470128 -
hypothetical protein ENOG41

MPRIQPSLLRRARRLSPHIASLLPACRDLNSAQNELRWLRSHADESRSRNKSWLLDNLCRRRGRGEPLQYVIGSQPFGSLDIK
CKQGVLIPRPETEAYTFHLTNLVKSGELLGQDVRKLDRGVNVIDFCTGTGCIPLGVFSSLQHSVDNLTVRGVDVSPVALRLAQ
ENIARNIRLGNLMEPTKHKRLDITRANVFSDGDMQQLAVTPWDILVSNPPYISEDIWHHGRGQLGYSVRKYEPRLALVPDKDL
PCPSKCNPADVFYARLLDVAELLKPRVVLFEIGDEEQARRVLQLYFNHPIAQKSRTEIWRDLPDFEGAKGAEIVLHLEESKEE
RRVPVKGDGLIRSILIHNLEWAE
FUN_010945 CDS scaffold_4 471194 471721 +
hypothetical protein EOG0926591L PF01193;PF13656

MTRYANKLNSFELFLLGEGEKKIEEKVFSGMSNTSDFVLMKEDHTLGNLLSEHLKMHPNVYMAGYKIAHPNVPELFIRVQTDG
TITPRDVFTSVCEKLIKQLEMLHQEFTREWELRRITNTGEQGNMQNGH
FUN_010946 CDS scaffold_4 472019 472538 - LSM5 RNA-binding
protein lsm5 PF01423

MASQLLPLELIDKCVGSRIWVIMKGDKEFSGTLVGFDDYVNMVLEDVTEFDYSGNHTKLPKILLNGNNICMLIPGGEGPEGAA
FUN_010947 CDS scaffold_4 473128 474804 +
hypothetical protein PF01612

MSPTIKNRLWNPAFGIRFSPKNDHQPPYPFLDITRFVHTPASGSGDVEKGDVEADEFSEGEEFERDAAEWFDQQDRLVLPTIE
TISKGTQVASDTDGDLVEASKREKALTDSVTAPLTSLDYTIDANLWDTARKSKAGSSQSFWSYKMYRQVQGNGEEQKVKVHYC
TSKHTMEHVCERYFSDEKVIGFDLEWFVARGPANSNPRRNVSLIQIASPSRVGLFHVALFPKDDFVAPTFKRIMEDESVTKVG

VAIKGDCTRLKNNLGINSKGILELSHLYKLVKYSKAGELDRINKVMVSLAVQTQEMLGLPLFKGDDVRSSNWMMRLSADQIAY
SASDAYVGLQLYYVLEQERMKLQPTPPRPAFVEQNLPIRFLTADDVDESDATSESGESEVIADVEVRLETPETRPTQSVATPT
TKATPPSENNSHDTRDPRILAAEKHAQQYRSTTRRQSTPPLSSLRTYYMWYDNQDLTPEAVATLLRTPPLKTNTVVSYILDAI
ISEGLPFSKARLRTEVLVHLAPQSLVSARYRALVDASQESEEL
FUN_010948 CDS scaffold_4 475043 476530 - YTM1 ribosome bioproteinsis protein ytm1 PF00400;PF08154

MGDVAQVKVVFTTTEQDLVLPDSKQQLLVPADIKRYGLSRILNSESMLDTSSPIPLDFLANGTFLRTSIEEYLATNGLSSEST
LTLQYVRSLLPPVYEASFEHDDWVGGIDLLSATSRAGLLVGGSNIPERVASASYDGLVRVWNPSGDAIAVSPAGRAGGHTQRA
NAVKWISQKQLASVGLDRKVIVWDYSESEDGFSGALKSSMELWGHEKEINSLDINGATKRILTASSDGKVGLWTSSKRTAPQA
DPESLPSAHSTKRAKLASAANTAQRGPLALIPVHDEPVTAAVFHPNDATVAYSASKDHTVRTIDLTTQREVSRLTTMHPLLCA
TALPGSSLVAAGSSARHITLLDPRESATTTSAMTLRGHVNMVVSLAPSPENDHSLVSGSHDSTCRVWDLRSVRMGTSEEGGGS
VSEPVYTIGREWLKGKKLPVAGDGAKVLSVAWDQSWGIVSGGEDKKVQINRGRDLFGPGS
FUN_010949 CDS scaffold_4 479937 482288 + GSY1 glycogen synthase isoform 1 PF05693;PF08323
GT3
MSEGEQQTRDIKNHLLFEIATEVAHRVGGIYSVIKSKAPVTTAEYGDRYTLIGPLNHQSAAVEVEEMEPTSPEIAATLQSMRD
RGIHIVYGRWLIEGAPRVILIDTKTAYGRMDEWKADLWEAASIPSPPGDDETNEAIVFGYLVAWFLGEFVCHEKKRAVIAHFH
EWLAGVALPLCKKRRIDVTTIFTTHATLLGRYLCAGSVDFYNNLQWFDVDAEAGKRGIYHRYCIERAAAHACDVFTTVSHITA
FESEHLLKRKPDGVLPNGLNVTKFSAVHEFQNLHQQAKEKIHDFVRGHFYGHYDFDPDNTLYLFTAGRYEFRNKGVDMFIESL
ARLNHRLKAAGSKVTVVAFIIMPAQTTSLTVEALKGQAVIKSLRDTTHVIEQSIGRRIFERSLKWHEGDPMPDEKELISAQDR
VLLRRRLFAMKRHGLPPIVTHNMLNDHEDPVLNQIRRVQLFNHPSDRVKVVFHPEFLNSANPVLPLDYDDFVRGCHLGVFSSY
YEPWGYTPAECTVMGVPSITTNLSGFGCYMEELIENSSDYGIYIVDRRTKGVDDSVNQLTSFMYDFCGKSRRQRINQRNRTER
LSDLLDWKRMGMEYVKARQLALRRAYPNSFSGDDEEEDFIPGVEQKISRPFSVPGSPRDRTGMMTPGDFASLQEGREGLSTED
YVAWKLPEEEDPDEYPFPLTLRTKQPAPHSPSDNVPVNGTQ
FUN_010950 CDS scaffold_4 484551 487034 + hypothetical protein PF01302;PF16641 ENOG41

MPSATTTTSTVPHRPTTPKLRPRSSSRAIRRPNSAASTPNLKSAYASHSRAAPPPLPRKGSLAQLTQGSLASIPDVSESYAVD
TVLSDSSQNMMPPTTPGRSQAVNVALGDIVDVPGGMHGVVRFVGPVQGKKGVFAGVELDQDFASRGKNDGDVDGISYFTTTIP
GAGIFLPAAKALPRRDSIPPFPMTPGAMGGLRAGNQNSLNYTSPTPGLPKFSASVGPGARAPSPQQKLAPRTSLPRADSPVRR
MQMTPGPRPSMTTPAKTPTRYGSPTQSRFAQSVRGTSGDPSKRPQRMDRKPSHGPRSASALGSISGLDDDPAPLGIQRTGTNG
SSGSVSSFAMKIRPASRINVNDEELDRLRSQIEDRDRQLKEQSATLADMESSLVELQGLMEQADMPAVQRNSWDNKDTAQLRQ
LLREKNDKIAMLTAEFDAHRADFRSTIDTLELASTETERVYEKRIEELMADVRELESRNLDVDSVATQLKQLEELVQELEEGL
EDARRGEAEARGEVEFLRGEVERTRTELRREREKASSAPPANGDHTVSSKELEQKDDEIRGLKAIIHSLSRDSIPGEPKPPAP
RPGSLMAEDVVANKIARDNLERQVAELQSLLEKKNSREDELEKELESIRQNGTAANRSSFRDSRDTVVMAQAMENRPVENLKR
SSTAIHKRVSTLDTMPESDDYSNATETSTLWCEICETNGHDILTCTNMFGPDGAKTNGESKAIKKASREELKPHTPTGDDAPA
PLTPFRLKNAGPPSPAVKIMPNPMESGPVAGKESGIMDPEKWCALCERDGHDSVDCPFEDAF
FUN_010951 CDS scaffold_4 487408 488936 - hypothetical protein PF11905 ENOG41

MGETTKIEKGKVSANRNSEARREQNRLASRNYREKRKQKLALLNELLEPSNLSSITGNGHIDDVPGPSGSSDTGAPPLQGQTL
SSAPLTNHLIPLDLNRFDGTGTYFVPQTWEDFTQESVPTVISNQLVPEFLPALNKGNPPSLGSHDQWEDVMPGLIQQSSAFTG
QPSADYMGYRAIEEVFEDSPDSDHSGSQGASSNDDGSLEDILYGVESLSIEQKRSLLRHLQQDIRGPKSPSSSQNILHQTPGQ
LQAIQFAKALYKTANARPSLFPAQYTMEPGIFGAIFANCYALGMGGVDEILHDDGCSVFSVTPDEGHHPSKLSFVKSRFLDVS
SDLQPIEKQLTFGHHPYIDVIPFKIFRENLITVLDQDPNAIDEGALCHDILSGGFTCWGAGRNSHGMGAGVPWDSRSWEPSIW
FLMKYRALAGEWDGELWKSARWWHSARGERIQISQGIDIGSNMFQGTPRR
FUN_010952 CDS scaffold_4 491049 492455 + hypothetical protein PF00226;PF00684;PF01556

MVKETKYYDTLGVAPTATEQELKKAYKVGALKYHPDKNAHNPDAEEKFKEISHAYEILSDSQKRQIYDQYGEAGLEGGAGGGG
MAAEDLFAQFFGGGGFGGMGGMFGGGGMNRGPPKARTIHHTHKVTLEDIYRGKISKLALQRSIICPKCEGLGGKEGAVKRCTG
CDGHGMKTMMRQMGPMIQRFQTVCPDCNGEGEIIKEKDRCKQCNGKKTTVDRKVLHVHVDKGVRSGTKVEFRGEGDQAPGVQA
GDVVFEIEQKPHARFTRRDDDLLYNCDIELVTALAGGTIYIEHLDDRWLAIDILPGEAISQDAVKMVRGQGMPSPRHHDFGNM
YIKFNVKFPEKNWTEDPEVFETLRKVLPPATQNAPPGDAMSEPASLEDLDNTTQNRVFGNSDGMMDEDDEDGHPGGERVQCAS
Q
FUN_010953 CDS scaffold_4 493202 494945 -
hypothetical protein EOG092632TF

MKFPFNVVHVSGNVLFAARGGKIHSFSLEDGSHLSTWKHPDVDKVDAAVKAISGDILTGNPVTKTHIAEEEDGNGPPAKRQRT
EEPKDETAPTKPEVQEDTQISEEMKSEGKKKGGKKSKNRQNQQRAKDHNISRVPDRPVITHLTSTPDSSHILAITGHDKAIWV
FENDGKASLNQISKRTLPKRPSDVVIGPDSQIVVADKFGDVYSLPLLYDPTLQTASTPAPAKPAYKPSANTTTVHSKRNLRAL
QEQQRQMELSTRTKNDSNSKAEGPDFEITLLLGHVSMLTALVIGESEGRRYILTADRDEHIRVSRYIPQSYVIEGFCFGHTEF
ISSMVIPASRGDVLVSGGGDEDLFVWNWKSNKLLSKISILSLAQKILPEITKVAVSGLYSLVYPHGGSDLVYILAICQDISAI
FSWQLTKNHALNHPAIIQLPGKPLSLSIKPANGDEAPKIVAALDPSDPTQARSLAVYSLTMTDEKLATSTIASVNDDDIENAE
LDVDEKIVRSLLYNTEPLRKQATEREEERGEEQVPEDQMMEEAGPAEE
FUN_010954 CDS scaffold_4 495309 496357 + 60S_1 60s acidic
ribosomal protein P2 EOG09264G1I PF00428;PF00466;PF17777

MGGKSENKANYFDKLKGLLEAYNSIFIVEVDNVSSQQMHEIRQALRSKGVVLMGKNTMVRRALKTFIPDSPEYERLLPHVKGN
VGFVFTNDDLKEVRDIILANKVAAPARAGALAPADVWVPAGNTGMEPGKTSFFQALGVPTKIARGTIEITTDLKLVEAGSKVG
PSEATLLNMLNISPFTYGMGISQVYDQGQSFPPSVLDVGEEQLLKTLSQAIATIATISLALNFPTLPSVLHSVVNSYKKVLAV
AISTEITWPEIEELKDRIANPDAYASAAPAAAASGGAAAPAEAEKKDESEEEDEDEEGFGGLFD
FUN_010955 CDS scaffold_4 497209 500549 -
hypothetical protein PF00488;PF01624;PF05188;PF05192 ENOG41

MVPPVFGIRHLRPLASPSASTSRSISVPASSSASASASSSVLKAATFVVSNAAAIVGAPSPKASAISHGHCHIEHSLYHGFSI
RSFQHPPSPHRPLQARGKKTTTTIKLSDLPQGLIQPKPVSPLPTSSSVSSPHDESTEALPLPLPPLPQDPPAYPTVLLQARQN
MLKFDNCVLLTRVGGFYELYFEHAEEYGPLLNIKVANKKTNAGLVPMAGFPFFQLDRFLKILVQDLGRHVAIAEEYPNSPTAK
VRSGGLMHDRRVARIITPGTLIDENFMDPYANNYVMAIHIDPGTKATNTVTDVKAVIGRVAGSEGGLGGQGPESAHPAPSAVG
VGRGAGQSQGFRDAVPLGLAWLDLSTGYFCTQEANLASLPTILSRLCPRELLLDQALQSCPDNGLSDLLAEDRHIISYAPRPH
DSSLFHPENWTPMLECALAEHEASAFSPAEVHAAGFLLGYVKDRLQGMSMKLQPPLRNKDMQVMAVDKNSMRALEIKQTIRDG
VFRGSLLHAIRRTVTKSGARLLNEWLSAPSTSLELITSRQDLVTRFIDDVNLSDSVILLLRRSHDSQRLVQKFTLGRGDADDL
LGLASTIDATKDIVNLLKKANAASRKSEHNCLTSLISRISLTQPLKLSQRIRDAIDEEGIELQHEVQDSEASQMLALAENVVS
NEGSQDDAASLPKGKRKRPVSIRDYYAEENGAWIMRPAASPNLTKLHTDLATLTEEKATLNETLRESLNAPSLSLKWTPGLGH
IAHIKGKDARNLADVQALSSSRSTRSFHISEWTLLGQRMDQVRAQIRAEEQAVFHSLRELVVKNLVKLRRNAAVLDELDITTS
FAKLAREQNLVRPVLNNTTSHTIIGGRHPTVEGGLFEQGRSFVRNDCLVGSPTDGRIWLITGPNMAGKSTFLRQNALITILAQ
IGCYVPASYAELGIVDAIFSRVGSADNLYRDQSTFMVEMLETAAILKQATPRSFVIMDEIGRGTTPEDGTAVAFASLHHLATV
NQCRTLFATHFHSVADLAQEEGLCSAEAGVVQTYCTNVVEDGEGGFFYNHKLQRGINRQSHALKVARLAGLPDRAIEVAKRAL
KQDNFI
FUN_010956 CDS scaffold_4 503412 504429 +
hypothetical protein ENOG41

MSSVNPFTPVTPPPESHSFPKLPAQRPLENNLKRKASMQEPFSGLAGLNTPTMMAPPPPPTSQPRMSSSPNRSSGPSSSNTPQ
PSESTTPELPSIAGANLDPKYLAMVSRIAAYYQQRCQAVANYQQQRCQAWANMHRQKCQDMMQASMLVVAWYVRDRIQRRRRR
EKRHFRSGLRRRTDQNKIARTEVVRRWVKQIPEGPESPNDPINVQLADREEAEFSMDRETQPDKDTKLFEMADNLIKSQYKKI
EVPIMGVLNFDESDNDSESDLDEPEPEQYDEMDEAEDLAEDDEYYEVEDDELYDDEDEIEYTGDGASEVVHHGTGTGSGSRNN
NLSETS
FUN_010957 CDS scaffold_4 507068 510410 +
hypothetical protein EOG092607QZ PF01074;PF07748;PF09261;PF17677

GH38
MGGGSENKGDDSSYPLFAERPVGVPKTSILKGRIEPFYKSGQYYKVNLQANMYNGRYSGKPHVQLSVWDAPDLSRPTFEEATS
HEFKETHTGTSFGPSWSTHWFKVVLRIPDETKDEELIELHWDANNEGTIWTEDGVPIQGLTGSGERIEWIIPDSFRDGEEHTI
YIEMACNGMFGNAPNGTTTIAPPDPNRRFNLSKADIVAINVPARKLHLDMWEIGDAARELPENSAEQNHALSVAMKIINTFEV
NNQESILKCREIAREILGPDVDSHKVYEGGKEPVVFGIGHCHIDSCWLWPWAETKRKVVRSWMNQCDLMDRYPESNFACSQAQ
QYKWLKTYYPAAYKRVKQKVKEGQFHPIGGSWVEHDTNLPSGESLVRQFFYGQRFFEAEFGSRCRTFWLPDTFGYSSQLPQLC
RLAGMDRFLTQKLSWNNINNFPHTTFMWVSPDGSQVICHMPPSETYTANADFGDLKRSIANHKTMRVDSSSLLVFGKGDGGGG
PTWQHFEKLRRCAGISNTIGGIPKIKMGLTVDGYFDRLNRKASEFPTWYGELYFELHRGTYTTQANNKYYNRKAEVMLRDIEQ
LATFASIKNKSYKYPTKDLDDMWEAVLLCQFHDCLPGSSIEMCYDDSDKVYAEVFETGKRLLKDLYESLNVSSEFSTCLNESV
AINTLPWHRKELVELSETEVGVACGDGQLLNLRTFKTQEEKPAVTVMEQSTDVYVLQNEQLRVVVESGVITSLYDVVNDREVI
EKGGEANKFVIFDDIPLYWQAWDVEVYHLDARRKVEYGKTKIFEQKPHRVTLVTDVKISENSYIKSYTTLSAALKGQPSRVDV
RADVNWHEDSKFLKVEFPVNVVNTEASYETAFSITKRPTHYNTSWDMAKFEVCCHKFADLSENNYGVSILNDSKYGFATAGKT
MRLSLLRAPKAPDANADMGRHSIRWAILPHKGSLSSTTVKAAYAFNNPLKPVTASRAVLESVSSAPIKLVNIDESDSLVLDTI
KRGQDDEDVSRDDGLRINKGQSIILRVYESLGGRSRGVIETSFDVKRVTKTNILEDELEEIQKEDGKIPITLRPFEVATFKLE
L
FUN_010958 CDS scaffold_4 510858 512179 -
hypothetical protein EOG09263OAE PF07714

MAQVLLDLFYSFGNCLNCFPGNPNLKINNRSFKILRLLGEGGFSYVYLVEDTSTHELFALKKIRCPFGAESVQQAMREVDAYR
LFSHVPTIISAVDHSVATERGADEATKTVYVLLPYYKRGNLQDMINANLVNHDRFPERRLMLLFLGVCKALRAMHDYKPAVER
MHMGREEDELNHGERNNTRGKRTEEEEEGEQERGLLEEENQVSGGRSIQHYAHRDIKPGNIMIDDSGSTPILMDLGSVAPSPI
PVTSQSLALQIQDTAAEHSTMPYRAPELFDVQTGMVIDTKVDIWSMGCTLYACLVGKSPFEMRSDETGGTLSLCVLGGDWRFP
DEGPAGVKRSNSMRPQQGQQQQQAPAVEISEPIREVVRKCLRVEAAERPDIHELIEMVEEVIEELPDDSH
FUN_010959 CDS scaffold_4 513484 518682 -
hypothetical protein PF00628;PF01426;PF13771;PF13831;PF13832
ENOG41
MAQHNDSNTSDPGAQSVSDPMTTQSSLDSAAKEAGADSAFPYGTRSRNRTGTSRINYAEDRDIDGDVYDYYHDKKDSDSKKTS
RQSSAAVNGDAPRGGASSRKAGTDEPKAAQVANTPKEQQSGSSNNVPTTTQQPAASQPARKRKVVGNPAGSTNPATSVNNSTK
KTAASGTPSRIPWPETNMLTFDNCKSRPVNGKMVADDGTALEPNDHVYLVCEPPGEPYYLGRIMEFLHVQNDSSKPVEAVRIN
WYYRPKDIGRKSTDTRMVFATMHSDISPLTALRGKCQIHHKAEIKNMELYRKDPDSFWYDKLYDRYIQKNYDLIPTRSIVNVP
ANVKKVLDERWKYVLVEQGRGKELTSAVKLCKRCAGYCASNDSVDCAVCQNTYHMNCVNPPLLKKPSRGFAWSCAACSRAQER
KLEARNTPNVNDPSFDAEDDDPMDEEDEEMQGLETNRTSPEEGDHPHHEGTAEQIYQASLWPYRYLGMHCKPEDALDYDDRIY
PRASTRIGPRNQANVLPWPGKPVEYVKPLEIKKNGKKDTKSKEALAAIEADKISRGKRPKWVQDMPPGYTARGEDFDDDDPRC
TATRMWIPPPEKVIKEKDMNEYMEKAKGMTKDLGLPERSTNLQDVAANKLFRAEFDTEHALKDLSETKKEAFKEPELTPAEQK
KFEEAVIKYGSELYLVRKHVKTMHYGMVTRYYYAWKKSARGKQVLENQAGRKGKKEAKRAEAAASKLADDVADNDDDSAFDTE
KANQKKQGFVCQFCSTTSSRQWRRAPNPVPGVVNDGGSKNSNKEKGQERVVALCRRCAELWRRYAIRYEDMDEVNKKITQGGR
AWKRKQDEELLKELQAAKEMGMMTPERAPTPSATPAVATVQEPPRKKLKGAASDKDVDNGHSDAASAAGSTTSKKKDKSVENL
AVPDIPKPLVLPCAVCGQLEPQGDQHLSCRECRLTVHRNCYGIMDNRNPGKWTCDMCANDKNPQVSIHYKCVLCPVEYTEHDF
VEQPKITHHKKKMSEKDREREREVQQARKAADFYRKKQEEMNRPVNPREPLKRTADNNWVHVTCAIWTPEVKFGNAKALEPS
EGIPSIPRSRYDETCQVCNKQDGACVSCHQCRIPFHAECARQHGHVLGFEMTSVKSSRRDQFSIVAINGESGIMSATVWCKDH
APTKTTIHQMQDIADESGLNALQLYVQNYKQADLALTGTVRKANLMTNAAKLSGTTTQPGLRRSSTTNIPNGGWGQQRNGDTG
DVASDSKQPGEKVCITCGIDTSPKWWPIDNSQERELTNGHYGTLGSEAQKFVEQRRFQCHKCRKAQRTAKPHPRRSPLMADAP
LPSAHGAGIPGTTTHPLRSPPQMMPAHRESNPSAAYAWPPPVHAPTVPPPPMQAPALVADHALGARPPPPAPYAPLPPQRPSY
SDWGRPASQQGSPPRHINGGPLIHNPPPMSTLSSLRPPPVVGPGPPVGSSPPAPPPANHHGGPPSQPYTNGLPPSPRRLNGPA
PPPRYTHPYQTHGHNAPPPPPPASHLPPPTLTNGAPPPPPRHDGFSHELHPQRPTYPAPHGSPPGPRNGPQPPSSRPASGASA
SPSLRNLLS
FUN_010960 CDS scaffold_4 522813 526183 +
hypothetical protein PF00270;PF00271;PF04851

MKSMRGLLYTTSVETTNEQNTTFATTSVAGHKRSISTAEEHHFRASTSRATVQFQRAVSLPSAPPRISASRIVRLDGRVGPSE

YSQRRAIAATPTSSCDPELELSHSTYSLPQQLVDNFASLGIKQIYPWQKSCLKGPGLLTGEKNLVYCAPTGGGKSLVADVLML
KRILEEKGTKALLVLPYVALVQEKVRWLRSVVQGLHFASETTIDDDKRIWRRRADENTVRVIGFFGGGKVRATWADFDIGVCT
LEKANALVNTAIEDCSISKLRAVVLDELHMVDDDHRGYLLELVATKLLSLEQRVQIVGMSATLPNMDMMARWLEGHCYETRYR
PVPIEEHLVYDGNIYPAGSTSSLIKTATQLNSRSTQTQAQMRPIRRIEPSTHKELRDPVLNAVVTLAHETAFAGFGALVFAGS
RGMCESDARWISRAMPQPHELKADVVDRRMDLLGELRSLNTGVDPAGLTTEERDLIAAAYDSGTLLVCVATCSLAAGINLPAR
RVILHNCRMGREFVGPSMLRQMRGRAGRQGKASIGETYLCSRENDLEQVVELMNAELPPVASCLNTENRRIQRALLEVISIRL
ATSRESIMDYFSKSLLSHTHSAKFVNDCITSSLEEIESMGFVTSDSLSMFTATQLGRAIVASAIDPDDGVFVHNELGRALQAF
VMDGEMHVLYTFTPVQEFGVMVNWQVFRNEMEGLDESGLRVLRLLGIKPTTILKLAQGATLRETTPEERQVARVHRRFYLALQ
LRDLCNEIPIHIVARKYDVPRGMVQNLSQTCQGFAAGMIKFCEQMSWGVMAAALEHFSDRLVAGARADLLALAKIPFIKSRTA
RVFWENGFRTVATIANADPTELLPILMQAQPNKIRLKGKDNEKYEEKLLVKAKVISDAASKIWKLQMQAEMEEE
FUN_010961 CDS scaffold_4 527117 528511 +
hypothetical protein ENOG41

MAGDAGNAELAGSTVTVHTLADAMGVPPPQVIVIDRDYQEEKEEDLPSTTEDLWVSSADGRYCSAPVPIRVGPLNNMQTFYVH
RDILVQAEWFRKALCGNFREASEQFIDLPEEDPAIFHFLVAFLYEGCFEPIRPAASVLEPEVDKGKGIEVDPEPAEASDSSSS
SSSSSSGSSGTSWRRRRRARQTRLGEAEETQEKHPGHHRPGCSCPRCLARRDFSRCWQCGAQRSREGNPLVPVPYGRGAPRRG
IHPPGVFPPPVSIPMPPQQSQPERITGEDLRTWFLAYELNLDVYICATRYLMDDFRKAVMRSCVDMLETAGWDAAQPKMMHLC
RKLYHNVPEVDDLLKMVMARVGFLQPLLWKRAPVETSEFLVSNPELAATILRETVMRHSQPMADLPSMEHVHTTAYEDEWQNL
GRAPARTRAYH
FUN_010962 CDS scaffold_4 528751 530197 -
hypothetical protein PF00561 ENOG41
S33
MSTPDNLSIPQADLVSRKSHVLPGQLCVTELFFKVPLDYTDIQGPSITLFARAVKKHDVPIFPPDDEESENSPKDEFGEPPKP
YMVYLEGGPGFGNREPQDHPLTRHALAKGYQLLLLDHRGVGLSTPVGADMLSLVSEDVEGRTRYLGLMRQDNTVRDCEAVRKY
LTASWPATKQPWSIFGQSYGGFIALSYLSMHPEGLREVFLTGGLAPVGKKPDQVYEATFQRVIERNEQYYEKFPEDVENVRQV
AKFIESKGGSIPLPSGGVLTVPRLLTIGISFGSHGGFDQVHSTILTLKTSLDQFGFFNRASLVPLEGWNPFDTNIIYAILHEA
IYCDGPGLASAWSANRIGRELKPFSWLNDNYSTESTSGCLYFSGEMIFPFHFETYPELQILRQEAELLASRDNWPALYDQEKL
RKNKVPVYAASFVEDMYVDYNFARDTAKLVKGTKTFETNVMYHSALRAKSDEVLQQLFSLRDDVID
FUN_010963 CDS scaffold_4 531232 533040 +
hypothetical protein ENOG41

MAYLHLAHTLADAFNALADEVQTLTDRKTVLEHKLRFAHEQFQYLADKYAPAAPEIADTLAKLQIPPYTPVDNSLPVPLPRAY
SSGSQHQLALVIRDGRRVANQLASLGEASKTTGTGSSRETESQPSHVATSMSTALEQDFTVQGKKGHLQCPFSKPATASGSGV
RDEDAQDTTPHHSTDPICAAMYEESTSQQARTNTNGSSAVKCPIRYMDKHSAEEIAHYVETHKHELPRSHEVCLRRYQRDEDQ
IKKIDSKYGDIVSMIEGLGQLHQPMLPEAEQDPNRPNDVAQPSNERVENWAQAVSVNSDPDNTQVPTREDGDRQSHFDRPLKE
VRVGESPSRPWGISIPVYDLSGQGDDQPLSPPPAPVCMPISAQAAASAQESIKKTPGKCPFDHTKLAAMGMPSPFKHDTPPHV
PEHTVSSSPHKEGGPPPPPPHQHQHQHQHQYQYQHQHTAPPQPAFINPDMTKGTNNGPQMIFTGPVFIGYPMDQAIQFMNQYT
GNQ
FUN_010964 CDS scaffold_4 533499 534718 -
hypothetical protein PF09335 ENOG41

MPADYNSTAQALALSPDRGSSPSPSRPPWSATSSSRRLSNPLMNRRRASSSYAGGGKTSFVRRIWGSVSLLGEQVLKVYLRLS
PLQRVLALVGVVAAGVLGILAIIYSHVFFKWLEPKAEQWRALPGGWTLAFLLVFITSFPPIVGYSTASTIAGFVYGFPLGWPI
VASGCTAGALCAFLASRTVLSGYVDRMVGRDHRFVALGQVLRQEGIWYLTAIRFCPLPFSLSNGFLATIPSITPLAFTISTAL
SSPKLLVHVFIGSRLALLAEKGDKMSAGSKAINYLSMILGAAVGLIVGLIIYRRTMARAEELARQEGLEPTGLSAEEGEAGYV
DSENSMLMDPEDAAVLMSDDDISLWGRDGTEGRYHDDDEEDEGDTNKRGNQRV
FUN_010965 CDS scaffold_4 535268 537549 -
hypothetical protein PF08590;PF14608 ENOG41

MVSDEHFEMCLPILQDATLEDEDKTDRLEELFRKETSLTGTSLDNAILDALWRFRDGGGTSASPPPIRQTILRRPSPASWRGS

PTPMSGSPRLAVSPLAPPGFVPSTFGRTISSTVSPFSSPRASPRLALATPVIPHSPNLNAYEFASDPTPATEILGEYQTENVE
WLVSDDAASVTSSVGTPSALNAAAPEFSSMSAQSADMSPYDMLRSILGQTKSDDEIEAALAQNGYDLSATIVSFMESQAGDNQ
PGGVSLEEPKNVLIGKSVDRPTTPLGQSKSGVICKFYMSTGSCLRADCRFSHDLSNHLCKYWVMGNCLAGETCIFSHDPTKLM
NKLALDGASTPPSKHGGLQLADLNSFPSLRPGTPDHLSGFAGSNSIPAVGITPPPGLKHFQGTSSPRARSRPSSRHQTREPDI
SAPALDDNDAFPSLGSASIKQGKKHHGKRGGHGHSHKENNAPNSLADIVKMSPSPTPVRPDLKRGLGRNGSSTVARNGENSAA
AQAIPSPKHIPWLETGERANKAYLKARQEAIKHGGLRNKFLQSAAQAWNRNDARAAKALSLRGQSENDLMRKAHREAAKELYE
ERNRGNSNGLELYVDLHGLHPEEAVEYLEKVLMENGRESQPIYAITGSGHHSKNGKDKVGRAIRNFLNEWRYAYREFSVPGDR
NNMGGILGIDARSWDKSLAKEGESDGKEEVDILSQGVEIGDGKVRLLVRDPPKGPSGRR
FUN_010966 CDS scaffold_4 539707 541266 -
hypothetical protein PF00646;PF02373;PF12937;PF13621 ENOG41

MPSAIVSNGTGHHDSLCDRRNDTYESTLQSDSIPPHPLGLKPLGNQYFFTGRNARRSIGSWRLLPDEVISLVLEQFDAVALLK
LGHTCKFLYAFCHSDEFWKPIFLQSPPPDSKDIRWQGSWRSTVLGLSSDQGTRIDCSNVFSDVLHRPFACSHVILNRFTSHIP
KANQIRRFENLTYDQYAEKWTEQPFVLTKCIQEWPAYEEWTIDSMLQMFAEVEFRAEAVDWPFATYHTYMKNNSDESPLYLFD
RRFAEKMGIRVGKKPGAAYWRPDCFGPDLFEVLGDERPAHRWLIVGPERSGSTFHKDPNATSAWNAVIQGSKYWIMFPPATQV
PGVYVSDDSSEVTSPLSIAEWLLTFHEEARQLPDCVEGICEAGEILHVPSGWWHLVVNLESGIALTQNFVPQSLNLVSEVVSF
LRDKADQVSGFDDKVADPFGLFVERMKSSYPEVLDKALDLADKKSGKKRKWDAAVGGIDDEQEQEGGDGFSFGFGGDGDDDEI
P
FUN_010967 CDS scaffold_4 542700 544125 + TIF1 translation
initiation factor eIF4A PF00270;PF00271;PF04851

MADKGLEDVPEGQIESNYDETVDSFDDMNLKSELLRGVYAYGFERPSAIQQRAIMPVIKGHDVIAQAQSGTGKTATFSISVLQ
KIDTNVKQCQALILAPTRELAQQIQKVVVAIGDFMQIECHACIGGTSVREDMKALQDGPQVVVGTPGRVQDMIQRRFLKTDSM
KMFVLDEADEMLSRGFTEQIYDIFQLLPQSTQVVLLSATMPQDVLEVTTKFMRDPVRILVKKDELTLEGIKQFYIAVEKEEWK
LDTLSDLYETVTITQAVIFCNTRRKVDWLTDKLTARDFTVSAMHGDMDQAQRDLIMKEFRSGSSRVLIATDLLARGIDVQQVS
LVINYDLPANRENYIHRIGRGGRFGRKGVAINFVTAEDVRMMREIEQFYSTQIEEMPMNVADLI
FUN_010968 CDS scaffold_4 545138 546776 -
hypothetical protein ENOG41

MAGPEDRRRPAALNLASARPGTSESSSSDGSLKPRTPRFAEATSVHSPVEARSPFADPEKSHVAQPQPADVGFGYIGQRESIP
VPMTPKTPLKSAMKVPGTPAHLKNPLSPTFREEDILEKREASTDKEQARDVKIKARVRLAKFALRGVNFSCSLIILAMLSASF
TIFNATKALPDQSKMPSWAKNTSAWPQKLVLAMACVSLVACIVVFISYCRGGHRRAEKVGTYYTMFAIGWFILALVLWVVTAV
IFQNSRNNSGNQDMWGWSCVNNHRSEVFGEKVDYALVCRLQNWAMICIIIEVVIEVLCILLYSVVFYRYYTKRRLFKSMDMRD
RARSDLYLAQLRSQSAPNTPGFGPKSPALSAYAMSPRHPPAAYRNLSDISENPTTFTPGTQFAEPKSQFAPQETGFKLQAPPM
KAPSATPKLNQSAFTPTEASPPAIPTVQVSQHGPVSTDEPTYEAVPIPGAYAGQAVRSPPPTQTHFGQGY
FUN_010969 CDS scaffold_4 549346 550995 + GCD11 eukaryotic
translation initiation factor 2 subunit gamma EOG09261TPN
PF00009;PF03144;PF09173

MSANGDPKYDDIESESDSGESVGHNEAGDEKPLKSALKKSNPAIAEPAAQKPPLPPQTDPKDLDVASLTPLTPEIIARQATIN
IGTIGHVAHGKSTVVKAISGVQTVRFKNELIRNITIKLGYANAKIYKCDNQACPRPGCYRSYKSEKEVDPPCERDGCGGTYRL
LRHVSFVDCPGHDILMSTMLSGAAVMDAALLLIAGNESCPQPQTSEHLAAIEIMKLDKIIILQNKVDLMREEAAQQHYQSILK
FIRGTVAGKSPVIPISAQLKFNIDAVNEAIVNTIPVPPRDFSIDPHMIVIRSFDVNKPGSEIDDLKGGVAGGSILHGVVKLGD
EIEIRPGIVSRDDSGALKCTPIFSRVVSLNSEANDLKYAVPGGLIGVGTRIDPTLCRADRLVGFVLGLKGRLPEIYSEIEVNF
YLLRRLLGVRTADGKQAKVAKLAKNEVIMVNIGSTSTGAKVAAIKNDAAKLVLTSPACTNIGEKVALSRRIEKHWRLIGWATI
AAGVTLEPSTA
FUN_010970 CDS scaffold_4 551378 553012 -
hypothetical protein PF17104 ENOG41

MTDAEKLELPKGAPVEDPGAHELAEESESDDQFTDAASGAGSPKISSPIPKTRVEKVDDQPSHGEVPGTEAYEMRGQDAQPDE

IAIAPETGASQNAPSTPEIKAPTTIVEEAPGARSRSHSIQYEERRKADASPDILVDSDGQVKEIQGESAHVDSNSDNSRPSPD
TTDSKLQEDGPSESPRAIAASLQDADDDDDDDDDDTQQDNDGDDGFGDDFDDFEEGNEDDDFDDFEDGFQEAETPAPTATQPP
QQSTLPFSIPDFDGLDPEAVVSALEPFLATLYPPEELDLPTFPPLSKDSVFFTARSASLWSQLVAPPPLAPPDWIRSRTRRLF
LVSLGVPVDLDEILPASKQKKLVLPSLNVPSLSPRTSTDSRSVSRLRQGEGNNSSTSVDTQGKPSASTRKRRGPPPQPELDLV
AARHLCQTTDEALDGMTDEELKAHVAKLEGLQEAAKEALEYWKKRTDEKIGDREAFEGVIENLVKHARKTRK
FUN_010971 CDS scaffold_4 554454 556661 +
hypothetical protein PF05064;PF13634

MSFSFGNASSSSAPNNTTSTAPASGGLFGAATGTPSFSFGSIGGSASNTTTPTSSATNMFGQQSGASAQKPGGNLFGGGANTA
NTSTTPSSGGIFGGAGSTTPATGGLFGGASSTTPAATSAPASGGLFGGGASNSTAPASSGLFGNNGNNASKPATGLFGGGGSS
TTASSGAGLFGQNNANQAATATPSSATTNLFGNAASGAATPAKPTFALPSTTPAGAPPADSSKPTGGLFGNTGAQASGGNSSL
FGGAKPASTSAPSAQPSTTAAASSSGLFGGAQSGSSAPPGGLFGAKPAPASTPAAGGLFGTKTEGAPPAAASQAGSTAPANST
ASLFGAKPAAPATTTPGSTPASGLFGGGATTSAASGASATTTAGGLFGAKPATSTTSTAAPATTSASAGSLFGGAPKPSTESS
TSTPAGGLFGAPASSQPAGSAATTTATAGATTTTAPAVGSTPAAASSLFGTKPTTDASKDAAKSAASSATPGALGASTTGPTS
QMARLKNKTMEDIVTRWASDLSKYQKEFKEQATTVSTWDRSLVDNGEKIQKLYLDTFEAERASHEIERQLAAVESQQDELEAW
LDRYESEVQDMFAKQLGPGEQLAGPDQERERTYKLAEKLTQQLDEKSRDLSKMVKEINDISGTLNKGAKPEDPLSQIVRVLNG
HLTQLQWIDANASSLQAKVAAAQKSSSNLGSHYAGSDNDAAESFYRSYMGRR
FUN_010972 CDS scaffold_4 557020 557455 -
hypothetical protein PF04568

MLRTTFRQTAAFRPVRCFSTTPRVMTEGATGSVRPTGGSGDAFQRREKANEDYAIRQREKEKLIELKKKLQEQQQHLDRLSKH
IDEITREQGGEKN
FUN_010973 CDS scaffold_4 560075 561116 +
hypothetical protein PF00244

MGHEDAVYLAKLAEQAERYEEMVENMKIVAGEDRDLTVEERNLLSVAYKNVIGARRASWRIVTSIEQKEESKGNSSQVTLIKE
YRQKIEAELAKICDDILEVLDQHLIPSAKSGESKVFYHKMKGDYHRYLAEFAIGDRRKDSADKSLEAYKAATEVAQTELPPTH
PIRLGLALNFSVFYYEILNAPDQACHLAKQAFDDAIAELDTLSEESYKDSTLIMQLLRDNLTLWTSSEAETSAAGQAEAPKED
APAAATEEPAAPAEEPKAE
FUN_010974 CDS scaffold_4 561589 562126 - DPH4 Diphthamide
biosynthesis protein 4 PF00226;PF05207

MASPEVATHYQVLNLTPTLLDTQHDSTPLIKRAYHRALLRNHPDKVTNSDPSSVCFTVDQITTALTILSDPSCRAAYDAALRV
SRPSEKRDGSFQIGVESVDLDDLAFDEDQECWYRPCRCGNEHSYEFCEADLEEVSDEGELVVGCLDCSLWLRVHFAVIEDEQE
SHPSNKTSKDKT
FUN_010975 CDS scaffold_4 562515 563145 -
hypothetical protein PF18126

MASTSQFIGLAKSLPAPLQRFFARYPPAAILPENTPKTRYQEERPNPFRFYKHPVTGKWQDPVYSQRRQAELVKMARENGVED
LLPETRKGTEYKLAHRVEHGLRVKGTGVGQKVKGHIHERHMIAKMETRRKAMLDMPSLIKRWKRVGKYGWTKFPK
FUN_010976 CDS scaffold_4 563493 565665 + PRP28 mRNA
splicing protein prp28 EOG09261JWS PF00270;PF00271

MPLDIEEILRQKKAADAAAAKPRFIPKAERERLAAEKAKKEEDEQKRKASEDAQKRREEEQKWAARSNGSSRHNDSTNGSSRV
PTGPKAMNQENGRDRDGYRDQGRGRGRGREGRKGDKQAADKQSAEDIEATLLRTRYLGPQVNQQSNFSAKKKRMRTTEKKFNF
EWDADEDTSRDNDPLYTRQAVNHNGSFAGVGGEFDDEAEERARKRARMIEQRDPENGKERAKGIMEDFFRARDKARQRADRTG
LGKRWSEKSLEDMRERDWRIFKEDFGIATKGGMIPNPMRSWQESNLPKRLLNIVEDVGYKEPTPIQRAAIPIALQARDLIGVA
VTGSGKTAAFLLPLLVYISDLPPLDELNKNDGPYALIMAPTRELVQQIESEARKFAGPLGFRVVSIVGGHSIEEQIYNMRDGA
EIIVATPGRLIDCLDRRVLVLAQCCYVIMDEADRMIDMGFEEPVNRILEALPVNNEKPDTEDAEDATKMKRYLGGNDRYRQTM
MYTATMPPQVEKIAKKYLRRPAIVTIGNAGEAVDTVEQRVEFIPGEDRRKKRLQEILASNDFAPPIIVFVNIKRNCDAVARDI

KQMGWTVATLHGSKTQEQREASLQSVRNGTTQVLVATDLAGRGIDVPDVSLVVNFNMAPKIDSYTHRIGRTGRAGKSGVAITF
LGPEDADTMYDLKQILSKSSISKVPEELKRHEAAQSKPVRGDGGGSRKDKEEGLGKGGW
FUN_010977 CDS scaffold_4 565898 569393 - CYT20
Valine--tRNA ligase, mitochondrial PF00133;PF08264;PF10458

MATNSGRGNPIAASEGIQDATSTPPAVPAAEKKEIVESATGAHATGQDAGAAAAAGAPKVKTEKELEKERKKAEKQAKFEAKK
QKAAATAPKAAKEKKPKEKKVEEEPLPEYVEDTPEGEKKRIRSFEDPHFKAYNPIAVESAWYSWWEKEGFFKPEFKPDGSVKD
EGKFVIVHPPPNVTGALHMGHALGDSLQDLMIRWNRMQGKTTLWLPGCDHAGISTQSVVENMLWRKEGKTRHDLGREEFVNTV
WKWKDEYHKRINKALTSLGGSFDWSREAFTMDKNLSAAVTETWVKLHEEGTIYRANRLVNWCTKLNTALSNLEVVNKELTGRT
LLEVPGYDKKVEFGVIVHFKYPIEGSDELVEVATTRIETMLGDTGIAVHPKDERYKHLIGKTAIHPFIEGRKLPIIADEYVDM
EFGTGAVKLTPAHDPNDFTLGQKHGLEFINILTDDGLMNENTGAYKGQKRFDVRYAIQDDLKAKGLYVDKKDNAMKVPLCEKS
KDIIEPLLKPQWWVRMKELAEPALAAVRDGRIKIRPETAEKSYFRWLEDINDWCISRQLWWGHRCPVYYAKIEGGAGDIPEEK
LWFAGRTREEAEEKAKAALPGKTYTLEQDEDVLDTWFSSGLWPFSTLGWPNNTHDLQTLYPTEVLETGWDILFFWIARMIMLG
LKMTGDIPFKEVYCHSLVRDSEGRKMSKSLGNVVDPLDVISGIQLQSLHDKLLQGNLHPSEVTKATKYQKTAFPEGIPQCGAD
ALRFTMVNATTGGGDINLDVKIIHGYRKFCNKIFQATKYVLGSLPKDFTPSTSGVVRGETLAERWILHKMNTAAKEINCALED
REFSKSTLIVYRYWYNELCDVYIENSKAIIRDGTPKERESAIQTLYTALEAALTMIHPFMPFITEEMWQRMPRRPEDQTKSIM
VAKYPVYHEQLDDPESERAYELVLGCSKAARSLMAEYALKDEAEVIIQAYNDTALTTVKDQSSSIKTLSGKGIKGVEILTPDA
TRPAGCVAYPVSTEASVFLHVKGRVDLDAEIAKAQKKLDKAKSSIQKQEKILNDPGYLEKVSDAVRETDEKRLADAKQELNSF
EETIKQFEQLKLE
FUN_010978 CDS scaffold_4 570059 571597 +
hypothetical protein ENOG41

MALRLALRAPESRLCNRISTTTRRVYSTSKQSDYFTVELSPKIKDPSKLLKAVRSQINITNPSRHDAAVVLATPQYAQWLGKD
EFMSEFVDMLSGSDGTGEFHTLGAVVDHIAPPVPNNKPIQGLSILRGNLDTILPGLWTKAPPKEWSDGGKVSALTMDLGNPHI
TIPLTNTTFQNHRTSTLIISRYDLSTGSPKLIEQTDTKHLQKVNIPFRKRLPSINDLGIWAPLVPLTQPRVVTESFGNIVRRV
DIEDESVPASNELEPAVNELHASKSDLVQSPMGIWAMITPPTFVQSGGSSPYVNPDPEATFADGQSITELTALTSDQLAILYR
QGGRLYQVLSGGGGWGAKKGLLSLDPEQTHFVVSEEEEMQRFIQAMEGGNFVPVSSKIQFFASTDSSASEPVGKKQTGVVFGT
SSKVEDETDANPSQPEARLLENHFGALSNVGIFVTSSDASMFSKPGFNNDWKLNVPNSRIYVV
FUN_010979 CDS scaffold_4 571721 572764 -
hypothetical protein PF03446;PF03807;PF09130 ENOG41

MSATFESLPKIGLLSIGDMGVGIAKLLVANGFTVATNISGRSKDTIERARDAEVELLESDVDLVRQSSVILSIVPPKDAEATA
TRITDALAGLETTKELYFVDLNAVAPSTVKAIAASVKRENLPIRFVDGSILGHPPKKTSTDATTESSSSNDSSDWYRPSIPIS
GSDGFSSLPFGKKLISVLNMKQISPDIGAASGLKMCFASLSKGFTAIATQSFTTAHNLGVLGALKDEIGTFYPAMLANAEKSV
PGMPPKAYRWVREMEEIALTFNEEAGWDKRMFMGAASVYKDIAENEVLGNEKTGKRKRGTTLDDVAAAAAEGLHKKQRPQ
FUN_010980 CDS scaffold_4 574057 574696 +
hypothetical protein ENOG41

MSSPPQHQHDTASASSSRTTRSSRDKHNNKKTEPIPTSTPRQPDEIEEADTTVPVKTYQPKQKQKRVEPSPDKDTDDAESVYL
SSDEDTSDEDSSDYDSDSEGSVEELPPRQLSRRATDRGVKTTPGSPFTITQTRSETTRWISTQNNGGMMRGARTINQQTTLHG
QNVNGPQPRPSNRNSISFNMSVNVDMSFNGLVTGRNLGFTGVGILP
FUN_010981 CDS scaffold_4 574950 576478 -
hypothetical protein PF00076

MADKMDRGLDEIIADTRANRPRNHRGQGRRREPRNDYPRDGVRKSVRDDSRNLDSEWVHDRFEENNNRRAPAPRRRRESPDQE
SKGAKLRVDNIHYDLTEEDLDELFRRIGPVVRLQLRYDRAGRSEGTAYVTYELKEDAQEAVKQFDGANANGQPIRLTLLPSRN
PFDTAVMPGRPLAERISSPGERRSHSPHRRYDDDDAARRGIDRYVPGQGPRRSPMPRQGGRGRGQGGRRPGARRDGRDGGRDQ
EGGRGGGRGNPRGKKTQEELDAEMADYFGGGDNAQAAEPADQQDAPPAQTQAPAQTQAQAATDDIDMIE
FUN_010982 CDS scaffold_4 577464 578630 +
hypothetical protein PF03452

GT62
MGAVRLKKTNPLTLALGAILCIFIIVFLVSPSGKSATARRFESITAEHHLSPPTSPYRKSKSRSGVPLKPPPVVHYNLNNVTI
TTSPIANRENVLILTPMSRFYPGYWDNLLRLTYPHELITLGFILPKTKEGNQATTELQEHIHKTQKHGREGDKFKSIIILRQD
FDPAIVSQDEAERHKLKNQKARREVMSKARNSLLFTTLGPDTSWVLWLDADIIETPTTLIQDLASFDKPLIVPNCFQRYYDEE
HKQMAERPYDFNSWQDSETALALAAKMGPDEILLEGYAEMPTYRMLMAYLAVEGGDRNLVIPLDGVGGTALLVKADVHRDGAM
FPPFPFYHLIETEGFAKMAKRLGWQPYGLPNYKVYHYNE
FUN_010983 CDS scaffold_4 579297 579865 - PEX4 E2
ubiquitin-protein ligase peroxin 4 PF00179

MPPSSSNSVAASRSASRRLIKELDTWSTEQKEEKGIERLGPVNEGDLMEWEAVINGRDIGHGYDEGRWLINISIPSTYPLAPP
KMTFVTPVVHPNIALQTGEICLDLLKDAWTPAYSVLECVRAVRMLLGCPETDSPLNVDVAALLRSGDVLGTRKLVEFWCRDSD
GRYEGP
FUN_010984 CDS scaffold_4 580483 582177 -
hypothetical protein PF03208 ENOG41

MSRIQIPLDVITSRLNFGDRFQGLRSGPLSGRFSNLRPVGEFLDFKRLSKPANFVEMQSRVNYNLSHYSSNYAVIFVMLSIYA
LLTNWLLLFDIILVVVGMWFIGKLDGHDLEIGTFRASCSQLYTALVCVAVPLGLIASPFTTLLWLIGASGVTILGGLGGRPQS
PEFAPQWGRPSRRSRRRTDGDSASRGRQGRDVRVEELDSDEDARGVGASSGTRFASDSLRFTGIDLGNRERGLVRRGDYQDSE
DSEDDSDSSEDEEFEQYLAELAVRDREEALVQSAMHRIERAKAKGRTDVDLNEEEMGALERRRKRDEAAKKKKSGKKRKDQRV
AVPLTQLEPSSRKKKAPPVSSQPRTQSRARQSSSNSNLTEDQDRSSRPSMGYFPPPATAGRPRSGTSSQRPQSRVRAPSNSRQ
PSDSSVIVRRGRNSSQAPLDPFQFQVPGAQASSPAGSWRQFSGAQRSSRGSRQINGSSEEESESEDESDEEREASVETTSSDD
VGSGAQIVVEESSPEPSKPEPVRRNPPRTSASSSTSKRKPAANGGRRKK
FUN_010985 CDS scaffold_4 584951 587099 -
hypothetical protein PF04727;PF11841;PF16457

MDQADIPALLARMASDEDASRKMAVFKLQNSINDPAFADVFISSGGLVVLRRLIMTSAGNTLAYSLQSLTRLLEVDMGWDIFE
GPTASDLVERVVELIVTNPLVNILRGAMSILVALVGHSQSTNQSDAANRTPGTFGFRALKPAVAVHPNFFELVIQQLQSADHA
LCANALMLINALIRDAVSGESTTNGGKANAGSGEDWAKFIKRLQDLGLIKAVHRLMQSSALQDLAHPMLEFQTLTKILLRKWR
EVDVDLERPEHRRALKGLHLASAPERAHVNGHSPGQESHEAPGKKGSRRHNPEKWRRLGFETESPAQEFDMTGFLGMMDLTDY
VRRNEDGFQKLLLEQAGKPTPERCPVARASFAVTMILYDHFDVDKTDLDDVRGYQLLEAKDHDRLFRPLLLQWSRLHTAGLYA
FFRVWKLTGAEQGDFEKVVELVRILIDCVVGGATRTKDVAEVEEEMQEYDVPRLREQQMGLLEMSFESTWGQHLQQVREELKQ
EALQFVKEQRIRCLLQGSWFSKPTPKRDNGAVKHRLFTPTPWRYAKLSHNRRYLHYADFEEKTVTAPSLDSLGEKVDLSTISS
VVSNVSAPNEDTRSTMSDLMGKDATPKTTTKITVYSFVNPTEAARGTDSKEQPIFTLWPLSHSLASEWLDGLLMLLNQAPITA
ETNKLVNLVSEYGLKIRLLNVRMDTAFDGPEPGAGVIPSRDGLDEDYFFEL
FUN_010986 CDS scaffold_4 587691 588442 +
hypothetical protein PF00642

MANFLASIFGTELDKVNCSFYFKIGACRHGDRCSRKHVKPSYSQTILMPNLYQNPAYDPKNRMNPSQLQNHFDAFYEDIWCEL
CKYGELEELVVCDNNNDHLIGNVYARFKYEESAQKACDELNSRWYAARPIYCELSPVTDFREACCRLNSGEGCVRGGFCNFIH
RKNPSEELDRDLTLSTKKWLKERGRDEKSASRSPTPEPTRRRY
FUN_010987 CDS scaffold_4 588851 591264 -
hypothetical protein EOG09262VQQ PF01237

MVMGLVSHGRRRTGSTASTRSSTDDPRGPDAEDDTLVMEPDQGNVLSHIISQLRPGADLSRVVLPTFILEPRSMLERITNFMC
HPEMLLQIPSIDDPVERFVSVVKFYLSGWHIRPPGVKKPLNPILGEIFTCYWDFEDGKRAYYISEQTSHHPPKSSYFYMAPDH
HIRIDGTLKPRSRFLGNSAASLMEGIAFLSLLNRGKDRAKGEQYILTQPNMYARGILFGKMKYELGDHSFVRCPELDLVADVD
FKTKGWVGGTYNAIGGVIKRESTGEVLFELSGLWSEEMYIKDMTTGHREMFFNALRSKPSPPTVRPIEEQEERESQRLWDKTA
RAVKDRNHELATDEKTKIEDRQREEAAQRAQENIEWHPRLFRRVQGGPGGLEEGEEDLEWVINANIDHAATPEKQAEQIMAIY
PIVKGQKSNQRNVIPPHSPSAKPAQTASAQPVENHIDDNLINFDEMPPPAKPDQTPAAEQATKQSEIQGLLSSTGKKADGPLI
DFTNDLKKDLPSATQQ

FUN_010988 CDS scaffold_4 593404 596945 -
hypothetical protein ENOG41

MENDIPTIQVPDGRSSRRDRLMGKLFGAKDRKGEEARSAANVESFLHSSSDNLTITNPPPPPPPSSLPKLAKLDTTSISRYPQ
ALGLNHAAQQNRPLVAGRPDTTKSPRRSPRPNKKGLTVRFDDTAPEIIGEGGDESEIPTQEISKRKVARPKQQPQQPQSSQPP
QPPAHRRAPMPPGRPPPPDSAASGSPVDDFRPKPLSRTQTGFSSIYTPDSDEESEAGLDRNSKSDELVSPVSAQQHSLSPGRP
IGARFLGPAARQDENRRSYIEIHSAQQREAEGRAFAEAARVASATSASSHNWDDPHSATPLSSSPIESRRRGPPSPEAQVSKP
SLEYSPAASLHSNSSSYSPPAPPAAVPASAPSLSGSPSGAQAPAPTAVSATSTPTSEVNQLSRQPSIAPQRAPPMMASRALSV
RVADGTSAAAEQALETFTSRTSHLFELFRLHAESVKPLSTCSLIEFGRGALWWFLKGRMGLEVAIRERPNSPQGQMQNDRDRQ
QAYANLAKAFWLCELVIPEIAQAQGIEIDAEVDDVSTSLVSALKKLAMSMKRNGFLPPEDAFLPQSIDKSIWVEYPPLSQDMI
ALLSGNWASGLTAMTSPMSKLRLLDSLPVGDTTDNFNYGRVPADAYLMEQGHEDQKLYFPCLLSMVRPQKSTGLVFILASQNG
HVQLVIQENKNVGPIWDDVRWRKETCTVDIRLRRGFMLTIQLVQQDFRTLWNMYDFGSKVNNSLYPRSDESMVFRSTLRSFQV
MDADPNYRQFPKEPTPYCEISLFEKLYKENGPAATRTWHLGFRIAVVTGPKTRTLSGVQHVYSPAQPVQFGFFRGDGDAPSLA
IKYENGKSKGRMVMVFSDDKARIKFHSILTGTALDHDEKIYSDVPLKNFSVAQSLREPLGMAPFSRMPWKAVRVVNDEYGGEM
PPTVLADHLKVVIDYQNGNVADRINVAPGELRVRLEVSNAKVFRVLRQPQKDMTISVSEAQVPKELPRNMSDALQLLRHNQTI
RTFEFNNLKDLHDFQYAMTGFEVIFDALAVTFAIQRRRMVVPIHKKWEAGYTRIQVIRQEDKQLQLLAFFEDFHHGHCMNFLL
KGTDVYESVQRSSKCGIKFVDAKFPLPRLPADKDGDYDDMAFVCLDLPDLPGEHDDISILFENESDRDALAQCLPAPIKGSKK
K
FUN_010989 CDS scaffold_4 600613 601931 +
hypothetical protein EOG092628FW PF01207

MATEVKRVPIPRRGVDYRGKVVLAPMVRSGELPSRLLALKYGADLVWGPETVDYSMIGTSRRFDEDSKTIEWYRLSSHGQKDP
PPDAKESIIYRMLPEAEKDKLIFQIGTSDPDRAVAAARLVAADVAGIDVNAGCPKPFSTSGGMGAALLKTPDKLCAILEALVK
NITPEFEIGISVKIRLLDTAAETEALVRRLCATGITGLTIHCRTTPMRPRERAIRGQLRMIAEVCHEYGVACLMNGDVENRDQ
GLQLAKEFGADGAMIAVAAEKNPSCFRSVADGGLAPWEEVVDHYVKTAIDVDNRFGNTKFLLSNMVPGKSKSYQPMNQSKNYK
SICEALSLEKHLESARIIDSKRGLDQPLQGKAAEKAAKKAAKKANAQTNTQPVKSKEGNDQKRKRRMSDNRPVKQVKTSEPAP
VVTAV
FUN_010990 CDS scaffold_4 602287 603145 -
hypothetical protein PF09769

MASRVVFSRRSLAPLATMALGGMALAPATVFAEGPSDFKRKPIYDDYDIPTANPAPVTPPPVAATPVEPVDDEERHHSPTPTE
RLAVHIGKGRLALYKYAVAAENKVNETMDSAFNLEQSFTSTIASLAPSRESGEQLMPGAIYVLVAAMAGSIITRNRSIILRAS
LPLALGIGAGWTVLPVTMRNISDLTWKYEQRFPVIAESHIRLRESIVNSVSFAKAHSQVGVRYVDEKVTDAREAVEGWVQKGK
FUN_010991 CDS scaffold_4 603652 606019 +
hypothetical protein EOG09260JJW PF01432
M03A
MLSSRGNRLWICNRCVRHTTKPPQRRWNSIASAVAQVISPSTPVDHTASSNHDDTVLRNLFDAPTGRTFPKFSLKKSQGLFKN
RYLTSPGGFQQFAQKNLERAKNIVNKVLSASSVHEYRAVVRDLDRLSDLLCRVLDLADFVRMTHPDSRFQEAAANAWGMVVQY
MNQLNTMTGLSDQLSQALSIPEVTKVWSEEERTVALQLKLDFMKSAVNLPKASRDRFVDLSSRISEIGSAFAQGMQPARKQIT
LPAYKFYGLYPGIAATLKVRQNITLPTLSSDAIGALQSVHDEETRKEIYLAQRTASRETVMYLEAMLKLRGELAELAGFESYG
HMALKDRMMAKTPASVMEFLLALRNNNTPVVQAELQELVLKKRDRLNLPDVPLQAWDKDFYMEKIRVDLRSRQRQEDQLNAFF
SVGTVIQGLSRLFDRLYGIRLVLRETLPGETWHPEVKRLDVVTDKGELIAVLYCDLFHRPQKSGNPAHFTVRCSREILPEEIA
EVAADHNSSGPSFESPEMAANDGMETSSADGVLKQLPTIALVCDFQPSENSKEPSLLSYQSVETLFHEMGHAIHSVLARTSFQ
NVSGTRCATDFAELPSTLMEHFAADPTVLSLFARHWKSDRPLPYELVAERIRLTKRFEGLETENQIILAMVDQAYHASTMRDA
EFDSTAVFHDIKARFAHGPRDPPNTCWQGFFGHLHSYGSTYYSYLFDRVLAERVWRVVFKAGENGGAINRENGERLKENLLKW
GGGRDPWTCLADTLQDERLAPGDEKSMALVGSWGIKDDHHP
FUN_010992 CDS scaffold_4 606620 608681 -
hypothetical protein ENOG41

MKKPWRSAANTAPTAQPVNIQQSNIRGRISGPVPISTSLDDEFPIRNPGTNMATPVQGEFKESQTEPTLSRNDFRSPTFPEHR

RNGPHAFREEPRSSLHARQPGTISRLQKAEPSNNVRHSFVSADTRDKERPQRKQSTLRGALGKLFNRKRKPSGNESSNNTDSE
TGSTPMRPGPYRRSTGEDGQDSVPSLSHRDRSAPRSRTRPFPGAESKRSASLPITEFDRALRSHSIRPEDVRAIESARNSLSA
DFGVSHARISVTRLREYNLAGLSPRPASSHGRESRQDQSDDNAHEIGRAITSDLPGVRRRSRSLSGLPDESHGHTRRRSDEIK
YWRESYGPTFMSPVSSNPAEGEGIAIEEIEQAQQVEDAPKQTEAEPFNFGSFADMKQVGGMKITHAANLETRINNLESRVYRL
EQALDRMCDAAPGWQSHQGPPDRPAPPIPIGGQTNTDLPDHQADRSRWDHRKSLGRSSGHPNYSKAGQRGSVLLDPHSSAILS
DSNKDRPTSTATIRGAASLPSLLKNTPEVFTMDHYTTLLALIHTERSAREALETQVKTLGHQLSILSHSVNTNSGFDPPPTAK
SFGDRSAFDHDDDDDNLTSLAIMTRSQSRHIDPEDSGIGTGHGDESEYSDTFETPREEGHSFGAFGEDLDKEDLAQTKAARTL
SLSQLTMGRGTQKSSQQVHRVI
FUN_010993 CDS scaffold_4 612330 614779 -
hypothetical protein EOG092617S2
PF04851;PF09848;PF13086;PF13087;PF13245;PF13604

MTVKREPVDIPTFATTQLALLEQELQTEINETSTLISNHSPTALQRAGLALTNLVVSGQRTGLGGRTVLELSPDAATGSPDEL
PEHGLRTGDIVLVAEQPAGSAKKREVKDLEKKGARGVVTRVSRGWIAVAIDEGKEEVGFSGRVWAVKLADEVTYKRMNWAMEK
LNKMKESEYSGFIRVLFGLSSPSPVAEDLSKDENVGDLEWFDPTLNDSQKNAIRFALLSREVALIHGPPGTGKTHTLIELILQ
MTKLGQRILVCGPSNISVDNIVERLAPHKIPILRLGHPARLLPSVVDHSLDVLTQTSEAGAIVKDIRTEMDTKQASIKKTKSG
KERKAIYTDLKELRKEFRERERRCVSTLIGGSKVLLATLHGAGGYQLRNDEFDVVIIDEASQALEAQCWVPLVAAKKVVCAGD
HLQLPPTIKSSNAKVKAPVKDGAAVTKGTTLEITLFDRLLALHGPSIKRMLTTQYRMHESIMRFPSNELYDSKLIAADAVKHR
LLKDLEYEVQDNEDTNEPVIFIDTQGGDFPEKNEEDDKDTPRKGRAGLHGDSKSNEMEAALVQQHVRQLVGAGVRPEDIAVVT
PYNAQLAILAPLKDKFPGIELGSVDGFQGREKEAVIVSLVRSNSEGEVGFLGEKRRLNVAMTRPKRSLTVIGDSETVQRGGSF
LKKWMQYLEDNADLRYPDASSLTHD
FUN_010994 CDS scaffold_4 615276 616069 -
hypothetical protein PF02656 ENOG41

MAGPSPTHLSPRPAGSPILPPNSSLRPSPVNEDSSADEATGFISHTPPLNYNSLNVTSTATSAPQIRKPSLRQRPSSQQNNGA
AQNPETTRQASSESGNTSWWQTQAEKFQSIELENKGSVARDHLAIERTFLAWLRTSLSFASIGIAVTQLFRLNTSLESEHGNN
SRSNTQTLRHMGKPLGTTFLGISILILFLGYKRYFQSQHWVMQGKFPASRGTIMIVAFVALAIMVVSLVVVIAIHPSEHEL
FUN_010995 CDS scaffold_4 618334 618574 +
hypothetical protein

MTIRLVKAYLLRAFGMPLPGKIDFHFSLAGCGAKGWELDVRKLLGLLEKATPAIALRKANDDEISQSGIDELLDDNKEI
FUN_010996 CDS scaffold_4 618849 620552 -
hypothetical protein EOG092621S9 PF00206;PF14698

MAEKKTDAPATNMLWGGRFTGGIDPLMHKYNASIIYDKALYREDVLGSIAFARANSKVGIITEDEFKMIEHGLLEVMEEWKQG
TFAIMPNDEDIHTANERRLGELIGKDTAGKLHTGRSRNEQVVCDMRMWLRDRIREIDSQLVAFLQVLTKRAEAEIDYLMPGYT
HLQRAQPVRWGQWIMSHAASFKQDLERLRQVFERVNLSPLGCGALAGNVFGIDRDAIAAELGFSGITLNSMNTSGDRDFIIEF
LSWNSIFTSHISRWAEDLILYSTSEFGFCRLSDLYSTGSSLMPNKKNADSLELLRGKSGRAFGQMAGLMMSVKGLPTCYNKDL
QEGWEPMLDSVQTVLDSLGIANGVIATMTVRPERMLAALDKTMLATDVAEWLVRNGCPFREAHHISGRVVALSENTETPMDKL
TLEQLQAIDSRFTADIVKAFEYESSVEAKSARGGTSRSAVLQQIQELHAILD
FUN_010997 CDS scaffold_4 620854 621605 +
hypothetical protein

MLNRRSQIGNPLTNGLLEVCGELGILVLAYSPLGQGFLGGKIKPVEDLPENDMRAKIHPRWQGDNFRKNAQLVDDIEALAKKK
GCTVSQIAINWLLSLSRRPGMSTIVPIPGSTKPDRIRENATIIDLTDEDLRDIDRLLASFTPAGDRYPPQHMKYVSA
FUN_010998 CDS scaffold_4 621859 624732 -
hypothetical protein PF01902

MVGSPGAHRETSLKHLLPNELAWRYQHLLLHHTPHPNSPPNSATLHHIKCPLLIKKLSFRSHFPASSCLPDWVQLPYSSAWST
ATVLSAMSSEKLNVIALISGGKDSFFSLIHCLEHGHQIVALANLFPGPAPDSSSAISGGQSPFRQEDATKAEPETNLQTPSDL

SSPVGFQRIDPGTWTPQPPDPATGDHDSLRQGQGSGESSDTDLNSFMYQTVGHEVLPLYADATGLPLYRLPITGCAVRHERDY
DATANAQDKVQESDETESMLPLLQSIIARHPEANAVCAGAILSTYQRTRVESIALRLGLVPLAYLWQYPVLPPPSAAISADTQ
LLIDMANVGLEARIIKVASAGLDENHLWERVSSETGSSRVKNALRKFGSAQGAAALGEGGEFETLVLDGPSSVFKKRIVVPEQ
GRRIVREGGGCSWLMLGGARLEDKHDAAAKPAVRIPNLLDPHFKTVFGDLPQPLNELKAAKASAISRRLTSLSQDAGTFTADS
EVLRWSVLPDLGLGEIPIQDETIQVVEKIRDLASGAGVRLSQVTSTIVVLRRMSDFPKVNGEYGKIFTRPNPPSRVTISCGDL
LPAGVNIAISLAAPTPKASPDRNGLHVQSRSYWAPANIGPYSQAIDVPVTAQDQPTGLRCISIAGQIPLIPATMLLPSTSEKS
HELQIVLSLQHLWRVGQEMKIQWWTSSVAYFPRANSSEIQRSAQLAGYAWKQAHGSPDEEGDGDNGPDLWDLKYNPAYMSLGN
DDKTTRKTLPDWEALTLRQQNEPETCIPPMFAAEVEELPRQAAVEWHAHNGLSKIEEASFLMCCLPELTLPGWKTWHSVVTTA
SATVIYSTAAYSHADRPHSASLDSLKIDMCKIMQESLHQLHPHGPETVQSKLTLVYADAAQIVSIWNNLGDNCDVALIPCRSV
WSSDGQSVAIVGLFETIFIRES
FUN_010999 CDS scaffold_4 626586 628422 -
hypothetical protein PF00096;PF12874

MMPQAVGQAPFYFYNPESKHDMRQHFAQQQMHMYPMVPTLPSTPVYSRPSSASNSQPPTLYSNGPAVMTPTASPQPMSHKPAI
LLENELYENSYFPSTPPLSTSGSTIGSPSACEVLQTPMNPMFSGLDGIDGFKEALEPVETAVLDWSNCGSPPMTPVYLQSQLG
KVPSLSNTASDLVSTPSLSPSPAPYARSVSTPDLDVDFCDPRNLTVGTGAINSTLAPEFALDEIKVEPKIAAQSFDFNPAVPH
GLPTFEDFSDFESEDDFVNSLVNLSEQPATASADITRPRACTGSSVVSLGHGSFIGDEDLSFDDNVAFQFPSHPSPPVSCASE
DCHKDKRTKKTHVKESTAGPVMNMAASATDSGNTQESSNQAAEASDSGASSGSEGSPAPLPAPANRRGRKQSLTEDPSKTFVC
DLCNRRFRRQEHLKRHYRSLHTQEKPFECNECGKKFSRSDNLAQHARTHGSGAIVMNLIDDDSHAYDASMIPPTAEDYSNYGK
VLFQIASEIPGSASEMSSDEGSDQGKKKRKRSD
FUN_011000 CDS scaffold_4 641781 642959 +
hypothetical protein PF00722
GH16
MRWISRVSAAAALLLATAVSAQTWSKCNPLQNSNCPSDDALGMSIDIDFSKGEVNSFAASGSPKYDSEGVSFTVARANDAPQL
ASLFYIMFGRVEITMKAAPGAGIVSSLVLQSDVLDEIDIEWLGSDNDEIQSNYFGKGQTTSYNRGEFHDVTNTQGEWITYTID
WTKDRIVWMAGGTVVRTLNAGDAEDNQYPQTPMQVKFGAWAGGDPNTNSAGTVKWARGPTDYSKGPFTMKVKSVIVTDYSTGD
EYKYKDTSGSWQSIESVGGEINGNENGDAMTVTATAQGTVATADGNVPVGGIAEDGSPATATQTGWPWTGSRPSGGAIPEGWR
LNAKGKIIPAENSAFVLQPLHNGIAVIPFLAGIMAFAGRFF
FUN_011001 CDS scaffold_4 643840 646975 -
hypothetical protein EOG09260MRU PF01028;PF02919;PF14370

MSYSSDDDRPLARANGHRLSSSKISRAEDDALDQPVPKQAAKMAGLSVRNGPLEDAMDIDEPATNGASKRKSRTSISKVNYKD
DESSDDAAPLAKRQKKQANRVPESDSDDEPIARARGKKLPPSYDETALPESSGDDDEPLSVKLAQKKRDMEKEAEKQAKVIRA
KERTKKPATKKAVKDESDDDVPLAKSSASKRRSNGTAAKRKSNGVKKEESDSDAPISKKAKAKPTSSAKKAVKAESKKASESE
DEEEYAWWNAPKKENDDIKWTTLEHNGVLFPPDYEPLPKNVKMLYDGQPVTLAPEVEEVATFWVAMMTPASSHHLENPVFRKN
FFEDFKEYCDKYGVTDAQGKKVAVKSLEKCNFDKIYAYWSEKVEQNKSKNMTKEEREAAKAKKDALEAPFTHCLWDGRKQKVG
NFRVEPPSLFRGRGEHPKTGKVKQRVQPEQITINIGKGAKVPEPPKGHKWKAVQHDQKATWLAMWQENINQNYKYVMLGADSD
IKGQSDFKKFEKARELKKHIDKIRKDYTKELKSEVMADRQRATAMYLIDKMALRAGNEKDTENEADTVGCCSLKYEHITLEPP
NKVTFDFLGKDSIPYRETAIVEPQVFKNLKLFKKAPKTTGDDLFDRLNTSQLNRHLTGYMKGLTAKVFRTYNASWTMSELLRK
LASDPRSRGTVAEKVKLYNDCNREVAVLCNHKRTVGAGHEQQMAKLGDRIKGLRYQQWRTKMMILDIESGYKKKKGAAWFERD
EDLDDEWVKEHQQFLLEEQRTKITKKFEKDNEKRKADKEKPLPEKELKERLQAVKEMEAKFKKENKTKKVEAEGRGVTVDKLL
KAVDKFDERIKTLELQAQDRDGNKEVALGTSKINYIDPRLTVVFSKKFDVPIEKFFSKTLRDKFRWAIKSVEDEDDWTF
FUN_012256 CDS scaffold_5 765 2580 +
hypothetical protein ENOG41

MAPKLLTDLPSEIRQQIFKECLKVDGGYVVYNAQTDKLTNADEARTPIDLSLRYTCCSIARDTKTIPLAVNTIHFSTSDNWRSL
AGCFNLVATAYYILEQDLVFHLAEFITPAMFAQIDAKFPRFRSMFESELANHNISNPVRDRPRSKSLIARMRPPLCPWVRYFF
KLYVDGPDVYGPFAHPSFADAHENDYMDPSCRLGRGSHDRWQEQSGDVRDALTYCLGLIAEQAPTEFDNHVYKALPHWVGKYQ
SQEFLRLKFNLWHIPSTEEVAYALALLNIHDFVWKLPEVWKYPLGFYQALGDDPDKPRPENAERGQYAAEYDNPMRLVDHFDY
RYREKTRFSATATAIRFLNRLPAEHRTQIRRLTLHEDSPSVNMPSLHAQGLAPLFKENSLLRVERRVSVFGCVHSFAVPGKDW

MTRHKPSPFYGPDFLPKLQSWLIDALAMRDLGIPLDSFIFTLEGGPYSDLCNEVFQACVHMGIAEGEAFNQCCELDLFRSIDS
MSVTADKFFLEPRFKEAIEHLVNKTSIFRSDFNPGVPVDPNALVEESIGFDDLEDLIERWEYQAGSFTCKMPTDLYYDVMLAS
KYDLQTREQYIESQGGKVTEQDS
FUN_012257 CDS scaffold_5 4159 5290 +
hypothetical protein PF01370;PF05368 ENOG41

METPRILITGATGYVGGSVLTTVLSNPFLAKLPITALIRTQSQASTLSSLPVTPLLFANLDDTAFLTEVASTHDVVIHAANGY
HIPSAQAFIRGLAQRKRQTGREVHYIHNSGTSNFGDRRVSKTYTEKRVFSDKDNVYAYEKMREGIEPYHQRTADVTVFELGEE
LDVKTYIICSPLIYGRGTGLFNKSSIQIPTMVRAALERGRAMYAGDGLGVWDHTHVEDIAALYALILAKVVKQEDVPFGKEGF
FFANHGKQSWLDIANEIARVGHQRGKLAAEPESVGLKQVSKAWFDGDEHLTELGLCSRSETRGDRSRELGWEPAKDDSKWIET
VAEEFEVALEVMA
FUN_012258 CDS scaffold_5 15295 16591 -
hypothetical protein PF00010 ENOG41

MADISFDYFDHPQNKQHLVPTSFYDPADFSGDSAHSPDSPLSPVFNNPYQLPVSNDWVNWDDKATLSPDLDALPKQEPFEGIN
LSTIPSRNSMELSPAINPHDLSGTIPDAVPFGRVGLNDIDTQPLFQTPIPSISSQPPHLQSNMMHSASNPSQMSSSLSSINSK
DANGRYPSRKRKSSGSGSSSSRSSTSPPPATRRHCSPPKKTAHNMIEKRYRTNLNDKIAALRDSVPALRVMVHRLEQPCTENM
DEDADEDLGGLAPAHKLNKATILSKATEYIAHLEKKNKSLARENSQLRNRVEGFEMIVMSRDQGPPAGVWA
FUN_012259 CDS scaffold_5 24726 25055 +
hypothetical protein

MITLASPSEASRKATKQASRPQKHPLVNLLQDPKQHLKEQMPKYQLCSNPSITITDAETGEEWDFVDHDELDDLANSDDNRTS
SEELEMNAREQEDLLYSRRWMN
FUN_012260 CDS scaffold_5 25532 25910 +
hypothetical protein ENOG41

MGMQMPSDPSFKWPAECTKPSMFSMLVKKVTTLIKGPSDEEKDRDLKESTVIHPEDATEPWELIPTIKDDPWTVVVKSCSMLG
EFKDGNGDRQSHYERTYQHEFDRQVVVADRLALREAKMRTAK
FUN_012261 CDS scaffold_5 26546 27745 -
hypothetical protein PF00400

MASEPRQYHPLTCHGHSRPVPHIAFSAFEKENEKEIEKKTDSFFLISACKDGNPMLRNGMTGDWIGTFIGHKGAVWQARLSPD
TCSAATASADFTAKIWDTYSGELLYTLQHDHIVRAIAYPPDNSDLIATGGFEKKLRIFDLSELSSTGSPATIPASAGFEIGEG
VHTGSIKFICWTQDPNIVVTASDKTIRWLDLPSRACIRHEVLDGDIKSCEMVSLDPQYASPTDIGGGLPVLAVAAGKTAYFWG
GRNAMDELKRFPLSYTIASVALDLKGRKLVVGEEPGTWAKVIRYDDGKELDVHKGHHGPIWSIAFSPDGKMYATGSEDGTIKL
WKNCEGFYGLWRGGGEKVAE
FUN_012262 CDS scaffold_5 29221 31635 +
hypothetical protein EOG09261B3Q PF00400;PF08324;PF09070

MTDFKLSAQLVGHESDVRAATFPSPDTVLTASRDCSVRIWRRTQASPPNFDATLVSRGSEYVNSISFFPPSSEHPDGYVVSGG
KDTIIEVKSPKASSSDNAERLLIGHSHNVCTLDVSPDGKYLVSGGWDGQARVWSPEKWETELSLSGHESMAVWSVVAFDEHTV
VTGCADKSIRIFDLRQSTAGEVSPSSTIYTPDVVRALCRVPNGHPSGADIASASNDGTIRLWKLNGQQVAELHGHESFVYSLA
SLPTGELVSSGEDRTVRIWKGTECVQTITHPAISVWTVAANPETGDIVTGASDSIARVFTRSPDRTADEATLKEFEESVKASS
IPQQQVGGVNKEKLPGPEFLTSKAGTKEGQVQMIKEDNGNVTAHTWSVSQQQWVNVGTVVDAVASTGKKVEYQGKMYDYVFDV
DIEDGKPPLKLPYNLSENPYERATKFLGDNELPLSYLDNVANFITENTKGATLGQTSEASGPDPLGTESRYRPGENTQPKVLP
QKDYLSITAAKNEAIFNKILTVNKNMISSGRKDAALNPSDESVLSELRAALESSKPVPQTALPLLVRILTQWPYSDRLAGLDL
LRCVAKYPLVAQFSDPTVGSLLDLAFASSLPQGETPNENAAMMGLRTLANIFSTANGRSIVSAQSDEAISFLERIVGVSSEPI
GPFNRNVLIAATTAAINLSVLVHRERLLTPDQRRRLAILLGTILSRDGQTDSEVLYRALVALGTLLSASKAEAANLGIKGWIQ
SAVGRSSEERVKSVAAECAKVAP
FUN_012263 CDS scaffold_5 32159 32974 -

hypothetical protein ENOG41

MRTSAFISVLLATIVSAHSVITYPGWRGNNLITNATFPYGMQWIYPCGGHNVTTNRTYWPTRGGAVAFQPGWFRGHETAFAYV
NMGFGDDGPDGGPANMTQPVVPMFQILGPTNNPFPGTICLPQVPLPKNTTVKAGDKATIQVVELAVHGAALYSCVDIIFAEPG
DKRIAEVNETNCFNSTDIGFAEIYTITTKQSGSDAYVSESGATQTLRQLNMAGWLPLVAAGAYALFA
FUN_012264 CDS scaffold_5 34200 35174 +
hypothetical protein PF07985 ENOG41

MSSSQDSSDWTRVVRKGRKNRRNNTESHSSRHAKPQTEGLRSPEDLEKDYRQHRKRWEEEAYSCPRLCELVTAKASHLTEIDK
AVHLGVGTFDPNDGLNLDGKRSTFAQLAAFEIMVEELEKITSGKIETFFQDPAFNASDKKFLENIGHTVLDDPKGTQMVDEKT
LFFGVHLYRPVYNDALKGELPALFVGTGWEDWGDIFADEHIENVERMHKAYERCDFPQERLDCAFSTTSIYWKPKSEEEGEAK
GKGIVIEEVEIENGEEKKESDEEEELVKKLDATTIN
FUN_012265 CDS scaffold_5 36259 37126 +
hypothetical protein ENOG41

MASSSPEAKPDRAAREAEYQALWQVCDRATRKHEYIYADDRLKRSLLEDACDSIMLKRQKVGKAPCGTDISAMVSLHLQREVT
GRLLATARMGVSTPSPNPMPYMSSGPVFNAPQDAYPANPSTFNGNPGPYNGNAAAYNVTQAPLHAIPQPQARVSPPDFDFSIP
NSTEELREDMDKVINTLREYARRIAALENKLDKFTQERDMWKAGTIGMTNADMSKAASVPPGMFHCPIEGIEGLSAVAYSHPR
PLTPRSQTQGRDEEGEVTYQPGN
FUN_012266 CDS scaffold_5 37771 39887 -
hypothetical protein
GH17
MQNYHGDPYESEPLDSHNRYDSSPRRPIPPPHSYSPSAAPYDAPPRSNHPSAHPDSSYSRVQGGYDDYSSHGRPVSPSPYAGS
AGYDSPRRPSPQQNDHGYYGNQGYNNYGGGGGGHMGYDHPNSHTTPGADNFGMSASGGMAGIAVNVADRNPRYSGIGAMEHSN
LPPPPSRTQQNPYGGNGGYGHGYDSPRGPPSAATSHFNGSDPYIDNPYPGYTNSRHGSDNLGVVNPNEIVDDGDDGLVYGKSQ
RNSMLSLSNSDRAKRGASATAAAVGGGAAAGGLLGGRGGSYEMNGAREKPYGYGSDDKDAGRSKRCKWLIIVVVFLVIVGAIV
GGVVGSMVNNGKKDDSTASSGESAKDDTKKNGDLGKNSAEIKDLLNNPDLHRVFPGMDYTPLNSQYPDCMHNPPSQNNITRDV
AVLGQLTNKIRLYGTDCNQTQMLIHALDRLELKDTKIWMGVWLDKNETTNDRQMAQMWDILDEYGDAPFEGIIIANEILFREE
MNITTLAKILDDTRTKLDKKGLKLPVATSDLGDDWTSELATDSDYIMANIHPFFAGVEASEAAAWTSNFWKTNNGDFWKTDTK
KNIISETGWPTGGGTNCGSAATCTKGSVAGIDELNTFMDDWVCQSLKNGTNYFWFEAFDEPWKERYNTKGKNWEDKWGLLTAD
RELKKGVKIPDCDGKTIQDYSMFSS
FUN_012267 CDS scaffold_5 43659 44240 +
hypothetical protein ENOG41

MSMNPESATPQQGEFRSKVAPSEPLTTKGHKPGVLVGNDAVPEFHAEQHPAGTAPQENTFQPNPETEIPAQAPGASGAISASA
TLGGATSADVHTGLGKPAAGQTSQELHGREKKDRSGLTGVGANPSDPIHDMGADRDHPTNYRGKGGENAVDYPGAEDREPVKA
EQVASERA
FUN_012268 CDS scaffold_5 46342 48722 +
hypothetical protein PF00172 ENOG41

MEIQFSIPINGGRSNSSAPSSGTDTERSANATASRAGTHRFALETRARHTRRSSSRARTGCATCEKRHVKCDETKPACLNCLK
WRGSCDGYGDAAATPAESATEPRPRSTASRSKATSNSGAKPRAASKIKVTTGSTAVVKARPPTIPEEPPINTICFTSSEQRSY
FDEWSALSVGFLSGGLGDSRLWTTTMPQLTLQEPSLRYAAMAVGALRKASNVEFEASSPGNELAGDNKHYLNAIVYYCEALQL
QAKARPTKEGLRTAMLASLLFICFEAQRGNMPAALKHITHGFSMLNELAACTDKAPSLVRIAPAPPALVQEILDCYKPLELQS
RSFMGSYKKFFFPPKPPAVAAKQAPASQMSSPHSVSSPPSQPGTPWASAPSSQQNPVGLPSPQSQASPQAVSDQQTPPRAPPQ
RPPGIAPFTKHSPYFRPKQTNISVIEDMPAVFSTLDEAHGYWTLLQRQLVQYIPLLTATTAQLALTKVRDMKELEMKLDSVRQ
NPRISKFLEESRHWLQRWSTSFSPLLQTAQDNRQNDEQSYLQAINFHIEFLILYIYTAMPRYSGVETAKDLTPQYRELNAIAE
TLIASRPNCGFAMDSGWTWPLFVSAFGCRDPEVKQDAIRILGKYPIRNALRDSRVFRAIALKNLEVEAMNAMEGDEDKQWLRL
RRRELVFEDLGMNIIFRYAEKNQATGEWELIEEVAAFMVGEDGQLNWHRQPISESVSILSGVC
FUN_012269 CDS scaffold_5 49500 50783 +

hypothetical protein PF08610

MDPSLKRDVPASAANSTNLPSATTNKQQKKPSLGRILSLPPKWLSTYEEFITKNAGQVSQIESALRSLTYIIPGRFRDAEIAS
ESIHSGVQLLSLYHDSLLQKAIARLPMASMPSAHARYTRYWTQRSGAYRRIAMFLQMIIYTEMLWEMTAKRRGGEKSRWKVVV
ILEALKAFCRLLLLRITRSRPLVTPVLPEREPIPEDAAADEEEEIAYRSESELMDDVSVNGSASGSSRDMKPAHEREWTMPRT
GMSLPSLPEGGDISSYLLGRVLTADDIKPATKLLNQLQGSGQGAEILQILSPLIYASAMAYNIRRGGDKKNWTPWLIGFAVEY
AARQLRDRGLRTTSLEREEWSKRGWAMGWWAMRGAAYENITKGVVGGVTKRMPSFIGGILEDYEYLWENYYFSTSA
FUN_012270 CDS scaffold_5 57102 58964 +
hypothetical protein ENOG41

MSQQPSPSPQQSHDANPQDVLAEARKNLQVLIDSGMSKELLHQLVDGGHGLSLNLSAMTAMQAMPTIPSQPQQTPFSQQPPNP
SQTAPTASLAPPPPMRCPPPPPVASNGPSIQAVTTTPNSISSTATAPSAEIKHEIISEDMNFPNPAMFLPPQAAFSHRPRISV
SSTSSGSSGHASIWSTNSGQSSMSWQSASTCNRSQAPLPIPPSSSLNGLGPMSAPVGTAGKTNIYWCTSCETSFKRKYDWKRH
EDEFHERWRKYPCPEPGCNRSFWGSNSFNQHHKQCHGCKTCPHAEKVVKFLRKRKYWACGFCSALHPARERHVEHVARHFESG
MTKGDWMHSRVVYGLLHQPLIHEAWDALVVSKQAEYNGRRPQFSWHPSKTGRAQGFLENENPGQLQDLLEFFSGDEGEAQWIV
SVAYDLADIVLTSAPIPSPQYSHASLGPQGFTHDPRMSFMPLPTQGHGGHQSLMPSPDPQQTQFNTPFRNTIMVPSQQFPSPS
RPSPDSSSHSPMGPSTSPLMPPPAPSPLEKRELPPPPHSQGHDAGESMMDFEYSPAPPVLFDDWESLTGSVVEQPGQQHDGAT
GGWGMVQYFNDPRVTS
FUN_012271 CDS scaffold_5 60193 61123 +
hypothetical protein ENOG41

MPRHSKRPTRKVGFLDALGQWTMGPSHRRSSRCRRHPRSTSATREARLANAPPDLNLTARQWRHYADVPPEPQDECDCSDCGE
EYSDEEQKPLVSAEPAQRICSKWSHSEGSAFTREPTAVHRDSGLNRILGRVEAETAEEQRLRHRVSYDADNDALFANIPRRDQ
RSAGEIIVHRHRLGDGEDYLAVETFGHGNSSHRDTDVQSVRSSQFFLNPREAPHVPQDAWPRSLQHVPESSQTRIQSWRDCVK
DGPEPEPSVFGGSNAPTVWPGSGETVVPDDSLTEVAVRSSRRSSRRESKGGGSRRHSGRK
FUN_012272 CDS scaffold_5 62042 64168 +
hypothetical protein EOG092614UB

MNTLDDDRLSASSADSDNDNDNDNDNDNDNDRNDVDETMEDADDNGDQDQDAEGDEDDNNDDEDDDENADADADADGEGEESQ
PPAADSNDQTGTQIKSEPDDSQSQPRKSATPAPSLDRRWPQPSAEYISAASYDIVPTMAAPQSTSVNAMAITPDLRYWITGGS
DGYIRKYDGPNTINGKLALTVAQRHPFVDSVTKAGILMSYWENEEPPPPGRGDQEHILSPVYSLAVQSQALWLLSGLESGGIN
LQSVRHDEGKRIHCLQQHTNAVSVLTMAQDEKSVLSGGWDKNIFDWDLNTGQTIRSFDGNAGQISALELRPASGAPIPEDDDE
PPPPTTMTTNNAAPIANGNFTDALTNDGVDDMTAEFNANPSNGDGDGAASPAHDSLFGGSDAGSLFGETKEDQPFGNDDDEFA
AVMPGHGHDSAMDHSADMAALDAGKDPEPEFEPEPEPVPEPVVEPPPPEPSQDASDAMDVDLVPAPTQATEPSQDALPKSEPT
SPSLLLTSHTSTNHDPTQTSNTTFLSAAMDGTLRIWDRRQPDPVARIGTRNGVPPWCMGACWSPSGNNIYAGRRNGTVEEYNI
HKAKRNWEPHRTLKFPGGSGAVSAVRPMINGRHLVCASHDIMRLYDLRESPLKHSSVPFLIIPGPPRAGVISSLYIDPTSRYM
LSAAGTRGWEGTSTEVLIGYEINATNG
FUN_012273 CDS scaffold_5 64657 68452 -
hypothetical protein PF13086;PF13087;PF13245

MPGDQTPLEIITQHVKHKNDLPKTGSVPWHMAPEFPHSSELMSIDPAPLPHLPASKDISEHDAKNAYLEFQYLTNRFEGTELL
RQAINCFRNKPRMMEGDRDNFYIYTQVSHIFSFLKSSTNSFKVHVQGYLLARSGPACRISFSTERSSTKVEWRQSNRLTAGTL
IALSPRDDNFNTQCYVAVVAARYIVGGLEPDPENNEDVNTPPRIEIFWSDCNTAVFDPSVEMVMLEAKGGYFETVRHAMVGLQ
HAAKFDSKFDKYIIHESNTEHMAAYLADTPGQTPSIPGRAEHLDPSQREAFERMTHKEMAVVQGPPGTGKTFTSVVALESYIK
TLKSRQKGTEYIPPIVVAAQTNHALDQLLDRLSAFEAVIVRLGGRTEDEAIEERSLYNLKANSQRLRGGVGGETARKKVLGRI
EKLLSECFPAFLIPAEEFLREGLLSDAQYESLDDDEWESAALVSRDDGTEIESRIAHWLQDFIETDTTYKYRPPQNQAEDPAK
EDAIDETQQENQKERLHGDFFPMKFNVTGALPGALSKQAAWAYRARRMLKQHQDLYKIIPQHRGMVYRLLREELIRARAQNRW
ENDVKVIKNEHIEIVGCTTTGLSKYRGMISALKPHILMIEEAAETREANISSALYPSLDQVVLVGDHQQLVPSVDVRELGCEP
YNMHVSLFERLVKLNLPYSMLRVQRRMVPAIREVVNAFYPKLTDHCSVYEPDNRPPIPGMGGKSLWWFDHEGQEMQNANDFSF
SNPDEADMIVGFVRYLVQNGVDPERITVLTFYKGQAALITEKLRRDLFLELKNPLKKWSVRTVDGFQGEENDIILLSLVRTAR

PGFTGNENRAVVATSRARCGMYIFGNSQTLVREENTEAYSTWRKVVDVFEGRRSIDNCLPVTCHNHGQRTNIKNANGWKHIPG
GGCDKSCEYTCAKGHPCQLTCHKFDQSEVKCLEKCEEVLKCGHNCESLCWQSCECPSRCDEPPKMKLAVRTSPVPARAPKAPH
GPRNTGFGASNGVIAGRGGGNSNNNSGRPNYRPQSRSNRTILKGPSHETSPTRRFESQPPSTFDAPSSKVQTSAHMAQTRKHC
LEPTSEQIMEGGYYAETIHQTQTANNAVTTTTFLESFTDASKERSRRKNWSPEKLAKKDKELLEDAKAKRRQKSPASIHISET
WHRTRTSENLNRQYEETVETHWSMKYSSCKEAKASQENSAKSGTEIGLESSVDDGVEDDEEEDLISFD
FUN_012274 CDS scaffold_5 70768 73544 -
hypothetical protein PF00172;PF04082 ENOG41

MDVDNEYSDAGSQRQPSAEHTPQGATAQVGGASSQGNPTPTSVHSSNTPSTASGPATANVTHTQNSKRRRGLGVVTPNACTEC
RKKRAKCDGKKPCGRCRAQKDAECVYEIPVRQSKENLRTEIETLRQKQRSSDQVFAALVRPELWEEVLTRLRGGQPIETISEW
LASAPRPGERSISSFPPRSEAPTMGPNPGFPGGGLGTLAAMSLGANHQQPQQSSMRPEMGQHSPWQSSFSQTGSTRSNSHPDV
MNWQTDMRGPPQNRVGSWAEGMNPDQISDGLPRYRGVEQILSPLNEPGLRTPTSTWTVITGDSVLVQHLLALYFCWEYPTFAS
LSKEHFLQDFQEGRNRYCSPILVNALLALGCRFSTQPMSRANPNDPYTSGDHFFKESLRLFAQETDHHSLTTIQALGIMSIRE
ASCGRDSESWYYAGQSIRLAIEMGLHRIMDEGDEDELAVQSATFWGAFALDHAWSLATGSLPQCSCFPHLPPKPAIIGDIEAS
LWVPYTDDGAPLQRSCQQPSNVRSVYKCFCELSELVHQSLYILHSPGKPLTARELLNMYTQYLNWYDRIPEVLRLGHNFTPAV
LFAHMYYHFAILLLFRPLIKLRIIGSKVSPRDVCSQAADAIQGLLRSYSSLYTLRRTPSFVPYFVLTSAIMHLAIGATTVGSD
TTEADMEKAVKVDPRVAESITQGISDLTEMAPCHQFAEQALNILRYLAAKWDIDVENNVDKLTAEEYDRLVLPHTGSLNFFSP
NLRDGDITCQWGAGPCMGERSPSVQKTGDNMENPLFWPFPMQGRPILPDGVELEHAGFAII
FUN_012275 CDS scaffold_5 76339 77898 -
hypothetical protein PF10104 ENOG41

MDRRTYEGPMDWEYQNSGPFDPTSPFTHAAKSNSQNVFGSPSKSSLRPNPFANLGTPSKTKPPQSSFTPQLSSRAAAPAFRNP
AFTTPRRPFDDVALSEASGAEDSPALTEVSDFPNDTPEADRMSDLNMSGMTSPSKIDKSFRYSKTPFSSKKHTPGRGEIRATR
DLSVSEFIRKRKRHNLDRDVSSITRHRWTESDSDSEDSIAPRRTRGKKKATKEAARGFLGSLLHMLDEHPNAPDNLYRWVKLI
VNFFLVSVFVYIGWSIVSTVKTDIENANEMARIEIMGRITECQTQYSINGCAKNDRPALRVACEEWSECMTQNPEAIMRVKVT
AKQIAEIINEFSEAMNLKAWVGTSCSILLIAKLTTSQGFFFAVLIFCAFANNFFLGGYASKPAPPVQSQPAAPPHDPSMAPEN
GPGFMWVPVQTPRMQRHMLLDDGTDTDSTPPPKMKTILAPPYTPSLRRSPSKTDRGRSPVKNRSPSKGHREPFA
FUN_012276 CDS scaffold_5 78348 79740 + IWS1
Transcription factor iws1 EOG0926390Q PF08711

MSERDSPIGEHANEPVNDGQEQEYEDAVAAADSDRDSDALSEVDEDQFEDYDPETANIEDRPVDIDEDVARTLKATKRKRADG
EAPKKPREGRRDKKRRDRDEDVDMDDAEDGGKKSRRSRRAAGDGERRQRSKPASPEPEKEENLTPAERKQRELDRMIDAAIKK
PSGLKRRKKDEIDLEDEIDDLLADLKVQMEQACQADNQAREAGQPALHKLKLLPEVTAILNRNNVQHAVLDPDTNFLQHVKFF
LEPLNDGSLPAYNIQRDIFNALAKMNIEKEALLSSGIGRVVVFYTRSKKPEPSIKRIATRLLGEWSRPILKRTDDYKKRQIET
RDYDYESAKMAQRQKTSSQFSLSQRPAVNAKDAERERLLAPSRGNNSARMVNLPSSYTVAPQSTFMGSQNSSHRPLGAGGMEA
FRKMAQKSKKRAN
FUN_012277 CDS scaffold_5 82007 85779 +
hypothetical protein PF00069;PF07714

MNGTQSPDAQKASNANTNGSNSNNSSLAAKKRKKDLKPIITMEGTNQPAGSHQLSAMAHSPSSSSVDDAAENTADEEDSEDYC
KGGYHPVQVGEKFKDGKYTVVRKLGWGHFSTVWLSRDNTNGKHVALKVVRSAAHYTETAIDEIKLLNKIVQAKPDHPGRKHVV
SLLDSFEHKGPHGTHVCMVFEVLGENLLGLIKRWNHRGIPMPLVKQITKQVLLGLDYLHRECGIIHTDLKPENVLIEIGDVEQ
IVKRVVKPESAEKENNRNGRRRRRTLITGSQPLPSPLNTSFNQSNLFPSPNTHSSLAGVLNEAGKSSKEASPKPSDDAQKQRE
KSADLLSREVSGISLDKSNSPSASTGEKRKAEDAHAFDVISVKIADLGNACWVNHHFTNDIQTRQYRSPEVILGSKWGASTDV
WSMAAMVFELITGDYLFDPQSGTKYGKDDDHIAQIIELLGPFPKSLCLSGKWSQEIFNRKGELRNIHRLRHWALPDVLREKYH
FKEDEAKRIADFLTPMLELVPEKRANAGGMAGHVWLEDTPGMKGLKIEGLEVGGRGEGIDGWATEVRKR
FUN_012278 CDS scaffold_5 86870 88101 -
hypothetical protein PF10058 ENOG41

MVAFWPWGGESSSTASFEKTLSTLSTKITDTQASLDKVRASSRRARVIWTLYLSFAYLVYAIVLLLVVGYNNLGAFEWGGLTG

GPVLIYVTRTTLAAYYNFRIESLSARLKDHQSERAKTIQKLKDATKYDSTMELIEKYGGADGKSKGKKKDGEQNADDTPNKQR
PNGPPQGAPGRTRMPPPPTANIQRPHSPMPAPNPMEPSAEFAPNAEFTEPPPPPPIVPGTPQPPPMPTGYASYQTGPPESHWY
DRIFDVLLGEDETAPKNRIVLICKSCRLVNGQAPPGTKTLSEVGQWRCMSCGALNGEIDEGKRIMNEVLDAAKDAPTDGESAH
EDIASDKGEPPMIEVGKSGIKEEDDDGPAASVKKRRGKGKK
FUN_012279 CDS scaffold_5 89157 92715 +
hypothetical protein PF01465 ENOG41

MFQRFKSAIDRTIAEEQARSQTAAQSRSPSRTRSTSSRKGDGTPAQRAKARKQAADAGDAPNPDPAVFEAAFVIDDESEPSRA
TTPMPPAANDDSKTDNGNAQNNGSDEKNADDSKDDASSGKNQDGGADASAARPEASKSQELSPEIRQKLRKLEKLEATYPELL
RSYRVAHRRATAIEPFEKALRENTPLTSIGDPEALVEYLNQLNLRSDMVMQELKKVSTDKEELQKKHNETEEKAKKLEEELAA
LKSGDSTQPKTDESEAHKDDKHEDTPPEVPEKSKSPVSSVIGMFSPKHKPQKSLSEVTESKDKSEEFFSYDDEIPQLQADLTS
KSEEIEKLKSEVESLQKELTTVKENSAGLVENLENATRELSESRDAAAAKESLQTQLDERNKEIESLNQRLEEVQKQLKQLEE
DKSTHTAKVDELEVSLASSDKRASELDAELAKASNAKNISKKLIDDLNSQIELLKKEKSESQTKIDDLTKKLESKPAPAVPTA
AATPAPAASQPATATGGGNKKKNKKKKGKGGAGGAAAPAIEAAVETPEPATPAEPEGNAELEAEIAKLKEEVAEKDTQIERLS
KRRKTEEDLREEIESLQENLLTIGQDHVEAKEKIKELEAEKLELKTQIADLEKKIDSSASDAEASTKLQSEMDSLKTEYDELK
EKTTTLQADLGAAQQLAQNRFKDLTELREVLQKTQPELKSLRQESATLKTTKEELAAKTKELKDMEKREKDLKRDVERVQKLA
SDRETEIKGLQEKLATETNAKLRLEDAQRVSGRDLRRSEAEKVEISAKAEKTEQELKTVQEELGKLRPKVKELEEQMHKLKRE
KTAAQEEAEFKTQQYSNAQGLLSSMRDQTAEMSVQLKESKSQAESLEEELAEVQRLLQERTREGETMRRLLADVDERADNKVR
EMRARMEAAVEERDRIEDESSTLARRKTRETEDLKLKLKDLEREVKTLTHERDELEQREKEWRRRREELESVEEKAEAEADEL
RTTASQLRTALDASEKQVRDVEKQRAELRRMLEEARQRYEKVSKDLKSVQTKLVANSSRSSFDSVRSGSNGSPAAGGTPDTVY
LKTILLQFLEQKDTKLRAQLVPVLGKLLRFDKTEEQKWQKAVQHIEVK
FUN_012280 CDS scaffold_5 93561 94513 +
hypothetical protein PF00106;PF08659;PF13561 ENOG41

MSGTQPASGGEHRDFSRDALFDLSGKVALVTGGGSGIGLMATQALAVNGAKVYITGRTKEKLDTVVEKYGKNIAGEIIPIQAD
VGNKEGIKKLYEEYSSRENCLCILVNNAGISSNSFQVEADSAQEMKENLFDNKDATFEDWNSTYNTNVTGLYFTTAAFLPLLQ
KSSEKHQGWSSTVINISSISGLIQKSQHHFAYNASKGAAVHLTRMLAAEIVSNKLKIRVNGIAPGVFPSEMTTDGSDEAQKSF
IPKEKYAEKVPAGRAGKDQDMAATVLFFATNQYLVGQTVAVDGGYTIAAGQ
FUN_012281 tRNA scaffold_5 95069 95160 - tRNA-Glu

FUN_012282 CDS scaffold_5 97079 98336 -
hypothetical protein PF04433 ENOG41

MQPSTTFDDNTISSVKLQTERTSSLPSIQSLPFHYSPSGPSNSTNNTTTMAANSPHNAQATSAMPPPAHPASTPSKMHINSLM
SPPDQAHDSFYYHTGKVDNSSSKNASGKPPMSPPISPCNRPMATTETPTNSIQDPVLYPSDEVPSSPAQGPLFPTLEDRDHSR
TIEEHMQKNINDKIFVEVQPPTRNDYELVASFQSQLNKNYELNPKAWMLSVNKQARDDNRRIAQAKARARPLAAKAAKISRPR
PDNRVQKPTQRFIRAQPTNSPKRPTPQLSANGTAEPKPRAQGPDKDFESLPDFCPPLSTLPDRSTILKVDWKGQPLDNSNDPH
RHLLHPDELTLASNLRLDCATYLASKRRIFISRLSCYKRVKDFRKTDAQQACKIDVNKASKLWTAFDRAGWFHKGFMEPFQNT
KLN
FUN_012283 CDS scaffold_5 107919 109608 -
hypothetical protein PF00646;PF12937 ENOG41

MPKRKLPPTEQPSEPQEVSQPRPSKRLRFANSAHNPPSRDILTTISSELLLRILSFLSSSELLDISPTSQRLYRLASDPHLWR
EHYYERFVLPRALRIPGFRLGGAGGTKLQYSSRRAVWADGRKGRAPEDEVLRESSKTESVDWKRQYKLRHNWARGKCAVEEVS
VRDAPDENGGQKRTLVKVVEGIAITADSEYGLRAWDLRTRRPIAQLGLHEENESDDNAPTSLAVDESGSGTVVDIVVGFADGG
FGLWRLNLERQELLRRYRHEKSCNGELVAIACGHPYVLTATTAGLISLYTFNRPDDKPSNDAADSVNDEPPLKATDKPPSTVL
PPPYLLTSLKSHTTHLPLALSIRRMSSSVSASIAYTFSVRDGWCIGIQDLLITPPSDGSGVDTLSSRLASTLPITASGGADSP
LTNESRSELTTLCYSHPYLLATLPDNTLVLYLCTSTAISLSLSLGIRLWGHTSGISDAEITPRGKAVSISTRGDEIRVWELEG
RGGGSSIEVRPTSRQPADDADSPTSAPRAEWEDRRNWVGFDDEMVIVLKEARDGRESLMVYDFT

FUN_012284 CDS scaffold_5 110185 111313 -
hypothetical protein PF07950 ENOG41

MDFDQSPDRRASQASFLSLQQLDPSPIDSPPADPSIDRRDLGPPPRGSDDGYEMVDADDMERSSATIKAPSTGAPGLSASTNL
TNGATGPGPIFYLMRIQKFSSYAMGLFTSLHLANVSLIPAVTRSVPGSETYLLMTREIYQTSLTEPILVALPVIAHIGSGIAL
RLLRRSQNMKRYGAATPGMYALHRSRTELEDQRRASSPWPALSYISISGYAFSVFFAAHVFVNRVLPLQVEGDSSNIGLAYVA
HAFARHPITSWVSYVGLLAAGCGHMIWGQAKWFGLSPTTKYIWGTSTAPAEKRARKQRRRRWVALHGASVAFAALWAVGGLGV
VARGGLMEGWIGKVYDDIFAAVGL
FUN_012285 CDS scaffold_5 111787 112340 +
hypothetical protein PF12171 ENOG41

MPPRHLTLPSAQTASAREAQKSFYCQLCSKGYSRMNDYEAHLSSYDHSHKQRLKDMKAMVRDPTAGSRARKAEAKADGLISIK
LPGQETATGGGGFKKGGFKKSGFKSAFTKVESGGPAATTTTEKPKPASTLEPAPVNPGLQKILSPESDTEDEGYEVYDPRFPT
D
FUN_012286 CDS scaffold_5 112426 115235 -
hypothetical protein PF00172 ENOG41

MDPGHANNGPAASDDSARRACDQCRTRKIRCDKDWPCSNCRTAKRSCTSTGVGQRPKEPRQRVLISSQYERKIDQIEVRLGNI
ESLLRDLSQRPVSTPGSNIQFPVTPAAFPGLDPASASTAAFDSSDDESVLGGDSVIAQQTTFASDLLEHAVERTSLNDVSPKM
WEALANLRQLAELQSRQSISHGPRFPLQQPIPKGGLGQLPMPPMDIVVNLLKRVRASPPGLFTFVCYFIGISDFSNLCRMVYF
PTDDFSDSTFIIVNVGLYYLFLEQHHLTVDNKPLKDEFASHLYTSRINLETALANMSLFMSTKIETVQALLMGTLYTIDVCRP
SVAWHLNCAAAQICQTAGFHRRDLSTRNPEEADIKAILFWYTYTTDKALALRLGRAPAIQDWEITIPRTFSFDGILSLETKAV
AGTWLNAATLQGQVYEQLFCPAALSQPPAVLAERARLLAAECRRVEAESYQIREMAISSLEKIGASPLVDVHLKGDEVQLLST
MTLIYRAVPAPEGSPTRFCQECIDTARKACHAHFACMDLVRKDPHARAIYVHWNLVLTPFAPFFVLFGYVIETSSQEDLQLLK
QFSASIEETSDASETMQKLSRLCNIMSNVAALYVEAKSQQVEDQAMVPIGDEFDVYLSQLGLMPVMDSTMGNAGDPNAEFGEN
TQVNQMADWMSGSRNLLGLLEQDISQIGGVQWPPM
FUN_012287 CDS scaffold_5 116038 120331 +
hypothetical protein PF00005;PF00664

MTETKEKDTPGGSQSGSGVSNDQDMAVTIEHNGDEKGDTKEEKKDTSFKYYLRVFTYNDRLGWILNSIAFVAMIAAGTILPLM
DLVFGKFINVFTDFATGVLSPAGYRSEVSKYRYCLLAIVCIRKLTIDSLYFIYLFIAKFALTYLWTILVSIAAINTTKALRVD
FVRSTLRQEVAFFDSPSSSIPGQITTNGNLINQGISEKFGITIAALSTFVSAFVVAFAVQWKLTLIVLAIIPVNLVVTIICVA
IDTGYEYAMFDVYSRSSSLAEEAFSTIRTAHAFWAFPKLSKRFTNTLEEARRIGHKKSWVYMILFPTEFFCIFAGYGLAFWQG
MRMYSEGEITQPGTVVTVIFAVLVAATALTQIAPQTIAISKATAAAQEMFEMIDRKSEIDALSQEGDKIPDFKGDIQFRGVRF
AYPSRANVEILHSLNLDIPADQTTALVGASGSGKSTIFGLLERWYMPSAGSITLDGRPVESLNLQWLRTNIRMVQQEPTLFSG
TIYQNVVDGLTGTPMVDLSEEEKKRMVVDACKSAYAHDFIETLPNGYDTWIGERGASLSGGQKQRVVIARSIISNPKVLMLDE
ATSALDPNAEKIVQQALNNVAKGRTMIVIAHRLSTIRDADNIIVMAKGETIEQGSHDSLIERGGTYSRLVRLQDLGKGSASSD
DEDSETDMEEPVAGLDPVLSRASQHATADISQDDGINYGLLKGLWLVIKEQRSLWFSGFILVIISLLGGGTYPALAILFSKTM
KAFETIDVSEANFFSLMFFVVALANFVIYAVAGWVCNEIGQHVMTVYRGELFDNTLRQDMVFFDDPNRGTGALVSRLAAEPTS
LQELLSMNLSLIMINIVTVLSSSVLAIAYGWKLGLVLTLCALPVLVGSGYVRIRLEYKFDDDTAGRFAKSSSLASEAVLGIRT
VSSLALERAVIERYSSALEGLAKEAIAGLGWKMLFYSFSQSASFLAMALGFWYGGRLVSFGEYTTDQFYVIFIAVVFSGETSA
MLFQYTTSITKARTAINYIFQQRRQKALHDNADNGPGASGSEKSSEKGIDVSCEGITFAYPRRPKLQVLKGVDISIESGKMVA
LVGASGCGKSTMIALLERFYDPTSGMLLADGEDIKMKDRRLHRRDIALVQQEPVLYQGSIRDNISLGIEEGVPSDDEIIEACK
QANVYEFVSSLPEGLTTPCGNQGLSLSGGQRQRIAIARALIRKPRLLLLDEATSALDTESEKVVKEALDRAAEGRTTVAVAHR
LSTIRDADVICVFAGGKIVERGRHEELVAKRGLYYEMVLGQSLDREA
FUN_012288 CDS scaffold_5 120866 122255 -
hypothetical protein EOG092634M1 PF00226;PF16717

MASVATVTLPLPALPSGWAADKDFKAIGKLTEATQRTIEPVGPHFLAHARRARHKRTFSEDDRIQAQESTKNVEDGDVSDESE
PEDPMMLQREAKDWKTQDHYKILGLSKYRWKATEDQIKKAHRKKVLKHHPDKKAAQGRTEDDQFFKCIQKATDVLLDPIKRRQ

FDSVDEEADVEPPTKKQLQKTDYYKAWSKVFKSEARFSKTHPVPSFGSADATKEQVEEFYNFWYNFDSWRTFEYLDEDVPDDN
ENRDQKRHQERKNTNARKKKKADDNARLRKLLDDASAGDERIKRFRQEANAAKNKKRFEREAAEKKAKEDAEAAKLAEEKARQ
EAEAAAKADRESSKKAKEAAKNAVKKNKRVLKGSVKDANYFAAGEPTPADIDSVLGDVETIQGKIDPDEIAALAGKLNGLKVA
DEIKAVWAGETKRLVEAGKLKEGDVKVLA
FUN_012289 CDS scaffold_5 122704 123206 + MZM1
Mitochondrial zinc maintenance protein 1, mitochondrial

MATAPAPALNAYRHLMRAARIAFQGDAPILSAAQLQIRNEFRQKASIDAADASAAIKHAEEVAKVLRQNVVQGQRTEEGKDTF
KLRIHEDIERGDNESIKTAGKGTVGGGCCGGGKK
FUN_012290 CDS scaffold_5 123477 124741 - SLT11
Pre-mRNA-splicing factor slt11 EOG09263BDA PF00076

MTEFPRLNSKSHHRQLEAFLCTKLLLYLFTMPPQIKQDLNRSGWETTDFPSVCETCLPDNPYVKMLKEDYGAECKLCTRPFTV
FAWSGTRANGRKKRTNICLTCARLKNCCQCCMLDLSFGLPIVVRDAALKMVAPGPQSDINREYFAQNNEQLIEEGKVGQEEYE
KTDEKARELLRRLANSKPYFRKGKTIEEGESSKGGNAAVGAGLGGPGPIRTRDSKAAAAAGARTGGKGRQVFPSAAQLPPSPR
DWAPPADKNIMSLFVTGIEDDLPEYKLRDFFKVHGKIKSLVCSHMSHCAFVNYETREAAEKAAEACQGRAIIAGCPLRVRWGQ
PKAIGTMDKDERTQMLRDARQRQGGSQGQRSIEGGSGSANHQALPAPVAAPPGASDTPAYAALTGD
FUN_012291 CDS scaffold_5 126019 127378 + ERG6_2
Delta(24)-sterol C-methyltransferase
PF02353;PF08241;PF08242;PF08498;PF13489;PF13649;PF13847

MVASSTASNLEREDHQRDADFNKAMHGTSAQARGGVAAMFRKGGSAKQAAVDEYFKHWDNKRAEDETPEERAARTAEYATLTR
HYYNLATDLYEYGWGQSFHFCRFSQGEPFYQAIARHEHYLAHQIGIKEGMKVLDVGCGVGGPAREIAKFTGAHITGLNNNDYQ
IERATHYAFKEGLSDQLKFVKGDFMQMSFPDNSFDAVYAIEATCHAPTLKGIYSEIFRVLKPGGVFGVYEWLMTDEYDNDNLR
HREIRLGIEQGDGISNMCKVSEGLDAIKDSVFEMLHHEDLAMRPDALPWYWPLAGELRYIQSVGDIFTIVRMTTWGRTIAHGL
AGLLETFKLAPAGTKKTADSLALAADCLVAGGRDHLFTPMYLMVARKPSA
FUN_012292 CDS scaffold_5 128310 129539 + PDB1 pyruvate
dehydrogenase E1, beta subunit PF02779;PF02780

MSRILRPAARLAASTRALRAPAAPSFAQSAIARAALARPVVFGSAQTRSYADGQSSGVKEVTVRDALNEALAEELEQNEKVFI
LGEEVAQYNGAYKVTKGLLDRFGEKRVIDTPITESGFCGLAVGAALSGLHPVCEFMTFNFAMQAIDQIINSAAKTLYMSGGIQ
PCNITFRGPNGFAAGVGAQHSQDYSAWYGSIPGLKVVSPWNSEDAKGLLKAAIRDPNPVVVLENELMYGQSFPMSEAAQKDDF
VIPFGKAKIERSGKDLTIVTLGRTVGQSLVAAENLKKKYGVEVEVINLRSIKPLDVETIIQSVKKTGRLLSVESGYPAFGVGS
EILALTMEYGFDYLDAPAARVTGAEVPTPYAQKLEEMSFPTEQLIEDYAAKVLRL
FUN_012293 CDS scaffold_5 130151 134028 +
hypothetical protein PF00270;PF00271;PF08148;PF13234;PF17911

MAGIDALLERGSEETGQRQPATQDPEALKRHLEELLLSPSPEFSPEWLNRLQQRWDYEADYTSLYKVAPPHTRTLTRFQRHGL
EGRVTGYKNVTVPANSATAKNSTSLSRKPANRADFVRGAAGFFPFAPGGLEGIEATAAFEDQVHTGISGGATETSSTGNKLER
VIQLGAEGGLLEKAPGMSRGIDFTKKKTSISQEDANAVKKVLEEDPEDAKVDEGGNEASTDVTTPDATPDENGEDGDDIDDIL
PVEFPALEPHGVLAASSARKAGREWAHMVDISHDMSNFRELVPDMAREWPFELDTFQKEAVYHLENGDSVFVAAHTSAGKTVV
AEYAIALAAKHMTKAIYTSPIKALSNQKFRDFRQTFDEVGILTGDVQINPEASCLIMTTEILRSMLYRGADLIRDVEFVIFDE
VHYVNDFERGVVWEEVIIMLPEHVSLILLSATVPNTKEFASWVGRTKQKDIYVISTPKRPVPLEHYLWAGKNIHKIVDSEKKF
IEKGWKDAHFAIQGKDKPKPAETTVATRGGNPRGNQRGGTQRGGPQRGGRGGQQQRGGNQQRGRGGPPRASHAPGHMGRGGRA
GGFTSVAQDKNLWVHLVQYLKKSTLLPACIFVFSKKRCEENADALSNQDFCTAAEKSHIHMIIEKSVARLKPDDRQLPQIIRL
RELLSRGIAVHHGGLLPIVKELVEILFAQTLVKVLFATETFAMGLNLPTRTVVFSGYRKHDGHSFRNLLPGEYTQMAGRAGRR
GLDTVGSVIIVPPGGLDDAPPVADLRNMILGEPSKLRSQFRLTYNMILNLLRVEALKIEEMIKRSFSEHATQQLLPEHEKDVK
LAQADLAKVKRDSCKICDVSMDECHQASQDFKRLTFELYKGLLSIPIGRRMFSQHRLVVFNWDGIRSIGILLADGVSNKGTTE
DPTLHVCAIKSLRDRRDATDQLPFIPAFRKYLHELPKSKKRVQTKTLHVPLSDVECLTRWVTKGIVPEIFQGGDGYQKAKDKL
QELCGSWDDRWEELDLSRIKQLQLQEIVEKRVELVKTISISPAEKCPDFLKHFAMCHDEWLIKDHISQLKQSLSDQNLQLLPD

YEQRVQVLKELGFIDDATRIQLKGKVACEIHSGDELVLTELILDNVLADYEPAEIAALLSAFVFQEKTDIEPSLTGNLERGKE
KIVAISEKVNDVQTRLQVIQSADDSNDFVSRPRFGIMEVVYEWARGMSFKNITGLTDVLEGTIVRTITRLDETCREVKNAARI
VGDPELYQKMQQAQEMIKRDITAVASLYM
FUN_012294 CDS scaffold_5 134345 135017 -
hypothetical protein EOG092656CM

MANRQMARDMQVEFQARFQAKQARREAQKAAKQDPILKKQIQDLLKKGETQKAYQKAKMLLSKQALAQQMDQMADMAELSAAQ
IQANNSMNRMTQMMAQSSRTMNVAQKNTNPEKTLVTLEQFKQQNEEYAMSNGIYQDAITQSTSTQVGEDAVHELLGKLADDAG
LELSKELNQATPSNAEPQQTNEPTAEEEDKLQQRLRALRA
FUN_012295 CDS scaffold_5 140135 141710 +
hypothetical protein PF00170;PF07716

MEQQGSPASQFFQSPAESLMSAPEDAYTSLFAATTPSAASTVNPMDMLTPKSYNDDSLLPMIKQEEGVASTPSPSPAPEKKTT
KKRKSWGQVLPEPKTNLPPRKRAKTADEKEQRRVERVLRNRRAAQSSRERKRQEVEQLEKRNKDLEAAIQQAEQMNARLMDEL
AQMRKANGLPPLSQELFSPQTTHQPTETTVSPNATVDPMALSPCLSPVPEESEEILQQEPAKETILEQSENTSPDLTQRPAAM
LSDLQCHMSAEVSKMFQSQSPTTLTQPTLAWLLPLQMTLFSAAVVLNFCQRPLTQIAISLKAGISLHPSPQLLTSLIWTLTRP
RSISTTASTTNCTTPSSTTPTTASRQSSRSQPTTPLRSSVTSSASTNLRIKSLRKILSSSPSLARPLTDATLVALRLVSEGRD
DRVEKSERETEDSRGQDELVRCFSEITLPSRELLLTLLWAIRTEEARIQREAGEVLDPETRSPLRVHQPSKKAALSVILEEEN
NRVQTL
FUN_012296 CDS scaffold_5 142354 143334 -
hypothetical protein PF07962

MPTLDSDPANGLDNYDVDDFSDDPFASPPPEAANKKRKEPDSGLGIDEEVDVKKRARVPNVKLDEERLLGPKGIPKLRQRAKD
LKIKGKGHEFSDASRLLSFYQLWLDDLFPKAKFLDALTMVEKAGHKKRVMIARNDYINESKPKDRTADDEEEDDMFGENDASK
PTEQEGTRPKTPEQDTGVPDDDDLYGATPRAAQRNSGPVVPIRNDVPEDDDFEALIAEAASHDAAPRARTNPTPAEPDDDDLD
ALMAEAESHDQTSKEKGQGSKDKETNNFDDEEAAMQEMDGLW
FUN_012297 CDS scaffold_5 143615 144410 +
hypothetical protein PF10785;PF12853 ENOG41

MAETATKQSTPSFVGTSKVVQTEYPLIDNDPHFKRVVGYARTSDYIAGTVAAAFAPAALYALEKFAPSHVGKGGFPKAMRLAG
GVGLLGGFLYFYQRSALRFYGATENAREIEMDMREMVDKVKKGEPLYGVSRLSPHLQGVAARQSRYSALFLSTIPWFNFVNHN
QHGVDTAKYYQQAERELEAERS
FUN_012298 CDS scaffold_5 145344 146645 +
hypothetical protein ENOG41

MVSAEQSMDLPVIDLDVYLNNPLDSEAVQEECRKAANALITYGALVLHDSRVSEEDNSTFLDILEDYFAQPEEALRKDEKPEL
SYQIGVTLENTEKPKCAVDEPCLDVIQRLDPSQRPLDISAHSPDPKCRFFWRMSEQPPFETEFPGLNAPNVVPEAPHIRDRWE
PAMNQWGTSMKSAVSKLAEMTAVGLDLPAQFFSDAGKYGPHLLAPTASDLVKYGEKNIILAGFHTDLNFLTIHGRSRYPGLHI
WARNTGNRIAVKIPPGNYLLVQAGKQIEHITGGLIKAGFHEVIVNEKTIETIEKRKCDFPDRPLIRISSTLFWHLGSDVDLNP
VPKLVARAKEVRERQKELGRDEGREVEYPPMKVGHQVQEELKHIALMA
FUN_012299 CDS scaffold_5 147209 149016 -
hypothetical protein

MGWMDKTNRKIAASPVGRWFQLEGSGHPRERKGSLFFTEIRAGLATFFAMAYIIAVNSSIVSESGGPCICPTYKDGACVPDEA
YQLCVAEVKRDAVTATAAISALATFFMGLLANLPVGLAPGMGLNAYFTYTVVGPSGSGPVPYELALTAIFIEGFIFFGLALFG
MRQWLARAIPRCIKLATSVGIGLFLTLIGLTYSEGIGLIVGAVSTPMELAGCESQYRDPDTGLCPSSQKMRSPTLWIGIFCGG
IFTVLLMMYRVKGAIIAGILLVSIISWPRDTPVSYFPYDTLGDDRFNFFKKVVDFHEIKHTLNVLQFNISGHSGQFGLALITF
LYVDILDCTGTLYGMARFANLVDPVTQDFEGSSIAYMVDALSISIGAVFGVPPVTAFVESGAGISEGGKTGLTAMVAGICFFI
SIFFAPIFASIPPWATGCVLVLVGSMMVGAVTEINWRYMGDAVPAFLTIAIMPFAYSIADGLIAGICTYMLINTVVLVIKVVS
GGRIVPPNYEERDGWTWRIPGGFLPPWLNRLAHGKKDFWREEFPPAANDTTVNQNAVPEETGSDHGGSEGGKVPETTLPREKE

T
FUN_012300 CDS scaffold_5 153846 155103 +
hypothetical protein ENOG41

MAYYRPSRQQARPYGYDDDYVAPDPGRSYDPYDDRNAPPPRKVRREPATDASPPRRYKTQRSRRPPSPGRPGYPDDPMMDDRR
AEGRPRRRYEPERRGRSAGPDRRSRDISPPPFGPPPDSPRRKARSARPDRDAHSPEREFRQSSREPRSGRDRDRERELPIRGK
RDARPDRYPRFERDAPPVHPYDRNAYAREPTSRAYPDDPRDQRRSKHRDAEPESPRRHGRSVPPSPRSKSQGRGRKSRYPDSD
SDSEDDHYGAKLGAAGAGPGAAAAASRRRPRSQTRDRRGRDPARSPNRGRPPTTQKNRGSGPAGRRSSMPASTKPRTAWWQNP
MVQAGARTAFTAGAQAAMKSGHESGPWLGPKGAKVATAALGAALVDGFMGQKHPNSTRQKLMRQGVDLASAQTTRVPEHHGHS
RRR
FUN_012301 CDS scaffold_5 155603 156172 -
hypothetical protein ENOG41

MKFVPTLLFPLSYGVLALQGVLQRSDPQFIHLTFHGGLASYSVAFPVDGSTRKTDHDLSVDVIHNSDYNALEQCFFAIEGQAK
LTSKVSTKDGSQHILDNSPRVIIAVDCTGSCVPMYGECYDTRGQPVGPCCNGMCMANRCQPWNSTLS
FUN_012302 CDS scaffold_5 194076 196650 -
hypothetical protein PF03033
GT1
MDLSRIKSHGLGHVAIDDSDTDNGPSGNVIDDGRVKVQFHDTPQALAQWCEHLKESFAGEGADSEHLLPPKPSKPRRTSTVAF
LPGVEKPAEGPRLNIAIHIVGSRGDVQPFIPIAQLLMKPPYGHRVRICTHASFKEFVEAQGVEFFNIGGDPEALMAYMVKNPG
LLPNKDSLKEGDVGKRRKEMMEIISGTWRSCIEAGDGMGDPVKAANVENVDDLFLADVIIANPPSMGHIHCAQKLNIPLHMVF
TMPWSPTKAFPHPLAAMSYGDADAKVANYLSFMMMELLTWQGLGDLINKFRRQTLHLDPISPMWGFQLLSRLRIPYSYLWSQT
LIPKPSDWDEHLNITGFSFLPLASSYTPPKDLLDFLEAGPPPIYIGFGSIVVKDPQALTNMILKAVELAGVRAIVSKGWGGVG
VGEVPDNVYLIGNCPHDWLFQRVSVVVHHGGAGTTAAGIAAGRPTVIVPFFGDQPFWGQMMARAGAGPVSVPYKELTAEILAD
SIKFALKPEVQEVAKDMAIQIGEEDGAGGTAQDIQDRLDIDSLRCDICPDRLASWLDRKTGAHLSGFAVGCLIDAGLLQSQNL
KLLRHRHWYVDEGAEYPLVGAVAAVSGFASAIGTATSDYAERLRNSPGRGKTRRSNVDDTQEGILTDPEEVKTGEARLRKGPR
GANQKDMEILARKMATKTLNGAEPVFLTPKLRRPVTGLHQKHKKAWRAHEGGRNGRVFYVTRVTGKYVGDLAAASARAPVAFF
YNVANGFHNAPSNFWNVEVRRRDEITGLGSGLKVAGKEFVLGFYDAFSGIVTKPYEDTKARGIKGLGRGLLRGGFGICGNLGA
GKSLSFPQVPET
FUN_012303 CDS scaffold_5 197248 198298 -
hypothetical protein PF01370 ENOG41

MTENRQRNVLVTGANGYIGSAVCRAFVRAGWNTYGLIRNPQAREELALSEIIPVVGSFTDLSFLETLFVEVNTFDTIISCTEK
IPGYAAHFGEVMACVKRLAEKSNENGVRPLVLWSSGCKDYGKTGLHGTPGLVPHTEASPLNVPEILKERTTNSAKVFNYTELF
DAAVIRPTCVFGYSNSYYGAIFDYAADQREKGSEVLRIPGDPNSIMHATHVDDCGRAYVSLAELDDRRAVGCQFFNVSGYRYE
TLEEVAGAVAKEYGFDRGVQFVPASEAEPSFPPGLHFVFSFSQWVGSDKLRSLTGWKDKRELFSKGIHTHRLAYEAYKGRGNA
NVAEIQKRIGGNFNAEN
FUN_012304 CDS scaffold_5 199112 200282 -
hypothetical protein PF07859;PF12146;PF12697
S09C
MLALSLTLGVPAMLYFSILGALVVAPSLQAHAIYLHKVTLTWFKDLNTPEQFGFAHHQVTPFYISTPDGVKLHIWHVLPLATY
ESHQEGLLAQGPDGGLVDKFEETLNFRLLKENPNARLVLYFHGTSGTMASGWRPDSYRSLYSADPVNTHVLTFDYRGYGESTG
SPSEDGIITDAVTIANWAIHTAGIPPERIVIFGQSLGSAVAIALVSELAQKTPSVHFAGLVVTATFADIPQLTATYRIGGFIP
VLSPVAKVKPLFAFFARQLSSTWDNMYRLGEFVKAAERYDITLLHAEDDTDIPMEHSIKLYREAVRAAEGVKDLTENEGALLS
RISSLEKGRGEGGSITVWPTSKGNIRLEILKYGVHDKIMAYPATGLAISRAFASVQK
FUN_012305 CDS scaffold_5 200826 202116 +
hypothetical protein ENOG41

MNGTETRRSTETEFVGWVPEDSNRGTWSLITSCLFTIAICTWTAIHPRIHVSRQLRHQHKFYQLVKAVLAPEMVCLESLQEFL
QARKAVRRCAATTSNEFKTVHGFYLCMMGVRYNSGDNGGYRTLWPEQYAWLLKNGLVSWQDHKNWGLAKEEIADKNKADGLVK

LAALLQVVWFTVQCITRSAHHLPLATIETMTLAYVFNGLVTYAFWWEKPKDIVTASVVNLPEMTPLQWEKFESLAMKNTYDVS
DPSQKQDNSIAWYVVPRDCRDDEVGVMEDPERTAEESVSTTEPNSQKIESTVTEVEADNVITEWDSSLYMTRYWPLLCLLGAS
FGAIHLISWTSSFPSEAERWLWRVSALVSVITSVICMQFRKMSLTWDGPLTVIKVASPLLYLVTRVAMAVEAFVGLRAMEPAT
YDTYALTNYWFHLL
FUN_012306 CDS scaffold_5 202179 203512 -
hypothetical protein ENOG41

MATDMDNLNRFVAQKNLERLQHYDITLPDPASDSNAIIGDKCKLAYSILKKWSDKNPDSIKKAIEAQVFGQKNPIDWNIAFLP
FMESMAVYLRDYGKILAACEKYAQCQDPEVDTTPEDLEDAISTYISAADEINNVAKLWGMKFMQICDLPRQGFEEQPKWSGPY
CGAFYTTDAQRSTPFIGLAFKGTSPFNLREILVDKNYQLVQGGLPTRSLGVIAKTLPRDEGTTIRAHVTGHSLGGSYSSMCYA
QLLIDASKFLPGLKMGDEYTFGAPRVWRIANSDDIVPQGVTIFNDKAPQDIASEINGPPPPGRKFTSWKEFKRDVIDAGPHLP
NSYYKSMVEAIKTTAKKETTEGNGF
FUN_012307 CDS scaffold_5 204726 204987 +
hypothetical protein ENOG41

MISYSSAIGRQQGKADIDNNGLARYMLKIETPAGIKSGNEPDLSLQYSQGTPNGIIGLSWVLGGESSIYLGAPKVVYGKVNPP
PPD
FUN_012308 CDS scaffold_5 205146 205725 +
hypothetical protein ENOG41

MGRQIEYGTTLDSRIVTQPEGKAREWRIKKQTDCHSNTIQYNYIPSPQTENTRDVNTSYLDSIKYCSNDVTDSPATRFVQFHY
ADRKDLVTHSIAGAIIIRANLLSSISIGINIGGEITVNRTYSLTYGQSATTNDSYLSQVAESSEKDGQAVNLLPTSFSYTAQD
TAPGDLFKTVPADPFQAESNVATCFP
FUN_012309 CDS scaffold_5 205787 209729 +
hypothetical protein ENOG41

MTVKTYTAERKPNPDVKPDAITDWTPSEENVQVPMPMWDPTSPDAKMPDFLAPDLHGNGRSDLIIPYQNARGMLEFFLSQSNG
AGLTAVQEVKQTNFPWVVKSKFMAMDITGTGVADVVQIFPNGQNLSFRNFPGVANESNSGSIGLADVIQTDTTYGFDNTIDWF
LLKHSGTGAVSLVRVWQEVLPNDLYDIKATSFRCLVTGDSSKGFKATGIDSMLGGPFPKRDANAPVLSILSCDINGDGTQDIV
LGKAEFQTPNMVFRYVVSLGDGMGSFAKCGDELTQTVENAMEPKGDGRFSVTNLNGGLYPSLAYVYQQSNDISFVCMSVVGRC
GAAVSPIVPYPVTPDPKTENTQVIPADLSKLSIVPVYNTGQPTDLLSSSNDSMGLVTAFSYGCLTDPDVYDSGVDWRNYPPTD
PGNYVVKGAPNYVVTSLEHTNDSATNSLAFEVNMAKTYRQVLVNSQGRGWLGFESITTHNIVDDIWTTEHYFQTFPKIGLKSK
VDTLNSDPKVDPQQSPLSSQKTDYDTPLSQQNGCDIYHVNKLFDKLETEGEQCRVQMTQFTYDDNGNVITKYVSEHQQGQIIF
QSWENCSYTSIRGITSLMTAMKLSSVETNRDLTAFQQGDVNFTQYTYNSNTALLETVKRWSDDTGDFLTTTHGFDDYSNEIST
MDPAGLTTTTTYGSYFNSLPIQQVEKGRGVNSIQLLAYDMASGELLAKRETSGRLTCTRVDGFGRNIEARMQSKVTRTLSTTA
KEFLPKVYAASSDFMKLLSDKCILDPVQQYSYDLFKSSAGNSYLTSSTLSYFNDTDIGQLQVLDVVDCVGQKRMQRSRQGVDP
ASSSTDKHVTWKCWTYDTRGNPLFESFNLQSAPWDNFEYQPGPSEGTTSVFDKLGRVLNKTRPSHNDSAINSVSSLQYSIGGG
TVLETVTGPDPNASATNVVLWSAPRTYISINGKEHIISAANQGALTSEFTYDVAGNLLSASDPQAKRETRKYNSLGQLRILDN
VYQRMQDPAKPPEQLAMTYIYNGTGQLEKTINANEEAIMFDRDSKGRPISKTGFDGRVLRYQYDDAGKERLSSMTVYPSGEAK
QLETKLSFEYDERARLSSRALTLADGVEYEIVFEYDWQGQVMTKSYPNGATKLNKYIGSLLSYSEVYAEGPGGQRETWLDGTF
EYNNATEKPNKITFGKADGQTDFQHTFEYDLQSCTLSHNLDQLGQSPSDTKPLVQKTYIYNGADQLAQNLDGMSNVAKKYNYE
GKRLKNSQIGDANVKNYVYDSSGNILTKADTTISCGSNPATGKRGAATAFNI
FUN_012310 CDS scaffold_5 209846 211138 +
hypothetical protein ENOG41

MTGAPDGKTVKREVPGNGGSLLTISDDYNIQIKKDGSEIITIKLPKQASTHASGYSPSSAALFTDVKGNVTHRFKSTDGSLLK
TITYGDFGTPRIKRVQPSDTSFDKTSTYESKSLDEGTELYDFGSRWYDPITGPFTTPDDILAVKDLARTDGLNRLAFENNDPI
NHADPSGHWSLSAVLGVVLGAALVVGAIALTIATGRAAAPLAAAAAGALASGGIAGGKFWGGYAATVLVNAAIGGATGALGAV
ATPANMVSATERLGQAVGLAMGTATENLIGAAATVGSKALISATSSLLTTVAHNAIENKFYGTHYSLFEGAGTAFGSGAAMGA
LGGLVSAGSHLVKGGESAVAQTSAQRLKKGLTGGLLIGVRAGFAVAKSEDLPQRAGKWVSKERMEMANLYRE

FUN_012311 CDS scaffold_5 211938 213633 -
hypothetical protein PF00383

MGVFKSLGLSPAKAAGAKALRRLFGRVLPGETDSASDTDQTGRGNSSNDNNTQREKNNKVNADSSSTKHQDTSAPTDNRQQED
ALTNTETSATTSDMVSPQKHVPQGIVDKENQPPNTAQLSDSLSRLKIGEEKAVATSPKPVVSTAPAAAAAEDAAPAVEGVPAA
PIYTDPAVIAERAMHMKFTEEALDMASQPTHLEMANRPPPSTKTARLALRTNETPVGCVLVHDGKVIARGMNATNVTRNGTRH
AEFMALGALLSYPPKNGPRTTYLKPKPENQSAEASDTSSVESGPADEGNEDGSKGHLYPYGQKCHPDARVDRSIIRESILYVT
VEPCVMCASLLRQLGIKKVYFGAVNDKFGGTGGVFSIHANSLPVSADGQTASAHPTPKPAQLPDGSGTLGVSYPPGGGDGGNL
ESGYEIEGGWGRDEAVALLRRFYVQENGRAPVPRKKEGRAARLAAMMEGEGTGEGTGESNGESNGEDNGEGNDDENGEETPTP
DLAATEPEPQVLDLPTSTQTLKETPAPLGDRTNV
FUN_012312 CDS scaffold_5 214722 216837 -
hypothetical protein ENOG41

MASNTNTLPQGFSVFQPTLGAQLQFFPAIGTRELDEMINAYMPGPASNQEKRASIALDYFEYAHLTGQTFKFYPIYSAADSPA
TASPLQDSGYGSSFNTSPATSNWDWSNVNTSSSSRRSSPKSTSSPQATDFSNLPGMKIMTKDGRDVTNSASRGSKTKEQRDHA
HLMRIIKACDSCKKKKIRCDPSHKKRGVSSTAAQPAKVTKKTKAVAPEPKATPVVVQDAFTQLTFPQQDFSADLDSLAAPTEQ
GVEPWEQFVQFPAEDDNYDFFNDPEGYFSPGSSSFLSEDSTKPVTPTTEQDLLRRPRDEDLAGLTDLAAYMPFNQTEANHDYV
DFNLYSPASIFSEDERMVPIEVSKQSISQPRSPAPNPLPPNESSSGFYSSGGELGGGDLNGDLFGSLSVGSSSAPQQPSTHGQ
LGREDVYRDPGAGLEYYSGTPESSLGSQLLSSRANSWSDRDAQSSVSEPLSSVDSQLSTNSNVRVSLVIPLLMEWQLTSEKVT
ISRDIIEARGVTTAQLESRPLETTATTTTLGTQSTETIAQSSYQPIVTEGTEDSSGCRTSENHTVTAQAVLQRVDAQGDLHQ
PASPDAEIHNVAEDISQPSPAPVVPAHQTIGLETQSDQPSMEALASIAAMLIASTVWSYVSQQFVLQDKYREQQTQPAVLSQR
KVNGIMPSIREKVCRLQSTTRSGKTSVGRMVSMNRSLIAV
FUN_012313 CDS scaffold_5 219321 222492 -
hypothetical protein PF10382 ENOG41

MTSGSIPTTALVVDFICLFTHDLKRKQKRWQDGVLKYHTFNKRVMVYDDRNHFIGDAHRQEGGDLEPGDEFELDRGAAIVQVS
DCTGQREQDLTELLDKRAKEVEKRRTNAGTTRTPGSTAAVTQTPRNDQNAPHFQLRHRPLNDLVGGASRIGRAVISPHSPFEQ
RRMAESPGQQQDSPSEDARPSKRRRREESPPRKMGHARALFGTTLTLTPFSSSISSARSQPLHDRAAARPKVAPPGSRDGTSR
RADRSSKSPPPSSNPEDTETSAPRQAAPRRVLTQRASLKELLAGNEHNRTSELPRTREPLPKNRTKASKPVKPRQPPPKDEVV
SLLTQKPVSPKHRSDAAANSSPNSRGHQESNADRPSRSRTSAPKLSKIVESDKGNEGSPAADDEAFLNWLAQSEDFPAVDQAP
VPPADNRPPRPKKTKGDPVPAIQRPAKESTRKVAQEPIVVDEDEDEDVEALPHPKTSRIRPESAVSKTTKRKETSTAEPHGTK
RTLSTDRASATETATDKPPPAKEPRTELRIRSRQRRGLLMMAQRKEGGRPGSAGRPVPPSSGAGSDTQKTSHTIARAASISEE
PQATERITTKTTASKKRQDSAKPSELHHSVEEPDDQSIASHNPNTEEVAREKDTDNESEALKEPEVELVPSDDAISVSELSED
RRSPTPRRRTNPSRRTRNRAAPVVFSDNEGETSAFDSPARSDDEEEGLSLASKAGRKPEPKPSSGPRITKMSRKSVKSREIIG
FVMPDEDFSMDGFNVGHAELVEVKKPSNDLKVAEKPPGKARDTLEAPANHKSVSDGLEAEQLRTNKKEPEKEQPPTKEKIPEP
TWSSANTKEPDTVPRSTNSRAHEAAEPVLQEKEREIEIVRPPTNAKQPEIGQPATRMQEQEASASETGSRGPKVQESSAKEKE
SKEVQPAISEKELETVQPLTKEKNTEAEAPSTSGKRLEAAQPPAHGKEQQEKQRPRIVNPATRGRKAARKQDAAGLPPQTLVQ
FEPATSSRIAPAQPPKKIAPLNNVPQSELPVFCRANGGAWSRHAEDLLGMTRPTRRMPRR
FUN_012314 CDS scaffold_5 223458 225628 -
hypothetical protein ENOG41

MPGRSMSTNSDHSNSPQSPRSDEIIGEFDLSGRNTPAIPSKRRNQPRDGFFDLDDIDSRTSYFSDDNEKSAPGTPSAPSTPRS
RSHVRPIAIDLPQLKRFTSAPARTPPEPLSARGDLPGGYFPLHEDPNSRVHRPHPFQHHPDTRMSRLISESGPIYADSPTSHP
AQSVVMSHSNTPVASYLAPGFHDHPVPMGKYYPSNYESRNGAQSNLRPPLSGSVSSNVGSDSSAIPRASSQSNPESELRRKLQ
QYQRDMIAQASMAANELISSSAKSGDKPGLSLNDLPLRDSRFATPGCQKPLSPRLLPMGSPGPVTPMDLEAIGAGDYMSR
FUN_012315 CDS scaffold_5 228500 229139 -
hypothetical protein ENOG41

MGIKDLAISYVVFSAIHLFLFALALTTIGLYGTDLHNANKQGKYSDSKWVYAVVVGSISAVTCVLYFIPFVLRVAGFVVAIWD
FILFVLWIALFGVFGKMYINEDAEGDGGVRRMKNAVWVDLASALLWLIATLAALAYWWKHRDTRSRFTGRARV

FUN_012316 CDS scaffold_5 232497 233304 +
hypothetical protein PF13350 ENOG41

MANLGPPFHPIAGVANFRDLGGHPLPIKSRPGYTIRSGLIFRSAEPSRLTEDGVSALQDLKISHVYDLRSRTEIERYATGTRE
WPGAQRVFTPVFLDEDYGPEAIALRFRNYTAEGSQGFVEAYRGIWETGTDSINTILSHLARPDPSPLLIHCTAGKDRTGVVCA
FILSICGVDEGTIAHEYGLTEVGLGDFREELLAAVMKSPELRANPEGAQRLLGAKPENMLAALEALRKQHGSVEEYVVKNCGL
TVEDLEQIRKNLIVPEESR
FUN_012317 CDS scaffold_5 233518 234658 -
hypothetical protein PF12298 ENOG41

MPPRVRGASTPLAGLDATISLSTRTSSSPFLRTFSTTPCREKMSKGRAQMFEWLNSGNGRNLAEPGNGPNYLGPHNDQPFPLN
PLFRSQPVLSDATREIIYEKVTAKGMSLKAVSAEMHIDVRRVAAVVRLKEVEKQWLSEGKQLATPYAKAVMKMLPKTHYNEGE
ENIPHEPINEIHVHNLTLQQLFTPVSESRHFTREDAAKAFHDNMLSADQRSPQPELIKMEQDIIKGIARPEAVAKFQAVTQAD
EDRVAKKILNERRREEERTKRVKTDRYEFRFKEINVDDVGRDGRSRKGTGWRYGAPFEDRKRGLVKIPTSVP
FUN_012318 CDS scaffold_5 235408 238042 -
hypothetical protein EOG09260FFP PF00072

MGATDIASRLRAKFTKRRHSTAGSQASSTRSITDTTSSSFGSRHLWDRDRSRARDGDGDHSSQRAVSNSRGTVSSRRGMTTTP
DGSHRQREDGDDVAKLEGSREVLEAAKSSSNSNSNSESAAKSSSEIGAEAAVPPANDNSTAPDTTSEPLKQPSPPIDQQSLDD
LDEEPDTTATSSSDQLPPHSDLHPPQPHPRLHFNQPPLLSPSLHPSEQPSPSRQRQANSNLDRIHEDSQDSEPPSPTARPRKV
ASLELDAGSELAGHNDDDVLSLDQAPNSPKSAVFVPLAAATSTTFVATSPGLPQAGTPGSSSRPAPPPRRQSLLSNRQTTLIN
TLLSPSANEPGYNQTSSYAPINGNMVTRKIWVKRPHASPTLVTVNEDDLVDDVRDMILCKYSNSLGRSFDSPDINIRIIPRDQ
HSERVLNPEEPMGRTLDAYYPGGQTVEEALVIDIPRRAPKASPRPVVPAYVGNIYYSEDGRPTETSDGYFPSVAAHPGAVGSP
HLPVAVPAHVNPQAPHSIAVLGTGHIPPIPSPGRSRAYRDRPDRPRLGRTHTSSPTLIANGSAVSLAAAAAANHANHDQPGMP
PAAPPLPTPPAPEPSAVRVSTPPVRPVSPRPTAKTKKSKKAEHPSLPPGMLNGGVPPINVLIVEDNPINLKLLEAFVKRLKVR
WSTAMNGRDAVKKWRSGGFHLVLMDIQLPVMNGLEATREIRRLERVNSIGVFSSSPDGVVTTENPDELDDKDRLENPSLFKSP
VIIVALTASSLQSDRHEALAAGCNDFLTKPVNFVWLERKVMEWGCMQALIDFDGWRKWKEISQEAEETAAAKKAAVAAAKAKS
KKNRLSMTKPG
FUN_012319 CDS scaffold_5 239536 240183 +
hypothetical protein ENOG41

MAPIKPLGEQPDKPVPRVPKTPSRTAQIQAQNRRREYLSRHPSYFDNLEHELADPVLYERLVKRFQSASEREAEGKAKGYGRT
LEADLQRGESKLKRLKDGDLESSGGDLEHPWEKPAADKAHGLLLWHAFLEERFVHGLDEDFDYKPVDADEDLDTMIRRDAQDA
WFDDEEPSWIDEDESSAQLPTRQGETGVQDF
FUN_012320 CDS scaffold_5 240872 241856 +
hypothetical protein PF04616 ENOG41
GH43_1;GH43_2;GH43;GH43_29;GH43_35;GH43_33;GH43_30;GH43_31;GH43_34
MAPLITNVYSADPSAHVFNGKIYVYPSHDRETDIKFNDNGDQYDMADYHVFSTESLDPPAEVVDHGVVLKTEDIPWVSKQLWA
PDAAEKNGKFYLYFPARDKQGIFRIGVAVGDKPEGPFTPDPEPIKGSYSIDPASFVDEDGQAYLYFGGLWGGQLQCYQKGNDI
FDPEWQGPKEVSGEGVPAQGPRVAKLTEDMHQFADEVKEIQILDPETGKPILGDDHDRRFFEAAWMHKRNGKYYFSYSTGDTH
FLCFATGDSPYGPFTYGGRILEPVLGWTTHHSIVEFKGKTYLFYHDCELSKGVDHLRSVKAKEIFYDDDGKIITTTAD
FUN_012321 CDS scaffold_5 242025 245376 -
hypothetical protein PF01764 ENOG41

MDNSSIISAEDLPPQPGRTLLPSPVAQAVSLATRSTCLAIRLGSRAGSYGLSAAKVTTLSSLELARSMVETVLSQAGRETLSR
SQSDMSTVEAESIIERSFEHLHHAMSQAVFWTSAGFHFTSTTFSMASGISQLLLSSLDQIFGSTDSSRAIASIVTLIRREFHD
PATGIHGDTVGMMDLVLGLCALAYLQRWSWKMVEEEKRRQDSEEIIWDVVVLNDGERVDIQDRITRTPAFTRPADSTSRDTSP
TNLQQAVIGSQSASFKEEEAVIDQLKDEMVKKLPPDTSVSISNTVSSTQTVTIDVDGPRPFPLPLLPGAEIVETKGLSSQAGR
RTSQILNDPPEASSYRVVYRLERSRTGDVSFQGSQDIPVSVIDVSKGEPAPEVPAKEPPQVPKKARTTGVKSPPPQRSRPVKS
ANDQTSRTSPQTIPKPRASVIRDEKGKFTRPTSASPPSSPVEIKTPQREANQKRPRAPVNSSQATNKTTAPRESTITTRRVLP

KRKSDSSTTSQTSEKKTGLRQALRDGSQSISNIWNKDSPPPESRPRPQSRITTVSKTGNKPMHSPRSSSDQKSIEKAELIPRN
PSRASYVSVHERRRDSMISQTDTYSIHSGYGTPTHSPSVSLSGAAFDSSWVQQAQNDGLLAPPPISAGHRRVLRKSPSLWSMA
SNDSQSSLVLSYYHQKSAYTASDALGVLRRDGTVNGIFPQAHVLRNITRYMRFSSASYGSAFLKFMGISKDMPLLRAWDSTHT
DVRHFVHHTESDTHSVLLASIVDPQGGTDSSGSTGTGVPLVHYISLDHDAKAVVLACRGTLGFEDVLADMTCDYDVLTWRGRG
HKVHKGVHASARRLLYGGDRRVLFTLREALLEFPDYGLVLCGHSLGGAVTALLGVMLSEPNPTGTGFVTAIEGPERTVGDELS
DGLLPVHSTLPPRRPIHVYAYGPPSTMSASLRKRTRGLITTVVHGNDIVPYLSLGVLHDFQAVALAFKNDQQQAKAEIRQRIW
QAFQTGVADKWYGGLPSVPSGDSSKWGHSVLQGLRAGMTNRKLVPPGEVFTIETQRVLRRDAFLLPEEDHIGKPAQRIVMKYV
KDVEERFKELRFGTSMLIDHNPARYEEALNKLRVGVV
FUN_012322 tRNA scaffold_5 246592 246666 + tRNA-Val

FUN_012323 CDS scaffold_5 247042 247623 +
hypothetical protein PF00583;PF13508;PF13673

MAQEGMFSANLLSPEVSAALPEGYTLRALRKSDFNSGFLDCLRVLTTVGDITEADFAKQYDDMLAAGSYYIIIIEDTSRGDKP
VVGTGALITERKFIHSLGAVGHIEDIAVAKDQQGKKLGLRIIQALDHIAEQVGCYKSILDCSEANEGFYVKCGFRRAGLQMAH
YYEGSKGKSQ
FUN_012324 CDS scaffold_5 248429 249311 -
hypothetical protein PF00106;PF01370;PF08659;PF13561

MSASFFSLEGQTALVTGGTRGIGQAVAIGLAEAGADIILVQRDTTSQSTKESIEKLGRKAFIYTADLSSQESVAGLVPKVLAD
GHQIRILVNCAGIQRRHPCEVFPDSDFNEVIQVNLNSVFTLCRDVGAHMLNLEPSPITGRRGSIINFASLLTFQGGFTVPAYA
ASKGAVGQVTKSFANEWTSRGITVNAIAPGYIATDMNEALLANPERLASITSRIPAGNWGSPDDFKGTAVYLASKASGYVSGH
VLVVDGGWMGR
FUN_012325 CDS scaffold_5 250328 251575 +
hypothetical protein PF00107;PF08240

MATERVKASVLHGEKDLRLEERELPAPSSNEVQVAVKSTGLCGSDLHYYNHFRNGDIIVREPLTLGHESAGTVVAVGSDVTHL
KPGDHVALEVGLPCEACELCGEGRYNICRGMKFRSSAKANPHAQGTLQERINHPAKWCHKMPEHVTLDLGALVEPLSVAMHAR
DRAALPKGSTVLVLGAGAVGLLAAAVAKADQAKTVIIADILKDRLDFAINNGFADASVVVPMERPQTIEDKLAFAQKVAAMVK
ETQVNGEAVGEVTAVYECTGVETCVQTAIYATKPGGKVMIIGMGTPVLTIPMSAAALREVDIVGVFRYANTYKEIIELLANPP
ANMPDVSRLVTQRYKGMENIEEAFKMAGKVRDEQGNLVIKVVVDFNEK
FUN_012326 CDS scaffold_5 252173 253318 - GAR1_2 H/ACA
snoRNP pseudouridylase subunit PF00248

MAPSTFKLNTGQEIPALGLGTWQSPPGEVEKAVTYALKDGYKLIDCAYCYGNEEEVGAGLKAAFEAGVKREDIFVVTKVWATY
NTRPELGLDKSLKALGLDYVDLFLVHWPLLLNPEGNDDKFPKKPDGSRDVIRDYNHVDGWKLMEKLPATGKTRGVGVCNYSKK
YLEELLPHATIIPAVNQIENHPSLPQQEIVDFCKEKGIHIEAYSPFGSTGGPVMTAEPVVKIAEKKGVSASTVLLSYHVARGS
TVLAKSVTPERITANKTIVDLDDEDMKALNDYSEDLVKKGEVKRYVYPPFGIDFGFPDKS
FUN_012327 CDS scaffold_5 254357 255473 -
hypothetical protein EOG09263J3H PF05739;PF11416

MAVASIQDRTSEFKSVLAQAQRKQSSSKVSSQRRSLLTDAQKDAANGHAGGPPRRSEFARKAAEIGRGISATMGKLEKLAQLA
KRRTLFDDRPVEINELTFVIKQDLSSLNQQIGALQTLTKQQHPKADQEGEHNKNVVYLLQGKLTDVSVNFKEVLEARTKNIQA
SRSRTENFISSVSQHAQPSIQQSASPLYGTPARNSPAPGAQDTLSLNPVGDQQLLMMEEAQPSNTYIQQRGEAIEAIEKTIGE
LGSIFGQLATMVSEQSEMIQRIDANTEDVVDNVEGAQRELLKYWNRVSSNRVLIAKMFGTLMIFFLIWVLVSG
FUN_012328 CDS scaffold_5 258444 266817 +
hypothetical protein ENOG41

MDGTYNNPYAQGQSQSPLSPQTPGGYQVNVNRTKTRKWVQAPTQNYDGDDWGADEFEDDEPPPPPPVPRVTTGLRPVGQRTES

FGPSPRLAAAVASSSRSSSNAPSLQLQTNHPDVPNPGHPEQSHNVSSPVTGRTGSPAPLSSGAGSSKAPSYHQQPDVRSSTPQ
STASSAARASPSIRPDFRHIDDERKLGGSPLSIAENIAPRPDDRSTPPINEPNQPGHGRGDGPAIDKVQKQPTFDAILAKREE
LGLGSQGLHDPPTLDSIASSEPASHKEETVIHSPADIDRKRLSVSPQLPDVARMSVFGADFFSSGSNPGAPASAQDPTKQDQP
SVPSQATDTPGNLATLEEKPRESEDNTSTPGPNSSTSLAPANDPRAVPPLRTPSPNSKGLFPSPREPASATDSKITPTEPLHP
RQPDYSPSNYEPNSALTQGSLNTFNSSPVKESDVLSEEIMRSLSPIAPTPSSAVSRPLPDNSQSLTPGDRGVTRNSSYTLSDY
DSYWADSNEKPGQDSKIDAEVQEKPKTETSPVHEQQQPTVQPLSSIVVSTESHPTAQPESPKSDSRRRFSWEAGFDSPPKIL
PVEPPSGTFNNEAPRPASPSPLAPAGVQSPKDEALDVASQTKESPRNAETPRIIIPSSGGISHQVSKASTLPPSQQPATLEPP
SPVSIENDDATPRTVENRRPSVAENKSLTQIPSNLTSASPTPDKSTKALPPSQFPQTTPWRDIMSLATPVQRTLKFEAGLATH
QAWESGLENWLSNLMTEHPEYANGTSSFSGAGVPTSQKGHASGPSLSHNQQPYYQQYLNASSPTTGPPPGRSRLGGSVPSGTS
GFGNSSNQIGTKGKEFMQSAGKMGKGLFSKGKSKLRGTGDKGEPTSPVQTKFKAERRASWGLDRLTRSRADDSSEKDVVNSRP
STQSYHAPQSSQSSSMLQFGSVPSTGESGKRLASRESYGPQLSQDPSASASTSASASASISAPASKENDDRSQWYIPTPIDES
TWDPFKGTDLAKGEVSEQSGAMLANQGHPQDGSSTQMPSSNPMSGGATPTPTSGGIRMVPPEDPAGPSPQSTSVYSSVISQAP
MANAPQSQTPTQPHDPSAMVQRHSSFVGLPPIRRGSTFDVNSRSTATEQRTTQPIAEHVSAEPEDFDEIARQQGTLTSEVTVG
STLVGTDSIMEEKFKKDADGSEPQSPPPQQSPRHSLQQSPPRGPQHPFQMHPVQPGPMPPQTQGYQPPTLPLLPPQLMGGNPV
QRLPPSGPWKLEESHLSEPLHRVTPKPQAVPPSGQWKLEESHLAEPLHMVNRNRAGTNASQLSQLSQRSQRSQRSQQSQHDPF
YGYNKETGDVPPSPSSSSTANKHYQRQKPSEVPPSSANRYPGLFPGHQRQPSGSKEVHPGPGSQFIRRFSQEGITRTGTGDTT
NEDRGRSKKASALLKDIGHRFRKSSTERRSTPVDHHGQQFHPPQQQQQLQPPHADVHHDAASVSSVTTDGTHDKKKSRASFML
GLRSKQSRERLSMPPPPRPDDSRPATSTEERRGSRLNPDFDPAAGQYRPAGPPRAATSNLANEMSQSATPPAKKRFSAFNTKA
AMTNVFNRPSSGTSSKPGTPTSSRPISSHLGAQQPVSPECPVHPGFERSSTAGPTFNQILPTPTGTPPVDANERRKRRGSAAG
LISGLLGHVHKSKESQQAASPQMQQPYDRRASGPQLGTPHPPALQTQGRRPSGPQLTSPVLPGGQLGSPQGPAPPLASPQPQR
PHQFGDQQSISREQSPMSSARPLSGGLRDNQHAQAAYRNSRPSPLGFGSTTPAGTPPIGSVTDFSRHGTPQTPFSVGQSFPDA
RAGRMRSISPAASAIRSAPTTQTSFNVPSTAPTTQPSFHHASQSSQSLDKLAHAKQEGTRIQRPVTDSPNMSFRHENRASHGA
SDNDMPLSPVSQMSKTGPPSVSSMATQSPRLNTTTLAAGRRQSSQLSQPDTPVVSAVSTSPQQQTFANRMGMSPPQQQEQFPS
GFQQQAHPQMNPQHGGYQITPGQQYEPNLRQPSAGQPMPGSHPQATSPGRPPTQMQHSDSGPPVPPKDGSGALQQPQIANHPA
RQASPGASKWKGLRNRMSAQVSNKPAASQGKGENDKLTASKILGAFKRGSKENRQSMVIPDLQAYQNHEMRPVQRQQQPQQAQ
QAPRPQQPQIPQQPQQQFAGQVPHGQTQSPRPSSKASGQPFGQVPTARPSSQFSNQSMGQAQTKRYSSMFTSPIMPQQEFRSP
SGAFASGSQGMSQPQPGHPYQSPPQMQGQFPPTQSPQAQYHSPPQPHGQFQSPVQQQGHFQSPPQPQGQFVSPPMQQGQFTSP
MQQQGRFQSPAQQQGQFQSPPQPPGQWQPQVPSPAGQFPPPQQRQASYPGQWHGGTIGQSPNTPSSYRSPQHSQFSSQPQSPQ
YGFPTGQNVHGEESPPVHVSTPTQVHSPTPVHAPSPRPSLLNLQAMVAANPEHDHTQEGEREHSDAAPRNDPNATHQGTPNMQ
SFNSGSPHSGRTSGSPRVGGNPPSAQHVSPRIGTEQNGSSKSRAPSLPMPVLVGGDFMGGHTAPGASSRFSPKAEESQGVSFS
PPSGAPGSVSPNSTRQSPSGGSDLGPPEPKSRGGPSFHAELEDTEDARKRTLRIDSQEEKIHYDPNADSDGEVPPQMSATSYP
GQEWNPYGMPEMGDWNEATDR
FUN_012329 CDS scaffold_5 267384 268005 -
hypothetical protein PF01931 ENOG41

MSTSRNHRTIINVLAKKFALQRPTMESPQKQKLVVVASNNIPKIKATRDGFTQMLPDSYDFRGISVDSNVSDQPFSDEETLRG
ATNRAQNAQAARIEADFWVGIEGGVETHNGSICSFAWIVVFGKDGKVGKARTSAYFLPERTCQLLKDGVELGHADDMVFGQTD
SKNKQGSVGLLTGGVVDRAAYYTQAVILALIPFRNASLDF
FUN_012330 CDS scaffold_5 268361 269471 -
hypothetical protein PF05368;PF13460 ENOG41

MTTKKLIVVIGATGGQGGSVVNSFINDPEWRVRGITRNPSSRKAKNLEARGVEVVQADLDDRASLAKAFQNANVIFAVSDFWG
IYNDPKNRNKPKPGQALNEWTKVQETQQLKNIIDEAARVSSLERFIMSSLPGPTKLSGGKYTNVCHHDSKADAEEYGEQNQPE
LWAKTSVYLPGYFLNNFLTHPMAQPTKKENGNIQFANNLDLDTKVPLISHDQDSGPFVRALIQNPPGKSVVGYRAWITPREFV
RIFSKVTGYNTELLHIPPGEFTFDCKGELRAELQDNFAFANEIGLHGGDDSPAAHPNEVNQPPQLESVEDWIREQDWSKVWGS
FUN_012331 CDS scaffold_5 272122 273643 +
hypothetical protein PF01554

MPSSERRRREGHQDSHSHSHSYDGVLHDETSPLLPRISEVSENGTAKSLTDVHSLLAEAKLLCQYSLPLIATYLLQYSFTVIT
TIVAGRLGAEELAASSLGLTTINIIGFTIFEGMATALDTLCAQAYGSGRYTGVGMHIQRMIVFMSIVMIPVGAIWLCSHWILP

LLVPQRSLALKAASFLRVSLVGLPGYAFFEAGKRFLQAQGEFAPGMVILIICAPVNAFLSWYFAVKLDMGLDGAAFGQALANN
LRPALLLLYVAFIGKKSHRCWGGWDSRAAFAFREWGPMVRLSVASTAVNLAEWLAFEILMVSTSYLGTRHLAAQTVLNTLSIV
TWHIPFSISVAVTTRFGHLVGAGALKEARRAAILYTGVFTVVGILDGAFLYVLRNPLADMFSDDSTVRDLATGSMVAVACFQV
IDSIICGTNGVLRGLAKQSVAAWVVLAVNYLAAVPFAIWLELGSPDLKLDGLWIGLGSGMVIIAIIECVYMKWISWQDCVNDV
KEREDDGT
FUN_012332 CDS scaffold_5 273774 274362 -
hypothetical protein ENOG41

MAGYAKARPQWTEVYPYKERLGEDTDASAPWLVDVGGGLGHELLGLALENVPGRLVLQDLSAVIEEAKSSAECKGAKTYFMRL
ILRDWPDAECATILSHLRDAMAKDYSRLLINETILRDVGAPWQLTSLDWTMMGMLVNRERAESQWRQILNDAGLKIIKIYYKG
IKSVIEAALA
FUN_012333 CDS scaffold_5 278714 280721 +
hypothetical protein PF06428

MSAAAAAGWGQPLATKSPASRRSLGHFRSLSSIAATPSYHPRTPSLTPDHTPIKSSSSTHLPIVSPNPPTDFGDEMSTIPDPR
SRAMSPADDSGVATPSHHPDLNDEVATLSNKLINAINHQTVLDDSLSAARHELDAARERIRMLETQNASQREMLAGDVWVRKH
TVEDEKKVWQAKVAAERQKRLDTELEKKKIEQELENLTAALFEEANKMVIAAKEEAKADHEALQRKNDQLKSQLADTESILKS
QQEQLVELKHVMEHMASERGDHNNPTAPSSPGLTKPDTRDDLRSSTEEPAASLWSASVEPAHPTSFSHLLQPVLRTDLSSYDD
FLILARTSRNRPGSRVSSGSVGALNALTGFGLGGSISSAHPSNASTTSLGTPIAGAGSAPQSPNTPASTVSAASNASATPLPS
LKETKFYKRVLAEDIEPTLRLDMAPGLSWLARRSVIAAIADGSLVVEPVPTTGSLVALTKPQFYPCALCGDSRKDPQYLRNHR
FRTSETDSAQRHPLCKYCLTRVRSTCDFLGFLRMVKDGHWRADNEDHEKAAWEESVRLREQMFWSRIGGGVIPCIPAPLSVDM
EKSPRNSHDGSVRSDHLEVPGFQTPPMTIERTRSDTTKSGPIEEPHTPPMQTDGARSVRSSVQSLDVKSNSGSEETKRLSITI
PTTD
FUN_012334 CDS scaffold_5 281838 284978 -
hypothetical protein PF02366;PF02815;PF16192
GT39
MARTRGKSPQPPVKVPTPVTEKQQPFAAPPPPQPQTTAVTTKSKNKNKNTKNTSYQSDGVEDNDVFLLPLSDYWVALALILVA
TIVRVYKIYQPTSVVFDEVHFGGFATKYIKGKFFMDVHPPLAKMLIALTGWLAGFDGSFDFKEIGKDYIEPGVPYVAMRMFPA
ICGILLVPCMFFTLKAVGCRTMTATMGAGLIIFENGLLTQARLILLDSPLVAATAFTVLAFSCFTNQHELGPSKAFQLSWWFW
LVLTGLGLGITVSIKWVGLFTIAWVGSLTLLQLWVLLGDNKNVSMRLFTKHFMARVFCLIIIPLTFYMAMFAIHFVCLKNPGD
GDGFMSSEFQATLNNKRMKDVPADVIMGSRVTIRHVNTQGGYLHSHPLMYPTGSKQQQITLYPHKDDNNVWLLENQTQPLGID
GQPINGTKAWDALPEPQYITDGTVLRLYHGPTHRRLHSHDVRPPITEADWQNEVSAYGYEGFEGDANDFFRVEIVKKKTYGSV
AKERLRTIESKFRLVHVMTGCVLFSHKVKLPDWASEQQEVTCARGGTLPNSLWYIEGNSHPQLQGDVEKVNYRNPGFFGKFWE
LQKVMWRTNAGLTDSHAWDSRPESWPILRRGINFWGRQHKQVYLLGNPIIWWSSSVAVAIWVVFKAIAVLRWQRSCNDYANTT
FKRFDYEIGTSVLGWALHYFPFYLMKRQLFLHHYFPALYFAIIALCQLFDYATARIPGAGARDTALINRIATVAFLVLSTAVF
MLYSPLAYGSPWTKADCKRMKLFNSWDFDCNTFYDSYDKYSEVPSISSSVVPTTSAAKNVEPKQEEAPVSQKQEEAVISGAPP
APDQAQVEHRVVAKEEKVEYRDQDGNLLDPEEVKALEGKVEFKTKYETKTRVVDEQGNEVQEPVEGWDQNLAGVAPPHPDVEG
VNSETVQGKDEGAAAPQDVAASKDGEKEAEEAKAKPASENQQDATIKEEL
FUN_012335 CDS scaffold_5 286219 287869 -
hypothetical protein ENOG41

MELTERRNSALEAASQSLFDESSTRSEDASVLLVLLSFFSPCEKIPLELFTRGSTPRKRWTIEGEVELVDATKVGLTSWLIDI
LADGQRLTRAFRELCQLAAVLKYPDETYHLNEDMSARVHRSLAPDALPFWRQQALIVAYRAIPWKYIEFPEPVVKSFLPHLHH
VAEAFHDCFDELPTATRTDFMLTLIEAFRFPDMAWKYFAIGQAELAAGRLKDTHLRLCIGQTKAVLGRLSGNMDEATESLQDF
ITNDPAAAVNKRISCEIGVAIIQRSLNSIQVADLSTAQKLLEDWNPLGDEPSPLEEILSFRKHSLLGRVKRLQGNFDESLKLL
ETAHEVSQKPSQLIFDEDLRDLTCDLADALRELDEPMTGEGYLRTEIMRRTERPDPLTGKSLLELALSEALFAQERYEEAEKI
CVDIESRVSLLKYERLRVYVILAKLSHIRSDFEVALSRWSEAMQALQEFSLVDGQVQTIISASMADVLDAQGHNWLTRESPRR
ASLNELAKPEGVPHWIAGFRQWADYLQSRGRHDL
FUN_012336 CDS scaffold_5 289086 290744 +
hypothetical protein EOG09264RIE PF01805;PF12230

MAAVTTNGDAAANVLDDIKPPAGVVLPPREIRNVLEKTAGYVARNGAVFEDRIREKERSNPKFSFLNPSDAYHPFYQWRLDEV
KSGRGTAIAAGRVGEPAAEAPKPQGPPKPADYQFSARTPRINRKDLDVIQLSALFVAKNGRQFMTQLAQREAGNPQFGFLIPN
HTLHNFFQHIIDQYTTLLRASGLGGEGGKLQEERIKELEANINDKFLVLNRAKRRAEYAKYVESERQKKEEEEVKAKEEFAMI
DWGDFVVVETITFNEADEHANLPPPTNLSDLQYASLEDRNNASISASLRIEEAMPDEDTTYNAYPNQPAPYPIAPQQPAYQPA
QMPPPVQPYSPVPQAQPQQPQRSAQEEEEERRIQERAEQQARKQQATAEARGGAPPMKIKENYVPRAAQKAANRQGAQTALCP
NCKQQIPMNELEAHMRIELLDPRWKEQKAKADTRYASSNTIHVDVANNLKRLASQRSDVFDPVTGQAISEDELARRKKAAIHS
YDGAMDAKSQAQLGHMQNVNVEDQIRAIHQKFADKK
FUN_012337 CDS scaffold_5 291830 292480 + SNX3 Sorting nexin-3 PF00787

MASDQDNSGLDAPGSQFHRPILQSMPDTRQQSFDEIYGPPENFLEIEVRNPRTHGMGRHMYTDYEILCRTNIPAFKLRQSSVR
RRYSDFEYFRDILERESARVTIPPLPGKVFTNRFSDDVIEGRRAGLEKFLKIVVGHPLLQTGSKVLAAFVQDPNWDRNAW
FUN_012338 CDS scaffold_5 293473 294786 -
hypothetical protein EOG09263HED PF00153

MSHHQPTSHIMSSRDEKPFHHSHSPIDHSALIQSRETGDVLPDDTPIQGNVKALPFAKSWVHMFAGGVGGMTAAAVTAPLDVL
KTRLQSDFYQAQIQAQREAQAQVIGRLNPARAALYHLNDTLQILGSVYRNEGWRALFKGLGPTSVGVVPARSINFYVYGNGKR
LISEHFNNGVEAPWVHLSAGVAAGVITSTATNPIWMIKTRLQLDKNVAAGGAQMRKYRNSYDCIRQIIRDEGIRSLYRGMSAS
YLGVVESTMQWMLYEQMKASLARRHNTIVRSGRELTWWDKTVDWTGKGFAAGSAKLVAAVIAYPHEVARTRLRQAPMENGLPK
YTGLVQCFKLVWLEEGVMGLYGGLTPHLMRTVPSAAIMFAMYEGILRLFHTPA
FUN_012339 CDS scaffold_5 295514 297554 -
hypothetical protein ENOG41

MGHTSDSEPAKRKRDVDDAGGQGRAQHAPTSLQQGVPGHIHYLPRSNASHLSLVQGDADTFADIIGLIGEYENVLDRHESLAA
SLGAKLTGPRLLRGIEKFFDGPIKTTPLQPFSNPISWLDVVSFARSNPKDFTLVQKADGSRCCQFVLKGSQVEIMEDDWRLVW
SGALDRFPLDHPLEEDETAELATLDILEQRTSILYKKADEVAARARILNHRLGQRKQDIRRRRSTHDTPPSRYQPTNSTGSSS
SQRTANFSAGYDLHADLLQQFLAASASPPPSRSTSVTGIPITTLSQPSPSHSTTHSQAHGPRYSGHAAETTVQPVMDSREAAF
RSLITQRTDQLGKGDVINPPCDRCRRLRVECVKHLTACSGCTKKHAKCSWKTVTDDEFRQLKRDMGLRDEMDVDGQSDSGRGP
DTLDPKDGHSSRLGERQEAGRTSGDRTPGTHGTDEGSRPPSRDNGHLPSTMEPSTASMHRFDLSAERHRLPFGMGQSARSDHS
NHSVETLAPGSIATGLASSLATPSNSTYRGSDGSLNPSAS
FUN_012340 CDS scaffold_5 299551 300096 + LSM7 Sm-like protein lsm7 PF01423

MSDRPSHRGGRGGQGSHPRGGRGGGRGGGGAGGAQQERERPKKENILDLGKYMDKQITVKFNGGREVKGTLKGYDALMNLVLD
DVHEVVRDDEGNDSTRSLGLVVVRGTLLVLLSPVDGSEEIANPFVQAEDD
FUN_012341 CDS scaffold_5 301146 303019 -
hypothetical protein EOG09261Q5L PF18101

MANDDKSRSKKALNVESPSFTPASVGSKKATFSSQAASAPAFTPRGLGSATPVGPQDGDSSVFNPATVREFTPNFDVNSTTTS
ANGSGQDGGMSYDPFTVPTMNQGITTPQYNPYAEDPGALGGHGPGYFPAQNAFAAPVQPLQHHLYFPAGPRRDDLMPYHRVPH
DFFLAEKDRIEIVNKLEATGQVLPNSQLPQLDNYHNLVPLDTTHRKNANIFGYPSWVYKATQTKSGHTYCLRRLEGYRLTNEN
AIRLVKEWRRINNGSVVSIIDAFTTRAFGDSSLIFVQNYFPLSKTLVEAHLTPSSTHGTRFQAKTPIAENVLWGYISQIANAL
QAIHSVNLAARCIDPSKIILTDKGRIRLSACSILDVVQYDAHRSIQELQQEDFIQFGRLLLCLTTNTLPIHLTNFNMSLEQLS
RVYSVELRDTVLWLLTPQQPPAQKGIEEFVRGIAGRITSTFDQNLQAYDKARADVMREVENGRVARLMMKLATVNERYDFNGD
QNWSENGERYMLKLFRDYVFHQVDSNGNPVLDMGHMLRCMSKLDIGTEERVCLTSRDEQTSFLVSYKELKKMLANSYGELVKA
SKSGRSF
FUN_012342 CDS scaffold_5 304894 306503 +
hypothetical protein EOG09262E4T PF00789;PF14555

MAEGDMDLGQLSTTQQEALDQYTQVTNQEISEAIPLLRRSEWNVQIAIAKFFDGEVADPVAAAQQEIPRATARHENLQESLFD
EAIRAPRASPRQRTDLAPRIVPQNPVTNRTPWLLGLLLTPFSLGWRAASTLFRTLIYALSFLPASIRPRAVTSRISTGFRGTN
GRRPLMPRDTASRFKREFEEEYGENELPFFEGGVAQAHDQAKKDLKFMLVVLMSPEHDDTESFVKETLLAPEVVDFIKDPSNN
IILWGGNVLDSEAYQVAQEYICTKFPFSALVCLTPKEGSTRMGIVKRLVGPMPPSTYLSELRAAIEKYGADLDGVRAERTAQE
VTRNLRTEQDSAYERSLAIDRERARQKREAAAAAAAAEKRAQEEAEAAKRLEEKREQWIAWRATTIVPEPPTSDKSVVRVALK
MPEESGIGRIVRRFPQDASLEDLYAFVECYSVLKEEGAAGAEKPGEYEHEYKFRIASILPRTVYEPSTTVTMGEKIGRSGNLI
VEDISPDSEDESDDEGDDED
FUN_012343 CDS scaffold_5 308044 309825 +
hypothetical protein PF03127 ENOG41

MKSMKGLGMSKVLGSIRRKASNVSNRASDGPSGTSPSGTTPAGTPEATAHNSVKAFCESGGKSKQDEVLFLPPIVDAAESSPA
AAAECARLIRKYLSKEYFSKPSWQYNAIMLLRILTDNPGATFTRNLDQKFVDTARGLLKAVRDPSVRQILMETLDDFQHTKAY
DENLTLLTSMWQKEKEQAYKNFGGPQQPMMSPGGYNTPPPNPHSQNYFARHHTNKRLPDPIELASRLEEARTSAKLLEQVVMN
TPPGEILDNELIKEFADRCSSASRSIQGYMTSENPTPDNDTMENLIDTNEQLQTALNQHKRAVLSARKQLGLNERSDSQSPSL
TPQIQPQQQPESQPETNGNNHYFNNGSGSGSGKGKETHSYPPPPGPPHQTGEGSSNRPYEESAENPFADPQPGNSSSVQPPSN
TATQYSEHERLAYEPFHPGFKPTESYLGRQDSAIGKVHMHGAGASTPSAQPPAQQRLDEVSDDDDIYDAPSGPPPKSKEPIYR
Y
FUN_012344 CDS scaffold_5 313669 315331 +
hypothetical protein ENOG41

MDTSHKAPTLPVPSLNHLSSELLALVFEQLREIDSPTLTSVRLVSKRFDAIATPIAYRFLTLNESLVAPDAEQRYPNVSRHIS
HLTNHVTIRSNLDPTRITEILSCIKRLMSVRWQYIDGDIPAEGLWSPSELRRLHNCNSNSTQLYIEDLPLRRDYNGNPLDTFT
RSIPSELLASLKLANPMPVLSTRLNSLKQLLVQSRQLKVLRYEDRGQGTCFRFSPGERMPPIVDLMLRSYDWSHTAEDVQKHW
DFSKIQSLELISVPIFNFLRSVPFRDFSALHTLHVEDYSAHLSDKREDATIGLNLLVKDHIEALEVLNITCHTRLFQLDSIAA
HRKSLQVLRLRDHVGFSEDDQKCPTLAPVDLAQLGQRLKFVHTLELDLDTRLCDPTTFLRAICVFPAVHTLTLHVQTIIHPLE
QVSPGIDRDREAAMQTFKLLVQEKERLSSHTPWKRIVLNVGGWKRVMVRRLGDVWRRRNEMGIYAERCFVYERDARDGNVTVR
EEVCVENTSRGGSPSP
FUN_012345 CDS scaffold_5 324751 326223 -
hypothetical protein PF00172 ENOG41

MADDSYRKEYPQQSRPEDTHSRPQSHYPPPPDTAQRPSDAPPSSMATSVTLPSIHDSRGYGPPPAAPGRGYPADPRYASPNAV
NGYPPPGHQPPPGQPQQYLPPLQPQSDPRSSAYPPPPPDQRGGYYDDRRPPYGQEAYSTDPYYAYYRGQPPPPGPPPPNGHPG
YRHIPAGVYEYPPVGHGSAPPLTQAAPRQRTSIACRYCRKRKIRCSGYQSAPGGKCQNCARMNQECIFQPVSSSSSTAFIPVS
ALPGGVPPGTQLFGAYGQPLAPGTVPAPPPPHAAYQHPSAPPPPGNYYAPVQSPTESFSSYGEARTDDGSQLAGRRRRRTSEE
QDEGYRLPPPRSAVDEDPRRRSPAEFSNHSSPGGVGYTPYQGARQSPRNPTSGSLPQAATTGSYAASAPGGRSPTGQNGSSGA
STPARQGQQSQGGNASIMSLSNLVDKSDIDKTMIDRLNRPAPGQPGARRDVSR
FUN_012346 CDS scaffold_5 340536 341680 +
hypothetical protein PF01209;PF08241;PF08242;PF13489;PF13649;PF13847
ENOG41
MTDVVSENQAYFGKIATEYDQRNADAIVQLERGIQERIPFISVKQDARLLDYACGTGMLSRVLAQHTSEAIGIDLSEEMVGVY
NAQAQSLGSSRQAYQGNLADPKDPSPTAFADSKFFGFDVAGVGLGFHHFDKPDLASKQLAERLNPGGVLFIIDFVAHKIDPKD
AALRGITHHGFTEDEIKKMFEDAGLTDFAYQELPEPITFKDCHSHDHGHEHGHGHGHNHKHNEDNHSGSGEHVHGHGKKKGSS
TTKRIFIARASKP
FUN_012347 CDS scaffold_5 343083 344156 -
hypothetical protein

MSTPRVFLIRHGETEWSLDGRHTGLTDIPLTSNGEKRVRATGKALVGPDRLIAPKKIAHIYVSPRKRAQRTFELLNLGLKRPL
PWTPHGAPDGTGLQCEAEVEVTDYIREWDYGDYEGITTPEIRKIRAEQGIKGSWDIWKDGCPGGEAPHDVTRRLDQLIEEIRE
RYHKPAMDKGSDQCGDVLLVAHGHILRAFAMRWAGYALREGPTFLLEAGGVGTLSYEHHRIEEPALLLGGAFVVELDGQD
FUN_012348 CDS scaffold_5 345597 345947 +

hypothetical protein ENOG41

MAVHQWTRQEKFKRGVWAVAFAACIFAGTITGAQLKTDNEKKEAIQEFRATSPNEQIAALLSQRKTLVSQKAVLQRKMDDFEV
RVKEREAEKAREKESP
FUN_012349 CDS scaffold_5 346302 348068 -
hypothetical protein EOG09263BGW PF00240;PF00443;PF13423
C19
MSSISVVVKHQGKKHDVEIDPSSTGEDFKLQMFSLTNVEPERQKILIKGGQLKDDADMSKLGLKNGQVIMMMGTPSAGGDALV
RPKEAIKFVEDMTEAEQAQQVGATPAGLINLGNTCYLNSTLQTLRLIPELQEALNTYQPDGNASSFMMTGTNMDLASQLSNLY
KKMGETQDSFPPMNFLNALRVVFPQFAEKSKTGQGFAQQDAEEAWSQIVQQLNQKLRIKSENTPETSFVEKYMSGEFTSVMEC
DEEEARNAGEQPIINKESFAKLNCHIDSSTNHLRDGIAAALKEKLEKQSEVLGRDAVYTKTSKISRAPKYLTVHFVRFFWKRE
TQKKAKIMRKVTFPMELDIVEFCSDELKKALIPVRDKVREIRKDEEDIERARKRRKKTHDQDVGDIPGGAGLPTEKEKKETEK
KEGKSADGDVVMGEEGETYKTDADIEAERNASILEAKKELNALINPELRNDDGANQSGLYELRGVVTHQGASADSGHYTSYVK
KAAPVDPKTGKKGEEDGKWWWFNDHQVSEVEAEKIATLAGGGESHSALILLYRAIPLPTAEGVLE
FUN_012350 CDS scaffold_5 348632 349364 + ARPC4 Actin-
protein 2/3 complex subunit 4 EOG09265CCT PF05856

MSQSLRPYLQCVRSSLTAALTLSNFASQTAERHNVPEIEAQTSPEVLLQPLTIARNENERVLIEPSINSVRISIKIKQADEIE
HILVHKFTRFLTQRAESFFILRRKPIKGYDISFLITNFHTEEMLKHKLVDFIIQFMEEVDKEISEMKLFLNARARFVAESFLT
PFD
FUN_012351 CDS scaffold_5 351412 351843 +
hypothetical protein ENOG41

MAQGDSAAQRSGPPASNPPFNAPSFCPQPRRPQCSNGGHQPEAASHVVSNANSWVPSAQQALHNALQASGYAGAQPGMNMVTA
LPAQYAGSSGPPTAPPNSPSDGVAHAIYPAMPYALDAMAP
FUN_012352 CDS scaffold_5 351876 354031 +
hypothetical protein ENOG41

MAAETVNGTIDGVVNNAALSTSGHISHIMASGATPSSNNSPEHVIVNGSANAATTNQASRGNHLANSNPGDPGGSGIGNRLPH
GTQLFATQHTHSGNSGSGGQTSFMSPPHGRSSASQPSSQYSARSRPATGGQQFYTPIGVSQTNAYNPSLPQSVSYNSGLSLAS
PSHTPHHYNGHRMSASQSTPNSGPNQHLTEPRNYAPRSSAHGVNSLPPPRFDLGLQAIPMTHESAAQDPFGSPERAEPTALIP
FPNLPPPAAQQAMLAAPMARHFTSNMSPELSKLVDTPSGLPTLETAMKPEFFPFTSGPGSAKASVAGAVRLKNIPFSTKRAEI
VAFIGRNSRMLPDVEEPIHIIMDRATSKTMDAFVEFVTMEDAMRCAEKHHQHAQAGRVSRLGERSIEVELSSQSALMQDLFPL
AHGVFWDGATPKVLPNNHQEPWENFKGFISPEEMVMLVKHVEVPHRSPFSKECPQRPYECLISTLRKFPWYAADHITISQRGA
VYKATIKLLRLLSRSVSQQDDPVNLTSQLFQRVVQTAMQCHGFTALQKDNIAFMVQMIPMEQQRRHHQPANANCWVHQYAITR
KPDAPLDLAEWYIRLIREQSTRDVLIRPIHERTVIQETLKETDEYWGYFFNEVNYIQGPQFDQMTLGQCAYLELSAIERILGR
ALSYN
FUN_012353 CDS scaffold_5 355574 357004 +
hypothetical protein PF00650

MAAEQEKTVPAAVPAENPATPETPLTKLNARLEDIFSKTLHKEMWGVQLTNIDHVPTKVVLQKFLRANNDDPVAAEKQLTQAL
EWRKKMNPTALVTQTFDKSKFGDLGYVTVHKGENGKETIITWNIYGAVKDNKATFGNVEEFIKWRAAIMELSVQKLKLDQVTE
PIPEGGEDPYQMIQVHDYLNVSFFRMDPAVKAASKETISVFSMAYPELLAHKYFVNVPAIMGWMFGAMKLFLAPATLRKFHPM
TSGTTLATELKGIVSTLPKEYGGQGASVREGMSVSLTEAGESAAGETSAEPSPTEVPEQTAAADSTTAEKTVVTRESAPATEK
TLTAEPIVAAAAPAPALAAESAPAPIPAPVEAAKAETAEQAVEQAGEKPIEEALEKLSVNPAESEKKDTLATDVEKKETAA
FUN_012354 CDS scaffold_5 361176 363640 +
hypothetical protein PF00856;PF17907

MALLLSSEGAFEVSTTDDSSSNVASATSTPPTTVADEASLHSDSPKRDVVHVALDPDSPEVAPTTSQPIEPTEIDSAANALPP
APIRSRRSRVSAPVYNLVQLSGTAGHGKRRAKGDIVADRRRRRKTISGPILGDDEGTSNTPQDATPETVRSGIDALGATQSAS

KLDSPRARRQKITEESSSSRRSSTRRTSVFAPIAATPITKNSKATKRSRKSMEKTSTPMSRELRRLQDTKEFAHIDEKPVVLS
VWSNGKFVDPKAAKSASRKKAEPEQAAEEPKEAEPEPVINTRKRRVKKYLAKGLYAGQDAPIDISKGLTVGEKKALAQLPELI
PSGRVNKTMPLPMFNGLRTLIEGRDFKLPYQVCNPLPPGQPKPDEWKKMTKNRFIGESKDYWRKSPHFHDYSSKCVCKPEDGC
GESCQNRIMLYECDEQNCNAGKQYCTNRAFATLTARRNKGGKYRVGVEVIKTSDRGYGVRSNRCFRPNQIIMEYAGEIITEDE
CERRMTEVYKDNECYYLMSFDQNMIIDATTGSIARFVNHSCNPNCRMIKWIVSGQPRMALFAGDKPIMTGDELTYDYNFDPFS
AKNVQKCLCGEPNCRGVLGPKPREVKQPKTDLKNAVKGAVKAGKRKLKELIGDEGDDGKNAKKRKVQPAKGVKRAISNAGSKM
AKGAANALKKGVSTVASTTRKAALGSKSPAKRRVSTGVLLKKTTTKRVMQTYSRTPQRRASERASSVGLVSASRTKSSSVTKV
STKATRTTASLRRTSSRVISPRQALDLSRDGEIRVVADD
FUN_012355 CDS scaffold_5 370381 371965 + PFA5
palmitoyltransferase pfa5 PF01529

MESTVVTHPDESDLADLARPGIVTTAETANAAAQTEDEIITRPRSARTHSHSSLSVITGIEPPASPHSSHSSNWSFLWSDEMP
EPSTSKRRSTRWIARGLPLLMLALVAYATYDIIVYCCVQGFMQKRGKTAVGVVLIALYSILLLLMVAAYIRCYVTVQFNPGFV
PWTAEREALEQERNQRASNGADVEIRSWAPRDDEPDSPGLESFYSKDIFVCELDGFPKWCSDCRNWKPDRAHHSTEYDYCVYK
MDHVCPWMGGIISETSFNFFIQFCFYCACFCIIIISTNGYLTSLRLQEAKSIDARLIVALALGGLFGLFSITMTVTALRFAFQ
NITNVDMYKKNQIFRLAVRIPTDTPPNDRFNTITYPLERPGEGPRAPDAAHTSGMTQANGAASARASAMAARDQQARRTFAIL
QAEPGENPWHLGYLQNFKSVMGDSIIEWFLPIRHSPCSRHDSMVSDYKFGPLVEELKRRYGLEGSDPEKRAAAMTSN
FUN_012356 CDS scaffold_5 373430 376157 +
hypothetical protein EOG09261801 PF00817;PF11799;PF18439

MSSSPLNRPLGASSPLLEGGRRRSQFTYRQFSQLASSNTSNPLRVIAHIDLDAFYAQCETVRLGIPEDKPLAVQQWQGLIAVN
YPAREFGIGRHCNVEEAKKLCPELIVQHVATWREGDDKWAYREDAAAHIATDKVSLDHYRLQSRKILACIKEALPLDLQKVEK
ASIDEVFLDLSSQTHLILLERFPELSNPPPYDDPTENLPLPSIAALDWQTDALIDLDEEQETVDPDWDDVAILIGSEIVRKVR
AEVRQKLGYTCSAGVASNKLLSKLGSAYKKPNKQTVVRNRAVSAFMAGFKITKLRNLGGKLGEQIVSTFNTESVIELLDVPLA
TMKTKLGHDTGFWIYNTIRGIDTSEVNSRTQIKSMLSAKSFRPTVNSSEQATRWLRIFAADIFARLVEEGVLENKRRPRTMNL
HHRHEGQVRSRSGPIPQGRIIDEGSLFELAKDLLLQIIAEGRGVWPCANLSLSVAGFEDGVKGNMGIGAFLVKGEEAEALRSS
IPDSRQSSTGPEPSAKKRRVEDGGIQRFFSKRPSTDHERTSLTDSHTHGEDSKAGSLPSDPTQKTLSFATKYDCEARHESDLA
MHESHASPWHESAFDVQVDQKQHSLTDVVCSRCKASFADPEALQNHRDWHMAKDLQDAERVKPTFAERQPAARNSAQKTQGTT
SRRSRGGKLEQGQSRLKFG
FUN_012357 CDS scaffold_5 379596 380663 +
hypothetical protein ENOG41

MSRSSRNAAPTTSGTGRQNEYFVPRDGIDREVISADICRYLGNDALVRPGHYENPQTGQAVQGYYITAYRNLTTAMIEDLKAD
SARWDSERRAQTSRNTSGGTIASRNVGVPPRHSSNSPVVQYRYSETHQSRQHHGPTEGPYQTDPYARDPGFDGPRYPGTGAPG
YTGAAGSYGQSYGASSSGAFAGYAQTQQSPPPADTRFSSTTAATMDPSYQASQSPYVAVGTNQRPRGGYDPYANQMATSSAAA
QQAYATAAPTQQGYTATAYPYSGQAPPAGYAMQPQDPFYGRGAYSGGNTRAAGFHMVELLTLKV
FUN_012358 CDS scaffold_5 383079 383667 -
hypothetical protein ENOG41

MIFPLSSDSTQASSPGPVANPPAQAPEPARYRVFHIQHSPGTHKIFIPTSSPRPAGYLYHVLESSNPEDSQPTMTLVSDIIET
LGQVGTIHDSRCVGSIASQELDNACATIKSHPRPFDPYEPTNAPGRPGRCELWILSVIDTLLGDGLLQPPPSEPTSRAHQPPR
HHHTAHCTHQRLYSYHPYQRRSRPALDRE
FUN_012359 CDS scaffold_5 385784 386597 -
hypothetical protein PF01328 ENOG41

MASTSEPLTKGTYAPSGPNDIRGPCPMINSLANHGYLPRDGRNVRVEEVLAGMDTIGLSKPLAAAFANPIFQERAPSKFHDDP
VVKKSLLQTILQTIRDPWSVLGKFGMRKPGQLDSEGHRVLNLDQLGLPNTVEHDISLTRRDHQQGDNLTLQKDLVEDLLASSK
DGQTLTANDLAEFRKKRIARQKEDNPGLQYGSFEHDLACAEIALILNVIGTGDSVPCNYARAFLQEERLPMQEGWKREESRLG
IIGLLTKRNGIKKIIGMEFKS
FUN_012360 CDS scaffold_5 391888 392174 +

hypothetical protein ENOG41

MAIHSHDIDEKSLALYSRTGGDAAWFHTPFDDNEAITEIWWGSIGGNGDAMGLRTSKGREVFLGFVGAIPDLDWELAS
FUN_012361 CDS scaffold_5 392180 392867 +
hypothetical protein ENOG41

MDNHRIYYDSMSSMGIGGLAFEDPSPVAQGFQPFDPTTIVPPMPDFRYCVEPFFFSSANLHNLSKITVCQIPDKRGISGLLLT
YKNGHKEALGEVRLNCLEPPIDVGKQDRVWLRFEYDPTGIIDEHPRLVEISFSPIEPIMRDGTDPAVVGINCLEVLFADELHW
WWSSLQCQVFYDVQGSLQPRDAEKWLLFDDYIVKSLHYQELSKSTDISQRYTHAYQGKWTSI
FUN_012362 CDS scaffold_5 396731 400532 +
hypothetical protein PF03810

MDPAAVRSLLATSLDPDADSRRRAELQLKQIEEQPGFLECLLDILQAEQEASVRLSTVIYVKNRVNRSWYNNEGYSPDPPTAI
IPEEEKARVRDRLLPILATSETLVRQQLIPVLQRILQYDFPARWPKFMDFTVELLNTNNPGSVLGGLQCLLAICRAFRYKSTD
SDDRQHFDKIVEATFPRLLAICNELVNQESDEAGEMLHLALKAYKHATWLELSPSLRQRDTNIAWCTVFLHTVSKACPTNAMQ
GDQHEREKHHWWKAKKWAFFNLNRLFIRHGNPASPGKGEDALAFAKDFTANIAPEILKHYLQEIEKWVAKTSWLSRPCLSYTL
VFLDESVRPKEMWTHLKPHLTNLVTHFVFPVLCLTEEDVEQFEDEPDEYLHRKLNYFEEASAPDVAATNFLVNLTKNRRKETF
EILKFVNAVVTEYEQAADDQKNHIAKEGALRMIATLAPVILGKKSPIADQVEYFLVRYVFPDFTSPQGYLRARACDTIEKFEQ
LNFQDQNNLLTIYRHILDCMADPALPVRVTAALALQPLIRHDVIRTSMQQNIPTIMQQLLKLANEADIDALANVMEDFVEVFA
TELTPFAVALSEQLRDTYMRIVRELLEKESKVGDDGELYNEYDDKSITALGVLQTIGTLILTLESTPDVLLHIEAVLMPVIKV
TLENKLYDLYNEVFEIIDSCTFAAKSISPTMWQAFELIHTTFKAGAEYYLEDMLPALDNFVQFGAPQLAQKPEYTQALYSMVA
DMFTDSIQGGVERICACKLAEAMMLSLKGQIDSCVEGFINIAMSILANQDVKVKSYRIHLMEMVINSIHYNPLLTLQVLENKG
WTNRFFSLWFGSMTSFTRVHDKKLCIVAISALLSLNPEHVPSSVSVGWPRLLQGITELFRSLPAAQKNRDEALRDDFHLESTY
DYGEEDEWDDDEANWNAEEEEETGETSESKDESTAYLNFLNEEAQKFSRAIDDVEEDDLGEDSVLLESPLDKIEPYQLFRGTL
MKMQQEQPQFYSSLAGHLTADDQNVIQSVMVKADEIAAQQVQQAQQAQLLAQQQAAAMAANLSAPNGGAS
FUN_012363 CDS scaffold_5 401399 403240 -
hypothetical protein

MPLFSNRGRELPEAILPIQEAKEAKIAMQQRIADSLSTKDNKLKNSDVLLYPTGMSDISHIHDIISLYTTARKRTVAIFGKIH
GYECKLYDATQSDLETLEEDLASGLQLWALFTEFPGNPLLGSVDLERIERIYDEYDFLFVVDDTVGTSVNVDVISHCDIVCTG
LTKMFSGRCNVMGGSVALKPNGGARWDIKRRLKDRYINTNFPLDTIVMEKNSADRFRRASYQSQSKCTTHRRYTSQAQFGWAS
LLSQGKPDTTTSREMKTSRKGTPAAAIAFHDALDVAKGPSLGTNFTLGCAYTLFAHYDELEWAEEYGVVKHLVRISVGIENER
FLDEVIKTALDAAERANVSSCVTLSLAYVRLIRDDPNDKHKFLAGSSLDISTRLELSIYVFPRGLNRSGGVTGYNPVAHNWKS
IFGSTTTSYLTEY
FUN_012364 CDS scaffold_5 404873 406544 + ISN1 IMP
5'-nucleotidase PF06437

MTTRYRVEYALKTHRRDQFIEWVKGLLAVPFVLYSQPTGVFGDGPSVTQMAEEAHRRYAEIMRDVELMIDDHISRQQDDTMLP
SKLRMLIPTAGPFFTRLPLEAAFKYQDRKRYISSRRFVAPSFNDVRQILNSAQSMAVTNGSLQLATFDGDVTLYDDGFNLEPS
SPVIPRLLDLLRKNIKIGIVTAAGYTSADRYYERLHGLLDAITESTDLDPIQKQNIVIMGGEANYLFEYSPSSPYKLAPVPRT
QWLTPEMASWSDADITRLLDVAEGALRDCVNNLNLPAMIMRKDRAVGIIPKTPGTRIARESLEETVLVVQRILELSSLGSSEE
RPTKHRPNSPPIPPSVASQSRRVPFCAFNGGNDVFVDIGDKSWGVTVCQQWFGSKENGGAIRGENTLHVGDQFLSAGSNDFKA
RSVGTTAWIASPAETVELLDELADLMQKKLS
FUN_012365 tRNA scaffold_5 407225 407319 + tRNA-Glu

FUN_012366 CDS scaffold_5 408629 410708 +
hypothetical protein ENOG41

MDYAGGLSSEEIRRIDRIMDLEWMEFAEGWEWHALNYVNPLPPMLPYPSYCVSRRCGWCRFTIKPGDLITARASGGTETVAFE

SCDSFNDNKFRATFRRCTSDHESCATIGYHVECSEIASSFGLDKRAFVQVARYSYEPSVQEDERRREWILNRLHRIMSQKFAD
LPPEILLMVNKHLVLHYAISSLSRVSESSQCTIEPSKDVWATHLDLDGIEYVASLSNTPQPGSRLLWREFQGREENHLFISED
HLGIRQIVNDPGAVTSAQGSSGWWRTLPITSKVLSFSDDGLKLRSFAAAPLNPTVCWPYPMAPDALKSMAYHVTKDSSSSLGM
TIEARMVALELNEPEITGYSACWYKDQLVDLHTHKTEESLAFYRELDEMAKHKLAKKNSDDSTASSSPHWTYHPLNPGERLGQ
VWLRTKKEADEKDTSEDERRPKTSDTVNETAVVLVTNQSRTLTMGRSNSPHSEWKCVAKESPDIPMLVYLSPRLDGISLLASP
TVEGSDKDPLPAVLVPGSETDPVETSEVSLDAVKEVQICREQSQKGISSVSGLLFRYKDGSQACFGRFRFDHAKEVLSVADSM
CFYLGTIYLDGGNRVLGEFCLSKPEYEDESLEWKSTLWKGSFPREIMLMMDNSMSVLNMQ
FUN_012367 CDS scaffold_5 411838 412558 +
hypothetical protein PF05042 ENOG41

MVEYHSDISQSTPNRLGNDYTKSTFAKACSDHSETPSAAIVNSKDEKLSPLQKHVQFWDRDNDGIINPWDVYNGFRELGFGLF
FSTGSLLIPIFFSYPTRLGHSWLPDPLFRIYVNDIHKAKHGSDTGIFDFDGNFSPERFEQMFQRFDTSGEGGLTADDLWRLWA
KDRCAADPAGWTFAFMEWWTTYVLLQEDGVVKKDDLRACYDGSLFWRIKDERERSSKYMDRKSFGMRNFFASI
FUN_012368 CDS scaffold_5 413598 414352 -
hypothetical protein ENOG41

MVTKEYPSLCVPDVSARDDFEGTVPIQSCEVNSHAMTKPQHRNIVKANTGMARPREIGLARVRIDVLSIAGTSRAQRLSKSPE
LSDNTSEDTQDELERPALEESKEQQAESAPPKICDYVASTQDSGETREASPSANPDRDQPDNPDGGNHDMNEDRTLQPQNNIF
DARGIGRPPRFACPYQAFEQFQVCFRPGPRIPNGGCASIQRLKQHLYRRHMRPYRCNRCWQSFEKKR
FUN_012369 CDS scaffold_5 416055 417429 -
hypothetical protein ENOG41

MASFSLYQLSNASRRLSLNLLRQHRNTLAVSRRQPSPIRLLSLQQPRFLHTGDDAKSDQSTQISSDIAAEEGTKLDSSYETSD
DVAPIKDESTQADNDVTRDQDVGDSDAPTLDLPILSSDPRDEPKVKGRPWTEEELAQLEGFIKRPIFSLDYIAIRMDRSIFSV
RGQLRQIQERKGIDILRRSRPRTPWQHPMDATPERVAVFEAKQDEVQKRMEHIVKTLMTLPKTDQWEEILKLKSASEWASAIM
KLIPLRTWHILAADHPPTEEEYMTITMVDRYKMIGVFAQIHQRKVYVGLAEVPFGVLAPSDSKDSQQQDATKKKPQEVNKKRH
MGSIVPLIPVSLLGSNQLTFEEKVEQKYVLGLARATLAAWLGANHVDASPALRQLYAWKDIISKGHPFGRNPCNPLEQRLRLP
STPEESAARKASLVEARKREAEETGKTEEELKKSETPKPFKK
FUN_012370 CDS scaffold_5 417889 418875 -
hypothetical protein PF18271 ENOG41
GH131
MRTAAFITLLASVVSATPFGQRSQVLARASETCPVVFDGRVPANASLTDFDTVNGGGWNPYNPGFVKGNNISWSEILQLPKTK
TKSRFDTEAGTIPLEVTISDKSIFMKQLGFRRAGLQFNKDSNEGSPGSKGVKTLHFSIMQDDKRPLNLSHEYLNVWHEKADFS
GNQFQFQAGQLIGQNGTAATWKLLDQDFKLLWETPMLKKVWQNFAITLNYEKNTIQAYYSKGCKPLKVATQPIARNLTGQGQF
QIGILKKPTGTDDVANSGFQEANLNEGLIYGGIFLEDSADGCVSL
FUN_012371 CDS scaffold_5 421770 424888 -
hypothetical protein PF00069;PF07714

MGIMAALSAPRAMVAPPQHQPQGPSTLVTDKSTSPSTFRPNQFQSLSSPRGYSTDLTESIVLEEGGESGSESAVEPVTPVSGR
QSQDFTLQSQDLQDLQNLQSYQTASATSSGVVPIAIPNSGNPNKGTYISQSVNNSRPPTTYEPSTPRATEPEPGSSLTRQSTR
GSISTSSFKRTMSSFFRRSNSHVKNDYTPSESATPSVVSAPTEPQTNGQPRAAARRRFSMNRSSATTRSNSPPSPSDNGLEMA
PAHRERASDKSLPSQGDFKKNRASTGLTLRGRAINFVGAHNTSRGTGHKRPEFTRRASSYDGSRPSTPLPPPAEGDETMYPPE
RSVWPLPPDSGTGAKARRMSLSLPDDFAVDVAELQNEFEYQRKFLGRHGKHLGKGAASKVTLMMRKGYPEELYAVKEFRGKSH
RESQQDYENKIKSEFSIAKSLHHPNIVETFRLCTDHGRWNHVMEYCSEGDLFSLVSKGHLKGDDRKKDRMCLFKQLIQGVHYL
HANGIAHRDIKLENLLITKDSKLKITDFGVSEVFCGTHPGLREAGGQCGRNMSGEIRLCSPGICGSEPYIAPEVLAKKENYDP
RALDVWSSAIVMIYLTFGGAIWSRAVPGELHYDKLVKGWETWYGKHPESDATISDTDYPKCYALDVGMSPPALRRLVLQMLNP
DPQKRISIEDVIHNRWLKNVECCQLESYDDPALLIDATKKDNTANGNKKIFCHNHLPPKGTGSHSLGKMPGQPGY
FUN_012372 CDS scaffold_5 435133 438126 -
hypothetical protein ENOG41

MASFKSLLIAYVLGGLTFIPLVIVSVLAFIFYTSPIVDDTANEPKYSPIVDKDDDTTALEAAKRSHKKDNRAHENDLDVAAGF
FAVCREYTPMGINAKPIERSTPVGSTTVAPSSPSVYQTMYRSIFERKPTPGPLDNNSNSTSQRPKNAGNVFYVVLRHGHLMLF
DDEEQVEVRHVVSLAYHEISIYSGGDVTPEGELFIKRNAIRLSRKQAGTELTSDSPVSKPFFLFSENCSAKEDFYFALLKNQE
QSFGVDGQVPKPLHFDVKNIISLVQKLHSTEENIHSRWLNALLGRVFLGVNKTKDIEAFIREKLTKKISRVKRPSFLTHITIN
GIDTGSAAPYFTNFKLKDLTVEGECVVEADVKYSGNARIEIAATAKIDLGARFKAREVNLVLAAVLKRAEGHMLFKIKPPPSN
RVWVTFQSMPKMEMDIEPIVSARQITYTVILRQIENRIKEVIAETVVLPFWDDMPFFKTEHKQWRGGIFEGDDATVPTDDAES
IVAAAGDVTAVSHLDGNPDLTEETKPLEKSQTVPIMEATPPPTGLFGRRLSRTGTNPQASASSTSVDSKGPGASPVLKPKISK
TSLQPIVGTDAAHADIFKPSTSPPDHATNYMAALHSRSHDASPKPPQTSDAPLESSSASQRSTRSSRSSSSANEALNNDVPED
TQKTPVAMGRRNTTSSTGSSHTDERPGSSAASLKESIKSQTGSLGRSFFTRREHPEVPVQVEEETDDSEASRDHQNNPLQRQT
TLAAVSNAALQAKQWGWNAYQRHKEARRQAEQASHLDLSQPMGRGQPLPPPGTPLPKPTNGMTRIAPPTSVPTRKPVPGHNSV
ESLETRHENHPEHEHEHEHEKHGEYRPPLPSRGRRRQSHAPEPDNGQNVFVVAAPEDSQPATPSGDDPSNHQVWASSDEQTVTDS
GTSHPPSMITNESSLVQEDEKSASPIEATPAASAAADDDDDFSGWMDNEPLDMEINDPVQPTAQEVK
FUN_012373 CDS scaffold_5 439714 445495 -
hypothetical protein PF12814

MASSLASDKPSLPAPGAAQYDPFITTTPPAPAPAPVPAQHRYSNYDHDFFVTGPASPESAKRALAAHLQETERRLEEAGKLGN
ALVLQRKQLTEQLKEIEKVNNEEELSQELRERMVGIEKEFNTLTRESARNLFPKQRVPSNEVNPNSPFAPENKGGRRSVSPSK
FESQATGSPTKLTVPNRKIRNQPSNRVHDIEFAAEISTSLIAQVRNLQGVLAERDEENKDLKAEKARLELEAEGFQQRFKSLD
ESENRYKEENWNLETKLQELAAQHKEALDREKKLTQSLNVIKTEKVTIQRELDEVKVHHARLIDQHAAAVKQHDIELGTVKRN
IAMAEGERAAMQRRIDDLTGQNQELARAFASQRRTVTELEPSIRASDDDLESSADHLTPEHSPPVSPIKGTPRHAMLETETMK
SSLSHAQRTIQSQRGLLHREKTEKLELRRIIQDLRDDLEKARSDAGEHKPHRKSRVKESKEFKKPPRLLGSFRSSRQEVITDD
GEWEDQHDVSPRASVSPMNSRSPLSNSTQTILPSIETSHPTDTEQFETANEGGESAFETANERGTETEDFQTGAEEMSGSDDT
ETEGTTRGFGSMKNAPRLPAGFTRHASSNSLHSTASTSGDEDDYSYTKTPTGTIGSQRSSRFRMSRSIFNRSRQVSEEPTFQS
SPVSFASSREGTPQAGGQSLFAELQDFGGSDDDSAGPGTPSRMRARSMTPGSIRRGSSPRPAVPPMPKVIMVDSGMMTEPVKI
VPRLSGLGTSQMSTTGAMTDDGDEHFAESPVSVVAAIRRASMHSSIGPSGDRDRRIPSWAYDSLADSRPISMVYSDAGAQHDP
DMEKNLALFPAPPSMLPILPPNLTMSSIASEEVEPVLEPEVVPTPPALTLTALSNQSVEPVSEPESPVPDLTMTSIISQELEP
RAEPVILPPALSLSGIVAHSLEPKAEPEVPLPDLSMTTIVTEQVEPIAEPEIPAPALSLSSISTEIVDPIAEPPVEPVMFMPP
PPALSMSSIIGEHVEPIAQKNPELSISNIRGENIEPISEPLVPAPELLMSTIRSEHVAPVAEPETEAVIPAIVAPSLPPAPAL
SFSAIQGENVEPVASKAPELAMSNIVGEVVEPIAEPVPVPVIPELTISTIHGEQVEPVFESAPELTISAIQREYVEPTDLPAP
DLALSSIAVQSVEPINEPEKVVPKPSLNLSSIYAEGCEPREELPSTPTTLVFSGLSTQNVEPVSEPAPSLALSGIISENVKPV
LEPLPLPPTLVMSDIATEGVEPVSPVYKTPTLPAFGFSNIESVETFPVSPRTPKRDGFILPRDMKSPFIEQDVPRTPENQRGI
DQGSSPLMVEDENGQSPSDTPEPATPDSERPFDKPLVIMNDQGAQTSLTADVIDAMFKARAQSAFSHQKSLSMASIGTPGTTG
TSGTVRIRRSQEMFETPSQHERDTDDVFNTSPIRRPGSSRSGRASIQDAPPLPANHRQVIEAARSNSSHGTQNNMGPPLWPAS
ALKQRPTTPGNRATSPASTARATPTQRTVRNPVSYGELGRQSPSKHTAASRKSSVSSFASELDTRFNMRPGEMGMDPSGFGPN
TDPRMIQAITQTMIGEYLWKYTRKTGRGEMSENRHRRYFWVHPYTRTLYWSDRDPSTAGRTELKAKSVPIEAVRVVTDDNPSP
PGLHRKSLVIISPGRTIKFTCATGQRHETWFNSLSYLLLRTNNEQANVEDMAENFTREDVDEFNPQFGQRAANGTRPGAPPSL
SSYNSRTTRNESPAVGMSMNIPTLTPKAQPQRPTGTLSKLSGYWKGSQLSGTLTSRRGRGSAHNVNIYEASEAHDSAEDVREI
IERQDREADRLENVRACCDGKHDVGTLHHFSKRGRHGNSTHTHSHPGLTHSHTTMSSLRSRV
FUN_012374 CDS scaffold_5 450231 450925 -
hypothetical protein ENOG41

MFCLGADTGGTDLISCSNITGSDTSYCCDHNTNCCNSGVGRFNVLPSDPEVWATWNRKATRYDVVGTMFSGQSTTETASTTTD
VTSSTASASFTTTSDASIPSSSVPDTREPSETPAGLSTGAKAGIGAGVSVGAIMAVAVIYLFWKMRRNEKAAKQGEQQPPSNN
NPMTDAWQQSQYVYASKGYQYQPQSPQELSGTPLQGYNGPAELPAHN
FUN_012375 CDS scaffold_5 451813 452813 +
hypothetical protein PF04488;PF05704
GT32
MLSPAFAHRKYVYTASIAAVIVIFLTVNYAYSGRIRPSRLPEQDALPTAPATLQQEPFPHKIWQSWKDDSENPTDRTVGFPHQ
WRAVNPDWRYERITDANNDAYVRDRFDANISDVFTSLQDPILKADFLRYLILLREGGVWADIDVFPHQPVSKWIPKQFQGSVN
LVVGIENDHHKQPIWPGSSYSVQLCQYSVLAKPNHPAIKTLVNQVAEDLKKLLESKQPGEAVSFEDVMSTTGPFAFTKVLMDY

FKESTGVEHTGDELDRLEEPRLIGDVLVLPKDSFGWLPQEHTHDKGDPIIFVEHLFIGSWRDGHPG
FUN_012376 CDS scaffold_5 453360 454350 + CWC24
RNA-splicing factor PF00097;PF00642

MADSTTEPQASAPVFKKRGAKGKANLRKRPATPPPADSDDSDYSSSEDESGQRVKRRKKTVTVTASSKDLATGKTDLQATVYT
ADRNVPITSTNDATKHSNWYDEDSQDALSAKNLLGSTRTATKDSQPDGTYKGLANQTTFIQKNPDAPNRAKGPVKASSNVRTI
TIMDFKPDICKDYKKTGHCGFGDSCIYLHDRTDVKQGWQLDKEWEEVTRGKKNLGGTIIASANRDKKEEKAEDEAEIAMLEKI
PFACIICEGPYREPIQTRCGHYFCEPCALKRYRKDPTCASCGASTNGVFNSAKKLKKLLEKKKEREDKKKKEEEEAEEEP
FUN_012377 CDS scaffold_5 455003 456149 + ATG3 E2-like
enzyme EOG09263WZ2 PF03987

MNYIYSTVNTLRDRYTPVSHKSTFRQTGQITPEEFLAAGDYLVVYKFPTWSWGDADSPEQRVSHLPPGKQFLVTRNVPCHRRLN
DDFAGDAGHEEALVNDGDDFKGATGDDEDGWLRTGGLASSQPLKVKEVRTVDDSGNVGDREVVEDDDEIPDMEDEDDDEAIIR
DSSADSKNSAHRTYTLYIMYSPYYRTPRLYLSGYLASGQPLPPNDMTEDIVGDYKDKTVTLEDFPFFANNIKMASVHPCKHAS
VMKTLLDRADAALRLRREKLRAGNSQTPSGMEGLVDEIGKLDVKGAQEAADKDEWEEVQETEIDDQEVAIRVDQYLVVFLKFM
ASVTPGIEHDFTMGV
FUN_012378 CDS scaffold_5 457236 459243 -
hypothetical protein ENOG41

MAARFTPEAFLPSGAYDSDVFLSLNSHLPYRNTSSIPSPPNSYPRAGTLRHQYPQHYLNPCQPQNQDYSQSQFQRGRIPPAST
LPLQHKDSSPLSLESLRAAQSVPPSYRPFPQQTESFDTFDARPATVSPTHGGPPPVPEFRFPASSTAPLPNVSSSSGLVGASL
SRSVSADAVAASQSSVHVVQRLVQQNQLIREAWEAERNHLEANRRRAEEVYQEERIIMDEVRDSWEAEKEGMAREIEELKERV
HRLEGENSALKAVTAQSIQVTGVVSPLASQRGGSGEGSMDSSYFQAPPGRLASRGQNPTIPTGLSMSDASSLPPGLDGASRRP
HYFQSSSARVSPTTQPESSPFIPLDPRMQTQNPVAKDFLSSPTEDVATPVPVIDVQEIDPKLEGIPLKATAVQKPTFAAGASP
PENPASPVASPPGPVARSLEQSRRPTNARASSKEYTLQALSAVESRRLTMHAGHTPNHSLSLFPKVNPTDPGSIIGEAATPTV
ESVINLDAAVVAEEEKNDSLSVPVQSNRRDSKVSFISVTEELDPNAPDLMMEPSGGDKALKGPLMLRNMPAKDELFFEKLHEA
LEPISQGQNALPAVMQGHGLGSIAGPSGSNAPTHMSTQAGPVGGDGSADAQRDADDSDGEGSGKAIEIDVPLKLKSTSNFGAP
FGAM
FUN_012379 CDS scaffold_5 463131 468265 -
hypothetical protein EOG092602FH PF00136;PF03104;PF14260

MDLFRVRLNCIDHYQATPSRYDPQLRNDIRPSQISKGPRVPVVRVFGSTETGQKVCAHIHGAFPYLYVEYEGGLSPDEVGAYI
YRFHLSIDHALAVSYRRDQKGDSARFVARITLVKGVPFYGFHVGYRFFLKIYMFNPIVMTRLADLLQQGVIMKNKFQPYEAHL
QYLLQFMTDFNLYGCDYLDASVTRFRSPVPEYEQGSSSLHKWHSRSISVDHITDETTLPRSSHCSIEVDICVQDIINRHKVKE
RPLHHGFTERTNPLPGDMKLVYSMAGLWKDETKRRKRKMQDPTEGSSPFPPEVLVSMSANARDSQPQGWLHEEEFRTEIQNLI
SEEKLSVDPEELTFETFAKPHPYEEHVNTTLESVEDLFPSKLAPALGLPFGMEAEQDLRSSIVVDEGKVRQVGEVHNDFYPED
SDEDAIAALVAIEKAAAKVSRGPTDQLPQEDLELAAALNINPDKFDKKTFWRSALGVCQSGLVTGELPDLPVNQELLDYAEDE
GFIGRTEKAPTLTSSNLTQLKRPLDDLLSSQASNKRTRLNQLSMVDEDEMDLVPVPSADANKNLASSFRGASILKGPPSLNKN
TSSVLKGASGSNQKLNFPTVKDQNDPNTKLRLSQMSQKSASQQTDDGRHTKHVSFDPSGFLPAEAELPQMALSSSMKSSGGRE
EDESHESKIQRAMSRFASQQVYLVSSLPPSAAAVASSLKQYGLPDVIYQDAYYSREKDVPGRMREYAGKEFRLESNTLPFLPE
FDPTGTAPANYGLKYETFDKTTAELRYERQRKKCSLRSWELTNPPPTYQEVEEWWTETERRANATEGFKISSTPRQYQSQIDG
PTPKNKHGFKYTPGKKSTSVQHEIQYMSTMSLEVHVNTRGKFVPNPEEDEVQCVFWAIKSDGTASGSQDSAGVQTGILLLSND
AEFTQRIQRQTNAEVIEENSELDLMVRMVEIVRNHDPDILTGYEVHGSSWGYLIERARLKYDYNLCDEFSRMKTESHGRFGKE
NDRWGFNTTSTIRVTGRHMVNVWRAMRGELNLLQYTMENVVWHLLHRRIPHYSWQSLTNWYKGGKHRELNRMLRYYQNRTKID
LEILDANELVSRTSEQARLLGVDFFSVFSRGSQFKVESIMFRIAKPENFLLVSPNRKQVGGQNALECLPLVMEPQSAFYDSPL
LVLDFQSLYPSVMIAYNYCYSTFLGRVTDWRGMNKMGFTEYKRQKGLLALLEDYINIAPNGMMYAKTEIRKSLLAKMLTEILE
TRVMVKSGMKQDKDDKTLQQLLNNRQLALKLLANVTYGYTSASFSGRMPCSEIADSIVQTGRETLERAIAYIHSVEKWGAEVV
YGDTDSLFIYLKGRTKDEAFDIGNEISKAITEMNPRPIKLKFEKVYHPCVLLAKKRYVGYKYESKDQVKPEFDAKGIETVRRD
GTPAEQKIEEKALRLLFETADLSQVKDYFQKQCQKIMRNNVSVQDFCFAKEVRLGTYSDKGAPPAGALISTKRMLQDARAEPQ
YGERVPYVVITGAPGARLIDRCVAPEELLSNPHWQLDAEYYISKNLIPPLERIFNLVGANVRQWYDEMPKVQRIYHATTLGKK

KTTLESYMKSTNCLVCGLKFSQEDNALCPSCRKNVPSSLLTLQTRLTTEERRLQEILSLCRSCSGLGPIEDVQCDSKDCPVFW
TRMRQASKTKGQRNASQPVIQSLVDSVEKLSLEW
FUN_012380 CDS scaffold_5 468611 470015 + SLU7 mRNA splicing protein EOG09262NNS PF11708

MPTAPAKPPPPSTGPGGAGAALKEENIYIPSFISKRPFYAGEEGDENDYLQHQRREEKNDKSQWYDRGRKAGPAATKYRKGAC
ENCGAMTHKKKDCLSRPRAKGAKWTGKDIQADEVIQDVNMGWDAKRDRWNGYDAKEYRSVVDEFNQMEELRKQATKDGNADEE
TEEGDKYAEENDMSKHQSTATRQLRIREDTAKYLLNLDLESAKYDPKTRSLVDAGATADKAADAFAEEGFMRSSGDAGAFENA
QRYAWEAQEKSGNTSQHLQANPTAGEFYRKKELEEAEAKRAEREKLLLEKYGGDQKAMPAALRNMAITESETFVEYDEAGLIK
GAPKKVAKSKYAEDVLINNHTSVWGSWWSSFKWGYACCHSFIKNSYCTGDDGKLAWEAAERQRTGANLVNDDEEETEEPSKEE
AEKNREEEQPRKRTREEMMNGVTEEEMDEYRRKRTVTNDPMAKLLGKDELLS
FUN_012381 CDS scaffold_5 470337 471056 - RPS9 40S ribosomal protein S9 PF00163;PF01479

MPPRAHSKTSRVPRRPFESARLDSELKLVGEYGLRNKREVWRVGLTLSKIRRAARQLLTLDEKDPKRLFEGNALIRRLVRVGV
LDESRMKLDYVLALKIEDFLERRLQTCVWKLGLAKSIHHARVLIRQRHIRVGKQIVNVPSFIVRLDSQKHIDFALTSPFGGGR
PGRVRRKKAAAAAGGEGGEDEEDEE
FUN_012382 CDS scaffold_5 471434 472109 + hypothetical protein PF01157

MGHSYGKRAGTRYAFSRDFRKKGMIALNTYLRQYRVGDIVDIKANGAVQKGMPFKVYHGKTGVIYNVTKSAVGVIIYKQVKHR
YIEKRINVRIEHIQQSRSREDFLKRVKANAEAKKEAKANGTVVQVKRQPVGPREAHTLSLADNPPQTVTPLPYETTI
FUN_012383 CDS scaffold_5 473870 477439 + hypothetical protein EOG09261A3K PF01608;PF07651

MAAAVRGTEHTKTDQELAINIKKATNVEEISPKRKHVRACIVYTWDHRSSAAFWSGIKVQPILADEVQTFKALITIHKVLQEG
HPSALKEAMANRAWIDSLNRGMGGGEGMRGYAPLIREYVYYLLAKLSFHHQHPEFNGTFEYEEYLSLKAINDPNEGYETISDL
MVLQDKIEQFQKLIFSHFRNVGNNECRIAALVPLVQESYGIYKFITSMLRAMHSTTGDDEALEPLRERYNAQHYRLVKFYYEC
SNLRYLTSLITIPKLPQDPPNLLAEDDDAPALPARPKQEIERKPSPPPEPKNDAPDEMNEFWKSELDRQNREYEEQQRILEQQ
QQQALLAQQQAQLQAQREFEQQQQQLMEQQQREQEALMAQQAQWQTQGRLAELERENLNARAQYERDQLMLQQYDQRVKALEG
ELSHIQNSLGQQMNSKDDQIRALQEQVNTWRTKYEALAKLYSQLRHEHLDLLQKFKAVQLKAASAQEAIDRREKLEREIKTKN
LELADMIRERDRALHDKDRATGSSKDEVEKLKRELRLAQDKADNLERSKGNELSTMLAKYNREMADLEEALRNKTRQLEDAQM
NLRDGSSDLEQLLRDKEEELEVYKAGMDQTLIELNELKMNQGDTDHALDGQIDALIMSNLDKINDIIDSVLQAGVARVDDALY
ELDSSMQAGNQNASPSYVLSQIEKASASAMEFATSFNNFIADGPNATHADLIKAINVFSGAIADVCSNTKGLTRLATDDKKTD
ALMNGTRQPAISAVQFLRGLQSFRLEGMDPLQKTDVVINSNNDVQMNLQKLNKLVESFAPGFGKLANKKGDLGDLVDQELSKA
ADAIAAAAARLAKLKNKPRDGYSTYELKVNDSILDAATAITNAITQLIQAATVTQQEIVQAGRGSTSRTAFYKKNNRWTEGLI
SAAKAVASSTNTLIETADGVISGRNSPEQLIVASNDVAASTAQLVAASRVKAGFMSKSQEKLEQASKAVGAACRALVRQVQSL
IKERSQEEDQVDYSKLGAHEFKVREMEQQVEILQLENALASARHRLGEMRKISYQEE
FUN_012384 CDS scaffold_5 479864 481061 - MAT1 CDK-activating kinase assembly factor mat1 PF04769 ENOG41

MEVLINAAKDEQKVAALNDVSKIATMRAVSQTSPNRLAEALSSPHMLKEMLDLFGHEYLPVFEGEIKPHGPLGTSDSRAKRPL
NAFMAFRTYYLKLFPDTQQKNASGFLTQLWGGDPHRNKWALIAKVYSFLRDQLGKGPVNLSAFLGIACPLMNMVEPSIYIEVL
GWEQTAPQGIVTFQQNTDIMKANLARLFNVHPTTEIDLLTSILSAGYFTDFSQVLLMRMWACQNGIMTTTSATAGNNVATTQP
LYESVPTTAEKVSFINAVRESRNLAAHDLFGPEYDAAFFGNRFVHSWEVQDLTSFQNVQISVADSPMESNTLYNFRMPSQCLP
QVSEFDLYDVIDTSIIDISSAWSIDQYLHEKQSKMQQQCKYFLFRNSDCS
FUN_012385 CDS scaffold_5 481504 482852 - hypothetical protein PF17043 ENOG41

MDSSFSFSPLWEDPAIIYKPEKALDALHAKILSIILRKIDLPKEGEKFYPKDVLRSVFFVINQVMTDLSIDNELLNGIRATHI

RLARYGSPLNIIDAALVRWYTGAVVVLYSHQSVRSPTGMPRHWIPGYRSNHPIANLGFMTILRGFEEWAHPKHPKLQAASLIS
KVALAILYATYTIGPHLEGFAFNHLSHMPISKSRELFLRVFVSISGNLYNDDEVFTTPPAFEFGAAQGNVRICQQGKKLLVRS
LSEQFYREAPDWHPYRRVPGSPWNNFIRNTELPVFSTNENPTPHAKTKFILPYNAMVLAGQFKAKYNLVRAFLRHPPNQLPEL
SEEVKTLQLKWFAQHSGRGYANIAPDMHTAESLVSVGNDFLYHTPAIQKPRADPSGFLTLPFMSTAVYAIERHRDPAETPVQG
LLVLTFNQELPDEVAN
FUN_012386 CDS scaffold_5 483254 484004 +
hypothetical protein PF00505 ENOG41

MPGTSKGRLREGPLGVLMPPEAEVPITMVYSQSQADIHIFLPENASLMLINHVADKFSRRVQQPVRVFHDKARSKYRLCPIPE
DVSPDTSTYGRFCFTRDQSTPVKVSDEDPTVGEGGCRIPRPRNCWLLYRQSKSQEITKKVEGITASELSRVIGRMWDEETPEI
QAYWYDMAEKEEFNHKQHYPGYKYTPAKEPDQELP
FUN_012387 CDS scaffold_5 484514 486747 -
hypothetical protein PF03372

MTFRITTWNVNGIRNPFGYQPWREKRTFQAMFEILEADIVVMQETKIQRKDLQDDMVLVPGWDVFFSLPKHKKGYSGVAIYTR
NSKCAPIRAEEGVTGILTAPKSTTRYRDLPEDQQIGGYPRPDQLDGIIDEATLDSEGRCVILEFPGFVLFGVYSPATRDETRD
DFRTGFFQALEVRIRNLVAAGKQVILTGDLNVVRSELDSTNVSETLRKEGIDLSDWMNAPVRRIFNQLIFEGSVLGERDQGRE
KPVLWDLCRCFHPERVGMNTCWDTKRNTRPANNGSRIDYVLCSDGIKSWFNYSNIQEGLMGSDHCPVFANLSDKVTVGDKECA
LLEMMNPPGMFNGDERLRDWSPKDHLPLSSKLIPEFDRRQSIRDMFTKKAAPPREPTRTDTPAEPLKNGNSSASGSLDKAEEA
SGSPTNASSTPRLGETTNSTKLSASQPSSKRPGTAADTTSRPFKKTKSFTGANDTKSKVAQGQRTLQGFFKPKAPAAQDGKAE
LVAANSTPSTTKKPAGSGKAPASAQRLSNTPQATPTEKSSPTVPLRGKDPDPSDRVFDPIEAKESWSKLLGKRVAPRCEHDEP
CISFTTKKPGVNCGRMFYICPRPLGPSGEKERNSEWRCSTFIWSSDWTGS
FUN_012388 CDS scaffold_5 487267 488029 +
hypothetical protein EOG092654RM PF02046

MSAVRFTRAATRATAQLRAPLQRRFASTTENEFIKERQHIKEHAVGTTELWKKISLYGVAPCLIAAGANAYWLWNEHWEHWNH
MPPLEERTEYPYQNIRTKNYQWGNGDKTLFWNDEVNYHNKDKAS
FUN_012389 CDS scaffold_5 488894 491356 +
hypothetical protein PF12862

MARYLNPAKIGLLALVELYVEGAVPSDAILPVLSFVTSHLVDHSSPNTSANQSERWSKAEKTVSLVISIKEFEKLLGSYPFLM
GMPGRKLWDQFLGKLWDINSLDALHKFFDNLSGLLAKTKEERQRLAELGQPVEEEEGIRLSANSPFGAFVRRSRLEYQRLRFH
DCTELWKHFVRYRQPTAPYLKRKIPGFGRLSFDNVLLVGEQEDWDLKSVMDLASVAYGDMLTGDQSGSLPVSTDDIESLLEFQ
IEQMQKFGNRIPLEIRHQFHDLLNDSYLIPSLTHYLKFLDAWRAGDYPTAFDYLHRYFDYTMQNRDRLFYQYALMNLAVLQAD
FGCYKEAVAAMLETVSTARENRDMTCLNFALNWLFHFGRAHPDLVQNLESNSLLGTGKESLAFLRVKARETGMFTLWSSVLLS
EAKLGLLNGDSVATAFESIVRSSHIIVERNMNNMFGSHLSLTSALWDRLGLSTLSSATCEVFLKCHARNAIFDDELKLTCRLA
LLLASRGKYDDAMTKLEDLEDNSLRSWKPSQYWHKYRGVIKLKRDLHHNNLEGAERLLSQILQSKTDDLEPDMAFLVDTLHID
YLTRRGDLQAAFAKVDSMMSQLENEKKDVVLKVKLLLLKASLLDKCGRPQRGFTTAVRAASISWGARLIPCLWQAIGSVSNIL
VSLKEFEAASQLLLAVIPRSLECETESLTAQLYSYLADANMGLAGNCEPKSAKRSEYMTKALAAVQKSFDHYSSIEDINMQCQ
MMAKKAMIMKLTGDMVLAADYAAAYVSLRKTAESLSLDG
FUN_012390 CDS scaffold_5 491554 492393 -
hypothetical protein PF08547

MRASQTLFSRGFFGRSMDELRRRTQIAVSFEAIKGATQPKPLYEFNTADSVRDCIFMTDKTIGGFSESNFDFHKSTDINNDPK
IPSAYARFHGNISTRLPSDRPNIQRTGFAGFRSPDQRPTAFGRSMWDIDPYIYLALRVKSDGRSYFVNLQTESVEPSDLHQHR
LFPKRPGQWETVLIKWNDFVRTNHGFVVEPQTEMLRQKVLTVGIGLTDRVDGPFELCIERVWATNDPSEVEVTEEPKAETVAE
GGQLRNKKGEKVRW
FUN_012391 CDS scaffold_5 492891 493903 +
hypothetical protein PF04280

MALRPAALRQSVLGAARVQCRHMSMPSKGKILREMQKAAYKDTKKTDTQSMSAIQKKANDEYFKGGGGPLFPGTFVSLPFSRY
PSGASNLFNYSWYRLRQFGFEYFSLLQMKLKSMPDWTTRPKWKIARSKIAPTAKNMYIEMLGAFAAGDKAAVNELCLGQFGKK
LIAAIDRRNPAERVTFELVKLNSTWAYPRVMAHQVHNVNPHDKEHSTEQAVVAIASTQKVAKYKKATGELIPGSTKVQDKMEY
VVVSRQISEKTFQAGPWRIWGTISPTTLEAFQEEQDWITREQAKRAGWEGPLK
FUN_012392 CDS scaffold_5 494645 496496 -
hypothetical protein PF00474 ENOG41

MNEQVRTLKEDLPSLPPTPPELLSDSVATNPIQLTLGSGILFSYPELATISGVQGMIVYALASSLPMLIFGYLGPIIRRRCPE
GFVLTEWTRQRYGVITMLYLSFMSLVTLFLYMVGELSAIGQVVNAMTGLKPLPVMIVQCAVTTIYTSLGGFRISFITDVAQGT
MVIGLVIIAAITIGAKTEIQRPLIEESGLTKSNLLGWQLLYILPVAVLTNDFFLSSFWLRTFASKTDKDLRIGTTIATIVILC
ILTLVGSTGLIAVWSGALSLEDAASSGSVAFFVLLETLPSWVVGIVLVMVVTMSTAAFDSLQSAMVSSGSNDLFRNKLNIWYI
RAFVVLIIIPVIVIALKAPSVLQIWLITDLISAATIPVLVIGLVDKFYWWRGFEVVVGGLGGILTVFIFGCIYYGNAQEAGEL
LLVQKGLYGNDWSAFGAFVAAPVGSLLWGCGALALRLSYQYMSAKIRGHRFDALDRPVDLRPSSTEEDVPTENLVSSTPGKFF
FUN_012393 CDS scaffold_5 498183 502225 -
hypothetical protein PF00076 ENOG41

MSTPEYRTPHAVASTNRTLRNAFSALNIHYSGDEEESESEGSPRSSSSRPLSNSKSMPSLVQKDNVAQPGYSSYPYNNTHQAG
EGNFDYPVAAEPTAPMMASQEIQPEQVLTGDFMSINQVVQTVAALPSNMDAFANIPREFDAQAVYPGDACLFVANLPQHVDDL
TLRVSLTKHFGKFGTVFVKVKRDRTRHMPMAFVQYTQIKHADVAVHQARGDMIHGRPIRVEKCGGNLSYIIFRKDTRPVHADE
ARSIFSRHGPVKKVEVLNYDAQTKLAVPPSLLVLYSRFDPKRDVIRDVGPNTPFIIMAFDPKMVQKRSDRPADDQTFMEMYEK
DCRSVFFGGLPHYADEAMVYRLAGICGNIRSVDLRISPDQDGGLPHPWAFVEYDEFDIPDRAMEEFNGKEVEGCILRVERKRT
KPTPEAVAVPQAIGTRLPDSGPRFPNPGSRFPNSGPSRVPGSGSSRMPGSVDDARIYPSPLRPVKRPHRRFDSRVASAAAAPV
NHRRVVSTAGLSREEREFLENCHPEDLLKPENVRPRPLPVPTLAVPNTKLDEVTPVQSPEKYPPIAESSNVVRFAPSPSTLPS
HTGSSPIPLQIPAPPAEFSPPNLRGFKDRLAADGHSRVTAFSPAAELAAMRCEVAAEDKANGKGHRRAVSMFITSTYPPPIDL
SESDDSTWRESGSSPDEQGSRKKKAKRCLIRRRHLSEENLKRNLLYQAKLKRTYASEENMRGRPISRETDQPRDKSGKSRKSD
ATERAPEGQLVVASQQAPEGQPVVAAQQPMYQPAMPYTHMVPQYPPPMPPMGYMPMPPQAMAPVPAPGGFTYMDMPQYPLPQF
HEPVQLYPGPSHHGYAGPGPSQVPYPTPPHPASMYQGQPQVPFQGHHQREYHEPMHVSQDARNHPYQSHGRPQYQGRRHASYA
HPQQMHYQGNYSGMPHSNFTPRGPRESYHQSRQRQEAERYRRYNS
FUN_012394 CDS scaffold_5 510360 511937 +
hypothetical protein ENOG41

MDKPQSPPVTLAPDPDVKSRDQPMDHIFKAVTWSEKEDERQQNERRLSLEAQGQDKVTRWMRDGLQVLYCDLLPDISSVVRPV
DKSGSYMDMLPWWNPDYESSTTLSTTERVSPIPQVKTLLSPDEQHTDDIKRERAFHDLLAKAGGRPWYSSELIDQVAENPENH
AELLEYWKQDATGSGKQEWMVFERQLERWNQFCRYQLRVRRNRDTFDEYLARCTKCLSKQSLTTPLHMKPDPTDQDPLSQWLE
YLCFEVSERKKYSWYKRYNQQYESAWQTLVDSKLLELHEDRDQIEAAGYTSVPDGERTKLRQAVEVASSNVLLAERDLLNPSV
RGPVAQRKLFETQAKLDSAIEAFDHLQRRGDAIKVFLQTTSAYREARRGALRHQMLLRWMRQQVPLIEEELGLPPSTGHVVVD
DDVLLETDSDESDDQKLNINSSRTELMDTLAGLQRNESKKRLASISAEVSRTNSNVPPKRFKHVGDDEDEAS
FUN_012395 CDS scaffold_5 512683 513692 +
hypothetical protein ENOG41

MTSTSSKSPSPPHTTKTAPPEIDGPVHPSLLQPRVAVVLNVPKPWHPWLFALRLFSILPALWWGLPSALQLLLRVLPGPEIVV
VVPSTTGGSVAMSVEADPRYALTETGLATIWCFASGYLSFFFTDCLMSRWLINYTPQATMVRLLTTNFCNAYLTAFVLSFTGG
FQDPRLLLPGWVGIATTLTMMYHVTHQKINIRKETSTSINVFSIACSITMVTLLVHLHQSTPDYPQMPIVASGRRAFDEASRV
ITQIRGSIEQHEDL
FUN_012396 CDS scaffold_5 514457 515224 - SUI1 Eukaryotic
translation initiation factor eIF-1 PF01253

MSIENLKTYDPFAEADEDTGETKQTQNYIHIRIQQRNGRKTLTTVQGLPKKFDQKKILKVIKKKFACNGTIVNDSEMGEVIQL
QGDQRKDVQEFLIDKKEGLELDAKTIKVHGF
FUN_012397 CDS scaffold_5 517408 518944 + TRM44 tRNA(Ser)

Um(44) 2'-O-methyltransferase   EOG092614DJ   PF07757

MEFQKKAPFEPEEFPPGSPPVIRERCGEVTWLPMCRHTCTFETQHFDKIMLNLVENPNLNSKWLFRADVLFDDDCQNPAPEAD
GAIQSHPRVLDFQGFSRQRTIVRRLIPRNTLRDAPLDQTCSFYQSISHEDEEADGEATLSRSLVVYIPHTSSAADLPFYHPKV
KGISQLHEWDVATNTGTISVHFALFEPETGEEEVDQVKLGRIAYHLLQTIHKHGHSTARGYVKRVHHDVIVPRERFQDRYSEL
KNKYARALVKSWLETTDPAKHVFEDLGIAAFLIELWKEMYHDGDFPGFVDIGCGNGLLVHILRMEGYVGWGFDARERKSWSQY
NSPFTGSLSGNSLQRLLLLPIVIPKEAASDDTRVVNSEDIHDGLFPKGTFIISNHADELTPWTPILAAISQCPFIMIPCCSHN
LTGDRWRAPPPRDKTKSKSMFASLVDWVTQIAEDCGWNVETEMLRIPSTRNTGLVGRENTRKVEEVDIHGILQKYGGVQGYYD
NVVKLLKSSPRGH
FUN_012398   CDS   scaffold_5   520336 520898 +
hypothetical protein   ENOG41

MSQVPYIEVYRFNDHIKSLQEKAIVEVLTSTPGEKQSRLGTYGLEKPCADLSDEVIRGLSGPLVRWATSRGAVIQDLQRPCFR
SEAFRLQKGSALWPGSHSTALLVPLSNRNAQIELIPRGSNEAITHNWDPRTVIHLNEMGLQFQGNGSVRFIYILFQTAPCPKR
QLW
FUN_012399   CDS   scaffold_5   520992 522152 -
hypothetical protein   ENOG41

MSQFQYRWIFEYPHVGLNMVSESPPKLHNLHEPSDGSNLKLDPQACSHEIASDTDTDDDLGDSKDVKHDDPGSDNNQHSAERG
RLHPHALHFLAQVPQSDADWSRYLCSNADVRSLCHRLKLPYLFPGFRAHLDWPRFFNECTWHLAQACNNPPLGSLFSLVFVAA
CHVALIDGCPREEVYNGLRACVRQCGISEDEVTPPMLDRMREGVITGIGILREYSKVVGCRANEIPLHAHNCLQVFQCCNPAC
IRLIKEQISSAYRPTTPLDSEYLEIPSLVYEVLGGRHSKWGYEVIYHILCPRETASIYVYGSVDMTRVMEFEERDDVVEWDGS
LDTELACVSSIADD
FUN_012400   CDS   scaffold_5   524928 525216 -
hypothetical protein   ENOG41

MADNNKNIENKKPEQKPLSEETREDLRRMTAGLTDQTTIETLSRAGMMAKNKREQSHFHRDKTVARVEAAEKKEGEAEEDKDT
GGGDKEKNKKQT
FUN_012401   CDS   scaffold_5   526391 529249 -
hypothetical protein   PF06011;PF14558   ENOG41

MFATQRPLTALSCLLSSLSVIPTVCAQDKAYINGTGDNGVFHERLDVSRTPSLYTGDFDDCLNDGSLFNVTGFDTAYYSDNLT
VSFHVYGSTNIRQESVMMHLSIEVYGEERFNKTYDPCAENVTSLCPLKAGQPIEAYITTSISANDIAGIPSIALNIPDLEGYS
RIRIFANSTQTEIGCFQATMSNGRTFSQPEIVGSILGAFTVFAVLASYATAIYGVQIPHIRMHYAHSFSILIIFETFQSIFFS
GALSVNWPALLPAWWSNFAWTAGMFASLDMIDAISPFAGVSGNASQVGSAGSVPINNGGGLAQQIFGRSLRSRSVPQLESIVH
SLTRRHDFNSSNPYDYDWAGEPRNPGMPLPGDYSGFPGVLSVVGIPRNDAFIIGLIWFLVVLAAVALFVATSKVVLDLCHKFK
LIKSDGFDFFRSHWVGYMAAGLLRTLFIAFFVMMTLAMFQFSIDAPPGPTAIAAVIFVMFLGFAGLTAYACSSRLREGKYEVV
SDTLRFEQGLILKKIPFVATTLESSIGEEEQAHKPRLFASLPIRRIKFVDKDPERPKVHQDEPYIKRYGWLSARYRLSRWWFF
TVYLAYQFVRACFIGGGRANPLAQVYGLFVFEVVALVIIIKLKPFEGSRNTTAAIWLLSISKIITTGLSIAFLRTLDLSRVAA
TAIGMIILVVQCFLAAAILVLIVLGMVSTWMSLSRNREDFPDKIDDLRVRYFEHMDDRAGTSKPKEDEVDELAELSKSIFSVS
NVRRNTQNNVFPMVEGSNSQVPSHPAHPLNRRRANSGGSSRHSVNSLPRSGRTHRASWSAKDFAEWDADMNRGDTSRMSRMRT
SSLRMQASRYSGSRPPMTPTRESAEFPRVSVNTMSPEDKDAIVQDKIEENAATPKRRKRTVSWADQSSICEEPINEAQKVDDK
PLEKIDSTGGREEEAKSKPEP
FUN_012402   CDS   scaffold_5   531962 534308 -
hypothetical protein   ENOG41

MATTCSIDQLLLASIPNLPWPDGNYTKTLQAVVLTRDWKYDDTTHNVPNVIKGRMPTRLLVNGRLPLFKNEPGYLFYEGRETA
MVMVVKGTMVVPVVLPTVILTKGAMLRPLAEQTANLLPSLTSESTPAPEVHDGGRPFSRFVHSLWQTLAKNTRQLQTLGMSMS
AIDACFAGPHLLENRNAILNAVLPPAREVLKDGTFSLKDLLDLRHVSGDWPAGRQCVYLRIYTHPQDRPDYRYSDSSQADINK
NVGFYIGSTNNAQRRNQQHEHATDNRSNSCYEMQHYRIARKSQKQHRHMIPLVVFDAIVPIHLLKMAEQTFILAFRSYQGVIH

APVNTGGSSGLVGLQRKGQFLSSIARPVMDAVAWPALKTRGCNMSSSLFEDQTRVELLCSPMVPGDASVRTFTTYRLRRNLYP
PRNPNGSKYVSINLHEVNTGIIRHVVFGLPFGSLEMQGPKPGDSTGGYLVFEVMDDKKAHPKAWFGVPSIGPFENFDRASSFA
VRYEWYDKDQQKWYSIPLSRWYWGLNGMKEHIAEGRTEKVLEPWRIAMDIVQAIEGIIYTEPLNDFKPQIRVGEVKMLQTDHL
GQQYSWITRPMRTMPAPRYATFNENFALMYRQFNTQLTHVGPTEPIPRDADVLYRAPGDNRDVVRCDSCLYMGENVKCERDER
ITDAWVCKCCFILYRPCSFTALSDALELWGQGPPYRGAGGSARLRRVPSGPHRFMAYHLTMTEEEMVTVKAVPKPLSEHLLEP
VDLDDGDGDDDVVAEVL
FUN_012403 CDS scaffold_5 537737 538479 +
hypothetical protein ENOG41

MAAQSLPSNLELEQFQWDETSPVVFPDTEAVMEQPDSPPPPPTLPELPPWQELQKKVDLQVQKAAEAAREHLWGHLFAHLPCS
EIDWSLDGGRAVCRVVDMPCYGKFFSDPSDMKKHLLSKRHCAHEARLALEWVGPAPSQTSTAPVSRRPGRGSGGGKRKAAIDN
GESELEEPSSKKQKKVATSS
FUN_012404 CDS scaffold_5 538609 540958 +
hypothetical protein PF00270;PF00271;PF04408;PF07717

MDRTSFRDTTAEDAFRLEDGAAHPLRENDLHGRRYFELLSIRRRLPASAKRQEFLDLYHQHQVIVVSGETGSGKTTQIPQFIL
FDELGSSKPIVCTQPHRPSAISGAEQVASELDVRVGEEVGYQVRFDKRISRVKTRLNYMTDGMLVQLVQGNGSFGKYSCVIID
EAHERTIPTDLLLALLKRALPLFPDLKVVIMSATPNVDIFLNYFGQGSHLPLSGREHPVEIRYLQEATPDYASLALHTAQHIH
QTTGDGDILVFMPSTAEIGDACGQLRSATWGLEVLPLYSHLPKAEQERVFRRSSRRRCIISTNIAEANVTVDGVVYIVDTGLV
IESRYNPRAGLKTIFTGPISRTAAEQRASRAGRTKPGVCYRLYTKEDYDEILLPTTPASIHTSDLSRLVLQIKAMGFDDVANF
DFIDKPDPEVFLRALEDLLAMGYLDDTGTITPKGQMASKLPVHPVWFNALEKAHELGCSGDMITIAAIQNTRHPIFVRGQGPC
STAGPAQRFSCFTSDHITQLNVVDAYMKKEDEAEDLRHEGVYIDVPGWCSDNSLSMRALEEFKRIRMKLRKSFADLFKDRPKR
FGLESSECDDNLRQALAWAFSHRVAFSDVGSGNDRYRVLHHNWQAGLSPESNLVGINHRWVIYDSFIMTNCQFLETVTAIEPE
WLRGCENFQEESWALKRGGKTGPYKMPLAKASFEEACGPGTNKQTDWW
FUN_012405 CDS scaffold_5 541251 542617 -
hypothetical protein PF00076 ENOG41

MATRKRPRDVEEYDKYQGLVIYVGNVHYDANPVDFEHFLQDNGMSCTLYWPEAGERSHGGWCWARFHHHDHASYAINNLNGTI
FCGRMLRTGHVATNSIFNPHTFFQDYNLLKYPGPSSHHYHRPAEHPGFYPSTKNLRPPAAPFRQSVNYEVQYPDCYPPKKTEH
LNHRHIPKLQDVWEADKPFLNTANIFAKGIKISGLAITESQDGSMDIEMTEPLPQLYWQPGRVLGPDYQERSTISHSERFIRP
ADMSTNILSATCRSHIVSYKRPPGVGLGWGDFDRFYEWQLLGRKVQKSMVRAQRFQQSNSPHMSFISELTGCLHIVRTDSTKI
ESRRTLKKPNLWQKLKNSKN
FUN_012406 CDS scaffold_5 543210 543543 +
hypothetical protein ENOG41

MTTINLNKTWRDVVFEQYQADLEADSPFPIRYLIFVHQWKKDIFINIPNKIEMMTLRRAVLDFARSRSREEDPTFGNEHISVW
PRKTFRERQEEEATKESNETNESGFLG
FUN_012407 CDS scaffold_5 543778 544489 -
hypothetical protein ENOG41

MDYPAPRIYPPIAMDTEQPVICIRYRAAWPSLPRLPLDVSRTQLPSSIHDRQGEIFDRVEKILQKNNISGPETEIELVVRRMN
PTVLIKSPWKKDSKTKWAQTVEQIVDVMKKHFQSAIHSGLHVEMIAPQNYETVYIGVVEEDVPWDDIRAVAKRHLDSSDATRN
YLNTLALTRRGFSKPEENPITVYIGCLEDSDETKWDGIIDGIESEFRERGWMDIVVHIEHNWPFTRMTVD
FUN_012408 CDS scaffold_5 544922 546140 -
hypothetical protein ENOG41

MAEEDFEIDIYGDESNDHQQDDHRGDDNNQDHHQGDDRRDSHGDQNMEEYHADNHHHAGDSHSSSHLQAHGSQQGTKRKQEDD
GRPVDQSATTSLMISELNWWNTDDDIRGWAREADCEDEIKDITFSEHKVNGKSKGQAYIEFYSPQASTATKRRIEQIIAESQG
AQKKLTLTYWATTNNPFKTLPKDAPARGKDQNRAPSGSYNNDRGGGHMGGNFGGGFRGGRGGGYNRGGMNQGGYNRNFNNNNN
MGGYNNMGGGYNGPMGGGGGGNFGFNNRGGMMGGGMRGGPGGMRGGRGGMMGMNPMGGNMGGMPMGMPGNMGMGMMGPNGMPG

FQGMPPNFNPGFGFNQNQGGGDWGNPHGAKRPRPE
FUN_012409 CDS scaffold_5 547405 547728 +
hypothetical protein EOG09265PUI PF00584

MSDQVQEILEVPSEFVKDGVQFVRRCTKPDQKEFLRLCQAVGVGFLIMGAVGYVVKLVHIPLNHALVGSA
FUN_012410 CDS scaffold_5 548055 550202 -
hypothetical protein PF00400

MAPGEGERSSRPLSADPSDEENRNESTRESSEAPAWYYQTEEDEDEDEEDPDYEDEPDEEDEDDFQGANDRDIEILMENGGDD
DDDEEDEDDIEGVNDTQRLVNIVRTGARTGLLTRRQLIALMQSPDLTSVLFQDNPGEDAEFLHNWPFNRRRTPKDPNRFPKVP
SEQGMKLMRSGAFGANNYNPRAKRPLAVRMLERELALGNREDQIRNNALINQSLLPKSRAEKIIHFDDPVYSGQFSDDGNFFF
ACSQDFKVRMYDTSSPYNWKHYKTVSYPWGQWTLTDASLSPDNKWLAYTSIQTMVSIAPTDPNDTGDPYTLDLDDGPPHGWHG
RRGFGIWSIRFSGDGRELVAGTSAASIVVYDIESRTVLHHVRGHSDDVNAVCFADKMSPHILYSGSDDTTIKVWDRRSMGDHR
EAGAFVGHIEGLTYIDSKGDGRYILSNGKDQSMKLWDIRMAMSTDRYNELDLTAHANTSGFDYRGGIYDDEDWDVHPHDNSLV
TFRGHKVLRTLIRCHFSPPTSTNSRYVYSGSTDGKVYVWNMDASLAGIVDVKKATMRTRPMDNRHRFYHFDEPGNWGTCVRDA
SWHPTAPLLVASAWNGYNMARGTCTLHSYNDNVDDEGEPPMGQSVNHLLKEQPELYQNSTYGVR
FUN_012411 CDS scaffold_5 553761 555621 +
hypothetical protein EOG092646CB PF01169 ENOG41

MKIRTTHSPLVLFLLPAVANALASNDIADLPAPGAAITEQTDGLPRAGVKRHDIPTKYAPVDGRDGKPHHGPFIEIDDHKKTP
EVALDVGSDTAAGSGSGAGAGAGAGAGTGAPAGAGAGTEPKTGTEAAPKIDSNPDSLPGLKGRPEDPTVVDGEKIPETNDGVM
FDTNRVHAQEGTTGTEGGVTERYRARKLEEDLAGEETFRKPSSPKEAPPLPHSEEEKIRASGGDKAKELGDDTGSAKAEDVVP
KAAKPVISNDDKHDYTAGLEKPADLPDRPVGQNKPVQDSTKTEPIDLSRGKTHDGHHDDESIIQPFHSFVLSFTMILVSEVGD
KTFLVAALMAMKHDRMVVFTAAFGALLVMTVLSAVLGHAVPTLIPKRVTSFLAAGLFFVFGAKLMREGMLMDPNEGVSAEMHE
VEQELAEKEKEMGRKRGDSVSAYTLEMGMNGNGRRSRPSNRLMSPPRSPSQSPVRDARSGSGAVTSIVQGATNLCSLLLSPAW
VQTFIMTFLGEWGDRSQIATIAMAAGQDYWWVTLGATCGHAICTGVAVIGGRAIAGRVSLKVVTVGGATAFLIFGVIYLLESL
YS
FUN_012412 CDS scaffold_5 556880 559634 -
hypothetical protein ENOG41

MSSKDHDLEVPADERPREKPRRSKSERKDHKDRDRDHDREKHKDPDRERRRHRTSRSSRSKLSEVSDTVSHTSSRRHRHRRER
DSDEHTHRHHRAASTSDLASSDMARTSTAATSILSDRISMPYPSFSKAHSKEAIVSRDSLSLPRQRSPKPSTSEPTDAPITED
SRSRKGSDSGKPDVPLSPPRTDLDADKTAVNEETPEDPAKEEYPKEEAPENETPKVETPREETPRIRTPTEELPASEAGLEQP
KSRVSSLSRSASRNEERSRVSRRSGVSSQATYVKSSSRRSGDKIRRSDSRASSSSHRSIHRTSSTRSHRRYDVDGASSPSSAQ
DSSPRTPTQAPFPHGYPDAKPETPSVIDVQDVDSNLHSKTATPASAFGAAPPPPPPPPPPALDVHDMPRVDYLLQNGGLAYTA
PKNFLSVLPRQNGNRPSNPPLVGTDTLFAPFHNLLDQYNTVLSKQGSIAVATGHRSVARRLLDRLENVFSRDLPPHGCTCIMC
DNPHEIHDGLNWGDVLEWVSGRIDLPQWPPFDLAEVGTKAAEISGDKPPRPQSPVQLDPDIAEEFREHYLRQSKKVRSAVDRW
LSNTGETPVPPPQDVDDETLSFAILTNLDPEDRPYFNAIMTGSKELKSSVRAPTPGHRPRTDFLIKTGLALQRLYRLQQVPRD
AESATYLVKNPHTHDLLVALSNINNSEWEILISGRFDGFLWSGADDDVPQTPHNESRGPTPASAYYNTRTMSPGPRASSSFSS
RNTTPFSVYSRGTTPASFVSVNTTGMPGSRQAVSNDEEMEMAAIAEIEREIYKGMETLEDAFEKLHKKAEVVRNALRQRGAAL
MQNLQNRRRIDVLSGPNSGNSQASGYERPAWAGESDRDPGSDDDWAFDDVDIMPDDSASNISSSRHRRPKRRTERRTPAPINE
EDED
FUN_012413 CDS scaffold_5 561348 563412 - MET3 Sulfate
adenylyltransferase PF01583;PF01747;PF14306

MANTPHGGVLKDLFARDLPRQAELEAESQKLPALTLSERHLCDLELILNGGFSPLEGFLTEKDYNGVVENNRLADGALFSMPI
NLDVDQAQIDQLGIKAGARITLRDFRDDRNLAILTVEDVYRPDKVNEAKKVFGSDDDTHPGVKYLFDTAKEFYIGGKLEAINR
LEHYDFLDLRFTPSELRAHFNKLGWQKVVAFQTRNPMHRAHRELTVRAARSQQANVLIQPVVGLTKPGDIDHFTRVRVYKALL
PRYPNGMAALALLPLAMRMGGPREALWHAIIRKNHGATHFIVGRDHAGPGKNKQGKDHYGPYDAQVLVQEHQEELGIKMVEFQ
AMIYLPDSDEYLPINEIPEGTRTLNISGTELRHRLRTGKEIPEWFSYPEVVKVLREENPLPAEKGFTVFMTGYQNSGKDQIAR

ALQATLNQGGGRPVSMLLGENVRHELSPELGFTRKDRDLNIQRIAFVASELTKAGAAVIAAPIAPFEEARKAARDLVEKSGPF
FLVHVATPLEFCEKTDRKGVYAAARRGDIKNFTGVDDPYEAPAKPDLVVDLEKQNVRSIVHQIVLLLESNGLLDRL
FUN_012414 CDS scaffold_5 573042 573810 +
hypothetical protein ENOG41

MLEDSDNCSTESLLLLPNIKRLFLSPFEPTFTICHPVVPDFQSGLQSLTIDGCVIRPAFLQYNLSRCKALQYLDLDLTDCYDI
KAVDLKVNLHNFGSVLREFGQDLASLRLRFYDCEEYAQGKIGSLKGLKRLRHLSAPVFVLVGVCEDAIPLPEVLPGSIETFEM
HFVDEYGLANSDTLGTKSAANDDRVVRLLNNNQLPKLQEVVYGCYMDGQDDRLTNFTQVEGWSVKNEAQLRRSFAREPFSRRM
ELVRGE
FUN_012415 CDS scaffold_5 574262 575714 +
hypothetical protein PF00646;PF12937 ENOG41

MLLCVPNEILAHIFSIIECEDVEGGWDTKSSYSALLSLSRVCKRFRVIAEFYLYRNIFLLEDDADEKWLQFARGLQRDPKRGL
TTRKFCSQRYDPMAPPNFLRFLTKHFLKFSLSPDRDRIPPGLRSVIQHELESRIYKRPYRSLRVLLLLLMPDLRHLELTITDL
KSTPFLLNGTFESDEDYGCYGFEANEESPSYKGIPNIFFFRHLESVSLRSYDDNLGSPIRGRTFSGILNHPCVKALRLDGFAV
WHCTCMRLNWGKAPSNLTRLDLDNCILQPMSLKRILEKCRSLTHLNMALTGFGDVKDPTHNFEDFGIVLREHGRNLVELSITK
PMWDPHIADPFNFIGPLKELTVLKHLSIGRLNFVGPIDDDTKKDITIHDALPQSLETLTIEAEVAGCSCDLYRVRQTEELVCN
MLTYEQLPPGLKQIHMKMPPGPEARGYEVNEAAELGGWRIGKKGLWVRRRGEGEYGVIQFITSATWAG
FUN_012416 CDS scaffold_5 576328 577059 + COQ4 Ubiquinone
biosynthesis protein EOG092643VB PF05019

MSFFDPYRHDLIAATGEATATPYFIYRLRDAMLADPTGRRILRARPRISSKTLSLEALRALPENSVGRAYVGWLDREGVSPDT
RATVRYIDDEECAYVMQRYRECHDFYHALTGLPIVREGEVALKAFEFANTLLPMTGLSIFAAATMKRSERQRFASIYLPWALK
NGARSKEVINVFWEERLEDDVSDLRKELGIEQPPDMRDIRKREREEKKRLKELREQGL
FUN_012417 CDS scaffold_5 577400 577895 -
hypothetical protein EOG09265HEP PF01984

MDDAELEQLRKARLEQLKAEAGGSGGGSSGQEQQQQRQQQQNDARQHILNQILHPEAADRLGRIRLVKEERAADIENRLITLA
QTGQLRQKVTEAQLKELLNAMSESKEEEKIVVSRRKAWDDDDDLDL
FUN_012418 CDS scaffold_5 578176 579574 -
hypothetical protein PF02558;PF08546

MQPRATGQIARKSTRWLRNSPNFGYTRLFASAVPPTEQKKPEAVPPELGSAPSRPWQSSSIQAGSVTPTSTSPSSQFYVPSVV
IPAEAAKSGPVHKIHILGEDVRSRFIAHALCSVYDSVETVPFREMPKSRYRNVEKVQPERTRKNAYVEKNAAMPEDDVVPQSD
RSHIDELIVTGRGFEAVKAIVSVKDRIDDKTSICLLNDGMGVLERVREEIFDGTQPEPNFYLGHMSHALGFNRNRDSVKQFKS
GRTVFTKADPVLNAEKELYIPAEQQPSMMHSLRRVKELNTTFSTYEHWLRFKLPSIMFTAAVEPVCVLLDLPYQGLLENRNAQ
KMMNQLLGEMALVTENMPEVQDSPDLIKFLRGEGLKKFCYRRITGKSNAPSDLLTRINKGLQTDINYQNGYFLKRARILGIDT
PTNQLMVQMIKARRIEQQQKLRSIIPVQEASPNDVRKKTQQRMLKNGLLL
FUN_012419 CDS scaffold_5 580301 580963 +
hypothetical protein PF04000

MTDVKDITPDLDRLDNQLDDLEEKLQPLLGNLEGMASQLPLLDKAKLFSLTAYAIESLMFSSLKLNEGNEAQTQAVLTELKRI
QQYFGKIKNIESPPEPETRTLTINQEATARILKADLGDNKTISNKLAEKIAEERAKALLKSVENRKRPAEESPVPSQPSSSAE
GKDKSKKKQKKNKKNNSKN
FUN_012420 CDS scaffold_5 581610 583470 +
hypothetical protein EOG09261CQG PF00152

MAVIYVDEEAGSDLPEIQGTEAAPFKTLLEAYYRHGAESEYQVKKKDDEEGYKPAAKSALKKAVAYAAQQKKKAEAAAKRAEK
DAQDEAARLAVLEEAKKLKITNDPSLPEPVAINLQETDPSKIGTLRKSKDEPTEGVKRVSVQGRVNRVAKQGGLIFVTLRRGL
NFMQCLLSGDLSKTYDALTLSRETSLEIIGELWEVPAGAHAPLDRELRADYFRVIAKAPGGDEAFTNLVPVDGDTNTLMNLRH

LTLRHEKPAAVMFVRDIVEDAFHTVYNELDVKKVSPPALVQTQVEGGSTLFKFDYYGETSYLTQSSQLYLETVLPVLGDCYCI
EKSFRAEKSLTRRHLSEYSHVEAELDFITFDNLLDHLEHVICRVIDLTLENPVAAEAIKKYNPDFEKPSRPFLRMRYSDAIDW
LRERGIKNEDGNDHTFGDDIAEAAERKMTDEINRPIFLTHFPVEIKSFYMSRAKDDPRVTESVDVLMPGVGEIVGGSMRIWDY
EELMAGYKREGIDPTGYFWYTDQRKYGSTPHGGYGLGAERFLAWLLKLWTVREACLYPRYMGRCTP
FUN_012421 CDS scaffold_5 583985 584555 +
hypothetical protein EOG09265RGS ENOG41

MPRQRSVGRAPSRPTAAAPKPAPQQTRPAATHAAPPAAAHPPAQAMPPQAAGSQGPGLFGQMASTAAGVAIGSSVGHAIGGLF
SGGSSEAAAPVQAQAAPQEQSWNQNNCAGVAQNFTKCMDENNGNMQICNWYLEQLKACQAAAGQ
FUN_012422 CDS scaffold_5 584760 586274 -
hypothetical protein PF00789 ENOG41

MFYEGTLQEGISTAVSQQKLVLCFVTDESDESTQWENEFLVDDTLSDLLKEHSIALRLKAGSEEAGYLAQIFPLPRTPTVVII
KNGELKEYITPGTSKEDFLRRVQTAFNSTAAPAAPAPATTQPTPAREQASPPQPQTETPAASEQQSTPPTAAPSTSENVRRIL
AERAARLQAQKEENERKVKEERARTKEKAKAEAEAGMDTDNARAHKQAEAVRKKKQKDQEEKARILKRIEDDKAERRLRAEQR
EKQKLDNLKGGDVAASLVSAPETKLSSTSKSGAITALQVRLFDGSTLRNRFKTTAHLKEVRHWVDESRGDGSQPYTFKQVLTP
LPNKNIDETEEDKPLGDLGLFPSSTLVLIPVKKFTTAYDDSQGIVSRVIGFILSIFTWIFSLFGGGDERTGHRGPTSDAPGSS
QARVQSFQNRRDQQRDQQLYNGNSLNFEPRPEEEEKDE
FUN_012423 CDS scaffold_5 586839 587999 +
hypothetical protein PF00856 ENOG41

MLIDPAFLPWAESKGVKLKGVEPRILPGRGIGIVATRDIKPNETILSVPMKALRTIDTVPKNITEALQGVSVHGILAAEIALD
KSDDFSVWKTVLPTREDLEAGVPMMWPSELQALLPKRAKDILDNQNTTFRRECEIVLKAFPKLTRDEYLYSWVLINTRTFYNS
MPKMKSYAHADRLVCMPTADLFNHADQGCKLAYSALGYSVQADRVYHQGEEVYVSYGPHSNDFLLSEYGFILDTNRWDEVYLD
EVILPKLNKTQRADLESINFLGRYTLDYQTLGCHRTQVALRMLCCTQRQWQRFFDAQDEGRSSQAEVDGLLLTALKDFLNIIG
KTLDDIDEVEGGSSSQRELLRRRWQQIEAMVTKTIDTLS
FUN_012424 CDS scaffold_5 590553 591228 +
hypothetical protein PF01753

MAACITCNKEEPAVQLRRCAKCSTTPYCSRECQKADWKAHKKICGKQADSFANANVHDPDEMSQSPKKGLEKSVPNPFTRLDN
GTYLHNRPEKDVYRLLIDTYRLRMDDMYNLEGQADGDSLYGGASDGLRGFQRFLRQASVRRGVLPSWWTPEKQQECEVLGMDS
SQWQNLTRTTRKQEIIDYYGDPRFPMQLRMLGEAVYLSAPGGGDGSQMRKMMAAMEGG
FUN_012425 CDS scaffold_5 591401 593401 -
hypothetical protein PF00172 ENOG41

MENQPKRAGLGRTSCTECRRRKVKCVYDDDQAECQQCNRHSLTCQPQNAESSLEERDEIIQGFSQFAGQVETRMIRMQTAIET
LARKLDSQTSHQAHCRSDASYRGASEPFETPQSRLTASLKGLLPDKPVLSRILEHSSKFTQNWMSWPLATVSYQQQDNDFLPV
KTVTLFDAGFPKDVESAFKFVDKSMSSSYQKPPNPGVVAKCIVWLCLSIKELPQRFKYSDNRAPPPFEPRFLIDNCIAKVEAL
YQVSSTPVCNIDFVQALILQGELFLSIGRPTKAWKCIRTGIENAMLLGVHQGRSSLDRGIWEELWIRDRQLSLFLGMSHAVPV
QLGPKMTEEEYPVAEKEAFRQIATVSGLLTSRNLMKEDTPSATITRTLQEMRELKAMIPEHWTKGHSRYVSYLPQAFTRAYIK
ILYHTFELMLNWPKCQPSNVDPQQVNDFEPSRKAILEATQEIIKAHEAMRAPEVEPNAVMRADFVDFLAFKAALVMVAVIKDK
KNPMPYEESEPLWKLIDDLGDRLQKTSKAYDDAFAKQACLALKILFCASEGDYIWCGTHEVAIPYFGRLETCKVISAKDLAKR
SRNGTRPSKIVVKMDSNHVVFGLPYERVPELELAADWTELGSSREYGWRTTFEFDCLTHEGF
FUN_012426 CDS scaffold_5 595074 596173 +
hypothetical protein EOG09263V60 PF02104

MNAPNTVLRSLRLSKQIPRCTRFTNAPPRRQFTSSIFRRKQQPSADDPEFVSILDGPPKLVRVGKRHGPGLIILAIIPITAFI
LGTWQVQRLGWKSELIAKFEDRLIRDPLPLPPTIDPDAIHDFDYRRVVAKGHFRHDQEMLIGPRMRDGTDGYMVVTPLERENG
TTILVNRGWIDKKHRDKRTRPDGLPTGEVTVEGLLREPWKKNMFTPENQPEKGMFYFPDVKQMAALTGSQPVWVEATMEPEFM
QMVDFEARGIPYGRPAEVNLRNNHAQYIFTWYGLAVATSIMLWMVVKKPANDVARRVARSKGL

FUN_012427 CDS scaffold_5 596208 597919 -
hypothetical protein PF00125;PF15511;PF15630 ENOG41

MSSLSPPGKAASQETPARTPSRTLNSRAISAEPYSGVHTPLDRSGARDLRASLRRGTPASAGRSNAPTPHAKAARRLLDQRRT
AMYTPGKNRRQSMLQQRETPLDILRMLGRTLAPKSQPIHSSSSSSPGNKRSSSPEQREEINMYDDDDDEDDDLLTRPPRLSLP
LDIEDASSTSSDLPPPILSQLDEDNFTMQSIEMPRRIANDSRLSRGSPGSERMSDYFNNEATEDDIGQQSDFFPGLLEDLQAR
AGQDDMTLEPIEADQTRRMTEGRGSDFNFEYPGGLEDGTVFQMSDPANDLQATSPIVDPSVAEAVADDSQDPRPDVMYGSGSD
AGGGDFGMDGWDYNDAGVDDHSSINEEPGPIAEPTVTTGMRHEPADDRLPRGLKAKKRKRVSQHGIEYPSLPPAFVKRVAQTA
LQSSGLSNQRLSAETLDALIQSSEWFFEQLGDDLGAYADHAKRRTIEESDVFTLMKRQRQIGPRSSMFSLAQKHLPRELLQEL
RMPVPQPAKQRKATRVSGRGDEGEVAEDAEDT
FUN_012428 CDS scaffold_5 598410 598815 -
hypothetical protein PF14087 ENOG41

MASGLFSNVSPWHIPAMFMGTAFTLGGLLPLRAPDRAMREYGLPEGIVRSEPAQLAFGIYGTRVAAYGVALWTFYLRGEYHVV
DTLMSLLFLWGAADCWICIKAGVPRTAVWRFVSSVMIGGYGYLGLTAKGSL
FUN_012429 tRNA scaffold_5 599060 599133 - tRNA-Cys

FUN_012430 CDS scaffold_5 600718 601557 + RAC1 Rho GTPase
protein rac1 PF00025;PF00071;PF08477

MAQPGVQSLKCVVTGDGAVGKTCLLISYTTNAFPGEYIPTVFDNYSASVMVDGKPISLGLWDTAGQEDYDRLRPLSYPQTDVF
LICFSIVSPPSFDNVKAKWYPEIDHHAPNIPIILVGTKLDLREDASTLESLRQKRMEPVSYEQALTCAKEIKAYKYLECSALT
QRNLKSVFDEAIRAVLNPRPTPSKQKKNKCSIL
FUN_012431 CDS scaffold_5 602621 603808 -
hypothetical protein EOG09265DRM PF00076

MKSKRAREAAEVDDKPIASVDSEAPAKKRKRSEDDGADVKKAKKEKRDKKYKKESRKERKEKRKNLQDLPEQDDDEEAAEDAP
MAEADDRPADAPVEADKPAKKDKKKDKKAKTDESQQKYDASADAEPKEEPKKSKKDKKKQNKASETTTTNDESAAGADAEAAD
GKGDRHIVFVGNLPFTATAETIKAHFASLNPVSVRCMSDPKDSKPCRGFAFVEFEKVWHMRTCLDKFHHSEFSDGVSYPRRIN
VELTAGGGGKTKQRQDKIKEKNRKLDENRAKRIERERMAKEENKGKGDTRKAGEDGIHPSRRAHIPGNRW
FUN_013551 CDS scaffold_6 1774 3629 -
hypothetical protein PF00013

MANPQPDIQSILAALASQRPTGTPTQTPPGAPNQAYPPPPASAQPAATYQPPPPNSGSYGAPGYNGLPAPSSSGNLDLSSIRP
VNSGTLSIADAIAQAKAYAAEKGVTSYDRPLVYASESRGPDRGYNRSRSRSPVRDEFRDGVNPYRDERRGGDRGSHGRGYGRD
RSYSPGPRGRNFSPRGSNGRERSPLRGGDDNSETIEIDSSLVGLIIGRQGENLRRIESESNCRVQFLAATDGGPHRLCKISGP
RHRRAEVKDAINRIIDDSGMGALNRPEKPRDPNKGGAAALREGEDHMQIMVPDRTVGLIIGRGGETIRDLQERSGCHINIVGE
SKSVNGLRPVNLIGTREAAARAKDFIMEIVDSDSRGDAPPPVKRLGGGGAAPGGRHDGPQRDAGGSGGPDKINDAVYVPSDAV
GMIIGKGGETIREMQNTTGCKINVAQSSGPGETQREIALIGSRDSIARAKLAIDEKVDAVRQKGSGPRGGGGGGRGHQDHDRP
SYSQPQGSSAPTNQPPQPAPADGSDPYAQYGGYQNYLALWYQSLMYQQQQGGQGAPPSGAPAPGA
FUN_013552 CDS scaffold_6 4774 6601 + HAS1
ATP-dependent RNA helicase EOG09261F73 PF00270;PF00271;PF13959

MDFAGSKKRKFKDANGVKASKERKSVTVPEKKSKKVKRAEPEPREEPEDDSSNEEEALKEDGDESDEADEDAEDSKPGEENEA
EDAEDKAEDKAEDNTDLPDGGKLTLPPVAGAESQSFAELNLSEKTMKAINEMKFTKMTEIQRRGIPPSLAGRDVLGAAKTGSG
KTLAFLIPVIEMLSSLRFKPRNGTGVIVVSPTRELALQIFGVARELMAHHSQTYGIVIGGANRRAEADKLAKGVNLLIATPGR
LLDHLQNTPFVFKNLKSLVIDEADRILEIGFEDEMRQIIKILPKEDRQTMLFSATQTTKVEDLARISLRPGPLYINVDEEKQY
STVEGLEQGYVICDAEKRFNLLFSFLKRNLKKKIIVFFSSCACVKYHAELLNYIDIPVLDLHGKQKQQKRTNTFFEFCNAKQG
TLICTDVAARGLDIPSVDWIVQFDPPDDPRDYIHRVGRTARGSNTKGRSLLILQPNEVGFLSHLKAARVPVVEYDFPKIINIQ

SQLEKLISSNYYLNKSAKDGYRSYLHAYASHSLRSVFDINKLDLAKVAKSFGFTVPPRVDITLGASMSKDKKHQGRRAYGSQP
RQGQGNKFTR
FUN_013553 CDS scaffold_6 9349 12766 +
hypothetical protein PF00400;PF11816

MAKKARQRISYVLENAKSSEGGHRLGVNGLAVDNDNAILYSGGRDGIVCAWDLNLDLKSRSDITDPTPANSEDKKPKHTTKFR
AQTHAHMHWINDIALAQNNTALVSGSSDLTVKVWRPYSEEDKNRTETIGEHADYVKCVTTPPPDMGANWVASGGLDRKICLWD
LNGAGKTLEIDVQGEEIAEKGSVYALRVGRNIMASGGPEKTVRLYDPRTGDKVSKLVGHVDNIRAILIDDAGDTILSASADKT
VKMWSIKNGRCMYTFSMHDESVWSLFSDDPSLGVFYSSDRSGLVAKTDVRGSLEDMDDGLSLAVAHEHIGVGKVVAAGGHIWT
ATNRSSINRWEDVDTGTNIQLPESVRHHRATSNASSRPHETSPATTNGIAKKEIPAESILRISAAASFPARSGPDPDSATITD
ATARKGSEAIIDSSEPEIKPIHAVAEETIEGQFGLLKHRLLNDRRRVLTSDTAGDVLLWDLIQCKPIKSFGKQHLEDVEHLVN
TVEAVAPWCSIDLSSGNLTVVLEPFNCFDAEVYADELDLPDNIEFREDQRISLGKWILRYLFADLIDEEIRRDEAHRQKLNEG
LEARQTASGGTTEAAPLSISLPKSAIPDWDNADQVTPKPNLYPNTPGLGIGLATPGPSIMSGPNSLPDVPENAATSPMTPIEK
RTSHVSRPSTEKEDYFTSPLQLLESAVKAASMVSTPTQNEADSKKSMDGDKEKDKDKDKADSAKSPSTPFGKKKFRMTFGTKK
LSRSASQATTEKPAIVEEKTEESESSSVHEKEKEVDDSFFGTIQKIHQEYERQLADNPDKPVESRVVPSLPSDTPVLKLPPGT
KVVIQEETTGGSANLYQGTVQDVGKDADLIEQKAPMWLGDVLLQNTLPFKEPVKVSFVLYPMDDTLPAIASADGNNRLNANRM
LRVKKILSYVAERIEPPLEEPEESPMKPEEYLELYCNEQLLSPATTLATLRTHLWKGGNDIVLHYKANGKKEIRPFPPPPEPK
PAEPEETQDGAAAEEANTNGQVPTQPQAQTQAS
FUN_013554 CDS scaffold_6 13062 13905 -
hypothetical protein EOG09263EDC

MSANPPTFQSALLAGALAGTTVDLSLFPLDTLKTRLQSSAGFFPSGGFNGIYRGIGSALVGSAPGAAFFFCTYEGVKGFLADK
DNTSAPGWKAPLTHMAAASAGEVAACAVRVPTEVVKQRAQAGHHGGSSAAALRAILSRYSSHGFVPMWRELYRGWGITVFREV
PFTVIQFPLWEAMKSWGRRRRGDREVTGAESALYGSMAGGLSAALTTPLDVLKTRVMLSKESVSVSDIFGRILREEGSRAFFA
GVAPRVTWISIGGAIFLGSYQWAINTMNGVV
FUN_013555 CDS scaffold_6 14612 15001 -
hypothetical protein ENOG41

MMSARSMMALRTAHTSKIILGRGFFTTSTRLGLKESSTQTDVDYDKHKQDSLRKQKEGSGHWKPELASDSEEAVKADRSDIDP
AKDIKNLQERTKKAAEETHKAGTSTRDNM
FUN_013556 CDS scaffold_6 17035 17646 -
hypothetical protein PF14618 ENOG41

MSAYYHYNAHPAHPAPVSHNHHGGRNRRAPRLSVSQQAQRQFRGVRSMKELNETASLTAFRTKFETSRSFDLEDDMEFCPGLL
TETDLVSISSASERSSLASNSPDSSPTQHPNTVAPGFSLNSTSPAFIPPAYQQQPSMKLHQPSATRGRNAIPIINPATGITMS
SPPQSVSPGRMQQSMRRW
FUN_013557 tRNA scaffold_6 22131 22228 + tRNA-Lys

FUN_013558 CDS scaffold_6 25362 26476 +
hypothetical protein PF03473;PF03476

MDVLKFGQSSSLGYILFIVGSATLPIFLLFILFRRPVGQSASKSLPLPADDEIVRLRVYPVKSCRGFDVKSTQLLRTGLDLDR
NWLFVTADKHEFITIRANSNMTLIITAWDADTDTLTISLNEHKIEIPAHPTTQWLKNNTELKKATIWGEQTDAWEYAESLTKP
ISEFLNMDVRLMYKGPTPRVLRGSGAPHRLGRTEATKFADMMPVLVASMASMNELNERLTQAGEDKIEIDRFRPNVIIRGSVP
WVEDGWKTLQIGEGEHRLDLDVVCRCLRCQVPNVHPITADKHPRQPWNQLMKYRRIDPGLKFKPSFGMLCAPSDEGHIEVGMK
FQVTAMTNDHFFISPMK
FUN_013559 CDS scaffold_6 28145 29536 +
hypothetical protein ENOG41

MGDDLVIYYNDSIDSDNLAAAMALFKATYWKPTVRVLWILEPRQVCFGLSMTMDQITRCKELIKQHFPSFENPFKTLLNGDIK
QQDIDDIKDLTKDDRKILEMAVKPKYGSINDATLHARLSALDLATCLSEWSNNNPIEVLVDYETLEHIENPVNLHMHHHEELV
NRTENELKEYYDILKKVLHFGRRTDNLRGWYNKCIWRLEHDRKLSDISVERLVLDKVLNRIQTAGSVRFFGGSSLRILQQFLD
RGVASKIKCHLQVGSCDMSANLFSNQFNIALNQQAAKIVLSRSAEFAEFTVVPSHTAQSIKYSALDLKKFGGHCIEKRILGFN
CHEEPVKIVTNQVSLEQQYPDKSYSMPDLTSFLCALVPGHMGSKPGYIEVDEQEGGTLLFKKSDKGIPMFDLDGVKELDEEQI
TTIFESLTRGEVLL
FUN_013560 CDS scaffold_6 30054 31072 + SSN8 RNA
polymerase II holoenzyme cyclin-like subunit EOG09262K8C PF00134

MSANYWQSTQCRFWSFTKEQLATMRQKLEEDNAELVRMFPLPQQRHLYIYFNQQLIRLAKRLTIRQQSMATAQVYMKRFYSKV
EIRRTNPYLVIATAIYLACKIEESPQHIRLIVTEARQMWGDLVAIDTSKLGECEFFMISEMRSQLIVFQPYRTITALRSELSL
VDDEVQLARSVINDHFMTDLPLLYPPHIIAMVAILLALVLRPNNSGPGQNTSGAAAAAGLAAAQQALMRAQGQQAQGGMPEPA
AVEPKEKRQQDRVSRVQKFAKWLVDSNVEIASMVDATQEIISFYECYEHYNDKLTREQINRFVKARGLDK
FUN_013561 CDS scaffold_6 32172 34616 +
hypothetical protein EOG09262PZ9
PF00036;PF00153;PF13202;PF13405;PF13499;PF13833

MRVTNIVEEIEEGLREPQNSRDARVEKLWASLEPDHTGELDLKGLRKGLRRIDHPMKNADDMLKRIMEEVDQNGDGKIQYNEF
RKFVENAERQLFALFRSIDKDGNGKLDKLELQTAFKNAGLTLSNRRLTEFFDDMDLNNDGYVTFDEWRNFLLFMPPHDHDSQL
HAVLDFYYSVVSVTPEGDTLVSEETLEGLGTDGFRSLFITLFGSLLRVAFPYDFPRPIPSPDRTSTSVSQPSTSNPDESPNQT
ENMATAAAVSYPDYDDAATEISQEIAETLTQHVDDGTHGPEHEHTTGASTAVHKKFRLTQFVPDPGYFLAGAIAGGVSRTATA
PLDRLKVYLLVNTSSRTETAGAALRQGRPLAALKNAAKPFGNAFRDLVRSGGVRSLFAGNGLNVIKIMPETAIKFGSYEAAKR
ALANFEGHGDPKHLSSWSKFASGGFAGMIAQASVYPLDTLKFRLQCETVKDGLQGAALVRQTAVKMYADGGVRACYRGLTMGL
VGMFPYSAIDMGTFELLKKSYKSYYARKNNVHEDDVKPGNIATGIIGATSGAFGASVVYPLNVVRTRLQTQGTAMHPATYTGI
WDVTRKTIQREGYRGLYKGLTPNLLKVAPALSITWVVYENSKRILGLS
FUN_013562 CDS scaffold_6 36376 38541 +
hypothetical protein ENOG41

MPAPYSDNLYSADSDDEPDALSPTDGYFHASSESSSSRSGQNVPHVPNVLVEDPTQLSRDAKIQEAERERRLLNTGGSAGRNP
ESTASSHENVSGSENPIASTPRSPPRASPLFAHPVDAPPAYSPSPTTTSPPTTVNNYQTFASTATMGRPEENRPLINHAPESM
SSPYNSAQQPSRWQQFKDSVSNFNIRKKVKTVLASLLIFSVVFMIFSSFTMQSNHGSHSKPVEDNDPVAPPADDGDDFDWNPS
RTCLEKPQHSAHFIEEIDIQSDRNLSIIQRMVKSNDLKGWSTHVSGEVILRPVEDSSPASIELQVISNHDNLRVKADFNKTTQ
VFEIKVPRRVDWSSSDKAPCIQIRITVSAHRETILDHLNIETQQLDVNLKEGVVLGVQNGVNIRSASGDITSPGIKTIKGKEV
VPYTLSSREIRIHTASGDVKGWYPLYDLLDIETASGDIITNIGPKPVNPQNVRPAEFRVRSASGTVKIDEPLTSAQKAARPDR
EFPPRDYTVDIITASGDITADVAASSLASFKSQSGDMKLQIWPVLDSSLLTINEKAKPTVITDTKSGDTHVTILEPLWTSLAT
IGETIPPVKPYDPTDGSDPYIVLPEGSELAEISMARPVLSVLHSKHKSVSGKIELYYPNSWEGMFFANSISGSQEFKGKDVTV
DHGRGSIPPHLISGRKGKGFSQLDISTVSGDQFALLGEEA
FUN_013563 CDS scaffold_6 39776 42954 +
hypothetical protein EOG09261I1I PF03915 ENOG41

MQAPSSGSQRAFAHRRSPGAEPGPPPNVPVRSISPALQARASSASARSVQSTGSRRMNREGSGSGSNMPLSQIEKSVTHLLVA
TKQLLETLTQWSRGQATDSQVSDVYVRLGYEFNMACRAFTAIHVDTSDLGNVPDLLRHILEATLSQEASAESLEKYLPRIRDI
IINLLHGLKRKQQKLRQKQARDRDGHAPPDRATSVSTVGSGNSGLTNMLEEGLENGFQPDSQPTDSLRQSGGSTNQSPRRFNA
QRESSRASAISEQSSLSSNTMQSMPVVPPYPEGESSIPTGPPAPPGPPGGELNIDSFPPPPPPPPDNTSSALAALQKGGDLER
RASRRYSQYQISKHLGGANGVPILPPQNSPVPNRGRKEVRESLRAVQGRQSIRHNRNTSSQSLRTTAPAEDSPVRIPDSVSEE
SSLAELPAKPDPPTVQTPDEAYAASATLSGPPTDPMFFSADAPETPKVEKPKVEPEVAPTPQQKPAETSYFQDSPPPTPTKDL
TLFLQYKSKVKKFVLPEGRAELTIGRLQLAFIEKFSWNTQHNGADLPEIYIQDPVSGVRHELEDLADVKDRTVLVLNVEALDE
VKKHIDDGIASLTEIVRDVKQTVTDQGAALQRVSDRQQEAANEMARLALSPPSTSHSSDGTKTQFSSGRKLDSSHLGELKTLR
RDLAIMRQTYSNFQADIQDSMATIRAKAANVKVATAKAAIPDIEGDAGYSYVTSGRKQLNSDSDRLVGKVDDLQDLIEDLRKD
VVHRGVRPLPRQLDSVGKDIATLTKELKRMEEYMTQEKPIWTKIWEKELEDVCQGRDELRLMEDLLVDLRDDLEKASETFTLV

EQATKEQMKDNGTGASAGSARQFSKGLINLGNSADQSAAKEGVLGEVRALQPNHEDRLEAIERAEKLRQKELATRQVNPLHRE
ITNFVADGKLKKSGGFEEVERARKAKDDRIRREVWQRQNGIIPENPIGEEGADAEADLEDEDAAFLRELEEDEARQAQAQADA
EAEANQPPPPPPKDDDDKQPDQPNGA
FUN_013564 CDS scaffold_6 43474 47521 -
hypothetical protein EOG09260JO5 PF04762

MRNLRNIRFGQCQRDNVTAACWDPEKDQVLVVAQPFEDSCEIELLRISGDRDISVSTAASWEAPNPSPDLVVDKVVSLQYLSG
SATTCLVFEGGDIVTVREDDFSDEGAHIEIVGSIDVGIAAARWSPDEELLIVVTKENNVIFMGSTFDPVAEIPMTVEDLNASK
HVSVGWGKKETQFQGRGAKAMRDPTIPEKVDEGLPSPHEDGATTISWRGDGAYVAINSVQEGSRRVIRVYSREGELDSASEPV
DGLESSLSWRPAGNLMAGIQRKLNQIDVVFFERNGLRHGEFTLRSPSGPVEAHERIRLEWNSDSTVLAVIFKDMVQLWTMGNY
HWYLKQEIPIEASSTCLSWHPEKALRFAATSTNTVVVTEHIFHTARGSCHPPHDNGAVAVIDGETVKLTPFRTANVPPPMSMF
DITVPAAAVDVAFGRDNTSFAVLHRKGIEVYEWPVKNGRSIKPKLSKKALFDEMASPGYNVLLRIAAVADAFHYFGYEEEKGF
IQRFVQAAGEGEVSVADANSREVLVTTTGYQDDNSFTGYGQDNSGKLFQISESGDEMLPVQFQTQLPWFEISKVDDEIVAFGL
SRNGHIYANSRLLAKNCTSFIVTPSHLIFTTNNHLVKFVHLSANVDDLEVPADDPETDERCRSVERGSRLVTAIPANMSIVLQ
MPRGNLETVFPRAMVVAGIRNLIDEKNYARAFSYCRTQRVDMNILYDHQPEQFLANVGLFLDQIPDVAHIDLFLSSLRAEDVT
QTMYQDTKRSTGFGADTIPSDLSPAPRGSAAKVNTVCDALLDALQSRKATNLQNTITAHVCKSPPALDDGLLLVAELMREDEK
IAEKAVEHICFLVDVNRLYENALGLYNLDLALLVAQQSQRDPREYLPFIQNLHSLPELRRHFEIDDHLERRIKALGHLQTMDV
FDELLAYTIKHSLYHDALRLYRYDPPRLRELTAAYAAYLESISAYREAGLAYESLENWAKATSCYRTAGATCWQECLYTAAQQ
QPPMSAEAMSDLANNLADALWEAKDYSAAATIHLEYLESIDMAVRCLCKGYHFADAIRLVVQRNRPDLLTASVDTGLADALGT
TTEFLADCKAQLKAQVPRVAELRRKAIEDPLAFYEGDRAGGMDIPDDVSVAASSRVSTSASLFTRYTGKAGSVGTAGTGVSRA
TSKNRKREEKKRARGRKGTVYEEEYLVNSIRRLIDRVSAAAPDAERLIFALVRRNMPERARAAEALMAEVSEACTAAVAEVFK
VPGAEAEQKNDAEPTWQATGGEAVLQDFVMGQGKKLEPPIVKGVKKLTLLGS
FUN_013565 CDS scaffold_6 48344 49457 +
hypothetical protein PF13136 ENOG41

MDVAYNQHAERSRRKNRSSSNLNHLSLAPLTAKLPLNDDELIADAIALHPHSPIHPIPSYIQGKSAPSTPRLLARSPTASRSR
SHTRTPSTPLAKSKSASHLAAGASRKTHSGRATPRRHKDDMSFSMRNRNDSDWLLRTGALMSSEARESKGQTWLISRQSSTSL
GGVDPDEDAFESELARERELTSRHASRRGSTVEDASPYASRFPSRTHSRSHSLTRPRSLLHSPLEFSMTADGSYFPNQEVNND
LPGPDFVNLDEKLEELEQDLSQDDEATVRRLVRKGQANTGTWFGNVWSLFSVEEDDEDSEDDSDETPQEGDSQLGRSRSWSSR
HLAGILTAPEERIPPPGTDEGGWRDAAWLLSVASKVMF
FUN_013566 CDS scaffold_6 53902 54488 +
hypothetical protein EOG09265DDU PF01627

MSPTEEKSDDIDFGDNIDMTTFEQILEMDEPGDCEFSSSIVFGFFEQAEETFEQIKEALEEEDLDKLSSLGHFLKGSSATLGL
IKIRDGCEKIQRYGKHEKLDGSPEDDSEVCLKHIKDAFEAVQTDYAEVEKLLKQYYESRE
FUN_013567 CDS scaffold_6 55413 56091 +
hypothetical protein PF10469;PF13563

MPPKPVRASPTHFVCIPLAGPQLARSLAAFRTEVTNPSGFGVPPTAVRPLGTMHLTLGVMSLKDEGVEQASEVLKSLKLKEFL
ASARTGKASAEEGLSITLKGLHAFQNAEKTSVLYAPPVDTEGILQKFCEQIKTTFQEAGLMVKEDRPLVLHATVVNTIYVKDG
RGRRREKLTIDARDIVSSYDDYVWLEDMPLDKVTLCRMGAKKIEGTDDEAYEVVAEVGF
FUN_013568 CDS scaffold_6 56802 58312 +
hypothetical protein

MNTSEKRRPGPGTSPAHTLRTGASSNNRNSMPAMPPRAAMASNANANPEETLDTTGGAPLKEKENAVTGSGSTKEKDRDKERK
ERREKREKKERERELEAALKEKDERIAYLEKEMGIMEREFHRELDKLSQNESETATFWQAKHSALNQQYLRTDTELRLLRAEV
EVREAEREELREGWEVLRRELKERDDEIRGLRGQVRGLKEWVSNSTRTDGQECDEVFGDGMARLGNGLQNWVIVNFRKAKINL
ENLDEATLAEVNDLVPMYEELASTHKVYLLQSIVSKILVEMVFDAYFVGLSEEQTQQFRQLEKLVSSYADSDEAINQWRSSTL
SLLRRDGSSLKESTSSVTEAILSRITRILDALCGTSPSEARDSGLRVLVNNSIELARLVAVQKAVLRVNMPTVLPHQRVMFES
ETMEDLGGEDEESLTGREICCVAFPGVIKHGDEHGGHLQYRNVILKARVLCSPEEG

FUN_013569 CDS scaffold_6 60371 61106 +
hypothetical protein ENOG41

MRGQRHTRDAIPAMIPSYYAPSLMLSEGDNNGTPTQSISMSTASSSTFMSSCFDRPTPQTSSVYTEPPPATTVPQSQPYVTSS
LLTCEFLGFGDCDFVFDLNDESRWIAHIADFHLRNVFPTVCICWFCDRTFRSSSHDQADAEACYRKRMHHIAKHFRNGLSGRQ
MRPDFFLLDHLYQNRLIDEEMYQRARAYHEASQVQIQNLYPAGWRPDHRNQEPAVVEASRSRHRGPSNLNRASQGYYR
FUN_013570 tRNA scaffold_6 62818 62913 + tRNA-Ser

FUN_013571 CDS scaffold_6 66147 68234 -
hypothetical protein PF01171

MTTAFRVFHHAPRPIQISEFKDAVDAVCRPRFPTARFARPQRIVLAISGGVDSMALAFLMTKAVRSFRGMKVADNPVHGVLAL
VVDHKLRDGSDHEASEVAKELRKLDIKASVSALSWKEEKRQGLNPRQLPNVEGLARTYRYRALGRYCSYHGSNSLFFAHHSDD
QYETVLMRLLGGHGYRGLQGIREANSIPECYDLHGVYKSGLLDDQLRSAPALSFRPALKELKHLRRRIRDELTLEKANLLDDI
PQDLIQSYPGSEEVRELSDVPFLKPLEIEDGGVMIYRPLMEFDKDRLIATCEANKIPWVEDATNKDPTLTTRNAIRYLVRNHT
LPKALQKPAILSLAKRSKERTELEEAEASRYLIREAVIKDFDPNVGTLLIEFPKLRNFNKRFKRRSLHPDNELRKDHRRLVMT
IAVRKLIDFVTPEYHLPPLSNLEKVVNTLVPGMTPDANTTPKAFTAAGVYFDPIVRGTSIKWLLSRAPYTSTQPLPIAKLYLP
PSYLSPPLNTEEEFTEAPEAFSHKGWARCKLFDGRFWIRIGRNRWPMWQVHPYRAEYAKAFRKALPPLRKARLEKLLKHYAPG
KIRYTLPAIYGVERKRDPYSQHISTTLTLLALPTLGIRVPGLERWVKYDVRYKKVDVSLLGHHPRREIRSRFVRHRPLFETGR
NHILRLMGSNGRQR
FUN_013572 CDS scaffold_6 69692 71348 +
hypothetical protein ENOG41

MVSTLEPPTASSVSRRGPSSVVSRRSQSDSRSKRYNRSHTGGTSHALHNDFPVFSSGDVEIIVRAHDTPPDAAPVQNRYLLHR
HTLTRSSGFFETSTSSQWSRARALPAGNELSRIGEDGEPDSVSASEVSSVSQRGSIHPPRKRWRYELGPKSGPNDIPMLVQRD
ESSRDTTQSLFGPASAPPAVSARDSRSQSRSKHSHSHSRSLSNSSASGGFFRSVANLSLSSNHPPPAQELSQADEDLLRDYDN
LFRIFYNYPPNLDGVNVADAYVQCKSLLNLADQYDALAVVGPRVDHHMLQFQSRLWKQIAKYPISYLRLGYLARSRVIFQEAL
IHVVGQWPVGERSLRAALPDIVLDIIEDKVDELEDTVGRIEGRLYRLGLTNSRGERVGPSNNYLDWLSISLFRQWLADNTSPQ
PPPDQRRRTTSRDGGRSVAALPPAAPPLNSVGRAYRQLGSNNPASFLGHDDCKRFLKLTPELYSRENLRRFEKRLDELKSMAR
EIVRPLMGSNLELDMGGTSRTPDSVTYLTCINVGNRDLPWVMEDQTTVGLVAN
FUN_013573 CDS scaffold_6 72227 74178 -
hypothetical protein PF07690

MAGNIVDQELLDAERAASPQRYQQRDSAEIERVISASTVSSSSSENSARRRSRSIGQYNSISRISTQNDLERHPTELSRIQTA
RSQHNATVGGSLRSRTASRASRKPLPNFGAGKPYPPPLPEQEQYVVEFDGPNDPLHSQNWPLRKKLITAAVLGFTTMTSAFTS
SIFSAATMVVAHEYNVGNEVGLLGTTFYVLGFAFGPSLWAPLSELRGRRLPILISIFGFSVFSIGCATGKDIQTILLCRFFSG
FFGACPLAVVAAVFSDMFDNRTRGTAITLFSMSVFTGPLLAPFIGGFIVESHLGWRWTEYLASIMGFTALILDVIFLEETYPP
VILIAKAADLRRRTKNWGIHAKQEEIEVDFKELVQKNFSRPLRLLFTEPIILLLSIYMSFIYGLLYLFLTAYPLVFVGVHGFS
SGQSGLCFFGMIVGQLIAGATVIAQQPWYIRKLAANNGIPIPEWRLPNIMAGGVAFAIGIFWFGWTGYTKDIHWIVPTLSGLF
TGFGLMSIFLQALNYLVDAYLMFAASAIAGNTFLRSLCGAGFPLFARQMFDGMGIQYAATLLGCVAAVLAPIPFIFYRYGAKI
RQRSKYAPTAPPAPASGSSAEEDEKENDNDTALAAVIARRDSVASGANKESA
FUN_013574 CDS scaffold_6 75664 77340 -
hypothetical protein PF00083;PF07690

MDSSQAPLIAGHSDEEELEYDVAQGSPAEAGKTEGGMPSLFVLALTFAAGISGLLFGYDTGVVSATLVSIGTSLSSRELTSMD
KSIITSSTSLFALIISPFSSVLADRLGRKHVILYADILFIAGALLQAWSSTVPIMVAGRCIIGAGVGAASFVVPLYIAEVAPA
AHRGRLVTLNIMFITMGQVIAYIVGWAFSTYGHAETGWRWMVGLGALPAIVQGSMIAFMPETPRWLVKVGRSGTAKEVIRRVN
GDTLQHNADVIIKEIELEVRDEYEAQRLRDLRASGRWKWLGGWELLISEGRNRRALAIACLLQGLQQLCGFNSLMYFSATIFT
VIGFQSPTLTSMVVAVTNFLGTVAALGLVDRIGRRRVLLYSIPFMIAGLLLSAYGFSFLSLAAGVNTTPDEPPATSGHEMAAL

TILVSIMVYVAAYALGLGNVPWMQSELFPLAVRSLGSGVATSTSWAANFVIGLTFLPLMDLLSPSWTFVLYACICGVGYLLVW
RIYPETAGLSLEEATALLDNGWGVR
FUN_013575	CDS	scaffold_6	77897	79200	-
hypothetical protein		PF01494;PF08491;PF13450		ENOG41

MGNRKIAIIGAGPAGCLLARLLHLAGTEVTVFEGEAHPNFRSQGGTLDLHTATGLAALKEAQIFDEFLKHARYDGQYMAIVDK
DLGYHLVRNADGKYNKIEERPEIDRSKLREILAQSLPEGMIKWGHHLKRVEGRTLLFNHTTVEGFDLIVGADGAWSKVRKEID
PSLIPEFAGIAMHELEVTDAENRAPDLTKLVNRGSVFASTDGNRTTIQQMGDGSLNIYCGYATDNEDWAKPENCGFDPYNLKE
TTDALLSTKYKDWDPRLKQALELADGRCTPRSLYMLPIGTKWEHKQGLTLIGDAAHLMTPYAGEGVNQALDDAMHLAKAINGA
ANKDDAALDKAIKAFEKDMFARILPIQELTWGLLQDWMHTPGAPKTVMAKSMSRHIKHRLPLVLQPLGLAAVHGYYFLKNKNL
IS
FUN_013576	CDS	scaffold_6	80269	81052	-
hypothetical protein			ENOG41

MFNQTEGVIQTWFDIWDVQGTKRGEIIVAKQGFADDPFHSNADSHYLTILSNPWFEDGQQTIQTWDLERMELTYECTISQADM
DEDHSCHTKNGIVYIFDGSGRISEYWAVDGRPISRKMADMEWNSDRSRKFSDGSRIVWAKWLTLYTNEGDVVQRFKVKQGKGY
CLEAGILFDRFIFSISWREKSKHATLMVYSKTGKKLTSSRVAVRGRCLRWFIDIAGRLVLIWDTCTCMEIVDFRGLLQRRRDS
QRYSYVESYEN
FUN_013577	CDS	scaffold_6	83445	84464	-
hypothetical protein		PF05368;PF13460		ENOG41

MSRHITVIPASTKAGRETIRTLLESESKPTIRAVYRDTSKAPPEFLKHSNFEAVKGDIGDGDSLDFTGSNTVFYIPPPTYDGT
DTGDWATRAGTNVKKALEKANIQRLLILSAIGAHKSSGIGVLRVNYISDELLKDSVPEVSIVRPTYFHEDWAHLLDAAKEENP
TIYSWVTPIDYKIPMVGLKDVAENCADALLAETAKPSPHYFKVFGPRAYGSLDLKEAVEEITGKKYELKLVEKDQLLDYWKQI
VPEFYAEEFKEMTTCGLADGIIAKNFVYDENTVRGKVELVDALRSLYKK
FUN_013578	CDS	scaffold_6	85210	86118	+
hypothetical protein		PF00596		ENOG41

MSPSATTASPTGQSILNTAKAQDGKVKRIHKIPEFSTREATRQWQLGQMAAAFRVFAKLGYADGSSGHISLRDPVDPDTFWIN
PYGVHFGLLTVSDMVHIDENGNRIGGAEKPVNTAGFIIHAAIHKRRPDINAACHLHSPYGRAWSTFGKPIEMINQDSCMFYND
LAVYANFGGVVFAKEEGNRLADALGATKKNIILQNHGLLTSGGTIGEAAAFFIALERACQAQLLVEAAVAPNGSQIKKTIVSD
EEAQYTKDNTGSPEAMYMQFEPEYQMLLKESKGDFLQ
FUN_013579	CDS	scaffold_6	86675	88587	+
hypothetical protein		PF00135		ENOG41
S09X
MPFVGNMSLIDAGRALLSLPQALLYPPPQATSAPLVTVRNGTYSGVYNSQYDQDYFLGVPFAQPPVRFSIAQGLNTTWDGIRT
AHEYPVHCYGYGWDQDGFEQSEDCLYLNIVRPAGLHNHGLPVAAWIHGGGLWMGGAADPRYNLSFIVEQSVALGKPVIGVSLN
YRLSAFGFLMGKEAIKEGVANLGFRDQRLALSWINENIAAFGGDPNKVTIFGESSGAESVAAQVFAFNGRDDGLFRGAIGESG
FGAPLGRYPGGFNATERPQGTYDSVVRSVPSCKKLAGSDSTLDCLRRVPSEEIDLALRSSGGQSWAPVLDGDFFADYTTNQLA
SGRFLKIPILIGANTDEGTSFGFRRVDDDDDLRAGIKVMMIPYGVEKTTGKIRDGLVDELLERYPNNQSIGIPSLETWPHVIQ
PGDGYAEEFGLQYRRDNAIFGDYVMQYQRRRANKAWAKHGVPSYVYRFNIRPNGQPPEAGVGHFQEVAFVFHNTNGDGYEPNP
FGGNGTYPADAKAMSKTITTAWINFINDLDPNGDSGPELFNGNKWPTYELSGGPNGKGVVFNINGTHIEVDDWRAGGMEWMAE
HALTVFGN
FUN_013580	CDS	scaffold_6	89142	90153	+
hypothetical protein		PF12417		ENOG41

MATGVSHDLTTQSSPRKLLRIGTGCCGSVWADADSSKDNSTPSCIKREDGDPHRSITNEHFIHQLVVQSLQLNPQHARNFRIP
LCRGFLNKEDEAWSLVLPRLPPGSKPCNALLSEKVQPPSQDVRKLLVSKFARGGSDQDAIINDKKNEHCLIRPYLGRRKKDWG
DTNRSTFFSLRNFPLHLDRMIELGLNVHSYAKVIAEGLAFLHWVARIDANDVEFVLARSRPTSHSHPNSPFDTTIFGPHSMWI
IDFDCCDPITMDETGAATAAECFWRNDPYYPRPGSADGFDTELWSAFKDHYLKVSEEMLKKEEESARGLPSLLISIIEQGPKL

AKGK
FUN_013581 CDS scaffold_6 90670 91257 +
hypothetical protein ENOG41

MFAKYLEKLANAGGPEATYEAGCHCGYIGLSVALSPPLPKHEVINCNCSICRRGGYLLVYPAYEKVTWHNDSDKRVSRYQFNT
KARDHMFCPKCGASIGIDFARVWPEAPRYGISVRQFNNIDLDSLQYMKLDGLHNAEPAVDLSGKEIDPKAGEAPASSS
FUN_013582 CDS scaffold_6 91964 95664 +
hypothetical protein PF00168 ENOG41

MSQFEQQQIPAAGQHYSAQNRVPNVRQFMDQLDQNKKARDAEIDEQLKQNKIHGETKDHKNDRAEALRKQKDIRTVRDPVTGK
DVGIRDTDIDYKEAVENPQLSIPNENLGKPATIATSSKQSGEEYRYAQDVTAPPDPVQEGATSDVPIRSEKTSVVFYKTPSVS
YEPMFEILEQRSNVLCIGIFAAIVFIGKMFGGRLLGLFPLGFCVASGVFLWTKDLIRQGRDLEWSSEQERGETATANLIPESV
EWMNTMLGIMWGLINPEMFAGIADTLEDVMAASVPGVIENVRVADISQGNNPIRILNMRALPDSHVQDIKDEQHRQNEKTTDP
EELAANEQAGSFYNLEVAIAYHAKPSGADIASKARNMGMQLVFYLGVKGLFGVPLPIWVELQGLVATARIRLQLTPDPPFLKT
LTFTLMGIPKVQAGCTPMIEKGVNILNLPLISNFVNWAIGAAASMYVAPKSLSLDLSKMLQGDDIKKETLALGVMFVRIHKAT
GLSKQDQRGSKGGGSDPYITISWSKFSKPQFCTRVIQDDLNPVFEESAGLLVTADLLKADEQLSVELWDSDRSSADDVVGKVE
LSIQKLIQHPGKMFPQVSKLRGVKADSEMPGELHWEVGFFGKTQFRKALRTDGRDLSLPKALADKPELQENKGAIDTKEEDAV
IHTPPDPLWPSGILSIVVHQIVGLELENIKGSNGKRKGREYEPARPEAGEVKEEQAKSLPSSYCTILLNDDLVYKTRTKVVSS
QPIFNAGTEKFIRDWRSAIVTVAVRDSRNRQHDPLIGVVPLKITDILQTSSESTRWYPLDGGIGFGRIRISLLFRSVELRLPP
PQLGWDIGTFEFVSDSITTTGYAPSNHAKIRFRTGGSSASVGKSSSTREADGMSFNISGADGNQRVRLPVRHRYSSPIFMEFY
PSGKRNPDAYAVLWLLELVDGEETDFDIPVWKCNHSVRLSQNYITEENYKSVPDIEMEQVGRVRFRARFSAGTDSDHIKFAST
NNDRETIEAWEACYAEGVRKQDVETEVPHVIQKLHEESLTHGRDVLAQADEKQKQKWLAKDGTDWSGAFGEDPSALMRKKSNS
ESSEEYDDFDEDDSDPDLGIQDADTEPTDPSEDGRPSVDTTGTNATSATNSSQASSNSKNPIKKIKSYRSDSRDMHRKHRGLM
QWRPMRNVQFAKDEAKFAVRKVAKLGALDGRKPDVETEV
FUN_013583 CDS scaffold_6 96270 97338 +
hypothetical protein ENOG41

MSFNSINPGNQSKKTHHTLLPSHITRFGSVSDSLESEDPLARDAPTRSHSQSPVKQHPGSLTPSGRSSLAVVLTRSPAHLEAY
RGVSIDEEDGFDAPVTSDLTPRGRPKSLFKAVNALKTSSPGGTQTGVSSDATSTTPARGRGRPKGSTNKSKGLQTAAGARQAR
QAATKPRPHPNGFPKRRGRPPKQPSPPPETIYYQVDAPIFPFLCEWRDCKAELHNLETLRRHVYIIHGDDVNCLWGECGMLEK
PSEFETDAGFKEHIEEAHLVPLSWHCGDGFNNNLGERGLPNTAEDVPDYLKDEHGNQVTPSIRDQQEEDLFTWRKNRRKLKEL
LIRMNDNLPDESDEEMGVERQAV
FUN_013584 CDS scaffold_6 98040 99198 -
hypothetical protein ENOG41

MRFSTLFASVGIVGSAIAGIVPRSYRVPAANGFPNPDASQEKQIAIQAGGKLPGAPLPTTLGDGSTTAFQLIAFNELFETAYF
CSLLYNVTNKIEGYKVEGDKFHEATHVLKTVLAQEEQHAIGALATLKSAGRFAPSPCKYQFPVDNLKDAILLAETFTAVVLGA
LQDANVVFASEGLPEVVRLISSVIGQEGEQNGFYRIYLGQVPSESPFLTTVPAAFAWSALQGFVVPGSCTEDIKKINLPIFPA
LQVNGGAIAVLDAEDQSLSFSATLEESHKGEDLYITYTTGQQLPYSVKAENVKWNGKTVKLEAEFPYHKLEAQGFTHAALTTS
NNIATPDDVPNFTLAAPGLIQVKNPLDGGKGSHSD
FUN_013585 CDS scaffold_6 101610 104690 +
hypothetical protein PF13374;PF13424 ENOG41

MIQLSPNERPIIFVCHSLGGLVCQQDALVTAKQRSEQHLQDIVNFTRGVIFLGTPHHGSSLAKIGELVSRSVGLIKETNSDIVQ
VLTRDSEVLARIQDSFQALLMTRSKDEATMIDITCFYEELPTKKFGVIVPKHSAILPGYISIGIHKNHAEMTKFSNSEEPGFV
AICGELKRWMKRIQQTQSKPKKHTRAAHCLIPYTFNPDFVGRSEILDLLKSQLEDLEPHSKRKGHRRAALYGLGGVGKTQIAL
SYAYWVQEISQDTSVFWVHASSVERFSEGYTNIANECKIPGHEDPTFDVLPAVRDWLESDECGQWLMVIDNADDMQLFFPQTE
SSKINLNTERRLGQFIPQCAHGNALITTRNMQVGSRFTKGKCPIEVGTMDEQEATQLLRQGLQQTDESEKDLIQLSSRLEFLP
LALVQAAAFIQENSVTVSEYLELLDGSEDDLIELLNEEFETVGRDSDTPRAVAQTWMLSFQQIERQYLFASELLSLMSLFDRQ
AIPLEFLEFYAKGNHGTESNIKMRLVKALGVLKAFCFIRAERGGDHTMHRLIQLVTRTWLVRKGSIAEFARHAFLSVSDFYPY

ADFEDVEWWAEDLVTCTTYLSHASSVLEAQIIETEEDSLLRASLLHRVGSFFNHQGRYSKAEKLRREGLTIREKLLGEEHIET
LVTASDLAGSLSNLGHFEESERLQRGVIETLKHLYGDEHEQTLDAMANLSVFLNKQEKTDEALALDRHIFEVKKRTLGAEHIS
TIRAMDNLAVSLGDEEGEEMHRQALERKIKIFGEEHPNTLYSKANLAMTLFNKGAHSEAEDLQVEALEASKRILGKEHPDTLR
VMNDLGSTWLLFGQIFLNERHLYPDVDMLGDAKRILEECLGLRAKLLGADHADTNNTRQELEECLEALEVADLIDAEAEAEAK
SKEQT
FUN_013586 CDS scaffold_6 105123 105489 -
hypothetical protein PF04419 ENOG41

MARGNQRDLARAKALKKKDGLKTGTNKSGTQMQRDNETAAEIMRRKQAAADARKAAEGKK
FUN_013587 CDS scaffold_6 109489 111500 + NRC2
serine/threonine protein kinase, AGC PF00069;PF07714

MPSQKTSNGTGQVSHRLRNFFRMNTGGSTGSASSDKDKEKEKEKEKGSAATTPDPSKSRHTKFFSNTVGRLRAHTVASEGNQLEE
AMSPTAHANPYFAHQGQPGLRHHNSDSVPPSPPDTPSLKVQGPDGAPANDQTTSETKEELARRLRRVASAPNAQGLFSKNDPN
NERPATAELGKDPLLTSSGALGLIETAKALEAEDKAKHANSDDVLAALPAPNLGMAFRRTYSSNSIKVRNVEVSPSSFDKIKL
IGKGDVGKVYLVREKKSSRLYAMKILSKKEMIKRNKIKRALAEQEILATSNHPFIVTLYHSFQSEDYLYLCMEYCSGGEFFRA
LQTRPGKCIPEEDARFYAAEVTAALEYLHLMGFIYRDLKPENILLHQSGHIMLSDFDLSKQSDPGGKPTMIVGKNGARTDALP
TIDTRSCIANFRTNSFVGTEEYIAPEVIKGSGHTSAVDWWTLGILIYEMLYGTTPFKGKNRNATFANILREDIPFPDHTGAPQ
ISNLCKSLIRKLLIKDENRRLGARAGASDIKAHPFFRTTQWALIRHQKPPIVPHAGRGVDTVNFRNVKESESVDLSGSRAMNL
KGVPLDSGLATPGGEIADPFLEFNSVTLHHDGDDDHHR
FUN_013588 CDS scaffold_6 112528 113929 +
hypothetical protein ENOG41

MSRLRPASEPRHRTRRSSDRAIPVPRIPQLSRPYLLEWSTQNCRQKELLNVEEFQEEMLAVERPGWRKLAIVRGECDMGVLIG
VELDKKRGGWVWEFPETEIRRRKPEEEDGETKEKDDDDGSDEDEGIRICRASLMVKEQLPILLLDGLPFRISSRPYRPSRVPS
NTPPDGKASSKRPPKRSGLEDSLWQALVDIRPIEDLVAELIYDMWLQKLDSLPPLGSEFVDVQWTIARALETNADITRSMERR
GEFSTITSADWSNLAERLQRRIQLSVTVALQAHAQQAQDDPKDSNSRSLDRIAYLGGLLLPLTVVSGILSIQSSYGPEGKSFW
VFWLASGLSSLLAILIIYADHLRTLDVWMEVAASEILDLDHNKRFQANRTPRRREYYSSGDPERGEAKLTTASDGGVYVVQHR
GDGTRGKAWRRGELGWLGAMKKMSGWYRWRGSPGMEFRMPGWERKVKNLVW
FUN_013589 CDS scaffold_6 114125 115625 -
hypothetical protein PF00587;PF02403

MNRTLSRLRCRALHPRLNPLLARSFADVKRPPSAPKPIVDIKHIRQHAELYQQTCVERNYRNQAQNPAKILELHARWQDLQRH
GRALRERSNLLRRQLANPATSSDDEDLKEVRQLTREQIQEEARQLKQDLSAIEKGESQAVTQMEELALELPNLTHPDTPKGSD
FEVMSYINDPPTFKESPEDKIWRSHVHIGSELGIFDFAGAATASGWGWYYLLGEAAQLEQALVQYALAVATRHGWTQVSPPSM
VYSHIGAACGFQPRDLNGEQQVYTIAQSAEDAERGVPEMCLTGTSEIALAGMKANTTLDSEDLPLKRVAVSRCYRAEAGARGA
DTKGLYRVHEFTKVEMFAWTAPDEDVAQELFDEMLDLQTEILSSLGLYCRILSMPSHDLGASATRKIDMEAFFPSRRDNWGEV
TSASMCTDYQTRRLGTRTRIDGKLAFPWTSNGTALAVPRVIAAILENGWDEEAKTVTIPECLRPWMDGKEKIGLGGRRSSVDR
V
FUN_013590 CDS scaffold_6 115966 117686 -
hypothetical protein PF00022

MASGTGSTLAHRSVASIRGGPPQGPGPSPHTPNRSSVISSYGSPSTIRADDEFIVVELGSRHIRVGFAGDTSPRVVLQCSPED
QRRAGDFRGWQQPKRSPGMQWSKEHEFWRYDLRDIELGLYQDKLERVLHDAFSKYLLIDSRPRRLGLVLGSGVPLPLLTATLD
TLFSRFQSPTISLMSTPSMSAISAGVRSALVIDMGWAETVITSVYEHREVKTTRTVRGGRALLDALYKTIEPLIPGEHDVTKQ
RAIRFEECEDIMCRLMWCKPSEFRSSQRQSTQLETVDEQEEIEAESAQAGVPTGVAQIPINTSRRSLTVELPFEKLAEVCDDT
FFDPSADRATFDDDELPLHLLLYRHLLQLPLDVRAICMSRIMFTGGCSNILGIKQRIIDDLTAIVNKRGWEPVFGKVVDKHRN
SARIHQQSSTTSPTEGSPSSSPGEDEQRKSPKNAADLKPEHDPIEAKVVRQRATVPQFKGQIRVIHTLGPWIGGSLLCQLKIP
AMAIIDRDSWLLQGANGASRPSEVDFKAQHRQSVQAGGYARGGGGHHGNWTLGVWGYL
FUN_013591 CDS scaffold_6 117982 120465 +

hypothetical protein PF00752;PF00867;PF18380

MGIKGIYQEVGEGERVALLKLAAESLEKQGRPLRIAIDIAIWQFQNQAAQGGTNPEIRTLFYRLVRLLACPVEPIFVFDGPYK
PAVKRNKKSGRGDSFANAQAKRLIRLFGCNTHDAPGEAEAECALLQKHGIVDMVLTEDVDALMFGCTKMLRQWSPASKRDTTP
THVSLFDTEKMKLDQHGLDREGMVLVALMSGGDYDPNGLPGCGIKVAVEAAKAGFGRDLCRLKAVDKEGTTAWRNSLIHELRT
NENGHFKRKNKALAIPETFPDFKVLRYYTHPVVSPGESIDEIREKVHLKREIQFEDLREFTREVFGWDYRVGALKFIKVLSHA
VLVQKLQALERHDKADYVTGIAGFRSDLKNDLVSQVRIQHIPSEIVPIDLSKEEDEKIVQARGGLALNSDDEIDDAENIEEST
AKRQPAPFNPSARQGVWALEILAKLRIPEVFADAQVVQKAAEKKKQQAKEPKTKATRSKPGPKTGMPAGALNKFVRASKPHTV
STVTSKALPKENEIITSPTRPTTSRRLRIPSPLEPSKQSASTSGSTVRQTTTPWTLASSQVTPRTRKPANEQQAIIITSSPPC
AADSPPPSPSPRPRTRVPASRDLPDPMCAIPTSSGSPQRLKDSALNARRARPPTSSQSSQPPKMKQMSMDMFTKKSKNKCSSQ
TSVSKSLLPASKQHQPKSQPRFDDDFDSDSSTDLAPLSSLISRSSASPNKRQQSSLLLDRNATPSPTPARKKKLLIPKASAVG
FYEEVEVDAEERDELMARETAALERRGVQAKIFRTSEVGFIDLTQDD
FUN_013592 CDS scaffold_6 122671 123523 +
hypothetical protein ENOG41

MFGSSQRAWGPGMIVLQILRVFTIISLLTAGASCWILIIKIDTSKGWFFFEALSLAMMSSATIFLIISELPFCRAYFANHWPA
FSDEHGLFWLGIGLLLIGTNLLGKLNSSDYGRDDLGLPFWRLIISAGILTCTFGVMNLICTFVFRGVNVNARKIRSNGSLADD
ESFGKPQSNYSASFTNEDRPKNTFKRFFWDRKNEPIPRRPTISQPITRERDLERNAPPPPHYEHQEPSSLSRQSHDSGEDRRS
PIVPEVRRPDTALHPINIRPHSPSMYSEAHMSRF
FUN_013593 CDS scaffold_6 124567 126498 +
hypothetical protein PF04818;PF11526

MSGDSAEVAEDYRMALEDLSSNMRFEISNLTVIARENTEHALAIAEVLQQHILKAPPNKKLPALYVLDSIVKNVGTPYTLYFG
RTLFKTFMESYAVVDQPVRRKMEEMLRTWKEPVPGSMDSRPVFTHELVRPIENALLKARAVSMPQAGMMPGRPRSGVPHRDTP
TPPGTRYPPGHPAQQYTSNGGQSLSNDIRNLIVATQAEASRNPQNVDVQTRLRALHELHGVVNSTSLPPDQLELIKRRVTELA
AVNMMAPLTQRPAPTPPVPVQARPMPPVSVALPPSAAPAPAQEGVTLDALFGPGALAALMARKSATPSSSTPTPQPPQVRPEP
PKPPMSNMSAMPAVAPMPAMSIPTIPGMTPMPTMSQTPTPALAANAAPWSLLDQLRKSGLLPPAPTATPMPVPVQGGINVAAL
KQPCQPFLIRALHNDLGQPCTQCGRRFRDDPVGKKQKIAHMDWHFRVHQRTNEAEKRGMHRSWYVDQRDWLKSREAVDVDHLP
TTEDVAAQASKASEAAKPKYILVPDASRRINTVCPICQDKFENKWLDTAQEWVWLDTVLVGDRAYHASCHAEATRDRENTPVL
GKRKAETSIASPKVRSLKTSA
FUN_013594 CDS scaffold_6 127050 128712 +
hypothetical protein EOG09261W2O PF00004;PF05496;PF07728;PF12002;PF16193

MAVDCPICNKPVKSSEINSHIDSGCESFIIDKEKAPTPPQSQTPQSNSQKRNASNFFSTPAPKRQAASENRTATPVNGALQPI
AGKKRTFEEGPGHGVASFKDAEETTNGENANGTSTWSPNTEQDGRAAKKTKTQRAAPLAERMRPRNLDEVCGQDLVGPNGVLR
SLIESNQVPSMILWGASGTGKTTIARCIARMVGSRFIELNATSTGVSECKKYFQEAMNDLALTGRKTIIFCDEIHRFNKAQQD
VFLKPVEAGTVTLIGATTENPSFKVANALLSRCRTFTLQSLTAEDVVRILQRAIKEEESVYPQTPLLDEAMVTYLARFADGDA
RTALNLLELALSLTTREGITQEDIKAALTKTLVYDRAGDQHYDTISAFHKSVRGNDADAALYYLARMLQSGEDPLFIARRMVV
IASEDVGLADNTLLPLATATYTATQQIGMPEARIPLAHCTVALCNAPKSTKAYRALNNAYAALREPGVAGLPVPLHLRNAPTR
LMRDMGYGAEYKYPPNYRDGQVKQTYLPDELVGRRFVEDRDLGTEVDPDLEMGGT
FUN_013595 CDS scaffold_6 129205 130278 -
hypothetical protein ENOG41

MKASTSLAVAAAFAPLTMARFLDFAQPERRDHPMPAEAVPAEAIPGNALPAELLSGHKKIVSGGHKISKTEIIIIWANPGAGA
ETTTINEKVTVTETVTKGGEAVATPAAPSQHTVTVGGPGGLVYQPEELHNIPIGDTVVFEFLSQNHTVSQSGFDKPCALLEGG
MDSGFMPNPNNTVSPPPQVAMQVMVDTPLWFYCKQGNHCGQGMVFSINPTAEKTQAKFKEMAIQQNGDGKATPITGGDAAPAP
PAPPAESKPAEAAPPAPAAPEATGVVPGKGVIGGDGSCVCMVSCSAGGFPAQAQGINSFGGMGGAMPYKLDSVV
FUN_013596 CDS scaffold_6 131712 132378 -
hypothetical protein ENOG41

MSKKQEKLTQEEIWDDSALIDSWNEALQEYKKYHSIHAKGGSVRELEPQNRAEAEAESGSEQPQVTETEEIELDSGTAEQNKE
IPSSRNESKEPIASQGTSSFPIQTVLGSVQDESLKKLLMSWYYAGYYTGLYEGQQQAQQK
FUN_013597 CDS scaffold_6 132837 134052 +
hypothetical protein EOG09264T8I PF00076

MAPELRKRKSASDIKKPNGAAKAAQPKRKAPEDASPVSLKKQKSVKKTVVTKKAATKPAQKSKSKPEEVKVVEEETTTLDIPD
ESSESEAEEGKDVQAIVKELDSDDEDITEGDSSFKQGQDVGKIPKVSKDVQKAAKSSDEEAGVIYIGRIPHGFYEHEMRQYFE
QFGPIVALRLSRNKKTGASKHYAFVKFEEASTAEIVCKTMDNYLLFGHILKCRMIPKEQVRDDLFKGANRRFKKVPWNKMAGH
KLEKPLTESAWANKVTKENSKRAKKAAKLKELGYEFDAPEIKDVPAPAAIENGESETKAIEDAPAEKEEEPKVEEPAEVVETV
VEEKTEVITEEKPAPKAKGKAAKGKATKAGKGRKAKA
FUN_013598 CDS scaffold_6 135449 137370 +
hypothetical protein EOG09263CUQ PF10375 ENOG41

MSSTVATNPTQETNSGSSKKKNNKKKKNANKNKAAEQPVSGSSDPQQGSDKETLEDEPETPTQPETPVNADAVNADEVVNEEA
PPAYSNGHAVEPKAQSNGLTPPPVDGEKEKPDDSDTSAKLEAMSQEREALRAEVEQLRKQLENIQETHSNEVTQLKSDLEESN
AAKENAEEEYQTLLGRVEKIKQTLSDRFKRDKAELEESKERIEELEAENEELRNNAVSSGDDVAKLKEELQDATRELNTLRSR
NNLSAQNWSKEKEELVRHVQHLKEEMETTANAMGEWEVIAMEERSIKESLVDKVSELEEQVTILRQNYENATTEREGQTTLIE
NLQNALREIQDARKKELRDMVETTEAQVQALKKQVQEADARAKEAEEAKQTLSQELERTAPYEKEVKEKNLLIGKLRHEAIVL
NDHLTKALRYLKKTKPEDNVDRQIVTNHLLHFLTLDRGDAKRFQVLQVMAGYLNWTDEQREQAGLARPGTSSNSLRLPMSPFT
RTPSSPSLSTDLFNESTSAKDKESLSELWANFLERSAQEGALETPSRKGSTSSAATGPVRPESTRPESKT
FUN_013599 CDS scaffold_6 137587 138758 -
hypothetical protein

MAIPWDSLRSLLIFFGPILLPKAISYYRSVKASSQARHAPIVPVPPKVRVALALLAFLIISYILKTLPPFAPENVFLATQSRL
QIPVDVLFNRVAVGRPDNVLTKQDEALRGRFVNLESRLLYLQFGPEVLANCPFCTSEEPKTFFYYALPQLLWPHIANLFAIAF
VTSPTFTGRAGSQWRPKATMAAMTIAALDIYFVNSYNYQANARALRLSEVDFFYWTARVARLVTLAAFDAALGWLLYLSATNR
AFVEPPSPVERIEATSRALLIVKSKLSALGIVKNTALRDEDLRSRSHAYWSHEVRLMGEVMEEREVVEGVNDALTNRIDVATI
TRDAEGYAQNVLHSLRGETADDDSI
FUN_013600 CDS scaffold_6 139443 140185 - GET1 GET complex
subunit get1 PF04420

MASLMLSIFVVEVVVNLVNTIGAAAINNLLWTIINFLPVSTAKAAGEQRKLQADYLKVRRDLNSTSSQDEFAKWAKLRRQHDK
LLEQLEKTKKTNEAARSNFDKILTVLRIVVTRAPQYFLPFWYATEPMFWLPYGWFPYWAEWILSFPRAPIGSVSIASWQLACT
GVIALFSDLIVGIAGLLLNAKQAKEAPVAAQKVAAEEKKKS
FUN_013601 CDS scaffold_6 140445 141429 +
hypothetical protein PF01239

MSRALDKSLKESLKHGDHHSVFLEISSVLIQPSNEPLEIELLGKSHILDANASVLRDDNAVAIPKLRLVQAFIVAQKLLKQSQ
ADSQRVSSDDISRSTAVILLMDPEHLTAANTRKRLIRDKLREQDLQDRLHLEKHLIDSLLTSRLHRHTKSPTLWNHRRWLIQQ
FRVYNINVPAENDLTRTIMVSGERHPRNYYAWCHARYLINAFILPLSSSQEGISRMIIATQKWCFAHHNDISGWQFLLFLLDK
QPAETSPVFRETLKLAASFKWRNESVWYFLRLVAARGVANTDKEEFEGLRKTLWETASEDSIEKKTLERAEQWPMASQ
FUN_013602 CDS scaffold_6 144811 146496 +
hypothetical protein PF00026;PF14543
A01A
MRPGSLLVQLALASSAHAFFPYSPSWRVDVGQKRMARSPKSVGNGGGVRMDIKQRSPQSSQPITERAAHEAARLIAKYAGGSI
PDTSTLLKRDNKYDVMEAAESTKEHTQGVDQDGTDYSYFIEVEIGSEKKTMYMLIDTGAGSSWVMGTSCTSKACEMHNTFGEG
DSDSLEVSKSDFNIAYGSGKVSGTWATDTITVAGMSIKYQFGLTHTTSDQFLEFAFDGILGLAINKGSDSNFLNALAKSKEVE
KNMFCVALNRAADGTNEGEISFGSPNPDKYSGKITYTSIGEDNDWAIEIDDMGYNGKKADIGGIRSFIDTGTSFMFGSPDNVK
KLHALIDGAQSSDGTTYTVPCDSNGNLTVTFSGVDYVISPKDWVSPPNKDGKCTSNVYGFEVVKGAWLLGDTFIKNVYAVFDA
DERRIGFATNAITDGSSDDSEDSSSSTKTMTASQATKTSDDTEETGITTTSGSKASAVPDMGLGKESVSSGTATADSSEPTET

KDSSAPSGPLSQARFTFIFCIVSFFALLA
FUN_013603 CDS scaffold_6 147506 149342 +
hypothetical protein PF01490

MTHARNDDPPNPHGEDRGLLEHGHDYDDDYFGVGDDDYSSGNASADLGKASGKVEADGQPRTPGRVRFDLTPEIVGVSNGEAF
GGFADRERRSSDDAYFDVEEVTSPGRAHRVPLLTDIEAPSVALANSMGDPSEQAEHEMNRPKSGLKSAFMNMANSIIGAGIIG
QPYAVRQAGLVGGILLLVGLTVVVDWTICLIVINSKLSGTSHFQGTVEHCFGHSGLIAISVAQWVFAFGGMVAYGVIVGDTIP
HVLVAVWPNLSNVPVIGLLANRQVAIAVFVMGIAYPLTLYRDISKLAKASTFALVGMVVIVLTILIQGILTPSSERGSFTPSL
LLFNGGFFQAIGVISFAFVCHHNSLLIYGSLKTPTIDNFSRVTHYSTGISMVFCLVLALGGFLTFGDKTLGNVLNNFPADNTM
VNIARLCFGLNMLTTLPLEAFVCREVMLTYFFPDEPFNMNRHLLFSTSLVASALVLSLVTCDLGAVFELVGATSAVAMAYILP
PLCYMKLTTRSWRTYMAGAVVVFGMIVMVISVIQAVQKMINSKDGPAQCV
FUN_013604 CDS scaffold_6 151922 153131 -
hypothetical protein PF00025;PF00071;PF08477

MAEIRRKLVIVGDGACGKTCLLIVFSKGTFPEVYVPTVFENYVADVEVDGKHVELALWDTAGQEDYDRLRPLSYPDSHVILIC
FAVDSPDSLDNVQEKWISEVLHFCQGLPIILVGCKKDLRYDQKTIEELRKTSQKPVSPEEGEEVRKKIAAYKYLECSAKTNEG
VREVFEHATRAALLSRSTRGSKHKKCLVL
FUN_013605 CDS scaffold_6 156476 157673 +
hypothetical protein

MQKLAKRVAQAQRQAGRRSQKAAKSEENNYKLRNRQAMRAAVSEVRQNLQDARRVRQEDWALGPIAPKRDLGFNGYGMFTEGV
RTDWSNYGLYRPRPEVLAKRCAWAGGLKQLNLAVSDRVVIMDGPDKGKIDRIKTINPQGGYVTLENYHRAISAGMFGNDSRSQ
PMPLSIGSIRLVYPIANPETGVTRDVIINELKAIPPNMKSPNMSFDRWEYGNKWDRIVPGINVVIPWPEVEAPEFETFDGDTI
RETVDNRTFYYNLLSPPMPETVIDELRNKFSKFRTRHEEWYVQQKEAEAAAKEAEKESIKSMQTPLQEFHEMQREKRAAEGEP
ELSTEMLEKLGAILAQRKEAAAFKKAGVSKVAAADASIPPSTTTPPAQ
FUN_013606 CDS scaffold_6 158306 158563 -
hypothetical protein

MPCDSTHTDTIPKGDTPPVTIDTKMLEPFIHDTSSLQDRLLMRSMAEDNKAIGNGAGYSGPKGSKL
FUN_013607 CDS scaffold_6 161533 161846 -
hypothetical protein ENOG41

MVSQNSQTPTNSTNERKPTKCRGLMLAPYIYDTDSVQEHLLCRTEANNTASESTNRMHETAEVSNHSDLEVHDLDAMMQEPPS
PKGRL
FUN_013608 tRNA scaffold_6 163710 163822 + tRNA-Val

FUN_013609 CDS scaffold_6 164459 165315 +
hypothetical protein PF02492

MSHSHDGHSHSHDGGFNAVEHGHSHEILDGPGSFLGREMPIVEGREWSERAFTIGIGGPVGSGKTALMLALSLALRKEYSLAA
VTNDIFTREDAEFLTRNKALPPPRIRAIETGGCPHAAVREDISQNLAALEDLHREFDTDILLIESGGDNLAANYSRELADFII
YVIDVSGGDKIPRKGGPGITQSDLLVVNKTDLAEIVGADLGVMERDARKIREGGPTVFAQVKKDVGVDHIIRLIKSAWKASGA
EEERRSKGGPRPTEGLEQLE
FUN_013610 CDS scaffold_6 165423 167925 -
hypothetical protein EOG092615SM PF04950;PF08142

MPVAVAHSHRPTTKVTHKAFKSRAASKHELRDRAKGRVADEKGNRKTPHQQVMSKFDRRNQAKQARLNKHKEHVKETSVFAGK
DGAPRHVAVIPLCAGTDLNAAIKQLNSSVDIEDNTESGNYRVTVDRFKQKLQYIPIERDLNACLDAARVADFVIVVLSANVEV
DELGELILRSVESQGMSTLFTIVEGLDKVEPAKQRQSVLVSLKSFITHFHPEQEKLYSIENRQDCANLMRSLCSTTPKGIRWR

DERSWMLADDIKFAYSESDSTVITGVVRGKGLKADRLIQVGDWGTFQIEKIEAAPLPKQIKKKGEEMTVDPEEAGKLLEEPSE
DRDDLVELAPEEVMMDDADDAMTTDATEKKGVLLDDHHYFSDEEAEARKIKKKVPKGTSNYQSAWYLEDVSDSESDMEDFEMK
DEAGEEEARPEDGLEGYVPAPATEGAPSEYPQSEMMIEPDEEEDAKQLEQYRAGKRNEVEDDMEFPDEIELHPHVLARERLAR
YRGLKSLRTSTWQEDEDRAHEPEEWRRLLQVPNYQSSKSQATREALVGGVEPGTRVHIHIKGISPIQEKTYNPKKPLTLFSLL
RHENKKTAVNYLFNLSSDFTKSIKAKEELIVQCGPRRMVIKPLFSQPGQTPNDVHKYCRFLHPGQSAIATFMGPLTWGAVPVL
FFKRTTAEEVEKNENEEGPHIGLSLIGTGTALPPSTSRVIAKRIILTGHPYHIHKRIVTIRYMFFNREDVEWFKALPLWTKRG
RSGFVKEPLGTHGYFKATFDGRINPQDSIGVSLYKRVWPRNATPVEGPLLEIEPSAVQADGDMMMDDQA
FUN_013611 CDS scaffold_6 168480 170187 +
hypothetical protein EOG09261UMG PF00400;PF08149

MEVDTIDVPTSKVRDNGALAVQGTRTELVPRHERERARRIREANKAYGRGKKVNIKAIKDKKLRSNMKRLEGKYQDAALKAKD
AEILLENTSGFLEAEDELERTYKVRQDDIASDIAVGTAQKRFELKLDDLGPYHFDYSRNGRDLLLGGRKGHVATMDWREGKLG
CELQLGETVRDIKWLHNNQYFAVAQKKYVYIYDHNGVELHTLRKHQEVSHMEFLPYHYLLATIGSVGFLKYQDTSTGQLVAEI
PTRLGQPCSLKQNPWNAILHVGHQNGTVTLWSPNTQDPLVKLLAHRGPVRDLAIDREGRYMVSAGQDQKMAVWDLRMFREVNN
YFTRQPASSLAISDTGLTAVGWGTQTTIWKGLFDKNAPIQEKVQSPYMAWGGEGKRIERVRWCPFEDVLGLGHDSGFSSIIVP
GAGEANYDALEVNPFETAKQRQESEVKGLLNKLQPDMIALDPNYIGNLDLRSDKQRRAEKDLDTPATDIAEEIRKRARGKNGA
LKKYLRKQRKKNIIDDKRLQVDEIWKEQQAKKDKKQLEAEADLGPALARFAKKE
FUN_013612 CDS scaffold_6 170389 171110 -
hypothetical protein PF01981

MNPGEQTGVIISTAVVALLTGYAFGIYTIRGYLIPPSLFEERRRNIHDPVESDESDIDEDDTVLDHAPNWANGADADKRQGLK
NVEKEKEPVLKDNGEECKLVLVVRTDLGMTKGKIAAQCSHATLACYKALSRAPSNSPLSKILKRWERLGQAKIAVQVKSQDEM
LELRRKARSLGITAEVIQDAGRTQIEAGSMTVLGVGPAPRSLVDQVTGGLKLL
FUN_013613 CDS scaffold_6 171512 172695 -
hypothetical protein PF09258
GT64
MGQHSRLLPQATMLKPSNSSFMEKTRPLRNRLAEKIEWVKLRTPKVVKSRAFRRAATITSAVFFALSFIVILAKLTNAGFFIP
KLVSHISRYPTCQSKGINGTRDIWEASQKKYENLMDDKFTIAMQTYRRPKELQETLNIILSEEVPSLLEIVVVWNDLENYPPD
DYVSKYGVPVRFRKSKRNSLNEKLWPDPDYKTQAILLSDDDVYYHPNDLEFVFQTWRKFGRNRMVGALARCTPVNTFGYHKYT
FCSSRRGEDEYNMVLTNLAFSHVSFLDYYSSNDTIMTQIREYVDEGFNCEDLAMNYVHGLLTGEGPLLINGHEKYVNFVPKVG
ISMKKGHMEARSACLNDFSEMFGCHPLVDESGYIQRGVLVM
FUN_013614 CDS scaffold_6 173631 174483 +
hypothetical protein EOG09264DMU

MSANKALIVGISGCSSSGKTTLARLLRDIFPNTFILHEDDFYRPENELPTKNGLLDWDCAEALDIPAMAESLAYIRQHAAFPP
TLDSKEDQNSVGKCPVSEASIAAQRAKVDAALGPDHPLRKNLRLCLLDGFLLYSPSMAAIKPNLDIKLFLRTTYEKAKKRREA
RDGYVTLEGFWADPPGYVDKIVWPNYVEEHAWMFEYGDVEGKFKTDVLDKEGIKVQSDVSADGDIEKTFEWTVDTILEELGKQ
V
FUN_013615 CDS scaffold_6 174970 179449 -
hypothetical protein
PF00035;PF00270;PF00271;PF00636;PF03368;PF04851;PF14622 ENOG41

MIDVEETVTLIPTDKALNRKQKAANKSKDASPVPDIIRPRAYQREMLEQSLKRNVIVAMDTGSGKTQVAVLRIQAELERSSPN
KLVWFLGKTVSLCEQQFNVIKRQMPSVPMTLLTGQLNIDAWSPEVWPRILKGTRIIVSTFSILRDALDHAFVTMDMLALIVFD
EVHNCVKNSSGRKIMMNFYHQHKNVGMPVPAILGLTASPIISSDLKEIEKLENTMDAICVTPTTHRDQLLKHVNKPHLIRSLY
DATKIPPRTPLMQQLQSEYSNMDIAKDPEVLKLRALVANDGKKREKLKNVIMKHDTFSQQQIRGLWNKSREILAELGSWAVDH
YISQRIKTFLDRIDAYSTFTDAWSNEDRTYLAGHLRRINIGVVDNSPPTAQTVSHKTSRLIHELLVAENDVVGIIFVKERTAA
HTLCELLNNYPPIRDRYRVGAIVGASSYGLQKQKVYEYSSGPGPQVLDDFRSGAINLLVATSVLEEGIDVPACNLVVSFDEIA
TLKSFIQRRGRARMQESKMIALLSSSTDIRAWEELEKGMKERYEDDQRELARLDEQARAEEIDSTYYIVKNTGARLDLDNARQ
HLDRFCQIVFRSDYVGQLPDYVFHKEETPYGGPILRATVTLPGGLPLNLRKFKSACGWKSERNAIRDAAFQAYVALYEAGLVT

DNLAPLNAKSEKVEEKTDLMPEPLFDPWVQIAQRWKSGCDKQFYLFEFTDDEFPGPCSFDVMLPALIDLPRKITLYPGNGSKW
SIVCDSFVDIPDEVSSKEPDQTSALLAMNFQHRWPVEDREHVIKMSIASTTIFPAPHVTRDDMGAFPFRGDGMEEAKKHRVLV
RDQYKTPFHYVGTIPSKPDFYDVQRPFYEYDKGPLNEEYLVLERWGLRVDLLHDLKDGQAKSSTKPYECVLPISYARVDRVKR
RVVKCGLFIPHIIHELEVQLIASELSSTLLKPVGIKNLQLVLEAVSSPSAREPVDYQRLEFLGDSILKFCTVTQAYSEHPSWP
EGLLNHFKDRLVSNERLQRVCLEKGLSKFILSKPFTALKWRPRYLDDILKEKPAPRPAPVGGSKTLADVVEALIGASYQDGGL
TKALKCISVFLGDSCNWHQEGVARDILFAAAPSDVQLPSNLEPLEDLIKYKFKKKSLLIEAVTHGSYATAMQEKSYEQLEFLG
DAILDYIVVTKMFQHDPPIPTGRLHMIKTAIVNGEFLSLVNLVNSIQRKDLEVRPDGELVTKTTALPLWKFMRFNSSEMANVM
VETEEKFESMRRELVIALWGDGHYPWALLARLHPQKFYSDMFEALLGAVWVDSGSIEECTRVLETFQVMDYLEFVIKEDIHVQ
HPKEELSKFADRQKMKYTTTLIEEPEPRWSCGVTIGERLVGEVESSLNKVEAMTKAAENAIRLLTEEMNAVNQQQDAMDAS
FUN_013616 CDS scaffold_6 180397 181496 +
hypothetical protein PF13540 ENOG41

MDLPVRTPSSDQTMVLYATGFNAWSQLTFDPSPADEEPDDVFTFTKVLAARFVGLIASEMSYTAVQQDTSWAIAGSSPDQLLH
REDNQVFLFDSPSAISGDGKVLAVQDSDCSPSNQVIVQYPSLAAWKASKSTDSWPCKASVRQITAYDAGFIIFLEDETVLSCG
DPRFRDCLGREVDESCPANEPGIIQDLSDLGEPIKKIAAAGYTAAALTESGGLYLWGMAAPGSQSQHNVFQDISELPNYFEVD
GDKDVQDIGIGESHAIALTTDGCIYVIGNNTNGQIGLGKDAQDPVSSWSKVDFTPPEGWAIVAVEAGPRSSFIVTKKVKQSQS
S
FUN_013617 CDS scaffold_6 181587 182668 - PCA2 putative
proteasome subunit alpha type-2 EOG092645G0 PF00227;PF10584
T01X
MADRYSFSLTTFSPSGKLVQIEYALNAVNQGITALGIKATNGIVLATEKKSSSPLADQSSLSKISDITPNIGMVYSGMGPDYR
VLVDRARKVSHTGYKRIYNEYPPTRILVQDVARVMQEATQSAGVRPYGVSLLVAGWDEGIEPEEENKTTEESEEKKVNRKTGG
IHKGGPMLYQVDPSGSYYPWKATAIGKSATKAKTFLEKRYSEELELEDAIHIALLTLKDNIEGEMNGDSIEIGIVGAPADHLL
GLEGVDGAVGPRFRKLTPQEIEDYLTSL
FUN_013618 CDS scaffold_6 183446 184448 +
hypothetical protein PF12906 ENOG41

METRPTWDWSSVGDTSARQSEPEPRAATPEPTTRWASPPKPDTTSNQESRSGPRQRRYGPRTCRICLDTEEPRFPEQSTTFGI
PTGSSRPTYVSDDPELGRLLSPCKCKGSQKYVHEGCLSAWRLANPMEARNYWQCPTCKFTYRISRLHWGSVLSSKWAQVALTL
WFCILSIFVLGFIADPLFDLWSDPIGTIGETVTSVVTDIEALRQPPPQVPTSWFEHFVKGFFSLGIVGLFKTMLTVSPFSWWQ
IRSSGVAGGGGRRGGTGRNRVENINLIFVLIGAFTFLVGIWKFVQKLSARVLKNVSDRVVDVGEDDDEDEDIGDGPNQGSKKG
Q
FUN_013619 CDS scaffold_6 185523 186589 +
hypothetical protein PF05637
GT34
MHFAYPPRKNSNPPPFKQRSSQIPPILRRIRKRTALLLLLGAIGFIFFLFGLKGETPYREHVPSGNPPVVVVTVLDNTQYSDT
YLESIRHNREQYASRHGYEAMIVQATDYDTGKSPRSWAKIIAMRHALSKYPDTTYVWFLDQNAYIMELDKKLEEQVMNPAKLE
SLMIKDWSIVPPDSIIKTFSHLKGEDAGLVISQDEAGLVTNSYILKNGEWAKFFIETWMDPLYQSYNFQKAERHALEHIVQWH
PTILSKLALIPQRTFASYGRNKEGNAYRDGDFVTLLVDCTVTGPTSCDKQSHFYLQQLKNKFGDASVKKLQ
FUN_013620 CDS scaffold_6 187858 189722 +
hypothetical protein PF00266;PF00282

MTSPAHIGTLSRLPLNLRGGIESVARGRHNVSWPLLNLDLLRNVLLFLFVWRWGRRAFWQLRGRGIIGSVVELYVNIRRILYG
YFLRAPGVRNKVQQQVQESLVKLSDKLVPKDQIRYLSLPKDGLPIDSVRAELENLANMDHTRWEDGYVSGAVYHGEEELMQLQ
TEAFGKFTVANPIHPDVFPGVRKMEAEVVSMVLSIFHAPPGAAGACTSGGTDSILSACLAARQRAYNERGITEPEMILPETAH
TAFHKAAQYFKFKVHLVSCPAPDYQVDVKAVSRLINPNTVMLVGSAPNFPHGIMDDIVALSKLAQRKKLWLHVDCCLGSFLVP
FLERAGFESQLFDFRLKGVTSISCDTHKYGFAPKGNSTVLYRTAELRKYQYFVSPDWSGGVYASPGMAGSRPGALIAGCWASL
MTMGEAGYIDACTKIVGATKKITEAIQSSPLSGELEIIGRPLVSVVAFTSRNLNIYDIADAMGAKGWHLNSLQNPPAIHVAVT
LPISKVWENLIADLEAVVEEEREKERVRLVEGKGAHGKAMGDSSALYGVAGSLPNKSVVVDLASGFLDILYKA
FUN_013621 CDS scaffold_6 191386 193440 +

hypothetical protein ENOG41

MLSAEPSSSPIAALAMDGGHVDGLHKGKTESIQIPHVKTTEKDDESIASGSTDSQSQLKKRLSADMSKEQVEDLVRKLTIDEM
KTRWFDEIFEKVKSELVEEHSPADTPSSSEPPSPRSDPAKPASHESTPRESVRGEPKKHESASSSFDSKNKSHNNPSSSRSSL
GSPTTSYISDSASSRASNRPSPPKPAIKESSPPAPKPRPCVRFSKAPIILNEGPERRHSEPRPASRPAPPPEPVKQGPALSAV
DLKWGTLFDDRGEPTKRLGQVLRGIANYLISEYSPHNSLVVTPEKLNAFYLEYKLDTETFPFQQIFDCRPHGALDNLETLYQH
LRCENHLIQRRPGGMPHIPSLTPAGFERWMTCQLRAFPDQEAKRLNHIMTDLPITADGVLVDDKPERLPKQISRHLLPATRHR
ETHDLVVDAIAGWIKHTEENESDYRRTSSEDVYKIKEEKSGRYRPDDYREREKYRRGSKDNHRQSPLSSSSRFVSRVGSDSTI
RPSKDSPVSCSSSHRARSPVSNRYRHSASAIDSSSSTVDAYDIPSSSHRNSHPASSSRRSRDKEYRYSSSPKSKSPIEATPRI
LPRRDADRRSSLIFEETGKDPSGITYDEYLRMNQRPMRNAVVDDGGHYRTTY
FUN_013622 CDS scaffold_6 194630 195922 -
hypothetical protein ENOG41

MDLAAIDEDTAAVLAAAAAEADEASAVSDQQFIFSTPDRKQPLSNSSSTATTPATNYFSYGNLSNGYRNEFRNRYHDDNDSAP
DQDIPGLRTPSLLSSPSVASSLGFSTGRVSPHKRDISGHSRLSSINDSPNNTSPSVRRTHRPRNSRDVLAQRLSQLAHELTSG
DDDLLDDSGVDILTAQLDQLEGTKMLKKTVSFSLPSTPSPRQAHQRLSLDMCSPGESIFSTPGSAFKTRFSDLGASLRRDPEP
EPEPEPEPPANMGMTVAQAKKVIQEVTQLNEEMSQLVTNLKARQEESDHIQGLLIERAERAAQRIIFLQNRIAHLEQELREND
DELQYLRICLKAVEIQMPAHPDKELQRCISSFKKDYQALKRKRVNRSSIASLSSLGSPYPGSPA
FUN_013623 CDS scaffold_6 198486 200002 +
hypothetical protein PF02518;PF10436

MSWKASERLMDTIRHYARFPATGVSLRQMVQFGEKPSVGTLFRASQFLAEELPIRLAHRVQELDELPDGLNEMPSVIKVKDWY
AQSFEEITQLPRPQLPSDIRNRLMKPSKAIGRNAFRLPAATPNPSIDEGEADGWGGLQNDNGKAKASARRYFAIVDDSSDWPA
DLHLYNQRFAQTLHHIKRRHDGVVTTMAQGILEYKRRRQRMQIDSTIQSFLDRFYMSRIGIRMLIGQHIALTDQSHHRDPTYV
GIICTKTNVQDLAQEAIENARFVCEDHYGLFEAPKVQLVCNPNLNFMYVPGHLSHMLFETLKNSLRAVVETHGMEKQAFPVTK
VIVAEGKEDITIKISDEGGGIPRSAIPLVWTYMYTTVDRTPSLDPDFDKSDFKAPMAGFGYGLPISRLYARYFGGDLKLISME
GYGTDVYLHLNRLSSSSEPLQ
FUN_013624 CDS scaffold_6 201246 202835 -
hypothetical protein PF12898 ENOG41

MPSNNNKGGYKPKAYEAAPTQFRCASGNEWKPLNMFSKAQQRNTRFLIDSGRKVDPENTGMICKEHNSTPALEHGCVGCDRTL
PYDAFSLNQRRLEDWRCLQCVAWDTVAEPSVVPIPKATGHVSVEEEEMMSQGYLAPVEVAQFFDEDDLPGAPITSPESLGLDP
NNEDDNKVFNEVVRGSSQTIHSSTTRNFASTTSSVGGDNMSTTSSRATLPPHLQRLLPERLASLSIDEDSVSSSSKVVLPQVP
QMASDFPPHLRGLKIAQRTATSSTTGSVSTATTLRKDQEEAAAARKITFNAWDPMGQRHTASKNPTVMSSSASEVSTTEESDQ
GAKIADGWDDIPPMKEASTRHEDKWGNGKENRISQADLRRQQQANFHTNNKWKKINDRYFTQPNIMDQAEDSPGRQRSGHSMQ
SRNMFDALSN
FUN_013625 CDS scaffold_6 211875 214284 +
hypothetical protein EOG09261EU7 PF00069;PF07714

MTVQNDLTYRGVSGQQLALEDRFEVLKEIGDGSFGSVVLGRVRTAGANVARRGTVVAIKTMKKSFESLAPCLELREVVFLRTL
PQHPHLVPALDIFLDPYTKKLHIAMEYMEGNLYQLMKARDHKCLDNASVKSILFQIMQGLEHIHSHHFFHRDIKPENILVTTS
GHNESGNTFRRYSALVTPPSTPPTYTVKIADFGLARETHSKLPYTTYVSTRWYRAPEVLLRAGEYSAPVDIWAVGAMAVEIAT
LKPLFPGGNEVDQVWRVCEIMGSPGNWYNKSGNRVGGGDWREGTRLAGKLGFSFPKMAPHAMDTILQTPQWPTSLSQFVTWCL
MWDPKNRPTSSQALAHEYFADAVDPMRPKSSASRILGRKQSDLSRSSKEAATTTPTSVKQSWFRKSLIGRSESTDLATMQAQT
KDSTAPRPAPVHASSAVEASTAKVRPAAGKRTTWTNGPSNVAPMPILPTARPISPIPDAVNARANNTYDDSYANGHGQGKTKK
LGRQLSVASSTNNYTEMHRQQAERALNGNSGLASPPSGQKESFFSHLRKRARRFSGRHQTPVSPAYDDDLEAQVGCGPWASNR
SSMVIDQSQQQAPIPKSEVYESLDKALRDVQNNLDSRPPVPPSHQISPTSTLKRHHSLPQHQARSVDNLIGAARGGPISSRTR
RAQATHGVHQYEAPDEEDELLDEALTSTQRAVKRMEQNQNKPLRQSASNIGLMNPYPTPSPSANGNQVLFADGHEAVTPRPLD
LNKKTDNQYKWPTPPYEESEWAASAAASIWAAGSRF
FUN_013626 CDS scaffold_6 216139 218239 +

hypothetical protein ENOG41

MSLNLPIRPSTSSQRNRDSRNNYFNSYTPPSSTEAINGPVRELVREQTPLNDRLIVGVDFGTTFSGVAAVYTSTPDDIEIIKT
WPGGNGITSDKVPTEIAYDFPPNAPQGTEPTVKWGFQFKPEESRLRCIKLFLDRSQKLPFYVSPLDTAAQLKRFNKNVVDAVS
DYLTQIYKHTMDTLTRRYGESFMASTKVEFVLTCPAVWSDAAKNTTLQAAERAGMGLRSEIQMISEPEAAAVYTLKAIQPNHL
NVGDNFIVCDAGGGTVDLIAYKIISLKPLRVEESAVGTGGLCGSAFLNYRFEEHVRGRLGQTRFDEMKAKKGKTWQMGLRYFE
EFVKRNFNEDEHQEVNVPFPGLPDDEEAGLDSGFLVMTAEQVKDIFEPVVKEVCDLVQGQVTGLRSKGGIVSGIVLVGGFGQS
DYLYRRLKNHFTSAAPPPYSERPTHANANSIQETGSIEVMQPVYAWTAVVRGAVLRGLEGNMVISRKSRMHYGTSYATVYDED
KHSVSERYWSPLWERWMVSDRMQWHIAKGEALSPLAPIAFHYTRNFRPGQSLIVTDDLIACDADEPPAAYTRDLIHVCTLTTD
LNAVPRSLFTRLTTTRGVEFDNLDFTLEMRVESAGLGFELKVDGVRYGRVEAEFH
FUN_013627 CDS scaffold_6 218785 219265 +
hypothetical protein ENOG41

MSSYPSFQWPNPQGVCCYRSFDNPNDHSIIAIKVPWLQWPNDPLEDVHSSWWVIREKSFAAVRLWKKILHEKLESETHYKHMK
ITSIVLMNEQLRLTIDDLGEGDDHLKLFYGFAADEIAKLNEVKGLDLGKDVVKWVGSGRDAVPVFRRIRKRNTALV
FUN_013628 CDS scaffold_6 219778 221149 +
hypothetical protein ENOG41

MTSSEHASALSKWAHSQGATINPSVEVTHLPETGLSFRAKAPTAPFDTIVSIPSSLTLSYLNTLPGRDDPKPFPAGFLAQTPP
HVIGRFVLIKHFLLKGESFWAPYIQALPQPDDHDSWSLPPFWPDEDAELFEGTNIEVGVTSIRANVKREFKTAHDLLAAESWE
LELLKQFTLPLYQWAYSIFSSRSFRPSLVLGPEDQQRLPEGVKLDDFSVLMPLFDVGNHDMTTKVEWVRNESINGCSLKVEKA
YQAGEQVFNNYSMKTNAELLLGYGFMLPETEELHNDYVHVRKRQPAHGEATEEYYISLRPIRDPSSLLARSKQSVQLNDGIPI
LGAFQHVQHDMVWDIFCTLAPPEQRSTLISEGTEQEQQEKFFSGQVSEDGRMFLQQTAAIIQHKVMQELERLLETDVEVVGGG
DLTRNQQLVLDYRARCRRVLENTLEAMNMEELFGEDEEGGE
FUN_013629 CDS scaffold_6 221525 222516 +
hypothetical protein PF00293 ENOG41

MTPSSDLKLLDLITAIDNAPLDFATNYAPYYRLFLSPDPRPHGFILPATVSLMPWPASFTIDHDNRTVTLSEPPSGSTLTEHA
NSAFQEAVDKAIGDDLFPILHKEHSEYFRIVGAREFVQVERFAAPLFGIATRGAHLTGYVRGDDGNIEIWVAKRSRHLFSYPG
LLDSTVAGGIKASDTPLACIKAESTEEACLPPDLVSSSVEPAGAITLANINQKSKLFHSDIIYVFDLEMPKDVIPRPGDDEVE
EFVLMDCQEVAQRMLAGEFKPNVCPVMIDFLVRKGFITKENEDDFEEIQKKLRREIPVPMESDV
FUN_013630 CDS scaffold_6 224521 227408 -
hypothetical protein PF00439;PF17035

MASPTPDATAPANENTSRDVTAEKEQKSEVNGHATPEKPKGTPESAPVNGNKADDHPVENGVNKDVEMTEASDETKPSQEPST
DEKNDPKTEGDDQTKETGDAKSVEEPNAAEEDVDMTDAVPAEKSGDQKPAEDATTKESKDVDMVDKPADTPEKAEGTDKAPAS
SNDAAAPASEAEVQPTSLSQLAIDTKEADAPKPSTEMSMQDAPVGDTSVSSKVAREREDDAIDEPAPKRAKTEPKSEEPADVT
STVPNTEATSAESAPEKEVSRFAGLTRWNEADFKNQTLTPFQRREFRKVLGRVKKTKAGGHFKDSVPKMWPQLAESYLAKIEK
PMDLSEIDRTLRDINGAYVTVGDFQNDLVLMYDNTRNFNGTLHDVTAAAFNAIRSIWEEVATIPQEEAVKPKQIPKPKPPRES
RISSLGDSIARKPSVGPGASPAAESVSSKPRLGSQEANTAATELRRASSATEGDRPKRTVRAPKSKDIDYTTKPSRKKLKPEL
QFSEEVLNDLMHPKNHAINNWFMEPVDAEGLNIPHYYSIIKKPMDLGKVARMLKSGDITNIKDFDKNVRLIFSNCYTFNGSVD
QGNTVSYVASQLEDYYNSLMKDKDSWLARHAKAHAPAASHGSDEEDEDEEADGDEVAAPPSADHSKEVRDLETKLREESEKLT
DLLCADSPNESMVAMQKSIVNLVQESLLKAKQALSAHRTKHPEKPSKKASKPSKPKPSGSAPRKPSGSVANPKKPSGTKKAVK
KTLTAADKDAIASAINDLDGAQLDRAIDIIKRDTGQNENTDGELELDIDQLSNEALLKLWELCKKVLPGFGKDTNVPSSPEVT
RAAPPKHTKASSTSAKPKKNKPMSAREQEERIAQLRDLSNLYRPGQEPGDNQPVLQAPTPTAESSDESDSEEE
FUN_013631 CDS scaffold_6 229949 231125 +
hypothetical protein EOG09264NJ1 PF04003

MSTKRKAPVKLAAPVARTSARIPNKSIIDESKASIAGPDALQPSQVETIEISSDNDSDEDISDAESPEEEQTAQETAVTNTRQ
KAAKQKTNGQSNDEESDAEGTSPSFGELLRAENDIIDVPSALNGAVVSQPSRNAIVPPTHQSLTTVLSQALRTDDTDLLESCL

HTTDLPTIRNTIERLDSALAGTLLTKLAARLYRRPGRAGNLMTWVQWTLVAHGGALASQPKVIHSLSGLQKVLAERAKGLSSL
LALKGKLDMLEGQMDLRRKMSRPGLHNGDNSDSDEDDEDVIWVEGEDDAARTPGRRRGLDTEFDDSDDDVPITNGFVGDSDDE
EDSAGEEDDSDAEESLDEDEVNHDDVDESMGEDEESDVEAASAPPSKLQKTAGSFGKRR
FUN_013632 CDS scaffold_6 231675 235890 -
hypothetical protein ENOG41

MTSPKATISNRGQENDDTLIEPVLSSPLSTPQTTSPELVSFQSLADQTSTPLSIMDDSCFLDIDKFIASGLEPSSVCSVGLDK
PTASSSQSPVITAGSPARPSDPSTKSQRNADPLGCTGTDADGGLAEQTRAAHEDNVSWSIENESKLQQTSNRQSYRRSQEYDQ
TKPSEAIAQPKVISPVGAASNTTGFAAQNSSAPSNTGEPSLGFRGPHSDQHEYRIDSSSRNPQYETALAFGGPLSVERLPASG
IATAQAQASSHCSAPQSATQAMSSTASTSNNSVPPGHQRHRNGHSSHDSRDNDFAPSHTTSLTSRVGGIKLDTSLVKPTPRPI
VRTAPDSPSLNIQHPTPDITSQARSGAYVGNIAQLEATAERLSMTSSIDDAIRDLHTELKRSDSRRSSILAASVKASGSIDDY
TSNPGSAIEQLRRHPSIASSIVSTNNAARHGGYSPGGYVLSPNHSFTGRLRSGSKNSAGRPDFDLDTVLSRHGPGKASVRSVR
STKVSLAEISESEPIALTQDVLDMADKTPVKNPEGESLLPDELRDANMPATDVFQNMMGDDDFMNSKNEHPHPDASTGLLADQ
ECRDPQRPGSSHSDTTFQQCQDAFGDFDGVHWVPEQDDPYSLPDDLEPQPQPRPRQRQMSHSENVRPQSYFDPESGQQMLYYP
ARVPAMLNLPPKLSNKPKANERNKRRSQVMSAMFEGNRQSKMLDVDAKAQERDTTRDSWLPDPLAGHRESFAALSSDGFGNLG
QEPETIRSVHSDQDVPPTVQPEANANAPIETLRRPQRLSRNELDKRKSRMSNLPPQLRASAYFDLPDNTQGEIEVKDGSAMAT
LESILDASANAPVSAFTDHERAGRLGKEVYGKEKKRASVAASTALQPEGDKKHARKRSSFMWLGKRHSHVSDDDKDHKAQSES
GLGPVNADTNANEHDGLARSVDGRSDGHSDDEQQAAKEEAEDLSDEEGYNGPPTTLLAELQLRKQQQKERTQRTFPGGMHATL
LEMDAVAETQKKARNNKRINLAWEDGQAQAVDEDSDDEDIPLAVIAARNQGAKNVMDLQRPIGLMERREMEENEPLSHRRARL
HGQDPRRPSMNNVSANHLPTTPHSAHQSVAQLSVQNASTEPDDEFEGETLADRKRRLAAKEEAENLLPKARPVSSAFSVELLN
QFEDPEEKKKKEEENKPKSGEEETLGQRRKRLQAEREAREREMSFNQLNASAEEAEAPIPGINGRMHQLSSVLSAHPKRETNQ
AQEERARMEHERRLLRERDAKMAAYRSQMPQALGGPSNAQSGGYRGGTFNNGLGGQNSQAAMSSPALHTQLFNQPALNHRKSA
VFSTYGAPMQQAPYGGSATNLGAMNMGYNGMPNGMSPYGAGAMSVYGGGMLQPGMQMPGQGGSTNRIEQWRQGVHP
FUN_013633 CDS scaffold_6 239687 241359 -
hypothetical protein PF07690 ENOG41

MSRTDEENPGVQAGRSRDSSMDVEKLGRQRPEVFKSIWAELGFGFSVFCSMLMAEFFVSGFHVILPPLAEELNIPAASQTWPS
SVFSLITGAMLLPMGRLSDMYGGYIVFNGGLLWFLLWALIAGFSKNYMMLIFCRALQGFGPAAYLPGGVMLMGKFYRPGPRKN
LIFALYGAFAPLGFFFGIFVAGLTAEYLDWRWYFWIGTIVFAVVSSVSLLAVPFDFHDRKTDIPMDWWGVGTIVPGLLLVVYS
LTDSSHAPHGWATPYIIVTLILGVMFLASAWYVETHKARVPLLPADLFAPKGMNRLLAVLFMAFGTFGLFLFYSSFYMELVLH
RSPLITAVWYIPMCVGGLIIGTVGGFTLHFLPGRVLLFISSLANAITMLLFALMPENPNYWAWVFPAMVCCTIGIDITFTVSN
IFLTTNMPSHRQGLAGALINSVLFLGISFFLGIADIAVAETASLGLRKSYKVAFWLSFGVASIPLLFIPFIRIGSAKSELTMD
ERQRLEQEAETQAQPQEPKTSMARGGEKTLGTLS
FUN_013634 CDS scaffold_6 247226 249233 +
hypothetical protein ENOG41

MATTDKPRRRFVPEPIETTFSTSRSSNRSDEKKTPRTMRPNPEPTPEPSPRSPSPGLNELQKKHQQRPKRRFAPQLIESSRRS
RRVGDAGPATKPTDKTDITPYTKNIYTATKHRGRRRDDAHESPSNGPRRRESEDENVVSFLLEIAAKEAERQMQEAALAAFPN
SHIREGNVDHFYFRESSGSDQSPESTSPVHDHHGHPVKHHHNVRRQSSDSNLGWWHKHMQEHVEAKQEQEQQQKDEDAMAIDE
KPGEMDTLVEEPAVEAPAVKKDEDVMMRTDTADLDAMDLEPPPDPMWTTSNTPRDSSSKRSSIGVSGPIGETHMPLLDTESYL
TAEQRSKASSPAPAASRHIGESTMPFIPSAPAVGSADIPYAKASQIPPDTGGFAATRNLARPFGYRPFGRGSPAPSFQKKRLG
VTPPMLGKDLVFRRCPSPKQTKLEPDHPFAERHAEEKFRDLSGQNGLWRGYCFRSDSNDNYLVPADLHAPPMLATPRPAATPG
DASKISSPPYTDEPAMIGAANGQANGVAQHRTRAGPPKGLHMLHGINERLQKEKAHAELEDKIDQEFDDSFVTQVYNYLSLGY
PAMARAFDGELSRISLMSIEDLERDDEKQLAQGHLVESEADKPRDQRCPRWHALQSYIKEWARQHPNLDSMDPVGWGVRERRG
SWAV
FUN_013635 CDS scaffold_6 249802 250569 -
hypothetical protein PF16815 ENOG41

MGTISIRKFIRWLPDEASEPTSTIVVTSPRLKRFVDLRILLPDNDPSWTGQDEPLPLSRLDWAIAGTTVSTKIPDPDGNLTSH
STFHQWISSRTLDSDAGFMYPQPNDLTLEKGSMVNPDTGIDTAYEELWHDATPTAVPGEPAVRALVLQTEDEKNGVRGSVVRL

GCYAQGLIRVGENISLERWEWKEGWKRTISMGDAELPIEKILGEETLKEGDTVGVGGREWKVIEESGKDGSKL
FUN_013636 CDS scaffold_6 251249 253257 -
hypothetical protein ENOG41

MWDSSDYCTSTCEDYPEDIPSYGDIGGIGVTLAFVLTAWMVVLVLIVYYLKAFDPKLDPFRKEGTQKLSKYPNSVDYFVNNLL
QKVLGLGNDSSNPVPCRSLRLGGIFNNCVLMFADIQSFTALAILISAYVAMGCDIVAYHWQYMIYLAWMASVTHLASLSFLRN
HLTNNFVKCIWRLTAMAVIQILLSVAVGLATTFGDNPWYREADQLIGIEVPPAEGGRPAICYFKQGVDVNSITFSSGVKMILL
LLWGFSIRIAKMFERFEGGLRKFAYRMHHKSRILRYGVDDGSTTQIWDPRARRKGQGFFGHLLEPFKIAILVVLSIQLDFLTS
FLAEVYWLLFTIIYITTRLVKFRIPSPADDNKWTFGQVLPLILLVAPIAIAIESFYTAPRTHTPQMTHTNTQQMNGPGSGLDP
VAGLGHVDSLAYRGVFFLAALSYIEVGIYFVLDQPDTEGLALPLLQIALSILLLQPLYQIYWVVCDVFLSKAIWSRPLKLTTY
DTLFLGSSAIKSHLSHAETESYQMAICHIVIFPMLHLSVLLFNVVFFSLLELEGWCFLL
FUN_013637 CDS scaffold_6 254232 255219 -
hypothetical protein PF00544
PL1_9;PL1_7;PL1;PL1_10;PL1_5;PL1_6;PL1_3;PL1_11;PL1_12;PL1_4;PL1_8
MMRSALISLIPAFLATACPVPQASESAGSVVSSGSCGAAEVDELVGYGAKTTGGSGDPVTVTSCSELTSALSNGGVIHIDGQL
TGCDIMNVNSDTTILGVGSNSGLTDSGFRIKKADNVIIRNLAMKNPPKGMDLIDIETSTNIWIDHNEFSAEGITGDKDFYDGL
LDAKRGSDFLTFSWNKFSDHWKASLIGHSDSNGDQDTGKLHVTYHHNYWSNINSRAPSIRFGTAHIYSSCYEDLPTSGVNSRM
GAQVLVETTAFVNVKRPIVTNLDSKEDGFAIEKDNLFENSEAQITQEKDFVPPYDYTTDSADCICEYLKENAGTGVINA
FUN_013638 CDS scaffold_6 255965 258518 -
hypothetical protein PF14295 ENOG41

MRSVSALVALAFASSFELVAASNCKPSSPAGAKTTDSASVTGGTATKSAPSPETTDGPVVITNAVTNGDFGGYDPSSDGGIYA
FQAGGNAKLLQGPGYQGDHTEERNCVQLKTNSGTPGQKRDFVSENPWIQQQLDNLEPSDFTVRFWYSIVNNAIADTCRIEGLY
GNVQFGATPYFPVVAEGNNWLEFVDSMPVTTTSGMIRFELNCIDGGSAEVYFDQVFVSNKIGDEWVDGISLFFPTSKHAATIA
PAQTSTAPAVDTATTSSDSTESSGLPATQSSQAQTTTDEASATDKTVESTSVSASAPTSAPTNPTASGPKLCAKLGTGAAGRG
CAKRPYSSTKGYKGYGGSKITKEQCAALCLSDTNCQSFEWLYQGSGCANTCNLLATNWADVPTNGGLDAAWAYDRSCIQEDEC
AEQPAGSLCVNVNADTPAKSCTQVKGKAKSCAKPFLTASTRGVCGLSNECRDLCAKYPSCKSYAATFGSCSLYDARSSEVAEA
GSELFWFTDMDCHACGQGNAYFDYISIGQDPANMPQWTCAAEDKTTSTMAVPTTTNPAIATTSDVESTTSASQASDKADSSTI
IPTTTTAPPSTTAALCANGVPSPGICSAANVVPSTATCGKRGWPRNVEPYGRSLADFPNQNTFEDCALICKLDRSCKAFAIDP
TRSGMRCMFSSDPTIDGVSNAPGVWSNLECMSCTLCGDAVTSSQPVSEAPTTMITSTRPAGEDSSSTQAATSASGEPTTTQTT
QAEPTDCAVPSASLSDDVTCGIPGQSGQQAQSARLLNYVIKPVGSLANCAAVCLQRADCQGFAYIKSNTECYPFRVGPASLGI
TYNPQGTEKYYDRGCFSCAS
FUN_013639 CDS scaffold_6 260067 261653 -
hypothetical protein ENOG41

MKFQFIDNDSTQKPGARKTIRSHVMKGKNLGRTIQGRGKRHAAPQLNDLYKGPDQSKKDFAKSLGGAVDNDGNALLFPEIADS
DTDRDTDTDTTYSINNPYSGKEYTYFSFPVQFTPSMRYYVYEFHTAICNIIYPYAFCRPTDEIGSPWFRYMVADQACLHCLLA
MAATYLNVLRNIPTPSLESIEATRHFSQALRLMNPRLSQPASAQKDSNIAIIISLAIHSNITQNLTASTMHLQGLENLLTFRP
GGISDIRECNRALLHKICRTDIELAVAQGTRTRFGNAGLTAAAAGPISARNPPYPLNQMSELLRQITRELLALCRRPGKAKMS
GLQYQDVLISMWQRLVDFAPLSGTRPQDLMDDVWQLALLAFLVTVTWSASYLKSVHCKLLHKLLREHIKSRALLAKGEEYRPL
WFWIVFVYGLSLTNGDCDTLVLGETREASNLLEIRIWEEARAKVKPFPWIGIVHNVPGRALWEAAVVTVPPQPSTVID
FUN_013640 CDS scaffold_6 262039 264903 -
hypothetical protein EOG09262A8G PF00764

MAPERVCLAYSGGLDTSTILKWLVLQGYEVVCFLGDCGQEEDFDAVKAKALKLGAEKMIIENVQQELIDDLVWPAIQCNAIYE
GQYLLGTSLARPVLARAMVQVAKDNNCTILSHGCTGKGNDQVRFELAWKACDPKMKVLAPWRIPAFFNRFQGRADLLKFAEEQ
KIPVSSTPKAPWSMDDNIIHCSYEAGILEQTDKEPPKDMWKRTVDPLDAPDKPTRFTVHFAEGVPVKLEVDGKTVTGSLEIFK
EANELGRANGIGREDIVESRFIGLKSRGCYDTPGLTILRKLHQNLEGLVMDSKVRIIRDRLSDDWAQCIYNGMYFTPEREFVQ
QAISFSQKQVDGKVEALAYKGNVIIVGRSSETSNLYSEEESSMDTLDMDWSVEDTTGFINVNAIRIAKYGERKIRDGEPLSKQ
ASGRQRKIGENGMVYVVLGHHEYEKQ

FUN_013641 CDS scaffold_6 271708 273430 +
hypothetical protein PF07690 ENOG41

MLHQHENKVTSGQVDSSSTEKVDYEKAVSFHEDASQEVFDEPATKRLLRKMDIKLLPFLALLYLLSFLDRTNIGNAKLAGLEE
ALGMTGKWDYNIAVAVFFPFYVAAEIPSNMAMKRFRPSIWIPSIMIAWAIVTTLMGITHNFGGFLATRMALGLAEGGLFPGIT
YYITLWYKRHECGLRMAIFFSAATAAGAFGGLFARGVMEMNGLGGLDGWAWIFIIEGLITFVFAIMAFFVMHDYPDTAKFLND
SERKEVLRRLEEDRSVLSDAFNMKFAKDALKDWKIWVHMMITIGIYTPLYSISIFLPTIVKSLGYTNEMAQLMSVPPYVVACL
FTIGGGFAADRHGQRGIYMIFFCVVAIIGFIILATVDSHGAKYFAAFLITCGIYPNVPQGVAWNGNNIGGSLKRGVGIAMHVG
FGNLGGAVSGFVYRKDDAPDYRSGHWTLVATTTMSCILSILMTIYLRRENARRDQTHKAPHEYTRAEKELEQDSGDNATFFRY
TV
FUN_013642 CDS scaffold_6 275098 283126 -
hypothetical protein
CE8
MARLSAWLLLPLVCGSSLAKEGSVVPAFVDSSSSHSTRFDSLHDTPLNGNVPVAPGGSSSFTPQVNPLVKRSSACKPRQPQGG
IGALILTASGTSSHGTTVASETAYSEASTASKSETTSIIGSVSLSEASSTIEITLTSGTSSGLASETSLSSAGGDTHFPSTAV
TAHDVSTSTLSNADAASTDAIGAKSATAAADRTTTDLTTVNPTTTDTVSASSVESSAGSDSTTGSVTSNVDTTLTSSVDSSAT
SDIATGSTTAGKTSASGNLASSSESSAGSDTTSGSATSDASTTLAASLESSATSEVSSGSLTASETSASGDLTSSSESSATLT
SSETSETLTSSGTQTSEASHSLTTEFPSSFTSSQQTSESVVSTSTDSPLVLTVAADGSGDYTAINDAIKAAQTSGYPTVTVLA
GTYIENVVIQATPTVTIVGEVSKKRDVSSPPQVVINNGGNANPALSFSTASAAGITWKNIKFSNTQAGGSGGAVFLRGSKNAF
YNCQFYAAGIAAITGSYASAIVANSYIEAPDKVLYNYPSLYLYGTTVTATNSNGLIVYNKGALAGSTLYNSTVVFDTCDISQK
SGSTNTRVFLAAGNGDGSVAVFRDTSIGGFITATGVYVDTKTQSKLNSYIEFGTTGDGSYSNHVADRSKYVTLVTDPKDLSPY
EISAFFKSAYPSVAVSSLDWIDQGVLSAIQASNAKDFKQGVETTTSATPTTSEASSETSESATTTSDSKKTTSESLTKAADSV
TTASDASSETKSATSTTEESTSAAPTTSSCSLPSAVPSTAFVVGPKSNPCASYDTVASAVAALPADDTTQYIYILAGTYQGQV
SVVRTGATVFRGETTDGSSSSNNKVTITARNAVLSSSGGSSGTATFAANKYEAKLVSFYNINFENSFDPTTNNIALAVYAKGT
KAAFYGCDIDSSQGTLYLDYGNFFFSHSRISGTTDFVWGQGAGYIYNSVIVSRGSTTGQAIAAHKYQGSYGGSRFVFDLCAVV
PESSSVPEASTYLGRDYSTKSNVAFVNSFLDGHIAGAGWKIASPTTFTGTFTEGNNTGPGWDSSARISAATIISDTSSFSAAG
ILGSDAWIDKAAIAPFKGWPGSVYGKDAASTTTTSAATTASSTTSATAAGATLTVAPSPTGNQYKTVASALAAIPNDGDDYTI
FVKAGSYNEQLTFTRSSGHVTIRGETSFENDFTQNEVLIWFKLGYSTGASRNEETPVLFWKTTSASAGLSLYNLNFTNTYPQT
SDTAALAADFFGNMAAYGCAFDGFQDTLLVNQGIQVFSNCYIAGSVDFIWGYSKAFFHQCYIASNTANAYITANNRKSSSWAG
GFIFDKCKVTYTDSYGSNFGTTSLGRPWSQYALVVYMNSFLDKHISPQGWATWSSSNPQTSNVLFGEFNNTGPGNWTSSRASF
ATKLTESQAAAYSLGSFIGSTSWLDMKAYSLVPSYAIDGSDTATDPETDDTWPVHPSDGTTPPKNAVTVSVGGEKSGSYSTLT
DALKSLPKDSTPQTIFIYAGTYEEQVPAINRPGPVTIIGYTESDPGKAYTSNQVTITQAKGLSVAGTIPAGRSNADTATIATA
STKIAFYNVKFVNSDNLDGATPSYVTLAASIYGNDIGFYGCSFIGWQDTLLNGATNGYQYYESCYIEGAIDFIWGYSKAYFKG
CTLAAKRAKSAITAQSRSSASAIGGYIFDQCLFTEASSATVDLQGQVYLGRPYSAYSLVVIKNSYLDSIIQPAGWKIWSTTDP
RTDHITFAEYNNEGPGSWEKNTAAREAFGFATLLTSDTYSLESVMDSTDWIDKTYWDSITTPQPSIVTPTPVPTAPYSGKTPP
AGAYIVSKDPIDGVTTYDTIQAAIDALPLSTKVTPTVFIYPGTYKEQIVLSRPGTTFFIGYSESPEDSSKNQVTITYDKGIDT
QAEASNSDSATFYATGNYFQAVNINFANTFGTTANYASLGFAVKSSKFASLYGCQVYGNQDALLINGNLFAYNSIVTGNIDMI
WGSGAGYFLKSTISPNRDGIALTANKKGTNLNSYGFVFDQCTVTPAGGATYSSIYLGRPWDQYARVAYIESELGSCVAAAGWQ
AWTKTDPRTANVIFGEFDNSGPGSSTSQRASFAKQLSGEDVAQFELSTFFTSTAWINMTLVDATPFDAGIAATQTFSSTTVYT
TQTVTTSQTALVTTTGAAVTITEKSTSTLDVGTTITPDPTTKTNTEKSTTTQTQQITQGDSVITVKSTNVVDVGKTVTPADVT
KTSTNVVAATVTSWITTSAKAVTTTVSTTAVTVTPKSVTQEETDFTTVFATSTSSPKAVTVKSTTTTTIGTGGTTTTSLKATT
VVSTVVTTSVKTVSKTTTLKCIPTQDKRDLGAAAVLKPRAAIVFGNPSFDLSSGLTRRDLDLGSINLEARAEALRTVTVTVVS
TYTTNVKTSTATVPGSTTTAELITTKTVGKTSTLKPVTVVEVSTSVATRFATVTIAGSTSTTTEVITKESGKTTTLKADTTTV
VVDATSVVTEKKTTTLPATTVTVYKTLTQTLAAGTVKVTSTNTVKKTSTVTLDQKTVTSWATAKTTLLPSSTVTERSTVSKVT
TSVVKSTKTNWATKTSKGAAACTN
FUN_013643 CDS scaffold_6 284715 286427 -
hypothetical protein PF07690 ENOG41

MGVKEDSKDLSRVQSREKEGETAIVSEKYADVTLRLVEEHGDQFGPLTPEKEKKLRRKLYWHVMGLLSAINLLLFIDKSTLGY
AAILGLFEETGINKAQYNNLGTFFYVGYLAAQWPGHYLMQRLPFGKFVAGLVFMWGATILLHCVATKYAGLVVLRIALGAAES

VVVPAMEMTIGMFFNRHEQSFLQPFLWVTSALAPVCAAFISYGLLWSHSAVLPWKLFMIVTGGLSVVLSVFVWFFYPNNPAEA
RFLTLEEKVHSIKRVHESSQSSIEQKQFKKTQFQETLRDPVSWLFALQAFTLMMSNNLTYGQQNLITTALGVDALGSTLVAAA
GGGFGVAICLAGTFALKWWPSNLALHGLVWCIPAVAGGTGMVAINWNEKLGMLACLLLAGHTYGNTYIIALGWATSSAAGYTK
KLTRNVMFMIGYSVANLISPQIWVPKDAPRYYGAWISMILISWVGTPAILFVIQFILKKRNKERKEWAAGLSEEERLAHEFGE
VEQLDENGQVVRRKVEIALLDLTDLENKFYIYPI
FUN_013644 CDS scaffold_6 287305 288961 -
hypothetical protein PF07690 ENOG41

MSTKLSETNFAPADGKETLELSPSKNTDREEGFSSEVNDPSYDTAQKSLVRKLDMTLMPMTGTILRKNLSRVLGAVLIYVASQ
AKLDSLEKDLVLSGNDYSTALAILNVGYMLMQIPSNMILTRVRPSIYIPVWVCLWSAVSAATAACNSFTHLIIIRFFLGICEA
PFFPGVFFLLSCWYTKKELALRYAFLYSGLVLATAVSGLLAAGIFAGLGGVAGLQGWRWLFILEGAAIFLLGLVAFVMLPDFP
ATKSQDWLFTAEEKEVAVTRMVRDAVSEEEHDQSVLHGLKLAVTDIKVWAFALLLLSNQSAYGFNYFYPAIVRGFNLGSRTIT
LVCTAPPYLLGAFISYFIAWHSDRKAERGWHISGAILFAIVGFIISAATINIPARWAASSVGQTPTKKACATAIINVTGQLGT
IWSPYFFDSNDESRYTRAMVLLLAFAVLEICVCFLLKFILRRANKKIIAEYEGTGRVPTLFTL
FUN_013645 CDS scaffold_6 289813 290336 -
hypothetical protein PF01042

MSLRTGVLTTDAPAPSPHLSQAIIHNGTVYCSGSFGMDPQTRQLAEGPYHQTAGALKNLDAILNKAGTSLHNALKVTIFILNM
DHYAEVNKAYLEFFTSDPKPSRTCVAVAQLPLKGAHVEMEAIAAIPEKSSKL
FUN_013646 CDS scaffold_6 290988 292240 +
hypothetical protein PF01266

MSRQGETIVIVGAGVIGLSTALRVQEQTLSQNNPPSIVIVARDFPNETSINYATPWAGAHYRPCPGNTPQLLQEAVWAKKTYD
TLDSWSEKDKLAAGVEFMPGEEFFESPTPEYVDVTKDVSQSAYAHLESSFELFSGSELSAMSDSLQLGFRYRTYSLNSPLYAS
FLQRRFQNRGGRVRQYTLASLEEVFSIEASVSVVINCSGMGFGDPKAFPIRGQTCLVRNPISKTVTRQNSDGSWSFAIPRPLE
GGTIIGGTKEPHNWNPYPSAETRQTLLNNAAKWFPFDSEGYLSSNTRDSDRFDVVRDIVGRRPAREGGLRLELERLKPGVVVH
AYGVGGRGLELSWGIADEVVTLLHKQSYFFLRAHL
FUN_013647 CDS scaffold_6 292452 294090 -
hypothetical protein PF00141 ENOG41
AA2
MKGSATLAFALVQFSAASQLVWPSKWDEVEDLLYMQGGFNKRGFADALRTCEFGSNVPGTQNTAEWLRTAFHDAITHDAKAGT
GGLDASIYWESSRPENPGKAFNNTFGFFSGFHNPRATASDLTALGTVLAVGACNGPRIPFRAGRIDAYKAGPAGVPEPSTNLK
DTFAAFTKAGFTKEEMTAMVACGHAIGGVHSVDFPEIVGIKADPNNDTNVPFQKDVSSFHNGIVTEYLAGTSKNPLVASKNGT
FHSDKRIFDNDKATMKKLSTKAGFNSMCADILTRMIDTVPKSVQLTPVLEAYDVRPYITELSLNKNKIHFTGSVRVRITNNIR
DNNDLAINLIYVGRDGKKVTVPTQQVTFQGGTSFGAGEVFANFEFDTTMDAKDGITKFFIQEIKPSTKATVTHDNQKTGGYKV
DDTVLYQLQQSCAVLEKLPNAPLVVTAMVRDARAKDALTLRVAHKKPVKGSIVPRFQTAITNFKATGKKSSGYTGFQAKTMFE
EQSTYFDIVLGGSPASGVQFLTSQAMPSQCS
FUN_013648 CDS scaffold_6 295074 300727 -
hypothetical protein PF00023;PF12796;PF13606;PF13637;PF13857

MEQEERSLDAMETSSNTMDNDSPSADLGLSIDTTEEYEASEKKFDIVAIHGLVGPGGKPWGSSKTNTWIQGLAAQMNWEVRLI
QYAYDATKITEATYPEEAISSEASTLFQKLSELRTDQQTPLPIAFFGHDIGGIIAKKALILASRYDSVHTDISYSTKLLMFFG
CPHRTLDTHQDIEILATRLDVLKPADSTATSLNMKCLARSIIDVNNSFLNTRVLTRANVINIISSKTDPAEEVFDKYTAALGV
PMEKVSLIDMSHNELNEEPEGYAPYEGFQTKRISSFPWQNDKLSRVMEAIMLQAPPIYPFKFNTTRGFGWITEHDTMIKWLKS
WGLALLHIHGTSNISNVAQHVYEGIVDRGKSTTFRAILYFKFDKYDIRRNSIGAMANSFLLQILSQIRESPNDKMPDIEPPDF
SNCWTDKDAFFFLEKLMRDITTHGEIHWIIDGLDQCDDSSGLFLSMVSDMAKNSEQHFKILVTTVDDSQIRGALSDFPSIDLR
EHPPTADVIESIVLEFMTELCHVRPQYRHVEQEIRQLLGSCGDDDLLRRFLLEWLLVAPCVSTKSALERELTSLAPLSPAKIF
ERCLQAVPENSRPWARKVLMWMVLSFRPLTPEELGSALSLGTSTDPGPDQETYGDLMWDIENCLGPMIILENGHVQFRHPAAR
DFLMTSHDASDRNQPWYAWNSSEEGHREITDACVGYLTLSSAQDKILAACRTCPVDQRIFEYPSDITPYAVEFWPLHYQRGHS
SVSPRIESSNIASFFNDETAPRRWAAARWYLSNPHIRSDRVFLSHLPIMASIGDDDSVKFRIESQEPDECDISQALIEASRYG

HSDVVKLLLKKSMISEDICLEAILAAGRSAELNTLGILLAYATENLHIAKWPGTLASRLAYLGSSEHLKTILQAGADANSTDP
DCSPPLHCAIVRDETAAFDTLIEHGADPAFINSNWVDTPPIVVVGKYGRAKIVDRLAKASLVEAQDHKGRNALWYAGGRGHHV
VFQALVKAGANTEVLTANVRELRPALIPISGAPYPKCLKSVLDAGLDINSKLREAHSFHALGIAARSGHVKICRMLLDHGTCK
EFDDDSALIHATRANNIEIVNMLLKEGHKVDVQLDDSPDHGTPLVAAARAGYQDIVTALLQKGASVNYAAPKEITPLNSAVLG
GQPTIAKTFIDAGADMNAVSDIGRSAIHSSYRRPECMKVFIDAGADINLPSSDGTSFYLASFFNQTNVVELLLEHNPDLETRC
PDGGFIDAGYTPLHCAASFGYNELLRLLLEKGADIEAKTPKGGTPLILAVATNNEESVKILLEYKLDIDSRDIGEGSVMFCLP
NPANLSVVRRLINRGASLTIRNNEKYTPLGRAVVNGDLPLVRMLSSQKVDLNAVAAENGTALHIATEKCNVDIVKVLVAKGAS
PDVANTWMYGSPLQRLFEKYYTSAENKDVIARYLINDAGADVNVHGGNMGSVLNAAILTGSIDTIKLILEKGADIGWQDLHGR
RPIHYAALKTAEHFRLLLESSGDDEERLGITTKTKSGLTVLHFAVATEKSDLVELVLSHTNGIISINEPDDDGWTPLLWACRL
CESWGTPKEISPEIVKLLLDRGADPSVRGRTSDDREWSPLKMARFHGASQEVIELLTASPSKKKEDDWKSKFHVSKKAARTTG
FCDLCLRHVYGILYFCNGCELSYSLCFKCYRYREEIHPYHDKWEERGPEFVDDDEEEEEKDEEEDDPKSSPGIEQVQDDDSEG
DWSNDEAEGDERAKGESK
FUN_013649 CDS scaffold_6 301459 302478 -
hypothetical protein ENOG41

MYLSTQILPIGVNAHTMSTDPDYDVDAEEEISFGIALAPKGEEGSEFRTQNDPLDLYQRENIIERKGAVDIRCSSVDIIHGLF
DPDGDDLCTLIVLEFRFDPRKRARRIAHVDIELRFSASEKGVADPQVFAIAPNGNLRFAQTTQTETTTTGGDVTLGAGAAGVE
LGSGLKYEREIAREMSYAASVTGSIDLRGRNYGKPNCASWTLLENPETKTGVPVSMRTAILLKRRDEGRFQCVVTVNAKADWR
TSMEWLVGTTKRDDPVLLDPTLAPTSDRYKDMEMKLGEIDLNAVCDITSANVIENSVKTSKIGNQSS
FUN_013650 CDS scaffold_6 302679 305910 +
hypothetical protein PF00023;PF00082 ENOG41
S08A
MTKVAFTTDVRGVVSDDDDKDYENDDFEEEPAKIPNHDEVLFAFESITNEARNMKLNLGRRENRESFLQKYGQFLSKSTKDSN
QTLLHMIANTVGHKSLTRCVIKFDKTLLYRVDDSSKTPLHTAIAKKNHAFIEVVLDEKKSSDDLDSLLRITCEHRRNCIHTAI
YHNLQHQYTIKLIKRASEETLRAPDQSGLTPLHLAVDYKYASEAQLKIVEALLAHGDSALDKYTKEPQNLSVYEYQDHTQRLW
RKQFEVPASARIGERKQVESREDQGQDGEHAGSRPKGGLEHVSGRSRISQQPGKDGHGGLHRGPDNFHARGSEQTDVSSSIHT
SNANGLHGAENGRTPVEAPEGFKPIQRRQTGMENVEKQAQEEKRKSEYAEKIRQAVKLHYLRTTLTINSKPASRDQLKAVKFL
HGANLNNINLCFDYSEAPPEIYEDSFKQSYDHINFDQVLRYVSFRRIELQKPPVSAVKARLTRNRTQTSNKGRGRDGLTVFFN
WLKGKGVKHILKVMVDDLKDPSHSDKALEDCLRPFEVEILDWTKVDLCPETILTACRNVRQLYLRWSGNRAILRAWSEPEGLA
KLEKLEAVHLVYSEEQALESADRITMYANDFEERLNESAAANPKRTLSNDEDDTGGVRRILVFRCKADVPGSERIVREGAFAK
AQSGINEMSLQSNRWLNCMDKFADELQNTCSEFVKPQNGIKVALIDDGADPYVESLRGKIWGGETFSRGFPHENGPSPYYRST
KGHGTVMADMICRVCPMARLYVYKLETHTSLNLATQTHGKEYIAAESAALAVRAAIDQKVDIISMSWTVKETVENRDGVNTFR
QAIKDALDAGILLFCAAADTGAIAEVEYPWSFDQRRIFRIGAATADGRVWGPTGNPQSLSFILPGHKVVSRNPHREGALPDDF
EERTGSSVATALAAGLAALILHCVNLAVVHGKEHPSTTAVSAEDLERLANHDDMYNVLRGIGLDEGQQRFIEVWRRFDRPAKE
LKEPMSDKMDALGIVARLARDLVPSGSS
FUN_013651 CDS scaffold_6 306163 307693 -
hypothetical protein ENOG41

MPLFKNFRSGAKVVDQAVNLDPHTWTLPSSYKPAQPDGKQTIIPDPRVFSNVFSIPCETEAQSIETLLAYPDASHAAVHLALL
ECFRNLRAGAKALDVEVIQPPSYDETKSPVPASPSEPTQLPASQKWDLLIKLAVTRFTTWWSGIELLLNHANAYSHHGGSRAA
LQLTKDYLPPLDILLVWYALMLNSEAYDAACDAQGANATRLKSICFPWPAIRDVIDMDKMQLVLPRSAQKLFTNITSQPCDIL
EYIQSPPAYADTGKVRIETDLFSEVKRHEDIMEESHKLLWIRSPALQGSLIRAGLEYLDFHLREPNAVEEEMVCHQSFGVRLS
WRTHRLFPRQYKAFLKEIGGIQSEPTQGQDLKRDAKILFDMDDPSPIQGHCHCWTCERIRDDIPEFVYTKPSATASSSTSLTT
MQKQISSLSAESLLQIQDDLGFYLAVEDARRSGLPLPTRPPTTTEKEADKIAKQKQKELGYRPGLNEYVEVLPDGRRKIRTHK
YASGMWGTTWV
FUN_013652 CDS scaffold_6 308289 309102 -
hypothetical protein ENOG41

MTVKSRLQQPVRVAILDCDYVVPKVAETWGPTYSSIFAHRLQAVNKTLGSDKILEISAFDIIKDEYPNPNDFDAFLITGSIKG
VYDKDTWIARLKSFIQETYQYYQHVRLFGACFGHQIISDALLERYGVIVEKDPKGYEVGIHKVALNPEFAAHFSHVLSLPDGD

GLRMQFAHGDHVRFKTSWPESWMSIGSTPHCTVQGIYQPGHVLTFQGHFEFDEEISTETIKYFFTPERGFMPEQTQAALDQIR
GKDDSEEAAKVLHAFLTGSND
FUN_013653 CDS scaffold_6 309400 310331 -
hypothetical protein PF00106;PF08659;PF13561 ENOG41

MASFDGKTIAITGAASGIGLAVAKLLASRRAQLSLADMNKAGLEAAIKSLPGDGHIITQVDVRDSQEVNAWIEKTVSVFGKLN
GAVNMAGVFTHGTCLRDETDDKWDFIMGVNARGVFNCLRAELHHIKSGGGIVSAASVDGQAGFANASVYCASKHAVIGMSRSA
AKENENIRINCVAPGSVRTPMMEGEGMAKAVEAEVALQVQKRPAEPHEIANVIAFLLGEEASFVTGAVYNVDGGWIC
FUN_013654 CDS scaffold_6 311147 312330 -
hypothetical protein PF03407 ENOG41

MVKTSAGDANDDVLHRKPTVSPSNPPSPPPPAPQTTDTFSANCHKKSRPEAYKQYGNGTESLALPKLMVELWKPLVHPITERV
FVTDKGERFEVPIDQVRWKKPLGKRVVIVDTDTRLDGKTENTMLNECPLDFTTLPGRTGGHLNHYIYAMIHGYDYRLVRAADY
PDRHGTWVKPAITKEALKTHDFVISLDSDAVFTHLDLPLEWLMNLWDYRPETLVAMAYDLDWEQDYDPQGNLILNTGFVIAQA
SQRTQDMFQRWEDCPRSIPGCDHWNFKWAHEQSAFSHYIRYEFNRTHDVKNIPCNQANGNEFTLDGNGECKGVFVSHNWKHKH
KTPELLSRSIMTSLARRVHGQFHHDIKDLYIDASKETYPIADGAT
FUN_013655 CDS scaffold_6 315664 316791 +
hypothetical protein PF00043;PF13409;PF13410;PF14497

MGDLPDEIRLKPNKDGSFIRPDSAFRSFVSQEPDSQFPAEKDRYALYLSPGCPWSHRAMIVRSLKKLENIIDLYICSLTMGKD
GWFFDDTPEAAKYDVLPKDPLYELETLKQLYLKANPNYEGRYTVPILWDKKTHTMVNNESSDIIRMLYTEFDHLLPEEDRESH
NPGRELYPERLRDKIDEINEWVYDTVNNGVYKTGFATSQAAYEENVVKVFKSLDRLEKILDNGPFLLGKTITEADIRLFPTIL
RFDVGYVPIFMCNLGTIRDHYPNLHLWLRRLYWDNSFRTHGAFRKTSEPWLEKYKTGYANARRRVLGITGPDIVPKGPLVLIH
ELEEGERLSA
FUN_013656 CDS scaffold_6 316927 318900 -
hypothetical protein PF00172;PF04082 ENOG41

MNPATGKQELSCKRCRYRKLRCTRTVPCINCATAGAGCEYEKIDKRRNPPSSEYTISLRNRVASLEAFIQELRDAPPPRRDEM
LGPAAYTQGAPNIPAAGPSQVIQNDTTARELLKPDAEGSLVYHGATSIFNSELLSTSESYPSPTQSLYAESNFDHVMDHFGIK
LEDDTVFKALQQFFRWQYPHFMFVYREAFLRDHFGERKGCKYWSSALLLSICALGLLMSETEGERNLSEQFFQAAESIVMVSG
LSRPSIPTVQSFLCLAFFEIGRGNVSKGWAFSGIAFRMAQDLGFQSDPMNWLPHDSTIISSEDIEIRRRIYWGSYISDKLISL
ILGRSVQLAFDSAEVDLLEFITDGPGMEYWRPVGFGDDPEELRVLSNIPYLKEQIRLYRIVEKMMTTVFSPRTPRTQTDVFTR
QTLLENLNLELLQWRDALPAFAKWNKWLTSVTITPAVATLHLLYSSIRIALNYESATTSNVASAREAALLSCTTAAQDILALV
RVYKMQYGLRHAPLILIYASIQASRSIEKFGIPEEQSYLVGCLAECSHTWSIAGQVQGPVPAVNVDAYHELV
FUN_013657 CDS scaffold_6 319614 320898 -
hypothetical protein ENOG41

MALAVTDPFIKLPPELRIQVLVSTRCKSSISRIIQASPVMLLQYLSHKKSILRQVLAAEFDAEMIQDAMAILSFPSHRGSGDS
PRHKHLSELIRAHLRAWSNSQLPDPLKGKDDHMVGQLNKLHGRILLFVEDYITKATASFPPQEYLCLPQIQPPSTEGHLMFKG
LKVTPRFSSANLTSSERKRFVKAFLVYDLLCKTSNVTRIPSKLRLRKVSNAEYEAVGCVHSYVCSLYGSMLAQCNDAYLPTAL
APPSLQTQLFFPIPDALYFDANVYAPNLRLLSRDLGDDIGAGFSTLGLDRLTNFLRYDMAKPDERKALDKKLKDVWKFGAPSI
SSTWDMHFVLLLASKQKYKNGCESSLYNQLSLKPKEKLRYKIGQQRAWVFFDDSRFYPQESTERPTFPSERFLAEQPAKKTFI
NDWFDNLRNEKD
FUN_013658 CDS scaffold_6 321790 322397 +
hypothetical protein ENOG41

MNAADQAHLAAMGLAIPSSEASALPLPLTRRQAIRDRATALRVHTLRGSVTPARYLLRHDGMRIAGKRRFGDARRQHKLMQKW
QNDEAEAYRRRVAALVDGPNPVWTFRGHLQWLPGFLASGEVPLQERKRVANHAEDTAHFAIEQAEKHHRLLKKWVWGYNTLAS
RRQHEGYARLARRAAIH
FUN_013659 CDS scaffold_6 332220 333607 +

hypothetical protein ENOG41

MATATHYTPDAGVMSSQIITYSVATALLVCRLISRRLTHVALWWDDFFAMLSWTSASLYFGFTIYWAVKMGLGHVKADVPLPA
ERVEEYARFGLFIAEFTYAMSLGFSKLAILGFYWRLFSVSNIRMGIRILQGCTVIWLTIRTFMTIFHCIPVEAYWNHNIKNAV
CNIDPAKFMFGTTLVHLMLEIAVLSLPVFQVKSLKLRPGQKVAVVALFMFGIFVCIASIIVLIEAINLNPKTTEMARDIRGVI
IWAGTEAYLAIISSCLPIIRPIFRKVISSSILSSKSNSTGPNPISGLTSSRGIKLTNVTRTKEIDDNSSQRQLAELEDGSSDD
MNFHGYPERVANHNTVITSCADERPNSHAHNTSVQGIQVKNETRIYYE
FUN_013660 CDS scaffold_6 334344 335307 +
hypothetical protein ENOG41

MDRLSTELLSMIAAEAAASGPQIDINTVFDVWDSDDTLKIPRTLSRDNSLAPFAAVSHNWQLAFEPFTFHTLVISPKRLVEAA
QHGYLTQRRLGYVRFIAVLIAFPLPRPWDTPIVFSPELDIRKDLFRARGDTGDVSSEDEVEGVEEDDVDGDGDYITSVIDFPH
PRDRGYDRVFAKIIRILFNTLKLAPVHENHQPYIDIRLGFPVPREYGLSRISGPEEMEANTLEGPWLTTVYLSMNHDGEELPE
LPSIASCSFELVSWSLCFEPHTACIIGSKMPCLKKLKLHLSDRELKDQGLRNELRNSKVSMGYTPESLTVL
FUN_013661 CDS scaffold_6 336603 337620 -
hypothetical protein ENOG41

MAAESGETGQTGPIVASLLVNMVIAGLFAIACYNCLEILISLLNRFKRHDGLYFWSMVVATLGILLHSIVVILRYYSLGPNFL
LCVLTCVGWYGMVTGQSVVLYSRLHLIVEDKSKTRWVLYMIIINFFILHVPTTVLFLGSNTKHSAHFIRAFNVYERIQLAGFC
IQETIISGLYIWETSRGLKPIFAVRKKMERKIMRYLIIVNILVILLDISLILTQYMSHFNIQTTYKPVVYSIKLKMEFVVLNK
LLLLVQHSDCNCMHVVGEPETPPPPDPSHIECQSIERNSSTRTGSDVPIQMNVAPIQMNHVQQYDTNASSQSGGRHQKANPLH
FLDYCK
FUN_013662 CDS scaffold_6 339431 341312 -
hypothetical protein PF00067

MFEISTFFSGVALGFLLYISIGVIRNGSKRPPLPPGPKGLPLLGNLSHLPPPGVFEAHHWLKFKDLYGPISSITVMGQTIIII
NNWKLASQLLDKRSAKHSSRPKIMMAGEMVGWENSLGFSPYNDRFRTYRKNMARIIGSKRAAAKYDRLQEAEVAHFLLHVLDD
PERFMDHIRKEAGSVILKIAYGYTAEPSKEDILIKMAGQAMDDVTAAGVPGAFLVDILPLLRYVPDWVPGANFKRLAKKWSSE
LDDLTEKPYAFVKHQHASGKQDNSFVSRLLEVGDSTEEARFTTKWSALSLYAAGADTTVSSISLFFLAMILYPDVQKKAQEEI
DGVISNERLPNCSDRQSLPYVNAIVKEVLRWHPVAPMGLPHTSTVDDVFEGYFVPKDALIMPNIWYFAHDPEVHHEPMRFNPE
RFLSTDGNQPEQDPHMYTFGFGRRVCPGRVLADNALFLNIAQSLAVLDIRKDENGAQPELLFTSGMISHPEPFKAAIAPRSPH
HEQLIRSLEQEYPWEQSDGHIMQRQAS
FUN_013663 CDS scaffold_6 342169 343597 +
hypothetical protein PF12937 ENOG41

MSSHRTLNTLPSEIQLQIFSQITAHDLSTIARTSKRLNEIATPLLWTDIELHEDGYHESRHELKVPPPARDPARRPYHSKQEW
GNGQGARMKAAQFFEILQIMHKENPNRLQQITQRVRLFCSVIDPSWRPVDKDNDSVDTEAIQVWHLLPYFSNLETLELYGDYY
YSQDAEEQVSEILGSPLKLRFLKLSGYMPPEVPAWLLKARDTLERLELDMLDRPISTYLSNDPHFTPLPCEKINTDDGSVSES
DYGSLADDMIIPRPLGNTFTDYGDYKLNLPKLKHLFLGHPAESCYTNDFATYSWSKRAESACYDDWLRILEASMKTLETLVLE
QRPAADYIEGDGYSEDMWMDERAWSHASEILLEMVETVMSSETSQLCLKRVYLYGIVAGLEEDGTPFLDFPAGKFMQFLKDRG
VECEARRGKWCFFDRQSGSVDWCPWNAAYEDEEEEEEDMDEDKDEDAEPKAKWDTVLAKV
FUN_013664 CDS scaffold_6 343824 344312 +
hypothetical protein PF00903 ENOG41

MSSFAVVKDIDHLVLTCNDIPATIEWYIKYLGMKSETFTSATDPSSPPRSALKFGTHKINLHQRGKEFEPKARTALPGTADLC
FIIEDGTDLQGLIEGFEKEGIKVLEGGKVVGRTGAMGGIRSIYVRDPDGNLIELSQYST
FUN_013665 CDS scaffold_6 346339 347094 +
hypothetical protein ENOG41

MKATFFSVFALAISAIASPVPEVQKDTRSVEQVNGAIHSAENIVSKVGVKQIVDEATKSPAKRAAISNPQELITVLKSGVSNI

NKKTTGLNSIAEKVKAGDLTKDAGATKAIPGFESVHFELTEIVTKLTGAAGLDVADSDVDTVLNLVVVLVSEVLTAVKTIVTV
TGLRPQLISILHSVFQILTKVLVLVIGLVSAIVPGLVAGLTPLLAGLGNGVLAPVLLPITGLLASLAV
FUN_013666 CDS scaffold_6 347676 348639 +
hypothetical protein PF00491 ENOG41

MAASRSVSITYVPADCGSIIPGKSKAPQAFRDVGIVSKLRDAAVPSVSEHHALKAPATFSATTFSAGGARNEDINISVCEDVE
RTISKNFKSSSKQPEFQLVLGGECCMLPSILSAFWKHADSQSQPKRVGLIYIDADTDLTSPTDPSSIGTFAGMNMAHLVRLSG
ALPRMGQFSRSSGEPVCDASNTVFFGTNMSLPGNKPEHFKYLFDNNFKVVSSASVAKDPEQRARETLEFLQDKVDIIMVHLDV
DSIDPGTFPLANVPNFTGVEFENMMRALKVLLGSEKVGGLTVAEVNPDHDPGLKMTDRLTSHIVEMLAARK
FUN_013667 CDS scaffold_6 348982 349954 +
hypothetical protein PF00106;PF08659;PF13561 ENOG41

MGNTYSLFFPPSPQFTEEDLTAQNGRVFIVTGGYSGVGFELSRMLYQAGGSVYIAGRSQEKAESAIKQLKEQYPDASGSLSFL
KIALDDLDSIKVAAEDFKSRESRLDVLFNNAGVSNPPAGSVSAQGYELSMATNAIGPWYFTQLLVSILKETAKSQPPASVRVV
WTASIVTDLSVPKGGVKMEEVINPSKDQQVNYTNSKTGNWYLASLLAHQIGSHGVLSVCHNPGNLHSNLLRHMPSIIGYIVSP
LLYHAKFGAYTNLWSGLSPGLQIEDGGRFILPWGRFHPNPRPDLLENLKSKEQGGTGTAADFVEYCDKIIADYR
FUN_013668 CDS scaffold_6 350173 350629 -
hypothetical protein PF12680 ENOG41

MASSTKYTARSILDAFYEAERTFMASPPDQRDFSAMAATLSQEVILKETSGLPFAGEYTGHDGFLQWAKSMPEYFDEVHVQNP
EIFEKEGSNRIISLGYLHLKVRKTGEQLKYPLCQVIQVDLEAGVIKSIMPFYWDVHHLNKAIGYTPDL
FUN_013669 CDS scaffold_6 351662 355469 +
hypothetical protein PF00072;PF00512;PF02518 ENOG41

MSSSAELQREREVQGYFQSWQIAQGSSLKVARSVDSINAPSQVNDAHAPQPSSDKALTAFAQLAVFRLDVKRCMVSLIDSSHQ
YILAEATRNLRLGSLEEESSAKKTGDKKDPEIAEYNSDLWLGTSILSRPEAVCEHCLHNTCIGRDPDGKTYSASGLIIPDCRL
DDRFKSRVYVQSEPGVRFYAGVPIFSRKGYKVGAYAVSDERPRDGLSIEDVKFMQSVAQAITEHLEWARDRVDRFKGERIVRG
LATFIEGCSSDDPILSKEDQPQRPSIKRPPGMTITATRRSSLHNTMRKVDPDSNQRAQKPTSPPRKHKPQTDGLSRMYHRAAD
NLRSAILADGVVLFGATASNVQHLTRAVQIEQGGATNADGLPTQIAAEPDAFSTESSDSETSPLSRPCKLLAYSLADKSKPLD
LEQGPSFSIGTLERYFSLFPKGKTFTFTEEGAGLSSDDDSASDGSPAPAGGKSKRRKEKIDHKELLKKIPGAKAVVFLPLFDY
VEDKLVGGCFLWTSVPGRMMNLDEDLSYLRAFGNSITSQVGRINTRKNEAAKTTFIASMSHELRSPLHGILGAAEFLKDAVTD
SYEVGLVNSIATCGKTLLDTLNHVLDFSKINNLGSGQLRKGAKHNKIISLSSDSMESLNMTASVDLSVLVEEVVDAVATGHNF
KKLPRKQLSQDDAVLGGKDSQGAPEPGTDHPVSILLDISPRASWMVKTQPGALRRIVMNLFGNALKYTALGFVLVSVRAQEIS
GDSRIDVLIRVTDTGKGMSEDFQQNRLFVPFSQEDSFQPGTGLGLSIVKQIVDSLGGTLEVKSEQDKGTEIEVRLRLEPVPEE
NPKPEEPMASMVEQLRGRSLVLLEASEDFHSKTITKQVEVRNEALMEIFSSWFNMKAAREVDTDVPKADFYLYCEPPSAKTLE
KRFEETAIDGRRNKKAPIIIISLNEEEAAKISRAQAKALARLSDVVEVIPQPCGPRKLAQVFSRCLNRIGEVRDDDDNRNLIQ
SAQTDGKTPTEQDTPDRADATEQMNSKLHQDKGNKPDWSETTTSPLPRGKEEQGRTKHDSRNNGQNNEKKESNEDFRKEVNNK
SSQDTRKQKKPEMPSKAHVLLVDDNKINLNLLTMFMKKCGFSYEEAENGEEAVETFKKSTIGDAEPGTLVKKHFDYILMDISM
PVMNGVEATKRIRKLEAEYKVPRTTVFALTGLASADARLDAMSAGVDLFLPKPVKFAELKTMIENN
FUN_013670 CDS scaffold_6 357465 359057 -
hypothetical protein PF01565;PF08031 ENOG41
AA7
MMVSFLLICLQAALALAASTSPANTSTALNNCLASKKVPYIRPNSPQWTQEVRPYNLRLAYTPAAIALPTTVDHVQDAVKCGN
QYQVRVSAKSGGHSYGSFGYGGENGHLVIVLDSMDQVTLNKYMSCNIQAGARLGHVASELYKLGQRAIPHGSCPGVGIAGHAL
HGGYGFASRTYGLTLDTFIGATVILANGTRTYAADGEGGDNQLMWALRGAGSSYGIVVELDFQTIKAPDTVTPFSIELDWNEN
QAVEGLIAFQKFAVTAPKQLNMQIYIGPSGQTIQGVYYGTRVALNTALKPFLGDIKAQISASSTGGWIEGLKAYANGQNLDQR
RPYNQHSTFYSTSLMTKAITRSQVKSFVRTLFDNIKDSDARHSWYILIDLFGGPNSAITTAGSTNSAFPHRDKLLLFQFSDHG
NYASHANNGFTLLKRFRESITKTMDDSDWGMYANYLDTQLENREAVEQYYELSLGRLRELKRAYDRNDMFWNPQGIRPATRG
FUN_013671 CDS scaffold_6 361366 361770 +
hypothetical protein ENOG41

MFSKLFLFALPLVFAAPAVKRTEGDITFYTPGKGACAGTHGVDDMVAAVGANLYDTQDVCGKTITLQGDAGTVTLTVVDRCEA
CKDTDLDVSPAAFEQAIGPKDIGRGKGTWVFA
FUN_013672 CDS scaffold_6 362284 364979 -
hypothetical protein PF07989 ENOG41

MERPPSRSSYGRTASRSSTSTTNTRATMHTAHSALSSSQAPGSPNLSQSQHSHRLYHATCQRPTSTIPRTTTRTPRLTREASS
ESTRQTVISSFLQEKLQQSRMAESDRSTTWSRSTDVNSSGEFSRSLGSPIKNLEADGHRPNSAAGSESNKKKGLGVKDMEQVI
SSLHKKNFDLKLELYHRRERQTALEDKVEALEVEKSRAEEMNDHLSQELEKRDKAIEEAVAMIVTLEARLEQYAKERSIIEQI
EAEGQYAAPTFDPCYEDPRPATQSPDMMKQMGSGKAINRMPSFLSDHSETTENLRNVYLGTRGSILSLAQVPEGSNEPDNGRN
TALGSPSLSVLSESSFVSVYGSKEHDSSGMQDVDEPLALDGADSLPAKASTINRPKTAFVKSHASPNRSTRSNSLSTRQAQFD
SINNVIEYNSPARKSDRSYASKKVEERPQGRDKDGADVFTGRSAMPVNHRRTKEEKRESLRRVITDAPGGVRLHDQNLPPTPD
TISTSTLRRFQNSSDTLSKHPTMQRSHGALSETSENEGSMGMHSGVQLQEPMRAVKASAHIIDLNNRAYAENRGRSIQRPRSA
DETTISNRRGNDWDTDTDDSDVDSLDSSLDIWMRESAKPNKNGGRTSPDLFSFPSSATKGGWSMEAMYGAGGASIGTDSEMIR
NLFPVQQELFSASLPPPPPNRRSSLNAQTVAQLKISNPSRPPANPKASKSGRKKGHQRRNSDDIQMRAEMQAQAPPAGEQKKN
HYPPISGQPAQNKFAKFFRRSSTTAPSPTPTSETGPTEPPSNGLSSVNGASGLPPWISRTGVADEDRDSATPPPIMRSSRHGR
HASVDVGDDSIMSQEPGTPGTPRMDRDTSEQVDSPRAGGKRRWFGLKNKQG
FUN_013673 CDS scaffold_6 369096 370704 - YOS1 Protein
transport protein yos1 EOG09264C3V PF07915

MRRFNLFLLATVQLAGARSPGFSIHEDLLTYPQFEVVFDSQYISEKDAHSLLDSQHPTYSADFAQSTLDQAREADERDNEGDN
QGQNGPSHTYELMKLPPHEYLCSIPIIQPPEPENKTANELAKAEEARELTRATASGWELLSQLEDSCLYFMSGWWSYSFCNNR
EIVQFHALPSIPNGQPPKRDPNTMDFTLGKVPAIPANAAHQAKLNGIENPPPAELQVKGDQRYLVQRLEGGTICDLTGRERTI
EVQYHCVPGMKTDRIGWIKEVTICAYLMVVNTPRLCNDVAFLPPEETRANPISCKLIVGENDQTPLLDQTIPVKEAEAQEPLK
QEGEEAKPEEAKPQDAAKEPVNIGGVIVGARNVLSGADEAGKPPAKLSPPRSYFSNTDTDGERFIEVVASGKSKEDGAEVKLL
STEELERLDLKPSTVKDMTERMKKLAGKYGWKLELVELPGGEKELRGYIDADEDELAKNKAKLKKEKEALDKAKAEKKKAGKE
GEEETEGSQEEFFKKKDEL
FUN_013674 CDS scaffold_6 375929 377988 +
hypothetical protein ENOG41

MNLENANTVQELQRQLEAANARNSQLEQFVQSQFRGDPTVLNQVAQNLSLAQPGFQIPSHGLSSARSSGLSRSKSTITYPTQV
KSAITERHNGLQAHKRERRAFSQQSGSALPMSRSVSNRSESHMPFSQTGNRIAPLSASMDRFNSPQNESSNLPLEGVSDRLPS
VQENQPLLNIGMDPDEFLKQWPETAQVPQHYDTSYSGLPYNMVPDNSFNNLSSNIPSSYPMSSACPSMISGPSAAEAASPLTR
QNSSFDSYGVGMERLESSQSQADAFFAPDLSPSSVNMYPTGKQYNSEQDLFGLGANLSAVNMQYTDSNGNTLMTSPESTDMER
SCSNTSMSSVRSNASNLERRAKEARERVLHSGKTIQLAPKPKEEVAKAEAVAAANKEAKLPVNKSNYQRPKHPKVYCTQCTEH
PDGFRGDHELRRHVNAKHEGTVKKFVCRDPATVGIESNVKAVNPLSKCKACVSGKEYGAYYNAAAHLRRTHFKPKTPRGKKGA
NGDEKRGGKGGGDWPPMNDLKAWFVEKKVKVDLNDSPMPEQDDVDEMGDDNMDGSGMPPQMDMFQGIGSSMAPFNMETGYDLA
VDGAADPAMMASVNGEPGIPAPISSASGNFGYSPFNGSPIGLADNYAFSEHATSAYGSNLSSTNTITPANFQDMSHMSMTDNA
LWAA
FUN_013675 CDS scaffold_6 379144 379737 - PSF3 DNA
replication protein EOG09264O2D PF05916

MSYYDIDAILTDAEKVPCHFEIDVRDLGHLDNSPHGLKAQTPLTLPLWLAEMLALASTPNSSAPLTLNLPPCLSTTVLAALKA
DPRAVPLRDQSPHFYGVGVKMLELFDEKEIAEVLRKTFVVRAGEVGLHARKADEAVGGNGEEFLRGLEEWERGLFRRGHEGVK
GAKEWTDKVKKT
FUN_013676 CDS scaffold_6 380674 381419 + SOD2_2 Superoxide
dismutase [Mn], mitochondrial PF00081;PF02777

MASALLRSTPALRAGLRARSTPLAAMATTSFVRSKATLPDLPYDYGALEPYISGQIMELHHSKHHQTYVTGFNNATDALAEAQ
HKNDAKAAAAQAPLINFHGGGHVNHSLFWENLAPNGKGGGGEPEGKLLNAIKEDFGSFDSFKKQTNATLAGIQGSGWAWLVKD

KNSGTLSIVTRPNQDPVTGTLEPLLGIDAWEHAYYLQYQNRKAEYFSAIWEVINWGTVSKRFEK
FUN_013677 CDS scaffold_6 382021 383244 -
hypothetical protein ENOG41

MSAPGSGPSVPPAPDVTAMNPERVRMLSEAPREKSPKPAPVVKREESLVPFRVTGANRDALGDWSNRSSRGSQKSDGGSIKRS
ISELMTEAKEKRAQQEKKPKIAPAKKYYTDIQPLGHAPLPDDSTHYLTSLLRNRNLNLCLQAAPNKKNDAPGKPKTYTFLISN
AGMVQHEKLSMLGHTRALPLSLHDQDISSSDGAITRTVIGELLEDVWPRLTKSDKYSYARQLRNLLHKMRNESKQGPNYPLGS
IESGTYSLLQDKHPDHTYYAIRPSPSQKQFMALLMSTLYESVPKAVAKALITQFRNDYPSVLTHGALCPRNIVVSNNTISWIL
GWDCAGQYPVWWEYARFFEARTTEANSDWYSYAAEIFEDEFPAELAAYQGIARCQQP
FUN_013678 CDS scaffold_6 384190 386710 -
hypothetical protein PF00534;PF13692
GT4
MSSNKPTRKFSTGATSHRKRQMSLLVEKDGHVNAPLQTLYLGISAVFADDHTAVIALAIHDTVYLNDFSIKHISLDEDMREGQ
DLIADHIINEVETYEHENFVKFIGAGLPVTLKYMSPSLCSRLWLDLDIVPVVLRPDHEAKEKNFWDVKRVDEQADSMARKCIL
NFGPSLVPHLQVGYRGIVQTDAGFRVHLTNLQNHKDTCSSATWGAMQFYANKLREKKTKIAFFSATPQGGGVALMRHALVRLS
RLLGVDVTWYVPKPRPGVFRITKNQHNILQGVSHPDQRISDAEKAAITDWIEDNAKRYWLSEGGPLRPPEEGGADVIIIDDPQ
MPGLVPMIKRLTPDRPVLYRSHIQIRSDLVANEGSPQNDIWNYLWSNIKDTDLFISHPIPKFVPHTVPKEKVVYLPATTDWID
GLNKHMNKWDTGYYAHIYNQQCRNQRMTELDWPNRKYIAQVARFDPAKGIPTVIDSYAEFRRRCDEANISDVPQLVVCGNGSI
DDPDGAIIFDQTMAQLEDHYPHLLDDVSVMRLDANDQLLNMVIANAHVILQLSTREGFEIKVSEALHAGVPVIVSNEGGIPLQ
VKDKVNGYLVTPGDYKTVAGHLMDLYTDHELHSRMSREARNGVSDEVGTVGNALGWFYLAAKCQELGCNPGLKGDEKWVNDMA
REEAGFPYTEGENRLPRHYTQRKEEQTVQENGE
FUN_013679 CDS scaffold_6 390336 392040 +
hypothetical protein ENOG41

MVPKASLGVLGVLALALGVANGHERIQPGHEYAKERAHMVFNAIHSAGREWGSALYHNGFGFFPATVPEGTMFYHGSRQNVTP
TGPEWLAFDIEHAENFARSFKYRPGGRRPVAPIPPPGKKKPDEDKGQTGEEEDRQELRRRSEYIKTGNGNDPLDKVADPKGDD
DPVVMRGYFHTYQTNRDLKVLLIDGMSAGKTDMGTLDSQDLVLRENNTDRGSMDEWHRARDLCKLASEWGFDGFVRIEIGIEI
IKCDFADSLDLVSMMRTELMDRIMGKAGLATFQFVRAVGERYDGVGANRLRIDFSSMVSGLFFPINISSTVAGRPDLKRLGSA
TLEELKDVKAYLTNVLRQPRRFIVDWQGVADLVIARYSKRLALMVHEPLQSHYFIDEVEAATLTWYNAPPSPDDNSMVDQREV
NKTSDAIEQCRLHYLRPALKARQQWSFEDELIYTSLDTVLGTICQTLFLVRNRLLEASGSDLDDYRIRLDNENDTEMEKAISS
GRAVIRALMSDLGWSTWKKPQPCQPDEVNTIAMWPFGTEEDHWHPGCRSIETVQHPADSYWRNRRPRMV
FUN_013680 tRNA scaffold_6 392313 392459 - tRNA-Arg

FUN_013681 CDS scaffold_6 393746 394994 +
hypothetical protein PF00042;PF00175;PF00970

MALTAAQVAIVKSTAPILKEHGKTITTTFYRNMLGAHPELKNYFSLRNQQTGAQQAALANSVLAYATYIDDLGKLSHAVERIA
HKHVSLFIKPEHYPIVGTHLIGAIGEVLGSALTTEIKDAWVAAYGQLADIFIQREGQMYEAAGEWNSWRKFKIVKKEAENDSV
TSFYLEPTDGKPLPKFLPGQYVSVQIPIPELDGLLQSRQFSLSEAPGTNHYRISVKLQGPTEEPAVEDLAAGKIAGLLSTRLH
NRYNVGDELELSPPAGEFSLDPADTSAAKKPLVLLSAGVGATPLVSILDSVLQSPTASRPITWIHGARYSGSTCFVPHVLDSA
KKHENITAKIFLEDVKEGDQYDFKGEIDLAKLQKEQLLQLDNADAEYYICGPEDWMVNVRAFLEENGVPRERQHLELFKTGDI
FUN_013682 CDS scaffold_6 396487 398010 +
hypothetical protein PF00172 ENOG41

MAETVHRKACDRCHAQKLSCKRYNDEACERCVRLKTECKSSPSLRYRKNQQQQQQFVNNGTTSSLTATSSGRRSPKRRRTDS
QEDPSLVSPDTDNSLSLTSKSHILPVSVPIPVPVSPDPVVDSTDFAFTFDQQLGFFTNPQYLSHSEFPGTRPVFWEPAPLYQH
QHQQPPEHSYHQLPDEVAPERDALHQQHVVPLSCAPEHSSSSDKRAPRTQSKQQQRSNRPRPRQITLRHDADLSPLQQHEVPP
IHWMAQLSDINARLLDLASALPSPQDGPLMGRPVDERFRSQGFPIEDMFKLTRRVADILEKPPSNNDGSKIHHSTIDGSDPGN
VMFILSTYVRLLDMYQRVFSLVHTELATQTDTGTTFSFWKLPAVTVGSFAVESSPFLQMSLTIQLAEEFLSRLRGSTARWSGA

GNATSVFAGVVDVSFQAFRDREEALAKHLVELRSEIEPLIDS
FUN_013683 CDS scaffold_6 406316 406777 +
hypothetical protein ENOG41

MCNYIYKELSCQHHYHLVESWCPKYIETERRCPPTIVSKQYWGDDICAACRERQQPSNHPWAKLIQRPESRLSYSPQVRAQ
FUN_013684 CDS scaffold_6 413884 415009 -
hypothetical protein

MGSSDIPPPSAPPWLVPASTACLSLGIIFWLIAYILMIRRSLATHATPAPLVALGLNLGWEVVYAFGVCEAPIETYGFMLWLL
LDIVVLYATLKTAPRSFASSPLIANNVALLLFLVFVAGVVGNGLFVWWWLKEPHRGYGIKWGKNWKGLEARDTTELAYWSAGV
AQMIFSVSALAMLLQRGHSGGQSYAIWFCRFVGTVMGLPVSCGLMWWYWPEAHGFVFHPLGVFITGTSVICDLAYPFLLAYVR
TREKVLPDGSLVDGRLEAETEKKQQ
FUN_013685 CDS scaffold_6 420834 422301 +
hypothetical protein PF01490 ENOG41

MSAYPQDVHNDIYPSDQGHDSSKTNSKEKVPLDNEKGHDIDLHSAHNVPVVDNGFGGGEGSKNFRNMTKWDTTFALLTNQVGL
GVLSLPSVLKTMGIIPGLIAIIGIGLLSWYTAFVLKQFFGRHPHVLNVVDMTRVVGGRPLAIASGIGLLVQVIMTAASASVTL
SIAFNTISSHSICTVGFIGVGCLCCWVLCAPRTSKFVSQSGIPCMVSVIAAAVLVMISLGIKNPSAAPVGWKKDIKIVANPDF
RSGLNACLKICYAYSGNINFVTYMAEMIDPVKDFGFALAWLEVVSITFYVVVAIAIYCLAGEYTVSPALGSASENIAKIAYGI
VLPAVLSTGLAFGHTGIKYVYVQVMKHFKLQSEMTANNVRSWSIWMTIVTVFWALCFILSNAIPVFDSILSIASATTISWFTF
GFSAVFWFHMNWHQMFASPTKILLTCVNAFLIAISIFMNAAGLWSSITELLDLFANDSSSIQGVFDCGSNALF
FUN_013686 CDS scaffold_6 423884 424385 -
hypothetical protein PF05676

MASDAITEPRQATREEMRDAKLPLAYRDSCAHLLIPLNKCRRDTWYAPWKCSDERHSYEKCQYVEFKKRVAKMDELRESKGGA
RSN
FUN_013687 CDS scaffold_6 425429 427434 -
hypothetical protein PF00884

MRPPAWIQPVLLASCLLAQSTNASFNYEPEDQAVLSPNNDAAQSKPKTNNGGKKNIVFILTDDQDAVLNSVSYMPKLKEHIID
KGTSFVNHFTTTAICCPSRVALWTGKQPHNTNVTDVNPPYGGFPKFVSQGLNENYLPVWLKEAGYNTYYTGKLFNAHTINNYN
SPYPAGWTGTNFLLDPGTYDYLNPIYQHNQEPPVQHKGVHTSDLISKYAHDLLKEAIDAENPFFVAIAPVAPHSNVNLRRQPG
NPSAPLMTIPIPLERHSHLFEGVKVPRTENFNPDSPSGVSWIHKLAQLNDSSVSYLDDFYRARLQALQGVDEIVEQVTKQLED
AGILDETYIIYSSDNGYHLGQHRLPPGKECGFDEDIRVPLFIRGPGVSSGSIENAVTTHIDLAPTILRLAGADLRSDFDGTPI
PVLSTQENKRHEHVAVEYWGGAIAEGEIGGFDGKGQIFAQNNTYKGVRIVHEDYNLYYSAWCNNEHELYDLKTDPGQLKSLFP
HDDTTADSALLGTTARQVLNRLDALMLVLKSCKGNTCIEPWKILHPEGGVTSLKDALQAKFNAFYKEQVKVRFDRCEYGYLID
AEGPQVGYEYREGLEWHHWT
FUN_013688 CDS scaffold_6 427773 429195 -
hypothetical protein PF01485 ENOG41

MDEPNDHAIALSMTQAVEADAELIATLIREEEQARRDFELARQLEQNPDAIPEAGDGADHSGLDDETYRTLQAFNVAAQQGES
TGVEAELRHEETHEASSVAGGDGTGVESATGDDSQSDADNQSYDTAPRDDVSQRDEEGDADYQPETTQVASPAPSELYVETKE
CLYCSDEFPIDMIFEAPCSHAMCQPCLIRSIRTAIKDESLFPPKCCGQAIPVDTTNTFIPEELLTKCDNKREEYETTNRTYCS
DKACSEFIPLRTIEAGIARCTRCETRTCLNCLSEAHEGTCTDDPESQRVIRLAEEHGWRRCEQCKNMVELTHGCFHISCRCGH
QFCYLCGRQWKTCNCPQWDERRLMARAGELAAARARVPAPAPAPVARPVQPVGCRHRNYRPFDRVEGEDECNDCGSEMRQYIF
SCPNCELVLCRRCLEARRNQRG
FUN_013689 CDS scaffold_6 430743 431472 +
hypothetical protein PF00498 ENOG41

MSDIIAWLVPTARNSLADKTTHLPSNIPRAVQTTSSPTLLSRLPNLLGSRPSRAIKLSFDKAPKRPGSFVLGSDPSTCDIVLP

SLPGIDARHCELSFDAEGRLVLNDFSEMGTQVWYDWESNGDQTDYTWILSSGAEAGFPSMVQRITVDIQGVRFQVVVNDHSDD
WDEYHDKVEDFVRQPSWAHGLSPGWDRGSISPVAPLFSNVPLFQHILVKALGEEPVGEVYLWNLARPWEPMVKAAA
FUN_013690 CDS scaffold_6 431664 433056 -
hypothetical protein PF17875

MAPIDHAAAKAEKKAKKEKKRAREEDAAADTERKHKKSKSVAAVEAPEASSDLKAEKKKKKSKKDKHTEDAVAQAETKAPAVE
DKSEKKHKKKKHHDAEVAVTEEVDTPVAADSEKKKKKSKKNHKETEAIEIAERPKKSKKDETAAEPEENASPADPDAMEIDMP
PPAKPSKSNSNIYQPPDIPANPQFPFFTQTVSLYEPLFPIGWAQPVTNCQQQHLSHLRNKYVPSLRGVLLDYKNVAFGENPGR
KGAATDDESPATVMAKGESAVGFGWITADVDLFVPSRGAWMEGSVNLQTEGHIGVVCFGKFNASIEARRLPPDWKWVPNESPE
AQGFEETASVITADDHGVVRQIHSTGFWADGNGDKVKGKIRFRIRNFDVGTSGETSYLSLEGTMLDKAGEKAVVAEEAETAKM
RKGKKGGQRVQRRRIPEFSMTRFAVEGEEQTEDNEAEKREVLALPEDL
FUN_013691 CDS scaffold_6 437287 437950 +
hypothetical protein ENOG41

MFTKFTTILAASSAALVSAGPLPRGDSGSASITPHDQYSSSIGVLGCKINTNRVAYWPGSVDCNNICVKVSNEGRSVYLLKID
SSGGAHDISYDAWNYLGFGESATKDPQQGGGIAMNYEYVHASKCKDILDNGKLPLAAANSMNYVASCLSEPKSWVAQNYELYN
INDPVCKHGVDEKCHLNLAVSNQPECPSGLGSVKDLNLKVENIAYGSGKKVAAQ
FUN_013692 CDS scaffold_6 440408 441634 + RIB1 GTP
cyclohydrolase II EOG09261UWT PF00925

MPSDTMTGSPAGSSSALPSFYSPKTEAADASTSHNEQSLEQSIDSLSLSPAPSTPASASVSAHPDPETPIGQEPPPSLLSPSF
TPPSTPGTSTPSTAGLRGVPVDSSIPSGGCGSKKPKLLQTLPEVECIVRARIPTVAGTEMFLHLYTNNVDNKEHLAIVFGNNI
RSKSLDAVREGETEMDRMVRGAYTGRLFPGRTTSGVESAAPQEQQPPQPSDQPPLVRIHSECYTGETAWSARCDCGEQLDEAA
RLMSLPGNKAGGIIIYLRQEGRGIGLGEKLKAYNLQDLGSDTVEANLLLRHPADARSYGLATAMLLDLGQPEVRLLTNNPDKI
RAVEGPNREVVVRERVAMVPLSWKGRGGFRSQEVEGYLKTKIEKMGHMLDMAALPQ
FUN_013693 CDS scaffold_6 442447 446969 +
hypothetical protein ENOG41

MSTTIRLRLVIRRHAVPDVKLVWPCVANDDLTVARLLALVNEVVPLESGDWGLEDYVVELYDGKGDSFELLHFQPVGKVLKED
DQVLIRSLSTDDLRRRRLSGRHQISYDGKHLIDGLAFGRPWLRAPRDRPALDLPPRKRARITHEEELDEDDDDGSYADEEDQS
EQLLLKDSVPPELEEPGSVRLRARFVDSDQLEEEDEDEDLDDENDFQPEDEDDAEGSDEDNLDADLSDELRLLQEDNAIVEQT
PAKEQETLLSGDNSQDPQALLPTHGFDVSWLDKITALRAAFPLTPVSTLQNELNQNKDLRKTYEKLSEANKPVISYDEALERV
LLGPGLSDAPSLNLDQPSEDEDPEDRILLLPGQAETKDTTRPLIQEIEEEPAPISTPRVSQNQTSRPLITEVANEEEDASSSS
SSSDSDSDSDSDSDSDVDMEKKSNSESDSDYDDPDDDSSDDSDVPAQTPSRPANTTQEDSEDSDSSDSDSDSDSSSDSSKGAE
ATKDSAESDSDDSSDSSSDSDSSSSESDSESEPEEISSKKQPQTKQPQAPENSEPKTNGQTVKAAPEPAQGPKPPGTGLAKTQ
KRNARKRDAKRLKAMQALDDTQSQPSVNGTASEEVELLARKRALLSAVSAEQGDDPSPPKDAPNKEARPMIVEVEEETPAVTT
SQDATPVEEEPAPAQRRHKVDMGAGRRLLLGALGLKNPKTKADEDKLRDSLMKNVKPLQNPRVQAEEAKVNDDKPAEEQPDED
PEAWRKKISYRAVECCHDDVVLSEPPFPFVQRWDPQQQYGSMRKRKRASQNFNDESYYEEDSQQWNGGEEWNDNRQGKKKKNK
KRKNKGGYDGEYDEEESPEGSFGGTGANGDEDIVLNYDDIPTKHRPEESQFTDTDDLPSLPSDISTLPGLTLSDMKPGMVATW
KQLLMSKATNWQPEMVPMTGLILSISEDNCMHVLLAKRDREGNDKEYDEHTGQRIYAKFEAPDLDEDEEDDGRREIPWAEMSD
PRLVQPAPPPAMLETSAKSIKLQAEQNKPVQNGNSTADNKVSARTDPDETMAEELDTQKVRAEVEADLKEQETEGKPSGTDKS
ISIPSGQQPPRLEFTASGNDSLISSFVQPAGLSKIDSPLHQLQESMQASVSQLQSLLSRDSGKGKKDNGEREKPEIDESDGAK
DAKDSEGESVPTVQGSWDAVIPDTIPSPDLPPLPTKDDALVDHRLLVVPSSAGSVRSGRQPPSVSGLDDLPEERIEETVEGGI
VDDPHGRTASPQVTNESRLRSSSPFPSLEGIFHTARQTQSPLKFSQMSVPRFIKSEKKDGDYEEAMRKLDEGEESDRSPDRNK
SLRSLFPNATQPESQKESPLICSFKSKGSKPEASEAKHVATPATSQKVKQESQFVIPEGSQVIELSSSPLSVQYTEDYAQDSE
DETYQDSPLPQGSGWVQKKHSEVNTRSRGKGLPATDAKKTTNVQSRTTRGQTSASLAQEVLTSAYSQIKGRRKTTRRF
FUN_013694 CDS scaffold_6 447845 448274 +
hypothetical protein ENOG41

MLPLNYIFTLLAMLSMGLCLPTQTPEPQPAPTLSPGLPDTTGLPVLPGGSFFEERQERNNANPSVFNPPVTAAPDQAEQLASM

GWAQTTYYECKTRDGKERCGWHIPVYKAGAEGRQAEIVHWKVVVGIALGLAMLIYGGLW
FUN_013695 CDS scaffold_6 448744 449519 -
hypothetical protein PF01282

MADNDSPVTLRTRKFIRNPLLGRKQMVVDILHPNRANISKEELREKLGSLYKAQKDQISVFGLRTQFGGGKTTGFALVYDSPE
AMKKFEPQYRLVRVGLATKAERASRQQRKQRKNRQKTLRGTAKVKGAKAKKEK
FUN_013696 CDS scaffold_6 449985 451072 +
hypothetical protein EOG09263VOP PF10294 ENOG41

MSSSSKQVDRFCHQYLQLQSDLDYPEASLLKTSQVQDAFYERLFADGAVRYGPPPRFQLRVLKELMSRIEASIDDWEEYGVSD
DLMSALSLFLANPLPSEATSAQQKSYVTYHLSELCEDNSEPSPQAPHITLLENHSLISGSGTTGLRTWEAALHLGSYLSQNRH
IVKGKRVLELGAGTGYLSILCANFLDSQHVIASDGSDDVINNLPDNLFLNELQDSIQVTPMDVKWGHALMGTEEEKWNGGRPI
DVVLGADITYDKSVIRALIGTLREVFDLHPHVEVFISATQRNEKTFQAFLDQCQANGLAVEVIQFPIPKWEVQQGPFYNDNVA
IHICKVSRT
FUN_013697 CDS scaffold_6 455884 456379 +
hypothetical protein ENOG41

MASTGAQSYTLALQKYMLLTEQHQELCDRLDQIRPEFNMSIRSHNSSPSTSPTRSSSSSLSLPPSPKRHSRSGRHHSRSHARC
SGWEDRNELTTIPDEETIYEISAEEQRLSEVNESIKRTLTELLNCSSVRADRAFRTWVQTRLMETEKELRSGRRRRSSGNE
FUN_013698 CDS scaffold_6 459001 460513 + CYP6
Peptidyl-prolyl cis-trans isomerase cyp6 PF00076;PF00160

MSVLLETSEGDIVIDLLVDHAPKLCENFLKLCKVKYYNFSPVHSVQKNFSFQTGDPLGPLSKDSDGGSSIWGHVSGDPAKRTF
PAFFHPKLKHLERGTVSMATAPLQSDPDTRVAGSQFIVTLGEDTDYLDGKAAIFGKVVEGFEVLDKINEAIVDEKGYPLIDIR
IKHTVILDDPYPDPPGLREPSSSPPPTAQQLKTVRIEDEAALHADDNVDEEELERRRRNREAQAQALTLEMMGDLPFAEVKPP
ENVLFVCKLNPVTGDEDLELIFGRFGKILSCEVIRDQKTGDSLQYAFIEFEDKASCEAAYFKMQDVLIDDRRIHVDFSQSVSK
LSDVWRKDTNSKRRATAHRGGWGGVDELEKRRQYRDEGERITGGNYRMVYGEEEMKGRLGRGGQKQEKDDAPPPRSQDDEGPS
RRRSRSRSPRPRDRSRDRYRKPRDDRQGDRRDRDHDRNRYRDRGHDRRDRGKERDRFGRDDYDRRPRR
FUN_013699 CDS scaffold_6 465551 467012 +
hypothetical protein PF00172 ENOG41

MSERDDDVTPQRKRIAVACGRCRKRKIRCSGDTGNGGPCTNCKNAGFEPCQFLRVASHEAPIVKSDGFSYSLEASRQYQARGS
SAMSSLPSTAPAYTDGVSSYTSDPFVYRAPTTYGYGAKPYCPLATWGHGYTDDQSYYNSMYQAPYSSVHDSDYAISHRIASGT
PGKSALCVDMEPNYAYSGGNNVTSLVHRPVPVTTDSGSLAYQTMMPDSTKSLGYQNLTPDSKSLGFQNMPGSVNIGDRLLPAP
VGRTALPNSMGSSYKNDSGSSIYSSSSKSSQASTSDTSPVSSTSEAPSSYTSYESSSMSSTSGCLPSYPANLASQYGRPSNDY
YSYTGSSDASVLGPADSMRQGPEMTYRYIDTTNSVTAAPPTTARRDMSTLNSSGISSFPLGHSNVHYMTNQSAAYMMPASDLG
SVGADAAADSYRKATGTLRA
FUN_013700 CDS scaffold_6 468590 469993 +
hypothetical protein PF01238

MQVPLLRLQCGVNSYAWGKKGNDSAAARFAAATPSDDLKIESDTPYAELWMGTHPSNPSRDLNTGRTLLDLCEDNQSLLSQTI
SSHYGNKLPFLFKVLSIAKALSIQAHPNKKLAEQLHARDAKNYPDDNHKPEMAIAITPFEGLCGFRPLTEIAHFLETVPALRG
LVGEEAAKEFAQVARDGEEGVTDEKKKFLKKAFGALMSSSAEDIAEQMPKLVGQAEKEGADFAGGGVPATSGEKLAELVKRTH
GEFGDDIGCFVLFFLNFVTLEPGEALFLVADDIHAYISGDIMECMAASDNVVRAGFTPKFKDVTTLVDMLTYNFAPIEQQKMA
PTEYPYATLNRNAYSSGSAVALYDPPIEEFSVVRTLLRGNGAKATFEPIEGPSIVICTIGKGRIAVGPTSYEMKEGYVYFVGA
TAELVLESEGGEDDEFTTFKAFCEIDDSEKQKL
FUN_013701 CDS scaffold_6 470852 472217 +
hypothetical protein EOG09263817 PF07039

MANGQKPARDELKNLQEDLGPYYQIPLKPRRSGTHSRENSFQESLRQSSNTPEPKRSGSKRQDPSSRDSTPNRPTKSRTKNHS

RDITSDVGTPKRSPRSEHNTPRPESVKPERHNREMSQRNRSGRASNRNNGNSHGEEVQIWELCKSQVGEIVSGINAENDSLTE
LVTMDKQVGTMDLEKIPSDSLKEMEQLCRTGVKHSEANMSALKNTIEQLKVLRAVVHAKEQQDAQAAGPGKRNARDSAAAASS
LYDFDGAGDSPVPSPIGGSARKYGDRARERERERERERERDRDSMPPKADSVEPQGSVGSGVGSGNNKSKVVFSKGDAVAFKP
KALNGDTTSDWILGEVAQVMGEGKSRRYKVLDIEPEDQSKQKEYRSSASSMIPITPESQASTLKDWESGKVVLALYPNTTTFY
KAEVHSMDSDGKVNLKFEGENDSSTLQQVERRFVIEYRA
FUN_013702 CDS scaffold_6 473403 474078 + ARF6
ADP-ribosylation factor, Arf Arf6
PF00025;PF00071;PF04670;PF08477;PF09439

MGGQFSKMMGKIFGSKEMRLLMLGLDAAGKTTILYKLKLGQDVTTIPTVGFNVETVTYKNVKFNVWDVGGQDKIRPLWRHYFS
GTQGLIFVIDSSDRNRMEEARQELHRIINDREMKDSLLLVFANKQDLAEAMKPQEVTEALQLSKLKDKVWYVVPSCATTGEGL
LEGLAWLSNNVKAPPTPAKK
FUN_013703 CDS scaffold_6 475438 476797 - SSN3
cyclin-dependent protein kinase PF00069;PF07714

MPLRPHIGLGFPAHAYQKRHVEPHDRSTGYQPKVRITDRYRIIGFISSGTYGRVYKAVGRNGKPVGEFAIKKFKPDKEGEQIS
YTGISQSAIREMSLCSELHHINVIRLCEIMLEDKCIFMVFEYAEHDLLQIIHHHTQQPRHPIPPATIKSIMFQLLNGCQYLHI
NWVLHRDLKPANIMVTSSGEVKIGDLGLARRFDKPLHSLFSGDKVVVTIWYRAPELILGSYHYTPAIDMWAVGCIFAELLSLR
PIFKGEEAKMDSKKTVPFQRNQMQKIIEIMGVPTKDKWPLLPTMPEYNQLNTLANSMASSHHGHHSHHHPHHHHGHYGSRNPP
PPPPGGSNLEKWYYSTINHTSAPGGTPPLASLGSEGYKLLAGLLEYDPSKRLTAAQALQSPFFSTGDRVSANCFEGCKNEYPC
RRVSQDDNDIRTSSLPGTKRSGLPDDSLIRPAKRQKE
FUN_013704 CDS scaffold_6 478539 479860 - ARP2 Arp2/3
complex subunit, actin nucleation center EOG09262MEK PF00022

MSNPPPIVLDGGTGFLKVGYAAQNFPEHQYPSIVGRPILRAEEQTDSDVVIKDIMCGDEAAAARTMLQISYPMENGIVKKWDD
MEHLWDYTFYEKLKVDPTGQKILLTEPPMNPLKNREKMCEVMFDRYNFGGVYVAIQAVLALYAQGLSSGVVVDSGDGVTHIVP
VYESVVLNHLTKRLDVAGRDVTRNLIKLLLRRGYPLNRTADFETVRQIKEKLCYVSYDLELDKRLSEDTTVLVEDYTLPDGRV
IRVGSERFEAPECLFQPHLVDSESPGLGEFLFNTIQSADVDIRSSLFKAIVLSGGSSMYPGLPSRLEKELKQLWLTRALQGNP
ERLSKFKVRIEDPPRRRHMVFLGGAVLANIMADKESMWVTKAEWEEEGTRVLEKLGPR
FUN_013705 CDS scaffold_6 480170 481018 +
hypothetical protein PF01177 ENOG41

MSELRFLRRNLRILVLNPNSSTIMTDGMANAIRQMSLPDSVEIYTYTAPPHSAPDSINDQEGIDRSTQAVLDDPKIEEELESD
KYDGVLVACFSVHCLVSKLTRYRHLAVTGIFEASILTSLSLMAAPDNAGKWGIVTTGKWWEDHLSHGVKNFLGQEKDGVNSKF
AGVFSSGLTAGDFHTVPPEKVREKLKEATRKLLNEGQVSVVLMGCGGMAGLEEIIRSTAIEEYGKADGNLVYIIDGVKAGVMQ
LEQMIRSKRIFR
FUN_013706 CDS scaffold_6 481180 482748 -
hypothetical protein PF00083;PF07690 ENOG41

MAGIRLRDVSGYLILLIAITTLGSLQFGFHLAELNAPQDVITCRKKSISALDKIKGLVYKPKDDDAVSGFMPHCIPMDEASFA
TISSIFMLGGLLGALASGPFSSKRGRLPAMRITGLLYLFGAAVETVAGSVFVMVLGRLFAGIGAGASTVVVPLYISEIAPPKE
RGLFGFMTQISINFGILVVQTMGFFLSYGTAWRWILATGVFIATAQTLGLFVVPESPSWLAAQGNAPKAKRTLQRIRGNGYDI
HEETETWDSDDRDASEEDGLLQADSASESPSKNRTEHLGFFDVLRDPDTRPAIVAVVGIMFAQQLCGVNSIIMYSVSLLADLL
PVSSSLLTIFVSVVNLFTTIACAPLPDRLGRKTCLLASIIGQGTSALILAISIIFGIKILSAIAVVAFVGFFAVGLGPVPFLI
SSELVGQEAVGATQSWCLAANYAATFLVAQFFPIVNSALNDLLGGHGWVYFIFAGLAAGSAIFVFWNVPETKGKKDVDEVWGR
TRRVD
FUN_013707 CDS scaffold_6 483284 484118 +
hypothetical protein PF12697 ENOG41

MSEVPIDEEKLLYFEERNNDQNESIVFLHGGGGSHVEWKLVAPQQSLSSYHLILVDLPMHSGSWDIGRPLTLASAADEVCKVI

QKHTHGGKAHIVGLSLGGFIALALTSRHPDVVLSTFATGAYPYRGMFKWFMEHPMAMSIVNSIQSIPGVLEASLRRQGIDYEE
WLAEAKKNHSPERSKAMNKELSAFSMEDIKAVADSGVRTCVISGGKMDQIDPVRDMGVILREGGQKKGVKNEAVVVRDAYHPW
HLQLPELFAAGIAAWVQEKELPEEFEIL
FUN_013708 CDS scaffold_6 484498 489266 +
hypothetical protein PF05729;PF12796;PF13637;PF17111 ENOG41

MADPLSLAASIAGLISLADLVFKTTYKFVRAAKDAKDEIQSLVDEINNLVSVLRRLEALTSDLEDEGQSFDPTLRNHYLNHCF
KTFNKIELRVKKASESFKKSKFDGIVRQLKWPFSSSETKELLAELSRHKETISVALLADSMRKIQLSLSKSDDIDKKITALGE
VARKIEINTMIAINDRKKRILDHFMKANPQLALQTSIRLRHSMTGLWLTESPTFIRWLETPGSKLWLTGIPGAGKTVLAGSVI
QEALSRSYASRRIGVAFFFCDYKESKTWDTVNILGALASQLARQNNESYNVLDAYYESLYPPKGLPQTADVDELRAQISQICE
TFDQTIIVVDGLDECDDLTDEVVDSLIQVAAYSERLSMALFSRDHYNIRARLEEEFEPIQIAAHTEDVELYVNAEVDKRIRTR
QLQLTSAHMKEEIRSALVGKADGMFRWVVCQLDYLCNCAHDQERREALGKLPPDLPESYRRLLERVNNCSVGVQNMVQMCLHF
MAVADPKLTIVELRQAVSTPAIGATLDESNIVPEYEILKRCSSLIRKSTNGKYFEFAHFSVREFLEDEKAISQTTGIEKYWIA
RSMTKFLLARQCLRFLQMENFDKMPDEPNKQVAATRQRDNSYPFYRHAAILWIKLTKDGLGDPEIFELAKSLFQPSKRAYFMC
WAVEVFKNVMYATGIGTRYEQRNGTEKHAWRIVMVPSFKPLHMAAALNLPEICNFLIRNGSDVNDKLDSATPLDLALMSVLAV
PGLPEMSGEKDQRHDLMRTPRDEFLPSSRRRNMTVDCLMHAGARSSDHLIPPNTLSVFSIACLFASIFHDLYPVFRCLCAYTI
PSVPEVKVLQEWLMATDTSNHVAEVSTRMLLKFLSSTGAYNTDWGAEMGLIVWNWAQECDFSLTRDLNLVGSCALMSDDNLVS
QIVSAVCSDNIELLKYCVEDRRLKNQVRYSGPVNGLLHLAVKHNAFNVFKALVNAGFDPYTYNDKGDLPIHLCERQGSLRPFK
VFKDLGISLVSQDRDGYNNLHYWAQDRPLNYEFVNGIFDLDAEEAIKGLQTRTFRGDTPLTMVFKSAGESPRSEHRDLDLNRL
CLQVLGFLQRYHREKGSDTSLVTGTSVSCLRKAFSEFDSMIGTNPTPLHKPNAWISLSQVQLLTKLYPEALNSRLNGRLPLET
YIRSTLDNRKDPEHNIIMSLFPDNLRDSDLSEKQSLWHFACYLPDNDRYSKARYMNTIDFGTMMEIMFQRGAMRVYEEQFKES
GLKPLLSNHQALLMSVIRDAILQTKYWDGMQDSDSISLLFQDVINRVNLDMIRLLVKYGANPHHRVNGQTPFEIAFRARVAID
LCSTEEGAEVLHNLLECCSIEEILKDSSETSNNSPLHTLATSEDATNIIWLMEALVQRGFDVNNVGSGFLATTPLVYHLQQSS
FQFAEELLELGADPSRDAVLACIVSDNLSFLQKILNHKTNPSAIFGWSDPVTFNIQHLDQTAVVKTGNAFHLASACNSVACLE
FILEHASEMDKASMSDEGLTPVHIVAYEGYVDAMKLLLKKGFSAMTESLYGFTPMHMAVLGGSLPTVQCLLNMVPLKL
FUN_013709 CDS scaffold_6 489883 491224 +
hypothetical protein PF00023;PF12796;PF13637 ENOG41

MFKDVLSCLLLRDFSCENSWQTWIHSASTTALHSAAWNGNKKAISLLLNRGADIDAADSNGWTPLMYPKDADIAQHLVSLGAS
MAAVCRFGSLASLINWFGDSLFDEAHPVSLSRLPREFLTVSDPPRFSTNCEEVSLTPEALDKLLKLKFDLLGEDEAGRSMMHY
ILGEEDLVDWMLGSDQDLSGTTPFPWHLEWCGFSDLAFLTSSFERLQKKVPADLFRKILNLEPSRGWSPLCHAAALNRVDIVA
NCLEMGADIDFEGSCFGSAVMNASACGSLDTVKLLVRNGASVTYMSKKGFISCFLVAGTEAVREWLICGRFRDQVRVASESDS
GCPQEEVPWGGYVEARVLLYGGRARWPEESTLDYAKRLSKMKKRWQGQVLRLYVEEASSSSGTDSDTGSEGDAGSNLEVEFKS
EYDLRSRSIHSQLRYAPYLVSDNGSGSESEL
FUN_013710 CDS scaffold_6 491793 492837 +
hypothetical protein ENOG41

MQAAEYASKRATATMHPSTGVDPTKNHGTYWKTALLTEERDWEKVPPPARDTFTLSDILGQTDPHLAVVSRTGLVAMSLQYYQ
LPGETECRQNFFFELNENVLFADEDGNRTAIHGFAMWMAPTADGKAELTVRFCVKSEPFMDPLLIFEFDVENSKKDPKENLIL
IDFINETGGKLYGYRDALLQWMMRLHNAQFLSWSCIGMDFLEANFAGDGEYMGDLKFDLIIHNKFKSDPNELGDNMKRIIDVK
KTPIKRGVWLDETFTRPLFYKLAKLPYEGRPAWGDAVNNASVDKWFEEIDDWFATGE
FUN_013711 CDS scaffold_6 492998 494792 -
hypothetical protein ENOG41

MATPAVYSSLDKEDRVDDKNPGLATDSPIPQTTDFKRLYKLDSWLKWYVIILGIYLAGAIFCVAWSYTGALLDNDASKRRVFR
TGDSVPVDQLYSGIALSALLAPAAVLVQFVVFDFRRLHLFALTTQQPVAIRDLDDIAEGVTLLTLPAVMKYSWWYGVMHTFLI
ITRTLLVPVGTLSLTVGPYGHYKEGWGVVGVPIARDAVGSNVTALSTAMGASPNLEFHPSLSQNDTFLSQTVFTFVGSLVSQN
ALANVDSGVLGPVPTHNLTFATNTTYDGLVFFNWTAACEAATEVNYTSYREDGNTIYNFTLPDDSVESIELLSQGSQRIRLWS
NASVHGTNKIPTSGTTYFLSATKSIPNINETALRNRGNDKSLVQTDGGDWISRTKCTPSIRWLVGSCTFNGTIMTSCTEAPGS
NQTALDTDALDALGVYMSAIPWYIFRNQIGIVDDTLDALYAIPTAEDFGHFFGNMAHAIVSISTAGYFGTSSVPTLSHITESV

YIVRCSILIAVTVMLFLSVSVSIIDIIRNRMKHLPQLPASFLAIAHAVRGSWWDNELSGYRPWDKMKTRSDGAEAVMFGVDTE
DSRFIRLAPSVAPIRC
FUN_013712 CDS scaffold_6 495397 495984 -
hypothetical protein ENOG41

MVNFITSFAILLTAASSALAAPQPLEARDDTSCMDNLPGNTLANVNEAVECINYLASLGDQACVAGVSGQSFCRRGNTQITGL
AVGLNSDQTSSSPCRDVARGAGLVMDRCTRADGKVRGQNPAWGNGHLMVDIRNVPQ
FUN_013713 CDS scaffold_6 497551 498448 +
hypothetical protein ENOG41

MFKLLHGKFVVRNGLQPDGDTIRFKSDNVDFVEELRRGSRGRTTMNEGVNIRLEAVDALEKSQELKGATAARDELLRRLGFTD
VRYSGNSPFTINSGDQEISGHVLSNGFDNFGRLIGFVYEGDGSVHGSDGSVISLDRSLIDESVNTALLSEGYVFPAFYDSLPE
NLREHLAMKSTAARIAKKGVWLRSKGFPEDPLIVETPILANLRKAVLWPKLYRRLEKYLESGRRENLDGFIRWLQEDPQSRDD
GILLIQSNPPESVRLHNVVEVSGSSVELKYCPEEFIIQSKPTNLNGSRP
FUN_013714 CDS scaffold_6 500340 501711 +
hypothetical protein ENOG41

MASYYPEGECYFVRSSLRYGPHWSANNQNLFYRTVLLEYYWNGWPATGVTQRSHPRSRWQHGTPSGIGGTRFGDWDRFWVGES
RNKSTWPMLRIHSHPNSLNWNECRDHQGGGPRKYILRPVEIQDSNTQWLWIDATIPLRAIYFPVAFNADQAFPNLCTYVVIDF
RHRDRFQMFSDTGVSRTIVPTSYNGLGWHDGLTWGFNIPDIIAYLSVPHKLRNPDYGLFNSKARGQELVVWTSSAPAWASSVG
VLNHPDFFREMDFARILSATFGVEIGWRPYQHNPFLRDMLVRITQLGLGFIPGVGPILSVAFGIGVQLLTDPDSFGRDNILDL
TFAVLESVIDSGKRSQKFIAPDWLAQCPCGGQQRGEPLTEQQREARRKLGEEINNRLTAELNAQPVIRSLLEQEQRSSGRVEE
VQDEATETTQDEETAEEAEEKPEAEESGKKQVTEENGDKHE
FUN_013715 CDS scaffold_6 502436 503814 -
hypothetical protein ENOG41

METESREEPATVMKHSLTTPTSLSALEKMPTEVLLMVGSHVGSIAFRVATFVSKRLRSVLLLRLFKSITFSGTLRKLAHDMRS
FLSGEFKHLMMTILPALKSVTIRFEPRSPAEESKDSHLSTNRIAVISEFISKLSSIDLISFDNQVRSNHSREIAFTEALGNTP
QWNGPKAVIFCGRRNLRLFSAIINQFAPNAVKAAQLPQYTAKRHPLALKSACPLLKGLRADVSAITSRSRTLACMSSNTLRAM
NRDFPHLEALVLDQEDAHIMSGGGRIYKKTYRPNLNEYVEKLITQLKVMPRLRRFAFTLQQYWLCKEYDCKLFNKGVRSLSGD
ESDDPLQATSEPNEWPYWLPTAAQQEEWHSELITRILEAVPLLKELCIVLDPLEYHRGTKTENIVTVRRVQLGYLEKPSQFPY
VLADID
FUN_013716 CDS scaffold_6 504491 505772 -
hypothetical protein

MAGTPTTPELATMPTEILCMIGDNLSVHEIKELTPTSKQFREIFLPKLCKHLKFSVNMKELTDSLHAYYTRKTASFRHLVHHH
TRFVTFEVTNFDDIFKMNAWLLGYGVKTIPIGRFLADTPSLHGAVFDIWLQNRKEAHKFLSFIRKGPDWAVGKFKAGALKGIA
APHASLTGRHYDELARSGTHLTSLRLNKYSFLRQDSSALTSLSDVLINRISKDFPQLESLNIYDKTRSGRYTHHMNDRDSYRW
CPKIDKVASSLRKMRRLRRFAFTLSEARFSENVIDALRTKLLAIIMKKGLLPVEARGLEGVYILLITYFAAHAKSLEEVCITT
WCPMFYRATLTNGAWNMSEESSEDPSQKYLFPNVLEG
FUN_013717 CDS scaffold_6 506174 507463 -
hypothetical protein ENOG41

MESMIVGDASLVCWGSFADAVSVMTYLGVHAEKLTGPYKPSLSSEHKRRLFGRVYNLDKAIVAFTGRPPLLNPRFCSTPPPLD
LSDEDLLAGGAVLEKAVSELDSRGWSLRGGVYPSSICRAGYLMAVILNEIIDISLGSGSNANVETIRNLKQRQLNVIAELPKC
LVYDPDDPADLPLPRREIRPYDKDLSDPPVETNVVSLRIFIRLTHLQNMFLLERLLLQYGCPDEGDVLLVSYEMITLTLMFWM
HKDRFRDIRRDMEWLLMAFAVPGGGILCLELLSPTFQGKHPKDSRLSRSSIVQQLSLLVGFLDWVHPSAPNGDLCASCKTIIQ
RVLDYHLNNIDPMSDLGSLDQFSSGLAGRLNFRFELLNTFDWLNAG
FUN_013718 CDS scaffold_6 508431 511589 -
hypothetical protein PF00023;PF00069;PF07714;PF13606;PF13637

ENOG41
MSSGFQYNSSFTFSHPGWESDPVADYAELALARVTAEAPDPGPIEDLTTFAQQLHTTGLPICEAADLEDLNDGAAIGSGTTMT
VFKCLWKSKNKAVAVKRVNLGIALGQSQHEAHSQKYSGLLKSLLREMRVMNHPWCKAHPNFVELLGISWDAVTSDGGISSYRP
AMIVELADPTTPQLRDLVKSSRYPLGLKEQGMMFDLLTDVAEGVTMLHAMKIVHGDLKPDNILLFRTKDRLVAKLSDFGFCSP
FTDIEYKIGGTPYWNAPECMPDAPEELRAFRKTITRDLYSFGLVMAYSLFSILPYDEEGEHDADTMTVQDWKLNQDASIFLYE
QIIVYVLDFHSTNSTRSGTSIETTSPVFRPKMVSSMGHGLQKRQFGKFDPFRLGQIPEAVQSLLLEDFEHRYSSVADGSPEVA
DLALVLSNCHSRGFGCPPSAAKALEYETLAAQAGSDSAQIQATFHGLVDGFDCSVTNLVRGFSMHPDTKSDYGDTPIERAAET
GSIDCVRLLISLGADIKPLADQELFSSVVITGTRNTIKDSPVSGTASLLSLLCRAVEEVSEDPTSGQTLINGRYMSGNENEGI
APGSPLELAVSVLNYDSVIALLNLGADPNIYNYLPPLQIAVSLREPLLALLLLTHGALQNTVDRTDGDTALHCANEVNGTEFA
EAPRNDIIPWQQFVKEGVEAIDVDSDESLTARVKACITILLHFGADLEARNSEGQTALVQAVRMGDYSTAKYLLQMGADIESR
DDEGNIALLGIESEEVQQWCRDKGLGIGTR
FUN_013719 CDS scaffold_6 511844 513350 +
hypothetical protein ENOG41

MEVLGAIVGCTSLALDIFNFATAELEGRGIDSLVLSLKHDVALLKDFARIFNAAAQSEQIPLTDKFLLNEICLALQPSLARIH
STIVRRKMSALTASPARKAVDKVHGFLYGKAEMQGISKDLFQWTERYHVRFGLLPVGLQTRLLAQGTDESLPGDIKALRETFR
RLTDRSRSTVVDGLRKSEGMVTLRPGRPSSRMFATMNQRTVIVEFKEHSLSPSMTYMNDFETDVAALAKLLSGTDPLLCRTLK
GEGYFHQPSRSAFAFIYQVPKLVVIPDDVTRPTTLLDLIMATRPALSKPGQIELIPPKHALEQRFELARKITSAVMYVHVMQY
VHKSIQTSNIVMFAKNDSSPANTEQFPKALGEPFLCGFETSRYDRGASDGQGDVEWGYNIYRHPKRQGLHPQSRYTMNHDLYS
LGVVLLELGLWRPLTAMGLRDPNSVNGSVKDHLKKLAMSGLPIMMGTKYRDVVLFCLDIDGDGQISNSTAVEEVLKKVEELAI
GMQ
FUN_013720 CDS scaffold_6 514100 515173 +
hypothetical protein PF04479 ENOG41

MSYNGTNYPPPTYETCSDGISERCPAELSLYGDTFTLGACVFFVVAHALALIPQLYFGIKARTWSYTLWLAIGTIFELIGYSG
RIVMSSNPWVYNAFVIQLVLLILGPTLVAAAISITFKHLVLWYGREYSFIKPVLYPWVFVGTDFFSIVIQAAGGGVSSAATNG
DNNDQGMLDVGSGLLVAGVVFQMANMIFCGGLMMIYIWRRHKAIKNGAAVRAGDKTATESESSTNGNVKVIRASDKKTKIFVY
ALTVAYIAIIIRCIYRIPEMQMGWGSTLMQNETTFLILDGAMILIAVWTLTIFHPYFFFPFLGKKHKKEMDAEKSRAEGPDAP
VMEENAQR
FUN_013721 CDS scaffold_6 515635 517368 -
hypothetical protein ENOG41

MFVYQGKMNWWTYAKDETFVVVLPNGPVRVGDSVYLFWKWTVDAKGVKNGNVFQTISVDSVTQTSATDVTFVLKGSWYSFTVT
TKGGYENISIVMRNPQGGVSDPMPLKREWKSDKELTGTTRIWTGKFKWMNFARDEHAIFIVPDGFGEGKPIISTWQWTKASNG
KTKEPSFRAEVQRNVTGLGTDTVKFSYKSYYDIKCTWDGKKDQLDVWVTEGAHSEDVGDMIRSAIIERSTHSHDFNPPRPPPT
KSECELYLPQPQASLPRLVCPLPFPKDLLETLAHTAAYVDQCGYLAKYAQDRFAALDADYHFSLEQIKALKIEIENINKTKGD
LTIDRDAAIAKSQKLQEALDKALKDGEGFQKEIARLNKVISDDKLKDAEALKVLEKTRADLKASQEQCSKLQAEIAHKDAQIA
EQGATISNLQKIIITLETTIAQLRAEVTVKTSENEKLAKANITLEGQKRDLENSVKDLQDELARAQQRIKELQETAKNHAAKD
KEIRDAKARADSEAAKSEEEAAKALAEVEKYKQWAYDNKVVIDLSKY
FUN_013722 CDS scaffold_6 519510 521268 -
hypothetical protein PF06985 ENOG41

MTLCCSVLSSNMQVNTNNEEVLGNVTHKILLFCAPKYQPLDLAKRQIRILHILPGQYGDPVRCTLHTMSLYDNPCYEALSYSW
GDPYICHVIEVDGQEATATRNLYNALRRLRFVDKTRYVWADALCINQADTAERTHQVGLMRNIYSSTTEGILWLGEFSGDGDT
GANSISQETAATAFELVRSLAADRHWNSQEHAQSMAREESDALAALLDLSWWQRAWTVQEAVLPKKATLYCGTMQLPLSEVKR
AHLMSVSHDRNGCCVTNPQCHDVLYKFWDCIEGFRVLQEESDKDILVRMALEIFRFRHASDRRDCVYAYLGLGSKALADYTIP
YETAFKYVVRSLIQESNNLGPLVRIAEHAETRSATLPPWCPDYGSSFLETYENLDQEFGWLYMYNWYQAGGRNGPTSRLSLID
SRLEQQGIVIDKVTKAEGPIYNTDTKDEIFSAWYQDRDPRVCCLGSYEEVGWCELMRDMFIVFSNKPRDYSRPRTRAGFEAMV
NSLWDPRSQLSVFQYQTFHTETDLVGHARVDVRVGDVLCVLLGGNMPFILRPLDDGGFGYVGQVFVHGIMDGEALQQGRELEW
ITLV

FUN_013723 CDS scaffold_6 521432 522930 -
hypothetical protein PF00249;PF13921

MSGQETSPQSPPANTPKHRRPWTSAEDATLRTLVGHFGASRGSEGRWKDIASGLEGRTAKDCRKRWLHSLDPSLRKGRWTSQE
DEILLSAYARLGPLWNEIALLIPGRKDDQCSKRYNDILNPSAKNRLSDWTAEEDNLLRQGVAALGHRWVAISSRIPGRPPLTC
RNRWRTLSRLSHNRQSRSQGTTPSSSSQMSPFDNGISPAAHNPNLGIENHGSTSDLMHFPMDTDIGHDQMGSSFLDSAFFDTF
TGPSPSPLRESDPSDETETPNDVSAPGAAPANTAPVPDLSSNIATQTQSDPKPWPQPPKPTQAQQHQNTTNASFMPSPSTWSS
LGTMLPPGTSPTDQNMWLGMTGNPPTPKNWTAPGPTDESNPLPQSFTEQFQVPAVPTSTQQTIHHHHHHHVHHHFHHYHHYPG
NGDQWRQLEQQSEAEAMQAHLQNENGPQNPG
FUN_013724 CDS scaffold_6 527880 529183 +
hypothetical protein ENOG41

MKLSVIHHKLLHHWFSAHAAMTFGPSPDGAFSPMRDVWLPLDLSNPASFNALMALSAAHLSRMQGFSQSEVALEFKSEAVRIV
QLWMQDPERAVSDNVLAAILRLLTFERYWGTEAEWIIHHKGLMNLLEARGGIAALSSNWRLELTTFLVSLMARPTWFDCSNQI
QELLTHTSELPLHPILRKGSDLRKLRSLWLLSFIQDMGTFRRNLLRTTAVHYSAFHEAILLLKAQRQDHELDTGRPELCSDLE
LTRLACLLFIGVFLQKSPFESPHDTGNCHEEAWTFQNLDLLNFFLETNMPSWHGSLENLHSILFFHFTTSEGTGLDPDYVRNM
TTIIASLTGGARYTVERCLLEILCRASGGEQNLFEEKYTPDILLSTISGQ
FUN_013725 CDS scaffold_6 531726 533469 +
hypothetical protein PF00732;PF05199 ENOG41
AA3_2;AA3;AA3_3
MAVKNGAYFDFIVIGGGTAGNIVAARLAENPNARILVVEAGIGTSKDNEEIRTPGLAMDIRGSKYDWAYKTTMIKRDDYERIE
KPNTRGKALGGSSSLNYFSWVPGSKGTFDMWEEYGGKEWTWEPLVPYLRKSVTYHDDAGLYAPELKKIGAGGPIPIAHAELMP
EMKPFRELLTKAWKSTGEPVSENIFDGTMRGLVHSVNTIYKGRRSGSFLAVADKPNITIFPEVHSKRLIIDQADRIAKGVTVI
TASGEELDLYATREVIVSQGVFETPKLLMLSGIGPKTELAKHNIPLLVDSPHVGQHLLDHPGVPFVLKIKDSYSMDNHVLRKG
ALQDKVMSAYANGHAGPMGSPFLEMIGFPRIDSYLEKSPEYRAAKAANDGLDPFCPYGQPHFELDFIPLFGSAFQWHFPHPNK
GSYMTVMVDLVRPVSEGGEVTLNSADPLQQANINLNYFNNDLDIIAMREGIRFSYDVLKNGEGFKDIVEDEYPWDMPLHDDEA
MKRTVLDRSQTSFHPCGTARLAKSIEEGVVNPSLQVHGIKNLRVIDASIIPVIPDCRIQNSVYMVAEKGADAIKRDHADLYH
FUN_013726 CDS scaffold_6 535872 539172 +
hypothetical protein PF00122;PF00689;PF00690;PF00702;PF13246

MSDPDIRGTKESDPEAQIQRITFQDGPERGREGDDAHRLGRTLSRSLSRRRSHSSSRSRIHPTSPYSGVQIEYRTLSIQVTEA
KQVEPTEDPKAAAGQKTDEDYFSKLQYHELQTEQLCQQLNVDVGAGLSESSAATRLERDGKNTLPHPKTNYIKRTLKYIFGGF
CSVLWIGAIIFFLCWQPLSNPPSNQNLSLAVLILIVIFLQAGFSAFQDWSTAKTMNAILDLLPSFAMVKRDGELKSLPTVGLV
AGDVVHLKVGDKVPADLRIVSHSGDIRFDRSVLTGESDEIEGAVDATDANFLESRNIAFMGTTVMNGNGVGVVILTGGRTVMG
RIATSTSGVKDSAALIQREITRFVMIIVCMTIVLALAILLTWVGWLRVDHHAYMSVPAMLVNVMACIVAFIPEGMPVAVALTL
MMVARRMKAVNVLPKGLSTVETLGCVNVICSDKTGTLTQNQMFVSSVAFVDKKFDSSDEFQHLLHGKDVSEPATALQRAALLC
NDASFDPTTNHLPLHERAIQGNATDSAVFRFAAYGPSGDSFRKTVPRIFEVPFNSKNKWMLTVFQSDDERGAYRVIIKGAPDI
LLAGCTKYWSSESNSVETLTRDARIKFQEIQDDASRRAERVIVLCEKFITPRAVAGTNSFSDEITHSAIQDLTVVGMLGIIDP
HRPEIPATVEQCRRAGTRFFMVTGDYALTAAAIARNTGIFSCQQDPDTIDTLYTGNQITESEKPKRKARKGDRAEIIKRSLLL
EGANLSRLSQEDWDIVCAYEEIVFARTTPEQKLRIVTELRERDNVVAVTGDGVNDAPALRAADVGVAIVTGSDVAIEASDLVL
LDRFDSIIDAMRLGRLVFQNLQKVISYLLPAGSWSEIWPVILNVWFGVPLPLSAFLMIIICVFTDLFLSLSLIMEKEEFDLLS
LPPRNHKRDHLINAKIYTQAYLFTGFMETITAHAMFFFYMWKYAKMPVSELFFLFEGYTEGYRGYTAAELTKFNNTGQCVYFV
TLVFLQWGNILAVRNRRLSIFQANPLSKAHRNPWLILSMLISLCIAIFVTEVPGIQNLFDTESVPIEFWLIPIPLGLGILLVD
EIRKFIVRTYPKSFVARIAW
FUN_013727 CDS scaffold_6 540159 541056 +
hypothetical protein ENOG41

MLACVVNAGFLPLAIFQRRDIEEAIDSLKTSTDANGDHLVNTDMDIWPVIGKILLVIPIIVAVCTLILPISAWYLKQYFSWQS
YRNVGADARMRKIRITHHIFVMLAKTDIYFIVCFVLIFCFPVLGGMRNGGTYFILNLVLLGVAVAIISASIMFSKSENRIGMC
FSIAMYLAMLGYLLYLVIDAQLDSRRRKGGSINSFTAFGCMSVLSIFMTTIFAILCLRNFFGGLKEHLGKHEEAWDPHGSAKD

FELHNDVNFELHNDVNFEYKSVPSVRDLDS
FUN_013728 CDS scaffold_6 542220 543344 -
hypothetical protein PF00264

MKFTSILALAGAITPAIAAPAKSDKTCSNPAKRIEWRELDAADQQGYIDAVLCLKTKPSRMGLKSSLYDDFPHLHFQLNSWIH
GGSPFLPWHRYFGVVYERALRDCGYEGPYTYWDWTKDAEGLRLSPVMASKNGFGGNGDAEKTEKTVSGSTLKCVTDGPFAKLR
PEYLETEPRKLTDGGHCLHRNLPEVDEPDAFATMAKVFGPEGVEEVQASGNWSHYAWSLEGGPHGSIHACLGGEMNPTTSPNE
PLFFLHHAQIDRLWWLWQKENSTRLTEYNGLYKNINQTEQHEVSLDDVLMMGGIDEDLTVGDVMDTTKGHLCYTY
FUN_013729 CDS scaffold_6 543975 545865 -
hypothetical protein PF01593;PF13450 ENOG41

MTTQEINVDVLVIGAGPTGLGAAKRLQHLNSVDWLIVDSNETPGGLASTDTTPEGFLFDVGGHVIFSHYKYFDDVINEALPKS
DDWYEHQRISYVRYQGLWVPYPFQNNIAVLPKEEQARCLGDLIDAAIQARARSPSDKPANFDEWNIRNVGERLNEIFMRPYNF
KVWAIPTTKMNSTWVGERVAAPNVKLVTTNAILGKAAGGWGPNATFRFPARGGTGGIWTAVADTLDQSKTRFGDHGAVTKIEA
DAKTVYLKDGTTVKYGALISTMAVDQLAKALDDARLQKSLKPLYYSSTNVIGVGIRGKRPDRIGDKCWLYFPEDNAPFYRATI
FSNYSPYNQPSEDVKLATKQLANGDKPASSEPASGPYWSIMLEVSESSYKPVNQATLLEESIQGLINTDLLNPEDEIVSTYVR
RFDHGYPTPTLERDAALSEALPYLYDKDILSRGRFGAWKYEVGNQDHSFMQGVEAVDNVISGGLELTLSSPDFVNSRKNTERR
LAGIKGARK
FUN_013730 CDS scaffold_6 546736 547726 -
hypothetical protein ENOG41

MAIQHPAARDDQFQAHAIGHRLRRDVTPEECYQCESAYQYVRQMQRNEKLCADDAFYDLYSSCVGCIEGNVNSGSPREYVEPN
LGRYVDYCAQFTALTKWASMGATSATSATVRSTKTTSAQRTDGTLMDEASITGRNVEQLETSVAGTINTNPLTTTEVAQPEDS
ASLSNILRKNSSSSSKAWIAGAVVPSVLFVTTAVSFAIWWQRRRNRTPKTPKSPYVDDVAGTSRFDKPELDSQVIPRPRSKEE
AITSTRTTVLDTVVLPVPAKMPENGLNRA
FUN_013731 CDS scaffold_6 548907 551629 +
hypothetical protein PF00069 ENOG41

MEAIGIAASVLSLVSSSTELVRAVQYLHCRMDNEHENYLRSLGKLEDVIDRIRTKLPKDNGVIPKDLGETIDLATKRALEDVG
STRQLLGRVLHDTGTTSSRAATWGTRRPNYTSKTTSARSRELVQVLQETATFVGLLDTFEGADHYALDPPFYDFGNSSRSIFD
DVEAIKPGGTSQVFRVGIHLSHIPDYGGESRKEVAFAVKKIYSESFQDYARERDAYRRMSLAQNPHPHIVPLFATYRMEGKYH
FVFPSANCDLATYWRTHPRPSNDKRNLGWFGWQMRGLADAVSTIHGQKGQARDVHGVHGDIKPENILCFGSNYNDRWPGFALS
DFGSSYFCTPEERDIPKGLKHTPAYRAPEIDTTNVGITQAYDIWSLGCVFAEAIAWFYDGKAGIAKLIDARLDEESNSSNRDA
FFYVKYDFNKRGGLSAKLKPEIQRLIISLRRSSRSSLFMDDMLYIVLEGMLKVNMSERMSAREILDALTQMCIKLENDPAYSE
SRGNSDEEMVHTQHASRRRLDNTVDANYYTNTTTQSLSDVQISLKPRFACPFHKAGILVSVHHRACEGPGWIDVNKVKEHLFR
CHLPKKYRGKHICRRCDTSFETDELLLAHQHQETPCPKRKPEAIYGMLSREQAARLRSLKRKSSKESEEDRWFDIYRIAFPSF
NRMLENVSPYHESNTTSLSTLNSTSSNGISQYKDYLRNRGAEEYAAKLAKMGISVTLEAAAKLLELQVKDLETFDETMREPVR
AYGIPTDADHEKAGNSAPSDLSGSSDLFGPIQLLARYADDEEH
FUN_013732 CDS scaffold_6 552174 553788 -
hypothetical protein PF00168;PF17047 ENOG41

MDNMASLKDTLTASGGAESAGFLNDIIAQLWPNINVAGGKIIKDVVEPMLDQMLPGPLANLRFVKLDFGPTPIRFSNVDVHKT
EFEGIKLDMDLDWDGKCDFELDASMVPKIGIEHVKMRGRLSILLCPLTNVIPLIGAAQVAFINPPELSLDFTDAANIADFSLI
DKTVRKVILNIISSMAVLPNRFLVKLDSSNDYFKTFQPHHGVLRLTIDNATEITGEKKSGAKRLLQKLVKDIPDCYCDVNVGA
EGEWRTSTIKNKHDPQWNETHDFLVTDYEQRITIDLNDEDLGGDDDIGIATTTVKQLLLNGGSQTLTLSHKGQPLETKVTIHG
KFFNFVGESNSISASSQNEGEICGLATVLIASVNGLNGQRDELKPSVKVTWGDKEFVTPVKSYSPGTDIFNPSFDTAFRFPIT
AEQLSNPHSFKLSLQNGTSEQGSVDIPFDSVTGANGMNREEEFDVGSGATIRARFSIRGLQLAE
FUN_013733 CDS scaffold_6 555807 556163 -
hypothetical protein ENOG41

MKISFVPLAFVAALAGFGEARNCTPKLDHCGHFLLDIGKYYSTISDELTRATKATCFKKNQINDALFSCGPNGTIKFKKYCKN
GCQVGAMGKHDYCWT
FUN_013734	CDS	scaffold_6	557454	558483	+
hypothetical protein		PF04366		ENOG41

MAAMGSNNNHLFELPVNEDLNTRPTGYECKNTHFPPPPPYSANSTSTQEQSTEMQINRKSGFVNRLHVLSAKIAGPVNDFANK
LGCEAFMPTTLDKECDKASRILTSFCDGTPLTTTSPNDSLSRRKAIVRIPRQVLKSAAGLAIFTAFRSGAQFSWGSGSGVVVA
RRPDGSWSLPSSFAVNTLSVGFMVAMDIYDCVCVLRTPEAVAAFTKPRVSFGGEVAVTAGPVGTGVYVDSTVNSNGVDEPIWS
YVKSRGLYVGAQIDGTVITTRDKANEDFYGVEVPVASILRGEVPARGPMWPEGYWRLNNVLREIDYGR
FUN_013735	CDS	scaffold_6	561122	561797	+
hypothetical protein				ENOG41

MVAFNTLLTLAATLVPLVTGAALPTHEDSSLTRRQYQDKSWDAYHRQNGAFARQGLCLFYLDPSPRDGAWACGVYCKNDRTKI
CTTVHPSKFTNDMLGANPDGERYAIGQCQCDTSAADFLATATVDFTARGLDKGFREIGSVTCEIMVNVMKEAVFASTYAIPGV
GPVATAARTVAKGVKLASKTQGGKDMWTDAVKESCNFRRDEELLNKGFGIFEGAPDEF
FUN_013736	CDS	scaffold_6	562277	563702	+
hypothetical protein				ENOG41

MPKAYAASAWPGAPPVSEDGFAFAEGVFFAQSSGQNRHRRATATELKEHFTSGNDKDHPAHWFEAQLIHYGLPPSKTKSVARM
RLFDAVNAGNLKVPANVSKLETKLKKEWTKNDREAKKGAASKPAAQVTPVKTETKKTSAPGPKRKANDDESNATAKKPKTVAP
KAVTPKAKAPAKTPAKAPAKSTAKTAAKDPETGPAPTLARKPQTARCVRGGASQAAGRGASTAATEPTKQPRTKQTARRGNSS
RWSTGGPSRQTARDPSPEAPRFATPQTARRSGAFAARGRIPAPSGYDDAPPPYSEFPDQDYSSDENNSSDDVDEVSLAPLGLL
NGDYEVESLDVSEQWDFDPDEFQLTLTISGNRLWGRFNLGVYEGVLLFEERPMRSSHDRVWFKWRGREDQGPVIYGDNNEGWM
EFLGDGRIEGWLGHQSLSFQARRLPGQGTRSSIDARTLQDEWNGYSYDLYEEENRARWR
FUN_013737	CDS	scaffold_6	564125	565032	+
hypothetical protein				ENOG41

MSAIRGFLNAFKARSSPITGIINLKPLSDSNARVGSAFIERCNPFRDIPKDARLENTLCEVRYIDRVLRDTLVPTSLPGDDYH
FCSKLLRSLETRRDLTWLVLRETDIRDTIGAIARRGGRRAPIPDEPHDIHSRAKALERHWSALEKCHTKLREWEPKYATQSQP
PLLEEEEEAYVLELSDEQAAHAEIEYREWRSDRDRKVSYLKLNPPEPAAFIPVERRDIQTDETWKILPPSGSEIGMRLLPVWT
PIIFQEVPLDWESPDSSLRSTLGERRRRDEFRRRENDRRERKYEFQKQLREEM
FUN_014608	CDS	scaffold_7	224	1442	+
hypothetical protein		PF01368;PF02833

MASRLSLGGFLAKARAALTAPAAQRKGPLTFVVGNESADLDSLCSALVYAYMRTHTPPHTLHIPLSNLPRDDLALRTEMSAVL
KHAGLTLKDLLTLSELPDLKSEETHWLLVDHNSLTGPLKKFSDHVTGCVDHHADENVIGKDADPRVIEPCGSCMSLVVDETRK
TWEEISHQDPEDSDAATENDKLTRLALAPIISDTINLTAKEKVREKDSKAVEFLEGRIHDKSFNRTAYFDEISAVKEDISDLS
FRDILRKDYKEWDGSGLKLGISCVVQDFNYLVDKAGASEPLIDAFEDWAKERELDVASIMTTSHPNGEFQRHLLVWGVTDQGR
EAVDRFVQAGDKLKLETWKEGELDHHGHKRFAWRQKELAASRKQVAPLLREALKKS
FUN_014609	CDS	scaffold_7	1652	3587	-	ATP25	ATPase
synthesis protein 25 mitochondrial		PF02410		ENOG41

MLTRPAAAALSCRQCQSAIIRAVVSRPATSSFRSLPQTTSRRLPLATRFYSDVKVNGTIIEKESSSPVPPSDALNSKLEATEE
AEDPEDVPWFLEVEPPRHPENQHAVQLPKIPEGAPEVLEPMVKYIFEDMGLDEISMLDMRDLDPPAALGPNLIMLFATARSER
HLHISSGRFVRWLRKNHNISARADGLIGPGELRTKLRRLRKKAKLMGTNTAIIPGGDNGISTGWVCVNFSSNSDSIDEAAKVD
DSGRFSGFGAPQTGTTVVIQCMTESRRAELDLESLWQTVLKKSLRENKQARGEKINSPEELDELLAAKVQLPKSESAQQWQAL
HKASQQRRAYTTSARRLSPEPSRIIHTSPVASDEIADLHDHSAQPTPDLEQVRKRFEHIQLVGLPLGQSQIYQFVRDAMTAPA
AEPASESRLTLVDQILLTAEERGMIIWTDEMFFTLIEGGLSSPSFGQPQLARAQKNLETLMVQKDCRFNNTQTSSLLSAYAKR
GDWPRFWDTFRRPSLFKKPRSSYVYKTVYEAMAHTNNQVFCIENLHWVYDEMINEPKPLPMFGPLYGSLKACIRVADPAAAKL
LESNAGDLRGHSHYGKQRLANREYLKMLKEVEALHTETSGYFARQRMYQGMNASKEPDLTEEQ

FUN_014610 CDS scaffold_7 4335 8655 +
hypothetical protein PF00940;PF14700

MFVRQTRRSLALHRSTALRSIGLHSRSLPSHITATNLGRRCVVSQPYQPRKRRGSESGPGLNHGRNLATAVDDYNFGHNLGAL
HNQLASSGLQPFQAPPLSYSDLRALDPTSLLTIPEPAAPRQFGKINNKGVPGEIEEMIPVFDACVRVGKLDRASLVLKRLNLT
GLLPGEEMIILHNQYLRASLDQLRTNPDRKQAERLHKWYELQIRNQNVPQTAETIACMLKASLISERGARLERLINRYMSMAP
DEAGLRVLSRADILSDQDLGVITEICPTYRVEPGSEEASYVSLANEEEGLELEDDLNEEMTAFASEDYPEVRPTPQRGEGLNA
LRRNLNLMAELQNVDISKLDPLEQREFQIRLEKDSIDTAIEKWRNSKKKMDKMGINTALSVKAEDKSLSDCMHRWLEAMEARL
KQEIIKVEESEDKEIKSEEDLERCIYGPILRMADPTRLAAVTILTLLNISALQGVDKGVVIQRLLNDVSRVAQDDIQTQFKEK
AAKERRRLRKVQFLAQEKDAQAAESNEPIAAKESTEANASTEVNESTEADASTETNVPVRQVIITDAYQPTEAKDPVLSSWSV
QVRARVGSILLKSLIETAKINVVKEHPISRERISQLQPAFSHMQAPKKGKKVGMLTINPELVERLKREPMGDFLAKHLPMIAE
PRPWTRINEGGFLVSKASLIRVKSGDVEQKLYANAAIKRGDMDQVFKGLDVLGKTPWRVNNSVLDVMIEAWNSGDAIANMPEL
TPDLQIPAEPTNSDPLLRRAWIRNVKQVENERGALHSQRCYMNLQLEIARAFRKQVIYFPHNMDYRGRAYPIPTYLNHMGADH
TRALLKFAEGKRLGEQGLRWLKIHLANVYGFDKASFDEREAFATDNLANITESVENPLNGSRWWLKAEDPWQCLATCFELKAA
YDLPDPTEFVSNLPVHQDGTCNGLQHYAALGGDTWGAQQVNLMPGSRPADVYSAVANLVKESIERDANAGSQLGKNCLGKITR
KVVKQTVMTNVYGVTFAGAKQQVCKQLDALYPEMFKETGIPNLVTATYIAQHIFNALGTMFRGAHDIQYWLGEIGARVCKALT
PAQLDQLQEEKDNKPAAKKTASGKNKELDELTSQFRSTVVWTTPLRMPVVQPYRKSITREIRTCLQSISYPTQGQTEPVDRRK
QLQGFPPNFIHSLDASHMLLSALKCDELGLKFAAVHDSFWTHAADVDILSGVLRDAFIRIHEEDVVGRLATEFEARYRGSLYL
ANIPSSSPAAMKIKEFRKSSKLSHKEEVLLEHKRNTLLRSGNPWDIEAAKKIVTPASIVEEMSASQEEDPEIKKETEDIGGLG
HIPESEGNLSSAVEEAVKNMDPADDKIAEMSHKLLERLKHTSFEYRVIQPKKDVKQNWKRITHFWRPLTFPDLPKKGDFDVKR
LRESQYFFS
FUN_014611 CDS scaffold_7 9034 10204 -
hypothetical protein EOG09263JFQ PF01588

MASFASQTYTPTEEAEIQQWLTTSERLKSPEDKSTILETLNNHLSSRSTLLGSKPSKADVAIYETLAPVVAKWTPEERTGEKG
HPHIVRHVDFVQNSPLFGLDVKDENKVQVDRDNVLYVKPPVDAKAEKEKKKKEAAAAAATGGEASLVDRTKEKVKEVVETAKE
KIATDKPKKEKKEKAPKQKAAPAPAAPLSPALIDLRVGHILKAINHPDADSLYVSTIAVGDKPGNEDYVEYEGQICRTVCSGL
NGLVPLESMQGRKVVVVCNLKPVKMRGIKSCAMVLAASPKIKEGEVDDHKGPVELVTPPEGAKAGDRVWFEGWEGSPEGVLNP
KKKIWETFQPGFTTTDGLEVAFDAGAVEQLGKTGRGRLVTESGGVCTVPSLKDAVVR
FUN_014612 CDS scaffold_7 10898 13666 +
hypothetical protein PF00176;PF00271;PF04851

MAAPKSPSNSSSARSNSRLPTSVPSSPPSEQQDDLEGADDALIKEEQEARLENERNEEKRRQAMLKRKKKKKAETKSEREAKA
RELDDLLAKSAAFSDILTKKTQVLGRVGSSLDGKTLGEHSLEMAQQPKCMINGTMRDYQLEGLTWMYEICSQGMSGILADEMG
LGKTVQTIALIALLREQENYLGPHLIVAPLSTLSNWMDEFHKWTPSIPVIMYHGNKDDREKIFRTQMLKHLKAGRPTTKFPVV
CTSYEMVLRDQHNLSKINWEFIIIDEGHRMKNADAKLFQQLRQFSSATRLLITGTPLQNNLKELWSLLHFLLPNIFTDWEAFE
SWFDFSDLEDEQGTEEFIADQKKQELVKKIHLILQPMLLRRIKQDVAAYLPKKREYVLFAPMTKEQTDLYNVLTNKKVDTRQY
LEDKVYAKIHGTESAEASTKSSRSSSLAAPKTMTLPVRESPRKKQVEPEEPAANAFSVMMAKRGRGRPRKNPKPEEAPVTPKS
GGKRKGAPAFLEPEPKSAKSTRQSTPVSTRGRPRKTRTYKDAGSDEEKMSDDEFEAKLAKEMVSDDEDLGDQVSLTPEERERA
KAFELAKKQISQKKLGNPLAQLRLVCNSPHNFYNPWIASRDLPVDDSIVTASGKMLLLDRLLPRLFQDDHKVLIFSQFTTQLD
ILEDYCRELRGWKVCRIDGSVAQDSRRTQIADFNSDPEYKIFLLSTRAGGQGINLASADTVILFDSDFNPQQDLQAQDRCHRI
GQTRPVVVFRLATKDTVEETLLNSADAKRRLEKLVIKKGNFKSMGQKMDLHEDIDPESLRALLLKDGQVYKASGGEEVLSDAD
LDILCDRSEAAYEKAASGQGDADAFRVVETGADSIKMARKD
FUN_014613 CDS scaffold_7 14294 19927 -
hypothetical protein PF00023;PF12796;PF13857 ENOG41

MNSSTVNARQRRIIQLSQQWGFSLPPNAPTPLTRPPHPTSFRTHGDDEVADGLLQRRSAEVAQLRPKSGLSRAFSSSNLKKGK
HWDTKEILNVLNSWVDSSGSPGVAESLIAKLAAAGVDLGGMQTQKQGLLNRRKSVEHGLDRTRLLKSAIERDQLDMVHVLIPH
ADPLTLDICLPAAARTRNARIVEVLLRYGANASNTAEGQDEFRKACSVPALSEIASLILRSDGRPEEICTSQALTDATRAQCF
DTVLHLSRSTADGNYNNAEALKIAINDQRRDIAMAIATGNKPPQSPGLDEAFEQLMNNTELQPRSKLDFAELLLCCGAQGPIL

ERSLEVACETQFFEMADLLASYGASIEHNDASALKTAISKGQTSLVGSLLNGSSKIDPALASSCVPLIPEHADSGTRQNLLYS
LLRRGANGDVLNDCLILAAKAGDVHSVELLLNPHFREPPSGKQNGQVKPRITNRHAVASPDYQNGEALRIAILAGNAEMAAKI
LFARPSNETLTAIFPLVKSLPPSDRYRMVEMFLRGALTGPALHKALQDAISEDPSQRDDALINLLLKYNADINYDDGAGLTQI
INQMDLGLFTKLMQNASPQTAAAHVNNVMSIDNHQIRYEMMSMLFNARASVAGQQVAEALLANISETPVDMSLLRLILEQGRP
DINGLNGQILQKAAQTPDPKVIDLILTLGKHNHDTISVCLEGLAKIPSSENKAWKLDVILTRSPKKINLDAMLVHEVQTLIED
TSGNASFKTLQKLLDRGADVNAFQARALCSAVKEAKDTIVDTLFGSERPPTAASLAFALPQALNILDPMTRLTFTKSLVESGA
SPREVNRALIHAISHFTTDVALICVLATYADASDGEALALAVAKESVEVLDLLLSQLKHTDETRDSCLDQAMKIADWTVRLNI
CNRLAKSGVSSQAASNALLVAARDGDLELGDVLIAHGASISTNGGQAIIEACRGGSVEVLDVLLKSNADTQKLTLERGFQAAT
EVRDLNKRAMVFQRLLKRGVSGEVVDIQLVSAAKYGEEGHEVLRVLLAAGADPNYSNGEAVVAATRSAFVGSLELLLGLWHEG
GNQKKASPPTLVRAMKASWKLNQDTRLHIVQDLMKAGMPAVDEVHIALSKAVGEDEPDERLIQVLLDSGASPFTNGCFALTQA
VQGVKVSALDLLLGSKIDEDEILKLFENGFSETQFSTWFSPNGLEVAKMLLAKGAKGVAVSSALVQVMKNSTDETEPLADEFV
DLFVAHGVDIDFENGEPLICAASKGNAHWTERLLACKPSPQTLTLAFERVFDTARSPDEVLKLFELFSGYRDGEVGLDVMTRS
AGSDPILIKAISQYPRSTELLQTLLDAGFYHDQTTTYRIHDSVEEPEEVTVLAWAIAQPQKKVSSALIKMLIERGANVNFETT
ISRSTPLMIAIQARRPDIVKELLLEGANADITDAMGRTPLAMATDIGGELSTRMMGNLLAFDQSKDDGSLHNSARELNFAAVS
VLVEHGHHPDFPSPLHDGRSALGEVCRHGAGSGKMTADAEKLMQKVMTFLIDAGSDLTLQSHGKSLLLLCFDSTDPIATTRSF
LKVAMWKEINKPFNHYTDGEFTYSPTMYVSKVMPESDTRDQLLSLLRANRAVDVYYANSGDQPDDAVGLPNDMVVQERERKAR
LRRLALETEDHAIALARAKEVASVEREIWNHKAEIEDARRRRLHNEDINATRQKADLEESLFHAAVKRRQLEQSKVTQSSLER
IQQVAAAELLAEETRQQKMIEWESKLNSERAGNAQALSSLRISEREELERIERGADERVKRRLEAQKKLVETQERLAKRLASG
PGGGADARRQIGYVEEIN
FUN_014614 CDS scaffold_7 22787 23823 +
hypothetical protein PF00107;PF08240;PF13602

MAPVPPKMKAVQINKNGGTEVLEFNDVAVPKAGEGQILVKNQIAGLNYIDTYFRSGLYKAPQFPLTLGREAAGSVVDAHSSVK
GFENGTRVVFMGTVGAYAEYSVVNASDAVKIPDEVPTDQAVAAYLQGLTAWTFIREAGQVKAGQWVLVHAASGGVGTLLVQLL
RIVGAKVIGTASSEEKLALAKKNGAEWVINSHDDVVAKVKEITGGHGADVIFDGVGKVTFDGDLEMIAAKGNLISFGNASGAV
EPVSLFRLTPKCVRLMRPVVNGYVSERADLEKYTSELFDLIKSKKLELTIHKVYPLKDVAQAHQDIESRKTIGKLLIKID
FUN_014615 CDS scaffold_7 23992 25356 -
hypothetical protein PF00106;PF01073;PF01370;PF04321;PF08659;PF16363

MPVGTVLVTGGTGYIGSFTSLALLEHGYDVVIVDSLYNSSEVVLDRIELICGRRPTFYKVDVTDEKAIDEVFAKHPAIDSVIH
FAALKAVGESGEIPLEYYRVNVGGSISLLRSMERNNVNNIVFSSSATVYGDATRFPNMIPIPEHCPIGPTNTYGRTKSMIEDV
ITDFINAQRSNLEKAGKEFKQWNGALLRYFNPCGAHPTGIMGEDPAGVPYNLLPLLGKVATGEREKLLVFGDDYSSRDGTAIR
DYIHVVDLARGHLVALNYLRENQPGVKAWNLGSGRGSTVFEIIKAFSKVVGRDLPYEVRPRRQGDVLDLTANPTLANKELNWK
TELTMEKACDDLWKWVSNNPKGYRQDPPAEMVEAVKASKA
FUN_014616 CDS scaffold_7 27036 30181 +
hypothetical protein ENOG41

MPPRVSRRNTRLTSREPEQQPTNESTNTLVRPHLPALQGTPSTRRQYTYGSGAEPPPKVGAGLQRMDLGSAVNQALANNADDE
EEFVRPAKPRAASARADDAGPSRDNASRSATIAVQQLATGRDDDSSRSFGLESDYYEDATIGSVPPLIVRTERQPAASNPNPR
AQRDTSVQRSLQPSRLREQPSLETQPRRPRDTQQTSLQIPRQTRQKSRELEEDEPEERAAPATRSRTTVNTPAPNKTRQSQPR
PRNPTVVEDSEESESSAGSDEDDIESEIQYGGNASKARTITKKSTRTVANPIRRQPAQQLSRNAAPERRDQRTSGGQSLFSRI
NRPASQSSSFEKARQIPHDPQERVWDIQREIQEAEDQIARERAERAERERAPQIEPWRQWFREQIAWLLTLWPFNLIWRRRED
DDFDDFDDDQYNNGGPTDWWKLLNPMTYLQSVVWLVDKIMDHFVNLLDGLSGVQIRGSWTERVVYAVLIGGVGLLLGVSLVSG
MGAVKPSLPSIPNFGLGGFHIPNPSGFVHNVGDMMPSFSLPSWPPWSRDDDEHDVFAETPINNIELTDEVKKAMKKLTSKLSD
QKKSLEKLEAKLPQYITMETVNGKPSIPQQFYHGLQDYLKESGSFLNMKKKGGNYEISTEKQWKAIVAGLGKDKDFKSKLTKV
VDSGIEEKLPAFWDTWFKNNNKILEPLIEKAMAKTKAAGSGAGFDKKVAQIVNEQLGKQNQTVVTRSEFIKHLGNELATHQAT
VRAELQELKSDMLTHIQESIRTAQLMAPQGTSDAEMRKLIREIVIQTISDEVLGVGAKAKIHEDWNTRLKHQVNFFAVGAGAT
IDEALTTGVWDPWKKGVATKEALKRGVSGVHPLPAAAALLPWQDEGDCFCSVHDIDSSGRYHGARISVHLGHLVVPTDIVIEH
INKNATTDPLARPRQIEVFARFEDKMERDQVFAITANWFREDTTNRNVSVPLSDQFVKISDFEYVGNELNDGVHVQHMKDEFS
NLGIGTDQIVVRSMGNYGAVDHTCFYRVRLFGNVVE

FUN_014617 CDS scaffold_7 30524 32962 - DBP4
ATP-dependent RNA helicase dbp4 EOG09260YMF PF00270;PF00271;PF04851;PF13959

MPPHARSGRAPKPQKNGRTEQRQQKRKREQEDLQQLEQRVNDLDPKSDEIKNFSHLPLSVPTASGLEASHFQTLTDVQAHAIP
LALKGKDVMGAAKTGSGKTLAFLVPVLEKLYRAQWTEYDGLGALIISPTRELAVQIFQVLRKVGRNHVFSAGLVIGGKSLKEE
AERLDRMNILVCTPGRMLQHLDQTAGFDANNLQILVLDEADRIMDMGFQSDVDALVEHLPKSRQTLMFSATQSKKVSDLARLS
LKDPEYVSVHEAAASATPTNLQQHYIVTPLTEKLDTLYGFIKANLKSKIIVFLSSGKQVRFVYESFRHLQPGIPLLHLHGRQK
QIARMEITNRFTAAKQSCLFATDVVARGIDFPAVDWVIQADCPEDVDTYIHRVGRTARYQSNGRAVLFLDPSEEPGMLKKLEQ
KKIPIQKVNVKEKKKKSIKDQLQSMCFQNPDLKYLGQKAFISYTRSIHLQRDKDVFKFNKLDLDGFAASLGLPGTPQVKFRKG
EDIKRIKNAPRVGMSSDSEMEEDGEKPKKKNEVRTKYDKMFERTNQDVLSSHYNKLVLDGDENADDDDGDFLSVKRVLKDDEL
DDEANNAFKSTAKVIDGLGGEEPFVVDSKRREKALKSKKKMLKFKGNSTKVVFDEDGNAHAIYELKDEDDFMGEGPAEEQRRK
FVEDETSRVREADVDDKALAKQKRREKREKRKAAERAERMGIVSDDDGDASMLHNADDGEDPLALMRSLPMGGDRSDSEEDRE
PPKKRAKKWFEDDSEDEKKSKGKGRVIKVQDEPETLEDLEALATGLLD
FUN_014618 CDS scaffold_7 33247 35323 +
hypothetical protein EOG09261FAX PF08159 ENOG41

MKNDKKTKKAAPAASQIADERFADFETDPRFRLPSKKQTKTTIDKRFSRMLKDDEFTATAKVDRYGRKVKSDSKKKALQRLYR
EEDEEEDEEEGGEEEDGIEVEDDEVVQRELRKAHEKYDPARGGGFSSSEDGSDSEEEESDSDEEEGGAQVDTEGDMQRFQDEQ
NDVEAGEVTNRIAIVNLDWDHVKSTDLMALFNSFLPETGGKIDKISVYPSEFGKERMQQEEVEGPPKALFKNSKNDSDDDSDD
SDEESEDGDERIKKSLLQEGDDQDFDSDALRSYQLDRLRYYYAVMVCSSPAVAQKLYEAVDGREYQSSSNFLDLRFVPEDVTF
DDEPRDECEKVPESYKPVEFVTNALQSSKVKLTWDMHPEEASRKESINRAFSGSRNEIEENDLRAYLASDSEDDDDVEEEEVV
EEKGEDEPKLSKKELARRKMRAALGLSEEPTKSSKNAPVGDMEITFTPALSESAPKKTVEEETTIEKYIRKEKERKEKKREAA
RARRAGQDPDAEEEEEEAVEVPAGETDDLGFDDPFFTAAEDPAASSKTSIRKADRLKKREAREAAEAENKAQKKQLEKVMADV
DVDQADHLDHFDMNEIVRAEKAKKKKGKAKKKALAKESRGGLQEDFSMDVDDDRFKAVFESHEYAIDPSNPKFKATEGMQKLL
EEGRKKRRNAEGGDEEPRSKKAKKGRR
FUN_014619 CDS scaffold_7 35509 36746 -
hypothetical protein PF08241;PF13489;PF13649 ENOG41

MTTNTTPDTDPTPDDNQGLLHGNAVIEAGGSDEDGEFSASDFDPEDSSSLRSSSTSISSSILEHSYQNGRRYHRYRHGRYPLP
NDEAEQNREDMLHAMMLEATDGRLFYAPVRENPQRIADLGTGTGIWAIEIGEKYPSAEVLGLDLSPIQPTWVPPNVKFYVDDI
EDEWLNGDDFDFVHLRNMIPILKSPVTLLKQVYANMKPGAWVELQDVDGQVHTDDNSIPDDWPLKRFTEIILQAFALYETNAH
AAVFGGQYLAEAGFVNIKHNFIKLPYGTWPKDKVMRLVGMYYRTACEEFFPAIGNLHFPQLGWGKDEMEVFFAECRQSMRDPK
VHAYGKMHFWSGQKSLDA
FUN_014620 CDS scaffold_7 39317 39977 +
hypothetical protein ENOG41

MPPHSNSPLLPISTPTAIASPYEQPPPRAPTAANHVQRFAITTFSLARFARGVSLIAYPRFGLWALDIAPDGSAYMLASLIGI
RDILLSGLLYTSDTTNAATDSAARREVTRALATNLFSDALDAFVLIFYAAWSDDWGNPIAVIVISAVMAIFEHLTLWSLSEDD
WAAHEYGSLGDDKKARMNAWLRELERCGPETYRPEQSVPPSSRASFRQYGAIV
FUN_014621 CDS scaffold_7 40708 42264 +
hypothetical protein PF07992;PF13738 ENOG41

MSVPRVKRVAVIGAGPAGAIAVDALVQEKTFDLIRGFERREGPGGCWIGDTTQPPTISNLAKLADRTADEQLPIPETLPTLLP
KSTQPRHEESSLYPYLETNVDTSTMEFTQEPIPEIRSERSIGMHGPETPFRHWKVMRDYIAGILHRNHYEDLISYNTTVEHVE
KTGDEWKVVLRKEGKKQDYWWSETFDAVVVASGHYWVPYIPAVEGLEQFEKTRPGSVVHSKHFRGRDSFHGKRVVVVGASVSA
ADIAVDLVDTAKSPIHCVTIGHTTNVFFGDTAFDHPKIQQHPSIAKVVNRTVHFIDGTSVADVDHIIFGTGYSWTLPFLPSLP
VRNNRVPGLYQHIVWQKDPTLLFVGAVGAGLTFKIFEWQAVYAARILAGRATVPSQEEMQRWEDERIQTHGDGPKFSVVFPDF
EDYFEAVRELAGEGEAGVGRKLPKFRREWFRNFIEGTELRKGLWRRWNAKAKADLEKDGVKARL
FUN_014622 CDS scaffold_7 43814 45530 +
hypothetical protein PF11951 ENOG41

MQPEPSFVATPSAVFDVPLDTPAPALAEELPIEDNTSILGDASWTTSSCTESDNDIFARSNSIVEENLSDMAVQNVEMTSQAL
TPTNESQYFEGTPLTVGLITDTSSLLISNWFEQVCTLWSGFDSDFNLNRKLALELCTSSQSVFSSLQSMSAGFLSTRLPHMKQ
SAMYFLQAATGSVLSEAEEIIQNPAVDNMPAGALFSLFCLGTTVCWVDARQLGLPFFRSARKILNRLNRRISSLSAKDLELLS
FFNRSLTYCEMLLAAVNQDDTALDTDDPVTGPQTDSSMQLAERYMDNSPHPWTGISPLASQLFAQAIRLCRSFRRNLKLPKET
GRDFQRALEEIQEAQRLEEKLLGLEFQNTLCVANTGDYRTPSLHLAQVAEAYKLAALLQLYQTFPDLVALRLPTDSVHANSRH
IPWEEWIIPLSLRLVNVLEHIPPNSGTRCIQPLLYITASTGLRYDTTTMLDISTSSFDTGIGSSNFQTDPFSMDMLGTSPPPA
NLISRMSLDISNARHFITGRLNMLESSLPPKPVMMAKELIKAIWDAYDSEAPGSASVHWVDVMEDNNLRSMFG
FUN_014623 CDS scaffold_7 45682 46036 -
hypothetical protein PF05899 ENOG41

MASFKHFAQAQSKFEIPPLSGGNTFIGDIHSTPEGAEKPLAMGLFRVNKGEPLTYTYKYDECKIILEGNFVIEDSTGQKVEAK
AGDVFYIPNGATLTFSSPDTGLAFYTGARKMGDL
FUN_014624 CDS scaffold_7 46602 47808 +
hypothetical protein PF11927 ENOG41

MIMDFTLSSFGPHEAVILLSIITSLALLGYKLNDIIQSRKSFRYKSKLPPVSSNYQPSDYKTPIPEPYPNWSIDKTKPLPYRA
FRYGPNYQVTMGLRTCPVEEWIELDNHYPKYHADKAARIKERGTKCVDTHPDAYPAAIELLQELADYLPARYPSLFERTAVGI
KNKWSGEDFNIIERPLAEDPMAICARLIQDDLAIMVERPDGQYYLLAGAILLAGFWRLSDKYGMSLSEIHTSGDVPHFKEKLE
TGMCKFFKRLRPETVYNRNNYFLQVDDSLAWSWSIGSEDAPSVSWSTAEKDRAIEHHMFRSERQSLRRLPKTGGVVFTIRTYF
HPVTEIAQEDHVPGRLASAVRSWDDKVANYKGKQKYGEVLLEYLDREHEKQLARGLDVEKEGEIRKYPW
FUN_014625 CDS scaffold_7 48090 49637 -
hypothetical protein PF11927 ENOG41

MLVFSESLHPNTVHTVLVVSFCASACACLGLLAHALVYKRKALNRYAYPSSPPPEPVKKTAPVDYTTVYPPSQRHALPEISPG
FGSDDVDLSVASKPLLRLDEDYRHANPSTFNFTSFSVGEIRRLGDFPDYAKLSGVPLPEPLKSFNVDKAMPRPYRPFRWAYHQ
TMSLKKMDADFWLEIENTYRERVAQRQSLYAKHGKDVLQALPGSELACKELMEMVIQFLCARYPQAFELHGKVFVNHILAVKE
DLDETEPLLFLLNHVPEDFALTLRDPATGRYCFRAGVICSSVGWKLSEKIGLGLPEIHAPVPDYKEKMEFSMDRFFTKMPTNS
PIQRGSWGLEVGQPLFIPSEDPEFKTREVQNPSLTEEDISLRVDWQTLRRLPLSGAIVFNFKALFTPMTEFRDEPHVPSLVLK
VLNEGKENIMKYKGTWHVEHITKPTLRKWEREQIEKGLIPKDWEVTTLAENPFFPGWERKWTVN
FUN_014626 CDS scaffold_7 50981 51362 +
hypothetical protein PF14087 ENOG41

MWDTFFYRGAFVISAIPGLIGINSMLRPEATLKIVKFPIPADPQSRKTCLSLARLQGARNIVVSYLFINNALTGDKKLMGMGL
LGALFMLATDGFVSKSLIGGNEWFHWGYIPVFVAFIGGLLYSE
FUN_014627 CDS scaffold_7 52091 53398 -
hypothetical protein PF00172;PF04082 ENOG41

MGKFQGRKLVACTACHSQKLKCSGDIPCHRCIQRNRECVYPKKDKFITVPESYLRDIEEKANINSQFSFLAGADDSTALSDTS
QFSPTTEASHIKELCPAEIFIQKLTDISALLHPYIQTESCQQLRVGEAGEFVPFRLKSEDGGSQMLVKLPSEKQAMHLLRIFE
GANSEYHWFLRQRFRDRIRQTYSQPTSYVDDRNWFCQLSLVLALGQALEKEPKQESEETNDPWDFNQPSTPLDLFGQAVSLFI
ISETLTLENLETLNLMAYYCHFTNRPKAAAIYISQSVALSRLLQLDDPEIYQPKISERQDSKSRCITKEHMLRLWWTTVCLDK
TLASELEMTPVDLSPSLELPLPSSEGLSPEDEEEFFDLELLLAEIRS
FUN_014628 CDS scaffold_7 54037 55484 -
hypothetical protein PF01266

MSKPSVLIIGGGVFGTSTAYHLIQRGYTNVTVVDRFAAPSRDSAGTDLNKVIRADYPNPYYAQLGLETLGVWKDPDSLFKGLY
RESGWIMGGHEETNQWLENAKDLAEKKGRQGVEYLDVERIREKWPALTGEFPGWTNLYSPQAGWVPSGQALLCMAKAAEKNGA
KYITGHAGQINELVYEDGTCKGAVAANGEIHRADKVIVAAGAGLPALVEGARTDVRAETSVICVMKLEPHEIEKYKDIPIIDD
FEQGIIFPPDENGLIKLCSVRLVTNYFNHVHSGASVLHSVGDYPFDGCPRELEVEIRKFVREMIPELADRPFVHTRQCWDGMA

SDLNFRICPYPDTKNLYIATAGSNHGFKFLPIIGKYVVDLLEDSLDSGLKNLWSWKFGKKPDDFQDPHPFPRRDLNELTGWKG
RNAPADGKLPWTWSRSRL
FUN_014629 CDS scaffold_7 58511 58981 +
hypothetical protein ENOG41

MNTPNRAAVVHPQYDAQSTQQLLALVQHLQVTSRTMAKQTPASDQKDSTIVTDIGDYLIQAAQAWAQMTDALVESRQVQQAVV
DAHEQAMVNNHLSDLSTVVSMDVDLTKLSKIRDLTAQFSRDVHQVWQNDAPCPGKSP
FUN_014630 CDS scaffold_7 60265 61075 -
hypothetical protein ENOG41

MPDFRPLFFIAKVLTCCCCCSRRKPEDARAADRPYGTSHGQRTGSSSENVSDYSEYSVPPAYPGGPMNLIHPDEFEHSDELDE
VHGRDEFGFDLQKSRSLVPCPPTPRPSSSVDSEPGTTRPSTAEPDTMRPSTTKSTNTKTISDEPVASPSVIVEITATDSVTEM
LLADEPSVTKLAGPVPIRDEPTATKPVSTEPEPEPEPEPVEAGPTDTKTDRPNFVNVDLAEAGPLKSDIEASEVNNGTTEASS
DAVGPTENKDEDKGKEKVSV
FUN_014631 CDS scaffold_7 61773 62558 -
hypothetical protein PF04117 ENOG41

MPSPIVAATLQAAGLSTISNLLAQMIEARQQNRPVSLDIVQLLRFVSLTLMTAPPNYHWQSFLERTFPAYPAGRGPQLGRLND
LEMQPTEDAPELKEGYEERMAQINKDREPQFSMRNTLTKWFVDCITAGAIMNTIAFLIIMGLLKGQGGSQIWSNIKTETIPII
VAGYKIWPVASIISFTFIPVHRRIVFLSFIGLLWGIYMSLVASRV
FUN_014632 tRNA scaffold_7 63336 63418 + tRNA-Gly

FUN_014633 CDS scaffold_7 64385 65356 +
hypothetical protein PF00657;PF13472 ENOG41
CE3
MHFFQKPALLLLPAAALAGAIPYEVRKDFANPKSGITLKKAFDIHAQEENGFLSYKGTQPGKVVQDGVKLRILSVGDSITVGY
GKGTDGNGYRKRLRKDLSGNEIVWAGTEKTKGNMKDGHFAAWSGKTVQYINDHVDPSLEQRPNLILIHAGTNDMNSNHRVSTD
GNHPQETTNRLKSMVEKMISKCPDATIIIGMITDVCDNKSYHFQRERTKIYRGHIAKLAAELSKDGSHVLAADFGPFDDTLLS
DCVHPTQKGYEILGDWWYDFIHQIPEGWIKDPVGPDPVRD
FUN_014634 CDS scaffold_7 66149 66809 +
hypothetical protein ENOG41

MPHHQRQSLSQGPFEGLEIDHEDQHLGREIRRMMSSTLSKATFTINMGDAYDYNILQENLKQLHQEFRMYISDIADGLKQSDD
NKNALDEYITDELARMVYGSNMIEVAGGGVDITLKLCKDVFQGKELPEEINERDEDYHDLKIHLLNKNLPSGHEAILRSRREI
VQHAEAARYMIDQVCIKGKDLSHKIISEAHGILTYKIDLGDTPWPYYSGVYRT
FUN_014635 CDS scaffold_7 66836 67241 +
hypothetical protein PF02661 ENOG41

MDESKVPAAMRDMIDELDSDIKKATEEGQIDPVVLASKYCHKFVNIHPFADGNGRMCRLILNALLLKYSGRFICIGQDGQDRQ
EYLRIAVAASVEEGSQSQETGAANYKQLASFTLRHARDSMRKVRQLFKRDA
FUN_014636 CDS scaffold_7 68666 70715 +
hypothetical protein ENOG41

MKCQWPSSGSGRVEKTCSRCLRMKLDCQVPEITQRRKRGKSTRVAQLEKKIDGIVSLLSANQRKGLSPLTPESPQEVQSHSQC
QPHTQENRFTANHDMINQIPDDSIRFSTDVELFPGFRISQQEASDRLDVYRRDYAPHFPFVPVPSSMCASELYVESRVLFWTI
LAVVSPLGDKVQMEFKAWFRRYLAEHLVVRQVKSIDILQAMLIYLAWNDFHFYGELQVTNIIQMAIGLVIDLRLDKHAGHFLG
GPKTLLGDAWTSMSKHPIKMKVHQTAADKRAVLGVYHITNLLSPYFRKSTIFHWSTHLASCCENLTEAREYESDIYLVSLVRM
QHLADRGFSAIPAIDPFDPTPPAFHAVTAMALDTVHRELENYFKLQPEIVKQIPGFRAHYNSILVRLYEPILIMKPSSLLSPD
TPLTEPFLRSEYLWKCLEAVQNTLENHVTLPPEKISILPVTVSCVLAFVTVTASRLIMAENSTDWDVKAARRRLQFQDILQRL

SDQFAQADEEAQRLNRRRRVMEDGSSVFLKSCFKVRWIRQWYLSKIPEEEQQLLVQQPQTAVESSSMLQSNPDWATNLQFDDE
FWADLLTGFDMEAFDRSLTTVAAQ
FUN_014637	CDS	scaffold_7	71060	71686	+
hypothetical protein		PF05241	ENOG41

MAQKAARDWIYLVIISLQLVGMICLEFTEFYPESIYSAPNAPLHFLANVKEQYLSFSGDPFFGDKFHGAWFRSMFFIEIFVQF
PLAIYIVRNLAAKKPSSGPVELAGLAYGCLTAMSSVACVAELLEMGPELVSEEHKRNLVWGTYFPYALIPGAMAVDMYTRLLR
RVSTDIKPKTQ
FUN_014638	CDS	scaffold_7	71829	72766	-
hypothetical protein			ENOG41

MIAPIAWAVVALGALTPLVTANPEDGLPSYHYGAPIPVECMNRNSETGEHVENEKHEIEWKPFPVCNETGRPLEFNYGTEGEV
NCTIAMIDDPFFHLLEFYIHSDAPLSCRIPARPAAEVEVVGEKVPEREYVPVVFALAGTLQLSHIHISTHMNVLLHSTPKHHT
HAHDSGVLDSAIAYSTSPLHHLQGSHTRKLVIGDPLPLSLSVRWFPTPALPKTEGKVEWQGLGGHIYASTVFYSLVSFGAGVL
CATMYTLGVILPRRLKGRALGGATPLGYGVNTMGNGWGYSKQNKRMD
FUN_014639	CDS	scaffold_7	73474	74463	+
hypothetical protein		PF01209;PF08241;PF08242;PF13649;PF13847
ENOG41
MTSSDTENALKTLREHYDSATLEAQMLYASKTMMPHLIKPLPTQMGISENTSKPVKFLDNACGSGVLTHAVQQALPKDILEKS
TFLCADASDGMVSVSKKRLDTEGWVNTEVKKLDATNTGLPENSFTHVGLGLALHVIPDPNAVLVDTKLILKSGGIFGATTFHK
DNTFWISDIRTAFASFPFEAKLPEVMKMQMHDQGDWANPAWIEEHLKKEGFQDVKVTAHNDSYTIESAEDYILQFGMMLGWLI
KTWWSEEVQREHSLKEVKELLRRHLVEKYEGKGWDIEFKVICMTGRVD
FUN_014640	CDS	scaffold_7	76000	80312	-
hypothetical protein	EOG092606UX	PF03177;PF08801

MSFPQTTPVRPVPGAFLNTPAVASRFQSGSDSVRRQLFTGNDSNQAVSHKSSAPVSSSRTVAATAPSAPRTTSNDNLIVPTAL
PPLRSENVPPVVKAARAINACLQSDGRYPDLDSYCRQGASSDYDLENTDSAMAPFHKTQMYPIPNQVFDHYNAGELQTLMGLF
AEINHAWVVIDNSLFLWDYTHPDPELIGFEDQPHTIQAVALVPPKPGIFVNTITHILVVATSSDVVLLGVSATATPSGSKTVS
LYQTKMQLPLRGTDVRVITGSANGRIFFGGSNDIDINELYYQSEEKWFSNRCGKINHTNPGWSSVVTLQGGFWSHKTPEHLVD
IVIDDSRNLVYTLSSRSTIRTYHMEGPDRLNKVIEKEKVHCLRDIAHMITQSRLLSDQVSIVSISPITKQEAAKLHLMALTNT
GCRLFFSATSAASYIYGSSTNLAPQSMQVQFIKFPPGQTSRRSTYPGGETITDFESTSLSYSRQGARFAPGYFLDFVSKESNP
NEDTLFVSGPETGRLKNTSPTSPFKYHESASWIDIGSRAEAVGLITKPFAAAPQPLGFGNELAVQFDDAPSEFAILTNTGVHI
VRRRRFVDIFASAIRGAVGDEGLELVCRRFINNYGRVETVTTALAVACGHGGDSKPGAARAIDQATEDRARSVFVDFGGQPTM
AETDSSSLTTDSINLSSRHDALALYLSRLVRQLWKSVVIAPGVSPNGGVTIGSTIPLSKLNTIQENLERLRRFLDTNRGLIQG
LSGPSDLQHVSSRQEEVALQAEHQALHALQKLMESISEGISFVLMLFDERVADIYTRLDATAQQQLKELTYEKLFSQTDGKDL
AKLLVKAIVNRNIESGSNVETVADALRRRCGSFCSPDDVVTFKAQEQLKRASDQPLQTNQSRSLLHESLRLFEKVAGSLTFAN
LQSAVEQYIALKYYAGAIQLCLVVAREKDRGNTALSWVNDGKPSGDPRANAFNDRKRCYDMIHDVLSHLDAASSSEPEMVDGR
LTLIATKRLEAYEVVNGSDDEVFHFDLYEWYIQQGWTDRILAIDSPHVITFLQRLAGTNIEHADLLCRFYTNRSRFFDAAEVQ
AELANSDFPISIKDRIRLLSLAKANANVSTTGVSRQQQQLLNHSVTELLEIAHIQDDLLERLRADDRIDPERALEIEEALKGK
IQGLSELFNDYADQAGYYDLCLLIYHVADYRNHMTISGTWSNLIQQTHDEVMSRLENSEPGMPSPPLPYESVTSKIQNIAHRT
SLDSFIFPIQDLLPELCRYAVAYQQDATIGADPTWPVQLFLTLGVSHDMIVRVLEGVFDSQDYGFSGSVRNRMIELIVYVVND
WVAEARRRGGAGKGGAIGPSVADLLKRCDAALPPPGHGNNAGGADLADVRRVLKLLKREVDGLSQRVPTGSLRFA
FUN_014641	CDS	scaffold_7	81173	82230	-
hypothetical protein	EOG09263100	PF00106;PF08659;PF13561

MDAITDFLHNVPQPLQWGLAGIGALFLGSKILSYLQLVLSAFVLGGTNLRKYGKPGTWAVITGASDGLGKEYALQLAAKGFNL
VLVSRTLSKLESLSAEIQQKYPGKGLQVKVLDMDFSQNNDADYERLSELISGLDVGILINNVGQSHSIPVSFLETTKEELQNI
VTINCIGTLRVTQVVAPILKQRKRGLILTMGSFGGWTPTPLLATYSGSKAFLQQWSNALSAELADHNVDVYLVLSHLVTTAMS
KVRRPSLLVPNARNFVKATLGKIGLGGYQTAPNTYTPWWSHAFMLWFIENIPGANGPITIFFNKRMHEDIRRRALRKAARDAK
KQ

FUN_014642	CDS	scaffold_7	82616	83187	+
hypothetical protein	EOG092655IF	PF03694		ENOG41

MDVIKSILPEAKGVLPYYMIILSIISIGNSLQTYTTLHFSRRVYNGKFVRNPKLPPKTDKFEPEDQINKLVPAQNDPKATDQL
TPLAGRLFGTWTLITCVVRCYAAYNLHIGPVYTIAYWTYIVALGHFASELFVFKTMTFGLPQYFPFTLASISLIWMPLVRDYY
VEYN
FUN_014643	CDS	scaffold_7	83337	84581	-	GAL7
galactose-1-phosphate uridyl transferase		PF01087;PF01230;PF02744

MPDQILDDISHRRYNPLTDSWLLVSPHRTKRPWQGQQEAAVASKLPEYDPKCYLCPGNPRAAGDQNPNYEKTFAFVNDYSAVK
QEQPDYDAKASSNDLESLLLQAQGVKGVCYVLTFSPKHHVTLADMTAEEIVPVIEHWTRIYANHLTPSNPRAAEADKLAISMP
KDAAPSPEEQYRYMQIFENKGAAMGCSNPHPHCQVWTTSTMPEEPGKELVQMTKYRNQHGGRNLLGDYVKLELEKQERVVWEN
DAFVIVCPWWAVWPFEVLVLPKRHIRSLLDFKPEERLQFAEAIQEVTRRYDNLFECNFPYSSGLHQAPLDGTPEEMESAYFHM
HFYPPLLRSATVKKFLVGYELLAEPQRDITPEQATVRLRNCGGELYRKSL
FUN_014644	CDS	scaffold_7	85614	87356	+
hypothetical protein		PF00249;PF00569;PF04433

MGVIRKKTITRGGEGGVKYVCDVCSSDITSTVRIRCADSDCSDFDLCVSCFAKGESRNAHNPATHAFRVIEQNSFPIFDREWG
ADEELLLLEGAEIYGLGSWADIADHIGGFREKDEVRDHYLSTYVDSSHFPLPERCSPHDCELANEIPREEFQARKKRRIEERR
DAAKNAPALQPKTKPTASVPSCHEIQGYMPGRLEFETEYANEAEEAVQLMQFDPGDGLNPRTGELEPEMELKLTVMDIYNARL
TQRVERKKVIFEHNLLEYRENTKLEKRRTKEERDLLQKAKPFARMMNQQDFEEFNQGLLDELNLRQAITQLQEWRNMRIGDLR
SGEKYENEKASRIQKAIPMGSMDRERLASAQRSKQPPPPEPPSGSALLIAPELPARLLPTPAAEVNGDVKPLTNGHADGLANG
QTNGQVNGVTNGVNGVNGANGHATPKQRYTAQPISGVQPMPMTQDTAPDLHLLTPEEVKLCEVIRLQPKPYLMIKEQILKEAL
KTNGTLKKKQAKEICRLDSQKGGRIFDFFINSGWVGKA
FUN_014645	CDS	scaffold_7	87866	88208	-
hypothetical protein				ENOG41

MDLPLQHKVHSSQTNPPGSNFATASLSIVSWVIGIIVTLGFVIMFFKCLMNRRAPRPTAPRPSIPGPPIPAPPQGIEGDVELG
TTNDRHDKTTKSSTVGDQPRVPPPPYCRRS
FUN_014646	CDS	scaffold_7	88619	88967	-
hypothetical protein

MGLNIYQGVGIILVFLTAIYTCGLIRHYCCNPKAQTKDEERGDVVINREENYEGIFVNPPQEQILHQPVPSYTPISLRSPHDT
ELPAYKPMGTPPPCYVPGLKDFQPDSTQPSPV
FUN_014647	CDS	scaffold_7	89408	90188	-
hypothetical protein				ENOG41

MPVPPYSELPRPGNIPEGWPLTGPLSSIPTRPGPPIPTKTPFTDKRPDHKANRLPLKEEPTPKEDKELMPGHNAVHGPAHAFP
SATSFDRPGRLGTAGVSHITGIPLRSPPIAGKTVVQNRQPEDEAYEYTGLLVFLIVILLGAAFGGCLLLMRLVASVSRRLGRR
YGWKWVKRADSADLEDAIYYWKDHPDVFEQYHLDLLREILASYGGSSYLPVDEKAAEMGRLDSTDCAPLAGPRPVHMAPSQSG
PCWPGMPGYR
FUN_014648	CDS	scaffold_7	91297	91873	-
hypothetical protein	EOG092658QH	PF00581

MRILQRIIEPFRPQARKMTTIASLKRLSAKSLSEKILEEVNATDPTFAVIDVRDNDYIGGHIKGSTNIPAHTLDAMMPTLVRR
LKDKKTVVFHCALSQQRGPSAALKYVRERDGLLKSMGEDPKGESGQDVFVLDRGFSGWQEVYGEDERLTEGYVKDLWDNY
FUN_014649	CDS	scaffold_7	92930	93797	+
hypothetical protein		PF12917;PF13023

MGSQPEKENGRVDLRPEEVIGDLGFTPMVKVADPWTVDKVVKEIPGGAPTEESSSPLPFFHMLERLKTTKREGWRRFGISRGE

SIADHMYRMSLISMFAPPSLAPKLDLPKCMKMCLIHDMAELLVGDITPVDGVPKPEKSRREAETMDFLTKNLLRNVAGGTTGE
DIRAIWQEYEDSETLDSHFVHDVDKMELLLQMVEYEKRGEGKVDLGEFAYVATRMTLPEMKAWGQEVLKEREAFWGSKEHVHG
EQGVNGGVTSERKGQQDNYYNKD
FUN_014650 CDS scaffold_7 95065 96442 +
hypothetical protein ENOG41

MSGSFNREGVHPGLFRPPISPSSSTSYGYASPYSTATLDGQATKRKRPCHDISRSQEFRFQDDSDRKGYFGTPVAQMRNEEMR
YQLAGQLDTPSSGPFDSGILGESMYSDTDYRKALGSKRTHDDIDHGAGPTQLFNLPPQPMQSQGWGSFAFSTIGGVVGRVWEF
CKTGSFRGFQAGGGKAFTMNSGRVEELDQTLFESQHLPGRFPPSQEDYFNQRQPDNADQGGNSGASTPTAPHPKRRHTDTKDE
LGRNWVMVSDEKPAEPEPKPEPPLRRPSYRPSPRSRNSGPSMTTGRRISAAPSARFSSASTPGQRRSTTNRPTSRLSHAAASP
TPRPASTASFASLAPLASPRIESPSRIPQPTRSTRRNTTISSPITKPSSHRRSHSNASVASGRTSAEASPRLTAEAKHLAARR
KMEERDADVRINAFNKQLQDMIRQGREALGTTIEVEGNWEDDL
FUN_014651 CDS scaffold_7 99287 100220 +
hypothetical protein PF01522
CE4
MRFSVLATCAGLLQLAAAAVPVGVAITKCTVDRNIALTFDDGPFAYTNELLDQLKKYGYKATFFMNGENWANINDYAATVKRV
VAEGHQLGSHTYSHPDLATISNANVRTQMTRLEAEFLKIVGKWPTYMRPPYFSYNANTLSVMKTLGYHVINADVDSLDWEYNT
PAETNTALNLFKKGITNGGSIALFHDVHENTVRNLIPKVLDAVKRSTRIPVTVGQCLGDPAANWYKTTKRTKRDFEDDEEVED
EKTVDERSLSEYIERSRFRRHAHHA
FUN_014652 CDS scaffold_7 105080 107464 +
hypothetical protein PF00172;PF04082 ENOG41

MTKSKASSPGSSASASATRRQHATARPGKARSLACRQCRDRKIRCDGARPVCDSCSRRGLCADQCVYPEIEHEGSIASSRSYI
RALQKRVQELEEKEKQLELVAHGHNAFVATTPAHEEWRNLSTSPSVVSERIARPPRHVDHALPPIHTDSGYSLPSFSSRAGSL
PVVHEPASYYSRPHHLMGASPPLTDDLEDAAPAKPSSFGSYSSNVKAALMLQKITLPPADLMDELLAEYFNIDWITMPVIHRP
SFYQRYHRLIAVANSRYRRDIPLEEATELAATYSLLLGMLAIGQLAKTSITESVESHASHAFEFHQQAKTLLLVDLLSVSSLP
VVQALIVHARFLRRAGAVQETWMLTGMALRLAEGLMLHVDLPGKAQAEREERRRAWCACALLVRMQNSPGSQTNPTFGTLPQE
IDDEYLEAFPHNPDRVQPCDTPSRLSFFNQTLKLYDDILQPIQDSVNHETGSVREPVSNILSDALKLDCDLEEWHAAIPEHLR
VKFPCTQPEAVFRRQATLLRMRYLETKALIPRVAIMKLISSNATQPTSIMAKSMLAGLFESCYAASVELEDIMARERRLVTFD
GPPESHSAIALSIIGMTLSVLVQHPLFRDIITNPAPVTEEATRCLTTAKQYTTSTHPLAERFVHTLESALHPPSRCVSPRGVL
DPALEPKTVGPVLSNLTEAPGAVEMALLEAWYMRRQDLASCGIPVTEIVSLW
FUN_014653 CDS scaffold_7 108453 110823 +
hypothetical protein PF00350;PF01031;PF01926 ENOG41

MTTAKAVKSEQGLGNRALLDKMDKLRELGISNMVPLPQMVVVGDQSAGKSSVLESLTGFHFPRSVTLCTRHATEIICRREETE
SIVVSIHAVDADEEQAKAFHRTATNLDAEEFAQIFKDAAKVMGIKSESGDGSTGSAFSRDVLRVEISGPNEDHLTVIDVPGMF
ENVTPGVTTKEDIELVKNMVQKYIQESRTIILAVVPCNGDIANQKILTYAKEVDPEGKRTLGVLTKPDLAVEEATKAVVVDLV
RGKRRDLQLGYCVVKNRSADDSSSSAEERTRAEKEFFSKAPWTKLSPDRLGIPALKVRVQQLLMDRTKSEFPKVRGDLATILK
ETETLLLKDMGQSRSTTEQQRIYIGQIAAQFTRIKHCGLDGQYSRENLFDTGTDLKLITRIREINEEFAKSVYDWGETRKFHRP
SDKEVTPAPSEEDEASEEAKAEPTDDRCGLYRRGISFDIPALGEQELEDGVLHDAFEWPEPNEEDIMQYIEREWLSSRAYELG
TFGGEMLPKNFKEQSKKWSGMARAHIANAILIVHHFISKVLDSCCPDPVIREELWNFLVDDLQTRYKRAVNHTEFLLKVEFEG
RSITYNPSFGNNLAENRSKGEDLGELVEKTTQTALQLCYETNIEVAKHVRNAVEGRPKPTNLATICQDIHDVLLTYYDIARSR
FVDVVCRQVIDFFLLDDTDGPLTVLSDQVVFHMTPEQLEAIAGEDMLSRDRREKLTRDVANLKEALKILRG
FUN_014654 CDS scaffold_7 111356 113835 -
hypothetical protein PF00083;PF07690

MPDYDEDMLPPPASGSQQDRTWGGNNAFHNFFNDFSHIPDPNLRRRLALSEIDKVPFGAYHVRAVLVAGVGFFLDSYDIFAIN
LITILLGVVFWGDPNPQNGFSGNDGYLPDRVNQAFKASTSAGIVVGMIVFGWLADGFGRRRMYGVELGIIILATLCCALISSS
PAMGSTGLLIFWRIIMGIGIGGDYPLSSVITSEFAPTRWRGAMVAAVFSMQGLGQLAAAIVALITTVAFKDAFIGAADESQCG
FECRLAADRAWRIIVGVGAIPACAALYYRITIPETPRYTFDVEHDVEKADADIKAYVASKSKGSFDVVHQVRSKRVAGRSLDV

PRASWSDLISFFRQWANFKMLLGTTLSWFFLDLAFYGLGLNNTFVLQAVGYGAGNSLFEKLHNQAVGMIILTCAGSLPGYWTA
ILSVDTFGRKPLQVFGFLLLTIIFCILGFAYKSLSQGGLLALYIVGQFLFNAGPNTTTFIVPGECFPTRYRSTGHGISAAMGK
IGAIIAQGISIPMVRQGSPESCKGTDCSPNMHRLLQLFALFMLFGTLVSLLIPETKGMTLEELSGEPRTSYNSGCNGSISLGS
PKLRAWNPFVGGQPAGFSYPRSRMSHFRKSDRVGIMTSPELMAETSRLRKPRIWRRHRKAKSSSGTDIALSTRSSGTGEDEIF
PAGPPASSSTPPQPPPTWGAGWGRIDRGGPPPLLNSVQLQDVGSLLTK
FUN_014655 CDS scaffold_7 118732 120406 -
hypothetical protein PF00561
S33
MPAVTMAENTTLNGDSRQHYERTQSQPENPFSALIPEQQIAIVPEFTLESGVTLYNLPVAYTTRGKLNEEGNNAMVICHALTG
SADVGDWWGPLLGGPGRAFDTSRFFIVCMNSLGSPYGTASPVTAKDNDPSKGRYGPEFPLTTIRDDVNLHKLILDDLGVKQLA
AVVGGSLGGMFVLEWAYFGKDYIRCIVPIATSSRHSAWGISWGEAQRQSIYADPKYDDGYYSFDDPPSTGLGAARMSALLTYR
SRNSFESRFGRNIPDPSKVQTIGGRPRPSTPSEAHFHIHNDGHVVKRTSISRQNSTTDAASSAESPALPDSVADPQFHGPTNS
SLTGGEMPQSHYFSAQSYLRYQGRKFVTRFDSNCYIAMTRKLDTHDVSRNRADTIADALAMIQQPTLVLGIESDGLFTFAEQE
EIAQHVPNARLERIESPEGHDAFLLQFEQVNNYVLSFFKEVLPDIMFKDGEAPEEASVGQITKSSTFGEAEVEDITAW
FUN_014656 CDS scaffold_7 121548 128905 +
hypothetical protein EOG092605VL PF11262;PF11732;PF16134

MPPGKRKRSDRHSVEGGRSSPHRPGDTALGQHDRDDNMARGRGRGPRGPGRRDSSQGQNRGPPNHQQNPSSTQRRPSSSHTPQ
PPAPPPPAPVIKRPTYAPPPLLRPPSPVRSNYRYNNLTDEKISAWAESGRNAIIQHGKQSREDVDITELSSLFQEFVHAVVEA
RLPPADAGACVKEILGDETTEIIKDSYAFAPHTLFLDSLSIVMDNEPAIYRPTLREFVLTSGVSSNLMRQVLEAEVLQRIGLI
RDNFVKLGIKQATNLLYRQANYNLLREETEGYSKLITELFTTSSVTPPPPEMAEQTFERIKALIGTFDLDVGRVLDVTLDVAA
AVLIKQFKFFVKFLRVSSWWPRSHLKFSAAVYVGGLPLWATPDYSQWTTTEDDEKLLEEKKRVRDIAFWDRAREVHMEAFFEL
GGRQVADSDSQQLTITNGAKGDDAAADIERQWVQETKTLPPPGNRTAAQLLGFKLRFYNSDARDQTDVLPANLLYLAALLVKI
GFISLTDLYPHLSPADEDMETVKEKEMKRIEEEERSSRGGPMNALLMAGVLPQGDDDNPNSSNLPRRELPKKVEAETKEAATE
TADNKPKLPEPLEQKVALLTQLLTIGAIPESLFILGRFPWIPEVFPEVLERIHRILHHCVDKVFRDSRPVVVGETGCPTKFVP
DFDQSGLPKGSVRLSRLTTRKSLRWPFPDKVDTNESQNYRYYWDEWADNIPVCQTVDDIFTLCGTLLNISGVNIGKDEALLTK
LARIGAKSLAEDKSEHNLTRWHDLLRRLLMPALSHTKANASAVNAIWELLRHYPMTTRYSMYAEWFEGQISRLPAMKAAFAKA
TSETRGTMKRVSLTNLSEMAKQLAKTSYSSPGIVFRVAFEQLESYPNLIDAFVECAKYFTDLSYDVLVWSLMSSLGKSRSRTQ
AEHALTTSKWLQALSRFSGRVFRRYTVLNATPVLQYVNHQLIQGNSTDLIILKELITSMGGIVDSVDFTDLQILSMAGGEYLR
RHTLIRGQDKRFENIKSSKRLIQALTESKLAAQLLINLAQYRQAAPFQVPEDEAHIKYLSSMIDDSHRILVQYLDLLWSNMDP
STFDDLVPSIPELIHSYGLDPSLAFLIGRASLSHRMHPWKQKKVDEAKEKAQVTQSTDKEGDVSMSDTPAASGGNETPSQEAV
QAEEQAGAQNSPAPNPATNSQQSQKSEDSSLAKSTLQPIVDAIQDTVRPEIWQKITPELYANFWALQLGDLYCPDKIYRQEKD
RLRAEEVAISRDRSDMSRRGQERKVEKRKELMQLQIGLSEENSEHLLRQAKWKIYLNKQFQTSFPEPRAKPDTISDILLEQCF
IPRMLLSKADAEYTYKFIKALHEGNAPGFQLMSLYGRLFNANRLRALIFTSTVMEAEYLGRFLKLILEDLSRWHKNATVPNEK
DKSRDQPRLGVYEKEAKGPPDQPRLGFALTFDDNDKPLTFVEHEQFRDLLFRWHKNLNSALRSCLEGTEWMHIRNAITVLKAV
LDFFPAIDFMATKFSSLLQTITKQESAAKPSPDSEVGGRVDLSVAAQGAMSELQRRKSKWVMVQAFRPGGAGAAQNEADKTTN
LRATAPEFKPGPSKQSRGKQTTVEEEDGEVKDGKDLKNSATDPTKPTGPAKDLLPAKPGSSRDSKRDHTPSGPRASNNNSGAG
AGAKHDSRPNTLPERPPHTLPSRPDVPIPAHYTPERFNQSRGHERRDGKEPRDARSRDSREPREPRDARDQREPREPRESRDQ
RDSRNFDPARPDRSRDFSERRTQDHGRESSRTDGSSRRGEHERDRTRDSKSGRGHDHGRLNETPASAPTAPAAAVEAPEPHIN
PERAALFAQDNERARAPDSDRATRGRKNAPVEPMDTINPERAALMGNRDNTPSRAPRNEPRDHNARVQSPRRGGRYGHESGQP
EGGREDRHDRNYPVEHRPSSGRDPRERSPYRGDRPMDREAERVSAPKGRDASSFHGTPSRVPEPEHKPPHQDDSYGRLNSIQN
VTEIPYGPRARGRGSNRGHGGPQGLPGRLDGRFAGAEGERPPTPDRPPPTGPAASGRGRRGFEQNNGPVTPSGTPSGPQNRMR
NAGPSQGLPSPASTNATPSGVHPERLAQIGAQVGAVPPPPPGPPPTHPHGHNHGHNRQSMPGTNTPDRGPGPGQRQTPGSYAG
SPAGEPNVPTGPASSNERVRNGGGRRQLAGINSTLQQAQANMPDVSRSNSGRSRHSRQTLGNSDAQVLTGGSPTATPAQERQD
PIRNESSSRAPANGEESSGRSEHDRSRRGDRESRSSRASRRGSREREREDRSPGREREGKEHREYRDRRSGAGEGGREERESRR
SNRDQSGAPREPMGPPPAGGRDLVQGREGRHRGDGQSGRSGEDWGGSGRGGGRGGQRDGGLRPEDRRDGREDRGRKRRSEEGV
GSLSSDREKRPRR
FUN_014657 CDS scaffold_7 130538 131591 -
hypothetical protein PF00388

MVAISGFRSLSTPVNRTRRLPTILGGVAFFIMCIIYLLLSIAPCMVYGNCYRGYSSAYSFDADLAHNPTWMAEIPDDVFLSSL
SIPGTHDTMTYEIGTEVLQCQNWNLTTQLEAGIRYFDIRARVRDDELHIYHANGYTGFSFEDVVGYMNDFLDRNPSETIIMRL
KQEGNGIGDNNTLSFEAAFNKYPLGDRLYNYSASEPLPTLGSLRSKIFVLQNFPAWRGGPYGVKWEGHQMILEDKWIIPDIYH
LSEKWTAIRDALELAATAALDNKVLYLAHISASVGVLPIEAAAGPMNRTVTGMNDMTGQWIKDFEDNEDTTRTGIVIIDFPGK
KFIDAILRWNKSYTKKRA
FUN_014658 CDS scaffold_7 138078 139486 +
hypothetical protein PF01822

MSPLSRHAQQAGLLVLIASSWAPLAAALDIEYCASYNTGETPRNTSIYQTNGLCHDFCIDDWAFAITQDKLCWCSNYAPVKST
QEDNSKCDTPCPAWPDEVCGGNGVYGYVSLGKVEPSGSRGPSPSSTKTEDETSATSTAEDESTTSSSRTSVVQTVTAGGTIRT
VTVAAPTETGGTTASENTNGSTTEKSGLGTGQVVGIVVGVIAAVIGAAALALFLWFRRKKQQNEQEAYQDDPSVRDSSSGIAR
PDMSMAGSPIASAAAGSRNSTLQVDPRMDPFKQGLYVRSASHESINTLRDDHDYSRRIQAPKVLRATNPDPAPEP
FUN_014659 CDS scaffold_7 141036 144636 -
hypothetical protein ENOG41

MPKMTISTTLLLLCASTLLLASPAAAQTFPYVPTEILMPDPTCVDGSVPCSASNLAYIFRQTASGNVEFRALDFSKNIDADED
SLERASNDTVLPFLDGSSKSTAFGAARTANGSVLVYAGDCESGSGDVWSFDYSNDEGEWSRRGLKNGGDTRRAPYFLGGTVAF
SSSLAPDMDQPTLYTYGGMCETPESGSEATNWQSNANYTKSMLRLAPHDSDAFTSYEMSVASSGGPKTPIAGFTLTGLTPSMT
NKSGIVTQQMSYVLLGGHTKTAFINMSTVAVWNLPAESWSYVNIQPPNVDMPKSDLAVKDTSSQNRRRSTNTADNVYSRSGHT
ATLSEDGSSIIVIGGWVGDVNTPAEPQLAILEMSEIYSGWQWKIPDKQPDFNGIYGHGAAVLPGNMLMVYGGWESSGSSSRKV
KRQASSGTLRFYNITSGSWGSQYTNPASSLSDDDNDAPKDDNKGSNSKRLGLGLGLGFGIAALIGIIIGALCWYKRRKRHISK
REEAVRALSQDPNYYNNEYPEMMENDPWGLDSGPWYGTGESPYAAGERSLGYESMRGARSGLPGLHIPHKSMARPSRGGYIPT
GPRPTSFPGPIHPIYEDDEEEPYHNRGLYDHVPTPTSEVPSDPFQTPVAGVGPNNIPHPICLTPGGGNRASATPSPESPRHDP
EVQDWVTEVDAAEGLLARMNSRRSQHQNVQNLDSAEGLLARMNSRRSQNQGSVGHSQGRSSPTRHNSTRSAALRDDESRSGSN
LSESARSAAESIKAKARQLNPLAPAFLAAGAEHPKPGSSSSSSYNTARSSFGVLQAEGPSLLMGDRAPRYDDDEPSSPSKSKP
RRGWLGSLRRVFSGASTPTSSREDMSGVRRSFDQEPVTGDYEPGLVGLRGELLRRKKGRQDWEGVEGDSHGAMMSGGAGPEPG
AESDWDIEKAVEQRLVQVMFTVPRERLRVVNGNEEGSDREDEVPMKPHKAEFVDPDNESQTSGGVSIREKRDEREFGHAGIRP
VVEPKRDDEINEKQPLVQDDQEQLNERRRLEEERQRLMDEKRTEEQRLAEEKSRQAQQKDKESTKREQPQPTSQEQQQLPLEK
LEHPKPPHLQPPQPLFQRSSRDSSPSSSRSPSPLPSPGHPSHSRRHTRGNSNNSVGGFLHPDLAPLEPRFSHETDRSSGVLLE
AQAVPLTRERARTRVLAMVDSFESLSRENSPSPTRFQ
FUN_014660 CDS scaffold_7 146448 149283 -
hypothetical protein PF02714;PF12621;PF13967;PF14703

MGNFISWLEDNLPSPNTSNSGGARGTQPQSLSGMVSTLVPVLILSGVYLVVFLVFRKSNRRFYAPRTYLGSLREHERSPALPT
GFFNWIGAFWKLPDAYALKHQSLDSYLFIRFLRICCTICFVSLCLTWPVLFPVNATGGNGKKQLEILSYANVNIDDSTQRNRL
YAHCFIAWLVYGFVIYTIMRECIFYISVRQAFLLTPQYAKRISSRTVLFTSVPKEYLDEARIRTLFNDSVKNVWIPGDTKELD
KIIEERDDAAMKLEKGEVKLLKLCNKERIKAMKKSGPEAEKVASAPSDPESGNLSARWIPAKKRPSHRTGPLGLVGKKVDTIE
WGREELKTLIPKADNAQADWLAGNYEKHSAVFVEFYTQSDAQAAFQTTTHHHALHMAPRFIGVKPDDIVWKSLNFPWWQVVIR
RYVVVYAIIAILIIFWAVPVAIVGIIAQVDTIKTLPGLTWIQDIPQVILGVVSGLLPSVALSILMSLVPVFMRLCARQAGCVSI
SQAELFTQNAYFVFQVLQVFLVQTLANSFVSSIATIVKDPSQVFSMLSSSIPTASNFYISYFIVQGLTIAVGVLTQVVGCIIF
NLLYKFLTSTPRSMYNKWTTLSALTWGSLLPVYTNIAVISIVYAVIAPVMLFWSTIGMGLFYLAYRYNILFVTETKIDTRGLI
YPRALKQLFVGVYLAEVCLVGMFIISKAAGPAVLMAAFLVFTILFHISLAKALNPLLYSLPRSLEVEEERIQRSLQGSELEDG
HVQNGEGAASNGASNGAGKSHGVSMFVPGGTDSVQKKGNFFSKWLKPWIYADYATMRQLVPHERNMGLEYPEEVERDAYFPPS
VTSQTPILWIPADPAGISKQEVLNTSKVIPISDEGCTLNDKNHIEWDTEGARPPIWSEKIYY
FUN_014661 CDS scaffold_7 153498 154902 +
hypothetical protein ENOG41

MVALRAAEKQKEGTQPLFVPSNDYATSYRSNLFTPDNGILDPQDLELLQHINCSYFSSGRPPTLLALKQHAQSLTNIIRKLAP
STTTRPVGTGDVDSKGWIFQKNEAFDWLNNLNSPYENDDPFHHDPLFALHNVVKSESEVHGIEHRCPLTQVEDFGPSAKEGDI
RRPYMTHHDLIMHANECLEIIDHEYSSTGGLMSILPTGFEDPEDKKTNHPLRESQLEAARNCLLGQWILHHQHLVGRIHELEI

NYANATDVLKGEALVPQQLLGRAGADGVSGGREIVYPQDKYILVNCGEDIRSYIHRLIDVAEAQIEQKEKIWKASGVIGERIW
NEERGGKVYAKGIVPIDLLTRFYRIKGKGHQSPLFVIPAAEQHPGVKSTRLMEERPTVVSIVTPTWPERVSAIEAKNKERLER
ADKLVSENQFLVRKMAELKDRLAVQNSEIKRMTDQLAWYEEHAEVVRKEPQD
FUN_014662 CDS scaffold_7 155241 156889 -
hypothetical protein PF00732;PF05199 ENOG41

MLSLIMPRNFTYVLNQLRMDTMAQCTSPTLSNSMTNPVTNVLEGFAQLGLPVIGDLNNGTATGAMILPSSMQPDNQTRFDARE
AHFNSASRRYNLHIATNQTVTRLVLDSNSAHNSSRRVMGVEFAPRNKSKARSISCTREVIVSAGAIFTPTLLQVSGIEPSDVL
KSLDILVKIDLPGVGCNLQDHSMVYANYYYHQTLMRHVMEKISTERHPSGTAAMMPLELGGVADPELKVYGTCNLRIADASIM
PLILLAHLQASVYGIAEKAADMIKSAKLDCRIGERLPFPPRSRPAI
FUN_014663 CDS scaffold_7 158975 159722 +
hypothetical protein

MSGCNMRCIFCQNYDIAHQRNGMDLTPEELGDWYIKLQEVGKVHNINIVTPEHVVPQVALSILHARDQGLTVPIVYNTSSFDS
LASLQLMDGLVDIYLADFKVWKPSTSKRLLKADDYAATARESIKVMHAQVGDLCFTGDGIAKKGLLIRHLVMPGKEDEGAEIM
EFLANEVSTDCFINIMEQYHPDAHVGKPKRAKSDGKEEKQEVRYADINRAVNAQEISSIRKAAEEAGLWRFNDPPGHDGFAI
FUN_014664 CDS scaffold_7 160344 161292 +
hypothetical protein ENOG41

MVQLPSMDARITPPPEELNIAIPKLQTCPIIPPSPQSLPDTPHPDTPSICSPSSRRRRRRSSSSKSRPTRESVNQQPYTNFRL
IFSPRPFESLYTDRVYLTSCLQQQAGRAADLMRQYCAVELQLQSLVGDNGRRKLRKQLALLKSKVNQAAEQEKAIFSRLGELY
VEIQSRETWAQRWTLESPSVASSVCFSPVPYGFTTPLTPLSGTSEHFAPMGYFGDVHQVYEPPYIRQEPTSCGLETVEEAAED
LFGPESSCDSAESETTPATPTDVVAPFVQEKDYGSELGEELSDERFVALRERRLSLPCLRNAWPEV
FUN_014665 CDS scaffold_7 161523 162291 -
hypothetical protein PF07933

MELLDPATGAALPNDAIQRILFVANAVHVYNIPPLTSTKGYSASTWTAEPQRHIFTARLRVVETAYESESSTNKLKVDAVLED
ASSGQLFAAAPYTAPGVVEPVLDSSRFFAVTVRDPQGRKAILGIGFEERSEAFDLSIALQEARKALGWEGEQSKPAATKEKEE
HRDYSLKDGETITVSFGGSKFGRRKQQDSSSGSGAGGDLQSFALPPPPAGPSSSGGFSLPPPPSANAVKQQKQSAKDLGFDDG
QFGEFA
FUN_014666 CDS scaffold_7 162890 164037 +
hypothetical protein EOG09262X8R PF01008

MATVDTTAAAPAEQPQASSTSDFDIVETYRGLLASDPYLTKPVAAIESLIALLNAHPSTTVFEILDTVKAHSDRLKASVANPV
PLSAGTDLFLQYLVSSLRQQDGSFEAVRQHLLRNGRLFAQRANAAREGIADAGWRLIHEGQCILTHGASRSVVGILERAVQNL
GAAKFKVIYVREETRVEESDRIVRELRSKGIPVAEIAEASVAYVMGLLRQVDMVIVGAEAVTSNGGIISRMGTLQLSKLATQA
GLPFYVAAETHKFARKFVMDQRDIGFKQEILDFSVDNKSKDIVDAVDFTPPDLISKLITENGIKLPGYVFEQLLDIYGSLNG
FUN_014667 CDS scaffold_7 164536 167828 - EGT1
Ergothioneine biosynthesis protein 1 PF03781;PF10017;PF12867

MPSITASSATPQRVKPTTAKPSSALPSIIDIRGEHVEINLKDQIASMFNPDEGPRKLPTLLLYNEKGLQIFEDITYLDEYYLT
NYEIEILKKSSAEIASQIPEGSMVIELGSGNLRKVCLLLQAFEELKKPIQYFALDLSLKELERTLAQVPEFKYVSCHGLHGTY
DDGREWLKHPSLTSRSKCIIHLGSSIGNFTRDGAADFLGGFAEVLTPSDSMIVAVDSCSNPAQVYHAYNDSKGVTHQFVLNGL
QNANEILGEEAFNTDEWRVIGEYVYDVEGGRHQAFLSPTRPTDALGSRVLPHERIEIEQSLKYSEAEKDKLWKLAGLTEMGRW
SRGDEYGLHFLQKSSMPFSRIPSLYAAEPLPTVQDWKALWKAWDVVTKDMMPDEELQEKPIKLRNACIFYLGHIPTFLDIQLT
KTTGNAPTEPATYYSIFERGIDPDVDNPEHCHTHSEIPDEWPLVQEIMIYQDRVRSRLQNLYNNGQDKISRDIGRAIWVGFEH
ELMHIETLLYMMLQSDRTLPPPHTVQPDFAKLAQQAHEARVPNQWFDVPEQTITLGMDDPEDGTDNSRHFGWDNEKPERQTKV
HAFRAQGRAITNEEYAQYLYNSKIEHIPASWSSVTNAYTNGATNGSHANGNSNGYSNGNGHHASQVPDSFIQDKFVKTVYGLV
PLKYALDWPVFASYDELAGCAAWMGGRIPTFEEAKSIYAHVNKQKRAEAERTLSKTVPAVNGHLVNDGVEETPPSNSSALVKD
SSSELFFDLTGANVGFRHWHPMPVTSRGNKLAGQSEMGGVWEWTSSSLERHEGFEPMSLYPLYTTDFFDGKHNVVLGGSWATH

PRIAGRASFVNWYQRNYPYAWVGARLVRDV
FUN_014668 CDS scaffold_7 169683 171444 -
hypothetical protein PF04082 ENOG41

MSSGSVQGKNYSPELNLILLGILTLTARFHPDLVRYVTHLPSQHGTNIRSSASKTNSRPLYASDFFATALTTALGPLRTVMGT
LTVERIQSFLMLGLFEWSQENAPSAWMYLGIAIRMAKLMRLEQDDRRMAILENQASDTKRFEKRSPEISIIRETRRRTMYSCF
ILDRLMSFGSERPLSIAVESMLIQLPCSEMAYDLSLEVYTGLLQPYGGSSQPQINDDSTLSRFINLVEIWGEISRYSSMGGRL
QETHPPWDPRSTFYKLQEKTNAFNEDLPGTFTLSRQNYYRHDNHQATSTYVSLHMLASVCQIVLNREYLPFLPLQSRGPQGPP
IGNQTVLNFAPDRFWEESAEDVFQASRNIIDLVGLCKEKLPLSSLSIFSIWLAGFMSVYAHHFPQIDTSHRLRSEQAESPHTG
ENLSMLEQGQAGTVCQALRRAATYLPSAQRYLDNLEAVDRCFTQAKNSTHGSLSLRLGEVGHGSELNQTGSQDKQRRGVYDMH
RTSRSPMSASPKTRNSHRPMLEEPPKPTSSTSKSQLSLLGIDEGPLAMDTPKLFVDNIETLESQRMSWVLNDLEAFSGAGSLR
VAFLG
FUN_014669 CDS scaffold_7 174677 176336 +
hypothetical protein PF06911

MSSGHNDPKLLYAVEGINAYHITNGKEQSLTPSGPQTLSLLMVPTSSGFVEPSGSGSDGEEDFYLHLHLPPELDLPLPATTQI
YHQPPTSYLIPRWDLGPDSGAFTRIEFPPTNSRPGVQEDVDTFETILAQCTAFLERAAPPQPPRPSEKAQAKAREAAGEQLPA
YNPADYNPGEGYVQGSHSSHTGGRIVLIDEEDGSVLGELTDSYQVVEDSKIKPGSKEPVEIALPTDGGQNISVQPVSQPWAEM
DMHPAYKKSTIVNSASKASRLIVTTSDVVSRTLQSQADNFTKNTKPLAKPVTFAPTTHAHIRRINNFSNKAATFSATTVGTIG
NMAQNLGATVTRRKDGRARGYDKDGNAIDTYKPGVLNKSLMAFNTVVDGIEQAGRNLLTGTSSSVTTVVGHRWGEEAGELSRN
LGGGVKNVGLVYIDVTGVSRRAILKSVAKGMVVGKVKGGGEIIVGGTDNKPNFETREGNGQGSHSTYGDNVSVASGYDPNGKK
PAKGHY
FUN_014670 CDS scaffold_7 176988 179386 +
hypothetical protein PF04130;PF17681

MLHEILLSLSGHPSPLLRTDATQPHALSGVSPAERQLLASAAHLSDVHIKLISYTAQVANSHPSTICRAVATAIDSIHLAAFQ
RKVLDVEASILQDDPDLVGAYNIVPLTAVIGEFKDWTRRMEWLWEMVQFILAKNKKGETCHGAQLMDRLRLELQSGYRDVQET
AMSLVTVAETAWLKQVSAWILYGRLPSFGGDDFFVQKVEESEEEYISRSSLLPAFVTPATASSMLFIGKSLNHIRVKSSVDSG
LRGLDHLSSKLQELSSLSFPLKSTSFSRAIIAVRISLSENTLQKLLPLAKVTEMLQLLRDFFLLGRGEFAMQLTHEADEKIRS
RWRRADNLAYEKGDGLKNVTVKEGEVAAVLARTWAVLASMQGQHAEEDEQLELARDLLRLNLTKSKATTNFGSGSGLSRDAAS
LLSESPFRNLLFSVPSLLSIQIPPPLDMVLSPSDIQIYSCINAYLLSMRRAHIRLTDLWKITSLRRQYPTAKGDREHVILLRK
RWTSRSSSMRSSWTTASAAIFFLAETEAYLQTEIVAGLWEGFHEWLTANDPKHGQSRAPTPTKLQDESGGADTDEEEYDDLWL
GNENDKPTAQDDTPKARDSTPPQDPQTLSTAHRLYLRTLIHRLLLTQPTFTQPLYNLLIHIDHLVAHLHRLHAIYTSIDLETD
AGVVDAFVDLESEERDVKRRLHDIESSVRSGIEDVVAALRALESDPIFTAEWEGDTAPATATEEDEDDQRDGRARDTDDAEER
GGFTAARVGGINRLLMKLDFGTWFGRADGHDDVEQL
FUN_014671 CDS scaffold_7 180331 181944 -
hypothetical protein ENOG41

MTPEPSLRRQRKVVITTSPAPSDSDSRRDSYFSEVSQSTAPTSFHSASGSNNKCLDVAQGNRPTTAMKSTTIERRFDTTSSSI
ATYDSVLSEMAPLCRDLPVGGEEVEFETEDEEEEEEEEEEEESGDEDDDDDDREDDQEDADKDYILSDPESLHIPPVLPPYRG
TSNERPCRPSTPQDFGRLFPSLDRLAVRHDDCTSDGNMNLRVDTVVPFGHTRRTLAVQLFHLRMHDLARREFSLRRYCRDSGR
EVCNSKRAYAPAPTSTVAAARPALQRSMSSAMRTMATPFHRPTSSNSSIFKFGNGTNASTTSDNASVWSGSHHTEKSDSPTPK
PKARMVPTNRIKLEFSNYARVDIARRGGRTNKRYEFEWWGHTYAWKRVIDKNLNVVSFHLVRDGHGAPVAHIVPEMRSPNQIL
DDEMAGAWVTPCYIWISDSSIIDAITDVADVIIATGLISLVDDCIKERWQAKKPSHRAIRRDTGDVNHANPRSFMHSFFQRRH
SEEHSSSHGALRFGRKPVAAY
FUN_014672 CDS scaffold_7 187710 191498 -
hypothetical protein PF00018;PF07653

MAAPFRVKAIYEYSSPHEDDLNFSIGQVITVTDEEDADWYGGEYVDDHGVKQEGIFPRNFVEKFEPTAPPRPARTRTKKEPEP
AQPTEDIASPPPPPVQQEAPSIPAPEPEPEIEEAHEPVHKHSAPEPAAPAVPEPVPSKPAEPVQATAPPPVAVPKPEPPAPAP

QAPAAASPKPKPSGPPPKAEKPSASSFKDRIAAFNKPAAPPVAPFKPSGLAGSGTGFIKKPFVAPPPSRNAFIPPPQQTPTAK
VYRRDEDPEIKEREAETQGQAERAGLVPSEGQGGEDEDQPKPTSLKERIALLQKQQQEQAARHADAAAKKEKPKKPQKKRESV
GHIDTPVEAEASPQEPHAPLERRDTQDTDARTSMDEPRQNRMPPPPRRRSSKGPAVEPVHDGNEADMSGAGDTTEGNDDLTER
DDSDGVARNAPRAPALPIHAGPPPEASTSRKEDSEEAEEEEEEEEEEEEEMDPEVRRKEELRARMAKMSGGMGFHGMFGAPMPA
PAPPPKKKKAPKPERTSIDETVEEASPTSHAAPPVPTMMALPGFGGSSKPEEPSRPEEYEPAARAPPPPAPSQPPRVPEHAEL
AEEEAESDITPAPHAAPTIPPRDPAGPPPIPGSRGAPPPVPSESRPPPPAPPADAKSQSEGSQSGDELSGGRDSDREGPVAAA
RSPPMAPPPVQPPHLPARSPPLSPTREEFSPTSPKSNASNRLSRLPPPIPGSAPAPPTQSRPPPPPPPGGLRRQSTQDSQAQH
TRGPPQAGEEEAEELTEYEGDYDTDIASSVPHKDALKSHARESSLEDNTILSPVSDAPPKLPPPIPTSTAPRAVPPPVPSQPP
PENKRQSIDTPRAAPPPPPPTDNKRQSIDVPRAAPPPPPPAGLLPPQPPQSPRLEEDYDPYNYSAPPGGHGYAPRTPKIEEEG
IFPQSPSVTSPIDRRAPPPPGVPAGRTSGRQSLDISRAAAGNRRSMDVHRPSMSMESGFIANDLDLAIQSGWWKQSNQVPPVL
QGRKDIYFEAEESTSTNGGQQTTITREIFILYQDYSQTFLTIRYDPYNASDVQLEQRHEPPPRPLRQDQMEEFYERFGRHIST
AVAAKKDSVVADGTPQGLVLELLKPYKDALPPVGTRAYGALVYSNMANASTQQNDVIRAGDIITIRNAKFQGKHGPMHAKYSA
EVGKPDHVAVVSEWDGTKKKVRAWEQGRESKKVKVESFKLDDLRSGEVKIWRVMPRSWVGWNSQS
FUN_014673 CDS scaffold_7 192798 193605 +
hypothetical protein PF04181

MASASKQPPKGILKKPKSIKERNADPREIAIEHAKIIQHRKDLEAEILDSLVILSEYPLAREPPHNASNPSPSDVADFKAHVR
LFQPSDYDDLVIERNVNELCGYTLCSRPRRNTGPGGQWKISNGNIMKREDLEKWCSNQCARRALYVKVQLNETAAWERAGIPD
IEIDLLDEDKSKETEVDRAARELGQLKLEEQRQAARDEAALALERGEPDASQSKSKKMNITLKENDPKAPSTASDKPEVYDDE
HLLVEGYRTKLNLEDKKKD
FUN_014674 CDS scaffold_7 193994 194556 - RPS17 40S
ribosomal protein S17 PF00833

MGRVRTKTVKKSAKVIIERYYPKLTLDFETNKRICDEIAIIASKRLRNKIAGYTTHLMKRIQRGPVRGISFKLQEEERERKDQ
YVPEVSALDFTQNSESGQLDVDGETKDLLKHLGFESIPVNVIPVTQQQVPERGGRRYGDRPRRD
FUN_014675 CDS scaffold_7 194972 196406 -
hypothetical protein PF11709 ENOG41

MGSRAVSPGGALLRSSRLFSLPKPLPEPQSTNLHIGDHKSPTMTRQYPQYQSITSPLSSREKGDWGFKRPFPLKSTLTTSTPL
IRVNQVDSIESVTDFASAADHTLSLEKFQEMRIPLSIPKGNEKSGISVRNDAWRKSVFEEDLDFTEYRKGRIDDKRWKFQGPW
LARMTEGAFIGYLNKKVRPKRAEFRQLLKKRLAESLNSKRATAAMEQGNTAPNKIQASDITEDQFTDYVRALRSDRATLYGLV
SKFLDLAPLGQPVGIIQTFLAAGENAAAESPYGKSGPPPSHPSAGISYLRTNSYMENHPVYGPQKQRTPTLARVVYPRQGPSP
AKLGVGGFVADSPPGDNEFNTRDSRHRVSQNKKMLTGITHLDTTTFGGAKAYVSPETATVDPSGKVVLQLREANTEAQLVARE
AQGRAKVYNATKQTLKRTSDEADPWRTERVANELIGDDTASTPENDAVSSSKNYGLDNDNQQ
FUN_014676 CDS scaffold_7 197224 198130 +
hypothetical protein PF01702

MVSLLKLATITEKCVEFLSPHDGTPMLLTPEHSMSLQNSIGSDIMMQLDDVLVTTSPDKARMREAMERSVRWLDRCIAAHKKP
ESQNLFCIIQGGLDLDMRRECCREMLARDTPGIAIGGLSGGEAKADYCRVVAACTELLPDLKPRYVMGIGYPEDLVVSVALGA
DMFDCVWPTRTARFGNAVTKHGVLNIRNKKYATDFGPVEEGCNCMVCKPIADGGMGVTRAFVHHNASKETVAAHLLTIHNVYY
QLNLMRQIREAIMEDRFPTFVRQFFAWLYADKTEYPEWAVGALKGVNIDLSE
FUN_014677 CDS scaffold_7 198375 199521 -
hypothetical protein ENOG41

MKGAVLSVLASGAAAQITSMPFAALGLGPHVFQNNAINPSDPEYSTCQQAVGIVQDCVSSVGGLDAAATADPSALVACACCDG
RSNAAPLYSVCSNYLEDEAPENTSQYEAYGTLYSACRLNARCTGGSGGSASDSRPTATSDDEDLSTITSATSPAEQTYAAACL
YMLDIFTSCTAEDRQFTELPPREQAECYCCRGSGSRRTWTDELDKYASTCADWARTGEPKTAYSVAKNLATFCDRFDDVCSPS
VTRTQDSGSSETDEANTTEDSGSRQTGASDNDASTSEQNMGPVTVTVPAQPSSTDSGNSASSARFGFGAVLAAVAALAIAL
FUN_014678 CDS scaffold_7 202395 205941 +
hypothetical protein PF00850

MASPNRPSLSVRRQSSARNLRQPEHDDLTQSLNQLSISTSPGRAPSRLSSEPASPFRPSGRRTSQSPAPNARALSRSPSRSRE
ASRDTPTLLRKASMNSLRSSNGIGPGSTPSRRSSVVHHMSPTQAKSPLSSTPPVLEKPRPTAISIAHDNLSDELQAHHGATST
RPTQMIVVLNDAVYGHRFSRPRTSRAALSTIVERPERIKAAVLGISAAYVRLGGRHSEGEYPLHPKREAEDIHGIPFRIQKTE
RRLSLLSPAVVNVHGTKWMEELKMMCDAAESKLAMGGKELQRPQMNRGAEKADKFHEGDLYLCSESLNAMEGALGAVCEAIDT
VFGPGPQRAFVGVRPPGHHCSASYPSGFCWVNNVHVGIMHAALNHGLTHAAIIDFDLHHGDGSQAITWQHNSRGNNAAKNAAA
WKKSSIGYFSLHDINSYPCEMGDEEKVRSASICIDNAHGQTVWNVHLEPWKSEEEFWKLYETRYIVLLDKVRNYLRNQAERLR
ESNLPPKAAIFFSAGFDASEWESAGMQRHKVNVPTEFYARIAQDVVKLAAEEGLHVDGRVISVLEGGYSDRALCSGILSHLSG
LAGDQTYAQEPDNVGRLGVEMGQKIGSLSGETSYSFEQKPSLDSVHAYDPSWWSPSLLDELEALMEVPNGPAKKPQSATIPTY
FSPTQASTAKVSDPIKMRRSLSNLGASMTRPPSPPPPEVPWAIAAHELSKLLIPSSRQTDSCKPEELNAEASKARRDRQSIIM
GIDPNPPAISSSRPTSRMSLRERRAKAAPPDPTTEKAPTSRRRTMGAAPISSDKAVARGIPSRTNSGEPAVRRTSRRLSEIPP
SLTTRDVSPGEADQPEVVEHARTVPNSPMATKSVASTNPPVKKARNSTAPRKEPAPRAPRGTKKATTTAGPSRPATATQKAKS
PAKASKKDGASGTGGDDIDSITSGMKKIRINLITQSQKDAKERARLEAEKAKGKESSITYKPATASSSTEHSSSAANHDQTSI
SASVETQDISPTQPAPGNARIPSPPTTASSSRIMSPSQEQYAFESTAQVTQLMSPPLSSPAIPSVSVHPSTDADDIFVPYQPE
GPAPEPVAQSEPLKWLPPNVPTPSEQTPVATPSPAKKSNLFQYTSGIPFAPRPGQGANEPKAAEQGVKPEPNPPNPIEEIPET
PQH
FUN_014679 CDS scaffold_7 206262 207302 -
hypothetical protein EOG09263UCR PF02811

MAFTLHSHSGEFCPGHAKDQLEDIVKHAISVGYKTIGFTEHMPRYDLRDLYPEELDNPQAALEALPPRHEAYLVEAQRLQREY
ASQIHILIGFEAEFIRPNCAAHVRKLAEHPIVDYFIGSVHYVHNTPIDYNKEMFATARDASSRKTEESLYEDYYDLQYDMLKE
LRPRVVGHFDLVRLMSEDPARDVRQWKGVWERILRNLALAREQDGWLEVNSAGLRKGLAEPYPGRIIAEEWIKMGGKFTFCDD
SHGIAQVATNYARTVTYLESLGVKEVWCLKRTSNSGVDGTTRATVEEVSVPLSAFRENFP
FUN_014680 CDS scaffold_7 207810 210975 -
hypothetical protein PF11915 ENOG41

MAVPWNSMGMPPSDADRDKIVVGPYDDSRYTASSSGLSYIRHPSSQPTNSTARLIPQPLLSPPIARVSPGPRSPSSPRSPASF
QSLNSPSSPGFPLSPFYPPSPGPLQRGQFPNSPTSDGFSSPRMSGATAATDPRLSTKASNMMETVLETPYFDTPPHTPGSWGD
ASRGTDNLDATEVSLNNGWRPWWLRRRVTSIFIGASIMLAVIGEVVMWWLSNNEVDSSLKGLWTFGPVVVVSIMAILWARVEA
QALLYMPWIVLDRRPTTVDETRRKQAHRTILLDYPSLGSFQALTKAFRNRHHLVVASIVIKLLLRAQIVLSTAVFHAEIYVDS
TTRLRARLGVLHAMVGVFLIISGVLLPMLYHAPSPRGIAPRDPTSPAGTAALLTSSQQFLSRLSGTGHADMNAVAGRLAGSWY
TTELTQPGRRPEEMFQLRQHGGGSGALGMNPSNRPEEATGTYQPWTQVTRTKLISIATSVALIVGICVIYNLKGNENGLEVDD
SIFIVWTCLPTIIFAALAVFWTRIDIDNRRLAPFLKLTNSKCRFQESLGLTYMNEFGLHTAGKAMKNQDWAVFLAKCTSMLGW
LMPIFTAGLFAVAQMAQRANLELGPESQFVSTTKSLSTAIDSDTIENVLLRETPKYPRWTWEDVALPAVSLINHPSEWPLPNT
ELVAKVPSLRGKLTCETISLYGGEGVDWQCVPLGGSTKQPICGSDQSRTALVASSCSQLSSKYSIKYIWGSCSDDGMISVMMC
NQSVVEVDVLTTFRTEDLYINLNAIPIVNASSETPSGVTADITSVYEALDNVGANNKTMNGLDAFFRTLVLSRLQTTLERIAM
PERQNSVSQAIRQLHGILAAQAINSDLIRQPLSSKLRARDDSPTDIPASVDYVLPRLMQGSVQTFVLIGLLGFTLAFGLLSLR
TASRGTLTKSPGSIAAQASLLADSTLWWRLPDGAEWMEDDDLARCLRRRTFHLGWSKGAAGTRSYGIGIVQDEGKAARPPMVS
QTSADKSGGLVPGRYISMAPGVYSYGDVATYEKS
FUN_014681 CDS scaffold_7 213611 216989 -
hypothetical protein ENOG41

MHAKNFIVALALASSSVVEANRPRMYIPRQVKREVENNAPVAPENQPAPVIERREPADSFIKSLIDKNSRKQASQEDGVNFDP
TLPLGFKKSGPSASAPALIIQPTTSEALPETTEPAVSTDAPTEADTGILLAPSGIVTSKTAAQPAEPAKTTAAAVKEPSTEAP
KKEETKPAATSDVEAKPATTEKPKEETKPAATTAPAAEEPSSVKTPEKKPVVETKTEAPVPVETVTEPAVVETKTEKPSATKG
LLDPVESILTSVLPVASDPKKETKPAAIETKTEEPAVETTEKPKTEPEETKPAAVPTTKEGLLDPVESIVSSVVADPADSKTT
EPKPVNTETKKVEPAKTDSKKETQPAPVETETAKPAEPETTSPAVEKPAPTTKGLLDPVETLLSSVLADPADNKTKDPTPDQT
ESISQPEETGTPTKEPQRDNSKPQTDGQTLPTFSPPADKTTAAPNAGETSKDGILDPVETLLSSVLPNQPDVKTTSPEESSAP
VDSQPTSAAPNGSEQTVSAAPSTEPETTGPPELIPTSILPDPTDILPVPTDVTSAVPEPSSPVTVSPVIPTTVSPPEQSESGS
PDLTSFLDPNTKFPQTGSVGPTDAPNTTVPEVTSGIDVKPEPTSEPINGTETIPNVTGEPQTTGAPEPTDVNTEEPTNITQTA

VPTGDATNEPSGNTTAPATEPATEPATEPATEPATKPVDTASGSVPTGSPATETDAETNGTATAEPTKPATGVETQSAPASEG
EQTSAVAPTDEQPSVAPTSQPPVETETKPEPEPTTLVPTAAVPVTSIRPTATLTNTNNWLPTTIVFEPTSIPAAPSQPTETST
STGLPANIPRVILPNDPNKPIPEGSVPIQLGFLFPFNYKFLARNTVAAAQLFKYLPKGLADAGGFSKDRILVSKIVPLDTQSQ
WGYITALAKIHYPENMIDSLQADLITPNSPLYNNDLEIVRNLTSVINTKIDIFGDSDTDNNTDNGNSDDGNSGNRDVFGNSGE
GDKSAKQKATTAGIAVGAVGLSVMYGAAMFLVARRYKRKKQLGHRRASSIGSSQRSSEMQYTGNGSPALMGGALMSRDFSAYG
ATHAEQGQVRPGGRDSHGSGRSGMGNSARTAYISAPVAAENSLGWN
FUN_014682 CDS scaffold_7 224489 225169 +
hypothetical protein PF04777

MARRQHLTLTFILVLGVFFTLSYFFSGPSRSILPKYQNDVELPLKEAPRSEFAADLGALPAGLLDGNSIAPKLENATLKAELG
HATWKFLHTMMARFPDKPTKDDRMALETFMHLFARLYPCGQCAAHFQKLLAQYPPQTSSRNAAAGWLCFAHNIVNERVHKPLF
DCENIGDFYDCGCGDKDKKEGGAETTAAAQAEGPDQELHKH
FUN_014683 CDS scaffold_7 225635 226599 + PRE3 Proteasome subunit beta type-1 PF00227
T01A
MEFGNAGVLNEDGIHVDMDRLKKGEVNLGTSIMAVTFKDGVILGADSRTTTGAYIANRVTDKLTRVHDTIWCCRSGSAADTQA
VADIVQYQLGLFAMTNGKPPMTQTAASIFQEICYANKDRLSAGLIIAGWDERFGGQVYSIPLGGSLHKQSYAIGGSGSTYIYG
YCDANWREGMEKDDAVNFVKGALREAIKWDGSSGGVIRMVVLTKDGADRHLYLPDTDYAVRHE
FUN_014684 CDS scaffold_7 231159 232735 +
hypothetical protein EOG09263L00 PF08613 ENOG41

MAAVIGDIISDTSYPHTMIANPSLQHHHSHDSSSSYRSRSRSRRSTTPRAQRTAELADFSSNHSSATWSHAPASATTPASAPA
SESTSTPVPAGHETAHVSATGGASRFPSPPPSSSPAAATGPAYADDAKSPSMASDARDSGPLGHASQPDPISHNSSAQQPSPA
SDDFQIPNPSFPLSGASSQPPVQTVIHIRDLAHIQSLASADLLSGNGGSGILNDPPLQQMKYEISGMPIGDIIEMVAALLTKI
TTTNDLQHDAMQRNVAHQQQANQSGDTSGSSISSLNHSVLAFHGKNVPAITILSYLSRIHKYCPTTYEVFLSLLVVYFDRMTER
VNDMVTKNEENRRQTRSQQPNATSPQDTPMRGEGMDVDESDSDLADDDDEEMADSTRPSRTSSHKGAATPTEYSGIAAATYFV
VDSFNIHRLIISGVTCASKFFSDVFYTNSRYAKVGGLPLVELNHLELQFLLLNDFRLAVPVEDLEAYATMLVEFYAREVVAKQ
GGTGNTE
FUN_014685 CDS scaffold_7 233436 234358 -
hypothetical protein ENOG41

MADSHIPSVPPPWTLKGDIYAFIFWTSPSQAKEGLPAMTYSPLEGQSDFAKGQKALGGLSMLQLIRYTDSPVGPYDELILAPG
TFGYEKEDENGRRLKGKGVKITRIYVSHKHTCYNGRKNWNVPKHLAKFDWTDNSNGSITAKVYPHDTLPTDSASSESASPDHT
PFFQATFKPIRYAPSFPFRTSWINYLGFDTTLVFPPLPEGAGSQGELPGTDQWCSVVPQQSTSKCMLGWFDVEQHRDEEGKLT
GEFENFWPGWSKWQIGIKMENSVIEFDHPETWEPPRTRL
FUN_014686 CDS scaffold_7 234852 236774 -
hypothetical protein PF00264;PF18132

MRIILAWLLFAAVTAQSYNYGGVDIDSLTRRQDPDASIVVKALPQTRNGTTPLRLEIRQMKADRYKWDLFILSMSMFQDVSQD
DPASWYKIAGIHGVPFEAWNGVEAAPGANQSGYCAHSSVLFPVWHRPYLALFEQELYRMANVIAGMFPNGTDRQPYIDAARDF
RMPYWDWAMPPPEGESHFPDVFWNATISQWGPRGVQEIRNPLYSYRFHPKNATAMIWSPLRDWDETKRAPNASESETDPTSDN
EKVNAALLSRLPEIQRRLYELLTSYKDFNSFGTKAWGATQNLSTADSIESVHDIIHTDGGLGGHMTYVPLSSFDPLFLLHHAM
TDRVVAIWQALNPYSWVTPMPAGENSFTTLKGEMQDSQSPLTPFFASVDGTFWNSDTARTTEAFGYTYADTDLTGKQKEDVRQ
DLQKKVSEWWGGSAAVGLQASTDIMMAGGISSTDYTTKWTIAVLVNMGAFPGSYAIHFYLGQPPADCGEQTSHYVGGIPFAGI
LMTNPSDSVIAAALPIESRLRERVIYGDLPSLSFKDVEYYLLERQKLQLCVMADFRRDVDPAQVSGLRVEIVRSSHKDGRGSF
KIDTS
FUN_014687 CDS scaffold_7 237849 241597 - ACR2
DNA-directed RNA polymerase I subunit RPA2
PF00562;PF04560;PF04561;PF04563;PF04565;PF06883

MAPSATETEWDHQFHTLRREDLFRNPPKDHTAYPALQLAVDPHLESFNGLFRDDGKPGLLAHGIADIGARTYLDGDDRASPDG
KNRLTIRYKDVFLQKPQVPPSNKMARNRQVFPAECRERHVTYRGRLSATLEYRINGGDPVEFTREFGQVPVMIKSNRCHLENN
SPALLVERKEESEELGGYFIVNGIEKIIRMLQLNKRNFPMAINRPSFQNRGPGYTPYGIILRAVRPDETSQTNVLHYLSDGNM
TFRFSWRKNEYLVPVMMILKALVETNDREIFEGLVGPAGSKSTENTFLTHRIELLLRTYKSYNLYSKSDTRAYLGEKFRVVLG
VPDTMTNLEVGTEFLRRIVLVHLGNVDVTEEQDKEKFKLLLFMIRKLYALVAGDCAVDNPDAVQNQEILLGGFLYGQILKERL
DEFLSVNVRASLRDYLRRNPGVAFTSEDFRKEFPNNIFRKANENLGNALEYFLSTGNLQSASGLDLQQTAGFTVVAEKLNFLR
FISHFRMVHRGAFFAQLKTTAVRKLLPESWGFMCPVHTPDGAPCGLLNHLAHKCKIMTDYVDVSHIASLAQELGIVDVSSAST
EENVVVMLDGKILGFCTPKESVRIADCFRYWKVEGTHGVPLQLEIGYVPPSRGGSYPGIYMTSTPARMVRPVKYLPLQKEDWV
GPYEQPYMSIAVVPQEVESGKSTHVEFDPTNMLSILANMTPFSDFNQSPRNMYQCQMGKQTMGTPGAAIRYRTDNKSYRIQTG
QTPIVRAPLHNTYGFDNFPNGMNAVVAVISYTGYDMDDAMILNKSAHERGFGHGTIYKTKKISLKDDSRTKATKSVTKAFGFA
PHSYVSAQYQGMLDDDGLPHVGRLIQEGDVICAWHTVTPDYNGKLVNLDGITHYEKYKDSENGFVEEVRLIGADSGNEPLQTI
SVKFRIPRSPIIGDKFSSRHGQKGVASQKWPTMDLPFSETGISPDIIINPHAFPSRMTIGMFVESLAGKAGALHGLAQDSTPF
KFDEENTAGDYFGHQLMKAGYNYHGNEPMYSGITGEELAADIYIGVVYYQRLRHMVNDKYQVRTTGPVVPTTGQPIKGRKRGG
GIRVGEMERDALLAHGTAFLLQDRLLNCSDYSKSWICRRCGSFLSVQPTVSQFAPGKKKAASIVRCRACAVKLDDAEGIDLTE
IQGEIWEDGHGNSWVGGDHTTQVVVPGALKFLDVELAAMGVKLKYRVDRGDEPRKGPMRPMALDGVRVGK
FUN_014688 CDS scaffold_7 244898 245772 +
hypothetical protein PF00025;PF00071;PF01926;PF08477;PF09439

MAGLFQRVYNWLMRMFWAMEMEVTMVGLQNAGKTSLLRVLAGGEFTLDSIPTVGFNMKKVQRGHVTLKCWDIGGQPRFRTMWE
RYCRGVSAIVFIVDIADTPLIPQAKEELHDLMSRKSLEGIPLLILGNKSDLPDKLSVDELIDELDLKSIRGREVCCYGISAKE
ETNLDAVVEFLMKYATRP
FUN_014689 CDS scaffold_7 248652 249783 -
hypothetical protein PF04724
GT17
MASRVPYRVFLSLIPLVCVVVLLSKQPFFDRSHIHRITDTDISVSLSLPRLNFSRSHHEYYASDAARSLCASHGYTVFNPHPD
APNGRRKIYDLFMVNTELDFLEIRLKTLYNHVDYFVVVEAPLTFQGGPKDLVIRDNWKRFEPYHDKMIYHQLEYPKDFKPLRH
WDREDLQRNAMFDQVFPKLTGEQTPTQGDVILVADVDEILRPATMLVLRTCNFPRRLTLSSKFYYYSFQFLHDGPEWPFPQAT
YYQGMRNTILPGNLRTGDAGIPLLRDLEKGTLANAGWHCSSCFSTIGQFLNKMASFSHAWMNHESFRDRDRIASAIREGVDLW
GRKENTFTRIDNNADLPTCLLEDRERFRYMLDRSGKTAGFSDYP
FUN_014690 CDS scaffold_7 250846 252108 +
hypothetical protein PF01212

MGDAVSEKPHNAWLGAKGPAALDLRSDVVTTPTPAMLEAVKSCSLLDDVFQEDPATNELEAYVAERTGKEAGLFVLSGTMGNQ
LALRSLLIQPPYSVLCDYRAHIFTAEAGGTSNLTGAQIQTVIPKNGRYMTLEEIQENVTLDDDVHGCPTRVISLENTLHGMVM
PLSEVKRIAEFAREHGIRLHLDGARLWEAVVSGAGSLTEYCSYFDTISLCLSKGLGAPAGSVIVGTKATLKHARWVRKSIGGG
LRQSGVLTSAGRIAIEQTFGKSPNGQDGPLKASHEMARKVDDLWTSMGGTIDEPTETNMVWLNLKATGCRVKRFIEIGADAGL
KFMSSRLVTHYQVAQNEEEVLRRLKIVFEKVLGEGGDSSTQQKIGKGSVYVPQ
FUN_014691 CDS scaffold_7 253451 255673 +
hypothetical protein PF01204
GH37
MPSPRHIAAALAASATTVSALYVNGTVVAPCDSPIYCHGEILEQVELAQPFSDSKTFVDMPAIRPLSEIQDAFDKLDKPLRNN
SALTEFLNDYFADAGNELEEVPEDELETDPKFLDKINNTAIKEFTQKVIDIWPDLTRRYDQDSKNCSDCPNSFIPVNRSFVVA
GGRFREPYYWDSYWIIEGLLRTGGSFVNIAKNTIENFLDFIEEYGFVPNGARIYYLNRSQPPLLSQMIKAYIEHTNDTDILER
ALPLLVQEHEFFMTNRSVPVYINNETYYLNTYNVSNTRPRPESYREDYVTANNESYYSPSGEVYSGGEELNFKTKEALYGNLA
SGAESGLDYSVKWVARPDDAIRDNYFPLRYLNTRNIIPVDLNSILYGNEIAIAEFYEQTGNSSASRQWREVAANRSFAMHAFM
WNETHWSYFDYNLTSKAQSIFYPTDNSTAEIDKENAPKGKQVFFSPTQFYPFWLGAAPDYLKNNPYAVLNAYKRVSYYLDTRQ
GGIPASNVESGQQWDQPNVWPPLMHILMSGLEKVPATFGIMDPSFIEVRRLALRLAQRYLDSTFCTWRATGGTTSDLPRIQSA
AEGADGIMFEKYADNATNVAGGGGEYEVVEGFGWTNGVLIWAVDEFGNRLKQPQCNFTGDSSNERRDTNSAVMLHARDAARVK
KFGNRKRAAEKAAHKRSSRLFHF
FUN_014692 CDS scaffold_7 256322 256880 +

hypothetical protein		PF01906		ENOG41

MVGFGSAKDQKKNHEEVADIPAHLSDLHCFTETDNCITTTMMDLPGYRITKVLGAVYGITVRSRNIAAGIGMVIKSMAGGELT
WFTSMLYSCRNDSISRVVQETKRRGGNAIICLRFETGDLGGFAQASAYGTACVVEKIEGANVEAPQLTH
FUN_014693	CDS	scaffold_7	257601	260385	+
hypothetical protein		PF00326;PF00930
	S09B
MSPLSPTSDRRYSDDSISSASTTSLVFDRIQEKTMMDASRDNNDSRALEDDDPLKEEDDLETGPFLGPGASLHREPMDRGLRR
ILIIVGSVFVAAWLIGLGTFLYFGSYHHESDTEHDPDAATRGSGKAVTMDQLFSGFWQAKSHSISWIGGPDGEDGLLLEVGAS
EKPYIVVEDIRNDKETRSPIDTEVKASKSRTLMKDNFFVYEGNQYSPEWNEPSPDLKKVLLGVEKKKNWRHSFSATYFILDVE
TQEAEPLVPGKPDARIQLATWSPKSDAVSYTQDNNLYIRKLDDKKNVVKITKDGGPEYFYGIPDWVYEEEVFGGRSATWWSDD
GEYLAFLRTNETGVPEFPVQFFIKRPSGTDPEEGEEAYPEVEQIKYPKAGAHNPVVDLLFYDVSKKDVFSVDIDGAFADDNRI
ISNLLWAGGKALVKETNRVSDVLKVVLIDVGSRKGKTVNTIDVDKIDGGWFEISHKTAYIPADPENGRDHDGYVDSYLHEGYD
HLAYFTPLDNPEPIMLTKGKWEIDDAPSAIDLANNLVYFIAAKESSIQRHVYSVKLDGTSLEALTDPETEAYYDASFSKGAGF
VLLSYRGPKVPSQKVISTPSSVFTYERVVEDNSELAERARRHELPVMQYGTLDLGGGVEVNYVERRPPHFDPKKEYPVLFQQY
SGPGSQSVTKRFSVDFQAYVASALGYLVVTVDPRGTGFLGRKHRVVVRSQLGVLESQDHIAAAKSFASRPYVDADRLAIWGWS
YGGFTTLKTLEQDAGRTFSYGMAVAPVTDWRFYDSIYTERYMRTPQENADGYDMSMIANATALGSNKRFLLMHGVADDNVHFQ
NSLTLLDELDLAGVENYDVHVFPDSDHSIFFHNGNRIVYDKLRNWLINAFNGEWLKISDPKPHKDGVEKEKREIDGVSLV
FUN_014694	CDS	scaffold_7	260846	266071	-
hypothetical protein	EOG092602BZ	PF00637;PF01394;PF13838

MPTLPIKFQELVQLANVGVDTQSIGFNSCTLESDSYVCVREKKNEAAQPEVVIIELKNGNNVTRRPIKADSAIMHWNRQVIAL
KAQSRTLQIFDLEQKKKLKSCTMNEDVQFWKWISENELGLVTTTSVFHWNVYDAGQDAPVKVFQRNDNLQGCQIINYRVNSDG
KWMVVVGISSQQGRVVGAMQLYSKDRGISQAIEGHAAAFGTLRLEGAPQDTKFFSFAVRTATGAKLHIVEVDHPESNPVFQKK
AVDMFFPPEATNDFPVALQVSQKYGVIYMVTKYGFIHLYDLETASCIYMNRISSETIFTTCTDDGSSGIVGINRKGQVLFVTI
DDNNIIPYLLQNPANSEMAIKMASRAGLPGADNLYARQFEQLFNAGSYLEAAKVAANSPRGFLRSAETIEKFKRLPVQPGQMA
FTLQYFGMLLDKGALNREETLELAQPVLQQNRKHLLEKWLKEGKLDCSEALGDLVRPYDMNMALTIYLKAEVPAKVVAGFAET
GQFDKILPYSAQTGYQPDYIQLLQHIIRINPEKGAEFASALANNEQGSLVDLERVCDIFQGQGMIQQATAFLLDALKENKPEH
ARLQTRLLEMNLMHAPQVAEAILGNEMFTHFDKSRIAQLCEQANLPQKALELYEDPEAIKRVIVNIPGSPNFNPDWLTTFFGK
LSVEQSLDCLDAMMKTNIRQNLQSVVTIATKYSDLLGAVRLIDLFEKYKTAEGLFYYLGSIVNLSEDPDVHFKYIEAATKMGQ
FNEVERLCRDSNVYNPEKVKNFLKEAKLPEQLPLIIVCDRFNFVHDLILYLYQNQQFAAIETYVQSVNPTRAPEVIGGLLDVD
CDENVIKQLLQTVNAQAISIDSLVSEVESRNRLKLLLPFLEKTLNEGNQQQAVFNALAKIYIDSNNNPEKFLKENDQYDTLTV
GKYCEKRDPNLAYIAYSKGQNDLELVNITNENSMYRAQARYLLERSDAELWGFVLSENNIHRRSVVDQVTATAVPEANDPSKV
SVAVAAFLENDLPLELIELLEKIVLEPSPFSDNQNLQNLLMFTAAKADKGRVMDYIHKLDNYNADEIATACIEVGLFEEAFEI
YKKADNKSAAVDVLIENVVSIDRAQGYAEEVDLPEVWSKVAKAQLDGLRVSDAIESYIKAEDPRNYLEVIEVATHAGKNEDLV
KYLRMCRKTLREPAIDTALAFSYARLDQLSELEDFLRATNVANIEESGDKAYEEGLFEASKIFYTSISNWAKLATTLVHLGDY
QAAVECARKANNIKVWKQVHEACVQKKEFRLAQICGLNLIVDAEQLQTLVKEYERNGYFDELISLLEQGLGLERAHMGMFTEL
GIALSKYHPDRLMEHIKIFWSRMNLPKMIKACEEANLWPELVFCYYHYDEFDNAALAVIERPENSWDHQQFKEIVVKVANLEI
YYRAIKFYVEQHPSLITDLLATLTPRIDVNRVVKIFQKNDDLPLIKPFLLNVQTQNKRVVNEAVNDLLIEEEDYKTLRDSVQN
YDNYDATELASRLEKHDLIFFRQIAASIYRKNKRWEKSIALSKQDKLYKDAIETAALSAKTEIVSDLLQYFVDIGHRECYTGM
LYACYELIRPDLVLELSWRHGLMDFSMPYMINMLAQQTKDLALLKADNEARKAKEQEKEKTDDNTPILGASRLMITAGPGGMG
SSPSPAPYGQANGFAPQPTGYGF
FUN_014695	CDS	scaffold_7	267235	267805	-
hypothetical protein		PF09962		ENOG41

MNRPNRPRNRQPRPPRQQQNNQRGGSSRPSRAPATPQQASGTIPTIQQVIPGASVFIILKEDQPTGEETKGIVQDILTRGNHP
RGIKVRLQDGQVGRVQRMGNTTAATGETGCQTQSTRFTMRYTDVRRDDEFATGLPPRSLADFIPDFDEGLGSAPSAVVETVKC
PFCEGFEGDEAAVTHHIDREHIS
FUN_014696	CDS	scaffold_7	268234	270206	-
hypothetical protein		PF07690		ENOG41

MASPTSNTERSEPQTISGDDEKTESRSNTPTLNEPTQDEKPKKSEDGDGELTTEEEETEWISGCKLASMMISLTLAAFLMLLD
MSIISTAVPRITSDFHSLPDIGWYASAYNLASAALQPLTGKIYMYFNTRWTFLALFFVFEVGSLICGVAQSSAMLIIGRAVAG
IGSSGIQNGALTMIAKAVPIHRRPSLVGIIMGFAQLGLISGPLIGGAFTQYTTWRWCFYINLPIGAICAVLILFVHMPDHRAN
RDESAMQILKTKLDFTGFVLFCPSIVMLLLALQWGGLEYPWDSATVIGLFCGGGVLFIIFVYWEHRVGSEAMIPLPIVRTREV
WASCLSQSFLFSTVMVASYYFPVYFQSAKDASPFTSGVNLLPSILSVIFAAVASGALAQRVGYYLPFATASAILSSIGFGLAS
SMSPYASTATWAGYQILIGLGRGLGLQMPIIAIQANTSADVTPIAMAILTFSQTFGSAIFITAANVIFTHELRNELVARLPDI
NADMIIDAGAGAVSEVVSGADLPQVLWSYSRGVRATFLLAVGTSCAMVLSSFGMGWKDIRKKPASSPVLDEA
FUN_014697 CDS scaffold_7 270544 272409 -
hypothetical protein ENOG41

MATSRKRVAKKSTSSRTAQLEEKLEDLVTILRASQPSNGHLHSAGPGDSSPLMNSSNMGHLTSRLESLATAAASSSSSESYPR
PYTVPPNYDSRSTANTHPNTSPSSIGEDPSTSFDPTPEEAEMYLAKFQQWLKNFPCMILPHDITAAALRREKPFLWLCIMNIT
SMCVEQQMKMKDQVRQEIATRIIINHERSMDCLQGLICYISWAATTSSPGRPFIITFCQMAVLLAYDLGLTKAPVEEQYFTIC
FKMWGGRPPPPRLRTQEERRAVVSLWFLTSVMSSFVGKMDPLQWTPHMSDCLEALERDKLCPSDELLVAFVRYQLVADEAQKL
LVRDVMGESSSAPTYVFRRSLLAKLQAVRDGLPANMPITPVLQAHSLATEIQINSVGLFMQNIPVNQRIESMYSCLQAIRSWY
DVFFGITPEDVPGAPFAIYIQLSQVQIALYRLTTSEDPAWDKELVRNTADLLVLLDQVVEFFTRLDSVYKMKASQGEETVFIM
GAKIMNNIRISWEPTLSRHLRNASSIPTNQGAGAPVPTPPPPSTVQASMEMVNINMIDFGDVTWMGDVFGPWEF
FUN_014698 CDS scaffold_7 273671 274503 +
hypothetical protein PF07798 ENOG41

MAVPSVAAAEMLPRFLLPRLSWGAPLSVSRATRPFASIPSRDQRRSFASIQAGPVPRRRSPQAESPIIKRAFHATTIRQRDHH
FDTLKFVKRLQGEGFTEEQSAAMMKVLNDVIEESIQNLTRTMVLREDAAKATYTQKVDFAKLRSELLSADSTESNTTRTAHER
LTNDIAKLSNRLRDEISRTQASVRLDLNLEKGRIREEAVGQELKIKETETKIEQEVAALREKLEQVKFQTLQWLMGVCTGFAA
LLLGAWRLLM
FUN_014699 CDS scaffold_7 275004 276194 +
hypothetical protein PF00389;PF02826

MADKPKVLFLGSTKQAHEEFASLCKIVEPVYPKSTERSAFIEETKSGAFDGVKAIYRTSKSVDITGLFDAELLDVLPESLKFI
CHNGAGYDQIHVSECTSRGIRVSNTPTAVDDATADITIFLLIGALRNISSSIFTLREGTWRGNPPPSLGHDPQGKVLGILGMG
GIGRNVARKARAFGMTVRYHNRSRLSPDLEDGAEYVDFETLLKDSDVLSLNLPLNPKTRHTIAKPQFDIMKRGIVIVNTARGA
VMDEAALVEALESGQVASAGLNVFENEPEVHPGLLKNKNVLLVPHMGTWTVETERLMEAWAMDNVRLAVTEGKLKSIVSEQKD
LQ
FUN_014700 CDS scaffold_7 277088 278491 - SAH1
S-adenosyl-L-homocysteine hydrolase EOG09262LYR PF00670;PF02826

MSAPAHKFKVADLSLAAFGRKEIELAENEMPGLMQTRAKYAADQPLAGARIAGCLHMTIQTAVLIETLTALGAEVTWTSCNIF
STQDHAAAAIAAAGVPVFAWKGETEEEYNWCLEQQLTAFKDNKKLNLILDDGGDLTTLVHQKYPEMLKDCFGVSEETTTGVHH
LYRMLKDGKLLVPAINVNDSVTKSKFDNLYGCRESLVDGIKRATDVMIAGKVAVVAGFGDVGKGCAMALHGMGARVLVTEIDP
INALQAAMAGYQVTTMEKAAKVGQIFVTTTGCRDILTGEHFEAMPNDAIVCNIGHFDIEIDVAWLKANASSVQNIKPQVDRFL
MPNGRHIILLAEGRLVNLGCATGHSSFVMSCSFTNQVLAQIMLYKAADKAWGEKYVEFAKTDKLDVGVYVLPKILDEEVARLH
LDHCQAELSTLSKVQAEYLGLTVEGPFKADIYRY
FUN_014701 CDS scaffold_7 279868 281107 - EIF3H Eukaryotic
translation initiation factor 3 subunit H PF01398
M67X
MADAPNDAPVAAPFQAVQVEALVIMKIAKHCSSAFPSVATGAIVGMENEGLLEVTNTFPFPTVDPATTDSHQNDASQIAAAAP
RQKNNIHYQNEMIRHLKEVNVDANNVGWYTSATMGNFVNLNFIENQFHYQSANENTVALVHDASKSSQGNLTFRAFRLSPAFM
SAYKEGKFTTESLQKSKLTFKDILTEVPVNVHNSHLLTTFLHQIPSAPVKGEIEQPSSLDDLNRNALEPPLYPSIDNLDLAID
PFLEKTCDLLLESIESHYTDLNNFQFYQRQLGREQTKITQWQTKRKAENAQRAAAKQAPLPEDEWQRLFKLPQEPSRLEGMLN
AKQVEQYSKQVDGFTANVSAKMFAVRENLMPK

FUN_014702 CDS scaffold_7 281744 282455 -
hypothetical protein ENOG41

MATADVQTAPASSLDVFSKRAAEVEVREVSLELAANHRYLIQSPYTEHAHLLDLDTLDNENELLARALSQFRVLRDDYATAPY
TESFNWPEVIEEVKRLAVESGKPFKATSFYIVAFRSRIKKETEYADLGVLDKGAHAEAVASGGFLKYWFGEPDSELANLATCV
WRSREDAKKGGTGPAHRKAAAATRSLYAFWKIDQHRLIIRDNAETWEIIPWQD
FUN_014703 CDS scaffold_7 283817 284876 -
hypothetical protein PF00153

MSEIYFAGAIAAFTVDVLVYPLDTIKTRLQSQDYLKTYSDSSKKNIWAVRGLYQGIGSVVLATLPAAGLFFSTYESAKRIIGN
ATPLPQPLVHSAASGVAEMASCLVLAPAEVIKQNAQMLQNDSHGVGRSGSSTSLQAFRQLTGDGVSRRLFTGYTALVARNLPF
TALQFPIFEHVRATYWKSRGSGSREPGLIETGLATGLSAGSAGSIAAFITTPSDVVKTRMMLSAGNQSENSTQGQSEVAAKME
GKQPKKGAWTVSREVYRERGVRGFFRGAALRSGWTMLGSGLYLGSYEMAKVWLRRGKTDVEDDGPL
FUN_014704 CDS scaffold_7 285449 286688 -
hypothetical protein EOG09263I4U PF07228;PF13672

MAAPYNRLATASLRTLAKAPTQSCSRLRAYPHVAMSSCPRSRLYSTSTDAPRFSYHVASSFIAKDRPYDPSTHVFHFNPYNRI
QPPRNRRPSARPESGHDAFFVSRINDSGSVAFGVADGVGGWVDSGVDPADFSHGFCDYMALAAHEHQTNSGPPLTARQLMQKG
YEAICNDNSLRAGGSTACVAIAGADGNLDVANLGDSGFLQLRLNGVHTYSEPQTHAFNTPFQLSLVPPSVAARMAAFGGAQLS
DLPRDADVTQHALRHGDILVLATDGVLDNLFNQDILRIASRVLVSTGAWVMTDAGGVRVADSLEPLVEFPEASEGKRTATLQS
ALATEIVTAAKRASVNTKLDGPFAKEVHKYYPQENWHGGKVDDICVVVAVVNETTPAVKSKL
FUN_014705 CDS scaffold_7 287473 288433 + SDH2 succinate
dehydrogenase complex, subunit B EOG09263X1F
PF13085;PF13183;PF13237;PF13534

MAALRSSSRLVGAFSRTAAFRPGAVFTRSMASVSEPPKDSDANLKSFQIYRWNPDTPSEKPRLQTYTLDLNKTGPMILDALIR
IKNELDPTLTFRRSCREGICGSCAMNINGQNTLACLCRIPTEAASDVKIYPLPHTYVVKDLVPDLTHFYKQYKSIKPYLQRDT
PAEDGKEYRQTKEDRRKLDGLYECILCACCSTSCPSYWWNSEEYLGPAILLQSYRWLADSRDERTAERKTNLENSMSLYRCHT
ILNCTRACPKGLNPGKAIAEIKKQMAFGN
FUN_014706 CDS scaffold_7 289962 290317 +
hypothetical protein ENOG41

MFFKAIINASAIASLAAAVPFSTNTTSACPTLCVDAINDCGVMYGGCYPICSPELYPTPPPCTPTTPTTTPTITPITPDITIT
LITPTPDPITNSTS
FUN_014707 CDS scaffold_7 290495 291984 -
hypothetical protein

MPFPFILPTTSAFSFSSSFSSESHPSLPLNASTYRGVVRDALKKHKRLPPSSQVSNLNTVISSVNGYIPYLLAVDDGLSNKGL
AIVTPKTPPAIEWRPTLSGEVVPGRERARVKISSLEHEIAFVLSTLGFSHVLIARSALQPLYSTSSEFLGAQERTNAVTTATK
CLLEAASVYDYLAGRGEHATATPPCPDVAPGTARALCSLALAEATLLAVLKDDPYPAIVAQDRNKNDKEWMFKAPEIPKVRAH
LYARLCLAASEHAAKASSLCSTAGSGSHKISSALLKYLEDLRRTCRARACRFFGIDAEIGGQVADGIGWVRAGLSELGVEIKD
NNKKGLSFSRLKKDFTEKREDRRVDKESAWGSDAGKMEEIRVLEMLDAKWNKVNDTMNTQAIPPINTLLSKMPSGREIHTLKP
YEVPSLDRDIFEAMRAPPEDDDTYVDDLSSDDDVKGGPSAPVGAFPGTVDDYSRSPSTPGNAYY
FUN_014708 CDS scaffold_7 292342 294191 +
hypothetical protein EOG09260NZ8
GT57
MAGPSPSPHKPRRKANTKKPGNGGSEKAPRTEALVRAPAFPLAAFLWPARTSLSQWEVLPVILMVVGLFRWAAGLWGYSGFSK
PPMFGDYEAQRHWMEVTTQLPISQWYFHDLQWWGLDYPPLTAYHSWLLGKIGSLIDPSWFALFTSRGNQDSNLKIFMRATVII
SEYLIYVPAAIVFVRRYSRLQGVTNWTAWLALVAILMQPATILIDHVHFQYNTVMLGFVMASMSSMLAERYKWAAVFFVGALG
FKQMALYYAFSVFSYLLGRCVYPRLDFTRLFGIALVTAVSFAILILPLVLGTLYDKSQGIDAHGDSKDPPSLPLFPQLVEILD

TKAFYWPIVEQLVQMVHRVFPFARGLFEDKVANFWCAANVVIKLRQWPTALLQKAALGATLGSIIPPNIILFLRPRKSTLPLA
FAATAWGFFLFSYQVHEKSVLLPLMPMTLLLAGKHGLNKDTRAWVGFANLLGVWTMFPLLKRVDLRIPYAVLTLLWSYLLGLP
PTSLSAYFQEGQAAWLQWGTALLHLLFYVAMGAWHVLDTFVTPPADKPDLWVVANVGVGAAGFIICYLWCFFKLIQESDLLPG
GSTSKKDKKSKTL
FUN_014709 CDS scaffold_7 297624 298995 +
hypothetical protein ENOG41

MVSKSLLVLTAALATAWAQTTTDEPTATAEESSTGTSSGQAVTHTVNVGASGHKFTPNEVKAEVGDIIEWRFYPTDHWVIRGD
YDNPCIPYEYIGVNRQGFSSGTQKVQAITDDGPRYRVRVNNTDPFFFYCGAPESCSKWKMMGVVNPSKNETIEGWLKNAEDVD
MQLRPGDPWPKEGESTSTGATSEPTADNSDEDDESSGSGKKSLSTGAIAGIAVGGAAVLLLAGALLYLCGRRGGFDKAYRKSF
RNSVPSAPPVVENPYSPHPSVADPWAAQKSPGLVAAPYGQSPPLSPHQSMPYGTHPGMVAQDGTPISYHEGYHQPPMAASPAT
TPKPHEQIAPVELPGSPDPNHSPLPAYNNNNNDGRGFSWNGDEQGYRPTK
FUN_014710 CDS scaffold_7 300264 300650 - 60S_2 60s acidic ribosomal protein P2 PF00428

MKHLAAYLLLGLGGNTSPSAADVKAVLESVGIEADDERLNTLISELEGKDIQELIAEGSEKLASVPSGGAGGAAAGGAAAAGG
AAEEAKEEEKEEEKEESDEDMGFGLFD
FUN_014711 CDS scaffold_7 301531 302722 +
hypothetical protein

MAFKIGDLLAQISGGDEGSSVPPKQGLVRSNTLPKRKADDDPRSSISKAPRVTTLSSTVAPRTTQSPTAVSRPNLPSRPADRS
IPSQRPSNPSNGSSKPSVLSSKPMNGGNRVSKPVAPSRPSPGPASASSAPPKKGSFAEILARAQKAQQSMGQVGKIQHKKVEG
GALKKVKEEPPAKVDPRAAKSMKQRSAPAPSGYSGTAKPGQRPAQRNGAPVSGTGRDPRNGKPLPPSKAERGKIAGSKSSGRA
APPEEETKKVKKAAMATTGYTGTARPRPGGDVGKKKKEAPRGGALLSAPRAPRPSRSKSRFEDDYDEDLDDFIDYDDEEDDGG
PRYDYASDGSSDMEAGMDDIDDEERQAERLAKIEDLKEERLEKQLKAEKEARKRQALEAMRGRR
FUN_014712 CDS scaffold_7 303959 305811 -
hypothetical protein PF10453 ENOG41

MSGYGYGPPPPPPPPSSGGTSSYGPQAPSYPQHGQGRGGHHGRGRGGHYNGGRGDYHGSPQGHYEYNGQPYPPHHNPPPYGAA
HPPAGSYPPQPPWGPEHGHPPHGHPQGSMPPGNYHPNYPAQPYPPSQYPQQPPYGAPQAYPYQGPPPPPNQAQWSGAGQPPGG
PYSNGRGRGGYNDRNGPKPPLNGPVRPGYEHEAMPPANGYGQPYPHDPRAVHYPPPQYPAYPGPPLPPGHHQDGYYGHNNRRG
RGGFRDGNTRGRGGHHGNDKSRHQKHGNNDSAHHKSETPTLGKKKKRKTNTLGLTPGMDSESEDDEGEEKVLTDLIGQETLNV
KDVASFLAERKKHFPTKARVEAKKNAELAQNGEDKASSLEKQADKLRRQLRKVESSIKRKREQGDEGDEMRDPTEESSDDEPE
VMSTRAHVAPPPPPPAKKADVSKHCKYYSTGGTCGKKGKCRFVHDPEVREAAMKDREANNGRLTIQQRLILNDKEQEDLTVLQ
SIQYLRKKGLMQPEEPEVKKETKSVKVDLPAHPPPSSLLPAASASLPPPPIKREPNLPRHNPPPSIIPSANGSSTQGVKPYQG
WNLRPYGSTGGKSSKSDDLP
FUN_014713 CDS scaffold_7 306551 307342 +
hypothetical protein PF03876;PF08292

MFILTKIADLVQIAPEDFSKRSIDAIEDNINAKYSNKVIQKIGLCICLYDLQWASEGLIGHGTGLVNVNTEFRMVVFRPFKGE
VMIGRIRSSTPAGINLRTDFFDDIFVPFEELPEGAEYNHSEQLWIWNLDEDRLFYDNHEMVRFQVIDEEWHDQAPAGPTQAED
SPLKTPYRIKASMAAEGLGVCLWWDGA
FUN_014714 CDS scaffold_7 307442 308377 -
hypothetical protein EOG0926431P PF01956 ENOG41

MAQAPVQTLHRDPQLFYWILIPITIVMVLTGVLRHYASVLMATAPKKQDEKAVREQRFLARGVALRTNHHVLSQRAFESRRDT
LTTNYEAGTYLKEPDRKGQPPANPLSDPSAMDGMMGMMKNNMAMIIPNTLIMSWINAFFSGYVIMKLPFPITIKFKSMLQAGV
QTREMDPRWMSSISWYFLCIFGLQFVYVFLLGSDNAASQVAQQMQAQQMPNPMMGGPGQDPHKQFVAEIENLNVVEHYSVLDN
VEERLLAGIKS
FUN_014715 CDS scaffold_7 308762 309557 +

hypothetical protein	EOG09265B95	PF06703	ENOG41

MANAAEKISVYNLADLKNTSDDAIPNYLNSLKFRQSHTLTDVRLALGYTAFGIAAACFLWDYKLGFESTKYYTAAAVTLYTLI
NGVLTLWIMFREKGVVYEGTSPSGEKISISSSTKKNVPIYNLTITVTDKNSKSTVHKISKPFSGWFDQTGQFVAVPFQELLAN
SVPLIGKRDPKRVTVSKELLDASPDVLDAILAANATGAEAASTPEVTEKKGGKRRKA
FUN_014716	CDS	scaffold_7	309831	313576	-
hypothetical protein	EOG09260SR2	PF12460;PF14500

MADFRQWALEFVLADNEGQQTAIAKKAANEIQTAAANTNPVARWVEAVQPWMPGGGNEAENETPDWTARAKALEFLSRTLDFL
SNDILKPSQVKLLVSFFGAMFEVDHKAGIMPSASALSRIVAMKSFQRHMGHDIIQKVCSLKDDFPRQVSKTRLEIYELIRLLM
TTPGVASDLQNTHGSSAGFMLDLIQLCRSERDPECLMMWFDILRLFMSEYTVSQDVLEEVYGVFKPYFPISLPRASQVAITPE
ELKLQLRKCFSANHLLADKVFPFLLGKLDQGDAVTVNVKLDILKTMQACLDEYSHPQKAIAPYANQIWGSLKYEVRNGEIEDT
IWGTLEVLKSLTRRLAGDSLRDYTLSVTRDCVPDLATTMYASSAGRLMVSVLSSKPSAFVLMAAPVITHIKENLRHPKAPVHT
QDLLKVLHIILETRFLLVDTNMAAEDREDFAAIDGFFKSLYQDTFKNPVSLGSKPDASYDDIKVATQAVPGAGVLVCQRPSKS
LDLSNNGASDISQRLLPESTCSEICSSLFAILKKSGPGYPRSSGGDELINDAAQALQRAIRYFPRGFDGLVDESLSIIRSSRQ
NGNVPDTVETIKTLGGLVAYLGSSELPFTPRDGFDHFIYSIRAFLSELFDSLESKSDPEIWCALVATIQSTIRYFNDACLAKN
PDKEFSIEDEPLQKIGSAYPALSLLGGETVTESSPEYHSQVPQSASVAEIRSEFLLVTLFIARQLYRRATKVVDTHPQTGKHA
LSLSDDFANVDEVSGHQYLHLISTLAGFVIHEFSETQQTSLKAESFAISLFRDESIRVPQTAPETQSSLENGSSWSWLASEAP
NVLAFGILQALQPSAVSRLFDIGVAQELILSGFAADTNFSRPVTLSILTILANKFKVETVPSLITALEQRSSSLSPASTSGND
ASRLEQITSIYALVAGMVRRYSGKEAKTLLDVVKESPKDPKPGTELARRLEMIVAPQQPLTKECYAIVKPLWTQKVYFQLVSP
MLRAATGKETEVQDQQVKANYSTAVLLMVKHMNFPIYEADADSILRISIAVAQNSGIGPETKAALDVIKNTLAEAPEKGKGHI
RSIIKICSSVFSHKPSPASADVETEGACGKLALEIVGSLPQTYDSQHLIPHAPQVQRQLTLVCGHRVREVRKTARLARAAWAD
LK
FUN_014717	CDS	scaffold_7	314131	315139	+
hypothetical protein	EOG09264BIX	PF06644

MASLRIPALRRLVAAPIRTTRAINQRRWAQVHDIRFLATTQPSQAVVEKYKEKLNQKAQKEGLQSIDQLKAAYADKIDAERRK
SGIEIPVGTIPQAPETPVVQPNADNPPGQDQPTRDPRDPPTTPPQHPPAGSDKPAIKTLNDIIDLPKARELPEKELTAIWRLR
HASSEQNLCAVIPMSTYKAMEDAARTAPQFVLPVPHPSQGAEIHFLQWTFDAASKTSTVLFTQLAEYKNRGEFAQPHTTITHH
LDLADERGLVLMQGQVLPDRGVTPENAKWLLMSLQRFYGGWDGEEVELTGERKDRAEERKKLLNWFAAGDSRFSVDKLLEEAE
RMG
FUN_014718	CDS	scaffold_7	315689	317861	-
hypothetical protein		PF04083	ENOG41
S33
MTQQQHGSSTATQEHHPKVVGKKLAETAVQFTQQAEKLSHQHPTGKDPGVAIEAQFVTPHDPVIITSDGKRLPGVPLQEAHKL
NVLREELQGEPESVEIKAGNETKEKISNVENANPKQKKVVELPSEQNGASLQKSNSNSTLRKQTPPSHTNPLFPPLPLYGPPN
MLRNLQCMFFRISAFFLSLAFLGVIVLGSLFTTIPMIWKNIWYRLTFRDPDSDRPFYEEEKRRAQARREQEKSWKQKSSNGRT
LDRMENAEEFPPTEGGPDPIICDVAYYARRVGLDVETFEVQTEDGFIIDLWHVYDPKEYTELEESLRSDRGPEVFQSQRKKLK
DPSQKPKFPVLLMHGLLQSSGAYCCNDDDSLAFWLCKSGYDVWLGNNRCGFKPKHALLEYNDPRMWCWNIRQMGVFDLPALTS
RVITETGFEKIGLICHSQGTTQTFVALAKEQRPELGEKLTVFCALAPAAYAGPLIGKMYFKFMRIISPGLFRLMFGIHAFIPF
MMQMHKLLDPRLYGWLGYKVFSFLFDWTDARWDRGLRNRMFQFAPVYVSAESMRWWLGRECFAKHKCILSTKEIVRAEEHMDS
KGDGRPVTPRTSSALEAHRKHPKGSTAWYNKQAPPMAMWVCGNDDLVDGRKLLRRFEKGREPHVNLVHSKVIPEYEHLDVIWA
MDAVDQVFKEVREVLWKTCNARDICRVPEGCEAVLPEKEKLSVKEDVVEECQSSSSGET
FUN_014719	CDS	scaffold_7	319573	323839	+	PEX6	peroxisomal assembly protein	EOG09260VTN	PF00004

MASSSKSALTSNRRRNRRRRQDKPAIAAKLVLDDHIKSDVGIVSEDIFRDLFPHLGNAQLYQGATEDIHHIAIAPWIPNANPT
DSPWSIVPIIESSALAPSTVRFSPSSASLQSFAVTLQQVAPSKLSSHSRGGIEILVLDVAPVPLETVFVSLESELTKRLENGE
GTFFRDHPASSKAKGADSTPDGRLVTSLRAALSSLKVVHAGDLFPLPLPPHPLTHVPPNPGKIMLCEPVAQGVLTESTKIILM
RGRVHTKQIRRVASMTASQQLNGVPEDEDDTSNDQFYSAAEDRDKSGMTTEDMESETETEPDMSDFDNEDELSDDSMDDMISL

QAPTLPTTNASGVSTLQPGTPMTVGTLRGRKTNGIATPGSVFSNFTATTARPDRPRGRLFKAQGLMEQIPTDLLHPKPSLEDD
EEARIYVDVTSLSRIGCFSGDWVRIEAAEEPPANGFGAFGLGSFGQVESEPAKWRPVRVYGLPEGYSQRPMTRIPSAKHGERR
LSFFESQIQRPASPTAYASPILLANLDDAPYLRLAPLKKASYQGKGTIPRFTSAARPPYARDITIHHIRTPISAERAFQTAVL
GGLKRYFAQKIRLVRSGDLIAVPVDAELGRTLQEMPGAGGSEVDDVMALTAGGQEGGKQPSKFDSVAWFKVGHIQIQKADEDE
DGSAEAFWGSVACIDSSSVAMHGSGFETSRIPGIKQSTWQYYLGLKKTPKKASESTPAPIQEPELPYVSPLRRQLRELIAAAT
SARAIHLKMPPVAILLTSTHRNIGKLTLASGACSDMGLHTYKIDAYDILSEGSGSGNDVKTEGFLRTRAERAMSCGPDCCALL
VQHVEALTADRIESTIKEILEDTRVLIATTNEVDKVPDGVRALFTHELEMTAPDEAERESILKSIISDRGVSLEPSIDLNSIA
LKTAALVAGDLVDVVERASIAQQSRLEQLSAKNTHDNMIITVRDVQVAGGPLARCLTKGDFEIAVEAARKNFSDSIGAPKIPN
VTWDDVGGLNNVKEAVTETIQLPLERPELFAKGMKKRSGILFYGPPGTGKTLLAKAIATEYSLNFFSVKGPELLNMYIGESEA
NVRRVFQRARDARPCVVFFDELDSVAPKRGNQGDSGGVMDRIVSQLLAELDGMSGGDDGGGGVFVIGATNRPDLLDPALLRPG
RFDKMLYLGVSDTNDKQQTILEALTRKFTLHPSVSLASVAEKLPFTYTGADFYALCSDAMLKAVTRQATAVDNKIRAINSDPA
TQHPISTAYYFDHYATPEDIAVMVQEEDFLAANDELVPSVSAGELAHYEHVRATFEGVREKEPENKPPVQRVVSGASTSSKGK
GKAVASGSSKGKGKAIALTSGDEFDEEDEVDDMNGRSKGKGKAPMGFQDGTASDDDGLY
FUN_014720	CDS	scaffold_7	324032	325573	-
hypothetical protein				ENOG41

MTNYLNDPALYIYTSLTAGSSHIVTATSRLETILRANRVPFKAIDIAVDTKARLLWGRRAGKDKSGRPRKLPALVQEGWVLGD
IVEIEEWNEYGELKEHVTIYFDEFTIPSINDKLPEPPLKRPIDLTGGLLASMTPSPVAKAVAAAAASAAAAPKPAPAAPPLPA
AKAAVSSATPKTSTEPKKEEKALPVRSVADEAAQKAKELRLKTLREKVHGKDGAKAKKEDTSAKDTGKSKESETPAAKIDGIQ
SPTSGSWAETDAGRNAIQSPTSGTWKESGPGSISSLKRASVEVSPDEAKAVEAKMAIKEEPGLEKKAETKSDDDDSDDDEDET
EEEEEEEEEEEEEEEEEEEEEEDTDEDSDEDSEEDDEDETDDEEEETKKPAVTPEPEAAAAAAETAEAAKDDKPKEESENTKK
EIKKDSHKDSKKDSKKDHKKDAKKDTKKSVKKDPKKDTKKDVKKDPKKDSKKDTKKDSKKDTKKDTKKEVKKDAKK
FUN_014721	CDS	scaffold_7	328227	328821	+
hypothetical protein				ENOG41

MQFSSIMIAAAGASVAAAQDYAPPAPVLDTTTLTSTTTRVITLTQCNPTVTNCPLRTTTSTEVVVTTSTAAPEVPTTSVYVPP
TTSVYEPVVNTTSHELPTIISTPTISTTKKAPSAKTTFYPAGNTTTKAGPTAPGSYTTTKLPVPGSSTDVPANPGKPGYEAPS
PSTVPTAGASGLIASSGFVGAAMVAAMVALF
FUN_014722	CDS	scaffold_7	337199	337616	+
hypothetical protein

MAVLQDCTPFQRYESQVYVAHNPENSRLSVVGSEPVLKCNSQSPKQPIWRRLKHARHSSTCTNTPVDDQTEREERRFLYGNNI
GSPRGITGETCCPKTWRTTFLPVAQLSNDKSTRQRQTNIHPRSLSEWPDPNATQA
FUN_014723	CDS	scaffold_7	339552	341243	+	MT2
Sphingolipid C9-methyltransferase 2			PF02353;PF08241;PF13489;PF13649

MAEKSGVTGADFEFVKTPAYPKPDFDKLEDVGVPTTRYPAIKNAPLPADGSGADTFNNYVLISILILVPWFLSRKVGGGLKTT
LFFALIVDLPLLAAWWLVISSISPRKNEKVRLPNRGVEHYLEFKKEADRLKYRGHNKIPMETFHEMYFDGDVDFKGDCLEVME
YRHDWASFRFTIGLIKFFLFGMIPEVIMHTRSQDEEQVRDHYDRGDDFYGWFLGPRMIYTSGIISDINREETLEELQDNKLTV
VCEKIGLNEGDSMLDIGCGWGTLARFASEQYGAHVTGVTLGRNQTAWGNSSMRKAGIPEEQSKILCMDYRDIPVPEGGYKKIT
SLEMSEHVGIRHFGSFLKQVHDMLDDDGVFFLQYAGLRKAWQYEDFTWGLFMNKYIFPGADASTPLGWVVDKLEGTGFEIKHI
DTIGVHYSATLWRWYRNWMSNRDKVEAKYGKRWFRIWEYFLAYSTIISRQGSATCFQITLVKNINSTHRVEGIETQFAITGAL
DNCRANLQSWASRNNVKTPSEYL
FUN_014724	CDS	scaffold_7	342103	343000	+
hypothetical protein			PF01575		ENOG41

MPSHLLTTTINDLLYPLPTESKISSPPCALPQGHHLVYFPIQLPPSRLIPDGADPDHSPGPPYTRRVWAGGEVIFRGDEMKLD
ARPAICKEKIEDVTLRGKEGEEKVFVDVWRKYGTGHEIEGRTEWDIEERRTLVFMRDEEGTASSPSRLLKYPQPPSYSISLTP
SPIHLFHFSALSFNAHSIHFDPQFAREVDGHRALLVHGPFTLALMLRVLNDQIGAGVAVHRFSYRNYAPLYVNERMSINIRKV
SKEDGAEGRWDVWIEGPEGGMAVKGTADVASK

FUN_014725	CDS	scaffold_7	343257	346461	-
hypothetical protein	EOG092612MY

MPQKPKAKPKAKPKAQQQPPQTEHQKIAEQWAKAERARAEQAANAKFGLPPAEENVEVLERRREALDKRRQKTYEKRWRWTAA
FWVVLLAIHALGIWLFTSGFLLTRLVLEDKSNCTLPPIENTKGSLNVDRGCWHPKSFDRAVVVLIDALRYDFTVPEDPAQAQH
FHNAFPYLYETAVKSPQNAFLRPFIADPPTATMQRLKGLTTGTLPTFVDVGSNFAGAAIEEDNLLMQLKDAGKKIAHLGDDTW
WSLFPGYFEPNISKAYDSFNVWDLHTVDNGVIDNIFPLLNKERKGEWDLLIGHCLGVDHAGHRYGPDHPAMGAKLRQMDEFIR
KLVESVDDKTLLVVMGDHGMDSKGDHGGESDDEVEAALWMYSKKPFFGRTSSDFAVPPPNAKVRPVNQIDLVPTLALLLGIPI
PFNNLGGPIEEAFAGTKGNDWRNLNAVSRVAAAGIERYQASYHEARGLTQGEGAESPAALWEAARAIAKNDRDAYVAFTKFQD
TTLSVCKDLWARFDVSRMIMGIVVFGVGLVLLLMYSSREEEDEYVIMNDAELDYAEKKLELMAFKENEPETAEVLHKQILKGL
WDPKILFTVSVLTGVGLYRQQPIEGLAALVAVLFMAGLSSSLHDAGRTILNVFPSTFWGWLAVIFTVSQSIGFASNSYTIWED
SILLFFITTFGVASAVSAFRIEARRERYLGIYHSVAFIILGRLASYSKLCREEQMPYCTSTYYASSTSSTSAIWQLAIPFAVC
LILPAIIKSFLVTSRSYEGLAPTWIGYVIRGGLFLSASYWVIDAADNGGWLEGRLAEGTPKTIGVYLAQIVLALAFIAGSTAF
VWAPPCVSLLQSAGAGGRPVVTIIGYGNAIGARYLLLPLNLLLGCFLLSKPMGGGALALMFWQILTLAELLDLNELKTSPIGP
IMLAVLGNFYYFKTGHQAVLSSIQWDSAFIPLFSIRYPWTPIVVILNSFAGQIIAAASVPLLALWKVGPKQKGVLETATRGIG
VFIIYYAVEALATMSWAGWLRRHLMLYRVFSPRFMLASTVLLVLDLVTILVTLTGLRSNTLSLGEVFGWAD
FUN_014726	CDS	scaffold_7	347201	349888	-
hypothetical protein

MAPATDELSAPGAVSYDSNTQVVGDGTWDFDKNTFLLPNLQGLNHETMRYNGMGNRFSTLTQYHGLVLAHAVLGTIIFLFLIP
FSVMTARFYSRRPGWAIKYHGQLNVFSGLLLVPLFILGYFAVGPERSLTNPHHGIGVAIFTLFLVQLMGGWIVRRVAKSVSLR
IMIHQWFGRAIALLGITQVPLGLTLYGSPLYLFILYAVWMFFLFILYFILSWRSASHRDYYMGGARSEMRSELAPTEDTRTRY
TESEYFSEYKSEPPKSKAKWLGPFIALGGLAALARGRNKNKNQDNGSRVRSRSPSFDRSQRPEVLPSRTGSASYLTGHTGEKY
SDVTRKDNSGAGAGGGIAKFLAVLGLGKLFSKKDRSRDEDYSEYSAVSTETPRRHRATASAPTMTDFTRTDFTSDVTPNNRRE
PDITRTSLLPPSGNRPPPPAMSQMRSAADGSYTETALSSPPPSVTPRGKRYPPNSGRSFFEESDYSSYVSPSRRPQPEKSGGG
FAKGLLGGLGFGWFAKKMADRRAAKEEQRLRDEEDLRSGTSLSRFTGGDGHDSPTRRDSRRPIERRQTGYPPTDTLLSTEMSE
STIEPRPARGNYVPPTEMSTLPPQSNLPPVAGRAGNHSRRSSEHVSMPAMPPDPHGILAPTETTASGISPGSRAQRSRQGRPG
DRDTSRLAPGEEDSRYERYASPASVSVKMKVHDDKDRNVTLRRLTEEEAKAARGRRDSASSISGLESPSYRRRYRRDSSQKRA
ETAAERRAEEEDAMPPLSPPNPSFAKQKRRGKDSAYYSGQPTQVQTPASLLPGASAQFGQTVSSLAESHGTWSGITPSVNTSP
PGPAGEGPVDGSAAAENRRQRRRLERRRASGPTAEPPATTNVDMFD
FUN_014727	CDS	scaffold_7	351882	353039	+
hypothetical protein		PF00150
	GH5_15;GH5_9;GH5_50;GH5;GH5_14;GH5_49;GH5_45;GH5_22
MNKGQDIADKNFQAHWKRWINQTDLDEMLSYGLNTIRVPLGYWLKEDLVDDSEHFPKGGLEYLTQLCGWASDRGFYIILDLHG
APGAQEPNQPFTGQYAPTVGFYSDYNYGRAIEWLEWMTDIIHTKKEYRNVGMLGLVNEPLNWDKAVDSLRKTYYPKACTIRKV
EDKLKVTSNNRLHIHMMGSLWGSGKPTEFLRDTSFTAFDDHRYLKWDTSVEASHDAYIKKSCSDDRNTDGPTIVGEWSLAVPD
DVEKTDAWNPQTQKEFYTKWLSAQVHAYEENTLGWVFWTWKASLGDDYRWSYRDAARAGVIPKDLDSLPSVC
FUN_014728	CDS	scaffold_7	353208	355194	-
hypothetical protein		PF00005;PF01061		ENOG41

MTVRKLSDLDNSMDSEQRPVAEKHLLNTTIKNFTWRNVTVTVKDRETKQPKAIVDNVEGIVEAGEICALMGPSGCGKTTLLNV
LARRPTNASSVEAEVNINGSHLSLAEFREVSCFVEQEDALIGSLTVRETLEFSSRLASSSSLSKKERIVRINNLLESFGLVEQ
ANTLIGTPIRKGISGGQKRRVGVASQLITSPKLLFLDEPTSGLDSAASLEVVKYLRDVAKRNNLIVICSIHQPSTSTFNLFDK
LLLLSGGKTHYVGPVNSVAAYYAEVGSPLPQYVNPAEHLLELVNIDFAQDRGEASRDLETLQAAWQNSRHASEVNTAIKSAES
SGGDWNIDTIEKRPSMFSLSMTLLHRSFIKSYRDVVAYGIRIAMYLGLAIMMGTVWVRLDPAQESIQPFINAIFFGSAFMSFM
AVAYVPAFIEDRLQYVKEHHNGLYGAAELIISNFFIGVPYLFLISILFSVISYWLSNFQPTAKAFFTWVLWLFLDLLAAESLV
VFMTSLFPSFVISLALVAFANGLWMSVGGFMVPPTILNVFYKYVFHYWDYQKYVFEGMMVNEFADRVYSCGDGCRCMYQSTLV
DQCMIDGQAVLNQYGYSNGHMGRNVGIMISIIAGYRLASWLVLVLRR
FUN_014729	CDS	scaffold_7	356307	358436	-
hypothetical protein		PF00172		ENOG41

MNSRISFHQTISNSNREDTYSGNDELSSNSNSPSSGVDGRASPCGSTSTTAESSRMSVTSVPAACLACRGRHLKCDGVNPCSR
CLSSGSDCVYVASRRGYKGPRRNTVQKPGKKHGVSPARTRTRSGESSVTSRPERSASLGSSLFLSPNMGDDMLNSPLSTPLLD
PALDASNSMESDMSFFGPYDPADTSTDMFTLFTSPQHEHVVTHVPALTLPERCIDSFYNHFHAAHPFVFPKESLLMISHESSV
MPVLAVMRWIGSLHIPACSERGRFFQEAHQLIYEPRRRKDGFLVQALLLLLIGLDGQGQQDQARQILSGLERISVDIGLNTRS
YATTYGRGIPMMEESWRRTWWELFIVDGLIAGVHRSTNFLLFDVASDVALPCEEFEYLSGDIPEPMYLRDMNESDFSGTSQEF
SSFAYRIQATQNLGRLLRIPPVSGPDDESLRNIQTMLTSWRVSLPKSKADALRQYGHFDEMLFQAHMITHATSILLHQPYSQL
NSILSQSNNQTDQYDSVSSRDDFNNHTKNIIQAATEISKLVTHRVSLLSHSHFFSYVVTMSSIIHLNRWAIFTPDTENNLRQV
LRLNIGALSRMSLVWSASERESSSVKSMARSIYQVKKQHRVLPQFWISMTHDEAMDRIAADDAMIHEFEDMQVVPGMMGHWTD
PGNKQWN
FUN_014730 CDS scaffold_7 361488 363466 +
hypothetical protein PF14479 ENOG41

MDPVSLAGLALGVASISLQVYTGCIQGIQLLVTALGYEDECKYLNLRLRMEQQRLFCWSEASGLLDLDAKNQDKVLNSNVFNL
HRQTVIDLLVQIQCLFNEFTEYQQKHNNLSAVVDKDGVLDAPEKDAKLSNYPMSEKKRNFIKKAMAGLKSKSSESLTRLRWTS
FDKQGFEKLLAKFSVLNDNMTNILDHSLQVEIRNTVQDTNRGVLLLHHKIADLSHLVLALKSQLDAGSRVGPSQMSKLEREAN
ADALRQLSRLAKFKAFNETIDPKSDSPAVFDEAAAKFLDLAKPGHQRNLFIPRYLIELDPEVDESDAPRCEAFMKTAEGKKKV
WIEWKDYDNTDTQPGSLSKTDIIERVRKLAALLNHSPKPEAFRTPHCLGFFDKADPNISEDDVDILDRRLGLIFERPSDDNLQ
TSLPPVSLRELLQDPKVRKPRVTERVYLAHAISNCLLYLHAVHWLHKGLRSHNVLFFRTRDDHVDYRQPYLSGFDFSRPGGSD
EMTDAPGDDAEHDLYRHPNAQSNRRRDRERSKKSFDIYSLGVILVELAHWRPVEDVLSIDIRRARGRPDVVRGVREALLEEDR
VAAVGADMGERFEDATRKCLAGGKDLGLNDGDDETRDEVAEKLSMKFYEDVVKRLEGVVV
FUN_014731 CDS scaffold_7 366670 369802 +
hypothetical protein PF00069;PF07714

MASHGQYPPAHMMPNKHHHHAPYGSPVAIPGGAPAGTFAPGTKIQVGSHRVVIQKYLSEGGFAHVYLVKLAKPVDGTDQAVLK
RVAVPDKDTLRGMRIEVETMKRLKGHRPIVTYIDSHASELQGGGYEVFLLMEFCNGGGLIDFMNTRLQHRLTEPEILNIFTDI
AEGVACMHYLKPPLLHRDIKVENVLITARGSSKRFKLCDFGSAASPRPAPTTVTECRLMDEDVQKHTTMQYRSPEMIDVYRKQ
PINEKSDIWALGVLLYKLCYYTTPFEDQGQLAILNASYRFPSHPVFSDRLKKLIASMLRENLQDRPNIFQVLKEGCAMQGRDV
PVQDIYSRPQSHARTDSPQPKAPKPEQTPAVGAVFTRAEPQKQVIPDVVPMRRGRPTASPAPAKVAQKPDASPMRKTEGDPFA
ALDSKAASKGFDELSSRFPTLDQFSLLHDQGTKFDFDVSSPISPTGNTSSDLNERVTAKLADEAFAAPQAESSRPKPVSRTHS
VTPVGHHAAVASPPLEPPLAKTLSAPSRPDISRAQSIISNNPELQAISSQSNSRYVSTGTMTSTPPPEKFSRDVERPIQREIA
EYSRSSSATRQADPSAAQWNAEEPRAAVSQRISAFQQPSHERQPSSSRPSLEGGRPRADFLEPAARASPAPSRPRPASTNLEG
TTLDFLREREAASRSSSRPPQSRSPYQEPADEPRTSSDMEFLRSLEDSERTKSSKRSSLTSLSGNKNILAGKFGDAFKRFEGS
SPNNGRIPSPERGMARRDLTPIAGSEATDGRSDDGHFHDDEDRMTPEMRREMERLKLQEEEQRVEAAQAEYRKRVAAGQRGPP
VPPKSIGGVSRAVSIQNRVQSLLNEDQKPSNIQRTAQGYGKYTDVPETGNKPEKSLPGVPKKPLMVAKTRVDSPQARGPSSTS
SAPPALTSKPPAKPPAPKKPVHLNSLPTGQRPSSPQKPTRAQTSTEELIEFPGQQMVDMTAKEKDDYLQDFSKRFPSLGSIEM
VEREIGGQNGGSSR
FUN_014732 CDS scaffold_7 370066 371539 +
hypothetical protein PF00400

MAKRKRQAIAQSEPASEPSIKKKAKGAQVLKEKPVKAKTSALKKSTTPATASNAPETIQIVVGSYDRILHGLTVTVGGKEKAE
FADTFLFNAHTSAIRCVAASPISAPVPGQTQKVMLASGSTDERVNVYNLSAHPPSRKNQEILAKVAPRPILENPKNREVGTLL
HHSSTVTSLCFPTRSKLLSSSEDSTIAVTRTRDWSLLSNIKAPIPKPQGRPSGDTAPFGGTPSGVNDFAIHPSMKLMISVSKG
ERSMRLWNLVTGKKAGVLNFSKDMLQEAGEGKHSTGEGRRVVWGSVDGADEFAVGFDRDIVAFGMDSVPKCRIMGSIRTKVHQ
FFYVALDKEGDATLLAVATEDGRILFFSTKESDLTQPESEDKKLPTAKLMGQIGGKAGGVEGRIKDFVVVPSAEDASTLYVAG
GSSDGRVRVWRVGVPELQGSVKAEEAKGELLGMYETQNRITCMTAFMMIPRPEGAEESEDELEDEEEEESDSDEE
FUN_014733 CDS scaffold_7 372985 374194 +
hypothetical protein PF07281 ENOG41

MADDGPPLIRPVPRRPFDLSLTTPTPPSENSSPPTPAPAPDPNIARFLQPTPEPASAGLSRPQSFMNLTSSTLFGIYSPTTSS

SRDRVFADRDELDTPWGTGAQTPIKRPSIDDATFDFLKDRSHHIRRRSSIRVHEPAGPQKHSPSGGALVLRGLLLFVLGVGYG
VLVTSRFHESDSLPPRATYNWGHLTFWGLAGVALGALFPWFDRVWENSFGDKGDEAVLETSASAKDDDPSTDWALAVRAIGAF
VGIVFAIRKVAWASTLQVSLTLALVNPLLWWLIDRSKPGFLLSAAVGLAGSVVLLGVNPDMMPAPTQVPFRHLVNATMAADGD
MPILGGLAQQKTLETGMWMLSVLFCSCVCFGNIGRRLTLGPSTTAKGRWAGVR
FUN_014734 CDS scaffold_7 374872 375377 -
hypothetical protein ENOG41

MTSNSISQLPTPEQREDITASLADLITAIESHSQWTPPNVDRGLFHVWDFVKRSHYIMTELDNIAAGRKVQHPEQIPKNDGVA
SGPEAALASYTDVCTRTITINEMIQNPRMLVMLGLSNVDFGSAIQEKSEAVKEAIKSAN
FUN_014735 CDS scaffold_7 379969 381757 +
hypothetical protein
PF00097;PF02190;PF13445;PF13639;PF13920;PF13923;PF14634 ENOG41

MSSEGTSQAAATDSASGPGPGPRQSRTPDPPTDSDHEPPDSGRQPTQVTQSQSSSANTSRHRDVAPRDFVRLFQCQICSLPLD
EPISLPCGKSLCRRCLPGTHMRANITYPAAPERLRGFKCPFEDCNKEHAIGDCAVDVVLNKTAQHVREEIERFKNESSTTELA
TKVMWPDPWAAAGVSSMRDDDEAYTKVIPGGKVIATYRLAEEGDLSYEADICYDEITLATPSPPPDADDTGLVRKAQDATRAE
MDCQVCYALFYDPLTTSCGHTFCRSCLHRILDHSRYCPICRRPLAISPLLSQTSSPSNQTIKRIIETFWLEEVNARKEALDAE
RVAQMQDYDLALFVCTLSFPQMPTFLHIFEPRYRLMIRRALEGDHTFGMVIPKRPQHAGDANFHELGTLLRIVNVQFFPDGRS
LIETVGLSRFRVLEHSYLDGYIVGKTERIDDVSLEDEEAAEAAEATPIETEDQQQPMQAMDTVKEPTEPTPTSPLPRSDSSSP
HIPTSVTEIDSMSTQSLMHYASSFVARMRQQSVPWLTERMLTIYGDCPEDPAIFPWWFASMLPVKDIEKYRLLGTSSVRERLK
ICCTWIVEWETSRW
FUN_014736 CDS scaffold_7 382334 384799 -
hypothetical protein ENOG41

MPNRKIKRPSHFCEWVVGTTIESVVGAIPQNKPPKKRDVVRVQVTTDDESEEDTVKITYPRTGRTYTPPPANEAKAEAKKVRF
NFGQNGPIKSAMKQTTTTTPMDSSDEASDSGPETESDSSQSAYISSSDATDSDIEYSVECVKHKRQKKKKHRQTQTEEDTEND
WDSDPEPTCDCLRCQKGRQILKRVGRKRSKEITPPSPETSESDEEESPNTKRQSGKNKRRCRKKAKPTEPDPNTSNSDSESEI
PAPKKRNQTKNQGKQRGGKKKQAAPQPPMTKKQRKAMNKKAKQQQEQEIEGETSDSPKEAEEETEPEEEPEKQNKGKQKNNKK
QDEKQANQKKQEPEKATHKDPEAVEQTPTDEAPEASKDKDQGKFRTMKGKAKAQPKQKDGVKKGNYPEGLPVPHMRRPQLIEP
IRAQVVQTERVIETPQDPAPNAYYDAEHNIMRVYQGPAYGNHQNNALYPERDAFTCPLPMGQPHPMQNPFYYNFNNTQQYPNY
PQMPPFHQGYAGIPPPEAYSHVPITQGMAPPPWYAMGPPPGVLPTGYYGHRSPETNKNTPASKTEKDKASQNNVGPPGSLTGQ
DNPYYKRKSQKNTWESHRSVSKENSQPPGSDKAGSNGSNGWNNGSGDNNGQNGQDGWSAPTQDNNQNGDGWNTSNNQAVDDWN
KTTQDSKPEEWVVSPPSGEQDQNQNGWGAGSNHSNKSNETVKRNGDHWGDDTFPGARGDWSEQPTISSVGQGADNNVGIWAPT
DVNGNGDGNQKPPSAEGEGRDNIMPGTWVDTPGVTMPTWGDPTAARDTNGEASNW
FUN_014737 CDS scaffold_7 385251 386070 -
hypothetical protein EOG09264E6Z PF01121

MLLIGLTGSIATGKTTVSSMLSSQPYNLPIIDADILARKVVEPGTRGYEAIVKHFGPTTPELLVQPSEKMPEDGPDGKGRPLN
RPALGRRVFGDSEERKKDRAVLNRIVHPAVRWEMFKSVVGCYFRGHWAVVLDIPLLFESGLDRFCGVTAVVAVHDPAVQMQRL
RARDPHLSAEDAENRVRSQTDVREKARRCEERGEGKGIVLWNDGSREELKEQLDKAIKDLRKASPDWWSWLLLGCPPLAVAVA
TWRFWQNLIINERWEEMKLNAKL
FUN_014738 CDS scaffold_7 387021 388541 -
hypothetical protein ENOG41

MASAGASAFKSPKIPRRPKTPETRQVHFGSQTKPDTSASASTSASAAFNEGITSYTTANSPSGGFSAGINGVTAPSTARATTT
TAATRATTSAPEEKGSNQPTNFPALGLGPNAQPQWHISADPTQSLAQQVPTGFVSPTPQQHQFLSPQFHPAPFINAGQNQLTP
SPITYIGIGPQSPAVNTANMGDYQNTAPPVHGMNFQPPVPDTTFGPMQHVYVPRFDNGLAGVPVAGYAGVQQPAVGGMAPQPT
VQGSYVVQQQPYYYQQPNMGQQPVLIASHQPQQFMPQVQQVAGVPSVGVAGGAAVPGTVPVFAGNVPGGQHVPDVTGVGRTAG
EEQLRQIQFAHANKMYEPQDFKPADDDPSRFYYVREVDGNWTQRNRFTIDHMGDSRWYVTDEGWFYAVRLPN
FUN_014739 CDS scaffold_7 389521 390977 -

hypothetical protein   ENOG41

MESHDFGNGQGAFQIYLDAVEEHTRDDRPSPALTDDPFTATDNPQDGTNAASSSKAPNQPRKARTSKQGIADASLREFKEIMI
QYRLDHWRARAQIVHVKGQINLTAFWSPPWAGFEVADRGMNMFDGFDRMWFVPFDIMTPLPAEKQEIADNLQRRRLELEAAYL
TKVERDKSFLTKLQHLIEEEVKETCQKLWEPNAYDPGREPARWDDVRARFQLTHLVTMIAQTRLAVGKEQLPGYYQVKVLDLR
VDGKITYQCVNLPSDVSMKGMLSRMKTKFPPDSEQSQHTINVFKHKLEAGWNHGDEAQRKKAQGLLRQLHDLVLVEKPESERY
SWVFKLRSNSHFYIPAGKLPDRLHTWTQLKETTYQELLQGVRAKKHPVFIRPWKIRLRRWWPEVDDLVNQAEVVFGGAGLSSL
QLDIVDELLASRTQEEVQRAAKTKQALDEAKLAIMENDCPSRNNNDDEFGPF
FUN_014740   CDS   scaffold_7   391558 392330 -
hypothetical protein   EOG09264NC7   PF02466

MSSLWNAFTSGNKPAHQQQQSQSQSHQQPTTQEPAHYAYDPTEGQGVESFLQSSAFADPSQLHPLAGLNRDTLEYISLEDSAL
SELPGGQSVLPSRGFTDDLCYGTGITYLAGLGIGGAWGLQEGLRRSAGQPPKLRLNAVLNSVTRRGPFLGNSLGVVAIIYNCI
NSLIGSVRGKHDAGNTVLAGALSGMLFKSTRGLRPMAISGGIVASVAGAWAIVRRSFFPIPEPAPAVSDELTL
FUN_014741   CDS   scaffold_7   394969 396239 -
hypothetical protein   PF04193   ENOG41

MTPPTANLNLDVEAISGICGSISIACWVVVFSPQIIQNFQRSSADALSIQFIIVWLLGDVFNILGAVLQGVLPTMIILAIYYT
IADIVLLGQCFYYRGFTWRDAPTPAAKPNTPAPGEPNERTGLIAHDRHEERRGSDWSGLSPAVPHIAEDATPQTPTILQTVVW
NTIVVLMVIAAGVVGWFLGQKASTGDKHGDNNAGGEDSLHFSLSGQIFGYLCTVAYIASRLPQLILNWRRKTTDGLSMLFFLF
ACLGNITYVLSIFAYEPKCKHDECRPGEARHIYGKYILVNLSWLAGSLVTLFLDLGVFAQYFMYSKPESTATPTRRRINDNVT
EEDEYDDDESLNEDNWDQRPLLQRGDSVYHP
FUN_014742   CDS   scaffold_7   397312 398609 +
hypothetical protein   PF00293;PF09296;PF09297

MSTNISGPGLPEIAALQTDDSMLTRRFGKEVVNYYAGGRINRFSFLRADTGFLRQAYNSPTARYLALHDLNPLVVDKKTPAYL
TFKDVEPLIGPDHFTLTEDEAIQSFNSAETRPLVVFIGMLEEGNEKDSISSSDHGDIKGHPYFAIDITPKGNHAEKATQFLEE
QEKKGLSLDKNPRAMNQSPEAAALYAQARSIIDWNTRSPFCAGCGLPNLSVHAGYKRVCPPADKKGGASSELRGDCPTRHGVS
NICFPRTDPTMIAAVVSADGTKILLGRQKRWPPHWYSTLAGFLEPGESIEESVRREVWEESGVRVGRVVIHSSQPWPYPSSLM
IGAIAQALPGDGEKISLNDKELEVAKWFSIDEARQALKNGTSSLGEPAPEGYKEGDLRLPPPQAIANRLITAVVEGYLTVAPK
I
FUN_014743   CDS   scaffold_7   399334 400190 +
hypothetical protein   ENOG41

MPGTAQPPLSARTAQRIHEQVTREVFRLAGRDPDNHMECFKACIKTIENTKVIETTDYAIWESQEPIASPLKEFSNTNRTTKS
GNFYTLSPTTVVAIAATLNLKHVIANSNVNNKHVVNAVSPSAVWAYLGIAINAWPQTGECQFFVKKSGEDSKSPTVGKITRGK
FAVTTNIFGDTTTPLMLLTIARFNGWVKSAPGAPCLKKRTVDAPQIIHRRQALSEGKEILEKLGNRKFKQNDPALIREVKAWL
SEPIILVLPEEAKNRLWY
FUN_014744   CDS   scaffold_7   400262 400943 +
hypothetical protein   ENOG41

MGKTGATLASLVSDEIDWIQLAQQVNDAESTAAVKKLVELSDKGAFLDMVSKMDVAVMSSLDLDLVRAKDRHQTRLNIDDDLS
YRNEIMANVAEAISRLERGSASQERLLRSKLCSIFDAADALILKENLPREISIVISTKANDE
FUN_014745   CDS   scaffold_7   401625 403286 -
hypothetical protein   PF00324;PF13520

MASTPPTDYGKEAVLDVSETRDVETGEVKNGGLKQDLKNRHMQMIAIGGAIGAGLFVGSGGALQKGGPAALLIGYLIIGIMLL
CTCLALAEMAVLYPVNGAFFTYIVRFVDPSWGFAMGWQYALAWLTVLPFELIAASITIRFWREDINMAVWVSVFLVVLMGIQI
FGVRGYGEVEFVLSIIKICACVGFIILGIVINCGGVGDQGYIGVKYWRDPGAFTSFKGFCAVFVVAAFSFGGTEMVGLAAAES
ANPRKSIPMASKQVFWRIAIFYILNLFIVGLILPANDPRLMGASGANTKASPFVLAIQDAGIKVLPSIMNAVITVAVLSVANS

CTFGSTRTIQAMAERNMAPNFFKYIDSKGRPLYCVILQIAFGLLAYIGAAPQGMEIFGWLLALTGLGFLFVWGSICLAHIRMR
AGMKAQGINLGLIPYKTPFGVAGSYLGLGLNILALIASFYTALFPASGASPTAEAFFSSYLAFFSVTLLYLGYKVCTRKWQMY
VRPAEMDLASGAVWLEEEEPKEPFVWADAPKKVLRSFF
FUN_014746 CDS scaffold_7 405740 408976 -
hypothetical protein EOG09260KGS

MESTASSKERTEFFQRLKPCCVKISQLAIREADGRASSRELGEQTGRLLELLNEQIRRNPLVLDEKLSEYVFFPLRHIFRQMD
QYPMPLIENCIKTLTILITHGWKTKLSAELVRQILSLLTFIIDGTPGSTPARPVAEETVLEAFRGLTALFNTAGLSAAASSGL
SEAESIPALGHGVTIMLNGVVDGATPQIQQEALRALQAVYHTVKEPAALASFLPGTVSLLAKVTSSPNRYKSAVLAKCFETVS
LVLTSVLADLRTRSILTKTETDQEGAEDNKILSPAWLKASTMQVKKALSTIMKLRSHESAEVRNALNKLCVTILDECHNTLSN
CANILVETAMMLGETENKMSLTQTSLQDLVNIYPELGEIVKTTTYNWMSSLSRLMQSADEDVKRDTIRNVLKGLELIKDLQIQ
SSTLDESMSIMLRDSIISLMQASGTSETNENMDVRLIDSGEASAEQGDVQYQPVLLPSESQKDIRHEMASLITFLGSSSQQAR
FAGTMLEQVQDSDNSASQVATFWLCFELIKASHRSSAEAEMFLNLSSVTDPSQDIDTIFNELYTTSVQILDRHTDVESIDWRL
EALALEATAYTAQRAGESFRPELIDILFPIATFLGSNNPNLQKHAIVTLNSIAASCGYANVSELIIQNVDYMVNSVSLRLNSL
DISPASINVLTMMVRLAGPRLVPYLDDVIDSIFGALDNFHGYPVFVENLFVVLKEVVNQGVRSDMLLLEHQARSKPDHRKVHK
KEPGLSELLDTLEKRRQRAALDETETEEEVKGHPKIPWKKEKNENDSNEAADPPPEPEKPPNSPTYQLLLRVANLTQHYLTSP
TPTLRRSLLELLATASTALAPDEDAFLPLVNAIWPVVIDRLHDPEAYIAVEACRALAGLCAAAGDFLSSRFKTDWAEWLRDWC
RNVKQQASASPSWVRPHGEAGTTWGKESDDSRVLIPLRHGDSLSGKPLTQIVAPSSGSLGKFASPARVWEGTVELLTALVSHV
QVDEEMFDDILDLLSDVLERNVVVREALEAVNRDAVWLSRYERGNVEWLPTPKMDGVEFTHMEISR
FUN_014747 CDS scaffold_7 409738 410736 +
hypothetical protein PF08538;PF12697 ENOG41

MSPSGPFQVTVHPFESKCRSSAAYEIGLTSAPNAIVFIGGLTDGPHTIPYTRLLAQRLEEVKELGFSVIEFRMRSSFSGFGTS
SLSNDVEDISALVKYLRGIGKEKIVLFGSSTGCQDCIEYANYAKHNNEPVDGFVMQGPVSDRETLDLIFPDPQPSLDLAAKMI
SEGKGGDCMPFDMIPAVLGAPISAYRFQSLASKGGDDDYFSSDLPDDVIERNWSRFNKPVLVLHSSEDEFVPERIDQAASNKK
YKALNPAVSRLSGLIPGASHTVDQPQAQEWLSKTVIEWLKTEVTSRAD
FUN_014748 CDS scaffold_7 411531 416774 +
hypothetical protein PF00005;PF00664 ENOG41

MALYNEQQVLFETGSFTGFRPLRELGANYLTTGPFKQPLCGNSEGWGPLSPHRYDFTPCFIDVWISAVSVFGLLFGSLAVWWL
LAKKQKQEGQTKNAHFYIKQSLLAVIIVDVIAQLIVQIVYMPHIWYGDFRVLTTFLTILSLFVIFAIQWIEHSRIRYPSGVAL
FYWLFLLISFGVKLRSLISQQIYDSNLPYFIVYCVGVGLSLVEFLVEWLWPRTSRPSGYEAIEEEEECPVEYANAFSQLAFSW
MTPMMQYGYKVYLTEEDLWALAKDDQTKNTGSRFDQAWQYELEHHKSPSLWRVLFKAYGGPYCVAAIFKVANDVAQYIQPQLL
RLLISFVKSYEEDNTPQPIIKGAAIALAMFACAVLQTTMVHQYFQLAFVTGMRIKGGLSSAIYRKSLRLSSEGRASKSTGDIV
NYMAVDGQRLQDLTQFAQQIWSAPFQIIICMVSLYNLLGWSMMAGVAVMIIMMPIQGFVARIMKNMQKEQMKNKDARSRLINE
IINNMKSIKLYAWGSAFMNKLNFVRNEKELKNLRKIGATQAFANFTWTTAPFFVSCSTFTVFVLTQDKPLTSDIVFPALALFN
LLTFPLAILPMVITSIVEASVAIGRLTSFLTAEELQPDAITIKPAPEQLGEESIIIRDGTFSWSRHENKPTLVDIDYTAYKGE
LSCVVGRVGAGKSSFLQSILGDMWKVKGNVEVRGTVAYASQQTWILNATVKENIIFGYKYDAEFYEKTVQACALLDDFAQLPD
GDETVVGERGISLSGGQKARVSLARAVYARADIYLLDDVLSAVDSHVGRHIIDNVLGTRGLLASKTRILATNAIAVLRQASYV
SLLKDGQIIERGTYKELVAQKGLVAELLKTAGHESGNASSEPSSSASSSKAATIIETDSGQAKEELEEAQEQVPEMAPIKTGA
GAKPRSSSMATLRRASTASFRGPRGKLTDEEIGGSSKTKQAKEHLEQGKVKWSVYGEYAKMNNLYAVALYLLMLIAAQTAGIG
GNFWLEKWSRENQEKQSNANVGKYLGIYFAFGIGASALTVIQTLVLWIFCSIEASRKLHERMANAIFRSPMSFFDTTPAGRIL
NRFSSDIYRVDEVLARTFNMLFVNAAKSGFTLVVISFATPPFVALIIPLALTYYYIQRYYLRTSRELKRLDSVSRSPIYAHFQ
ESLGGISTIRAFRQQQRFELENEWRVDANLRAYFPSISANRWLAVRLEFIGAVVILAAAGFAIIAVTGNKPIQSGIVGLAMSY
ALQITTSLNWIVRQTVEVETNIVSVERVLEYAALPSEAPEIIAKNRPPVSWPAKGEVDFVNYSTRYREGLDLVLKNISLDIKS
HEKIGVVGRTGAGKSSLTLALFRLIEPVTGHIGIDNIDTSSIGLLDLRRRLAIIPQDAALFEGTVRDNLDPGHVHDDTELWSV
LEHARLKDHVSSMDGGLEAKINEGGSNLSQGQRQLVSLARAMLTPSNILVLDEATAAVDVETDAMLQSTLRSPLFANRTIITV
AHRLNTILDSDRVVVLDKGEVVEFDSPAELFKKQGVFYGLMKQAGLEVE
FUN_014749 CDS scaffold_7 424357 426349 +
hypothetical protein PF11001 ENOG41

MVRDWTRESFSRHPHHPYLPERFVGDGVDDNRFPEAQDMHRRQPPMQIADALRRDCGISVRHYFLAVVMDDGSPQTFSGPSTS
GPRSGYEKQFFDMEKYLRAMDRLDSGASPVVDDFAFDGYKQSMDFPRNRMMDRRRMSQFDDWEAPARQGRKRPRARHPIIDED
EVPMTVSTTIRKGIQVGNPTELWTFYEQRFKNMQQTACKLIAKAWVKAVEPKKQSTHPYTGSDEKAPDWWPKPWGPTKEDKVR
HKEPDHLYKRERVHLLNHILRMIVEPNKTQHPDIQKLNLNVKKLEEITIEALSGFFADKDNPANAKKRPFLNEIFKVAKQQER
YKDGMIDGTTEVFVMADDKMLDSYASGNDDDGPVPKEEDDQDVQPNKLAAVPGLMPSSNSHSSGASLHGTPFLGGELPVRGNQ
YNHSMMQDMAPDQHGFVENNAMPVNGQPAVHPNSGNLGIDMGVPPAAHDSGRRSSIFSASADYAGQNGTTIYTQQWQPGSTAP
NSTSMYTFTQQPANPPATAFVSPGVPMNQAQPYMNASFDGLTRGYDPNQPTMFRTGSVAQPSQVHPTPGYDYMSQDSRGLPGV
KVDDVPRHAMH
FUN_014750 CDS scaffold_7 427911 428887 -
hypothetical protein EOG09263P17 PF00010

MAEVLAEQPDLSSLTGSPGQKRKRESGSPDLARNKRPAPAAHMSADPASFIDSAMESQAVNANGVNVADFNALQQAAQADHDH
PEAPDPTSATSTAQAALGMYPTLHVPPSTEEQFAAQAATDPEHGHDPAFNPDVTPTVDSIMEPPQVAPPQHQHTPPNGVPHQP
PRYNPAAPNPKPTVGSEEWHKMRKDNHKEVERRRRETINEGINELAKIVPGCEKNKGSILQRAVSFISQLKENEQQNIEKWTL
EKLLTEQAITELSASNDKLKQECERLYRELETWKRVAQNAGLEPPQPKEENTTANASS
FUN_014751 CDS scaffold_7 431683 433058 +
hypothetical protein EOG0926107R PF08513;PF10607

MGDHESSQIRHNEHLLLDQPLLRLPSELLRKNFRTAHFTIEKDTAALKTLLKDSATAAVSGRASQQDVLRNIDAMVSRMRGLK
RKLNASAAEEARLHTQTAARISHLDELYKMDTVEDVKYETWSRKRLDRLLADYLLRHGYNDTAKELAEQRGITDLVDIDTFVA
ASRVRDSLLKQSVVEALAWCTDNKKELRKMESKLEFMLRFQQYIELVRSQSSAKLTEAIAHAKKHLIPYRATFPREVQQVCGL
LAFPPGGASTAAPYGDLYKPSRWADLANLFTTTHNQLLALPAVPLLHVALSSGLSALKTPACHTDPMHSSDSPSAQSTSDIAA
AASTLGHGVCPICSTELNELARNVPYAHHTQSHVEHDLRLLPNGSVYGRDRLEIQARKNNLPSDQVKDLRTGDIFPVESLKKV
YIT
FUN_014752 CDS scaffold_7 433680 435194 -
hypothetical protein EOG09262K67

MPPPQLRGFGGGPAVAAPYHQGGFPSHAQQQGGHLGGNQYLNANTQLGPFSAANSNAFTTAGLNGQGFADTGFGSQSARMGFH
GPAAALQQPQHGQVHQNMLMEHPGIRTHPNKGRIREVWKHNLHEEMAVLRDLVDKYPYIAMDTEFPGVVSRPMGGFRGKSDYH
YQCLRTNVDMLRVIQIGLTFFNEDGETPPPRPTNDLKLGTAAQRAATNAPFPCSWQFNFKFSLKDDMYNEKSIESLQQAGIDF
NALERDGIDPHEFASLLIPSGLVCFDNVKWISFHGGYDFGYLTKLLICLPLPNDEVDFDHKMKLYFPTTYDVKHLMKHAIRLH
NSGLLTPSDPSSTEILQKFEHKSGLENIAETLKIKRVGSAHQAGSDSLLTGKVFFSMRDKIFAGDIPDEHVGKVWGLGFPDSN
SSLVMSMMNQQSGNDGHTNGNGPSTPNTTNAALATTPGPQGHNGTINTGPMTPGGGGGVFGNFAFGGNR
FUN_014753 CDS scaffold_7 436048 436495 +
hypothetical protein PF08122

MLATRVLRAAAGGPKPNITGFNMRAFQASTGQPRYDPWERNEAWRYQGPYTRWNRLKSGFPGLGIATVAFTAYCGYEYFFLED
EHHHGEAHGEEHH
FUN_014754 CDS scaffold_7 437398 438976 -
hypothetical protein ENOG41

MQPLDPQGLVEDRAAEEEHGQFPIEYHSRLTGPSSQASYGSSVRASTARRRAAVTNKPRNNNLSETTSLRLSPAALKTPSAAQ
STPRSSPPLAPLSVTPPTTAHHPPSSSVRTRDPGLHVEPTSPKLQSPLRHSINSADNNLDVESQFYKISSSQPRPLSVDMSFT
GDKRPAASKTRSSLPAVVEEAPRNGFSTWFNRSNAPSPLSTTAAEPRRRSTSSAKPSTPTSSRFNFFGSLTASPTACPSEEQN
EALMNLDIEKGLFPNGLPVEGTAFSPSAFKNLQMNATGLLSKFQAAYEQRTTEFRELQAERDIQNFNKSEAEIRVQQLEKQLE
EQAILMAERDAMIEYLLNRLANEKDRADEATNGEKENITSAASIVSEDLGVDEDRLKRWRKSTSTSSDDESVDEASVFSRSRS
PTICTNISEISPVTQPKPVTLEPPRATGNAAARNTNNTQMGAFQKLIRGMSSEKPKPAPATCLNCQGQTASMAWDTVSLLKDE
NKGLKVRVEELETAVEGALDAVNGLGL
FUN_014755 CDS scaffold_7 442163 442850 +

hypothetical protein ENOG41

MNQPKGAGRAGGTILQAFLFLFTITNFFTLILNAATAGLYWDKQWGYIKALLVQGVYDEDYLSTAGSPDVFYTDVTSLEPGKF
MKDSVRPYLLLAVVAGITWVIASIALLFLFTKIRHFNHRVARYVFRVIGVATFVFLVIFYLTWAHTYNFDFPFTLDWPLGKAA
VIMAHINLLLGIFVTLLAAVLDTPKDWADHAPTGPNSRRTSQV
FUN_014756 CDS scaffold_7 443230 444976 -
hypothetical protein ENOG41

MSGRSGSNSHRRHSREPLDDFPPLPIPPRLPSLRALAHAQRDRDRDSPSPTSSNRPQSRHWSAASRRADRIRNLENRAPGPGS
GSGFDDFERPMDDGWPTSRRTDRMRAATAAENTRPSNLEDLDRHLEEANSHLRALLDLQHHPSLTPPLMPPNFSPPLRPSDLP
DSNRRHKRRKLDSDRLAPALRGFRYGKYGQVEQGDLKMEIVSCDGGMFSNELSYAAENILKNDTSVYCTKGNRCNIVLRHKGG
TVFTLQELVIKAPASSNYSHPVREGMVFVAMNQDEVLNRTARYQIQYAQPTNEPTHDEDPRVLQPIPTEIISVQHHENGTTTT
RSRPAYPYRTNADDYEPRTPQMPEEFASNLPDFQVTTECSDDEDDDFDGPRFLRRAPNRIGSLPFETLDSDSDEGGNPFDPEY
LDDHHPSHWRLYSSAANTSGSGFDPPYSRRRERERERERERDRERDRDHDRSNRSFSLTEAWEAHASATQDAVRAVGGGQLLS
PHARFNIEKKKSKCTIRFDPPISGRFILLKMWSSHHDPTSNIDIQSVLARGFAGPRYFPSVELR
FUN_014757 CDS scaffold_7 447484 449258 +
hypothetical protein PF00899

MDNSNQDQGQPQAEPQAQQLPNPSVQEEQIPHIDASANINVNGNGIPQLPGNLLNPMNPQALSMLADPSLMADPSLMAMQMPL
ADPSFMMQPGMGLPNGQNGFSLPVAPPATVSADEVALYDRQIRLWGMAAQAKIQSANILLITIKALANEIAKNLVLAGVGSLT
LLDSATVTEADRGAQFFIPGGEDVIGQNRAQVASAALQKLNPRVRVHVDTEGVKTKGPSYFAAYDIVIATDLDPESFNIINTA
TRLNCKAFYAAGCHGLYGFIFSDLIEHDYVIQRDLGNMPTSIGPESRTRTIVDVQTRKEGPKTIESVTKRELYSTWFLASDLA
VLPAEYTQSKRRLKSVTPALSCLRALWEFMQIQNGRVPSNRDDLKLFTQIATQKHKALGLPSETLRPEFLRSFLQNLVSEISP
VAAILGGQLAQDVINVLGQTQQPIQNMVVFDGTTMEALMYPLHPEGSLGASQLTDHTVPNGGAPMILGGMDALPLGLDPTAMG
ALPHHNNPIMIPTGLPQNGNLIPMPDGSLADPTQQFNAATQVPQQPMQGHTAQVTAEQTTQETPANPGASESKE
FUN_014758 CDS scaffold_7 450162 454393 -
hypothetical protein ENOG41

MPENDSPAREPSTRGRGRGGRGRGGRGRGGGRGGSTAALSKAATAKAGRGGSRRGRAKNFADSRVQAAYERQRHLKANYQAVA
HALKPALQELAERTIDDALQNPDKYKLVQEFIPAMEELQANLDAKLAEHKRRLDRDLQLAQHLHEADNYVAEQEFQNGLDEIE
EQFYESQENRARILNSLYDKNLPVDIADDQYEYKVISDEHYDEEFGIYECYQDGHLVPYPSRVQGTSMWRKGRENEAAATAAV
TTAATASSKGRGRGRGVNKRRAQDQPDGQPTPKKNTRSNADDGASVAAPAPAKGLLASAAEAEATPEGTPAESESAPGTPEPP
ALVNGITPAAAARAQSKLPARERSPPLPKNMGDEDPWGYRIYNQKPSMREKGINSRLFCPRTFEFNDWEIGFRDSTNDSSKGH
THAKRGKYLNTPNSNGLHVDHWCNGYDFSTTVPEDFDQDTVKRFGVHPKYGVIMKDAPVPEVEKTPHVMPGKPVVYIANPSGR
IAHASRSFLKTTNHRRSDDNPYRAKIGASMRRFCKLEEVDPEEISISEYVRSDEVLRSRSLGTAAKELEVRPVVSRVASESQE
PESEEQEPEGEDAEAPQPETGFTDLAVLAYAGAFLETEESNKAPKPAPKPARYDAIRDVFTDSKPAPAPTPDTSSSVGLDVLA
ALSNVVSTSNESAPIREAEPVYDTAPATELAMSYEPASVPEAPSMLEPYGTQEQPSYRDAMSNGMGPGPVSSHGMLEHDPRSS
VSQTNELPPVTPSQAPIDPRYNPSPVEVPQGYMHQPRAQMSMQPSGPVHGHPGEPMAYPPIQEHNVSQHPPVRHPDYPPPHPS
QGPPVQDQGAYLSHSSYPSTDPRDAHMAAGRPIDPGYSPRRMSIYGAPEPSYSRAYWSQPPPPGPAPAGPSNAPPGPPGPAPG
PVSSHYSSPAPPPARIPFSHNGSAEPLPPLRPSRGRNQSLQDEALLEPSLRSSGSYYPPGPPRSYHRGYPGPEPHGQPPLQPI
ATDRILPNPQTAGQGYVTSPHQGYAHQVLSPTYANPPPMPPHMAQSPQDNPQGLPSSIHGHRSTPSGSSDAGNKYRKLQPAPV
PAHRAWPNKPELKTIPYDHKETGSAAALPSSGPTQIRGWSNGDDSSSKADGQAKPARPYKSVRFAEPAIGVPPTAQKLNKRKH
SDTQPDEDVVAVEPSAKKSKKNDDVALVIDEDGNSLKRRLRPRKPKTTEAEPIATKAGPAKKKPRAAQSRKSKTVEATKTKET
AVKKKIKIVSSSTSNKTSDQDVESATSKGNSSSAKEPRVKGESSDDVQIIKSQRKVITIDSTDESS
FUN_014759 CDS scaffold_7 456738 457767 +
hypothetical protein EOG09263W48 PF10354

MGKKRKLSQQNAPNARPKKLPQKNKSDQKKTPNGPAKKGKGKQQHQSDDEPTIPFEPHHRILLVGEGDLSFAASIIQHHGCAN
VTATVLEKDAEELLSKYPHVEDNIAVIRGDAPNTKEADKEDKETSAKESEAGETSQGNQNEGEDTNGESDSEEDDYYDSDDPN
APPRPKRKLTPNNKLLYNIDATKLPNSLIRARFDRIIFNFPHVGGKSTDVNRQVRHNQSLLVSFFERAIPALAPGAAIVITLF

EGEPYTLWNVRDLARHAGLQVERSFRFQARAYRGYKHARTLGVVRNSKGEVSESGWRGEERASRSFVFKRKEDIAPVVGKKRR
KGDDSSDDED
FUN_014760 CDS scaffold_7 458352 461247 - SET2 histone methyltransferase set2 EOG09261B14 PF00856;PF08236;PF08711;PF17907

MEDDEYTTSKMEEIKLEEGTNGAQVKQEERTPMSVTNGGQEESRSPSASHDGVKSRSGSADTPSSNRPSKLSRKASQKLAASR
EPVLFDHLPDMTAESCNFFQLIPDCLYGSKHLGSTDNDALDCECREEWHDGENIACGEDSDCINRATKMECSAEAGNCAGGCQ
NQRFQRKQYANVSVIKTEKKGFGLRADSDLQANDFVFEYIGEVINEPTFRRRMMQYDEEGIKHFYFMSLNKSEFVDATKKGNY
GRFCNHSCNPNCYVDKWVVGDKLRMGIFASRKIQSGEELVFNYNVDRYGADPQPCYCGEANCVGFIGGKTQTERATKLPVATV
EALGIDGGDGWDTSVAKKPRKKKPDEDDEEYVNSIPSRSLNEDDARKVMAALMQCKEKWIAVKLLDRILRCDEERVIHCVMRM
HAYQILKTTLNTFIEDHNVVLQVLDILDKFPRLTRNKIQDSKIEATIEGLTRSEHEDVASKSKYLLDEWSKLEVAYRIRRRKF
DPNAPAANSFEERRGAGRDEEVAQSSKTASPQTIDAPKGPRNSMPQRNVAFFQNGGRPRRPPFNGGLPQGWFTAKDAAGNTYF
YNKQGATTWQRPTQPVAEPAVKAPSKAMKEQLAIQSIINQVTERGTPKHTSVSTPKTADTPPKEVKEEKWRSLPVEKQMKIYE
NTVFPHIKHVLDKFHHKLPKEELKRFGKEIAKKLVSSDYRNNRVGDPNAPLSEKQARKMKQYVKDFLDRAVKKFGDQQKPRAG
ENADTQMKGDQGPSAAGSSTGSVAGGLEGTSLVKVNGTPADGLDAATASDNEGSGSLGSPERKRKRESEVEGPLSISPSDGPN
MKRLREDELDAPSPPPPPPPPPQSAMEGVVNAEQQALREQEEALMRENEEAQRLEDEANHTKDLEDATRGAEKDLLDASNELS
RLNHEARGIGSQKTSA
FUN_014761 CDS scaffold_7 461893 463220 - hypothetical protein PF07478 ENOG41

MQRIVSSSMHVIQSLTTAAGRQFRHRSFLINLKASMSSVSRARVAVLYQSLDPPVIDGIQKPKKPGGYMDSGADIAYNLSLSP
NVDVICPHNDPNPSEQAGWSFPDTEDGILEAVKKGASHIWANTILFSSHPLQVSAHLAEHQDHVKVVGQGPLIAERYDDKEFV
NNLLRKLGGFTMPRAWTINESQGTQGILEKLNLPFPIVAKPIRGRGSHGVRVCRSLKELIEHGHALFKESPSIMLEEYLAGEE
ATVTVMPPISRGGRYWALPVVTRFNHIDGVAPYNGAVAVTANSRAINAAEGTQAAYKQVAEECERVAQELAVTAPIRIDVRRF
KNSADSKFVLFDVNMKPNMTGPGRPGREDQASLTLLAAEALGWDYQELLRRILGTASTLRTLRDLKLRESV
FUN_014762 CDS scaffold_7 465731 466813 + hypothetical protein ENOG41

MEPFNDQEKRHLLAEIIKHSQLDVQYLENLVRHIEPNWMQMQLPNGRNMAQCMETAQNMYIGQRGTKRKASEEESSTQNNIDG
QLPSDQALSLLSQPFPAQNSPANFQRQPAMTPGPHLQQHQQPEQPPKKKKGRPAYAGRDVTSQRPFNPRPIAPKPSAQILQNS
HSVFRTIAPAPQAVLPPRAPGSLDNTYRGLSRAPVEFQNTHGNLDTYRPSITPSSGMLQPKQAAGILDNIRHGEQMSNRPRSL
SEAVPRVQKQESSPNKRSSSLQPMGTDDQSRIMQSRASDQMQETKPKSDKETTNRRQRKT
FUN_014763 CDS scaffold_7 467621 469183 - hypothetical protein PF00202

MSAFRAGMAARPAVQAARAAACRSFATSRSMRMAAASEKTFFAGEPEAPTVKTAIPGPKTKSHMDELTKVFDTRSANMLVDYT
KSKGNYIADPDGNVLLDVFAQIASIAVGYNNPALLKAASSPEMVNAIVNRPALGAFPSHDWADLLKSSLLTVAPKGLDNVFTA
MAGSDANETAYKAAFMWRRQQERGGPEVDFSTKELDSAMNNHAPGSPNLSILSFKTGFHGRLFGSLSTTRSKAIHKVDIPAFD
WPQATFPKLKYPLEENVEENKKIEQASLDEVEHLIKSWHLPPCAVVVEPVQSEGGDNHASPDFFRKLRALTKKHNILLIVDEV
QTGIGATGKFWAHEHWNLETPPDIVTFSKKAQAAGFYYGDPAIRPNKPYRQFNTWMGDPARAILFKAIVDEINKNDLVNHTAR
VGDRLFKDLESLSKKYPGQIENLRGKGQGTFIAFDSPKRDQFLAEAKKLGVNIGGSGASAVRLRPMLIFQQKHADILVDTIEK
IVKQ
FUN_014764 CDS scaffold_7 471030 473972 - hypothetical protein PF00654

MAGQEAEGSEPPSPRTTRRRNPLQRNASTGELDERTPLLTASRSRIRISEQSSTRQQKGSLSRDHSYIGAYHGSRNHSRHPSW
SSRLVNALSDRHESSMAESKGTIFPDERMWYDQFTSTDWVHDAIADSYRVKELRSRKDFWGRVRIIFDGAQGWILSALCGFLV
ALMAYTVDVAESTVFDFKEGYCSKAWYLSRKRCCAEEACEDWVRWAHVVNSPSGDIWRSFAIYMAFVLALALIACWMALWTKT
VVPSAYQLTTLDENLAVDEPTQTYDDLNPSESSTPQPQPDPKPENPPMVYYSAAGSGVAEVRVILSGFVLHGFLGARTLIIKM
VALILSVASGMSLGKEGPYVHMAACVGNILCRLFSKYDRNDGKRREVISAAAAAGVAVAFGAPLGGVLFGLEEVSYFFPAKTL

FRTFFCCIVAALSLKFLNPYGTHKIVLFEVRYLVDWEYFELGSFIFVGIIGGALGALFIKASKYWAQSFRRIQLIKKHPLLEV
FLVALVTGLMSYWNALTKLPVAELLLNVASPCEGSSTDWEERALCPGAIDEIPPILFELFVALLIKGFLTVITFGIKVPAGIY
VPSMVVGGLMGRIVGHMVQWAVLRVPDWAIWGDCAFSRDGSCIQPGVYGLIAAGATMCGVTRLSVTLAVILFELTGSLDYVLP
FSLAILVSKWTADAIEPNSIYDLLTSMNSYPFLDNKHKPIFTEDLADIVPRIRKERIIDITNSPVVPATSLRIKLELLHRAGE
LDGGLPIVRHGILVGLIPAPDLGYALDQLEDEATNLCLMDHVPSIDEDEGEHDPTDFTPYIDPAPVALDIRSPMDLVYELFAK
LGLRYICVLKDGKYAGMTHKKTFVRYMREHEKDI
FUN_014765 CDS scaffold_7 475610 477461 +
hypothetical protein EOG09264BOA PF04000;PF09368

MAKKRKASGRSNAISGPKELDPSEARLGPITTYEDVADSEDEYFMNRDQILLDEAPDSKRRRKEDDDIELSDEEILGYEDSHS
EEEDDRNDQPRKNKGSASKDAVSDDEAGQEEEEGDSGWWGSSRNDYYNADNIETEADALEEEVEAKRLQQKKLSKMAEEDFIF
DGDEWLAEGQEHGEGEETITEVLKEVEITDDMSPEERYTILKNRYPEFEPLVKEFQNLQPTLVDLQKSAQGLSGQSLEAVKYW
VAGCYVAALASYFAILTSPARDNAKVQKTIDPTELRDHEVMETLMNCRETWQKVKDLKSTKSFDGDAISDIDDDALMQGVENL
EAEVKRTKKSKKPSKQEKAAAEKLARKLEKVKAGEAAVADLYDLPLPGKKSKKSKKAVAIEQDDDNSDFGEEDVLDERTAAEK
AARKKSLKFYTSQIVQKANKRAGAGRDAGGDMDIPHRERLRDRAARLNAEAERRGQKNSKLGADLDDNSDDEDTTTAKTVRDN
EDEYYDMVAQKSKKNKEDKAARYAAYAAASKADRVVEKEELGEDGKRKITYAIEKNKGLAPKRSKDVRNPRVKKRKQYEAKQK
KLKSMKPVWQGGEPKGGYQGELSGINTAVVKSTRL
FUN_014766 CDS scaffold_7 477774 480228 +
hypothetical protein PF08700

MADIDTSKLTSSAQIFSPNNTLPQIRTIHKSLHVAIEEKSTRLRTQVGSSYRDLLGTADTIVHMRDDNDVVQELLGKMGGRCG
RAVISAKVTGLANFVAKENKYATSIAARLRLLDACVLVVGRILKGSGGLGDNVKKGDRLVLSTKVLVLSRLLIKSLSDDATDT
SSRQAIETARASVSKLRRRLLRNVEKVFERAGDETEREDVQKALCAYSLATSSGARDVLRHFLHVRAEAMGLAFEAEENNRKR
SADDVVQGLRLYSRTLLDVQALVPGRLSLALSGLKSHPLLADPGLKQLEGLRLDIYERWCDEEIQYFTPFVRHDDLDGSQARE
MLAAWSEKGSEVLLDGLKKTLNTMSEFKSIMELRTSVLELWIRDGGKARGIDPSEMQDSLREAINSRMLEVLDTKVGKLRLVG
SEVSASLGRWKDGITDEHVSIWNEEGYDEALAGGAAPFVQEVVSRFYGRNDAVSKAAHSYKSWYHVIDDVKEVVEQLRRQRWD
NDYDEIEDEETIEARQKILSKDDPQMLQERLDATLDKSFKELEDQIQSLWDERAESSRNGKVAMYFLRVLRDIRHQLPERPSI
KSFGLKAVPSMHEKVAVAVSQPTLEEFSTQGLSQRVVVGRPLWDGEPALPNQPSPEIFQFLRSLSLSMSDEGADLWTKAAVSV
LKKHLTEQLCKMWEVAVVENSVDNKEDVKDEKSNEVDAKEEKDDDDTEDENAEETDEKKASAEEELPKPEDATEEQKPGLSAE
QKTDVFTQWLFDIALLRRCIGNTESAGSSFQNLEDNVYRHSKLDEAAKHRILKSANDFWQRASLLFGLLA
FUN_014767 CDS scaffold_7 480722 481635 -
hypothetical protein PF00378;PF16113

MAEESTLQLEYRGRVAVLTICNERKLNAFNSAQYYDLAQKMREIATHDEVFTTVILAKGRYFSAGADVGVSREIAGDGKAVHK
QVLQGFVAQNLNITHAFATHPKVLVVGLNGPVVGLTAALIAFADFIYCAPSTFLLTPFSSIGLVAEGGASRALALRLGPARAN
EALLMSRRIGSQDLERCGFVNAIFDEGDDVKFRARVLQEVDERLGDHLIGDSLIGIKRLIRKPELDILHTQNVHEVFAGLERF
MTGVPQEEFAKLASGAKRHKL
FUN_014768 CDS scaffold_7 482343 483773 -
hypothetical protein EOG09262BVA PF06870

MGSEKRKRSGDSTKPKKKVVIDAPPSSATVSSVLRPKFCPPVIATAVGFQVPKNIPFHSYAPKNGAKSKGKQPESAAEKDFLL
HSNAHRSMDYTVKSDEPRGKKPALNHFLGIYDPKTGKLEVVEAKKMTMRASVRARQGAAATSGQRDNSQSMLQLKTDLGQTFG
TKKAKKAIQENVLNAIAPQKKAGEEPAKIDAAARAMLNSVGEVTSTMSSREELQAVVDAAKPVPIANMEATEIQDVYDPKKII
GADILKLVPVREWQEKVQHKESIQVPSRFVAARVNRVAGNEEAVDRLRVLRYFFFVLLFYLHSKPGKARGTRTIAPREKLREF
LAPAPEAVIESIRRKFSDRGEMRKFHVDLLITHCCVFASIIDNFEVDTQNLRDDLKIDQKTMNAYFQEIGGRVKHVTNKDTKQ
QVSVARLTLPLNFPKQRQFAPKRR
FUN_014769 CDS scaffold_7 485499 487784 +
hypothetical protein PF00069;PF00498;PF07714

MDEDTARASQESPAAYLHLVDTRSEHQSQQVFPVWEREIFKIGRDPRANTLAVDNDLNVAVSRNHCEVYVVVYEPTINHVYVR

DRKSSNGTFVNGQLIGSVIQDQPPPRHELTSLQLEECKLFASKYHVNNHCLGQGAEAVVCLANDVQTKKQLVCKLINLDKIQG
KNSQEDIRRKFQEADILRQLRHPNILPYVDAISSPHSLYTFTELASGGDLMSFILRHGTVKELDSRIIIRQVVRGLGYLHEKD
IVHRDLKPENILLAYSPKIAYHRVMLSDFGNSAVPRRSRMITNAGTPGYQAPEFAAGGQAHTAAVDIWSLGVVALLLLASDNH
DTELNKMGQGDIIEYLTRLFNGLQTKPSLEGIEFVWNCLKVDPLQRINAYGAKTHAWLCSPKKNRELFKRMDSRTLASWKPQD
KLKPMPWVLPDLAPSNPPTPTRSPGSSISQYFTTTPTRFETTTTKSRDRVLEIAAEPDPGTATRKTTSSFSVPPCPSGTLRES
ATSLKKSQPPWQGFRKPNGSANTEFPSKRKRVPRARTHDTANLPLPGLERHLRSPTNTNPRYRQNVLSALERTKSKFLTDSIS
PVPRTPLDALTTSALVPPSSPKKRKSKRDVRLKGSVGANMRHPPQLTFR
FUN_014770 CDS scaffold_7 488102 489035 -
hypothetical protein ENOG41

MTGNCYSPNSVLSDIIRELDPNAPPAAPLDLLSTVSSIFHQRSERLRREALARTDAEIEAGFVATREARIKEKLEELEEEEEE
EEETSEDEWDEDEYEDEIPSREDVEAFINGMIDKEATGPALMRAIGETREDHRKRMETLEDRIQGYQLSKFVLEDLRGDPPRG
QETGDIEQVELEWLERLVNKSQAEGIQALPVWPEVGGNSSNNSGEEEMPKKWQDFLKGSAKRKISTW
FUN_014771 CDS scaffold_7 489906 492998 - ATG1
Serine/threonine-protein kinase PF00069;PF07714;PF12063

MAARQESSTSSGSRKSGSRAVVGQFNIGSEIGKGSFAQVYLGWHKDTKAAVAIKSVELERLNKKLRENLYSEIQILKTLRHPH
IVALHDCLESTSHINLIMEYCELGDLSLFIKKREKLATHPATHDMARKYPSAPNSGLHEVVIRHFLKQLTSALEFLRSKNYVH
RDVKPQNLLLLPSRPFRDQRSRPVMQASQDSLIPIAGLASLPMLKLADFGFARVLPSTSLADTLCGSPLYMAPEILRYERYDA
KADLWSVGTVLYEMSTGRPPFRARNHVELLRKIEAAEDVIKFPREVTISADLKALIRSLLKRSPVERLSFENFFAHQVVTGEI
PGLVEDDIPKPARQEPREVRSAFHGGSPSLSSRSPRQTGYESPRDGIASRSPRDQHSRSPQAASPREGRYSRQSHDSQRPTGN
SPREIGEGLGIRRPVAHHAATAPVQQRIQDRGNGRDKVSPPTSLLNQARRDRALSNPSITEEEKAAQDVALEREYVVVERRHV
EVNALADELAANERFGDPSSQRTGPMTRRYTQQGAPTSTSGAIATPSSRNALVTQGRHDRKSSYEKALSASPGSASSAISKAI
QDASLRLFGFKVPPLRGTPKGPSPPLYQAFPTYPTPQAPVGLLGDGKNTHGTDEDGRAAQAIEEFATRSDCVYGFAEVKYKQL
VPLAPSADHILGGLEPEQLENEEDGLTVEAIVALSEEALVLYVKSLTLLARAMDIASVWWSKKSRGETGTGLSAAAAQSIVQR
INAVVQWVRQRFNEVLEKSEIVRMKLTEAQKQLPEDHPSHPSNHGTESIASSAGGATKQVYLTPGISAEKLMYDRALEMSRAA
AIDEVTNENLAGCEISYITAIRMLEAVLDNDDDSGSDARRLSTGKEDERDAVKETSGGELDSDEEAHVRKMIKMITGRLAAVR
KKQQMIAEANSKTLQSYQQSTRRRSGDITPRSVPSHASS
FUN_014772 CDS scaffold_7 498927 500292 -
hypothetical protein PF00202

MSPHAEKAVSRFHATSTEKAIAIEEEYAAHNYHPLPVVFSRAEGINVWDPEGRHYYDFLSAYSAVNQGHCHPELVKTLAEQAG
RLTLSSRAFYNDVFPKWAEKVRSVFGYDMVLPMCTGAEAVETAIKIARKWAYKVKGVPQEKAWIFGVSENFHGRTVSSNIPKS
RSNVANMLQMTAITLSVDPESRENYGPYVPNVGAFNPTTGKAIRYNNIADLEEVLEAHGKETAAFICEPIQGEAGVVVPDEDY
LAKVQALCTKHNVLFICDEIQTGIGRTGRMLCSQWANIKPDLVTLGKAISGGMYPVSCVLSSKEVMLVVEPGTHGSTYGGNPL
GCAVSIRALEIMEEEDLTAKAEKLGNMFRDGLKALNTPILKVIRGKGLLNAVVIDESAANGRTAWDLCLLLKSKGLLAKPTHG
DIIRFAPPLVISEEELRKGLEIISEAIKELPNVERAKGH
FUN_014773 CDS scaffold_7 502007 504083 + GPI10
glycosylphosphatidylinositol anchor biosynthesis EOG09260UJK PF03901
GT22
MSSATVSRKVLDEARNKRNRSVFLRDIIVIRLINAWWIATFFQPDEFFQSLEPAWDLAFGSRSGAWLTWEWKHQLRTSLHPAL
FAGVYLIVDFISSRILPIGILRAAILVAAPKALQAVIAGLGDWYTWQLAVSIYGANSNVSFFALFLQLFNPWQWYCSTRTFSN
SLETTLTVMALYYWPWELVSAARTTKENPKPTPVLKSLWSLRASLCLAALAVVLRPTNVLIWATIVFFTLTRISLQGSSPLTI
STVFALIREAILCGSLILVISIASDRLYFGFWTFPAYNFLNFNLSKSLAVFYGRNPWHYYILQGLPLICTTSLPFAIMALYKS
SAFASSTSQSNTLKTLAYTVFTTIGALSLISHKEVRFIYPLLPALSILSAPVAASFFTFQPDATTNNPRPRPQIRNKHYLLAA
LGVNVFLAGYLSFFHQTAPLNVLTYLRHEYERIHPDSVQLAQTSRFSVGPGKDEELFALFLMPCHSTPWRSHLVYPGLRAYAL
TCEPPLHTEPNTRERENYRDEADRFYDNPIPFLTSELFGPEKPLAVPRYIVGFDGIEPWLQDFVKTPEAQALSLTQVRPVWKG
FNGLFNEDWRRSGKMIVWDTGIYDNAPPAKES
FUN_014774 CDS scaffold_7 504471 507711 -
hypothetical protein PF00620

MTSAASNPNQANQPALQINNAASPPSKRDLKSWWKGFKLPSKHQEPQETRPQGIFGVPLRQSITYANVAISLIDENGQSYIYG
YVPIVVAKCGVFLKEKATHVEGIFRLSGSEKRIKELKTIFDSPDRYGKGLVWDGYTVHDAANVLRRYLNDLPEPVVPLDLYEK
FRDPLRGATKQAVGDVEGPQFVENFNEKAAILQYQRLITELPPLNRQLLLYILDLLAVFAAKADENRMNSQNLAAIFQPGMLS
HPAHAMAPEEYRLNQCVIIFLIENQDHFLIGMQGTAADEKTKKEVESGTPSGPLTPQPTRKTSLGRSASNASAGANSVRRDGK
LRRNRSVSSRNSRNDGSSTPNSPALTATPTTGGLARSNTVPSKKSPAIGAGRFQRSDGSGPRSPAHLEPLTQVNPTESIEQSP
QSTRSSDFISGGYLTPTKAPVTGRSHERLLEVPQESSTPSKERNLPNIFRSTPNTDTGDKRQPNKLKKKRIPGSLNPSAQSSA
ASLTYTHPNAASSPGAELANPMEHAQQYESGTQALPQQPAPILEEQHREALEQQQELHDRPSHGQNPQETSLAHPASLTQQKS
DVSSEHTPRASQASSNQSLHPDNTLKSKKSPPTSLHSSFNEGSDLDQILDEQSVSTVDQAEKERKKRWRLSRSKNADNGSSAG
LTSPRLALGQNDNGDVSNLSISSSSHRPRKSESSDRPYMVSEGQGSFDPGRESDSKGPIGWIRNKYREAKESAEQRRNKSPPP
ADRPGLGASSFQPRGKSLEIRRDEPTQHASLAAIAGAPAVASNPAAAPGPALGPAPTTAQIPQTATVSMVQPEPTSPVQPTHP
QAQAPPPAQVQAQPQPQPTTPAQPPVQEQTQSPVEPKLQTESQKPQSPQTPQKIISMLDVDTPPAAATTSTPVPTVASVTTQP
QAEEPKHAQEQPQPLLSQEPKAEEKPAQATEQPAVISPVQPEQVQPEQQQVKSESETPKQEQ
FUN_014775 CDS scaffold_7 511445 511830 + TIM9 protein transporter tim9 EOG09265L8N PF02953

MDMLSAAEQRTLEQRMQKRQVKEFMGAFGGLVEHCFMSCVDDFTSKAISTRESGCINRCVQKWMASQQRISDRFQEHNAQLTA
QMNK
FUN_014776 CDS scaffold_7 512347 518006 -
hypothetical protein PF18378

MAPVADRIYFPSLEQCLTGERVLLSWKLIATALSDSSGQRTTSSAVIEFLSDDYVHRLLEDPASTFAPADEATKKVFETKTAP
INVPASSDGRPDIESIKKDAEWLSKNAKINLVAALRVVVIEHQSRPARHLSGPLSSQDAKNLQEAAGLNNGQGSGLLLDLGSA
AALDAEEIWTEFEKPETRQRRLFDTYLTERRFFMMSADYANSIKLYERLPTFASVDLDLAKTYRLKLPSRDDAEPLLPTYLQV
LTDSMSRIESGLKAVTDEKWVTEEVELDWLRTLLTEAIHALSVVFQIVDSFGDDFAPSTAVNQWFSLMEVYRFFDAVQPIHES
IAPLVLPLKTLSAAVSLILLKPARSLTYLSEREEDATQADTSYDTYLLSSDVLEQVHKSILNAAEADCESASPVIFAWTLLLH
RMNVSYQSRTEKRDNLLQQNARETFEAGGVVRPVARRNSAGSIFSIESSKFDGFLENATSSKDLVVVEQLASQVTAQGRVFDV
VSNMATALGPSDEGSMTPLLSARLRNVFLELLKVSYPIVGYQSEPVSSLLSILSAGRNYWDISNKENLLEKQDILASMVNDDL
ALEFFFQQALDRYPYEFLPFITLCRTLASATSLRESDRSQMILNLLRATPTLTFVLPDYFQEYELAQEDENTNTFCLLQDIPI
ISLSPSWRGRRVEDDAYRIPAGTYGRFVTDTGRVVSMEYPHSTISLLGRRLEINLMKEGYQSELGMLQPEETAEVISLFATLI
RMDHLNAAGGESTGALVFSDNDILHEAKKHIDAGSGRDLLTVVCETMDYYMQVDLAESEDVVVTILNSCVQFLDAILPIYPSR
VWSYLSRCELLSSESRAGKLTKVVGSLDLVQERLEFLLSCLRLFSNLIDTAMTSAVQRRAGIHTTSRNKSDLNPWLGTADKVL
AKVSYAIAQAGVDIFENTSTWRFASDTCRSSVLREVVPILQKVIMYSYSLGDPSTSENLTSCLRPAASYVIDCFVSPSTGSLR
FQPLLSSLITAFTTADSTLYPRRVEMVQAQLNSVLEFSATLLRVANYLEQSSSMIETYLFKSSTLLARLCAVSDHFRKPTMWL
LESLVVNAGKSSSEPPSLLGYLGPQISKSFLQLLSILGKPFELRGEVKATWKFFSAILRNRQQWMSNCLLTGQTPREAMKKDK
KKTDASSSSVFATGLAKLGKLKDLEISEALVILDFVASAQNFWPWTVFTLQKDTAYLDGLREYVRDLKSSSITVKSDPVRAAF
DARLAAYIAETFAMQLYHSRHLGNSGTLAKSLVGDLDYYLRDGVEVGGYNKSLHNNFARNFSNKYSDCPVDCFKRTILEPREL
GKSYYYDLERADQMLRFDPGWKGRKDDGFKTEMERANTNLSLVDAQLALFHAWEFLLVELSTCMPKNDTVEKQMLQVAQQCLN
ANQGVPGPEHIFLKLVGSRASLALVLIQRLVKSSVPVKDINQLLSTLVGTMNGVEEPWGTESISYYRTLLKALFVTLRAYQIS
GKKATESQDAFEGTTVTVTQTVLNILDRVVGRGFRALVSLIHDNENSVSPEDLGLLTAILQACISLPNMDQSQTQVLNIMAEH
QVLHAASSLYSWADRLADQGDPIYGELSMLFLLELSTLPMVAEQMACDGILSNILSANLTKFMLKSNISPYSDSPVVQRCYGI
WAKGLLPLMLNLLTALGATVAPEVAYVLNQFPHLLEASVDRFEAPGASRTQSRASPHYLTLVAISEVHSLALLTRVLAALRVN
NNRDIPDVEWDSANLLENVDFWLSTKKLLKERLMPLGQREMDWRGMKPATGAYDSLLEEKAVAQLDAIRDVLSEEADN
FUN_014777 CDS scaffold_7 518484 520820 + TRP1 bifunctional tryptophan synthase trp1 EOG092610LD C26 PF00117;PF00218;PF00697

MGSSDIIDHSPHQADPSPPIPTASNLILIDNYDSFTWNIYQYLVLAGATVTVYRNDKVTVDEIIAKKPTQIIISPGPGHPETD
SGVSRDVIRHFAGKVPIFGVCMGLQCIFNVYGGEVSSAGEWLHGKTSPLTHDSKGVFSGLKQGVPVTRYHSLAGTHVTLPEDL
EVSSWVAKPDGSPGVIQGVRHKKYTIEGVQFHPESILTEGGRAMITNFLRMQGGTWEENTQFQKSGSANSDDSAAPKSKKNNI
LQRIYANRKAAVDAQKQIPSQRFEDLQVAYDLNAAPPQIPFVNRLRQSPFDVALMAEIKRGSPSKGIFALDISAPAQARKYAL

AGASVISVLTEPEWFKGSIEDLRAVRQALDGMPNRPAILRKEFIFDEYQILEARLAGADTVLLIVKMLDSELLQRLYNYSLQL
GMEPLVEVQNAEEMTTAVKLGSKVIGVNNRNLESFEVDLNTTGRLRSMVPESTIICALSGINTHDDVLMNKRDGVNAVLVGEA
IMRAPDASVFISQLCSGTEPPAKSDVASSLYVKICGTRSPEAALKAAESGADFVGICLVPGAKRCISHETALAISEAVHTYPS
TRKYETQVAAVDNKAKDYFEAAAQRLRSPRPQLVGIFQNQPLSEVLEKQKQYDLDLVQLHGDEPIEWASLIPVPVVRCFKPGQ
VGIARRGYHTVPLLDSGSGSGKLLNVTNVQAVLESDPELRVFLAGGLNPDNVAESVKALGPLADRVIGVDVSSGVEEDGKQSL
DKIAAFVKAAKEIR
FUN_014778 CDS scaffold_7 521441 523009 +
hypothetical protein ENOG41

MVAQIFKSRIIAAAGPLPGQLTVENLRRWTSLRKGVFIEDFDETVTHLLCTKEQFDKKLPKVRKALKLGKGIHIVHCDWFEYS
TVKNKKLPEADYSMRSLVAKENAKKREKARIEKGKRNAEKFVNTNLFHLYRDRLNFVYQVDITRDNEFTGEFGQKYSLCLWES
NAKPHLYWFTAKFLKHKGSAQPVYHRPSPHEGKWRAQMGLFVDFFKKKTGIDWQDRVSLAQTMPSSYFQYEPPSRGKPIGRRL
KHDIEYCREINAGIRGLPWPPLEEEERKTDSVEDESDDGPRALEEDEDEVMVSPPDSPCAPKDESGADPKESGDDIEMASPPD
PEKLTDENDSCSSFKIPREEREDIPPSEFEHSQDEKAAIANSETHEGEKKAESPPESEEIQEADKPPDAPMDAETKSALTTPA
SSIDKKDLSHAADEGAAVVPGDVGEMGISFEFETRESL
FUN_014779 CDS scaffold_7 523328 524476 -
hypothetical protein ENOG41

MARLSHCTVPSINRSAIDIASAELGNLLLRLQKTVLHTDSDRERRLRSSEFERARVASNLEYARNSLTKLEHDALGIKAPGRR
AEVQSDLNGKRELLELLLDRLEDLRQVAIEEDEDDGSTDGEDILSEIIPTPSDSMVDSISTGQPTESSGQDDDAESEPPEATT
IPVTTATVPAPASTTPSDTSTTSQEHKQQPPQTTQNLRSRGAPSSPSTPAHSTARAALFANRSKPSTPQTSTATAEALLDHQR
AEQEALSESILQMASALKSSSQRFSTTLEADKDVVARAGEGMDKTEQSMEAAKGRMGMLKRMTEGKGYFGRLLLYAYIALLAV
ACILVVFVLPKLRF
FUN_015549 CDS scaffold_8 791 3008 +
hypothetical protein PF00172 ENOG41

MTGTNSRIPMSYSFDLRAPIIRDSTTTVLPTTSATSPGFSCNSPEEPTTQDGQDHDDAQSQGSGKRQRRLTTTDFTRRKRAVT
ACQFCRVRKTKCDNVRPTCGFCVRHRAKCIYLDADSEMGDEGVEDAVSNRQILDRLDEIKEILQKTNDSPQPFSDIEPILTNT
EAPQTPAAGSPWTRFSAGAQYETQKASKVQPYESERLPFAALRCESLLQWPALCNIIPSDARDVDSFPLSPGYKESSVQKPNS
TGKGIDESSFVTLCRKFLAHVHPRNPVLDGQQLIRHARTTEENGLKWDTSSCLVLLSCALAEYTGFWIKPNEYLVPPTREQLR
QRDDPDQGPRAEPYYAAAQKRMGLLGNTIEDIQCFVLATIFEKFAFRPLRAWYYVQQACSRLEIHLRQRGERPRVPFRQNNPA
DYHLEQRLFWTCFRAESELVFELGLRPSALESFSYPEPLPTPPEGLLAGTGQTPRSEGSHIDQDEEQRYVDERGWYYYLSEIS
IRRTVDETLNLLYRNGEEYWMKHPTYLIRQYHESEQQISQWHYHLPAAVQFENDKLPEEEFASALQGRASLWREYTLRPIFYY
MLHCPQDDPVFPQAHILALKELDICAKMIHRLAFQGRHGGTWLISRKIFLSACIVLAAASNPHRIHPPDEWQMLIELAIHTLE
RWAVDAVDVGQMAEILRHMYHQV
FUN_015550 CDS scaffold_8 3396 5919 -
hypothetical protein PF00004 ENOG41

MDTPQPHEVNVVDGDDSSSIVSFEDIPPRSRPRYRHSPHMASMPRVCPKTEILKIDGKVPDVLHVVKYLGWQNNVIECRQSPV
AFKNVPPYGFNGDINLVVDDDATGDEKPVIEIITKVLPRSRSPIPIHRRSRGPRDQEDRPITEAFENIDYRYDNLYPDEGQDP
EVPGPEISRPFMIIHSDHLINALKAVVAYYPYVSFDGKPVKINAPYRVLYHHRQELARYKDHQPLAHDSDYAATTSKHIDVLL
KFLDDNLGDEIRREEERHQLATPRATFDLFWLLLKPGEVFYAKRYDIWTPYVISSVTMGQGHNSSCDVYNINCWMLESNGTKV
NRFMFSYNLSPWLGEQAIGSLSIIPAAFWPEDLEAQGGITMREKNIALGKLYWELLKRPTYMEYEGLLVNSGPSNRQCRGPTG
FMCGRVICDAPGFDTFYNQGPDNMRAGRYHRIPVYNETAPPSKDHLPKDLPRCSCDTCMANRPNYSTESPYHGFEDLDPTQDS
PPDSDLFYLVCSKTMPGFLLGDRRWGHLNIANLKQLKTDKEAFKYLVLDDEIKMTVKALIGKFAADLGDGKLSPWGNDFVKNK
GEGRIFLLHGAPGVGKTCTAECVAELTNRPLISLTSGDLSVDSFNVEANLSYFLELGQRYGALVLLDEADIYLERRRSKDITR
NGLVSVFLRALEYYRGVLFLTTNRVQSFDTAFLSRIHVALHYKSLTHEDCERIWTHNFDRLSRDSNGLIHVSGAAREFVWNSQ
DVRSLKWNGREIRNAMQTALALAENEAQEENSERVTISEKHLRAVVKMSRGFRDYIKTAVPLEGFEKTLNDSDEE
FUN_015551 CDS scaffold_8 6877 7848 -
hypothetical protein ENOG41

MGAPEYQDGDAVTTFWCSPECQAAHEPTHQEYCYNMQRRKALLRTAKLLKAALLAYKEVVYDIHLTKIEHDEDSGTLVLIHTP
NRIERHLFPSHLTRIENHKEAALLVNQCTMSISLLGPMTRGLLAGIVSRMDVAIVEIRNPPLPIRFHPPDGIMTDRVFHTIVE
ATLDSSGERWLIDITGCQYGFRDILLPLKKYITQNNCSSYELLQPYGHTETTDQDELPRSPFFILTGGPNEQQLADIEIEKGY
RRHFATLVRALFHQGLTQGSDAHFAAILDDLAHRVITHMSSYQPHLGAYQERTTH
FUN_015552 CDS scaffold_8 8443 9954 - VPS30 Vacuolar
protein-sorting-associated protein 30 EOG09262FJB PF04111;PF17675

MNCQKCRQPLRLDGSLEDLNPAAYDVLVSSTSPQTLKKSSVTSPPITQPQEQARKSLYETVSRNTGPPTFKRNHGGHPRDSSM
SFVLLSESQMTHPSQPPESHAMPTALRRASSARSNGDNVDAPVGNEMDRVNRLFEILSARSDIDHPICVECTEMLVEGLQKKL
EVTSRERDAYVKHLKEAKANKPSEEDMKAQEEALRKAEQDRATAMEELKKLESEKTSLDEELVALEEESRQLDKEEEKFWRER
NEFATKMGEFQAEKDSINAKYSNDSQLLEKLQRSNVYNDTFCISHDGSFATINGLRLGRLSNKPVDWPEINAAWGHALLLLVT
VADKLSYRFDGYDPQPMGSTSRIIRYEVPSPSSSRLGSRAVNAPPKKHVLELYSSGDMPLGLTFMHRRFDNAMVGFLELVRQL
GAYVHRQTDATGTPLSLPYKIDGDKIGDVSIKLGIAQDDGWTKACKLTLTCCKFLLAHASNVTSNARNGGN
FUN_015553 CDS scaffold_8 10589 13520 +
hypothetical protein PF10384 ENOG41

MEPPTKKRRQGSSAQKEAQDESDDDELASHPQEIRMRRDPDIQLALKRANADHKLQATMAHIIEKYSRDFEGIGDEIDMNTGE
IVVNNGHLRNMRDEGDVEGLWMEGDSNIDEDEGVLLEDLTDGYSDSEEPVKGVQHSQSDNQNNQEPTFAEQGAPTSQRTHSEV
AREALQTDMDTAMDPSQISNAMLHTSESHFGPPSYGPGSSVGFGPTPGFGPWGRMHGFPMQAWGRDDIPPYFNMPPSMPGPWF
SGGRYDFPTHNGQTSIWSRNRAKRTKRAGSMKASSKQATSRHPSSSRTEEEAVADDHHKSMSEEQPRDNQEDLAPDRTINDTD
DDDDLMFSETTEVASTPEASLVLPTKETSPAKGQVHRHSQGALEETNGNAVKVPQKNDEEDDSGRRRSGRARKQTEYMGKISW
DDAKEWQKSGQRLSVELYKADPVIRKDFESVDHIGDECASSSEELHDNALPTKVLERGTTSQRQVVPDSQDTATPFNSSAPEA
PERKTGNDRHYASDVPTVPSMELSDDEAPLVLSRIRAPKRQVENRKPPPPLMPTQDRIRSAEPIVYVASSSPKPNQRAAPIVE
PTVGGMQGQTIEPLKRKRGRPKGSTRKAQNNNTVSSTKVITRGPTVPQQHDAGAPKKSNPANPDIEVGAKDHSTYATRSHGCP
DRDEETLQKLDHDDTETEERTVTHHLTSEPKSPASYRSQARTSDETIKLRRSWEKLQKSNNSTDESTELNEDISDDQEEQPVP
LTPSGLLQEPSTILEGPMEAPETFVIIGDEQSSTASPSSPVHVEEPPNYSDPPDESRSRLNDNSSDYAPPRDEAVHDDESLPE
LPRDTMVHGASTPRKPKDTRTPVTEPPSSSHKPHTPRHSSIRTTRAPSSRRSLLSFVSDSESDTGESRDELARRVKSTSKTAS
VRPSTKRIWRASALTREIQRTPSRKRFHEMSSPTNTVKTPGGTLRTCGIDGYQCGRDFCFTCI
FUN_015554 CDS scaffold_8 14140 15436 -
hypothetical protein PF00069;PF07714

MARVYADVNQSMPRSYWDYDSVNISWGALENYEVVRKIGRGKYSEVFEGINVVNYQKCVVKVLKPVKKKKIKREIKILQNLAG
GPNVVALLDVVRDAQSKTPSLIFEHVNNTDFRSLYPKFNDIDVRFYIFELLKALDFCHSKGIMHRDVKPHNVMIDHENRKLRL
IDWGLAEFYHPGTEYNVRVASRYFKGPELLVDFQEYDYSLDMWSLGAMFASMIFRKEPFFHGNSNSDQLVKIAKVLGTDDLFD
YIDKYEIELDAQYDDILGRFQKKPWHSFVTSENQRFVSNEAIDFLDKLLRYDHQERLTAKEAQAHPYFNPVRDPEVFKQHLAA
ANGAANKS
FUN_015555 CDS scaffold_8 16604 17231 -
hypothetical protein PF12720 ENOG41

MPDLALKKNQDMPQSNNGSTTNAHVIPNISPDDLVAFHESHFSHTAVAAFGSEFIDSPPQDQIQDDAVDDTWEEEDDNLGYYP
DGVKRTLTDAQIEIFRHSELETLRKEKERAKQLGSKETAPSSEAMDLSDDTPTSTQTKNMSSSLPASFQSNKKRKKKKGLQRP
RPEPKPDLRKRTWDVVDKGLDSLEYD
FUN_015556 CDS scaffold_8 17454 18043 -
hypothetical protein ENOG41

MDTALPLQEDGENHPSQDDIKPSVPAPEETNMSNIQKHPHASRRTPEPQVDRQANNSDPPDTPGALEPFDWDEFEARYEAALQ
EADERERЕILKEADALSKYFKIWAASASAHDDERAAKRLQTRRRFVNLAEDKMERKQQHYDQVVRAFESALALLKSQ
FUN_015557 CDS scaffold_8 18585 20036 + ATG14 autophagy
protein 14 PF10186 ENOG41

MDCDICHRIHDTERLPFLCTVDARNSLLDGRINNVELLLQNESLQNEINELLDETSTPNKFHKESLQAQQRTAEDRTTQILAA
ADKLRNDIKTAREEIQARKAALSRRKSDIAAVSGGLVERRVKQQKDIETDIGRLKYKWSRSAGDMARTRSFLCMEAARLYGLE
RIKEGSTGKYEYYLGGIPVVDLATMNSSTPEMISTSLSHICHILMLVSHYLSIRLPAAITLPHRDYPRPTIFNLSASYRSGDP
VFPSQAGVGNPSHSGDTESQRVSRPRPLFIDKPPALLAKEDPATFSYFIEGVTLLAYNIAWACSTQGVSIGDRTHFEDICNMG
RNLHELLTNHQNVGGNLYPLSSNKKDPSASKNDTDAQASRMGQYSHGTTFYYLGGAEGTEFSKTFKLPGPIKLADKLKKKLLS
EAPAPDWEVLDDDAWKVEEELVDTNQPNKDLLGSDKNSPRRGTSGWMKVKNR
FUN_015558 tRNA scaffold_8 20340 20414 - tRNA-Val

FUN_015559 CDS scaffold_8 20861 21835 -
hypothetical protein ENOG41

MGWISSVLGTDKSSDPLSKLDPSLRDFLEKESPLKYPSNQPTNPPPANQQPRNADAAVTAAEPQKPVVPSASLYQDGRYAHLW
KNYQPLAEIEAETATDHEKLMSVLEAYKERKEAIGKAALENCAEYQEEWVNCMKHGSWEDQMQMCRHQVRRFERCYMMQSRFL
RALGHGSVVGRSPEVEEDIQMHADSLYQRMIKHEEAVEKAKAEGLPVPVFQTTLPKAKKDAVMPTEELQQQWKEQLDKLPAEE
RAVEEAALRADLQAKAEVAGSVQKIWDTQAEQRKQRQAEGTSTFGDYIASLFGKSGK
FUN_015560 CDS scaffold_8 22502 23660 - RPS11A ribosomal
40S subunit protein S11A EOG092653NM PF00366;PF16205

MKRDDDKATELTVQSERAFQKQPHIFQNSKTKTKSTRPGKGGRRWYKDVGLGFRTPKAAIEGSYIDKKCPFTGLVSIRGRILT
GTVVSTKMHRTIIIRREYLHFIPKYSRYEKRHKNVAAHVSPAFRVEEGDQVTVGQCRPLSKTVRFNVLRVLPRTGKAVKKFSK
F
FUN_015561 CDS scaffold_8 23711 28255 +
hypothetical protein EOG092640PR PF08317;PF15577

MAPADATLPATRRTRRSIGAHTDVNKAFEKENATVDVTSSLAASRKKSRSKSLGPGGLDALMKANGNRRASLAVPSRAPRSIL
KQTMPIPEIPPMKPRQPDLIDFGEPTKEVSTPNSADNSGSKIAVKTEEEQQAAREREERERRDARRKSLANRRVSFAAEATL
HTFHEVEFNQDSTTSTDSTRRNSAFSNAAAQQKERDDEEKKQRRSSGLPPVNFHNSEDDTATTLYSSDSEPADAVEEVADVEE
DGDDDSSESDDGTMMTVDDVTGTTAASDRSVDSDGESSTLDEALRLAARRAGNQQLQDEEDDDLDEDEEYIPMFGWGKENKNN
IAAQDQENLPPPARTQQAQAMQDTEDATETNMSMDMDMDMTRAVGGIIKPQPAQQYDPDEDMSMDVTRVIGGIIKPQPPQEHN
PDEDMSMDVTRAFGGIVSQPQRTSAHNDDEEEEEEDTPMEEATMEFTTAIGGIKRPALEQDEGSDGNEDMSMELTTVIGGVLS
QKKRKSIAASRRRTVNRAENENDDDDAPMDMTLAVGQILSKPGTDHPEDHEDADEDEGDATMGMDMTTAIGGILSKATGGSRN
LGKRVMEEEADSADSPDDVLQAAISKTAVDEQAKPTTPSNKQTKTPSPGPSRTPRSALRSITGTKISTPQANPRNSRTPSPAK
ATTTPQSTKAKTPTSAKPATPQLDSVVSEKKATPRSTQRKPRSASPKRPSSVRTTRASSRTPSPVKSTPKQGLFQNNFRNSNR
TPTVILTPQRSLSGIGADRAGLGSPQVAALFDRRGSIGDMATNFVPGKRGVMFEDPKEMTQEIDREAQEEEDKENRRKILERE
ADGAEATLNLREMIDSLSPKRKPLRGRKSLHVGSAKGLLGKRPNELDDDEDEPEDNDGVKRLKGHQGSPVKNVRLQQPPSKEE
TTGRLNYSFRQSLQPNTATPTSSSPEKPDAATTPRHQGRFKEVEDDRAGHQVNFDENPIRDAEQLEDEVEAEADQDGDRIHLQ
DFLNMTSIRFMELTTTKRRHTQAPGTLDSGFLDDGEEDLSLERCVVAGACTVPMLELYQHSCRELKKYISEGRRIVKEIETDT
FEENPPLFKEYMAATPEIKTLMDKQFMNVKNHARLLSKAMWYEWRMKLQEGLKEGLLGIDDGMETDKELLDKQKSLLDSVLPA
IVARYKSLLEESNNLEEIARELADCDPADLEAARDELASLDEDVEYKKKRIAELREQFQASEVEVEDLITQKQNCVDEIAESE
KIREECRGWTSTEVNSLKARVDSIEKQCGWSVTGISGTVLSMAYRREIEIVFDIAAFQEHQPNSAIDLWYIADSREQNALPKT
TEKEFFLQSIRDYVRALPHSRAGVAHLLKSVQNAWDKANLVSSQVVRLNATFPTTVEKTSDSSIAITTSLLLVPLETRLEIKL
DLQGQSSAEALDVMIAPDAKVLYGEHFNVPKVTEFLTARIGKAVGAGEEQWSDVMVELHGRLIARGRKAG
FUN_015562 CDS scaffold_8 29057 30500 +
hypothetical protein ENOG41

MARASDLSWVWGWLPLDDEYEWDSIFTIDEAFDNKYYNITFAAISVLYKILKAISIILVPIAILLGHWLASLAVLILSVVRLS
ILVLILKVFGDGFESHQEVVRPFFSTRWNIGECLSMSSLLTYFPMIRFRACQAIEIVREHLVSIRDELYEITGIDFRYPGLPD
GDITHIQPSDTRAERWMRQLVQEDRIRRRRGIENERIGGHDVIGNQQESLDGGPETPAARTNPHDSDEEMGSFEPGAAGDPGV

SSKEEQTPLASTIYDAQMADVWRPNEEESKRIGDAAQDLFKDLMDQDDEDSSEDTKPEREPSMSPMREIVTEDQQANLEDTTM
QDMQSSAPPAITAGLHLPIQQPEGPRVQAQPFISPESRPIGQGMSPREEAKLADMLVAAFEEHGPDTPTQDAQPPSVRPQPLT
SADPLLEAFRARLKAAETEPQPDKYYAQVPKDRDPLDPVKEATADQRAN
FUN_015563 CDS scaffold_8 30656 31583 +
hypothetical protein ENOG41

MDLAARSLSDLAAATGIVIGNDASNQAASPSGKDSSHKDTDAQKTDVHITSSQKTSTHETNMQESSVSDPEVERQTAMTLPEE
QSGPNSQQPQSASASILSAKSQPSLVQSTPIQTPPAQPSPAAASTAAAQQHTPTTNNSPTAAGPINTQQQPSICPVMHSGLIL
PGGNPTLSNPTATATPAPKKSGPDPEKVAEFHRKKQAQLKKQIPKKPVNIFMQTKRPAAVPSTPNIPQKNPNSSTQGRSPSLT
AIDEGDKNTKAGLDLLFGVPGSNKKKQGLQIIDINDIPDHQRTRIQKDDAGRKDEEEEE
FUN_015564 CDS scaffold_8 32390 32617 -
hypothetical protein ENOG41
MPAAMEPTQQAPMTSDNGDIVTQQPSAEPQPDMTMRGGEEAGCEICCGLCACDEGCC
FUN_015565 CDS scaffold_8 33737 35480 -
hypothetical protein PF12927;PF13758

MSEVSDFDKQQRQLEDDLSKLQNALKHWQTWHVEYEMLKEEVEAVCKNGETELKTIHAGFEGELLKDRELDEIFGERTHRSGE
QILNILDRRIDYVTTNITSLQKQISAAETKLDESQPDFTEDDGLPITEIIEQLDDDDNVISYKLNQPDTALPKIQDALQKAGI
EDLEDGNSASKIESSEQKEAEYSQESPSQHTELETTTLAPSATKGVAFAADTKMQDGPKPQVSRNAKRVEEIMNHAKEQEKIS
NEQAYIPEDEDEEDAELRRQMLEYSGEVGAVVAELQLEEGDSDDYEYEYSDEGFGDDDDDQEDKYGRYTGRVVTDDYRQRMLE
LEKKLGIKSRFTEQPEDRDGDASSDDEEGIGRIVVKPASATTTSSSSASKPAPTKSNIKEKQPEQPNGKKGVRFASNLDIAPE
NEPTVLPVREAPVKEKEPIVEPLSDKIVERSSTAKAPDTSKIKSTRKPSRFKKARDTVPPGPLDVPTKFLDQDRPTAPTGPEG
TTLADTLVERESTRAAPHPPDEFDDELIYQEVADEHHKLRKKFIQREGGFLKEDESPIQPLEEQDGGREPVSRFKAARLSKY
FUN_015566 CDS scaffold_8 36143 37217 +
hypothetical protein PF04828 ENOG41

MAECNESKTLEAKCYCGSVHYTIDVPVSKLPLLTHLCHCSLCRYGSGAPCIFHAPLPDGVKPKFVEPSSRSNMTSYAIGKKIG
TWNFCSTCGCHIASTGPPENEFWTVASSTFVNASDFFDVRKHIFSNSTKDRGIAEALTHTGGKEFIDWNPSDGSPEAKIVESR
VEVGKDGEERLRVECECKGISFTIPRPSQEIKEDKFYSQFVSHRDDTKWLATFDACDDCRLHNGTNVVGWTFVPLSICEPRIE
DDLLIGSAKTFKSSDNVVRSFCGTCGATVFFSHACRRPSENHHVVDLATGIIRAPEGVMAEKWLTWRARLAWADSGKRFDSDF
TEALQEGMNKWVLKREGLVEDYNIG
FUN_015567 CDS scaffold_8 37798 39431 + MET6
methionine-synthesizing 5- methyltetrahydropteroyltriglutamate--homocysteine
methyltransferase

MASKGRSYNDAIDALNSLQTPFDVVEARRKAGIRPDAVSIKEMQTYLHRIGYTPSDLNRLNIVHVAGTKGKGSTCAFVDSILS
QYQHTRGTLRKTGFFTSPHLIAVRERIRINSTPISEDLFAKYFFEVWDLLESAPKDDAKKLMAPRPIYARYLTLMSWHVFLQE
GIDVAIYETGIGGEFDATNVVEKPIASGISTLGIDHVFALGDTVAKIAWHKAGIMKTGSAAFTIEQVPDAEEVLRKRASEKNV
DLKVLPVDPRLSSVQIRPDATFQKRNATLAIALAETALEKVGISLPPRTDTLPQEFIDGLEKVVWRGRCEVKKEDKVTWHVDG
AHTSDSLKMSSKWFKDETSGRNGPRVMIFNQQGRSEAVDFLESVFKATRREGQPAFDHVIFCTNVTYAASGYKRDFVNRQFDP
AEIDKMTVQQRFADKWMSLDPTATVKVMPTIEQSINYARHLGEDLPEGETVQAFITGSLHLVGGALGILEKADAL
FUN_015568 CDS scaffold_8 39479 40958 -
hypothetical protein

MAARRGIGTVFRDYNEALRQLSTLYSNRQATNLFDKSIAPNASSVPPKDLNALAIPEMREWLRRAGYEPKDLARLRHIHIAGT
KGKGSVSAFATGMLRQYETVGTYTSPHLVSPRERIAIQGEQVSQKLFAEAFFELWERLSEAAKKDGKSLTESEGPGSKPFFFR
FMTLLAWHIFLREKVDSVVLECGIGGEYDATNVVPPEAVSAAVITQLGIDHVAMLGDTVEKISWHKAGILKPGVRGFVRRMDE
KPGVRQVLQQRAAEKGAELIELDDASVERWGGVAGRLPGDFQKYNQALAVLAVRQHLGMSVDPKTAFLNLPEKMFTGLKEASL
RGRCEIIQQGHIGWYLDGAHTKDSMYEVAKWFASNIGNDESAVLVFNQQERDATQLLATLLDTVQEVTGRENIFSHALFTRND
QEGPSTDEEARDLSVQTRVAKMMAERAANCEVNTFDNIQDAVTEARKVAGSHQKVLSTGSMYLAGGVLRALEPEGLL

FUN_015569	CDS	scaffold_8	41542	43584	-	MIC60	MICOS complex subunit mic60

MLRTSLQSVRAFGGRPVVAAAARQWPIAATRRASLAGQRFYSVEKKSEIDPTKQPVLPASQTVTSERIHETTIDDVGETKAAT
TDVPLTPPPPPPAPAPIKKKGFFRRLKNFVFTLLVLGVVGFAGGVWYSRVSDNFHDFFTEYVPFGEQAVLYLEEMDYKKRFPN
ITGRSKTQATGDAVRVPAQSGASWRVADAGQAGRHSSAGPVSAAAKKAEEAVKPKPKAKAPVEAKPVAKAVEEPAPTPRSDSG
FKAPEVNEPSKFPPLKPIDLLSLEDAKEPVVQDLVHMVNDLILVINADNAHGKYGTSVSKAKDEIAKVGGKLKAMKGEFEKKA
AQQVREKIEEFDKAATDLIDRVENTMISQENQWRQDFEQEMKKVRENYEDRVNVLLEREKKLNEEKLQNQLAQQALALKKEFT
KDVEKQVEQERESRLGKLNALSSAVSDLEKLTTGWNDVIDTNLKTQQLHVAVEAVRASLEDDHHPRPFIRELVALREIASDDP
VVNAAIASVNPSAYQRGISTTSQLIDRFRRVANEVRKASLLPDEAGVASHASSWVLSHVMFKKQGLADGNDVESILTRTQTYL
EEGDLDSAAREINGLDGWAKTLSKDWLGEVRKVLEVQQALDVIATEARLQSLRVD
FUN_015570	CDS	scaffold_8	44852	45753	-	YPT5	GTPase Ypt5
	PF00009;PF00025;PF00071;PF01926;PF04670;PF08477

MASRQPPAGARGTNTRFAQFKLVLLGESAVGKSSIVLRFVKDQFDSYRESTIGAAFLTQTISLDENTTVKFEIWDTAGQERYK
SLAPMYYRNANCAVVVYDITQSASLDKAKAWVKELQRQANENIVIALAGNKLDLVTEQPDKRAIPTADAEAYAREAGLLFFET
SAKTAENVQTLFTAIAKKLPLDQAGPRHARPGQRPGVSLAPENSNTNVSGPCSC
FUN_015571	CDS	scaffold_8	46639	48896	+
hypothetical protein		PF02383

MAQLNTPKLPYRDINVKIANDSYTFTSPSSPDAPALVIDRPTGDVRLSEGSAASAKRTTRVSSIAGILGIIQLRLDKYVIFIT
KAQPVGRLKGHMVYKVAATEILPMRERLIHDPDEDIFIQLLKNFLASGPMYFSYSIDLTNSFQRQAQADTSKPLWMQADDRFF
FNKYLQGDLIDFRTRGARSQPGAQPGVDPYILPCIFGMLEIKPTTFKGNPLTLVLITRRSRHRGGTRYFTRGVDDEGHVANYN
ETEQIIILNDSSSGLGGYAGSSDMQSGKFGAGAGQEMQIFSYVQTRGSVPTFWAEINSLRYVPKLQIRGVDAAFSAAQKHFDE
QIRLYGDNYLINLVNQKGREQRVKQSYEQMVEKLVSSPKERQEADLLTEEKFTTIQPEGKRQEFDRLHYVYFDYHSETKGMKM
HKAYALIEKLADALEKQGYFRAIDTPSSVDGSLDARSYQTSVMRTNCMDCLDRTNVVQSMFARHKLDRIFEDVGFMPRGSSFR
DEDPAFEDLFRNLWADNADVVSNSYAGTGAMKTDVTRTGNRTKVGALQDARIGITRYFRNNFLDGPRQDSFDLFLGAYRPGST
NIGTTLVFTDRRPVLIQAIPYILAFSVFIVFIGLFTRRDPEASALPLRIFLFFWMAIAAWSFYFVWNHGMLYVNWPRLNPRPF
AVEGYNEHFAKARKDSVIGTYVAKHERGLSTARYLNAEEGKKRIE
FUN_015572	CDS	scaffold_8	49700	53351	-
hypothetical protein

MADPRAASSLSAYGRRDGPVTPEHPTRFSYDTAERGEPSMNALLHAPLSPERPLAFSDDETLPRYHHKSRGAVVVPSGLQAGR
PKRARSKRPYKTQPSSTFLLQEPVATDDDSGNQRRAHRSKSRHKGKDVQEPYLSNGTRDLGLGISSGSGPRARIVSPDTSQEE
LHITKRNEAQRRGRRAHASQPGAALDVDSTQIVNMALNLSESRRIASRRNISRGNPPRLAPVQDSQAGSNLRAHLQQQRKAVR
GASPIPNQSLTPRLPGVRSSSPLRPSPFETTDASYRYHFSTSTLARAQKAREHLELMAQYRRLLQVLPPLKTGYDRPSASSPP
GSPTEGKMHAFESREFMIPLGREYNPLQYIRNRKVRARERMVIDGEKQGFADVESVKDWVNEASERASRELLSSDGSILPPFP
GAEEIDSQIATDSATKAALRVRRPRVDWFFEACDLIADAYWLEQDHHKQLIEDRNLNKIYPQTGETLSRPISGQTDELGIGIP
PFSTETMEEMNGKANSNESNLSKPEIELLEPSHRDRARQKLHEMGSFHRQTGSVHHRRFRRRGSSSDDSASEDDMPNEGKARR
GTISSRETELLEKQMLEMLARDMRQQRLSHVQETEAEHMQPEDSTSSEPKPQYQLATSREWNMMDSKDSENRPIHNRASLEIP
SLPKLGRNTGGGSQRGRNSKEHTDSPQPMSPELAPQYSLSAPPTGLQLSAPSSRSSSPSRNPFTKVKQIFKDKTRDEPEEQAL
ETEASSRRPSIPDPLSPTETKPLERNPSNSRPTFESHKHQRSVGSIRPRGDDQAGLRGMFKGPRIDTVIRGGVSRLGDMLWKK
DITMEGTTDVLSSDESDNDQPKGRSRISLNLSRTNSRRNKPEPQHGAKHFLDSMPQFQPMAETHGQSATDVKKLQPSQSRVDA
SKPPAPDTSGISSPSKPQQVEKVTIVEPAMSEVESCQESVKDGPRIMEREADEAAPMVRFNEPDNFRARKWSIANQAIPQEGQ
LSKREIARLRTLILTSGIKAMEISRRAQELKKPLPSNDLQEFISSPQSSPVGIPWVDIAKLTPKKSLPFDDAVPCSDHFRIAR
QTLDVAIQTSIEQWQTSADKFTAQTRPKLEERIWCVRSRIADELSGMTREASDQADETGKDLALGQLLKVKHVTDLTDKMMRN
RRRRFRWLRRGMWSVVEWVLVGFMWYVWFVVTIFRLFLGLGQGIWQGVRWLLWL
FUN_015573	CDS	scaffold_8	54395	57341	-
hypothetical protein	EOG092609JB	PF00133;PF08264

MLKSWKSSLRLPRSRFPARPTPKLEPNYLKQCTDDLYAWQAQNRSGEKPFTLHDGPPYANGELHVGHAMNKILKDMIVRVKVQ
QGRRVTYRPGWDCHGLPIEMKALGSHTTKGLTPVEVRDTARKLASKTVVDQMKGFRALAVMSDWEKKWTTMDREYEIRQLRVF
QNMVRRGLIYRKHKPVYWSPSSRTALAEAELQFVEDHVSTAAYIRFPIISDWSSIPELADFKGNLYATIWTTTPWTLPANRAI
AIHDQIKYSILQVGDDGYLVASSCVGNMKRFLGDFQVVAHSIPGSSLKGLEYKNKLRGQSAPPQPIITADFVTSSSGTGLVHI
APGHGRDDYEVCSKIGIEAFAPIDDGGFFTEEAYPDDPEKLTKASSVMDGGSHAVLELVGDDVLGSHKQRHSFPYDWRTKRPV
VTRATAQWFADVGSIKDDALEALKDVRFVPRGGRNRLEAFITRRSEWCISRQRSWGVPIPALYDENGNAVMTTETIDHIISVM
EERSSSAWFSDDPDEPAWIPPNMEGKYRRGTDTMDVWFDSGSSWTETEGPADVYLEGSDQHRGWFQSSLLTYVATQTSKETTG
KASAPFKTLITHGFTLDGKGKKMSKSEGNMIVPNEVIKGLLSQAYSRGNRLKLDPLGPDAIRLWAASADFTSDVLIGPNILRP
VNASLIKYRTIIKMLLGSIYPEARQSPLTKLDQMALVQLSDTMSEVMQSYNDFEFYRAVSTINRWVATDLSAFYLEALKDRLY
CGDGGGVLEPILIGFLRMLAPITPMLVEEAWSFRPEWMTEDTSLISPARQLYDDPLVDPMRLTLPQDVVRKDQSIITEVHGAV
KATLEDARTAKALGSSLQSAVYLEVEDGKAAAVLGRYMDELHDIFVVSSVQVNEPLPENPAWAFQKEFEIQDAKVNVHVLPPK
QEKCSRCWRYMAPKEDALCGRCEEVVGA
FUN_015574	CDS	scaffold_8	57671	58557	+
hypothetical protein				ENOG41

MAPVRRYLRISKYSVLECRIYLDNPALAQSWLLNPRNPVLPKVIESIRPLVLPKLREEKERIRKKSKKKSIKDVIVSDEFEVS
IFLTETSTRHSLLYKTKHFRDRLPPKLESNSSKLIGETDSVPVDVEDEYRAPVLQEDDDEVRLADIPVAETTTRRSKRQRGIQ
DPEQDGNDEAFEDDETASAIEIDSDTEAPPHKRHRARTNDGLDGNEDKKKLAMDVSYEGFAIYGQVLCLVVKKRANAAASSGG
GGKTRPEGQATMENWISSTQVPAGEDIP
FUN_015575	CDS	scaffold_8	75423	76767	+
hypothetical protein		PF10568;PF17171;PF17172

MLDLHVWGSAFGLPSIDPECLAIITYLHSSLPVSAWRLIPSNDPSISPRNTLPALYDEGVWVSGFGPIVEYLTDKSIGDNLDA
GLTDIQQADRIAYAAFLSAHAAPLLDLSLYVSAANWSAITRPAFSTLLSFPLTWTVPPLLRAEAVKRVEHLGFADMDTDFDPN
GGLHLSSGRDALPETFRRHIPVRTKKTIHEEMTPEQAVLIRLYGITEDCLTVLDDFLEGGPEEKMHGFFNGTEISSLDCLAFG
YLALMRDAPVPRSFLKDWLMKKTPRLSTFVDGMKTRCLDKSKLLPLVEPGNNTVLRIGSRTLDSILHNVPGLGDEYATEVRRR
AEGGITGIDARSLMLALSLLATGAALGYGYHTYKALQPFGSRIQVWYSHKNYSKLSEFGALGSMLGSVLGGPVEAPRPSHPSN
QARFVETDTDVD
FUN_015576	CDS	scaffold_8	76965	78349	-
hypothetical protein	EOG092620OR	PF06728

MALRGKASVFAGAALLRLILFLAFPGLPDLLTGRVEISTPVTSFKRLQEGLFLYTNNVSPYDGGVFHQAPLLLPLFSLLPDVK
SWPIFTHLLYIAVDLLSADALYKIAESGVAGNSKLFTSPRRANKFGSAAIAAGFLFNPYTIATCIGRSTGVFTNCAILLAIAK
AIQGSPFNAMVAISFASYLSMYPILLLPPLVLLAYDCQVEKRRIACITKFAATNVAVVLGCVVSLLGMSFLLANNSWEFLART
YGIQLTLSDLTPNVGLWWYFFIEMFDSFRAFFLGVFWLHLAAYPAALSIRLRPQPLAVLTILLGIFSIFKPYPSLADASLFLS
VVPLFRHVFPLMRYAFVTTSTLLYATFLGPAFYHLWIYAGSGNANFFYAITLVWSLGQSLLVTDLTFAVLRDEWEIERPEMVG
KEIRQI
FUN_015577	CDS	scaffold_8	78821	80801	+	NOP7
mRNA-binding ribosome synthesis protein nop7	EOG09261L4M	PF06732

MPRIKKKGKAGAAKNYVTRNQAIRKLQISLPDFRKLCIWKGIYPREPRSRKKVSKSSTSSTTFYYTKDIQYLLHEPLLQKFRD
QKVLEKKISRALGRGDVTDAARLEGNASRPEKTGKPRYTLDHVIRERYPTFIDAIRDLDDCLSMLFLFANLPSTSSVPAKMIA
RCERLCLEFQHYLIVSQSVTKSFLSIKGIYYQANIQGEEVLWLVPYKFNQRVVGDVDFRIMGTFVEFYMTLLGFVNFRLYTSL
GLKYPPKFDQAKDEAGAELGAFTLEGKTLVGADEKEQKTLEAAEHKPDPKVQAAVNKVIKKIKGNDDDESTTTSENQVEGEDQ
EAGVIDKFEPAVSGGDILPQPSNTGNNPNSLFSNLTIYLSRETPRQPLEFLLKSFGCRRVGWDAVLGDGAFTTNELDPNITHQ
IVDRPPIQASAEDEADGDDNQTSQKLAANRRVPGRTYIQPQWVWDSVNDGELKEPHLYAPGADLPPHLSPFVRNVQGAYDPTV
PLEEQEPEAEAIEAQSDGEDEVDDTAEGMDVADSDEGEGEGGEDDEFGGFSADEQEEDEEEDDAEQRQDELEAELTGGAVKPK
TTNAKAKAKEDARKALSKKAREEAEDLERAKGMLSKKKRKLYEQMVYTNNKKSAEDQKLRAKRRKFEKEKATKGKAKA
FUN_015578	CDS	scaffold_8	81112	83079	+
hypothetical protein		PF10680		ENOG41

MEASATTWELDSDEIASIASEDLHEHRPNRWTGAKSTWRTLTEEDRLLWQSMKQIKDQDLAVHLYNAFALKRRGRNPETAQDL
VVRTESGEEGVWAPPKAWTAWPLKHKHVPKECFTNEQHDEDDHFTFRREEEILPSTELEEEISATILRTAKRRFRRRKSKMAK
PSIEEDSTPLYNESGNSTPDPLSRESSAKLETDEGKEEDEDEKMAVDEQDGRRTRRNPKTYEPVESTNDEASYALLRPPTRHI
LSQLDNTLKILHNSRITGMNYLSDSSTEEESDNQSQSGQKRSRGRPRKILHEVDTSASTSPAGAGKTSRRGRPRKVHIPHAGE
SHEEMLQRVARESHRKIPSTLEDRDAAFEEWIRKGDEIIERERSRSLSLKRATSQGAETEEESVPGTNAERKLLRWGLRDWSD
VLGAAAIAGFSGDVIARATRRCADLFGEGMVVRTLKEVPATNDKGTTSVEYRPEPIQLSYSDEEANDESDDAADLIQRRIASR
QASLAYSSRSPDSVRSPSRHSTRSPGPSSAARSPSSRSNSAGGMLFCPIPSCDRAAQGFARKANLRRHMELIHQGQTEELDSD
EEVAGGVHVDGFLKPIVPGRGWRGEDTIQRKRKRFYGDKPGMSRETSYAEEDLSS
FUN_015579 tRNA scaffold_8 83331 83421 - tRNA-Glu

FUN_015580 CDS scaffold_8 83775 87366 -
hypothetical protein PF00225;PF16796

MAPGVRAATANSSRSSVRAGSRAPPTRYGSSLATRNGAVSPTGSIASVNTVNTIGTKRKERDFEAESSAEETNIQVVVRCRGR
NEREVRENSNVVVTADAVKGKIVELSMGSNALSNRSYNFDRVFSQAADQNMVFDDTVKPILDEMLSGYNCTIFAYGQTGTGKT
YTMSGDMTDTLGMLSDEAGIIPRVLQTLFNKLELENAESTIKCSFIELYNEELRDLLSSDEGNKLKIFDDTSRRGHLSTIVQG
MEEKHIKSATEGVKVLQDGSLKRQVAATKCNDLSSRSHTVFTITTYVRKPNEHGVDALVSAGKLNLVDLAGSENIQRSGAENK
RAAEAGLINKSLLTLGRVINALVDRSSHIPYRESKLTRLLQDSLGGRTKTCIIATISPAKSNLEETISTLDYAFRAKNIKNKP
QLNPMVEKKTLLRDFTMEIEKLKSELIATRQRNGVYLSNEAYEEMTAQSESRRIVNEEQAAKLETLENNLRNKVQELFSLQST
FMGLKKDHEGTKAQLDDTKEVLDQTEIVLSATRKSLAEETKIRKAHQKTEQKLTEVGGELINKLHKTVRDVGGLHAKNKRKSD
LQSINRNTWTTSQDQVADVTSMVERRINEFQEEQQEHIASVGQRMENFVDEELRKLSTTQTFLDEHLSTFTQSKKDLLQSKQK
SKEDMDEVLEEIKVVRDTVKERMGESLQSISHSAERIAADMLNEMTAFHGQLHNSYSAIGKDFKSTFEDLVKHITAQRAECDN
LKRQLQAATNTIVLQNATISSRIQDALVEERRQAVDERQKLMTQITALINTHADAQESRLHDRASQIQKSITATSTNLEQAVD
TYGEGMSTWDVKEGELLDEVKKSREQLKTKLKDDWTAANDHSSSIQATAKSVHAETVRVVDEQIKDLDVQMEALDDFVSRAKT
ENGHHHESHSVSVQSLSNTVEESFGNISSHFKSTFDRVKNLGEEMELDLGDLQDGLEPLNDQLCQPLANLREDITRAALQEYQ
PTGETPAKVQYHYPTDLPRTEDHDLIISRIDEVSTPTKDRDASDRDGTMVFADLDCPQKMMTSPVRPPPSRMSNASAPEHIGL
AGSLREVNPNVPNHTTGSIGFDPRASIISMPPERTLPLFKRSTRVTRSAKKVGVRDPIISEGGENMLPTALEESLSRRKSPRL
N
FUN_015581 CDS scaffold_8 88033 89510 -
hypothetical protein

MEVDSDNGPLTLFASGATVEKDVWPTVRSDIIARLDKIAHNEFPIPKLPPPPPAHQPPVEVRASAPASPQAESSQETNKETAA
TESLPATQVIDGALPKQLADMVIDITKHLETFETHPPHTIQRLAELILRPRAHYRAVAPYLHAVDRVVQVTSSTKTYPLPPPI
PDMSSMHLNGEDPKDPAVSVSWSNPTTAALGTDEALGGALLTPIPWLTRRSPESSQDTPGAQIHSESTETIEGPNGVGSIETV
SVSVNGIPSTGHARGVTQGELLRQEQRAGVVPVSQLSRAQEVVHEGERMTMDEEEDEEPHEEEDEIPHARGPEEIGMSDTGPQ
STTTSFMSEGSMGMQGIDVEAAVGRKHDDDEEGKVDVDADGDAESESEARSPGAESVGSKREADEDLESETHKKLKEEEEAED
DKKADTVASDVKEEEKKGEEAKAPASETKSEADDNKMDTE
FUN_015582 CDS scaffold_8 90033 91221 -
hypothetical protein ENOG41

MCFGSKKEANNDPAPRPAQRPASSQSQGDVKKSKYNPQNDPNQYAGESSYSAPPGPPPDRKQASDFAPPPGPPPGQSSSPQYS
SPLGSPPGKAAAQYDAPPYSAPLGPPPGRGDYAPPPGPPPKHDWEVAVPDTSLFPPPPAIFSGYDRSPTSNATEEEANAGEDW
CQSYPLTAPINLDQTGKTALQAGNIRLMEPVGFSGKLTWMEPGHWQGWSKKNSPDRCIIGYPPLYSVTEHDPVRLGQPKTIYY
EVKLARDSPEVFIALGFTALPYPSFRMPGWHRGSLAVHGDDGHKYVNDRWGGRDFTQPFKRGETYGIGMTLRSVGGPKPQVDI
FFTRNGTMTGGWALHEETDAEQDLPVTGLEGFHDLSCAIGTYDGIKFEAMFEPSKWLYNPFQF
FUN_015583 CDS scaffold_8 91837 94322 -
hypothetical protein PF01699

MKEASPDPPNIDGSRSGAQPAAPTAQGLNEHSNPEHKEFNEKQNGSSGSVNGGDRQGPSRNNSSLLKALSAGDVPSHTSSQAA
RPGSVIETPNKAEEPSQENPNPTSKMNRFFAEVKRSVKISIFHSWLNILLIFVPVGIAVSQVDGINGGIVFGLNAVAIIPLAG
LLAYATESVARKMGDALGALLNVTFGNAVELIIFIYALTKDEIRIVQASLLGSILANLLLILGMSMLLGGLRFREQVYNSTVT
QMSACLLSLSVISLVLPTAFHASFRDNDLADRQSLKISRGTSIVLLLVYVIYLMFQLKSHAYMYESTPQHIIDAELAPGPAAG
LLDSSSSDDSSSSSSDSSDSDSSHGTMRKKMKKVLRNRRHRRSSTASVDTADTKGTRSASFTTTNTTPQDEVSEATASRHRFP
RFPSYEGSESAIEEEDEEEAKRSRRRRRYRRHRHRKSKKQARAAKKAAGVTEAQNELPENAPQDNSECEPRRVDFAVQDDLEA
GPSNTGKRPFPLRGLSVKNIAPVFTEKSTVASTPVPHVRYGIRRTNSLPDRLQFRAPGAMLPSSVPPILTAPGAQMIAKEQEN
EEDHLTQSGAIILLLISTALVAVCAEFLVDSIKEVVETSGVNEVGIGLIILPIVGNAAEHVTAIKVAYKNHMDLAIGVAVGSS
IQIALLLTPVMVILGWIMDKDMSLYFTLFETVCLFVSAFIVNFLVLDGRSNYLEGALLCAVYIIIAVVAFFYPNEQQASEWGG
GS

FUN_015584 CDS scaffold_8 97261 100367 -
hypothetical protein PF00069;PF07714

MPPVAPPRTSSNHQSGSSRRSQYSNEKSTNSPRRNESTRTGSRGDSAAAENGHRSSKADAAARASSRDGRAATTSMPVRSAHS
TPSKPVHDATDSLTKAIAAAQDTIDRDRNHAAAQHSVEDAAAPPPVVATGDHHEERRGQRSRHDHSRSHKGNTKFGDFILGNT
IGEGEFGKVKLGWKQDSSVQVAIKLIKRDSVGSNPSRFAKIYREVAILRGVQHPNIVRLIDKVETDRHIGIILEYASGGELFD
YILNHRYLKDNAARRLFAQLVSGVGYLHKKGIVHRDLKLENLLLDRNRNIIITDFGFANTFDPNEELSKEEELNLTDKEFVKR
MGLDRVKMNGSRKGDLMQTSCGSPCYAAPELVVSDSLYTGRKVDVWSCGVILYAMLAGYLPFDDDPANPEGDNINLLYKYIVT
TPLTFPEYVTPHARDLLRRILVPNPRKRADLFEVARHSWLSEYANLVEFITSSTTLPSEVPDSGVSPDDYAEAPTIARSSSVR
EASKQKQSHPPVVGGLAKTHGSVDPESETTHRSTPKDAKRRTVQVEYVAPTTKTQRGADAAPSKPSSHSLQQQVPVDVPEANT
EKPLPREPPMTKDNPSRTQSRRPQSSHKSAVPHRPARDTRATSDNAFMTGPTNTARPRTQGSMQSSASMGLQSHGNYGQPAPP
TIADTNAHGRIQQPQNPEDEENIAKTVGSVPPKVMKMSTFQNETKSTGRGHKRSNTLGDLGNKIMGRSGSIFGGRSKKRPEQQ
QQPDKSRKYPPVSMPTTMMPGEEAGPRPSMESKASRRSFSLGLGKKRSGSVQGSSDKKDRRFSLVKAMGLGKDQGSVTGSEAD
SQQDLPIQHPRAEQLRGYSAHEEARHSEPYFDAPYEQYPQRDTAQSSPVYHTRHANAQQQDGRRPSAIPTYIQGSHLNTGSDS
SVDVRRPPTDSRSRPYQADFSESEGYDGRPVGSSRDDRSSVLQKSHKKFADAYDGENYRGHEGSSGAAKRVMDFFRRRGKARG
GEDR

FUN_015585 CDS scaffold_8 106100 107670 - GWT1
Glucosaminyl phosphatidylinositol (GlcN-PI) nositol acylation protein EOG09264FWI
PF06423

MSESGSYKQRKEDFVSNLSGGSVAEINYVTSVAAVAIILWSVLQARQSFFEPYTVLAFAVDFLLNVGAILLSITLYSKSPLLL
NLLLIAPAILIFVLPPNSTSRKKKLKVPPNARSKENSGQLDILSTKPFLTNFRGCMMIVTCVAILAVDFRLFPRRFAKVETWG
TSLMDLGVGSFVFSAGLVAARPVLREKAIGRTTGRATPLSHRIVHSLRHSIPLLVLGLIRFLSVKGLDYAEHVTEYGVHWNFF
FTLGFLPPFVAIFQSALKWIPSFAALSLLVGVTYQILLETTSLKAYVLTAPRTDLISMNREGIFSFIGYLAIFLAGQDTGMFV
IPRNIPPKSTASPGAQRNTLLMTMAVWGGVWTGLYLLSTNYHYGFGLAVSRRMANLPYVLWVVAFNTLQLLGFAVIDTIFFPT
FYNAQDAKTEKEAYMQATSRVIRAYNRNGLAIFLLANLLTGLVNMTVNTLDATPVLTMGILVAYTATITGVAVALDAYNISIK
L

FUN_015586 CDS scaffold_8 108506 109730 +
hypothetical protein ENOG41

MTTVASEARPRPSIEPIRTSGPGLNVRSLRSRIIQKSPSWTPSPLSPHTRPLPQLREREDDDTKADQLEPRKRDLPKLESGLR
NVSLRPIAPLSLPSPGPSTLPSRRSPLSAPLLAPPVFQRPTLADETRSMLLQESDAERGGESPYVDCERRQSAPGGPLHARAM
HLARQREHHRRRWIAASYRPREPEIFVLPVELCRLSQLSSSDAEASLPYPSERRLTTRVAVHSPGRKSIILTRTFDLDELRAT
LPVMSPLERHKENRRASVVTLQPPSVSSRASSPGISHERRHSHGVLPRVDAKHSPAYERRGSRQGCGPLAIRLSYARTYLPVL
AAVILSEHVRPGATVELPLPHPHAWGDTVAHVYTGRVTLTEPIKQNILYLGGSV

FUN_015587 CDS scaffold_8 115760 117565 - GT2 Type 2
glycosyltransferase PF00535;PF13506;PF13641 ENOG41
GT2_Glyco_tranf_2_3

MSESETQSIGRSHLPGLAGLLALPTPSFNFWYSTTFWLYLFVGLWVHRYLRLLVHCVSHWTYKSKPIPSKPTFTSKDVTVVIP
TIHNAFEELRPSLESILACEPAELILVTTHDKRKDLQRLADSLVFPKVRVLDTPIANKRLQVCEALPKVETPITIMADDDVTW
PSTLMPWILAPFEDPQIGGVGTCQRVRREHDGSWSTKAWNWLGAAYIERRNFEISATHNIDGGTSCMSGRTGAYRSEILSSHD

FLHGFKNEKWRKWILNADDDNFVTRWLVSHQWKTWIQYDKECEIETTLENSTKFLYQCSRWARSNWRSNWTSMVTERYIWTQQ
LWCTYALHFATFTSLAFVVDPLLLASCWWGTADWEIQNRRYAFWTQFIFMFAFTKVVKLMGLFIRNPSDIMFLPVSVIFGYFH
GLIKLYALITLNMTSWGSRADGDANDEQRLAPAPQPSIVLKTPPGKGSLIRYNARQKGRQPQAQQVAWEKSDYAAYDSSTSYV
PIRVHTPMAMTPNNAKLHANETSINSQCWVI
FUN_015588 CDS scaffold_8 129331 130744 +
hypothetical protein ENOG41

MAQTSSSLPGHLHGHGFGHGNQYGHGHAKITKPRSRPAVKPILKKLQSHHSHSEKNSLDLDRGWDEQGQFGYSSYNVDGDDSS
YTAGASALPTPAARARDVSFSISATDLSNGGARSNKYSHGHARSTSGTSHASIATSASGGRNGSFVHPFQQTPRTSTPPLSYA
NSLASLDNTTGPRDYSPTITENEDNIDSQSLQTSTRAYHSGSRPRRPSLASQRTSSLSDVNQPLRITTNGRAAPGISARKPHS
IVTRSRSDLHLNTGSTTALDSASSTSPPSGSFVSPQMIAASTSSNAMSPLRSSLDMSGFRLRSRSEVDTVTRQENVREARRKF
EEKERAKEEKYAREQIRKQERAESREAHRFNKNGRKGSFGTSRISCDRVSSSTDIRPSISRKTTGTGVGLGLTTENEKVDFGT
RSYDTVPTGQTPGARADDVHFQSTHRTKTTKRKTTSTWTAFVLWLRTRLLKLGRR
FUN_015589 CDS scaffold_8 131959 132868 -
hypothetical protein PF00378;PF16113 ENOG41

MASPSLPDSYATLNLPTLQLFHHPPSSKEVTHVIVIKLHRPEARNAFTDTMAASLSHALNTLSVDPRVRAIVLTSSDPKNRMY
CAGMDFNEEHALGKDAADHRDSGGIVTLPMYRCNKPVIVAINGSAVGVGITMTLGANIRVVSRDAQIGFVFGRRGFSMEACSS
FFLPRLIGTSRALHLTTTGAVYPATHKLFDGLFSEIVAPEEVLPTALRIADEIAANVSGVSARVMKDMIYRGASSPEEAHLLE
SKIFWDLFTGKDAKEGMNSFLQKRKPDFTGTMDKNAPKIYPWWTPVDIARPKL
FUN_015590 CDS scaffold_8 133797 135881 +
hypothetical protein PF14420 ENOG41

MVYQWEPHKDVCYQLYINERRSLEDIMVHMKNVYQFTPSKRAFQVQFSRWKFPTKQRLKHKDDRLVKRIHELWKRNVPQSEML
RILTDEDGFDINSRELMRVRARNRWLLRVRNGDRARSGDAEPDDENLENDTSLDDGDVTTLLPDQEPRDLDHPTPYAPENSVQ
EDPNSVPTTPKPRKISKRQSRRNRQHLTESEHIVRFPSEMTLNDSKEALGLDATTYAEARECFARICQEESIVKKTQAGPGRW
DYVKNRLIHERPHLQQALWLSKEDLERKQLALDVICTDVTKRRRNQDTRMTLVDAKNELGLNPEESHEIRAALHEVLCDAKFT
CKSDGTPEQWEELKKRWLEKSERMRAIPLHGNDESSRRKVKAVEIVARDVIKRRRDEKRSKTTKSGEAPKQKESTKRKSQNDK
PQHTVQDSHLTAAQPSTYQQQAQRAMENPPAFRMSDRGAPGSSSPADPMMDQPMDSNGYEPVHEPVDSRIAYTTTGQPPAPQR
PGQVQLPALGMPASQGVLPQPHRMLGSSAANTVPMDAQYGSSMYLGNQPQANYMPQQYVQHQFSASAPNTMFSPVPSVPAVPS
SFAVFMRLHPSSTYPANTSLWISTMSSQSIQELRQVAVTKFPGALCVRIEGIIKDPKGNELPLQIQGDDELAAYFDHMQSGTP
TFTVQLVQHGSI
FUN_015591 CDS scaffold_8 136851 138803 +
hypothetical protein PF01565;PF02913

MASLQLRAQRAALRSLRTPTTASCRTMRLPASFSRSYATEIEPERPNKNTPTRQSNETKVGRTFQGQVMGSIGSRLRREREQR
EKYEEWRNMTDPTRNWMVTFVFLSGVGISYWLGTYWPRDPEPTSTLPLNKSKAPKHNTKLENMQAAWADFVKIVGQDNVSTAE
NDLEHHATSSWSSHQAGPEDRPFCIVYPSSTEEVSEIMKVCHKRKIPVTGYSGGTSLEGHFTPTRKGISIDFGRMNKIINLHK
EDLDVVVQPAMGWEALNDHLGHEGLFFPPDPGPGAMIGGMIGTGCSGTNAYRYGTMREWVLSLTVVLADGTVIKTRQRPRKSS
AGYDLTKLFIGSEGTLGLVTEATLKVTVKPESTSVAVCSFPSIRHAATCVSKVVAQGIPVAAVEILDDNQMKCINDAGMTSRA
WTEAATLFFKFAGTPAGVKEQVAQVQGLAKQSGSKTFEFAKSQEEQDELWSARKEALWSTTAVKKPGDHVWTGDVAVPMSKLP
DLIEATKEDMAKSGLFASIVGHVGDGNFHTILLYNDAQRAKAEAVVHRMVKKAVEMEGTVTGEHGVGLVKRDYLPHELGESTV
DAMRQIKKAFDPLCLLNCDKVVRVQKPKKGEVDEW
FUN_015592 CDS scaffold_8 139736 140138 -
hypothetical protein ENOG41

MSTTEKKNTTEPVDKEAIRELINELEHKLDALYTLSDEVNRGILESKDALESVREKRVKVLRERLDRIFQQWNIGSLTSNHHP
YVTSELCAFDAEYSFRSTQNQSQNPGPGPGSSQAQTQAGINQQGQFGLPW
FUN_015593 CDS scaffold_8 140511 142100 -
hypothetical protein PF03676 ENOG41

MSLFTAQLYPGRALGFLVLGATLHDVLTRLKAEPQRFPKIDLLYSPGRPVTEPVGVELPGNGIRLRFDGPEQRLRLIEVTDFT
KNHITFKERDLVKPFAGTPGSPSPGDSASGPTFRHIYHKLLGPTYAGEFIPPSSGHDNGIYVLSYPGVAFNFALNPSEYSPEK
DVVSTFASASSQVATSMAVFSGDSWADARPTLWTEILPSIKSTTVIPRGKDVYPDEVSLVRLHGAGKIQLFRKWTSNSFWITL
GETTPQDLLMELGPPNAIYRKNDQKMVIHKTRTASNAKGCPDAADIGRPEDLTDTDQSSMHEGSDASDNEEAVDDQIGLNTNG
ECFYNYFYLGFDILISTRVPPSQTPPGSKVGEAEGQNGIISHSPDRLVATKLVLHGNIPGSYEFNRHRRCRWEIAYLDSLNDG
NGPTDSETPFPQIEDRMNAAWASAFPRNDSQQRQRGMVLNRGWGDSPGSSCEFLGGWEESGARRAETNGDSTTTLYGFPGLVF
EVLKNGHVNTVTVF
FUN_015594 CDS scaffold_8 143031 145006 +
hypothetical protein PF01425

MAPSDFADYPKPVEAPAATYNPTVENNPTFRGIPLVVGANLIAKVGFLQQYFWDNAHFGTIKDIPALDNIPYRFHPTVTPLGP
AEPMLDFEPKLLKSQYAESKARYYTASDYHEMYKSGQVTPLQVAEALLPQISRPDGKYSDGWVDNHGKDHLVLEAAKASTERY
AAGKPLGVLDGVPIGVKDDTDVKGFHNHIGMKYDPSIETFKEQKESSWPVKKLQEAGAVVIGKNAMHELGSDTSGCNAAQGTP
TNWLNKSYYPGGSSSGGSSTVSAGVVPIAVGTDAGGSIRIPPTFNGVYGLKTTHNRTMFMNNTMCITGPIAATVADLTIAYRV
MSQPNPDCHIQSRFARSDPPPKGQKKVMGIYRDWWNKADPRVKEVCDRAIDYLANKCGYEIIDITIPYIPEAQLAHSVVCITE
MAESARRRTPNPADWLSLVGPANKVVMSVGTRTPAADMLKYGALRELIMQHLAYLFQKHPGLLIMTPTSPLIGWPIVPGDQAY
GMSDTNTTIRNMLYVFLANLTGTPAVSAPVGYVDPDQGEGKLSIGLMATGEWGSEEQLLAWATEAEEYLHNATESGRRRPDEW
LDVADLVKQKA
FUN_015595 CDS scaffold_8 145160 146301 -
hypothetical protein ENOG41

MDKLINAAKDYLDDDKDKRQEHQQQGHGQQQGYGGQDQGYGQQQGYGQQQGYGQQQGYGQQESQYGQQQQYPPAGGIQSQGGG
GFSSFLGGNVDFDNAKEEAHQKAGSSGDKDLFGSILGSLGQKQNKLADEDLDEEDAIKQHKNTYNNDDSQQDSKSLGTAAAMQ
ALKMFTQGQGQSSTGANSQTAFLGLALAEASKLFDNKAASGKVSEGTSKESVIQQAGEVAFKMYLKNQAGAGGQQGGASGLMG
LASKFLAK
FUN_015596 CDS scaffold_8 147250 148889 -
hypothetical protein PF00320;PF00989;PF08447 ENOG41

MSHGPPPPPPPPQGAPHPNNFNNFYGFNLDIGSLPDMTGGQMGADGQDNDLMAMLDNSILGGITDIPMNIESADDSKMSGSFG
NGQSTTEESVARAENQFNTAANNVPGAGPLPAGAFAQMNLAGASTLTEFTKRRNWPAKVVEELRDFLQILDANGRIKYASPSI
LQVTGYSVEEIQDVFLKDLIHPDDQGVFVAELNESIASGNPLRLFYRFKKKDAEYAIFETVGHAHIAGAKFAPNPNNQSPFCQ
AVFMMARPYPTKNAGLLDSFLEHKIENERLKRRIAELRREEEAENDEAQKQWLQSQEGRSDMTPSEGTGVSSGTFYRPVSERG
MTEADRAALNKALTRENLEGSVAGGRQDSLKDKMARYEGASHTDTIEMLTGLRYIEGERSRGITTGNASPTLIKGDAGIAIPM
ERDPRTGDKKKKLKTSEEYVCTDCGTLDSPEWRKGPQGPKTLCNACGLRWAKREKKRTSTSHPPTVDQSV
FUN_015597 CDS scaffold_8 151602 155580 +
hypothetical protein EOG092606WZ PF13476

MSRIDKLSISGVRSFSPSVREAIQFNTPLTLIVGYNGSGKTTIIECLKYATTGELPPNSKGGAFIHDPKLCGEKEVMAQVKLQ
FRSINDRQHVATRSLQLTVKKTTRSQKTLDCSLVVVNNGERTTTSTRQAQLDEMIPERLGVSPAILDAVIFCHQDESLWPLSE
PAALKKRFDEIFEAMKYTKAIDNLKVLRKKQVEQLGKLQNDEAHNKVNKDRGDRAEKRMKALQAEIEGSRETCESISSEMEET
QDKIRNIRETANSHLAIVQNLNTKREQLEYREEAVKELKATIDELPDDDGKLEGDLAHYEDRMQHLQDEADQNKAQYNELQTE
LGKSRKELSAKLSEQGKHQSDKDKYERQLKIRMETIQEAAQSYGFSGFDGDLLDHHVKSFNDRLQKLLSEKKRDLERLQKENS
AELDRATGVITELEGRKAARIQDRVSAKQRMGAIEKRTSVLQNESSQIDVDEGAKAVLDGQMQDLEIRFQTAQKTFENADWDR
QISDENDKLHQLENENDRLGRELVECTRLASERAQLDYRKKELGDRKRKLDTLTSTWKPKLDKILGSDWQPETLDSKFQALVK
DQSKIVSEVQKCREQIRQKQQKVDFKLRTSKESHEKKSKEAATCQQQVVNALLAVRDNATVEDYKEEIEVTESQVEELRNDLS
LFDALVDYYTKCKRLLDTKRKCLLCDRHFDDNQAASMERLSKRIDKHLDPKGKVDTEKDLKDTVANLEKLRSVRGSYDTYERL
TAELPSLREECKVAEAEFDALERQVEEQGSVVSAEEEKQKDLEDMSKTVLSIAQTVKDIAESENQVDRIMSQQMSGGVTRSPD
EIHELQAGLSDQMRNLKNRIVKLTTDRQRTKDQLNSLELEKSELRNKISRAAGQLDKKQDLQNQIQALKEEASHQRDVIQRAD
EELETIEPSITEARSARDDVLRRGRAKEQVIADARDAVANSVNEMKMMDADIQDYIDRGGPSNLASNERSIATLEKTIASTEK

EVTDLTVRTNKLKQDIDNGDRKKKNIKDNLNYRKNLRTIDVIRQEIAELEDRNADEDYERLQAEARMQENHYNRLLAERGSVM
GSMKTKDEELGRLLQEWEMDYKDAKRKYRESHIRVETTKAAIEDLAQCSSAVDKAVMQFHSMKMAEVNRIAGELWQSTYQGTD
IDTILIRSDNESNTGKRSYNYRLCMVKQDTEMDMRGRCSAGQKVLASIIIRLALAESFGVNCGLIALDEPTTNLDRDNIKSLA
ESLHMIIKARQAQSNFQLIVITHDEDFLRHMRCSDFCDSFFRVKRDERQNSVISRESITKIF
FUN_015598 CDS scaffold_8 156766 158587 +
hypothetical protein PF07690 ENOG41

MAASPPTPERRTSSDSTLDVERDGAHAANGHSQDPTAAEATSEGAETTPAGSNPSPGAPEAGDPEAGRTKSETILVVGALCLA
LFLAALDTTIITTAVPTIANEFHSSQGYIWIGSAYLLGNAAFVPTWGKISDIFGRKPVLLAAAVIFWIGSLLCAVSNGMPMLI
ASRAIQGIGGGGLIVLPNIAISDLFSMRNRGMYFGILGMVWALASAVGPILGGVFTSQVTWRWCFYINLPISAVAIVILVFVL
KLHNPRTPVKEGLLALDWPGSILIIGGTIMWLLGLELGGVSFPWDSATTICLIVFGIFVVGLFLVYEWKVAKFPVLPIRLFHT
QNSVASYGVGFTHAFVFMSGSYWLPLYYQGVLGASSLLSGVYLLPYVLSLSFVSAGAGIYIKKTGNYKLVIVLGLIITVIGFG
LFINLEPRANWAKIIIFQIVAGIGIGPNFQAPLIALQTNVEPRDIGSATSCFSFIRQLGTSISVVVGGVIFNNKMESQYPRLK
EELGSELADRLSGSNAAGSVELVGSLTGHDGRVAKTAFWKSLQTMYIVYACFAGLSLIISLMIKQVNLSKEHKEHKTGLKSLK
RRDDEKEDVDAAEGVTTGNEKTTDN
FUN_015599 CDS scaffold_8 158933 160506 -
hypothetical protein PF07992;PF13450;PF13454 ENOG41

MALADKLDVRRIAIIGAGPSGLAAARYLLAEKNFSKIRVFEQRATPGGVWNYTPLAREQGFSVPRTQPSFTPDQALWPNDNGD
VEFMSPIYDLLETNIPHSLMRYSDKEFPQGSSLFPRHSVVLQYLKEYAQEINPHISYQTQVLNIEKPDPSRSQPWTVEVLDLK
ANKVIKDEYDAVVVASGHYNDPFIPDIPGLVDFDKAYPGAISHSKFYRRPGDFKDKKVIVVGNSASGVDVSAQLSTVAKQPIF
VSEKEKPTVIPPAKEPWAAGVPEIVEFLPSQRGVRFANGQIENDIDAVIFCTGFHYSYPFLKSLDPTVVVPSGGHAAHLWEQI
LYTADPTLSFLSVPQRIVPFPIAEAQSALIARIWSGRLNPPSEAAMEAWVQEQHEKKGEGKAIHVMAFPEDVDYINRLYELSK
TATKAPELGLENDGEGKRPPYWGLDKRWVRERVPKIKLASRAAGDKRHELRTLEDLGFDYEEWKRTVEEGEKLF
FUN_015600 CDS scaffold_8 161373 163614 +
hypothetical protein PF00172;PF04082 ENOG41

MASASDMKASIHRFRLRRVSPSPRDVGRVRSASPPVSPDQRLQQQDATQGEAQARRQDQQKQSQTVVAGRSATGRPIAPACDR
CRSFKKKCSRTFPVCSLCASAGQRCSYTNPVANAEAQVHQLRSRVQWLSQYIEQNILPPGRQGVIEGIETGSDLGIVLGHGPR
NGMGNIATSGTASSPLSRVPGQMASPLETIGSLPSIRGLDHPSTGRQSVEIRLEERDPGSVRTTSSRLSYGESTDGSVTKRRL
IARQALPPDIAPSRFVDAFFRHVNRAYPFVDQTRIRRDLELLGDASPADQDPPMTLLYLVMAIGCTSLVRAGQIPSDTARRFD
VVYPDIIQECLSNETIESVQILILLGLYSLFDPAATSAYFIVGIAARQAMVIGLTRPDEQPRTAVETELRHRLYWSIFVLDRM
MSASQGLPAAFTDEHANVPLPGLTVEEFASPDRAVYARNLQTSRHVIQLRQLEYRILHAVHLQQNSETARLAPADRRTVLNSL
RSEIEDWYSNGCLMSPMEADNLPIHSSITWLSAQYYHLLILLYFPNHFNSSAAAVTRQEQLEFAQKQLQANSALLQQRQLPLN
RVSLSRLLPVCLLLMHDFVASYRADGASSSAREEVVLLITLLEAFPEGWTVAQEAARIMQQFAGVLTGQGGMATAYYGETIEE
LIKQCIASFTGLLHQFLGKATCFQSIEYAQETQEVGRMGQIGATQQSSTNSLWLNGTDSSIGGVNEEVVLGYGWGSWDLDFL
FUN_015601 CDS scaffold_8 166445 167870 +
hypothetical protein ENOG41

MYTVATRLESRKSLLGADTGFGYGGHSYNNSYGDDSYAPSPVPSPQPIPQPAAFAQPNFADAGVDPAYNQGFAYREGLTPSVP
AQSMKNGKIDFMKSRWPASFMAVSLLQAILCICFEAYVFGKFQVNLGTHISAPQVQSQYKTIPTFLTLFIFGFLYTLVVVWDA
LRQKNTIQVIGVCFSNLALMVYTTIQVDQISEAFDILEQSNALKRGMTAAELWHDVKPYLVAIPAIIALATLVMGFLSWKIYQ
EYAWDILKNIGADYRMKKRFLHYQIYIALLKFDFFFFLGFIVQFVVVVADKDDPEFALTIVTIPITILILLAAAFFTRRENKI
GMVCIIILYLGGLSYFIFKLVRIYQPGHRQNYEAVRRSLTAFAVITILLILLTITNAIVCMRNFGKGLKPHLQSLSRKRKVEE
KPDINSINMQDVKPQIPSRMTID
FUN_015602 CDS scaffold_8 168687 170082 -
hypothetical protein EOG09263QPR PF10607;PF13445 ENOG41

MAEADNDHVSPFAPLMVELARMRNRTLKSAIDDVDQVIELLTNAREQIAQEQDATRTGMAMMTLQNPVKARFERITTDLKDVT
KAQKSFGKALDKALPHRELPMETDAMADHPRLINRAIAMHLLREGQFSVASTFLKEATDHPPGREAHTISQTDEDGDDDMESE

DDLEDEMAGLHSEHLQNKFAEMYSILSQLKYHNLLPAINWAHANGVHLEARGSTLEFELIKLQYVWLFKGPSVNGLPDDPATN
GLGGAINYARQNFPRFQNRHLLEIQQLSSAVVFAPNLAKSPYSHIFETESAFEDVAMSFTREFCSLLGLSAESPLYIAVTAGS
IALPRLIKYTTYMREKKTEWTTENELAFATPLPEFMIYHPIFVCPVSKEQTTQDNPPMMLSCGHVLCRESLNNIVKAARYKCP
YCPTEGHLKDAIQIRL
FUN_015603 CDS scaffold_8 170867 173615 +
hypothetical protein ENOG41

MAFRFPDPRLRVDDELSAGTTAKGSVARTATTSNSSTTRESPPNGIPPLEVSAYNEVADSQPLPSPRRKNLVFADPFAFRYLE
EGSNVVVVERRGILRGYESYLVEQWACSRKPPTLLIVTYTGDEKHSVVVGVLSVPVDESLWPPRLRFYFDQLQDSHARPKETE
LGELMVTNLSNFPSALTVIMVPEGDIRKYRSSFIVNEDLKRLGCSGRSGMTLSDPTEATQLKFLQLYRTSDRVPVPQAVVELI
KLCQVTLYMFDKLGHQYIDGLLCDKTEMAINNWWIEIGAEHYNFEPTDGILGPSTVAALLGMFMGARNRLHWYGAPVSKDVFD
LENTKRGIGHFQKSQKLEKTRRLDRQTLLKLYIVSAKAAAGESWGVQRAVKSTLTEVGGKRGELVMGMVSGKDKGGLADIETL
DIGTFASLAYGERAKWLWHGKPRRSVAELPHHSPELSSAVPWKEEEANPNPKRVYSAPAENELDAKRLDETPDVYSGHPPGSA
VSVADGSGDKEASRKNIFKGVAGKMNDARSGFGRIKDVVGGGLRGHNNRPSVSTRDDFSESGPRSSSQGLTPGTTQPPAPTQG
VGRTFTWNNKPQEYLAAMKRADAEGGAGFPQLSHFASTSTVDIKQPASAPVETQKREQDGELFDIGVEVRKDVLSKAPSAVGS
LVDEHDLQGPVLDAELNDDLSKRGLSRRHSIQLSQCPNMTVLNESRWPRRMSFGDAEEAVLEWEPLIDVADAADDLANIEAFN
DLAQHFNQCLEEMKHRIEPWVIEKISAVEALDERYAKDKEEMHALYYQLNEACQRMRITSHELLAEERSHLLEGLKEVEALVA
RLDYEINALLQKVVDVEDGIVTFERQVEDMENRAEALKAQLETEGWLHWMFRTVTGIGTGPDITRSPS
FUN_015604 CDS scaffold_8 174032 174377 - TIM10 protein
transporter tim10 PF02953

MSFLGMGRPQPTSEQKIAAVESEMRMMADTYNRLQQSCQKKCIPNDYREGELNKGESVCLDRCTAKFLDTSMKVSEIMQQQGQ
ALGGGQQGGGGMF
FUN_015605 CDS scaffold_8 174832 176319 -
hypothetical protein PF00171 ENOG41

MATKLHTVPFLINGSDHTSERAVDVVSPASGEVVHRYHSADVKDANAAVEAAAEAFKSWRKTRPGERRDLFLKAAEIMEKRRD
ELRKYSMSETGSDATWADFDISTGISHLKEVAGRIGTLEGAIPTVSDPNTTALVLREPYGVVVAIAPWNAPYILGTRSVVFPM
AAGNTVVFKASEVSPRTLWAIADVFREAGLPDGVLNVIFHERANAAAVTAALIEHPEVKKINFTGSTPVGRIIGKLAGENLKP
VILELGGKAPAIVWEDADLDLAALQCTLGAFMNSGQVCMSTERILVHKNVKDEFEKKLSATIEQVFSSKGDAPILIASAPVAK
NKALIKDAISKGASLVHGNPDVEESSKTRMRPIVVRDVTTEMDIYKSESFGPTVSLMAIETEEEAIKIANDTEYGLSSAVFTS
DLQRGLRIAREIETGAVHINNMSIHDESGLPHGGAKASGYGRFGASAGLDEWTRTKNITFRN
FUN_015606 CDS scaffold_8 177064 177334 -
hypothetical protein ENOG41

MPESDKPRQSVKRQLKPAPQRPGQDVHCPRFKSEVHFVIPGSMPEIEILRIRTTDVSQPFLDPNSMQQS
FUN_015607 CDS scaffold_8 178710 181122 +
hypothetical protein PF00172 ENOG41

MDGSMMAGGAASMSPTSPVSSRHGHGHGNSNGTNKRKRSSMVGIESSPGSNLDDHDAEQEKKRQPGVKRACNECRQQKLRCDV
VQDPFQSCSRCNRLKLECKIESNFKRVGKRSKHAEMEKEIDRLRRSLARAKSQGFTPEADDDMDSPIAASNPTYTHTHTRNPS
LMGSDEAVSSLLHLKRGGNYNMPRYSHDLENVKLTEEDVNNLFRQFWAFYHQFLPFLNPSQAPDLFYQQHPLLFWSIISVAAR
RYNPPNANDLLSNLSGPLTRFLWSTIGEVPSNYYVVKAMCLLCTWPLPTSTTSSDPTHILCGVMMKTATGIGLHRPNNIQDFS
RVSVELNKEQLNDRVTTWAVCNIVAQSVGTGYGQPASTLYDWTLASRAGEESAFQLSRDLEARLQIERFCDKVSKEMYSNASD
PRGVAGDEHRAMLMRVYRRDYAELNASILSQGLGPIVSLHLRAAALHMRLAGFFDSSKTPGYMDDLMGLWRATTAFLDEILEV
DQATSPRDGVGGHLLLHCSNYIGQMMVAAGFALLKLMRSFFSKTIDFQRGRTLFHQTIRAIRATSVVNNDLQWRLAELMVQMW
NGARLEHNQHNFNSNDNSPIQMDDSLQLKVRCRHSMSLVFDSVWRWREDFQASGRGTIDSAKQPTNPDSANESSASSTHMDST
LVPSHTLPPSSLLSTNNTHNSNPNSNGALTPGATGLSAVSSAHSMMAGVSYGDAANYDFFDPQHWMLDGLLDFNYSYVPPLEG
A
FUN_015608 CDS scaffold_8 191281 192999 +

hypothetical protein ENOG41

MVEQQYIAPGLPQPDGLSGAPSLHDSIPTTETSPYHDLTSYSSVSSPFNSISGQCDPNLLTPISVVGSPPLHGVSKIASQYPP
PPSTPNQHPSPPGSSRMYHQQQWAGQFDVNSQSPQASSPMTSQAPVAGGEFLHANYVHDGRRTPGPPEPYMGAFGVSNGPEPQ
PMSNPYYVNMAPPVEHQDHIMMRDNHHIPMGMHHREMAPAPLLAEHHPPQYRRRSPEETGLHGLPSDVPRSMTGSPRRRSALH
APGRVKKRTTKRSGASRSAAAEEPVDEHKNCFGEEVPPTLKSTCPDEERCIFESRWEHRNQKGQDMWESIQSDYKSRFQKCPG
KEMLQMKFKRGRSKYIEWLSRDEDLLREAYKIVEKSRYQMMLETFHELGGSRNMRLNASDIEVKVVNDLKLEEGIYMESHGDL
NIRRRRKSVQTRKRTGRGDEHDEMMSVGSHNTHEDEVINQVHGLPNIKMEEDGPGGHMMSTHMWDQQMKMEPGAMPPQNNRMQ
PLMRLSPTQTMYGGRRGS
FUN_015609 CDS scaffold_8 195840 198953 +
hypothetical protein PF01532
GH47
MRQDHLVRLRQQTVDMFYHGFGNYMKHAFPEDELRPLTCSPLTRDRENPAHIGLNDALGNYSLTLIDSLSTLAILAGGPEDAS
GTGSKALSDFQDGIADFVRYYGDGRPGVSGQGIRATGFDLDSKVQVFETVIRGLGGLLSAHLFAIGELPINGYTVQPAWATQA
DDPLELAPITWPNGFKYDGQLLRLALDLGQRLLPAFYTDTGIPYPRVNLRHGIPFYTNSPLFRHMEGKPETASGEITETCSAG
AGSLTLEFSVLSRLSGDVRFEQAAKRAFWAVWGRRSEIGLVGNGLDAEGGQWIGPHAGIGAGMDSFFEYALKSHILLSGHGMP
NKSTTSRQSSTEWLDPNVLHPPLPTELQTSEAFLDAWHQAHASVKRYLYTDRSHYPYYSNNHRTTGQPYTMWIDSLGAFYPGL
LAMAGEVDEAVEANLVYTALWTRYAALPERWSVRENTVDQGLGWWPGRPEFIESTYYIYRATQDPWYLHVGEMVLKDIERRCR
APCGWAGLQDVRTGELSDRMESFFLGETTKYMYLLFDPDHALNKVDAAFVFSTEGHPLIIPKRSRQKPTRRHSLSKRVVSKND
TIDESFTHSCPVPTVPGLSGSATAARPDLFSVALFVDLHNTPNVHGPLEAVEVFDRKKGHVTRYRAKTNHTMFPWTLPPTMLP
HDGVCAAPVERVMTWIEFPPGDTASSLVSRFGTSLVWYSHNGPTVRNLDGMQLQLEQQKGGKVADRSWRITHAASRELGRHEN
VFFHAEHVRSYKDDAFTCIRRKDIVEIDLLLDTPEKSNASAPAPAQGVNVSSEILPSHAALPSESLLKSLLRAVSSVFEPAYT
DLPETETDGPGPTILRWTAYTSTGPGAFPLPPIIDTPMVGSPSYNSRKPVANFPWKTVFLGGEACGAPLPESSSRQHQIIVIR
RGGCSFSEKLERIPSFPASPHTLQLVIVIDEGDDSGEGPDEIVRPLLTTAQVTPRGMGRLNGVPLVLMRGAKGDYERFGEAKG
VGMRRKYVVESQGMLIENAIVL
FUN_015610 CDS scaffold_8 199643 200380 -
hypothetical protein EOG09264SSI PF00156

MSAQEPLPSTNSSSAINNTTSTAVASQDASGRQPSSSAAGSSELSGAKISLRKSLRSFPDFPIPGINFVDIMPLFADPTAHAT
LVDALELQIAEAFPTKPDVIVGLDARGFLFGPGLALRLGVSFAAVRKQGKLPGPCATAEYVKEYGKDLFQMQEDAVREGQKVL
IVDDIIATGGSAKAAAELVQQLKGEVIGYLFILEIPGLNGQEKLGTASVKILLEDA
FUN_015611 CDS scaffold_8 206299 208213 -
hypothetical protein ENOG41

MPSFFQFTQGTESHVRPNDSAPLLGRFRAVPPRPGVVSRRRSQLGLFSNTADNRGSVHILGYGAFPADDNDTDSDYDLEDDRS
LWQRLWQRWVLDIWVDPRQSAVKRVVDKWWSRYAFLVLMPALLAVAWCSIPFPQYRIPDDHIGDGDEDRLPDGSKTPGHGAAR
VQVNFWFFLFVYYGFYNLTALIWITKVFNLYSLNWWPQSLGFPLTVSLIAILSIAVPIPMYYNDKARYLLVHNTAWISWTFII
MALPVTIAFIILTTNERHIGLRHSLSETQRIFTTSWWTGEPDTFNGRDRRRRNVTADLFDADATQVEPELRPQGVRMRRLWLP
ASFVRFLWFCVALFIGLMAYVIGEAYAEIYLRTLPHNNLETVVYVYGWVATVHLLDALSGWVLGIREGERVGSYPLSWIFKLY
FMLTYQMYVRALYARLRSPQQFITLQILSSTGLVVITPIMMTSFVHKLLTILGLNSQSFGSYQKLQTRNVFIRFLAENVSMVA
FLGSILVLHFGANKDVYPYFAFDTEGEDYNFGLTFYASSVTWACELVASLAVRGLIRLFFQIDVGLEGKLDLAVWPELMPTCV
AVMLHVLQNMLFSIIRLQFQ
FUN_015612 CDS scaffold_8 208909 210223 - PEX2 peroxisome
assembly protein (Peroxin-2) PF04757

MTDSSFVQAQRRVAARRQTREAEAAARIAAQRQSSRPRANLERLPYPLNRIGSAWDAVSTREGTRPAFRVGQVDAELLDVELL
DILKDQVCEGLKYFGGGHLHDDWSAEIMLALRAVLFKLTVWDHDATYGAALQNLKYTDARNDGLILKAPSKVQKSLYGLVTVF
GNYAWTRWEDWLLEHDDGYDEPSPRLKRLSKLTSALSTVHSAAACVSFLVFLLHGRYRTLLDRILRMRLAPPTSQVSREVSFE
YLNRQLVWHAFTEFLLFVLPLIGIQRWRRWLTRTWRKTKDIIRTGPQEEGKANGEYAFLPERTCAICYQDQNTATSENEVMAA
AASSGVIGSAQTDITNPYETIPCGCVYCFVCLATRLEREEGEGWTCLRCGEHVKECKPWSGDLVEPSAKHASAKTVAFSDDIT

GGVLVDEQDEESFVESLSTTSE
FUN_015613 CDS scaffold_8 212783 214567 +
hypothetical protein ENOG41

MMKEEDLVNDQDDATESTPSPDLAGPGIPFPQMIASTPCSTIHPFDLYYANFHVTHSWLPPRKKLDDLCKTQPENLRFLVATV
TYIGSLYLDEVDKTQLQQNAYQMSHDTLSPTIWSVQALLCLSITAFGSQHGNVGKIMFNKACVLASCLGLQSKEFADRETDPV
LAESCRRTYWGLYTHEMLLCLRQNQLCSTLYPPGPSFVVGLPCEDWDYQAGKIPVPVTLEEYDRLGPSREYSSWAYFVDLIRI
YDSHVAPFLHDCSQAARHNLKYANQRIDSWRFRINDRKKYRVWEDGMFDMILYHALVVSFGLEIRMQAHLYGHWDQHIAASLL
HRVATEHDAQSPLVLQAPLRLMALLGSQLVPEKFSPSCLLDLEHAASPLCHAVLHGAGQPDYRSRVLFLAEFLKKSGEFWPRS
KALSDVFMEALGRGGHEHGYMELSQAMSAVGDTPASTTEGGSLWLRSPTVEHMGGWSGLPSYSLQCEPETGDVAWGGDLTQVP
GGDMTASDITAGAGSSVSNVYVEAGCLGDKSAGGSVPVKVEEG
FUN_015614 CDS scaffold_8 215731 217537 +
hypothetical protein ENOG41

MGSSRQRELRPEDSWRFVEGENDSFDTSILPSLGESSPPTSSGNIPSQPFSQGNLSFASQDSIRDFGADPDDEQVILREPFRP
SLSRASVSANGERVFRSPDPEFHMPRIDTASEGSSRSERTIRGLGIEYGGNEGVRRRGNPPPSSPPKQRNARARYETEDEYHR
RQYREQPPPTVGNQIAQSIPAAIYNTLAWFLNVIGLAFRYAQKPFAILLAIYLCFGGLIIAQNMATRSIAASLSPLCRIPGAS
LLNLPFCPSLDTIVPAGGDGSPVEFDDLMNVQSKFEQVLEKSSDGVSLPFEMKRSETAIRDLRSLVRHSDLQARDELLFEFDG
YIDTARQTSSDLQKFNTHVGSAVDAVISINRWTSRYLDTLSPETDDGKSISSPSPILGWASWLFHPFQPTTDHIFNERVLLDK
YIEHTALVSDRIATLILEAQAVLRLLTKAEDHLMLIYDISSRSSADTASRRENVLWNIWTLVGANSNQLNSLSRQLALLRQVD
AQRSSAVAQVNALILELESIQAGLGDLRDRVAEPEVLRNGIGSGPPIPLSVHIETIDRGVERLEDARSRIRAAENDRVRDALA
RGGVRNEPLLEGKGKRGEGN
FUN_015615 CDS scaffold_8 218158 220017 -
hypothetical protein PF00097;PF13639;PF13923 ENOG41

MERRQYGPVDNHPKPHGSSDIAYKERISTNITTLTSTNADSSNGLITGLLYVPDLDNDRECDQQQYQFIPRNVTRRDNLPPTN
YNLIALAPWFSIDCTLAYLASARLDPIRAFIFYKPNNSSNQPQDADSPVWNLEDGGAWMTANKYPIYAISGQEGQNMMRQLSY
YSGSVDDIPYGENISTLYGPNPKDYVRIWTELSVQDESDIPPLWAFFLIVIGALLAIFAFVSITMHLVQRRRRISLRKRVESG
EVDLEAMGIKRLTVPATHITSFPLFTYNADPDSTAPPPTPSSTRRMRSSRGFDQRSTRSGASVRSKRSNIGASGDNAATNFQP
SCHICLTNFEHRVTIIRELPCGHIFHPECIDEFLSKNSSLCPMCKHSMLPRGYSPRITNGMVRRERALRKLRQRVDLEDLSEE
GDNTKMKDWSKRLFRTSPSPSRPSSSPLVTLKLPKFRRQPVTNTATNVEGRVETPPVSSTLAQFQEPEPEPEPNTRPPAPAQI
PKTRRPRPRLLNMLPTQPENSELNTDPTPRRGSPSSFARQRMREIAAKNAPFDDPDSQRAIWLRALSKVFPGYF
FUN_015616 CDS scaffold_8 224169 227199 -
hypothetical protein PF07217 ENOG41

MFTFRSHSLLVGLVILVALASPAFAFGAGNIASISKVEGQNWRHGDITDALLILSQAKALNGKKFNKINVSRVYFGNWLRDYS
QAIDVGTVKSVSAEAIRLLLCVLGFMTFGYGSGEFEVTAERLGCYRPEDHIDNPKDYAENVDARQYDERLRGPVDEDRELAID
PETGMKNYIANERARIMTSARHVKKLFTGTIELGRRYKDSQRKADKYEALRLMGTGLHCLEDFFAHSNYCELALIELGERDVF
PHVGRNTQIEIEGARGPVYPIVTGTFGGVDFLHSVTGEVSDKLTQNEIEELEGTLQQGAKSDTSMLRDLLDKIPDGIFGDKHQ
SDRVDELQNNAAAAQMENTTVSPRDPEEFTIYIQNVFKQVMPAIEFHDDIMKSISGAMEKIPVLPKIIEQLEEQLSVFIFSII
APFIVPLIQQIRNELKTGSDEIIASSEREQHIVFHDDDSSNPTHSMLSKDHFSNILNEIAGRNAAAVVQWVVPQLMDAIDDES
VDVDRLLDDIVNGVMHHPAQRDLGNRKVQEGRRRSYEGVKEWWNNMGDRQRDDYRQKLTREGVQNGENHKEGVVDTGHGHGCA
GKLQMRKLYGGGPETLEDKIAGAAADAIFKGATGAISGMVEQNTGYKMPSSSRKEEKEEGGLSGFLSQAGSILGGAFGSDETK
RQSSSHREDDGSYTRTEVEYGRHGDRYGQAEYSQTQRPDGSQQSEYRRYEQQDSSDGRHTGGYGYGYEERTETHQSYSGGYEQRT
ERHEYSGSTESHGRRRDDDDGGYGGRRHDEGGYGGGSSYGRQEESSYGGNSGYRRQESNYGGNSSYGRQEESSYGRQEQSSYG
GGYGGSHERRDEGGYGGRSGGYERREEGGYGGSGGYERREESGGYGRREEGGYGGDNYGRRDEGGFGGGSADEYLRQSQGRHG
RERRGSNEYGDNGYGGDRRW
FUN_015617 CDS scaffold_8 231153 232691 +
hypothetical protein PF00505

MARPKKSAVEKQQQQQQPAPVPIPPQHIQQYPQHPQPIAAVPQPAMPMPMPPPVGVPQPQAVMPQRVVEADSFLRVRDSAVGR
LTTILELLRSFTADYVRQTNLLLGEPTAEGSQDNLLANFEHAAQQLIMPIPELAPPVEEKKERKKRTIDPNAPKRPLTPYFLY
MQHARSIIANDLGSEAPKGAVQEEGQRRWAHMGPQEKQGWNNAYQYNLRLYNARVHSYKHGNPLAKNMTDEEALKYAEDFNVP
MSEIKDAAQNEAPADDQDAIAEQLQAVAAAPAIDEPEQAETPAKTPKKAAGGRKRKTATPAAAAEEAKPAPASPDKKRRRTST
KAAAAEPQEEPKKSGRKKTKSS
FUN_015618 CDS scaffold_8 233466 234298 +
hypothetical protein ENOG41

MPSDTPSEADSMAHHRPPALHRTLRLVAIASFLPAFPLCIAHGVLSNDAAPAVGLVPLAFSSGGSLFLLRRRERDDGLAHKLS
HPVMAFAFDVVLAAACMIVLVFTWISKGQLASLSMLAAYATIPLLVNFVIHLLIALESFYTGLAVHSIVQWLAWRTLPPDCPH
CDHRLRPDFPELPWLDRLRERRHGDYSALFVDEENRYHDDETEETLQRAESAAQEAEAQPEVVDVRKKNNRRNRTNTPPSRDE
PASPWSAS
FUN_015619 CDS scaffold_8 235317 235951 + SYM1 Protein
required for ethanol metabolism PF04117

MASFIRWYNARLAARPLLTQSVTTAFLFATGDVTAQQLVEKKGVEKHDLVRTGRMALYGGFVFGPVATTWFAFLARRVNVPGN
KKAEVLTRVACDQLGFAPVMIGVFLSSMATMEGKSAQERIDKAWWPALKANWMLWPAVQVINFSLIPLQYRLFFANIIAIGWN
SYLSWVNSQ
FUN_015620 CDS scaffold_8 236893 237952 +
hypothetical protein PF08213 ENOG41

MLPSSMRRVVSSAVSSTPQTGVISVLASATSKTTPVFVRNQQRRCSSSKPSKSDNGANEISAGQSVPAATSPKSGNEKRKRKS
NKDASDRAASMRKLPSVPNTHHMSQEALGLSSFFSLHRPISVTQTMPRAVTDEHFASIFASRTRNNKMADTDSTLSSAIEQLE
GPMAQMTIGGHEGQEGMHKVDIKNPDGTESSMYLQIDTMSGEFLPFRPPPLPQPQAAGQADSAVADIEAAEDVPQHRMYKALF
TIEESIDPDGQVRIMAHSPQIVQDNQPRSFLERLALRQLKFDEAQGRRDMHAISVKRQRKLRMKKKKYKKLMKRTRVLRRKLD
RT
FUN_015621 CDS scaffold_8 238703 242068 +
hypothetical protein EOG09260ETR PF05127;PF08351;PF13718;PF13725

MQRKAVDSRIPALIQNGLQEKKRSFFVVVGDRSKDVIVRLHYIMSQFDIKQNKSVLWAYKNKLLGFTSHRKKREQKIKKEIKR
GIREANTEDPFELFVSLHNIRYTYYKETDKILGQTFGMCILQDFEAITPNILARTIETVEGGGLVVLLLKGMNSLKQLYSLSM
DVHSRYRTEAHDDVVARFNERFILSLGSCNSCLVIDDEMNVLPISGGKGVKKLPPPDLDNPKTESQIELEAMKEQNEGRQPVG
PLISLAKTVDQAKALITFTDAIAEKTLRSTVTLTAARGRGKSAAMGVAVAAAVAYGYSNIFITSPSPENLKTLFEFVFKGFDE
LGYADHADYSIIQSTNPDFNKAIVRVNIHRQHRQTIQYIRPQDAHVLGQAELVVIDEAAAIPLPLVKKLMGPYLVFMASTING
YEGTGRSLSLKLIKQLRDQSRTASTAGEGMEITDRSTGKTSKTEEFQAGRKLREITLSEPIRYAQGDAVEKWLNTVLCLDATL
PKAKSNINGCPDPTQCQLLNVNRDTLFSFHPVSEKFLQQMVALYVASHYKNSPDDLQLMSDAPAHELFVLVPPVSEDSARLPE
PLCVIQVSLEGKISRQSVLNSLSRGQRPSGDLIPWLVSQQFQDEEFASLSGARVVRIATNPEYVSMGYGSKALELLVDYYEGR
FANLSEDEDQIMEETMTRVTDAELANANLLDDDIKVRDINKMPPLFAKLSEKKPERLDYVGVSYGLTQPLHKFWKKASFAPVY
LRQTANDLTGEHTCVMLRPLENSEDRSWLGAFSRDFQKRFLSLLSYQFRTFTPITALSIDEAAKLGAQLDSVEVQPLTKTDLD
IYMSPFDLKRLESYANNMLDYHVVLDLVPTIAHLYFTGRIKGDVTLSGVQQAVMLALGLQRKDIDVVAQEINMSTSQALAMFI
KMMRKVTKHFAGLVSDAVGAELPKVERLGVSRENASGAHDDEIVDERYVPLATTLDDELEEGGDEAMRELKKKQRELIDSLPL
DQYEIEGDTPAWEEAEKQVLSATKQGKSNPVVSVKSAKQKRKAGGQTAAEVYEEAFGEKKKKSKRVKKSA
FUN_015622 CDS scaffold_8 242465 242936 -
hypothetical protein ENOG41

MARDFVAHEKLKEWEAQFAREYFEKKPSPPRKKPEGFMFLIRQDMMGDLESVTSIDSLESADTDYGEGSNQHSHEDSYDADSE
NSTQDNKNFSDDASNETSQVDPEDLFYGDATEGTPDVEIPERKLVRAYDEPAAPILILFFPMMDLGRDGDFVE
FUN_015623 CDS scaffold_8 245440 246415 +
hypothetical protein PF05175;PF08241;PF08242;PF13489;PF13649;PF13847
ENOG41

MAEQKDRWSTNAYQHSASFVPKLATKVVQWLDLQKDDVLLDIGCGDGILNAEFAKILAQGSGSMHGVDSSASMIEAARELCKD
SQNSTFDVLDATQLVSKPELQKGAFTKVFSNAAMHWILRPEDTRAQFFKGVYASLAPGGSFVFEMGGFGNVSEMRTAALSAVG
RRIGMKAALAADPWFFPDEAWVTKALQDAGFVVERAEREWRPTPADKGGVEGWIRLMASQFLDAISDDKEREEAVQECVGVLR
EVCRNPSGGETISYVRLRGVARKPE
FUN_015624 CDS scaffold_8 246904 248180 +
hypothetical protein PF08325 ENOG41
M80
MPEHDALVLSYSHLAKLPRANDALQTLKKVASLVKPIMRARNWKVRELAEFYPEQHNLLGLNINRGAKICLRLRHAGDKNQFM
PIESVVDTMLHELSHIVHGPHDAKFHALWDQLRDEHEGLVLKGYTGEGFLSEGRRLGGSRIPPLEARRVAREAAEKRRARPGT
GSGKRLGGSAPRPGEDIRRVIADAAERRSRILKGCGTDNLSETQIRNISDNATKNGFRTQAEEDEANDAAIAQALWELVQEDK
SVEYGNSYIAPTADNPTGNGGGSVIPHRGPDGSSERSRESPGWTCSTCTLHNPANYLCCDACGMERSEMSPRTQQVKSNRQSR
SPVSQPAVIDLTTSSPPQDRQKSATTSRHSRPRVTGQEAASSGSQIWECSFCGTVMEKKWWTCSTCGKIKDNSR
FUN_015625 CDS scaffold_8 251619 255880 +
hypothetical protein PF00072;PF00512;PF02518;PF08447;PF08448;PF13426
ENOG41
MSPPEQPRESSQTRAIRPAAAVHDTGFPLPCSESHQNQIQAPVPTTLDNARPRATFDLVNDIKGHDATPRPFAGGRFLRDLGD
ASRSPSQSPFRLAMPSISPGQLAFSAMQYLPVPTLVLNNLKTVVLANEAMGRMLGLITEDSDEEDTVPTIESLRGQTLSQVGV
DILQDGQPVWATWDAFLDSQVDEMGVRIAARDPRRPSQSGGDETPTASTMPLPERAPSPPTRKTQDSIVEVVITRKNGTKAAF
DGRYNPKESDCQVYAKMIITIWELEDHQTFFTLTFTSTESPSSTLHSTKRPIARPSILEAAERKSIAHSNPSSVASSRDSNSP
SFHSPGVVTMSSSPFPPMGPPSVATHSSTPSLLQKMLLMKDALLNNTQMPILAMWKDGSVTFPNTAARKLFEKEAPLDTSLNG
FDLMRYWHIYTEDFSERFVVEDMPICKLLKTEKPFSGFRVGMYDENRNKRIYDVLGEAIRDDSTGEFLAGVVTGRDVTVMTEE
ITQIKERDEERFKLICDTMPQLVWTATPEGMVDFWNTRFYTYTGLTPENSLGKSWVNAFHPDDLPEANRRWQHSLDTGDSYVT
EYRCQSKDGNWRWYLGRALPQRNKETGEVEKWFGTCTDVHESIQTKMSAKRTRQQLLSVIAHSHVTIFTVDPNRRVTMLEGAL
IWNNTHEENHDGTRWFIGENMYTVFNRLTAQLADGERPEFLEPIELLLDGKATEDVKEHGIDDRWYRTRFLPMFGKRSQEGDA
TTDTYIEGVIGVIMDVTELRVREEALEKQSKEKRRAMANEAAAKEANRLKSQFLANMSHEIRTPITGVLGMAELLSGMQLDAE
QQEYVENIQSSATSLLTVINDILDFSKVESGRLDIEEVQFSLSHIVKEVGRMLQFAVERKNLDFQSDIGDSIENDLVVIGDPG
RVRQILTNLLTNSIKFTNQGYVRFSVTAEKETSESIEVRFTVEDSGIGIQDDVRKKLFQPFSQGDASTARRFGGTGLGLTICK
NLLDLMHGRISLESNVGVGTQATFWIPFNKPSGPAEAGLANAGAIPDRLQSELSLSCNSSEYDQIVGTPPPFDGIQANNAGSR
RTRFGISSTSSPDQELPASERAKIHVLVVEDNPINQKIATKTIGKLGFQVTAAWNGKEALEYLASVQKGIKQKPDIILMDVQM
PIIDGYKCTHLLRHHMPYKPLVQDVPIVAMTASAIQGDREKCTKAGMDDYLAKPVRGVILEKMLLRWCHSRRRTSATPDPTAS
DCSEMSEHCDNADIPNIGIDDNDMPQTSEEINGSPITPRPLTTNGYQNEPSPFDSTGLAVQVRRPEGETEWSNRLQETKLIDA
AGGSSSYRRNSYHELQTGDSLTEENVNKLKSENQSTTRR
FUN_015626 CDS scaffold_8 256699 260558 -
hypothetical protein PF00168 ENOG41

MSGRDDAEEHRRYTAPYSARHPIPTISKYREERAARQDAAVGKPDPSEAAGSNDYQFQQKSREDAGPHLESVEDNKDDNSKKD
DSEEKGNGEEMTDTSQVGPQSSDPKQRRKEIKKSKRERAEREVTDPVTHLPVTIHDFTDEALKEIDTNEPPFGTTQRTATGSE
NKNKTDEQLRDEHRDIQQSHDAMGELFPPPDFDALRLELSSISKRGVTFGLAGVATIVASAFSIERLVREGILQGGKTKSSLF
VGASLWLILTAATVGAVWFLVVSVREWISKKVNNIFEDEVWDAHRRGVVKASKKNDAETTVWLNSLIGSVWPLINPDLFASLA
DTLEDVMQASLPRLIRMVSVEDIGQGSESLRILGIRWLPTGAAARAVDEEGQLMTREQSENDTEEKDGENSDSSATIQDGMEA
EEGDFINLEVAFAYRTRSSSKSLKERTKDMHLYLAFYLPGNLKIPVWVDLQGMIGTMRMRLQLTPDPPFFSLCTLTFLGQPKV
NLSCVPLSKHAINIMDVPFISNFVQSAVDAAMAEYVAPKSLTLDLKDMLAGDDFKKDTNAVGVLVVNIKRGYDFKMGDSGIPL
IKEGSSDGYVSVGWAKFGKVMWTTRVLENEMEPVWDETCFVLVTPQELNIDERLRVQLWDSDRLTADDDLGRIEVPIKELMKD
DKSNGKMSHREDGFRALKSGDGMPGKLEWSVGYFSKTRIQQCQFEKQTHDPEIRTMDQLKEKVRESSDRKLRETMFKEGKKER
DTSELEQQTAQELKAKQDAMIISAPPPDGYPSGLFSIQVHNITGLELERLNKRKAENDAEATDEEDSGDGLPSAYCTIILNHR
KTFKTRTKPQNSKPFYNAGCERFIRDWREAEVFVTVRDARLKEDDPLLGIIHLPLGEVFRERSQVMGFWPLTGGIGYGRVRIS
MVFRSVQLQAPRNLLGWQYGTIEVQPTVTVVDCPKELRSSKLKFRTSISSAKMYTEDGDDQGTVTWKSKRGRSLALAVAKRYS
SCMSIAFREKGFFGDDTAAFAVLWLKDIADEEETELELPIWKGNFQRATACCLEECGERLGTIKLKLTFWSGLGSAHSRWASR
DEHLQNVVEVIETARDNLEVGQQEREAGIVESDDSDGECSGQRARRAGSIDSNSDSSSSSSDTEDEDGQVSSVPDGSADHKQG
PIDQLRDYRRRDKRLHRQHRGLMQWKIPRTAKWIKNKMYRVGDRASGVFEHSSKQPGIETEV

FUN_015627 CDS scaffold_8 263419 265201 +
hypothetical protein PF01794;PF08022;PF08030

MGSHIGFLELIKKQFTASKLFFHFLFWGFHWGIFAYGWWKQAADARLAGLNTLKFSVWISRGAGLVLSVDCMLILLPVCRTIM
RWVRPKIRFIPLDENLWMHRQLAYSILLFTCLHTGAHYVNFYNVELTQIRPVLALQIHYAQPGGITGHVMLLCMLLMYTSAHA
RIRQQSFETFWYTHHLFIPFFLGLYTHTVGCFVRDTPEAISPFAGDQFWEHCIGYLGWRWELWTGGFYLLERLWREVRARRET
KITRVVRHPYDVVEIQFNKPSFKYKAGQWLFLQVPGLSKYQWHPFTITSCPFDPYVSVHVRQVGDFTRELGDALGAGAAQAKL
YDDVDPMGMYEVALQNGDQMPALRIDGPYGAPAEDVFENEIAVLIGTGIGVTPWASILKNIWHLRNSPNPPRRLRRVEFIWVC
KDTGSFEWFQTLLSSLEEQSNEAARMPGSSGVEFLKIHTYLTQKLDIDTAQNIVLNSVGAQMDPLTELQSRTNFGRPDFPRLF
TTMRNGILDRTYLNGLESHIRTTVGVYFCGPSSAARDIKTACKAATVPDVDFRFWKEHF
FUN_015628 CDS scaffold_8 265641 268478 - URE1 Urease
PF00449;PF00547;PF00699;PF01979 M38

MHLVPKEIDKLVISQLGLLAQRRLARGVKLNHSEAVALIANNLHELIRDGNHTVSDLMALGATMLGRRHVLPSVCTTLHEIQV
EGTFPSGTYLVTVHNPISSDDGDLRRALYGSFLPVPDNSIFPMAATEDYQLDKQPGAVVPVKTKKITLNEGRKRIRLQVTSTG
DRPIQVGSHYHFIETNPQLEFDRIRAYGYRLDIPAGTSVRFEPGDTKTVTLVEIGGNRVIRGGNNLASGVVDLSRADEILARL
QEAGYAHKPDPAGDMAHIDVFQMDHASYATMFGPTTGDLVRLGSTDLWIKVERDETVYGDECKFGGGKTLREGMGQATGRHDA
DTLDLVVTNALIVDWTGIYKADIGVKEGMIVGIGKAGNPDVMDGVTEGMVVGSCTDVVAGEGKIVTAGAIDTHIHFICPQQVP
EALASGVTTMLGGGTGPSAGTNATTCTPGAHYMRQMLQACDQLPINIGITGKGNDSSPEGLRDQVNAGACGLKLHEDWGCTPA
AIDACLSVCDDFDIQCLIHTDTLNESGFVESTIAAFKNRTIHTYHTEGAGGGHAPDIISVVEHQNVLPSSTNPTRPFTRNTLD
EHLDMLMVCHHLSKNIPEDVAFAESRIRAETIAAEDVLHDKGAISMMSSDSQAMGRCGEVVLRTWNTAHKNKVQRGWLLEDEG
TGADNARVKRYVSKYTINPAIAQGFGHVIGSIEVGKFADLVLWDPAWFGTKPSHVLKGGHIAYAQMGDPNASIPTVQPIIARP
MFSPHCASTSILFVSSASIETGAIASYGLRSRVEAVKGCRNIGKSDMRHNDLKPKMRVDPESYTVEADGEVCVAAPAETLPLT
QQFYVY
FUN_015629 CDS scaffold_8 269149 271183 -
hypothetical protein EOG09261EMF PF10198

MPPGPSQKGTGKKNSAIPKQSRNTTPAPVATASLPPQEFYDPDYLNTRVILFRNLTYDDIVDQSASNATIPDSKSLDGMIERL
KSLSNIMEKRSTFYDRGMRHLADERKKRPSEDYSNRADGEQEGKRPKHKRKKPDSLAPQEGNVERSSPMRDSKNRKHDRDPSS
PLSPTHAGSPSAMDVDKKAKTEEKEGEEEESSSEDEDAPPRREQPQAMTFGEDPSTFPDPTIYEIRATYPGMPDEERKEIYSV
ATYPPSDLADLIAGDPPDKDFSNAKPNSQINFSTFSTYVEPFFRPFSEEDLAFLRERGDRVTPFVMPKRGKRHYTEIWAEEDG
AMSIDTPQQGRDKLPPNQPRGSIDNMDDDVGETDKLSVGPWLTRLMQTLRPEARAQSADDKPTTNGTTNGDISMNGDADVEDK
PASSDDKPNQQPAAFMPESSTEAWKKASHPKLEYSQVDERIKQELRHIGFLPLDGFEAEYDGHFDDEVAARLRLLQNRLKEQM
LINGARKARLTDLVRERMAYQEYQTILEDLDGQVQAAYLKRTRTMGKSKKTKRPGGAGGGSHFVGGAAGTARPGIGDLTKTLM
ERRKRWIDTIGSVFDDESLRKVPRITDPESSIFKPVDMSELMNKEKEQWDEEVEED
FUN_015630 CDS scaffold_8 273436 276115 +
hypothetical protein PF08241;PF08242;PF11899;PF13489;PF13847

MASHGGNMLSLANVQSLLAGIVATALSTLCLTSIFSSKSQDEAPGLIKSFFLFFYSSFIKPHQGGKKGNQQDALESFYKKQAG
AYDATRKVLLRGREDMLALVAAQIQAKLTDDAPKNGNKNKRIWVDIGGGTGFNIEAMGAFVDVPTFFSSVYLVDFSPSLCEVA
RKRFERLGWKNVKVVCEDARKFRLEDYESGMPSKSIPPRSPALSYFDKPRPDFGGADLITMSYSLSMIPDYYSVIDSVISLLS
PQGILGVVDFYVQNQVDFAFRNYTGGLVDRHVNFLSRSFWRSWFDLDRVGLEPSRRDYLEYKFGTVLNVNTRNKGLGAIPYYI
WLGCHKKPFSSSSLPHEIVERIDALVTESPYLYPANHGDALTRAIERSAPEIRSKAFLTAVQNLSSNLPLPSFFYQNHHWRIY
YDEQLPKHTQFKDEYIYAFTWEDTRVDERLLKLGADDKVLAITSAGDNILSYLLQSPARVHAVDLNPTQNHLLELKAASYTAL
PYEDFWKIFGDGKHPHFRELLITKLSPHLSGRAFQYWLKNVHVFQNSSGYGLYDTGGSRHAIRVFRWIARIFGLQKAVKQLLE
AKTLNEQREIWRRKIRPALLSKLVCNLVVSQESFLWAALGVPKNQLAMIEVDHAESDLVKGPKPAAKNTRSHAIWHYMVNTLD
PVAEETHIAADNPYYYVCMDGKFSPKCHPDYLSPRAHAKLSRPNALDGLRIHTDELEEVIARITPGTLTVAVVMDSMDWFDTG
SRAAAAQITKLNRALAMGGRVLLRSSALTPWYVKEFEAHGFSPKRHGARLDGACIDRVNMYASCWICTKSENLPPPTPEMDRA
GGSEITSLTI
FUN_015631 CDS scaffold_8 276481 277160 -

hypothetical protein PF05328

MASIARSSLLRQTAMASRLAAVPATRTTFMPMPSIRSQLKGVAAFHNTARRSAILPPGPQRIEGGVNDPAPIPEPNAAHGSYH
WTFERLLAAGLVPLTVAPFAAGSLNPTLDAILCSVLLLHSHMGFQQVVIDYIPSRTYPGLRKIFNWLLNIATVLVGVGLYEFE
TNDVGITEAVRRVWKA
FUN_015632 CDS scaffold_8 277608 279568 +
hypothetical protein EOG09262JRP PF00579;PF16714

MAIHATISKLGPSRTTIYSRCLNAQRSLQVRNVATTYLKKVAEGEERWKERAEKIQNGEIPHTWDVLQERGYIKDVAGSPDKI
KEIMRVRRIGSYVGIDPTADSMHVGHLLPLMPMFWMWFHGYPAVTLIGGSTARIGDPTDRLESRTILSNADISKNITKIHVQL
TKLWKNVHILKQKYGYEEDWAATHRLLNNNMWLGNLTLYDFAKRIARHTRIGPMLARDTVKRKMTEGDGMSLGEFMYPLLQGW
DFWHMYNKLGIQMQIGGSDQYGNITAGIDALKTIRETEEAPHLKMPSTWDHEPVGFTVPLLTDSAGNKFGKSAGNAIWLDEFK
TTAFDLYGYFVRRSDEEVENLLKMFTFMPLEQISKLMEEHRAAPQERKAQHTLAFEVLSLIHGSQKAVQEAKQHEFRFGTKLP
RGIVNEPTADTGIVTVNNAPRSDIQLPRSIMGMSPAKILHAVGLASSSSEGQRLLSAQGAYIAAQPGQKRGLVPGNLSWTPMK
AWFPEETAKFLIDERMLIMRKGKHNVRIVELISDEEWKKSGQIYPGQPGTGLLRRMKEELKKEIEAKGRSVSDKELTEIATRK
KDQLNVANNSDIELPNKHDVRERRARLTWDRRDK
FUN_015633 CDS scaffold_8 279698 281034 -
hypothetical protein EOG09262H34 PF06218

MIQGIFYARFFPQEGPKIVAQSPAGCITPVEGSTKSPLIDFDVLHEYIIPRQAFCNRYLTINTPDGKYTVLGYPVVIPHTKYL
RNEFIFNFGLVLDADVDHVPYERVVRGLASTFSEMEKQNEYLSQNAETKGSGRRPIDSLLEIVKEDLDNYGECMIPVDEANTI
NMKLFPHHMDPPRVRGWHVPVPKAKFSDIMDPTWDLTLQKVISHIDGVSDVRRIAHAASVSLELAKTALRHLLYYDTILLLDM
FFFTACYAPRPGIHDFIRNVDGMVDECAAYVSHGRGLVSNYHLIKLMSTFAPGKSVMEWLKGHQDTGFEVLRFVDVRRFVQFG
VIKGCIYRVHKFVVSKQYLAALASGQSRPVPGGDPLQKYTDGCHHFDQIITEQNMTDSEIMEKLKKLPVPKGDLAVFYR
FUN_015634 CDS scaffold_8 282329 283727 +
hypothetical protein EOG09264JQ1 PF00240;PF00627;PF11976

MADNPEPSADSQITFKVKTSSDSTHSITINDTATVLDLKTKLAGEDFENIPVERQRLIYSGRVMKNDDALSTYKIKHNNTIHM
VKSAASNQQPNQTPTANTSTSTPAPTNMASGTANQPFANLTGARYAGFGNGLPGLDMFGPDGGMGAPMDEARIQRLMSDPNVQ
QSMNEALNNPDFINMLIDSNPMLRNLPNAREIITSPFMRQMMSSPEMMSQAMRMQRSMQGGEAGAFPAPGATDTTPQGAASGG
NAQGQQGQANPFANPFLSGMMGGQQGGNAPDMAQLMQQLQGLRSLYGDVGQGQTAAGQTGQTGTGNTPSQGATDSSADAQTSG
TTPNQGGAQQGSTTSPPPANPFAALFPPPGAGAQPNNPFGMNPEMMQQMMQMLGGGGLDSAPAAPADNRPPEERYAEQLRQLN
DMGFFDFDRNVAALRRSGGSVQGAIEHLLSGP
FUN_015635 CDS scaffold_8 284331 285496 +
hypothetical protein PF00107;PF08240;PF13602

MRGIQITEYLKGPEQLKVSDLPDPKPTDDEYLIQVHAAAANFFDILQIQGKYQNQPPFPWIAGAEFAGTVIATPKDSSNPKFP
VGSRVFGASQGAFATKIKAIEDTLLPVPDGWSFKEAAGLFVTAPTSYGALVVRAGIKKGDYVLVHAAAGGVGLAAVQVAKAFG
ATVIATASTQHKLDIAKSYGADHVISYNDATWPAKVKALTPDSRGVDIVYDPVGLIDLSTKCTAWNGRILVIGFAAGKIEKVA
MNKVLLKNISIVGLHWGAYSIHERETIPKVWNGIMDLVKQGKLRGTEYTDEEFVGLERTGAALKALGGRGTWGKVVIKIPEEG
QSKL
FUN_015636 CDS scaffold_8 285723 288920 -
hypothetical protein EOG09260DUW PF03810 ENOG41

MSFAIEVPADANPLSLQSLYQTLQSATSTDYAQRQTAGQQLTSWELQSGYYSSLQAVYLDKSLAYEVRFLAIIQLKNGIDRYW
RLFNHVKNSIKPDEKSLIRSRLFQGSIEEEHTNLALHNALVVAKIVRIDYPGEWPDAMGNIIELLRSSRNGNQRHLHGILEIL
LRVVKELGTARMRKNQTALQSVTPEIVHVLSEVYSEKSQAWMGFLTGGPGGQEEVNLAMLNSLLALRVLRRLVIMGYERPHSD
NTVEQFWTLSQNQFGQLLNLVSHDSAVPPNYQEVVGKHLLQFTKLHIDMAEQHAASFVFLPNSIPLVQSYWELVAKFAEVFDK
SGGIRQGPSETGSAKSKVEGPVLERLALKGLLLLRSCVRIGFQHVQTFRWRSPETKAEQEKAKNLIKSELLKPDLVIQIVNSI
ITHLFVFRKSDLEGWEEDPEEWEQQEQSEGNAYEWEVRPCAEKLFLDLLTNYKDLLVPPLLSYFQTAQDPQADIATKEAVYTA

MGLAAAHVHHVFDFDAVLASTIVNDARLQGGLCKVLRRRIAILVSQWAPIKLDDSSRPIVYQIYRHFLNPEDETNDVVVRITA
ARQLRWIADELDFNVDAFLPYTSDVLSQLINLVQSVDVDETKLAILESIRILVTRMEEQVSQFGDQLMSALPTVWENSGAEEY
MIKQAVTSIFAALVMSMGPDSQRYQHFMLPLLSEAARRGSDLHLHLIDESLELWNNILMQSYAPLAPELINIAELALPLLEYD
SETASLALTAVESYILLAPASMLEDRLRRPTLSALSNILDSKSREQVRMGTSCIENFIRAAVELGGSTGVSVILQDMVEIGFM
NKILGNLHDAWESHQTTGPNRKVSKLSTITETDYFAILGRLALAEPTLFSQMLTNMGSLDQVWAWLSSEWFSHMSSMDHIERQ
KLYLLGLTRLLELSSPVQELVLSKLQDYFDMWINVISDLQDGVANGTDTLIWGPLETTEYDTPKIIAERNMEAKDPIHTVHAF
DFVKMRLQDLVNRVGGEQAFQEQWAVNVDKEVLDKFQQMASGVQQQQQQQQ
FUN_015637 CDS scaffold_8 289564 291064 +
hypothetical protein PF00587;PF03129

MIRSRTARAAFNTSLRRPWTCPQCISRSRFYSGKSEKREPPDHRKLGTKQELFISSIYSPGSPIFLPNGSRIFNRLVDFLRKQ
YVRYGFEEVITPTIYKKSLWAKSGHLENYADDMYAVRGNTEPPSAQHPGCCGSEQKDLKPDEEEEGDYGLKPMNCPGHCLIFA
SKNRSYRDLPIRYADFSPLHRNEISGALSGLTRVRRFHQDDGHIFCRPIQVEKEIKKTLDFVKVVYTVLRFGSNYRLALSTRP
KDHYIGTEEEWDQAENALKRALDASGMEWGVNEGDGAFYGPKIDIVLTDSDGKEHQTATIQLDFQLPKRFELEYQAPAPEYEA
RGETTTDASLLDEYGPVRPVMIHRAVLGSVERLMALLIEKYNGKWPFWLNPRQVAILTINTAEPVVEWAEQVRSVLVGNNDQL
YEQLENGTLPSQDNAFRPTGLSVDIDDSARPLGAKIKEAREKNYGEILVIGQKDVENKRVSLGKEMLSPEEVRERFKTMVDTF
A
FUN_015638 CDS scaffold_8 291324 291825 -
hypothetical protein PF00010

MSSPNKSPANGDAGAADEKPRLTEEEKKQNHIASEQKRRQAIREGFDRLTELVPGLEGQGRSEGLVLKRTVDYMREQLSERQA
MVDRIEQAGGDVDEKHKRALYQQAANPGRTRG
FUN_015639 CDS scaffold_8 292501 293365 -
hypothetical protein EOG092643IE PF02545

MADTPSEPPPDYEKSTATQGTPQRPLRKGPIPLELPILKHLNGKRVILASASPRRKALLQQFGLTNLEILPSTKPENLDKAAL
GPYEYVAATAHQKCMDVYTTALEVHLKSIPDPDLVIAADTVIVTKDGRILEKPRSEADHIRMLKHLRDQRMHKVLTSIVVIAP
REDARHPGYVIDTYTEETKVYFLSESEGLPDDVIEAYVKTREGADKAGGYAVQGVGGMLLVEKIDGSVDNVVGLPVRKCMAMA
EKVIFRQEEEDFEGEGEEED
FUN_015640 CDS scaffold_8 294420 296740 +
hypothetical protein PF00439 ENOG41

MDSKRKANGAATVDNDDRGSKRRRLTGDFDLSKGESRESTTTYGLSFLEHIRKTADKAGRLVATNFEELPPREENADYYEQTR
MPISLSMIEQKLNDGEFENLSELEGYFKRMISNAKEFYPRSTEIFEDAERLRKAVSNYMTKKNPAYHVRGYQAVPTPFPDDDG
EEANEDNEDEEMNDDENEDDDEPEEEKEDEDEEEDEEEEEEAPSSRRRTITLKRRTPGRTPGRRASTRGKETPKPAAPAAKPD
HVYEDVPYKGLSFQQAQEKLIEELIRREDPGYDGPYFEAFINLPPRSLKEYFKVISDPMSIRKLQKAVKGFHGRGGSTGVSDF
KSWAAFEEKAKLLWTNAYFYNEEGSEIYLVAQDLEKFFYDQLKQAQAVVTEPSQPKIKLKVGGSSETPTPGPKKITIHVGGQR
DSADSPAPAQSKEAITNGQTVNGTARTSTPAQAVNPQLEKARSTSLSAVPSPSPSVQSALKAEEASRASPAVTSQPPSAAPNQ
ATPATPAAAPVAVPAPVPVAPPQPINNPLVNGYMDQKHPRRPGKGIDDALIESMKIQVHPTLQSHSPVLATIRPNPKEMEQAA
TLNLPPHLTRILAVTAIPSHLQTRQYSLWTLVNKQPLKPIHHQAPGQLPHERAFEAMLHPGLNVLESHLIAAIPRDERVPGGP
EVELEVFTISINVLRN
FUN_015641 CDS scaffold_8 297190 298664 -
hypothetical protein ENOG41

MLARQAVLRQAALPTTRFQTQRTVASQIRPLNRRISFLPWRQRQSEPIPVYFSKPDPSTNANRVNPVYHGSANQVGGEEVKRA
LRRAKWTIFRYRLRRAIFTTIVLYACWQIFIAVVFDPIFDWADAEWEALSEKEKEEAEALHDPDEPLIFLPFPFTTKEVKQPP
YRGSDPEWQTFVKVSQDKKLLQEIKNGLANEIKRGVERNPAFNKLLGGEIAVRKYWLDIIFPPAPPPIHYVSGIMIDEEGIFW
GDRPIDSIAASFLAKALYPKALAMGCWTFVSFLTKQVALDISKAIGLSEPDPPESGWHQVPVGNLPGIIPPPAATRAAVQHDP
KAHTAHLPPVFNEIISAAFGPDAKLDPRLQAASMAGALAFLRHWKPTKSLPSRGCIRVDGIVELKGKTAVMAVLVAGYYDPKT
RQYVAVQTMLKHLMQLRQRPAPG

FUN_015642 CDS scaffold_8 299079 300242 +
hypothetical protein PF12955 ENOG41

MKLNIGLSAAFAGIAAASQQPAADGTKVYILRGSHHIEPTTSITPSEARLILYQRLAPEGQDFSFRDLTASDDEGRVVSLMNK
YGKVPAPLLSDEKTATPRQLAITIDGMPSFEYESFFGWLFNTQPDFTTDISKENGEHPEVFANVLAKMPECPLSQIAALDEKC
FNGQSAYASYSRSQDGDHPTTLLNELPKLQKLAESGELQTILVFLPVDVDSEDKSDKDQELRRRQAETVISSIEETAESPAET
STTPAKPSNPIKAVCFTSEDSCMEATGNCTGHGKCLNKYGQSKDSCFACHCQVTKSESGSVTHWGGNMCQKQDISVQFWLFAG
FTIALLTILYMAIGMLFSVGEEKLPGVIGAGVSRSK
FUN_015643 CDS scaffold_8 301329 303894 +
hypothetical protein ENOG41

MQTGPSPQRLNGVLLVRSALPRKPSVYGQIIRLDQSAIVNLLKAGGLTNADLATDTTCIPTEVDPTTADQRKPSTKERDSSLT
PETTEWSIPDTYNSYAPPQCICRQTSGVAPPPPASDEELLKLYRKELQALHPFVVIPDGVTAAKLKATRPFLMSAIRMVTSYR
SLRSMRAQMYHLMKYIADHMLIRSERSLDLLLGIIVIIAWYKYHCFMHAQLNNLIALASTLVGEMSLGFLRIEPMRYTKYIQQ
CLAVLEQEREYDTDMRLVTLVRVQHLAERIAQLNAPDDPTEEIVGLPAAPLSAYVSAFHNELDRIRNGLPPELTNDRVINMFL
NTAMLRLYEPPILDAPKIISMSESLTSPTLGAASALDIFYQSRNALKKFFDDWLMVPIPEFYNQTTPVVAQLVYGMTMLGRWA
KYTAPIPLTKATTPMPQDTSARDPHDEIYKADSAYSASVSPSVATPSVTTGRDSHETSPISLREGTDPRLPAAVAALRSQIQT
QPGLSLDVTGILSAICARFEEANASFQMASTEPDASDHNLWSMSAIKVKITRAKLERWAEIVAAGTEALKIHDEVRDTAMIDS
STSEIGAAGPVAAMGQDMQPDAFTMPDWNPDMPWASEMLQGVDPSIWFDGYLDWGAVMNSMGNLGHPN
FUN_015644 CDS scaffold_8 317139 319102 +
hypothetical protein PF00750;PF05746

MAATTCSVSGIENLLGRVGLDTPIPEFPGADIVHNPQDIFRVYLADTLEKLLHCDRMVAYDAIQTSNVTGMGDLVIVAPRLRL
KDVRPEDLVKDLLQRLPRTPPFGCPLRDGIRLQVYFSPNTLPRLLLSYISDRSASYGYDTSLGLSDPASPDGSKKKVIVEYSS
PNMASEFQVSHLRSTLIGTYIANIYASMGWDVVKMNYLGDWGKQIGLLAAGWERFGSEDEFAKQPLRHLLEVNHKIQDLFKPE
MEECKIAKAKGQDVTEMESQGLYAERDIFFKKMEDKEPGAIAVWQRFRDATVRDYTDSYAQLGVTFDEYSGESQVTAESIAEV
EQLLKDKGIYEEHEDSWKIDFSKHDAKGLSLAVLRYRNGTTSYLLRDLAAVFDRYKKHKFDKMIYVVAMEQEMHFHRVTKTLE
LMGRQDLADRIQHIPFAKINGLPEELKGAALLSDYIDGCRSMVRTGLDEDDEEASYVDKSENSVRQLSLAGLFIQDHYHKRTT
SYAIDPKKMISLEGETGAAIQNCYARLLKKLQSGPSSFDYTTLDHASLEEEDYAELLRILLQYPDAVHGSFRTLEPSFIVVYL
LRIADQLTATLDDDDEKDWTGLDTASEARYALYENARQVFENALRLLGVSPWSL
FUN_015645 CDS scaffold_8 319781 321683 +
hypothetical protein EOG09261TEQ
PF00515;PF07719;PF13176;PF13181;PF13374;PF13424;PF13432;PF17830

MASADELKALGNKAIAEKNFDEAVAKFTEAIAIQPDNHILYSNRSAAYASKKDWEHALKDAEKTTEIKPDWAKGWGRKGAALH
GQGDLLGANDAYEEGLKHDANNAQLKSGLASVKKAMEAEVGGPQDPSGGLGQMFNDPQLIQKLASNPKTSGFLADPSFMAKLQ
SIKDNPSNASEIFSDPRLLTVMGVLMGVDLEMREREVDPNAESQDSPMPDAPPAPKQPEPKKAPEPEPEPEPELDEEALEKKKKK
EEADKEKALGTENYKKRNFDEAIAHYTKAWETFKDITYLNNLGAAYFEKGDYDKAIEACTKAVEEGREIYADFKLIAKSYARI
GTSYERKGDLEKAVENYNKSLTEHRTPDVLNKLRAAERAKTEAGKKAYIDPAKAEEAREEGNKKFKEMDFPGAVAAYTEMTKR
APDDPRGYSNRAAAFVKLFEFPSALEDCDTAIKKDPTFIRAYIRKAQAYFGMRKYSECVDACTEAQRVDQEHHNGANAREIEQ
QQQKALSAMYSARDNETEEQTRERLMKDPEIMGLMQDPVMQSILQQAQSDPAALQEHMRNPGVRSKIQKLIAAGVIRVGR
FUN_015646 CDS scaffold_8 327723 329091 +
hypothetical protein PF00042;PF00175;PF00970

MALSYQQTRLIRGTIPALTDHGERITTIFYRNMLRDHPELNDYFNTVNQANGRQPRALTAVILSYANNINHITELIPKMERMC
HKHCSLGIKPEHYAIVEKYLIAAFAEVLGPAMTPQVREAWMKAYWMLAKMLIGREAQLYRDFGKWQGYRKFRIEKKVEESDDI
YSFYLVPVDGKRLPPFQPGQYVSVQVPIADKGYVQSRQYSLSEAPRPDYYRVTVKRDEGLHMTRSGRYLGGDALNPGVVSNLL
IDMKDEGDIVELTHPAGEFYLDMSNTSNVPIVLISAGVGVTPMMSILNTVSERQPHRPVSWIHGSRRSVPFYDQVRRIARNRP
SFRTNIFKTHLAESDVYGVTYDHDFRMDLAKVDKEDLYLCNSSTEYYICGPEQFMLEMAEYLKAQKVDAPRMHFELFSTGDME
FKVDTLSIGSASRATSINSDEARCPSSGAISTNGATCPFS

FUN_015647 CDS scaffold_8 329700 330512 -
hypothetical protein PF00096 ENOG41

MSRRSDRSHRHSRSSRSHQSQQDDIPIDPNLTSEASYPGYTDTQGFASYSQAGPSVYATASSTPETLDPSMLYYMTQQPATVN
PNEVLLSSGSGYATHNYPSSYPTAEPTQPEASSSIAQVTTPHHCSECDRFFGGNRDLNKHMKTHNKPVKCKADPNCNVTKAEQ
RDMDRHYRTSHRAYAASKGILTEERICGFEGCTSKFTRQDNLLKHWKKFHNYEQ
FUN_015648 CDS scaffold_8 331239 332954 +
hypothetical protein PF14441 ENOG41

MTTDDIISLKSRQKRRLYEPIVLYKALTEITHEQGALRPAEVPERPRTEEERYHHFLHKLASICDSTKGGKTVTSIAILDEEE
KYKYVFACNQISDRDLSDTRDLLTTVLTSLYDFHTFPLDKKNTVESEILKKILSFNSPRINCYLNTLKAKNIVECLEYCQMQT
DTANTSVEEGLRSLDRAIEDTTFKNMGNNEYFETYSTIQHALERFLTREVKSYIDKRATNGRFSEGRSFVCWSELRHSLSRLQ
SYKKTVQDLIAAEGEWPHIFQELEVVSVKSSQTEPNPLSKKSEKASNIIGRMCNNEASRQRYCDLAQNLQRMFDLDDRIQTQC
AKPTFKPYVHCEILVLEWIMALNREMAQKHHPGVTFFNDWSYIGSSKPTCQLCRYYFDTAGQHNGIRTRAGHGNLYVNWRFPD
LHESDGTFGQTRRQSIFNSMIEKIRQDAFGILVTRDSGGKRHDSSTHPLMSVRYTDVQTELSVRDLPDVDELGEDLAMRLSLD
SPSNSLEESDFDDEDGGTIL
FUN_015649 CDS scaffold_8 333065 333935 -
hypothetical protein PF13409;PF13410;PF13417 ENOG41

MSSEQYVLFDLPSRDPVGAWSLNPWKTRFLLNFKGIDYKTEWLEYPDIKPTLEPHVPANPATGTWTIPTVKFPDGEYIMDSNK
ILERVEKDHPEPSVHKDSPVLAKLFSIMPKIMSALQPVYFYTVPTNILSEKSLPYWHETRSKVAGKPLDEFHKEKGGQQAWAN
AKAPIQEVEALLKENSEGPFFLGKTPSYADFVWGGFLIFMQRNNIIDEVYKISGDSQLHKDLLEATAPWHKRNDH
FUN_015650 CDS scaffold_8 335350 336193 +
hypothetical protein PF10601 ENOG41

MATIQSTTPPDMLEKPDLPQRPTTPRVIRTVVHDEGLPEVVHSNQPINATEYYDAPIPVDSETDKRYNEAPIPVTTLDKYEPA
PALPVRPSSITPQPQQPYPYSPGGYAQPQHPSFVAQSPQLPPYNSNEVVTYVTPLDQLGDHPKFVDCPFCRHRAETRVKKISS
KMTHVSATVLGFTTIAGAAVPYAGKWHSHTAHYCSNCDHKVAIRKWGSSQMKPLGTPDYLREVSRYGPAHSPSMSSSSTRG
FUN_015651 CDS scaffold_8 337067 338546 +
hypothetical protein PF13450;PF13738 ENOG41

MEESTEHHEILVIGAGLSGINTAHVLNQRLPHRSYTILEAQSAIGGTWRFFRYPGFRSDSFMTAFGLPWHPWKHKQKMAQAGE
IVEYLEEAVDAAGLRDKIRFRHKMLACEWRTDEQNWKIEVDADGQRKTFIANFVISCVGYYAYDKAFPTTIPGLKGFGGQVVH
PQWWPEDLDYSGKRVIVIGSGATAITVVPSLAEKAGMVTMLQRSPSFVISRPTTSSLDSCLKSLLPFSLAYWLIYWKDVFLEV
FSTQFLLNFPALGRKVLMGEMQKALPKDIDVNVHFNPRYNPFQQRLCMCPDEDFFKALHQDNCEIVTDTIETVTKDGILLESG
RKLEADIIVTATGLYFQLFSGIAPLVDGQPIEVGSHYAWRGCMVDSVPNMGFVMGYVTTSWTPGADIMAKTLISVIKEMEKTG
STSVMPVLDEKDRSKPQKLPVSATSSYFVKAADRMPKVTDEGPWYGRVNLAKDWWAWLMGDMSSGLLYSGGQRKKDI
FUN_015652 CDS scaffold_8 339584 341084 +
hypothetical protein PF00067 ENOG41

MGPLVRIGPNEVLSTDPVVLRNMSAVRSNYTKGDFYSSGRIVPGVDNVVSERDEAKHKFMRVKMAPGYSYKENEGFGFEAGID
RQLLNFISLIDRKYLSTTSESRPLDLAEKTQFFALDVIGDVSFGEPFGYLTKDEDLFQYNEINASSLPVMNVVSVYPWLGRVV
HRWPLSLLLPREEDQVGFGRLMGFARHFVRKRLAEGITMRKDMMQMHISNGMNEEELIQQAFISIIAGSNTTAHALRMIILSL
ITNPNAYRSLIAEIRKVTSSVSSPISWAQTQTLPYLQAVVREGLRTWPPVAGLGFKQVPPEGDTINGFFVPGGTQVGQGFYAV
GRSRLVWGDDADVFRPERWLLAGEDRLRDMVAALDTHFGHGKYSCLGKPIALMEIHKAVFELFKRYDFAILNAERPIKTQTSV
FLFASDFWVTITQRDDEEN
FUN_015653 CDS scaffold_8 341351 342806 -
hypothetical protein ENOG41

MSFSAKRKAKVIKIADEDSNENAPSVAGGDSAGSDEPVKPVFGAKAGRKPFRQSGLRKSFNPADSEGLGDAPAANDDEDDGPV

VVRPAISRSGSLKGKKKSNKSSRLSFGGDDSAAGDDETAELFTPKKLPLGRALENSAIKRGLGTKGLPMRTIGDDDDRPKYSK
EYLEELQSSTPNTPQRPSTLPPDDADLMDLDASELEGAVVVDSSEFSQTQPTAILTEAEILEKKERRKRLALEKDFLSVEDDE
DPFGRKKKDDTRLIAEDEDLGEGFDNYVEDGGLSLGRRAEKERRKQDRKMMEELITAAEGHTSDSSSDSDAERRIAYEAAQTR
AGMDGLKKPRKDPSQDLLQAPPKISPLPSLTECLARLQTTLKGMEEEMKGKQNRVEKLKKEREDIVKREGEVQALLDETGKKY
QEAMGQGKVVEGKGVEIPANAPVEMVGARGLETLGTPSRKPEEEEI
FUN_015654 CDS scaffold_8 343003 343476 + SMG1
Serine/threonine-protein kinase smg1 PF01423

MAPAQPELKKYLDKRLFVQLNGSRKVIGILRGYDVFLNIVLDEAVEEKDGGEKERIGMVVIRGNSVVMLEALERIGGDDRRGD
R
FUN_015655 CDS scaffold_8 343649 344184 -
hypothetical protein PF00581

MGEDAPQDTPQKQWWEEFPEPKAECPRTDPSIVAKLIEVNAASGKNAQRDFLLVDVRRTDWEGGTIATSINLPAHTLYQTRPQ
IYQLVKQAGIKRIIFYCGSCGSRGPRCAGWMQDYLDEVEETETKAEILIGGIKGWQKAYDGQLMDSYDKKAWEKKE
FUN_015656 CDS scaffold_8 344880 347506 -
hypothetical protein PF00933;PF01915;PF07691;PF14310
GH3
MASVDIQSILSSLTLEEKISLLAGKDLWETVPISSKGVPNVKVSDGPNGARGAAFAGGTSAACFPAACLMASTFDVNLAKRIG
TALAEETHSKGARCLLAPTMCLHRHPLGGRNFESFSEDPFLTGKMAARVVEGLQDKGVSATIKHFAANEQETERLTVDETISE
RTLRELYLRPFEITIKEANPWAVMTSYNQVNGHHADSNEFLLKNVLRGDWGWDGLVMSDWGGVNSTAESLEAGLDLEMPGPTR
WRKTEDVIAAIKVGKTSEETINERAKRVLKFLERLNCFKDPSIPDERAINNPKHQALIREAGSKGIVLLKNQDNVLPLSKDKV
KGKKIALLGHAKIGLAHGGGSASVNAHYKVTPWDALHEALGDSVEFSYAKGAHTFRMLPPIAENVVDLDGNPGFTFKIFEPGN
PEPIEVRPAHPDSEVSVLSGFGFSNKDVTLEGTFTAQETAKYYFTASGLGPSKLIIDGKVVFEQKENSKDAMGFLFGGDNPPE
FKVSLEAGRKYKLAMHTSPPAAKDGEDLDLGILEGRCGLRVGYMSEAEHDRDLLSEAVDVAKDADYAIIFTGHEPFWETEGQD
QVSFNLPKDGSQDRLIAQVAEANPNTIVVNSTGVAIAMPWLEKIQGLVQTWFPGQEAGNSIADVLTGAQTPEGHLTCTFPKKL
EDCPAYGNFPGKHIDGRLRVTYGEGVFIGYRYFDCLSADKVNFPFGFGLSYTTFDYSDLEVKESSGDYTVSVKVSNIGKVVGA
IAVQVYVGAAQTAKGDPLKQLAAFEKVTLKPGESTKVEIPVRARDFAFFDESTKKWVVKGGDYKFMIGRSAGDIALESKIAVE
EKSFDP
FUN_015657 CDS scaffold_8 348782 350230 +
hypothetical protein PF02535

MSAHSEVPRGESWASIPTDLILAELKRRKDDGEKPECGSRTKGSYDTSAHVFALILILTLSTLACAFPLFSRRTMRGRGQKTV
IFYCQHIGTGVLLATAFVHLLPTAFESMTDPCLPDFFSKGYTPFPGFVAMVSAIIVVGIESYLTARGAGHSHSHNHGYFDSDD
EHESELPMMDAAGLSERRHGPRPPDIRLENMESQGLVAGVSPLPGSSPLVGQEGKKLNDDFNDDDSDLDLDMEELEPAGSSST
RHRHGPYASLKPEGAGAEEEPMTPMTPMSPGPQNPEEQQRKLLQCLLLEAGILFHSVFIGMAISVATGPAFVVFLVAISFHQT
FEGLALGSRIAAIQFPRKSLRPWLMVLAYGTTTPIGQAIGLIVHRMYDPKSAGGLLVVGFMNAVSSGLLLYAGLVQLLAEDFL
TEKSYKILKGKRRLRAYMAVCAGALLMAAVGAFA
FUN_015658 CDS scaffold_8 350801 351809 +
hypothetical protein PF01814 ENOG41

MSGQRILLFIVAQALFAGLAVFLANSPYLDMSASPTGSQPWADGPMKLVTTPQYETKKTDLFTTGATHMALLHNAIIRGFNSI
YLQAPHVKDADKADFIGYSQTWFKFVKSHHDDEEDNLFPKVQELLGDEPVWDETHHEHESFLAPLAEFNTYLSNLASPTDLDG
AQVIKLMDAFKEPFEHHFHSEISTIAALSNHPKAPKEDTPEGAAAGLTFKTWGKKTVTKAGVLDVVPFFLLNLDRTAEEGMWA
NWPPMPAPISWGLTNIAGSWYGKWWKFSSCDSQGKPQELYALRGLKQ
FUN_015659 CDS scaffold_8 352615 353050 +
hypothetical protein

MVSEHQSRPKYAGFSNYTHDPRAIYIDSLGRSPPRMSKGDDLVQTPSAPSGVVQNAIPKDSLGPSPAAKATKPPRLILPVVLM
PLDQTRPRVAPVHRARL

FUN_015660 CDS scaffold_8 354616 355517 +
hypothetical protein PF13460 ENOG41

MASNRHVLVLGGNGKISRLLTVILLEKSWTVTSLIRNPDQIDDLKKISEGLPGVHKILVHDFIKVDSQEKAAAILDEVKPDSV
VFSAGPGRGTDQDTIIRIDRDAAIFFIKASVANKSVGHHLQVSYLGARREQAPWWTAEDWEGWKKVNSTFLGPYYEPKTAADE
ALVTEARKRDGLTAVSVRPGGLTDKDEGGVLLGQTKQARGMTTRASTARVMALVLEKEDVKGHWLDVLDGEEDAETAVDRYVR
DETECAEGEPVMKN
FUN_015661 CDS scaffold_8 356319 356718 +
hypothetical protein ENOG41

MGLIAKLKFKLRKVRAKYLARKRLAQGAEPIAHGERLAQVHRVEGTEHRIERGIDVMEAARRSVYGAHMLPQTTDFGVSPEEA
RALHKKQQDEDRAKKTAKESDVQEEAGI
FUN_015662 CDS scaffold_8 358371 359107 +
hypothetical protein PF04145

MHPAFATALASLSEEGAGNCTENPMLWNWNTIGTCFISKAWYMDSGGKFGITCFGAFMLVMLLEFLARVSREWERHRFARGAP
NSQEPYAPAAGIELAVLQPAAAQPAAAQPDDIQPVASRPASATGPNNGKDANDANNDANPIPNFCPSFVEHAFINAILHTVQC
VITYILILMAVSFNGYIIISMFIGAYVGALCFQRDPLTETRNPNAYQRAFQQPVVCCV
FUN_015663 CDS scaffold_8 359916 361752 +
hypothetical protein PF00172;PF11951 ENOG41

MFRHLMSSKVRSNAGCWTCRLRRKKCDEKRPVCDACGTLEISCFFDEEKPEWMDGGPKQKAMAEKIKAQVKKQASQRRDRKYM
EMLEAGTRNVTLQNEGQNQQQPHDAAMSAASDTDPASGHEFGSTPASSNTSGASPPDMPWHSHFAVPTEDSSSSTGASTEIHF
IMIYLDYVFPYLFPFYRPPILAGGRGWVLDVLQSNKSVWHTAVSLTSYFFSIVMANGAVEHQECTNRMVHQLQSQLEIGLREL
QREMTAVNHKVSCAGVRDRLLVLQAILQMLIFEVSTSNKDHWKMHLDAAITMFQQILPQSEKWGETLEALYSYRWPPPEMGIR
RPFSTNQAALRFFTANVLYIDVISSITLEQAPRLQKYQDNIMPSCKTYTNPHEVRISGSLFMEDYVGLNNWVVQIIGDIAALD
AWKKEQKKKNSLSINELVQRGKIMEDAIKGGLSVIEAQIQGPGPFFDPVISVVANPTQNYGYGVDTKQAPGLAVNNMIWLQAA
LTYLHVVISGWQPSCPEIQTSVSRMTELLTTLPSNICLRTLVWPFCIAGCLSPPEDEDKYRAMVERLGPVSVFGTIREALEVM
EKVWSRRDQIDESWDVAKCLQILGHGVLLI
FUN_015664 CDS scaffold_8 362227 362566 -
hypothetical protein ENOG41

MAQERSLGTLAMAKRFDSDSSDAESARQPWCPPPPTAPVRVKRKEVPQPLQHLPPLPPVPVSPGRMNDSGPKSPLERVWSKRK
TLLERVEGWWDLDLLEKRQTLFGGKRVQG
FUN_015665 CDS scaffold_8 362623 363246 -
hypothetical protein ENOG41

MQPALGALGHTWTAMRAMEFISLITIIGLTANFIGEMVNADYAAPSALIGTLVVACISTLYIAISYILYWDGMLPLLLSTGAD
IMLLVACIVVACTVGKPVSYLSCPKFPSDGNTANFVNSLFHNVYHSRGNTFAWVDPDKASCYQIKSIWGLSIALCVLFAFSAI
TSGCLWKRTKGASRPAPKDIEG
FUN_015666 CDS scaffold_8 364446 366230 -
hypothetical protein EOG09263WB5 PF01417

MDINDLKNTVSNLTLYDLKAGFRKAQNAVMNFTEMEAKVREATNNEPWGASSTLMQEIANGTFNYQTLNEIMPMIYRRFTEKA
AEEWRQIYKALQLLEFLIKHGSERVIDDARGHISLLKMLRQFHFIDQNGKDQGINVRNRAKELADLLSDVDRIRTERKKARAT
KNKYTGVEGGATFGGGFSSGSSGRYGGFGSESAGYGGGGGSSGGPGNYGGYSGGVYGDGGGFGGQSDDWRGDGAASRSERFEE
YDEFDEGERPAASSSRAAAKRPERTPVKKAAEPLKKKEPEVDLFSFDDPAPASSAAPALAPSNTAAAPAQEDDDEFDDFQSAA
PAQAAPSNNFMQSPAVTSTASSTAQFVAPQPQSAPQQADISQMVSMASISPAPSASGTPGPNYSAFGIPAAPAAQAPKPAAFQ
QTGPNYFSSVQAQTAKTPTTSAFSGMGSASNAAPSGPTTMANMKPVTSPSAKPAPAAGGDAFGALWGKASGGVKKPETPKTGP
AMGQLAKEKSSAGIWGAPAAPSSSGSAHPKTGGSAMDDLLG

FUN_015667 CDS scaffold_8 368173 369107 + CSR1
phosphatidylinositol transfer protein csr1 PF00160

MGNKVFFDIEWEGPVFQNGKPTSTVQSQRGRINFNLYDKEVPKTAENFRALCTGEKGFGYKGSSFHRIIPDFMLQGGDFTRGN
GTGGKSIYGEKFADENFKLTHDRPGLLSMANAGPNTNGSQFFITTVVTSWLNGRHVVFGEVADEESMNVVKALEATGSGSGAV
KYQKRATIVDSGEL
FUN_015668 CDS scaffold_8 369527 370735 -
hypothetical protein EOG092646WF PF05158

MPTPAPAEGDAATAKLGVWKQALYDRCRESGTDMFSQDDLLRLDVIPNRDLMLLARVVQSLTDDKLFITMREASGQVLWKWRD
SEEAHKYKQCSTDEQVMVYSLIDDSGGDGIWSQTLQKRLNMHDSVLKNALKQLQTKGLIAPFKNVEHPNKKMFIKASIRPSDR
ATGGPWYTDQNFDEAFIEDLQRVVYDFIKRQSSYHSTHGGGAARTQVPKKGVVKGGVEKGKKRDASHMDEPPAKATKISPTAA
VKKDALLPLPAGYTGYPTVRDIARLLSSSGITHNTILSEHDVQKLVDVLVWDNLIESVKVAGKIGYRVSRIAKQSLESWAGRD
DPTGREGGPEPIVSAFTEAPCGRCPVFEICEEGGPVGPNNCEYFKRWLGVE
FUN_015669 CDS scaffold_8 371898 372393 +
hypothetical protein ENOG41

MNESLKKNLPDIFVMLSAAEREEIDREVTAFMAEFGHMFGGHLNEGISNDDASNSGTSSADPSTNETNTQGTRKGTQEDASSA
ESSARRDRFAHLSPELASHDRAIERYNFLKRHCPWSQDEAFLNMMCSLHSHPQTMGLPPSSTEPLADESPEKEDEGSPCGS
FUN_015670 CDS scaffold_8 372993 375117 - RPD3 histone
deacetylase PF00850

MGDDIRVELGSVALNGSSPKKVAYFYDSDIGNYAYVTGHPMKPHRIRLAHSLIMQYNLYQKMEIYRAKPATRGEMTQFHTDDY
IDFLQKVTPDNMDSFMREQGKYNVGDDCPVFDGLFEFCGISAGGSMEGAARLNRQKCDIAINWAGGLHHAKKCEASGFCYVND
IVLGILELLRFKKRVLYIDIDVHHGDGVEEAFYTTDRVMTVSFHKYGEYFPGTGELRDTGIGQGKNYAVNFPLRDGITDVSYR
SIFQPVIENVMKYYQPEAVVLQCGGDSLSGDRLGCFNLSMDGHANCVNYVKSFNLPTLVLGGGGYTMRNVARTWAFETGVLVG
KEMDRTLPYNEYYEYYAPDFELNVRSSNMENSNSREYLEKITSSVIDNLRQTGPAPSVQLQDVPRKPFGGMTDEEEAELDDLD
EDENKDVRMTEHRWDKHVEHDNEFEASDDDEMAQAHGATRQNGNKRTFTDYRKGEMEIDNADAPPAKVTNGASADEPAEEQAG
EDAHDVNDDTIDDISAPAPGEKEAPPKESEKPDEAAKEPEASKVDGDGDVGMEDSAAPEETTIKKEEVEPEAPAEPSAPTEKA
SKDEPAAQEATDSATKEPTTEPKTIEETSDKPAAAPEKAQSEQPDDAMDVDTEKDKPEPPIEKSKSPVN
FUN_015671 CDS scaffold_8 375696 376844 + RPN11
multicatalytic endopeptidase EOG09263QB7 PF01398;PF13012;PF14464
M67A
MERFRSLLGGGGMGLGGAAHGTDNTNLIDNSETVYISSLALLKMLRHGRAGVPMEVMGLMLGEFVDDFTVKVMDVFAMPQSGT
GVSVEAVDPVFQTKMMDMLRQTGRPESVVGWYHSHPGFGCWLSSVDINTQQSFEQLNPRAVAVVIDPIQSVKGKVVIDAFRLI
NPQLLMLGQEPRQSTSNLGHLNKPSIQALIHGLNRHYYSIGINYRKTALEENMLMNLHKHVWTEALEMDDFRHEGCKNKDRLQ
QLVSLADGYEKRVKEETELTKDQLKTRYVGKLDPKKHLEDVGQELIEDNIVSVSRQMIDKEATMPRKEGQAGPKGQVNGEQMD
VEEEL
FUN_015672 CDS scaffold_8 378008 378617 +
hypothetical protein ENOG41

MNREAVSEVVDMIALRFSHIPYAVSGLAAMVYYGYEARPYKVSIVCPEHTRENQKCWAKAQGMIPIPRQRHVWGVATSDGIIQ
QIRVRFPYDFNEMHALKIGTAGSNPVSMLSLAGLADELARTYVNELKHSDHDRQENLAHEMIWILKRIIQCRLPEHALRPERI
PHLIRETFWLPFTLSYPESVPLFAKAGWSIPNEGWY
FUN_015673 CDS scaffold_8 379092 381868 -
hypothetical protein PF01083 ENOG41
CE5
MLLILRYLILFVTFVTCQEYVQPGNASCVSGAHIIVARGSLEPRGPGIIGAVAQQVLQRIPHSQMISLEYPAIYNPYQPSQME
GVAALSVMLAQYVTVCPKTKIILLGFSQGAHVIADVMCGASSIGFLPTKPSPSAMTNNIAAVVLMGDPSTTKGQPFHVGSSVG
KGIFPRQKPEGCQCVSDKTVSFCDAGDPFCEAGGHDLRTHMNYVTAYGHVAVDYAFYPTNISAMISFMLVLSFLMALASTNST

TDAITCAKGAHLIVARGSLEPEGPGAMGILAEEIIKLIPGSDMEALVYPALYNEYVESQTEGVRTMNSVINNYVKNCPDTDLI
LMGYSQGAHVTMDTMCGASSEGFPGTLSQPSYITDNVAAIIAMGDPSLTEGQPFLVGTSEGSGIFPRNLPIGCNSIASKTVSI
CDKGDPYCEAGGKDLSVHLSYIKNYGEFTVGHVVKAWKSRN
FUN_015674 CDS scaffold_8 382554 383110 +
hypothetical protein ENOG41

MSSSDLDDMLQAPDATVRAILRALCQDSGTRSRALSYFESLEAINDSSETRKRKAEDELSICVQCDEAFYTNDNNDKEACCYH
WGELEVDYDADIWADHDENCHGTIDTDSMRAEYPEGFVWTCCDKPGDEAGCTWGRHEADPTKSRRESGEEPIDSDDYEDGDEI
FUN_015675 CDS scaffold_8 383390 384005 +
hypothetical protein ENOG41

MSLTLEQEMVHLAPEQHVRAILLALLDDRSVKARTLNHYRALKAADNPVTGLKRKAMNDLFVCVQCDQAFTHEDNTETSCRYH
PGELEVDDTEDFWADHDEDCHGEIDTPEMREEMPDGFRWTCCDKLGGRKGCTKGKHQADPAKSQRGGNVPSGGDLRKNNGEHL
PVSDEDTEEEEEGDEEH
FUN_015676 CDS scaffold_8 384647 386886 -
hypothetical protein PF00069;PF07714

MSTIQQLKNFIRHGKQARAAAPEEAPRKTEQQQAPTVQHKTASDPSNTHYARAQEPGNEYGDDDYARSKSKKRVDDEKLAKLI
AEENASKSKFPRYPGLERWELVDKMGDGAFSNVYRARDTTGQQGEVAIKVVRKYEMNSMQRSNILKEVQIMRQLDHPNIIKLV
EFSESRQYYYIILELAPGGELFHQIVRLTYFSEELSRHVIVQVAKALEYLHEEKGVVHRDIKPENILFEPIPMVPSKHPKPKQ
PGDEDKVDEGEFIPGQGAGGIGRIKIADFGLSKIVWDNQTMTPCGTVGYTAPEIVKDERYSKSVDMWALGCVLYTLLCGFPPF
YDESIEVLTEKVAKGQYTFLSPWWDEISKSAQDLISHLLTVDPEKRFTITEFLAHPWIAGNGPTPRDEMKKADGMLRAFDATK
FEESGKRYDFRSPGAVNLREVFDVGYAVHRQEEEGKRRAQIGPKGTPARFLGGLNEEDEDDDVMQIDGQDNTAAKPNAATQAL
EQSMRKANIRDQEQQQQSRGRERERAERGYGQHSATVAAAARQQVRERNRQRGAFELNLDNATLLGKRNKKVPVMGV
FUN_015677 CDS scaffold_8 390306 392009 -
hypothetical protein PF07690 ENOG41

MSSDVEMEKVPIPVPFPPPAAVDDVDSTLGKDVDAHVHYGDAISPLEKGLLWKQDLRIIPLSGAIYFLSFLDRTNIGNAMILN
SSTNNDMQRETNMTDHQFVISLMVFLVSYAVFEVPSNMLLKKLRPSRWLAFLMFCWGAMTILTAATNNFGSVTAVRFLLGVFE
AGLFPGLVYYLTFWYKHDERSVRVAFILASATLAGAFGGAIAYGIGHMNGTAGLSGWRWLFIIEGIPSCLAAFLVLFFLPDYP
ERAKWLSEAEKDMAIHRLYYEGSKESHPTMNWAEAKETLMDWRLYAHYAIYFASGPAFASLSLFAPSITVGLGYFDLKAQLMT
VPPWAIAYVCQVLVAWSADHFNARGVHTAMAAAVGAIGFIISAALPADAYAARYGGLIMATAGSFACMPPMLGWLSSNVFTTA
STGLAIALNVSLGGGIGQIPGVWIYQTSQREGGFTTGHWVNAALLLFVSVVALGLRMFYGWKNRQLREYAESRAVPYRCYKL
FUN_015678 CDS scaffold_8 394163 395039 -
hypothetical protein PF06912 ENOG41

MVRAVDRGVRNTVVRDEETPLLGSSLSRPQKKSWKERLQHDVSCSWADAVLLACYVVTGLLDSSSIQVWGTFVSMQTGNTVYV
GLGLASFATSTPGPRLYKSALSIASFCLGSFLFARFHRLVSPRRRWVLCASFTIQALLTSAAALIVTLRPPGPNTDSLAWNVL
VPIALMAFQSCGQAVASRALRQNALISLVLTSVYCDLFSDKELFALDNVSRNQRAAAPTLLLVGVVAGGLFAQSSVGIAGALW
IAAGLKLCMVIAWFLWRSEEETEDD
FUN_015679 CDS scaffold_8 395833 397930 -
hypothetical protein ENOG41

MSRLLTFAAGLALAAAQLTSSSLENHVDTLTLGASFNPVKEAYWTGLPHHRRTPFAVSPDGKTAFLAYLDASGTGVHIQGVDP
KTFAAVGSPVTVKNGKEAGGLVAHNDGFALLTNEVVSGESKDPLPVIYKYTNGKQAFRTLLGGSGFSGNALASPDINGDLVFS
EKAGYYAAYIVVTSYSGDASGHFGDAIRYITPEGKVEEIQGASSSWGCSHNTGIAFEAADEPPFASLCSEDQGAIWLNTETQG
MSNNGVKVSNEHVINGASNEPMGGMSGSYSSLARFIGADSYIFSWVSRGAIDLTLNDWMGEGYTHAENRTNNRNVAIALFSDK
KTLIGEQASSKVGAKDGDSQVNWVTDGANDCSNAHVATFDASTALVTWEEISNPVCEFEAMGCKGKFAGTKFQAVDSKGKKIG
SAITSEDTYVAGDMVTMSDGRICWPYVNMAWSLSGPTQAADVKKISFACISNGSGSGSGSGSTTKASTAVAQPAATKTPNAGA
AKPSTAEAAAGAEVSSAESDVPEGAPRPSFATVPDSVTDVLSAPTEVTSSAAAAEKPSSVVIKPSAPAAPVDGDNEPTTTEAP

TKAAPTASKPTDGEDDCDEQESGNAPVATQVSTEIAAPVETIPTSADGEDDCNEEASGNSPVTTQAPTKANKSSGRRTRTRTR
THTRRPSSPFSKKECAAKTVTQTVYVTATAAPGY
FUN_015680 CDS scaffold_8 399828 400924 +
hypothetical protein ENOG41

MMLKYLTKPTRKNISDALLILGVIGSVISVILASIVFIAGTTIIRGDPDHLLWKPLALVSFEGIIPNNDTSHFLVHLNWFASS
FGWEHPTAPKGLPKVGITSSGLRYHSNIVNDLNQIATELRLPEDTFNCPKPGPYEDPCGNIFFEAWRSYMASGGLPISSWIVW
VNIYVALVFSIMDILREWVIPNRPHWMKCRCIIGKRWCPLPKGSKEEIEQFDDYVWDKVRLTYWAISAAYFGIAAGHTCLNSV
FFVRYLGYFEERLPDGISMHGRRRLASEILLWVAFGIKTFGALCMAVRWKLSRRPKGWMDGQSLGRLERSKPNGADGGKYTD
FUN_015681 CDS scaffold_8 401385 402510 +
hypothetical protein PF00596 ENOG41

MLTSKSFRTAPLKRVISRQASLSFSSSASKSRIILPSQKLTTKPYSNFQPSLRSASFATSSEASAPTPEEPVNQNFAAVATGG
IPYPGIPKIEDPYQKRQWQLEHMAGAFRVFSRMGFTEGAAGHISVRDPVDPNTFWINPMGVHFGMLEAGHMVHINEDGQVIGG
NRVAVNAAGFTIHAAIHKARPDVDAACHAHSKYGKAWSTFGKPLEMISQDACIFYNDHSVYSDFGGVVFEDDEGARIAKALGP
KNRSVILQNHGLLTAGKTVDEAAYLFSLMERTCEIQLLVESAGLPRQIIGNEEAEYTYRYNADPEALYTEFQPDFEYEVWKSN
GKLKSGVN
FUN_015682 CDS scaffold_8 402698 404739 -
hypothetical protein PF00326 ENOG41
S09C CE10
MSTTTTKKTAPYGDWESPISVDLIVSKTRRLDSPRANPKSGRAFYTESREDGSTTIVEILKDSIKDILPSEYSAENSVYEYGG
STYAVLPDDRIIFSNKGDTVHVLNPGSKEVEKLTGHSNLRYSNFEANLTSPWVLANQEDHERDTPDGVRNYIVAINTETAEVK
RILDTADFYYEPSFSPDGSKLAWLEWNHPELPFDSARLYTAAWNDGTISDISLIVGKDREGVAEPRWGPDGSLFFGKEVGEYR
RLFRILPGSDEHVEIKVNGIANAEFGGLRWFQGSHTYAPLSDRHLVASPFILGKSRVILIDLESGRWKDIGDSQRLAEVQLDA
VARLSNTSVLVVGSGETNAKALYRIDTEGSGQITQVRGSTDDGLPREFCSKPILKTIRSKGQPERELYGFLWFPHNPDFQAPE
GDLPPLIMTSHGGPTSYTGPGLDPRTQYFTSRGYAVLAFNYKGSCAHGREYREALWGNWGLVDSDDAAEFADNLTSKGVVKLG
GVGITGVSAGGYNTLRSLTRHPSTFAGGVCLSGVSDIKRLDDSTHKLESDYTDHLVLQKGVDKSEKDRICHERSPLFEAHKIT
APLLLLHGGSDKITPLDQAQEMANAIKKAGGEVELIVVPEEGHGFGQPKNVRLWLEEEEKWWRKTLL
FUN_015683 CDS scaffold_8 406131 407337 -
hypothetical protein ENOG41

MDDPGWSWPAWKFGMKRDDLFHTLHEKYNTFTFNLQDPEAFHHDVYEISRDADTVDDFHRLLDERKQQRIHELHESLESLAVE
IIANPKLMDSEHWQYALQLFRTKSFDSIVRYFASYLPEQYLDHNDRDHDAISIASLSSFSETNSVKTESTTASSVDDASSFFD
DEPILTKGPLSIHTDIRSSSVVQAPPSPPHSEAAHGDESSVSSPTSMESHEYSTPPSRSMSFSGSETAAAISDLTRSLVHDED
ETSQFDDGDFAVASDSDCAESQSSLDVMDDGEQSNDKYEDDLEEEDDFPTTQFPEDASDGCDFYDDTPNFDDTPTPRQESIAP
CYIEYKSVAAWRIPSPRRSPSPSPRSHHPYRREGSSLKDIRRSPEESMSKIQKPMHEQRKRPTFRKRLD
FUN_015684 CDS scaffold_8 409791 411666 +
hypothetical protein PF00069;PF07714 ENOG41

MAEPRLGLSEVVSEERTEPFSQSEIDARFKGIGVEEPVPTTSSSLVSLDELHVEDHHHRQGIRILFRPLANFPKSRSVSPGSE
SLQWLALRAEVASPEQPEHKVLAVTQTSKDIKFTVRIPGETQDDYPRPPLWCELYYDPASDKVIFLNRSDLPISLSGVSPTPP
SSPYSPSHVVNPGSAKALRPGTWRITLRDMEVLDFRVLEKRPVILYQPQQQTIVEDVPSESSTPTVNSSGKRALSPEYDEKRV
KRRVSDPNTPGDDGVVMFLRPSADPLVFSLPNGRESKELSSVNGHPLLDAEKGETAAISGVCEVDEYELTKREPIASTSLSAV
YTATHSHVPNNIVTVKVLKTRVANPNDKALVHERTVIRQADMWLRECRGQEDLQHKSIVRYYGGDARFLSLYMEHIDAKDLTS
MPRWRNNTNDEFIGDRNDAARILGDIAGALNYIHGRKLVHNDIKPANILYSPERGAVLCDFGLSTPATATNSPTVGGTPYYVP
PEFIGRKQRGPASDVWALGVTMLYVLRKITYPDSRAHRRHPRPLYWLIAGVNSPNMPHKQYGNGQPAMKQMRDWLAEIFDARK
SLNPKDRLERIVMEMLYPNPNQRITMAKVVQELAVEQVAAPAG
FUN_015685 CDS scaffold_8 412175 412890 -
hypothetical protein ENOG41

MLALKTLLGLATVQAVSAHFGLVFPPWRADTLSEEGEERYNQWEQPCARVDFNKKNLTDWPLDGGALTLDLHHEWSYIFVNLG
LGENSTNFNISLTPEFLNATNSGTLFIDKLELPSDVKPKDGDIASIQVVTVGDTGNALYNCADIRFKENAKGPSNKTGDVDYV
KIKQQKGNGTEEDSSSNSTSSGDSKDNAAGTMGVNTMVMTSVVGLAAAFVMGLGL
FUN_015686 CDS scaffold_8 414431 416185 +
hypothetical protein PF09732;PF10312 ENOG41

MDPGRKAMIAGRRSPTRRDVTSPRHDPTNRITKSTKPSGSKINPKYLTQDEQSRQFVADEDKFVLKQSKKKADIRIREGRAKP
IDYLAFNLRYIDTDRDIFDDDDADIEIDVPAPGDVVASLDAEQIAELESDIASYHVLETNATNREYWRALQTICKDRKAKLDP
HGHERRVVSSVSDDIDKILAPKTHDQLEALEKQIKAKLQSNEDIDTDYWEQLLRSLRVWKARAKLNQVYEAIQETRLNQLKER
DPTKVKASGQPTSAPKVTFGSQPVPSASEEKVSPAPVSSSRHVGTSAPPGTERFSQVDNVDFSQATKALYDREVARGVDENEE
IFTSEEAVTTSKPQWADKYRPRKPRYFNRVQMGYDWNKYNQTHYDHDNPPPKVVQGYKFNIFYPELIDKTKAPTFKIIREHGR
RRGESFAAAGEEDTCLIRFMAGPPYEDIAFRIVDREWDYSAKKDRGFKSSFDKGILQLHFQFKKIYYRK
FUN_015687 CDS scaffold_8 418528 420575 +
hypothetical protein PF00250 ENOG41

MDTPFSGDEQASSYTNIITGHGQQSPCVSELTGMEPYYALSSGPQDQPSHLSSTGPLSLGSTLSQHQENQQQTSHPTHDATVY
ASMTSGFPHPPRSHLQVWPSPPPGSDDYDNYSYQSSPSSGSAPMSCYNPSPISPRTWSSPDFPLPQSTDPLQQNSLKNLRICT
PTSNEGYPVRGPRGLTPFSGGGMNQIFDNEIDGLPQNYSPVTIPSSSAGALSCSGSPQEPLLGTPVLMKARHKSPAGVPEYRT
NSEVVQEGKDLQAAAAGGAKTDEPYAKLLYRALMSVPDHAMTLQEIYQWFRENTDKDIKKDKSEKRPGKNAEGWQNSIRHNLS
MNDAFVKREHKLASDSASRATEQGSGSTKTGEAKKATEWVLEDWAVLNGVESTTKYRGKNTSRRGMGRKYHHHPYDDRFSQRH
SPIAAKGGSIVKGEFLTNDMRMRERQYTQEVNMAPRSHHINLIAPPHPHPMRRATTMPSMYQSPQPQHRGYEGLMMPRVERMP
QPVIKQEYSPMTPDSSGFGFVLPEPSLIHAHAVNSSASNSNGTTAYTLPSGTQSMYVGSSPCEYPYGMVDVTGVYQGSGHNSP
TGAGASASASGGVNERLIGIGPNDVYSWNGQAL
FUN_015688 CDS scaffold_8 427341 428970 -
hypothetical protein PF09302 ENOG41

MSQIKSWRTLPLDSSPDFPVLLVSFHTDTSAYTIHVTDMANMWSETLDRKAIFMRGWNENTSIDPSDTPDNMAKLLGCLNTAL
DSSHPRHNEASIRLDRDSRSDAGDHDLVLNITYEIPGLQPLKWPMYLKKLPPSSIAKHLVLPLIQAHHAKDVEIEFLTRSLGN
KDAVLNKLLDKLEAVGTGMEHVFNALSGKKKISRAAAAEKVPGLAPFDRRRWKSGLAYEEDCPNDAESLVQNVFEKGGLQFEP
IIDSVESPRLDQWWQDFKGASSAAHPPQYKAAVAKDKSPTPQDPAKARIEKDDDDFQVQSTPPHLLPQGKSTHAKETLVADDA
STEGESIASAGSVSPGPGTRKPNKPVRKIGALGGKKQSTPPLSPNPVQSHAQKKSLSQQHDDSETASESEDDDKPATAEVEKS
APSTSSPPRPAANRSGLGRIGGNKTERLVEEQPVTLNPEAGEPSTAAVSHRHSNKLGAIGKHKDNINEAMKTTNEDNRRSRTA
TKEVTAESRPRETSQERADRRREELKKELEKKAAAGPAKKKRRF
FUN_015689 CDS scaffold_8 430324 432200 +
hypothetical protein PF00875;PF03441

MPPKRTKRESSSPPAKKAKTSKTNGSSTSDALRQPHHKAGEAEENGIAIRKFYPPEMSNARARAYNNNELPRPIEVLISALEN
TAQERKSIDVKKAVVHWFKMDLRLSDNRALALASDKAKEAGVPLIAMYIISPQDYEAHLRSPVRVDFMLRTLEVIKHDLAKLD
IPLYVETVEKRKRVPDRILELMDQWGAGHLYANMEYEVDELRREASMIRDFAENGKAFEVVHDTCVVPPGELHTGAGKQYAVY
TPWYRSWVAHIHENLDLLELYEPPEKNPSSARKDFAKLFDVEIPDAPKSKRLDGEEKERLRSLWPCGEHEAKKRLDKFCEEMI
GNYQKKRNIPAEAGTSSISVHLASGTISARTCVRTARDRNKTKKLNGGNEGIQTWISEVAWRDFYKHVLVHWPYVCMNKPFKP
EYSNISWSYDNEHFAAWCEGRTGFPIVDAAMRQLNHLGYMHNRCRMIVACFLAKDLLLDWRKGERYFMEHLADGDFASNNGGW
GFSASVGVDPQPYFRIFNPLLQSEKFDPDGDYIRKWVPELKDIDRKAIHDPYNRGAGSKAKKNGYPAPIVTHKDCRDRALAAY
KEGLASGE
FUN_015690 CDS scaffold_8 433101 433439 - CRP7 40S
ribosomal protein S21 PF01249

MENDRGEIVDLYVPRKCSATNRIIKAKDHGSVQISIAKVDENGRAIQGENHVYALCGFVRAMGESDDSLNRLAQRDGLLKSVW
SGQR
FUN_015691 CDS scaffold_8 433867 436549 +

hypothetical protein PF02178;PF09494 ENOG41

MVSSNAFGTSPLGDTRRQPLHVNSSSPDLPSLRDVLTTKTTFQPSAHSGSGAAALSIDGPSGFISARQLYPATESAPKVVSTP
STGLPWTLSHSGEVTGLAEDVSGNGGFIAGDNETPKEREEHIEVIKITGKSTRKPRQQKVSNQAAPKRTAKPKPTTKKQSSKA
ASKDHSPAEDNDDDKQKKVKPTKPRKKTGTMSSHFPPPKESKDLEKAKQIDVNEPLHLEQAPARRLDWTPPAQKTIVDLDSDS
SVFKNLGSSEADQRLPVFKNLVDGYSCMEGPNESISHSITNASDEDSSFLKKRKRIELLATMGTDAPAMAYDKSPTKKATKKK
KPRTITELATAAYRVPSQPDTEPPNASILDHFSTTNKEAGSLADEQTKNAKGKSTSRRKPSKAPKKKVPPKPVLLSPSAALAQ
VANQDFVFGTSSQLAREESPTVLRDLQAALRQSDQNHDMDFAMPLNSDAIESPQQRSNLWNAAARDAEGDLFDVEVINLADDT
GLSEVGHVSNPFGYQLGADDSVICVESRVLDDHIPPAKPRNDIPSPDEKDLVDSRASSPYFSDSELSTSTNVHRPPLAQTEVS
QANQMTTAEEPESLPELPAQPPRPNYEAFTDIRLAREIKRFGFKPIKRRSAMIALLDQCWQSKTRMGQASLHTSTKLSSPTKT
TRATPPATTSSPKKPRGRPRKNSINAPEPQEPPPSAQPPETPKRPRGRPRKDSLSSIPGAASPSKPKSAAKPQRVVASPRRKK
ATAKSVIEIPDSEDNESDEFASSPDTNAEQMFSSPPPLDMSLTTNDGTPLTATQSDQEALLFDHITNAVTSAPRTTNPQEPSW
HEKILIYDPIVLEDLAAWLNTGELSRVGYDGEVNPNDVKKWCESKSICCLWRISLRGKERKRF
FUN_015692 CDS scaffold_8 440693 443654 +
hypothetical protein PF00172;PF04082 ENOG41

MSGYLEGMALGFGFNVSEGLDSAQQSTASPSSNTQQDASFHTQPNSFQGNVTVYQTTGPDAHTSPIHLPGSNTPALNPRSCVT
CRRRKVRCDKQMPCSNCRRAQIPCVFPAPGRAPRQPRRKDPNAPTKNSSQREIELMKRLRKLEGIVEELSGQIEVESGGKGPS
STNSPEAHVGQTTSFDASAHNAQRQSSNAGSHAGNIGRIDDSPKGGSDATSEQSETARKNMHKKFGRLVLSDTGGSQRYVSHG
FWSKLNDELDSIREETQKLTDEDLDESDEDETLDTSPASALSGNHNAWIFGYRSADVDLDKYWPLPSHIPFLWSVYQENVEPL
LKVLHIPTMEPIFRDARKDHKQLSPANEALVWSIYYAALTSLEPDEVRANLGLNKEDVVVQYRFAVEQALAKANFLNSLETPI
MQALIIFILVVKGQDGSRFCWSLTGLVAHLAQGMGLHRDGSHFDLSPFETEMRRRLWWSLLLLDLRSADELGTDLIIQDFNYD
TQMPSNINDTDIKPDSTEAPEPREGHSDCSLALVRFEIAGFGRRLVRLSSASMSFCPQGMPYKDTSLAERERMLIEVYNRVEQ
KFLKHHTGDSDPLHWMAASIARIIMAKMTLVIYQPVLFPGSEGEGTLSEEAKERAYVAAIEIAEYNHILNNDSRSKQFRWLFK
TYTNWSAMAYFLIASCRRPWTPLVERGWEAVNGYEGRPGQLVRSAEYATVVMPIRKLYMRAQRHRAAEIARLRGNPEEARRLE
FEERTNPRYQARFGQDADGAANKMEEIREKWKVLVRSDGSNVPISTPAELSPVMPPSMLQPKQEQVPSRPRTPSAYDVPATLD
LSDTTMGYMNDLMADTTPFPMVNFWPLQDVGSVQIKAMGTGMTPMSNTSQLPQYTIQPGDTGQQFSPQTMQFPKDDNPPPCLW
GGQFPSVNSTSDNTGLDDVDMLGDDFNWHTWSQNVRGL
FUN_015693 CDS scaffold_8 443877 444831 -
hypothetical protein PF02661 ENOG41

MGPYAFMRALTERTRVEPYNIIEVLSRMIYGSNMISVAGGSLPTTYRLCRVVFEETSQPPEEITQNDLEYEEIRNHLEFRCIP
VSHGLILRCYRETLQHAQTAKYLIQKIFIDREAFSEATFKEAHRILTYKIDINQQTPWELYSGNYRTWSATYDTRFVDPVHIT
TAMLSMIDNLIVEMGSREVPYDGITIQQLSKEEVWERICYASKFCQHFINIRPFLDGNGRMYRLLLTTLLLRAGICPAVYGLH
NMDRESHSRAEVSCYRHDLQGLGEVVTETHHELALFILNHTYGQWKSTDAQMMTFLQATRRDGAMFPH
FUN_015694 CDS scaffold_8 445460 446559 -
hypothetical protein PF00293

MTSRNQSSSANKPPPSEPRPSSSVLLVSPVNEVLLLHRVHTSTSFASAHVFPGGNLDPYHDGIIPAEDSPERHHDGLAYRIGA
IRETFEETGILLAKKDNELVNLTVEDRDTARKMIHGNQVKFLEWLKSVGAEPDLDGLIPFTRWITPATNTKRFTTQMYLYMLP
QSQSDMPSEMLIPTPDNGIEHTAALFAPAESFLARAATNSIILFPPQYFLLSLVARVFQNSKESSDGQRTPQITAQRENLLSF
LNRVPTAETEKGQAHKTAMIPWSEKVMSPHNLFIRQQDKRIVLGLDKPGPELRGSERGGDWEYVVLVNFGKGGPTNVEVRRRE
DALEEERKVKADSEKL
FUN_015695 tRNA scaffold_8 447404 447488 - tRNA-Lys

FUN_015696 CDS scaffold_8 447834 448853 + RPL23A_2 60S
ribosomal protein L23A EOG09265CQO PF00238

MAKISRGAPGGKLKMTLGLPVGAVMNCADNSGARNLYIIAVKGIGARLNRLPAGGVGDMVMATVKKGKPELRKKVHPAVIVRQ

SKPWKRFDGVFLYFEDNAGVIVNPKGEMKGSAITGPVGKEAAELWPRIASNSGVVM
FUN_015697 CDS scaffold_8 450243 454673 -
hypothetical protein ENOG41

MDRSRRMPQRRRPLGASASPTSTSEASFDLNPLTSPVVSHTNTTTPQGVGGRRSAKPLQHAFTFSSPSSSRNSNAKKRSTTMD
NYPIPDEGEHGPKKGGHSLRKRARVDYTFEHIDDDVVVPNSTSSARGKKRRSEVNFDTDDFYSNDSKRRGASMGADTPSNRRR
NPTRKSSDLKAYHQAALQEDDNDVQDTIEVGAYYSDVDDSELRDAASNNSSPQNKMPSKDSPKRSPQNESTKDAFAAPQVGEN
QHQLETKETLSVPQVEQISSDKVSSAVEPTRTSIEEPVSQPTEPQAEKLPKVEPEPESLSTTHKSPAAEGVNEVKPPAVAVAD
AQSEPAATAAPDSNPVEVEPEVETEAVAAPVSEPVPAPTIAPLPITADENNKSEPVHSPIVPASEPVPVPVNGVAEAPTTEPL
PQDPSEPALSLINTNNNTDTDTNIKQESSHLITNIYQFPTSTDKLIDLHQDDKMNAMGNKEIDPVQDEHPTQDVEMTDAAPLD
TQELVQEPVQEPASKPAQESMELTQEPEQLSQMPEQEPVEEPTTAPLPPAASSPSASRARSASPPKPPSKPPSASPAREIEEL
RQSVENVASPQNVTSNDVPTPVEVNGDSTKDEKVNNETVENNKDKESAQVPPTADEFPIPDSPEIPLTQEEPPTISSPTELKA
PTPPPTETIETPASSAPSDTKASPQKPVAPRIAMESMPQPTPVGRWSHLKPYVDGEFTLYPEKKGRSDEDGANDDTTPEGKDT
DMEPMVDDQYDIVDGVGLEAPTPALNTPTRGSPVADSTDPTAFNSPAPGGDDGDDADVSESQEVPERTRYYKYRKLRDPEEYI
SILENYEDMSTEDLYELLEAVNVSMVQWQDEWNDLGAVVDDYENSLRRRAADAKYEARTRNLNQHGLNYEEPEFAVKGYKSRD
KEGLNETRYLQGQDRIMAAAYGFEYDPHPSKIGKQNPETQQVGIMTRGRSLRNQPRQTAKATETDEVVGKRQRKPVQLFDPAT
QDVSRSSTPVPPTRGGRRRKNANVEDEPQTNLSVSFSTEAASDNEASGPKTRRKRGRGKNAAVAEEPAPTPDAEEPVQESPAK
PTRRGRARPAVKYEEADPNEFVDDEPQEEEEVQEKEEKAPVRRALVTLKLPRAYYTDVTPENEITDNGDSRPTTASSEASGHT
VESSYSFRPKRQKRFRDDPNEAEESAQAPPKKKGKRTSGGTTTTEVPSNASTPAPSVEPAQVPNSRKIQKIKVVRSGQDAKNG
GTPAPQAPHPVAVPATGPPSAPPSVAGDDGDDTPKDYKSMTKSEKMSASMKNRWANGNMAGAVEKRKATLAAKKAAQAAAEQR
TGVAAPNKPKGKATVKREPSSLKMQMQPDQPQMPPQQPPMLHQPPPMHPHQHELPPHQYHPHQHQHQHPPHPLHLQQQPPMHP
AHPQHLPPPPPPPPRQAGNPPPPFMHGHPQHHSHGHSHGMPGMGYPY
FUN_015698 CDS scaffold_8 456074 457630 +
hypothetical protein EOG09264NDD PF10310 ENOG41

MASKKTKPTAGDDVDIDELLSGLDRDKKPKKTTKSKPTSAASKALADQDILADLESELAEQPSRPHTPRLKDATRRSTATPPA
GDPRKSTDSARSLKATFTPSATSSELHENEKKPTFEPVQQEAAPQASGGGWWGGILSTASAAMKQAEAAVSQIQQNEEAKKWA
DQVKGIRGLDVNTLRTYGDELRHRALPTFTNILHTLAPPISSHERLLIHITHDLVGYPSLDPLIHNVFGHVMAQVEGGDLLVI
QRGQESHSRRSTDSITGWHDGPWWRQTDTPRELGLIKGLPEGTKLCRANAESHANEYFSANGGVEAAKHKATEDVSEANPVRT
SDLFLAVQAISVDEDKNLFARTASAEKEKDSSGVENQDENEELICFAVFILDPVHDIEFYTVSQSVPARWVQWLDTPAPLTPR
SGEDGDVTDANIPEEIRDIIESGGVDPREWVAEWLEELLNLSIGTVAQRYVARRMGVGEGGLGRGKRRMEDLVQDNAGEAARA
GVI
FUN_015699 CDS scaffold_8 457901 460013 -
hypothetical protein PF00390;PF03949

MFKLSRSSPVHRFRLYPSTMRYLNLTNNYATKADHSKKSKFGDLPLSTTGPINCALSGTSLLNTPYLNKGSAFPPDERREFNL
TGLLPQGVQTLEQQCKRAYEQYSSRHDDLAKNTFLASLKDQNEVLYYKLLQLHLDEMMSIIYTPTEGDAIQNYSRLFRRPEGC
YLNIRDPDHVHHNLAQWGNPEDIDYIVVSDGEEILGIGDQGVGGILISVAKLVLTTLCAGVHPNRTLPVVLDCGTDNKELLDD
ELYLGLKEKRVRGEEYAKFVDTFVQSARKLYPKAYIHFEDFGLTNARKILDRYRPDFACFNDDVQGTGCVTLAAILSGLHVSK
QKLKDLRMVVFGAGSAGLGIADQVRDAIATEGNMSHEGAAKQIWLIDKSGLLTSETEGVTDAQKIFAKDASEWKDKDADLLSV
IKEVRPNVLVGTSTKPGAFTEDVIRAMAEHHERPIVLPLSNPTRLHEAKPEDILKWTDGKALVATGSPFPPVKGPWGKDGSEI
TINVGECNNSVVFPGIGLGCVLSRAKHLTDRMLVAAVEGVSSLSPVLKDSTAPLLPDVDSVRDVSVRIARNVIQAAVKDGEAT
QEGIPEKEEDLEEWIREQMWDPVYRPLKKVDLEGATREARGELKKAGTVHRVGHT
FUN_015700 CDS scaffold_8 461002 462838 -
hypothetical protein PF00083;PF07690 ENOG41

MESTVDERTKLAYQNRWRTLAKNPKLIVIALFASFGGFEYGYQQGVLGQSLVMHRFKENFPSIVNSSSATGWLTSILQLGGIL
GSLSAGVFGEVYSRKYTMFMACCWVILGSYLYIGGTKDNASMLYAGRFFTGIGVGTFSGVGPLYNAELSSPELRGFLVSFYQF
CTILGIMLSFWIGYGSNYIGGTGQGQSNLAWMLPSIIQGIPAVLLALGIWWLPFSPRWLVKQGRDEEALKTLSYLRNLPIEHE
LIQVEYKEIKAEALFEQRAFAKNFPNLAAKEQGSVWMAQFAQYYQIFRTKDNFKRVATAWLVMFWQQWSGIDAIIYYASQVFE

SLGLTGGTQALLATGVTGVVFFVFTLPAMAIIDKVGRKPMLYVGSIVMLISMVLAGIIVAKFQHDWETHSAAGWVAVAFIWLY
VGAFGATWGPVSWTLVAEIFPLSIRSKGSSIGASSNWLNNFAVAFYVPPMFETLEWGTYIFFAVFLACSMVWLYFCLPETKGA
TLEDMDRVFGSHTGEEDAKMLDEARRDVGLGIELEVDALKAEKALVEDGEIDTRPSHHENA
FUN_015701 CDS scaffold_8 464549 465659 -
hypothetical protein PF02661 ENOG41

MPNHKRKSLSQGPFEGLQNENNHTGKTPAPKRQRFDALFDDPDDYILDNHLGRSIRGMLSPLSKEALVINMDDDAYEYNILQK
SPKELHQELRRHITDVKEGLEQSEDKSDLDEHIIDELARMVYGSNMIENVGGGLDITLKLCKDVFRGGKEPPEEISERDPDYE
QLEIYLSNNDLPCKHADILQSYREIVQHAKAARFMIEQVCINGKDLSEDIIVQAHGDLTYKIEISEHISWNVYSGRYRLWNVR
CGSQVFMDHSKVRIAMRDMIEELESDIKKATNKGQIDPVALASKYCHQFVNIHPFGDGNGRTCRLILNTLLIKYGGGCPIVSI
GQDGRDRREYLRIAVARKVWRSSRFSHYNMPVIACLG
FUN_015702 CDS scaffold_8 466488 467790 -
hypothetical protein PF00082;PF05922
S08A
MVNFKNLAFAATTLLGLVNAAPTTAKVDSSKIIPGKYIVTLKSDIAAADVESHLSWVEDVHKRGLNKRAEKGVERTYNGKYGF
HGYAGSFDKSTIKEIKENPDVAIVEEDREWVINWVEEEEEEAETLAKRALTTQSGATWGLGTVSHRSRGSTSYIYDTNAGTNT
YAYVVDTGVRTTHNEFEGRAQAVYTAFSGDNADSVGHGTHVSGTIAGKTYGVSKKATIQAVKVFQGSSSSTSIILAGFNWAAN
DIISKSRTARSVVNMSLGGGYSASFNNAVDSASRSGIISAIAAGNDGANAANTSPASAASAITVGAIDSNWAIASYSNYGTVL
DIFAPGSAVLSAWYTSNSATNSISGTSMATPHIAGLVLYGISVNGVSGVSGVTNWLTSTATSGQITGNLRSSPNLIGNNGNTA
Q
FUN_015703 CDS scaffold_8 472940 473501 -
hypothetical protein PF03992 ENOG41

MPIYLSMQRVRFSSPDAYEKFKVLFADTRRHLMTLPGFLHLTWWEHPDDRSWYNECSFWTSRGALYDWHKNTYHKYCKEWAAN
GAIMEDIITNFELVGTRLLRVCPVCNHTQDKKYNLAEEQAVLHEQCPQCGFHFPVLEETPSSFAVFKDVPGIPMPEGAPKKVD
GSKVDGRQTDGNKESSTCPM
FUN_015704 CDS scaffold_8 474053 476669 - MAN9
Beta-mannosidase B PF00703;PF17786
GH2
MAPRTVTPIDKKWQFKQEKEDDSAYLPVAQFPTNVHLDLLHHKKIPDPYIGKNELKVQWIGETVWVYKTTFSSPEIHDGAEAI
LAFDGLDTFATVELNGEKILETENMFVPERVDVTKNLKRDGENELRITFDAAYLRGWKLVEEHPDHKYVVWNGDGSRLPVRKA
QYHWGWDWGPALLTCGPWRPINLEVYESHIADLYTESEVEKSLKKADVVVHAAVEGTASKVRFEVSLDGKKVAFETTHVKDKD
ATTTFHIQDPALWYPVRYGKQPLYTIKATLLEDDNEIDTISKKIGLRKLELVERELDGQPGTSFFFQVNNIPIFCGGSNWIPA
DNFIPRITKERYRDWVKLVADGNQFMLRVWGGGIYEEDVFYDACDEFGILVWQDFMFACGNYPAWPDLRKSIDREARANIKRL
RHHPSIVLWAGNNEDYQLRESENLTYDFDDHDPESWLKTDFPARYIYEKLLPDACKDLHPNAVYHIASPWGGKVTTDPTIGDI
HQWNVWHGSQQKYQDYDKLVGRFVSEFGMEGFPDIKTIDAFLPQGKDDPERFSTSSTIDFHNKADGQLRRLGLYMAENFRFTL
EPLEDYVYYSQLLQAEALASAYRLWKRQWQGPKKEYCSGVLVWQTNDCWPVTSWAICDYYLQPKHSYFTIKREMAPITIGMTR
REHKHPKNKYTRVDIETKVKVEIWGSNLKLEDVTVDCVVKAWDVETGKETYSQTVVSDLVLQSNRSTEIKTLDVPVEKPNADL
EVKTVVAAYFYQDGKQIARYVNWPEPLKYLHFQKPKNLKTEISEGCKSVEISAEVPIKGLAVLSGDDGIKFEDNLVDIVPGEV
VKIGVTGAKKGTVLKTQYLGMQVHD
FUN_015705 CDS scaffold_8 477539 478779 -
hypothetical protein PF00025;PF00071;PF01926;PF08477

MTRPRRHSGASEESSGTAREQELGSMYDYLAKIILLGPSGTGKSCLLHRFVKNEWRVLSSQTIGVEFATKIIKVGTGSRRKRI
KLQLWDTAGTERFRSVSRSYYRGAAGAILVYDLTSHASFRNLQPFLNDARALASPNLSLMLVGNKLDLTDTLVDTSLPPPTPT
SVTSNSTLTSGMPGAGGSSYTSTATTRDRGASISAGSQQRATVAPEGREVTRAESSRWASTAGISVVTEASAFNGEGVDEIFE
RLARIILTKIELGEIDPDDPASGIQYGDSGGWNTASDGGSIKSSMTGATVDDGHSGLRRRRKKNRTQNWGLREWEEVFTLSNR
RRGGGCC
FUN_015706 CDS scaffold_8 479951 481306 + LYS1_1
Saccharopine dehydrogenase PF00682

MQSANNPISHHTSHTMGQGAASSNGGDNSCGANCVDNHVGHAIGDGDGVVRVSGGETGNDGGHVVEASVTDVGVNDLNQTTTT
YDESDDESSLADSSGSISDGPSFGGTSIRSLGSMSNFSIIDTTLREGEQFINGNYDTETKLKIARALDDIGVEYIEVTSPAAS
PQSKLDCAAICKLGLKAKVLCHIRCNMDDARTAVETGVDGINMCIGTSTQLMEHSHGKDLDWIAAKAKEVIEFTQAHGIEVRF
SGEDSFRSDFSEILKLYSLMDRLGAHRVGIADTVGGATPREVFDKISTLKQMVGCDIETHFHNDTGCAVANAYTAIEAGATHI
DTTVLGIGERNGITSLSKLLQCMLQMGHDSVLAKYKLEKIPALEQLVAEAVNIEIPWNNASLAAYL
FUN_015707 CDS scaffold_8 481812 482716 +
hypothetical protein PF00106;PF08659;PF13561 ENOG41

MKIQGRTFVISGGASGLGQACVEDICANGGNVAILDMNEENGQELVKKLGPSAKFFECNVLETESVTKAVQGAAQWAKETGKP
LGGVIAAAGVSTPATMLDRNGAAFSLDDFDFVINVNLRGTIDLVRQTLEHLAKVDPEGSDGERGVVIMVASSAAFDGQKGQVS
YSASKGAVTAMTLPMARDLARYGIRVVTIAPSLFDSRMTSVMPEKVKKSLEGAMEFPKRAGQPKEFAQLARQGIENVMLNGVV
IRLDGAMRMPSKM
FUN_015708 CDS scaffold_8 483161 484300 +
hypothetical protein PF00389;PF02826

MAPTKIAILDDYQGVADPFFKKLDPSKYKVVSIKDTLLPYNHPDSSQSNKDALVERFKPFDVICTMRERTPFPRELIEQLPSL
KLLLTTAFRNRSLDLDTLKERGIPVAGTVDKPRSGKPVLAGTGSTTQHCVTLILSLARGIARDDAAVKEGLWQTGLATDLSGK
TLGVLGLGRLGSAVARILNVALGMKIIAWSSNLTQEAADEKAREAGLPVEDGEGEKTFKVVSRDEFFRNADVVSVHLVLSDRT
RGIVNKEDLTKMKSTALFVNTSRGPLVVERDLLDHLKAGRIRGAAVDVFEPEPLPADSEWRNKNWGRDGSSQVLVTPHMGYCE
EDTIKSWYEQQVENIERWAANEPLHNVFT
FUN_015709 CDS scaffold_8 487408 488434 +
hypothetical protein PF00230

MVQFTRADTGMSGLPTEEAVADRRAGSPIPNRVRNAIVIVLGEFCGTFMFLLLSFIGAQTALVTNNPTNSTAPLEPFSLMYIA
ASFGTALAVNVWIFYRVSGGMFNPAVTLGLVLVGAVPPLHALAIIPTQLVAAIAAAGVTDGLIPGPLLVTNSLGNGTSIAQGV
FLEMFLTAQLVLTVYFLAVEKHRSTHLAPVGIGISVFIAHICLTNWTGTSINPARSLGPSVIAGFHGYDWIYYLGPFMGSFLA
FGCYKIFKVLEYQTANPGQDDDDLERGSKHHFFGHHEKEPISHSQTDTLEPKDHGVAPRNDSVIDGQMSNA
FUN_015710 CDS scaffold_8 488766 489342 -
hypothetical protein ENOG41

MSTERSLFKTVVYGLWSFQFGFLLIAETYQLLVDSFSSRLLLLLLPFTFELFLLFTTASRIPILAPWTKRFHRFFYETRDWAM
VLPVLSILWIFGVLMVASLVFTDLIGSGRGPINRTLVFIQSYLGFVVYIVYIGGIISFIPLPFWAAFVGFQACMRVKNLRSPE
EEEAMGLTTKDDDDEWNDFQDNRGN
FUN_015711 CDS scaffold_8 491244 493647 +
hypothetical protein PF00172;PF04082 ENOG41

MSSVKATKKSAFSCEPCRRRKVKCGGEQPICNRCAARSDDCIYKLNPTLSYTQRLEERIKELEDQLAAIKSPPSVAASNHSSP
GVWNGHDLNRHPDEHAMTRSFMGLKIDDKGGITYHGPTSLFNLPSDPHKHKPDSISSIDSDAHRRERLVNNAWHQRAMENLSD
IPEPFQYLLNVHWCWIQPLFNFIYRPAFTRDMQSMGPYYSHTLLNAMLSHSIRWGKSDPSTKQLLDQSYDGGAVFAKHARSML
FDELSRGVCTIPTVQTLLLLSAGECGHGNTTQAWIYSGIAFRLIDHLGICVDGQRYPGSVHLTDEEVEIRHRLYWSCYFWDKI
ISLYLGRSPSLQHTQVSPPQIIMDDSAENELWVPFDSPHGSDWKYPPATAHSTSCFMSACRLSVIFNEILIHMYDPLLENTEA
EMQECLQTQDPAMRLWWEQLPPHLKIELSAMPELAPPSHIVTMNALYHTFRILLFRPMLTWQVYPEDEGPHPMQNHLVECVTS
ATSIIAIFDLFCRTFTISHCVLSLSYSVYIAATIFLLQVQATPEDQQAVRKLSFCVRALHQAKTVNPVISSALDLITREMANL
GLDPGFSTPQQQPPPPMPQPMPDVQMPTDIPITETVYPASFEIPAQQSPYEPEQLFQTPWADNMNPNAMAVDRGVFEALSSFE
PLSVRVGAIYESGNNPQNFG
FUN_015712 CDS scaffold_8 494331 495222 -
hypothetical protein PF04909 ENOG41

MTSPNLAQRIPPDSWDSHMHIVDVENYTLDASAKYRPTSHTLDQALAFETGVGLQNIVLVQPSIYGLDNSCLLDGLRALGTER

GRGVVAIDPEVVNEEELRSWHGLGVRGVRLNILSNELAIDAAELERQLMLYADAVRPLGWVVQVYVPMDMVTLLEPIIPKLNV
KFCIDHLGQPPLNKYAGADPYDIPGFTSLINLLKNGQTYVKLSAAYRMSTLPDYSDLDPIAKEVIRVAGKTHVVFATDWPHTR
FEGLDIKPWMERVLSWCGDDEHLKERLFRGNAEDLWDVQRPSGKADQ
FUN_015713 CDS scaffold_8 496126 496794 +
hypothetical protein PF01625

MSYLPPFLARLARPFTQSTRLSIAPDQSAASVIPEGAQRCTVAAGCFWGTEHLYRRHFADKGLIDAKVGYIGGDLENPSYRAV
CGGKTGHAEAAQIIFDPTKVSYAQLLEFFYKTHDPTTLNRQGPDTGPQYRSAIYFHNEEQEKIAREVTEKANKQWWKGGIVTE
IAPAGKWWTAEEYHQLYLHNNPSGYECPSHFLRPFPPLE
FUN_015714 CDS scaffold_8 497140 498223 -
hypothetical protein PF01027

MTVRQGPARLAQRLPELSKTAFRNTASVRSFHVQSKPTANFFTSRVTSAATRNAFQRTSRAGGRSYNQQAAGAATAPQSTSRR
LLVGGAIFGGTLVAINAVFNRETREDGGMPVYEREYLNNTFLHTGLGIGIIGLTARQMVQTGFVYRIMVTNPWVVGIGGLALS
FATMIGTRSISPDNYIPKYALWTAFNATQAAFVAPLLAFVPPALLGRAGLYTIAMMGGLAVVGATAKQEKYLYIGGPLLAGAA
IVAASGLAPLVIPATAIRTLAFTESIWLYGGLAVFGGFTLYDVQKVLHHARLAQAGVIRRDPVNESISLELDFLNIFIRMVQV
LMMQQNRRK
FUN_015715 CDS scaffold_8 498854 501833 -
hypothetical protein PF00027;PF00646;PF13516;PF16643

MRRGRSPSSPSPYRALAHHQQASAAATSSDSISLIRSFNVETNPTRPVRPSPLTASLIRDMPLDLVDRIRSFPLFMSAPEEFL
VAIGNHLKPQIQSPNDHIVTEGDEAKAMYWLVRGVVAVTSRDGEAVYAELNPGSFFGEIGVLMQMPRTATIVARTKCLLLVLK
KEDLHAVIPKFPEMEKAIREEAQERLNLLKKKRQESGRLVKSPQGDFSAREPAPGEVSTGERGAIKDGAVVNNKKRKSPSPGI
MEDPAAGSAIGSGQINIRTTLKELPLFSTLPADILHFLGLSVQPKSYTPFTDIVRQGLPGNEIFFIVRGEAEVIHDEPVYDDN
IQNKAKAIPKRPRLKAGQYFGEVASLGLSRGRTATVRSITSVECIMITGDVLDEFWRRCPSDILKQVKETARLRYQKQSDDVV
MVDVDVKEKVNKSDPPTPTTPTRASLPDLTFTTPSKPGSPAKDDTSNIAPKDPDPFLSVNMENIRNRRRHSLAPPVPPTDSST
PTTNGARSRLSEVTPIKFAFSDASADDDDVPAKRVRTTLPTRPITMSKPVLSDEILIYIFKHVDIGELFRLRLVSTHWRKLLT
TSPKVCQDVDLSIYNRKVTDEVLIKVLAPFIGTRALSIDLNNCFHITDEGFGALWRSCGKNVKKWKMKSVWDVSANQILEMSE
NAKGLEEVDWSNCRKVGDNLLGRVVGWVVPDPPPSKSVVISSSAARSRSKQQQQKHAAQHVLPPGTVVGCPKLKRLNLSYCKH
ITDRSMAHLAAHASNRIESLSLTRCTSITDAGFQSWAPFRFEKLSRLCLADCTYLSDNAVVALVNSAKNLTHLDLSFCCALSD
TATEVVALRLPKLRELRLAFCGSAVSDGSLESVALHLNDLEALSVRGCVRVTGRGVENVLNGCGRLNWMDVSQCRNLESWIRA
GGVSNWGFDDRVGQRSTEPTSSETSGQGQSEDDGPAPKAMSVAMLTSQNFMPRSAFGYRAKRARRPVRFVIEKGFGSLR
FUN_015716 CDS scaffold_8 506853 507910 + LYS1_2
Saccharopine dehydrogenase PF00682

MCGTENGSVGAAPHPAQNANNPATSVYSQVQDFLSNTNRYKIIESTLREGEQFSNAFFTRENKVKIATMLSDFGVDYIELTSP
VSSQASYDDCKHICSLGLKAKILTHVRCNMDDAKRAVECGVDGVDLVIGTSSFLREYSHGKSMEMIQKTAIEVIEYVKSQGVE
VRFSSEDSFRSDLVDILALYKAVDKAGVNRVGIADTVGGATPRMVYDLVRTLRGK
FUN_016450 CDS scaffold_9 581 1485 -
hypothetical protein ENOG41

MGGRGGGRRGRGGGRGGGRGGHNGGVKRSGGNCYNCNSPYHQRKDCPTRDRPRHGRASRQEALPAAPPVAALPAPATTATPAA
PVPAATLAAPALAPTLVPAPPLSLAPSELSAVIFPLDPVEFEAEVLDTPMSDLPAVPQANEGSAAVVQDPEQLEAARLMVIQA
VSRATTLSGDVCREILTSNNGDVNVSIDRGIMLATVSRVTGMTLEASEALLRASAWVLGDAETVIAQWFPDGNIPDNLLRADV
IAARRERDATMAEPR
FUN_016451 CDS scaffold_9 4239 5356 +
hypothetical protein ENOG41

MSGEDATRRFIRAMHQVYGDFDKDADTWTPPDNPGAGGHKGRYLWTDAFGVVNFITLYEETTDNKYLTLAKRLVQTVHDVLGR
TRDGKSSLPGATDAEPLKGGLRIGKEREAGSDGDGQYHHYLTLWMFALNRLSWATQDSSFNDLAIQLAKAIHPKFCFQSSGGL

KMVWKISVDMNKVLVPTEGHLDAAAGFVVYRMLQETAVQQGRKDQLLKQEIGDYEEVMDQRGSMYPSGDPLDLGMGLWTCHFY
PHEDWSQNFTQQAMDLVDDLLDGKSTDMSGSASKRLAFREFGACLGVQCVEPDEPLKEQVSTLIDFWEEHIHQHDGDGLKPIS
QVMYAAAVIPGAFRRGFIAGSEP
FUN_016452 CDS scaffold_9 5890 6067 -
hypothetical protein
MSFSNLCKVAPVDAKAPTTINTPVDMANPKCTCSCGYNNIVKFTGGNFWEPCNDNHNQ
FUN_016453 CDS scaffold_9 9997 12604 -
hypothetical protein PF00152;PF01336;PF09924

MASPETSRPASAVLSIDTRPSAAILSSAFSSPRSASPSRTIRETMRDSAVELRNSARSSMDGTTTTTNGAVVPSSRTSLDSGR
TSMSSDWPVHPGEINTIAQLDTLPIGTEVTFRARIETQRPISKVLDFLLLRDQTHSVQGVLARDASNADFITWVRKINPESLV
QITGTLKSPPEPIRSATHSNVEVDVESVHLVNPAQNLPFSNYKPPETLRNRMNARILDLRHPSNQALFRVRSMVSRIFRNTLE
EQGFVEINTPKLQPAATESGAAVFPVNYFGRRAFLAQSPQLAKQEAISADFGRVFEIGPVFRAENSNTHRHLTEYTGLDLEMA
IDTDYHEVIQFIDMFLKEVFRTVYASRELEVIRKRWPSGEFKWLEETPIIPFSEGIQMLRDDGRDVEEEDLSTPDEMRLGQLV
REKYGTDYYVLDKFPANARPFYTAKDPEDPKWTRSFDIFIRGQEICSGGQRIHNVDELRANMAAAGMSEDGMEDYLTAFELGA
PPHAGAGLGLERIVAWMLELGDVRYASLFHRDPKSLPTKAPGLPHPEADTTKPHHADSPPPIEKLIANYGDATNTSWLDDRFQ
IWRHSTGAAVGWVQREKFAMITGDPLCDRSQYTQVIRDFIHYITVDLRLTPFWMLVSYEVQKILASEFRWRSLSCTEEQRVDA
DKHNSAQIDGLAAKARRVEREGVKIHEVKPDEDFISRANPAIEAWKGTRKGKGKQVHLTEVRPWIDQEHRRYFAAEKDGKVMS
LVVLAKLAPRHGWQVKWALDFPGAVNGAIEVLVSFALQNVSGKVTFGAGVSEKLTPGEHVGGIRAKFLSASYRSIVDSLGLRR
KASFRSKFGALGEEIYICYPKHGVGLRDLQNVIKFFTD
FUN_016454 CDS scaffold_9 15465 16626 +
hypothetical protein PF07859 ENOG41
S09X CE10
METEFTPSESRPTTPEPPGYVNPLESSSRWYLIARAQAIRSAASLGFSIANRTDPPAPSPTKDIWLDATLSKADLSRADRAKT
KIKAEVWMPPRPALGARAAVINFHGGGWILGQGTDDARWAGAIMANLDAVVFTVNYRLAPSCPFPTPIEDCVDAVLQIKHRAA
EFGIDPDRIILSGFSAGATNALASWLILNDPDRWGYKFPIEPPKIAGITLYYPLLDWTISRPEKRLTCSKPEFTLPKGLTDLI
DASFIYPIIPREERCDPRLSPGLMPDELLKKLPPIHLVLCEYDMLLAEGLKFSNRLKAANKQFTLRIVEGERHAWDCPPPMSP
KESVAIEYQEAVETMASWFGQKCETDTESMRSMRLKRPVLKRPKYLSFKTWSGR
FUN_016455 CDS scaffold_9 18381 19190 -
hypothetical protein PF08718 ENOG41

MSAVYPPGGTIVQSLKRTFADVPVDEANGNAVSTVEFLEASESLTTIFDAIGGVAFGPVKKDILGNVEKLRARHAAAPAESAT
VQDLVRNELKTGKHTATEGCLWLIRGLDFTKQGLEHNVQNPSVELSDSFRDAYGNTLKPHHSFMVKPIFSAAMSAVPYRKDFY
AKLGDNPEKVQADLEAYLASFSKVVAILKEFINSKEAKW
FUN_016456 CDS scaffold_9 20025 22933 -
hypothetical protein PF00614

MADHKKHESSGSMSSMLDKLKLSDGLHDAKVHLVHQKHKIGKLANLFNKNHRHDEEHEQRCDDKRTSIAEQNRFKSYFPMRAG
NHVKWYVDGRDYFWAVSIALEQAKESIYIADWWLSPELFLRRPPYAAQEYRLDKLIKRKAEEGVQIYVCVYKEVEQALTCNSA
HTKHALRRLCPKGSPGHGNIHVARHPDHNVFENFGDMTWYWAHHEKFIVIDYEIAFVGGLDLCFGRWDNHQHPLSDIHPTNVQ
DEIWPGQDFNNNRVMDFQNVSSWQDNELSKADYGRMPWHDVAMCIQGPTVYDVAEHFVLRWNFVKRDKYKRDERFDWLVLEKR
ENEDEDLIGVQRPKHPVGEYVKHPFTPLSTKNLENRGTVNCQLVRSSADWSSGILTDHSIQNAYIEIISKAQHYVYIENQFFI
TATGDQQAPIKNTIGRAIVDAVVRAAKEGRKFRVIILIPAIPGFAGDLRDQAATGTRAIMDYQYKSICRGEHSIFGQCRAEGV
DPEKYIFFFNLRSYDRLNKTPAIVKAEKETGISYQQVQRAEAEEIMGEGIHGSYDPEDSEGDEHMRGDKKQKELDEDIKKTME
ARKKFEAAMPKEVVHTTSSVAHHAMAGTGEGLKGESWFEDDPESEIKNWIQEELYIHGKLLLVDDRIAICGSSNLNDRSQLGD
HDSELSVVMEDTRLIPSTMDGKPFEAGYHAATLRRYLWREHMGLLPPQPHDASQDINAMPPSVNPNNDIYDQDESYKFVEDPL
SDELWEKWTGQADKNTELFRHLFHADPDDHIRTFDDYDRYLPPRGVKAGHIFDQFMPPEDAREKLSQIKGHLVWFPLRFLEDA
EMAEWGLQVNSVTESIYT
FUN_016457 CDS scaffold_9 24297 27742 -
hypothetical protein PF00067;PF00175;PF00258;PF00667

MAESVPIPEPPGYPLIGNLGEFTSNPLSDLNRLADTYGPIFRLRLGAKAPIFVSSNSLINEVCDEKRFKKTLKSVLSQVREGV
HDGLFTAFEDEPNWGKAHRILVPAFGPLSIRGMFPEMHDIATQLCMKFARHGPRTPIDASDNFTRLALDTLALCAMDFRFNSY
YKEELHPFIEAMGDFLTESGNRNRRPPFAPNFLYRAANEKFYGDIALMKSVADEVVAARKASPSDRKDLLAAMLNGVDPQTGE
KLSDENITNQLITFLIAGHETTSGTLSFAMYQLLKNPDAYSKVQKEVDEVVGRGPVLVEHLTKLPYISAVLRETLRLNSPITA
FGLEAIDDTFLGGKYLVKKGEIVTALLSRGHVDPVVYGNDADKFIPERMLDDEFARLNKEYPNCWKPFGNGKRACIGRPFAWQ
ESLLAMVVLFQNFNFTMTDPNYALEIKQTLTIKPDHFYINATLRHGMTPTELEHVLAGNGATSSSTHNIKAAANSDTKAGGSG
KPMAIFYGSNSGTCEALANRLASDAPSHGFSATTVGPLDQAKQNLPEDRPVVIVTASYEGQPPSNAAHFIKWMEDLNGNEMEK
VSYAVFACGHHDWVETFHRIPKLVDSTLEKRGGTRLVPMGSADAATSDMFSDFEAWEDTVLWPGLKEKYKISDEESGGQKGLL
VEVSTPRKTSLRQDVEEALVVAEKTLTKSGPAKKHIEIQLPSAMTYKAGDYLAILPLNPKSTVARVFRRFSLAWDSFLKIQSE
GPTTLPTNVAISAFDVFSAYVELSQPATKRNILALAEATEDKATIQELERLAGDAYQAEISPKRVSVLDLLEKFPAVALPVSS
YLAMLPPMRVRQYSISSSPFADPSKLTLTYSLLDAPSLSGQGRHVGVATNFLSHLTAGDKLHVSVRASSEAFHLPSDAEKTPI
ICVAAGTGLAPFRGFIQERAAMLAAGRTLAPALLFFGCRNPEIDDLYAEEFERWEKMGAVDVRRAYSRATDKSEGCKYVQDRV
YHDRADVFKVWDQGAKVFICGSREIGKAVEDVCVRLAIEKAQQNGRDVTEEMARAWFERSRNERFATDVFD
FUN_016458 tRNA scaffold_9 28960 29032 - tRNA-Gln

FUN_016459 CDS scaffold_9 32045 33800 -
hypothetical protein
PF00097;PF12678;PF13445;PF13639;PF13920;PF13923;PF17123

MSSHAERHGHLDATAGREVVYCHACAGEWYRDENGLTCPECHGEITEIIDPANDPRDFDHHSSASTSPEHHPYDSDPEEADID
EHAQHGFVFRRSVRDGPNHPHHHDPATAPTYERFWDMLNGFGPRAPGGNGSFPSSPGEHEQPQEPNFGPRIHRTTFTSGPLGG
TTSVTIVSGPIHATSRGPAGAPGGETFQEYAQPPFQLAERPGGLRVTAISMTIGANQRASFFQNMLRDIVPPPPAHNHGGEEG
AGGPPPGLARSLQDILNLLNPANAMHGDAVYSQEALDRIITGLMEANPQSNAAPPATEEALKNLERKPIDKQMLGSEGKAECT
ICIDEMKEGDMATFLPCNHWFHEECVTLWLKEHNTCPICRTPIEKTDRSGNNNGGNGNNNNNNNSGDGNQSQSPNPGPNSDQP
NPFGARASFTFTPNQWTTFDSPHSHTRSMRFSRPPSQSQSRLNEALRTISNRQQERERERERGETSGFSYDTSRLQRRSSH
SPTSPRATAPSEHGARMRQRSPSQSSRRSAADSDQQRRQSSHGPISWLRDRFGGGGPGNGPPREGRRE
FUN_016460 CDS scaffold_9 36000 36452 -
hypothetical protein ENOG41

MGGGGKVPYPKHVWSPAGGWYAQPANWRANTLIAGVVMAGIVAVTWKFSAGREQWAHRPEQGQWYASRHWSKQLKQWDEEDKG
NASKSE
FUN_016461 CDS scaffold_9 36767 37919 +
hypothetical protein EOG09263CGP

MKRVRIDRWGGQLLQAQARSIGSGISRQAFLQTTRRQASTAAASQANEETPTFANPPSPSPERAAESAKLAALHSRLSLSNKI
PLETLARALVVPSADKNPSFNNANLAFLGSTFINYHVLEFLVCKWPRLPMAVLYEALRAYSGKESLHQVARRWGVEAAAAPGE
EVDPGLLQWAPRDADMQSRWGSVRQESAFTDRFKPRRGISSRVVYDNAFGDPVNVEKMSSEEGYKYYQHEAYSSFVQAVVGAV
YTHSGQDAARDFVKAHVLARQVDVSTMFEFKLPTRELSMLCAREGFEAPIARLESETGRHSRTPVFIVGIYSGSEKLGEGGGP
SLDIARWKASMNALKAWYLYSPGNKVRVPSDMYEPDAKRWKAPHIDIGEVI
FUN_016462 CDS scaffold_9 38235 40701 -
hypothetical protein PF01735

MRHYRGSLKSLNRVTTRLNTHQRIPRRRLSHRQHRHLWTSPSKNGAKQEAQQKSTFPVALSGKQSRQRTHEDSPKDEKQDKTQ
DGESDTDQSAWINFARRFETFCKLNDVQFSSFSDKVVSYLLPEWSRLIPGYVRKLQRELSMSPGSLADEIWQDAHDPLINPEI
RYSAKVRVSPNLCDEEKEFLSRRKRVARIGLAKYLGLKEEDVHPDDVPTIAMCGSGGGLRALIAGTGSILATEEDGLFDCVTY
TSGVSGSCWLQALSLTSFNKGNIRNLLEHLKARASTHIAYPPVAFQSLASMPTNKYLLSGMVEKLKGDPKADFGLVDVYGVLL
AARYLVPKGDLGVNDRDFKLSNQRQYVQYGQLPMPIYTAVRHEIPNLPEAAEQGPIEAEIAKEKAKKEAWFQWYEITPYEFFC
EEFGAGIPTWALGRRFNQGCDVPPEDGFHLPELRLPLLMGIFGSAFCATLSHYYREIKPLVQGLTGFGTIDELISTRDDDLVK

VHPIDPAKIPNFAYGMHGKLPETTPTSIYDNEYIQLMDAGMSNNLPIYPLLRPGRDVDVLVAFDASADIKTDNWLSVADGYAR
QRGVKGWPVGIGWPKPGEATSQVVEELDEAQAKSTREAEAKLREAKKEQDELRQEAHEEGKQVMAENDKTKFEPGDQESGDLG
YCTVWVGTNTERSTEPPPPSKAISDETSWQLMEPDAGIAVVYLPFISNDKVPGISPGTTDYLSTWNFIYTPEQIDNVTALARA
NYDEGKQQIRDTIRAVYQRKKKLREEAEKAQRQDRYRSRMRRGEGIRLGEGDHFS
FUN_016463 CDS scaffold_9 42194 43500 +
hypothetical protein PF00076;PF05383

MSEAEAPKPVEAVQPEAEVPTTQPTEEKVEEKTEQKAEESTEKSEDNSEEKKEILKTTAKHDDENPRNNRKFDPSTREVTDDP
EAIRKQVEFYFGDWNFPQDKFMWESCGGSENKPMKVKTIHSFKRMRTFQPYSAVIAALKDSKFLELSGEEGEEVVKRKTPYQP
MNASKSKVEAATVYVKGFGDEAPNTQFDLESFFAQFGEVKGLKLRRTNEGLFKGSVFVTFADEDEANKFIKIDPKPKWKDHDL
KIMSKRAYCDEKNDLIRQGKIEPNQSNPRKFYEGRETGKGGRGRGGKDSRGRGQDDWKQRRENDQKNGFKDRRGGRGGRGGRG
RGRGRGGRDGGRDRDQSKDEVKHSSNDTTPRIQSTADESADNKKRSRDEDAPSGEPAAKKVDVKAE
FUN_016464 CDS scaffold_9 43885 44829 -
hypothetical protein PF00459

MADIDLQAVHDELVSVAYEAGAMILAANPAELDTDTKLNSVDIVTEADKGVEKMVSTRLSSSFPSISFMGEETYKPGVRLGSE
PTFVVDPIDGTTNFVHSFPSACISLGLAIDRQPAIGVIYNPWLDTLYTAIKGRGAFLTHGRGKEPRRLPLARSPRPIEGLGSS
LVAVEWGAQRDGPNFDIKVEAFRKLTATPENGGSMVHSLRSMGSAALNIAAVAAGQVDLYWEGGCWAWDVCAGWAILNEAGGR
MVHGNPGNWDPELEARVYLAVRGAPSGQKELIEEFWGVLGDKKLDYSV
FUN_016465 CDS scaffold_9 45386 46116 -
hypothetical protein ENOG41

MVSTAGGIVIAIIVLLVAGAVGWVVFTQLRARRLGLPPPSLASYLPWHKEDSGYGIQPAPSGIAGWFNDKIRKFKNRNNRSAA
GAYEQSGGARGRRGFGPLDPDEAWDSRVGNEADQYGYYEEDVGGRGRDTGYGGGYNMNLAATPGLSGGQGFDEEEDRGRRASR
SPASGPGGRNPFDDDAGSSLRGVSPRPIDTGVAKPKNRSGSAESSPTERRSVFRENM
FUN_016466 CDS scaffold_9 46894 48515 -
hypothetical protein PF00328

MVRIPRDDAPSEANRSDASDADAERGRLLSGNDDEAEESYADAERLENWHTEHKRRETRRWSYLVMVTSTVALITVLAIWVHN
NTLSSGCEYDGSCNDISKLWGQYSSFFSVPSEVDSSTPDDCEVTIGIALSRHGARYPTAGKTTAYSATIARLQKSVTNYGKGY
EWLSDYEYNLGSEDLTDFGQDQMIDSGKAFYERYIGLAEKTEPFIRASGSDRVIMSSHNFTQGFYASRGESGYDYTDDILVIP
EEPGINNTLSHGTCSSFEDNDEVGDNNAQTAWGNKFLPPIRDRLNRNLHKAKLSLQETIYLMDLCPFNIVNTPDGVGQSRFCD
LFSIEDWRSYDYYMTLGKYYEYGNGNAMGPTQGVGYVNELVSRLTRKPVDDHTTTNRTLDGNPETFPLDRALYADFSHDNTMV
SIFSAMGLYNSTSKLPKHHIVPAIQAHGYSSAWVVPFAARMYVEKLECGATNEEKGEEFVRVLINDRVMEMESCGGDEYGRCK
LEDFVESLSFARQGGHWNKCGIKG
FUN_016467 CDS scaffold_9 49383 51039 -
hypothetical protein ENOG41

MTHAGSHGAHGSEGASWPSWLSLCCAPLVSSEDRRPRRSKSMSTSTSAHTANTWASDSFSTLNISWPVGRLSDDQLLIGQVDE
KRSYPVIYDQPIHHPQPVAMPEHRKMSERSTRDSVSSKRRRFWSLSPSSRRPLISAPSDFRHVSTAADPFLDYPEPTPSRASN
LRPAKQLPHRPYRPLELSIYQSDNGVSPIMPHFEPRPANPAPSSDYSDCVSDSQPEEVKPSLEHQKSFSDSPMSFHFPRRQAL
GSAPSSSHGEDEIPPLIPPKSPGRPRAYSSPDVEKVKERVANAMLEVEKLQKQIDDVIERQSLYVPSRPPTPHSVARTMPGMS
SIYGPGDEPLPPTSSDLEPMPSIPALPPAAPSFAQRLNSDIIERPHTAPIRPPRTPVRTPITPPRRSRTPGEQRSPSTPPSRP
RRDDMPPPPPLPLVLRPPLRKKKSFSRVSSWLFPGSQHNRNMSFDSVTNAPRPIRGNQGFYQVSLGGASEHRRSTESVDTVST
WESDDENRTVPTTCSVGSTAAVSQDEPLISRFATSTFGRNDTRPQRRSVGVAM
FUN_016468 CDS scaffold_9 54564 58015 +
hypothetical protein EOG0926025H PF11707;PF16201

MGKRDFNSGDGAAAFRKRQKVVHEAPTSEEVTSSDQLRNLLSFDQDLRNARHGLQSFKNLLDQIVHEEGDRRANLDILNQYLE
TVKPRDTSEDAVFLNDIMEMWSFAVQVNNDGVMSSVAVVLALLLQILSGSLQLVKHGLGICQTLLQEQQLKSLAKNLSAEKSK

GFIISPTLRLLREATCLDGGSYAKRIFRARNFILTSLGRNLEIGHIGDTQEDVRKASVRTNAVRFFLSLLKFLGSDGRKELVS
QKELLSHVTYMIKSDPAYLVVDILDSLKLYLLKDDKIPREVKFRCFNTKSLVRFLALYTYTSDGEDINEKSTVSYKAHQLLIY
ICTTPTAGILYPSTGLYPKESEDDPTPSGRAKAGTQGALFADKYKQDIPVYNFVLAEFAQKLRPWSSLKHSELLVAIFTAAPE
LTAKYFLSNRSFTFEPKLSMTWIGYAAFLFNTMQLPLPPRFGDRSRSAKAPPPTSILLDNIIPLPINQKVLIRCLSSKSNLTS
FFATRILIVAVEKLAEALKMHEEVSKGNDPVWAEASRRLIDAFCQRIPDMKEIVRSYKGIPAENILHKAMASRLLRLYYEAIP
QVALAANFDVSPFFTEVLKSLNKESEQHEDESLRFMELENLVSIASYSPGMRWFSRIDNLIDGAGSSPYTALLRLLCDKKRDL
PFQQLKKVLGEVAVENQMVTKASLMSPLFEALQTTLADAKAKPMEKVWSYVDNCINRCASSPIKYLDLLETYAQEDGVSSGSE
TALVNVTLVEQLSFAMGSANSDEQAALGQFLCCYFSTSYKFKSGKQLTKALYKRILEQFSSSKVKMKKLKEEKSDDADADEDM
ADADTVDVNGSKDGSTIDSSKLEAMLHVPLPEDEDTTALTKWAGKNAEDLVEDGWAAALIRLLSSPHTNIRKESLTSILKMAA
KLKESSYEEKEQVWLLLCELAESSRDQVDKGPVPSAFTAFAIQALDVLKNPLHPLYPKINSFLTRSPVWSADKLPLAHDILHG
EPSEDDKYYTEITWLLTYLLDSLRTPSDLSIFHRKRWFEKILALGANPYLRINLRTRLLRLLYRATCIEGGSTTLTTRFGILS
WLDSQRAAVEGGEEADVMVALMRRVWETCDQEKVKVWSKGGVEKLLAMHDL
FUN_016469 CDS scaffold_9 58544 64249 -
hypothetical protein PF00063;PF00173;PF03142;PF08766;PF13632
GT2_Chitin_synth_2
MAMSLPQLGGAGGPHTQPSLPSLPAHLQSDTHLTAHLASRFHVSHPTARLSSHALISLNTYTNSSKGPDGGKEGSAMAGAEEI
ADRAFLRLGHRSENQAVVFLGESGAGKSTIRAHLLTALLNKSSTPLSTKLSLAAYVFDSLTTTKTATTPTASKSGLFYELQYD
TSATTNPVLIGGKLLDHRLERSRIADVPTGERNFHVLYYLLAGTSEAEKSHLGLDGGSATGTTQKRWKYLGHPTQLKVGINDA
EGFQVFKNALRKLEFPRAEIAEICQILASILHIGQLEFETTSQTSVTGDDSGGFSHEGGTTITAVKNKDVLSIIAAFLGVSAA
DLQTTLGYKTKMIHRERVTVMLDPNGARAHAGELARTLYSLLVAWILETINQRLCAPEESIANTVSIVDFPGFCQQTPTGSAL
DQLLNNAATECIYNLTLQNFFDRKADMLESEEVSVAATSYFDNSDAVRGILKPGNGLLSILDDQTRRNRTDMQFLEALRRRFD
GKNAAIEVGSAQAKLPGSNFMTENTSAVFTVKHFAGEVDYPVKGLIEENGEIISGDLLNMINGTKSEFVARLFGQDALQTVTH
PNERTTVMQATVSSKPMRAPSVMSRKTHRTGRPSTAYKRQQQEAMEELDQQSQAGESKKNAKMTLEQGASGQFLASLDNVQKA
VTDPGTNSYFVFCLKPNDRRIANQFDSKCVRMQVQTFGIAEISQRLRSADFSLFLPFGEFLGMTDAETILVGSERERAEMVIE
EKQWPQNEVRVGATGVFLSERCWMEIAQLGEAVSVSGRYGGLPSSDAGDGLTPAESMAFGASKEHLVSGGNTPLMYGEKAKGG
YFTDDTRSEAGVSAFGGGDMFKNLDTREQMAERGNEKSLEEVEEYRDKPSRKRWVALVFFLTWFIPDFAIRLIGRMPRKDVRM
AWREKVAINMLIWLMCAVAAFFMVGFPMLICPKQYVYSSNELSSYDGDKGSKGAYVAIRGFVIDLNAFIPNHYPGSNLVSEDT
LLNYAGKDISALFPIQVSALCQGKDGQIPPEVTLDNRNTNITGQPQLLASRDIDVNSVYHDFRYFTNDSRPDWYFEQMYTFKH
VYLKGRMGYSPKYVKKLARDSSWNVVTIHGKVYDMTKYLQGGLRLKAKAGKPTPNIPGATDFMEDSVVQLFRSAKGQDVSKYW
DNIKLSPVKKQRMETCLNNLFYIGDSDTRNSTRCQFATYFILAISVMLASILVFKFLAALQFGGKNVPENLDKFVMCMIPAYT
EDEDSLRRAIDSLSRMKYDDKRKLLVVVCDGMIIGQGNDRPTPRIVLDILGVSETVDPEPLSFESLGEGMKQHNMGKIYSGLY
EVQGHIVPFMVIVKVGKPSEVSRPGNRGKRDSQMVLMRFLNRVHYNLAMSPMELEMYHQIRNIIGVNPTFYEYLFQIDADTVV
AADSATRMISAFIDDTRLIACCGETALTNAKGSFITMIQVYEYWISHNLSKAFESLFGSVTCLPGCFSMYRIRAAETGKPLFV
SKEIVEDYSTIRVDTLHMKNLLHLGEDRYLTTLLLKYHSKYKTKYLFSAQAWTIAPDSWSVFLSQRRRWINSTVHNLAELIPL
AQLCGFCCFSMRFVVFIDLLSTIVQPVIVMYIVYLIYQVATNPSVVPITAFLLLGAIYGLQAVIFILRRKWEMVGWMIMYIAA
IPVFSFGLPLYSFWHMDDFNWGNTRVIAGEAGKKIVVSDEGKFDPNSIPRKKWEEYQAELWETQTQTARDDVRSEISGYSYAT
KAQGPFSEYGGGYQPSRPGSTAGFGHQNMSRMSLAHSEMPGNRASQFGGSQFFSPEDLVGMPSDDALLAEIRDILKTADLMTV
TKKGIKQELERRFNVPLDAKRAYINSATEALLSGQL
FUN_016470 CDS scaffold_9 68690 74135 +
hypothetical protein PF00173;PF03142;PF08766;PF13632;PF13641
GT2_Chitin_synth_2
MASRMSMYSMASEALGGGPQAAQVSTTTLLNAIHNIYLSSQPYQLDASTSLVVNTWLTAAQAGATVDATLAARAWEHARRRAE
DGCVILGSLHQSTPSLLVPFLNTFPFAIPASIYKSLEALQPFLRCVTPYNASAPRQIALGVTLTLSLGGNVTGASLALSQGGI
DTENGLLNIPAEAGYRAFDVFYYLLTSASTPAEREFLGLKSPSAYALLARSGTYEPPSYLITADDGAAADDFRQALKEIGIKG
SAHRNFISTLAGLLKLGNTLDYDADSDDFEEICEDVSGLLGMEPEVLMQQLSTEDRRTLVGGLYEALVDWVISKANSAIAAQM
LRIRDGDESIDGRGVRTPTSNEDGDTVSITVVEVPEPSIGRALAMRTIFDDTIGINAEMIEDGVEVHPVGSSVVREMQQAVSD
VAPDLGIMTGPQGRDRQHDLEKREVILEKVAYGSEDDSFVKKLLFPVEGEGISLGRAGRFDLPALLSASRTWFHLSLHPTDDS
PANLATLPAITSAWSAGTVSRQLRSWRLPEWANRRNRNLDYTADFDVDEFVGRYGALGCKDGKDGIETWMLERGWSNGEVFIG
KERIWVREGPWWEAETMLDIKPAHSLQSMGQNPFTSGFDTSYSANPPNGSGFFPPPAMDNSLNGSNDQLMHTRNFSQGNMSQV
TLNQHQNLNPQSAPSIAPSAMRNVQTTGDYGLGTKGDTYKGQVYYNEDGEFTGILDGELAKNKKIESKPLPFGRRAWIAFVWA

LTFWIPSPLLKFIGRMRRPDVRMAWREKFVLFFLIILINGMVVFWIIGFGKLLCPNANKAWNVKEVATHAEDEKSFWVAIHGK
VYDITDFWQQQHSDTSIKVTKQNMLPLSGMVMDNYFMPPLNRACRGLGIKETTQLTFNDTITNPLAQHTSGFYTRDRTSALHD
PDWYWKKFQPKIKEYYHGNLVWKKSKVKNEGENEQHMWATYGNEVYDLTDYLHTLDVNDNFDSYKFLNEDMVDLWKNSPGTNI
KKDLDLLIANAKNETVSANLKNSWQCIQNIAYKGILDFRDSARCQVNNYLLLAFAIIICIVTAVKFLAALQFGSKRRPSPQDK
FVICQVPVYTEGEDSLRKALDSLTALQYDNKRKLICVICDGVVVGQGNDRPTPKIVLDILGVDPKVDPPALPFKSVGSSSEQL
NYGKVYSGLYEFEGNVVPYIVVVKVGKESEQSKAKPGNRGKRDSQILLMSFLNRVHHRAPMSPLELEMFHQVNNIIGVDPELY
EYLLMVDADTCVEEDSLNRLVAACAHNAKIAGICGETALENDEKTWWTMIQVYEYFISHHLAKAFESLFGSVTCLPGCFTMYR
LRSVDKGKPLIISDAVIKDYSVCDVDTLHKKNLLSLGEDRYLTTLMTKYFPEMSYKFIPDAQCKTAAPESWSVLLSQRRRWIN
STIHNLVELMQLKELCGFCCFSMRFVVFIDLCGTIILPSTCVYIGYLIYILATGSGPIPYISLAMIGAVYGLQALIFILKRQW
QHIGWMIIYILAFPIYSFILPLYSFWNQDNFSWGNTRIVIGEKGNKQVVAVDDEGFDPRSIPLQRWDDYALANNLPGRRGGYQ
EKTDYSYGDNYELDEIKSVYSAGPQGSVLTGMPGRNTYMPPQSPAFNNGRASTMGFQDSPMQHRQSMMSMGTGVHDMRSQSPY
QDYPGQHPSVSNLRGQANLSPATGGGHSRSGTALGFSSGARSPMPDAMRSQSSFDFQHGHAGPNDMAIVESIRSVLCEVDLDT
VTKKQVRALVEQRLQTELVGERRTFMDRQIDHELENM
FUN_016471 CDS scaffold_9 74744 76043 -
hypothetical protein PF02518;PF10436 ENOG41

MRLRHWLDNINILCVSQTSHGRPPLSSKSLLSSANFALSLLPVRLAHRIQALRNLPYIVVANPNISRIYNNYLHSLSILLPYW
HATREGRPISNLEDEIRFTNVLAELVATHTDTIPILAKGFLECRRYISPEEVTKFLDQHLRARIGTRLIAEQHIALHFSSQPH
FDPDASPTPCPDDPSYIGVIDTALRPAQIVESCAGFVADICELRYGVRPLLYIHGEPDTTFAFVPMHLEYIVTELLKNAFRAT
IENKSNEPVIVTIAPEPALTEEPSTTSWSNSSTKDSDLPSKDSRNATNNDAIVPLDDNAPGVTIRIRDRGGGIPPEVSPNIWS
YSFTTFSDDMDDFPGDGNGGDGLSAISTASTGGSSIAGLGYGLPLSRAYAEYFGGGIAVQSLYGWGTDVYLRLKGVGNLGKK
FUN_016472 CDS scaffold_9 76756 77138 -
hypothetical protein PF05922 ENOG41

MPSYIVTCKDEASDEQVQSAKQHAIDQGGKITHEYSLIKGFAVQYDDDKIQTLESHEHVKAVEPDGEMKTQ
FUN_016473 CDS scaffold_9 81155 83002 +
hypothetical protein PF00067

MEVVNATSSGFSSVLAGTKYANVALPPQVEYVIEAVSNAGVWTWVFTFIALCVAYDQIAYIIRKGPIEGPAMKLPFIGPFLDS
MDPRFDGYHAKWSSGPLSCVSIFHKFVVIASTRDMARKVFNSPAYVKPTVVDVAPKLLGHDNWVFLDGKAHVDFRKGLNGLFT
RKALESYLPGQEEAYNTYFKHFLKMTKDAGGKPVPFMHEFREVMCAVSCRTFVGHYISDEAVTKIAEDYYLITAALELVNLPV
ILPYTKSWYGKKAADMVLAEFSKCAAKSKVRMAAGGEVTCIMDAWVLSMIQSERWREAEEKGEGHTVEKPTPLLRMFNDYEIS
QTIFTFLFASQDATSSAATWLFQVTAQRPDVLDRVREENIKVRNGDPNAPITMDQLESLTYTRAVVRELLRWRPPVIMVPYVT
KKAFPLTENYTVPKGSMLIPTTFMALHDPEVYDNPSHFDPERYYSGDAEEKGSKNYLVFGTGPHYCLGQVYAQLNLALMIGKA
SVMLDWKHHATPKSEEIKVFATIFPMDDCPLTFEERKW
FUN_016474 CDS scaffold_9 83828 84457 +
hypothetical protein PF00581 ENOG41

MASRRALFSSASRASAAIQPARPILIAAARSASPSLPSLAFAPFASTRSYASKAQAKAKPIADLTPRDPKPWNFEDIKNHVEK
SDQGDVVLIDVREPVELFETGKIPGAINIPITSAAQSFHISDEDFEDMYGFQRPAKNKELVFYCKAGVRARAAAQLAHHAGWN
KIGDYAGSWLDWEAQKGPVEKVKKPY
FUN_016475 CDS scaffold_9 84676 85674 -
hypothetical protein PF04893 ENOG41

MSGSGYDAVVDVDDEGDLGHTDLQEDLEFHTSNFNDTNPNTRKPPSSGSGLPPPATASTGSGSSKRFLWSMNFYAQFFDVDTS
AVLSRCWAALFPRANFLDVLEGNPDLYGPFWIATTVILILFLGGTISQYMSDTGKGPFLYDFKLLSGAAGLIYGYTLFIPMAL
FLALRYFGSESANLLECWALYGYSNLIWVPVALISSSPISILNWVFVGIGFGISVAFLLRNLYPVLSATDRQVSKVLLVVVVV
LHAGLALAIKILFFAHGSPVASKPDEGKGSRMF
FUN_016476 CDS scaffold_9 89984 90879 - ANB1 translation
initiation factor eIF5A PF01287

MAAPNDAEHEMTFDSADAGASLTYPMQCSALRKNGFVVIKNRPCKIVDMSTSKTGKHGHAKVHLVATDIFTGKKYEDLSPSTH
NMDVPNVSRREYQLLDISDDGFLSLMTDDGDTKDDVPLPDNEVGQKITKLFKEEEKDTNVIVLTSMGEECAMEAKEAPNQG
FUN_016477 CDS scaffold_9 92659 94744 +
hypothetical protein EOG092621GA PF04129

MWLDRLAGGPASSPGPSTPQPGGRVYPKRTSSTLSPYVTSQRPGISPRGSSLSLVSNDSTSSLLSSSRRPNGSNLRQASTIET
GPDSLEVLETLLAGLSSDRDQADKKTVINQDDINFDALFGGLSLKELATSEPPDTTTISNRKPKTAEEGENDRTKLEDLHRSI
EACDDVLNSVEINLANFRNDLAMVSADIESLQTRSTALNRRLENRKQVEKALGPLVEELSLPPEVISKISEGHIDETWAKMLA
ELDRRSTLFKKKSETQTSNAAKDLEPILEKLTFKAIERIRDFIVAQIKALRSPHINAQIIQQQSFLRYKDLYTFLHKHHPTLA
NEIALAYMNTMRWYYLNQFSRYEKALGKIRLHILDKNDALGHEDATRKATVLSSNRAPGPPHDAFNLGRRIDLLKTNNQTALS
SYLAEEDQTTHYLEVPFRNFNLALIDNATAEYTFMATFFSPALSYSQISKNFNYVFEATFELGRTLSKTLIGETYDALGLLLC
IRLNQHLAFELQRRKVPAVDGYINATTMLLWPRLQVIMDRHCDSVRHLSNAMPAKPSRAEAARLSAAPHVVTQRFGQLLHGLL
ALSTDAGDDEPVVSSLRRLRSEVETFLTRHAELFGDKRKSGRFLYNNYSLVLTIISDANGKLADEQQEHFEKLKAQYQETS
FUN_016478 CDS scaffold_9 95037 96391 -
hypothetical protein EOG092642UD PF00566

MDSPRDSDNKSPLSFSRTSSNTNANTNTDISQGPDILSHPKAADIIEACKWRDIGCLRSLAEAHGGFLSDTLRRQAWPILLGL
DAPSDEKDDLDLALAQEDSNTWKQLPRHRDEDQVQLDVNRSFIYYPDNQSDAELSLRKSELSSLITEVLRRYPYLCYFQGYHD
ISQVLLLVLAPAWRARVLARLSVLRIRDFMLPSFGPTTAQLRLLPDILHVADPKLRRHLAGVEPFYALAGTLTMYAHNIETYQ
DIARLFDVFLAREPVFTVYMFAQIILDRRNEIFEIDEPDMLHVILGKVPTKMDLDELIIKSVALFEQHPPETLHYWRHISNFS
ALKTARDIETCAKQTMEEGSELFEKQVNELHWQEVRNRVKLALWRYRRPVKAVGMALVVGALAFYLRRNPILVHRIASFFSR
FUN_016479 CDS scaffold_9 101114 105608 +
hypothetical protein PF10433 ENOG41

MAFQSSVLRDGEWVTQTVNFQDALKASSTTPKVASDPSLKAPECGILSRTIIESPVVHWILPVCLRSRSHNDIAFIGDRFVQI
SELRDDGQVHEVIRKADFGTRIRNAIVLGGPPKGDIPGAAAASTKTEDADVPMKDVAEAKETKEAKEAKEAKEAKEAKEAKEA
KEAKEASEGKEAKEAKEAEEKRVLPPQLLVLMLETGDAVFLFIQERLDGTLRFVATTFQSPRNLQFLGYHFSIDPSSRYMSAG
SAEGGFIIYELESLSHMSTQYESCGSLKPIKSTRVRLTQGVIHKMEFLYPRPEDDYHIILLLIIVRREVSKQAHVSRMVVYDW
ELGDELTAVFRSEKGTPLPKEHRMPLMIIPLKVNTAFLAVSEHSIGIVKNAFTGQTSFDTLETHSPQQTRLHHGAAEPLWTTW
ARPFRINIYLEKMDVIYLAREDGVIAHIEIDSRDLVPTVMTLGTISTNITTAFTTAYDVFSDVLITGGDSGPGGIWKLIPRKD
PQQVSVIPNWSPVVDLVTTDTNPSWRSDSKRKDLSTKRKVSSNSRPKSDRIFCTSGRGPRSSLTELRWGIQARIGLEFDHDQH
VRQSWMFPVEAQGERGFYVILSLPHSTDVLHFPADLSNANALSPEACPFDTSSRTISAFQTEQTVIIQVSETSTYLVAPTQSS
RHNHGEVCGKSSLLAEHACCTDDIVVISSHGANASQLDVLLVDQMNLTHSTSIDAKGEITCIGLFKASAQTYIIAGSVTEGLP
LLTIYSLAGLEISSNVTQPDLVTPGQEGHFHMEAYTSVLAIHDTAEKVILVLGTRSGHLVTILIPGEDIAHYSLSIEQIGISP
TNVFPASSLFDGKHAFFACCDNSLSIMTDFSPKGSRFKTKTRIWATDSNEPSMSSPPVHAAFCLEGSLSGYQRHMSLMLLSGT
RVLLVDIWPHVGPVPRSILLGGTPMRVIFSQIWKVLVVAHLKDDRPTLSFIDPESGVNVATAANKDKQPSDYISGLGHPGDRI
FGLYEWTYVKDGKLFPFIIVTTQHGRLMIVSVTALKPKSDDGPTRKLQYWTRYKKKGFAEPIYTVVGDDVGLLFCVGKVLHWE
VLDLAEKKLKPMKQFRLDSPATTLRVEGTKVCVLTAQHSLQVIDLNVESENSDPSIIHSDRVTRFTGHVIEMGDSEEAPGKWP
VSVISTAQAGFAGVWIPWSQRHKEFEVVVTGSLPTSIRRFRRGHTRPFWSAVDRQRRYNTLFSTADQADILGVSIDGSLHQFS
LVGLDLWRFLRLIQNLAYQDKKICPFVRNSQSLRDSDPGMDLDPELEPQRFREMMHIDGDLLKRCLDMSALEELVLIGDGIDL
FCEYLDGIDDGIYTEGFRETGSQGRKKYIELGYEILEYVLTLAI
FUN_016480 CDS scaffold_9 106425 109784 +
hypothetical protein EOG092610ZY PF00009;PF01926;PF03144;PF11987

MPPKKKGNKKNQQDDWEAELGESIAPPANANGTDGDANANDDDDDAGGAGGLMATLRKNKEKRKKKGIVEPEPVPEPEAPAAE
EPPAQAPVEANIDDEFALPEKKGKGGKQNKQAAKKPEPAATDDAADGGRILTKAEKEKLKKEREKQRKKEQAAAKKKGGAAPK
AAPEVKEKEEAAPVEAAPAPEPAAGGKKKKLPAHLALLQKQQEELKRQREEAAKAEAEAKAQAEEDERLAAEEEKRREEAKAI
KKQKERERIEQLKKEGKYLTKAQKEEKARNERKLQQMLAAGIQVGPAGEPSEKKSKPADNRRKKGRAQQKIDEEKALAEAAER
ARQEAERALKEAEEKAAHEKAEAEAKAAAEKKGAESDVEDDWEAAAASDGDVKDSWDADSDEEAEKANGTSEAKAEAESGDES

EEESSDDEAATAAQLAEEKRKREAAERREKAHQAALAARSKDNLRSPICCILGHVDTGKTKLLDKVRQTNVQEGEAGGITQQI
GATYFPVEAIRQKTAVVNPDGKFEFKVPGLLIIDTPGHESFSNLRSRGSSLCNIAILVVDIMHGLEPQTLESMRMLRERKTPF
IVALNKIDRLYGWKQVANNGFQDSLALQSKAVRNEFETRLEKTKIAFAEQGFNSELFYENKSMSKYVSLVPTSAHTGEGVPDM
LKLIVQLTQERMVGSLMYLSEVQATVLEVKAIEGFGMTIDVVLSNGILREGDRIVLCGTEGAIKTNIRALLTPAPMKELRLKS
AYVHNKEVKAALGVKISAPGLEGAIAGSRLMVVGPDDDEDDIEDEVESDLAVLFNRVEKTGRGVSVQASTLGSLEALLDFLKD
CKIPVANVGIGPVYKRDVLQCGVMLEKAPDYAIMLCFDVKVDKEAQQYAEDNGIKIFTADIIYHLFDAFTKHMDEQLEKKKEE
SKMLAVFPCVLKPVKVFNKNDPIVIGVDVTEGQLRINCPVAAVRNNPTTGAKEIVKLGRVTSIEREHKQIPVCKRKEPSVAIK
IEMGGNQPMYGRHLEEDETLYSLISRASIDTLKEYYRKEVSNEEWQLIIKLKPLFDIS
FUN_016481 CDS scaffold_9 110614 113405 - HSP88 Heat shock protein hsp88 EOG092616YZ PF00012;PF06723

MSVVGIDFGTLKTVIAVARNRGVDVVTNEVSNRATPSLVGFGPKSRYLGEAAKTQEISNLKNTVSSLKRLAGRQFNDPDIQIE
QQYVTAPLADCNGQVGAEVNYLGKKEKFTATQLVAMYLSKIKQTAGAELKLPVQDVCLSVPPWFTDVQRRALIDAAEIAGLRV
LRLINDGTAAALGWGITKLDLPAPEEAPRRVCFVDIGHSSYTVSIVEFKKGELAVKATTWDKDFGGRDFDRALVDHLAKEFKG
KYKVDIMTHGRALARTIAAAEKTKKILSANQQAPVNIESLMNDIDASAMITRQEFEAMIEPLLARTHLPLEEALAQAKLTKDD
IDVIEVVGGGSRVPALKERIQEFFGKPLSFTLNADEALARGSAFSCAILSPVFRVRDFSVQDIISYPIEFAWEKAPDIPDEDT
SLTVFNKGNVMPSTKILTFYRKQPFDLEARYAQPELLPGKTNPWIGRFSVKNVKADGKDDFMICKLKARVNIHGVLNVETGYY
VEEEEVEEEVNEDPDVSLSEPYTASNDFPPVHDQDSASSSSASVGDDSRAYPAKRPRLDDEEETDSSRSAFAVPEHLEESIYE
PRSLTSVSQKAMETDKDAPKKTRKVKKQVRKGDLPISTGSASLDDSTKASLLEKEAAMVMEDKLVADTEEKKNELEAYIYDLR
AKLDEQYSEFASEEEKQTIKAKLEATEDWLYEEGDDTTKGVYVAKIDEIRAMAGPIVQRHFEKVEAERQAALEKAEAERAAKK
AEEDARKAAEAEKANTDQEMKDADAPQQETEGSADPQ
FUN_016482 CDS scaffold_9 115379 117796 - hypothetical protein PF03142;PF13632;PF13641

MALLKDWPLENVIYTSIVGVVLLMACLEWFLWLAAFVYCLVKVFQKAEHWSINVLCIIVGTAFVLLRVIFLPIMVVTLPLPDT
VTKLWPNEMVSFLQWFAFWAFAILLTVPWLFCIYQVVTNQLGRTKRMKLVLDDVTAPKVVIVMPCYREEPEVLLKAVNSVVEC
DYPPACIHVFLSFDGEEEDELYLSTIDKLGVSMKMETYPKSIDVTYRSARVTISRFEHGGKRHCQKVTFKLIDRVYEEYLKQN
DNLFILFIDSDCILDKVCLQNFVYDMELSPGNSRDMLAMTGVITSTTKKHSIITLLQDMEYIHGQLFERTVESGCGSVTCLPG
ALTMLRFSAFRRMAKYYFVDKTEQCEDLFDFAKCHLGEDRWLTHLFMIGARKRYQIQMCTSAFCKTEAVQTYRSLVKQRRRWF
LGFITNEVCMLTDWRLWKRYPVLILVRFMQNTIRTTALLFFIMVLALITTVKKVDDLPVGFIAVSLGLNWLLMLYFGAKLRRF
KIWFYPLMFILNPFYNWLYMVYGIFTAGQRTWGGPRADAAAADAHTTAREAVEQAEKQGDELNVVPETFKAAHEARRAASNRK
STATTELGRTRSVVRPPDKIDGKFSARQKTASGTYAHPDEIDTSTEDIEMGKTTSHVCSKPQECADLGVGTQRRMKLLMDEEN
IRKYETAQQMQGRASQVSDMEGIQSSHGFSASPMRRAEISGDMATSPSPVQYNQVVQDEPVEPRRRTSPERSIGISLDGEQVR
LGSNVSFARRMTRGRLKKSR
FUN_016483 CDS scaffold_9 119004 122806 - hypothetical protein EOG09260BZ0 PF02854;PF04050

MDRARKRELRTLNEKAWDGESDLFPVAKTLDSSLKKNTAFIKRLRTAITAATLSTFLQEIRTLSLHKYLSEIISACYEGLCRL
KSPGEIEAGVEIVSALHQRFGPNEFTEYLAWLLGKGMATPDKGILKTLAPEVREKEEKERIIRQRALLRVVTELWLVGVIRTL
DDVSRPDDATKGAAGKTPELKTRTNKGTGAEPFPLEVLKDLLGHDREHANLPLLVIFVKSFSWDILGVKPTGADGRKTVEEDG
TTEGNEAEDGNSGNEASPGADQPFTEPEMRDRFRGILKKYFDDVKGHITRDQKSIQSQARRNAEAYVKSGEVFEDRQANYEKQ
VKAQERLVSNAQVIADAIGAEMPDLKDSDDSFAASNGSIGLVKTGDYLRSMGDGAGIWEDEDERRFYENLVDLKGKVPAILLE
DGKRKKTDTDEQVGKKVDPSEASEASKTAETADDQSTSIANRTIGAQVDALLARLPELTNKDIADQTAIDFCFLNSKASRNRL
VKALTEVPKGRSDLLPSWSRLVATLGRYMPDVPKGLVDYLDAEFRSLQRRKEKDFLGQVRLSNIRYLAELTKFGIVPEHVVFH
CLKVSLDDFSRMNIEILCNLIENCGRYLLRNPETAPRMMSFLETLQRKKSVQHIGQPERMLIDNAVYYVDPPERLAIEQKERT
PMELFVRKLIYIDMTKRNYSKILKQIRRLHWEETEVVAILEKVFSKPGKVKYGSVHLLGILLSALYRYHPAFVVKVIDNVIES
MTFGLEQNDYRFYQRRIAEVKYLGELYNYRMLEHPVIFDTMYKIMTFGYGGPPVPGKYIPLDPPDDFFRIRLVSTMLETCGMF
FNRGAAGKKLDYFLSFFQYYIFTKVSLPMDIEFLVQDTFALTRPQWKLATSLDEAVKAFQLAVAQDQKSAGVDKGIDVDDATS
DASSDDDNVDNDEDGDDNEDSASEEEEAEDVEEDSDDESTFDEEAIVVTRQEEEVDPEDEADFEREYAKMMAESLESRKFERK
QLFDVPLPVRSKNREATSTEGGEGESPPGHTMAFSLLTKKGNRQQTRTVELPSDSTFAIAMKNQQQAEREEQQRIKNLVLNYD

LQQNEDQDGGNERPATFYHNRLERSNKDKSQRSRKLQLSDVDW
FUN_016484 CDS scaffold_9 124514 127286 +
hypothetical protein PF00173;PF00174;PF00175;PF00970;PF03404

METSTTTLLQQERIPENSEPISTHTHTHSLPPTPPGTAKPSRIGSFHDLQELSSVESPRLDHIKPYPLPPKFSPKSVLKEDLK
TPDNFVERDPRLIRLTGVHPFNVEAPLSDLYDEGFLTSENLHYVRNHGHVPRCEDDDILDWEFEISGLVENPIKMNVRDLIND
YQQLTYPVTFVCAGNRRKEQNIVRKTKGFSWGPAGLSTALWTGVAIGDLLSAAKPKRGARYACFEGADKLPNGYYGTSIKLNW
CMDPNRGVMVAHKMNGNPLPPDHGKPVRIVIPGQIGGRSIKWLKKITITQEPSDNWYHIYDNRVLPTMISPEESANLPEVWKD
EKYAIYDLSTNSAICYPAHEEKVPFTDAPASYKVRGYAYSGGGRRITRVEVTLDKGKSWRLANIRYPEDDYRNAPEGDTLYGG
RVDMWWRETSFCWCFWDLDIPLDELKSADDIMMRAMDESMNVQPRDMYWSVLGMMNNPWFRIVIHKEDHALRFEHPTHATLKI
KGWMERVKEAGGDLTNGYWGEKAPGEVQEVVVKEPEKQICMTNPQINRKITIEELKAHSGEEEPWFVVKGEVYDGTPYLSGHP
GGAASIFGAAGQDATEEFMAIHSENAKAMLPTYHIGTLDEVSRAILSGDATKTNDDADREVFLQAKTWSKAILDKKTSISPDT
KIFSFKLNHEAQKIGLPTGQHLMMRLRDPATREAIIRSYTPYSDGSDCGRLDILIKIYYDTPQRKGGVMTQALDALPIGHWVD
FKGPTGKFVYHGNGLCTINEKERRVRRFIMVCGGSGITPIRQVLRAVMHNPKDTTPCLVFNGNRSVNDILCKEELEELEAANP
SRCRVVNALSNPPPEWNGLKGFVNQALVPEYMDLPKASGEGDELVLVCGPPPMVKAVEASFLGMGFKSDDFVFF
FUN_016485 CDS scaffold_9 127609 129190 -
hypothetical protein PF01699

MPRDRSSSRSHRHRSYNSPDMRQVSSDDRTLPVPNTYSEHKRGEKRSHGNSKMSRHIKPAGESGRKGFHPIHFSKISFRSTSR
ASLLCNFLWPFVPAAIAVRYAMPDNHITIFALAYIAMVPCANLIGFAGQELSRKLPHVLGVLLEITIGSIVEIILFMVLLSKD
MFFVIKAAIMGSILATMLLCLGACFFVGGMLKEEQEFNEAISEAGSGLLLTAGVVLALPTVFEYGVGNGESLTAANLDHKTLQ
ISRVISVLLIIAYLVYVFYQARTHHGIYAAVFEQDEHNDRDKHKDQAKAKLTLTECIIALVISVGLVAWTAVILVMQIEYVIE
RGDVSDAFMGLILVPLVEKLAEHLTAIDEAWDNQINLAMSHVLGATLQTALFNAPLAVVVSWCLNKGLDLNFAVFDLVMLILA
ILTVGRFLQDQKSNYLEGFLLIILYVAIAVAAFYYPNPTGHGSEGAAEGSSEGGH
FUN_016486 CDS scaffold_9 130932 134404 -
hypothetical protein EOG09260DXP PF00270;PF00271;PF04851;PF09369

MTKRKEISDRIGPNAEYSLPGAKRKRDGDEDSTAESLTSSQPSSSKSKRGGKGGKSRPTLQKEASSAVVPCAVEWPEEFKQVE
RTHRALNLVYTFCTTRKHLATTFDTIKSAVEAHIKRELLVDEIASMVAIRPEGLFFAYVDENMLQLDVKGTERDEVFRTGKSF
RSQAPAHDASVGGYTGMDSLDKQHDRDLEPAGREVLFLEFIDGDLKRQVPGKAGEPTKPNRKLRDEQLRMPVFSQKQMTNLIE
RRNQKFTNAINVFLNKCSEDGIDPLETLKEQTKAYFPAPSAQEEFAPEKAADSIPESIPKERKTVPEIVQELKESPWYTGQIV
PDGHRVFEKQEAVYGDLNFLLSQDLVNALYNAKGITQFYAHQSEALNSLHDGKHVVVSTSTSSGKSLIYQLPVIRALEEDYNS
RAMYIFPTKALAQDQKRSLKEMMSYMPGLEETMVETFDGDTPMTERNEIREQARIIFTNPDMLHITILPQEERWRSYLKNLKY
VVVDELHYYNGQMGSHMSFIMRRLRRICAAVGNRRVKFISCSATVANPEQHFKTIFGIENVQLIDYDGSPSGRKEFLCWNTPY
KDPGDPASGRGSTKFECARLFCALMLRGVRIIAFCRVRAQCELLVSTIKQELENLGRPECTNLVMGYRGGYTAQDRRRIETEM
FQGQLLGIVATTALELGIDIGSLDCVMTWGFPYTIANLRQQSGRAGRRNKDSLSILVGDGFATDQHYMQNPDELFTKPNCELQ
VDLENMLVREGHIQCAAYEMPIRPKDDAKYFGKDLPKICMERLIKDDMGFYHCHDRFRPVPAKYVAIRDTEDDHFAIIDITNG
RNVVLEELEASRATFTLYDGAIFLHQGNPYLVRDFQPDKGMARVERVKVEWTTIQRDYTDIDPTETEAIRTISNSRSHAYYGT
IKIQQNVFGFFKVDKKNRVLDAVQVDNPPVIRFSKGMWLDVPKKAMSILQERRLHIAAAIHAAEHAIMSLLPAFVISMPGDVR
TECKTAVKEFAKQESQRKRPARLTFYDAKGGAGGSGISTKAFDHVDQLLRDALKRVEDCHCERGCVECVASELCKQANEVMSK
AGSQVILKTLLNVEIDMESLPMGPELNIPIGTETVVLAEPVPYRAKEAAFENRNIGDTNE
FUN_016487 CDS scaffold_9 135037 136605 -
hypothetical protein

MADDAPDASPKNEPLNVETKEDAETRATRRELKQSSISDPPTSGPEDAANTSDAPDNDLKEQVASPKKKRAHDQLEGSKDAEE
NDANSVASSDSAKDRALRTEPEKKRHRDEDTDLPSTIASSEATKDTEAGKSPTKKSQGQTSASAFAASGFGKLSSGTSPFASL
GASQSGSAFGSLAAGKPSLSSFASPPSSTTAQPAAPPKLTFGSSGGASPFAGLSTGSNGSPFGGSTFGSALGGTKPLSSFAAP
GAEPLKSEKPAKPFGAPDSESEEGEEEDEEREEIEVNDGEAGEATVVSVRAKMFYHDKEAGWKERGAGMLKINVPQACVEYDE
NGAVIPGSFDASALEMDEEAAGESQGHKVARLIMRQDQTHRVILNTALVAAMKFQEKASLKSVGILFTAFEGEQSKPVSITMR
MSAANAKLFMNEIGSIQKELQNS

FUN_016488 CDS scaffold_9 137718 138756 -
hypothetical protein PF04893 ENOG41

MSASSARGHYANPPLEDEDDLIDPDDADLNDFDDPLATNNARQPLTGNIGSSSSRPLDEGYLTSRIPGEDRRAPQNTIDESV
WDTLRRDLLAVWAKLREVLYPRYLLGGTMFDNEGGLRGAYSSIRGAGLSGTREELTGLASRMVDAEALLQSNMTPGLRDWDLW
GPLIFCLLLSVLLSITARSEQRDAVFSGVFAMIWLGEAVVTLQIKLLGGNISFAQSVCIIGYTLFPLVLAAMMSALGLHWIAR
IPIYIVLISWSLAAGVSILGGSGVVKNRVAIAVYPLLVFYLGLGCLCFIS
FUN_016489 CDS scaffold_9 139439 141541 -
hypothetical protein PF08447 ENOG41

MEHTFLTIHNLHPDANILYASDSIFEILGYSPQDVINKSAFEFFHPDEIPYARSVHSRGVLLDKAAVLHYARLRSRDGRWVSS
ECCFSIVHDILFACISIYRRDAKSERRALDAPQVRRLFSRSPRDPRYHMLQHLSPKFRLPPVEREPRAALILNRFTRTLTIMF
ATDAVASILGVPADQVQHKSFYECIRETCLDDALKCIESAKANDSIAYLRFWSRDPRRPEDFEEEESNVDEESTIDGVNGRNE
SEGPRHLRNGTTNSPYPDQNGSGSGLMAPNQQRRAESRRSSDSEGGGVKLDGAMDLDSNSNAHRGSAGPSIKVEPDVDMQDGI
TSNEISGESRTTSSNALGSSSRATRYQTPLTPQSPPPPEDNAPQVRRSRSRPARQLAPSVELEAVVSCTSDGLVVILRRARPQ
IPSVEPPQIPSTFDHGVFAAPWAQQPVYPQYPHEAVHNFQAPYAPHHMPLRSGIQAAGGPPMDQLMQSIRDVAVFAWALVGIN
GNIASHGHGQPSGEAQPPDGLPIWDPEAGDASYEGPYNQAAQRWAQEAQRQQPLLHQQDSVSTEFSMEGTADYSTAGLGYQNH
ATNGHGNRWYSQPSAHQRNQEAFAQNSRQHHQDPSNMAASSVNQHPSQWSDGQHWLGNPQVPTTSAEDHHNPTNGWH
FUN_016490 CDS scaffold_9 145908 148004 +
hypothetical protein PF00400

MAITDENAFELPKLRSSFSLENDTEFLENDNVAEFFDVKFCPYQPLDAQPVFAAISKKHVVICTLSQTADNNPCEVLSVIRDD
DEEASACCCTWTKDPVTGAPYLCIGGVDAKVKIYDVVNGKLYRCLTGHGGDVNDLATSPADPSIIASASGDTSIRVWSLDPVH
ANRPCLVILAGEGHSWDLLSLAFHDTGRYILSAGHDQIINLWTLPDLPTEAITTPVRVHYPHFSTSAVHSGIIDCVAFYGDYI
LSRACHDNVISLWRIEGFSSANPPPPQSMAPTAQTTVPTNYDEASRLTRSAFVPTMSPQCPSQYTMLLQFHTPNCGPQFFMRF
KLHFVPDQHPVLAFCNAGGNVFFWDFERLLAYREFMEMLKDPNRDRSKPVPHPSWMRLVKGRPPKTDSNKGRNAGADKDIPSA
FRADAIRLSEEIGDYNAETLETWASRYSQEDPHEPLKAHKTESSSANFVGRQAAWSPGGEWCVVVGSSNQALILQRWANKGST
SRASASASASAPPSAPPSAPIQNGTS
FUN_016491 CDS scaffold_9 148040 149249 -
hypothetical protein PF04090 ENOG41

MDSLLDPPSFSQQIRSNKRKRPETTDNDFPSSLPQSTTNPLSHSPGVVAQFAIAGLSETEENPSRRIRDFPHRGISSNEAFSI
EAESDEDPETEGDEAARPKSKKSTSRKRGGHFDVLLQSIHYFLDRGEIAKASRAYGLILQLRPSGLPIDIRHHDLWAIGAEIL
MREGEETLQQDESSHENQPKSTKRWGRAANMNKVKAYFDTLIQQHPYDYKTPKVVSALDFWIALFSCEIYNTHTEHILALDRL
ESEIDEDPRQESFGNDESIASDETDSVETGRLHKKYELRVQALSIMRDITKRMDVLMQDMPYSRNQHYLRLRAMASFYIADLI
LPISPVSQFQAEEGQKARLMEQESARNHLERIITYGGELDKAAWNALNPSGDVEEDTSIPLYSSLPIRGL
FUN_016492 CDS scaffold_9 149638 150460 -
hypothetical protein PF04716

MRPTARVFARYLEAGTPTGLTGLWTHATPRSTLLYLYGTTLSKLQSIPETSMYRQSVEAVTKHRMSLVEQMIPPGYNEWAVRA
KELVSKNSSQFRVASGRVDGSEAHTVKLGDKIFVVGRKHEAGDIRVEEWNGEEDEGPEYEGIRTQKEREDQVTWAERKPLEDH
EKIEWEDEPQMTADQVHELEQKIGAGLIEEVIQVAEGELKLVETMEKSKVWEDLEEKPAPGQWTYFDRI
FUN_016493 CDS scaffold_9 151065 152393 -
hypothetical protein PF03114;PF10455 ENOG41

MDFGNFGTNLGKNLSNLGASITPFASRTFQYTKEQFGQTEDKTQLPPDYIDLEKRVDALKQAHQKMLAVTSQYSNEAYDYPPN
IKETFQDLGRTVSEKVSLLSSATSPAEAQAALVAPPAAKPQPKTFSHAVARASLASSQLLHQHHTGAGEDPLATALEKYALAS
ERVGEARLAQDAQIQSRFLAGWNTTLNTNLTFATRARKNVENSRLSLDAVKAHAKGTTFKLGGHAHGDQPHEEQELSPEAQEE
IEKAEDEFVTQTEEAVGVMKNVLDTPEPLRNLAELVSAQIEYHKKAYEILTELAPVIEGLQSEQEAAYRKSREESS
FUN_016494 CDS scaffold_9 153138 155178 -

hypothetical protein   EOG09261BP0   PF04768

MSWPRVLKQAPRCGHCAPSTDSRLITSSHSIATPIRSRYRGFASQSGNSISLPCLGKGATRRMSAASTALAKKKAIDRDFIVS
VLEVSATKRDAKGYLQKYTGKNPKSLTDAPLFVQGDPQRETEPLDMAPPHVAIMKLRVPQEIDATTLAGVANTLSQLRKLGLL
SVVVIDCGIDESRMTFQDQAFRLCEAIDSFGEHGARVVKHAFVCSEPVGQKGRPQTTDIRLDDPAFINHILHHRMMPVIPSVV
SRDETRPPQPVDSNQIVLALTRYFAGVQSNTTDTSAEADAAPPKPIALVERIILLDPLGATPMAGRPGASHRFINLEQEYEPI
LKELMGPDGSPLADDKDLRASITAHAANLALAKDALAMLPHTSSALITTPDAAANLMKTSFEPNEATGSDSFFGIGGIVTTRR
KKNPLLHNLLTDKPVFSSSLPMARAPTNSGREPTVGASGGSTLLKKGMPLRVFPHPRENPWVPPKPGSPRLRLTDKCIDLPRL
VHLIEDSFGRKLDVDDYLNRVNENLAGVIIAGEYEGGAILTWEKPEGLDDHTAYEQGRYVPYLDKFAVLRKSQGSGGCADIVF
NAMVRGCLPDGVSWRSRKDNPVNKWYFERSLGTSKLSGCNWTMFWTTPNLDNQSPILRDYESVCRGVEPSWADNKHILD
FUN_016495   CDS   scaffold_9   156907 158260 +
hypothetical protein   ENOG41

MIITSITTAEVRVVLRVYFLVVLALTGFFVRRVLHGRNKLRILGGDSILATRALNSLDGLDLLLAPLKLRALPSGAFGFSFLL
FLFVLGKAADLLTSYLVVNHLIQSRCSFGQGLVFDDGTKLPMPPFNGRPFQVVQNAQLLAVNNSCPYGIYKKLNTDSSFCPEK
RDILGSWNCNIVNDQTPQSWPSGTNSSDIAEDMNQKGLIYNNGYIEQTQWSSGFVNHLVIWSTSASLDSDGSLWSVKAAVETT
YKSEDDIQLTPLTCSVSASGAERTIKDMASGTALKEWVPTFQGLMYNGFNTSAIQDVQDALAILLNTMTMISGGNNYLLAEPE
AGQDKTQGCLARAAYVPTVMVVIFLVVAGLGITAFVAWCITSLRLFRSPSELEALPLSAEEWAAFAAREYLSTANNNQDVVVA
PVETSELKSFRVGFSQPNIGRIGLYQRRQGNLLNQ
FUN_016496   CDS   scaffold_9   158578 159558 +
hypothetical protein   PF01488;PF03446;PF03807;PF14833

MAPTLLWIGLGNMGRGMSKNIVEKGNLDGPLLLYNRSSQRATDFSAKFPAGKTEVVGSLADGVSRADVIFSCIANDEAVQETF
KAMLEGNVKGKLFIESSTIHPDTTEAIAKDVVAKGAEFVAAPVFGAPQMAEAGQLVGVLAGPAASVARAKPWFKGVTSSKEID
LSDKPYSKALTLKVLGNTFILNMAEQLAEAHVVAEKTGLGTEAIHQIVDSIFGGPYTAYSQRMLTGDYYNRDEPLFAVELARK
DARHAMSLAESVGVRLRNVEAADAHLAMAYEHAGSSADMAGIYGAIRKESGLKYENDA
FUN_016497   CDS   scaffold_9   159970 160346 +
hypothetical protein

MAQGYAVFIGLVVIAALCVASWFLAPKGENQVSVYPSRRCDSSQAACDNTADPFSLQTLAILCYSGHRELLPNVGDYVPRSTS
PSYCAEKKRYPRRAPPLDIHL
FUN_016498   CDS   scaffold_9   160561 161575 -
hypothetical protein   PF04161

MPICIECRHPVKTLWTAYSGAGDKASGHNIRLTVCRNCGRFCDKYVEHDFVVLFIDLVLIKPQVYRHLLYNTLMRDDDRLDPS
VIRLGTLLLLFDVYLTWARLEKQMVPDAIPGASNLGKLSQQPIVFQYLFFLIFCALSTAAFHVSIRFLTSSAFSPLNLLGILP
RYTRPNSVSTALLVSSSTKLFPILMVIWDYDVPASARSLGWAVVANNVEALRILLDCNYVIACLLAIAGAASRWVVGRAVLLA
AGLADVDSIGESGVAADGKALWALLMYAKEWAGRLAVG
FUN_016499   CDS   scaffold_9   162179 164060 -
hypothetical protein   ENOG41

MTEPRRSARPSPHRSSSALAPANTSDSTSIRPRNRRLASALDDDSDTATSREASRSRRGRNLNVHDTAASPSPARSYGLLTAF
SPANSRSASPLPSERGGAPHRTNSAANLSNFFNDSLTQSWASIQGFTSSLLSGDNGAARRSRSHNRAGSRPGTWGRDFSGSGS
MKKTGGAWGPAPPQEPNLNDVGAGSLAERETALKAARRAMVLESHDGVNGGLDVSGNHKRRNSDESTPEQPQTQEYLVYIHKV
EPSDTYAGIILKYQCREDAFRKANGLWTRDSIQVRKWLTIPVDACDVRGRPCDPPSWHNAHSVDLLAPTPAVEENSSAVHDDF
FSRPINGTPSATQPSEEDKPWTHVRWVKIDSIPQPVEVARVARNAMGYFPPRRKKSIRTVSTFSTPRQSLDLYSNPPGSAGTS
SRRQSSLGHRPQLSGTVMSSPGRGAISEGGETLPAWMRRPGGVGSMGMSTRAPGPDKDYLTTWTKKHLPGLTLEGIPSMSVMG
SETAHFGFKSGESSSIVENPSGEGQEIGSTQQGNGLDKAAAAVETWLRGALSRKPSSQLDRIRGMPEGSRPERNGDLIELTDT
GSDDGRPSGAPPISLMESLQIKSSSRSEGEGGARGRMVAKGDKDD
FUN_016500   CDS   scaffold_9   165689 168874 -   CTF1ALPHA

Cutinase transcription factor 1 alpha	PF00172;PF04082	ENOG41

MDVDMSSGSGTPQAQPQQQQQPESQLSAPALPAPSTSTSTSAGGVSFRRQRASRACETCHARKVRCDAASLGVPCTNCVAFQI
ECRIPNPKRKKTQGSGSQTNKDSDSDRGDANEDPSPRPVAPSSTSSLTPRAPSVYHSNNGTPPTNWTEAQARKEEVDSGTYLD
LVMKPKFTRAPITEAGRVAYLGESSNLTLLVHDRQGSADVVHYPLPENVRGSRARLTELDNVEIDILHQRGAFLLPPRSLCDE
LIDAYFKWVHPIVPVINRTRFMRQYRDPKNPPSLLLLQSVLLAGTRACNNPQLMDANGSTTPAALTFYKRAKALYDANYEDDR
VTIVQSLLLMGWYWEGPEDVTKNVFYWSRVATIVAQGSGMHRSVEQSQLSRADKRLWKRIWWSLFTRDRSVAVALGRPVHINL
DDADVEMLTEDDFIEDEVDRASEYPPDPIHVQFFLEYVKLCEIMGLVLSQQYSVASKGRQRNAIDLTHSDMALADWLQNCPKI
VYWEMPRHHFWSALLHSNYYTTLCLLHRAHMPPGGSSRLPDPSPYPSRNIAFQAAMITSIVENLAAHDQLRYCPAFVVYSLF
SALIMHVYQMKSPVPSIQQVTQDRLRSCMSAMKEISRVWLVGKMVYALFESIMGNKVLEERLQRAEGKRHRNLRQSLSHLEQQ
QNRQAEAPKRKYDDMAIDFGTTTPQPQESYERSRPQTPSAVKVESGSTMQPPPVTSPNARQSAADTFMGGTNSRPQTRPATPF
NPSFSVPPTPPDLYLVTRNSPNLSQSLWENFQPDQLFPDSAAMPAFPNLSPTQTHSNLDHNAMGSVPPNNGQGGMHNPQAGQY
QQRGNGMMPQGFQGHSNMWQPNMDPNLPEGQSPDSWSTASGPGQAVPTTLNVEDWFQFFGINGTDPNHINLDMPLG
FUN_016501	CDS	scaffold_9	171237	174440	-	MCD4	Glycosyl phosphatidyl inositol anchor synthesis	EOG09260KDB	PF01663;PF04987

MSGFSRIGFMAIAVAFHLVYIFSIFDIYFVSPIVSGMRLFNVERTDDSPKAPADRLVLFVGDGLRADKAFQAHPEPYPESDDD
LVPRPLAPYLRSRVLEHGTFGVSHTRVPTESRPGHVALIAGLYEDVSAVATGWKMNPVNFDSVFNRSRHTWSWGSPDILPMFQ
HGAVPGRVDAFAYGAELEDFSKDATELDYWVFDHVKDFFAAAATNETLNTALREDKVVFFLHLLGLDTTGHSYRPYSKEYLHN
IKVVDQGVKEMVELIEGFYADDRTAFVFTADHGMTDWGSHGDGHPNNTRTPLISWGSGVATPVLHPDSIAPGHDEYSSDWNLD
HVRRHDVAQADVAALMSYLIGAEFPANGVGQLPLNFLSANIKEKAQASLVNAQVILEQYRVKEESKRNVELRYQAYGPLSNEN
LGPEQRIAHIRSLIDAGQFEEAIEESDALMELGLQGIRYLQTYDWLFLRALITVGYLGWMTYATTTVLSLYVVQESIPPQRTL
LGSAFFSSVLVALYSSFVVSKSPPAYYLYAFFPVLFWEEVYARRASVAKGFQALFGNVKSGGAVAALIFNVVLYFGIIQSLAI
GYIHREVLTGLFVLGAFWPMTLGVSFLKSHVFLTLLWFISCLAMSTFTLLPAMKVENVSLILIGGGLMTVIGLAYLVLEDFIL
SDFTTFKSKSKRLHTSRTLLGTQIGLIVLAMLVTHSSATSLQAKLGLPRGNQVVGWVVLVVSLLMPLVYRLQPNSHYMHRLAI
IFLTCAPTFIILTISYEGLFYVAFSITLLAWVRLEYAVHAFSKGKDQNETAVAEQQKRELGTFRPLTLSDARIALFFMVLLQS
AFFSTGNIASISSFSLESVSRLIPVFDPFSQGALLILKIIIPFFLISANLGVLNKRLGVAPSALFMVVLTVSDVLTLYFFWVV
KDEGSWLEIGSTITHFAIASFLCVFVAALELVSAAFIAGIEVEHEEPVAANGMKPKTNGKVPSRTLAG
FUN_016502	CDS	scaffold_9	175314	176827	-
hypothetical protein	PF12539	ENOG41

MPPRARAKPAAGLAGLLASDSEPDLDMFDQSELQAAVRTMSTTKKPRGRPPGTASKVTKTAPRTTRQNGADKLAPLPEAIAQP
ATASKINGGATKGGRKTKQLAPDETVTFEDIGVPASVPATETKKGLRGRPKKLNGDTSILAPESAVKRRGRPPRQPTTPFEEI
PETQAEDVMELDAVPEEDEDRDSVVGTTEPLESWSSYDTSDVALRRRLGELTKKYEALEMRHRDLREVGVREAERNFDRLKKQ
AEERTAAATQLISELKAELAAQTALAKEGEELRKQLEASEVKAENLEGNIESLNNSLSEAEAEIKTLSTKLAAVRSGNANNRV
PGSAIKASGPANRTAQAEAAHAAQAIAQAKENLYADMTGLIMRGVKQEDIGDTFDCIQTGRNGTLHFKLTVDNDDTSDNIEDV
QFTYQPQLDAGRDVELMEMLPDYLTEEIVFPRSQAPKFYLRVSKSLAEDLSRQ
FUN_016503	CDS	scaffold_9	177400	178806	+
hypothetical protein	PF00004;PF17862

MPSESAPWLRWVLGMGERRKLGDVAMDILLFAGMMTAGLYVARNFLNPILSNIADPDKEKHEQARRQAKAHLERLNRHKKDGL
DYGDDSDSRTGPRPEDLVLNEYENLVALEMVPPEDISVGFDDIGGLDTIIEELKESIIYPLTMPHLYSHAAPLLSAPSGVLLY
GPPGCGKTMLAKAVAHESGASFINLHISTLTEKWYGDSNKIVRAVFSLARKMQPAIIFIDEIDAVLGTRRSGEHEASGMVKAE
FMTLWDGLTSSNSSGMPARIMVLGATNRINDIDEAILRRMPKKFPVTLPGTEQRRRILQLILQDTKTDPEYFNLDYVARITAG
LSGSDIKEACRDAAMVPVREYMRQHRESGHAMSSVDPRQFRGIRSDDFLGREGGQIKLQPKRRSSGVEELIAEEQD
FUN_016504	CDS	scaffold_9	179738	181127	+	MET2	homoserine O- acetyltransferase	PF01053

MSAPSPTDSGKSANPLKRIDNEGHDLPPSPAPSSPRNGRKRYALATELVYTDSKDQYGASSVPIYQSATFKQTSASGGQQEYD
YTRSGNPTRTHLERHLAKIMNAQRALAISSGMGALDVITRLLRPGDEVITGDDLYGGSHRLLTYLAANQGIIVHHVDTTTVDS

VRARLSEKTAMVLLETPTNPLIKVVDIPSIARLAHEVNPKALVVVDNTMLSPMLFNPLDVGCDIVYESGTKYLSGHHDIMAGV
IAMNDTQIGDKLFFVINSTGCGLSPNDSFLLMRGVKTLAIRMEKQQANAQAIAEFLESRGFRVRYPGLKSHPQYDLHWSMARG
AGAVLSFETGDPAVSERIVEAARLWAISVSFGCVNSLISMPCQMSHASIDAKTRAERQMPEDIIRLCVGIEDPNDLIEDLSRA
LVQAGAVTVTLDGFHAAGVAKELGATPLVIQ
FUN_016505 CDS scaffold_9 183093 184578 -
hypothetical protein PF03348 ENOG41

MGALLSLPLLAVPSIGTIASFAASCCGAATCSMVCSACGKCGNSIATRIAYALLLLVNSIIAWIMLTPWAIKKLQKLTLDYVT
INCPTGECHGWFAVHRINFALGLFHLILAGLLFGVATSKNPRAAIQNGYWGPKVIAWLAFVVLSFLIPDNFFLFWGNYIAFAA
AMLFLLLGLILLVDLAHNWAEYCLAQIEDTDSRVWRFVLIGSTLGMYIGSLAMTIVQYIFFARGSCSMNQAVITINLILWLGI
SVISVNPTVQEFNPKAGLAQAAMVAVYCTYLTMSAVSMEPNNECNPLIQGQGTRATSIVIGAIVTLLTIAYTTTRAATQSLGL
GNSNGIQLPDDDEHGLVTQQPSARREMRAEALRRAVEEGSLPADALLSDDESEAGDAPAGDDERNRTQYSYSVFHIIFFLATA
WVATLLTMNFDESTKDGDFATVGRTYAASWVKIVSAWVCYGMYTWTLVAPVVLPDRFDFS
FUN_016506 CDS scaffold_9 185368 187267 +
hypothetical protein PF06813;PF07690 ENOG41

MHQDQQQSLFQARIIASIAATILSLACGTNYVYSAWAPQFAERLKLSSTESNLIGLAGNLGMYTLGMPIGIFVDHRGPRPAVL
GGAIFMLFGYFPLHQAYHRGSGSVMLMCLFSYMTGLGGCMAFAAAVKTSALNWPNHRGTATAFPLAAFGLSAFFFSFIGAVFF
PGDPSAFLELLAWGTFGLTFGSFFFLKVHHEQAEYEAVPASDETPSIPSTDPQQLRAGSSDEPKPYRSSRAEDEPGMLTNSSS
NSTYTSTSTNTNTNTITSSHAPAVASSSTAPADAPDRTGSHGISFAPEFGLVVAPENPEDLEVGETSPLISRPGQPPLENSDI
NSGGPHHVDIRGLALVRSVSFWHLFVIMAILAGVGLMTINNIGNDAKALWKQYDKNVTDEFLVHRQQMHVSTLSICSFLGRLL
SGVGSDFLVNKLHASRLWCLVVACAVFIFAQVCALNIENPQLLGLVSGPSGLAYGFLFGVSPSLVAEMFGVRGLSQNWGFLTM
APVISSNIFNIFYGKIYDQHSVVGPDGERFCSVGLECYRSAYWATLISCCAGTGITLWAIRHRHNKYLRAMDKGDEED
FUN_016507 CDS scaffold_9 188032 191465 - BST1 GPI
inositol deacylase EOG09262KN8 PF07819

MKRRSSGSADDGDESSLDSDIPDPDPNSKTRTSPARARRPSSLNWKPSNDRNGRNGQPREEPRQTTALKPTSLAAPSTPDMAL
ARTPTQSPSPNRRIPRPRFSIVVLLTSALGIVILISILRSLVTSQLDPKGCRMSYMRPSYVRFTEFDTEHTRFATKYSLYLYR
EQGIEPPDKLLGIPVLFIPGNAGSYKQVRPIAAEAANYFHNNLQHDHGALDAGIRSLDFFTVDFNEDITAFHGQTLLDQAEYL
NEAVRYILSLYSDPQRATREGHLPDPTSVIVLGHSMGGVVARAMLVQPNYQTNSINTIITMSAPHARPPVTFDGQIVQIYDEI
NDYWRQAYSQKWANNNPLWHVTLVSIAGGGLDTVVPSDYASLESLVPPTHGFTVFTTGIPTVWTSMDHQAILWCDQFRKVITK
TLYDIVNVHRGSQTKPRADRMKTFKKRFLTGMEPTMEKDLPLKEATTLLTLSDDSNSILPAGQRLVLSQIGQQPKPRAHLLPI
PPQGSPEVKRFTVLTDASLDEAGENGKVEVLFCSVFPFQSGQATSLYPFQIDLSTDDSGSTRLACKNAASDRILLPASTSSSR
YPFYLDREREIVPFSYLQYDLDQLPDHQFVAIIEKSVSSTRSFLIAEFSDQSAFSKKEDASLAKLLLSGITVDLPANRPLTTE
VNIASLQSALLAYKLEITSPHCQAEKVLFSPLVRQYLDQPYESKYFVNAQSVDVSLHGVAPYLPPSLGSGAEKGVTFQVWTDP
SCGSSIHLQIKPDFWGSLGKLYMRYRTVFAAFPMLTVALVLRKQFRVYDSSGIFISFSQSLDLCLRQSIPLLLVSLTLLSMSM
EGVPATWLGRWWYQAPAAPLSIDFHRHDILIGTDDPFFWFLVPLIGVVCIGVCAMVHYMTKAFTHVLGFLYWALTLLPLSSGA
EDDGVARTPAFVPSTPKRRMITTAILLFLVWTFIPYQFAYLVACLVQLFTVIRALRINETAPSDANSNYYHYAHSVLLLMLWI
LPINLPILAVWIRNLAVHWLTPFSSHHNVLSIMPFILLVENLTTGKMVPQVGRLFGYVTSLLLFATALYAAMYGISHAYMLHY
LVNIIAAWLVVLHSTKDPFSLSGLGAIFESSPTEEHKKSKASL
FUN_016508 CDS scaffold_9 193722 196879 +
hypothetical protein EOG0926213Z PF01119;PF08676;PF13589

MATIKQIDGRTVHQIQSGQVIVDLCSVVKELVENSIDAGATSIDVRFKNQGLDLIEVADNGSGIAPANYPSVALKHHTSKLSS
YSDIATLETFGFRGEALASLCALSIVSITTCQQGEAPKGTKLSFEPSGALKGTAVVAASKGTTVSVERLFHNLPVRRRELERN
IKREWNKVIALLGQYACIQTSLKFSVSQQPTKGKRIHLFSTKGNPTTRENIINIFGAKTMTVLVPLDLTLEMHPSTGGPALQI
DQKHKSSSRDVRVVGHVSRPSHGDGRQTPDRQMFFVNGRPCGLPQFAKAFNEVYKAYNSSQAPFILADIQLDTHMYDVNVSPD
KRSILLHDQNQLLDNLRTSLTALFDSQDYSVPTSQLLPFKNRTEKQSTTNTPFKPLSITPQKPLDDSQPEVESDEDSDESSPV
AKVPLDAVRRVGGSKSLEKNISSQHLISGWVERGSTSRPKDRGTTASTFAQNQSPSVRPSTRREEPSLFIGSPSLSSDSDEND
AGKPLPVRDFNKQLKVNSVTKTGSSPTKKKSSELLENEPQIPATKPLSRGQGGSSHTSPVKTPREKATSEAATIVIGNEIIQD

AASTPDRHANNVAVSEDEMEVDNPKPSFGSRLTQIYAAGSGATDRKISFRKEQAGEGSDGGEISDSEGTDTNTDSDTFNANKD
GKAVSEPAIPVAGSDRPGKQTETNDLSDITPFTFAEEPATADHKTQSLKSSVRKKESTAQLLQHLRTDESLIKLGLDSWRNSL
SPSQDSTTVDDEVTDLAAANAEEKLSLTIARKDFLKMKIVGQFNMGFIIATRPASTGSEEDLEVAQKNELFIIDQHATDEKYN
FERLQEIQTVQSQRLVHPKRLELTALEEEIVLQNIPAIEANGFKVHVDMTGDEPVGSRCEVLALPMSREVTFSITDLEELIAL
LGEESSESKHIPRPSKVRKMFASRACRSSVMIGKALTHGQMETLVRHMAELDKPWNCPHGRPTMRHLCQLDSWDAKGWRDDNL
QGPSVSWRAYLNGE
FUN_016509 CDS scaffold_9 197078 198962 -
hypothetical protein ENOG41

MAPSQAPFLYSAVHSDSRFPEPKFDPKAVTRASWEPPKQRKKPDGPLVSFNRHPDMHKVLTYRTNEYASMSPRSKSFIKWLRH
VQSGLRVLQLVSALGLLALMILIDKVPSLEGWVMRITPGVVILHCIYGIYHLSRDAAGRSPASSASYQLFCGVTDLGVAPLYA
FGALSVRTKSEAWITRLSDQSLMNTFKPVLFYTFVAAGGLHLISLAIAGWLGFMFRKISLMPPDMNPLESHLTARPMHKRNKS
SVMTASTMDSDARLSTPRSARRESLVPQEGIHDGIEAPRAIPFTHTRNGSSMTIGTRDSRTKFPPPPQYQRDPGSPRRERRDS
AASRATTRTTATRSIYHDAYSEIPLDDQAGSRPSSSYNRHRQPRPARFTETWMPTDSLISRTNQRNREMEAAKTSAANRENKS
YEALAQRYAHDDSDSEYGDENNPSYDLGVNGDELRPHPLRLNPPAAQQERKSPPRAKTPFFPFKNSLTDISTNARRVSVSRDI
ADEKPALAPRNRQSSIQPESEFYARSYGELQSATPPIMIGSDNRKVSTGNDYSQNTSTAYGRRNVSGKMVEEGRAAGNRYSFL
DGRNPLAERR
FUN_016510 CDS scaffold_9 199575 201060 +
hypothetical protein EOG09261UOJ

MGKVPGVKVDMYESLPVPFGLVRHGVAPDHPEVKNCQDKFDEIASQPNFTFIGNVSIGHPGNSSKHCTIELHNLMQHYDAVLF
AYGASEDKKLGIPGESTLSGIHSAREVVGWYNGLPGCSGLDLDLSQAEEAVVIGQGNVALDVARMLLEDIDVLRKTDITEKAL
ETLSKSRVKRVHVVGRRGPMQASFTIKEVRELMKLRDVGFLPFNRSLVPEDLKSLPRASKRLMEVLVKGSSTPHETASKSWSL
DSCFSPKHFLGNQDAPSKVASTEFDITELAAPFDPKSSVKATGKTTILPSDVVFRSVGYKSVALPGFAEIGIQFDDRRGVVDN
DGLGRVTRMVSDTHAAGAHNERVPGVYCAGWVKNGPTGVIASTMQDAFTTGDAIVHDWLSGDRFLNASNHDSVTGWEGLRHDA
GPSTCRAVAWDDWRQIDRAERERGQKNGKEREKFTSTDEMLTVLE
FUN_016511 CDS scaffold_9 201132 202234 -
hypothetical protein EOG092649VG PF00766;PF01012

MLPTAKRVLAQRATGLMARAHAYSLGAQRQRLQSTLAILEQKDGVLNHSSLSAFTAAKKLGGPVHGFIAGNNIKPVAEEIAKA
EGVEKIIAVDNSAYEKGLPESYAPLLVENIKKGGYTHVIAGHTAFGKSVMPRVAALLDSQQISDITAIENENTFVRPIYAGNA
IATVESSDSVKVITIRGTAFAAGPLEGGSATVEDGVDSKPESPTEWVSEDLAKSDRPDLSTASRVVSGGRGLKSKEEFDKVML
PLADALGAAVGASRAAVDSGYADNSLQVGQTGKVVAPQLYMAVGISGAIQHLAGMKDSKVIAAINKDPDAPIFQVADVGLVGD
LFEKVPELTEKVKSS
FUN_016512 CDS scaffold_9 203114 205287 +
hypothetical protein PF00249;PF04433;PF13921;PF16495

MDDSATYGSPGNGASPNSNPILAQPPLQPESGAGDSEDIQMSEGLATESNIKQDSTTPAPARGDEEMNDAPADDAKTKEDGAA
ATDGQESKSKEVVESAARDHLIAQTHAIVLPSYSTWFDMNAIHEIERKAMAEFFNNRNRSKTPAVYKDYRDFMINTYRLNPVE
YLTVTACRRNLAGDVCAIMRVHAFLEQWGLINYQVDAEQRPSHVGPPFTGHFKIICDTPRGLQAWQPSADPVVLEGKKSQDTD
QKAAAAAPAKGEQNLEIGRNIYEANAKNTLISKTEGKTNGETPATNGATGADDATKAPIAKVHCHQCGNDCTRIYYHSNQTDA
NPKAKYDLCPNCFTEGRLPANHNSNMYVKMENPTYTSTLDRDAPWTDAEILRLLEGLERFDDDWGEIAEHVGTRTREECVLQF
LQLDIEEKYLDSEAPISAPTGLSMLGPQQGQLPFSQVDNPVMSVVGFLASLADPASTAAAASKSAEELKRKLRKQLDGDKADD
DSQANGDSKAKSDSMDIDLRQETTTTTTTTTTTTTKTNALASIPLASIGARAAGFASHEEREMTRLVSAASNVTLQKLELKLK
YFDEMEAVLRAERRELERARQQLFLDRLTFRRRVREVQEGLKAAAAVGGEQGVRLAQEAMSEGDRLSFQAVPGAQAVPPPSSD
GQIKSYEA
FUN_016513 CDS scaffold_9 207090 209045 +
hypothetical protein PF00755

MAPTKRANSSAASSGYVENHSKGPMLRWQDSLPKLPVPTLEETAKRYLKSLHPLLSASEYEASKKAVEEFVRPGGVGSKLQEK

LVAKREDPNTKNWIYEWWNDAAYLSYRDPVVPYVSYFYSHRDDRRRRDPAKRAAAITTSALEFKKQVDAGTLEPEYMKKLPIC
MDSYKWMFNASRVAAKPADYPVKFSPEENKHIIAIRKNQFFKIQHEVNGKQLNTSELEQQFKRVYELAQRVPAVGALTSENRD
VWTDARDILLKASPKNKDALEAIESSSFVVCLDDAAPVTLEERAHQYWHGDGANRWYDKPLQFIVNDNGTSGFMGEHSMMDGT
PTHRLNDYVNDLIFGNKLDFSDPSIRSNLPEPQVVKFDITKEVQSEIDRATKDFNDVISKHQLAVQAYQGYGKGLIKKFKCSP
DAYVQMIIQLAYYKMYGKSRPTYESAAVRRFQLGRTETCRTVSDDSVAWVKSMSDSSIDDKTRVDLFRKAINAHVEYITAASD
GKGVDRHLFGLKKLLEPGQEVPAIYQDPAYSYSSSWHLSTSQLSSEFFNGYGWSQVIDAGFGIAYMINENSLNFNVVSKGLGS
DRMSYYLNEAANDMRELLTPTLEPPKAKL
FUN_016514 CDS scaffold_9 209429 211293 -
hypothetical protein EOG09262KZ3 PF01213;PF08603

MAANSQMHNLTTLIKRLEAATSRLEDIASSTEPPADAAVLNQVIPSPPNPSLAAPPPPAASDSKATTPAAAKPEPVAEPLPES
IEEFDAFLNTSVDKYVKLSHQLGGLVAEQAALVKSGFQEQRKFLLISTKAKKPNLSGPDLPVYESLIKPINEALMAVTELKDA
NRPSPMYTQLSTVSDGIMVLAWVTIDTRPYKHVDECLGSAQFFGNRVLKEQKDKDPKQIEWVQSFYQMFRDLADYVKQYFPTG
IPWNPNGQPAQEVLKSLSAGSAPASAPTAPAPAAGGAPPPPPPPPPPGPPPVLDIKTDDAPAPASSGGFGAVFSELNKGDAVT
KGLRKVDKSEMTHKNPSLRSGSTVSSGQRGKSPAPGKKPKPESMRIKKPAKKELEGNKWTIENYEKEAEPIEIEASLTHSVLI
SRCNNTTIIVRGKANQVTVENSTRLSLIVDTLVSTVDVVKANNFALQVMGTIPTVMLDQVDSAQIYFSKESIGTKVFTSKSAG
VNLNVISGEDDDYKEVPLPSQICSYYDESKGDLVNEIVAHAG
FUN_016515 CDS scaffold_9 212173 213698 -
hypothetical protein ENOG41

MADVAETPEIELPTDSKEAVVVEEEKDIDQTKSEDAEEHKNGDEGAPGTEPEPEPEAEAEAEADKSPEHPPLAIIEGKASDSS
GEEAVPIDFFTMGMFIIDDIDFIPPTPPVKDILGGAGTYSALGARLFSPPPKSASVGWIVDQGSDFPPSLSTLIDSWSTSALF
RHDGLRLTTRGWNGYEGTAEKRAFKYLTPKKRLTAQDLTPALLKSRSFHLICSPNRCRDLVSEITSLRKKVVPPETYTKPIFI
WEPVPDLCTPDELLNCTNCLPLVDICSPNHAELAGFMGDSGLDPETGEISTIAVERACEQLLASMPLQSFSLVIRAGEKGCYI
AKNGGRKRGRDPKTTKRRKKDYVRGGLQPDTDMEALFAGLLQDADGIVAREEIEVDPGVEKWVPAYHTDPSKVVDPTGGGNTF
LGGLGVALARGESLEDAVAWATVAASFAIEQVGVPTIGRDAEGREAWNGHNVEDRLREFRKRL
FUN_016516 CDS scaffold_9 214659 217416 -
hypothetical protein PF00493;PF14551;PF17207;PF17855

MDSFMLQDEGVRDRIRQAEEFLDPNDPQVRSYRSDIILMLQKNQRRLTVNLDHVRNHSPDLAQGLLQQPFDFTLAFDQALKNI
VQTIPQARPDQTAKDTIYYCAWAGSFGLNACNPRTLSSHLLNYMVSIEGIVTRCSLIRPKVVKSVHYNEKKDMFHFREYQDQT
MTNGVTTSSVYPREDDDGNPLITEYGFCTYRDHQTISIQEMPERAPAGQLPRGVDAILDDDLVDSVKPGDRVQLVGIYRTLGN
RNTNHNSALFKTMILTNNVVLLSSKSGGGVATATITDTDIRNINKVAKKKNLLELLSQSLAPSIYGHDYVKKAILLMLLGGME
KNLENGTHLRGDINILMVGDPSTAKSQLLRFVLNTAPLAIATTGRGSSGVGLTAAVTSDKETGERRLEAGAMVMADRGVVCID
EFDKMSDVDRVAIHEVMEQQTVTIAKAGIHTSLNARCSVVAAANPIFGQYDPHKDPHKNIALPDSLLSRFDLLFVVTDDIEDT
RDRHVSEHVLRMHRYRQPGTEEGAPVREQGGQSLGVSASNQTESQGPTEVYQKYDAMLHSGVTITSGRGSNKKPEILSIPFMK
KYIQYAKTRIKPILTQEASDRIADIYVGLRNDEMEGNQRRTSPLTVRTLETIIRLATAHAKSRLSNRVEERDAAAAEGILRFA
LFKEVVEESRKKRRKTQTVDFASSSDESSSDDEDDDGDIANATQSNRSTSRATRNSSRLQARETSDTARSSREPDVPEDDSQS
TLRRSSRRTQDQSQSQASFASSIPASQLPSSNQLESQDGDEDLASGAAALAIDDTPISSERLTTFRTALGQLLNTNLFEDDAA
ELDAVVEAVNKKIGNRRDAFDKGEATKALQKMGEANQIMFTEGDLVYKI
FUN_016517 CDS scaffold_9 217916 219867 + EXO70 exocyst
complex component exo70 EOG092612J3 PF03081

MSVGLSGNAHTALDEEARAEVDVLNSRLEKTTQLTKKIQSCLNRLESTGKSVQDVAGPLNGETRRLQILGNNVDSVLAAIDRL
RQPADSKNDEEQIIRVGPEKAGLSNYLASIKRLDKALNEMKASNLRANQQTMADLTRLIKSGNSQLESHFETLLRAETPRSVE
PLHFITKDKPFPTLPQDKIARLGLVYSYVIESHHNGSSELAHIYAEVRGPYLSTSLANLAAASVNTAKKKSPDAVYRAGTNGI
GTYSQAMEGLFVSEYDNVCSVFSREDWGVVFQSTCQAAMAELARTLRELNAHIKNHLNTDCYLAYEITEIISALSGKLETRTG
ELKGALAAALKPVRETAKSSLAELLDETRRKISMLQMLPSDGAPISLVSETMQRLQTMVHFLRPISSIMISLGDGGWNSNAAA
SGRSTDAIPSLASFDIGADGKEIFSHYCTDTIEMLLSGLDQKSRVLMKSRAVAGVFLANSVVIIGRMVQNSELSGLLENKLDI
LEQWRKKATAAYTDVCKDLSVHLFDTVHTNRAHRPTSGPVDSTSIVKGLGSKDKDRIKEKFTQFNSAFDDMVSRHKSYSMERE

VRRMFGEDIRQKLQPLYERFWDRYHEIDKGKGKYVKYDKTSIAAVFSSLAS
FUN_016518 CDS scaffold_9 221484 225214 -
hypothetical protein PF03372;PF04457;PF04928;PF13563 ENOG41

MEKTEQTSLYGFDSHETALCVIPPRGLWLSVDRLRSLYDKAYSAWPPHVNLIYPFVRPEVLGNAAQALEGLVIQNRPHLALNV
ADCFKHKNRNTIFLGSNSDSTTTELSKIRDIIYNALGRSHGAQNDRFVPHMTIGQSEDAEAAPHHFLLEKARLLAPIEWEVKE
IAILIRDGALAQGGSGPRPMRLWGTLDLESGTFHRITPPQSFNDHLDEMSTIAFQPCYHSIQSSGAWEMAESTNTSPDTNEVI
LEQLVVASYNVLAEFEWPPQSYRHPALVENLLSARAAADIVVLQEVTDHFLPDLLANKDIRSRYPFVTHGPPDQSGIGPLPSL
LNTVVLSRFAFEWHYLPFHRKHKGCTVARFPGIGTRDVDDGKFKPWILVACHLSQGLTDGAVATKKKEVQKMLDYLSATFLQH
PCIIAGDFNLATSTYTIDAAKKKHDISAQTVRHLRDIDRAISDSGFLDTWLATRLESGESSDIANEKRNVLDSFQGEQGATFD
PLTNTLASDLVGNGLNNRPQRYDKILIKANGHYNPHGFNTFGQTTFESPEQGGPTYASDHWGIRCLLVKSSPSDMSRSCVPEM
KIALQQPPPGLANSESLEDFLEGRGCLPNEQERTDRVEAIKTLEMVLKDATSSISENDSRSGPAFIVVPVGSYGLGVWTSSSD
IDCLCIGPFSSKTFFALAVQRLKRATTLGVRILRRVKANSGNMLELEVNGIKVDLQYCAAASIVERWPEVMKRPASDPAFALS
FQALAKLKPVRDLFYLRRSLPDMVQYRMAHLFIKSWAQARGIYSAKFGFLGGIHISVLLVPVCKALAYENETVTPADIVVTFF
HYYSEFDWKNSMVFDPFFHKELRYNRTFREPLCLIGWHAPALNTAPIASIPTVATISSEFGRAKRMLSDGDCIWGKLLGIEPE
TALGSVQAPAATEFLHAFKTYIKIDAHYWGPSQEKSGRFIGWLESRCVMLLVDINRKLKHLLARIWPSRFLDVSAGAETSGAE
YHGCYLIGLAWDDDSNKEDAKEVDMAMQTVLQDFEARICRDEKYYDSQFCWMSATVARAHDLGTLEVDQSLWGEYGGETDDDE
SDEELDEEEDFDDAEEELKGHKSTGPGSRAAVVGKTPGLGKFRTAADVLNRLRWDPNFDSGDYIIGYEDRFLGARERAVEHWK
TEQTDEEFIPQHRILYFKRREDNAILWERRTRIDEIFGSGVKIDR
FUN_016519 CDS scaffold_9 225981 226995 -
hypothetical protein ENOG41

MEKSAAVTEPEPSSPIAVLTGETPEHHVTLPPYSGPSIPSSAVSAPASQAIAAHKGPARFPGLPRVDYRLYSPPLFKLSNDYK
TLSSRAEYLSAKPEALIALIRAQASVPPKPQIHIRGSRGRKVDFDIKLNLMSLLVPDDDNQRLDYLRCVGDGELAYRGGLKPD
ALPEVGDAGLDEWCRRFVQDPASVKLFTLERVVTNLDTAWLEGQFRSLVAALNYRGVVDVSFVVTHSRVVIQNPDKVNQFFTS
VTSLFSGKHKYEVVKAVWPFATAKNGEPDRRCIVQSEETWWREWRDPIKYAISQKRHGWVTVEDKLEALMEGKGHETGIIDWG
PENMG
FUN_016520 CDS scaffold_9 227495 230127 + SEC23
GTPase-activating protein S23 PF00626;PF04810;PF04811;PF04815;PF08033

MDYETLKEQWSEVEDRDGVRLSWNVFPSSRMEASRLVVPIGALYTPLKEKPDTPLLHFEPVTCKQPCRSVLNPFCQVDVRARV
WICPFCLSRNQLPPHYKDITANAIPPELHPANTTIEYRLSRPAPAPPIFLYVVDMCQEADSLASLKESLVMSLSLLPENALVG
LITYGTMAHVHEIGYEECAKSYVFRGSKEYAAKQVQEMLGLSSSGVRPGMQPQPGRPFPAGPASRFLLPVQQAEFQLTKALES
LQKDPWPVANDRRNLRCTGVALSVAVGLLESSFQNAGGRIMLFAGGPATEGPGMVVGPELREPIRSHHDIDRDNVKYYKKALK
FYENLAKRTAHNGHIIDIFAGCLDQVGLLEMKGLCNSTGGHMILTDSFTSSMFKQSFVRIFEKDGDDNLLMGFNAVLEVLTTK
ELKVTGLIGHAVSLNKKSISVGETECGIGNTCSWKMCGIDPKSSYGIYFEIAGQGPATHQQAPQKGMMQFLTYYQHSSGQFHL
RVTTVARNLSSPAGDPAIAQSFDQEAAAVLMSRIAVFKAEVDDGPDVLRWVDRMLIRLCSRFADYRKDDPSSFRLEKNFTLYP
QFMFHLRRSQFLQVFNNSPDETAFYRHVLNHEDVSNSLVMIQPTLDSYTFDQDGGQPVLLDSASIQPTHILLLDTFFHILIFH
GETIAEWRKAGYQDQEGYENFAGLLEQPKEDARDLITDRFPLPRFIVCDAGGSQARFLLSKLNPSTTHTTGPYGGVGATTAQT
IFTDDVSLQTFMDHLMKLAVSGTN
FUN_016521 CDS scaffold_9 230534 231241 + RPL12 60S
ribosomal protein L12 EOG092651LN PF00298;PF03946

MPPKFDPNEVKVIHLRATGGEVGASSALAPKIGPLGLSPKKVGEDIAKATGDWKGLRVTVKLTIQNRQAAVSVVPTASSLIIK
ALKEPPRDRKKEKNIKHNKSVSLDEIIEIARTMRYKSFAKELSGTVKEILGTAYSVGCQVDGKPPQAIIEAIDSGDIDIPEE
FUN_016522 CDS scaffold_9 233349 238111 +
hypothetical protein PF13181 ENOG41

MSPHAVTPPQSNLDTLSTFPNNDAATFNNHHPRNVPAPYQQPFAIRTSRPHRHTIPYGSALYPPRQEHHLRRKTPNGTVDAGY
DGSPSLFSHGPPPSKHLVLPRSATATAFPLTGPPIRQSHYNGGIDYRRPSSVGNTGLSDDLQARPGSSTWAYGSDTALHHGAL

PTNHYGLHQAEGQYIPQGQPEAPGVFPNLYQPIIRAHEYNVRAFCPPPIVMNESLPFGQPGMHTPWNYHGRPGLVSARHVQPQ
DLRSFQQHCGSDLARRSSFASGFDSETQHHLGFDSGMYTQSYLRPPHMSSQQGFREKVLLQAHRVYVDLLAYAQTIRKPQTSK
GAAGHNTSPRLLVYPKPPKPSLRISEAALGQDTTLFKSSSENDSYQDFQSRGHVVPYFQGPYNSYSSTGNSTLFAAAKSILDM
LSSLCEQSTWSWIDGIMLGGCLHYGLDQYQEALEWFSRITALDSSHVEAITNKAATLYCLGRQDEAEKHWIRAVEVQPSYLEA
VEHLVGLLYKKRSKEAVEIIDHVQRALRVSPAQNQQAGPHDQLVKPPGFASSGYAIHGKENGRVLALIHAKGTMLYSLKDVNR
ASEAFEEAVLISVGRQMTSIQDLVRRIQAALSPTETPVSHRNARHGTRPLLLPPERARHTAQLVFGGTGSLPGLQHVAEGSTK
RAAVQTTSNSLLSLAKIFQDSLSSGQGLPNILRRPSGVEDILALYYLSLSLQESPSTANNVGILLAGVQQIASNPPTPNVDVP
AHSSIPGIVPGSGLALALAYYHYGLRLDPKHVHLHTNLGSLLKDIGQLDLAIQMYEQAVACDGTFDIALTNLANAVKDRGRIN
DAITYYKRAVSSNPEFAEAVCGLSTALNSVCDWRGRGGVILKAGKYDRWHVDDKGLLIDARSFGHDSGLVKRVVRIINRQLSD
ASHWGQGVLDEKKIASFARQIKDLGLVTSFDPEQALRGWSNKPWEGSRVIRLIERVARATQWKWYQDQHVSGSQAHTQYTRLR
LPSGLSVPSAPTVLPFHTFTCPLSAKDIRVISQRNGIRISCSTLRLPWLPSTVYPPPPPPNPNLNIGYVSSDFNNHPLAHLMQ
SVFGFHNPSRARAICYAITASDRSVHRQQIEREAPVFRDVSSWPADKLVEQIIKDEIHILVNLNGYTRGARNEIFAARPAPVQ
MSFMGFAGTLGAEWCDYILADSTAIPPETLRPWRGNFKITDVFKDDTEGEEEDWMYSENIIYCRDTFFCCDHAQSCDASERSV
TWEQEQRRRWKMRKELFPALSDDTIIMGNFNQLYKIEPTTFRTWLRILAQVPKAVIWLLRFPELGEANLRRTAKAWAGEEVAS
RLIFTDVAPKSQHISRARVCDLFLDTPECNAHTTAADVLWSSTPLLTLPRYPYKMCSRMAASILKGALPKSNEGQEAAAELIA
VSEEEYEQRAVELATGLSYTMSADGYGQGDGRLADIRKLLWESKWHCGLFDTKRWVNDVETAYEQAWQRWVAGEGGDIYL
FUN_016523 CDS scaffold_9 243126 243972 +
hypothetical protein PF00249;PF13921

MNNTAPTQQRRGPWSQFEDVYLMDLVRTHGALNWVRIASTLGTRTPKQCRERYHQNLKPTLNHEPITPEEGLMIERLVADLGK
RWAEIARRLNGRSDNAVKNWWNGSQNRRKRTDRRRAAQDHHDSYYPTNRPGLSISTPAMPMPGAQLSPLTGTAMRHPMHSWIE
APLPSPCSSDSAESEMGSNYTTSPSHQSMQLAVPVELPPLRHSGLPTAGDRLPSFDRLAPPPHAYTDRPEYQPRLPPFAPQAQ
LPTAPNSPVQQQQQPQNRKAQDSRMHLSALMD
FUN_016524 CDS scaffold_9 252461 254019 -
hypothetical protein ENOG41

MQRIMADQSWLRFLGGNRTRQHDLKYRRSEAALQDLYKDPPKSKEMHAHRLKKSMTRLPSWQGVSQANQATTAAAEAEYSLDE
VDKLFINAGQQIWHNPSIDQVAETLQVAVMTNPASEALSAEYRSHILLLCEAYGTRHRKIAKLEKELAEAKMALKDEASRHKS
IEEHWMVQDARYKAEVKRLEMFIHRMSDQGMEAVALARAGRLLRGRAGAATGGQRAINSDVADEDTSNGATEATVKNERNPTL
LTRQDTVLSGTRTMDTSNEIRLSRGFRVQDHLKKKKKKKAATGARRLETHADDDRKTRPEQGREELQSRSASMLSIGPGGRG
TADMPPHKGSARRERDSTEESTVMEEVGIIDRTSSTSTSLTQSQSSQDTVTELRCDRRSHQNNPRHHRQFSFVAGDDITAVRL
GGPRGSRIALGQHIQTSQAPTTSRETGRFCEEKRRSRSAEWIRTIEQRWSDIDDNPGASDLRL
FUN_016525 CDS scaffold_9 254436 255099 -
hypothetical protein ENOG41

MEEDMAEHASEGFCSIKPLRIGNLCTESPSSSVLDDALSVQQEPRSSTRPRRLTEAAPIIAAPKASARPLPEVPIKKPADHGD
IEKQVQAHEYTSLSDIQEEIEHTDTSITSTTSFLGRSDSPRSVKARVLELNDCGIMPLQPALKRDTSSTILESLVDYSEQSGP
GQNRALSTSTGNMGYLETDEPEQTSEMKSIVAEAEHSFRTGWRLSESEWIKGEV
FUN_016526 CDS scaffold_9 256589 257056 -
hypothetical protein

MASGYGMNGGVGRCFPFWQEVMGCYVVNTTAADDSGKKKCGLVLEDYYECLHHKKEHARALAMQAAYARSESATARDDAPSVK
QIRSLGLIDKEDDTKKVLGQS
FUN_016527 CDS scaffold_9 257511 260379 +
hypothetical protein PF08314

MAAHVTLGAAKMVLLAVHFATIADIDSLTYLIGQHAAILRPELVLRIILTYLPETLNPAVYVPLIQAIASGESPLQTSSDYQL
DTSSVQSITEDQASRKVKRLHLLSLKNTDVPLALADEADPLTSFLLLRAYRMDEEAGMLPQVPDLVSPFLQHSQDLHTWMIST
VLPLVRRNYEYYPQRPIRYSLLEFQNLPDRTAIDYLLAETGAVDDDFGLAGRDLSGLVGPWLHSESRWKREAFDQDVSNETDA
APELSCPGWEQALEWLASTAATSWPVALKAIESWDGPSDIDLGNDAGSWLQENQQQYLDRSYARAVLASAYLVSDADVEALEA

AFRICNKIRSLLDEDSDITLQSAVKNLAPVPPFDIDALGGAKAATYLRNDLLQSSNPLTTPKEESLDLLPALIQSAYILTHSG
VPCTVRKAGDLAFIQDQQEQKSQLGRLIRNLPTRASDDDGCWIRGRKEILWLHGWGNSPKSTGDETLQGIFGNVTLEFIETEL
LKAMLSRSRYNLARTLYDHEGPDAPLLVSAVLNTAQNAALGAFDNASNPNRNRGGLKRCDEILKAFPRTLGTNHPVRKRIEAL
LKATHALSDYRLVLKQGEPFSPVVLRVHSDPVSIIEKVLEQNPKAYTRLQEFEELGSNMIHANLPIYVRPGKQQPIPEDQDAV
KSLVTKRIVAMCIKAALREDDFETAYSYVVTRLGADENVHKSQPAGISDEWSWKAALDAGKYVRTERSQKPTHLGTASGNPEI
RHLEQRIECLATSLRIAPPSQLQEVLKTFRRCEEQLDAAIKEEAAREAAWDTAGDLSGLPGSFDTPDPDKAYPPRNISASATA
RQAEEAPMSLFDLSRATARVASRNLTVLSSLQGSFTSDKTNPESESSESELHEQNRTRKRDQLREAATGTLVSGVGWLLGANP
NRPTSGQQ
FUN_016528 CDS scaffold_9 261968 263356 -
hypothetical protein PF00076

MATTAMDYEAANGDRYEDEAPRYERDNRSASPRPRDDNEGSRRRSASPGGHGDSNMKDVSASRDDDDGAINPGSNLFVTGIHP
KLSEAEVSKMFEKYGDVEKCQIMRDPHTKESRGFGFVKMVTSEQAEAAKEGLQGEQIEGRTLSIEKARRARPRTPTPGKYFGP
PKREPRPGRYDDRRRGGYSGGGGGYGRDDPYRYRGYDRNYERRYEERGGGYGGSSGGGGGGGRDYDRSYRDERRYEDRGGYGG
RDYDRGYERRDRDEGYGRDRYGGGGGGGGREERDRYGPRGGPGGSGYERGGDRYERGGDRDAARTRDPAASAGGAGYGESASR
SETRDAYGGAPDRNIQ
FUN_016529 CDS scaffold_9 264671 265479 + ABA4_2 Short-chain
dehydrogenase/reductase aba4 PF00106;PF08659;PF13561 ENOG41

MSLKGKVYALTGGASGIGFATAKVLAQRGATVSIADIDPDAMSTAIAYFEEQGIEALVSRVDVSQRPQVEDWLDATVEKYGRL
DGAANVAGIIGKHHGLRAVAELEDEEWHKIIAVNLTGTMYCLRAQLNRIVDGGSIVNVASIHGIKGRLQTAHRFAKHGAYDAS
KHGIVGLTRAAAKENGHREVRVNAVAPGAIYTPLMKKAWDMHNRPDDAAFDEPTAFQRQGTSEECANVIAFLLGPESTFVSGS
VYEVDGAWI
FUN_016530 CDS scaffold_9 265591 266223 -
hypothetical protein EOG09264ABT PF00583;PF08445;PF13508;PF13673

MATFRRFRPDDVNKFSKCNLDPFTETYELNFYLQYHAKWPSLFQVCEDMDGNIVGYIMGKVESSPEAYKFSEHYLPWHAHITA
LTVAPEARRSGIAKILTDQLEVAADAENAWFVDLFVRSSNHRAIALYKNLGYSVFRVVKDYYGDHATDPSRASEDAFDMRKPM
KRDKDRQHIRDDGETHMVDPEDVW
FUN_016531 CDS scaffold_9 267150 268335 +
hypothetical protein ENOG41

MPSSPHTPRHSRNSSAFDSFSGSPQARRLSKSSVQEPSTPFRNSFNQQDASDLGGNGMGNLADELADAFSDSGDEGEYTDGDA
NSDMFGAEGQGQGNDQIDEIEDTEDTNSPKKQGSDRAKAMNLTLLSPRRKHQRQTSNYDGSEYGSESDLDSPGMPPGLVSKMD
AVESLARRGTENYGGPADDVFRRVTNALRDLGSQSSVEASASRLITAHSALTTHLNHQSRQIHNLSFSLLSPLVAPPDPETID
DLIPLLLNLSDAMPRPSTSAFNSLTSLHTLTTELVQNLNYLSDTLHMSRQTTTMATRRLKSAKEIVAEMRREEELREEGERWL
TRGNWGERLEKRECAGVCGDVIGGFEEVCNGWRERLLAQAEAQA
FUN_016532 CDS scaffold_9 271003 272936 +
hypothetical protein PF00400;PF12894

MAALPEAAGMAALNHQSAKRAIDESKSHTATPRSSTPPRSDRARLEPRHSHDNAPNRALRSNVRKSEGARASPFDIDAVDSAL
LREFQRPQRESTPGASPHRKRQRINGDRFIPTRSGQDLQASFSLLHEDGSPATPSKQKKRTPHGELHFQKTEEANRTFSHLLR
AELFESSVPQAATPTLTPTQTLPTSSHIPANDGTRAHTPPTNATAPSLPSSLTPSTPHKNLFSYMSPRHHNQVAGHPTPSKTP
QSRHGPNLDTRSEIYSLSPVRFGSQQMLLSPRRQPRAVSKVPYKVLDAPELADDFYLNLVDWGSANILGVGLGSSVYMWNAQT
SKVNKLCTLDDDTVTSVSWIQKGTHLAIGTGKGLVQIWDAEKARRLRTMTGHTARVGSLAWNTHILTSGSRDRLIYHRDVRAP
DQWLRKLVGHKQEVCGLKWNCEDGQLASGGNDNKLMVWDKLSETPLWKFSDHTAAVKAISWSPHQRGLLASGGGTADRRIIFH
DTVKGSVINEIDTGSQVCNIAWSKNSNEIVSTHGYSQNQIVVWKYPSMTQVASLTGHTYRVLYLAMSPDGRTIVTGAGDETLR
FWSTFGRRPGTREDGDNGGRLADLAVIR
FUN_016533 CDS scaffold_9 273787 275488 -
hypothetical protein EOG09263JCT PF10998 ENOG41

MDDDTKGASRTAQGLKSDNISTDSEIILTPDHESPSSPNNPPADSALLDTPMLGATTPRGSMHSRNPSFSGSSSNHEDWDGLP
PLDRLSVLDLLDNFALPQQLEKLQKGISAQTDKVRRSREAIKTKTQSARDRMVEEWRRRVPEADEQLDRYRKQMQRRVDKLGK
RWNDTKVISAREKVSFIFGVMNIFVSGYLIGAYPEYFHLWYTVQLLYFMPIRFFTYHRRGYHYFLADLCYFTNLLLALSIWIF
PGSKRLFTASYCLAFGNNAVAIIMWRNSLVFHSFDKVTSLFIHIMPCATLHCIVHLFSPDEQKERFPAIWTIKNSPPGSPTAY
ANVVSMLAWSSLPYIFWQVLYYIFITVRRRDKIAAGRPTSFTWLKRSYAKTWLGKFVLRQPEHMQEATFMMIQYSYALLTMLP
CPLWFLSRWASAGFLMVVFTWSIYNGATYYIDVFGVRFQKELEAMKAEVAQWQNSPEYMPLSPPLNPRPDASSVPRQAQQGAA
PSASTGQIPSMNEVNREKDLMEGTSLSKDDVNRPVSVDKIPLLDETRSSSATGVAGNSGDTTRGRRTR
FUN_016534 CDS scaffold_9 277434 281411 +
hypothetical protein EOG092606CY PF02463;PF06470;PF13555

MGKLIRLELFNFKSYKGHHVLLFGDSYFTSIIGPNGSGKSNSMDAISFVLGIKSSHLRSAHLKDLVYRGRVLKTAKINDDGSA
QANGDANGDADGNDKASRGDPKTAWVMAVYEDDAGEEQKWKRSITNQGASEYRINDRVVTAQQYNESLESENILIKARNFLVF
QGDVEAIASQSPQDLTRLIEQISGSLEYKAEYERTQAEAEQAAENQNFQLHRRRGINSEIKQYREQKKEADNFQNKTDERDAA
IVTHSLWKLYHFQKAMEDSFAAIQDHQENLKELRRNVESFEKRLEAARKEQAAAHRQVARLDKEIKAKERDIEDKENSLVPIE
EKINESTQAVETLQAAIAKATKERDEQAEVVRQVQKDIESVEKARQVFENDYKEQMKKQGREISDEDRREYNRLRAQLMSRTG
SNQAKLENLDRQRKADEVTVNNLKGKVDSIAASIEKIEAELSSIEERRSAAQATSKDLSQEIEAKKKEFNKLQSERVRTNQKR
TELEEKLEDVARKLREADDGRRQNDREARLKEMVTSLKRMFPGVRGRIGDLCKPKQKKFDEAVVVALGRDFDAVVVDSEKIGV
ECVQYLKEQRFQPMTFIPLDNIKVNAVNTAVKGFSGARLTIDTIDFDSTVERAMSYACGSSVVCDTLDIAKHICYEKKIPVKA
VTLEGYIIHKAGLMTGGRGPESKSKRRFEEADVQNLQRMATKLKDEIDRLPKADRRGSQEETLQIDLSGLERRLAAVKDELAA
FNKNHASKKRELDNQRRQLRELEPKYQEQASQLESTTATCEEFRNAIARVEDEVFADFCRRLGYSDIRSYRDSQGKLEQEVSE
KRNEFEVQKQRLSSRLQWEQQRYETATARIERNQAHVRKLRNDIKSYARDKDAIENAMREEQEELEALREALDENKSELTEKN
QKVSEAKLEVQKRSKDIEAHLKDINSLETVVQKNSASKAALLRRCRLEQIRIPLAEGTLENLPNDDRLLNQDPDAMDIDDEED
DEEMMGMALDDHGITIDFSGLDEELKASDDPSVEESLSEKITNLTSELEKLNPNMRAMERLESVESRLRVTDQEYEDSKTAAH
EAKEVFNQVKQKRYDLFNKAFSHIQEQISHVYKDLTRSEAYPLGGQAYLDIEEDTDMPYLSGIKYHAMPPLKRFRDMEHLSGG
EKTMAALALLFAIHSYQPSPFFVLDEVDAALDNANVDKIKKYIKDHRGPGMQFIVISLKAGLFQDSDSLVGVYRDQEVNSSRT
LTLDLRQYV
FUN_016535 CDS scaffold_9 282070 283314 +
hypothetical protein PF02002

MDLAITLIKSVARAFYETRDILVIDALILHEALRDDDLAYLMSTNTKELHKICGKLREDRFLVVHTRSELREGNPRPSNKTWY
YIDYRATIDAIKWRVYTIDKEVQGTTQVASEKKEYFCSFCKAEWTAMEVLDNVGPEGFLCHRCSHVLTFEADRTSTGHEQSTR
LNDQFKFISELLPKIDAVHIPECDFDRAFAKARPVKRDETHQRAQTIAVDSGANRPMAVKGLTNTGPQSIAVNISTSDGPTEA
EKAAEQARKEQIAKQNALPSWMSNSTVTGESFSANATPGTASVAKKESSKDAGPAAPAVANAQIDDIFEKLKAEIAQEKSEEE
EDDEEEEDLFEDVPAAKKVKLAGPDTHEQKEDEDSEEIEFEDV
FUN_016536 CDS scaffold_9 283596 285024 -
hypothetical protein ENOG41

MSPSPSRFPPPASTPQPDESQLPKGQCRYILMIPELKGQRCGCMGFDHNKSLPGATCNCGHLSCFHVANADTPTPSTNKSEID
AIQQRVRALEELANPQDRDRLSHVVRRISELEETVEKNKDETDTELKGSYRNASAAWAFVTQLQQHIKSLEQTLRSQGEQLAR
TGSEVDDLRNRQLELLDSDECLEERIEKLESAEPLLSPPQDPDPSDATVNTFHLSPTTTVPSQLPLNSTHSLSIEPTASTERP
RSWTVHVSLMPSKDTPFPFEKDTTSYKRCLSRGLHRMLAVEGDDAKSVTEAVSRAFEPLLKGRPWVPLQAKLCDAERLQGLPM
LRPLEPRLIHGPYDRAFLKQHCAVLDASGKMDSLYIAMEHDSLSWNFLKRSPVYLDGLESAWERDSVLDKKDEQDQSSRDHHG
DEGDESPPAGNIVGALTGLKRSMSEISCSSLDDTELPLAKLPRTCKTSQLEMRRGVETAR
FUN_016537 CDS scaffold_9 287351 288245 +
hypothetical protein ENOG41

MGNFGTAVNSLLGTYTKCLSLLKGFPKDEDVGRLSETRSTLSTSLRSDRARVRREYASRLSQDGSRFEKGDAPARSALRRIVR
KLTNTLADVVRSFQDPKGQPINCESLLALSTGSSLDAVRAMNDLSSRVGSTSSSSSRGSIVSRGRQRSRRHSQKTGIKSKRCS
TEERSKREKTRSKKPTSQSRSRRRTSSDRLHRQHGSRSRQPPEIKGPHNRVSIVTIASDSTKLGEIRRRLSKQMPLEEYENRV

AYPLYAYHAQEAPVRKKWWNPFRRS
FUN_016538 CDS scaffold_9 289062 291047 +
hypothetical protein PF08492;PF17004

MPQDPAAALSALLRQSSIEDHDEALKIANAALKANKNDVDSQHTRIIALLKLDRFDDALRAIADGSPALHARIALENAYALYK
TGKLDEATSVLQAFGLEKKRSLQHVAAQVAYRAERFDEACNIYSRLLDTDPADEENDISINLRAAFAQSSWLGYSVIDKISVQ
DSDGFELCYNAACAHIANGSLETAADLLQRASRLCDASDDLTDEDKQAEMRPILAQQAYVFAKLGKLKEALDLYNSLSSTKEE
DPDLALIIGNNLVTLEPKDENPYLLERRFSTLTAKARNAKLFQHQSYILRRNRLTVDLQILKGAGVKRRTDVLLAEATHPSTG
AETSILSVLNAAASAQGTSGKQLLKNLQGLAQKRPHDVGLVLTIVQIQLNEGKVGSAMSILESFLQRLEKNETDDSQNARFSP
GLVALTVTLLRTQGRESSAKAELVKAATYWQSRPANSAISLLEEAGIELMKSSNAQDLQLAGTSFQKLIDERKGSDIAAAGLV
ASFAPSDPSRVEKHLQNLPPVDSLIEGIDVTALLSAGVATTASKAGSSALKRSAPSGPSEKTRKRRRKIRLPKNYVEGTKPDP
ERWLPLRDRSSYRPKGKKGKKKAVDSTQGGIVKEEETLELVGGGGVKVEKAPPPSKKKKKGKK
FUN_016539 CDS scaffold_9 291555 295389 -
hypothetical protein PF00169;PF01369 ENOG41

MFDLAPEIITTAPTNDFNGPKKKSSRIGLTARFRRSTDLPREDINTNNGTGRQKTLRKSFTDDRRRPTLATLEEPESPRPSTT
ATTSSPNGTILTPPNRPSESSRSDASSNDRLSHQSSSYQSPETTPKKSGPFFRLRRHKKAPEPLFPFAHLQQQGKAPPSISSA
SSLGISSTPRPASAQSGRTAGGNTTKGDSDLRSGQSPATTLVAPGGIQSGHSSPTRGDLLRGRSSTMSSMGRDSNDDHLLPPT
TRTSSSTGRKSFGDLFGLSRLRQNSELSRQGTMTPTTPGSIGSKNNSLQLARDSFVLPERNEDESPAKYLARVEEVASRGIIA
ATLSKSADPFMTAVLRSYMRSFSFFGDPMDMAIRKLLMEAELPKETQQIDRCLQAFANRYHECNPGIYSSPDQAYFIAFSLLI
LHTDVFNKNNKHKMQKADYLKNTNGEGIFDDILECFYDNITYTPFIHVEDDLDPTSDRYASHKSRRKPLISSTVNDPAKRAVK
EPIDPYTLILDGSLDALRPNLKDAMELEDHYNYLGSATSLNLKDLQKTFFRTGVLQIVSARSRPDAFMTEQTASNPADAHPGI
VDIKVTKVGLLWRKDAKKRKARSPWQEWGAILTGAQLYFFRNTGWVKSLMHQYENHIKAGHDGTPLIFTPPLQEFKPDGLMST
YAAVALHDAAYKKHKNAFVYVRQGGLEEVLLADNEEEMNDWLAKLNYAAAFRTTGVKMRGVIGGNYDGQSRRGLRRLDSADAA
TLIQTPTGPVSIARSRIDHKMAEDISAARRDVMKQKIAEADEHVQEIQKQLEHQLRNARHLLILAPIQPRTREQLLSAAARIS
AQLKWTRQDMWKHKCHRDILIQDLEEEHPSSTFTPTKQQSTRFSQRTGSPTPRVNVGRRGSRSTHHSGTEADPRTPTEPQVVH
VPNTAEVTEDVDSPLDQVFTTPPQSATKRRHSSRDLSFVRPEAASTRHGSISSIAQSPIASLPSTPLAKPLSAQDESNKPPLR
KEDDGNHDPDADERDFLEQAGLLEQRPSRDPTDKATVSTGADVPGESGTPTDKGDRSKIRRSLQRTLREGAGHLSHHRGSRKG
KEVAGSGEESTQEHQLSRGTGSFVVHGKKASVINFGDGLQNMTHDEKIRARKSSQQQDPPLSPMPSGPEDEDFYSAVGVPVEP
SERRESIASASTATARSFRELHRKYSTAQAARSTSAGGRLTIPSDTESEVAVSFSDGRRSPLPPMETETDEESDGTGVRRGRS
IKKDDLESRDSDSESLQDIEQLPSRPVQPVNA
FUN_016540 CDS scaffold_9 297774 299448 +
hypothetical protein PF00246
M14A
MRLPLLAASLLLLQASSAHAAGIDTHRAVPDLQNNENNNNDANKRLFPFLSKLRDKAIETVFGRHPSKNTPPPVINQLRAQYL
NQLVLRFNVTTSHEEGALADAAARLFLDVWAFTDDYVDIRLHADEVRPLLSLLPKTLHASHSILIPDLAAAVYKSLPIGGNPA
YYDPKPAPPVLKVASSPGDNLFFQDYQSLPVVMRWMRLLEAMFPSYVKYINIGKSYEGRDIPALRVGIPNTAPDAPRRKTIMI
MGGSHAREWISTSTVNYLAWSFITSYGKERMITKLLDEFDLVFLPIANPDGFEYTWHIDRLWRKTRQQTNLQFCRGLDLDRAY
GYEWDGSQVQRDPCSESYGGEKPFQAVEAVHIADWARNQTWNNNVRFVGLIDLHSYSQQILYPYSYTCSVDPPNLENLEELAA
GIAKSIRLSNGESYTVASACEGAVSAREFSGGRYWSRIESGGGSAVDWFYHEMRAHYSYQLKLRDTGSYGFLLPKEHIVPTGE
EIFNAMKYYGDYLLGNNGIESITEAEEDDKVEMHENQPVQDSEQNPTVGQELRRRRLRR
FUN_016541 CDS scaffold_9 299732 300366 -
hypothetical protein PF01124

MSLVLELPAEYGFVLVAATSTFFINTLHVLLTSKARKRSGIKYPVAYASNELAEKDAEAFKFNCAQRSHANFTENQISFLGAL
LISGLRFPVASAVLGAGWAFSRVFYAIGYSAGGPKGRMGGSIGSFLCDTSLKCMAAYTSIMFAMGN
FUN_016542 CDS scaffold_9 301113 302970 -
hypothetical protein PF10391;PF14716;PF14791;PF14792

MRLDNLPRIFLLPTHLKPEELHDLEERIPTLTYDIREAEIVVGKISQQRRAEFELRRAKFEFASVEEPQKESHQVDSPVVADD

PGGSPDPKRRRIKEQPEVGTDIVKVVKLSWLLDSWEKEELLPVDLYLIFQCNRVLPRETTPAPVLPKGSTSPASSILERALLE
QKAQSTSTSPSNQHKRRHDVSTTISQNAPSLLHQTTSEHDITLPVMPEFLRTTYSCQRPTYMNPPNEAFVNILTEIRTIRQLR
EDEVGVRAYSTSIASIAAYPYALKNAQEVARLPGCGDRIAELWHHWKETGESVEVREANADPKITVLKLFYNIWGVGAATACD
FYRKGWRDLDDLVEFGWNTLSRSQQLGVKYYNEFLLGIPRDEVATIAAAILEHARLIDPGFEMVIVGGYRRGKQQSGDADVVL
SHRNENKTLNVITQIVVALEKAQLITHTLTLSTHNSDRGQRPVSWKGEKSNSSGFDTLDKALVVWQDSSKKDAPHRRVDIIIS
PWKTVGCAVLGWSGGTTFQRDVRRYCKRVKGYKFDSSGIRRRADGRWVDLEGTSGGDEAPDMETAERRVFAGLGLQWRSPEER
CTG
FUN_016543 CDS scaffold_9 304248 305520 +
hypothetical protein PF10309 ENOG41

MDLDIEMDDAVDTVHDAPILDVGRDDILQPDEPEEPGEVAEDEPPTDDLKTLIPTKIHIKGVDSLHTSDIKAYVKAHFGDVDR
IEWIDDSSANLLFPSEPTARDAIVALSSVPVADATALAIGETLPAKPFEGKPEISLQVRFAIQSDKKEVGAALRSRYYLLHPE
HDPEERRRRNQENRSRYRDRDDGYHRGGEGRRRRASDDDVETFEASMYDDAPRPTRRRRDSDIEERPRSYARENQGKELFSGR
KTQRDRSASPRRDNDGDDLMGERASSSGNRTRARDLKDRISGSNNSKELFPTKKSIKQFGGGNLDTLERAIGSARLKDEDRPK
IVSVPNARGDGNSFNIRGMASQQGSGEGFAIKGAASANARELFPTKLGSTNTGKELFGGGRSKQRQKAEDLFS
FUN_016544 CDS scaffold_9 305802 306750 -
hypothetical protein PF00175 ENOG41

MSTQRSGHLERTAQEPRDESHLDVRLERIDQVNERIRLYRLKLESGPIKFLAGQWLDTYIPGNPKPGGFTLTSTPRAASDPKS
PYLELAVQESPENPPAAWLWQSPSDITGSKLQVRVGGSFVFPPSEIPLNDISHVVFVAGGVGINPLVSMMGHIAEEGYNLEVK
VLYASKLPAQGLKGVLFLERIRKWFTEKELIGDLKVFTTGIYDGHIESRELDVHERRFTVEDVKEAMGEKNNSVVYVCGPATM
TDEIVDGLTRDGGMDKNRVMLEKWW
FUN_016545 CDS scaffold_9 307203 312370 -
hypothetical protein PF01532;PF01602;PF02883
GH47
MAGPLRRGRLWIGVAFIWILCFWYWSNSDSGFKLFGNGDPLAGKGATAWTRRLPKYPIPKEKLAALPKITGDVKVPKIQATTP
VESAAAKELRLSRLAAVKKSFEHSWSGYSNYAWMHDEVTPLTGKSKDPFGGWAATLVDALDTLWIMDMKDEFTKAVAAADGID
FTRSPMATINIFETNIRYLGGFLSAYELSGRAHPILLKKAIELGDLLMCAFDTPNHMPVTRWEWKKSAYGQAQSPSREALVSE
LGSLSLELTKLSQLTGNPRFYDAVKRIGDQFESTQLNTRLPGMWPVVVDAYTPAFHAGSDFTLGGMSDSLYEYLPKWYLLLGG
QLEQPRRLYENFIPVAIKHLFKRALTPSDKPVLISGDYQVTDLPGEQPKYTYVARGQHLTCFAGGMVAMGSKIFNRPADLEIA
SQLTDGCIWAYQATQTGLGPEIFNFISCGGVDAKDSSDCTWNEDRWLKAIEEQHAPDFRAPPLRGSDWKKPTVHDVIKKHNLP
KGMIDVSDARYILRPEAIESIFIMYRVTGDAQWMEKAWTMFETIEKVTRTEIAASAIDDVTKAEPTKMDSMESFWLAETLKYF
YLIFSEFDVISLDEWVLNTEAHPLSRPDVKGLPLLLLGIVKQFIRNVRAAKTIADERAVIQKESAAIRASFREESHDPNIRRN
NVAKLLYLFTLGERTHFGQIECLKLLASPRFADKRLGHLATSLLLDENQEVLTLVTNSLKNDLGHSNQYVVGLALCTLGNIAS
IEMSRDLFPEIETLVATANPYIRRKAALCAMRICRKVPDLQEHFIDKATQLLSDRNHGVLLCGLTLVTSLCEADEEEGGEEGI
VEKFRSFVPGLVRTLKGLATSGYAPEHDVTGITDPFLQVKILHLLRVLAVGDAETSEQINDILAQVATNTESSKNVGNSILYE
AVRTILDIEADSGLRVLGVNILGKFLTNRDNNIRYVALNTLIKVVAIEPNAVQRHRNTILECLRDPDISIRRRALDLSFTLIN
EGNVRVLIRELLAFLEVADNEFKPTMTSQIGIAADKFAPNKRWHFDTMLRVLSLAGNYVKEQILSSFVRLVATTPELQTYAVQ
KLYINLKKDITQESLTQAGAWCIGEYADALLKGGQYEEEELVQEVKEHEVVDLFSLILNSAYATQVSTEYIVTALMKLTTRFS
DPAQIEKIRRILQYHQTSLDIEIQQRVVEYGNLFSFDQVRRGVLEKMPIPQIKEESRVLGQAPTKKKAANRKSRVIKPTEQDL
LDIMDAPAASPAAPSTTNTDLLADILGGTSSPPPSASSPPPQQSNVSNIMDLFGSAPVPSTASPAPPSSNLDLMSPVSSAPQP
QASQAPAGIPCYNANDLNVTFQIQRNAEGMIQATAKFINTSGSANLSNVSLQAAVPKSQKLQLLSISSSDLGPGAEASQMMRV
SGCKGPLRLRLRIGYTHPTAGQVMDQVNWTESS
FUN_016546 CDS scaffold_9 314122 315094 -
hypothetical protein
PF00036;PF08726;PF13202;PF13405;PF13499;PF13833;PF14658

MADSLTEEQVSEFKEAFSLFDKDGDGQITTKELGTVMRSLGQNPSESELQDMINEVDADNNGTIDFPEFLTMMARKMKDTDSE
EEIREAFKVFDRDNNGFISAAELRHVMTSIGEKLTDDEVDEMIREADQDGDGRIDYNEFVQLMMQK
FUN_016547 CDS scaffold_9 315939 317125 +

hypothetical protein PF05739

MWRDRTNLYISYRQSYAHHPTQRRPYGPGALASGPLADTYTSSYANDDRRGLLSAGAFEDDGDAVIEMDLLPPRWADVSDEIT
DLLANIATKGQSLEKLHQKHVLPGFNDEDAKRAEEAQIEKLTQEITKGFHDCHRCIQRIEQMVRESQHAGTITRAEETMAKNI
QISLAARVQDASASFRKKQSAYLKKLRGMGGFGGLSPGERSGTPQPGSYLDPSLQESDADRSFSQSTLQATQQQRVLHSNDAA
IAQREREIEDIAQGIIELSDLFRDLQNMVIDQGTMLDRIDYNVERMNTDVKAADKELVVASGYQRRTTKRKIILLLILIIFGM
IILLVIKPKKHG
FUN_016548 CDS scaffold_9 317307 319842 -
hypothetical protein ENOG41

MHRIPTQFRIRQSAYTRYIRTLTQHRSQYNTELAPALTRIYEKDDRLISDREAGGGTRQHNLQQETSQWPPKPELPFPLGIRL
RGSEQDTPSGLNSDLDAISSWERRILTSRQDRGDQGTWKTFLELQDRDQSHLLTKTGALFLRDNILEAALTNSDRMEELFKVA
GQLLETHDFNWPELYLKVVHFYLAHADYETALGWHFKLMPMFRPDLDSFGALLASCAIDFTPKLQSTLTKIYVFSPYRELYDY
VVPALFESGQSHAARVWRKRFNLFNDHPKSPKSMPFLDFLSHYFSAIQLTKEELAVLNGDTSVGYGVGTSVRKDTRLETNSSF
FSDRFTARWFASSWTSSEFAINLMHKIGLRTISYYSLQSIALREDDAHGVADRLKQLRKLGIEIRSGAYCNAIISFAERGEDY
LLRTLLHCDIHPEEFGNPETRSTLLASSAQRQDWGLERLLQEVEGVAATPGPSKDNIISKDLNKHLSMALSMKHLAKVRNVVD
RMDSLDVTMEQKNSKALLSRIFEDIWYHPKLTRQGTLDRAIHLSLRVARHGVAVPVRYWQILLYNLGRLGRFNDLEGLSYEIC
ELYRPEPGGLIPVHRQDQPPKPDSKTKELNGESETWSEYYSFADGKKKRSHFKEEFWRAEIGPCQNETNPKQKYHKRSKNRND
PNYAFRIPADLPFTNRQHPIQKIFDISLQRAIVRWGFDQTLAQQPIQPSLANTKPVGIADFDLACGVRLLAVLRDKGVYVDPQ
IVRSTVINRLAVARLPMRPRARARDDRELSPANMKRLVEGAWGSQILPNEAQLASEIEKQKPKLWKSYPRLFEKSYDNDDRIS
SKPHASRTKEKDWF
FUN_016549 CDS scaffold_9 320238 320702 +
hypothetical protein PF02238

MPLVNAKNPVPQNQRFYQNAYKNHTRLWKIGPRSRILMTPYLILLWGTLGASFYGAGRKVLGYNSYFGN
FUN_016550 CDS scaffold_9 321022 321844 -
hypothetical protein EOG09264XOC PF01086 ENOG41

MADRFPSLEDFDSGAQTDIKDPTAEPSTDDFLAREKALLGDDADQFATNDDAAAFASNDDDLLGGAGNNEQSTFESQFPDLTN
PEAGTGVSGGTAITGGPSVSYNSGYQAFAEEEEEPEVVKEWRERRDNQIAKRAEQFAAQREETIKEAQQNIDDFYENYNNKKE
KGIAQTRKEAEEFLASREDTVSGGTSWDRIAKLVDVSGKGAKGGASGSGKERFRELLVSLRKDEKAPGATGY
FUN_016551 CDS scaffold_9 322518 323896 +
hypothetical protein PF04818

MAYNDDSVLARLSSLNESHDSIATAAQWIMFHRRHAERTVQLWMQRLKDSSSTKRLSLIYLANEVAQQSKIRHKDDFIIAFAP
VIAEAVSVAYKGAPAELQAKLKRVIDVWRDRSIFEAPIQSAIDARIGDLDKARGMSKPGFGGSPFGSTSTATSVPSEFAPLVT
AHQKVNKLSTPLKATITSADQEYEKQTDPSTPVPSAPVYAARLNGLLKTLANAESAVAECVKAREGLVSGLESLLNANRAALE
EEKSAAAQLTSRKAEIEDKKHQVEVGIMRALGPADSNGTPGQGDSSVTPPEPDRPEVEALTPPAFEPFEAPTPEALTPEGGPV
AAPSMSPVPVPVPAAAAAAPTSQDEEEAPSYQSLPISTNGSNKRRRVDTEEFPDLGGDDGIDADVAEMLKGQPQS
FUN_016552 CDS scaffold_9 324680 327077 + MDR2
multidrug-resistance transporter mdr2 PF00005;PF00664

MVSGVARRVLLPRLAEPSQVSLATSRCQLGASFHKSEALPLQSRLFHASSTRQAALLPFSIATFRSSANNSLARNQPSLAHNR
PSQFGPVSHALRQFSTAPRLLQEIQTKVEEGAKKPVELEANRAIAQDEHKQEFTKSEKAARAAQVNLAAKLSKEGKQAGKAGT
DEIVRLIKIARPEIRWLGIAFVFLVISSSVTMLIPFSVGRILDLATKGESEDVRLFGLTMNQFFFGLGTVLTIGAMANFGRVV
LLRIVGERVVARLRSQLYRRTYVQDAEFFDANRVGDLISRLSSDTVIVGKSVTQNLSDGLRALFSGGAGFAIMVWTSPKLTGL
LLLMFPPIAVGAFIYGRAIRNVSRSIQKNLGTLTKIAEERLGNVKTSQAFVGEVQEIGRYNNQIRKIFALGKKESLIAATFFA
STGWAGNMTILAMLIVGGGFVRSGAMSLGDLTSFMMYTAFAGSSLFGLSGFYSELMKGVGAASRLFELQDRKPGIPQTVGVRV
KSAQGPIKFTDVSFAYPTRPAVKIFNGLDFEIPSGSNVCVVGPSGGGKSTVASLLLRFYNPTSGTITINGQDISGMNVKSLRR
RIGMVSQEPVLFSGTIAENIAYGKPQASRFDIISAARRANCNFISDLPDGLETQVGARGSQLSGGQKQRIAIARALLKDPDIL

ILDEATSALDAESETLVNEALAGLLRGRNTTISIAHRLSTIKRSDQIIVLNSEGKVAEIGSYKQLASDKDSAFSKLMEWQMSG
GEVSKDGGSTASRAHITEAEELEDDLERDDEEENIEHEEESRDSKNEEKRP
FUN_016553 CDS scaffold_9 327938 328891 +
hypothetical protein EOG092638EN PF00561;PF12146;PF12697
S33
MSTETEGTFEVANASLHTKTWTPEGPVKAKLVHVHGFSDHVNWYNDVYRILASHGIQVFGFDQRGWGRSVKQPSDKGNTGPTS
RVIADIAAFIESKLPSDAPVFVLGHSMGGGEVITLAADPQYAKLVSQVRGWLLEAPFIGFAPEEVPSAFKIFAGRLACKILPR
FQLKHVLDVANLTRRPEVAKGFKEDPLCHDTGTLEGLASLLDRTGALQSGSVKLGKEVKSLWLGHGDHDKACSYQAAIDFAQK
QDIEDKTIKTYVGGYHALHVDLCQEEYAKDITDWILERSQDQKPIEAKL
FUN_016554 CDS scaffold_9 330101 331466 +
hypothetical protein PF01545;PF16916 ENOG41

MTPDNSNHVAHHPQAIHLQSLERARSSSRHSTTRNGSSTAFEPATADENETIARNVSHMTDVESQLTATGRRQPSCLDHDPYG
LSRGYKTESDLEQIKANTSRKRDSLPGRKTVSGEVGPKTMARKLQGFYQNQNAAIDRMLKSVEEHRDEARDQHGEDQVKFRIA
VWGSFAANVALSGVQLYAAISSKSLSLFTTMADSIFDPLSNLTLILSARAIRHVDSRRFPAGKARLETVGNIVFCFLMIAVSL
IIIAFACQELSRGVQEKEFKIAAVISVCCAFATKFVLFLYCWALKDKYSQINILWQDHRNDLFINGFGILTSCGGAKLKWWID
PMGAIILSVLISCIWLHTAFGEFLLIVGVTASVETQQLITYVCVTHDDAVVGIDTVRVYHSGPRLIAEVDIVMDPTQTLQESH
DIAEALQTKLEDLPDIERAYVHIDYETTHKPEHAFKKDM
FUN_016555 CDS scaffold_9 333720 336447 +
hypothetical protein PF01501
GT8
MAAQGAGEHVYATLLLSDSYLPGALVLAHSLRDAGAHRKLAVLVTLDTVSADSITQLKRVYDYIFPVPRIRNDHPANLYLMNR
GDLHSAFTKINLWKLTQFSKIVYIDADVVAYRAPDELFDTPHPFAAAPDIGWPDLFNTGVMVLEPNMGDYYAMIAMAERGISF
DGADQGLINMHFGQRYHRLSFTYNVTPSAHYQYVPAYRHFQSSINMVHFIGSNKPWFTGRDTPSGNNPFGEMTGRWWAVYDRH
YRHQESSPDNASSTPQYVQYFTKGEWHPQPVHAQSSTSEHHSSGSAEGTHHSAPGHEHHASQEEQGSGHHHSGGSGHHEGSHH
GGGYHDGSDSGSAHHDHHSAPPSGQPVSAPGQHHEQHHGQHASEHQHDHHEEPKNEPPPITMHDWDAQRHPPPADSKPEAMNF
PSTHYEMSRDTAPFIPPERYPSPPKNMWYEVPKEKPAPRSKPAPEIFPWERNRPRPTRSFIGEPEPPAPEPEPTPGESGSDPS
TGSTTQLSVKTEGLTEPSEVTTSGSGAKNESNASTPIVQITPSDPWTSYTRTNAWDEVPEIERYVDRLHGGHRRGKSSTSTIG
RVKSPSTGAWRDDRSNFKNRGLKLTDFPSATERPSLPVTPAPIQRHSFWAGDDGEHEDEAAKKLPEAEGVPTQSDWDPLAQLQ
KLAKQQSDALLKKLGGEEEEGKEGVSREIPTRALPFGSGDAKSPTYVAQSAAGVLSPQPVKGERSVGILRDMSSGRLEPESTS
QSTTIPEPSYTGPGAAWEKDEDIPQYETPLPPKEGELDALNA
FUN_016556 CDS scaffold_9 337274 339989 -
hypothetical protein EOG09263X0V PF00097;PF12678;PF13445;PF13639

MRLGWYAGASTALAGGVVLSAFYQRANFYSAMVYLAQSNFCLLVLVNFSFLVYSSFVYGLTRMFFGPLRAVEVEQLTERAWFA
ITETCLAMTIFREEIGAWFLVMFAALVTGKVWGWIGDGRVEVLEQQPPANPRLFHLRLSVSLTLSFIYDIYILRYTINTVIQQ
ARPNMMVMFLFEFAVLATSSWRTAARYALSLTEQNIQEAQKRKRLAERRQEVRQEREAIIRRREIAAAAGEEVSDEPLPNEDD
IDEMDIEVPGWSAKGEFVLWLDLITDLVKLGIYIVFFFMLLAFYGLPIHIMRDLFMTARDFIKRLGALLRYRKAIQEMNRYPD
ATQEELEREDTCIICREEMRPWDPENNPGAMDRIRPKKLPCGHILHLGCLKSWLERQQVCPTCRSPVTLGETTPRAGQNRAAG
LRIELGGRRPANQPAANPGDAPAQGQQNGGAQPEQRPAGPRIFNIGPLRVGFGANGQQVRELAQQFGVPPAAPNPQGLVPPTP
GAGTPIVAGQAAPATPLPTGDNLQNVGNLISQADQLIQREMQSLQLAHQELQTIHLLQAELYRLRQRQQHPEQQPNIQTQAIP
PTAVPLPQPLIPQQFPMTSQMSMPPGFPQFPGIPPRHPSPLMARHGAPLNAVPIPAGSAELPEGVVIPPGWSLLPLQRLDGGQ
ASTQPSPQVGPQATVADNINVPQPTSTVRQPSPIGSRNQEAQPVENPSSTTPTEQRPPQLTPQFTRAVEPPQVVAPSPRIPNW
GGSAQLFGNGSRLDHADPVTQNEPQAEGSSSQIAPVEATSQPSPTAEHTPQGTPPSHSDEDEGSGIDSKEKGKARAVTVEEVE
DEENVKD
FUN_016557 CDS scaffold_9 340455 341336 +
hypothetical protein ENOG41

MKPILTPTLLAAIRKQPNLPRNTWYFITATTLSALNRPEELPEVFKNATGEGSDTTGNGVPSRDDQLRISRRLREALLKASAV
GGMPKSINALMSLKSTTPEDLLDEPGTGISIRHKDIYDTAPAKVLERGQTFFEAIYGKISRRIMGQLDWSGAPDLGLLARLTY

GYVLSNTDVLTPAETSFVLIASLIPQDVNPQLKGHLRGALNGGASVDEVRAVRDVVIKICEASGMKRLEEDAIGGWGWRSEVA
NV
FUN_016558 CDS scaffold_9 354468 357057 +
hypothetical protein ENOG41

MTALTETSFAMFPHHSDAEDFARYTDSNVIFTSGPMDVSIPADAFGFQQQSGSQDAFASSMGFVEPSLYAEAPNYMLNSRASP
SMYTDESSDMRLPSSSLSTASAASTTSSAIGSPQSNHGQMGAMPEWAPQGLGLQPTIVGSDYIGSDFASFPSSGMDELSFDFP
AAKAFVDPSLIHPELTRPPMGIPPYDSHFQQTNANPHHTYPTSPSASSSSPQPHMMRTNSSSPFLQTTNFQSTFPSSPYGAPM
DNHSQTQNHNHIRAPSVTQFVPPTPGEYSSDESKEKQRCTYPDCGKTFKDLKAHMLTHQTERPEKCPIQTCEYHTKGFARKYD
KNRHTLTHYKGTMVCGFCPGSGSAAEKSFNRADVFKRHLTAVHGVEQTPPNSRKKSSAGANSNKKLTGYAPDATGKCSTCSQT
FSNAQDFYEHLDDCVLRIVQQEDPAEAINAKRLAEVENDKDVHQTLEKNNLPTTTDTVMANHDDEDADSGDDDDDSSPRSNSS
PATRKKGNPANGVQKSRGVTHSRGGVPLGTKTRSRKNRRDYPSSWGFDKGQMTMKKRVMAVFDGPRRLAKDDMMLSTDHEVRI
KLSDGKSYVTDLDVQTLKRAAGFHGATEEEKGPWISDDPTAEQLKEMQQMLTANTTTTTL
FUN_016559 CDS scaffold_9 358417 360069 +
hypothetical protein PF00324;PF01490 ENOG41

MGIVHANNREPRGLDPVPILSQEGLNHVEARPTVAHDEVFGDITEDGPNYRDVGWLGTSALMMKTQIGLGILSIPSAFHTLGL
IPGLICLLVIGGITTWSDYIIGTFKLKHREVYGIDDAGGILFGRFGQEFLGISFALFTIFASASGILGISISFNALSMHGTCT
ATFVAVAAMIVFLCASIRTLGRISWLAWVGVISLMAGVYVVTIAVSIQDRPSAAPKMTLEDEWKSDYKLYNKPSFTEAMSALS
TMVFAYAGTPLFFPIAAEMRDPRHYTKAMLLCQSVATATYIIVGVIIYYYCGSYVASPALGSAGKTIKQVAYGLALPGLIVGA
TINAHVTGKYVFVRVLRGSRHLTANTLTHWATWLGLTFAAALLAYIIASAIPVFGSLVSLVGALLGTLQTFQPYGCFWLYDNW
SAGKQERSLKWILMVIWSSFIILSGMFLMIAGTYGSVIGVIDSYKVSGGSAAFSCADNSNSV
FUN_016560 CDS scaffold_9 360519 361422 +
hypothetical protein PF10294

MSLTWRISVNGEADSPEDYLSTSLGVIFPDDITNQHGDAEHNLSYASPHLPKPLVIDLADPVKEDDRKLFSHYLWNASLLLAE
FIEADSLSIPLEKHREAQDIISFSVKGLETLELGAGTALPSIMGGLLGAKRVVVTDYPAEPVLKTLSTNVARNIQPSLSPAGA
ETTPFSAVSVQGHSWGELEDPFSASAAHSFDRVIAADCLWMPWQHENLHRSIAHFLKQTPEARCWVVAGFHTGRTKMSGFYDA
AALQKVGLEVERIWERDCNGEERVWDIEREDDITVRKRWLVVASLKWISIS
FUN_016561 CDS scaffold_9 361920 365610 -
hypothetical protein EOG09263OZR

MTSDGSGDGPSITSPGLTSREVGPYVLRTLLDEVPLSADGSKDDIKINCVDYLDANLYVGTSASELLHFVQIPPDPNDRSGQP
VYILASRLCPQYVETPGTPNSRPGVQQILLLPRVGKACILCNWTVTFYSLPELSPVFGTTLVKNCSWIGGIDLNEPLFDDGSA
ERGGGVTILLSLMRKIQVVRVGEDARAFKKIDFSGSTLSVRRDSIACVADSKSYALIDVEQQLKIPLMSISSLEETTSPSEIG
HAQSIGADTTSGLLRSSSSTGNRTSSEAPSHSRSTSLGGSILDSIRRQDHRGNEGEDFLGRNTSPQPSISPRPSMERASTPNP
PSLDKPLPAAPSPSGTPSQSTDPRPRPGSVFLKPHITSPTPDEFLIVTGTDPLEPGIGMFVNLDGDPTRPTLEFTRYPKEVVV
DSPPLDANSSQPSSMQEEDGYVLASMTREKDGLEYGLEIQRFDAGSDPNPQKFWLTAREADGVYGIRSLAGSEETRFDEIVQK
LSQRRYTPFSVSISPGTPDPSSSAKTPDSRTALSIEHLSKEKELFDRNIDSQDEESLPEEWEANRNAEGEEFARQLAKAQARL
AVWSGSRIWWAIRNPLLIQLDAALEAACVGDIFNPAELDRQAIFTTINSIRGREPQSELEFMTYGYIRQKAGILLLTNLLKSP
EDKQFTEKDMKALEEVLIESGLDPRVVLSLIPSVRNEIIEGSRGIWIYGGVRRTADAYLRSREFQMTSKDSIGTLEPRTMHFL
RRFLGTWRKKKGFGSVADEREVFHTVDAALLLVLLEIDQHSPKGLGKGGVVRSELYEIVDKGVDCFDRAVDLLETYHRLFVLS
RLYQSRKLSGDVLATWRRIIEGEQDVGQELREGEQRVREYLTKISSQALVQEYGVWLANRNPKLGVQVFTEDKGRAPKFEPAQ
AVAILREEAPDAVKYYLEHLVFGKGHTTYINDLMAYYLDVVVYDLESSDESQTLVRATYEAYRALQPPKPAYSHFLRDNAPDD
NEVWHSRLRLLQLLGEANDYDAAAIRDRISKLPDDLLVPETIILAGRECKHDDALRLLVHNLGDYDTAVSYCLRGGASTTGAA
PSQRPSFEEQRRLFNVVLHELLTLENISDRVEQTGALLERFGGWFEIDDVLSLIPDDWSVDIIAGFLVGALKRLVGERHEAML
TRALSGAQNLRVSYDRVAKIDEKGPSIEASN
FUN_016562 CDS scaffold_9 366138 368411 +
hypothetical protein EOG09261OXD PF08172

MTDAAVEAALASTTEAPTPAPTFEGENKFQHAISAWRTIDLTTLVSNLDNTASDIVAYQRDSTVQRKELAQKTKEFRKLDDAA
KLTEIKGLLKSYQTFIDLLTNHSKSVNSAFLQTYASFSDAPDPYPLLEASVDSMLLSEDTLPKITEENQHLQENVSNLSSQLE
ETESKLQNERKVRRELEENLESRVKEVETSWNAVLEEKTDNWEAKEKALEDKLEKQERLLTEMRASYEVNQRLGKNGDDQEAH
RNHVSSAELEMLHADLDRTSSRLAEVEARNEQMRLELAQAKSSAQQQPETSLEDDPGYMRVRSENQSMIRKLDAARVEKEGLK
RELDGKLRSIEREVNTLKEERDTLKSKVQKWSDYEDIKQELEVLKSIEFSTGDDDEVREHLEAKDAEGNSLEKLLLARNKKLG
DELTVLRVSHNDLQSRLEDLQEELSKTNAELERAQKLNQKLESDLETIQEEGANAFPSGASVAGTYVTRAMGRKSGRISPTSS
IISGMDPRMGGGEPGERVYGGGSGMLPMVTAQRDRYKKKNAELEEELSDTHRTVSQLRQEVAALQKDNLNLYEKTRYVSTYNR
GGGAATSSAYGANPNPSTVSIGETGNPGIAMDRYRQAYESNISPFAAFRGRESARAYKRMSLPERAVYSLTRTVLASRTSRNL
FAAYCVALHLLVFVTLYWLGSSDVQRVTHLESAAAAAAGVAGGSLGSPMDGAPKDPAK
FUN_016563 CDS scaffold_9 370067 370970 +
hypothetical protein PF11937 ENOG41

MQLSTFVTSVLAVTASAAPTYPTVTVTDAGSAIDALGSLSGYFNLVADKVKAVKSYGMAPVCDLSQAKMPLDGQLPPPAKGLK
LAHVAIGRGTQNYTCDTSKPSSAPVATGAVATLFNGSCVAAMYPDLIERIPGMAVHFPLSDANKLGPASLAESGHHYFTADGV
PFFDLRTPNQDIGEAPCAKNSSAPAPSLSAKGQLGENAVPWLRLTTIEGATHKMKEVYRTTTAGGSAPATCQGMDAEFEVQYA
TLYWFWAGDIEEDDA
FUN_016564 CDS scaffold_9 372394 374956 +
hypothetical protein ENOG41

MDSNWIDAIDPALSQRRPSTAPPSCDEEMPALLELSDSHDRTQAMSAVTWPTNEIPVEIFEIIISYLTRLEVRCLRLVCKEFE
AKVSAQYFRNVVVPFKSELYSKLDRDENGAFKRTSSALLSNGMRIFQSFGPHIRRFALSLELDEDTLSYPPVKPSQEAVPSFW
GIYRWPHGTYHRYTDLEGLEQTADETEAMKEALKCLVKVTNLGLCCDAGLGFLSGPDHIARGATTLHPVFATQNWRHTGSESQ
ARKQPIVTMSDLNEMVKDFRKPVFENPISFKRTVLQKMASDAGYSGAQINEAVLMLLETEGTNINTIDFDERATIINEMEARR
VMAQNTHRADFEPATAEVANHPLIPASLTRAQKEMLLELEWAHRAMIQSYVIGLIDNARDNCFNNLTTITIAKIPSSHVYILC
RHELWQNLSSLSNVSLGVIADWRRVSKSAPGCVEDDTVSPVEAVGKVYQLLNNYIGAQRHIESLHFEWICGGEFAPGTYQRNH
HILPAPFFEQPQLMTRIDSPKTNTDDLLALPFIKHLSLKNCWASPHVLLQTIRNMALSTLEKLDLESVSLSGPPTLTPQAPLQ
QAGAANNAHLLNMIGIHPAPPGVLVPPPTNIANGVVAPFPFNATAQQLAGVFGAAFMPPPPPNNQAPPPPPPPAPMDILQQPE
VLSWAGIIEHFSPSIKIRDILSARDELSLSIPSQPALDQPDPLAQHIPAPQRLRSDEKKYTLKCLSFKSCGYVTVDSPALNTR
SILPHGAQGLGANGNPHSQDLSPLMQKCRDKLLCRIVPCLRNQELLQLTTAFGMDMGWANIYDERTIYDARADGVEQPGKGRF
SGTVDAASIPGARDSEAMVSLDF
FUN_016565 CDS scaffold_9 375888 376812 - RPL17 60S
ribosomal protein L17 PF00237

MVRYAATEIQSSKSARARGAYLRVSFKNTRETAQAINGWKLQRAVTFLENVKEHKEAVPMRRYAGSTGRTAQGKQFGVSKARW
PVKSAEFLLGLLKNAEANADAKGLDTGALIVKHIQVNQAPKQRRRTYRAHGRINPYMSNPCHIELILTEGEEVVQKSDAVVER
EHLNSRQRGARLRKAIAAA
FUN_016566 CDS scaffold_9 377473 378680 + NOP1 Small
subunit processome complex component EOG092648VW PF01269;PF08704

MAPFTPRGGGRGGRGGARGGRGGFGGDRGGFGGRGGARGGRGGGRGAPRGGPRGGGRGGPRGGRGGAGAGQRGGAKVIVEPHR
HPGVFVVRGGKEDGLATRNTTPGESVYGEKRISVDESVQNDDGTTTTTKIEYRMWNPFRSKLCAAIAGGADEIYIKPGSRVLY
LGGASGTSVSHVADIVGPTGYVYAVEFSSRSGRDLITMASKRPNVVPIVEDARQPARYRMIVPMVDVIFADVAQPDQARIVAM
NANWFLKVGGGVLISIKANCIDSTAPAAEVFANEVQKMRAESIKPKFQLTLEPFERDHCLVAGEYQRYKS
FUN_016567 CDS scaffold_9 379437 381146 -
hypothetical protein PF00067 ENOG41

MENVSSLAASPIGVDYQLPQFVSEGHCTGYYITITIFLIAIWFFLPKKQTSHLEIPFYKASKTKWIFDAETLIKDSYTKFRDR
VYQIKATEGVQVIVPARLVGELKGLPEDILSATEAVSDALQSKYTKFSPGHNGELLALLVRTKLTQNLTRVVPLLKKELEHLL
ATEFPVCEDWTPVKWQPFSLRAVARLSGRAFVGPSINRDEKWMDTSINFAVHVFTACVKLQFIPEWARPVGQHLVSELRQIRK
DIKIAKEMLKPILEERLRDMEFSNGGNAPDDMIQWLIEALPEEEKADLTTQAELQLIIAAASIHTTNNLLCECLCDLAAYPEV

QEELREEAYRVLEVDNGWENKESMAKLKKLDSFMREVQRLSGNITSFIRKVMKPISLSDGTELPVGTKVLAPQAGIALDERYF
PDPERFDAFRFYKLRQESAEASNRWQFTSLNDTYINFGAGKHACPGRFFASNEIKLVLAHLLINYDIRLKAGEDRPKAMSVVM
AKAPSPDTELEFRRRSWAA
FUN_016568 CDS scaffold_9 383511 385986 +
hypothetical protein PF00168 ENOG41

MADFQGLGAKFDGFQSSTSGSSSSPFVASDTSSHTSISLASNGCLPPLSTHDTTKSQHHMNNFQQFVDDLQSAAKRAFPNSAY
SRYSEVQVLLIRWEEDELEVEWELSELQRVFHRLYGFTTDKFLIPTQNSHRKLNHKVLSFVEEHENEDTLLIVYYGGHGVINK
ARQSTWSCKGDKSYATVEWSAIQTLFEMVKCDVLLLLDCCAGASAAPLTERPTNIKETIAACGFETWTPRPGAQSFTHTLIEV
LKEWASQLPFSAAMLHSEILTRLKHAQPDRSSQGRLIESRKTPAYILSTSDPHAASITLGRMFEEESSSVSSSRAIPESDLGS
LSERVQSDSYISAEDRIQALLAVGAKGQRAAPHVLLTIALEEEQVFDAASCARWLKQFPLLATHVSVESVYHSYSTLLLLSVP
VVLWDLLPEHPATQFVGYVKSRNLLSVDMAKDGAATPIPIARQVTDDKPGDSFDCASPLRSVSGLSSLDSGYDSCLSRPASLG
VALRHNRLPMDGTMGSVASQALASKRRNKLSSAELLGPDRPTSTPVNTRFNPKQGSSLKSRRSKRLVVSVGEAELNNDKIGHT
CVLWYGYDVQQSQPTKRTTPKPIWNEDFRFEVHDSPMYYHLRVSVWGAQMLELIGELHVDLCELVLRGQLPELRTWQSLSCNG
AVVGRIRIKLACMDANDYEEQQTAATKVSAAKRQQQRQKLERQQSDDGQARVHQMDFQVW
FUN_016569 CDS scaffold_9 389884 391735 +
hypothetical protein PF00175;PF00667 ENOG41

MTAPAIRIDGIVNPAAPPHPELIMAEGAPSIAPSLTYSDDSDSQEDEPVIPLERRRRASTRLIAQNARDIQRITGETPAEYMD
RCCGGACCMGDRNNVEYEQVTLPDNDAFKSLGLRLENIPANLTGIIDMPEQTVSFTSIPRPTSSPSDSAISLGYDSESSPIAT
PPKNNYLVPGPGYEGVDTSIQPPRFVQPHPPHNVYPARIHNTRELTKPGAEKRTFHFDLDITDYPPEEVFDFKVGGAIGVMAP
NDEISVDEILDALMIPRFQRDKPVLMKTTKGRWPTVWGDDKPRELVTTRRELLTWTTDIQSYAPTKELLRVIAEHATAPNEKK
ILMYLCSNEGASIFSDFRGGPHVTLSQILNAFPSSHPPMDQLLSVQAQLMPRFYSLSNDPTESYTLREHAQRRLIEIAVTVHD
SMDWRRGCRAGVGSGFFERQAHKFMRAQAAGERAPQIFIPMFKGLMANPLAKQFNSDGPMLLIGAGVGIAPFRGFVQRRLKQA
NCANKVWVLQGIRDSLVDEIYSGEWGVHEEEVKRVVQSRVGTGKYVQEEVMNQSDLVWYIINSLDGRIFVCGSSKGMGEGVEE
ALCDVAMEKGNLEREEARNFWKLKKEEGKYISETW
FUN_016570 CDS scaffold_9 393454 394072 +
hypothetical protein PF00881

MAAKLSANALIELAKNRRSIYTLSKNLPISTQRITEIVNQTTLETPSSFNSQSNRVVVLYGAEHDKLWDITADTLKAIVPAET
WEGTSKRISGFKAAAGTVLFYVDNTAVEAMQTKFAIYADRFPPWAVQSAGIQQYLLWTALEAEGLGANLQHYNPLIDEKVAET
WKIPSTWTLNAQLVFGAKTAEAAPKDYAPLEERVKVFGA
FUN_016571 CDS scaffold_9 394462 396192 -
hypothetical protein EOG092639H5 PF02666

MAPTLGFGPGSSKLGSHMLRNSFYTHGRWTNCYSPSASRHMFLASTRSRCLSRPSVSSIRTFASNSGKRPRFSQRLGEAMRNT
KIQWYRIPVGLGIGFLGLVQFYKVSSREKERLENEDGQEGSRAPKKRARIRPDGPWQVQVMSTLPLKAISRLWGRFNELTIPY
YLRVPGFKLYSWIFGVNLDEVAEPDLHVYPNLASFFYRTLKPGARPLDQDPHTLICPSDGKVLQFGQIQGNDIEQVKGMTYTI
DALLGKNTPAPSISGTSGTSTPATSQGDVSEEESLVKQHEEFAQVNGISYTLPDLLTGTGKQAPAAKDESMPASPGTVSEVRA
ELALGERPWYDLISPDNTTSLYYAVIYLAPGDYHRFHSPTNWVVDRRRHFAGELYSVSPYLQRTLPGLFTLNERVVLLGRWRW
GFFSYVPVGATNVGSIVINFDKELRTNSLLTDTAADRAAEEAARRGEVYHGFAEATYEAASPVLRGHALRRGEEMGGFQLGST
IVLVFEAPSSKTDENNRHVGWDWAVQKGQKVKMGQALGRVIE
FUN_016572 CDS scaffold_9 397157 399530 +
hypothetical protein ENOG41

MASMPSATRPQDDGPSVASTATTDEQTVNLQVLSPSVGVNRPLLFPNLAANTTIKQLKDKIRQALPLRPADDHQRLIHRGHAL
VREADTLVDIFGADAIRTSEQQTIHLVLRDMTDSPSSTPNAPSAPPSLAAPVPAPAPARGPSPTASAPPTHPPRPQPQQPQQP
HGQPFGYQMPPGWRNSVPPTSNPFPQPRMPSPSPSHTPEQAAAFQHQHQNMTQWLNSIQREAMARALNQNQRTRAQMGMRGVG
DPNNPGNTGGNNSGRASPAPGHTVYREHVGPNGSTYQFETVIRTVVPGQQGNGGASGSMSPADVQNLFRNADINQATSAMASA
MQRSASSTSLHNRHQHGMTAPMWPMSQPMTGSGRGTPDLSLNRLSTTAAPGSSQTRQGPEVYILSSPEGPRALLLNNTSSETY

ITPRLRTQTSLPHLRQAAALSSAALDAQSQLRARHHHHHHHPHHHHHHHPRSQIAPQPIPQLQAQGRPLNLQPPQPFQQQAQN
LAPQQQPAYPAQAQGQNQAQEPDPAAIMAPLMHRGNPPMAALPPLLMQLWPQIWLLFRLVLFVWFFTSPNASWSRWFTIIVIA
VLIFLLSTGIFNGVPDHVWQPLGRHLENLIPAEPRRRQAIAGEDQQNQQPRQNPDPTEMARRLVAQHQGPESWLLSQVRRVER
AGLLFLASIAPGVAERHIANLEAAARAERERREAEEREAEAAAAAAAAQNEESHENQDQAENLGEEATTSTGTEHNVEAQTEQ
GVQDNNQRNEPAREQLIPL
FUN_016573 CDS scaffold_9 400371 401459 +
hypothetical protein PF01088;PF18031
C12
MSGGWNTIESDAGVFTSLIENLGVKDVQFEELLTLDPSELLSLQPVYGVIFLFKYPTDQPYATPEGPRDGSFDNAASENIFFA
AQTIQNACATQALLSVLLNKTKDVEIGEKLGEFREFTMVLPPEFRGEALSNSDLIREVHNSFARSSPFADETDKTGAEAEDAF
HFIAYTPINGTLYELDGLQPAPISHGACSSEDFATKVVDVLQRRIARYDTTEIRFNLLAMCRDLRQRARDFGDEELLAREERK
RRDWMFENALRRHNFVGFAGEVMKGVVRSKIAEGGDAAYEKWVKESLERRKNAEQAMRGRAGGGDGDGDVDMGA
FUN_016574 CDS scaffold_9 401917 404235 -
hypothetical protein PF00860

MATSPEQQGVAPTPNQEDVDIVQHLGRSRYFGFRDFLDRVDHYVTTSAFGYFFTLSGTGHPQAIVGATFFREVRAGITTFATM
AYIIAVNAALLAQSGGTCVCDLTDRHACDKIDSYVACKEDIRRDIVTATAAVSGLASFMFGFLTNLPVALAPGMGLNAYFTFQ
VVGYNGSGPISYRLALTAVFVEGLIFIFLALTGMRQWLVKLIPSTIKTATGVGIGFFLTEIGLSYSAGIGAITGGGKATPLAV
GGCPQEMLDEVTGMCTEGQMSSPKLWVAVFCGGIVTAFLMAFRVKYALILGITLVSVLSWPRNTPITYFPHTDEGDSRFDFFS
QVVMWHPIERTLNQLDWSFGGSASQFALALFTFLYVDIIDATATLYSMVRFCGVVNPRDGDFPRSTLAYCTDAFFISVGALLG
SSPVTAFIESGAGIAEGGRTGLTAMTTGLCFAAAVFFAPIFASVPPWATGCTLILVGCMMIRQITQINWRYIGDVLPSFVVMT
FIPFSYSVAYGLIAGVFVYAVLNGLVGLVVWATRGYVEPREYDLKEYWTWKGSGRAPWFIRAIRHGRNTSQENDEDQPGDAGH
AMRDLDYDDRRENSSSRTASSGKDDQPTSPSNERWER
FUN_016575 CDS scaffold_9 406649 408507 +
hypothetical protein ENOG41

MFDVIWVDPDRELVGEHRAKKEIKRELKNKEREKEQENSILSRSISVASTRSSTESRFAFLRPRNGKHGKGKEKTPSGLLTPS
HHSSRSNSGSYHRSALMANLSNVSLGAERAHRANTPVSTDASSSHVVKRNGVSQPRQSDLGNPDLCSEVYGCSELEGDHPPTP
TGSMGDRNFPIPILQPESPNSLVPSLNLAKEPKAPIVTNFRPNDPDSWRPPEEWEYIDRQAEEAQMLENQEDVEMTVGAEKTV
QANPCTDFEAVKHQVKIMAASSPSMILARIKDIWTVTDERLHGELNIELKRWMLSVLHCLDIEVRDSAGVACVATNMSDNLGV
LALYESEITAIYLAALHSRREVYSLTTASFQNKELPNIHPVLVPSMTPSTLPIDLHSFTLVYSLSLPVVCSSPEIPGVLKTIS
SRLRVGGSLQLTLIDPLPCAGTLGHRMRTWLQEHLLINLERHFKCTNPSRLFPEWMGDAGLRGQGSTLTTSKFYAVSASIRSQ
ADDSDPFIERSPSEREVKAEVRSIIGRLLWMEVWGSFVTGDKWWWEHEGCVNECLQLGTVWEYHMIDGVKEGQEDIPQEVEDL
ENSEH
FUN_016576 CDS scaffold_9 408991 411509 -
hypothetical protein PF00023;PF12796;PF13637;PF13857

MAQAVSRMAPTALEQDLINTAARNLNEVTAELEALLRICSRESGPEGNKMNKARKRKWLVEKSDIKKLQQRMSQAKETLHFAL
NSSRVSNDIRFQAEMRQIMMNMQTMHTSSPSPPLQVPQLPEIIESPAPVQQLQDDHTPESDAVVMNNGHDSNLKHMNGSVQIN
SVVSTQRIPSDSACRRRCHFSESQYQSSGWMHSLLGSWLVRCKSSSRKECSDPECHCIGSTALRLEYLVPKWFLIRTFKICAS
YSSLSGLTCALRPFRSIDNNSMVWIDQKGSSQVVRRSVAVYGIYPHDTDIRGMGLIEYALKDGLYSSIEDLLDIWKNILTEVG
LPRRVAIAAANRLRNNKLNDNESYLVQKVLSLSRDISEVATTKVHEAVFQCAGLQEALQEQPWAINTIDDTGDSPLYLATRRN
QVRSMEFLISGGADVNQQSYNGLTPLMVAVWQQDVESVKLLLKSKSNVNLCNEEGTTALHWASMKVNPEVTSLLLAAGASVKH
RNMYGDTPLYSLAGSTNTTHQDIEAAIEMLLAAGSDLEARNNLGITPFLLSIHHNKLEFTRGLVKSDCSVHVLNDYSQNVLHL
AARFASLELLWYLSVLDLSSINPYQEDSFGDTPFDDLVRNSHATGEWDLGDSRRPSLAEQEAFVELYQGVRDQALQNDIQNLE
RVLGALQEQDIAVAREHLAMLIRKEKRWERENLVSWYRALDKRIQHAEWELATEDIEGHLLDLQEELATPVWEIPSKYGYLWE
FDDGESVSEEVSESEDRSEKLYDSDQQVVHEDDAQAA
FUN_016577 CDS scaffold_9 413403 414881 -
hypothetical protein PF00001;PF00002;PF05462 ENOG41

MSSAEHLSNSQLRALSIIERTCSVFSLVGSLFIIATFLSSKAFHKPINRLVFYASFGNMMTNVGTLMSRSYIGSPNSVGCQFQ
AFLIQMFMPADAFWTLSMAINVYLTFYFKFDARRLRKMEIPYLIGCYGIPFVPAFVYIFIKNQEGHRMYGNATLWCWVSMEWD
IWRIITFYGPVWVVIAVTFFIYIRAGSTIYRKRQELDDFSSTDRDLTYGAGDHLGTIKTTEVSVTTEVVTPNAIHLQPMGGRR
APDPSDPSSNAYSVHISAQPSSGDVGDEVLPIERQQTRASVQVPAQQQQRSGGNPARRRNRELSNAAWQYTKCALLFFTAILI
TWVPSSANRVYSVFHTKNSSVPLEFMSAFVLPLQGFWNALIYMVTSWSACKNLATDLRMGRRPDVTELVGGMAPEVGANNART
MTSHHRHNLGQFRRSNKFETESMTELANESRPTSDDERRDNVRV
FUN_016578 CDS scaffold_9 420560 421029 +
hypothetical protein ENOG41

MTKGQRAKIPAKDKKTSKKSRPKSKSKSYRLKTIEDPQKGQGRDSIRNTLIALHNTVFNKRKDIPAEVQEFCYRVDILHTRVL
EFTLEAGVDGQDGECMDWQWEPTIPVHLVRTVYEESCYPEGAVARPWDMGNSQIPKVM
FUN_016579 CDS scaffold_9 421636 422515 +
hypothetical protein ENOG41

MEDVGSRTVVAAATAAEDPPIPPSTYNHTTIPNNTGSSDQSREPSLSSRPSLPWTHYSDSSIWIDKDSTDDHHSRFSHVHTPE
SSYGTRPPSVNEEAMRYHRRRQGKYKIPEERFSPDDRYRRSHRGRRGRHAHSSHRQPQRNERRNSANTGCLRYAKHAETPADI
VLEPQPDKTKVVGDKLTKKNENNGTTWRRISQGLGLGKSKKNEKPEEHPPVKAEDVPRRKSKWKFWAKQPPGDDAEASDAPET
SDPNNEPQFTVTSDSRPSLLESHEEAPISIQLHTVMRRLSQGE
FUN_016580 CDS scaffold_9 424776 427242 -
hypothetical protein ENOG41

MEAVAYDNHNHSSYARIHQNSHHQYPHSSTSPLPVPGTHGNSRYRHEFEGEGSYPIHDALPPSNLIHVSTSFPMSEVETVFSN
NQAPSTQASVIDMSGPPPPSHPPPPWKPGLDNPPPPPPPPPPPPLYLRPPTVPSPSSPHTSSRPPFCLPPPPPPPQPPPGNFI
ANTNLRPPFTYSLDTVPPPQRLSQQTLPATPPRMTFSSAPNSPSDAPTQTQTPNLRPPRGFVSFGPPHSAHQSGNKSNLAIRL
APKASTQPELRDTTSTSQSSNHTIPRLIDDEVSFSDSLSDEDSLVLRNSRQFPPLYPTASNLNPQGGNRKTSMTTRITNWISE
YERDRAPKDRLDLSTSLDPVWETTRGSRSHRSKESAPPEDTLELLWVKLKDQRVKLNDIKSQMAKKRKRLRELRRQRDDADNT
FMGVVRPMLVAQQGQIVTGPATLERHLAELQRLRTEYQASENDYEDLEITLDEEEEILNRLEIRFFSLLAVGQAVPLEQPPED
DTKHEEDKNIPNDLMGISPDGPSEDLHPKYLDLMAAVGDLENAKEELDELLFLKEQHEGELKMKAAAGMELNEAEIEFFDEFP
LEEQEMRNSITSLQRQVTDLRKLCEEKGVMQKHLSSRVAYLLNPENGYEDIELDDVSVILRSRTDLAHPTFPVLLSQPEHVMA
DEFPLTPRDALKAAAALPVTDPVKPSRMQLASKEYSIDRLMLDHGEGGKGDFINRWLLHQLRLSSVNALLLHTTFTKSRGLKI
RDLDRWQSDVLHYWWNDEGAELPEDMMQLVTSEHSNDGSRIGTNQLSRAVTYTPGVSGGRQSLLTPSSVAHSTW
FUN_016581 CDS scaffold_9 429793 431608 +
hypothetical protein ENOG41

MYQTSRPGWSEWSRWIWNPDYQQYYRQRVDAAGNSDIQWQSEYESAQNREVPRTPVQVPAQVQQMDQMDQVTQQFGSVNIQSP
EQAAGDEHDNNEYVTGGSSSRIKSKSSRSHSSSSKSKSKPSKSSSGKGKATAQEAIDEVDEHGYENRHSQRRPSASATTQQTE
QTYYDPNTGQYYILPQATQGDSASNQPYPSGSAEGEDPELQAALAQSRQYSRDQYRGGESSSAAYTPYAQDEEEDPGPAVAST
NQEHIVGTGGETETLDPRYRVAQSHMFQPGEVFKVLWPEPAGGGSVVEETVYRDRYGGQIFVHFRRFIVVANDRGHCTCIPIS
TYGKKGCKKSGVKAEQHGIVVESSNRNPTALPGEPELGYPPVRVHIHEPDERIAKESRVNYSKLTTVEHNVKVLFIGRVVTSD
WDIVTEAVNTSWAKKTHVKKKHRRP
FUN_016582 CDS scaffold_9 432728 433642 +
hypothetical protein PF00106;PF08659;PF13561 ENOG41

MAEAQLEDFPSLFSLKGKVAVITGGSRGLGLHAASAFLQAGASKVFISSRKAAACEEACKALNALPNLAPGAVAISVPADSSK
FEGVESLLAQVKKHTDRVDILLANAGATWGEQFDTHPDSAFAKVMDLNVKAVFNTIRLFTPLLEKSASLQDPSRVIITASVAG
LGVGTIGKQGTYGYSASKAAVLHLGRNIAMELGPRHITVNSICPGFFPSKMSNGLLEMSGGAEEIANSNPMRRLGKPEDIAGV
VVYLASRAGSHVNGETIAIDGGALWQRGELMVDSKSKL
FUN_016583 CDS scaffold_9 433770 435180 -
hypothetical protein PF07942

MAGSEDGHHIEIVPDNSAWAGVEDNAEDPEEVRVIFSALDSFFQYAKVSHFNVTHLRRQAFYALPQEQWKLLAAPPFNFLDTL
EKTDDAIDENAELARTIARVGLQSFRMMTTDPEDPEPKMPQEWAGVAKHSDVDKARSTIRQFYRDWTEAGAAEREASYGPVMK
ALAAETERYLDRAPLNVLVPGAGLGRLVFDLVANGYTAEGNEISYHQLLASSYILNYCPAAEQHTIYPWVHSFSNHLTRANHL
RSYRVPDIHPASVLAATPNAGEMSMCAADFLCLYSNEDHEAQYDAVASVFFLDTAPNLIRYLEVIRHCLRPGGVLINVGPLLW
HFENNAPGTHGQDGEAEHDPKSSSGIADPGSFELADDEVIALLEKMGFVVEWHKTGVHAPYIHDPDSLLQSTYRASTWMARKP
LEQGSS
FUN_016584 CDS scaffold_9 435773 436248 +
hypothetical protein PF16093 ENOG41

MATDVIELSVPLPRSLDTRIYLRLSTKAKSIVLFLTTATQDELATPVSMGSFVYALPNRLDQAQPLSTTLYSSEASVEFTTRL
AKLLARKSQLPVYVTNSISFANAGMGGTVEEEMEAFKAIVQVVSERLQLSGPKSTAAW
FUN_016585 CDS scaffold_9 438205 441154 +
hypothetical protein PF02985;PF03810;PF13513

MSSWEPAPDSLQQLAACLKDSLSGFDKSAQKQADLMLQQAKNSPDINNYLAYLFSSSSPPNGLQFSEQDFHLVRSAAGIMLKN
NVRSEWKSIPESNLQLIKLAVPMCLQDKNSQIRNFAGNIATEIVRRGGLLSWPELLPQLLDLVGNTSGQTSNEAQEGAMSALA
KICEDNFRQLTKEVNGQRPLNYVLPHFIAATKSPLPKVRAGALTAINVFTPRESQAMLNSIDDLLQHLFVLASDDNVDVRRQV
CRAFVHLVETRPDKLQPHISGLVDYIISQQKGDDEDLACEAAEFWLAVGEHDNLWRALEPYINKIIPVLLECMVYSGEDIALL
GGASDDEEEEDREEDIRPAFAKKALARKANGEVSESADQAQNGNGFEKLSGMDDDTEEGEIDDYDDGDDANPDERWTIRKCSA
AALDVFARDFQSPVFEAIFPYLSQHLKHEEWPQREAAVLALGAVADGCMDVVVPHLPELVPYLISLLEDSEPVVRQITCWTLG
RYSSWAANLEDTDKDQFFLPLMDGILRHMLDKNKKVQEAAASAFANLEEKAGKILEPYCGPIIQQFVHCFAKYKDRNMYILYD
CVQTLAEHIGPAMAAPELSGKLMPALIDRYNRVSDQSRELFPLLECLSYVAMALGDAFAPYAEPIFLRCVNIIHVNLEQTLAA
AGNPIIDQPDKDFLVTSLDLLSAIVQALNDDKSAALVKSSQQSFFELLSFCMEDPSDEVRQSAYALLGDCARYVYPLLQPYLA
TIIPILLKQLDMDSVLDEDMDSGFGVVNNACWSVGEISMQHKEHMGPWVQELLQRFVEIMTNPRVPKALNENAAMALGRLGLE
NSEQLGPHLSTFAEEWISIMNEVDATEEKATAFKGFSMIVGRNPQAMEKELLNYFTAIARYRDIDLKSPVRQELHDVFQKVID
IYKQIIPEFSNFVGQMQQRDRQNLESHYTL
FUN_016586 CDS scaffold_9 454895 456830 +
hypothetical protein PF00172 ENOG41

MAGPGGGPPRRSHTKSRKGCDTCKRRHIRCDENFPQCRNCTKHKIRCPYNDVQVPDAERSTTPDKPDLMWTPQIEAAIAEWQT
TGIFPLPSLGIYPAPMPHLYSVEDLRLIYHVATLYYQLATIDANNFTLWTRHIPTLLRIGATTPYVMHALLAFSAMHIAFLTD
CPLVGSMAYEHRGIALKGLQEAIGSFSRETSDAILAASLVLSWQATDWRSWTQLMQGTSSVIDAMDPWKHESQFGDFIAESST
FPTAPPSPGKDHRPSQPRPEDLEAYQRTLEQIQKVEAHLKYHKEDTTQIQHLIGFLRGSRKISPTLSIAQQFERLQPLRKWLF
WMPVGYLQNYHGSPNSLVIIAYLYTVALLMERLFPEIGAAYFGSLSISPVEEIARRLMSINVSGVGSEMQTPLTLMEFPIDTV
SQFRSRMGWMHPERTPSFPQFNPRNFTIQEEVPTHESYLYGNPAFSYSTEEMPMLNASGPPSAVSPLVLSPYPSQQYLNVPSP
AYNGAYSPASSTFEGSVAYSDTEEYGSWDMNTLQGGPSSSIQDSPQNYGVGFVTPVQAVWI
FUN_016587 CDS scaffold_9 459442 461093 - SSH4
Rsp5p-dependent ubiquitination, sorting of cargo proteins at the multivesicular
body PF00622 ENOG41

MGLSSNPSSAFTGVVIGLVSSFGSIVLIALVIFIFWVSGCASTGRIILDRLGRPGEYDDEQAFARDEAEALEVMDDMSRQEYL
RAKAWVTANPPESMQTDISLSQYLAIQEKGVSAWEFEPELEIANCFVEARTEIEFFDSECTVMSNLPVPKQNDVYYWEAKIYE
KPENTLLSIGMATKPYPLFRLPGYHKYSVGYQSTGARRYNQPFGATPYGPPLVQGDVVGVGYRPRTGAIFFTRNGKKLEEVVH
GLKAQNFFPAIGANGPATIHVNLGQAGFVFIEANVKKWGLAPVTGSLAPPPPYGSEQGSILLEAGTKDGNTYPQGRQHSHSIT
ASSYNTRNPSNDLNPSHGRTRSGNFRVLPPTSPGPVRSSTDISLAHFVPNEENGEASSNAGPQQETHDHNPLHLHLEDATNPP
PEYQSPDHSGSEDGRYGSDSEEDTPLIRVMNRSRGNSSTTVLPSRQTPSPRQPPVPSYSDAMRQGAGRDRSDSARLPPPNRTP
SNRPRSSTSA
FUN_016588 CDS scaffold_9 463016 464174 -
hypothetical protein ENOG41

MAPAIIRSLRSASSLTQHAPRAAEAFSTLSEHLLEGSKVLLARENKDDDKNDRHKPSVEPESGSFDPHDISNAGFFFLFALIG
VVFVCTGIWFFFWAKNGGFQFKETDWDDYKSTVLRRTGPNGTILSNATRPTNLGGGSVYKDVDDDNTTVVTESTTLTGITAGA
SDIGAREKRRRKQEKRDRERAKKGKKTSRHVGEAGVEDEEAELAAKKELRSYRHERPARVGGLNKESEGSEWDGSTNPGTESN
VSTNLLSDRQTTPTTTPTKSKGGIRKVYSTADRRENREAERMRAEARRLREAGRAARRDFSYQRAESYTRADSQVSESLLDGS
QVTRTTDDSGDLGTKSYHHPMPELRELERERAREERRARRAQREYRRGRGEEC
FUN_016589 CDS scaffold_9 466335 468189 -
hypothetical protein ENOG41

MSNHVCYVEDAEEDSGSSVLEGIQSTRRYAVSEAPGPASPPKERPNTGKSRGETRRYSSRRQVSPPSEGLSDEEPPRREERPR
RDYEREAREQRDKDRRRKERKQKEAAEQQQRRVRAEAKPPKEREPKPVRRPRPTSLTHTKTEPVVQQYRRGRVEDPSCYGVQQ
PAVSGMRPRAQTRPHSYYPGQTAPPISNAGWHHAQASQPSFPVGSFPPPPYFPGGQSPSVVGVPPSPGPGAGTPGFFDMPSQQ
ASAHLRNRFERPASSMGFRQQQPSPSNVGYPQDDFEEEEQPDPRLIRRASRSKRPQQNDEDRRRMPPPDSIPRPKSAMPPPTT
PFRPPPQSLTRQKTRTPSRPPPSSRSRVGFVDQGYEDEDYNDSPGLFADSPEANFDRRTALARTRRSSVAYEHHGADLVPART
RRGSVAYEQRGIDIVPARTRRDSYYEDRSLGGGGVSLDEDKYLDAMRYQDDINGGPAMPLTAETLRKASNPRLGGGSSRSSGS
HDDSEYKRSNTTGLTRSSSSDTDNVTIKVSGSAVVRVSGAEIECGDGGEITFSRPTGSSRVGSDRASSYYQLEDVRSRVEQKA
LPYRPHTPSRSDSHHRGYSGNHAPYDPSFAMDDYIY
FUN_016590 CDS scaffold_9 474375 475272 -
hypothetical protein PF03372 ENOG41

MTQLDIPSTASLRMEVLAKHQRMPLRILTFNVRYATNNREHNEKPWDVRRPKLINQLNFVTAGHESPFICLQECLNHQVNEIQ
EDLGSHWKHVGRGRGEKPEDGEFSPIFYRNDVWKNVRKEVRWLSKTPEKPSRGWDAVLNRIVTMGEFEHRVTGTRVVVFSTHF
DHIGVKARQHSAELLIKFAKEWGQAGDKKASAVLIGGDFNSEPEDGAYKTITASGSGISDVSDLVPKHKHYGNETTYTSFGEG
WQPSRIDFLFIQEPRTAHVKTFGVLANGFDDGVRVSDHRPVVSDLDIVV
FUN_016591 CDS scaffold_9 480471 481263 +
hypothetical protein ENOG41

MPTESEMRSRRDSWPPTQMRLKPTISTKNRPLPSLDDIDDDPLTYFLTPAPLLDDDDMDDVLLDFDAGIEDASSPRPFVRSVS
PSTLDGLRKPGLRPMSPDTSSEISESNNEDDYDDDDEDYVSFSPSKHGGLLSLRDLFANSRPAVRPKSPAFNQSTNNSLLSPT
ALSSSPKLRGRSRGRGRHGASSRGHSATRRPGQLWREPSPDVWSIEEETEEEMMSEVGSSVGAHSDTSDDEAESERRKGKKAK
SNKKVRFVLPAQEV
FUN_016592 CDS scaffold_9 484438 490651 +
hypothetical protein ENOG41

MSSSNPASPEEAVDEAVLDPEPEPETPLKPSAQDENDGKETPKSAPPVAHRGRPRAKSTREPTLLHDFLLGRPSASRQAAERQ
RRMSLEAVKAELRHEMKQSQVKRIQPPNGVRDRVKKWQKANAAAMENTPDDAATEPPDIAFEDEEFQSVTEEDRVRIKMRKKL
KRPNNKTKESLATGDEATPDETPTTPTPAAAKPPPKKRVVSDEHWRKNRKHIPKKTSPAKTPAPAKIPKDFVLRTAANPRVAS
RVKAWAEMVEIPDPPAPRSHKSSKSVGAKPWMDDTDSEFMSDSEFTVSNVTSSRFTKARSAKSTPGADDGIRVKPIRRGRNND
DGIRVKAVRTTLTDDEGIRVRPMSDISSNAGTSVRTARLRLKSAPSSRRPSMSSVKREMKKSQSDKSKIEVIEDPSEVSTNKF
IEVIEDDSELPKTTKTTDKSGLFGTAPIEVMEDDSAIDTPTKKRSVSAHKSKPRPKSYQPPTTKQKGKETWVPEEESTIEPSQ
LDGSDLSSSILAKSIADIPGEIPWGNSAFTELDLPLRARGGPLRSRPIRPARPAKPERNTSFKSMPKVFKKVVEGANKIIHEM
NEHPPPRDTVPNNPRSIEKWLNTTVDPFVEEPKSEPATTPLSEHVPDSPSSPETVKSHRRSSRTEKRRVSSSTMSKPDTADRT
VSSEKLPVTPEPAKEPIKEKPTPSPVSKPGPVKETAKEPPPEPEPPKKSTPKPKSEPKPEPQPEPAAVKEPAPEEVSPLSSPA
PSPEPEAKTKPKRQDTKAPTKAPGGSGLKRTKATRTTSSPVKSSPKNSLFGMLKEAFTGESGNSLPKVKPYQSREERPYDDEP
ESMYTDSRYNDSRYDDSKYDDYHYEESRYDDELTYDDSTENWTETELSKAEKSRSVRSPSPLEEKKKPQSPEQPREPIREPAR
EPEQNRDDRSALPSARMAGPRLPPPTRGQYELSTILSEEASSAVESELTTDVTQSTLTQSTGVTKETRRSNSRSNSKSQGSGL
KRRLTRHSDLVSVLSLPDDKNVPSAITSNRSRPSMRKSRGGATDVTNDDLLKEFTEDESLYLRELKTLVDGVIPVLLSQVVNG
DNATDLFGTSTPVTKADALSKSVVNMGVALEKLRMAHRKAPIHDIRRLASWGHGVVPIYHKYLDAWRLGFQDLVVNLAPAAGS
PEDEDSLVGAMPRNESGDIVDATGERVDVAHLLKRPLLRLKQITKFFKCVDSILGTNDTYDLLSDFEDLQEKARRRHREEMAR
LTDEDAINTDTTRSRDLRTLAPMEAVYIEPSRQVSAKDFFSLDLAHSNGQRLECQIELVHRDNQRYPEDKGDILIRENGVKGR
SYLLFPPVPIELISARTGDGNFDMVIMIRGTHNDRPWHELLTLTTDNEDQILDWLDILPVSPVPPREPEPSIIDDEEPYSQSR

MLDVPVGVRGSSRKYSAQSLRNSHSRRTSAAESPPASPTTPKRALPARYRPRSASPITPSPIKSPDQYDLQRTPTQYDYGYED
RPMTSQSMQPDPLNIRKSPSSSSYRDDGAPPPPAHRTFSPSPSQQAPSKPRFSKPPPLEPPNSSRVKRRTSSPLKHEYLPSDQ
SSISGTDITGSYENTEDDYTESDYSYDSSDDDIESVDLPETELGVSIRDEHPARDIIREELEHQLETLEEEGSLHDYHQRHEL
HESVVSGSIDSLAPSNSASQAGLHGQKVAPGDNATRYVATISRWSDKGMWKDISGGNLEVIVTPGLIEAYVVHSGAEQGDKPM
LALDLTPLVLIRQSTALDLEIRSSVRSDCQLASSHGGGNFRFRCHNGPDCYNLYMSVHHARLNNQKFIELENEARFRSFGEHK
PPPDNDDTSSRRRSWFGRKNSYRGSVRAPAQSHDGASTTPSSTLSASSFLKRLTGRGLPFSLARSSVNRNSRYGGTNTPGSSS
YGGNTPRSPSISVENSGRGPASFGTEDLKIRLHLLVAAAKWEDFGNCRLSIRRPPPGWHQALRADHGLEKRVTVTTIPRKDSE
QPKILLDAVLGSGCFTAMGSRGIVCGVWEEMKDSNGVVGVVPATGPTGGNVKKWCFQCASVAEASWVLRLVHQEVMTI
FUN_016593 CDS scaffold_9 491567 493401 -
hypothetical protein EOG09260K29 PF00149 ENOG41

MTRRIVRTAAQLAGAAIFTFLVIAFLDRNYRVLPNSIHSYMPSHHPGLVVTDVTIVTCSSVNPFSSCDLGKEWHRIDKELYLG
KTWTTSAYMYIMRKHEGDLTADDRVVVDVAVGRLQPQPKGETDDSWESRPSGIWIKRSAKKKASDSKQAVTDLDVLFGDDAVE
ARDGYAVTGTPLLLNTGGSLLSVQLSVRRGPPREVKKPTPRIRNDGRYKIMQIGDLHLSTGVGVCREAVPDSYNGGKCEADPR
TLEFVSRVLDDEKPDLVVLSGDQVNGDTAPDAPTAMFKIISLLIERKIPYAAIFGNHDDEQTMSREAQMAIMETLPYSLSIAG
PADIEGVGNYYIEVLARGKTDHSALTIYLLDTHAYTPDERNFPGYDWVKPNQIEWFKKTAAGLKKNHNEYTGRHMDIAFIHIP
LTEYANPELPRVGDWKEGVTAPIYNSGFRDALVEQGVLMVSAGHDHCNDYCSLSLMGEGETKVPAMWMCYAGGSGFGGYAGYG
GYHRRVRLFEVDTNEARIKTWKRLEYGDIAARVHEQIIVDGGKPQPITPVS
FUN_016594 CDS scaffold_9 495134 495984 +
hypothetical protein PF02037;PF18592

MAEYSSLKVPELKKLLAEKGLPQTGNKADLIARLQENDRTSEPAEKPAENKEDEISYSDDEAPAEPAAAAPVAASEPVAEVPA
TEAAPAEAAKAEETTVEKPAETAEAPAEPEKSYAIGLQSTAADDEAKKRAERAKRFGIEEDEDAKKRADRAKRFGLDEKELAT
TLDSALPERSRKRGRDRTTEGEGNRPGKRQSLDRRNGGGGRRRGRGGRDGGRDGGARKEKTSTRSGILDDPTEKAKAEKRAAR
FAGN
FUN_016595 CDS scaffold_9 501961 506338 +
hypothetical protein ENOG41

MGTEPGVISNAPKPLNRSRRREEIPRPLPKPSSISRPSARAPQPSTTRRPSIPQPVPRPSLNHQAQSWGSSSTSVSTRSTANH
RGGSGSSSSAQSMDNPRSNPNVLRRKKSSLSGDVKTTRTGSSRTDSSSSSQHKRQGSLDPAFGFHLDRELTSSPAEIQIAEVV
EVKKPSKSPVIYPELDRYRNIRRPSDSSDNRIEVPFRLATHDLPPPTPASLLFSGTSGSPSTRFSESPGPGSYSRDTTPTSIA
SQSPGLVAPLRTQNKNRLQSPILNRPPVTRRAGGSFPNEVDAITVDPHGLAAVRESLTSSSSNSTVREATIKKKTKNLPPPPP
SPPPRKSSQYFREETSSPKSVRKVSRPVLRSPSPERSAQSSASPRAVPPSRPSRDGTPDMQSQLFNPMPVIHSNLSTQSLPTT
ERRGSESAATAALSPPKPQYTHGGRTASTSQLPIKGELYAQPAAKATAESKKGKGLEPPRMTSTPSPNVATSSFSRFPFFSRK
KHANGTQAEKNEKEKKTLRKGPVAGTGHEGYGRIGASKRRSGSISNMTRNSPGSQTTQEPRSSNDSFFADRVNPVVIAGGAII
ENRNASSEIYRSVSSQSLATEESRFGSSQSLATSQDQPRNTLWPSPMSRTPHPAFGARRPSESSDSEGPTMKSTLAFRRSVHR
LRSSPDDPLKLPQPINTSGYASSPMTSFDTSVMSDESHFELQREISHEAKSSHPAPKKLVKRPRSPRKWNLFGRSTQTQQPKQ
SEKVSATVKPVEKKPLAFYAMMDSSEQEDSEPMDIQDVLRFAEVYGKSPSVGGTPELSQGAPEMRSSTNSPVRPAEPAQPPRR
ESVEMKTKPRLPVPQQTLMSKKPTLQPPSTSTTAGRRSRLPQVGRIPKVVSNRTEHVSPMSFSRPFRASMQLAPENLEVYDPE
SIAKGPSPSRPSTPVPDLTTDGSTAGSTNNLSSRDSNPPALSRVEKEFLAFSPRKDSEGTIGTSSSSCSGILAFSGSTAVIPQ
PYDPPAEDEVWDEYDDLLGEDAGKALPSATSSRGTPFHLETYESKLASKEKPLESPTIVFDKKASRQSKATTISSTCSADMTE
RLRAAFQPHPSPSTPFSVSKFVSGYEDRNTNTEAPATGEVSRRSSRSSRKTRRSDASSSSEDGSPLAQVNLRVGSMTVSKWLT
FGHVLFSDVRHELVPVEGSLKRHSILVIDGLGNDDWSFYAAETYPAATFFNLSPRAPLPEELKSSSSSFPLSPPNHHQIQYVS
HLDKFPFAPQSFTAVVYRFPVAAPEAYYRSILTEARRVLKPGGFIELSILDVDLNNMGNRGRRTIRRLKERINEKTPDTSLGS
TADLIVRLLGKVGFTTIKAARVGVPVASSVTRSDSNADKGKATAEKKKDPPSLSEMMSDNSPLADEGITKMVSRVGRWWYTRC
YENAAQNPSGKSIWSDKSLLSESEELGTSLKLMVCCARAPLERITSV
FUN_000389 CDS scaffold_10 2734 3193 +
hypothetical protein ENOG41

MSSVAPHQGSAVGVAVEARQEAPELSQNRVAPYSHHNVFARIQSRGANNGHVAGGQPREHSIASLEAGMARYQPMHSVVTVPS

NSNLAGIGAQFKGLFKGAPD
FUN_000390 CDS scaffold_10 5198 7540 -
hypothetical protein PF00172;PF04082 ENOG41

MGEGAQRREVSYRSACTECARRKQRCNREWPCSGCQKRKVADKCRFKDTNQPTADRVALDRQRKNRTNNKTGPDSGDELDIEG
TRSDDIDTLGYMPSNLIFDLTSEQETTRSASQEFVKDFRSFPQLERALRVIPARPFTDTLVQNFLSNVNYHYYIVYPPSFIEQ
YQLWWTARTENRPLSVQWTCLLLTICACASQYTDVELQRNLEVGLGDPIQRISERFHEASRELGSAISLGHGHIINVQQLLHS
CYWFKAEARFVECWHVLNAAIREAQELSIHQEGKAGPISEFDLEVRRRIWCILDTWDWQISTLLGRPMIIDRADCDASLPSLG
LEGCQYSPLLHMKMQCSVIRQLSRRFGSPKNVMTHADVQEYQSIIETWIATFPPPFNVQHPDRSSDASCPWMVLHRHILHTVA
FSMLLQPIRGFLTRPFAIRSIEAELQIRSDGINYCLELMASLRRFFKYVYPRDAKFHFVLFCIFDISTVLCSAVLHDEHHTLP
RRDDVYKAIDEAHAMLQELRMVTKSAKASYGILTRIVQRLPRPTTTHNMASKSPVPSRPQAPPEAAASPQTLSPGSVSHSQNS
PPAATFSPSPVIIPTPGAVSPVYTLGGVPVVGGDFAPPIAYAATAPCSDWQPQLYMQNPTGFVPTDLPPEGPVFANISDEELG
ELANLWNYQSLDFSFMST
FUN_000391 CDS scaffold_10 11051 13461 - GRC3
Polynucleotide 5'-hydroxyl-kinase grc3 EOG09261DRB PF16575

MSSHKKRRIDGVDATKPMSAIAALAARRREAAASSTSAQQSSQCDEKPPTSNVNYFSPLQKGTSQVGTPSTPKKSAVKTPRSQ
RGTESPAAHPKSHEMASRPPGPITPESGTVQRAIPYSSFRLSNQNYRVKKGGVVELRLSSKERFPIIGSFGIRVIQGEVVLAG
ATLYPAETIEWVHAPHCHAVPVLRTKEQTRLELHPDKNAQGLRQLGRLSPLFRKMWNEPPEGDSTKSNKEATFQIIQTPEDAP
KKCIIQELDSLAAWNRKLSPLVTTSREKPSLSTLICGPKSAGKSTFSRLFLNRLLTDRSHRQALRGVVVMDLDPGQPEYAPAG
TLSLVFVTKPNLGTPFTHPGIRDAASNVVRCHSMASVTPASDPDLYLACAVDLFDTYNKSFAGVPLIINTPGWILGTGLDLLC
ALVRKMKPEEVLYMSEDGPSETVDALRAATETTFTELPSQQSEFTSRTAAHLRAMQTMSYFHLQELALTTTKQTDITQRLKWN
PSPLSFRSPLLVRYSSKKGILGLVSYDYQCPPELLADTVNGLVLAAVEIEDVKAFSGFTQDQVSADTSSKLTKLDTASKDEAL
QIVSTSPEGIPFIPNYNDAALDPRYSRTIGLVLLRGIDTKSHTLQLTTPIPAEEFKHIKSQGRDIVLLHGKFDTPSWAYTEDL
YERAGADEGNDTILEVTDEDTDDDRSEAEPENVDRVSDLTEVPWVEVLKGSQRRPVGSRVWRVRRDLGRNNA
FUN_000392 CDS scaffold_10 14006 16053 +
hypothetical protein PF08208 ENOG41

MAPKEPYKVHSLNKHIENTKKGLSAEAQFNRRPGNRAATIKKESSDPNNIFANNDGDDDDESGSGSDSDSSSDGPADFISKLT
STTKPASATPRRRGKDDEIADSDAERNASKKSTSVKKTAPAKSKPQSSSDSSSEDESGDEKTKAKTNGTSPTKASESTSSSSE
SGSDSDSDEEDKKAPKPSTKKQDDSASISDSSSEDESEDEADAKAKPALNGKAAPTPDSSSEDSDSESEDEEQDAKPAAKPAA
KPVAPEPGSSSSEEEDSDDEMVDESMHIEDREGQLALPNFIAPDFVLRKGDDGTNGRDVAEICNKANLEGKQFWYFTVPSNVP
ISVVQNLEIPMNQSQSTDKLFSHNGEDYGVSFESIAPKGNIQIMIPSDRPQYQPATKQIDQVMQVKKITQLGSTNSVAPVPKP
APRAQPTGLKARYQPIGVNEPMGSIGDDEDVEMGDAPVLSAKAAKKEKKRKSKETSDKKQKKGQSIPEPPSSRTTEAPTPDTR
KNKRKLAASEDDAVAVAEQLQEETKLAASKSKKQKTNRAGSPDLGSEPASAAAKKYTPVLPPAIPTAGTPTPKSASTPVTSSK
KQKKVKEASVPAPRQSVVPIPSIPHSSPPRSSPARAPASQLPASPSQGKEKRAKKRKDKDGKKTPSGKKETPVVPPVPLSSSE
FUN_000393 CDS scaffold_10 23451 24866 +
hypothetical protein PF01569;PF14378 ENOG41

MNDFSATLSGKPKLQLLWPEHVNLPFLNTVPHRYRIGRRRTKSKSRPGNEEITSLKTSFNAWDGVRDLQKHHWRTSDLQYAVV
GLLILFSFWIAPPAPGVKLLAIIGSVWVLLMPATRQFFLPSVTIWTWLLYFFCSRFIPNEYRPHIWVRVLPALENVLYGANLS
SILSAHQHSVLDILAWIPYGLCHFGAPVVCALLMFVFAAPGTAPVWARTFGWMSILGVTIQLLFPCAPPWYEMEHGLEPAAYG
MPGSPAGLARIDAIFGIDLYTTNFSTAPVPFGAFPSLHAANAVLEALFMSHCFPQFRTYFIMYAGWVCWATMYLSHHYAIDLV
VGGLIASCFFYAAKARWLPRRQEGKITRWEYEYVEYGDRNAISDEEYGEYFGMGLLGRPRADSSDGWTVASSSCSSSSGTLSP
TLSEEALPGMLMDMDNNGQLWDGNAPARDVELSEVVVVR
FUN_000394 CDS scaffold_10 26658 27964 -
hypothetical protein PF00069;PF07714

MDSAARQQPQVQPCRYKVGKTLGAGSYSVVKECVHIDTGRYYAAKVINKRLMAGREHMVRNEIAVLKKVSMGHQNILTLVDYF
ETMNNLYLVTDLALGGELFDRICRKGSYFESDAADLVRATLSAVAYLHDHGIVHRDLKPENLLFRTPEDNADLLIADFGLSRI

MDEEQFHVLTTTCGTPGYMAPEIFKKTGHGKPVDLWALGVITYFLLCGYTPFDRDSDFEEMQAILNADYSFTPIEYWRGVSSH
AKDFIQRCLTIDSTKRITAHEALQHPFVAGFINAEGESQNLLPNIKKNFNARRTLHAAIDTVRAINKLREAQSGMMDGARSKE
PSRGAARQQPPTNKKGDSAISMGSNQPKDSGYGTQTETDVVMGNTSASNVPSSLQPGNNGNRVIETSKGLWSAPAPKR
FUN_000395 CDS scaffold_10 28972 29646 -
hypothetical protein EOG09264PDD PF00472

MLQRHFVLAPRLRNVPAYLTSRPFAPRFKRYQAFDTGLDQEALSEARSWFQSFSPTQLPKGNTTYARSSGPGGQHVNKTETKA
ITAYPLGQLLPVLPKSLHPGIRKSRYYTATNDSLTFQAQDSRSRDANAEDNRRKLIEEVTSIYKDVIPAETSAEKKKKHEEIG
RRFHETRIKHKKFTSAKKQSRRGPSD
FUN_000396 CDS scaffold_10 30574 33219 +
hypothetical protein PF00076;PF13893

MTEPEAELQATANLSPVSPSPVHSATSLAVPVLQETVETIDAMVAAAAAAAADANGSINKAPNLEPISGAGGDDDIVDDDSLN
DPYGEDDTDVAAQQVPQPQVEQELPDSNDDYAKTFDSPIGTEEGEDGVVEPQDVSSLPRESNEISLSSDHLTNRTSQVSHVVH
ESSLSAQPGQSATSTTSNASSEAQAAGVANTFTSQPSNQQTSSPSTAKPGASNAASSASSASPSADIQRLVADLTAPSNPNTH
YAPSVLGEHSLESTIPSSAFSSSASLPPRPPLSQAVPQTYASQHHPPSINASIPSNLAAPPTPGQPSTYVATGAPVAIPDALG
EFPVPLGSGLQAPIAITSMNAPPYPPKPALYNTDQAQDSEYERQWNQFLADENQYIAEAKWDRFPEGSRIFIGTRSASGASKN
GAHIFATGNLSSDKVSKQDVFDLFHRFGRIAQISLKSAYGFVQYHSIEEGQRAMDNLQGIEIKGRRIHLEVSRAQDKSKKDKT
RSPDRNKTRDSGRRNERQGHQTRDDYRSSRGHSPHRNDYGRDESYGRDRGFYDNSRGRARSRSPGYGRNGKDNNYRRRSPSPY
GRPRHEAELDLPRRYGADVPDVQVILQPDVNRDFVNWVEQAFKSQGLRSEVMFLHPRIPKDMVVQRQAAEGVHAVVDLDIRAQ
SSGKIPVQAFDRSAGSNNVRYDQYENLDPATAAKVILQAKASGGPPNYGQTYRGNGYGSAPYGGHPPYQAPAASYPGPPPPAQ
YGQQQPPPVNVTDIANMMGQLDPASLQQLLAQVQGGVQNPTPAQGGTSAAAAGAPQPDIQALLGSLGGNGAAQQHQQQQHPPG
PYGASYGHVPQPPSNGDAAVQVQNIMAQLARYRQ
FUN_000397 CDS scaffold_10 34045 35808 -
hypothetical protein PF01794;PF08022;PF08030 ENOG41

MGWPYEFLTLTDEEKHQRRLSLDYYAYIAHLSALVPALLFLVIRLINRVRKGRNSGYLQVPGSPGVKANRQRWISRVMEKGPI
VQWWLGEDVVFMGRHWGQRDEWVLGTAWTVWLLVLSVRGTGKDYLHLTKRVGIVATSQMPIQYLLALKALNPYAYLLHSSHEH
LNRYHRVLGRIIYSLLILHAILYNIFFIESGIWFKRFFAPVVFAGVAGFTVLHVLNGTAMARVRQASYRLFFIVHLFGAFAIP
PLIYYHAPSSRFYIAEAVGVFVLDLAARKITTITAPAVIEAIPGTSLVKVSAKLPAPKIAKYTARPGSHIYLNVPAASRPGIG
QFSAPYLVFEFLYNPFTVASTNQETGELTFVARTRNGPMTNRLHHLSSTTNAGGSTEKVELNIEGPYGAIGKTFNDLINSGIN
RILIVAGGVGATFAVPLYHAILAENSTTNVQLVWAIRSAGDATWAASTPTGKSLLDDDQVQLFLTGDMGVANDSDNAAGVEMN
NLSQQSTRPVVRNSKRPDLQKIVDDTFQKGQQDSVAILVCGPVEMSRELRKSVTPWVMKGRRVWWHNESFGW
FUN_000398 CDS scaffold_10 36510 37648 -
hypothetical protein PF08551 ENOG41

MPPRINIPPVTRILLIALLGQSILSAAIRYRQWTANANIVIPYLTLVPQLSIIYPWTFITSTFVESNVFTLGIAAVTIYHGGR
YLERAWSSAELAKFVAIVSLVPNFLTLCVMVLCFTLTRNENWTLTVIAGTIPVQIAFLVAFSQLVPAHTVTLFRGVISLRVPR
FPLIYIGAVFLLSLTPLLTRAALWLAIFGFLTSWTYLRFYKTVFPDLDSAQDASLRGDASETFAFAEFFPGPVKPFVAAIADQ
IFLVMVAMRLCTPFTPADASRNDSRIQRTAPGGARAEAERRRAIALRALDQRLHAATSNQPAQKPAQTNRPTGPTVQSQPQPA
TQTAMTSSPGPMLGETKFEPDHDDAKS
FUN_000399 CDS scaffold_10 42937 44741 +
hypothetical protein PF00400 ENOG41

MQLPQDAAAEAADSLQMGIFGPLDAHPQGRIEADEEEDAEGEEIIEEEVTRKRAPQQLSNVSPAKRQRLSNGLENGADAPAAV
TVPASAPTATTATGTGTDSTTAAPAPVTTPMEIDNQPDNHAYPSPLEGEQAPEPMVRTDGPEQGTQVDKVEELAPETTFIRLV
YDGQNQIQGNRGATPSPSSPSGQDNAPILLRCEWNPRDPSILAAGGTDALARVWTVPRAGPIEPGQDHVSLQGHSLIDRDVPR
DTITTAMSWTSDGAAIAVATDSRNHASINVWSAEGAHLQSMELSEPPIIKMLWNPNNTALLAISPDKGGTLITVHYPPAGNSL
SYVLPDHNIDATPFDAAWTGEAEFLLCGGDLMLCLQCTETTIVQARKFETKDDDLFSQVLFDGRSRLAATSSDKGTLDLWDES
GQRRSISAHQGTITAMQWQPLPENQSGPDDERLIATGGDDCAILIWNARMPESKPKCFLTMDSPIMRLAFTPDGAFIAGATST

QILIWKVGSYAVPRASWSRPVHPGWLSPKASAETDEEDEHCLCWDADGHKLAYGSNSRLAIINFRR
FUN_000400 CDS scaffold_10 47830 50215 -
hypothetical protein PF00200;PF01421;PF13574;PF13582;PF13583;PF13688
ENOG41 M12B
MVSLRSIATAIAGVALFFETSLASSVKRNAVNYISFIDEPVINTPSHRIRADSHFDLLFSLHNGQQKIRLKLEPNHDILHENF
AITHIGADGAVRSVETVNREDEKVFKGHVFIQRVGREGWTNAGWARITMHRDGKEPVFEGTLRIDGNHHHIHTGTNYQQVRHE
DDPILSASEARDDVMVVWRDSDIMSFNPTELKRRGASAALCDSDTLGFNSKFHELQDFDTFGAANTKSLFGRQSIDTGTGNDG
SSVDLESTIGSVNGCPTSRRVALLGIATDCGYTSTFNSTEALRKNVIRMVNDASEVYEKSFNITLGIQNLTISDGSCPGSPSD
TAPWNQKCSNSVDLSDRLNLFSAWRGQNEDSNAYWTLLSTCNTDSAVGLAWLGQLCRPGASANSNTGGRNETVAGANVVVKTS
AEWQVFAHETGHTFGAVHDCTSQTCPVSSDAQSCCPLSKSSCDAQGDFIMNPSSREGISEFSPCTIGNICSGFRRNVNTECLT
DNRNVKTISGQQCGNGIVEEGEDCDCGGEASCGDNPCCDAKTCKFKGQAECDNSNEECCTDKCKFASSGTVCRSSTGPCDPEE
KCTGSSATCPKDKHSDDGTDCGDSLQCASGQCTSRDEQCRANYQNTTSSSVKACTNSCLLSCQASGDGFCTQRNQNFLDGTPC
GGGGRCQNGSCEGASTWKEIENWFKNNKNIAIPVGCVLAALLLLSISCCCFSCIKRRIARRRAAKQPAMGAWPGYPRGPVPNQ
QGPYMYAPIDNNNGWQQQRTRSMRYA
FUN_000401 CDS scaffold_10 52284 54817 - ACU5_1
Acetyl-coenzyme A synthetase PF00501;PF13193;PF16177

MATAVEQPQMPQELRTSAPESLPKSAIPAAVASPVPAPAATETVSLRAEEAPAPAQETKAPVVAEAHLVDTYHPPQRMFAKHP
HRPHLANLEEYKRLYKESITQPEKFWAERARELISWDRDFQTTRSGSLLNADVSYFNEGRLNASYNCVDRHAFKDPDRVAIIY
EADEPTEGRNVTYGELLREVSRTAWVLKQMGVRKGDTVAIYLPMIPEAIIALMACVRIGAVHSVIFAGFSSDSLRDRVVDAGC
KVVITTDEGKRGGKLIGTKKIVDDALKQCPAVENVLVYKRTGSEIPWTSGRDFWWHEEVEKWPNYFPPEPVASEDPLFLLYTS
GSTGKPKGVLHTTAGYLLGAAMTGKYVFDIHEGDRYFCGGDVGWITGHTYVVYAPLMLGVSTVVFEGTPAYPNFSRYWDIIER
HNVTQFYVAPTALRLLKRAGDEHVRGKFAHLRVLGSVGEPIAAEIWKWYFEVIGQEECHIVDTYWQTESGSHVLTPLAGITPT
KPGSCSLPFFGVEPALIDPVSGEEIHGNDVEGVLTFKQSWPSMARTVWGAHNRYRETYLDVYKGYYFTGDGAARDHEGFYWIR
GRVDDVVNVSGHRLSTAEIEAALLEHHSVADAAVVGIADELTGQAVNAFVDLKEGVESSDALRKELIVQVRKSIGPFAAPKAV
YVVPDMPKTRSGKIMRRVLRKIVAGEEDQLGDITTLSDPSVVEKIIKAVHDAKRK
FUN_000402 CDS scaffold_10 58441 59618 -
hypothetical protein EOG09263OQH PF00004;PF13177

MALIVDKHRPRSLETLTYHNELSDRLRSLAQSGDFPHLLVYGPSGAGKKTRIVATLKELYGPGVEKIKIDARVFQTTSNRKLE
FNIVASVYHLEITPSDVGNYDRVVVQDLLKEVAQTQQVDQSAKQKFKVVVINEADHLTRDAQAALRRTMEKYSPNLRLILLAN
STANIIAPIRSRTLLVRVAAPTHEEICDVLAISAKKEGWPVVEGLHKRIAEESGRNLRRALLMYEAVHAQNEKVTDSTVIPPA
DWEALIGQIAKEILEEHSPARILQVRSKLYDLLTHCIPPTTILKTLAFKLIALIDDGLKGEVIQWAAFYEHRIKTGTKVIFHL
EAFVAKFMRIVEMYLMSMDM
FUN_000403 CDS scaffold_10 60019 60880 -
hypothetical protein EOG09264T3S PF00252 ENOG41

MKIANTSALLNAFQGLRLCASSPLQQLHAPLRQSSKTLDAIRRPQNVRAFSTTPSMLGNWLEPNLNRKKKMAKGRPRVATGGS
TKGTTVIWGDYGLRMTDHHRRISAKQLKMAEDTIKVRLRGQKYRLYKRKNCNVGVYVSGNDMRMGKGKGSFDHWATRMAVSQI
LFEIRGRLHEQVVRDAFRLAGNKLPGQWEFVKKGEPPMVGITKLDGVTLEELKRPRRHIAPVELLEASSPTTETLVGSTSEAS
RNP
FUN_000404 CDS scaffold_10 61213 64549 +
hypothetical protein EOG09261NW2 PF00004;PF00533;PF08519

MPSDIRSFFGPKGGGAPKPAPRRAEELAKSKRTKGRKVVEDSDDDEDEAVEVQKPTTKPAPKRKSKEEETKGVAISADDYFAS
AKNSKPTSSATPKKPAAKPDIPARSSPRGKPTASKPAAPAKNGKAPATRKSTTSYSRHNADQDSDAYMDDGDEDDEDIFAADA
KGGSKRKNDDYEEDESEDEELPKPKRIATRGRPSAVKKEEKEEEVKPAAKGRKRKSLSEDSEESEEEAPRKKAATAKPKAAAK
PRAPRKKNDEPEDDEIKDILDSVATVRAPTPPPKDANAKFDWRKNAFGGGNASAQPAAGAADLPEGADECLTGLSFVFTGVLQ
TISRDEGQALVKRYGGKVVGQPSSKTSFVVLGEDAGPSKLAKIKSIGIKTIDENGLFELIRKLPAFGGGGKGAQKAQEKKKAE
EDKVKKQVAEMEAEEKARKAEAAKAAKKAAAAGGPPVKAAAAPPVQLLTSKYAPTQLGHICGNKAQVEKIQNWLRNWPKHKKY

NFQRRGADGMGGERAIIISGPPGIGKTTAAHLAAKLEGYDVLESNASDTRSKKLVENGVSDVMNNTSLLGYFAGDGKDVDATK
KKIVLIMDEVDGMSAGDRGGVGALAKFCKKTQVPLILICNERRLPKMKPFDHVAFDIRFNRPTVDQVRSRVMTICHREGLKLP
PSVVDALIEGSNKDIRQIINMISTAKLDQTSMDFDQSKAMSKAWEKHVILKPWDICQKMLAGGLFTPASKSTLNDKIELYFND
HEFSYLMIQENYLRTKPMALNGKGYNQRELKLKALELFDNAAESISDGDLVDRMIHGPQQHWSLMPTHAVFSTVRPASFIAGQ
LMGSNFTSWLGNNSKYGRLGRSIREVHSHMRLRSSGDHNEIRQQYLPILWTQLVDRLQKQGNEAVPEVIDLMDSYYLTREDFD
AVQELGVGPMDEEHVKIETKTKAAFTRTYNAMSHPVPFMKASNVVAPKAQPKEVPDLEEAVEDDDTEAVEAPEVDEEDDEIDF
KKDKYIKKPKAKKVTKKATKAAADDEEEDEDKPKRGRPKAKAKGKK
FUN_000405 CDS scaffold_10 65345 67850 +
hypothetical protein ENOG41

MQGSASYPAALGSSKWIASFLARHQYDDGIYARTSTRPSSPQEVKFRDFVKKSKAELKNVFSANEKKKSEAGVDELRNANDTG
PERKNKKLSWAKIFDTKSGNKLHKQAKSAPGGIHVPHQLDPITEVGESRETQADLVSANTSSRKPVGENHQCSTSRQTDGDDV
SVLTTCTTIIQQANLQGVSEGNARDAATNDESTAIGGRNCTSNPSTHLSSGNSNNTEDEYPSQEESDNTSDGCEKDVRPEENA
RQEETARQEENVLQDENPGQEQINNKDLTSKSSSKFSLRSVLSMFMGSDGSDEEAKAKSDGTRKLKRKGRLSPRLRTLLIGKT
KSESSTKSHSQSSSQDLNQSDAQLANQIVEQDRTFKGLDWKLPVGQNEMGQLIREFSGDLNTATQEMVHKTPVSPKTPSLQSY
PSKFDISIDQNLLPRPAMAPALYSHDQGHNQQVRNEPTSKDALVAKAVHMENKQAQLVEIKTRSDRTMNQDTRGDANQNQVAQ
EVGRSGKEPVERDTTNDVHPPRTWQHGGDPAEVETVEEAYARHRSSLAVVDRIRSVGLLGPNVDETKSIAADEPKELDEQRLL
REMTEDFELPPHKVAFNESLIAATNNSQVIPSIEHQNTNLGCEGQKTPEPKVEQTDDASDQTELILTRARSSDRFVPEGLYYQ
ESVDGE
FUN_000406 CDS scaffold_10 67871 69028 +
hypothetical protein ENOG41

MVSELQGFEEEELPEDFTPGQPATEALMKELEIERLGGFFTTLQNVRNESLEQLIAARDPRAVVTWRDEVAELTSNGGMIRND
KAEQLYQPAPSTAGSDGALSAMTYKPSRAEVKAALAHPEAPVIQGCKPRRVLTECDRIQEYIHIDSLAKNKYTSQMQPAMSRN
EIILDEWDRADAVACIHKHNQQQKLKQQEEESEEESEEESEEESEEESEEESEEAARRRRLKIYRSVERHIVKGRRLAVKINR
EATAAFQMCEYLDEEYERLRGQILRYAEDCGHEPTDNLQHAVDLIRKTQQEEEITYRKYEGCESADPESPESVVAAHYGRDYH
EIAGNHVDKTVDPANEFF
FUN_000407 CDS scaffold_10 70081 70474 -
hypothetical protein PF08649

MGGHEKTYFEQQREALIGEIAMSFEHVLANINKLNRSLEAVTAVGNEFSSVEALWSQFENVMDKDGKAEGETEQADQTEHHEE
EGDTTVKHEGA
FUN_000408 CDS scaffold_10 70923 77615 -
hypothetical protein ENOG41

MNGPDHPVTARLTDLTELRSNGGFQPGRDFGDPLRREPPRGPKALIDAPSGPRGGGFGGEFRGGRGRGRGRGWSSRDDSRDRG
RDRDIDFRDRYHDDRSRERDRDRERDREWREPRDFRSRRSPIGRARSPTRDFRDRDGPLGVDADRSRRGSRDGGPPSAGSSSS
DPQFGMAPYPRGGGFHRGRGRGRGDWSSERGRGRGPYMDDRGDRYPRSRSQEGRWGRDRDDRDRMDRMDRYADPDTRRDIRDD
RDPRDRELFRNKLEARANAGHDSGLSSKEVSPPPAAPSASAFGPVPNRGLSVGEGYVSASAATKIPPTAPRAFNDRPPSAGHD
PTMPSVGLGKGMMHDGPSIPVGPRAQQPPPPRPSSKQWINPNLKKGPDSPKLMRSPSFVQQRPPLRRGSSQYDHYHDDERRPR
SSDAKSEPHYDNRARTNYSAEPGEITVKSERESQSARASMDRDTRPANASRRDSYRSGPSPTMENMPRFGQESELRARDPREA
PKEAPRRKRSQPIIRAVRFEVPPKLAPVEQNSESDDDDDMADYFDMEIGKTEAELSKLEKPKLPTQVMARFAALSHGSMVKII
GEGEGLLKMVEKLPEAVNKRVIEKIHDPTPIEQAKKEDVEMADAVGSTDKVDSEPPKELHQEVQAELDPGPEHSIQVKGSEEP
VEAAPTPESSAQVGQETTERPVEHAAKAAGEKIDDSSHEEAAPNLDETPDQSHVEKSDEQRIENPVEKPVEESIGKPAEETEE
NHAEKPVQKAENEDTEMFVAPELEPKSGDMDIDEPPIEADRVAHESPPSPSNKKAMDVPSIASETAPTESFEPQKTERTPLGA
CEDSHKEKSDKDKPVDITAKANGVQPTQTLVEKPTEALPEPTPGKPDDAVSEKPAELFTEPRPENLTENAEALGHRQVAIEGA
PVSVVCPNVVLQTTETASKPPSTPSQVDDDETESEDDSFMNLDTVRQYMTTPPIDSLPDYSCMPWDKDSAFLKTLDSSITLDD
FVVEHLDKMHLEKSAEQDHDRKIYKDNYERYLDFTMSNDPAAVKSRGKFSVSTGIEITGTVTPEPKHEGSGRGRRFATERDLE
RVLQASMREDEERKERELRMQQEKYRTDKEAIIPNMIWNQEEKDNTKYLDKSGFTPVDRLVSAWRVLPPVNNFTEEEAGLFEK
RYLEAPKQWGRVAEAIPHRDFGSCIQYYYMNKKDLNLKEKLKKQPKRRRKGKAKQRSSALVSELGNGDGETEENHDTGENGER

RRPRRAAAPTWGFEQPPVDTENSTPNATPGRRGGSAKVDHPEKVDGRKRRRGPKEKEPKAAKANQTLAAAPGPGKGRSRSNSR
ALNTDGPSTPLPAAESHRLPGQFEQHPAGMQLPFSVQQQQQQQPQQQPIQTLERQQSVAPSSISEVMAAPSLRPDPPPQPAMA
TFNLAHPPSERKAPTQASSYWSVSESNDFPHLLRSFGSDWTGIAAHMGSKTAVMVKNYFVRQKDQGKAEWEVLVQDADRKRAI
GDKLPDPPQPSTGGRGRRYDSSAAASRPLAVAPGIDLHGETSQPKMEPSVQPPRGQPFGMYGSPIAQAPVTQQPLVQPQQQPM
PIGQHSTTQPVTQTMSPGQRPLRAPVRQFGFPDGERERERGREQSVRVPLPQKASSRPPGSEPREQRPLAAAQPLQPARSEAM
IEHNAQMERQKMDIQRLEQEQLELARQSERRVKQEPEVTPQPHRYEPFGHRQQGSLGGQPLARPPQEPGRPPSYAPPMQQQAP
VQPVRNLMGEQTPVRSPQMNTPSSRPMSALQHRPSSGSMHEQYGSATPQAGTPVQPPAAAPRPAEPRKGGLNIMSLLNDDPPP
QPKRVSDVTSTPTRPSPTPPPQAMGRTLPGPAPPSQMRRGEAESYSPYGRGTPVMPSLKPTYADSPQPQHIGSSRASIGVSLE
AAAERDYYRQNPYQPSSHSRTNSPQSGHRYAPPGPPGPPGPPQYQNQGYPTSYAAAGQPAHAGSPGGQYALHPSASRAREVPQ
GGREASWPPPQQPPSSMQQPPGWASQQPPNAQPPPQQQQQQPWPTQHPSSKPQTPAPSWAPSQPPSQPPPQQAHHMSMRDDGR
NSYFASGPGMPPQQHQMQGRYPPTVSRGPESVPPPQPYSRYASTPGPGPPREIPGRSYTPISSYDSRGPPQPPPGAPVYPGHD
PRDPRGDPRGDPRGDPRDPRDMMRGGLRPHEYERHPDHYRR
FUN_000409 CDS scaffold_10 78748 79948 + DYS1
Deoxyhypusine synthase EOG09263H5H PF01916

MTSNSDAPPSAATEAVLVKSEEMPADAQKVEELDFNKFKGPITAENLFEGMRHMGFQASSMCEAVRIINDMRAWKDPETGDKT
TIFLGYTSNLISSGLRGVLRWLVEHNHVSCIVTTAGGIEEDFIKCLGDTYVGSFSTPGAELRRKGLNRIGNLVVPNANYCAFE
DWVVPILDKMLEEQEASKGTEEEINWTPSKVIHRLGKEINDERSVYYWAYKNNIPVFCPAITDGSLGDMLYFHTFKSSPLQLK
IDLVEDIRRINTIAVRAKRAGMIILGGGIIKHHIANACLMRNGAESAVYINTAQEFDGSDAGARPDEAVSWGKIKIGADNVKV
YMEATACFPFIVANTFAKDIGKSDK
FUN_000410 CDS scaffold_10 80221 81119 -
hypothetical protein ENOG41

MSIRRDAVQRDFGLRPGSRAVPRPQLSIEPAEDVVTSPPEMNSPPHSSDDAREPYVQESTPLTPVKDTCDDDRSSTISPTVNT
NGLLPIPKNRSPERRGSDDSQSGKESPGSPETPSLPAHFPPCPRERPTRQEIALWHEGCMAVQRDDGTVDGFDIVDWLMTRKG
GVLNPWPRLDEGRMVVESMGREIIDLDAIHRQQEEEAEVARLKEERRQRNKRRYRPRGQIEKEKAERRAAQEAQQAQDLAKKT
SSAYDSEQGQDMADTDEPPAKKARVDIES
FUN_000411 CDS scaffold_10 81494 82449 -
hypothetical protein PF00248 ENOG41

MAAKLAKDLTLKGSSTAMPKLVYGTAWKKDRSADLVYFALKHGFRGVDTAGQPKHYNEKGVGEGVQRAIKEGLIKREGLFLQT
KFSPPGNQDENAPYDFNAPLVDKIHQSIQSSLTYFTVEGEEPYFDSVLLHSPLRTLEETITAWKTLETYVPHKIRNLGISNTT
LPTLKALNDAVTVKPSVVQNRFYPDTSFEVDLRAYCREQGIAFQSFWTFSANPRLAATKPVKMVAEKAGISEIAAYYSLVLGL
EGVTVLDGTTTENHMKDDLEGIEKVATWAETDGTAEWNSALKQFKQSIGEV
FUN_000412 CDS scaffold_10 83387 84665 + XYD1 D-xylose
1-dehydrogenase (NADP(+)) PF01408 ENOG41

MAPYTAKWGILATGGIAECFTKDILTDPAARDVNDVQHEIVAVASSSSVDRARDFIKKIDGPSSAAVYGSYAELVADPNVDII
YVATPHSHHFQNAMLALEAGKNVLCEKALTVTAAQTKKLYEVAKSKNLFFMEAVWTRYFPLSIKIRELIQSGAIGTVYRTFAD
LSFNKNTDSQDLDFPDSNRMVNPDLAGGALLDLGIYSLTWVFQSLYHVQPEAEKEAPKVVASINKYRTGADESTSIICQFPKH
NSVGIATTSLRIATDVDGLYTGGPAIRIQGSKGEIQVTGPAFRPTEYKVIKSDANGKVEVVNCPIPTDPKRNNWGHGMFWEAD
ECARCLRDGKKESASMPWSESIVIMEVMDSALKQGGVSYPDLITTDVFDPNSPLNTGKK
FUN_000413 CDS scaffold_10 86197 87040 +
hypothetical protein EOG09264DIM PF08241;PF08242;PF13489;PF13649;PF13847

MASASSATPAGDQDDRPLRQPSSQAQTNQTSAPTESESFSQSAAEEAPETYEATHVHAVYEAIAPHFSATRHKPWPRVASFLL
SQPPGSLGLDVGCGNGKYLRVNPSVHLLGSDRSPALVQLARTELRRPREDGVEHDPRVSDVDVAVADGFALPYRNGAADFVIC
IAVVHHMSTRERRQAAIAELLALVTPKAGRVLVYVWALEQASSRRGWDASSDQDRLVSWVMRKPKGQEPGGTFQRYYHLYKEG
ELEEDVKAAGGIVVETGYEKDNWWVVCSRPS
FUN_000414 CDS scaffold_10 87926 88863 -

hypothetical protein		PF00249		ENOG41

MSGRRGMHPHATDQPPVKKQSKWSPEEDALIIELRGRNMKWEDISKRLPGRSAISCRLHYQNYLERRSEWDEERKNKLARLYE
RFKPEMWAKVAEEMAVPWRAAEAMHWQLGEADMARRAGVIPFSLAAVNVEGNNSHRSSPSRSHIHSQPQVSMPRDLGAPSPRV
VYNRPPPMPASSRTMVPRRESIPPPPPLSMVPDPAEAQYVPGPGLAPIQNQPPPRTAGMLPGVAELTGGVSPYSTPAAVPNMG
PITGVGQGPLYSTLAGYSMESAGSKRRASPDEYPHEANQRRRIT
FUN_000415	CDS	scaffold_10	99050	100121	+
hypothetical protein	EOG0926505R	PF01302;PF14560

MADVPLVVISEFAQSERRITPSWSISQLKGKLETVTGVPPSCQRLSLKPTAGAEAIAIEAPNEDDTHLSNFPLAPYAELHVID
TRPAASRINLNDTSGVDKYVMPEEEYEKKTDSVLAWKKAQKLGRFDPDAPSHEEAKLQALDREVATRGIAVGRRCRVGGEDTR
RGVIQYVGEVKEIPGGLGSWVGVQLDEPVGKNDGSIAGTRYWGEPSELKHGVFVRPERVEVGDFPALDDLEDMEEI
FUN_000416	CDS	scaffold_10	103430	105060	+
hypothetical protein		PF00097;PF13639;PF17123		ENOG41

MALFISILACTLTGVLADDTEVSTWAETEIPEWATINGMQLSLSANPGLQFAVIPLTSNLGLNDSTGRVIDIIHITGNMIATN
SSNYNKVTDKDDIAYLSCDEPKGDSFLSPNKMLNTLMQLDPHLRAIVLYSTSNTWCAIDPVEKLPFTSILSMADGGEATQALN
LLNGTSRGEVVKASIFGNNTDNTIPKDESKGGSNTAAMSVLYAITGLITLLFLVIITTGAVRAHRYPERYGPRGALGGRPRQS
RAKGLARAVLDTIPIVKFGNQTPTKPDPELELENTEGNDAATQRTASRLSEARRSDGAPAPGGETNAVPAATRASSSPESAED
ETSDHLGCSICTEDFTVGEDVRVLPCKHQYHPACVDPWLINVSGTCPLCRYDLRPGKGHASAEADVGDSSTLPPPLAMEGAEN
DAPQSSHRNRLSRLFDINRLREGTVEEQMEALRQMRAETNENNNQETTEVTEAADGESQGQSARFAARLRDRFRIRTRAQAVE
DHESQRGS
FUN_000417	CDS	scaffold_10	108751	110457	+
hypothetical protein				ENOG41

MAPKPSLEPREAYLPKPVPIGGDIMSVMVSLAACSILAVFLFQRITAVRSWFRLPFVAWLVLVIYVDSYGFVFTSAILQQVFG
VNSSFNICHGAILLCLICYVTTKILIYVFLVEKAHVIRSSTTRRRNSKLYLFNMITLLGGYGVVVVLNFIYRIARIVNGECFI
GMKSISMIPLIAFDAVVNVYLTILFLIPLKNLYSFKNLPKTHANSRLRSVAFRTFVGACCTLTSSIVNLTVLMVLNGEPGWVC
LMCCNSDVLFSAIVVQWVTSRDSAGSSSQPASAAVTDGSYIGRRASSAHPRSSLHASNVPNVPRDVDDETSLVGHATVTSLTD
DPGFEMTKVHSGATSGVIVTTTIHRQSRPTTQRKESSDDDDDIGIESDRDFPVRSVAFAPGHTEVAEPPRTRIQGGPRPHHHA
KAPSFSRPLNK
FUN_000418	CDS	scaffold_10	111368	112535	-
hypothetical protein		PF11790		ENOG41

MIANKLTILTATAALLEPALALGHRHAHHQHAQKRGDWVVATIDGQVVSWENNYFGPGGAAPAATQPAAASQPEAPSAPAPAA
AQPTTIIKVAKPSQAAKSAYEPASQPKKEETAAKASQKAAIKEKTQSKAKSSSTQSKSSGSTSGGSAGFSHKRGVCYNNVDLA
NTFAGDCKNCGWGYNWDSSSGGLKGLNFIPTLWNDQPLHTDRFADNCAQALSDGAKAIFSFNEPDNAGQADMTPAYAAKAHVK
WLNPYAGKALIGAPSISNSGLPMEGVEWLKNWVSECEKLGEQCHYDFCNVHWYSEVEYGNTLFDHLKASHEACGGKPIWLTEF
APTGSDEAIADWLKDAIPKLEALSYLDAYSYFKVETGMLMTSETELSSYGSVYASA
FUN_000419	CDS	scaffold_10	116652	118205	+
hypothetical protein		PF00172		ENOG41

MDINQQFTPGILSFTDQPEFDYAAFLQADDITFGEGAQTQQQVPSDSSDKATPPSTTSGSSSELVAQPSAQKQRLERRGHTKS
RRGCYNCKRRRIKCQETRPACGHCTKTGLKCEYPSMPQITHQPHHQIPLFSLQDMRFFQHFLTQCYPHHPLKQEDIWTHEIPC
IAHNHEYLMHAILGFSASELMRNDASIVSSAMNHRIKAIRAIKKRLAETARSSMDYEEANAMVATCYALTFQSVSLDDGLAEY
LTFIRGIMIVGMQMLFRGIKPIFENMMEPDQDALLEPLMQGLPLIPKGYTDSAMEALTNLKPLCTQPVEVEYHHHLVKMVEKL
YINSWDAYKAYTSQYGWWMLLPHDTFQALIDPDNQVMMLLHSHWVAINEIMSPISGQERNVSEKYPPQRDGQPQLDPGFARWL
KHINANIDLEHQIYNQWPMWVEEQLDHDITFFGRA
FUN_000420	CDS	scaffold_10	119455	120406	+
hypothetical protein				ENOG41

MSTKTETMTTSSFELGHRPSGHLAPPPDYETTEAVELSRLSPATSSTNSLPEYTTLYNNENGDSASSSSTSGFYPTKQLQIQA
PGFALIRLPLPPQPDPIYVFNVAATGELDDAEYVSIRPTRNSGSCFLARANDPTQAALCTTTYRFGPGKPPKIRFVGDGSQNG
QAEEIEISCKGVLTRSTIMRTHLGTFEWRYSSRAERRSAKASVGEEIDCLLILDQVMKVAVAGGKQEERRRKVGQFVRSDGLR
TQGSRRSSAGNGGRLMLDLREWLDRKNEAIEMEILAVVSCVSMMKKEVDRRRMHQTMAIMGGASGGS
FUN_000421 CDS scaffold_10 120573 122672 -
hypothetical protein PF06772 ENOG41

MSYIASARRRRPQEFFLPNGKKIIASLPEDLESLREEHGKRNDVQVEVVIHGSPEHCDYLRETRDHHENRRAHFRQRHGPAFD
EWEDMQSQLDIVNGHLERLSTNTSALSANFDKFGYGAELRTYDDDDPSPAADPSNMSTSDTLSVGSGGGQPRLGETIKLFKKP
VVKQWFHKGLLWRASENTEIMAIELFFDLLYVGIIHVNGEHVWAEPTGKELLRFAITFIMSWKIWSDITLILSWFETDDVFTR
LEILFEIACLLGFSTNMTYAFYPGEHNTYTMLVSFYVAARFRGVFHFLYTGYLLPMVRGVMFCQALNIIVPAALWIASIHVEM
PSRLGLIWVAIILDMCGQSILTGIYQYGRKAAHDKRIVKYLSGIFEFFPAVNIEHRVERTNAFVSLVFGYSVMGPMFQSHGGY
TVDVFLGKAVLGLCQAFVFNWIYFDVDGSNINIHAIRRSVISSSVWHYAHLPFIMGYILASAGLSKLVWVADTPDANPEHLTE
HYIERSDPELADGIRYFYSQGLAVALFFMGVISVTHDHRHPATRRLSKKTRLANRLLVCLILFLLPLAKSLNSLNLISITLAL
SVWVLLLELFGKSCRNDPFIGEKQGCSVRYRCKCSKKDLENANLNEKEQIPSTEVLELGPREKTAVPDVQE
FUN_000422 CDS scaffold_10 123317 123701 +
hypothetical protein PF04627

MTAAWRAAGLTYNRYLAIAARVVRRSLKEDKRIAAERRGEMDLRFSKWQNGKQGEPKNLAAANAAIAAENAA
FUN_000423 CDS scaffold_10 124329 126942 +
hypothetical protein EOG09261IFQ PF04112

MDDIGNGIQDDIARISLDHQYEYPPPPLPDIATAGIVAVDITDKFSEAVKTLAPGELVKDGAFTLFESVSGLEIMDPKMDSGC
VESAEELEELYDVARPLLPEEVLGIIDQLLCHEMAWHQGYPLSQTLFTSVYAEALLTPTPRTVEDAHFVRNGSYGSSGQSDML
KILRAYCLGLLKACGYVNERIRSEHYYEEEDFVTNTYSRTLLAEIQPQAIRQAIQEARDLLITLSTGVSDDLREALDQRLQLR
LYFLEATECPKHVKEPEVARKPWLEGLKVLPAIKSSHTLGKPVDEAFSAKLQRKLASTMPPRPIVQLKFEDAFGHLSRLFTDG
VELIDVLNYTDSQCLQNFVLQFQAKKPQPLVFVRTLLQTFLFNEMEVLGSMSIRQIMDDDFSIVSMPASVQLDRNNDEIEFPQ
DPRFIVAQQMELFRQRAAQSFLDIFRTFCQNRCRVRRTLCHIIRDWDNLQLDAEEIDQIIQVKLNEKPMRHFTGANSQPVDTY
SLPLSSWTYLYKIRQMEWIVQLGFELEVYQPDELAGMYWYLNYLAKWRVQHTERLKSFIVRRVEESRAPSQPRNPSVDRQLER
SLAFIRLMLLDAAVTWELSDALSCLYTALMRLELVKVPPRPYSNDELRFELRMRPFAVIGLPPCPGFPEFLLGTTQPESSTVD
ILQYAERALKGAKQGLEVLSKLSAADSFSVGSHDKWQTSKKNALKACIATGLAITAVLKASKEDASNLKLRAEVPTPDKCYHD
WWIVPRLVPV
FUN_000424 CDS scaffold_10 127284 128433 -
hypothetical protein PF00153

MSDNAEKETTPQIDTPVTKVEITPNKVEISTAPETQAIPQNDNVAHALAGAGGGLLSMILTYPLITLSTRAQVESKKAESKFS
EAVGNIIAREGISGLYSGINSALFGISVTNFVYYYWYEWTRGFFEKAAAKAGRAGAKLTTVESMIAGAIAGSATVIITNPIWV
VNTRVTTRQQEKKQDVEAGESKPAKAPSTIGTLLLLLKNEGPQALFSGVIPALVLVINPILQYTLFEQLKNTVEKRRKVTPTI
AFFLGALGKLFATAITYPYITVKSQMHVQGSGKKEGSLSALSRIVRESGYSGLYRGIGPKVTQSVLTAALLFAFKDVLYEQTV
RLRMAQAARRRVAA
FUN_000425 CDS scaffold_10 129204 133896 +
hypothetical protein ENOG41

MPKRKSLGVDNDSFAVRWHSFADMNTPSADIMSSPDPLNDTVEQSVVPPPSTRRVVRSSQRSSRFSSMGLGTSPRKQTFELEV
GDNKAPQKLLVTVETEEPSATAGPSSARRKLFQDSPYLPISRRREMATTTTTVPLKGAIEDDNNATPRKRGRPRKTNGTPLPS
AGTKRKSITPMHGSPRRRQRTTKDTDTDTDTPNKSADSNAQPTPGTKRRGRPPKNRLVDPPNATEDEQNARGISETSNRSESQAL
DKIEVAGDASDLPSLRADDSQADNDVTLVITPSEAEANQLRANRTANISAPAQQSEPDSDIWMATLDDEPTPRPGSRATQNAP
APSSPDHGRADDSSDFGDYGFGPAGSDVSSADEPQSDTRPGNEDTVAAGEDFSMIFMDSLPSLQASMRSSVREIAENDIGEET
SLIINNTLESLRQSLQDRDRPNVNKETAEQDKEPQPSAPAPAEPRDELRQSTIRPPMTFSPNRNLSSIWRQTPRRMGSSPLRR

QVLQSNARDNPDAEYRGSPLATQRQDSSHLEIDNSSLYDDSFSEIPDAVLEAATPRRPRAMAKKAPSETEDVEMGDADLIQFE
DEPQQAEIQQREQVVEEQGAEAEQEAVEDQESVEEQEQQEAEPPAASVGSVISRSDGGRLPTPDDTPPNIDFGNNTAPKSTSV
SQSPARSASSRNSPPRNSSPAPATQAQVPPETIEIEDEVVDQAVETTGQVTNGAIVEVEELADNDDEPIMDMTIMPEPDDHSV
PDGQPLEETIMEDLVEEEPEEAQEIPEIVVPQQPRLSLEAEMQQPEVTPLNQLTSPIQEPQSLPDVPPERVRRPTLSPIMRAG
RALQSVTSDPPTPEARDHHLRSPFRRSASRELGPRVTQPNLRTNASPGKSRPFTETAQIPKGDDVYDDPFGTNTRHTGQASFM
EALERRSAGSSPRHRRTMSRESAASSTHFQAPSEGGMSWVDREGPISDNLRGDVPLEAFARSTARPPEPQSISHGQLDGTVDD
ADDETEDAGDDMDLWEFEAQRSSPHAARQPSPARKPESPIPRRSTLPSPWRRQTNKGTQRPPTTADQTRNELEENEEHLEEET
APQPDNMDVAQDEPSQAEEYSLVTQREVPQESDKAPATAKANRFDLSTFFSSPAAIPGMLADKLSPLKKMSVFGARPTETEPQ
PMETAHTLPTSSMFPQLPQQELVPRGQPRAGFFSPIRPVQPLARRGLSEEPENDEYSRSDSRSNMSSPSRQATASPRQDERDA
SMQETSEQQNDGEQQEELDEEGTEVLEEEEGEDQVSEPLPSVPQKMNFTPQRRNPSQSFFKASTQAAAPDPAPILSSSATTPP
RMQLTHADIQKWQQQTSNASEDSSERSTASTLLRPLPPKNASPIKSSLRSPLKPHTPGRVVEFTSSVLSPIEQAKVRHQRRLS
NSSAASQSSTGAQRPRVRALPHQTANKENNTIPQIPIYNEKLPTKQTHPEPLSQTVWTRRHWLLLDDLLQQRRQGPFRLHFER
YSDQYLGKTVASQGESMTLERWHLDCVDAFKAQVGGWDEAALVKRLFALILGEEKRSRGAGKPARVMFH
FUN_000426 CDS scaffold_10 134292 135680 -
hypothetical protein EOG092630YS PF01399;PF18055;PF18503

MAAAESARVQEAQKLAKSDPRKAEAIYKDIISKAPSTTSDAATREYETALISLGELYRDEKNTQELVTLVKESRTVFSSFAKA
KSAKLVRQLLDLIKEIPDSTDIEISVTKDCIEWATAERRAFQRQDLEVRLVTLQMAKQSYYEALGLINNLLRELKRLDDKLRL
VEVQLLESRVYHALGNIPKSRAALTSARTSAASVYTPPMLQANLDMQSGMLHAEDKDFNTAFSYFIEALDGYHSQDESTRAQA
ALQYMLLCKIMLNLVDDVNNLMTSKQALKYAGKNLEAMKAIARAHSNRSLEEYERALSSYRYELGSDAFIRNHLRRLYDAMLE
QNLIKVIEPFSRVEIDHIAKMVGLDTQQVERKLSQMILDKVIIGVLDQGAGCLIIFDETHRDESYDAALATIEKLSSVVDVLY
TNQASMLE
FUN_000427 CDS scaffold_10 136278 137065 +
hypothetical protein EOG09264IDN PF01025

MFRQAIATSSRALRAAPKVAVPRLFIQSQFQAAPAFALRSVQPAVSRWYSDAKETEAKPAEEAKTEEKPAEGEKESDPLAELK
KELAAKENEVKDWKDKCMRTVADFRNLQERTTREVKAAKDFAIQKFAKDLVDSVDNLDRALGMVPKDKLNAPDRPEGLEDLAN
LYEGLKMTEDILMNTLKKHGLERLNPEGEKFNPNEQEATFMTPQPDKEDGTVFFVQQKGFKLNGRVLRAAKVGVVKNK
FUN_000428 CDS scaffold_10 138288 139200 -
hypothetical protein PF00025;PF00071;PF08477

MASSHQYHTSYFDTNEQIRRQQRTRSSEGTVSTTMSSSTGRESAATHLTEAPTFSKKIVVVGDGGCGKTCLLISYSQGYFPEK
YVPTVFENYITYPIHSATGKTVELALWDTAGQEEYDRLRPLSYPETDLIFVCFAIDCPNSLDNVLDKWYPEVLHFCPYTPLVL
VGLKSDLRYKKTCIDMLKTQGLTPVTTEQGMAVAKKMNAQYMECSSKEMSGVDEIFEQAINTVVANDRKTIEAAAASGSSKES
KSSTPGVGQIKRKKRRCPIL
FUN_000429 CDS scaffold_10 139987 140641 +
hypothetical protein EOG09265QTV PF03931

MDWKTPSKYITLVSGDGFEFVVLRDAALVSPIIKGMLDVRSQFAEAKEARCVFQEMSAMVLDKVVEYFHYWYRYRNSEDVPDM
EIPVELCLELLAAADYLGLDQYAAQPCIATTDCFDTNLIQGEYGHEVRSYEILTQIWA
FUN_000430 CDS scaffold_10 141558 143297 +
hypothetical protein EOG09262LI4 PF00149;PF05011

MAANTFETQGVRVAVEGCGHGTLDAIYASVEESCKQRGWDGVDILIIGGDFQSVRNAADLSIMSCPVKYRRLGDFPKYYSGEQ
KAPYLTIFIAGNHEASSHLWELYYGGWVAPNIYYMGAANILRFGPLRIAGLSGIWKGFDYRKPHHERLPFSGDDVKSWYHIRE
FDVRKMLQVRTQVDVGLSHDWPRAVEWHGDHAELFKKKPFFKQESIDGTLGNVAAEYVMDRLRPPYWFSAHMHVKFAAVKVYK
DAEPVAQESKEVLVPAPAPAPATEANPDEIDLDMDDDDDEGSKAEPVAEVKKSESETKEAPEASNEVSDELRAQLPASFARPQ
PKKTPGQPVPPEITNKEVRFLALDKCEGHRHYLQLCEIQPFKPETSSEYPPSQESPRWRLQYDPEWLAITRVFHDSLIVGDRS
VQAPPDLGEEHYLPLIEKEREWIEENVVKSGKLDVPYNFEITAPPHVPGAPEIVNEQPEEYTSPQMAAFCELMGLANIWDATD
TERRQRKAQGPPKTDQRFSGRGRGRGGGRGRGRGQGRGRGGRW

FUN_000431	CDS	scaffold_10	144030	144993	+	MOB11	Maintenance of ploidy protein mob1		PF03637

MESPCSPPHTASMFRYLNPKSLLTMQQLETTIIPVSLSGEPLMFVDELTLADPGTLVNSNQRTRNQFRPRVGKGGSASYQLRQ
YAEVTLGGGSLRKVVKLPEGEDENEWLAVNMVDFYNQINLLYGAITEFCSPQSCPEMKATDEFEYLWQDNENYKRPTKMPAPA
YIEQLMSWVQANIDNEQVLPSKIGVPFPKSFPALVRQIFKRMYRVYAHIYCHHYPVIRELGLEPHLNTSFKQYVLFVDEHGLA
SGRDYWGPLGDLVDSMLKSD
FUN_000432	CDS	scaffold_10	145477	146521	+	ATP5	ATP synthase F0 subcomplex subunit OSCP atp5	EOG09264KDO	PF00213

MFSRQVLRAARVAAPQRALALRAAPVRSFAAAASTEVKPPISVFGVDGTYATALYTAAVKTSSIDAAADALNRLGALIEKDPK
LAAVLSAPTLTAADKKAIVQELEKQINTKDETVKNFLATLAENNRLGLIPGVVEKFSSIISAARGEVELTVTSAQALDKRTLN
RLETAVSKSAYVGQGQKLKVTNEVNPEIVGGLVVEIGDRTIDLSVSSRIAKMNKLLTDSL
FUN_000433	CDS	scaffold_10	147182	150682	-
hypothetical protein				ENOG41

MVVHDGHEYLTEEERRLKEDRDRTKYWKKWGPYVAERQWATVREDYSHDGDAWSHFPHDHARSRAFRWGEDGIAGVCDTHGYQ
NIGFSFWNEEDEFLKERLFGLSNPQGNHGESVKEAHFHVDNTPHSYMKFLYKYPQKKFPYKDLIDENARRGKEDKEYQITDTG
VFDEDRYWDIFIETAKETDDPDEILFRVTAWNRGPDPAPLHIIPQVWFRNTWSWGREPEEKKPSIKYVDENVAKSNHWSLGER
HFLCSPSPGVGSSGDDVMPQFMFTENDTNFKALYDQKNKQPYVKDAFHRYIVDGEKEAVNPAKTGTKCAAWFNFNEDGGVNPG
ECAVVRFRFTRRDDNYLDEEDFDDVIESRKEEADEFYYRLSPLPMADDLRNIQRQAFSGMMWTKQHYHFIWDHWANGDPTMPP
PPASRKDIRNSAWKHMHCDDILSMPDSWEYPFFAAWDSAFHCIPLAMIDPDFAKKQLDLFTREWYCHPNGQLPAYEWNFGDVN
PPVHAWATFRTFKIERKMYGRQDLDFLERVFQKLLLNFTWWVNRKDADGKNVFEGGFLGLDNIGLFNRSEPLPTGGVLEQADS
TGWMAFYCLSMLNIALELAKHRRIYEDIASKFFEHFIFISDAMTFRTGHKDEKSLWNEEDGFYYDAISWGGPWIQQLPVRSLV
GLIPLYATITLEPELINRLPSFKKRVDWFIENRCDLAERNMASIRKRGKGNRILLSIVSKDRLEKILKRMLDEDEFFSDHGIR
SLSKFHKENPFSMDVNGQKFCVGYVPGDSDSGLFGGNSNWRGPIWLCVNFLLVESLQRFFLFYGPDFQVECPTGSGDYMHLGK
VSEEIQHRLQHLFARTDDGRRSINGGDDRLDFDEHWKDYLWFHEFFDGDNGRGLGATHQCGWTGLIARMIHDTGVNCRLPHTP
RTPSIAMSHYFDDIFHRSVETGVPHSGMPRIRRSSTARSIGARSDFDDDTRSVTNSIYHDDPDRARERTEADAHMHSYVSEQL
ERYKDDKKDGNYEHDDEFETKA
FUN_000434	CDS	scaffold_10	153342	154537	+
hypothetical protein		PF01255

MSDAISSFLRRVIVESPPGEWAVRQLRELLIGALKQGPIPQHVSFEMDGNRRYARNHRMETVEGHHRGFEALARIMEICYKSG
VKVVTVYAFSIENFNRPKYEVEGLMELAKIKLEQLTKYGHILDRYGARVRVLGQREMIRSDVLEVVDKAVARTKHNNKAVLNI
CFPYTSRAEITSAVKSTVQEFLAPTPPRSTPFSPSRIRQKILSRQLDGREGLPTIPDVLPEEVEPLDGDDAKKDSDGDSSSPT
LQPDSPPPRLRARNSAASLSGLPNPETITAETLDKHMYTANDPPLDIFVRTSGVERLSDFMLWQCHQDTQIFFVDTLWPDFDL
KDFIWILLEWQWRQKQKDRDEAVVRPSSVTA
FUN_000435	CDS	scaffold_10	155467	157746	+
hypothetical protein

MKSAKAPATENSSHRRSQPVRQTRTNPPRSIANLGRSGGAGRDSIGGAEQPIDIFPAITHFADAITALPKELVRHFTLLKEVD
AKLFTPEDQLFRLVEAATNAPVSEARPNNEASSANAPGSAPMSAQNSSSGIAFNNSVQSVPSVDESNRDAVFDPSNLPRRHLF
RQTALKIQEMLVSLEEKNHVISTANEALQAQLTRIEDVWPHLEGEFSDEAKWGSTTHWAYPENRFSKASHVDRTRRDGAAAIS
AAAQALADEAAARSDARKQAVQAKKNSRNHNNNHNHSNAHNHDSEGDDHDGKHKPEAPKKTAKSRKTVTATETANVGLGISTA
ATNNGNPPQKRRKVEKTTNGAAATTERAMSSVFGNAAPKAKTTSPRATPAPEAPKKRKALPSGSGQSKKSRNGVTGLSNSVNS
SPVITELPEPKLPPVRASPVPVPTPNPARTRSRQNSIQTVNTDSNKARPTSSASNKPNGIAASTPAAGAPAASALRNGAEPKT
PKETSAPIKTENPKKEVEKTETASAPPVPTPTTTATISTPTTAATTTAVTTGSKKDTKKPEENDRKSESLPPVPQTSTVTTKS
GRASKPSTPALATFQEAVQPRARSSRNNDGTGTGKKNQKKAAPTIHAAVAAQLAEEDTNSSMQGDEDDGDVDADEPTYCYCNG
VSYGEMVACDAVECPREWFHLECVGLKVAPTSTAKWYCEDCKERLKAGGKKANGR
FUN_000436	CDS	scaffold_10	161937	163825	+

hypothetical protein ENOG41

MSRPESPNSNSDKTARSSFSSVRENDDGLAQTFTRSKISSYTQGPEEVFDESPSPSVEIAQPSFQAPLTQPHSRLHGFWFPAD
NFRGWKQIPIKGKSASRSCEDLHKLSMTWSSPAPPAVAEKASPNSYQTGTSPLERLPSEVLGAIIDLLVIEIPPNGLTPRNAD
LMALLLTSRSIHAATLNTLYRHITIPHSRIFRKFLATITEYPALASIVRRLDFSHFNPSTIFSTASERAQTRNLTSETLYKCL
ELTPYLQEFLAQEYIDEDLGPDVIKKLFFEMPRLQAIDFCGCSSPSFKNSFTSLLQAQWPDSLTLTRISFHKCLNLPGSVFEA
ILPRLERATHLDLAGTRVTDKALQSLPETARLTHLNLAKCRELTSEVVVKFVTTHPAIIQTITVLSLATNASSHLLLGKADVD
AILPRLPPTLRSLSLKGSRMDPSQIPLLTPLVQHLEELAIGRGLDLRDIHQLLYQAQEWIPHSLRYLDISDLDTIIGSASALL
TPASAPLQVIELEERAYERAAKAKKNLERAGWTPKEFGSRYWLVRLNTDGASLDSGARWWKLGAESWGMRKIPVAEADVGGMY
GTFMFGRRL
FUN_000437 CDS scaffold_10 164538 167329 + VPH1
H(+)-transporting V0 sector ATPase subunit a PF01496

MAPKPDTPFRSADMSMVQLYVSNEIGREVVTALGELGLCQFRDLNEDVSAFQRTFTQEIRRLDNVERQLRYFYAQMDKIGIPL
RKLDLDVERLASPSTSEIDELAERSQKLEQRVSALNESYETLKKREGDLTEWRWVLREAGSFFDRAHGNVEEIRASTDNDDAP
LLSDIEQNQGGPDAERSFSGMNIGFVAGVIARDRVASFERILWRTLRGNLYMNQSEIPEPLIDPTNNEAINKNVFVIFAHGKE
ILNKIRKISESMGADVYNVDENSDLRRDQIHEVNNRLEDVQNVLQNTQATLQAELNQISQSLSAWMVLVAKEKAVYNALNNFS
YDSARRTLIAEAWVPTNDLPLIRTTLQDVTNRAGLSVPSIINKIQTNKTPPTYLKTNKITEGFQTIVNAYGTATYQEVNPAIP
VFVTFPFLFAVMFGDFGHAIIMLSAALAMIYWEKSLKKVSFELFAMIFYGRYIALVMAVFSVFTGLIYNDVFSMSMTLFESAW
EFKKPENYTNTTSIVATLREDGHRYPFGLDYAWHGSENDLLFSNSLKMKMSILLGWAHMTYSLCFSYINARHFKKPIDIWGNF
IPGMIFFQSIFGYLVICIVYKWSVDWLGTGRQPPGLLNMLIYMFLQPGTIPEGEELYAGQSVVQVILLLLAFVQVPILLFLKP
FYLRWENSRARAKGYRSIGETSRVSALDGDDEDANGHGNSFDEDGEGVAMISQNISEEHEEFEFSEVMIHQVIHTIEFCLNCV
SHTASYLRLWALSLAHQQLSIVLWSMTLGPALKMSGVVGVIMIVVCFTMWFFLTIAILVCMEGTSAMLHSLRLAWVESFSKFA
EFAGWPFAPFSFNTLLEESEELKDYLG
FUN_000438 CDS scaffold_10 167566 170515 - YSH1
endoribonuclease ysh1 EOG09260RRC
PF00753;PF07521;PF10996;PF11718;PF13483;PF16661

MASKRKASAMNAAAAEEPVDPSDELMFLCLGGGNEVGRSCHIIQYKGKTVMLDAGQHPAYDGLAALPFYDDFDLSTVDVLLIS
HFHIDHAASLPYVLAKTNFRGRVFMTHPTKAIYKWLIQDSVRVGNTSSNPTTQPVYTEQDHLNTFPQIEAIDYHTTHTISSIR
ITPYPAGHVLGAAMFLIEIAGLNIFFTGDYSREQDRHLVSAEVPKGVKIDVLITESTYGIASHVPRLEREQALMKSITSILNR
GGRVLMPVFALGRAQELLLILDEYWGKHADFQKYPIYYASNLARKCMLIYQTYVGAMNDNIKRLFRERMAEAEASGDGAGKGG
PWDFKYIRSLKNLDRFDDVGGCVMLASPGMLQNGVSRELLERWAPSEKNGVIITGYSVEGTMAKQIMQEPDQIQAVMSRSMAG
ARRMPGGDGEKVLIPRRCSVQEYSFAAHVDGVENREFIEEVAAPVVILVHGEQHNMMRLKSKLLSLNANKTAKVKVYSPRNCE
ELRIPFKADKTAKVVGKLASIQPPQSIHPDQSAAPPLVTGVLVQNDFKLSLMAPEDLREYAGLNTTTITCKQRLTLSAAGVDL
VKWALEGTFGNIEELPEMRRGKNGQSNGHGDHMITDGEKMKQEDADEEVASLVAAYLVMGCVSVRYRTNGEVELEWEGNMLND
GIADSVMAVLFSVESSPAAVKRSSAKHSHSHELPDVNPHHSASPEERLERLLWFLEAQFGQDNVAPVTIPKLPAVEDTEEKIK
KDEADDAMKTEEDGEDTQLQERQQKEIDRLHKIGIPVPGVSIKVDRMNATVWLEDLEVESNNKVFADRVRAVVERAVEVTAPL
WG
FUN_000439 CDS scaffold_10 171210 173178 +
hypothetical protein PF05236 ENOG41

MAQPQPQTQVQVPPRAFSPPQHSPSPAASQSSFALPPQKRARTDGPSSQPESPYATSPYAASPGATATPPAGAGSPAFAQSPA
LPQPYATPYTNGHTTPGLNLPDVRPNSTPPIQQTQQLQQPIPQYTNATMTPVPVPTPGQLMTPTPGPGVMGPPQRPAERPTKD
YEYDVTDSLAGTGIDLRAEEQYMNDLYATGFDEARTGFAHQPPGPKSSFYGAGPANQPAQSVPDQPQDQFAAQQAERAWSESS
MRLALQRTQEISDPFLLVALLHRRADKIAREHHLALNLDLKNNSQTMGKMRLPEQFPAPKVTVKVTPGPDSTMVHTTGSYIPH
DAFLVDQLALMSIATKQRLRELVEDAHVVATNRQKTSHGDIPEEWTPAAAPMNAEPLEPIEKPADAVNGVDGTHVESANGDST
NPLKRSSDAAGLTNGAPPVKLPKVSSYMTTTMRDLARQERDWEEARLRKRQKRKDGIPDSGATTSRAGSVAPGTPGAVAPEAP
KAMTKKEMKKNQQMKAAEIDSHQSQNTTSSLFAGFGGKSGLFGKKKAGKTYDWMTRGSGTSTPTRNAPGAGKGPGGLGAAPAP
ANMAMTTEGRNRLGTWREDKEKGRNIQLRDWVSVLERDGREGKALQKAYLYLDASNPK

FUN_000440 CDS scaffold_10 174174 175506 +
hypothetical protein PF00795

MAIAAIGQICSTASIKGNLEQCVKLVASAARGQAKVLFLPEASDYIAPDGQTSLQLAEPQSTSPFVKGLQEAAREHNVAIHVG
IHHRDEGQDQSKRILNRALYITANGDINDATTYDKLHVFDYGGLKESATVQPGTSLTAPFDSPIGRIGSLICFDLRFPEAGLA
LAQPGPHSAWKNKPAQIITYPSAFTLRTGAAHWETLLRARAIETQSYVIAAAQVGRHNEKRASWGQSIVADPWGNVVLKLKGV
KEGEPQGTAEDGAEGGIGFVDIDLAHLEKVRREMPLQRRIDVYPEL
FUN_000441 CDS scaffold_10 183753 184059 -
hypothetical protein

MEDPGHVMEEEADSTTSFLTKEVPRDLARLYYTVQQHGPEYRFDAELDAGRISSQLPPPWIVLTAQGIGASFGSTTGRRETID
SLKGLPVHFSVRVATCLA
FUN_000442 CDS scaffold_10 188948 189687 +
hypothetical protein EOG09264V51 PF04699

MSIIQQHTSATLGDAWRTINIDALNEDSSVNFDTSTLHPPQPEIDEAEVRQLSGQIRQLLRGGDAEGALRGSLETPVYNGTDA
AKDAHLHTIIEVLQSIKASDMSPLLKSIYGSEGGPECLDVLMKYIYKGMATVHPGTASRSPNKVTPQSTGGFSQIAGRPGSNE
PATAAMSVLLSWHEKVVEVAGLGCIGRTMTDWRKV
FUN_000443 CDS scaffold_10 189828 190954 -
hypothetical protein PF08241;PF08242;PF13489;PF13649

MSSCTVSRLAMRCSLRTLSQPTNLYHHISNQRFYAVQAAGAPRFQVFNRHTKWLQKERAGANSEQSREADYLKDEVAIRVSER
LLDIKRRFPRVLDLGANSCNIARALVQENPDPDPNTPESPPLATRIDELVAADSSHSLLHRDADHEFNNKLNITRTVVDDEET
IPFEPASFDLVMSSLSLHWINDLPGILTQINNVLKPDSPFIGAMLGGDTLFELRTSLQLAETERRGGMSPRVSPLADVKDIGG
LLQKAGFKMLTVDIDDIIVDYPDTFALMQDLQAMGEGNAVLGREMGAISRDVLLAGDAIYKELHGNPDGSIPATFRIIYMIGW
REGENQPQPLARGSGDINLKDVLEQK
FUN_000444 CDS scaffold_10 191776 194892 +
hypothetical protein PF01602;PF02296;PF02883

MSSSGTGFLGRSSSSNANMRGLVQFIADLRNARARELEEKRINKELANIRQKFKDGSLSGYHKKKYVCKLLYIYILGWNVDFG
HLEAVNLISANKYSEKQIGYLAMTLFLHEKHELLHLVVNSIRKDLLDHNELFNCLALHAIANVGSREMGEALNGEVHRLLISP
TSKSFVKKKAALTLLRLYRKHPDIVSPQWAERIIHLMDDSDLGVALSVTSLVMTLAQDNLEQYKGAYAKAAARLKRILIDGEY
TTDYLYYKVPCPWLQIKLLRLLQYFPPAEDTHVREMIRESLQKILNLAMETNKNVQQNNAQNAVLFEAINLIIHLDTEHGLMK
QISTRLGKFIQSRETNVRYLGLEAMTHLAARAETLDPIKQHQEIILGSLKDRDISVRRKGLDLLYSMCDATNAQVIVGELLHY
LQNADFAIREEMVLKIAILTEKYATDVQWYVDISLRLIAMAGDHVSDEVWQRVIQIVTNNEELQVYAAQNALQYVKGDHCHET
LVKIGAYILGEFGHLVADQPRCSPIEQFMALQGKLTACSSSTRAMILSCFVKYVNLFPEIKPQLVHVFEFHSHNLDSELQQRA
CEYLTLVNMPTDDLLRTVCDEMPPFPERQSALLSRLHQKHANTSDRRTWVVGGKDANADARELSMGTGALKRTFSSNVPLNGK
PNGQVATGTNGHSNGVADLAGLDLNAPTPSEPKMLKAPNLASAAHLSPGWEQGFNKLLVRSDGVLYEDGQLQIGVRSEYRGQM
ACLITYFKNKTPATISSFTTTLDLDENEKENLTWDVKNLPDSTIVQGGQSQQVVMFEAKKIFEKSPTVRISYLAGALQALTLK
LPVSIHKFMDPAELTAEDFFKRWKQIGGAPREAQAIFGVSGKGDREITESFVTKTVEGFRWRVLDMVDPNPKNVVGASVLHTS
QGGKFGCLMRLEPNYGQQMIRLTIRATDDSVPAVLLKYMQERLAAGISTGPDRFEPPSREQISDSFRNVMVT
FUN_000445 CDS scaffold_10 195284 196204 -
hypothetical protein EOG09263BE5 PF01168

MSDSQDEMRIDPSRAQALVSQLSSVKERIAAVANGRKVRLVAVSKLKPVNDILALHQAPTSHTHFGENYSQELTQKAALLPKT
IQWHFIGGLQSSHCKSLGKIPNLFCVSSIDTSKKAQLLNTARTNLLSSQPDLDKIGVHVQVNTSGEEAKSGCAPGEETVALCR
EVIETCPSLRFLGLMTIGAIARSKATTAETENEDFVTLKEQLDLVAKELNLDKDSLELSMGMSEDFEGAIRLGSSEVRVGSTI
FGQRPAKADAKIKE
FUN_000446 CDS scaffold_10 196448 197573 +
hypothetical protein ENOG41

MATDLKTGRRRATSSAATRPGHTRRKSRAVIQPKSTHTSDEFLQDFLDPTFDPATFLNSTLPPLQQRSIPGRTGDVAPLAELS
TQAQTLISQLNAQTSRLSTTLTHLTDDILRSGSRLAYEVELLRGETLSLQETMNETLHDDIKKFVPEGLQEAIEAKNAAVAAA
KEDRSAAPSTTAAAVTNDGANPDDPEFIRQLQTLTLVRARLDSVIKTFGDAMEFVFPPSEISVSSSFLSVSAPEPGSAQQSSE
EKGKEVLKNIRNEISDLLNKSDDPIQGIEKAAQRIEQLKELNRVWKDTAEEKGRNKFIEGLAKTVEDKHRDLMKEMEQTKEKT
KVESRSRKGSVTRDAAATAVEDTKSPGGFGLISQLHKLRGGL
FUN_000447 CDS scaffold_10 198235 199922 +
hypothetical protein PF08598 ENOG41

MAANEAAALTNMANRSDRRSPAPASQSKRDRKRQALIERLSSMTDKFHRERDMTYRDQLQKIQFDINLVQRFDPYDPKVLEVI
SELQKEHTNTQGPPVNAEDARSLLDMAGIRFSDFVDEVEDLVEIRDFQLAQSKNEYDRRREEYRNTYDYKVETAKREHRALTN
TLRDRLVNTLTHKKNRLNREKEVLEINDSNALLLNPNQFSLTNPASPGGPHGKRATRLRKDADDLQMYSDNKKRKRNAGDDDG
SPAPTRRALDNHNTTPLWQSEKARAAAKQNGPIYSIDKLFTDKELSLNYNTAALAAHQYILRNRVSGGGSPEDSDSGNGEANG
DQDADSQPSAPAMERSVSHATRSTRGGNLLDDKILGLEGITNFEVHNLDILHAHEPPKMPPPVPQQYLKPYPRTADQNFPVPL
SNDDITSDLTIMGFFKQYDAAHKPGAHLDRPTGMRRVLAAVSIPYQHSRYVAFTSAPREDPEYVRDSLGLPALSSLRDQPSPA
HAGAATSVAALSSAAVPMSRQSSAGGVAMSRQGSSSTRGKGRKN
FUN_000448 CDS scaffold_10 200301 201150 -
hypothetical protein ENOG41

MAISSTIPRGDVTATLNFFNPPSDNSTPYNHVDTPPKGLPQRNFTDNPQPTVIHDIRGHESKYHLDRDAFQVIQDVPPSSEVD
FVDDDSIKENYYPELEQLLLNNVPGSNRVFFFDHTIRRQTPNATRAPVTRVHIDQTAAAVAQRVRRYFPGEADELLKRRYRLI
NVWRPLNKKPLESFPLAVASSASVEDDDVIPVEHRYPDGYRGWTAAIRHDPEQKWYYLSGMTGSERLLLECFDSESLKPGSNI
KGRVPHTAFEDPRTRAEAEGRESIEVRALVFGP
FUN_000449 CDS scaffold_10 202161 204187 +
hypothetical protein PF09441 ENOG41

MPSPASVNNATGPSPSPPAPSPAQTQPPPQPGASGAGSAAPPTTRPGLPDRSVSENTVEDAYVDFILFCNPAVPLSTDTTSLR
EAFRNPPRSGGKSFSTFAIYELVRKFYNSEIRTWTELTTKLGVEPPDPNKEESAQKVAQYGVRLKKWMNSMHVKAFFEYLMDI
PNDYWTRIPTDSNPTSQPIRDGVAIEDDMALRALFPHIRPKRGRKRPVDDDTATSPAQRSHLAPSSAVEAVSGPMSMSLSADP
TRLSAAPWTPSDASQQTPLFRWPQSAITPNSRKSFWDDALEPQSAVTPSKPRLTTQRRGPKNVSSAWRPGVPNGGVKPRGRPP
MNRTPIDASLPPFSTTATTTAPEEPVAPICTPPVSAPGADSGQSENSFSIPSITSQPHPTPAPARPSRPSISLQVPERPSGSV
RLATPPPVVVINGPSETQAPPPHTYVGTSSNFARATNEVTTSRQQQAEDTMSQQKDVPRLYFERLEERTNVDEVIGYMMRSLF
GANWLDAEGNPTHTCSTTEAMAIVNSVLEDLYRKAASPDMFLINLAAMTGGGYLLAGTTKLKRVGVQDGAMKYTCEWIYGLGH
LRGQYQMEIAVPVEMLEDSKEPSPEATSAESASKLSADEWQAKYEKLLEELDKKDKTFMDLRMKVTDILRGVPKK
FUN_000450 CDS scaffold_10 206784 208925 +
hypothetical protein PF00083;PF07690 ENOG41

MAPNRGEDDIERLSRATNEYPSSSMIEHARRPSEAYVNLHANIEAKIKNPLWGLPRARLLADVDDFCRKKELEQYRPLIRKGA
LVAQDPTGYEDIDGDEKLDDTEIQALRDEILHKWRVPFVLYLTVATCSIGAAVQGWDQTGSNGANLEFPYAFNIGSESIHDKL
LVGLVNSAPYIGTALFGCWLSDPLNSRWGRRGTIFFSANFCLWPVIGSAFCNSWRQLFACRVLLGIGMGTKASTVPIFAAENS
PAPIRGAFVMSWQMWTAFGIFLGTCANVAVTKIGHNAWRFQLGSAFIPAVPLMCLIYFCPESPRWYMKKNRYQDAMKSLLRLR
NHPIQAARDLYYIHAQLEVELDFIGEAHYAKRFIELFTIPRVRRATVASFVVMIAQQMCGINIIAFYSTSVFRDAGADDNQAL
LASMGFGLVNFVFAWPAIWTIDTFGRRSLLLFTFPQMAWTLLAAGLCTLIPGTGGAHMALVAFFVYLFAAFYSPGEGPVPFTY
SAEVFPLSHREVGMSWAVATCLFWAAVLSITFPLMLARLHAIGAFGMYAGFNIVALVMIFLLLPETKQRTLEELDYIFAVPTR
VFMRYQVTKVLPWWIRRWILFQREARLEPLYQFDTVGEPEDNHSSDFGYENDKAKVELKDTIGLTSGVQTTR
FUN_000451 CDS scaffold_10 213073 215737 +
hypothetical protein PF00924

MGDGIQLTPVKSNASARSGMRKADMPQPSTMDSNSPTEDEKLTGQGHRGRRRRMGDGLSRSGTGGEETTLNPMGRLYYKIIGF
SVVTRYLVYVVPVGLLLAIPLIVLAAIGKKDGIPVGKWNGDGDRAGDDGPPLFKIFLWVLIMWLSCWVAKVVAWFLPSLFMFF

TGVVSKGTRKYATVLGNLILPFSFFFWALASYVTFKNLWMEEDRGNNYVPWVRTMGRVLGALFVSSAVFLGEKAIVQLIGISY
HQRSFANRIRESKREVHLLGLLFDASRTLFPLHCHEFADEDAIINDSIEVMLRGKKGHKRNGSATPMKLIGEVGKIGDKVTSV
FGNLASEIAGRQVFNPNSAHSIVIEALEKTKSSEAMGRRIWMSYVVEGHNSLVLDDFQEVLGPAYKEEAEESFYMIDGDDNGD
ISLDEMVRKVVEIGTERKAIAEGMKDIGQALQAFDKILLVVVLLVVIFVFLAFFQSSFITTLTTAGTTLLSLSFIFAVTAQEF
LGSCIFLFVKHPYDVGDRVDISGTRMVVNKISLLYSVFHRLDTMQTVQVPNIQLNNIWIENISRSKAMHETVEVNVSFDTSFE
DIELLRLEMEKFVRQPENARDFQPDLSISVGGVGNLDKLLLYVTIAHKSNWHNDSVRASRRSKFMCALALALKKVPLIAPGGG
GEPLGGPTNPSYSVTVTDEFAKNSRDEAAKATDEARMVPTSKQENTEESQAQAAEGFNSRPPAAGLGVWDNSDNHTLGSHDDE
VNRNRDIEHLRTDLKRESTRGRRKAGEAIPSLSLETAPRASITLTQASPRSPHRGPFDEEAEAGMGPTPYHSNLSAGPSQGSQ
GYQPYAGSSNQYNVANPLQRPGQGPPQGPPPQGQPPAPQR
FUN_000452 CDS scaffold_10 216079 217205 -
hypothetical protein PF01738

MLIKESHVDVPTSANGKEGTMRIFVFHPIIPGYPNARFPGVSLFSEIYQVTGPVARFARQIAGQGYIVAAPSSYHDFTGPEPL
KYDAEDTDRGNKFKIEKTLESYDADSYATVDYLLSLPTCTGRIGATGMCLGGHLAVRAALDPRISACVAYFATDIHSRTLGPY
DAPNTSSTAPPNSNHTIDLFNKFKGEVAMIFGVKDTHVPDAGRDLIRVKLREAGVPTSFYEFAWAQHAFIRDELSKGRYDPAI
TKVCFEVLLELFGRVLKTELGAKDGEVPPPEHVC
FUN_000453 CDS scaffold_10 218084 220866 + SEY1
Dynamin-like GTPase that mediates homotypic ER fusion EOG092610TN PF05879
ENOG41
MNGHFSAVGEAPGAGSYEHGVQVIDEDKEFNTHLNEYLNVTDVAASGFNYHLISVFGSQSTGKSTLLNNLFGTDFSVMSETER
RQTTKGIWMSKNKRETSAGTKMSDNILVMDVEGTDGRERGEDQDFERKSALFALATSEVLIVNIWEHQVGLYQGANMGLLKTV
FEVNLQLFLKDKQSTPRSLLFFVIRDHLGTTPLGNLRTTLIQDLTKIWSSISKPQGLEGSRIEDYFDFGFAALPHKILQADKF
TEEVQKLGSRFTAGHRHGKPGLHGDQELEGGLFLPEYHRRIPADGFSVYAEGIWDQIVNNKDLDLPTQQELLAQFRCDEISRE
VLIDFDVVVTPLEEKQSEASKLGVPTVLPDLGLAGNDARQKCIKAFEVQASRYHKGVYARKRHELEGKIDTRLKALYQGQLAA
AHKAGVTAFGEAVANKVKAGQKAGGSYEFAEIVANEKRKTLDIFGVEAQSLLIEGVSWTNFESQLKLFEKELDEESAKLRKEE
MRRLATRVERWVRSRLGDAVGLEFNKLGSGRAGGGAPETGEKPDSEKDLWDRVWKVFTGIVKEAEVRFAERAKSFDATQEEVE
IGTWRLRRKSWNALREKIEEEVMEGNILLKLRENFEDKFRYDEAGVPRIWRPSDDIEGIYTKARESTLTLVPLLSRFRLSETY
GPPDLPGFVGPQPNGVQASDEEDLTPIGGIDEEDGKSLEEEMTVLSEGKRQDLVVRFKKTADGVYVEAKRGAIGGVAQVPWYF
YGLLLALGWNEILTVLRNPFLCLLILVIAGGTYVAYTLNLLGPMLSMGNAAMTQGVEIAKQQLRDFIANSDTARQAVGMPARE
DSDNISMDTLDSRGKKANTTSTEKDDIDNI
FUN_000454 CDS scaffold_10 221043 222063 -
hypothetical protein EOG09263OWL PF01351

MTDSPAEDVPETATRSSTTYIPPSVHGEALLSGSSFNHFSAVPPSLLPSDPSDPSSATPCCLGVDEAGRGPVLGPMVYGVFFL
PIDLSDSLLREKHHFDDSKVLTPAVRSDLMRTLCTPGSDLHDSCGWATASLSARDIGANMYRPTNAYNLNAQAMDATVDLIKA
VYALGVNIQEIYVDTIGQPAAYQAKLQRVFPTAKITVAKKADSLYACVSAASVCAKVTRDAALEVLFEARADEDEKDGEGMAW
GSGYPSDGRCVGWMKGNMHPVFGWGPECRFSWGTAKDMLEAKGNGVKVEWPLEDDGETGRLTDYFTSGDKDKESSDLGNWFGT
STGLEAF
FUN_000455 CDS scaffold_10 222960 225109 +
hypothetical protein PF11699;PF15624

MAPRGARRSEVAEPQAIYKLGERGRKTGVELIDTGNRDEYGMQPLDDLLSSPEKETGSDQRYDDEDEGPNNDQYESGDDEDED
GSEDMDIETIPGPGPQTVLRGKMQYPIPRSRSPVKTTLMSSPRRNPNLEHLSSPTRAPYSNDRDATVTRKIDFGAKPSLRGKA
TANGVNGGTHDEEDADETEDAPEASEEGDVADDMDLLDKSLELVDDLTRHSSEHESEQESEPEPEPEPVKTNKASAKDKSQRAEP
EPRKPGKRGRPAKAKPVEEEPVEEEPVEEEPQEIQEPGKGRRGRKPKTAGQPKKSAAPESRKRPAPEEPEEAEEHEEPDNEAS
DPEPEPEESPKHAKRPRTEASKPSKPAKSAPAAESKPRGRPGRKPKAQTAGDVGDTSIAAIQHRPPMPKKRGLVSVRHNPDEV
KTTRSGRHSFRPLNWWAGDKVVQEEEEFKDVSGRDRFVLSTIKEIIRAPQEEPPAKPRTKARGRAKSKPVREVEVVEPEEWEL
NPGVVNAEVILWEPEHETNPPGDEEPVEVMEDRVAISGEAIQTRVVQNVSFRMAKTLTTPFMGAGVVDLPAGSEKRPKNSRKM
HMVFFVHTGKVLVTVNEASFRLSAGGMWFVPRGNYYSITNDYDTDSRIFFTQGCEISAQPAEPDQSQMSMMA
FUN_000456 CDS scaffold_10 225584 228314 -

hypothetical protein   EOG092613R2   PF00076;PF10443   ENOG41

MIPSRGLATAALFAARRPLISSRSLGCGFAAASLRQRLGQNAINRPVSRVWESTVVNPEDAVPPIAPEEGADKSGHITTQSNE
AILFFDNIFPLKLSNVLMRPWKSDNDVSDLLRRFESSSLGILDPIRLVKTAIPEDLPIKVTEILPRLKDGGAYVKFMHDSNID
PAEIEAQLASRLKEKPIKPWFSIFRPVNARLVQGTPWLEDLYRYPTSFIKIEFVPPTPGTTPEELPEETLYSLFRKYGKIADI
IPQPTDSKVTPRYANVMFPVTRDAIMARNCMHGFIVNEVLGGGKNGTKLRLSFEKRVKAHSIWNWLTNHPRIVIPVFAALVAG
LSVLIFDPIREFFIKLHIQHSLEFKDSRIYKWFKKQSGNFGLSTKPKGGDGLEEVWNHRRDAIDKVQGWLDETSDTFIVVTGP
KGSGKVEMVMDQALEGRKNVLKIDCRRIVEARGEAGTIKRLATAVGYRPVFSWANSMSSMIDLAVQSTTGVKAGFSETLESQL
TKIMQTTTGALKDVAVSSRNKKDSDYKLSEDAWLEAHPERRPVVVIDNFLHKGEENSIIYDKIAEWAATIVQNNVAHVIFLTT
DSAYSKPLDKALPDRLFRTVSLGDLAPQVAKNFVLSRLKDQLAADEKARKEQGDEDTEKSPIPRPNMFELDQCIDTLGGRLTD
LEFLARRLRTGQSPKQAMEEIVSETATDIVRIYLLGKSSEIEDKKFSSQQAWHLIKSLAQTPNLRYNEVTLSAPFSSAAPAAA
SNADAAIDSLVSAELIAVKTHQGRPMTITASKPLHQAAFSVLLQDRVLRAKMDYDVLNDSSKAEARSIEKVENELALLGSLPR
QTAETAGRINFLLRKLEDSQAKITKWDKEMTTLKKLLSEEF
FUN_000457   CDS   scaffold_10   229388 230039 +
hypothetical protein   EOG092656IY   PF11326   ENOG41

MGWLWSSTPSSEDKSAVPVTETPPVTSTTTTTTSDPSTSAPSNSNTDPEIQKFIDLFEAEKNAKPSTTEDQQQQTSSSSKIPS
WLSLKKSTRSSAATPADIPPRDALSEALLPTDMSCRQAFDQAWACNSMGGQFNAVYRYGEMRSCSEHWDDFWFCMRTKSYSPE
MREKAIRDYYRAKEFTKYGPGKPSSEDVWESREDKVPEGTVFNEPVEPRN
FUN_000458   CDS   scaffold_10   230675 232898 -   HSPS1   Heat shock
70 kDa protein   PF00012;PF06723

MAPAVGIDLGTTYSCVGIFREDRCDIIANDQGNRTTPSFVGFTDTERLIGDAAKNQVAMNPQNTVFDAKRLIGRKFADPEVQA
DMKHFPFKIVDKGGKPNIEVEFKGETKTFTPEEISAMILTKMRETAESYLGETVNNAVVTVPAYFNDSQRQATKDAGLIAGLN
VLRIINEPTAAAIAYGLDKKVEGERNVLIFDLGGGTFDVSLLTIEEGIFEVKSTAGDTHLGGEDFDNRLVNHFVNEFKRKHKK
DLSTNVRALRRLRTACERAKRTLSSSAQTSIEIDSLFEGIDFYTSITRARFEELCQDLFRSTIQPVDRVLTDAKIDKSLVHEI
VLVGGSTRIPRVQKLITDYFNGKEPNKSINPDEAVAYGAAVQAAILSGDTSSKATNEILLLDVAPLSLGIETAGGMMTKLIPR
NTTIPTKKSEVFSTFSDNQPGVLIQVYEGERQRTKDNNLMGKFELTGIPPAPRGVPQIEVTFDLDANGIMNVSAVEKGTGKSN
KIVITNDKGRLSKEEIERMLNDAEKYKEEDEAEGKRVAAKNGLESYAYSLRNTLSDPKVEEKIEASDKETLTAEIDKVVQWLD
DNQQATREEYEEHQKELEGKANPIMMKFYGAGGEGAPGGMPGGPGGFPGAGGPGGAPGAGGDDGPTVEEVD
FUN_000459   CDS   scaffold_10   235557 236373 -
hypothetical protein   ENOG41

MSDIDTEEEQSPNPTFWLEFRTFDDGLSSQVNSSSTLTAFPQFAQLPPELRLRIWSCLVQPRIIVVCCLQRDERLDQRLYELN
QRSHGRSVPVLLHINREARYLALQHYELAFGWRISKLLSDTPVSEPPRVWFNFALDAVYLAGELEAYDIYGFNSAMVYFLRRE
DTHRVKHLACVLSELGYAEQESDQVFGCLWHVVDGFPAAKKVLLVVGEKDEEALKGSKLLSTDNVMQKIWNGFLGGSATETSS
RMADKSILMIEEHDLPGFIARQ
FUN_000460   CDS   scaffold_10   237110 239341 -
hypothetical protein   ENOG41

MISLARKAIKANQQRKQAQTNQAPMELQETGSDATKQAPQQPQCRHMEAYESSSGKCVACTSEKKAARIYRWKIILGLVAPFA
LQALDSTIIASALPWIASDFGEISQLNWIVSAFNLTSAAFIPFWAQMADVFGRNASINAAIILMLIGSALCTGAPTNAFPVLL
LGRGFQGLAAAGLNVVVRTILADRVSLQENAKNWAIFSLVGGVSYGLGPVIGGYLTNADWRWCFAINLPIAAVALIIVFFVLR
KELLGPQPIPELNETAETGRRTKFVARLKTVDVGGQLLFILGFGLVILALTWGGATYSWSSPAVIVPLVLGIIFAGVFCYWEY
LLSPGNTMAEKLPWQRAMLPWELISNRDIGLLFYCECATGIGMYAVLYFCNIYFIAVRGYDSDKAGVQLLYFVPGLGAGVYIC
SFMCNKWPRMTFPCIFLGTLTEAVGLGVLAWAIYANRLSVIYGMMALVGCGSGMRFMASPLHGIGLFRHLRASVIGLMAVAIP
FGGTIGLTIMSTVFNNTSGLDSHSDFSSVHSSSGGAANEQAVHQAKMGVVWAFVAITPPVALAFPITWFIGNVKLGQGPPGED
GPTDIVVKGSYLLKLVRGQEKLKIEKNAYRLNSSHSGTWSEAVASDQSVNASRPLQGSPQV
FUN_000461   CDS   scaffold_10   242583 245273 +
hypothetical protein   PF00755

MTQVRTFTPPQPLGDRLSDPTSAPKPAGSTVPRTLPDIPVLAQPRRNSPPRLSGDYTRVPMANYNESRKDGGITFSGQDKLPK
LPIPELEATCKRYLEALKPLQTAREHAETQHAVNEFLKSDGPELQEKLKAYAQGKTSYIEQFWYDSYLNFDNPVVLNLNPFFL
LEDDPTPARNDQVTRAASLVVSSLEFVRAVRKEELAPDKVKGTPLCMYQFSRLFGTARVPTEEGCQIEQDPESKHIVVMCHGQ
FYWFDVLDDNSDVIMSEKDISINLQTIVDDATQVPIQDAAKGAVGVLSTENRKVWSGLRDVLTSEPGSNNADSLGIVDTALFV
LCLDYTEPADAAALCQNMLCGTSEIEKGVQIGTCTNRWYDKLQIIVCKNGSAGINFEHTGVDGHTVLRFASDVYTDTILRFAR
TINGMAPSLWASSSPDPSKRDPESFGDVSVTPRKLEWDMIPELSVAVRFAETRLADLIEQNEFQTLDFGHYGKNFITSMGFSP
DAFVQMAFQAAYYGLYGRVECTYEPAMTKTFLHGRTEAIRTVSSEVVEFVQAFWADNPVDAKIEALRKACQKHTANTRQCLKA
EGCDRHLYALFSVWQRSLDDDLDSGFNSNGYSSPGDGYSDQAGSPVGSPENESLPSVDGVEVRPTRDRGYSVNSRSRDQNPLP
LLFADSGWDKLNNTILSTSNCGNPCLRQFGFGPTSGDGFGIGYIIKDEGLAICVASKHRQTKRFVDTLESYLLEVRRILRIDN
RKMSTGKVSRAREVEIERPKLVSRIKSRGRPITAVESLRSATGTTSPTNESSTFSEDDEMGGYGFFDAGMLLQALKARNEQFE
STDTRASDRAAAAQARRRDIGKKLRLSDY
FUN_000462 CDS scaffold_10 246366 250785 -
hypothetical protein PF00806 ENOG41

MDELRFRSQQSPRGNDQSMHSFVSPTRNASRMPAPQVNPHAGAHDHRSGLPRRFTTDSGRVPTLSSLGAQRVPESSQDYNAMH
KVQLIEKKKMEYERIREQRRRFELEMQKLDQQQRREALELAQMEEEVGRMGGHQSEPTTPPEYRDNSGFPTFLSRPSRYSMSS
LTSPPGLFNRPVRSGSQLASPQSGIRQARFGFEDTTTQMPSRSVPTTRRNSDDEKEEAVRQDPSSHRSGNAINRYSMPVTRSR
TGMYDVGLDQTNTTRFLFGDEETNAGGHSGPDANFPTLVRHDDQILSASSAALDLALSPSPNPESTSSSRGWSRVVNRHRPQQ
SLSSINGTSNIVSDLVGLASRPTSLRHSIDLKYISENAVETGAIMSPSATPNMATPPKLQSSFSSSDVPTVKSPGTSSSKTNN
HAQQHFHNHNASLGRIPAGAVHRGHSRELSSENPAVSREQHNYPSIQSALQASAAPFGPSTTAAVPPSAMVNPPAGAAAANNN
YNNVFYSANGYGAPQGVPQGGIPQVMSQGAPHQPGGYNVSMLANSMQQMNINGANGSNMYQPQNYNGYNAGPYNQGNQPRDSQ
ARVIQHRRQLDNEAMSRYQNTPLDSFVGNIYELCKDQHGCRYLQKKLEERNADQVHIIWVETNQHVIELMTDPFGNYLCQKLL
EFCNDDERTVLIQNASQDMVRIALNQHGTRALQKMIEYVSTPQQVHIIIEALRYRVVELIQDLNGNHVIQKCLNKLTPPDAQF
IFDAVGGSCVEVGTHRHGCCVLQRCIDHASGDQKLWLIQRITEHARILVQDPFGNYVVQYIIDLNEPLFTEPIVLTFKDCITQ
LSRHKFSSNVIEKCLRCAQPPSKDLIVEELLRNQEMERLLRDSFANYVIQTALEYATPHQKHRLVEAIRPILPQIRTTPYGRR
IQAKISAFDNRGSAASSGQVTPADNTQGQIPMRATHARGIPGSGPILQGNGMPHSGPMPPMRQNMPVYSPNPAMNGQVPTAGG
PVQQPQFGQMTPGNFAPNTAANGAANGSAGNSNLAGQVSGNSGPTSPTNGNGNVSTGQGEQPQWV
FUN_000463 CDS scaffold_10 256492 260130 + RPC2
DNA-directed RNA polymerase III subunit
PF00562;PF04560;PF04561;PF04563;PF04565;PF04566;PF04567

MPTVDDGFEALLEPFYNGKKLTDPISTKEDKFQLLPAFLKVKGLVKQHIDSYNFFVEQEIKDIVRANRTIRSEVDSNFWLEFT
DIRVDSPRRQDWTDNKSHSEVTPMECRLRDMTYAAPIFVDIQYIRDKQRIVRKNVPLGRMPVMLKSSKCRLAGANNTQMEEMN
ECPLDPGGYFIIGGTEKVILIQEQLSKNRIIVEADEKNNIISASVTSSTHERKSKTYVTLKKDRILLTHNVLVEGIPIVIILK
ALGGLSDMEIMQLVAGSDGRYQDEFLVNFDEATKAGVFTQHQALEYIGAKVKMGSRRGTFGPQVRRNHVEEGLDALANLVIAH
VPIEGLDFYPKAIYVAQMTRRVLIAAHNPKLVDDRDFVGNKRLELAGQLLSLLFEDLFKQFTSGVKMSIDKFLKKNNRAVPLD
AVHMISNHANNIGYGINRAIQTGNWTVKRFNMNRAGVTHVLSRLSYIAALGMMTRISSQFEKTRKVSGPRALQPSQWGMLCTS
DTPEGEACGLVKNLALMTHITTNVDEEPVKRWIFTLDAGVEPIRNFSGAEMHREGSYIIHINGTPFALTRYPKRFAQKFRTMR
RRGWISPFVGININTHFNAVHIATDEGRICRPYIIVKNGKQKLKPEHLRLLQMGKATFDDFLNRGIVEYLDVNEENDALITIY
EDQVTQSTTHLEIEPFTVLGAVAGLIPFPHHNQSPRNTYQCAMGKQAIGAIAYNQFNRIDTLLYTLVYPQRPMVISKTIQLIG
YDKLPAGQNATVVVMSYSGYDIEDALVLNKASIDRGFGRCQVFRKYTTELQKYPNGRRERIGDPENEGDGKIKRRIAKHEALD
DDGLAIVGYKVNSGEAMVKKETPLDQTSTGIGLDRGPAEFRDSSVSYRIADPAYIDKVMISQTEKDTTVIKVQTRQTRRPELG
DKFSSRHGQKGVVGIIVEQEDLPFSDSGLSPDIIMNPHGFPSRMTVGKLLECLTGKASIVHGRPDYGFGDAFRSHPLEEMSQV
LVDHGFSWEGKDYFTSGITGEPLEAYVFNGPIYYQRLKHMVQDKMHSRSRGPRAILTRQPTEGRSRDGGLRLGEMERDCLIAY
GASQLLLERLMISSDGTEIDICQQCGLFGYKGYCHTCKSTREVTKMTMPYAAKLLVQELISMNVGVRLQMDDEFPHPR
FUN_000464 CDS scaffold_10 260361 261681 -
hypothetical protein PF08423

MTDLLQILPSFPLRPFAALIPTIEQQALTTTDLLTLHPADIAKQTRLPILDLKRLIAAIQASLSDDLSPQQPLLQPAEEPKVI

STLDEGLDAALGGGVPVGVITEITGESGAGKTQALLSLCLAVQLPPPHGLGREALYISTEAALATSRLAQMLNSNPILQQYGD
PETRPSLDAIHSAVTPDLETQDHILDFQVPVLLSRHKIGLIILDSVAANYRAEFERQGSHGSNMAARSAELVRLGALLRDLAR
RHNLAVVVANQVADRFASSSTPRHAPPRSSGVAYESPLASRSMPPPSSTNLPGTPSSSLPFALQDPDGPPPPPALMLDHQQRW
FTGWGDDPHASYSLKTPSLGLVWSTQIACRIALFKRPVYGRIRQAAPVTAEDDSDLAAPTLRGWRRWMKVVFAPHAPQTGQGL
DGAVEFEVTMGGLKSIKIQKAKKQ
FUN_000465 CDS scaffold_10 262494 263748 - RPL15 60S
ribosomal protein L15 EOG09264L0C PF00827

MVRFGAALADSAAKSARASASLNPQATIFPRKVGYRWFFLFSENLRPPTNHQDLDQAAKMGALKYVEELQKKKQSDVVAFLLR
VRCWELRQLNVIHRASRPSRLDKARRLGYKAKQGYVIYRVRVRRGGRKRPAPKGATYGKPTNQGINQLKYQRSLKATAEERVG
RRCANLRVLNSYWINQDSTYKYYEVILVDPQHKAIRIDPRINWIVNPVHKHRESRGLTATGKKSRGLNKGHRYNKTQAGRRKT
WKRHNTLSLWRYR
FUN_000466 CDS scaffold_10 263993 264844 -
hypothetical protein ENOG41

MPPRARKRKASATESNGTEDAPPRKTTRAPRKSGARGTNQRRDKNFMRATNASSNKRRKVFSAEAKSQIKKHGADFVKFINKE
TNTRIPRISVDILKKDISPDLAGVLPWMSSSSALQRQGTQTYPRMILKALDNLQRDVRTYEDLVKQDNKIRPPDWMRWQQDTK
DLEEMSKHGLAMASKMINHFIMPDLHELPPKPSEGAGGVEHVAWDLIEEVLPKMSDDIWGKIAQGHLKAFTEVLKALSAEDEE
FUN_000467 CDS scaffold_10 265819 268613 -
hypothetical protein PF04468 ENOG41

MSSGPLKTGPSSRLGQGSHPTAPGNNQSLLLEKLHARTSTPDSEALASSDDEGEHRYESATAASHSASQPPRPVRRPSWLNET
SQTLGRPRRGSIASNSMSPTTSHPSTPSGETGGAWGSHSASSVMGRGSGAPGFSWGTAIWNTERKDPPSRLSEVLPSPTSTMP
PGGGNSFFSSEMQTSPVSRDPAPNSQIPFAIPLHPTPKTYRSQSYSVGQLDPESTATPPSMSSSAIMGRARYPALQHRPSRPS
MLSEMANDGSMLGKVKEVEDDDDESPSESMQSSFHQSDAKTIEILARENAMLRQQQQQTQYNNARIRPRAVTGSSYPANGYPI
RDTVPEESDYAIDELDEANESGDMLAKRALSRRMSEYGAGSLRSPFENNRKPENANLKKALWSSSLGFSPSDISQSRRHSFAN
MPTRQGSISSIPDATALEPSALDMQQSQEYPPGYPDPAAFSATSHASQYFGVGGGANIGNAYQTGFANQYGSPYGLSTTYGNR
APSPHRNVYSIAQPRHNQLLHIVLFKCARADVFYIQEGTGLNVKPGDLVIVEADRGTDLGTVAKDNVDWQAAKELKEHYAEEQ
YKWLMMYSQNAAVAQEGSSAGLLASSTGLQGSAVGGMGPPSQHHAQEPNAGELRPKLIKRLAQNHEIQALRDKEGQEAKAKRV
CMQKVKEHALNMEILDAEFQMDWKKLTFYYFADSYINFNSLVTDLFKIYKTRIWMSAINPASFASPTLGIQAPSGIGPGAVNA
NRGAGGNDRRQNQQVQEPHQASAFTGGARDGRGFRPAPTFNQPFGNEGRLAQPSGYPPSNYPYGQFANPYSNPRSNVGYGAGS
GAMPGAMEGYPVGVPQLGEYTSMRQRFPNPQSGPSPAPHSQATSPLAPQNDWVGSFQGLSLNTH
FUN_000468 CDS scaffold_10 277242 280531 -
hypothetical protein PF00400 ENOG41

MADSSLTREFFQGPITSLAFFHDAEYLLAGEDTHLTLYDLQTGHALRVGSIRVFSAQPIHGIRLLSNGRVLVWGAAQIAVVSN
IEGFIGNMTDAQMFVKKAAAPDWIYDVAPSPFDDSSAVLATAHNEVVHLHLNGDDAPVVGSIVSPSRPILYAARLKWLDQDTV
LMAGGTIFGEILVWRCYVSESHSELLAILNGHEGSIFGVDISEELPCQNGTKLRLIASCSDDRTVRIWNITGRDGSHEKHDFA
APRETGFGSKAGLDDGPRAEELRPVATIMGHLSRIWGIKFAQLTEGETLPESPLSLYSFGEDSTSQRWQLNLDISSEKVSGSL
KHIETYAVHDGKHLWAHAVVNKGDQTLIATGGADSKISLITQPASLTPAASVSTHELLGLGFDEIAVTLPNLRTLKSVRGKEI
FSRYDFISDDRMLVTTNWGRLLLGTFRPALEWREIELEDELNVEFQTCYTLQTIGDGAAILGTTSGKLYYFSESQGVKVLGSV
PGKIFNISILSSSVGGLTELIVHIHGTSDSQYFSIDWRTGAVVATADIRGIDKRFVAISAARFREDLIAIGSRHGFLALLRQT
GNEFRPILDFRFNTRDAITGLVPLPTTDGQEAPLYFLATSRDTKYRIYEIEDLGDEVRLHLQHEVAAPSGADIEGGWFTQDES
PELILYGFKSKNFIVWNETRREEIASVDCGGAHRTFTIAHDSINHNRVRFGYTRATKLYIFSQHNPIHRPLRCGTHGREIRGL
SASGRYIATGAEDTTVRIWEYGDERCGTKNSGGLRCVASTKLHISGLQKIHWVGDDYLLSSAGFEEFFVWSVQRLDSQYKGLG
ILCEAILEDKSPAADLRIMDFDACKVGNGSIIITMVLSNSTLKTYRYTPGEGFHLLACMSYTGACLTQVRHLGVDENGLSALT
ASTDGHLTTWEVSFGGEVASHALVQVAPVHQSSIKCLDLQPTPEGFLVLTGGDDNGLGVTTLVSMSDESDKRRYTTSSRGIVR
KAHAAAINGLVLVQRGGETFGITVSNDQRVRVWRVQVNNVKLVADSYSGVADPGDIAVIGESDGEKVNVVLGGVGAEVWSW
FUN_000469 CDS scaffold_10 281122 282142 -
hypothetical protein PF06687 ENOG41

MGVGRFFCVLLPFVLTVGSIIFLLVGALAGVADKSLYIFRIDVKNLSISPADVGNIIDNLDLKDFKLDTRDAPVIEERADKIK
DNITAELLGLDKYYDINLWGFCKTDSDGKRKCEKPEFDWANKKLNTSYLIGADKNVQIELPDEIQDALKAFKTVTKWTQVVYI
AAFIALAAEIILGIFSNCSRIFSCLTWIVAGIASTLVIAAVVLSGVFAGTVVGAVEGTAKFYGVKGNINGRFFACVSIAAAFA
IAAGLFWMFTICCCKPESRSRDKKNRRSDGEKLLGGATNKHGSYAPLGDDHEMQTGYYNHNQSQSQYGAPRYPSGTARSDLAY
EPYSHRA
FUN_000470 CDS scaffold_10 285106 286736 -
hypothetical protein ENOG41

MAAKQAGILDPRLAGSTTLRFVSEPEAAALATIKDLSKRSSIKAGDTIVVCDAGGGTVDLISYIFQSTDPFVVKECVKGDGDL
CGGVFLDEAFIRFVKRKSPKGTWWPVTKTEERKFLNDEWEHGIKPQFENQKRSWSLALPDTCVNPSSQGLMRRMTLDLTSQDL
FSVFSSIVDKIEVLVRHQVDAIQSKYHEKPKYIILVGGFGRSRFLFNHLQERFRRIVLQSRGNKPWTAICRGAVVQGLVRHNP
STNLGVDVETRVARMSYGIMYSSPFIEGYHNEADKTWSHVNQNYRVNRQMAWFLRVGEDISEKRSVSHSYVRYFEVPSFQGHE
KIYCSASRAPPVRYGDSDDIHQLCAMKWDKIVDVDTLPKWTNPVGVAYPRLDYHIKMDCEDGTVNFSLHYQGSKVGEEVVEVQ
FN
FUN_000471 CDS scaffold_10 286856 287292 -
hypothetical protein ENOG41

MTANKPLEDAVIIGIDFGTTYSGVAWACSREPEEIEIVTSWESELNHCSDVEKAPTQIHYGHNSRDIKWGYGISPEHEPLKWF
KLLLLEPKDIPQGAANSTQLKEARRLQSKVGKEPVEIVACFLRKI
FUN_000472 CDS scaffold_10 288048 289417 +
hypothetical protein ENOG41

MHDRTPHAAAAARYPHQSHASGPLVDVSSPEGGPGQHGNTYVQGFGNLADPLAIDGFLRIQEGRVNQFLQVSDRRAEAQRSAL
QQAADRLSNIANNIGAIVNQWITHPSTNQGSDLSHGLQKAYKRLADVESQHLQLKLEHEAIKKQLQEANAKVDEAVKARDELR
RLADGVNWTGSAKVSDQEIRSKWKQLEYNIRSLASVLAKCQTKVPSDKLTRTRLDEVSTAWRKLLVVDDYKNFLIGAYIWTIV
ESVFTTGRKFCSGGHNIDLKAMRAAFLESASEVDNPSRPGPTLRHVARWFAQGTALFGHFFGRDQHAFRREARSEVDRLKLFC
NITADKSGTDFHQEMKAILEAALDLDEMLMSSKAIFLVRWPQDGQSKTLQRFDANQMESLAHTNELSSKTIVRFFVSPMLIKI
GNADGCNYDSEMTLCKATVACE
FUN_000473 CDS scaffold_10 292081 293780 + PZH1
serine/threonine protein phosphatase Pzh1 PF00149;PF16891

MGNNNSTPGGSNSKGSGSAGPDGPLQSYPSFSRSDTKESSRSFRSLGSKIRGSKSDSPRNSQVLSNGDTATETKTEERRSSRH
GRSSSSRLSRSELPPLNTSASDMSAPESALADSAIGDDQPPPSPVQGNTKGGAHDVSAAQASGEVDHVSDQPPSVNAGATAHM
QAPGQSILVKRENTINPVNSPSAESKTDGNSNVAMSEIKDIDLDDFIKRLLDAGYAGKVTKSVCLKNAEIVAICQRAREVLLS
QPALLELDAPVKVVGDVHGQYTDVIRMFEMCGFPPNSNYLFLGDYVDRGKQSLETILLLLCYKLKFPENFFLLRGNHECANVT
RVYGFYDECKRRCNVKIWKTFIDCFNTLPIAAIVAGKIFCVHGGLSPALVHMDDIRNIARPTDVPDYGLLNDLLWSDPADMEQ
DWEANERGVSYCFGKRVITEFLAVHDFDLICRAHMVVEDGYEFFNDRVLVTVFSAPNYCGEFDNWGAVMSVSAELLCSFELLK
PLDSSALKSHIKKSRNKRQHMLNSPPAMVQPQSV
FUN_000474 CDS scaffold_10 294438 295485 +
hypothetical protein ENOG41

MQRHAFRSVCSRTRPELEQVSDAFRNYARQFSTTQFQAADGPSPSTPRPPRSPAGGPKVIDIKSMPLRGRGGLRGRGEARGRG
GLMYRGRGGLARGGSSAQDRSSTPVRTSSPTRGRGGFGAGRGRGGRGGRARGRGGRTNDRRPRDEEDKDKKRIDRNEFNTLDP
DEKSFDDNIRFGITSEYRPSLTLESINEFAPLAPNSASGKSAAVLQSLSVLGTADHVGAPDTFQPKYYASEIETNGVRFFADP
KDKEATEKYLQEKLKAEGKESTGPIITDTEEAIRQVITKRAVQGQHETPKFASDPVGLSRSWHLRAETYSTNDVNSFETKLTS
LLAKKPAAGKGGKARA
FUN_000475 CDS scaffold_10 295565 296432 -
hypothetical protein PF01765

MAMSKANGTLRTCLRQLVRSEGPAPLRSIAATPRVFSTSPSLLKKRKITAPSNSPNSANAKAAAGSMNKAEFDTAKVPMPDPE
GPLDFTAVIAAYAPIDAHFKTQLAGMIHGGRFNPTNLGSLPVPMKDHDGNDVTFPLRELAQVVPRSGRAISLLVNDKEYIKPI
MSAVQASREFNQQPQRSEDNELELLLKVELERKDDVERRIKETVQHWKDRIRNARSRHEKTLKDWKKSGTVLPDIVKKAEKEL
QKVQDKKMKEIDQEEAQTIRQL
FUN_000476 tRNA scaffold_10 303787 303883 + tRNA-Val

FUN_000477 CDS scaffold_10 304843 305329 -
hypothetical protein

MPTPGKHEYKVNKYYKVEIREEFGAWLSDHIELEKLVLADLEEGRNLSDVFLLGKKDRKNENAHLIHNVFESESVGVVVLDDL
KFGRARFGSQIIRKVPAILCILKRESSGVLRNQGGNIQLLMGTENFIFTRNVNGLVLLHLSIVGIAAKLDKCAASSCS
FUN_000478 CDS scaffold_10 316050 317776 -
hypothetical protein PF00171

MALLQRLAAPSRGALRQSPVISLMITRRASNVAPPKLKDPSLLKQDVCYVNGEWVKAGSGKTFEVHDPSTGKLIGTCPEFNSR
DTQLAIAAAADAFPSFRAKTGRERSKLLRAWYDQMAANAEDIAKIITWENGKPIADAKGETTYAANFLEWFSEEAPRVYGDTI
PSSVPGNRVWTIKEPVGVCGLITPWNFPAAMITRKIGPALAAGCTVVAKSPAETPFTSLALAELAHRAGIPKGVVNVVTSHEN
TPELGELLTSDPTIRKVSFTGSTGVGKLLMKQSSGTLKKLSMELGGNAPFIVFDDADVDAAVAGAIASKFRSSGQTCVCANRI
YVQRGIYDEFVSKFTEKVKSFSVGNGFDQGVTHGPLIHDRAIEKVEAHVKDAEKKGGKITIGGKRLNDLGSNFYAPTVIRDMT
PEMDMASQETFGPVAGLFPFETEEEVVKMANNTEVGLAGYFFSRDIERVHRIAESLEVGMVGVNTGIISDAAAPFGGVKESGF
GREGSLYGIGEYQITKMITYGGMGKPLQS
FUN_000479 CDS scaffold_10 318370 319334 -
hypothetical protein ENOG41

MKFPTFKRSQQEHTDKPAKAKNYRVLFRKWPRVSRKGTWWLMPLELIGIVPALVIFGISQPNLYRTDMWQIGWEHDPPLNSNP
ARVLYAYANYQPQPKIALIWTRTFTNFNVAISIISLFFLLGKLTAFIMRVWYPIFATFINTSMVALYTVCVYGTIGPDYTDSR
YPAPAAWYYRIGCDIAKPYGKYKSCMIARYSLVIGVYMLAIYLCNLAFSIYAMLPNKINDVSDDDDDEDGSTTSEPKGGVWEM
HNMKTPSIRAMPYTPRTQAFHTLDRQLPLRSQQNRYYG
FUN_000480 CDS scaffold_10 323717 328297 +
hypothetical protein PF00072;PF00512;PF02518 ENOG41

MQRLPSFNSASSPSASASSNCPVADSTDQEKEGHKKHPPPTSTSTLGSVPTFTTPSNTTTTSTTDAELDTDVSVLAAAAAKAA
GEAYNRVTITGRKAYDSRRISEAPAWRHELSIPNLASIAPFSDFATSFSPASTTGAIRQSSLSTAGSPRGLSASTRGLPQTPI
PAKRPSAPFRGEPVMHPSPIKKARTGASPPRKVELPNQAANLEPSARGKQQGPLSPLFFSHTPARHVHRPASFPATDPAISML
SRMREDPAGGVTTLKLPRASVSSISPGRSESTPGSWGSSMDAPAHTTTPDSRSLPPGLQMLQGVGVIEFLEQDERPTFLIDLD
NSANASRAGLHILYYNASLRGAHEVLQYLSVDQEDASADTDFGRFKSWIMTQSKARVSADTHKYAGITWTLSTLRRRFRFVSA
QTCVATPRPSTALPLIDETAASETRSAGHSPQMPVTPDAELVDAPDYFGDAEPIDANFGRADVDAIMEGGDTRLHPDEFTNQV
FQSQPARSIFDWTRIPVSDALPPHIKFARSVDWAATPLGPIEDWPADLRSMSNLIMGSPHPAAMYWGPQCVIIYNEAYLDLAG
QKHPKLMGSCYMDAWPEIWDEIKPVFKSALESGQATMKHENQLFINRHGFLEEAFFSWSIVPLVGGEGEVVGLYNPSFENTRR
RVNERRMLMLREIGERTAAATTVAGFWPQVQKGLEFNEHDVPFALIYSARDDTESEVSSLHSGSLIHSPQLVLEGSLGAPENH
QAALSHLDMRQSEDGFAPYMRQSMAAGGVPIVLSEENGNLPTHLIDGLHWRGFGDPSKTLVVFPVIPTTTGESVIGFIVMGVN
PRRPYDDDYKLFIHLLSRQLATSMASVVLFEEEIKRGQRAARLAALDRQELSMQLYLRTQEAVESEYRFTRMAEFAPVGMFIA
NFAGKINYCNDMWWQISRHSRSEDSVDTWMDSVRDEDRPALEDAWDKLLREKVTISVEFRFKCSQQSDGNTIDTWVLMSAYPE
RNQEGNLKLIFGCITDISSQKWAEKVQNERREEAVEMKRQQENFIDITSHEMRNPLSAILQCADQIANNITIFDSHSVKADVE
NLLDGCLDAANTINLCASHQKRIVDDILTLSKLDSNLLAVTPIDEQPVRVVQRALKMFESELVAHDIEFDFNVDESFEQYGIK
WVKLDPSRLRQVLINLMTNAIKFTQGREKRAITVSLSASKHVSEVTRKGISYFDRVDHQRTAVMDINNTDEWGHGEEINIHCT
VEDTGPGLIEEEMKLLFQRFQQATPRTHVQYGGSGLGLFISRILTEMQGGQIGVTSRRDIGSSFSFYVKCRRSLTPPQDYEQI
TPFKIARKSHAPAIPQKPELNRQPSRTTTTTEDTTQLFDVLIVEDNIVNQKVLQRQLRNCGNNTFVANHGKEALQTLERSRFW
AGKETEGVDISVILMDLEMPVMDGMTCARKIRELEREGTIVQHIPIIAVTAYARPEQIESAKAAGIDDVISKPFRIPELLPKI

EELVGKYKNLSVSA
FUN_000481	CDS	scaffold_10	329571	331511	-
hypothetical protein		PF15406		ENOG41

MAEEQKNIEVPQETKPEIAAPTAGISAPAVEATAETKPVEETPVVAAETAPATEEKPAEETKPAEEAAKAEEKKEEEEIKPVE
EGHLNHKAQGLSFPKNLIPSKEFFFFGTEAVEPKALSHYLKSEKSAETAHSNIAWASETGKGLLFVGDKKNPSSVISLADATE
PEIDGSHKFHLTSKGNKHTFKASSAAERDNWVAQLKLKIAEAKELATTVTESETYKTTLESFKPTPVKKEEKASEAAKEETPA
EAAVAVPATEEAAPVTEETPKEEETKDERKKDVKSEEPKRRSASRKRTSFFGFGKKEESKKEEVKKEAEAKPADEVAVETPAL
EETPKIEEPVKPVEEVAPAAVEEPAKPAEETAPEAVPAVAEDKPAESPKEKPTATKRNSFFGNVFSKKEKKTPELKPTEPEAT
KEAEAETTAPVIPPVEATTPLAVDVSNPATVPTETTETAAATSPAPEVKKDLKEKRKSSLPFAFGKRDKSPAPAEGEKKESPF
SKLRNTIRGKSPKPAEKRAEENKEETVQEEPTEAKTEELKTEEAPKIEEPAKPAEPEAEDKPKDAAPAPVVTAAA
FUN_000482	CDS	scaffold_10	340323	342789	+
hypothetical protein				ENOG41

MAGRKSKARPWNKNKQNVPGNIPVNSTIDTNSEWNTNAGWTQTTTANYQHVHVPLTEVTPICKPEDRGRYGPKESRERLESQG
YHPLVTSMTKEEVEASYEEKRKKSLDKPDFIEYACEGKEQGFEGRYTSDKNLLIKASESSFERRDETPELTKEVFNGTHPQGL
LIAQHLASLGIKQLEKLALARPREWDTLSKGMAIWDFTTKSFHGEPSGHVFLCLFPKISQGAFSKSPAHRESRREEKLTRKNI
VKQIRWIYFLCGPTELPAWHPEEKLKMQTEGPSAEVWRCRTTGTKPPLVVINMARSIPDINKLDHMMRDYVDQPKHLDYYMSK
QVALKNMFGLLTSIFNRRTNIRVLHFQKTPLLDRRLVACILRACPHITTLGIYQCPLIHFGDVICLLDLIHEVNLNRSSQSLP
LVEALDFYPLYHAGMPFKLPINDEPTKAEKDRGMEPAARETHGYGITWTYMPNEVLQRGIFAILLKTVLKARKMGIRLLMDKD
AAFMKYLSDIPMLPGIIFQFLDGLYRYLDLKEAGSEDNTAMKRAKYDMILAVHAGLQSPYRNTLDPSDDVTRRVTSLFCRSCG
YKMLDIFFCSNKIGLPPHRRTCAGCEFQACLDKQSDHQKHHDLNLMGRFLEKDWDPKGFNVDAPVHDDGQELLFLHTRKTTND
LPLMVLLPNGEFDRPSFQEQLICEHKENNDCLQGLPTLQSLLLQKDAEKADAKDHALHVDACRALSIIMNHHDFELAMHDKNH
GVGGHFCEGQGWRPLSGGFRDKGVSHTFESATKAAEDLKARADIAEGRRPEQDAAGFW
FUN_000483	CDS	scaffold_10	343322	343692	+
hypothetical protein		PF00444

MASFLRAFSLSVRSLTPSNAMGAFATRANWSLFASPSTTPISRALGNGLMQQTRGMKVHSSVKKRCEHCKVVRRKAGKRHNGY
LYIICKANPRHKQRQG
FUN_000484	CDS	scaffold_10	343928	344355	-
hypothetical protein		PF05347;PF13233		ENOG41

MALSIRLLTFRRGYATRPGGSRLKPTLSLDQFIQRGRVLAFYRTILRGTKKIADPTTRAESRKYARDEFERRRNVTDASHVRY
LLSVGKTEWEGMERYIDGM
FUN_000485	CDS	scaffold_10	344652	345562	+
hypothetical protein		PF00583;PF08445;PF13302;PF13508;PF13673

MTDTNGTKEPRRLVGRRRQRPENPIETANKIEDDEFLTHYVKPSSEGWSEWKHPQNEATYTMSLIRPTCMSDGDLEACYILVE
ETSGEDYRTSSLGWHPATKKKEMRSPDLRYILVKDSKDHIKGFTSFMPTFENHEPVVYCYEIHLKPELQGTGLGKKLMGYFTD
VAENIPSVEKAMLTCFVSNKSALKFYEKLGFSKDDYSPRERKLRGGKVVIPDYVILSRQTTSKRVEASRAHEPGLR
FUN_000486	CDS	scaffold_10	346713	347418	+
hypothetical protein		PF04749		ENOG41

MAQQYSQGPNVQNQEWQNNLCNCSPCDSCLLGTFCPCILLGKTADRMRDPTMQTADTCNSDALIFCAINCVTGCGWIYSMMKR
GEIRERFGIKGSGMNDCCVSYWCLCCALIQQDNEVKSRLSQGPITQGYQAQKEGMHMPTSPPAQQQQYHDQKPNPQQQAPQPD
YQLPQHGYQSPQPQGSYPPPQQTYNPQQPQY
FUN_000487	CDS	scaffold_10	348417	350262	-
hypothetical protein	EOG09261T6U	PF00478;PF00571

MSASVLDYTTALEVLKEYKQKDGLDIHELMDTTKHGGLTYNDFLLLPGYIGFAASEVTLDAPITKRITLKTPFVSSPMDTVTE

HEMAIHMALQGGLGVIHHNCSPEAQADMVRKVKRYENGFILDPIVIDRNTTVGEAKALKEKWGFGGFPVTADGKLGSKLLGIV
TNRDLQFEEDLDQPVSNVMVTDLVTAPEHVTLLEANKSLSKSKKGKLPIVDKDFNLVSMISRSDLTKNQHFPNASKLPDSKQL
LCAAAIGTRPEDKLRLKKLVDAGLDIVILDSSQGNSMYQIEMIKWVKSEFPGVDVIGGNVVTREQAASLIAAGVDGLRIGMGS
GSACITQEVMAVGRPQAAAVYSVSRFAARFGVPCIADGGIQNVGHIVKGLALGASTIMMGGLLAGTTESPGTSFVSREGKLVK
AYRGMGSIDAMQDKKAGNGGKDSQKSNAGTARYFSEGDSVLVAQGVSGAVAHRGSINKFVPYLAAGLKHSLQDSGMTSLKTMH
ESAEAGELRFELRTASAQLEGNVNMESYEKKLYA
FUN_000488 CDS scaffold_10 350782 353730 -
hypothetical protein ENOG41

MPSDNYNFGDVFQAIYIAKQKPSPPPVAEDMKVVLQSFPPLGKVTPIQGTKVTLTAVLEIPKFRANEPWEASVWHSVDGSDWK
DLEVASITETGVPQTLQVLDDSMARFYFRSSFSFTASVQFTLKFRHSPDADWRWIRDEQGLNDGLIVNASNRISSSDLSDLIP
DLNSQDWSIKPRLSQSPRTSLWSLEAVIPAANGDESTYRDISVGTPWGSFVRWFSLVRLWSPWLAPRHGHSQFNLDKDAILCC
FLSPQGQNLVFLAVGGVSHVLPVFRSEPNGKLHVHIRNDGLSEEKAVILVSVGDDFDCAIASVIYHARDMVAGTKKASDEWSQ
ELSALKNDFKPEWLEYWFDGLGFCTWNALGQRLTDQKIFDALDKLSEHNIQVSSLIIDDNWQSIDYRGPSQFQYGWNDFEAEP
KAFPKGLKSTISHIRQNHPHIQHIAVWHALLGYWGGIAPDGKLAKTYKTIEVTREDADRRNLPLGGKMTVIAQEDVNRFYDDF
YRFLSDAGIDAVKTDAQFMIDTWIEASPRRDLINTYLDAWTISTLRHFSAKAISCMSQFPEALFHSQMPTNRPTILVRNSDDF
FPEIPASHPWHVWTNAHNAIFMQHLNVLPDWDMFQTVHEYSGFHAAARCVSGGPIYITDVPGEHDMDLIEQMSGHTPRGKTVI
FRPSSLGKAVDPYIGYDDDLLLKVGSYHGASHTGSPIMAIFNISSRPLTELVSLSAFPGVVHDLEYVVRAHTTGKVSHPTKVE
SPESLFTISLPVRGYDILSAFPLTRLSSKKHGNVVISNLGLLGKMAGAAAILMSDVQERENGRALIDTRVKAFGILGIFVSTL
PSLTIEGDFLITVQEKVIPLHTVAISKVDTRVLEIDIGKAWKELGLETRWGNDVEVKVYFSL
FUN_000489 CDS scaffold_10 354176 355826 +
hypothetical protein PF00675;PF05193
M16B
MASRRLALNLSRGLRNRAGLSAAAPFTRGFATPSTVGKTQTSTLKNGLTVATEHSPFAQTSTVGVWIDAGSRAETDETNGTAH
FLEHLAFKGTAKRTQQQLELEIENMGGHLNAYTSRENTVYFAKAFNSDVPQCVDILSDILQNSKLEESAIERERDVILRESEE
VEKQVEEVVFDHLHATAFQHQPLGRTILGPRQNIRDITRKELTDYIKNNYTADRMVLVGAGGIPHEQLVELAEKHFSGLPSSA
PQTSAYLASKQKADFMGSDVRVRDDGMPTANIALAVEGVSWNSEDYFTALVAQAIVGNYDKAVGQAPHQGSKLSGWVHKHDLA
NSFMSFSTSYNDTGLWGIYLVSDKPDRVDDLVHFAIREWMRLCTNVSAAETERAKAQLKASILLSLDGTTAVAEDIGRQLVTT
GRRMAPNEIERKIDAITEKDIMDFANRKLWDRDIAVSAVGTIEGLFDYQRLRNTMKPKF
FUN_000490 CDS scaffold_10 356096 356858 -
hypothetical protein EOG092650VI PF02291

MSSSQPQTNGVPASSAGTSQTVPNTQTTAASSSQPAAAASQNAPPPAPSTTAPRPRDSRLIELLLTSQGVTAYEPRVPLLLLD
FAYRHTSSVLSDALHLSGDPYVTQAGSKPSANAGAISAPAGDAAVTANAVKLAIAARLSYQFRGGNAGGGISKDYMQELARER
NKVALPKIVPSEWGVRLPSERFVLSGTSWGLRDVWDEEDDEEEDQGGDAMEGIEGPEPEDIGGDGVEGGTVEDMFGEDVDEEM
AEEG
FUN_000491 CDS scaffold_10 357627 360242 + HIS3
imidazoleglycerol-phosphate dehydratase EOG09262E98 PF00815;PF01502;PF01503

METTLPLPFLVSIGSPNGQATNDGLTRQEIALLGAPFYETASKDWGRPSAGTNAHMDATSLSNPDDVIALLDGGVRTVFVTSD
SYSEYTEYGARVIPTVSSLDISTASENGLLVKDFDISSSDVDKFVEEASAKKIKSLYIKPAPKTDIEKFIEIAKKANAIPIIP
STGLTTDKNDSSRLLLSKIIASYWKSDRSDGLIPTVVTDDAGIALGLAYTSEESILEALRTQTGVYQSRKRGLWVKGLTSGDT
QELLRIGLDCDNDTLKFVVNQKGRFCHLQQFGCFGDLNGISALEQTLKSRKESAPEGSYTARLFSDEKLLRAKIMEEAEELCD
GKTKENIAFEAADLIYFALTKAVGAGVSLADIEANLDAKSLKVKRRTGNAKGKWAEKEGIKTDAAPVKPSQPAVEKPADGRIA
MERVDASKISEADLVEKLKRPSQKSPDAILKIIQPIIEEVRTGGDKAVLSYTHKFEKATSLTSPVLKAPFPKELMDISPETIE
AIDVSFENIRKFHSAQQEEKSLQVETMPGIVCSRFSRPIERVGLYIPGGTAVLPSTALMLGVPAMVAGCQKIVFASPPRSDGR
ITPEIVYVAHKVGAESIVLAGGAQAVAALAYGTESVTKVDKILGPGNQFVTAAKMHVSNDTNAGCGIDMPAGPSEVLVVADKD
ANPAFVASDLLSQAEHGVDSQVILLAVGLSEQELQAIEDEVHKQALALPRVDIVRGSIAHSVTVQVKDVEEAMRISNEYAPEH
LILQIKDAEKAVDQVMNAGSVFVGHWTPESVGDYSAGVNHSLPTYGFAKQYSGVNLGSFQKHITSSNLTAEGLKNVGTAVMQL
AKVEELEAHRRAVEIRLNYLKQQ

FUN_000492 CDS scaffold_10 360636 363108 -
hypothetical protein EOG092636Y6 PF04571;PF08235

MQYVRGLSESVSTAWNSINPATLSGAIDVIVVEHEDGTLVCSPFHVRFGKFSLLRPSDKKVEFKVNGVKQSYSMKLGEGGEAF
FVFETTDRIPQSLQTSPLVSPASSPASSPPLNPEQSESGLQEPEAFELDATESKLRRPLPSVLYKKDNDNGLITPLSTSPELS
SARPASGDWSSRVPRPHSDDILRASARSRGLDGNSSADDDEHVASERSSSPPPLAPGEALERARALSKGLSAASIPTKVTDSG
DLMLDMNGFKGNEEDILRAEILARQILSEELDGNYDIGALIGFDEQGNLWIYSSEEAKQAAMNKTIEASLQSHRRATAADAIS
DPGYQSDSSDATASPILLHRRAESDAGPMDLQTPPRTPPSSSGHAGDPNRNYAKTLRLTSQQLKDLKLQPGANSMAFTVNRAT
CKANMYLWRHETPVVISDIDGTITKSDALGHVLNMIGRDWTHSGVAKLYSDISANGYNIMYLTSRSVGQSDTTRAYLAGIVQD
GYRMPPGPTILSPDRTMAALRREVYLRKPHIFKMATLRDIRNLYGPDRHPFYAGYGNRLTDQISYRTVDVPRNRIFTINSNSE
VSLDLLTLNKLKMSYVNINEVVDHYFPPVSTLVKGGGEEYTDFTYWRDDPLAMADFSASESDDDDDEDGPGGPTSEYGDDEEE
YDDEDGEIDEDVDEIGEGLADSYISRASMDEFAEAASVLGGSVDEERLKASMTRDLKEAYDEDDEDEIYEDGEEGRQDEDEGD
GTPDAAPIGQRRQGQLKEDVLAQHITGATEAVAAAPNRQ
FUN_000493 CDS scaffold_10 366607 370192 +
hypothetical protein PF00041 ENOG41

MSWELPVLTTLILSVLGFWWIYSPRASYQKALLYLLLCSSFTVFFDPRRLIEYLSPATVEALLDFRIHVLLIHHSKMVLTLGA
VVWLLHRSWQTLWKPVPDLINILGVDVPEPPDVSLAGIRSDAATLSWTRPTSNRPVQKYTIQVNGVHVGESPGNEVAITVTGL
KPDHFYNIRVVAVGPNNFQAGSQVLRLRTFGKDGRPQLGNSRVPTSFPNNDHSHPKNGDEDDDSDSQKKPPVPSVEAAPVLDG
NNASTSDVSATGPSQRRNTVNRRHSPSVASMDQPQIKLPPVDGPELSLDELNRRFESTRKEVDETLAQYAKEEAELQQQEEEL
KQEKDAKRQALKEKEEQTAQLKASVRTTMEQMRTAEKDRARKEQQLKDKENKKSKIRDTASKLENEMERMKKEREGFEAQKKE
LEAKRDKDGQELDDSNAELQEKCAELEAELKEKGKQLQDLKAAREQLPGGDDEQWKENDLIVRREWDARRKELHNQLVAETKK
LHNLDQQIRFLKEEQHQLALYYSSQPDPTAMEFEPAASQDALESGNINSLSHINLSPPPNQFGPPEDSFSTGFSRAGFSQILF
MDHNAADNADDHQSEADFRANAGPLSPTAHALLPSGIFDETFEDAEEHLPSQFLPDSVTAAEEDNPQSPLSSDGRSLNMFSSP
HGSSHNLPFPQYNDVDSHSLNINSSPQQAPLASSHRLSNLLSNFQRNRGPKPADVEGPAIGSLKAGQSQSFPRSTDDHDMPGN
KRRISFSWMNRHSGGESNRSSMAPTSRPFSAKRLNPFASSAGALTGDNDLPHSRPASITSIDLPRPSTDSGSIWNIPGDFSML
GTKGVWSPNEGRWSSRNPSRRPSLGAVPTVLETTLATANDEILDDDDLLDPQTSPSQVGVIGSRPPSHRASMSRHLNPAAPTF
RTPFFTRRERDASPDKDKSKGKEKEKRGKSKEVLTPTIEVPPNLDDSPSDSRMSRDTYSVHTQNSISESHESLDLGMTASNST
TDNNAASHKDADSSVVKKLFRKGSSSKFSTLSSRLSKDSVLFKKGPGSSTFSEKNMSAEHRSSIGDADEIGEDLSQLGRSYES
VTSSPSLGQGSFKSKEAPKEGRMGMGTWRFSMKKKSKEPASKEKESMESDRPADE
FUN_000494 CDS scaffold_10 370592 373195 +
hypothetical protein EOG092612AK PF04100

MNGTYPEASALDAVDYDAIDHLNLLFSHPSAISSISEVSKSLQNHQNALSNDIATLETNQAYGSDSSLERMQSAQAELAQLFR
KIETVRSRAIETEQNITSMTADIKRLDGTKKNLTLSMTALKRLQMLTTAYEQLRGLAKTRQYRECAGLLQAVLQLMKHFNSYR
SIEQIATLSRGVAELQRELLEQVCEDFEMAFAKGEVGARRGTLVEACLVMDALGESAKARLMNWYVNTELREYRQVFRGNDEA
GSLDNIGRRYAWFKRMMKTHDDEHAMIFPPHWRANETLAAAFCDGTRDDFKGILERSMRRTDGNKIDVNLLLSCLQETLDFEQ
SLEKRFATSSRASIDTLSSVEEKAHSFHGLISVAFEPYLSLWVESQDKQLAAMIPKYRSQPLIPPDDEFSPQAVIASAIELFH
FYKLTLSQCAKLSTSERLLDLAKILAKYLDEYAQQVLLHILQSGGPQGPPLQDVVLVLNTADFWHINTNQLEESIKKRIDSEL
VSKVDLSSQSDAFLGVASASVLALVRAVELDCEGVWREMKNTNWSTMESVGDQSSYVGELVKHADGKAAEILAIISKQQYARA
FCDNLVEHLATGYITSIIQCRPISEVGAEQMLLDKYVLTKALEKLPMHHASFSGHETPPSSFVRRVQHCMNRLDPLLKTLQVR
PSPPEGLVQAYLIHIADRSDTNFKKILDLKGVRKADHAHLIELFGIHRDSTPNDKLVASSPLLTPLMTTPSLGNTVPLGVMNP
SVAAAVSPRFEAGSLGEKLLSAARDISQSSTSTAAQTGLEKATINENLRNFGKFFKRDIGSLGARFGKRDGSVGAEEGR
FUN_000495 CDS scaffold_10 373961 375158 + CYS9 Thioredoxin
reductase EOG0926386D PF00070;PF07992;PF13738

MHSKVVIIGSGPAAHTAAVYLARAELKPVLYEGFMANGIAAGGQLTTTTEVENFPGFPKGIMGGELMDNMRAQSERFGTEIVT
DTVTTLDLSSRPFKYSTEFSPEETHTADSVIIATGASARRLNLPGEDKYWQNGVSACAVCDGAVPIFRNKPLFVIGGGDSAAE
EATFLTKYASHVTVLVRRDVLRASRTMANRLLNHPKVTVKFNSGATEIRGGEDGLMSHLVVKNNKTGQEEVHEANGLFYAIGH
DPATTLVKGQVDMDEDGYIKTVPGTTHTNVEGVFAAGDVQDKRYRQAITSAGTGCMAALEAEKFLADHEDDQRADVRPNLN

FUN_000496 CDS scaffold_10 375456 377017 -
hypothetical protein EOG09262MJW PF01704

MEAIKQALHLGKSNDAPAEPSPEALNELKEKYTKAGQEQVFTFYDSLSSAERGTLYQQLSGFDPAHINEITHRALNPPKTSDE
PDRLEPLPESATASILDSSADDISKWYDSGLDLISKGQVAVVLMAGGQGTRLGSSAPKGCYDIGLPSHKSLFQLQGERIVKVQ
ELAAKKSAGSSPVVPWYVMTSGPTRGPTEKFFQENNYFGLSQDNVKIFEQGVLPCISNDGKILLETKGKVAVAPDGNGGLYNA
LVVSGVVDDMRKRGIQHIHAYCVDNCLVRVADPVFIGFSAALNVDIATKVVRKRNATESVGLILSKNGKPDVVEYSEIDKATA
EELDPKQSDLLRFRAANIVNHYYSFSFLDSIPQWAHKLPHHIARKKIPSADLQSGETVKPEKPNGIKLEQFVFDVFPFLTLDK
FASLEVKREDEFSPLKNAPGTGEDDPDTSKADIMTQGKRWVEAAGAIVVGDKADVGVEVSPLISYGGEGLEKLKGTEITPPTL
LWRE
FUN_000497 CDS scaffold_10 378474 379434 +
hypothetical protein PF03167

MTRSKAAQEATIQDPVGDDGVKEEQDISSSPPQPATFKGRLQMSDFMFKSNESSNGQTSPIRRSPRFTTPITSTSASSASAAK
TKRTKERDAEDIEKEIKPKKKRARQASGYAPPSTYAHLNGLPDAIASNLLVLFIGLNPGIQTARTGHAYAHPSNLFWKLLYSS
GLTPRLCSPTEDRQLPELYSMGFTNIVARPSRNGSELSKQEMDEGVEILHEKCRKYRPESVCVVGKSIWESIWRVRHGKPVGK
AFKYGWQDESENMGVIEGEWEGSKVFVSSSTSGLAATLSPAEKERIWAEIGTWAQKRRAERELEAKEESK
FUN_000498 CDS scaffold_10 379641 381130 - ARP4 NuA4
histone acetyltransferase subunit EOG09262MOO PF00022

MAQQPLPTSMQPTDIYGGDEVSALVLDPGYCYTRAGFAGEDVPKSILPSFYGHTTGDNPKDLFGDECLIPRPDFEVRNYMNKD
SVVEDWDVAARIWENMLVKRLQPERQTPPSKNGLNDDPKAEGQDGEGDVAMDDAEAAESAEKPLGENPLLITEAPWNTPKARE
KAIEIIMENWGCPAFWLSRTPVLAAFAAGKATSLVIDVGGANTSVTAIHDGMVLKRSIQKSPVGGIWLSSQIRNLFEASEPKV
ELTPTFMIENKTPVDALAPPSVRLRHFPFQINDSFRAYEEERVLTEFKESVVEVWRGPGRYSVSGNEDYVKTQPGRVFEMPDG
YNQMWREQRFRVTEGMFDETVGYHGSDSEQLTKAQTIPELIRSALNAVDVDLRGNLLANVVVTGSTSLINGFNDRLNNELMAM
YPGLKIKIHAAGLTSERRFGAWIGGSILASLGTFHQMWISRKEYEENGPNVVEKRCK
FUN_000499 CDS scaffold_10 381662 383614 +
hypothetical protein PF00152;PF01336

MSARYLRQASRPLIQSISQTTSARCSHALSCGNSSRTLWGSQSVAYGRSLATSAARSQDGLEGYSSFKQQRRTELLAEKESED
PLFEETHPRLIHHAERKTVPEFHEAFRDALDDSKYISVCGRVRSKRVVGKNLIFLDIVNEFERLQVMLNRSKCMVEEEARSLK
FGMLRNLIEVGDHISVVGIPVRTKAGELTMEAKTLPELLSPTMEQIPEKLTDPKTRMQERHVDMLVNRQAIDVLRLRAEITKH
MREYFHSRKFLEFQTPILAENAGGAVARPFVTRATEFKDKDLALRIAPELWLKRLVIGGVDKVFEIGPSFRNEGVDATHNPEF
TMCEFYSAYSNLEDLINQTEEILCSLAQHSQDLISTQLTSLPSIDMKQFVRPFKRVEFVPALQEALGLRLPKLSSPDALPELL
AILKLAGIKVPGEVPTSLAKLLDRLAAVYLEPMSFTEPILIMNHPACMSPLAKSFLCPQTYQLVSARAELFIGGRELANMYEE
ENDPEEQKRKLLDHRNLVNKDDGSIAIEHNEASEGEATVEEALDEPFEDEDGDAAPLDQSYVKALDYGLPPTGGWGCGVERMV
MLFSGANRISDCLSFGTIRNVVGLSAAEENKTSSEEDKKLEEDKQASPKSS
FUN_000500 CDS scaffold_10 383921 385461 -
hypothetical protein PF10287;PF10290 ENOG41

MKVTNLSILAYTGLATALTQQCSGSAVNEGGNWFCGVIDQILYEGFSSSGSFKAVTKMGDDGSCDQEPFSYDGALGPLSEDLS
VHIRGPFNLKEFAVYNLGSSEKKRDSAPSPHLHGHRHFHEQRKKKRGDWVTATIDGQVVSWENTYNGSPATQAAPVDIPAAPT
DAVNVVDEKKPMKLKADPLPGLDKIKSKVENVKGKVDQVKSKATSKAKEYTATPGGHWKRTAYYNAQRRVADNVIFMGNYGGE
GSGVFDNTWGNSLSYLNANGNGGSSSPKILKDVFIPSNKEFSIFSSEKCDESCGYSRVPDVAYKGFSGSNKIFLFNFKMPFDG
NTGFNGDMPALWALNGRIPRTGQYSGCSCWKTGCGEVDIYEVLATGDDKCKSTFHLTNGAGSSDYFKRPADKYIKVAVVFCER
TSSVAIKQLDDTFDFGSSLSDETVRDWIETMSTPKKGSSLFQLSISV
FUN_000501 CDS scaffold_10 387189 389271 - ROK1
RNA-dependent ATPase rok1 PF00270;PF00271

MDVLKVLSRGTKKTQKNSQNSSNAQQKLPSAGTSTNPQLYHDQVRGQKRKRTKNEPEPEAHDELPEVDFFAPKPEPVAKAAVE

TEEPVQVPKPTRPSRLLSEDECRQLLRSHRLKITLLSKSEDQSKVKKSKKKKKAAVEVKKDGKKQLFPQPLDSFSELRNAYGL
SNKVADNLVFQGYRVPTEVQMGSLPLLVHPQTALKDEDGLEAGVDFLAIAPTGSGKTISFLIPAINNILRRRSQQSLSDIHEL
EAIIVAPTRELVHQIVSEGQKLAQGTGLKVVSMKKHTQLSAEQVDMAEDGSEDEEDKESDSEDEDENKADDKPKQITKADILV
TTPFLLLKFLTSGPPSTQKVLPTVRDLILDEADVLLDPLFRDAMMSDWTACTNPDLRVSFWSATMGSNIESMVTEKLTSRAQS
LGITPKPFVRLVVGLKDTAVPNIAHKLIYTATEQGKLLALRQLLHPTAADDSGPPLRPPFLVFTQTIDRATALHEELQYDIPL
EAGGAARIAALHSGLTDSARSSIMRKFRAGDIWVLITTDVLARGVDFAGVNGVVNYDVPGSSAGYVHRAGRTGRAGREGGVAV
TFYTKEDIPFVKMVANVIAASEKQAGKTGDEAGVQKWLLDALPNVGKADRKKLKERGVEARRSGNKAKITSKSGYERRKENNR
RGAIEGSKKRKLQANEDSADDGEWGGFDD
FUN_000502 CDS scaffold_10 389969 390596 +
hypothetical protein ENOG41

MPTMLGLTNDRSPHGLNAEESKFGGPSSQRGMELTPHQQNGSIVSGIEGSDFFNGGGKQKEHDADEESDEENLKTIKGRNLSA
VKEMETLLWRALCDKPKTALKYIAKDAVMSNRFLFGDPVPRTADSDPSLEEEIKHCEEWLAYKMHDPQPVEIGLMAAAIGYKV
TLFRQLDQGDGNFGMQTVQATCSSSWRQLASGDWELTSMFAG
FUN_000503 CDS scaffold_10 391277 392178 -
hypothetical protein ENOG41

MVSSSDPNQTFIKRSKSYACLAPILNGPSKSTPLQEADVVGESDIDSSCGIDPTSENSPKDRNGSGSHDQSSSLSRGSTEEVP
DMALDKTINKDDPAADHTSSSSRSSETSESKPSFTVVATSHKVTSKSDYAQGQDHPISSSVEASNSNAEPPKVPGQSISNSQV
DQSKSNNSLIIEDLGIQDRQSGLGRRPDTPIPESLRDEGYPSAQDEDEEPVDEESYSSYVHEENYGKEEDYEQEQDHGHDPTA
SAHSSPDSIVTMIANDRLFAIDFTVYHTD
FUN_000504 CDS scaffold_10 393332 395135 -
hypothetical protein ENOG41

MTSKKKGTARRTGPFLFSSRTRTTKDVPPIMKLLSELGFGDSADKALLNEFNTARKFHMNRFIEEKILDDKDMLEWSSEPCKQ
KIRRMVKDFLVRQNHGPRFWPDEPSAANTKKYHYSQDSQQIIKTLMMLFFRQLYNRRSHNIKVRKEKRRTNLFERGDAPDEPI
ALDLSSDESDYEYQVFDTPASPISGESSLKREPSDSPELPSLEEIRHTVQSQTMPSDAVTPLPSVVQDEHVNPEPAQESSQPS
ATSPVLPTKRPLSQSEASPLRTKSPRLSGFRGPTADPFSRDANVRLESVARAVSPRQDLRRSSRKSVPMPVASMGEGSGTNDT
QELSPEAERNTTDEIVCGFKTAIRVVPPIGEDSSTHIPGNVTERAATDQPVKLGSTPLSQNETTATSGRDIEELSSISYDTAA
ARRLLSSHLRGHNTDTPTPLQFSFSFHDGSHGDPFNISAVEFFTMTLKQFMDVLPMNDKEMITGLCIRQYGPKFCLRQVYLYN
EDVFGNIRQQFLRCIESDTRDVKNHGKRLDYEISIEPLRE
FUN_000505 CDS scaffold_10 396268 398253 -
hypothetical protein ENOG41

MHLTTPYIDMASLARGKSRKFGERIFKTSKATSIASLTDLRKELGYGHPGQDQDIAFKRAIRHQIEQFKSTEGIPGYRLTKWT
DPSHKRGLLEITRDFLDTQGKGVEYWPDRCHLDTSRPLRYHKDSAKIHRLMIKVFWRTAKEYKRHKTSSVATAQLLSQPTNTI
DRPIKAQDNSDGTDHRVLHNTPDIRGQTADNPIYLDDMPPFVQGVSETHAPFAGQPMAPFCDERFESNGPENSEHIRAMIGIP
FDPNIQNFAPPTQETMATGLDYTLDLLQQDEQRAQNNGKRPAELNDDTTPQPSSSQPQHLPTKRARKRIARLDSAPEEEVAVY
DETSSSSMQSSQHSNWSALNQPTRGSPFKGPLPRHGSRSVGRKTFEPERTPRVEAARRAERDAQATVSAIEECHATTTDTGSA
DGVQEHTADESQIQPTLTHPEPVGDMALIQEPTPAQEATPVQESSAPGVDFSQAASQNVSDSRRNPRLKLQSKALMASRIELR
SSISNCMSLEIWRPSSSIFDMSLEELTAALVPNRELGSLHLLFKSLIPGRQRGLPAPALRNGDSDFRFFKLECLDIIIEEFER
SRSLPKAERPELAYSIHISRALDFY
FUN_000506 CDS scaffold_10 400278 402353 -
hypothetical protein ENOG41

MHRFASKIYISVANTMEPVEAQGSRPKVLDRLFKTTKTSTIPHLTELRKALGYGNPGQDQDIAFKRAVRTQIESFISPSTKLP
AYKLTKWRLPLHQRGLQEVTKDFLDTKGKGPEFWPARHNSVNTRCLEYSKDSSRIRRLMIKVFWRAAREHKRYKPSNSTSAKL
LSQPSDRDTSPLKPLDTPSDDKHQVLNNSHILKGQSIDNPIDVESMQSRTKTSGPSTDDHFAAFGMSLNLTVHTQRQDTPEES
SSSEPFSSSLLVPDTAETMMPIPEEADSACRTDPYEVPKSPPRATETAQINGKRPVESDSNDSNRQAKFPRQDPSTSAKPTRQ
PPKGTRGGRKTQSHRPRVRVSQRQTKSIYRPEAATEEQIQAAENPTPTPTPSPPPAVVGPQRRSNASQGKQPSATTNDSTSKT

SAGETQSQSPTEAIRAALTRQAERQTTEAAIAAGVDQQLDAHGTSTTGLSAKAQGKLPDVPVRQTPTVQAGPSREREAESTAR
PEASAARQQGSRPGNLEPEDIQRQVLNGREIVIRSDIANGVDFRSWKPSSFLFHMSLAAVARELGLRHGRTLHMSLRGRTSNL
NEEFENGDQDGFESFKDECLGAIMQRHSGAASTPVLQRRQFWIYFSDKPISI
FUN_000507 CDS scaffold_10 406443 407169 -
hypothetical protein PF08510

MSLSSDDEDDILSDAQSASSSGEDEDSPPRQLSQNLFAPPFYGRPPTPLPPSPSLTSLLRPSRPTTPDASDDDAIAPVPRAAP
KVPTYEYYGFVLYLFSSLTFLIYLLWGYLPSPFLHALGIYYYPNRWWALAIPAFIVMTVVYIYVALAAFNTEMLTVPLSSVET
VVDGAGKLAEIDAKGRLRGSRNRERKVHGDGRLRWREIWSEGTDAVMDIPLAGVCEVLYGEGREDGEDMYVDDET
FUN_000508 CDS scaffold_10 407646 412534 -
hypothetical protein EOG09260EOI PF12816

MADSEDLSLAGDVGLDNGDTINDAPTLAGTDVDADADAGNDGLLEDSDGPDRHLTPVPIGNGSVTNRYREIMQQQHQDSSDVS
SLGGSSLHGLPKRAGSPIDSVLSGPDDTPSVQGSFVSSPGSSVLPSVASRPGLSSPSPSFRPFDRRFQSRISSSAINTPRSSS
PAFPFLSSHSRNVSLSSQFLLDQADLETPTPPWEVVRWTKLRKLNGHAFSEAGKRSFGSPTCLAVSATIVLGTSKGIILVFDY
NQNLKMIIGPGTKAVESGPITAIAISADHTTIAGGHASGNIFTWDTGRASRPFLTIPHLEPSQLQNRTADGHVPDVAITHLGF
LGTRHTALVSADNKGMAFSHLATRGTGALGRTVKTHRILGRYPDAPMPPGKTVKPSTVLAFAPLPLGNVECSTDTLGLTAMLT
PYLLVIVSTTPVAQTQHKSARPKDVPPHSAMTGCLAWFPAVKLKVPDPQTGSDISKVKLAYCWSNILTVLDVDEYPREDKDQP
ASYRFKARSRWKCEEAIVAVQWLSRSVLAVLTISQRMIVLEDRSMRMTEGFDLVQKYIYHADLFSEQLHTLVEQLDENDSSMH
GVVADAFYMSFKAYKGRIFILGFNEVSIGALSNWADRLIAMMENGDYLGAIQLATSYYTGDADKLTVGLPEDPSLRHTMVRDK
IMEIISASLKYAFGQRQKDPESYDDEHMKQLAETCFTACQAVDNQDFLFEDMYDWYEDADTGGIFLECLEPYILEKTITMVPP
TVVKDLVQHYVSRGWESRLEEMICHMETATLDLDQITLLCKQHSLYDALIYVWNQALGDYITPMIDLLSLLIPLMVDGDFTAN
NPADDFFSINAFKIFPYLSYTLTGRVYPNDEAMSEEMATKAKSEIYWFFFSGRTITWPKGSGNEFRTIPNSESQPSFPYLRMI
LKLDAPSFLSALNEAFEDPFLNDSTDQHMNGSKGDMPEEQIFGQTINRQYVLSILLDVMNPNDFAPEDTIYLDMFIARNLPKF
PQYLLFSGTTLSRVLTGLCNYPGLDLAEDAQLSAEYLLSVYHPSDMPELMPLFKKAGFYRILKRIFKTDKQYGSLAQTYFEDP
EDQELVFDCLRECLRPQTGLTGRQIQEVLTVVKEHARRLLELDAALTAKTLAEQGLELHQHMIGSAEDAPDLQHTYLKTLLEP
EEPTADDKLVAARDLVERYVQLMCKFEPSHVSDYVGLVQSTDLRLEKLLPTMEETGVIDAAVVLMAREGQVKDAMERLVKHLK
TLESAMHGLLTGAEEQHSGPSLGYAAEGLLEALQKYVHVGIWLCQGQTKSSTKKSKLVQKAPKTATEALSPDEALWLSLIDAC
VQTTKKLSPALSAAAEQNISDDEFDEEKLVALLRSLVQHTFTALLTTTSSQTTSQSGSKLLSNAGSSLSFLRILRAFLTQAAA
SSPNLADLRGVLASIFSAYAYEESILKLSNRLLERSLFVNVNQSVELRQRGWRPRGSTCEACSRRVWGPGVAGTAVFEAWEDK
QAVEEKRRKERQANAAERAKGDDGETDLKAKGKGVDTRPSSMLIETNANSGVGSNKDKPMGPLVVLACRHIYHQSCLDELQER
QQNGVVGVVEREYRCPIDG
FUN_000509 CDS scaffold_10 413088 414021 +
hypothetical protein PF12572 ENOG41

MSSIGPQLPPHLSKRKRTPDDEASNSSKHRRSESPAKNTDEIDLEDSDDDYGPSAPLPPKAPVGPALPASNKDEIDLESDSES
DTGPAPPKPTVGPAPPPADISQRPATSPDSDSDSEDDYGPALPGSVKANKPTIGPQLPSAEPAPQRDSWMLAPPTASGYSERD
PTKMRNRKFASKPASSSGPSTVSTIWTETPEEKLKRLQDSVLGRADKNTDTAPKLGKSQEEEERNRRIAANIESQRGKSLYAE
HQGRREKEGQRVEEEEDDPSKRGFDREKDMALGGKIGTAQRRELMNKAADFGGRFSKGSFL
FUN_000510 CDS scaffold_10 414846 416282 -
hypothetical protein PF00097;PF13639;PF13923;PF17123 ENOG41

MVAVNQILTEITHVVIRAAAAEDPSSTTSHAAAETTDSGNEKKDNGGGSNSPLLFFVALGFGVVFTNLWIIVGVKYCFRYNAR
NRQMRMNEDGEPITLENMPRPHRRRREKKLMTMDEVNDKFPMMKYKTWVSDRAKEGLPTTGGVSAPPTRPNSIQEADGIVPDF
SAKVRDSTDEQPATTVPKTNGQGETTGASNATEASAPEIAATAVVPETEKPKETPKTTEGQTSTEQTANNNRLQRISSEETDD
EDDHIDGALPPECLENPGDTCAICIDTLEDDDDVRGLTCGHAFHAVCVDPWLTSRRACCPLCKADYYVPKPRPAPDAANEAGA
NNGSSFDPRNNPRFGLPAAIRSAWIRGPRNGRNETPAPASRRRRTGDAAAEQEPVQQPEQAHQTPEVTQTSNGGVLSSVRGAL
RFGRRRQAQDNQNGSETVTPSQLEAANRTPAAT
FUN_000511 CDS scaffold_10 420702 421672 + CYP3
Peptidyl-prolyl cis-trans isomerase H PF00160

MPPTKLPESGNPLVFLDITLGGEPLGRIQIELFKDVVPKTAENFRQFCTGESKNSAGRPQGYKGSKFHRIIPNFMCQGGDFLN
NDGSGSTCIWGYKNFEDENFTLKHDQPGLLSMANAGPNTNGSQFFITTVPTPFLDNKHVVFGKVVDGMDVVKKMEATKTGYAA
PDRPNLDVVISQCGEM
FUN_000512 CDS scaffold_10 422592 424152 -
hypothetical protein ENOG41

MKFFHASGLALASTTLISPALAHSWVEYAFKLARNGTMFGNIGYPRGYVPRDSTNPPFTDTIPQNILPQAGQPAYSGEEILNK
FPLEKKPQFPMLEAAPGDYIAIMHLENGHTTLPHNQPNKPRNRGTIYFYGTTQPKEQEKLFDVHLLWNKQGTGGDKRGKLLAT
RNYDDGQCYQPNPGELSTERSSELAPQGALPTKELACQSDIKLPDNLKTGDIYTIYWYWDWPDLNKDKIDMEATKNGKFPWAG
TFMRGEKDPNGFTMDAIARNESYSSVIDIKITGPDAEPFAAKNLQTLEWVDNQNIYSAGIEDQMKSNFQVDIDGNKGDDTIPS
STAKPTATAPVGGGAGNGDGVATVTVTATVKPSPIISTVYVTMPAESLAAPKPEPTEAGPSTKTITTTAFVTRSKTSHPSDAV
PTPPTASTVYETLYTTRPKEQPTGGDQQQQPQPSTVIKTIVTHINTPTSSGEANTPPQHTMPGGEFREDPSSANSGAPSQTQS
DGPPIPTPFLRRRQNWVFGNF
FUN_000513 CDS scaffold_10 428044 430714 -
hypothetical protein PF01285

MSSSLYHPRPVLSSQRYTPSPDYLQDARRTYHNNSRLPLREQAGNAQSHSFNSMVSCYPNTVGISPSVPVSLPAPMIPSQSFE
CLYRVPTPRNQPRYQRRPRNGVNPLYFWPAFRQYRNRQAHKDTQKDKGGVWRRPELEDAFVDSVLLMPHMGRRKFSMGGKLHG
RNMLISEYIFTICVAILGSKEIFRIDNSNDSIEQMGRKQVSSHMQVVKKFFEDLRCFHFLFPAEEKKEPGSTNSEDYYDEEEQ
ESFKSNPVLTALAEGRVPDVKPNYEYFSQLLALQSLISVRPKTAEIYVSSSEVKFKDEIAYDAQDNALDAESFPHLNKYNNCD
DSPSVLGKDVLLHEYTRSLDRTTSACVKTVTRRWQKDAPEIYETLDLPTRDEECLLLEMCATLELHEHARFPSGSELTGFVEV
AITNPNLQNHRWKCVTRLTRPSELHGDDKKQGVYTNETGIHRRGCSDSKADCDCHSRPRQDIHVPFPAVEWASILSMAVQYPD
VEHQRKKDKRSKGDDRKKHDLDRSGSKRKRSEDDGDAASWARRDLTGSDLICKVAMYQELWSCAPDSNRWVRQGIVFWRFNTT
NQWYKYNPVFKPAGTSWRWLTVNDPMSRYHQQKALVYPSTSMSRDAIMSPTPSINQHMTAAMNETFSSAWDPSVSLAQVPTVP
ATNNGGLTLFESFSGGLATPPPTAGLQPSYPTSFDHGMPPSTGVGFMPSTCSTANDSHPVAGHGHSQTAGYYDAQTTLADLKP
VISTVNPFQSPTTTSGLDLSSSLGYDNPDSLQGWDMPALDGWSASTGGSEWGSHHKPEASGDQTALWTQSQWAMAGADRDGSP
RPMKRRRGDAIDSHVPVTMAAAGGW
FUN_000514 CDS scaffold_10 431746 434577 -
hypothetical protein PF00076

MASSRIFIKGLPPSISEADFRKHFSAQGREITDVKLIPQRRIGYVGYKTPEDASKAVKYFNRSYIRMSKIAVETARPISDPAL
TKGQSAWHSKAASTPSTTIKGNEQPAERESDSSLRKRKRDEPQPADPKLREFLHVMKEGREGALDDGVRGGIGDGVAAVAATA
VPEEESDDEYEQIPTRREKQRRIEAPENPLVSQSLPSQLKEARVVSDVQAETPAGDEPVEPQEAAAKSNLEQQEVVAEDDDWL
RSRTNRLLDLVDPDDLERTPAQGPSTTETDHAEGGEAMELTSVDPPATADVAMNETTMEASGGEPAKDDSLEAIRRTSRLFVR
NLPYSATQEDLRETFERFGTIEEVHLPVSNSGTSKGFALVLFTDPSGAVEAFQAMDGATFQGRILHIIPASAKRDTGLDEFAI
SKLPLKKQNMIRKKQEAAATTFNWNALYMSQDAVNASVADRLGVSKSELLDPTSADAAIKQAIAETSVIQETKAYFTANGVDL
EAFKSKKRGDTAILVKNFPYGTTIDELRKLFEESGPVLRVLMPPSGTIAIVQFSQPNYAKSAFGKLAYRRIGDSVLFLEKAPS
DIFRGGDQLDQAVSLKDRPAPTVQNLSVDDLLSRGDKPEEDLETTSLFVRNLNFSTSTSRLAEAFQSLDGFVSARVKTKMDPK
KPGQTLSMGFGFVEFRTRSQAQAALKVMDGHVLDDHALAVKASHKGHDAAEERRREDKAKKAAGQRTKIIIKNLPFQATKKDI
RSLFGTYGQLRSVRLPKKADYTPRGFAFADFVTPREAENALNALRDTHLLGRKLVLDFAEAEAVDAEEEIAKMQKKVGGQVNK
VALQQLTGKGRSRVNIGNENDEMDM
FUN_000515 CDS scaffold_10 438370 439041 +
hypothetical protein PF01221

MADAHVPKAESPVAREKLEAQIKSADMTEDMQQESIEVAQEAMAKFTIEKDIAQHIKRTFDERKGPTWHCIVGRNFGSFVTHE
TKHFIYFYLGHCAILLFKTQ
FUN_000516 CDS scaffold_10 448038 450170 +
hypothetical protein

MNSLKVPESGLQLEGSTGKDASLPSQAFAVTLSDNVIEDMIKCVQNGDGIQLSLGANPTLLFGSKSHAIEPPSDSNTYDLYLT
RPFESTRTAEKIPYTGSLFEKPSGATSKKLKTGKADNTTDGKPAKSSSKSAQLGRDSDIEALQNGLAAHAASREKTRVVDRLPA
KKAAIKSKSKLLDSTPRSIPQSPGQVSLGSLTPNPTTSQPLDRAKGDRAMLVHELAVQDRSFDYLATKWEGKVEDMEPTLRKV
ADDLDDNNKWSLKKKSWRDLDVWNYDYDTQELRQKAIDNAVRQYDKQRVTISEPEWDRLLPKEERGTGKSLSRVQAKITKTVS
APSINVEMADGGTTPRESGDSQDANRPRVGGEAMSRSTSNPLSTKPKKPTAQEAQVKRLLGKSKAKTAASAASSKPASKVSSA
KASPTKSRPTAAPKANGGRVLSQAIISNSDTSEDDAAPMVKPKPKAVPKPAPKMRDTVVAKPRPVVREPMKQQITAKRPREDD
DSSSSSGTPLSKRIKPKQPLPAPRLKHRPSDASQNSRGTITSTSMKSKNTSPTKSSPLASSPPTNASDLENEAPGASIVAKKR
KVEGFSKGGVTKRPAASRFSDDLVQKAQAFKLFYQKYETLHHEISALDNPSSRKLGHLMEMRDRLKTMKMEIYSECPPERS
FUN_000517 CDS scaffold_10 451215 451998 +
hypothetical protein ENOG41

MTSRRSQFVKEVRPGAYFQEPAYVYTVHPAQTQDPFRNVPARTTEDYSHMYPPSSTATNVFIPGCCRHRRRASRYSKTCESDG
STKSYASYQDVQPAQPMDRLPPRIALKQLSDFLHEASIFYNTQLMDFTREHQRQGHDTSNEALRQWLWNDWTRSRDNPTRENF
TSTKASITLLLRQVETAIATPWLENADLNARFEFSYRALKSSCDEIVRLSGKVMSDWQTCRFLAVELKNARVYANPEGPVLRQ
LFVGWEKSEPW
FUN_000518 CDS scaffold_10 452712 453864 +
hypothetical protein PF10294 ENOG41

MGIEALDFPQMWERPSYTQLADILQSLELSPPIWNHKRRRSEIIEEQESLASQRKAEVTRYLSSIIKSPLSWIDDDDEKEVLW
TQASKRMSERCGRTAMGEVIRRWPFGEGNDVFELIIREPALTGDSLGFKTWGSSYVLSQHLPRMAETSLFRLFDETLGQPRPD
VLELGSGTGLLGLAAAALWKVPVALSDLPNIVPNLRENVAKNTELVKSRGGSLTVGDLTWGGSEDEVDQTLFGQPHQFKIVLA
ADPMYDDEHPALLASAISDHLALGSDSRAVVMVPRRDATTERLLESFRQAMLDLETPLFCEEEDELAGQDDWVEDDDAGNVRC
WLGVFSRGGSPPSQVGDAVPI
FUN_000519 CDS scaffold_10 454624 455915 +
hypothetical protein ENOG41

MTGKPVVYKPDEIRFALSLMEKDFYNDAISNAFRERFNRALTDNQIRYLRNKYGKDPDYGAPLCNKQSRKRKERQPDSGAGSSA
SPGSEGSPTETKRAHQGSFKASAGPSQHPQYQVGSLPTQSPVKFEDMKSPSLSSAPLFHEAPMVQMGNLRSPPTNSYSLFSGA
PAQGGFGVIPPANPWQTQSRANFNTNFSPINTRSGQQQIKSPEQGSPGGCNAALYQTPHQSPNVQPSYASYPRQQLTASSHAP
VSSSQQTVVMDMPLSNFSQLSDFVYPQQASPTEEHKQEQDQKPEMAGRDDVAPVYCEDYFEPAQQAVEQSEPQPTQDQDEETP
EQKIPQDRKQSAASGTSGDSNEHQVAKSGSSHDSKN
FUN_000520 CDS scaffold_10 456810 459249 -
hypothetical protein ENOG41

MALEAPLRSSTGSLQFPFVYPQTDMSAPTENESRRSPFRPFSSPPPATDSSSLRPNSVNRMSSSSYNVMSPSDIVGPSPVTSN
GTETTEIEDDVSEDIEKEVSDRKATRRSELLMLTTNLPESVRQSSNEEAISVIHAPESFSSWAAPAAAAPPAMEQKGPSPRTS
PKSEAESLASLNGGQEKTSTHPVRPPILTDVNPVRYSIDSATPRAQDLQSMLDDGARLRSSSTSSLEKIDEQTEAEGDDEDYE
SEAMPIPQQQDEIETLRTTLAECWTLCNSLSKLSSINRNRALGKNGIPDAHEKAWRTCWKLCQRLYHSRDEDVSNFNVKVNLD
LCRDFCQALFDVRQRNDEAADSVLRVSFELNNHLYSAQDGTLPEQFRERTLDFYIALCHRLMKQRSDLAEETDQLLRACWALA
EMLFNLRQNRRDGRPPDEELLSSTVQACWELCDVFRDSWTQVRPDRGTPRAGYAFPQHLNDQSGRQSRGSNPSSVHSRHDSVK
SGRQEEKPRNPAVPETPVTEFEDTPISPQSRSPTMPNILVLGTPSDNSRGRWSSSASNLSSYSKSSTRTSSTATTTAASEDAN
VTRAKVLVLRAAMNLGFNRDTIHEPKAAAAALQKFVQDLPPGSFGSLISHSTLLSQYKNSVLTDAIIPRHHALPARGKRVTAQ
EMAKSIQMMMKSSQRYAYLRDLFKVVFQFPVEEVDTRRNVSIVV
FUN_000521 CDS scaffold_10 465369 465769 - RPB10
DNA-directed RNA Polymerase II subunit L PF01194

MIIPIRCFSCGKVTGDLWERYLQLIADPRKTDGDAMDELGLKRYCCRRMIMTHVDLIEKLLKYTPDGRSEKKQNLGVQYS
FUN_000522 CDS scaffold_10 466099 469088 +
hypothetical protein EOG09262E3Q PF00400

MAGHKSPRTKNDPTKKRKRDVTENDSRTKRLRAERKANKINGIKLENDSGNGVVAEVNGISGALAEAGTREIEIVRQFDDTEA
GWRVSKPMGGRMLDIDPVLTENEQFLIIAYNTSIQVYSAADSLLVRRIPITAAETSDDKAAEPAHIVAMRQAKQNSNIVWVAT
SDGRVCEVDWTTSKTPEFFQTQSKTATAMALVTKKVSGKVQEIIFVAESDKPGRIEVVAYPASTTESEHKVLFVMKKPGNGLQ
LLETSEDGHLVGAINDRLFFGVPSQRQFDNLASFDYEIYTFDIPDLVSALDLRVYPRPVTSGKKSRQEAAPVLDIIVGGARGS
IYLYHDALARSQALAKSGSDKEPIQAQNFHWHRRAVHALKWSRDGNYVISGGSENSLVLWQMDTGKKDFLPHLSGSVENITVS
ANGSSYVVHLDDNSVMVISTAEMKPTAYIAGIQSAAVNVTTPKDLLVQRTWTTPASVQRPIPAVISPTDQSRLHVCVGNGRQA
TMSGGFSAPLLQTFDLETFRSVSKQALARTQPTDVNITNKGHPIEEPLITHLAFSADGAWLASVDTWEPSQRDVDNVISDAKD
QFIQERREVYLKFWEAQEGEEQIALVSRINAPHATNRNEAVLDLASNPVSTCFATIGTDGNVRLWRPKTRSQNGVVVKGPNGR
EVFTWSCSQIIAVGDGIPQDGVIDLPESGVVQEPQGSIAFSEDGSTLFVAFGTAGSGVVYVIDAASGDVVKTLEGQWKGQLRS
IRALSPFVIILSDELRVYDVVSDELRYGVVIPQSKANALLQLAVDHTSSHFALALPANEGSLIAVFQPEDIEPLLVRSTPQRI
VSLVSAPETSGFIALDDAAQVWVVAEGSDPSSLATVQPLEDLRLEGIDVNGNEADLLDEDEDMASDVDDAEAEPVTEPEDVEM
EDDDSHPSVIQQQHLADIFDAAPAFAAPPIEDLFYKVTGLLATKPPSAS
FUN_000523 CDS scaffold_10 469581 470802 +
hypothetical protein PF03171;PF14226 ENOG41

MSFTSIPILDLAAAQDPATKPQFLKELRHALMEVGFLYLKNVGISDELFQKIIELGKGFFDIPEEEKLKIEMKNAPSFLGYSR
LSAEITAGEIDHREQIDLSTEHPIPGPGAPLYRNLLAPNQWPSEDSLPEFRKVYTDYMERMGKISIQFTSLIAEAIELPSDAF
NKYFDEDQQHKLKIVKYPDAKELGIEGNVQGQGVGPHKDSMLTSYLLQATSHKGLQVQNVRGDWIDCEPIPGTLVVAIGQGLE
ALTQGVCVSTTHRVLSPAAGSGARFSIPFFQGVRMSAEFEDLEKVGVGLVPEEVREQRRKIVERNGGRIDDVEFTFRAGGASK
TLGEATLRNRVKSHPDVGERWYPDILASIREEQAKAKQQKEAAAPVVKTPPKAVEAH
FUN_000524 CDS scaffold_10 471032 471446 -
hypothetical protein PF04419;PF12907 ENOG41

MGNGAKAQQKRERNAKDKNTAKSQLKVNEKACDIQCQICKSTFLKTTKAPALKEHAENKHSKTIADCFPNVQVA
FUN_000525 CDS scaffold_10 472716 474211 +
hypothetical protein PF01565;PF08031 ENOG41
AA7
MTQTKFSSLRSKLVQGRLLLPGDEGFEESLQRWSLTCIKPAAAVAQPRTAEEASEVVKFATSNGIKFNVKGGGHSTSQTSCAP
SPEGMVLDLSLMRDVSVDTEANTVSFGGGCLWKDVDEALWPKGLATVGGTVSHTGVGGLILHGGYGVLSGIHGLAIDVLLSCQ
VVLADGSIVTASSTENSDLFWALRGAGSSFGVVTQFTSKIFPQGDIWGGMAFLPPSALPAVIKFLNHWGETNDGTQIFLTGYT
YAPPGPGPNAPRPLVVLVQMAQMGPNPEEDGQKYFAPILELEALMKQVGPMPYPMINQGADEVFAKGSRYLFGGANFTLPMEL
STAESIRDKFTGFVEDTPGTEGSVVMIECIPQKTIRTIPVESTSFNSRGNYYNVGINYKWMDESLDAKIRQFNRGFQKSIREL
GYDDKALSDGIGHYLNYVSTDTISAEEAFGSNSKRLRGLKKRYDPNNVFDKLWKLVGEVEDNNWIA
FUN_000526 CDS scaffold_10 474806 475759 +
hypothetical protein ENOG41

MASALSITSTNKISLEEFKRVLNQYPPLIKKVSDEKGAKGGQRTLQELDNYRYNDALDAFNSSAKSRPMKLDDIKNLVEWKLR
HGKFRPTLMNLVSSNDANDAQEIVKQALDAYKKDADIEAALGVLTKLRGIGPATASLLLAVHDPTRVIFFADEAFWWLCCNGK
QSPIKYNAKEYRMLCSKVDDLRNRLNVQASDVEKVAYVLMKQPAQPDQSHDVAPPKEAKQITAPMAAKKEKKRKANSNAEIVQ
DATHEQPSLRRSKRVKI
FUN_000527 CDS scaffold_10 476398 477835 +
hypothetical protein PF07350 ENOG41

MAPKGHRHGPSSSMFAQITEPIPLPTRFARIKRDLIEGKESQIKSSWIRLLVKLNTEIDIIAKAGSDIYPSIDFNDLKESSVA
DDFSSRLKDRGVAVVRNVIPHDLATKWKADTDAHLGDLQAQRLPTHDPHLYGVYWSPGQIKSRAHPNILYTQRFLMNLWHSSD
PEALVSPNFPISYADRMRVRRPEDETCSLSVYVDGGSVERWEPDGYGSAATYQRIWDGRWEDWDPWESSTRLKVTSDLYNGDG
SCSVFRMLQGWVALSEVPFGHGTLLLCPMIRLTTAYLLLRPFFVPKESSPASPDFLTPGNWILEEPPSSVIQGALPSYPQELN
DALHPHLQLHRSMIPIPKLDPGDYLVWHCDTVYALDRLRRPDIPPTTIIYLPACPLTQTNALYLAHQRKGFLHGEAGPDFTGA
PTGDVNVGGEQAVREVGEAGGVDGMRAMGLLPWDEDEAQDDLEYAVLEMANGVLFPEKYDGTY
FUN_000528 CDS scaffold_10 478288 479114 -

hypothetical protein   EOG09264XKX   PF05529;PF18035

MTLYYTLVFMLLVFEMALFMLLIVPMPFNVKRKIFTFISENPVVAKIQYWMKITFVFILILFIDSVNRVYRVQLELAAASEQS
KQGGGAAVMGHERLEVQARKFYSQRNMYLCGFTLFLSLILNRTYVMILEVMRLEDKVRAYEGTKSDTKEAEKLAVAGKPGELA
RLRKELEQKDQDLKTLKKQSEQLHKSYDELSDKYAATQQTGESRKGI
FUN_000529   CDS   scaffold_10   479858 483341 -
hypothetical protein   PF00856;PF18264;PF18600;PF18601

MASPHLVAIVAGKPDTIDLTGDSDSDYEPHSLVPLNRNNTTTARFTPNFSISSERPSSTNAPVSPPKLNRSTSSTAVSQDKQP
SASAEPPRPASSVLKLSQKVTTITANSDDAQSMAKLSSVTASNLASRQVTPTPEKDASKSTQQTKPASPKYQTPKKQEWDVAA
IAEKLTQFRQDIKDGHSRMTSYIIESTKPNERRVLTGKNLFAGLTNKPVPSKKGETMRIKSKEHTKLKNNKREDHYSIVRIQT
DEERVPRYRFHHVEIKKNILTPNTMLTFVPHLRDLETSEEYKYNVWLQELEDIDRKSGFKPMSREEKVRIAVRSEFASTVLLY
LGTWLDKLSLPGCNRSALIRYMASREPDDAITPRQKSDILNSHRPAESTTPPEANKAANMFTEAFRRVFHQGQPPTKQIELRD
VLLLDESVDSIMETKPTAKDGSSTQHENEHDAVEYWLGTYSILGCLICFSHSCEHGEYDAQNCKRTLSILPRLSDTLKRRRRL
PYSAADTVNGNGPLSPLCKRNCYRSSPRPVAASIQRPWDEEDHFILRSTVATADRSKVTKDPFCLVAQLLDRDCSDVFNEYQK
LNVSMSPVEPANRLQIRTVSWYDRFKKTLIGDWESHTKTHEHQKRELSEPCNHEGPCTSESCICVQHGVLCEKFCGCTVENCA
YKFTGCACHSQGKTCLSKGKERPCICVQLNRECDPDVCGRCGVVERADPENADNEVLHSTGCQNCSLQRGQAKSLALGESQLE
GVGYGLFTIEDIAQDDFIIEYVGELITHDEGVRREARRGDVFDEESNISYVFTLLDNEGIWVDAATYGNLSRYINHASESDKR
GCNITPRILYVNGEYRIKFTAMRDIKAGEELFFNYGENFPNLTKKLLDHKVGGKSDQGKKRSRRPNGEGVARKNPKTDKKKPG
KGKKRAQAVLDEDDMLADTLGFAPKPLRKRKKGPFEEDSEEEEYHPTGTDASIMETPSDGDSDFGNSAARLRKRARQDGKGAA
SSHNGKPRGDLKTRGKRGGARPGSGRPRKYPRKLTSAPMLEHPSASESQDREDPEERQIVAADTIVPAALQRPQPVEINDSME
YVTFSEERTEESRLLQSQLEEDNDEDDDDDDQDVVVRSRVDRGVRNRRPTAKLRDDGDWISGSQ
FUN_000530   CDS   scaffold_10   485455 486743 +   PIL1   Eisosome
core component   PF13805   ENOG41

MHRTYSMRQTRAPTASQLQNPPPPPSSTKSGRLFKSSFGHALRRNAGGAFGPDLAKKLSQLVKMEKNVMRSLETVARERMEVA
QQLSIWGEAGDEDVSDVTDKLGVLLYEIGELEDQYVDRYDQYRVTMKSIRNIEASVQPSRDRKQKITDQIAQLKYKEPNSPKI
VVLEQELVRAEAESLVAEAQLSNITREKVKAAYTYQFDALREHCEKVAIIAGYGKHLLELIDDTPVTPGETRQAYDGYEASKA
IIQDCEDALTNWVTSNAVVSSKLSTRARTLSQRRRNNIRNRGEGHDLSGQDVPLNDGSSWTARDRDLDSDEEEEEEDARGSIL
DSDGGLNGESRGRTQEAVAA
FUN_000531   CDS   scaffold_10   486983 487367 -
hypothetical protein   PF01423

MDTEKAREYLSSILNKNLRVYTSDGRLFWGALKCTDPDKNIVLAHTYEYRQPSSREREKAAEKADGETVKVDMTSRYLGLVVI
PGEHIAKMEVEEFASQMRNQNII
FUN_000532   CDS   scaffold_10   487754 491459 +
hypothetical protein   EOG09260B3H   PF00752;PF00867

MGVTGLWTVVQPCARPTNLATLNRKRLAVDASIWIYQFLKAVRDKEGNALRNSHVVGFFRRICKLLWFGIQPVFVFDGGAPIL
KRQTIQHRKRRREGRREDAVRTAGKLLAVQMQRIAEEDHDRRRRDRERDISAREEQQQEVIPDASQLVYDDEALLSQNERQKG
RTFRKQDAYHLPDLDGGGIAAMGKPDDPRIMSVEELEEYARQFHDGEDINLYDFSKIDFDGEFFKSLPAPDRYNILNAARLRS
RLRMGLSKEQLEEMFPDRMAFSKFQIERVKERNHLTQRLMYEVGMTGTDLTLGVNARVAGEKNREYILVKNEGAEGGWALGVV
SRDKDVGEAHKPIDVDAIQVQYQSKEDEEEEEEDFEDVPIEGLNRLPKPSPAQVASYQAAQDIAARRRLIYGNHRQESSPQGE
ESLFVGGNIGNTDVLFEQPPEEALHDDEEDDLNRAIAMSLRNQHGVGQESDHEEEFEDVPMEAPKWTQKSVDAQKPITAKGGS
MIAHIVNNRASAAVPRRQERDTAADSDSDEDMQTMLAKARIKKKPQPKPKFVPVVENKKNPFDGPLPFPKLDWGSSLFGKKKA
PEAGPNKKSEVADENPTVLNDEEDDMAGGFERELQDENAPRPLPPWLTDDTDIRESLKKQQETEREINNADRQAAEEEERLYR
RKMQDQLIHIDSSSDDDSDVEVLERVPSPKKPAQKEPATIDVEDETEKIKEASLQNEESESTEKASTLTHEREDALEQVADKA
EEPSPEAEAQGLADRTVEEPTAHSAQEDGDKPQESESPEPEFEDVVPTNVVTEAAAEKSPTPIFEELPSIGVPAVEGNQELDI
DDDGLFDDVEYDEFSDPGDEELMAQMAEEAEEHARFASELNNKTAEQNKEDYERELRALRNQQKKDRRDADEVTQVMITECQA
LLRLFGIPYITAPMEAEAQCAELVRLGLVDGIVTDDSDTFLFGGTRVYKNMFNSNKFVECYLVGDIEKELSLSREQLISLAHL

LGSDYTEGLPGVGPVTAVEILSEFPGKSGLENFREWWRSVQSQTRPKDADVSTPFRKKFRKSQGTKLFLPPGFPNPAVYDAYL
HPEVDDSNENFQWGVPDVEGLRQFLMATIGWSKERTDEVLVPVIKDMNKRDREGTQSNITRFFGGSVGVGAKEAFAPRQKAQG
SKRMAAAVDRLRANVADEEPRGAENGGKRKRASRRNAAAPVDEEEYAEEVGDEGNEESISRGRGKVKRARAS
FUN_000533 CDS scaffold_10 492086 495507 -
hypothetical protein PF08101 ENOG41

MAGLFSRLKGKDGKSKKKGANDSAQQLPQKPQWTDAWARTYVEPEEVHELIRCCTEELKARGLDHPFLLLPYRPTSDPSAVRS
FIRHFFESHANLRGEQLTQELRMTEPMVVSGVAKWCWSRIQGGIVGWDAYELFKVGEIDSNMARDSFKTFIPISVENGARQRI
IFDFFDLLAAVAAHGKSNGLGGRKLSRMAAWWAFEHKDTNKGFEGGYDAWLRAADATSHMFFAFLRSLSPEQNVTGITMLPRS
LQKLLQETEYPPQRPSLMMSTTNKVVMIVDTVSPTPFALLRRANHFQYRDSDRALQEFSEYDDPVQALTDECRRVLKAISGAN
QSQASSSKHSTSLRDASWSRFEDIGFSSTLDEDDDDDDSALTYKRQPPALRRTPASGNGLGRPTTPSWADFLSSGFVDENQPA
RSNLLLPPDKVLPPLETQRQQSSQSHRPRLEHDHSLEPGELASIATFLLDDSFWWVWMSSLAPEETAERKSAFGRCAVIETKI
TSGRWLVMEEMVAGAAPEPQEGAYIAEKKGLFSWTKRGKGLGRRKSTANKSVENIGQGTSKASIGPDTHARIQAKAAQLRAQE
QQGKKLAQQPLYQRRGRTDAELMAEKTNSVLTLQPTIIGEASSALKWVSKYDKGTIKDLYMANNNAGRGTMVSPIPSESMNGD
AASNSGTQAPDQPPQVPVKDVASPITSPTFSPTSNETTRKPLPQPEHPVEVQPQPEPVQAPTPEPVSEPVQEPTPQPVLPVSP
LSPVPPPKDEAPQDLARENMVSPTPSSPENKKKKLQKAEKENRGFRKLFRKNRSSKLPDDAAANVHEFLRQTQATPEPQAQKP
LTPADTPEPAATIQRVPVPQPAQTDEADMPTPTPAAEFVTPMEEPVQRVDTPQKNQDIAEPTIDQSQIALNAKDSAPAQKAVP
QFNQGPLQDQPAFAPDSDDEDDATPPPIPRSPRHSPQIGGPAQPEEKLSHSAGPGVQDRWAQIRKNAAERAATRPDDQLRPSY
SNSNRTGDGDDDTSGEETIESRVARIKARVAELTNNMEGTNGPQANATRH
FUN_000534 CDS scaffold_10 498987 500150 -
hypothetical protein EOG09264GMT PF03167

MSTLKRKAGASAGNDSKKPKANGNIASFFGAAPKPTGTGASAPAPAVKFDKAKWVASLKPEQKELLQLEIDTLDESWLAHLKD
DLVTKDFLDLKRFLDREISSGRKVFPPRNDIYSWSRHTPFSNVKVVIVGQDPYHNDNQAHGLAFSVRPPTPAPPSLKNMYIAL
KKDYPTFEPPPNRGGLLTPWADRGVLMLNTCLTVRAHEANSHSNRGWEKLTQRVIDLVAQKRTRGVVFMAWGTPAGKRVQKID
RVKHLVLQSVHPSPLSASRGFFDCGHFRKANDWLVTRYGPEGEIDWALGPGTSTKAPATEADAKEAKSAEKKAEAIEKTKSEV
VKKAEEDKENDAFDEDEEALEEALRLAEEEEKEKEK
FUN_000535 CDS scaffold_10 501534 504183 +
hypothetical protein

MRQSIVWVLLLWQVTTGLGTNVKHYDETYKDKNLEGAGQNQYIASPKTDVVVDQSQPAGPQPGAELVDAARIELTKAPQPLHR
RKRRPSGLVGTALRYILKAIPSGPATAPSQSQETTVNGAVAKAVDFLEQAAQQNNSDALYLLAEINFFGNYSYPRNLEVAFNN
YHQLATVYGNSTAQYMLGVYYSTGIGNVVERDQAKALLYYTFAAIRGNARAEMATAFRHHAGIGTTKNCEAAVKYYKRVADKA
IAWYRAGPPGGRSWIHQSWRISDDDGGIYGEGASASSSGMNAFKASPGSDANAAIDDVIEYLDLMSQKGDSKASYNLGRIYYD
GQRGLERDVDLARKYFVLVALRYWKKDGRLSENPKPGIEKIASKAAGYLGRMYLRGDGVPQNFERAKVWFERGITQHDAQSQH
GLGLMMLHGYGQKENVKRAMELFKSSADQDYAPALVQMGQLYLDQGGQEDVRIANNYFELAGRHGNIEAHYYIAEMIHHGVGR
EKHCPASLTYYKSVAEKAEPLVSSWGDANDAYEASDYELAFLEYLLAAEQGYEKAQTNVAYMIDSVQNGVSISSLLGQPREKS
SLLKNPSLALVYWTRSSRQSNVDALVKMGDYYYHGIGTEEDITKAVQCYTGASDYSQSAQALFNLGWMHENGIGLVQDFHLAK
RYYDHALEVNDEAYLPVTLSLLKLRLRSAWNTLTHGPIHSIQDEPKPKKDRSFSEWIANFLREDNQYYEEEHYTGGDLYDGAL
GDGQGTDDDDGVLESVLIIGVTFSLVFLLWWRQRLQQAHQQAEDARRREQGLPPNPRLDGPGPDFAGWGAAGGVVL
FUN_001175 CDS scaffold_11 3832 4978 +
hypothetical protein PF00098

MDSFQQQQQQQPRGACYSCGSTGHQARDCPTKGPAKCYNCGGEGHMSRDCTEPMKDNKSCYKCGQPGHISRDCPMSGGSGQAT
ECYKASLFVLQTTAAANNSSYGNNYGGGFGGGAGKTCYSCGGYGHMSRECVNGMKCYNCGESGHYSRDCPKESAGGEKICYKC
QQPGHVQSQCPSN
FUN_001176 CDS scaffold_11 7516 9227 -
hypothetical protein PF00173;PF00441;PF02770;PF02771;PF08028
ENOG41
MSSPTDTFSREEVRSHTTDESLWCIIDSTVYDLTDFVDAHPGGETVLKQVAGQDATTAFYNLHRHEVLTKYKDLAVGTIEGEK

PQVITPQPGDLSKVPYAEPLWLAEPFRSPYYKDSHRRLQRKLREFVDSELYAEAQECEATGRYISQKMIDRMSELGILHMRIG
PGKHLHGVDLMGGAVKGEEFDYFHDLIVAQELARAMARGFADGNMAGMTIGLTAVLNFARDEAWKNKIANEVFSGKKKICLAI
TEAFAGSDVAGLRTTAEKTPDGKHYIINGTKKWITNGVWCDYFVTGARTDKGLSVFLIERGEGVETKQIKTSYSTTAGTAFVT
FDNVKVPAENMLGKENKGIQVILSNFNHERWYMVCGSLRLSRSIIEECLKWSNQRQVFGKSLIDQPVIRQNLAKMIALIEANQ
SWLETVTYQMCHMPYSQQAQHLAGPIGLLKMSATRAAHEVADESVQIWGGRGITQTGMGKFIEMFHRTYKFDAILGGAEEVLA
DLGVRQAMRNFPKAML
FUN_001177 CDS scaffold_11 11587 13394 +
hypothetical protein PF01266;PF01593;PF13450 ENOG41

MDAYSICCTSPAARHTSKIYQDLWSTRPHTRVGYDQDKILRKFVTMDSLKQRWRGDLTSKPHIGVVGAGLAGLRCADILIQNG
FKVTIIEARNRVGGRLHQEVLPNGHLADVGPNWIHGTNDNPMLDLAKQTNTAISDWESTSCVFNESGELLSLKDGEKYSTIVW
DIIQDAFKHSNSNSSDIDSKESLHDFFAQKVTERVPDTEADHERIRSIVMQISEMWGAFIGSPVYRQSLKFFWLEECIEGENL
FCAGTYKKVLDEVAKPALEGAEIKFQTKVDRISYRSNSEEKAKVTTESGQTLQFDEVVVTAPLGWLKRNLDAFEPALPARIAK
AIDAIGYGCLEKVYINFPKPYWLGKEGDDRKAEGFVQWLSPNYVPDLNPRRWNQEVVELASLDPEVSHPTLLFYTYGEQSQYI
TSELLKILDPQKKDEFLLNFFKPYYSRLPHYSESNPDCKPSGFMATDWLHDELSGFGSYSNFQVGLEEGDADIRTMREGLPDH
GLWLAGEHTAPFVGLGTATGAYWSGEAVGKRIAEAYYGRSK
FUN_001178 CDS scaffold_11 14578 21326 + ATG2 autophagy-
protein 2 EOG092602MO PF09333;PF13329

MATLFQSFRSSSMPKRLLRYALSRLELLDAEALDIDNLDLAIGRNTVLEFRDVGIKLKKLNKLLQLPDTFRLQKAKVLLLRVT
IPMDFYTSPIIVEVDGVDIALQVIGAESKPARSSGTRTPEETTAVPNTVDLAQSFLETQPRSERRKLEDALAAETQDLGASIA
MSDDGSEDDLAYGTGQALSLPAFLADFLQGIVDRTQVRIKGVNFQLDVEVPVEPNTTTTELVSFQVALDGIDVEGVTTASDNA
EGSPTIVPKEGKRHVSLSNVRAYLISEANVFSALARSPSVTSPSLASSPAMTRNPPSRQTTTLSLSSLHEESVTASQASIRSE
EQESSPRPGFPQVEPLVLSQHSINQRLAGSHQSLQSDNSLAGSQQSLRDEYSVDHESPEHEYPLSDSEDALGIPYEFSSPQDD
DAEDSPVTPRASMYHDFNNSPNDEPVFHSTFLPGEHGSHSTALENERPQWTIAEREARSAPTLQTPATQANLEASRRGVSNEH
LSWTSSSESFGARSAEDLAQSHMYTHEEAESMYMSAFSQTNAQSAAFLPAVPSHGNAPSPKPQDDLIEEVEQLSEPAKPVEPV
EAAVTPPTTAHTHVANEETEPDTIDAKPDMPEAIPLQISDVKPPSPELQEAPATVPLEQESKPFIPGAWEDDDDDDDPSGNAI
APSTTLRHSMYRKDDPSISLDASDSESSEPAFSRACLTDADPENPRTPTRQQEGAATPKGPTRLVKEILNLKTISIYIPSQHQ
HLHVQPASPESVAQLSQSMGRSTYPQAPGAFSMHGGPSMQQNKAASGASKIDETLEVNLSPINLKFDASLGFLLAMVVGKLLD
AVKDKKAPSVESTKQETSSKKMPNFKITLEEIKLDFINRLGGISDTPERYMDLDPSPFFDQEVLLNTTLQNLVISIFETEIST
PPVIKGKLATQPASLTKIDLQKFRFGYANGDIISFDKGKPMSASVRETFLSAGSDIGIEILQSGGKTKIDVQTLPLVFQLDLR
RLDETFSWFGGLSSFLNMSASIASSPAPTPKPAAPTPKPRGVRFDTPVDPNDKSVASQNKINVRIGGSYVELIGKDCSMLVET
SSIKAVSRDEVIGLGCGMVRITGPYLKNSTADPPINTEITTVRVEFLTTPNDSDLEKLLELIIPSKHQFDGDNDEIMVDTLLR
QRRKGSVLRLSVDGVNVRVQNMSQLSILPNLGDEVAKLSTVAKYLPEDDRPGLLTLAKIQKVALSLDFGGKLGHLGADIKDLH
VGHITVPSLVAIAIHDLSVKRNRSEELVSTATYGAKEISLRGPVLMARMIGDEIEPVIKLKMQDLCIEYRVPTIMDILELGED
ATPQDFEASLAASVANLGDHAHHALTGQPGSPGEKGKGGKPMTLDIGFRDCLLGLNPLGQPSKMVIALTDAHLVTMLPKDVET
NAVFTINKSSILLINDIAEVATSELPVTQRPRASSSTSRQVADMCARGYVDICYISSAKLVVDVKELDDGEKQLCVELKDDLL
VLETCADSTQTLISLANALKPPTPPSKENKYLTDVVPMQDLLASISAEAFGRPEGEYDFDQDFAGAQEMAGSDSEADYNTDSP
LQVQSQFYDEEPVAEELFDATSSSIISHGSYRSGPQMKDTNEGVLLTGFGSASQQTVDSDDLVIQEDYYGSGASKDSKAKIWN
SKKNSYDRAPSDLVKRSPVKVSVRDVHVIWNLFDGYDWVHTRDVITKAVQDVEAKAYERQARAGQVQVYEEELEDEEAIGDFL
FNSIYIGIPANRDPQELSRAINEGLNDNATETESVATTAFTSATNRTARARQPKSKRLKLKRSRHHKITFELRGVDADLFVFP
PNSGETLNSIDIRIDNLDIFDHVPTSTWKKFATYDQDMGEREMGTSMVHLELLNVKPQPSLEASEIVLRATLLPLRLHVDQDA
LDFITRFFEFKDDQVPVHTSKSDVPFLQRAEVNNVAVKLDFKPKRVDYAGLRSGHTTEFMNFIVLEEARMVLRHVIIYGISGF
EKLGKTLNDIWMPDVKANQLPGILAGLAPVRSLVNVGSGFRDLVEVPIREYKKDGRVIRSISKGATAFARTTGTELVKLGAKL
AVGTQYALQGAEGMLSDPQQAYEGWDEDDMDPEEQRQISLYADQPMGVISGIRGGYRSLARDVNLVRDAIIAVPGEVMESSSA
TGAAKAVLKRAPTIIFRPAVGVTRAIGQTLMGATNSIDPNNRRRIEEKYKRY
FUN_001179 CDS scaffold_11 21889 22907 -
hypothetical protein PF01501 ENOG41
GT8
MADSESRKRAVDSPKVWTSLITNLDYLPGLLTLEHSLRAAKSRYPLVALYTDSFPPEGHTALRARGIAVQHIPYLLPTKGKDY

SNDPRFYDCWSKLAPFSLTEYERVVQLDSDMLVLRNMDELMDLKLDPPSIAETGDKTVSKRVFAAGHACVCNPLKKPHYPKDW
IPENCAFTSQHSNPDAAQTEAADPSVGPLGFMNGGLQVVNPSQGLYAQILAHMEADAANMDFADQSLLSDLYRERWVPLPYIY
NALKTLRWKGVHDAIWRDESVKNIHYILSPKPWDEINEKGEWTGKEESHKWWVDANRVRKELEKEKGVPDDGF
FUN_001180 CDS scaffold_11 26942 27610 +
hypothetical protein ENOG41

MPRITAPVSKLTRSLAESQRHAGIALMPKYAELLGASSEQSQHSESRGLKTTHRPTPQPSLSGRRRPLMQSFHSSTPPRAPVN
HVDSTVLPKIHATPNFNEPLPRMPLLPDNYGAYHSSMSDAADLYSKRPVIFAVDPDVVVPGAPMATMDAVNMDSVDVKFVHEQ
PAQETKADDSDRSTFLRDVFGGMIQDILPGRTLSKN
FUN_001181 CDS scaffold_11 28238 29424 - GLE2 RNA export
factor gle2 PF00400

MSTLFGGASAAATTSNTVGDLKQDVALSDPPTDTISDLSFSPAPNGPDFLAISSWDNKVRIYEIAANGQSQGRHAYEHSQPVL
NCDFSKDGTKVASAGADKNVKVCDLASQQDVVIGTHDQPVRTCRFFLNDGNPMVVSGSWDKTIKYWDLRQQGPAATVQCQERV
YTMDVRDNLCVVGTADRYINVIDLKNPTKFYKTLQSPLKWQTRVVSCFTDSAGFAIGSIEGRCAIQYVEDKDSSSNFSFKCHR
DPPANSVTNVYAVNDISFHPVHGTFSTAGSDGTFHFWDKDAKHRLKGYPNVGGSITSTTFNKNGSIFAYAVGYDWAKGYQHNT
QNYPIKVMLHPVNQDECKPRPSLKKR
FUN_001182 CDS scaffold_11 30993 32084 -
hypothetical protein ENOG41

MAGANKKMFGLSHVVPVIMPLALGSAGYAAYRINWGPLIKAFLTGPGRTSRILLLLFVVFNWKSMPFAWTYRVFYVIFYHTFW
RKSPNLGPEALFKPMISTTHAPLLEIDYNLHKSNSTYFADLDVSRSHLVTYLCRASYITLAQNTKTKIVLDPKTDKPIKGQMS
IILGAVECSFKREIPAYKSFEMWSRILCWDRKWLYIVTHYIPKGAAKPTSWLDPTFKKVSARGPQDAAGGWEKKIYATAISKY
VFKLGRFTVNPAIMLGGASGLLPERPGGWTSGEQQLGDLSADVSDVDLDVPGEWDWRRVEAQRRQGMEYAKHFQELDNLHDLF
DGGNEGALGKFTP
FUN_001183 tRNA scaffold_11 32751 32841 - tRNA-Ala

FUN_001184 CDS scaffold_11 33023 34061 -
hypothetical protein PF00701

MSAPPPKGIYVPVPTFFASKKTSNYNAITPPIDIETQVAHSVYLAKNGIKGLVVLGSTGEAVHIHPRDRHAILSGIKAGLEKE
GFKDYPIIAGTATNSVEETVEQLKDAQKSGSQWGLCLVPGYNAGAATQEGIIQWFTAVADQSPIPVMIYHYPGVSNNVKVAPS
TYATLAKHSNIVGCKLSHGDVSVAAQVASNPSIDHTHFHCFTGLGQQLLPVISVGFAGAIDGSAGFFPKSLVRLFELSSKTQP
TDAEIQERRLLQFKVSSMEELVVKFGTVGIKDAISRLRGFGDTDGTRLPLVGGIPGGDAEWAKWKGVIGAVDEVEKSL
FUN_001185 CDS scaffold_11 35067 36240 -
hypothetical protein PF00288;PF08544

MESFIIKTPSSSANIGPGFDVIGLALSVYLELHVTIDRSKTKSEHPLNCKITYEGEGEDEISLDPQVNLITRVALYVLRCNDQ
RAFPVETHVHVKNPIPLGRGLGSSGAAVVAGVALGKEVGGLKHLDNDRLFDYCLMIERHPDNVGAALYGGFVGTYLKPLTPED
VARTEIPLSEVLPAPAGGEDTGKKPPNPPYGIGHHIKFPWAKEIKAVAIIPDFQVATHAARGVLPANYTRPDVVFNLQRIALL
PVALGQSPPDPELIHLAMQDKIHQPYRQTLIPGLTEIVETMSPKTDEGFLGVCLSGAGPTILALATHNFEAIAAKIIAKLREH
NEKKDLPCQWMVLEPAEGTHVIRS
FUN_001186 CDS scaffold_11 38920 39396 +
hypothetical protein PF07249 ENOG41

MKLSGVLSALALTSAVSATTVSYDTGYDDKSRPMTAVSCSCSDGKNGLITKYGWKTQGNVPTKYVGGVNIIEGWNSANCGGCYRL
EYKGKKINVLAIDHAASGFNIGLDAMNALTNGQAVKLGRINAQVYHANPSDCGLKK
FUN_001187 CDS scaffold_11 39870 40936 -
hypothetical protein ENOG41

MAAIVEDSLDSLLTGDLSDFTQQRAAILEPAYKSYLLSTDFREDSPRPSSTYLLPSSEVVVLISRCPETETATDLHITKLSDT
FADNFIGYCPEEKCVVFKGERGLIDDFIDFTESVAGEKEVTVILKAPEQIRLPLGGLEHGLKYEEFVARVEVLECALWIGNSP
SEQEEGGIRFTNLATDPKLPEPVTSKRVPTPDYVHVTDDNSLSLEGLSNINSTNIRSPMTGASSPLSDPPPSPLIHTPSSPLS
DPPSDIDEISLHNWDDLHALSSSSSEGGETPPSLREWE
FUN_001188 CDS scaffold_11 42163 43504 +
hypothetical protein PF00646;PF12937 ENOG41

MASLNMQNLPNELVAHILSFLDSESDLQRRIYLDPAKLNRPVSSGPNTPLKNASLVCRLWRSSVIKILFRYVVWPFWRFYKPV
AQDVASQIEVLNFIRRNNLSSSVENFTILIDLPLGSGQNRYPDGQFWGVLPPVSHQPETPMSWNLLWSVLNQEDRDAEGSDTR
TEEDRAQCFWDNNWLWHTIFDVIDPSRITMISSIDILASLLSRTVDLSSHWAFNSTYYVLSLSRPSRSIAESKRVQTLPSHIP
GPTNRIPCDLFHIRDWSSVLVNEGSFAPVYSTYEFFHYSAATLLPAIFDSSDPSFDVLCANLHSLSYIATFPLSHHISDHLIP
NCPPVEHLYVQLMPKNRDFWESSSLSRVNLSDLWLECDTSYALLVRQMLETVPQPGWQKLKVFETGDTPAEGVWKMNTYDDHI
NGSNGWREESEGVFVKDDIESSDEQTMVEES
FUN_001189 CDS scaffold_11 43983 45651 -
hypothetical protein ENOG41

MPRRDTSDQEVRLLQGHNRSSTDSGSSTSSSDDTTCEPGPSSTARPNVVGARGTAEPPSDASDLDTKRHDPKNTKIHSDSEWL
ELDNMDPNDLRRPHYHRHGDGKSGTPLLYKEDDPERGRAGYSPPNDGSKGSREPSGDDLPYHPRESMGRPSLSRRSTMRSRSP
QTAAAVAEDSTKKRYTYAGFFLIISLIAFCVQTELSAYIQHDLGWDKAYCMMYFTHGSWVVLWPVQLLILRFQKRDVPWPVFW
KRHKQQLRTTAIMIEKQTLDVFHISIQHRARPVRYFVRFTAIITCSLTVAGLSWYIAVSLTTPSDLTAIYNCSAFFAYVFSVP
LLREPLRLDKSVAVLIAIGGVLVVAYGDTKEGGESVEAGNRFLGNLVIGVGSVLYGLYEVLYKRYACPPEGCSPGRGMIFANT
FGSLIGLFTLTVLWLPLPILDWLNIEKFEIPAASTCWLILLAVLSNATFSGSFLVLISLTSPVLSSVAALLTIFIVAIVDWMI
TGEPLSFAAIVGGAMIIVAFLGLTWSTYREMKEHAAMEPVPDFSDSDKDGDIDSDED
FUN_001190 CDS scaffold_11 46489 47603 -
hypothetical protein PF00248

MPLVTQNVKPRVILGLMTFGPPGSEQLDARIFDPETYNKALDIFQSKGYNEVDTARIYVGGKQEGWTGSQTNWKERGLTVDTK
VKYPVQPGENTYDKVIESVETSLKELGTDCIDLLYLHRPDRGTPFQETLEAINKLHKDGKFVKFGISNFTAYEVAEVVMICKY
NNWVRPTVYQGMYNCLTRSIEAELFVACRRYGLDIVVYNPIAGGLLSGKIKSMDIKPESGRFSDQSKIGTAYRQRYFRESTFK
ALKAIEEATEKNGLSMLETALRWIIHHSGLKVTNGNDGIIIGMSNIQQLEQNLELVEKGPLPDEVVKALDQAWLYSKADTANY
WHGDLEYTYDVHEALFGASAK
FUN_001191 CDS scaffold_11 48618 51047 +
hypothetical protein ENOG41

MRAQLARHARRILIQQVRSPSCAVYSAPLRRRCAPRTINNGYSRRFFLDGLFAKAPREIRQPEFEPGWQKIMIWRSRMLDNLR
PPPAEELIQAWKSFFEGKLSSRMALNGTQAMQCRRLLEYLIKEREFPTQAKIEISEILKALTALESLRPRERTEHHLDFARTL
WSAINNPNLQKPNWVTSGSIWSQYLTVLCTFGGSKEALEIAYAKWNDVMEFCKKKTKNPILSLADGLARDGCEEDLVELINYA
EKNGYPYDKHLQRVLVTYYAQNNRVSETKQWFEKPLSSGSPSNKIIPLIASFAARNNLQEWAVPFFLEIGNKLNEKPEQHYWD
SLLQAILIIGKGLPEVEKMISHMDSSQEAIKTTTATINSLLRAALGMNDSLLAEEILSLATDRGLPLDGESHLILMEMRLHAG
YLPGVHAAYKKVTHHEPWHVQPDLWWDFGRLVNEYMMTLTTQSTPNFKIIGEIIEMCEENQVLLEPETVASLCIRFLENEQHY
EVMDILSVHSFQFSAAEREVIQDAFTQFCVNRETSTSRAWNGYQILRQFFQDLSFEHRVQLMEAFFERKRPDMASHVFGHMRQ
HRNSDYHPKRETYISCFEGLSRCPDEEPLDIVHNMLKMDTTVEPNTQLYTSLLLAHAACGKTTQAMDFWQAITASQEGPSYAS
LEAVFWALERTPNGASQAHIIWKRIKSMDIDVPPAVYNAYVGAVAGSGEEAGVKDLVMRMSLATAGEPDAMTLGIAFNALPGQ
TLQSNFKSWAQARYPEAWASLESKGKRLNKDSLCQYKLNRVIRA
FUN_001192 CDS scaffold_11 51235 51924 -
hypothetical protein EOG09264XLN PF01084

MPPQLPTASTASTIARSVIRPFSTTAATAAPGRHPPSGGHANRLAGLNASNSKGGLGRGGRGDAAARLLERFKTRTNTYTMEK
TAQMEFLKNQKMSNDFLKQMPRRWEAGDVYSPHDLSPVEMQKWRKKTVRNNDVIDALGISPLDMYKNFSLIEHFTSTSGMINH

SNLTRLRPVNQRKVAKMVRRVQGMGIYPSVHAHPEMLRDQFFSRHK
FUN_001193 CDS scaffold_11 52212 52819 + IMP3 Small subunit (SSU) processome component EOG09264Y0W PF00163;PF01479

MVRKLKHHEQKLLRKTDFITYKSDNGHRDKAVMRRYMIQKPEDYHKYNRLCGSLRQLAHRLSLLPPENATRRKHEELLLNKLY
DMGVLSSASKLSAVENSVTVSAFARRRLPVLMTRLRMAETVQAATKMIEQGHVRVGTETVTDPAYLVTRGMEDFVTWTVGSKI
KRNIMKYRDQLDDFELL
FUN_001194 CDS scaffold_11 53602 54557 +
hypothetical protein PF08241;PF08242;PF13489 ENOG41

MAVLSTLKELIECLTGPYLFMGIAMRFLPGTLLEVIRNKDFRTLFSPSRFKDVWFGNFWAFIGPQVKANAESRVIPLLEGRVR
NGRMGDEVVGEPINGVVLEIGAGTGMWADVFAKVNVGVNNQGDADGELRRRKGGNGLTRVYGIEPNPKSAATLRQRVKEIGLD
DIYEVVPVGIESLDDPEAWDGRIEPESVDCIVTILCLCSIPEPEKNIKLLYQSLKKGGRWYAYEHVRINENRGVLLRAYQWVT
GLVWSQVMGSCRICRSTGKSLREAGPWEKIDLAQPEGEARFGILPHVVGTLTK
FUN_001195 CDS scaffold_11 54659 57746 -
hypothetical protein PF00176;PF00271;PF04851;PF14773

MPPAPPDVIEIPDDEGTASESEDNISSFSKSAQKRKHETEIEEKVEWTGDEDDLPAKPKKRRSGRKSTRKKKGDDVHAENETP
KDDYELAGLPDYLIERRRKFDTKRKLHHDNALMVPPDYSGIHFEETRRLGELEERPKFSESSGIKPSRPYKDIELPQSAGLIP
ASIAQYLRDYQVAGVSFLHRKFVYQEGGILGDDMGLGKTVQVAAFLAVAFGKTGDERDAKRLRQIRQYPHRWYPRILIICPGS
LIVNWKNELDRWGWWHTDLFHGSHKDDALSTARAGLVEIMITTYDTYKNSRSSINMVQWDAVIADECHRLKDRYSETTKALNE
INALCRLGLTGTAIQNRYEELWTLLDWTNPGHFGTLAEWTQRVTKPLTVGQSHDATKAQLSLARTTADKLVHNLLPQYFLRRM
KSIIAKQLPKKTDRVVFCPLTDLQREAYENFLESADIDLLRSLSEPCCGDKKRGWCCDRLLSSGVRWQNIVFPSMIVLQKMAN
HLTLLVPQTTDLEGKHESDLNTLRTCLPEDWKYVYDNRDRIKNLVNPEFCGKWKVLKKLLKFWHSNGDKVLVFSHSVRLLRIL
QHLFTNTSYTVSYLDGSLSYEQRQEVVDTFNSDPTQFVFLISTKAGGVGLNITSANKVVIVDPHWNPSYDLQAQDRAYRIGQT
RDVEVFRLISLGTVEEIVYARQIYKQQQANIGYTASSERRYFKGVQQDTDRKGEIFGLANIFTYHNDSGLLQEIVNKTNIAEA
KAGVHLVDVNMEQAAKDGEDLAMVKNEETDAEDGGMSQLATLLTAENQQRMIESKKAKKPKSDAIQAILTSAGVEYTHDNNEV
IGTSKVEEQLSRRAVMTTYSSGDVDGQSALFAESDDEQGDGLHNMYNPPEEVMLRHFCEMAREFGFTNATDFALVVESWSQEA
RRNCLDTFYKRRAAKLIQEGIIKEEDMKDTAQVDTKHDIDAKEEVKDEKKDAKLTLKEEDIKKDDTIHTLVKTENTKMEAVDT
KMEGEKKIKTEAQGSSIRTSIFLSDDDDDDEL
FUN_001196 CDS scaffold_11 61870 62696 - GPI11 Glycosylphosphatidylinositol (GPI) anchor assembly protein PF06699

MPLVDPVTMSTTLVKSGPATQQPATKAAVPAIPLVNSPAALPASVAHQLLLAGLFYWRFDALVADPVSTLQTGLPVVAAIQAV
YLILSLPPAGSSGSSKKPRPGEKRKSDGREAKAIPTAVISLLLALILTPALHLLLVLFGAPFLTHVPHTFLCCAHIAVLAIYP
VFYVRGSDPVPLQAVVGVSAPFDQTFGGFIGTVVGAWLGAVPIPLDWDREWQKWPVTIVVGAYIGYIVGSQILGTVFFGKRWE
VTPEIKEE
FUN_001197 CDS scaffold_11 63964 64278 +
hypothetical protein ENOG41

MSPIVVRSAARAVQRRQFSLLTAMRNAGRAMESHPFERLPITQQPAKPDYAKMFKRVGSQALFFFPGFAVILGWPLAAQYAFD
GRL
FUN_001198 CDS scaffold_11 65175 67403 - ATG7 Autophagy protein 7 PF00899;PF16420

MAAPLQFAPFISEIELSFYSALFGSKLEHDKLDDSARSILGLYEPRLEEPESSCRMQILGNALTSSRTNAPSPPLGTMRAEGI
IRNVNTLEDFKNTDKPAMLKTAGRQIWDAIKDGSIYSVPSLLSSFIILSYADLKKYKFTYWFAFPALHSDPSWKRSGPAERLN
SKESTALVDRVGTWRYSVDSREHGFFLVKKVHGEVQDTDDPDSSQGLPFRWEVASLREFENGFFDQVAEEDRYVAFVDPSTYP
EGPGWPLRNFLVLIRQRFHLTKTKVICYRDIQAKRHEARSVILPIEMDPVENLEITEMPKVTGWARSSNGKLQAQQVNLGEYM
DPARQADGSVDLNLKLMKWRIAPELDLDAIKNTKCLLLGAGTLGSYVSRNLMGWGVRKITFVDYGRVSFSNPVRQPLFNFQDC

LEGGKPKAPRAAEALKEIYPGIDSEGHTLFVPMLGHPYIDESKTREEYEKLEKLIDEHDAIFLLMDSRESRWLPTVMGKAKGK
IVMNAALGFDSYVVMRHGSESHAEGQTPLGCYFCNDVVAPADSQKDQTLDQQCTVTRPGVAAIASALLVELLTSLLQHPLGKD
APAPQPTPGVVPERDPPEHPLGLVPHQIRGYVSTFQNIVIRGQSYDCCSACSPKILDAYRNDGWGFVKKALQEKDYVAELSGL
AEVQRRAEEMAAQVDWEEDDDLVEDGDGELI
FUN_001199 CDS scaffold_11 69154 70462 +
hypothetical protein PF00383;PF14437

MLIGVCGSICSGKKTVAQYLVEHHGFKLLHLQPHSQPRPSNSSLGAESASPEEHGAESPSQSTDSTAFKGALTSTALTTKNGY
QSSSETTLTLRDGPQHVFSTAEELLDFVTKRWRSRFVTTDIPTEAVLDVFLRRPFFLLLSVDAPLTVRWRRFQDRARQRHAGE
PDMSLEDFVTESDTHLYDAEKGLSPLISRAVVRLLNTSSSLAHLYATLGKLDIPNPDRLRPCWDTYFMELASLAAQRSNCMKR
RVGCVLVGKERRVISTGYNGTPRGLQNCTEGGCPRCNDGNSSGVGLSTCLCIHAEENALLEAGRERIREGSVLYCDTCPCLTC
SIKICQVGISEVVYAHTYSMDNDTARVFREAGVKLRQFIPPPNGLIHLEKMELY
FUN_001200 CDS scaffold_11 75464 78966 +
hypothetical protein EOG09260W9L PF00069;PF07714

MSFSNSSGGTLALPSPTHAHHIDVTSAVRTLRRSISRSPSKFLTRSNSQNSLSPESPHQTSPQSPCRRFGATPQNQQLLPSHT
RSAPPHINGAPHSTAFTPFRPSVRLSLRSAKAGKTSPSRPLTRARASPKSPLKRALNMAPDSGNSLPAIPLASVIDASGQENN
ALGATSPISVSPVKRRSLTLDVAESSPTSFLKSLDSNNDGQMVTNNGSLKRSDATMNLDQPSQGSPVAKRRSLHGISGLGQNE
DQNIFGANTTSSQSFYIHEDSSTEYEIAGTSGSPFRDPVPSPTPATTNVPKRSSSLRKSTLQQRYGERGSWGRRSGERQLAQM
SSEYSPVRSRPRLSLDQFVPPPVPQESPFVSTTPTSKPPPTTFFGDKAHQPHPLSKTLTTSSSGNSLTEEAPIYAPVARMPDR
PRPHLFSRSLPLNATRPTRPNDMSKAMATPSNSQLWVGAFNSTGLISKVNRNPEEDADKKMAPPDTPCKKHSNPFATFPPPAG
SAMKKRGNNRNSFGGLPSTPFGNSTGPNSFGRPGKGMSIFQRGSASRSARRGSVLSLDGDEHKLFGETIDFSTPMEGDAPPTP
TKNTLTPSLSKVSEYSFESPHDNHSPTANRTLHSTTPVLGTSIPREATSSPLDGRRTPQTPRESLLPLDTSRLSISQIGDGFS
DNMPPPVTPTAGRDLRSSTSLLVTPVNARTSNIDIDASLSSRFDKVEQIGKGEFSVVYRVTQADHQMTFGNLSTTPTTSPTKG
RVYAVKKSKHAFQGPKDRDTKVREAEILRTLSFSEHVVQYFDHWDYNNHLYIQTEYCEEGTLDKFLGTVGRGGRLDDFRIFKI
LQDLCLGLKDIHDSGFMHLDLKPANILVTFEGVLKIGDFGLAQSCSSAEGVDVEGDREYMAPEMLKGKSCQSADVFSLGLIIL
ETAANVVLPDNGPTWIALRSGDLSEVPSLTWNPSIEVQRDATGNPTEIMHSDDFTSGKTHDPSNLFGPSKRSELLQPPEFMVN
ATHSSSLDLIVRWMTAQEPSERPTVHQVLELEGLQWVGHHRAAPATVYEGNWGPDEELIPISIAEGGDSDTEMTDV
FUN_001201 CDS scaffold_11 80143 81578 +
hypothetical protein PF01266 ENOG41

MTIPTLLPGQAGLPSIESTKSYWHRKPSEKLLGHRTTAELPSTADVVVVGSGITGAFAARELVKGDCKNVVLLEAREACWGAT
GRNGGHCQPLIYAAKAPVAQFELDTFNFLKDLVSEHNIPCDWETVGGVHRVPSQEILDIVTRHLDRLKKTNPDLADDIKVITD
QEQLKKLRVSGASAAIYQPNAAKLWPYKLVSWVLESLLEANDSSVFNLQTKTPVEHIQRIGDSWALHTPRGQIAARKVILATN
AYTSHLIPKLSGYIVPVRGQVAALTPSDGSSPLEHSHVWWTPDGGDDYLVQRPSGELITGGERLGSSGGQVGLSRDDSINPVI
AQRLRTSLHEAVKLRAPEESEDTSLQATYEWTGIMGYSKDGYPWVGQLPASLGGEDSGLWLSVAYTGHGMPVAARCGIAVAQE
ILGIDDGVKLPAQYRIDGGRVDRARDMKIPSTLLDEVRMMIGDEN
FUN_001202 CDS scaffold_11 81900 83735 -
hypothetical protein PF00501;PF13193

MPIESRFSVSVPNCSIQQWIFGSPSGPLPDQKAFIDADNPEQRYFTYSQARLFAKRIAVGLINNGLKPGDRVLLFASNSLFFP
TIVMGIWMAGGIFTGANPGYVSRELAHQLQDSEASFVVAAEGGMEVALAAASQVGMKASQVFLLDSTWPDSPVETQPREGSRH
WTELIASRPEGERFEWTEPNDSNDSVCSLNYSSGTTGIPKGVEISHYNHVANSCGVTLFHKLHPDYEARRQRAAALCFLPMYH
AFSQGYFITSFPCERVPVYIMPSFDFPKMLAHIQNFRITKLLAVPPILVLMSKHPLARRADMSSIDMIASGAAPLAKDTQREI
AGMIPGGESVVRQGWGMTEATCTALSWDPNKAPSSAAGELTPDSKARLIHIETGEEITAANTPGELWITGPTVMRGYWRNPSA
TQGAFVTDSEGTRWLRTGDVAYVEEYAKGSLFHIVDRVKELIKVKGMQVAPAELESLLLEREDIADAAVVGVMVNGEELPRAY
VVKSTNAKDTSEKDITDWLASRVVKYKQLKGGVVFVDAIPKVPSGKILRKVLRERAKREVSNGLEVRAKL
FUN_001203 CDS scaffold_11 85688 86724 + NUO24
NADH:ubiquinone oxidoreductase 24 PF01257

MASKLTPLIRSAIRPVCRAARPQSRAAFSLTASRRSDTLMVHRNTEDNNPDIPFKFNEKNQTVIAEILKRYPPQYKKAAVMPL
LDLGQRQHGFTSISVMNEVARLLEMPPMRVYEVASFYTMYNRTPVGKYFVQICTTTPCQLGGCGSDVIVKAIKEELGIEQGQT
TADGLFTILEVECLGACVNAPMIQINDDYYEDLTPASVKNLLKSLRAQATASDPSTVNVPKPGPLSSRDTCENSAGQTSLNAE
PWGTETTRADL
FUN_001204 CDS scaffold_11 87016 88747 -
hypothetical protein PF01344;PF07646;PF13415;PF13418;PF13964
ENOG41
MESFSLLKRRTTDLLHNVQQNLPSMPMPSVPSVPSATHQKKSSMKGTWERIDAPDVPRSSHSLNIVHGTAYLFGGEIEPRKPV
DNDMHVVTLPWSSAGADYFKIKASPAKPDAQPKKPEVQQPVQPKAPEPVKSDEKPDEEEIEKKLDEVSLAEDDEEEEEDHDEE
EDESSDEEVESKSKGKQPAGPEKPELGDVPASRVGHATAVIGSRIFLFGGRGGPDMRPLNEGGRVWVFDTRSNTWTYLDPAPV
VPGGAIVPQPAPRSYHTATSIDRPRDFAPKRPKEPETWGQWALGDTSKTGIPQAPVVGNVAEDAVDEESDGYGTFFIHAGCLA
SGERTNDIWAFNVHDRTWTELPAAPGPARGGSAICVSKTRLFRFGGFDGQSELGGQLDFLHLEVETFDDRGTKGEVAVRARGG
WQTILESDPNTDSTEIHAEPAQIWPEPRSVASLEAITSGAGYEYLVLTMGERDPSSEGHEGAGKFLNDVWAFQVPPLGMTAAS
VTAAMWQAVGRKTGEGKWTKLDLEPYDDDNSDEMPAARGWLASAPMGELEESGILIWGGLGEDNERLGDGWILRVGDS
FUN_001205 CDS scaffold_11 90016 91327 +
hypothetical protein ENOG41

MDEIYHKIAEFNSRRIVQTHDVLSFKYSELWSRDDTGIVDYHEGVTTIPDVASWLAMETRTGTTGPESLILRLIWPKFILDDN
CVELPEDSKNQILEKFDLELAYNYFSSCVAGVNAFPEIVKSEATQQAYTFCYAPKLASIWSHTRFRPPSLRPGVTYGMILAGD
KEQHYLKKLLKSKSKWSLSTASHAMFPAFLFAFMFHGEIEVTQNNMKPGIQQIEARTGYSDFKTERKHIAAGELGTLSAQMSG
FAVRLASTERKDKTLQKLLEFVQQRLSLGQSSNPEADELMKNHVGVLQKRLMMQAMDREYTLKRVQIQIDVLNNLISENYSLS
NYIISISTLRDAASMKTLAFVTMFFLPGSFISALFSTDLFDWDSAQADGGDIGIKTTPQFRLYWAL
FUN_001206 CDS scaffold_11 92366 94763 +
hypothetical protein PF19031 ENOG41

MASRSASGTANPAPGTVVPAQLGFLAIFNPTLGNTDETIEDQIVYYASVTTQAAHNKRRRARGQPTQNISPEERNERLRQIGL
AQGMASFSRGFASGSTVDTIDTEKSRVILHELEPGWWILASIDLTKLPLPPRLATKKSEAAEERYEYSSREMKPASLLLRDLL
RAHSIFLMHHDSSLSALFVRNKRTKFITILSRYWDLYLSTWNVMMHGNPARDIFGGIPIAASGELGVGVGEEERGSGERAVLE
GLVGRIEGLVDLVVSKFGDYNPESPPPSDPYGDPEQWLGTGREPGPDDGAIFLGTGALSRKSVRDVTHWMEDLYTWGNHAYGI
IESPTSVRRAKRRRAADRQKAAQDQTRAPASELTKSSTGEQAGESSDKDQPQIQVTEDGKLEKMVSYLKLGYGTYWSLPGGTG
SGDSPTKQPDSPQQDPEPAQDTTKLARPSLAERTPSYEAAGHYLIGLKGEINEDYSGTDSASSDDAGDGANNSRTVLRTVNIE
LESEALDRPEAITVRDFEHPASALTQSQVVGNMIPGYDSHDLNKAKKLRVVVYVNRPFMFTFLFNLRTDSLAMDSLYRSLHHQ
LAPLRKPLLLSTSYRPERPGTDAGSGSNHSGNNIYDLVWDPVSLTVHSSIPNIPEVYDDTEPWSRPDALNTHLHLVNLYIGTR
SRSTALERTEKSSRGWWIVWTRLLDRQEDAATGNLSTIHEGGAGPNTDQNTSQDEGERKYSGPDPRHPDVAKEIFLIRRASDH
VGFRTDSAEGAGKLAQGIGVDTRRYVEELLNLL
FUN_001207 CDS scaffold_11 95917 96796 -
hypothetical protein ENOG41

MKSFTLASAFFAAAAVAQPHANPHGNHHRRHHQNDKRDVVTEVEWVTEIEYVTKMVDATTTVWVRPEAEPTTAPQPETTQPAP
KKVAPKKEKKPAPPPAPTTTLVTSVYTPPPAPEPTTEAEAAAEPTSQYVAPVEPTTQAPAPKPTTQAPKPVVKAPKKEPEPET
EVAPVQQEKAAKPASGGSSSGGSDTKHGEFTYYDIGQGACGEDDTGKDDSINIVALSHLLMGELSNSNPMCGKTITIKANGKT
CQATVADKCMGCAINDIDVSRKVYEEIWGSLDSGRTEVEWWFN
FUN_001208 CDS scaffold_11 99253 100252 -
hypothetical protein PF10294

MSSPPSSPLMDPVTISEDIAPLPSFKASGVATVDFDGVLSSPLKVHEDVRSGCGGQTWPAGMLLGKHMLRYHKDRLADARILE
LGAGGGLIGLAVALECQLQNQLLVTDQLEMYELMKHNIELNNLQDKAKAMVLNWGEDLPAAVLEQKPDVILAGECVYFEPAFP
LLMSTLKALLELNPAAVVYFCFKKRRRADMTFVKMAKKAFKVEEIFDEDRPVFQRQGLFLFSFTSRPTTSQTKATKNQSQPQL
SK
FUN_001209 CDS scaffold_11 101753 104465 +

hypothetical protein PF05345

MTSFILAVLLLTISGLTSSQPTIDYPINSQLPPVARVDEPFSYVFSRYTFRSDSKISYSLGDAPKWISIDSKDRRLYGIPTND
TVPSGDVVGQTIEIIAKDDSGSTLLSSTLVVSRNKGPSLKTPLLEQMEDFGDYSPPSSLISYPSTEFRFTFDAATFEYQPNMI
NYYATSGDGSPLPAWMRFDAGSLTFSGKTPPFESLIQPPQTFDFELVASDIVGFSAVSVAFSVIVGRHKLSVDNPNITLNTTR
GKKLEYSGLAESIKLDDKPVKIDEIDVSTAGMPDWLSLDKKTWDIEGTPGKGDHSTNFTITLRDSYQDTLNIYATVNVSTALF
RSTFDGIQVEAGKDVDLDLRPYFWDPDDIDLQISTKPKKDWLKLDDFNITGKIPVSASGDLNISVTASSKTLDDTETEVLNLS
VIPFESTSSSTTQSRTSSTSTGTSTSVAPTGTSSEPDVQLSDSDGSLTTGTLLLAILLPLLVVIFLSTLLVCCLLRRRRKRQT
CLSSKFRHKISGPVLESLRVNGGSTAMREADKVEIIAAAGKQQRRPIRTPHSEMDSETLVMASPTLGFMATPLVPPRFVAEDS
NTSVSRSLGTPNSEDERRSWVTVGTATAGRPSRDSLRSQRSNSTLSQSTSQLIPPPVFLSDARRRSFMGGNDAADSSLNGLPS
IQSQRALFQQGSDYYTSGNESSLAFASSHLSSPRLLTRVPTRVPDAQLGSHASVGDGEGPSIGATQSLPALRRPELVRLSTQE
LLGEDGGPSSRPWYDLEAPRGLFSDPSFGSGENWRVYESQRDGTGASYHQLVDESPFHPLRPSTAMSSSRDGAQPGERASSEL
ISPSQWGDAQNSIRGSLASLRQGLGHSMSKLSRLSVDPLSVPGSRNSKPAGNSSVNWRREDSGKSEGGSYAFL
FUN_001210 CDS scaffold_11 106531 108659 + PTC2 Protein phosphatase 2C 2 PF00481

MGQTLSEPVVEKTSEKGEDERLIYGVSAMQGWRISMEDAHTAVLDLDSAKSHSSKLSFFGVFDGHGGDKVALFTGQNIHNIIF
KQDTFKSGDYAQGLKDGFLATDRAILNDPKYEEEVSGCTACVSLIAGNKLYVANAGDSRGVLGIKGRAKPLSQDHKPQLENEK
NRITAAGGFVDFGRVNGNLALSRAIGDFEFKKSAELSPENQIVTAYPDVEQHDLTDEDEFLVIACDGIWDCQSSQAVVEFVRR
GIAAKQELDKICENMMDNCLASNSETGGVGCDNMTMVIIGFLNGKTKEEWYEEIARRVANGDGPCAPPEYAEFRGPGVHHNFE
DSDSGYEMDPENKGRSFGVGGYRGRIIFLGDGTEVLTDSDDTEMFDNADEDKDLASQVTKNPSSAEKDTAPVTAPGAAAADNA
SADTKPADVTVTKPTDKDAAEEVKKEASAQEIKKEE
FUN_001211 CDS scaffold_11 109639 112165 -
hypothetical protein ENOG41

MSLAIKSIWRARSRCSAFSGPFPKSRAPLPSRISNPYAIRLESTTAYSDGTNSTEPPETALVALPEKSLEVSEQQPEQVIANL
VTDDTHESRNRAFKDAFRIDAPRLVEDKDAMKLFKPVIGKPLQYPNEQANKASVQEHRRDARRQAGLGVVWSHFDKTVPQWIE
CFGLMKRMTPQWSERANMSAVRIILPKTWDIEVNNQNLEYVDSATGVLQKLRAVVDRNPSAIVLRGQSKILVKVADEIVRYCE
DAEIYELGEVAASDYKTRQLWPTIENAPNGGASLPDDHNDNIWVHKEYQPHSLHVPYEQIPRPPLWTHDNFEHYISTLCYGKV
PPHLAIRFYGQRRVNGRYIDTDGIRIKLIVDAFEDPAAKPFITVPVLKMAISMMAFRGGHRASANHLVQLGEELGIPMDTDIY
NIMLEGYTHKRDIGFFYRFLRRMQARYHHPNIRTWLLFLRLVRGEDERRQVIVAMYQLGMFNHQATRRGIADVMASLDAYAAF
KAGTPLRCFLDEQKERYGEGWLAIDGLNSIATELLRFHRPEDPRIEDCKKLVSIHTESGHRVELKTINIFLSHAAKSRDWDTA
LWAMSLFKEAGCEPDQETYGALLSLAVKTRSPHALGTVYFYGVLHRQLQKGSRNLLSQVLLRTHKDPFWKKLEHQPGIFPRQA
IPDILANKIPRLWVVMSRVERVILDKWAGYVPLKPLEYALGLSYRSNDLPMHSQISAGKPIQVQDLIVKLRRADGQPGKINVR
LKGRFDPAHMIEGWDESLADSNHPSIVQEEEPDSWPDVDDDTPKLLSAGPKTNPKSPGGGQDGPPEGPDSTPSPEAALKELEK
KLEEARRRDSI
FUN_001212 CDS scaffold_11 112590 114134 -
hypothetical protein ENOG41

MLRVKSSRRPAGLKPTDYDHEIALVDHDAAATPAGLLDLEDEQEEEQQENRDEQDGGQKKPSSNTPNNAHTNEHDDSHTEGES
QTHPSSNRIENGQPSTPPQTRPSIEVQEPTPDGFHGDHPHVKPRKPHVERETAIDILYENERGGFLCGIPLFSSQALGGLDPP
AWTNGYHKASPSNIHNAQVPDPTWEWAWPEWRINYQKGVDEHGWEYSFHFSKKFSWHSGKWWNSFVRRRAWIRKRVRKRPEEV
SADPHMLNTDYFTIRPASHKSPKSRASVISSRLSRSSMSQMSAADADENPADIDNVDDLMRALRQARIDREKLDAVNNYMEHA
TDLQNLQHEMHEIMSLFVFQQSRRILLSRLMEIHDETTTQMKKTNTSELRERNQALKDAVHHADEEVRKLAYWSDVKQMAESG
EAKEAVKEDKGWDESWEGVDQSGGAHPMREKAMVNGKKEASET
FUN_001213 CDS scaffold_11 115590 116108 +
hypothetical protein ENOG41

MSKSRTPLILGGAAVSGIGYYLYAAGGNPRAAEKKAESDAHSVAADIKSHLPGRSPNAEGELKGAGAAAGAKIDSVAAEADRQ
AGVIKSNVESVAKDAKAEAMRVVDKFDHRVEAEAAKAKGGISSWFGGGK

FUN_001214 CDS scaffold_11 116482 119275 -
hypothetical protein PF06424;PF13181;PF13432;PF14559

MSGRRDFLNQAAPENYVAGLGRGATGFTTRSDLGPARDGPSEDQIKEALAKRAAQLGLAPDKKGKDKEKDEDEGGDEERYQDP
DNEVGLFAGGVYDKDDEEADKIWEWVDERMDRRKKQREAREQAEKDEYERNNPKIQQQFSDLKRALATVTDDEWANLPEVGDL
TGKNRRSKQALRQRFYAVPDSVIAAARDSSEMGTTVMDEGTSSNAGGSDAADGTMTNFAKIGAARDKVLKSRLEQAGSDSAAP
GTSTSIDPQGYLTSLNKMQMSEAQAQVGDINRVRELLQSVVKTNPTNALGWIAAARLEELAGKSVTARKTIDKGCTQCPKSED
AWLENIRLNSDSPNAKIIARRAIEANNTSVRLWVEAMRLETIPSNKKRVIRQALDHIPESEALWKEAVNLEENSDDAKLLLAK
ATELIPLSVDLWLALARLETPENAQKVLNKARKACPTSHEIWIAAARLQEQLGQGTKVNVIKRGVQVLAKESAMPKREEWIAE
AERCEEEGAIITCQNIIRETLGWSLDEDDDRKDTWMEDARASINRGKYETARAIYAYALRIFVNSRTMWMAAADLERNHGTRE
SLWQVLEKAVEACPKSEDLWMMLAKEKWQAGEVDNARLVLKRAFNQNPNNEDIWLSAVKLESENGNGEQARKLLEIAREKAPT
DRVWMKSVVFERVLGNIEAALDLVLQALQLFPAAAKLWMLKGQIYEDLGKTGQAREAYATGVKAVPKSVPLWLLYARLEEQAG
LTVKARSVLDRARLAVPKNAQLWCESVRLERRAGNTAQAKSMMAKAQQEVPKSGLLWAEQIWHLEPRTQRKARSLEAIKKVDS
DPILFVAVARIFWGDRKLEKAQNWFEKALVLDSDYGDSWAWYYRFLCQHGTEEKRADVVTKCVLNEPRHGEVWQAIAKKPTNA
RKSCEEILKLVAEELEQ
FUN_001215 CDS scaffold_11 120333 122940 -
hypothetical protein PF12751 ENOG41

MDKSSAVSDGTSGVGRTSRNVTDPSATSSSATTGSQNSKQSSANNSVGPSPLASRDSSPTRRPRRTASANRLPGNRSRKNSQT
DNSPSRTTRPSLSSAAPSRSLSSTNTPTLTPSQEQQQQQQQQQQQQQQQIQAPTPQKPGFNADLKDSPRWPVSPRLRSPPPQ
FSNRPAIPARRSEQDLPSISVQRPTPSPQPMEQQTISESEMEELQYPSGMRTPARVALETVQEVSLPNSPNPPSGSALVEKVK
EKLSSTDNYSDTALPDGRALRAKNSLVGQESGSDTSNNKAETRRPTSVPPPIITRQSSAMSNKQMKSKQDGSIQTMTVETETV
PSVPQVALTTAQKSEGTNGTLKTKQSTETIKPKKDKKKVTRKQPPVNAGNASSKADVFEATIASAVDEANTSDSEETFVYDSN
PPDGNDRAGRRFHSRTPSATSMASQADRQNLRSIYGIMESGGPAHGPKKTMKFVNTFTSNGNESLAVETEDGKGSNRSGGSGS
TRGTTRHHHHIGRWGRQPGNGHASLFDNESPFPNAARSKLAGPNSRNSSGPPSPRNAHSNAPRHFGHKRSAMQMSSSYDMDDT
TGADDERTPLLGSVRSGRSGRSRRGPHNLRQAESQTFARRSSYLNRFAACLVLTMMFMLVITGAIGFMFATSQPMTGIEITAI
HNVVTSDQVLMFDLTVKAHNPNIVVVTIDHANLEVFAKSEYTGTDSDWWARPFFPHGPDDLRVSDDPENDPPLDDPDPDDDLRP
NVLLGRITEFDSPLTFEGSLFHQGTSSSTGEMQLEYPLNNTVGGSRRWERIYQNEFDLIVKGVVKYTLPMSARIRSATVSGRK
AVSPNSSNDPSNSTKVEPSN
FUN_001216 CDS scaffold_11 128672 130595 -
hypothetical protein ENOG41

MSAATKKPPAGGVGRVSHTESNSSTSPSPSRSNSRSTTPTNNGHTRTRSLRTTAPVSARAAIARRDTLSAAESDARAEAAAAV
EDLQKRLENEEKSSLQYKRQVEVLQSKLDEAVKESAKLEEKAHEYEEQIETLSNEKREMTRQMREMESIYEAERSSILKEKEE
MASREEEMQAMIQRLKDSLAQRNNTAEESRPSRQHSAGSSPSLENGSFAPPSSIQRSDSRNSSKLILQKDKLIESLRLELAEA
QIKLVESENQGGGRLHEVERLLMEARMANARLMEDNESYQLLLQERTLKGDFGQNDFSYMGSNSNQDALAALEGKAAGSSLAD
ELSEATEGESVSDGDRRLEAELKSMKEQNKALTLYINKIIERLLQHQDFESILDQSSDLKSLPDTNKELPPAPADKQQPGATI
LQRAKSMAVGPTKPRPRPISVMQPSTSSNHTDPDKAPSIPIGLSRSTSTRRSPRPQSDQYTGAASLVSQMYRGPDGPVSPPLG
TPRHSQTFFSPPNNSGNPNAAARVPSGASVATSGNFPGMRSETSSLSGESGELSMSTPPSQSPPRSHNDRHTTSFAGGKPRPL
RLVQENQEAVKDNKRSSWYSPFTWGAKKEEQQVPSGNPIPE
FUN_001217 CDS scaffold_11 132422 133005 - RPL30 60S
ribosomal protein L30 PF01248

MAPQKKSKKDANSINSKLALVMKSGKVTLGYKSTLKSLRSGKAKLIIIAGNTPPLRKSELEYYSMLSKAPIHHFSGNNIELGT
ACGKLFRCSTMAILDAGDSDILSDQQA
FUN_001218 CDS scaffold_11 136244 137948 +
hypothetical protein ENOG41

MAFHQPTRQSVQRVVRAPVDEQEIPRREVPITQEREPEQSQTWVLFAPTDVTTTSYLTETDHSLKTPGRSRVGDLGSLNSAAK
SEAESRQSASAFGLDEEDDAELDSLDGHLPGFRSIPAVQYQPQDEQQGSGPVFPGHDGLGSFHLDQPTLGAEAQDHIYQFERF

NPRRQYQRRESFDLRQLDVEHDQVHEAEKRQRIEAWRLEHSRVLLGEIQRETRRRRFSQASIQKRPSSNVPSVPSALAYDGES
DNMTWHDEDAIGTSSDSEGFFSKVTRRFLRDVVGMDERSISVFLGETVPEDDEDLSSTPRASQTLAWESRQFASREEWQLDML
ERMSRELGSLVSHISHHPGAFSTYTRVQQMPLPYAGLPIIPESNSSHSATDNIRPTEHEHASFPQFKPTIQTSPQPINIHGRS
EPSAEATQASRQDTPMSNTFTQEEWEKDLDMKLVFRYLRSRFMPRSSNPAPSTPAHLATSNSQDTAAKLARVRQHHPLVSRMR
PPAERRTFKAITPGSPVAIRHPSSCASQSTRRSARRSSVSSRHYWDIGGSLGTGSVIASNGPMGSWGEV
FUN_001219 CDS scaffold_11 139914 142731 +
hypothetical protein ENOG41

MATTPFDPSHFTPNAFDIYTDAVAIKAPTPSLPGGACGFVDLNPGANGARCGCRRFWSRCPTGNLVPDQSQWCMCNHHACYHE
EGSRDVQQPEVGIPGQENERPRTGREPLSPVVDIAIKTPPVIPGMDFSSFNAGAFSFIQRTPDQRLSDQRISADTGQFSRIPA
SQNLGASASIPDTLSWGGLLQSQSCPSAIPPIPAQCLIASQTASTTSSAQAKYLRPFAGKGLHTLNGLTAEKPPSPTQANTQD
VPPTSVPQNQAPPADSFTWTSQDYKPPDTPRASTPTQSEHRATAFNAAVSRRAFKNLSDIVSGHDQRLDRLETVSFTAAGHEE
CHEKHDHADIRMTDLESRVEEVEKLVNDNKSVLNDSNSVTSRRGDDASVISVSTSTTSRPTHSQELWSQVQSLQAQVVQLQSL
MPSPNHPFEIEVVFLPFPLKKVWQEAQQFKNEPQISNDDWTQLPMTHSTATLRSEMSLYPDWMVSDQDTKWLLPKACADKGVI
DRRLRSRGLIKTISVKGSDARSVTMAMNAAFGHLFREMNIFSRPQTTDPRSSSFLGLQSTWVPLRKIHKDSRLRFLSPAEMMT
PAVWDVQFLSQVMMRASEPRLFVTHPDAYLQDFQAYEMGWTWQMLREMSPVAPDPSASLDEGKPYEECWSWVEQLDEPPSASN
SMSVRHDKAPTSSSPSQTFYPALQSIKSASPSMTRAQSPLLSRRGSRPPHIRTSSMPMSAPIQASPALSRRRVASYGQSRRSS
PALHANSQSAIMKRRRTRSPSHRFTPRWTASPSPMPLGIHDRQPARGTTPFAYATPYSNAPLQERPIRAGSAAPVPAQDGFDD
DSDFNIEIYESGSEDMEDAESVNDMEIVTHNQALIEQVSQGWQLPEDEPWPGIEDQREASDGENVDPHQTDPGSNVSSQPSEY
PSTQNAWPDNNAAEFHIHEDDDNRS
FUN_001220 CDS scaffold_11 144068 145647 + SPE3 putrescine
aminopropyltransferase PF01564;PF17284

MSSEEITHPTIKDGWFREISDMWPGHAMTLRVEKVLVHEKSKYQDVLIFKSTDFGNVLVLDNVIQCTERDEFSYQEMIAHLAL
NSHPNPKKVLVIGGGDGGVLREIVKHDCVEEATLCDIDEAVVRLSKEHLPSMACGFDHPKSKTHIGDGFKFLNDYKNEFDVII
TDSSDPDGPAEALFQKSYFQLLHDALREGGVITTQGSESPWLHLPLITRLKKDCQAIFPVAEYAYTTIPTYPSGQIGFMVCSK
DPKADVKTPLRSWTKEEEDAKCRYYSAEIHKASFVLPKFAQKALE
FUN_001221 CDS scaffold_11 145945 146956 -
hypothetical protein PF00795

MRTVLSRLALASRSLTTSVSPLSLFQRSNPSIIRKMSSITEPVLKQRVKLSLIQLASGSDKKANLDSAASHVARAASLGAKIV
VLPECFNSPYGTDHFPQYAETLQPSPPSNDAAPSYHALSVMAADNKVYLVGGSIPEYSPDTKKYYNTTLIFGPDGALLGTHRK
VHLFDIDIPGKITFRESDILSPGNKVTLVDLPEYGKIAVAICYDVRFPELATIAARKGAFALIYPGAFNTTTGPLHWQLLGRA
RAADNQLYVALCSPARAETGYPAYGHSLVADPMAQVQVEADEKETTVDWELDPEKITEARKNIPLNTQRRFDVYPDVSEGKIQ
FEEP
FUN_001222 CDS scaffold_11 151361 152479 +
hypothetical protein EOG092638XA PF00400

MSVILCTAGYDHTIRFWEALSGICSRTIQHPDSQVNRLCISPDKRYLAAAGHHTVKLYDIKSTNPNPLLTFEGHTGNITGVAF
HCEGKWMVTSSEDGTVKIWETRTGSIQRSYNHGCPANDVVIHPNQGEIISCDRSGSVRVWDLAENNCSHELIPEEDVSVSSVT
VASDGSLLCAANNAGNVFVWNLIQSFDRTQLVPVTHFNAHKEYITRILLSPDVKKLATCSADHTAKIWEVKNIEPSTDLEPKP
YPLEATLTGHQRWVWDCAFSADSAYLVTACSDHYARLWELHSQQIIRQYNGHHRGAVCVALNDYSETR
FUN_001223 CDS scaffold_11 152904 153253 - CON6
conidiation-specific expression protein PF10346 ENOG41

MTDAQVAGGHKATINNPNTSEEAKEHSRQALENEFNGGEVTGAEDNKDKNPGNVAGGLKANSTLNNPNVSDEAKKNAKERLDK
EDF
FUN_001224 CDS scaffold_11 155657 156266 +
hypothetical protein PF10639 ENOG41

MASAEEKPPVFNYILSFVLVGLAWGFTTPFIRRAAQSHNPPTHPVLESPSVQSSWLKSKLYGAFFAVIDLLKNPRYAIPLVLN
LTGSIWFFLLIGQAELSLTVPIVNTLAFLFTVLGDWYVDGKVISKDTAVGMALMLVGIGLCVQSKR
FUN_001225 CDS scaffold_11 156922 157718 -
hypothetical protein ENOG41

MDGLNEFRTMRVAEVLSDFRTLQYYIAAAPVEPEDTADYYTEGWAALRQCALDGQHILECGADTSVPTPPGGEQEQMKAELKQ
VLLDAYARRHEGQKIYLRQAAAQRWVEYRNQVLQGGVPNSSNQSQLRACDRQLRAELSAIADEVIYAELKASDEGMGRWTAED
PSLRSVLRWLRARR
FUN_001226 CDS scaffold_11 161045 162072 -
hypothetical protein EOG092645QN PF00227;PF10584
T01A
MSAGAYDRHITIFADNGRLYQVEYAFKAITAANIMSVGVRGKDCAVVLSQKKVPDKLMDPASVTHIFQLSPSVGCVITGSIAD
ARAFAQRAQGEAAEFRYKYGYEMPADALAKRLANISQVYTQRAYMRPYGVATTLISLDSEYGPQLFKCDPAGYYIGYKGTAAG
PKQQEALNHLEKKLRNKEHAEGSWEEVVELAITTLSTVLSMDFKKGEIEIGIVGGPRPDGEEGTYSGFRTLTEDEIDDRLQAI
AEKD
FUN_001227 CDS scaffold_11 162951 164133 +
hypothetical protein EOG092644DY PF10180 ENOG41

MSGQTPAWKKLGLKLKQPSDASEPSLGHPTSRTSTSTQSSTKRKFDAPPPSNSSQVAKRPRQDEATTSWNAPGQLKRQKSVTF
ADAPPNNHFVRTPSRPVKQSKAKSKGPAKKPKPQVPTDLKPALEYLRLWKTARDSWKFNKNHQSNLIKHVFDADGIPASDIET
FYDYIQDLKGFVRMRLLENAREVRDQDQSDGAKVFPEGTKDLEATQQRYEEVLARILENHPVGTKRKGFTEVDYLSDSEEEDV
IIRRLVKRMRAELVIDELSDGEDTEASIASSQTITASDNNVTSTTDAKTVKIADGVPGKRRRKLRVNVEDSDSSSSESESDSD
SDSDSDTSSSGSSSSSEDESENEAPARPYRQPGEEDSSDSSSSDESSSGDSSSEDDSDSE
FUN_001228 CDS scaffold_11 164341 165307 -
hypothetical protein PF05721 ENOG41

MTTDTPGLSPDQLDTFNRDGYLILPGALSSSTVKSLLDETHNLLETFSLDDHPLTRFSTGEKRDHVGDDYFLTSGDKVRFFFE
EDAFDDDGKLTKPKARAVNKIGHYLHALSPPFARLLDHDTSKVSPPAVARSLGFKDPRCLQSMVICKQPEIGGAVPPHQDSTF
LYTNPPSAVGFWYALEDATLENGCLSFLPGSHLWAPVEKRLVRKAGNVGTEMVDNDGPKFPAAVGYGQAAPDGQHEYVPGEVK
AGDLVLIHGNLLHKSEKNTSQKGRIIYTFHIIEGQDRDYDDKNWLQPPNEGFTKLYA
FUN_001229 CDS scaffold_11 165816 166549 -
hypothetical protein ENOG41

MTRVSVYTSALAAFVAATVMIVIPIWLPNWVTYSVTSATGEIVERHIGLHKSCSTLDDPYCRPFPPKDLCQGGERYFCSMWRT
VGFLASFATLLCLGGLVTFVVIMCGGKYKRETGWPFAAGMLTLISVMEFVAISIVAYLYDHDDQFNIPGWSLDTSFYLSTTAA
VICLLTATGIAFSAYLLPPEEGYDFLSDPLDA
FUN_001230 CDS scaffold_11 167231 168590 -
hypothetical protein PF05004 ENOG41

MMNHARVKALKGDGKTTSKKAIKSGRASAGLTPRGSPFASLLTSPAHSAAPSRAASDESEDDFEYDDTMSVASGSSLVDTNED
GTNTFDAKQFMEELQDRKHNNSDARTQLLELYIKVLRHRFAPATHEWLDESANELAELFIRGANRGMEARERLLYLQAYLLTL
SSSEDANIYEHAEGTLKQIIADDDDEECKAFGIYALCFTVLYGGGGETEALELLDYLYAIVESDGDNIDSYDNGFIVAAALRG
WAFVASHVDDFSYAATMALDGFYEQLDSVDVGVQAQAAACIALIFEAARNHEEETGESWDLPVNPQKLTGRMSELAKQSSKSV
SKKDRRDLRDSLMSAITSLEKGVGPGYSSAGYTPQKGDKRPTSKPNEDGVIEFGYRHKLRLGNYVAVIDSWSLSSRIDMMRLL
FGGHLQKHIFDNPVVAECLSDADFSDQGPVAKKSPKK
FUN_001231 CDS scaffold_11 171441 173685 +
hypothetical protein PF00498 ENOG41

MAVPEYRDEVSITLSIADPSPSFSFPTRRIFLSKKNPTIEIGRTSKRNGAFEAAKSNAWFDSPVMSRKHALFTLDADKQKLYV
KDTGSLHGTYKNDVRLETNVNHEVASGDKLKFGAFIERPQDKHYPCAMIVRQTYGSINPDLRGNSFRVPEDSDIESLISDEDQ

VNNSYEILRTNKFVPARMANSSFGHGPIDLTIDDHCPFSNIRDGTEAFDNPPHILEIPSPKLPEPVSVSGHEFSDLEEEPEEG
ENDLSPNSMDTDDSYGGISEDCQSIDYAMSSVAGDSIVDEEEDFAEHDEFEEEQVHTQEPAPQGNGHFEEATIMASAMSEKPE
KTPAQPLANSMSTQTRDVPQQLDSANLIQLDKSHYTWKSDLAPMMSINGTSPLKLPPISEAVFDTYNHEMITHSTAEVVGRKH
GKYEFFAAREANAAAARAQPQPRPIDPVRRAPALEQCLLRAEMENKTQVQPADEVQQTTDEVQPRASDLVSSGLRYLATPPEH
VETTTTSVEPELDDSSAWAFQESKKSSGIEVTTETTEKVVAQENDADVVEDGDAQPAEALEAAELVKPVKAVEPAKPVEAAKA
AEPVTIPAPESTATKRKAEEISQLTTEEQISNDRQSRRQAHRRSRMQSTARPRHTAALGPPPTKRLRRMAEAVGYVALGGAAV
MSVLIATAPAL
FUN_001232 CDS scaffold_11 174040 176162 -
hypothetical protein PF11559 ENOG41

MIDTDNLRTASLYINNQLLSRGLLRDGQSIDFAGAAFNGDESGATMGRIVSVLNDLILRRDRDAEHRESLSTTMRTLRADNLK
HTNDIARLTEKHTEAKRKLDIAEASEAALKTQMKSADAAIRGLKEEVARTKGLVAQARAACATDVRRRDRQIDTLKKQLAEAG
RARGSRANPSVTSILVTGDVGEEKSTGRGGTTAADDYDLRNETNAFLANLAQNLSEENEAILSIMRKTMQQLRDMSGWNNENQ
DNLVIQQQGWQDMASELDSVLEHMRTILTNPSFVPIEEVMVREEEISRLKDGWVKMESRWTEAVHLIDGWRKRMAANGRPICD
EELQMGLRLSPVRVRDVEETRHASALRLSAVAEEPEDEMDVIDSPCPSRCSPRVQLVPESEPEDEVGRESDIESDYDENIPID
DYDVEEPNVQILQQSTAAPLYHPDPDSSPLPEPPQLSPLKDSASAGNRGSQPRKHGKGDFTTIAEENTWDLATEGGSIPVRYD
QEPRDRVSSSSSLDEVLLVKSPETAPQVNEMKMSTPRHQSSHRSSDDQSETQTSPSRSPRRTASRLPLPRNIDPAPQQSPLTM
ATIAAKLAASEREADAARVRAKLRAARGTRGVKKPTITSSQPDQATESTNETEKTNDFENVDPVKRDPVSADDQLKPEKRRRE
RRMSKSASRRRSTLSPWELESLMNGSVQ
FUN_001233 CDS scaffold_11 177328 178270 -
hypothetical protein EOG092655SO PF12923;PF17799

MSPVEETGDQFSVLPIKIPATPSYPETAIHEVRIRKNAPKIPTANDSRSLFLKNIPADSTEPHFRAVFTQLVGAGRFESITFE
DESRTLLPIDPAQATKVAGFAKKRKRGDVEAEEREREEEAAQLPQIWPRRVQRSSSTAIVLLADEKSVQLVLKAIAKLQKSKK
YPTWGSDLSDDVPSLGSSWIASHLQLSRVDKPATQKAVHAFFTAFNRKEKEAIELAKRLRNEPDEDGFVTVTRGGRAAPASRN
EAEEAKRRMIERDTKKKSETKDFYRFQMRERRKAEQMALMKRFQEDKRKVEAMREKRGKFRPET
FUN_001234 CDS scaffold_11 184498 186200 - GLN1
glutamate--ammonia ligase EOG09262MXH PF03951

MAAPITSRTETLQKFLKLDQKGMIMAEYVWVDAEGGTRSKSRTLPEKEYKPEDLPVWNFDGSSTNQAPGDNSDVYLRPCAVYP
DPFRGSPNIIVLAECWNADGTPNKYNFRHDCVKVMDTYADDEPWFGLEQEYTLLGSDNRPYGWPAGGFPAPQGEYYCGVGTGK
VVQRDIVEAHYKACLYAGIKISGTNAEVMPAQWEYQVGPCTGIEMGDQLWVSRFFLHRVAEEFGAKVSLHPKPIAGDWNGAGL
HSNFSTKAMREEGGMKVIEEALKKLEPHHVECIAEYGEDNELRLTGRHETGSIDSFSWGVANRGTSIRVPRETAAKGYGYFED
RRPASNADPYRVTKVLLQFSMA
FUN_001235 CDS scaffold_11 189863 191105 -
hypothetical protein PF01940 ENOG41

MRLLYALPATCALIYRAKSRNSLTPAGIFAATLTAAAHAYHPWNLPFALLCVFFLAGTRVTHIKEGIKANYTLRSKGTSGGEG
PRTHVQVLANSLMASVLSVLHANQLRKREAAFADPNTPDPTGSLCFSWGGDLLVVGIIANYAAVAADTFSSELGILSSGQPRL
ITSPTLRKVPRGTNGGVTLLGLGAGLLGSMIIVTASMIFLPSCSEESSRTPAGGAPWTMLERRKFMGFMVIWGALGSVLDSFL
GGLFQRSVRDVRSGKIVEGEGGNRVLVAESATDAAAHSNVKTEAMQSEAKEKLGGSAIVDDDDANAGVYDHKDKHRKSSFGDQ
RPSRAIENGWDLLDNNDVNFLMAVTMSVGGMAVASWYWDVPIQSVMGV
FUN_001236 CDS scaffold_11 192652 194773 -
hypothetical protein ENOG41

MSPGRTHEDLAPLADGVESLAASLEQMFQDKFSQLIFGEVGKTQQQQSTEVRFTGLLPLIAFDLVASVGQRYSDLGSPQSNES
NQLIRSGLSNDTSTNMFDNPSAYCSPLSTFSHRQRNQLIDVWFSAHPLTFLISKTLLLREIREDTCDEILMAVMLADASFVVG
EDSTVARGHELLQWAKTQLQMRPCYHPSEDNDAVYSGVPTRVYKGVTTAQTLVLLAWNALSSHEFRRAICYIEVASKLVTEIK
DCMSKDASQPNSSRINGVDVLDVEKEVVSNLWWTTYSLHIWTSIQAGVLPKTTPSTFDLESLPVTSTSSVTIQLDLVSENFNT
LQKQKGNIREMRPLAQVVSTVAYLFSQTSDQAALKHGVGICKEAIRSFGTNANDPKEKGQDDDRLWQLLVAFHHNMIIQFLFP

KNGSFYERVVVPAETVDQFCASVEQIIQSFSPTTRQVNQNSASKFQQPLSKALCTLLDTCSRALNLLRDNLGLSLDHGYFRPE
WDNRLCSVASSLYTISKDERFYQGTTLRSVRKQIKSCARAFGSNSTTNALGLLEPSMNSVRSMSHSPQSNVRPPNGFIANNEA
LVDMPSPFQAEACSRVSSSMPSSSGSSTSVSTHSFTPFEDMNKWPVNETFSHGIMSTNMNSPPIVQQGIQDGTPMQNMWYSQA
PPMMNFEAAGPVSLQHSQWAWPPTSGEEAPYMSFQTLDMDET
FUN_001237 CDS scaffold_11 197055 200094 -
hypothetical protein PF00763;PF01268;PF02882

MVAAKLDGNAIAKSIREKLCAEVTEKQKLNPRFKPCLKIIQVGDRSDSSTYVRMKLKAAAEAGIDSQLLHYPESITEAELLDH
IRQFNHDPSIHGILVQLPLPKHISEYTVTSFVADEKDVDGFGTKNIGELAKRGGKPLFVPCTPKGVMILLKESGIDLKGKNAV
VLGRSDIVGSPVSYLLRNADATVTVVHSKTQNIEEYVKNADVVVAAIGQPLFVQGSWIKPGAVVIDVGTNFLPDATKKSGQRL
VGDVDYAAAVEVASAITPVPGGVGPMTVAMLMQNVVDATTWYFESQKLRKTIPLPLKLEEPVPSDIAVSRAQTPKEITRIAAE
VGIAPHELEPYGAYKAKVDLGLLQRLEHRRNGRYVVVTGITPTPLGEGKSTTTMGLAQALGAHVGRLTFANVRQPSQGPTFGI
KGGAAGGGYSQVIPMDEFNMHLTGDIHAITAANNLLAAAIETRIFHENTQKDGPLYKRLVPTKNGERKFAPVMFRRLKKLGID
KTNPDELTEDEIHRFARLDIDPETITWRRVLDVNDRHLRGVTVGTAPTEKGQTRETGFDITVASECMAILALSNSLAEMRERL
GSMVVATSRNGDTVTADDIGAGGALTALMKDAIKPNLMQTLEGTPVFVHAGPFANISIGQSSILADKLALKLAGTEPDEDHDE
KAGFVVTEAGFDFTMGGERFFNIKCRTSGLAPDVVVVVATVRALKVHGGAPPIAPGAALSPVYREENVDILRAGCVNLKKQIA
NAKSFGIPVVVAINKFSTDTEAEIAVIREEAISAGAEDAILANHWAEGGKGAVDLAHGVIAASKKPKDLKLTYDLEGTVQERL
EAIGKKMYGAEKVEFSELAQKKVDTYTRQGYGHLPICVAKTQYSLSHDPDLKGAPTGFTIPIRDVRMAAGAGYLYALAADIQT
IPGLPTAPGYLNVDVNVETGEIEGLF
FUN_001238 CDS scaffold_11 201553 202744 + TIF34 translation initiation factor eIF3 subunit EOG09262W7C PF00400

MRPILLAGHERALTQIRFNKDGDLIFSVAKDQQICAWFSHNGERLGTYHGHVGAIWTVDVNPTSTMIASGSADNTIRLWEVKT
GRLIKTWEFPTAVKRVEFNEDATKLLGVTEKRMGYLSNIVVIDINPDLNAEQTDEKALTIVCDESKATVAGWSALSKYIIAGH
EDGSVSRYDAKTGDLLDNVPVHELNQPIVDLQWSPDRTYFITACKDKTAKLISARDLEVLKTYTADTPLNSATITPKKEFVIL
GGGQAAMDVTRTSARQGKFEARFYHKIFEDEIGRVRGHFGPLNTVAADPTGKSYASGGEDGYVRVHHFDKGYFDFNYEVERER
INRMQ
FUN_001239 CDS scaffold_11 203298 206874 -
hypothetical protein PF00176;PF00271;PF04851;PF09110;PF09111

MAPRSRAKAVDTDASMSDAQEHRQEEEMEVDETPDYTDTENPSTTASSVAGEPTGDGRRRRTEVNQLRRSIFGKKHDRLGESK
EDDTIRRFRYLLGLTDLFRHFIETNPDPKIRDIMTEIDRQNAESARGKKGAGRQGGATSERRRRTEAEEDAELLKDEKHGGSA
ETVFRESPPFVHGTMRDYQVAGLNWLISLHENGISGILADEMGLGKTLQTISFLGYLRHILDITGPHLVIVPKSTLDNWKREF
AKWTPEVDVLVLQGAKDERQNLINDRLVDEKFDVCITSYEMVLREKAHLKKFAWEYIIIDEAHRIKNEESSLSQVIRLFSSRN
RLLITGTPLQNNLHELWALLNFLLPDVFGDAEAFDQWFSGQDRDQDTVVQQLHKVLRPFLLRRVKSDVEKSLLPKKEVNVYLG
MSEMQIKWYQKILEKDIDAVNGAGGKRESKTRLLNIVMQLRKCCNHPYLFEGAEPGPPYTTDEHLVYNAGKMAVLDKLLKRLQ
KQGSRVLIFSQMSRLLDILEDYCVFREYKYCRIDGGTAHEDRIAAIDEYNKPGSEKFVFLLTTRAGGLGINLTTADIVILYDS
DWNPQADLQAMDRAHRIGQTKQVVVYRFVTDNAIEEKVLERAAQKLRLDQLVIQQGRAQQAAKAAANKDELLSMIQHGAEKVF
QSKGPTGNMASKDGEVGDDDIDEILAKGENRTKELNAKYEKLGIDDLQKFTSESAYEWNGENFANTKKNINMTWINPAKRERK
EQSYSMDKYFRQTMYPNPKADAKPKAPRAPKQVPVHDYQFYPPRLRDLQDRETAYYRKEIGYKVPLPDGDEENLEEREAERAL
DQQEIDNATPLTEEEREEKEKLSLQGFGDWNKRDFQQFVNGSGKYGRTDYEGISNEIDSKSAPEIKAYAKVFWQRYTEIADYP
KYIKTIEDGEERTRRIGHHQKLLRKKMQQYRVPLQQLKINYSVSTTNKKVYTEEEDRFLLVLLDRYGIDSEGLYEKMRDDIRE
SPLFRFDWFFLSRTPIELSRRCTTLITTIVKEFEDVPARGSNGVNGKSKREPDDENDEDSILGMAPAKKKAKNGVKNKALDNV
KSVKSSKNSSATPSRASSVASTVSAGGSAKGKKGKKK
FUN_001240 CDS scaffold_11 207737 208469 +
hypothetical protein ENOG41

MEPMRGVSGSMGKQPAQQESPEQLLDVFKAAALSVTKLYKISAAGQSKARADGYQDCLDDLLQFLDKEGMGLGDGEGWKIRKW
ATERLDGRESSSLNTESEDEVDKAETASSPELTRTAIPPHPANQVRTDSAPPSIPPVAEQPTPIVVPSQDNFTFQSSHPYPNI
ATLDLSDSRPPDDVPAPTRPTKTRLAGGPSRSCPRGPGHLVQRAGSKRKLNFEEIFDLGSLSGKDPFEKGGKRGRHA

FUN_001241 CDS scaffold_11 209372 210099 +
hypothetical protein PF00076

MSDSGKQDEKPTGEHEIDHDREHDNEHEQGTEMSGAEEEEITAMKRRVAEMEEEAKKLREMQATLEQQSADLADDKESIDARS
IFVGNVDYSASPEDIQSHFQSCGSINRVTILLDKFTGQPKGYAYVEFTEPSLVAQALVLNESVFKGRNIKVTPKRTNVPGMSR
GRGRGGFRGGRGFHGRGNFPRGGGYRGGYRGRGRGFAPY
FUN_001242 CDS scaffold_11 213006 215834 + RSP5 NEDD4 E3
ubiquitin-protein ligase PF00168;PF00397;PF00632

MTSRTDSNRPNLRVTIIAADGLYKRDVFRFPDPFAVATINGEQTKTTTVSKRTLNPYWNESFDFRTNEDGILAVQVFDQKKFK
KKDQGFLGVINIRVGDVIPELSPDADDQMLTRDLKKSTDNLVVHGKLIINLSCNLSAPARGGQASTARPSLGTGPSNASNLSA
PPPENRPGSSLSGPNGAGGSQVNLAHRPSSINSVGGASAPGPNASGQTRQSNQLSPFEDAQGRLPAGWERREDNLGRTYYVDH
NTRTTSWNRPTASGAQEQRNDREAATQVERQRHQNRTLPEERTGSNSPTLHAQQPQPSASPATNGGAVMHTGATSPGTGELPP
GWEQRWTPEGRPYFVDHNTRTTTWVDPRRQQYIRMYGGQNNANGQIQQQPVSQLGPLPSGWEMRLTNTARVYFVDHNTKTTTW
DDPRLPSSLDQNVPQYKRDFRRKLIYFRSQPAMRILSGQCHIKVRRSHIFEDSFAEITRQSATDLKKRLMIKFDGEDGLDYGG
LSREFFFLLSHEMFNPFYCLFEYSAHDNYTLQINPHSGINPEHLNYFKFIGRVVGLAIFHRRFLDAFFIGALYKMMLGKAVAL
ADMEGVDADFHRSLQWMLDNDISGGILEQTFSTEDERFGVMTTEDLIPDGRNIDVTNENKKEYVDLMVKWRIEKRIAEQFQAF
KEGFQELIPQDLINVFDERELELLIGGIAEIDVDDWKKHTDYRGYTESDEVVQNFWATVRSWDGEQKSRLLQFTTGTSRIPVN
GFKDLQGSDGPRRFTIEKAGEITNLPKAHTCFNRLDLPPYKSLEMLQQKLTIAVEETMGFGQE
FUN_001243 CDS scaffold_11 216822 219779 -
hypothetical protein PF00076

MTEPENFEDDLFADLYDDNDAAKPGSAPAAAAPPAPEVQPPTNIHNNDNDEHNGMQQHQETGDDEHMTQDQDDDDDDVDFNLG
GGGYGGSGHTGQADMHDDAPTPPYGTVHKASAKEDGKMFIGGLNWETTDQSLRDYFSQFGEVVECTVMRDSSTGRSRGFGFLT
FKDAKTVNIVMVKEHFLDGKIIDPKRAIPRDEQEKTSKIFVGGVSQETTDQEFKEYFAQFGRVVDATLMMDKDTGRPRGFGFV
TFENEAGVDACINVPLEIHGKPIEVKKAQPRGNLREEEEASRRGKFRKDGDQSSQGSMGQQMGNNGMTPQVMAQYFQRMQQYF
AMMQSQMAMSRGMPMNPAMWQNMMQMQQMQQQMMGRGGGGANAQNMMQNMNPQMMQQMQQMQQQMMQQQGQQGGQDAASGSAS
PTASASGGGGRGYDNNNNYNQYQQAQGGRRGGRGGYGGHGGHGGYGMGGGGGAPTSWEGMYDDVPQPNSNQGSGGFNRAGSAS
ANSDPQHAPPANAPTGPKNAGKPGANYRGGGRGGNRGFHPYSR
FUN_001244 CDS scaffold_11 221229 222941 +
hypothetical protein EOG09261YLQ PF00566

MSIARNLSNSSRPRTPPSSSSAKIRIQEASPPPKTTPGKNGSPPYKNYINFLSNTNDDWKADEDDMMGYEDDDGDDFGLPSLS
SMKRRTRRIATQNKPESSTLSPTTEGPPNALSARRYSNSADIAIERPAPTYPMPKKSEGKILRPQYKEILKDPANALHLINYP
TVPANATPKEADAINSRITRINKFKKLLQASTISLPDLRSLAWSGVPQEVRAITWQLLLSYLPANSERRVATLERKRKEYLDG
VRQAFERGGNAATTAANTAPAGRTRGLDEAIWHQISIDVPRTNPHIELYSYEATQRSLERILYVWAVRHPASGYVQGINDLVT
PFWQVFLGIYIGDPDIESGMDPGQLPKSVLDAVEADSFWCLTKLLDGIQDHYIVAQPGIQRQVAALRDLTARIDGNLSKHLER
EGIEFIQFSFRWMNCLLMREISVKNTVRMWDTYLAEEQGFSEFHLYVCAAFLVKWSDKLLSMDFQEIMMFLQCLPTKDWTEKD
IELLLSEAFIWQSLFKGSAAHLKGQPAREPVTNLQL
FUN_001245 CDS scaffold_11 223513 225355 -
hypothetical protein ENOG41
GT90
MWPRFKQWLSNWPSRLVFRYLSTLFLICFAITNYILWTSSQGWDVTEPKGPVTMGPKHPIKKLMADARARHESLLSKRSHDLY
DAAERYRARRGRHPPPGFDKWMEAALASNSIVVEDYFDRIYKDLAPYWALDAHTLARRASAWHWVVKVRNGVATGVGDATDRV
PWLELWTNLVEEFAKDLPDVDMPINYMDEPRLLVPFDELSKFVDQERDNRRIAPMKEVVTKFKTLSKLDDEKPQPYDPYWYNS
SANYWELARVTCDPNAPSRNVQQVSDFKAPVEYPSNWDPEYAYKGYIKNWTAAQDPCLQPHLRQMHGSFVAPLSLSTSTELIP
LFGGSKLPMNNEILIPGAMYLTADEFYSGGEKMGPAWHAKKTGIVWRGDASGGEPRADVWHRFHRHRLIQMLNGSYVDSVEHQ
GVKPKTFQLPNPDHYNSTHRTSNTIGQWLMQISDCGFKRLLCEKVGCDPVAPYYRELKHMTMKEQYRYKFLPDTDGNSFSARF
RGFLRSSSMPLKATIYAEWHDDRLTPWVHFVPFDNTFQDLYPILEFFTDEEAGGDTAARFIAERGRDWASQVLRREDMRLYTW
RLLLEWARVCDEDREKLGFIRDLIEPRKDSIR

FUN_001246 CDS scaffold_11 228688 229605 +
hypothetical protein PF01058

MLSSTRSAASMALRVKPTTAIVPFRTTAAAFTTNSRRDASALQTHSATGVGKVRREVPLPSEIAPKGALQYALTTLDAVTNWA
RQSSLWPMTFGLACCAVEMMHLSTPRYDQDRLGIIFRASPRQSDVMIVAGTLTNKMAPALRQVYDQMPDPRWVISMGSCANGG
GYYHYSYSVVRGCDRVVPVDVYVPGCPPTSEALMYGIFQLQRKMRNTRITRHWYRK
FUN_001247 CDS scaffold_11 231197 231630 -
hypothetical protein

MEPGLDSKVPKENLRGRDKAFITIRDSQSKLSSLKNAGIDPAIGDIYEACGIVCANQDAVEARQVSDYNKSYARKITRHEIIK
WMKAGTNGEIVAKEEEDQLSGFLGMELFLQWPREIF
FUN_001248 CDS scaffold_11 233092 233488 +
hypothetical protein

MVPAEAQNETDARLPPGLLTSTLVMSLKTVRPIYKSDFVDEETLETLYDQVLVEIYERAGVTSFLHGHLPNYHKATRKMLMSG
HGLLGPDYSRSPPNYRTFRRQAERVSEVEKAKLSPAVLEGCLTRTTFR
FUN_001249 tRNA scaffold_11 236105 236195 - tRNA-Glu

FUN_001250 CDS scaffold_11 236415 237133 -
hypothetical protein

MDAVSHHQCPYKSPDYYYEERLADGQSPYQHHRYKYRYQHPRSQSPPQRRRGSIDRNDPGPTERGSTSNGGPLGYGPPGYGPP
QLNFNGHQLPACIEDEELTKEYVLSDVSRSASQPAGQDADGPEDLVFGEVHPLFQMYSELSKVANPPYLPSRAFEASLKKPLR
IGLLLWML
FUN_001251 CDS scaffold_11 241865 243666 -
hypothetical protein PF00962

MADEHTMSNEEWEEVSQDIPSLSDPFLQQYLTGRANLMSQEQKSRTDASFRASLSPIAKRASDIVDCIRDQENDSIWTPQVEE
ELAQAGNECIFPGMMFMLAKDRMEKTNLWKIVRRMPKGALLHAHMDAMVNFDFLFDELLKMPGMHMCSDRPLNTEESREDAVP
SFRYRTKADTDGSIWEESYKPDAFVPLPKAADEFPHGGRSGFLKWLKGRCTLSVTDTHEQHHGVDAIWVKFGKCFLVCATIIH
YEPMFRIFLRELMKNLKDDGVNWAELRFTWPLNYCRDKQEEPEKDYIHMFEVLREEIDNFKKSPEGKGFWGLTTIWTCLRSWP
TRLIIENMDCCIATKIAFPDLIAGYDLVGPEDLGRPLSDLLPELFWFRKQCAMEGVNLPFFFHAGETLGDGTDTDANLFDAIL
LGTRRIGHGFSLFKHPLLIDMVKEKRILIESCPISNEVLRLCGSVTAHPLPALLARGVVCSLCNDDPAMLGQDTAGMSHDFWQ
ALQGWKNLGLAGLGSLAENSVRWAAFEDQNQTDWINDIKQASLGTNVKAKRMQEWQIEWEKFCLWIVEEFGDEFGDEKEKEKA
SDA
FUN_001252 CDS scaffold_11 244865 245360 +
hypothetical protein ENOG41

MKSFTLVTLLCTVAIAAVIPRDTVFDGHCCFTLQDVATSATVQQNSDGNLLLNAGKTNGFYCIDLSNSQDILRDNAFNACFLS
PSGALKCVDPTPGFQSWTLQKSGSNTLLQHDGGSTFNSCSKTSTSAKGTVLYGDKHTDATACKKVTLKAKNFKGTCKSLQG
FUN_001253 CDS scaffold_11 245523 247541 -
hypothetical protein PF00441;PF02770;PF18158 ENOG41

MEASGADKGFFQKPPVLNNQFHDDVTFQRCFKLFLPRQIVQQVQAEMASLGDDVLSDQVFAWITDAEHNKPFLKGSGRDVFGQ
WKGDLVTGEGWRGLQNFGLSRGFVAAGYDTPYGAFARAYQFMRIQLWTPSCANVGCPSAMQDGAARLLQTHLGSPELVMKLTD
EQKKVFENAFSHLTTRDPQHAWTSGQWMTERTGGSDVSLTETTAVHMPTSEIQLASKDEKIPLGPWSINGFKWFSSATDSRMT
VLLARTSRGGLSTFMAPMYKHDASATTATGLPRSNGEQLNGVRIQRLKNKSGTQSLPTAELVLQDMRAWLVGQEGRGIQEIAT
ILTLTRVHSSVAAVGYLGRGFAIARAYAQVREVGGGKGTRMKLTESSLHMRTLSKISSEYRGLMLLTIFTSYVLGVSEHDIAS
DASLSPALEALTPSAQHAAPLIRVLTQLTKAYVCKASVQLLFSCMEALGGVGYLANEEQEYLNIARLHRDCAVLPIWEGTTDV

LSTDFLRAIKHTKAGQDTLNALEDSIKKGMAFQGKVSWPEGLNPLDVWKSLRQRIERESLADLMGDAREVLWAVADLLVSVLL
YVDAGSDGDQVSLDIFHRFLEGKNVTTKREKVSAQEQLAKDFAVVYGGARNKIFAKL
FUN_001254 CDS scaffold_11 248543 249566 -
hypothetical protein PF05368;PF13460 ENOG41

MMRIAIAGGSGLGYLLASHISKAANAYQVVVLSRSSRPEYAALDVQVMVVDYNDENSLAYALHGVNLVICTFSGGEQVNLITA
AVQSGVQFFVPSEFEGSLEKRPENDQLDPYSYSSQARQHLRRCARSSQMKWTVFSCGIFMERFLPQGLGSLAIGYGSNLANVG
SYILDMNTYTAECVEKNPRGHTVRVCMTSVYDVAQFIVAAIDLGPANWPREFTMRGDRLSLRDIVGQCSRTLNAAFSLSQWQA
SDIAGLMSGFYQQGDYAKVAFYQQLQATVEGRYDFGHTSLNGLVNVQPRSFRQWLSDQFTG
FUN_001255 CDS scaffold_11 252096 256267 -
hypothetical protein PF01968;PF02538;PF05378

MAATSQRGIRIAIDRGGTFTDCVGESNGKEIIIKLLSEDPANYKDAPLEGIRRIMSHFLGRDIPRGEALDTSQIDSIRMGTTV
ATNALLERKGEKIAMVVTKGFKDCLTIGNQSRPKIFDLAIRKPDVLYEQVIEIDERVTLEDYAEDPERTQTEAEAQVGTKEAE
GKTLVRGLSGETVRILKKAEQEDIRSKLQSVYDQGIRSIAVCLMHGYTYPDHEALIGRIAKDIGFQHISLSHELMPMIKLVSR
ATSVCADAYLTPAIRKYIDGFQAGFEGGLGTRSVKEEKGAKGARCEFMQSDGGLVDVEKLTGLKAILSGPAGGVVGYAITSYD
EETKTPVIGFDMGGTSTDVSRYGEGRYEHVFETTTAGVTIQSPQLDINTVAAGGGSRLFFRNGLFVVGPESAGAHPGPACYRK
GGPATVTDANLVLGRLLPEFFPKIFGENEDQGLDIEASRKVLQELADQVNQENNKNLTVDEVAYGFLTVANETMTRPIRSITE
AKGHDSSKHRLATFGGAGGQHAVAIAESLGIQQILVHRYSSVLSAYGMALADVVDERQEPDSLVWKDDDKTVNELKKKMEKLK
DQSRNSLKDQGFQEDEIAFEEYLNMRYRGTESALMIVRPTPEEAKEHFDGKEWDFGQAFVKQHRYEFGFTLDERDIIVDDVRV
RGIGKSFRHQDDTVDKQLKELKQQKVSDKKKLDSQQVYFEGGRKKTPIFKLEDLDTGDSIPGPAMLADGTQTIVVTPKATAII
LKTHVVINLEKEGAKYESAKSSEDREVDPIMLSIFGHRFMAIAEQMGRALQKTSVSTNVKERLDFSCAIFDATGGLVANAPHL
PVHLGSMSTCVRRQAEIWKGRLEKGDVIISNHPSYGGTHLPDVTLLMPAFDEKGENILFYAASRAHHADIGGISAGSMPPHSR
ELYQEGASIRSEKLVSGGKFNEKRVVELFYEEPAKYPGCSGTRCLADNINDLRAQVSANQKGISLIETLIAEYGEETVQFYMV
HIQNNAEQCVRRLLKDVYKRFEGRDLSAVDFMDDGSPIRLKIRIDPEKGEAEFDFSGTGPEVYGNINAPQAITFSAIIYCLRC
LISEDIPLNQGCLKPIHVKIPPKSILSPSPGAAVVGGNVLTSQRITDVIFKAFQACAASQGCCNNLTFGFGGNQDGTEAVKGF
GYYETIAGGSGAGSDWEGTSGVHCHMTNTRITDSEIFERRYPVLLHEFSIRSGSGGRGQHRGGDGVIRDIEFRIPLQVSILSE
RRVYRPYGLNGGGDGECGLNLWVRNVEKANWETSLKQFHTKEDAGEVEYEERRINMGAKNTAAMKAGDRIIVCTPGGGAWGAE
GSESVARKKIDHTEGWRKGSGNTRDETALQA
FUN_001256 tRNA scaffold_11 256889 256972 - tRNA-His

FUN_001257 tRNA scaffold_11 257069 257151 - tRNA-His

FUN_001258 CDS scaffold_11 259931 260206 +
hypothetical protein PF01428

MPPKRIRCTAASCREPAQRIVGDCTFCQGHFCGKHRLLEDHKCTGLEDCKKQSHERNAAQLESERTQVIRGV
FUN_001259 CDS scaffold_11 261033 262274 -
hypothetical protein EOG09263R4M PF00633;PF00730

MRSSRISRDASKLFDRTAEPISPPRRLTRSALAKFAFTSTEESKSTLADIEDSGGPARKRRRVVVTKTEISTSAQAVKPEVVD
EALDDIPSPPAKARRVRKPARKAFDATTGETKTEPPSDWEAVYDTVRKMRAPGGRAYGAAVDTMGCERLADRNASPKDQRFHT
LVALMLSSQTKDTVNAVVMKKMQTELPPFEPGAPPGLNLNNILAIDPKLLNEFIWAVGFHNNKTKYIKQTAEILRDKWDGDIP
NTIEGLVSLPGVGPKMAYLCMSVAWGKHLGIGVDVHVHRITNLWGWHKTKNPEETRTTLQSWLPQDRWHEINHLLVGLGQSVC
LPVGRKCGECDLGLEGLCKAADRAKVSAGRQLKAEKIESEAQNDTLVKTEVSQNIVIKEEGVV
FUN_001260 CDS scaffold_11 263427 264873 - FDH1 formate
dehydrogenase (NAD+) EOG09262PIH PF00107;PF08240

MSTVGKTITCKAAVAWEAGKPLSIEDIEVAPPKANEVRIQIYYTGVCHTDAYTLSGKDPEGAFPIVLGHEGAGIVESVGEGVT
NVKPGDHVVALYTPECKECKFCKSGKTNLCGKIRATQGKGLMPDGTSRFKCKGKDLLHFMGTSTFSQYTVVADISVVAIEHDA
PMDRTCLLGCGITTGYGAATITANVQEGDNVAVFGAGCVGLSVVQGAAARKAGKIIVVDVNPSKKAWGEKFGATHFVNPTELG
DQSIQDKLIEMTDGGCDYTFDCTGNVGVMRAALEACHKGWGESIVIGVAAAGQEISTRPFQLVTGRVWRGCAFGGVKGRSQLN
DLVADYKKGDLKVDEFITHRKTLGEINNAFETMKQGDCIRAVVDMQKLEPKI
FUN_001261 CDS scaffold_11 266158 269483 +
hypothetical protein PF07575 ENOG41

MSSRFIVPDSSPPVSPAPSTPDKSGRGTGFSFLADNPSTTPAGPPPSSAASFTPAGAPSDSYMGSSFMRGMTDTKPLSFGVSN
NSSSGRNLFGRTGASSNPLGRSIRGRQPSGLSRQFSAADEEEEDQEEDQDAEGDVELPPPRGNLFRMSAAPDERLEDDEVDAE
IERYIDQDIAGEEDERESYGEDDHAFEREESEEPDMFLNMRHDDRPYGQPMIGDESDLMMLNTPAATKRVRQEAESIFRQTSA
HLGMSGRQEGFHFASIAKNLYSQHEHAPVVEPADMILETEELVCRLYEEGVGPEEDVEKMDNSLSNITFRLTELWNKTVNGLP
QPEGEDFATIGPGFNSDPFEKAAYVAQLILRMHHTRFGTNTDEEKTPPLPEILFDWMHNNHNLYPDQVREVSRHIPSPACHPL
FWQTVRCSLLRGDVGSASHLLKEAGWQNVRRGPRSDYAYTGKALENVRRFAAATSEVLDQCPGTRSEWDIWNSNWTLFRIQAR
GSLNRMTLFAEGREQDVQDLMDDEFAPQQQSLSTMARKASSQIPWDIYENLQTVYGIILGNHEAILEIAQDWCEATIGLFGWW
DDGNQRHKALRLSQSQLLRASSRFAGSEDYFDRLATAFHMVVQSDLNPNVLNPVEVAVASAFEGNVNAVLGFLRIWSLPLACT
VAEVASLGEWLPAPNGPNLFPTDSLDMDDLALLGVTQPGPDELDGIKDTTLVLYARELAGIEQLSPERDGWEMAIQVLGRMDS
PERSEETVGELLRDLLATLNENSGRTVDKIWTILNDLGMINYAEETAETFAEILSKESHRYGEALWYYALSHRSDKVREVLNL
LMSYSLVQSTVYPSEKDLDQDLKDLLRKRTETLEKRAKQDLEAAQLLGRMLSGYATLRKFYELRDAEDLENISPTKALKLKKQ
AASALVAVISSSDDNIRGGLYDETRDAVVSEDFLLALLGEATVFINQSPAVVTLPQIDVLLKAIEDLQTVGDRVYSACVDFFN
LVLASAQGLKGSTPQDLMAKSTASLGGSFMMTGSSVLVNHLHKSISAGTKVNRGWDWRKGWLATSKGEDVLRKLRLGLAKDLA
TLWLEDADGVVAY
FUN_001262 CDS scaffold_11 269899 272464 +
hypothetical protein PF00330;PF00694

MVSMEVASRSGGAMTQLLRRSGPNAIRSTTVLSNNTRRSFSSAKPLPPTYEKLYTKYTDVRRVLGKQRLTLAEKILYSHLDNP
EDSLLNNTDNGRNIRGKANLRLKPDRVNMQDASAQMAILQFMSCNLAKPAIPASIHCDHLIVGSKGAEDDLSAGIQTNKEVFD
FLESAARKYGMDFWPPGAGIIHQTVLENYALPGLMMLGTDSHSPNAGGLSTITIGVGGADAVEALVGAPWELKAPKILGVQLV
GKLSNWASPKDVILRLAGHLTVRGGTGSIIEYFGEGVESLSATGMATICNMGAEVGATTSIFPYTKASARYLDSTRRSQANKN
IEALEAFASNSSDPDARWQFKADEGAEYDELITIDLSTLEPHINGPFTPDLATPLSKFKEVVKEQDWPQVLSAGLIGSCTNSS
YEDMTRVESLLKDAKKAGLKPAADFYITPGSEQIRATLERDGTLETFQEAGGIVLSNACGPCIGQWKRQDGVEKGTSNAILTS
YNRNFRGRNDGNPDTMNFLASPEIVTAMAFAGSTTFNPVTDSIKTPDGKDFMFSPPHGLEGPQTPFESGNAELGVLSQAPDPN
TKIAISPTSERLAFLEPFAPFPSSDLSGLRVLVKVTGKCTTDTISAAGPWLKYKGHLPNISTNTLNTAINKETGEVNAAYDLD
GSKHTIPELGQLWKERGQEWLVVAEHNYGEGSAREHAALQPRYLGARVVLTKSFARIHETNLKKQGVVPLTFENEADYDKIAA
GDEVATVGLYEMLQNGGKGDVQLRVTKASGEEILIPTKHAVTKDQAGFILAGSALNLLSKGI
FUN_001263 CDS scaffold_11 272945 274664 -
hypothetical protein ENOG41

MTASTHVGGTCAVLTCEKIILLSSRHYCDEHSALEPTAASRDPSATNMAKGFQPSNRSFVAKRGRGKFMFTPSRHFSRQDPPS
QDASSPDTSAPVTQSPKANGQEAPPSSLVRDNSIDTNDISLHLRLERNGTSHVGSTPNSAQIKAAQDVAKADKGSSASQVNSH
TVSNPKTRLEMELQAETEEVINQPLGLDSATKKMSSCSNIGSETLPTEDLKMDAHQNGQSVHATDGQHLAGTAQAASEGAPSN
EGFKKTEPSLGLDSQPRVPSTAPVNRMQFTSEDGVKFKSSPRTSKPKKDNTQKHLEHHYPETRTSSKAKLLSTPTKRSAPLTS
KGLPVRKQQFQGSLPEVSAEIDDEVNMADVSDIDGEAKPAKSVPLSFRAKAEARKKRLLVEFDSEAFDSLIYRQSSLQPPPYV
TVSSRIVKYNPVFGDQKPLYLPVNPAIHHMHKRSRSWYRKKCDEIRRRPKRKEWFGKVVERQRWLQALEMKQQEKIKQAQQDG
TTPPYRPPKPRGVKRILDFGDLPEEELPEYVRSNPAWLKACAWFRECEDKATARQRHVNNKTKETESYFQSLNA
FUN_001264 CDS scaffold_11 276570 279174 +
hypothetical protein PF00069

MNFLKSAVASAIAQGPPFPYNFGDKVDIDESIWTLYNGTKREDGSNCSIFSFDITTNRSQLPLAKNALKKLRTLRHPGVIKLL
DAVETETYIYIATERVVPLRWDVRRKSLSPETIKWGLHSVARTIKFINEDASSIHGSIKVGSIYTSEAGEWKLGGFDVLSSLK

DDESIIYTYGSLVPDAGRYSPPELARGGWDVIKKNPHTAVDAFNLGTLIFEVFNGDYNGADQAGQTKSIPPSMQSSYKRLCNA
NPKARISVGAFLDQGNRNGSFFDSSLIKLTEGIDNLDIKTPDEREEFLTGLDELSDDFPEEFFKLKVMPELMKSAEFGGGGPR
AVSVVLKIASKLPKDDFDSKITPFIIRLFGNPDRAIRVCLLDSLPLMIDQLSQKIVNDKIFPQLITGFTDVTPVVREQTLKSV
LVIITKLSDRTINGDLLKQLARTANDEQPGIRTNTTICLGKIAKHLGTSSRSKVLIAAFTRSLRDPFVHARNASLMALGATAE
YFTDEDSACRILPVISPVLIDKEKIVRDSATRTMDIYLQKIKKAASAMPESVLPPPQTNDGSAPRMSTPQPNENTAGGWAGWA
ISSFTNKISAAAGEIHAESDSRAASPGPAPSPNSEPKKPATSTASSLHRQAVKSPPATLSRNSSHTASVVADSFLPADDGDDA
GDSWGDMNDDFDSFDSPGQSSKQPATTTASAATFDDGEPDFAGWLAAQSQKKPTKALPKGLSKSSAAKKPAAKSATKPIAAKK
IDMKPKETDDDDGWGDGW
FUN_001265 CDS scaffold_11 279597 280735 +
hypothetical protein PF09495 ENOG41

MAQGTVKKLGRATPAKITHSKRQASKVHKPKKTKASVDKVHKKFTSGMTARTEALLGERAGHLELLGQGKKKTDKKTTQKGGS
KKFGDDLIDVYEVIDLTEGIQDTEELVDDDDPEDEVTDGVTKYLITRLLAITQDPPKDITPYLMPDVSLPVVEADQQLFKSTV
TSMLERAQRYAMK
FUN_001266 CDS scaffold_11 281864 283476 -
hypothetical protein PF00282 ENOG41

MSDLFNLDVQRTRLSRRHGPEPGSAPAPTLPELSAIQTAEAKLPRPDSEDYLRGVSQEDVVRHIIRDIIPACNGQGSSSRYYG
FVTGGTLPIAEWADNVVSRLDQNVQVHLPAQTIATTLEDAALEMLVSLLRLGDWRGRTFTTGATGSNVLGLACGREAILEERG
QSIGEVGLLAACLSAGIRELQILTSAGHSSLSKAASVVGFGRGAVKELRLSDSEPWRLDLEALERELQKKDTASVIALSAGEV
NTGRYALTGVEEMRKVRELADRYGAWIHVDGAFGIFARALPEEDDYKILRNRVEGIELADSITVDGHKLLNVPYDCGMFLTRS
PAILQSVFTNPNAAYLSTGGASTIPSPLNVGLENSRRFRALPAYAVLLSEGRPGMANLLANMTALSRQVAAFLRGSEHYELLP
DDGANFNEIFMIVLFRAKKSSLNDELVQKINATRQMYVSGTSWKGEKAVRMAVSNWMVDVARDFRVVTSILNHVAEGKVFDIN
NC
FUN_001267 CDS scaffold_11 284025 284331 -
hypothetical protein PF00173 ENOG41

MSETYTTAEVGKHKDEANGFWLIVENDVYDVTKFIDEHPGGAKILKRWSGKNATKAFWKYHNEHVLEKYGKDLKIGAVGESAK
L
FUN_001268 CDS scaffold_11 284888 286110 -
hypothetical protein PF01435 ENOG41
M48C
MTAPRLLQNSSLSINRPAIFLIHRTAISSISRRRTFSQCHYRPQQHSERELQERLQAARPLVPHRAARKFTQAGSSRNSRLTV
IATVLAAIGFYFYNSQTVPVTGRRRFNFLSDSMVARAHSKAAAQVIEQVRAQGGHFLSEWDPRTILVRRVMKRLIPVSGMTDL
NWEIFVIADNRTANAFVLPGGKVFVHSGIINVCRSEDALAAVLGHEIAHNTASHAAERLSAAWVGNLTAGSLFFLAGALPGLA
LFGLWNVVGGYYLQDLLFYLPMGRKQESEADYIGLMMMAEACYDPRAALGFWQRMHALQHASGEEAPEMLSTHPSNENRIVKI
SEWLPKALEKRAESDCRGTAAFADRFRDAIRRRSTTVVV
FUN_001269 CDS scaffold_11 287535 288207 +
hypothetical protein PF06172

MGEFVNSEPDLGTIKPSFTSSSEPENPRSKSLIQALSLESHIEGGYFRQTDTNPTTIPSPYSSEALSHETLALSGPAREGYRA
DTRRLSTTIYYLLTPRHGQGNFHRNRSRVIHTLHRGRGRYVLIHPDGRIESFIVGNAVERGERIQWIVEGDVWKASYLLPNEP
DTGNESNREETEGLLISETVVPGFEYHDHAFLTQQGIRELLNEEQARELDWLVRRSE
FUN_001270 CDS scaffold_11 288621 289990 -
hypothetical protein PF13489;PF13649 ENOG41

MAPAVMAAQPPSHGSMPNSSPSSLHHFQFTFNCSASNSGGNSFIEPDPDAQGITIRDSESLTDSVQNFPEEFGRTYHAYRAGS
YAFPNDQLERERLDLQNEALVKLFGERLYFAPLSRRAPPVNILDIATGTGDWAIKMGDLFPETEVVATDLSPIQPRKVPPNVN
FYVEDSSEPWDYSQPFDYIHTRVTSGCWASFKEQIADQAFQTLKPGGWFESQEYDAIIMSDDESLPPNGPLATWFREINEASE
LMGRPTNVAAHVREAYVQAGFVDVQERIFKMPMNGWPKDERLKELGRMWERNFLMGLSGFSFTLFNRVYERTPAQIEVALVDV

RRELIDTRIHAYIPIHVIIGRKPFPGEWPVPRVPFT
FUN_001271 CDS scaffold_11 294701 300089 -
hypothetical protein PF00225;PF12423;PF12473;PF16183;PF16796

MPPAGGGNIKVVVRCRPFNSREIERGAKCIVEMKGNQTVITAPEGKGVKDGGPKAFAFDRSYWSFNKDDPNYAGQSNLFDDLG
QPLLDNAFQGYNNCIFAYGQTGSGKSYSMMGYGKEIGIVPLICQDMFKRIDELKKDKTTKCTVEVSYLEIYNERVRDLLNPST
KGNLKVREHPSTGPYVEDLAKLAVNEFQEIEHLMDEGNKARTVAATNMNQTSSRSHAVFTLMLTQKKYDTDTKMEMEKVAKIS
LVDLAGSERATSTGATGARLKEGAEINRSLSTLGRVIAALADLSTGGKKKKGTGQVPYRDSVLTWLLKDSLGGNSMTAMIAAV
SPADINFDETLSTLRYADSAKRIKNHAVVNEDANARMIRELKEELSLLRSKLGNGPVPGGAAGGGLVSGETYPEGTPLDQQMV
SITGSDGVLKKVSKAEIAEQLSQSEKLLTDLNQTWEEKLLKTEEIHKEREAALEELGVSIEKGFVGLHTPKKMPHLVNLSDDP
LLAECLVYNLKPGTTTVGNVDTNADHQANIRLNGSRILHDHCTFENAPDGTVTLTPSEGASVMINGKRITEPSQLHSGYRVIL
GDFHIFRFNHPMEARAERAEVPERPQSLLRHSITASQLQALDRGSPSPSPRPGHERSFSRVSEFGDISRPESPSIFQRSGRES
DWSLARREAAGAILGSDQNLTSLTDEELNALFEDVQRARAERVNGREDGDDSESSYPIRDKYLSNGTMDNFSLDTALTMPSTP
KQGEPDDRLKEVREELQSKLEKQKEEYQDQLKSAEAANVEIEEIKQEKVKMEAALKELKEDMQKQLNQQRKQFEEKIEKMDPL
KMPKKSPTLSEEEIEMAKKTVKTWRGRHFVKMAEAVLQNASILKEAQVMSHELDEHVVFQFAAVDVGHVLCSSYDMVLNGLTG
EGDDVALEEAHKPCIGIRVVDYKHSVVHLWSLEKLHDRVRQMRQMHQYLDQPEYAQHLSLDNPFVETCMPSYTLVGEVDVPLK
AVFECRVQDSTLDVLSPYTSHVVGIIKLSLEPSHARAPTNTLKFNVVMHELVGFAEREGTEVHAQLFIPGISEEDGITTTQMI
KDFDEGPIRFESVHSMSIPLFAPQDVTLRVAIFAKVSTMHLDKLLSWDDMRDAVPVREDKAKASRINESQFYTQEKHDLLSRI
QIMELNENGEYGAVEVTQTSELDTGTFQLHQGLQRRIGINISHSSGDALPWSGVTAVRVGKIRLVDSAGKTPDMGANEPDISL
KLSQSPIFRENANGTKSLTIYAQWDSSLHNSLLLDRVTQDKYRVQMTISWEISSEKLAEPMKFSQKVCVQILSRTFVRQTSMF
SALWQNVRFVRSSTGIFTLAMRPAPVKKVGDLWRLNSQHDYVKGEENLTSWTPRGVSLVSDFLMARKKKRRAADMSVTESVLA
KLGLDGANTLPEKKEPEPEPSPELKPIIPSDDDLLNDTPETSQTPSLDDEEQPPADEPQLQLFIEAQQPETDLSNLEQREIRE
PHVDEQDQPVKEEPKKEDTEEASSETPEATEVEEEETITVEEPLLKPEYDEDQTYLLTKCLKLWKKYPDPSNNILSPTNMAPP
TDGLMTDGPAQPTLIATVIRVPKNPKVLKGGYLMIPNSDSTRWVKRFVELRRPYLHLHSATDGDEVGLISLRNSRIDSQPGVL
GLLQGSDDSGAQGQDGGTDFTPGHRRTASGRVISTIWTGSGPGASSGGPGLQRLPERMQSAVFAIYGTDNTWLFAARSERDKM
DWIFRIDQTYMSSNDSVPGSGIASPYPGSDF
FUN_001272 CDS scaffold_11 302584 303035 +
hypothetical protein ENOG41

MNKYWIPHQDIHRKVITQELQYYLGPQATVRPYTLEGEDGFLITTPGSCLTDEQIDDICRKSKDLWDRQAAAKVQQNPEKLLK
RPLHQPVIVSRGSSRDSHRRYHDRRRQDDD
FUN_001273 CDS scaffold_11 303636 304106 -
hypothetical protein PF12658;PF15490 ENOG41

MSRGPVSSQLHLLSNLPAVPIGDKVRFLGCVTSYSTSSACLFLSHLYPRDSNATAVVDVSLLLESLTAEQTSVGEFVNVIGYI
TEQKMTRDKKQPYQESKQVCVQAIVLWSTGPMDLQKYEKLLDSK
FUN_001274 CDS scaffold_11 304962 307086 -
hypothetical protein PF01433;PF09127;PF17900
M01
MSWFSSLKKPAILRTCQVKITHNRNLRRTFATVNMAANRDPSTLSNYSAWRTRHTTASFKIDFEEKALKGSVILQLESQTDKE
SNEIILDSRYVDISTIRINSTESKWELKEYNAPLGAPLHIYVPGGVAKGELIDVAIDLETTSKCTALQWLTPAQTSNKKHPYM
FSQCQAINARSIFPCQDTPDVKSTFTFKLTSSLPVVASGVPVGDHSATPGEEKLYEFEQKVPIPSYLFAVASGDIATAPIGPR
SIVATGPNELEECKWELERDMEKFMEVAEKLVFPYKWGAYNVLVLPPSFPYGGMENPIYTFATPTIISGDRQNVDVIAHELSH
SWSGNLVSNASWEHFWLNEGWTMYLERRIQAAIHGDAEFDFSSIIGWKALEDAVELFGEDHEYTKLIIKHEGVDPEDVYSTVA
YEKGFHFLYYLEGVVGRENFDKFIPFYFTKWSGKSLDSFEFKQTFLDFFNNLGDEKTSKNVAEINWEEKFYTPGLPPKPEFDT
TLASQCYDLATKWKDANFTPSAKDLENFTANQKLVFLAEVQQSGELSADRAQLMGKTYDFLSSKNVEILSAYYLIALKAHDSA
IYQDTATLLGRVGRMKFVRPLFRALNKVDRQLALQTFEKNKDFYHPICKGMVEKDLGL
FUN_001275 CDS scaffold_11 312771 314933 +
hypothetical protein PF15456 ENOG41

MAHIAKCLVDIDTTYITKQASQSPNDSKLSPLPPQEKTFTWRMQQPDPSKKYQLFPKERKLPVLNSSKAVDPDKATMGPSITA
SDKTDNQQTTLNGLKKRLNQHSLARRRKISVPEVGPMTTVQELSMDSPTIPGRPPFHERSISAPGNSSRNYHLTDTFLFASSD
DEGPDPQASMKGETEADSRGATPTSPRHLAPLVIPRRGGQSPLLRRQQSLNCFPVKNEPLRRGRTSESSPQSCTSFTPNSSMP
DLTTPKSASTGSTTPIPVSAPLMEPQAESPQLSDGRYTPPVDSIRAGHRRGGSESSVMDRGRPRKRRDQRTNNGPIIQRTESK
SRITSEQKAFEKLPVGVKHTEALEKIEMPELALLQKQAFEQVGRFEVLRAEDVEALSKELRHLDEKTEYLRRTYTALRSGRKN
LHSRICQYLRSPRVAKFSNESMLKQEEALTELDASIDDWVNKLEQAENRRTRVRQKLLEHVAAAACLPLGGPSMTREPVVSGP
SSSGIGNISTPPRSPSKETAISPRTASSPSPQRVVAQVPSTILEQPVIEEEATKESADRVTSTGSLNRSDVESIRIYAGDDVY
ALLADVEDEITKMGKQPVYEPTPPNNLIETKRIELHRQRSHELLNGLSGEAHAMKKRDFSECRAISPLASPPTSFATKQNEQT
EDDLPLLTNVVYRP
FUN_001276	CDS	scaffold_11	316551	319824	-
hypothetical protein		PF00787;PF02194;PF08628		ENOG41

MSSTAFPNQNAPQPGASLTNPPKPKAIALNGSPLPTLSQTQDDDKPLLTDKPSDANDLANKMTTTNDAMNSDSPITNTTRTET
TNTRTATEDGTPIAKVIHFFATATPESLAGVAVGFAAFTYFTMGRLGLVLIGALGGVAGFISWEARNPELAKAVRGERGIDVI
DRLLEAKNNQAGEKREDSSDDEEPALKNFDEFRPETREALGGLVDAIVRDYVKWWYSPIVPTDHSFPLACRKTLTSYLLSVSS
HLSRKRPADAFLDLLTNSSSIIIVFMSELASAFSELPPDSSMTAADAVYNYLASNPDCHLANLLNQRQQASKFKMVAEDLLGF
LDRNSYNCDPARVFLREILANSVLEKTLHTCSQADWINGWIVYLLEAGEPNLNQAIDVGMQRAPESSVAATNVFADLDGNVGN
VGIAKPGRSSLETERARRKEPLGHKKKLSKAEEEMDEAMQEMKRMNEMIAEEEARRKRASVVSFHDSPKDSNNAEKRLSGAPD
LSAATKRDVSLPSATSTEGFSSKSDIVQTPVTPRSPMDSSSPESSPRRSDGTRFTSFDQIVPPGQVTDEGEEDAPKKAPLTLH
NATITLHDDTPGEPNRIRSKPNWDYWVQVEPSATAYPGWMIVRRYADFETLHEILRRIANISGATAFTELHKDLPDWKLHTRE
SLRGELERYLRDACWYQSLAESEGMKRFLEKSKGHTHSESKAGFGWETMGKGMLDALTSGPKGAMEGGKSLVGGVTGVFGSTF
SGLSRKSTQSVDLTANSSRLSISTPPSQLSGVRSPVRSARDSMDSQRSSIVSLQPGKMPPMERRPSYQSQSESDADIRTSRSE
CKESASASASALPSREHSRAPSLAQLRSPSAVSLDFTKLPPPPDQIADDYGLPEGNIDMQGGKGQQDRKGSQQIPTPPPSVTD
GKRASLKPGKQYTPLSEPETRVAVELLFAVINEMYTLSSAWNIRRTLLTAAKSFLLRPGNPSLLTVQSLIQKSVIDANTSDSG
IAEQLRKIRENMMPTEQELAGWPAELTADEKEKLRVKARRLLIQSGLPAALMGVMGQAATGEALGRVFDCLQIEEVARGLLFG
LILQVVRIVTH
FUN_001277	CDS	scaffold_11	320948	321503	+
hypothetical protein		PF02229		ENOG41

MARTSAAKKRAADSDSEPETVSKRVKSGTSVESDGKDDDGNPYWELSNKRRVGVSDFSKKTFVNIREYYDKDGKTLPGKKGIS
LSIEQYNAFLKAVPHINAALRAKGLVVEGDIADKPDTALIPAAKVKKERKKSPKANIETTSEEED
FUN_001278	CDS	scaffold_11	323551	325349	+
hypothetical protein		PF00083;PF07690		ENOG41

MRISWRLGSGYVESVDDYIGVLVPLEEAHLYSQAAKAKGAGHGETREEGEMDDLEMKEEREGMLGGDAAEYTVDGLRKEMREG
RRGQWTTYEIKSKLMNKAVGDIGMGSYNWQLFVLCGCGWFADNLWMQGVSLCLPSLSGEFGVDEKTVRYTTSALFVGLSIGSF
FWGIGSDFLGRRIAFNTTLLITSVFGIMSAYAQSWGSVCFFFSALGFGVGGNLPVDGALFLEFLPDASSALLTLLSVWWPFGQ
LVSSLIAWYFIGNWPVEQGWRYFIVTIGIVTFAMFVVRFFVFHLFESPKFLLNKGRQHEAVAVIHGLAYRNGTKTWLTSELLD
LVACEDGEDLAHVAEKKIPAPSTMGFLREKLQSFSGERLRPLFQNKTLGMATSLIWFCWATIGMGYPLFNAFLPQYFARAHNE
ASDNMQSETDTISSDTYRNYAIASVMGVPGSLLAAYLVDHPSPFLGRRGTLASSTLLSAVFLFLFVFYGTTSTAQLLMSCVEA
FAQNIMYGVLYAFTPEIFPAPVRGAGTGVASFLNRITGLMAPIIAATVPGDGATTPIIMSAVLILSAFIGICLIPIETRGASR
L
FUN_001279	CDS	scaffold_11	325595	326725	-
hypothetical protein		PF00226		ENOG41

MSGYLSMLGWAFLPNLATGWLQSIYYGFTIRAGDPKPAPGSARYNSHRRTIHILVVAAYLCYTIFEADYEQRRISSYYADLGV
PLDAADREIKTRFRRLAAMHHPDKAGKDAGDSAAFFMHLKVASDTLQDTAKRFAYERFGPEIARWQKCVTIRDYVTRGVVSGI
LPHYVVAAASIYILGLFGYMEFGKYYRWLVLIALCVFELHTVTRPDFPPFVNAINAILTQFTSSPPYLPFQIISLARRLTITF
YIALNQIGPLIAFMITGPEKSEEDDEKAMRQSLHRLEMLTKQLDGDAVRLLDMEMAPFKGDTDATSNLQGKMREWLVQNTIRA
DPMVRDAMGTSLRKRRVDAPAGAKGNR

FUN_001280 CDS scaffold_11 327563 328519 +
hypothetical protein ENOG41

MQLSLILAFATATSAACNGHDELCRRKYSDITFIGTHNSAFVGKLPVHNQYISVAEQLDLGVRFLQAQTQDKDGDIQMCHTHC
WELDAGPLQDYLEEISEWVGKNPDEVVTIFLTNIDALPIEKFDEAFSSAGLKDLVFRPKTKLSRDEWPTLQKLLEDRTRLVVF
MDYNMDEGRVDYILDEFDYFWETPFGESNSSFPTCEVDRPEKGDPTQLMGIMNHMLNHDVLGIVIPNQADAKKTNSEYSIQKQ
IDLCEDNWGRRPNVVLLDWVNVGEAMDAQISLNGL
FUN_001281 CDS scaffold_11 329688 331264 +
hypothetical protein PF03188;PF16010 ENOG41

MLLRKLGHALAATAALFPVTSAKEKDEGGQSTFVSPDGDVAFAIDIGENTNTEVYFSLRVKKNRSWGAVGLGSEDMPGALFLM
VYKSRTTNNVTFSPRLAYGHYEPYYWDEMDYEILNTTGIIDDHMVATIRCKSGCRSWPSHGGQKGYLDVYDHNSKAVFAFGPK
EDYYSDDTDAPLKYHAGYGSFSLDIKRTHGKSELPSLSDSTKDVGSELIYATKAKPNWASPLHGVFMVLSIIFLMPIGVVLLR
SGGWVKWHALNQSIATLGVFAGFGIGVANSFYYQRSRSFDDPHQIIGFVVTGLLLGQFGLGVMHHTQYRRTQAPTKYGKFHLW
VGRIILFLGTLNAFLGFTFALNRKFGMLLALLIIFICISSLILIYGRRYMDKRRLGPRGPGLAGPQQYSAPPWREPPPQHMGY
PSDPPPGYQPPSNQAGLGQMSPALRSPSPWQSNDKDDEADLNLGREQRPREF
FUN_001282 CDS scaffold_11 331904 332648 +
hypothetical protein PF04115

MSVKVNVGNLSLRIGTAPLTQEEFAPFGDVVSNPRPSLLPSKHASEGGSLPYNGTTANQGTAIRYADVSKPQDLLSQAPSSNG
RLIMSQFVCEARTLAPASDDASQSEFAVNILERHPFTSQTFAPLASTASSYLVIVAPSLPPSPQDDGLPVPSGEGLPGRGLPD
LKGLRAFVATDRQAVTYAAGTWHAPMVALGKKETTLDFLVVQFSSGVDIQDCQIVTFEGQDSKESDIKVRVPRGGRVTAKL
FUN_001283 CDS scaffold_11 333077 334152 +
hypothetical protein ENOG41

MIARFEAAEARNKLEDLSGIQLEPGENPYNALIKACNNDPAEVQTLYSVHRTKRNGQQAEKFLSSDFKELVIDQTLLRLEDPT
VEPGFRDNRNCLVIWARPPDHVIRLASKVNELLKKAAPSVWLMPSHRMHLTTLEVAFSKTPQEIAAFVTTLRPAIPDIVNYPY
HHRSRLVKPMVSYDLSAFALSFLPASGEAALSPPLTEPVVTEGITQGDDYTYHHLRRDVYDKVQESGVVVGSRYQVPSAHITL
GRYLNHDDHDTPEKRAAWVKAIDEVNAWLEKEVWDNPDSEYNAGEWLVGHERGLDARNGTLWYGGGKTIMLGEGF
FUN_001284 CDS scaffold_11 334489 335068 - RPL44 40s
ribosomal protein L44e EOG09265JX6 PF00935

MKGHELETTTPNLEPTILEGVTCSALEVLSDDSIFSLKLSSGRATRYAEIWVNIPKTRNTYCKGKECRKHTQHKVTQYKAGKA
SLFAQGKRRYDRKQSGYGGQTKPVFHKKAKTTKKVVLRLECVKCKTKLQLALKRCKHFELGGDKKTKGAALVF
FUN_001285 CDS scaffold_11 335603 336649 +
hypothetical protein PF04733

MDPYSTEGELINIHNHFHQGQYQEVIDFDTSSFSPDNALPARILVLRARLALGQAEDVLDDVKGDSQPELQALGALAEFKLGK
ADSAVQTIEKLASSAADNTTVQVIGGTVLQAAGKSEEALALLTQHQGSLDAVSLIVQIHLQQNRTDLALKEVSAARRWAQDSL
LVNLAESWVGLRVGGEKYQQAFYVFEELAQAPSTSSIRSLVSQAVCELHLGRTEEAQAALEQALEKDANNADAIANLLVLNVI
SGNQSDEFAQKLRSVKPDHQFLTDLEEKSALFDKAATKYSPKVSA
FUN_001286 CDS scaffold_11 337492 337999 - MBF1
multiprotein-bridging factor 1 EOG09264MN3 PF01381;PF08523

MDDWDTTTKIGSRTRGSGAAQRETVVRGKAALNAAQRAGGLTTEKKYSSANAGSAPEGQRMTKVDRSDDIIKPNTIGKTVGDV
ISKARQQVEPKMTQKDLATRCNTTQAIVADFERGTAAPDQKVLGAMERVLNVKLRGSDIGAPKFPNKKK
FUN_001287 CDS scaffold_11 339723 340809 +
hypothetical protein ENOG41

MTQNDQVLQRAQIKTTWRKRFHVSPFNSRKGSYSILAKDPLGPGMQGFRGLDISITLSSSKGQPKLVANLFSEGEAIDPYRIS

ILGRVGFVSSWFGSVLTILPRFMMQSTILFFMHNLHFWYRPEPLKDSIGRSANWIEKILEQVFREYLKYLVQRSTAPVTILYM
PGGVAEASEETFISHSTCGPGDSACEIKIKVLTPIFYSRFVYYAHDSEAIFCEVAESCTLWTDKPEQLTRVFLKKGSPPIHAS
NLLDYMHFQLIKNLRRRPDKIERPLTSTNGHSSSVKGIDIRDFRMSSMDAFVLEQGDKELKNAYLRSVVRLFVADRIAMSSVG
LLGMMELIGRVGVSWVLALLITETILGFS
FUN_001288 CDS scaffold_11 341928 342454 +
hypothetical protein PF04828 ENOG41

MPLSGHCLCKAVTYKVDDEPLLVGYDHCDDCQRQSGSTYSLVAVVKKDKLTISGPVKTYSGSKGSSGNIVHRLFCPECGSPIA
HDPDAAPEIIALKAGTLDTEIKKNLKPDTEIWTVSKLPFCQEHLAKPFSHMPE
FUN_001289 CDS scaffold_11 342705 344044 -
hypothetical protein ENOG41

MRSISSLLLCLATLLPPQALAESGSPIAIRKVAPGSHDKLLREHLAFAPLHGLTPREAAAAAISFLDEQDDRLLSLNGTERFY
RPAFAPHSEESHESLLRRAAEALALLQRRSSCPKGMNSCSDIDSEVKCCQEGTYCVNVGNTVAGGIACCPDGASCGGGVGACP
DGATSCSAELGGGCCIPGYVCQGLGCVPSAVATQTRTATQDQTTVLTTSQETTSEGSTVEPTTKTETEVETTGEPTTTSEAGT
TTESETEIESGTEDSTVTVTPTATGSATGIAPYRPTGTSVTSSDDGETQTGCPTGFYGCLATHGGGCCRTGRNCDVHDCPAPS
TTIVSDGATVVVLATDAPPAPGPSSTCADGWFLCGRDAGPVAGCCPDGYDCGTASCFTAQASETGRVQKEFPKADAAAYEKPA
ITVVMVVVLCVALV
FUN_001290 CDS scaffold_11 344833 346912 -
hypothetical protein PF00588;PF08032

MLIPTQLRVVALNSPLARLHPATRGFAPIFVRAKGLSAIIRGAIRSERVQNGPDSFDARNVYHDTGRRKVGGGRLTRPKEPPH
RDRDDFFAKFSARATDSSGTRNWRQEQKLKKKLRKKEEEKKEREEREELGIGRRTRRRRFADPENEFGNRSFVHRMKYGDLKE
VADNLNVKQPVRPRSFPEAQKERTFGSRDGDNDVARHERHERHEPSNEKFEKLNQQFNRSIRRPEDPQQRFGRFDRQDRQSNI
SGRSSNGEFDGSQRRFDRSQPSDFRHDNARQDRQNDQGDDSVDIKRGKKKEMMAMTLKYTTAASQFLYGRSVVKAALEQNRRK
LYNLYIHANENRKYNTDTVTLTRMAKKLNVPIKIVPPEEQRIMDKMSMGRPHNGFVLEASPLPQIPIKSLGNLEESPGRLGFH
VDLDHQTKEEATINGTDTFIRRSNDVMPKPFVLLLNEIVDPGNLGGIIRTASYLGVDAVGITNRGSSTLTPVVLKSAAGAVEE
VSLFAVDDPVKFIQDSQKAGWKSYAAVAPPDRKLVRKHGSKFVSTDTIEAQSPLNEHPCLLILGNEGYGLSKPIKVAADYELS
VPRFVQGSCVDSLNVSVAAGLLCHSFVKEPVTVAPQPVEPTARLADSVASSDDAESRTIATKTGAKLIEAESDNSSEVKTTGE
ESEVDAATEAEQKQPDDQKLNESDDLVF
FUN_001291 CDS scaffold_11 347470 352751 +
hypothetical protein PF00176;PF00271;PF11496;PF15446 ENOG41

MDTENSPYEPLEGLVNQDDNAIGRSLEWLLADLNGNNGSLPANADMDTGRQPTARGKMNARDEDKEQDIPQIEDDESSIFRLS
QPSYRFEVAIPEMPIEKRNEYSAVHSNIVEFIFGELPSSRGIIKYHVEFTDGRQELISTAELVTLEIGRVALARFSQGLWPHE
LDDMSARKRKYITDDEWDSGQESAVSDRMEIDEEGEDDDEEPVRRKRSSRRLRSRENTKQLSRSPAVVVDENDLDDLQPEPLG
ATRSLRTRQPRQLTRVSFKSGINSQDQDELLGDAPLVSSGEEDDDFMPMVVSDLAPKKGRPTRSRQKSKRLNTASMRQKARSQ
LRQKSPDSDIEFEAPRRSSRATRNTLNMRDDAQMDEDSFYIADDRIPGAPKIISIREVFQPLPADSEFASMHMDTCHTCGGSR
QRGQMIHCQGCTLSYHKNCLGYRSAREHMVTKIGQDDFVMQCRFCIGVPNKKDDSAPRHSMCQACKSPGRSCAAFSEKKTSRQ
EEKLREQNNGVDPITQVPLDLLNNADAVLFRCTSCHRGWHVDHLPGARGGTIGTDLKSERLKDHSIDWQCKECANARHKIHRL
VAWRPTPTELAKRGPRPMYAEVREDDKEYLIKWESRSYAHCDWKPGAWVYGVAASTMRTSFGKRDLEQDLMKLSEQEAIPDEF
LMPDIVLNTRLGPSVPRPKTKKDELENLANVSKIFVKFQGLGYDDVVWDTPPSEDAGDIYASFVEAYYEYVEGKYFKSESQHK
IRERVKAYKAEPFEEISEQPAGLKRGKLMGYQIEGVNWLLGNYHSGRSVVLADEMGLGKTVQVVGLVTSLVQDSPKCWPFLVV
VPNATCPNWRREFKQWVPELRVVAYHGGREPQALAYKYELFPNGSTDMKAHVVIMSYDSAQDPATKALFKSINWAGLVVDEGQ
RLKNDQNLLYGALRSMRIPFRLLLTGTPLQNNKRELFNLLQFIDEKQSAEELDAEYAVLDKETLPKLHNKIRPYFLRRTKAGV
LKFLPPMTQIILPVTMTVIQEKLSKSIMAKNPELIKAMFSNSKMNKKERGSLNNILMQLRKCLCHPFMYSEAIEERHHDPTVI
QRNLVEASTKLLLLRVMLPKLQEKGHRVLIFSQFLQQLDIIEDFLNGLGYDFRRLDGSISSLEKQRRIDAFNAPDSPIFAFLL
STRAGGVGINLATADTVIILDPDFNPHQDVQALSRAHRIGQKKKVLCFQLMTVDSVEERIMQIGKKKMALDHALIESMDDDEL
AGDDLESILKHGAQALFNDDYEKKSIHYDSAAVDALLDRTDDTESKPDDGASFSYAKVWSNDKSGFDAGLATEETAEPEAINS
SVWDRILAQREAEAQRQAEVNREVLGRGGRRRQAINYKTGALDNRIPGDAEAEPSDSSDDFAGGDSGEESEEEDPSLAKDAEA

DLQNLQASNARNARPQQVIGEQQKASRRSRQETPSNHTKQQKQQPTWQIRNTQNPMQNSYRQGSGPVNTGMPIDFTRYIKGNY
LYQGKSTDPVPPERVIPTQPHAPNNQIVDYRNIRLRSEAEVRVALDWVQRSEPETEVRKQYKAVLLSRLRSFTAKDGTQYK
FUN_001292 CDS scaffold_11 353707 354863 +
hypothetical protein ENOG41

MDLSQPSSNNHSHVVGGNGYPVQVSYGGWQQNTQVSQLSAPRGMTSGNWRGSQPSANPINSTVPGTKHSLMSSPTHPSPSNSD
SGPVSISDCLNGYAYCVKRPDGLYTRLIPADMVPTLIELPATQTSAQGMVLLPDLHMQPPQGVPGMNQPVTFKTRIDRIVATS
PTQQRRTKIYCDKWIHDGTCAFTQQGCKYKHEMPFDKATQQSLGLFHGFPKWWRDHQEELQKHRNRDLPASSNQAILQPGWRD
DGNEDVPRTATVLSPIGPERNKKSSKGNRLSQTREGMSTAPASEKRSLTGSETEWFMASPAIQGWNGSSPRRSSVSAGHYIR
FUN_001293 CDS scaffold_11 355939 357508 +
hypothetical protein

MDTVAAVLAAFTPTQAAAHSVKKYDTAIRDHVAAVTSLFANQREVISANASQILQNIDPSIDSITFQAILLLSLQTLNPAPGS
ERSTLLDEILRFLLNFNPLQIRYVGSEFRRLLEYIAAGTLFTTSVTVEALAAAILRLDPTGSMFTSTHLTMIKIAYSTSWIGP
ALKVLDCDLTFYPGMAGQKDAKLLCDSSLHPASFISVDTGLTGDVKSSAVLEYNHLAAQCYMSRRDWTKAYRALERVITHPSK
DKGVSKVMDEAYKRWLLVGLLKDGKEPSIPPYTATIAKNTFSTLGTPYKNITTQFTTTNAAQLKADIEANRQVWEEDGTSSLI
AEVVAAYPKWQIINLRDIYARVSISQVRLSTLSAETGEILADDDATTRLVRDMIDSGLLKGELQPGNNGGELYLHFHDDNEAM
TEAKFAQQIAQRYHNIESLGNQYKAANERLGNSKEYVKHAVREQKRADKDPADPGVGFDSLIEDEDLMTGITPTA
FUN_001294 CDS scaffold_11 359321 360198 -
hypothetical protein PF06687 ENOG41

MANVHRRNTAFAASTLYFLSIPFLILVIIGNTHINSTLNDIYFFKLDVSQVIPISVDNSKLLNSVARSLGLHDFYQVGLWNFC
EGYNDEGVTYCSNPKQWYWFNPVEILVSELLAGAQIALPAEAVTVLNLLKIGSRIMYGCFMAGICIDFVLIPLSLLVIRTRWW
SLVITVLAFCTAILISLAAIIATVISVAAKVALTAQDQLNIKANIGIKMFVFMWLAAIFTDVAFLLHAAMGCCCRPNRDKPQG
PQEEMRAAHPNLRHRRGHRSSSHSS
FUN_001295 CDS scaffold_11 362152 363487 +
hypothetical protein ENOG41

MVLWSSVLAVLALTSSHVAAQTVDLSASSDLTFIGGTRTQKITRQTGPPTGAYSSYASKITLQGSSDTESDELTSTLTGLFPT
DATDSTTITSNLTETGTGTATANGTSTATTKAKPTNTQPCNNYVELCKRKYGNITMVGCHNSPFVRPGNSGSNQELEVETQLN
DGIRFLQAQIQFPSNSSVPHFCHSTCDLLDAGPITDWLTRVRKWVDDHPYDVVTILLGNGNYSHPDLYVPYIRETGILKYVYQ
APYLPMALEDWPTLEDLIIRGKRVIMFIDYVSDQKKYPWLLDEFTQMWETPFDPLDRDFPCTVQRPPDLSEKSAKNRLYLMNH
NLNAEFNVFGAEILVPAVALLNETNNVTGYGSLGVAANNCRSDWGRAPNILNVDYYNYGGFPGSVFEVAAQMNNVTYDRDSCC
GTTSLAPHTYQAASMTALLAALMSAYLMF
FUN_001296 CDS scaffold_11 365843 368456 +
hypothetical protein PF11915 ENOG41

MAYQDDQAEEPFRTYEPYQPAGSATASQRPATRQPHLSHRVSAMTIYSDDNSYPQLHGRESMVSLDSYAGNSSPPQQEYDGHS
YAAGSGWSASFGYNGAGGSYAPVSAAGPPPSRFPSRAKRAYGSSLGRTIPEESTEDEAYDLSLLGGAAPMGHSGRYDPIKEDE
DDVSPGFDLSAALGPMTSHDEVFVKKLQAQEASGMLTGGLGQGFHADVKMRDAELLSSTPSVQRSLSRSFSRRKPPAHLGRFE
TIRQRGQDEANRKGEVIEVILEEGPGADLSSIEGLSTVASNDFRRSTFPTKEGKTEIFYPQPNWKPPTMRWPYLTLLITISVG
LAIAQEYLFQRFHKKPIVKFRSPKDIDAFVYFAIKFLPTVVAVTFGVFWQFTDFEVRRLEAFYQLSRQQGALASKSINADYVT
SYSFWRPFRAIKLGHYAVALSSFASTLAVSLVPTCAAASIILTPSRKERMENPSDMKEIFVASVWSRLLTVTLVLCAAMGCGL
LYVLQTRRSGLLSDVRGIAGLASMAVVSHVLMDFKDMDTAKHKDIHQKLKRRRYMLRNSSLAPYDGTSTKVETDSEQDDGTHL
SEHPHPLMLRPMGCIPFIIGLILFAALIPTFLFTPAQIVTDKAAWVVTALAVILKLCWGAMETSVRMMEPYYILSKRHAHSKV
LTLDYTALPFAYMPLRALLNGHFLVFLVGFGSVMAEFLTILVTSLATVDGQDFITGYGINKYNKGKWKDDKEDGEILNSGQET
IRSFYITLGATLFILIYMFVVSNMVFFRRRHPFLPRQPNTIASILAFIHQSKMLYNFVGTSKLNNTEMAKKLDDGKTYGLGWF
TGRDGQTHCGVDQEELTSSYKHGVDYTTMNNPWNAQWDVL
FUN_001297 CDS scaffold_11 371791 374115 +
hypothetical protein PF06011;PF14558 ENOG41

MWSCIMKPLAVLASVAALASPVSGEKILYSNSLNSCQKDNGFKATLFNVALTPNNGSAFVKAVATSSIQGKVIFDIRLSAYGY
QFLQQTVDPCTINLAGLCPMVSGKIPFKFNLPVGKENLKQVPGIAYSVPDLDATVRVYVNMSSTGESVACVEADFSNGKTVDL
KGVKWATAIIAGLGLISSAVVSGVGHANTAAHVASNSLSLFGYFQAQAILGLTSVGLPPIVSSWTQDFQWSMGIIRVGFMQDI
FTWYQRATGGTPESILYNVRQVSVEVQKRSLQAASTSIDLFKRSAAMMPRSITEPIGHAAGALAKRANIQTEDGTYIVYGIQR
VAFKSRIETTNLFMTGLTFFCIFVVITCLAIAAFKGVCELCVRQKWMAPDKFLEFRNGWMTVLKGILFRLTLIGFPQMVILCL
WEFTQQDSPAEVVLAVFFLLGMTFTLAWGASKVIRIARRSVAMHRNPAYILFSDPQALNKWGFLYVQFRASAYYFIVPVLVYT
VVKAMFVALAQKNDVAQAVGFIIIEVGFLIAASVLRPWMDKPTNSFNIAIAAINFINAIFLLIFSNVFGLPKIVVGVVGVVLF
VLNAAFALILLLMLIVSTALVFFRKNPDARYQFMADDRASFMKSQTQVNATTELDALAATARGDKAGYSNKHLDLDEEDSVSS
ESLRRRAEMNNQSQTSFQRGDVPPRSPVNPAMPLFPADGQRPASPFRNASPNPYQQSTSNLSQQRSAGDSSPGGYRSQNNASP
WQRGAGYEH
FUN_001298 CDS scaffold_11 375826 377662 -
hypothetical protein ENOG41

MASWTLPYRVEAVDRDPGFYWQDIDNGPNDGSNDFFGQFLNFDGEIPSSMADPHGHNPSMPALANGLILDHPTESTASASSGV
STTEDEFDFFSCSSQVDATIPSQPASSAVASASHEVDPRSLALASAVGLTDEKPPLVPRVSMSDPELPRVDGISLHSSPGKRV
PISQPSSPTPPNTMARKPNKFVEALSSTIRKASKLRKPKKLVAMDRPGSPTMDNPPRALRLQHHEYGNGNDAFPPSPTCGPDS
TNFVHGFCDDPFSEIPQPPPNNSLRFFSTNGIHTPAESPGVKTEPGLYHPDMAGQVPQSSWQHQQHPHSHPHPHQHPHPHPHP
HPHPHPHPQAHVHPHPHQQPVTVAGTAPETWPGQEYMAQNPHQSGWWDLNLLNQNGEYVVADPQQQKNASLNLAMHAQHAELP
YEYQVHDPNSAGLMIHMPQPRNASAPSHDLSLNTQTYLPPPPIPPTSERHRPPRAPSSGARHLSCSPIRKTRQPSIPPTPATA
HSRHSSNGSVASARSASGRGIVPGTPTAMRKQRRSRDASGSSGGGSEIGFVNFTPSDGGLLMTGVAPSGSSKTKARREREAME
RRRRLSEAAMKAVQAAGGDVDKLMEQGFAF
FUN_001299 CDS scaffold_11 380415 380904 -
hypothetical protein ENOG41

MELDPYKDMNTTKSRNYHPRNTQEDFACALDGPFNPFVPIAVDPTTLSEGPGDASRSMYRPNANGGFQVDEGYCEDEDDFSWL
QPLVLRRTTGTPDGIKKRYELGYRRSADAASRCRNTIHSVPRMRRRDKKRSRQSQPHSTASSVRGRSDSQTSQMVFSTQ
FUN_001300 CDS scaffold_11 388022 389589 +
hypothetical protein PF01987 ENOG41

MASQQHYYPPPPGATQSSPAPQPQQQQHQYALPPTSPPANRTQFYPPPPQPQPQPGQAVNYPPPPQHTPSPQVPHQPTPIHYP
APPQAQQQTPSPQAKHHSHSSSQGYYPPPPTSAPAYQQHFQHNLPQATPPPPQNQYHAPGQQSNLAMRPGPTPPVATPPAATP
QSTQQNFEPPPPVSSLDENQYPIEKPKQEPEQQPGTAVAMTVSGAPEAGQFVGAVATSDDVGTFNGGSYRISHRDTNTILTIQ
LAMGCPMTGKPGAMIAMSPSVTLKGQIKFSMKKMITGGDMSSSTYIGPGEVLLAPHSLGDITTLRLTGKEAWSVGHDAFLACT
QGVIKDFKRQGLGKAMFSGEGLWVYKISGVGLLWVSSFGAIIHKTLVEGEKYIVDNGHLIAWNCKYVMERVASGGIISGIASA
EGLVCKFTGPGTIYMQTRNSRAFAAFMGGQQYQG
FUN_001301 CDS scaffold_11 390774 392706 +
hypothetical protein ENOG41

MSFEKRIRSRMAQSVISTDTTADDSFEEWYSAPSLSRREYSEENFASSKFAEAMNNPDWRRRRDGFSVSDVTDTTNSTNQASP
HSREVPNNSTEGAYISPMRNGNISPWTFIGNSNQFQPCLCGSETDIKDSNVSSTNQEHGCLDHDNVQPADTISGLCTIRLNVD
LAGGHYKDRQLRVLQVSPLPSYAASTQKHRTAVQNIDREAPIAYVQRVVKNQRRTTQGTDNPQVERRRTPHHIFPTEAGNESE
LDKERTDRARQAVLKDPAVANIAHAKSQPKVLSGLSSPTEQTRKSEELERRKAAFMKILQKLQQGSKHETENTKRDTNRPFIG
HGCPWSPKSSQQPSQSTQQSSDSGIGSLYSDPPRNKDSSNDSGIRIDSDVLTHGLNPRAREFLSFKRSFPAAPQSQGIINFPE
DEVSKSMELSQSETRKEDTVRNTVKTAPHNMDDISVPVLQSDIGANSTESTSVPGGTTADTTKQDPGEFNYSSKISPPAKFGF
PGGACGPPVRNILTPGVLPGLGLGILPTPATFGNTPSLAMFGPLLQQYPVASGHFMGTSAAPQILFNSTLGSSSFQARPHPVP
KPTNPDPIQQQQYEEYIEWRKAHEPGYALACKGRQQRRAQRGSATQHVSMHHASCEGAQMVQ
FUN_001302 CDS scaffold_11 394067 395600 -
hypothetical protein ENOG41

MQRYLQEPQSSYFIASSNIDTPGTRCTGIYPDMSCPPHQSSQVRQGSPMSYQPSSAASGPQSPNWTDNDAHVSTLGPSTPSDV
AVLSPNIVGSGSLSPSVSLYGLSSVPQGCVNPAVIEPSQTVSGIKEEQEINAFTLDMNADFDGATPNSIYQGDSSSSPSTFDP
SSSELYPQADTKAAYPDPDNMDLDVNFENEAETIDGDDGDDEYRPNCRTKSSRNAKGGNRRGRTRRLSSTTNASRGKVTKSRT
VTHGTTARKLLSSCESILCLHCQKVFNDDVSLQKHINAMHTRPFICVFHFAGCNHAFANKNEWKRHVSAQHLNLHYWLCTTGA
CGYSSTPLKCVLGTATHCRVFKRKDLYTQHIRRMHAPEEVAKADKKNKTFPPDWLAQEKKLQEEAFRQRCKLPDYMHCPAKGC
TKVFNNGHKTWDDRMEHVAVHLERAAKNEEPHVVFGGTNDEALTEWASHPNVRVIVSTSGGWETCQPLKAAKTELSRLTSVHD
EGLDEDAEGEEC
FUN_001303 CDS scaffold_11 399794 402332 -
hypothetical protein PF00096 ENOG41

MDSFSSPQDQRGGANVSPQAAQQQSSNLAALGQQRHQTTNPFWQNWAGDDPWNPLAFGAPNDGQTRSQLIGSANFQTSYQDYR
SKPLLSECDTNPEDSAYGSRLTHSIGNPSAYGEDMDPDVQNLEPQNADAQLVNRNLETLQLQCQPATNDAHLYQDQWVRPHPP
ASVATAPVGGERRWICGECQKRCRTRSELRKHELKHSLPWHCDVTGCSREKGFTSKNDLDRHKRTVHSDRTVSGRTFVCNIGN
CAKKSKVWPRADNFRSHLQRIHAKTYSANDDLSEYVHRPVPSQDLEGVGGSAMAYLQAQEQLPGLAHPSTILSFRVSYGERRA
SQPQSGANNLPRGPPSLSFDRDASSLATVREGDENFIHPGIIHGRSALPTNQWPVSASEEDAPGDDVTASEAEEPDDVALETM
DGVQQGERTTTDVSRNSGDSGSQQADVRMVDADEAQRTPKAIPLHEKESENPSDVSPESAYDDLLDKIPKELIASYLKKRFAG
LGEEASKLDTVSSKSQTHPHKCQDCDKVFPRLCELKKHQKRHSKPYGCTFADCKKTFGSKNDWKRHESIQHYQLETWICDCAK
SDSAESCGKVCYRRESFRNHLSKEHEITDTSKLEEKVEGCRKGRHCDAHFWCGFCQETVEIKETDNTWAKRCDHIDDHFSGRR
VPKKDISEWIHEKDHRIVPSAPQVDESGSSSGSSPSDPISPQSTVSTENSGEDRRGRKKDMYMWTCCNCAVENSFSNNTLCCE
CNQRSCKNCTWHAIPSTSDS
FUN_001304 CDS scaffold_11 406038 406634 -
hypothetical protein PF00173 ENOG41

MAGKFEPKVPVNLDPPKDDIISREELAKANGADGNKCYVAIKGKVYDVTGNKAYLPGASYNVFAGKDASRALGKTSTKPEDAR
PEWQDLDEKEKGVLNDWITFFSKRYNVVGVVEGATNLD
FUN_001305 CDS scaffold_11 407020 408636 +
hypothetical protein ENOG41

MSVSAVASLRPLRRRCWQHPASGLLSVQLRSRTSTVNFYAVVDLRPDQRRFQTSNSSNNRHDGDLEARKNQLPKKSQPTQPQI
TFRQFAGRALAAGLRSLVVAMSPTGIKTAFRQSPGATTLGIFILGVVSVIGAYTVYLYFNYFYHYQFTRYPKPIANSLRRALY
YTNISPDSELALKYYKRAMDQCAEHGLDPYSDEVLGIRIQTSHWLQKIGNYSGCLQVLEGILADCNRWIRVMEQSVKEGKVND
DGRLVKPASETPEASSTHVANDTTSPNEQEATDGEFVPESLWHKRQRLLTKTVGIAIKLGELYADEHVLDPDNSQKHLIWAVE
TALKEFRRRKEEGIKPGEGDWLSPEQMGGAMESLGRDYERKNQFHLAIPLFFQALRLCETPCHRPIIMNNLAVSFAQHPIFVP
PTSEPSEMIKELHDPAMPATRKECLEAAQNWAKNAYKHAKDVSGDERTDECDEACAVALCNWGDVAAMLGNNDLARKKYKQCI
EMAGKLGFPHAVKQAQSGLAKLA
FUN_001306 CDS scaffold_11 409079 410743 -
hypothetical protein EOG09261PJZ PF04098

MPAPGDQHTAVTNPFFEEPRLRVAEWAAKDIATISAKLDKQLGPEYISARAGPGGTKVHYLTAEKCITLANEVFGFNGWSSSIQ
NIQVDFADENPQTQRVSIGLSVIVRITLRDGTYHEDIGYGSIENAKGKAMAFEKAKKEGTTDGLKRALRSFGNVLGNCIYDKD
YVKQVTKMKAEPVKKFDQDNLHRHADFIKRDVVRQEPAAAATSEAPAAPVVKTEPVPPLPVAESFDDYLGELDEADFCVPEDG
HPDEIMLSTSILGQSANEKPANRTAAQQIQQQTGRSNSTGPPNRAPQTPIHGQQRPTPPNGAATLSNRPQGGPSGRTTPNQIG
NPRPPPNGPQNMPETVGFFSAKAVNQLPESTLEGSSNARLALPQGQQAFNPKAESPSIRKTPGIDHTSSKPVARNGQHVAPTN
SQATVAPATRSNTNSFTPVRPSMSSSQSARGNVINPSLDQTRRIGAPSGPGSPLANRGSYRPPAMKRPPPADGRTALADLPAN
GNGGTAPTVASGLEAKRQKTG
FUN_001307 CDS scaffold_11 412291 413295 - STS1 Tethering factor for nuclear proteasome sts1 PF08559

MNVLLSPQPPVFPHQHENPRLSPQRSLPPFHSMASRKRKADEDGDETMSPRSSPTISARPLARPSKKVRSNEVIGRPLALPRL
LETLDTSQLRTVLERICETHPDIGHEVVSQAPRPSVPSALQVLQGYEAKLKDAIPYGETSPEYTYYRIKEPLVALIDALSDFT

PQFLPPNENQPTKSLEFLDEATNIIHRLPNWEPQTYRHHKETAYEEISKAWALVINEAGKRAGGFNLHTGGWDQILSRHNEQS
GDRLASAINAMSTSVGWIGANANPGAGGPSDPNSILNQLMSGTYGAPVRVGPW
FUN_001308 CDS scaffold_11 415266 416535 +
hypothetical protein EOG092645U1 PF00076 ENOG41

MATKEKKEKKSKTPRSEPKTENPEKTIEIEKVESTEAAEMAESEAAASSSKRKIELEEIEVDVDAPEPPSKRAKRALKKGKSL
PSKQDSDDEKKGDKDGKDKKAVRSEHSVWIGNLPFHVTAMELRKWLVDNSGGVITEEMITRVKLPTNKEPGRDKSVKPANKGF
AYVDFTEIGPKVSAISLTESDLNGRKLLIKDATSFEGRPKKEPESTEENANEGKTSKEQKQDVNASRKIFVGNMSFKTTEDDL
YRNFEKCGEIEWAKVATFEDTGKCKGFGWVKFQDPNAAAWAVKGFVKIKEAVETEDDFKDEEEKDKSQQKQFKTRKWWVNRML
GRELKVELAEDDQVRYKKRFGKDRKAFPDKDSNPRGRPPPRKYDQTNGAVRESNDAHGEVAKPLKEAEDISVARLTGAVVKHT
GTKMTFD
FUN_001309 CDS scaffold_11 416806 417564 +
hypothetical protein PF01323 ENOG41

MDTICPWTYLGKRRLDEALAQFRSSNSSSSISFKLHFASYQPNPNRPETIPDRAAYALHKKHNDNQEAQKIFEEHMLSLAQPL
KLPIAFTGPTGNSFPGHRVIQQVQESQGAEVANRLVDAVFRLYFAEGRHPGADDMLVEACVEAGIDEGVAKSLVEDKSRGERD
TKEKIRSTGMDIDAVPTVVIEGRRRDLTLTGLKEVSEYIKAMETISKESS
FUN_001310 CDS scaffold_11 417752 419732 -
hypothetical protein ENOG41

MASSAEQLFLAILRSDHIKDSLLPHLSRNDLCNIRQSSSACCNLLTKRIFTRLHISFSASTFTKSARVAALARIGHHIEHLTF
YFPHSDATFLPPLIHPVSGNEICFLYTPHTSMGSVLSRPKYANAELGDILTQQYPPLFHAATNVPSFINAMRSLTNMRHLTIR
CPGQDPRERYRRDIVDYALISLRISLERAPLEKLHKLSLSQLHPAAFNYLRHVNGFGTVPSAGRRWRQINKLSISVDAWDFYG
TSPGRDHLKILDEYVRAFAPNLEKFAFTWLGRKGPCPVALASDPLFSPPRASKKLFHEVTSPMSPLPETPGKGAIYFPKLRYL
QVRNAVMNAPQLSNLINSHRPTVKEFDFESVVLADNGNWDDVLAPIDTDDSWSRSNSMAAQSEYSLVTSPSSEHLPSPSAAVT
AASRELLDMDLGGFPFGDIENEVVDDPTGEITANEQFSAPVYESDAGFSTKLRKKRSRRRRKHRSSVEDAPETPSPSPKSSQ
HSLSRRHKTSRPRLQPFVEEQILRPSTPVPNITAPILINDPQPVLLQPTTYDPTARKNASRDPDEGISPVQRNIEQEEAHRLL
AEDAVARVSALQKAKAAVLSKLSREFCHSKKPRVGEANACRFLAGRDIGTAFGPSMVMEDRRALESRSVLVPLMFSRS
FUN_001311 CDS scaffold_11 422171 424067 -
hypothetical protein

MADEEFDPGEVIYLSSGDEAAAAAKKRPLEDDSTPEPKRLRSHARAAKVPRIEEQSVKGSKPTEESTPVGKSSAQSITSGDSA
PEDSGEIPDYKQGRASFKIPNLVDKKGGSWLKRFKDWVKAFHALNPGKCDAITSSLTYSAYGYYIDHHAGLKPKKRKTAKQVA
KELESTGELQALIGNLHSLSSCGPNQPTLDSWVTRQHRQEPVNKGKNSRASSVSEGEIDERDEDGSESEAEYEPTLNKTERIE
EDAREEYASGMNIPANGNSTPKVSDSQSLPPINTHRNVPTGDNALEQQRRYFPSAMDPAQMCLLCGLNTHLAPSCPSLICSSC
GSLEHSKICCPEKERCNKCRQLGHQAGQCTEKLALTKEEGLACVFCSSMDHLEEQCTQVWRSFYPDVSTVRKVAFIPASCAVC
GSDRHFSSDCNAHRNAVSNPTWSVKNRDQYIDPECGSVPIEGEAGGQQNARTTRAPDLKIRGHAARTTNIHYSESDDSEVEFL
GHKRVQKPALGQIRMASNIQMPNMQNGGSRRNNRQNGVPVQPPLPPGPPPGLPPMRGSFGHPPPQGAPSGQFPSLPRKPPIQD
YRNVPPPSRHGSQGPGRSHASNGPQGSKPRGGGRGGRGGRGRGRGRGRGR
FUN_001312 CDS scaffold_11 426305 427065 +
hypothetical protein EOG09264F60 PF04061 ENOG41

MPERNAAGGAGARRRRSSSILQVYHEPPETLEQISDQASLPNLNANWTNAKGAWTIHFVLIVCAKIIFDAIPGVSQETSWTLV
NMSYMFGSYIMFHHVRGVPFDFNSGAFDNLNMWEQIDNGAQYTPTKKFLLGVPIALFLVSTHYTHYDLTYFTINLLAVLGVVI
PKLPFSHRMRFGLFSGPPEE
FUN_001313 CDS scaffold_11 427816 429292 +
hypothetical protein ENOG41

MRYNAFAFMALVNGLAQANRFDWQSESKADVAKHEHKNAADPVFKVEVNEGHRAGNKGQVEVGKLKKEEEKAEEEAKKMEEKL
QKKEDELELKAAQARGSRHHRHTHSSYSPDPTYDPVTRTTGYHRHHSGHLQKGPYTLDGEKFDYIHKPKVHFHTSYTTHAPTL

SPGCVGDKCARKPCKECNGNWAQPGCWHCKAHGATGEEVAEQAAKTTKHAPEETKAKEEQEDKKEKDPYAKTYTHGDKTVIVP
KETKAPEATRPEAPQIEQPHKEGPKVIVIEEPKKKPEEEKKLEEKKEEKKKYPDEKPKHEYPKEEPKHEYPKEEPKHEYPKGK
PKKEDICDKVHHHGQPSICDKIKHQHHKVDEPKKENICSKPKGCHPHHQAPAPAPAPHHEVKLVPAPVPAPHYPEQKPEIQPA
PIPAPAPQQPEQKPGEVQPAPAPAPAPAQPEGAKPEEGKHGNAPSVPITVPKSGATYNRVSVGIAVFAGIASMMLL
FUN_001314 CDS scaffold_11 430053 430924 +
hypothetical protein PF01596;PF13578;PF13847 ENOG41

MKDNSTPLYTSPQLGQKVTDYAEQHSTPLPKHISDYHTDISANREDSYYMSSIFQSQYNTFLAKSIRATRVLEIGVYVGFSAL
VWADAVGPSGLVTGLEFEPEYAELSKKAFAANGVDNVEILVGPASESLPKLNPTEPYDLVFIDADKTGYPGYLKQLLELSKPG
NSSRILRPGALIVSDNVLRRGLVADDKALGDDELKGDQLKNVLAVRQFNDLALQSPRLETFVLPLWDGVNVSRLLD
FUN_001315 CDS scaffold_11 431190 431907 -
hypothetical protein PF06687 ENOG41

MGLGRFIHHIGAFFLLAATVMLIVVSITAPVVNDIALLKVNLNGNSRGGNGVNFGTFGYCVTRSSGSDSCTSARIGYDPSNAV
AGTNFSNAGSDTAKALTYVMVLHPIGAGLCFIAFLLALGAGIFGSLMSTLVSLLAFFVTIIALACDFAGFSIVRRRINRDTSA
SARWSVGIWLVLAAAILCLIGTIAVFVTCCSGRRRRNRENKKMAAYNSSPTRY
FUN_001316 CDS scaffold_11 432802 433627 -
hypothetical protein ENOG41

MSASKTYRAAQLSAKAERLATTHLKNVEIGKRSIRLYVSPAPVNFAERRSVLRALEQYGPVEFFKLMPGQGSAFISLMKESEA
AAKAVCSSPVNITVPSSCKEPRSAVKVDGLKPRFEKQSSQPSNPEDTKFVIEITESPAYNHNGSSSSFLARVWPDFVTENRTL
ASTTLRQSLPNSIAAKGLSHWAIDFGKETTMDSRKIDRVQTRNWIPTKIKTPPREPVPRAVGEWERRHMQHRRQSEQKTRTTS
EDQDI
FUN_001317 CDS scaffold_11 433996 436071 +
hypothetical protein PF10744 ENOG41

MATPTAAKHASQQGRTPSQLAAATPPVSTPFSNPAHVVFSPRGPRSSPQQFKKSPAASTLMAHASNAPLNFDSPSTAAAMGAL
GIPSGLDMGLDNVGVGGLGSLGALASEDDKLKRLETIIATLDQKKGLVSEAGLERLAQRIGLDCLSEDHTAPDGRKQRTLVIA
GSAIQLEITLHNNIVENISLAFPESARSVTDHVTRASEILLRDLQLLPNQSPLTKTLDKFAVNLERLAVLDKLSIIPGLDCHE
ALAGIYVSLERLHKWDVARLRDEPGMGGKPDSELSTMAMCTRHGYPVMHSKDRVGLALQYWKALRLVQPTNTKMASFTSSHEP
VWSLLIGCASTGGMGHMPVRVSEDWISKDVVKAEPSMDPKRPNLDWQEPDNVVLPHSEENKDASMEMLQPDLSTARVPQVMFV
ATFDPPVILPQNDWLRLHSYANVNANPLFGYSPTFDSLFFPIPPGSAQDPSELRAISRSRDVRIYDKDQKAIIKPHQNTLYIY
KQIYSQVVTEIPFSHPQQLIEMLPLLRQYAFVATLLENSFGSQTNEVEGLPKQGNTAAPVPKSNLTTRDELADFMKSTSVTDE
HPSAVSEVKLDVTLWVHPIPHLQVVFPFRNSTANITLKVLEDGIVEVVEENVIPRGDDSRMKGKELTRADLGKLLEYMEDLCK
WAEWIRTRLA
FUN_001318 CDS scaffold_11 436270 437541 -
hypothetical protein EOG092631ML PF00551;PF02911

MILFSVAKSRSLLDVQIPPWRRLFSQSVPRNSSDPLRILFCGSDEFSCASLKALHEEHRHNKKLVESLDVMVLPPRRMGRGFK
TLREVPCKLLAENLRLNIHQRKTFTGWDLPEGTNLVIAVSFGLFVPPRILTSAKYGGLNVHPSLLPDLRGPAPIHHAILKGYD
YTGISLQTLDHRIFDHGTILSQTSRPGISIPPDCTVQELTSLLAPIGAQMLIQGLRDGVYVPPRQNTGWRAEELSDEQLVHAP
KVTKADGHIKWTKWTGDDIVRRVRVLGSVWTHAVNKKGDKKRLIFQDAETISSKDIGNHGAKVRLLEDTGVVLETLVWDQGDG
SCAIRTLDDSVIRVKKIKEEGKSQRDAMAGLRGYLADD
FUN_001319 CDS scaffold_11 438269 439119 -
hypothetical protein PF04252 ENOG41

MAASGKTFIVEHLDPELGPWSELEYLAIARETQATHGSFILSSLPSTFQVPTDLASNPAFTAEQRGVEELYAANKSRVCLLDP
SAALDLSPEDGENFDAFLFGGILGDDPPRDRTSELRKKGFEGRRLGPKQMTTDTAVRVTRIVVQDKVPLDQVPYLDFPELKFN
EHESTEMPFRYVQGEDGKPIMPKGMVELIQKDADKAVDDLF
FUN_001320 CDS scaffold_11 440224 442192 + KEX1 Cell death

protease EOG092623WR PF00450
S10
MVVKSMIPSPRRWAALVSFCLAPALAVAVNDATTTKAAAADYYVRDLPGLPTDGPDVKMHAGHIEVTPDTHGNLFFWHFENQH
IADRQRTVIWINGGPGCSSEDGSMMEIGPYRLKDQDHLVYNNGSWNEFANLLFVDNPVGTGFSSVDTNNYIHELKEMADQFVK
FLEKWFALFPQYDRDDIYIAGESYAGQHIPYIARAILDRNMKNPTTAWSLKGLLIGNGWIDPIEQYPAYITYAIQKGLIKKDS
DEHKQLQGDLRNCERMMASDVGHVDYGECEAILSNMLRLTKNGDGDNACVNMYDVRLKDSYPSCGMNWPPDLVHLTPYLRKPE
VTKALHVDGIKKSVGWTECNGAVGGAFNARKSKPSVDLLPALLKEVPIMLFSGAEDLICNHIGTENMISKMEWNGGKGFEVTP
GNWAPRRDWTFEDEAAGFWQEARNLTYVLVYNSSHMVPFDLPRRSRDMLDRFMGVDISRVGGDPADSRIDGEKGPKTSVGDAK
NSTKTADEEHQKQLDEAKWAAYYKSGEIVLVIVVIGVIVWGYWIWRERRRGAAYSALADHDLSSRPNGHRAKSQPGDLESAAF
DESELDDLHVTTPGTSNSARFPANHDSSEKFVTKYAE
FUN_001321 CDS scaffold_11 442577 443629 - DRE2 electron
carrier EOG09265040 PF05093;PF16803 ENOG41

MAPATLMIDPSDDFVMPVTKSVQSTSPPKRTLLLAPPSLASNSDALTSVLADYDRSVTDLQMLDRLSLGLVTLPPSTYDLVLV
LSDASSILGESLALMNRTVLGPVAESLKPSGRLQSQDGNSLEESTLSKEAVLAGLISSRGGFEKPDYGENDGAITLKFGKKKS
QPAPLADGSVPLNLKRKPAEAKSKPVVPAGVGFIDLEDDLEDDDLIDEDTLMTEADLARPINIPAECQPKAGKRRRACKDCTC
GLAERLAAEDADKRATADKKLESIKLATDDLAEIDFTVQGKVGSCGNCSLGDAFRCDGCPYIGLPPFKPGEEVRLLNNDVQL
FUN_001322 CDS scaffold_11 445891 447600 +
hypothetical protein PF00098 ENOG41

MSDWNNDQGGSAHPNTFEQENWTNSGQAENTSANNAGFGNDDFDNSDGFVGEQPTGNEKCFGCGETGHRRAECPNPQEMTCRF
CKQPGHMIKECPDKPPMICENCGDEGHMRKNCEKPRKINRDHVADVSADDAWNKIKQAAIERDVDDAKEAVEEYIKAVDGDIT
YRQLQEALIDQGIGLWLIPTERSLIQVFTNMDLQGHIDKKYTISYRFVEQADRPRELEGWPKSRDELLSRLDDAGEVVDRGVP
LCLNCKELGHISKFCTQEKTERSDAVKISCFNCGADGHRVRDCPEPRVDKNACKNCGKSGHRAADCEEPPNPANVECRKCNEM
GHFAKDCPQGGGSRACRNCGQEGHISKDCDQPRDMSTVTCRNCEKQGHFSRECPEPKDWSKVQCSNCQEYGHTKVRCKVPPVD
EADGVGVAGDGDGDGGWSNADAVGGGDGYGNQNTGGNDGW
FUN_001323 CDS scaffold_11 453070 454945 +
hypothetical protein PF00170;PF07716;PF11785;PF11786;PF11787

MGTTTEASAGGESKSPKQSNSSPARPETTTDTKAADPPVKLEVANAEQAKPLAPPPRPGQQQGNTPDYFAVQAGGSLSLEPNP
FEQSFGGAPETPGGTKLPSVAALTSPSSLLPGSNATPFNWGGGSLRTGPLSPAMLSGPANDYFGDTHHLRGGFPTPNESSLRT
GLTPGGSGSMFPAPSPNSQALFAQLASGGATPSTLDFHRTAMSAAAKRDQNGAAPRQAQQTSQAQQPQQPSVTSQPQEMPNGA
STNKSEAKPASGPFDPHDNDAANGLFMLAQGRNGAQNANQFAVTSGAPGHAHPAPVAPQNMNTSPQMSSINGGSIGSARGMSE
GSMMSDESEQARPNTRGRGKKNPPATNGRRKADEPPAKAPANKKSKANNSMPMDMDMSDDESKMKYEDGGSKSKMTDEEKRKN
FLERNRVAALKCRQRKKQWLANLQTKVEMFSTENDALTAQITQLREEVVNLKTLLLAHKDCPVTQQQGIHGAFLSQVVEPFNP
QINPYGMAAPMPNQQVMAGQGVQRRFS
FUN_001324 CDS scaffold_11 456236 457942 +
hypothetical protein PF08202

MSNEHGRRLSKRLAAAATDYEHDDDFAFVRKSKRPKTDKSDESKPEPEPEPEPEPEPKAEPVKKNAKGRPPKQRAAKVPTTNGTI
AEEAPAENEMNATTKPATRKSSRRKASVDASEGRQINVPKRPSTRRSTRRSGDSQDEVVPQAAAAAAAASAPEPDPGPQPEVV
PEPAPAPRVNGASKKRGTRAKSTRPPPDWDKSPQRELHAQSATIALPMSDTPIINRNKEMRKKGGNSNRRSSLGNRGRRASSL
IESGQTAIPHREVNPADFYKHIEAEGLTEPRRMKQLLTWCGERALSGKPPHGTPNSNAILGARAIQDQLLKDFAARSEFSDWF
SREDDAPKAPVVLKPNPRNMELDEKLAQLEINIKRLQDEKRAWQAIRKPPPEQPPLFPEDEIGPIVLPDFDLLESDEGKIRGF
LADDKASFDAVRSQTESRLRSIQSSLEFQIDELADNVHKLEQRVVVAGKEADKVLSVSALRLRQREEREKASAGTRDMPVIEV
LRSLGNILPEGGG
FUN_001325 CDS scaffold_11 458505 459214 -
hypothetical protein PF03937

MTSLISRTMRPAALRRIVSMPVRPLTTTVSRPSDNVSSSELKVGELQGAKFRVEPLRRVGEDDATKRARLVYQSRKRGTLESD

LLLSTFAAAHLPTLSPELLDQYDLLLDENDWDIYYWATQKEQLSTTNPSSAQSPSTTDIDAKPESDQITRHPPSGEWAQTVGN
FKPAYRPVPARWKDSEILEKLRAHVRSRSVDGGEGGGMGFMPPLEPPEVKK
FUN_001326 CDS scaffold_11 459614 461129 -
hypothetical protein EOG092624KK PF00579

MADLNPPKTAVQDQDINPWSVEGAQGENGEVAAIDYDAICSKWNTSKIDHALLERFEQVTGKKPHRWLRRGLFFSHRDFDKIL
TKYEHGEPFFLYTGRGPSTGSLHLGHTIPLEFTKWLQDVFDVPLVFMLTDDEKALFKDSLSFEDTHKYALENAKDIIALGFDE
KKTFIYSDLEYLGHHFLMNAYEFSKLVTFNQVRGAFGFDGSANIGKIFFPAVQCSAAFATSYPEIWSDDPSPVRTKALGKIQC
LIPMGIDQDPYFRLIRDNAHRMKNPSPKPALIHSKFLTALQGAGGKMSSSNPNSAIFMTDTAKQIKNKINKFAFSGGRETLEE
HREKGGNPDVDVAYIYLTYFEDDDEKLQKIYDDYKSGALLTGELKKMAIEALQSVVETFQDRRKAVTDEVLKSYMKPRRLQWG
GNPNPKPKESKPKGEKKKENDEKKTELPDRTAEKSA
FUN_001327 CDS scaffold_11 462220 463023 - ERV25 vesicle
coat component PF01105

MALQQTLVKYICSVLLLVSAASALKFDLIAQAESSKRERCVRNFVGKDTLVVVTATVDGYKGDGMVVNLYIRDAVGNEYGRPR
DVVGESRTVFTSHADAAFDVCFENIVTGSQRINNPTRHVELDIDIGADAKDWSAIQATEKLKPVEAELRRIEEITGELVTEME
YLRAREQKLRDTNESTNNRVKWFGIATTWLLIGLWAWQIMYLRAYFRSKHLI
FUN_001328 CDS scaffold_11 463403 464288 +
hypothetical protein PF06220

MPKFFCDYCDVYLTHDSMSVRKAHNSGRNHLRNVVDYYQQIGHEKAQSVIDSITSSYAAEGQAHANPMLPQNQPGQGFPPPPF
AFPGGIPPPPFPGMPGAPPGQFPQGLPPPPGGGRGMPPMPPFPGPNGMPVPPNGLPFPPPPGGFPFPPPGAPGGPGASGGAPP
PFPGMPGMPPPGQGFPPHGFAPPGAGAPGHEKR
FUN_001329 CDS scaffold_11 464840 467927 -
hypothetical protein ENOG41

MLSAHSIPSPVMMAPPADYPPQAQYETSPTSQLQFKKHKILPRPRSERTSELPIRVSSPLTRHDLATNTSVSGNGKHPSSPRT
LKHQSRRISSGPDLPPTPPRHSRQPSENSSGKTNSPAATDIALRTPQPSHLRPPTTPPNQKSPPTPDVTPPHSTSRPELLRPI
ASDRAGSSTTAGESQSGSFTTAREEPLSSEDEDKPVVNQNSRPAVRKIFDPSPRIDRPENLDLALGNLAPHSEGSSTSRSPAG
DWAHNDSDWGSVSEVEQEWDHNLQRLVTVKKRPEALIMPRLSPNRARVVEPNIIIPTQAAKAVRGIPLYQTTETLTETLPRGT
SSRDIPPSATSTTIATSDPRRMSAISTKSTVSTVVEAYLLDTVPKRQRTLRHVRKQTILRDRTNESSTSTANSLKSDRSHTDP
RGRNRPTAPKSESQSSNATLNSISSSRARREVWKAGGIPVVVVPGRLSSHKTKSREPSLRSHSSRKSSRTTSASPTLNDRSTK
DEPPIVRRQLRGRSYSMSESSDERTMDYPPIVPARSSSLSAPTSRNGSRASSLTTESMKLHNALQEYLKEKKDTPRVPDLRFS
EETPVARTFSNASESHRRSSSERHDDFLNVKGYGSQNTPFSLASVDTNGTNPVVSEALAVQMYPHQNSSLLMIDHSTKPSESP
DETQSEAEEDLDVPMPTDSSEDIPTTPPQPKFSLDDVDSPLRNPRAPPEPPNHPPVINLIPATPSGTTPAHEKMAQMGNYFES
MNEKPPRRPSVVRRAFNRTRRHSVDYTPSTRRPSSFLSRTLSLSRTGRIRGMPTMEREPAYLSSDDSPVEENKLHPFWRPQWS
SEDSDECDGYCDDDCDLHWSDELGDQTYRYPPVDNRPKGPQRSFSSRMKRTFAVLPPRDGSYYTSYDWPTTERRTIGRTPSGN
LRVMRHRSSLESLRRNYNDDERPHSADGEVKRSFWRANNIHRRANKEKRRLSLGSKLEELQNLPRKFSERRREKRSQELRKKI
SGPKEVRDGVGEVIRSSAARDHYKPFDRTPSR
FUN_001883 CDS scaffold_12 1301 3223 +
hypothetical protein PF01544

MSDHEESAAQSGYDTPVPELDDHRHQSGSTRVERPRRGTFDSLYGARQLEPEPPAPPAIVVRDFEEAIVDEEVGDLSPTARRS
RRPTVDSTDRSVSPPNSVKAFAQARRRERDMSFSEPNKPDIEDVASRAMSISSKRSYRSKPRTIDNDAASLSTNTTAEEDVCF
PVKESYHKDHLYIDFDYLENFINEQAAERAAERAAERNAAAAEKQSFEDMRTQQNESRLQRMVTVDGDILEPVSDDSMTREKT
NQTAQTHAEGSIAEKQPAPVDPNRFSFFSSAWESTIHAADFGDLVLPGEDLRGLFELPDEDEDGVWWLNVNAASKEEVQSICK
AFGIHPLTIEDIITQEAREKIELFPSYYFACFRSFSVVQEPDGIEYEPFNTYVIVFREGTLSFSFEPNSHAAQVRKRITALKD
YVSLSSDWICYALIDDIVDSFGPVIRQIELEADAIEDEVFVMREDDSNSFLRRIGRVRKNCLALLRLLGGKADVLRGFTKRCN
ENYKVTPRMDIGMYLGDIQDHVVTMATNLGHFEKILSRAHSNYLATVSINSIAQGTHTNEVLSKITFLASILVPLNLVSGVFG
MNVPVPFATAAANNLAPFFGIISFMVLLCIIFMALARWKRYI

FUN_001884	CDS	scaffold_12	3761	4103	+	TIM8
Mitochondrial import inner membrane translocase subunit tim8		PF02953

MSAQQLSIDNADLEKLNDKDRLELRQFLANEQQRSQIQAQTHSLTQMCWTKCVPGNIKNPKLDKSEETCLANCVERFLDVNYL
TMKHLNGMRN
FUN_001885	CDS	scaffold_12	4683	5671	-	RPL23A_1	60S
ribosomal protein L23A	EOG092657YR	PF00276;PF03939

MAANKKVQPKGKGNKKAQDAAKAALKGSNSHKKVKARFSTSFHRPKTLITSRAPKYPRRSIPHQPRLDEHKIIVHPLNTESAM
KKMEENNTLVFIVDIKSNKAQIKLALKKLYDIDCVKINTLIRPDGTKKAYARLTPDVDALDIAANKLSLV
FUN_001886	CDS	scaffold_12	6157	7033	+
hypothetical protein	EOG09263PWF	PF11968

MVSKKQTRLKSLSSGRPPTVKSSRSMSRKASRSLINTHHQLEKQRRRAAAKGDTVTETRLASEIAKLGGLDHYQKASLQGQSL
DRGGDTSRVLLEWLPVAELKKRAQPFHMLEVGSLSTRNACSTSGIFTMKHIDLNSQEPGITKQDFMERPLPKDDSEVFDIISL
SLVLNFVPEAEGRGQMLLRTLSFLRPAAEPATGSDEPFPCLFVVLPRSCVDNSRYFTETRFEELMTILGYVCTKSKMTQKLAY
TLWKRIGTVSEKRSDFTKKEVNPGRTRNNFVMTLKASTSHSN
FUN_001887	CDS	scaffold_12	7469	8760	-	UTR4
enolase-phosphatase E1

MTLNLNEFDVLVFDIEGTVCPISFVKDVLFPYALEALPKVLDQEWDSPEFAKYRDAFPEEYRNSRSDFEAHVRDLVKRDVKIA
YLKSLQGYLWLQGYKSGNIVAPLFPDVEPFFNEATQAGKKIIIYSSGSVPAQKLLFSHTNSGKSDMTPLIADYFDTTNAGPKT
EVDSYTKIISEHPEHKDVNRWIFLSDNINEVKAAVGAGMRSLPVVRPGNAPLPPDEPLSKLAITEFKNSEVASLGV
FUN_001888	CDS	scaffold_12	14794	16705	+
hypothetical protein		PF00069;PF07714		ENOG41

MECLKDKFVGGALLKGRYQTISPLNHGSFGMVFKAQDLVTNEPVAIKCLTKRSACPDADSDFAIDEKSEEQALHSRLGTHRNI
VNLIDSFETDSHIYLVLEFCGQGDLYEAIRKGHGPLETEHVRQFMIELIDAVDYMHSKGVYHRDIKPENIFLTQDGAMKLGDF
GLATTEKWSYEMTVGSDRYMAPEQFDSAGAGYSPAEADIWAIGICLLNILFSRNPFTTPTEADPLFLDYSRDKQSLFDVFPSM
SQDTYEVLVQCMNLDPRRRSLEGAREALLRVVTFTTDDEDDDEYCGAESPNVATANREPLRTPSIQSPAVDTGAFPWAKALHG
TTHLGRQLSVIPDDESYTEDLFPKSEATTTDWCSANMQTPMSSVYDSHLGASMKSLAIKPTARFNRSSATAGSLPINMAKPMP
AMSMVFGRRQETVSKSWSDMWDEDEEEEEMEQQRQMLQELNARTWSQESKEEKKVEEEEEEEQVMKTDIVQPEPESASHTHDI
KGDVDDDLVADGFFFQEAPTPKPQQITITPHYSHSPMSKPRSSLDKWAALGERRRGQTATLDSIKSVDFSKPRRHNFGFGYNK
SYEAGVWDHSNYHHKFGAAKERSNSKECPWNKGRDWNWRRDRRTDLGDVEWVGGW
FUN_001889	CDS	scaffold_12	17936	20176	-
hypothetical protein		PF01490

MSPARNPTSWDDYEGGASPRGSVTDSMLEREQALVDDEGDETGDQENGEGFSRPRRRSSVTYQLAAIADIGGVNSFRSFARSW
QRAAGFPEVIPRRPSFVLAPEQDTEDLQYSRSHVQGSSQQQSGLLRQHLDASSSHANGESSSAIESSPRSMRIPHRDGETKPL
LDVEAGSSPVGSPRSSIFAVPPHLAAPDIVGSYGSFRDSSPFGTMDRSSRHRISFSEGSGWGVADEEEEEGEGAAHGEHQPIL
VKEVKQGNKVVLAVEGQSTLPQSVFNSINALIGVGLLSLPLALQMTGWIPGLFLLTLTAAVTSYTGKLLAKCMDFDPSLITYS
DLAYISFGTRARVIVSALFSLELVAACVALVILFADSLSLLLPGLASVNTWKVVASVMVLVLNALPLRLLSYTSVVGIFSTFC
IVVIVIIDGLYKPHYPGSLREPATTYLFPENWLAVPLAYGLLASPWGAHSVFPSIYRDMRHPYKWGKAVNVTFSFSYVVDTCL
AVIGLLMFGDGIKDAITSNILKSKGYPDALKIIMSIFIAIIPLTKIPLNARPIITTLDVICGVHDQHHHHHDQPHSQPTRSSV
LVTKAVRGLVRVFVVILLLFISIVFPAFDSVCAFLGAALCTLISIILPISFYLKLFWQDVTMHERIVSAILLVVFAILGTLGT
IWTFLPKHLIGAD
FUN_001890	CDS	scaffold_12	21940	22816	+
hypothetical protein

MTTPPDAALLATTSDSLAPLIPILNGFAHRHKNQHSSSHWWSSFSVLRRAVRNLVNDLNSRPKKVKSGGVKRDVYPALARAKW

MMRHVVPGAFVTFSQLAADNQHAPLGLLLLSVLARTSTLLSQLVPDHDHNPTISTSTEPMPSELSKHQTSDLRTDDAPNAGVD
MGVAISRDELLSTQKKTKPVPKADSRSKDLKLKEYETKTTHTDTKKISSKSDTMDKPKKKTKNSDELSSLFGSLSSKNGTTDK
PKKKKKKGDAFSSLFDSL
FUN_001891 CDS scaffold_12 23324 25375 - RCO1
transcriptional regulatory protein rco1 PF00400;PF08581

MSMYPHRAMGGGPQGNSTRLNELLEQIRVEFDTQMRATENYEHQIAAQVSEMQLVREKVYQMEQTHLSLKQKYEDEISALRHQ
LEAARKGGIQPGIPGPPQHAAPSQQPPSIAPGNGLFSGIMAGGSQGGLVPPQPGQQQGQQGQPQGAQQQQQQTQQGHAPQEQP
LGPQHQMGQGPPGLPVPPPHPNAQQAPYSQNYPPGPVSNGMGPQPQSTASPGPGRRGIGRPPGAVGPATPQVNTPVPYPGSAA
SPQVSHPTPEHGRMGGPHAPAVGNALGDLEVDSVAPHNKKTGQDWYAIFNPQVQRVLDVDLVHSLNHESVVCCVRFSHDGKYV
ATGCNRSAQIFDVQTGEKVCVLEDHNASDMSADLYIRSVCFSPDGRYLATGAEDKLIRVWDIQTRTIRNHFSGHEQDIYSLDF
ARDGRTIASGSGDRTVRLWDIEQGTNTLTLTIEDGVTTVAISPDTQFVAAGSLDKSVRVWDIHSGFLVERLEGPDGHKDSVYS
VAFSPSGKDLVSGSLDRTIKMWELSAPRQGNQPGPKGGKCVKTFEGHRDFVLSVALTPEANWVLSGSKDRGVQFWDPRTGTTQ
LMLQGHKNSVISVAPSPQGRYFATGSGDMKARIWSYRPYS
FUN_001892 CDS scaffold_12 29009 30098 +
hypothetical protein PF04116

MDVVLEVVDTFIADYAYSYFHPKPPAPYDFPSPSNSTDTSAKAFSTWTYKPATQFITLEPAEEAYMSAWDRDNPLRQALTLYL
ITWIFGLLVVYFIVATLSYIFIFDKRTFDHPRFIKNQVRLEIIAANKAMPVMAIITAPFFLLEVRGYGKLYDTTEDGPGLWYNF
FQFPLFLLFTDFCIYWAHRWLHHRLVYKYLHKLHHKWIMPTPFASHAFHPLDGFTQSLPYHIFPFIFPLQKMAYVALFVFVNL
WSVMIHDGEYLTNNPVVNGAACHSLHHSRFEVNYGQFFTGFDRMGGTYLMPEQWMFERNMKMSEGRWKKEIEKVDELIEEIEG
KDNRTYGSSSTKKTQ
FUN_001893 CDS scaffold_12 30659 31209 -
hypothetical protein PF09435 ENOG41

MSYLLYSISFFALVLGTVLFFTRAHWIPHVQHLRPRLPGADYIYSRLPNSFAGDIEAGLSSNTFDLSGNVESGDSRAGLDDAA
KAEVLAIMKKRRMNFDQARKVYMENRFKANGIGADGLPRDPKFPITTIFDSELDSPIIITIPTLQSSMLPTSFRNYTTSKR
FUN_001894 CDS scaffold_12 32055 32887 +
hypothetical protein PF01778

MSTAQELSNISSDLIWEIVRDNNCFSAKSKKNGGVQFSRDPLNLTNKTSRKHAGFVNDKALGISSGEKGAIVVTSKKAQPNKP
AQNLVKTSYSGSKSNRKTYQAVANQAAKNGYRADLRSAAVERASALKKSNKPVKPEPEQKLRGNKAKKAAAAAEEN
FUN_001895 CDS scaffold_12 35643 37132 +
hypothetical protein PF00056;PF02866

MVKAVVAGASGGIGQPLSLLLKTSPHIDELALYDVVNTPGVATDLSHISSRAKTTGYLPANDGAKAAFKDADIIVIPAGIPRK
PGMTRDDLFNINAGIVKGLIEVAAEVAPKAFILVISNPVNSTVPIAAEVLKAKGVFNPQRLFGVTTLDIVRAETFVAEITGRA
NPQELTIPVIGGHSGETIVPLFSKASPAVQIPEDKYDALVNRVQFGGDEVVKAKDGAGSATLSMAYAGFRFAEKVLRAVKGEK
GLVEPSYVYLPGVSGGGIPGGEAIAKETGTDFFSVPIELGPNGAEKATNPLEGITEKEKALLAKATEGLKGNISKGVSFVHNP
PQK
FUN_001896 CDS scaffold_12 37508 38339 -
hypothetical protein EOG09263RY2 PF04051

MSFEPVMPPFNSSDPSANFLSSSCLDFLLIELVPMAYRVTHERDSIASESNGAPADTASTSHAVSSSAAGIMAGTATRKDEEE
DMDAVHYRLEMLGYRVGQGLVERFSRDRPRFNDTLDVIKFLCKDLWTLVFGKNIDNLKTNHRGVYVLTDNLFRPFSRMSTEAG
GQAIVRAQPFLWFPCGVVRGALAALGINTSVQAEINELPGAVFQIKTIPNKP
FUN_001897 CDS scaffold_12 38914 41308 +
hypothetical protein PF00586;PF01071;PF02769;PF02843;PF02844

MADLRILLIGNGGREHALAWKLSQSSRVEQIFAVPGNGGTAGCPKTSNVSSVKAEDFAGLVKFSQENGVNLVVPGPEAPLVDG

VEGWFRKAGIPCFGPSKEAARLEGSKTYSKDFMKKYNVPTAAYENFSDYNKAVAYIDSVDHDVVIKATGLAAGKGVILPQTKE
EAKAALKQIMVDKEFGDAGDEVVIEELLIGDELSVLTFCDGYAIRSLPLAQDHKRIFDGDQGPNTGGMGCYAPTNIATKELTE
LIDREILEPTISGMRREQQPFRGVLFTGLMITSKGPKVLEYNVRFGDPETQTVLPLLSADTDLAEIMLACANGYLDNCKLTIE
NKFSATVVLASGGYPGSYPKGKAMSVQTPPAGCNIFHAGTTLDSTGLKTSGGRVIAINAVGDSLRAAVDAAYAALDKKVIEFE
GAFYRKDIAHRAFRSTAQTSMTYAQAGVDIQAGNDFVEKIKKAVASTKRPGADAEIGGFGGEVDLSQAGYPQAPIIVGAIDGV
GTKLMIAQAMKKHDTVGIDLVAMNVNDLVVQGATPVMFLDYYGCSKLDLSTAADFVQGVADGCRQAGCALVGGETAEMPGMYQ
NDDYDAAGCAVGTVTADIKLPRKDDMAQDDVLLGLGSAGVHSNGFSLARRIVSRAGLSYTDPAPWDQSTSVGESLLTPTRIYV
KSLLPVLSHIKGLAHITGGGLVENVPRMIPESLAAEIEFGSWEIPPVFKWMREAGNVEPIEMCRTFNSGVGMVIAVDPAKADS
VTQALTDAGEKVYRIGRLTRRDQGEACLIHNVDSWKAETAVPSKRKDIGA
FUN_001898 CDS scaffold_12 41984 43630 -
hypothetical protein PF02891;PF14324

MPASPPVIQEAVNANDPVRLQQIRQSVHNSISSSQTGSSPSRSNFPISHGHASSTPFGLHSMSFPSTHSSLSNGQRSVVNFSS
SVTLTFKPSPFYQIEAVAGDIKTCDVMSQHRNTITYPIRLDDHPALHKCVNDPSYRVMVFCAGDTSGMQDVAFPHQSELKVNG
GEVKANLRGLKNKPGSTRPVDITKALRLRPKYTNNVEFTYALTSKNFYLVIHVCKITSVEQLAERIANGKKISIDSVKQELNA
KAQDPDVVATSQVLSLKCPLSYMRLALPCRGLSCTHLQCFDATSYLQLQEQGPQWQCPICNKSATFEQLAVDAYVKDILEKTP
KSQETVTIEPNGEWHLKSIDDSSQGHTNGNSSYANAFDDDDDDLVISEVNSIGHRRLETPKISTPTISTPVTAGRDVSSSAGP
RGIASTSAKRPMAAVIDLTLSDDDDDLPPPPKRQNTSMNGYSGNLPSNSPISPGDLGYL
FUN_001899 CDS scaffold_12 44613 45075 +
hypothetical protein PF10338 ENOG41

MAKSARASTRKANNRRLVKNVFGPAEAARNERLSAKLLELAKQPKPESSDVNMNTQEEDANDDAKEDVQGNEVTSMDVDSKKP
SSGRIEKKRVDKRKQKASKIVFKSYGGRSKGKKKSS
FUN_001900 CDS scaffold_12 45469 48401 +
hypothetical protein

MVSSHNGSPLRTLLLVAANLLIPVSIVVFALGFFPYKPFLPGLAEFESLDFGSPPDAPFDRLIFMVVDALRSDFVYSDASGFD
YVQSLIRDGSAMPFTANARSPTVTMPRIKSMTTGSIPSFVDLILNFDEADTSSTLASQDTWLAQIKAKQMGKLLMYGDDTWLK
LFPNTFDREDGTSSFFVADFTEVDNNVTRNIAPELENNDWGLMVLHYLGLDHIGHKAGPKSSNMFPKQREMDGIVKTLFEAME
SKPHLDSTLLVLCGDHGMNDAGNHGASSPGETSPALVFMSPRLKKVSHRLPAPAQPKDEFDYYSMVEQSDLAPTIAALLGFPV
SKNNLGAFIPDFLPFWHKTSDQIQILVRNARQILNIITAAFGSELFDAQSSVDPCALEQTEINELACQWRRINKEAHVLAAGN
KLDQKWLDDMSQWLRRAQDLMSSMASNYDMPKLYIGQAIAAVAATASTVVLVSLGTHRDGQILPFSLMTLSYGAMMYASSYVE
EEQHFWYWSSSIWLVIQGVLHIRRRNSLADIAWVFVALVALRLTRGWNQTGQKFAGSPDIVKSFIVTHPQLLWAIITFGYILM
SFRLLARLKSLPSLASTSTTSILLMSAYSFKLGFTSEDAPELVVGFARSLNDMFVGQSLLWRARTAFILLGVLFGYGIYRSFT
GGRNGQLQSAYLFHHLYTIFGITQSRATNIPLFLLSDILFHALQATDLSVTGITITAILLQYTTFFAFGGSNAISSVDLSSAY
NGISGFNFFAVGFLTLVSNWAGPIFWTSAANLLLLRKYHDGQRNAFWQYITLQTVFVSATVALVMAACTSLRTHLFIWTVFSP
KYLYCMAWSLGQHLLINIGFGGLLFWLGSRNWHHLASQ
FUN_001901 CDS scaffold_12 48628 51752 -
hypothetical protein EOG09260LRX PF12719

MPARTTARSSRTSAARSSAASRTSPVAEPVDTPDNALRTKVCAVFRDAQRTTATHRKLVVNLRKIQESCAYEPTNPDQSRADE
FDEDAFNHEFIRCVSKIMPIKKSESVGEKSIKFTGLFLQHASAKDNELLGEIDQDASVMPETPSTRLTSQLLEAILPLLTAKD
KVVRFRSTQLISHIINSLDAIDDDLFQKLRHGLLKRIRDKEAMVRSQAVLGLGRLAGNQTEGCTNSDDSDDDDTGLLEKLLEV
LQNDPNADVRRSLLVNLPILPETLSVLLERARDQDAATRRAVYSRLLPALGDFRHLSVKMREKLLRWGVRDRDENVRKAASRL
FRERWIEDCAGAPPQEDGVPAEPAPPNFDGLLELLERIDVVNTGVENGVALEAMKGFWEGRPDYRETVEFNDHFWETLSAESV
FMARSFNDFCRLEGDGKYLNLLEEKLPEVTKLAFFLERYLHVLTAAVKRVAEADDEDEEDTVEQEFIVEQLLQIALTLDYSD
EVGRRKMFTLLRQAISIPDLPDEVTKLAVETLQSICSPDAAGEREFISIVLESVADVHDTIVDDPPADDAEDSFHSARSEVSG
QSTPTKGSKRGGTPELSEEEAREKAVREIVVNMKCLHIVQCMLTHVASNLQDNTDLVAMLNNLVVPAVRSHEAPVRERGLVCL
GLCALLDRSLAEENLSLFMHFFTKGHTALQITALHILTDILNVHGAQLLSSTPGLLKIYVKAVKGGGKSPEVQAAATVAASKL
LLGRVVSEEEACQDLLKSLVVAYFDPSSASNQTVRQALNYFLPVFCFSRDENQNLMRSIALDAIHALFNVRESLEDDDVDVEE

DMVSMTTIGACLVDWTDPRKCYRPTSLLESDKKAVNGDVHLDFAMDILEKLQGNITKEEKKLIGTLLGKLHISPGSSEEKLRE
AYNEVSIAVEEGLLSDATSRSALFKLHVSLGKIVNALDEQQPAHRRTSRSTSMITDHQSPEEEKTVMEEPSIKEEDEESDGTV
VPKEEKDERESLVDDLLSDGEDVEMTDV
FUN_001902 CDS scaffold_12 52040 54260 +
hypothetical protein EOG09260XQV PF00076

MGASKNGRKRPHTEAEELDPASFEDRDNGAVKASKRARVEERRSLFVRSLPPGATSESLTDFFSQHYPVKHATVVVDQKTKES
RGYGFVTLADAEDAAEAKKALNNQDWNGRRIRIDVAEPRQRNNTTGELPAHKVRKEELQRPPKLIVRNLPWSIKTSEQLSHLF
RSFGKVKFADLPQDKGKLKGFGFVTLRGRPNAERALEAINGKEIDGRTLAVDWAVDKDTWEKQQPEENAKPKKAQKKQEDDEE
DEDDQTSSEGSDDEDEEEGGAEVNRDDQLDADLKNFFKNHMENLEDEEDDDDDEEDDEDDEADKQAEKEVQSLAPKRMTDNTS
TVFIRNLPFTTTDEQLKDFFDHFGKVRYARVVMDKVTEKPAGTGFVCFYDVDDAKKCIKGAPRPDPSVAAAKNSILQDESADP
DGKYTLDGRLLQVAQAVNKQEATNLADSSLAKRNEKDKRKLFLLNEGVIDRGSPLFNLLTPSEMQVRQASAAQRKKLVQGNPS
LHLSLTRLAIRNIPRNMDSKDLKELARKAVVEFAKEVKAGRRQPLSKEENARDGKDAKEKEHERKAKGKGIIRQAKIVFESGQ
GQKMPEKDGGKSRGYGFIEYTSHHWALMGLRYLNGHQLENEVGKKQRLVVEFAIENAQVVQRRRANEERSRQLNPEHNKAAKA
EGQPKTNAQHTRDDKKRGRKGDRGDKVHKGGKDRGSKQAEEEGGRKPESDAEMKHRLIARTRVMRKKKALSRGKK
FUN_001903 CDS scaffold_12 54481 55471 -
hypothetical protein ENOG41

MLGPLDLGFAWPSEQSMTSIAFSSYSPLALRDMPQPRPNDAPSDIDTSVFTFEDAFEDLLAESQGKELPDIKSRYEQRKLLRS
MYPDGEPAYFWLHRLRSQGLLEDSGPAKLVKSLANNNWDQLHEELDRRAQEVWRAAFGEDEKNDTLRETKREHALSSEERNEE
RSCWERENSAEYEIEDLRAELHGHRNLRRVPNDFDELFSAIQSSFASDRSPWDSFMKLITEDHPTAPSSSEDKQLQKPGSDEK
QVVTKDEYVDRFGYLHTKTLVKTLDGNGNEIGSHSHYTVRPAPKDNEHAQIGDKDKDKDGQESGTKVDGTAKAKTGWLWK
FUN_001904 CDS scaffold_12 56618 58800 +
hypothetical protein PF05653 ENOG41

MVVDSWAQDASNNFSHHGHSGKPGDKIQEWSSLIGIVTAIVGNVLIALALNVQRYAHIRLHKQRSRNRKRAKEALKRPHPGDH
GGSYGTIGGGSDGAGYQNGHGTITDDGNRMDESCETDPLTGSFHSEASQSTDSKEDDTSSSYLKSPYWWLGQVLITLGEMGNF
LAYGFAPASIVSPLGVVALISNCIIAPAMFHEKFRQRDFWGVVIAVGGVVTVVLSAKQEETKLNPHDVWDAITTLAFEIYLAV
TIFLILVLMWASPRYGRRTILIDLGLVGLFGGYTALATKGVSSMLSSTLWRAFTTPVTYVLIVILLGTAVMQIRYVNKALQRF
DSTQVIPIQFVMFTLSVIIGSAVLYRDFERTNAEQAAKFVGGCLLTFFGVFLITSGREQSHDDDEVLSEDDDFEDTIGLANQE
GNGTASYQDGQGFSTPKSQSRRSSHLSGLNYPDAQRALKAAVTPHQDNNNLPSAKALGSQMSPGRETMASGFPWNDFGGPSTP
PGRGVRAYSTDSVAPPGTAGSAAFASHPTTPMRDALATDGSLLPRPQASPSKPGGTGSRSRTFISPSPLSSTVTAVVKDAFLR
ASDDPSVRQSSMRRIRSTIRAGLFFKEDDENADPENNSRSNTLPAQSAAPVSVENLPSALDEAERPAGEDDLVRRRSRSVSDT
LGDFFGVRRKKRHDGYFASDVEDVTIGGATPGQDRTDRQGDCREV
FUN_001905 CDS scaffold_12 66605 69032 +
hypothetical protein ENOG41

MWAATMPYYSEYQSERSRRDTNQNAQANASNDAHGYRDVSPHTRHSPIDLDEYMVPAIEGPPSPERIRQLSKQMKHASHLNRG
HRAVSSGSSSFVNSERGWEQGFENMSITRHSSQRSTTSGTSSSRDRPDSVQVLGKNIFHRRAKSSKSGRSRRESSAQSSSGSS
LYSTELSSETSLTTFKDAIMPTIFARRKSSRDETALQKKLQISGPFNFQHVTHTPREQVANSASRTPGQEHPQPNFYSDRVVE
HEVSPRSSRASNLPRRLIKHARSQEQLRTSPPRPVRPPRPPLEPLHSPTLGAPIPPPRGSSRQPSLQDGDALAVARFDRPLTS
GGFRHPQPFSPSEIMERPPATSHGYAMAPEQAVLPVDDHRFSHAITTPDDAAWPLPVTASYEPPLPDVPEEEEHMVNIGRSPK
SIISTRSSLRASQSVPLLRGSQTLENNAAQHSAGHYNGQAHSIRESWEDDIDYCYEHEADANFDYEWERPSLEGGREHAPGVQ
VALFEDDELRELPIIGTAESSPGMLSASRFDMPALSPASQASPESAFEAVTPSTGVAVTNNFSLPRGDKVHRPSNLSHCRNDS
RASSFKESYGFTLSPSLLIPGDYHQQMLLNETERQSYSGEDDVVHGIVDPNHPYEDVSNAASHTSLQMSHQRASTSTTATNST
SLSDSTGERHVSTNSTWTNLTRLTSSTSLNKWVESNNTIRESQIIKSHDDSDEEETTPPASTEKDTVPELTPFPSVPVTKKPH
HKSHASESIVRDDIPNNTKSVELPKTRRPRARTTSLSTQAPPVGQYALFPRTYAKGNGDHI
FUN_001906 CDS scaffold_12 70893 72950 -
hypothetical protein PF00010 ENOG41

MAGDHNEDQSFYDFLIEEPEMIAPAPPGHFPHQQPISSPNRTSRDAPPRPEPTEIETHHHANPNANHHSGPVTLPGPVLGMQL
PGTVPESSRAQSRAHLEIDLDIDLHAQQHPHGSHLSHGAHPQAHGIHGHGHGHGAHSAQSPAGLRSAGFASHIPPASSGPMSM
DWSMYHVTPNLQLNHPQFDFEIPGHMGVVGHSNHLSHAPTDPASFSYGSNLVSPSSIHPNSAHFDTGVPSQWDDSMSHDASTP
KVRTPVHHVASNPWAEIDEPTGDNNATPASRPRKTPRPRRQKKEARKLSDASQGAMSSSTGGTAPSISDAASPSSASQNSRAS
IGSKSVSMASTASTTSSRQSKLRSASRTSKNSRDKPNDTPEDRRTRASHNLVEKQYRNRLNAQFESLLSALPDQARHGESGSG
NGNEDHESDHANDADRRVSKGEVLEMARKHIQALERERNQLELENFELQGSLRRLKESNSESATSSSGQENSVEPDTNKDHKR
ERDDDDEKEGS
FUN_001907 CDS scaffold_12 77934 79856 - POB3 FACT
complex subunit EOG09261WVT PF03531;PF08512;PF17292

MTAIESFDNIYLDLSKESGKCRFAETGFGWKPAGGGDTFTLDHNNIASAQWSRAAKGYEIKIVQRAKSGIIQLDGFQQEDYDR
LAKVFKNWYSTVLESKEHALRGWNWGKAEFSKSELTFSVQNRPAFELAYSEIGNTNLAGRNEVAVEMALPDTGANAQLGGARS
KGSKAAAGRDQLVEMRFYIPGVTTRKEAEGEDAGSDAGNDEQEKNAATLFYETLIDKAEIGETAGDTIATFLDVLHLTPRGRF
DIDMYEASFRLRGKTYDYKIQYEAIKKFMVLPKPDEVHYMLVMGLDPPLRQGQTRYPFVVMQFKKDEEVTIDLNLNEDELKSK
YQDKLEPHYEEPLHQVVAKIFRGLGNRKISSPAKDFITHRNQYGIKCSIKASEGFLYCLEKAFMFVPKPAVYIAYEQTQSVTF
SRVSGAVSALSTFDITVLLKNGAGSSQFSNISREDLKALETFFKLKGLRVKNEIDEDANLLAAALDQQMDDSEDEVAAKADRG
SADEDEESVDEDFRTDSESDVAEEYDSAHESSGSGSDESNVDEDEERDDDDEDAEEERPKKKKKTG
FUN_001908 CDS scaffold_12 80699 82583 + ATG15 Putative
lipase atg15 PF01764

MTTNRRSRLSGFRCASTGRVTAALLLSFLAFSPSSAHNNIGDRFHDDVLLPPGPALPVSPEVPAPAEHTFSLRHIYHHGTHLH
PGLHRKRDVFHDESRVYLAAEDEYGEYDVSRLKAKSRSQTIQRLVDRRPSVVDPMVAESRQRGYAAVLDASAWTMDQVSSPDI
KDKDTVLTLAIMTANAYVKDETETDWEDVGERWNRSADFGWESDGLRGHIFVDDTNSTIVIGLKGTSMAVFDGEGTTTNDKVN
DNLFFSCCCAQQGQWTWHQVCDCATGTYSCNNTCVVQALREENRYYGAARELYSNVTELYPDAQVWLTGHSLGGAVTSMLGMT
YGLPVVTFEAVPEALPASRLGLPVPPGASADYPQMRENTGTFHFGHTADPVYIGTCNGATASCTYGGYAMESACHAGYECVYD
VVADKGWRVGIGTHRIRSVIDDVIKKYDGVPECRRTPECRDCAQWKMYESNGTETTTTSSSPTSTSMTTRTRTRTSTCETPGW
WGCLDKTTPASTTTSTTSTSTSTCKTPGWFGCKDKTTTESSTTTTVDATPTTTCHTPGRFWGCRDEVEATTTAEPGQVTNVPV
TAAPTGTTEDVPSTSVPESQRCLTRSWFGLCKEWAVEDMEFAPDEM
FUN_001909 CDS scaffold_12 83243 85844 +
hypothetical protein PF05764;PF08265

METESKPSDMGTPLSSTLSSLSDSDSDAEVSKTAGPNTDTNSQKNNEDTVEWLATTRKRRSTAGNRMKSMLANEEPDSDLELL
FAEDENDQGFSDAEENGSDVQMDSSDDEDENDNNDDDLEGEKELEKQAKERRAAQRKRKAQEAIPAKFRKKVRIDPTTRTPAP
AKPAPRPKKKSERTSWLPSAADLPTRASSRQTTRLSKEQLHQQMVEREARRIKQLAQMQKKAAKLEAMKKPPMTQEERLREAA
IVEKRNSKSLNRWEEAEKQREEERRAKLAALHNRTLKGPVITFWSGIGEWMGRHMVIEEPAKEKRKRGEKAKGKDKDKEKAGP
TEDKKEDGQNGTPAANGEIANTKEGSQPSQDKPPSIASAEPLAASDSKDTNPATPTKPVEDITKPTDAPSTSETSDAPPAVPK
TEDIPSPMTMPPPPQPPPTPPSSLAAPAPPTSTFVPAPSPPRAQDPPPSGLAAPPPPPPTPAGSASTTNSFVAPPSGLSRPPD
SKPSGVLAAPILAPPPGIGISGNAPPMMGYGNPKSNVLAPPNTSQGAMPPTLTATAPLHNTPSTISPPPSKPSTGVTPISSTT
NIQPKTITPALPTKSTSPDTPAVTQAPDSQTQPPQPSTGARNAIVFQNFDDNALKDRSIQTQIIFGRKMNKLSKPSPAPLCVI
TNHPARYRDPRTGLPYYNAYAYREIQRLTRGEYRWSHILGAWVGSGTYAARGVPERFLNPDKKGPEKKSTPDEGIKGKDAVQA
PDQAQDPQTNTPDVPSGIPTTGHKMTGVGQGASAPPNSEPNQTIQPKIAPPAVTQPTKTTNTTAATPIAPNTTPASEPKAQAP
TNPQSTTTGNPPAPLQTAP
FUN_001910 CDS scaffold_12 86560 88224 -
hypothetical protein ENOG41

MQRDIRRGRDQWRGCYSFRDIVCDDVSVSASATCAGVGAGTSSSSCSSTGTAPSYPNKRHGGLKMGHTYYYYYELDGSVETHD
PSLPSTTACPYLPGQTVNTLIVPVEQALRQRSASLTSMRATDLKTMNPESKYITPRPAPSVPANHIPYRVGSSPSLNHKSSSR
SLSPAPAAWKRLFGRKVNSEGERGRSPIRNDLSGTAEDIDDRCTTPSEGTRTRDISPESLRRFLSDDLPPRPGSNLSERPSLV
IPEEVVEDNENDDDDNFATSAASDGQPYVTRLSPPPFKRTISSESVRSDAANLSSLPLIPSKPSRKDLLEVSGVPASAVLPSR
PRLETLRAPNPRINPATSFFLSPATPNFPEDEGTNFFDLTDDEDDVVSSSNSDILAFQPVPGMAPTTDSFECYSLPESQEQGR

KIVESQSAYAKVNSPSLLSRGDSGNFLGAPIDTGLDDFAAELGWMVDVIGSKAN
FUN_001911 CDS scaffold_12 94059 95247 -
hypothetical protein EOG09262V3O PF04116

MGNDSSILADGSAMAVPGTSFNHTLFETALPPLPTFSLEVQPDLFPWVSDFWLNLLLPVVVYWVLSLTFHAIDVNDWFPQYRL
HTPEEITKRNHVSRFEVARDVILQQIIQVVTGALLALSEPPEMIGKSEYDVAVWATRIRIAQRALPTVLGLVGLNATAISKSL
LDTHPLLAGAVAGGYYPSLVGANSEPAFASWEMTLAKTIYYFLIPAVQYFIGAAIIDTWQYFLHRLMHVNKWLYVHFHSRHHR
LYVPYAYGALYNHPVEGFLLDTLGAAVAFKVTRMTLRQGILFFSMSTIKTVDDHCGYAFPWDPLQIVTSNNAEYHDIHHQHWG
IKTNFAQPFFTFWDTLLDTKYRGSKTNKPGQKKAKVEEKAQ
FUN_001912 CDS scaffold_12 97187 98011 +
hypothetical protein EOG09264U81 PF00838 ENOG41

MLIYKDILTGDELISDSYDLKEVDGIVYEADCAMIEEGGLNVDIGANASAEEAAEDVDDQVVKVNNIVSSFRLQETSFDKKSY
LTYLKGYMKAVKAALQEKGAPAEEITAFEKGAQNYVKNVVLAKFKDFEFLTGESMNPDGMVVLLNYREDGVTPYITVWKHGLT
EMKV
FUN_001913 CDS scaffold_12 98303 99608 -
hypothetical protein ENOG41

MSKYKDIMKNGWHPEKPGSSLKGQVKGLVGRGKSEDEDRGSHVSVPLSQLKDPSSFAPPPKRTGSGLLPPPPKAESAPRKVIT
APSKYQDPRAPAPQEPVYAEPEEEYEEQPQPRGPYRTNTTGLSTDNLPKPPGRRDGADGRSGQPPSYQSAMAGVGGGGRAAPP
SLPPRLPPRTNSGSTVASSPAASPAPTGNSLLNQGAVNRLGAAGISVPGFGIGRSSPAAEASPSPTQPPRPSIASPPPAPAAS
HMSELQNRFSKLGTSSSANAAAPPPPSEGTTWAQKQAALKTASSFQKDPSSVSLSDARAAASTANNFRQRHGEQVAAGAKTAN
NLNQKYGLLDKAQNSYSRFQGNQTQTQNEDTPSAPGSPGLASVVGKKKPPPPPPKKKPALMGASAAPSNDEPPPIPMATRPQF
FUN_001914 CDS scaffold_12 100117 101204 +
hypothetical protein EOG09264DY4 PF05859

MAAQQNSDYELLTEHFGYPPVSLLDDIINTVNVLADRALDSVERLLLSIPPQSLGFSNKHASKDGTPALPPDEAAKLEIEHGT
HQLETLLNASIDKNFDLFELYTMRNILTVRPDDQPYMRLAHYEGLDFSGPEGPDRPTTESVTALRRRLHASQRLHTALESERA
RNDALLGKLRSVLGVVPGNVKAEEGQAQAPDGSAFGFLRDKTSLQAAGADKPIATTTEFTLSQLQALRALSTSLRTLLPDLGP
TDADTSMEDASSSSRTWRRERVEYVEGASRKYLETARGLELGDQGEVRDGEWQGEGRKITRSDVEGLEQVAAMLGQKSTTTGE
ASGEGEPMDQS
FUN_001915 CDS scaffold_12 101445 104007 -
hypothetical protein PF01535;PF13041;PF13812 ENOG41

MYASRTACSRCSSQFRSVPASAKRLGAVASYSTTAEIPDTPRTRTRTRTASQSANTSRESKHPHNGPRIGVNAFKQKQKPDQD
PALALFKEIVSRADHEATPSTEAASGPMPILNELEIIARLKEVMSRGVHPDERYNIFRDEIWPSIVALEGQIPKAIYTAVTQL
LGIECAYMFQNGLYKNSHDIAEKYRAFGDYGLRLRNDLILNMCVRFLQHKNGSGERTAMRLEILKMWQHLSQLKRPSEANKEA
RFALPSVTEVLKDTEYSRGGAPAQAGTQASRALASLFLQLPPPQAIEVIPSLLATLAIASDNRYSGSDYKTQLSPLLILVRAV
LSKYKIKQADIDTIVSKSSRISPERLATLKEYVSQQWALASTMIMGESHAWVKASSQGPDPGARNSLNNFHKQLRAAYRARDR
GAIGSVWDAMLVALRDNPGIKPQLAHDREFLDFWIFVWCAVRRYPRVQETLDLMHSLEVEPTVRTYTSMMHGWKMCKDIGKIE
ALWSQLVKSGMKLDAVIWTERVSALIELGQHQKGLQALAELVENWQLAVKQGKQSQAVEPSIEAVNAAFKSLLNADPKAAHDL
LAWAGRQGLAPNTRTYNILIRETIRMGYHKNVDELLKSMQREGIEPDSATFTILLEAVIGRMEDASAEEQVAAIKAVFNDIEQ
AGLKPNLETYGKMLYALDGLPNCSDDVIAALQQHMRSNGFGSVLTPHMITILVERALRHDPPDINKIRTLLKDNNLTKVSTGD
QTLWERVMTAHAIGGDVREAMSIFDDLADSGRPVTSLSCLKELMQALLEKGDTESAKRVVDVVLKYKLHDRESKSANERYWRH
HFWYLAQHNGLIDSQKLRQYARS
FUN_001916 CDS scaffold_12 104367 105286 -
hypothetical protein PF02798;PF13409;PF13410;PF13417;PF14497

MASNGLPHVKLFWLEKSRSQRILWLLQELNLPYELEIFHRNKKTMMAPPELQKVHPLGKSPVIQIRPAGAGEDAEPITLAESG
FITQYLAENVPEGKKLVPQRWKEGMEGKIGGETEAWMRYQYYLHYCEGTLMPPLVMALVVSALKSPQVPFFIRPISTILANRI

FSAFIFPNVHVNLKMIEGHLATSGGKYICGPQLTAADILLSFPLIAAKEDLDNFGTFENGSWKSEFPKVAEYIDLIENEEGYK
KSVEKIIEIDGEFSATLRPGQ
FUN_001917 CDS scaffold_12 105939 109228 -
hypothetical protein PF02791;PF10537;PF15612;PF15613

MVRASVACLSTTSSLLNEYHQVLFKRKPVRFLPAQEIEDENTEVWHIPQTGEIFTSYEDYLDRLAIISALHLGIPCLPILQRR
FNDQITGHSGLTFFEALKSELAGGKEVEASFPEALKGPILRKVQFQTVSRLDNLVDQIYDEFKHDYYPGEEVTVTMKGGDRAH
GLVRDKTTFGPRALPDGTHSLPTTRYLVDLKDTEQDTIVTDEHICRDRGIFTKAMLRSFIKKTVTRDAWNGAPWLVKHDYASQ
YHIDTRVPPHLRYDTKVMERKQLQAQKRAHLNHDTNGHDLTSALHSGPVRLPELKPAPKSKAKPGQPGSGSKGLKWPLNMPVH
GGNPFSSPSDYGIQTPIQREPTPPPPPPPPPKYPIEDLQLEPRDDRKRPPLKFLCRNPPVKVENDDMPQSEYCDKIEMESIGP
LLETWDTLNVYCEVFKLDSFTFDDYVETLCVASEEVPIQLLDEIHCSVLKILVDSEADGGKVRITLPEVDEEDSDEDSDEDAS
ALPTPEPEARPVGRATRSSLAKAEAERLAAEAAAAEKESEEETKHRAEQLLKDYDWIEHLRKRDFTNGGWERIMVGLLHQLSK
NERRHEVCEELLHLLVPAGVDATQETVKQRYAQLDVNQRVKALQIICMLTTETKAMRGYMEDCSETMTTYRKERIEWQRQRKQ
AIEDLRQLNEQKKILLPENMPPSPPKEEDGDVKIADPEDSIISRGGDGEETEDDPSARKRRRPQTAKQRKQEEEEARKKEKAA
KVAKTPKPPAQSKQFTKLLKDIQKKEEVIKKCEEEIAVIENDLREADCPRTRVLGKDRFWNRYYWFERNGMPYGGLPDSSTAS
ADYANGLIWVQGPDELEREGYIDLPAELQEEYKEKFNMTVPERKAMEEANTSVFNAHQWGYISEPAHVDELIKWLDPRGFNEL
KLRKELLSFKDKIAKHMESRKAYLSKEEDGDKKEENHKRMSTRIRDKSPEAPNYRSLQWENTTALEELGHLHSDPPPPPRSRK
QTRKREAVNDAPAPAAKSRRR
FUN_001918 CDS scaffold_12 111079 112694 - ALG8 glycosyl
transferase PF03155
GT57
MSEHYPTLAQTALVAAAFKILLFPAYKSTDFEVHRNWLAITNSLPLEKWYFEKTSEWTLDYPPFFAYFEWILAHVARLIDPLM
VKVYNLDYESWQTVYFQRTSVIITELVLVWALQTFIDNAPLKSRRAAQVAALSIILSPGLLIIDHIHFQYNGFMYGILVMSLV
LARDKNELLTSGLIFAALLCFKHIYLYLAPAYFVYLLRAYCLSSKSIFRIRFLNSIKLGLGIGTIFGAAFGPFAAMNQIPQLL
SRLFPFSRGLCHAYWAPNVWALYSFADRILIHIAPRVGWAVDKNALQSVTRGLVGDTAFAVLPEISPRTCFVLTLFFQVLPLI
KLFSQPTWETFIGAVTLCGYASFLFGWHVHEKAILLVIIPFSLIALRDRRHLGAFRPLAVAGHVSLFPLLFTPAEFPIKTIYT
ITWLVVFLMAFDRLAPASNKPRIFLLDRFSTLYIAVSIPLILYCSLLHQIIFGKSYEFLPLMFTSSYTAIGVVGSWLGYMVVY
FTA
FUN_001919 CDS scaffold_12 113871 115256 +
hypothetical protein PF03388

MKVSFSLAALLAASLSQAQYLISELSFGHAGRISPPEARGQIPNFVVQGNPNTPEVLSNRIILTPLAPGNQRGAVWGQQPLLR
NQWIADVDFRANGPDRGRGNLNIWLVRNGPATIGSGSIYTVGKFDGLALVIDTSGGSSGMVRGFLNDGNTDYSRQSNVDELSF
GHCPYNYRNLGRPSQVKLRQTARTFRVELDGNLCFESDKFSIPTGYQFGVTAATPDNPDSFEVFKMVVMADNSDSGPVRDPPK
QNQNQGQNQNQNQRVPPVRDTSNSNNKRNDGEYADEDPDIFQTSKAQFMDLHNRLQNTNHQLASLQRTSSRHQQDEKRHEDL
TALIGQLRADMRKLDDITALHSRVMELEKDIQGLRKELNRKLQSTERTFKDTLSDHHNSLSETVMAKTPGHRFLIFVFLGTQG
LLVAAYVVYKKRRSSMPKKYL
FUN_001920 CDS scaffold_12 115639 117483 -
hypothetical protein PF00557;PF05195
M24B
MAPPITPPKPVCGEQRPQARERRSMSYEFVLEDEFDALCVEVKKSDDDCGGKSSSGNKYPAKLHARKVVKELGVTDGLLYLPG
EPTRLYEDSDQSPPFKQRRYFYYLTGVDFPDCAVTYEIAMDRLTLWIPYAEPRQVLYHGPTPDAAEAMRKYDVDDVRYTAQLS
KFLHGQLRPEKTLYVLHSDQAPKLTDRPRGQTQINCSKLRPAMDEARVIKTEYEVALIRRASRISAAAHRAVAERLLTMRNEQ
DVEAVLLAACTSRGAHAQAYPIIAGAGVNASILHYGANNQPLEGKQLIVVDAGCEYQCYASDITRTLPVSGSFSKEASAIYSI
VQRMQEECIARVRPGTVYYELQLHASDVALQGLRKLGLLKGNFEEIQKAGTVAAFFPHGLGHHVGLEVHDVTGYERLLMRDNF
HVEGGKREMVTATALVAMHRMASSSPPTKPRQSLQPNMIVTIEPGIYFCRPFIEGYFLNNPSHAKFINKDLLETYYPVGGVRI
EDDILVTPGGYENLTSAAPKGDEMLDVINGSFEKI
FUN_001921 CDS scaffold_12 118424 121515 -
hypothetical protein PF00172;PF04082 ENOG41

MPLDVGDNDAGQSAHDWHDDAPSPSASIKSDNELDEDGNPQPGASSQPIQKRRRVTRACDECRRKKIKCDGKQPCTHCSVYSY
ECTYDKPSNRRRNPAPQYIEALESRLQRAESLLHKFMPDVDLADPNLDPAIQQEFRNRENARAQASKARQPPPPPQPEQNEAH
LTSMIDSVGQLDIDEKGSLEFHGNSSGAVFLKRMKDHFRGMLGSSSKAPFLPRAERPAGLESPSLSSVGTPFSAVPSYPELPP
KDVARKLCYYSLSCATCLVRIVHVPTFYEQMDKIYERPLDNLTKDETQYLGLLFSVMSLGCMYNNLDDNDPGSMPYQDATEEG
LKYYNAARSVLHDVTDCRDLISLQALLFIILFLQATSNLSSCYSFVGIALRSSLRIGLHRHLQHEKIDPIEQEVRKRVFYVVR
QMDIYVSTLLGFPLLLNVDDIDQPFPLEVDDEFITHTGILRPPPGSPSFFQAFNAHSELMEILSKVTKYVYPTKGLGQSVTKD
NKPASTYLISYGRIKEIEAELHSWFERLPQHWRPSGEGPIETVRIRHLLRFAYAHVQLVLYRPFLHYVSPRLSQGNKVDELSY
ACAAAAISVSRNIVHIGLEIRKQRVLSGPYWFMLYTEFFAVLSLVFYATENPEKPGSGEVLADARAGRQMIAALAEKSMSAER
VTSALKALFDQLPEQLDAGTIRQAPSKKRPAPTGRPSSVSSHHTPIPASMSSIGTTDFTRASQMSSSSYPTTLSQGTMDLPGG
GIAAFQDNTFSANFGDFMSPDLQRSTPESTSSAATSTQRYPYTGQQMGMHNPVNKLDSLMFPSEDPFAYPNQPMMELGFPGKT
DQPTTMGDQNQDMQLFLTGTFDDVESQIFGQPPPYMMHQQGQPMMSASSHQPMFASPSAIMGMQPGQPMMQQRRPVSQTHAIH
QGHAMSNRRAHAQRHQERQIQQMFTEQGMQADWGGFFGSGRGGFQGM
FUN_001922 CDS scaffold_12 126508 128112 +
hypothetical protein PF01399;PF10602

MSESSPLTAFFGQMKNLGGIVVQEQPKLDLDLYIQNYTGRTRIDRLIQIGKSSVPLCIDALKAAIAEIKKGSDVALYVEAWGC
IRVAAPDDPDAQKDQAWIDRVERENKMEAARLETQLKQYRHNLIKESIRMGNEDLGLHFEKTGYLEAAAEAYNRMRQDVTTTK
HIIDCGIHLVNVYIARRDWTMVLNNLGKIVGVQSGEEERTYQPYTKLVSGIALLGLKHYKDAANNFLQVDFTLPPAQYNHIAS
PNDIAVYGGLLALATMDRQELQTRVLDNQSFRSFLEHESHVRKAISLFVNGRYSNCLAILESIRNDCLLDIYLQRHVSSLYSQ
IRRKCIVQYFIPFSCVTLESLNQAFAQEGESVEMELVSMIREGILKARLDIKEQLLIADQPNPRLEMQKQALEVATKYEEEAK
ERLRRISLIAAGLEVAGRAKGPGLGGRGIDEQWYGESKAPGQPGAVEA
FUN_001923 CDS scaffold_12 128437 129528 -
hypothetical protein EOG0926420U PF02840;PF08799

MDFAALMNKELSKSKKSSGSEEKKYLKRSEIEAQRKEAYLAEQKALEAEREAKAAAKRKREEDAAAENAAREEKRRKLAEDMR
KRREEQEAEEERARRKRLGLPELVKEKSEDAEDGVHASEDIPEEELVEKLRGLGEPAVLFGENHSAKLRRYRKLTTVMTKGPI
PTTLELVDEKDMKVTTELPKDKDGRKWLFRQLASYFTMVLTEYERAMEAEKRDTSASKTAYNAMVQTRENMKPLFRKFEQGEL
DDDILKPVTEIVQALQERRYVDANDGYLRLSIGKAAWPIGVTMVGIHERSAREKLHDGEKGHVMGDEVTRKYLQSIKRCLTFA
QVRWPPTDLRQLMG
FUN_001924 CDS scaffold_12 130038 130632 +
hypothetical protein PF06487

MPPHIPVYTWSDCTLNELALELAGAKKSALPSPSVGCRLVFQSVFPDLRNTAAVTNALPKYGVKDLGSVVLGGGSGADTYGDI
SMDGTTRGSDDKTKTLSDCRFVVGDYITCAILPPLSDGSVAPASSVRREQASGPHEVRGTHRGGRDNGSGRRNSRGGRAGWRD
DVGGDFPMGDWRRGERLPETSSGRSRGRGRW
FUN_001925 CDS scaffold_12 130826 131218 -
hypothetical protein ENOG41

MAHLNVKPDPAYLKYAAMMKTRHHYFRWTPRTARLTFIYVAVVPAIMGYIAYKTDGLWDFRAKRKGDLIYEK
FUN_001926 CDS scaffold_12 132241 139348 + PRPF8
Pre-mRNA-processing-splicing factor 8 EOG092600NM
PF08082;PF08083;PF08084;PF10596;PF10597;PF10598;PF12134

MSQLPPPPPPGWGPPPPPPPPSSSLPPPPSTPAPPPPGYRPPTDPQMAKFAQKKKEWVKSQRNRFGEKRKGGFVQTQKADMPPE
HLRKIVKDIGDVSQKKYTNDKRSYLGALKFMPHAVLKLLENMPMPWESAREVKVLYHVNGCLTLVNEIPRVIEPVFFAQWAMM
WTFMRKEKADRRLFKRMRFPPFDDEEPPLSWAENLEDVEPLEPIQMELDEEDDEAVYEWFYDHRPLLDTPHVNGPSYKAWNLT
LPQMATLFRLSRPLVSDVVDKNYFYLFDLKSLLTAKALNVALPGGPRFEPLYKDIDPNDEDFGEFNAIDRIIFRNPIRTEFRV
AYPYLYNSLPRSVHLSWHSHPQVVFNRADDPDLPTFHFDRRINPISSRTVAPKNVEVSHEDEIFGAGNIEESEDDAFELPAGV
EPFLADEDIENENTSSAVELWWAPYPFDRRSGRMVRAQDVPLVKQWYLEHPPSDRPPVKVRVSYQKLLKNFVLNELHKKKPKA
QNKQNLMRSLKQTKFFQQTTIDWVEAGLQVCRQGFNMLNLLIHRKNLTYLHLDYNFNLKPVKTLTTKERKKSRFGNAFHLMRE

ILRLTKLIVDAQVQYRLGNIDAFQLADGILYAFNHVGQLTGMYRYKYKLMHQIRTCKDLKHLIYYRFNSGPVGKGPGCGFWAP
AWRVWLFFMRGIIPLLERWLGNLLSRQFEGRHSKGVAKTVTKQRVESHFDLELRASVMADLMDMMPEGIKQNKVNTVLQHLSE
AWRCWKSNIPWKVPGLPAPIENIILRYVKSKADWWISVAHYNRERIRRGATVDKTVAKKNVGRLTRLWLKAEQERQHNHMKDG
PYVSSEEAIAIYTTTVHWLESRKFSPIPFPSVSYKHDTKILILALERLREAYSVKGRLNQSQREELALIEQAYDSPGTTLERI
KRFLLTQRAFKEVNIDMNDNYSTINPVYDIEPIEKISDAYLDQYLWYQADQRHLFPAWIKPSDSEVPPLLVYKWAQGINNLDR
VWQTENGECNVMIETELSKVYEKMELTLLNSLLRLIMDHNLADYITAKNNVQLTYKDMNHVNSYGMIRGLQFSAFVFQFYGLV
LDLLLLGPQRASEIAGPPQSPNDFLQFRDRETESSHPIRLYTRYIDKVWIFLRFTAEESRDLIQRFLTEQPDPNFENVIGYKS
KKCWPRDSRMRLMRHDVNLGRAVFWDMKNRLPRSVTTIEWDESFVSVYSRDNPNLLFSMCGFEVRILPKIRNQNEEFPVKDSV
WSLVDNTTKERTAHAFLQVTEEDIQKFNNRIRQILMSSGSTTFTKIANKWNTALIALFTYYREAAVSTVDLLDTIVKCETKIQ
TRVKIGLNSKMPSRFPPAVFYTPKELGGLGMISGSHILIPASDKRWSKQTDTGVTHYRAGMTHDEETLIPNIFRYIIPWEAEF
IDSQRVWTEYSQKRLEANQQNRRLTLEDLEDSWDRGLPRINTLFQKDRSTLSFDKGFRARAEFKIYQLMKNNPFWWTSQRHDG
KLWNLNAYRTDVIQALGGVETILEHTLFKATGFPSWEGLFWEKASGFEESMKFKKLTNAQRSGLNQIPNRRFTLWWSPTINRA
NVYVGFQVQLDLTGIFLHGKIPTLKISLIQIFRAHLWQKIHESVVMDLCQVFDQELESLGIETVQKETIHPRKSYKMNSSCAD
ILLFSNHKWNVTRPSLLYDTKDVIEQTTTNKFWIDVQLRYGDYDSHDIERYTRAKYLDYTTDSASIYPSATGLMIGIDLAYNL
YSAYGMYFPGLKVLVQQAMAKIMKANPALYVLRERIRKGLQLYASESNQEFLNSQNYSELFSNQTQLFIDDTNVYRVTIHKTF
EGNLTTKPINGAIFIFNPRTGQLFLKIIHTSVWAGQKRLGQLAKWKTAEEVAALIRSLPVEEQPKQLIVTRKGLLDPLEVHLL
DFPNISIRASELQLPFQAAMKVEKLGDMILRATEPQMVLFNLYDEWLKSISSYTAFSRLVLILRALHVNPDKTKLILRPDKTV
ITHEHHIWPSLSDEDWIKVETQLRDLILNDYGKKNNVNVSSLTTSEVRDIILGMEISAPSMQRQQAAEIEKQQQEQAQLTAVT
TKTQNVHGEEIIVTTTSQFEQQTFASKTEWRTRAIATSNLRTRAKNIYVSSVDNDLDDITYVMPNNILKRFITIADLRVQVAG
YLYGSSAPDNDQVKEIKCIVMIPQIGGLRNVQLPQQLPQSEFLEGMEPLGVIHTVSGSELPYMSAMDVTEHSKLLDAHNEWDK
TNTVTVSVAFTPGSVSLSAWGLTPAGYKWGAENKDTQSDQPQGFTTTMGEKRKLLLSPRFRGFFLVPDDGKWNYSFMGNAFNG
MEKRPVHVKLDTPLPFYSDQHRPVHFVNFAELEDIWVDRSDNFA
FUN_001927 CDS scaffold_12 144521 145461 +
hypothetical protein PF13921 ENOG41

MAQHHQHPSPQMVMDPSHLGHHRLGPPQFGAPGQVMAPMYQTLSTPTPPNHPQHSVPPPGSKRPRPSDLDLSVTGMADLDHSD
LSGMQQTPLGAAYAHAAAVAVSQPQHQPIHHHHHLPDSEPPSKMMRRDDGLGGAPSVVGQIGMPDPAPRPRGPKLKFTPEDDQ
LLIELKENKNLTWKQIADFFPGRSSGTLQVRYCTKLKAKTTNWTDETDQKLRTALQDYENEKWRIVANKVGTGFTPAACRERA
EQLMVEDPATLAALSQSLSPAVASNAESEEPLYSHHQLQ
FUN_001928 CDS scaffold_12 148450 149857 -
hypothetical protein PF02558;PF08546

MRSIFFKFNQSHRLKIATRPRRIWPLASVASVRMYSQRPSWLEVCCNDVRPTPTLFAWNLSNLASYDGSDPSTSSASGDSDQR
IYVLGVGNLGRLFASSLAQAPDRPPITLVVHRKELLEQWIESDGIEILRQDKLEKNKDFDIEWWTENEPDFGPVREVADGEKL
KNLIVLTKASAALPQVDRVRGYLDRNSTVLFAQNGMSKLWPPHGSLYVSHRYHSNHEPNFLACVTTHGVTSQGTFKSFHASQA
DVVVGAVLPNSISLGKTAYLVKQLVEAPYLEARSVSRGELWILQLEKLVVNAVINPLTALLGCKNGALFTESDGVLARVIDQL
LSEASQVLQLLVAHESSAEIIGLQEGRLPDEPETTIDLSLKSLHERFSHPQLKDMIYRVGHKIGENTSSMLQDVRAGRSTEIR
DFNGWLVEMAAFLDPGLEVTGHGTLVELVEAGRTLDVDQLGSCFNGSGTDAGK
FUN_001929 CDS scaffold_12 151108 154591 +
hypothetical protein PF00176;PF00271;PF04851

MAFEDTVSDNREVDKAALLDRMVAVAQQSASRDDEDKEEQPNTEGLNEEDALRNLTGTVRDQNDLERDITLQANAALNEAEDK
KDQNRIEKLEATKHRLQLQLDKEKKRLEKLAHSNVYQTRNIQKEIAKLDEEIRQVSNDISDFQARIKKRHEESSVDDSTKSKS
TRLPGESHRDFLIRTGKITPFAKIGGPRPAGIQGQLADTILDAEEEAAAEQLGGELEGPASHQRLRRPGFADELDSEPEPPQP
APAVESEFSLRPRKKRRVQKERSPSADFEPEASGSESHDDDVWKQGNEDDLIREQRRQNKAKAKAAGQEQIDLSKIDDGNEVH
FKARLNDWVTRRSRARRARKQAVDVSTDAGESDDEEEEWFKPAPDFPDYNFGDDLKLPGDIHPSLFGYQKTGVQWLAELYKQN
VGGIIGDEMGLGKTVQLIAFIAALHYSKKLRRPVIVVAPATLLRQWVSEFHRWWPPLRVSILHASGSGMMNPKFEDEYDLDHF
QPLATKSEKAAQRIVNGVVKSGHVLVTTYTGLQTYAETLLPVEWDYAVLDEGHKIRNPNAEITVTCKELNTPNRVILSGTPVQ
NNLTELWSLFDFIYPMRLGTLVNFRAQFEIPIRQGGYANASNLQVMTAEKCAEALKETIGEYLLQRLKVDVAADLPEKTEQVL
FCKLTDGQHKAYETFIKSDEVSAILNRRRQSLYGIDILRKICNHPDLLDKTLGKKAGYDFGNPKLSAKLQLTKDLLQKVMIPN

GHKTLLFSQGKQMLNIIEKCISECGISYVRMDGETPVDQRQPMIDRFNESPDIHVFLMTTRTGGLGTNLTGADRIIIFDPDWN
PSTDLQARERAWRLGQKKPVKIYRLMTEGTIEEKIYHRQIFKQFMTNKVLKDPKQRSSYDLSDLYDLFSYNTGEDASANRSEL
FKKAEVELPSGDGEQEQTSEPAQNVAGGTDVESKELRQMGLVAAMEDFREDKSVHDEKRMLDGIFAKSVNNAYDHEAIVNGPQ
KAQADISVLQQEANMVARQAAAHLRQAGAEARRIPIGTVTWTGEVGQAGRPGANRRRGGPSSAAIMSNLADRQGLDTGSGGSS
RSGTPGADKNLKSKDFVAMIKTFINRHNGRVPSKMLVDHFNPYCPGKKQSDEFKAALDKVAVLNKTGGAGRGIWTLKPGVK
FUN_001930 CDS scaffold_12 154944 155619 - RPS14 ribosomal protein S14, S11 PF00411

MAPKKTTTRAPQENISLGPSVRDGELVFGVARIFASFNDTFVHVTDLSGRETITRVTGGMKVKADRDESSPYAAMLAAQDVAA
RCKELGINALHIKIRATGGNGTKTPGPGAQSALRALARAGMKIGRIEDVTPTPSDSTRRKGGRRGRRL
FUN_001931 CDS scaffold_12 155898 157341 + RPL7 60S ribosomal protein L7 EOG09264P3R PF00327;PF08079

MGATVPTNDQILVPETLLKKRKSQEKARAERTDAIEKKKAANKEKRQVIFKRAEKYVQEYRDAEREKIRLHRVAKAEDSAFVP
AEAKLIFVVRIKGINKMPPKPRKILQLLRLLQINNGVFVKVTKAITEMLKVVEPWIAYGYPNLKTVKELVYKRGYGKVNKQRV
ALTDNAIVEENLGKYGIVCVEDLIHEIYTVGPNFKQASNFLWPFKLSNPTGGFRPRKFKHFIEGGDLGNREEAINALVRQMN
FUN_001932 CDS scaffold_12 157662 158422 - hypothetical protein EOG09265G5K PF01521

MARIHCANAQSTATSFIQLARRSYTTTPQTSTPLEFLIPRCTPSSRLRTPHITIAQSRTYAHPRRSWDVKSRETATRQFSTTA
IRQKTRAILNPQQDEDGNEMMLEITERAAKRLNKIMEKDGNPNLALRIQVESGGCHGFQYLMSLVTLPAKDAAEWSSIVNEDD
TIFQYIPDDADPATTSEEGPKIILDEPSLDLLKGSKVDFTMELIGSQFKIIDNPLATSSCGCGTSFDIKM
FUN_001933 CDS scaffold_12 159165 161866 + NIP1 Translation initiation factor 3 subunit c EOG092616QN PF01399;PF05470

MSRFFRGGDDSSSDSSSDEEELYSEEEEEEEQQDSQEDSSEADSDDSSSDDEGGRGINKFLKDASSDSEDSDDEVRAKVKSAK
DKRLDELESSIKQIENGQKNGDWTLISAEYEKLNRQVAKLPGSRPPKPYIRILAELEDFMNESLAKQKVTPKKMNAIQARSLN
AVKQKVKKTNKEYQTQIDAYRADKDGFMESSDNEPVAPAPKPKKVSLQVDAAPTEEGDDDGFATVGKGGRTLQYTPESIFKHL
RTIMESRGKKNTDRTEQIKTMEKLHEIATTPYQKIRVLLTLVSTRFDVGSGGAAAMSVEHWKAAEKELSSLLQVLEDNHDYVV
VENAEEWDDDDKPPTLQEGEKHIKVPGSIVSYIERLDDELVRSLQSIDPHTSEYIERLQDEGALYNIIFRGLLYYEYLRKDDS
LEIPQDSVNRIVMRRLEHVYFKPAQVVKTFEENCWKVVGETVESVITPRDQAQNAGNLVNVLCNYLFSNSDGIIRARAMLCQV
YFLALHGEYYKARDMMLMSHLQENIPNFDVQSQILYNRTLVQVGLCAFRKGLVYEAQNTLQEICGSGRQKELLAQGVMMQRYN
QVSPEQERLEKQRQLPFHMHINLELLECVYLTCSMLLEIPLLAQTGSSPDVKKRVISKTYRRMLEYHERQIFTGPPENTRDHV
MQASKALAAGEWKKSIDFIHSIKIWDLMPSAEEIKTMLSKQIQEEGLRTYLFTYAPFYDTLSIETLSTMFELDSTKVAAVISK
MISHEELAASLDQVTSTVIFRKGVELSRLQSLALALSDKASALIESNERTLEQKTQGTSNAFERQGGRGRGGQRRGGGQGRGG
ARAGGNTQRQAGGTQFTGGALGAAVRG
FUN_001934 CDS scaffold_12 162751 163377 + hypothetical protein PF01814 ENOG41

MSESKPQEEAKPVENAETKTEEELPPLSDHEFKIYNRAADHMEYFHNNFRRSWNLLWNACTNNRRPQGMSLKQFIMEGLQFAE
HLTMHHNIEETYIFPVLAKKMPEFRGGRAELLRQHKQIHAGLDHFEEYLKKCRTGDEEFELSVLKTKMETWGEVLWKHLDQEV
ETLGANNMRKYWTKEEVMRLPM
FUN_001935 CDS scaffold_12 163756 164603 + RSM10 mitochondrial 37S ribosomal protein rsm10 PF00338

MMRQSIRPLRAFSSEVSWIARRTQASLAKPGDLIPNKVEESQPEKGPEPRFPRSIQALHLKPLKREAEHGIPSCDLQLRSYSV
QPLEFFSDFALRAAYYLGLPAYGPVPLPRITERWTVPRDNFIFKKSQENFERKTLRRLIQIRDGNPETVQLWLAYLRKHQLYG
VGMKANMWEFSELGVGKKMDALPEAEKGEIDAKWSHLGQTKTIGTVEKVEELLNQRRFREAAGLRVPPTTSV
FUN_001936 CDS scaffold_12 165142 166403 + hypothetical protein PF00579

MLKSAITKPRVIFSGIQPTGIPHLGNYAGALRQWVKLQESHAEDRLIYSIVDLHAITTPQPADVLRKSKREALAALLAIGIDP
EKVTLFYQSSVPAHSELMWILSCTASVGYLSRMTQWKSKLNISDKSNMNDKAASNLKLGLFSYPVLQAADILVHRATHVPVGE
DQRQHLEFARECVTNFNAAYGNHLVSPQTTTSPVQRVMSLQKPTEKMSKSSKSPKSRICIIDSPEEIKAKIQAATTDSIPGIS
YNREERPGISNLLDIMAIFDPEGRKAQELGEQYNDLSPKQLKEMVSDAVIGGLDGIRDRYTELLKKGDGYLDSIEAIGAEKAR
KSAEETMQVVREAVGL
FUN_001937 CDS scaffold_12 166729 170216 -
hypothetical protein ENOG41

MAEDPAEVERLILSLYQPNPPEVIAQTQATLSRLQGTPEAWGLARILLEKPDQQVKFFGALTIVIKLNNESSSLSPEDASELL
LLLINHYLDTLRSQSSPLVARKLASALATFFINFHQLWSRFIRHLIFCLVSGQSCLPNALDEGPDTVSLMERMEPSQAQAALW
VLTNVFDEVAKINLNSVQNIGIYGSILENSSDAVALMSRSMSPQTTTEEAHEDAIRCLQSWVWFAQKSSSKDTRLSEPIGPLI
TAVITSLTVDELYSVSVELMVDLLSNCLMFLSDTHFANLAQLFDSPWSQGRYEKLVQGDFEFDSLQYGLLLLAFGEAKIERFM
QSDDSRSQKLLSNLCGLLAARGYVIEEDRIFVPALEFWLTYAETLTDLTYSDEESTGTWVPRARSHLLQAVSNAWQKITYPPA
EEFHSWDSNERISFHEARKDVVDLLQSVFTLVGPQLVSTFADLVLKALSDSSWPQLEAAAFCLGGLADCISETNRGDDALSVV
FKSSLFAILRSGQSEIPPKVQQTCVSLIARYTEYFERNVSELHPVLNFIFGVVGEHAMANAAAKSIHRLCGSCRGYLHTEANS
FLNEYQSLVSGRRLDCGASEKVVGGIASVIQAMPDSNPKYSACGRLLEFVQTDVQRSLDLLRSYDGSPMPCCAVYTCSYAAPD
ESPALHTALKSLRCLASIGKGLQAPAELSVDLESSRASTRAVDPHLAQLQGSIMSMISEIQEAFNTSGEVVELICSVLRSGFS
ESEPGPFVLPPQSVAQYLTNQNLQTPRVGLLVKTACSFVSSLEHDGLDNQQDMLNSVLLWVIGLLKQLPNYEVDTELTQNGID
FVTRLLNKRPITLLRLQPSDAAEFFFLFTLQVLDGKEPLPKASAAEFWATFVSIKYDSQELNDVAQQAMQMLGPLLSRTLARN
LGGNASRSELDKLSEPLKKLVSRYPMAKSWLEAGLTHETFPSTKVTPQDKSMFLKKIISLRGAKATNQVVRDFWQSARGSNFA
YAS
FUN_001938 CDS scaffold_12 170450 171286 -
hypothetical protein EOG092643NE PF08698 ENOG41

MTDISDDQVDELLRKAEQRLKDGSASAIVSSTTSNAVVAGDAATTKDVKQQNSSNSSSKKDGLTVRAPPQPQLGLKAKEKSTA
GPEWFDLPKTNMTPEFKREWQVLRMRGILDPKHQKKNLRASAPEYSQVGEIIAGPTEFFSARLTRKERKNTLLEEVTRDLDSH
KFTDKYAGIQKQKTSGKKAFYKNVVAQRRKRN
FUN_001939 CDS scaffold_12 171894 172959 +
hypothetical protein PF00125;PF00808

MENNPNQAGRTRPVYDASQGGHYGASALLATQGFAPSELYTGPWANAHQGLTGQYKDILTTYWQHTISHLENDNHDYKLHQLP
LARIKKVMKADPEVKMISAEAPILFAKGCEIFITELTMRAWIHAEENKRRTLQRSDIASALAKSDMFDFLIDIVPREEASSHA
KRTQTAVAQPVPAGQAPMSGQHTMTQAPNQAHPMGGDYMAGHGLAPDQDYRNQPNMYPDQSVPNPQAAYGQTQPPTMNPYGNM
GDMYPYSAMPPQQTNMPNEEFEQ
FUN_001940 CDS scaffold_12 173778 176570 -
hypothetical protein EOG09260NY2 PF04091

MPRRPPAVDDYASAVPHVILASSDAEFLDQLIPVLKDATHSNRTPLLTQCLSRYYEEREAEIERIGLTRHEEFLDSINHLQTV
RAETVTLTQEILSLNESIAESTKKLATQKEALVNTSAVRQNIADATSALNDSLTILRAVNNAHELVRRKNYYAALKSLDDLQN
EHLVPIIQNRYSTQHRLADVIQKSIPASQKAISEAVMTDLNTWLFRIRETSQFLGEVAFYHTEMRRTRQKKRIEDDPFLANFK
LNSAIELVSDENEDFDVLNNEELQVDFTPLLEALHIHEALGQLEKFRSEYGATRRQQKDLLLPGFIELLADDEQSLSSLLEGI
AGFAIIEKATMQRVPHLRSANDVEELWESMCTAVINLTSKALSDIEDAEALIKIKTIIALFIQTMEGWGYTVTMLDNFLLKLF
DKYAELLKKRFSVDFQEIVSTDDYMPMAINTPEEYEKVVNVSWFTQEKPTDQLTYPCVLPFSQMYPLCCIDIRNFLNQFYFFS
DDHFQHPNVIDETLKKSLDELLTEKVCKSLVERLSSQYLGQIVQILINLEHFEIACQELEQLLIRARSSTSAGGPVKLKATDS
FRNNKKTAEKRIFELVNSKIDDLIETAEYEWTASTVEKEPSNYMQTLTRYLSNIMNSTLLGLPREIKELIYFDALSHTAEKIL
ALPLSPDVKHINPNGVAALAQDVDYLVQFVSSLENGQMLRENLDELAQTIALLQTDNQDEFFDISTRNKKYGRVDALNGPMLL
EKLTPITTSPVRSAPLANFSSRFGINRT
FUN_001941 CDS scaffold_12 185226 185529 -
hypothetical protein

MKTREKVSYLQLSFSDLQRHTEIVPEPQDCFPSVWPPFPCPEIDHDTATKGREELPPDGPGGSTFDSLAFNDTGQVLLSFFSL
CFLVLRRRVQRAGPGDA
FUN_001942 CDS scaffold_12 187440 189848 +
hypothetical protein PF04059 ENOG41

MSSRADHRVYANMSSPAEGAADSIQGTPNTQITVFSPAESSQGTSMQGNNYVGQSQASEDPFVDSGRAQGSTLSATASTFQPR
GVRSKGKNAIVFYPQGSPTVAGALSQDMDISHRIEVCDTPTPSIIDLGNFITELTQKGVRFHGGRNLETTGGHVYVVFEDLRD
AAWAFYAIRKAPKGWFTAYVKIRPERLDLGLVRFSELRQLSIEVNVLNFAIIDPAHVDEITQRALNVYGQLFALIRNLSFPNG
AFRGVAQFCKAADAFIAFKAFGNGITTGGVVITLSKPEDIGSALTSATDQLASNMQGMSFQQALRGIDRSRHQSFFTMNAPQG
NPFAAAGALHMPFHPQHHQQPFTGQQFGPPVMNPNSLISQRDAGLGNLNGFGRFDPCRGAGRYGRGSRGLNNIVDINELVAGR
DVRTTIMLRNIPNKVDQPLLKKIVDVSSFGRYDFMYLRIDFANDCNVGYAFINFVKAEYIIDFFQARANKRWNCFRSDKVAEI
SYATIQGKDCLVQKFRNSSVMLEAEHYRPKLFYTIHSDETKLVGQEEPFPGPDNQSKMKRSVENAEHVGLFTPTAGQYFRDEQ
RRRHSQYDRGTRLAELEEISYGSSVAPYYGRGRY
FUN_001943 CDS scaffold_12 190272 192810 -
hypothetical protein PF07738 ENOG41

MPSLGPALLRSLALIAAWTQAQTEAQSHEPNAIPDPIDLTCDARTINYITHTLPQACLTSSWTSTSMSATPSDANSTSDASPD
SAQSDAPPPNTQSESPSQPQPSASTATPAKEEPEDAAADGDAKPFMSFEDWKAMMLKQTGQDPKDLHRNAKPRDRTPPDMGYG
GLGEEDEISLNFGSYMDDTGEQKERPSDLDQAKNEGSGKDGRVAIHRNKDAGKTCKERFSYSSFDAGATILKTSPGARNAKAI
LVENKDSYLLFECSAKEKWFIVELSDDVLIDTVVLANFEFFSSMIQTFTVSVSDRYPVKREEWKQIGVFQAENSRAIQPFLVE
NAQIFSKYVRIDYLTHYGKQYYCPVSLLRIHGSRFFAAWNEGRDEDANEAEEAEVTPQALPSGEESTKPQELESPPEPAKPSS
MGLMPFCGVNTTSRLLFEPLFCSASLNQTTQPISNHSSSVEVTSAVPTTKSPDERARKGTSTPHTLRSEHPASEEPTASSSST
AVSPSATPAVSPTSPSSISSSNIESASNSTASAASTKTTATPSPSSTPSTQKPSPANTVNAKKGSTGTASGSSASPTVQEGFF
KSISKRLTIVETNLTLSLKYVEDQARHMSETLHRTEQKQLSKTTLFLENLNKTVLAELRSVREQYDQIWQSTVLALESQREQS
NREIVALSARLNLLADEVVFQKRMAIVQAVLLMSCLILVIFSRGVPLHHLAPFSDQAGLASYDGASSAARVRAMHGSAYDGED
AVLLAARQRGQYTPTSRGDDGATGLHRGPFADDIHHDRVECEQLSPPPTPRSSGGFSSSSDLSPPSHDTQPNVLRRSIAQPTN
SRKPLPALPENPSSP
FUN_001944 CDS scaffold_12 196754 197300 +
hypothetical protein ENOG41

MQDVIYNYFNGIKNEEATAQALEMAMFFANEALLTTAASLSQNRPIFYNPGTAVMKPKKDLAAVITVTTLIGLQVVALCALMW
FILRAPTWTEILDADALAQIGGQLKEWGEPRPDLTKINGIVGVDETRHQAETSSVRLSTGQDAASHSGSLHLTLGGEGLINRG
VMERMQADTTTAVQA
FUN_001945 CDS scaffold_12 200430 201091 +
hypothetical protein ENOG41

MKFSAFFVIASAAFAVAAPGVATFSQERAALNESAPSRDREEGRADLREPEREEGREGEREDDREDRAGRGRQDGRLNRGGLA
FSQVDLNYLLRVNQLNLGKLQVLSQRNNFNVVVFQDLFASRDFSLQSLLQLQQLSTMLAIAETGIFDQFELSRLDLGNLNLGL
INGIAGLNLAQFIDAALKPQITIISKEVNNNIIIAK
FUN_001946 CDS scaffold_12 201388 202488 -
hypothetical protein ENOG41

MTKLFTTVFIFRRPGWDDLMIVFSLVGSIVASSLVQTSADLGLGRHTAAVASEPGGTQRMVKTKILQVVGYPFNIMAYSLPNA
AILILIERLVGKTKAISRRFLRVVVAIQIMLALASVIIIFVQCRPTKMLWDQSAQGECWSPHVFNYTSYVVSGFTVFTDLVLA
IVPIHAFWKLQLKVQEKLEITFMLGLTFLSAIFTIIKATYLDTFNDRTDPLYNVVTLIIWGLIEQNIVIMAASVPTLRPLLRL
IKDKHPMRSAVGYIKSGSGTDPKHNASVSEIPLGNVQQRAEQGSEPSWETHSSSEELNGRRATPPFE
FUN_001947 CDS scaffold_12 203697 204946 +
hypothetical protein PF00891 ENOG41

MAPSDIIKRMQDAAVSYDKQELGARETLLDLNRQLLAELETPTDFIQRIWFATASLGGCLEVAANREIFQLLQGAENGLTTQA
LSEKTGIAGPLLERFMRHFVAHKVVRFSKSTGWHATALSNTLAQENYQHSISFCQRAAAKSFCHFPDHFKQANYQHPGLTDGP
YQYAHNSSLPFFDWLAANPPFVNLFGSFMSVYRAGNTDWWTFYPVEERLSSGFDTNISDVFLVDVGGGRGHDLLSFSNSIKPP
GRLILQDLPEVIVDVTDKSVFETQKHDFFTPQQVQHARAYFLHSILHDWSVEHGVQILKNLKPALKPGYSKVLINEIVLSEEN
PSVPATSMDMMMLGHIGEACERTEETFRAIVAEAGLEVVDVYSNAASPESVIEVMLPCSDRALEESKL
FUN_001948 CDS scaffold_12 205679 206552 +
hypothetical protein PF12697 ENOG41
S33
MLFKQLLAFGALANGLATRQEKPIWLTLPPTPDLPAPITHRTTPINNVSLWMQEYNKKADAIPIVMDHGGLGYSAYFGSVISR
LIKDGYYVIAVDRRGHGRSTFNKNDVFTYEGFAQDTNDLLKSHGISEYNVVGWSDGAITTLAALIDPVMSEPIKKAFIFGGSA
NPEQTNATFSSTDIFSEFVSRCRTEYGELQPHANFTLFGTKVATMEATLPQFTDEQLGSIDGPRVIIVGAEHEEAVNRDVPEK
LHKAIKGSHIEILTGVSHFAPLQDPDQFTKAVEEWFEAPIP
FUN_001949 CDS scaffold_12 207104 207548 -
hypothetical protein ENOG41

MTSLINFTALLFIFVKIVGTAPPEHIFQKPEDFQRTDDDVENAHRHNANIRAWYDVHRRNRRWDIYLGLWLAKLAVALHTMVV
TNGHFPGSLAGWSAYVVVYFFLAEVLMIVESFWGISCVVFGWYNLPAMWFNLLTPSHFEVRFLV
FUN_001950 CDS scaffold_12 208136 210830 - MCM2 MCM DNA
helicase complex subunit
PF00493;PF07728;PF12619;PF14551;PF17207;PF17855

MSSPLHPSSARPNRKRSRTGGSDGPSPAGFGSSPMPSSPPAEFNMAHGVEDDDDIEEEAQIQDDIDELDEMAEDDVDLFREGY
EADYQERENDMYEGIGIDDEGDYDEMNLADRRRLEAQLNRRDQAVRREIPNIYLDGDEDDGDIDLTAQPRRRRHHYDEDPDDL
MDTDIMAEELSLEALADVKASNLTEWVSQPSVQRTIRREFKAFLTSYTDSSGSSVYGNRIRTLGEINAESLEVSYEHLSESKA
ILAYFLANAPQEMIKLFDEVAMDVVLLHYPDYERIHAEIHVRIFDLPVHYTLRQLRQSHLNCLVRVSGVVTRRSGVFPQLKYV
KFDCSKCGVTLGPFQQESNVEVKITFCQSCQSRGPFTLNSEKTVYRNYQKLTLQESPGTVPAGRLPRSREVILLWDLIDKAKP
GEEIEVTGIYRNNYDAQLNNRNGFPVFATILEANNVVKSHDQLAGFRMTEEDEHTIRKLSRDPNIVDKIINSIAPSIYGHTDI
KTAVALSLFGGVAKVTKGAHHLRGDINVLLLGDPGTAKSQVLKYAEKTAHRAVFATGQGASAVGLTASVRRDPLTSEWTLEGG
ALVLADRGTCLIDEFDKMNDQDRTSIHEAMEQQTISISKAGIVTTLQARCGVIAAANPIGGRYNSTAPFSANVELTEPILSRF
DILCVVRDTVEPEEDERLARFIVGSHSRSHPLSQAEQDSMEVEHDTQAETQATNGGRKAEGEIPQELLRKYILYAREHCSPKL
YHVDEDKIARLFADMRRESLATGAYPITVRHLEAIIRISEAFCRMRLSEYCSSQDIDRAIAVTVDSFVGSQKVSCKKALARAF
AKYTLARPGTQKKAGGNSQARRQTAVVG
FUN_001951 CDS scaffold_12 211377 213133 +
hypothetical protein EOG09262E7W PF02207

MSADAPSASAPAQDAPQQQSRERKDSFSGKSQTSETSQTAAEFIRSQEALEADAREALPYSIDTCTKILGPLRQAVFACLTCN
PPPAKEGDDWTPAGVCYACSIQCHGEHTLVEIFQKRNFTCDCGTKRIPSTSPCTLRLNEATNTRGGVHSEEPDVNNKYNHNFR
NRFCGCQCDYDPFQQKGTMFQCLGLGTTETGGCGEDWYHPGCLVGMGPNWFEKMEKPKKAAKENADEGKLTTITEGNEEQNS
GEKSGETNGTAQPAAEEEEEDEDVPMPPGFPEEDAFEAFLCYKCVDAHPWIKRYAGTSGFLPAVFVNQADSETKAEAAPVKEE
PATATEQAPTDEPAQDSRKRKAEDDAEESTEAKRVKSETLPEDAKPAETSSTDTLPCKLKNLPPAPQGQFSLFMKEDFREAFC
RCSSCYPNLNPHPQLLEEEEVYEPPLSDGGSQAGGNGGGSHGSGSLLERGESALRNVDRVRAIEGVMAYNHLKERLKPFFQQF
AESGQAIGAEDIKAYFAKLRGDEQGIQEAAAGAKDGEGGGSGDGDQRKEQSGY
FUN_001952 CDS scaffold_12 213549 214710 -
hypothetical protein ENOG41

MSFQAPPSPNPHDPPLSSPSSPAPVPSPTSTARHYQQQQQYQQQQHSPQPQHQWQQQQIAMMNGMNGTNMAAGMPVPTPAGHQ
AELNYIYGMVEELSRQLAENRRVTEDIVNGLGRVRNRARAQGVSNSQVIENAADDLNGMCPISHSLKPQLTYPGQEQNIDALV
SLLTESLEKAKFSRDANAKLLSQYANAMANLLRQFHEYKTKHVSDVAAWHRSYRKQLADARDENARLREQIWQMQAHAGRANE
SLREYRRKYDEDEGRWNKLVDEKAHRQELRFWKRMAMPELDDDDPYWSDDDDIIDPAEKQRLHELDLKAAQEQLDSQAEDSDG
QGPPPQLPLPGMGIGMGMMGGIPMQREDSGASHGVPALPPRPSSAASSTGSTGQ

FUN_001953 CDS scaffold_12 215403 218059 -
hypothetical protein ENOG41

MEPSSKKRKLAPKVNTTPSKPPPPTQFPHESPQQHYPAQEAAALSERHDFESFARHLQDAAMLIQRQTERHPYTDVSVLLLRW
EEDESVDEDLAALEQVFQKHYKYRTERWHIPTVPNPSIKLGVQIASFLENARANHLLIIYYAGQGYVSSDGQLFWACNAREDA
AKLKWDGVRCLFEDAQSDILLLLDTCAVPDPPMAGSHGVKQAIAACTSDRSPDSTERSFTSNLVEALQKLSSGRPFTTQKLHE
EVLSLKQQQQLVQTPRQTNGSTSNTHSSPHHPVFMPLTPGKGHSIALAPMPTRPRSESQNGVDTEGQGNREEQLIDPESVVDL
RFEEHRVLVCTTFVGDASPDMSFFSQWLQSTPPLGDKIAVEGMFLGPPTMLLISMPHSIWNVVQHDKVCCFLGYISSHNMIHL
YHRLVGSSGIKPSAREVEDGRILLEARDHAYGTPARVRRDEGHDITFHSPAVRETPNSVERKEHLPQTSPSAPSYANPAGGHG
KPKDEVEDSAEMQEAAEQLKALSHVRHRSDETQNINRPRTILPDGMPDIRPEGETEEHSNDDPLATLRNANAAAKPPRRSLPK
QDTRCNHCSHAPFKDSSSLRKHIAAAHTRPFPCAFSFAGCTSTFGSKNEWKRHIASQHLCLQYYRCSSCPQSTAEGKGNEFNR
KDLFTQHLRRMHAPFQIKRAIAKGDSKLQSEWDTHVKDMQVSCLVQRRLPPQRSACPKQGCQSIFEGPSSWDEWTEHVGRHME
KGEGGRLGVDGLLAQWALDEGIIERKPDGEYRLSASNGMGGGGLTGGMSGSSAAVPPVFEQKESTLTSTSTLEPSQPAEESLI
MDTKPSEDRMEVDTPE
FUN_001954 CDS scaffold_12 222200 228366 +
hypothetical protein PF01734;PF11951

MDSGGVRRKDTTKGPPLRVLSLDGGGVRGYSIFIIIQELMHRTFVEIEGRAPKRHEIPKPCDHFDLIVGTGTGGLIALMLGRL
RLDLETCKELYVRLTRMVFQTDKTIAGIPYRSTLFKASKLEEAIKAAVQEHTVKEREGNDGSDSPLTNPLNAAMYSSAGAPRR
NQSNASTVSFSARSPASQMAYRPAYNSRYGDPNARLYDHRETRTKTAVTAVYKGSPRGSPAALLRSYDSRKEPPPEFDCKIWQ
AGRATCAIGLAFKPITIGQSVFHDDGVGTFNPSPEALDEAVVNEWPGREVGVFVSVGTGRRPKGSDSNSQVWYEGFLGDFAEA
RRKLISKIEGCEKIHEYMMREHLGRRNVSIDAYYRLNVEVGVGEFGMNEWNRLGEISTGTRRYMSREAEQKMIQGISSKLAKI
HRARIRHARAPEGIPELVKSTSTTWEMPMAFELAGDIPTTVPLPHSPASRSSFESGSDSLHIRNNNHNGSPRSSNERMHAETS
VPHNVPNSPPLGPRGPSASAPALHPEDRLAVSSPTPAQYREGVSGQDLIAIVSPDEYPRPPPNMAAPPPTRIEPPPLPPKTPL
PESQQHQGRRVPAAAPPYPLDDDEPPPVNMARKPDYRGSALKVNHSSKRHHPIASRPKPPLVVDVREGPYSHLNPASIQDALY
YLEDERSPSSSATSPSTSVATSATQPRTDLWLPYKIQSSANGDDDDTIAQSVWTITAKSTTKAKLNYSKDRVTEILGSQDHDE
RYNMLPPVDGSAVQLEYLPMKPTKLNKELVRIHLQLLSRFKCAVDGNPDPANEFMNFWIPCTLQDPLLLQIVLFTSSCWLSET
RYLPKALQYAHKHQVYRMLNSQLTDQQAQTSDTLVLGVVQMIADSWYWGATHDLKAHLSGLRGMINLRGGLSQLGLRGYLAKM
VLIHDIVMALAHEIKPSMYGHPGFEFRDSRTMPFKTAFNTPFISNWQSFRECSNSLQLHPSTAQILDDMRSLFSAVLALPRNP
SSEHVQRVLSTAHWFHERILDLPEDTPALQSPRPSQSSRASSIESPGSSAGSLRTDKSSSPFVLPDMVYRVIRKVALIYCKAI
LNRSPISYACSEEDIQAIWLSIWQSGLATWKSVLGIFAWVMIALVTNCHKIGPGRLIKTLTMSTMMSIGMENWDVFNHIAKTS
FRLQRWLAGGRDDTNDLMGGEKVVDKYGFGMTGALPPVDIPADVESSEDI
FUN_001955 CDS scaffold_12 228646 230105 +
hypothetical protein PF01565;PF08031 ENOG41
AA7
MYDFTPLRGNIPNGDVLLPGDDGYEEVLQRWSAACVKRAAVVILPINSREVSAAVKFAVSNQIPMTVCGGGHSSSGASSSEGM
VIDLRKMRKVKVNAEAMTVEFGGGCLWEDVDTALERHGLATVGGVVNHTGVGGLTLGGGHGFLTPRHGLTIDNLLKAEIVLAD
GTIFEASDDTNQDLFWAIRGAGAQFGVVTRFVSRAHRQDKVWSGTLAYPADKLPDLLRFANVLYERPNPDGHCFALGIGFGPD
ETSRVVSAIPLFHGSEEDAKQCFSGLLEIKATANNTEMMTTAKTNTLLNSVMDHGIRRLMGSGNITMPLDIEPMLQTAETFWE
FCESRDGMGKSVLAFEFFPTDKIREVPQDATAYANRGDYYDAMTSFAWENPAYDSEIRQFNRSLCKRIRETNGYSATAGGHWS
KGPVGVYINIEADSISPKDAWGVNLHRLRELKKKFDPNNVFNKWHGIAEDTAGTG
FUN_001956 CDS scaffold_12 232487 233588 +
hypothetical protein PF01408;PF02894

MAPIGVALIGGGLFAKQAHMPAIMKADNLALKAIYSRSLKSAKETAALNTRSGQEPDLYSADSGSGKSFNDLLAREDIEAVII
ALPIPSQPEHIRAALAAGKHVLAEKPLAPTVADGQKLIEYYRGLGGKATFSIAENFRYKPSFDYAAAEASKLGKLQHFSVRVF
FYMSEDSQWYGTAWRAKPEFQGGFLLDGGVHFSAATRQLLTDPAVDVTAFTTQTQPHLAPIDTVNAVIRLKSGVSGTYQQSCG
SKMSTSVFQFGYEKGSVVVDGDKVTVTPWDDEPVVKEFERTSGVTEEVEAWAKALAEGKPDKRQSVEEALADLEFLELMFESG
LNGGQAKKYECQL
FUN_001957 CDS scaffold_12 233991 234732 -

hypothetical protein PF03061;PF13279

MGNKASPAELKTRKREDYRFFLDYRTRWNDNDMYDHMNNSVYNFLFDSIINAYLIENCGLHPPTAPQFGMCVHTHTDYFSSIA
YPAVAELALRVNKLGKSSVTYETALFEKGKEEVKAVGEFIQVYVDRETNRPLKDGMASTLREKLQELVVDDDAIKAKAKL
FUN_001958 CDS scaffold_12 236095 237640 +
hypothetical protein PF00849

MAASSPKLEASPSEDNIFANSFQDLDIHSDKGLTPSPAPSQPSSDPPQAATNNNKQQNQRRPKQKKKQDPPKVSDSDEPLITP
VGDPWPRPYHFEGDGLRRVVPYHFTYNTHCKERWRNRPLLEIYESEFRDRPLEYYRQSMIRGTIFVNGRRVGPDYILRNGDLI
SHTLHRHEPPVTEDPVQIIHEDEDMIVINKPAGVPVHPAGRYNFNSVVEIMKSDRGPAFLPYLCNRLDRLTSGIMFIAKNPTA
AEALGIKIKDRTVRKEYIARVMGEFPDGEVVCDQPILQISPKLGLNRVRANGKTARTVFKKLAYYPAEGENDVNERPKTPEQV
QEEKLRPWVNKKGYSIVRCLPVTGRTHQLRVHLQHLGHPIQNDPIYANRRVWGVDLGQNDADATQNTDEDIVSRLSRMGKDEV
AEAVAYYDAMVDRYEEKRAEKLTGELCDICETPLYSDPGEHELSLWLHSLRYEDAGGAWSYVSPLPRWALPPEGMSGPTSVGG
MEELVEAVKDENPEIS
FUN_001959 CDS scaffold_12 243113 244169 +
hypothetical protein ENOG41

MVRSSSAEPAKSSKRKGTRSVSTLTPSQLARKRANDREAQRAIRARTKEHIDRLERELEDLKSKQSRDQTVQELLRRNQALEE
ELMRLKENMGVSMTSSPYSAPVYDDNLSTGSGAIPSPRGSPFPSGDYSPIPDYGQQYVPLPNNCESWASTVPCPVPSNVSSPS
SSADDYSAGYIPTSVPTSLLPSNNTSSSSISVGHAHKDVIKMEYEDVDSHDAGFRLSNPSLQPVSHTYMAQQQQPAWNMYPVY
YPQAQSSVH
FUN_001960 CDS scaffold_12 244282 244858 +
hypothetical protein PF11905 ENOG41

MITAESLAHTLGPPQIDVRSLFRGHDSHGQIACSPHPITDLIRSLIDTAGMTRLTERIAIFAPLQTMICWLVQPTPERRARLC
EDYVPRERQLTTPHPQWLDLLLWGSLREAAIERQDLYATDEFQRVYFDALRLVNWPYQPLDGLVTDPQTGHVGLTDALMAHAM
NGSNWRLAETFAQRYPELCGLVALE
FUN_001961 CDS scaffold_12 253350 255897 +
hypothetical protein PF00933;PF01915;PF07691;PF14310
GH3
MADINVEEVLKKLSLAEKVSLLAGIDFWHTKSFPEHGVPSLRLSDGPNGVRGTRFFNGIPAACFPCGTGLGATFNQELLEEAG
KKMGEEAIGKSAHVILGPTINMQRSPLGGRGFESIGEDPFLAGLGAAALVRGIQSTGVQSTIKHFVCNDQEHKRMSVQAIITE
RALREIYALPFQLVVRDSQPGAFMTSYNGVNGTACSENKKLLNDMLREEWGWDGLIMSDWYGTYSVTDSIKAGLDLEMPGPTR
WRGDVLNFAAACEKVWGHVIDDRAREVLKFVKKCAASGVKENGPEKTLDTPETAALLRKIGNESIVLLKNEKSLLPLAKDKKT
LVIGPNAKVATYHGGGSASLAAYYAVTPYDGIAEKLGSQPAYTIGAYTHKLSPLLGSQTKGEDGKAGMSWKVYNKPPSDSSRK
PVDELWLAKSEMHLVDYYNSELEDLWFANLEGILEVEEDSTYEFGVIVCGTANLYVNDELVVDNSTKQIAGDAFFGTATREEL
GRKDLKKGEKYKIRIEFASAPSFTLKLDNPVVGHGSLRVGASKVIDAKQEIERSVALAKEHDQVIICAGLNSDWETEGSDRES
MKLPGALDELISAVAAANPQTAVVMQTGTPEEMPWLDEVPAVIQAWYGGNETGNCIADILFGDANPSGKLSLSFPKHLTDVPA
FLNFRTEAGRTLYGEDVYMGYRYYEFADRAVNFPFGHGLSYTTFAFSDLNVLSNEGKLTTSVIIKNTGSVRGAEVAQLYVRPM
QAAKVNRPVKELKGFAKVELDAGESKTVTITELEKYTAAYFDEEKDKWCVEAGEYEVIISDSSAVTDKALKGSFKVDETYWWS
GI
FUN_001962 CDS scaffold_12 256057 257168 -
hypothetical protein PF10067 ENOG41

MDPKKPQILPYTTLLRRIYRAFGFNKGYNFVLWFLFGGAFLAFTLSRFIYLDFDGRLCPSEPPNNRPYGSRGAVPGECYHYRR
GVGKAGIIMHLAGILPAAVLVVFQFLPVIRHRSLIVHRINGYIVLLLSTVGMIGVFMIARHTFGGTLEMQTVTGAASILFITC
MLLAYINIKKLQLEQHRAWMIRGWIIAAHVVTMRLIGIIMAQITSRMDPYYTPCAVLDSMFYHNKPAVEALYPDCIGFYTGET
PDQRVIIKGTSGGRPDEIAASLNSAFGASAWLALLIHIIAAELYLRLTSAESERLRKVSYRWQQNAGMKDPGNAGLTAQRLGD
AEPWAYPVDDQTLYDDGESFR
FUN_001963 CDS scaffold_12 258557 259363 -

hypothetical protein PF13093 ENOG41

MSSRTAAPTIPSLKQSFITSQTTLLSQPLAPSRSWQETNDASDEAIPERVVQEVLYNLNHTIQQHCRRVYPPQASRNIAEQIN
DVYTKDAERKVGDPTDTEGGIGRELDLTENEAIEALPASWHLEKDINDHPMETKRYADAVQQLTELNDQRKQLREQVARLKRL
QSIVEPLQTDENGAGIQENLLTRNGPVEKELERMRILLARVGGRVLSLPEEAPNGEAKEVSLSEMGAQGRKRRVDQFLADDKV
FPS
FUN_001964 CDS scaffold_12 260986 262766 +
hypothetical protein PF03055

MALNGPGVYHRTREHEQEDASNITKNILAQSWKSWPNEAAFDRLEEHRGPLRLTVRGTFPSWAAGSLYRTGPGQSRVEDTARG
THFTTHWFDGFAQTHRFDIIPSEDDETQVWYSSKRQADEWIADVKKKGWRSGMTFGQKADPCVGIFAKVMTVFEPKLGNHNVA
LLANVPGVPEDEKTEVSNGVTGPLGHRVNTSNLFVSTDYTGIRRIDPSTLQPLGETTQYDLHPSLSGPCSCSHAQRDPDSGDL
FNFNLAFGRVPTYRIFRVDAASGETEVLATISDLNVPPAYMHSFFLTENHVVICIPASHYAWRGLKTQWEGNIIDSMKPFDKE
RKCKWLVVDRRHGKGLVATFSTPAAFFFHSINAFEEKVKDEDGAERTDLFFDMAKYENMDIIKGFYYDVLMDRDDATKKYWFK
NDRYKNCAPTLTRYRFRLPPKPTPDTTFSASAEEVLAIPSPHAGELPTIHPLRNGKPYRYVYSASLRGLTTSVDALVKTDLDT
SEAFIWTGPEGHTPGEPVFVPRPGAVEEDDGIIFSLVVDGVNETAYILCLNGKTMEEMGRAQADFAIGQGFHGIHLPAA
FUN_001965 CDS scaffold_12 264103 264752 - ISU1
iron-binding protein PF01592

MFARGIRAVSRRAAVAAPLARPTILPARQISPAVAVNATRSYHEKVLDHYSRPRNVGSMNKKDSDVGTGLVGAPACGDVMKLQ
IRVDPETQKISEVKFKTFGCGSAIASSSYLTELVRGMSLDDAGKVKNTEIAKELCLPPVKLHCSMLAEDAIKSAISDYYTKNP
NAKVSNLSGTGMKLPEADAAAA
FUN_001966 CDS scaffold_12 267740 268124 -
hypothetical protein ENOG41

MYAASMNVPETVIMLVENGANLFITGENDFTVLDLVGMRGNWDLIWQIVDFAAASYPGLVPELFGSLLPLLEPADAERCYAAH
DSSGLKGCIKFWSRVISTLGSPNFYFNDGTTLMHMTDDSNLPEL
FUN_001967 CDS scaffold_12 269408 269855 -
hypothetical protein

MAELLGIVAGGAGLASLALQLVDGGQKLRHRYKNAKGMEVNVLWLSEDIELIGKQLIQLEASADDIMQEQLGPIMMGRCRDRS
AKVADRLANLAGDLPVNSSRIQMIRTTFRSGQWKGELDELQALVTGLKQDISQYTTPPLILMEQD
FUN_001968 CDS scaffold_12 270445 271534 -
hypothetical protein ENOG41

MSSNTVQYTLALRQELRSHLYTIRASIGLGIMAELPEDSKSPIPSSVFDSDADDTNFMSPVLLPLTVSIDDDDAGQLSAPQTN
TPAELSEPRQIISSGDSSELLQAELRRPVLAGTYFNEPAYLVQLRLHLSLSSGNRSWLSRIQTVDIHVLVEDAPRDDGQNVSD
DSEDSDDDYEEEEFPHPAIVKAFPGPSGWEGSPQSAEVTITNGIGMQVGYNPASISYNIGESRTWTKTGAIKVKVAHKGPGQN
KLHVRVEESSVDKTGVPDYLLVPFIIRHRSRRFRMRVGIHARFGFWRGKLAEVVPILGRADEPLFFDPRVMRRAMEKGRRGVN
GEKVVEWRGALDEVALQGYSSLVDENAAQE
FUN_001969 CDS scaffold_12 272114 276131 +
hypothetical protein PF12770 ENOG41

MDVPLEQMSKMSISPHYWGIHTSLYSSLPLEAVKQLERAIWFKISGLTNEARAIFDNELKSFAAVPVVAIEHADLELEAGKWG
RAWRILNSKLLQLREAKEGLESPEHRLMLLTWAMLGTRHQGDVTSSAIEIERTQHWLAGVPVADYTDIQAGCIRRYVIASLFT
RLCSEYNNPEAEHIPLHERTDASGEKMPWGGLQLLRRSLTQRGMFNEANALFRVELNRTPPEDRGPVVNEFLEAVASIPLSRG
RDYIEAVVRLQWATTHIQLQVQESAAEEFDKSAAAFQRFCGDFGIRNKEATPHMQAMAYERLSCIDDPIDKLERTEELAAHME
AVDGTKTGYCLAAAADLSRKYYQVTGMELFRTTYFDVQKRLDTYDQTVSEDICDLVHHHVDLISVALASLIDRDKALDWVDAF
FKEYQHFDAPVVRALLRRSQASLLRSLRRLDEAGRVDEEAIRLETSSPSVGNWMHTRQGYRAPTRRSTTSSVQPDPQNQVQDD
DGEETFYWGWSDAIGDQAKMQEMSIQLVLKWLLEDITTGNSALEEFREMTEAALKAIVLPKAVDINELAEERYMRLCAWLGKP

PVHQKNRRLFCLVMLKFGRQMHFSGLKLWDLRLSELQSLLELEESLPQIIRDKFPGSKGSWLGQMALTYMAPLDYSADFTSAE
SGERLSNAQAYNDHALAEYRRNKDLVQVALHQRSGAQICMFMIRRIRQLEQQAASSSNTRGGIVDNGPKKAENDSAFLSVFAE
AEVHDIRNRGISQAEEADEIFSLSELHASSSSGLEGITHRQSITAFNANAFTILTAIELLLAAPGEPSDETITKVWQWVQKYK
ARSLARTIGVRISDPPELVSKIKACPDAARGYEEMRAMEQRIKEAQGMAKFNLRRQLDTYRRTMRETHDLMRQLLDLREAQPF
DLSDVARIKAQSGTPIVLVDWFYLPPYFTGDTGRLLLLTVKERSKRNLNIKPTMDVLTTKVGDVIAWQKTYLTPEKFEDMPEE
NLNTKEARKAFDSMLGGLIAPLARRVKPGELIVLCPSSSLHRLPLHALAIKGYEALIHRNPVVYTHSQSLLRSCFAATDLARP
LPARINARFISGIAESESVYHKAGRTSIQTLARRFRTDPMIDRTASKKLFLDAAGQSRLLHLHTHCGWESKDPLDHKVELPMS
CAQPKGQRPVESVTAREFFDIQVSPGTHINMIACQGGVMDVQLGDEVMGLVPALMCSGASSTVSTLWRIQDTHGSRFAKVFFD
SFLKQSASTDGNTSKAGGSLIVNLAKAMQLAADAMDEMKEAPLYEWAGYVLHGCWQFPLSEQDITSLQTGGM
FUN_001970 CDS scaffold_12 277341 279899 +
hypothetical protein PF00443;PF00627;PF02148;PF13423;PF17807
C19
MACQHLDLIGLTPPNPSQSVYREDCTQCFDSIDDPAGLDVCLQCFNGGCAGDRNHNRLHYALTQHPLALNIRRTRKIVERDEP
PTKMSKLAIAAETEEDRYDTALTVKCLDCNTELDRTHPKLAPMVDGILKANTFSRKEEVKAWEQELTSCEHILMLQQAPAKKI
EQNDLSHCYACDLPENLWLCIECGNLGCGRKQMGGVDGNSHALAHANESGHGVAVKLGSITPEGTADIYCYKCDDERVDDNLG
EHLNHWGIVLAERQKTEKSLTEMQIEQNLKWDFSMTTEDGKELKPLFGPGLTGLKNLGNSCYLASIIQCLFDMPSFQDRYFRP
NDDLPIVPEPAADIETQLRKTADGLLSGRYSKPDADVAGEGITHQKGLAPAMLKHLIGRGHGEFSTMRQQDALEFLQHLFKLI
TRSPHPDGKDPTQPFRFVLEQRLQCLGCHKVRYSSNEQDNIFIDVPLEKLPREEGSEGPDAYKPVSLKECLDNFTGAEKVELT
CSACGSKDGFTKRSLFKTFPDTLVVNARKMTVVNWVPIKVDVPVLVPDEPFNLDEYLSRGLQPGEEQLPDEPEVKAPAFEPDA
AALAQLEAMGFPRNRCERALHATGNSDANAAMEWLFGHMEDPDIDAPLDLGAQSGGANTADPEKIEMLGAMGFGAPQAKKALK
ETGGDVERAVEWLFSHPDDQGTFDDDAPAEAATPKEPAGSADLPATFQLRSIVCHKGTSIHAGHYVAFIRKALGDANVPTWVL
FNDEKVVEAHDVDEMRKFAYVYFFKRV
FUN_001971 CDS scaffold_12 280290 281259 -
hypothetical protein EOG09264BJC PF02301

MSSKEASKSKDKDKSKVHKLSLKGSARLVAEFFQYSIHSILFQRGVYPAEDFTVVKKYGLNMLVSADDQVKAYIKKIMSQLDK
WMVGGKISKLVIVITDKDTGEHVERWQFDVQIFQPVKKSKSSKSASKDQENAAPAGSTPTAPEKTETEIQAEIAAIFRQITAS
VTFLPQLSGDCTFNVLVYADADSEVPVEWGDSDAKEIENGEKVQLRGFSTANHRVDTLVSYRFTD
FUN_001972 CDS scaffold_12 283586 285439 + DBP3
RNA-dependent ATPase EOG092625P1 PF00270;PF00271

MAATKHLLADSEDAIVDRPSKKTKVTDVDEKARLKKERKEKKKDKKRKTEEQETPADKTGDSEAERAERKKAKKEKKKAKKVK
AAEESAETTEAAEEAPKEKKSKKEKKVPVSYESSDDASSDSAYVQTMSLSNVPQAEIDEFLSKNQITITDPKTETVTLRPVLE
FHQLPATNLLEKKPSPFANYKAPTPIQSASWPFTLSGRDVIGVAETGSGKTMAFALPCVEAVSDIKYKGTKAVIVSPTRELAM
QTYEQMASVAALNKLKCVCLYGGASKDDQRNLLRCGADIIVATPGRLKDFMSDGTVDLSQVTFAVLDEADRMLDKGFEEDIKQ
ILGACLPREKRQTLMFTATWPQSVQALASSFMVTPVKIAIGSGGKETADGSVELQANTRITQRVEVLEPREKEFRLLQLLKEH
QQGKQKNDRILVFCLYKKEATRVENFLSRKGIRVGGIHGDLRQEQRTRSLEAFKSGATPVLVATDVAARGLDIPEVKLVINVT
FPLTIEDYVHRIGRTGRAGKTGEAITFFTVEDKSHSGSLVNILRGANQPVPEDLLKFGTTVKKKTHDMYGAFFKDVDMNAKST
KITFD
FUN_001973 CDS scaffold_12 285465 287148 -
hypothetical protein PF01943;PF04506

MSPSPEKESPPAPSMIKGASLLIILQLASRLITFIANQLLLRYLTAPLLGLSTQLEVYYLSVLFFARESLRVAIQRRDSGSQA
KEESQAVVNLGYLAIGLGSFVSLGLGWMYLAYANEITLATPYLVESLYLYGFAAMVELLSEPCFVLMQTRLQFGTRAAAESIA
TFLRCIVVFGSAVWASKHNDIGVLPFALGQITYGVSLLLVYLVSGYRLASSIGFSLLPKTIASKDNRFWASMFDRSTIGLAGS
MMAQSVVKHLLTQGDTFLISFLASASVQGAYALANNYGSLLARLLFQPVEESSRSYFSRLLSSVTPVKQGGKPVQEVREAKQN
LQTLLRLYILLTSIIISLGPFAAPPLLAIVAGKQWAGSGAGDVLAAYCFYIPFMGLNGLTESFVASVATEAEVHIQSVWMGAF
SVIFATSAFLFMRIYPLGAIGLVLANIINMGCRIIWSGAFIKRFFKRHGTDFKIKSLIPESTLGVSITTAILLKQLKVADNAD
QPIKSLVKIAGSAIPLLLLILVLERHFILECLNSVRGRKAAKQ
FUN_001974 CDS scaffold_12 287847 289141 +

hypothetical protein ENOG41

MGKNKKKNKNKTKNGEQQGEGSKNAAASQKGTPTTTEDVQAAPDMDCPPYSLSPPPNIPLAVQYHRNMVYHLLQANITLEDKE
RSVSDAFVTTRNASERAAMERIATFFDLNENIRAFIRKGVQSGHITEQDIESFVTEQIVPFEGPTSYAAFSQIQTYLAQCGVP
FVHHRTSVSSGSAPGCSVRSYMAQKVPSEIIEVNESPNTYKITNAEDMVDNPVHKSDKSVEEEVEVAESDQPEHSNPEDTSSI
NANCSGYQAQKKVDDRSPVSDVASKEKVDEGDGHGSQTTEAHQLDDLDAQDTEPAVVGEDESCGKEPRSDTIGESDYDAYERK
TLESCEAAVEQSTFHKQRAQEQLEHATSKISQCDEMAQHAAALRRLVKQRREMENQMDYHEREIEDLKVSIQEV
FUN_001975 CDS scaffold_12 292085 293874 +
hypothetical protein PF00083;PF07690 ENOG41

MNAIRRASLSKPEEAGKAWPAIAIGLFVAFGGVLYGYDTGTISGILAMPYWQRLFSTGYTDSKGNPNITTGQESSIVSILSAG
TFFGALSSPFMTDYIGRRPGLMIATWVFNLGVALQTAATAIPMFLAGRFFAGFGVGQISAIIPLYQSETAPKWIRGAIVGSYQ
WAITIGLLLAAIVNNATGKRNDTGSYRIPIAVQFAYSLVLFGGMLILPETPRFLIKKDRHDDASRALSKIRRLSPDHPAVQAE
LSEIKANHDHEMSLGTSSYIDCFKPPILKRQFTGCALQALQQLTGINFIFYYGTKYFENSGISSGFTISMITSAINVASTLPG
MYAIDKWGRRPLLLWGAVGMCVSQFIVAMSGTFSTGQDSAGVIFVKSLAGQRAAVSFVCIYIFFFASTWGPLAWVVTGEIFPL
QTRAKSLSMTTATNWLFNWAIAYSTPYLVDYGSGKANLQSKIFFIWFGCCFLCIAFVYFFIYETKGLSLEEVDQLYDEVSVAR
KSIGWKPHDTWEHRNSVAGAGGKMFNEGHEQGEVSHTEKNEV
FUN_001976 CDS scaffold_12 300894 301929 -
hypothetical protein ENOG41

MSESRSPRPNRAAALRANERILEPELLWKAFEKAKPRMCGQAAKKTIEAMEFHRQCPNYESPSKHLFYIRIKGKGRIDDPELY
YYPYLLVLQAIWPYSEHVQTIAKAFARYWGVPDMWRGPEHYPMLNDRDREMELFMGLKPRETEAREDESSAGNGEDNEEDEED
VKQENEEKREQEQEDEQGEEQDEEQEHEQEQQLEGEQNEDDEEQEEQQEEGDEDTIPDISDIDAITDINKLDKLESILEVREN
KLMYQIQETKERVRKVQENKRDLYRKKSSASKKAAERLQVRLDAERMVSDHWATKEREYIKKLERSSATSSVISPPPEQESER
PAKRQRAAGQKE
FUN_001977 CDS scaffold_12 302626 305518 -
hypothetical protein ENOG41

MDPLSIASGVAGLLTVSAAVIKTFDTVKNSIENCPRTVFWAHAETKEINWAIKRLKSLIDDPDSVPKAARMSVGIHDATVTIS
ELIKTCDNLITTLKPLNEDGKVVLTRWDRVKWLRVQDDVVKYIRQLQAHKGSVTLILNILQCESDVVIVESQRALELKIDAVL
NSSEATQRRIEGLEALFNKFIAENSTLIPSNRSSMAPTLHRWREDDATETDSVATSRKYDPDEWDGFHASAERSLSFPTSSVP
ELEMPKLKRSTSAETSMKSESNGMTSPPFREFEATLEKTNVYKRVYNSTDAFSIHSTHGRSMTGSVMTSFSLADDSSILSAFP
IMSRTELQHPEFYTQMSGNQRISRDLMRAITTRSVVKSPVEPPPGAPFSLKQFRSPPNCGENVMGRWHQSRTKAWRPKEFRTE
IQFEVPVIFVTTSKIDKGPIDGADVFFIDGTRKSLDDTWTDLEPPPTGTFTKTGRFIKNERATWLSLLSSLQKMENESQNWTR
QQIMGLRPRPASATAILTSIAGKHTLNVALQREKKSWDDMPPSINRPYATTSMSSLIEMLAMLGIYWKDFNTFHHVYQAEGNG
FLVKGHKISGLGIMFHFQALEKAVFEGNRTIPTKAVTRLAFGQLPTIFTEGYPENKSSNKLYEWPNVGLGSRTEIAETLSSFG
CGRKAVNYFLRDGAVTSHVFPVTFELIGMIGQVFHIKDLCFRYIPNPCYCVWSTRGFSLTKLLAAYQRHIQEDADMANGQLSL
HVNTIFAPKAGIRPGDGTPQNYTWQAQDALHSVLDELDESLKDYTAPKNGNPRGSLGQEILGAHFEELLSQMNSASSTSAFVP
ANASNKEDSLMKEYFQQILPAVLRSASMAFFNLKNADSEFQVVKGEAAQTAEYAGYLRAAKEVWCTFVLRMICWLMLHEFHRD
DVQIQKGNLFGSDMPVYIS
FUN_001978 CDS scaffold_12 306699 309615 +
hypothetical protein PF00648 ENOG41
C02A
MPSSSSSSSSSASPSFAEIASGVADLGDRSKVTPQQAIDKFWKQFTTKNPGKATTVIPSNTYTDLAAKRGNQVTTTTTQASYE
DAAATCRAKVEKIVKECRRINKKYRDPHFDIEVDLKLYRNDCLHSLSNVENYQPGEDLRPQSVKRVGDIFEKPCFYIDGPTAN
DVRQGRDGDCWLMAALCTLSEKPGLIERLCVAHDQDVGVYGFVFYRDGAWISEIVDDFLYLIKPDYDEGYFDRILFDDIERVD
PDEAYRRIFQSNSSALYFAQCQHPQETWLPLLEKCYAKAHGDYGAIEGGFGGEGIEDLTGGVTSELLTTDILDKEYFWKEELL
KVNQEFLFGCNTGIWGRGWGERKGIVELHAYSIQKAVEIDGKRLLKLKNPWGKGEWTGPWSDGSKEWTAEWLQKLDHRFGDDG
DFWISYEDLLRKYQAFERTRLFTDDWRVSQLWTTLSVPWALDYHDTHFSFTLSKPGSVVLVLAQLDDRYFRGLEGQYMFELGF
RLHKAGHSDYVVRSQTPYRMTRSVNVELELEAGDYEIRVKINATRNQDILPIEKVIKDNAKSRREKLLRIGLAYDLGHCKGRF

VETPEEKAARKEHEAKIKQKKRDKIKAKLLKEREETHYLATRQFERDEHKRLKRKEKLALKKAAKKARQEARKAERAAIKAEK
AAKKAAEAEAKAKLEAEEAAKKEKEEEAEAAKEEKIESGEEKAESKPEEPMTPESTADEDSNEKEGAKEAEVAEPKADEKGAE
ADAEEEEEEQDIESEPETDVESVGTLAALADRELWIHVDNYTGTISSDSDSDNESTTSSSSSTSEAEKDPWNAVVVAGIRIFH
TGLGEGEDNSDINLKVIRPIPFGKDDEEVDVEKKCSTKVLDVDDSAKDATLVGDANEKIRFIKGDGAKRRTATTLF
FUN_001979 CDS scaffold_12 311150 313885 +
hypothetical protein ENOG41

MAEPEDIHARKPSGINIFGITGSGYHHLMPHEMADLHNSGGVPPSPEAKMSSPPETPHSFHNLLGGHHPAGSSSSSPRIADSQ
GTPEPYSEIWAPSMPRGDEKKDMPRTMEREVKMGEGGKLPKIVEPRGDAMDDEFDDEIFYKKFAEPPKGCSTTRDIHQPRSSW
LSITIYVLSVYSTVGSGIWLVTAILQPRWGHKIASGASLSPSTATTLAALLAKTIEMSFVTVFVSCLGQVLTRRAFIRKAHGM
SLAEMTMRNWVIQPGSLITHFETLPSSSLTLLGMLSLTATIAAAFYTTASDAMVSPKLKSGSWEHKELAGYVRASYANAAYVR
EDCPYLFNITEDIHAAESCMNVQFSGQSYRNLLNYMNMWTNLNQNGTEISSDLKKRPGGTTLLFDNTTMYSSWIETEHGDVEA
QFEETGRIINNVTLALPHPGVYGASTLKVNGILQPDDLAGVGEYTVRAGVVSPSVNVLCVDMEKKELAPLVYTTWPNAKVNNT
DIPGQKTGWPGWTGEVPQPLDGKNKDYYLNRTVVDDIFRWGPKYERRPPVFQMYPFDFNLLTNATVYAADAIYTLGKTAENKN
YTVCQLRSWVSPNCSTEFNISGIAGASMKAHCEDDADENAYRRSFPSDQGWSAPSLDWKWLADQWRLSMDLNGGAVNNNASNA
RILTQLALHEPKMPASYPSLAEALAVYSSSLIMISAIDTPFRHYWDQDPKAYPENVIPAPGFPQPFNASLITQEYTSGHTQPW
QNIFYVVLVLVFAINLFCLGYFIMRSGLVTDFTEPQNLFSLAINSPPSNSFHGSCGGGPEKRHLVVPWKVAYAPSANHYFFQD
NSPGKETSEGLSTAREYGEPRVSSYKRLSMSRTWL
FUN_001980 CDS scaffold_12 314588 315728 +
hypothetical protein ENOG41

MEPIGAAYTLTAAQFAGQVKQRGGNVAVRRAQVDCVLASFEEAKKYMSKDGIEEVHKYLRTGEWVRDQTNAGSHPAYKRQIGD
AVEQMYEELSRHWGKFIPSDKPFYPRLADPDQERLLIMGDYRWRDFQVGRQASNNTIHVAIPEAKGHAAALSDNNNTNNKQKV
LDGYLRAAAQLSAEINTESQLKGRDREVQLLQDQLRDSQQRNADLQRQVQEQTKLRRHAEHQTKMSQNRLKERQEQLQDAQDQ
LHETQQELQDMHHRYRESQMLLSKYEEKAKQVHHHISQSQSFSNRALEAASKAQSLLALGQQQSNKVLNLLSDPIEEFSAAAE
SSGLAVHDGQRTNKRAREN
FUN_001981 CDS scaffold_12 316389 317364 +
hypothetical protein ENOG41

MSSPPEDSHPPGTVSRRLPLSALVSECDHALDINMFRAKWENDSNDRKRRWRRTQVYMVPRSFAKAKEVMSDRAINELCEYLK
RGTWNSTARNASGHPCYKHFKGTSPCPSPFAGPYYPYPFFIIQALFPLGEAVKGMYRDLSDLWEMDLDPTRPFYPRLADSEREF
SLIMGQKPSPQVPGPGPVTMSSADQPQSPKVKAEQDSGEDNHTLKRKAQPEMLSNNNSPDNDREQLTSVRSDIKQETSEFQTQ
LHKDTTGTQEIRRHSIKAQKHANAAFKLANTLVEDLGRTTEHSDGAISEEERPSKRVREN
FUN_001982 CDS scaffold_12 318742 321524 -
hypothetical protein

MAMNPNVNMANMNPMGGPVGGAPMPMMNNGGVNPQAAAAAAAASRQQANDNQRSVLNTYIYEYFIRMGMYDCARSLLSSDQQV
NVLSKDGANRRRDENGNVINGVGDDPMDTDSKDDIDSKLPEDLPPPKLPMPASDTSFLYEWFSVFWDIYYAQRAKSGNNTINQ
YVQHTQQQSRLKQNQQQELLRQMRPDMATQQYQMMRNMQNGGMMNMKQGNLARAAMANNQNNPQMMLQQAKQNQMQRDPSGMD
GQNRPSSPASGENAPSPSKRQRIDGAPFNPNQPGMMPNGRPGQGMPGQQMPGGANTAAAQQTLLANGINPHSLHPQQLQNFAA
APPAAQQKSIATYSQNLQQHHGTQMGGKQMPNAVPQGQGSPMMAQGPDGTALNAFYNPGEMGGPGGIRAGPGGPQNGGGSNHA
LQDYQMQLMLLEQQNKKRLMMARQEQDTVGGNGMPREGQPGQPGGPGPNGQFPDTSPQAMRPGASPNPAEQMKRGTPQMNNSG
IPSPVPEGGQSRGSPNPAMNFMGNQMDPNMAPHFFKQMDGNMGGQPPMNGGMRPPSSHPGQPFNGQMNQQQMMAAARQQQGQP
GQGPQGNPQQWQQGPNGQMVPQQMQQQSPQIQGTPQQRSMPPPQAPPAGANGANSRTTASPQQAAAAPPTPSQANKAAPKKKK
SETAKDKRAAANKKANQNLNNAGATPSNDNNESQDTPAPATPIQNANAANFNKNGQAGNTGQNGQPTTGPAANAPAAATTAPP
PQPPAPQHDPNQMMGMDNSFGPLFDGGGMELANPLNSGDVLNDFDFDSFLHDQDGDNSAFNFTEAFPMGDEIGAD
FUN_001983 CDS scaffold_12 324451 325196 -
hypothetical protein EOG09264OM7 PF04051

MAANKASRVGEEIWKTRIDKVNAELVTLTYGTIVAQLCKDYESDYAEVNKQLDKMGYNIGLRLIEDYLAKSNTMKRCANFRET

AEMISRVGFKIFLNITPQVMNWTSENDQFSLVFEENPLADFVELPDDGRAQDQLWYSNILCGVLRGALEMVQMQVEAHFVSDV
LRGNDSTEMRVSLIRYIDDELPPEDD
FUN_001984 CDS scaffold_12 325620 326834 +
hypothetical protein PF00400

MSAEKRPADGDPSSSQSLVKRQNVNSSTGALARLNASSNALVQSAPRTSGLQAPVMELSGHSGEIFAAKFDPTGNLIASGGMD
RSLMLWRTYGDCENYGILNGHRGAILDLQWSRDSEILYSASADMHLASWDLTSGTRIRRYVGHEEVINTVGISRRGEEILVSG
SDDSTIGIWDPRSKNAVDYIQTEFPITAIAVSEAGNEIYAGGIDNDIRVWDLRKKSVIYSMLGHSDTITSLRVSPDSQSLVSY
AMDSTVRTWDIRPFAPTERHIRTFDGATMGMEKNLLGASWDSEGKRIAAASGDGTVMVWSSETGKLAYKLPGHKGTVNCAEFS
PNKEPILLSASSDRTMLLGELK
FUN_001985 CDS scaffold_12 328381 329740 +
hypothetical protein ENOG41

MDMNRSDMERLRADLIQRSRMERGGSPNGGDVDLDVNVDVEAQHSIQEPRSSPAPLSRLLSRREPASMTGQAQLAEIESVKSQ
DSNTNRPAGSRRFAVPGFLRIWGRNNTTPQSDDIRNATASPAPLLPIAEPLSPTRPEPVATPSESHRSRRQRRQRTPPQDPLE
AHLSEILGGSTIRRHRRRREYHERERDRHRSRRSHQRHPKVFLGCIPWVRSRRMRAQILRCLTSGLFLIILVTVYLALSMTHK
VNTREMNIMLILVVLFAAAFFFHGLIRLCLLIIKSNREAAERANRPPRYRAPRYAVPPVPIPVVLAQDEEAVGMESEATKIQP
PAYGRWRETVRVDPDRLFWQRNEAVDLSQIRPNTRAGPRPPSYVSEDGISYVVEAQPRSTAPTTEVPLPTHPSELGRVARVPT
E
FUN_001986 CDS scaffold_12 332162 332934 +
hypothetical protein PF00098

MSSLSRRACYKCGNVGHYAEVCSSAERLCYNCKQPGHESNGCPLPRTTEAKQCYHCQGLGHVQADCPTLRLSGTGTSGRCYNC
GQPGHLARACPNPVGPTMGRGAPMGRGGFPGGYGRGGFAGGPRPATCYKCGGPNHFARDCQAQAMKCYACGKLGHISRDCTAP
NGGPLNTAGKTCYQCGEAGHISRDCPQKNAPGVGEIPQEVDIGSVPAPPVASIAPVA
FUN_001987 CDS scaffold_12 334637 336101 -
hypothetical protein PF01231

MLPSIPVLAEYGISPTHGFLPDTLPLTRLPDPYYNKWEAIAANLQALILSRRLRGVIDRLPVLSTIGLEHDAEWRRAYSLLGF
MAHGYIWAGDSPSDRLPASISVPLLEISDHLEVPPVATYAAVCLWNFKPLFVDEDIDNLENLATLNTYTGALDESWFYLVSVA
IEARGAPIIPLMLTAIAAARHGDSAAVTRCLRTFAERLTDLTNILQRMHESCDPTIFYHRIRPFLAGSKNMAEAGLPNGVIYE
DGSGTEEYRQYSGGSNAQSSLIQFFDIILGIEHRPTGESRDPSSDTDRGRAANRHNFIMEMRRYMPGPHARFLNDVSNVANIR
EYVESHHSDKQLCLAYDACLAMLSAFRDKHIAIVTRYIINPSKQVRARSRSRSPEVTRNKLNLAVASRKNKENQKGTGGTALI
PFLKQARDETGEPAVEEWTRRFMKRKMQTEGRNDFFLGKTLQDDVSLTEDVEIKGLAGSWTMDDEIGGICQY
FUN_001988 CDS scaffold_12 337462 339230 +
hypothetical protein PF05743;PF09454

MPVQQHVLNWLYSVLTSEYHDVNRAYNDVAQALDRFPSLSPRTDVHTFSNGANALLLHLSGTLPVNFRGTTYRFPISIWVPHA
YPREPPMIYVVPTETMMIRPGQHIDPQGLVYHPYLVGWAEFWDKSNLRDFLNILTDIFAKEPPVVARQQQPRPPPAQANPTPP
PVPPLPPGMGSTSRPATQDPHPSEQPRPPPPPPKGPQTASPAQTQSQNGPPLPPIPGRSPAQSNRMSRYDSAPPLPPQVGSPN
AQDPRLPRPQEQYATRHIPPAGPPMAAQPGYTNDTQSFAPPSQQQWQMPPHHQQYQQPPQPPAWTAQAAPPPQSPAKQPPPPP
DLLDEPLELSLPNTTVAAPPIPPNPEKDALLRQLAQTLYSIRMRSRQQNDSSMAGLQAQRTAMLSAIPAFQAEGGQLTQLSNV
LTSNSNSNILHDALHKADAVIEGSKSHPVPDVDELLVAPTVVANQLYTLVAEERAIGDAIFMLGRAVERGRITPAVFAKMTRTLA
REWYLKKALVRKIGQGMGLAS
FUN_001989 CDS scaffold_12 340201 341395 +
hypothetical protein PF02116

MSTFDPFTQNITFLAADGNTTISIPVVLVDEMRRMMVNTVINYATQLGACLVMLVVLLVMVPFSKFRRPFNALQIISLFICSC
RMVLLCIWNISKFADFYPFWSHDFSHIPRSEYAPAIAANIISLLLVISIETMLMSQAWTMVKLWPNMWKYIIAGLSLVISLMT
ICVRIAFTTVQNKAVLDTIPPLHMLWLIKWTVIMNVSSISWWCAIFNIKLIWHLISNRGILPSYKTLTPMEVLVMTNGILMII

PVIFASLEWAHFVDFEAASLTLSSVAVILPLGTLAAQRIAASNANSAHSSGASNGIRYGASGPSSFTGFKAPSFSTNRSGATD
RPHVSVYARCEAGTSSRDHINPEDVELGKIDSDHVRVDKAFLQREERI
FUN_001990 CDS scaffold_12 341927 344125 -
hypothetical protein ENOG41

MSTHSSSRKNSPRKFVSESSDTRSLTSSKFAQLLSKFEVLEAVSSVGSPPPPSKTKPASAIKVTSTIEQTSSSASKPSSNAST
RIRERGSTGSSHVSSGRFVPSSQETHRTRRVKTFGTQSPVISTRNADSKARQKSVAERIIMFEAADQQAQTEASSPTRTSIPR
SPSKLAHSKSWQTIKTRRSAAGPRADDSPITPNLVRIAKTFSPTPHTVPSPRTLLASDDPFGTWRSPLNRLDDSWTSFKRNSL
VMSTPDASPSYIKSQYTEVTPSSVQAVSTSRFKLDDVRDDAFVENNNVTIEETRHHGWVRNARKIASSLSNTVSAGSSSLRRK
SRGSHDAPSQPPSNNSGLMSFRFARASTPSRSSLPRSRISNFRKQFDELKDQPVASVGALETKRLESILTKNEAPNSPSKHPL
LGYSDSSESEILNAPKFRSKSYPPRWSRATVESPQTPVGRRKTEKYISPLKQKIDLFESLDRQAPSLDSTHTIDTGKQPVLNI
NKRKSSIVGPLRTFKGTLRQISTSYRRTPSEWSTTSSRDIAPSQLRGSEDATVVQDGQAVEFANDEPLSPRSIDAIPGQPVLG
QTFLTNEIISPLLLEKPSSLPRNSITQAGFNMDGEAGFGVAPPPLFTEPQRRFSHTKHGLSRAANRFSLPDIEKELELIELLE
DDRQTVPAHSPFVSKVVCELEQPRPVHANELKRLVSLCKEKIRKISGGRSE
FUN_001991 CDS scaffold_12 344821 345062 -
hypothetical protein ENOG41

MPPKRIQVEPRGRRAPKGFIGSTYDALTSPDNAAVVRSIAIFGAAVTFLSSSWGEILLPP
FUN_001992 CDS scaffold_12 345791 346639 +
hypothetical protein PF17110 ENOG41

MESPGPGGFLPAPAGGSLPSPAPSSASSYSNIQGLPTPRTKALKPNSSKEFMVRRYAEEQLLLVTRRYVKKFGNPEPGDTVVG
YARFGEVCRDLDSITNVLWKSGTPSLQIPFLLRLTSDFTRYVRSFPPAPKASFAILRKLDHCFASLLCGQDIETHETLPGFEN
GLRGGMTTTEMIRCRSLVDQCRVLMVEVMRDPAEEDEEDEEAETDTDTDAEEPGIKGWGGVEDDDEMMLQLDAARVFEKTIVQ
LNERLGDLEPLQMSAD
FUN_001993 CDS scaffold_12 348547 349450 +
hypothetical protein PF00722 ENOG41
GH16
MLNFIPIIAGLASLVSATSAPTRPDLKLIWKDEFTGCQGCSPKTNNWNTALNINSNNELQVYSTSNKNIQLSGGDTLQLVPWK
DGKGEWTSGRLESKKAFHADKNKALRVEASIRMGDSARKQGIWPAFWMLGDALRHGTGWPLCGEIDIFERVNGDLTGFGTVHC
GHEGGGPCNEPEGLGQRVTIPDNEFHAWSVVIDRRASSWQDEKITWLLDGEPFHSITGKTLNDEGTWATLAHSPMYILLNVAV
GGTWPGNPNKATEAGYKNMMEVAYVAVYETTN
FUN_001994 CDS scaffold_12 350387 351594 -
hypothetical protein PF00734;PF02015 ENOG41
GH45
MRSYTLLALAGPLAVSAASGSGHSTRYWDCCKPSCSWSGKAAVNAPALTCDKNDNPISNTNAVNGCEGGGSAYACTNYSPWAV
NDELAYGFAATKISGGSEASWCCACYALTFTSGPVKGKKMIVQSTNTGGDLGDNHFDLMMPGGGVGIFDGCTSEFGKALGGAQ
YGGISSRSECDSFPELLKDGCHWRFDWFQNADNPDFTFEQVQCPKALLDISGCKRDDDSSFPAYKGDTSASKPQPSSSAKKTT
SAAAAAQPQKTKDSAPVVQKSSTKAAAQPEPTKPAEKPSATKPAVKPQTDKPAATKPAATKPAQPVNKPKTTQKVRGTKTRGS
CPAKTDPTAKASVVPAYYQCGGSKSAYPNGNLACATGSKCVKQNEYYSQCIPN
FUN_001995 CDS scaffold_12 353553 357460 + RPB2
DNA-dependent RNA polymerase II
PF00562;PF04560;PF04561;PF04563;PF04565;PF04566;PF04567

MDDYENESDYDYGYDEGITPEDCWTVISSFFETKGLASQQTDSFDEFTQTMVQDLVNEYSTITLDQHNPPAPPGVNIALRRYE
IKFGSVMVSRPSMSETDGTVTNLLPYECRDRNLTYAAPTYLKITKKVSVAVDKEIPLHEMDDEQQAEYKRTGENPTKLVWEVE
EDAGSIKAEDEPSEMIFIGKMPIMVKSKICHLSSHSDEDLFMLNECPYDQGGYFVINGSEKVLIAQERSAANIVQVFKKAQPS
PYTYTAEIRSALEKGSRLISSLTMKLYGKGDSARGGFGQTIQATLPFVKADLPVAIVFRALGVVSDEDILNHICYDRNDSQML
EMLRPCIEEAFCVQDREVALDFIGKRGNRDQASLGREKRVRVAKDILQKETLPHISQSEGSETRKAFFLGYMVHKLLQCALGR
REPDDRDHFGKKRLDLAGPLLAKLFRGIIRRINTELSNYLKRCVESNRNFNLTVAIKPSTLSNGLKYSLATGNWGDQKKAASS

TAGVSQVLNRYTFASTLSHLRRTNTPIGRDGKLAKPRQLHNTHWGLVCPAETPEGQACGLVKNLSLMCYVSVGSPADPLIEFM
INRGMEVVEEYEPTRYPHATKIFVNGSWVGVHADPKHLVNQVLDTRRKSYVQFEVSLVRDIRDREFKIFSDAGRVMRPVFTVH
QEDDYENNITKGQLVLTKDHVNRLAQEQAEPPANPADKFGWDGLIREGAVEYLDAEEEETAMICMTPEDLELYREQKNDEATL
TEEEKRAKQEAEKREQEEDRNKRLKTKVNPTTHMYTHCEIHPSMILGICASIIPFPDHNQSPRNTYQSAMGKQAMGFFLTNYS
RRMDTMANILYYPQKPLATTRSMEFLKFRELPAGQNAIVAIACYSGYNQEDSVIMNQSSIDRGLFRSLFFRSYSDQEKKVGLN
YTEIFEKPFQQTTLRMKHGTYDKLDEDGIVAPGVRVSGEDIIGKTAPIDQENQDLGTRTQSHQRRDISTPLRSTENGIVDQV
ILTVNADNVKYVKVRVRTTKIPQIGDKFASRHGQKGTIGVTYRQEDMPFSREGLTPDIIINPHAIPSRMTIAHLIECLLSKVS
TLEGMEGDATPFTDVTVDSVSELLRKHGYQSRGFEVMYNGHTGRKLRAQVFFGPTYYQRLRHMVDDKIHARARGPVQIMTRQP
VEGRARDGGLRFGEMERDCMIAHGAAAFLKERLFEVSDAFRVHVCEICGLMTPIANLSKQSFECRPCKNKTKIAQIHIPYAAK
LLFQELQAMNIAARMFTNRSGVSNR
FUN_001996 CDS scaffold_12 357721 358050 -
hypothetical protein EOG09265K5D PF15786

MSRASKLTLLGTSLLTVCTVVGVHFQQKFEQAAMHEGVVRDMEQQRIKRERQLDFDLQKQLEAEYKREQTVHDSIAAAEKGLP
NR
FUN_001997 CDS scaffold_12 358517 359176 +
hypothetical protein EOG09265PWR PF03179

MSAQNSAGIQQLLNAEQDASKIVQKAREYRTKRVREARDEAKQEIADYKAKKEEEYKKFEAEHSKGNEQAEAEANQEAEKQIK
SIQEAGKKGQAQVIKNLLSAVFDVNPVPPTKS
FUN_001998 CDS scaffold_12 359343 361193 -
hypothetical protein PF08216

MASVDDIFKSSGISGKRKLDPIRDPNEIYKSAKINANGSNRHAQVNDAPDEDEDMEAGPAPPPADDEDFGPELPPDDDEGRFF
GGGITKQESEILDYVEEADAGRAPEKIDAAWLRKTLTNLERHINKNAELRAKFEDQPQKFIGSEADLDADIKGLSLLSEHPEL
YPEFVKLECIASVVGLLAHENTDIAIDAIEIMGELTDEDVAAEDEQWNVLVDAMMEADLLSLLVSNFSRLNEDDESDRNGIYH
ALGLIENLCSRQSVAERVGEDEKLLQWLLQRIQRKEDTVSQNKQYAAEILAILAQMAVANRTKLINLDAVDLLLQLIAPYRRR
DPDKGGDEEEYMENIFASLTCLADEAAGKAKFIDAEGVELCLIMLKEGKKSKPPALRLLNHAAGGNAGVDVCQKIVEAGGLKG
LFTLFMKTQDHRLAEHLVEIFASMLRLLPANSAERIRTLAKFVEKDYEKISKLIKFRRDYVARVSLAEQQNDAEKATTSEDDR
EAAELEWLSRRIDAGLFTLQTIDTVLAWLVAEDTGAARKIRQILAERDEKISVIGRTLKEQLDGLDTTEENQDLRDMLSTLIE
FVQ
FUN_001999 CDS scaffold_12 363192 366243 +
hypothetical protein PF04212 ENOG41

MQYDQDQEAYPPQRGHSRSRSGKGSNDRGRAKPPSQKAMLHRALQKANTAVQLDNAQNFKGAREAYAEACDLLQQVLQKTTAD
EDKRKLEAIRRTYTSRIDELDQMAPWQEEETKALPARPESLAQHSESESVLRLDDDDDEPNDTAVFDTATAARIDSRSPQPRI
VNSPPRDDNQGYKRRSAQNKPKPIVTSLTPEPGLLQSSFSRSPVRLRTPEHFLPQRPADPYMPAPLSPRRPLSPAKEVNDMDE
PVRTDFSMSHDQQNDHAQEHNVPQTHFREDSMNSWLDPIDESGGSTASSVHSRTSSLGFRRKHIRGVSGETEAEFDTALDAAI
EAAYDDGYEPMSPMDHRRMVSMDAGEEAVANAMRKVELARQKVRETEQEVYEMERDNRSQQQYQSYEYQGTPNDFYNDNSSDE
EERILDEIARDYGLESYRHRPPPPRESDSSGVTSRTWHSSQGSNPPTGATSLSTVTELPPPITHLIQGPAAPPPTQSLPELPQ
RPGSSAQSVRNRRLSGQNPKQLKIETSKLALPMQNYADANQAKSAPLSSQNMDTQAETDTKAASANHRQPSPPLFEASPTDMT
GSRPTPSPFGQLGSEKGDDDITGSPNTRKLRKNFSSSSLRSMKSRNMSLTHLEEGSDLSPGTPGSNPFGSLNAPSVPALPTPL
ATSFRERSETNAAGLSLFDDHFYSPTSPGSPNPLISDPPAPLEPCPTDFMLRPFWLMRCLYQTLAHPRGGYISSKLFVSRDVW
RVKGVKLKNIEDKVANCDFLTAALLKIARVDTYDADAVLEEMQSLEGILEQVQTALSRKLGNEVGVHGSGALFKDASTGDGDA
AAGVPRSASVSGKSSFSWRRLRSKNSAVGLGGSYMSRVNTETSKDIPSIASLPMTPTPTNRPPKRDLAQVQFTGPNAMYMSSL
ARLFDAAQAIDQIARQVEDPGLRHADKTQVGLELCTRHAAEFFGFYVCRFVLSDLGLLLDKYLKRGSEWVLT
FUN_002000 CDS scaffold_12 367019 369167 + GUF1 Translation
factor guf1 mitochondrial PF00009;PF00679;PF01926;PF06421

MSLAWSAGRAWSRQCLSQARSRPSFTPSHILTPRIYANLRRYATKPTPAELEARIAAIPIERYRNFCIVAHIDHGKSTLSDRL

LEYTGTISASDANKQILDKLDVERERGITVKAQTCTMIHKHNGQDYLLHLVDTPGHVDFRAEVTRSYASCGGALLLVDASQGI
QAQTVSNFHLAFAQDLALVPVVNKIDMPAADVPRVLQQMKDSFELDPKDAIMLSAKTGKGVPDVLPAVIERIPHPVGDEKKPL
KMMLVDSWYDNFRGVVLLVRLFDGTIKAGDNVVSLGTGMKYTVGQVGIQYPHAIPQATLSAGQVGYVYFNPGMKKIQDAKLGD
TFTFVGSEEKVEPYPGFEEPKPMVFVAAFPTDQSDYSRLADSIGQLVLNDRSVTLQKDHSEALGAGWRLGFLGSLHCSVFQDR
LRQEHGKSVILTEPTVPSKIIWPDGSEEIVSNPALFPETSNPKVKQSQLFEPYVTATITMPEEYLGRVIELCEANRGEQKSLE
FFHTTQVILQYEIPTAQLVDDLFGKLKSVTKGYATLDYEDAGWRESNLVKLQLLVNRQPVDAICKVVHSSQVDRLGRQWVTKF
KEHVDRQHFEVVIQATAGNRIVARETIKPFRKDVLAKLHAADVSRRRKLLEKQKEGRKRLRAVGNVIIDQSAFQSFMSR
FUN_002001 CDS scaffold_12 369312 371371 -
hypothetical protein PF00300;PF01083
CE5
MHSSTTLLLLATATVNAIGIRDDCKDVHIFLAKGNNEPYPGRQGKLAGAICSGLKSCDYEDIQFQNALEDPFCDSVTEGVKNG
IKQITAYNKKCPDSKLVVSGYSQGGQVVGDILGGGGGVFFQNCVEPDLQGLNPKTLPGSKIVAAMVFGDTRHTKDQPFNVLSG
AGKNGLFPRPAGMLQNLASYGDVFRNYCVETDPICAQGDEVETHLNYFDVFTDDVAEWVKERIGEDATTTTAATKTSTRAKST
AKETSTKDAETESTTEAASTTKDASKDSTTKDTTTAAASTTADSSSTTDASSAEGRATAGEASSGAAAPSSTDNAATSKEWSL
PDPPLTEKGEEQCAHLRENLISTFSDKVDNIDDVAIVVSPMRRTMQTAMLSLGWLVERGVKIEGNADWQENSSKPCDTGSPIS
SVSPSFPKVNFSSVDPLWPDKTSPSAERYWYTKKSILARGQRALEDLKKRPEKLIFVVSHAGFLRLGVAGYWFFNSDYRVFDF
DGQGIKQQEGTLAGGMGLSLTEPVELGLDLPEEDPGYDAEVKE
FUN_002002 CDS scaffold_12 374002 377652 -
hypothetical protein PF03098 ENOG41

MSFNEKFQAGESYGDSKEDPSSLLNNPEKLVADLMKDFAGVRSQASPAQLLGLVKELLQKGQPLDDKKGTTELLIGILTALPA
TSKARTALTNKLIDTLWGNLQHPPLSYMGGDVKYDVVNSDKPAHKHNCELYDTIEFKVPGTDVLLREQVPQAPDGLHQYRMPD
GSFNNILEPNLGRAGTPYAKSVKSEKRLHGVKPDPGLLFDLLMARDETTFQENPAGISSMLFYHAAIIIHDIFRTNRTDMNKS
DTSSYLDLAPLYGSSLKDQHEIRTMKEGKLKPDTFHEKRLLGQPAGVNVMLVLYSRFHNYVADILLKINENGRFSLSVPPNAS
EEDKAKAIAKQDHDLFNVARLITGGLYINICLHDYLRAITNTHHSASDWTLDPRVAIDKQFDGDGVPRGVGNQVSVEFNLLYR
FHSCISKRDEKWINNFFLKLFPGRKAEDLQDVSWTELGQALLIFEQNTPKDPSVRTFDGLERQADGTFKDEDLVRILKDAMED
PAGTFGARMVPKALKVVEVLGIIQGRKWQCASLNEFREFFGLKRYDSFSEINSNPDIANILEKLYTDPDMVELYPGLMIEDIK
PQRNPGSGIMPTYSVGRAVLSDAVTLVRSDRFNTIDYTVSNLTAWGYNEVQQDYKTLGGSMLYKLIQRGVPNWFPFNSIAVMQ
PMYTKKANEQIAKEIGTFDQYTLDDPKAPPKVAVLTSGPAIKQILSNTKQYVVPWLKPLNTLFPGKKDFGWFMLAGDQPQNYT
HRANFSKAMSKIPNMHNAVHAFIEREGTKLINKETFTLKKGLDQIDIIRDVAIPLNTQLLADLFYFDLRTEENPDGKLGVAEL
YRSLLDIRIWGVNNNDPAQAWNRRRRAQEGAKRMIETTKTIVAEADAGRPRGIGLVSAVANRIGARSYLKKDSLRSCGLKLVE
ELLAQGNNVDQVTDNLWLTAFGGIGVPVTAFYEVLSFFLRPENEAIWAEVQAIAQKGDDATLHAYVAEAQRMTSSQRNVRVAT
APGEVQGQAIQPGTAVVLMLGEAGRNPKEVPDAGKFNPQRKKEDVSAFSYGQHECIAKDVALAFVTGLVKLVADLKELRPAPG
QMGTVKTIQVGTEKAYLNDSWSYLGFDASTWKVHFNGHGKGKFEGERVPTKSTPIQEYYYLLQKRKDEILGN
FUN_002003 CDS scaffold_12 379429 381288 +
hypothetical protein PF09994 ENOG41

MPHAENENTESTEPKKLILCFDGTGNTFSGSNADTNVVKILRKLDRNNKNQFHYYQTGIGTYDINETSVNKGIFGEFGSNISK
TIDQGFGTTFDAHVIAGYRFLMRYYETNAKIYVFGFSRGAYTAKFLARMVNKVGLLCKGNEEMVPFAYRLYQRYLQGEIKDSE
HAQCTSKHSSSEADAPTNCDTTQLLGTKPEDVSEETQDASNEIEAFSQTFCRKETNRAGEEENIKVFFLGIWDCVASVAVLER
KAKMPVPVTGTATHVRHAVAIDERRVKFKPALLAQDIRAAVHSHDDDEDIKEVWFPGNHGDVGGGWPAQPDEQEDEAEKPSFW
GRIKNAWLTRKAGKASQNLNKDRFQMSDIPLEWMIREVKKCGEMDKSLQAGVHWCHSLKPFERSMADPAKRREATHGFMHDCL
AFGYGTAFFKVLMWKFMEWLPFLTRWELDDTTDANVKLLGWKPVTFPPNRGSYRDIPRGAVLHQSLLDRLQEFSSYTPGNNHG
DKSSACFKGRNKVLVYNEKEIEGLSEDAKAQKLSEAHFKIHSKGNDLVWEDDKRHQTYDFNPHFKGALHPAIVQ
FUN_002004 CDS scaffold_12 381516 382633 -
hypothetical protein PF00172 ENOG41

MLSPATSSKTSTPGRARASCEPCRSRKVRCSGEYPVCSKCTASNRGCVYSLQKRAGRPKRPYVIARAPASPARRDDRGPSPAH
SNTARITTGTEERLSHHEPNTDIPPFLADFESFAPAQSHSDILTQTDINASCACLSILYLLLNRLGVRTELIVPDDLAILRNT
FERATDVLECSKCPMRFSSVLQNAGILGILCVCIAESYVRFIKTIGAKAIEATDKGEKLKVALNPLGQSHTADDTVLVPIEVS

PEQWKSLMYNVVKSEIFGIENHRDKSFVAFIERLEERQTRWHTLPTAPDCPASYQSACSSSNELPLCLTITKAARKVLDPIASTLE
FUN_002005 CDS scaffold_12 383056 383446 -
hypothetical protein ENOG41

MAAWDLKCFHDMIEEANESALRAEPPLKPSNSVAKRNDERYGSLRPRLGMQRPFLPMLNYDEGGTWDLNYYPSYDPALFKWKTIPKRTKEKPIGHMPKKPIGGVSIKRKSTATEEAQQMEKGKSKKRKR
FUN_002006 CDS scaffold_12 383652 384262 -
hypothetical protein PF13095 ENOG41

MPTLKPLPDCEGPKLERFTNDLTKHDFKFLEYLGSGCHSVVVKAEIDGKIYVIKLFFPVYVHEPNFELDPIDEDYFVEREEKERLTASEKIPQHVVDSLRVHATSFYNECRAYGRLKELGREHLAGKVHGYLRLYLRQIDEQAQAAIKNTIPEAKWPTIQVMEMMDDEVDLPIMAIVSPTTEVLQAI
FUN_002007 CDS scaffold_12 386274 387892 +
hypothetical protein PF07690 ENOG41

MTFSQQSPHEMWERVQLSDKKVDDQGDTASSGEEQGQSVTSIIWNADDPYAKNEGSSQDGERKDIQPQRGLSALDRVLSRTSVTDRDPGPPPDGGFWAWASAIAGHIVMLNSWGYNSSFGVFQTYYTTHLDNPASQISWIGSVQISALFFIGTLSGRLTDAGYFRITFAAGTLLTCLGVFMTSLSTTFWQLLLSQGLCTGLGNGLMFTPSLAVVSTYFKRRRALAFGITATGSATGGLIFPSMARQLLGKIGFAWTVRAMGLVQLVTLVMANLLLRPRIPPRQSGPWIDLASFKEKGYFFFAIGIFFTFWGTFFPFYYLASFAVSQLDNPLTYSAALNVLLVVNGVGIIGRSLPNAIAHHFGSITVLIPVVFISSICLFTWPAVSTEGGLYAWASIYGIVAGASMSLFPPALSDLTTDVHKTGVRMGMIFTTNSIATLTGPPIAGMIIRRSGGHYLGAQVFAGALLFLGGVFIFMAKRFRVVADQDRAT
FUN_002008 CDS scaffold_12 388706 389069 -
hypothetical protein ENOG41

MAIVSNNTPLTEGDAPPPPNVHDVEKPVQNSSGETKTDKSTETAIFHLKAAWTTETIDVSLKVGDAGIAFMIVLSFLVLVSLTQDSAGHTFMIASSLLALVSILVALGDSKESAGLRSGN
FUN_002009 tRNA scaffold_12 389682 389756 + tRNA-Val

FUN_002010 CDS scaffold_12 389990 390811 -
hypothetical protein ENOG41

MRNSLARSLDLIPMSVQTLLVDYQRERILDQTYNPPSIIPVESNREVLSEAVRRLAQRHGIREITLLASVDSAIFQPSTETSCGPTLESFTFALCDVLPSGEWVATGENPLSAEEEEYQRQEVREGMADGHYCPPGCEIENSFKSRMKEGIMDRFLLAAAQAVCHMPKIRKLSVRHVFDPTDFEEDISRAGFSFSTATGLMVLGKTDVPILSHEALVAWKTAMKTHDIVFDRCLTNELDVAGRYANEFGPEFQWDE
FUN_002011 CDS scaffold_12 392467 393977 -
hypothetical protein PF08240 ENOG41

MTTQAAYQAEKVIGHSDNAVTAQDVASYREPGAGESGETMKALAWISKNKVQMVDAPKPKIIEDRDVILKVTGSTVCGSDLHLLHGTVVQLSEGDILGHEFCGVVDQVGSAVKGIDVGKRYVASFQIACGDCFFCKQKLSSQCEKTNSNTTERAMYGGRTAGMFGYSHFTGGFAGGQAEYVRVPLGDVNLLEIPDDVPDEKALYLSDVLATSYNAVKDTAVYPDDSVAIFGAGPIGQMAGVFAIKEGASQIIFVDTEPRLSFIRDHWPAEHKDKLELVDYKHLSHGVTNKPTVVSRLKELTGNRGPDCAIECAAGEYAKGWMHWLEMAVGIETDTSEIINEMIEGVRNFGRCGVTGVYVGYTNHFNIGSLMERGIRLIGNGQAPVHKYWHELLEMIQKGELDPGQMLSHRVRLEDLDKVYYKFEKREDGMQKIFVETKFSFPQDPSTPSLTTY
FUN_002012 CDS scaffold_12 394382 396057 -
hypothetical protein PF00070;PF00355;PF07992;PF14759 ENOG41

MAQEYKLKGVSSLDLSPGSKQEVEVEGIENGKVLLVNTGGKTQALGSNCTHYGAPLAKGVLTSDGRITCPWHGACFNAKTGDI

EDAPALDHLPVFQVAERDGAVYLTGEESAIKSSKRQPKVKCAEFGSVQDDHVVVVGGGSGTLGVVESLRENGYKGGITVISNE
GYYPIDRTKLSKALMTDLSKLQWRNKDFYEGGSIKFVQGEVNNVDFSGRFVTTSEGEKIDYTKLVLATGGTPRRLPLEGFKNL
GNIFTLRNVHDTKKIVDQIGEKGKKIVVVGSSFIGIELAVATAGDNDVTVIGMENVPLERVLGEKVGSGLQKALEGKGVKFYM
GASVDKAEPSASDASKVGSVSLKDGTKLEADLVVLGVGVAPATQFLKNNSGIKLEDDGSIKTNDDYSVPGLKDVYAIGDIATF
PYHGPGGEGKHVRIEHWNVAQKAGRIAANHIVNPGGKTEHFIPIFWSALGAQLRYCGNTMASGWDDLVLEGSPAENKFVAYYC
KGETVVAMASMGRDPAMAQSAELMRVNKMPTKTELQEGISVV
FUN_002013 CDS scaffold_12 397509 399793 +
hypothetical protein ENOG41

MERPDFGPEPHISEMTCCCGRIDCALLKKNCSILETVEKDVHTAAQLGQALLARHEAYMADAERDRLDLSSRIERLEMAKQEL
EAENALKIEENRNLLDQLELLNSTVSESDTKIKALEASLLSSQQAVRRLESAALRAEDAERHISLLELEQDDLYHELRSTKED
ARTHAQRCKEAQRGIMDMQDQLERMEEEAREERQRHAEVIGRMERQRQVEKQLNTAAGRLKGAAASKTLQEPKPGNPVVNHFV
RDLLQDNANLQLGMAELREMLLNSNDEIQSLREQLVTHQPIADSSTTLKDELEAYEPPTTPRISQELHIHHHYHVTAKADKHK
MRRKRASLNSSIFSASTSSGTSTPRSSARWSLGPALPALASINTKEPNSVISMPKQRWSMLSEQPSEIASSVPSSPRSNLRNS
MFDSNFGDSDYPMSPATSFDPMSPTWRTHRKGPSDISAFSLQAPSLQLDPGTPPQNSRQFNDDVIHEEDENEDEEVMSRIETP
DLGNTPSIDESSIVESSEDNRDDYMPRPRIHRAISHESIMSLTGGLDIHTLKARPSQLTLRPLGGAEAVVTGVTAQPTLYRGA
AKRSTAALRDTFAGLPASRTVSNPLNRPGSNRSASPAPSDTPQGSPAGGIGKWVGWRPWASGNSENAPPKPAEKERDRHINRT
PGINQPGSIPGFHQYWAAQKRKGAPAQVTTVDVDREALVEGLQD
FUN_002014 CDS scaffold_12 400006 402060 -
hypothetical protein PF00172 ENOG41

MSEPPAKRRKVRKGTRSCWECRRRKIKCQFSKEDDPICIGCTQRETSCISQEYVPTPPSAPQDKRLAQRLGRLEELMEKLVEK
PLPSPESEDPALERFTQHTADVFESSVVDNAQQILDQPDAAIVTHKFPSRSAKATHVSAQLHALFPSQQILYAISQQSPGARF
VLAMFYSQQDQIDGKPEPLSSLAQIPPITSHPAILAKRLLQLAVCLQQMPPFFDGSTLGLSKSPRETMEEWIAVTGRLVTSDD
DFVGCLEGLECLILQSFYQSDAGHLRKAWLTGRRALNMAQLMGIDRKSAKSIKSVDPSFSNKHRPSPPVIWFRINCNDRYVSL
ILGLPIGSRDNSFAEDNALVSDAPSDKLGKVYAMMAGKIADRNELKGSEAYVLTQSIDMELEKAYKMMDDGLWWKLPDMAICC
GTADASGDGMSVIKLQIRHFSLLILLHLPYLLRDRGQRRYEYNRATCMQASRDVLARFIEYRNCFTTSVSGRHVDYSALVAAM
TLLLGYLGWRWPGYETEKEHDRDLAEKTQERMQEMGALNNDRLCIEAAETIRQLLPIVQRQSGTSGENVHLNVPFLGTVNINP
GPTPPASTASMIASNSTTPSGNVDMSQFPLSFNSPNLGSENCDLSTTCAFTPDVLDSNWPCFTADPEDWALQGVDTTYWTMLN
SSIA
FUN_002015 CDS scaffold_12 405195 406044 +
hypothetical protein ENOG41

MHENCSHPRLYRVLYCSVHILSHTVSGSTKACKPEHQGFVSQQLQTPGNWKIDKSSNFEPFLNRQKLSAKSGFFALDIEGYIS
RKPPVVEQAAAVSIDSINGEDGKILFNVNIKGPSVDQGSKQYPTAANQDFATNLLKFVTHDYWQYNKETLSGERVDLMRAVAI
IQDSGITCQDYVVVWHKNYADVRALRHLFSQTGIQDILPPDDHVIRLNYLFRHNLNIPKGMMCSLELLFSIVFPMHPLRFTHH
DALIDSRKTVLMALFAEKLCKGEYTTTIRGSGL
FUN_002016 CDS scaffold_12 408377 409826 +
hypothetical protein PF00724 ENOG41

MPVRYTSKDVRPEPLAQELHLYPSGRVAKNRFLKSPMAESLASWDPEIISKRGIPTDELIELYRRWGEGKNNFGIVVTGNIDI
NLNSVGAAASPGIPVDAPFEGERFEKFKQLAAAAKKDGSLFLAQVNHPGRQVPYKFNPVAISASDVQLDPKMGMTFGKPHAAT
KEEIAQIIEGFAHAAEYLEKAGFDGIELHAAHGYLLSQFLSRTTNKRTDEYGTQTVENRLRLISEIANAIKARVSSTFIVSAK
LNSVEFQDGGVTPDEARELCERLEALGFDFVELSGGTYERMALSWEKESTKQREGFFLEWAETITKALDPAHQMRIFIAGGLR
TVGAMVDALDVVDGVSIGRPAAAEPRLASDIIEGRVKGAIQPAEMVENDLGMGMGVAGAQFAQIAKGFEPLDASDNEVVQIFG
QDMGAWYEKLVQDGDKNEFVRAIQYSGPQVPYGKVKA
FUN_002017 CDS scaffold_12 410140 412339 +
hypothetical protein PF00172;PF04082 ENOG41

MSTPETDLTPSNRRKACDLCFTKKIKCDMLKPDCSNCILYNTSCRTSIIRRKPNSARVRAGAKQQEENNRQEGLETRLARIEE

QLQIVLNAAKDAQTARPQWPDDHSSPSDSAESVLNNSANFEEMAKRGFAWKFDPVNPAVYQGPQPNTLELPPLDQILPIVDHY
FTTFNGVIPLFQQSEFMKLLTTWYKQPETRDRASWAAIQTVMAIGYRTPQLSLRDSQSVHIEKADQCLRNAQTVVSELVTRDE
DLLGVQILLGIVMLFQNSRDPKPASVLIGTAVRLAHRLKLHSQEAAMQFPAAEAEQRSRVFWIAYTLDKDICLRAQTPSCQFD
EDIDISLPALAPPDSVGLIWTQNGQVHFNYHRRRVELAYIQGKVYDLLYSNRSSKVTAQERQRRVSRLQSMLDQWYERIPTVF
HIEHVTATVGPSQLVQMTKMHHAFLLTEVMIHGIYSHNAEWVKRISSFSRATITSVGNLENRGFDGAGKCQDQAPPLPEGWNH
CVDVSRGCMKLFQEATPTECLIWQCSCSHFSALIVLLANMILNPGHNSIHIDQHLSVKALDLFDKLLKIIDDEAFRALRSVVG
ELSQRAQTAVAVYREELKGLGKDVFEALNVDDVAVREVDFLPPGDMFEGLDGPFVGLDGPFIGLSGELEQGFGDGMNFMDGGM
LDMEFMR
FUN_002018	CDS	scaffold_12	412668	413592	-
hypothetical protein				ENOG41

MSSTANFPNAWATIGQSLTTPNPTLQTATGNSNIAMSQTTFQAASSIIKRWRDFDFGNLHEPYGDLLDNYNVPVVVDEQSSVP
ATNITDLKKALTGHIVHRLRQPIATGVALLASRCGEARRDVTVLTDEPLAHRRRSALSFVTSDGERLAVSCAYTSNQWTSREL
EDSESSQSYPLLRLATYCHDAGTRYGFIFSDHELVVARVSGTSFSCLIEWQSIPWEAAGPGVLTVNLSLFLMVMMALHNSHRP
ILRHLQPFPINFWYRFQNIDGDWVNRHHLSRREVFHHLPGAITELPNGVPFEDAHITE
FUN_002019	CDS	scaffold_12	415641	416191	+
hypothetical protein		PF13095		ENOG41

MPQLKPLPDCEGPKLECFIDDITTCDFKLLGPLGSGCHSQVWKAEINGKVYAIKMFFHLGAHEPSFLMDPIDDDFPDPDEPEP
LVASSKISQPTIDSLRLHATSFYNECRVFGRLKELGREDLAIKAYGYMDTCNLISATTRSSDISYHLETAREDIWPL
FUN_002020	CDS	scaffold_12	416232	416895	+
hypothetical protein				ENOG41

MQHDDLRIPMMAIVKEWVHSFEGTKWVQLIGGDWASRESVKRDIGHLPRLLRNLRELHKSGIVIRDLKWQQYLDGQLVDFSFA
WTIPHIFGPESGLRPRWTFESMAAWDLKCFQTILNEFDDRAEISVPRLRKHNLVAWQSDDMYERLRPRPQLYGPALPMLVYDG
NFQPMVHHPPFDPAKFNWRAVQKLTKKGATRRVTKTKAPQRRALREASKERRTK
FUN_002021	CDS	scaffold_12	417038	418008	-
hypothetical protein				ENOG41

MTLVPVTLESANAHNSCQFLFPTRRGDYLIQVSWPLCWNDRAPVNDDTIVSTVYIVDGNAYFFTATDIARRLEFTHQTRAVIV
AVGYPNKTCVFDKRRNGDLTPASSDGAYDIPPGPDGKPSLGPFGGASDFLDIIKKDIQPYIEKILFPNVPLGAGPKALFGHSY
GGLFTLNALFTKPESFDTFIAASPSIWFNNCSIVRDQEQRFNERDPSTGRAPRLLVMFGGAEQTLIQLPGESDEKFEKKQKGA
MLRKMKDNALALVARMEESPHLRNVWCWEFEGEDHGCAAPCALQRGLLRFLIDQRAG
FUN_002022	CDS	scaffold_12	420178	420789	+
hypothetical protein				ENOG41

MQLRQIFANRLMPVAFALPWVMGMLISWIPFTKISLYNAPDFLILAALISTLEVLSIVAVFIFTSLRPSTLETEPSPSTTLKE
KFFRAWFWFLFISPFITGTMVVLMASDISCDPEDDLDMHQPICQVGIRLIKATALCRAGIIATFLFATSSYLEQRPTKRCTDS
DSA
FUN_002023	CDS	scaffold_12	422307	423867	-
hypothetical protein				ENOG41

MSTPSLSALPEEIQVQILGYVYKYSLIDLRLVSHTFARLITPIIFRAIRVRAYRDEPQRFIHIAMTEHLRSHVREVTCDTWTG
SCPMNKLRELTDKFFQAVPFISMFPRLDTLNIRLEHDRSHIDHNTRSALLETLFHCLEGTWNMDDYVRRNTMSTPQPSTCECY
PQAQTGIPLPIKTLTISNLIDVSGIKVLPAFQAVMNSGALKDLRISVADGGDLDDLDDSEVITTTQKGSQDIHHDLLDIVLTP
SIASNLQVLSLFSSTPWGWFPNMDFRLIGANDLPNLKVLALGHFEFSQWWQVEWIGSLGIEKLYLDECTVLHSDHNRASIMPD
ACCTICQDEASELSDKGYFAQRVEGNAWREGSTYHTHTRWRHIFEHWANTMPNLRVFKIGQGWCLDDGAWKAIDPTYEGDFYE
YVEEDHEAIFQSFQDTSHQHFECPLPLTTGTGIRRYGSEAVQGYGAVFPYVEHIEGGGYTYTDSPDDGKEFLNVSLGEKEKDK
NAFDKFLTVVETRRKGLAGQE
FUN_002024	CDS	scaffold_12	424533	426051	-

hypothetical protein PF00296

MSESQEAPKVKKQLIINAFVESCSGHQSPGLWRHPDDKSWDFNNISHWVKLAQLLEKGKFHGIFIADVLGSYDVYKGPRNPDP
AIISGAQWPVNEPLATVPAMAAATKNIGFGVTVATTYEQPYHLARRLSTVDHLTGGRLGWNIVTGYLDSAARNLGHTEQPSHD
ERYAIAEEYVDVTYKLWQSSWRDDAVKLDREKGIYTDPSLVRLINHSGKYYTVPGPHICQPSPQRTPLILQAGTSKAGKNFAA
KHAEAIFVAGHSPVTVAKNIAEIRTLAKEQFGRDPKAIKFLAMFCPIIGKTQEEADAKYKEFVGYGSEDGALALFGGWTGIDL
AQYGDDEELRYVESNAIRSAVESWSKSIPGVPKWTKHTVANHIKVGGLGATVAGTPERIADEMERWVNEADVDGFNLAYALMP
ASFEEVISDLLPVLRERGLFWEDYAVDGGTYRENVYGRKGVARPPADHPAAKYHWTKDD
FUN_002025 CDS scaffold_12 427176 428917 -
hypothetical protein PF04082 ENOG41

MRLNSHGVGASYVGSVHWAAVLDSISELRDHYEEQEEARLLATNDHLFLHSPGPRLLYEPVQTTKADLLASVPARPVVDRMVA
RYFNTQGVVPSILHSGRFLQEYDKFWQDPPAMPCIWIGLLFSAMCVATLCQHSIDDPTDPEIQGRVRVFREQTLLSLILGQYT
RGGEYVLETLINYLICESFMSQEDETGLWLVQGIIVQLALSQGYHRDPHNFPSITPFSGEMRRRVWAVIVQLDLRLSSQMALP
CVLKAQQYDTAEPRNLLDTDFDEDTIDLPPSRPETEVTPVLYSLAKGRIDKMMGLVNDIVNDTKEHPYTEIVELDRKLQEAEA
SLPPIFKWQPLSQSFMVAPEIIMHRVLLQLAIQRLTIWLHRKYLAPSYTHTCYQHSRKACVQAAIKVLEFQQIVLEETQGDGL
LYPTGWARWMLLSSRPRAVFLLGVSILCYYVQLTKSRPDVSLDESTGSKIHGLLRNAYPLWLHSTTVSREARRAIDYLSLRLG
LQEQEIGVSLADSTTDTPQDTDMSLDQLTWDAYEGKLPK
FUN_002026 CDS scaffold_12 430132 431850 +
hypothetical protein PF07690 ENOG41

MAKGQAESDKTVCNHEATTTVSFDATAQGHIGHDKEPNTDKQEETISEDIHAANMKTDAEKDAMEAGEVAPTPDPNIVDWSGP
DDPENPRNWSKAYKLTNIIVISLSVLYTNLATTMFAPGAATMQREFGFKSNTVEVLTITMASLGFALGQLFIPPMSEVFGRMP
IYRTSSILYMGFTAGCARSTDVAEFLVFRLLTGMAAASYMSTGGGTVADLLPKEERGVAMAIFTAGPLFGPVLGPIVGGFVVE
NIGWRWCFYLILMLAGAVTLSTFLYMHETSSINILKSKAARLRKETGNPNLRASGDKQTPIKQLVLHALTRPMKFLFTSPIVA
LISLYIAFNFGVTMLLFATFPTVYENTYQWSVSVSGLAYIGVGIGCAIGVITFAKLSDRLLNAEGGNYHAERRLIMIMFVSPM
FPIGLFIYGWTTEYKVHWVVPIIGTAICGPGAVIINSSSQTYIIDIFGPQAAASALAAITLLRNLTGAFLPLAAPTLYANLGI
GWGNSVLAFITVGFIPVPIYFYLYGASLRKRFPVEI
FUN_002027 CDS scaffold_12 432569 433999 +
hypothetical protein PF02771;PF08028 ENOG41

MATTFESSDPAVYKEYEAQWSTLPTDAEGWIKRAKDVAEVLAKDAPARERANKSPKAEVALLKHSGLLKILGPAKYGGGEQPW
SVGFRAIREVAKKDGSVGMLLGYHLVWSTTANVVGTPEQADRTHKLIISNNYFVGGAVNPRDNDLKITSNGDKLIFNGFKNFS
TGGVISDLTVLEGVYGEKEEHIFAIVPTQQPGIQFKHNWDNVGLRLTESGGVNIENVEAPWGDALGWDVEAKKPDPSILAIPF
TSLFLPTIQLNFANLYLGIAAGALEFAKDYTLKNTRAWPFGGDNKEKATDEFYILSTYGNFFAHLRATEALAEKVNAEADSLY
AKYSQDRSAVTTEQRGEFAEWVASLKVVTTDTGLKITSGVFEVTGSRSTAAKVGLDRFWRDLRTHTLHDPVAYKNRELGRYLL
LGEYPEPTWYT
FUN_002028 CDS scaffold_12 434298 436244 -
hypothetical protein PF03098 ENOG41

MAGRFQSPRSSIRTPREEPSRHLPNAEFQGTAADCPIKNSKFIASRNSDGDFNDLAQPKMGCAGMRFGRNVPRKYTTPPTQQE
LLTPNPRIISEKILARPEGQFKPAEIVNLLAAAWIQFQVHDWAQHFLVTNGDKDVEIPLDKADRWSERIMKIPRTKKDDALSQ
QDIETPAYTNECTHWWDASQIYGSTEAETKALRAQCDKSYPGQLHVTREDGVQFLPRSDDGIPKTGFRQNWWLGLELLHTLFA
LEHNAIATQLHLSNPSWSSDQIFDTARLINCALMAKIHTVEWTPGILQHPALQIGMNANWWGLLGDKLWHVFGRVFDNKSEVI
SGIPGSGVDHDNVPYCLTEEFVSVYRLHPLIQDNVAFFSIKDGQHKGTLPIKEVAFESARKPFDEDKSGLGLSFADVFYSFGV
NYPGAIRAHNMPNFLRDLNIPGDKDFPHGRHLDLGTIDILRDRERGVPRYNAFRRLFHMAPAKSFLDLTGGDAKLAAELEDVY
DGDLEAVDLLVGTLSEPLPKGFGFSDTAFRVFILMATRRIKSDRFLAGDGWCPEVYTREGINWVQNNTMKEVLCRHFPELAAT
LHNVKNAFAPWTKIGQTEAYAGPETNKAKN
FUN_002029 CDS scaffold_12 439719 440316 -
hypothetical protein ENOG41

MRFSTVIATALAAFSNASPCLAGAPEPVICGGNGYVGATNTPWYKKSQAEGTIEGCFNSCTGGCKAISFDTQYKTCYFYTAEV
SKMRLYQVTNSPMTFHYFDRACVADAVGKTCGATGYVGATNTPWYKKSQAEGNMKNCLDACRGGCKAVSFDVKYKTCYFYTAE
VSKMQLYTVSSPMSFYYFDRACGHEVTETANV
FUN_002030 CDS scaffold_12 442953 444996 +
hypothetical protein ENOG41

MDPLSITSASVALAAAVYKCTIEVKKIVGTIADAADSLSDLAEEAQLIQGALQGVEDALQDNQEAISRYKVEEVFSIAVKGCR
ATLACIKQEFELLFNRSDWKARFMVLWKEDDMKKLLGRLDRKRESILLLLQLLNLSVREVQALVAKKQSALTVAKEDITALIP
TYWSCRDTILDSLDKETVDSIYADWENRESKLSTTEFDFDYELINTRAYRRALAQAQAKRGRELPPRRRIVTPAREPNGGIVE
PVEDLIDLTSEPSQQIASMPSHIPPKSYSDLAGLQFMPITADEGSASAGSLETSETAEPINMRGMTDHLRESDMSPESRDKSP
QNRMPGIRRWHQTTTKEAVTEIRQDETQHEEALTGRLAQTMYRNSNSLSAGLSIARHLPSSKSHDSLDIEYFDALKGHGGPSG
KRRPGVRVRITPSKRKGVDTKHHHDITETSPNSRIPSDELLDGSPMGSSKAPNIGDSILQKSPVDIEIDRGHRQAAKILDELR
RVRIRPAKNVGDSKYEPYDSGYIPDDDMRPRSRNIARRSRPKAKQSHRSKSTDFTETNAGSPPNINNPRLLKVVEDAIQRLIL
PELNALKRETEQSERGFTEADGSPLHVTSALADGLEGNEKEQDNKKKKKKKAGSSSRRLNRIRERDRDRFNREEKRRNRS
FUN_002031 CDS scaffold_12 447714 452414 + ZRA1_1
ZEB2-regulated ABC transporter 1 PF00005;PF01061;PF06422;PF14510

MASTYMGGASVQPAPLPGLENVTTNNQTTTSTHDTQAPVNPSSDTTPTNYSQDHMASKDETPARSTTEDEEDSELERRHSIVR
DLARQYTNQSQMSAISGNPFTADENSPLNPRSDKFRALAWAKAISKLHSETGFTNRKAGVCYQNLNVFGYGQPTDYQKNVANI
WLDAASLPRQLMGYGKTRIDIIRDFDGVVKNGEMLVVLGPPGSGCSTYLKTISGETSGIYINDDAYFNYRGITAHEMHTRHRG
EAIYTAEVDVHFPHLTVGDTLDFAAHARAPRSIPGGIDRETFISHSRDVVMAMFGISHTVNTKVGNEYVRGVSGGERKRVTIA
EASLSGAPLQCWDNSTRGLDSANAVEFCKTLRLQTQMSDTAALVSIYQAPQAAYDIFDKVVVLYEGRQIFFGGTKMAKQYFVD
LGFECPARQTVPDFLTSMTSPQERIIRKGFEDRAPRTPDEFAAAWKASQANALLQREIEEYKRDFPINGPEAEEFRANRRAAQ
AKNQRKKSPYTLSYWQQTKLCVWRGFKRLVTDPTITLTQLFGNFIMALIVGSVFYNIDQTTASFFQRGALLFLACLSNAFSSA
LEILTLYAQRPIVEKHDRYALYHPSSEAVASMLCDMPYKILNAFTYNLTLYFMTNLRREPGPFFFFLFITFLVTLCMSMIFRT
IASASRTLSQAMVPAALLILALVTFTGFVIQIDYMLGWCRWINYVNPLAYAFESLMVNEFHNRKFECNLFIPTYPDAAPENRV
CSTVGAVQGEPMVSGDRYINLSFSYYHAHKWRNVGIVIAFTLLFLATYMFFAETVSAKKSKGEVLVFRRGHRLAKNRVDAESS
PAGRVAITEKEGYGEGQPSNFKSTSVFHWNNVCYDIKIKSENRRILDNVAGWVKPGTMTALMGVSGAGKTTLLDCLADRTSMG
VIHGDILVDDKLRDASFQRKTGYVQQQDLHLSTTTVREALNFSAIMRQPKHIPVKQKIAYVDEVIKMLDMQDYSEAVVGVLGE
GLNVEQRKRLTIGVELAAKPPVLLFVDEPTSGLDSQTSWAILDLLEKLARSGQAILCTIHQPSAMLFQRFDRLLFLASGGKTV
YFGDVGDHSRTMTQYFEAHGAEPCPPEANPAEWMLEVIGAAPGSETNLDWPQIWKDSSEFAAVQQHLQELSHHRVEKSQDEDP
HLYDEFAATFTTQLKYVTTRVFEQYWRTPSYIYSKAALCTLVALFIGFSFYQAPNTALGLQNQMFAIFQVLTVFGQLTQQQMP
HFVIQRDLYEVRERPSKTYSWQVFILSQIIVEIPWNTLMAAIMYFCWYYPIGLYRNAEPTDAVAERGALMFLLFLVFMLFTCT
FTDFIIAGCSSAETGGNIANLLFMMCLIFCGVLAPPNSFPKFWIWMYRVSPFTYLISAILSTAVANTEAVCAQNEYVVFPPPE
GQTCGEYMQAYIKSAGGYLVDKSSNSTSRVQSHYDERWRNFGLMWVYIIFNVFAALALYWLVRVPKNKKGKKEKKE
FUN_002032 CDS scaffold_12 452616 453735 -
hypothetical protein PF01636;PF02958 ENOG41

MFNSLVTGSQAPRGNFDSEKPLPLTPDEVTSEWLSEALGVAVKDFKISSIIHGTSSKVFVDLIFGDGVETEIPSRVVLKGGFN
PVIRETVPQMLPTYRREAEFYYHVAPQTTMLLPKIWFAGTDTVNGQGIFIMSDVSSEATFGTPLEPWAPERVGEALKQLASLH
AKTWNTKEEEYPWLFGKDGSKLENPVRNIILALLEPAPWGARFDEKVRPPVAKELLDPDRIRKAFQALWKHADADQKYYSLIH
GDDHVGNTLIANDGTPGFIDWQGLQYGPSVLDLAYFLTGALTVEDRRKHEASLVDTYLEALHKEGGPKLTREDLWDDYRRYSM
QGFLWAMTPQTMQPDEVVFAMAERYSACIVDHGTLELLGV
FUN_002033 CDS scaffold_12 454666 455873 +
hypothetical protein ENOG41

MATLDPGVNIYNTSVVPPPIGQESNFDRPLSDVQLATITVFAVTYFLATVSLALRYATSALVVKEWELDVVLITLAWGTALGY
FISVCFMMKYGWGSHAWDVSLADMVQYNKYLSPTTLTYMWSPTLTKLSILSVLYRISPARGYQISVYVIAIALLIYTITFTVL
LSGPCNPKDVGSGVCLNNLAISQVVLNIATDLSIIILPLPTLHNLQIPFRQKVVVGCILSIGSAVLIASIVRAPYVHIMATNP

DFTRKQAEAGVWSLVELNMGIVCNNLMRLKPFLRTYLPRVLTFLGLTAKSKPQGENPSKSSGNWRGRPSHSYQLNSLEHEDPH
HKSFRKEPSVDEAAFGDLDKEPSRHNGSTDDILRS
FUN_002034 CDS scaffold_12 458913 459897 +
hypothetical protein ENOG41

MYNPSGTQRLEATVPRDIVYALLGMASDRAELGIDPDYTCTYAQFCTNTTKALIVQDINMLLRLNCQGPNTLSHDLPSWAIDW
AAPFQRTLIPSDPKYEAAVGTQARVRFIDDETGPVLVISAINVGTVLVNEHAIDSKTCLVKPKGWPGRGRIRPTTRSLTVILQ
VEKAARLTMVIKSAYATCQERREAVWATATTGLRMVQGTGPRRATAEESHAYSSAGLKSDMRRLSSFRGRSDFSHTSRISAAK
YATEQKFFFVTMRGRLGLGTEDIKAGDLVCVALGLHMPIILRSTGNGCYRLVGNAYVHGIMNSEALGKGGAATEMCII
FUN_002035 CDS scaffold_12 461394 462669 +
hypothetical protein PF06985 ENOG41

MPYPELLKGFSDHILQLNDRIDRLSRILSNARRQFTSSSSDDPGFQNQQLQDTSQRCHRYAEGDAKFVSIVQALWADCAKKVD
GSAHFAAQDSNSEWKMIHRLMIQALDLSKLLIDIWEIGIWNLFEPVYTSGTQLRERLFSESMKETKEVRLQNAWQMEFRKSNY
NVFKSPISGPKAQREIQSVESRFRSPTQQLYTPLDPAADEIRLIFVSVGTSGQLFAKMKHVSSGTGLPYIALSYVWGGNHKPD
LLLVNGVSVNITESLDAALRSQVILDYSSKGVPVWVDAICIDQENIQERNTEVLRMRKIYAQSLETICWLGKSAGSSNQAFDF
LATLATNSDSKPTEQQRIGLCQRSFIVEPSQGDYGPEE
FUN_002036 CDS scaffold_12 464135 465028 -
hypothetical protein PF06912 ENOG41

MPDSGSDNPEAGPKGESKSPVASRFTTEIDTRWGDILLLICFFVAGLVDSAAFNMYGCFVSMQTGNTIFVGLGVSHQPENLPG
KAWSRCFLAIVCFAFGALFFSSFHRFFGPQKRWVLITSFLIQALLTGLVAILATTGAVYNHPHGRETVHEDGWIIERVQDSFP
ATDYAAIIILAFQSAGQIVASRVLKYNAMPTIVLTSLYCDMMSDAKLFTAPINDNADRNRRATGAVLLFAGAVSGGFLSKSWV
GFAGALWIAAFLKLCITFAWVLWKPKSSNK
FUN_002037 CDS scaffold_12 466224 467037 +
hypothetical protein EOG09262SWJ PF05891

MSNPANSQPDSLIQAEKCKEYWESVDSDNNGMLGGVLSVMPSVSRIDLQGSRTFLARLGIGIKSGRQRIPRVLEGGAGIGRIT
EGLLLKLADQVDVVEPVVKFTDTLRGKPGVGEIHNVGLEKWEPSEGAVYDLIWIQWCIGHLNDEELVQFLERCKSVLEKEHGL
IVFKENLSTWGQDKFDELDGSVTREDEKFQQLFKKAGLKLLKSDMQRGFPVVKNRQLLPLKMYALRPEQ
FUN_002531 CDS scaffold_13 564 1407 +
hypothetical protein PF13883 ENOG41

MKILHAIAGFAATAQAFTPASNIFGDNDNADTNAPHKIPTVHESVVMARRILALTKLATLSTVFPSGRSNIEPETDSNGLEGV
PIGMMDYVADCEDEGNPTILEIKIATTFRNVRAGSNLTLSMNWVPPYPPAKRISLLSRLSAYIPFLSSYDYNSRSEESLSVPD
TVPYSAANLPRFALFGYLEPIETTPVSALKLAACFTRKHQDAKYWLPGNVIHESGWARLVVTKIYWVGGFGDRARIGWLPVDE
WKSVTRDEWESIKLPGEERGWSEWSVNAAEL
FUN_002532 CDS scaffold_13 5285 9948 +
hypothetical protein EOG092605ZA PF09497

MGAHPRPPQRSVSSSSLPVQRPPPQRSLSHQQQFMAAASPVRKDAPFIDLTADAGDATPNRYHTTPRRGGSRLRLELSHNASS
SSLPTSESPQSLTPSRIPNSDPFQAMVGSPAGSASSTLMPMPTRRPVTSQPRFAPRITATAPAPAKKDARPKPYTVEIPSVAP
RYFSANRPETAVRNPVDPFSKGLNSGYADFFPWNGAHHEDEWSSEAITKGTWDKVNLNVPETSSAKLAIFPALKQKTGLNALS
TIFMGVLTQRRHRGQINAPSTFKPPPRVTLTDTKREVWLKDLANPAISLRRLSRTIPHGIRGRTLLDQCLNKNVPAERAVWLA
KCVGANEIRGLKRKGVNGAFVMGGELKWARDWTVFVEQFVDAVVAGFGENDWKNRVTYAIRLATNLYSEQLLDRDHYLDWVVS
GIENSLQSRIPMWLLIAQIYWKDLLRSRKYGRRLVFALLSHLHVISNDPDQDLLVQLSSRLSALLGSLIRSNPESFINPGAWS
TYKDTLHACLRSDDEQARKALHAINSRNSRLVVSSTASPPAGRSQLVKLLDAALKESSDNHLAAACWATSENKSLIMKTVVEW
ATSFHRPGLAKVYAATRLIRQWSQFRVNPTTPILETLDNIAANDKARKNLVYHLVTELVRTGHFSVSQYMQWTIARGGYHCAA
EIDPVSGPCSSRLLVELPLHVLSEKKRAERGNLLRRAGNYSVAEEEQDITNAIRVVKHVVGLPLLSNDPLSGRKPMSLKKLLQ
RMGGSSTALRSSIGAHLRDELTGQFIMKGHSLSLTMFTAVRDIMEMAEDYAMLSDILGACAKTADSDVLASVADTVHSNLQIF

CALDSANELFNSLIERLRMMNEEQRLVPRPLLAALSSLAQRMAGHEVIASQLRQELVQNDRNNAIDACSPVSDIVATPSHQAE
NNVAEEVERTLSSGTRLDPPTMNKLFRMIIPALERGWEKEDDTRRVFATLLARIRVFDAHHFDKLMTDWTSHIRSISNRSSLS
TLFPLLIITGCLTMPIIMSTASPPFANIQNLAPESSRGPATYLQELLQLVIMPLPQATGLVAEESYRFHTEQKCAKFEQSKGL
LNLVRNALLEYSTVRNHVNGTEFPLDNLECQESLLETLRTLVLVDSSAVSNALGIKSLPAEAVGLVRKVTTKLLIPGDSGETQ
ISFDHILQIANELTLPFCQLKLNLDLSLPQPSVIEGQDQSSSRFEIFARAMDRAIEAGNIMWTSLLPCLSDDITQHLKTQAQT
GFLDLIPSSKAPEFVDTGSRQSLRMAENLLEVVEAIISGQAPPKMAQLSLGMVEKLTDLWEIVAAGPQERPNCHAAVLQHWLP
AMLRFVNLHSLSSEPPSAPLPTASATRPPIPPVHDVRARIVLVLCGLLLELETLPPATVGSLVQQVFDIAILLVDALPEELRA
NCARATLLTPGGMPNQGTSSDPRLYYLFSSPPPSPSDNLMLSHREKAATPQSAAARGMGAQYGIGPVVQERLSPFILRRWEVL
SEPTPNVGENDTSLSLGLFEAIKLQ
FUN_002533 CDS scaffold_13 11254 13927 -
hypothetical protein EOG092620CR PF00611;PF00620

MNEYHPPSANEAFSPSQERDRSGSMSLGNIPVSPQGPPSDHGGAAVDDAMNKDQKSDADTTSPTQTSGPSGVNSEQIKQVSDV
LSSEIGIVTLLNRLKQSIASAKEFALFLKKRSSLEEDHANGIRKLTRTTQEVMRRPDHRQGTFGNAYEAMASTHERMAENGIE
FAKELHQMHDELMELAGVAERSRKSWKQNGLSAENKVAEMEQTMRKSKAKYDSLAEDYERVKTGESRQSGKMFGLKGHKSAQQ
HEGELLKKVQAADSTYHGHVQALQAEKAQLVSTTRPEAIRALQDSIKEIDAGISLQMQKFASFNERLLLSNGLSVSPIQGPGA
QGSSAQRSLRQAASSIDNDKDLNDYVTAQHRSVPPNTGEIKYERNPALNPPGSSSQHNPGGPVQQPAAVVQSPVSQTQPQGGF
SGPQPLGPGSRTSTLGLGPITGITGGDPSFPPTDDPRPFSQHHNRSVSQGNMPVQQGTPGQQFSGRGPNQQQAPGQRYGNGGS
ISSSGPPQLGALPFQPGGSPPQQPVPGQAPHERSGSGVNSGQGPPGAARSPPPYGASSHPPPGPGPSGPAKPMFGLPLSRLYE
RDGLAVPMVVYQCIQAVDMYGLNVEGIYRQSGSMAHIQRLKNMFDTESSNPALDFRNPENFYHDVNSVTGLLKQFFRDLPDPL
LTLEYHDSFITAAKQEDDTVRRDSIHAIINSLPDPNYATLRALTLHLWRVMDNSHNNRMNCHNLAVIFGPTLMGTDPSTAIAD
AGWQIKAIDTILQNTLQIFDED
FUN_002534 CDS scaffold_13 16794 18968 -
hypothetical protein PF01485 ENOG41

MIAAALRQSPIIAVDMANSTTTTPLTTTSVSTGVNNGADTNTGATAPELSDLDYFITVLQLPDGRTENQIEDDLVSKANALGI
SSARVADKRITSSVESASTVYNARTFSMLSAGSTSTALTTHSSIFGPSTPDLALSSARQSKDLSFSQYDRYLSVIDPHHNHSK
TTRDSQTPDATAQSILSGKTKRSLFSVRSGFRLRWKKKASPQPLQVILTCASCRADFKSSKSLHSISCGHTYCDNCLRTLIHT
AMSDESSMPPRCCAQPLPGFVMRDLLSRDAQQEFLKAIIQYSTPWQARIFCSNPSCGGFIPPHQKLDPKYPSTVTCRKCNTRV
CLMCKHNAHPTGKDCPEDWELDQVIKEGGKPGWKRCYKCQNLVPLEEASTHMTCRCKAQFCFTCGGVWDANAGCPNDCDGEEE
MDRRRTEEQAQLAEYEDEKAAQEAAAAAASAERLEAEERTRNNADFSNLAEMHRQELGRFLEFAEQGREHMRARFVEQKKSMM
KRHAELKETMKEKHVRTVGQLEDRQVAAEMDLRNTLEASERSVRIRLKHMEAYCDGLGRNSANSSPGSNSQPHRVVTERDLRE
LGQQYNIRDGMERSHQAKINVMRDRQAKRMEELIDRQEAEYETLLERNRQELVELDAQAVLEEETLAHIFTARKSKLVRRWEL
AIEILRKELEDQDGVKYAPIPTPTWPEQTSQVLLSSE
FUN_002535 CDS scaffold_13 26683 27758 +
hypothetical protein PF09816 ENOG41

MAAAGLIDPTKTGNYPVILGDALLGKTSNEIFTGIKYNHKPTLSSDSAPNLARIKPSIPGKTASYDLTYTDNDAKYAFTGTRN
KDSGQYVLYFDPSREAFILDLVDSTFNMNVTRLPGNADADSLRRQYPHIDSASNGASKTNIKAEKSASDKSNAKARAKSTTQK
RDVKRKPEKKQAPKNIELSLPVPQSNEQSKPAEPKKRTPAPEEEDEDEDDDDGGLLVEYPGADTNTARRTDFSPAFPSVRRFD
EFMDQRESEGDDADGEDDDDPDFEFKLPSPVNNRSQYETGPDPMDVDGEEEGEEGPEVDLAKELEDAFENLENSQQESPDGDE
SEISEED
FUN_002536 CDS scaffold_13 28022 28552 -
hypothetical protein PF04828 ENOG41

MASEEYPTSVSGGCLCGSVRYTLTFPSGHDWKRSCSTCQCSQCRKNGGSLIAWLHSVPASSVEFTSQTTLSRYHASPEAARGF
CSNCGSWLFWRNEKSEKISMSVGTFDKDDLKTWGRQLSYSEVHLWSEDAIEGITDHLPGEKWKYDDQGEGAERIA
FUN_002537 CDS scaffold_13 28915 31636 -
hypothetical protein EOG09260TPT PF06978;PF08170

MAPGSSTGAQPGPDSRKRKADTPASNGRREKRSVVHSARSIPAQPAEAALKDGELDLQAFVAAHEFEIRALEQSMATSKAVAS
SRAFQKVPRGLRRRTASHNPKRVPRRLRKRALKEMKDDNTPVVEARRRKPRTTRAKIRSETARKLGILAARKRKASLAMAKAK
QDGDASKDKALAVIGRPPRPKLRRNELNEPPKPKSKFRKRQLNKTWLPTHSWHAKRARMTDPLEPLWRFAIPLTPNEKIYRPT
HRAQGDRGAVVWEMSYMSTIGLYGKHAGIERVLKRIGVVQDSCWNDKGKKWRIGTRSWTGVLSREIQSGQQNQVDRKMIGPCT
ILWNPEPSAQEDSQELKQESRQVFLRIHPSAFLELFNDLLSLIKQENPRLYIEDLRFEIGSLDLTGPGSTEALLGILRTYPVK
DKTKTNHTEIFQGLAGLTNAATLPAGAVLGFSVQDPRLQYPPRRLEVPEDPESQLQLLEKIASWPAEENLNPYAIFDRDTRHA
ASCFPSQKQIDRRRAKNAPGTSLKLTADDPPIPVILLASRTDRGTQKQGTWTLLAPWKCIQHLWYSLVHYPLSSGGNPRFAGL
NEIRQVAFERGQTWFPGDFPGTKAGAAWELEERRKRRAAWEKRPKSKRTAWESLDLGGGRKGEIGDGFASDFELLFNAGDSQT
SEENNTEGAMDVSQDENSASTQVPKQQEPRIYQLRQISKDVFNQAVTSPSTPSTIPINALLGVRISLISRGVVTPCARIYRLP
SRPTTDPQSSEAEVPATIPPGSLSTPSSLPYDLRSQWLSQARPSLSTKNKACRNSTPRDLESLKRQLAAELVSKPAPYPFTPA
NKSDMNGHPLVPNVEDLVGFVTTGSFSLTEGHGMAIGSIAIDKVLDDVKENAKEGRYCIVRNAGESVGWLAKWEAV
FUN_002538 CDS scaffold_13 34349 35791 +
hypothetical protein PF10358 ENOG41

MGFLKPEVGKARKPKFDLHLKIYDLNNVPLVSGQAFVKWHLSHSMTAEHRGRTPKCPIANHRVEWGFVTAVPSIRISIDRNNN
LTECPMEFEVIQEFGLTEKVTLGIVRLNLSEYVEESEAFVKDVASPGRMRSNSMGVSPTRGGTSRPRRDSDVVEDGIVRRYLM
QDSKVNSTLKISILMIQVDGERSYVAPPLKTAPVFGGIGGIMSEAIEDDVGPIAAAVPNLSKPRDAAELQDLYRSVLAASWSR
QPNEHSAEEVIEDIFNGGNGWKTKPHNASPVTDGEDDDDDMGPRDTIRARDTRRITHNLLHPHHHHHHHSNRTASPSHNHQNN
GPAGSSHRRTPSNSSDKSFSTVTVTPSNRRKGVRIHEHHLDHARSMASMTSTMTLDSDPMREVSYKGTREVREDDMRNDLIAW
RLPGDGNQVM
FUN_002539 CDS scaffold_13 36082 37923 -
hypothetical protein PF00012;PF06723 ENOG41

MSDNAGAKGPEPGDRVAIGITFGNSNSSIAYTVDDKAEVIANEDGDRQIPTVLSYVDGDEYYGGQAKNFLVRNPDNTIANFRD
FLGQEFKSIDPTHSHASAHPKDVSGTVAFSIKDKNEEETSTVSVSEATTRYLRRLVGSASDYLGKKVTSAVVTVPTNFTEKQR
EALIKAANDADIEILQLISDPVAAVLAYDARPEAKIEDKIIVVADLGGTRSDVAVIASRGGLYTILATAHDYEFAGVHLDNAL
MDHFAKEFQKKHNIDPRNNTRSLAKLRLESEATKRALSLGSNAQFSVESLADGFDFSVTINRIRYEMVARKVFEGFNRLIEGV
VKKAGLDVLDIDEVILSGGTAHTPRIANNLRGIFPETTEIQAPATGVSAINPSELLARGAALQASLIQEYEAADIEQSTHPAV
TTVKHISNSIGVITVGADGEDVFTPIIAPETAAPARRTIHIPAPKEGGDVLIKIVEGNTHIKVTKPEPKAKEENGDKDEDDSD
FDDSDEEEEETREKIWKIGNPLAEAAIKGVKAGGKVEVTINVLADLGVTVTAREVGGKGGVRGNI
FUN_002540 CDS scaffold_13 38274 39065 +
hypothetical protein EOG09264XJC PF15985;PF18311

MASDAVFVLPGDIIDPSLIPSHPKKPLRLGPGLRHVPPNDIVPTLAGQLVTDRQKNAIRVENARGKYTPRVGELVIGTVQRSA
ADLFYVTLSDYTAPALLPQLSFEGATKKTRPQLTAGALVYARIALANRHMDPEIECVSSSTGKSEGLGPLTGGMLYTVSLGMA
RRLMMPKSVQEGRVVVLEELGNAGLQFETATGRNGKFWVDSENVKTVIAVGRAVLETDEKRLDVEEQKKLVRKIIRDLS
FUN_002541 CDS scaffold_13 39474 41421 -
hypothetical protein PF00096 ENOG41

MDTLRCLSGRKSHAMGNLETRGVVLKRQLSDGGIAGVVVGVIVGIALVVLCSYPFIIRRIRRRKQTSLHGPPDTNDPETGEVP
ATGAPGIDDNQRRLSSQDSFKPSGENTRGGVDGSVKDLDWTAHDGFLQQTNGQGQPDYVPRIDTNLPSYGFGNAQPTANDSIP
RSAPFGSDENHHEEYMPQSIGDAPRAVLNGTSADYYSPSVPSEAFGMFSTEEPRPQPERSWSRGSSLRYNLKQMFSRKSTRDQ
SLNSPTSQSLAEYLESVPRAQDGSLQQITTAGRPTESPTDMTAPATDSLPVPSSTQALGSPIALPSTLPKGAVQSPPDSPPTT
FNFNASQSPPSHPAPGTVNPMDIMPASTETEVWHRNDYQLYVSQSSPNQPPPSAGPATHEGPVDSPSPLTLPPSDPHPQPVPI
VHSPTPTQSQIAFKTEETDDNQDISMSEIPTTNHLSPLPDSSVRHPSYPSDASTPLPGPAFTNPSSLSTPATQLDTPSPHSGT
SSDYRHSVSPGNAVSNPSPSNGSHACDEPGCNQVFDQPHKLKHHQRYHTKDHKCPYPNCGKGFGTKTHLQRHVNDRHERKKKF
HCAVQGCDYSRQGGKGFPRKDNWKRHMTKIHNMDQRHLPEPVEVDQEMGGT
FUN_002542 CDS scaffold_13 43606 46039 -
hypothetical protein PF13086;PF13087;PF13538

MSAPVGRSVHTSGNKPLEPLYALSVSEYVNRHENGTIIEQRNEETILKRFNDLRTSFKAWPIAQIDCLANPFTTTWLLLVPVP
KQSEDLIFPALTDNFRIEFEERIKLSNRTYSLVNLSATRVKNPYEDMESTVGPEIVKCAAFNVQVPRAVKNEEGDQEILELMN
HMETASTLDDFESLTLDQTSQKNIFITWDTFSNTYEAELAAMHRLTGDCQLESRQISQKCKAAFEMILNFEGSKKTIVNLHSI
FPHLRNPRHRDYRVRYSLLRRFRSFNKDHMAAFDGLKAIPNGLYFVNGCPGAGKTEWNMVVSALIQSVRRPGAKKRYNPILFL
VDLNKTVDDAADRYWNLAKECGLHLRIIRMHGWPYEMKNSERLGKNQAQDCDQQTDFTRKFLTTASIEKTLNAGRNPNKAPTL
DEAAWDYYEKHKHGGFVALKKVLSRMEANDALDHDEWKCLRNEVTKLYTAVLKQADFIATTPVAAYGGFSKLFKPDIIFVDEA
PHARELTTLIPIAFFEPFAWIFTGDVNQTRPFVKSGDARDALKKGLEVNPYSEQLRLSLMARANRLGAINSSLLINKRAHGNL
HKLPSDLFYGGKMSSDYPSSMQFPETVSYLRRHLEAFGSGRRLNANRAIIALSASKEESHCHSFWNPVNHRWVLAEVKKLLQD
PSFRTITDSQQPGRVMIQTPYSVSMRQYAYVVKGWPIEWQERTEVLTVDKAQGNQADVVFLDMVRTTKPGFMDEAQRLNVAIT
RARQAEIVLMHPAMTFRLRQGKRVPTDYTSKVWEDAVANDRLFVV
FUN_002543 CDS scaffold_13 46753 48694 -
hypothetical protein PF00145

MPIERIDLEAQSETDDVLFLSEESSGTFERRRRLSSCTIGDEDTSSHSNTSARLLEQGELELKAMRLLNTTIQAGSFLGVRQF
FSGKYLVEFILVKILVRCRSTDAIKIRGTPFLRNRSALAKLPKKPNEVCMLICRDSDTNNEEVTDISPSQVVQVHPLLITNTK
YPNFNRKLEVCYAGQGSRRKQVEESLVRVDSSEVSRREYRISNEILRNRWRGTTAKGGSWNPFSGGHHIDLESERSNEGDRSH
GQQYTIFDSCSGAGGVSRGALMAGFRVRYALDKAPEVWDTYQANFPGTELYKMSLDQFLSSAQTSHMRVDVLHFSPPCQFFSP
AHTHASEHDDANIFALYGCNQLLKKLRPRIITVEQTFGLTHDRHEEYFNGFLGDFTQHGYSIRWRVIRLCTWGAAQDRRRLII
IAAAPGERLPTFPQATHSQEGGNGLLPYNSIGKALRDIRAGDDLHDLDNVHYFDPPRAPYDPERLAGTITTGGGYFYYPDGSR
KLTLREYASLQGFPKSHQFLGTKTSIKRQIGNAFPPNTVLVLYKHLEQWLLNEDRMKPFTNNQDAILIEDDSDTPPNLTDDSE
DRPRHFPDMMDGNIVETFDIQQHRRGRDPWAVEDMVIDLT
FUN_002544 CDS scaffold_13 50300 51761 +
hypothetical protein

MAARTLRKRKVASREEASVKKRKTGRKAKDQVENEYLDDLPHNLGPVRAPRKDIETIPTPPEDLKETQDLKKSAKVEEVSPVA
KSGMKAANQGSAQVMELATRRSLRPRKSAAKSSYFESDDETITKLQVKPSSVKKEVKDEDLQVAIEAPVWKTVKKTKDNPYGL
TPGMTPFPEWSAPSAEECEEVYRRLAKVHGEAKAPEKIPAPSLEVSGCGEVPSVLDALIRTRLSANTSNRNSSAAFRGLVATF
GTVDKGIGKGSVDWNKVRVAPLPTIVESIKTGGLAQVKGKDIKAILELVHEENTKRREAFMQEKKGGNLSGITGADNKTQGQK
DLEILKTEQDILSLDHIHGMAPDEAMQTLTKFPGIGVKTASCVILFCLQQPSFAVDTHVHRISGWLKWIPPKATRDQTFSHLE
VRIPNHLKYGLHKLFVQHGRSCIRCRANTSEGSEEWSKSKCPLDGLMERTGKRQYGGKAGLKKQLKEEDSD
FUN_002545 CDS scaffold_13 52472 58872 +
hypothetical protein EOG092608AE PF12726;PF13086;PF13087;PF13245

MDLHEQLSQWYEKIQCFDEECHLLCPRINDEDNENYKLVDDPDSSITQEEKNSRIKAGKERLEVTYWNCLIFGFDQSSQGKWG
EQLSERLNGCLKHCADCVYNWHMQRRELLQQFQERWNEEVVVDIRSMLEQRDVERIDLSLRWAKGFIEKIISAGSTFKKSQLG
EHLPEVHISIYEALCCMPYMEDADRRMTFQYVFQRLQGKSGLKLGTKEPLPGMTYFIFDLKNQDRRKWATDNYRNLDSNSMTL
EQFDWAVSSGLTRAIEDISKKEPQAVDSWPEMEQFWEGFNEILRTLTADVILQRLRNLELNGTISHIYDLLFRHIQFCPAESI
LVMTITILTNFLKKAPRAFWDVIGDARPNVIADLIFASPGYKNLLRQSLEDCWTGYDGSVSRGPFPTSWVDPWLQSVGRDRRY
NACEILMHTLFESLAKDETIGEPGRAACIRAGFDALQLTISTFVDPETKIGAGTTHLYACCAFNLVMKYKDIILRNLRAPGQE
KEGWVTFQVANAAKKAMQAAMKLDMKVFAEEYIACLEGSQLQSAVTRDSKAFWQGIIEMFDLSTEQVDLARDIVLSLEPLIGV
EQIRALKSDDKLSDSSKNFNKSLAATVDILGNLLGRMSELDAATDLNTFFDDRLAFQGTITLSTHGEAGLAEAAAELLKSWTG
EISQSGAFEQMSQLHPEQTLSSIVWALERVLKPPNPWGPIRPMLNMSRDILRGLTDPMSGVLRVKTLEPMSAAKVLRWWSEQW
RFVSTACRNIEGWSRYIQNAVMTEFCREIMELAEALVAEDGLITSAISKALNKSEKDTMSQILIPAKQHFGGMENMIRLKDKW
LVDVTVRVLCKILTRLRENNHEINPASRKLITDACLPTNVPGKFVRSTNMTDQQRAELLQALGQTDDEIQIYQLGVAQTPLAQ
RPKDGIKKQSKLDAWSKSGTSSSSTATVAPSRSNREDVLDLSRSIDSPILRQLEAKKAKDQAKAKTKAQAMIKPRTQTAPDKK
EIAAIKEARQKAKLEKEKRDAAVIANAKALRGETVPGEGSGLHSLGLIGKDHGRSEIMVNSSEEESDEEEEDVSDADNELAAL
STGGQTALEEANKRRMAKALQDKMRRPVKKVRQQRSVKDRRARVIPPMDRLHNTILAWDIFHGGNDPPNGPAASEVATKYSEP
RTYQDTFFPLLASEAWRSFVTAKDELTSQPFGMKIASRASVDSYLEATFTMPVVQSRERGVSEGDILLVSESEQPLIDQTARH
CLARVHRITYKKELIEITYRVASRNNPMTQVLTPNVSVFGVKITNMTTIEREFAALESLPYYDLMDEILNAEPSPILQYGDEK
INNCMNNYALNRGQATAVLGAHDNDGFTLIQGPPGTGKTKTIVAMVGTLLSEQLSQSGNQGIPVGVPLRPNGAPGAPKQNRSK

KLLVCAPSNAAVDELVLRLKAGVKTISGKTKNINVLRLGRSDAINAAVRDVTLDELVKARMEGDQTKDKAKADRDQLHENAGK
VKEELGKLRPQLEAAKLMDDRTLYNKLSREFDELKRRQMSYGKQIDADKSSGNSVAREMEMRRRQVQQEILNNAQVLCATLSG
SGHEMFRNLEDVEFETVIIDEAAQCVELSALIPLKYGCYKCILVGDPKQLPPTVLSQSAAKFGYDQSLFVRMQQNHPRSVHLL
DMQYRMHPEISLFPSREFYEGQLADGQNMHELRQQPWHKSALLGPYRFFDVQGVQERGHKGQSLVNTKELDVAIQMYDRFSNE
YRECDLTGKIGIITPYKAQLYELRNRFRSRYGENITSIIEFNTTDAFQGRECEIIIFSCVRASSTGGIGFMTDIRRMNVGLTR
AKSSLWILGDSRALVQGEFWRKLIEDAQARDRYTNGDVLSMFRKPLEKAKPGAYLPPPPQAQDRDVPMRDASLGTSSLPSSRP
NSPKVEGAQQPTAPTTHIPGIGGGELAGVLPRSAGPPVIHTSSSKPPGEARKRPHDGPDSNQPVPKRIASDKARGGGLMGKFG
NKSHRPPKAPTDPSAMAVMGLAPPERPPAAAPTSPSLSSGPQASTGPRIPTGPSNPGPQYSNIQQRRPPHPPSSRKKGKPSLF
VPKKR
FUN_002546 CDS scaffold_13 61468 63913 +
hypothetical protein ENOG41

MAQVIGLDPEASVTSGSGASCPRTTIAPALTSSASVSASTVPNANRYSYQHLQRPGSQAQAQARTRPRAKSASNLLGGPEKDK
DLHFHDDTFIDQQQEEDGEPHDGLAELIDFLRNHSPPSGNFMSIPDGIPPEDRGRWYRLRKLGKRSKSLSKSPQTIRLPDSAV
SGTTIGGHRHIAISIPLDASPFGRTPRSQYPIYQHRSVKPLTSQYAPTRAVLNDKGVVTVLRTVTEDRESSPSVSPANSQLLL
STIPQRSPNSSINGHSTPAPINSSNQSTAPEYLNVLATPPPRVVTPDQVRQANEQGRGFQSNEQGNTHAGSPNNHDRIPSRSP
SLTGGRFVHLGDLSIDTMMSQSTKTEAHQVPTSPARPVSQDGSYQPSLSKSIMTTSENDPVVAEARPVEARPVSARESRIRLS
TKKTEEPSVTVITKSPLRVQRQGSRKQTTKERSSTPEAKLSQSRRDKVRDKKKKDMEAASLKRRSMISISMDPQPDSEQETIK
ELPKEKAEEPPPLPLRSPQRQSMCPIMVVANVKPSPPPSAVVPASLKRQTTANPEDLTPAMKSKPDEAVPKQEDDTSSRAISP
SPASYQNGSPTPPQSAHSSPTHKVDSHDRTSLSRRREWSAARELERKRKEAASNASPRPRARRAVTMREDTGDRAPTVENEVL
RRYEAYREYRIREMERRIRRLERNGDVWLRALVPVLDNLNRTLANSQDGHPGGAKGWISDDDRSNFQATSKMRPSSRGRMMTR
TGTSEREFLEQLVRTKEELEAGSVSDDMSGFDTIEPLMRELAGRSRLSFEARSLGMDDGGLLHSLSE
FUN_002547 CDS scaffold_13 64681 65500 +
hypothetical protein ENOG41

MSKPWIFVCPSTRGIGYALTRHLLQKTSLPILATARLQHDPESVKKSLLEGLTENEGLTKRLSIVHADVTDEGSLIDAASRAA
ELFPPDRHHLRLACAVPGVLKPEKNVNQIDAEASLGQFRINTIGPLLLIKHFDKFLPRRSSELEDSPDSDEVKMPPHSVWLSM
AARVGSTSDNRAGGWFSYRASKAGVISLGKSYDIFLRGRSGDKAISIAYHPGTVKTDLSKGFWESTKEGKLFSPEFAVDRLVK
VATGLPLEGRGKCMDWNNKEILP
FUN_002548 CDS scaffold_13 65747 67008 -
hypothetical protein EOG092612CC PF00533;PF12738;PF16892;PF16893

MLVSLTVGKVDAGVTVLLTPDKRLIEFPSILLPPNISSGSIVDINVSQNATKEAAADRAFRALQDSIYNSFGASEPTTPVLRC
RNATQTSVVLEWDPIQLATADLISLSLYRNGQKAGNIPRPLQMHSTKISGLAVDTEYTFHLVLRTSAGTFISERVPVRTHKMT
DLRGITITTGILPSAVKDSLTSAVERIGAKIVDGVRIDTTHFVTTEGRGQQWEKAVESNIPVVRPEWVEACEKSGRILGVTKF
YLDAMKPGPPAEEASPPPPPEKEEKSLPVPPAHNGEQTSPSEEKLDEKVKEKEEEDAQKNGGKQSSSGSSTEGEEEVEIEQKM
KTSPQEEQKVRFEDKEEQKVALRPANNGESSKPEHDDKQGASDDDAEDESNAKTAPTPDGASFQEVEL
FUN_002549 CDS scaffold_13 69175 69896 +
hypothetical protein EOG092652Y6 PF01176

MPKNKGKGGKNRRRGTKENDDQRRELTFKEDGQEYAQVVKMLGNGRLEALCFDGSKRLANIRGKMRKKVWINQGDIILLSLRD
FQDNKGDVILKYTADEARTLKSYGELPENAKINETDSFGQGEDGEAYFEFGDADSDDESDDGAAGNKKEVDIDDI
FUN_002550 CDS scaffold_13 70657 72638 -
hypothetical protein EOG09264U78 PF00568

MPSILNDDDKDTVKRFIPKQSNKIQAVAVARLYVAYPNRSKWTYTGLQGAVALVNDLVGNTYWIKMVDISPSNRGVIWDQEIF
DTWNYNQDRTFFHTFELEECLAGLSFVDEKEAKQFKKKMDEREKNASRATKSTPFGGGAQPAHKHGLLGGLFGHRHSTHISSQ
HTAPTPPESPRMPHNSIQHHVVNSTPNLNGSRPSEFALLDAFDPLWREHFGADLQDKGLTDDFIKENQEFIVDFLREEQQKAN
QPHSTPPPPLPPAAKAPSPTPTANGNEGRGSRAPPPPPPGGRPQSDGPPAPPAPRKGGPPPPPAPRRSGKAESPAKDAAPERA
PSPPRPKFGVPPPLADAGKFARQDPPRAVPATPTPGPPPPPRPAKTPIETQKNESHRFGVPPPFPGSRVPPPTPSRGPVPPPP

PPRPADNHPVPAALPPPLPPKVPASSAPPLPPPSSRPVPPPPAANNHPPAPPPLPVTSAPPPPPLPPTSTNGAPPPPPPPPPP
PGGSGAPPPPPPPPMPPGTGAPPPPPLPPVAAGGDTGRSAMLEGIQRAGGINSLKKVDRSQIRDRSGAQVGSGGDSGGANAASA
AAGAPGGGGGMADALAAALQKRKEKVSKSDDESDNDDW
FUN_002551 CDS scaffold_13 73932 74181 -
hypothetical protein ENOG41

MTGTEQQKEGEKQFSIQPIKDEAKDKVSAFNAHPGPAVPKDMPQEEGTKEERKAKMEALNKK
FUN_002552 CDS scaffold_13 75307 78170 +
hypothetical protein EOG09260AZK PF02732

MSASNAPQPVKLSLPLEYQQTLFQELRAEDELVVIARGLGLMRLVNNLLHSYDAGGNNLIVIVAADERENGWIGEALAEHAAI
SMSPKARGLTVVNTDFQSVGAREKMYSGGGIFSITSRILVVDLLTGLLNPESITGLVVLHADRVVATSLEAFILRVYRQKNKI
GFLKAFSDNPDPFTTGFSPLATMMRNLFLRKASLWPRFHVTVAQSLEGKKKAEVIELEVPMTDSMREIQNAIMECVEVSIHEL
KKGNSGLEMDDWNLDSALLKNFDIMVRRQLDPNWHRVSWKTKQIVNDLTVLRAMLNSILSYDAVSFLQHLDTIHAAHSPPPGS
TRQTQSPWLFLDAAQTIFDTARRRVYSASAKDAAREDNIDSLRPVLEELPKWALLAEVLKEIDRDLYFEPPVRDDSNGTILIM
CSNTDTCRQLRDFLQTMHVKPKTEKRSSEDDEDEDKPSAAFMMRRRLRNYLKWKRQFAQVSATLFSENQKALNGATDTRPGFA
GSRGGKAPANKRRRVRGGGNVGVSMGRAENGSIMQYIEKPGEVADLMAEVRITEEEAQQKEDVVADPLDNMEEYFKLYDMQDL
VVIHAYDGDQDEHVLEETKPRYIIMYEPDAAFIRRVEVYRSSHNDRNVRVYFMYYGGSVEEQKYLSSVRREKDAFTKLIKERA
SMSLVMTVDPAEDPEEAFLRTVNTRIAGGGRLAATAEPPRVVVDVREFRSSLPSLLHGRSMIIVPCMLTVGDYILSPNICVER
KSISDLISSFKDGRLYSQAETMFQYYKSVMLLIEFDQNKSFTLEPFADLSGSLNSVAPTNMSSDLQSKLVLLTLAFPKLRIIW
SSSPYQTAEIFESLKTQEEEPDPIAAVRAGLDKDTRAEEQAFNQEPQDMLAIVPGVTPQNIKNLVLKTENIREVANMTVQELT
PLVGTTAGRQIHGFFNRNIMEEDD
FUN_002553 CDS scaffold_13 79989 81726 - MRS2 magnesium
ion transporter PF01544

MQSALCLPVPSRNLLRFLRCQSKRAFSNANHGRTSMTTPVLCCRARTSDTLTTRKQFTTLGTACQRTVTVLKPTLFHRDPTAR
KLQDRHQPWTSQRCFSTSRGARNLFKGQDPNPENPPTWERFLKFAGRKDDKVLKPDDLPNHDEYSDGSSIFNNRRTLAAKAAS
EPRLRCTEVDEHGNVILVDGEFKKTELIAKFGLLPRDLRKIDSSNLPHILIRPSAILLNLLHLKVLIKHDRVLLFDIYGSKTS
YPQSAFMYDLQGKLQQKNTQASGALPYEFRALEAVLTSVTSELEADFEAVREPVMHILSELEDDIDRSKLRMLLILSKRVSTF
EQKAKLVRDAIEDLLEADDDLSAMYLTEKTHDLYRGEDDHTEVEMLLESYHKLTDEIVQEAGNLVSGIRNTEEIVRAILDANR
NALMLLDLKFSVGTLGLAMGTFLAGLYGMNLENFIEETNWGFAGVTGVSVVFSLIVCWYGLTKLRRVQRIKMMDDERPRIPRG
QAYFPDDRSALGLLDNRNREMLRRINMQKAVSQKKKWFS
FUN_002554 CDS scaffold_13 82379 83488 +
hypothetical protein ENOG41

MGLTPIRIRSKRKFPSRGKPSPWQTITSIPDDKISPSKKMKRSLSNVLASRSTRWRPAIQALPAEILESIFLYSANVALPRSA
PIIGSKLSGRATLIRFIIWAFQDTWEQSFGDPEKFAAIDKNRVSGNWQLQSTILCLPWITAEFILQAQQTWVEKYARDQHYRH
YLPRLDDDGDPFIYGHSHHFEGGVGHFNSQECFEADYQEVLSWKPFERVGSWGGCDVHPKVRVPTILITGPWDEKNLRLLFWL
RRGGLVYGLEEHDRSWEIQLDCLRNAFIDAPEPNVFITNLIDLTALCQGLPRDIAREQRRRIDQRLKWGADSVISKEILRQVY
GTIGMFHDGFGTSSSPK
FUN_002555 CDS scaffold_13 85670 87395 -
hypothetical protein

MRLAFTLLDSSTHIVEAIWPLSSVACRNELGSRAILPLRTFIQETLRRSRTSYSTLQVALYYLILIKPHVPKHNFTMEQPEDR
HADRALQCGRRMFLAALILASKYLQDRNYSARAWSKISGLHTQEINQNEIAFLHAVNWKMHIVDEVFQRWTDIVLKYTPPPSG
PPSPGGVSQIVSQQVVDWKRIILGLNPELTNLATLIPANPAIPRSSDLCALSPRSILNMPHQAQISCGYESAEATPTPKPYSA
PMAMEPAAVGYTCSRAAPSLGMLPTPRLTPQSSGLCTPAASAASHMLNKSSAMGLAMAQANNVNTSQYLDRLPAAMTSSPQAY
CPTRRSSLATSVSTASSPESMISDSSRSSRSSSISSSSSLVSATLSSSRLGMPSRFRASKSCNERLSQKPTVPSVPEDYDVHC
DTSSPESYTGPVGKLVDLSLESSLARRQQELDDMARDSDAARALQELHNYRAAEIGPATAPRSGTKRTRTASMDNCLQENVRE
ILGGRYSTQHSVWPDTLVRTRGITSESNLQIPVRPSLPGTGKRVCCSAEAAQGYEISSVHPAIGLRGPGLLKGILN

FUN_002556 CDS scaffold_13 94596 96582 - SUB2 Suppressor of the cold-sensitive snRNP bioproteinsis mutant brr1-1 EOG09262E4Q PF00270;PF00271;PF04851

MSHEEDLIDYSDEEIGGNETTATTSNGKKGELAAGNNVDKKGSYVGIHSTGFRDFLLKAELIRAIGDCGFEHPSEVQQTCIPQ
ALLGGDIICQAKSGLGKTAVFVLATLQQVEPVNGEVSVVVMCHTRELAYQIRDEYNRFSKYMPDIKTGVFYGGTPIKTDMETL
KNKDTCPHIIVGTPGRLKALVRDKALRLGSVRIFVLDECDKMLDQPDMRTDVQDVFRATPPQKQVMMFSATLSEEVKPICRKF
MQNPTEHYVDEDTKLTLHGLQQFYIKLEEKEKNRKLNELLDELQFNQVIIFVRSTVRATELDKLLRECNFPSIAVHSGVSQEE
RIRRYKEFKEFKKRICVATDVFGRGIDIERINLAINYDLSNDASSYLHRVGRAGRFGTKGLAISFVSTDQDQEVLKEIEKRFE
VALPEFPKEGVDASTYMAS
FUN_002557 CDS scaffold_13 99767 100721 +
hypothetical protein ENOG41

MKAALRIIALTSSAIAFDGRYGTGGSPGNMSEQAWQSAFENPNATGTYKFEAYNVSERFPPNKTVDGWTATIRVANITDDPGD
DTPYPGIDISVQAPEGMRVPTLNSSRTNSSDWHVCIAFWGPSRLKDEATDDAQHDDGDCSSFLDEDCLEALNAEAAGYYWNGE
KCTMLPHMPVKCEKYYKDYGPTYGGVPGANLSQFESGKTLISQRPQTAGKYNAMTQEEAYDYAVRGVWTVMINWGRRDPDTYS
LDDVLQPSLLCLRTRNITGGSEDPNAGTRTTGYVPMAVFSALMATLLLAY
FUN_002558 CDS scaffold_13 101859 104593 -
hypothetical protein PF02558;PF08546 ENOG41

MAPVSPRLKILSVGGNPVSAFLSWRLQATNACDVTLVWKSGYEHVAQYGISFKSPIFGNERFKPRHVVRNPEDAASAREGPFD
YVVLCVKALPDVYDLASVIDSVVTPQHTCIIVNTTHTLGVEAALEERFPTNVVLSLVSGAELTQLGQSEFEHKGPADIWIGLA
NNPNNIPQTIQEDMAQALAMTLSSGQVECKVSPNIRQQQYERVIGPIAFHPISVIFETPNHASLLEKVGVRDMVSEVIDELLR
LADANGCKFDPDFKQKTIDEMSKASGESIMWQDYVARRPMEVETYLGSPVKLARDSNISLPRIETLYSILHNLNLVNRSRPKP
GDPNANIIPPASPSAAPVPRMPSQNSHRPMMNGMPNGNGMPPRQPRPRNSSNFSQGGMRRGPPPMNGGPPNGYGRPPPMNGPG
SRQPSRRGSLEETNLEEFSHLVLYDDIPEGSEPSVSGDPADINLRERELALRQREMAIREQEIRMRRGPPPGPPGGPGPRRGP
HPMRNSKQVFDEDDDDDDDYFDPTANPGPPLIDPDNFDMMSVTSKKNRKAPGPSPGQFRRNPDDMPPPTRGGRFRPNFGRNRS
SQVGHSSMTSENILEDPLMSCSSDRYGSVDRGAMNAGSRANSLTASRLDEMQYGPGPGGPPGMNGAYPRRASQSPGNPYTPSM
RGGSRRGSPPGGYGPGPGMNGRPSPPDGVRQPVPRHPPGHGNSVAPQQVEQHIGVSALHQTKPRNVRSLTGSASASAGSGEFD
SEQSANSSQGSLPPRPPIGVR
FUN_002559 CDS scaffold_13 113176 113932 -
hypothetical protein PF01088
C12
MSTTPGVSFRKDGTKTFIPLENNPEVFTKLIHNLGVSEKLGFYDVYSIDEPELLAMIPRPVHALVFITPAPMWARVRENDPGS
KELSYDGSGPDEPVVWYKQTIGHACGLIALLHSVSNGTARSFINTNSILDKILKETQDLKPLQRANHLYNSIELEKAHMAAAV
TGDTVAPASEEPSGYHFISFVKGQDGHLYDLEGGWGGPVDCGTLDDGEDLLSNKALEVTVKRFTKAAEGNLEFSIIALATVPD
GA
FUN_002560 CDS scaffold_13 116263 118766 +
hypothetical protein PF03169 ENOG41

MAPSLFRKRHEAEDIIPIPGPLETVSASGRDYLENLQQFEKSHKFDPNLPIDDLTDVDAAIATGNAEKGIEIEHALMEDNSPY
PEVRSVVRNYDVDVPANTIRAWTIGLILCTIGSGVNMLFSLRNPSVTVTTYVVQLVAYPLGLGWDLIMPDREWTLFGLKFNLK
PGKFNYKEHVVIVAMSNAAYGGGVLYATDVLLAQQIFYGQKFGWAFQILFGITTLCTGYGLAGLARRFLVWPAAMIWPTDLVN
CALFYTLHDHSGSDPTKTNGWKMGRYRWFLIVGAGGFVWYWFPGWIFKGLSYFTWVCWLAPHNVTVNKIFGGMTGYGLMPTSF
DWTVVYSGYLQSPLIPPFHAIANVLLGIIVFFICISMGIHFTGTWYADYFPVQSSESYDNTGAIYNVTRILDNNFQFNETAYKE
YSPLFLPTQFALSYGLSFAAVTAVVVHVVLYHGHEIWRQFKLARNQEDDVHMRLMKKYRDAEDWWYATLFIVMVAISIGVIAG
WPTGFPVWAYFICLLIPVLWLIPIGVVQAITNIQLGLNVLTEFIIGYMVPGRPMAMMMFKNYGYICMGQALYFAQDLKLGHYM
KVPPRVMFSSQLVASIWSAIVQICVMNWALGHIPDVCAIDQPNNYTCPGGRVFYTASVIWGAIGPARIFSHGSIYSSLQWFWL
VGAVTPIITWLLARKWPRSIWRYVSTPLIYGGAGMLPPASVYIYLCWGMVGAFFNRFVKRRYTGWWLQYNYVTSAALDCGLIM
STMVIFFTLYLTSASPPNWWGNYGALETMDWKTTAVSKTVPKGSFIGPSTWN

FUN_002561 CDS scaffold_13 119865 120499 + RPL37 60S ribosomal protein L37 PF01907

MTKGTSSFGKRHNKTHTLCRRCGRRSLHVQKHECSSCGYPAAKIRKYNWSEKAKRRKTVGTGRTRYLKDVSRRFKNGFQTGTP
KGARGATAEKA
FUN_002562 CDS scaffold_13 121058 121967 -
hypothetical protein PF09807

MASTSRIPHLLGPYISLPPESSLNLVTSVLGASANWLVLRHVYSYLRGSTDGDESAKDTGVVLLSFMRDGAFWKEGATKLGLD
LDALSRAGRFTFIDGFSGLYGEAKTGVPGTRKERTLRSTKLADIKREIEGAIADLRASRKILVIDQLDALLAVTDDSTTSLTL
QDAVLSLRSLVHSTLLTLSADVPLVATQVTTLEREHASLVLSSAHEADMVMALRMLDTGTARDVSGVVRITGPGAEGLGGATE
YLYHVVADGGVKVFERGT
FUN_002563 CDS scaffold_13 122999 123688 -
hypothetical protein PF00085 ENOG41

MHTITLRTGLRPLCNKAQTIPRSFQRALISSTTSRQAKNQIYAPIRNNDSFSTYLSLSTSSRTPLLTLWTASWCSTCKVVSPL
IRDLVESGVGEEEGGVSLATVEFDSPDIMSGSPNLAMTYMITSIPTLLSFDGGEAQTATKLSDARKLADREFLKEWIRTEARR
HGGRGGGGGGSSIFGGLFGK
FUN_002564 CDS scaffold_13 124735 126979 +
hypothetical protein PF07690 ENOG41

MQSEKPNVPRWRRFLGGGRPTGARPDESYGPSRWSMGVLNDKKTIEVPGSVLLLASHRNEPLGLRNVHARTSHSSIPAGFPVD
TRRTSASSAAAAAAAAASVAESHAPADESDGKKKTSDGAIILEPQPEESGNDPLNWPAWKRDIALLSLGFYCMLGGGTTPLIA
AGFTDVAETYDVDVERVALTTGLYMMGMGLGSVVFSPTAILWGKRPVYLGSAILFIGTSLWAAWSPSFNSLLAARVFQGFAVS
PVECLPSATIAEIFFLHERAYRIGIYTLLLLGGKNLVPLVSAVIIGGLSWRWVFWILAMIIAFGAVLLFLFVPETFWDRTPHA
RPKHPSKRPSFLRRLSSRHAVPQTGGEGLLQGEPGAHFADAGHTQDGRQSPAALHRGRDLHVGFAESGDAAEEAAAPVTPVSP
QAVVHSSDKNPSSPGDVGSTTGSESISASFKLGPNLENDKLDASRLQGPPKIQAYTHNLRQQPPKSFVQQLKPWHGRLNSDSW
LKVMVRPFILFAYPAVLWSAAVYACSVGWLIVISESIAMIYRDKDSYNFTALQTGLVYISPFIGGILGTGVAGKVSDIIVKIM
SRRNGGLYEPEFRLVMALPILISTCIGLMGFGWSAEEKDHWMVPTFFFGVVSFGCSLGSTTSITFCVDSYRQYAGEALVTLNF
SKNILHGLVFSLFVTHWLHGDGPKKVYIWIGIIQLLLMLCTIPMYIYGKRARMWTVRANLMEKF
FUN_002565 CDS scaffold_13 128649 131571 -
hypothetical protein PF08238 ENOG41

MAYEGGYGGQQRPYGGQAPPRHTPRQQVNAPPQQYGASQQYDEYHQDYGYGSGQDFGGQRQDQNYGRGPPPQDQYGRGGTVMP
GPRGGPIPRPQTADPHRGQQRPPRAGPMPGPGRGIPNGHPGQGGIRPGPYASNLDPTARRHPMNTPPMSPRDGHFGHKHVPQE
RGRRPDMPYEQQMVDQMSNMDMNQRPIVPTNGRTSSDAQRSQGEYGRPPVNAGRGPPPRGYPQGGPPRQNPGYGAPDGYGNYN
DGYNGAREPPAGGFGPPARSRTMPVDDMRQRGNMPPPQQMDAMQYSTPAGRGIPQRPSTAGGHRAPPQRSYTAGPQGGPGGHY
SGEYDDRNAYHKPSTASYDEVYDAYFDNQRNSHPEYGPESQQNRVSLPDFDATPAPGQRSSFEQSMRPSNEQPQRGAHQTAVL
NHAKSQPVLRQPQTAVFEMAGDVPDVPAVPPVPPMSNYQAAYDQDYNQIPARGPSAPAAMNGARTSGPMNPDGLPSHPAPIRP
GLMQDSMVHLGNKPAPVRNYGVSTPAPQPQPQPQPQPRQPQYQQQQRSPPRNNSQRQPARDEGPVTTQELEHLRMLIKNNSSD
QDSALRLAKKLVEAADVLAPKMPDPKQRTRARERYLIDCHKILKKLANAQNPDAMFLLADCLGRGLFGDSDHKEAFTQYQSAA
KLGHPAAAYRTAVCCEIGHEEGGGTRKDPMKAIQWYKRAATLGDPPAMYKVGMILLKGLLGQPKNPREAVGWLKRAAERADGE
NPHALHELGLLYESAQPNDAIIRDEAYAYSLFLQAADLGYKFSQYRLGCAFEYGLLGCPIDPRQSIQWYSKAATQEEHQAELA
LSGWYLTGSEGVLGQSDTEAYLWARKAAIAGLAKAEYAMGYFTEVGIGVPPNLEDAKRWYWRAAAQDFPKARERLEDLKRGAG
KNGARQRERISRSKMERQQEGDCVLM
FUN_002566 CDS scaffold_13 134026 134497 + CBP4 assembly factor cbp4 PF07960 ENOG41

MAAKQPSSRWWFWTKVLMGGAVVAVGGPAFTMWLTPTEEELRSRYNPELRKKSLENREERQQEFDDFVTRLKEYSKSDKPIWI
VVKEEEERKRKAAAAAAKASQKDADTRREEMRREAGLDAK

FUN_002567	CDS	scaffold_13	135337	136008	+	EGD1	Nascent polypeptide-associated complex subunit beta	EOG092653SU	PF01849

MSSAEVQERLKKLGLSARTGTGKGTPRRKVKRAPARSGADDKKLQLALKKLNTQPIQAIEEVNMFKQDGNVIHFAAPKVHAAV
PSNTFAIYGNGEDKELTELVPGILNQLGPDSLASLRKLAESYQNLQKEKGEDDDEIPDLVEGENFENEPKVE
FUN_002568	CDS	scaffold_13	136304	137647	-
hypothetical protein	EOG09263CG2	PF02137

MTSQADLVAQAVLQQFYKLPSKRKPAVRDNGVHEWVPLSGIVAEKDGILTCLALATGMKCLPASKISQSNGVGIHDWHAEVLA
MRTFNRFLLDECENILEKDDESSIIQRKMSTSNLNDTPLQLFEIKDGVKLHMYCSEAPCGDASMELTMASQEDDSAWETPTPA
DAKPGGDVQISLLGRACFSRTGIVRRKPARGDAPPTLSKSCSDKLALKQCASLLSAITSIFIDPTNAYISSIILPRSQYSDLA
CRRAFSAEGRMNLMAGKKWLGGYSFREFSVATTGLEFEFSKRSVQTRADKISASNLAATWSRSGFEENIIGGVIQGRKAFKTD
GASQVSKRCMWEAANDLASRIGGASSSTAICPGGETYRHLKQGQLMAHRRQVKDEAREMALSGWIRNDGGSDFQLEKRSNH
FUN_002569	CDS	scaffold_13	138010	138763	+	RPL24	60S ribosomal protein L24		PF01246

MRTYEDTFSGARIYPGKGKLYVRGDSKIFRFQNGKSESLFLQRKNPRRIAWTVLYRRQHRKGISEEVAKKRTRRTVKSQRGIV
GASLDVIKEKRNIRPEARSAARAQAIKESKEKKQADAAAKKSEKAKLAAQASKGQAVRQVSKQGAKGSAPKVQAKTR
FUN_002570	CDS	scaffold_13	139189	139812	-
hypothetical protein		PF00578;PF08534

MSELKAGDSFPEGVWFSYIPPSPETSEFTTCGTPVPFNASQEFKNKKVVLVSIPGAFTPTCSGSHIPSYLEHVDKIKAKGVDQ
VIVIAVNDPFVMSGWAKANGITDDKILFMSDKDAKFSQTIGWNIGERTGRFAIIVDQGKVVYAARDEEPGSIEKSGALGVLAQ
L
FUN_002571	CDS	scaffold_13	141679	142515	+
hypothetical protein		PF12917;PF13023

MSRESLQKPTAGSENGEPWTVEKALSHNDLVNISDSPISFFHLLGALKTTKREGWKRHGINPESVADHSYRMGMVAMFAPQEL
DQAKCMKMCLVHDIAESVVGDITPFSGVSRIEKGRREASTIAYIANRWSGPYTAEIEKLWHEFEAGETPEAQFAQDIDKIELL
LQAVEYERESKKEKDLGEFMGVARKLRTEAGKAWANEILGDRERFWQGRQHLRGEHAQQGGLSEEMTKAHDAYYG
FUN_002572	CDS	scaffold_13	144731	146185	+
hypothetical protein			ENOG41

MGLLSLSYTRPKYVSHQPFRNTSSSDSLSDAEKSDATDNSSSSRSSTGIPDSLSFDRIIGGGTCPPMTVRDFMTYLIYVEHAA
ENLQFYLWFQDYEKRFNANPTTDVKLAPEWTRAMQDEAVIKIRKEQSDKMRKESEAAAIFKGSDFEKKVGSQDRNMSAVDPFA
TPPQSSKGDDSSIYTASCTMKSLNAMSHQSQASDAFKMAGAQQPFTIQPFREEINRVISTYIMEGAPRQLNLSSSEQKAVIVA
LSKTTHPSAFRSVFKTVDSSLRHQAHPNFIRWSICNGNPARVFFARSLGVGLIAAGIVVGLILTLSNAGRGYRALAAIALVLG
ISTLVAAYKGMCVVLHGMHHRHVRPWELFVDSENMEDAQKQSFDSFGSQNSYEDEPWVVKYEKRNVVRKVFDREVWIQEPALR
QIQDTIFVQAILASVLLGGIVTAIFVAVPGGHFF
FUN_002573	CDS	scaffold_13	147206	148714	+
hypothetical protein	EOG09262IP2	PF03630

MTPASEHEDAIAQELQAPAHPLDHKRRRTMTSTDEIDSTIIRPGSVRINVKGAFIVDPDTATPASASGASAGGATSHNGRMSP
THPETSDIRLPYHTAIVSHIAIDIGGSLIKLVYFSREVDSTDPGGRLNFQSFETDRIDDCVEFMRHLRDNQLVNGSQPGELCV
MATGGGAYKYYDKIRAALEVDVSQEDEMECLIIGLDFFITEIPREVFTYSETDPMHFVVPQDNIYPYLLVNIGSGVSFLKVTG
PRSYQRVGGTSLGGGTLWGLLSLLTGARTFDEMLEQAAHGDNANVDMLVGDIYGTDYGKIGLKSTTIASSFGKVFRMKREAES
AAEDGRSATPEDASFNSADVSRSLLYAISNNIGQIAYLQSQIHNLSNIYFGGSFIRGHRQTINTLSYAIKFWSKGEKQAYFLR
HEGYLGAVGAFLKRKPKNWGRKGSLEGMDDIAELRRNLREGAPNSSTS
FUN_002574	tRNA	scaffold_13	150687	150778	-		tRNA-Pro

FUN_002575 CDS scaffold_13 151391 151688 -
hypothetical protein ENOG41

MPRNGDGSSDNGPFEAADHNIAHGVGKPDSDRVDRADKTADLPEGKDEHGQGLQDAPASGGQSQGIKQGKDVGQGN
FUN_002576 CDS scaffold_13 152614 153865 -
hypothetical protein PF00645

MGEYRIGKAPKISPFLMIALRCSTSVVCSAEQFAHIVLELSPNNRATCKDTECKKNGEKVTKGTLRFGSYVVIKEHGSWSWKH
WGCVSGQQLENVRELCQQGDGSFDCDLIDGYDELGEHPDVQEKVRRCVNQGFIDPEDFKGDPEKNKLGEKGIHLTEAQKKKKA
AAEAAATGDEEKPKAKGKRGRKKAADDEEDEDEPQPKKARTSKTVKADEEEEPAAKPARGRKAAKVKDESEDETAASAPAPAP
APAKRGRRASAQKAKDESDAEEKPVPVKKGRKAATKKVKDESDDEVLAPAPAPAKRGRKAATKKAAAPEEEDVAVEEEEQEKT
APRTRARRSRSSKA
FUN_002577 CDS scaffold_13 154487 162365 -
hypothetical protein EOG092600SD PF00454;PF02259;PF02260;PF08064

MAANLHLRAAVPDHGQPAGKFAAGPPPSTLAAQLVENISASTKSSKSDENSELKGFFAIIQRVKDDPTLLKTPEDRVEHNHML
IYVYSRAVLEGIRLDDPFLDRTQVRTEGLKAISFLRFSIKETPAVLKHRVGEQEYMFRGREPLWVWLLPQLLRLLGHSQCLEL
TEAIEGFLQYTMLIIAQNWTLWDMAPSFLFYLRTITSHTLQQLQDPLVVPSVEESFKSLSLPPQIALSQFVDKQSPFTSQLTY
SVDRMSQALQQLISFCKVAAYPLTASDDAFNNVTSFSESAVWLIDVLGDMRVIQTRYGSNFPASSLHVLQLAQEIERALRRRK
GISASVHKKAITLMILLCGEIATSLNPMPMLDIDEETRHTYCMALAIITAASTENTSIGRLAVSGIVDESSMFYTSLPEGTDL
WRVIQTLRQINFDPRPKLLSSNLHPLSFEDSAVQEIIETLALSYEPPETSSQDRLKRRKIAQADSSPTAVLMRLLGDVLGLAD
VDDDFLNLENQVLGAFPKADTSRQCLILDLLSRISCVADGGNETVNDTKAAVTETACSICEHNAPPVRSNLHGATVHKSRIEA
LFTKLIRLPALAESRRPRVAAMLAIRRVILHCEDTELLNLETSGMGQWCLQSLNSSVRELRIAAGRVLATFSLARPEPILSNM
VLPARPAEANGARNPRVNQAQAASTDGKFQTNASNGSSPMKTQDLISRNRKNSIAFLKSISDKNQPNLTETFIMAWGQLGTVV
WEHELNLVLIKLLEYLGSNNNIVSACAFNELLNLADARRVTPRRLFEPFWPNLAYMTTKDMVQRPQMSRTIAELLQVSVNELL
LLIQTHALPWLVLDKQKDVIQKIAEARQESEVWLPLIDPANLAATLALLLVQDTDDIASFAKSRLDELSTHFQTEPLVNLLQV
EPVLTVIELLKAAGDADETKKAPVRKALDTMAKMMIPANKETRAKKSDHTARFIHSQLLGLMACLFDVINDQSLPDPERRRYI
RAMEEMIRVSRGYASTARPQMSACLLSTLAQDALREASFSCWASMLTHLEETDVEALLETTFFVVTRYWPFLSESTALLAQQM
LKSLVDEFDPLVAKYIVKLPSLRHIPELRDIETKLDQHRPATLAVEEVLEAFAERICHENSGVALQALTELIPYLQENQALLH
TSEISLQSDIGVVALMRSLLDCASKYSGFPGDIARLCTEAMGLIGCLDPSKIETVREQRSIVILNNFATNEETTDFVLFLLEE
ALVPAFLSTTDVKFQGFLSFAMQELMGRCDIKAACAMENSGMKGGNDIYRKWVAMPETVREVVAPFLASRYVVAPMPHTEIEY
PLFRPGRPYPSWLRVYVLDLLRKGQTPFADLIFEPLARVIRVKDLSIAEFILPYIVLHTLLGSRTTQQERDDILGELLAILQY
QPAETASYQEKEDMRRYCHLVFRVVDYAMRWMQTRRAAGRLTETDRERLAQVQEALDLIPAELIAQRAVDCNEYARALFHLEQ
HAHKMEQRKKEPGERTRLLQKLQDIYANVDEPDGLDGISAHLQVLDINQQILSHRKAGRWTAVQNWYEMQLAENPVNTDIQID
LLHCLKQAGQHEGLLNHIEGMHTDASTDNKIMPYAVEAAWVTGRWESLTKFTQRFRGDPIEDFNISIATLFEKLRAKDKPKEL
IKIMKDIRVKISSSMNAASTSSLQACHDLLLKSHVLTDVEIIIGTKAGDEVARQKTAALLERRLEIIGAYMNDKQYLLGIRRA
TMELTRPTFTDLDISGLWLSSSRLARKSNSLHQSFNAILHASKLGDDAATIENAKLLWREDQHRKAIQVLQGAIKSNKFMTQT
GTATSTSSSKLSPQQKLLTARAQLLLAKWLDSAGQTHAGALREKYQQPPKTFSTWEKGHYYLGRHYKKILEAEKPLKADDQSD
NYITGEVARLVIENYVRSLNSGTKYLYQTLPRILTLWLDLGAQVDRAPEGKASLSRELHRRRVEQLNLLHSFLDKYIHRLPAY
IFYTALPQIVARIAHPNSNVFDRLTHIIAKVVEAHPRQALWSLIGIMTTRQVSERKARGTQILQTLRNISKKVEGSTTDFKHL
LRMGEKLAEQLLLACQNGDFRGNKTVHASLGRDLRFNHKCTPCPLVVPVESSLTATLPAVSEYVKKHKAFSRDVVTIDSFLDD
VLVLSSLAKPRRLTTRGSDGKSYMLLIKPKDDLRTDQRLMEFNGLINRSLKRDAESSRRQLYIRTYAVTPLNEECGIIEWVPG
IKTMRDILINLYASRKIYPDYTVLKQLMDEACLSDGKTRIFTDEVLGRFPPVLQLWFTQQFPSPSAWFAARLKYTRSCAVMSM
VGTILGLGDRHGENVNLEEGNGGVFHVDFNCLFDKGLTFAKPERVPFRLTHNMVAAMGIYGYEGPFRKSCELTLSILRQQEET
LMTILEAFIYDPTLDLQKEKRAHRRGDVGVKLQPQSVVDSIKRKVRGLLPTESIPLGVEGQVEELIKQAVDPRNLAAMYIGWC
PFL
FUN_002578 CDS scaffold_13 162683 164670 -
hypothetical protein ENOG41

MSNRRIVVSIDFGTTYSGVAWADTARPDVQHVLTEWPSAGSSKSSPKVPTELRRVATGWQWGFQIPESAKRIRYFKLKLDDPA
QITKDGESAKDLTKVYLSCLHEHFVSLLEKRLSSSVVKSTPMDFVVTVPAIWSPKAKQSTEQAAAMAGFCGNQRIQLISEPEA
AALYTLKTLSPSTLQVGRKFVVCDAGGGTVDLISYQVTKIGRVEVKEVTEGTGGKCGSSMLNMRFRRHLKQTHGDKYWTDERL
VTALSEFESFKKTFSPKGEPLTLKVDPSLGLRRNRYTMTQDDMKTKIFEPIMKDVVCLIKEQIKMAGDGVAAVIMVGGFGQSR
YLKSRVRDAISSRTDVLQPESGWTAVVKGAAMHGLSRYQPAGTRVEVASRIARRSYGTCLMTKYDMMKHNPKEAFWSEKEQET
VVSEMCWFIQKGESYPEGKPSRIEYQCDLPVMLGHVPQTEIDIYSNNNDGKAPVHLDETTQHIGTLSLDLRKIPETVKRTAKI
RRMGWHRYYCLKGAIEAVYGSAEITYTVKLGGITHDMISVRYER
FUN_002579 CDS scaffold_13 165520 166895 +
hypothetical protein ENOG41

MDPETARHENSQKQAEDHTTKQGSPQTPALKDKLASETHLRRFTENVLDSRQQEIENRESEKGQLAVEAETLRRQLAVAQRQL
AEAQDQIFRLQPRRRDITEAEAREAYKILVGNVQRWVENRAAYAIEDLEIGRLAARASSPEGLRLVSLLREQSRRCLNVTQSD
EFHITGVIMNYLHVTLFSKSFYCPLDDTEGDGTSRWVDDLENTMSRLPRDTAHCREWRSETLTALTHQPSFRTRRQRFLNFVV
DDLTTLLSIIAPKSPPAELHSSVRRSIVEPAADLAHQLHLAPSVFSLKWPARTAATRLEVYQCLNLANGGAILDLSGTKAASQ
ARRGVIYLFDIAPGLFVERVEGGRKLGLKAIVKPTVLVTNSGGDVLQGPTIMSFLWNNTPTSQGTSRSSSRASSSRRPAGSVI
SASSRQRY
FUN_002580 CDS scaffold_13 167067 168174 -
hypothetical protein PF06094 ENOG41

MNLSADEAQADTGQPLTKYYFAYGSNLHLQQMKRRCPGSKFIGSGKLADHRWQINERGYANVIESQGHWVEGLVYEINPRDEA
RLDINEGVSKDAYQKKYMTVTLRRAEAALYRRPVSWIVNNGGPDHARVLAQQGEGQRKAINHKPHLEQNILVYISPRYIVDSE
PKEEYINRINSGIVDATSLGVTNDYISNCVRPFIPAPTGQTSGDKEATGPAKATAPPKPKVVRKVPNPAKKVKQPAREVKRAT
PPRGRELSPPNNRTPGQRPLHLQPQVVQGQRLTRNAASNVPERLGRPSQSRSSAPPLPPRPLAPHYPGNTLQVPVSGRRRPAS
EVGAPPPPLPPRPTRRVRSIPVIVTHQEGYGGYWYR
FUN_002581 CDS scaffold_13 169221 170308 +
hypothetical protein PF12224 ENOG41

MTVSKLRHYNFGVEIEAVVKPYGGADSFTNVDWYRQLAQKLRNRNIEAVHDDCSKYSKHPEYYGGKWFVTRDGSLKRERPMVC
MEVVSPRLDTKQHVSGILGDFWEAMRVHFSPQRDISCGGHVHVTPVSGQNKFSLRGLKKIAFASAVYEEFVAAVLPKARRENQ
YCRPNSQSMGSGLRETLIRFGKSKGSLLQVASEIKSTTSEADLCYYMQGNRYVLWNFQNIFPNPKTGKCTGTVEFRGGNQFLS
TKGTLAWVAFVMGFITLALEEDLLNKLTTYTSPDDPKFQSRLEDWWKRIRQAAKQSKLSRYLPLEYIKMHTR
FUN_002582 CDS scaffold_13 170636 172933 -
hypothetical protein PF00289;PF00364;PF02222;PF02785;PF02786;PF07478

MRSTLRANRRLPLKPLPRFLSTSASSASVSSVASSSSAPKHTPINSVLIANRGEIAIRINRTAERLGIRATTVYTDVDAGSWH
ASSGFQSLALGPANAYLDGEKIIALAKQNGIQALHPGYGFLSENSKFAERCEEEGIVFVGPPATAMADMGHKARSKEIMTAAN
VPCVPGYHGADQGEQELLEHAKNITFPVLLKSVRGGGGKGMRIVLTEEEFLTQLRSARAEAKASFGEGGEVMLVEKYIIRPRH
VEVQVFADKWGNTVALGERDCSVQRRHQKILEESPAPDLDLATRHDLWDKARKAASAVGYVGAGTVEFILDKDTNKFYFMEMN
TRLQVEHPVTEMVTGLDLVEWQFRVAAGEKLPLSQEEVEAQMNERGAAIEARIYAENPEKGFIPDSGKLVRAYLPTELQNEDV
RLDWGFRSGNTISEAYDGMIAKLIVRGDTRERAIAKLESVLRSYEIVGVATNIEFLKRLCETDAFVAGDVETGFIDKWREELF
KPRPIKNEVVAQAALGMINFEHRNSGPHGLTLGFGETNNIGERKLNFKILDGYSKEEGEVVEASVTQTGHNLYNVAVHRKGDE
TPQVFTNIACQPEPEGEVMKLESYFPLERIQSSVVPQHTDNDTKVTVFQHGVKTDLVLLPPKWYEKALGLKESSASVAAPMPC
KILKNEVVEGQIVQKGAPLVVIESMKMETIIRSPQDGIIKKLAHKEGDICKAGTVLVLFEEGETKDGES
FUN_002583 CDS scaffold_13 173653 174205 +
hypothetical protein ENOG41

MTTNPASRKTTNLVANPNVSLLVHDWVSHRPPTHGRRPSGGSPGPEHRSSLASLLLNLNTSALSSISATIGGAARLVPSGTEE
EKYYREQHLENNTFDESAAQPFQRDSGPVEDGGRNCFVAGEEVRVVSVDIKDVRISDWKGTVRDWAIVPEGNLVNGTQQE
FUN_002584 CDS scaffold_13 174658 176472 -
hypothetical protein EOG092624SJ PF03547

MASWKSFSVPSFRLTQLAQDAYELPLQTLANTHESHPSIGHLCLLVFEAVLEVVCVSLPGYIVARLGHFDAEKQKFLANLNVM
LFTPCLIFTKLASQLNAEKLSDLAIIPVIFVVQTFVSWAVSYVVAKLFRFNRRASNFVTAMGVFGNSNSLPISLVLSLSQTLK
GLHWDKVPGDNDDEVGARGILYLLIFQQLGQLVRWSWGYHVLLAPKDKYPEYREEIAEEGQRYHDDENHDDYQNAALIDGLDG
ETEDEGDSHSIDSQNYDPAGRTPVANASRVSLAVSSDDEYLPKKPHFKNNQEQTDVVAPLNGNEGSMDSFPRVPALEDQEEPT
GIADRTKSAIKSPFIRLGKATSQTLSNWYQKSPAPVKSCLKVTKRVAGKFNNFIWEFMNPPLWAMLIAILVASIPALQRLFFE
EGSFVQNSVTNAVRSSGDVAVPLILVVLGANLARNTMAKDEALDPEEERIGNKLLIASLLCRMVLPTAIMAPMLALMAKYVPV
SILDDPIFVIVCFLLTGAPSALQLAQICQINSVFEKTMGRILFQSYVIWILPSTLILVMMALEVVEWAR
FUN_002585 CDS scaffold_13 180959 182978 +
hypothetical protein PF00069;PF07714

MSASDSIPATPEDWKDLSTPASPGSEDSSEPSTPLESPGHDSPSEPPRRVPSLRSVSDPRNMNPNSTAMGVLGMARRPVQTSS
SFAGSSSASVENSIMAKARALHQQRMQKGMSPAGNPGTPSGSSPTASIANGFPTNLGLPPNMQRPHAPHVNSAPAITKPSLSE
RRAMGGGMGMKLSDMGRPSPVTATGKKRGPPGKLSDITGDAPGSQQSNGTGGGQGSKMDDFKKYIDTEKGWVTFDGAATITRT
GVNFANGQTFSISLDEVEVLTELGKGNYGTVYKVKHAKRRVPRFGQGLSRKPLPVQYSQSDPSPIGSAEDSAEGLPDTTDGTT
GTVMAMKEMRLELDDAKFTTILKELVILHECVSPYIIDFYGAFFQEGAVYMCIEYMDGGSIDKLYNGGIPENVLRKITYSTVM
GLKSLKEEHSIIHRDVKPTNILVNTRGQVKICDFGVSGNLVASIARTNIGCQSYMAPERISGGGFAQAGNSDGSYSVQSDVWS
LGLTIIECAKGAYPYPPEVSSTIFSQLSAIVEGEPPAMPEDTYSDMAKDFVKSCLHKIPMKRPTYAMLLKHPWLVEFTKPQTI
TEEAEEGDGVDTVAEAVGKIDLSSSTADTEVADWVNGVLQREKDGLKEDGPLKPALHNAPLDSVSPMSSPKDA
FUN_002586 CDS scaffold_13 184045 184956 -
hypothetical protein PF06687 ENOG41

MAKNAPLGLAGLVLLAVSLLFLWFIILSGLTSTTPFDKTYFLRADTGGISGAREVTQWNFFYICGAGNNDCDGARAAPVIGRA
WDSNPRNAPSSLVGSRAGDTTSNKQFFLWRFGWVFILITLFFETLAFFTGFIACCGRLGAGISGIMSMFALFCSSVAMSLMTA
TWVLARNAFHHDGRSASIGRYAFGFAWASWATLFIATILFCLGMRGDKSSSGGYSGRSWRRRRSVRSSHGGGYEGRRVKDDYS
FUN_002587 CDS scaffold_13 186849 187866 +
hypothetical protein EOG09264AWW PF03099

MPPGLRLKAIPSSAIKSTSFRIGAGTSAGHVLVNRWSSTYTAPTSELPSLKLLVHRHLTGSGPDAFVPYDTANNEQEELRSLF
LEWKSLSEEQQQHRGHIPRPHLLSFEPTPTLTLGRRQRPLTSEQTSYFKSPLYVSLPHRRNPVNEQSFIPEVKQTSRGGLTTY
HGPGQLVLWPVLDMHSPLYAHYSVMSYANHLEATTRRLLACLFGLETYTTRDEPGVWVRTPAGQPERKIAAIGVHHRRHVTAL
GIAINIDVSVDGPDVVNPWARFVPCGLEGKLVTSVAAELAMKDGIARLGGWNTASLAAVWAKIFEEGIVDDTKRSIDGEGSSQ
FRRAAS
FUN_002588 CDS scaffold_13 189216 190761 +
hypothetical protein EOG092624X0 PF01565;PF04030

MDPLVAAELAKFDDSVAFRARPQHVHHTWARTFSSLPELFIQPESLPEVEKVVNLARRCRRRLVTTGCGHSPSNITCTSSWLV
NLDNFNRVLSVNKDTGVVTMEGGIRLYALCEELEKHGLTMPNLGSINEQSISGAISTGTHGSSLRHGLMSEDILSLKVTMADG
TTVYCSKDIKTDLFRAALLSLGAIGIITEVSFQAVPAFTLKWEQSIDADHKMFESWNRNLWTQSEFVRVWWFPYTRRAVVWQA
EQTNEEHRDPPQSGYDGSIGYYVYHNLLYLAQHVPRILPWVEWFVFGMQYGFRNGTTSSAVQPSRKALLMNCLYSQFVNEWAI
PLHKGPEALRRLSSWLNHLTPDDPDYVPHNIPFSADGLYVHAPVEVRVSDTTLTSNVRPYLDITVENGPTLYLNATLYRPYLM
DPPCHERYYEAFEWLMKDLGGRPHWAKNFRTTRPEIEAFYGKQLESFRAIRNDADPQGMFVGPWHRETIMEDGEGLEFEEVEI
RRGKNRTGGVTTFGII
FUN_002589 CDS scaffold_13 191061 191860 -
hypothetical protein ENOG41

MVDKIFLSTVAADALFLSSGVMELVFSLVVQSKMKDMPTDGEDVTRNLLYQRFPLTAGIVNAIFILVTFAVTLPGLVMPARSF
LKVSGYMVTVCAIFTMCVAVFLWVMTLRMSEQFFNIYIEQEPDVQSLIQDSFQCCGYFNSTTPAFVMDSTCSSPASAALLRGC
ATSISSFANLHIDGIFTVLFGIVGIDAIFILCIACLLKDRKERERYRHIDEKAGYRQI
FUN_002590 CDS scaffold_13 197965 198981 +

hypothetical protein PF00319

MADITDQHDQTSPTELDDSQNVGNGNATESRGIKRQRPSAGDDDDDDDEKGSRERRKIEIKFISDKSRRHITFSKRKAGIMKK
AYELSVLTGTQVLLLVVSETGLVYTFTTPKLQPLVTKSEGKNLIQACLNAPEPTPGNENGVDGGDQVESPEEPPNQHLPPQGN
RPGMPQNPHMPNNYMPNMPMDPQQALAYQSYVQRNQAYGSGIPPQPGMPANSHHQS
FUN_002591 CDS scaffold_13 201705 201903 +
hypothetical protein ENOG41

MPLLHEESPKSGGKRVAFRGQEHATRKPPASVKTEIKNEYKQTAKALQSSTQQDGGGGGEAPGDE
FUN_002592 CDS scaffold_13 202473 203127 -
hypothetical protein PF04145 ENOG41

MNFPRHDMPGMDMGGDSSGSSSSSHGSSIMTMVFQTETRTPLYANSWTPNNAGSYAGTCIFLAVLAIIARALVAFKAVQEARW
LDREAARRYVAVNGKIPLSEQIASSPDARRMTLSENGLEETVVVVERKRAAMRPWRFSVDPVRASLDTMIVGIGYLLMLAVMT
MNVGYFLSVLAGVFVGSLAVGRYIPTVEH
FUN_002593 CDS scaffold_13 218426 219862 +
hypothetical protein PF00096;PF13894 ENOG41

MSMEGASFSSRRPAAGALPAMTLPPPTADVPSMAKYPFYPSFTGNSSESFQCSREGESVGYSYPPSYSPLPGPFPDRRISSSP
SILTPSPGASDGLSPSLSSVNTGSSQGSQAPGNMYQYAQLPAAWATPSNSSYTVSSATQAQPALGQQHFPGRNHSIYNQGPGM
HQYNHPRSSQSPATGGDGLPAPPYDQVHQPFQTAVSGGGGQTTPPGLSTSQQTQGAILTSQSSVATQPPTPSSASAHVDSYTH
SRPPSTPNYYTSSASQPSSYPPYGHQPSPTQHSSSNGGPVPRGLGSISGQPHGAGMAPPGPYRSYAPYQTLPTMGGSVLSNIH
QPGSQMSMIPGMPVTAYGHPMLYGGQGQPPPTQSERPFKCDQCVQSFSRNHDLKRHKRIHLAVKPFPCTYCSKSFSRKDALKR
HRLVKGCESKANENPGDGNGADRSGEEKDSPEVKREQ
FUN_002594 CDS scaffold_13 225427 227231 - ACU9 Malate
synthase, glyoxysomal PF01274

MASVDSVLQGVSVSGKVDDIHRKILTPEALAFLALLHRSFNERRKNLLERRKARQAELDRGVLPDFLPETKHIRENPTWKGAP
PAPGLVDRRVEITGPTDRKMVVNALNANVYTYMADFEDSSAPTWDNMINGQVNLYDANRRQVDFKQGQKEYKLRTDRKLPTLI
VRPRGWHLEEKHVTVDGEPISGSLFDFGLYFFHNAFETQRQGFGPYFYLPKMESHLEARLWNDAFNLAQDYIGMPRGTIRGTV
LIETILAAFEMDEIIYELRDHSSGLNCGRWDYIFSVIKKFRNNPNFVLPDRSAVTMTVPFMESYVKLLIQTCHKRGVHAMGGM
AAQIPIKNDTAANDKAMEGVRADKLREVRAGHDGTWVAHPALASIATEIFDKHMPTPNQLFVRREDVTIGQNDLLNMNVPGTI
TEEGIKKNLNIGLGYMEAWIRGVGCVPINYLMEDAATAEVSRSQLWQWVKHGVNTAEGKRVDKSYALKLLKESADQLAASAPK
GNKYHLAAQYFSSQVTGEDYADFLTTLLYNEITQAGPPSPASKL
FUN_002595 CDS scaffold_13 230314 234176 -
hypothetical protein PF16797

MVSTVRSPLSEASNRINSNYPTQEGQRHKPRYDRPNSSIVNNTARTNYGHPTAYFSGRPPTQQQPTVSTPQEQATLKPPSEMD
DERRHSAASYDSSGSGRSKKNYKTHIGPWQLGRTLGKGSSARVRLCRHTGTNQLAAVKIVNRRMAYLVQDSSLAALSKWDNNL
PEQIDGEMRVPMAIEREVAILKLIEHPNIMKLYDIWENRSEVADLQIKIADFGMAALHQTDTHQLATACGSPHYAAPELLKNR
QYRGDRADIWSMGVILFAMLSATLPFDDPDLRVMMARTKKGQYEMPSFLSLEAEDLIRRMLQVNPDRRITLKDIWRHPLIRKY
AYLDNLSDINSQPPDIRKGFQYTPVPTKDVDPQLVRQLRSMWHMFSENDLKLKLTCDEPNDQKAFYWLLYHYREQQLEDFKPE
LAHSTSDYHHMKPGTWKKRVSTCEFAQPSGNGHGRSISRFTVISNATEPEPALNQEPFQTEQNGSYTGQPKRLRSGSGARRGR
GTSTRNSTTGRLQSSRGSLSSMHSSRQGTPHARSLRHKRGVDFTHVRKRSTSSSNRNRSGSMPPYVTEQGSTYQIEMIRSHTP
ELPSLPSGVLPKLADGQEPQQRSSGQALRITLGSRYASEIFKEELRHFSSNIAKDCDEAFKSSLIEEDSIAGSLTEPDHTQYE
STPFSFTIDAPEDSAPDLLELSNKSFSNRPLPPLPTPTPSYNGNSLAPTPTGSRPVTGESYSEELRAEQVKVAQPVVLTRHAE
RRVVSAPTYTQKHKRSAALPSINEAGAAASDKARIVSAPPHTPTKKGVDRIRGIEYLSQVENSIRVVHSPGDESPVKIPEPLN
VRKKVPEPEQRNSAPAAQLREPDSASQRSVDGGTTATTLSLEPPSHDAQAVTKKKKMSWFKRSSKVESEPSRESVEWQDCTSY
LTSSDGKRSDSTSTEAPTKKKTFHFSFWKSNKQRDSIMSIARPDQKEERPSLEFNTMGKTHVSAKVSQSKWHESGSGPVRNIE
VKQNWLARLFRVKPATDHICMTISRRRARQEVTILLREWRKYGIRDIQVDKQRNIVFARIAAKNCEWYLDMIAAPELTETLVL

NLKEVSFAAEVITVIEHGKKQPLSIIRFTQERGAASSFHRVVDTMNMIFDARALVVADRNKRKMMIKTLNS
FUN_002596 CDS scaffold_13 236068 239655 +
hypothetical protein EOG0926071Q PF02463;PF13476 ENOG41

MAPASSRRRRRSDTDNTDDDDDETHYGHRSYADDIGSSKRPRLDQDGDHNATPEQPHHGKVNGASNGNNMDDGFQPGAIVRVS
VQNFVTYEKAEFLPGPHLNMVVGPNGTGKSSLVCAICLGLGYSPKHLGRAGSVKEFVKHGKDTATIEIELYRRPNHRKNYVVK
VQIRREQNNQKWWLNGKETSHKEVQRLIRKLKIQVDNLCQFLPQDRVVEFAACTPVDLLRETLRAAAPEEMLIWQKQLQELDK
DKKELEQATHVDAETLRNLENRQQGLQTDVDRIREREEIIDQVKNLKSALVFAKYSEARTRHKDAKERRKLAERSLRRLEHDA
GPSLQAVNEKQLYAQQIDEATLARKTSLKNAEDAAKKLARDASTASENLKEFENSLEAERKGFDVKRKELAQSKSRITALQAD
IRNQPEEFNPSEFNQKIRGEEHNLRELEREIREVSSQREEVKNKGKTINNEIRQVENNIQSLETQQGQQLNFMRKNFPELATA
WTWIQEHQGDFEKEVFGPPIVSCSIKDERYSDQVQSLLQGDDFTCFTAQTKNDYKKLSDELYRVQSLSVVIRSCAQPLNAFSR
PVSMDEASDLGLDGFAIDFLEGPEPVLAMLCAEKRLHQSGVSLRDHNDAQYDRLVRSGRVNSWAAGTQSFIVRRRKEYGPQAM
TAVTKNIPPGRFWTSQPIDAQEKQEMNKRLVELNGERDILKQQYHELQDKIQAIEDRRTSTHDNITRLKSEKNALQKEYQKWQ
SLPEKIESEERSKIAHEQSMQDARKRMVEIRYDWDEAVLRRAEIVLRHKEAIENIRRAHQALLEAEIRGIEAYSDVEGLKGRN
AHIMQRLDAEKEILQQATDDASRARDEGNRLSDEVQQVLENEPEKRDLFSQLCENKSSQDIQDEIGGEEAKLEMVHVANPNIL
REFEKRAEEIDRLTRKIAGSNDQLQGLSQEIDGLKSRWEPRLDELVSKINDAFAYNFEQISCAGEVRVHKPEDFDAWALDIMV
RFRENETLQQLTAHRQSGGERAVSTIFFLMALQSLAQSPFRVVDEINQGMDPRNERMVHERMVEIACREHSSQYFLITPKLLT
GLRYDPKMRVLCIASGEHMPREGRKLDFKRCLNIQKGLTAASA
FUN_002597 CDS scaffold_13 240480 245010 -
hypothetical protein EOG092608GK
PF01344;PF07646;PF13415;PF13418;PF13854;PF13964 ENOG41

MAFLFKSKKHPERSQPASRDAGSGSQGSIQSASARTAEKNSLQHRATPTGSLNSIDNEGSNGSPDHSHGHGHGHGRRGGSADQ
PIQQSSDTSVRNGTPMNGPNASLYPWSQRRLTYTSTHPSPFPRYGAAVNSVSSKEGDVYLMGGLINGSTVKGDLWMIEAGGNM
ACYPLATTAEGPGPRVGHSSLLVGNAFIVYGGDTKIDEADVLDETLYLLNTSTRQWSRALPAGPRPSGRYGHSLNILGSKIYV
FGGQVEGLFMNDLSAFDLNQLQMPNNRWEILVQGETSPKMPAARTNHTMITFNDKMYLFGGTNGFQWFNDVWCYDPAINKWSQ
FDCIGYIPAPREGHAAALVDDVMYVFGGRTEEGTDLGDLAAFRISSRRWYTFQNMGPSPSPRSGHSMTTVGKSIVVLGGEPSS
ATTSVSDLGLLYVLDTSKIRYPNDAQQPAQQRAHGSRRPSASEGSRPHPARDGSNGPTDSRKLIVGGASAPANGQRSPPNNGD
AENPQSATASTKLPRASAMPPSGPPPQGPMPARPTVDVSAVARVRGQSAERSGASGSPRTMHSGSPITREVINETESPISNGR
RTPVQQNARAGSRQDQVNGDPSKAKPAQQTRPQGAGDAPPDVAPKPAMTPRPASPPVPVRQPSNPLSRRSSGRNSQTVVLLKE
LDSARNRNAWYASELELARKAGYVPNTSYSPLLDNKATETFDDEDRPLIEALLAMRSELANVQTAVDKQAVIAAKQIAEAEKQ
RDAAIQEAVYSKAKLAAHLGASSASTPQLDGPRDDFEGRSSELSRKLATALHIQKDLQSRLDSAKSELDSEKKARQLADDTSN
AAQKRMAELESYKQQTSTEVERLKAELHLAQHEAREHSVAHAEVAAAVELLRIEKNDVQQKYEEAIGSSKDHSETFDSLRAAI
LASEDSTGLLESKLAEERSQREKIEAQLNKLKFEHEARTAELVATTQRLRDAEELSEKHAAEARTNRQAVLAGLDKISARDVS
GSSKADAERIAALQNQLTTSNELVKKSQQELEAAVHKLRGAEERIAGLEQYQEQSSREGVTIRRQLQSALRDTQSLQAANTDL
KNQLAAQQLETNAMTVQHNALKDILSERGISPTASIRARNLPNPRTNSPDLNRVRDLESQLATSSAAHEETKQAFAIQIQESE
AAWREKLSQLESDYQSAVHYVKGTEKMLKQLKDQLSRYKTENGRLKTEIEELEDRVQSSTTSTSPHWENEKAELEARIRSLET
ELQESSAQMEKRLLGLQTELQETNHHRETALKNSEEVTQKLSNHRKDLEQLQSENALLEQRASDAEQKVALLLDQVEHSVDNY
RRRSRQVPSLNSEMAASANGLGLGHSRQESSEGESIYGGTSGNGGLEARNSAALDSLANELETLRSQWEATNKNYRLSTNFDF
DTTSAAKKDNDTATGVGLSESLADWRKRLDTEEHHSDGEKPRHP
FUN_002598 CDS scaffold_13 248711 253253 +
hypothetical protein EOG092609RF PF14538

MALRSDVNGTTAPTAEPNGAASAARPRMNGRSTSNDATRSDDDDQRPLSAPNRRNGSIALETRDDIEHHQKTRPSKPILLRSK
SDYAPRPMEDPEPTEDDIPEWGARHGFEDHYQSEDIISQLANNWYMYFTDKRHETTGKPKPLQYELQDWRQRDRLKTVSAALA
VCLNIGVEPPDQLKTNPGAKLEAWTDPTIPPIQKALENIGKSLQAQYETLAIRARYKQYLDPSVEETKKFCISLRRNAKDERV
LLHYNGHGVPKPTASGEIWVFNKNYTQYIPVSLYDLQHWLQAPTIFVWDCSEAGNILNNYHRFVEKHEEEEEEAAERDPNYTK
TNFRPYIHLAACAVKENLPTNPLLPADLFTACLTTPIEMALWFFVLQNPLKTNISPERAKKLPGRLQERRTPLGELNWIFTAI
TDSIAWTTLPRDLFRKFFRQDLMVAALFRNFLLAQRVMTVYGCHPQSYPKLPDTHQHPLWETWDLAVDMALAQLPMLEKKESE
GFDYEYQNSTFFTEQLTAFDVYLTRGDAMAQKSPDQLPVVLQVLLSQQHRVRALILLGRFLDLGPWSVQLALSIGIFPYVLKL

LQSAAAELKPVMVFIWARLIAVDLSCQQDLIKDSGYSYFAQILKPSEGLPVVDSDEHKAMCAFILAMLCKDYKNGQMVCNQTE
IMSYCLTHLQNEENPLLRQWACLCISQLWQDLPEAKWRGIRENAYVKLAYLVRDPCCEVRAAMIHAMTTFLGIPDLTEEVARI
EESIAWTILDMGNDGSPMVRKEFIVFLSHFAIRFESKFLVAAYEQLVEEKEYLIFPPQDDGQEHKMGLHYARPDNRNKDGTIK
PMAHGLSHNTVYMALWKLALVLSVDPHPEVQREATIVVDFVHNALLSSKVGVQTQLVMAEIRKRTARTAHKHTPSASQRNNAP
GTSNSQPLPSPGLLRRTASLLFPSLVTNEDKSRPTTPTLPRSPSLKLTPNQIPVPAEQNDRPSSLAQYNVAHEPYSGAYKERD
LMKPPALPLKSKFLEWSIEYFREPQMKPSEADEPGSTEYNERLWRRARNETIFRETQPLKQQAGSHKWNNQLGIINNGAQPAK
MTFHQFEDHLAVADDGNTVYVWDWKKQGRLSRFSNGNPEGSKISDMKFINEDDQAMLLTGSSDGVIRVYRNYESDRAIELASS
WRALTHMVPSNVNSGMVFDWQQVTGRVLVAGDVRVIRVWYAAYETCVMDIPARSGSCVTSLTSDQMTGNMFVAGFGDGAVRVF
DTRNRPQESMVRKWKDESDRQWIKSVHMQRGGQRELLSASRNGKVKIWDIRMDKPLHSFQTTRDTLRTASTHEHLPVFAVGTS
AHAVKVFNLDGHELSNVEPYSSFLQQNRSSPISATAFHPHRPILGCAARGDQHINLFTCEKSESLHPS
FUN_002599 CDS scaffold_13 254085 255958 +
hypothetical protein PF00152

MADAPAEQAAAPPPAEQAPAVSGEAVEGGEAGPSKKALKKAEAKAKKEAEKARRAAEHEARQSAAKAAAGNTEDLAKDNYGDS
THLTKIDAERVKLRNLSDEHLGKTIKLRAWIQNARMQGAKMAFVELREERDWAIQGVVAASSEGTPVSKQMVKWIGSLNLEGY
ILVEAKVVKPQEPVKSVKVSNYELHISKCYVISPGPQVLGMGLAVANRPVTNFDDEDAGGPVEEVKEGVDNLSLENVPGASMA
THLNNPVMHKRAPVQQAIADVRLAVRKLFVEYLEARDFVQFEAPCLIGAASEGGSNVFGMPYFDKQAYLAQSPQFYKQYEIAG
GRKRVLCIGPVFRAENSNTPRHMTEFTGLDLEMEIEDHYHEVRDMLEGVLLHIFRGLQERCAEQIALVRTVYPSEDFLLPKPG
KEVRLTFAEGQALLRAEGPEEYRNVSDEEDMSTPQEKALGALIREKYSTDFYVLDKFPEGARPFYALEDPENPKVTNAFDFFM
RGQEILSGGQRIHVPETLEARIRTKGIDPKSQGIKEYVDIFRSAGVPPHGGGGIGLDRVVAWYLNLPSVHLASYYPRTPKRLL
P
FUN_002600 CDS scaffold_13 259232 260476 +
hypothetical protein PF00170

MDFTGQYNFANPQPYHQFMPIPPLTPSHSHSAGSDDFNASPPPCLSVSLPEHYSSNVEPYKHDLLGIFNVENYDGLSASQNDQ
FQSFDYTTAQGFNANPHQPASGFPGPPTPPGYTSQAQAHQQQGGSLRNGSPDEQSVARGGSEEDENLTPAQSRRKAQNRAAQR
AFRERKEKHVKDLEAKLAGLEAAQQQSSLENERLKRDLQKISTENEILRATSRMSHGSISPEPATGPLRFKPTDFYSNVLQNH
TNKSPSHRIVTSDDGERLLAAGATWDFIISHELFKKGLVDIADVSERLKNCARCDGQGPVFSERSITNAIEQSVASGTDDLL
FUN_002601 CDS scaffold_13 261269 262619 -
hypothetical protein PF01066

MVYVRQKELPALREYKYSGVDHSLLSKYVLKPFYTKFVIKCFPMSMAPNLITLTGFSFVVANFLTLLWYTPTLDQDCPSWVYY
SWALGLFLYQTFDAVDGTQARRTRQSGPLGELFDHGVDACNTSLEVLLFAASQNMGQSWQTVAVLFASLLTFYVQTWETYHTK
TLTLGIVNGPVEGVLAIVLVFVFTGYVGGAHFWQQSMFHTIGVPSTIGIPSFIYNLTFTEWYLIQGSIVLVFNTVESSLNVIR
ARRARGDRSRGALLGLVPFFSIWSMVVAYLYLQPKIRECHLIPFALFAGLVNAYSVGQMITAHLVHLRFPYWNVLGLPLAFGV
IDSLGPIFQRYLGFGWPSALGDSVYQVAFMFMMLGTAVGVYGSFVVDVIVTICDYLDIWCLTIKHPYVENEDAQTNGTKKD
FUN_002602 CDS scaffold_13 264040 265660 -
hypothetical protein ENOG41

MEDLSSPISIASSTPTAPTSASKVTYTTAGTPYNPSTSQPLQPPARRGRSLKWPAGLPAAGLSLLPKSVLATLPLKAGSRSSP
LQQYSPQTPYDRASSPLNDTDHRCINMSAQVPRLVPSATPSPLTSLFSEAEQDTFTDVDPSEESDEECELGMDPLMNMTVKSL
HNLASYPNPNQKRAQKALLRGLKPALGLKTTTRVDEPLAPITHSFGLQDASNNNNNDLSSVLQQPQAEKPTLGRSFDDVFWNS
HVNKSPSIPLGTSRSFNRAVGSNTARAAGTIAPGAGAPRPLTAGPPGQRQYRPSTFESTFKALHTNIPSQDVKQDEDEDVLII
TRQTLRQVGISNNSLAQNIPSFEATASLASQTAEGLEGLSANSKTTRPSVPTTAATRGSQYPARGTSSWGLGTATTSVCWTSS
PELSQSRYTSGPRFRAPKELQAYNNRINHAWYYGADFLVATPSEIALQVQPPKGKGQFGAIGDGRPSKKSEFHRRMEIEDANR
MSVTDHARPLFNMAHANVQQFIEGEWNKSQGRRATVIQATK
FUN_002603 CDS scaffold_13 267543 269723 -
hypothetical protein PF06687 ENOG41

MLRPGTPLTVLLFAAFALLLLAVISTPIIEAIPLSDFGGVTYGVFGYCQQGKGCSGIGVGYDTAKLVGDDSQAFDLPSGVRNT

LSAILIVHPVAALITLVMTIMAAVSHLHAPSHSTRYLLILFIFLLIDFLVCLLAFLIDVLLFVPHLAWGSYLVLAATILVAMS
GLVTCAMRRTLVSRKDRQRRIAQNAEMSGENYYNRTAQDKTTAEPEVSRQPTLPTVSGGNGVHDNLPSFTTFEQKSRSDQVSE
EAIPLTRRTTSNRSPAPPNEVASVGEVAAFSRNPQRQGSRDQFGNPIDTATDPYATRRGPSPSMSNRGTGAGGGYRGGRGGYS
RGGYDNYGAPVRGRGGYGPSGRAGYGPRGGRGGYGQPNRGVYAANGMRGGLNAPPGYNNGPYDRRPSPAQTFAHDVQPSEPSN
GYSSTNPSMPSVNTYTAYSPESTLPRAESPPPLPGTEPATVGGRAVEMDASPAVQHHDGGTYGQLRDSDADVAGMVGLQQGQI
PQRHDTYMSEGSKYSTDEQYVPPRSAWAQGDGRNSPRAPSPLVTGQPKAELTERRTPPVVQQPVATGNSNYYEDVDPRFASDA
PPASNNLQPPPIEPIYEDIHANNAGTRSPAESEHSTFTSISQRGINPRWNPNPPPLPYQQGPPHRRPAHQQQQRQDMLLDTPD
FQLPSSRTGRGPGMVPGSAYPPGGF
FUN_002604 CDS scaffold_13 272562 274539 +
hypothetical protein PF03109

MRCRHRTLASSLRRLQSGGPFQAMRPPSPEELGAPRRAKEYRRGRRWARRLLLTCVLGGTIYLGDRQIYASGFGRSLHTFGTG
LLVAFDYKLNFRPQPITGGTVQDLHNRNAERLFNLLRSNGGLYLKIGQAIAMQSAVLPPEFQKMFSRMFDDAPQDDWSDIEAV
IRQDFGKSVEEVFGVSFTGKEGLGLMERKARASASVAQVHWARLADGREVAIKIQKREIAKQISWDLWAFKTVAWIYSKWFDL
PLYKLVPFITERLELETDFVNEAKNSEKMRELVNAEKALKGRVYIPTVYPEFTTKRVLVTEWIEGVRLWDKKAITSRWLGGHG
NGSPGAGRPLPQIDLEAARLELRTRPFQENLKPERQELWRGRRGRGGLGLSTREVMTTMVDLFSAQIFKWGVVHCDPHPGNIF
IRRLPNGRAELVLIDHGLYVYMSSKFRNEYATFWKALMTFDNKTISQVTEAWGIKAADLFASATLLRPYEGGDKRTHNGIMKE
LEGNTPAERHYAMQKRMKQGIREILTDEDKWPQELIFIGRNMRIVQGNNQYLGSPVNRVKMMGEWASRSLFEDPQLPLSQRLQ
NRWRHIIFKTVMAASDVAFYIFKLRQWLGLGGGMEDEMEKRMKDVAQDFGIELQHDVFEG
FUN_002605 CDS scaffold_13 277732 279361 +
hypothetical protein ENOG41

MNNEQPRRTSFGMLLRRSKSGDLGKGGRKAQALREAELERQRQASTRIAPKLPEFANHSEQLSKSFGPELRPESPANVPRSAD
YTAIRPSAEYPRGYGAAAHAAVPVPPLPNGGFDPYARTESMTNRGRYSYASSAISTVNSPRRVRRRKDPTPLNILVIGTRNSG
KTSFLEFLKTALALPPKKRAKKGDDEVPQNRNTPSGNFIPHYLETEIDGERVGLTIWDSEGLEKNVVDLQLREMSAFLESKFE
ETFAEEMKVLRSPGVQDTHIHATFLVLDPSRLDRNIASARNPAINGQQNGHLGARVLGSLDEDLDLQVLRTLQGKTTVIPVIA
KADTITTKHMNVLKRSVWDSIKKSGLDPLEALGLDDEDESSITSERIAEEDEEEDEAGHERDGAQTPESQEFPLQGHRESPAA
SPSSKRLSTSSIRRHKAQEEAKEKEDEIPFLPLSIISPDLYEPGVIGRQFPWGFADPYNEEHCDFQRLKEAVFSEWRAELREA
SREQWYEGWRTNRLKQRDVPYRHR
FUN_002606 CDS scaffold_13 280329 282555 -
hypothetical protein ENOG41

MNTRSAAGRSKRASAQNENREPRRSNRRNGPVQRATSPTKNIINQPVPGPGRVLLNDPVARRNPPPQARDDISIPSNPLKPDD
ETEFWDEYDYAQPENSGEEPEDVNSPIEKLQLGLEITTAQQRILELSIPDLARAADSLFDCFQGNEADDVFNGILAIKRRAFH
TIRGEYDALDQSSTAPFIDFAHFVDDTPTQNQSVRVTQIARTNVVTAYDKLYGLENEEASAIFPFLECLNRVLPAFFTPANNM
FQNPQSTLHLRTWLFVEDLSHRKDEKDFRELLVSYFCKDKEILKDLDRSGHKGEGGLPSYPELFSGGYFRDLGGVEGVDHDVD
DLCSARIAQIKKIIDDNKEDGGISQLRKHFPLHNLVEDLLKCFESLYNVLAFEESVRAGTEPPQHGQQEAAYSQGNDYGSESQ
SIVRVGSQEAEPSLFVGPKSIQALRGGSRATSTVPASNQQLAGFRSGVPRDYHEDSNADLLGGSPFPPASSYRLATKGGRERE
RGQKRPRLSQDDEDGEDPFETDTRRVDPARRDELQRQMPPPPRPQPVPRHPSRHVSECSFSSGPVHPLSVQESSQDMGLRSVS
QAPLSSRPFNAIAKAASQMKKEVRMADPERQGPRQRVPWSEHDSQVLCDLIKEYGVRWSSIETMSERFEYPRNQQAYRDRARN
LKTELLMIDMPLPPNFDAVALSNKEIAKIRGVGKNPFRKEADIDDDNNPINTELAESTPF
FUN_002607 CDS scaffold_13 283092 283699 -
hypothetical protein ENOG41

MASSLPTFPRIVFTIIEPISLVAGFAGAVIDPAWFIGEQSPQKNDGDASPNSIIIAWQLGNLYLLLAFIGVAILSTTTENRVV
RSYLVALWLADIGHVGFSSYGIGRDRLLSPPQWNAMTWGNVGMTLFLFLTRTAYLTGFFGPDHVDKPAKKTA
FUN_002608 CDS scaffold_13 284240 286050 +
hypothetical protein PF00206;PF10415

MLRTVTGRAAASGLMKSALPRVSQAAASTCSLAPQLRLQNKTRVGGLQLSRAIHSTSVRMSQTRTESDAFGEIQVPADRYWGA

QTERSLENFRINQPQDRMPPPIVKAFGILKGAAATVNMRYGLDPKIGAAIQQAAKEVADGKLLDHFPLVVWQTGSGTQSNMNA
NEVISNRAIEILGGTMGSKKPVHPNDHVNRSASSNDTFPTVMHIAAVLDIEGELLPALHSLRAALQKKVDEFEAKKIIKIGRT
HLQDATPLTLAQEFSGYVAQLDFGIKRVESSLPDLRLLAQGGTAVGTGINTFQGFAEAIAEEVTNMTGTEFKTAPNKFEALAA
HDAIVQAHGSLNTLAASLTKIAQDIRYLGSGPRCGLGELNLPENEPGSSIMPGKVNPTQCEALTMVCAQVMGNHVATTIGGMN
GQFELNVYKPLVIRNLLHSSRLLTDGMRSFEKNLVAGLAANEEKIASIMKESLMLVTCLNPKIGYDMASKVAKNAHKKGLTLK
QSALELQALTEEEFDTLVKPELMVGPSPYKG
FUN_002609 CDS scaffold_13 286949 288698 +
hypothetical protein PF00249;PF13921 ENOG41

MATIEPRLIHLLNEPTRPQLNHADLPPLHSLAFPSATDRSLAPIESDTNHRGDRPLADTQSVINAVDLINLTNDDISSEPRKE
GNAQDGKIVPASGGFPLRVASSDSDAIEPTNSISRLLDDGPELLEDVSNKKRQRSLHVKDDFMQLPQPLKKQKAAQQAPVMPP
IINGLHEPPPHAALFPPISSDSFDSKDGSHMKTMPDFTQPSAQSSTQASQEAESVSVASKTRKRTSKPRRKWSEEETNHLLLG
VNRHGVGKWTSILEDADFKFNDRTAGDLKDRFRTCCPEELRKSNRSLELDSPSRFDRGSKQKGKADVHLERILIDGNGASTKD
TASAMQQEVDTTPKQKKSRAHRKKMEDLVELGIHGPFKKSHRRERRPFTEQDDMEILQGLEIHGPAWTKIQRDPRFHLSSRQP
TDLRDRVRNKYSSVYQRIEKSNLQVREMSRGNDVMEPSVRIPAGNSFKPRKATTLEPQMNQSTSREDLSRWPAQQRIETGEIM
GAGHVFEFGETMPPQFMGGEMDISRLLLDDAKLSPAIPRFGIEGIPGLSPPANTPQKSHNKEGMQHPSRLSTVGPASISRGAL
H
FUN_002610 CDS scaffold_13 289353 290374 + RPS4 40S
ribosomal protein S4 PF00467;PF00900;PF01479;PF08071;PF16121

MARGIKKHQKRLSAPSHWLLDKLSGTYAPKPSAGPHKLRDCMPLIVFIRNRLKYALNYRETKAILMQRLVKVDGKVRTDSTYP
SGFMDVITIEKTGENFRLVYDTKGRFTVHRIQAEEAEYKLGKVKRVQLGRGGIPFLVTHDARTIRYPDPLIKVNDTVKIDLAT
GKITDFIKFDTGAVVMVTGGRNMGRVGVITHRERHDGGFNIVHVKDAIDNSFATRESNVFVIGQDKPWISLPKGKGVKLTIAE
ERDRRRAYAISH
FUN_002611 CDS scaffold_13 292273 293965 +
hypothetical protein PF01565;PF02913
AA4
MEKAIKRIENEVGIEGFISTDPEDLLAHGYSEWSTVNPDTLPVAVAYPRTTEQVSVIARICHEHRVPIIPYSGGSSLEGNFSA
PYGGISVDFAYMDQIIQFNKDDMDIVVQPSIGWQDLNAKLLAMDSGLFFPVDPGPSAKIGGMIGTNCSGTNAVRYGTMKDWVI
NLTVVLSDGRIIKTRRRPRKSSAGYNLNGLFVGSEGTLGIVTEATLKLAVIPEQYSVAVVTFPTIRDAADAAAGVMQAGVPVA
AMEIMDEVQMRVINLGGATKPRVWKELPTLFFKFSGTKDGVKDNINRVKKIAAANKGGSFEFARDAAEQQLLWSARKESLWSM
LSLRKNGDDVWSTDVAVPFSRLADIIEVSKKEMDELGLFASILGHVGDGNFHESIMYNKNVPGERQKVEACVKNMVKRALDMD
GTCTGEHSIGWGKKESLLWEVGPETLEVMVAVKKAFDPEWILNPGKIMDVPWENTLYPGSNTAVTPNRVVKDLV
FUN_002612 CDS scaffold_13 295458 299099 +
hypothetical protein PF05183

MKIMKSIPGMGNGRSLKLQLSPKYQRLTVFFPIPSVAGGNRTERQHKVVIDFSSMKDVYQTAAVDNCCTLVVPLKTPPQYYWK
TPDIHSTFSDETKNWSVMESWNRATNIVQHAGLPMMYPVTLHNDFQDPEFVDIGRWTTLGFVLDGGTEIASQVNQQLVSILDD
FNITTKVQDDFKTVHGTKAKMWEYIDHQAATEGHSALQMLQYSPDSIVHLPFEIRYQLEVCLSRGYLNEHMIGREFLERLANM
KPTKARLCLESVADNAQVVIDPMAILEESQIDGSIIMSRIPSHCCLIRKAVITPTTIRYTTPTVEMSNRVMRRYKHYEDRFLK
IQFTEEMEKGRIAVNKDQNDEIYKRVLRSMYKGVRIGDRFYEFLAFGNSQLRVNGAYFFCPTEHLSCDDIRRWMGQFSHIKVV
AKYAARLGQCFSTTRELHAISAPATQQIPDIERNGYCFTDGIGKISSFLAQLISEDMILDVIARPSAFQFRMGGCKGVLAIWP
NDTKVMEVHIRESQKKFESDSKGLEIIRSAAMATATLNRQTITILECLGVPIASFTNLLDHQLRSYELAMEDNDVAIDMLTKF
VDENNISDLLRAGFKTDDLQEPFVVNVLNLWRSWSLKLLKEKARIQVQKSAFVLGCVDETGTLRGHSSETEGSEDKAIDRLPQ
IFIQLSDATHYNKTQVISGVCIIGRNPSLHPGDIRVVEAVDCPALHHLRDVVVFPSTGDQPVPNMLSGGDLDGDDFFVIWEPT
LIPRRWNYPPMNYLAPKPIELDRDVTVDDLRNFFVKYLKNDKLPLIATSHLAFSDDLGPMSPKCLELAELHSKAVDYPKTGDP
AVLRRDQQPRKWPHFMEKKNSYTSRKALGVIFDKVKGKQVKFDPIWDSPFDQRIIKKFELKRDTLKSARKIKTQYDTSVRRLL
SQHALKTEFELWTGFAMSKPAVGSDYKVQEELRREYNNLKAAFRQLCIDTAGSKNAEKLEPFVAAMYKVTDEEMKIALYEHHR
GVINNAGTLLQPRKLEPKSMPLISFPWIFHKEMCRIANSSNLELRSPLAAGPRQGEMPGFGEHLEPTVKNDATPANQEVPEVS
ELAPEIHSHLPDGTVLHRGQPLAIFPLEDDAIPEGDDDFPNGISGSGQSSSAGDDFEMVSDEQPDLSSENGMEVEVPSDDIVE

DETDSIYSDELEEEDAEEAIDRLVSLMGEADAK
FUN_002613 CDS scaffold_13 301814 309120 -
hypothetical protein PF00063;PF02736

MTFNANANGTAHRRSNPFARTPSASPATNTNLRPKSAIFSSPSSTPALSTPLGHSRTQSNGSLSAITHGFSGSAPRQQRSDSK
SGTPTSTTFAPSFIKTEEMKRHDTVKGIEGENDFSGKRYVWLKDPQAAFVRGWIVEEMDGNRLLVQCDDGTQREADAESVDKV
NPAKFDKANDMAELTHLNEASVVHNLHMRYQADLIYTYSGLFLVTVNPYASIPIYSNEYINMYRGRSREDTKPHIFAMADEAF
RNLVEEGKNQSILVTGESGAGKTENTKKVIQYLAAVAQADSTIKHKPHHSNLSQQILRANPILEAFGNAQTVRNNNSSRFGKF
IRIEFTRNGAICGAYIDWYLLEKSRVVHINPHERNYHVFYQLLKGASSSLKREFLIDGLTVENFNYTRDGHDTIVGVSDHDEW
ESLMEAFNVMGFSDEEQRSILRTVAAVLHLGNITVVKESRTADQARLAPDAKEQAAKVCKLLGVPLEPFLKGLLHPKVKAGRE
WVEKVQTPEQVRLGLDALSKGIYERGFGDLVNRINGQLDRTGMGLEDSHFIGVLDIAGFEIFEQNSFEQLCINYTNEKLQQFF
NHHMFVLEQEEYAREQIEWKFIDFGRDLQPTIDLIELSNPIGVFSCLDEDCVMPKATDKSFTEKLNSLWDKKSTKYRPSRLGQ
GFILTHYAAEVEYSTEGWLEKNKDPLNDNITRLLAASSNKHVASLFSDCAESEEDHATGRSRVKRGLFRTVAQKHKEHLHSLM
AQLHSTHPHFVRCILPNHKKKPKQFNNLLVLDQLRCNGVLEGIRIARTGFPNRLPFAEFRQRYEVLCQDMPKGYLEGQAAASL
MLEKLGLDRALYRVGLTKVFFRAGVLAELEEQRDALITQIMSRFQSVARGFIQRRAAYKRLFRTEATRIIQRNFNVYLDLAEN
PWWQLIVKMKPLLGTTRTATEVKRRDAMIKDLNERMQLESQNRMKLEEERRNCHAEMVRIQQTLESERALALDKEEIFKRLQL
REAELEEKLAGAIEDQERLEDELDDLLNAKNRAERDVETYRSQLEQAATLIAKLEEEKKGLSTKISEVEQSMADITQRQAERS
EQELALQDEIKMLQSQLSVKERKVQDLETKLVKLDQDSELKLNAAERELQSTKLRETQLTHENEDIQRQLNELSKTSTDYEDL
VRQKESELALLRTDNKNFESERQSLEDQKKSLTTEKEKNAEKYREAQAELVAMKSRQSQLEREAEDAKSLLEARLSEDAQADQ
NRQVLESQIKDLKDELYKTQMDLSRERQSRDDVQLLGEHRYQELKDEFDRLNESKIIIEKELYAQQDTLRRTMEARTTAEKER
DEARQEIRRLRVAKTQAEEARMQAEVAGERQASKAALDRENSLRKDLDAAQERLQWFEGECAKLNHQIEDLNKLILESGEFGL
KNDQAKERLERELTTVKSRLTASENDNRALLNKLQQKGLEIARSSSRASEASRGQIMSLQREKARLEEQNTKLNKQLGDSQLA
IASLEKRAEKLQLSLEDLSHEVAREVKSSRNAEKATSTFTAQLAEANRTIESERQLRTQAQTTIRTLQTSVDTRDKEVNELRA
QMLDVLRTVDPEVPIPPQSDGADENVLIQNFDLVRKVEELQQNLRVQTAARTNAENQLSEMRASRNDPASRPKLEEIQLNEAP
FQGSPTQKRAAKLHARNYSNTSTPTRRFQELDHLQDSTRSDRTIDTLNFNNRMDLKAEVEELQNQLQLKEMQNRHLQSQIDRS
TPVPENYDDQSPSLRRIQKLEMVNTRLHEMLDDSNKKVSALERTIRSGQLSLRDVQTRTHEEILEVLNSQEDSRRALVHSHKD
AVAELTDIKGHFERLRHDRAKAEVELRDVKSDLQEMTLAREQEAQNRNQLLQEFADLQIRLDAETSKFADVSSSLEMYKSRAD
EYFSKLEQAEIAVLKASRAEQFAKSQAKEAEDTYAEVMAEREKMDASIEDLQRQNQRLEEKVEDISTDLAAATQAKKRLQHEL
EDYRNQRAMEIEDKESSMEQTRKKYQAEFATLTNELDLAREERLFKQAEIARLREELDELRSKWDDEVLNSSTWSKEKARLES
TLADVASSRDEAVNAHNEAQGKVVTLLSQVRTLRSSVDEITAERDHLLREKRNVEARLEEAKSGLEDLVKGDSPSLRNAANMD
KEILELRSHLAQQEDIAAAAVEKMRRAEALVTEVQKDVMVERENSIQLQQQKASLEKVLNEAQLKLVDLETKGYSSASQDVKF
LHKRIQELESQLEDQETERSKSQRSVRNVDRIVKDMQGQIDRKDKQNTQLSEDVSRMRDKVEKLLKTIEELQSSESQNQLSAR
RAERELREEKEKIARLERELAGLKNLRNDKGSGSVMGSVRSRMGPWRVSEGDDASTVDIPRRKSSLSRVPSFTKGFL
FUN_002614 CDS scaffold_13 309869 313469 +
hypothetical protein PF00642 ENOG41

MSRYVYGHGAAQGYQTSQPYAYSANESYNPALYHNDAAGTQPAHGQGVAEAYEYNQTAIPGLGMRFSHDTANWQPTLPQNIPD
TQIDSTSAMTTGVTTKPDDNDASSHSITYNSRSTIAMDVDKMEEGELSEGELEDIYEPNGIDGPDAENQGSQPPDSTAAKHHT
TKNLQNNHDAEKRASNSKQLGRERSGSYSPYLSPREIESNGSENGGSNTRNSRVANDLKMTESATVMKTTNDNGGEYSSSPRP
DARHIDSIISESKKRAEKAILHLWPLNVRYHNYIEEGIDRALLDELFEEMGLDLEATTANQGEAKRPSMSKVAKVPSNTLDQE
SPSTVASTATTGPRGVEQMKDKSEERKDRIARLLAAKGSKTTVGDTDNTKPTYPMSLLPGNTKTDKTKSQSEKSKLIQQKMEA
LIKAREANAKAAPQAHTPSEPHLPLPVTETSRSQSLARADSMNIDEQTEVNVEVADPATAPSIPGLFLSSNAPSPVPHQRKRP
VAADLNENSTAKSHKRPFGQTRESRPFLIDVSDDEDDAEMEIDSPELRPSSIQRPMTPGSRTASFRDHPALPDSASRRAASSP
KTAGTPTTSANGMYDLESMNKKIEDMKRKIAEAEARKKAKQSGNNSPLPQSGSPSKEGSVDVPTPPAPAVRRAVSTNTEVVQG
SPASSVHHSPRAIAKLPKARDQSVIARPSLRARVASERLPILEARRRAQAEQLKHLYSEAARLEKEIQNSLAEEERLKEDAAE
NELNPSTPNESELEDFQRDSLDMVTNAQRSSQETNSKETLTGLAENEKMNLSYDEVEPDASMDEASSIESTNNKETIDEPLQG
ELRELENHRTKETTVPGSHSALATRSSITHNTVNISGDPAMNGAEEAEEDVAMEEADTSSEEEDTDDYEPTHVGIRLPDPQSP
ASWHSSPFHAPLDHGVLETSDTDMQGMATTTPITQPISTGDGDTESETHREAEAQKALRVCEKIDKTTFVPYETPLQYFRAYR
FHPQYSGSVAGGLRSLTYSNKIDVTREVCPDQLIEGACPRGSECQFQHFENMQVPDDQILLQLGAYGNCEAEQHDQYVEGLRE
LLTDFRKRKVKDFDAISNGIIEYRAKFLGDKTKILPLGAVTL

FUN_002615 CDS scaffold_13 314865 315928 + SOD1 Superoxide dismutase [Cu-Zn] PF00080

MVKAVSVLRGDSKVSGTVIFEQESESAPTTITWDITGNDPNAKRGFHIHTFGDNTNGCTSAGPHFNPHNKTHGAPSDETRHVG
DLGNLETDGQGNAKGSVTDSLIKLIGPHSIIGRTVVVHAGTDDLGKGDNEESLKTGNAGPRPACGVIGISN
FUN_002616 CDS scaffold_13 317056 319714 +
hypothetical protein PF00565;PF00567

MSKTFIGNVKSVLSGDTLILTSPKNPNNERTLSLAYVTAPHLKREGDEPFAFQSREYLRDLVVGKPVQATVLYTIPTSGREFG
TAQLQDGTNLPDELVKAGWLKVREDAGRKEESDEVLDRLETLRQLENKAKAEDKGLHVGVGGIIDVQNDLGGPEFLKEWKGKT
VDGIVERVLSGDRVLMRLLLSEKKHVQPMTLIAGIRTPATERTLPSTGTTQPAEEYGNEARSFVESRLLQRKIKVEIVGASAQ
GQLVATLIHPRGNIAEFLLQEGLARCNDFHSIMLGERMAALRAAEKQAQSKKLRLHKHHVAKTESGNQDAIVSKVIGADTILV
RNKAGTTEKRISISSVRGPRTTEPSESPFREEAKEFLRQKLIGKHVRISIDGNKPATEGYEAREVATVTEKNTNINLLLVENG
WASVIRHRKDDTDRAPNYDDLLAAQEKAKEEKKGMWSGKPQKAKQYTDLSENTQKAKIMLATLQRQKKVPAIVDFCKAGSRFT
ILIPRESVKLTMVLGGIRAPRAPRADGEGGEPFGKESLDLANRRCNQRDCEVDIHDMDKVGGFIGDLYINRENFAKVLVEEGL
ASVHAYSAEKSGNAAELFTAEKKAKEARKNLWQDWDPSQDEEYEEENTAETAPETEVSIDKKPTDYRDVVITNIDANGKLKIQ
EIGKGTAALESLMSEFRRFHLDSKNNNPLKDAPKTGEYVSAKFSADGQWYRARVRANDRSAKKSEVVYIDYGNSEKVPWSSLR
ALDQPKFGAQKLKAQAIDASLSFVQLPTGADYFEDAIGFIYELTENKRLVANFDFIDNKENLSYVTLYDTGSSGELPGPNDSI
NKEVVAGGYGMVPKKLKAWERSKAFESTLKHLKEVESQAKDQRLGMWEYGDITED
FUN_002617 CDS scaffold_13 321421 322952 +
hypothetical protein PF00923

MSSSLEQLKATGTTVVSDSGDFASIGKYKPQDATTNPSLILAASKKAEYAKLIDVAIDYAKQKGGPIDQQVDDALDRLLVEFG
KEILKIIPGKVSTEVDARYSFDTEASVNKALHLIELYGEQGISKDRILIKIAATWEGIKAAEILQRDHGINTNLTLMFSLVQA
IGAAEAGAYLISPFVGRILDWFKASTKKEYSKEEDPGVQSVKTIFNYYKKYGYNTIVMGASFRNTGEITELAGCDYLTISPNL
LEELLNSNEPVPKKLDASQASSLDIEKKSYINDEALFRFDFNEDQMAVEKLREGISKFAADAVTLKSILKEKLA
FUN_002618 CDS scaffold_13 323456 324707 -
hypothetical protein PF00149;PF12850 ENOG41

MITAPQRRLLVLLSTFFFVFSIFYCCSRLAALTVGPVEVEDEVLRPQVELLRADNPMSYGMYNRPRYDGIDLIRNLPSEYVPT
AKNGRRLVVVGDIHGMLDPFEKLLKKIEFNPKTDHVIAVGDMINKGPKSSEVVARLMELKASAVRGNHEDRVILAWRGLSSQQ
GVAAYLDTKDAAKHRGEEAYLKTARSLSEAQMTWLRNLPVIISAESMALYFVHAGLVPGVPLPQQDAWAVMNMRTLRFPREEF
RKKEIEKKRKQAEKQRQKELAKAQEDAQAKFQQRSFVPGEEGTDSHAHAARAEPAPADSAGTVLSTDEAAAERTNPDRDVWIP
IDGHEGERWTDLWNSEQRKLRGPDRRSVIYGHDAKMGYQEDSYTFGLDSGCVKGNALTALVIQAKEDGGWKHSTHQVMCKKGW
W
FUN_002619 CDS scaffold_13 326009 326476 -
hypothetical protein EOG09265I72 PF10203

MPSSCKELREALAQCLQESDCVMVERNSAADCLREPLVNTLPLKCRQLKKGFGECKRGMVDMRKRFRGNMPVAYRTMEQAEEG
QGYQLYAGRPAFAGGVKKTDGNEPIPQDWREVENEKWKAEQAAIEQQKK
FUN_002620 CDS scaffold_13 326784 328142 -
hypothetical protein ENOG41

MNPPQSPPSQASSHFQLEPIPAAILADKEVKRRDAIALLGACKTGCKVIDEEVMLGGFERGSVVGISAEDEELGVQLGLQTLA
HSLCEGTVTSGLLITPRPASVMLAGLRDAVKSELEAKWCTKDTIRMKLRECLEKVMLSCVFDMNGLWEALADLDCELVEEKDD
ALQDQGATNVRQHDTTYQFDEIQDSQDDDDVAFSPIHQASQPRAEEPYNKTQQHPDVIVITHFSSLLTGLFFHREKSAAHAAL
QLLNSHLRDLSRNLPSKPLILLLNSTSAVSSGPALATVTAASPAKQSQVDPTLRSIFNPPPLTGYSARRTKPNFGLIFTQLLD
LHLLCTKVPKTRRDAEGAVRQLPGDEIEMVWIVEVLLDELGVWEGHTGARSGREQRWAAVKVEKGRIGQAIDEKEPEAKIPNI
SDVRLAGGFGGRRV
FUN_002621 CDS scaffold_13 329063 331160 -

hypothetical protein PF08316 ENOG41

MSTAQSFQSGHTSSGQSPGLTLNLSSNNPFRNRAPSPASADLLFPSKPASPFDDPPARPLSRNPFLDQPPVDLLSQPLRSPGA
MSSHSDSKSLSAEEIFNSVTLDDASNDRARQQATRRPMNGPPPRRGGENPPPENGSHRPSRSQEEALRARRMQGSGPRPQQSS
PQRRAPPRRSRRNSESSLVDFDARPITEEEKRMIEAARRREYERQRRGEGKERGDRSDRDRDRDRRERGDREKTSRPSRKMDI
IDQLDATSIYGTGLFHHDGPFDALNPHRNRQNARRAPMQAFPKDSLNNSLGGAGPLNARADHSVLMGNATDEAFRDFSAPSKA
RSKEPAIFDASGRDQIVHGDESVGLGTSTFLEGTPAARSAIQRHQAEQAQENMESGLQRKKSLAQRIRHINKGPRDYSQSGRP
NGEYSRITPDAYPTAASTSSDNNPFFAEFGKGEEGFSVRPRDGSMTSNSPPGARRPSAGAVLERRATTDAATTLDDAPAKPTG
LMGRMKSLKGRKPRPEIPSNGPGVPGTAV
FUN_002622 CDS scaffold_13 334862 335848 -
hypothetical protein PF00106;PF13561 ENOG41

MAVILIVGATRGLGASITKKYAEKSENTVYGTTRSSEGPKDSPSGVKWLSDIDLMQSNAGQKLASLLDPAQPLDSVIITAGYF
ATEDLTWDKGPKWEEEQRMYTTSSIAPVFIVHQLIHKGLLRKGSKVVLVSSESGSITLRHPKEGGGNYAHHASKAALNMVGKL
LSLDLKDQGVIVSIVHPGFMRTEMTKGVGFDKFWDEGGAVTPDKAAASLINWIDNLDISKTGEYWAPRGPRDIGTAEVVLGED
LATPLHLPW
FUN_002623 CDS scaffold_13 336840 340280 -
hypothetical protein EOG09260K4B PF00566

MFSNLTNIVQKAQQLIDPTQGLNLSSSDRNPSKSSLFQSQFRLPASQTPLYEINAELTIPPSNATHGDKDQDRGWHYAGKLHL
SEHFMCFSTTPTSFVQSASTSTSTAFTGQTHGGGPSGNGFTFPLCAIRRVERLNSQNFQFALALTTWNGLLADTPKDKDSKDK
KDLREQRITIHLAGTRQSCERFCDGLKKGLRAGVGNVAKLRRVAAECYSEHLLRTEDRKNASPPDAGLGMLFRYPGDPKKLRD
RAKMRLWAEYLRDNGRNFTLIRQPTFHKLIRVGLPNRLRGEIWELTSGSIYLRLENPALYTDTLAKFEGQESLAIDEIEKDLN
RSLPEYPGFQSEDGINRLRRVLTAYSWVNTEVGYCQAMNIVVAALLIYMSEAQAFFLLSALCDRLVPGYYSTTMYGTLLDQKV
FESLVERTMPILWDHLVKSDVQLSVVSLPWFLSLYINSMPLVFAFRVLDVFFVEGPKVLFQVGLAILRINGEELLDAADDGAF
ISVLKAYFSRLDESAHPKSENPKLRAVTRFQELMVVAFKEFAGITHSSITELRLKNKDAVLNNIENFAKRTAIRNLGPESKLL
STDELGSLYDRFYNILYERQQRDHMIQQEKLRRARSSRMRASEIFATHHDEVEKGRVGLGPSTSLMDYDAFREFLASMSKWAI
SDSPGATKYSNYDDRQNMFHSARRPSELMSPWGGGPEPADHEFLQRLFKRWDVNGSSALTLQNVVTGLAQIKGKRDIMGTITY
FFELYDDDKDGKVDREGILRISEALLFLSRRGLEGTLSPGTSSVLESDSASGQESQASLPPGISTNERFLGSVSAFIRRCFEY
ADPASGHNDTALAKPDDEDSRNAFAIGDEDEDEEDEEDLLGPESPTGTPTKAKKPSLSSESSLSTADNHDAASRRRVSKAKSE
AANAALDPAHPLHLTLPTFRMVVLADELLEQFFESSFPASFHIIEGLPNTTSSASSLTTFSSLGFGARPPIPPPVGPPGAGRG
LRGVLDNIVTDGMRVATEVRRRMEEAQKELEKNALPGQRPEEDEEDDDDLGVEVGVRKGPSGSSTDIERRSVRSSDRDLLDGA
DAEAGALSKPQEQSLLDVNPNEGRPRAGTATSTGSTGGSAVVEFEG
FUN_002624 CDS scaffold_13 343923 345211 +
hypothetical protein PF00069;PF07714

MAATVAPSHAGGLPSSSGIPAPSHSANSHSHAQDAPSGPTSPTPSPLSQTRKFNLRDFRRVRTLGTGTFARVCLVRPAATAHI
PLDHQLNPEVYALKILRKPEVIRLKQVDHVRHERAILADVAGHPFITNLIASFSDQDSLYMLLDYVPGGELFTYLRKLRRFEE
SVARFYAAEIVLVLEYLHEQQGGIAYRDLKPENLLLDQEGHIKLVDFGFAKRLGYREDNRPVETYTLCGTPEYLAPEVIQNKG
HTGAVDWWALGILIYEFLTGYPPFWHQNPIEIYKQILEKPVVFPQDPPLSEEAKDIIRSFCTVDRSRRLGNISGGAARVKSHP
FFHDTNWDDIFNRRQKGPIQPPIRYPGDAQCFDVYPEDDGSREPYGREMAEKYDPYFADF
FUN_002625 CDS scaffold_13 346040 347749 -
hypothetical protein

MSCVAMAASTSVTKSTTTTSTSTSQSKLSSSSAPSTMTPPSIDLLYLTFNCAKNLLDIPVFGSHLQTAFRQNATGLPEVVVLS
LQEVAPLAYSFIGGYFLNPYLSRYEQAINIAAQHIIDDISSRNSETVNTTPTALPSKPYTLVRAKNVGYTAILLFAREPSRLK
NIQEAEVGFGAAEIGNKGAVGLRMLYEADGGSSELTFVATHLAAMEWNLPRRNANWAAIMRGMAFENPEVVVNSYKTSVTPSP
ASTPPAEDEPERTRLLDNEHNEQHSQLQQQLHNISVFRPSSFLFVAGDLNYRISTTSPPPSATFPSLDPESENYYPDFFRLDQ
LTRERNAGRTLHGLSEHEVRFPPTYKYDVLPPRPGTREPELDVPWKFAVHRYPGWTDRILFLDVPSWLKKGNSQDPKFNVRAY
DCLPVLRMSDHRPVFLRIDVPLISPSDMAPPSDLDRGVSKDPRARLPMEIDPEAWERRAAARRKEVMAGWSMYLWSTKQGVCI

LATVLAFGAGVYWLYRRF
FUN_002626 CDS scaffold_13 350053 351342 - HHP1 casein kinase I PF00069;PF07714

MTTMDLRVGNKYRIGRKIGSGSFGDIYLGTNIISGEEIAIKLESVKAKHPQLEYEARVYKSLAGGVGIPFVRWFGTECDYNAM
VLDLLGPSLEDLFNFCNRKFSLKTVLLLADQLISRIEYIHAKSFIHRDIKPDNFLMGIGKRGNQVNVIDFGLAKKYRDPKTHF
HIPYRENKNLTGTARYASINTHLGVEQSRRDDMESLGYVMLYFCRGSLPWQGLKAATKKQKYDRIMEKKMTTPTEVLCRGFPN
EFAIYLNYTRSLRFDDKPDYSYLRKIFRDLFVREGFQYDYVFDWTVYKYQKNAQAIAQAAGQANPEDEEKARASRTNAATAGQ
SAAKPNAIPSTRRKMLERGSGAGVDTPDTNRAIGGSDRM
FUN_002627 CDS scaffold_13 355681 357904 - hypothetical protein ENOG41

MVRTPYYDGNHPPPLFVAELDSEPGRPGDAPSHHRPDGTPVFEMSGEPVLPQHGQDGNQSSLSNKCPSSLQALGASKPTQQGQ
EDQNKPSKDTSTASQSVMANPWAYFGPESSDDKLKETQRPQTDNRRPSEPVYRPYPGDSDDSPPEVPHGTRPSTNPSSPDLKN
EDYAFYPAPLKLSHRPANATSPPAFKAYVPPVSQESSDLEPKPLGRPDRTDSQSPTALNAYRPYRPHSTQPLASSVPTSNTHP
ATASSNQNVPPASPSKPPEQQPPGTVSHQITSTTPPSHTPNPPASGATLPKPAASLPASPKPQTQQSEPQPQRQYGIPNAAPA
QPMPPSTSTYLPSPVTPSVQLSSVVSSPATPATNAGVPFTQPQYAAHVAVQTYAQTPASLVDSPSSVASPGPSHHAGVPFAQP
QYATPVQAHNFGSNQSNITVAQAPASVMPTSASNSSQSTPVQFNYGVQQPTYSSPVEPAQSTLNQASPHLPAPYQQPITSGPK
VQQTHAQQPNTRPYGTPVPTVIPQSPPPPYASQDTNFGAGAQQSGPGNPTMYQPSPTGPYPPQPQYSTQPTYAPATSPPLPSH
SGNSLQPPALPPRPSSSQGFAPTSGFGAGPAGYNPASYPRPPQTYFAPPPALPPRPGLGKLSGGGGKLFGSSSADKWLKKTGQ
VLESTLAPYLQGQSGQSASYRPGSNGLQGQQGQPMQQAPGQYPPQGHHFHVPHLAPQYRGASESGRPPQGPSAPGY
FUN_002628 CDS scaffold_13 361063 364164 - hypothetical protein ENOG41

MGLLSMQHHPQHQQQGHPPSQHLQHSRPPSVVHQQHHTQPHQPPQQQHPSAYSSTHSLPQGYQTSAQPPSNQDNLNYYNHPSP
YSTPGATSGYTSADTSDMMAAAQMPRPPYPPMSYHTPQSNSPASVASPSQHDQHRSIYGQPPSQHLQQSMYYQPQNQYQTMPP
QAAASPYAQHAPHPQQSMTSQPTMMMSQTAPQNQMTQHAAQHAHAGMTGSPRPKIEPQVPLQLQKPQSSPMQQAHHPQNGGQL
QSPGNTTPGVNPSAAPGPIPATTPLVVRQDGNGVQWIAFEYSRDRVKMEYTIRCDVESVNIDELSPEFKQENCVYPRACCPKD
QYRGNRLMYETDCNRVGWALAELNIPLRGKRGLIQRAVDSWRNSNQDPRLRSRRVRRMAKMNQRKQVQASPHPGAAHMPGPSG
PSGMPGGPGPMGPGPAAMGKPGLGSMGQPMHHHQPGAHPDGTGQSGGDEVGDGNSYEHHHHQAPTAQNPSSNDDVRPAHVFTG
YNNQPYPTSAPMSHMAPHSGGAIPAKRHSKANADEPDDLFPDIPEAKKRKFILVEDNVRGSRLRVRVTLEGVDTNEIPDSFRK
GASVFPRSYFPREMQSPPPSATGSRFFGEDHDDDGTEETEGRESSRRGVNRTGKEMVKVPMGESSGEIAIPRMRKSTRGREVR
LNDLGYRMAWLQSRVFAGRTVFLQRALDCYRNKTRAAIESVMQDVKTVAPHYETRAGKRRWNERVRRGAKEDDE
FUN_002629 CDS scaffold_13 376554 379257 - hypothetical protein ENOG41

MTDRHRYPSSGRAATFANHARTSLPSSIGYTSLYAGDMHVMPTSTRQQPISTTPRGYVTTTSTTPTPNTTTRTYAVTQDPRSH
RPTTRDVTRSQRSSTLDSTSRPPVIVMTTQKDRANGTSSHSSNARPGSPLRDDYRSSDGQFYTQPASSIRSHSTARSYADQPG
NDTYARNSRDRSNSLVRDDPYRNSRHSAIYQNNPRQSSSNIDYGDDGYQYTNAGELVRYDLDHSGPARTRRRESFDRSYYRPN
INYNADQRTFNVDTSPDLSRNYITTSTTSVPNNRQYDNRGGPPPSTRGFDKIRGYESPRDFVPMPSKTPEPTSVKQDTPLVAT
TETTRRPRPVSLYQEGPPRSSHHDDYYRSREDERKMREFRDQPESEHTYEPERAYEAPYFQDDRVTSRGFGIRTDTADEHDDR
RERRPEQPKKRSDEDLPRDADYERDDRRRGRLDSKDTRRERRESKKGSDDDEKERTRFRDKVATGLGIAATAVGLVPAVKDDD
KRDKDPGRRKGSDDERERERERDRDRDRRRDSMKSERPRILERDLSRQRDQSRQRDQDRDSRETSRRNGESRKSTEAVISSSD
SDEAKKSTRRRRDSNAFNPNDTSELRHLKDQLAALKTTDKEKEKETPVVVENKSRSSSPTRETRTATEARPFKDSWSPPDSKP
SGPPEEESRGRESQQTPPDDKQVRVVSPPRDKKDEKPLKGILKQPKVSFPEEPNPIREGVAPHKEDKKAREAPAGAKWTKISR
KIVNPEALTIGKERFEVRDDFVIVLRVLSKEEIQAYASATQVLRERRRRDDDGGDTDRDRDRDEDDRRRHHRHRRHRDDDSSD
YEDKDRDRDRDRERRRRHRDEEEYDTRSRDLDHHHHHHHRAQREREPVLEA
FUN_002630 CDS scaffold_13 383759 386506 - GUA1 GMP synthase (glutamine-hydrolyzing) PF00117;PF00958

MSETLDAAPPHATFDTVLVLDLGSQTSHLILRRLRSIGVYSEMLPCTQKLKDLSWKPVGIILSGGPSSVYSPDAPNVDPLVFE
LGVPVLGVCYGCQLIAWRVDPNNVAPGVAREYGETSMAIRKIGNHADRLFEGLGDSLNVVMSHFDKLVHLPDGFKTIATTKNS
EFAGIAHDSQPVFGIQFHPEISHTERGTDIIANFALKICGARADWKMDNFSEREIVRIRKLVGDKAQVIGAVSGGVDSTVAAK
LLTEAIGDRFKSILVDTGLMRLNECEQVKETLDKHLGINLTVVDGSDLFFSRLAGVTEPEAKRKIIGETFIDLFEIEALRIEK
EAENTPFAGKVEWFLQGTLYADIVESLSFKGAASSTIKSHHNAGGLPARMQNGEAHLKLLEPLRELFKDEVRAFGRQLQIHEE
LIGRHPFPGPGLGIRIIGEVTRERVEIVRKADHIFISMIREAGIYDEVTQAYAALDTNRAVGVQGDARAYGYICVLRAVTSLD
MMSAEPYEFEWSLLKAISRRIVNEVDGIARVVYDTTSKPPGTIELE
FUN_002631 CDS scaffold_13 388940 390518 -
hypothetical protein PF03051
C01B
MKVTMGAQPSKPTLHEKAVMERLRSLQLQEDDEYVEISSDAEKGTVGPLTREAQGLSVHVLESWQSAILKDPKNKLALTALSS
ANPRQVLISLPTEIADQQIYNVKIPFEGDPITNQRSSGRCWLFASTNVFRVAIMKRYNLESFELSQQYLFFWDKLEKANWFLE
QIIDTAGEDLEGRLVQTLLGDIVSDGGQWDMVYNLVEKYGLVPQTLYPDTWNAQSTGILNSVIKTKLREFALKLRGLINSKNG
VSATTLSSAKDKMLREISLIMTLLLGPPPSSEEAFTWQYNDKNGKSHQLKATPKDFAKNIYSSDFRITSSTIESMISLVDDPR
HEPLSLLSVSRLGNIVGGRGVSYVNVDIDTLKGTCIKMLKAGLPIFFGSDVGKFSDSKSGIMDLDLIDYELGFNTSLLGMSKA
QRLTTQESQMTHAMVLTAVHLDEETGKPVRWRVQNSWGTAAGDKGWFVMSDAWLDEFVYQAVVDPRFCSKEVRDVLKKEAIIL
PPWDPMGALA
FUN_002632 CDS scaffold_13 391385 393927 -
hypothetical protein ENOG41

MERAKCVRKGWDRTGQNGSTGHSAAEGATQRSVWHKSSNPAPVFKSPVSFCVLFVANLDFEARVPVPFSIFPSTYRESESHTQ
TVTETKTHEEVEIKPQQPQAGREGQYSSVSVTAEQVPPRGEQRFSEEEVRITREEERYRRPGVQKFEHEEEHFTIREDSRRYV
RFYNFLGPLVLILPIFYLFADRARRVRNGLRNHCSPEVDTHRHPYYSTPIDLAEREYRQRYRPAQAFSTEDPTSHSHSHSHYQ
TQDNFQANNYTVEGRPAPQFYSSEKTEINNFTVDGRSSRPQYNQTEKTEFNNFTVDGRSSRPQYTSEKTEINNFTVDGRSSQP
QYRDTKTTQVNNFTVDTPVARPSNKKDVRFTEETVETTKVDNKSKMGYYDDDGHYNSFRAGAHKLGDRIAHGGRRDIEIDIHE
GPRPSADCLPNTVSIPCHHIRLGDFLMLQGRPCQVIRISTSSATGQYRYLGVDLFTKQLHEESSFISNPAPSVVVQSMLGPVF
KQYRVLDMQEGQIVAMTETGDVKQGLPVIDQSNLYSRLSSAFESGRGSVRVLVLNDGARELAVDMKVIHGSRL
FUN_002633 CDS scaffold_13 396794 400140 +
hypothetical protein PF05670;PF05833;PF11923 ENOG41

MKQRFSSLDVKIIAHELQERLVTLRLSNVYDLSSKILLLKFAKLDNKKQLVIDTGFRCHLTKFARTTAAAPSAFVARLRKFLK
TRRLTSVRQVGTDRVLEFEFSDGQYRLFLEFFASGNIILADADLKILALARTVSEGEGQEPQRVGLQYSLENRQNFGGIPPLT
RERVQDALRTAVEKAATATASSKKQKGKPGGDLRKSLAVSITELPPVLVDHWLHTNNFDTTIKPDEILANETLLADLVKSLQE
ARQSVEELTSSEACTGYIFAKRRERTEGAEASDESKTQRDNLLYEDFHPFVPYKLKKDPTIEVLEFTGYNETVDEFFSSLEGQ
RLESRLSEREAAAKRKLEAARNEQSKRIEGLQEAQALNFRKAAAIEANAERVQEAMDAVNGLLSQGMDWVDVGKLVEREKKRH
NPVAEIIKLPLNLAENLITLELAEEEFEPEEDDPYETDDDDSALGDDESTSAAKGKQANKALSVEINLGLSPWSNAREYFDQR
KTAAVKEEKTQQQASRALKNAEQKITEDLKKGLKQEKALLQPIRKPMWFEKFVWFISSDGYLVIGGKDAQQNEMIYKKYLRKG
DVYCHADLHGASSVIIKNNPKTPDAPIPPATLSQAGSLAVCSSNAWDSKAGMSAWWVNADQVSKSAPTGEFLPAGSFMIRGKK
NFLPPAQLLLGLGVAFKISEESKAKHVKHRLHDANSTGGDEASAVATSQPADRAGDLNELEGEQSAEPSDNESEDEQQDEESR
DNPLQAFGKDEDRNDEVEKAEEGLSDLKISDDTADESHSQEVAELDENEAVGGEAEEGEDDESARPDDETAGKPVDTRPKNNG
PSSSKKGPPKRGQKGKAKKIASKYKHQDEEDRAAAEALIGATVGQKKAEAEAKAKADRELELAAAKERRRAQHQREQKETAEH
EEIRRVMMEEGVDILDEDEASQMTVLDSIVGTPLPGDEILEIIPVCAPWNALGRYKYKAKLQPGATKKGKAVKEVLDRWKAAS
TKKGVVDETARDTERMWPREVELIKALKPEETFNVVPVGKVRVMMAGGTGGNAAGGGGGSKKGAGGKGKGGRGGKGSKRS
FUN_002634 CDS scaffold_13 400337 402784 -
hypothetical protein ENOG41

MSLKPDDRSRKDRSRSRSRDRRSKSDVAPERPSYNDNREPSYIYPEDDLNDRYSRPGAPQASGALPYPEEGGIYPVIPGNPSQ
YSYDSQPPTYRTASPPRDSPYRSSHDRVDSSLPGAFPDDRRREKDRDPYIQNAEPRAKEDEDKFSFLPQKYMRKLTGSSGNDK
ERDKDDDGLAYGKPPVPVRPKDSSDLAYGKATGAPSTASQVMTTYNPPPGSEDPYYAQQPPYGYSHREPASPSLGQYGSRKTR
EDARYDDTYGSGANIMTAEPRSRDDEKHRDRRRDRSSGPRGSSDRLTVDSSSRKREKSRDRSRSRRRDKSPSRDTLGVDKHHR

HRSRSRDGHRSRSRDKHRSRKDKSPQPPSERMSGLSINTGLTVGGLAAGGLAGASLANAPPSPMLESYYGTYQDCSPMPSPLL
LASKHSSDDNKALEALSPLGSDNEGDSRRRSRRARFHDSEDITTQLAQALKGSRRPDTEPLIEILPSLTHDQVMKLRADYKAL
VKTGSERKGVNIAKHIRARLKDEDPLLMKACYSVALGMWESEAYWANFWYQGDKTRRELLIESLMGRTNGEIRYIKDAFTDKK
YDNSLVKCMKEELKEDKFKKAVLLVLEERRMEEYDHYGRLVPIDYALVDQDVADLRRSVRSEKGGETAMINIIILRSDSHLRA
ILQEYERQHRSNFARDALKKSGNLVGELLAHILNGVINRPVRDALLIHHATSASRKDGLRRELLISRLVRYHWDPHHMRAVKQ
AFRERYGRGMRDAVRDATSGEWGEFCGELCIARMPDDVRKFDKVSYSVR
FUN_002635 CDS scaffold_13 403749 404940 -
hypothetical protein ENOG41

MPQSRFVHCFGSPSFNPDELIQDADRQREALNHLLKNDLFNASRVLFQLPDPDPYTYHAVASVKLAQVQSVVNLGGVNGLHAW
YRNEDGSPREPPPQADIEAYTSIFRPSTATAAFLKNLGTNAKKDSIRREVAANLESKRYLHPGLVTKLTIPKSKKPPSANPYL
DFWMWSCHSLEWCGPCLASERVPMSHHVLPIFMHHFGCATPTHESLAILKILSDGRPIVDIGSGNGYWSYMLRRYGLTVHAVD
NMQSEWRVNWVNDTVISDGVKWLRKNSNGEGMVLLLVYPVVGGGIGGGTEGGFTRNLVSAFGGDTIAVVGTQNRNGYTGFKGV
TMDEFMAKEHEDWTMVVKVALPSFPGKDEALYVFQRGERVPQE
FUN_002636 CDS scaffold_13 406311 407815 +
hypothetical protein PF08241;PF08242;PF13489;PF13649 ENOG41

MDPEPHSQTQAFPPSTRGDTTKHDGKPNDSERSEARDQANAKVSISVPSGTGIAGQRIPDGSTELSSQAVKPFDIFKDVYGVS
RTPGSPSSDTESQHRPITSPLTHPNGVSMFPGLSVTEQEAMSRQAFAQAEDNIVADSDEYSSIGDAGSDAGYGSDNMSTASTS
IGSSVRDYMFENGRRYHSFRAGAYNFPNDDIEQEREDMKHAMVRLLSGQKLHFAPIDENPQNILDIGTGTGIWAIEMGEQYPS
AHVLGIDLSPIQPTWLPPNVHFMVDDVESPWLHPQNHFDYVHSRHTVQGIKDWPQLFSNALQHIKPGGWMELQEIHHYPHHAR
TGDAVPPREHPVAQYWTYINEGLAQLGVDFPAAAGGKLATKMQAAGFVNVTERVFHVPLGTWPKNQVLKTVGLYWRTILTDGL
QAIALGPMSRGMGWSREQIEVFLVDVRRAYSDHTALLYMPMHIVYGQKPY
FUN_002637 CDS scaffold_13 408962 410484 + CMT1
Alpha-1,3-mannosyltransferase cmt1 PF00891 ENOG41

MAESHISSYSQSLDTSLMVQLARKITKETEKLDGYFKANGLQDLGFDVNAPGDLPKLPDDIQRSRMEISSATKQLELLVRGPR
ETVRWGIWSYLDTLSLQILNSYGIPNLVPLEGTIALAELQSKAGLDRINLARALRHAMTNKIFQEPASGFIAHTAASKMLAQD
ASLQAWVGLNGEDFLPAGAHTLEALRADPEATSLTRTGFNHAWGTVDKEPMFATFGKDPPRGKRFAAAMASLTGGEGYEVRYL
VDNYDFSEINNNKGTLVDVGGSHGFVCVDLAKKWDKMKFVVQDLAKTISSAPEPICADEEVAQRISLQVHDFFTEQPVKDADV
YYFRWILHNYSTPYAVKILKNLVPALKPGARVIINDHCLREPGVENHWDEKVMRSMDLLMLTLLNAQERTEKEFEELFRAADE
RFVFKGVSRPEGCRMSIIEAVWQP
FUN_002638 tRNA scaffold_13 410705 410787 + tRNA-Gly

FUN_002639 CDS scaffold_13 411352 418836 -
hypothetical protein
PF00106;PF00109;PF00698;PF02801;PF08659;PF14765;PF16197 ENOG41

MENDIAIVGIGLRFPGDASSPEELWKVLERGESQWSEFPKDRLNIDGYYHPSSDRQGSISFRGAHFIKGNFASFDASFFSISA
EDAKAIDPQQRILLEASYEALENAGIRKEDIDGSDAAVYVGSFVKDYEQVCLRDPDWQPQYAATGNGIAIMANRISHFFNLHG
PSMTTDTGCSGSLVSVHLASQSLRAKETSLAIAAGAGMILTPNTMMPMTALNFLSPDGKCFTFDSRANGYGRGEGIGVVVMKR
LSDAIRDNDTIRAVIRATKVNQDGHTTGITLPSKEAQVANINSVYESAGLDFSQTGYVECHGTGTKAGDWRELKAISESLCTV
RDIDNPIVVGSIKPNIGHLEGAAGVAGLIKGVLTLEHAKIPPNINFEKPNPDIDFKEWKVKVPTELLDWPVCGLRRVSVNCFG
FGGTNAHVIMDEAPRYLSARGLRGNHNSFEAITSSRRTSNTKASFEPQLFVFSSHDKLGVRRIMESHIPYLESQGEDDTAFLH
NYAYTLGSRRSNLEWKHAVVAKSREDLIVKLQNIDESLFKRTSKNKQPNICFLFCGQGAQFAQMGKDLLPFEAFHDALEAGSR
YMKNKLASEFDLLEEILKDEAETRISDSRISQPATTAIQMALVDLLDSFHVAPNYVIGHSSGEIAAAYASGALTKEEAWEVAY
YRGLVASSLAIEHTRTQGSMMVVGLSVLDLDESYDDKTYPCEVACVNGPRSLTLSGRKENIEGAFRELSAKGIFCRILPVKVA
YHSSDMLKIEKEYKSALRLINPRQHRKSVTMFSSVTGEEIDGHELNNKYWTLNLVSPVVFMSAISTMLQLPSDKSPDIIVELS
PSSTLRSPVLDIITFFGFPTPPRYSTILDRKVHGAMSLLGTMAELWECGCKFSMEKVVTRGSYQIPLKPLADLPPYPWNHTRS

YWHESHLGEATRFREFPRQDLIGAPTGDAISFEPRWRGFLRIAENPWIQDHQIQKTIIYPAAGMIAMALQGAYQATKGQENVL
GYEIINMRIERAMIVPTTAHGLEMAMNFKSVSCTSVHSLQGAAFDFCIYSKQLNSSWEQNATGRIEVRYMRGQWKIAFQKHRE
EYELLKGSCLEPVVPRQLYELLDIVGMNYGSLFQNIIHIRKSKGACVTKIRIPDTRSKMPAKFEYDHLIHPATLDSMFQTPFA
IESEPMVPSFIESIFVSAKVSSDINKEFDGYSTATRVGVRDASADISMAQSGWEQPSVIIRGLRFTGISGSSREGDGFLPNNR
TLCTQVSWLEDITCSRTENVEDFVKRLAHKLPGLSILQVGGTSDTTQKIVSALTSPEGQRPWLSRYTLAEASPDDRIFDIASV
FAGTAAEPFIEKRPVDGSEPLPDYDLILVCSRSEIDIAKLSSHLKRPGYIMENCPDGNPDLDETISHDYNEVDGKKVEIKLRV
NRKNLAEDWLSVPDVAILLPDNPPSETQWLAGKLIMERQSSVQMRTMQLNEFLSDPSRLKNKIVISLLDFSAGSNAGASIYNW
TETEFNAFHSVHQVVKGILWISRSAHMDPMNPKGAPVIALARTLMSEEPLKTFVTFDLSMSTPLNSSSVVQNVNKIFLQAFVS
DQGSGPRELEYAERNGVICVPRLVPIGPLNDVVENGISSEVTKVSFHSYPRHLKLHVSQPGLTDDSLVFTAGTRYAPQPGEVE
VIFESAPLDFIDLETAMGKTLESEVGSEICGRIGRIGRNVSGFMTGDLVSGLVTSGSVQSNVNIDSRFVSKWSSRLSFSHFVR
AFHCFDKVGRLGPGKSVLIHAGASGVGMAAIIVAFALSAEVFVTVMGPNSDKQRAFLEESGVKKANIVHADSDLFTTVVCNRS
GHGVDLVYNATQRHIEDNFKCVRRGGIVVQFSCRLPSPPPVHVLSSDVSLVNFDLQNMLKYDADYVAELVNELSRLVESSSDD
LGNDLPLPEPINNIDINRIKEAFGLIEKNPFFGLVSITGGPDATPQVDMAIKKTIKPLHQALSGTDTYLLAGGLGGLGRSICE
LLVKNGARHIAFLSRTGTSSEASQRFFKDLCNKGIDSRVYKADLCDAEALTKVIKEEICQEMPPIKGVFQCAATIKDSIFSNM
TYSDWTEAIRPKTVGSDNLVRAISANSEDPFFIFLASSAGVIGSRGQANYAAGNCFEDALALNLRLEGKHAVSIDLGPVLGAG
MLADDEEILDILRASGFYGIRHEDFLTMITHAITAEISPGTPMPGRVIMGIGSGGLVRQNQPADPYWSRTALYSYLNLVDTSN
PDLKVVDASNDFDLKSLLSCCSSTDVAAEIVTTGLSIMLAKAMNMLPEEIDTGKPPNVYGVDSLVAVGVRNWVVTNCGVEVSV
FEVLSERTVAELAREIARRGGFGDEDK
FUN_002640 CDS scaffold_13 422237 424227 +
hypothetical protein PF07690 ENOG41

MANEYRMSLSYPNPSLGQIAVTENALEENAPMTTEVIGLRKHSITGPRRVVLVISLLAGLLFSSLDTSIVSTSLVTISHDLDD
FTNAPWIVLAYLLTYMGFAACVSKLSDIYGRRNILVVSWVIFVGFSQGCGSATSMTALVVCRAFQGMGASGLYSLTQIGLVEV
GPSHSPSLIGAMIGATLAVAFVLGPIIGGAVSQLSDWRWLFNMNIPFGLFAILAITNLWPHEEVAPFFSWKAFASIDFLGSAT
LLTSSGFLVFAVQQAGSDTFAWGSPEIISALVISGVSWLVFVWWEVHLETRPLRNIEPIFPIRLMLQRVYSAGLLVTLFTGFP
YISLSIVIPERFQIVSREEVLTAGLHILPMLGACAVGSFLGGAISSRRNNTSMTLLGASCLQLLGVGLMSMLTGPDSHSKAQY
AFQAIFGLGVGLSFSAATIMTSILAAERSELASAQGAVSQARVLGGCIGLAVCTVIFNSHVNEYLQDHLTSAQLASLHRSPLT
SLQLPGHLRDLVRDVYAGAFAEEIKVMMLVCAVMTALSLFSLERNPAPLERLTAFPKDQFSSRRGSESATEMTDISSIRRSV
FUN_002641 CDS scaffold_13 426099 426501 -
hypothetical protein PF07876 ENOG41

MANRVHRITMFKLPSKDEQAKLLDQYHKLNASQQKDGKPYILSMVVGAADEDARSQGYTFVSKTEFASMEDMKYYDEGCQAHQ
ALKDFVKGSLNPQGVMTVYFKPQAVGGADHTS
FUN_002642 CDS scaffold_13 427999 430894 -
hypothetical protein PF00096;PF00172;PF04082 ENOG41

MAATEQNGIDILCDAAGSDMLLSSLFSLAAPVSVADSNQRAQPPLQQEQHELQHSLPPASPSKHQIFQQQLDPALQQHQGETP
QRIASTLPAKRKLSDASASSPSHVCHICRRVYERADHLTRHLRSHENARPYQCTRCPKRFNRADLLTRHETTHDRDGTAKDRP
FIRRSDRAAEACLNCAASKAKCEDQKPCSRCRSKSLTCQMPVRRGNQYRTSESQAGMSPSDSSMVASTTGNDSQVFTAGEATY
ALPQSAIAHQGHAVDATVDYSVTSFLGAPRLEDSPVESLFFAATQSIFPEIEFSWDVDFGSYKIPGLGVTARNPQSGNVGKRT
SRQGRKDTVSVDAPLQCSVWLYEPEANGQAGSSVSRSAIVSPYLRDSMFAMVLANNPTPHRVPTFPSAELLGYMIETYFKSED
PKSEAFIHRSSFDPSATSQELFSAVVSSGASCISTPAIWQFGLALDELTASVIGKRVSMDHGVAMLESSCLAVRDITFLQASV
LHLETGQWSGFNRQTEVAESFAPQLLTLLRKTGKLSFTSDLTTHTPNAGDSSETLESKWQTFTTIESYKRLAIRAFLHDMQTS
IVSWKGPTLAYGQLDFALPAARDLWKASNSQIWWDLNLAKGSFPPTGFPRLSDVRDCQFVMTEQNTWVDTESCCKAALHGLWG
QIWTYRNAVAQCHHAAPGRPGSDVPEWARSLYQELYEGLRKLSTQLKSAPTPKPELSLLSDFFMMIIHVSLDDVQRLAGRKGD
EESRRAMQLLESTWLPGPESRFAAWHAGQVLRHAQECIPTTLRGFNAMVVYLASLTLWAYGLLSQRTANGDQGMGQEVLPLNE
PETRETTMFLELGQGTPSLSSPEGLRLQLEPVSNYVAVLSLARLIFRQNYPVASEAMPPLVESLCRQLGDLQGGLEGYVVTKT
L
FUN_002643 CDS scaffold_13 440092 442969 -
hypothetical protein PF00387;PF00388 ENOG41

MSLFCGLIRRRSAKDDKPRRARTMSIFGNSNFQAIGALKGMVRFTTAASPAPMYGWEQPPAQKKPPIDECSSRSSDQQAPIPD
IPDIHITTEMHEALDRTYHDLRGSEPRLSKAKFAEFLRTVQGESESAVSELAKEDYSLGDFKYTWVTDFSWEATGPLPEKDLS
KPLTNYFISSSHNTYLVGNQLASLSRSSPEAYRTVLLRGCRCIEIDVWNGDTIIPTTRGRATKIDHKRGLSGESFPNVATTVI
DKVEGYLGDKTANRSRSTSLGSRNSSPRSSLNQVSLETRESGDRLDVAKPLSPRTRQSFPKDEPIVTHGWTLTAPCGFREVCR
AVGESAFVDNDLPIIVSLEVHADEDQQEVMVKIMKEEWGEMLVDKAFDDTGPRLQLPKLGELRKRILVKVKKAPAKIVVPPST
MDLPAIYAHDEDASGSEDEHPLQSTGPSLTGSNSTFPSHEKNTSSKVRICENLGKLAVYTRSQHYKGLATKEAKIPAHIFSIS
ENRILELHQKQHRDMFTHNKGYFMRAFPAGRRFDSSNPDPSLFWRGGVQMVAMNWQYLDEGMMLNEGMFADEKGWVLKPEGYR
SSNKSAETQGEATPGRTMNLRITVFAGQHIPIQAGDAADVGRSASTLRPLIKAELHVDKPTDAERDAYMQEHKYKDKTDVGKT
DHPDFGPHGSTLQFLNIPKVVEELSFIRFKVEDDSTKLGSSPLLAWACIRLDRLRPGYRFIRLMDSKPRVVPGGKLLVKIEKT
LV

FUN_002644	CDS	scaffold_13	444112	444482	+	
hypothetical protein				ENOG41		

MGFGNEYEEQVRAWGFERVFTWSDSPNTHYPPHSHAALTTHLIIEGSITIWYPQEKDSQKQVYGIGSRIDVDAGRIHEVQVGG
SGCTMVIGE

FUN_002645	tRNA	scaffold_13	444775	444875	-	tRNA-Ala

FUN_002646	CDS	scaffold_13	448547	451828	+	
hypothetical protein				ENOG41		

MGNEQSAESPRGHRKLSKPPHCSQASAAGLPYTATAVTPHREHFSNSYLVGSLPPAPNKASTTRRVAPGLGIAVPAGDASSPI
QSPTCRDPMRDLKSRTRSIQSEQSPILPAFVNSSRTSSIAQDSGYRTLTRADSMPVAVRRGSASYDTRAAEAKTLLSAKEKLP
REPIISKSNKHSDETSQDVTDDTNNATQSAGSSISRTNSDVSLYMPMRRRSVVQTPGIATRAHHSNPPVSAKSSFRKSLPATP
SQSRHNSIESGMARRMSMPTTIPSQAQSPDRAVTPVEADYKQLGGMKFGSLRITNGAPGTSPMPEDDVKQQGASESALVIPRA
EYFHEQAKNPLRLMNQGSTMTEANELMTGEKSLTARVASCVTECSSDAEKSRTGDGGMSGNGNAVSFPVAEVLDVREDPNAKP
DPKKTQLGLENKMLKGLSRSDSGFIPSPSSEKTHRSASRVDSGYSSNVSLRSLPSLRSGVTDKNTMASEKLDVDMSGICSPSD
SNSTRTSSSVPSQTRNRLPIHLEVPLSSTDDDTSSACLASPSSPASRPFSSFSRGSRMRLASLKSNKSFEPRVSNTATAPIVL
SKGDFQEQLPSPAADVSRGGSKIYRFLNGSRKKGSLKKGEVAMIEQDFTTDPLGLKTGSIPKPHRPSLRKEPSKDTLHTIMSV
GSQDALGNTRRGEDVARGAKKTVGKKMSRRQSWRQSIAQIFGSRSADASPALSSKPEPEPTPRRPSTAMELTSRSFNAAPSPR
AVSSWSSRPKPRKAASSSGSLALQTRKVVNKSSIQESRDKPELAHLRTNVSAPNLSANHRSSLSPSALEMDQTLRTKTSPPVS
MQNRGSKPPRAKSQAQNRAMAPSFPINSRPSASRRLSLPQDYGRMTPHSRQISHGRTESRSSSRGMTMSPTAGLSSQQVNSVQ
HSRVLSYSSNGTQQSGPAAQQVKHHRRVSAPAQPRQIANMPRSRSNTTLIQIQQQQMHQQQMLQQHQQRRPRTSVQSWAPMDM
HSLNQQLQQNYMMQNQALVYGAPNMVQQYPNIYVTSPVPNHPVHGRQSSQGGMYVQDPPFRVLHSYNSPAYRNVPIWSQ

FUN_002647	CDS	scaffold_13	454659	455754	+	
hypothetical protein				ENOG41		

MYMTRLVKLLMVALAVVCVAQASWIDLNKIHLRERRLVRRASPSQSDDSQPSATAPDSKPSATQADPASDPAPTDAADPTSDD
SAKTDAEATQATNEQTKPSATDAEPTNNDSAKTTAADPSPTNASPTSKPAEKSSAADSKPSETAAATQSEATDNAESSASATD
AKTAAQSTTNSPAATSTSSNNQNSDDASSTASENNDEASSTAAEKNTRTTARPVTSTHVAVVTRTNSDGDLETMTTTSVSTST
PGLSDGGDDGSSSGMSAKTRNTVIGVVVGIGGAIVVGALGLVAWRIWGRKKHQEEADGLMDFNENNSRPSSNGPYHSVEKTEY
GSVGSSGPQRSPFQSTLDTYHQPTPVNASSNF

FUN_003099	CDS	scaffold_14	1333	2064	+	
hypothetical protein				ENOG41		

AGVFFDLIQCLIQRALLYATQDVRECYILVKNALLDDHKKKVILILHSQCGIEGGMIIDWLLDKMPLDKLQKLEVYTFGNLAN
HFSNPYRGNSTRSSVIPHIEHHHQLNQHYLDDMFPLEKTLQRARAATEGDFMHRKVRVRTDGTMKVTTKAELPPGFVVTGDEG
SKNASMRPDRVPMMGDLSRLWKYRNGQLPDSFKSVLTDGM

FUN_003100	CDS	scaffold_14	2315	3556	-

hypothetical protein ENOG41

MSDSKHEDVDKTSNGEEQAATGEKHQIQHDSPDAKRTKKEAGQTTLDDVVIISDDSKDNDSAAEKEEDEPKSKSDAKQETNGK
DSKPEDKQKTEGKDSETSGKQGTNGKDAVQPSEEADVPSSILEKGIIYFFIRGRVNLEDPESVDDIARSFIMLRPIAKDARLG
DGPIADSGNTRILALPKKTLPESGKERYMVFVEKSGASYDEIKNSFLSADEYETKTAGTRRTPPAKPVGEGVYAITSTGRESH
LAYLTTLPEKLEEVQKELGLKEKGSFIISTKNPQYPGPQNAQLPQGPDFPKEIIDEFRSLRWLPSKPSHFDYPNAQILLIGES
DGIEKAVEPQKKDQKDGKEEPETVLEHLEDDDVKRMKHLADDQSAAIYADLHVQAKDYPKMQTTF
FUN_003101 CDS scaffold_14 3819 5835 -
hypothetical protein PF00172;PF04082 ENOG41

MFSHPLHGAGPSDLSRSGSGSGRPLMAAAGPSVGNTNARARPCDTCRVRKTRCVKEEGQTRCVLCTFHNQPCTFLRGPTPRQR
RQNREKEREKQTDARDGDENQGISTTSPVAGFETGASPSSRHGDAASTPASVESNQQFTQIQQVMPFEENMPEPSRPSILSNT
LGLDLKTHAEYIGPTDYRDPVLLDLHRPNLLNQEAPPLSTTSTFARRLDYQTVFLVHPDESTASEKMRIADLDAIEATVHPLG
RTLVDLYFRIVHPSFPILHKDVFISKHRLSHRHFAPSLLAAVYLVALDWQLYDSQLAGREVESIPDPAALEELAERTINQDMR
RPKLSTLEAGLLLLQRNRKIVESGSHTHPMSNRMFTAQIVAMAQDLGIHIDCSSWSIPAWEVGLRRRLAWALYMQDRWGACVH
GRPFLIQDNDWDVRPCTAPDYPELGQMDPEANPDHTSPIIVGWDLFIRHIELTQILSDVIRTFYSAAATRVGGTLDQMGVVAA
VELAKPLVFRLREWHANLPARLQLQNTQLRELCANGALHLAHAAVEIALHRALVRITTPDTPASLYEVLRSTARAKLQSAIEL
LGSLRPEHTAAFWGSAAAYQAAEIGSLAGLLWATADSFDEMAWCASRVDELRWALRVRGAAAPFAREALRLLERDIGGLGMVK
TANDSIS
FUN_003102 CDS scaffold_14 7149 12639 +
hypothetical protein
PF00289;PF00364;PF01425;PF02626;PF02682;PF02785;PF02786;PF07478;PF08443

MTELDTAINGVEDLPNGSTDSFPLTISQWRSAQIRGDGIERLLSLISIERAANSTTNAWISLASSEQIKAQWTSLQTLCADSM
SLPLYGVPFAAKDNINAEGFITTAACPTFSSSPATSDATVVAKLKAAGAILIGKTNLDQFATGLVGTRSPYGAVPNSFDPSRV
SGGSSSGSAVVVARGIVPFSLGTDTAGSGRVPAGFNNIVGLKPTRGALSTHGVLPACKSLDCVSIFTLTVGDAETVLSVAEGY
DIHDAYSRERPDPDIAPYNEFGGSASLTKLSVAICASPDWFGRDGHSPAYDKALAKAEALGWSLVPVDFSLLFRLARLLYNGP
WVAERYEAIRGFIENAAAKEMDPVVRKIIIRAQSLSAADVFEGEYLRQELTRQIEREFVRFDGLLVPTSPTFPTLGELVKNPV
EENSILGTYTNFVNFMDWSALSIPAGFRPDGLPFGVTLISTRWQEPFLLSWAREWFGDEERLLGATGVRACESRIAASSAPSN
YIPIAVVGAHLTGFPLNKDVVTRGGRFLGVTKTSSSYRLYSLNIAAGPKKPGLKRVPTDEGAEIEVEVWNLPKTSLASFMLTI
TPPLAIGSIELKDGSWVKGFVCEPCGFDEAVDITSHGGWRVYHAFLEKGSKFLTNGVRKSAISTILVANRGEIAVRIIETIHK
MGLRAVAIYSEIDADAAHVSKADLALKLEGSSVSETYLDMEQILKLAVQSSADAIIPGYGFLSENAEFATAVEERDMIWIGPT
PQQMADLGLKHRARAIAVEAGVPTVPGCDTLLTSLEEALKEGERIGFPLMLKSTAGGGGIGLSYCKDRQSLATTFEGVQRQAE
ANFGNGGVFIERFIQRARHVEVQVLGDGTGRVIAAGERDCSLQRRHQKVVEESPAILVPESIRAEMRAAAIRLASSVKYRNVG
TVEFIYDIDSREFYFLEVNTRLQVEHPVTESVTGLDLVECMINVADGNATHLFDTELKVSGASIEVRVYAESPLQSFRPCAGR
ITALEFPPSLRVDTWIQLGTEITTAFDPLVAKLIALGSDREEAIENLAKGLAATRIEGVQTNLEYLRQIIASPMFKSGDYTTK
SLDSFQFHSSSFEVLQPGALTTVQEYPGRTGYWNIGIPPGGPMDSYSFRLANRLVDNPSDAAGLECTLGGPSLKFHCDAVIAA
VGGVAQVTIDDVTVSMNQAIGVEAGQILSIGVIEHGHRVYLAIRGGIEVPVVMGSRATFELGKLGGFGGRKLQARDILAAGPA
DASVSEAQAISPVPVPTHPKASWTIRVIPGPHGAPDYFTPESVRALYLSNWKVHHNSNRLGVRLTGPQPEWSRKSGGEAGLHP
SNIHDAPYSIGSVSFTGDEAVVLGCDGPSLGGFVVLCVVCSADLWKMGQVRPGDTIRFDPVDVATAIGLERDLDKAIETLSPL
EALEGIPSDEAALVQKLGRIPAALGTIKHDGQRVTVLQAGDRAFLLEFGDASAFNLRHNFEISAFCENHEKKPIPGVEELTPG
VFSLHVTYTRGLSPELMLSRLADHIRSYKVPSQVPSRSVRLPIAFDDSISRAAIERYTATIRPNAPWLPSNIDFLQKLNGIPD
LTEVLSAAEFVVLGLGDVFMGSPCAIPLDPRHRLFGTKYNPSRSFTPRGAVGIGGQYMCIYATDSPGGYQLVGRTVEIWDADL
VDTEAWFFRRFDRISFYPVTEAALDGTARSELVQISDGVLDLEQYEAWLTMNKDDIAAAADQQASAISNAPFVDELVLPYQVE
PVVNGHSPSLDSLSLRGERVKATMPGRCYKVMAKEGSVVDKGDVLVCIESSKMEVEIRSPVGGKCVAVLVQAGDLIDVEDDLV
VVE
FUN_003103 CDS scaffold_14 13437 13842 -
hypothetical protein ENOG41

MGLLAKLKFKLRKRLAQEYVFTGSALASATDQLAVNMNSISFQQAPSGIDRGRNQSFFAMNAPQGNPFAAAGALHMPFPSQLH

QQPFSLDSSLAPLS
FUN_003104 CDS scaffold_14 17739 19023 -
hypothetical protein ENOG41
GT1
MKILLHSHFPAGHAYPMQAVAQSLIRRGHQVTWLTSASNEARVLAVGATFVPTSAIAIIDEPLIAANETGILDKEYSRLQIRL
LAQVTDYRRVLRDFKADVLLVDVMPYGARALCELGEVPVYATLGVIPMYMSSWGAPQAVSGESPATSWVGLIWNHILHLVSQW
ILLPLLLRPIINAQRRELGLKNLPYGEPIESFTYSPYLHIQASSPSLEFKLLPKPPQQREHTKFVGPVVTQVPTSLAQLPPEW
DEIVAHPRVVGITQGTLAMDPTSLIIPAIEALSDDPDLLLVVASPHVIEITSRIGEPPNVRFIRWIPYHLFLPQLCLLITNGG
YGSITQALSHKVPLICAGQTEDKKDTAARVSWASAGIDLKTDNPSTEQVRRAARTILDDPGFVERAGHLGDELNELGGAERAS
ELLEELVESRDR
FUN_003105 CDS scaffold_14 23689 25839 - ATM1 Iron-sulfur
clusters transporter atm1, mitochondrial PF00005;PF00664

MIPQLLLRSSRTCPQRSLALYRLSSVSQRPGLTQTFWTCAPRRERAPTDSKTKTTATTKPTAVFPAQKQPTKQNDPLATVDKS
ATEQRKADWAIMKEMTRYLWPKDDWGTKLRVGLAVSLLIGAKVLNVQVPFYFKSIVDSMNIDIAAVGGTATTVAGAMILAYGA
SRIGATVFQELRNAVFASVAQNAIRRVACNVFDHLLRLDLTFHLSKQTGGLTRALDRGTKGISFILSSMVFHIMPTALEISMV
CGILTYNYGAKFAALTVLTMVSYTAFTIWTTAWRTKFRRQANAADNKASTVAVDSLINYEAVKYFNNEKFEVARYDKALQQYE
KNSIKVATSLAFLNSGQNLIFSSALTGMMYLAANGVAEGTLTVGDLVMVNQLVFQLSVPLNFLGSVYRELRQSLLDMETLFNL
QKVNANIKEKPDAKPLVLSKGGEIKFENVDFAYHPNRPILRNLSLTIPAGKKIAVVGPSGCGKSTLLRLLFRSYDVQGGRILI
DDQDVRDVNLESLRRSIGVVPQDTPLFNDTVEHNIRYGSINATHDEVVAVAKRAHVHNTIQSFPEGYDTKVGERGLMISGGEK
QRLAVSRLLLKDPPLLFFDEATSALDTHTEQALMMNINSILREKGRTSVFVAHRLRTIFDSDLIIVLKEGQVAEMGTHRELID
RDGVYAELWSAQETRFQEDGNEKDEAEEETDGQKKN
FUN_003106 tRNA scaffold_14 26832 26904 - tRNA-Gln

FUN_003107 CDS scaffold_14 27519 29178 +
hypothetical protein ENOG41

MDRLEALLERLNETLIPHAQRYPLQAILVTTIILFITTRLFTGSSSSSRKDGSKTPPLAPFWVPLFGHAPRIFLSPSSALTRF
RNRYAQGVFSIRLFQSIHSFVFRPSLVARLLEQSESVADKEYVARRIMMTNFGLSKQDLAAYDKAAPEVYQITKEYLSGSHLD
TLAKATLRDLDDNAADAISFNSYPTDQMDWERQANAELLEDTGDEKIMAVDFFELMKAFIARTATISVFGTDFVEIYPDIWPH
LWIFNDAFHSLAMGVPVWAPFPSSQRARIALGRLLTFMREYHTELDKFLSDEEPATKWQDFHTISPLVRARTEVYRKHGLPID
VRATFDVALLWATTVNSTSLISWSLFELYQDPVLLSQIREQITPFVKIVQPKNDFGGAVWIPPQVQKLDLEGLVTKCPLLQGV
YLETLRLYGGGWSARYLKEDVILKGKEDSFVLKKGTFAHIVNDLHHSDPRSFTDAKVWQVGRYLEDTVDKKGVKTQRAEPYTV
RASDGTLTIVYQPLRTRTRGE
FUN_003108 tRNA scaffold_14 29673 29757 - tRNA-Asn

FUN_003109 tRNA scaffold_14 30299 30384 - tRNA-Asn

FUN_003110 CDS scaffold_14 33147 34320 +
hypothetical protein PF01145 ENOG41
I87
MSSTEGMINGGSSSANPTKAPVMEGNGGFMAQHKMAVEPPKKEDLQRSYATVVENDANPKGWYGSMMNALGTIIGTMGAVPCC
IICPNPFKEVNQGNVGLVTKFGKFYKAVDPGLVNINPLSERLIQIDVKIQTTEVPEQICMTKDNVTLRLTSVIYYHIVSPHKA
AFGINNVKQALMERTQTTLRHVVGARVLQDVIERREEIAQSIGEIIEDVAAGWGVQVESMLIKDIVFSQELQESLSMAAQSKR
IGESKIIAAKAEVESAKLMRQAADILSSAPAMQIRYLEAMQAMAKSANSKVIFLPAANQTMGNALNAAMANQTGESSARALDN
ENDFGGQDPGFQQAMNARVVENI
FUN_003111 CDS scaffold_14 35603 37613 - NOP58 Nucleolar

protein 58 PF01798;PF08156

MSLFVLAETPAGYGLFKATDKKMLKNEELAAELGRPEKVVEMLKLKKFVKFDSAATALEEAASLKEGKVPELLTQLLDDLKSE
KKASLAVADMKLGTAISNMPSLNISPVSGSNTMDLFRGIRGGLPNLIPGLLEENFDRMALGLSHSMSRHKLKFSADKVDSMII
QAIKLLDDLDKELNVYAMRTKEWYGWHFPEMAKILNDNLAYARVILAVGMRTNIADSDLSEILPEEIETSIKAAAEISMGTEI
TDEDLDNIKLLADQVIVYSNYRTQLSSYLESRMRAIAPNLTALVGYLVGARLIAHAGSLISLAKSPGSTIQILGAEKALFRAL
KTKHDTPKYGLIYHSSLIGQATGRNKGKIARMLSAKAALGLRVDALGDAEDDADEEERAILGLSNRIKLENHLRKLEGKPLLP
KGANVTPSGEIVGAGQFTLKETRRYNGDADGVADDEETNEATPAKKLKKAKKLIEEVDEEMKDAEDDSDDEAATPGKPKKLSE
ADYERLAEEAGISVKKFKRKYERGDVELNADGTPKVISKKELKKLRKAEEKATPSKSQPAAEETDGKKKRKHDDSEDEEPKKE
KKQKKKKRHSEA
FUN_003112 CDS scaffold_14 38079 39336 + VMA6
H(+)-transporting V0 sector ATPase subunit d EOG092638RC PF01992

MEGLLYNVNGGYVEGIVRGYRNGLLTGAAYNNLTQCETIDDLKLQLGPSYGDFLASLPPNPSTSALAAKTTDKLISEFRYVRA
QAVGSLATFMDYVTYGYMIDNVALLITGTLHERDTRELLERCHPLGWFETMPVLCVATNIEELYNSVLIETPLAPYFKGSLSH
QDLDELNIEIVRNTLYKNYLEDFYNFVNTHPEMAGSPTAEVMSEILEFEADRRAINITLNSFGTDLSKQDRNKLYPNFGKLYP
EGTLMLSRAEDPEGVRLAVDGVHDYKSFFDAISVGGGPSGPGNMGGGAGETKTLEDMFYQKEMEISKKAFTRQFTHAIVYAWV
KLREQEIRNITWIAECIAQNQKERIGNYISVF
FUN_003113 CDS scaffold_14 39555 40562 -
hypothetical protein EOG092643JW PF00312 ENOG41

MPPRIPALPRFGTLNLCLRPAAKPATPNFLPIVQTANLSQREKKRKAKQDPYRWAQAQQRKAANVQRREELARERDEAWGDPV
KGKTTPFIESLESAGQEATSRVPVDGSGNPLAEAHELPTSPELRNYFLTDSELTEAVKHAYTLTKPMIGVVESQMEPGRGEDK
TKQHEQRHQKAIEALRRITSLSNSSAKDRFHANVRRIVEEFGRHNTDLVLQGKPKSIHPNKVDMPPRSGPDTGSSEVQIAILT
TKINNLSQALQINRGYKDKHNKRNLRLLLHRRQKLMKYMDRKERGSERWTHMVEKLGLTPATWKDQISL
FUN_003114 CDS scaffold_14 40932 42040 + RPS0 structural
constituent of ribosome PF16122

MAPSNLPALFNATSQDIETLLAAQCHLGSKNLQVHMENYLWKTRADGVNVINVGKTWEKIVLAARIIAAVDNPADICVISARP
YGQRAVLKFAAHTGATAIAGRFTPGSFTNYITRSFKEPRLIIVTDPRTDAQAIKEASYVNIPVIALADTDSPTEYVDVAIPTN
NKGRHAIGAVWWMLAREVLRLRGTIYNRETPWDVMVDLYFYRDPEAEAEDKVEEEKLPGADEEGPAAIESGFQNSAGADWETP
AAGFAGATGAAAPGWDGAAGEEWGAAPANTEWAASAEAPKESQW
FUN_003115 CDS scaffold_14 43205 48897 -
hypothetical protein PF00617;PF00618

MEALESSTSADPLTPASGLSAPPPVPVPIPSPPPAATTVKDPLSTKSKDQSRSRSGSGAIAVAAPNVTAAVAALKKKENTSPG
FSTPSKWPSVRSTNTARMPTTIVAKPLVPLSERRIHQKKPTTRSLKDSDSSLDNQGSLSRWEITPDGGSAGREGRQFTVANVG
NNGRIYLRPTVRPAYQRTPQPQFVFPITPPSTAGLDAISSKQLQDETSELHVSQWTPTPEPSPYGGNRSYFDHDSQYPLRPVK
HRRALSDSTVHEVSAAKDSEPGAFKIVISKPLEEYRPRTMEDFDSTSDPLLDITIPSWRIGVPRFTGRGTPMIRGSSYAPTEE
FRSSSASLLSRPQGVLNSSHPDITSSRRPSSMAVPMLRISRNISAMTQTPSTTQPSRTIRPSYQSNSKVEPAMFDDLTFKPAC
DDRTLVKYSAATGAVIAATPPRLVAEITSPSFLDYELISDFFLTFRAFLEPVDLLRMLIARLRWALARDDETGMVVRVRTFVA
LRHWILNYFMDDFVVDYNLRQTFCELLNDFVDELSQCNRSRKVQLKILGELKKCWRRVCALYWDGPNFDDTLDSSVPITPGGI
AGHRDPSLDPSFWDRDDGETPRIESLLPLRKSPAERTSFIADVSRAGHVGDSVVVEHRPSTPERQTARDSAEQQTSPISMTSM
DIISCSFPSKIKPAQPHQTNPLGAHPVSTSGYHTGPVATTPRALVGKRVRPQASHKRNNSLTDSLREHSTDKLSFKDQEFMMT
APYGGSLVRGNLLPPGQAFVDVEPDEFSTSLVPSPNLHPESRSLVKERIPAGAMSGHGMKKLLGTVRRALSTRGQGMSPTQGS
LVSLGNIGPRGAVTNRIPGTAVVHQSRHRPNGARPTVRIDLLGAEIVEDFKKAVRDEAAAEADRRGLPPPMSPNPSLTPTGDF
HYSAAHMDTTTYDHITHEHRHRPVSDMAITTGSKSIVIVDDTTPFDPSLLHRIPNSNASVEAFADTFKLTGGDPTPPSTPPAG
NGGNTPRRSSYLLNQHVVRPSLDEDPLPPFVPDFETLAPVTSAPMSEDGSRVSYSTAARSTRNRPPVSYQNHQRNKSGRTHRS
LNSILRRRSSFTNGLARRSTVQSFDATTVSAQSIAEPEPEEPMPPPLRILRRRPGGDLRAATNVGELDPVTLRRSQSVGSLTT
YSESIRGSFMQSPRVASFGRGSFGQGSVVSIEYVQPRSDAFSVGQLADKPKRELSLFSTHSSKPAMRPSFEKEAQKLAQIPDD

DDDGGIESALAKLEGKLPEKKSHKSAVSLQAPPQTIGEVMLASEQSEVDRKRNHHEQELMVRETFMLDPEDSEPEPLIEPQDQ
KLSPERPTTEARSFLTGSSRESYSSVPILDRDSAKGRPWTNMSILEGSDEETTPRPHKTVLYTEDGGDSQHASYDFIEKTRSI
EGIKPGNTAPSVDEDQSFLDDDSDLSSEMSAEALEYDDSEYGDGILPRVKLPAHPLGSPAPPPNRKPPSPPMTLVQALEMSPP
HCFTVPELHEDQVWGQKPLPPTPETTPTAAYVQTADQTSADALRQVTKVNSFESNKYSAHLPFILAFDSEILAQQFTLIEKDA
LNEIDWKELIDMNWKDAAHSDSRSWVDFLRNSDAHGVEVVIARFNIMVKWAISEIVLTQGLEERVRCITKYIHIATHCRRYRN
FATLAQLTIALSSNEVARLTKTWQHVAPQDLKTLQDLEKLVTPMRNFYNLRAEMEVGSDQGCIPFVGIYTHDLLYNAQRPSEM
AGSPTTPPLINFERCRIAASVVKTLLRLLEASTRYKFQPIEGITERCLWMSALSDEDIRRHSEMLE
FUN_003116 CDS scaffold_14 52243 54670 -
hypothetical protein EOG09260NC6 PF00004;PF07724;PF17862

MNRPYRPHPGQQGAQPRAARLHVETIEDKTLRTKFVYNNKCAVSPEDFPPNRDGSDHHILIRGGQPVGEYVVTATPIPGFPRG
GISLTDAQRTWAGITMRDEFIGEIYNPFSARSDAYLGTVELSITFRTQNKKTDVPYDEEELSKLFIDSYQNQVLAPGQRMLMD
HRNIPLLVVVESVVLTGLDTSSEAEQKNQDPNARGILIPQSKVFFHAKTGDGFKLKPSVNKPNASAIFAPSFKFDDLGIGGLD
TQISTIFRRVFASRIFPPGIVAKMGIDHVKGLLLYGPPGTGKTLIARQLGKTLNAHPPKIVNGPEVLNKFVGQSEENIRKLFA
DAEKEYKEKGDESGLHIIIFDELDAVCKQRGSGSGGGTGVGDTVVNQLLAKLDGVDKLNNILLIGMTNRKDMIDEALLRPGRL
EVQLEISLPDEEGRFGIIKIHTSKFAKNDILDHDVDLAELARLTKNYSGAEIAGLVKAAAASAFSRHTDANQITVTKDIEHMK
VKWSDFLLALSEVRPAYGASEAELEAALSQDIIHYSPDIQRILNDMLGVAHMVKEDSEKLTSSIIFHGAQGSGKTALTAHIAK
LSDIPFIRMITPQKLAGYRDDFAKSEFIHKTFSDAQKSAVSVVILDSIEQLIGWNPIGPRFSMTVLGTLSALITSPPINGHRL
LVLATTSQPNVLKELDIAKDFEKDVRVPTVSNLRELQTVLHSSSGVPAGSIEQALARIHEHAGGDHVGIGIKPIIAFISEARM
NQDSAAFADKFSNLVLTKMQGSWAQ
FUN_003117 CDS scaffold_14 55024 56181 +
hypothetical protein PF10197 ENOG41

MPLHLLGKKSWNVYNADNIARVRRDEAAAKAAEEAEEQRMQEIDAQRRLAILRGEVPPPIEDDEPPKDEEPPVRDRDALQRTS
MRRKRKRPGEDDTDFEMRLARENDNSALVRIESEKKSTSSAPIVDHNGHIDLLGDEKSRTHAEKNEEAEREAKKKKQSYEDQY
TMRFSNATGKDGLLKPWYSQSDAAAPDASSKDVWGNQDPNRKERDAKRIVSNDPLAMMKKGASKVREIKQERKRFQEEREEEL
KQMRRDERHREKRRRRQHERDRSRERHRSRERGEERSPSRHERRHRSREGRHSHSGHADSRSRRHERHDREGSRHERKRYEKS
EQRKERHRGDDEPHKRDSRSSERVGHERRRHHSKN
FUN_003118 CDS scaffold_14 56715 57321 -
hypothetical protein ENOG41

MADPKPIKLNPPTKYAPSTKNSPPVDPPTLDWITTTWSVTHSTLSMWRDARNVRITYKLLPGTADGRPRIDDLVEYEPLSKDG
TLKTVQGIDTQASSIGWDWRGKGWLVFVNTHWELLGWGEAVTSEGVKERWAVTWFAPTVFTKEGVDIYCDRKEGLSEETYVRI
REALKGLETRKVAEMVEAHMQPVEIRLPWVEGSNE
FUN_003119 CDS scaffold_14 58148 58824 +
hypothetical protein ENOG41

MDATENDVEVSDDDLMAQMGFSSFGAASPPSKRRRYNPNADALLPSKSSATGANSTALGTGSNKDEISLDDDNEGNANPEQKV
PAQARPASLPQRPAPAQATPQGQHSGAHGARGNKFTGYYDRLSNENPWEKIEKVRGLTTRGTWITRNAGNAATTDANLTSMDY
LGKRLTPYYDAVAHIQPEADFDIKYRVTWYIM
FUN_003120 CDS scaffold_14 59980 60804 -
hypothetical protein PF10241

MSSHYTPVGYSMPLPVPSKGSQYPTYSQYSVSPPECDDSVSSASGIPSYSNGGYSATSSGYQYSGGYGEYESTRSASGVDFQE
YMQDRFANSFDPIPLDRSMAMQAQTSGKMNAKHRELMELQKKAQARLAKTRERFQEGMRDAHEVRSDLEWTQKKVGSLKTKAS
RKHGKEYSKARARFPSPEN
FUN_003121 CDS scaffold_14 66874 68077 -
hypothetical protein EOG09263OD3

MSFRSKIPETPRVISPSPTPSERDAADYMGPVTRSAARRRTPTPQPLEEEPDEDLPEPPEFRRARTRSRSPIDTQAVTRLTRR

TSTAAKAGKMSKDTIPEEQTTSNGALSSNGKGKSTTSNGHLAPPQPTTPVGWSWRDFSRSPSPLGLIPIHRHWRTFVHKHEVP
RKVLHVSIGFFVIWLYVTGTQTTSVTPWLMSALIPIAATDWLRHRYASVNRLYVKVLGALMRESEYSGWNGVIFYLLGAWIVL
YGFPKDVGIMSVLLLSWCDTAASTFGRLWGRYTPRLRRGKSLAGSTAAFLVGVGTSYFFYGWLVPTVGPFPGDENFLFKGFLS
LPKTICEAVGVSKEQTTISGALALGVMSVWSGFVASASEVVDIFGWDDNLTIPVLSGFGIWGFLKIFS
FUN_003122 CDS scaffold_14 69570 71530 + SPB4
ATP-dependent rRNA helicase spb4 PF00270;PF00271;PF13959

MASEKVKKRSPRAWDALTPPLAEWIRDAVATMGFSQMTPVQAATLPHFLGNKDVVVEAVTGSGKTLAFLIPLVQKLLRLSEPT
KKHHVAAIIVSPTRELAAQIHTVLVNLLQFHEASAEILPHLKGDEKRPSTTVPAIVPQLLVGGTTTTAQDLSFFLRHSPNVLI
SSPGRLVELLSSPHVHCPQSSFEVLVLDEADRLLDLGFKPDLQKILSHLPKQRRTGLFSASVSEAVGEIIRVGLRNPVKIEVK
VKMKDGGILEDRKTPASLQMAYMVKPASQKLPALAELLRQLPIKPQRSIVFLSTCAAVDYFQHVLPAILPEGFALVPLHGKHP
AKVREKNFNRFLNSVAPTILLTTDLAARGLDIPQVDLVVQIDAPSDPKAFIHRSGRAGRAGRKGLAVVMLHPGREEDYVQFLE
IRKTPISKLEKPTVFTSEEDAVAATKKMRDLVLTDRALFDKAQKGFVSWARSYGAHQATSIFRAADLDWADLGNAWGLLRMPR
MPELKSWKGDKMCGLEIDWDNYAYKEKTREQARKAALEEERSGEKKDKSDETKRKRKNNEAWSAKHEKEDERVERREKRRKRR
EAEATSKMTDEEKFKQMELNELIAEVRRQNREKAAAEAAAAKKDKEDEFAGFDD
FUN_003123 CDS scaffold_14 72660 74040 +
hypothetical protein PF00172 ENOG41

MPPRTSGSGSGSGAATAKLRRAHRKSRNGCWECKRRHIKCDENRPACSNCVISERTCSFPAQPTPAAAPTPPEQPVSSFNEPL
SASQVESPADSISSTAASHRPSSIPPPSYLAPNSGFTSNIDDGAHSTSLPSFTESFAAADLSESPLSTPLPDALPTPYFTNKH
LILLHHFRKSLLFDVDNVDIIIDLALDWSSEAPYALDQLLAISADHLAMAQVESSAESAASWRRIATELQTRALTHFNRDGQD
TANHESRFVPRFLFSSLLSLHMLYDTLTQYRATFHVFIERFCNSVCLHRGVKSTSSPGYQTLIRSPLAPFFLGVRDAAELGDK
GDECAPLITLIENSDLSPSAVEACSTAAHTLQWAFNVHRNLPKSVNVQAASAFPVVLSDNYIEPVRKHRPEALLVLAYYGVLL
HRCRHVWLFGDSGAFLIHLIADHLGSYWKDALRWPLEELERDQG
FUN_003124 CDS scaffold_14 75208 78986 -
hypothetical protein EOG09260ABY
PF03810;PF08389;PF08767;PF18777;PF18784;PF18787

MAASIEELDVLVRSFYEGRGEQQKAAQAALNQFKEDPDAWLMVDKILSDAQYPQTKYLGLQVLDNVILTRWKVLPREQCQGIR
NFIVQFIIQCSSTEESLRQQKTLLNKLNLVLVSVLKQEWPHNWPTFINEIISSCHSNLSICENNMIILRLLSEEVFDYSAEQM
TSTKTRNLKQTMCAEFSQIFNLCQEVLNTATQPSLIKATLETLLRFCNWIPLGYIFETPLIETLRTRFLSTPEFRNVTMQCLT
EIGGLQTGGPGQPNSYDEQLVKMFTETLTTIANIIPVSLDLKSTYPSSNSRDQEFIQNLALFLCNFFGMHLNLIEKLPNRDFL
IHGHYYLIRISQIDDREIFKICLDYWLKLVQELYEEMQQLPMTDLNPLMAVGAGMSGSGAPNPTMLNNYPLRKHKYNEVLSNL
RVVMIEKMVRPEEVLIVENDEGEIVREFVKESDTVQLYKTIRECLVYLTHLDVVDTENIMTEKLARQVDGSEWSWHNCNVLCW
AIGSISLAMNEETEKRFLVTVIKDLLGLTEMKRGKDNKAVVASNIMYIVGQYPRFLKAHWKFLKTVVNKLFEFMHESHEGVQD
MACDTFIKIARQCRRHFVALQPSEQEPFIEEIVRNMGKITCDLTPQQVHTFYEACGYMVAAQGNKHQQERLLADLMAIPNAAW
DEIIKQATVNPTILQDAETIKVIGNIMKTNVSACSSVGSYFYPQIGRIYHDMLQMYNATSTLISEAVARDGELATKMPKVRGL
RTIKKEILKLIEVYVDKAEDLQAVRAQMVPPLLESVLVDYNRNVPGARDAEVLKACSTIITKLSALMEDQVPTIMQNVFECTL
EMINKDFSEFPEHRVEFFNLLRAINLHCFPALLKLDNNQFKFVIDSCSWAFKHDNRDVEAAGLNMALELINNIAEKTDVQTAN
AFFQRFFITILQDVFFVVTDNDHKAGFKTQSMLLMKLFYYINPADGSQPKIQGPIYSPDQAQAGTDNREFVANFVANLLQNAF
RNLQANQITSFVEGLFTLNTQYDKFRLNLRDFLVSLKEFAGDNAELFIVEKEQQEQDAKAADMERRQKVGGLLKPSELDDEEL
FUN_003125 CDS scaffold_14 80344 81859 -
hypothetical protein PF00266

MEGRKVPRAPELSAESPSSSSAGFKTYSAAGTGSASADRISLVSGHLSSSSYTAASPAQASVSSQARRLSTSTATMSSQPEHA
TLLIPGPIEFDDEVLKSMGHYSESHVGPGFVNTFGETLTLTRKLFQSTDPSAQPYVISGSGTLGWDIVAANLVEAGENALVLS
TGYFGDGFADCLRAYGANVTKLDGEVGGRPQLPEIEKALSEKKYKILTVTHVDTSTGVLSELKNLAATVRRVSPETLVIVDGV
CSVACEEIAFDEWGLDGVVTASQKAIGVPAGLSISFFSGRAVAAALENRKTPIPAYFASLKNWTPIMKNYEAKKPSYFATPSP
QLIHALHTGLTQIFTKPLSERFQGHIEVSNKVKKAVTDLGLKVVATKPEDQAHAMTAIYLPDGIGAADVLPKLAAKGVVFAGG
IHKAIAPKYIRFGHMGVSALDPSRNHIEKALSALKDSLVEAGYKA

FUN_003126 CDS scaffold_14 82607 83649 -
hypothetical protein PF12697 ENOG41

MIGFHLSYQWLHYTRPSGPPSPVPEGLERHWVNTPQGRIEVLSNTSSITNKFNGPPIVFCHGGMGCAWVWTEYMKYLAARGVQ
CYAVSLRGHGESWYPSYFRMVFATPRSALASDLVAAIEWVQIREKIEVMLVGHSSGGGLSQAVLSDGLANVKALALLGAVPGF
GSMGVYMNWWKMDPWFTIRLIFHLWHSNSPLSHPKLIQRAFFGDKFPLSAVIPFQRHMNRYESYLWPFGMMYSFTSPSNILSH
IRNDGASGEKVLVMAGTQDKLMTVPVTEKTATFYREAGNDKRDGVRLRFVEGAGHHLQNDVQWEDGAEKLFEFYKGIA
FUN_003127 CDS scaffold_14 84581 87148 +
hypothetical protein PF00172;PF04082

MSSSTRANPTRPNNARKRVPKACVRCRDQKIRCSGSQPCQQCDKRGSVCQFDDESRKVVVSRGYINRLQERLAALENNSEQHS
LLEATAGRSRSPDLSDRIPLSTKSNEISEQVIKASTSISPYGITEAQNQQPNGEGKAPLTNPLAFHTYDFVPSVTGNILFMGT
SSNWSFNKRVLTMTNERVKGRPLSMHNLHFAGTEGKVFDLRWDGTRKLTAQDVPDMSALPTKDFALYLINSVKFHCGWLYSLF
DEDIFMERFRLFHENPSEYARAEPLWFVHYLLVLAFGKAFVVQSTKSRRPPGGDFFIQAMKLMPDFNFFDCDIIEEMQVLCCA
ALYLQSVDHIQQAYRLVCSALRHGLEHGIHTEMQSNSLDAAYVQRSRHMFWTLYILERQLISLLGLPLSIADETISARFPDFP
GQPQKLEALKIHVDFCRVLAKINQTVYGLEGKLDCRYLGATQSVLKSIATVTERLNKSFEIQASEGMAGISRISAHLHLMQHQ
CIILTTRPLLYTFLLSRLGHLEVALMHWLQSESVKGLVQMCTESAQQILRILSSLSEQGLLETFLRFDQDAAFTATIALLMAA
AIDSSLLPDHTPWTQRAYGLFDEMNSRGNLVANMVAAELKQLEDLLKGFLTSNDSRPVVATQGSNTPRQGFMNDIDNSTGSVT
DYTEPFSLESDDDFGLGLNYELSAEQLLNIANSLDIDSLTWPWPEDPMAEDVDGA
FUN_003128 CDS scaffold_14 87582 89590 -
hypothetical protein PF00324;PF13520

MRRESIHDVNIPAGMTEKSRGSMTKDDGLDLAASRSIDEVQAIEMQEGKHGQFHRSFTPRQVHIIALGSNIGSGVFIATGKAL
ATGGPGNMVIAYFMVCTCVWAVLQSLSEMTIAFPVSGNYIDYADRWVDPALAFGAGFAEWLGWTAIVSAEAGFFHILVGYWSG
GSFPLAASLSIFMFACLVIFLLPNTVFAWFEYVTSLIKIFLFLIIITLSLALVLGAGPNGYVHHGETWTDLPAFLNGFSGFAN
CALLATWAVGDQVFIGIMGGEAESPRFSLAHATKLVPFRVNFIYMLSVVFITLLVPSNDPRLLGGGGVAASPFVIAVTDSGIP
GISSLLNAGMICGVLAIAAESVYLSSRVLRTMAHQGLISTRLAHVDDKGRPRLALIITSVVAVVLGYIQLSSGGLTVLNWLIS
ITSASFFTNWIIISFTNYRFHCALKAQNDKLFSETYAWRSSAWPIAPAWLMLISLMLLVCCIYSGINPVGGAGFSASNFFQYM
IGVLVIFGFTAGYKVIFRTPWRDPATADCLTGRRHLSVEEIKHLDEYYAKPKWRRFLTYVQLW
FUN_003129 CDS scaffold_14 96043 97938 + INDA1_1 Amino-acid
permease inda1 PF00324;PF13520

MESKSSSDNPTNVSQEKGTTVTEHPGDVQTGGGFAFQEESYWTRNGLTLESFQRRKDTGDNELDRRMKPRHLQMIAIGGSIGA
GFFVGSGKALYTGGPGSVLIDFIIIGAMIFNVVFALGELAVMYPVSGGFYIYSSRFIDPSWGFAMGWNYVAQWATVLPLELTI
CAVCIEYWNDELSPGIFIAIFLVAIILINIFGGIGYAEEEFWSSCLKLGATVAFMFISLVLVCGGGPSNGRYNEYWGTRYWRE
DPGAFKNGFKGFCSVFVTAAFSFAGTELVGLAAAESENPTKALPGAIKQVFWRITLFFVLGLFFIGLLIKSTDDRLNISGYSN
AKASPFVLVGEYSGLTGLAHFMNVIILVSVLSLGVSCVYGGSRTLTAMAQNGYAPKMFAYIDRGGRPLVSVAVHILMGFIAFV
NLDPKGGEIFDWLLALSGLSTLFTWGSICLAHIRFRKAWARQGHTLDEIPFRAAFGVVGSWISLGLICLVLIAQFYVAITAPP
GESGMGTVEGFFISYLALPVVLVFWAIGYLWKREGWLSIDKIDVDTGRREHNWDEINAYRAKVATWPAWRRFLNKIM
FUN_003130 tRNA scaffold_14 100949 101031 - tRNA-Gly

FUN_003131 CDS scaffold_14 103480 105761 +
hypothetical protein PF08427 ENOG41

MAHFSIATRPPSTLSYLSTFSSPPMEASPLTRQPQPEVFTPKIIQLYDSLFKDDYDDEEKTDGFWTEFFLLRPDRAALRRILN
ELSPSHVLSLDVRTQELFARGIAALKGSHSLAQLHSLDTLSVFLSCLLSKRYPHPSSDIMVLLAGLDQIDAVFTDFVSAVEAI
IRNSKELEVRYKAVEVLLAVTAGAYQTTLLTYLIQRDLFPAVMKFIQDAETSDQVLQPFILLGLLANYNKFEFQNPYQQRLND
FVNEAVIQKIVGAVGQACQTVRSRYITIQDDLPEGWTFSATLNMIGLGAIAPGPKPVKKPVYDVETAKALFTKLPEEEAAVLL
ATYDFTHANKLFCFNLVTQAAEPGAEQPIASYVSLTSYLLQHAYLSPRATYYAHLNLMVFRLLIEDPTICKRICSDESRVAVR

LCRQRQPYLPLVKGERVLAISLLDTMIDGVNHNLRRRLDVSLYTLSFGIMLRIISYLSRTRTRLEYHWPEFFRSLLSIVRFLA
TYTADLKDLPHIDTLLDHVVNLIALSLSAGEAFLPSPADYDDLFYKVVESGEVLTKFKENYRLGNRNSNSIDTLINVSAHYNQ
MLSEGGANRRKPSLLTTQEVTEVIRQGYETLSIQAKEGLDSWERYREADERTLLKKMARATVGDVRVMVER
FUN_003132 CDS scaffold_14 120401 124083 +
hypothetical protein PF00617;PF00618

MFSDQPMRASLQVAPLTIAKSRTESSGDASSTSENDNTLYSQSQMTPPATPNGSQEDLSPSPQYIPPQVFHNFLRAFYPFNPS
YVMSDSSVTLPLNEGDVILVHSIHTNGWADGTLLVSGARGWLPTNYCEAYEPDDMCNLLKALLNFWDLLRSTSVNDKEIFGNQ
EFMKGIIAGVRFLLERTNCLNRESTFIQRNDALRKCRKSLLSELSSLVKTAKRLQETQRLEIAPVEDVNDIIDEMILKAFRIV
TKGVRFMDVLEDDRRARAPSAVTVMDTVLEESYVPPTPPADQSSFDEQSQRSTSINDTDSQAAASVAASESSETTQTSTSIFN
KRLSSLSGRTGPSSHRLSQGSLSQAQAHRLSATISHRVSLAGPSSVSRAHHLVTERLNRSHDTFLSHLGSFIGRLHLQSQSRP
ELASAIRQSALSGGELLAVIDGVYDHINSSSEALARARSTMFARIQDLVFSARDTLVSAASEDADLIMPHDNTMLLASATGCV
RAAGDCVAKAKSAIERIGDFEFELETSSLGIDLSILDIDTEERARTPSVSEHNDAVSVAGSNRTSNSSNGHARRLTVVAIDKP
LPEVPQVTTPTDEQTVHPSPTSSRRSSVADDNVSSVASSISSLRPSLPPLPKLSTTLLANEEYSPVDNNDNDFNSSSRFDSMV
ASSAGSSATYLSRDSEVSIVSQSSTRATTPEQNLAPQKQPSLSNLSNGGSSEDVDVESRLMEKTFAHELMFNKEGQVTGGSLP
ALIERLTTHESTPDAMFVSTFYLTFRLFCTPVRLAEALIERYDYVNDSPHVAGPVRLRVYNAFKGWLESHWRDETDRDALDLI
IPFAEHKLASSLPSAGRRLFELAQRVSGEGSLVPRLVSSMGKTNTSIAQYVPADTPLPIPAITKGQLNLLSAFKMGAAQPSIL
DFDPLELARQLTIKQMNIFSSILPEELLASQWMKNGGVAAPNVKAMSSLSTDLSNLVAETILQQQEVKKRAQVIKQWIKIAHQ
CLELHNYDGLMAIICSLNSSTISRLRKTWDAISTKRKDMLQNLQDLVEPSQNNKVLRTRLHDHVPPCLPFLGMYLTDLTFVDI
GNPATKQMCLGPESEEDGNGGITVVNFDKHTRTAKIIGELQRFQIPYRLTEVPDMQDWMSSQISHLRDSEEGNVQVTYYRKSL
LLEPRETASRPPVDSSAASIVGVGGGRPDLFSWISRDRGTSTPTPTQI
FUN_003133 CDS scaffold_14 125228 126844 -
hypothetical protein PF05978;PF07690 ENOG41

MSTHEKENPIAQAASESADSDTPGFRPKGWMYKTVRIAGVDLWYASPKVQLLMVSIVCFLCPGMFNALTGLGGGGQVSNEAQD
HANTALYSTFAVIGFFAGTFANRLGLRLTLSIGGLGYCVYAASFLCYSHTQNMPFVVFAGALLGLCAGLLWAAQGSIMMSYPP
EQSKGRYISWFWVIFNMGAVIGSLIPLAQNINKKAGPVSDGTYAAFIALMAIGAVLALMICDADKIVREDNTKVIVMKNPSWS
TEIKGLWETLSRAPWVVLLFPMFFSSNIFYTYQNVNMNLAQFNVRTRALNNLLYWLAQIFGALIVGYALDVSSVRRSVRAKIS
LVVLFVLTFAIWGGGYAWQKDQRPREIAMNPANEDDKIDWDDGAHKFLAPMFLYFFYGFFDAAWQTCIYWYMGALSNSGRKTA
NFAGFYKGIQSAGAAIFWRLDGLGKPFDTIFAATWACLGASLVIAAPVIFMKIQDSVSVEEDLKFSDEKIEDVIVAPTDKKIA
LTEA
FUN_003134 CDS scaffold_14 129502 130240 +
hypothetical protein PF01521

MFASRSITRIALRRGCTLRQFSTSRPLAVSYHSYTLPTHTSTPPRNDDVPETSITQPDPLPKVHETRPPPQQPQHPQPPKQQE
PAPTQNAAPAASASAPKLSETEAQKPKAARPRPKLRPRKAAMKLTPSAVEQLRELLDQPEPKLIKVGVRNRGCSGLAYNLEYV
DKPGAFDETVEQDGVKVLIDSKALFSIIGSEMDYVEDKLNQRFVFKNPNIKEECGCGESFMV
FUN_003135 tRNA scaffold_14 131026 131137 + tRNA-Leu

FUN_003136 CDS scaffold_14 131658 132147 +
hypothetical protein PF01035

MAAITKSKRPLEEPQEERKVKVTKTKKQASPTSPTSPADFTTIMASQLALIEASTRTPFEKSVWTLLCQIPRGSFSTYGIMAA
HLKSSPRAVGNALRRNPFAPEVPCHRVVATGGALGGFKGKWPKDGEGITLDEKRKLLRGEGVRWDDKGKVIGTPFGGFT
FUN_003137 CDS scaffold_14 133021 134610 + TCA9
Succinate--CoA ligase [ADP-forming] subunit beta, mitochondrial EOG09262GXD
PF00549;PF08442;PF13549

MFKLGRSRAVASALNASKLQFAAPAARFPGVQQRRALSIHEYLSADLLRQYGIGVPKGSVAKSAKEAKEIAEKIGNDDMVIKA

QVLAGGRGKGTFDNGLKGGVRVIYSPHEAEMFAEQMIGHNLITKQTGAGGRLCNAVYICERKFARREFYLAILMDRQNQCPVI
VSSSQGGMDIETVAKETPDAINTNYIDINVGVTDEVAREIATKLGFSEQCIEEAKDTIQKLYKIFTEKDSTQIEINPLSETSD
HKVLCMDAKFGFDDNAEFRQKDVFEWRDTTQEDPDEVRAAESNLNFIKLDGDIGCLVNGAGLAMATMDIIKLNGGQPANFLDV
GGGATPAAIREAFELITSDAKVTAIFVNIFGGIVRCDAIATGLIKTVESMNLRIPIIARLQGTNVEQAHQLINDSGLKIFSID
DLQTAAEKAVQLSKVVKMARDIDVGVEFTLGI
FUN_003138	CDS	scaffold_14	138827	139901	+
hypothetical protein		PF01636;PF03881

MPHTKVDPAIIEALGLDPANSAINSHGGSGFASTFKITSEKDGKPLNYFVKTGTGKEAEVMFRGEHASLNAIADSVPNFCPRS
HAHGALAGRSGQYFLATDFLDLGSSAPGGSGKTLASKVAQLHTTPAPIPEGYDKPMFGFPVPTCCGATEQDNSWRESWADFYA
NNRLRHIARQAVRNNGSDPELEEVAEKVASKVVPRLIGDDVVKNIKPVVIHGDLWSGNHSKGQIGGQGGCEEVVYDPSAVYGH
SEFELGIMNMFGGFTNHFWKEYHELVPKAEPVAEWPDRVKLYELYHHLNHYAMFGGGYRGGAMSIMRKLISKYA
FUN_003139	CDS	scaffold_14	140063	140787	-
hypothetical protein	EOG09264IIZ	PF03357

MGGNASKVTAQDKAILDMKNQRDRLHQYQRRITVLTDKETDIAKQMLAKGDKKRALLALRRKKYQESLLAKTDAQLAQLEKLT
NDVEFALIQKDVVFGLQQGTKVLKEIHAEMGGIENVEKLMGETAEAIAYQQEVSDMLGGRISNQDEQEVEDELEALEAELTGP
AKLPSVPVANLPEVQKNQPVEAQAERAKQPAMLAA
FUN_003140	CDS	scaffold_14	141535	143812	+
hypothetical protein	EOG09261YV6	PF00004;PF17862

MTLGAKRPRLNLRQGLDRDVYMVMKKLEDANDGKPFKTTTAVYEAIKRSNSSLSRQKKRPLEDSIDRVLQFRKEELGDSSDSE
AELEEPPKPDDDRFLLNRQMTKHWHQNTTTATEVETATPPTSEAPRAIKKRRVQQDGEERVKDGVVTPKGALTDTDAAPTEKQ
DKAPLKKTQKTSRYKVEHPSGPIELGGVKEVQEDIIEQIHWLLKWPELYAANGWRGLPGLLLSGPPGIGKKSLIRSTAAKLDV
PIVYLTGCFEDLERMERSLNDAIEEAMRVAPCIILIENLDWHMPKPGGSGHSEGHRRNVIQFMRQMQRIAAEQDKDRHILAMA
TTARITDVDPAVLKTGLFERTLQMRIPDLGARQDILKLVTRDMNLAEDVDFAELAKITHGFVGADIINVATLAQRQPQRRMRL
VDQYRYHLLDISQIQSTQPQPDLMELAGQQPSAPYPEPPSVPLAQEDFKKAIKDYTPSLRKEGFTVIPNVSWEQVGALQAARD
QLNMSIIGPIKDPELYRRAGLGRPAGCLLWGPPGCGKTLVAQAVANEAQASFILINGPELLNKYVGESERAVRELFNRARSST
PCILFFDEMDSLVPKRDNASTEAGTRVVNALLTELDGVQDRTGVYVIGTTNRPDMIDPAMLRPGRLNVRLFIDLPTPDERVDI
LRAIYRTNHPDAAAEEFERLESIARDPRCTDFSGADLGGLHVKAAESALRRCLQGAKTAAEIDGVDWEHALSTTYRSVQNPET
YRKLEVKLGKS
FUN_003141	CDS	scaffold_14	144755	145631	-	CYC1_1
iso-1-cytochrome c		PF00034;PF13442

MAGGDIKKGANLFKTRCAQCHTVEKDGGNKIGPALHGLWGRKTGSVEGYAYTDANKQKGIEWNDDTLFEYLENPKKYIPGTKM
AFGGLKKAKDRNDLIAYLKDSTK
FUN_003142	CDS	scaffold_14	146346	147137	+
hypothetical protein			ENOG41

MASRAIIPRFLLPLQGPLWRGLSIPLSQNSIRIRFASSSSKGKPIVLEKPARFNPPSHGSRLKKNHVPKHYGPELTASEVAAQ
NKKHYPGMMAPEGTWEHFFWHSRLLHTFITMGTLFALGVFTFFMNYAYNSPYKELVPPISDLWTHPLHFFVAWKNVIVLHEKD
KAEKARDHRTNHLDDVAKRRLFMKMHGIETKDPVTVVFGKGEEDPKPEQTAVEDELVQKTEVQEKVEEPKKKWFGVF
FUN_003143	CDS	scaffold_14	147299	148365	-
hypothetical protein		PF01138

MASQINLFSPAELAYLHQSLSLHPPIRPDGRTPTQFRPLTAETGVLPGTNGSARVHFSDGTEAIVGVKAEIEKTGGSEEQRDE
EDRTRGDWLELAVEIPGQRDDEASTIFLAEMLREALLADNEFAKKLRINRRFHWRLYLDVLLISPPLSYPLPLLSLTTHLALL
ATRLPRLKSEGDEDPMFDDDWEASTFLYPRDGADAAGSRPSITLLVVAIGDNIIFDPAKEELAVAETALAVSVAEVRRVATEG
DMEVDKKSRELKLLSIRTIDPPSRLTPPGVPHTTNGTAQKGEAQTAEGVWKAPLGGTKFGVMDSIIQAVLEKGGVADEVLEGL
EGVELT

FUN_003144	CDS	scaffold_14	148877	151273	-
hypothetical protein	EOG092638AP	PF04376;PF04377

MEPSGMHIPEDALLQYEYITPIGYFKSSRCGYCGKRDKSYAYHAVTTSLSPAFYQTLLDRYWRRSGTLMYRPDQRRSCCPHYT
IRLDSSQFKPSRDQRQTVNRFNKYVMGEAYMKEAARLYPKSRDQAKKRDNHFDLVERIHEAEEANLKNPPEAAHKLVVTLEHD
DCTDEKFAVYQNYQAVVHKESPDEITKSGFRRFLCDSPLRRETMVTSDGRKRRLGSYHQCYRLDGKLVAIGVLDLLPECVSSV
YFLYHESIHQHAPGKLGALYEIALSIEEGYGWWYPGYYIHSCPKMWYKIDYSPQYVLDPVSLTWDPLDRTVLDLLDKKPYVSL
SLEKEPASREIEQGSASPAKDTNESDKAEDKGSDSAASDEDDNNWLFATGMPGIPPISTVADWDMDHIALKISPRAPLYETSD
LVSWDEQGVEEYPGMRAGVAELIAAIGPDLMDRVCLDFSRRH
FUN_003145	CDS	scaffold_14	156321	157073	+
hypothetical protein	EOG09265BJ3	PF00293

MSSSASGDSGGGRSMESRVGRSKQRYNTKGERLVAGIVPLTPDQNYVLLIQSTRRKGWVLPKGGWESDETCQEAAEREAWEEA
GITVQITYDLGDIDEKRAPKSSKDRSRYHFFEGTVTSEYDDWPESHKRERQWFTFTQAWEALSTRPELQEALQRSTVNRQ
FUN_003146	CDS	scaffold_14	158260	158941	+
hypothetical protein		PF01423

MENLSIADAPPQGRGAPQPLPQLPPQMFTTAAQLLDLTDSTTPPWYQFIPVSEKLMVALRDGRKLIGVLRSWDQFANLVLQST
VERIFAPSPESAGSDRPTGLYADINHGIFLVRGENVLLLGEIDLDRDDDPPPGFELGELDVVKKLAEEKKAVDKAREKARVKK
LAKQGFEGENIGEIVL
FUN_003147	CDS	scaffold_14	159808	160731	-
hypothetical protein		PF17119	ENOG41

MAAPIRSSLSRSGRTSVASIRSLSNARPAKAQVQPVTVREPKPSLPPSDATLLEDYLRRGALSRTYFDATGVRWVGSGDDGPK
DPNKAKLGKTLRVLQERLPTLLLHPLPHDILAPNIALHLFPSTHPHLPTVSGRVAYNAALWTSPIAWNRVPILGNVCIEVLAE
RMTTEPLTFLPRRAGAIPEQLVVRWCEKRKGSDSSSNKGVIARSLPFGRGVDPDKAFTGLFVFDFDAEGRILSHTIEQAQAGG
DWEAGVGAKVVGLTDWLLGGCKNPGTPIPMFERVRRRRSI
FUN_003148	CDS	scaffold_14	162031	163093	+	YPT1	GTP-binding protein of the rab		PF00025;PF00071;PF01926;PF08477

MNPEYDYLFKLLLIGDSGVGKSCLLLRFADDTYTESYISTIGVDFKIRTIELDGKTVKLQIWDTAGQERFRTITSSYYRGAHG
ICVVYDVTDMDSFNNVKQWLQEIDRYATEGVNKLLVGNKSDMSDKKVVEYSVAKEFADSLGIPFLETSAKNASNVEQAFLTMA
RQIKERMGTTTANNTKPSVQVGQGQGVGSSSNNSCC
FUN_003149	CDS	scaffold_14	164483	164844	+
hypothetical protein			ENOG41

MASVESQVSKQSETPLQNDNENAEQLATLAEGKVADAVKGTSSRDKPRKDDIKIEDYASDLDRKKAEQAEAREETKTQRKAGV
DVDGSLGQGRLSNEDNSSV
FUN_003150	CDS	scaffold_14	165148	168147	-
hypothetical protein			ENOG41

MSTDNKLLGSVVAFEGHVDTISTQLRLLPTSPNILILPSIQNYISNRDLKNRSDVRVYVKKIHEATVARYEAAQAFLHGSTAS
GRRLVFINGGTPSARTLCIRAIMDYETDGDYCRAESLFNQLVKDGVVGLDNPVRDWRRQDMYQPKDKGDQEQEFEDPITRAMR
AADALDRQTASLQPSNELDLTLTPRPRSNSLPLYGYEDNFGDDAPFFVFGAANKDEGGSTADEAPGCSYSPQACRFSARHYDR
SAGRIYHDISNLQSPSPRASFLASPSCVGEAYGLMAAPVSPALEGLSPRSDVLSIPSTDNVVYGEASLVDMRSPGHSPTLSRV
KSLDRIYPASPRYRDLCVSAATSGLARQLESDPGPELLKAHRHSQTITTNPKELVSNRRSYIEGPQTVIVRTNLPIIKVAPVP
LEKRRKPARASYVDRGTDAEHISGPKETFQPVLPFMEDLVVHFREEVPEPLLNSVTHGLRMGQFPAAIYSPTSETDDGNALVP
GTPESNTITDVSCAESQCEESAVTDQSILSDEYDPFAYNQPVWPNKKPIPSVPKLKVARPPTPVRTPPPLVSDVDSKFHDLRI
NGQETAITVQNSLRSVLQEHFPPETQGYRQFQFSLLPELEGLWNPVFWETGSNDSRGSCHRIDQILAIGAQRGVKRDYTCAIT
GQLEKLGTKCNGMSRSGRLDFRYLLANAMQAYTTQPLASQAKDNPFANSFLLATLIIPHLETYLALHAEVRYLLLEYPPEHLA

TVLALQKLVGVELMKVAQIVDASSKSIPFTHLRGNSITGPEQGPVGRFGKTFPQNSGSGYDGTVSKANFLITSTATDAEIATF
IATISRILSKTSDFYVPHEPPKKPSPKKSKPPSITGTFSPFPRVPSATHSPPMSPALGASLGAALSGSSAVPSRAPSIAETVK
TVKSTRSKQSRSYSKRKPSAIDARSILTMYVDDSDWDQEDRRIMPLLEDKAERRKGNSHKALKFLGLS
FUN_003151 CDS scaffold_14 172270 173551 -
hypothetical protein PF00400 ENOG41

MQSADRDHFFETDAVQASRQRKAAKASNKNGNPVIMKSKILAAVPDPAAPLTCVFIAESAGAVRRINLETSETKTTYRGPKAP
VTCLATGGQDNKTVFAGSWDKDIWSWDIETAKPGRKFSGHTDFVKTIICATVSGKHILISGGADKKIFVWDVESGKRLHTLQD
PTTTMLAVQHLAIDPVLSDPTTVVFASAGSDPHIRRWKVTLDSYEQLPESFSDRPDAERLTIQEHETSVYRLVFDQMSEDADL
WTASADGTAKCLARSRNFVADDTLTHGDYVRGVLVTDQWVITAGRDENVKVWDRSSGKLYCTLVGHYEEVTDLVLLQDTDGTP
RKVCSVGIDGTVRTWPLGKSELDEVLVKIQEAAEPKKEEEEKDGNVLTAEEEAELAELMDD
FUN_003152 CDS scaffold_14 173919 176541 +
hypothetical protein EOG09260S2Z PF00400;PF12341

MDSTITSKPRPRPAHTQGTTRCAYTPDGSRLVTVGSNNTIRLYKTGSDGEPINIDDCQEQNMAVAAGDEFFVVGSEDGTVSLY
SLETNTFERFLTRTVLPIRDVALTNDRQWCAVASDELSVKIVNTKDITQVKHLREHARAVRNVSFDPQGRLVALSGTDGIVYV
YSLTAEEPELIRKVDGIIGAIDGDSETSTRVAWHPDGRAFAVPTPVRDIQIISKNDWEKQRTFANGHLADITAIAWSPNGAML
ASASKDGKVLIWETKTQSVIARYDYSNVIDIVWHPTKNILSFTTTDGEVYIYPDFLTDQFSPLLKLATQPAPFIHDPLAELSA
NRRPPPVNGQKQQGLPTRPRRESLGSLDSFLEGGDGYGDDDFVEDDDGAGYTIGQGQKRARDGDDGYGTSNKRRHMLEPQYHE
AFQPGATPWRGNRKYLCLNLIGFVWTVDQDSHHTVTVEFYDHELHRDFHFSDTFLYDKACLTEHGTLFSCPPKDDAPAVIFYR
PHETWTQRYDWRIELPKDEAVTAMSLSESFITVTTSANYVRVYTLFGMPYRVYRPKSTPMVTCASWRDYVLTMGNGPMGADGN
TRLLYTIENVKRDEICQNEDTVALPEGATLKSVFFSDNGDPCIYDSTGTLLTLLHWRQPSRASWVPLLDTKLLDRLASGRKNE
SYFPIAVADNKFHCIILKGGDQYPYFPRPLLSEFEFSIPISSAPKTSKRKAREGSEDLDMADGDEDKDEDEDGSSETRKLEQQ
FMLHNVKAAQLRDLVEATSSSHSQRSQLSRLELEIDKTLLQLLAVECREGEERGMRALEMVQLMRDRTGRMMEAAGRVAERYG
RTILGEKIREVGEKRIEGLEDDDFP
FUN_003153 CDS scaffold_14 184376 186349 +
hypothetical protein PF00447 ENOG41

MATAHMAVRTETFPHSNPIPHSESMGARIIPIPTPTIKMSPPSNGDPMEITSPAPSSTGNNNSDSDANGKSNDRPKNSPAPDS
NNVANNNANNSNSMPAPPPAAAAVHQPKIVQTAFIHKLYNMLEDPNIQHLISWSASAESFVMSPSADFSKVLSQYFKHTNISS
FVRQLNMYGFHKERDVFHTGNPDTTLWEFKHGNGNFKRGDLVGLREIKRRASRHALVHRESNYTKPAASQPGTPAEPVPIPPD
SADARLLNIEHTLFDLSNRLQRSEEAAHYMHVKSQAAMDTVNRLLHFNQELSRTMLSLVPAESPLSREVITLQGEMQRQAEVL
RTLDEPPQEAPYTGRQQYFANVENAPVSPRQLAQDDQRRGPALGVPPARGQNFYKPPVPSNLSSSTRRPYGSISGSSTTQSSP
LRTGPPPPPPAPHPLSNVETVPGPGSLARRHTAADIRAHGWQPAPSPFSTGAPSGAWPPSPSRVAPEDQRIRDSFSTYSLQAA
SQTHPHSRPTTPPPPFANGAGGGSDTFGGWSWGSAGRENKNLAVKDHSAPPTRRGSMAHILNPSDTAERSDEDEDPRGDDDRK
RKRMQ
FUN_003154 CDS scaffold_14 186878 187499 -
hypothetical protein ENOG41

MAPEPPPPFPVATNRDAIANTLSLLLASRTSIGKSMPLTRNLPRKRRTLPDDNDDDLTRGARPNEGLGYVPEKKDVQKFANSK
EEKMLRGRLNKDAKGNLKKKVEESESEDEAGRSALGKRKRPRKEKEIEPGPESENQTAPVTPEDKEKNENGEEAEVDVNMKDA
ATQDEPSLGDGQTEKKRKKKNKKKKQKTANAVAEVEAEA
FUN_003155 CDS scaffold_14 187972 189253 -
hypothetical protein ENOG41

MAPPVSHRRLVAAIVYRTLYLLLYVCLLGLLIATPADAIHRSFQNRQLYNIWIVIAAYVLAIVIISFVFITRLYINKTDLGSI
PKGWVPIEKGDLRSTVYKVIALGLGRSASIAYESRPRLQTDNTAPDDETVETRVKLGFDGPSGPAEELVVVIPRKQPVWGDIE
HYGWSSPNAPDLPNLEYTTVFSELPNLIEGKALTLAPPDPTSDTNPPLLDPDAVALLQRTANMSLRDYIVHLADLGVIEMDST
TVEFLSHYEYARFSNRPISNAQFKELMHLFAEILRAMEPLDPDILDEQDNTSSPSTDGDDDARSRLGTSRSNLAPSDASISSS
TRIRRQPSTNTWNVYETAPNSVRSGFTGPGSISRRLSNISLARSRRHYPMSQPSTSSLRSKSSASSGSVIRLATRHDSTDLPY

VLSLRDTTASY
FUN_003156 CDS scaffold_14 189776 191746 -
hypothetical protein PF09531

MAPTTVRKAPYKDFLQPALHRRFTSTATVLLLVSYLLAVVLDGWKSYFWSWFPIGPVGVRTAFIFTCGLAIVVLRIANYHVGL
RTTGPGFQTLRNTITQLQTYETLFWYGFSSLLFSHVFLWAMPKPANLSWITYFSGDRARLNERPLFLLAYMVSCAITQTFNHY
RKDTDRLVLASSKGKNEKQPDALKLLITAVPATFGASLSGAASALPVALILYYAILRSFIWGWALMFLRIFYNLPKTNMLPPS
WPSDLWLLFRCIEAGTFVHLIWNIGNFAFSKFMVKEPLKNGKPLTSESKDPNGSLLNGLKSKKLSIKAFAMWELAIIAQGFET
RRQAIYEDIDRKDGPMWSQVYAICMEVVKSIETRIDTYGKVPDAPPAAPAPEPKQRVSAPLRDDPIFNSRGASKTMLNEVEKR
LGQVARSPGESPVSKLSPIAKKTWKEAKDRVLTKEQQELVAPNHIKSQFENWTLQMMEVDAVAALFQQDFRTQFAAAVLGTPY
AEPTLCINAINVLHHLSVHSLAEDQFGNVHRDVPSIIRTFTSVIKKLEAFRLQFPLHWTDVSGKRISPEVEEVVDALKTALQQ
VLVKFEPYSSDLRLTLTDIRLAKEASATAKKSEMAEVAR
FUN_003157 CDS scaffold_14 192357 193192 +
hypothetical protein PF05739;PF14523 ENOG41

MSYDLESGRRGGGYSDDPAFQELQYDLKSKLQTLLSSNRKLANDVNVLGTRKDTPRLRERVHNSMDKTRDMCREIGEGVKRLQ
TWEELTKQQKYEQTKVSSDFQAALQEFQSLQRKALEKERASVTAARAAQEGEHAEGPQSGNQLEQLQQQEQVSQLAPQDEVDF
QEALIIEREEEIRNIEQGVGDLNVLFRQVAQIVNEQGEQLGTIADNVENVRDDTRQADVENRQAARYQKAARNKSCCLLLILA
VILTIVILAIVLD
FUN_003158 CDS scaffold_14 193322 194384 -
hypothetical protein

MSLLRRTAHSISPHARPSLSAINTTRRISHPAILSSLTSSPYANRRNFSVASALGETVTVTADALSWAHSAGVPWYVAIPLLA
VGVNATIRFPLQLYSARLREARKPLDPLITAWTRRHMMDRSLNDPELPERVRSLRIAGAVEKSRRRILKTWGLQRWKSMAPLL
GMVPFVTISEALRRKCGAPLGWISHKIGLGNTGLGPSSSMFDQSLVDGGLFWFTDLASADPYYGLPIICSCILVWSIWARMPK
EQLQALLRIQPPGSKMMVTSRLQQLLGRVMLMMPLLPLLFADLPSAIFLYWGTSFALTRVNDFFIQRLVPPKMPDLRAPKPLK
EFPFVLKDQSRIAKTSGKKNN
FUN_003159 CDS scaffold_14 195477 197222 + ALG11
asparagine-linked glycosylation protein EOG09262UB3 PF00534;PF13692;PF15924
GT4
MASLATLVPLAILIALPLIGGLLRRVLGWSLRKRTEGRHAHLLALMTQEDKDARAKDPQGTAATKLVFDVDDNLQSTLSSQKD
WSGIVGFFHPFCNAGGGGERVLWAAIRATQDRWPKAKCVVYTGDHNVTKDAILNRVKTRFNIELHAPTITFLYLSKRDWVLPS
TWPHFTLLGQSIGSVVLAWDAFSLLVPDVFVDTMGYAFALGLCKFLFPKVPTGAYVHYPTISTDMLDSLDSTAASGTQGAHAG
KGAGAQGFAKKNYWKLFALLYSWVGSTVDIVMTNSTWTQGHIKSLWGPYRKQKSKTDPIAVVYPPTAVREMEREVEVSEESEK
RREKAIVYLAQFRPEKNHQLIMRSFATFLKTKSEASKGAQLILIGSVRDDSDSKRVYELRLLANELGIKDSVKFHLDAPWSDV
LDWLRRASVGVNGMWNEHFGIGVVEYQAAGLISVVHDSAGPKFDIVVPIDGQPTGFHATTEEEFAEGYEKALSLPDPLAVRLR
ARESAKRFTEEEFARKWTVQMARLVSLAKQKA
FUN_003160 CDS scaffold_14 197422 199469 -
hypothetical protein PF01019
T03
MKIPFLLLASSIHYISISASPIFEEFFSPSVGTRGAVASEAQECSYIGRDLLARGGNAVDAMIGTTFCVGVIGMYHSGIGGGG
FMIVRDKKGNYEAIDFRESAPAAAFEDMYQGNVNGSIYGGLSVGVPGEVRGFEYAHKKYGSLPWKTVLQGAIKVAQDGFIVNA
DMARFIEKTIRDHHNFFVEDPSWAEDFTRNGMSFHSQRWDQGSQVAGQLIPEGEKMTRRRYARTLQAIAEHGADAFYTGAFAE
SMVKTIQETNGTITLDDYKNYDVISREVLHTEYKGYDVYGISSPAGGAVSLNILNTMNGYTHQDEDRNTTLHIYIEAMKFAYG
ARLHLGDPDFVDGVPELEHEMLNATTAEKIRRKIDPRKTQKIEKYDPDGIYSSDGHGTSHVVTADGDGMAVSLTTTVNLIFGS
FLMDPLTGVILNNEMNDFSIPGVPNEFGFAPAEANFIRPNKRPLSSCTPLIVSNKDGSLFAVIGAAGGSRIISATTQVAWRVL
TSPSWSIKDAVREPRVHNQLIPNTLLVEKKFSSYDVPSLVERGHNITWVDEGLSTVQALTRDSAGVFEVAPEPRQKNSGGVTL
FUN_003161 CDS scaffold_14 200517 202146 +
hypothetical protein PF05958;PF09445

MVSAQAEAVPNGNSQVNGNAQQVNKRPNQQNNRQWKQKKTKREKNITEGSSEEVLKYDIQALLKERAVEYGEGTEHGPEVLPE
EGTETEVEILELSSTGDGLGMKPGSNQVYVVSFTVPGDTAKVKVFRHLRRDTQTLADFISITKPSPLRDDARISCKYFASCGG
CQFQMLEYPEQLKLKKRIVEKAFKNFSNLDPNIVPAVQDTVGSPLQYNYRTKLTPHFDGPPGFRPSKRGKNPRIPFEKCPDIG
FMQKGRRKVLDIEDCPIGTNAMRLGMTRERERMQKEFANYQRGATILLRENTKRVPKDSEELEGEVEPYTVRVENEDTVDIKS
CITDSKATTTEYIGPYTFTNPAGSFFQNNNSILPSFTGYVRDNILPPAGTGMDIKYLIDAYSGSGLFTVTLASLFQHSIGIDI
AADSIAYASRNAALNGMGEDRCKFIAADAGELFKSVTYNRDETVVVLDPPRKGCDADFLNQLRKFGPRRVLYVSCNVHTQARD
VGVLVRGEGEGERYEIESLRGFDFFPQTGHVEGVAILNRVDTTA
FUN_003162 CDS scaffold_14 202324 204485 -
hypothetical protein EOG09261W1K PF11701

MAEPTEAPSQAPPAQSLEDQTLIVFARLMEGGQEDNETCRDLDELTKLLNDDYEARQKDKSRETICKVIDGDCVDTVLCYLDM
RQPEIVRGHATLSTSAYLKAAGDDGSKKLSTFFFDRVRRGTYDDYIVAFCVAAATFPIVPDLTAELFLNEDFLPSLGTLMRRK
WKSRKVETACLEMLNAACMNSLCREAINKYCIEWLEEIVDQDLSEAVRSMNADPNLQSDGGSISMRRHSEQVQYLAAVILAKL
RAVPAKPAPGDNKSRIEPAVTSIEDLSGMFTKMILRDEDHGRKHSIEGLAYASLQPKVKESIVSNPELLQKLVKTLSEAQPRS
PTTYGALSIFVNLTKYLPTLTEEEKKMNQLKAYANAAGKLGGPDPLNDDEHVAKRCKLVFDAGITPVLVTHSKNGSPASLGLV
ISIIFSLSVDRTLRGKLAQQGAVKLLLVSWMSLPQTEAASRRLAAQALARILISTNPALVFGGNRDTPIIAAVRPLVSIIPPD
PAAQTRDLLPSFEALMALTNLASMDDDATRRSIINTAWNQIEEQMLASNTLVSKAAVELVCNLVQQPEAIALYAEETAKARNR
LNVLLALADAPDAGTRSAAGGALASLTNFEGVIRGIINRDRGVKVILGMCVDDSEDIRHRGVFVVHNLVTAEGEVGELAREKV
KSEGGVETLTECAKKSRSNDVVELTVQALKALLGDQS
FUN_003163 CDS scaffold_14 205199 206276 +
hypothetical protein EOG0926369X PF03850

MDAVDVSEHYEVGTREETPSLLTIVLDTNPRAWASLNNVLPLSRAIANILVFVNAHLAFSNANQVALIAAHVDRAEWLYPTPP
KPSRDASGDVAMNDASQTQTQTSANKFPQFAQIEAAVLAAIRKLMDQTKEEDLSATTTQISGALTLALCHINKAAQALCAPTA
NLEDSHKGSSNTAPPTVRGRILVISVSDSEPSQYIPTMNAVFAAGHTQVAIDTLSLTGEPTFLQQACYNTGGTYLAATHPQGL
LNYLMFGLIADTEAREALIAPTHDTVDFRAACFCHGRVVDTGFVCSICLSIFCETPENSECFTCGTKLSLGNYGAKPAVVPRK
KKKKRKIVNGGGSREETGSATGTPRP
FUN_003164 CDS scaffold_14 206642 208550 -
hypothetical protein PF01112
T02
MSDSLDDDTSFSSGSTPYGTDGAFDREMKVAHRTKAVPAIFIHAGAGFHSHQNEHIHLQACVAAVEMGMRFLRAGASATEAVE
AALRILEDKEITNAGYGSNLSIDGVVECDATVVDHLGRSGACGAVPNIRNPISLAKLVLDMSNKPLSLRRVAPNALVGEGARA
FAEEHGLMIYPNEYMVSKNARDRFLRWQEDLKRAESKGQEPKALPETVDTTATCPPCQYDTASPTKPHSSLPRDQRAAIIAGT
WNEGQPDSPFAGTPIQDAPSPDQSTSTSYFRATSTSPPTVGRSSFTRSPQEGATQTYAGVVSGAQGVLSSPFRPSLSRKSNSL
AEGFKPVLPENASIRHKHHAHASNKVDNTSQRTPTGNRGSGPDRQSSSGPRGAKRPLSPDEKSQPHSRAVHDRFHFGSVNEDI
ITDTIGAIAIDDRGQIAAGSSSGGIGMKHRGRLGPAALVGVGTAVVPCDDEDEDQVAVAAVTSGTGEHMATTMASQRCAERIY
HGTRRGKGGANIQDDDEDAIMESFIAEDFMKHPGVKNCHSAGAIGVMVVKKTPSGYYFYFAHNTDSFALASMGGSERQPVCTM
SRLSDGAKIAKGGRKVRFE
FUN_003165 CDS scaffold_14 209603 211250 + ARP3 Arp2/3
complex subunit, actin nucleation center PF00022

MANQTPAVVMDNGTGFSKLGFAGNDSPSFVFPTAIATKGPTGGAGGSGSGRPAVANKPSFLTGGAGAGGHLSGKRGTEDLDFF
IGDEAIAASGGPGYGLHYPIRHGQIENWDHMERFWSNSIFKYLRVEPEDHYFLLTEPPLNPPENRENTAEIFFESFNCAGMYI
AVQAVLALAASWTSSKVQDRSLTGTVIDSGDGVTHVIPVAEGYVIGSSIKSIPIAGRDITYFVQSLLRDRGEPDSSLKTAQEI
KEEYCYVCPDIVKEFSKYDRDRTRFAQHVVSHPNGRQVTVDVGYERFLAPEIFFNPEIYSSDFLTPLPVVVDGVIQQSPIDVR
RGLYKNIVLSGGSTLYKDFGRRLQRDIKQLVDARIRASEVRSGGARSGGLDVSVITHKRQRHGPWFGGSLLGQTPEFRSYCHT
KAEYQEYGPSIVRRFALLGGPGGS
FUN_003166 CDS scaffold_14 212706 213949 - ADH1_1 Alcohol
dehydrogenase PF00107;PF08240

MAAPQIPEKQWAQVFEKTAGPIEYKQIPVQKPGPDEVLVNVKFSGVCHTDLHAWQGDWPLDTKLPLVGGHEGAGVVVARGDLV
KDVKIGEKVGIKWLNGSCLSCSYCQNADESLCAEALLSGYTVDGSFQQYAIAKAIHVARIPEECDLESISPILCAGITVYKGL
KESGVKAGQSIAIVGAGGGLGSIAVQYCKAMGIHAIAIDGGEEKGKLTKELGATAYVDFTTTKNLVADVKATTSDGLGPHAAL
LVATNEKPFQQATQYIRSRGTVVCIGLPANAQFSAPVFDTVVRMISIKGSYVGNRADTAEAIDFFRRGLIKVPFKTVGLSELN
EVYKLMKAGQIVGRYVVDTSR
FUN_003167 CDS scaffold_14 219338 219642 -
hypothetical protein PF00403

MADTHTYEFNITMSCGGCSGAIDRVLKKLDGVESYDVSLENQTAKVVTALPYDTVLQKIAKTGKKVNSGKADGVEQSVEVAA
FUN_003168 CDS scaffold_14 222171 223059 -
hypothetical protein ENOG41

MDQLFPQFTRLPPEVRAAIWEHTLPEGDGGAALYMYNMDWWAQYSPPGVAFHDMTTQGIQKLSRTPRVQVPIPTCAAVCKEGR
RVVEQWRKKNNLGWYFREETKGDILVRPFDAERDILYVSRLKWESFQLLAVDWENDDEHAAVVRIMESIKYLALPAFTAYYSI
STIAALLPWMKNIKTIYVVWNKLPKAHTIKRPPPDVAEIADIKVTLDAPVQPRWELDTFLKRENTVELHQPDEDTGRDFVEEG
ELAEWLDDIDDLWNTTEVDPEIWDEDEEKLKTPQVHVTVKELPTWY
FUN_003169 CDS scaffold_14 223676 224393 -
hypothetical protein ENOG41

MENPNVCLPARIQVPIPAVYDVCRDARPVVHRWMAKNNIQWYDRLETGEHILVRPFDVQRDILYVPQNDWDIFEGFVNSDERD
PEEHQSMCNKIINLGVAAHTMTQLQCAEGIVRLMLRAPNLNKIYTIFSDLPRVRTVTLHLPSYREWWGNVPVQQRCELASQPK
PNETVHVHRLDRHEHLDHVEKNYLLNWQKEMNEVWRTVMDEFPDIAHSATGELKAPRVDVSIMEVPTWDTVR
FUN_003170 CDS scaffold_14 225012 226442 -
hypothetical protein PF00108;PF02803

MPEKKKSPVYVLGVGMTKFIKPRGKVDYTELGFEAGIKALLDAQINYDDVDQGVACYCYGDSTCGQRVFYQFGMTQIPIYNVN
NNCSTGSTGLNMARTLVEHGAADCVMVVGFEKMAAGSLQSNFKDRENPIGTTGKMMAETRGVTNAPGAAQMFGNAGREYMEKY
GATAEDFAEIARINHAHSPKNPYSQFQQVYTKEQVLQSPMIHEPLTKLQCCPTSDGGAAAIVVSEAFLNARPHLREQAVEIAG
QHLATDAPSLFSESSIDLMGYEMTQRAMKEATQQAGISPRDCQVVELHDCFSANEMITIDALGLCDKGKAHELVRAGDITYGG
KYVINPSGGLISKGHPLGATGIAQCAELVWHLRGWANNRATPNTRYCLQHNLGLGGAAVVTVYKRADGGVAQAVNSTMVGNRN
KLGYNPAVEAKGFTQEQVDLVRSKKNRSEWALQDVEKKVEARF
FUN_003171 CDS scaffold_14 227199 227814 +
hypothetical protein ENOG41

MRILLLSSSNHNSARIIQSALSRNFTITFLTPQTSQIPHHANVTLINGLPASQHDLEIALQTPNPAEAVILAFYNTHVLEAVT
HTLVRALRAIQSAKVNEEKEAVALPLRLVFIQSNAMQLEVDGYKKVDKIVRESGLPFDLAQCPRLAGELSATIRASPEEGRVA
AWLATVKKARMAHFPVDKARENKSNGREESGVGDIKLG
FUN_003172 CDS scaffold_14 228371 230869 -
hypothetical protein PF00172;PF04082 ENOG41

MTAGMPEMTTAHSTTHDGGSAPVTASQSILSSTAYDAPLSQQQTPPQHLHQQTPSQGLALTQQQQQQQRSVKRPRPVKSCTEC
RKRKLRCDRLCPCSQCQKSNRTCKYAMDHDSANLSEGSDSEMPEPARPAKRNCNPGIFNSGTPLPSNDSTYGPIRNGDTGAAP
SLEELSMRMERLERQLMARSPVVSEGSGGRLIAASSETIRGLTVKQGALRTRYHGQNSPRVLLNLFDEAKEFMANNSNNNGVR
DVLLNFKRFHKALIDEYRKSLSPITVFVDSMMPVQKRMTDILPKKAVCDRLVASYVDTSETIYRIVHLPMFMEQYNLFWEGKI
QSEYFLPQLLSLCSVASRFETKSKGLGNERSDGVHIPTACALVRTWLDSLRGKQLVEFEVLQVEVLLLHAQRMITPRIQDSWT
SLGSIVRMAMTMGLHRDPSEFEPRIPIFFSEMRRRLWFTILDMDLHISLASNLPCLVREGDFTCRPPRNLDDIELFPDMKELP
QSKPIDVVTDNQMQVYAAMTLGVRMKVAHMLNRIDTIRDYQEILEVGAKLERFLDDINYIFPRQGILSDAQKSKQWRSRVILD
MHVRRPLLALYRPFAIGASDAPGQISRAYLRSSMVIMKYLDELDPMLAHFQDIAEMYHQVLKKDIIQAALSVCFYIRSAVRPA
SDSTGLGSQALRMSPDSSDDFPTYNPENLVLWSPSRLISTVEKTLDLLVRNISGRDTKDVVCLAVVLECVSKPEVKTDEVTQS
LRIVLDRCLRATNMNLDGVPMGSTGVPVDSFQGDAYRHHVPFMYQRNSIGVPAALNDFDNWMLWEGWE

FUN_003173 CDS scaffold_14 234536 236642 -
hypothetical protein EOG09260TDY PF04910 ENOG41

MSTRQLRKLQKQRELEETQILAEMSSGESDKENEPVAPKPRVSLFAALGGDDDDEAQDEDEVEQQQPEEDVTEVKQPSTSGKS
KKKKNKKKKSKAKAAAPEEDEDEIDKAMRELNIKTSTPANTSTSENTAAQTRRINELLSINPYHLKANNEMRSLFGRDVIESA
AAEEEQERNRRPMQREVDLETFLRGPPGAPKLPEVSLRRNVFIQGREHWPKQSAGGLTMKEVAKAEDGSFTEYAYVHEQSYDA
LQAFFFTCVQIGDPQRMIVLLKEAPYHVSTLLQVSSIAKQDQNQALAAELCERALFTFGRVTTNAFRQNIEQGKARLDFRRPE
NRQFWLAGYHYLKSLVRKGTNRTALEWAKLLYSLDPSDPYAMRHFIHWLAIRAHESQWLIDFVEELEKTTDNRDSIYLRQTLV
LAHLQLGNTARATEELEKGMRRVPWLYCGLFQELNLDTPPSIWGINADSNSRSFWVKLYIYQAKDIWNNAQAIPLLEKTSKSL
EKVDTSVLPSSDSPPDQGVTRLVYLEGQTSLMGLAPREYLEMQPNYEFDPLPPPEEENIFTGEGTKLPFAKQQRGQQSAVETR
LNNLFERLAAAGQGGAPGAAPFGDDGEDDEEDAHLAAVQALDDEELMRDLAEHERGGGDAPGFVQRMMHVLGIIAGPNADDYD
MPDEADDDQEHEHEHEPDQDQERGPNSGLPGAWPGSQ
FUN_003174 CDS scaffold_14 237700 239679 -
hypothetical protein PF09532;PF12701

MSEFLGSRISLISKSDIRYVGTLHEINSDESTVSLENVRSFGTEGRKGRPDEEIAPSDQVYEYIVFRGSDVKDLRIEDHPNIK
ENKPPAVPDDPAIVGARARPGAQGPNQNAPPGGPAAFGPGQGPYPPNNFYGGPPPGNWGRGGAPGPGPGPNFGNMPYPPPPGW
FPPGQGFPGGPGGPNGPGGPGGPGPWGNYPFPPGPGGPGAPGPNAPVEAPANQRATSGSGPSQDAKPAPIGPGADRAKPATPS
GQAAPPTEPKSLAQGVRQPSHAPTPPVESKPSVEEVKQTAASLAANGQAKPEDISKATPTGPRNHRVNPVIPLTGSAAKPFSL
PGAGGDAASKAPAATAPPNVQDATNAARAAVAVAMAQLGNNSGNAMDNLTNKVNEMRVNAVRGGPASRGRGRGGRPAAAKVAV
PDSDFDFAQSNAKFNKQDVVKEAIAGSPLETPENPAVAEQPEISTTTDDAAVAYNKSRSFFDNISSESRERTENGGQKPGGRE
WRGEEQRKNMETFGQGSVDGAYRNYRGRGRGRGRGGRGYGRGGRGGNRPQYQTTPAQQ
FUN_003175 CDS scaffold_14 240443 241258 -
hypothetical protein EOG09264R4M PF05008;PF12352

MSNPLDTDAGSELFSSYEAEFKLIQADLNQKLDQIPELAGEPRKAAISQAERALEELDEQLSAMRLEKQNIPTSSRTKVNQRF
RNYESDTDAARRKLTTLSSDRSALFGSRYTDDPAGSSDIHMEQRQQLLSGTDRLDRSTQRLKASQALANETEAIGAGTLADLS
RQRETIEHTRGIMLESEGYVDRSVKTLRGMARRMATNRIITISIITVLVILIIAVILSKFR
FUN_003176 CDS scaffold_14 242861 244541 +
hypothetical protein PF01593;PF13450 ENOG41

MSSSTDRFRRKRVAIVGSGSAGIGALWALNKTYHDVYVYEASDRFGGHTNTVDFKKGKFSTKVDAGFHVLNAATYPNFTKFLE
KINVKTTPTDLTFSLSRDRGAFEWSGSSPASLFCQRRNLLSPRMWRMLFDIFRFNQFALDILSDDEDDISSIEVLNKATNCTC
KDITIGQYLELEGYSNAFRDDYLLPLAASIWSTSPDKCSLEFPAATFVRFLWNHHLLNTIRARPQWLTIEGGSKSYVDAVMKG
FPPNHLFLKTPVRHVTTESNGQVRLHLENGSSALYDHVILATHGDEAFDIIKSSASEQERSIMSSFKTSQNEAVLHSDLDMMP
RRKKAWASWNYLAVSSPSSRKGNINQVSLTYNMNILQHIPRNTFGDVFVTLNPLQRPKPAKTQGRYYYSQPIYTSSAVRAQKL
LKHIQNTRGISYAGAWTKYGLHEDGFSSGLEVAQEHLGAKLPFEFTDSTYSRGKIPSLSLIDHLLRLIILVIQVFVVQILERL
VGSSPSPRQRLVNGNRVRFANGKHR
FUN_003177 CDS scaffold_14 245106 246833 -
hypothetical protein ENOG41

MERTVEGRHNNSHSAHELERFHTPKEEFQTQPHHDYQRDESNPLCRSNSTPTPGSPQQSDKALHHRRPSRAIALEKLRNRPKK
LRKALVANILRWFASVLFVVAIYVILWYYSKLDIMSTTTKREFNALIIGLSLGLGMSITISLEAMAVEIRYWIISLRHWPDRE
TELILKAKDLTKIIQLTWVSKSRSLRSYAVAFIILNIVSQIALAMLGLVYSTNTADSAAILHPGNVTIPDMSNIVTNRVLADK
SQNNSQALGALRYTANSYGTIALGPVWGGMDDIPKPGTLWDNNDPLAFCGGKSCIYVFQESASRPKKYDLVVSTNRSVEATGY
CRSWKVAKGGNGNEISITIDDDKKTEVNITAINGANQTTFMFNAAAPQGQTWSEVTAFEASSSQAWFYRCNVSVAPVVNAIRK
EHYIGNNVTSLATSAIALQGYGASSLGPTNSDRQFQSYPAESSYGQPLNGSTEDMGQLMSTFAIGVIAVTAQASTGIVVPGMQ
PENGVVLQITKWMYVHLILGLSLGVQLLLGVGIAILSNLPSR
FUN_003178 CDS scaffold_14 248110 249044 +
hypothetical protein PF04442

MNVAPRLARSSARAASSQWSCFFCQNTRPKLQRNAFNFARNASNNARTGQPYTPTMEEIRAHYSKKNRTVAYYVLSTILGFVT
LTYGSVPFYKMICQTTGWGGQPVRAHGGPGSGDEGDLASRVVPVRDAKRIKVTFSASVSDVLPWKFVPQQREVRVLPGETALA
FYKATNKSDQDIIGVATYSVTPAQCAPYFSKIQCFCFEEQRLNAGETVDMPVFFYLDPDLLNDMNMKGVETVTLSYTFFKARY
DNNGRFQRPMSA
FUN_003179 CDS scaffold_14 249535 250053 + RPL26A 60S
ribosomal protein L26A EOG09265DAV PF00467;PF16906

MVKVSPIVSSSRRKSRAAHFKAPSDQRRVIMSAPLSKELREKYNVRSIPIRKDDEVTIVRGSNKGREGKITSVYRLKYVVHVE
RVTRDKANGQSVPLGIHPSNVVITKLKLDKDREDILARSKVGRELAANNKVTA
FUN_003180 CDS scaffold_14 251320 254018 -
hypothetical protein ENOG41

MRHIVYTARELLRLRHRPPRKEISRKLVSKVLEDRELEDIIRTPLVISPSLSLIIEEGNSSDSDEMHRRPAQQLDGTDSDRKY
RGRSDSERDRAPTVAPADLATQKSEGFQKFYNSVVSPTHVRVTAGGRIVPNTRGPHSPTSKWSRERSSIDGQNQARNMNGSQP
EGVAYPVFPPSWGHFAPMVPPHAPGTLPGMALRPEGYHFIPPPMGYNLAPVSYNQFSTSGLPMRLPSQAGPYGKLPDAGMEQA
RNIHLSPAEQFDNSRPFLYNGQWWIANGGALYPISMPPQMSFAPPAVPSPSAPRPSGDFNVAPARYGRPSSRQPGPLPLCLQN
DPSRSASPPVSSIRPSSITQKQLEVLRSQKRYHEDQLRYNKHQIDAGDMEERLQNICREIEHFERMLATQLEFESRKYPKVGS
PSGPPSNSSNDYGNSNSESDGSNAVNPLHPSGFDNQSSVDKTDTRLASVQSRLPTTAATSAKNFSNASKNDDVQQRKPSSLPI
HAALAPVFQPRLDLSTSAIAPDEVEEVDEGAQIRQYERMYKNAEGWRTFFRNKLHADKSMGPPYLVGKLKPGVQPENARDTDY
VYDRELTPDEEHARFTYWGRAPFIFENGLPLYDGKNFYPPCVIKEEPISAGEYVPQNASGSNEPKTSPMKPNEVDPFHAVSEA
TRSSARYGFDDATRSESLHRTDDSLHASSTSEVPRSESHAAHSGSRYLEFRSAINEGPRNPSEKLRAGVSDDSGDEEGNLLFK
GRRVDRYTSKYPKDIWSTMRKNGKTSANVVAGQVSPMTAQGVLPHYAGHATASLTPAVTTNSRGNSARLGDVNASPTANITVG
RREENRPPLELLDQQLRGVSLQDKSHHGFSTR
FUN_003181 CDS scaffold_14 257676 258560 +
hypothetical protein ENOG41

MAPLTPHWHQPSHPDIQEVVIVNPEEFTTKSISRVSLPPFAVFAKFDFPPCTPASEPTYATVQTGRDTHLNLNSDLLYINHSC
EPTLLFDVGNLNILVGPKGLKVGDELTFFYPSTEWHMAQPFDCLCGTPSCRGRISGARDMTQAQLDGIWLNGHIIQLRAEQLA
RSEDPTANALRDAVVHAEKVLESARLALRSYTSNAHAQNGNGTFPSKYTNGTLGKAAVLEHRGPTSRELSGEMGGDTVRA
FUN_003182 CDS scaffold_14 258935 260906 -
hypothetical protein ENOG41

MAINIFYGRQRRFVSRRLTYLVLVLIVFAYSTLTLLSPNAAQTDVAEDAAKADASSKIKTSVDGIRHSLKKGVTELNPFRGPA
HPPPTRAQDSYQGTSWWADWNWLSVPFSSSLTLDEDRALLPPLQDRPFIYCYYDATVKKSREEKDADSDLLLTWRRAWWAQGF
RPTILGSSEATGNPMYQELQRLEVQPELKADLMRWLAWQSMGTGMLVDYTLLPIAHTEDPLLVFLRRGKYPAMTRWKGLESAL
FVGPKEEVTKTIRKVMDSPTLKTAKSVIDASTKNDFKVDKAQTALVHYSPEIIKKQYPKVHDSVTNGRARGLGALNRLINAHL
HAVWQSSFPEGIEVLKPFPEHTTEMVTPALKLADSLAFCPDNPIPSSCPPGISSCAHCAAGTTSMKVTTPEQYQNTSGAFAIG
TVPHPWTLATLTSLKERVDVSWIRKEAPRDPWLETITKGLLGSKVSSNSRIMHFKQAVADEHAPTHALWLSAEADIPPDLEWY
FGFRIPKGLGETYMAMSGKIEQGPMPGKVKADKPAKAERAAKADGDTRPEKPQPRPEDTPKGKELTPKEMQAKEKALLEHAKK
IVAFKKSTVDTRLRASLEAWNLADTEAWKFARAFLARRSMERIEWEKQESKYSGGAGSEKGRNAWSRWKDSKERE
FUN_003183 CDS scaffold_14 262445 266117 +
hypothetical protein PF00179

MGLRQFSLDLRVAIEALDSFSNVSNLRKGDSDGEIAFTYTPEAEHEAELPPIDVQALSKDADSYPNHPGFMIFTSSETPTDLE
KWLEETSSLTDGTTIADAVTLISSMLATKLSTPNQSTSQKPPYYGEESDVDLLSDDEDSFFETEFDEIDIDPLLPGEFASENA
TEADDPESMARLKRHLREAKQKGFCISIPPEEIRQSSFGLFSLSIRVCKLGIPEEALEVWGLGPIEYIVMLCKLPSTYPHVLD
FQHLPPDQTRMHFRVGKCSSPRPSYKSMRQMFAGVDDPAMPINRDEEITGSFIPLYMSLSLDALLNRLFPTLLRLRQSEGISW
DEARELEYNLSLHRHSRDMSDHAQDMIVCQDRDSSDDWFEPEFLRHDYLSRNADEINIVLIAMQFGLQRLIKCTKYCLVCHRR
AKGGFEAVKPFVCENELCLYQYLSLGFGQSIEHEIINNPYVVDLLVSFFYAAVTRSRLREFPRGLGLKCPSLAFTARASTPVN

ADAYFKTGVLRFESKDLTTCRTIKAGHWLLLFVPGANTLDSLTPVMRTYREKHICCVESCIDTDFTFKVMHTFTSLDNIGQET
PENSVNQVTQPTPGGVKVLIYKYGQDIDELEDNVRDEALNFMTSCIPPVQTMRQYLKEVPGRRLSSWRGMKGSTLRLLNWIVS
SNRSHIVQDSPVPGASATEEQTQNGSLVGGMNNHWMQFRFAQGSPEKELIFFNELQKIPRSRIGRSYQTLFAWHGSPLSNWHS
IIRTGLDFSNTLHGRAYGNGVYLARNFETSRGYSNRDTLKGWPSSALKVASAISMCEIVNNPSQFVSTDPFFVVNRIDWIQCR
YLFLEVQALQLESAVPLSSEGLFPDRVQDEDYIEQDPAYQLTYGRQQRIHIPLSAIPKSRRRAFRQGQQNGTGSSIDHPIVLD
DSDSNGSPNLNDSSSLESPETSDDIDVLLASDDEGDDLQFVGTRKRRRTSTDSGLGETQQGPDDVTPFQPGQLDIDSLPKLAE
PTWASSSPAALRALNSQIKDLQRTQSKTNLATLGWYIDFDKLDNLFHWFVELHSFDSDLLLAKDMASHGCSSIVLELRFGASF
PISPPFVRVIRPRFLPFAQGGGGHVTIGGAICSELLTNSGWSPALSLEKVFLEVRMNLCERDPPARLERVHGYRGNRSSGNMD
YSMFEAVDAYRRAAVSHGWQIPSDLQMLQSMSNITET
FUN_003184 CDS scaffold_14 267202 268588 -
hypothetical protein PF00156;PF13793;PF14572

MVRNIVVLGGNSHPQLTENVCQILGVPASNRILGKFSGGESRCEIKDSVRGKDVYIIQSGSGNVNDNLIDLCIMISACKTGSA
KRVTAVVPLFPYSRQPDWPYNKAGAPLSQISGASKDYTFESVPPTPRPGGPKSAGLPNGVNNLTEKLSKTALANEGATNGANG
NNGVFSTPRRSDTISSSTSEARGHHAENSTSSQRNAYTTHDYENQSNISAFQPKPGYKQWIAQAGTLVADLLTCAGADHIITM
DLHDPQYQGFFDIPVDNLYGKALLQNYIQSQIPNHHTAVIVSPDAGGAKRATLIADSLKTDFALIHKVIPQNSDFAAFENSCL
ATKERRPIRFTEHRNASMMLVGDVSNRICILVDDIVDTGNTITRAAKLLKKEGATQVYALVTHGVFSGDAIARINASAIDKML
VTNSVPQNEHRRLCPKLEVLDISAVFAEAIRRVHHGESISVLFQHN
FUN_003185 CDS scaffold_14 271419 271931 +
hypothetical protein PF03874 ENOG41

MKILEAQSAVLTNYEVYQHVSEQRERYKNAKRRGPPNLETVVRELLQYLRTNPGPLSQEPLPYTEGCISTLLERLRPYNLAKG
EVVMIINLRPASVAALNTVVEEMSERFNEEQQESMVNIIAEVLGQFPAAEEGAEEEGADVSMNDAAA
FUN_003186 CDS scaffold_14 272011 272683 -
hypothetical protein PF00025;PF00071;PF08477 ENOG41

MDPEVIKILLVGDEKCGKTTFLSRLSAGEKANPIPMLRDMDQPFIFGIQTGAKKFQFEFFDTSCPDNWRTLDPDAIVICYDIS
QRLSLINMQRYWKDEIKRSFQRIDTLPLIILGLKRDLRSEQDPNGIIYPQEGYQVAQTLRADRYVECSAVTGELLKLAFEDLC
KTAMTLTTGADSQSASTCAVL
FUN_003187 CDS scaffold_14 273138 274779 +
hypothetical protein EOG09263UAJ PF01416

MSAESNYQSWTKNGLIQKVKDLEAELRNRPAPGPEPKPENQPENEEGDEAKRTKPKGKRKMDPSKYSTRYIALKLAYLGKNYG
GFEFQAMANAPSIEEELWNALTKACLIFPEDERIVDFDCCEYSKCGRTDKGVSAFGQVIGLRVRSNRPLPKKRRRLSEAGEAM
VVDEQAGENAEEEPVEETPSFDDIKDELQYPRLLNRVLPPDIRILAWCPSPPPDFSARFSCRERQYRYFFTQPAFTPEPSAGG
ARNGWLDIEAMREAAKRYEGEHDFRNFCKIDPGKQITNFVRTIYEADIEEVKDVSSALPYLSGSQYVPAGGLPNDGTVYPKVY
SFNVRGSAFLWHQIRHMVAVLFLAGQKLETPDVVTTLLDVEANPCKPNYVMADEMPLVLWDCIFPGLDAAPVDPAAKKTDGLD
WVYLADDPALSKPGPFGVMDGMWAYWRERKLDELLSNQLLQMVSTQRSKDPSRHVTRPEPSGKQANSVVVYEGGNGGRMGGRY
VPLLQKVKLQSPEEQNERYAIRKGYASSEDMRNQKGIGKRKVDEAMDE
FUN_003188 CDS scaffold_14 274886 275522 -
hypothetical protein PF10181

MLSTSPRLLIRHPSPTTAEFTVTTLRPIPPALHTLLIISRILLSIFALLLLHARLTLHPLLAYAPPSLLKIIPASYLRAPTST
AALAQNIPLSVLVPASIAVLWLSSRRGYASESILVMRGLGVQTSESPGSYLAGTATRFIPTEKIQDILVNEAFLGFEVRYYLI
VIVEGEDDVVVVFPGLLPRRKIVEEVWRGVRRCLYEQRGEDKSLG
FUN_003189 CDS scaffold_14 277066 282077 -
hypothetical protein ENOG41

MSVPVAGSSRNRTVSLSSGGRSATRSKTSQVPDSTTMPHVTDLKYFDPCAATASMFLYAQGSSVVCCHHDTLTIERRFSRHAD
EVQLLAVDNQSEAGGGRFVVSYDAGQTAIVWDLMTGDEISRFACYDHLTVAAWMKNGNVAFGNTKGSIIMFEPSTSEHVSSRT

IDQIAVTALAPSADCRTFAIGYQNGSLFVATLQPRFTILHNLTTSRGPSPIVNLAWHASSSRQKSDMLAVQMRDGDLRVWSVA
KKYSADDPAKVVRNLRKAEISMDGPNWMGWSKNGRIIQYSDSQTQSWDVRTKHVTHDPIPTLDLVRGLAVYGPGATLFTLGPN
NTVQQFDLNSPAIMVANVQHPANLLPPSPPVSEETGDRSATSATTIASESETSSIPLDMNISESDEDHLSPFARLTRRQAYDS
GNEPYESASPVSSRSGLSSLSKSSAGSSQTPGRYPGSMRSRGLSENTYISAGTSIRSSTIGNSAHGNTRDLDTYSMGYSLGTT
SVPSMASSRSRRRPSRLRNEVPRSPDDNKVHDLFKFTRARLSDIPYKHAMGANSARLTNDDLRRQMLNTIFGWNKEVEDLIRD
EMSRHPQGSPSRILLAKWLGDIDPNIMNANFENMTSSDWMLLALSGIGGHTSQQKLGHTYVQRLLEAGDVHAAVTIMLGMGDH
NDAIEVYISHKRYMEALILTCLSAPSVWERQAAIIRKWGEWAVQHGQQQLAIRCFACTDQESTEPWTSPSAAQLNFQTLTPSI
PEILSPPLSPPGVQRGPQRSIAKTSALKLITSFGDQTQKSKFFAGDGGQTPIAAGVTPIADSAGSPAFSHASNNEATTAFLRP
SNNSRFNTPVSARGRGRLPSIGEIPSDLNREALRSAELSAVPYDYEPRSGHARTPSGNDNMMMGTALQRAATASPMMMREHAQ
RVVQPSEGDFPPPPDQATMLKMQQASRNHRNGSRDRIPVGVNLQLHPGPAHDDITSPEQSVTSSTRYHWPTRRRGPASVTSSV
TSASSAGRSLRHQAVRNREDYIHSLDAANHYSKRQRSRQRSHSRTRDASRSRPGSRERTARSREPSEERGRVSARNWPKAKRS
PTSPIPMSPEDLLNLSTPRHGQVAEPNTVRKVSGSTAKPKSRNSSRGSRRRSPEGRSRPPALTLRGRSQGRESSAQRSPSSPL
PLSAAIYHYQGSEDEEDYRAAMAAQEEFRNKHSRSGSRGLGSPALSKQDASEHRRRANSEVANFDGSISVHGRAASTEHAGDL
KQMKDERQRKKEQAARELEERRKSLAKRAQTPSIPHPNEFSPALAITVETAEPKPLPDDIPPRSATEPPQRSMYARSGPVIGL
PATPKAMRLIIEGNHDQSGSTPRPDVPSIPAGFSQRYSPETSPQQSPEREEPVAEPSLTLLPSTVYQPPARPPIPRSMSAPIP
DEPGQRPARFARKNSVGRIEEVVPGERRRSHEDPMPPPPPPPAPPVLKELRHLASPPPPPPAPLQHAQRHQQAGAVASGMIEI
VMDDDLSSGNDQASEASPPAPASHRSHNRGRSIGDSSSISGRIHKATERLRSASRSRKEYVRSPPFEAPYESIPMPRQMKSPP
PVSFNPDVLRSPIDSRNKHLSTGLNRNEMI

FUN_003190 tRNA scaffold_14 290474 290558 - tRNA-Asn

FUN_003191 CDS scaffold_14 290948 291620 -
hypothetical protein PF00383;PF14437 ENOG41

MDLSSLTPLTSLLTSQLTTSTYNNTGTAKMISDQDLEHLRLAVSLAHEALKAGDAPFGSVLVSSDNKVLQTDRNRTVTGSNGD
GRADSTLHPEFTLARWAQLNLSDEERAKSTVYTSGEHCAMCSAAHAWCGLGRIVYVSSTEQLEAWKAEFGVGAPVAPLSINQV
APGITVEGPVEELAKEVYQFQVENWTAKGFKSKSKA

FUN_003192 CDS scaffold_14 294824 295289 -
hypothetical protein ENOG41

MFGSYSSISSMCASSAPMDIASRNIYRSQDSACAFPSWPRRDSLSESDREERPTSYLSDDDLFLSDPFDDDAQSVSSAGSSSP
GAMASPPNVITDFELIQAERERQAALQRECLRVVALEKERRRAAARKQRRSSSHKKSPKTKLTSINEEAIE

FUN_003193 CDS scaffold_14 307082 307826 -
hypothetical protein EOG0926510L PF03357

METFKSLFAKPDPQAQMRKCNALMRQNIRKIDRDIQTVKQVEYKTKNLILQADKRAQRDPSRAKQAQQEVRDFARELVRARKT
SARLITSKAQLNSVQMQVQEAFAVRKIEGSIRASVGIMKDVNSLIRLPELAGTMQELSVELMKAGIIEEMVEDSLPADGDMLM
EDEEADGEVDKVLGEILKDRKQPALPVAPVPEPQKPQEEEEEEEDPEAMMDQMRNRLEALRS

FUN_003194 CDS scaffold_14 308949 309808 +
hypothetical protein EOG09265BE5 PF04969 ENOG41

MSDTVYPEVLWAQRSSVADASKNFIYLTISVPDVPKDSLTLDLQPTKLTFTGTSSTLKKKYHVELEFWGEIDPAESKINHTAK
NVEIKLQKKELKEEYWPRLLKDSKRVHFLKTDFDKWVDEDEQNEAPEDDFSQFGGMGGMPGMGDMGGDFGGIDFSKLGGGGAG
FPGAEGVGDELDEEDDDDDMPALEGDDKKEAAPAAAPASTEKKD

FUN_003195 CDS scaffold_14 310643 312364 -
hypothetical protein PF00076

MSGKLDQALDDITQAQRRSARRRPNQRRGAGRPAAAAPVGGIQKSTKPVRGAGAKPAPAKATPTNSDSKIIVSNLPKDVSEQQ
IKEYFIQSVGPIKRVELSYGPNSQSRGIANVTFHKSDGASKAFQKLNGLLVDNRPIKIEIVVGAAQADKVIPTVKTLAERTTQ
PKAQPKSAAADKHNAGAAKGAAAKGAAGKKRRGRNSRPAKKTQEELDSEMADYFDASAAPEASNNAAAPAPAPAATGDADMDE

IAVCNVVLDLHC
FUN_003196 CDS scaffold_14 314408 315537 +
hypothetical protein EOG09264DJ8 PF00642;PF16543 ENOG41

MPPKKGKEPVAKKATKTVEDRTFGMKNKKGGAAQREISRMQANLKNSGSAEEKKKQAEREAREREKKAAEEAKRDLEAMMNKP
AQVQKVPFGVDPKTVVCIFYKKGNCEKGKKCKFSHDLSVERKTEKKNLYNDSRGDEEENKKAETSADWDEDKLRSVVLSKKGN
QQTTTDKVCKYFIEAIEDGKYGWFWICPNGGDKCKYKHALPPGFVLKTKEQRAAEKALMDKSPLKTLTLEDFLESERHKLTGT
LTPVTPETFAKWKKDRLDKKAAEEQARKAKENTGRALFESGKWRDEDDSDDDDDDDDVWNLQKLREETEAVRSKKEEERLLSS
YDTPNNGTTGQATPAEDTPAETATGS
FUN_003197 CDS scaffold_14 317385 318692 +
hypothetical protein PF00153 ENOG41

MTTGAKLAHQDVTGHQSQLSSLSPATTSAISMPLGAGGRRDGETTALTATGAAVSAVPKNSPSNRFVKRYRTEIAASSSSVFS
TLAAFPLDSVKTRMQTYHYNGFLDCVRKTYHAEKLGGFFRGVTAPMASITLVRTVSFSIYQRAKYSYSAWVKRNFGFDIIGHV
NRPGTYPNLYSVACFGAAGATAGSCITFLACPFELTKLSAQVSVLLAERGSTNKGSHAVAASYQNKGTLRTMANIIKHRGVFG
LYTGLKLHLLRDTLGTAIYFMVYESGKQIGNTLAGDHPNSNKVAVVAAGGMCGLVSWAMIYPIDSAKSIYQRNSLLYSKGEKV
EPAPKIEFFKRHMYRGLGVSMSRSCVVNAIFFSSFEFVKKHIHQMDDEN
FUN_003198 CDS scaffold_14 319955 321812 -
hypothetical protein ENOG41

MPPIVILDSDDEDEDGSFSPPPNIQAPFSFGPAGIEAQTEAANDSSGGVSRATTSTNPSFFQNIYNEQNDAARGYVPEPTARS
HDQDQHSVSSSEMTTPAPFKRTVTGLIEPSSMPSARDQTTKRGQTNGNGPDEWTQASTPGRRKAPMAVMDDMWDVPSSPDERP
VRPKIKVKVKSSKAQSDSTSDSRHTQESDKRGKTPSSKSGRSDGSPSGKKRRRVSHPEPSFQGSNEVDLVAIPFSYENEGEYH
ESQPAPTPSMLPPTLPVNQDDSFFITTNPLTDAQKLEYESVQLPSSDSQNHQLPLVRQFEINLASSGDATNINTPRSHAHYLM
STAPPPPSSVMDPNRAAIGQATGNRWDSSPDVISAIDSPPKQKATRQLEQIRNGDSPKPANDETTDEQQPEQPELPAMQEEAF
DNYEPDKVTKTKKSRGRPKKKAIEEPITMTEAPSPRPEVVTPVTKPKKKRGRPRKSDQTDTRDESPKIDPSASTNDASLSEDA
VRPEKRPKIDIDESESKDELADESEMEPSPKSKSRESSILKESDPNILAKSVSRLDDDAASKASDAATEEKEEKPEKRAITSR
SSTPSKGLSAIINKPVYRVGLSKKSRIAPLLKCLRKE
FUN_003199 CDS scaffold_14 322413 324828 - MET26
homocysteine methyltransferase Met26 EOG09260LBU PF01717;PF08267

MVQSAILGFPRMGVNRDLKKATEAYWGGKISQSDLLAEAKRLRLAHWKIQQDAGVDIIPSNDFALYDQVLHQIQDFGAVPERY
TKDGLDPIDQYFAMGRGHQKEGVDVPSLEMVKWFDSNYHYVKPTLQDNQTFKLTDSPKAVAEFKEAKEAGINTRPVLVGPVSF
LHLGKADRGQSVEPIDLLEKLLPVYEQILTQLKEAGAETVQIDEPVLVFDLPQKTKAAFKPAYEKFASLGDKIPKIVFTTYFG
DIVHNLDLLPKDVYGVHIDLVRNPEQLEKVLGAIGPNTILSAGVVDGRNIWKTNLKRAIETVETAIQKLGKDRVIAATSSSLL
HTPHTLASEKKLDPEIADWFSFATEKASEIALIAKAVTEGPASVREQLEANAKSIKARADSPRTNDPQVKERQSKVTQKDYER
KSEFTTRISQQQKKLNLPLFPTTTIGSFPQTKEIRVQRNKFTKGEITAEEYDRFIEKEIEENIKIQEELDLDVFVHGEPERND
MVQFFGERFQGYAFTTHAWVQSYGSRCVRPPIIVGDISRPQPMTVKESKYAVSVSKKPMKGMLTGPVTCLRWSFPRDDVHQSV
QAEQLALALRDEVVDLEKAGIDVIQVDEPALREGLPLRSGEERDAYLKWAVQAFRLSTAGVEDATQIHSHFCYSEFQDFFHAI
AALDADVLSIENSKSDAKLLRVFVDSEYPRHIGPGVYDIHSPRVPSEQEIKDRIEEMLQFLKPEQLWIDPDCGLKTRQWKETK
EALANMVNAAKYFRAKYAK
FUN_003200 CDS scaffold_14 326603 329764 +
hypothetical protein ENOG41

MPSFNLSKLLCCCKIPKRSKQNSESTANDVALQPIPVLAPPTEEEHQERSEKNRQSLIGLPDTAWAIPVRNSSFESGSFIRQH
VDDDDSDSDQDRAQPTKKSSTAFDTVRNKLIRTIAHSNNGDHPSHVSVGNSEEEIARRAELKRIMHQRIQDELKSDESDEHIE
SKPTNSIRCMASAVDVALPNSGPRDAIEFGVSKSSSRNGHSTQLDSDQVDQDTHQDSRVSQQGENRPQRKFSQKSESINGADD
WRQDDVHSRPSPSYPKRHVSASEDSDFTHSQKSFQLSNGTRRLDRILGPDSSFNSRHASSGDGHSALGVWLIAQGLRSRDNST
LFLDEEDGLEPTEDTKPTDYNTSSLKRAEEDHAREPRETPANGNPNIPHAPTIIDIPNTVETYSRSNFNAKNDRYEISSLSGE
LPWGPAVTALLNSFTDHTPSSDPSKSQPSPMRSQQNLYKLDPKDLDSMELSPFKWRSHTSSQDQDNANDHNSKSVNTGVPQIR

SQSYGNIHSAEFPVKAPQDTTSLAQSESASFLQREAELGSIRRRFSEALALKKPEKRVVTRFRKEFSESSLPPTVHKSFRSKI
QLAIPSHLRAKSDSIDLRHKEGSSAPPKGQNYLLDPVERLGGHEREASDRSKGRSNLSIRPHMNRLPTEEHHKPGLKRRESAT
DLWQRAIRLEVERRHSSSIHLSTTKPSQRSQSSNRSINDLGAARNSTSSVSDTRREVSQLTPTTDELSSPGNSKWLIERWVAT
MRPRIGESAGDMRLARLAGPPKSWSRFPSHNREERNKNATDRDRVHPRDFAVKHVTSEGQTRWATDAPLDEAKQRARTLPRSF
STKFGELVKSKMGRIIPSKACMEYPEMGIRPSESGYTELQALGREIRNMKGEPHLHVPDREMSKPQSSRSLGDRVVALMHEAI
GQQHSKHDDAAEVAEYPTVPLTPSVVRESIVATDVFVTPRSRFSNDAMDGEAKTGTEAESNKVKDQKTQLTVPESIKDAPPSN
SSTSIKDVGPHTQLSTPEKGST
FUN_003201 CDS scaffold_14 330145 332059 -
hypothetical protein PF07690 ENOG41

MAASESSETTREPSPYRGRRRSSSFSDHIHHLDEIEAQSRYEEEAADRGLGALHNIRSRTRSRTNEVLVTWEVDDPENPVNWS
DTKKKIVVLCTSVLVINSTMGSALPSMAIPDITKDFGITSEEQSVLPISVYLIGYVLGPVVWGPLSEHYGRRDLSLVTFCLFT
IFTMACALAPTWPALLVFRFFCGAFASSPIAIVAGILADVYNDPRTRGRAFAIFMVCTTGGPILSPILSGFAGPNIGWRWVFW
IALIIAGFTLVLIIFLPETYGPILLSRRARKLRKQDPSSRAIAPRDLETTDLRQLLTVVLTRPLRMIISEPIVTTSCAYLALV
YSIFYMSFQAFPIIFQDVYGLSPGVTGLAYLPIFGGAALTLPIFWTWDNTLARATERNAPWVQREEYRRLPLACLGGPLFVVS
LFWLGWSARAEISFAAPMLAGALFGMGFMLIFMAILNYLTDAYEIFAASANAASSTTRSLFAVVLPLATNPMFRQLHINGACS
LLGGLSALMCFIPFVFIWKGPSIRSRSKFCIALRERKEEMQRKEEEARARKERYELRSEEREKEKEVV
FUN_003202 CDS scaffold_14 332961 333570 -
hypothetical protein PF01920

MASLVQRRMLSKADEEAADEVEVRREDQDKINRFSRLHQREILLEEELSTKTKEKEELDDLSTELELADEDEKIQYKIGDAFF
HVSVEQAQEMLEQATEKLEEESTSLDEKLSSIREEMTKLKVELYARFGKQINLET
FUN_003203 CDS scaffold_14 333883 334109 +
hypothetical protein ENOG41
MALGKKPYPKATVKKIIKAHSNHNLKKNADVTIFLDYVLFMETYEFDEPSYTATT
FUN_003204 CDS scaffold_14 335150 336641 +
hypothetical protein ENOG41

MAAQAVQPSKSAKKKAAKALERTESPAPSVASATADKNSDDSFESPYIKEIQKNIRNVNKKITNASKTDSLLSQNPGKSLDEL
VESRIINNDQKAQILKKPALQAQLAQYEEQLVQYQKVDEQYRTRAASDKAEWEKNLEKAKADAVAEAKAESTKALHDNLLVLS
QFLRLAAYRREEAQDPDSDESQAIEGVLLAIYSGDENAVQSMLKLVNGSEDQIFSVPGEQLQTSFSKVKALAQEYKTHFDEPQ
PAEGDAEHVKEVATDPTIAHAAATEIEAGDTAAPVVTEPSSTSNGLANASVADDAANAVAESHWDTSNDLSASQEWVDVKPAE
AVEADAAAPTRVANTHSWADDHPETTAPADPNDGFHQVQRKGPRNEGGNHRGRGRGEHRGRGGFRGDGRGRGRGRGGAGRGGR
RGDEA
FUN_003205 CDS scaffold_14 338047 341071 +
hypothetical protein EOG09260JPV PF00012

MASRVSPLMTLLATIFLFTTNVFAISAVLGVDLGTEYIKATLVKPGIPLEIVLTKDSRRKETSAVVFKPSRNGPQKGEYPERA
YGADAMALASRFPSDVYPNLKTILGLTTQDSVVQEYAARHPALQLQSHPTRGTAAFKSKSFTDDVEAWMVEELLAMELQSIQK
NAEVAAGDGTTVRSVVLTVPPFYTADEKRALQTAAELAGLKVLGLLSDGLAVGLNYATTREFPNVSNGSKPEYNIIFDMGAGS
TKATILKFQGRSIKDIGKFNKTVQEVHVLGAGWDRTLGGDSLNYLILDDMVKQFVESKAAQRASVAAESVKSHGRAIAKLTNQ
VEKVRHVLSANQNTGASFEGLYEDIDFRYKITRTQFEEMAAEHAERITVVINDALKAANLDIIDIDSIILHGGASRTPFVQKV
LEKVSGSPEKIRSNVNSDEAAVFGAGFRAAELSPSFRVKEIRIAEGGFYPSGVKWEAKEGKTQHQRLWSAVSPQGAAPKELTF
TNEKDFTATFYQQIGSDEKDVKTLTTKNLTATIAAIKQKYPSCVESEVRFKLGVKLSGENGEVEIAKAAVECEAEVKEGLVDG
VKNLFGFGKKDQKPLKEGEGSEEEMKDDKSSSSTASPESATASAADSAASGSTEEVKPEAKKREIVGIPVEITIENRGIPALT
PAEISKSKDRLKAFAASDKARLQREEALNQLEAFTYKIRDLLEGDAFIAASTEKERTKLAELASETSDWLYADGAEASKDVLK
SKLKVLKDLVTPIQKRVEETEKRPEYLTALKQTLENTQGFVDKIKGQIAEHESWHKSASESASASSSVESPSTEAAQEKATGD
FDGLEDEDPAVAEARKMEQVIREKGPIPPLYTLEDLKEVIDLHKATLDWLNNLEAKQSKLAATADPVLLIKDLKTKRDKLEKV
SVEAALKGARKVEEKNRQAKKAAKEAKKKSKKSKTTSGEASQETVQLNAEDFMKDGEIDKEQLEKLLNDMKKENEKNEKSKKS
EGEKKQTHDEL

FUN_003206 CDS scaffold_14 342145 343554 +
hypothetical protein PF00026;PF14543 ENOG41
A01A
MRSLAFLAVLLVVSISSTAATPQHKGRKGFSLEAAKNSNSKPDFVREWVAAHQKWGKAVSPETLSAFSLLDDDGRVDVKPLGA
DEIYIADVKVGTPPQTLKMALDTGSADFWVQSTDTIYRANPEGPWAPRYKPNSSKTSHRLRDAEWEVEYVDGTHADGIVYLDT
VRLGDFELKNTAIQSAQIVAPRFERETELTGILGLAKSLPSNIDPPTPSLLDKLRPLLDDPVFTVDMRRNATGRFDFGYIDES
RAKDNISWMATREDSPHWDVTFDTTAWAGLRQVWMAHTFEATIDTGTTLLFLPGDLASMYWYDVPDMRVDPRLGDSFTFPCKF
SNDLPDLMFKVPGTERILKIPGPYLSYSTTDNDSDYCWGGLQSAESLGVTIIGDIALKALYVAFDLENNKVGFANKDLDDIDE
W
FUN_003207 CDS scaffold_14 344626 345286 -
hypothetical protein ENOG41

MTLTDEELDRDWQPSGRRPQSTIARSFSAELMDIFRIENSLTDLDQQVHDKKQTVDKNTEELASLEARIREMEDRLRRSVGGS
TQQRSPLPQVQTQTANQSQTQQQQPSSLDAPTDDSKARSRPGTARAAQQAPSSGNMPPTPGASEGEYYVVTRDDLQDGPR
FUN_003208 tRNA scaffold_14 347582 347654 - tRNA-Thr

FUN_003209 CDS scaffold_14 349524 351204 -
hypothetical protein PF00230

MSLDPLPTIETTLTSSSPSDMSSSIQSRSARSTPAINASAFNPPEANGSSNERVPFMRDNSPEADSLPSNPALTPVITHGDNP
SASNRNSEADTVVPLQPVISQDPSRVRPGSRTGNFVTPKTARFSQDGGEPLQYSGGSMKSSRRRYRGEDYDFDQAADYPTVSD
YERYWRNEGRNDRYTARRGPHPPPTFMNRRRAHPHDSDEEFYSSDDPRMPPTGGRRMMDEEMGYPHRPFHHRRASTLNGFNIS
TGKLEWSNLSPKEKSEIMRLPLTQWMNSNFKNHFVASLGELIGTTMFLFFAFAGTEVANIQAQTDSKTTTGESTGSLNVSKLL
YISIIFGFSLMVNVWVFFRISGGLFNPAVTIAMLMVKAISMTRAICLFLSQILGSMLASVMVRYLFPETFNVRTTLGGGASLV
QGVFIEALLTAELVFTIFMLAKEKHRATFIAPVGIGLALFIAEMVGVQFTGGSLNPARSFGPCVITGTFDSEHWIYWVGPGIG
SLIAVCFYWFIKTLEYEMANPGADGDDLNDPTKNPEKRAEIQSNAAPSIAFGGGKTPSIRS
FUN_003210 CDS scaffold_14 353858 355195 +
hypothetical protein EOG09265DVA PF03256

MPSRREIYIPPPPASIHQINRDHRIKRVPLQELAFKKSSGALLQQRKRVFRTVPSPDKENVEACYRAIDHLSVTFEEWYQERM
QLLRESRELRTNINNILLAAQQPTDRSRLSMPPRDSDASSSDAEPDESILFNRARPMAAPAGPSSDATMAYNDDQAAEDDADQ
RTLSHSPEEYEDENSIGEEEDDDADEDEDDEEAAEHDQAHEAATLFDPATAGLKEISNLARFTVSSHKPGNGVEELKSDDLKL
FWQSDGPQPHKLTMYFTKRVGIRDIRFYVDYNEDESYTPTKVVFKAGTSENNVIEFATMALENPSGWQQVPIAGAGGGPDGNT
LVAWVVQMQILENHQNGKDTHLRGIKIYSFDNDSALGPGRDGNPVEDVLDLMDTATSHINTTSTRSRLSNFGAPNFESGEGGL
AIPEFMREPEIR
FUN_003211 CDS scaffold_14 355814 359063 -
hypothetical protein PF00249;PF13921

MSGNGNSSSKWPDFRLASTLWSLMPGSQSNRPDKQNDEDRDGNEGHKNEQQLKNEPQEELPEELMQSLELTQDEVGTENRPID
EMSGFDNYDGDAQAGPEDDVQDQDFGTLEMFDSNATQAPYTSQLDNASQEEQAPEAPMLEDIPSSQSRKHKRDKKGRKQKGIN
ESESPQLPAAEESSRKSKKSKKKSTVPVEIPDSLVENTSTIFPSQPLETLAADFDDEAAAPATQTKRKRNPSDSTDGKQRKKR
RSRDEAAVIESQLVEPGTQDGQIEEADTKESQAASFLRTRDAARPAAIYDDVAEDAPQSPSAARLEVRDARSREGSAAHDEMD
IDASVKQNQDQENELSSFSAINGQENTEQQELEQVARDVWNAHINGQNQPNTQDSSVHTEEMEIPTSAQRPHTSNDVYDVPGS
PVQASNPPSASAKRTRSGKAKKAKPTFFEKSPSPEVPEGDLPSPSAMTPMPRKRTKKASNRRKSEANRALSSQDMEVRDDDDL
YGNPATGRRNRMAGFTQGRFSDEELGRIAQAVESYRVERNMVQHELNAMIHAPGGTTAGDEHAALWARIFATCPDRHRQKIIN
ITRKKFHNFVARGTWTSEQDAELRDLIEANGTKWSKIAGIINRHPEDLRDRYRNYIICGDSQRKDTWDEDEEGNLTQYVMEAM
AAIDELRIIQPTRELLKKPYEELIDWQNISERMGRTRSRLQCITKWRAMNIKTHGKDKLVSHAPDASISFRLEKARRQIAAMP
EEERYRMIMAISGSSASVESKIPWQRLVDKQFRNSWHRPTQMLLWRRLKQSVPESEQKTVRDSAQYLLNLYAQTGELPNVEDA
LFDDAEEMEFLNSIPTTHASHNNASANGQNHMSAEFVNEADEEESEQPQGEYGVPDENINPDLPIAPMEDFNPAPMASMDPVA

AVADLAPMEDTEMVDPALSAEHAEPTEPEEPVDAPMKEEPAPVAKPANTPVFDEPAPPKATRVAKRTGMGKAKAPKPPRKSAA
RSTPARATPSRRSARRAAPSQDPIEDDGDVQAGAPAEDNSEVDEAQTRKRKTPSRFRSAGVPEVSAAADDSDSVMDDMEDLPA
RVM
FUN_003212 CDS scaffold_14 359761 361811 -
hypothetical protein ENOG41

MILRHSGRLVRRLNQPVSSLNLARPVGPQIRYLGPRCTNSNVVSKLRFSSQARKRKAEEAPEYNAEAEAETQKGPKSSSSEPS
LLGKMGEAAATTFASIVVLGLGFAAAGYIYHKFYKMLVLQKMNHAFEPGDPVLDLAAIGKDVPHQPGGSGEHWIYRPEQQRVD
DIVMGRVVGHYYLLIGDKGSGKSSMLIDAMRKNEGDGVAMFEAHADLEIFRIRLGKALDYEFHEDYIGGYFSERGPRETTALL
DIERALNKLEKVAMQKRRERGKPLVVIVNQMHLLRHDDDGRDVIELLQQRAEQWAAANIVTMVFNSDDYWVYERLKQLATRME
VLPVTDLPKQQAISALQRYRQKYWKESIDAAVLEEVYERVGGRLSFLNRVAKSRDMMGTCERIKEIEKKWFLNQCWILGPEMD
DDVMDQQKWAAAAMCLAKALVDKEKDMEEHETYDPIVGHVLPTYPFHIAQQIMTRCDFIRDLDRLNLFSITSEADVRASSVPM
HRAFREICAEPNFEKHLEGTIQRIADIESLGRTRELVAKDLVLGGQYEIEHNRKGITVKLKEAEKEDD
FUN_003213 CDS scaffold_14 362838 364154 +
hypothetical protein ENOG41

MPPYAAELETWWLGTGYEALKQLVNDESDDLNSRQCGFAQQMQTRLLAFDNDQTIQASLQTTWPAIRAARGVDYDANSRKNSK
YIASNIFRKPKDGGIDFERAMDGLGYLDAIEIRRLRLLEATHAAMETILPTESQLKMLQELDRTSTANFRLLHAGFRACILIK
ELTRDSECRKEIPQIMGMLNALVPSDVFLEDEDDVDPTPHSPSLRDSVRFSVFEHLMSESPFSPQLQEIVKMKLSSWCEVPGY
PQARDAMVRYCEEFKKLEGVCLAIMNDLERKSFSIGSYHVASPTSSTLFDTETSRSSGFSANATQGASAIFTTVTPNDNQDSA
PTTPLSPWVLSTSHPLLKGMPGREMSPAKTDGALFSQDLSAPPVLSYPDSLSKTEFFQHPFTRKLDMSPVEQAALGLILPREI
DSRDF
FUN_003214 CDS scaffold_14 364970 367522 +
hypothetical protein ENOG41

MSDAQVKKVSELDKEAGDGRGGADPEEKDMVVPRRVLAGGEVEDWRPMSEYEPDENDEIASAITPLEASKDSDPTEDSDETTK
GTGTAARFACVDDAVTFRNQQPPSLPDPGTPSSRQSVRKKDLIGDSDQADRQVHNDSGSFRLSRQLSRICKEEMSVFDFATPR
PRGKTVDCTPQSLATTDTRSPLARFPIAVEPTHRLTPGTLTKRVVGDSGGKLLFEDTRQARLIPSPSDKYHQGLSADLWEADT
ESIISELQPSPLHINRRKHRANTDESDRRWPRLFDRGYAGPPTTLEMLRISAPTERDDSFDPEVFGDDDDTPTEGDRTPRGLG
HQEQGLLQATPAPRGLQRSASTIVTPTQPMKRLEHKASFNLEAMGQPGFTPEHRRESISRGTFPRSGMMLNLQSTQERLRCPP
QESNGIQMSKSITEKTSKVRRLPDLPDSGRHERAFAPKEWMRAIAATPRKSRTVSRNDGRIENYGEASSGADWPKTLFQPSAT
MDEGDVERSTSATTRDFSQPHKLQKPPSRARLTPSSTYRSSNQQLDHSTRASSLIPPGSEADVPDPRTPSPTASQTTAVPRTP
SSSISSIFRKRIRSDYNPPATPRTPAEPTLAWSPFANCEPEPPCASPWVSGNGEGKREKEKRAAYFRDKVEREFQSRQSPKRS
SRKDSLESAANGDSAVQNNVVTDDRTSKESLIQWKQFLEDAPEPLFSSPRPPVPPLPSESQLNLALDRPPRQTQTPSRATARV
EVNPAATYRDKKPQGLTVETRKLRKAMREGAPTPSRNKTMTPASGSSFRSAFRLEGRRMSFGRPAADHESQGKGRETETFPRC
K
FUN_003215 CDS scaffold_14 375225 377402 +
hypothetical protein PF00172;PF11951 ENOG41

MVHSASSSCADSWSTDYVDRDDVEMDNNWEQGSDDILTIPKLEPVEDDLNMDDLKAAPLAPASMDSAKIDPKPKRPRGRPRKH
PLTTAVSASKVTKGRSKTGCITCRKRKKKCDEAKPRCMNCEKNAVVCEGYHEKQIWKSGKEKAEEERLRQEALPIITMQPIFH
GVETVEDKVFWKHYITHLSNVLTVEGEARNAFKDIILHLANEHQGLMHSILAVSSKHIDWDTPYGAKLLADHPQSNREALQQR
ADYHHDESMKRLYEDMGRPLDKDDPEYKTILSARYGQMLCLLLQTRADGNPRGEHRVHLQAYQTLIQHSPPEDPALYTFITEF
FQYHIFADDLFWHPENMTARLSSEDWEPVAPIQPPRLIGVGDGLFQYLSQITTIRNTIRANISAGVDPVVDYTSLYQAAEIDA
AIREWTPNWPWGDSRDRVGLLYKQMMWVYLFRTIYPPSVSSMRRSTFSTLPISASTTVAPPPPPPRRASMMATVSRGNGATGA
TDVHMASSCPTSRNPSRTNSMHEQDSSTCQERASSPPPSRRPAYHDRRITLAVEESLTILESFKPSDPSQTLLLIPCLVIGTA
CFEPAQQERIRTAVRAVRGYTGLRNCERVMELLEKVWACMDQGDWVSVWDWQGVVRRMRLDFSCA
FUN_003216 CDS scaffold_14 378583 379796 -
hypothetical protein ENOG41

MAQTQPPTFINLPPPTSRPETPSEMPGTPTSTTTSLSALSTTAIKDGHRGHFPQGPLSRGHQHNPSTQSLEAERADRISRLAG
LERVSTLRAPPQANAQGASSPSPQTTPTSTTGFPPNYPATQHLTPAYFDSNGQPVAVTKMSTVGTASATESHVGDGDDTRTTA
GEHDEDMFSTDTNYREAESVASMGGYPDAMDEDLDGANARSVAGYDDRMSDDGSASLVGFGEGAGSTVSGPIYHRRTAPQAWG
LERTNSGLSDARRERDTHMGGDTPVSVSALQERRDARMMDGVATDPNPGQAPENDSFVDTTFRGPVSASQPQSQTAAAQEAAE
RIVQRLDRGETRAPASGLVGPGRGDEPLGKFYFEGEKRDD
FUN_003217 CDS scaffold_14 381040 381457 -
hypothetical protein ENOG41

MRFTKHVLPIAILLTGVIAAKDEQSTTAVEKAKATDDKASATADDSKTADATEEASATATGKDDKKESKTDKSDTKSATKEKS
AASQTTADGPTITWDPPAPKTTVPDINAGSPSPMTPGVVVLSLVLGITSLGMALL
FUN_003218 CDS scaffold_14 382704 384373 -
hypothetical protein PF07690 ENOG41

MASKDDQDSTPRRHSLSDECDTNSAADEKRLVAKLDFYIIPLVMVLYLFSFLDRVNIGNARLYGLEEDLDLSSSQFQVAVSIL
FVTYLLFEVPSNLVLKLFTPRRWIAFIAAAWGIIATLTGLVNSYGALIACRLLLGAVEAGLFPGLTVYLTFFYTKRELALRVG
YLFVSAAVAGALGGLLAYGIGHMDGLHGMSGWRWIMIIEGIPSFLLGVVTYFALPNDAETAYFLDENERALMRLRHAREYGNT
ASSREFSKKDMMCAFKDWKVWAFCVAQFGVDTMLYGFSTFLPTIIRDLGDWTVAETQLLTVPCYFLGAAAYMSTAFLSDKLQQ
RGLFCVIFGVISVVGYAVLLADVSSGVHYFGCFLVAGGLYVVVGLPLAWLPTNSPRYGKRTTGTGMQLTWGNAAGIMSAPASD
KPRYIRGHAVTLSMVALGTVIYGFMWWWYRRENERRQAGLMDDKFRGMGEDELAELGDESPRYRYTI
FUN_003219 CDS scaffold_14 385697 387278 -
hypothetical protein ENOG41

MQLRRFLLLGMLLGAEATQLVARQNTNSDSAAAPSTDSSPASTAEADPTSDDSAATTAAEPTTAAQPTDDSSTADGSTTDGSS
DGGSTTAEDVTVTKTVTVTDANAKTVSRQTTVMKTITSTVVVTSTAFDTKTVTSSDAETATTTTYVTSTKWANEKRALDLAPR
TIGGVELVAPALPTYTTTTSAPPHFNAQIEGEHYGLRAFRRGLHKRATTTTTVTVTDGGGDSDTTVFNTISRTVISTVSSQTK
ITKTITETEQAGASTTVTVTSTLVVTSTRVTTGVVRTATVAPSGEYGPSGTGGASSDNNSSSNSNDGGLSTGAKAGIGAGVGV
AGLAIIAGLLWFCLRKRRNAKNKAEYDDVFGASEVPVGGRGGGGAAAAAGTSPHMSQTTAAASTLAPSRNTTKSEGYRGTALG
DGRAGFAKPQQFGRSYMPVSPETNYSRTAGSDQAYSATTPENNYAHPVGGSPNHNAAEMYSPANTAELASDSAATKWHQNDAA
EIDGNQVSSARGTAPPENVYEMPAQPYR
FUN_003220 CDS scaffold_14 390086 391916 -
hypothetical protein PF01794;PF08022;PF08030

MSYQMQDWGEKPSERSRWTPLTRMLLSGEMTQEKQQELSSREKFDRWMINEGYRRFFVFVFMLLHALIFSFACVHYSQKESLK
TSNELFGFTFVIARSAALVLHVDVAVILFPVSRTLISLLRQTPLNGILQFDKNITFHIVTAWSIVFWSWVHTIAHWNNFAQVA
IKYKLGIYGWLVANFVSGPGWTGYVMLIALMGMVLTSTEKPRRANFERFWYTHHMFIVFFFFWSIHGAFCMIQPDVAPFCTSV
GASAVGVFWQYWMYSGFIYLAERIAREVRGRHRTYITKVIQHPSNVCEIQMKKEHTKTRAGQYIFLCCPAVSLWQYHPFTLTS
APEEDYISVHIRCVGDFTKELSKALGCDWSKKREPGGNDSSKVVGLTGRDSEIDPAIRRVLPRVYVDGPFGSASEDVFKYEVS
VLVGAGIGVTPFASILKSIWYRMNYPQKKTRLAKVYFFWICRDFDSFEWFRSLLLAVEAQDLDHRIEIHTYLTAKIKADDATN
IMINDANADKDTITGLRSPTNFGRPNWDMIFRGIRKLHSPAEAGVFFCGPKGLGSSLHTYCNKYTEPGFSFVWGKENF
FUN_003221 CDS scaffold_14 394133 395079 - UBC1
Ubiquitin-conjugating enzyme E2 1 PF00179

MALKRINKELTDLGRDPPSSCSAGPVGEDLFHWQATIMGPSDSPYSGGVFFLAIHFPTDYPFKPPKVNFTTRIYHPNINSNGS
ICLDILRDQWSPALTISKVLLSICSMLTDPNPDDPLVPEIAHVYKTDRPRYEATAREWTRKYAI
FUN_003222 CDS scaffold_14 396575 397961 -
hypothetical protein PF00441;PF02770;PF02771;PF08028

MFRPILRQCARRAAAPLRPCATRTFASASGPTFNWEDPLASNNLLTEEERAIGETAERYCQEQLLPRVLQAYRDESYDPKILE
EMGELGLLGATIEGYDCAGVSSVAGGLITRAVERVDSGYRSGMSVQSSLVMGGIFEHGTAEQKEKFLPQMAKGKLLGAFGLTE
PNHGSDPGSMETVAKPHPTKKGYYSLSGSKTWITNSPIADVLLVWAKLQDTGKIRGFLVERKDCPPGTLETPAIKDKNGLRAS

ITGMIHLDNCPVPDINMFPDVEGLRGPFSCLNNARYGIALGVMGALEDCIARARTYALERKQFKGNPLAKYQLVQKKLADATT
DAAYGTLAAIQVGRLKDEGKATPEMISMVKRQNCDTALKNARVLQEIFGGNAASDEYMIGRHVANLYVTQTYEGQSDIHSLIL
GRAITGIQAFV
FUN_003223 CDS scaffold_14 399767 401575 +
hypothetical protein PF04172 ENOG41

MAADTSDFLQLMQAIKLAANKNSSKLVRAWFYVPLGITIILAVNFGVDSIFYKFSVSFPASVTCMVLLFFALIASESLLGTHK
TRKLVNIIDVPAGWSLRWMGIFFTPSFVLLPLSPPIGIIEVMIIIAIFVIGFFIMMAMAAWITRSLQLALGVSRRSRSQRAEE
LGRQTPDLSTGDLNSPGISRRTSVSRAGSQIPLMSLSAPNSEPTSAAPSRPQTPPQLRLQEHHYANGQAPVNAVEALNPTTVL
TQIPLPPPKAELWAARISANLDVAIFVGLFAFIGIPLYYSTGYAMPLHLAVNVLAWFGALSVTPKWKEYLHPVLVSSLATVLV
IWALAEIRGDSLTTTLHAYRTGAKYIQLWRNATSKQDLLPGAGDVFGTLLDASIVALALPMFQYRRELRTHFISIVIPNVAIS
VGSLLAYPPLCYACGISAARSLAFASRSLTLALAVPATVNLGGDVNTAAALAIISGILGAVLGARILRWLRVPDDDYVTRGVT
LGVNSSAIATSVLLRTDPRAAALSSLSMSLFGTITVLFTSIPPAAAFVRGLVGL
FUN_003224 CDS scaffold_14 404445 406296 -
hypothetical protein ENOG41

MASSNPFRKSAAITVDSASFPNSTSSAASRFPALDQIETASTPPPPTSFQQTGSSTIDSKSNITKKVRVLSPPPLSPDSPEWA
FAAPSIAQQTANQRNEDDPFDATSTDDSDREMVAAAARGYAPNGGQGIGNPFSKTLRSVDSPVAEQKLEHERKEEGHALKAAN
TARRSLDVNAFKRLLMTGNTESDKSSPPKIRQSMPPPAKNTKDRPSVFRRISHDDSRDISPPENALQEASLISEQASDTPEDD
IDQENVSDSSASAQLSSKNSKKPPPPPSSRHGKSIKLDLVGQASREPLATLSSDMNKPLPPAPVRRSMEEGGESPFDREAAGK
VPEAEGDVGLASPEPSGGSRKAVPAPPPRRGHARGESKVYASGLGLTQQQFVHNEENLSRSSSMRSRPEHNRRDSHAAAPPPP
PPRSHHTSRQSTQIPSGVASSLTDLSQSSSSPAPSLDVDMSTTSTPSQLRRSSTVELTPPRDTQSGTHQPVTTTKSWAPPPPP
ARNTSVRRPASIRSVDSSSRRISFEAKPHNQMAPPPPPRRQRGSSRGSVDGPRRTSLDGVGKAGSSQVAEEEPDNVAATVGSG
PTSPQSANEPGKSVDILADLDALQREVDALRGKLG
FUN_003225 CDS scaffold_14 408528 409575 -
hypothetical protein PF00538

MPPKAAAAAPKKSGHASYQDMITDAIVNLKDRKGSSRQSLKKYVKANNTLNVTDNMFDSLFNKALKAGVDKGVFEQPKGPSGG
TKLAKKQPEAKKPAAPKKPAAEKKEAAEKPAAKKPAAKKAAAPKKAATEKKVAAPKKAAAEKKTTEKKAEKAEKAEKPAPAKK
AAAPKKAAATKKADKAEKPETALTKTKTGRVTKTTKAAPAKKAAAPKKAAPKKAAAKA
FUN_003226 CDS scaffold_14 412460 415735 +
hypothetical protein PF00626;PF04810;PF04811;PF04815;PF08033

MADYAQYHALGQGEIIDPNDPNRTSQPSAQQFQPPIAPSPYQQQASPYGAPQYLGGQQAPPPMTGSPAPAPGYGYAPPQAQAP
PGQAPPSQDATLAAQLGGMNLGDGHGTARRKKKDRHAYHTVEPTGSSQAFNGMPPQGTSATQFLDSVPGGPGFGGQFGSPQGT
PQMQSQSQFSAPVNPAFGPGPVAGTPGVGEGLGTASVSTSGPKGVSPDDMPSVPASRDAIQQYYLKNVYPTFERHVPPPSTVS
FVAYDQGNSSPKYTRLTLNNIPTTQDALQATGLSLGLLLQPLAPLQAGEAEIPVLDFGEAGPPRCRRCRAYMNPFMMFRSGGN
KFVCNLCAYPNDTPPEYFSATNPQGVRVDRDTRPELHRGTVEFVVPKEYWTREPVGLRWLFLIDVTQESYNKGYVEAFCEGIR
VALYGGEDQELDENGEPKRRIPEGAKVGFVTYDKDIHFYNVNPALDQAQMMIMPDLEDPFVPLSEGLFVDPYESKDVITSLLT
RLPDMFSTIKNPEPALLAALNSALAALEATGGKVVASCSALPTWGPGRLFMRDNGNHPGGEIDKKLYTTEHPAWKKVAEKMAA
SGVGADFFLAAPSGGYLDIATIGHVSSTTGGETFYYPNFIAARDSRKLSLEISHAVTRETGFQALMKVRCSNGLQVSGYHGNF
IQHTFGADLEIGVIDADKAMGVSFSYDGKLDPKLDAHFQSALLYTTASGERRVRCSNVIASVTETSKESGAREQGIRECLKFV
DQDAVIGMLAKEASTKLATTSSNLKDIRHWLSEKAIDVLACYRKHAAQQHPPGQLVMPERLKEYCMYLLGLLKCRALKGGVEN
SDRRVHEMRMLRSMGALELSLYLYPRMIPIHNLAPEEGFADPETGHLKMPPAIRTSFSRVEPGGVYLVDNGQQCLLWFHSQTS
PNLISDLFGEDKDSLKSLDPYTSALPLLETHLNAQVRNIIEFLRTMRGSKGLTIQLARQGIDGAEFDFARMLVEDRNNEAQSY
VDWLVHIHKGVQLELSGQRKKEGEEHTAASLSNFAGLRPAYW
FUN_003227 CDS scaffold_14 418095 419121 -
hypothetical protein PF10021 ENOG41

MAPPRSSRVRPSEVAADTKRNFIPLVKKNYGEVFPPYSYLYRQPIMQLAIQRQKPTTRPPSFRIEVGDPVMKALTYAAMDTQQ

SEAMGGPRVRIPFICAANERRPGGDWETGCVGYEEKLCRRSNLSATLNTPWPNSPEPNNYPIPSQGGILSDAVVVCRGPHDRY
DRLESWFDLPVVSVPPTRWPKLKDNGLKYSFAEEREMTRDKLRGALRICLYNGYDRVVIGDFGLGNGYRNPPQELAELWRDVL
LFDPDLRGQFAYVVFVFEDPLQSTMRHIMDEITKKENKGKSRTGSSSSSGSSKSHSNAPADREIFEHVFSASEVDRVLRQPDP
RYGLNMITT
FUN_003228 CDS scaffold_14 422057 423762 +
hypothetical protein PF00152;PF01336

MRAFRASRVPRLGKPWPQIRPYAVKVPGECQKDIKTVAELKAWGPTSHVPDVEVCGWVRSVRKSSGVRFIDITDGSSMRPVQV
VVDKNLATDMRPGAAVRLKGTWVDESRKQSDIEEISQASSEDETSTGSEQPELKVSEVEVLGGSDPMTYPIQNKYQTPESLRT
ISHLRPRTPLNSTMLRFRSDSTAMLTQFFFRERFQQTHPPIITSSDCEGAGEAFAVKASSPDEFFRDPKYLTVSSQLHLEALA
QALGNVWTLSPTFRAEQSDTSRHLSEFYMLEAEMSFVDNMEEVMNLAQRMLNSMASGLKELNAAKELEQNRFDSKEPSERLAF
NDLIDQKQLDRRWRGMLTTKTWPRITYSEALEILKPIADQFEHKPTWGSGLQSEHEKYLAEKIGYDKATDAYLPIFITQYPRD
IKAFYMRQSSSSPASGLTVDCFDLLVPHLGELAGGSMREHRLPQLEENMRALGLEVPSKRSDKGKELAWYLDLRRWGCPPHGG
FGLGFDRLLSYLTGVSNVRDVVPFPRHYHRCDC
FUN_003661 CDS scaffold_15 360 938 - CWC21
RNA-splicing factor PF08312 ENOG41

MSDNVGLNTPRGSGTSGYVQRNLAQIKPRDYGAPYPKDLDSLRHKQRQPDKGILEHDRKREVEVKVFDLRDTLEEEEVDEDEI
DRRCDELRQKLFAEMNSGRRGGQRKTFKQHQVHEMADAKIKESERLRKALKISADYEEGGHWKRQEERLRDALEKEEEKDGGD
DRKSEPRD
FUN_003662 CDS scaffold_15 1328 2582 +
hypothetical protein EOG09264WF4 PF07065 ENOG41

MPHVPASAEADSQGDAPELVFPPVTKDHIQNCSYESWFPKYRSSCLKSRIIPLPPSFVEYLHEDGIVLADDDDENQDEPEEEW
HASSNTSARPQVKDPDSSDDEEDEPERLPPNQRFPETHNLIKEKIAELGGSVAPKLNWSSPKDAKWISPHQNTVKCTTPNDIY
LLLKSSSFVSHDLDHAFDDCTPRPPTKPYAPILVLRPFFTPHVALEFRCFVKHRTLIGITQRDLNHYAFLEQIRPQLWRKIKT
FFREKLRFTFPDASFVFDVYIPENSFEKDGLGRVRLMDINPWAPRTDSLLFGWQELLEMDVKNPLYGTSSNDAGADVSGDDTV
TEGEEEDDDIEYRPEMSPEFRIIEKDDPAAYNFSSPQYSAHKLPKEVVDASMGGQGGLMEFAMQWKEITEGRGDGIWEQASGA
RQ
FUN_003663 CDS scaffold_15 3333 4356 +
hypothetical protein PF12511 ENOG41

MGKMKAGRSTRRHTPLPNTSTRSSNRIAAKKQTKGTKQAGSSQEKVPSAPHEPTSSQETLKNSTDSSNQNNFSSFSSYNTNDS
HSAEQVEGTLTLAHRNPQVHGTGVTSSNPSNNTVGSDTPQLTAGDVALEIYGSRILIDSTLGKKLAGEASLRPIRQRRSDMVL
NMERRSNVEAFLAHLTGVQVSRSCKNCSKGHGPWSECIIYDGQMCGSCTNCWFNASGSRCTFHENNQNSIYAPAPLYHPQSAG
MPTQPFQYAQLNPQALPQGILPMNREATASLYVAPQMRNQFALSIFQSLGIGSGE
FUN_003664 CDS scaffold_15 4380 4785 +
hypothetical protein ENOG41

MIGNVVSNVAAMSQGERKFAQVERAAEELGIRLAELHEFLATPEGNAFVTQLSGMAAPQQISSGDDQSQEPLSQEPSSQNNAH
QEHASQDTSSHEPSSQEQSAQQTSSQEGFSQGNIEAHPMAFALLNTPPYEN
FUN_003665 CDS scaffold_15 6713 7970 -
hypothetical protein PF00025;PF00503

MGCGMSTEEKEGKARNEEIENQLKRDKMMQRNEIKMLLLGAGESGKSTILKQMKLIHEGGYSRDERESFKEIIFSNTVQSMRV
ILEAMESLELPLEDQRMEYHVQTIFMQPAQIEGDVLPPEVGSAIEALWKDRGVQECFKRSREYQLNDSARYYFDNIARIAAPD
YMPNDQDVLRSRVKTTGITETTFIIGDLTYRMFDVGGQRSERKKWIHCFENVTTILFLVAISEYDQLLFEDETVNRMQEALTL
FDSICNSRWFIKTSIILFLNKIDRFKEKLPVSPMKNYFPDYEGGDDYAAACDYILNRFVSLNQHETKQIYTHFTCATDTTQIR
FVMAAVNDIIIQENLRLCGLI
FUN_003666 CDS scaffold_15 15429 16312 +

hypothetical protein PF01713;PF08590 ENOG41

MSIPMHRLGGRAFAHDDDDIEQEYDRLRDLARAEAEKRNDCFARSRQAYEDGDGAGAKELSNQGKAHAARMDDYNQQAADFIF
RENNASGRVEADSIDLHGLYVEEAERILEERIRADQRNGQTHLYAIVGKGHHSAGGVQKLKPKVEELCQELGLRYETDEDNTG
RIIINLQGGEPVPPSHSGGYGGHHGGQQHHQPQHHQPQHHQQEQQNDDVGQVLPWILKKLEKACCVVM
FUN_003667 CDS scaffold_15 16690 17479 -
hypothetical protein ENOG41

MARPMDAHDDIAIAEIVIYTFILIGALFLCKTHGFGRTAGWLYICILSLARLIGSSMLLATINDPSNTSLYVGWLTLNGLGLG
PLILILLGLLSRCFDSIKRQGGRGVLPIYQRAIQLLMLAAIILISVGGANSDYELNGSSPSVKYNSECKIGVILMILVLVLVL
GQFFLALHSQSYIADGEHRLLIAVGASLPFVVVRLAYTCALILGGHKQDVWMYLGAGIIMEMMVAIICEAVGFTLCKAPKPTE
SQEMINIKDSIEA
FUN_003668 CDS scaffold_15 18612 19682 +
hypothetical protein EOG09263L9T PF08433

MPLIIVSGLPTSGKSTRSQQLYDYLSKRIADSKYRLHLISDESLSISRSVYDLSSLPAHTRSANASEKDARATIYGAVKRVLS
DKDIVILDGLNYIKGWRYQLHCESKAVRTPSCILQIGCSVDKAREVNEARLKKREAEASKTADKEEATPSATTSCDTIVDSTK
PYEPGNWDNLVFRYEEPNPMTRWDSPLFTLIWEDDEAQTNKVFSDLWDAIAGEARKVVRPNQATIQRGREESGDYLYLLDRET
SDVVKRIVEAQREGDDVDEVRIPSGSVELTIHLPAGNKVGLPQLQRLRRAFMGLNRGGIGLEAVGDMKSSRLRDTFVTYLNDS
FEKDE
FUN_003669 CDS scaffold_15 21401 24097 + CDC48 AAA ATPase
cdc48 EOG09261YV6 PF00004;PF02359;PF02933;PF07724;PF07728;PF09336;PF17862

MAPQPDEHHPHKKVNLTDPSGAEVKNEDDVATAILKKKKKPNQLMVTDAVNDDNSIIALSEATMDQLQLFRGDTVLVRGKKRK
DTVLIVLADEELDDGSARINRVVRHNLRVKHGDMITIHPCPDIKYAKRIAVLPIADTVEGITGSLFDVFLAPYFREAYRPVRQ
GDLFIVRGGMRQVEFKVVEVDPPEYGIVAQDTVIHCEGEPIQRDEEENNLNEVGYDDIGGCRKQMAQIREMVELPLRHPQLFK
SIGIKPPRGVLLYGPPGTGKTLMARAVANETGAFFFLINGPEIMSKMAGESESNLRKAFEEAEKNSPAIIFIDEIDSIAPKRE
KTNGEVERRVVSQLLTLMDGMKARSNVVVMAATNRPNSIDPALRRFGRFDREVDIGIPDPTGRLEILQIHTKNMKLGDDVDLE
QIASETHGYVGSDVAALCSEAAMQQIREKMDLIDLDEDTIDAEVLDSLGVTMENFRFALGVSNPSALREVAVVEVPNVRWEDI
GGLEEVKQDLKENVQYPVDHPEKYLKFGMSPSRGVLFFGPPGTGKTMLAKAVANECAANFISVKGPELLSMWFGESESNIRDI
FDKARAAAPCVVFLDELDSIAKARGGSMGDAGGASDRVVNQLLTEMDGMTSKKNVFVIGATNRPEQLDPALCRPGRLDSLIYV
PLPDEPGRLSIIKAQLRKTPIASDIDFGYIASKTHGFSGADIGFITQRAVKIAIKESIAADIERQKAREAAGDEMDTDEDAED
PVPELTKAHFEEAMQMARRSVSDVEIRRYEAFAQQMKNAGPGAFFKFPEAGAEAAGADGGNSFGDAGNDDDLYD
FUN_003670 CDS scaffold_15 25048 25590 -
hypothetical protein ENOG41

MSNNAPSTPVKVPSSAANHTQATLDPDLRSQINTVLLRDGHVSKIQDALLHALNSHSTNWPTAIQSHALALLRSGEVTTFPAL
LSRVLEDVRHDSALNPISSSSNGTSAKPATNGDAPKTNGAADAKPSLAVPESVVEEALRVTRESLEAVCDIEDEGTA
FUN_003671 CDS scaffold_15 38951 42115 +
hypothetical protein PF00005;PF00664

MATTDDTATISFRGAGRDDLPLADYILRKTQYLYALILLTAFISCAAWYSVVNSKKQEDLVQPTVKGPGGKPLPITKRKRARG
DGDRKIGPGFGRTAKNVFRYLAFVVFMTYVGSAGFMFYHAFWYEDPYQWSNEGLPWAGEWSVVHVTGSLFFYLYILISLFDWR
KGPNIVHFIIWILGFIGEIIMFSTTAVAAVDCHFFRSVKTSGSDKEGNCVDYWTKIDLVLYFVRILHLLALVGVFSLAWIRKA
RDTDEKKAEEANPAESTPLLNGNRRLYETNGQAVNGQIPNGRDTNGRRGRGRTMSNAHKTPSFPKKEEPAAFYRPEKLPHKTW
WEYLKGYSLFFPYLWPKDSVRLQINVLICFILVMLQRLVNVAVPMAVKQVVNDLEEVIKDVQDGERLSMDNFPLKDFLILGIL
WILQGQSGLLGSLRSILWIPVSQYSYRGLTTAAFNHVHSLSLDFHLSKRTGEVLSALNKGSSINQFLEQVTFQVFPMLFDLFL
AISVFYYYYGPLYAEINLVDTCWYLYMTIKMASTRADQRREMTNADREEEAVKNDSISSYETVKYFNAEDFESRRYQGKVAVF
QNAEAKVQMGMMMMNICQTVVFNLGRIIAALVCGWQVAMGMDRRTTGDWFMVVSYLTQLQGPLNFFGTFYRTVQQAMISGERL
LELFKIQPTVVDTPHAVPLGKDFQGHVRWNNVSFSYDRRKPALRNISFECLPGTTTAFVGESGGGKSTLFRHMFRYYDCDEGS

IELDGKNVKDLTINSVREKIGVVPQDTTLFNETLMYNLKYARPSATDEEVYEACRAASIHDRILSFPDQYSTQVGERGLRLSG
GEKQRVAIARTILKDPRIIMLDEATSALDSHTEQEIQDNVWNIGQGRTLLIIAHRLSTITHADQIIVLNSGTIVEKGTHEELL
AAKGRYASMWEKQIRAERALNVAREAQLKAARALRKAKMGPKRNSDGAIDSYNSLGSSGSISGSGSQGHEDDTHTSSSSCSDA
ESSHSEEHSMERK
FUN_003672 CDS scaffold_15 44910 46408 -
hypothetical protein PF03798;PF08390

MSGSEPFPLLNTANDQLHQRRPADQANSYVSNTTATQDAINNARKRRKSSLLGGEIRGDTGAPALASSRASLEASDNHSNGHS
DGRKRLSKRRRARGLIGRAKQTMIKHTFVLPAVLLVFFLVGYAINPTESNPIHHFIFLSYKLPQDDPNEPAQYGKGRWDIAFV
GFYTIVLSFTREFIMQELLSPLARYYNLTRGKKARFMEQVYTALYFGVLGPAGLWVMSHTPVWYFNTHGMYEGFPHRTHLAPV
KFYYLFEAAYWAQQAIVLLLGMEKPRKDFKELVGHHVVTLGLIGLSYRFHFTYIGLAVYVTHDISDFFLATSKTLNYIDSPLV
GPYFGVFMVAWIYLRHFLNLKIIWSLLTEFETVGPFELNWETQQYKCRIAQVITFALLSSLQALNLFWLFCIARIAWRFLSQN
DLQDDRSEDEDDDIEEEEEMPASALKTNGKANGFVNGHTNGHAISKIPEDAKN
FUN_003673 CDS scaffold_15 48218 50558 -
hypothetical protein PF08733 ENOG41

MAAAAALDMVSATITALPSLCTTTLLPDSGVMTLGLDGVPVPLTEPAVYQVPCPTPDLLPRAEYSVTVAASGDESSLTRHDPA
DSSPKFSDFRDPFYASTFPICYALAATTVTAYMLVIMLFVTPRSFLDGGIVYLGRRSAFTHSSSSSVTIGGRPWLQKVAALTV
AISLTIASADTFHAAKSQYMWGIQNANQLQDEVMNSVELKVIRLVSDTFLWLAQAQTLIRLFPRHREKIIIKWVAFALITLDV
VFSAITSFKYSENGINTTARPKNFVHPVPALSYLFQLSLGLLYAAWVVYYALMKKRYSFYHPFMKNISFVAIISLISILIPVV
FFILDISQPDFTGWGDYVRWVGAAAASVVVWEWVERIEALEREEKKDGILGREVFDGDDTLEINASEFPWLRNRKSRKGGSSG
SSGDGDRQQMSSSAANGWPTVSSIANRYRGQSNGAFAETTDQSTQRGTSRGLRPNIWPARPAPAVTPISRTDTASAASTVYAV
RYQAPSETTSRTPDPLPQPSVVDLSQQSSTPPSQHMHDESSSSNQAPASVQNGHVVSSPSQQSADLEANSTNSPAQGGWRSLA
FAGPRSGHSGEDATREMTQQSDHNPVAEREGRTNSSSRWDLRARLEDFAANQAEKLRDRMRTAPNTESLPVTVIPAPPRRGAA
LQQVLEEEELNTAEQGSPGREPFTSQNRSTNSPSNASRDATGEITQLDRSRSAFSGSQGDGPIPPNNPPLWRGVRPRMAPDED
YYDDSDDGSLSGRSSLDHRRQDSDSSGLHRTS
FUN_003674 CDS scaffold_15 53404 55664 -
hypothetical protein PF03853;PF09532

MATQFIGLHMRVVLRDPPGSELTGTVGNVQAGSGLTLTNVFVSDTKQWLPHLQVHAANIADLAEVKNDRPATYTPAPAESIAA
PFISQPPPQPAFVDPAILSMGRPPAALTPTGSDSKVPLEDGRAELQVAPMSATVSTPGRPTPDHDLTGSVSNLAINEAVQEMD
NGQPIEGQAPRSAKKKNNNNRRSRQSKQGKGQRAEDDGQAIEGSPASGRGKGWRQTPILQSTSSFQPFHSLKKSSKGRKGILD
NGWASEDVTEEMGEFDFENNLAKFDKRTIFDQMRKEDQIDDASRLVSHNRKPKPGTAGGKNLHYTENVLDLPSSTKKDADFWN
SEADDGLNEAERLSGRDVRSGQGNRRADSKSGPSRRSQSRKASAVAISGGLPLSRVNSGQGHPPGLYLVPSHKRLETVSALQM
LNLENIAANEIGLTEQLMAENGGRGIAEVALIALSDPAIKVRFGLAGANPSSSATLTSPVVVILAGNNKSGIRAIAAARHLRN
KNVNMLVCLVGIEREKDLLEDLRQQIQLYRAFGGKILSKADLFEHLRKASSSGSPVSVSLIIDALLGLTISFEELRTGDQAAV
YELMEWANRNEAFVLSVDVPTGIDPTSGKVAVIDGSRLFVKPRYVVAMGAPKRGLLEAVTPPDEDDPQNMNNAGHEEEWRLFI
ADIGLGSAVWRKAGTKIRRGIDFDEKWLLEMKYRDGQQDGTDSDEE
FUN_003675 CDS scaffold_15 59914 61930 -
hypothetical protein ENOG41

MAGFPPVGAMPQNIPAPMHHTQAPQTRPSMPQQNGAPAGLPAFMPANTQPYSHPPDPPMYLRRQDPAEIKNARSGEPPMYRAL
RLEKTPGASSGGKARDTEYDWETITRTDKNMSQSRIKERIEHLDKTTRSVSKKKQDKNETIQLQLDRAQADLTKEDRDFRFYY
KLAQIESEWRAADDPRHRDDRSVRSSRKHRSHSKRSKSPRLERVAINAYFQRMPADGRHSSRTLDTHGSMRQPRQIQQTPAQP
RMGGLPSVTLPQGFVPPPPTAAPAVAAPVRPAPPPATDPRLNGQLRPPMGEQNVPRMPQAHSPRQGPFNNAQQSVMPPAEPGN
AVPHNAGVVAPVPAPVPAPAPAQGQVPVRPPPRLPAINIPIDRPPHGPLPGAKAPPQMPSSGPGAPKTPFEAGNRSPKKTAPE
SGFNIEEKVKVYHSSGRSSSPSDGDGDEWSEDESEDTRPSSIGSGSSPQRGRGRSPNPKRMPSDHHENIIIQDPRNLRKNAEY
MADDRSSQHSDRQHVRFNSPGRRYRDESCSPRVEYHSPREQPWKGEPPRIIQAARLSVRHVRGTAPRRDDSHDIRPSRTDKPL
ERARLDDGHRERDVRRDNDRFKHIEEDLRRRREFKERREDRRPDDYKYVDPEPRWSDRRAREYMSHREPRYSRQHHEDREAYR
GYSDRRD

FUN_003676 CDS scaffold_15 66432 69037 -
hypothetical protein PF00888;PF10557

MQPLASETTSPSRRRPRLDSSSSASPPLGASKRARFEMTTAKARGKLPETIDLTQPPSAFKPYAGSKKLVIKNLRAPTSRDTQ
VAEYYKRTEKELEDSLEAVFAGRTPDVPLERLYRGVEDVCRKGDPAKVYQMLKERIDAHLQRIVLPRIQKNGHASNLDTVKSA
LAEWKTWNTQTILIRSTFSYLDRTYLLQKNLASINDMAIQQFRKMAFPSQTQAYKTSVGVKLIAGVCDLVENDRRGIDQIEPA
LLKDSIMMLYVLEVYIKHFEPYFLEQSERYFKEFGEAWSTSSLKDYILVCEKLLKKEDYRCIQFNLDSTTEKQLMDSAHTHLI
TNYSEKLLNGGNLAKLLADREVESMKALYDLLRLSGIQKKMKDPWGDYIQSTGSTIISDKDEGDQMVLRLLELRRSLDLMIRD
AFNKDEDFLWGMRDSFGKFMNDRKAASCWDTGTSKIGEMIAKYIDMLLRGGLKSLPKELLSDVKDRATAEKEGQASTGDEDAE
LDRQLDQALELFRFIEGKDAFEAFYKKDLARRLLMGRSASQDAERNMLTKLRGECGANFTHNLEQMFKDQELAKDEMESFKQW
CQGSAERKNPLDLSVMILSAAAWPTYPDVRLNLPDEVATQIERFDKYYKNKHTGRALTWKHSLAHCSLKGIFAKGPKELLVSA
YQAVVLMMFNSVPADGFLAYEQIATGTGLSGGDLDRTLQSLACGKARVLTKHPKGRDVKPTDTFTFNKTFTDPKYRVKINQIQ
LKETKEENKATHERIAQDRRFETQAAIVRIMKSRKSMGHSDLVAEVINLTKKRGSVEPAAIKKEIESLIEKDYIEREGNSYVY
LA
FUN_003677 CDS scaffold_15 69846 79033 - TEL1
Serine/threonine-protein kinase tel1 EOG09260090 PF00454;PF02260;PF11640

MASSHGFNVMNLARDVKAGSVRDREKAVDELAHLLNPWNRATNLSDLGDKSYHEIFEAIFSFVLREKPIFYDKKKSQTTINAT
TSRLSKCADAVRMTVGRGNPKIGRKTLLAIVDHITQVLPGPNDDFVPPLLQDYIKALTEVLSRPAHVEILARKEGHPWELCVG
FFLSVAQFLLPNEGDVSTLALARASPALVRSSPAPGSAGYGRSGGRSTPSTQSQRRVAPGEGGLLKDVLEGLYYLVVGGNAPL
LRQFKDITPVVLRVLSLKQVSLGSLQTLAFAIINTIFSATHADDLEHASSLVQTLVPLMSYWWRSEKVSQDEVIRALRIEISR
SILLTHLHIEHLALRSSDETIRLDLEDLVENIWSEYSRRGEAFRLQMSDITFALSSLPTYGLQLDVFGLRPHNVYGEGHWAIV
QNLAFLEGIMALPRLKRQGDDNGEQDEQPRKRQRTRQDNSSRIRLKLKAVDVSICRTALQLIPFLLAHNSLSREELLDLLPDL
ISLANDKNPVTASWALIASASCIAYSKGCHDQVDMWHQLWRFAIRSVSLPGTSRAAAVLLHQLLEADVLPYHTISQDINNMVT
TADVSGPSTLSDASISLMFHVLNLRNAKVPSASQSTCHHIIRWVFLRWNPNESAFASYNSLHVQPIHLVNLIRTCCGTTTLEL
TSHPAAPGGPLTETWSSFKEIESFIRYILLSEDVSNNPATAGSCSFAKPTNSPPLADANTRYASQKLTLELFYPKLGELMELC
TSWTKKMNEGGIQISFDRFQSLVSACLAGTLLLPLFGDINSTQSSSVESTLKEILEKGMNSALTSVEPNAFVDSILRAVRPIM
PDLNTTSLNRALTNNPGLLQFFSRVWSSLGEQKDQTHHDNSIDLMDIDEDFDSQSSRASSIHAPMVVPRFNIQMKLDTQAFYT
ETRTRLHFLSIINDDIGQMGLIPDIYVEHLINLPDDDLLMCQNLLLDIFTSDLVVTPDNALAIIERLGEYIGQPDYQCAEVVL
STCIGVIDGLHPIWLNDKRHLSDRVGDLYYHFIKVCLTSNIFSPRAQTSMVRLLFTLLRTNTEYGKDQGLDSPRTCLLYILKK
GPMLVKFAISRKIASVFDLFVLKLHDEVFVDVLDSLPTNPLDTTGIAFRLLVLSNLACRWSTLLRRCTYHIFETPGKILDSTH
YATRCLINVSNTLKLESPKALFRLFSRQLLYTWLERDPIEDIPFSIFGFATLEDLLKSAQSEAIGLTVMRGQDEAFAEVCRNL
GNSESDLVRQNFTTAIAYSMIFGDSNGGEDKERGEAHIKKLLGSQVYMDSIYINLVDIAALFFDLIDQENSLERVFARYKLDY
AGEIMDAVKAISHSPAELPANQQPMFKAKYLIHELHRLCQNTEFQFNDLWTPPVVVSIARKLLNTVHPALGPLHACSVLRKVR
VLISLAGPVSWDSYPLEMLLNATRKFITDSECADDALGISQYLLGQGAKHLSNVPSFLAGYALSTLASLRVFLESSQSSTTQE
SQFKATMSKAEKFHGWFSKYLEEYDSPMFKDLAQRSAFKSITQSAARIRSSGNAERGTAESKLLLDILDDGGADHQLLNDSSR
QLALGLLCGDFSIPASIKDDIIESDHDAVKHSTAVWKSCDAENLSEEYLAWAGRVVGRAFSASGEIPVNILRESHLTRYQQIA
PGSNGSEMGILYLLQDLTSNPDSVTAGLAEAALRSIVSDAAILEDEPLTVACQKSLTESLLITSQWGSHRSPPSDKAPVTPPS
SPDQQDVWSVEITSKEWLPSLSAHLAQCVPESIILSVLAPILARVEHFAESAFPFVAHLALYFPINQQHSPKRPFSVAIKSWL
KCTDPKAKENLKLLINMLLYLRTQQYPKESSIADRSYWLEVDSAMVASTASRCGMYKTALLFAEYVTPETSRSSRRSSAAKEV
DMSDTLLTIFENIDDPDAYYGLPEEPSLSKVVARVEYENDGPRSLAFRGAMYDNHIFYGDPMAQSDEQALVRALGTLGLSGLS
NSLLQTQQNIESSPAALEDTFNTARKLGIWNLPAPSSDHHAVTVYKAYQSISQAADIANVRTAVHEGFSRTMRSLAALDLNAT
SLRKRLGALASLTELDDVIGVSDTAEMDGLIEKFKTRSDWMRSGLYGSVSQILSCRGSTMSMVSQQNTLRTNIKLSAAAARHM
EVEAMITASQIYRYHQATQESLRISTGLTKLIPTCAALDIHVDAAVNIETANSLWDYGQMSTSIRMLQGIDRDASLKKQTLPV
SRSDLLSKIGYQVSVARLEEPHDIQKNYLEPALKELKGKGQGRQAGAVFHQFAMFCDQQLQDPDGLEDLKRLQSLKKAKGDEV
SELKTLVSGTKDTQLKTRYSHVLNKEKQWLDLDEQELRRVEQTRSEFVRLSLENYLLSLIASDEHNNDALRFTALWLERSEEE
TTNKAVMRHLSDVPTRKFAGLTNQLTSRLQDQDTSFQKLLLELVYKICVDHPYHGMYQIWSGTKAKAQQKDDVAVLRVRATDR
VAKRLAETQSVANIWLSIDKTSKYYHALAMDRNPNRYKSGAKIPLKESSPGHNLINCLIKYRIPSPTMHIELSHTKDYSKVPI
ISKLEPTMTIASGVSAPKIITAIGSDGVRYKQLVKGGHDDLRQDAIMEQVFSAVSSLLKLHRTTQQRNLGIRTYKVLPLTASS
GLIEFVPNTIPLHEFLMPAHERYYPRDLKGSQCRKEIFGVQSRTVETRISTYRKVTEKFHPVMRYFFMEHFMDPDEWFLKRLA

YTRSTAAISMLGHVLGLGDRHGHNILLDHKTGEVVHIDLGVAFEAGRILPVPELVPFRLTRDIVDGMGITKTEGVFRRCCEFT
LDALREEQYSIMTILDVLRFDPLYTWSISPLRLAKLQKARHNDETPMDDEQSEAETKKGKKAAGHVNEPSEADRALEIVRKKL
SKTLSVTATVNKLINQATDERNLAVLYSGWAAYA
FUN_003678 CDS scaffold_15 79959 85108 +
hypothetical protein EOG092604ML PF11894

MAEITALDALQAFHQGLLSLREGRVEGAEALGNEFLVQVFETELGKLWDKPARKGDNRNAVKSGKVVIDGDEYSINDNFQQDV
LTFSDEVELDELESTRCLLESQEDPDTLGRSLLECAIIRFHQQRKYVLDIVRLLLELEGLDDDLADTEALESVKLYVAERLLQ
PSMGADATKRIVPRCMASMKEIKVWLQKIGDKIAAAQTLGQTTTGGISEQMETIEFSRVSLMQQHELLGVILCRSIEMRQGST
QDFLDFVSVLQKVDRYDALLVHLIPSIGAYISVFGSAEGGYDLIKARELNGKLFPADSPLWPLSQLQAAFRAWWLAEYSGFYV
DDPPEAAIPPNTDLDEEDRQRSKAFLESLKDGSFDFLLSIAADVKSPDWHDPVRAGMRKWLQRKSPALAPDAIPFADFFQLTL
MLQLEVFVDGFISNLPDVLRKLRVEEDEQRQSSQTHEQDLDLERFLLVIAYAYEGRPDAAMNFWSDPDSNLAGFMHWASRRAS
TPLVTAFCEMLQAISENEECASAAHEFLMDEGHHSSGKMRKSQSLTWAQIFRELDFFSAKIRQKPTPPQTIRYRNEKMSSDQA
ETEPESAMMLECYLRLMSKLASESETTRQFLLHNPNYSLVETLYELASSPIPPRLRGCTFMALRALMARKSLQEGILMWNCLD
TWITGGYASHPITRQAQQSPIVSMDRIFDEISNGFEDPESFIQLLLSLISPAIDSSPLNDGLPFPENLGSASRMPGIEVYVDF
VMGLVFATKANDLQDVNQTRVLRLSCLEFILVCLNTFNEDLIIMANETNINVDGVIATTDLATYVRMHPFARVMEWMFNDKVM
AALFNTIHQEPVDVGNAAPDSPLILGILRAVEVISKVLDLQATYLELVRPIIKLQSNQRRPPVSNAAFASFEDGLVTRLNLVV
DLGNYCGIGHPDLTLACLKLLEKMSSSSKITAVWSGSNRQMHRNKAIVALEANGEHEAISRSFVSELITPLETGREADSPAYV
TKIYILDFLYQCLQETPRKPTIAHLLLGFKCGIDSLSVDAKGAFASRTSLFHTLLRLLLEAPSGDAQGMRQWLIAIKSRVMRI
LHILWGSPLSAPVVVEELRENEILFHLLLRETVINPDLPWEGENVAMVQFPVTDGAVALIDFLSLRSMGLEYIAMELCSIAQN
RMPSVKRRIFEALNGQIIGEGNTPISIPTIFDLYDFLLPEGVWEIPLPPLQYYKNIDLSACLENDADSNPIYNLERVKEVLLL
KRGEAQLSGALIAVQDLAAIEREEGMIIEYLISSNRQKQLTTQSLKVLKTWTKLLLIMIESNDFKGSAQTSFFLQALQSILPS
LEAFASERPDEAFELARLAKVLLFKLDLSASADDKQGSAIGNLVSDKLFQLFQICLQAIGKWAGTPELRSVYYGICYRYLTGM
ADQGSLISNRPKTIKTIQVYGERLIHVICDDAYGGEAQCQTAALILLNALVSLGRQESDDHVVETLNRLNFIGIMVDSLRNIM
REWHEVFTSGTPEQQNYQNARLALLLELAQTRFGAKYIIHSNLFRSLEDSGLFGADPELQINNTNPRALEQHYYLLAQVVRII
GAALVSRGSHNVVQGRKFLTEHRMLVTHTLKRSAGIGSGNADEGLDQKIEELAEGLMVIIAATRFLEFENEVLPEPKQQSHGL
FH
FUN_003679 CDS scaffold_15 85998 87793 -
hypothetical protein PF12861;PF13920

MEQEFLYMDSLLSYMLQGNADLFLDSANAYYSPPITISMSTTTRIVSPPDFAGAGAPHPNTQPRSRSQRTIDLEIDGISASAS
ANASGSSGNSSSSGGGASRSHIRQTTTSSSLPLTYLSLPPLISNSRLRTYRTPSPLTTTQRPRHSHSPPSQNIQTPPWGEPAS
ASSTHIELPPIGSWENGHPESLHNADLENSLLASEIEDNFLADLADGDFSSPSSYPPFTNPDSRPSQSHSVSLQSTVQASTPH
RALRNTTASTNTTTSTTATAAPRELSSTNCILHPFGPERTTTQLSSASNSTGESQASLPVFDSLEENDFFFDSPASSFSEAMP
PATRRSTTAAAARTGSAHASKRRRTSTTAAPAAPPRPTSRQRKSPATRKDMDVEELFGTSPTRSPIDIEPKAEFDTIDLTENN
EVFEEVRKPEKDDRVKLAAFQCVICMDDCSNLTVTHCGHLYCASCLHQSLHVDVTKGKCPMCRQKLDMKPRESYNSKTKGYWP
LELKLMTATRKGKRKANTMS
FUN_003680 CDS scaffold_15 89609 93079 + MIP1
DNA-directed DNA polymerase gamma mip1 EOG092603KJ PF00476;PF18136

MLISAAAQPLRGSTPSFIRVARHQAFRRHALRAHVRWLAAVADNIKSNPKGTLALPSEARYNEIGVQQLSSHIFDQIFPDGVK
PPPQELVELSRDHLRRHDLLGKNTDKSEPIAFDLPPLQGRTLDEHFHKLGVDCAEPYLAYAKQFARANPPPKPRKWIHQSGWT
KYYPDGRTEKVDAPDEEMLSFDTEVMWKVSPFAVMACASSPTAWYAWLSPWLLMETTNDRQLIPLGDPNKAKIIVGHNVGFDR
ARVLEEYSVKQTRNAFLDTMSLHVAVNGMCSQQRPTWMRHKKNRELRDQVASKTPDHDLAVLMTSGMHDEEELWVERSSVNSL
RDVAKFHLNVSIDKALRDDFSELDREKILERLDQLLDYCAADVSVTHRIYQIVLPNFLETCPHPVSFAALRHLSSVILPVDKS
WDAYITNAEATYHKLNDAVEERLISLTDKALDIKDKPEAYTNDPWMQQLDWSGQEIKMVKGKRKNDPPRPAARQKKPGMPKWY
KDLFQSNDAPISITVRTRIAPLLLRLAWDGHPLFWSDKYGWTFRVLRAEASKYTAKQMIECKFDEKEIHLRDDHTHTYFKLPH
KDGPTARCVNPMAKGYLAYFESGVLSSEYSYAKEALEMNASCSYWISARDRIKSQMVVYQEDLPHQGMQAETGHKGGEASANG
FILPQIIPMGTITRRAVENTWLTASNAKKNRVGSELKAMVKAPEGYCFVGADVDSEELWIASLVGDATFKLHGGNAVGFMTLE
GTKAAGTDLHSRTASILGITRNDAKVFNYGRIYGAGLKFAATLLRQFNPNLSEKETLETASKLYANTKGTKTNRKVLYKRPFW

RGGTESFVFNKLEEFAEQDKARTPVLGAGITEALMGRYISKGGYLTSRINWAIQSSGVDYLHLLIVSMDYLIRRFNIDARLAI
TVHDEIRYLVKDTDKYRTALALQVANLWTRAMFSQQVGIEDLPQSCAYFSAVDIDHVLRKEVDMECVTPSHPTPISPGESIDV
STLLEKGEIARLDPDIVPDAKHAPRLEQIKYTPRVPIMQGIQESAQESIPFLKAQIVSDDAELRELVKDVQKAKLAAAPKKDK
PLSKRELQSVLPYRAYPKLVPMEEPVSVSEALKVKPSAYETRKMTWPKYSGKSWSQNSRM
FUN_003681 CDS scaffold_15 94148 95997 +
hypothetical protein PF08568 ENOG41

MVSVDEAIQRLKETRPPTTDALTYLTIVETNLSPEVLPTLEEILEDAELTSDIGWDLVEMLISVPGSQGCLETVARLGNPREV
IIKVLEVLDSKAESSEAGDASASANFITLVGMLGILHRRLQVKAPSRFLHTTLQTVYRAYNPQGAETTAAVIDLARSLSGRKR
PPLPTRQSSTKLETPFQDSDISKSAPDPEADAGQAAEGEAELVSRLLQSFVTSILEAYVNSNRLEWAARHLEYYNPERIVPGK
PTMLQAFKQVDELQAKDALVGQLVSVARDLGLAKLPSAEVKKALQSPFAVNPLEVEPDPKNPEGIKLSTGGFLCLTAYRMFAS
DIFDADYEQPDAYIFPEHHNLLKRFLGDDPQTQIESNPGTVEALLVIALWLHDQKRIIGPKGTDDKDVTFMSYHHLLTLISVF
HPSLRVRNAATVLAGHILHEDPHEEDRLGILEDLLENCMFSSLKACAVTWLREEIIAARKAGSTGRFADPDCLDTLQYTLFED
LESLAETDLEALWEFWVQFAPFHLQVANFALFLFGGQDFKHLVPAGMAAAVEHRYASPLLAAAKRLREAIDKKEIDGQGQEDE
VSMQLGILMDTLERVPLQ
FUN_003682 CDS scaffold_15 97457 100919 +
hypothetical protein PF03033 ENOG41
GT1
MDSQQEDPNKLKLEVSEQHHNNDDGQQSTTLITDTHGDVAYPAPIPRPIQSPESPLSPRSPKENIDPGDGVKQTPVQSEPAHL
PTADASISPKKSPAQKSRPRPTKSWQTEQPRRAHHHRSYFRPGPARAGTSYLNKAIWEAPEDPRLSSSDSSVASAGEDEPQTS
QQESHHSEHKKKAGEKASGKASHDEGKYGHFSIGNQNYQTSGKVKKDGRLRITVNETAGTGYLTKALGAAVNKVTPAKAEQAD
RVSTLKIPESPRRSSSSTAVADPRPQARLNIVIMVIGSRGDAQPFLKIGKVLKEDYGHRVRIATHPAFREFVEKDSGLEFFSV
GGDPSELMAFMVKNPGLIPTLETVKAGDIGRRRAAMATMFHGFWRACTNATDDESDRQNLKMMGEKDPFVADVIIANPPSFAH
IHCAEALGIPVHLMFTFPYTPTQAFPHPLASIKNSNVDPGYTNFISYPLVEMMVWQGLGDLVNEFRVKTLSLDPVSTLWAPGA
TYRLHVPFTYLWSPGLVPKPQDWGSEVDVAGFVFLDLASTFEPPKELEEFLAAGETPIYIGFGSIVVDDADKFTQMIFKAVDL
AGVRALVSKGWGGLGGDDVPENIFMLDNTPHDWLFPRVKACVIHGGAGTTAIALKCGKPTMIVPFFGDQNFWGKMVSNANVGP
EPVPYKHLNAEKLAEGIEFCLTEKARDAAGAIAERIAEEGDGAVNAVKEFHRQLNLRSPSLLRCSLLKDHTAVWELKNTNIKL
GALAADILVDNGLLTWKRLRLLRHVEWNDFEGPGEPVTGIAGSIASTVGEAFHGVASVPYHWAKRTRSISKKKNQRNAKKSKK
SGNSITRHNPADSDDPVDAASLRTETTAGDTGGQYVGDIASGVERTATAIAKAPVDLSMALAQGFHNAPRLYGDNTVRRPIRV
TGFHSGLRAARHEFAYGVYDGFTGVVRLPVRGAKENGPIGFVKGTGMGLTGLVLKNLSAIVGPIGYTLKGVVKQAERSKTPQK
YIRRARIVQGQREYKAVPEDQRKQLEKEIMEGWHIMDELREKIQKLEKQRGIAGQIDRVLLDTEAIFEDVDVAKRSLEALKQG
KALEDVVDPDRDRGRDGGKSERNRSMSIRRSFNLTKKSREEKRLTSLADAEESTKTGGADEGPAEMDRLTVPAR
FUN_003683 CDS scaffold_15 101257 102334 -
hypothetical protein PF12656 ENOG41

MSEQSKPPAPRIAIKFGATSNDNNSKKASKPNPPSSLGKRPRSHALGGASDSEDDDYEHRGRHEKITGFGVDGAETERKAKDS
RMEKKDYVIARQPNHDWRSEAKAQRKSKNSLPEEARAQQNNTTVETEPADQDKGLKWGLTIKEKTEDDNKDIGSESKEKPVSN
DDDQKKPSPKRTADDEAMDALLGNKEEDEKIIHPTEADAYRRDIQGAGETSTLDDYEAMPVEEFGAALLRGMGWDGKPRGTVK
EVKRRPNRIGLGAKELKGEEDLGGWNQSGKKNRRPRLDEYRREESKRKEGRRHEDSYKRERERERERDRDSRHGHRDRDRDRD
RDRDHDRDRNRDYDRHRSHRDYDRRR
FUN_003684 CDS scaffold_15 103904 108704 + EAF1 chromatin modification- protein VID21 EOG09261DG0 PF07529;PF13921

MTEVGAADLSRLLQTKRNECSSIVTSRKRKLRELFAVATQSEGLPHPVLTNPDAPTTTPAEWQFLQANDIIQGKTLNEASIPA
RPTISLELFKKSLAKAIFIAPEPAPKRTLEDASKPEIANVQDKEQSNGTYPPPKVGDAPPKRTPSPPPSTDPATTDKAGSLPT
PTSQPVPLPEDAIVEPTSTTSPVTQTAADTNANSKSKAQDIPNASEVSDTPAPRQPQVTFESGTKGENAGPGASTAEKTSRGP
STSSDTRGAALTVKPPTDATRAPDALSSPGSTAQSATTPAVHDDASTDTSPEHEGPQFVEPAEEKQTGDEVEEGVNEKDEKET
SDDVGATLSQDLQNRVLEAPPDSAEAQLLQESIRSSVAAEAAATAASPSHQTEPVTSVPVSEDVDMSGADDVIVQPTALNANE
QVKEDSTQTKSDIDIARANSFENSDEVPGIKEIPDSQEEPPEQMDVDVPEPKATVQAQTQSQEVKSDGSTQATEKAPSTPTPA
PISDVATVPVESAPKESPQKVERAVTRVSSGAMRPKSISEIVGATPRQTPTLEHTSTNKSLDNQLTPLTSTPKSPSLRHRHIS

AHQRQRSRSQPSTVVFGKQSKKTDERSIVASQRDPIIPTEDYYTPLFVQGFAGSSSWMQPIEKILYTANKTVTTPDANLAIQD
HQACKVLRRVYHLQQHDKWSLRQPKRCPEPTRPPSHWDVMLQEMKWMRTDFREERKWKRAVAKNLAYACAEWHESTPEERKLM
QVSAVIPPKAQPAPDVSMGDVEGENQLTPDLISSGDVESIDNLDDLTEDFPETIAPSAIFSLQDDDVVFGLRRTAAADQLLEE
LPMFGKPLQVPKFDLTGPEWDPDAHWRRPALPLSKYVEGHMKLVSDGPPRKRSRYDYQNEDSDDEGEAGFVSSDPLPSLPLPP
ATDEVALFNPEMKHIRDRLYVGHQFRPPSEYPMPLQSFFECRSPSQWTINEDDELRNLVREYSYNWSLIASMLAPKSRFTSGA
ERRSPWECFERWISLEGLPADMSKTQYFKAYTGRITAAQNFIAQQNQLVAQQINPANGAVTPGRRRPPSTPVRVERRRNQKHL
TLLDAMRKLAKKRETAAQKQQHTASQNAANKKVNDPASQRTNKTPREYSILRHERDQALAEKMAQYASRQEAQRRAALQARAQ
GQAQMAGTPGAAQVGQNSAQAAAAAAAAVAAANGMNGVGRLNVPNQLAVAAAAAGQARPRMPIQSPAANGMGGVPSQMAGGLV
PPNQMTSAQQAQLQAMQGQHNRMPMPNPQPDVNLMMRAQRISEQQRLAQMQHAQGGPGTPGQGANGSQQSPPLNMRNGVNSIN
GANTMNQQNFINNAQAMMAQFNSGNLGSPQANGLHMPAGPAGSIAPRPQAQLPPSIAAQLVQLEAQFRTKNPTLPPDQIRQMA
TEHLTRLMMAQRTAMNSAAGTANGQGGLAASIAATTSPHQYAALLRQQQQQQASQAAGSPGQQHQQLHQQHQQAKQQQPQQQQ
QGQGQAQVQAQQQQQAQQQQQRQASGSATPSAG
FUN_003685 CDS scaffold_15 109237 111426 -
hypothetical protein PF00999

MASARLFGMGFQLLKRATDEESDPEEGDSAQKELFAAWALFISIMLLIIAFFTSYMLQMKKVTAIHETVISIFAGMVVGLVLM
IASGDSIRNMISFDYQIFFNLLLPPIILSSGYELHQANFFRNIGTILTFAFAGTFLSAVVIGLILYIFTLLPGSLGMTFVDAI
SVGATLSATDPVTILAIFNTYKVDPKLYTIIFGESILNDAIAIVIFETAQKYKKGEAANLGIVSFFEGTGIFLLVFFCSMFIG
FIVGVGTALLLKFTYLRRIPKIESCLIVLIAYATYFFSHGLHMSGIVSLLFCGITLKHYAYFNMSRRTQLTTKFIFQILSQLS
ENFIFIYLGLALFTDNNLSFQPPLIIVTILAVCAARWVAVFPLSKAINWFIRYRASRRGQEVGDELPYNYQAMLYWAGLRGAV
GVALAALLTGDNAPALRATVLVVVVLTVIIFGGTTARMLEILGIRTGVVEEVDSDDEFDIETFGGGLQVKRSGTGIGYNPRRN
GSVALGSLEAGQSASYVSGASSPHTGGRPVSKSRKNSIPERSDLLRHNDDSDIGSDIDVSDLPPPARRSPLPRASTAPPSRVD
SAYATPTTETPGGQPITATNALRQLWSSEDPASVFHHLDEDFIKPRLLLQGDGSRGGHGGPSWVGYSE
FUN_003686 CDS scaffold_15 112554 113254 +
hypothetical protein PF00025;PF00071;PF04670;PF08477;PF09439

MYHLAKGIYLYATSKEEYSVILLGLDNAGKTTFHEQVKSMFLPNRPDPKLKTVPTVGQNVSTIPLQDMYLKLWDVGGQHSLRK
LWESYYASCHAIVFIIDSTDIGDGNIEHDENVGRLEECRLVLEDVLQHSETEGVPLLILANKQDREDCVETIRIKEGLVKKVM
EGDKGSAVRDSRVLAMSALTGDGVREAIEWVRTRVQWNKESRPPVMR
FUN_003687 CDS scaffold_15 113472 114408 -
hypothetical protein EOG09264398 PF01138;PF03725

MPREAEPSLPERTFVTQALDEGLRLDGRKFDEFRPLELTFGDEYGVAEVKYGKTRVLAKASAEVTVPYSDRPFDGVFTITSEL
SPMVAPSYEVNRPSLEEVLLSRLLEKTIRRSGALDTESLCLIAGQKCWSIRVDLHVLTHDGNLTDAACLAVVAALRHFRKPDS
SIEGENLTIYTPAEREPVPLSWLHSPFCVTFSFFGEDGSQVLVDTNWLEEQLRISHCTYSLNKHGEICQIAKLGGTSLDAPLF
IQCAQGALNRSKELSDLVDSKLAEDAKRRDKGGLMAELTAENDR
FUN_003688 CDS scaffold_15 114633 115572 + CWC15 complexed
with cef1p PF04889

MTTAHRPTFDPARGKEALRGPAYHQRLLPAHTQLKYRQAGQGGDADEPTRDLAAELLAAEAAHFTKKNGAPALIDDADEDDET
SVSNGAKRALPSAEGEGEDLEAKRRRILAETRAIDADDSEEDDDDDSEDDEDSDDDSDAELQRELDRVRREREERKKKEEAER
LKEEEEARERDIALGNPLLNKQDFNMKRRWDDDVVFKNQARGTEDKGKKKEFVNDLLRSDFHRRFMSKYVR
FUN_003689 CDS scaffold_15 115623 117520 -
hypothetical protein EOG09261HZD PF02782

MPFLARSNSPDQSSDFKPLYLGFDLSTQQLKALVVDSDLKVKGEAKVDFDKDFGHKYGIKKGVHVYEETGEVYAPVAMWLESV
DLVLDRLAESMPVPLSHIRGISGSCQQHGSVYWNGNAYEILHHLDPRLPLVVQLPQALSHQWSPNWQDQSTQAECDAFDAALG
GRQKLAEVTGSGAHHRFTGTQIMRLKKDLPDMYAKTAHISLVSSWLASVFLGAIAPMDVSDVCGMNLWDMSRQTYSEPLLELA
AGSKRDALNLRKKLGEPCLDGAAVLGSISPYFVDRHGFHPDCQITPFTGDNPGTILALPLRPLDAIVSLGTSTTFLMNTPKYK
PDGAYHFFNHPTTDGHYMFMLCYKNGGLARERVRDQLPKPENGPTGWENFNKAIENTPALGAAKEDDRRKLGLYFYLTEVVPN

IRAGTWRYTCEPDGSDLQEVKGEWDKETDARVIVESQALSMRLRSQNLVESTRPGLPAQPRRIYLVGGGSLNPAIARVIGEVL
GGSDGVYKLDVGGNACALGGAYKALWALERQPNETFDELIGKRWTEEGNIQRIDDGYREGTYQKYGNVLGAFEGLENKILTEQ
ALESKDGRRRSQESA
FUN_003690	CDS	scaffold_15	119959	121113	-
hypothetical protein		PF01545		ENOG41

MTADAWHSITDLASDILTLATVTWSLKPPTDKFPMGFGKVESLGSLGVSSMLLAGGLYMGWDSGISLYGHFYPEAAHGILEHV
GHGHSHSHGAAALGIPSMHAAWLAAGTILIKEWLYHATMKVARERKSSVLASNAIHHRVDSLTGFVTLAAIMGANAMENAAWL
DPVGGLLISIMVIQAGAGNTVAAFRELADQGIDDEIKSSVTKQANKALKNVVEGHEVALREVSGIKSGQNYLVDLEMTVPGAW
SVDTTQQVEDAVRTQVGGKVRGVRRVRIRFSPKEATAKARFDDFIPGTVVLPPETEEDASEAEGESNHNHEHHEHHDHKSSGH
VHEPNGSHEQGDGLRNRNKH
FUN_003691	CDS	scaffold_15	123060	124263	+
hypothetical protein		PF02146		ENOG41

MSKPLLRIPYTDLPLPSVIAPASASTLPGAIAALHRFFVAPSPGDLPASAVVLTGAGLSVASGLADYRGVKGTYKVNKTYRPI
YYPEFLKSHESRKRYWARSFLGWSNLQKASPNNGHYAIRDLANLGLIRSVITQNVDSFHPRAHPDLPTLELHGYLRAVVCTSC
KNEFSRNEFQEKLATLNPKWAELLERALESGALDTEDPVERRFKGLKVNPDGDVDLPDAPYTTFRYPSCPKCLSSPPKNADGH
QHVVQVDTDGAWKLPSTAGILKPAVVMFGESIDNHIKHAAEEAIDNAGKLLVVGTSLATYSAWRLAKRAQDRGMPIAIISMGG
IRGEDKFFAGMDPNQEGEQGVRVALSTDDLLPALLSALRNDVAPSEAPTQASLQASFGNRGVFKDMLS
FUN_003692	CDS	scaffold_15	124314	124958	-
hypothetical protein		PF17254		ENOG41

MSLRQLSLRPGYIRVASFVLRTSNTSGCGIRYSSTAPATIVDRSFWRNLVPKPLRKENRQLRKKSKEWNPATFFIAIFLLIGS
MSIQMITLRNSFDRYMRQSEARIASLREVVEKIQRGEPVDVEGALGTGDPAKEADWEEMLKAIERDEAIRKSKREKPERSEPA
STTSSTSTQNVESVKAAPTNAKPGSLGNFI
FUN_003693	CDS	scaffold_15	125281	131614	+
hypothetical protein	EOG092603JK	PF03568
	C50
MASLQAKADAAKTALASTSTCTTATVVTLKELLLPDIETSYTASQANSRTASKTTTNSKAKRTAGSKTQTAKSISEQLSSKDR
AALATHVINICIRSLNEAAKPTTPATPIKQQASQGDSKKTSGPRTLRRSLSAPLSPLQPRTLNRVATSPNIAAKAATHAATQS
TGCLAAVECARVAFTCLRSITGPMQVAQTDFQLENGMSAFISKLLALGFQDQALKELRILKRRLDAGVNKSTKPISTEGQTAA
QVILELLDYGDKIPDNLLSMVTGCQIQVLRWIAHSKKPAHIEAVLPLLEESHVSSPITLLVRSGAKNAKETQKAARQLASLSQ
TLLSLAPSVSSKEDAVATELRLSPSPTSAFKLQVLCFKIQLRWWKMAGHRGNVEDDILSPFSRCVRAYTRRQPPTNGLTYDLI
AASFHALMDLVGPHKSQAKASFDSALTSIYQLLGVAAQTDRQNDAACHWFQKLKDILVPDGESAIRMFSISARLLAALLKQSK
HDTKTQQLVQEVTESLEGSLSGNITDLNELLESLSLVRRSAVGFLVSNSNGSEVKDDTLKLILEHLKTFITKFPRFGRRWLGA
PPSRDASAKSVLQFDQRREVLMQSINQILDGALVVINGDIQADSITWKHMDEVLWDCNKLIDAVLDPTISRARTEQLSGYFAK
ISSLYFARYNQIRKDLGKSKQVNKEILQCLSRSIEIIQDRPMAQQEKAHMSTKLELFADLCKAANRSEDAIRTLRSICTNMIE
EGALVNVTTALDSQPPSIAWSADDKAEILSRTLRSIAKLDHSWNDWAFFLPEGERAAVLEHLMQIVGDTAARAERLKLHDPSV
QALLRIYTPDRFPIRRLRVLLQLLFQTIGEENEMEQISNLAGETIQQLDNKDYGDDGSLLRYLPHLRALQKSMSALAVTSAPL
PVFALREAVVSWKSMTESCKSRNDVYERIDNPDGLLTHLQSASHFASLRGENSLQLEILELSTPLSKILAEPTYNNVILNHTL
LASQHLNIGHFSEAKQTLDETKKLLDQADGASRGLIAGFHLTQAEYYSGIGSIDEADSSLAAAKAIYNGSASSWALSKSQVNI
SLALSSFLDSVLALKRGRIQDALTCVKSSVRILSHDWSKIEATASRAASTSVMNVSTASLDSVKADAKLGQVIGPRFWALAFP
LLRGLLHVSSVYAHLGMYQETIYYAESAQKIAESTGSSLYRAQVLAWTGSIYHRAGKLTKALDFGNEAFNELPEDISASRVQV
ACQLGGLFRDSGDEDKARELLQLAEETAQRLGDHRKVLSIQNEVKKTAPVAAKSRATAATRATRAATRTTRAKTAANPAPRTR
KQEVIVKVQPSAAEVLKLPKDAYQASLMASVILSRALGFISQKDWASALSTLEDARELPKLFGALSQEQVVTAISLIGHSTEQ
MIHDPVFSVIQDSTISFPAIAASDKTKASQGQTPPRKGRAGATERKNSKETSVPAFAEALRQAQELLLEAHASTLSTANSTMV
HRISALLQNTVILLSATSTTQSRVLAGSGFATFSVDLARNVTWKREQSTLQHKETAKPGDIDSAHNSRRSSLGLTAEMAQFQN
NYIDMVPHNWSVISVSLSDNHHDLCITKFRAGHSPFILRLPLERANSRDADSDIFNFENGKDEMMEIIRLANETSHSASRDFS
VKGAKAAWWAEREALDDRLKDLLSTIETTWLGGFKGIFSQHERRSDLLARFQKSFQQILDSNLPSRKQGPSKKSAKTSKVALD
PRVLDLFIGLGDPSDPDSDYDEALNDLLYFVVDILQFHGERNAYDEIDFDAMVVETYDALRGYYDAAKTGSDRAEGAHTVLVL

DKALHAFPWESMPCMEGLAISRVPSLACLRQLITESQPSASDDNSEDAPEGHYVSAEQGTYILNPSKDLVNTQSTFQPIMKGL
GSWTGIVNRAPEEAEFEEALSSSEILLYFGHGSGAQYIRARTIRRLEKCRPATFLMGCSSASLTEAGEFESYGPVWNYMMAGC
PAVVGTLWDVTDRDIDRFAGRSFEEWGLFPKGTFKENKRVKGKSRASSEDESESEEDGQVMNNVSLAEAVGRSREACKFRYLN
AAAVVLYGIPVYIKHRGEE
FUN_003694 CDS scaffold_15 136704 139973 +
hypothetical protein PF00172;PF04082 ENOG41

MSGRAVSNSQHAQQSFGSGPQLYRDVAELHAVPSPSHGALMDPSHFDDFAFAYQGLPDQPSLVSLADHAHAHASQSPTAFPQH
QAMSGLAHNGLPFGTLPAGNRSQSMDGSDAPPDRTSPASNALEDSTTDEFGLASRSRADATDLGGKPKEDKADATPAWSELKT
KAGKERKRLPLACIACRRKKIRCSGEKPACKHCLRSRIPCVYKVTTRKAAPRTDYMAMLDKRLKRMEERIIKVIPKSDQEVAS
SVTRAVVKPAIPGTVPSNKPTKKRGAEEAFGPDLEAWAKAPSKPKIDGDDRPSSLQVQEAEENKLQHEGTEALPSKEIQEHLA
EVFFDNIYGQSYHLLHKPSYMRKLKNGTLPPVLVLTVCAVAARFTSSPLVNSSGPEFLRGEEWASHARDICTRRYEWPNLTIL
TCLLILGLHEFGTCQGGRSWALGGQAIRMAFALQLHKDLEYDPSGRTGPKKQLSFIDREIRRRIMWACFLMDRFNSSGTDRPM
FIREDTIQIPLPVKEKYFQFDMPAPTEMLDGQVPHPASPNDGQLADARENMGVAAFLIRAIALWGRIITYLSQGGKDLDPNPM
WEDESQYVKHLNDVVNLEASLPSSLKYSAENLDVHKTENTASQFLFMHICLQHNILFVSRAAMSARKQQGVHDDFFSEASKRT
FSAANQISELLREAEQSRCFVSAPFAGYCAFSSTTVHILGVISRNPNMKPTAEANLTTNVKYLHKMKKYWGMFHWMVENVRTQ
YRNALDAMRAGANLQDRAAQSSFLQYGDWFNRYPHGLSDAEFMDPATHKRKDSGADGVLEAKPELQSVEEYFSTLPTPQSVEH
KDTIRAVGTKRKQSAKKQAGLPTQSGQHLESMQGTDADSVSGAQERRFSGGLGLPSNSYNPLAVSNAQNPAFSTAMSPMSPAN
MTAFSHHAHTPTFFPPELLAMNFGQGANGNIDPLDRQLVFGGYSLDASTGLGGGQDIMSGLDWDAVASGAHTDGGLQGRRSTA
KAGMNGQAAGMADGAGLSGPEASSAWFMPFNMEPPEMGQDPGFNMGGIDPFTGVFGGGGSGLATPNALGGLQQQGP
FUN_003695 CDS scaffold_15 140547 142524 -
hypothetical protein EOG09262POL PF05178;PF12936

MGKSTEDPKNTKRTLLDDSDSESEDGGAAIATTGFKVNEDFARRFEHNKKREERQRLEEKFKNTGKQPDNDDDDDESSSSDEE
EDEDGFLATEDLDAQISATLQAIRNKDPKVYDKEVTFYKPDDPAVTATEKEKKEKPVYLKDYHREKYMRGDTGADDADEDVPM
TYNQEQDVLKNSIVAEMHAAANEDSDDEDGGFMKRKETEKADSNGVHPSRKAAMAITEVDVANADKNPETFLSNFMSARAWVP
PDGSNWKAFESDEGEDDDDRADQFEQAYNLRFEDPERSNEFLRSYARDVAAAKSVRREEKTGRKRQRELERERKEAEKKERRE
EKARLKKLKLDEAQEKLRKIKRTAGNAGKDLTDEDWIKFLDDAWEDDKWEEEMRKRFGDDYYAQQDDAVASEDEEQDGEKKKS
KHPKKPKWDDDIDIKDIIPDFEDDEKKPNISLSDVEDDAQEDEEDEEDEDGRASKKRKTDHKKARKESQKQARQERAKLEALV
DSKLELTDHAIFKQSSHAPFRYRETSPQSFGMTARDILLAPSDAALNDYAGLKKLATFRDQEKKRKDKKRLGKKARLRQWRRE
IFGKEYERDGPTYGFERLISADEDAGGAVIEPASDKKRKHKKEDGEEDNVVGDVGERKKKRKRSKSKKNATTTTAEE
FUN_003696 CDS scaffold_15 143204 144031 +
hypothetical protein PF00025;PF00071;PF01926;PF08477

MADSANAPKPSSSVKLVLLGEAAVGKSSLVLRFVNNDFQENKEPTIGAAFLTQKCNLPTRTIKFEIWDTAGQERFASLAPMYY
RNAQAALVVYDLTKPTSLIKAKHWVAELQRQASPGIVIALVGNKLDLTGDSGAAADGEDGEEGDDSGDARKVPTEEAQAYAEE
EGLLFFETSAKSGHNVTEVFTAIANAIPETSLKSARGAGASNATSRAGEEQRVNLGGPKDVGAKDSCAC
FUN_003697 CDS scaffold_15 145196 148169 +
hypothetical protein ENOG41

MASTITEAQAELIRSLAPDDIPIKLRCAICSKLAVNAYRLPCCEQAICESCQSNLPASCPVCEHSPLSAEDCNPNKSLRTTIR
VFLRTAEKKREASRPKEDKDSEPATPVEAPKQNLPTTETSAIETPIEQISGVDAPSSVQVDSADKVDEGAPAPYVVQESITHH
DDTPATESPRQVEAGDAQSAPAEGSTEDAPIEHSDEQTLTTQNGEGGINGAGEENEQQMDAENDEDQDQEKPDSESMNGNFPN
GSFSSSGDFNQMQMMMAMQNGMTPNSFGGFPMMGMPGMGMDPMTMQNMYMNGGFQGMGMNGMGGFGGGFGQGSNNNWNGSQSW
NFDQNNYNQNGPGMGTGDFGGFNSGFQTGYNQGNYGQFNDYRRNTFGRGRGRGRGYYGGYGRGGYQFGGNPNYQDQTQGQFPQ
IGPGQNGQNGVDQGDGQQQVDESGQPIQGDSTEGPADAGQADGSSEAIGNADAATGDASSTTRNTGPGNSDLAQGNVSGAVRP
VAAADVPLNAPTGPKAMRQGLPNTSLHHLRARGYQVGSEVPPSKPTGVRDSPADRGRSRSRSSSPTPITADAREKDKDRESRH
EQSKERPRDHDKRDRDRDRDHDQTGSASRTVSRSRSRSRGHRSSRRRRRHRSESVEDDGYDDDSRRKKHRSRRHFHDDEESSR
SKDKDKDEKYTDRNRSASPAESKRSSHRSRRDRDSDKRRDREKDNDDRKKSSHRPSHRDRDYDRDRRREKDRDRSDKDRKDKD
RKDRHRDRDRRDRDRDREREKDKDRESGRDRDRERDRDRDRGSRYSSRRESIDAGDSKPISGPRGHDTKVKTGSSSRTENSGK

DPHTLEREARDRERLLKEAQRIAGMAGWKRSRGDDGDDAGSRKGRRTGSSTSAAASRRSEAINGGDEEERMQRLEAEREGDRW
G
FUN_003698 CDS scaffold_15 149042 152345 -
hypothetical protein PF00454;PF11522

MSWDLLQRFLESDVFNSNPFLPVSYLSRYADHVGIHYVLCQKLRQFPYEDIEFFLPQLCHLIISVHNESMALEEFLMDLCEES
VTAALLTFWLFQTYLHDLSSNPQSDSFQTCRRVYNKVQHIVFGLADTARHEKITENVLPVTVLSSFVLASIALPMFPQWAGPL
AVAQARKPQPITNETQEPRENPKPTRARTVTVGNSRSRRARDNGRTFSAPDPKGSRDSSPKPARSPRPPSVIIPPQRSKTPVG
MTRPSALELRSLEAKLSTSSLPLPSAQPTTRPTTPNSAGNAQRPVDNATRRHSHHAKAVLTVSDMSPVQKMRLLRQHYFRSQT
QFLTALEGISNRLVLVPKPARMSALRAELALISQDLPAEIDIPVICPPTLVNGSPGKSRHHRIVRVNPAEATVLNSAEKVPYL
IMVEVLRDDFSFDPDAPENDRLLNSLLGGSARAKRIFDLSDSPRLSPTVKAPEPLLDSVFEPASGDLGSSPLLKGIDESPVRV
SSRPAQNHTAQRLPSGATTSSSTLDLTTPRSSGTGTSRSNSPGPVSRRMTMPIQRSAPADQPDFGALATHMRTASQMLAQLEA
TSGKRPKQEVAAIRAKIIASMQSLEEKSFDVDEQGPTFDTIIAKASTTPIPVHNPDLEEDVQIDPNLNASAGRERMENDIKTG
GVQRRGDRDDPSAAVFGEAWEAKKERIRKSSPYGWMKNWDLVSVIVKTGADLRQEAFACQLIDVCHKIWVDAGTDVWVKRMRI
LVTGESSGLIETITSGVSLHSLKRSLTLASVESGQNPRNRIATLRDHFLKAFGKPDSKEFQAGVDAFKKSLAAYSIISYVLQL
KDRHNGNVLVDSEGHIIHIDFGFMLSNSPGSVGFEAAPFKLTHEYVDVLGGPLSPDFEDYKRLCKQAFQALRRSADNIIDLVA
MMGHDSRMPCFAVGIAQVTNTLRQRFQLHLSAEEAEHFVDDLVAKSFGSYYTRLYDTFQYRTQGIY
FUN_003699 CDS scaffold_15 154155 155142 -
hypothetical protein PF00808

MSTDDSYAPKSPDLSSFYSSGPTQEELHHPQPDSHFSPGYEYKAESPTYARSASFSSYVPPSLNFNNNSGTYDSKYRPQVDYE
QASSQLRSTRPEYQSYRSRHSSIQDPYVYSSPDKRPSSGNGFEDTFASPHTQSQHHTPARPAPYGQAYPEQTPLSSAAAYDIS
YAPPVGTGTVERASTFGAIAFDTDMPPRKAAPPAAPAIDPSPVRTKFPTARIKRIMQADEEVGKVAQQTPIAVGKALELFMIQ
LVTKSADVAREKGSKRVTAPMLKHVVEADEQWDFLRDIVSRVENEKEGSRSKAKQESSSDEEIEEPKKRTRGGRRKKAQ
FUN_003700 CDS scaffold_15 157577 158931 -
hypothetical protein PF07859;PF10340 ENOG41
S09X CE10
MARWILSDTIHGSQGPTVSPLVIPHYISCQSNLFNIVIRLPYEFITDRYLTHSTRQLAFTQTATVFEDLVIRCVRYAFANIPA
KVGRVFFGKHVALPFIHWRMLRHGYIRSPVHYREYNIGKGDIKIKGTWIMHQPEHPPDFVLYYVHGGGFLMGSSYFYLEFLMA
WHHLLVEAGFNNPAIFALEYTLVPDQVYPRQVLEALEGYKHVLEVVQDASKICVSGDSAGGSLILSLLLELGAQAGNQDKKGM
KVNVRGGLSDADPPHLPLPRMATLISPWITLMSNLHYSSKSDFLDKRTLWKYAHEYAGENMVQQQPASPGNCVDEGLWRAASP
ERGYFIVFGEEEVFAPDIEEFLKRQAKIGIQAEGQKFDGGIHAWPVASLFLSSTEDKRLQGLRTAVKEIRRRMLEWGTLNGDK
V
FUN_003701 CDS scaffold_15 159653 160355 +
hypothetical protein PF12239 ENOG41

MGENPPEEAPFPLTAVDRWVLSQTDEEFHKHDWEELKEIIRTNNLSVLKRKPSDLRRYMAWTAETKAEYGSMANYLLVNRLPQ
EWGNPPFTPRSTVPFEDPSDYRILINDWPYGLEPDIRHIVVWLRTTIPTDSETGDMTPESRALVQSFIEKTFIDALGPNGESK
VLWFKNWVALQSVRALEHIHVLVRNVDYDTLERWTGEGPKQKP
FUN_003702 CDS scaffold_15 160415 160904 -
hypothetical protein EOG092658ZO PF05620 ENOG41

MAQKAKKDRAKSNAAALNNLHIGSLIVHALFLLSHFLFRARSIWLYVLLSAPAVICEYILEASGRPKYDPTTRALRTAGEDLS
SPGLTEYMFDVIWVTWAAVIFVIIFGNKAWFLWLILPAYGVYLGSGLLGMGKQKMAAFQGAGDGAGAAPQGNRRARRAA
FUN_003703 CDS scaffold_15 161645 162106 + RPS20 40S
ribosomal protein S20 EOG09265CFP PF00338

MSHQKNEKDVGDAPKSHRIRITLTSRKVQSLEKVSAELIERARSKGLTIKGPVRLPTKNLKITTRKTPCGEGSKTWDSYELRV
HKRLIDLHAPTEVVKSVIVNIEAGVEVEVTIAA
FUN_003704 CDS scaffold_15 163592 166129 +

hypothetical protein PF00515;PF07719;PF12895;PF13176;PF13181;PF13432

MAPTPTVASGFLRQVIYYHLDNASYHNGLFFAERLLAQDPRSSESTYLYALCHLRLGDFRSAYDVGKPIGYRGVHLGCAWVFA
QACLALERYKDGISALEKARSLWASKCSLGKHSATTRTALPDAPAVLCLLGRLHRAYDDKKKAVACFEEALQLNPFMWDAFAA
LCDLGVTVRVPNVFKTSDTLVHSFDVETSPLISREGSVPIVPDIPMKKSQRNATMDVASDPFSSSHSPTIHELTNGGSGQEVD
KSEFMQKIQEGRMRYATSTNSSSGFDGMETPPGTMSSVTTSSRIGHSHEPPQAPPRRTRHAQAIDQAQEAPPRMNYRIGSRRT
RAQDKGHQEQSTENVMTDASNAAPSASLRSSIPAAERKRTVSGHPAQRTTNSEEPATTRRSARLNMFRPSSKANSGAAGMGTS
AVRELKKARPPISRIVRPGSSGSSVGRVVSGNRKPLEEAQADIDHSELPKAKEIPPPPPVHKTSELDAQKLEEGLRWLLDLVK
KLANGYYSLSQFQCTESLQHFQSLPMSHQNTPWVLAQMGRAHFEQASYAESEKFFRKMRVQAPSRLEDMEVYSTVLWHLRRET
DLSFLAHELVDSAWLSPQAWCALGNAWSLARDHEQALKCFKRATQLDPKFAYAFTLQGHEHVTNEEYDKALTAYRQAISADRR
HYNAYYGIGRVQERLGAYDKAYTHFHAAQSINPNNAVLITCIGTVLEKQKQIMPALQAYSKAVDLAPRAAQTRYKKARALLAV
GQLDAAQKELMILKDLAPDEATVHFLLGKLYRSMGEKQLSVRHFTIALALDPKASQQIKEAIESFEDDVEMDDSMM
FUN_003705 CDS scaffold_15 167395 167762 +
hypothetical protein PF02969;PF15511

MLGGKGGKGLGKGGAKRHRKILRDNIQGITKPAIRRLARRGGVKRISAMIYEETRGVLKTFLEGVIRDAVTYTEHAKRKTVTS
LDVVYALKRQGRTLYGFGG
FUN_003706 CDS scaffold_15 168108 168716 -
hypothetical protein EOG09265GGX PF04628

MSYYFAIVGTQDNPLFEHEFGTSKQGGDGQSRFSDQVRHLNQFILHSSLDIAEEVQWSHGQMYLKCIDKFFNNYISCFVTAGN
VKFLLLHQPIIPTGTSSRSSTAIGANPTSPATEEAIKMFFTEVYENWVKAIMSPFYRANMEVRSPVFRTRVAAAGRKYL
FUN_003707 CDS scaffold_15 169134 169526 +
hypothetical protein PF09784 ENOG41

MFGAFRITNPLSSGLLWKIPWRLSKFQKRRHRLRLRAVDNVVATVDAALAKKGQTLEALERWKAEMPTEAEMLPKDKYTMFDR
KAKRYRKGIHKLPKWTRVSQRINPPGY
FUN_003708 CDS scaffold_15 169920 170567 -
hypothetical protein EOG09264G04 PF00076

MRPGVRNTVERLDRPSAYYQNRNKRRRDRDDDHDQPSEPQADPLANATTLYVGNLSFYTTEEQVYELFAKCGEIKRLVMGLDR
FNKTPCGFCFVEYYTHQDALDCLKYIGGTKLDERIIRTDLDPGFEEGRQFGRGKSGGQVRDEYREDFDEGRGGVGRAIQAEER
DEYAEGK
FUN_003709 CDS scaffold_15 171004 173731 +
hypothetical protein

MPKVKSYSAAWLSSNAPGHRLFEPSSEASKSRSLESAYSSKKKTIPGPRRTIAHRGTEVFVAVGREIRWGDLAYLKDEWANRY
SKRGSSQGIRIKREDSTQPFDDESLESAAGLRTIKTPVADDIRQLIISPNSNFLAILTAHTVHICVLPDPSHLTSEDAGPLKP
KIFTLGPTTHVTSRSPIVSALWHPLGVSGSCIVTTTADAIVRVWELSTTDRWSFDTPTTSVDLKKLVDGTTLDQDFSASAAAT
NTAFSPDSFEMEVAAASFSTKGAGGWNPMTLWVAMREGDVYALCPLLPQKWAPPPTLIPSLSVSIVTTVASIEDDPDVGEQEK
LLAQQQLEWMGELDSQEPTIMEGPIGEPVTEIYTRPTRPGIIPRLQGPFELQSDPDTGDELDTSVTDMLVIGKKTETEQLMMG
EEEDLDMDDGDQEGLSLTVLCLLSTSGQVRVYLDLEGVQAQWLPPKNKNKLGRLLAEAETPSLLSFQAIDTMTPVEVNEDSWP
VFSNDVTSRYNFFVTHHAAITFISLTSWIFRLESELNGDSEAGTDFRIGLLVNSQSTRDRIYSQPTADIAVPLAACNALRDPD
VGYFLLSATPYEPIALTFDTPEVDLAPIRQESPSYEREASMAPLDFYEPRPVFNPSHTFDEGSALPTLLERLRTSRHKTVVNQ
EVRLSPLTLQIFTDAHKVLADETHRLGMAAAEVFRRCELLQSELKQQVMKANEVKGKIDTINGSHRDGEADNAMYDRRVSAAR
DRQDRLTERLENLRKIVGKATTRDLSAKERAFVEEVKSMEASISAPDYAELASGKSKATPAQAKQLWKRLEDIKHLQADLTAE
VEAMNKATGATDGPATPSAELRIPQEVRRAKLQQVQGLLSRETALVDAVTARLERLQTSV
FUN_003710 CDS scaffold_15 175143 176355 +
hypothetical protein

MAPHLMDSKGFILQNCRVSAESSDADFEVLTRIDLSRQWHAFVLKTEGGKRTKYLEEKAPRPERALELLHEASARLVDQYVTC
HGYDLPPGATKSSSGMRGGSVKPDVIACSSSDSEAFASDSDDSALPPRRSHRRSHRSGRAAGEAANTRPDRAEADSGSDSDEP
TSRRTPFWGGSPPRPAGMPSHGHPPPPQPWGYSMPIQAQASAPAPVPAPIPGIRPGPSMVPPPPGRSISIPPMVGKSYPARLN
ITWPGNGKRNMLVNVSPTVHYLQQVAVNEANMRPTGFVNSSSAPYLPLPNRSSNGHALLRAMVRRVTLDEDDTYEIAAFGNDL
SALFRSTSSIPKFEIEIINANIVPIFPPMPPPPRPASVASSRSSVEIAD
FUN_003711 CDS scaffold_15 178154 179042 -
hypothetical protein PF05241

MADLKHPYFPVDAAIPGYLPNSTPVAELIATFGAIVGAVIGLTLWQTTRTAKPVRPIDKFAAAWFALCGFLHIAFEGYYLVYR
YQLPGMSSLFAQLWKEYTLSDSRYLTHDIFTVSVETITCLAWGPLSFLAVVGILRDWHSRHVVQVIVCTAHVYGVALYYLTNW
NESRVHGVAYSRPETLYFWIYYVGFNLPWAIVPLVLLQDSWLQVARAFAALEERKRE
FUN_003712 CDS scaffold_15 179474 180665 -
hypothetical protein PF04303 ENOG41

MATCKPLSRHIIRSVRLSQTRLMTTSTPASSFPAWFARGGTSNGLVIHRKDLPPESQWHKILPPAMGSPDPYGRQLNGMGSGI
SSTSKIVILGSPSREDVDVDFTFVQVGIRDGSLDMAGNCGNMSSLVGPAAWDSGLLSAQAKAVEQDENGLQWATVRFLNTNTN
KVMSSKFQVEGEPLKYAHQGEYAMDGVPGTGSKVIMSFIDPAGAKTGKSLPTGNPVDVLQLQDGTKIKASLVDVGNPGVFITT
ESLGLADHMSLTPAIVESNPELKKKLGEIRRAGASLMGLDPNTESVPKIVLLFPSSGSPDVDIRCLALSMGQAHKAVPLTLAL
CLGAASQLKGTLASDLIGGQSKETVSIGHPSGKVDIGTVIRDGKIESAQLLRTARVLMKGDVYY
FUN_003713 CDS scaffold_15 181062 181686 -
hypothetical protein PF06094;PF13772 ENOG41

MSSQGDSQPSQDSTMTLMQENLSEVLYFAYGSNLSTKQMRRRCPYSTPVGLAYLKGWRWIINERGYANVVQLPIDSDNDETPE
AEDNRQPSTKGKGKEKQKQKPEEGVYGLLYLVPADDEESLDRYEGVPSAYQKFQVDVKWASANKEEDETLRALVYVDEMRVEE
DVPREEYIERMEDGIEDAVENWGMDEDYADRVMRRFWIEKD
FUN_003714 CDS scaffold_15 182477 185255 -
hypothetical protein PF00069;PF00536;PF00788;PF07647;PF07714;PF14847

MAMLASKASFPAGLGASSLTPVPSQPTMAPPRRAPTMPSSGAYGSPTESEFNESDPAESVKNWDEEQVCEYLRSVKCGEYEKI
FRKNHINGENLLEMDKDVLKEMGVEKVGDRVRLFLSIKKLRTRAYANEKKRNRDSFGGLDIHITAPHDSPRMHTSRGVPTSSS
NKRFSRQLDLHGALDNGMNSSRPNSPLPGTDLRNLRARGKTYVTSPVNAQGQRIVITNPDLPANRLVTTHTRNNSSMDGSLMA
ALPQGQEVIRVISTGGVTKVVKISACNTCEEVMRVTLRKFALREDHERNYCFWVLAGIDPDPGQCRRLGDTELWRIIKDQRRP
ERNRLILRRVPAGEPGEAELQRAAAIAMEEEQQKHARALETVDKRSQLKVQKMLGEKWNDQLQSPLSPISYQDRERNLHNAAK
DLERPPSEIESRPARRVGALRQFGGLRPPSELIASDLTSYFPDHTREDIDRTARLSMRRSTRLSKVNSRLSVASSLSFASSIQ
DAPPIPTIADSWLNANSQLAKVKSRDSHIRVPQNAYNRDSVTSSVLDTLQEESSLEPNRKSYVSFTESGSDSAALSVTDPEGN
TTATSYYDGDNSTGSGSFQELRQALTNDGDDVDEELQSFLAGESWGDDKWMKGALIGQGSFGSVYLALHAVTGELLAVKQVET
PAPGANSQGDTRKKGMIEALKREISLLRDLRHPNIVQYLGCSSSADYLNIFLEYVPGGSVQTILNSYGALPEPLVRSFVRQIL
TGLSYLHDRDIIHRDIKGANILVDNKGTIKISDFGISKKLEASNILNGANNNKHRPSLQGSVFWMAPEVVKQTSYTRKADIWS
LGCLVVEMMTGSHPFPDCSQLQAIFKIGGGKAAPTIPEHASEAAKEFLAQTFEIDHNLRPSADQLILSPFLIPIT
FUN_003715 CDS scaffold_15 188293 189946 -
hypothetical protein PF00324;PF13520

MSESILPGPGPHPLKARDEQEKSPSHLGEVETIRNGSSDPERNEDTHRGFKPRHSQMIAIGGAIGTSLFLGTAQVLRIGGPLF
LLISYGVLSILVYCIVTGIAEIATYLPVPGGTMAYYGHKYVSRSMGFAMGYLYWYSLGILIPYEFVASTLLINYWGTAVNGAV
WITIIYVVIVIVNLFPVRVFGECEFWSAGMKVLLILGLIFLSIVLFFGGGPDQDAIYFRYWKDPGPVNTYIVEGDAGRLVALL
QSFVLASFAFVLAPEQLIVTAGEMQSPRYNLPRAARRYIWRLIVLFMPSVLGIGVICASNDPRLAAPGTARSPFIIAIKNASI
PVLDSIVNALILLSAITAANAFLYSASRNLYSLAKAGNAPAIFKRCNRYGLPYYAVLITASLGCLAYLSLSNSSLTVFNWLIN
LTNTSGYISWICCGIIYVRYRNAITHHKLESPYRSRLQPWGMYFGVIVSIILLLINGFTVFFPSQWSVGDFFTAYIGIPAFLL
IYFGHRLYHRGDGWVRNPEDVDMYEGMDEVLAAERPPPVRGNITRRLWASVE
FUN_003716 CDS scaffold_15 190609 192091 +

hypothetical protein PF00202 ENOG41

MAHPFVSNSNILHRSFADQPERVVSASGVSLFLESGREILDASAGPAVSCLGFGRPEIAEVVANQMNQLPYLYSGARFTCDVT
EELASMLLQGQPGGLSKAIFVNSGSEATDAAIKLATQYWHERGMPQKHHVIARNRAIMETRLEHSVFLATNLEGPCTATGSLI
MLKGNESDEDYVKRLADQLEAEILRIGPENVSAFVAETVSGTTLGCLPAVTGYFVAVREICDKHDVLLILDEIMCGMGKTGTM
HAWEQEGISGPDIQMIGKALGGGFVPLSGVLLRNKIFDALADGSRGLAHGHTFQAHPVACAAALEAQRIIRDEDILTNVQEMG
KVLERLLRTNLGHMEFVGDIRGRGLFWAVEFVQDRRTKTPFPANMRLCHQIVDKALDLGLNILGNLGETGDIHVDHVIMSPPY
VVTESELGRMVGILRESIQTVMSEVIDIQGARL
FUN_003717 CDS scaffold_15 193132 194173 +
hypothetical protein PF11951 ENOG41

MAFECPALMYAVSAWASSHLALRDEKFRADSLRHRGHALAQLQQSMKQSELSTEMCLAVTMVLCSMESISEATDAWYPHLTGA
AAALAWQSEGSLAVVDPKQAVQATFEGRWLLRNFAYHDIMMSVSMDCRPLVRGFYWASEDDTLADPYFGFASRILYLISETSI
LNADFAEAKLGSQTRGYSFAERSQKIESELQSWVCPSGHDGSPLALLGEAYRNAALIHLYRTLARYINSYSGILKAKLKACVE
SICKLSRQVSEGCLVECTLLFPLFMAGGEAHETSEIEIIREKLGEMIKWRKFRNVEACLDVLDEVWRRRMDGSRREDQDKVDW
LDVVKQRGWKLSIS
FUN_003718 CDS scaffold_15 194514 195099 +
hypothetical protein PF12973 ENOG41

MATTTQTVTMISNTSKQEELKERADQAAVPYRGPRLPAVPDDLVVPGIFDFECDERLWVPQAPDVWFRPLLLSVSGGYFVNIL
RVRRSGILSRHRHAGCVHATVLKGRWHYLEHPWWATEGGYAFEPPGDIHTLEVPEDVKEMVTMFHVTGAYIYVDPDGNPVGVE
DVFSKLDKARKHYEAVGLGASFADQFVR
FUN_003719 CDS scaffold_15 196441 197568 +
hypothetical protein PF00800

MASQHKPVVSFLGPVASYTHQAVRQAFSESTWEFSPAVTIDDVFDQVQSGQVQAGVVPFENSTNGSVSFTLDNLADRTNHYPD
ITVDGETYVDVHHCLVGHKSPVPAVEDIAEGSGTCTPTATDPSPSKPRSRPLASLKHIQRLYSHPQAFGQCTAFISTYLKGVE
IFEVSSTSKAAEIVSKDTTGTWAAISSELAAKLHGLDFLGKSIEDREDNTTRFFVIGKNAAGPKDLEKTEKAVAEKGSKSLVS
FTVPHTSPGALADVLSCFRTFDINLTSINTLPSLVKPFQYIFFIEFEGHKYADPEDRVNGALEKISRVAESWRWLGSWERYI
FUN_003720 CDS scaffold_15 198702 200136 +
hypothetical protein PF13469 ENOG41

MALRPIFTATHPRACSTAFERVFMARRDILESVHEPFGDAFYYGPEILSDRFRNDTATREQSGFSQKTYKDVLNEVMDAGKDG
KRIFIKDMAYYLMAPDNKPTKVAPSLGEEEPGNPTVLPLEVLKQFQFTFLIRHPRRAIPSYYRCTVPPLDEVTGFYDFMPNEA
GYKELVRFFDFLIKENIVDKDNLVVIDADDLLDNPEKTIRLYCEKTGIDFKPEMLEWNDEDCNYATIAFQKWNGWHNDAIKSS
ALRPRTHHQKTMTTESEDKEWTAKYGPEAQKVIRKTVEDNVADYEYLKQFALPI
FUN_003721 CDS scaffold_15 200620 201449 -
hypothetical protein PF12829 ENOG41

MNTVPTSRLGQLPSYVRIWVRYGHFGKKKDGRGFPPPEGHGEDIWVFGHRRTNQIIYSFDKTLNGFHDLKQLPFNGKKTKPAK
LRKDYWSPFAHISFPAGQGSIGRSVFQKLRELKHLHEVAWDDDFRYKNPEEFSEADRKRVAKQQEKGNMDHRPIRTREERGVA
LNAQKPNSIADIASVLGGAGRGNKIVLSEDAEGAEKKLLDVKVNWANDQDREYAQKWSGNVTHGLFEKPSYISNEPEKKEEKA
PEPVAE
FUN_003722 CDS scaffold_15 202311 205086 + HSP98 Heat shock
protein hsp98 PF00004;PF00158;PF07724;PF07728;PF10431;PF17871

MNSRMDFTDRAQKAVEDAMALAEQYGHSQLAPVHLAVSLLDPPPDPSKDQQNGPPPTSTLFRQVVERAHGDPQLFDRALKKTL
VRLPSQDPPPEHVSLAPQFHNVLRKAMELQKVQKDTYIGVDHLITALSEDSTIQGPLKEANIPKPKLVQEAVQAIRGTKRVDS
KTADTEEENENLAKFTIDMTEMAREKKIDPVIGREEEIRRVVRILSRRTKNNPVLIGEPGVGKTTVIEGLAQRIVNRDVPDNL
KSCKLLSLDVGALVAGSKYRGEFEERMKGVLKEITESKDVIILFVDEIHLLMGAGSSGEGGMDAANLLKPMLARGQLHCIGAT

TLAEYRKYVEKDAAFERRFQQVIVKEPSIPETVSILRGLKERYDRHHRVTILDSALVAAANLAARYLTSRRLPDSAIDLVDEA
AAAVRVARESQPEIIDSLERKLRQLMIEIAALEKEKDEASQTRLAQARKDAKNVEEELQPLREKYQSEIKRSEEIHQAKLKLD
DLEKRLEDAMNNSEHAKAADLKYGAIPEQEAVIKELEARKAAADAALNATAPTDSGGAMVTDIVTADNINEIVARWTGIPVTR
LRTSEKEKLIHMEKVLSKVVVGQKEAVQSVANAIRLQRSGLSNPNQPPSFLFCGPSGTGKTLLTKALAEFLFDDAKAMIRFDM
SEYQERHALSRMIGAPPGYVGHDAGGQLTEALRRKPFSILLFDEVEKAAKEVLTVLLQLMDDGRITDGQGRVVDAKNCIVVMT
SNLGAEYLVRPGVKEGRVDPGTRELVMTALRNYFLPEFLNRINSVVIFNRLTRKEIRKIVDIRLGEIQKRLEDNGRKVHIDVS
EEAKDYLGNSGYSPAYGARPLSRLIEKEVLNRLAILILRNNIRDGEYARVELIDGKIVVLSNHPDSELGEDEDMVDEEDAVDE
MLDDMDQDIYD
FUN_003723 CDS scaffold_15 205430 207723 -
hypothetical protein PF00172 ENOG41

MAPTPSSDESPASTSGPGLTRAPDSPQSKIRRNVACISCRDSKVRCRASPVTGQPCQRCAKLQIPCVYDRSHKRVTRRSKLEL
LEQELRSIKEVVQPKSNAESPSVSQRNGTVFPTKPSQLPPATNGTVFSLSSNLQSPQQPQSFVPSATLLEKTEPTKPRMLGGK
LVSGQDIDWYFEKYLQCYHPYLPILRKKDPDECFEASTTLFWTIISTACRRYAKDEQLVTLLLDSLNRDVWVLLQAITLDLES
IQALLIICTWPFPSIRFVTDPSPNFISSALNACMLLGLHTGRGSHPSFLIGGRQHMTCTDYEASITWVFCSILAQRVSTGNGH
PPPFLQHNDTKCKDTIKDTLAPELLTFFELQKFSNRLHTAMSAQISANNGVPETIVKMWEAEFELLRPLVTRVETDFSRFIML
VAQLEVQAYYYISPPDQRPNFTLNTLRTYNTSQNLINTALTLESTCQLLTHSPHWVYRALVDASCILLSTLHSTAAPQHLSSS
DAEVVAAQVLSLLKTCSVRDNDLPTRGSIILETFWSVRHLLPKWDIPVGAWPDRIGAATSYWCLTAFKNALQEAKNITDGTQK
GIDAFQQRFPGNNNGDNNESNVNANTDINGQEQTMDSAIDPLQGIDWSMIIDDFGWIGEGPVFLGPA
FUN_003724 CDS scaffold_15 208132 209723 - PBN1 protease B
nonderepressible form PF08320

MRERVTFIHNDYTLDPEELDNQEAGLLGPQIESVRQDKLTIPFDELPSELTDILQEYEALHIRWASPVKSETLDPFTSRISAG
LHVYVTPESPNSCNPTKLCGWLQRFGPLACSKPEAFTEFKQPATSTAPNFSFHQALEDLHSFITVSSQEFCPELDTVCNARLR
SLLTATSLDLSFDKSTKALVASALWPLRPQTVAVPASTERRVEVGIFVDDRSQPNIKENELGVAGVLSVLGDKKKPSPTIFTF
PARHRRDESVFTSRFLTPTGLHPTLQLSFSSNKPPSAEGECAPYAFLTLPKTIFTDRYQLGDDLFLASKNLTALRYTTLPVDL
EAPAYATEAWGSSILLELAPPTSEEGQPWNVEIPLHLRYLKPSVTGQAEAEVPYPAVFWACSSGEETLENPFDRLHIGYDNLF
PRDTAFWHATPQPEGGSRLMNRVTVPVLKLEGIDTIRSGTAIAVSLGFAWVLWKLVGVMLKFEKPVLKKIAPKRSKQTKKA
FUN_003725 CDS scaffold_15 210661 213793 +
hypothetical protein PF00046;PF00581 ENOG41

MADHQPYVTIRSRRPVEGPGSRSTVPPMAASSGGDYYARSATHDTSRTSFHVKHIGQFAQRLEDSAARAFPNRGRSSARYSKV
QALLLHWNSDDLFVIPELEDLEKCLSEDYGFNTDIFAIPSENSHLELMMRIGQLIKDHESQDTLFVVYYGGHARIDESRQSTW
CATREANSPWLQWSAIQTLLERSLSDVLILLDCCAGAASATFPSGASITETISASSWDAIAPDPGRYSFTNALIEVLQEWRVR
AFSAAMLHAEVLARLKHPRPITINGKYFEARSTPVHFMMTSNHKAPSIEMSRMSRGDNLPSPELLPIPAMVHETGRAADSTTP
VSRNDYMFTEPNEDTPHVMISLALEDDQRLDINAWEQWLGAFPAMAKYVKVQGVFKSHSTMLLVSMPVSIWDLLPEDHATSFV
AFIRSNNLMTQKPRNQSVPTLVPANRYPVENDSASFISGVSGTTFVPTELTGLTGQMGAFRDPAYGRQTGTVVRSTLPPIQQL
SPLHMPSQPGSPPQSLPQRSMRPMHSTTSLTTLQRQQSSSSLGGSGNLTRQMIMNQQQALRRTTFGSDVPEPKKFSPHVEKRL
EEYYQTEPLPNDGQKAFFASNLGVEPWHVEVWFHHRRERDAFSQRFAALRVEDSKAGAHEGPRMILPANLSELLDISLPGQSL
LLDLRSSTEFQRSHIRGAIHLRAPQSFLRPASLDMIERAFPDAQSRRTFSRWQQARCIVFYSRGLEYPWECPSADTLLEKLWA
CGWHGRCFILKGHYREFSDSFGKHVYRAQDSDAEKGPPKEAPISEGMPSNERELAALFARLETEDQTLHLSSSPGHNEERTTA
LVEQEKALESEFQNHFPALFKKAQDVHGVGRSDDETFVTKAQMVEYLDRGLTKIRDGQASQAEATVYEPGHSKLAADSYFDRT
NVNRESDEYVEVPRGDEVASDGSPQMTSKGKEIRAVSSPDEITRRGRGGGLLNKVFRRT
FUN_003726 CDS scaffold_15 223114 224485 +
hypothetical protein PF00046;PF05920

MSMLATASPSPHPSSFGMPRPWETNRCPDYSLRPRTENDKVALPSIRQAFPELQLQTQPPHDLNTKPPSTGPPLGAPPLTAAS
PQYIHSPNSSKRRRLSMEREVETERVRQVPRLCYSPDRASPRQISPHLPIQGGSQENWAAPTRTSPFLTNGSNSHSAPMEASE
RAESRPTLPSLPPPRSLERELPVGRGTAPSDGYRPPQQSMSHSRTPISEPGVSPYRENGYGYPYHHPTRYQSLSTGSSHSYDR
TPFTPGTYNTPYQDFVRFGDMSSASLSGDNKQRKRRGNLPKETTDKLRAWFVAHLQHPYPTEDEKQDLMRQTGLQMNQISNWF

INARRRQLPAMINNARAETDAMTGARGGDLKVLATTERGDFDHSKREPAGPLSDGEGATYDEELEALSQRRPGTIGRGSV
FUN_003727 CDS scaffold_15 230616 233349 +
hypothetical protein PF00172;PF04082 ENOG41

MPPDTSEPDPSGQPESAKTEPLACVSCRARKLKCDRTKPACTRCVKVSNECVYPESRRKPNFKRRNVKELEARLAQVEDYLKE
VNKNSSEEKTDDGSPVQPPQANVNFFDMDFTPGLGQTINNLPDPDFSSFTSPNIVQNPQESPQSLNAQLMSLGMTEPLPPDDV
MEELNNIFFHNQYRMIPIIHPGKYLQSFYGPLLRRPPMCLRYAIWALAATGSTKFDEYHDVFYRRSRAYIEADMMKGHGDAFI
SVQHAQTWALIAFYEAKHLLFTRSTISVANSVRLCHMIGLDRLDGDPLGMPPALGPPLTWAELEERRRIFWGVFAADAHCSIS
TGWPSLINSEDIATRLPASEEAFVEDRKEDAPFLDEAIQGAQYTGFAGTILTCEIFKIILRHVHRSKPDARNDDLMHGSFWTR
HRDLDNTLSSLFMFLPEKFRLPENVRDVSALHLNLNLHAAIICLHHAAVEKADKYNLPDHVKQGSVTRLRAAAEEIVNIMRLT
ANGTLFFKSPLSALSLYCTTTVYVYLGKENPVNGLSTIDLSNFRLIIQSMEAIARVHTITRTFLQQACLDIEKNGLDSVIQVP
SLRRYRNAFGPSRSRIPMLARSSVAEHTGASPILFASNFTQEKRMDAEKSRLDKVASAMSLGKTCYSAVLGAAARNVEPADRP
DPETNKRKRTNESPGPEIMANMRSSDPLNLNLNPNFRGTGMWPASFSPRMASGAISLPDRTNSSAASSPANQGSNQGSYTKTV
SGSSHTSPDVGLGNSAEENRIDLRAFQERISTPIWQSTEETLFAQMAANTIPLGDGDPWGILNAVDLSWDVEGLPAGS
FUN_003728 CDS scaffold_15 233754 234628 +
hypothetical protein PF13640

MFQYVIGLLAIILVFSNPISDFLYPDRATRINDVSPRPKFNESLLAIDPPNATAPECAPDGYVARILSREPLIIYLENFLNEG
ERKHLLDISEPIFVPSTITNNGEATHRDSSVRDSHVALIPRTDPVRCIESRARALQGWRPDLWIERMRTQRYGPGGHYNHHFD
WSSNTRGWGRVSSLMVWVEGSDSLKGGGTEFPLIKRPAETEWCRFIECPDDMEKDRGTGVTFKVVPGNAVYWENFAADGRGYE
ETWHAGLPVQEGTKTGLNIWSFGRI
FUN_003729 CDS scaffold_15 234732 235429 -
hypothetical protein PF01161

MTAYIELAASWLFKNSKGRDARAFFTTPAFAEHPEPTLAVTSPDCGPDGATLGKDYMHGDQHKFPELSWDPHSGVKEWLLVSE
DPDAPLTTPICHGIYLGIPSEKTRVVESDFEPVEKSSTRLAGGFYYGASRNGSIYIAPRPLLNHGIHRYWYEVIGLNEPLDEK
FKTSKPNRDQVAEAINGKVVVWGRWMGQCERRWE
FUN_003730 CDS scaffold_15 235907 237397 -
hypothetical protein PF00026;PF14543 ENOG41
A01A
MDELDRSTYKVSIMKSIQLSSLLFSLLSLTEGISLNKRDNGLEPRVMSVEIQRRTISDPISNDRRRLRKRDGTIDIGIDNEQS
LYFLNASLGTPPQDFRLHLDTGSSDLWVNAQGSKLCSTHANICSESGLYSPNKSSTYEYLNSDFNISYADGSGASGDYATETF
RMGSVKLEDLQFGIGYVTSDNEGVLGIGYKSNEAQVGQLNRDAYDNLPAKLASKGLIASNAYSLYLNDLESATGTILFGGVDQ
EQYTGDLVTLPINKINGEFAELSITLQSVSADSETIADNLDLAVILDSGSTLSYLPATLTSDIYDIVGAQYEEGESVAYVPCD
LGNDSGNLTFKFKDPAEISVPLSELVLDFTDVTGRQLSFDNGQAACTFGIAPTTGDISILGDTFLRSAYVVFDLENNEISLAQ
SNFDATKSHILEIGTGKHPVPTATGSGSSDNKENAAASLAPLGGDAAISMVAGAFALGFAWMLI
FUN_003731 CDS scaffold_15 239573 240038 +
hypothetical protein PF00137

MMMQNTLCAIMAQILSIYGLVASVIMSNNIKEKMAIHTAFLQLGAGISVGLCGMAAGFAIGIVGDAGVRASSQQPRLYIGMVL
ILIFAEVLGLYGVIVSILMLTRSTDGVTECRY
FUN_003732 CDS scaffold_15 240218 243219 -
hypothetical protein EOG09260HS3 PF00637;PF05131

MALDLQDGFVAANGVPDLEEPIFTIERVQLQFSVAADFVAAQVANNVIVLALSNGRILRIDLERPEDIDDIDLPKKPSEIGMI
RRMFLDPTASHLIVCTALGENYYLHSQSKHPRPLGRLRGVSIESVAWNPSLPTASTREILIGASDGNIYEAFIETSKEFYKKE
VKHLKNLHKLPDGPITGLWVDNLQNNKSDLRRVVIATQTRLFHLVGRIGFGHDGSGSVYTRLFESEQPVVHELSRTTSGAPSS
LAVSPDPPDSGPYDDDIPDRAYAWLSYQGVFHGKLLNQPVDSNLGTKVFSESHMLSRAQILSPENSERRVPTTEAIDAIALTQ
WHIVHLVGGRVITTNRLTGKMVSEHDVIGQGQKPLGFSVDIQKNTFWLFTSDEIFEIVVRDEERNIWEIMTKLKEFEPALQHA
RTPLQKETVAAAYGDHLAKNGHWLEAATVYGRSNKPFEDIALSIIDNNQPDALRKFLLTKLASLKKSAVMQRMMIAGWLIEVF

MSKLNTLDDTINTQAEPSEHLNSTESRKLLESVRKEFRDFVDKYKNDLDRKMVYDVISSHGREGELLYFADAVNDYNYVLSYW
VQRERWNEVLNVLKKQTDPEVFYRYSSVLMTYVAPELVEILMRHADLKPRNLIPAFLEYNRTFSGGPNAQNQAIRYLNYAVYQ
LNSKDAAVHNTLVSIYASHSSKDESGLLSYLQAQGDEPRYDPDFALRLCIQHHRTLSCVHIYTSMGQYLQAVDLALSHGEVEL
AAVIADRPMSNPQLRKRLWLAVARKVISQSNGIKTAIEFLKRCDLLKIEDLIPFFPDFVVIDDFKEEICAALEDYSRNIDNLK
KEMDESSQTATNIKVDIAALDHRYAIVEPGEKCYTCGLPLLSRQFFVFPCQHSFHSDCLGRKVLEQAGVGKSTRIRELQTQIQ
KGLVSGTQRETVVAELDALVASACILCSDLAIKRIDEPFITHNDNVNEWAL
FUN_003733 CDS scaffold_15 243976 245393 -
hypothetical protein PF13489;PF13649;PF13847 ENOG41

MAKKRRTKQRKSTRGRHNQSQTSTMSSEDSYAVQSGRQSSDGTPSEHANSDGNNTAMTSIMGTDEEHHHDESDLDEDVDEDDM
ISIYPASQYPRVSASHRVETPFSPDSHAVDSDMAASTRSLWAQDLDYREIHGRRYCREYYMPNDDIEQWRMSLQHQVFMHVLD
GELTLVPLQNPEHILDIGTGTGEWAIKMAELQPQCEVVGTDIAAIAETKSVPMNVFFEIEDAEDWDRHPDMYDLIHFRLMEGA
FRNWSFVYDNTFFSLKPGGWIEVQDFVSAEGFTQFLSQFPDNSAMHELLRDLLIASEKSGRPRGTYHLEPRLLMEAGFVDVRV
TEYSIPITVAEASAGKIWLISCLDSFEAHCLRLLTEYMDWDPEKCKQACEAAARDLANAAKDPEKSKGLQIKMRIIIGRKPLD
APPTDLAEFLGRSYSPVTELNGNSPDPTLRPDDQTTAGLS
FUN_003734 CDS scaffold_15 248819 250368 -
hypothetical protein
PF01073;PF01370;PF02719;PF04321;PF07993;PF13460;PF16363

MTASSDQPRTNGDASVGINNHSKQSDIWKHAPVLIGTTKFEPRSDVKNIMITGGAGFIACWVVRHLTLTYPHAYNIVSFDKLD
YCAALNNTGVLSESSNFTFYHGDITNPAEVVDCMERYNIDTVLHFAAQSHVDLSFGNSYGFTHTNVYGTHVLLESAKKVGIGR
FIHVSTDEVYGEVKEDDDDLLETSILAPTNPYAASKAAAEMLVQSYQKSFKLPAIIVRSNNVYGPHQYPEKIIPKFTCLLNRQ
RPLVLHGDGTPTRRYLYAGDAADAFDTILHKGQIGQIYNVGSQDEVSNLELCGMLLDRMRIPHDTPEQLRKWIKYTRDRPFND
RRYAVDGTKLKRLGWEQKVSIDEGLKITVDWFTQFGETWWGDISHVLTPFPTVSDGEMVPDDARSMRDEPLESQDIRQGKLPE
QHKKDDMSGGCRETQQNGEDTSGLHQNNGGGYQITA
FUN_003735 CDS scaffold_15 252259 252748 -
hypothetical protein

MASVDPPYLRFRMFYFFTKRIICEDPPAMRDLFDEDHIIFLAGLSLSGCYIRIQRTFFLLIKKLVRGPMDELQADYEDYIRSR
SQHPVEFQNVTNDSAGKSPEFLLTAMILKVTNKVKEHILDQMPTPMPSPPAPLRDADSITATPAGSPAVPQGAEDRASA
FUN_003736 CDS scaffold_15 253511 254759 -
hypothetical protein PF04427

MGFTKPPVQALKSSNKLKRKELFVQYKKHQNKERHEERHRRRREEAKDPELKAARLAKRKPATIDSKRVWDDIDDDSLGVQVD
LEKLKRRRIEEAEAAEQAAHEAAMRAEEELDEDDDVDSMLASDEEMGEERQVALEKKRQRRAQRDNSVAPSTTSTNLDLTPTS
LALKFPSLFTDIPAPEPKILVTTSINSTLHEQAHVLCEFFPNSSYVPRSAHRYGHKYSLREICKYASNREHTAVILLKEDSKK
PTGLTVVHLPSGPTFHFSITNWIEGKKLPGHGNPTNHYPELLLNNFKTPLGLLTAKLFMTLFPPKPEFQGRQVTTLHNQRDYI
FVRRHRYVFREKRQTEKSIVGADGKEVKGVEGIRAGLQELGPRFTLKLRRVDKGIGRAGSEGDDATQWEWKAKMEKDRKRFNL
FUN_003737 CDS scaffold_15 255030 257028 +
hypothetical protein EOG092629WJ PF03914;PF07540

MAQNGTKRRKIAHDSADHETQDQNDSRSAQKSFFKNASNWNLEQDYETRPRKGKKQKESNRLPIKTADGRIEQAEGQDDETAS
IESDTEWLEGREDEAEEEPEEEEEEEKEPEIPEPQQILAAQEELAKIAMQMNENPEEHVGAFKALAKIGQSKIIAIQMLALMT
QMSVYKDVIPGYRIRPQTDNGPKEKLSKEVRTLRQYEQALVSGYQNYIKELARCSKLEVRTAKDGQSLATIAITCVCTLVTSV
PHFNFRIDLLKILVTKLSRRKINQDGVKCLEALQTLFKEDEEGRPTNDAVALLSKMMKAREFQVDESVVNLFLSLRLLSEFSG
KASRDHVENDDAPLKRKKKEFRTKRHRKALKEQKALDKDMANADASVSHEERDRMQSETLKLVFATYFRILKLRLPHLMGAVL
EGLAKYAHLINQDFFGDLLEALKDLIRHSEEDATEGPGDEEGNEDEEGDDDIPIRNLTREALLCTVTAYALLEGQDAHNSRND
LHLDLSFFTTHLFKSLLTLSTNPDVELTRPANTTNATTKINVQTTTVLLLRALTGILLPPWNIRSVPPMRLAAFSKQLMTAAL
QLPDKSCQAVLVLLSDVAHTHSKKVRSLWNTEERKGDGRYNPVSDSVESSNPFTATVWEGELLRKHYSPKVREGVKILEKGMS
E

FUN_003738	CDS	scaffold_15	257305	259164	-
hypothetical protein				ENOG41

MSDRGYQDPSKVSDAKALQLLFKSKSGAKGQSNIPKKRAGPSMQVARPAPVTPSGSTTPSTKDRFYTPTLLPNPLDRKAGAIL
GSSASDFLGRRDATPKLSSNLVKGQDHKGEAEVPSTASTVPKAKEVSSSPAQKESEKPVVKLPPTAAVILEAPKPDSNFIQGQ
SEKAGAHATSTNPPTVSKAPERVSNLDLLCGLATDSCQASIKASTAGTADHPQTPAQNINPSGRESKKKSLWELFQNQKSDDE
SDGGVSDCQAKGVAIHDSRTAEPLKYSPDKLLELKVNAKIGVLPPDCIVKRRNNSGKADIAAAYKSAASFITNSTPSVSISQA
EMTESTVLRPAAPQTSSQQAKLDSNQTPTVTRGEPAQVEDLITLEEVQIELKSEAAEKVPGTASLDHQRWMSGLTYDDLADLQ
TSSEHTKLDSIQALTEAKSESMQAKDLVAFKEFQGPQESHGLRPQAPGFVPRLLHAPSATSMPAQYATPAAIDLDGKEATQHA
STKTFGSAGLIMVTVTPVAHLSNGLLITGPPTSQLMAKSPFNDTFSTELPLRTADDNEEVVRAPRKPTKGLGASMWAK
FUN_003739	CDS	scaffold_15	260553	261430	-
hypothetical protein		PF08228

MSAPASQGPRKKVVHQLDTPFSTVPWPSILTEDQDTILELLCDLLSPIGQYRQTQIKRSKGKRSLQKEKGAKKTAHDSEQPPV
PPMPEIEASIDVGLNSITRNLQLCSRQDPESMGDEPDRQYSMVFVARGDHASTFNCHFPQMIGAASRKISPERKIRLVGISRP
CSERLSSCLGVPRVSSVAICMDSPGAGALQEFVMKKVAPIEASWLDVSPDAQYLAPKINATETTVGPKRARVEQGRQIT
FUN_003740	CDS	scaffold_15	263678	272035	+
hypothetical protein		PF00443;PF12030;PF13423
	C19
MDPASRSPVLPDRAASAEASSTRPNPFDDGNISSRKRQRTSLSGSPTNSHSTVNPGHDSSRSATLDTEPDLPRPGSTAVDSHP
DSVAPNTSDPGSPVCDPLTEPPSSMATLNLRNAPENDSTSSPPPPNVAQLVAAETTACGFKDQVKESVEDAEVAMVPPPQNLD
TPRSSFSHSASPPIEVITVQSDDDMASDRQSIGVSIVDDDSVMVDPINEFPFREPTETLEGMVVRLSNYIETQSSIDAGVIYK
LQQWLGRYLDYIATANQQLVVDSCRLNLVFWLTFPRIITAMAAHKPPFLVDDLLRHPTKAIFLDYARLSAWFVNQDIHMIKEF
IAEQGGRSQQTFELFCPCYLQNLHSITNPLLLSDDQYEHFGALSTAFLKAYQDSPGGSLSRLLQLIRALFQVIPTYPRLTNDL
APICLVASDIMEDSCNFVTPEIASKSNQKLLIGHTLLDLIYERLDIMIDKSPTHLIADSVLMIVQALSNMINIALKGDHAAAT
LMLEEHRAEFPAVPLRYTAEAISYEHRFRFLIKLIKSSQMQLRFQGMTQMCKELIAFWKRHYESKADDGSHYVEHIAEYLIRT
GFIDYLLGANCHPEIIVEGANIIGFIIVTRRYRRMHIDRVWEGIASRQDPRTADALARMVIQIAHLFDDDGFLLLCEKFQTLP
IDAFTPVIRMLWEGVMKHMADKCSLDRPLVVQPYNLCLRLLRDSSVCASGSQVMYPDIQQAAMQKLRELLRCGPNTEGQQQLY
LGCLQDLANKSATTLGSLWCLFIALRSRNLSQEMHWLVEHHDLTRLVIEELEHAIGAGQAAGILAVLSCSVNQPRKEFIANLI
QLEPLSITEDLGVKLWGMLVGPISLSPDDRRVGWEILNSLHRRNNNGNPFLQACLTYHLPALSSEYFCEGMLDFLRIEILPRL
NDNLNLALDDPEAVATSGIEQLWRLILEAEDETLVDRAIRTLAVDIYIDSRFFNSISTQRAQSVHLKLASRCLEQLKSAAQEL
RNASQGTNSDDQVVVTAITQRQLRQQERVFTRSLKFLRYILEAHQSKPSLSAPDLRTLIPQASHEVQGDSAGLKYQSFDGDQQ
TDIKPLVIGKANTAASLLASLRQETGFENYRIYYRGRPFLPSEQDICRSLEDLCVHDGLILVRREEGGPALSNRVRPGASPLE
IEISAHFDEMWEYLSMDESLAQEIYYFLIKLPTDGHFMRLIGSETTSSKDIFPSGQPFKSLYAVHALIEYAETLLPSQLISHL
NDATTGSGPDPVILPEALKTPISFIVQAISDEDIFNGASVLLRLKLASTLLHAVRQFLDRVGTLRGSAPPQGLPLPDPNRLVK
MLSYAIDCPGDIPSPVIAGALVICLRLSMLDDKFWAELSTNPDFNDILHRLLLIDSRQTMRSLSAKLVEELFTVMSTATLISE
TDYETSVRSRECSMAEYFWKVTSAMVRQTTVFPSQCEELLKLVYFLVVKINARSPELLNIEKFASQISQLLLEHTTTETIGSS
SVDDSLARGLSSLLHICLQLDNSVAQSKTLPSNLAMSLVWKHLYPPKGQRSGQPVPKVILNEETRAKLSDVLFTLVKHDQKKM
VVVVEALDQQVPFFEDEEDDHYIYDLTYHFDRHRALRASCGYAGLLNLSNTCYLNSLMTQLFMNTTFRRFILGCRINDPANSQ
QLLSYTQKLFGHMQESYRRFVDPSNFVHSIKTYDDALIDIHNQMDVDEFYNLLLDRWETQLSGHDEKRVIKSFYGGQLVQQVK
SKECEHISERLEPFSAIQCDIKGKGTLAESLQAYVDGEVMEGDNKYKCSTCDRHVDAVKRACLKDVPDNVIFHLKRFDFNLRT
LQRNKINDYFSFPDQIDMRPYTIEHLSNPKSDIEEDIFELVGVLVHAGTAESGHYYSYIRERPSSSNRPSWVEFNDDLVTPWD
PAQMEYSTFGGPDHRPIYDTNGILYDKNYSAYMLFYQRSSSLRAEQEKMLALAIPAPLRVGVPDHLADHLSDENTNILRRHCI
YDPSNVKLVQVLFRQSRQHCRSIGRSEKNSSINSFMAMRNQEHGLQDLAMRTLNGNLDQVVTRTKDTPDFLSYEEIIDDAVTS
CERCAFSFYEYFNQHPSALRMLLQRNPDQLVRSKIRNLFKVAVTKISKALPNVYDPEIRYKLVHDADGDETLSEPDAPHRSVI
DGVMMIFQHLWKFFHIHIRAWDEYFGAVLDFAEMGHRETGYVLAANFLASAIRIISADPLQELSGNWARMLQGVIRRNNTTKP
TSYVSIIKLVNHLMSQMRPQIGAEYVEDPTERVSQLVEAFSWTSEEVDLIYSSPNGSYTSLFVEKLLTLDQEHAGCNNIIRIL
TRLDNSMDEKVLGTLKLCIRGETSTQAMDPFLRASITYLESTEQLSNAKDMIEHVYMQAKSLQNTEGVYFVQLFRTALDLHQQ
DREFCKAVRTFSRGLIPKWVPFLLASPEEQVRRSTDDFLVCELGKIDVGATSDRDVTLDDQVSIKQMIKQIGLMCLLYLRDHH
VRRRAQMSREIADKFLKVIEGCAATAVATTPDTQTELDVEFLTLQEDVLNPFRRLIVDELEEDGSDWEGSCGSSEQLDDIVEM

NIPGMNEINDLEQMS
FUN_003741 CDS scaffold_15 274262 275831 +
hypothetical protein ENOG41

MSLKDRISSPLEAGTSILDAHHLPPHLAPALEHVSSRLARKSQHITLVVARRDYQLPSVVPPKGLLTPTTPSPLSPGLRLNLS
QGPVSRLKSLVRTGSSTSLRSMNSPRSALSSPGQVPPLEASSPRFRWPLSPSTPMSPPPMTPCTASSTTTDTMSSMMSASSGF
GMRLIHTNELHPRSEKILNSILTKTEKKFAIGTEWLSPAVRASSCGLTNQLIHSSIIQNEVLFCSEGLTVLSLDRLYSLKSAL
SSYSKTKSHLRLEDAVDELRRIILATNGAKVSKTAILRSYDWLSVSNWALVDLDRMYRRAYGGPEQYGGISGLTSFAEPAIQP
ISDSQERYKEEEAEEATIGIAVSKGKTPSPKPLLKLQTNFTPGPILKPKPKKIEVPAPVSVPAQAQAQAQAPTQTEVEEEEDG
DRTARPTDQLYFTMQQWEPPSTIDQILTAGPINRSAFSPLTPTPIMSPASPRFGPLTPHDYDDISPTTRGEWGFMMADNAFQS
GRKAGVEIF
FUN_003742 CDS scaffold_15 276058 277210 -
hypothetical protein PF10253 ENOG41

MGLVDYSDSDGSGSEAEVQAPVKPAPKSSASSKKPFQKVVDRSNPGKIVVSLPQLVNDKPQAEEPPAKRTKTAGGGRFSGFNS
FLPAPKNANKPKPVASSSSTARPTFQFKTSAAPGFSRETGGDLNNSNDNADGMSLPTPKAAAQPHIPEGQKPADEVKLVGKPL
MFKPLSVARNPQKKKKLNSGLAKPAPTPTHEQAQLKPAEATPEATPAVPKKVSLFSMHTEEPSEPVENSSSGTYEPMFATAES
SLAYDEGAYGDYASHAQAGPSATTLLGSESLDTVVEDLNLTAAQRRELFGRNGLGNQAAKKVINFNMDKEYRHNEEIRAAGEE
QTHNPVRAIQGGGKHSLRQLVQNAQSQREALEDSFAKGKNNRKEAGSKYGW
FUN_003743 CDS scaffold_15 277600 280185 +
hypothetical protein PF16978;PF16979

MSVIQLEELVSYQLRTSYLDEIADGVGERLLNVNDGFINSAPFKAAGWRPNSSHHKRTHSPPIPTAIASEYFQAPKQAGLTLE
DGEEDGGMLTAGGSDTMGPGMATRRRRRREQMEEEDSSDLSDESDDDTDQRAAQQIKFAKMPVRHRAGSSPLQNTNLRQVGAV
SPRAPRRGSQSALVTVQERPRRDTVTSSEISSENETDIPTVQRHREAARAAARAAKLQARILEEPSPGIQRADTSLLPEEEED
SDEASDLSDNYAESIDSASILDGVENAINASPTRQVVGTPPKNFVRQSTIRKSKPPTHPIVLGALPPPRPMSMIRPLSVAQPK
SLLSAALKAKDKKSSIPFQKFAHFSGQGTQGSIAVRIYAPFSKTPSKPFQVLIRPRVHDGQGAERVVTVADLIGLSLYRYNEE
KLEPPLPKGKLNINWWTLRMVEEGGEVDDDFPPFERTKPLTSFTTVNNAAARGGGRMRSNSTAYDDFALAEASEEEYLENKSL
TPQEDEEEEPATSQDSGGGVPLTPTEPDADATPRGTPGPAVNPFLAERPRQNPIVTTTYRSNAPPADIPQAPAAAPNTSRGQQ
KLLRIHIMSSDVAPGQMVTLDVMTDTYLAEVLDMVCRKRQLDKANHVLKLPGSGAVVMLDRPVSSIGNVSDLELYRRRFATDG
PLTITGSPGSSSPKMLPLADQAMQKRSKKSQTPMVGSHPLARESLKQDELSNASYKKYTVWRKQPMRIVGMSERILVIDGEYI
HIMPASGGKALHDGSGKTTTVHFSNVVGCKVLRKHPTNVKLVVYKATESKRYDFEARSALEAAEIVEELKKGMPK
FUN_003744 CDS scaffold_15 283386 284010 +
hypothetical protein ENOG41

MKYSAVTLLAALAAYSQAQSISIPGGDEVSSIIGDVSSRIDDASSIIEDVSTRVGDEVSSRLGDISSRISTATETGASTTVTD
ASTATESAETETETGSNTVDTSALASVTSALASASSAIEDEIASLSSDLATATGTASEAIESKIAEATSRAGAAASSATGDNA
GAMPTAAVAMGALMGGAALFANM
FUN_003745 CDS scaffold_15 285845 286898 -
hypothetical protein ENOG41

MMGETEKSPRRRHILSSINPDKPKSRRRSRSPESRSPVDVKREPSLDATSEHTDAQRNDKDDEGDTTAPRRSRIRLKDHHRSR
RRSRDRHRHRDYDDDDSHGRSPVRHRRHRHRRRHRSPTPKNPHEPEPLDPDAAFRESLFDAMADDEGAAYWESVYGQPVHVYP
NERVGPTGHLEQMTDEEYASYVRQKMWEKTNAGLLEERARREEAKKRKASEDRRAQKLQEDMEYSIRRGEERRERRRWEQRWE
DYTKAWINWEGTPTEIAWPVEGGRIEDVDEPTVREFFVNGLNPQEIGEKSFVAKLRDERVRWHPDKIQQKLGGQVDEETMKGV
TAVFQIIDKLWTESRPKT
FUN_003746 CDS scaffold_15 288357 289701 +
hypothetical protein PF02535

MPNSSFLAGALPLSMSLSQSQLRLLSSVGVGILVGTSLIVIIPEGIEAATAPAEAAHMHRVRSLVRRTPWSHAVLTRGLPESI

VTISTGSIEKRNDELDTEALVRRIINAAAGNGRVKRADIETAVEATGDDTPATPGAGDTTKDSTTDNKGSETAESGKDGQQSH
DGDHEAEHEKEEAHEHEHEHAVPTFEIGFSLTLGFLLMFLIDRLPRHATESLHSAPQTRHISLDNLNGDSASVDEEADGFLGS
LTPTPRRARSLATTTGLVIHAAADGIAMGASSTTSDMKLGFIIFAAIMIHKAPAAFGLTSLLLKQGLSKRAARGHLIVFSLAA
PFGALTTWTLFTLLGGGKVESDHWWTGMLLLFSGGTFLYVAMHAMQEDTTPHTHDHGINGYADSSAAAQRKLKGPQMRDTLAT
MGGMLVPLLTQFGHHH
FUN_003747 CDS scaffold_15 292984 295217 +
hypothetical protein PF00400;PF00646;PF12937

MAGYQRPMSFSERRGSALSDDLTLGTSTNPMVNHRLEKLPQQQRHLRGPPTPSMSTPAADFDQQLTGSPPPPPTPAASPGPSH
FQPDWSDAADDEDFFLAKVRQHFKNCSGPQRTRVLADLLNLCTSQQLSFVHQFVSPLLKKDPFTSLPDELCLRILSFIDDPKV
LARASQVSKRWRDLLSDDMTWKNLCVKHDYGRRLSEVYTHAPNFSSRPSVQALHGLDADMTSSSSFPGARSYSYTSGTRSFEG
SNTSGRPRLRTYKSHFKQRYLVEAAWRSGGTSTTRNITQEGGVVTSLHLTPKYIIVALDNAKIHVFDTEGDSQRTLQGHVMGV
WAMVPWDDTLVSGGCDRDVRVWNLKTGACLHTLRGHTSTVRCLKMADANTAISGSRDTTLRIWDIRTGLCKNVLVGHQSSVRC
LEIKGDIVVSGSYDTFARVWSISEGRCLQTLQGHFSQIYAIAFDGKRVVTGSLDTNVRIWDPSSGECLAILQGHTSLVGQLQM
RGDTLVTGGSDGSVRVWSLKEMCPIHRLAAHDNSVTSLQFDDTRVVSGGSDGRVKIWDLKTGHLVRELIAQGEAVWRVAFEDE
KCVALALRHGRTVMEVWSFSPPEDMLYDRPLSLQQRVLEDDPNRPLSAMAIDYRTSQQTLASSSRDASTQDVEMNDAGPSTAP
LQGGTFFHDD
FUN_003748 CDS scaffold_15 296287 297735 + PDA1 alpha
subunit of pyruvate dehydrogenase EOG09262Q8D PF00676

MLSRALRLPRAVPMRTKLVAPTYTAIRSVTTDAASASLSHSVPKSDDEPFSVNLSDESFETYELDPPPYTLEVTKKELKDMYR
EMVITRQMEMAADRLYKEKKIRGFCHLSTGQEAVAVGIEHAITKEDDIITAYRCHGYALMRGATVRSIIGELLGRREGISYGK
GGSMHMFAKSFYGGNGIVGAQVPVGAGLAFAHKYNGNKNASIILYGDGASNQGQVFEAFNMAKLWNLPALFGCENNKYGMGTA
AARSSALTDYYKRGQYIPGLKVNGMDVLAVKAAVKYGKEWTAADKGPLVLEYVTYRYGGHSMSDPGTTYRTREEIQRMRSTND
PIAGLKQKILDWEITSEEELKKIDKEARAHVNEEVAAAEAMAAPEPKPEILFEDIYVRGSEPEYIRGRIPEENHYFQ
FUN_003749 CDS scaffold_15 298360 301214 +
hypothetical protein PF04675;PF04679;PF09414

MAPKQATLGKFFGAKGAPPQQTKLSFSSKPKKEEAKHEEEANMNTKSGSDSEKETKKRARSEDKTKPELTPIKKEEVDDESDG
PVTKRARRSRKRVEEEDDDEMIEETVKKEKHASPNKSKVASTPPKVKSPKRSKATPKAKAAEEPTPEENDESAKEEASTASAS
EAELDDEADVEDKPEVAAKAREKVQTKFKSKTKDPYPDWKPGTPIPYAALCTTFSLVEMTTKRLIIMEHCSLFLRQVMRLTPE
DLLPTVLLMINKLAPDYAGIELGIGESLIMKAIGETTGRSLQVIKADQKEIGDLGLVAVKSRSTQRTMFKPKALTIRGVHQGL
MNIATVTGNGAQGRKVDGIKKLLAAADANSTGKVDITKDKGGPSEAKFIIRFLEGKLRLGLAERTVLVSLAQAIVAHEADAKG
KVPSTSDLEKGESILKTVYSELPSYDVIIPAMLEHGIMKLRENCKLRPGVPLKPMLAKPTKAITEVLDRFEGQTFTCEYKYDG
ERAQIHYVAKDAPQELNEASQGAAKEAAAGVASIFSRNSEDLSRKYPDILAKLHTWVKPDTKSFVLDCETVAWDVDEKKVLPF
QQLMTRKKKDVKVEDVKVKVCVFAFDLLYLNGEAVVEKALRERRELLETAFNPVEGEFSFATHMNGQELDEIQVFLDESVKAS
CEGLMVKMLDGTESGYEPSKRSRNWLKIKKDYLSGVGDSLDLIVLGAYYGKGKRTSVYGAFLLACYNPNSETYETVCNIGTGF
SEQVLEDLHTQLSEITIDRPKPFYSHSSGGQHQPDVWFEPRYVWEVKTADLTLSPRYKAGAKEGVDPSGNKGISLRFPRFIRV
RDDKKADAATTSRQVAEMYRKQESVTKNKGPSVDDDFEY
FUN_003750 CDS scaffold_15 301874 304865 -
hypothetical protein PF00654

MSRIMSGASQNDPYALASASASASASASASSSSHTPNSNSDDRDELDFLHDDASDDSGRGRSRDRDHVLGDDPLQSNLSAPMTFK
RRQGPSLWSAPSRLLSAITGSRPHASSRGPSPAVYSSSNTPPRPEAVMFNEASKDGVPHDWYVEGPGRRVGYEDLTAIDWIFE
YTKERQRLRMLYSSASGVIGYVRRLIDASQVWIVLLLTGMLVGTVAAVINVTTDWLGDLKEGYCASGPEGGHFYLNKAFCCYG
YDQGSKCDGWKTWGDALGVHSKGGKWFIEYFFFVSFAMLFAYVAALLVQEYAIYAKHSGIPEIKTVLGGFVIRRFLGLWTLII
KSLGLALAVASGMWLGKEGPLIHVACCCANVFTKLFRNINDNEARKREVLSAAAASGVSVAFGSPIGGVLFSLETLSYYFPDK
TMWQSFVCAMTAAVVLQAFDPFRSGKLVLYQVHYSIGWHGFELLPYAILGVMGGIHGGLFIRLNMAIARWKKSNRWIPGPIVQ
VLIVAFFTALINYPNFYMKVQTTELVSNLFSECSQVLDDPIGLCRTGAASARTIVLLVFASVLGFFLAAVTFGLQIPAGIILP
SMAIGALTGRAVGIIMEIWVTNHPGFFLFGSCEPDIPCVTPGTYAIVGAAASLAGVTRLTVSIVVIMFELTGALTYVLPIMIA

VMISKWVGDAFSRRGIYESWIHFNEYPFLDNSENNESIPDIPASQVMTRIEDLVVLTATGHTIASLTTILEMHPYRGFPVISD
PREAILLGYISRAELAYNLSASTQAPRLLPPETEAFFSHQPMADPRTTLDLRPWMDQTPLTRASHTSLHLVATYFQKLGLRYL
LFSDRGVLQGLLTKKDVWYVLNGAEETRRTMGLAPGGAGHETGVTDRIADDNGGGESSGLLHGDGADDGDSIQGEEPML
FUN_003751 CDS scaffold_15 305634 307618 +
hypothetical protein PF05536 ENOG41

MAENTQSTASQPTDVNKIRSLLKAKDDTQRFVGLALLKSLLDSSEQLRQDEQTVQGLWSSLSPKFLDRLLRTGSKPSTQNSKE
MLDLAVSVLYIFSILLPDQAKSDAKFINRIPLLVNAVLYSSEDTTKLILQLLHTLASSQQGAQEFIKVEDFSSLTEIAPSHAQ
VLDIFCFAWLNSMTTIEDPSTLVRQIDDTIQSLVSSFTGTDAVTLLEFLGYFLRHANSSILPQHPKWLKVVVSYIQNLVTSRP
TPEARAAYTNATASILQAFPSEAPKLVFIDDKKDDKAFSYLLINLLLIDIRSSAPTLLEQLNKPEYPKVSTRLISAFDVISIF
IGYLVQCLEDESMETFFMTPENLLKLRKGISETMSLTAEYLRDRWDASVAGAMGLHPDARTGTIDTSAGVHHTLAWDSMRDNA
DDDMFILSAVRALALWLREEENDILRKEATGLMDMFMDLYKSSSQHKLDFRSPVLVALEGVTTLPKGRELLLANEGWTILTHD
LNSILQHASQTWGEQEVARATDIVRILLPIVEQESNGVPEAWMDLITSVAAWDVPDSGLPPQVQEAVISSLQLCCAVLVAANR
GMRQRYKHSIAAIYGIASQLAKQVSQDNPEREMLEDVLATLGSLTQE
FUN_003752 CDS scaffold_15 308044 309481 +
hypothetical protein PF00070;PF02852;PF07992 ENOG41

MVTHYDVIVLGSGQSGNPVAKAFANAGRKTAVIERTALGGTCVNVGCTPTKTMIASGRAAYMTKRGKDYGVHEGNGNVEIDMA
RVRQRKREIVEQWNAGSVRGLDAAGVDVIMGDGSFVGEKKLKVLLNNGGEKEVSADQIFINVGERPSRPDIIGLDDVDPARVL
NSTTIMELGEVPQRLVVLGGGYIGLEFGQLFRRFGSEVTVIQRAKQLVPREDTDVAKCLMDILQQDGVTVHLSSTVNSISASK
DTKTPFAVSVKTPGGETEVSGTHLLLATGRVPNTDSLNLSEVGIKTTPRGHIIVDDKLQTTASDIYAIGDCHGGAAFTHMSYD
DSRIIRTNLIPEAMSSTTPAMPTTKASISRVLTPYVMYTDPQLGHVGLHARDLANSSREVKIATMPMSYVARALETAEPRGMM
KATVDAKTGDILGFTCLGLEGGEIMSIVQTAMMGNLKWWDLEAAVYAHPTLAESLNNLWGHWE
FUN_003753 CDS scaffold_15 309536 310142 -
hypothetical protein ENOG41

MEEQQHIDELLKRYLNLLDEYSQLRQSLSQIQSDVFHNIARANFAGERGMRYGQDHYDERMQATRLLAIEQNASQSPSFSFFP
APDEDNVADKEETSSAEKPDNASTPEAKEASPTEKKRKQNKNPLQWFGLFAPMPLRTAQTHSVKAVEDIIPRLVSVNAEMLHV
EIEVRRARKRRAKAEAAEKKTSQTAAATQPQSTAV
FUN_003754 CDS scaffold_15 310529 312055 -
hypothetical protein PF07690 ENOG41

MRLLRWPHKDKPPVLLKVRSSAGLIVTTCSFAIFTDIFLYGVIVPILPFSLQDRIGISTKRVQYWVSIALAVFGAALLAGSPV
WGYLADRIHNRRVPMLIGLIVLCGATVFLCVGRTLALFMIGRALQGISAALTWTVGLALVVDTVDKEHIGKAMGWISTACSLG
ILVAPLLGGLVYGKGGYYSVFAMCFGLLAVDIVLRLIIIEVKDAKRWLDRAEPIAEADPERVVTEEPKIEKSKDNIAQQTGGQ
TKDVSDPSRSPIETLVKLLGQPRFLAALWGTFVQALINTALESTLPLLTHEIFGWDSIGAGLIFLPVILPSFLGPVVGMVGDR
YGPKWLASFGFLFATPFVVCLRFVDENTIQDKTLLCGLLAGVGIATACIFGPLMAEITWSVQGEDGTEGAGQIAQAYGLYNMA
YSGGSLVGPIMGGMVKDSAGWATVGWSLGIVVFVSAIPTVLYTGGPLKVKLRGWG
FUN_003755 CDS scaffold_15 316174 317240 + CDC42 Rho GTPase
PF00025;PF00071;PF08477

MAVVATIKCVVVGDGAVGKTCLLISYTTNKFPSEYVPTVFDNYAVTVMIGDEPYTLGLFDTAGQEDYDRLRPLSYPQTDVFLV
CFSVTSPASFENVREKWFPEVRHHCPGVPCLIVGTQVDLRDDPSVREKLSKQKMQPVRREDGERMAKDLGAVKYVECSALTQY
KLKDVFDEAIVAALEPPAPKKKSHKCLVL
FUN_003756 CDS scaffold_15 318996 320324 +
hypothetical protein

MKLLNVLALAGLATACAHDHDDKEWTKEELDELEQKWGYEWPFAGINTFAHLNHVKCLTEPTEPFDIAIVGAPFDTAVSYRPG
ARFGPRAIRSASARQTSMRGFNPRAGINPYQNWAKIVDCGDISITPIDNNIAREQMTQAFKQLGRRKTVSALAPKARLVTLGG
DHSLALPALRALNEIHGKPIQVLHFDGGLIVPFVNYLVKNLINTSTTAHLDTWNPAAYPSWWGATQFTHGSMFWMANQEGLLS

NSSSERSVHAGLRTRLSGNDFADNEDDTSQGWVRFTADDIDDLGTKGIIDGILKVLGTENPVYLSVDIDVLDPAFAPGTGTPE
PGGWSTREFIRILRGLEGLNLVGADVVEVSPAYQNGGEETALAAAQVVYEIISSMVKRGLQDGGKEGNGGNAAAGKDEL
FUN_003757 CDS scaffold_15 324538 326899 +
hypothetical protein EOG09261ZFN PF00076;PF10378 ENOG41

MQDLNGPVGQGAPNGPANRFGHASKPSNSDTGFSHGAFNSNISGFADRHPRRGNIPSINTQPQPMVQQASNNGVDMATPGTAF
DMQFTPLLPSQLLLGSPFQPGTPAAFASPQFQSIPGFQQQQGNNQQQNGISSPVQQTISPQSYQGIVSPSTYGAPQFYPPQSP
TGGFGMQAPLPPTSPVSMGSGVVTGTSRTVYLGNIPPDTVAEEILGHVRSGQIESVRLLPDKNCAFISFLDASSATHFHSDAI
LKKLCIKGQDIKIGWGKPSQVPTSVALAVQQSGASRNVYLGNLPEEVSEEELREDLGKFGAIDTIKIVREKNIAFVHYLSIAN
AIKAVSQLPQEPKWQAPRRVYYGKDRCAYVSKTQQQNAAQYLGIAPGYAHMLTGADRDLISNALAQQSVAAAAVATTAGGINN
LGNRTIYLGNIHPETTIEEICNVVRGGLLHHIRYIPDKHICFVTFIDPTAAASFYALSNIQGLMIHNRRLKIGWGKHSGALPP
AIALAVSGGASRNVYIGNLDETWTEERLRQDFSEYGEIELVNTLREKSCAFVNFTNIANAIKAIEAIRGRDDYRKFKVNFGKD
RCGNPPRQMQQSQSPRGDGVSSPPPNGSQNSGSPTNGNTPQQSAALFNANSNPLTMYLNQVSHQAQQQQHHQQQQIAAQQNAL
FGTAQPSPSDLDLAMSQQVPVSGHGQSASISNGYPANSAAPGATTIGGLLAPGRGQHSRAVSLPVLAPGFENGGNASNGIPGA
ETEQRRGHHYQASYGGMGSGFGLAIQGGLNGWVEEEVAN
FUN_003758 CDS scaffold_15 327566 329218 -
hypothetical protein PF00328

MSTLEPRPPYSEAEIKNLYPPQLRLQLVQILLRHGERTPITARFTSTGLAPFWPYCSAARRLTSVILDPSATDTHVDPADNPG
LTALEWKRRLETFGPDDAPILATGRNGELDAICDMGMLTDRGRETTYNLGQRLRHLYVDQLRFLPEKLAFDTDTAAIYLRATP
IPRALESLQQAFYGLYPPSARASGLHPPTILTRSPADETLFPNDGNCRRFAALARAFAQRCADRWNDSDEMAYLTKRLGRWMP
EEHPHVAVDSRPRLSGIMDTINATKAHGQATRLPKEFYDREVKRIIEKIGVEEWYAGYKESEEYRTLGIGGLLGDVVSRMVGS
AERSTADGEYELALNKKVSATTPQPVRFGMSGCHDTTLAATLASLGAFNEEAWPPFTSHVAIELFRHVNAGATPPPEAPKPTG
FLSFFGLGSKGASHAGLPPPGIGRKKTPDLTEDEKERLKGYYVRLRYNDRPVTIPGCAPTGKHLDGDESLCTLEAFKEIVDKF
TPINWKQQCRANIKDPAFPSKPEPSGY
FUN_003759 CDS scaffold_15 330757 333160 +
hypothetical protein ENOG41

MATKPPNEPLPDIPSPDIQDDDDSIICNQDEAEAILQPALSDAARHLTSQNTPAIEKALALDKSRAGLFDVPIDKHGDTSPDL
SSSPETTPQVPQIATPERPPAKVSPSQQYRNALPSPWQAQPKPTTKGSTSRPSGSILGNALAPARNRSRSAGQEALRRLQKAW
PSLSPPTHLLPSMSNSFFSSSHDKASASTSGVSSHSSPAARQHSPHIPSDALGVSQVRPSPSPQQARGLAPAPRDPNAASASV
RPRVLRRVTSDESHLYHSLSRTSSLGDDEQFQDVREMVNIRFMALKDSLPDVPNFKMPSLPKLYSQARKSTHSLGAFHSPEPS
PTRAQPPKDFDVSSKDGSAVLDRVLESLTGDLVIMGGYRGSVLRSAEPPHQQVWAPVKLGLNMRKVNLEVGLEDEDEERMEET
IKPDGMLKHIGPVDVSRKFIKRLRSCDNARSGKLRIWDYGYDWRLSPHLLSRKLQEYLQKLPSNQGGTPAGSRGALVISHSLG
GIITRHAVNQRPDLFSGVLYCGTPQRCVNILNPLRHGDVVLLNEKLLTASVNFSMRTSFVFLPEDGFCFVDRNTGEEYPIDFY
DPQEWIRWHLSPCMQPALPPRNRPQSSSFSSFLPNSLRTRADSKSEKWPPSPTAAARDHNPIAPQLNGSGANVEESKPSDLQR
KRYLDYLTRTLAATRKFRSELAHLPRHQEANAYPPHAVLYGKSIPTVYAAQVTGREGICYSDAYDDLLFRPGDGVVLAKEAML
PEGYSIARGGRVCTERGHITMLGDLPAMGKALEALVRGRRKGIGMGTGGDEAK
FUN_003760 CDS scaffold_15 334038 334826 -
hypothetical protein ENOG41

MSSHTVYAPPSPTSRTFHNFEQLPAELRVKIWNYNLPPARLVPMQCGSSWSGSSSSFSWSGCTSSAIIPANLHACAESRSEAL
KYYGLGFGFARGPGQVFLDPDRDILYFGPRDGYMAADSQFQTCMSMCDPEELALVRRLAINDALFWVDTTYRSMTAASLTVEL
LRRIQSRMPGLEELIFVPRAEETPESLSYIEPTMVYVRMAQQIEVAIQTLRQQTPDWTPPKWRILPLSAFQSVGAA
FUN_003761 CDS scaffold_15 339248 340000 +
hypothetical protein ENOG41

MSIGTKTEISASNFEADVESVRTEPSFRIYRLIDSARLGLTVLGLAAALTILGVSADALAVFHATYVPEDWLLQLWPANFDMR
PTVALVVGSALVTGANLVSLIAGKLPTARRNAAVKAAITFVPPAIALVAAIIAMSFFYGVNASTTNDSLQSWTCRWKDVTMNV
QPHFGTLCKQSRAGVALSVMLVPVEAIILGLAAYQFTLEKRFDLGARGPSRKAGSPALS

FUN_003762 CDS scaffold_15 344295 345597 +
hypothetical protein EOG09263274 PF04670

MSLSPPIYNLARSFDEADDTSASGTSPAQLNLQRLFEQSISGASASKKDAQKPAAPGPNSVGAAPKGKPRLLLMGQRRSGKSS
ISSVVFHKLPPNETLFLESTARIQKDSMASFMDFQVWDFPGQIDVFENPGFDIEAIFSEIGALIWVIDAQDDYLEAVMRLNTT
ILFLQRTYPNINIEVFIHKVDGLSDDYKLDIQRDITIRIQDELSDHGFENAPVTFHLTSIYNHSIFEAFSKVIQKLIPRLGTL
ESMLTNLCRTCRFEKAYLFDVLTKIYIATDSATADMASYEICSDYIDVIIDITEVYGTWQRSDEGRRRLEGEPWSAPIDKQIG
CNTAESCLVLHDGNKPIMLREVDRYLALVAIMKEDSYDSMPLVNMNVEAVVEGLTEFFNITKPRQQ
FUN_003763 CDS scaffold_15 345780 348386 -
hypothetical protein PF12894;PF12896

MAQRNQLAPFSATEYQDRAPDGYPVSCPTLDLSATWDPVDKNILVYRPPGQVVSKIHQVGAPGQKAPDAQAVTWRSDGQFLAV
GWSDGFVRLMGLENNKAAHHIKVGESSGNKITHIGWASSSIAGKSSSAVSQALKDGLGEDPTRNGGGLPLDLPRELTFLEVDT
VLPKISPLPSSSAGSGEDALVFTLRTGIDFLFQPPKPEEYDQVSVMIIGTSDGQLQLSIYDSFIIGSFQCPQINPSSSSQLIL
HASHPQVSTQALLVADKSEEPEEVHLVPIDLPFISSHPINLSLLASKLTTLQKLLRYLKQAQLHMQVEWRNTRELPTRFLRSI
EGDLENLETGPRSIVPALYHLAVTGHAYEPVREWLVESLAERGHKRWDKAVVSGLEGLRNLVHENFLPALDRCAIILSRLRGL
AQFHDTRDDIGFTLQQTSRLMDIVQCLQLIGHKVLINVMDELDSFTAFSTWLRFQIDRLASSSSASEELTEKEATMDIAKVLS
YIENYLIDSPLRLFFDEMTREDYEADWAHIEGGPTLLHVLDQALVKWENGQPSMKALPRVEFLVSYATNWANRTFKGIAEAKR
RSVRLGNPIRLSAGRVVSHRDMRMSKSKNKETNVVTALASKEAKSEVQIFSVGINIINGISTNRPPTSCRIDLGPRTLIDLKF
LNDETLVILSNSQDNGPQVVCVPIRTERLTYADYDPNRLSETPCVSVDGFTAYSFPEKSVSRPLRMEVNDRNNVRGDMPPRVC
VLESNRTTIKTFTFP
FUN_003764 CDS scaffold_15 348994 350787 -
hypothetical protein EOG09264V3H PF00928;PF01217

MVVLAASICTRGGKAVLSRQFREMPRSRIEALLASFPKLADSGTQHTTVEQDNVRFVYQPLDELYMVLITNRQSNILQDIDSL
HLFAQVVTSTCKSLDEREILKNAYELLSAFDELVTLGYRENLTISQIKTFLDMESHEERIQEIIARNKELEATEERKRKAKQL
EMQRKDAARSGRGAMGGMGGMGGMGSSGGMPRSPSYPSYNPPSQSNTSTYDSYEAEKNKSFSKPLAPKAKGMQLGKKSKTTDM
FERVRGDMGGEIDDSPLVTPAPAQHAEAAEPRLSSTLDRDAIHVTISETISAKLSREGAVNSLAISGDLVLRISDPSLTKIKL
GLQAVASHGVQFRTHPNVDRNLFNGSKIIQMSNTARGFPVNNAVGVLRWRASPKVDDASACPITFTVWINKEGAKYNITVEYE
LTGSDALNDVSVVIPYAGSEPVVSSFDATYDVSGDALEWTIGNVDEENPNGSFEFEAESGDENDFFPMTVRFNKSTPYIDVDV
HSASLLEEDEEVTFSKEIKSHADNFLVE
FUN_003765 CDS scaffold_15 351552 354155 +
hypothetical protein PF10336 ENOG41

MTEIEMDIDMAKPVLDGKIEDDFIDFDTDMVDSNHDLQKNDDNLESMDREMQEDEDAVNHEAYETNGITGEDVDFDLHDVEDT
ANSPEHVDYEVTEAPELGTQGPIQTAQPPDEGVPENEENVEILDEIIVHGDAVSDDHESTHEIDYEFEDNAESEDSHQDEKTA
AASRATRSEPNGTAGAAEEQDDHEPENDATATGQELSDHTGDENNISSHNDHNEPRDPKEEEAEPDEVATGEAEAEDVKADEY
NVENTHASDDNDEIHDGEDANSHEETTTLASGQNDETTEAVEEAEDHDYDVGEHVADAYDEHATTDTDHVDHSATGEDSNGKT
DEGFPAITVQYKGDEFPMFSTTTHGFFANTSVLDEPLEKLIAGLRSELENEIAEDDDLVLQVDELGLELVETTQGETMSNVTL
RQILEIFDLLVKNQDPDGSRPLYAYLFTKPNAEKRFEYLVESATAGKGLDEVIHLFETPMTAGTSMLETAATIDGVHEELDEF
DSPIDDEHHSEIDEAENEDEYLEGEHANPDATGSEGQEFKDQEVDDHEVNSQQANNQDGHYEDDQDNINVATDTQIEISVNAS
AVDLEDPETAVDSLTAADEDGLEQNARADSEIDPFANFELDEEAEFNGDVALGENTETQDEVNVDAEPEPLHAQTNDTSTTTT
LQEEEEASSFNVDLGVASATVETAEKSKTDDDDLDEIDWRDEHEPEDQAPSTPSAAGKRARGDDDDVDAEDEQDAKRRRP
FUN_003766 CDS scaffold_15 357333 358968 +
hypothetical protein PF05653 ENOG41

MIEDKYIGLALAMTSALAIGTSFVITKKGLIQAEERHGFEGDGFVYLRNPLWWAGIATLGLGEVCNFAAYAFAPAILVTPLGA
LSVLIGAVLGSYFLNEELGTLGKLGSAICLIGAVIIVLHAPPDEEIETVDEILHYAIQPGFLLYAFAVVAFAVFMIYKIAPVY
GRRNALIYLSICSTVGSISVMSVKAFGIALKLTFAGHNQFSHPSTYVFMILTAVCIVTQMNYFNKALASFPTNIVNPLYYVTF
TTATLCASFILFSGFNTNDPVNTLSLLCGFLVTFTGVYLLNLSRGDPHGQRLTAGRGGSDATGTDMVSGLQTRLSMQARRSGD

PSRHSMSSQRGDREGLIRAYDEEEAAGFALTDLASDSDEDVRHANGNGKASYDENIELDNRHKSGDR
FUN_003767 CDS scaffold_15 360723 361650 - RPS3 40S ribosomal protein S3 EOG092647L7 PF00189;PF07650

MAHPGSQISKRRKFVADGVFYAELNEFFQRELAEEGYSGVEVRVTPTVTDIIIRATHTQEVLGEQGRRIRELTSLIQKRFKFP
ENSVSLYAAKVQNRGLSAVAQCESLRYKLLNGLAVRRACYGVLRFIMESGAKGCEVVVSGKLRAARAKSMKFTDGFMIHSGQP
AKDFIDHATRHVLLRQGVLGIKVKIMRGSDPEGKAGPQKTLPDAVTIIEPKEEQAVLQPVSQDYGAKAAQAAQAAQDARVAEE
EGGDEVQAVEQ
FUN_003768 CDS scaffold_15 362421 364829 +
hypothetical protein EOG09261ABB PF06602

MEARKIIESVQGIYGGQTTHGTLRLTDFHLVFCAPIDQSAKPSESSSHKPKVRERWIPYPMLCYCALRPVPPGSRQAPSIRLR
CRDFTFVTFNFTDSEIAREAFDFIKSRTCKLGTVEKLYAFSHKPLKHEREVGGWSVYDPKAEFRRQGISEKLPDKGWRITHIN
KDYTFCDTYPAFLVVPSKISDNVLKYAKEFRSRNRVPALSYIHPVNNCTITRSSQPLSGITRKTNVQDEKLVAASFNALLPPG
SEESTPLSSQPDVSLGNSSLETELSETERYEDELISRSVAVYDSSGKRQVYGAQQSNLIVDARPTINAMVNQVQGMGSENMDK
YKFARKIFLSIENIHVMRSSLNKVIDALKDADISPLPPNRELLHQSGWLRHIHDVLDGSAIIARQIGINHSHVLIHCSDGWDR
TSQLSALAQIMLDPYYRTIEGFIVLVEKDWLSFGHMFRLRSGHLNHEDWFTVQKDAFAGSKVQPGENDGRGDAFQNVLSGAKR
FFNQNKDDPDLAAVSETAPGKVVDDEATSPKMVSPVFHQFLDCTYQLLRQNKTRFEYNERFLRRLLYHLYSCQYGTFLFNSEK
QRKDARAAERTSSVWDYFLCRRAEFTNPDFDASIDDHVKGQERILLPRLKEIRWWHQLFGRTEDEMNGGLNAAAAAETDRQTA
ISNLQYPSVIQADADPRSSTSSSKSGPPSVLTGVETAHETLTPESRHTPIERSASTEPSGNAFAAIRDGIAGLNLGKGMLGQF
TNTGESASSSSSAPAPARSDQELREMI
FUN_003769 CDS scaffold_15 365654 367644 -
hypothetical protein EOG09260VTA PF03483;PF03484;PF17759;PF18262

MPTISVDKYKLYEALGQKFTTEEFEDLCFEFGIELDEDTENDERPIVNGEQEPPQLKIEIPANRYDMLCFEGIVTNLNIFRGR
IEPPKYRIVEPASGKLESITVKPEAEQVRPYVSGAILRNIKFDKSRYESFISLQDKLHQNLARNRTLVSIGTHDYDTIKGPFT
YEALPPKDIKFIPLNQTKEMDSAELMNFYENDKHLGRFLHIIRDSPVYPAIYDSNRVVCSLPPIINGDHSKITLDTTNVFIEI
TATDLTKLDIVTDIMVTMFSMYCSEPFTVEPVQINSDHNNQTRVTPNLKPRVAEVEIDYLNSCTGLTESPESLCKLLSKMSYT
STPSTKDSNILEVAIPPTRADVLHQCDVMEDLAVCYGYNNLPRTAPSRSATVGAPLLVNKLSDIIRIEAAVAGWSEVMPLILC
SHDENFAWLNRKDDGNTVVRLANPKTAEYQVVRSTLLPGLLKTIRENKGHSVPMKIFEVSDVVFKDESQERKARNERHFAAAW
YGRTSGFEVVHGLLDRVLLMLRTAFLTHEEGLSGKSVDFEVKENPSKPDGYWIEELDDATFFAGHAASVYLRLGGKERRIGEF
GILHPTVLEKFDLKYPVSTLEINLEVFL
FUN_003770 CDS scaffold_15 369206 371538 +
hypothetical protein EOG09261AH9 PF13646

MADAPNSGDELYPIAVLIDELKHDDVLLRLNAIHRLSTIALALGAERTREELIPFLDESVEDEDEVLVALSEELGGFVEYVGG
SQWGHVLLSPLENLASIEEPVVRDKAVESLNKICTELSSQQVEEYFIPLTIRLAKADWFTSKVSGCGLFTTPYNKVSPPVQEQ
LRQQFGLLVHDETPMVRRQAATNLAKFVKEMPATIVVDEMIPLFQHLAQDDQDSVRLLTVEVLISIAEAVPKEQQASHGVLLT
ALRNLIEDKSWRVRYMIADRFEKIAKAVDEEVVSRDLVPAFVKLLKDNEAEVRTAIAGQIPGFCALVDRNVLLNDIMGSIEDL
VSDTSQHVRAALGTQISGLAPILGKQETIDHLLPMFLQMLKDEFPEVRLHIISKLELVNQVIGIDLLSQSLLPAIVQLAEDKQ
WRVRLAIIEYIPLLASQLGVQFFDEKLSNLCMGWLGDTVFSIREAATHNLKKLTEVFGVEWASEAIIPKVMAMGNHPNYLYRM
TTCFAISTLASVVSMDVIAKSILPMLDKLVTDDIPNIRFNVAKTYRVLINVLRRLPDEGTLYDLEKQGSEITPSPRGSELIQQ
RVVPNLEKLQKDDDVDVRYFATTAAAEISGPIAGGEPMNTSP
FUN_003771 CDS scaffold_15 372009 372818 -
hypothetical protein PF08241;PF08242;PF13649;PF13847 ENOG41

MSSNEKPQHLEPSKLGTKQYWDDLYTNEITNHAADPSDIGTVWFDDSDAEAKILEFLDGLLDPSDPDSPVLSHDTTTFLDLGC
GNGSLLFSLREEDWSARALGVDYSPQSIALARQIATTKDDLEKPVEFEEWDLIAGPYSPVLNGEQTEGWDVVLDKGTFDAISL
SDEKDAQGRRLCECYRERVLPLVRKGGVFLVTSCNWTETELRGWFEKTSEEGFQVVGRVEYRSFSFGGHKGQTISTLCFRRV
FUN_003772 CDS scaffold_15 373789 374755 - TIF6 Eukaryotic

translation initiation factor 6 EOG092644O2   PF01912

MAVRAQFENSNEVGVFSTLTNSYALVALGASENFYSVFEAELQDIIPTVRTTIAGTRIIGRMTAGNRKGLLVPTTTTDQELQH
LRNSLPDSVRIQRIEERLSALGNVIVTNDHIALVHPDLERETEEIIADVLGVEVFRQTVADNVLVGSYMSLSNQGGLVHPKTP
IQDQDELSSLLQVPLVAGSVNRGSNVVGAGMVVNDWLAVTGLDTTATELSVIESVFRLGEGQGPSNINTSMKDTMVESFY
FUN_003773	CDS	scaffold_15	375747	379279	-
hypothetical protein			ENOG41

MRNFLGKHSNRSSHALGEPRNSSKTPAASTPTNNSQTPTGGSAATPQAAGHQRQPSHQAQQLQQQQQIQQDQHYQVQQHQQLL
DEADSASHGAHFGQSHSVAANANAASANASANNPSSINPHSALSSSHNTAFSSSESFDSQQPPPPSHYLQNQQPPLHLQHPQQ
QPQGSLPLLPSPSSASAGAPPPPHPYSSPSAASSTTGANINQHPPSLQQQQSSNLSNVAFDPHQQQSEFNDAGLGRSQSIRYS
AATPYNLQTSSVEDLNRYGQIPPQQLQQQQQQQQQAQAQAQAQAQAQAQQQTAPAEKRSTRKLIKGIFSGSNRGASDGQHHHS
ISIGHNNNQSYDNTGGLARRPSKRVQQLPEGQRSPLPQLSSRQGLDDINEHYTAHQSNKSSPSQTSRISLHNTIRQVSGEVDQ
QQHQQQQQPYEDAAYHQNQSQAPLQQQQQHQQQPQTHIQVHTEDQQPQSGYETNVYDQQQSPLNPSQHSPSQQPYQFSNAQQQ
GNYHQGGNQGGDLRSAPAHLGPSAQFQNPETVSQFSHESPIADSDQRSAKLQSATNSPAVNHALYIQNQGSTPSLPPAQQTTT
QPQQPGMAPPTGGPPSARRPDSDKLRGQADVPGGPPPTYRPANSMNNMNPLPPVPPQSAGQSPAYRGDRPSPYEGQTVDQGRD
SPQPVSAVPAEAGENDKSFKDLLTKYKNVKRLYFDGKGQIEALNGQIVHLQNAVANQRMSQSRTALDDSEYSTRFNRLNGAIN
NLSFNIRKDWRCVPQWLDKYVSTEALKTGKAEMTAIGRAVISRWIMEEVFNKCFHPGLDAQLSQSLKEIELGIRSNTHTLTSQ
EEFDALTNKIVSWRMTSLDGLHRQLNSPAANDNRNAFIAKSTASLTSCLYQFLCNPPPPGVDGSASMIVELAVGIAANLPLES
RDVAITYPLPGEIVQPHVMEVEKAALPVLEAQKGEGEGDDKDGDDEKSGKAKTGTLNNAAPPESGEGNDASKSARKDADKDTF
VAALPKDSNRVRFAGFMALEVRGRQVLMKAPVWTL
FUN_003774	CDS	scaffold_15	382484	384244	+
hypothetical protein		PF00067		ENOG41

MLILSPSFMLGAAALLVITHIIIRLTSATAKVPGPFVSNFTSLVLKWHELNANRTMYIHELHKRYGPVVRVAPNEVSFTSIDA
LKEIYGSGGSGYDKTEFYDLFQVYGKRTMFSTLVKGDHAKRRRMIGDRYANSNVMKQAPLAGIQERSKRRQTYLEVSSDYEEQ
NRLRRKTQLTKTALHAYACDCVTHQLFHPYGSNCLQNKDDEEMMHQVAADDSLQNRLVQYYSPILHRLGSSILTSFAKPRLIP
LADNFVIENAKENGAADFTVLNRLKEKDSVLDTIDMAAECLDHMAAGIDTTGDVLCFLMWELSQPRSTKYQRLLREEFRSKPD
AHFDELPMLDAVLNEGLRCFPAIPMSLPRYVPHGGRVIDGFSLPEKSVVSCQALSVHRINDHVFPEPDKFYPERWLEAEGDAD
RRRHQWAFSSGGRGCIGKHLAMAEMKTLLRDVYSRYETEPDESMKSESMAMDDQLISSRPLGKRCLLKFVPVKG
FUN_003775	CDS	scaffold_15	385928	387252	+
hypothetical protein	EOG09262M7B	PF01873;PF02020

MALVNVRRDVSDAFYRYKMERLQTKIEGKGNGIKTVVVNLSSVAASLARPGSYVIKYFGFELGAQTNIDPKDDRWIINGAHDA
PKLQDHLDGFINKFVLCKKCKNPETDVVIKDDRILLDCKACGQRTDVDIRLKLSGFILKNQPKKGKKDKAERRAAKKARQNGN
AANGTNGQNGAGSDEASENGSNDNGDEDVGSDDEFQKVAAEAAEAAQENEVKEHEWAVDMSEEAVKARQASLPGEFKQKLNIG
DDDDEEGEGGPTIYDELGDWIQSQAEEKGSVDKVESIDIYVKAKELGIEGKHRSVLVLVQTIFDKNIVAQIPKRASMLKQFVT
SERHEKALLGGTERLVGTLAADHPEMFQSIVKILQLYYHYDLISEEVVTKWGSKASKKYTDISTSKKVRKAAEPFLTWLAEAD
SEESSDEE
FUN_003776	CDS	scaffold_15	387605	390284	-
hypothetical protein		PF00625;PF11957

MAAQEIDNPAAPAITAFTTLLEEFLSQAETAKPTSAIEPALRKSDFDDLSGRVSRTADDVSSPAAEGAASTIDKGRQFAIIET
AARNIFSQLIATTTIDSPDYVKVWNLLDILSILSDDGQCDPALLFWLAEELLDSQTIAGCRKIFDFLESRRERITANHFKQKQ
LIILRTCNELLRRLSRAEDTAFCGRVFIFMFQSFPLGDKSSVNLRGEYHVENVTTYEATRAEDESKMVVDEPEEQLKEQAESK
STPKSADARKADKEKPLSTDELYPLFWSLQEYFSQPKKLFETTNLTSFKEGLAATMKVFQTVHNDSRRSLKRKRESGEEDESS
NTFNPKYLTSKDLFDLEISDLSFRRHILVQALIIMDFLISLSIQARERLTEALPVNASVNKAVMYSDQVLSDEDAEWASDMKK
TIADYLRQGADGPYFYRMVETVLARDKNWVRWKIEGCHPIKRDPVSPASFLEAKANVQKMATSKRLRSVPMGSVSLDFLKEED
EETAMSRLKAKERYELPELDAFKRKIADDDFEIDMPTNDQTKAAAIAGKASKSWRALRIAARSKLAALDKIEDPKKIDIVFEE
LTEVDDIEDTAEPTANEEDMPSNREPIIISGITGVGKSTIIRKLMGERKGVFAPVVRHTTREPVEGEVNGKSFHFVKQQEFNQ

LRDGDRLIESGTRDGVDYGTSFKAIEAISESGKVPIIELDIEAAQYAKDMDFQARYVLITTSTPELLKQRLEASGKEESAIQD
ILKKFSTELESRKVDELFDTSIVDDDDVEAVKAIGNYIYGESEGKQGLSEDTAMKDEEEDTTKVAETKEATMTDS
FUN_003777 CDS scaffold_15 390867 395411 +
hypothetical protein PF13086;PF13087;PF16399

MPTVKRLKGSGAAKAQKQNEPKPKVNDGRPTPAEVEEEEHQFVQLARKHWLKAGKKPAKPKVKNDVLKQSIWDVLEREGFQYK
SLLLLESLQTLESYLWPGYTEEASNFHVLLIALIANVKHREHLATWTLFEDRPADFSSLFRRLLSMMLDRTLSVTLRTQLLCF
LIYAFQSLDCTLVRKECAPLVSIGIWHNLSTDSSREASLDQLPHLRKAWKAAHKRYDAADEPNKARLRFERSWLYTLLLDFLS
LLYTENRKADQILYCERFTEFLADLQGQLPTRRYVNTLIQDLHVVPAMRLSPMFNDEENTLLRDLQALLSHFTFFDINDQTGA
QYSITEAYDKHCASLAKLQRIALKHFREKLTVLALSNYGAINQRHELQALLEPLTDEELLSLVSLLGFRIAYPESLDLPMDRK
LLLEVLLSNFERRKTFQEAARDMSLTPTEETIFDNSFQQAETYDGSHPMALPKLNLQYLSIGDFLWRSLILYRCESFYGVRKD
IETALRRLRPEAKGSDETNFAGFSKMAMPISKPAILEVVPPLVGDDKPSTVRAEVSFDVRRLGEGVRREWDSLRQDDVVFLIA
VEPPLAKSISNGGDNLSESERLGVITVRTAEIHQITDDKGRHVREGAGKLDTKRRIQLKLDTHAYSRDAERAAAGKPDVYGKV
NLLLRRGRRENNFKPVLESIRSLVLSDVPLPEWLHEVFLGYGDPAGAHYKNLPNRERKVDFRDTFLDWQHLAESLPGKIIDPG
DDVSGSFGPPYVLESVEKQEEPQGSKPSKKRRRDADPALIAEIETLKVSSYNPPNNGPYPVDNPKLNSVRFTPAQIEAITSGT
QPGLTVIVGPPGTGKTDVATQVINNIYHNYPEQKTLLLAHSNQALNQLFAKIVALDIDERHLLRLGHGEEDLDTEGNFSKHGR
VESFLENRDRYLLEVRKLATSLGAPGAHENSAETAGYFNNVYVAPAWNRFKQIAYAEQSTAAEIVAAFPFHSYFSDAPQPIFH
PESNKEQVLEVVEGCYRHISKIFSELADALPFEILRRDRDKANYLLTSEARIVAMTTTHAAIRRGEIASLGFHYDNVVMEEAA
QVTEIETFLPLAMQKPRNGKLPLQRVVLCGDHFQNSPVIQSLAFRHYANLEQSLFSRLVRLGVPTVTLDQQGRARGSIASLYQ
WRYPKLDNLPEVQTSAEFVKANAGFKFDYQFINVPDYKGRGEAEPTPHFIQNLGEAEYAVAIFQYMRLLGYPAEKITILTTYA
GQRALVKDVLSHRCARNPVFGMPKAVATVDKYQGEQNDYIILSLTRTSRVGYLRDVRRMTVALSRARLGLYILGRREVFEACP
ELRPAFDLLLQRPDKLMLVTGELWPTQREVTQELGAVEGEVPMEGVEHLGQYVFEMTNTKIKQLEAEQGGLPETIMEEPEDEE
GGYGNNEDEEDDEEEGVEADILEVREGE
FUN_003778 CDS scaffold_15 399158 403793 +
hypothetical protein PF02854;PF12152

MTSPANQQNPIQANNTPATTASSYASAAGAPKKSAQAPIVATGSHPPAVVGASSSSQNAKDASSSPVNGKPAVTPAVPAVTRS
SANLNGSDHSRKSSVTMAANGPNSFVANGGPVSAGKPGIQFGYDSPAMANSTPQSSSAAPIPIPGGGNARVPSPAHSPSPIPQ
PSASGGRPPSGLQQAGGNTMTFGSLGSDGERHMRQGSTPTNPNAQPGAHFRRESGHSAQGDGAGRGNFQPQGGRGRGYNPHGS
NYSSQMGFPPTNQFRNGPGQGRGMPPAFQPQGRNLQYPNSPQPARSPALVPSLPGTPNMQPATMQPGMTPQYHYQPPMPQQHQ
QVQYPLSQVDKPFSKNNKKNKGKRGDLEKSVQVNHPQESSRDLHGHEFNQRRRFSKRLDQPWRETDTSSSRVPSFSYPKKPFQ
PLSLNESTPALDLSPGSGFFEYMLTLKKQNYGYPPPQVDGFGRPLSMQYNYANMPYMAVPPQANSPSYGQNYVTPYTQHGHNM
SRTPSQPERPPSASQPNAPVVVSSTPQPQNSSLKPTANSFVKVPKKSAAIQIKNAAGEVIDTSSFKTPSSPAPSIQQSKTPPV
IASSTPPPKPSTPSHGRTDSHAATKTAKQIQDELKEKIKQATQAPPKEDKPKPVEPAAKTDGKPAEQSEAKLTETKAEPAPST
KAEPESKPEPEVKTPEATPKIEEPKKEAEKPAEPAKKDETEEEEIERMIREMEEEDARREKAEEEHRKKVEAQKAIAKEQAEK
DRVKNAAEEDRKLREQEREMERLEEEKEKRRQQAGSSAPSVAELLSQARSGKDTEPTKVESVTDKLASMKIGGDKPADSAAPK
HEKRGAKPAALNLAPLNTKPVEPAQPSAALQSLKSARFLKVIEQDIYPAGIKSPNPALNAAVQKKGKTFKYDANFLLQFQKVF
TEQPSLEFGQQVKSLIGDDRSRTNTPGGSGRGSRGPSSGIGSGFPMGSFNAPTASRPLPSNSTSELRFALSNQQRPGIGSMGR
GPSNFPTGGSSRGPSQLPNSPRGSRRRGGGSQRGEGVREAQAAKTMPLTAGQELKPIAQTANGWKPTSIGKNANPAAANPSGH
LEPDMVQRKVKAALNKMTPEKFDRIADQILLIASQSKDESDGRTLRQVIQLTFEKATDEAHWASMYAKFCKRMLETMSPDVRD
ERIKDKNGNVVSGGNLFRKYLLNRCQEEFERGWTVNLPDKPDEEEDAAGKKTAEAALLSDEYYAAAAAKRRGLGLVQFIGELY
KLGMLTERIMHECVHKLVDYKGVPDEAEVESLSKLLRTIGGNLDSTEKGRPMMDAYFQRIQTMMDLPELPSRLKFMLMDVMDL
RKAGWHSKETNKGPKTLDEVRAEAEAAQAAKAQEAARTNQRGGGRPPVGRGDARNFSAGYAQQTSNQVGMDDLRRLKGSANRT
SSGNISLGPASMLSSRSNSGRRMGPGGYLGPRGEDSATSSRTGTPPTRENTSHSNAFSLLANMENDHPASPPSTSASPAMSKA
VPDSDKKESE
FUN_003779 CDS scaffold_15 405131 409720 -
hypothetical protein EOG09260375 PF00136;PF03104;PF08996;PF12254

MSHRARLAELKALRESGKKVFDTYKVADVDNLYDEVDEDGYKKVVRERLNQDDFVVDDNGEGYADDGREDWDRVQAYGSESEE
EATLRGRPSKTAKKNRQEEQAKRAATDKDISEYFKGANRAQPKPKVVKTKEDDDFLSNLLGEVDNNIPAPAPHTSRVERSGGR

RKARALSPAPEPVPKKTKTLDTRLPSPLPPAPDDNGFFPSADDDILETADVPMSDPAPSSPAAKVAQRKAQIKQEPKEDDDDD
DDDMMEVAHAGTIATTSVNLASSRPIKKIQKAESYPTPASSSPVKPNGAQVDSASWNALTERLNVVTSSPAETKTIGKIDYKD
AIEEDGSLNFFWTDYTEVNGSLCLFGKVLNKKTRSYVSCLVKVDNILRKLYFLPRQHRLQDGEETGEEVEMMNVYDEIDTMMT
KMNVGMYKIKACTRKYAFELRDVPKEAQYMKLLYPYNKPEVDPNRPGETYSHVFGSNTALFEQFVLWKNIMGPCWLKIEDADF
DKLKNASHCKLEVLADHPNMVSVLSESDNLDAPPLTLMTVSLRTAFNEKDNKQEIMAISARIYEKVSLSDTTPAEKLPCRTFT
VIRPHGQAFPMGFDHLAKKRNRGLIVLKKQEADILAFFLAQLDVADPDVILGHQLEGVDYSVLLNRLYEKKIPGWSRLGRLRR
SQWPSSIGKTGGNVFAERQILSGRLLCDLANDAGKSVMFKCQSWSLTEMCSLYLSGDNRRRDFDNEVALKTWAREKQGLLDYI
THMEADTHYIAALALGVQMLPLTKVLTNLAGNSWARTLTGTRAERNEYILLHEFYRNKYICPDKQTFRSRQRAEEAQREEEAV
EGKKKDKYKGGLVFEPEKGLYDKFVLVMDFNSLYPSIIQEFNICFTTVDRPATNGEEDEVPEVPINQDQGILPRLIATLVSRR
RQVKSLMKDKKATPEELATWDIKQLALKLTANSMYGCLGYTKSRFYARPLAVLTTFKGREILRSTKELAESNSLQVIYGDTDS
VMINANVDNVADAFKVGNDFKKAVNEQYKLLEIDIDNVFRRILLQAKKKYAAINLVEKDGKFIEKMEVKGLDMKRREYCALSK
EISQHLLNEILSGDDTEVSIARIHEYLSDIAGKMREQTIPVQKYIIHTQLGKAPNDYPDSNSMPQVQVALREMAKGKTVRKGD
VIAYVITGDSKSSEPAPKRAYTPGDLKADPTLLPDVEWYIGKQIFPPVERLCANIVGTSTSQLAEHLGLDIKRYSSFQTQQNS
SSNDLEIYPLDSQIPDEVRFGDCARLSLRCRKCKTSFAFEGLATSPNHVSQSGVICGSCGSSISTLSVVAQMEHAIRTQTSRY
YEGWLVCDDSQCGNRTRQMSVYGSRCLGPKGLARDCLGRMRYEYTEKAIYTQLLYFTSLWDVDKAKAKATSTEMSRQDRENIL
ALAEHNRVRFGNVKGIVDKYLDKCGRQWVAMDTLFTKLGFKPMA
FUN_003780 CDS scaffold_15 412788 415090 -
hypothetical protein PF01535;PF13041;PF13812;PF17177 ENOG41

MADRLLHGGACLRRALQLAPRQRLEIRPASAFQTRSFQHSQRMFNSTTTPTTEVTTTAQDASSIADAITKDAPKSLDAPADEA
IWKKKETLKMERAVNKELQYLNNDPWKVAEYVRRALEAKKFDEAHLLVQKGSKDMQLVVPWNLLLEYLLDQQQVKAAIKLFNE
MKKRAQFPNAQTYTTLFRGLAKSKHPKTAVFEAVKHYNALFKDSRLEPNSTHLNAVLSVCNRAGDLDSMFSIVDTLNESTRAP
TSYTYTTVLNAIRFNTLDGLENLTPEQKEFNLKNGFQRAKSIWSEVMSKWRQGRLIIDEELVCSMGRMMIISSDTNEKKEVLD
LIEQTMNIPNLSKVKKMSAEEARKTNPHTGQVVKKDKNGVFTSPGNNTLSLILKVVLQTKLNSLGIKYWNFILRQYNLEPDRD
CWLRLFSILKQAKASGHATEILDIIPASITGPDFYRIAFETCVRDNINPNVIKNADRALDSMMNHLEVPDAHLMRLYLNVSQS
THYHLRARAREGDVPGAKRAYGVQITDALDRLWVPYRKLHDHYFKRVKAVTKEQKKALYNDQREVIALARIMYGSFNKVIQQE
MLPEEDLKEIRPVGGRINREIQAFYANREETEPNLRKTKGRGTAEEDMSAHNEAMNMVNFWDTTQAGKPRSPKQTDQGRLHRD
NGPGDGYRRDESSRFESKPPRPAPHSYKIYPKNSQSPPGRRIPEQDLRKRKGGTRPVRQPVYGGYPGPKPNWNAIRDDRKFR
FUN_003781 CDS scaffold_15 415549 416644 +
hypothetical protein ENOG41

MAKVATDFEKIINEGRERKKNEALADRIFSKNRRQSAPSKLKSAPGQSLASRVGVKKQRTSTIGTRSTQPAPGNVNGEWTHDL
HHSVSGDLSSRISAPNAAHKRVNNRRAARLSAAVDRMDTSSDAPQQVNIVKGKGSAGNGMGMTIRGLAGPFTILAQNFAPGTT
AADIESAMTPIGGEILSCRIVKSNPFLLFELAFASREVGERVIETFNDKTADGRIIKVYPKLGAPSSNTITSSNAPRNAPSGP
RGTRGNKPNANDNFVDGSMGFPDLMDTTTTNGSSRNNQLYSDKIVGGNRRGRGGQRGRGGR
FUN_003782 CDS scaffold_15 417851 420785 -
hypothetical protein PF00069;PF07714

MDPQWQQYPSANSPGSSRRHNGNTQAPRDYTNQPPPQGGYKYDQFHGGAPVHSAAAPPASAPVGNPNSAASPISPSQMRDGNG
DVAMHDAHDAHAGIKYPMRPLHQAHMSGSRPVNLHSPQESSVAQRYSPMEALSPTSPYAPKSSSNSQFTSPTSQRQSPTRQPD
YSQSPYFPGARQHQQGQHLPPINPYASASHQENYPSSAVGNLDAAFNLDPKSPRRPVPQQVTRGPVPEFKQLSSPADLQPKVN
SQPPFRRANPEGGFISPLQALTVQLPATYRICNPSFKYESSRNPRRVLTKPSKGTKNDGYDNEDSDYILYVNDILGSEEAGHK
NRYLILDVLGQGTFGQVVKCQNLKTQEVVAVKVIKNRTAYFNQSMMEVSVLDLLNTKLDKNDDHHLLRLKDTFIHRQHLCLVF
ELLSVNLYELIKQNQFRGLSTTLVRVFAQQLLNGLALLNKARLIHCDLKPENILLKNLESPIIKIIDFGSACDERQTVYTYIQ
SRFYRSPEVLLGLPYSSAIDMWSLGCIVVELFLGLPLFPGSSEYNQVSRIVEMLGNPPNWMIEMGKQAGDFFEKRQDEFGRRT
YQLKPMEQYARERGTKEQPSKKYFQANTLPEIIKSYPMPRKNMKQSEIDREMNNRIAFIDFVRGLLTINPLERWSPQQAKLHP
FITQQKFTGPFIPPMNLKSSSLNRSPAPGTQQQQQAEALSKQRAQAAAAQHQAQHQAQQQAASAAPTPYANQYAPQGHTQPQM
YGNNSMYSPGGNHANMPSAYGQQQQHGQYGQMVMSQPPQQMPQGQYAQMPQPNIYQHQQGGMRPARQQASTMEQQQSGIPAAI
QRVASHLDPSQPIRLQPSPAYYPPTGESMASGSIDNRTAAGRRGSRVQQGGRGNRDFIRNLEERTLEEGFMGNQNTWH
FUN_004147 CDS scaffold_16 1922 4808 +

hypothetical protein PF02187 ENOG41

MTDLNAQVAKQARDPAATSPAPKNKLTEDIIVNLGPATVVDALNTATGALRTSLDNASSKERDFINRTALASYTIWGWFEELQ
SWPWPSEPGSAGFLMPSNEERKRRSMQLTVPGDDEDQWMGSLLARDVSKYEQRLAQIQRDLSDLDLEDIKVHVLNHHILPLSR
PGTPLAEFTGAGQLSRSSYNRMEDHTAVITAIVLKALPVLDKLTRLLHTWNLRLCALRRIPTVLLALEDAEVALKSAFKAISI
PTKDTPKKDDNTPLQDSALTRNTFDVMKGHLGELIVCPGRSLDYMLDCLEGLPDTLPSDWLDRMEAVESSYGEWVVVCERKIR
HTERTAPPEPSGPPRSPSPVKYNTASDKSTTLVDSLTDDESDNDDESSVKAGAVVVPIPLLLPPKKVAVTDSDNSAMEPTKAR
VPSGVSESETVVPEWYRRDPSSESEPESPSFLPIEEVDESYEDENHTFDGTRDVMPVQSQLPTPSIEQDFNSSIMSTQYPETP
DHSFTHHFDRDISPELPPLRPLPLPRQKIDPSRTLTVTQPSSSYFGGLSSDPLEVSASPDIPRTRIREAEYTQASPPSTPPML
PMDSRESSLAPLDSPLLPDRNIDDLDMSNMRIEDSFNEDFDDSFSIAEYSTTFDRRSSVGDQHLQQQISQIIDSIPAKIKLTN
DSSSRVNLNPPDVQLPRIRKRPSIEPFRRSASNLSSRAGTPSFTLSPAKNTRVRNRGHHEIKVYHLSRSSGEAPIKLFIRCVG
EQGERVMVRVGGGWADLGEYLKEYASHHKRRSATANAKVEVLPESPGASRSNMGSSPNGRPQSAVELSPMSPLSVRKTRRSIG
AVGSEAPKLNTKTYAFGPALGEEDPSEVFNRSRSNSHLDWNEDESSFLGLAGPTGKKVEMSEENKAWVASVKEKVRIASGERS
RLPSTSSQFGELGRIGGTKRLFLKSEDRRESKGGRESRGGRESRGSVR
FUN_004148 CDS scaffold_16 5407 7479 -
hypothetical protein EOG09262I5I

MVLAKSKNSVGLGNALMNDRFGKGKGTDRKRTSAITRTNHATGEQYLVNEKKDAAWVKMRSVTEQGDLDEFLATAELAGTDFT
AEKTNNVKIIHTDQKNPYLLSAHEERDVLGKHKQHKGRLTVPRRPKWDSTTTPEELDARERESFLNWRRGLAELQENNDLLMT
PFERNLEVWRQLWRVIERSDLIVQIVDARNPLLFRSEDLESYVKAVDPKKENLLLINKADMMTPKQRKAWAKHLKEAGIAYRF
FSAELAKAENEAREFDDSEDESPAGPSSESAQEDQGEGSDQKEQEGAPLTQDFVQQSQIDKEANSAEEADDIDTQILTVQELE
DIFLKHSPADAGSDHKLQVGLVGYPNVGKSSTINALIGAKKVSVSSTPGKTKHFQTIHLSERVILCDCPGLVFPNFATTKADL
VCNGVLPIDQLREFQGPVGLVTQRIPQPFLEAIYGIHIRTRAIEEGGTGIPTAAELLRAYARARGFQTQGLGQPDESRAARYI
LKDYVNGKLLFVSPPPGIEDAADFNRGLYDEEHLPEKRRAALSAAMDQLSVIDPASSEPFLAMDDDAVDLASTIDVPLPAGPK
SKKIDKGFFGPSSSQGHVSRPFNYQYSEQGKEDPLAGKHLSGRKARTAVALENGLDPKEVQMASSKKHFKGNPKGGKGKRRAA
RRAGDDDD
FUN_004149 CDS scaffold_16 7912 9193 +
hypothetical protein EOG09262VVF PF04180

MPRKWIDKKSAQHFTLVHRPQNDPLIHDENAPAMVLNPTQTKKGSSKSKDLDDLASELGYEAEDVRANEGEAASYGVYFDDSE
YDYMQHLRDLNTGGGEVVFVESTATANKGKGKQKQSLEDALKKLDIEQNAGDILDDEILPSKNLTRVTYEAQQDIPDSIKGFQ
PDMDPRLREVLEALEDDAYVDDGDDDIFKELAKDGRELEDYEFDGADFDEDDGWESDATAKPNKEYKDDNIPQLVPTEQPEEG
PSQDWLEDFKQFKKEQKGGKVPVAPSQSEMQSNWTTTTHGGRRKKRKGALTDNSSYSMTSSSLVRTEQMTLLDARFDKIEERY
NEDMEDDMQSMSAVSTMSTVQGPVRGDFDNIMDDFLGNFTKPGKRTSKKTKAQTGLEQLEEIRKGLGPARIRGRIK
FUN_004150 CDS scaffold_16 9573 13815 +
hypothetical protein PF03399 ENOG41

MFSTFGQPTRSPSPAFNPFATNSENKGSAFGIDSAGDSLKTKRTKRSSAFGDQSDNEAKRKANNRFKNKDANLSDGGGRNKNR
KSDEKKNNTDPKKNPFNTATFPRKQAGQNTRGNGSTPFVATRNESRPTSSSSANSDDEPIPDTYHSDDPQAKRVYEQLRKDGI
HPPQWPSQPGNPKNKAEVTKFREKYETYRNKARASLTKAGLIDDPDKRKTLQDAIDFKGICEDMCPEYEKITRINEMDVHQPE
KNPKTTYANTRRMVKKLARSAAGQEAPLPMDVRSVPALKRTLDYLIDDLLRDDGNLPVLHGFLWDRTRAIRRDFTFFSSLTPD
EMKIQVYVLENIARFHVTALHLLTRDGKTPEDFVEQQELEQLGKALLSLRDAYDDCNDQGIRCENEPEFRAYYLIFHAYDSNI
IETLQRQWKPALWKDSNEVRTAVLLVEALQNTQDFHGPLKDAPSLAASAAYQSYFRIVEDPKVSYTMACFAECHFPRLRRAIL
AAINRGLARPRETSKDVTAAVLNKFLRFDTIEQAIEFAELHNIEFSQCDEDPSDASRQYAKLDSRGSLPHLRLQHQFSYSLVE
KKRGSKSLPELIHQTTYQSANPPKAAVNGSSQESLFVPETKPTTDKPTIPTGPKATTSNPFSSFGKPSESGGDNKPINGNSGT
TINGTASISASGSQQTPPQKTPFASGLASTAKRPAVNPFAPTTSAFTPSSVGNVSHLPAPTPEQLPGSGNRPILNPFASSFKP
PTTSGEQAQSSPFSQFSASSEKDTATGTTFSSLPATPAAASTENTSSKGITADKPQLHAPTISSAQPAKQPSINILPPTPQLS
SSPFTTVTQPQKPSTSSDSSATSQATIPSTPFNLDKTPTLTGEQSKPATGIFDTQPPTTSTPQFSFPSIGTSSLSHPASNGDG
FQLSTTPATTQITKEPIPSPEPPSPPRDLLGDFTRWFVTGDGGLMDEFQAFMLDNILQGVYRKYVKDEEARRQKEEEDQALAE
AQKFRVYSLSVRYFYRWKEIARDRRLSQLRRSGREQMRAYYQAQRVAQAKAQREAARQAAREQAEIAELNRPEELKDLLKHKK

PSKRRQAAEEDALLASGVLSGINNEEEAVARIVRRVPSATSSISSKQSSSSLARSGSKTRALRQQFGDQSATFRRSLPPLVSR
NTESPEPSNRVSRVSERWRLKAMGIVQMPDGTAIPERLANEMQYGKKQSTGSMGPPSSGFNRRASISGLGHSEERHRLSGSHG
TVDTTESDSSAKNKRKRATDDDGEPAQDDLAKISSHKRVMSDAQTLIDELRAMREEMEEGATWFKDQNDRMQSELISRGSTPW
DQDI
FUN_004151 CDS scaffold_16 14213 15636 -
hypothetical protein ENOG41

MFVLSIVPFFLFWTPVSALQVTPNSPCSQFCLDRSDPNSYETRGDEIVCDDDDFKRKAAGQKFQRCLACLQDSAFKQGDESDQ
DWFLYNMRYSFDYCIFGYPNATGISSGPCVTSEACGPIGNALKKGITEPNDREQYDYCDTDDRAMLGDAVDKCQACVKADSSQ
TTISNFLVALNAGCQQRPKPGTPIALNDTVFSEGTISIVDPSASVAPGDNHDLPNTSIAAIAVGAAAFLLVSAGCIFMQHRKR
KKLSVRNRRSSLSFRCQTHVTPRTPGFHDVPPYMDEERNLSKSALWNPSSAVRNLHDTHKLGIVTTAIPHPPPTKAPLHTPYR
EDASPAESVSSCTNAPLLSNSAGPQCSTPGITSPLFSPGFSITTPRSATFPRECQDSNNPWVQQPSNGSGKGFLRKKQSRDSQ
APSETRNIRIVFDPPPKKARRS
FUN_004152 CDS scaffold_16 16825 17947 -
hypothetical protein PF04616 ENOG41
GH43_30;GH43;GH43_3;GH43_8;GH43_31
MTSRLADTEIEAQLNDLYAAVPASQKVLSSEDMPVMPVSSSLWGRIIFFILSVSLFDTASALPANTSSPVVRPFVLPPPKQHL
SRRHKGRDKSFSPRLDVNFPDPCLVQDQDDHWVSFATSGGGYHIQVAVADDLFGEWTHLQQDALPGDGWTSGRNYWAPDVRKL
EDDSYIMYFSGELPEGGHCIGVARSHNSTGPYTMDPKPFACPRDEGGAIDPAGFFDESTNKRYVVYKVDGNALNNGSGTPIRL
QEVSTKDGSTPIGKPVDILDRIASEDGPLVEAPSLVRTEQGKYLLFFSSHMYTGDAYDVKWAMSDRVEGPYTRGSSPFLKTPA
LGLKGPGGGTSSENGQFMVFHAWCGKGKRCMYAIGYGLDYE
FUN_004153 CDS scaffold_16 19338 21053 - CBF5
centromere/microtubule-binding protein cbf5 EOG0926273Q
PF01472;PF01509;PF08068;PF16198

MSKDVAVVKKTTTEEEEYTIKPQATTPSLDTSQWPLLLKNYDKLLVRTGHFTPIPNGCSPLKRDLKQYISSGVINLDKPSNPS
SHEVVAWVKRILRCEKTGHSGTLDPKVTGCLIVCIDRATRLVKSQQGAGKEYVAVIRLHDKLPGGQAQFARALETLTGALFQR
PPLISAVKRQLRIRTIHESKLIEFDNDRHLGVFWVSCEAGTYIRTLCVHLGLLLGVGGHMQELRRVRSGAMDESKGLVTLHDV
LDAQWQMDNTRDESYLRKVISPLETLLTSYKRLVVKDSAVNAVCYGAKLMLPGLLRYESAIEHHEEVVLMTTKGEAIALGIAQ
MSTVEMSTCDHGVVAKVKRCIMERDLYPRRWGLGPVALEKKKLKADGKLDKYGRPNDATPAKWSSEYKDFSTSDASAAAAAAA
PAKASEAEDTPMEEAATPSSPAADSKDKKRKKHDGETAEEKAERKRRKAEKKAAKAAKKAAKGEAMDSDSD
FUN_004154 CDS scaffold_16 21612 22727 -
hypothetical protein ENOG41

MNRKACGPFRQAVEYKNLSSDIQSYEYCDTWPVQDTPDFDGCTDCLRAGDNHYLANFFTLLQAGCEQKPQPGITVSVDGGLFS
QDIVNITEPTPVASVDPDWLDQGPISLGAKVGIAAGGVVFLLFILGFCIIWRGKRRRRAFLRQLENRPHTKGSWPKRGWPTPL
HISNDNDMRETPLSQKPFRSWDESPVSARSEKTFSRYVSPYGSQFGSPVSATELQLAQWPVMNPNQQAYPPMPNHASNPHPYP
EMFPSMYKPDPSPSGSQIGVALGGDDSSLNTPQSKGKDKDESYEMYPVDVRSHGAIGIYQPEHLEGLYSQPDYFAHGDVQQGQ
IYQGQGHEYQDTYDDDFRGRRP
FUN_004155 CDS scaffold_16 24812 28076 -
hypothetical protein

MRIGCLQFAPQVGDVDNNLNRADAVLSRANPDDLDVLVLPELAFSGYNFKSLQDITPFLEPSGSGITSLWARTIALKYNCVVT
VGYPEKVDVSPKWPTGPEYYNSVIVVNGDGETIANYRKSFLYQTDETWALEGNRGFYDGFIPGLGNTSIGICMDINPYKFEAP
WHAFEFAFHILEVESNLVIVSMAWMTREDPRRFTRMPNEPDMETLTYWVTRLEPLIRSDNQDEIVVVFCNRCGNEDEVLYAGT
SAVIGIHDGEVKVYGLLGRGEKELLVVDTNNSPYAKLVYRPESGGSGMEAPGHLEQSKKPDNDTSKKNDPKAAPSDKPLTDQT
SNKSLGSPSSKEKPKSPTAPLPPPPQLPENLALNSHDTSTRKRHPPDITIPKESSVLKMNSARSPGNDSINIPTPSAPSPTPM
AIRPRLIIPESPPILPHQYPPDHPISAASLRSERSIRSVKSDQSEASTKTIRTNPRPPEESTPYPDSGAPLSGYPENFFPSEK
IIYGGHVTIESAGPFSPNTPFEDISPISPEQYYWRPSDNMMRTPLSAGGWTPGTPIGRKPEPFPWPAITGNAHPPDRNNNKER
MRDNATPRDFRHLHSQSPQSSSSSNRTTKSERSARSVSSVNSKGTVQGSVKENKALARPSSPKSRNASRSRMHKRTGSALGDR

ETNAAISLHLEGISLRAESASRMRAGSAAGTPVGDRVQSPPSRNSSRPRIAEAVNVQPEQHMIRIAASPSILKEDQQNQYQSL
NRATYHHRSNSFVSNRTPIPGNDRSATPKSAIEMARPASRAASRGRTPGPKSLATNGVIESVSPVAERASSADSTRNDRLHSR
VYQEGVGNRPSRTGRTSSAHTARRRSDEGNTVEFERVEAVICPSCPVHGRRSSANGEQGTEHPASAPQSPPKSSPVPRPASSA
GPSRHLQPEPAEPRRASSGDTGQLVFEKPILDLNNGTQSCSQNEAAEASDCSSFAETLQTISTPGRSPATPSFNPSTPKAMIL
NTNDLEIEPLFGGSLNGPEKTPTSRCAEVRDTSSVSPKPVA
FUN_004156 CDS scaffold_16 33355 34306 +
hypothetical protein ENOG41

MTTGTIVGIAVGCGLGVAGLAWLLFMYCRRSRQRYGAGIKIESPPPDAPMNVRSTYGIQKSSYFQDDAHGRRPPPIHDPSRSH
MRTMSNAQYHDSVERDMMDSHLHVSYHYAPHSKTNGPNAALPTHPAYIPRVVSKLPEPHIITHNRKTHAPDSYALQTYLNARD
DFGIHHGSANNVSSSDTISSRNPSPSRQEKLHEPAASVAPQQNPETARPRLPSLSFPSLHRLIIPKKQVVPPPEVNLLSATPI
SALSADPHRELRISKPLAVLDPRFQDKPLGGGPVMAEGAPATFSDEYLKRREANKSPMLSGNSRVYG
FUN_004157 CDS scaffold_16 34525 35704 -
hypothetical protein PF01694;PF04511

MADNVMAAYWALPPVSRNLITATVLTSIGCIVGLLPAMRLIYHPSYLWMFPPQIWRLVTCFLIEMQPINLLFNSFFLYRYSVQ
LEMGNPRFPRKVDLVFYILFVCTVILMIDYLAGLTSFMYMNGLILAMVYTTTQDQRGQKTQYLVLTIPAQALPICMIVVTALM
AGPQKALVEIEGLLAAHLFDFLTRIWPEFGNGPRLLRTPAWLERLVQTPRVTARGFGTAVRPGNTPSSGRNTGVNTGPLPDSW
RSRGPGQRLG
FUN_004158 CDS scaffold_16 36271 36650 +
hypothetical protein ENOG41

MRAHDAMQSDEANDAAWEAARGGVAGAVKWGIGAAVLGAAGYIWSPLYRSMTIQFKVVYLQMCGMVPGAMIEADSRLRAYEHRV
RMQRRLMGDRAKWERYEEEFIKGDGK
FUN_004159 CDS scaffold_16 36871 38031 - RFC4 replication
factor C subunit 4 PF00004;PF08542;PF13173

MSSQKPEERGESSNSVSKAALKASSNGTTNYELPWVEKYRPVFLDDVVGNTETIERLKIIAREGNMPHVIISGMPGIGKTTSV
LCLARQLLGESYKEAVLELNASDERGIDVVRNRIKGFAQKKVTLPAGRHKLVILDEADSMTSGAQQALRRTMEIYSNTTRFAF
ACNQSNKIIEPLQSRCAILRYAKLTDAQVVKRLMQIIEAEKVEYSDDGLAALVFSAEGDMRQAINNLQSTFAGFGFVSGDNVF
KVVDSPHPIKVQAMLKACYEGNVDSALETLRELWDLGYSSHDIISTMFKVTKTIPTLSEHAKLEFIKEIGFTHMKVLEGVQTL
LQLSGCVVRLCKINMDPKRFRV
FUN_004160 CDS scaffold_16 38378 42258 +
hypothetical protein EOG09264SET PF00172;PF04082;PF04900;PF18477
ENOG41
MGVQKKTRKFAEVKRVIGRRDARLKENKLKAELAQKEKEKKTINGELIREAPQMPSNMFFQHNTALVPPYNVLVDTNFLSHSV
QRKLSLLDSMMDCLYAKCNPIITSCVMSELEKLGPKYRLALRVARDERWTRLECDHKGTYADDCLVDRVQKSRIYIVGTNDKA
LKQRLRKIPGVPIMSVARANTTHCLVLQTIESFLDLRIPLTPAHYLTQMDSSPNDNDQSSTSEARQKRSRVLLSCAPCRNSKL
KCDREQPCGQCSKKGRASQCAYAPKPERKRPAKGMSARLKRLEGMVREMMDSEGTAPGTHASAGAGAGLANGTATGPDVQGHV
VHSEKGATYVGATHCMAMLEDIEDLKDYFEQPEEAGEDQILDDEIDPTEVMLNTRAGPRNREELLSRLPERKVADRLLSRYFA
SMSPSQHIVHRPVFTRQYSKFWQDPNEASLHWIAQLFMMLSLGVFFNNFINSSELEGDSPLPAQDRIRHYKACAGWALVWGKY
TQPSSATLPAFLLYVESSFLFNRAAQMNCYVLSGVCIRLMLKMGLHRDPSKLANISPYEGEMRRRMWNMAIQVELLVSFHMGL
PSMLQGIETDTAVPRNLDDHDFDEDSTELPLSRPPTDWTSMTYPIHKTQILRVFGQIARQAHALTPPSYAEVAKLDNLLHETW
KSVPSFMMVRPLDECVGDPPVLLIQRFGLTALYNKCRIVLHRRYLAEAIPQREHDYSRRQCLEGALSLLNYQHIIWEACKPGH
VLSQHGWFVSSLAVHDFLMAAMVLYLVVKNENYSEVGSDYDWMSQTAPTPTKDELICMIKRSWLIWADVSRDAVEVKKTADTL
ATMLAKLGAPVEEPANALEQAVPSVNGDKEYSSLGAPSVDSSTLGSIGGDPALLSTLGLGSSTGSTSDQTPLDLNLAGDAGTS
MNFTNMGTEGLQFDPAWMDTAASNMDWRYLDISLAHSHDAGRNEDTGQTWIERAPPLEQMDIMGSGIWIPDHPEST
FUN_004161 CDS scaffold_16 48579 49776 +
hypothetical protein PF05368;PF13460 ENOG41

MSNPDKTIAVINASGRQAASFIRVATAVGYHVRAQLRNLDGVVAAEVSGNPNVTVFVGELYTRHQPSEANRDVTKHGPLAGVG
VNYDLIASLFRGAQLAFINTTFYGDEIQIGKALADAAKRAGIQHYVYSSMPDHGAYNPEWPSLPLWAAKHEVEQYVRKLRIPA
TFVYTGIYNNNFTSLQYPLFCMDLQKDGSFKWQAPFHPDAKLPWLDAEHDVGPAVLQIFKDGPSKWNGERIALAYEYLTPKEV
CRLFSKGVGRPVRYVQGPIEIKVRIPEGYREQLVGLEKLFDPNQKDPRKQPPYFGDPKVEVSCPSEALELWEGPRGIEEYARE
MFPIEEEANGLTWMLDDDASDDEEVHNTATHVEQLRIHDGDDDSDDDNDDGLVIKGRKRDEEEWLA
FUN_004162 CDS scaffold_16 51596 52151 -
hypothetical protein ENOG41

MPTLHEKKIQETSVPVGYFVENLPITPSKRKRGQTFQTNPHRMAQAAAYMSAKFESNSEGKDFKLCWKDKGGLTVGAEFVRFK
EGVTKAQAIESAIVNWDKCERARVETYNTELIIALARMRIVRFAREGTALPPYIPQELRVNNRTIKCNLISDEFEAHYNIIKA
VHEGLKGRKIGRPNHMII
FUN_004163 CDS scaffold_16 52382 52838 -
hypothetical protein ENOG41

MSDTPPSRDLLTAEQMAVDTELVASRAAGQSTVSAAEFTRLEKMSFNDLLQEWCNTRPATARHNQVISSDSDDSFISLPQLLP
NKLGQTSVPVGKLTDNLPITPLKRKRGQTMQDDNHWVANAPAFLRVEFKSEPEG
FUN_004164 CDS scaffold_16 56770 58950 -
hypothetical protein PF00854

MAPAGDSLAEPITVAGLRAEVLDEKRASISAEKRASIATDPRPATADAGAVTPAGVVTPDGQFPTAEELDTLRRVPNKIPMKL
FSIAFIELCERFSYYGCTVVFTNFIQQKLPEGSTTGADPEQPGALGMGQRASTGITTFNQFWQYFMPLLGAYIADQYWGRYKT
ISWALGIDIIGHIILIMSAIPPVIGNQGGALGAMIIAIIVIGFGTGGFKPNVNPLIVEQLGEQYMHVKTLKSGERVIVDPAVT
IERVYLWFYFCINVGALVGQVTMVFAEKEIGFWLSYTLPTFMLCLCPLVMWLNRHKYERRPPGGSVLGQALKTWFLAQKGCWS
INPVATWKNLHKDDFWEKVKPSNFSHETRPKWMTFDDAWVDELSRGFNACAVFCWYPIFWLCYNQINNNLISQAAVMQRHGVP
NDILSNLNPFALLIFIPLNDFFIYPALRKAGFRFTPIKKITAGFFTGAAAMIWAAVVQHYIYQKSECGKYASGEDCAAVEINV
WAQTGSYVLIALSEVFASITSLEYAFSKAPKNMRSMVQAVALFMTAFSAALGQALVGLAADPLLVWNYGVVAILAVIAGTCFY
VQFRDLDVHEDDLNALPEGHAGATADEEAPLGTKEAN
FUN_004165 CDS scaffold_16 62202 62962 - ERG1 Squalene
epoxidase PF04622

MAKNKSKSKSSAAAASQGSGLNKLLLVLGLLTALLSSVVYFVEQNLNQFYIFDLDHLDDLSKRAIAKHGEDTRSVVQYIVTEL
NEKVPEHINLKEEWVFNNAGGAMGAMYIIHASVTEYLIIFGTAIGTEGHTGRHTADDYFHILSGTQLAYVPGEYKAEVYPAGS
IHHLRRGDVKQYKMPEGCFALEYARGWIPPMLFFGFADGLSSTLDFPTLWDTTRITGREMINNLIKGKL
FUN_004166 CDS scaffold_16 63524 65556 -
hypothetical protein

MASPASLAATSEVDPPSSPGQPEAKSFVSRAMTEDDDTQMANATPDPQEKSSRGKTKSSQNKDVANQPTIGKIRHLKKEDGEP
LWREDIQYDFLKAVFDNEQCVFTNSYEPKRLQRQCFADLYIDTMSRSSKTSKVLRDKLLSDREAAKGMAMVCLLVNMGRMNTT
LNFFPEMRAQLRTYHAIPSLQARQDPHAYKQLQDAPRLKSILKGGAEDREEPNSLDKIKQLDTPRTNPVNLIFVMCNTAQKIA
ELHFPRHRELHDLIMRTEYTSKSRANAFLWLMWFYLESDFTEEGCEENPFGPGVDYGLDVANQGVPELVEMTPEEKAKENVDT
EEEIEFGRSKQKMRAKILEMDQALLNERDTKRGKMRPSFADEGPAILPRIRPSKHESDMDSTRSTPPPRGLGRGLTSNRRGAP
LKYQIFEGSSPAHNVNEGVVARKPRPPTAHQLAVERNRSQRVEYILDRGLRRQHHKSRKLRRQDGAVFRAYQRLQVQEDPFED
SDDETSTPRNLTYGDKTPGPFREKGVGGLCLLTKEKDDFGEEVSAYAGALRRATRRLARWGPRSSELGVIAPVKRPRPKKPEE
DDADLDLDDDMDKQNGDLNGDANGDVTLGDVTVGDMTAGDITMGDVTMDDAEIEDQTVLHDDEDVDDADRTEVMGDSDVD
FUN_004167 CDS scaffold_16 66486 67303 +
hypothetical protein PF08058

MATPVRTTTIGGPPVTDSPGTWRHPRLNEIARRRNATTFSEKNVRQIAYNVIALLGFWSAQLLAKLNIGSQVVPSSFRIYLSW
AWFILNLIPFINIGIACLPLIRPKDDLSDIPLTPAQRKLLGLDPSSAAPTPDAKFSTPPRYSRTPSIGGSVGSRGSYGSSPLS
GRGSPLVQGAAGSPLGSPLFQKSVNSFGNGRRSSFGSTSPFAASSSANLFSDPTSPGSAGGKRTSVGLNSKWLYEKGRRPSGN

AWNL
FUN_004168 CDS scaffold_16 67715 68919 -
hypothetical protein PF00107;PF08240

MSAHVTVPQTMKALRLVEFHKNYTLLHDVPVPKPKPDEVLIRVAAAGFCHTDLMVYHGITQVSLPFIGSHEPAGTIVGLGSDV
PEIWHIGDRVGVTNFMDPCQGCNGCKWAMQSLGSLDPRFCDNRTMCGIIRRDGAFAEYMASWHGAVVSLPGSIGFEQAAPLMC
AGATVWHAINQADITKGETIGIIGIGGLGILGIQFAKARGYRVVAIDNHEVGLKLASGVPSHLQPDLILKLDDPETIQKISDF
TNGIGLKATIVCTSDDAANDWAAQRLQPRGVLVAAGFPEHGLKFDPMNLILREIFVKGTVHGSMDETREMMEFVVQHGIRSHL
TLLMMEEAEDIAAKSEAHAFTGRPVVKIGMH
FUN_004169 CDS scaffold_16 69297 70454 -
hypothetical protein PF01557 ENOG41

MASSALQRLVRFVPRSSPSKILIGQPADKDIDVGAALRKGQEVAVNVWSGSSVLSPGSSTGTTETIDRVLSPLAQNEIGTIRC
VGLNYRKHAAECGLDPPAIPVIFMKPATTIVDPWPARGTIPKLSQVDESGDYEAELAVVIGKTAKNVSEAEALDYVLGYTAAN
DVSSRTQQLNQSQWSFSKSFDGACPLGPTLVLKSLITDPTKLHMRGLKNGEVYQESGTDDLIFSVPKIISWLSQGTTLPPGTV
IVTGTPAGVGMGRTPKDALRHGDEFAVEILPHIGTLTNIFEDEKSGFMTKL
FUN_004170 tRNA scaffold_16 71047 71118 - tRNA-Gly

FUN_004171 CDS scaffold_16 71976 73031 +
hypothetical protein EOG09264T1U PF04190 ENOG41

MASKVDRIVARLQAKIEEGDYYEAQQQTRVAASRYIKTSNWPAAIDILSNVSQALLRAGQGGSGGDLCAMLVDVYKQAELKPD
ATAKGRLLTCLRLFEAGEPTRKKFIGEIIAWSAKFGEYPAGDPELHHVAGSLYAEEHDTYEAEKHLILGTKDSPEILTKMEYN
WYKESEPHQAALFAGRAVLPYLLVGNVRAANTCYRLFTSSLSNDNPNLGVQDVSSSAGDIRIFPSLPLLNFLGFLLLAIQRGA
PELYRALVSKYATQINEAGSWAEALEMVGEMYFGLSKPRQSNPLMDMMSGFFGGGGGQPEKPKRKQVTQRQDAPAAEGLD
FUN_004172 CDS scaffold_16 73123 73691 -
hypothetical protein ENOG41

MADRTRTTTNGTGVPRSRNGASSAGVRRNLFQSQLTRRPTAGSTSTSAETLHSDVDILSDTSEIVVRDKHGEIELGDPPTPMM
EDEDELPLDHRQECEKERQRLAEAVKHHQMHRHAMTEQPEELLEAVRASLRAKVAALAEDNWMFEPEEQPRG
FUN_004173 CDS scaffold_16 74370 75078 -
hypothetical protein EOG092654VM PF05255 ENOG41

MEEERLWKFRRPEWLNTVWARNAGVYSAGAIFSIAFYIMLDSAVWSKSSRNGSDIHVKFVDWLPFIFSSLGMLIINSVEKQRL
SADSFSYSGNGVAWKARVVLFLGFAALAGGMAGGVTVFVLKFVVPSVAMPALGMGIENVLSNALVGVSSVVLWISQNMEDEYS
YNLSL
FUN_004174 CDS scaffold_16 76974 78153 +
hypothetical protein ENOG41

MRPINYLPRPDLLVSVFASVSLYGGLAMAQDTKDDPKNNDPSAVPEATNSKNSDANKPTNTAAEPASTETKDNAKPASTKDEQ
PSKTEEKEKSKSTAKEDQASTATGEGPTKTKDVQSTITSIASSGTEEAMPTLTRYKPIPTYPAPSVPPTNNAPFMRHSSAPDG
TVFIAVGAILGALGLAILLWRLIVGILLHRSVERAAKAQHDENAKTGFPAPPAPFYKYTDTGSTMSLSAGRGVRRTTRGPVLS
STPSASNLFFSPTAAASGNGAGNRGSAFLPSGFYAAGTSSPGGQHDNNISMTNLRPDSRGHFGTPSRHTLNPSPPESPSFQGR
RDISNSTLNLNRPPSQRAPSAFLEDLLADDPSSLPPPHMPASTARRSSYGSGSPGAHNRI
FUN_004175 CDS scaffold_16 79213 80188 -
hypothetical protein PF06428 ENOG41

MVMTMAAPTLSADSTPCCPGCGYSLPLDHSQVQLLEAQARIEDLENQVRLLNEKATAAVDRWADYEDELANLRAQLPSSPTQK
QLPATPTNPRMSFLQSGTSRLSSFLSPRKSHELPHIDTNPSRPSNQRSISSQSHRPQQLQPPPSPSAEDLLEALNREQCLRKE

AEGKVAATSQEVEELSAALFEQANEMVAEERRARAKLEQRVDELERRDQEKRRRLDKLEGAMTRIERVRLLLAEAEEDVESSK
KTVKEIEELERQQEEEERKEKEQSKPKQPQYDNRDSVYSVSVDEHPERRSLEGHSNKEQLNDNIGGERATMTSRA
FUN_004176 CDS scaffold_16 81274 81955 -
hypothetical protein EOG092653O3 PF14382

MAVDDIPSVALPGNVLGPVTKFAPGAGTHVYEGNVVSSLLGRVTVTPPTKNPGPQKRLNKITAPTTEELATISVSRHGRKREI
LPDVENIVLARVLRLMPKQAIVVIQQVGDTVLQTEWQGVIRVQDVRATEKDKVKIYESFKPGDIVRAQVISLGDQANYYLSTA
SNELGVVMATSEAGNDMVPISWKEYRDPETGICEPRKVAKPS
FUN_004177 CDS scaffold_16 83928 88455 +
hypothetical protein PF00005;PF00664 ENOG41

MPDPVDKELQSSDTQAASLSPTNFEKSSDSDEDKIREFGEKDAERSEKIDEISEKDESRIADLEEIKTGATEASAITPTTSVT
QAQQKKPWHKKLNPLRWGSPPPVPEKPVQSREHDAGFLSKLTFQWMGPLMHAGYRRPLEENDIWTVNPDRAVEPLTLQMKASF
QKRVERGDKHPLFWAMHETFKGEFWTGGACALYTSIIQVISPFTLRYLIQFAADAYVANKSGGPPPHLGKGIGLAFGITLMQI
TQSLGTNHYIYRGMTVGGQSRGVLIGLIYEKSLVISGRAKAEGALQSNAPGAKDDAEDKKKAKKAKKAKPDASDGTGWGNGRI
TALQSVDTYRVDQASALFHMVWTSPILCLLTLALLLVNITYSALAGYGLLVIGMPFLTKAIRSLFHRRRAINQITDQRVSLTQ
EILQSVRFVKFFGWEKAFLERLGDFRNKEIHAIQILLAIRNALNAVSMSLPIFASMLSFICYSLTHSGLTAAEVFSSLALFNG
LRIPLNLLPMVLGQVIDAWGSVQRIQEFLLQEEMVEDMTIDTTGDDAIRLEGASFTWEKSHKEESEKSEKDKKQSQVQTPQHE
PSGDDTSTLVEEREPFKLQDLSFGVKRNELVAVIGSVGSGKSSLLSALAGDMRKTDGHVTFGASRAFCPQYAWIQNTTLKNNI
IFGKDMDKNWYKEVIQACALQADLDMLPNGDMTEIGERGITISGGQKQRLNIARAIYFDADIVLMDDPLSAVDAHVGRHIFDN
AILGLLKDKCRILATHQLWVLSRCDRIIWMENGKIQAVDTFENLMKDHKGFQSLMETTAVEEKREEAKKPDDGEQPTADEKKK
KKKKGAALMTQEEKASSSVSWSVYAAYVRASGSILNAPLVLFLLIVSQGANIVTSLWLSYWTSNKFNLSTGVYIAIYAALGVV
QALLMFAFSVVLSILGTKSSKVMLRIAVTRVLRAPMSFFDTTPLGRITNRFSRDVDVMDNNLSDALRMFLLTMGMITSVFILI
IAFYYYFVIALVPLYIAFVTAAMYYRASAREVKRFESVLRSHVFAKFGEGLTGVASIRAYGLQDRFVNELRESIDNMNGAYYI
TFANQRWLSMRIDLIGVLLVFVTAILVVTSRFSINPSTGGLVLSYILSIVGMMQFSVRQLAEVENAMNAVERLYYYGTELEEE
APSHTVEVRKTWPEKGEIVFDNVEMRYRAGLPLVLSGLTMHVKGGERIGIVGRTGAGKSSIMSTLFRLVEISGGKITIDGLDI
STLGLHDLRSRLAIIPQDPTLFRGTVRSNLDPFHEHTDLALWSALRQADLVPANAASPEEARRTNDPSRIHLDTPVEEDGLNF
SLGQRQLMALARALVRGAQIIVCDEATSSVDMETDDKIQATMAVGFRGKTLLCIAHRLRTIIGYDRICVMDAGRIAELDTPME
LWKRGGIFRSMCDRSGIRVEDIHGAKEEMGVVTGESSQH
FUN_004178 CDS scaffold_16 89047 90623 -
hypothetical protein EOG09264HU0 PF01509

MRLPRKTLKMAADKVREGVFAINKPCGQSSAQVIRECQQAFNPSSFFKPLIDSEVAKRISESGKEFARRKLIKKASQVKIGHG
GTLDPLATGVLILGIGSATKSLPQFLECTKTYETIVVFGAATDTYDRVGRILTKRPYEHITREKVEKELETFRGRQMQIPPLY
SALKMNGKPLYEYAREGKPIPRQIEGREVEVKDIELVEWYEPGKHNHRWPTEEAEAAERNLAEQVWRVKKQQETNKKLSPEEK
QQDDKAIAAHESFKRSFEERQDALIKDAPSKRSRKSKEPPMMSGALGQLPQPTYSNKGSNLVPESPDTSTPPPWSDQGPPAVK
VRLTVTSGFYVRSFCHDLGAKLDSAALMAELARTRQSDFEVGNDNCLEYEDLAKGEEVWGPQVARMLESWSKNKPQPPAGNKS
NKKKNEAKGNGSPKWKQDKQQSHNKGEKRRRSNSPNETESPPRKAIALESQEDSKPVKEPKSETPKSETANRSEDEKSWNGIE
D
FUN_004179 CDS scaffold_16 91723 94239 +
hypothetical protein ENOG41

MVGRPRRASTSSVDTEDESQRSWKQEKSVLRFDPGGVDPDTTFEIKDAVVLNKDGHALENALDVATRGPYIIRGLLHIEEQSQ
RARLIMKVRSLTPFEVRKCGQFSIGESDSGSPVIWMLGTCGWFELNPAPAYLPIYRKMQEAIRLYYRLMAIYRDKKPKKAKKS
KKDALKELYQVFHEYAASIGDGSTLEEVIARCDDHASFLIAQCIQDQSEVEWFVTPFYKWLINRHQDLYNKELERMNNPPQPQ
RSPSVEGSNPPVSQILTSRTRNGSSAPSTARDATPDIIEPEDSLPRRRYSRSRSATHRPENSVVDLFSSTQISRDGSTRPVSV
HSSASIPSVRMSQAPISVDAKDDAFRSVLDALEWIRVEISNSKGGRLNISAVANKFWSNFRFPTYKGDIKPSYRVPIQEVLHY
NVHGLLQVLDKDKYDGEFYSWLQELAEMPFNPVAIKPSEFPFYVIRRKPSSNNNPSKPAPPSQAGAASRNLDEGFSTPPRSSP
AGKSLMRPGRPSGIKSSLRLATTSKKRPHSEVDSDSEDEGSELKRSHYFSGEEETMGNTSHMSLSEDEDAQETSEGPVKLFLR
ADNIPTTVPRGPGETWVCEEEDCGYVVRGGDIQNCRDRIRRHFNEHEQQMDRVNLARTEATRGHLPVNHLMDKLKKLGEKSQS

AEQLMVNEIVLPQRIKRKLLI
FUN_004180 CDS scaffold_16 94753 97332 -
hypothetical protein EOG09260PI1 PF09088;PF09090

MADYDRRPGGYRKRRYRDDDDHYDRRNQRRRVDSAPLPVRVRRQLLSIADSPLRRWAEEVQSIAHMVAENHDDENLRKTFLDL
VTQILLEQPLKTPFVAAVVHIINTLKPEFVDVILARIGQITADKIAQGEWRDVKLLLKFLACLQSLMEGDGLFPILEELFSRA
ADLQTASSDDTIGTEIVKIILLTIPYIMVSAPGQFQQKAAELMEKTDIIASEPHALQALVDPYHPEGKEESPGTSLSVCMLLQ
QQLQSEAAKNWELSCLPRPWEMPLEDIEAQDKLSNAPKHALPAIAIPAAVIAGPRPLFPEIYFSVYSQQEVPSVPAVTDIAAS
LIRDGLLDTINILDFNRNVTARYLMDLDCYFADGTFVKRATPFDELKNFPDGKNTWKPEDVAVDTVFSQLFQLPTPERKLVYY
HSVLTEACKLAPAAIAPSLGRAIRYLYRNSPQMDLELSYRFMDWFSHHLSNFGFTWKWAEWVDDAQLPDAHPSKWFLKGALDK
EVRLSFAQRIQKTLPEPYLPLVGPEKEKDVPDFKFQNSETPFSKEGQEIASLLRRKAPDEEFQPLFESIQTQASEQSLDPVVA
STDVFMTAVCWVGSKSLSHVLACIDRTKGRLLEAGNSSEAARAQIISAVMSYWHAHPGVALSIIEKLLNYSILTPFTVVDWAL
VASTPANGTDGGDSLTEPHIFELVFNTIFKVTRRSRDVVAAPETDEETRIKEIKSTRDLFRAMNDALVSWAGGSKDELMEGGD
GSSDREAMIRRWGQRWLRVFKRMGAIEEAFVIEVSKNLGKDKMATDGVEN
FUN_004181 CDS scaffold_16 98850 100332 - GSK1 Glycogen synthase kinase 1 EOG09262KXK PF00069;PF07714

MSAHRPNAFNSLRMGEVIREKVQDGVTGETRDLQYTQCKIVGNGSFGVVFQTKLSPSGEDAAIKRVLQDKRFKNRELQIMRIV
RHPNIVQLKAFYYSNGERKDEVYLNLVQEFVPETVYRASRFFNKMKTTMPILEVKLYIYQLFRALAYIHSQGICHRDIKPQNL
LLDPNSGILKLCDFGSAKILVPNEPNVSYICSRYYRAPELIFGATNYTTKIDVWSTGCVMAELMLGQPLFPGESGIDQLVEII
KVLGTPTREQIRTMNPNYMEHKFPQIKPHPFNKVFRKADANAIDLIARLLEYTPTERQSAIDAMVHPFFDELRDPNTKLPDSR
HGTGQLRELPALFDFTRHELSIAPSLNQKLVPAHIRPVLASQGLDIDHFTPLTEQEMMAKLD
FUN_004182 CDS scaffold_16 109677 111723 -
hypothetical protein PF00300;PF01591

MDTLLDEADVDRTAEIAANAPRYRRKSSTFIDAIHDIPHGQDLAPAQLYSTMSGRLFHSGRIAIVMVGPPARGKTHICVSMAR
YLGWLGVKSRIFHLGDYRRATVGPDGSIPDDYFFPNASPASVILRQKILKKCREDIYSWLNHDNGQVAIYDAVNPTANGRRSL
AKEFAKHDVQTLFIESFVDDERILRENARNVKISSPDFAGMEPDEAAKLYLQRIEMKIPVFETMNEKELNYIKMINAGEKFFY
NNVSFNYLAHRIVFYLTNLHIKSRKTFFARAGTTAEEDSYKADAPLSQEGRDYAQKMSEALLKHREQERLTNIEEGGPDVPLP
PLTVWTSTRMRTVQTSDVLKEKGYKVRQRTQLSQINPGVCEKMSERMIRQIYPDEVEKHELDPYHHRYPRAESYHDLAVRLEP
IILELEREQHDLLIIAHESVLRVLYAYLMHCSTTDIPVINFPRDEIIEIIPAAYQNEAKRIHIPGLDPQIVPGSPQDIKIPVP
SSGVVSAQLSPIPSGIGTPAEHVERPPEKVINTAKDMVADKVNDED
FUN_004183 CDS scaffold_16 112819 113996 + TOM40 translocase of outer mitochondrial membrane EOG09262M2J PF01459

MASAAALDPVKTALLSNPIASVLGDALNSFNERRAKLGLSNPGTIESLAKEVQRDVFLNNYMFTGMRADLTKIFSMAPLFQVS
HQFAMGERINPYTFAAMYGTGKVFCQGNLDNEGSLSGRFNWRWSDKFVSKSQISISPGGQDMAQFEHEYTGNDFSASLKMLNP
SYLEGGVTGIYIGSYLQSVTPKLALGLETLWQRPALTQGPECAVSYAARYKSADWIATAQLQAAMGTLNTSYWRRLSDKVQAG
VDLSLGLVPSAGGLMGGGLQKEGVTTIGAKYDFRMSTFRAQVDSKGKLSCVLEKRVAPPVMMTFAADIDHFTHQAKIGLGVSI
EAAPEELQEQQESLGSQPPPNIPF
FUN_004184 CDS scaffold_16 115162 118010 -
hypothetical protein PF00443;PF13423 ENOG41

MKEFPRKFLSKREKNGRRSKSATPAPVVPTVPDSTSNKNKPRPLSAGAFMALFKNDSAKQGAKAEAEKEKDTAQIEDILRRLD
ELNITDVTADHVTDIMATKFADHDPNKTVEFIDMEQKAAAGIITPYDPSVDMVGAENRGNVTCYLDALLFSMFAKIDAFECML
KNDFPEEDNRNKLVNLLRMWVNMLRTGKLVHTDMTKLIQDALADCGWSDARMLEQQDTSEAFAFITETLQLPLLSLQVDLFHQ
GKKDKDDHKVVYERLLNLAVPPDPEEKGLKLEDCLEEYFNAQVDVKRDSEDGKKLGVEEKSRSETPTLKHRDTIRIITEERGE
ASTPTSPLVNTPLEITPTSEKGIPLLLTDPNVKDGNAPDDEEEGEVTTVEIVKNKPSMRTRSTSVIQRVVLDEDGRPSTPDNV
TMLQKAMRKGSTVVKAVTIPAWQFFRLIPWHALSSSEPRNNTEVALNFEQRPVVGICLKRYAMTESGQPKRHNTFIDIPDSLR
LPHFMLADEPKAEEDMNVLNTDYKLVLQSVICHRGDSLQSGHYVSFARVAPKLLKDNRRHNFDPPPDYEEAQWVKFDDLQIEN

RVSYVEDIRSALKEEMPYLLFYQIMPMVEVSPPSVDETETEPPSYNDSKVSIELPPTPSRSNRLGSLEGGYFDSTPTLENSQL
LSSKPPSIRLSMEGERPRRSEDIDRNPGSLAGDSRRPSAVWTDSTTHTPNSHSPAVTPNEESTASRLSRAASRFTLVNRSRPS
SQNGENRISFSMSRLGGLMRPSKEPLAEPKDEPNGIGMSSANSTGILTAEISKESKESKDVVDGTEQLAEPEPEQHHHRHHGH
KHGHKRVKSKDKVKNKSGDQPERECTVM
FUN_004185 CDS scaffold_16 120665 121832 +
hypothetical protein EOG09264K2W PF00281;PF00673

MTTVRDGARMASSLGRRHLPLRTGSVCVQRFASTEAKKSSALADLDNASSFATGSPDEAAIQAFNSREKAAETGKKLPGNRYQ
YHPPKYYRGPLHPVQVPKSSDPTARDFVPGPFSFPRLKHTYESTIAPDLIAMTYQHTPPGSEVPVSNKGNLREWDESSPYHKN
RPRRGPRGGGSSRLGLVERPIEWHNVPDIEAITINSFAPMGAQNKEYLHVCRAVIQAISGAFPEVTRVKHGVVQWGVRKGDKT
GCKVTLKGAQAYDFLDKLITLVLPKIKDWPGIDATTGDSAGNLAFGMKPDWMAYFPEIEFNYDMYPARLMPGCDIFIKTTGTS
DRQGRLLLEALGLPFYGKARH
FUN_004186 CDS scaffold_16 122098 122740 - FES1_1 hsp70
nucleotide exchange factor fes1 EOG0926448Q PF08609 ENOG41

MDDKRLNELLKWSIEQSDATRNDPDAPAPTTQLTPELMASLMGGPSDADLMKASMDIITSDDAEQVSLDDKLIAFDNFEQLIE
GLDNANNIANLSLWTPLLDQLKHDEREMRKMAAWCVGTAVQNNERTQERLLAMGGLPLLVNLATQEDEHNDVRRKAVYALSSA
VRNYQPAMDLFADELTKRGHKTDKVDATSMEAVDEVVNGLREKIGKA
FUN_004187 CDS scaffold_16 125385 127017 +
hypothetical protein PF00022

MEEEVAALVIDNGSGMCKAGFAGDDAPRAVFPSIVGRPRHHGIMIGMGQKDSYVGDEAQSKRGILTLRYPIEHGVVTNWDDME
KIWHHTFYNELRVAPEEHPVLLTEAPINPKSNREKMTQIVFETFNAPAFYVSIQAVLSLYASGRTTGIVLDSGDGVTHVVPIY
EGFALPHAIARVDMAGRDLTDYLMKILAERGYTFSTTAEREIVRDIKEKLCYVALDFEQEIQTAAQSSSLEKSYELPDGQVIT
IGNERFRAPEALFQPSVLGLESGGIHVTTFNSIMKCDVDVRKDLYGNIVMSGGTTMYPGLSDRMQKEITALAPSSMKVKIIAP
PERKYSVWIGGSILASLSTFQQMWISKQEYDESGPSIVHRKCF
FUN_004188 CDS scaffold_16 127446 128111 -
hypothetical protein PF00472 ENOG41

MSAFNGIVMTPLKLLRTIRYSTPPVLPTRTFHNTSIFLAKALPPRPKPPPESEIEESYVKGSGPGGQKINKTNSAVQLKHIPT
GIVVKSQATRSRDQNRKHARELLAQRVDELRNGDQSRSAIVGQVKQKRAASASKKSRRKYKKLEEEKAATAAEAEAQSDTTTA
SPEGRDLTTDDANNYQTSQPLDTTSKAP
FUN_004189 CDS scaffold_16 128646 129056 +
hypothetical protein PF11779 ENOG41

MAVLGSFRKWLSLKQYQLEVTFSVYMFTPWEKFFFSSIVFLLFSLTFIAACLYLPHHISFLVGRAWYYINGEHIDVAEVVQEA
VKDMSASALSGTAASVVDVTKETVETVVREL
FUN_004190 CDS scaffold_16 129394 131870 -
hypothetical protein PF00400

MSIYPALRFFLADHLGVLVLTYVKSESPKGVTKLLNGHKSTVKALTFLPEGDQDENSFLVSGSDDKTVIIWKASRNSDEFRPI
HTSTEHTAAINCIAALRIKGAQKWIIATGSADASVKLWNLENDQLTLLQSVKTTPKYFPMCLSLSYAGDEDDVLVLASAGTRD
IVQIFTAEVKSENVQFDLQATLTGHEGWIRSLSFAKETCAPDSDLLLASASQDKYVRIWRFHQGRELPAAAASGSDPSSDAYL
PGKSPSNKAHRLQSAGKDFSVTFEALLLGHEDWIYSARWQRQDDDKLQLLSTSADNSLAIWEADSSSGIWVSMARLGEISREK
GATTATGSTGGFWTGLWSPDGKSVACLGRTGSWRRWEYVPAEDFWQPCVAISGHTRAVTGITWAKNGEYLLSTSSDQTTRLHT
RWDRPGNETWHEMSRPQIHGYDLNCIDSVGASQFVSGADEKLMRVFSEPKAVASMLNRLAGIGGGMDVQNMPDAANMPVLGLS
NKAIDAVEDDQEIQPIDDRDREAMDPASIVKKSHLEIDHPPFEETLSRHTLWPETEKLYGHGYEISCLAASHDGTLVASACKA
SSTNHAVIRLFETNRWTELKPPLTAHSLTATRLRFSSDDQFLLSVGRDRQWAIFERAPEDEAAYKLLQINPKGHTRMVLDAAW
APASSTAPVFATAGRDKQVRVWAAKPNEDGKLQFSQTASIPTASPVTSVDFVPQVIDGRFVLAVGTELGRLSLCLIKEDGTDV
TEKPAYEDYCLPKAVHQLAWRPIVREGAERPFEVAVAGEDSSLRVYAFSQAYLQVSADP

FUN_004191 CDS scaffold_16 132565 133322 + URM1 Ubiquitin-modifier 1 EOG09265K1R PF09138

MAESTSAPKLNIDVEFSGGLEMLFSNKRQHALTIPAADQDGKPVDIAYLIDHLCQNVMDDSRKDLFVLDNHLRPGILVLINDA
DWELEGEEAYEIQSGDNILFVSTLHGG
FUN_004192 CDS scaffold_16 136040 137627 -
hypothetical protein PF00096 ENOG41

MALTAQSSDAPNWGRWSQQLPGDYTMMASPHMLPFDSRATTTGPLQRPVMAPYMVQPPYSSGPVNSLTAPHYQVPNPYQFGGY
QGPPTPPHHSTPFKMEYNDRRPMGHDNDHGRMPSYSREMKYTYAEQAPSPARSDSQASTVRSSGTNPSMGSKTITSNETLNPG
DQINFETEVDELMKAIQRKVDLQVDTVQQPLTPGMSPVSEASFESQGTPGPMDSKTARKRYRCDGPNCQKSFTQKTHLDIHRR
THTGIKPYNCDFPGCDLTFSQLGNLKTHRRRHTGERPFACDKCDRHFAQRGNLRAHLQTHQGLKPFICILDDCNKTFSQLGNM
KTHQNNFHKKTLKKLTMKFANIIASGEEVSEADRELFEYFAAHYKNSNKGIKGRGKARTVADRKAKTSQSPPANTMTAVPQYP
LPQIAPTPVTPHGLPVSGSLASYSVTRGQPGPINHMSRQTHTGGYEVYDIQGHHHIQPPNNNGMLYETGSTREMGYHGRMY
FUN_004193 CDS scaffold_16 153962 154211 +
hypothetical protein ENOG41

MCVLTCTKYDCAQCQRAISGTEQLKECPESSQGIKCETTNECITENVSADQCEVCEMRRIEAEVAAAAEEEEAKRRQMRLKQ
FUN_004194 CDS scaffold_16 154387 155373 -
hypothetical protein PF00153

MPDSDHAGLSPATIESIAGLSAGTVATLTVHPLDVVKTRMQIYRSAAPDAVRPTTVSILRALTSTPHPVASLYRGLTPNLVGN
ASSWASFFFFKSRFERALATWQGRLGGRPSGGDYFIASALAGAATTTLTNPIWVLKVRMVSSDRGSHGAYPSMLAGARSILQT
EGIRGFYRGLGISLIGVSHGAVQFAVYEPAKKWYHARRRERHGIEREHMTTEATVGLSSLSKFVAGAVTYPYQVLRSRLQNYQ
ADERFGRGIRGVVVRIWTEDGLRGFYRGMVPGVVRVMPATWVTFLVYENVKYYLPQWA
FUN_004195 CDS scaffold_16 156717 157845 +
hypothetical protein PF00076

MAQNRHWEQDKEATVYIGNIDERATSAMVYEIMLQMGPIHNIHMPRDRVTQTHQGFGFVEFRTPTDAEYAANVMNGIKLYGKS
LRVNKASADKQRAAEVGAELFIGNLDPMVDEKILYDTFSRFGPLLSLPKVAREESGASKGFGFVSFADFESSDAAIANLHGQY
ILSKEVSVQYAFKKDGKGERHGDEAERELAAQAKKRNIVPEAQPVPQAFLQPPPVAAAPPAGFDPSAMGRMPPVGAPPQGVPG
GFAPPGRGPSPYNAGVPPPGAPPRGSVPLPPAPSGLPARPPQSQAGYTNPADFHPGSFRPPSGSPAQGFPVAPPPGFPAPQGA
PGAAGAPPPGFMPPPGFQPPPGFRR
FUN_004196 CDS scaffold_16 158165 161478 -
hypothetical protein PF10345 ENOG41

MSYRPFHGHGQGQSQQGSQQPLPVPGMNPPSSYMPGGYQQYTNPQQQQFYSMPQQQGRQQQQQPYLQQQQMAQFGGMQTGFQG
YDTAGQAYGGHQMQPAAAQYTPPANVWQQQQSTGGQNMMYQQQYQPSHHHQQQQQHIYQQHVASPQQHVQPRRQSGHVPSPQQ
RPSSIPQNQGYPLPQAQQQRSQHQQYSQQHTSQQQYQQQHAHYQQQPQVTAPQPSPQPAPKQVASPARPQARQIKQNQQPRQD
ASKQQQETIRQPAQKPTIIDVDEPASETVAQQEPEQMVYVNLQDIQKLPLLPPVTMDQTQGGPTSHTGKTTNWDAQYESGTVK
PMDIMLSSPQTQPLPPPQPKVQASHHQPAQTQPQMATPRAKPSPLSSVVNSPAINAHSPSITKKSPASTPKSRPLDTVHLMVA
VAEECFDKARGSVHDVAMSLNATRIDEYQKLISTGLACLEACLQSNRLSPRQEAKMRLRYATAVLEETENPMEAETAVTKGIS
LCEKNGLADLKYCMDYIRLKLLFQRNHKAALNAADRQIADCETYKHIQWVYAFRLLKASFYMELGQTADASALENIRSIQVTA
SSRGDDALSVFAYILEGLALLKASKDGNIEKVQACIAQVAKYQCEPTIHIMQLDILTLLLDLVCTINHSGLDLMLDKLKMLQD
RMDACTDWHSVKSDFLIPVQKQPATGHTISSDTAGIIRSGEGVPEDFLVMSFMTKVELNSLIFTLTGLVNMHKSSAHGRQTAD
FLNEGVKALETWDKATAGFRYGPSISLQDAIAQREWRLEAHCYLTILLGLYAASHCQWDLVKQYIVKLQDIMIPSTQGILRLL
AIYLSGVFDQGTGELDAAMNTFQKPDFDLNQRGTGVKAAHRELAMLAGLNRLWIMQHPSYRNDRATLDLIEELRPLCLNHHNV
DLRTAWHNVMAAIGTDPPQQLNQQKQHIQSAMAGSKSTKNVLGAAVTLAIMRSRLFENVVGDQALKSALAAAKQAHRSGNVLW
QSVADGMLAHSYEVQGKEEEAAQEWARATREAQEAFAGSF
FUN_004197 CDS scaffold_16 162246 166120 -

hypothetical protein PF01841;PF07653 ENOG41

MAPAAPPLPTRFPCWCRAVYSWGGESKRDLGFIEGDLIECLNAGDGSWWVGRLYRDRRTVGSFPSNFVELLPSQFRPTTKSVP
APVPNAPPSTAPTKSRTFRKPFEAYAKAPHYTSAKQPEIFKETPKPKERQNSGASFAPSTHETERGPSPAPPQSYNHRGPSPA
PPQAHDHRAPSPAPSPAPSHHYGSRAPSPAPMHSYHSRAPSPAPMHSYHHSRAPSPAPPHGYDARGPSPAPPMHQSYGYASRG
PSPAPSFHRQIVPYRGGQDRGNSPPPPPPPPHRQMTRGGSNDLHRRSIDISRHGSNASFHPFSPSRQNSNNSYRPPQPPQPTI
RHGSRGSFDDRSYMPYTPGRNSPVPPSPAGGMTPSPLREAMDGVMEQLDVLGGGLGRPGQAPSPEPPADPWSPESFDMLSHRS
NRRDQERSRPKTSMGIAQDEGYETYSGESSQEVSYQSGGREDKLSSYVDRMEKRFQQMHRQNSRTSEPDDDQPPPPPPKNVPY
ERPKSSMGRSIEPERKLRARKSAYEIGRGVARTLTTKTSSTNSSSGNQSNTSSSTQSTSRTLWSGTSAGAFSTTSAGSLARSG
KRQRAQSALGARDLEIDRPDSPFTGVTYHSSHASDSPAQQRPQTQVGFHDDPSLELGGLVQPKPPKRNIFRKIFDTAKTGVAS
SRGGLVAGGLGMDSPKSPFARSMPAGASPMPGMGSTPTNRDVATEMGLSGGVDWVQVRRDVNRSNSLSTIELTERRERCQMMD
HPALNPVDELYEGVEGDEGADGEPVQEPTNYQGINLSQVDKNSRFISGLPPAITAVQLATTYVCRPYRSDVQRLRAIFTWVSE
KICWEEDFEGEIDTTRVIQAKRACAEEYAVLVMEMCSAIGIHCEIVRGYLKSPGEVSEINIMPRPNHWWNAVLVDNEWRMVDC
CLASPSYPRRGLYSNANIIADPWWFLTRPLEICWTHIPEHHSQQHIVPPVAHETLLNLPCACAPFFRNGFEMVDYNTALTRIE
DLEMVHIKFSVPCDVEIAAEVEVRGYSRDSDGDVFESGDIVKKRALAQAEWFNGIKRYTVKALLPGDEGQGTLKIYAGKRGLM
HSIKDIPHPLAFALPIVHTGENPPYEFVTRHPTPHAQRHDIYVVQPQCQRLALNNTFVFAIRQHPSSLGGSALTPSSNPGGTS
PIPFARPSSALSMNASSVSGSTPSSATGTVAGKKPAKLAIQTPGGKILRLMRKEDRKGIHVGGRSLSGSETASDGGTWETIIK
CSEKGVWRGLVLADRTARWCVFAEWVCMG
FUN_004198 CDS scaffold_16 168467 170510 +
hypothetical protein PF00069;PF07714

MADEGVAEHYQVLEELGRGSFGVVYKGIEKATGETVAIKHIDLESNDDDIQDIQAEIAVLSTCASSYVTQYKGSFLRGHKLWI
VMEYLGGGSCLDLLKPANFSETHIAIVCRELLLGIQYLHNEGKIHRDIKAANVLLSETGKVKLADFGVAAQLTNIKSQRNTFV
GTPFWMAPEVIQQDGYSFKADIWSLGITAMEMANGEPPLCHIHPMKVLFHIPKNPAPRLEGNFSKDFKDFIAQCLTKDYDRRP
SAKDLLRHRFIRNAGKVEALQELIARRQMWDANQNRQKHPIYYQETLQTMTPKDEQQEWVFDTVKSVAPPPKRPTIKQHRKPS
SIWNAEEAMKKLDVKDGPLGGTSPAPGTVRKSTVRRAPSLAQETSMRPGSPRGSIAPKRPLQPDMSFGNSGSTMRLFRRVPSD
GSNGSQGNRPVSPDDVFCDENLPRPTTTPVEPYGKEAVLGRRLYNKAVEPSLAELHAQTSAMQKREALAKLSDAFAALDAVDP
EGAYHLMTSMVSLISQDNKLSATILRQPAQKIPGDGTPQGTVVVKNTTPASSPTKLVMASNNPHLRSHRRRHADTPTMYEKNF
DPEKLATENKYPGQEVRSGMEHCKQLSDVLYSRWVDGLRIRWPAV
FUN_004199 CDS scaffold_16 171053 172618 - RAI1 decapping
endonuclease targeting mRNA PF08652

MSARFSVQPIGRFAGASQPVKRPKEFACFSYDDNHEFHLDDSSLKYYYTPQLGADLSKGFDTFQKLDDTGDDHLDSLLKTIAA
HEQETGKKIDANIVTWRGMMTKIMAAPFDQMDGFEMNATLYQDCIFIEENNAYKIASRSNEGNNKRRRGPPLEVMQFWGYKFE
TLSTLPAPWAETSRDFIENRENEVVNNKAQYCSVVRTGIGKSVLCLGGEVDAIWDSKPEEKGSPINWVELKTSAEIRNPGGME
NFKRKLMKYWIQSFLLGVPRIVVGFRTQDGILVEAREMETHRIPDMVNADPNPKWNADMCVNFAATFLEWLTENINDEGVWRI
RREPQSPTIELFKVEETGHGDILSDEFKNWRIKLALGPSDAS
FUN_004200 CDS scaffold_16 174083 179063 +
hypothetical protein ENOG41

MVRTRKEENQSTTNTPRTSLERKSKRKRKGQAQDSEPELVQQSPARKRRKLGKKKEWWNIRRVINEEYLANPNSGEINHRCLV
EWETDSDGETYENEWIPYKDLNAEALEDWEKEKHKRERERTEREAQRRETRNRRGLRDLTPRRGKSETPVVCNKQKQPKLDAS
THDISRFDSHEQDVQPNRSSRIKSEEPVPSFISASSVDAESVSSYEFSRAPTQGVAVVLSKPDNFDPSEYQSVTTQNSSQRIT
ELEDGDQRVTFASQLSQDTIPDSQDLSGNWDYQDCESQPAARPDCVPRSPEIPEDPSSLAEDNPGSSRQVREVVCQKEVHNRE
SIARSPEREAFQSANVPVDLKTEYPDSENPEDNLPPTDFGEFTGAAHHLDDQTEDIQSLKETHDLEGDNTENRTTTNNQILDG
DNLGSTGGIRDILSPENESFETANSQASHSSNSRDLPVNQDPYGQDSVSVNQGSGDGQRVVQDHVFDSSSRLPFEEETLGSQQ
PGVKRSAPSHHHPPRDRDERPGLQFEKGDVNPQRPDKPTSVSGVEDLSQAQVLSSSSKEIDIIVPDSQGDSSLSTDLNIITDR
TSTLVSKTQVTSLASQAEVVSDSAATGSDIIPSRQPGHSHLATSERGILLDSSLPSDQSIRHVVGEAAASLRSSPGSHRTQKP
QIYYSQPQRLYGSLDISSSLSSAAGTLNKTARPRATIETQASQEETFEASQLSKSQPEKNSVSVQRIGSSQRSPSPTTIAKTD
SVLSKSKTGSDSKGLEKPRLTSKQKMERPGSQTRDSAVDELKSYIDFGRDSALTHVGDSLEENPGGTPYEAITEKELVPGASA

SSHAAVLQSQPVYSMDPWKPQALGTNSDTPVSSIAPAAIMADPSTTAVARMREELSRRFAESDVSLTRSIISQDEEDSLLPAT
ISPTVISIVNEPVASTHTLNFPNQGSVPTEVESSGHSITMGQVPVEQVSDVLSESSEQNDFYVQTKVTLPMQASRRSYYGEVI
EDHKDEIQAFSRCFTGETPEEPSEELIKKIDSLFYRLFEICDYPQDVIGSVLETLPSSELARYCCDANPKFSFLFELMAALDG
KEKEILIVVGTKELMRLLFKLAEAAKVECSAENINQHAKFASFARISIALTSEDFDPFSFDVIIGYDFHYPSSLIARQLCDNT
VRKSPLVLRLVTTYSIEHVYGPLDGATALEVKNAILASTVSASRYLEEPERGYYEPHEVAEIFANYFNGITDTLNWEPQGIPD
DVLDIFENPGTQTQLPFTGDALYGNDLKRKHFNDEGDGDAKRMRTLPLRDLPTDTNNPPMPIAVRQMLESASLRSGTRDRKAM
ISVPLVTLETIQEQIDEYKRQTAQAGEVEVELKSYISRLDKELKDHRKTLSKIESSNRAALQDRTVAERERLRTEGVAAAAAK
VAQKEKEKQQQRIDELESVIARLNQNPELAKREEALSEVKGQLQLSESRRTSALSDVDFMKSRYQDVDAQALRLANENKSLKI
QNEELEQKASENLRAIHAQNVAIERQELLEQIASLQAQLQQRDAELYNANQRLASVSNGRNTRGGSMPRSPRVPSGMSPRPRA
AFSASRGTSPIGPGQPFVGQQAGNGRWNHLQ
FUN_004201 CDS scaffold_16 179576 180545 - COX6 Cytochrome c oxidase subunit 6 EOG0926522L PF02284

MSAFIRVARSVRAAPIRPAVQPLVARSAVAHFTTSIPRRSEHAEETFEEFSARFEKEFDGVQDVFELQRNLNNAFAYDLVPAP
SVIAAALKAARRVNDFGTAVRIFEGIKTKVENKGQYEQYLEELKPLREELGVLLKEELYPEEK
FUN_004202 CDS scaffold_16 182561 183688 +
hypothetical protein PF01151

MSASPATFYEQLPLPTIDRPFGVHLWPIFDKAFTAVVGYSANDFKFVPFETPMSTLKSTSIFIVIYYAIIFGGREWMRNREPF
KLKGLFLIHNLYLTLISGTLLALFVEQLLPTVVRGGIFHAICDAEGGWTQPLVVLYYLNYLTKYLELLDTVFLFLKKKPLTFL
HCYHHGATAFLCYTQLIGSTSVSWVPIVLNLLVHVVMYWYYFQSARGVRVWWKEWVTRLQIIQFVIDLGFIYFASYTYFTSTY
FPWMPNAGKCSGEEFAAFSGIITISSYLVLFISFYFATYKKQGKAPTGRQSLRRMSQAPLPDPHLVQTTPVKKSNGATATGSK
TNGSVRSRKA
FUN_004203 CDS scaffold_16 184477 186481 -
hypothetical protein PF06985 ENOG41

MRRFLQIPTRSLTRRLFSQSICQASSGLYKPLNPRTSETRLLKIPPNASSEFELVTVSLDDEPKYAALSYLWGDPEKYGQVTI
KGNTVKIPDNLASAFLCVLSDDSFRSQSHVRYLRADAICINQNDLDERSQQVQLMRRIFQRAHVTFAWVGPKDYSLAFETIKT
ITKDIYENTPKENAPYKTNFDLEWLRRHPMLCIDRGPDLPNNHLNDPWSAVADFLQHQYWQRVWIFQEIVLANQLVFISPGEA
NLSWSMLRDTPQSVISVANDKKPEFLPDAAWNLLSNPMSWGKISWLFLAQARPKIPDPDGFKGWAVSTFAANLQATDDRDYIY
GLLGVSGILMTPDYSSKNTTSQVNTKYMAGWLKAAHTQQTTHAHSSLAFLSLAGTGKYGHSDLPSWVPNYRKKREASSSWCYS
ASNFRSPDEGEPTSSPAHHASYPYVVEETHSLFSWGKDMGHITLSTKCRHDSDPEILASFMSFANSFTTRNSQYVTGIPAAQA
IIRLISMDKSKNVSQDLVGNAMNLAMLITYFNMSESQTVTKTWSSNWDHDLLNMAFSPSDLAPLKVRLNVLEELSSWSLERRR
EAVSGIMPVLYNYRFFETNRGYLGAVDFDVLEGDRLCILWGYKEPVVLRPDATDRYTFVGPAYAVDVDTKNTIPSSPSQGQWF
ELR
FUN_004204 CDS scaffold_16 188020 189941 +
hypothetical protein ENOG41

MRNPFRRRNKKDKAGSGGQSSDFIVPIEFRPPGAGRPPLYPPTRHSAYLLAHLPPKVLERIFTFVCPHALDETYDTCEGSAST
SDICRAWRPSAVKILYHSVRIDPVHYCRLEEWLAEKRKKTSRFDRNGVPEDPAQARLRLLRRTVRDDPTRIGKSVQYLKLPYM
LRESSQVELAQTIAVLPNLRYVDLPEGMFCDDPHYATLRLEVQARCPNLRKMTYMGGSERSLAMLASGQVWPNLEVLELNDLA
IDAMTLRAVLGSLTNLQALKVSNTPGLSDEVLSSSDGLPTLPPMKELVLKDTPGVTLKGLIEYLAWQETQQALTVLTLKDTGV
HPASLQEIFTMASSLKTFAIQTNVSEQFPTSANSRPLASRTLETLRFEVSSSSAGAFSSVTMGYYSYLASSILGGGFPRLRRL
YVHDESFPDQLQGLPPPNAAFAGGHVRAGSNSSTKSTPGFAFPSSNLSPKSANLPVPRRPMSNIQPPSKRFSSNNPFAPRNPS
SAAPTHTLEVFTKSDEFGKWNFVSVDPMIRSAAAPSRRPVSSYGLAADVTGPGWDRGDARRSVMVGNGAGVFLAVPGQGGDDG
AFNGSSDPWRPQSSGGSPRRSRELW
FUN_004205 CDS scaffold_16 190800 191346 -
hypothetical protein ENOG41

MELRPKIEAACNTDMDAVAFLYEDKIFPPTYMVDLLLLSFNTYCYRDRVTGKSCDLQLAEWRIHRGSGKALECEDCFLAPLRI

ELEAGISHNDEDASEFEEMTSSCNATGYDYTKPAPYATTLSTESWATMVKSALAIPTPWYSI
FUN_004206 CDS scaffold_16 192385 192730 -
hypothetical protein ENOG41

MEDCETLNNAPKEQVHDQFSQWVSERSTERDGARADDPLAVYLQDIDIVSMLIRNASTPSTNTLPGSRQALKVTKGLSFVSCW
MRNVNQEDEAETDFPLSKDAPRNTQAGFTLA
FUN_004207 CDS scaffold_16 198393 199551 +
hypothetical protein ENOG41

MSLAVTQQPQRELDAPKLDTALAQRVQTQSQPRSQIQSLSVEPPIYSPSADADSSPESTPTTMIPSQRQLSAAPALPAKSSLR
ASRCLDGLLTHKLTPEGQAPFSTPHDVYLSSEEDASSSADDFSDFEFDSGSDESQSTRRGSQEDTAKVVSVVFSGKPSVIDLP
RRSVSPSIISEASSRPSSRMSISSRPPSRLRRTTTLPVTLPVLDRRMSFCTTSSGSVMHPPRTSSMGTRLEKQRPDFLSIDPF
AKVEPEVKEEATERPKTPKSPGVFKRTLSLVKKRSRPSLNTHFASQSKDNLSLFTPTFPMEQVREEPGTDSEERAPKPILQKA
PTYHEFAQRRMSSAPMSPMSPMSEPGSPMTPNGTRSRLRQGLAAAARRRSVKV
FUN_004208 CDS scaffold_16 208509 210858 +
hypothetical protein ENOG41

MAAQQATPVLVLGIDFGTTYSGIAWCRAGENNEIKFTTNWKKRSFAQGDKQKVPSTIFYHHLNDEDPEWGATTPQDDTVLRWF
KLLLVDEKDLPDHIRHSVQLKTARALMLKANKTSVQILGDYLRQLWRHCRQNIVRAEGRRMMNVSAVHLVVTLPAIWPHYVRS
RMTEALGHAGLLEITRAGQTTLTFISEPEAAALTCLKENADRFNVRVGDHFVICDAGGGTVDLITYKIEKLGPLIMSESVKGD
GGLCGAMFLDERFLKLLKETIPFEAQKRLDKQTFHRIMESDWEGGLKSSFCKSSGDLPIQLSYDGPVDTNFFPSKFSINVSEI
RENVFDPVVEQVKQLVQKQVDAVKHKYKKKPKFVILVGGFGRSLYLHEHLQDAVGNRIDVLQQDGEKPWTAVLRGAVLHGLAR
TNHTSSITVAVASRVSRHSYGTLVNILPFDAKEHDAKDRVWDDGQQEFLAVEQTRWFVEIGDSVSTYDPVCASFWQDLTSPDQ
DLQIDIVTSDASTPPTRKDDSVKPMCVIKSSELPKWDTIPVWTNEDGQVFHRITYELRMISDGSSLEFQVYYKNKSIACGSVA
FDSTGQNDTDDEDDVQMGSRRSLYNYTNDNNIVDMTDADSDSDYVEAEE
FUN_004209 tRNA scaffold_16 211800 211922 + tRNA-iMet

FUN_004210 CDS scaffold_16 212552 214991 +
hypothetical protein PF00326;PF00930;PF01738;PF01847;PF02129
ENOG41 S09B
MEQLAQQYIDSTNVTPRWLPGGDAFWYKQNISPAKCQFLLVDVRGKIREVAFDHKQLAEALREKTNEEIDQFSLPFNWIEPVP
DESCVRFRFDNRKWQFGPNSQLEEWEGQFTTEPDHLLQKEVISAHSDTKVTVDFVNRTGKTLRVFWIDWKAKAIPYMSIRNGE
TKRQITYVGHVWRLVDVSNEKSRAVYSAPDEGNHVAIIEHLSDSIPEASPPSDDEDSETDEEPTEPENGPCLYVRDFNVWYKN
ENDEAFQLSEKGNEESPYDKRRLYISPDKQHAVAWQYTPGQDHKINLVESSPEDQIEPKLHTKTYLKPGDRVRVDRPRLFNLN
AHSEVAIDDSLFRDPYQVSNLEWNPNSQEYLFYFNERGHQHTRLIGIKVDGSVRTLVEEKSRTFVDYTKSYFKLLRDSDELIW
ASERGGYNHLYLIDLITGSVKNQITKGSWMVNFVESVDKDHRRIWVRGYGLKDGEDPYHAHLARVNFDGSDFKVLTDGDGTHS
WSFSPDKRYLIDTWSRVDCPPTTVLRESESGEEIMTLEKTDSKELEDKGWSSPERFASPGRDGKTLIYGIIIRPVDFDSTKKY
PVFDEIYAGPHNFRVPKAFSTLSDRRRWADEGYIVVVIDGMGTNWRNKSFHDVCYKNLHDSGLPDHIAWLEAAASSRPWMDLS
RVGIMGVSAGGQNAVAGMLHHSDFFKAGIADSGCHDNRMDKLWWNELWMGYPVDEAYEKASNITHAKKLKGPLMLIAGDMDDN
VDPSSTFQMVNALNKAGKNYEFLFIPGGGHGCGGEKYGLARQREFFKRHLQQESEYRVDMYDTGS
FUN_004211 CDS scaffold_16 215466 216806 +
hypothetical protein PF00226 ENOG41

MKISYLSVGLLALFSPLAAAWSKEDREIFRIRDEISKYETDPAATFYDILGVPPSASLDDITKAYRKQTRNLHPDKVRQGMRS
KAGKDKKAGAKTKPPTNAEIKAAVKKAGEAQARLSLIANILRGPERDRYDHFLSNGFPLWKGTDYYYNRYRPGLGTVIVGLFL
VVGGGIHYLTLYMSWKRQKEFVERYIKFARDTAWGGGLNIPGVDAAPAPAPAASPAPASDEEEAPPLPQNRRERRMQEKAAKR
DGARPAKKTRRAAQASSGTATPTPAAGPTGARKRVVAENGKILVVDSLGDVYLEGEDEEGEVHEFLLDPNELAKPTIKDTAVF
RAPIFLFNITAGRFLSKKPTDLEIEIPAEDEDSDVPQPTPSTDSAGEDFELLDKSTDSLTKAKASGASQGKASKRKGKKR
FUN_004212 CDS scaffold_16 217743 219425 +

hypothetical protein PF07690 ENOG41

MTSSNQPERPNVSGDSITETFPSQDSAEDTKVRNSVESTEEMGSGNVVLQSSKYETSFLRRILTPKNCRWNDESPPALSMRHC
CLYAMASGITVANLYYNQPILNKIAATFNVSYEESSQVATLLQSGYAAGLIFVLPLGDILERRPFIIALVLATATMWIGLCVT
NDFTVFRALSFVCGATTVTPQLMVPLVGDFAPAHRKASLLAIVTSGLMLGLLMARLLSGVVANFTSWRNIYWLACGLQYLMVA
VLFCFMPDYPSTNPDGLNYLYALWSIPRMMVTEPVLIQACLIAFSLSAVFTSFWTTLTFLLASPPFDYTSLQIGLFSLTALAT
ILSIPLIGRLIDRYVPLLSTIGGQILALTGAIVGTFTGNFTVAGPVIQAIGIDVGMQTAQVSNRAAIFSINPKARNRVNTAYM
ALAFAGQLTGTAVGNRLYASGGWRRSGGCSIAFVGLSILIAVARGPREKGWIGWTGGWHPRREKPVITQTEDEEQSEEQDRKE
ER
FUN_004213 CDS scaffold_16 222016 224291 - SDH1 succinate dehydrogenase flavoprotein subunit PF00890;PF02910

MMASSMAMRRLAVGPAFTRAARFPSRAFSTTRPVARVITGNGPLRAKEASPFVSNKYAVIDHEYDALVVGAGGAGLRAAFGLA
EAGFNTACISKLFPTRSHTVAAQGGINAALGNMHEDDWRWHMYDTVKGSDWLGDQDAIHYMTREAPASIIELENYGCPFSRTE
DGKIYQRAFGGQSKQYGKGGQAYRCCAAADRTGHALLHTLYGQSLRHKTNYFIEYFALDLIMQDGECRGVLAYNQEDGTLHRF
LANHTVLATGGYGRAYFSCTSAHTCTGDGMAMVARAGLPNQDLEFVQFHPTGIYGAGCLITEGSRGEGGYLLNSEGERFMERY
APTAKDLASRDVVSRSMTMEIREGRGVGAEKDHIYLQLSHLPAEILAERLPGISETAGIFAGVDVRKQPIPVLPTVHYNMGGI
PTRYTGEVLTVDEKGEDKVVPGLYACGEAACVSVHGANRLGANSLLDLVVFGRAVSHTIRDKFNPGDALKPVAADAASDHIEV
LDKVRTADGPKSTAEIRLAMQKAMQTDVSVFRTQESLDEGVRKMKEIDGMYSQVGMKDRSMIWNSDLVETLELRNLLTCAIQT
ATSAANRKESRGAHAREDFPERDDENWMKHTLSFQKEPHGKVDLAYRKVIANTLDEAECKPVPPFKRVY
FUN_004214 CDS scaffold_16 225463 228634 -
hypothetical protein EOG09260DUR PF00787;PF12825;PF12828 ENOG41

MAAEAGDTPAVVEDATANPPSTVDLPVRSKQDATPPPGQTLTGKQEHYLKRELISEQVKWEINELNSTTALQRFGAPFRSDHG
EVPPEESELPILRYIFVHHVRDFPFLDKAREKEFWQDKLQVFLESFAGKQISSSEDRLEETKRRKLAIKARKLVELMMVSGIG
TSSGFEERIRFSEIEIVDSNAIDTGVMHTLPEGNYINGWDVNVAGVRVTQVKRTIRKHKHAEFILRIKKKGELEHYVGRRYSD
FAKLHSRLRQELPGRVVPEMPKKNKSDTTTTIAFTSMFSNGNESDASSISSVSTRPNGYSSMLTPGHRRAGSAASFRSNRSKG
LNSPRIPSPRVSTEGGRLSPAVRQDSDKSDKSETVVLWRENQRISLRAFLRSVLHNPQFANTKAMQDFLSGEPITPTDEDVED
ILRRKEVDEKRMEEQKQFYEIARKRAADLDEYMEQFRRDIVEHNGLTMLFKEIKEKETIQDLSIQYRKFAEWLRIEIAAVIYH
LFLAEDNSPELFAQARRIHSLIPYSVIKNVIRIANPAAVMSGVLDIFLAQPFGTRSLMQRIFSLTLNDGIKSFQKSIDILASK
IDDSVFSGKLYKYTHAEEHIKVAIREEAEADDVDLIVAIMRSDLIEPGLTGEQIQRLYNAYVAFNNAVENIDEELRQGAQLFS
YMKQYLKLCTRQRDKEMMLQLIEEPVTLQLFRDLFTIFYEPLVRVYKSANVYNSVTDFAVFIDDMIQVVDKCREQDASADPNQ
TVQAFIDLCQRHEHNFYKFVHEVHTHDNGLFTQLMGWIEGILEFLRKGPKNGTLNVNALFEGGVSAGILDKDKAIAEINSLIA
WQEARKKWHNDKTRQKMAAEGHAGSDMMPTAMAFNSSDFGLDSEDLEDMAYDDGSEDEAEAEAEDELDPIEAERRRRAKRQDR
LRRSAGEPVKPPVREVHKLTDNFLVMLREVLSN
FUN_004215 CDS scaffold_16 230665 231795 -
hypothetical protein ENOG41

MASAGFTSPRDSTNSVSSVRSSSRHQLKRSITELASPVKLGRREKKERDRYNEDRAVHAHHISISHPVSANPQYQGRASVDIM
TRSEGVTPYMSPDQSRRPSLMLSREEENKALNVPAPPEPKTKEKILKDQAKTSSQVEGLKQSLVELGSFSTSTARRLDETYYA
VLEKMSTLQSTVSALKELAEESQSIHRNFEKDACEIENDITSQIAAMGQFKEHQENIESLTTRVQDGRARIRALSDRVDIVRE
QVELWERLDKESQDKTRLRLRAIIICITVVFLAVVVLFFGAQYVSRHSSLLGGDNSSTPRVAESIIQSHKGAAHPSLSLRRPE
KSGVPYSKRKQVHTHAEEELRVFDEL
FUN_004216 CDS scaffold_16 232717 233189 -
hypothetical protein PF03879

MSDTEKTNLTPAPAAEKTLGMRKNGKQWHAPKKAFRPTAGLKSYEKRTQERALMAQVKAKEKEMKDEKEQERQRKIAAIKEKR
AKKEEAERYEKMAEKMHKKRVERLKRKEKRNKLINS
FUN_004217 CDS scaffold_16 239615 242438 +
hypothetical protein ENOG41

MATAFGPERLTRPTPGPAFDSPSDRFASSMNSFTGPQSVESQAQLDEPPPNPPFVFPAKPPAPASAPSSFSRATGRRPMSAIE
TPNFSFGFSAENAEGQSRSALPDFSFNPGALLSPDRENNFLLTPPISPHSPRNIASSQRPGGHGHRRGGSEFVGGRIREGNSI
AIMSTSPTKSESGLVTPTFQPPRRGHRRGISGAISTNDLPILQPPVFDGAGRGNSAPTSPTTFGQPSNQPSLPLDEKVIPEPE
SAPEPEAPKETEELVSVPSPSGSPKQPKARVGFSDTLEFIPRPLSLVSNETSSTVTARPGHSVSGSISSIVSAASHNGRDSPS
PLSQTSTREISDSRPSTAGAILERTPDQANTPSSPKRRNSIPTLLNFAEAPKAEDPEPSPTRKRWSFFVRDSSAGNTSPGKRP
QSAGSIDLLTKALPVTGHGIDPVGENAEITPVKSKSSKKTKKKKKKVKGWAGAILPRKPKSHSKRSKSEGGRPPTPPAPRVAR
YEGEDAMYGEPELLEISTPTLTVTESPNIPQDEAPKRSADESSYPMIDLDAALGPFNTPLPLNPEWEAAQRAAGGAGKRRLHS
AQGMKGFSGPGMHYHRRAESAPDLPPFDPGRAGMHRYNSSSTMADVFEEDEEDDDDNARSGTTDDSSEEETDSDSDATPPAAV
TRDPLRDSQDKLSIHSSSQPNQAMRRTTSSLSDKDQISASTVRGERSRSSLHESVIAEEMPSVIFRSPFMPAERTEATDSTPS
SLRRFSGTKDLSPGEVSPLHLPAPANVPTSPYAMSYSSSFPSPRSPMSVEVQRISTAPSSVTDDNNFRSLLLMGEPGPEVRVS
VDYDIPSLTSSNSTMTRESMFLPSQRQSQPTLREPRPVSVSSAAFGRRRSSLASLSRLISSSHGERSKLSMEVTLDNDENHKK
AKSSRTKKLGRMMQFWKPAKEKEGEAK
FUN_004218 CDS scaffold_16 243342 244058 -
hypothetical protein ENOG41

MGFLDWNDGASVLSGRSRRHKSSRSHKKRRSRSRSRSSSRERGRGGFADMADSFFGGDRYGKHNSSRASFFGLGGNNSRSSFF
GNSRSSYYKRSPRQGFVQKTYKQLKRLLRDLVHWAKRHPWKVFFVVIMPLITGGALTALLARFGLRIPPAIERMLGVASKAAT
GDSSGLVGEAVRMASGFGGNSAVRVERDTHSYSGGGGGDTWGGFADMAKKWM
FUN_004219 CDS scaffold_16 246321 247246 -
hypothetical protein ENOG41

MATSVRSFGRCRFSGCPPVLDVKLTQPGQSRNKNPFKKRPSRPPYWGQLHLVAFFGAIVVSRFIMEDPNLTEAPGAAQCPSSQ
SAELKDSSGRPHCGQPIAGATPLGTPVGASEDDAESPGSTPPPSSAFKMAEGSTTNSGASIEPSSPSTIRGVMTPKPGSADIS
DGNADLEGSSSVSHEEAVLNLEDKLRQFESQLRERNESSSVLTVPYPSLKHKMQDELYDPVSKKMKPASQVSPEMGSLSTEVA
GR
FUN_004220 CDS scaffold_16 248383 250037 -
hypothetical protein ENOG41

MAGMDKPPEEKRLLFINTNENNFPPRRNQHNSIDSEVRRHVMLDIGKARRKPSKDRQFVTLTWQTQKHTGKQESPKRSHYGHS
KTPEECQATISTPTLYALAVFEKEWGEDSFSAYGFTLIMATGKNAMSCTYSANTFWSPFAFRKSAFLKHYQQIFSSPDVLIPL
YRRSSLELKSIALERSLMTIQCIESRLTSSDASWATSDSVISAVLALICYNFSNLDFDQAMVHISGIWMVIEARGGISTLENN
QDLLLMISWVDITAALLHNTKPLFPLPMNLDAFVRPGLNMLPGPLFGLLNGDILCDERFLAVLACMGDLNALATFLQTEQVTK
GDAIWSDEEQMGLLLNPIAHDLLNQQQLEPPKPDTPYEIILESLRLGAIIWIIQVKRRCRSYPGTAQARITTLLRTISNDTEG
DIPWNCSADLQVVRLWLLVLCSVSEPSGQDYSTLMRIIASEMKELNLVTWGQLISIIRRMPWIYLSEVPDLSTL
FUN_004221 CDS scaffold_16 250716 251701 +
hypothetical protein PF00043;PF02798;PF13409;PF13410;PF13417;PF14497

MSAENMITLYCEGTPNPLKISIALEELGLKYNARAIKLFEHEQKEEWFLDINPNGRIPAIVDTDEHGNELKIWESGAILQYLV
ERYDKDHKISYPRGTKEYWQMTSWLFWQVSGLGPMQGQANHFEMSAFGDYPYALKRYVNETRRLYRTMDKALAENPSGYLVGG
HLSIADIAIWPWTTAYKYSGLSQIDEFPHVKNWMYKLLERPGFEKGRNVPSPHIYLQLNELPDEELKKLGRERSVWVQEAMKR
DAE
FUN_004222 CDS scaffold_16 251943 256691 -
hypothetical protein PF00005;PF01061;PF06422;PF14510

MLGNTVDGDALRRTQSSMRQQQQQPISQQTVEGGQHNPTSSSSSESGVVQEGKWGERGQEDVTAQEAMQEFESLRHNLMSLHK
TRTADTHASRAQSRRTSTAARVRSEKHEHDYDDDGSKTDVEAGPEESEGFELGRFMREGHFEKRSEEEGSLKRVGVVYENLTV
KGVGSTTTFVRTVPDAILGTFGPDLYNVLTRYFPSLRIGKPPTRTLITDFTGCVRDGEMMLVLGRPGAGCSTFLKAISNNRES
YAAVEGEVSYSGISAAEQKKHYRGEVNYNGEDDVHFATLSVWKTLAFALTNKTKIKEKADIPVIVDALMKMFGISHTRDTVVG
DDFTRGVSGGERKRVSIAETLASKSTVMAWDNSTRGLDASTALDYARSLRIMTDISNRTTLVTLYQAGEGIYELMDKVLVIDQ

GRQIFSGPANKAKQYFIDLGFECPERQTTADFLTAVTDPTERRFRPGFEHRAPRTPEELESAFRNSPHYQELLADVAAYKESL
HRSDYQDAKRFQGAVQESKSKHVSDKSPYTISFPRQVLACTKREAWLLFGDTTTLWTKLFIIISNGFIVGSLFYGQTEDTASA
FSRGGTIFFSILFLGWLQLSELMKAVSGRAVVARHHDYAFYRPSAVSLARVLLDFPVIAVQVCIFGLIMYFMTGLDVDVSKFW
IYMLFVYTTTIMVTALYRMFASLSPEIDTAVRFSGISLNLLIIYTGYVIPKTQLLKDYIWFGWLYWINPISYSFEAVLTNELS
DRIMECAPSQLVPQGPGVQSGYQGCAISGATVNAQSVSGSDYLQATYNYSRSNLWRNFGVVIAFAVLYILVTVLATEMVSFTQ
GGGGALIFKKSRKAKEQARKAEAPVDDEKVVGNGASTSSGVVADPNPGSEDEALEQITDSESIFTWRDIEYTVPYLGGEKKLL
NKVSGYAKPGVMVALMGASGAGKTTLLNILSQRTSIGVVTGEMLVDGRSLGADFQRNTGFCLQGDLHDTTQTVREAIEFSAIL
RQDKSVSRADKLAYVDKIIDLLELNDLQDAVIMCLGVEQRKRLTIGVELAAKPSLLLFLDEPTSGLDSQSAYSIVRFLRKLSR
AGQAIICTIHQPSSVLIQQFDMVLALNPGGNTFYFGPMGENGEDVVKYFSDRGAVCPPNKNVAEFILETAARPHRRPDGTKVD
WNQEWVESEEAQKVLEEIDGLKRVRSSATEGVVSSNKEHSEFAAPTWLQTVELTKRMFRQHWRDPSYIYGKFFIAVIFGIFNG
FTFWKLGYTMQDMQNRMFTCFLIVTVPPTIVNGVVPKFFTNMALWQAREYPSRIYGWFAFCTANIVSDIPAAVVSAVLYFVLW
YWPTGLPTESSVSGYTFLMTLLIFLFMTSWGQWICAFAPSFTVISNVLPFFFVMFGLFNGVVRPYASLPVFWRFWMYYANPST
YWIGGILAATLDGTPVECSSEETAKFDAPPGQTCQEYAGAFADSAGGYLLNPNATSACEFCPMSSGNDYLASLNIDASDKWRD
FGIFLAFCVSNWMLVYFFIYTVRVRGWSFGFGTIFGGLGKLVDLIKKPFQRKKEE
FUN_004223 tRNA scaffold_16 259457 259577 - tRNA-iMet

FUN_004224 CDS scaffold_16 260272 264361 -
hypothetical protein EOG092606AJ PF00443;PF00917;PF12436;PF13423;PF14533
C19
MTSFHRRLCFRSGLNADRRNLLQTHDINSPNDMVMDTDDYIGVANEPEKVAIINPDNLEQSEVDQLQDLPMANDHEAMREICL
PPLIDEPKILGDYEYTWTVENWRSLNKKEHGPVFQAGGFPWRILLFPHGNNIDQCSIYLEHGFDADSVPDNWSCCVQFALVLW
NPNDPSLYVHHTAHHRFTKEEGDWGFTRFVEHRRMFNVPWEGSSRPLCENDTANITAYLRLVEDETGVLWHNFANYDSKKETG
YVGLKNQGATCYLNSLMQSLYFTNKFRKAIYEIPTEADPSMTNSAYTLQRLFYQLQTSDQAVGTTELTKSFGWDTRHIFEQQD
VQEFSRKLMERMEDKMKGTASENVLPEMFSGKIKTYISCINVDYESSRIEDFWDIQLNVSGNKHLLESFEDYVQVEKMDGENQ
YFAGDQYKLQDANKGVIFNSFPDVLHLQLKRFEYDIQRDTMMKINDRYEFPEFFDAAPYLSEDADKSVPWTYQLHSVLVHSGD
LNAGHYYAFLKPEKDGWFYKYDDDKVTKATMREVLEENFGGEYQTSNGYPRAPVQKKAPIMRQNSAYMLVYIRQSRIDDILCP
VTKDHIPLHLRQKFEEETVQREARKKEQREAHLYMWAKVITDYSFQQYGGTDLCQFDAKPEDPAAPKFYRVRRAMTMEEFVAQ
VASDMGEDPRRVRLWLMVNRQNKTVRPDQPIMDLRPTVDETFSRSAAHRDASLRVWAEVAEEVNADGEPIWPSYQSQPNGVIV
KNDTILLLLKHFDIDAQTLRGVGHVYISKDKKVEELLPMILKKMGWGEKLPAEEKLLLWEEIKPTMIEPLKPKQSLKVAELQD
GDIICFQRTKAGVEKRAGEKTSQETSNTSDHFEDAREYYDFLENRRTVKFHPHPARCDQAQYPPFDLVLNTKITYDTLSERVG
AYLDVKPTHIRFWTVNASTQNPKTPVRRGANPTLRQILSPMGSTALNSTQRDDAFYFEVLEMSLTELDTKKSIKVNLLSEGIT
KEDTYDLLVPKTGTIDDLVEVLIRKAQIPSETDGGRIRIYETSSNRFYREPLREHPVINLNEFAKIYAERVPQEELNADDTHF
IHVFHFHNDVSRVHGVPFKFLVIEGESFADTKKRLEKRTGIKGKSFEKIKIAVVRRANYSKPQYLNDDDVLSSFIQGEDDYLG
LDHVDRTRTLRNGVGDLFLK
FUN_004225 CDS scaffold_16 266315 267832 +
hypothetical protein PF02518;PF10436

MPSIALRQSRRCKLTDPRPYLANIPRTSYTKFQFRAGTNASWRPVSILDEYGIFFRYDFWLQKLTHDAFSWVAKEARPISLRQ
LMVFGRSLTESRLISSANYVRTELPTRIAHRIRDMQRLPYVVTMNPHIKEVYDLYYHAFDTFRKVKEVKTLEDNDKLCELISH
NLKGHLTVIPKLAMGILECGGLMSPQDLDNFMNTILRSRISRRVIAEQHLSLTETYNSPNFSPGAKLSESDFIGEVFIKCYAK
DVVERCSKAITILARTTNGPDVQIPEITVDGHLDASFPYILSHLEYIIGELLRNSVQAVIDRHQRVHSDPDSVKPPPVEITIC
ENQQHVIIRICDRGGGIPRAELPHLWSFSKGPQSQRRLENLAQVPKMAATMQELHVEEELGRADLKAPPYQSSLSSLTSRPPN
LRLGMGLPLSRVYAEYWAGNLDLHSLEGYGTDVFLQISRLGNKNEQLTTRASMDAL
FUN_004226 CDS scaffold_16 268554 269919 -
hypothetical protein PF00735;PF01926

MASATAENASPIGIANLPNQRHKIVAKRGAGFTIMVAGESGLGKTTFINTLFSTTIKNYADHKRRHQKQVDKTVEIEITKAEL
EEKFFKVRLTVIDTPGFGDYVNNRDSWMPIIEFLDDQHESYMLQEQQPRRQDKIDLRVHACLYFIRPTGHTLKPLDIEVMKRL
CSRVNLIPVIAKADTLSPADLAKFKQRIVSVIEAQNIKIYQPPIEEDDEAAAQHARSLMNAMPFAVIGSEKDVKTGDGRIVKG

RQYSWGVAEVENEDHCDFKKLRSILIRTHMLDLIHTTEELHYEAYRAQQMETRKFGEARPRKLDNPKFKEEEEALRKRFTEQV
KIEEQRFRQWEQKLISERDRLNKDLEQTHAQIKQLEQELEQMQGSAVRSHGRR
FUN_004227 CDS scaffold_16 270653 273459 +
hypothetical protein EOG09260E2O PF00400;PF04003

MKTDFKFSNLLGTVYCQGNLLYSPDGTHLFSPVGNRVTVFNLVENKSYTLPFAHRKNIARIGLTPRGNLLLSIDEDGHAILTN
VPRRISIYHFSFRSSVTALSFSPSGRHFAVGLGRKIEVWHVPSTPDASAEGELEFAPFVRYHTHTGHFDTVSHIEWSSDSRFF
LTTSKDLTARIWSLNPEEGFTPTVLSGHKQAVIGAWFSENQEMIYTVSKDGAVFDWQYVKPINRIKDDDKMEDDDEDSDMRWR
IVQRHYFMQGSAHVRCAAFHPETNLLVAGFSNGLFGLYEMPDFNMIHKLSISQNDIDFVTINKSGEWLAFGASKLGQLLVWEW
QSESYILKQQGHFDAMNALVYSPDGQRIITTADDGKIKVWDIQSGFCIVTFTEHTSGVTACEFAKKGNVLFTSSLDGSIRAWD
LIRYRNFRTFTAPTRLSFSCMAVDPSGEVVAAGSLDSFDIHIWSVQTGQLLDQLSGHEGPVSSLAFTPNGDSLISGSWDRTAR
IWSIFSRTQTSEPLQLQADVLDIAVRPDSLQLAISTLDGQLTFWSVTDAEQTSGVDGRRDVSGGRKLTDRRTAANMAGTKSFN
TIRYSADGSCLLAGGNSKYICLYSVTTMVLLKKYTVSVNLSLSGTQEFLNSKLLTEAGPAGDLDDQEASDREDRVDSSLPGSK
RGDPSARRKAPEVRVTGIGFSPAGTAFCAASTEGLLIYSLDQDIQFDPFDLNMEITPASTLAVLETEQDYLKALVMAFRLNEA
GLIKRVFQAIPSAEIPLVVADLPTVYYVSRLLRFVAAQTEESPHIEFCLVWIKAIVDKHGAWLSANRAKADIELRVVARAVSKM
RDEIRRLADENVYMVDYLLGQAEDKSGKKDVKTLESGKTVDVKLMDVDGDEQSEAGSDEWIGLE
FUN_004228 CDS scaffold_16 273518 275009 -
hypothetical protein PF14027 ENOG41

MATPFKIQLEPQNSGLLGMKLGQSEASKATDLLQKDLENHHVFFNESGFHDHIVHQVLTLYGTGATTKDLDTAYNANKAYQLK
AMKPKSDVVDKLEHGSDWSEYLGKGRNYATFFRFFQDEIERIGWQDTLKEYLFKDDARGRDMQSRLFAGILHPLIQLLYGMEW
SQPMIIASALAQTAVHRDDYKEFLTAAAEKAQASDAPPKMKTIGGLYEDAVKNEKLRKSSHWDDSNRIFDGVFTRAKDEMITL
AARVRIGEEELDEKIAEMVHHSAFVASAAAFHPPHAPRFEFILMHHITSTPFFLTLKEQSWIPVSTKARFLENKVRMDLLQYI
ARGSPALRSDLLRGYEPKDGEKLVGKPEDLFPRIHKVVDDGHTVKLARALMLAQRITKPYENKEWVKIKGDDEWLRAVYLLLD
ANEEADRQGGTMWVRSSGFDEAWEEIPKAKM
FUN_004229 CDS scaffold_16 275849 276928 -
hypothetical protein PF00956 ENOG41

MHPDSFKLKFKMSAGDMHDEQVKEALITYEKLQAIEDDFEDVELEILRQQDKLTKDLYVKRQEVIANIPQFWPLVFEQSPPEV
DEYIQPSDSELLLNALTGLSVERFELPNGDPRSISLKWEFKENDWFEDKVLEKKFYWRFHKDGWAGLVSEPVDIKWKEGKDLT
NGMLSLAKKVYDEEKAGKKLGETEASKKLHKLMEETGMGGVSFFCWFGFRGRKVSPEESEEGRKLEEEKRKARKEGKYDEDDD
DMDEDDDDDEYEYEIFPTADDLAVFIAEDLWPGAIKYFTNAQEADDMPSDLEFEEMDEDDSDDDEAPALKKRKA
FUN_004230 CDS scaffold_16 277854 278688 +
hypothetical protein PF03932

MPPLPAKYPRQIPLEVTIYGPHNALRAANFGAKRLLLCRKGSSPVGGLTPTAQELRHLKSKIHIPISCVIRPRGAPNPSTPGE
SHDYVYSNNELVQMSESIRQLKETSVMNAIRGDSFVFGCVRRSHEETTQNSRDEIVIDSAYCRYLVNMAKPFGCVFNRAFDHF
AERGNSEAVIAELITLGFAGIMTAGGPGLFSNHVEQLDAMCHHTTNIQLIVAGGVCCPEIQKLRKRAHNHDRLSIWLNGDCLR
PRDHDDPETTDMNGVMSLIDQLGLQTTD
FUN_004231 CDS scaffold_16 279127 281291 -
hypothetical protein PF00245 ENOG41

MVKINAIAALAASIAAVSAQTYQRLGTCPTLGCVLPPDQSDFLPGQLFDLRVEVHAPVNGSEAAHNGKPDEKFTVTIAKKGEK
AKDFAKAFGVSEPKLETWKFKWYEDLFAEDEDKPSIVNVASKVYRKIALYEPGTYTVTLNYYNGEKTTAEWTVRELQPKRKAK
NVIFFIGDGMTTNMITAARLLGHKSINGKYQTLMKLDEFPVLGHQMTHSIDSYITDSANSASALYTGHKSTVNAMGVYADSSP
NPFDDPKVETIVEVFKRVWGGAWGAVSTAFLADATPIALSGHTRLRGQYGPLIDQALNGMTNYSWTQHGGPDVFFGGGAENFF
AGKGSYQGKDYYKEFQKKGYTVSLNKTSLLKADKSKRALGVFCQSNLPVWLDRNVYKDNLEGRENNPTGGKGDASDLPGLKDM
TLKAIDVLATRGKDKGFFLMSEAASIDKQMHALDYDRALGDLLELDDTVRATVEKLKKLKILDETLIIVSADHGHGFDVYGSA
DTEYLAQQEDDRDKRRAIGTYAQSGESQYTKKAKGINYGTGANFPTNWEPRYAIAAGVAAVPDHREDYKVKKAGPREAAVELK
DGDYYANPEDSPKGFLINGTISTDNAQGVHSLTDVPVYALGPCQETFSGTFNNVDIFYKIANCLGLAQGKKQGY

FUN_004232	CDS	scaffold_16	283140	284560	-
hypothetical protein		PF01702

MFELLKPAAAEAGVARLGRLAFAGNRRIMQTPNYITVASRGVVPHLTPDNVTKHTTFDAAYLAIEDFLEKSQPPVLQLPPGTP
RNLQSFTAFPSDRALILGPRRFPAVTTPVGNGAHHVSIFTSTGFRNLTIPEFAKTIELTQPDIAIPPADLFHSSSTPTSKRQI
RMVERTEEWVDEFFHLSDPKGRLKEMGVSIFAPVLPVEYPIQWDYLRYLAEDVRDCLSGLAVYDVNLVPELVNYPSLGSLPRL
SFGPSKSPQDILRQIALGIDICTVPFANTASDAGIALSFTFPPPESSDMQPLGIDMWSEEHTTSLQPLVNGCQCYTCTKHHRA
FIKHLLNAKEMLGWNLLQIHNHAILTSFFEGVRAALNESAEKFDELHKKFLTVYEPEVPAGTGVRPRARGYHFKSIAGQTKIN
EPSWQAFDSVDASPHPEAIAEPLAAVDGTLPVDEVAPSLEK
FUN_004233	CDS	scaffold_16	285464	285975	-
hypothetical protein

MASAARFEQELKTVGTIKRLATIDKGNPVIARLREELVKSAEREDLESLAQKTSEEHKHELREARLDGVASCVGFILLMLLIL
SLVGVAVPFVSKDEKGPPKPESKSLEEKVTPRSGSTRIYCGKANKADPETSELKCTCHRCPLELDNTRVL
FUN_004234	CDS	scaffold_16	287440	289441	+
hypothetical protein		PF03815		ENOG41

MVSHEDDEESQPLMARTDSIDVGVVPVSGSDTDRENAHPDAPLDTGTTLEVDDSGPSTPRFIQDETTWKRWNWVPYPIRRASK
WVAKWSRGPVNPQPFRIKPLFPAIQEYPLVLVERIIPKPKHRFWLVFCYLSIWFVTFALVKRRDTMASEIEGWGTPQTIGCGV
SYWTAGNSCGLDGSDCRPFNGSGFPFRCRANCEGYHVLNPRAVGDQELIYAPMVVGGPGTNGSAEPVYRGDSFVCAAAIHAGV
VSNQDGGCGVVELVGRNENYAASKHHGISSVGFDSYFPLSFRFVPDVKCSAIDSRWDLLAVSTVFAGVLSLFTNSAPLFFFTI
FTAIFWTVGMALDPPPSRTVAALFSNLIGKFLPAMFCAWVMYDKMGVRRTLKGLTAQIEKTILWLGGCWVGGMENYTLSFIPI
SRLNKHDLNQQPGARAALAIIIIVLVVIVSWQVWFFRQEGRLIKYLKLYALLIAAILISLTIPSLNLRIHHYIIALLLLPGTS
MQTRPSLLYQGLLVGLFISGIARWGFDPVLQTSEALQGDAQKGSKLPAINEPVIHLANGTDTLSNITFTWNEPPRQLYDGISI
LVNDVERFRTYFGDVDAERSFVWSRNSTLDLPEYFRFAYMDGSESGDYTKAGTWTADGEWNKMKPGPSRVKARSLDQDKLVSD
AL
FUN_004235	CDS	scaffold_16	290141	290771	+
hypothetical protein				ENOG41

MPGAKGRKPPPPPPPPFPPLPPVPPKTTPTGFEPKESLDKDEPAPEGCVLYAHSNSNPPDPCPYHYKPVYYFFYDGLRNPAVLQ
VVLEASEPPKLRDAKVLGYSLAPRGRYTSLIEGQPGSGVLGRAVQVQSAEEEYKLGWYQTSAYILAPCLIQFIDGGEPREVAG
LVFIDAGDQRAWRTMRFDYRAWKSQMGTRLPRNWQRSALEPSS
FUN_004236	CDS	scaffold_16	291168	291933	+
hypothetical protein		PF02230		ENOG41

MPPRIPTEDDFSPLSSTLPHTLHFPSPPESTTTFLILFHGLGDHDVPFASFAKNLNLPGVLAISVRGTSVLPAALLGGDRPGY
HWGDDLTVDPSTGDIADDSGFEKARKLIMEKLIGELLIEKCGWEMRDIIFFGFGQGGSLALGLAASLNKTPRVTDVSEGESTS
PGNMKFKGAISLGGALPQSMVSTVTNRSKSSTSVLVCQLDEDAVDAVKREFDDVKVVQWKRREVSMPMNRDEVLPLMQFFADK
LKSEW
FUN_004237	CDS	scaffold_16	292013	294723	-
hypothetical protein		PF04130;PF17681

MAFAARLGALTGELVEAVTSSSTETNPRFKAQRDAALQRLKSHPYLRTNQFEVEHQLDGLEERFRVNGRDALADALVERREQL
RQIHSNFHPEVLYLILELSDQPTYYSKLSDLDALKTGPSDSELELRWEDIAKEDGWEDDASMWKTIKYTDSSDDELYEDDSKS
ESEASTDLSEVPLGRTAEDLIVNPEDMTKLHEIRGAQGWRTEKPTDASGHARKVPVTEFQIVREVLFMLQGLDTTLFGPNGTV
NPAFQMAHLKWDTHKALLSYFSEAGRQLGILRDFVSKSQRASHIQVLQDTVAKRLDDLDRKSTGIESRLVAPENNVVVSLLSI
KGELATSLEPLYSLSNIIAQIQNVPNQGTFRYLELLFDDASMAQLSGKLDVYEFLARIFVECFNVYLRPIRLWMEEGKLIPGD
KIFFVSQAPSQVSPSKIWREQFRLRRTADGKLHAPSFLQPAAAKIFNAGKNIVILKRLGRWISSGSEWTIQEPPLHYDTLCPK
GLELAPFSELFDAAFDAWIQTKYNTSSTTLRNTLFEECGLWSALDAMERLYFMSDGAATEAWTSSLFGKLDALDPNWNNRYSL
TSVAQEAFTTLVDMTRISIYISPTGLKVPLLKARDSVKSVLPSTKVNYRVAWPIRMILSEDSNTQYDAIFTLLLQLKRALYVL

HKRKILENYWIDHDNWDERALYYSLRNNLLWFCTTLQTYLATLVLAPNCAKMRQDMQDAHDVDAMMRVHAAFLKQVVDEACLG
SRLTPIRECFLDMLDLAIRLEQAQTVNMTKETERMQQFSRLSTRNLSPTPGTPGLKSKYVDSSDEEDDAERDESRTLKMGKPF
MTVLKEIKSDYDRHLRFVCGGLRSVARATSDAQSAKWDILAEMFQTGCRDERPGYA
FUN_004238 CDS scaffold_16 295108 296457 +
hypothetical protein PF01793
GT15
MTVARPVRALIAGGCILWCFFLWQIFAPSWSLRGPGDRYSNFERDPMLDPTDEPEGVLHRTSPRYAHDAKKTERIDATLLALV
RNEEVDAMVMSMRDLERTWNSKFNYPWTFFNDKPFTEEFKRKTRAATKAKCNYEIIPKEHWDMPSWIDEELFQESAKILEKNG
VQYASKISYHQMCRWNSGLFYKHPALKDIRYYWRVEPNVHFFCDVDYDVFRYMHDNNKTYGFTINLYDDPKTLPSLWPETVKF
LAEHPNAIHENSAVAWVTDDIRRPSTNRKAQGYSTCHFWSNFEIADMSFWRSKTYEDYFNHLDRAGGFFYERWGDAPVHSIAL
GLFEDQSKIHWFRDIGYQHIPFFNCPNSPKCKGCVTGRLTDGEAWLHREDCRPNWFKYAGMG
FUN_004239 CDS scaffold_16 296718 297917 -
hypothetical protein PF00400

MGFPDKPSAHLLGANGPVHALAYSASPGTYILTGSADRSIRLYNPFPSTSGPGDRHSVVPQGRLIQTYAAHGYEVLSLSVAAD
NERFVSAGGDRSVFLWDVSTAVTTKRFGGNVHGHTARINCVSFAGEGDSIIVSAGFDTTVRLWDTKSNSFKPIQVLDDANDAV
TCLAIRGPEVLAGSVDGRVRSYDIRMGKVTTDVIGAPVTSLSLTRDGRTMLVGSLDNKLRLMDRDNGSCLRAYTDPGWKNEDV
KLQALLGGKEKYVVVGDAMAGEPAPNGHGRIWAWDLLTGKLAAKVTVPWGPEGFEPKKKAVGRDGKEKARSNVVSCMAWREDG
WGDQFCVGGTSGVVTVYGAL
FUN_004240 CDS scaffold_16 299614 302513 -
hypothetical protein PF00172;PF04082 ENOG41

MGVLPSIDSRPRLVFPNTPPESTSPGASTYSTNSSADRPWISPAPTSPSMNNYDKSLTTEMPADQKMNGEQSRPESTTQAAPR
QQLPSLSSIFGPPPPAGRPAHSPLSEHNNSYSTTSPLDRTRAPSGDRHHSTSYFPPTLSPSLSQPRTYDNKFEADRHPAHGLS
RSYSGSASPRFRNGEHARQESVTEGQPGRWSVHQESRSEYSLGSRDGSFRPPQDQFRLHFPSPKERVIPTYNDQGSSQGAPIP
PPTPSTTATDGVPSKDGLGPKIWTGTHFLPRFVRATEVPGEGMCYFYDDGSHCKTVIDGEAVNAHWGVTKAGKPRKRLAIACV
TCREKKIKCDPDYPRCVQCEKFGRVCKFKNAPRGGHNTSPSTPPAELDDVRTLNGPTIPNESENSSPVSPRTTLRPQSPESGS
HKRLRSGYDSYVSMGEPVHALPPVEAPRPHVPIQPPAIELPRISEDVLNQAWRTDPSLSDPQSIRSVISHFFVHLDSTMIVRF
IPENVFKAWVVNPGHRKTPEDLMILYSILAVGVALSGGPKAIAFEYASVAHYAQKLTTANCIQLVQTRVLLALYYISISRSSE
ASEMISAAVASAACLQLNVEIEESKEAGLVTFPFGMNKAGYCEARRRTFWSLYMLERLDGRFPDHLAMINAEDIYTRLPADGQ
SFEREMDGFAPVFNPYVSGVATAQDRELGISAYLVQMVHIWSNDVCRIYRMARRANVMETDLEPSQRILKRIQEWRNALPSRL
AFTASNLESAALAGELGPFLTMHLLYNHAMIKLSRHTLAAGRLSPQTTSHNIQTCYEHATQVLDMAKALVRLHRGGQTVLSGP
APVLAMVLAEAVDVFTASGRLSHLSDIIENVHMVQGVVQAMFAIWDNVRNAQEAIDGRLAMLHRIRERGSQPVSPMEGYRVFY
SSEGREEEKILRWQINNPMEKLYPRDMDTIYYLQ
FUN_004241 CDS scaffold_16 315724 316545 +
hypothetical protein PF17648 ENOG41

MISQLNSIFHSFSNLTPRSQRLAIAAAAGVIIGIPVFRIAAEDYRGYLALGPGGPPHNLIGWIGQILLKPLKRESFHTSCYDE
KACEKAGPNGHIAFLSEKDVPVRKAPRPMIGKWTAPSRQLTDMANHSLIDRYQSFLSSLASSSSSKLKIATSLAERRGPALFV
ASNKPSHPIAKRTGGEIGHMHGSDGSTHINLAPKDAKLVLERGWGQRHPLSGTVLYLGNVMVYAPRNEDELEIVKNITRAGVK
FMLGEEC
FUN_004242 tRNA scaffold_16 316939 317063 - tRNA-iMet

FUN_004243 tRNA scaffold_16 317367 317487 + tRNA-Met

FUN_004244 CDS scaffold_16 318346 319156 +
hypothetical protein ENOG41

MGHSNSTLQSHQLSAGGIEKGIPPVIRVSYESGWRKYACFARMKLFLSGPNGEPMYTLNFVKGLYGDIILHSGSSIDSPPLAA
SGRQSSLRDDYLVTLPSLSGNGPQEEILRRPKGLKGRFWFAIQVGHGPDQHVERFEWRRSRGAEVKSVGQSRWGGYKLVRLGS
TNTKEEYSSSEEDLHDGSTNGEGYTSDGKEVVAVWGSTNCLKSLSGVGEFQFRGSGATGELGQLWALMAVMSCMSIWQKVQRD
NATAGASSSSAASASVAASA
FUN_004245 CDS scaffold_16 319639 323430 -
hypothetical protein PF01734 ENOG41

MSTSGGISLSQFQARLTVLQSQHQSLKAADLEMRATQSERCRSSLEKAVKSLFQVINLVRKHDKRLVAWHDRSPHLKSALRLI
GIDRRTEHTVNKTTAKLKNGVHKVETQFEYAGQVIGESIDDTTRFQKDFSQISRSGVEENLQAMRAAEAELKQELEDLKYQIQ
NQDVECSGAMAELRQQIQRLEELTADHDSARAWKNTWGAVTVLAAAGAFAMPFVGISVGIVAGVKTLMNSEQQEALRVQKEQH
ELLVQSHDSELKSLELQMAQSGRHHDSCAYYMDQIKQIQSANLTIRQESSRAFKTLCEVQMALEKARLNIFEISQELQECQYE
KTRRGMAARLNGILQNILEFPAKAKLKAIDQKLIEQATSGIRAISGATAEVMAIVVPIDLGFYQVQYCLSIARLRCQLKMASY
NGRTIHSLTKGNNATDQASADIIALHGLGGDWNRTWSSFGYCWLKHTLVPKFPSCRILSLNYPDMLSTLSAHPPDINGLIQDI
LRDHRQENRSKSPIIFLGHSFGGTVLKQVYVATHPSNTDDPDYKALHTQIRGYVFFGTIHKDRDMSRAKLEVPEFWRALSRGA
SGTLGGHSHELEKAMYTTFRVNHAFRRLGGENLLINCFYETKGPGGLSRRVLITQEESTLVARPVPTMPLDLDHQSLVCFKSP
RDENLARVLDALDSFMIRAMDVETRLHRSSAPESRGLCLLSLDGGGVKGLFSIIIIDRLMQETRRLEGPGAGHKKPCDYFDLI
GGTSTGGLLAIMLGRLQMDTQLCIQAYKSLSKEVFSRKFKVPFLENFRKASNVALSWPWFDGDKLKEAVCRTVKENLLPSDSA
MLRQSGCTVEDLTLITDMKSTTYSFVCAVPKYEEKVRRIRSYEPLDQQTNAPAERFKIWEAARATSAAPMYFPHIEAGGVSYF
DGGLESNNPVIEVIEEAKQEFPDDKISTVISVGTGAYQASDASAGLTGLMNYMINMATSTEKHHKAVLEDPRFADIRKEGYFR
LNGTLELGAIDLAAVERMDEIERLAEAYLSSNEGQQQIGMCAERLVERAKHQ
FUN_004246 CDS scaffold_16 324020 325771 +
hypothetical protein ENOG41

MGSLVRFPAEMSQGPRDNLLEPLIRAELLTQKLQLCLYISTTSNATSVFLVDADGFPGSDNLSTALAEHCHMGLDKLVETHGA
FYSIYMKTKPMIQDTGTLNALQSTFGVPGEKASFDRHLRKLTNATEVCRKQCDELDKSLQAWRASVDEFSRRISKTKDIMNQE
ENATNKKIGTLKTEIDDQIYNQDRAQANEDAARARREDLSRQKNKLTNQIGTIIQSAGSADIGGKMLMAGGQMAATWILNLWA
SNDLQSAQRNMQDQLERGQEYRRRKEEAEVDIQKLSTQANELKQCIEVLQPVQLLLHGIIDCLRNAAIHHNEMLAKMQSFLES
CSESKEIATGISEGLSFGDTLDFLNEIQLMRQGALEIGHWSLIYSKVIQQTMMKGFQQNETGFSHEDVLFTIQRSRHQAIESG
SAKLIAGQLKQQHEAEVEAIKKDHDEVVGKLKRKNEKEIKKMARSGKMRRLLPFSKAS
FUN_004247 CDS scaffold_16 327981 330940 - QNS1
glutamine-dependent NAD(+) synthetase EOG092610LP PF00795;PF02540

MADLITLATCSLNQWVLDWEGNLKRIRKSIILAKEAGATLRTGPELEITGYGCLDHFLEADVYEHSLESLLAILTDTELHGIL
IDVGLPLMHRGCRYNCRAIILDGKLLCLRPKIYLANDGNFRENRFFTPWNRPRYVEKYNLPPALQKHQGVRQVPIGDVVLSLN
DTTVAAETCEELFTPQAPHINMALNGVEIFTNSSGSHHTLRKLDERIALISEATRKSGGVYLYANQSGSDGDRLLYDGASLIM
VNGSIVAQGSQFSLDDVEVVTATVDLEEVRAYRFAPSRNFQAVQAPVYERIEVDFSLGHEDLDLLRAPTAPRPACYHVPEEEI
ALGPACWLWDYLRRSRASGYLVPLSGGIDSCATATIVFSMCRLVVEAIKAGNEEVIEDVKRIAVYSDKLPETPEDFCNQIFHT
VYMGMEKQSSKETRQRAKDLAERIGSYHTDMNIDDTFHATKNLLTQGTGFEPKFKVHGGSATENLALQNIQARSRMVIAYYYA
QMLPTVRQRPGGGSLLVLGSSNVDECLRGCDCSSADLNPLGAISKRDLKSFISWAAKNFDMPILEEFIHATPTAELEPITENY
VQSDEIDMGMTYDELSRFGRLRKESKLGPYGMFLRLVEEWGGEGKLSPREIATKVKRFYHFHYINRHKQAVATPAVHVEDYSP
DDHRFDLRPLFYPPAFQGWSFQKIDKRVEAIEKALEKKQKKAEGGLQ
FUN_004248 CDS scaffold_16 331569 333750 -
hypothetical protein EOG09262Z14 PF03109

MRFVDFAIDLAAVLNASRGVAAKHVALRGRQIDTYSRTSSVAAALRKRNAQRSSPTGEKAQNAAGASDEVVYEQAAPAARETK
PITETPKQNAPPEVTRTERAPFNSPIPNFRPSPQQQATSEPAKEDDLDLPPGIDVNIFHTARGSKVLDSLRKQGRPGAPPARP
GVGGPVKPHPMRDWPPRPPPLEESYEEEPVKPQEPVFKPVEPVEPVEPAEKEPLVEKFVQKKQLEEKPVEQKPVEESSVPEPE
KATPLAPTADKKIDEDVIQAAREIAGETTTPSETPYALRESKVPSSRISRIWNYSGLAAGMLGGAMTEGFSRAFGGGGEGSVL
LSEKNMERLVAKLSRMRGAALKLGQMMSFQDTKMLPAPIQQVLQRVQDRADYMPAWQRDRVLVANLGPEWRELFSDFEEKPIA
AASIGQVHKATLKNGKRVAVKIQFPGVADSINSDLDNLSILLTATKLLPKGLYLNKTIDNARLELGWECDYERELQCAQRYRE

LLGSSEKDVFMVPNVYPEASGKQVLTMDFMDGIGVTRITSFTQEQRDWIGTQILRLCLREITEFRFMQTDPNWTNFLYNADVN
KLELLDFGASREYPDEFVTQYVQLLAAASRSDKAAVKELSESLGYLTGHESRTMVEAHTKSVLTLAEPFLASAPDIYDFKDQT
ITERVKALIPVMLHERLAPPPEETYSLHRKLSGAFLLCAKLGSKVPCKSMFEEALAKGGYSR
FUN_004249 CDS scaffold_16 334245 335598 -
hypothetical protein PF09751

MDSSGSGTSNALVRKRTDTDLMPPPPQAKKIKRPKKVLDEDTYTEALSQIIARDFFPGLLQTEIQQEYLDALESKDSAWISSA
GRRLQHVMTPGRRNAPLSSQANSFNAGDRTPSTYGGDTPASVTSNVPDPQPRLGANMSLTKFQDTYTSEDNESFYKLVDKQNQ
KKADKYAWLWRGNKLPSKQMIKQKEVEDRLSQTRSLIDDGFKKDLLAIKDKDDRPARPDSWNANPRNSLMFRPDGLEDGVLTV
SQKAEESSKMAPKSIVYENTRMPQPRITPRPPSPTMSAVRDAIAGKPRKDDRDFSIVSGGETPRVNGYAFVDDEDDEYEPILP
APIINLGPGDAAPNPFRLQEQRDRESLHERMVERISQSKKESSRNGLTGRVDKTPVPKFPSSPRVNGGLTPAAQRLWSKIGTP
GRGSSGSSFGNSKPMTPRGSLLRSVKRPGSTSGKK
FUN_004250 CDS scaffold_16 336572 338313 +
hypothetical protein EOG09261M78 PF00149;PF08321;PF13181

MAVELKNKGNKAFQAGDYPSAVDFYSQAIKLNDKEPTFFTNRAQAYIKTEAYGYAIADATKAIELNPKLVKAYYRRGLAKTAI
LRPKEAIDDFKTCVSLDPSNKDARLKLEECKKIVRQLAFFAAIEVGDEPSAAEGLDLDSMVVEPGYDGVRLGDEMTQEFIDDM
IERFKTGKKIHRKYVYQIIIAVKKLVYDEPTMVEVEIPSDVKLTVCGDTHGQYFDLMELFRRNGTPDEKHWYLFNGDFVDRGS
WSTEIALLLYAYKWLRPNQFFLNRGNHETDDMNRVYGFEGECKAKYNERVFKLFSESFSALALATLIGKKYLVLHGGLFSDDS
VTLDDIRKLNRHNQRQPGQAGLMMEMLWTDPQEENGRGPSKRGVGMQFGPDITKKFCEKNGLEAVIRSHEVRMDGYEVQHDGR
CITVFSAPRYCDSTENRGAYINIGPDYKLQYEQFDAVPHPDIKPMAYAQSSLMSSLM
FUN_004251 CDS scaffold_16 338514 338949 -
hypothetical protein

MTQNSSQGSQKSIFQQKDLEKSHDLLACTIKEPPFSYVHLQLVSLSGSSSVALDNLSLKSYCTTALRRCLGMVGTAISMDILK
IEADHAWVRIPRPDLGSFATAITAWQGTTDDGEQVSLQLRQCSDWLGAMVGADGQDRLWNA
FUN_004252 CDS scaffold_16 339182 339667 + SPT4
transcription elongation factor spt4 EOG09265BBJ PF06093

MSSSNYVSTGQARYLRACMVCSIVMTYARFRDEGCPNCEEFLHLQHSQDQIESCTSQVFEGVITLANPAKSWIAKYQRLDNYV
PGMYAIKVSGQLPDEIRSTLEDEYRIQYIPRDGTEAENDA
FUN_004253 CDS scaffold_16 339918 341071 -
hypothetical protein EOG092657UN PF10357

MPKAEVGSTKYVANRMKAKGLQRLRWYCQVCEKQCRDANGFKQHTMSESHVRQMLLVGEDPKKYIKSYTQQFQADFLQLLRTG
HGEKPVHINHFYQEYIRNKEHVHMNSTSFASLTEFAKHLGREGICRVEENDKGIHIAWIDRSPEALRRQEALRRKEAQDQGNE
EIEQRMIREQIKRAQATAGSREEEKEEDNEARELKRQEGEKIKLSFGAKPAASEKKSSESPVPDTTTPEAESSKTADTSTDKG
KEPEAAPAKPSGFGGISMKLGGKPQTKNVFAQAKKNAFSSGSKKPAKIEQPKKMSEAERIMKEEMEKKRSRESAGFSFGMGSG
KKQRNN
FUN_004254 CDS scaffold_16 341614 342472 +
hypothetical protein EOG092655M5 PF04502 ENOG41

MSERKVLQKYYPPDFDPSALQRRRGPKNAGPRIQQVRIMCPFTMRCTRCGAFSNRGLKKNARKETPPDEKYLGIQIVKLSFRC
SSCSSEIIIKTDPKNQDYVVVSGAVRNNEPWRNRAAEEESVEQRLDRLEREEAEAAGEEELDKMEELEAKNQDAQREMAAADA
LDEIRHRNARINRSEKEGVDFVDTIVRTEDEERERQEKEDEEAAKAAFAAARAQVESSAKVAEPEETPIEPQAPAPTFKRAVK
KKKDHAAALGLKKKSS
FUN_004255 CDS scaffold_16 343014 343224 -
hypothetical protein ENOG41

MPARSDYGSVSMSMSPPPPTNLSSYSRFMHDHTKRQMEASGASPPPTNATAQRARSSGMTNGHTSPNYS

FUN_004256	CDS	scaffold_16	348888	350061	+
hypothetical protein		PF00070;PF07992		ENOG41

MADVKTIVVLGAGPAALPVIRQTMVNQVMKRKDLKMVVISPNTHFHWPVAMPRVVVPGQISDDKVIIPLEPTFSTYPSDKFEW
IQGKAISLDTTSNLVSVELNSSATVREVNYHTLVIATGSRTRDGLIWKDIGSTEATKAKLHEIQDQISKAKTIVVSGGGLTGS
ETAGELGFEYSQHGTKEVIFIYSDDLPLAPPATDGVRKQTLKELKKLKVKTMPKTTVISATPSGSDTILEVRSADGATKKITA
NAYLPATGTIPNTEFVPKNLLDSNGYVKQTTHLKVEGQKNIFVIGDAGNLENSQLGMADKQAQHLFKNLPTHLDGGEIPEYTP
ANKPMFAVTLGRSRATGQMGTMKLFSIMIWWVKGRYLGTDYADGWAAGKRTMMTVFEK
FUN_004257	CDS	scaffold_16	350594	351657	+
hypothetical protein		PF00106;PF13561		ENOG41

MSFLLNMIHGKFTTIPSQEEDCTDKVVIITGGNGGIALEAARHFTQLSAAKVILACRSLEKGDHAKKDVEETTGKQNVVEVWH
LDLASHDSIREFADRVNKLDRLDVFINNAGLLVFKRELIEGHESMLSINVISTALLTLLVLPALRQTSNRFNIIPHIVIVSSD
AAFEGRLPVQEPNFIKALDEQPSVLEHYSKTKLVQLIFMTQLAKAIEASGKGYIVVNGVHPGFCSTPLFDNTPWPFNLIFKGL
LALFGRTPEVGSRALLAGAFADESLNGKFMSNGAFHELPKIMQGDEGEEMCRKVWEELISILEEIEPGVTKKV
FUN_004258	CDS	scaffold_16	352225	355532	+
hypothetical protein		PF08689

MDSRAAAAEAMRLAIEYWSQFVARCISQRLDTEKFENYVRLVHDQHPLPPALVADFFLRPQPSNDNSLDPRIPPYLQVLTRLG
YVDAPSILKALYKYSSLHAYAQQPNANDGKETEKETEQSDDKEKDEQKQKVTRWKSSYWAEEVLFYGITKSVVEGKAIRDSKT
ALDMTRIISKLMVLFTTAFAADMLEQLHTAQVRDEMESSRAALVALLLRMCENDILVGAVSKPIAKDVRKEMSASLASFVPTL
QLVPQITDKLEQFRTEILASSDPADKKKQATNAAMDELLDSAVGLDNFVVAEIPVSNTRAGLYIYLNAALIARPLLDDHALFS
YMSNKYQGDIQSSAIDLILASFDILANAVFRNEGQKDAHLLRSFLINKVPILLYQLLPPGFPGTSAEFCITEALSHVDTSLFP
TASLMFDESRNNNPYTESIREEFCAACVLHGLVQREHVERILGEISLSYEPSLQKHSKDKLVQDCLSDTEKIQGLVRELDKMD
GNVGAVCQALVEVLRQSCHSKETMSLKVLCSQLAAKPQSLDVILLFEKLPNILEPLCQLLDNWRYEDDQEEYQPVYEEFGAVL
LLVLAFTYRYNLNAVDIGIASPDSWVAKIIGRGHIGRQCNELTQRENDHLNGWIHGLFDTEAGGLGDELMSSCPPQEFYLVVA
PLFQSIVVAYTHGYLNDESLKGGIEYLVDTFLLPSLVPAIRFLADYLWIDQKEQKSIIKILQLILLPSSISGEASTMLSSVKN
LIAKPLEHALRTYQRRDPKNQDIEPLLRTLKDSIPLSRRTGGTDLNELESWTVTPPTGLSSAIKLTIQGLVHWSIHPAMNSMP
TSYTHRQILAALKIMGPKRVLHVILEEIRQHTEAGSASIVYDVAISLICAPDAAKDAPAVVDANGNMLPPVQRQRSLRDLLKV
EAEGCRKLQKKDPALAEIVVRLHRRVEAQMIIPQPPGILQTAEMSLNLDGDTAALGDAMAAAASGVQGDNMSVDNLTLDVSMG
GVPSDLGLGSANDGGSLDPSGDAAMFEGFDTQDMDNFDWDDIGNSF
FUN_004259	tRNA	scaffold_16	355743	355817	+		tRNA-Val

FUN_004260	tRNA	scaffold_16	355929	356019	-		tRNA-Arg

FUN_004261	CDS	scaffold_16	356282	361772	-
hypothetical protein		PF01388		ENOG41

MSTLPVLNISSIIFRYSGLVGNLIAISSAARVPPGSPPLRYIYDQLLAIQSELQDGLLSLQEERALSADRNFTTILKCARACA
RDCGTLLKAAKTNLERETQNGQGGQFLQLVDQLFVPSYFGNLQGSINEYTRPSTRHLANNIICHVHDQLRDIENRFSVSLGCN
AFEELQRSTGLVDAQLGQIPCGTEAWDEVCDRLQHILFKLHHLQTAVPMLCALNHSERSLRQSRISTALEGTFSWVFQEQTDD
VINAQSSLRRWLTSDQSLFWVTGKSCSGKSTLMKFVSEHQDTRTLLQKWAGHRELLIVNHYFDAMGLPIQRSFEGLLRSLVFG
ILSAKPSLLKNSPSSLVSASSALPATFKFGHIFSSITKNAVPVKMCFFIDGLDEYEGDHARLCQLIEDVAALSYAKIIVSSRP
RGAFEDSFGSSTETTLSLEEVNRGDINKYIKFHLEGSPLQKNKDCSKYKDLDLESLSAQAQGDFLWAVLVTRFLLEDPSPLAD
AYNGAENVEDIPHNLSGLAKIVAESPNLQCYGKAAEYLTIAREAKRCLAAEILFFHNLDFANDMYAFMTPTDLLSPHDEDTKE
LHHQIRRDIMQTCRGLLEVVDGQVVFVHRAVLELIHSEEFCNFLLTKVRKGFDSDLSVFKASVSWFKRKRFRSGEFVDLQPEP
AAVSLTYVQSLSKFHKKLGSTLPDFQDTFNHPVDVYWMTKAVNIRGGFHNVCKHERWLDVIKDLGYSDDTAVSVSDPLKKLYK
RWLHPYEEHLHRKKLEEHDPMKDIEDLVESLRKCLEYARRCDQKENTVATTWALLDNVEESLLSMTARNQFNISDVSMARGIY

RHLVIESGIKRYIANKLWVDPGYCDNKYSQKYRSPLCSALGAAPHNLHPAHLSPESMDRSLFKSLLALGHEPNKFHHTGETPW
VSFVNTHIPNTLSNSPPGLNKININLINEISILMLEHGARPGDHVELLDKKSRTWKVPIWFKFLMLAHWVPAFQQATFERVFS
MMLERTPDVPDSRALMELKSDGETYEWIPFPLWTICADNFALPNISDSSPSDAGFVFRIFVGSCEKNLNNLEVSERSRKYLSS
YFLPDYPELGRQILMLIPGSDSRTIGFTTITSVTRDGNTESISGRNSTVATSIELPEGIEVESRKEGAVCNGARAEVDAIIDE
VGSIQSIDEDIQSHNSSIARTPGIIAAERQIGDLLSRHPDLRFIIDVSSELMPKERLERNIRRSLKHLYLKLREQTEKHIEIQ
VLTTRMLKGRGSRTRISRRTVDGELASPSPRNSNEEDEEIRPSFRKDRAYLNNWILKTELSARPDEKPQQPADAADDTKLTES
DDDMCEERESDSEEFQAVQVAEEFLMKGPPFQAFLAHLGLSLLPDTLRQVMQLAAWDTIELIDTPAEVSISDEIKTSVEKLTG
SPWDWWPLKPPEVPLRRGYVRMNWACHCGKPFWVHITPSQAQIIDRMVKNPTTFAEDHCKATHARRNTFQRVTAWFKWNSGTL
LPVSTPVSQDSQQPSGVGGNNRIQQGVATTEQNNIPPVTIDDLRILFGVPMGLKTLHVIPIPVNQPDSNVFPQLKAEYRRMRG
RWRAWFSFWQLSHCNFVKFESIAHNIVVACGQDLPKASDLDYDYTPKPPQASIPPVHPHIFEAAFNSCTGGKCRNPSPFHDCY
EFEETTYIKRIPKKKTPLSPGAIDMPIWGLEARHCISCFHVIIYHLLILVPPFVLWGWWMSRHPDDIQSASVPMTIVLAMISM
FWSATGIMKQFRGEP
FUN_004262 CDS scaffold_16 362961 363468 +
hypothetical protein ENOG41

MSPKDLFTKYFPRRKKQRDTEKSKPTPDEPIPRRAYTAPVHIQKTPTNKASSAGPPRGRYHVPATTDGGNYPVGNMYGGVSDD
TAGNSHGHGHRDRGNWGWGGVEDGSGSGGHGYGGHGDHGGSSSGGSSSGGGCSDSGGGSGGGNSGGSSGGGGDGGGGGGGGG
GD
FUN_004263 CDS scaffold_16 364857 366393 +
hypothetical protein PF13640 ENOG41

MASVHAGGASGSGAGSGRYDDNDNQSVASDLSTLDFKMDFPTDLMKLLEAINSEGSFAFNTKLDIPSSFDISVNGVGAIPLPL
SEAHARQIITQARQAPYGKGSETIVDTAVRNTWELDPTQFTIGWSKWPGLLKQTCNMVAEKMGINTPIRAELYKMLLYEKGAM
FKAHTDTEKIPGMFGTLVVSLPSPHTGGDVVLKHCGEKLKYSSSKQEASCAAWYSDVHHEVLPVTSGYRWVLTYNLAIDQSLP
RPSAALKRSELRPLRHCIRRWLARDPSSRENPFVYHVLDHEYTEDNVSYRALKGPDIARVAALRQACKGLPVTIFLALLEKED
RGNVEFDPSDLQYNYRGATGEYDDEDDGEFHYLDEVLETDYRLKTVRDLQGKVVAGQMTIDDKNITDPCAFEDMSPREEDYEG
YMGNYGPQATQWYRLGAVALVPHESLVDFLTQSDSSYHGATP
FUN_004264 CDS scaffold_16 366471 368063 +
hypothetical protein ENOG41

MLDLVEPGLLYMESRETELEDASVLPNVVNAALRHREYQLVDEIIAKLFKILPSNVFTSLRQWMIKPDGEDKAMERFSKIKGG
LSVALTKPYKLGLKFQAISHLVPLPRDLAVDALPTPAPIMDWTREMLHAFVEGDGPRDVTRHDGSSIISMALYFDDPILFLRE
CVVPIFEKRLSVPALGFRVLSQLMNMSTNGKLPHQEGIRLYRTMARLLITSQDFTLLRDEKAVQEEAKKSRPSWITVPPQAKR
EIGTAVSWETLKDFFSKLCKVSTELDNLKDQFLVKVTLQASKPPRPELYLLWLPFLRSIIPLLHENSIPLSTPSCQKFFSAVV
QAVLDVCLGPQPARPADWSLPSVQCDCRDCEWVNGFLNHPTEMTGRFAINKKRRQHIHQQVDRAGIGCKYETRRDTNPNTLVV
TKTTRPQDVKRQDWQKWRVYLVEQFSRFKGDELKTLLGLDFEKIEKLCTGQTGQASFSRRPATGEKRRIEHAAEVIDLTSD
FUN_004265 CDS scaffold_16 368852 369107 +
hypothetical protein ENOG41

MTTLQDHRFQDPEFPEQNPSGKIVILNGFPGTGIFTILQNLKKFLPGGTTFLLDNHLLIDPVAAIIPDRSNRHHEQDQRAVIW
N
FUN_004266 CDS scaffold_16 371713 372906 + GNT1_1
N-acetylglucosaminyltransferase ENOG41
GT8
MAKLLAELTPPFPPSFMASRRYRWAAGLTFAFFVFFVLSNNGFLSEKPLKFHGSIEVDWSRFAYTQYVTNSDYLCNSVMFFES
LDRLSSRADRVMMYPSHMFEANNGSSVDTQLLIKARDEYRVKLVPITVQHKDNKDDTWADSYTKLLAFNQTQYSRVLSIDSDS
TLLQDMDELFLSPPAPVAMPRAYWLFPEKEILSSQVILIQPSEKEFARIMAKVDSASENDFDMEIVNYLYRESALVLPHRPYD
LLTGEFRADNHKRYLGSDTEPWDPATVYNEAKLVHFSDWPLPKPWIETDGELRLQSQPNCTTLSDGAEDCAARIIWNSFYTDF
KRKRKEICGLSEVILPKEEYEE
FUN_004267 CDS scaffold_16 374134 375126 -

hypothetical protein   EOG09265M98   PF06102

MAFKRKTSSSFGLERRVRPRREDEWVEEPESQGSSSEGDDEVEEEGIRGVSDDDDDEGEEDQESEEGSEDESEPEQDAPKIDL
SSVSFGALAKAQASLPSAGRKSKSKKSTDEDASKTETPAPRKSTKSKDDPKPKRSSKHAPQEQTSKKPVSRRREILPDNRRQY
RDPRFDPLVGRVDEEKASKAYAFLDEYRDKEMADLRAQIKKTKDSYEKDNLKRQLQSMESRKKANMRRQEEERLLKEHRQKEK
ELVAQGKTPFYLKKSEQKKQLLVNRYEGMSKGQVDRAIERKRKKVAGKEKKELDFLQRGSRPRG
FUN_004268   CDS   scaffold_16   375747 376851 +
hypothetical protein   PF03031   ENOG41

MTGYKEPVVPNGSSFRPMNIPGLTLAQPRQSDAPKNAPKGPKKKSKAAADAKIPKGPKAMIQAPPTQRRSSSPHKIPSRASGG
VPEPTPQYMAQAKLSPERLPQPRRILIIMDLNGTLLYRPNKRRPFNFVERPHAKTFMKYCLDAFHVAIWSSARPENVNKMVEQ
LLTPEQREQVLVVWGRDSFGLSEGDYNAKVQVYKRLTTVWTNPRVRAAHPQAHKGGLWNQSNTILVDDSLEKGRSEPFNTLTL
PEFSGLSTEMPNVLPQVHDYLNELAYQADISRFVRQSPFKLDPVYVLPEDAA
FUN_004269   CDS   scaffold_16   377227 378841 -
hypothetical protein   PF00043;PF00647;PF02798;PF13410;PF14497

MAFGTLFTRENNCRSTAIRAVAKANDIELNIVEAEKGNATVEHLKANGLGKIPAFIGEDGFALSECIAIAIYITSQNEKTTLL
GKTKQDYASILKWMSFFNSEVLPSLIAWFGPLKGDAPYNKKNVDDASKASLKAFSVVEEHLIRNTFLVGERITLADLFAAGIA
VRGFQYFFDKQWREENPAVTRWFETVRAQPIFAEVAEKVELLETPALTNTPPKKPEQPKKEAKKEVKKEAAPAAEAAPASDEA
PAAPKAKHPLEALGRPSFPLDEWKRQYSNIKDHNEAMKYFWDNFNFEEYSIWKVDYKYNEELTLTFMSNNLIGGFNNRLEGSR
KYIFGCAAVYGENNDSVIQGAFVIRGQEHVPAFDVAPDWESYNFEKLDPTNPEHRQFVEDAWGWEKPITVNGKEYKLADGKVF
K
FUN_004270   CDS   scaffold_16   379189 380198 +
hypothetical protein   EOG09263K05   PF00380

MSTFTHSLRQLRCQGKLFRAPWPQPALQRATFPLGVRSIASETSSTIVEPKTDPFAVIDSSSKISLPFQDVQHARAVPVSASY
FSREPQFNEMYLRIARLLTKYYTLPTLPPSAAPPTAWVKLDEMRSKLGEPIKASHYAKVIRVAKRLNQIETSLFPEEVRVALE
DFKRDINPFMNVPHEVTVDKFGRAIGVGKRKESTARAWVVEGTGEVLINGKTLSQAFGRVHDRESAVWALQATERLDKYNVWA
LVEGGGTTGQAEALTLAVAKALIVHEPALKTALRKAGCITRDPRTVERKKHGHVKARKMPAWVKR
FUN_004271   CDS   scaffold_16   386698 387936 +
hypothetical protein   ENOG41

MDDSNRMRRQNEPPVHSNPNQRYSLNDPSQQRRSLAGISNDRYRPASLAGPSSSGRGMGAPGNYSSYYSESSASFSTANMPGT
AIAYGSEYGHDSRQAQGFGGYSTAATMMYNVGQPSTQNTVYDAQQFSSRQPAAMQMMPPDVASSYFGSETGASAGPSLQQPAQ
GSSGSANVYQQSNAMNYASNSMSTVSAMPQATNTADVSMTDDHDYAEGALEEKWVSYQRQLGSIFQEIVNGSLESASETLLSV
TSWLLSQVADLGLNLDDTNLHADRIQLWNDFNHAWLGLGQRQIDLMTSSQQLSKTQSLVPKAMIKKMGNELIRLCDGIERHGL
VDYQYGVWEDQITAVLEDCLDLYDASEEGSDSGNR
FUN_004272   CDS   scaffold_16   394849 395856 -   CNB1   Calcineurin
subunit B   PF00036;PF13202;PF13499;PF13833

MGNTTSAVLDNLVQGSNFDREEVDRLRKRFMKLDKDNSGTIERDEFLSLPQISSNPLATRMIAIFDEDGGGDVDFQEFVTGLS
AFSSKGNKEQKLQFAFKVYDIDRDGYISNGELFIVLKMMVGSNLKDQQLQQIVDKTIMEADLDKDGKISFEEFTKMVESTDIS
MSMTVGKLFEAIVTSASAMSYAVMIGNWDLGGLSDADMILQTSSKRSPTKGCIQGRRVKGSSLPWTYTVHSPTRAAGGNG
FUN_004628   CDS   scaffold_17   3036   5419   +
hypothetical protein   PF00018;PF07653;PF14604   ENOG41

MHLRHLQVHRRADGLFGFGNIAHEIDVSSDGSKRHVQKRQPQEVRTVFKTMEATFEGKIGGYKTVGHEPKPTGSSNDDDETDS
KKEAEEKKKEEAEEKKKEAEEAKEKAKEQAEKEEEAAKKKAAEDKAKQEKAEEEKKKAEEAAKKKAAEDAKSRSKSEDDDAKE
SSTAKPTRTRAQITTSNDPSSVPSVLSDPTGSQSIESILAKATGSASASTTIDEVADMSGASSLPSSSSSAAPEASGGTSAGT
KAGIAFGVLGGLLAMGLLVYFIFTKRRKQAEMERLENDDEKLHGPMAGNGGPPMVAAAAVRRSMSPEPIETASIRTDAKAPRV

SLRPVTQFLPNWNGLEKEKRASKGAGLTLAVPAAASSPATGPLSPRTVGGSAWERPSTAQSVDPANPFGTQAERVPSPVQEET
ASALRASPSPMSEKSTMAVAAAAAAPVSPQSPSSPQGPATANGPAVAAIVARKTSIRKAGGPQQLDLTLPNAPMAAVPASPAG
TEYSSSSAPSGGPDSPSAGAAAIAAAGGPANSAVHRVQLDFKPTMDDELELRAGDLVRLLHEYDDGWALCIRLDRSRQGVVPR
TCLSTRPVKPRPAQGGPRPGPPVKAGGPPRGPGPNQPQGQRPMTPQGNFGQARPASPAGPNPNGSRPQSPMGPPGRPQGRPMS
PGPRAQSPGPRNGPPSGGPMSPGPRSQSPGPAGGRSQSPSGMNRRHNPPGPSPMNPSQDPRSGPSSSGSLSRKPVPGQAY
FUN_004629 CDS scaffold_17 6351 7671 -
hypothetical protein ENOG41

MSASRSGAAVPAVSANFEESLASKPFLFVVGPEAKEFHVHKELIGRQSPVLNALVNGNMREAREGRVVWADLDVDTFVRFAKF
AYSGDYSEAQPVLIPAPELDTEGNHSPLKSYTHDGNGYESSSGQDSDDSRSTSSDSEEIRDDNDSDEDEDLDDDDDSSEGTPT
GHHSPAGDEASSSEASTTVDHMPTRNGGNRPPFTYTYTYEYCEWKLPAAVGEFIQSYQSNNDQQNGVLQLAEDGGLSRRKRRC
PDQSYMLDVTTPIGKRFEAMRNFARPPYNSSMIHGGAPAVTSLQNTQEGKYSYLPVFLSHARLYILADKYGVEDLRRLSICRL
HCILYYFYFDQYHVPDIVALAQELFENTVEDDIAREIIVEYFVCFIEYIRDTPEVKELLRKGGDFPAALVSRVAMRL
FUN_004630 CDS scaffold_17 8355 10158 -
hypothetical protein PF12660 ENOG41

MIAGMRVRSFKAWKTLWGLGAQLPLPDSNEEDGYRNMNERIQAFSWAKEVDTGRGLLAYCNDVEEVAVMAVQLFSQAKEGDPS
CEETRWDIQEVGRFDGRGRHIKEDAVDITDPDYVPHGSAFSLKWSPWFNSQGKNMAILAYLAKNHVGFRKITILGNWERGQPP
HIEVEKTDMTAICMFLSTDAYIEWEDLIVYDDDKPIVRGVVADPFNVKPFQVSFVGDAEELAGAHYTWECSTTYSKEDEIVSS
NPISGLLIHDQGITHTGSVPYYSIARLSATSRNQDWFQTNLPDSEASVPKWATRIRKHTTRLVARAVALEGLDSDSDDSEDDL
MDDDAAQLQVPESRYRIWGMVHSPGGGTTAVLVSRYSTLHPERRALCKLMFSRRDEERGEYDAVTPTKPLTTEGQVWEWMYGN
APEVLGTTATRKISPELNNSLLREQFRDIAASQHCVFCDAALRLEEEEAKCENGHLFARCASTGLAIMAPDISRICAVCELRC
LKVAELKRVVETHFGPGANVQASGEVCGGCGGKFVA
FUN_004631 CDS scaffold_17 11309 12533 - GPI8
glycosylphosphatidylinositol anchor biosynthesis EOG09262M0W PF01650
C13
MKLSILRFHAIVAATMFATIAFAEHTSNWAVLVCTSRFWFNYRHLANVLSMYRTVKRLGIPDSQIILMLPDDMACNPRNAFPG
TVYSNSDRAVDLYGDNIEVDYRGYEVTVENFIRLLTDRVGAEMPRSKRLLTDDRSNILVYMTGHGGNEFLKFQDAEEIGAFDL
ANAFEEMWEKKRYHEILFMIDTCQANTMYSRLYSPNIIATGSSKLDQSSYSHHADNDVGVAVIDRYTYYNLEFLENNVKDLNS
QKTVGELFDSYDFGKIHSHAGVRYDLFPGGEENARSRLITDFFGNIQNVEVDRSGNLTLEEDLLALSKKIALLQQREAEAAKE
SVGEKVSAKAPASAPTETARKIQKAKALTDDNWWTKKVVGATALAGCGLLWTLGSFLEG
FUN_004632 CDS scaffold_17 15953 18179 +
hypothetical protein ENOG41

MGRTPAYIFVVRHGNRLDAADKKWHLSSPTPYDPPLTFGGWLQARAVGNQISSILEQAKDEYETTSKGTKKRRRFKVVIHSSP
FLRCAQTSIGISSGLAQTPHESPYRPNDIFVSATGPPKHFRSTILRLDSFLGEWLSPEYFEQITPPPGAALMLGSAKAELLRR
EDYSALTSAPAPTPMPHRGSTASLWNGSPAASPSLGPLRSGSAVEGSTGSAMAAALTGQLATQMEKKGYTVPKPNYAISSSGK
IPDGFVAHARDECVVVDYQWDSMRAPLDFGDGGQLGEEWTDMHKRFRRGLKKMVDWYATTDAPDEMVTQAAGASQNDTHDPSI
SEDEDVETVVILVSHGAGCNALIGAITHQPVLMDVGIASITMAVRKQDADYDTLLAASASETPDSEPHVSPHQMYDIRMSAST
EHLRSTTSTPVSSRSTSADGFPAAIRGRNPSISSTDGPGPLIGSFVYSDPIIPMGSRSASASATVGSSIRRTSGSQRPLNRGP
ALTTATGEPGSRSNSPSANTSSFGLWTPGPSSLRLMDDGSDDASDKEDFDSVLPNFSRFKGIPEDNKGEAQASTAMSTSNSST
QSSFSSRFELKSTNSLSMSLPPAAITSLSSFSFPPSGETTPKATPRGPILSAPIRIKTDILHDFDKPAEELHIGQLGDGLGGL
WGLPRPPGEAERFRDMSLSKRRWTTTERA
FUN_004633 CDS scaffold_17 19048 20110 -
hypothetical protein PF00153

MSSADTPDSAIEARVVDKLKTIAPAPEPKDDSKAGASSGLAQQLRAFAAGGFGGLCAVVVGHPFDLVKVRLQTAERGVYSSAI
DVVRKSIARDGLRRGLYAGVSAPLVGVTPMFAVSFWGYDLGKQIVRGVSEVPAEGLTIAQISTAGFISAIPMTAITAPFERIK
VILQVQGQKQLAPGEKPKYNGGVDVVRQLYKEGGIRSVFRGSAATLARDGPGSAAYFAAYEYIKRKMTPIDPLTGKPSGQLSL
SAITCAGAAAGVAMWIPVFPIDTVKSRLQTSEGNVSVGSIVRELYGKGGVKAFFPGFGPALARAVPANAATFLGVELAHQAMN

KVFG
FUN_004634 CDS scaffold_17 21504 23260 -
hypothetical protein EOG092621CK PF01545

MASSYALPTSSLPHAHQNHMHSHTRSHSQSSLNNLRRQSTFSNGSLPSVPDEDHNHDVSHDHADGNGNHSAHSHNPSIHKHGH
RRSRMSNTSATIAREKLPPAPLNTLEGWTEERTPGGKSILTPGPESANMTYSPPEFPHDHHDHHHHDHSHDHSHSHDHSHSHD
HSNHYGHDHGHPHDDDKGKRSLFTRMILPYTTRFPILNAILIEKDSRRIFYFMALNFSFMAVQAFYGYVTDSLGLLSDSIHMF
FDCVALMVGLLAAVLSKWPRSQRFPYGFGKIETLSGFANGILLMLLSVEIAFEAFERLWEGTQTKRLGELFIVSTLGLLVNLV
GMMAFGHHHHHGHDHGHDHGHSHSHDHGHNHGHDHGHGHDNENMHGIYLHILADTLGSVSVIVSTILTSFWGWAGWDPLASCF
IAVLIFLSSKPLVYSSAKRLLLSIPEDTEYNLRNSLGGILNQRGVVGYSSPKFWRDDHSASPSGGKLLGVVHVVAARGAPLED
VRDRVREYLLREGADVVVQVEREGDNSCWCSRRGIPAPPATPTLASAKAF
FUN_004635 CDS scaffold_17 24827 26333 +
hypothetical protein EOG09262Z2S PF13692
GT33
MSAAFIVSVIAGALSCVFLVWLIRGLAILIPTRYQPSQKAEDDHIQILVVGDVGRSPRMQYHALSVAKHGRNVDIVGYKETSR
HPDLIGNPRVTMYALPPQPEVLQWGTLPFFLNIPLKVLWQFWGLFSTLMYDAPAAKWIIIQNPPSIPTFHVALLVSFLRGSKL
VVDWHNYGYTILAQGKWYVKPLVPVYRWYETGFGRYLGDINLSVTDAMARQLKEKPFNLKRSVLTLHDRPAQVFQPILSTAKR
LAFLSRLAETKDIAKDIVDGAVRLIASSTSWTPDEDFGLLLDALVSYASSAEASPILAIITGKGPQKELYLEKIKTLQEGGKL
PGVRIITAWLSTRDYASLLASADLGISLHKSSSGVDLPMKVVDMFGAGLPVAAYSAFESFSELVKEGQNGCGFETSSELAEIL
ARLLSFSGQEELARLKKGAVSEGSLRWDQEWDRVVGKVIGLVGEESI
FUN_004636 CDS scaffold_17 26568 27165 -
hypothetical protein PF00179

MAASSAPAASLLKRQLKEMQAGKDIPGISCGLVNDNNIFEWEVMLMISDDCKYYGGGNFRARLSFPSSYPHMPPSLTFQDPIP
FHPNIYENGKLCISILHPPEEDEYGYEAASERWSPVQTPETILLSTISLFHSPNDESPANVEAARMFREEREGKNKDFRRRCR
KCVRESLGED
FUN_004637 CDS scaffold_17 27676 28390 -
hypothetical protein PF12171;PF12874

MSDSKKGAYGGAPSGDTDFRKTYDLDEYAAKANEREAKEKEEAKARYEAKLAGKKYHKPLTGDETYTSARRNVIDLTQQIGKT
QLVPGGAGVGKRGRGAGFYCEPCDLTFKDNKQYIEHLNTPQHLINTGQTTEVKRATAEEVHERIEYYIRRRDELEKEKQTSLH
ERLQLREEEAEKEAEERRKKRREDNERRRIKKEEEARVKMEYGEDVRVDGEHDEDDMMAAMGFTGFGTSKK
FUN_004638 CDS scaffold_17 28886 29845 + DPH5 diphthine
synthase EOG09263JW5 PF00590

MLYLVGLGLSDETDITVKGLEVVKKASRVYLEAYTSILLVEQSVLESYYGRSITVADREMVESNSDEILRNAQNEDVAFLVVG
DPFGATTHTDLVLRARELEIPVRTVPNASIMSGIGACGLQLYNFGQTVSMVFFTDTWKPASFYDRIKENRQIGLHTLVLVDIK
VKEQSLENMARGRLVYEPPRYMTVGQCAQQMLEIEDERKEGVYTKDSLAIGAARVGGKTEKFVAGTLEELCSTDEELGPPLHS
LVLLGRRTHELELDYVRQFAVDKEKWDRLWKAEYGKQL
FUN_004639 CDS scaffold_17 29927 30594 - BLI3 BLI-3
blue-light-inducible Bli-3 protein PF01243;PF16242 ENOG41

MSFSNANTGDAPADPYKKANAEEVPLQTKIEDLVHFITKEKFGMMTTRGSGSGNLVSRCMALAATETGGIDLLFHTNTESGKT
DDLDADPHVNVSFINASGEWASIAGNASISTDRSLVSKHYSPTLKAWLGDLGDGVHDGSENDPRIGIIRVKTVSVTYSLVSKN
LLSRAADIASSAVTGKPASPNTLREISEGDVRSWRASRE
FUN_004640 CDS scaffold_17 32624 34089 +
hypothetical protein ENOG41

MTSNTPATAYPWECRDKEDQAKAAFNHPLRRANQSLTEENMKLKRILRENGISWSSVALSHMNQHNPLKRKTRSSMTAQELGH
PYLPMEVILRILKFAMKSPFPIVDPLSPLTPENMSDKEKTRGNQIAINFLATCRALHVEGTRYLWESNNFTFTTPQAVRTFAE

LPVKFRQGITHITFRVIARYYDDQPCRRHKLDRCYHGDLKKDHVLKVQQRPTESPLIRGGFRCYTWNQIVDFLEGLRAPFDPI
YRNRKQPRPRMLPSLVSLRLDLVNFSDTLLPFSGSELHDICSHELGCTLNELQVTGMPFDDTGMKASAEMSGMLKDEGLYLDG
PASYMAHSRHLQPLSGKKWCARVIRAWKPKDDDDEDSENDSDTVHGHSHSHVRLGALPPAPTEEGHPTSNRDEESVIWKRVPV
SRDMEKRDWVQFSRSSGYEISDVDWDSDEEGICPCCGESHPGSSFLEILVDEDLAD
FUN_004641 CDS scaffold_17 34474 36292 -
hypothetical protein PF00394;PF07731;PF07732 ENOG41
AA1_3;AA1_1;AA1_2
MSRLYSVLFIALASPLTALPEPISSKFQKQGPCSGNTPTTRDQWCGFDIHSDYYSVVPDTGVTREYWLEISEITFAPDGVPRF
AQAINGTIPGPTIFADWGDNVVVHMTSNLKTSSNGSSFHFHGTHQNYTNSHDGVVAITQCPVAPGSSVTYRWRATQYGTSWYH
SHFGLQTYDGVYGGLIIRGPASANYEEDVGTIMLSDWSHKTVNQLLHDVQREGPALMDNVLINGTNVYGNDTDKNQTGERFRL
DFEEGKTYRLRLINTGIDTLYKFSIDHHTLKVIAVDFVSVEPYETDHISLAIGERMDILVTANQASRASSFWLRAIPQLDCTR
NVNPQNALGIVSYASNSTTPTTKGRKHEDHCQDEPYESIVPVVPRNVGPPEMEVIQHANRSWNSDGIIKWALNSTTMIAEWGE
PSLLKLKNSASFTKSNAVIRVPKRDVWVYLAIETEQDISHPIHLHGFDFQILAQGPGPYNPNVTLNTENPPRRDTALLPAKGH
LVIAFLTDNPGVWLMHCHIGWHAAEGFSLQFIVREDEIPSLMSDHEYKDIEEGCRAWNDFSHKMKMEQDDSGI
FUN_004642 CDS scaffold_17 37566 38988 +
hypothetical protein PF15463 ENOG41

MPPSLKDKGGRLLAFANGGPRTNEAQSGVDVKPTKSEPPQNVPTAPIPRPTSYPREITGLGGRNTAPPKQNYPFSQPPPHHSP
VRSTISSPDGRIKADSSDRRVDIFSGSQLDENFMESGLTTPHNEPSDPVKLGPELTRDLKKTIPSHRLPDRNRFHRPPPTSDL
FTVGHDLRMNVVSRSQQHNADHMGDGFQDDVNVRHGKLNGHHGHGRTRPDSPARQDSKIPMREVRIRKSHSGRSEAYDTNQAR
NISPRRPETQRQTQWQTHYKNESSRQPTVHHLDDEDAESASQEEHTTPRPKPAKPVVQRTLLESSMPATTNFMSNTSRLDRKR
RRPSPEYDDVALDSMTFASLRQQPFDFDPSKDGQKGTGVNADNLEDKLEHFRHLGEKEQHDLFSNMSMENWETSGEWFVHEFS
ALMQKLLDSRRQKRKIIQEFEQEAANREEAVRLKTQAIDRKLSKMKQDGQRVVEDKTA
FUN_004643 CDS scaffold_17 39326 40264 -
hypothetical protein EOG09261ONU

MAPHFIDDKTEICLPFILSQLKLHREESPDRPFVIGLNGIQGAGKSTLVKALSKALESQHVPTLVCSIDEFYLTRQDQVALAE
AHPDNALVQHRGEPGTHDLPLLKAFFEALLRGEPTKLPKYDKAIKGGKGDRLPESEWLPVNQPGQEKIQAIILEGWCVGFRPL
TREDVEARLNMSNRTLKQHKLEDLLFVNEKLSEYDSVTDSFDAFIQIDAEDLGYVYGWRLEQEDHLREERGDPEAGMTSVEVV
KFVDGYYPAYELYTDRMRKGVLANRPGRQLRMVVGRDRKVKHVRRV
FUN_004644 CDS scaffold_17 41271 44186 + FOX2
bifunctional hydroxyacyl-CoA dehydrogenase/enoyl-CoA hydratase fox2
PF00106;PF01575;PF08659;PF13561

MADQLRYDGQVVVVTGAGGGLGKAYATFFGSRGASVVVNDLGVTSKGEGNSSKAADVVVNEIKAAGGKAVANYDSVENGERII
ETAISAFGRIDILINNAGILRDISFKNMKDEDWDLIYKVHIKGSYKCARAAWPHFRKQKYGRVINTASAAGLFGNFGQTNYSA
AKLAMVGFTETLAKEGIKYGILANVIAPVAASRMTETIMPPDVLANLKPEWVVPLVAVLVHKNNTEETGGIFEVGGGHVAKLR
WERSSGLLLKADDSYTPGAIIKNWNKVVDYSNPQYPTGPNDFLTLLEESMKMGPSEQGEKLDFTGRVALVTGGGAGIGRVYAL
AFAKHGASVVVNDLADPEPVVAEIKKLGGKAVGVKASAEDGEKVVKAAIDAFGRIDIVINNAGILRDKAFSNMNDELWDPVLN
VHLRGTYKVTKAAWPYFLKQKYGRVLNTTSTSGIYGNFGQANYAAAKCGILGFSRALALEGQKYGIYVNTIAPNAGTAMTATI
MPEEMVQAFKPDYIAPLVLALCSDKCPTPTGGLYEVGSGWCGQTRWQRTGGHGFPVDVTLTPEEVLKNWKDIVTFDGRADHPS
KSQDSIGKIMANLENRSKPKESSGETNYLKVIEETLKKEGKPTEYKYEERDVILYNLGVGAKRTDLKYVFEGSDDFQVLPTFG
VIPPFNAEMPFEFDNIVPNFSPMMLLHGEQYLEIRKFPIPTNARLVTRGRLLEVIDKGNASIARTSTTTVDANTGEDVFYNEA
NVFLRGAGGFGGPKRGADRGASTAANKPPARAPDVVVESPTHDDQAAIYRLSGDYNPLHIDPAFAKVGGFKAPILHGLCSFGI
AGKAVYERFGAFKNIKVRFAGVVIPGQTLVTEMWREGNKIIFQTKVKETGKPAIAGAAAELRTDGKSKL
FUN_004645 CDS scaffold_17 44481 46425 -
hypothetical protein ENOG41

MGFPGLLTSTSTSTDRTRTPSVRSKSSDSNLVQPEYIYDEAQLPCPPPERPDLRELNSSLEALAAVFPDIQIEVFRELLSNFD
GESRLALVADALLKNRVTWVKGRWRVPDKETAQPDAIPKTEVFKSKEYKRAVQNLAWHEFKGLSRSTINAVLAESNYSYLEAR

QTLVTLSSKSWRFTISSLFFRRKPVATGEAENHPLVSWRSTGQGSITPTIRATGSAELDRELYDTLVAPLKEKDRRDREDRDR
GLAVILNNEEAEKADATHECVCCFISAAFEEFTHCNREGHLVCYRCVQHSIKEAVFGQGWQSSINPETGTLKCLSSDGDGCPG
HIPPDHIRRAMEEEKMGAEILHRLDCRLAEHSLLASNLPLVRCPFCDYAEIDDIYTPAHESALRIKSDSIYNLFFLVLCIGTL
PFVFPFVLISSFICLLLSTQQSFGDYISVEWSKALNRHRRRRRGTKFTCQSPSCGRMSCLSCHKSWKDIHVCNESSLVALRTQ
VEQAMSMAIKRVCPRCNTSFVKNAGCNKLTCPCGYKMCYVCRADLTDEGYRHFCDHFRPEGDATPCKECDRCNLWESEDTEQV
LQAAREEAERKWKDMENRELSGTERAFLETGVAANRMDVSVERVLFSGRIPALSEVLDLVVETLFV
FUN_004646 CDS scaffold_17 51323 52403 +
hypothetical protein ENOG41

MNRDMKLDVESVMADQDKNSRYEGYFAYRPLSNLPTPPPSSRNSSAAQSPRTTLDDGEPLMPRFRGPAIHLVNLIPSSASLAT
ASVPLVQAILSRANLPIETVALAVCILDSLDSRFARRWRLSCPLLSGYFSSTSKRHTLPPTPIMPQRQQLHIDSVNPELIILA
ALVIAVKFMEDPQEASQYYCKAWGRGMWSPEQLNTTERCIMENLNYRIMPLCDEDCLTDAMVDMQYAAQQPQWDINEHMPADS
DDESDDSNNHNNYVPSHSRSKTLGTGSAVLGLGLSLTPVDTPTAEASHSTTPQPSRLCNDYFNHFQARED
FUN_004647 CDS scaffold_17 53228 54331 -
hypothetical protein EOG09264ZXA PF01243;PF10590

MNDGTFPEISMFILGIKLANRNFLHFEDVMTPPPADSAVRTFFPLEHPIPSYASKSVHSRLFSTAKMALHSADTSKLIFAPAG
HQATGQAEQFTKGTLVRSQLNPTSPIPQFHAWFSRAQENDSGVDHPESCTLSTASLPSGRISSRTVYLKELDNHGFVIYTNLG
TSRKAADIATNPRAAMLFFWESLQRQVHVEGRVEKISREESQTYYDTRARGSRIGAWASRQSQVLEPQGDDDDGRKQLEGWVK
EVEKRFEGQEKIPVPEFWGGLRIIPDRIEFWQGRESRLHDRFVYEREGEGEEWTLKRLSP
FUN_004648 CDS scaffold_17 54769 61597 -
hypothetical protein
PF00117;PF00185;PF00988;PF02142;PF02222;PF02729;PF02786;PF02787;PF07478
C26
MALVSQPPASARQIGAEDDGLVCLELEDGSSFQGYSFGAQKSIAGELVFQTGMVGYPESVTDPSYRGQILVITFPLVGNYGVP
SREAMDELLGDLPAHFESSEIHIAGLVTASYAGEDFSHFLAKSSLGTWLKEQGVPAMYGVDTRSLTKRIREKGSMLGKMRLET
RGLTNGSVNSQADDALAALPTDHFEQVEWVNPNTKNLVAEVSIKAPKLYKPPTSVARKHPSGRTIRVLCLDVGMKFNQLRCFL
KRGVEVLVCPWDYDIASAIDEFDGLFLSNGPGDPAMLDNVVKNIAAVLEKNEIPVFGICLGHQLLARAAGATTKKMKFGNRGH
NIPCTSMVTGKCHITSQNHGFAVDTDSLTNGWKELFVNANDGSNEGIFHTEKPYFSVQFHPESTPGPRDTEFLFDVFINTMAN
CAEKPELLKAPVSFPGGTIEENERLHPRVSVRKVLVLGSGGLSIGQAGEFDYSGSQAIKALKEEGIYTVLINPNIATIQTSKG
LADKVYFLPVNADFVRKVIQYERPDAIYVTFGGQTALQVGIQLKDEFESLGVKVLGTPIDTIITTEDRELFARSMDSIGEKCA
KSASANNIDEAMHVVKDIGFPVIVRAAYALGGLGSGFANNEEELMDLCNKAFAASPQVLIERSMKGWKEIEYEVVRDAQDNCI
TVCNMENFDPLGIHTGDSIVVAPSQTLSDEDYNMLRTTAVNVIRHLGVVGECNIQYALNPFSREYCIIEVNARLSRSSALASK
ATGYPLAFIAAKLGLGIPLKEIKNSVTKSTCACFEPSLDYVVVKMPRWDLKKFTRVSTQLGSSMKSVGEVMSIGRSFEEAIQK
AIRAIDFHNLGFNETEALMSIEDELQTPSDQRLFAIANAMHEGYTVDKIWELTKIDKWFLRKLKGLSDFAKRMTSYSTTDITT
SPSILLQAKRLGFSDRQLAKFWSSNEIAVRRLRLEAGIQPFVKQIDTVAAEFPAFTNYLYMTYNASDHDVNFDDHGVMVLGSG
VYRIGSSVEFDWCSVRAIRTLRATGFKTIMVNYNPETVSTDYDEADKLYFENINLETVLDIYQLENSSGVLGAMGGQTPNNIA
LPLHRAGVKVLGTSPEMIDNAENRYKFSRMLDRIEVDQPTWKELTSFAEAQEFCNAVSYPVLVRPSYVLSGAAMNTVYSEKDL
KNYLDQAAEVSREHPVVITKYIENAKEIEMDAVAKDGKVIGHFISEHVENAGVHSGDATLILPPQDLEATTIERIEEATRKIG
LALNVTGPFNIQFIAKDNDIKVIECNVRASRSFPFVSKVMGVDLIEMATKAIMGQPFQAYPPTDLAPNCVGVKVPQFSFSRLS
GADPVLGVEMASTGEVACFGVDKNEAYLKALMSTGFKIPKKNILLSIGSYKDKREMLPSVQKLEKLGYKLFATSGTADFLQEH
GVQCQYLEVLGKEEDRSSEFSLTQHLAKNTIDLYINLPSNNKYRRPANYMSKGYQTRRMAVDYQIPLVTNVKNAKILVEAIAR
HFELEVSKRDYQTSHRTIVLPGLINIAAFVPGIATASNDDLQTVTKSSISAGFSMIRVMPLGVDGAITDALTLKTAQQNSRRG
GYCDYNFSVAATSDNADQISHVTGEVGSLFIPFNHLSGNISKVAAVTAHFDSWPTHKPIVTDAKLTDLASILLLASLHNRRIH
VTSVTNKDDIKLIALSKAKGLQVTCDVSIYSLYLSRDEYPECERLPTAADQKALWQHMSTIDVFSIGSLPYRLATSLGHKGDP
HMGISDALPLLLTSVAEGRLTVDDIKDRLYTNPMEIFELHDQSGTTIEAEIDRPYTVAPGTFWSPFVGRTMRGSVQRVTFQNT
TVCLDGEVLPVSPQGKDMSSHIAPPMSPPVKPTTSIAQATDSPQARRMSMLGGFQPLNRLRGVDTGAVGATLPSPARGAAPVE
ELAPGLQLQPLVSSSLQQLLAQPSSFKNTHVLSVKSYSRADLHLLFTVAQEMRLGVQREGVLNILRGRVLTTLFYEPSTRTSA
SFDAAMQRLGGRTIAISTSHSSVQKGETLQDTLRTLACYGDAVVLRHPEESSVHVAEKYSPVPVINGGNGSKEHPTQAFLDLF
TIREELGTVQGLTITFLGDLLYGRPVHSLVYLLRHYHVQVQLVAPKALALPPKVREQLVASGQLLCESETLTPEIMARSDVLY

CTRVQRERFPTEEEYQRVKNSYRVDNASLKHAKSSSIVMHPLPRNEEVAEEVDFDQRAAYFRQMRYGLYCRMALLALVMADS
FUN_004649 CDS scaffold_17 63353 65044 -
hypothetical protein ENOG41

MYSAGYGFSNNAAPSFNNPAPQQQSGAQPAAQQMMYNQQQQFASMTPQGGFNPGANPQMMGAGPGGMMPNAGMQHMAANGQMA
GFQQQFAGNPYGQVVPSSVAPQNFAPNYMMGGGMQGFPMNQPGMPQNPQMMQRLQQQQQQQQAQAQAQQQQQQQQQQQQQQQ
QQQQQQQAANPAGMGQVSTPQRPPPPSAAQGTPNNALPSQQGQFPTPQPPPQSQTPTNQQSQQPQSQQQQQTQQAPTQAQGQQ
QQQQPVSAGLSTPQTPTFSSNQGPAVNGTLTAAPLSPGTESRDKERFALLLDINHELLYESIQIQATQQELKKESAAAGNPAD
KKPTDEETQLQQDYLQCMRRLQANLSYMAALADRKPEVKVPPCPAYLTAPALTLSLKLRVPAVGPEGADNNIDPVADREERNN
SIRDLYSRLQAVFPGIDPKREPAYRMNTQGGQKPGMMGSQASPTAQRTPRITNMGAPPMP
FUN_004650 tRNA scaffold_17 66064 66136 - tRNA-Arg

FUN_004651 CDS scaffold_17 66895 68145 +
hypothetical protein EOG09262K9A PF00155

MAPFNLESCARPNILALQPYRCARDDYKDDGTNILLDANENAYGPSLSADVAGKTANGVEVDLLGLHRYPDPHQEPLKQQLCE
LRNTHAHTDKTLKPENLFVGVGSDEAIDALLRCFCVPGKDRILTCPPTYGMYSVSAQVNDVALVKVPLLEAPTFSIDVPAVME
ALTKESNIKLVYLCSPGNPTGSVLAKSDVQQILDHPTWNGVVVLDEAYIDFAPEDASLAEWVTEFPNLVVMQTLSKAFGMAGI
RLGAAFTSPPIARLLNSLKAPYNISSPTSALASYAVSEKGLAIMRDHRARLLEQRDRLIKELPNIPGVGRLRGGTESNFLLYE
MLNTAGEPDNTVALAVYEKLAEGKGVVVRFRGKEHGCQGCLRITVGTDAEVTRFLESLKTTLAEVRGS
FUN_004652 CDS scaffold_17 68354 69822 -
hypothetical protein PF03663
GH76
MKFFALASLASVAIATKTPPSSLDTANPDSIRDVAGTLAFDAMKYYSGNTSSVPKDLGDLQDPYYWWVAGALWGVMLDYYHLT
GDYSYNDVVIQALLGPTNLGTGKDYMPAEHADEEGNDDLFFWGSAVLSAAERNFPQPNKDLPSWLDISINVFNELVGRWNTTA
CGGGLLWQIYPSNPNGMTYKNSVSNGGFFQLAARLARITGDNKYLDWALKIWDWSWEVGFIDHRNYHVYDGASVEDNCQKTTY
HSFTYTSGIYLYGAAVLANYTEKPEWVDRSKRLLEGTDWFFSPFPNATNVMYEAACETVNTCNDDMSTFKGYLSRFMYLSIQM
QPALKEHVHAHLLPSAKAAVQTCTGGKSGRECGMRWYNSGFDGNPGLGQQMCALEAVQGLLLSDAPAPLKGDDVKVVRSTDWA
AMKKNESKIQSTPTSSSETSSATAAQPTKSEDAASLPRVDLVLASVSIGSVLMFLGWA
FUN_004653 tRNA scaffold_17 72962 73066 - tRNA-Ser

FUN_004654 CDS scaffold_17 74255 75003 -
hypothetical protein ENOG41

MSPRSKSIWLALLAANSFAYASEEPPAVTTAPIYLPYYDEDSWSLVRGSVISSDAEAKETTYTIFCPDTDDSDPPECDLSLEF
PFVLVEGPDTVRFHGTHTSRLTANLECNLQGTTRATCSGYSSFNEGYSDGVHTGPTEVTWTSTYSGSEVEWGVLTMGELPHDP
DPVTAVSSTPTDFVSMPIATNGDNAGASLDVSFKAALLAACCTLVVGWTL
FUN_004655 CDS scaffold_17 78572 79305 +
hypothetical protein ENOG41

MARSLALLAGALATVAAQTTTVEFLLPMFDPQPLDGSVVAVKGGATTLAIHCPDAKATNCGLAGTSTIVGGPSTIEYSYTMHP
EVDGFDGGEIKQNMGCVLDPKKDVATCSVHQVAVLSGITDVTSMTDSVSGYKSLLMPITVTAGVNKLNGNDDASATEAAGSEA
TSADKPKDTGAKATDATDATTASSDAAATPVSSDNAAGPMITQNAILAAAAVVGGAAMLL
FUN_004656 CDS scaffold_17 79772 80519 -
hypothetical protein PF01741

MFWLSGLWRTSRPNYRIMPGRSHDEESGSGESESLLGRGHKRVKRFWEGFVDFAFQDDILKIAVGLILAAAFTDLVKSFVSDV
LMPPVSILLPLNRNMEEKFAVLQKGPNYNKTTGYNTLHQAQEDGAVVLAYGSFVGQMISFLVLGIALYGLAHLWQLASSEPII

KHTKKCKYCRQRINEKSIRCIQCTSWLDGREDRN
FUN_004657 CDS scaffold_17 81291 82310 -
hypothetical protein PF06687;PF12351 ENOG41

MLSRFFVLLPLVLSMVAFILSMLCLFAGHKEGFMEEYSVARLNTSMIGHNVLDTDSDASSNDNDEDDGFFGKVTDKWNEVKDD
VKGKINDITGDVADKLADTIGISEWYSIHVMATCDGQYKPNATSPGAGYNVTNCTNSAPEKRFNLTEMLDKQLEVGPFKLNLA
DINWPDDIQDSIDLLNTALLVTFVFYVLAVGFSGLAMVASAGAFFLFARRGVNAVNVILSGLAALVLLIASILVTVAGKKGVD
KINDVGDDVGLSASVGNKFLALTWAAAALMIIAAIYWVMHLCLMRRERKRQWKPRKGSY
FUN_004658 tRNA scaffold_17 83269 83364 + tRNA-Ile

FUN_004659 CDS scaffold_17 85011 86223 +
hypothetical protein

MPSINSLLTMALAGSASAAFQGFNYGATFTDGRVKAQSDFENEFKTAAGLEGTSGAFTSARLYTMIQGGSINQPISAIPAAIK
TKTSLLFGLWASGDGFANEIAALKNTVDQYCGQLDDLVAGISVGNEDLYRISPTGIKANENPGTNPDVLVDYVKQTRAAIKGT
CLESVPIGHVDTWTAYANASNNAVIEACDWLGMDAYPYFEDTKNNPISEGANLFKAAWNEVKAVAKGKEIWVTETGWPVSGKT
YGKAVPSTKNARTFYEDVGCPMFGDINVWWYTLQDSAPQTPNPSFGVIGTELTEKPLYDLSCDEKSKKGTLVSRSNDASGNVE
HRFVSPSFATGNYTNGTVPVVPGTPTSLVPTPSSTSGNGGSAATPLPGSGAQQLNSMGAAAVAFLLAAALL
FUN_004660 CDS scaffold_17 86740 87168 -
hypothetical protein

MLYPYFLVATEKADAAGKQLGERPWYSNHHQQGGMTPNNCTFRVVTGRVENKKNKTKAQNRQAEKLADKTTDKQLKFGIS
FUN_004661 CDS scaffold_17 89373 90803 +
hypothetical protein PF02535

MALTRRSVVCSAVCLALLVGLVYAGHDHEHVETVNNVNIDNMSLEELDTQLQTCPIVEQLNAAKHAHHAAAPSSMTSRLFAVL
FPGSPAVNALLATLYISGPPNFLLALCPTNIDPASLSVMVAFAVGGLLGDTLFHLLPEIFVGEDHDEAVKFVLVEPNRNLVLG
LGILVGFMTFVAMDKGLRIATGGAGHDHSHGHGDAHAHSHGEDKAVSSGVDATDNLVKSRKKGNEDKGAVVANAVEKPEKEIN
PSVKLGGYLNLIADFTHNITDGLAMSASFYASPTIGATTTVAVFFHEIPHEVGDFALLIQSGFSKRAAMGSQFITALGALLGT
LIGIAIQEFGSPSSDVPMGRNEGIWGTSLTWGDMLLPFTAGTFLYVGTVAVIPELLETGPNKAKELKNMLIQFSAVAIGAGIM
LYISWHD
FUN_004662 CDS scaffold_17 96526 97993 +
hypothetical protein ENOG41

MSNAASDNGPRSTRPRPAKSGIDIKLDQHWSSKSYTSGSKVTGNVIISTQRDVAYDGFDIFFTGTAYTRVDFVSQYSSANYAS
RPFMKLRMPLSPADFPSNRVFEAGRTYTIPFHFVVPHQLTLSACNHPVEHEGIRDHHLQLPPTMGFWERNDQSPDMAHIEYGI
RAVATKNSEEEGRTLPLDSFHMVKVLPAMPEDAPLDICSKDERYCLSKTKPIRKNLFSAKQGELTTTASQPSAIMLSGNFLNA
SGTNLKVNFEFSSTSKDVTPPKVNSVSAKLHATTFFSSAPMTQLPNLGSRIKHQSTPSLTYSTTTPVPMRPDTRLTWEDEAPG
SRRDSGYDSAFWNEGETSESDDTQRNKKASKYNVRRYATLDIPFNLPTSNSKLFLPTFYSCLSARAYILHLTLSVGPMNTNIN
LNIPIQVAVENTYDHIDDDELPSFDAAIAEAEEADVDLHLQPRLMQVPDARFQGNSVLPGYEDMRMRAQVAVA
FUN_004663 CDS scaffold_17 100328 101998 +
hypothetical protein PF00282

MEAQEFREAAKAAIDEMTDYTENVAEYRVVSNVKPGYLRPLLPSSPPTDPEPWSAIHQDIESKILPGITHWSSPRFMAFFPCA
SSYPAALAEIYSNAFNGAHFNWICSPAVTELETIVMDWLAQALGLPECFLSGGSTHGGGVIHGSISESLIVNMAAARDKYLAS
VTAHLPPGSEEKEEALWNLRRRLVALGSSGTHSSTKKVAQVLGVRFATIPVSEADGFSLRGEALAATIENLRARGLEPFFLTA
TLGTTDVCAVDDFEGIAQALKPTFDTSRDIWVHIDAAYAGAALVLEEYQHLAKAFSSFHSISFSPHKWFLTTFDCTAVWVRHR
SWLVQALSIKPPYLRNQFSDDELVTDYRDWQIPLGRRFRSLKLWFVMRSYGISGLQKHIRNGVDLAESLEDKIDSRRDIFSVF
TPARFGLITIRINGESEQQINDRTEAVYEVINAAGEFYLTATVVNDKFAIRVCTSVTKVEEQHVQRMFDVLVQTAETEIAKGH

KWLECNL
FUN_004664	CDS	scaffold_17	103450	108247	-	NTE1
phosphatidylcholine and lysophosphatidylcholine phospholipase	EOG092603EH
PF00027;PF01734
MASDPSNAAQMAGDVAETVAYALSSSSPSIVSTMPAVTAHEPYTWFGLFGWLIWFLLHITSTILYWVIRIATINVPSFLFHLF
STSWTVTMNATTLMFIMAAVVSAVSWVVRYRILNMYSRLPPEPQRKEPDIDLFPDTHEGGTKSGLANYLDEFLSAIKIFGYLE
RPVFHELTRSMQTRKLIAGETINLEEEKGFCIVVDGLVEIFVKSNRSPRAAAASRGRLDLDSSDEDDIAPGQQTYQLLTEVRN
GAPMSSLFSIMSLFTEDVRLRPNDDQEDSSLHPAKSSGLRHPTFEKPHLRRVPSDVNMSMPGTPQSRNDMEADEKKPEGLGVS
GLETDQAAIPAMSLDGDTPPPTRPPLPTRATSGSAHPDIIARATVDTTIAIIPASAFRRLIKIYPKATAHIVHVILSRFQRVT
LAQAFNYLGLTSEVLQTEKSMIKYTVCQLPNILRGDALERLKEKFKRERERTDEDDDTKGIALHNSASTHRRRSSTTLRKEAM
LQSMTKLRTTSVVTSTIAPPRERTSPHTPSPGDLLSNIQLARGTSRISPADQRSVSGASMRHQGANPKDDRFFNDPFDSRRLG
RMTMDPRETVDEDNLFRASILECMFKSLGLDANGSASREPESMEGSPRLVSFDHRRSKFYSQNNAFGFMPPYDGSVDGETESV
TSGGTALHASVSAQGLASDMKDEVEIVFFPKGSVLVEQGERNPGLYYVVDGFLDIGTTGEADAHNILQSNGFGSSQPAPPSPD
PSEMGSFPNLARTEAARDQAETQQSTSKKPRRSIALVKPGGLAGYIGSVSSYRSFIDVVAKTDVYVGFLPRASLERIVDRYPI
VLLTMAKRLTNILPRLILHIDFALEWVQVNAGQVLFHEGDESEAIYIVLNGRLRLVQERKGGSGGVTALAEFGQGDSVGELEV
LTETARPGTLHAIRDTELVKFPRTLFNSLAQEHPNITIKISKIIASRMRAIVDETSKFSYKDASTNPKTLMRKSNMNLRTVAI
LPVTAGVPVVEFGNRLMNALAQVGPANGATSLNQAAILNHLGKHAFNKMGKLRLSQYLADLEEKYGLVVYVADTNVNSPWTQT
CITQADSILLVGLAEGSPAIGEYERFMLGMKSTARKVLVLLHAERYSRPGLTRAWLQNRMWINGGHCHIQMAFRTNEMPVHAP
KKLGQALKERVQVLQAEIQKYTSRKVHHTPYYSPDSPYKGDFHRLARRLCGKSIGLVLGGGGARGITQIGIIRAMEEAGIPID
LVGGTSIGAFVGALYARHADVVPMFGFAKKFAGRMASLWRFALDLTYPSASYTTGHEFNRGIFKALGDTQMEDFWLEYYCNTT
NISKSRAEFHTSGYAWRYIRASMSLAGLLPPLCDEGSMLLDGGYIDNLTVSHMKGLGVDIIFAIDVGALDDDTPQTYGDSLSG
AWAFVNRWNPFSSHPNPPTLAEIQGRLAYVSSVDALERAKTMAGCIYMRPPIDDYGTLDFHKFDELYQLGYKYGQEFFNKMKE
QGVLPLVEETEAKKALRRTMAPRRASI
FUN_004665	CDS	scaffold_17	108766	109924	+
hypothetical protein		PF03029

MPFAQLVVGSPGSGKSTYCDGMHQFLGAIGRACSVVNLDPANDHTNYPAALDIRNLVKLEEIMKDDKLGPNGGILYALEELEH
NFEWLEEGLKEFSEDYVLFDCPGQVELYTHHNSLRNIFYKLQKIGYRLVCVHLSDSFCLTQPSLYVSNVLLSLRAMIQMDMPH
INILSKIDKVSEYDELPFNLDYYTDVDDLTYLTPHLESESPALRSEKFGKLNEAIANLIESYGLVRYEVLAVENKKSMMHILR
VIDRAGGYVFGGAEGANDTVWAVAMRSESSMLEVQDIQERWIDQKVEYDQMEREAEEEQARIQEEQAMELDQSEPFAPAGGMD
PDFGDMTVPKDSGIKVVRKK
FUN_004666	CDS	scaffold_17	109934	110519	-
hypothetical protein		PF01900

MVRIKERYLLVNIVYLPDPAKAAKSDLPDFVLNHQPTIEKLTPGALIRGIRAEVASLYGDCGSGALMSCSVKYLSLATSTFIL
RCSRAHYQMLWSTLTFMDHVPVKDGRPCIFRVVRVSGTIRKAEEEAIRQAKRFILAAKEDTDSQSSLGISFRHQDEMVLDVND
ASSDEEMDDAD
FUN_004667	CDS	scaffold_17	110852	112974	+
hypothetical protein		PF00994;PF03453;PF03454

MASGPLKAALLIVSTTAAKDASADASGPILTQVLKDEGDGKWEIHDTKIVSDDVLAIQRQITTWTDSPDSVNLVVTTGGTGFA
TVDSTPEAVTPLLHKQAPGLVHGMLAASLTVTPFAMMSRPAAGVRNSSIIVTLPGSPKGAKENLQAIVKTLPHACIQAAGGNS
RAIHSGGIKKLEAEAGLTGGETHSTAAGGCGHHHHGHGHGHGHGHGHGNLVRHTKPGTTLSNDPALGPSRRYRESPYPMLPVE
EALSLIEEHTPEPEVISAPVNQSIINSVLAEDVKARENVPAFRASIVDGYAVVAPKDGQLKGVFPVTAISHASPGEIGALNEG
ELARITTGAPLPPGATTVVMVEDTILKTMSEDGKEEKEVEIQVDGVKEGENIREVGSDVQQGTVILNKGETISGVGGEIGLLA
SVGVSEAKVYRRPVVAVLSTGDEIVEYNRPGDLKLGEVRDTNRITLMSAAREWGYEVVDLGIASDKPGTLEETLRDGFRHADL
LITTGGVSMGELDLLKPTIERSLGGTIHFGRVAMKPGKPTTFATVPVKDNSGQRVTKTIFSLPGNPASALVTFHLFVLPSLHK
LSGASTKGLPRVPAVISHDFPLDPRPEYHRAVVTVGKDGLLSATSTGGQRSSKVGSLRSANALVILPSGKGKLEKGSKVEVLL
LSAVQAA
FUN_004668	CDS	scaffold_17	113283	114083	-	VMA16

H(+)-transporting V0 sector ATPase subunit c'' EOG09265G5C   PF00137

MSLYYGVGGIGSFLTIVGAYMLFTGSGESFDIGGFLESVSPYTWATLGIALCIGLSVVGAAWGIFITGSSILGGGVKAPRIRT
KNLISIIFCEVVAIYGVIMAIVFSQKVENVSGAELYSANSYYTGYALFWSGITVGMCNLVCGVAVGINGSGAALADAADPSLF
VKILVIEIFSSVLGLFGLIIGLLVSSKAPAFGEKK
FUN_004669   CDS   scaffold_17   114439 114911 +   TOA2
Transcription initiation factor IIA small chain (TFIIA 13.5 kDa subunit)
EOG09265ER6   PF02268;PF02751

MATGAQSFYELYRRSSIGLALTDTLDDLISDERINPQLAMKILSNFDQAITEALQKNVKARLQFKGSLDTYRFCDEVWTFLIK
NVTFKMDNGSQSVTANKVKIVSCNAKKPGEGQ
FUN_004670   CDS   scaffold_17   115478 116264 +
hypothetical protein   ENOG41

MPSPSNWSLRSQWMFFAVASGACAAFNGVFAKLTTTQLTTNLSDSIAKLIGLSAHEHIVEYLVRGIFFVLNLTFNGVMWTLFT
QALARGTSTTQVSIMNTSTNFMVTAVLGALIFSEVLPPLWWAGAALLVAGNVIVGRKDETKDAGAAAAAEGPSYVPLPTEDDG
AQRDDDEDVIDLGRESD
FUN_004671   CDS   scaffold_17   116465 119162 -
hypothetical protein   PF00571;PF00654

MNVRQSSFSLAPPGPGLERRPSVVSRLSSVFSNADQEAGTAARTGAGLIEEEIAEIKRYEDFTTIDWVQDAAREQAKRKARRK
QAAGLYDKGQPGWRYQLWRSYDAAQAWIVVTIIGIAIGLNAALLNIITEWLSDVKMGYCETGFYLNENFCCWGEENGCDQWHR
WTGFEPLNYFIYFVFATLFACVAGTLVKSFAPYAAGSGISEIKCIIAGFVMKGFLGWWTLIIKSVCLPLAIASGLSVGKEGPS
VHYAVCTGNVISRLFDKYKRNASKTREFLSASAAAGVAVAFGSPIGGVLFSLEEMSNQFPLKTLWRSYFCALVATAVLAAMNP
FRTGQLVMFQVEYKNDWHFFELLFYVLIGIFGGLYGAFVIKWNLRVQSFRKKYLKEYAVLEATLLAAGTAIIAYPNAFLRIDM
TESMEILFSECGRGESYHGLCEPDKRWWNIISLFLATFLRLFLVILSYGCKVPAGIFVPSMAIGASFGRTIGILVQAIHEANP
TSVFFSACKPDEPCITPGTYALLGAAAALSGIMHITISVVVIMFELTGALNYILPTMIVVGVTKAVSELFGKGGIADRMIWFS
GMPFLDSKEEHNFGVPVSAVMRTSVVSMPAHGLTLGEVQSLLADDRYQGFPVVEDKHTKVLVGYIGSTELRYAIDKMSRTSPL
SETAKCTFAPSSANLSSTSLNNIHGDSSHSSTLDFSRYVDATPVTAHPRLPLETVMELFQKIGPRVILIEYHGKLTGLVTVKD
CLKYQFKVEAAENPKDDHRIEEGQEQLWALFQRAGNWFSGRVSRYSGGRIRLTSTSGGERPLGRGQILDGDEEVLDEGVELES
RR
FUN_004672   CDS   scaffold_17   119917 120598 +
hypothetical protein   PF00808

MPYNTTAIPPRKEPTGQNQLPLSRVKKIIAQDPDIGLCSNNAAFVITLAAEMFVQHLAEESHTQAKLDRKPRRNIQYKDVASA
VAHHDNLEFLEDVVPKTVPYKNIKAKAKATQARLQGDNTNQKLEFPLANGAGKPLVNGNSVAHHDRQDDPNSQLELEFRQASG
ANRDGDVAMTG
FUN_004673   CDS   scaffold_17   120860 121825 -   LEA1   U2 snRNP
complex subunit   PF13855;PF14580

MRLTADLIRDSLSYLNPLKERELDLRGHRIPAIENLGVAGPHDAIDFTDNDIQVLGNFPLSPRITTLLLARNRVSSIQPSLAK
AIPNLANLVLSSNNLTELADLDALASFPRLTHLVLSDNPVSKKENYRYWVLWKCPSVRFLDFEKVKESEREKARELFGTEEEP
TALASKIQGIKTTTFNTSTDGSDAPSKLSRIKLTDAEKKRLQERIKKATSLQEIIALEKELNEGRLPSGIHGDAMEE
FUN_004674   CDS   scaffold_17   122349 126978 -
hypothetical protein   EOG092602OP   PF00069;PF00400;PF07714

MGQGFSLATPSVGSAGIDISELSDVHFEKSIGNARFMKSIRGRHENGLVLVKVLVKPYADVKLQQYKKQIIEQSNAIDKIPNT
LGFQRIIETETNGYLVRQFLYSSLYDRVSTRPFLEDIEKKWLAFQLLCALRDCHARDVYHGDIKAQNVLVTSWNWLYLSDFSS
AYKPVMLPDDNPADFSYFFDTSGLRTCYVAPERFYASGEAPPKKAKMTWAMDIFSAGCVIAQLFLESDIFSLAQLYKYRRGEY
DPVITHLSTIANKDLADMIAHMIQLDPEKRYSADQYLEFWKGKVFPHYFYNFLHQYMELITDPSSGNSPMSGTSKNLGEADDR

IDRVFYDFDKISYFLGYQLEKRTSDELQPPPRLSLSHFPVRLNIPNHNHTVSSELEPPEDDGTLIFLTLIVSQLRNTARASSR
IRACDVLLAFSERLTDEAKLDRVLPYLMTLLVPDETDLVLIAAIRTITQLLQLVQTVNAFNSHVLVEYVLPRMEIALGSRSRV
PSPLVRAAYASCLGSLATTAQRFLNMASSLRADGSMPITDPEVEPGADAKANFESVFDNAGRQLFEALESHSKQLVEDPDISV
RRAFLASVPELCLFFDHHSNDVLLTHLNTYLNDRDWTLKCAFFDTIVGIAAFIGSTSLEEFMLPLMMQALTDPEEYVVQAALH
SLAQLAGLGLLSRPKVWELVDLISRFTMHPNIWIRESAAEFLSMSARYLPPADIRCNLMPLVKPFLKVEVLSDFSELNILDAL
KRPLARNVFDQAVTWASKTERGIYWKPLQQLKPLLFGSKGSTSSRTSRDLMQSSMGRVARNDEDEQWLTKLRNLGLKHEDELK
LDALREFIWRLSKIKARDTSTSDGTTSNGVISLKSLGVTPQTVFFNDAPLRDPNAVVDLEPPAEPYTIADALLDASMTIDDST
GGKKRVNTNRRVQSVGPRSSRRLLSPTSAGRTHTRAPSAGQVMQTTDNGPSQGEPPSRNAVRHQASALSLLDRKDKNKSIAQT
GTTDANAFGEVEGPFAQTSTDQQASSEGNEGDTTGSRVSKHSYAGNDPSIQRMLDNMYVDNFPRDVIEFGPMVTPIKRSKASR
ASVQPGEEPWKPVGQLVATFSEHKSPINRVLPSPDHVFFITGGDDGTVRVWDTARLERNITHRSRQLHKHGDNTRVVALCFVE
NSHCFVSCASDGTVHVVKVDTVSVSGVVRYGKLRVLREYQLPDGEFAVWCEHFRQESNSILVIATNRSRILGIDLRTMSLLYT
LENPVHHGTPTCFCVDRKRNWLCVGTSHGVVDLWDLRFKMRLKGWGLPGKGSIYRICIHPTKGRGKWVCISGGTGQGEVTVWD
LEKTVCREIYRAGGSKDGLKGYEPWDVDEDKREGMLGRYATNIEPNEIANADRGVRAMVVGTATAEDSRDVRHAFIVTGGSDK
RLRFWDLSRMESSFIYSGLSPDLPKPTYTTSHPTTALTLNTERFPRQAPTAPNAGSGSRAKSSTARPPRSTVISLQQQQLLQS
HVDSILDVALLEFPYIMSVSVDRMGVVFVFQ
FUN_004675 CDS scaffold_17 129635 130799 -
hypothetical protein ENOG41

MDALKNLVNNVPDWLQRLDDLSGQIDRRQAELAAVAAAEGKSAETKSLRNQGSTESLKPKDDPPVVHAEVPANEDILEDGAGD
NTAQTPVKPETPKVHTPSPGSIRQQQEIIKAAQARARAQVRKKPKSPSMMSNEDAPAAYRTRSMIIVYYDSYVQSFFDDLVRF
VSSSRNLMRKAKMAARVAQIKKLAEQDVSEDGSHDDALPSLRYMSSRRFGPMSISRPGAGDQPPDVYDKLDKGLEFVQSMCEH
GAHQFLRDGDCNDEISKVQKRLTEVLEMAKTEMERVEREEPELAKETGEMGKVRTRRPISMRRDMTVGLKESSPTPTKEENKL
EPAKLEAAEPIIAADPMAPMAVDPNIMEADEGIDVEMELPKLQYRSTRAMRSRGP
FUN_004676 CDS scaffold_17 142447 143401 -
hypothetical protein PF12766

MLNVEVGFDDLNTTYQLRSQQPHDGQVQQQGSRNIPQLSLPMSPVARPIQRTTQILSHLQHTMSAHHPIPAAPWRSAFLSHVD
KMESPTFMLSTLHHHGSSITPRSRTIVYRGAWAEIPVNPKNQAPLNPSLYESDLLTITTDARMEKVPELSTDGEDVPQSGGGG
PVEAVFWVVETKTQWRLRGRAYLLGQDIDDPSASHVRQEIEKHMRLKNNDDSGSWNWGKEITAHFGNLSPGMRGSFRNPPPGT
KRDEKPAPGLGLGQKVEDLDDEIARRNFRVVVIVPEEVDQVDLSDPEDGRRWNYQLKDGSWEKTELWP
FUN_004677 CDS scaffold_17 147435 149875 +
hypothetical protein PF00172;PF04082 ENOG41

MDLSQRALPRHTHHQPPPSLSNPSPSHLPPQHSTSTYSPGPVASHPTLPLPGTHPPSSALTPGSASTSPASISHRLPSASAST
TSRHPTGDYLPDADDQDASTPVDGEEPPKKKQKRNKPTLSCHECVERKTKCDRGRPHCLACIKRQTECRYAHVANLLEETTRS
AANGRRMTKPPKKKSGSSGKSPIPNIADRGMSNDPRATSRGAVALSIGLLSNVPYSLPSASNVFGIGSEHPFANYWTCEGGLP
EVISVLPDKIQADILLSRYFECVDPVYPMIHRQTFYADYEHFWQMNQQEKTDTDPSFIALIFVMLALGTQFVTSTTSPQERRQ
TAEFYASASNQALRIASYLSSASLRSIQAMVLLVYFLINDNHASDGWAFAGILIRQAYAMGLHRDPNIVTPNATLFEKQQRRK
VWQAVLLQDTFLTVLLSLPPSATHTDVSVEDLLDDGSSIANSDPTDTAYIRGSWTLANLVQETICSPRSLDLPICTTARHKSK
LIADFRAVYRSFPDVFRSWDPDSITALAKTNKRVVRQTLFLTSNYFHNLMLVHASESPDVPVNVRGTLEAAHDAITAFFLLFT
LLEIEARVWWVFNHRAFLEALCIGNVLKEAAREPGGADMMARDPLLVRAKADITRMIQIMQVMGEDSEVARTRVQVLSDFLA
FUN_004678 CDS scaffold_17 150836 152720 +
hypothetical protein ENOG41

MRGLAQAAMVLAPTLVLAHGPHHPPPPPPPPPMGFLPSALDVTPSAFVTVKLPLSESESYPLTLRFDVEESEEVCGPASITLN
GEPLKQDAKGRGVGSFLVNNETTISADWDFECIGPKEFPFGQSMRFNVKALDDMALTQESSFWMTFKQTAPVRISDVGNAAYV
WSLSMPFSKDKESSSDDKSASTSPPIWEYPDRDFQDPEEELQVELMELNALHKQILQLEELVKEREASVAKKLGKQYPPPPPS
TMKKIKQCDGVQCVLHTVGEEVRHSAHRFYDSIFGHPPPPHGPHGPHHGPHHGNGTHRGPHPPHHGPPHPRPPPHHGHPPFPF
PGGHPPPPMCAPCACDPHHGNPPPPPHHGDKPHHDRPEHGHGGFGAEDHEGHPPPPPPHHPIIIILGIVTIALALFCAIAIAY
IHRRVARLSPEARRAIRRAFRQTRDERRKNSALKAAYRAFVTRWIENEDDEKEAMLSGERRRRSSSCSSVTMEEEIASFREAA

HMVEGIVAAEEGHHSHARSYSYAAGPPVPPRPSHHTAATAYPGFMETDENLPAYDDDERDSSVVADGCRYTPGSSDYTPSNSG
SNASDVLGDTKN
FUN_004679 CDS scaffold_17 152969 154190 -
hypothetical protein EOG09262X01 PF01026 ENOG41

MCQHTHNYDEPQQSKPEPAGDQPPFPWHLPICDAHCHPTDTMASVTDIPSMRAAALTIMATRAQDQDLVTDVASNHSNPDLKS
LLQGKSEATDTCAIPSFGWHPWFSHLLYDDSADTPTFQSISGSETNNAAKQAHYNAVLQPVPSPDFVASLPSPIAISSFLNAT
ESRLSTSSCALVGEIGLDKAFRLPEPWQPSDHVERDSTLTPGGREGRQLSPYRVKIEHQRDILAAQLRLAAKARRAVSVHGVQ
AHGILYETLAATWKGHEREVITRRKRRLVASGAEDFSNDDDDDGSDKPYPPRVCLHSFSASVEVLRQYLNRTIPARIFVSLST
AVNLSTDASRNKTDDVLRALPDDSILVESDLHIAGKQMDSALEDMYRHVCRVKGWNLDEGVKKIARNYEEFIFG
FUN_004680 CDS scaffold_17 155622 156387 +
hypothetical protein ENOG41

MALLNPVYAFVVPFLFVVTVPLAVFAGITTTIAFSVLILRVLAVYLDVALSFVPHYLGGRKTRPYHPNTYHPHHQLLEPSPAT
LYTNPIPASAGSTAFESSLRRRRHRRSSSALSTAGTTTPISEKGLGLLPSIGPERDFEGVGGWRLGGDDETWTTINSRLELPD
KQHHGRNHHRSPSGGPTTPGEGGYLMMKGRNRSPGAKRLASPNSSRTRTPSGPRIAFTNKSFIDSYFPSLKTSPKGLKKLPTQ
GVVDA
FUN_004681 CDS scaffold_17 156751 157405 - SPC24
kinetochore-associated Ndc80 complex subunit spc24 PF08286

MLLSEEPAILIHHTIENFNIAPDKLAVSRVTESLSTLQQARDLRVREAESSLKKLSRQLATHTSRHDDLVASHSSADHASNIA
RLDTLKFRTAKAAADAETDAERLALTAADLKARLRELELQGVEGDAAANARRRDPVDDEVLLRLKVYRSLGIDIERDERDGEW
SKAVIRNDRKGDVHVVNMDKKFSRFFYANYFWQTL
FUN_004682 CDS scaffold_17 157851 158473 + FIS1
mitochondrial membrane protein EOG09265A4E PF14852;PF14853

MVTELPYALDAETPLNASELNVLKAQYEREGEMVGIQTKFNYAWGLVKSNQRNDQQLGVRLLSDIFRISPERRRECLYYLALG
NYKLGNYGEARRYNDLLLDKEPANLQASNLRQLIDDKVAREGLMGVAILSGVGVAAGVVGAFILRNARKR
FUN_004683 CDS scaffold_17 159662 161618 -
hypothetical protein PF00735;PF01926

MTFDLVQHIKARTRSVHFDRNLIFMATTPLAGSSPKASRDSKRTISFPLSQDDKTPSSPSSPRPSTVRHYRSMLDVDIQDPPT
LSPMKPMSFDELYLYSATSGPAGDERIANVKPNRDHLTIEASTRGNRRNTSSDYSPSQAPPCVSASDSIIFNITSPGSESPAL
APSASPANVPSQPPPPADNRNIVRRKLTGYVGFANLPNQWHRKSVRKGFNFNVMVVGESGLGKSTLVNTLFNTSLYPPKERKG
PSLDIIPKTVTIQSISADIEEAGVRLRLTVVDTPGFGDFVNNDESWRPIVDNIEQRFDSYLDAENKVNRMNIVDNRIHACVFF
IQPTGHSLKPLDIEVMRRLHTKVNLIPVIAKADTLTDEEIAAFKSRILADIKHHGIQIFEGPRYELDDEETIAENNEIMSKVP
FAVVGANNEITSADGRKIRGRAYPWGIIEVDNEEHCDFVKLRQMLIRTHMEELKEHTNNQLYENYRTDKLLAMGVSQDPSVFK
EVNPAVKQEEERALHEQKLAKMEAEMKMVFQQKVAEKESKLKQSEEELYARHKEMKEQLDRQRLELEDKKQRVESGRPLEKEG
KRKGFSLR
FUN_004684 CDS scaffold_17 165013 166380 -
hypothetical protein EOG09262BHE PF00762

MALRRSGGQLCKSISQASTHRLALPQIATQKRYLATPVHPVTQDATGSKGPTAMVFLNMGGPSTTDEVGDFLSRLFADGDLIP
LGPLQNYLGPLISKRRTPKIIKQYSAIGGGSPIRKWSEYQNAEMCKILDKISPETAPHKPYVAFRYADPLTEEMYEQLLKDGF
GNGKGGRAVAFTQYPQYSCSTTGSSLNELWKWRHRLEGKTNKESGDGTITWSVIDRWPNHSGLVEAFAQNIEAKLAEYPEERR
KDAVLLFSAHSLPMSVVNRGDPYPAEVAATVYAVMQRLGFSNPYRLCWQSQVGPSAWLGPQTSDSVEHYISKGQKDLVLIPIA
FTSDHIETLYELDQEVIGDSGHPETVKRVESLNGSPVFIQALADIAKAHLAAGQACSKQMGLRCPGCKSERCAESKRFFASQQ
AGLA
FUN_004685 CDS scaffold_17 166886 168921 + MUS51
ATP-dependent DNA helicase II subunit 1 EOG092617RY

PF02037;PF02735;PF03730;PF03731

MADKQNWRKEDEDEEEQELDENNYKAQKDAILLAIDVSKSMLEPPPPSDSKKADRDSPVQAALKCAYHLMEQRIISNPKDMMG
ILLFGTKKSKFQDSVDGRSGLGYPHCYLFTDLDVPAAEDVKVLKVLVEDGEDEDEVLTPSDEPVSMSNVLFCANQIFTTKAAN
FGSRRLFIVTDNDNPHASDKQAKSAAAVRAKDLYDLGILIDLFPITRGDEKFDLNKFYDDIIYRDPVGEANMTEVRTSKSGDG
LTLLNSLISNVNSKQTAKRALFSNLPFEIAPGLRISVKGYNIVHRQTPARTCYIWLDGEKPQIATGETTRIAEDSARTVEKAE
IKKAYKFGGEYVYFSPDEQKSLKDFGSPIIRIIGFKPRSLLPVWASTKKSTFIFPSEEDYVGSTRVFTALWQKLLKDDKMGVA
WCITRANAQPMLAAIIPSRERSDADSGTPYLPAGLWIYPLPFQDDLRNINPPSEVLRSSGELTTQMRTIIQQLQLPKAMYDPL
KYPNPALQWHYRILQALALEEEVPEKADDATEPKYKAISKRAGGYLEEWSESLEEESGKVANKKSTKRETDDEDMDRPVKKSR
SSSEKATGSSFSMAQLKAAIEGGTIQKMTVVQLKDILATKGMSTAGRKVELIERIEQWIEESS
FUN_004686 CDS scaffold_17 169309 172832 +
hypothetical protein PF00172 ENOG41

MATAGGRIDSSSILTPPSAPVSGSQWRGAQLTPTKLQLNTPRPLTSSIKSVAPPSPTCAFVSPQTPGQRHPKIPWATLVASSP
QKEPGADEYNSRLLRLARRRPTCEQRKTMVDFLASLGLGGAQTPAASSNSQCDSVEELQLKLRSILDAKATPTRAENVALTIE
LRATVRFQLPVSESENGTLESLNNIDPSLGGAQSVGMNGNRDEGQLSRVVFANDTLMNQPQDDPTLQRSVAKHIISIISSTDG
STWTVREVSRVTQGWTFTYLCKDSYQQWTRQNAKNPTKTIVGEFSQREPDPVLHARPAFDCRGSITIAFNRGSRSINVKYNHT
PLHKTVAELAAHFKPAPRQLGPGAQRLLEQQQKAKEKTPRKSRGEKKDKKERKKRDSTIKAQDENGNPRKRKKKNNGTSQPAD
SVDGPIIPPDCPGAPAIDGQNQGGPSYMNGGEASGNPGQQGFNDYPQGLMGDASATTNGTSSQQANGQLASVAFPVNVSAAEA
ARRKEAATAMLSNAGVDPTTLSPEQFGIFANQAPELQRESLNMLVKYGAERLRIVHPGNKEGSTQASSSTQSSQTTSTGPRTT
KELVPQSSAQSNANTDTETAVAQAADDTSAPKRKRKKMGKSRTACFPCKARKAKCPRERPICTECADHGTACEYAPQKPRNKA
TKPKKSEAIVVDDDKDDGDEEDEEVEPQEDAEAEGSEQEDDEQQDTSQDYSYPQMNIGNMVTNTDATTQDTQSTQNNYYQPAS
GLVLPQPDPNSTSSQAMGTSVSQRGNYSHNLPEVTQPMVHPAPPIPAPAGTIAPSETRRWTAFGSGSVGVGGSNTTSKTRRSL
PSEPSHTNAQNMPANPQPSDWAQTSNATMPATTMASVSPQMTYDTRSSGRTRQKNQSLTNDASQQAAAVSNTVMQQAQARQSP
VAAAAMMAQTRKSPYQQAAVPRTTSRTSQRNQSRTPVTDQARGYQPPSDMAQQQNPLGSTHYDTSHISSGSGYNDNGRYSSTS
TSGSHQPPTTMSTSYQTTSSTANQWSNSSSRNDRSYGTSPSYQTPNIHAQTTASKPTAASRQNFNMRSNTQQHIRASSGSYNQ
QLPQQQQQGYASYSGSTHQSQQGQTSNQQQPSSWYFQNSHNPSIHPGGQSSGYNYEPWSGV
FUN_004687 CDS scaffold_17 173261 175239 -
hypothetical protein PF07690

MSDLIRDAPLGQLIRFVTRNKYLQYPEEKPDFKLPESWVAVINNPDAIIEESSPHDNTVLTGTALASSASSTVAAEEDPKLKA
ENENEKNEKSEKNNENDDIERADPQPMRLHRSRSPQETQAYTVDRLEADEEHDVEKVKSIPVVPKRTKDGSILVDWYFSDDNE
NPHNWTNNRRLGVSLIICLYTFVVYTSSAIYTSSTEGVMRAFGVSQLKATLGLSLYVLGYGIGPLIFSPLSEIPRIGRNPVYI
VTMFLFVIISIPTALVNNYPGLMVLRFLQGFFGSPCLASGGASLGDIYSLMALPYAMMAWVSAAYCGPALGPLISGFAVPAKN
WRWSLYESIWASAPIFILMFLLLPETSGANILLRRAERLRKLTGNQRFMSQSEIDQRHMKVSAIAVDALIKPMEITIKDPAVL
FVQVYTAIIYGIYYSFFEVFPRVYPVYYNMNLGQIGLVFLCVLVSCMIGVGLYVSYLYFYMDPRIAKRGWPIQESRLVPALPA
ALGPTIGLFLFAWTARASIHWIVPTIGITIYGATVFIVMQCIFVYIPLSYPMYAASLFAANDFFRSALACGSVLFAQPLFDNL
GVAKGTSLLGGLSVIGIIGIWLLYFYGGKLRSLSKFAISDHVE
FUN_004688 CDS scaffold_17 176934 178674 -
hypothetical protein ENOG41

MSLDSEEKTGSLSAFFQEYEHGQAQQKSIETGQHVEQQAKLEGRNPALAKVLPDLNTVSVPVMVDALRRLRMPRQKGNSDLPT
NRYLRILNIVRYLLRNRKYPPDALIYESLMAAMADPQGSSTGVRKLLDDMSFQNVPLTADVCYLALEALSVHPDYILRQEVVQ
TMEQHWFEFTMPAKQSIAVAMLREGQFELALNKFDQLLEDPGYVDLWVYDIFILECGREDFFDEMLHILKKRKFARGSDSAFR
SIQLMALDMFSQGFHHGGTTFVWRDVVNIPIHSPSNGIIENVLATGARHGDTELASQALQKLAIRGKPRQQHNDAIVEAFANS
GDVTGALTLLNLQQHNGRVLLRGTTLPVLRALLKNRNLISEAEAAIQSMRKEGPVPREVLMVTIEALARTRTSEAAMHLFNDM
HLLTGKSPRLSDIGLLLRHASQTATQYDLAVAYNSELAKIDTTSVSIGKKSSKNDQDNDDATVPKTWMDQVEAGPAFDVIIPA
CAQIGDFELAFKFIDYAKNAALDYPAWRFASWVEPFIKLALDAEHPGVWKVVDLLDQGHDAPAMMVRKELQRRRIQKRASS
FUN_004689 CDS scaffold_17 179203 179793 +
hypothetical protein PF01423

MLPLGLLNAAQGHPMLVELKNGETLNGHLVSCDTWMNLTLKEVVQTSPEGDRFVRLPEVYVKGNNIKYLRVPDEIIDQVSESQ
KGQQGGFRGGRGGQSRGDHSGRGDRGRGRGGRGRGRGRGQ
FUN_004690 CDS scaffold_17 180505 186180 -
hypothetical protein PF00109;PF01648;PF02801;PF18314;PF18325

MRPEVEQELAHTLLVELLAYQFASPVRWIETQDVFLGERTAERVVEIGPADTLGVMAKRTLKSKYEAYDAAKSVQRNILCYNK
DAKEIYYDVDPVEEEPEPAAASSSDAPAAPAAAAAAPAAAAAPAPSSGPVAQVADEPVQAVDIVRALIAQKLKKPLLEVPLSK
AIKDLVGGKSTLQNEILGDLGKEFGSTPEKPEDTPLDELGASMQATFDGNLGKQSLSLIARLISSKMPGGFNITAARKYLESR
WGLGPGRQDGALLLALTMEPPARLGSEGDAKAFFDSVANKYATNAGISLSTATAAGPAGGSSGGMMMDPAAIDALTKDQRALF
KQQLELLARYLKIDLREGEKAHLNSQKSEKVLQAQLDLWTAEHGDFYASGIEPVFTPLKARTYDSSWNWARQDALSMYFDIIF
GRLQAVDREIVSQCIRLMNRSNPKLLDFMQYHIDNCPTERGETYKLAQELGQQLIENCKDVLDVAPVYKDVSLPTGPRTTVDA
RGNLNYEEVPRTSCRKLEHYVQQMAEGGKISEYGNRTKVQSDLSRIYRLIKQQHKLSKTSQLEIKSLYGEVLRSLAMNESQIL
PKDNKGRKVLKNSQSKGKVETIPFLHLKKKGLHGWDYSKKLTGVYLNCLEDAAKSGVTFQDKHVLMTGAGAGSIGAEVLQGLI
SGGAKVIVTTSRFSREVTEYYQAMYTRYGSRGSQIVVVPFNQGSKQDVEALVEYIYDTKEGLGWDLDFIVPFAAIPENGREID
SIDSKSELAHRIMLTNLVRLLGCVKAQKTERGFETRPAQVVLPLSPNHGTFGNDGLYSESKLGLETLFNRWHSESWANYLTIC
GAVIGWTRGTGLMSGNNIVAEGVEAFGVRTFSQQEMAFNLLGLMSPTLVDLCQNEPVFADLNGGLQFIPNLNETMTKLRKDIM
ETSEVRRAVAKESAIENTIVNGAASEVLYKKKTIDPRANIKLDFPNRPDWKTEIEPLNENLKGMVDLEKVVVVTGFAEVGPWG
NSRTRWEMEAYGEFSLEGCVEMAWIMGLIKNHNGLIKGKPYSGWVDAKTGEPVDDKDVKPKYEKYVLEHSGIRLIEPELFEGY
DPNKKQLLHEVVIEEDLEPFEASKETAEEFKREHGDKVEVFEIPDSGEYIIRLRKGASLWIPKALRFDRLVAGQIPTGWDPKR
YGVPEDIVSQVDPVTLFLLVSTAEALLSCGITDPYEFYKYVHVSEVGNCVGSGMGGAAALRDMHRTRFLDQPVQNDILQESFI
NTMAAWVNMLLMSSSGPIKTPVGACATAVESIDTGYETIMEGKARVCFVGGFDDLGEEGSYEFANMKATSNTVDEFAHGRTPK
EMSRPTTTTRNGFMESQGCGIQVIMTAKLALDMGVPIHGIVALTTTASDKIGRSVPAPGQGVLTTARENPGKFPSPLLDINYR
RRQIERRKKTIKQWQESELEYVHDEIDAMKAQGASFDEKEYAADRFAHIEKEAARQEKELLRSMGNNFWKSDPSIAPLRGALA
TWGLTVDDIGVASFHGTSTKANDKNESSVICQQLRHLGRKKGNAVLGIFQKYLTGHPKGAAGAWMMNGCLQVLDTGLVPGNRN
ADNVDPVMEQYDLIVYPSRSIQTDGVKAFSVTSFGFGQKGAQAIGVHPKYLFATLDEKTYAEYCAKVDARQKKAYRYFHDGFI
NNKLFVAKNNSPYSDDQLSKVLLNPDARVSEDKKSSELKYAPDFMKKSEKVVSSAKAKETEQVMEALALKVTNKNSQVGVDVE
DIAAVNIDNDTFVERNFTANEISYCRQAPSPQSSFAGRWSAKEAVFKSLGVASQGAGAAMKDIEIVKGENGAPAVSLHGEAAA
AAKKAGVKDITLSISHSDSQAIAVAVANF
FUN_004691 CDS scaffold_17 190291 196657 +
hypothetical protein
PF00698;PF01575;PF08354;PF13452;PF16073;PF17828;PF17951

MYGAGQGPQTGISTPRSSASLRPLTLSHGSLETSFLIPTNLHFHASQLKDKFVATLPEATDELAQDDEPSSVPDLVARYLGLI
AHEVDEGEDDEQGSYEEVLKLVLNEFERNFLRGNEAHAIAASLPGIESKKLDFIRSYYTARAVCNRPIKPHASALFRAAEDGD
AEIYTIFGGQGNIEEYFEELREIFKTYSSFVGDLITRSAELLQTLSKNPKAEKMFPKGLDIMNWLHHQDSTPDVDYLISAPVS
FPLIGLVQLAHYEVTCKVLGVHPGMLRERITGSTGHSQGIVMAAATAAADSWDSWRDITSSVLTMLFWIGTRSQQAFPITSMT
PTMLRESQEHGEGAPTPMLSIRDLPQAEVQKHIDATNHYLPEDRHISISLINSPRNLVVTGPPTSLYGLNAQLRKVKAPVGLD
QNRIPYTERKVRFANRFLPITAPFHSNYLAEATAMIDEDLKDISIDSSDLGIAVFDTNTGKDLREEVKGNIVPALVRLITRDP
VNWEKATVFPDATHILDFGPGGVSGLGVLTSRNKEGTGVRVILAGTVDGGMNDLGYKAELFDRDEENAVKYAIDWVKEFGPKL
VTNKSGRTYLDTKMSRLLGLPPIVVAGMTPATVPWDFVAATMNAGYHIELAGGGYFIGPMMTDAITKIEKAIPAGRGISVNLI
YVNPRAMAWQIPLIGKLRSEGVPIEGLTIGAGVPSIEVAQEYIETLGLKHISFKPGSVDAIQAVINIAKANPHFPVMLQWTGG
RGGGHHSFEDFHQPILQMYGRIRRQENIILVAGSGFGGADDTYPYITGDWAKKYGYPPMPFDGCLFGSRMMVAKEAHTSKAAK
EAIVAAPGLEDSEWEQTYKGPAGGVLTVRSEMGEPIHKLATRGVRFWAEMDQKIFSLPKEKRVAELNKNKDYIIKKLNDDCQK
VWFGRNKEGKACDLDDMTYADVLRRLVELLYVKHQSRWIDRSYTVLVGDYIHRLEERLTTTPGKASLLQSYSELNEPFEVIDR
ILAAYPDAETQIINAQDVQHFLIMCMRPTQKPVTFIPVFDDNFEFYFKKDSLWQSEDLDAVVGQDVGRTCILQGPAAAKFSTE
IDQPIKSILDGIHESHVEYLARDLYNNNKATIPYIEYFGGKLVDPEMPLDVEGLTVSYDTYKNTYRLSSSAAATLPELDSWLS
LLAGPNRNWRHALLMADVVAQGQKFQTNPIRRIFAPSRGLFVEISYPNDPSKTTIVVREQPRHNQYIDVIEVKLVGENKIQVN
MIKETTALGKPAALPLEFTYHPEAGYSPIREVMEGRNDRIKEFYWKAWFGEEPLDLDADVTAKFDGGKTTITGEAINDFVHAV
GNHGEAFVDRPGKTVYAPMDFAIVIGWKAITKPIFPRTIDGDLLKLVHLSNQFRMIPGAEPLKKGDEVSTTAQINAVINQEAG

KMVEVCGTLVRDGKPVMEVTSQFLYRGTYTDYENTFQRKVETPIQLHLATTRDVAILRSKQWFSVDELPQNIDLLGQTLTFRL
QSLVRYKNKAVFSSIETRGQVLLELPTKEIIQVGSVDYETGESHGNPVVDYLERNGQPIDQPINFENSIPLSGKSPLALRAPA
SNENYARVSGDYNPIHVSRVFSSYANLPGTITHGMYSSASVRSLIETWAAENNIGRVRSFHASLVGMVLPNDELEVKLQHTGM
VSGRKIIKVEVRNKETEDKVLEGEAEVEQPVTAYVFTGQGSQEQGMGMELYASSPVAKEVWDRADKYLLDAYGFSITNIVKNN
PKELTIHFGGPRGKAIRQNYIAMTFETVAADGSIKSEKIFKEIDETTTSYTYRSPTGLLSATQFTQPALTLMEKASFEDMKAK
GLVPNNSTFAGHSLGEYSALAALAEVMPIESLVSVVFYRGLTMQVAVERDAAGRSNYSMCAVNPSRISKTFNEAALQFVVDNI
AEETGWLLEIVNYNIANMQYVCAGDLRALDTLAGVTNFIKIQKIDIEEVKDNIEEVKGHLREIIRGCAEKTLAKPTPLELERG
FATIPLRGIDVPFHSTFLRSGVKPFRSFLLKKINKTSIDPSKLVGKYIPNVTAKPFALTKEYFEDVYKLTNSPKIGAVLANWE
KYNQDETATNGVEGSDSSSGEYEGSGRAA
FUN_004692 CDS scaffold_17 197394 198839 +
hypothetical protein PF01979;PF07969
M38
MTGLKKQVFVGTFISSKNPKELDYQHDTIVCVDGEGRIVKVDTEGGSTEQIGKRLRETLDWELSDVDVHIAKPGQFFFPGFVD
SHVHASQYPNVGIFGKTTLLDWLEKYTFPLESSLKDLSKAKRVYGSCVRRTLSHGTTTAAYYATIDVAATNLLADLCLEIGQR
ALVGRVCMDNPDMCPSYYRDESVEEGLEKTRQTIRHVQKIDPEFKLVSPVLTPRFAPTCSSESMKGLAKIQKELDLPVQTHVS
ENEGEVELVAKLFPESESYTQLYDDCGLLTTRTILAHAIHLTEAEAKLIAQRGSKISHCPCSNSSLTSGSARVRWLWDKGITV
GLGTDMSGGYSPSILEAARQAALVSRHVAMGMDEEKERSKLTVEEVLYLATRGGAEVVGLADRIGGFEEGMEWDAQLIGLGSV
DEDGVAEDDGNVNVFGWETWEEKIAKWLFNGDDRNVKRVWVRGRTVHVR
FUN_004693 CDS scaffold_17 199452 202516 -
hypothetical protein ENOG41

MSEGLPFKPLQPARPLSQVHLPPGSAEEAIASVRAFGTSFPQAAGSQAAQTSTTLQLPTVPVTEQSQQPQTGAPPAKKARRRQ
PTKAQEQVSTSQLSIPQGPQQVQHPQTEAPSSKKAKQPSKSLKRSIDSVEPSTSQEGPPTKKPALQASQPAQPQIHAPPSTIS
LPAHQPAQPLQIQAPRSDPVPGAWWEIPGFSEAFKQFHRDRYGKDGSLKACVIKGLVDLAIREHKRIHAAAAAQQAADNQGVA
NPAALEQDTAPVAAGLQALEDLPGGPSSEMGAENQQTAIDPPIDPQLQEQYDASSMDPRAHAMLVGGVKLSQVGLGPDPALPK
DDVDGRFKMIQAELKNQAFKIPIPPVSALPRQPPKEFVGSTYQMPPGVQDLRLEEVLRRRNEIILNRQKQVDLGRNNLAAKGT
RERREEKEKNLKKIISRLQVEMNWWRLKAVSLGGNAREWDAIPDPLKNKMLQDMDGKVCQAEKAQSVDIKKIKGLEHSARNTG
NARLSKQDIAKKENEVQAIIAAIEAKDREIQEAQSQGLEPPEMNLDEFVEMTFPDDVIHVDTTTAPGATTGDAVEAPTPIDDN
LNDSIHANTSGYININGVPIETSANIGENAGAGGAIGYANTTDVNSATDVGNPINGSMALNNTMDGNALNTDNMVSALAMN
NGSLPPNNWAYQPYAQIDNTPAMSMDFQGAPLNNYDIQDSGMQDSGMQNNWVPDGTVMDNGLQSDAFDDFDPQSTDFLNLGSQ
DNGFMQNSPQDTLFATNDMQNTNITTNGFQRITSLDNCTQGSTSLDFSIEDDAPFDTSLFSNASIDNGTQNNASSDINFQDNT
SVNYGVQGNLPINNSIYGAASYNNVIQDNASISTNPQGNGNATNKQKPNRVTKASKPSRASKSNGRTVKKPENVRGEITGADL
PRVANPTPNNVFNPTILARARLDHVDYRDPYAFGGRPPSASQQIITPRASDFANDKYLTYENSFTSAGNQSLFNADYEMLPSD
PESHEY
FUN_004694 CDS scaffold_17 204570 207487 -
hypothetical protein PF00172;PF04082

MLSNPLQRFSPYQNITSSNISPDGNVQQGTMSGTGLESLGQSHQYPIQPLSQAVPLSNAHLERPGPQVKNRQHPYGIHPRNAS
TSGPIRRRISRACDQCNQLRTKCDGQHPCAHCIEFGLGCEYIRERKKRGKASRKELAQQAAAQAAAAANGQTLDESTSENGQS
GNKGLDSSNMVLEQQSNERHPSTSSKSSRDPGDDVMQHTQGLEGLDPLGNISEQPHLGRSSLDGEHIENNGGLDLNGFGSMAH
GYETQGLEGPVLNGQSYAANGRGNMPGYAEFPYSMQAQSPPNFANNPTFRMGNSPLGYSMGKGTSPGWGISMASPPGQYQSQV
PAPAFNNSKLRYPVLEPLVPYLNNLIPIPLACDLIDLYFASSSSAQMHPMSPYVLGFVFRKRYFLDQTRPRPCQPALLASMLW
VAAQTSDAPFLASTPSARAKTCQKLLELTVYLLRPLIHTAPSDAPSPVADGVALGGLGVAMPGSISLDATSGESGPFGAAGSL
DDVITYIHLAVVVSASEYKGASMRWWTAAWGLARELKLGRELPPGPSPAAQENMDTDTADDGEGGISGSGYVGEEEREERRRI
WWLLYIVDRHLALCYNRPLFLLDIECQGLLQPMDDARWQSGDFSGHSNSTTDPNLLGTSPEGYGADMTQAHGPQYECRGHSIF
GYFLPLMTILGEIVDLHHAKNHPRFGTGFRQGHEWNAQTAEITRHLEIYEQSLQAFEHKNLPRPAEERVDAQNEGNERSGVPD
ANTPSAHSVHTNGSNRLTESNIQTRIVIAYGTHVMHVLHILLAGKWDPINLLDDEDLWISSQGFITSTSHAVAAAEAINQILE
FDPGLEFMPFFFGIYLLQGSFLLLLIADKLQSEASPSVAKACETIVRAHEACVVTLSTEYQRKFSKVMRSALAQVRGRVPEDL
GEQQQRRRELLAVYRWTKDGTGLAL
FUN_004695 CDS scaffold_17 211365 214114 - NIT4 Nitrogen

assimilation transcription factor nit-4 PF00172;PF04082 ENOG41

MEEPESKTAVQPGPSTTAQGSTRKKPRRGADSASQKRRCVSTACIACRKRKSKCDGALPSCAACSSVYGTECVYDPNSDHRRK
GVYREKVDSMKARNSTLQILIEAILNAEEDDVLGIVQKIRTCNSLDAVAEDIIKLQQQPLQQQPPQPTIPLANDDSDDYDQS
NDNYTTDQPEQGERDLARKMGELRLENGNVRFIGGTSHLIYLSDPTHEGEMEPNFEDYTAGQDPITSWTRVTQDTQLITHLIN
MYFNWHYPYFTTLSKTLFFQDFLRGKAGIGRGTAYCSSLLVNAMLALGCHFTSVAGAYGTPGDSRTKGDHFFAEAKRLIVEND
EYEKPRLVTVQALALMSVREAGCAREAKGWVYSGMSFRMAEDIGLNLDVGQLDKERMSDYEIDARRITFWGCYLFDKCWSNYL
GRLPQIPKSSFNVSKFDVFPDEDAESWSPYTDSGFDQSFKQPARTRAIALQMSKLSEISSDLLAFFYHPSHIGRSSGKTVELK
KLSELHRRLEEWRKELPREFEPKDGQLPNVLLMHMFFHLQYIHLFRPFLKYSPTSSPLPPHVSPRRICTANAGAISKLLRLYK
KLWNLRQICNIAVYMVHSACTIHLLNLPEKTARRDITHGVKHLEEIAEDWLCARRTLSILSVLARKWNCELPEDAAFVLHRAD
EKYGTYSTSDVPSPHSQAGASPLSDGGLGMKVMGEYSPFPPYNRAQLARPMQQLQQQQQQPQHQPQRSVTDAMLTSAPLSNTI
LSQQQGIPLNRSNAGFGPEPMGGWGSVPVTSSMPSYQQAFAPINRNSMANAGTSVPPVSNPPTSNRSMRMDGQEWFLNDSARW
HQSFEAWQMANNGQDNSVFMFGDSNNQDTPNSSTEGSGNMQAETNDQIAALDGLGASLSRGGWLPGLD
FUN_004696 CDS scaffold_17 216272 218000 -
hypothetical protein EOG092651FJ PF01479 ENOG41

MRRPTKYYSLTRPKLRQSWNKYNLFNIARAAGREPQVNSRATFFQQKWAAKSKTRGYHGEHVSEKKWVRLFSRRLQSAVDLPP
EYLAANDGAEQAAGRGSGLTTSNVTAETYSRVPKGSTTVRSPSHPAQRGRAFGNVNDMLSEHFQNMTPYMQMTFAPLERRLDT
AVFRAMFASSVRQARQFIIHGAVKVNGKKMVHPSYALNPGDMFQVEVEKVLYGTGEQKTAEYTTMESREKEMLDQQEESVRKH
GLKKRSKVLEAVAKEGEEAESADTKAPGKHARKRVEREVQLHRVRNLRTTAREILKGDMRNLSAKQKKDLRLFRDTAQRFLSL
PEARELDARDLLHQIQAQITKLESVDTFRKADSTLAKAANAEGSAEASAEESAEPQAEKSNSKLSKEEREAKKLQFKNNVLEK
GLEGITNKADRDRAMNILATENLTHEETRRLINILRNDSENPIDPSKPYVTPWRPRPFMSAFAFIPRYLEVNPNICAAVYLRH
PVARKGMAEVPTPFSYLTNQLAHNWYLGRG
FUN_004697 CDS scaffold_17 218337 221282 +
hypothetical protein EOG09260N2T PF00004;PF07728

MLSASSPITFPHSPPPAKRAKPHDHHSPPKPKARGPFLVEDDESDSDSNGEPTDSTRVAKRLMTEDPREKPSDTIAHDQTTTI
DLVDEEEPSSTPDVQAQEPTRLSQRPIFTTPIFSNLVPQSFKATTCNGKSVTIKERKANSAPTYESMVAARSKTKEGRAKRSY
YGVDIHNLMSAASAEIANQKQTQPQRPNVPVQSVEPQLPNSKKPKRTRLWTEKYRARNFMDLCGNDNTNRRVLGWLKKWDPVV
FPGAAKSKPVIARRPGAQQQEEEEKPHRKILLLTGPPGLGKTTLAHVCARQAGYEVMEINASDDRSRDVVKNRIRTSLGTESV
KTVSNRKDGDGPPKLAKPACVVVDEVDGVVSGSGGSGEGGFVKALIDLVLLDQKNASGATTENYGRRKKKGDDFRLLRPLILI
CNDVYAPALRPLRHSNLAEIIHVGKPTMESVVTRLKNVFEKEGIPCEKDAARKLCEAAWGMTSGIDARRGAESTVEGDLRGVM
VVGEWVAGRFRASNLKGKGSLTRQWLERNVLQDLTSGAGGARGLGRGGVKDIVSRLFQEGGGFPKQAMDLSQSKTQHEQPQAE
LGFGEYQKKHAMERLRQMIDTSGEISHIMTEVFTEYPNREFNDDLYLTKPNQAYEWLHFHDTCQSRLYASQEWELAQYLSQPV
LACHHLFASPKRYLPSMGYDRRWGGDAEDDSAPPLPFSGSRADYQAFEAERQNRTQLQALQGQLPPTLMRSFRSAEDVAAEFL
PYLARIVSPDVKPVVVGGSQGSTASVRRESERAMVKRAAEVLAEVGIELQKGRIESESLTNRTPQYVYRMEPDLDVLATFETA
ASLLLPSAAPTRYAVRQVLDQELKRTLVEREARARQSRFKAGNPLGHAEHLSTDKIMASKKHALEASSQDTTLIKRDFFGRII
EARPLAEITGNPPQQQAKHEEKERKVWVTYHEGLNNAVRKPISLQEFMRGL
FUN_004698 CDS scaffold_17 221476 223190 - CYB2 Cytochrome
b2, mitochondrial precursor PF00173;PF01070

MVLKGGEVAEHNSAKSCWVIVHGKAYDVTEFLPEHPGGQKIILKYAGKDATEEYEPIHPPDTLDKYLDASKHLGPVDMGTVQQ
EKKEVDPEEEERLQRIEQKPLLSQCYNLFDFEAVARRVMSKTAWGYYSSAADDEITMRENHSAFHRIWFRPQILVDVENIDFS
TTMLGTKTDIPVYVTATALGKLGNPEGEVVLTRAAAKHNIIQMIPTLASCSFDEIVDAKAGDQIQWLQLYVNKDRAITKKIVQ
HAEKRGCKGLFITVDAPQLGRREKDMRSKFTDPGSHVQEGTDTDNSQGAARAISTFIDPALSWKDIAWFQSITSMPIILKGVQ
RVEDVLKAIDYGCQGVVLSNHGGRQLEFARSAIEVLAETMPVLRERGLENKIEIFIDGGIRRGTDILKALCLGARGVGIGRPF
LYAMSAYGEAGVVRAMQLLKDELEMNMRLIGASTIEDLHPGMLDLRSLFQHSAVPSDNLSSTVYDPLSVPAQRPKAGPKQDAP
KAKL
FUN_004699 CDS scaffold_17 224084 227259 - BRO1 bck1-like
resistance to osmotic shock PF03097;PF13949 ENOG41

MAQSPMISVPLKATNEIDWIEPLKGYIRDTYGDDPERYAEECATLNRLRQDVRGAGKDSTSGRDMLYRYYGQLELLDLRFPVD
EQHIKISFTWFDAFTHKSTAQYSLAFEKASIIFNISAVLSCHAAAQDRAEESALKTAYHNFQASAGMFTYINENFLHAPSSDL
SRETVKALIHIMLAQAQEVFLEKQIADKKKIGLLAKLAAQAGYLYGQALEGVQENVNKAIFEKVWLLMIQIKTNLLNSMAQYY
QALADDEANQHGIAAGRLQVAEAQAKEAERIARNFPSSVPMSSNLSADCGGFLQEITKRHHSTVQTQLQSALRDNDYVYHQEV
PAEASLEAVAKLPAAKPIPVSELYAGQDIQRITGPDLFAKIVPFAVTESASLYDEEKAKLVRAEAERVETANDEMAASLDYLR
LPGALQVLKGGFDQDILPDEDFRQWCADVSDQDNPVALFDSLRSEKDSILMILDKSTKQLDMEEGVCEKMRSKYENDWTQQPS
SRLTTTLRGDIRHYREALDEASRSDNQLAGKLRQNEMDFDEMRRAAQSGEVDQLFQRAVAQARARGSNATSPAGIEPNLLDDD
FEEGPSVVDQINRVEDILKKLNLIKRERNQVLKDLKEKAHNDDISQILILNKKSISNYETQLFEQELEKFRPHQNRLLQANHK
QSALMKELTNTFNRLLQDKRVQSEQSKYEAIQRQRSSVINRYKRAYQEFLDLVAGLQSAKNWYTEMRETVESLEKNVDSFVNN
RRSEGAQLLNQIEQERSSSKNSQAEMERERLRGLMDRMSIDPSKSSPQPQTQNRPTPPSQYQQGQNPRYPQTNYQGQYQAPNS
PPAQQQTLAQQQAYQNFSPPPTTQSFGPPPINTFVQPAYNPSQYGRTPGPTSPPPNQTSFNIGGYRGPASPPPNQTTFGQTQS
FGGYGASATPQTQGGYVPPGFVPPPPPPGPPPLGPQQTFHYGNQPNSAHPNSAYPQSAHPQSAAPQQQQQNDPWAGLNAWK
FUN_004700 CDS scaffold_17 230681 231947 +
hypothetical protein PF01238

MSTPAASFYQLRCGCNQYPWGKQGSNSLSARLCDKTPGWDGDGKKNFKIDEDKPYAEMWMGTYPVLPSYVASTGEDLQDVLDR
YPKELLGEKITSKFGHSKLPYLPKVLSIAKALPLQVHPNKEFSSKKHKEDPGSFTDGNHKPEIALALSEFEAFCGFKPVEAIV
DILRQKPLRHFLVAGGATVADEKLSQEGLKQVVKTILTSSDEDIKKAFQAIRELPDSAFTGLNSAIPQVAKKLEKNFPANDPG
NLVGLLCMNYMLLQPGEVIYIPAGGIHAYIQGDIIECMARSDNVLNTGFCPKAERDNVDEFCSVLNWEPTTKSQTTLQPETYP
GSKEGKTQLFKPPTSEFNVLATDLKSGEREALSEGGPKIMLATRGSASVKANDQSFELSEGHVYFIAQGVKLDITAGSGGLLL
HTAVAE
FUN_004701 CDS scaffold_17 233090 235493 +
hypothetical protein PF12706 ENOG41

MSTFNGIVSEFPDIRIDFFRRNAGAQPPLACFLSHVHSDHLAGIESLRSPFVYCSAATKEILLRLERYPCRINYAKGVLESTQ
QTFKHLRKVLKPLPLETPTSIELRPGREIQVTLFDANHCPGAVMFLVEGDNKAILYTGDIRSEPWFVNAISRNPNLIEYTSGI
KTLDKIYLDTSFTENVPFQTKAQGIAELLRKVSQYPSDTIFHLQAWTYGYEDVWLALSKALKSKIHVDDYKLRIYGSLKSNTP
DSRFDTDTHLTPESPALTGHMCGNMPHPGCLTSNVNVRLHSCEKGNLCEVAQRPTTISIQPIVAHLPTGEDLAEVGVGGGGDD
LQREAELEFLDQASLATLFNTFLSSSIASTDMSDILEGTLEKIVSTGRNIPLDWGIGTLNAHSAEEVIMMLVERLRKNPKNQK
QRDKCSLPRTIYFPYSRHSSLPELCHFVEAFRPLDVWPCTVNNAEWLKNGVTIGGMFGPSCSGELFEHDLLMQDFSAKHTSDD
GHQQHGSQTTIGPDSVPSSPVRESQGASQKRPVNIDHRFVSPLLETQASIRHGRVVSEAECPKEHLEAPSENSTQQSLVVPTI
EQAEDVQQENHIHPESLIDSPSNSPAPVSCRRKRTVSDISVGENGGNRRQMRQITEESLPLGLSDEASIARRDAYWHMVDYMD
EGTWGTFQLISTSNEYTANEPEL
FUN_004702 CDS scaffold_17 235608 237028 -
hypothetical protein PF00172 ENOG41

MSSRRTHTKSRTGCTNCKKRKVKCDEARPSCFHCTRHGVTCSLASQPSTSLGISSVDSSSDSRSSYFPNLSPSNSTPTPTLSS
LDNHHLVDSPDPFGSYSHPSPTQQIAPFPPHELWARDCELMHHYCTVTADTLCVRKDLAYVWGVAIPRLGYQDPFVMHGILAI
AAAHKAYLVPANRKTYLPLADYHQTLGSEGYRRYLQHYNMTNWMPVFGFASLVVLHMLTLPTRMDNCALEDPITNLIELAGLL
RGIKTTLEPAMGRIVRTEFAPVVFGIWVLDSDNEAERCPSLENSSLPTDTWAALQRLRAFQEADIPASGLQQYTAAIEGLETS
ARLFAAAGLHAEVAAAHFWLYIIDDSILIDLAAQRPHALLLFAHYLVHWAVFEKGFWFTRGWSRQVMAKIEEGLSGRPNFLEM
MHWPKQMVAEAVV
FUN_004703 CDS scaffold_17 237662 238680 +
hypothetical protein PF04479 ENOG41

MDCNPNYKDAVWAFYRFVPSKEANIVFVVLFAITTLLHVFQLWRTRTWYLIPLVLGGVCEVIGYIGRVLNTNEEPGCWTMGPY
IMQSVLILIAPALFAASIYMILGRIILLTEGEHHSLIRRKWLTKIFVFGDVASFMLQSSGGGLMAIADLSKMGEKIIVGGLFV
QLFFFGCFILVSAVFHIRMHRDPTPKSLEPRVRWQTYLTTLYVTGILIWVRSLFRVVEFIEGNDGHLMRSEVWVFVFDGMLML
LVLVWMNWFHPGEIGLLIRGERSITNGLELVKMGGSGRKRLDTMESLSSEHNV

FUN_004704 CDS scaffold_17 239114 240881 +
hypothetical protein PF00067 ENOG41

MTVLGDQLTEVTFTRVLLCGSLFLLVSFIVDLLTQPRYPSEIPVLGHSSEKWFSRIRNSLAYFTQHQAWIGEGYEKYGKNGLP
FIAPAPISRPPDVILPRSQIAWMMEQPDRILSASHAHDKILYTEYNFLGKDLAAEPFANRVMHKYLARNLSSAIPAVEDEVNG
AVEHALETMMKAAAEKDPKNVDGEGYTKVNLWDLWLAIVPRVTNRLLVGNPACRNPDILKSMVSFTDNVVMNSFLLHGFPQVL
HPIIGRLITIPNYLHWRKASRVLLPIIEQRLKHMQRQEAGDPEVKNYTPPEDFITWDIRLAMAEKKPFELDPVVISKRLLPIN
FAAIHTTVLTGQSWMLDFLSSPPSVLDALRNEILEHKPSEGHWTKQALSSLVRVDSSIRESQRVSNFAANLIERQVIAPEGLH
HPDYGWTLPRGAFVTVNLQGTHHDDEIYEDAMSYDPWRYSRVREEWEAKSAEEKQEKEEEAKKIRGLGMVTTSDAHLAFGHGR
HACPGRFFVAHELKLIMAAFLLNYDIKTLEKRPKPQWMGATIIPPLDACIEIRRKKST
FUN_004705 CDS scaffold_17 240902 242114 -
hypothetical protein PF01212

MPSLTTPESPFTPKVQAKLLASREKASRDFRSDVVTVPVEEMMTSILEASVNDDIYDPEGDPSVKALEARLVELTGMEAALWA
VSGTQGNQICLRTHLTQPPHSVLLDHRAHVHCWESGALPVISQASVTTVHAENGVHLTLEDVKKNIIADGNIHFPPTRVVSLE
NTLSGTILPLADAQAISNYVRSFPVPEGQKPIAMHLDGARVFDGVIGEGVDLKAYAACFDSISICLAKGIGAPMGSVILGKKP
FIERAKWFRKMLGGGTRQPGMMAAAALSALEYSIPRFPSVHAMTKDASARLEAVGYKFTLPVQTNMILLDLEAAEIPPAAFVE
YCAKEDVSVFPMARLVFHHQTSEVAVDKLVTALTRLMEDKRNGVTLNDGEAHGGYS
FUN_004706 CDS scaffold_17 242923 245292 - MAK5
ATP-dependent RNA helicase PF00270;PF00271

MVPPSKKRKLPTATGPASKRSKNISKKKSGGKESRRAVDATALAWSSVGEDFGGLEVIEGVDVVKDGSRVQFLVAGDKSNIID
PQQEVVDGDQSFEGFGDDPVEVGDINPGEAGSGQGGLESEKPQGKNKGKAGQGKAEQEKVKKNKNKDKLDGKNSRPKKEDEQL
DKASKVVQKSSARGGNTFGALADANDYADQEDVDMAAWVPLNLSPQILSAIAKLKFTKPTLIQEKTIPEILAGDDVIGKAQTG
SGKTLAFGIPMIERWLELQEQGVERTGPMAVVLSPTRELAKQLGDHLKALCDGLPSAPYVCVVTGGLSIQKQQRQLEKADIVI
GTPGRLWEVLSGDRALQSKFAKIKFLVVDEADRLFKVGQFKEAEDIIGALDGKSPGDDAESSDEDEDEDDDDEEDDDRNARQT
LVFSATFDKDLQTKLAGKGKSTGNDEEKMAYLMKCLKFRGEPKFIDVNPVSQMAQGLKEGLIECGAMEKDLYLYTVLLLNPGR
RTLVFTNSISAVRRLTPLLSNLNLTALPLHSQMAQKARLRSLERFTAARNSILIATDVAARGLDIKQVDQVLHYHVPRSADTY
IHRSGRTARGESSGVSVILCSPDEVLPTRRLASKVHAERSSGAKREHFIQTLLIDRRMASRLKPRVDLAKKIADTVLAKEKAH
SDDTWLRNAADELGVEYDSEDLEAVNASGGKGGRGGGRRRKEQAAKSLSKAEMGALKAQLREELSRRVNLGVSERYITGGRVD
VGALLREGKNGGIFLGNTDGLGFDL
FUN_004707 CDS scaffold_17 245950 252232 -
hypothetical protein PF07926

MAAADVDIGYVAGHLGLDQPVIASLTTEPTVELVNIVLQAVAAKAHEFDTLYAEKLQTDIELENAVRSSETRSQTFKATADKA
LKDVEEARQQLKDEETKRQSLENELQVFKSKKSDYDAEIKALNDKIETLQSSNRTNLSIIESNNKRDQTITEELTKQHQRNVE
LSREITALQQSEQNARGQLNSAKYREESLQQQLDLARKNSEWLENELKTKSEEGLKYRKEKGARIAELQRQNEDIQSQVDSLK
RTEQQLRDRLDAMQAKADEALVKVQNQEGTFAQTIESYKHELEAQKRLVEMSNQLSKKHQERVHDLEAEKERLRDNYENELRR
VRAELEQERQTTSEMEERINQLQSEIDELQARMEQHAPPPESAPQTPRANGSLMRPSSPFATPSSMRAKGAITTTQALDQLYQ
VKGQLATEKRRNAQLSEELDNMMTALEAKAPEIQDLQTEAEQLREEITRMSELSQNSFEERDKAKRAARKAESALATSQSETN
ILRTQLRDLGTQINMLLFQIHAMEKGTDQLTDEETFRLQQLQKGEISEEALSDMSDTHQFITQKLVVFKDIQSLQAKNEELLR
ITRELANQLESEEALAEKHQAKQDHDMVEKLQQELSHMTEEARSIKKTMESFKTERDMFRRLLQQRGIHGDEASLMRNSIAGG
ERLPLASIEATEQTEALNEALRKLQSEYDNYREAQDGVRKDLRDQIDALSAERNSLQTDKVKLHAEARLESERREMLHTNFVA
LQSENRELQKRAQTLSETAAKQDIRTQQVAEELIEARGLLDSVRNETANLKAEKKLWKDIQDRLSKDNENLIEEKNRLNNLLA
TQQSIENERNMADSEARRKAQAKIESLEQELSDAQRKLTYEAEETKKLQLRKEFESKESQKRIDELMASLSQIREEHVAVKTS
RDHLQARVDELTVELRNAEERAGRLQPRPTPRPGTIEVSEQQQQLENEIQELNVEISDLKRDLDMANTHLENAKTQAEQFKEL
SQANEEALEDLRGSQEQYRQEMETIIEEKDAKIKELSQRAEDLSAELSRSNTELSSLRDSQGEVARRYEDEKSILQEEVNRLK
EESARHLEATRFHQQDLRAQAEIASKAQQDYEQELVKHAEAAKLVQQLRTEYNTLKSEAASLKSEADSAKVTLAQSESSWEER
RQQLEQEMAELKTRREDVNSQNKILHQQLEALTTQVAALQQKRSGGEEEDERMSPVPLGDASEGLRELNSYLRREKEILEVQY
DLKAQEAKRLQQQLEYTQSQLDEARLKLDQERTQAAQSGRTSMTHQDLMEKLNELNIYRESSMTLRNENQQLKDQIGEKNKKI

EEMEARIQPLEAEIDTLKTQKSFLEDEIKQIQEDRDRWQKRTEGILTKYGRVDPAEMEQLKEKITELETERDALKQGQEPLKA
KITELETTMESERNNWSTTRAKIVEQAKERSRKLTGEKNEAIQRANQLQESLDKATSELEGTKKEAEESRNQKSELEQQIQNF
QKEVEQLRQQAQTTQSSPTPAQPAEASASSDVVAQLEQQLADARKELESINGQKQGAEQQLESLRNELQTAISERDEATKKLQ
EATANASAQTTDQTPAQPAATNSTEQPVASISDEEKKALEERVAAAEAKAAEFEQKANEVETRIQTTIKERSDRMRDTLNAKL
RESRSKMEEEYKKKDEDLKLKFEQEKQIWLAENPTARNADATKTEPVDQPPATPAKAEAPAVPATPSSVGQTDLSQLDDTGIR
QFLATNPTVKNIVAANIKKKLEIESSKLKAELESTLKTEYEPKIAAARDQAQLMESKKSTLRINMAENKLRAANAKISVVEKA
VSETPQKPVVEVWEVAKVAVAPPATPAAAKPTPARQGSVAGVPPSPSPATPAAGAKAAQGTPAATPAGQQQAGSESKPAQAAA
AAAAPATSGIPGPKTSNPFAVPANAATAAAPNPFATKNENGVAPAAKPSQQQTQAPQQQGQQQGQQQGQQQGQQQGQQPVRSG
IPMPRGGGRGRGGAYVPPGQRAASGPQEGGHGNAGRGGRGGRGGGRGGMNPSANDFQPGNKRPRGDSEVGGGAKRARGAH
FUN_004708 CDS scaffold_17 252997 254818 -
hypothetical protein ENOG41

MVLVDTMKHLHGLSWIGLLGLAVLTTAKDVSYVTDLEIFTYLAPCVSSAISYNVGMQTYSTMCGDSETELQKCICSTRMDEVA
SSISTDIEYSCGSSATEDLSSASKLMQKYCNQEEHITFFSPTKNIVEAYITDLPELAYLPPCAQSGLSYAVVNIGSSRCPEAA
ELNAPCVCNKKDVVRDITSTLKSAVKYSCSNNGDVTSAQNFYSEFCQMNDGTTSFAPPKGPPGDMSYYITAMPHFKSLNKCAQ
SGIASAVMEQSSWLCGDGPQELASCICIKSGMSKIISSSLTERVKDYCDSTHIANVTSAIDVFRYYCSAAESLVVATVSQSIF
QSYPTASSGAGSGTTVPGMTGSAGATATSSSNTAKESDPGHEDKEDDESKTSIAPIAGGVAGAVVVAALGAGLFLCIRRKRQR
QAKGEELSNNADGPPEYTGHPELMGNSAYIKGHTAPPIVSELSSTTSFKPELGGGQGISELPPNHAPTAELQAHLAKSNWGHS
PAQSEYVSPVSPAPAYSGLHEMGFQSGPVEAYELDSNIRSR
FUN_004709 CDS scaffold_17 256383 257112 -
hypothetical protein ENOG41

MLRGTTSNVPKVPLFGVPKRRKQRQQQEQEQKDKEEKLRLQALEEAEFATKAETSDFLLYYLERSPFKDQSDQISIAWENLPS
LGELESFARLDKTAGTQACRRLFKAVVIWDDLGLDLWETLNSTFHPESDEEDSDDDLLDEISEEPVGWDHASPRRSRSRASSR
TSSSETHNSERSRQVSSERQDQRERSRDSRKTASARDQSVNGRLPPRRPMGLQGGMISEYYDSPSRYRVPKIVRVL
FUN_004710 CDS scaffold_17 262856 264416 +
hypothetical protein ENOG41

MLWRRSRLTYDPKQFPNYRPCPETGSERLIKECPNVFHFIAFTIGFQISIFSIILEWFCMMLRSPLQYSRHNDWILDHYKASP
TASHMLWGAFITAWSSHFMFRIGKAVCKVIIDAYDDPVWALYEIWDLGVYSQFFLIRTIFFILTTIFNFVCFWAIFLFNMSTY
SFVVSLALLGAVAMWQLHYYFFVYSPEVTEEVSYHSPEKEVLSTPDKPGRCFRAPTPSTGSSTSSSRSSPEHTPPHLRPRPSN
KPPGAAYVKVSPKAIQKLHDEGVFVPEPTRVPDYSYLEPFAGKRPTNFSRYDAKRHFRRVREIERKKGIISNSPDPYKDDPPS
STVITKTAWIYSEMDAVNRRLRFITEDVTEMNMLVNTFREKGSVRSLPDASHAIPLSPRTQRRNATLAEKDRIGEVVIDYTRE
IDSLSFRVFSHKTRTEEALKRIDRLVAHAGDAASRAIRKEVEDCRSRVDFAFEGAEAAVMSSSRIADLCIDRKVKLEAQAKKL
RRMDRTQARHPNISIIDDYYG
FUN_004711 CDS scaffold_17 265644 266196 -
hypothetical protein PF14856 ENOG41

MHFINLVAVFIGLAAAAPTATPTKDNMIAVSTTAPSAYEDPYTYWAAGNHSFSTCDKKTYNAHQAPKSTKRANYRDCAALLST
FGTRNGTFSIPAASDDKREYGVGDGLVNIVKSGSCAFAVRADKGLKVGDDDVVWIMQKAVLEYSAGTEMAARGSVKCAADEAG
KKGGLYWQVHGIESAGY
FUN_004712 CDS scaffold_17 268203 269279 +
hypothetical protein PF17100 ENOG41

MKTASQPSAPHNATSKVKRLVGKIRRHSHPPSPEASSTSPSSSANPSRESSSCPIPHIGKHSQRQSRRGSASEEEERAHDLEL
WNAAYDVLKRDSTSSGLVLAYESIVSHALPDTLRPGHNGNGNGLPTESERRAELMMMIAKSGLNREVKETSQTDSGDVEAREN
LIHTRSTIASLLDDQPSAAVAWAGFCSLTPLLLEPLLRHEDIRQGFVHIAHTIPHYMTLHRVLHPSSWTSLPEFQRQQPQVHQ
TLLTLYRRILEYEMNIVCAAASAWNMAARNVVDWHGWKAMADAVRDMDAELMGYVEKNGTDEARALMEVQRKLEPEGGGRGEL
QDDTSPPNA
FUN_004713 CDS scaffold_17 269568 270828 -

hypothetical protein ENOG41

MPIPQSISVGIELEFMVALQIPNSDAVTGEARWACPTTPEAFLGLVMGEYKDIEPSCIHKVCELIANSGVSVSCSLIPPSPIS
PAQIPGTAILPLTDNSGDIRAWNNESVSGPVSKTDFWFIVPERHITRDCVSKSGMTPSNKYDWYGTELNSPILTRPEEFSQGL
PTLRKCLAAVQGGMVVGLNSGCGLHLHVNDAGSMQLETALRLASLVWLLEDSLLYPLCHPFRSTSPYSARISVESRIAMERGE
PTVYGEGAALVEALGEVMRQLHWRKKVDKGLLGSMKRLWSETSLASLGIALRKFDEGSLHTTTRCALVVSKYDTIEFRYPEST
FDVDFIAGWADLVRHLYAVAMRPQVEFHQILCRVYELVTRDQMPGWSAMLGAIGFQGDASRWQRHINEYGDTLSNLDKQGILQ
NIGQ
FUN_004714 CDS scaffold_17 272178 275359 + HIR1 HIR complex subunit EOG09260U6R PF00400;PF07569;PF09453

MRMIKPSWLSHSGEQKDFEVYSCHVSPDGKRLATAGGDGHVRVWSTESIYNADTPKYNKPRQLCHMSHHLGTIHSVRFSPNGR
YLASGADDKIICVYHLDKGPPAATFGTNEPPPVENWKTYKRLIGHDNDVQDLAWSYDSSILVSVGLDSKVVVWSGHTFEKLKT
LPAHQSHVKGITFDPANKFFATASDDRTIKIFRFTSPAPNATQHDMVNNFVLETTISSPFKSSPLTTYFRRCSWSPDGNHIAA
ANAVNGPVSSVAIIERTRWDSEINLIGHEAPTEVCMFSPRLFHTSKPDASAADGAATSLVTVIASAGQDKTLSIWNTNTSRPV
VVCQDLAGKSISDLAWTPDGQTIFASSLDGSIVVVNFEEGELGWVAQPEENVKALQKYGASRKGMGIAEDVDGLILENQSKEG
ESRAVESRMGALMGDFSESTKESTPVANGSKPNGTSAKPSTNGQAEPENEPEKQPEEAANKTAERVKELKSRVTIGKDGKKRV
APLLVSSSGTGTSSLPQTQLVGTTSTNKNTQNDTPQTIIDMSKPYDGLPKGGIAAMLLGNKRQINLGDDEDEEEPVAKRVATG
PTPIVTDGPNGVEPAALVPVQNGVVPTPEFLRPAVMNVSMSFAQVRLAVPKIRSHILRPLDRGVLQGESSLEEATKTPENIIL
EAKNDATHGHPSHVIVSKRGALIWEEWLPRDVILVTATKHFWAVACEDGSVHTWTSAGRRLLTPIILESQPVILEARDHWLLC
ITAVGLAHVWDMKTQSSPNPPVSLGPILDIATISLNQHSATAGPGVTSAHLNSTGHIIVTLTNGDGYCYAREMYTWQRLSEAW
WAVGSQYWNSNDSSISALQSTAVGPDSKDRQDKKSSKATVSSGIIPFLERHTTNEFLLKGRSYSLQRIIKMVVQRKESENLES
SVSIAHLETRIAGALQLEAREEFRLYLFMYAKRLGAEGARVKVEELLNSLLGGILEDNEEDEEEDGHGWFSKEGDICGWDRKE
LLTGVVMILGKYRELQRLTLQYARILDISLEDGSVDVEQMDVEA
FUN_004715 CDS scaffold_17 276437 277287 +
hypothetical protein PF01793
GT15
MAKALRFTLLWAWALTLLHFTLFVCGSALPSSADAPLKAALVTLVQEDDISATLFSIQQIEDKFNNRHLYDWVFFSVQDLPER
FKELTSNATNATCIFEVIPEENWNMPGWTDHSQLSDSHEINSDCNPKTFKPLADIRQMKRWSSAPFANERRLRNYEWFWRVEP
GASLSQDIPFDVFRYMRDNGIAYGFNRAVLGQANLYHLSRIKSFIDKHPELLHEEADITWLIENGTSLAIQSSSSDDSYEGLV
DEGSDKLRSRII
FUN_004716 CDS scaffold_17 280060 281691 +
hypothetical protein PF04142

MAILEAFPKAAPASQGGFLGLSKKQWALTTLTFQNSALILIMHYSRVMPPSGDHRYFTSTAVFLNEIIKLAVSLSLAIYDTSK
TLAPTTPATVLFEQIYNSVFAGDGWKLALTAAFYTLQNMLQYVAVGNLDAVHFQVLYQLKILITALFSVVLLRRHLGPKRWLA
LIVLTLGVCVVSLPQAGSSSSSSSIPLRHMTDHFFPRSLHELGHVPTDNSQAGNLAKRSATYQGIDHDLPPLDPLMNYSVGLV
SVLVAATVSGLTGVYFEKLLKESPTQASVWIRNVQLSFYSIFAAGLGGVIWQDGEGISEHGFFEGYNWVVWSAVVLQAAGGML
ASVVIRDTDNIVKNFATSISIVISFLISIMLFQFEVSATFVIGTFLVLLSTWIYNGSDRAIRRPPPIQIHSFEKPAIEPFQTP
RNLANRLTVNPMDSPMGLTTSRPSTPLLPRTPSRSNFKRDD
FUN_004717 CDS scaffold_17 282299 283757 -
hypothetical protein PF00132;PF00483;PF12804

MSLHVPMAHRTKDQGATKAVILVGGPSRGTRFRPLSLDVPKPLFEVAGHPIIWHCLSSVARVPNKQIQEVYIIGYYDESVFRD
FIKDSAKEFPAITIKYLREYEALGTAGGLYHFRDAILKGRPERLFVLNADVCCSFPLDEMLKLFNDKDAEAVILGTRVSDEAA
TNFGCIVSDSHTRRVLHYVEKPESRISNLINCGVYLFSTEAIFPSIRSAIKRRLDRPSRLVSYPSSDNLENSFVLPDDDDDDE
EKKSEVIRLEQDILSDMADNKQFYVYETKDFWRQIKTAGSAVPANALYLQKAAQSDHSEELATPSANIVPPVFIHPTAEVHPT
AKLGPNVSIGPRAHIGAGARVKESIVLEDCEIKHDACVLYSIIGWGSRVGAWARVEGTPTPVGSHSTSIIKNGVKVQSITILG
KDCGVGDEVRVQNCVCLPYKELKRDVTNEVIM
FUN_004718 CDS scaffold_17 285944 286255 +

hypothetical protein ENOG41

MSPLVARVARSAVVLRPLPAAQRGLRTSSILRDSEPYQAKPPKQNQNHNKILVAGALAIGGGFWWFYRGGKPILDASGEPKAPTK
FUN_004719 tRNA scaffold_17 286891 286963 - tRNA-Thr

FUN_004720 CDS scaffold_17 287131 288391 -
hypothetical protein ENOG41

MKGFKLCWKSYGDDGVVNSFFLEKGVPCTNLLKKVEGLGAVRAVTPDLLTSEESLLALSFILLNYFMVRKQLNPKSNDDAKNQGSGDSTTKFKPLEWIQRSERNRTRAVIFSSWMYLLQLRYAHTACTLVWVLAYRAYLADFEWAKPYVLSSIIVAIRLLLFTLGTVGGLCPLYHIAKAQAQYIRALIHLELSPEQKTKDESSVPQELPASAPPSSVQAIDTKTAALWSPVPPMTPVVSSPALEKLTQTAALRQTSPYLETTRESPYEREKRAMKTAPERHTPTPAFLLLAPRSDYTHPMTTTPQPTSEAGQQGPQTTVPHLSRNPSAGNAPPSYTDSTTNGANNVSRQEAFDALTRRLNAPVVTRQDQRTFNTKPASSPTGRVPVGQAFEENAAKSSDSPAQSEDWEYVHHEEGKSSS
FUN_004721 CDS scaffold_17 289280 289739 -
hypothetical protein ENOG41

MSVFDPNTWQNIHSGNPNIAMYYEGGDSGAQNMGGLFPGDSSLGSLSSIASPAISGTASTHRNDNDPQALGGTRLSPWPGSDMFQHTQDPGALGDIGELFQNRAGPNPGLDLNMDPGEVDDIDWQNWDETLRTLNRPAMDQMGAGAGSWGGM
FUN_004722 CDS scaffold_17 289763 292134 -
hypothetical protein PF00172;PF04082 ENOG41

MASNTPKTPSSGASPGSLHRATNPATTGRQAGHATIPHDRPVALMTSGAPLLNPRSCVTCRRRRVRCDKTMPCSNCRRAQCDCIYPAPGRAPRHTRLAPTGPATGQSEREMDLLGRLRKLEGLVYEMSGQGTASENDMEVSNGESPLASEKSKNAKDVVQAATEKVKRESENTDFSKINKQLGRLVLHDGDSTPRYVNSGFFVKLNDELAEIRNEMEIMNEEEAEFEEDTTPEQSPPQNLGIEHDHHSFLFGYSSADVDLTGLHPLPAQGSFLWQIYLENIEPLVKVLHIPTMSRLMTQVRRGEHDLRPGDEALVFAIYYSAVTSMETQEVEANLGASQSHFISQFRFALEQALAKSNLLNTTNMAMLQAFVIYLTVVKCHDDSKFAWTLTSLCVRMAQALGLYRDGQQLGLSPFEVEMRRRLWWAIVSLDVRSAEEMGSDLIIGDKTFDTQLPSNINDADIDPSFTETPTPRQGRTDSAICLSRYEITALSRGLFAAIAQMRPVDPKEVEKSLEEREHMLVEVYERMEDKFLKHLIREDDPIFWVASLISRIMMAKVGLIIYQPVLFPGTGPEVSYEIRSRLWQSCIEIVEYSHILNIDPSCRQWRWLFKSYRQWHAIACMLLELSKRPWGVTSERAWEAAQILEYDHPIDGSNNTNHTAAWMPIKRLFTKAKRHRESELVRLRADPEAARRLDREERIKPVLERIGPAPGMETRMSELRVRWRKIFRPGDFSDDVEYQSILPSADQPASHSQTR
FUN_004723 CDS scaffold_17 293717 294332 -
hypothetical protein PF01042 ENOG41

MTLATRRIAPVLSILRPAAHIPQINIQPRTINYVHSRTFSASIPYYAAMEHVFSPKAVAPIGPYTQAIKANGMVFLSGQIPANSQARLIEGTIAEKTHKMCQNAKAVLEAAGSSLDKAVRVTVYFQNMDDMKEMNEVYAEYFPHKPARSACESLRLPAGASMEMDIIALE
FUN_004724 CDS scaffold_17 294574 297074 -
hypothetical protein PF00172;PF04082 ENOG41

MDNEINVRSTRTRARKREKISNACIGCRNSKTRCSGRHPCERCIRRKETCVFEEKEKKVAVSVDYLDQLHARIESLEAQSRSVQGLDSNTEALSSSSVQLNARPSLRNPPDSPLNETGTPSRSESSSLTNTLVTSGPQIKVHRYGHAAYLGHSSTLSFSRNVRNLLQQSSPIADPGSVSMERQDISYTRTLPPIGLDLSSIALPKLSYAEYLTNTVIVHIGSLYSIFDADIFLQRMRRFYDDRDKGLEADASLWHIQMLLMLAFGKSIQSREHSEMGPSGMAYFTLAIEAMPDIRRLYEDPLLSIEVLCLAALFMHATDLLQEAYVTIGQALRISVTKVLNKKFPEALRSSNFEYQRRLWWTVYCIDRKCAAMLGSPSVMRDDDISIPFPEIKPGDESQNAFAIHAILSSHLGKILDAIYGVHDEQRNFLLEVKSILSRIAETYVVLRQHLPLDVQQPGQPISRESATLHLLLHQCVILTVRPVLFSLLKPLLALGAPVPNARHSSSPFESMLKMCIESAVQILMIMSILKQQMMCDIFLPYDIDALFSAAFALILIDIIRPANELLWDLPQVMNLLGEFV

SRRVAPAQAYRSDLVQILELHTKLRGNNSRQHQDTGNMFNVSLVPGLDEYAITHPGQLGISPDPLWSNIRDGNGVGDPAHPNT
ILSVIDDLNVEGFDILDTAMLDNAWMWDMDDMSTNI
FUN_004725 CDS scaffold_17 298048 301000 +
hypothetical protein PF00350 ENOG41

MSSRTTHSLSSTRMSHSSRRRGPRVKPDPEDNTSMPDSGTVNFGIRTAPPAPSSYGGSTSYIPPDQAFYHHRSDAPTPSERIT
EPEPLLRELRMRPNAAAESPTNVPTDGSFFQTSFQDIGKKLKACNDTLGELQQLGVDHDVPLPELVLVGDQSAGKSSLMSGLA
NLDLPRSEGTCTRCPLHIRVSRSHDPSCRVSLRKAYSYQPPPSGRISEGDVTDRDPFFPWRKLPSSATHEFKTMIDYSEIEDV
LRWAQIAILNDDKSHELFVPGSGALAVNIPIDRAAEAVAAKFSPNIVALEIKGPELPDLSFYDMPGIFQNPADASDDYLVSVV
RNLSKEYIQHPSAIIMCSMPMNSDAENSSTFGLIRRLGALNRTIGVLTKADLIPEGGNHEQWLAIMRGQAHIAGLGYFITSRP
QGKDLDELKKWEELVFVDHSLERWPATFHGFSERCGVEKLKAFLSEKLGQEFAKSLPHIKQKLRQRLTDIVEQLEKLPELPDN
VELEVQTSVMEFGELTRTQLKPSEFSKHFVPLPDNFRDCLLDMKPKFTLRDSSDIPVVDISDDESDAGSVAPAINPHTPSKRR
TMAPPITPSKRVRMEQTPAGTPSRSLQPEDSRASVANSPAPRRQLFPAPFTPFSGVGRGFRTLRQVRDEIKSKTRAGVPDIIT
EEVYNDMCREAIEPWSGPMKAFLDHVMSLLDDMLDQALAKSFASLNKRLIFRECRKILTEYLNDRRRETAGALDLVYRLETYG
LFTVNQEAFRRYQKDEQILLTRYRHQMRMQAAGYGDGRAPVPWESLTEEKRVLDEKRRENELTKLGPDSFERELEVVAYVRGY
YRLAALRFADAVALHITNGMIPHIQRNLPFHLDRKLGIAGADAKSVYERLMEEDSDTAARRGTLRGEREKFVRALDSIEELER
GAGSSTLSEEENDDDMVDVPLMSGALPAEEI
FUN_004726 CDS scaffold_17 304201 306087 +
hypothetical protein PF00083;PF07690 ENOG41

MALPKFLQPIGSKVPAHSDRVPLDIAQAEAPRFEKVIWYKEPHLRKLYFLIIFLLIASATTGYDGMMVNTSQQMDKWKEYFPE
EKNESKLGILINMYNIGSIVSFFIVPYLADNWGRKPTIMLGCVIMITGSFVSAFCNGYGMYMGGRFVLGFGNSLAQMSSPMLL
TEICHPQHRGPLTAVYNCLWNLGSLIVSVVGWGTASINGHWSWRSITFIQAVPSIIQLCGIWWVPESPRFLINKDKSEEALQV
LAKHHAGGDVNNATVQFQYREIKETIQADKSVTSTRYIDFFKTSGNRWRLAIIISLGIISQYSGNALFSNYMDVIYEGAGIKE
QNKKLALSAGKTILDLSISIGAAFTVDKVGRRPLFLTAISGMVFAFVLWTLTGGIYENSQYKTPATEEQPDGGVAYSNTGAGY
AQIAFVWLFGIFYDIGFSGLLVAYALEVLPFHLRAKGMMIMNITVQAILAIGNQTNKLAWNNMPNHWNFMLFYTLWDFCELIF
VWYFYVETKGPTLEEIARIFDGDDAVAHIDLAQVEKETNLTRHESNTFEDYPPPKSLA
FUN_004727 CDS scaffold_17 306694 308035 -
hypothetical protein ENOG41

MEQRPPTTLDSKAPTIAGINGTLDRFWSSNGESVQDLVHWSKPKGVDKIMGLVFYGRRQSVSILDCYLKRNLAKNGGLLDGVI
FVERTKDPHDLALLKKLIESESSYEKWSVEMSGDDNFSSGFGNSYDRVEDHVMYVKIDDDIVFMEDTVIPSIVKKRLEHPEYY
IVSANVVNQPLLSWVHWNLGAIRPYLPEVDSEGKAMPSKQSGQKPLDWNPSNLPPWNGPEGFSLDNWEPNDTTHRWLPLDKGG
KDHILDTTPIEATEYHPMGRGWTQWTIGAQEHFSFFENLWKKKLSAYKFETWDFQYERMGIQFVALLGRDINRAKPIEADDEN
HFSCNMPRALGRHAVADGRGVAAHYSFGSQRAGMDRTDIIDRYRSFARNNICTGPMLWAPGDD
FUN_004728 CDS scaffold_17 312833 314228 -
hypothetical protein ENOG41

MVRFTPLATLALVPLAQARFDWLYPRDTIAQCPPCSLESEPKDCSQTVTVTKPSGPYETVTVSDPNGSYHTVTVTEQDGPGKT
VTITQNYQNPQTKVVYISQGETVEPAIETITVTRPGKTVTVTETETETSEHIVTKKIDSYPGPKTVTISNGYPAPSAGSPDYN
GGVVTKIVGGDGDHKKDPDNVHTVTVIKDGHIQTLTYKDGNSNTVIKTITQDNGHEEVVTVTGNDQYEVTKTITMENGNKVIE
TITAENTNNYVVTKTIQQGGKYYTVIVKPEPTVSTITKENGDCITTVVEVDQTYTLTAPAKYGTATVTDDDCETYTRTATYTG
KPEVEVIVYDPETGDSTCTKEDGKPCNPSYDNGGDGSKGQYEDPTNTKGQDGSGNYNTNAPVYTKIRTYGNGDVYTEVHGAEP
TGCESAAVSTSIATVYNTVIVTVDASPSQAPTAERRAANKVRSPLSVRW
FUN_004729 CDS scaffold_17 315315 315937 +
hypothetical protein PF15454 ENOG41

MGACASCLGRGDGNNYDEEEENRLLYDDANGMQYGSFGDQAINGENDTLEAQRENEALQRVIAKTSDNMVDIFDIAPQENTNR
GATTPFAYAGQGARLARYQHLVSKLSNQGDNNPASGVKVDWLPEDETIEMQKNGPASIKTLESDEGPLVGTFADAAAAMQ
FUN_004730 CDS scaffold_17 319947 320337 -

hypothetical protein ENOG41

MCYFDQTRWACGYWRWGHFRQQCNKEYRMGETCGLKLVYETRTERDVCKLCHDTEKKQRRYDKMYRDVQRWQREGNRNATIER
TCAEMQEVLGQIYRMREEHDHRLQSLGQVSLKHDVPFLSFMSIRIE
FUN_004731 CDS scaffold_17 328979 330001 +
hypothetical protein PF00646;PF12937 ENOG41

MKPVATSPEQLTSQVHRLSFASPRLENAGCLAPGEDVNRSYPAMFVTTDARSAASPIAPSVPQEVACLTFAAGDVFPGSSLRT
SAVGCTRLQSALLLSPSLEDLPNEVLFHIMGFLDVNDLLSTSRTSHLLRDISQAPILHHYRLQRARINLPSRLSSPSRPTLAD
LIARSIFMTHTSIVSRKLARSLVSIRLSRRLAARPSAAALVERSVLPKECVPGMASVHVAPAIVAKKKAIEKERVKDGLRQWI
ASRWKREVKERQDGVRKWEESRGIGRVWRLRRFWERVSRGEERDSAW
FUN_004732 CDS scaffold_17 330298 331360 -
hypothetical protein

MSDVRSLLRQQRAARRIEHPYAAYSDAGKLLCTLCHEHIKTESLWDSHVRGEAHKTRLIKAQTASSANARTSQQQTSQKRKHD
DNEEAIDDKDGDVEMDDGRRKRNKTDGVDGDGDKDNKDQTLTPPRLTRRTSTTPSQGVEIQIPSRPATPAHRDGSSASTPGGQ
LNNLTSQSATASLPSRQASSLTTDERLTSAAAAAAAAAAVDEAEWAAFEADIAATTATYDEDATISAPAMTAEEVAAADAQK
EEEAEKRRAQADVDLEDEKEEATRALEEEFEEMEELEARVKRLKDKREALRKRGESFSQENGTEKPTSLGKENLDTPAIEEKD
EDDDEEDDEDDDWDGFRFRTA
FUN_004733 CDS scaffold_17 332053 332850 +
hypothetical protein EOG0926587S PF05983

MAEQQQEGHSLSSMFPNPPPFWQDFTTDKISRIQELRNSYIAEHGGDPLTVRVPDVPGDLVNLQPPAEPADGRWRVFGDQYML
DDKLPTLEDQGITNLPSSSQSESKDAKRFDRAFELKRLAKSLLLNFLELSGTLSHNPSHAEAKVQDLRTLFINFHHILNEYRP
HQARESVIALMQEHLDRTRAETMAIRIQVDKARRVLDGLGSLSVPDVPKALGQNTEEEDLEALAKQREADVWAATDALFT
FUN_004734 CDS scaffold_17 333352 333520 -
hypothetical protein ENOG41
MACGSSKCLYDRNGPGSAATSIAESIIKPSEATGTKTKCIECGDYECCCIPCTIL
FUN_004735 CDS scaffold_17 334500 335146 +
hypothetical protein PF07366;PF12680 ENOG41

MQPPGVISPCQTEQEQRNIDLCKEYMRIAYSPTENKGATSVQHLCHPDSWFWAPATFPGCETPMDYADSHSTVMASVADLKII
RFDQPWAKDRHVLLRYTAQGSHCGEPYKGISKTGRHVQWSAAAIFEVEDGKIRSFTKDWDQKTMQIQLGWAPVHESDDPRWNS
KALDSPEEARKHNQ
FUN_004736 CDS scaffold_17 335709 338776 -
hypothetical protein

MPITVILGAQWGDEGKGKLVDGQCQEAQLCARAAVSTMQAETEYLLINVKGGNNAGHLVRVNGVSYSFHLLPSGLINPRCMNF
IGSGVVFHVPSFFSELKELEKNLTAVHDRILVSDRVNVLLDLHIAVDGLEEEELGDAAIGTTRRGTLPPGFHGGLSFSAFVTS
SQAFPNLNGGENPSIGPCYQTSRARTGIKMTDVFHPEVFEQKLRRLADGFQKRFGELLEYDIEAELARFDEYRQTLSKYVVDG
VAFMRSAQESNMKIVIEGANALMLDVDYGSYPFVTSSSTTLAGIIGGLALNPKNITETVGVVKAYTTRVGAGAFKTEDTEEIG
TKLQEIGREWGTSTGRRRRCGWLGRHNLKTLSNLVVVKYSNSINYYDSLNLTKLDVLDTFETIKIAIAYKIDGEELDSYPADL
EILNRAEVVYHEMPGWQKPTTNARTFYDLPRAARDYVEYIEKFVGVKIKYIGTGPDREAMIQRA
FUN_004737 CDS scaffold_17 340092 342783 -
hypothetical protein PF00628;PF07500;PF07744 ENOG41

MSGKRQRLAKSPKQSQLVRDQAPPKRDRRRDTTHSAAPTNSNVSGEAEPRRSVRATKGQHTKSFDELEPATVPKKRQTKKTKK
AKEQEQEQEQEQEQQQEQEEEEEDELIRCVCGATEQDEDSGEAWIACETCGAWQHNVCVGVSSFDDEIPEHYWCEQCRPDDHK
ELLDGIAKGEKPWEARRKAHEEESKKKKRGGRKGKGKRHSETKEEDKTKAKSSPVPVPDATKDKKETKTGKRKAREESHDADG
KSAKVRRISENGATPVPVSYTPPDDLAKSISDLPGSRTGPAKAMSKSISHVLTSMQKQGELDLEEGNTIESMTETFSLQIERA

VYDSYPVTKGQKEYNQQIKSLSFNLKNNPELCHGLVHKTLSPAALATMTSEQLASSEMKKQTAEMKAKAEKQSILYTSETGPR
VRRTHKGEEVVDDETFVNDSAVPLPPGPPRRPEPQAVKKEPTGGDKADLASHPQQQDDKQRSPSHPDFDINEVFSSVKSPTTT
QNRRPSALVTGSNGPGVDPDVDRMLQDENESPPYSPTEETQDPDVIWQGSLAMSSIADFPATAKYIGGANFASVGPWSKLIPK
RMTVAGRIPEQSAIEYLCSLRYSNSTDVIVVSVSPVSPFSHTEFHNLLNYFTSKKRYGVIGDKTIGNVRDTYLVPVPAGEDNY
PEFMLNLVDNKIPKVRTEPILLAVFVYHNEPDQLKHLKDGTANQQDAKALGTPVPAAHAQRSNSTASAGPAFSPATPQAPQGG
FQGGFPQQSSPGQWQSTTPVPIPQPPYTRPPVPPQAPTPAPAAAPAQMLEAQRHQAQQAGVAMAHELLGPWITSPTVQFILPQ
AYQMARKEWEVVRRVLERDPKARDDLQHLGNLLEKETAADKTGGAAAA
FUN_004738 CDS scaffold_17 343996 344935 +
hypothetical protein PF00227;PF10584
T01A
MSRRYDSRTTIFSPEGRLYQVEYALEAISHAGTAIGILAKDGIVLAAERKVTSKLLEQDTSAEKLYILNDNMICAVAGMTADA
NILINYARQAAQRYLLTYNEDIPCEQLVRRLCDLKQGYTQHGGLRPFGVSFIYAGWDPRRQFQLYLSNPSGNYGGWKATSAGA
NNASAQSLLKQDYKEDCTLKEACGMAVKVLSKTMDSTKLSSEKIEFATVGQTEDGKIYHRLWSADEITALLKEHDLAKNEETE
EK
FUN_004739 CDS scaffold_17 345168 346245 -
hypothetical protein PF01569

MAPAHSYGSSGGPISFLRRYWKTTHAPDYVGFLFLLTGWILTVLFVNPFHRMFFINDLQISYPFAVHERVPVFMNFVYALFIP
LGVLIAYNIIARSSAAKHEVTYLSFLISIVLTSFITDIIKNAVGRPRPDLLDRCKPAIGTKANTLVTIDVCTAEDGHILQEGW
RSFPSGHSSFSFAGLGFLSLFLAGQLHVFRYSAGGRDLSRALVCLLPLIGAGLVAISRCEDYRHDVYDVCVGSALGMSIAYWS
YRRHFPRLSSTKCDEPYPRPGVDTQPGWQRITDDEEAARGTDVGFEMENLRGSRR
FUN_004740 CDS scaffold_17 347131 348829 +
hypothetical protein PF00397;PF01846

MLKSTHTPSAAALAPLPPGWSEHTAPTGHTYYYNAETKESTYKRPGVQPPLQPAQPQAPAYPGYGSIPYLADPNVANAYMAQF
NQDNNGQRHNNSSRGGHGGGRGGFEGRPRPQPVDKPRRKEPIPGCEPWILVYTKYSRRFAYNPVKNASYWRIPEKLMPAILEL
DKARLRQKAAGENQEKEHKQDEKREKQAVPQKNDAPVAGLDDDSEYEEVEVTDDEGEGEGDEEHPSKRQRTADPDADEDVPME
FTEADFAAQLQAMGDDYGLEPGDYDDGNAEEWPEGAEGVPLSEDDAKYLFKDLLNDFNINPYSPWEKLLEEGKIIDDLRYTAL
STTKARRDCWDEWTREKIAELKEQRARQEKRDPRIAYMAFLQEKATPKLYWPEFKRKYKKEDVMKDHKITDKDREKAYREHIG
RLKMPQSKLKSDLTALLKAQPVHLLNNKSLSTGLPDPVLTDIRFISLEPKIRDPLIEAYVSNLPPPPEDLDAAKDDEEQRKQR
ESREKREKALEERNRVVEEQKKKRDREVAASKARLRDEERELEMAMKVGKQGLQSQLASMKVAEKEP
FUN_004741 CDS scaffold_17 348925 350703 -
hypothetical protein PF07690 ENOG41

MSDREALQPTKSSKTPSSLPAAYSVEQNKERSRALNQKLDFALLPLLSLLYLFNGLDRGNVGNAETQGFTTDIGAEPDDLNEA
VSLFFVTFVVLQPVSAAAGRYIGAKHWIPFLMFGWGAVTIGQAFIKGRGALIATRLLIGAFEAGFYPTAVAYLSFFYPRYDFC
VRLALFYGQYAIAGAFSGSISYGIFHLRGGGLKNWQYLFLIEGALTCFFAIIAWLWLPKGPGSAWFLRPDEREFAVERMKQDN
AEFVQHEYSEDGIEENRLSKRDFVETMKDWKLWTVLLLNICASVPSGAFSVFLPLVVQGLGYESILANLMTVPPFVCGALGLY
AFALSSDHNKERGYHILGGLVIGIIGLILTVTISSSTGQYVSLCVLLSGVYISAPLTMAWLSGNTPEPGKRALVLGVNGFGNL
GGVIGAQLYRQRYKPHYRFPFYVTLGFLAIALIGYLSYRFMLAAVNKRKQAILRTMSPEEIESERLNDRRYADKKLTFMYGL
FUN_004742 CDS scaffold_17 352019 353577 -
hypothetical protein PF00293;PF01008 ENOG41

MAPLKHRDVATSFIFRFPAEGEAKKPQVALFRRSGHVNTYQHKYAGISGSVDDTDADPLATAWRELAEETTLTHDSLRLFRKG
KPFSFADESIGREWTVNPFGFVLKSESDGGRGEAGIKIDWEHEGYEWFDPDAINDSDDFEGVPRILESLRRVWFNVDLGEVAG
NTLSRGLVALQEDHESGARQLATKALDTYIDVIKKLDSTDQVQWWNNIRFAGWHLWKNGRESMGASILNVVLSALDIVQDGIP
PSGPLDKSSIDNIVRALGDYARDRQNTTSRTGAMFQAFIEQHLSGGGPLKILTLSSSSTITSAITHVMGNTSRPVEVHVLESR
PLFEGVKMAQSIASCANNSKAKTRLIIHTDASVGIAAKDIDIVLIGADLIDKTAAVSNKVGSLPVVLTAKYMSPKAKIVILSE
KEKVLPFAPPGQEENDSCEVMQAWGDLSSAVREVPHSLVTVKNVYFEWVSPDLIDHYITEDGVTGRAGMSNYAQEVEKKADQY
FTNL

FUN_004743 CDS scaffold_17 354138 356943 -
hypothetical protein PF00172 ENOG41

MKQPDDSGPTANTNATGPQSARPRASVSVSASASHTPRRLPVNPRRHKVAPEHRKRVATACNACNVRRVRCSGEHPCRQCQSS
SRDCIYPLPVEKVSITRSELEDLKHKVDLYERVLRDTVPAAVSNAVPDSASRQELSDLLGWTASNHDIGSSAVSLAAQTTPTI
DAAAHIGRVPPELPQLQYPSIHRQFQQQPPHLHLQAQLSDTRIQEPSYGLSRTHSIQLSHPGATPQNRQDDEENLVSTEGRLL
HDPDGYARFLGETSGATFLDYLKEFMTTVLPLAYQSLRPGSDGSAFLSSLGRYQTYDSRPLHDRDVDPTWFPTPPEMDSMLSE
LRYFIQDGNCHWPSGGIYWWGDLSSAPTIPSMERDSANLRLAKCRHLAFYQTAFAVVSQAMIMPPQDISIDPHLSENYFSRAR
MLLGNPLDITRFTPNEVSVLTLMGFYLIEMNRRDAAYICVSNAMHISIMHGAHRGWLDESGKRVFWTLYVLDRYLSCLMGRPP
TIMDDAIRLPLPCDAPSMPPADGLKAHVQLSRISCHIVCNTYRISPSEDAARPVRSLEGEISMLDEWCLKLPPTVQLASNGLS
DDPATCLLHMHYNQLIILTIRPVFFALVKKSFAEKLVSRQCSLSSHPQLPLLKRCIASAEHNIQLARQILLVNHPRKLLQAGL
HFIFNAAIVLMLQQLVEDLCPSSRSEKARSLDLDFVIGRFEDESRVGSNYGRDCATVLRDLRVLVQRLPIPVDMSTATRSVNM
TQTTTTYDPIANRTHVDMGQQWSDKLTSEALQQPILVDQGHILYNELVSWIDVDWQSNNGYLI
FUN_004744 CDS scaffold_17 358818 360548 - SID1 PAK- GC
kinase Sid1 PF13434

MSPHSEVSFNGSEAPVSNGTTNGHNGTNGTNGITNGHNGHVNGNSNSNGNGAAPAARHGPRVHKERSPYLESAPADSEFDLIC
AGFGPASLAVAVAIHDAIAEGKKLRPDGAAPKVLFLEKQTKFAWHAGMLLPGAKMQISFIKDLATLRNPRSEFTFLNYLHRQG
RLVDFTNLSTFLPARTEYEDYLRWCSSWFDHVVSYNNEVLSISPESKEAGAVKTFTVQARNGKTGQVQSFRSRHVLVAAGGQP
SLPKSLPAKHPRVLHSSQFANYAPQILAKQNAPYRVAVIGAGQSAAEIFNNVQNLYPNSKTYLIMRQEFLRPSDDSPFVNSIF
NPEYIDNLWPRSVRARETLLTEARATNYGVVRLELIEHLFEKMYDQKREISDDETQWPHRILAGREIASVDTKGDALEIKVQR
VNDGPLDGFVDQETFDVDLIVAATGYKRSAHVDMLKDAWTMLPKTVSGRNESPKGVNGWNVETQDGERKLAVGRDYRVKFSPG
TVADDSGVWLQGCCEGTHGLSDTLLSVLGTRSGEIVQSIFKQ
FUN_004745 CDS scaffold_17 368033 382683 + NPS2
non-ribosomal peptide synthetase nps2 PF00501;PF00550;PF00668

MDLTGLSILNARPSRLPGPNLLHQLVKKPGQHVALDYMSNGRQSTLTYSELHEAAASIATRITTASGNVTGQFVVPVLIHQSP
LLYISLLAILKSGGAFCPLNIDAPPERVKFILDDVAATVVLTTKELASEIPNNTGATIIIVDENEEYQGSSRSPGVELKHRVP
QPEDLAYVMYTSGSTGTPKGVGISHDAASQALIAHDRHIPPFSRFLQFAAPTFDVSVFEIFFPFFRGATLVSIRRVEMLDDLP
GVLRAMRVDACELTPTVAGSLLRRRENAPDLKLLLTIGEMLNAPVVDEFGGDDERPSMLWAMYGPTEATIHCTLQTAFSSDSS
TGNIGVPLDTVSCFIIEIPESDSHEFEIKILPQGEVGELAVGGYQLAAGYINRPEQTNSVFVDSPFGRIYRTGDKARLLPNGK
LECFGRLSDGQVKLRGQRLELGEVEQAVLRTSGCHSTVAAIVQSILIVFCAVDEGVTEFAILKHCKDWLPQYMVPGEVVLMSE
FPRLPSGKVDRKRLKAEYEQRKADMSESILDSAPVDTMESELLAVVSQSLNFKVDKSTSLALIGMDSLKAIKLASALRTSGFG
IDSTALLTMKTASEILSAIRRQLQRDIANPQQPSTNLSLDFEDIVRQDATLARIRGSVEDVIPCTPLQAAMLAETARNPTTYC
NELELSIPLGYGIDYISETFRELSQQNAILRAGFAAAKGNFVMIFQRELRTEQICVVDQLQKRLSLSSPQDFISPLILQIQRS
PVEDRVRVLLQLHHSLYDGWSMDILLSDWSKLLLRQQVSQHPSFREVVRFHHDMQTGDATRVFWTEHLAGWKRSPLPKLRDKL
MHSNQTLSIRRRLSLSRSRVDTGMQKLGCSPQVIFQASLALLWSGITGMRDITIGSVTSGRTIPLAGIEQVIGPCIASLPVRV
DLDNVSVCLDLLNSIHSSNRAIMQHCTFSLSELKKLVALQPGESLYDVLFVYQESLTSSERTQNVVRETSHLDRLETPLVFEV
EPREDGFELQVTYQEDFVPPKTAEHMVNQLELLIISMLENPTTKVKTTLKEVSCSPSIHNIRPTMPQKTPDLARLFEEVVLQT
PDSDALFFAHSLDGGLQTKWTFQELNAVANRIARYLHCSGVGVGEVVAIVMEKSPTLYASILGIIKCGCGYLPILPSTPPARI
REILQQAHTEHCVVDNVSRDQCPSLPGLSTITVDTNSLHDISTDNLDVGVDGTRLAYVIYTSGTTGTPKGVAIQQHSIAANIE
HLEALYPKCLNSQGRLLQACSQAFDVSVFEIFYTWCAGMCLCSGTNDTILEDIERSIRDLGITHLSMTPTVANIVNPTNVPGV
EFLVTAGEPMTQSVHEKWQRQLWQGYGPSETTNICTVKKMTADDHIEHLGHVFPNTSVVVLSPESPDVVPLNWVGEFCFGGAQ
VAQGYLNMPEFTSQKFIQHRQYGRLYRSGDMGRMLPDGSLLILGRIDDQVKLRGQRIEVGEISSMVTSSGLASSAATVLVQRG
ETSPQQLSLFFVPNHDPSSFHVLKINSETHQSLVAHLKSRLPSYMVPTYLIPISSTPMTSSGKIDKRRLRDCFHRLSMDYLEE
ASRMSLDTQNDGDWSQSDLKIADTLVESANVSRNDFGRWTPFALLGIDSISAIDLARNLSSKLGSRVAVSEILRNPTVAQLAR
YLDGKISQQEETPVDSLEGFFPVTFVDSMEKEFTKEAKTIREILPCTPLQEAMLSRGQRGYYNKVLLRLTTSSGAMRSYWKTM
SYRHDILRTCFAATTDSRRAIAQIVLESWEIPWKTFDVSELSFDGAIEQHLKSLPDPVDSRTPPISLALLRYRGSEFLSFICH
HALYDGVAMERLLKEVEALAVGEELPPPVPYSQFLRLSTRLPDDAEQFWQRHFENYKPSAIFPQTTTSEVDQSTCTTSMDIPL
SDLQLRIRDLGSTLLSVCQASWATVLAAAYRRTDVCFGNVVSGRTLDIEGLERLVAPCFNTIPVRVKFPEASSNIDLAKHLQK

MNTELIAYQFTPLRLIQRSINRTGKHIFDTLLLLQKPLQDIDKNVWTLEGDSGDMDIPLVCEIVPCPGLNSLVVNLHRDMSIV
TEEVATAMADVFKLTLRAILTTPHASSLNKKDLPDTLRSIIDQLVPRTEKDNTTVTPSNKKEEWSELELQVRHVLATLSGVSD
QHIQRRTTIFQLGLDSINAVQVASVLRQRGFVVSASDVIECPSCSKIAAKLLENSSRADAGYVLKYDLDRFSRQVLTEVVDQL
PKSVKIEAVLPCTPVQSAMLASFVQSGGDNYLNAVRYVVTEDIQVANLERAWQLLQQRHPMLRTGFVPVQHAESSFAMVRYEV
GSIETTLNRLHLVGDELADLIQMKADASKSMLSSLHHPPWSVILAQTSQTNTMSLVIHHALYDAPALHLMLDELLQLVKGNQL
PRPTTIEPAVSAILGMALNDKSTEKEFWEAKASKIVVNKFPIMTPLRVEERHVLVDSMVGSLSSTKLRTATQASNVTIQAAIQ
AAWTRVLASYLGEDSVVFGVALSGRTIDETKDVAFPCLNTVPVVASNVPSNADLVSYMMSYNQHLHKYQFSPLNQVQKWLGHP
AGPIFDTLIAYQKMPDHNTSFVPWKLVNEEAKVEYPVSLEIEPTDNDQIRLCITYYSDILPREQAQVLMKQFDASLTHIACNA
AGFEDEVMKNSLSLYSILPASAPVLDTPVQLLHQFVEKGAVSHPEKIALEFVSEFGGDACVKQQWNYRQLDCMGNKVANMLRN
KVTLGGIVAVHFDKCPEAYFSILGILKAGCSFVALDPSAPRARKEFIIEDSNAPCLLTRCSKDLDFETKTTVMEIETQVLNDV
SEEHVVFHPNTSPSDTCYCLYTSGTTGTPKGCEITHDNAVQAMMAFRELFKGHWDEDSRWLQFAALHFDVSVLEQYWSWSVGM
AVVAAPKDLILDDLTAAINRLGITHIDLTPSLARLTHPDEVPSLCRGVFITGGEQLKQEILDVWGPQAVIYNAYGPTEATIGV
TMFQRVPRNGRPSNIGKQFPNVGSFVFKQNTNTPVFRGGVGELCVSGKLVGKGYLNRPELTAERFPTLEQFNERVYRTGDLVR
VLHDGCFDFLGRADDQVKLRGQRLEIAEINHIIRTDVVDIHDAATIVARHGSSGKDVLVTFVVDQKSKKGPLEILMDQEGLAA
QAKEACRAKLPGYMVPTYILSLPYIPLSSNNKAEIKDLKRLFSELAPEKLLELSHATTAPVSQGARKIVGQLLESIAQFSNMS
KNDLSSSTSIFDVGVDSVTALRLSSLLKSRGLKAASPALLLKNPVIGDIANSLAKNTSNRQEKLVREVKQSIQAYGHRHRGMV
CRLLNIEPADVEYVAPCSPLQEGIISRSLTSSEPGAYFNTFELKLHESTSISKLQVAWDDLVRTESILRTVFVPTPHGFLQAA
VRKSTLAWETYTVQSDESIPAYLAEQKKYWIQRNESSITQPLLFIYMETPTSRLLTVHIFHALYDGNSFDLMMKRVAANYSGI
HAPDAPSFLETLSYGPLAKYDNCRGFWEKQLEGWAPVSIQAHGNGEPDAVAVAEREMSTASLEAIRSSQNVTLQAVVMALWTS
VLQNFTQQQLTVGIVVSGRAIDLPGIENTIGPLFNTVPFYCRAIRHESWQSLLRRCHDFNASVLDFQHVPLKDIQKWCSKGKA
LFDNLFTFQIEETAPANESSLLFDIMDSPATPDYPLALEAVYAHSGNLRLTLVAQGHIASSQVLNSILDDIERFANLAASSPE
SEVPVPEIEQKCDPDVNNERIQTHKSANFEWSQEAQMVRNEVAFLADAAPSDLNENVSVLELGLDSIDVIKLSATLRRKGIHL
SPSHIMRHKTIANILAESSDLSLKPSTSADDASLRQVKTRLREYLESVGADLGRVESVLPPTHLQESMVAGMIQSDFESYFNH
DILQVSDHVDTTRLINAWKELIQRTPILRTGFVQVAHQDLDMTYCQVIAESFDADIEITTVQNLDELHQLTATATAKAREGAG
LRQLVQLRLANMGSHRYMVLSMAHALYDGWSLSLLFQDLQALLEGRLVTRSPVEPFLIKVLGSTNQKAKDFWTQYVEDASPSI
IPTSETSSRPTENTLRRLESASKIGLSEIETACKRLSVSLQVLCQACWAITLARQTKKLDVLFGTVLSGRDFDGADNLVFPTM
NTIALRCILHGSVFEFLRYLEENMIDIRDSQQYPLRKVQSATKLNGQGLFNTLFILQKSPVAESSDRPLLTSIEASSATEYPL
CVEAEAMSDTLLWRLALQPQCVWDGGPESILETLDTVMGFLIQPGSSEILSFEEHGVSICGMAPVMLDDGSKSGSINSTDLSP
EEDREWSHVEAEIRNILHQVSCVPVASIRISDNLYHLGLDSISAIKVSSLLRKNEINLRPQDLVKSSSIAEMAQLATQAQAIP
SETLETTEEWVPPEDINLEVLLRARGIAKEDAEVLPALPMQVYMLSAWQKAEGSVFFPEFPCQIKTSVDLNGIQTAWDKLVSE
LPLLRTCFIPTQSPTIPVLQVVLKSYKLSLTQHPPMEKSNQAAQPLVRAHITLQEDCIWSFKLQIHHALYDGVSLPALLRRFS
QLLHGSVVVENNGFAQWKNVVVRQASDAAQKDRRAFWAAYLHGTSSSSIAAKSQADVKNRISHLNESAIPDVSQIQAISTQSG
ISLQAWFLAAYARVLATRNNTPDNGSVVFGMYLANRAAASDSLPQVYPTLNLVPLRVDSPIELPLSSVARNIQRDIHQITSDG
RSDVGLWEIDQWTGVQVKSFVNFLSLSNDTGSVENSVTVLSQEITDNSYHHHVPAQFEAARQGSFSVDGIPLAVDVEASVDKG
RLAIGIFGSQQQISREEAVLVASSIADTLRHATE
FUN_004746 CDS scaffold_17 383007 385554 -
hypothetical protein PF07350 ENOG41

MATPTIDLPPTTCEEEVTAPILDNAQGLASAIGAAAVAAVAVGDGLTHGLGRVAYGKPYDGHISEEDGQFHDVDHKGAREAKQ
QKSLREAQEKWDAATMGPVTRVETVIAAEGGGGDGRGEREVWEGGGGGGGGGGGGGGGPEGVHLPPRKGADVAGQQVRAALSR
SRLGRSNPSSSSSGSLSGASPYNATNTAVAAAASNNSPNNLNFTQTGRVTKSRHRSSLSAPTAASLVNAAATESARLSYEAKL
TAALAANVAGTMAGSQSSTTTTALPSLKSNGLSSVPQKPKTNGTTPILSTASSMDKDAPVISFWGPEPIALPSRFARIKRNLI
SGHEAELEASWARLITALRKEVSHIEDLGAHLIPSIEFGDLDDSVQTARFGHDLRRYGVGVVRKVVPRADTDTAVRETVDYLD
SKRHIKALQQHDPACFDFFWTPAQVRSRAHPNVLSAQRFMMSLWETNPDDQLVTRLPITYVDRIRVHGNGENQSNSLNVPPLE
PPQSADDWIQALQSSAGITAQVDNGSLERWEPDGYQRAGTYNHIFHGQWEDYDPWKCTSRTSVTTDLYNGYGACTIFRMFQGI
LALSTVEPGMVRLLPSPKLATAYYLLRPFFTTKNPPPENRTGPEWEAYLAPENWKLQTEPDSIIHGAVPGHAQRITETWHPHL
HLRNSMITLPTLQPGDYIFWHPDLPYYLSSNNYGLKTPSGSKSEVSMLVYIPAAPLTQTNALYLARQRKAFQRGHPGPDFDST
GRGIVEEDAETRPGEKEIAEVGGPSSLQAMGLAPWEVAGTRSGSPPSEKSKSKADEDVEMEGDGDAKSLTSTSSISRAEAEVV
RLANIILFPDRSMLGYSV
FUN_004747 CDS scaffold_17 386820 388452 -

hypothetical protein PF00732;PF05199 ENOG41
AA3;AA3_2;AA3_3;AA3_1;AA8
MTTEYLPASASSAYDYIIVGGGTAGCVLASRLSSYLPERKVLMIEAGPSDFGLNNVLNLREWLSLLGGDLDYDYPTTEQPNGN
SHIRHSRAKVLGGCSSHNTLISFRPFRHDMDRWVAKGCKGWDFETVMRNVDNLRNQLNPVHPRHRNQLTKDWVKACSEAMGIP
IIHDFNHEISEKGQLTQGAGFFSVSYNPDTGHRSSASVAYIHPILRGDERRPNLTVLTEAHVSKVIVENDVATGINVTLKSGE
KHTLNARKEIILSAGAVDTPRLLLHSGIGPKGQLEDLKIPVVKDIPGVGENLLDHPETIIMWELNKPVPANQTTMDSDAGIFL
RREPKNAAGNDGDAADVMMHCYQIPFHLNTERLGYPIIKDGYAFCMTPNIPRPRSRGRIYLTSADPTVKPALDFRYFTDPEGY
DAATLVHGIKAARKIAQQSPFKDWLKGEVAPGPKIQTDEEISEYARRVAHTVYHPAGTTKMGDVERDEMAVVDPELKVRGISK
LRIVDAGIFPEMPTINPMVTVLAVGERAAELIAQEEGWKPKHSRL
FUN_004748 CDS scaffold_17 391348 392878 +
hypothetical protein PF00171

MTFSSEIHTFHSGRPQPESSSKTNTFVSVDPSNAKPLATIYTTTPEQLNSAIAAAQAAFPTWSQTPAPRRAAILLKAAAILRE
RNDELALTETLDTGKPWSETSTVDVATGADVLEYYAHIAAGSFPGQNTRLRADAFVLTTHEPLGVCAGIGAWNYPIQIALWKS
APCLAAGNCMVYKPSEVTPLHAQTLAQIYIEAGVPPGVFNVVYGDGIAVGAPLVAHSNIAKVSFTGQVSTGSKVASQAVKDMK
GVTMELGGKSPLVILPNADIEEAADTAMVANFFSTGQVCTNGTRVFVPDTLLSQVEQAIVQRCREGIRMGLPQNGETNFGPMV
SAAQQEKVKMYIQHGRTVDKAKVLFDGSEEPSMAQPTSDGFWVHPVVFTNCTDNMRVVKEEIFGPVMCIMPYSTQGRSQQEWV
SELIARANNTPMGLAAGVVSSDVGLAHEVVQKLDAGITWINTWGESPAEMPVGGWKMSGIGLENGHEGIAAYMRTKSTLVQLG
KGACKGVFSKL
FUN_005051 tRNA scaffold_18 2303 2415 - tRNA-Leu

FUN_005052 tRNA scaffold_18 2494 2606 - tRNA-Leu

FUN_005053 CDS scaffold_18 4539 6402 +
hypothetical protein

MDTAIRLLMSHDISYTSRQALCIELHIQAEATLKNAGYFESLQVAKAEFAYTKKINTSEPMICKGVASETRSAATEGHALPSF
SIPVDRRAVLDHHLSYSALSDQRISQPMFESTEDDSKKRPRDVFEENERFGDPRASLTSNNWGPSDKTLERWRKVYDFIRKDE
SLEFPHKLRNKVTQYMAFFSVAASVHRCHGGLVVGPNPLDTAHPPEWKGDLRSFNEEFPKCVHSFQNAVINSYIKNLPKFLLE
RSWTGSNSSVLPESTLDDNNSAIAGNQPYSPIPSSLFETKSLAVHPPYRFKLALERCGKTEGPPSSIIRRQQAEQLFEDTVPK
LCGWLLDKVAWVEFLQELDEGVWIAASLETARGDRVVQPERDRFIETFGGKGDAGESSNDETGGIKLQMASQQFMIQQMQWLG
NITRGKVAGSPYLDLNEAEGHQIKVSQRGKVQLRFKSKNDSMVTTNIKLGPIIAPLEKDDKRTIHFTDLGIDIRTATGLTIKV
PINNSACTLPLSAMKSHQNGTDFISLWKEVRGEARPEDRVSSLLKSSDEDADYPPELCVSSTGNMGEPPRKLLKTIQEPEKND
ALWLLKGYIDLRLPDGGDFWTGNKEDFPQGTDDVTGFIE
FUN_005054 CDS scaffold_18 7106 7435 -
hypothetical protein PF01423

MSFVHLNPRPMLEKLVGNPVLVRLKWGEVEYKGTLVSIDSYMNIQLSGTEEYIEDKPTGSLGQVLIRCNNVLWVRGADAGSDA
DAAMTG
FUN_005055 CDS scaffold_18 8533 10327 +
hypothetical protein PF01490

MAEFANFDGTPSSLGSETSKRKAAILDRHLPDGYSAEPDVAFGPANPSASGSASNDNNDTTGDPVESSLLLQGGDMHRDMFKI
KARANSLRRAATFSHHTPPPLAAEDELSHSEQREPGGFRRYHLQRRARQRRMSGYTIVAKNFVDFLDLYGSFAGEDLEDADSE
DESAIDDREDGDVEQPPELRPLLGRRRSTKRMRKTGDATTTKTFFTLIKAFIGTGIMFLPKAFRNGGILFSSITLIVLSLVNC
GCFRLLLDCRQIYGGGYGELGEAIVGPRFRSLVLASIAISQLGFVCAGLIFTAENLYAFLDAVTANHREFMFNVPSLIALQLV
ALVPLALIRNISKLGPAALLADVFILIGIVYIWYYDIAALSQRGMDSTVKLFNPRDFTLTIGSGIFTFEGIGLILPIQSSMKR
PEHFPNLLYLVMFIITIIFTSVGALCYATFGEDTKIQVISNFPQDSPLVNAVQFLYSIAVLAGDPVQLFPAARIIETSVFGER

ATGKKSAAIKWKKNALRTLLVGVCVGISIVGASDLDKFVALIGSFACVPLVYIYPAYLHYKGAAQTTRAKVIDIVVMVVGFIA
MVYTTLVTVYQWIDSA
FUN_005056 CDS scaffold_18 12686 13733 +
hypothetical protein PF00106;PF01370;PF08659;PF13561 ENOG41

MNKFSPILRTLARQPLQRQAFPRPTTRYVPVAIRAFHSTLPQHNAALGQPHLLPEFSLKDKTVVVSGGARGLGLVQIEALIEA
GATVHAVDRLPSPVEDPKSNFSQVSDRARELGTSLHYHQVDVRDVPDLNRIFEDIANGAGRLDGLIAAAGINHETPAIDYSME
ETERMMSINFTGAFMTAQAAARQMIRLKQPGSIVMIASMSATIANKGMLAPVYNPSKAAVVQLARNLAMEWGEHGIRVNTLSP
GYILTQMLQNLFNDYPGRRERWPKENMLGRLSTPEEYRGAAVFLLSDASSFMTGADLRMDGGHAAW
FUN_005057 CDS scaffold_18 13933 14557 -
hypothetical protein ENOG41

MGSSSSKVAKGAARKYPARAPGAAVPQAVARQPRAEAPKPKSKTLGNSAKDDAVRADAMDPDFPTGDFSRRLHQMGIADSNPT
YSPSSTAATPLGPSAPPGPSFAPSRSNPTLVALSARDRLQDEAEEDFANMGREGRRFLDMRTLVDAMKFRDRGIPEKEIESRL
RIQPGLLSKLGQEKTFSHVTSPN
FUN_005058 CDS scaffold_18 15149 16121 -
hypothetical protein ENOG41

MARGSSSAALRRGEDGPKYDLTTLANPIRPTSGPRVPMFNLEQVYDIFNDTRWGNQQSDAVGEQNPTERRQNNKTAQSRASKQ
TKRNPLGPRAGSGVSKTPRLHKQPGTQLKRSKNPRGKNPIDVVARGASSTPSSPSDPAPYYEVARAPNLSRPIPDFDPALIST
CPKPLSPSEMLDGEKFFLYQMATARHLAFNGVSGPYQIGHCLCDVHKATGNLVKFARACTAPNGERYWRHLMTNASIKAQDGS
RMAGKPVALSNGALVWMADEEGKIIAPEWTFNEPQFLPGSSDDQEMGQYPKQAQQGTNPIHPGLKTTSKDPFWW
FUN_005059 CDS scaffold_18 16256 16539 -
hypothetical protein ENOG41

MNGISAPGEPQVVRNGLQSPPINTSKDMASRLASKPHDAQPNYDVMSTGSNMKASGNLIDATDDLANIGDNNAS
FUN_005060 CDS scaffold_18 17979 19014 +
hypothetical protein PF02698 ENOG41

MDIFPDKVSKAADAANIISEFVAHEQVCTLSDLHRALFRSSATKKQLNVKTSPVSVAVVVLCGSAILSIVDTVISSVMKLVEA
MADEPVVLVITGGIGHSTQLLYDAVARHPKYNSITGQVQGQPEARVFQTIVEQFFHLEVEKGSLITAGKHPTQKFTILIEDAS
TNCALNAVFTRKVLDQHGYTSPYSIVVAQDPTMCRRTVAAFEQVYSDKMDDTPVFAGWPTFVPRVAVQDSIAQNQVGGLSSYW
RYDIAEFHNNQANGLWGMERLMSLLVGEIPRMRDDQNGYGPRGKGSIVHVDIPQYVEDAWMILGDTLGQKMR
FUN_005061 tRNA scaffold_18 19670 19742 + tRNA-Thr

FUN_005062 tRNA scaffold_18 19828 19900 + tRNA-Thr

FUN_005063 CDS scaffold_18 20361 22568 - NOG2 GTPase
required for pre-60S ribosomal subunit nuclear export and maturation
PF08153
MGTGKKEATRRERQGKGGDGLNNVRTKGENFYRDAKKVKHLNMYKEGKAQRNAEGKIVKAASFQSRDIPNARIEPNRKWFTNT
RVVSQDTLNAFREAMDEKARDPYQVLLKSNKLPMSLIRDGKDTENGIKQHKAKMTVETSPFAEVFGPKAQRKRVKLGVSSLDD
LAGDTEKSMDTYRERLEQQRLLSGTSGDPETLGDTDKPLTMSIEPVFDKGQSKRIWNELYKVIDSSDVVIHVLDARDPVGTRC
LSVEKYLKEEAPHKHLIFVLNKCDLVPTSVAAGWVRSLSKEYPTLAFHASITNSFGKGSLIQLLRQFSSLHSDRKQISVGLIG
GPNTGKSSIINTLLKKKVCTVAPIPGETKVWQYVSLMKRIYLIDCPGIVPPSSTDSPTDLVLRGVVRVEKVEHPEQYIQPLLN
RVKQHHMEKTYELKGWTNSTEFLELLARKAGRLLRGGEPDLDGVAKMILNDFMRGKIPWFTPAPKTEGEADGKVGNREGRLGE
MPRKRKQEETVEDEDASAEAQLQREAEAAEKAEQESPDGSDEESEFEGFGSDTEEARSRKPSFGATVDGEDSDGESADNDVIS
IGGSEVEDEEQEEEAPTPAAKQSKGKKIKGGPPNKKRRT

FUN_005064 CDS scaffold_18 22906 23815 +
hypothetical protein ENOG41

MSFSLTTSCPRAAARQFLVASRAFSTSAPALEQVPPESPSYIRLPTPPQSEEKKLPRVRGHLPVPREIFPRMEGDRKIRADYI
KQTVPKPAHKQKPKNETQEWKQTMANSRRQNLETGLKELWVRRNRRDAVQNERVSHKFLENHRAVKAPEREDDRLTRSTVLKA
VLDTKTYPDPERFIHAHKSRVKVKAIEEAKREARRDALMELYINASNFIVNESELKEEIETIFAEDFFHKQGFDIGRYGSAQN
TWDVWGKPTSIGNMLETTTGVSTKIMDFYETEYDRSVKRQKRIAEEFTGGKME
FUN_005065 CDS scaffold_18 24130 27529 -
hypothetical protein PF00005;PF01061

MAPSSLRSLLLGAVALWPGLVMASQNSTYSQADQLRAQLALMGDRPDGCPPCFNCLLPAHTCAQYAGCNEFNGKCACPEGFGG
DDCLEPLCGSLPKGKDRPMRSGASCDCDEGWTGINCNVCTDNKACNALMETGEGGVCYQNGEVVNHNYQICDVTNEKITSLLG
KQRPEVTFTCKQEDQTCDFQFWVDAIESFYCHLSECDSSADWEEEKNTTSYKCNKISCSCIPDRMLCGKDGSIDLTDFLDQSI
QGPGRFECTQKHGGTHDCAFREPEMDALILALIGDSSILLSCHAGECLYETEVPGYKRPVKKINTPLIAGVIAGCSLFLVAVI
ILTWYLSRRKLSYGAIRLEDSDDESTKLMTDHKPASLYFDDVAYTLNGKQILTGIRGICKPGEVTAIMGASGAGKTTFLDILA
RKNKRGQVHGNFYVNGEKVNDNDYKNVVGFVDQEDTMLPTLTVHETILNSALLRLPRDMGRAAKEQRVVEVEKELGIHHIRDS
LIGSEEGKGRGISGGEKRRVGIACELVTSPSILFLDEPTSGLDAYNAYNVVECLVTLAKTYKRTVIFTIHQPRSNIVALFDRL
VLLAQGRTVYSGPFNQCQSYFDGIGYECPPGFNIADYLVDLTMHAGSTQSVDNGTVGLDNNSVGPSSTRAIKSVASISGTTSG
DDDASSSRPQNRRRDSVKVRQDRELFTRRKTVDTAASSDAGGEDNGAYRLHQNPVDSTTATLLEDPHDLPPAAATGTDLDIMT
RAFIHSEIASSTQDEIQRAIRIAQDANGSNANGYTVEGPNVHVSAVGKGYARVGYLRQFIILSQRTWKNLYRNPMLMLTHFAI
AIVLAVFSGYLFYGLTDDIPGFQNRLGLFFFLLALFGFSTLTSLNVFATERLLFVRERANGYYAPATYFAAKVLFDIIPLRIV
PPILMGSIIYPMTGLVADSTHFFNFILVLVLFNMAAAAVCLFIGIVCKDGGVANLIGSLVMLFSLLFAGFLLNHDATPKGALW
LQTLSIFHYGFESLIVNEVLQLTLVDSKYGLDITVPGAAILSSFGFNNNAMWPDIRNLGIFAAVFIILSYAAMHFLLVERR
FUN_005066 CDS scaffold_18 29199 30221 +
hypothetical protein PF00155 ENOG41

MDEYETTPGALNVAETCAASVSIDDLVGMCKDPKAQGPIDTRLKRTYGPIPGSNALRERIAAQCSTEDTKLAAEDIVVTQGAI
GANFLALYSLVGPNDHVVCVYPTYQQLYEVPRSIGAEVSLWKLKEEESFVPNVDGLINLIRKNTKMIIINNPNNPTGAPIHNS
ELGRIAQIAKEKGIILFSDEVYRPLFHGGASGQIDIPKPATSLGYERTITAGSMSKGYALAGIRVGWIASKDKCIISAIMAAR
DYTTISVSQIDDQVARYALSPAVLPSLVDRNLTLARNNAKLVKEFIGRYEAVCSWVEPTAGTTAFVRFIKNANP
FUN_005067 CDS scaffold_18 31913 33450 +
hypothetical protein PF00026 ENOG41
A01A
MKSSLVGASLVSTLGLVQAQTIQWNIEGRHPQPHLARRAKTTYEEVISNDRSEGGYFTEVTIGNPPQNITLQLDTGSSDVWVP
WNCADICEDDKNGKSGCPLGSFDPDKSKTFKHVAPGMFGITFVDDSYVKGDYFEDHFELDGAVINNLTMGLAIKTNISYGLIG
VGYAKNEASGSTTDVIYPNLPVAMYQAGYINTIAYSVWLNDLDAKAGSILFGGVDTAKYVGDMHRIDIQKLDGHYYHFVVALT
SLVATSSSGSDVLTSDSFPIQAVLDSGTTLSYLPQDLAHEVWEEVGAVWSPYYGVAVLPCAYGRNQGNFTFGFAGPDGPSISV
GMDELVLSMTSDAANMPVFESGAYKGENICTFGIQNDTNDLYLLGNTFLRSAYVVYDLVNNEVGIAATDFNSTKSKRVPFKSY
GATIPSATASSNQHRATEKPTVPGHNFTAAEGFQESDSNGNDDKDNAGSLLNPPTTPLALVVTTMSFMLAGGGIFSSIF
FUN_005068 CDS scaffold_18 34028 36670 + MEF1 Elongation
factor G, mitochondrial PF00009;PF00679;PF03144;PF03764;PF14492

MWRYTSILLIPTPKKKVSNGASIIGNPLLNFFAEVQDCKMRATWAARWLNCRLLLGTQQPSCGSHSFTISAAAPAWESRRAFS
QTKRSCSAAQEALKKAQEDAASLTPEYVAANMSAEEAKRLSRVRNIGIAAHIDSGKTTVSERVLFYTGRINSIHEVRGKDSVG
AKMDSMELEREKGITIQSAATFADWKKMENGKEETYHFNLIDTPGHIDFTIEVERALRVLDGAVMILCAVSGVQSQTITVDRQ
MKRYNVPRISFVNKMDRMGANPWKAVEQINTKLKIPAAAIQIPIGAEDEFLGVVDLINMQAMYFEGPRGTKVRVTDQIPGPLQ
EFAKEKRQALIEKLADVDDEIAELYLEEQEPTNLQIKAAIRRATIARTFTPVMMGSALADKGVQPMLDAVCDYLPNPSEIENT
GLDKSQGEKTVKLVPYDSLPFVGLAFKLEENNYGQLTYIRVYQGKLSKGTYLFNSRTDKKVRIPRIVRMHSNEMEDVSEIGAG
EICAVFGVDCASGDTFTDGGLPYTMSSMFVPDAVMSLSIKPKRTGDADNFSKAMNRFQREDPTFRVHVDAESEETIISGMGEL
HLEVYVERLRREYKTECVTGQPRVAYRETIARRADYDYLLKRQSGGPGDFARVAGWIEPNDKPDENHYESQVVGGHIPDKFLS

ACAKGFDVVCEKGPLLGHKVIGAKMVVNDGATHVTDSSDYAFNLATQMAFKKAFSDAGGQVLEPLMKTTITAPNEFQGNILML
MNKRNATIHDTDIGSEDFTLICDCSLNAMFGFSSQLRAATQGKGEFSMEFSHYAPAPPHLQKELVAKYQAELEAKRTK
FUN_005069 CDS scaffold_18 37065 38192 - LYS12
homoisocitrate dehydrogenase PF00180

MSFRTLRIGLIPGDGIGKEVIPAGRRILEALPASLGLKFDFVDLKAGFETFEQTGAALPDKTVEVLKNECDGALFGAVSSPTQ
AVKGYSSPIVALRKKLDLYANVRPVKTVMTAAKPIDMVIVRENTEDLYVKQEQTHETPEGKVAEAIKRISEKASFRIATMAGD
IALRRQKIRDAGASSIHKSPLVTITHKSNVLSQTDGLFRATSKKALADPKFSSVAVEEQIVDSMVYKLFRQPEDYDVIVAPNL
YGDILSDGAAALVGSLGLVPSANVGEGFAIGEPCHGSAPDIQGQNIANPIATLRSTALMLEFLNEEEAAAKIYAAVDGNLEEG
KLLSPDLGGKATTEEVVADILRRL
FUN_005070 CDS scaffold_18 40174 42596 +
hypothetical protein ENOG41

MSQVKNLRAMFENKGDTSPPDRGRSSGASTPVQGSGTGNSGTESPVRPLSKVRTSFVAIEKDGRIGLRRDPSHESSVSRRRLS
MDTDAESVSTIPDKPSASLEDAPRASKPFFQEAIPESPRHLIDPPKTPTEQMGAVTDEPSVNPDKKVDAEEPSPSLLPAEPVT
AEPVAASPKKENKPPAAPAGANGKAKVENTKEAKEPKQTAPAATAKASAPKKTTTTRPSTISTKAPNTKPPAKSPSATRPTAS
ATHKPEPKPAPKAASAPKKETKPAPKTTPTATRPSATSATKKPQPLKPTPSDAPFVKPKPKSPTKPVHLPASLMAPTASSGAK
TGRQVQPSASSQNLSVPARPASRASATGAGASTGKTVKRQPSNVGRSRPSLGPPPKKASDPAHPKKEAPVDESFLARMMRPTQ
ASAQKTSEKVPTTPPKKTVKRPTSSGADATRRESSMKTPGSAKKPARKAAESKSVTGSVVSDETFKVEPVVEEKVPKPAKSES
PEVPEIETEVSQTNNAPEGLKKPEPGPVVEDAPVEVEEPAPEESQAEVDPKESSKEEQEKKEGVRPIAPEIPKEEKPVDVLET
TATEEPIASPRETPADESEESREQEPATESKKPIENDDIVPLVEEGSTQPPVVIEETNKASATDPEPETTEEMIPLLEKDAES
AETKTAENKDNKIDIINRNDTKEDEKAAEDVEEKDTTGEKIDEEKTEDQESVTKTE
FUN_005071 CDS scaffold_18 42936 43437 -
hypothetical protein EOG092650I8 PF00410

MPSVTNIANMCSHLQNASKARLGITSVKNCKYNLQLALALHRSGFFSAIYRSGPHPPTLEQMVSEPPVRVTNANVARMRLWLG
LKYWDGKPVLGKANAISTPKRLMTANIAELARLARGFPTKVDGGVVPGLNLGECMFVSTSKGMLEVREALARKQGGLLVCRVS
FUN_005072 CDS scaffold_18 44261 46072 - NIC3 Kynurenine
3-monooxygenase PF01494

MVKPQKIVVVGAGPVGSLAALYAAQRGHEVEVYELRPDLRDPSTIPLNFTKSINLAISERGINAMRHAGQPGLLDHVMSTTIP
MRGRMIHGRGPTGALFEQSQDYDVKGRAIHAIDRAGLNKRLLDILDNMPNVKLFFNHKLTGADYRECKAWFEVADAKSSEESR
PKEIDISFDLMIGADGAHSAVRYHLMKFTRMNYQQEYIDTLWCEFQLKPVKTDETADPMAKFRISPNHLHIWPGKDFMFIAIP
SDDGSFTCTLFMPSKDFSDLENNPASVPAFFDSHFPGVTDLIPGDELIESFNTNPHLPLISLKCKPYHYGSSCVIVGDAAHAM
VPFYGQGMNAGMEDVRILFSILDKHAQIDESNDPASESSKSEPAFQRSLALAEYSAVRPADAHAINDLALQNYVEMRSSVLSK
RYRLRKYLEEMMSVYFPRLGWQTKYSRVSFSNEGYLDVINKSDAQGKILVRSFLTLVASPFLVSAAVFAYRYRRSFSAMIKAV
RQLPS
FUN_005073 CDS scaffold_18 47234 48612 +
hypothetical protein PF00557;PF01753;PF15801
M24A
MTADPPAKKECMGADCQNEAGSLQCPTCLKLGVKDSFFCSQECFKRNWGIHKTMHKSQSNILHHLKAPKAISPDPATGYYNPF
PNFPYSGTLRPVYPLSPHRTLPQSIPHPVWWQDGNPRYSRSLTNRNKIEILDKKGQDAMRKSCKLAREVLDIAAAAAKPGVTT
DYIDEIVHKACIERNSYPSPLNYNNFPKSCCTSVNEVICHGIPDQRVLLDGDILNIDVSLYHEGYHADLNETYYIGDKAKADP
DTVRVVETARQCLDESIKAVKPGTLIREFGNIIEKHAKKHNCSVIRTYCGHGVGKLFHCPPNVPHYAKNKTVGECKPGMTFTI
EPMIALGKYRDITWPDNWTSTTIDGKLTAQFEHTLLVTEDGVEILTARQPDSPGGALPMPGTENGETQA
FUN_005074 CDS scaffold_18 49584 50220 + ERV14
COPII-coated vesicle protein PF03311

MMSGEAWLFLLSVLINAVNLFLQVFFTIMYSDLECDYINPIDLCNRLNTYIIPEAAVHGFLTFLFLINGYWVPLILNLPLLGW
NVKKIVDNTHLLDATEIFRKLNVHKKESFFKLGFHLLMFFFYLYSMIVALIRDESS

FUN_005075 CDS scaffold_18 51302 52892 +
hypothetical protein EOG09262A6N PF00400;PF12894

MADFGEYPPNMAPQDALIVREQAEPDHAVVPYSAEDLSRPKAGPLNPFKDENNVLKRKSVPTGHAEETFLSEHTFRSKHRAIE
RRGGPEREYQTNAEQKAEAARIRAGRESKGSATIAEGPGSYVGPWARYKKAEYEVVADDEELASDEEYEIVEEEEEVVDSGTI
VKAPTKALERRKEAEEMGDETTTFHGSAEYDYQGRTYMHVPQDLDIDLRKEVGSVTNFIPKKQIHSWKNHSKAITALRFFPSS
GHLLLSASADTTVKIFDVYHERELLRTYSGHSKAISDICFNTSGTQFLSSSYDRMIKLWDTEKGVCVSKFTTGKTPHVIKFNP
DPEHANEFLAGMSDKKIVQFDIRTPNEVVQEYDHHLAAINTITFVDENRRFMTTSDDKSLRAWDYNIPVPIKYIAEPDMYPMT
RAAPHPSGKYVAYQSSDNQILVYGANDKFRQNRKKSYRGHNNAGLGIDLDCSPDGQFLASGDSGGYVCFWDWKTCKMYHKFKA
GNQAVTTVKWHPQETSKVVTAGLDGEIRYWD
FUN_005076 CDS scaffold_18 53125 54637 -
hypothetical protein ENOG41

MGPYTFIEGNTPGSVTDWSSHQNNSGNDVFYFSFTQLAMQLVLSADHQTMISCQIIDTYWSFSRKAIDPLREEVRSIMRTALG
DDSCNVREFDLQIAILQMLEAIHEFELGQGSSKPEKYERGYAADEVSDWNEVINNPFGLDVSTIQDTAYSILRKTPEELTAGI
PKEWRVIHMESVLRHDLVRRFWNYQESLKTDLKSCGTSLRDRIPPHSKLGGQVRAQLSQESILQDMISPRVTFHGTPLQSVGS
IVRHGFKMPGNIVEGKVVASPRSGIAFDRGIYSSQEAFYAISYARGQREMTPVGMLPSMRLFVCATIMGRTYTGSDGRHGPLI
EGYDSHFDGGFEYIVHDERAIIPCYVIHLDLGSEAAKQALKDVQTDPEEFHNLAKTKKRPEAKSTAPGDIQREKEARKAAALK
WFPYGFGPAMGTRFVIEEVGEISDDEEEYGDWQADKHGYSNSYLEKNTVWDNEDGEYYDENGNLVKEKRRLMDQYQHATRAYK
NKRGA
FUN_005077 CDS scaffold_18 55368 56588 -
hypothetical protein PF00856 ENOG41

MDDNNSQPPDEPTLSPTLASLSIEDIPILDLQVKLELDKGSNGTKPCDDKETEVTEAQPKEEIVSESIPDEFHLPHGNQTPLD
HEQGPFDDIATEYGDTSGSEPDFTSSSEFDFGSDDEDEETTDGLIRHVTAHRDKPFEYVYRFPPSKPLESIESPEHIAMEPAG
LPLTPSPTTDVLFQNDYFIVKRSFIAGWGAFAVKDLEFGDKILVERALFTADNNTLYKEFDKLDQASQDIALGLHANSYCKFQ
SIKAVWTTNCFSTGVWDEAGLFPIASRFNHTCHPRQTVVYHYNKAEEVLEMKVKTDVIKEGEELTISYGCGLTPADLFYRYGF
RCRCGGCSGWTEEDERGMW
FUN_005078 CDS scaffold_18 60989 61983 +
hypothetical protein ENOG41

MALFESANCDRNATAPTASDAGVAGAGILLSFMITALLAIILSSSVIFAEMRGRDSVIRRKLLNGYSDSQIMQGIGIQSVGLA
KMNSLVPYHFFLIWMLSLLSMATHNTTLLALKQDYRRDWVIRWLRQFLMFVNLALSCVSGVFVLEGVSKGLDDKTLPISCVWE
VEGKGAASNAGLSYVGTIVVIAGNCIVFGLATWYLHSKQQRFYKITQIVGLVLMTAIAVGAAIRIILLSQAFGHPSIELKDNG
ETVWSFGQLLSMLLLILPLVNVIEIYRGEIQVPGPIRDERAKVYDGELQDNPQGSRTNLFQK
FUN_005079 CDS scaffold_18 62748 63577 - ALTA7 Minor
allergen Alt a 7 PF03358
AA6
MAPKIAIVYYSMYGHIKQLAEAEKAGIEKAGGSADIFQVPETLPEDVLAKMHAPPKPTDVPTLDDPSVLEGYDAFLLGIPTRY
GNFPAQWKAFWDKTGKQWASGGFWGKMAGIFVSTASQGGGQETTAQNAISTLTHHGIIYVPFGYAKAFGVLTDLSEVRGGSAW
GAGTFAGGDGSRQPSAKELELAQIQGENFYQTVAKFTG
FUN_005080 CDS scaffold_18 65126 66564 -
hypothetical protein ENOG41

MHLNVPIPRGWDATESALQPRETSSSNGGGGTNNTVVFIVIGSIAGVVVAITLAVCITKARQKKKSTESKKKGGFLERIRGRS
GQGNYEQTAGEDGGESGRQSHQLDPTSTSTNNRQNRNSSNNGTNAGATVDRNTSVRSVLTLPAYRQSASHNERVLGREGERDG
VDVIIDLPTAEAEEEARNEEMETLYQIRLARRQALAEREERRERRREARLRSDYRELEAIRAETRAANEDNTIAELRTTVDQI
KDNRQRSVSSVSYADVGVARHDGTRIRANSSESERVGLLSDAASMQSGHQRGRSYSSAASHDDDFASLAPTRSQGASIRSGSA
DGRAGSSPELVEADLGEEAMPPPEYEDIPLTDDRSSNHGPPPEYPGPERSSSQRTQRTTERDLGSDWHEMDPASDGQSNSPPS
GQGNGSAPQLPSLRIPQLPEIVIEPSSAHPRDDEQRSPHR

FUN_005081 CDS scaffold_18 68598 69996 -
hypothetical protein ENOG41

MAARPGEENVATLFADIHYFYGPDTVKPRHHRFDKGSYVYLFENANERRCRIEIANQPGTGDQDAFEGYLDQTHVRYSYKQQC
NVTLTGPEAVADQNEWHLPTFDPQNQNKYHYKLHSLDIYFWTQADALQFVNGVRRVAPPSHVEVLDEPGPPPQPAPMSSVVQQ
LENVAISDPQYGSANAPSFAPPPNVPAPPPAPPSEAPANFAPMAYNPAAPAAPETIQHREKTPPPDEDPLNPLAVAVAYDYHK
QPFTPGMPPPSQFPPGGVTSPGLPPPQFGQHPGLQRAATMPAQALGSPYGATFPGSPGFVSPPPPPQTQPPAQTQPHSQPTPP
ASNPGLPPPPPGGFTQYNYTHQEQQAPTQPGAEYSVHQQVYRPTQTELANSEIYSFTPKTKEPRGKLEENAGRLERGVTGMLK
KFEKKFG
FUN_005082 CDS scaffold_18 70556 72532 +
hypothetical protein PF00817;PF11799

MSSTDQAEEQPPLQPTEEPSGAEKETNHVSRDTPETAEDDAFKTLKYSLLGPSLLKAGQDTVDQTKVSEIIYNASKGSKFFNR
EEERDKVLTQKIEQIIARKAQLEKRDLTRDLRNADRLIAELELTRDLTQHIVHVDCDAFYAAVEQLDRPEMKDLPFAVGGGVL
TTCNYVARKFGCRSGMAGFVAKKLCPSLILLKPNFQKYNAKAHEVREVLVNYDPRFESASIDEAYLNITEYCQEHQMDPAEAV
EQMRREIHEKTNITVSAGIAANAKLAKICSNMNKPNGQYVLSSDRPTIMAFMRDLPTRKVNGIGRVLERELLEIGIKTCGDFY
EHRQYLNPLFGDKTSEFLFTCYLGLGRTKIQPAEEYERKSVGTESTFRDMSDPTQLREKLRATAEELEQDMKRAECKGRTLCL
KVKLHTFEVLTRQVVLPRAICLADDLYNYALPILNKLEQEMPGMKLRLMGLRCTHLVSTKKPDAMAFFGFRPRRTDSEDRPQG
GTLKRKASDDDGEWEQWPGAGLVLDDPDGLLEGSSSNQDTPDQSLGRRHGKEIAPNPTKENATEEQWWDCPICSRPQAADERH
FNEHIDLCLSRQTIREAVQADGPSIKRDPTPEIKKPRTTEKKRGRPKVPDPKQRQLFFG
FUN_005083 CDS scaffold_18 72695 75055 -
hypothetical protein PF02386 ENOG41

MLDSITSRWKDLREEHPRLDAVATQAGRLVPPINFITIHYAYFIFGSLFFSLIFWLSSEPSQSIPYVDCLFLVVSSFCDTGLN
TVNLSEITTWQQVLLYLLFIIGSALWVSFWTVMTRKQAFEKRFEDIVRIEREARKRRAALRRAKPLGRFLPFTKARTTPVSTE
TSNTLTGLGTVQRTATEKPDPNDLFKTTAPMRRAVTAPPDSISDSDDNDSNTTQVAAPARNAPMTPNSGDHIQFADTLSPRSA
GRDSVSIYRRNENVEPNRRPSTSDSVNSDESEDFLLHWKKILGSHNTSKRGQFYDLSSDEREALGGCEYRALKILAVTVPLYA
FMWQAVCGIALGAWISINRPSAATVNAINPWWCGIFLSSSAFNNAGMSLNDAGMGAFQDAYFVLIVVGILVLAGNTGYPLLLR
FFLWCSLRVLQLTTEPKTLGPWKETIEFILKYPRRVYTTLFPSGATWWLFTVIVTINTIDWVAFEVLNIGNDVVEAMPVSDRI
IAGWFQAIAVRAAGFAVVSISGLYPAVQLLYMIMMYISVYPVSITMRHSNVYEERSLGIYEDDPQVLEAENGNGNGNGNGNEN
GNHLLPTISEEPEKPALRRRVTAAAQKTVKKSMTFHGVGVRPPPKGPDDNSRISFIGQQIRGQLAHDMWWLILPVIVIMIIET
DHFLEQPLVYSVFNVLFEVVSAYGCVGLSMGLPGKSYSMAGGMHRGSKFVLCLVILRGRHRGLPVALDKAVRLPGEKLVREEE
EDSKIRRTKTMNRIASRESRVM
FUN_005084 CDS scaffold_18 77177 77999 - FPR2
Peptidyl-prolyl cis-trans isomerase fpr2 PF00254

MKAALFLSALASAAVGVVAEDLKIDVTLPVVCDRKTQKGDRVQMHYRGTLKDSGKQFDASYDRGTPLDFVVGSGQVIRGWDEG
LLDMCIGEKRLLTIPPEYGYGQRAIGPIPAGSTLVFETELVGIQGVPKPEKIETKVVEGAETAAEAISEAAESAASATKNVAG
KVAQAVVDAADAAKTIIADTDDAPEHEEL
FUN_005085 CDS scaffold_18 80564 80903 +
hypothetical protein ENOG41

MSSPYTVQIHQSTKEPSISSSYSYSSGSYSSSTSTTPRSLSPGDYREYGADKHTTQVASSGRNLVINHNKVSYEKDSPAPKYS
GAYTRI
FUN_005086 CDS scaffold_18 81972 82438 -
hypothetical protein PF02755 ENOG41

MADTEQQPKLVDESPISPVERRNSLEAHLKHRPERSELVEKNILPASTAAPGLQAHQKELEKHMLEDKLNDKISHRPDPEALI
KEGVLHDDPRTVAQDEAAKKYEEAIEDEYAKREGGA
FUN_005087 CDS scaffold_18 84026 87815 + MYO1 class II

myosin EOG092608L0 PF00018;PF00063;PF06017;PF07653

MGISRRPKNKGAGASGGAKPKKATFETTKKKEIGVSDLTLLSKVSNEAINENLKKRFEGHEIYTYIGHVLVSVNPFRDLGIYT
DQVLESYMGKNRLEMPPHVFAIAEAAYYNMKAYSDNQCVIISGESGAGKTEAAKRIMQYIASASGGESGDIKQIKDMVLATNP
LLESFGNAKTLRNNNSSRFGKYLQIYFNAQGEPVGADITNYLLEKSRVVGQITNERNFHIFYQFAKGASQQYRETFGVQKPET
YVYTSRSKCLDVDGIDDLAEFQDTLNAMKVIGLTQAEQDEIFRMLAAILWIGNIQFQEDQGGYAEVIDRSVVDFAAYLLEVTP
DQLISGITIRILTPRNGEVIESPANPAQAQATRDALAMAIYSNLFDWIVERINKSLKARQPTTNTIGILDIYGFEIFEKNTFE
QLCINYVNEKLQQIFIQLTLKAEQEEYAREQIQWTPIKYFDNKVVCDLIEQIRPVGIFSAMKDATKTAHADPAACDRTFMQSI
NGMSHAHLTPRQGNFIIKHYAGDVTYTVEGITDKNKDQLLKGLLNLFQHSGNQFVHTLFPRPVDQDNRKQPPSAGDRIRASAN
ALVDTLMKCQPSYIRTIKPNENKSPTEYNSPNVLHQIKYLGLQENVRIRRAGFAYRQDFDKFVDRFFLLSPATSYAGEFTWEG
TTEAAVKQILKDTSIPKEEWQMGVTKAFIKAPETLFALEHMRDRYWHNMATRIQRMWRAYLAYRAESATRIQRFWRKKRTGAE
YLQLRDHGHQVLGGRKERRRMSLLGSRRFLGDYLGVNASTGPGAQIRNAASIGTNEKAVFSCRAEILEAKFGRSSKASPRIIV
VTTNKFYIIAQMLVNGHPQISVEKAVPLGAIKFIGASSARDDWFSLGIGSPQEPDPLMNCMLKTEMFTQMQRVMPGGFNLKIA
ETIEYAKKPGKMQQVKVLKDSQLPVDYYKSGAVHTQPGEPPSSVSRPTPKGKPVPPRPITRGKLIKPGGPNGRPSRIQGNRTA
KPRPGAGRAVPQPPAAVSNIAAVPAAATQSTPRIAPRPGANAAAPIPAAHNALPSHTRNASGAGRAPPPPPPPAVAARPPSPP
KVMAKVLYDFAGQRENELTITANEIVEIVQKESNGWWLAKNPQTAQQAWVPAAYVEEQAPPAPRAPPAPPRSKPTPPAPPAKR
PAANRKPAELQQRDSGMSLNTPNGGDSRSSTPTPSLGGSLADALLARKNAMQKEKEDDDDW
FUN_005088 CDS scaffold_18 88231 89521 - PFA4
Palmitoyltransferase PF01529

MAGLNDVPFIKGLAVPSVCALITFLGYFSQFLFHYSTSLEPGPPSRRETIIFNTLLFTLWLTYYRAVTVDPGRYIFKDRVIEA
DGQRWCNKCAAPKPARAHHCRHCARCVPKMDHHCPWTRNCVSMTTFPHFLRFLVYTNISLWMLGYFLWQRFSKIWEHRKLPAY
LGPSLSGLIGLSLIAIVNFFTTVALGIMLINTIRSWIFNQTMIEGWEQERHEALMDKGPKDWWDITGPDGEKVRFERLEFPYD
IGFFDNMAQAMGTRNILMWFFPLGGNPAIAKDGTSSGWEWEENGFNRVEGLWPPPDPDKIRRAARGWPAANRDYAEELRQANM
SSSEFKAEFLKRKADDEKRKRHLMAELEEVDDYDMYDDEEYDREFDQGLGWVNSDGDRLRDYGVDEEESEAEVADEDVPLAEL
IRRRKVLQRDISDD
FUN_005089 CDS scaffold_18 90880 93049 +
hypothetical protein ENOG41
GT90
MSLVWVGSRLFRGSSVLHAGFGFLYGFDIIVDSVAFAALMISATFWVCETSPLTPSAIITFLVGTAKACHNIFNYGDYLHPTR
LHEITPLYFIAFGMIIFTYTHGIQSVIFIRRFVLMALLVGLVVGTTVFIAKSDPQTFKRHPVNDFIYRANIRQDRWMQEASTS
GSLSIAHKIYKERHEGRDPPPAFPDWYDLAKDTVVIDKFDQIDQDLAPFRAIPPNKLRQRASILSQMHGIHTIGIKDGNASRL
PEVTGHDAEVLDGLVVTINKFAKFLPDMLFPVNLGVTPRILPSWETANGHSRADLTPIVNLISKRAVKEDDADQVKEAGSAET
PVPAPLPALPDYPHDKEPSMISPANYRQMQVEACPPGSRTRTNPHWNIGEFCAECVRRHSKAQLMVNWERSLQYCFQPDLKYL
HGMSLSSPHAEPIRDLLPLFGFSKSEPFNDILIPLPQEDDDRADIGWNFDRRYDAMFWRGQTDNGVISDQALRGNHKFRLLHM
LGNQNPEDKVSMVLPTAKDASVYRHEKVPITEANLVLPSSVGMNNYTGCIGPNCELLHQTYPIVNEAQDQEPLEYRYILLLDK
DHGPSPDLLRVLRSKSVPFISTIFRTWYSDRLIPWLHFVPIDTRYQGLHTTLTYFTGTQKKAYLNGRDTDMKGLAKDGAWIAQ
QGAKWANQALGEKDKEVYLFRLLLEWGRLVDDKRDEIGTSKNDKGELKSSPWTKNQKW
FUN_005090 CDS scaffold_18 98775 100803 +
hypothetical protein ENOG41

MNSVAHSMAEASPAPAAPSMQHQHQHQQPQPPPSPSPSQPTSVSETAPTPSLPSISSPSAQSASLPAVLAAATAVASVSAHPL
APNPDELPPLRQPSPTSPAHYTFEQHQQQQHHHHPHHNLHPVTPPLRKDTCSSISTQATSATLASTETNNTSYSADTSPNLHQ
SIFSIKDGSDVSNNRRTSRRRTGPLSQQSRERAALIRKLGACIDCRRRRVACHPSHHNMTWEDVVTKFHRSHSPTIQDIAPSL
AAAGRPLSPAPAITNVQALFHDTQEMDIDSSTPGSNLPGRPPLSDARIRTPLPSGPRLEKSLSLPGIESFKNDLQSNVGRMLS
TPSRDRYSSVQVLFLFWQDDEEVPIIQTAVRELAEVFDKYYRYNFQVQAIPSSSDGCKSSWRWLSRALNEFVEDRDERDVLKI
VYYAGHTYLDGNREMVLASSRNPEQASTIRWGGIQQTLEEACADTLIIMDAAYYPSSRMVRQQGVLELLAASVSEEHYYDLDR
CAFTRALAEQLRVRAARLSPLSAAELHANLFSQYAKMVQDKYPEKEVLTSFFPSPLHMMMSGNSRLPSIFLSPVYQNSPTRGSF
SSYENSPQLHLSIRLDDDNVDLESWNEWLRLMPEGIRDVKVEGPFRATFR
FUN_005091 CDS scaffold_18 102649 105019 +

hypothetical protein ENOG41

MGGVVSAFSSSSGEDSVAARQALEKQLADREETIAVLEEDLSKKEKECTTVSKDAEDLREKIKELEAQSSLALDETHARIAIL
QEELKKGGDSSSEQLRSTKESAEKNAKELEDAKTSLTATEEKLKGLEQERQSIADELAGLKAELAEAKEAREALEAALTKEIA
TLKTQISEAEEKHQTLTKAHSTLEEELAAASSAAEQGKQALTGSEDKFTTLQSSHAKLETDLAAAVTALDEQKKALAGSEEKY
AALQETLDKLQEQSDSQIATAKKDLAEAQEKTNALQQTHNKHKADSENELSELKKQLSELGDLQTKYATLEETNKSLEKELAE
LKEKVADLEKTNESLKSNSSSELVAAQKDAAEWKEKHGSLQSTHDGLSKDLETAKKDLAAAEEAQKKLTADHTAALTKAQGDS
SAELEKVKKEAADLEAKLKSTTDEHDALKKERDEQAEKLKTVTSDHEAIQKKQEETEAKLKAANEERESVEKQLNEKTSKLAE
LEKEIEEAKAQVAKAEEDLKTSQAEKKELETKIADLESSAKNSQESESGLNAKLKEAEDKVKSLESDAAKAKESESSLKSKAE
EAEAKAKETEASLKSKADEAEAKVAALEADAKKAQDSESALKKQLEEAQAATESEKKSSADKTKSLEDELKELKEKFAKSEEA
AKKVEALEKEKADAVAKAESLQKEADEKTKTLEAEKKAAEEKAAALEKEKTDAEEKTKTAQSAFSNALEKVKTIQGEKKEALE
KVKALEAEVKELKEKSATTNGTSEA
FUN_005092 CDS scaffold_18 105601 106432 +
hypothetical protein PF09430 ENOG41

MRFTFETSAILALLPLALADSLTLYLPSNPNPFSIPANTHATLSSAGVHFSAPLSSANTFVFHNVTPGSYLADVHCPTDAFHP
LRIDVQLAEDGEEGIVRAWETYRGNDWGNKGEVVQVKEGSKGRGFELRAIGGKNYFMERPQFSVFAILKNPMILMALVSLVLM
VGMPKLMDSMDPEMRAEFEAQRKNSPLSAAMTGQSSGDNPLTNFDMAAYLAGSKPKESTPANNGGNGNTKNQGVRR
FUN_005093 CDS scaffold_18 106784 107867 -
hypothetical protein PF00397 ENOG41

MADFAPPSGPPPPKAPEVPAGWAVRWNEQYKEWFYVNIYTKQSQWEKPTAPVFPADDAAPAGPPPGYDQGNAPIPTDTKKNPY
EDHRDQSAGGPSEDEDAKLAAKLQAEEDARARSGPGGPDVPAGYGASNSPYPQSNSPYPQNNSPYPQQQGGSSYPTELPPRER
GAKSGGGFLGKLLGKGKQMAGGHQQGGAGGYGGSQGGYGGGYPQQQYYGQQPQGYPGQGPPMGYGAQPHYGGGYGAPGGYGGY
GGAPGYGAPHGGGFGGGGRKPGGGGMGMMGGAALGAGAGLLGGAMIANSFDNDEQDAYQDGFEDGADFGDDGGD
FUN_005094 CDS scaffold_18 108872 110257 -
hypothetical protein PF06772 ENOG41

MNCNELQEHSKADCFLVKKPRALQWFYKGQLIKEKEEERQAGRFELFLDLLYVAIVANFSDELAEHPDGAHLAKYILIFAPAW
HIWADLREIMNSYYTDDLLQRLVILWVMALLVLYANNANDADEDITAMRTTAGAYLVARFTTLNVFLVTSFAAYQHRTQARIM
AGFMFVGLLITIPLFLEDISVRAKAAIVAVGIFYQEATWALTLSPWIKAKLRLTYSTAVDIAHEIDRMGAFFIIIILGEFVYSI
IVGNKTGIGLTSGYAKAVCTLIIAFVLNWVYSSGDGSIQATHPIRRSAWTAFGFFLLHLPLSASFLIGGHIAAASTAIEEFED
GQRWLLGGGLGVGMFCLWVYGMLYRVEGECTLFMGQTTRISMRLIIAIILVVIPESHNHLNAESFMFVIMALFAFLLIWETIG
GLSRTSKFFEPWTNRNPPPEEDDREGLAGE
FUN_005095 CDS scaffold_18 110858 112361 -
hypothetical protein ENOG41

MKVTHKTTDIQLPKAVLDTTLNDLYRQAGSEYRNKRIGQVLSSFSALVPPSSKITPNHKYIYQGGPFTSSKGSRTEKEDSATA
IKYLSLVNQRGAFECGTAPVIFFYMDGDSKKRDHDRKQVAKTSATLPDYQRPQPVFCEGPDSIPINETGIDLLACKVVHDSLE
RHSNVVPLETHWFLNSKRALADSGLPTPKCVIITVKGIPKDAQSCCAPCTGSGLDSFVIPGDCTGNRGTWLKEQSQRIYDALK
SHPLPFVLKSQATFGGAGTFIVKTEEQRQKIIDNLGKGFLGRLLSSVNADNSHLEPATMLLSDMIQNPIGDYGMTVFNNKKGQ
ELVFLGVSKQMIQDGTAWVGSTIDYRQQSELRLKFETLTNQILEWLQSYEYIGPAGADVLEDADCFHVVDLNVRTTDSCYLPL
LRKHFTNRELYIASSFSTDVQQRRDEFLDMFKAEFQDGRMCVISWYEDQEKGSSLAELVIGGGEGDDRLKELMDRVNEMSKSV
TF
FUN_005096 CDS scaffold_18 113983 114829 +
hypothetical protein ENOG41

MEYYCKKSWQSGHFIGRVKSHDELIENARKLIQDFQNARSEIGDDFPSLVETAIELEELDKSVALVQQEESLRTAAVIREIKG
AIRSVHYQSWEAVRVVGKKNKAVDKNGPDAMVPKRYIHSFRPMPKWVISTKDTLDNAILCAQVGIIGNEKDGFSIRPDKVFEM
NGKTKDVLGIHMVLALRLWDEPTCIGGHGTYPDDESKGEGAGQHGTAVEGIDDEKHDAEESSVYDNITLDQARAMAGEVGVQG

WTEAARLQPRRMKR
FUN_005097 CDS scaffold_18 115061 116764 -
hypothetical protein PF01053;PF01212

MAEELSKNFETLQLHAGAEVDPTTKSRAVPIYATTSYVFDDSAHGARLFGLKEFGNIYSRIMNPTVDVFEKRIAALEGGVAAL
AASSGQAAQFLAISTLAQAGDNIVSTSNLYGGTYNQFKVFFPRLGIKTKFVDGDKPEDIAAAIDDKTKAVYVESIGNPRYNIP
DLEAISKAAHEKGVPVIVDNTFGAGGYFIRPIDHGADIVVHSATKWIGGHGTTIGGVVVDSGKFDWGKNAARFPQFHEPSEGY
HGLKFYETFGNITFIIRARVEILRDLGSTLNPFAAQQLLLGLETLSLRAERHAQNALALAQYLEKSPYVSWVSYPGLESHPYH
ETAKKYLKRGFGGVLSFGVKGGGAGSEVVDGFKLISNLANVGDAKTLAIHPWSTTHEQLSDEEKRSSGVTEDLIRISVGIEHI
DDIIADFEQSFKAAADATTKGEKKDIPVGDKETEAPLAP
FUN_005098 CDS scaffold_18 118038 118568 -
hypothetical protein PF01284 ENOG41

MSRIAFLSLRALQLALSIASIGLSAYVVNDYNQRSRNSAPSPFTYLMVSSIFSIISVAYLSLTPLFVPRIYHQYAAVVVESVN
AALYFAGFIAIAVFIGSLIMCEGTVCSCARADAVVAAGQFTAWITTTAFTAKDLFKKAFQEPKKDDEGREMGQA
FUN_005099 CDS scaffold_18 121269 123536 +
hypothetical protein PF00501

MALQTQKDYDALYKRLTTVPPPGKPYGIPVPGTERPNRTAAYRHWAVGDGPLVTALEPGINSTHDVLERSARKWPNSRCLGTR
LWNQATQQWEDKYDWISYGDFDARRKNFGAGLVEIHKAINYSPEKYGVGLWSQNRAEWQIADFGIASQSLYSVSLYETLGPDT
TEYIINHAEVACVVCSLPHIPVLLKMSPRLPGLKLIVSLDPLEQGELASHTKASVLNEIASHHGIQIYSMAQVEEIGAKSGRA
PRAPSRNDICTINYTSGTTGNPKGVLITHGNAVSAIAGGRTNGNVSSKDVHLSYLPLAHIYGRLIDQIAVAEGAAIGFFRGDI
LGLVDDMKILKPTGFISVPRLFNRFNSAIRTATIEADGVRGALSRRVIDTKKANMRLPPGKASNTHFLYDRIWTPKVKAAVGM
DRVHSMVSGSAQLDPDVQEFLRAAFANHFAQGFGMTETYAVGSIQARGDFTTGNIGGPMCCVELCLESVPEFDYTVNDTPNPR
GELLLRGPVIFREYYKNAEETEKTLDADGWFHSGDICEVDKMGRFKIIDRKKNVLKLAQGEYISPERIENVYMGSTNLINAAY
VHGDGTQSSLVAIFGIDVENFAPFASKILQETIAPNQVADLRLAANDPKVKAKFLKVLDGIGKKHKFNSFEKVRNAHLEIDPF
TIDNGLFTPTLKLKRPQAAKAYREHIDRMYEELAAQEPVGKNKL
FUN_005100 CDS scaffold_18 124318 127455 +
hypothetical protein PF00400 ENOG41

MPHAPISILNSLPVPCDEPPPKRRRVSTGLQRKRGVKECLLSQVTPLVERAVGHLSRDVYHVNALAIKTVTELSINKFFRRRW
DETGGYLSQQDLDVLAIQAHDVVRGLAGGSEFRISPLSSETPEPQLLPPPRSTPKSQPQGQTAPTIVNTEATRKSEDRDSPIF
YDALTSQPSHQPELPQNVERKPYKPRERREPKPSNRYYQISERPYLSAAHRKAIEEGTKKPVAVNNELIAQPIVYHVDFSPED
IAEIIGQISQNENKHIPATRESMMEQIRLRNRPLIPIPGRTIRDMSAFASDLWAGKVAETPRVLSFTAEHVTEETKKRQGQMR
RTSRLSSLLMAREMEGNQGFQRTREYLNFQAEFKRLCEDDLQVVAEFTNCAGDITTGAWVSRESFLCGTTTHSDTHNQQYNRP
GNLLLCSTGKGTLRSFPDHRIPRPRVEKGGNSTEAMRQSQDPWLYSSVVSCDYSAEDDLAYTSSFDKTVKVWKVDPKGEHMEA
MATWQHTGNVNFVAAAEDGSGRVATAADSPTDAVRIYTITKGNESNSTYQTFSCSRTDADGSEKWAYFPATMQWGRAKGCQHL
LLVGYSPRSLTGDDLDIPEDKRSSGEIILWDAEQGVRIPVMTASTANVFEVAWHPTLPLFLVGVTPTGMNNVPRNVRTQVHVF
HRDKDRLNGIGFCQHQALDCYASDINELTIVPNSPGHAYVTAACTDGKVYIWDTAQGDKPIHTLRHGKPIEEYYGDREKEDTG
VKFTAWNTSLDRFYTGSSDGVVKVWNVRKLKNPFVRDILTAPGPIAWGGFSPDNSKLAIGDATGRCFILSTDERDIPESHMMT
LPGTTRRRRRPIPLIPHPEPAPPTPARDDDEMLTDDELSDANIADYSRQTYLDSQCLKLTSNPVIGVVQGPRYQSTNLYRREA
HYDEDPSAPLLAHFERRQKDSEAASLGRRRRSVRRLRTPSPPDERLQRIHAQNASKDLDVNHLEDSEVKELVKSATLLSIEED
WGFIYEETMSSDEDA
FUN_005101 CDS scaffold_18 128139 129210 - YHM2
Mitochondrial DNA replication protein yhm2 EOG09263HD8 PF00153
ENOG41
MAATIRTDLPAPIGDKQLEKKPIKFSNLLLGAGLNLFEVTTLGQPLEVVKTTMAANRGDSMAAALGRVWARGGPLGFYQGLIP
WAWIEASSKGAVLLFVASEAEYYARVAGASEFGGGILGGVTGGVAQAYATMGFCTCMKTVEITKHKMAASGVKPQSTFQTFAE
IYRKEGIRGINKGVNAVAIRQMTNWGSRFGLSRLAEGWIKSLTGKKEGEKLSAGEKVIASALGGGLSAWNQPIEVIRVEMQSK
KEDPNRPKKMTVGNTFRYIYETNGVRGLYRGIAPRISLGIWQTVCMVAFGDMAKTYVEKLTGESVTAKH

FUN_005102 CDS scaffold_18 131139 132877 - CIT1 citrate (Si)-synthase PF00285

MASVARLSNAALRASLRSTPINGSVFNAVRCYSAKTQTLKERFAELLPEKIEQVKALRKEHGSKVVDKVTLDQVYGGARGIKA
LVWEGSVLDSEEGIRFRGKTIPECQELLPKAPGGKEPLPEGLFWLLLTGEVPTEQQVRDLSAEWAARSDLPKFVEELIDHCPT
DLHPMAQFSLAVTALEHTSSFAKAYAKGINKKEYWGYTFEDSMDLIAKLPNIASRIYQNVFKGGKVAPIQKDKDYSFNFANQL
GFADNADFVELMRLYLTIHTDHEGGNVSAHTTHLVGSALSSPFLSLAAGLNGLAGPLHGLANQEVLNWLTEFKKSVGDDLSDK
AITDYLWSTLNAGRVVPGYGHAVLRKTDPRYMAQRAFAQEKMPNDPMFKLVSQVYKIAPGVLTEHGKTKNPYPNVDAHSGVLL
QYYGLTEANYYTVLFGVSRAIGVLPQLIIDRALGAPIERPKSFSTDKWAELVKKL
FUN_005103 CDS scaffold_18 136607 139115 +
hypothetical protein EOG092629WA PF00076;PF04818

MASAVADLETGLQAMLNLKPPGVSGSRITSLTSLCVSNIQAESVLIQKIFTHFKKTPGTHKLGVLYVVDSVTRKWLEQAKAQA
QTINSSAPDGTYAAGVNRVTELMPVLMNDILQSAPEEQKEKIKKLLDIWEKGQTFPTPMLESFREKLNAPQQNESTTPPGSPP
PNVLGSQHGSASAPATTSAPQAPSGTSILEALANIARQNTTAPPANPLPAPTAPYNLPPAAPPQPVSSATPPVSYPPASQPVN
VPSLPFGLPHMGAQNGYAGSPLNNPSNPPNPVNGAMPGAAPGLGLDPSTQQQIMLIKALADQGIPFDKIPALIQSMGGAGAAA
AAGALPAQQPAPYAQGPQIPGQTAWGGPSGKPEDSRMPGYGHTRSPPRFHGRSRSRSPDRGWGGRGSPRNGRDRPDYARDSPN
RGRHDDRRGGEYRQRSPHGHRGRSETPLHEQGSADKWVEYDPTLPSGHIKVYSRTLFVGGVTCSESELRGIFGRFGTVQTCIV
NKDKRHAFVKMLSRQDAVNAKQGTEDSRNLEVPLRTRWGVGFGPRDCSDYSTGVSIIPIHKLTEADRKWMLTAPFGGSGGQPI
ETGLSVEEPDIEIGAGVSSKAISRRMQTDKGGSNGPKSTRNRDEDLSRWRRSRDNGSNGHNRRDDHRDLAQDGQNMVASFPFG
IGTLPNGMPNFPAGFTFPDPTNN
FUN_005104 CDS scaffold_18 152918 156342 + PMA1 plasma membrane H+-ATPase PF00122;PF00690;PF00702

MAEEKAAGAPALDTNIETGGFDEKRGQAPPPTHAPKAPVAEDEEPDEDMDALIEDLESEDGHAFDEEEETQPGGGRVVPEDQL
QTDSRVGLTEAEVINRRRKWGLNQMKEERENMILKFLMFFVGPIQFVMEAAAVLAAGLEDWIDFGVICALLLLNACVGFIQEF
QAKTLALKAVVLRDGTLKEVEAPEVVPGDILQVEEGTIIPADGRFVTEGCFVQVDQSAITGESLAVDKHAGDNCYASSAVKRG
EAFVIVTATGDNTFVGRAAALVSQSAGGTGHFTEVLNGIGTILLVLVVATLLIVWVSSFYRSNGIVDILRFTLAITIVGVPVG
LPAVVTTTMAVGAAYLAKKQAIVQKLSAIESLAGVEILCSDKTGTLTKNKLSLAEPFCVAGVEPDDLMLTACLAASRKKKGID
AIDKAFLKALKYYPRAKSVLSKYKVLDFHPFDPVSKKVQAVVESPQGERIICVKGAPLFVLKTVEEDHPIPEEVDAAYKNCVA
EFATRGFRSLGVARKRGEGAWEILGIMPCSDPPRHDTARTINEAKRLGLSIKMLTGDAVGIARETSRQLGLGTNVYNAERLGL
GGGGDMPGSEVYDFVEAADGFAEVFPQHKYNVVEILQQRGYLVAMTGDGVNDAPSLKKADTGIAVEGASDAARSASDIVFLAP
GLGAIIDALKTSRQIFHRMYAYVVYRIALSLHMEIFLGLWIAILNRSLNIELVVFIAIFADIATLAIAYDNAPFSQTPVKWNL
PKLWGMSVLLGVVLAVGTWIALTTMYANSEDGGIVQNFGKIDEVLFLEISLTENWLIFITRANGPFWSSIPSWQLSGAILIVD
ILATLFCIFGWFVGGQTSIVAVVRIWIFSFGVFCVMGGLYYFMQGSTGFDNLMHGKSPKQNQKQRSLEDFVVSLQRVSTQHEK
SQ
FUN_005105 CDS scaffold_18 156844 157494 +
hypothetical protein ENOG41

MNREIPGYYYDNEKKKYFKIEKTHTAPSSAAWSSDVVKRRKVEHEAQKLAERQAHQVKKHIKRHFVARDTVSSALLTREIGLP
YVAESGRGRVEDEDLGAAAWARGLVAKGNIPFAPSFARERHANMPCFYVSGEDEKTGIGASYATLDEETLVGSYIPTDENDEL
VFEAGRSFNLLT
FUN_005106 CDS scaffold_18 159986 161213 -
hypothetical protein

MPPFAGSVDKDEDVPMAGNPPSESLAGIEEAAPLYLEYITSESTRNDLLSIMPGNNLTTYAFCAVAEGVFQDPCHDSIVPRYL
IWSYFKAESDAAKLEILEAITEYFQDPAPGKSRETFIVSPFDDNINFLVRYRAHSHGWSLAILEPSDLIRDVPAWLVHLLDRI
FDIMRLRIPYFEPKGFRWRFVHFGTDPSNTLFPLMFPRIEEVRSDCFWEFAGKESRSQLYGDGLNLVDCSESMLEDQFAGDEG
EGSQVQPRSRDRSRSPPSLRRSSRLKRRRINYSEVSDVNVDLRAKSSHDDKDDLHSDCEQESHLTSVEQSETVEFSSPEDAVP
DAYGKAPVKTRSTPPVPAFTEADLVFPLTQPDELKLAVLCAALIQVAQLQTLRLSGSWSL

FUN_005107 CDS scaffold_18 162853 164020 +
hypothetical protein PF00096 ENOG41

MPVVKPFECPECKKGYGSQPGLREHVNTAHKHKRHRCPYGCGKSYSQWNGMNAHVKADHENTVYPCPYKEKEGCKAKDFKQKR
SLNDHIAKKHKGEEGKSYKCPDCSKKYTQSHNLSKHVKATHEGVRLPCPYKEEDECEADFATPDGVKQHVKAIHDEWPRFPCQ
ANDCTKTFKREDYATSHYNRVHVDAPLPYSCGFCGKKFQFLPSKISHEEKHTQHACPRNSCYQGFSCIEEALEHAKDHQHRSD
QQLYECPLKNCRLAIIGKALDKPCLVKHWNMHIKREHISNELELVYKEAKQPLFRDIPILGSIMANNHSILLASASLDNAVPE
PEEDADEDDQGDEEEDKILVSNDEDINILEQNKVWWGM
FUN_005108 CDS scaffold_18 164238 165090 +
hypothetical protein ENOG41

MSLDSRCASCHSDMRVRQLIKRFDSPKIDPKGGLGALTKTFSTAISKTWTCTKDYERRVRQPVRKGLDRKVVIIDNEFDPITH
ELYETAIIDRVSGETLLNTLIAHTEETKSAVPSRRTQGEKVGIISQLWKKKVYGRSRGLALLDVHQVAKRLQECGITPETIFL
AWHVSCADLQILTGFLAKGGYRNILPKDRNCFPLIPLFRENVPKGCFPLRFEIVYPVMFPRSDLCGHNHRALVDCKQARNICN
GLDDFCEPVESRGKEWQPAKFVKEAQRCIGDYLA
FUN_005109 CDS scaffold_18 165622 167663 -
hypothetical protein PF11905 ENOG41

MASGPDTPGTVQSPAGFGPGPDDDSASLKRDADAVNPSSPAGGDDGAKRRKKAGPGSRGVANLTPEQLAKKRANDREAQRAIR
ERTKNQIEDLQRQIQELTNQQPYQELQSVLRAKEAVEQENADIKRRLAGIVAMLQPIIGQAGIEQAYVSPAQTYAPPVQHSVT
LAVHNNHHNNANAASTPGSVASPPSHDPPPSQGHWHHQGHHAPPQHHHSPHPPPGPPSTAEAQAASQLTQQRRNLQHGLDMGP
ERLGLEFLLEPSQRVNRIQNGVNGAQDTPQFRHIPMKHDWTGVNQERALHSRSASWGSQGKSGQPSQEQQQGQHQPPSHHTLS
AAHTPESSHSPGYSPAMSAAGGPVPRSEPSTTDPATPIKNCPPTCPLDSLLLDFLSERRQRAADGLPAQDVVGPRYPSVSSLL
NPANSAYSHPLSKVFTDILATFPDISTLPERVAVLYIMFLVMRWQIEPTEENYDRLPKWMAPCPSQLTIPHAAWLDHIPFPKM
RDYLVRDHNPLLHPFDNFFVPFTTTLSLSWPYEETDTLLQDPDSDELMINPVFERHLRNLDNWKLGDAFAKAFPKLVGTFNLS
QSTSPRPTSSSSR
FUN_005110 CDS scaffold_18 168998 172534 - NIC96 nuclear
pore complex subunit PF04097;PF13634

MSLFGQSTNTGGSSLFGGGNTGGTTGGLFGQSTAQNQQSGATGGGLFGQNAQSQQQQQSGTTGGGLFGGLGANKPATTGTSLF
GQPQQQQQTGNTGGGLFGQSTTQPASTTTGAGTGTGLFGQQPAQTQQSGTTGGLFGAQQQQQKPSLFGSSLAQPAAAAKPSLF
GQPQQQQPQPQQNAPSMLGASTLGGSTLGGSLFASQAPNGQSQSQPAGAYFDSLFAKSQKADGKANMEDLPSLELGLGDLRHR
LKKLQSKPSDKPLGGKAHYLLAASGVDPGVAAKDLGLLDVQAGRTERTTHGYAPSELDVETYLSNLQTKTTLSMIADGIDRSV
RDFDNFLEDNVTMEWEAQRKRIYQHFGISPREDVATSGRESQSAFGRSRRKSQAPAAKTGRASVLGRSTLQKSVIGTPSRIGA
HATDFSDVDRSSESGGLDGRVSSDDRVLRERQGRLSEKIRNLNTARLLKRPYPILSELAAVEKKSHEPHAPHLVEAYLAVMEI
VGENPDAETTLNNATAREREFADMYLNDNPNSAKAVEMRKRILSGANVFLEKQFLREVESLIAKHPHEAKLGGLPDIVSKVKA
YIRLRSARKDLVPDNTELQQVQGEFVWAIVFYLLRSGHVSEAAKYVNDNTNQFRGIDRTFATYLNNYAASEDRRITNRKLLDR
CTNEYIQRSRNAPENSIDPFRMACYKVIGRIELGNRNLDGLNTDINDWIWLQFNLAREGDKTIEMAGESYGLAELQSSIREIG
LKHFPKANSEDSNGSFGMFFYLQILSGMFEDAIAYLYPFSYVDAVHFGLALEFYGLLRPSDAMSPSNDLRSYSTKNLPQINFG
RMIGYYTRDFRAADVVSAVDYLTLICLNQDLGGEAGQRHGSLCHEALRELVLETREFSKLIGDIRPDGRRIRGIIEERGPLIG
LDAEDDFVNTVTLQAASFADENGRTTDSVLLYHLAGEYDTVVAIVSRALSEAVSLEIGEDPMRLMPVKPRAGGQEADAGSSLS
LAAIDDPVELAKTMMSMYERDAMFYRRIQDQNKVACRVLLEMSGIKGLVESGQWAQCLDRIRSLEILPLDAAGDGSTIRAYAS
KFSGLSQPVSINVPNLLMWTIICCVRQREQLTSGQFSGNEGTRRLMVDQLKQMTLDLTTYTSQLRYRFPPHLHEALARASAE
FUN_005111 CDS scaffold_18 173072 175091 -
hypothetical protein PF00370;PF02782 ENOG41

MDRPPQVLDHYIGVDVGTGSARACIIDETGDIKALASENIKLWQPETGYYEQSTTDIWQCICECVRRVVSESTVNPSSIKGIG
FDATCSLAVFTHDTDEPLPVTGPDFTNDGNDRNVILWLDHRPVGETEKINNTKHKLLKYVGGKMSIEMEMPKVLWLKNHMPPE
LFARAKFYDLADALTHLATGNETRSYCSVVCKQGFVPVGVDGSVKGWQEDFYHEIGLDDLTKDDFKQMGGVNGVNGTYVSAGE
PVGTLSRLAANQLGLPMGIPVGSGVIDAYAGWIGTVGAKVDLGDDELNAAVPHNDLAQAFTRLAAVAGTSTCHLALSKEPVFV

PGVWGPYRDVLLPEFWMAEGGQSATGELLRHMLDIHPAYNETCALAKAEDKHIYDFLNTHLELMVEKHNAPSISYLGRHHFFY
GDLWGNRSPIADPNMKGSMIGLDSDKSTDNMALWYYATMEFIALQTRQIIEQMNNAGHEISSIFMSGSQCQNPVLMNLLATAC
GMPVLIPRYVNAAVVHGAAMLGAKAASHAKEGSEPESLWSIMDRMSKRGRLVDPSTNEDEKALLDAKYEIFLDMCKTQQEYRS
KIDKVASKKARVNGAPNGA
FUN_005112 CDS scaffold_18 177392 180224 -
hypothetical protein ENOG41

MTDQGEKKGLWSMLRRASSRRHSTKPPPKDLEPREYHRPRRASNGDVGHAPFRQSIGSAPDLTYDSQRPLTAEDESNMRRRPK
RGQPEPEPEPRSSPIRSKSSGFLNKSRASNGSRPSNGRPVRARDPQSAWPPYGISMNDELPHQHALELVARGAPSHKGHKRPI
PHQPEEFYALYTTSSGNRGDGKSSDNTDVSNLHDSMTQLMTLRLIGHGYADRPWETLEQPSFAFFFGHLPGTITLNEWASMSS
VLPPTIALRDAGVMPRTMSLESIFDRLQELRHGIEDDDESLLYRVLYKRILRDPEKFLNPHKTLDKQITDLIMVLSRPDWIDF
SNPRNQVATRFIFDRSPGNEEQFHKFFHQLLLSLELELRIQSDQHSDWAKEKLTSQIPPSIQWNLALARRWQDFVRVDDVGKT
PEEILLRYKLKKRQIRMLRRFAQMMRWPNLADTLDNMKQKDSELALDEVSSDAFAFFSGLVLPGQTFPFLIMNTLLDLDPDSA
TDNLALLSHMHPHCGFQYKNSYTYWSATSIVGKVLAPTCHAVAGWIGPARPTKDLDPTQIARIRSRKPIQRMSPQDVESMAER
SDPLGPAADAYPVEDYQPVRPKGGPEDAIDTVRIELLSLKPADGPGDAPTAAGLSRLFDATVQFAIDGVSWPLKLMYDVSFVA
AWPCSDGPHPLFFDYVYTSIRVDSIVGVRDWGGLYGGPQLHSVRSSPGPGMMRGPYRDMFSQSFNHNHAPLNGFMQTGALEEE
DENDDEKVLVVEAFGVRDNEVLARAWCSHWGLSAVVADVRRTCMACAIREAYAATLTVVILVEGQEYPNNE
FUN_005113 CDS scaffold_18 181333 183750 -
hypothetical protein PF02656;PF09359 ENOG41

MRFGRTLRESTYPPWKDKYIDYAKLKSLLREDVADDDNQPWTEEDETRFCEEIFNNQLEKVAQFQEQRFNALKERVDAAFDKL
KELAPVESSEDDGAPQKGEISASRLRTLESELDEITNEVRELKKYSNINYTGFLKIIKKHDRKRGDRYKVRPMMQLSLSQRPF
NSETGYSPLLNKLSIMYFAIRQQLEDGGEQLPPLDLESQGETRNGERYIAHKFWVHPDNLLEVKTVILRHLPALVYSEQSAKE
LDGSDSPSITSLYFDNKQFELYGEKVNRKSEATSLRLRWYGQLATRPEIFVEQKTVDDKGGSQDLKFTIKDKYVKSFVDGEYN
PEKAIQKMKRQSFAPEKIEQFQETVNNIQKYVKEKGLSPVLRANYVRTAFQKPADDRIRIAIDTDVAFIREDTLDPSRPCRDP
NEWHRLDIDESEMTYPFKKMNQSEVNRFPYALLEIKLKEDSQRKHPTWVEDLMSSHLVHPTPRFSKFVHGIAALFEDYVNNLP
FWLSDLESDIRKDPQKTFEEEEQRRAQRHEDVMAVGSLIGAGAKSGSYKPTQSSPVSKSYLAERMSKDSIAQALNRRTSTPAN
GEEAGESSTQQRSEQERSYGTLSSVIPGFSLSKYSRAKRASQQPLPEGVVAPTEWIKNMGELKVEPKVWLANERTFLKWQHIC
ILQGGLAVGLYTAAGENTLASVVGLLFVLIAVFAGIWGYAMLRIRRTMIVERSGKDFDNMIGPMIISGSLMLVLILNFFFAYR
EAFGKFKDGQNSLGELLSEDLK
FUN_005114 CDS scaffold_18 185420 191866 - GLT1 glutamate
synthase [NADH] EOG092600T9
PF00310;PF01493;PF01645;PF04898;PF07992;PF13450;PF14691
C44
MGLNEDFDDRQIQTEAEQQPYIPYEYQTENNDSWAGALPVKQGLYDPSYEKDACGVGFACHIKGKPSHKIVSDARNLLCNMTH
RGAVGSDARDGDGAGVMTSIPHRFFIKNFEKEEDIKLPPLGQYAVGNLFFKPDEETLQESKRQLEDVAESLGLRVLGWRRPPV
DSTLLGPAAKSREPIIAQPFVVLASAYGTGNAPEMTDPEKFDERLFERQLYILRKRATQSIGLHNWFYLCSLSNKNIVYKGQL
APVQVYSYYHDLVNADYEAHFALVHSRFSTNTFPSWDRAQPLRWAAHNGEINTLRGNKNWMRAREGVMQSDIFKEELEQMYPV
VEDGGSDSAAFDNVLELLTINGVLSLPEAVMLMVPEAWQGNQHMDPKKAAFYEWAACQMEPWDGPALFTFADGRYCGANLDRN
GLRPCRFYVMDDDRIICASEVGTIPVEPETVIQKGRLQPGRMLLVDTQAGRIIDDKELKEAVSSRYDFRAWLDSELITLPKVV
EIMEQALDLAPKLDDKAIQADPLLLSYGYTHEQVSLLLAPMAADEKEALGSMGNDAPLACLTQAPRLLYDYFRQLFAQVTNPP
IDPIRESIVMSLECYVGPQGNLLEMDASQCGRLLLPSPILSIEEFNAVKNMSNKYSDWTVKTIDLTFPKNQGVQGYIKHLDEI
CNEASAAIEARDRVIVLSDRNTSADRVPVSAVLASAMVHHHLVSNKWRSMVALVVETAEAREVHHMCVLLGYGADAINPYLAM
ECILKLNREGLIKKKTTNETLIRNYKHSCDGGILKVMSKMGISTLASYKGAQIFEILGLDETVVERCFRGTASRIQGMTFELI
AEEAFRFHERGFPTRETILPSGLPESGEYHWRDGGEPHVNDPTSIANIQDAVRTKNDKSYEAYSRSEYEQIKNCTLRGLLDFK
FEDCTPVPIDQVEPWTDIVRRFCTGAMSYGSISMESHSTLAVAMNRLGGKSNTGEGGEDPERSQRMPNGDTMRSAIKQVASGR
FGVTSAYLADSDELQIKMAQGAKPGEGGELPGHKVSKSIARTRHSTPGVGLISPPPHHDIYSIEDLKQLIYDLKCSSPRSRVS
VKLVSEVGVGIVASGVAKAKADHILISGHDGGTGASRWTGIKYAGLPWELGLAETHQTLVLNDLRGRVVVQTDGQLKTGRDVA
LACLLGAEEWGFATAPLIAMGCVFMRKCHLNTCPVGIATQDPELRKKFTGTPEHVINFFYYVANELRAIMAQLGFRTINEMVG
HVEVLKMRDDLRTNKTANIDLSLLLTPAHKLRPGVATFNVRKQDHKLHVRLDNKLISESELTLDKGLPSRIECDIVNTDRAMG

TSLSYHISKRYGEAGLPMDTVHVNIKGSAGQSFGAFLAPGVTLELEGDANDYVGKGLSGGRLIIYPPRSAVFKSEENILIGNT
CLYGATTGTCFFRGVAAERFAVRNSGATAVVEGVGDHGCEYMTGGRVVVLGSTGRNFAAGMSGGIAYILDVHGDFHSKLNGEM
VEASGLEDPAEIAFVRGLIEDHHHYTGSERAARILVDFNRALPRFIKILPVDYKRVLEEEAAKAAEAKRAEYNLPAVSGVQHK
KSEKVAKLQDLEEAVGDNAAEKKRALVLDKTRGFKMYKRRQEKYRPVNSRLKDWAELSSRLDEDELKYQSARCMDCGVPFCQS
ETGCPISNIIPKWNELVFQNQWKDALNRLLMTNNFPEFTGRVCPAPCEGACVLGINEDPVGIKSIECAIIDRGFEMGWMVPQP
PKVRTGKTVAIIGSGPAGLAAADQLNRAGHLVTVYERADRLGGLLMYGIPNMKLDKRIVKRRTDFMASEGIIFKTGVAVGEEG
HPSLNDLRASNSAVVIATGATVARDLPIKGRQLEGIHYAMEFLHKNTKSLLDSELADNTYISAKGKNVVVIGGGDTGNDCIGT
SLRHGAKSVTNFELLPQPPPERANDNPWPQWPRIYRVDYGHTEVRQHTGKDPREYCIMSEEFMDDGSGKVKGINTIRVEWTKS
PSGGWDMKKVEGSQQFFPADLVLLAMGFLGPEARVLGDEIEKDARKNVKTAPGKYSTNLEGVFAAGDARRGQSLIVWGINEGR
QAAREIDLYLEKYTNLPVTGGITKRTAQEIFSQIKVEA
FUN_005115 CDS scaffold_18 198084 199190 +
hypothetical protein PF05841 ENOG41

MFTTLPDFTPRDSHSLWYRSSRSPYTPPGPLDDLSGDTHNIQHGANQARQRTKLIERSALARLAADEQYMQRRRLNVQNYGSG
WLKPPGVPKTLHQMREEKREQEEHQEAMRREQLAQELAEAEAEGMPGEGTMDDVQLDGAQDLDDEIPDADEEGYAFGDDDDEE
DDEEEDEEDGEETDDEALRAERQNDLMQARMRMGDDAFREALVRGEPDANDMYGDTEGLEEEEDNGHLLDEEDFAQDDDLGMD
MDADLDEDIPEAESGGYEHTDSEAEISSSEQESSNDDEDDDEDDVDVSFAPRSATLPRPMSPTFQGRSSMAHPRASMDLSNLL
SQDGSSFMQSSPAGRRARHG
FUN_005116 CDS scaffold_18 199582 200242 -
hypothetical protein

MTLLSRWWQTASSEPQQSIRDQKLTAHHRFGASSLHQRDSRSALFEGYTGDAASRRPVSASPNRGYGYGGYGAPSPSPLGQGG
FDASRPGSFRSATPNKRGQYSDAVLNELESQNDAQVEGILGKVKVLKDMTVAIGDEIRESSALAEKMNDSFDSTRLRLRGTMN
RMLVMAQRTGVGWKVWLIFFAAVMMLFIYVWLF
FUN_005117 CDS scaffold_18 200985 201453 -
hypothetical protein PF09649 ENOG41

MADNNATLPQDPSQAQGATFAEEKGKGKAPAEQMTEDTAMDEDDEDDSDDDDEEVPEADEEDGLEEIDPSNIVEGRRTRGAQI
DFAKAAEQHPADDEDEEDDEDFQPPNEDTKMSD
FUN_005118 CDS scaffold_18 203492 204116 -
hypothetical protein ENOG41

MRITTFLSTICAAVLVAAEQRTAQVYVQPILSSPSKPQILAEVAYDAASLVSTEIVSYEAPELPETASLVRVGIYDPKSSRWI
AGTTAASTENFDRGYAPTILLSVDESGDVRSVVLKGVRVDAGQTRDFGPKAVVIAEKKGAQPELNKPVVLSPNGRKAAEEEPK
SLLQKYWWLIAIVVVMSMAGGNEK
FUN_005119 CDS scaffold_18 204645 205746 -
hypothetical protein PF00134;PF08613

MLKTSPALSASANASPTAFRYATSSPQHSSKPSPALRRRPSTRSDTPSSADAMKSSPIQRPRQYVANDSGIHGSPEVQRPPSS
ISQKASAYPAQSPAPSRPSQDQTVQQGQALSGVSPTKRRSSPTPTNPDAVAATNSSPQTSKRAKPATPPPKVLPDRYELCAIE
DMVELVAHMLAELIATNDAIRISNGGLTRFHSRTAPGISVRDYLHRLARHATLTPPLLLAMVYYIDRLCAMYQEFTINTLTVH
RFLITAATVAAKGLSDSFWNNTTYARVGGVRVAELKLLELEFLYRVDWKIVPNPEVLVAYYKGLVERTPGYVLESDGSDDEDE
DEDDDAEDGENEPLDN
FUN_005120 CDS scaffold_18 208619 211759 +
hypothetical protein ENOG41

MARAQSHPILAQLRNARTLPEQTDALRALKNEIIGHVQRKEQWIGLGVLEPIVRTLASARSPAKPNGKDSRYPLLQRPLSEDE
NVRLQALQLVASFANGGPAFLAPLHAARAIPSLMANVSPFTNAPQLVVAALKALTDIADASTLAAPSSPIDVESLADTVFSVQ
YLDSFGAMLSITSTNILLQSQVSLAAGLISRLCREERHQQALATSGVLDALATRLASVAVLRGEVVPGADASAKNDGLFEAFP
EPAPQTLRLDSILEAIGAILGDSKYRANRLANSPSILAVFPPIKFEPAGNPDIEQTGPNSTRQHPVTAMEYMLPINLPRNQST

SPYGSVPPGGSSDSQSSSRSSLSKFSSSAIWDSPRFQTTGSSPEDTEEIESPFIPWLVRLVRRSGEYERLLASAILAALIKGG
VASKGLREASLGLLVVPLLIRMIAKNDKDVSDVNEVDNTVKRTILERAPVVLARMITDSEYLQKAAYECEAVPMLSRLLKHAY
TPVKEGTRPHYWSPHADVDMEVENNSPVAQLGQEGQNELLVHRLKVRESTLKAIAALAGGKEDYRKAFVAEDVVSYVVESLSE
YPRRPQSAKERGSEKPSAETTRKGETAGYGANPASVIVAGCHVVRMLSRSVSILRTSLVDHGVALPVFRFMKHPDVNVQIAAT
AAICNLVLEVSPVRELLAENGVVTVLCEHAHSQNPALRLNGLWALKHFVDAVGPDLKKACLAELGSHWLQQLVCEDTEDMALQ
LAKEREASGLDMDDDVDMQPSDEPHRWIYGSNGMLRELNAADSSRLRQVEDKLAAVRESELNPVRRARNDDVAIQEQGLDMIR
NLIGRPRSGLSPETTNETTEMIDFLLHEFGSERLFEILSSKLRPKVHRPFSRRVTSGREPRMFHPQSKIIVAVIYILTHIAAS
ISRHQQMIIAQTDLLKLLAQQASSKDREVRVALCHFIINLTYKDDDGEAQACAIRAHELRKLGFYSKMEALTKQDGDLDVRER
AKTAAYQIEQAGY
FUN_005121 CDS scaffold_18 213140 214127 +
hypothetical protein PF01036 ENOG41

MPAVIFARNDALDVNPQSGDARLSDAGSDWLWAVTAIYIVSFLAYFALSFKPRNNERIFHYLFTIALFVGAIAYFSIASGIAY
SVIPTQRNLGRALSYQIYFAKYINWVVAFPIIILALGLMSGVSWATILFNIFLAWIWIISYLCSAYTTTSYKWGFFGFGTFAY
LMLAFQTLHPGRTSAARLGLSRDYLMLAGWVNLLWMLYPIAYGISDGGNVVGVTGSFIFFGILDVLLIPGLAFAFMFLSKRWD
YGALNLHFTQYGRVNAGNGVFPEKRAPVAPAAEQPVPATATAAV
FUN_005122 CDS scaffold_18 220928 222621 +
hypothetical protein ENOG41

MATPSSIPAEPKRDVINRIHRVTRENRSLWYQMTVLQQPERARACGSGSKANSDRRPVDPPPVVELRIIEGPSVEEGKDITFD
YNANFFLYASLEHARPLARGRVNTPAAGNPPILTGVPASGMAYLDRPTEAGYFIFPDLSVRHEGLYILTFSLFETTKEERDYD
LEPADGDLPPGVDYRMEIKTEPFSVYSAKKFPGLMESTQLSKTVADQGCRVRIRRDVRMRKRESKPGAGNSNSGGNGFERREE
DFGRRRTITPASEDPHSIRNRSHSNSSEHRTPYTDASRRPSMVDSYPPPPPPPPPSYEPAPSASRHLDFGDSSAAQYPTPRQY
AHQPGLQITPGPPNGSYAPTAQSPYSKTDVPYGYVNRNIPPSCPSPAPSLKHELYDRRQSTSTYVPPSPSVYSTEGHHRRDSR
PSYPPTPVAAPHPRPMHSQTSLPALKIDQLVSPVSPLPPIEPQTGPAPELPPINVGGKRKHESVFAQSTRPLHNGQRQVDPHY
GRSHRGYSPDHDQGWYSRADGQISSVQFNRYYDE
FUN_005123 CDS scaffold_18 224034 225468 +
hypothetical protein ENOG41

MGRPKSRRALSASRFQEGSMNDRTSAAPPVQFLGPEERAALERPALTNIKSNNSRPMSDDFSDKQVKRGRLLGQVWDEVRGRL
GFKRDNEEERGARKRSRSRKRDQSEQRGRQRERENGSRDGAVELKTVEETPREDMPSREEILANYHNLMASGFFSSHAIQSTR
QPPPGSVQTSHHLSAPPHPTSLRDALSRATTPQDGIPRAPNYIAPSPPKSPTKIKHLQKHHLRPDGPGAATYEELVASGFFTP
PISDSAPPPTPIGAAPAPTVYTGRMSRDYLQPPRQTRGSRAATPSASRAPSPIRSPASASSRGTKRAAADSNSEDEGESASRD
PPTPKKKLRKSASRDITLPKLRNVASRRNMLSSRRSVSGPHGASKEYNTLARRVLGRLPGGGGRGSLDIDRRPASASRRSIED
SRQPLQEVQYTRILRSRKSAAEGLSVVPNANRGIPKVPNIPEKFAAYDEDAENTAPAIRSRY
FUN_005124 CDS scaffold_18 226660 230938 -
hypothetical protein PF00023;PF01833;PF12796;PF13606;PF13637;PF13857

MTPAPTGPGSPLIAGAAMSGGDFNDFPDEELGASNLFDFSNSPDTLQSFESLGGNDAKTFLTPQELTGAPLPDSPNGSYQDSS
SETASSKRTATPTSKAAMHPAEAMLEEDEMKVEWGNSFFNDDDHFVFGQNHDDASMEMYPFGEPGESILEHPYDMDTASSSPE
GLNTEQPALTSQMPTVSSKAPIKDATPKRKKAQGHQKASSQYSVSSAMNGLKTSASREVSPMSNMVLSHGSSPAPLFNSPSPN
TQIDFSRVVNGGVGAQHVWPNKIDVAAQPVPDSLPMHFSGQMNQPMPMPQQMPMPTQFNFNGRCQLKIMPTPLKSRVETQIPI
KMTLWPLPPGVTKLHLPPHTISKPKLLSKPTPEKSPDMLELHVSLVCTSAMDEPGVKEAALKRAASHPQGYLPPLPDNNSSQP
KGGDVRICQGCITRERKRAARKKIKKPEEEKVWLEDEERRVIVFNTQEVKDWQPPSGVMESTVPEGTMQVDAAMRIACYCRHH
GEKMGFNVIFTIKDFQDRVVAQAMSNPIMITDDHKTHPIAQVAAQQPAIPEPPMPSLPQASIENNVLLPPTTNGSFRMSPSNG
DLSNMHRSSQASYQSKASSTGNLGRTLSRPASPTLGGPMAKKRKSSASRVPNGLTMTRLDTSPSPGADVNQLSNATSPFTPNL
AAFSQADPMFGQQAMAFTTGPPTPSNELPGFYANHRSASMDNMAMAQLYSAPASGHPSRAPSPNGLRNSIVGNPQNQFAQALA
STFSVPTGMTPLRAPPVIHKIIPNEGPTVGGIEVTVLGAAFFQGLEIWFGNHKATTTTFWGESSVVCLLPPAPGPGPVPVTFK
NQNPQAGQAFLNTAKQPPTFNYLDDGEDKLIRAALSVLGHKMSGQMIDAMDVARRILDNGNGSHWSGGSDGAQGGSSSGGAMF
SHAPYAHLESQLLKCLDLIDLDDSMHRTRLDLKRPTGHTMLHLSCSLGYHRLVAALLARGANPDARDRGGFTPLHIASIHNHP

EIVRRLMLNGADPTIRSVSGLSPSDIAQSRAVIHAIRRSQRHARSRSSGGSGSLHSRASSATSLRSLWEPMTKIHSHEEPISP
DSSEESPEYTSGDFEDEDPDEDSYLSMRRPSGFRQDRERPNLRRQQTSEMEEELAPPATTMAALKEQFQQQVHQFQQFQQSIA
MQFQNFPHFPQMPQMPQMPALPNMPGYQAPFMRRVQQLMPGMAGPRADGEQPQRWWDSSSKAAAPAPPAYEEIFPQESLDQKH
ESAARAAVEAEADQKCAALFDQTTSTETESAEITEVTEVAEIKTEEPGVLKIGRKNAITKEQQEQFRRAHEVKMKRISSDHNL
FFIWIPLLLVMICGMLYSYFPWLFTFAWAFVRSLYQNGLTKTQQLLQQNLPERVLEGVEV
FUN_005125 CDS scaffold_18 235687 237351 -
hypothetical protein ENOG41
S28
MRLFSRVVTAVVLLQQAYAFSPRQVTHFETSPKSRLVTRDDDTTGVIGGTFDQLIDHDNSSLGTFKQRYWCSLNYANGTNPPV
VFISPLDAEAEQVKFWLHDDYVIGGMIARRIGAVMLLLEGRYFGQSSPYQQLTTENMKYYAEDQIMRDKIYFAENVKLPFAKN
GSARPAKVPWVHTGCSGQGNRVLFTQKQYPGTFWASWASSAPPQAISDYWRYFDAAKAYLPKNCTADVEKVIEHLDNVMLHGS
ADEIQQIKEDFGAPDLKHNDDFMNLLNYGPQSYQGASLRIHDTWEFCDYVENAVGVIDKSKLPGSGGVGLDKALAGYARWTKE
VWIPGKCEERGPWKGVTNTGCFNFGDADSLVYANKTLASSGIAETLQFQWLLCNEPEEYWQTGSPEGTPTLVSRLVNREYFRK
TCARYFPPGPGGETYGLAKGKTTESWNAHYGGWSDPSPYVKRTVLVDGLYDPWRAAGFASSQRPGGVLGNSTHIRHFLNPLGN
HCTDTFRNAGTIWPEVKAIQEAGIHQIEKWVAEFPKNR
FUN_005126 CDS scaffold_18 238615 239717 -
hypothetical protein PF00075;PF01693

MVKKRNAPSGGFGAGSSSKMRKAAKGTKFYAVKTGREPGIYLNYEACQAQTTGFPGAQFKSFLTREDAQAYIAGNETPSAADG
PPRFYAVAVGHVPGIYATWDETQPQVTDVTGPKHKRFKTQEEAENFIRDNASPETCRRLGISLEKPQEYTGAAFEPVEASTKK
VKAEPTPAPKAAPRAEPSREESGNLLKIWTDGSSLANGQAGSRAGLGVYFGPNDKRNLAERLPGEPQTNQRAELMAILRALQI
APSTQDVQIVSDSQYSIKCVTQWGLGWEKNGWKNAAGGEVKNQDLVRGVLARIKERDAARSKTSFQWVKGHASDYGNHRADDL
ANEGARKSPVV
FUN_005127 CDS scaffold_18 240715 242096 -
hypothetical protein PF00383

MDATQDNSAAGPSSEVCQTPEPVEASAPPKTEHRGPSEILSNPLIDDLKEGHTRRGVLIPLKTTQEVRQDHTITHAYITRAPT
KQANDVITALRNMRPDDNSANPLPHLRRCSKPGDLPAHLKTQFMNDSAVGRQIHTAKSTWIYIIIGEVKDITREELAEVLSIV
EGAETDLFIEKIPIPLLAPTSQVQAAMWSSQFWPTVYRKNNPLGPHPSIVARGTEDIKDDASVWMALAHRVALQAKETGIGEA
VGAVIVQREGGKVELVGVAGDARWHQECGLLEGTSNPMTHCVVRAISMVAQKLVRHERRAAGLPFTAPNLEYDAFQDGPLLEI
EKQCFEQEHPNKDGYLCHGLELYVTHEPCVSCSMGILHSRMGKAVFATHMPRSGGLSSDDRPDGGGRGLGLFWRRELNWSLMA
WEWERDGVPNLPPLDPITHV
FUN_005128 CDS scaffold_18 248628 250849 +
hypothetical protein PF00300

MHNLLGVSPPSPALPAQHSSAAQQTSSNSLPPTSNPSSNPTSNPTSDPSSNPNNPNNYAVLPPPADGDHDHLPPPANAPSSLA
VALRSLNDLTETPTYARSVTSTAPSSPRIPPLRQNSGHQTPRVRPHATTLNIPGMTRSRVSPDGRIPSRDVAAKLVIVMVGLP
ARGKSYITKKLQRYLSWQQHDSRIFNVGNRRRVAAGRRVSVHPKLQPEPEHMDPPVHAASILLNGNPAPFGPEQAEPEKLDLN
DAAQSEVDQSATFFDPKNQTAAAMREQCAMDTLDELLDYLLDGGGAVGILDATNSTIERRKNITNRIKQREPKLGILFIESIC
QDPTLLEANMRLKLSGPDYRDKDPQKSLEDFKKRVAAYESAYVPLGEYEEKHDLQYIQMIDVGRKLIQHRLKGFLSSGISTYL
ASFNLAPRQIWITRHGQSVDNELGRLGGNSPLTERGHCYGQALHNFITYQRKHWLIEQKSKKAQATFPPVPGDNTPPYPEMYR
EIEDKNFCVWTSMLDRSVDTAEYFEADDDYDVKNWEMLNEMNSGQFEGMTYAEIAQKEPEEFAKRAQDKLNYIYPGVGGEGYL
QVVSRLRDMVREVERIEDHLLIIGHRSVCRVLMAYFMDLSLADITDMDIPLGMLYSIEPKPYGLDFHAYRYNEDQGWFDELPN
YKPTKTARNSIIKPQH
FUN_005129 CDS scaffold_18 251707 253261 -
hypothetical protein PF00400;PF04158

MRTEALTLDVHMRSAGSPPKLPASRTATVNKINFTGPFDMKIKALSRSVSAQQAAGSDVTKQPRNLDSALHPFERAREYQRAL
NAVKLERMHAQPFVGQLGRGHVDGVYSIAKDPNSLEHFASGSGDGVVKVWDLADRDEIWHATAHENIVKGLEWTRDQKLLTCA
ADRTIKLFDPYNTPSEAAPISSWLGNGAFTSLSHHRSKNSFAAASSVINIYDLERHTAAPEILKWPTSVDTITDVAFNYVETS

ILGSCSNDRSIVIYDLRTSTPVTKTVLKFASNRLSWSPMEAFNLAAASEDHNIYLFDMRKFDRALNVLKDHVAAVMDVEWSPT
GEELVSASWDRTVRLWNRDRGHSRDIYHTKRMQRVTAASWTPDARYILSGSDDGNVRLWRANASRREGVKSARQRQALEYNEA
LIERYQHMPEVRRIHRHRHVPKVLKKAGEIKAEELKSIKRREENERRHTKKQFERRRGEREKMILAREK
FUN_005130 CDS scaffold_18 253432 254573 +
hypothetical protein EOG09263PXH PF01000;PF01193

MDGMDYDPMVMDGEPEQPQVKISAADHTHVDFELSKTNLAFANAVRRIIQAEVPTIAIDLVEIETNSSVLADEFIAHRLGLIP
LDSKGVDELNYSRDCDCEQYCEQCSVTLTLHAKCTSDEIMKVYARDLVVDGRHMNGVGSPVITDPEGYGCLIAKLRKGQELKI
SCIAKKGIAKEHAKWMPTSAVGFEYDPHNKLHHIDWWFENDTDPAVEWPKSKYAEWEEPPQEGEPFDYDAVPNRFYFEVETSG
SMEPDQIVQNGIRVLQQKIGGLLKGLDPRKYGGDEAEFDGPRSPDMNMDGGTTPWQDGGYTTPYGGGGGQTAYGGGMTAYGTT
PYGGSSWQ
FUN_005131 CDS scaffold_18 254964 255789 -
hypothetical protein PF00153

MRTGNAPKNMVGTFVHILRHDGPLGLYSGISASLLRQMTYSTVRFGVYEEVKTRLTRRNEGRDPSFMTLVALAAGSGFVGGIA
GNFADVLNVRMQHDAALPPAERRNYRHAFDGMVRMAREEGPKSMFRGWLPNSGRAMFMTAGQLASYDVSKSLLLQYTPMEDNL
KTHFTSSFIAGLVAATVTSPIDVIKTRVMSSAYDHNILHLIRDIHRTDGLMWMFKGWVPSFLRLGPQTICTFVFLEMHRKAYR
KVKGLDTNL
FUN_005132 CDS scaffold_18 255822 256073 -
hypothetical protein

MATSQEAMSKTTETAPVRTGKATADAKKTTIRYPFWFGGSASSMAACVTHPLDLVKVTLHKCLVIA
FUN_005133 CDS scaffold_18 256701 258220 +
hypothetical protein EOG09262N0U PF03147

MRLYAQGMRCLRASAPRAAQLSRSSILLRTAYAVRTYSSAPPTGSPGSVTIRDQTIKTDPQWFNVPDNVLDATTRKLHLLKDH
PVSITRQIIQANFPEPTYKYHNEFSPVVSTAQNFDSLGFPANHPGRALSDTYYLNSETLLRTHTSAHQADTFRANQSAGYLVS
ADVYRRDAIDRSHYPVFHQMEGAMSWDRTKVPNGDVAAAVWKDFEKLPVHGVKVDDPNPPMHPETNPLQDAHHTAAEAEAIGA
HLKRSLENMVVDIFSRAKAAAIKDDPNFVDEPLQMRWVEAYFPFTSPSWELEVYYAGDWLEVLGCGVVKQDIYINAGVPNQLG
WAFGIGIDRIAMLLFKIPDIRLFWSKDKRFLSQFEGVTDNLDNLKRFVPFSKYPPCPKDVSFWLSSTTAAGGNTKGTFHENDV
MEIVRNVAGDVVEDVRLIDEFTHPKTGRKSMAYRIVYRSLERTLTNDEAVAFHEDVRKALVKELGVELR
FUN_005134 CDS scaffold_18 261122 261884 +
hypothetical protein PF04847 ENOG41

METSPQHSRSSSSASRRSNLTLDLSNIPPMTPPTPPSNTLLFTNLTDPAIFLPENLQVIRDLITHSAPIHAFAPLKSFRRIVV
SFFDEQAAIAVRQVWDNEAIMGQQCRVYFGMPTPVDKRDEHLALPDAGKLFFISPPPSPPHGWEMRLEDAPNKLVHAEDLADA
LAKLHHRPGPMDEDQDSPVTPPDSALPGRTRSRSSTLIYKPEDSASTMPAVIVDDMTDEPEEVSPVEQSKPILAHTARPPVEL
MHHA
FUN_005135 CDS scaffold_18 262256 263836 -
hypothetical protein PF09995 ENOG41

MDCRYPSYFKDSRIIGGHRFKWTSGHKTTEELDHYLYSYDKLATDALDRLDLISPPTTKGWKCPHGSGPGQRDIYELLKKHAN
CDEILGQLWEEVSTVPEWVDWDQIQRGQQVVYQFSGQILFGLMYKSLLGGMGAYRIVETLSRTGGFGINVTRRRLLETLQHFM
EVVDDLDAIKPGGRGYISSVRVRLLHASVRRRLMQLEHQNPGYFDMEKWGVPINDLHTIATTSAYSAAIVYMALPRQGIYLSK
QQTGDYLALWRYVGYLLGTPVDWMATPQQAKAMLESIAVSEVAPSANSQILANNILTAEARAPPLNMPREVLAAHAYRLNGDE
FASALGIEKPDLRSRLFVWMQCTILFFISYSYPWMPAYMQKRQEQRFKDFAYFMIHNHKAGGLGESSTFEFQYLPRLGMLTEL
GVRKDFEAERTTSADFSKSMTISRRAVLLVGTILGAIAAVSLVRLVGVSNIPRHLLSAVLAQQSLVWAD
FUN_005136 CDS scaffold_18 265624 266659 +
hypothetical protein ENOG41

MSCTCRATPLKIFVQGLSQVHKVDASPSLLRLPIRTRPALYQNNFALARQARSLHFSKALQDASGQAARDDDPFSHIRSEDEN
RAVKNDDTFATDESPESIPWKAQPGTNNEAQKPRRRRRREEPEDFTTLNYRRNQSVRQQLEDDNEANEYNGNPNRRMKGPSSL
QPQPKESYWKIQKAALKEKFPEGWRPRKRLSPDALAGIRALNAQFPDVYTTEALSNKFEVSPEAIRRILRSKWQANPEEEEKR
NARWLRRGKDVWEQKAALGIKPPQKWRREGITRDPSYHDWKKEAVERNRVREERDDMEIRGDYTPRPRTYTPRSRIPRTGEYT
PRSVSRTGRGRS
FUN_005137 CDS scaffold_18 267193 268306 +
hypothetical protein PF00106;PF08659;PF13561 ENOG41

MPTQYPRPPQRESWFAPLSIDLFLKVLNTTLLHPFVCWIIPLCFRAQTVKWEAPPMVASIAWATIVTLFWMANVVNQRIAHGI
PREVDLSEEVIVITGGASGMGLLVAEVYGMRGATVAVLDVNEMENTEARGVTYYKCDVGDKDQVAKVALEIEKDLGTPTVLIN
NAAIVIGKTLLDLSLDEIDKSLTTNLLGPFYCLKTFLPAIIRGGRGGTIVNVSSVIGTVGAAQLTDYAAAKAGLTAMHRSLTA
ELRESHPEIRTVLVTPGQVSTPLFYGVQTPNSFIAPVVEPVDVAKEIVAAIDSGKGATVAMPLYARWVDWLNVLPVGVQTIAR
WAAGVDRGMKTFVGREGQKLE
FUN_005138 CDS scaffold_18 270675 272481 +
hypothetical protein ENOG41

MGQWWVDSRIEATVTRSYVASQLLPDEIERLDRPVAFGGEDLTERTYWESIRNDAPRLFLILVDLGVPDQIFGVVDDGWDDAE
LPIALEDVDRLLLTATRDEKVEKKFYQRQFHYLLKPLQRGNHVAYKDNEVVPLDVVEKLSLPTHKSHGNDKVRLPNIPGEVFY
RRRYVLGNGPGAMPVQDFLDAVHGIKGLQNEHMISYWASYTQHGYGYVLFTPAGDFTLKSFLATTPSQFKHLAKARRRELVMN
WILCLVDTLCDFHGKNQSHGYIKPSTIFFTNQNHVFYSDSTRLTPDNVILHTDKSSFDRERYDYAAPELWSRSAGATSPTGRF
PSDEHFGMVQTYDPSGSPTAMFIAPNPQLSGQQADIFSVGCIILELLSFLVKRTTSKFATFRSAKHKTAGRGGAVLDTSFHKN
LGQVEAWMSGLAKDASRKSSSSKDGANILKGVTPMLHVVTGMLAMNPYDRPPAIEVQQRIYQILTEVCDISEPHCVHQYAHDL
ESSFGGLQIQTVGDGMMGVSPPGGSNTTYGTPRTYEHSRNGSSGGYSQVSRTTGSSETDMDAIHSVGSVGLQQIRTQGSWPRN
IAYTQHTGVAQYPVGQWDTT
FUN_005139 CDS scaffold_18 275054 275563 +
hypothetical protein ENOG41

MRSPISLLILLCAFMALACAAEVHISNNRAPTSQFATHGVASQLPSRDRAIYAQDRARQIALSQMIGSSAKISIVETASLNVK
AFAHGGMSFLFGLASSSNDVLDYINNGTNSVFSRSVTSEEGSGASGRKHVAYTSIALVLVMLFFAVWM
FUN_005140 CDS scaffold_18 276200 276487 -
hypothetical protein

MCIISGAVAVGIATAIVVVFGGFTIDGHGSGTTRSSQLHETADIFLGIDVVPPPPSSGSTRLLEKNMTALSRVRRNH
FUN_005141 CDS scaffold_18 279288 282281 +
hypothetical protein PF08558

MAEPGQPELNAGMSPTGIKKEPTNEADILEGLGLDLDSIMQAATKRPFEDESNGDDPSKRVKTEAEPPSENALDLPQGAEEEL
EDGLALLVQNALSNVSDFVFGTSNDPMEIDGITDHNLPPPPPPPSPPSVSFFSDPQKYLRKASRHALGNLALSVLLLFSQSFD
DSIKPIQDADSQHAKSFRDLQASFAQIKQIYSSGPILSADDPDLHDHESRTILDLANLAQFGIWLLQTTPSYSEAHQHFHAIF
RGQVSDLGSDALDLYLSLKTQVAIDALAAKTQEQSKEQVLEEALINGMEEKLRGLHNGFDLTPADSEFITSVRSRKTSLEGEA
STESAVLREKYKVDDLLRQVSTCVKTRLALMSDLGSRLGHPMRSSEETAVDLLGDTTGDPNLDLDDLSSFFEKTASGLVQDAL
AGLTEDTTATPAEANPQPEQTPTVTETIEPPPAVEPTKSPEKEPTLTPQTNGKVECTQQDYKELEALVAQSTDHYVKTTLHGL
SPVPYQPTVPTSTTQTYLNQLQQQQAQNPYYASYTQSIPEPQPPPQEPGHQLPPHQTCSSALLYDKARQAALSKNASHTRREG
IHSTRRPWTQDEEKALMAGLDLVKGPHWSQILTLFGAQGTVSDILKDRTQVQLKDKARNLKLFFLKTNSEMPYYLQAVTGELK
TRAPTQAARKEAEERARMNSEEDQAKIQGIMTLAGGLQNSPQGQGRAAGTPASAVGSGVMTPAQNAAQAASSQQSQAHNQAAA
HSYNPATSSGYPPSTLPQSRPSVISATQQSPHQAAHPQSQMSMSPQTPRSQQQQLPQQHHSAMHSPAHAQAAAQARSHTSTPV
GQLHHSPPPQHTPTPQPATQHHPQHFPPAPHTQLTFPSPPPANATPNMHAHGQVPVPDTQTQMDQHMHDNAAEAALLQGLQA
AVAESM
FUN_005142 CDS scaffold_18 286096 286762 +
hypothetical protein ENOG41

MSRRCYVETSPDGRPQLVTIKRSRSYHHSHRHPCDYYKVSREEWRTLVRQNELLDEANQAYASQNETLRSRLHSSEAETHRLC
HVVIPSLNDQIAKLSHENECLRRAAEKPCDPPVHTYHFELEKLRAKICRLEKENKTLRDDNGDLRFRVRELSKQIDQSISRRV
ADLAKQIEYWTNQSGFWKKKYDELRERHLGLITIVESKTEKTEYYDDVLKRRRVF
FUN_005143 CDS scaffold_18 292092 293301 +
hypothetical protein ENOG41

MFNGRLSSSLPQPTNFTLPFVVQDHDYEYEFDIESELRDDLQKANTELNKANAENDKLSAQNDKLKATIEQLTRKLNESKTSL
EETTRKLNESTISCRELKAQIEGVRSEADVLREDNRRLNTDFEELKGDKRRLNNECEELKEEKEALKEENERVKFEHHDLEQR
FIGLQAKHENLTMRHNAVSSQAPSSAMTSPMSSPMPSPLPAPLQEGRKNPSRPSSKASSKASSKVSTKSSSKREPREDRHREQ
EDRHQSEREESSREEQTRERRHREDRERRDRDREDRGRPQRSEKPKDKERFEAKERLSKRFDERPAVAPRQASFIEPWGPGGW
SPRYDAPPSDFDAYGHMATGRKAASVVSSGAYSNVPRTTSIVNGHFSSDSSAYDDERYEDGSYHAYPIPN
FUN_005144 CDS scaffold_18 293799 294630 +
hypothetical protein ENOG41

MKLYPSIPDDLAAWVQQQPVFFTGSAPTHGSHINVSPKGLSDSHFAILGPNQCAYIDRTGSGCETIAHSYDNGRLCLMFMSFG
PAPRIARFFCRSRIVEWDDPAFPDLVRRISKGKRSTFHGARAIILADVFEAQTSCGFGVPRVKRGIYAPDETSEKNLSLEQVL
REGVDGKVNELAVFEERPTMDMWVAKRVENNTLLEYHKETNVLSMDGLPGLRAARRSVGETLWFTDAKAHARKVFAQSEAIAV
GFVLALLLYVVMVSVGAISASWPRIQA
FUN_005145 CDS scaffold_18 295687 297840 -
hypothetical protein PF00076;PF03467

MSSQAPQVLSRRANGGPQTSSNQTSEPTKGKGSHENSSHGKARGRGSNARSTEANKHANESETPKPTKTKAHNEGEKLVIRRL
PPGMTQAEFVSILGSEWELGKGKVDWFSFAGGKISTDPSKPSRPGRAYVHVMKKDDILPLSDAVRTAVWEDAKASFNSPSLIG
PPSVEFAIYKKVPSNKKRTDARQGTIDQDPEFMAFLEELANPAPPKETVEGEEGDDLGKVETKVTTTPLIEYLKEKKANKAKE
SSSSKNSKHGQDSRSAKGKNSSKDEDSGKKKGKESKAEKGEKTPKETVKILTKKATSEPNAETSKTNAKSQPNDSSAQETAPK
SRRAGILAAARILQRDLGLSPGSAHRKARQDAAKAEADAKTPEKESTSTAEAKASASESPSTPVKSAEAEAPEPKGRGAANAN
KQQQSNRRTRGGKNAEKSNQKEKGKENSKEKERVKESTSSQPAPNPPIVLLKKKATDAESNPPAKPANTTTQAPAAQGGRSKA
DNAAGGSKGGPNKGSNAQKKPPTVSPDATRGFVKHANPSQGVTEALLKQTLSAFGTITFVEIDKRKGFAYVDFAEHEGLVKAV
TASPIPVAQGTVQVLERKDKKPASANANAATTAPAPVAGSAAPEKASGRGRRGRGGGNKVKDSGPNNQGTAPPPPPAPANAGG
FUN_005146 CDS scaffold_18 298379 299738 +
hypothetical protein PF00149

MASGVPRPGPANLGPNAGLDEWLEEAKQCHYLPERAMKELCEKVKEILMEESNIQPVCTPVTVCGDIHGQFYDLLELFRVSGG
MPGESNVQAPKTSTTVITSDDIEPPTEITNPKLRKKIKSSGENATSSGAEETEAVEAEEDPDSTMADQTESGVTVNKSSQSAD
TRYIFLGDFVDRGYFSLETFTLLMCLKAKYPDRIVLVRGNHESRQITQVYGFYEECQQKYGNASVWKACCHVFDFLVLAAIID
GEILCVHGGLSPEIRTIDQIRVVARAQEIPHEGAFCDLVWSDPEDVETWAISPRGAGWLFGDKVATEFNHVNGLKLIARAHQL
VNEGYKYHFPENSVVTVWSAPNYCYRCGNVASIMAVDKDLNPRFSIFSAVPDDQRHVPANRRGPGDYFL
FUN_005147 CDS scaffold_18 300066 301203 +
hypothetical protein

MPKPKGSKSNPIQNAINEWIGSGQASKTETDQEWNAQLLNSAPKRFTIYEPMVLLPTGSFTSLPWTTTLQDAGPDSCQQLWSQ
ILAQLSTQAKEPLTHLAVNEGIPLHKAGEEADENVRRSPSGLRTLCGDFGSSDASADEPSEEDFENTLWVSTKQNGIFQTWAP
SWTMFSRGNVKEKARLLDMGNKTLQGSWAVDLYAGIGYFVFSYARLGMRVLCWEINPWSVEGLQRGAKANRWSVRIVQGEDLA
LLNEDIVNGGEQIIVFLESNEKALQRIQSLQGNGMVQDIRHINCGFLPTSEPTWRDTWDISSPASETWLHLHENVGANDTETR
RQEIQRKLDDWSQGTDRAAKAEHIELVKTFAPGVWHCVFDVHVYKI
FUN_005148 CDS scaffold_18 301367 302031 -
hypothetical protein ENOG41

MRTGVLLSVAFATTVFAHGGAHSQKPVVDANADWMTKHMAEEHHVQGWDADSFFTLHDYNSDGWWQAAELMRTYGLFDESNKG

MTEKKKDEVRDVLLGLLDKDGDSSVSRQEWMDFIKSGKTLPDLNTGPGHHGDDEYEYEIHHWEKYHDDNTKLEDLTHPEDIEH
FKKHDEMEDAQERLEAMQKLSIVEANIPQKFRRQ
FUN_005149 CDS scaffold_18 302699 303894 +
hypothetical protein PF05460

MSRQIEQALLSLMPTYGSDLPPALVELAGSLLAQSRHQASTLKADEEIARTYACANIACDRLKITLDLPPIEPRPPIPPRIYK
RLYTHLDNILRGNSTPTRATPGRTRTPGSRFRDADNSPASGSRPLPSRATPTKEQSLAQFRTPTKGATGTPTKSTRKKEAFAG
VNLHAWIQPTTRWVCAETDHKKLAPTILAGMENIITPAGRRTDDEWVLQNLTALFAAIYFFVTMRVKALASGEGIDREGYVPL
RKEILALLARARNEVIVKDLAEEDAWEGWSTVKSKDFDDAVAKVNERAWLTGDWYQGIADVVKLSQRSHLEDVEMQDEEAIPK
MQIKKADTMFQDKYDFLSEAKRTKYMHWKEDMFAKIALSTTQGAAMEVDT
FUN_005150 CDS scaffold_18 304007 305411 -
hypothetical protein ENOG41

MAVLNLVTLALALSSPAMARPRPQFGNGGDAQTPGAGLGGALPTVLPSGGSGLIPSGLPSLGGGNGGAIPTPGSGFNLPGSGS
GNDAPATTPGTGGGFFGGGRGGGIPGLGNLPFFGGGNGGNGGNGGGFFGGGGNTNTPPAAAPSGGLPGGNDGNTGGDASVPAP
IQTPGTGGVQGASSGFRGRGRSRNQDSPSTPGGSGNSEGGNGDGLSGSQPSGAIPTFVAGPGSGFGEGGNGGNGGNGGNGGNG
DATEAPVAPTLVPTPGSGLEGPGTAPSGVIPTFVAGPGNGFGEGNSPGTPSAPIQTPGSGNQPGGGLGGSQPSGTIPTFVAGP
GNGFGEGGNSGSPDTPVQTPGSGNGSGNDNGNGNLPGGDQTQPSGVIPTFVAGPGNGFGGDNGPVATPTPGAGLGGGNNNTPD
VPETPVQTPGAGSGSGSQPGGAIPTFVAGPGQGFGGGDGQNGGFQKRARSSQ
FUN_005151 CDS scaffold_18 307078 308093 -
hypothetical protein PF00106;PF08659;PF13561 ENOG41

MSDKAQSRINAIGKQLLPPINKVAPGSSTNSPLGIGRATAHQYAESGARALYLCDFDDTHLESHKKEINAAFPKVEIHTRRFD
AADEDKVKEVVDDAIQRYGRLDVFFANAGVVGRTTLFSDFSKDEFMSILNTNTSSVFLAAKYAAPAMMQTSADKPQASGSIIG
TASVAGIRSNAGSTPYSASKAAVVSLAQTISYQLAGTGVRMNAICPGLIETGMTAPVYEAARARGSEKKIGQLNPMKRGGHAD
EIARVALFLGSDESSYVNGQAWAVDGGLSAGHPFVPGKLG
FUN_005152 CDS scaffold_18 308424 309577 -
hypothetical protein ENOG41

MPSVIYDTPTALPLRKPAPFVIGPTNKAPFRPTNKIPPDVIETAQATPIEEFDAKKHVNFEFPKRTYTMKEWGYENQGVSPIA
ASDPFSLFTEAAVKQVRRELLSEDVLRTCQYASTFTKNQIRGYTQKQAPFAHALWKSPEVQQAVSKVAGIDLVHAFEYEIGHC
NLLFSDGEDEKKGASDNVGFSWHYDSFPFVCVTMLSDCSDMKGGETAVLKGDGEVLKVRGPTMGTAVILQGRYVQHAALKAIG
KERISFITAFRPKSPFVKDEMVLTGSRPISNQSELVYDYCTYRADVLEIRFREHAKKLRELQAAGIKFNPDAVREFLQEQKEM
LEATLLEMVPIYDVVEVE
FUN_005153 CDS scaffold_18 312814 313341 +
hypothetical protein

MHTGSATRAIQALSQTFACVGREYVSMIAGEAKSPRVYLKNAFKTTYIRFGIFFILSSLRVDIILPYNDPTLVGIVNGVDGAG
TGAASPYAIAMGNLGIGVLYHVVNGLPVSSIFSSGNALTYYGTQSLYSLALKDRPPNSYSSAHPSVSLFTV
FUN_005154 CDS scaffold_18 313963 315473 -
hypothetical protein EOG09262Z0M PF03367

MATEDTTKQTPEEFFQSIGDKVNKLAPAAVDAQNADDDERAVEEIESLCMNCGKNGVTRLLLTAIPYFREVVIMSFSCEHCHT
QNNEIQAAGTVQPKGTHYELRLTDLADFSRQVVKSDTSTVKFIELDLEIPAGRGQLTNVEGLLTTVIEDLEMGQEARKEQAPE
VYVKVAEIIAKGRAMLMGDSFPFRVWVDDPAGNSFIAPDLKDGVGKWEKHEYARTAEQNAALGLSDSDANAQQPQNPGLTADG
EIIPNEVYSFPATCPGCMHPCTTHMKMVDIPHFKQVVLMSTVCDDCGYRSNDVKTGGEIPEKGEKIILEVSDGADLARDILKS
ETCGLECPELKLQVNPGTLGGRFTTVEGLLTQVRGDLHSQIFEADGSGQGGDSLASDEKSQWTAFFDGLDAAIRGEKPFTIIL
TDPFASSYVQPLVDPPAPDPKIHRENYTRTDEEEEELGLKDMKLENYGEQEEDKKGKEGKETETAAER
FUN_005155 CDS scaffold_18 315740 316301 -
hypothetical protein PF04032

MAVSELPGTIDFLTDAAHLLRATAPETSAHLMSQRGHLLQQYGVILSDVQRQHVCGGCGIIMIPGQEATLKLDARKSLHRKAK
SKKSSGNSTPQPTEDLQGPCKILHCDNCQRDTKIVMPPPGPAVRRKTAQSKVKKVSTPVDQPKQTLNASSKKRAKNRKAGLQA
LLSGQKQQTTNPLSLSHFMK
FUN_005156 CDS scaffold_18 316689 317389 -
hypothetical protein EOG09264X31 PF05907 ENOG41

MFALYLTADLQGVTNLRPDDTQDNPFWYMFKVQCTSCRETHANHVGVNRFETNEMSGSRGEANFVWKCKNCKRESSASVKAGP
FPYEQTEPAKPQKILEFDCRGLEFTEFRAEGEWLAEGVETGTKFTGIDLEEGEWFEYDEKSNEEVSINDVKWEVRRS
FUN_005157 CDS scaffold_18 317729 319922 +
hypothetical protein ENOG41

MQALWSRAGQVHRCGCRACESVVSSLGRRVTTAARPRKVTFADIFTACYSSMFATAAIVDAVRKEDRRQELDRQLEETRRELA
QLQLRNLEASQTTNGIDSAQNEVTLHQMDQLWGAIKSLYTNRPYMKEIYNPATIRIDEFLNRVHTEEYECPDQATMDALRRTD
YDRLEQVIMREETDVGIRQRTPLNTKQLHNAGRTLSHLVQQLLKRANAHDKSNLPSPSFDEAMQLANDKTSFRYLVENDPERV
IKNRVDLNSDLRKVVGSTLSLKEKVGRVCYNLLVSAYPADMHTYNTLIVAFDKHGYKYLSEPVVNSFYYYRRLRPTPSTFVAI
LNHYKNSGNHGRFLRSLASLAGLDGETGAKLRRRLADETYDGAVDERRKSYKVQTWTQTGDYFWEHAPLDKPLIEAAIQGLLH
MKLFDQAAEFFVTCMKAKVVLSTHVIRQLFDECIVALDWRSAVRLIQGFTDCSITWPSMLLGRDQDTSYLISRVRVLLDLAGL
RDASGEVPTSTLDNLSISRYDFYKFLSDLASADLTSQIDMQGSSAQTRDSASDPVSASKRRLLQIEALWKEQDLVGKTTRSIE
SKLLYPEFSPEFRMSMAFHIGNAAADQTVELDGEIMEVMSQLPLSERVERGITECKSFQGSNNSRAESGNIIEEVQELRRDTP
EEQGKAEAGRFLRDEGLDIISAHKATTVKRMNANAAERTLRARPMRLTAWPVTEQQVGYLQRASFG
FUN_005158 CDS scaffold_18 321285 324989 +
hypothetical protein PF00023;PF03009;PF12796;PF13606;PF13637

MKFGRNLPRNQVPEWAGSYINYKGLKKLVKAAAEKARNGEKVDPAEFFFALDRNLEDVDFFYNKKYAEFCRRLNLLQNRYGRT
VDVVATLDQDEVEEVMGALLELRSQFRNLQWFGEINHKGFVKITKKLDKKVPDLVTQGPYIDTKVKVKPFAKQANTTRLLDEI
NKWMSVLSEAQTFDDTMSEHSTRSLGRASAKGMLSLPQAQLDALDQAVRNDDVPALEAGLKTSNVIEEGAQPLMLGLLQRSIS
SRSKTCLAFLLSNLKSLDEPDDINARNCIHRLVIHIGRTKSVPEGEQDSKSRPVPAGSQFSNKHLEPGLATSKAPKSLNQKES
LLLTKDDEAVQLFIFLLEQLRPEQRVALKSRDSFGRMPLHYAAQFGIVVVCQIIMSKMQQWDQFHVKDGIDTPEWQDNDGYAP
LHLSVVGGHPLTTQALLQGENWEGSSPYKAEIRKSISKSGAVLAIATKSNYSVIVQLLIDAGVDINWRDKTDETALHVAARFG
HEECARTILKGTTDQKADLEATESTYSWTPLHVAAVDGKYNVAELLVEAGADITRLDSSGWTAREHAALRGHMDIARLLETND
VEIESPPTRPVDTLQPTPSDMSSIGERRSNGNGINNGSRAPDAAIKSFGHRYLTNESLVLVSLGSMDMRKNIEPVSLDRVPLT
EAHNTQLDTALSIVVSATGASGEPTIIDLPVHDSIATEPVVFTTTDAGKVKLLFDIVPTYSGNDKHKVGRAVALLSSVRQTLG
SSRMNLQGDVCVPIMSSSFDVIGTVNFNFLVITPFHHPNMEITSRQTYWKKLESTMVIGHRGLGKNLTSNRSLQLGENTLPSF
IAAANLGAQYVEFDVQLTKDHVPVIYHDFLVSETGIDAPVHTLTLEQFLHINPDKNRDSKQQSRKKPAPTGEPGDFRARSNSL
TPKRSQSMGFAGSGPDELDERMKHTKDFKDKGFKGNTRGNFIQAPFATLEELFRELPQETGFNIEMKYPMLHESEEHEMDTYA
VELNSFCDTVLSKVYDLAGERHIIFSSFNPDICLCLSYKQPSIPILFLTDAGCCEVCDIRASSLQEAIRFARRWNLLGIVAAA
EPFVNSPRLIKIVKENGIVCFSYGTLNNDPEMVRRQVKQGIDAVIVDSVLAIRKGLTSGETPTEPVNGENGTNGTLKPDHELK
EKLDELTINGGGP
FUN_005159 CDS scaffold_18 338366 339779 +
hypothetical protein PF00096 ENOG41

MELMELVENEPTARPFQCDWQSCTKSFNRKSDLQRHYRIHTNERPYACSIPGCGKSFIQRSALTVHIRTHTGEKPHQCQHIGC
GKRFSDSSSLARHRRIHTGKRPYKCAHDGCSKSFCRKTTMVKHQRRSHQQGMNPNDIDDCSSDSDDDESPSTPQHSSMTWSPH
EMVSMNQGAPSGPLHRASSYADFESQVHGHHMPQHYGNRQGIPASVPHEYHGTPVPEQHAHVQLVHRAATMPRQAYYVTEAGN
PGVATMTSTVPPHYQLSQQVERPPMELPYSAPGIATSIQSSPSTFSATSVPSPMIPEGFYVHQPGSQPAYATAESQPAMIQYQ
QPIQHHMAQPQQPVVSQAQHIPSTAGHYAPPSAHPQQEQWPHYDPPIEVTTIGQLPAYGTAVYDIYGPKMEFEDPSLQLPSSR
LASM
FUN_005160 CDS scaffold_18 341273 343908 +
hypothetical protein PF00004;PF01434;PF07728;PF17862

M41
MAYNAMMLPNMAATTTELWPSITNIAMAPWRHMTSVTPSRSVPSKNGVEQTKVADAQRVTELQIPEFLLQVDFGSLSRSRFSP
MTPMTAQTTLSIMDRVGLRAANPILRHPQFQPRVLTQSSFLRSFSQSSSGILSSPRGISTLGAFSVQRRAFGSSNGISRNQLA
TLEESANRNPGNANAQNAFYQLLLRANMPAILVERYQSGRFASNAATNDAYQRALTMLGVNANQAANAQGAVNGNFAGTQWPT
YEQGIANAAVAGSANGGNPMGHKGEPIHVIVQESTRSTIFRWFKFLAIFIVTTYLCFALVTIAIEAFSTFRRGGPSSKADSEV
KAEKQNTRFSDVHGCDEAKEELQEVVEFLKNPEKFSDLGAKLPKGVLLVGPPGTGKTLLARAVAGEAGVPFFYMSGSEFDEIF
VGVGAKRVRELFTAAKSKSPAIVFIDELDAIGGKRNPRDQAHAKQTLNQLLTELDGFDQDSKIIIIGATNLPKMLDKALTRPG
RFDRHVNVELPDVRGRIAILKHHAKKIKVGPDVDLEAIAARCPGRSGAELENMLNVAALRASRAKASVVRKQDMEWAYDRVTM
GSERKSMVVTEKEKEMTAYHEAGHALVQLFDKESSNTLYKVTILPKGPSLGHTAQLPQMDKYSYTAAEYMSNIRVALGGKMAE
ELRYGDDKVTSGVSSDLERATDLSFMMVTLFGMSSVLGPVEYGRRYENLSSETKAAIEGEVQRTIRKSYEDVRKLLTDKRNEL
DLLAKALVKYETLDKAEVEKVIRGESLPGRIITPKGPMTLPIPQQAPTPPGLGGVTHPQPPETPAPPAAADTSNTEG
FUN_005161 CDS scaffold_18 344789 345976 +
hypothetical protein PF01073;PF01370;PF07993;PF13460

MTKVLLTGGSGFIAAHTLEQLLEKDYAVVTTVRSEDKAQKIRNAFPDKAKAGKLEIVLVPDIAKPDAFDEVAKTPGLDAVIHV
ASPFHYNITDPKKDLIDPAVIGTTGILKALHATAPGVKRVVITSSFASILDEAHFTDGSHVFSEKTWNPVTIDDISSSFMTAY
RASKTLAERAAWDFVAEKKPSFDIVTVCPPLVLGPVSKHLATLESINTSNERIVKLLRGGWKDEIPPCTPVPLWIDVRDAARA
HVRGLEEPNAGGKRLFATAGWFSHREIVDAVAKNFPEFKDRLPGPEVKGGELPPKDQIFKIDTQETEKLLKIDWISIEQSVTD
LVKSLKEIGI
FUN_005162 CDS scaffold_18 346261 347611 -
hypothetical protein ENOG41

MSTKTKGCSGHSWGFIAPDVQDPPLCISLCRERFLKELVPGDETIERVCEVLQDRDWMEKEQPFRALYCCDAQACGVDNLGEG
GKDPNVNWLINTCHDHGYHSIIDPGPPQPYHICDPESLNDDKDCQQVKGTDKSQDASSQTTAGASTAESTLQATTKPTTLETT
SVPIQSSIPETTYSTTTDPSTLATSDPSSSNSNETNKGMPLGVKVTIAIISVVGLLAIAALIFCLLRRRRSRKTDIRRLIKHP
SSPPPRADSPTPLVSPTISHTDPDGVPLTPPARLPERRFLADEPNGFPTSPVYSPTSSKLSSRHERTLRIYSANQVPMIVMTA
PDGGKVRDDGSLSFSDGIITPPPSAHIAPLGYNTQTSPPRPPRSRDASFNMLASPGPPPTRALPSTPPYRPSTPTSPPRKGTM
PL
FUN_005163 tRNA scaffold_18 350173 350255 - tRNA-His

FUN_005164 CDS scaffold_18 351188 354620 -
hypothetical protein ENOG41

MENTDSDKPRPVAPPEPISPALSSGAPKPPTSGAASPARVPTPDSALVIKVKSPLVESAPVNVATTGISAAPAPRAASPPAAM
PPSPLPKASTPAPVTKDRAPSPANAHSPSTANGRSPVAAPSAAISASPRQVTFPLSWVVALSFSILKPPQTHQNIHVVLDCFA
IMSSRPQPVKQTNGRKISIGDADVESELSEPASTVHSPSGVDFNELEDEIVVGARTNGIRKSSPLRPASEADDEIMGDAEDEP
VASHYPKRKRNSIFHDLSERKMEPVRSLADERQPQATTTKSKPPRLSTGSVKGVTIGYWRDSEAPTVDLKHAVIGFIDVRERL
RTRIQPTTLSGETISDEYPLPPGPGGSWVTFDKIVFLDHLVGLDQLQVKEYVKIRAEAIGTDELEGERVAAEKAAVKEAVRRA
NLNSTAEHGTNLPQVAYGVDLPEHLQQNRDAKRRRTSGGFAPANPPPSNGPVIEHTPIQPAPGGPQPLRHTVDPLPGTRPTRI
LIGHWAKSNSPDPRDRHAVYGILGQNDMFRVKLVRETRDGRFVEGNFPAGAGALWIAYEEVAFEPHLKSLARNEIKEYCRVRQ
YQIDMGEKEGERTSNETKAVYEAQARAAGTNYKAPAPLSIAPALPRLPGDYGEESDQTGRMGYGGHELRQSRRVEAARAEARQ
SYIDIDTPPQPAPAPRQTTGRTSLNNNAIERTSALAEREIARVELAQERAHLQAANRERAAAAAAQAAQAAAANIPGVAVNGR
QQLHERDEMQRLNKIWARQESLRLRTNAEDAKMYAGVKYERKTQGPFTGKLVSQGTIINIDGEDYIEYRVLAKPSFF
FUN_005165 CDS scaffold_18 358879 360241 -
hypothetical protein EOG09263AZP PF12928

MAFDDDDNPSTLAQPAKSTNGAATEDVDDDAPDVNLFLSMFDKHGVSSKAIRKGEKDFESHGTRAQDGLLEASRTVLDNVLGY
TRIHREDSWVRGWCFPDWWAKVEEAGEKEGLWLRDRVVMVEHERGTWQKDIGRAVPAHKERLGTGKVWLLPEEAIFLIERGTM
DLWWPNRQLEDILPLADGKPKTDMGPDDYDAGLPLSLEAAYSLFIGNEGERGKITLPQYQVYSHLKRAGFYVLRAPPYEAPAP

THQSKTLWQWLFSFFNSESQPKRNPSGPLVQPGLYRAYRPIYDQLAILPRHKPLPTPPIVHPPQDPWRVFFHVWKSGGAPFSK
KNPPPPDFRFAVVDASDTCVPTLEQIEALLESTPYDPPNEAWKGPGGRMYQRLKHGHRNVLVAIVDRGLVNFMRFGEGAFGEE
RLFKRFDNQGGQRGGPKTGRGGGRGRGRGRGRGRGR
FUN_005166 tRNA scaffold_18 360484 360582 + tRNA-Leu

FUN_005167 CDS scaffold_18 361360 362725 -
hypothetical protein PF02752 ENOG41

MSGPASVRVSGPPNSSFLVGYPGISATLPRIEGKVEIRPGQGFSMPVPVSLVRICLQRRETIHPDADSIAKRHLGAPRRETTD
LVGKEQLLFRCSSGKEAERIIAMDLPFVLFIPFGRGGEESNRRIPPASLQLPSRTAETYYELVVTVQQGHSNQYKYTFPIPLQ
RYDTLSTFGMYNKPESKLVTNDNIVHLGINLPRWSYGPSDPITVYIKLSPNLDWLSKAKRVTIEKITLTVEEEITFNPEGDEP
TKKINRISKQVQPVKTKMPEAGYATNVGLTFPSKDLRDPEGVVRRGKPQFPLYEVTSFTTTSTLYKIEFYLVIKVNLSGARDV
MIRQPIVICPLDQQACKEEMDAIEQAAKDASHVDPNNPMLPAPTVILANDRDSLRALGLCMVGGQKKPFIE
FUN_005168 CDS scaffold_18 364618 366583 -
hypothetical protein ENOG41

MARKNGGNTKKDSGRRIRRQTEGTAIAPRLGQRPGRSPANREINLRLRRSQRLQSARPQLFPAPPHMGEVAAAEGYSAQSQGE
QTAASTSSPDTSAQMSSAAHEPSRARKPIPPDWRPPLAIGESLGDNLSRLEEAERSVLQWEAIVENARITQPLADLSGLEQQL
HKARQRFSQMEDTDENLIPVDELETTKRQRIQQRIGLLEASLERSECPVGDTNIRSAIRAYQSGRIKCWDKWTLLYAGNVVDF
CPSYESFTLDRDERLDRYHSMYGDGWLWYEAPLAPNGNYQPEQLMAATWAQPSGSCSALTDYHSHAWSIHMGFQRVKGFHSRF
ISRLNDPSPMDTGKVLPYETRMREHGSPGRGTCFVEDDSTAPRVCFHMQLDSGATHPSLHKSDMAYLAIDRKTYPAQTHTTVA
TANSTTVAALYEIRVDICRHNGESLVGEEPVFPNARRQIGGIVPVMVLVESTTDQSEPLAEWYKEALKNGEDVSEEAMAARYK
GQDESRLSGMLPFQVCYFSGAPGAPTFWFGEDRRDVLGADRMPGHQRWERHKKAYIVKRPAEFEDLDRPTVIFEHKGNGIRLV
DMDSKEDKGTSILSVERQVTLKAGETPQKKTLPGKGSSSKSGSSKRKRRQQSWLGR
FUN_005169 CDS scaffold_18 367228 371727 +
hypothetical protein ENOG41

MAGPMDEGSASRIPGLNLAGNPPSEDAPALASSSPLARRSDRIRDLLEIILLTFRFEFERLPPEAQQSYTRFQENKNYNEFAA
TLKRLVLGRRLDDFMGGSANCLIAATKLFDSRHPHLGLKKLRDDLESGRKRAGSGVGSRRRSPKSPRRAQKKALRETRNEQAG
ESSRRTSRANLASTLTLPARIRTIARPEGRLLHWTRVEASPSQAGPETAGPSSDIPSIHIDPAEPADNLEGQSAPTKKPLIPG
FVISTRTGPVDQGSNKPAVERSGREKLRQEQNIAWLKDYVEALGGPKYDFNLYPYKGWKIEPPEILLHRARNFLADEDIDRTL
NWGQKIAELTEYFSYLLHNWQGEDWGAELHEVIDMLRTHWTFEQYHYGKPRLNLKFDSMWPNYSKRLPPIPGVETTVERPRSE
MGERRLLLNKIRPAIDVHYKLPGDVLEKYIDLYAFEAPYLWGSAPRSQSSLHELVKIENNAFEKCVFSGMPTTDKLIRASEAK
FKSESDRKSFSPSALQAFATLRGSQRAALQQTLNHLDNSENLAVCNIFRTLILPPPRIPEKPDAGILFPTMQVADVPEKESRD
PFSYTVAHRWYLEAERFWKNWSYEHAVKECYGHKRWEEREEPIMDLPKNWMGPFVYDFLDHDMKKTLELLRRCETLLRRISLQ
NERVPRPFLADMTQLMLEGLSGRPWDETGLDFKGEDFVSGSQDLAHIRPLEEDFLRLLGMKSIHSNTVDPKINHDGISPLSAI
FEQRVKDIMYPSGSANAGSYDFANFMVSLNEVCDGPVKRWRFSNEEALEELELLDQRGVIKLNEDNSVSCSVPNLHPEQRVRW
LDTDDDLDAYMQAVQATRGGQPDNDDDDSDDSDDSGDSDDEDEIMTISTDVSVPETEDLGNYMFGLPKIENLRSWVGLGTVSG
REASEKDMSRNHGFFQRLCFRLGRTIRELQERMERYRRQLTPERQGRNRDFLDTMVSQWESDTRTRPELPQYNPNVPLDNYAR
PDYAEIMCVSQPADEFGDIGYPEITYGCQNRSYRFTVDVVRKAILREAYENKSMLFPNRIDRSVDEKGLRLRSPRRREPVWSF
AHPDRRGKAPRFWDINRWPLHLQSESTAESIRSGTFYRPAPSPLAPEPTKAPRIPVRVATPPVSPISSVLPTVQRKVHREVQP
EATTMAPDMTESMDTSESQVHHFPGLGEEFTVTFQDLADSRRTFTTGSSQYLLGDTPLQKKYIEDLIKSGIETEEPRTWTQRL
SALFGRKNVPVDPSALPRVNPRDIPQSKPRSEPEVSDDEPAEGEGSSVGSTDVEMDEAAMQLWEETQAGPSSERRSFRAQAPS
PIVPTFLTEPDSSSVSPPEPGTTQAESSSAGTWGQGLSGALPKRLRLRQVTTEIQSHPVELEVIEPESELPEEQEDQESDQDS
LFDL
FUN_005170 CDS scaffold_18 372112 373093 - RPS2 40S
ribosomal protein EOG09264903 PF00333;PF03719

MADRGASRGGGFGSRGGDRGRGRGRGRGRGRGKNEEKEWQPVTKLGRLVKAGKINSMEEIYLHSLPIKEYQIVDNFLPKLKDE

VMKIKPVQKQTRAGQRTRFKAIVIIGDSEGHVGLGIKTSKEVATAIRAAIIIAKLSVIPVRRGYWGTNLGQPHSLPCKESGKC
GSVTVRLIPAPRGTGLVASPAVKRFLQLAGVEDAYTSSAGSTKTLENTLKATFVAVSNTYGFLTPNLWKETKLIKSPLDEYAD
TLREGKRY
FUN_005171 CDS scaffold_18 373719 374789 -
hypothetical protein EOG09263FGN PF00581

MASIAFCRSARAGIPQWGSKVFRPSLLTRRNFSSYLVTPKELHEALKKNPPSPISTDPRVIPLCASWFLPNDDRSGIQTFREQ
RIPKARFFDLDKVIDKRSPYPHMLPNPKGFAAAMSELGIRKEDIVVVYDSKELGIFSAPRVGWTMKVFGHDKVHVLNNFKLWV
DQGLPTESGELYSIECYPYQIPEMEENRVASFQQVKEIAQDHNKEGAEGVQILDARPYGRFTGEAPEPREGLSSGHIPGSINI
PFSSVLDPETKAFLPKDQLKKLFAEKGVDPQKPIISSCGTGVTACVIDTALEEAGVGSPDSRRVYDGSWTEWAQRVQPSENLI
VKTTKE
FUN_005172 CDS scaffold_18 376870 378610 +
hypothetical protein ENOG41

MYSYPTPSPEFTAQSQSQDGLHNRPVANYPWAVQGGLGAINTNVRPQYGYSESPLPMSSSTNGYPSYDMALLSQPTFSAHPSF
SSAGEWPYPRPQHGSSSQTFVPATKTEANKVLIRRLGWLWLLLKARSKLRSLGRRHQRRHSASDYTSRTAGHNNGHYLGSSLS
VSFADDAALGNLVTGDEADLFSTIDPTNPVWFDNALGIITGDGNSLFPVPADAPRQATVLFEEDVPELEPGQKPTECQDETVP
LVNLVNQPQQGIIVPGQSKKRAFDCVELDDSSTDEAEGKSVDKKRSKTTSTAPRFACPFYKHDPERYENSRSCCGPGWESVHR
VKEHIFRSHTLPEHECQRCFQCFKTERMLSKHLRASAPCEVQSRNTQEQEGINASQSKALHARARKCNPGADLKEVEEERWND
VYKIIFPGGGQVPTPYYDRLQKSNNDDFDTNLMSDFEQRMRGKVGEMKNLQPTLVPLVVQVACNALMESMQSCRQKPQDGSSQ
CSSSIVSQAQEFQSFLDGGLQMTGINENFLRCLDKQFFVQALRDSGL
FUN_005173 CDS scaffold_18 379229 381031 +
hypothetical protein ENOG41

MSGIKHDDEMATWDFSAPEELISFLKNAPMLSIEASDSDENKKMCRTESSETSWSTGGVSTDSGCFCSAFNTNESCTHVPSEE
VYGSWFDFSDPANLLPLRPDQWIAELYGSQQNPQAVYSSQVCGSPTLESVPETRVSLPASSGPSDCGYSRETSADSAETCWSS
DQASSLSAVTERKKHQLVVQIIARLQEWLEVMLRRHGAQNGQTASSSGSTPVVGAPSRAGEPSNSQNRRKRQRSGSDDGFGED
DKTPDRTQKKRGKTSDSTETRYVCPFFKHNREKYKTSQWKSCCWPGWTSVHRVKEHLYRRHMLPKFRCNRCRQDLKSAFNLNE
HQRANTICQRQSEEPEEEGIDEEQERLLRVRKRKNGKASQVAEEEKWVEMYKILFPHDDPIPSPYPELCPLQPEQDTEHPGAN
VLDNFEDYARREFSRRMRPRIESLVDGILEQTLTSQTITDVANNVLQGIMESFRESQSQETCQPDSQKSPSRSPSPHGLPVES
SNRVPQEIHTSETGPYSNLEIDLDEILNSLDANQSLEFERWGIEDEGINTFNHFGLHVEQYNKANA
FUN_005174 CDS scaffold_18 381347 383007 -
hypothetical protein PF00069;PF07714

MNQSQLDPLPTDLPFRIVSKTVGRGAYASIKKAIPLDAPTPVFAVKLIHKGYAVKHGRISTKQLAMEVSLHSHIGQHPNIIEW
FASGEDDIWRWIAMEYAEGGDLFDKIEADVGVREDIAQVYFVQLISGVSFMHSKGVAHRDLKPENILLSQDGSLKLADFGMAT
MFEYKGQRKLSSTLCGSPPYIAPEILACGRADKKLPNAAKYSPDLVDVWSCGVILFVLLVGNTPWDEPSQGSWEFQEYVRTSG
RSTDALWGRVPAEALSLLRGMMSIDSSKRFNFTQVRQHPWYTRHNALLNSDGRVTDPINLATQMLENLHIDFNHHPTSQPSSS
DNMDIDTGLNVGKFSSTQPETPIADKDWDWERPPLRSMASPASSLPHTHDASRAMLDTLADEPSMSQFSQTPGPSMTLTQQAR
RFRDICPPESLTRFFSAVPPAHIIQMINDALHHLNVPTTHISPNPHGNPVAKIKVKALDGRQQSLHGEIQVDRQPLPDGTEVL
DIRFVKVKGDPLEWRRFFKKVVVLCKDGVYVPES
FUN_005175 CDS scaffold_18 383892 384540 +
hypothetical protein PF00179

MEYNMEDSQNSAPGSVQAAKLNSGHKGPDSQSTTKRLQTELMQLMTSPAPGVSAFPGADGNLLSWTATIEGPDDTPYAGLTFK
LSFAFPSNYPYAAPTVLFKTPIYHPNVDFSGRICLDILKDKWTAAYNIQTVLLSLQSLLGEPNNASPLNGEAAELWDKDTEEF
KKKVLARHRDVEDE
FUN_005176 CDS scaffold_18 384972 385513 - RPL31 60S
ribosomal protein L31 EOG09265EOF PF01198

MSSKKPSGKTQRSAIADVVAREYTIHMHKRLHGVTFKKRAPRAIKEIKAFATKSMGTSDVRIDPQLNKKVWEQGIKGVDYRLR
VRISRRRNDEEGAKEKLYSYVQAVNVKNPKGLATVVVEE
FUN_005177 CDS scaffold_18 386211 387402 +
hypothetical protein PF00615 ENOG41

MAEHEPVTTMASPSPQPDMTRNRLPTLFEVLSRRTLPPVDLFSFYIYMRDQQRSVDYLDFWLDVAQHMSLCRHYVRELRRSVL
IGTPEAQSKRSSAILENIGDLEPRTAGPSMYATEKEKNQDAQMSAFLREDQSHDSPQSASAPVRPSPQFSNSHEVTTESNSPA
HTVARQDIRASAEKILYTFLLPGAEREITLPGSITAEVTTAIEEYGRDDPEVFDVAKDYVFQAMERDAFPGFLRMKALGNLIP
PTLIMRLILGLIAMFAALWAAFVLIFLDYSRATRCWLILPFTVGVYFLASYQYSLDPIMALVGYSEYTPFNFSRIREPYVRKL
IAKRAIMVLAVTVLIDAALCVLFILVPGKRL
FUN_005178 CDS scaffold_18 387885 388766 -
hypothetical protein PF01399

MEQTKALNALEPFIALSKSATSPRAAADLVTRATSAPNTFLFSDLLQTPAIQNLADSEFASHLKLLQIFSYGTYSSYKTTEGL
PALAEVQATKLRQLSLLSLVRDRQNLSYAALQEALDLPGAREVETLVISAVYAGLLHATLDPARAVVQVSSVAPLRDLAPGAI
PEMATALKKWSDRCTTTLDGLDLQIKEIRTIAAVRQREQRLADEKLQQLMNEQQEGIGPRKYDLPNMTRDLGRRGFNKRSVMD
TGNLVDNDSMDVDEAEEEKKRANKRKL
FUN_005446 CDS scaffold_19 410 2738 +
hypothetical protein PF00067 ENOG41

MIEHYPSQALSGLTLAIATILLLAWARWRSRPTLPLPPGPPSEFLLGHSRVIPKENAAAVYAKWSKEYNSDIIHVRSLGRSTV
VLNSADVARDILDKKGANFCDRPRFTLLKIMGWGKTLTFLPFGERWQMHRKLLQTSFSNTNVRQWHNLQITEARRTIRNILKK
PKTWETSLRRLAVAIVLQVSYGTQVLEDDDPYIQIANDAMYATGNGGVPANSIVDLVPFVRYLPDCIVRDWSLRFARQWRWAI
KKLHDVPFAAAQAEYSDHHRNTSLAHHLLREYKDKEFRGQEQQWSLDDIKGAAGAVFIAGADTTWATCVIFVLNMVLHPEIQE
KAQQELDAVIGTDRLPDFSDRPALVYIEHIVQEIYRWSPLAPLGIPHKSLHDDIYKGMLIPKGISEAKVTWWGGYLISTRTVV
YANAYAMAHDERVYKNPHDFNPDRYEAGEPFPVGNFGFGRRVCVGRFLADNSVWITVATMLSVLRFCKKMSSDGKPIEPRVRF
TNGGTCHPEHFDCVIKPRSLEAEALVKSD
FUN_005447 CDS scaffold_19 4801 6509 +
hypothetical protein PF02133 ENOG41

MAVLKDLEANHGLHLSEHSRNNTENGESASVSTAGNAKAGVVSRWYRGVLDAGVEENGVRPVPIEQRTQTQHSNLFTVFFTCL
LCILPLPTGALGTAVYGLGLRDVSLIIIFFNIVTCIPPAVISIGGCKTGMRQMIQARYSFGLYLGIIPILLNAGTVTGFTLVG
SIVSGQAIAAVNETANISVNVGIGIVCTLSFCLAFLGYRAVHVWQRWQWLPNLLAIVIAVGYGGKHLMNQADHDPASVESVVG
YGSLMAGYFMTFGGTVSDFTVYHDPKSSKLKIFTYVYLGLITPSTPLLILGAAIGGALPNVESWQTAWDNYGIGGVMAEMLAP
AGGFGKFVLIVLALSVVGNMVLSNYSVALCLQMLVPAFAKVPRFLFIVATLAIMIPMSIYAAAKWEESLVNFLSVIGYWAGCF
DAVVIEELIVFRNMDYHSYDPAIWNQVRRLPTGLAAIGASLVSIGLVVPSMDTPWFTGPIGERIGDLGFESAFVVTGIAYYPL
RTLEVRLMGHV
FUN_005448 CDS scaffold_19 6801 7599 -
hypothetical protein PF01872

MSELTFPASSAAMLESRLPPSTTASGTSRPFVTLTFATSLDSSLSLAPGVRTRLSGPDSKAMTHYLRSRHDAILIGVSTLLAD
SPALNCRITGATSQPRPIVIDPHLRWAPSSSDKVLEVSRLGNGLAPFVLTGVSSQNLPAENVALLEQHGGKYIHVPTTLGPNE
RMRFDWHDVFRALHHAGLSSVMVEGGGQIINSLLDPQYHELVDSVIITIAPTWLGQGGVVVSPDRLQDPAGVPIPAARLSNVS
WHPFGEDVVLCGRLHA
FUN_005449 CDS scaffold_19 10284 11624 -
hypothetical protein PF06839;PF13532 ENOG41

MKAFLTRPKRKPSPELQKEIAEPEESTEVKLALLSSLHPQLDQETLLDILLAHDGSVSEASASLQVQAPVKKGTRVIGYQQSL
KQYATPSDSTSLDTTPKKKKLKSKAGSTLHLYDPEDVAEQTPCTIIHNFLPSEEANELLQELLEESKSFEKITFQLFENVVSS
PHTSSFYVESYDEIERQKTEYHYNGGRLTDVRRITPQLIKVKPKVQEAVNCEIQKRIKTRYPGGKKLKYQSPKLWVPNSAFVN

AYTGPQQNVGWHSDHLTYLGPRAVIGSISLGVAREFRVRRIIPRDSDTKTVQEQDAEGQISIHLPHNSLLVMHAEMQEEWKHS
IAPALSIDPHPISGTTRINITYRDYKANLHPRFTPECRCGLPCVLRAVTKKKENFGKYFWMCYANSPGQENCGFFQWAEFDDD
GNPLFRKGAGTKT
FUN_005450 CDS scaffold_19 12033 13193 +
hypothetical protein EOG09262VOC

MAAMLEFRTQGYNPYAVKYSPYYDSRIAVATSANFGIVGNGRVFALALTAQGVQVEKTFDTNDALYDLAWSEINENQLIVACG
DGSLKLFDLGIDDFPVMNFHEHKRETFSVCWNPITKDTFLSSSWDGTVKIWSPTRNHSIKTLPIGNCTYSTSFCPSNPALVSA
VSSDSHLRIFDLRTPSSAKYHLTATIPVHASGPQPSPGASAPAEILTHDWNKYRDTVIATGGVDRVLRTFDIRNPNGGPLSIM
QGHEYAVRRLSWSPHSSDTLISASYDMTVRLWNDGSNQQQAPAMGPTIGSQMGIMNRHTEFVTGVDWCLFGMGGWVASVGWDE
RVLLWDANMLMGQRIP
FUN_005451 CDS scaffold_19 13580 15673 -
hypothetical protein PF00270;PF00271

MFRSSLRRCATQARSAATAALLTQTRTALPAVRRQVLTSQSSIAAISRIASITRAYSTEAVAEQNESAPSVEAEPQVRFADLQ
GIHPNLLKPIINDMKYDTMTPVQAKTIQPALKGTDIVAQAKTGTGKTIAFLLPLLQRMIEEDSTLADRTARRQARSDDIRGII
LSPTRELAEQIAVEARRLVAHTGLVVQCAVGGTDKRSMLQQTRRQGCHLLVATPGRLNDLLQDPGSGIDAPNLAALVLDEADR
MLDVGFERELNEIIKCLPRPEEKVRQTMLVSATIPDSVIRLARNMVRANDFEFVQTIPENESLTHDKVPQHIVPVSGWAQVFP
SLFELLEREAAKIRETPGAMPLKAIVYFNTTALVELAGELFYQQRQNARNDGSPYFSSFVMQSKLSQVQRQRAADRFREARTG
VLLSSDVTARGMDFPNVTHVIQIDTPRERESYIHRLGRTGRQNKEGQGWLIIPNSSVRNARKMLQGLPIQQNSSLTSAETDVA
GGETTPHHESTKALFGTVPRSLLATAYMSMFGMANDKVSMAEEVNEWTRLGWGWEKPPSVSHSWAQKLGLANIRGMQLSEPGQ
RFGDRSGGRSDDRSDGFSRRESSDPFDSMASNVRRDDFGRGRSGGFGRGGSSGGRSGGFGGSRSGGYGGNRSGGYGGGRGDRS
SGGGRSGGYGGRSSF
FUN_005452 CDS scaffold_19 16223 17260 -
hypothetical protein ENOG41

MASHLRSFAPSLDEIKIDQITESRIFNVVSMLPNSLPGPIRFLRRSISFHTLRPGVTISGPQTKPRPLSEADVSPTMAQPLQT
DMIKHSGGFVESQDLNPQPMSRVLYSEAPEDEPVMRAASGVHWKFARQGTNLVNISIDGGKAAVAEEDIAFERKAFVDGVTYL
LKALPQELDEGELRRIQCALPEQVNQSDMALARAEPDSNRIHPSQSRSIIHRGVQMTVVNLIFFLSFLMPYLLLLVRCAARLE
RKYKISEKVVGHGVDLVNTIGKQSALLTEAIGQMNDGKVAQALLEVFVWTVDGVTQGISDGLGEGLSMVGSKPKMSG
FUN_005453 CDS scaffold_19 18207 22160 -
hypothetical protein PF03941 ENOG41

MAAMRGPRIPTGSAAWVADERASALEIVDVEVDEFTYAAHNEFSWLNEHMANIFNENETNIAETFKTPGRLRGKTPRTARKLI
TEPRVPLSNVFGGTPSSSANRFAQHLLLQSPQRELVNQAKSSSPRRHAPSPKTTPLTSDEPVEDAPEQPSNDAEAIHSDQDAH
MALEEESDPIAPAKSQPAMQGDSGYFGSQDVNPPSFDPLEDDDTSDIDPVALDEAIAFPQPSPSRTSLAKDPIKTSSPIKTQQ
ATTDVDMEEAQAEPQEDENAIQDDSQSQSDRHSPSPVRPGFRNSLQFPSLPAREPLTAGKSIGARASRTSHVDPKRTSYLGRN
TRGRSLASHAEQSASDESDQDEMDVDDFPTVPQQPKNAEELAIHHNKTYTQMLQDRISVLGKSQPNGSRPSKSIHSLTNSQQP
QTVSQSVPEPRSPSPRKLETTTTPGAFPEDDDDDWIVPPTTQQPVTAPSPTRPSLSKSRTADVMEGIHGKQTLGSFDLGEDRD
NIRSPARANKPQRPAYLGHSKSVSVPAVPLIPASDPEEGSPLKKTISVTNASVRPATAVAPSSSSPSKSPTRLFRDSPLKHLK
NKMSSILSTSKGLIASSAALSAEGKSSLLSPSRAQLGTFPDPSSDSIVPKASIDSHRSGDGVTDQQSTAPRPVAKRTRASVER
EKEEKRREKEARVQAEQMEKLEKEREKEREKARVFSKEQEKLANMEKQVVARKEVETPAPKATPKPTRTSPRRAKPQEEPEAK
VADQDLDMGDAPALVPPPSAHRSAIPGQLSRPKEMRRIAKPGREPAAKTKAAPTVHIKVNTGSQLHPGNSVASGYQDFGASTS
SQPPQTISKASKASTQPKQSAPNFGASTGSNGRPRALDLAAQKKAKIERDRAALKDDKKKQEAIRRQEEERQKREKNAQRQAA
QDRLAIIEKAKKTPAPPPAVRSQPNGPPQYSLQQDKSASVQREGPPRPPSRMNSGIFRSQDDMGRPVNANLTNGSKSGPKRPL
RDANEDGPSRPPPACGPQYQAKDAKRRRTSDDYADELDMEQPPNIKGPPVRPSSALKRDLQTKTLTSGYVSQASAQNLFKTGV
PQSQMKGGNPMDMAQIHKGTIPFAPNPNPAGPSYKTPGRPGPVNGMKSAAKSVPRSSPRFQNGEAIELPEIQTDDEDEDDDEP
QAGTVAAWANSPYIRDALFRQEDIDPASIFGPPAPLNMEEVFDKSKDRHHKFRARTSSANWSGTDRLTEEDIRKNLAARDKLR
REGGWSYELSKDMM
FUN_005454 CDS scaffold_19 22774 26534 +

hypothetical protein PF02463;PF06470

MRVIEVIIDGFKSYAVRTVISGWDESFNSITGLNGSGKSNILDAICFVLGITNMSTVRAQNLQDLIYKRGQAGVTKASVTIVF
DNRETKKSPIGFEEYATISVTRQIVLGGTSKYLINGHRAQQQTVQNLFQSVQLNINSPNFLIMQGRITKVLNMKAVEILAMIE
EAAGTRMFEDRRDKALKTMAKKETKLVELRELLKDEIEPKLEKLRTEKRAFLDFQQTQNDLERLTRVVVAYDYLRYQDSLSQS
AADLEGKKQRRRDLEESAARLKSEISHLEEDVKKVRAQRDKELRKGGKASALEEAAKKHSNELVRLATILDLKKSSLAEEEEK
KVAVEKTVSELEATLQEKTAAFENAKARYDAAKEDLEQQNKDAESKEELLQTLQTGVASKDGQENGYQGQLQDAKNRATTAAT
EQEQAKIKIAHLEKRVKEEEPRAKKAKDQNADLLRDLDGLKTQAQKLEKELGRLGFEPGQEEQMFKRESELQQTVRNLRQESD
KLKRQVANTEFNYADPVPNFDRSKVKGLVAQLFTLDKEHTQAGTALEICAGGRLYNVVVDTEVTGTQLLQRGKLRKRVTIIPL
NKIAAFKASAQTIATAQNIAPGKVDLALSLVGYDHEVSAAMEYVFGNTLICADADTAKRVTFDPNVRMRSITLEGDAYDPSGT
LSGGSSPNSSGVLVLLQKLNGLTRQLSEAEGALRELQARISKEKAKLDQARRIKQDLDLKSHEIKLAEEQISGNSSSSIIQEV
ANMKSTIQELKESISEARIRQAKATADIKTIEKDMNDFDNNKDAKLVELQKALDKLRAGLGKNAAAVKTLQKELQGAQLDAEQ
AGVDLSTAREQLQEVEIAIKAQQKDIENLAKQKAELQETHDTVQAQLDDERAKLHQFDDELRALEDATRSKNSRIAEESLEMQ
KLGHLVEKFHKEQQGAAEKVARLEKEFDWIADEKDKFGRSGTPYDFKNHNIGECKSTLHNLTERFQGMKKKINPKVMNMIDSV
EKKEVSLKHMIKTVIRDKRKIEETIVSLDDYKKKALHETWEKVNGDFGNIFSELLPGGSFAKLDPPEGKTISDGLEVKVCLGK
VWKQSLTELSGGQRSLVALSLIMALLQFKPAPMYILDEVDAALDLSHTQNIGRLIKTRFKGSQFIVVSLKDGMFQNANRIFRT
RFSEGTSMVQALTPADMK
FUN_005455 CDS scaffold_19 26914 27823 -
hypothetical protein ENOG41

MTDLIPRLQGYLTAAGRDSAVAQRLSDMTTMLSGIRIRDGMVATKHFVCNLDAVSLILLTVTLVAFLVPVLILFPPVPVDRSD
VLRQTHSRAGVALKDMDIGPGRSPERRLPGTEPEGKGAVAQSLLVFPVQPCCGIELERDVASFKHSEFDRLYTLAQRDSEVGQ
KDVWQAISREHFPLLANIEVDLWIPDPNKTSRLLGHIEGSFLVLKFPWTDSGLQGILQQVAAKISHGLKAIPEKELLLPVSFP
NEEEIRARGYSFAEVTIGGKNTTGLNMDVELPAELARYLGAERQIGIFRVDQL
FUN_005456 CDS scaffold_19 35177 37493 +
hypothetical protein PF00172;PF11951 ENOG41

MGDYYHHFLSSMSGAGMNPATSPEEMLDPRMHGAPIPMVSAPLPGPYQQLGYFTGFPDPMMFNQNKSQKARRKSAAGTPAAVD
HVKHRRTRSGCFMCRSRRVKCDETRPICERCKKGNRECVYPDPPSSKGSSASSGKFKDTATSAQQTSPTSSKSEDDEDTEQES
KLEMIVDEDEDTEDQTKSSVLSRQSKSVSPSTATATPSSLAAVYSSSEFPIQMGEADWSHLPPEYQQGLSFFVENLNHFNYCI
PLDSDGFFTKILPNLATRHEPLLNALVGFSAYHITLRNPQGKLQDFLQYYNKSVTQLLGLFKRREKPNVATLLTILQLATIEE
YFGDWVNLMGHQKAAFEIITSIFTPQTVMQTPVGRMCLSWYARFDNFVALMGGFPTDLPREWFQAMLDFYQSGITSNPDELHW
KIDAWSARLRLISYDMSILFARGSRGQISQEDFVKEHEHLNQQLIEWKEKRDPALQDPKYLIKDFPPAETLDPDDFVNPYTVG
ILYDFPLFDVTVLCAEWNSIMIMHKCQSSGMRPDQLFADLNRHAYRTCQYFETLEFWPSTPRGVLVLIQACIAIAALFCPQDA
RHHMWFRRKFALLETMGYIHPLPLRTKMAELFREPSCVNWWLPNEEGFSPVLQEIRNFADERNAAAVTAQQESVREVRHIFAK
MSLMDDANAPSSSSLDSASGPSVDSSSAKR
FUN_005457 CDS scaffold_19 38014 39548 -
hypothetical protein PF03637 ENOG41

MSLAPSSPRLPSPPPAAEIQMAPMSPSGGPAASLHTTPQEQSQLEASSRRRIHPGTKAADMAAGPPLIPLQELDSAFQLQEHL
AALHCYHTSSNTQPITRGTALQLATPPNGVDRTIWLYELCRFLISQCNSLIVGFLFDTPPCSANTCPEMRASEWQFLCAVHEQ
PKSCCAIDYCCHTLDWAANVVTDQKIFPSRFVVHNDNHSKNVGVKNLVNVFRRLHRIFAHAWFQHRGVFWSVEAETGLYVLFK
TVCDLYDLLPAENYKLPPEAEGLEAPHTEQEPEKPPPTILKPSSHQRGPPAEEDNMHPTRTNTRRHIRSSPSTGSAVTTVIEA
EEEEPAGVSRKLKDMHLTAPSPVEEEPEVADVPVIVEQSVMGESGAQSHSPPAEKEEDDDDDDDDEDEEIDDDADQTVVGSVS
EQDSANDDDTQTDEHEPAADSLEPAQSTDTGVAEDEPTRENASVDSAPDADAPESSEDAAKAIDTKSDDEPKKDEK
FUN_005458 CDS scaffold_19 40096 41458 +
hypothetical protein PF02492 ENOG41

MDFDDDAPPELVDTTANDVDEEITVKVPITIVTGYLGAGKTTLLNYILTAHHGKKIAVIMNEFGDSLDIEKSLTVNKGGEQVE
EWLEVGNGCICCSVKDTGVNAIESLMSKKGAFDYILLETTGLADPGNLAPLFWVDDGLGSTIYLDGIVTLVDAKNILRSLDDP

NGVVEGHDDHGHGPVMTTAHVQISHADVIVINKADMVTEVELNQAKERIQSINGLAKIHVTERSVVPQLEGFLLDLHAYDQFN
ESDANAKGHSHLDPTISTVTIPVGRLGPGQLDTVDRWLRSVLWDSKLPEDEKEGDFEIHRSKGRLVFANGEVKMLQGVREVFE
INDGPLGDETPKEGKIILIGRNVADVGFEDSFKKALS
FUN_005459 CDS scaffold_19 42653 44190 +
hypothetical protein PF04153

MAGAGPQNAGRQLGGNVSFAQSLSGSQPATSLDLSEFPSLSNNAQMSNPNQSSMWSTAGSRNLGGPSPRNQATPQQGGQDDMF
SPTSSRGQGGFRFGNQGNIGQQTQSSSVDDFPPLNQTSNGEMGSDRTASLMSSLGFSSQAAGAGPSASNRGNGLLNALSANSR
ANEARSPPGIGAPGSSRPQDGKPPGLEEPRQKSSKEDAVPDDASNAADYNNADYSATNYVAQTMQKRLDTEDVIDPLEGMPAS
DKWGLKGLTTLMNNYPDYHAMVVGMDPNSLGLDINSQELFSTQIYSLFDDAPPRPVLSNGRFRLPDCYNVTNVQPIESKIQSF
NEETLFWIFYSCTADVKQQMAAVELHSRNWRWHKKHQIWLTKDEHMTPQILSPNHERGYYVVWDTNSWRKERKELTLHYGDLD
TTLGQAQVVP
FUN_005460 CDS scaffold_19 44856 45485 +
hypothetical protein PF02845

MADDQISLPYLLAILVVSGLIIRYLFFGGPSPPQPTRSPEAFLRSREIAVERIQQMFPQADRRSILWDLQRNGGNIQSTTERI
LAGRLDTPPVTFQPPPPPGQSAPSSSAATASRQPDKPAQPDLITRYNLKDKLDTASQEEQDAKGKGWSSNRDERQASLQRRRD
EMILAARRKMEAKLAAEKAAQGS
FUN_005461 CDS scaffold_19 47394 50277 +
hypothetical protein PF00226 ENOG41

MGTPRDYYADLELPPTADAAEIRKQYRKLALKYHPDRNPGREAEVNTQFQIIQTAHEILSDPEQKAKHDASRGRGRFPGASGV
KGNPWSNVSAQYPPPPRRNNANPRAPPSGAQRWQRFSTGVPPTAKQYTASDAESKKNAARAFENMRKGTSTKANDQPRPPPPP
PPPRTESARQRQEASFGARKTGFHPRTTVGDEPPVSSSNYSSRPASERYAQRFGSDIPQQAPPPTPQRPPPTAMPDPLSQFRD
RDSTADPRQSSPYASNGGEKTNPFDGIPINRAKSTREATRQDQSASENEAFNRQRSASAPKGGKAEDVPLNNKAPEQPSIPRD
PADRPKAPLKKTRSGFKSVPLGPNQQATEFPANDKPTDSPGGPTMYGNPFSTTSSRPFLKSSNKCRGHHGYDSLGNHCPYEIP
IKPSQPSPSGRQDSTHQLTRFERQQQELLSQLINNASPDSPAKKPKLQDRDSKIPSMPNRANKVYSSSFSFPVNDDTFARTSP
DHHRFSRRSTDDINTSFADEDDAAGWEFNAGGAEQDSPSRPRKPGRRSPGKRPPMSGKSTPSFMSDAERSDSAKPESAFNPAG
WDFGPQTFVPQPSKSVSPTRSTRTNSRKPKASKKSSHANLVDDSSSEDEVLEWRGRKVQEEPLAAESPQAMDIDTPPAPSSMP
PPRPPKVPSPQPAQTTPSPQPAQPVSQPPQPVETNQAPGVVPAPHAARNINVEPTRPEWRPGNVDTVNGNEHGPESPRKAFNP
NNMGSEDSEEFQASFADLRNVAPFTQQSSGLKSFSDLKDNLPFESKAAPQIPMKLPKVHPLIFPAAPAAPQIPAAATINGVKP
SALSWETYVKDFEHYLKQWDTFNAQVVDHFATRKSHIARTREEKGYSFLENRGDADIQEYYNWLEQDMDVRRRWAAACEEHEH
RFREFMAFRMKMK
FUN_005462 CDS scaffold_19 51466 52086 +
hypothetical protein ENOG41

MSDAAVKPNAWTDEAKNELLLRIIAQLKPEGKSINWSEINMEGRTVKSLQNQWTFFNKKLEAFKTQSNDGSTPSTPAKKATSA
RKRGPKAKKVAPDDDEVYESPKVTPRKRRAPKTPESDSKAVKKELSEAVVKDEEMDDDRDVDGEV
FUN_005463 CDS scaffold_19 52751 54543 +
hypothetical protein PF06963

MASTTGVGDEETAPLLATSPTSSTGVINSQYQSLSQISSSNDSDVSNTSNTDPGWTLSQSARRLYISHTLSTWNSRVFEFGSV
LYLASIFPGTLMPLAIYSIVRGASAITLSSWVGSYIDRKDRLNTVRLSIVSQRLVVAASCVIFFILSRVESPSDELRVGLLAA
LIFMACVEKLAAIMNLVSVERDWVIVVARSDTTALRTMNSQMRRIDLVCKLLGPFFIGILDGVSTETAILVNLAMNCTSVIVE
YFTIAKVYYQVPELQHPKTTPPIVPQNEEQQTRQNAWVSFKKAIQKTSSDLRLYFTHPAFAPSFSIALLYCTVLSFGGVMVTY
LLASGYTSAQIAAMRTVSVTLEVLATWIGPWVMKKIGPVRAGLWFLSWELGCLAIGVSIFWRYADNVLVSTLGLVCGSMLSRI
GLWGVDLSAQVIIQEEVEAEKRGAFSAVEASWQNVFEMCSYASTIIFSSPSQFHNPTALSVTSVFFAWLLYSSFVKKRRGHLV
HWPTCMSPEKQQATIDELFESGPSRRREGL
FUN_005464 CDS scaffold_19 55719 58930 +
hypothetical protein PF12812;PF13180;PF13365 ENOG41

S01D
MNGANSSARAKRKAPGSPGEAPPSKKPINGKHSPNDNTPDIIEFDDHSDMTDELHPHAMYIGTPGSLGEWQDTIQKVVRNVVA
IRFCQTCSFDTDSALTSEATGYVVDSEKGYILTNRHVVGAGPFWGHCVFDNHEEVDCYPVYRDPVHDFGILRYDPKAIKYMHI
DGLELRPDLAKVGTEIRVVGNDAGEKLSILSGIISRLDRNAPEYGEGYSDFNTCYYQANAAASGGSSGSPVVNKDGCAVALQA
GGRSDGASTDYFLPLDRPLRALQCIQNGNPVTRGDIQCQFLLKPFDECRRLGLSPHWEAAMREKFPEETNMLVAEIVLPQGPS
DKKIEEGDVLIKVNGELITQFIRLDDILDSSVGETIKLHLQRGGQDVEVEIEVGDLHKITPDRFVSVAGASFHDLSYQQARLY
AVAVKGVYVCESAGSFRFDNTDNGWIVQSIDHKKVPDLDTFIQVMKAIPDRARVVVTYKHLRDLHTLNTTVAYIDRHWAAKMK
LAVRNDETGVWDFTDLGDPLPPVPPTRRSASFIELDHMPHQGIADLIRSFVHINCTMPLKLDGFPKNRRWGMGLVIDADKGLV
LISRAIVPYDLCDITVTIADSIIVEGKVVFLHPLQNYAIIQYDPSLVDAPVMSARLSNEVLTQGAKTYFLGYNRIGRVVHGST
SVTEITAVAIPANSGAPRYRAVNVDAITIDSNLGSTCNSGVLVAPDGTVQALWLSYLGERSHHTSRDEEYYLGLGTKTLLPVV
ESVQKGINPKLRILSVEFRSVQMAQASVMGVSDEWIKKVTQTNRSHHQLFMVSKRTFERENHPVSLLEGDIILTLNGKICTTI
SDFDLMYSHELLDAVIVRECEEMHLQLPTVAADDMETNHAVSFCGAILHRPHQAVRQQISKLHSEVYVSSRIRGSPAYQYGVA
PTNFITHVNGTPTPDLDSFIAATREIPDNTYFRLKAVTFDCVPWVITMKKNDHYFPTMEWIKDDKEACGWRRVTYEGSEVFKG
EATDGVVPVAQDADME
FUN_005465	CDS	scaffold_19	59541	60505	+
hypothetical protein	EOG09264PK5	PF03398

MAPGASLITKLKVQLKLTIARLRMVQQRDEQLGKTARRAMAQLLEAGKEDSARIRVENIIRSDITSELHEMLELYCELLLARA
GLMEGHTVDPGLEEAIQSLIYAAPKTEIKELATVRTLFAEKYGKDYVLAATENTDGKVNEKVVKKLSVTPPREELVVGYLEEI
ARAYGVDWPKREAVSPPPELLDDDDDDDDQGGQKQKVLETPLAADSKDPVLNTPVKKLAGGGPTSPLTVTPPRMTTDNIHPKV
TLGSVELKPSKKAEPAARKGEPDGSIPDLGDLERRFAALKKR
FUN_005466	CDS	scaffold_19	61248	62092	+
hypothetical protein	ENOG41

MESSRILLNTLRYTLGLTNLRDVSQLTQVSQSRLEQRFDAISERFSTRRMSSIYMRWLIHQDFTVGERLQSQVVEQWVSFVDS
WGLEVEEAIALWAKTMDEDTESDEETKQLRVKSNSLRHEIESQFFKLKNPEPIPPSGLVVAVPPRRNHNIKTRGQIRESDEYA
NDIKTKSALENWPTKDIPFPSIETAVDTDTESEESSETASLDIKREHGEETAKKKARLDTGGSIPSLVLNGSGTISSPIDLTM
FUN_005467	CDS	scaffold_19	62326	62814	-	APS2	AP-2
complex subunit sigma	PF01217

MLSFILIQNRQGKTRLAKWYAPFSDEQKIKLKGEVHRLVAPRDQKYQSNFVEFRNNKIVYRRYAGLFFCACVDTNDNELAYLE
AIHFFVEVLDAFFGNVCELDLVFNFYKVYAILDEVFLAGEIEETSKQVVLTRLEHLDKLE
FUN_005468	CDS	scaffold_19	63780	67316	+
hypothetical protein	PF10334;PF13515	ENOG41

MAKSPIMKTVTKRLRELAIVIWVDTETNDLWKQIIKCSIASIGSVVAVMTPEAAAVLGPSTFLAPMATVFAHPGQRLGTMIET
LLMMLLGTLFGLAWSILALWLSGYASGTNETAPNAICAVFFTIAAFFHGYIRSASPRMFLFVTFFLIANIITLLGGYTSVSSE
VITHTYYPMMVGGGISVVVNLSIFPELSSSYLGLSAIDALCETMDTMTRATHWFITPGADSEEDSTTTALTMTNTVKSVPEKP
KRKKGRFRKWLSRFPNPFKQSQHRYHASTIPVGLTTIRSLTSKRGSLRARLTHCKAAQKEVNYEISVSALPLSSMKPLSTSHM
SSLVQNTLAIIGACENKFIVLQNNDNSDDESDSDMTGPSGIKRMNTFDDYLQRVEESKPLREIEASSASLLESIIERIREPVQ
EFEASLKEAVRLVVVCVAYCYDVRRLPSGSPAPRGIHLQELDFRIDLFTEAITNFDASCSMELRRSGMDKSGYSMDFMPRMET
FLISSFVLSLRQAAMYVLDMLRHVRKTVEQRQARNDRATIWFPKHVDIRQWLITGGESDGFVLPEAARKEVRRGKSTTGHKSK
NKTHQSNKDTKTKPAKGKDEEKGIRFAEPVFQTREDRNVEQPQEKEQPQPQQSSKILKIRGMAADALEWIQDSEHVKYAFKLT
IAILLLSWPAFVESQMGWYSAYRGIWAPMQLFLVFEVAIGTSFHVFFIRLCGVVAGSAFGYASALVGDRSLIAMVFFLIIGIM
PSFYVQLGTRYVKAGMISTVTMVVVALSAVNGTESAYHYFYKRLCAFIIGGTTALLIELILYPVRARDRLVESLAASVKQVQN
MQAAMAVGLDEPIKPNFRNPDLNKRFRRATNKARGALAAAETFLPFSVTEPRLKGDFKPLVPVYKEIFYVLHQIIDRMENVVM
LRREYGSSILEDLNPQVHAYRRNVAASIMLVLFTVYEAFITWTPLPQFIPSSRLAQLRLVNHVREILASRSGTQTPAGEPPSV
FDENGELAAQVAYLITQKRFLSWNASTSGQMEIIEYLEELVQLVKILVGVNDFRSGLLKTPTLSNYRERKHLTRMPLSRVPTA
DSRTTGVPAEEVPTVESRATGLQRTQTIRAGHARRRNHRREDNEKEADSDSEEDIPMSLQRVGTRLCEDAAVVRRRAMSVSKD
FUN_005469	CDS	scaffold_19	67521	68536	-

hypothetical protein		PF02269

MAESRDSKSYKYRQEISQMMYVSGETAEPPVETTSIIEDIVRQQVIELLRNCTELASRRGSKSISINDLIFQIRHDQAKVSRL
RTFLSWKDVRKNVKDSDDKGADADIAAGDDPVAAGDNTTDEAAKKNKKAKVGLPWEPSSYYNVEVPEREEEEDEEEEEMNYIT
LQRLRKADERTKAMTKEEYVTWSEYRQASFTYRKGKRFREWAGFGIVTDSKPSDDIVDILGFLTFEMVQTLTEMALKVKEQED
LVRAQNGGDNVGNAKKRKHEGALFDFPSEGKTPIEPRHVQEGSRRLQQRPKKSRAMLNGTRIAQHTPLNIF
FUN_005470	CDS	scaffold_19	69213	70394	-	PPM1	carboxy methyl transferase for protein phosphatase 2A		PF04072

MPAPEIPNLLNSLRGSRGGRGRGRGRGGHASSAVTHDATIQGTDTDASVSRLSAVDLGYLHDPYAQYFVQSGDGPVARRLPII
NRGTYTRTISLDTLIESFLDGDKDSEQGSGPKQVVSLGAGTDTRPFRLFFSESRPGLVYHELDFEVVTSKKLRTVQATPKLRN
ILKDAAQLTEHSWSAKPTGCEYYCHGQDLRGFSQPKTPKEDDGTEATTKEIPEISLPGLRTDIPTLLLSECCLCYLTATEASD
VINFFGSRIPNLGTIIYEPIRPDDAFGKMMVSNLAARRIQMPTLQMYHTPEDQRARMSRAGFGKVYHMTIEDIWQNWVSADEK
RRVDSLEGLDEVEEWKLLAAHYIVVWASKGEGFESWDSVRGCT
FUN_005471	CDS	scaffold_19	71514	72644	+
hypothetical protein				ENOG41

MLSSAGSLLMRRDNMPKPDLGYELQQLPDWSRFVFLANFILFLPVFVLINYTFEKVFPVLAIVEDEKPPAYEPLPVEPLANDD
MPKPASTSSVPVAGQGRPVTSSFRATWRLLRSTGGFRAIFRGLPCFLAQAVVTALINGITYTVIPYSFVLSNLIGSLVCVQLS
TAWVHIVITPPSPLKFWSRLPPFKTTFAATWRPTLIFWAASQIVNLGTFGVLYLFGGDQTTEIPQLQGLGSVFGILLVAVLLQ
VAIQIPAYVVLVRVQASLLPADADTIIPFDRSFNGRIEPVVVGGLGYATVRDAWSSFSKSAWRRIVMLSVKIVAVTFAALCVI
FAVIIPQVILLAAMGTDNGDGNQDIKL
FUN_005472	CDS	scaffold_19	73327	76267	-	KLP1	Kinesin heavy chain		PF00225;PF16796

MSSANSIKVVARFRPQNKVELESGGKPIVSFDGEETCTIASKEAQGSFTFDRVFDMGCKQQDIFDFSIRSTVDDILNGYNGTV
FAYGQTGAGKSYTMMGTNIDDDEGRGIIPRIVEQIFASIMSSPGTIEYTVRVSYMEIYMERIRDLLAPQNDNLPVHEEKNRGV
YVKGLLEIYVSSVQEVYEVMRRGGNARAVAATNMNQESSRSHSIFVITITQKNVETGSAKSGQLFLVDLAGSEKVGKTGASGQ
TLEEAKKINKSLSALGMVINALTDGKSSHIPYRDSKLTRILQESLGGNSRTTLIINCSPSSYNDAETLGTLRFGMRAKSIKNK
AKVNAELSPAELKSLLKKAQGQVTNFESYISSLEGEIQMWRAGEAVPKERWATPLTTDAVARTKADARTSTRPSTPSLISDSR
SETPAISERAGTPSLPLDKDEREEFLRRENELQDQISEKESQAASAEKQLRETKEELAYLKDHDSKVGKENEKLTTEVNEFKM
QLERLTFESKEAQITMDALKEANAELTTELDEVKQQLLDVKMSAKESGAALDEKEKRKAEKMAKMMAGFDLGGDVFSENERHI
AETIEKVDSLHDLSATGDNIAPDEFKALKARLVETQGIVRQAELSMYSTSSSESDSRRRQELEARLEAVQAEYEEILTRNLGP
EDIEEVKARLENAFANRQTAQSQFVEELKEDIAQKAAENTRMKTLIEDLQQRVKAGATAPMANGKTIQQQIAEFDVMKKSLMR
DLQNRCERVVELEISLDETREQYNNVLRSSNNRAQQKKMAFLERNLEQLTQVQRQLVEQNSALKKEVAIAERKLIARNERIQS
LESLLQDSQEKMAAANHKFEVQLAAVKERLELAKAGSTRGLNSPGGFSFANAGSRIAKPLRGGGGGNDAPSIPTIQNLQGQNE
GNTSSGSSSKRASWFFTKS
FUN_005473	CDS	scaffold_19	77669	78295	-	UBC2
Ubiquitin-conjugating enzyme E2 2			PF00179

MSTAARRRLMRDFKRMQTDPPAGVSASPVPDNVMTWNAVIIGPADTPFEDGTFRLVMQFEEQYPNKPPQVKFISQMFHPNVYA
TGELCLDILQNRWSPTYDVAAVLTSIQSLLNDPNTGSPANVEASNLYKDNRKEYTKRVRETVEKSWED
FUN_005474	CDS	scaffold_19	79279	80628	+
hypothetical protein		PF00108;PF02803

MSPAQLRSAGRLAQLAGHVNGARQFSTRPALRKEIQDAYILSAARTPTAKFNGSFLSVSAPKLGAVAIKSALEKSKVPVEKIT
DVYMGNVLQGSVGQAPARQAAIFAGLPKEIEATTINKVCASGLKAVALAAQNIQLGLSEAQIAGGMENMSQVPYYVPRASGLP
AFGHVKMEDGLIKDGLTDVYDQFHMGNCAENTVKNYQITREQQDEYAIQSYRNAQKAWADKAFADEIAPVTVKSRKGETVIDT
DEGFNEVKFDKIPTLKPAFVRDGSGTVTAANSSTLNDGASALVLGSKAIAQQYGSGSRVLAKICGYADAATSPIDFPIAPAKA
VPIALERAGITKDQVAIWEFNEAFASVILANSKILGLEGAKVNPLGGAISLGHALGSSGSRILTTLLHQLKPGEYGVAAICNG

GGAATALVVQRVESV
FUN_005475 CDS scaffold_19 81159 82857 +
hypothetical protein ENOG41

MPLVLDFLTQIRNFIRTQNGDELRAWLQVEPNSPQQYHNLASELRSQFRQQGLDNIVERTLPQEDDVPEGQATVWPGFVAFMK
DYMAFWRDVNYDDLLGAHQLLSGLVNSCATAFAHPTYGAMLLKTSMSLSETLARLTMSLNKRPDLARRLRAVDEDKSIAESSA
EIIQKIFTTCLTDRSSGRYAKPEGKKVGVYMFANLVLKLLFACRRTHLAKMIFVNISTISPPLSLYPAAQRVTFLYYLGRFNF
SNNHYLRAALCLEEAYLQTPSQLVSHRTNILTYLIPCNILLGRFPSQVLLQRPECQTLAPVFFPICLAIRSGNFIQFQHHLAQ
HETWLFEKGLLLTLGNRLRPLLWRSLSRKTFLLTYVPPTDASSRKAATLDLADLHTLGVYLQHRLEGWLPAGPNSLGRSQSVN
PLLMKALENNAQNPEAASTLAPPPGGPKSLRPNEGIIWGNAEVTFEDVEMTVATLVQQGLMHGFIAHGQGRFAIIGAKAKGSP
VLAGWPNVWQINRERRYEDYDPDEVPGWVKE
FUN_005476 CDS scaffold_19 83614 85215 -
hypothetical protein PF03856 ENOG41
GH132
MRLINLSAALLGALSAPSMVAGKQHGHRHNGFEHLRQHLPVQSSILEKRGGQCQFPTDDPNMVAVTPDAKNAGWAMSPDQECK
PGSYCPFACKPGMVMNQWDPDSTYEYPASMNGGLFCNKNGEIEKPFEGKPNCVSGTGSIEAVNKCGSKMSWCQTVLPGNEAML
IPTLVTSSATLAVPGSSYWCSTAAHFYINPPGTGEEGCIWGTESEPIGNWSPYVAGANTESNGQTFVKIGWNPIWEDSALKSK
LPDFGVEIQCPDGGCNGLPCRIDPAKGEGNVGSSLSAKGAGGAAFCVVTVQKGSTAQIVAFSSGKGSSGGDDDDDEETESSTA
EAEPSTTAEAKVTTSEEPVVSTTKEPSTTAEPTTSALPTTTSSTSTTTTSVETTSSAETSSTTEQETSSDTTTEVPTSTIFTS
ATSKTTKHKSTKKVYPTVKPGIFRENGTSTYTSSESEETAAATEVDSGAAPTQSKENEAGRHQGNTAVAGLVVAFIAAACFF
FUN_005477 CDS scaffold_19 88465 89298 +
hypothetical protein PF00190 ENOG41

MKFTTALVSAVLAFTSFAEAAPRNTRRTDSSKPLSLTAQLRLADTAADRFALLPEDDDFVFDFNKKQDNPGKGGELIAANRAN
FPALVGTGAGMAFGRIDPCGMNTLHVHPRSAELQVVTSGRLITEMVPENGVLDKDGKRRVIRTELTANMMTPFYQGSIHTQFN
PDCEPATFIATFATEDFGTGQIADELVSLSDDVIAATFGQSIAGEDIDKVRQAVPKSIALGVDKCLETCGLKKRSI
FUN_005478 CDS scaffold_19 89560 92126 -
hypothetical protein PF00009;PF00679;PF14492

MIILSLLRNGPAVKGSGRCLARQWRNKELMNLSRRAMHQAHHDTQLHNIRNIGIIAHVDAGKTTTTERMLYYSGVTQRVGDVD
SGNTVTDFLDLERERGITIQSAAITFHWPKHQALAPGEHAKTINLIDTPGHQDFRFEVDRCLPILDGAVCIIDSVKGVEAHTE
RVWGSAHEFKVPRIVYCNKLDREGASFKKAVLEIGTRLKGWPLVCQIPWWEKEDFVGVIDIVNRVGYRWKSEREKKRYDTEEL
KEKLSSSNKDLLPEIETARQALVEGLADFDEAIMDEFLAENENIDGALIKEAIRRVIRSGDGRVIPVFAGSSFRHIGVEPLMD
AITDYLPSPDERPSAEVRVGSAKHRLTEVLENSPKSARNQVASISSVFKVFNHPKEGVISFVRVYHGTLTRNASSFNTNILTH
EKPMGILQIAANKTQDIQSLGVGQIGALRGLKKARTGDTLVTMNANKAIPENFRHVKIRPPEIPPPVAFLQVEPYGNVAAQQL
QIALENTTREDPSLRYSRDPKTEQFTIQGMGKLHLDVSLYNMKQKYKIDADFGPIEVDYKECVTEPTRPQYTVFDRPVAGKPG
KASCTVVLSPLEDHHHDTLLESCVERDGNIYHVAIPLTDETSTLDFDPEEARAQLLNGAIAGLARGPRRAAPIHGCHVTITLD
TEPGACETPTGGHFSTAARTAVQAALRDAFEKEKIGILEPIMHVNITCPEVVAGTVQHDVTAGAGGQVLEVNDRSAESTGEDH
IDVSKIYTPPDPYDSITSLRGKKSSARVVEIIAKVPYKEMLDYDNHLRSKTAGRHSMTMSFDSFAKVVGHREKGL
FUN_005479 CDS scaffold_19 92620 93274 +
hypothetical protein PF08507 ENOG41

MELSDIFRIVNLAVGVITVLGGIASIFHLGLQPIVVGAYMIVFGLVIALLEFQIPPQVSRHASFMFSFVGRGVFYVFIGCLLL
GNFILSKIAGSIIGIVGLAYIALEFVPSIEPPSNMREAEDAGWGAEQV
FUN_005480 CDS scaffold_19 93714 96947 -
hypothetical protein EOG09260SJV PF00400;PF04192

MPHKDADHPVVKRQKTAAPANASKAASGSSRIFAPFRTVGLVSPTSVPFTSIPLGKTTFQISTSVGRSIQTYDLRRGLNLVFI
TRPQTPADITATLAWKEKVFAAWGGSKKGESQGIWVFQRGKKVDELALPSDLNEPVKQILILGIWTIVAATTRIEVFKTATLE
HYTTIHTTPANNRGNEITGGIVSMPTFLNKIFVGRKDGWVEIWNVSTGKLIYTILPPAPDCGSVTCLEPSPALSLLAIAYSEG

TLIITNVLTDKVTLRIEAGSSEAQVNSISFRTDGMGAGNDGRKDGVMATATAATGDVTFWDLNGGGRVMGVLRSAHNPPSYDG
PLVRGGISKIEFLPGQPVVVTSGRDNSLKSWIFDETPFSPIPRILHSRSGHAGPVNCLEFLPTDFEGADAGNKWLLSGGQDRS
LWGWSLRRDGQSTELSQGNIRKKAKKIGILATNALSHGPTTTLEDLKAPEITCMASSLNRDGGIGAMPGNQPIWQKSRDRKKT
QDAEVSGMTGWESVVTAHKNDPYARTWFWGRKRAGRWAFPTGDETNVSTVAISPCGTFALVGAESGGIDMYNLQSGNHRQRFP
SRLTPGQARQLKMQQLRQADDIVQMHAEATRQFPVGTGKHTKRITGLVVDSMNKNVVSCSLDGKIKFWDFLTGNLLEQIDWAP
MTFPTGCRYHPASNLLAFSCDDLSIRVVDMETKRTIREFWGPQGAINDFCFSNDGRWIIAASQDCVIRVWDLPTSHLIDAIRL
EKPCKALAMSVTGEYLAATIENELGVTLWTNKALFKHVPTRQISEKEIGQVSAPTVSGENSHGMLEGAFEEEQAEDEDTVVAP
TIEQLSSELTTLSLVPKSRWQTLLHLDLIKERNKPTEAPKAPEKAPFFLPSTTGKENPMASKLQGESEAESNSRITRVEKARF
EEVLSTKLRAGAETGSYDEFIEHLKSLSPSSADLELRSLSIGDGDEDSNELLHFIRALTARLKTRRDYELTQAWMTVFLRLHF
DLVIENQDLLAALSEWKSYQEKECNRLDGLVGYCSGVVSFLRSPRT
FUN_005481 CDS scaffold_19 99325 102173 +
hypothetical protein PF00884 ENOG41

MPSLVSYLFAVFFVSLLFTKLLHLFIHIHSIAVIDFVVYLPTFFLQDFFLVLFARLLLRRERTILSLIGYVLGCFLTLITFVA
AASELGFYYRTGGEVEWSDAGDFANDEGLNVLISESSSVIVSGLIILIVAWLPQNYLYRVFGDIVSGIGKRIASVSRYLRGKI
RPQPQHQQDPENAEPFLQRVNSESTVTNSGHPSPNLSRNGDEKEVEERTQKRRCCAFIPSWVINAVVLLFIGITWVARPDQPY
NHIASSLPLRLSDSVRPKLGLCASQNEFPLRQLIEKSRWQDPKGNFKGWAPSRENNDLVKKYRENPPAWLPNPIPSGFLKWNP
DRYKDEHDKKDGPSNLCPNTWQDRGFYNPVNDPMRITNLDQEILAPIQEALSNNSVKIRHIALILMESMREELFPLQQGSDIH
RFIMESNDENARDEVNGRVSRMTQNFEKITGKPGNYQSANGSAYDPPAKPVWNDQTKPGFGGVNVVGGLTSSSVSTKSLAAAH
CGAWPMAVNMFEESELESYQPCIPQILELFNKVKGNTTKRDDKPEDKPEDKPQKKRDWWGFGGTAQAKFHEYQWKPAFFQAVT
DHYDRQDKFDEKIGFDFKVTKPRLDEEAKDNPEMEEINYFGYPETDLREHIRKFISDGIAEDKRLFMSHFTSTTHHPWGVPKW
FEKEKYMGKEGGNHQDLDKYLNTIRFTDAWLGELMQMFEDTGIADETLVVFVGDHGQAFKEDFSKTGTYENRHISNFRVPISF
RHPSIPRVQYEANVTSISILPTILDLLVNSGSLNKKDSEIALDLAHDYEGQSLIRPYQKTQDGRRAWNFGLINPGGGMLTVTS
ADAPWRLAVPLKKDLEYTFTDLGKDPLELDALDKWSIKSMIKAVKRQYGNDAATWVKEADEVAHWWALERRRLWGYNPDEDK
FUN_005482 CDS scaffold_19 104517 109649 +
hypothetical protein EOG09260FMW PF00328;PF18086 ENOG41

MNKNNPLDPDLNAASLADTDDSPAQAQAQAQEEPEPEQEPSVSSSVSASGEPSAADFSSSPRRFHPHSHPPSSSLTFGYVSVE
QHNIQPNPPRQSRALSSTSASLLANSSAAAITRKSRGAEPVTRDAYATARRASFTAAPSSTTAVSSLPIRNPRPLARTRRESM
TSDTASEEQTLADSQLSGPGEEPHQLRTRLPSTNENSHTVSSEASNAAYHRLSSSSDYQSRRLSGTSIYSLASARGVLSGSSS
AHGSELGTPPRSVPGLLSTGKSTATGQSEAEVSNITVTTSSNQPGQSVVGHPNQHHLTPRDLHSQPLDFTKRAIRHDNMQSST
SGLRQGPDRSRSRAKRRFSGSTATSSHSPSSDRGPHHREREEVKPSRWGVIGVCALDVKARSKPSRNILNRLIANREFDVVVF
GDKVILDEEVENWPICDYLISFYSDGFPLDKAIAYVKARKPFCVNDVPMQQILWDRRLCLHLLDKIEVRTPKRVEVTRDGGPQ
YLTPEMVKHIKDVSGVSLDPIDPSQTPPPQKVELIEDGNTISVDGQTLRKPFVEKPTSGEDHNIIIYFPTEEGGGARKLFRKI
GNKSSDYIKDLNVPRAITEPGSSYIYESFMQVDNAEDVKAYTVGPNYCHAETRKSPVVDGVVRRNTHGKELRYVTALGAEEKE
MASRISTAFGQRVCGFDMLRASGKSYVIDVNGWSFVKDNDDYYDHCSNILKDLFIKEKMRKGGVTPPMPSPAPSDTDPFTRAS
NAFKDREQTQSGTNGVRTSINSIAGTTDSQPDDSSRRAPSGTVTPLLPPDSGLPSKTHSTLATPAIPPTPVDVILPGISSAPP
TQQSAVAEPPSEQASIVPEPPLPTHSWKLKGMVSVIRHADRTPKQKYKFTFHSEPFIALLKGHQEEVLLIGEAALGSVVQAVD
LAYEQGIEDRAKLRSLRNVLVKKGSWPGTKIQIKPMFRKKKTEEPVISEELVAMTEEKKDITEEAGDSSREDKSHQGPKRQDS
LSGVTMSKFTAAEERLVLDKLQLIVKWGGEPTHSARYQSQELGENMRNDLMLLNRDILDEVHVFSSSERRVTTSAQIWAASFL
GKKDIPEDFITIRKDLLDDSNAAKDEMDKVKKKLKGLLRKGNERPAQFTWPENMPEPSEVQTRVVQLMNFHRRVMDHNYKKLQ
SGAVTSLNAISNPSTEKLSGENSSSSIASSLSQANTLNQIQSRWCCGEDAELFRERWEKLFQEFCDGEKVDPSKISELYDTMK
FDALHNRQFLEWVYTPPNHMLDEYTASGASSVPGGSNGTKDGKSKESEDGKTSDDKSDKSHHHSPEGSDKVDAGSRSASVKKL
FRRRSFLNNLRHFNEEAPPEQYFRLYKGTKQTASTADAHNEPLQELYRLAKVLFDFICPQEYGISDSEKLEIGLLTSLPLLKE
IVQDLEEMQASDDAKSFFYFTKESHIYTLLNCIIEGGVETKIKRSTIPELDYLSQICFELYEAEMKSGDGSSPHDAPTFTYSI
RITISPGCHVFDPLHVQLDSRHCIGCAPRRSLTPHADWLQVIKTLRAKFNQVKLPKTFLAVNLSDAFTFEDLERQGSDSDVLE
MKTAPSRGITEQPKSPEDEGSETLATGAGEVMIS
FUN_005483 CDS scaffold_19 120546 121738 -
hypothetical protein PF03473;PF03476 ENOG41

MKVTQLWYYPIKGLRGIQVQSAKLGPQGLQYDRRFMLYKIEKSGDFSKIQLSGHPECSLFAQEVVGDKIRVKYLIPEVPLVPW
KPEQDTVLEVPIEPDIKELSKADVSLHQSRVIAYRMGPKYDAWFTACFGFDTALVFIGDGRRPVLGTFSPKAQATPPPWPMLL
LNHLLGKKATEDDWITFTDCAPYLFTTEESLSNVKARLSTCDVDMKAMRPNIVLDGETAWDEDFWAQLTINGAHQVALTKMCG
RCTSLNVDYSTGRTAKGERGTVLKKLMSDRRVDTGSKYSPVFGRYGFLTNNPGGDTIISIGDSVEVTKRSAERTVWDWALADP
KIAKYYQSSSDTRSSIPIVLSFWLTAAALLGLLPCWLLLA
FUN_005484 CDS scaffold_19 125086 128164 +
hypothetical protein PF00005;PF01061 ENOG41

MSLSSYGVDDAKLPQQPFLPGAKPDTPSFLDTTACLCALRALPGLDKLAEAGANFDNGQAAWQCIGNQTQGVYNITSGKWYKA
KNPKGSLDLPMEDNSNPPVGGDLVWDSKSRSLKPLKDKNTLTVYDRDCTGTNRSSFSTSFYRTTYQLLNDKLPIDAAPCWRPG
ALPLQLQNVSSWQKYGCKEGFLCQNNTINSLPQSCPPLEKCTVARLSGMTCSLNGTNVGMGVFEPIICQAGHYCPLEEKGMKK
IRCPAGSYCQPGASTPTPCLSGSRCPEGASFERYLIPVGVLVIIDFLLLVGMFVLMFRSRWQRTSKAHAGALKRHKTMRAVKA
TITGRGYKELPEDQDNEMAPMQPGYSRYSPYGQDFREGRTRTGFEAALSRNSVYSSEQEQKEMEANPQLKAFVESMRRAAEAT
NFGLSFRYSDLVFHPKKSPRPILQNVTGSIEQGQLVAVMGGSGAGKSTFVNVLMGKTSNTGGIVAVNNTPGKMKQYKKLTGYV
PQDDIVLPELTVYENIVHSARIRLPRNWSKKDVEAHVESVIDCLELSHVRDSRVGSVGKPVISGGQRKRVSIGMELAAAPMAI
FLDEPTSGLDATAASSIMRTLKAIARLGISIIVIIHQPRTEIFDLFDNLILLGNGQTIYEGPQMESQTYFESMGFQFPEHSNH
ADVITDIITGNGREYNSAGDVSKEALISHWAKMRQSKSEEAYERRSVRSTMLGDTGMRQALKKRGAPYIKQGWLCLCRAMLQQ
YRARAALFAEMGLAVLAGALLGLANNPKNGIMFVGLFHEPYEVLSTAIDFFSAPQFALLIAIAIGLVAGAPGVKLFSEETLLY
RREAEAGHSRLAYFLAKVISVFPRMALGCMHFTVPLFLLSVPIIDWGLAFLTNLLYFYCIYGLASIVSMVVRREDAPLFATMI
ALITGILSGSAPPLASVKDWHMEWLWRASPATWLAELYFGQLVAPFAYLFQVDIAAEATGYHLDRKWLNIGVLVAIGTAYRLI
AFAGMVLGHRLRR
FUN_005485 CDS scaffold_19 131023 132362 +
hypothetical protein PF00583 ENOG41

MATYTTMEGPQHIAIRELDLRACHPSTVVDRVGAVPVLKTSAAFFTFLRANLVLHSAMTSATSTPQFGPSVPFSSFVPPSPSP
LAASTPYFGLDNDDDVEPLSLEVLDKPQDKVEGMRLVADSIAQMRQRASLNLVFHPLCLAGLSATLAAIYHFGGVTRDSGLGM
TVSCGVIMSYLMGIRYLANGYISLAERLRWNWLRADDGEEDTLLAARLNQDIIGTLVLRLEPNPTSSHRRRRSLSLRGGKGVI
RAWTTNLNYRGKGVGKDLLQEAVRVTKERCGKDAEVGFAQEHANSAMLLPGIFNAPFRRDEVRATRALDGILSNWDFVKKRR
FUN_005486 CDS scaffold_19 133028 133752 -
hypothetical protein

MPTVPTRGGPSGSRPTFTGGKVVPFQKKGQVHRGASGKTVLGGRRHRKILRDSITGVTKPAIRIDTDTVKHRRLARRGGVKRI
SAGIYDEVRAALKSRLEAILQMCVIYVEYRNAKTVTVHDVIHSLSRFGRPIYGFDPDTYDGQNKSKMQS
FUN_005487 CDS scaffold_19 134680 136807 -
hypothetical protein PF00320

MEEDGAQLLQQTGLDPISRPDSTSTSASASLPHVLPGISALAAANAATEATSQLRASAAPSPAMYATASPAATSGGSGSTMPT
CQNCSTSTTPLWRRDEFGSVLCNACGLFLKLHGRPRPISLKTDVIKSRNRVKTMRPDLANKKKQQQQQQAAAQGFPTTDANG
FDTTGQAAAAAAHAAQAARRASQRSNGHDGADSPISRTGTPSMYAHGLSQFMVDDPYQTGFVATGEGRASSPTNGDRKMDAPQ
THEQLIAHNSSLKTRVSELEVIIELFRGRLSQLEQQEAVVRSGQQLNQLRSQLEQTQESEAQLRRQLEDSHRRESSLKRRLDE
LELELQAFQEANSAPGERPAKRPRTIEEPTKTQAEADIEAAAEALLASEASRPSEAVESAPVVDAPEPTELPQHPESSKEGSV
PKTEDEIEAADVPIDPNMPVIDDVSQPAATEETTKTEDVEIPDAPDASEAPGAPEAAEAADAAATADASPEATEGTETPAPAT
AAT
FUN_005488 CDS scaffold_19 138419 139767 + SVF1 Putative
cell survival pathways protein PF08622;PF17187 ENOG41

MFNWAKQQLANVAGTQEPIYGPSAIKSVATEAEKTPYTEISRNDLKWQAMDSTSVETHTFYIFGDNGYIALAQLIYSNVAGIR
TTCQFNAKVFSSDPSKPHLWASTPLNNHDFNEDKTSFYADDCAIELSEDGTYYTIKSMNDPNAIVNLKITRSTPGFQAGTTGT
TLYGTDLENPWGSMRHAFWPRCVSEGTITTKDGPMDFKGKAFFAYALQGMKPHHAASRWNFLNFQGPTYSAVLMEFTSTPSYG
STLVNVGGIVKDGEIVIGGVDNTATHTQVKKDSENDWPEPSDAKYTWVGKTKDGKAVEASVEGSLGERVDRVDVMAEVPGFVK

TIVAAAAGTKPYIYQYHPKLSLKMKIGDEEIVEEGTIFTEATFIS
FUN_005489 CDS scaffold_19 141075 141615 +
hypothetical protein ENOG41

MVACTYHNASSYAPVFPSPLNPTNHGPENKYATAPGRIVRRRRSRRGPPGSHTLTQRLLRVKAADAWRGHVLSAQVVQYEYAE
AAVMGHKAPPKSRNCLYSMAGLKNLGLNFSLSDAHRIASNIRLPDLTCLRTRRMLLAMGLVGLLPALKVRDMLRAAETL
FUN_005490 CDS scaffold_19 141940 142838 -
hypothetical protein EOG092653VU PF10173 ENOG41

MMRLYLLPLSTRRTLLYAKRLEANTAPQGRTYVDKGTAWAAKTWAQWEKMESGWKRKVVDYGNYAFRRIPYEEWGLKSVPPLS
VRRRGKEIEMKEKVDLCFPSSVIPPNKAEGILKTLATERQALHKKRLVWCIVGMPITIPFALVPIIPNLPFFYLVYRAWSHGR
AISGGKHLQWLLENKLLRLAPSEKLDRLYALHAPPAEEPDNKERTLLTQKEVQTFSDTLDMPALEVELERAIWQVEQAVQDPD
SQSPPKESSPGTAAEKPGSTETVDKKDQ
FUN_005491 tRNA scaffold_19 143569 143659 + tRNA-Ala

FUN_005492 CDS scaffold_19 144422 147692 -
hypothetical protein ENOG41

MLLLHQVGSLKTGEVVRYTITYIPSQDRILPSPEKLFLRVRNTSAIALRAAFVHGPYTLSAAAYPAAFNPNEKFEYPERYGVP
EFEPMVKAGGSWECELVVPVTIRQSAGLGSHGGFGKAPEHDHESASWIVEVSSQILFSTSAAVGFEVVLARDKKSLSLSNSSP
VVGGQSQVPQPGKISDYQQGVEAKEGHHSAQPRGVFSRAIQLKVEDTAALWNTPRLPGWDDKGEGRAEKRSGADQHVESVVKT
GDPETKGDESKPENPKKKQKKVHMVVLTHGLHSNLGADMLFMKESIDAAVKQAKIDAKARRARERAARKKSGNGLVESDSHDT
KDMDKVEGEDHEDDDDEEVIVRGYSGNSTKTERGIKYLGKRLARYVLSMTYPDQPFLPTGKGMTDAITHNFDKHDPQQKPVHK
HSTIHLSKDGAHLHKAERPYKITSISFIAHSLGGLVQTYAIAYIQKHSPQFFDLIKPINFVALATPFLGLSNENPLYVKFALD
SGLVGRTGKDLGLTWRAPTLARSGWGAIVGNLGETAHKKVYGETQPESKPLLRILPTGPAHTALKKFRNRTVYSNVVNDGIVP
LRTSCLLFLDWQGLGRVEKARRDAGLVETVVGFGWAELTGTNQARQKQLGNGSAGQSGTSSPQTHENMREVPQPPSTAMVDDD
RASLRSVATPYGDEVPSDSADSTNSGPLAGFFNFFKSNEPPKPPSVSQRQKKIYSKSQTLKLDESADGSVDTTSEAASTKQSK
VTTGHDGEDEMTAPPRTTIFESAGDLLNPKVPDVEYLIDPSKRPRTIFHDRVYHPADIPGPPLKRRPTTLVRRKTGQRQNSIG
QASTSSADSSPYPHVNHEDSSLSAKDYDDTANTNPDKDIHEVIDTSNMRVEEKIARAYHKDLSWRKVLVKVEPDAHNNVLVRR
MFANAYGWPVIKHLVDAHFSDSAASRLRDEDEHGRERARNMNEPPDEHGAELKKSRDNLASMTRTDSESREAMDKVSDLPKSQ
QSQRGSDASTHQRPKTERADSVTWSERDWHDSDCDSDLDFGGDLPAPTSPSALHQQDKGKEAQGPSGGWNWAEKIVGKGASRA
KSPVHGAKDE
FUN_005493 CDS scaffold_19 148575 148953 +
hypothetical protein ENOG41

MIDKDIKYFSLSGDIPIGGPSTWDIIGWDQRRVVSVTMDGEQDDESLAIEHFSRHSDQLSPDFHRTYVSHNGEINSTYTDSKN
DPTCCVHYPSLHDACPPEEVQTVRRDKLEELERLGPDADLVA
FUN_005494 CDS scaffold_19 149081 149882 +
hypothetical protein ENOG41

MSWKEMNLWMRLPCNPNIVPFDQVVVDELEGRIVGFTSNYVPGGNLEENKSRVFKLKWLQQLIKVVDELNLGHGIAHQDIAPR
NLLINESTDSIMLFDFNFAARINCPSSGEGESYVEERNDIKGVIFTTYEIITQDDSLRSIPHEDQNLDNLELKWVTHPEVKLD
HPVESYQLMLKEWRERRERDSRSGNVPRLIDWPAMPKPPQKTISLKTVQGQTTSVTVDNWYERRQDIRGRGDKVLNWERPPQR
LLDNGIRVLSTGEILNC
FUN_005495 CDS scaffold_19 150365 151674 -
hypothetical protein

MQQGCGLDRTQNLHYLPGSYHEPLQYASSSENSVDYFERQQFPSGQPNSGPTTATSAWYSGVDMQNLITPDDMVSLFSPDREW
SQSDIERLSAPMESNVTGPDSQPTCSCRNSSEPVLGQVPEGFLLWRQNEQGPNTCRIPSAPSHSELDSHRLRRRFSETGHHGP

GPGLHDSAAGMPHSTLQGHKLHQDSQEWPKWESRGLHESMSIATASLCVDEIEQLVKDEFPTQAQDKPPRSGTTLEQLLSTHD
IGKVFSRSKEQSLSPLPSEVAPDKVTNQYSSSDFAKFRLDTAPLVIAN
FUN_005496 CDS scaffold_19 153396 158247 +
hypothetical protein PF00005;PF00664 ENOG41

MPFHDSEFGLLYMNRDRFDFNLHFEQLFFSILPCTLFIVCSLWRAITQSRKPSVVYAPTFQLTKLGAIITYVGLQIALLIQVG
MGSFHVTRIFIASSVVNLVAALAMVMISYTDHNRSPRPSILLNTYLFLTLLFDIAQSRTLFLLSESTPEITYSAVFTTSLAVK
IVILFLETKSKNKWITWDRDEHSPEETSSIFSLGVLSWLNSIFLQGYRSTLSMDDLYPLDVALHSKQLHDRFLTNMDYSKLKG
DSYGLVKVLVKTLLVPLLLPIPLRLALLGFTFCQPFFIEKLLDHLSRPEIEANVGYGFIGAAILIYLGMAVSAALCWYLHHRM
RAMTRSILVTEIFSRATQARIGADDHNAALTLMSTDIERIRLGLRMLHNVWAGVIQAALAAWMLYIRLGLVFLVPIGVVVVCF
TLLGVLVNFTGDSQRAWMTGVQKRVGLTATVIAGMKNLKLSGLASTVSSFVQKLRVEELAAGARFRKIFILAAMLGFTPMLIS
PPLTLAFTRASLDTSKVFTSLAFLMLLTNPLSQIFTSIPELVSGIACIGRIQAFLECETRHDFRQVLSDRKKNLNFTEIEADT
GSSEIETAPDSTMISIKNGNFGWKENVFVLQDINAHIEKSSFTMVVGPVGSGKSTFCKTLLGEIPMSRGSVVLSMRCPRIGFC
DQTAFMWNGTIRDNILGFSIYDEKRYDSVIQATALSVDFAKLTQGDQTNVGSDGITLSGGQKQRVSLARALYLESDLVILDEV
FSGLDADTELHVFQRVFGPGGLLQQRRTTVVLCTHSVRFLPFSDHVIALGDNTIKEQGSFKELAEQGGYVQSLGIRDSAGGDG
SEAVSETAASKERTEAQKDAQGSPDGSPGAEVNLDDPNRATGDKAVYKYYFLSMGLLVAFCCFFLAALWGFFTNFPTIWLKFW
TDDISSETRSHSHAYYVGIYALLQVSALISLLLLGITLFIVSIKRAGANLHHDTLTTLIQAPLSFFTHTDTGVITNLFSQDLN
LIDTELPDALLNTLFCVFQALGQAAVMPTSSAYLAISYPLLAVLLYSVQRFYLRTSRQLRLLDLEAKSPLYTHFMDTVRGITT
LRAFGYISDEVSKNARLLNRSQQPAYLLAMVQEWLNLVLDMIVMILAAVLTTLAVKLHSNSGFTGASLVTLMSFGESLSGIVI
YYTRLETSIGAISRLKTFNATVKPEGKEGEDMVPPENWPQHGSVELNNVSASYQMGSESEGEPNLALDNLCLSIKPGEKVAIC
GRTGSGKSSLVSLLLKLLEPLQDTPNEGLLIDGLPLNNLDRDALRQRVIAVPQEAVFLPDGATFKENLDPSGLSTLDECKAVL
IDIGLWDFVQGRGGIDAGMTSSTLSGGQRQLMSIGRSLLRRRARTPQTPGPGNMTEKIKDQGGLLLLDEVNSSVDQDMELIIK
RIIETEFDAYTVIAVSHRLDMIMDFDTVVVLDTGKLVEVGNPRALASTEGSRFASLVRAGGK
FUN_005497 CDS scaffold_19 158852 159719 +
hypothetical protein PF00550;PF08659 ENOG41

MSMVFRGRMFEDMTFQEWEMTTRPKAQGAWNLHNASLAVKCPLDFFLLFNSLSGILGQVGQANYASANTFLDAFARYRAGMGL
PCTSLDLGAMEGIGYLDENQDLLRKMKGTGWRPVDESELVEALNVALMPPSSPQEYGDAFLLGVAPTVPLGSAESSTRLSNVR
MAAYHNIGRGQSDALPANDGLRAFLSSVKKDPSILNSHESVNTLALEIGKKLASLLLTGDVDLDTSTNTAAMGLDSLVAIELR
GWWKLTLGFEISTLEMLSMGTLEALGKRTADGLKGLYDN
FUN_005498 CDS scaffold_19 161374 163833 -
hypothetical protein PF01485 ENOG41

MRLGYDRDVADGEARLPLVRRRPLRPESHDWYGPSDVVPYNQYPMPHRTRIDELQRPNGRTPRGYSRSMSLDAYPPTMPMPPA
HLMRASTDVYKKRPQFSVYEAGEDDEDDERWPAMKPRGRHPEPARPRQVVRRRREPTPESTPPTDSESESESESEEESDDQNQ
IEVVVEEPEDKHRRHHRRHRRSPSPDDYLSSPDERRTPRRRNRIPSPSTSDEREIRYRQASSERGSPDRRPALRREVSDTPRY
SLPTRFNSVLGASRPPVASRRTAKVVESRELVREGRPRRLPIATIETVEVSRESSRRPPSIARSGFDSSRNSSPDRPRLIRDC
KGCLDEVPVSKCPKLDCGHRMCHSCLKRRFKQSMTEAEHMPPTCCTDAHIGLEHVDKLFDSTFKRIWNKKFVEYSLRNRLYCP
SRRCGEWIRPADIYRDRETGRKAARCDRCNTKVCVACNGRWHFASKCPRDEETAMFLEYVRAEGQKRCYRCGASAQLREGDNH
ALCRCGAEFCVVCGEKPKRCECPWFEPDAPESDHEQEVTHRGEIQVFREDSTEEESRVARRPRKAKPTRPPIYDEEAIMRQQQ
EERDDELVRRSHYHGKDYEYDIGGAPEVPRSSDPYSGDSPRRVGRRVVGGAPPSPSRADFERGTPRSGGYYAGRMDRRHAEDR
YAPDSPGMRPPPMGMPPPVIEEGYRGGVGPLIVERDPNTYDDDDSYYSHSSRSRKGTRRSENRKSSELAGLGPRTPGLNRIDV
WRTFVEPGDPEGEIAAAAAVTA
FUN_005499 CDS scaffold_19 166194 168409 -
hypothetical protein PF00172 ENOG41

MSASSTGESLHSISLNAARPFTVPARDSFSLELPISSLTAMSADASLKSPVSYKNQRTPSFSREGILSASQKSRHLSQSSDNR
PDSMSNGAQKVSSDEGSNPLKRRNTETGVDYPRRRATIACEVCRSRKSRCDGTKPKCKLCTELGAECIYREPGIKLDAGDKLI
LERLNRIENLLQMNMVNTTANGLNLTDNSPGMSNGTGLDGDNMMLSANAASFGSIPNGGFGTWSAQPTGTNISTMPKVHTNAT
MHLLQWPLIRDLVSRPCDPQILLQLEMAREPLHTLTKTPCVDLSNTQAYIEAYFERVNIWYACVNPYTWRSQYRTALSNGFRE

GPESCIVLLVLALGQASLHGSISRILPHEDPPGLQYFTAAWSLLPGLMTANSVVAAQCHLLAAAYHFYLVRPMEAWNLLCTTS
TKLQLLLMAPSRIPSHQRELVERIYWNALLFESDLLAELDLPHSGVVQFEENVGLPCGFEGDESEQVGRDELWYFLAEIALRR
LLNRVSQLIYSKDSIASTTSLDPVVAELDYQLSQWYESLPLPLQFPFTRNALQDPVQTVLRLRFFACRTIIYRPYILAVLDNE
QAVLDPAVRENCHKCLEASIRQLEHIHEHHAGHMPYIWQGALSIVSQTLLIMGASMSPSLLSILLTLVPHREALDQMINDVVI
EVERYATLAPSLSLSSEILKEAEVRRRAFLGTP
FUN_005500 CDS scaffold_19 171550 172994 -
hypothetical protein PF10282 ENOG41

MKFFGRQKPSKNPFRDNSTTLLVSTLLLAAPITVFLLHCVYQTYYRSGAHWSHNHDILELSTHHEPDVLLYVASYSGLVTTLN
LSVAEYRDTPVKLETLSTTDGCAGSPSWLTLDWYNGVLYCTDEGLKDGKHGSLASFATHENGTLTPLAKTSTVLGPVSAVMYG
EWDYGLAVAHYGGSAFTTWDIQDPANLTSLNVQQFSIPEPGPDPSRQEASHPHAAVVDPSKRFVLVPDLGADLIHIYGVGFGD
LGLSKLDPLVVAPGSGPRHIAFVVKETKTFMYLVTELANTIIGYEVVYGGEFIRFKEMWNSGIHGEGKDVPQGAAAAEIVVSP
DREFLIISSRNESTLQVPDFDTSNGTIPSDPLVSFKIDSETGHLTLQQDIPCGGRFPRHFSINKAGTLVAVALQSDSRVVIIE
RDAKTGIMGDFIAYAELEGEVTAVIFYE
FUN_005501 CDS scaffold_19 173835 176873 - CHO2
phosphatidylethanolamine N-methyltransferase EOG09260EZT PF04191

MSTAADLPATGPGLRQRQSGVDPPAQQDKPQAEQGHSRQSSDVPVPSDKSSKTYGRTPDGTVFIVPTTHDMVSQLLDPRQPKN
LSDAIVLTILGLHILAAYFLPSSSKRIVFAAVFLFWRACYNIGIGVLLQIQSNHRRLVTWARRWKLFENPSTGKNPRPWLYKL
LKNELETKIPEDYEFEKAPIEYNTWLVFRRVVDLILMCDFVSYCLFAIVCGHTPEGENPLIGFSRWAVGIALIGFNLWVKLDA
HRVVKDFAWYWGDFFYLIDQDLTFDGVFEMAPHPMYSIGYAGYYGISMMAASYEVLFISILAHLAQFAFLVIVENPHIEKTYN
PPPPRKRTVSGGQSDAIPTDMKSLEGAFDQQTLTPAQKDEPAPVHNLVGLSNLDLFRVPDFAVILMPFYVAVLTLVTPSTAVW
QAAFVFHALAWRVWYHLGLGLILDQQSKNKMWTRHFLKFGESAGEAWRQWKGLHHISMIMCNTAFVAACWKMYSPPEDWAYGL
VLLKHVLGASLVALQLWTAFSVYDSLGEFGWFCGDFFFDQQAKLTYKSIYRFLNNPDRFFGTAAVWGAALITWSRSIFLMALF
TQILTVYYISYIERPHMQKIYGRSLRQEAGLTKFIKKSLPPPVKGWQESVDKVLDDTSQFVEDFIDTARPKFAAGVKTIVRDT
SALFNMAPARLTITRIAPDLEGRDPKFYSLSVQGTPVNNAPIVEKYTGKESLTGRFPKPVKTMAFEYGAPLRVKWRAPANHSK
KDWIGLYMVTDNRSRETTEVSSLGRWAPTNAGSYDALTADVSIVVEEHPVTSTEITETDLVEGEVVFEGDKLWWTQGVFEFRY
HHNGHHHAMAISEPFEIRISKFDEEDVDLGAKGLYEQAVEAALLPVVQNCLDRDPDIAPNQPEEPFGGHVERDTKYAKRIVYA
IREMFGIEFAPPVVAADGSVRKLAWRICNAKEVLAPYSMSLSRGTTTPAIQDFPSEKA
FUN_005502 CDS scaffold_19 180295 181306 - EIF3G Eukaryotic
translation initiation factor 3 subunit G EOG0926489S PF00076;PF12353

MAAAVANQPKHDWADDDDIEETSTDLPEPQTISNKDGTRTIITFRYDDDGRKVKTTRRIRFTTHTETVNPRVADRKTWPKFGL
SAKDPPGPAPDTTSVGENIIFRPSVNWRKAEKDESADANAQSMKDKLKDKQVKCRICNGQHFTARCPYKDTMAPIGETGAADV
AAGMGDEPSAAGAGAAGAKKGSYVPPALRGDRGAGERMGSKFGERDDFATLRVTNVSEMAEEQELRDMFERFGRVTRVFLAKD
RETGMAKGFAFISFADRGDAVKACAKMDGFGFKHLILRVEFAKKAQ
FUN_005503 CDS scaffold_19 182377 182740 +
hypothetical protein PF12734 ENOG41

MAFKQEEPPAYGPPPGAPQPTYGGYQQDPYGQQQGQFQQGGQQQGYYQQGPPQMGYYNQQQGPFPAGQGPYPPQQGPYGQPPP
QGYYPQDDQRGGGGAGGGIMTGLLAGLACCCCLDCLF
FUN_005504 CDS scaffold_19 183004 184376 -
hypothetical protein PF01494;PF13450

MASTKDLQIAIMGAGMGGLGAALALAKRGFKHIDVYETASSLGFVGAGIQMAPNMGRILDRLGCWDDIEKEATCVAGSSIRQG
ATNVELAHVDMPDIKGKYGFSHLCGHRASLAGHLYEACKKESAITFQFATSLVEVESFAPKVTFKLQPRDGEAFTREADILLG
ADGIKSVTRSQLLQQVNATPEEAETGQAAYRIMLKREDMAHDPELLALIDSDKVVRWVGEKRHIIAYSIANKSIYNLSTVQPD
DNFASAPSITYTTKGSKKIMLEVFETFCPLVQKMLNLVPEGEVCEWRLRMYKPLPTWTQGSVALLGDACHPTLPHLSQGAAMA
IEDGSTIAEVLSLAPDTQPETIAKCLKVYEQSRKEWTSNLVEMAYLSGRTLHLGEGKAKEERDRMFKEHKTSGSVPDKWTSPD
VQKMIYTNDCVAKVRSEFETAFAAA

FUN_005505 CDS scaffold_19 185300 185930 +
hypothetical protein PF04828 ENOG41

MHLDALCACEAIHLRITRPNEASYLPHRAYPDLTYPYCSTDESITSNPSDEKWWIKGDKYLAGTCICESCRRASGFEIQTWAF
IPRANIFIPSTDGSGSEVALDFESLPTGALKSYQSSQGAVRHFCGGCGATVFWRDATDSSVVDVSVGIFRADEGARAENWFHW
HKSRISFAEEVQNHRSGPLAIAAQGLLGTLSMGLKGSSEGGLD
FUN_005506 CDS scaffold_19 186533 188875 +
hypothetical protein PF02333

MVGSSLVGLLAVVFQICFVKAVDVDVAVSAYTGEVESDWTAVYYSNRNPLLIGNDGGPDEGGFHVYDLNSKTPLKEVAAKTPG
RSKLVTTVYDVNKKDILVTIAQPDSIVRVYEMPKFEQIKDADFKVLGDWSALCSWKSPAGNDYVYLFGKGQAAQLLLQETKKS
FEFVQIQTFPTDFETSGCAVSRSESRMYISTDDDKSVYAFSLKESTKAPKITKVGKVEDDVTGLAVYVPKKGSDYLFVAQTDK
ITVYDSSFKLKGTLSLTGQKDIEVQGLNIYQGATSKYPVGALTYAIESEDVNGFAVSSLENALKNLKIEANTKYDPRKVKTDT
KTEPICKTCGGNGYCIKDKKNKCDCFAGFAGSTCSSFTCTDKCSGHGKCVGPNECKCDKGWGGLHCSFLLVEPTYETESRLGD
GDDPAIWISPDGPEKSRIVTTMKSGKEAGLGVFDLAGNLLQSFTAGEPNNVDMIYGFKAGDRKVDLAFAACRADDTLCLFEML
PNGTLTNIPGGIHPVVDDYKVYGSCTYKSPKTGKQYLFVNEKSARYLQYELASTSKGELQTKLVREFQGGSGGQVEGCVTDEE
NGWIFLGEEPSALWRYDAEPDSKDKGVVIGKVGDGKLYGDVEGVTLVYGSKPTEGFILVSCQGVSAYNVYRRASPHEYVTTFT
LVESSDGQIDPVSNTDGITAVGTALNKDFPHGLVVVHDDANQLPNGKTSAEASFKLVSLEKILGSKVFGKKGLLDQVDKNWDP
RK
FUN_005507 CDS scaffold_19 190320 192123 +
hypothetical protein ENOG41

MKYSFVTLAVVAGAALAAPPPSAIDNFGPDFFTPFCNLDYKGKPCEELVGKGDKNADAICKAGREHFCGPQKRDAVPEPQPDP
VADPMPWCTWRGQPCWKEKMKAKREAIPEPVTAPQPDPVADPMPWCTWRGQPCWKEKMAKREAIAEPVAAAQPDPVAEPMPWC
TWRGQPCWKEKMKAKREAEAIPEPIAAPQPDPVADPMPWCTWRGQPCWKEKMAKREAIPEPVAAPQPDPVADPMPWCTWRGQP
CWKEKMAKREAIAEPVAAAQPDPVADPMPWCTWRGQPCWKEKMRMAKREPEPVAAPHPDPVAEPMPWCTWRGQPCWKEKMRMA
KRDAVPEPVAAPKPDPVAEPMPWCTWRGQPCWKKTKRAAAPEPAPEAENEPRWCLWRGQPCWKKTKRDATPEPWCLWRGQPCW
KAKRDAAPEPWCMWRGQPCWKAKRDAGQALSNALHATRSLDTRSADAPSTAHLPRDAAHKAKRSIVELANLIALSARGSPEEY
FKSLELETFFPDAAPNATAKRDLNTLQEDKRWCMWRGQPCWKAKRAAEAVLDAVDGDDGATGPGGPDSHYDTRDFKSENFAAK
RDLIAIKAAARSIADMSEE
FUN_005508 CDS scaffold_19 194003 194823 +
hypothetical protein ENOG41

MASTHNVSTGLEKGQRSAPYREGIHVLYASNTFHTTSKAMILNMHNFFQQQRLNAITSVEIIWEFTPWILPDISEDLRVAPPL
SDIETFRQFLKAIPGLFLNIKKLYVSLQGDMIPKSRGQNSEYQYSTKERIELVEKNIIWKVDEMVPYLKPHVDFSIAYPTSTY
AVQRSLALRQGWIVKQRQETGEVERHWRPLVKCKPRTGYWVCLGDKDVRRQRARGVIGHQHMPARVSEKKYDVFFRS
FUN_005509 CDS scaffold_19 195599 197339 +
hypothetical protein PF00387;PF00388

MASELATRIGDLNPFSSKNRRIDDEDFGEEIDNNTVAGGGHSARRVVTDLRVSSALREFLVKEKMLSKKEAKVESEDSSRELL
ELVSKPHIRVPAELTDRSHPLPEYFISSSHNTYLMAHQLYGSSCATAYESAIRTGARCVEIDAWDNSDDRDEPKVTHGYTLVS
HISFRLVCETIRNSADQEAVDAQKYGFRASPILLSLENHCDPYGQMRLVNIMQDVFGDRLLSKAVRHIGHEEQAGSDQHVKLE
DLGAKIAVIVEYHFTDEPSSSDSSSDDSEDEEEEKAARKEYKDKRKKEEPSIIIPELAELGVYAQSVKPMDNSWFEEGSLANG
PHHHLINVSESGLASHLPEHATHIARHNAHHLMRVYPKGTRISSTNLKPVPYWGIGAQICALNLQNFGTSNQLNEALFSGTDG
YVLKPAALRAGGNGRLSTGRSKRLRLHVAGATDIPLHGDSEADSIKPYLTCTLYHPDNIKGDPPKRKTEPYKQHKLEFLHRGP
NPPPTEPLWDETLTWEYEDNELTFLRMLIKSDDSWTRNPMFAVAAVRLLYVEPGWRFIRMLDLKGHETQCSVLVNFEIIDA
FUN_005510 tRNA scaffold_19 197621 197693 + tRNA-Gln

FUN_005511 CDS scaffold_19 199086 199878 -

hypothetical protein PF01596;PF13578 ENOG41

MAPSPTALQALEASPRVHGLLAHLHAESEAQEKTLSQSWFYFKYLFGFYFTGKTWSTSADAHMSDKFVALEQDKCLFMYLIAR
SINAKNIVEAGTSFGVSTMYLALAVGQNVNAMRARDDKVAGRVIATEWESSKAERARKHWSEAGEEVEPWIELREGDLRETLS
EEGMPEEIDMMLLDTWIPMALPALEIIKPRLKRGAIILADNTKMAKALYKEFLDYIHDPKNGFKTTTTAYSGGLEMIVYLP
FUN_005512 CDS scaffold_19 200541 200910 -
hypothetical protein PF12973 ENOG41

MEQFEFHPATTSFNRVWSTIEPGVDELVLNEDSQTGRRTTLQRWQPGARNQQDIFIHDYVEEIFMVEGDLYDQNLQQGWEKGA
YAYRKPGMRHGPFKSERGCLMFIVCIPVDTNGKEAGDRV
FUN_005513 CDS scaffold_19 206887 207411 +
hypothetical protein PF10224

MAVNELPNAPAEHPHAIARSDSSTSSSPEDNNEPIIHQPVPVRPGLPQRKSSGPLVVPRDSSAVGPLEPDFGPDDVRAMSPRR
TSEDLDRIGKAAREEMRRHAKALQDSLLTIFNRIEAVREEHDKLDNNNKFLQKYIGDLMSTSKITATSSQRKK
FUN_005514 tRNA scaffold_19 211434 211506 + tRNA-Thr

FUN_005515 CDS scaffold_19 211766 216434 -
hypothetical protein EOG09260NHB PF12584

MEQPFSTSKVTVEYFDPHDVYKLLAPGLIPRLPLRNLNWQSHAGPLRSIDTLHVELLQGDDPGSASSPHTLRRSGSTLDDGFQ
QQNFGGQASSADQIDTQSLPSRAIGSQRRHQIPGLRRTPYLKVLLVRCDDNETYKTSVRAEIREWIKTQIPSSSNPKRNSKQE
KHDAFDYLILHVVLPNTVASTQPRTSSRSQDTSEKSTPRWRTGSTPLLEKLRTDFSGLGKTAPDRVAQIRIGINDLPYDQLPR
VVPAVPTGYSEDEHDAENAWAELIAKFKSLILTSFDLRVTQYEEDIKEKDGQRSLPGWNFCTFFILKEGLARGFESVGLVEDA
LVGYDELSVGLDSVVNEQADEGSPTRHGGAMLSYTEDLKEIVKTALAKAPGGNTEDEEAVDLQSNETIKEQFDEIPISATKKA
YRDKILANDVSVFDFRCYIFARQMALLLRLGNAWSSRDDLLDKLKEQQDSVLHGVAPLAPPPKHTEETENLASLGEICRRTLQ
FIPAVSQIMRRDILTAVASDKSDEDDSPIDPNLSEVIDNVVASFAFSVAQQILAQTASKSLPIPPSTLAPPQSQEQKSSIPEP
KTMMHPARTTSLHVSTGPAPGTRPPLSPGVFPGPGMPKINNDQESQFLKAGLEDLAARRAELYMLSRSILDGLGKKRGWSDGW
KEAPIIAEAEADFEEISLDDQPAASGISEEIPPLDAGVNNNLLQTAIDNPENFYRLYEILTDKALRHYTVANHDHAVQASMAD
LAVLNFYLKEYSTAASYFYRATPFFGESGWTSLELSMLVMYLHCLREMKSKDDYVRVALKLLTKSCAAEKERLEQRSKRVSRL
GKPEPADAMSMKGVVGNLFDLASSLSSQVKVHLSNFFTNIELAGAPEYFDKEDKCSLTINLWCLLPDEIKLDGIDVRATASEP
GPTKELLFSRKGAIVLQPGQNSITVECTSVIPGKYKIDHLGLFSSNLFLHFERDINQPPPQTTDIFKHPEVVIFHRDGALDVQ
LVATRHTSLDKNNTLDFTLNPGWNSIKSCEIRLRPTTGGLRMLTLEAQVIDSDVKFAKKPEAGGIIYFNEIAAESSITLRFPY
SVEHDIADVSAKVDVAYTLESGQQYTFAKSIIIPISLALGVNVQDVFKHNALYSRFNVSTASHSPLRLYKSELMPSDLFESEF
GVPPADTTMVFSKQPATLLYRIKRKTNVRSSKRAARTMYLKLYYNILQTEIEEALVKSIFDALDDPSLASFSKLITSLVVREA
KSLQPIDLERAALLGAVPTAFLADVPWDKYFAGIGRVPGTQVDATEKISTAIRKWQASNPQVQISAAQPDNPCTLLIPVEIPS
LPILHTADIELQTPVTELMDQKPGAVPTVAVNQMLPATLHLRWTQIWDTEMQHKKDVEYSYEVAAAGDTWLLGGRRKGHLVIP
GSKKDPLSSTSDTEAEIPLIMIPLREGWLPYPGIEIREVKDGVENQAQTCEVDFRNLGESIRAVGERKGVTVSLDASGPGGGP
LVLESEEPKRERGRIVA
FUN_005516 CDS scaffold_19 216816 217983 + MRI1
S-methyl-5-thioribose-1-phosphate isomerase PF01008

MSSLKAVKYTRGQLQVLDQLRLPHEFHYDDVSNRTEAFDSIATMRVRGAPAIAIVAALGLAVEIHNGSITASNAEDTISQIEE
ALDYLKESRPTAVDLTNAINQLKSTIRAVGSTATKEQVITAFIEEAEKIFEKDLQTNLSIGDYGAEWLRAQAGASPDKKISVL
THCNTGSLATSGHGTALGIIRTLQSKDLLQHAFCTETRPYNQGSRLTAFELVFENIPSTLITDSMAASLFRNRKQEKNIAAVI
VGADRVVRNGDTANKIGTYQLAVLAKHHGIKFIVAAPTTSIDLETETGDGIKIEERKKEELTQVTGAVIKPDGTVDESSKVRV
ATADQRINVWNPAFDVTPAELIDAVVTEKGAIEKGPDGNFDFSKILPERWAKITGA
FUN_005517 CDS scaffold_19 218064 219359 -
hypothetical protein PF01546;PF07687

M20A
MRVATVASFLASASLATAFDWQQVLGDWDQQPSSDAIASNDDAPSYRSELLSLHKSLIETSSVSGTEHDVGVWLEGYLEKKGY
TTSRQEVEPFENTPEGQPRFNVLAWRQDGKKTFDPKICVSSHIDVVPPHIPYGIDDGEVTKETMITGRGSVDAKGSVAAQITA
VEDLIEHGKIDPHKLVLLFVVGEEVKGDGMRRFSEAIETKELPYSLDAVIFGEPTELKLACGHKGMLGCDVTTKGFPGHSGYP
WLGKSANELMIRAFAKVLDTDLGSSELFGNTTVNIGRFHGGVASNVIPEEAKVGLAVRVASGKQADGHVVVHDKIQAIFDEVD
KDAFIFDCTHGYGPVEANCDVDGFEKITVNYGTDIPNLKGDHTRYLYGPGNILVAHGARENLTVADLETAVEGYQKLILHALK
Q
FUN_005518 CDS scaffold_19 220636 222427 +
hypothetical protein PF01989;PF04412

MGSLEDDAWTGTAYVQGRASARLLASDLELSFWGGVEPQTSEIIDRHHPLSGQHLQNTILAIPGGRGSCTGSGVMLELLLNGK
APEAIIFERREDILTLGVMIAEEVFSQSIPVVVLDKEDFRHLIRMNGQMIYVDDGYVSTHPPSKYRKGSVIDTESGLILETTP
ALEGIKLSTLDQELLRGDYGEASRVAIRIVLRMAHLLGATRLMDVTQVHVDGCVYTGPATLALAQRLRDWGGKVRIPTTLNSL
SVDQKRWRALGVDTTFGEAADALGKAYTDMGARPTFTCAPYQLESAPKLGEQIAWAESNAVVYANSVLGARTMKYPDFLDISI
ALTGRAPKGGPHLGVNRLASVCVKVMGLEDTTGLDDSFPPLLGYYVGTLSTSRIPVIAGLEKLGLSTDDLKAFGAAFATISSA
PMFHIVGVTPEATTLEAVVAPDFTYVEVKTRDLSTCWDKLNSAPRTQPIDLISLGNPHFSLSEIRKLANLCEGRQKITSVSVI
VTCGRSVYKLAEQAGLISQLEDFGVQILTDICWCMVTEPVIPAAAKTIMTNSGKYAHYGPGLTGRGMYFGSLAGCVEAACSGT
YEAEKPGWLQIKGLI
FUN_005519 CDS scaffold_19 222483 223538 -
hypothetical protein ENOG41

MFSVEVYFDMLLGRDYGSLAHFHFLKTLRLLQARINNPKDPTSISDATIMVVVILGLAAEMIGDRTAAENHATGMARIVDLRG
GLEMLRFDNPRLPAKVCRVDIGLALRFGCKPVFFDKDMSWNPYLSSQDFVRGKRKHPDTNHDMEAFLKTLDPRLSNVWRDLEE
FAKLSNIASQTGRKLQPNIFSEAMVSILYRLLALSPESASENAFRLGMMTFAASIFFRWRDMKQRQAYLDDSFTDALIELKKA
ATRPPSTVLLWLLMIWRTNSVQGGGDQAIEGWILEVMDGLAICSWSELHNVLKSVLWVDCLFDASSKRILEPTLEKAARKGAG
VDS
FUN_005520 CDS scaffold_19 224398 225520 -
hypothetical protein PF00106;PF08659;PF13561 ENOG41

MPCQKNQKPSPTVPSREMSPLPDFWLPVFWKNQFRTTIELPTREKYPQVEGGTAIMTGSNTGLGFEASKQLLSLGLSHLIMGV
RSIEKGNAAAEKLRKIAPSARIDVWQLDMQSYDSIQAFAKRCDTKLSRIDFVILNAGLSPSNFELVPDTGHEVGIQVNHISTA
LLTILLIPILKTKTSGHRPPRLTTVNSLTAHLCKFPNKDERPLLASFDDLKITPWSSQERYGVSKLLNQLFVVKLCEIVSPDD
VIINMVDPGLTKGTNLGGHHELGGVAGALAGAFFAICGRPVDRGAASYTNAVYGHGKESHGCFLMSCEIAPLAGWFYTHGDVL
VDQVWNETLEELDFAGAKDIVSNI
FUN_005521 CDS scaffold_19 226687 227774 +
hypothetical protein PF00291

MTVVTLPEPFASIPRENFLFGASPLQPLPRISAALGGKVNVYAKREDCNSGLAYGGNKVRKLEYLAAEAQAQGCDTLVSIGGV
QSNHTRAVTAVATKLGLKAATVQEHWVDWEDPGYEKVGNIQLSRLMGGDVRLDPSTFGIEHKTTLAKLTDELKSNGRKPYYIP
AGASDHPLGGLGFARWAFEVEAQEKELGIFFDTIIVCAVTGSTFAGMIAGFKLTQKNGSPARKIIGIDASGKVQQTFDQVLRI
AKNTAAKIGLSEDDITADDVILDPNYNAKIYGIPDETTIEAMKFGAATEAFITDPVYEGKSLAGMMDLIKTGKIASGNVLYAH
LGGQLALNAYSSL
FUN_005522 CDS scaffold_19 228006 228795 +
hypothetical protein ENOG41

MGDSRDTKAAVLSALTDFFSISPKSPKDARNHLLETSYVIQSTPDTIEQTTLGELFLSDTKQKGHLEWAIYEPQELFIHEKLA
AAWTGWSICADDGTIISHKKGILGLAYTDGRWKISGLASTERSLETPDPDDESTVTQEIMKPINALLDDFSHPDWDVLKQWFL
PNAGVTLYRPPAEPAPMTMEQSIVRLQGMIRSGITIQEKLHDIQVRKHGDIALVWAPFVVEINGVPKHDGVNAFTLLRREGRW
VFSGCQDYGVALQ
FUN_005523 CDS scaffold_19 230322 232048 +

hypothetical protein ENOG41

MADMSYEDTQHLLDSITSTGFDHLLNDHKDDNTTEEVDEDTKVSMLMDPSNSNFQNHQAQHGIENEGLLASNSFEKEFMDACK
AANARIPNSSGSDAQLSGLDNSGSTIGEDVVQGPVSTALTPATQFSSPTNPKDYLPKSFQTQPYPPLSNQDQSYPVPMGQPFY
DQTALQQMQPFPVRQPTGLGGRQIRQTQYPQQQLFVSVNNQLHQPLPGQVSHPAFQVSQPHKPPSQAPPRKGPAPGEPTRHKI
PANPANIKAHTLEQQSLLSELEHFTHEYQGYITNPVQARHIGNIFLSLAHHEVKVTPPDIDPTFPRTHEAYRARVQELFMAIV
DWSNPRGWRTKMGSKLAAQWVEEVMAYRKNVGLSVEPSDMLDHQIEPPLDRMPPVNEQWKNVVHRQLSNIEIEILSSKILDNK
VLIHSALRSSWLSRITNSPVAELVRKENNKAGNDKKRNAAHRQNGKGAKRPQNEESEDEEEGRKTKRKRN
FUN_005524 CDS scaffold_19 232524 233460 - PDX1 pyridoxine biosynthesis protein EOG09263MIB PF01680

MTNDSSNTGAASNGEAKSSFTVKAGLAQMLKGGVIMDVTNAEQARIAEEAGACAVMALERVPADIRKDGGVARMSDPAMIKQI
QEAVTIPVMAKARIGHFVECQILEALGVDYIDESEVLTPADDESHVEKSTFGVPFVCGCRNLGEALRRIAEGAAMIRTKGEAG
TGDVVEAVRHMKTVNKQIAQAKAALAEGGIIRIRELARELEVDADLLRQTAELGRLPVVNFAAGGVATPADAALMMQLGCDGV
FVGSGIFKSGDPAKRAKAIVRATTHYKDAKVLAETSTGLGEAMVGINCDSMKPEEKLAGRGW
FUN_005525 CDS scaffold_19 234527 235292 +
hypothetical protein PF01174
C26
MISLTVGVLALQGGFAEHIDLVRKAAEYLSSTEGISKTKFHCIEVRTKEELDQCNALIIPGGESTTISFVAAQSGLLEPLRDF
VKVQKRPVWGTCAGLILLSDEANATKKGGQELIGGLAVRVHRNHFGRQMESFESGMNLPFLNDDKPFPGVFIRAPVVEEVIGS
SDDGRPPVEVLAKLPGRVDKMKSGVSQANTKDDSGDIVAVRQGNVLGTSFHPELTKDERIHVWWLKEILNQQ
FUN_005526 CDS scaffold_19 235377 237407 -
hypothetical protein PF12796;PF13637;PF13857 ENOG41

MDGDGFSVISLTSLGSSISHRVLSSAEELNELNTALEVPDERLSSFATRLYQLHQHVGVLETALNSASAISGRLQTTISQSLG
SCDVVATVLNKQVMRLQAETLPLLDEVFLVVYSDAVVAFSRLFAFFAKVLSIKVRDDQDSALDTKSGREIFDHVNRACQKASQ
TKDIIFTGSQNDSYASSSKGPLPGDIEPPPYEPAQPSSAQSPSCESPSGSSGFAKGLSSLTNSFKAMTAGLWPKPDPLSTALC
QAALRGDVQQMSGLLAQGANINGRNGEGNSPLGCAILANQEDAVRFLIGSGADTNGRDMKIPPVFLAASVGSIGVAKMLIDQG
TWNVNAASWSGQSYFVDVCNSENLEGIQLLLDNGAKPNTTNTSGRPVLAQAVKKGNLELARMLLQHGANPDTSDLSGNSLLSI
AASQDRMDMMKLLLESGTNASSKNLSGLSVLADAIAKRKLDMAELLLKHGANASAKDLVGHPILVIALRDSKLSQNEKIRVVR
MLLDHGASPNVSDGTWGVAAICFAMETGNTDLVKMMLAAGSNTKKKMSSGETLLLYAIDNGKRDQAKLLLEAGADANAADKKG
RTPLMQAISRRDTELIRVLKAHGADMNAGGCISPQELAQSMGDLEILQILGFGSSQRGPARAHSPPPPGYDAAMGKV
FUN_005527 CDS scaffold_19 237931 238260 -
hypothetical protein ENOG41

MSARIARSSMALRPSLPIQRGFRTTRLLRHNEKEPIGPKSQGKPMSKGDNSQIVMLGGVGLAIGLGFFFLMSRPEKAKEQPIE
GVKSKPVSPK
FUN_005528 CDS scaffold_19 239279 240773 - PAC1 protein with putative role during mitosis PF00400;PF12894

MSRTLTSRQAEELHKSIIAYLAANNLQESANAMRTELSLGEDAFDTATAKKYETLLEKKWTSVVRLQKKIMDLEAQNNALQTE
LNSATPTSLSNRRGDPSSWLPSGPPRHVLQSHRTPINCVAFHPIFSSIASGDEDATIKIWDWEFGELERTVKGHTKAVLDLDY
GGPKGHTLLASCSSDLTIKLWDPSNEYQNIRTLPGHDHSVSAVRFIPSGAPGAPLSGNLLASASRDVTVRIWDVTTGYCVKTI
RGHVDWIRDVAPSLDGKYLLSTGNDRTVRLWDISVPNPEAKLVMIGHEHFVECCTFAPPAAYSHLATLAGVKKAPPASSTAEF
MATGGRDKTIKLWDGRGTCIKTLVGHDNWVRGLVFHPSGKFLLSVSDDKTIRCWDLSQEGKCVKTVEGAHEHFITSLRWAPPI
IKDKGPTEEANGDVGTPKKAAAAPQDVQIRCVIATGSVDMSLRIFSR
FUN_005529 CDS scaffold_19 242585 243838 -
hypothetical protein ENOG41

MAESQLPGLPGFDKHNIQPWTVEVVVSMTVLALVSCGLRLYSRHLKAQKLWWDDYVILFSMSWNLVVVGFIFAMHANGMGIHA

DKVPMTQIVMMAKWLVVAEVLYAWNLGWTKLSLLLMYYRIFRVPYFKKMAWLVGSFVFAWVITITFLFIFICIPVAKLWYPDL
PGRCINQVGTWIANAASTIFTDIVILCLPLPPIWKLQLGKSEKLGLTAAFAIGSFVVFASAYRTSVLFTYTSTDPSYTLAPTV
GWTEIEMSAGIVSANLPTMLPVLRLVAKFTGLSSFASTVRSNVRPSGDKSTKGLKSHENTGAGRSSNGSASTNTFYRLPDDND
STDLIKGPKFTETSAPVNTKLRPDVEGFELTTTTYNAKVDGGNSSSEEILQGIRVHREFQQTTTRSP
FUN_005530 CDS scaffold_19 245098 246161 +
hypothetical protein PF12697

MAAKSTIKVPHLGGIDAGYRVSGSGIDSSKPTLVLVNSMCTASSLFEAQFSAQELTDKVNLLAIEPLGHGATSSKSEHFTYWD
SAIMNLQVMEALGVDKAFALGTSAGGWIVVRMALLAPEKILGVLPLGTSMDYESAESREKGCWNPKTQLGPFYENWHSTTPTP
DFVVDDVWRGLVSSVGFGSNPSPETLAFWDETLKQVYRGDEGRKKLRIAVVNLFERDGLLLRLRDVKCPVYWLQGTADPVFGT
TVPAEHIKLFTSSPEATLEFVEGGGHYLSATNPKEINEAILKMVNKYA
FUN_005531 CDS scaffold_19 247527 248253 -
hypothetical protein ENOG41

MQQGTIHLNGNATLVVPVSIPTSSVWTIWSTPASTVATAISSSRAGMLLARRHQGMSYSGVSTYQKGVSCPFCKAYYQAASGV
VHHLERGCCPNAPLDRDTLYQEVRRRDPNGFVCNKLLEWHGTVSYHATSLAFNSRYGQYECYFCGDLFRQLSSLNQHLASPRH
QQELYHCPNRKCLKEFTTLAGMVNHLESESCRFLRFGAVQNGIRGFFSSDRLIAF
FUN_005532 CDS scaffold_19 250098 251124 -
hypothetical protein PF01504 ENOG41

MKIRSSRISGSIAKAISQRHGKSKKGIIKTIIAFFRLFRLLLTRYRSIDFLALRKEVWQLDEDEYHESFQTDGEDNAELIPIG
DLGYSGSTFFTTANEKYLIKSLPRRFEHDFFVKELLDPYVGHMKFQPHSLLVRITDLVVSSQASIGGMIGAAPTHHIVMENLL
YGKPKGDEGKKWETYDLKPNDYFFPERDIADGALVPDSVIERLVDEFPDKVRVTHQAKQELISLLFADSALLASHNAVDYSLF
LVRYPQGMEVPVVESDAGRWRRGVDDVDGKWTYRCVVLDFFWAKHAFQARALTKIVKIFNKVAHKGPMSITADPEEYRERFMK
MVDEMVVEG
FUN_005533 CDS scaffold_19 251663 254378 -
hypothetical protein PF06738;PF12821 ENOG41

MLSEQNDSGSGSGGRPSQVHTPLRREKKRVGFHSSKESSGESSDSTTHQQQTRQQRQDEGVFSPAGSPKLAPGELPPNAPDAY
ELSLALTKILSAEQEKKRQVQNLAPDTPDVGPKRPRPALRRNTSYDDPNEREKADQAESRTTERQYRSMADARQRADRLAVSV
GSYSAPGSRRGSLDLEEVPSFKPLIEDYDNPKGSPPAGSNTIEDAGYFGLRQRIANSDRFQHHAAAENLVRSHTRKGKRDLFT
QHPNGGPASGTATPVLQQAYAHDYVPRPDQYRGGILSSLLKLYHDDRTPGSGMNTPKDTGTPLMSPRNSPPISRPGSSNGTPP
IRSPPRSRPTSGLFNYHKNRHSTSSLALTELMKSSSMFAAPASANISDKWAEKIKQQPPPPKKRDKARITIHIAGIIQRHRYL
LKLCRALMMYGAPTHRLEEYMTMSSRVLEIEAQFLYLPGCMIISFDDSTTHTTEVKLVRVPQGIDLGRLRDVHNIYKEVVHDK
IGVEEATKRLDDVNARKDKYNVWIRVFLYGVASACVAPFAFQGRFIDLPIAFFLGCIVGILQLVLAPSNELYAHVFEVSAALI
TSFFARAFGSIQNGKLFCFSALAQSSIALILPGYMVLCSSLELQSHNLVAGSVRMVHALIYTLFLGYGITIGASLYGMIDSNA
TSRSTCDNPLGREWYFLFVPGFTMCLCLINQAKWKQTPVMVGMALAGWSVNSYCAEYFGGNGQISNMLGALTIGILANLYSRM
GRHVENGYLDFVDWWKLRVRPRYTKKRIDSDSWSLPTLNDPESRPGTPEKHQEPEKKPRKVGYSLAAAAMLPAIFVQVPSGLA
AGGSLLASITMADEITKNGTKVASDMNTMGNLEGTAFNVLFKVIQVAIGISVGLFMSALIVYPLGKRRSGLFSF
FUN_005534 CDS scaffold_19 256268 257240 +
hypothetical protein PF17784 ENOG41

MSTSTYTIPDRIVPMRVIVCGVHRTGTLSIRNALWQLGFHDCYHMQTLFNNPTRAPEWVRAMEAKYADKGTFTRADWDHLLGD
CQAVCDVPAAFFGPELAELYPEAKVIIMNRDPEKWYESVLNSIYLITTPKGFLAKLSMIYCFLLDTNLQYMAKYSKSMRSLVQ
KYDHGNDKEKAIAWYKAQYQEFHDRIPADRYIEYTITEGWGPLCEYLGVPVPEVQDPVTGKTVEAPFPHLNDGETFRRNSKVM
KKKTMERANQNLLAAVGRLALTGVVGYAGYLVWKTRLGGRV
FUN_005535 CDS scaffold_19 257680 258250 -
hypothetical protein ENOG41

MKFNGWVSLSLDNRICVDNPREPWTIATVGELLTECRKKAMMDGKGHIQEDLKQKAKEANLEAVPKSPSGLIEQLAQVVNSEI

PRISFKFFTMHNIAWSFLTDLKRALTAGVDPEFLTYVHSEDQLPFVVGYVSSTAAGHGSSDVRERSVGNDRFLDVATEAMDEF
LYQGKGN
FUN_005536	CDS	scaffold_19	258440	260122	-
hypothetical protein				ENOG41

MTNTAKAHGHDADGAVGAAALDTLKKRKKSNGNGKGKGHIRAKNKKTQEKQDPSTKKYVIRVRDFEAMAKHVAETNAVAVPHR
TAVALERGIWGVLNASGYVDSSLADIIYSSTIILSEAHRERCKKGSPLVLLTLISLRSWRKSAAISNPSWKLFFSNQMISTKR
QMSRQTTPPRACLTSSASTPFSEEFLNAPHITATPTPETQYTVEEEVTWEDAFFAFTTLLRDYAYLSQEIHSLWEKYANGELD
LATVALATNTAFEFAHSMEMDIKKVMDKFGGTTVFAQLCFNAACEGLGIDKERNSRGAMYNLAAYDVAKVLTLNSMGLFKSLV
DHNRDAVISGSYNGAFGWYEERLGAGGGNNTERRNRDKTALSEIFPGFHFLNTTPGQGDVEDELVRGMGAALKEKKENPRLWI
SWALQMYLDIVQGLGESVGRGYEQFKQESLKIQKALVDLPKTAERRQVLQVATRWNHDPIYETNQSMMEFGAMAHSDDNAAFP
FLRRNPIYCGLLIHHTRSMLHVNGVKAAAPSRGLMTTTQLYQALR
FUN_005537	CDS	scaffold_19	261533	262472	+
hypothetical protein		PF00701

MGSPKASQLRLGDGVYVPTLAFFTPEDEVDTSATEKHIIKLLESGVAGIVVHGSNGECAHLSPSERTTIIRVARDTIFHEGSG
TRVPLIAGCGAQSTRETTELCEDAGKAGATHALVLPPSYYGGLLPDDLVIQHYYAVADKSPIPLLVYNFPGAAAGRDLSSDTI
LSIAKHPNVVGVKLTCGNTGKLARIAADCPEGFWVGGGSADFILQGHAVGANGTISGLANLCPKACVRITELAEEGNWKEARQ
LQAKVAKADWTAIKTGFVGVKAALGHFSGYGGEPRRPCVAPSQKDLDTIAQGFQEVMDLEVTL
FUN_005538	CDS	scaffold_19	263490	267058	-
hypothetical protein		PF01546;PF07687
	M20A
MSTTCGHSSTYVDCSRAAANAGFVRPSPEERTLAMAKTGRLARGAYLSKEDAADNEYSFPGPLVLPGDHLTEQPEEQGQTFKD
WFEMEATQQRNGIISRRNKLYVVRPPSMTRPSEEMRDWNKPVLPPDSPPGTERWNSTLKMFKQLIVYLCAFYNTMQITQYPKK
ISFLPIEQRKRVHFVDTPQTQVIGLKTRDRRKPFEIRYRPSPDGVSKIQLNVSDLRDALMRSIPSTVHSFVMIVDHDMYEDGE
GAYVTARAWASDGVATVSVFRYNPSLDGLAGIDPLHRWPASHCKANVDERFRVLNKTKGWKKRNRLSSKAFGKPPVKSALDLA
IQAAKQAGQPSTREELSNQWFARVVFAVSREATHCFGLGNCTYYSCLMQGVSGIRQTGEIPPYLCQACYSKLGWELVLLQHGF
EGGIEREKTWLEAQYAALKAFCNKWNKIPQFAAFVAWLEKRLEDRKGDNDDAAWPSNQEASVIDWPKTIRDMVDIHREPAYNS
GSQRKPPDTPGNNVEPIDQHSFTSKKILHQKFFIPTSSSGILLATVISTGSGFGFGADVVLPTPLCLYSPPVSPYKSSLSNLD
ISNSAPNIFKMVRKSLAVLATVAGASGFTLGPFRTQKPIGQNEHDFKCDLPPVLSPGNDGLPSASELFDSKDAFNKQVKRHQA
IVRVPSICYDDLGDVDKDERWAPFYDLHDVLAKTYPAIHKYAKLEKINKFGLVYTFTGSDASLKPYLLAAHQDVVPVPDPSTW
THPPFDAYFDGEWLWGRGASDDKNSLTALMSAIETLLTETKWQPKRTVILAFGFDEECSGPRGAAKIGEFLTERYGDNGIPFI
LDEGGSGIQLIDDTLYVLPSVQEKGAIDIWAELRVKGGHSSIPHPHTGIGIMAEIISALESKPYEPAILKGSPVYNHLVCLAR
HSPDSHTKLHDLLKKDDLEKLKDELLHLDPMAKFTIQTTQAVDIIRGGQKINAMPEVVSLGVNYRVAPQDSLDKVQEQFVKNI
DAVVQKYNLTLSAYEDDAKSQNIKPGFDYDGHLKLTAFRNSVTTPVSPTSGPIWDIFSGTIQHSFAFDGGKVVPVGEIMTGNT
DTRHYLNLSPNIYRWTPSRQRGSENIHTVDERIRIESHVDIVKFYYDLIRNFDAADF
FUN_005539	CDS	scaffold_19	268320	269721	+
hypothetical protein				ENOG41

MPPRFHSLPPEIQDMILEDISREPSTAPYATVCKSWQNFFERKNFRSISTKIKRFEKPKVTFRADRVFTHSIFGLWDILSLWD
PENRITLELSVYAYDDWAPILRQERIFERDLAAYKVHKESGSREPYRYDNPHGPYIEQYRLWGLPYADHRIRGHWNACRHDLI
GWKDIELTTARRADARPYYIEENAILPRVPIISKFLIRNTQFRRIYPGTLLQMFKSFKNLEDVTIERWASVDAGDETDWTRHA
SVAFASELPASVKTLSINGRKCAVFQDWVSRDVKLRNILAKRFRQYSKELAHMSVTDVIDAKDFLDLFRVSVSEDDLESLIRW
PDLETLTLSTDLFETQNRVKIEKLLCSAARAARKMPQLRKLEIRSSRHAQCLFRYNVVEAIAEVTWSGPVVDSTDIMKEWEKT
SAWRNNIDVINDF
FUN_005540	CDS	scaffold_19	270048	271819	-
hypothetical protein		PF00083;PF07690

MLGQRSIKVNGADCGIESIILGIITSIGGFLFGYDTGQISSMLLFRDFIDRFATGYDYDKNEKVFVPIIQSTMVSLMSIGSLI
GALSGAYTSDWWGRRKSMTFGVALFIIGNVIQITAMESWIHMMMGRFVAGLGVGNLSVGVPMFQSECAPREIRGAVVASYQLM

ITIGILVSNIVCLGLKKIDHSDASWRIVIGLGIGFSLPLGIGILCVPESPRWLASKGDWEGAQISLGRLRGMKNKLDHPLVQD
DLNEMRGILEKEKQVGQGGWLECFNPKSGVPKLVWRTFLGFFIHFLQQWTGVNYFFYYGATIFKSAGIEDPIQTQLILGAVNV
GTTFAGLWFVERFGRRWPLLIGAIWQAAWLCVFASIGTAINPETNSTVGIVLIVCACMFIASFASTWGPMAWVVIGETFPLRT
RAKQAAIATAGNWLGNWMIAFLSPIADAGIKYAFGFVLVGTNLAAALLIWFFLFESRQLSLENVDLMYGQEGLKAWNSHKWVP
PGYITRAQRDEAVFRGNDKGRNSESHSGEDSRVEKIDA
FUN_005541 CDS scaffold_19 275052 277629 -
hypothetical protein PF11443 ENOG41

MASSSESDLPWFLKSSYPVLFPFDDAVRLPQVEFEECIRQMIADRHKRSLLLDKREAHFAAMADADESETFTLVSNSTSAEIR
QAMEATNLLSHASQDSETIREYPAAQEPEAEEAKPAQSNPFMEGLISYGNQEPSRNLENMMFTENGDLAYRSSQDALVDLFQE
LEEVVSGPRLKDLLTRAWQENPLATLRIIFNACSIHLGKSSRTVFYRCAGWLAQNHPLTLVANLCWLSRPVINKKAEKKEDDD
LVLVEEENDDDSAKFDVKNGVAHGYWKDLLNILAIAANDKLTVVDDPKEVLNIENPGIIRGKSINKPRHRDGRRLRGLHRGDR
GRGRGRGRGRGRGSHSSGQEMREQNRKVDPKELRRETRENRHETAQVLFKTDPVYRALHLTIARLFAEQLKKDIQALRGDDKA
KRSISLCGKWAPSPDHFHDRHTFIVSTIAEIMYPRASIYHPMLSPRDDHEKYLRFAREQYRKDTSALRKQLEIVEREISVKKY
ENIKYDRVPSLAMNQYSKLFIENDAERFGKYLDKVAEGKANISGATLLPSTLIQKVRMGGSSDRSGANKLLDQKVAEIEAKVL
DGQWNTLVKRIKDSGTMDSCIAVCDVSGSMNSPVFKDKTTPIDSAIGLSLLLAEVAKPPFAGTFITFSENPRVEEVDLSKTLR
EKYWAMHRSEWGMSTNFVSVFEDLILPMALQNKLKQEDMIKRVFVFSDMQFNDASSSDSRYYQGWASERPSSWSTSFERIKAK
FEKAGYELPELVFWNLAGGRAGYTGSGGDPTAPKPVKADEDGTCLVSGYSQGLLKVFLEGGGFEEEDEEVVVEKDEEGNVTQK
TKKVKMTPLKIVQKAISHKAYEMLKVVD
FUN_005542 CDS scaffold_19 278328 279951 -
hypothetical protein ENOG41

MTASDEGRGSSGLLGRLPAEVLNQVFYQLHNIDLKTLRQTCTYIKDATHLRLNRLFLSPNFRDIEVFRAVADHQTFRFQVTEI
IYDDARFDPAHEMEWPSWYDQEDDIGEVTGVPEWYRHVYRQNRVMIRRYKPHHSQVKEAFEHLLSPAEAFKHFKQLWQEQQET
IATNGDADALRYGLLRFPNLRRMVVSPAAHGKPGLPLYLTPTIRSLPRGLLYPIERGWPLTKMHSNFPEARDWDRSEKDRWRG
YCLVSRIIAQHLRENPESKLADFAIDTNQLRTGISCRMFDDAENSEIKDLNTILSHPGFAGLDLSLHCGNQCNMNWCSFRSHH
LRNTLASARDIQHFSLFTNIVYVDDFDDSDDSDDSDDSDGEKGDHFISLHSIFPVNDWHQLRHFGLSRFIVKKDDVIKFLRAL
PPTLESVELSFFIFMPDSDDYHSLVHDMHEKLGWRERDAENQPKIVVRVDIRFAKVHGAVMMDVSREVTSLMYQNGENPFEQD
EGCVADPYIPQSDTPQGNGRLLDAFDPSFEEPNVSRLVMQAG
FUN_005543 CDS scaffold_19 280570 281084 -
hypothetical protein ENOG41

MDSWCFPPFDPEPVKQTDDVHRPSATVIPIFFDHNSCGGGLPTGHTRHRPKTRKHSTKDAATWERHMGETRAFARRHHARSPC
HDREEHRHTYIQSITSSKTRFASIPYSCTCGCFHIHQTTCLDCDRIYEASTLLDPYRGSPYNRTYKL
FUN_005544 CDS scaffold_19 282196 284460 +
hypothetical protein ENOG41

MSALPPDELRGPTSEEASWDILSSRSDEVASHSSDFNGIGTAFGNLDSHPSDMSLPLSADSDEVTLAFQERDDILKAKLASVE
TQIDYMTSTANFPNLSSEEIMSRREAREQARADLVRLKEENQANRVRYDKLREDLINSTDGNLTPAQVLAKIKELDRVEVDLA
ESNAALRRMSEAQGDSSSGSDGDDEASSEEDLSDDSDESSVHGTTSESDSDEMESQFARSGGNSAADGGNNDASPANQGTASA
DQGCPTNDQNETVSIPSDDQKYTINVSSVFNANSNVDSRRVLLEKIPQSTTLTQVANAVCGVGGLVRISVLENIIKSGTAFKL
ACVEFRDSTAAEKYVANVKANGLELVDANGRFVDIEVKLINNTSSYQSRHGHPHNYGVGYYSEHSGRCITLDNFPTSAIWYLF
TRFGTKYIIRAWFSPNENQMNGKLSVELASTFEAGRLCGMILNRGFLPYHGPVAQMGFGPTPSDRDVEELTSSVSNTIDYISP
KHLVDTWNVQPFNLFTPATNFVYGAPIPRRAVTSRTSSMTVEVSRIPSHYSGMSSVVPHILVRYEKTSTDLQDIPMTNITLTF
VDEEMKYIVVGGQIFSREVTGDIYTKVDGVELVKLKQDNILKTKWAPFWKHYCEVNGLPDIRKYAEYGRIAGIRRNVNKKFGF
ASSWNPERLTAIPTQIINYLNPKSRKVVSTAGAPNRK
FUN_005545 CDS scaffold_19 284935 286582 -
hypothetical protein PF00385 ENOG41

MESFQQQLEPDESEKRKPKEHKRSRIEVRLRSKPRDYVPGSGPPLQPISLLPPQDSTAYILERIILPSPGVAADGLPLPRRMT

YIVSWTDLPAAQMLVPAMDVLEYVSPRKLEEWEFKNTEEQLEEEAAEKKKTEAGQVTQPKRRGRPPKHSTIETAVVAVVEDEE
AVPMKGAMTIKTPTKNRLKDFEGLSEEEGPARQLQWEMTGNSAETDDQALDEHRGTVEDSESAFSGTNMDPLHSTTVDSHAPK
KSHTKGKHFESFPLTGTSSRQSTPQITSIRSKSEGKKKLDRPSKKAQPSHGLLSGVRGTGSASDWTPQESLTYSNSGVETPDP
EPETQTARLIEEAISIQKKPPKPKSKPKTSIPKSPEPVPQEEPVREDPEEADEPGWEVKRLEDMELYDVEGQGLVRYFLVRWE
GDWPPDQNPSWEPESNLPKDLVKAYLRNRKRKRSDKPVTKPVSKQPSAAPSYAPKKKSMKQTTLSWGIPAKQFKSVSEAFAGG
EEDEPGMPVAADPRQEDDDDDELFVVEEPPAKKNRAKNWGANGWGADDYS
FUN_005546 CDS scaffold_19 287312 288025 + PAM17 TIM23 complex component EOG09264HN5 PF08566

MASSLKTSVLRMPLTGLVRSSQKASFSTLRPASCLSASPFRPQCFTPVVAKTFSRTYADKPQSTELPPLDWNSFFKLRVSRRR
YQLLFSITNGLFAGGAGAVFLSTGLAEPIISQIPLDPFMTLGLMTLAFSGLGWLSGPSVGNQVFYLLNRQWKKQMTQKEAQFF
ERIKKHRVDPTNSSASNPVPDFYGEKISSVSGYRQWLKDQKAFNKKKTANFV
FUN_005547 CDS scaffold_19 288229 289674 -
hypothetical protein PF00581;PF00899

MMEEIDNLRREIAKREVELADLRSQLAAAESQARESKEAWKWPLDNHEYERYSRQMIVPNFGLQGQLRLRNAKVLLVGAGGLG
CPAAAYLAGSGIGTIGLVDGDEVEVSNLHRQVAHSTGRVGMSKVQSAITYLKDLNPTITYNAHNTHLTPQNSEDIVSGYDLVL
DCTDHPTSRYLISDICVLLSKPLVSASAFQTSGQLIVLNNPPGKGPCYRCVFPTPPPPDSVVGCGEGGIIGPIVGTMGILQAL
EAIKLISRGDLETHGEAKTPMLLLFSGMADTPFRSVRMRGRRKTCLACGDENRLTLDELKNSMDYVQFCGVKQPVQLLKPDER
VTAKEYDELSKLENKDMLLVDVREKEHFDLCNISGSVNIPISRFMSARGEATPEGWPSDLPHSTPIYVVCRVGNDSQIAAQKL
KDLGLGNNGERFIGDILGGIKSWKDTVDPSVPFI
FUN_005548 CDS scaffold_19 290080 290695 +
hypothetical protein PF15341

MAPQPPGLKKPSARTLRHQRITGQIRPQAPQKVFRDDAAVTDSFLSSKRDKRLIKHSSFVSRIQSARISKSTKRRRPSKKLAA
NLESLADALPELEEGAEVEQGKVRHKSLKSKRGALKRKERVVKGEMERFGVSMARLTQQDGVPVQKVEAEEKAAPAPTANRWA
ALRGYISSTMEQNPAFAEKN
FUN_005549 CDS scaffold_19 291805 292743 -
hypothetical protein PF02902
C48
MPFRQRMARHFGDSLAPEKPYLSYYDVLLTAEDIKALKHDWLTDNNIAFWEEYLERETLPKYPQARIVLLRPSMTFLLMKEPD
LRSVQSALPDFSKVTHIFLPINDNRNVSVAEGGSHWSLLLVSTLDGVAFHYDSLGGANYSEANVATRKLATILGRPLRFINLE
DCPQQENGSDCGVFVCLLMRHLLVKRLLCANAREKVSMSMGGKMVDSYGGRKEMMRIIENLRKEGERRRSRSASPFSNKTPPR
IE
FUN_005550 CDS scaffold_19 295619 297848 -
hypothetical protein ENOG41

MPRRGGKSGPRLKGKRHSGRIRAIGQLDTTPNRHDQSPRGVQPQDSTPALPNQDANSELPNSEIPSTNEFDDDLNEDDLMQLV
EAEAAPSAQPPVDFSWSEIGSTRRSTPLREDEPTFLDNQDSLLDEFSSPKSGLGKLPNTQPLPSEPDQESKEPPLVDFSETAS
TVRRSSPYYSLAASQIGDDEALNILENNPLAPLPSTIPDSIQALGLPPFPKSATMDHLPEDELYDVTPPKSTAELEAETSRKE
EEAPEAATERSQRTKKPNSSQNSKTKGRPGKASKSHDALTRLLEEELDPSGEDERQEQASSPPEKLPAKLPKRKNSALKGKET
PKRKPAPAITQDVRTSEQRPKERKGRRQRAKTPIQFDEETHEIKEATPRKAAEQQPRMPIVSNLRKSHAASVSPIASAGKGTP
GSKRKPAPKRKPPAQDASQKAPPATRKSDLKNVVPQDKPHTAEERAVPATKVAAKPAPAINKRITRAKVKEEKAPVSQPLKAQ
ETPGKRNETQGSTQDPIVLSSDPESSLFSDDDDAFVPTEDLQPKEKRQSARKTQSVMGELLVDPIVDVEPVGHAHREKAQPST
RPQTLPQKPEASHPSRPPEEQISRQKTTMTVKPDSKKIVEEPLPSTRGNRAAQKGRREVLSARDTNILVQQDTSQARTLKRPA
TTVDPIIDVSQPPRKISKSSRSFSISQAGSPLPLETSPAQLVGGTSPSAMEEVVSAKVPKRNQQTEPRRSQRLRRTAE
FUN_005551 CDS scaffold_19 298979 300164 +
hypothetical protein ENOG41

MSNPVHASANRDAASKSSLSVVLRKFSVLAQRPEPKKYPPISSIPRTSAARLAQAAEDHIIDRDHPAPKTYLYLAYGSNLAAE

TFLGVRGIRPLSQVNVSVPTLELKFNLPGIPYREPCFANVDYRKLPEKPKLPPKVPVPPFDPPQPPHSEEIRWDEGLMGVVYE
VTEEDYRTIIRTEGGGAGYKEIVVPCIPLPPKMSIPEKPFPDIPRPFLSRTLFAPQIPDKDLPDDPRKHKWWYRFLLGPQREP
GYAQASARYLNLIKDGAKEHELPDGYQRYLHSLQPYTITTLRQRIGAFFFLFFTVFFFGTVILLSKFLADKRGKVPQWLAATM
TVMFTVAWMVYDNVFKPIFGNGERTEEKDERKTHGRKRSLSAMLNHFPSTDEEKVELLGEYN
FUN_005552 CDS scaffold_19 301316 302637 + GLD1_2
mitochondrial glycerol dehydrogenase Gld1 PF01210;PF07479

MASLGGHSKKHKVTIVGSGNWGSTIAKIVAENTRANKDLFEEDVQMWVYEEDVTITKDSKLYDETTGDKPQKLTEVINKHHEN
VKYLPGIPLPSNIIANPDIRDAVKDSTILVFNLPHQFIANVCKQVNGHILPYARGISCIKGVNVTDDGISLFSEWIGDGLGIY
CGALSGANLAREIAEEKWSETTIAYDPPALDNSRAPTPRTGSPNPTIGELPEMQHKDVRGRTSKTKLTAMPADYPPLDQDCFR
TLFHRPYFHVQMVSDVAGVSLSGALKNVVALAAGFVDGRGWGDNAKAAIMRVGLMEMVKFGKEFFGETVHTATFTESSAGVAD
LITSCSGGRNFRCAKKAVEKGISVQEVEKQDLNGQKIQGTTTAEEVNSFLKARGLESEYPLFTAVNAILNGQAKVDDIPHLVQ
DS
FUN_005553 CDS scaffold_19 303553 304438 +
hypothetical protein PF18142 ENOG41

MAEIDSTVPQTSMYPTQNFGHYMNGADQSSMENGPGQKKHKNVRYRFLSPTEWAILARGVGGIIDIEHNKPVHPTSSLWPPNG
LPQGLYRDCVYRRTISSYSFHFIGFVRAILFVLQLMIGASITALSSGGFDDTAITILAASNTVIAGLVALLHNSGIPDRYRYD
KSEFQKVEDHIREVLATGIVRADQSVQEALAKCYDRFDHARRTVEANMPSAYAPSQSTPSSRRGSQPQHQPLPPIPMPIPIPA
RAPTTVASTPTHTRATTEPITPPRKKSLNTEAAQEEKGPTTKPSE
FUN_005554 CDS scaffold_19 305201 306764 +
hypothetical protein ENOG41

MIMISSPPTIPRRVVQLLVLVVVVVLFFQTRWLHWPRTNRDTEIAIKGPVPEEQYLERLRQKIGLSDQTSWTAWKVRVLEQDS
EYTSVTKIDTDFHSNQPRIIDTKTPERRNLVARQELSLPIVGGPKPGQIDASDFVFAISTSFDRVVRDDYAVAKDWARWMTGG
NQHGNGASFLLVLDQGHYQQVKKLTEVLVSLGIDAHVTISEGQISTAKRYFNMMESVKKYSATLAQKGQMKKWYGILDEEIFL
PNLSYLQERLFAYNSEKELYIGLPSERGDWAIGEGFMMTYGGGAIFLTRPAISKVTHLPCFDGVEDSPMSKKRWDGILQDCLA
KHTKMKMHIIPSFYSPKDNDDYSSHLDSYETGIQPLALHDYGERHHLTPSQAHLVTDVCGEACFLQRYRFRDNWVLVNGYTIT
EYPDGIHLSDMPLSTAGKASSSRRSVIGPVKLKERDTDRKMLFWTGRRNVWKLMDSVTTAEGDVWQAYVKRGVIDEPALKKRW
GEEVGDVKDSVIVLVWESAKRL
FUN_005555 CDS scaffold_19 307174 308005 -
hypothetical protein ENOG41

MSGHLSTLLHHISSTHSKDTMCVLENLNNNTEWCSIPNDVPNITEESEDSYWPNLEASIDSNEIEFKDLSIECVVCRSNVTIF
PKDHVVDEEVGESHRAVILPCGHIFGASCVKRFFDMKTEEGARASCIKCRAACYHPHPDCGHPFYGEPMPSHKPGMAFTPHVL
GKGGKIDDCCKMCTYKKAIYDITAEIQRAPEFPEDMKKSLGVVLTVDGKSYTTRRVVMSRLQEVYFPTSVADLFVEYKDRLEE
KEDGQDYWISGYLSQCKVKLYVLAPQN
FUN_005556 CDS scaffold_19 310203 310839 +
hypothetical protein ENOG41

MCIIFLTPVDCTGPKKITGKAKESFWPQLASDIERYNVKFRDLAPECGICREDVTVLPFDHKLQNGKGETLRGLILPRGHIVG
ASCFGKNVRYCLQTKTKICCPACRTVFVHPMCGHYFHGLYMPSNKPDVDIIPPTINNGGIIADYCDECAINNTVDELADNLYN
LLPEESKGRYAVSLAYGGETFPLPLMRNCLEMRAPAAFRSFFAQY
FUN_005557 CDS scaffold_19 311864 312587 +
hypothetical protein PF00097;PF13445;PF13639;PF14447;PF14634
ENOG41
MPIQEDFWPSLKAFAENNGVASLETLGLECVVCNEPFQYRRRDDDDAGSPRRPRVLPCGHILCGRCLLAYYETGESRCPTCRT
ELIHECGHAHTGMPMPLTRLDRLPPILSQGGGMPKGCSPCGIVGLLKLFDQELSNSPDIAEELKGEYLGIGIKLSNSDEYCSR
DVTGPVLEIEPPAAIKQLINDIVDSAIESQRENEVWLEADFSSMKIRVLHFKPELLSEVEESPAEQETVSQDDN
FUN_005558 CDS scaffold_19 313353 314385 -

hypothetical protein PF00097 ENOG41

MPGPKYPITATYYPELASIINNDPTAIERLELDCIMCKDRMSNPEDRTCGAFIFPCGHMFCPFCVKEYREYNLSNGIPHKCPV
CHFDLSRKICGCPIDDDTLFKPTQQQDENLLAKLQEEISSLKGCCDSCHVATLIHGLRQVALYLHDPAQMIKDGHILRITIKK
CDTEMRIESEKNKVVKQLPVSSDLLELFEIAGDSLDTAYNGPRLPEDESSLFNFELALCKQCPENQGDHCRFRRGEMELVKKK
LQAPGCDRLVLAREAAVMLAKSFTSDEAYKEYMSETRKLQREEFAFLEAIELNENNQDHQDHEISRLYALAAIPVAAGLLAGG
YQFGAFLARHT
FUN_005559 CDS scaffold_19 316663 317373 +
hypothetical protein ENOG41

MKSSLFLASSLLALFPSCLQALAIQGYPENVRFGEPTFVEGGNTTTAKRQVATPPDNCWVQGLGQSSYWFLPVYSGDDVYACI
MARKRMTGSSWVCPRDDWWPDSYVDEIVSAGQEQIFKEGGGKTTEKGVFKARFEGTTRKIEDKEGMSAMLDMLFRYSLPNCDT
SYSLCVTKSRHYYYKYKGDTIGIVHRESDCRGWFGIGGDTYAYCPGGSHCQKD
FUN_005560 CDS scaffold_19 318452 322342 +
hypothetical protein PF02714;PF12621;PF13967

MSSTDSDASASATDSATGTAASVTSSIVSSLLSSAISASETLSASGTSSDQPTRTSSGGLAGVSPESTSAADKDKGIGIVSFL
TAVAVAVAIFAVQILLFLILRNKLARIFKPKTYLVPERERTESPPNKPLAMLKTLWHYGDREVIEKCGLDAYFFLRYLKTLLI
IFLPIGAIVMPILIPMNYVGGLGQKVDVKDDDDEKNEGTPTGLDTLSWGNVSPKNSSRYAAHLLMAILVVIWVCTVFFFELRV
YIKVRQDYLTSAEHRLRASATTVLVNSIPAKWLSEDALMGLFDVFPGGIRNIWLNRDLSTLLDKVKERNKIHLALEQAETDLI
KDAKKAQLKQQKAEEKKRRKQLKLGAETKQERADRNARDDAAAQRMAQSASGTAAGDQHNVPHSVDAGVRESQYETYNHDLEV
DHEDHNKKKGFRVPLLGDPLAKVGHGIMGVVSKAGNNVDDTLETTNGFMGLSQSTEPRTTSRNSDRVRERASVDEDELNSPSD
TLRVLNNQSRNGRASADSTAAINPQPNREHFGNAGNTVRKLENIDEIYSKEEATWWQFWKPPPGSYASPVPQGGVSAAFRQKK
TNESKPLWQKIKYALPFMSPEDEGEPMEYPTAYNTEYKEDAEPAEWGKYLKKKDRPTHRLPLFGQSWLFGVPFVTKKVDTIYW
CREELARLNLEIEMDQQHPERFPLANSAFIQFNHQVAAHMACQSVIHHVPRQMAPRMNEISPKDVDWDNMAFSWWQEWLRSGF
VFVIVVAMIFLWAIPVAWTAALSQLDNLIRSNQWLSFLKDNDTVHNIAKAVAGVLPAVVLGLLLVLIPIILDFLARFKGAKTG
SQRAEFVQVFYFAFLFIQVFLIVSIASFFAASLDELVHNVQELQTVRDVLDLLAYNLPSAANYFFSYMILQAMSTSSATLLQL
GALVMWYVIAKILDSTARNKWSRNTNLRQVKWGAFFPIYTNFACIGIVYCVIAPLISIFAILTFALLWFAQRYAMLYVTRFEH
DTGGVLYPRAINQTFTGIYFMELCMAGLFFLVQDEKGKNVCTPHGIIMIIVLILTVVYQVLLNYSFGPLFRYLPITFEDEAVL
RDQAFQRAQDRRLGLLDNDELAEERDSSDYQEKDQQPQNGQGIEMRKFGTIRRPVKQVGTWAKDGGNQLRKLAGVNKDKDKSK
RASEYRRKHRQKDIEAQLAIGEALFGGIHDEIEDLTPEERDALVRHAFQHEALRARRPTVWIPHDDLGISDDEIRRTQAYSEH
IWISNEGTALDSKVRVVYGRAPPDFSEVDLINL
FUN_005561 CDS scaffold_19 323115 324682 -
hypothetical protein PF00561;PF12146;PF12697
S33
MLRLQSSSAVLPISRLSSITAFNTITTPTRSMSTDGTVRPPHGGRVADAPDEIKHQAELKPSHAFLFPLGYKDAAYQWWTSLA
PQAVERNLLNLMPHLREANDSITNIDTPERPDPYGTRVWRRTMVHLSGKNRALNEVTVERVGEKEEDALVMIHGYGAGLGFFY
KNFEPISRMKGLKLYALDMLGCGNSSRPAFKIHAKKKEDQVMEAEGWFVDALEEWRKARKLEQFTLLGHSLGGYLAVSYAIKY
PGRLKKLILASPVGIPSDPYAVNASMPEPSTSTIQNEIIQDQQSTTDQNGTLKKHKPASNVLRRPLPGWFVWLWDQNISPFSI
VRMSGPLGPRFVSGWSFRRFNHLPQAESQALHDYSFSIFKQKGSGEYALAYILAPGAYARRPVIDRIQEVGRQTITKPDGTTV
KENGIPVVFMYGENDWMDVAGGLASEEKLKEARQKALENATEEEKKRENGSAKVLLVPKAGHHLYLDNPEVFNDMIRKELEDT
RKSEQKKQ
FUN_005562 CDS scaffold_19 325431 326706 +
hypothetical protein PF03907 ENOG41

MSDDVDSIVKGASAPSQGVKDVAGTSPILPTTAKQGDPLHHTPSSPSMIYLNLLILEASLRAQFLELRARKRHHTFFLTLLSL
WIAGFGYALFLAPREDGRGVGGSIYWAVEGAEKVCFMGGIITAILVWATGIWERGIRWPRRWFVISNRGLRGFNCKLIIIRRT
WWAEALSTIGFFLTYGLFSHTASSSYRYVEPNLLREVEKELQITSDTHPTLILPHEDEEKGGHEEDLAPGGDYVKLLLLAKPF
SATFRENWELYRTEYWEKENERRALLRQKVKERDHQLVKQQYGWLWWLPGRQPRRQEPEKTPPRHISVERERRRRGSSVRRAS
TSSSRTPTPIVEEDNSVSRRPSSGSMSARRKRLSTSSKPKRPGTDSRSVTPDFPSPLARESSATPTPDSGPEGGKVLRNSSSR

STVGSKERD
FUN_005563 CDS scaffold_19 328134 329899 +
hypothetical protein PF07690 ENOG41

MFFNWDKFLSPKPSLDVPALETVDLNDKEMERRIVRKQDLRIMPWVCITYLLNYLDRVNLGNARTLNNDSPAHNIVEQLNLTG
QKYNIAVALFFVPYVLMEFPSNILLKYFSPSKWISRIMVSWGIVTICTAAVSTYGGLLAVRIMLGLAEAGFFPGIMMYLCFWY
KPEERATRMAIFASSVAVAGAFGGILATGISFLNGKAGLAGWQWLFVLEGIPAVIVGVLVWFFLPDYPQTVSWLSPEERAFAV
KRLGPYAPSMNDKHWDSKVAKDTVADPFFWLFAIAYFFMANSLNAFGYFAPSIVASLGFKGYKAQLLTVPPNVFAVFIIVGNC
LHSDKTKERSKHVMGGLAFVATGYLLLAVVKHWGARYFAVCIIACTNAAVLPFVAHRTATVRGSTATALATGGMIAISNTGGI
TAPFLFPSSTGPMYSMGNWTVFAFLIAAACITGYVWYVFGAHSGYRTGDATETGHFEVLDVGDKDPNQVMDQAMENEKAKDKI
KAMDMA
FUN_005564 CDS scaffold_19 331002 332193 +
hypothetical protein PF02661 ENOG41

MAPKLERRTLAEAFEGLEIPSSRAQHQTDSNLGHSIRLMLLSSLRGNGSIRIRSAPSKESTLLLTQDDGHYEDDAEDESHPDE
DNIELKNYLDDIADALKKQKSKNDSKIAATFNERLSQMVYGSNMIEMVGSGLDITVKLCQRIFEGQDVDDAEINDRDPIYQAL
RMDLVSKNKSSGHEDVLRSRREIIQHAKAAYYMMNEVAIKGKDLSEEIILETHRLLTYKIDSPDKIPWSEYGGSYRTCPVQRG
FHSFPHQAGVPKAVRDLTNSLKADIATAEASGEIDPVALAAKYCHKFVNIHPFLDGNSRTSRLILNTILLKYGGCFVWLGMDS
KDRDEYMRISAEGFSAEAMDMQREDMDDLPEDFKPKHHKKLATFTLKHARDTMTKVLRTLKRKD
FUN_005565 CDS scaffold_19 332967 333701 +
hypothetical protein ENOG41

MGGGPISPSSKLIPGAPLTLRRATIDDLDDITWICVNGSPDDPGTDYRFPYRDKYPEDFWKWTRIEHEELFERPDKLVILVVT
APVLDNGEVVHQPVSYGVWDLKVTSDFISGDHGINERRDVNKKHMEAYAKALPAGLKKYFKQFGSEQIPLWEVVTHPDFRRRG
AASMICEWGQKEADKDGKSLSLLATPMGKGLYSKLGYHVLGSVVVQVEGEEERVVVDAMVKHNK
FUN_005566 CDS scaffold_19 335682 336813 +
hypothetical protein ENOG41

MADNGFGSGLGGISQTTITVVLVFLAISLYNVIELNFIIFGTFKRHSGLYFWSFLVSTWGIAVYCCGFFIKYYASSALGYLAS
TFISVGWVAMVSGQSLVLWSRLHLVLRNRFRLKAVLYMIIINGVLMHGAVIPMIYGSFSPNTDMWKQPYSIMEKIQVTVFFIQ
EIIISCFYIYETVKMMRLERTMGNKRGSRRLMNHLVYVNILIIILLDITILGLEYADQYQYQTSYKAFVYSTKLKLEFTILNRL
VEMTTGNKDASSGPRSHTAGTTTGNKTNIALDTFISNTVDKPPGDISYRAYVMGGDERQTPRQTPRDDEVVMTTEVIVQREER
EDDGMSMGGKSSAESTIGTSKGHHYESEGLSKSSSELHLATRGF
FUN_005567 CDS scaffold_19 337737 338952 +
hypothetical protein ENOG41

MPSYQNITFGVELELMTPLPDSYRMWRFISPSAASRFNMADLLAKRTSLPIAAQCCHPPDDRCTICATVPKDNQFRGDCVLQF
PEILSCEEFVSERCFIFKTEFLELAHPLSKERMWDGVEITTPVFHSGELDSGLATMNTALTNLRQLDLQISADDSCGMHVHVG
VETGMTILLAQKIATIVILLENTLLLRLVAPPRWKSGFSMPICENSSLAMDMDLHKSLEDPTTLNQHVPCMDAMKPGKWNNWY
PQHIYKMLYGVWGSTILADLSLRLKKARVHRCGFAMSLRDHNVSVSVSDRGENLEGSPTTVEFRYSQMTFDHELLRNWTEILARI
VVIAQADAEEFKSCVGKIISINGRDDKDVWKGLMMDVLGLGHRVPQWEEQLKRFEKGEYVSHLDEQLLLKSI
FUN_005568 CDS scaffold_19 339692 341530 +
hypothetical protein PF00199;PF06628

MAPSATDTSFAAHVASQFQSYETDRQASSKETLYTTSNGVPIAHPYETQRAGENGPLLLQDFHLIDLLSHFDRERIPERVVHA
KGSGAHGYFECTDPLDDLCLADILSEKGKRCSVSMRFSTVGGESGSHDMARDPRGFSVKMRTDEGNWDMVFNNTPVFFLRDPA
KFPHFIHTQKRDPSTHLTHADDSFMFWDYLSQNPESIHQVMILMGDRGIPANYRQMHGYSGHTFKLINKAGEWVYCQIHLKSM
QGVDFVTQEESADYSPDHSQKDLYEAIQNGDYPKWTLEVQTMTAKEAEELWEKQKINVFDLTHVWPQKQFPRRKVGEFTLNEN
AINYFAEVEQIAFNPSHLPPGVEPSADPVLQSRLFSYPDTHRHRLGVNYQQLPVNATRTGYQFGNFQRDGQMAFYNQGARPNY
LSSIDPIKFRSRAVDLDKTHGHFTGEAITFLTEIRPEDFNAPRALWRNVFDEPARERFINNVTGKMKLCKQEEPLKRQIAIFR

EVDPEIAERLEKSTGIKGYDGISNMAFNGTHNGMTSDGKNKLANGIVDKNKSPAARQGAPGSGAHGSLVTNGKSNGVNGH
FUN_005569 CDS scaffold_19 342337 343534 -
hypothetical protein PF01063

MTAFPPPPVNTIDWSNVGFKVREVNGHIESTYSRSTGKWTPLHFVADPFMRIHGMAPALNYGQQAYEGLKAFRTPNDEAITIF
RPNRNAKRLQHSAEVVSMPHVPEELFLDAVRAAVALNAEYVPPHDTGAALYIRPQLYGSSAQLGLSPPDEYIFAVFVIPTGVY
HGAHPVKALILEDFDRAAPNGTGNAKVGGNYAPVLRWSDKARDEGYGITLHLDSVRHEEIDEFSTSGFIGVKDNGGQVTLAVP
DSKCVIESVTSDSIQELARSYGWTVEKRAIKYEELKEFTEVFAAGTAAALVPIRSITRRVGGGEDVVTYIKDGSEEPGPLFEK
LLMHLKDIQLGRAEDSFGWRYPVGEKDKEIAGAPGGRNGDATTVDQMD
FUN_005570 CDS scaffold_19 344255 347190 -
hypothetical protein PF00982;PF02358
GT20
MTVFVCSLFLPKTIHFTLPGTPPLGADPTRASLSGSSNPKPTKAATLPPKPKPAAPTGGRPAPLARQPSLFQKEDITPPHTPT
EETDSNLFANEDGFRIQFPHGASSSIEHAKTWGSRANQPKSRASSPPPSASLSENNRTMQKARELGRQGVLQPRSLVRSDSHD
RVFASAGWMVVNADQGNGGLRNAADAAARDGKIDDITWVGTLGMPTDALEGTQQKEDIEAVLANEHNMLTVFCSDKDFDGHYA
HFCKHILWPVFHYQIPDNPKSKAYEDHSWKYYVNVNQAFADQIVKNWKRGDVIWIHDYHLLLVPGMVRKKLPEAKIGFFLHVA
FPSSEVFRCLAVRKELLEGMLGANLVGFQIHEYGRHFLQTCSRILSAEATPDGLQLEDRFVDVIHLAIGIDPVSLREHLDAPD
VAKWLQVLQERYKGKKLIVARDKLDHVRGVRQKLLSYELFLNKNPQWRENAVLIQVALSSNEKSDLEATVSDIVTRVNSSWAN
LAYQPVVYLKQDIDYAQYLALLTIADALMITSQREGMNLTSHEYLFCQDGKLLPEKKHGSLILSEFTGTSSLFAKNELAVNPW
DYRQCADAIKQALEMGEEEKDRRWENLYKRVNGHTGSHWFTEFLGRLDIVYEEQHKRDQTSVPRLSIPALSNKYLKAERRLFI
IDYEGTLVAWGPVNQIIPISPQRTLDVLNDLLLDERNTVYVMSGRRPEELDRVFRRVPNLGLIAENGCFLKVHGSDSWTEMAD
LEHVKDWKESVRPIMTYYLERTPGAEIEERRCSLIFHYKSAEDYESASRQASDCASHVNDACESQRVHAIALDGCIAVEPIDW
TKRTAARQVFETLKTEMSSTQEHKTPVDFLMVIGDGREDEKVFKWANRLQDDGSVQNVVTVSLGNRNTEATATLTQGVSGVLS
CLQRLASLDS
FUN_005571 CDS scaffold_19 351165 352807 -
hypothetical protein PF00400;PF12894

MATIVPPPSKRQKREELERSQIQQDVTAIASGPAGSFKARFLDGDGKQMADVIEVPLADASEKNLSLLLNTLLAREKEEFLPY
RFRIHIPDTDIIVDQYPTDLLQLLRNHGIENPFETTVTLSAEPQAVFKVQPVTRMSHKIPGHGEAILAAQFSPKNSNRLATGS
GDKTARIWDTDTGTPKYTLSGHGGWVLAVAWSPDGARLATGSMDKSVRLWDPETGKAVGNPWTGHSKWVTNIVWEPYHLWRDG
TPRLASASKDATVRIWVVNTGKTEHVLSGHKSSVSCVRWGGEGLVYSASHDKTVRVWNAEKGTLVHTLSSHVHWVNHLALSTD
FVLRTGFYDHTPVPDTEEGKRSKAKERFEKAAKFQGRIAERLVTASDDFTMYLWDPAQSTKPVARMLGHQKQVNHVTFSPDGS
LIASAGWDNHTKLWSARDGKFINTLRGHVAPVYQCAFSADSRLLVTASKDTTLKVWSMASHKLAVDLPGHQDEVYAVDWAPDG
KRVGSGGKDKAVRLWRN
FUN_005572 CDS scaffold_19 353231 354347 -
hypothetical protein ENOG41

MFGILETVQVPHSGIYNSYEDLFKQLSDGMEKEGYKIVKARSHRGKVGGADVPGNDIVRCDLVCDRGGRPYRCMATKHKTTTK
KTDCPWKAKAVHRKTMGGWVLTITCDQHNHEPGTPEPPTPEAASEDETNMMDDLEGEHSLDPAATPLLTPWQDEGPQPDQETQ
AAIQVAGVSNAVLRLTGETFHQFKSEYRKMSHPDRVAQLSHLQLRVAAIYAVQNEDLQRQKRQEAQDKRHRQIEETKRQSSVQ
KQRARQRRQQVVEQNHQQQQQQQQQQQQQQQQQMHQHIQQQQLPQQTTQAQVQAQAQAQAQAQAQAQAQAQAMMQSQLYLPQN
PQQAAIAVPTMDMPQFQHYVAPANKRMRGRTSQGGQQTQ
FUN_005573 CDS scaffold_19 358261 359632 +
hypothetical protein PF00441;PF02771;PF08028

MVDFKLSPSQLEARRHAQAFANTVLTKASAEYSTQKDQLSRFQATRPFYREAVRHGLIKAQVPIPLGGTMESLVHESIILEEL
FAVEPATSITIVATALGLMPVILCDSPSLQEKFLKPFISGEGEPLASLMHSEPNGTANWLQKGGPGLQTTARKVGNEWVINGE
KLWPSNSGGWDYKGADLACVVCRVSDDPSKPQDPNVDPATQIAVLLVTRETIANNKKDAYQILGEPELAGHITTSGPHTRFTG
FRVPHENLLCTPGLKAQGLVETAFAMSAALVGAMAIGTARAAFEEALAFAKSDTRGGFKPIIEHQSVADKLIDCKIRLETSRL
LVWKAVTTLEDEALEWKVKLEMAMQTKIYTTDVAVECVIDAMKAVGMKSYAKDMSFPRLLNEVMCYPLFDGGNIGLRRRQMQR

VMALEDYEPWAATYGSSKVDKSRL
FUN_005574 CDS scaffold_19 362190 365060 +
hypothetical protein PF00488;PF05190

MDVNTERFRSMPSRTASRSSSSYFQSSDSQAHLDHTAARSSLAPRRPQTSLSEYSGYDHSGNSTRPNISRPSTIQLSAPSRII
FMTTACPPNNPSTLFSLIQDLIPETQIDAMERSAWSETEGLEYIHNLAFKDDIKPLKVATQGKFYAISSLAALIIVPTNADVQ
QVEEQINQVLMIKNFLESIPELHTALGPANSVLLTKVRELCRPEITSHSLNTIRQTIEADVTYMKSALDLRNQRTFAVKAGIN
GMLDVARQTYKELTEDIHQHIDVLNELHGLDANLRYDNGRKYWLRLRAADFDDRPLPDLLINVVRKKDKIECQTLDLVKLNLR
LSDTSNEVVIRSDKVVQDLLKELRSDVPHLFRVCESVALVDMISSFAQLATTRDYVKPDLSTTLALKAARHPVLDKTMNGSFV
PNDYYSTEQYCFHIVTGCNMSGKSTYIRAVALLQIMAQIGSFVPAEYAAFSIIHSIFARVSLDDNIESNLSTFSVEMREMSFI
LRNIDDKSLAVIDELGRATSNRDGLAIAIAMSEALIQSRASVWFATHYIDLTKVLTDRPGILNLHLAATTSTTEDGLPHITML
YKATSGATRGEEHYGINLARAIGLPQSFIDKAEEVAKDIRRRRETTKRSSESTRLVARRKLILNLQNALRRAKDSGSKEALPG
YLQLLQEDFVARMEEVDNM
FUN_005575 CDS scaffold_19 365172 366373 -
hypothetical protein PF00459

MEQHELNDIYAFAVQLGKDAGDMLMAAAQRRIDGNGESSSAVSYVEKENSVDIVTKTDNDVENFIRTTVLSKYPDHGFLGEES
YSAGASRTYLVEDKPTWVVDPLDGTVNFTHLFPMFCVSIALVVKGVPVIGVINAPFLRQFFSSCTGRGAWLNETQRLPLIRNP
IPPMPANAPSGCTFSCEWGKDRRDIPDGNMSRKVESFVNMASERNGRGGKGGMVHGMRSLGSATLDLAYTATGSFDIWWEGGC
WEWDVAAGFAILEEAGGLVTTANPPSDPETADITRAHLGGRLYLAIRPAGPSETETGRQTQERTVREVWKRVRELDYKRPGV
FUN_005576 CDS scaffold_19 367384 368440 -
hypothetical protein PF01261

MAHKPSICTMSLGRCFAGHSLPHKLDMAAKYGFQGIEVFYEDLVDLAKSLPGGDTPTNQITAARTIRDLCAARSLTIICLQPF
MHFGGLLNREAQAQQFEELKLWFELCHALETDLILFPSSFLPEEQLTDDMGVIAADFTVAADMGLEQQPPIKFAFEALCWGTR
INKWEQSWDIIQRVNRENFGICLDSYNILGRIYADPAALDGKTKDCESVTKESIKRILTDIDVSKVFLVQVADGEKLSSPLNE
SHPFYNAEQPSRMSWSRNARLFYGESSYNAYLPCKQLLRAVVQGLGFEGWLSFEVFNRRFCDKDQIVPEETAKRAAESWEKMK
IELGLRTADDVQPRLQAML
FUN_005577 CDS scaffold_19 369145 370194 + QA3 Quinate
dehydrogenase PF08501

MSVQQVSSEQVSVSGDSLQSNQVSQLQRHGYLFGKKLTASLSPFLHNVIYQDLGLKWGQVRLDSADIPGFLELAQHPDFYGAS
VTMPNKVAIIPYLDELTEECRDVGACNTLFIRERDGRRIFCGANTDVIGIRDSFYQNIDNPDAVFHNKPALVVGGGGAARSAI
YALRKWMKVKTIYLVNRDEAEVTAVIADCAERGYGEGLLHVKDVSQAQALEAPGAIVACVPDFEPETEEEIRARAVTETFLDK
EQKGAILEMCYNPTPFTRLGGIAEDKGWKVILGTEALIYQGLEQDKYWTGKTLEEMPIEKVHVAIAEKVAQRAEAKL
FUN_005578 CDS scaffold_19 370746 371193 +
hypothetical protein PF06094 ENOG41

MAESSKPRPLFIYGSLRARPLLAWALTGDAANTEPISAFASPATVRGYARYTVRSRDYPAVIKKDGYEVDGDLLILEAKSLRK
KLDDFEGEIYTPTSVDVVLEDGNIAEADMYVWAGDEDALTDEPWELDTFAKERLEDWLDLLRAWR
FUN_006106 CDS scaffold_20 557 1908 +
hypothetical protein PF01488;PF03435;PF16653

MPEKKILALGSGMVAKPCVDYLLRDKKNVLTIACRTLSSTQNIAAGRSSAKAVALDVASPELDHHVAEHDLVISLVPFVHHAA
IVQSAIKGNTNVITTSYDSPALKYLTTLCVSRYVGLPPVSWSPRGALLSQQISATFLQDGKVIEISNKDPMNKAVLYHVLDGY
SFLACPNRDSVPFRQAYGIAEAHAVIRGSKAWLNEGLKWSHIQQQLTGAASATEVDLLAKVDEVCSFRSPEERSKILAGLKWM
GLFSDEVAAVRDSPVGTLRSTGQDMQLPGRTSALELFGEPGGYSAMAKSVGLTCGIAIQLLLDDEPASNKPGVIAPYSREICD
PIRVRAEAEGIKLVEHTL
FUN_006107 tRNA scaffold_20 2204 2278 - tRNA-Val

FUN_006108 CDS scaffold_20 2539 4093 -
hypothetical protein ENOG41

MPSTRPPNAQSPIVDVDGALDNIDTIRIPEPHGTYLQTRGARAARRIYKAMGVWPWDIVPEAPPKMWAINLLEDLALVADHVA
RNPGDTLQDLQSELRKSLARDRKPSYAGKLMAQDVHAAKKRFVRTMPQSPDAQDDTGDDVGTAERRSGRRRRTANYSAPDSPI
DPSRSRRDTSSEESEEHMASSITVASEFPPSPPVPRNIKHLKRPATTIPTPIEKRVRHSSPEGSRLSAAQSLMMFSSPMATFQ
MATSFGPNATANATRSLEPNNLQEALKPATNVFVAAIDAHVRTIRSSLAIAQQEIFPHRSRSQEAMSKLRAAQLGVQAIQDDI
AESQDRLRRLVEESVAEDALAPQLHALQENNMSLPPEIAQAIRSYESRRAAKSEEIKTQESAINAKRDELKRAAEELDMAEKN
SGGIEADIRDLQAAVTRGTEAARFANMFSVMVQLGPEGLANIEQIYPEIGSLLESLLASRNSPSQQNHNNNHHVHNAVDADEA
NGL
FUN_006109 CDS scaffold_20 5127 7277 -
hypothetical protein PF02837 ENOG41
GH2
MTYPRPDFERNPLHWESLNGQWDFLFDDGDSGLNENWQRKGLPEKVNVDTSANRKETGQNDSVVQKIMGDAHSLIQNNVFASS
TQTTNSKTKINVPYVFQSPASGINDRGVHEVMWYERAISDLRSAQEKEKGHKLVLRFGAVDYEATVWVNGEYVGGHRGGHVPF
EIDITDAVDAAKDATTHRVTLRVYDSAYDLTQLRGKQYWAAKPETIFYTPSGGIWQSVWLEVVPSARITDSSYGTVIKSNDIH
SGQLRSTIAVKGRRIGKDYSVELESSFAGIPIAKSDRKALPRETSSVGVELNMRLSEEQRSKLPKSVTETAPLDNDFAWRDGV
ALWSPEHPQLYDLVIRLYDDAENVIDEVKTTTGMRNINWTTGDGTWRLNDKPYFQALCLDQGYWPETFMTPPSSESLKLDIEL
AKKMGLNGCRKHQKVEDPLFYYWADKLGFLVWGEIANAYAFSAEYVDRFNAEWTEAVKLVINRPSVVTWTPVNESWAYPNLED
NVDQRNHIRQLYYLTKGLDDTRSINDNCGWQHVLTDLTTFHDYSDGPEIEKTCSTLEGIIGPKAGRGVFLPPIPGVDEGSTHK
PGAPVMNTEFGGVNIAPAKKEDGDSLDWGYTTATNADDLVERIERLVNGVVLGGHCCAFVYTQLADIEQEVNGLYTFNREEKL
DASKVKAVIDKALGAYYNRVK
FUN_006110 CDS scaffold_20 7878 9764 -
hypothetical protein ENOG41

MAPTSRIAFLVLSFLLSGHFAAAECDISGNYTIIAAPDDQDGVQSCKDVDGSLSLLFDKDPSSWPSDKATVDLGDISSLTGDL
VIYPHHDSKKTVVTASSLKTIEGALTIWNTGTGKDHTIEEFDLVFDALEEVGKDYVITEAFAKLSLEHKTDITVGGMARVYDT
DVEELQLNGVYTVNGDFSIDSNSDMKELDVPALTYANKGLIVKGNNALEKVSFAKLKGVKGNMQFNQNGALQTIYLAALESAA
TMSITANGNDATVLLPWLSSIGNSTTTATSDFSGLGKIEFSSLRTVNGSLTFESNSFEDLTIPLLKEMNGAITVEDNPSLTTF
ALPRVTYVDELDISSNDKLTNITANALKSAGTIAIKGSFTNVEFFNLKKVTGDFKVSGDESMDCSWFDANIKSIVEGKYTCVG
DHDKKERKPSTGGIEDTEGNPKDYMSSPDDNGGNGGSGGGSGGSGSGSGSGSGNSDGDKGGSKGGLSTGAKAGIGIGVAIIVV
LLIVGAVMFWLWRRRQTAAPGEKRLGSSGSGSTKKGIFDGQQMGVQTKIEATNPSPSPSIGTLNFGRNSLIESTGGFTEKSLN
AWKTIRRVSVSSGGSSTAKEGTSILKG
FUN_006111 CDS scaffold_20 10784 11679 -
hypothetical protein ENOG41

MTDKKGPGKADQEMDSSDSAPTELPPSYDITLSQSESQQQDQLQATEDFPTLVLDGIKIYSLYAPNRILYQLSNAPYDATRKI
YAIEKLRYRMTNGGSEQKLKHTVDHIYDFRHGISIESYKQITILGQTSRKRTYPNMIMHRCLNGCHTICEGGQVKLTPDYVLK
AESPLKDRLNQGKNNTTIWKDEKGEVVAIETKLQRDENGSIQELPRLAIKARIEEKLYDLLVTSWCAHVWKDAQKDLKEPMSW
DKFKRIASTMPNKSAGIYGGAGIGGASLGF
FUN_006112 CDS scaffold_20 12863 13627 +
hypothetical protein PF13302 ENOG41

MSEQPVGPITPSGTASAPSRGSLDGRYTSLIPLEPSHADPSWKHLGGEENGHLWTYMFGGPYLDQQQWREAVEGFSKTEDPFF
YTVLSGPRDDPKSEPVGQMSYLNIVPSHRRIEIGSIIFGSQLKQTCAATEAFYLIIKHAFEDLGYLRVEWKANHLNKPSLAAA
ERLGFVFEGIFRKHMILKGRRRDTAWLSITDEEWPLVKKAFEAWLSENNFDTNGKQIKSLKDIRQGLTKD
FUN_006113 CDS scaffold_20 14901 17422 +
hypothetical protein PF00172;PF04082 ENOG41

MEDNSSHTVQYRSACAECHRRKQKCNREWPCNHCQKRKVADKCRFIQATTAGSPSDGLTPNSDKKRSRSREDDIEPDDSEPDE
DDDDVGLGAMGYAAGPLFESLTIDAKKDKKPLGELTWVHPSTCPQLKHAIDVLPGRPQMDALVQSFFNNMNYHYYIIYPPTFL
QEYQQWWERRNRNQSLSLQWTILLVMVCACATQHLDVEIKPIVEVELSEPAEKLTVEYHKAATELGRVIPVGHCHLLNIQWML
HSIYWYKSEAKFVEAWHVIGAAVREAHELGLHRTAIAEGLSEFEREMRRRVWCILDCWDWQFASGLGRPTIIDHSDINVEPPS
LTLEDFSPSPLLHMKLQSELVTQLAQRWGAPKNITSASDIQEYKSILEDWIRRFPAVYDVDNPDTSKDYKFPWIVYHRFYIHT
MAYLMILNPMRAFMAQVYTRNSPADLLAVRKDAVFYSLKNLDTTTRWADHASHRDARFHFIIFSLFDTAAVLSTAVIKDQDDT
IPRKDEIADSVDNAIRLLKRLKALSKTAKTSHDILVKMVQKMPRRSAPPRETVNRKKQRVAPVSPAAPATNTRHDVVIKPEDG
YHPEHVSQESSPSYYASSEGAPSGSTPQSTHSSYDNHENTGYVMNNQVPAEGYAKPQVAYNMDVAHGHMPPQQVPSPSAGIDG
IVQGGLYGMPHADHTGGGMVPPTYVDYHPQDLNIDYGLGNITDVDLGDFSQLWDWRSLNLDFIQSST
FUN_006114 CDS scaffold_20 17935 20012 -
hypothetical protein ENOG41

MSPVRLFPFLLACLFGASIALQDDQRQPESWCITYLSTYLVSVSDGHTRPAASSVGEAFFPETSEAALTFSTDQAIPSTFPVH
DDLVITTSAPSPTTGILVPDTNGRSVIFRIIPELQDTKRDLQVRAVGGFVGSQSEICAAAAIFNLVQGRLLDGTWPIYYNGEG
FKELRGQPGALPRDAVAETFVDEDGFLQFFSSVLPNGEAGFCQEAESGQVYITFTASPPSCQSIRLSITGVDECRDGDITSTP
SSRPASVPPLAESTSALLGTTSRFNAAASVTTKAIQKGSSAIDVPTFTPPFQDTSQVKPVYTSSVRFYNSSSSVIFPTHPALT
QTSSVLEISTPVTDFPLTSRLSSWASNLENLPTDALSTNDLGTSKEPPSTFEPTTTATPTTTNAKNTIGTSSGIDSTPSSVVN
SSTTSGRSYSFETDTSTLSDLSVDASSSFDSSPTSLSTADTRLSTTTTVDTTTSHTSTTTSSEATGTRACPEGLSEPLTLFDG
QRPSDNGVEWLVLPFLITIYGQASDMLYISPNGLVTLGDSFGADSFSNVALPAENVGPLAIFPYWIDMIVPEGSGAEVTYQIE
DIPNQPGVLTIDWCLFSTSDGTEPNHFQLVVSEDQLTPFTFFYYTINPGGSYATIGAQNRGSDQFLQYYGPIPDRTFMEVSTF
GDEEIRIGQF
FUN_006115 CDS scaffold_20 20776 21568 +
hypothetical protein ENOG41

MYSKSLVVPALFAAFGIAQEDVQYETRTATITQCFTRDGLRPPATVTVPGPTCVVHKPAVTGEAIIVEVQAPDCHCGCPTCVH
TLEYTTKYPAFCSTGIFEQEYIITETYSGMEAKPTKNEHDLPYGFTCDVQTCTTCGPEPVTATITYPVSDRPYMNEVAPTEMP
MAKPGKEQPEEQGNAAPKVTGDNNRFQSSVKPSVVPESPGNAPPGESTDRPVIVSGAMSQHLGLAGAGIAFFALIQFLFL
FUN_006116 CDS scaffold_20 22540 23910 +
hypothetical protein PF12776 ENOG41

MSDEDADVLGPDGQPPARVDRDRRAPRFSWTPAYEATFFRSLCESVQLGLRENSSFKAEAWERAALALQESHGAYPAKSHLIN
KSDNARKRFRLWRGLREDPEFVYNPVTRTVTATEEAWTAHIEREPLSRALRGRPFDHEDFMEVLYPDVIGSGGAPKRIMKPRR
RTDGPMTDDPDMPGTGILNLTSDPPPRPPGLESPNSRPMLTQTPTTSSTGSATQPRPTSTTIPPRGPPTVANANALTPPDETI
TQSRKRQLPTSSTPNMFESTPPATTIPMGQSAPESPGKRRRTSSNDGSRALSTSVLNSSMLPLAVRDGPSAGSPSQADSQNTN
GTNGPVMEEIVEAVRSRNTLRWQEEALDIFFRDFADEDLDLQVKISEGVLINECKAMVFCKMPSRVRQHWVKRFKETPLRQ
FUN_006117 CDS scaffold_20 24806 28504 -
hypothetical protein PF00072;PF00512;PF02518 ENOG41

MASPACVQVPKKSIVSEATRERETFRYDDSLFTNVRFNDTGLPVPSNELSTSPDVVLTALARLAASQTGAARSMISLFDQTHQ
YIIAEASSITPLIPSLGQDGHPETLWHCGTAIPRSHSACESTLCSDEIQSDASGPKANHDLPVIVVDDVTLDPEYSTKPYCRP
GGAARFYAAVPIRTDRGVNIGVICVIHTEPSLPWEDSHSRLMRELSRAVTEHLETTSLKSHQRRSERKSWGLTSFVEGNTAPL
SQVSSTGREYAEHEMRGDGMARSRRPKINDLQQALGGMSVRNSTATADSRTTNALHTKEVIMCDPQARSKFAHDPGQLLETPR
QIFSKAASIVKDSIETDGCLFLNADMLEFSSAGSSTEWIDEDLATRRPSTFSYNESEDAYTTGSSDDRLRPSCQALGSSEHGA
AHIALPQALLARMIRRYPRGHVFNYDTDGNLLQEELSDDSIRHPPNNNDWGNLHNRQDHRFRVQQREASILLETFPGARSIAF
VPVWDLRKKRWSAGGFIHTYNIKRTFTIDLDLSYLRALGMLAAAEAFKLETLAADKAKTDALGSLSHELRSPLHGAVLSVELL
NDTDLTIPQQNIVRTIETCCRTLSDTIDHLLEYSKVNKALPQEATRETQADGSGINRSSSVRIASKPSRKVVQLDVLAEEVLE
SVYAGFNFQQATLAQSSSQTPRRPGHTSIRRMDTMQAMEELQAPLKKKGNLSTKIGDVDVFLVIDSRPSWGFYTQPGPVRRII
MNLFGNSLKYTQCGIIRVSLEQTSAFGADDNDCWVTIKVSDTGVGISEDFLQNGLFKPFSQENHLSPGTGLGLSFVKQITAQF
GGHISVDSRIGVGTTVTVRLPMKLGDDCHHNVCEIRQREEEFDAQTEKLRGLRVKLVDFSKDSVTGGGSVQRTIESPDQLVQD
MCKEWLNMQVNTTSETPCFMPDLAIWSEEGFERPYVPQDSVLDIPNIVLCANAVTAHQYATGTKKIPRPGVFEFISQPLGPRK

LAKACSLALYRWTVMQTSTQSPTTASTEPESLSIVTSTAPSLTNPSSPFPASEDETPTQPKDYFRASEFLLVDDNFINLKILS
SYMKKLKQPYQTASDGLEALTAYEADPGRYSCILMDISMPVMDGFEATRRIRAFESQQGLRPALILALTGLASEEAQREATVS
GLDLFLTKPVRLKELGPILRAKGVLEEESNLG
FUN_006118 CDS scaffold_20 32106 33306 -
hypothetical protein PF00144 ENOG41
S12
MGDLPHLSDHIGSFSLSSIHPADPAYTPLLYRLREETFQNRTTQVRHGSYLRRFTRDTSFNIAEAIKENSGIDLNNFMRRYGL
TGCVVVQNGAIKLEEYRHGNSQASRNDVQSVTKSFVATALAIAQQEGKLCVNDPVSRHVEELRGTAWADVPLLALSSMSSGVV
EQSEEERPADVPNPMYATELYPQTDTEAVINWLKTSRKVTEPWEEFHYYNPNYYVLSTAISRATGEPLNEYISRTIWDPAGMQ
YDGYIRTTGAGHVDGHGGLSITLTDMARFGCFILESFRDGGKGPNVPPGWFQDISDAKNSVGARALQPDNFVSNFGYESGWWT
TGRADKEYKMGDDGAFAAIGMYGQSIYIVPKLDAVIAIQSGYPEHELDLFTKNAELATAILKALRDQ
FUN_006119 CDS scaffold_20 33934 35081 +
hypothetical protein PF03417
C45
MLKVHCSGTPYEIGHQHGTAAKDRVVGSIAFYKDLFQESCSMNWEAVRQEAQQYIQPLQKLSPRYVEELQGLADGAGFELVDI
VALNVRTEITFGLYTRVPSAPIQTDGCTSAAYRQPNGEVLLAQNWDWQPEQAPNLFICHISQPGTDIPDISMVTEGGVIGKIG
INSSGVGTCLNAIRARGADNTKIPIHLALRTALESHSAREAADKLYEMGTAGSGHILVSDPSEAIGLECTSIGMKEINFDGNG
TLIHTNHLLLDHPGVDEPGWIPDSTDRIKRIAQLLDERLATSTIGHSSFFDFFKDEQGYPTAINRDQFSPGNGKATLFTINMD
LAKRKAIVTMGRPTNWTERIELSPGLA
FUN_006120 CDS scaffold_20 35578 36361 +
hypothetical protein PF12352

MNIAYNSALRQSKSLRAELSNLNNKPQASPSEIGNVSASIASFTKTLDEYQSLARQEIVPKKQEEGFERVKRFREDLSDFRTQ
VDSLKKVRDDAQHQANRTELLGRRPYNATPENPYANATTTNTHSAFQPRHPPQASALTTGSPDEMREAHAFREQNFFANTNQA
LDDYIARGQAVLGDLGQQREMLKSTQKRLYNVANTLGVSGDTIRMVERRAKEDKWIFFAGVVIFFLFCWLVLHFLR
FUN_006121 CDS scaffold_20 36529 37424 -
hypothetical protein PF04263;PF04265

MASKSFEWRPAKLLQDYNIGQDFALLILNQPLKNGVNLRKLWKNSSVRVAADGGANRLHKLSSFHGKYSNLQLIIGDLDSLTP
QVRDFYSSQPSPATVIHDADQESTDFAKAINWIRKEYPEGIDIVALGGIGGRVDQGLSQLHHLYLFQNDPDYATGRLFLLSGS
SLTFLLKAGSHQIQVREEGEEDVFGKHVGIIPLKEAANITTKGFEWDVENWHSEIGGKLSTSNHILPESQVVSVTTDKDVLFT
VALKEGDGDDE
FUN_006122 CDS scaffold_20 38065 39209 + MIA40
Oxidoreductase EOG092651BA

MYRTTMRSASRPVIASLRSNTIRAAPRRFNSTATPADKSRSWKSSVARLGLAFGAVYYYNTSPIFADEAISKTVPAPAAFSDD
DLPTVDSVLEEKRKQIRAKSEKPAESSKPSEPQQSNTQAAAADGSPAALEEEANQQGAFNPETGEINWDCPCLGGMAHGPCGE
EFKTAFSCFVYSTEEPKGMDCIEKFQGMQECFKKYPEIYGAELADDEEGAPTPDFGDEQPAPDARKAPATDKAPATDFKSLPE
DSSAAAEPDQQATPRATSNEAEKETLVNSSRNVQDVATPIEKPHNPELWQDMHKTEVPVKEVFVEPSQAHDATAANAEIKIIE
QEREAKKKAEKKQ
FUN_006123 CDS scaffold_20 39695 41991 -
hypothetical protein PF01168;PF14031

MDYSLENHKAYIGKHVSELPTPSLVVNLPVLKTNVETLHRDVEKLGIGFRPHVKTLKSLEVTRLMMADGKYRGIIASTIPEIK
GALPLVKEGILEECLYGLPVYPGILPRLIELRKSLRIMLMVDNEQQVEFLEESASSKQPWDVFIKLDVGSRRAGVEANSAALN
RLVEHAQKSSAISIYGFYCHAGHSYGGRSRDEAEKTLNIEVSSVLAAAKLLPSDRQLVISVGSTPTAHVVESLKASMPENIKL
ELHAGNFPCNDLQQVSTGLVTETQQSVSVAAEVCSVYPQRNEALVNAGVIALSREASAFSGFGRVVGSPAWGVVRLSQEHGIL
GTSEGRKVDEDFKVGQKVQLWCNHACIAAAAFYVYYYVVDEQGIVRDTWIPWKGWITHPQQLESTLLFPSLPRLTIMSYADVAA
SGPKQSPEEAAAPQPPQVITDESASTASLVDVDMPSVHTVPNDFLEQEIKTETQAARIEREEEAREEKRKRDEAAAKAKRADN
WLIQQFSRLSDGSATGLVIANFATVVGLGAYLGYKGWGLYEKGKLDWKAVTLGAGILAGVTAAEGAVGRYLYKGKKGGS

FUN_006124 CDS scaffold_20 43496 45080 +
hypothetical protein PF07690 ENOG41

MSVPKQEDDHIGDSESTHTRVSNQDPEKQSNTEIEIPDAPYSSFSLWQKRLIVLAAASTALFSPMTAQIYFPALPAIAKDLGV
TTSQINLTVTTYMIFQGITPMFIGSLADSGGRRPAYLVCFTIYIAANIGLALAPSYGALLGLRCLQSAGSASTVALCFAVVAD
VVTSAERGQYIGLTAVPSVLGPSLGPIIGGVLAEFLGWRSIFWFLAIFAGVTIVLLALFYPETCREIVGDGSIKAPLVYRSVW
QILVSRRKRSGSEKNGLSRQGSRASTVKKFKFKFPNVLDSILMLSEKETGFLLGFSSICFAGFYCIATAMPTLFKEIYGYNEL
EIGLTFLPMAAGSVIAAFIVGTFTNRNFKRHCENQGIPYERSKQLDLSGFPIERARLEIGFPLLMLAAATLVCWGWAVHARAH
IAVPCVLSGILGVGVVGFNNTVNSLLIDIHPKKAGTASAANNLTRCLVGAGASAAIVPMIDAMGTGWAFTLVGGLYVLGCPIL
ITVMVRGVKWREELRVKEEKKQKAKEAQE
FUN_006125 CDS scaffold_20 45588 47145 +
hypothetical protein PF00067 ENOG41

MGRMAYLGKTLHRKYGPVVRVGPNEVWFNSKEAFHAIYSSGSGYEKSDFYLATALSKPHIDWHLNPEFQDTLDLLSERDVRRY
RLQRRLIGPVYQTSNLLQYEAAIDEVLARSIAKLKTLNGAQIELNEWMHIIAVECLGAVVLSWSPGMLKNGTDNGSGAHAYHG
WRRKSVFGLFPMAAKLEFLHKSIGRLFSTIWGVNFQPPKDFRPFFPDVGKRVSRRVNAATKSKLNKDDRKDLLADLIQLHRTK
PNFTELYLRKMAVTNFGAGHETMASTLTSILALLGSNDDVQAQVSLEVLGTSNPAEYSTATRLPETQSLIKEAKRLYPVISMS
LPRKVPSEGLHLHGYYFLPNTTVGCNPVALHRNPDIFGSDSDMFNPARWLTADPDAARAMERVNLSWGGSSRSCPGRHLAELV
VFKVVPALVKEFKIDVDLPPEDKNRSYFLSMLIGVKARFIERDAGLSSDEE
FUN_006126 CDS scaffold_20 47345 48194 +
hypothetical protein PF00561;PF12697 ENOG41
S33
MVKPIVTENALVKGSRVAYGVYGTGNPVVLIHGTPSSSLIWRNIVPKLVDGGYKVHVFDLLGFGLSERPWDSAVDTSMTGQVA
ILEGLLELWGLEKTHIIAHDIGGGIAQRFSVFSPESVLSLTLIDVVSFDSYPSKRTKQQMQNGLEALNKTDSDQHRAHFREWL
LSAVKNPDKLEKSSLNTYLDYISGPIGQPSLFEHQVRHYDPKHTLEVAPRLGELEKLPVQLIWGADDAWQVVDWAHKLHDAIP
GSKLMIVEDAGHFSPEDQPEKISELLINFLGKH
FUN_006127 CDS scaffold_20 48285 49254 -
hypothetical protein PF07859 ENOG41
S09C CE10
MPVQYDTEFAGALALIKSGHPPAPPETALEMRHNNHALFLKVFPKAPSPDIVEQTDYTVESYDGVQILLRRYATPKVLRAKEP
QPAILVIHGGGFVSGTVEICGGTNAAIALEIERPVFAVDYRLAPEHPYPAAVEDSFASLKYLIDHAKELNIDPFRICVKGNSA
GGGIAVGTVLMARDRELNPPVAKLMAIYPELDDRTCHPADTEFLKLATWTSQHNQLAWKAYVGEDKAGKPDADVSPYAAPARA
QNYNGLPSTYVDVGTLDLFRDEDLELVRRLLEDNVEVEFHLWPGVPHVFEFLAPGTRWHQRAKEAQNDALRRV
FUN_006128 CDS scaffold_20 50206 54265 +
hypothetical protein PF00122;PF00702

MAPLVDNPQIKHAELLRPLSFHFHAYIWPFTIIWPIFFAFYLKPELYEKYIGAEEWTVVWVGTIITLQSLVWLSTHWSVDLEG
KFKASKAKDIEDAELIKVIPIANAGSAEICKLVRDKAGGKLNTSFLFQKRRFLYDPTTKSFTTLKYDIDTEPKPSIGHFQTSK
GIQTQTELSRIEQHYGKNAFDIPVPTFTELFKEHAVAPFFVFQIFCVGLWMLDEYWYYSLFTLFMLVVFESTVVWQRQRTLNE
FRGMSIKPYDMWVFRLGKWTEVQSDELLPGDLASVNRTKEDSGVACDMLLVEGTAIVNEAMLSGESTPLLKDSIQLRPSDVPL
DAEGLDKNAFLWGGTKVLQITHGNPDQEKPKLSSGVPPPPDNGAMAIVMKTGFETSQGNLVRTMIYSTERVSANNAEALFFIL
FLLIFAIAAAWYVWDEGVRKDRKRSKLMLDCVLIVTSVVPPELPMELSLAVNTSLAALAKLAIFCTEPFRIPFAGRVDVACFD
KTGTLTGEDLVVEGIAGLALGHEDEIKDTKEEDGAHSTMTAVTEASLETKLVLATAHALVKLDEGDIVGDPMEKATLTSLGWT
LGRNDTLMSTNKAGSTHGTVQIKRRFQFSSALKRQSSVAMVHGNDIKTGHKIKGTFAGVKGAPETIQKMLKVIPDDYEETYKY
FTRKGSRVLALAYKQLTIDTELGSGKINDLKREKVESDLTFAGFLVLHCPLKDDAKEAVQMLNESSHRVVMITGDNPLTAVHV
AREVEIVDRDVLILDAPEDNSNGDKLVWKSVDDKVSIKVDPTEPIDPEIIRSKDICVTGYALAKFKGQVAWNEILRHTWVYAR
VSPKQKEDILLGLKDMGYYTLMAGDGTNDVGALKQAHIGIALLNGTPEDLTRIAEHSRNTKMKEMYQKQCDLMKRFNQPNPPV
PAMIAHLYPPGPQNPQFQKAIEREAQKKNVTPEEYANSQGFDFETITSPGAQALMDAGPHANRQGEAAKKAAGFADKLASGMM
EAELGDDEPPTLKLGDASVAAPFTSKLRNVVAVPNIIRQGRCTLVATIQMYKILALNCLITAYSLSVLYLEGIKFGDTQYTIS
GMLMSVCFLSISRARVVEGLSKERPQPNIFNVYIIGSILGQFAVHIVTLIYIARLCDRLAPRSEDVDLEAEFAPSLLNSAVYL

LQLIQQISTFAINYQGRPFRESLSENKGMFYGIVGVSGLAFACALELFPDINEGMKLVPFSDEFKTNMTAVMVIDYAACWIIE
VSLKKFFSDYRPRDIAERRPEQLEREAVRRAAAQKKKDEEEEKKRLEKVAEFERKVEERRRKIEEWRAGRT
FUN_006129 CDS scaffold_20 54834 56187 - MDH1 Malate dehydrogenase, cytoplasmic PF00056;PF02866

MFAASRIQRRAFSATARDLSKVTVLGAAGGIGQPLSLLLKMNPRVTDLALYDIRGGPGVAADISHVNTKSTVKGYEPNAAGLK
DALSGAEVVLIPAGVPRKPGMTRDDLFNTNASIVRDLAKAAAQAAPKAKLLIISNPVNSTVPIVKEVFKAEGVYNPKTLFGVT
TLDVVRASRFVSEIKGTDPKDENITVVGGHSGVTIVPLFSQSNHPDLSSNAELVKRVQFGGDEVVKAKDGAGSATLSMAMAGA
RMADSILRAVQGEKGVIEPSFVESPLYKDQGIEFFSSKVELGPEGVEKIHPLGKLDANEEKLVEAALVDLKKNIEKGVAFVAS
NPPK
FUN_006130 CDS scaffold_20 57326 58901 +
hypothetical protein PF06221 ENOG41

MSIAQLSQLLPLPDEELKQVLDYASTLSTTEAAEHFGNLLGDSPQAIEFISSFNARRQTSAPGSSSYSNAAAPPEPESQNIEA
VPKTKRQPKKKKANIHTPQARQVNDYAAPAGTTYSKKDLSLDYIPQRSSAPSSNNASRSNTPKPDPKPVAKPPPKQHASAGYL
IGEGPSKPRPKSTPVSRSSTPKPATSTKISIAGGMPMAGASTAISDLDAAIRALEISTNPTLENSKSRKCNCVATRHPLQGAA
PNCLSCGKVICMKEGLGPCTFCGSPLLTPDDVQAMVRELKDERGRERMAANRDANRRADVAKTPAPFTQPRGNDGASLSDAEA
KARAHRDKLLNFQAQNARRTTVRDEAADFDVGGALTGTGSMWATPEERARELKRQQKVLREMEWNARPDYEKRKQVVSIDVVG
GKVVRKMAAVERPVTPESDEDPVDNGALGETSANKNSGRGGGAFSGNPLLGALIKPVFDAKGKGAEVEGRESRKKKGWQRVQH
DLNNNEEVILDGGVQGHDRSDEPACG
FUN_006131 CDS scaffold_20 60320 62096 +
hypothetical protein PF08528 ENOG41

MSPNQPLAEIALDNNRIAPPPPFKTTDTRLDAPTETAPATVTGARQGGDFAVPPPPADALSRASHSNPIASITPTPTPTAAPA
SDIAVDSTAGTTTAHAVVVAAIAKMQRKSSVDRDDGSTQSHPHSNTDSQETTMTTSTDPAFTPPASDASNCAPGPSSQDSQLL
QLSEIAAAQHRIATDSTNSRKRMADGEVKSRTDSQSPVKGHSRNPSAVSRTSAAGGHIGELSNELKTRLSYAMIKVNKGWQSN
SLEEVENMASQAASPVSSTSTIHNRQGSSASPRIPMSKPPSSSAPLPQPYGSASQRRKSNTPPSSSWNKPALAPPAPIRPSVL
VPNSHANPRRNSHPNRTPNMLTHSHSASPHTPGQPSTVPHGPKSRVMGDPVLMSPHQNVREQDAIETLLFMSSPGNSANLKHA
FSPTGSPGPNVGMARPPPPRHALPSGPRKPLPTSQRQPQQTRKAPYDKSPMAPPPGSPMDLDSPQQHHAALNRVAPRRRTIGS
RSQLRGTLSLPSGIGLGSGKTRKPLRDEDIERMLDKASVEAADSSDDEEILIPRRAVAGVMQS
FUN_006132 CDS scaffold_20 62866 63735 -
hypothetical protein ENOG41

MMKSRRTQRSKSLPNNQSPKRSFFSIISRRLSLSSRRHANSVRLYDDSEDDDTIQVIPQEVPPPTQSRFSRRASRFWSTSAAN
QFDDDDSSPQSPTYPAYGGAYVPRHAASDFSKTASNRLTMMAEADETTLCSYNFRTNRTTRSFGTDDELDVDHRERALTALTA
RRSIALSSESSSESTNDYTLFLADAATGSDARRRRSAAAWAELEHRATAASRRTSGTDFPMGRPGRTQSSYLGLPGSAYDSSR
PASSVAVSITEYIRPAQTGRVW
FUN_006133 CDS scaffold_20 66086 67995 -
hypothetical protein PF00291;PF00581 ENOG41

MTASEHLNVFKGPESVRNYFDPEQSPPLPLVEIPDSLNPYRQDGVRIYAKMMTMHPATNVKVMPAMNLLEKGVEPGKTKTIVE
SSSGSTVISMAMVARAFHGIDDVHAYLSNKTSETKLRMMQFFGLNVTLFGGPAQPTPVDERGGIRAAARKDAESGSVCSPNQY
INDDNWKAHVRWTGPQIHRQLPEINVIAASMGTAGTMTGLGTYFKQAKPSVYRIAVCTNAGERVPGPREYSLMTEVEFPYLTS
HDALEEVGAPDSYSLSLDLTRQGIVCGPSSGFTLKGLFQRIEKHKQENTLSNLAGPDGEIHCVFMCCDLPFQYLNEYFQNLGP
EKFSPLRNERLRNVDLYRYDDAWELDSLTALSNYFTIHPLGTHDTVLSLADQIDKALTPSANTQILDFRCSSDYDAFHLPNSV
NMPLDALRNGPSSGSPFADPADDCAMLEELWLELESLFNIKDKTGNRNASAEALMSIFRGKRVLTLCYDGDSARVANSVLRAK
GVEAECIRGGYSALAKLQMPCNDTCEVVSVPVSVEV
FUN_006134 CDS scaffold_20 69847 71635 +
hypothetical protein PF00082;PF09286
S53

MVPMIFFTALALAGFSTAKFVKVEDTSVLPVGWEKIDQAVDSSHTLRLSIALRQPDIDNLKTNLRRRDCTSGKYTQNHLTQKE
ALALRDPDQKDVGQVLGWLKSKGVAAKATPDKDWIHVKTTVKAAEDLLDTKIGFYKFEDQKPVLRTKQYSVPESVADAVNFIH
PIANFMRPKKELTTPDPALTPSMLESLQLEKRATPCSRAVTPDCLREQYNISYPAYNGSSQIRFGIAGFLEENANYQDSNDFL
RIYAKPLYEARYNFSVQLINGAENSQELFDSGNEAALDVQYGMALGYPTNITYYLADGRGPTLDDDGEKLPEEYNDNEPYLEF
LDYLLDLSDDEIPHVLSISYGDNEVSVPRKYAERVCSLFGLLAARGTTIVAASGDGGAKGASNSTCRTNDGSDKDVTMSVFPA
TCPWVTSVGGVTSGVEPFEGAEFSGGGFSQYFPREKWQDSSIKSYVKALDGHLNGHYNASNRGVPDISISATSFITRLKGQAV
GLRGTSASAPVVAAMLALVNDARVRKGKEVLGWLNEVLYSEEVQAALQDVTNGESRSCDFRKGGSPGGWPAAKGWDAITGLGV
PSDFQKLFDVLVHI
FUN_006135 CDS scaffold_20 72455 75409 +
hypothetical protein

MPTIHVSRDSQDELQDIANGLLKARKVIVVTGAGISTNSGIPDFRSENGLYSLISAQFDAAAQQARLNEGSSDDKPTDLDIRR
PAKRRRIVHDVSAKVEEGPVGMKEEIEVQLEDPEPDHEKIADTIQVEGHPDDEEQQVRSDGMPVVNPRTTRSTIAVAQPPSSP
LSSPPPEGLNIPPPSVFRRTRRSHLTDSTIPPSSSPLSSPPPVLFDPFSSGTSSEDAPSRRSSTSPSEVDDTPPSNPINLSQA
SFGSGKNTLPNMKGKDMFDASIWSDPLRTSVFYTFATTLRQKVRDVEPTSSHRFISHLRDRGKLVRCYTQNIDQIEEKVGLST
CLHDGPGSRGRFSRKSTANINQLNRMVDEVNAMTDTNSSDKSQQSSDNEASQQSQCSQPNFESTVPASQAESDQCATDEALTA
VRNLRRDLPKSGVECVFLHGSLELLRCFLCGRVCSWDDDERQLETMSGQQPECPHCVGATVAREERGKRALGVGKLRPDIVLY
GEEHPNAHLISPIVTHDLALCPDLLLILGTSLRVHGLKVMVREFAKAVHAKGGKVVFVNYTKPPESSWGDVIDYWVEWDCDAW
VSDLQDKIPKLWQTPEPPKLKKKRESSGVAEDAEKTETKRPVAAYPVALRDTKATGAYWSSRIVKELHRITGHDLLESPVFTP
PAIITTTETLPPAHTPLKTPQGKPDRVRTRAQRSRKSAPGVLERSSMPPSTLNPNHGRTLRSTETRAHLLGDEMAPSNEIITH
ERVLLHGSSIASLVKSRARERKRKKIDGEEVSLPSKRTLGSLRLAPLRPQPSDPRDIIPFDRLPTMELPPDTPEPYTETGQLQ
SISPVMHHTEPLMPVSHNTRKQTHIRRSQASFMRGQDLAASPAPMLPPEMSHGLLVSSHDRCNLVMKHVRSQREADAALALSA
LSQGDARPVITKEFAIPPCTGVGIRKSARLIHRSGTPTST
FUN_006136 CDS scaffold_20 75970 77429 +
hypothetical protein PF00266

MGSIGQGSSQLPVRGKTNTSVFGSAIKKEFMFDPEWRNLNHGSFGTYPQAVRTKFREYQDASEARPDPFIRYEYPKILDENRA
AVAKLLNAPVDSVVFVSNATTGVNTVYRNMKWNEDGKDVIISFSTIYEACGKVADYYVDYYNEKVTHREIELPYPLDDDEIIK
KFEDAVKKIESEGKRVRICTFDVVSSRPGVVFPWEEMVKTCRRLNVLSMVDGAQGVGMVKLDLSAADPDFFVSNCHKWLHVPR
GCAVFYVPQRNQALIQTTLATSHGYVPKLANRITPLPPSSKSPFVINFEFVGTLDNSPYLCVKDAIKWREEALGGEDAILEYI
WDLNKKGSELVAEKLGTTYMENSTGTMRNCGMANIALPVWTVEGKEGEVVISAEETQTAFQWILNTLIGDYKTFVALFLHGGR
FWIRTSAQVYLEIEDYEWLGGVLKEVCERVGKKEYLK
FUN_006137 CDS scaffold_20 78152 78533 +
hypothetical protein ENOG41

MGNSSSSSSNPSEEAKDKKTLYERFQAGKKSVPLSDEDILKYTGKTRDELSTWADTQPGVGKNQLAGSVTAGPISGLGGVAMA
DGLGGWGPSAHPNDKNRGMKFPPTRENEAKDTTEVTEVVEDKK
FUN_006138 CDS scaffold_20 78786 79799 - STI35 Thiamine
thiazole synthase PF01946

MAPPAAVSPPSRSAELATSTKLPVMSKNINTKTVEEMLGQWDDFKFAPIRESQVSRAMTRRYFQDLDNYAESDIVIIGAGSCG
LSAAYILGKKRPDLKIAIIEASVSPGGGAWLGGQLFSAMIMRKPADAFLREVGVPYEDEGNYVVVKHAALFTSTIMSKVLQMP
NIKLFNATCVEDLITRPSEEGVRIAGVVTNWTLVSMHHDDQSCMDPNTINAPLIISTTGHDGPMGAFCVKRLVSMQRIEKLGG
MRGLDMNLAEDAIVKGTREIVPGLIVGGMELSEVDGANRMGPTFGAMALSGLKAAEEALKIFDTRKKQNDL
FUN_006139 CDS scaffold_20 96843 99341 +
hypothetical protein ENOG41

MSTVKFQCPPYKLFEPDYHPQKPIIVGVDDRLESPDTIALRYEEAARVETGGDPRGDSPPRLLEMATYTKAQLPSMHAGYDVA
RYQDAGYEQYSTQAYPTQQQSDKFAHLNQQSFASNSAAVAQYMPSGPTVLSCQPATGIFGTKVYVKLSSQYDLFSLSTPIPTW
FLVFGSEKCSAQDVSRDIQEGSGFIYTCSGDAPQFLVTNCANSNVPLSLVLDGPSGEEISRIPVGTFQYLEGSGDDITRSTKM

PKHEDAAPATTIDQPSTSPKGEPQLPSEPGTNTYEYPPQQGQYANTFPQANSDMISTYNRSTSFTDPHYHRRPTTGWSPYPST
LGSTGRGPGGLDTSLVGRPPLTPLGMSSPSHSNGAPQLVRTSTITANAGNNTSYHPISLYSGKAVLKISGKLESMAENWTSEE
WANRRRIVLFRKTQKGSTVNATCQSVSVNERPTNSICISCIWWAEKGECYVTSVDTIHLLEQLVAAPNRFSVEEKNRIRRNLE
GFHPQTVSKAKPDSEEFFKIIMGFPNPKPRNIEKDVKVFPWKILESALKKIIGKYSVNPSTPVPPPMMSQTNGGYAPLPTPPG
QSMAPAHPDAHTQYSLPQHHDSIPSPRSLSGSQPSWTPYTTGPGYSTAASRTLSPGLRHHSPQQPPLRINTTPLPAVSTYDSR
SGGYGTSGLHTPLSHHPPTATPPRWDPTPATYPEGYTSLTSQTAQPVYGAAGYAEPAPRA
FUN_006140 CDS scaffold_20 102709 105705 +
hypothetical protein EOG09260JT9 PF00493;PF14551;PF17207;PF17855;PF18263

MATPSSDAGFMMSDAPSRAAPTPRRNMGFPSSSSARPRGPPSENMGAPSDDEGDGFADDQVPRSSRIPNSTEISRVEDRIGLL
VQEHFESFIENFAEDPISAPTPSAPTPSAVTTDKYYVAQIKGMRTYSLSTFYVDYRHLAAWENGSLADGVMRQYYRFLPFLTA
ALHNMIAKYEPMYFREHRQPTASSNLTTSAASHLGSASQSESSHRKNEHQQTDKLFSIAFYNLPLVSRVRALRAANIGQLLSI
SGTVTRTSEVRPELSVATFTCEACRTVVPNVEQTFRYTEPTQCPNSTCQNRVAWQLDIRRSTFVDWQKVRIQENSSEIPTGSM
PRTMDVILRGEIVDRAKAGEKCIFTGALIVVPDVSQLGLPGLRPTAIRDDRNAPRGADAGGSGISGLKALGVRDLTYRLAFLA
CMVNPDTSTSGQSAASGAADVVNALTQNTANEGEQSVEDAQAAVLASMNPSEIEDLRAMVHGDHIYSRLVQSIAPMVYGHEVV
KKGLLLQLMSGVPKSTAEGMQLRGDINICIVGDPSTSKSQFLKYVCSFAPRAVYTSGKASSAAGLTAAVVKDEETGEFTIEAG
ALMLADNGVCAIDEFDKMDIADQVAIHEAMEQQTISIAKAGIQATLNARTSILAAANPVGGRYNRKTTLRSNINMSAPIMSRF
DLFFVVLDECNEQVDRHLAEHIVGIHQLRDEAVEPEFSTEQLQRYIRFAKTFRPEFTDEAKDVLVEKYKELRADDAQGGVGKN
SYRITVRQLESMIRLSEAIAKVNCVEEISSEMVVEAYNLLRQSIISVEHDDVEVYDEEPEEDSETLLRAADAASGRGQEHDAV
MEEDDTQPGRSSVAPERRKQTITYDNYIKMVNMFVQHITDAEVGNLEGVNGDELVNWYLEQKEDELQGEDDYHREKALANMVL
KKMVKENILMALRGEGLTDGDASSSTDPSQIMYIVHPNCAVEEV
FUN_006141 CDS scaffold_20 106800 110637 -
hypothetical protein

MMATEAAVDKAADATASQSQPSPDAENVDSVRSNLNGSAGNDDYENKHITSVEDQLTSADASASGGSDTEASRADASKTKDGE
KVHGRAGSTVKKPAFKAVSVNKTFLAAKATANSAATKAAEKQPAGSSTPPTTSATLSSSRPRLIAKTGSSTRDSSPRFSGGVN
GGKPGSAPDANAVWNKNRPVPPPEPKKFTDEELKKYGIHMASRLNEDDAQGQNKWADIDDDDEDWAPEAITWGDGTKTTLPHP
DEHPPPPSDSGSVASKGKPLDKPRSPAPPVAVSSPLTKSSNLAQGKGLILKSGSQEKPTLVAKPPAPPAPVKSPWAPLPPVEK
ASPIPEQASLARPPHNKGMTPPPPKEFAADDFNRWRDSSSHGGRELFNSHSGRYEPVHDNRRGSMRMEQHPRHHPSLLQRPPQ
PDYPAEPSGAFQTNRVSQEPPFARRRGSSNVSGGSGSFMQRMNKGGEVPLAPHELQEGRRPSFVGSVDGLVSPNLSASAHGPP
RGQHHSSWMPPRGSPNPAFAQPHHPAPLPEQVAGPPPPLQPASEEVEYQKKLMRERVELARKRRQEEEAREEAERRERIQKKL
DAMGPAPEKKTEKKELAEKSDDASHPTHIQQRDHSSAANQTPTQLQQDGDHSATASSARQQPQDSDAKVGHTQGTAPAARRVS
HSQEAKPSELWGAAAASRQDRAVSNWGTGSQPRSNVWGSPNNDRGLGNGTFNTDIGRAPLDNRPGSQGNKGPSPIAPPNNPRQ
PSQARPQPPAPIGSGPSRYGSSGPHPPAGSDVASKWITAVGEKDKQLSASRLAERVERDRLLAERGVSLEDAQPVIKDTWRQV
HAPGDGTRRAIGPQEAQPAGPWRSPGDDAKEDPNNVGVVGGGVPGQAGASQNRTSRFFPTRDSHDRINENEPSRSKSPTPPPP
TMEGHPAYEGDVMKPHVSLPKPHPVVKLPPSMAASQPPSNRVQFGWAHPPSFKDVARGPVSPNRAPLTAGESSQEKWQNRINS
LLGNSSRSPPQRSIGVDPASKSALDHVAHHDSATVSLPRNNVSAPARFDKSLLSPVTKPMAEECFEEQEMGSLPQIRLPHKAP
EAAWQPALAPNKPLPKKFAVQPTVMEPYYFSADVVGGGNVMRIMFPNMQEARTVTIPFSATRGGRGSQGRGGPRNRGSGYERR
GGKRDVSSSRGEHPPTSSGRGGRGSYRGRGSDWGRAPSNPASLVA
FUN_006142 CDS scaffold_20 112053 112720 -
hypothetical protein EOG09265FTN PF10791

MSFVTRRALSTLIPPKVRESPFGYGRKPPVLRLIPLLTPDFQKQVASPKAIGAAPDAIRMQRVVSFYEKLPRGAAPEVKAKGL
LGRYQAKHFGKNPTGKPIIHLIVFLVSIGYVQNYYFHLRHHKNNAH
FUN_006143 CDS scaffold_20 113017 113741 + NUO21
NADH:ubiquinone oxidoreductase 21kD subunit PF04800

MAALRSQSAARMLRSAAAPRVALSAAPRRFQSNVTQASGTITGPVTSEPDYNIEADKATSTYTPVPRSVQNGSEEILPAAVIS
GAPMELQARTVRIYQEAKPATQSGDWRGRRWRMDWDILPKGHRWENPLIGWQSSGDFMQGTHINFSSKEDAIHFAEKQGYEYF
VQEPNSRKFAPKAYANNFLYSARKLKHIRTK

FUN_006144 CDS scaffold_20 114074 115256 -
hypothetical protein EOG09264JK1 PF08513;PF10607 ENOG41

MTSSASTATPVKHAFDRRVHDVKSPKSDINALILDYLTMEGYPNAAANFSKEANLEPHQDTQHIIARQEIQNCIHSGDIKTAI
TTLNEFDPQILDGDKALHFTLLRLQLIELIRACNATGDIQPALTFATEELGPKAPTNPKFLEDLERTMALLLIPSDAREPQLA
ALLEPELRREVADSVNRAILERQSRRREAAIRQLVRMRVWAENTARDKRKNLPDRLDIGLNGEEPDSPRPHPGNGHDPMITT
FUN_006145 CDS scaffold_20 116530 118365 +
hypothetical protein PF00485;PF14681

MSASAILEVNGGLESHVTVQKRAYYSPPWADVSIIGVAGSSGSGKSTLSQAIVKKLNLPWVVILSMDSFYKTLTPEQSKLAFA
NEYDFDSPDAIDFDVLVDKLRDLKAGKRAEIPVYSFAKHSRLDRTTSIYSPHVLVLEGIFALYDPRVLELLDMGIYCEADADT
CLSRRLVRDVRERGRDIEGIIKQWFGFVKPNFEKFVEPQRKVADLIVPRGIENRVALEMMVQFVEKKLFEKSRHHREALSRLE
AASKDSPLSERVVVLDDTRQLKFMNTILQDIDTDPEDFIFYFDRLASLIIEQALNNAHFEAKNIVTPQEYEYKGLVSTGEVCA
VIVLRGGSAFEPALRKTIPDCRTGRLLIQSDYSTGEPELHYLRLPDDIADQESVLLLDTQMATGGAALMAVQVLVDHGVKQDR
IVLATYSAGKVGLHRLTSVFPEITVVVCNMLDYQQERWVEKRYFRC
FUN_006146 CDS scaffold_20 119256 121380 +
hypothetical protein PF00036;PF00070;PF07992;PF13405

MAARLARAGQNRAITSSRCFKPSLAPRPIYSSQSRSFVKLTGASPSITKGSPATSRTTRQLPSQFFVRALSGKPLPQGKSRAL
NFCYRLAAWVGISISVIGVGIVGFFIYDASTYFEHPTQSDIDVSKIALQPRSGGEKNLPIAEVFIDDDDSEEKRRLKDKPRLV
ILGGGWGGVALLKELNPDDYHVTVISPTNYFLFTPMLPSATVGTLELRSLVEPIRRILSRVNGHFIRAKAEDIDFSHKMVEVS
QVDANGKDIRFYVPYDKLVVAVGSTTNPHGVKGLENAYFLKDINDARMIRNQVIQNFELANLPTCPDEERKRLLSFCVSGGGP
TGVEFAAELFDLLNEDLTRHFPRLLRNEISVHLIQSRGHILNTYDETVSRYAEERFARDQVDVLTNSRVKEVLPDKIIFTQKQ
EDGTMITKELPIGFCLWSTGVSQTQFCQTLAAKLGKSQTNRHALETDTHLRLNGSPLGDVYAIGDCSTVQNNVADHIVTFLRS
LAWKRGKDPETLQLSFADWRGVAEDVKRRFPQSINHLKRVDKLFKEFDKDKSGTLDFDELTQLLKQVDDKLTSLPATAQRAHQ
QGQYLARKFNKMARMHEGLNANDIREGDVDAAVYKAFEYKHLGSLAYVGNSAIFDLGEGRSLAGGLWAVYAWRSVYFAQSVSL
RTRLLMAMDWTKRGLFGRGLYPFPFSQTSLTINRFNEFLNLFL
FUN_006147 CDS scaffold_20 121615 122985 -
hypothetical protein EOG09263C55 PF07767

MPVLQSKNATGGDAPKQFNQPSRKGKKAWRKNVDVTEVQKGLEELNKEIIAGGVIKEKSSEDLFTLDTKGDAGLPKRFNKHIK
KGLKADEIINARSPVPAVSMRKRPGDKTTNGILPVKRQRTDWVSHKELARLKRVADGEHQNTVQVQDATYDIWDMPVEPKAKD
PENFLEEEVKPKAPKSMKKEPLSLLESGKQVPAVLKPTGGYSYNPDFNEYTQRLEEESEKALEAERKRLEKEEQERIKQEAAA
RSAAEAEAAEARANLSEWEEDSEWEGFQSGAEDDKPSAKRPQRKTQAQRNRIKRRKEEERLAKHKAALKAHRRQAQRIQEIAE
EIEERDRNKALVLQKDDETDPIDELRAEKLRRKQLGKYKLPERDLELVLPDELESSLRRLKPEGNMLRDRYRSMLVRGKVESR
RHIAFHKQAKRKFTEKWTYKDFMI
FUN_006148 CDS scaffold_20 123512 124442 + RAB6B Ras-
protein Rab-6B EOG09264JHE PF00025;PF00071;PF01926;PF04670;PF08477;PF09439

MSQAGGSYNNPLKKFKLVFLGEQSVGKTSLITRFMYDSFDNMYQATIGIDFLSKTMYLEDRTVRLQLWDTAGQERFRSLIPSY
IRDSSVAVVVYDISNAKSFQNTKKWIDDVRAERGNDVIIVLVGNKTDLNDKREVTTQQGEEEAKKNNLMFVETSAKLGHNVKN
LFKRIAQALPGMEGSDAAAQASNQMIDVKTNNSQQSQEGCAC
FUN_006149 CDS scaffold_20 124854 125756 -
hypothetical protein EOG092641UM PF06026

MSSAANLVESAKKLAAYQAVNDHLDASYKFVGIGSGSTVVYVVDAIVSKGPEFYKEMTFIPTGSQSKGLIRAAGLNLVNLDER
PVVDGLPVPLDVAFDGADEVDEDLNLIKGGGACLFQEKLVAIAAKKFIAVADYRKQSPRLCTTWKTIPIEVLPLSAPDVLTRL
RAMGSANPVVRSGLPSKAGECVTDNGMWLIDAPFSPLLLPKDIRSEGDGRGKDGAWEVNALAEELVRTPGIVEIGLFHGFNGS
EAVKLGKQLQAQKPIAAYFGLANGEVQVQNAA
FUN_006150 CDS scaffold_20 126764 127629 +

hypothetical protein PF08552 ENOG41

MSRFTRSLRVPRPKSLFGITSLQTGVELIALALVFNKITGVYGLLAILTGYQLSLLQLSTYVYSIAVLVGLAILIPHVRRQSP
FECLALAWLYIIDTVINAAYTAAFGLDWYFSTQLSETTESKKTDLPNFVAEGMQGLRKEAAMHGKVVPQETAASMLLIVGATL
IRVYFSFIVMAYAQQVLQKYMQLMILEGPGVDDQEGPFAEDLPDGEGRRGRLGRLMVSCGRAYWLDTRESDEWAPNLGGPNKP
VSAGTLSGEV
FUN_006151 CDS scaffold_20 129631 134698 +
hypothetical protein EOG0926047G PF00258;PF01077;PF01855;PF03460

MDAASQHQIKTPEEAVARIAYLSSDVVVSVQPAIGTDSEFSSHLNRYASRKDSSLVAQSADTVPEIIPVRHNTDPLLSVYTPI
RAGKLVSVTTTSNILLPSVSHLYKLANYPVVLHVSLQPRTFPDYSVITAVRNSGFTFVQSETLQEAQDLALTAHALAVRTGKG
VVHFFAPSTSSKDKPIAIEDAAVVRDVLDIESVRRFQAGSAPSSQTGIYADDGHIAVSSDHDAPAVNHGTAASGNLTVPPSAN
ASKAPSAGTSVKSSQDSEASTPVAPSSVATTVETAPPQVTSEDIYKSAGRIWAQLKASVGREYSAFEYSGPPDAENCLFIFGS
DAGAFAQAIDEARPEEIFANTAILTPRLYRPWIGSRLVEAIPRSVKRIAVLEQIHRKTTKWGPLLIDVLTSVKSGPGGVEAIV
GHQLGYIAPDVVIQALRGVLQNLTAEKPIQNLEVGKKEAWKEPTGYDLQTPQLETAYTKILDQLFGKRAYVANAIKSSTTGLS
PNVAASPEFGFGSLLARKQRRGQFVSQVKDAATNGSFTTDAPKSWLARWAMNADDATKSTEIADDVIARLETDGSSLAAELLS
AKGLFRKESLWLTGSDAWSYDLGNSGVHHVLASGENVNMLIIDSTPYSERAAADANRRKKDIGLYAMNFGNVYVASTAVYSSY
TQVLQALLEADKFEGPSVVLAYLPYFGETDSPLTVLQETKKAVDTGYWPLYRWNPDNEKKGEPNFSLDSELIKQELKKFLARD
NQLTQLAAKEPKFAAALVKDFGSEVRAQQKRKAKDAYNQLLEGLLGAPLTILYASVNGNATTLAKRLASRGRARGLKTTVLAM
EDYPVEDLPTEENIVFITSTAGQGEFPQNGLPFWDAIKDNTGLDLAAVNYSIFGLGDSHYWPRKEDKIYYNKPAKDLDRVLTN
LGGKHLIDIGLGDDQDPDSFQTGYQEWEPKLWTALGVDKVDGLPDEPPPITNEDIKLASNYLRGTIVEGLNDPTTLAISASDQ
QLTKFHGTYMQDDRDIRDERKAQGLEPAYSFMIRCRLPGGISTPKQWVQMDDIANELGNETMKLTTRQTFQFHGVVKSKLKPA
MQAINRALMTTIAACGDVNRNVMCSSLPTQSKYHRQVFACSQLISDHLLPSTTAYHEIWLTDDDNKKTQVAGDAVQDFEPLYG
PTYLPRKFKITIAIPPHNDTDVYAHDIGLIAIKGEDGNLAGFNVLAGGGMGATHNNKKTYPQTGRMFGFCKTEDVHIVCEKIM
LVQRDNGDRKNRKHARLKYTIDDMTVPVFRSKVEELWGKKFENERPFEFKSNVDTFGWQKDEHGLNHFTFFIENGRIEDTPEF
QMKTGLREIAKSLKGQSEFRLTGNQHLILSNVADEDLDDVKQLMKKYKLDNIQFSALRLSSSACVAFPTCGLAMAESERYLPV
LISKLESCLEENGLRQDSIVMRMTGCPNGCARPWLAEVAFVGKAYGAYNMYLGGGYHGQRLNKLYRQSIKEDEILAIMKPLLK
RYALERNEGERFGDFCVRSGIIVATTDGQNFHDNVAEEEEDEE
FUN_006152 CDS scaffold_20 135343 138955 - CHS2 Chitin
synthase, class 2 PF01644;PF08407;PF13632
GT2_Chitin_synth_1;GT2_Chitin_synth_2
MDPNMSRPPDYSLPPYDDEDLHHTPTGYSPAAVRLLTSEEHYDSHTYVPPSPPSQNQTQTPPARPPSSPSVSPSTSPSKTTPS
PLEPPVAFPPPLSISPVNITMSSEREERTFRSSPVQQDDVRENISNENQEHPSPNPPSYASSMAESQTLLPKRPIIGGQTAKL
QNKNRTSVHVAFADLPRDLPEIPDGISDRRRVHKEQQHLGLDTTPPVPPRPLSRLRDVNSHDKLPSIRSPRNLNYQPSVRSSR
SGSIFDDAPSMAPPGGSYVSYGMHDDGSPQRPWTPSSRVSGFTRSDLSRPPPSDGMYEPSDLNGSPRPGTPSSRYGGSPRRPL
PPAPLFSNSRQPVPPIADDATISIPLHDTYDDDVFAPESDLSDARPHPVDRSSYMSSESQDTLNEGDMEDYDKVEHYGPAPTG
AQERRGVRAPQMSRKEVQLINGELVLECKIPTILYSFLPRRGEVEFTHMRYTAVTCDPDDFVERGYTLRQTFGKTVRETELFI
CVTMYNEDEIGFTRTMHAVMKNISHFCSRSRSRTWGETGWQKIVVCIVSDGREKIHPRTLDALAAMGVYQHGIAKNFVNNRAV
QAHVYEYTTQVSLDSDLKFKGAEKGIVPCQMIFCLKEKNQRKLNSHRWFFNAFGKALNPNVCILLDVGTRPSGTSLYHLWKAF
DTDSNVAGACGEIKAMKGRLGANLLNPLVASQNFEYKMSNILDKPLESVFGYITVLPGALSAYRYHALQNDETGHGPLSQYFK
GETLHGQHADVFTANMYLAEDRILCWELVAKRGERWVLKYVKGCTGETDVPDTVPEFISQRRRWLNGAFFAAVYSLVHFKQIW
FTDHTLARKILLHMEFLYQFIQLMFTFFSLANFYLTFYFVAGGLTDPKVDPFGHNIATVIFHILRYACVLLISTQFILSLGNR
PQGSKKLYLISMIIYSIIMVYTTFATFYIIIHQLTSKDDKIEMGDNVFTNMIVSILSTIGMYFIMSILYLDPWHMITSSAQYF
ILLPSYICTLQVYAFCNTHDVTWGTKGDNVMKTDLGGAVGKGETVELEMPSEQLDIDSGYDEALRNLRDRLEVPESPPSESQL
QEDYYKSVRTYLVLTWMIGNGILGMAVSEIYSARGIGDNYYLRFLLWSVAALAVFRAIGSTTFAVLNVINMIVEGRVRLSLKA
PRWMGGLKERVNDKMSSVSSNLRS
FUN_006153 CDS scaffold_20 141100 143050 +
hypothetical protein PF00083;PF07690 ENOG41

METIKETQGELRSDPTPQDGRTPEAKTRSATAVLQNPLANMTHDELVADAENLAQDNGLDSYRPWFSKGALIAKVINTPKGYE

AIEELTEEEKELLRNEDEHRWKSQPKMLYFLCALCAGCAIVQGMDQTVINGAQHFYFEEYKIKDRALQGLTNGAPYASAALIG
CWLNAPLNRYFGRRGTIAFSCLLAFVTGIWQAAANSWTCLLIARFTLGLAVGAKSSTTPVYAAECAPKAIRGALTMMWQMWTA
FGIMLGFASSLAFQNIKWPNQYAPWRWMIGSTALPPLIVGLLIFLLPESPRWYMDRGDFQKAYLSLRKLRRDDLQAARDLYLA
WKFLEAEQNSKEGRKTLKELFTVRRNWRAAQSSWFCMLMQQFCGVNVIAYYSTKIFTDAGYTRSQALLASFGGGAINWIFALP
AIWTIDTFGRRNLLLVTFPMMSACLYWTGSCFGIQDNGLRLPLIATSIYIFMAVYSPGLGPVPFTYSAEAFPLHIRALGMASA
TSITWSFNFLLSFTWPMMEKSFKPSGAFYWYATWNVVGFVFTYFLLPETKALSLEELDIVFSVRNRDHVRYYSKRMGWYAKRL
MGRSPDPMPPLYELENSTVGSMSGEAVKQTNSSSV
FUN_006154 CDS scaffold_20 143901 146810 +
hypothetical protein PF01979 ENOG41

MDEKAHLPPYSSVSLPAPLGLHPGQQRNRRLLRRSRGIRLFALACLTFIVFAQWRQLAPRNKTTLSIQKLNEDLETCKKFRVK
PQDPPGLGRDKNARYIDGGKPTLIKNATIWIGEPLEGTSQEDAYAGKGWEWVQGDVFLEKGLIQRVEKDIKTASLPDDTIVYE
AHGRRLTSGIVDTHSHAGVYPLPSLEGNSDGNEMSDNITPWARAIDSLLPLDPQIEVIKSGGVTTSLILPGSGNNIGGEAYVI
KHAVGKPDGRKEISAVDLLADPDRNWRYMKMACGENAKRVHGRVGNRPFSRMGESYEFRHAFEQARDLIQKQDDWCAKAESQG
VESLDEYLPGEIAWEALTAALRGQVHINTHCYTIPDLEAMVDHTNEFKFPVRAFHHAHQTYLVPEILKRTWGGRPPASALFAD
NMFYKTEAYIGSEYAGKILHENNLTVVYVSDNPVINAQHVIFEAAKAYHYGLPYHVALASVTSAPAGLLGLGKRLGKVKPGFD
ADVAVWDSDPLSVGATPVQVWIDGTAQFEDPIELMKSQEAAVSVEKPAEIVEGPSKLDTVLFTGVKKVFLEDEKTYESQDKPV
NAVVHNGKISCIGACEREFQAVAATGAKTIALKNGYLTRSFTAVGGTLGLSEIDAEDATNNGPNPLIFSRAIDGLALDSKKLH
VASRYGVTRAVSAPVFVGGATHFGTSVGFLTSALTTLEEGAVFAPDLAVHYTLDLNVRGSTSYSAAFGGLRNKLLTAATTPEP
ASNPFSEAAYLKKVISGEQVLALTINSADGIATALRIKSEIEGVLTTAGSSKRVGGLKLAFIGGAEAHLLAKELGEAGVGVIL
SPLQAKGESWDSRRALTGAPLTNGTTIDALVDAGVVAAIGLQEDWELRDLGFAAGTAFKNGGGRLNEKEAIDLVSSNIYKILG
ADEKSAKDSGHFMVTEGSPLEIGSRIKAVGHGRGDVSVFI
FUN_006155 CDS scaffold_20 147530 148448 +
hypothetical protein PF02900 ENOG41

MSKVAPVIALSHGGGPMPVLGDPKHESIVYSLKNRVPKILKLGTPDQPRAIILVTAHWSTAKPTISSGASHKLLYDYYGFPRE
SYSLKYPAPGHPEIANEIADALRGEGLTPELDDERGWDHGVFIPLLLVNPKADIPVVQVSVLESEDPEEHLRMGAALRKLRAN
NIAVVGSGFASFHNIRAMMMLMNAGSEQQAQFRTLSDEWNNTLTEATLKKDTDERWKALKGWRKFPYADMMHPPNGGEHFMPL
LVCAGAAREGEETRKYNDEYVGVEISTFYWGSEEESKL
FUN_006156 CDS scaffold_20 149033 149937 +
hypothetical protein PF00117 ENOG41
C26
MGQHYKPTIDAQVVKVFVLETDTPHPDTQTERGSFGEILHRHFSEAGSKHHPPLGVETEQVFVVTEEGGRMPKVEEFEGYDGL
LITGSMYDAHGDNQWIHDLLDLLKQLWIKRPDFHFTGVCFGHQVLSRLLGGKVGPSPTNDWELGHNAITLTPVGKRLFRTHDD
KVYLHQMHQDQVLEAPTVESSNGLLKPDTDVHVWGTSNHTSIQGLYIPNRLFTSQAHLAFDEDMVKRQIQMRIDSGGIKDLEH
ADRAAETADLEHDGEQVASAILRLFRYDDDGMSWD
FUN_006157 CDS scaffold_20 153076 154531 -
hypothetical protein PF00069

MATCSLKSLRRPSLTLISGARKTSFRFNGLISKAATRGLATVTAQTTQPAVMMEDHNTRLINMGYNYNYIEHVENLEQYRLGG
FHPVSLGETINNRYLVLNKLGHGGYSTVWLSWDILKQKYAALKIVLADFGEDSTEVDVLQRLEAKATKKHPGRSLIRHVTDNF
HFDGPNGRHTCLVNDPAMMSLRLAKDASFTRLFQPRTARAIAAQVVQSIAYLHEGGVVHGDLHLDNILLQLPDRIKRLSPDQL
HERHSHPTTVPVTRLDGLPLDENAPRNAVLPIWLGQKSEDVALADAKILLGDFGESYLPSQEKRQYSNAPIRYRAPETQFPDV
CQPLSFSSDIWSLGCLLWNVVGQRPLFDAWTLSEDDILQDQIDLLGEIPEEWSAYGAKRSEYWVEDVEEEASSQATTQGGFTW
DVRFERCVQQGRKESGMKPMSKDEKEAFIDMMKKIITFRPEDRITAKDLLESRWMKQWALPELKKLAAS
FUN_006158 CDS scaffold_20 158552 159971 -
hypothetical protein PF13002

MPHRVANFIRTSSGNIAQIKKVARSRTNSPYTSDGEDRPQIHAVKMTELADAVKKHHRISLPFGKSHKEQPSDVTIDWSIESP
PVVFHGNTEESTGALITGQMFLDVKEEVVEVGSFAASLKLHIYQKRPYQGHCTDCQNQYTELKSWQFLALPTTLRKGRHPFPF

SILLDGHLPATMDTPLVAISYEFKADAYVTKSVHSASGSVTPVRFERTVPVKRSLPENEIPHHSVRVFPPTNIKASAHYNNII
HPTGTNTVSLRLDGLMSRNDKAKTIDFWRLKKVTWRLEETIKTVAPACDRHAPAAEDPEKKSLPRNEVRVLGEKHLHDGWKSD
FTGTDGKVELEFDYFANQYKSHTKELKYACDTKSAEGVEVTHSLLIELVVSREYAPEGKPQLATQTGTGRILRMHYGVVMTAH
AGMGVSWDNEAPPVYQDVPPSPPAYPAVESPVEYESLEHLDAHRLSSLSNSSDDGSQ
FUN_006159 CDS scaffold_20 169703 170759 +
hypothetical protein ENOG41

MFASGWSPQDKMHIYRLLPEVRTNHTNRRVPSKHKLNKHDITFGLWVILRCIYPDQKLPALRLALMEKYPGIDLSAFTGPVPN
AACMAPEEHTAGKLELKVVTPCFEKKMLEEDDKINPSDLSLKEEEYFLGETCTTGHGFLVTNFLLDDTVSQIPKFGPHLDQLL
STEPDKSSARRLMEVESKMRGMVLGEPHSASYSKFLKSRSSNAPVDVSTYGPDITKLCASPSAVYEADIESLASDESSPEDYS
AQTALSNKRFLELKDLITNVTTESKRGVSDEMRKMHTETTKQIQNMHTEMIEQFTKNRKTLVDIGEQVAEIRAHIELARIDRK
DLDEVEARAINLEQFILSL
FUN_006160 CDS scaffold_20 171919 173122 -
hypothetical protein ENOG41

MATEHGFLVPPNFKEEPPSDDDMNFASIIWGLSLGVTIFNCGKAMRQTQAAIRRRNRLTLYIMFIWLEVCSSTVLGIMVWLYL
RGIIKPSFQYYFFIIVFWSIQVQCLIQIIINRVSLLMVVRRNGTVLKWTCFGILLAINISVFCVWIPARLQISEKYIHVNEIW
DRMEKVLFAIMDAALNFYFIWVVKQRLVANGLQKYTRLYHMNMFLVAISIALDILLIGMMSLPNSFVYVQFHPLVYLCKLHIE
MNMADLIGKVVRAGNNQADGTHDYSSRSRTTVKTGTRPAGASRFGGGLTRTHIELGEEDEIELKHRENLEGIKKTVITEVIRS
PNSEDDEIMEHHERAVSEASSTNARHEPYSIEHAK
FUN_006161 tRNA scaffold_20 177332 177422 + tRNA-Glu

FUN_006162 CDS scaffold_20 178088 179460 +
hypothetical protein EOG09262TUR PF12519

MREFMDYVHSAFYEATGWNRDNSYAALNVTSDALLNFQTPRGLRLTLSALASPNFATSYQLGSVGIVDGSISYLFSSVPLRLL
LTPQSETVNLPELLRSYRPLTELPHRSNPLLQTPKDKPESSLLYGRLYLPQSLLEALFVKRLSPALQVQLSSVSAQHLKNGGT
VLGLAQYDVGKYALEGLASSDGGLLGFRGVYNFGGDAEKPSDQAVNDNGNADRERIYGRFSTGGEIYYGTLNKSGGISVGTRF
ATLPAHKGTPLSATLTLNPLMGNISASYAVVAGQHCSLATRMEFNVFSYESAWAIGMELWRKPFTRPVLDDEPVIDDKVVLDD
KPEVKRPKERSFQAKLEWRLDDPEPVVVKPPPPPPSPPKEEIDPEKPGEERYAGVLKTRLDQNMRIGVLWEGRVKSLLFSLGS
SIDLRKLDKPFRTLGLEIQFSS
FUN_006163 CDS scaffold_20 181498 182533 +
hypothetical protein PF02535

MADADTCSGEAVDLGRRGLRIGSIFIIMASSAIGALLPIFLARQKTIPVPKMTFFICKFIGTGVIIATAFMHLLVPAVENLTD
PCLEDRLGGYDWAEAIALMTVIVMFFVEMLAARLSNADMEHNHSMEIDHELDPAMDFIAKKQPSNPDIENGDRMGPGYAPGGD
SHLAHGREHKEGDAQGGLAGQLLAIFILEFGVVFHSVFIGLTLGTIASDELTVLLIVLVFHQMFEGLGLGSRLAVAPWPSNRQ
WMPYLLGCIFALSTPIGIAAGIGAKPNNANDQKLTNGIFDAISAGILMYTGLVELLAHEFMFNPYMRKAPIRILLLAFACVAF
GVAVMAILAKWA
FUN_006164 CDS scaffold_20 183255 184055 + RPS19 40S
ribosomal protein S19 PF01090

MAGGVTVRDVDAQKFITAYSAFLKRQGKLPIPGWVDTVKTGPAKELPPQDIDWFYVRAASIARHVYLRKTVGVGRLRKVHGTA
KNRGSRPSKHVDASGSVDRKVMQALEKIGVLEQDEDKGGRRITQAGQRDLDRIAQTTAEAEEEEDDE
FUN_006165 CDS scaffold_20 184894 186274 +
hypothetical protein ENOG41

MNTPQSAASSSEEVEEVLPVFVSAISNPSPLIYFHHIIDSETTRLTVQVLGANISFVFSHRRAKTINRRSTIERQLENKTTGF
ISIVSAKSQRVVLAAAESGHGLGRYGDVLDKKKGILPNSIWTRRVVAMGKTLGLNMRRPFDNPGRRAGNTEGIFLGSHVEVKL

AVHGICVLLQTFGITKDFNNVTMKQLNQLGQARWEDGSRPVLEVYFSRKFCKPCKTLVQKLQDATGVTIRLVWKHRLVMKKYI
IRPIDRNRKPGEPLRAQEFEPQDYDFGDILPDDSDIEVISDDESAKEEVEKIDLTHIRSTSPISWNQEIDDFLDGLAYRVGQM
EDCPEGARSAIVEFASIRVNTKKSPNATKVSKPLPATPVIEAPADFLEGDERQRQRHTFPRAQKRLFRGPRDRNDSRARSASP
RRRAQIVRDRSPRRYNLGPLGRYSTEAPQERSSAKASSRAHSIR
FUN_006166 CDS scaffold_20 186654 188865 -
hypothetical protein PF12825;PF12828 ENOG41

MTSTNLAPEQVHALFDILTHHETYAEIQGFKDPDAVTGYGYPFSRATVEPQTTQSAPPSTGWRSWAGTPVSSRPGTPKQGAAK
DVKETPSDDDDKKPSSSPILQTMLTRFILKLPGVRDLPREFWSVRVQGLLTRLGEAELSESYDKGALGTRKVLATGSSGLLEM
VGRGVLGGVKKANRPDKSSDKKVEYDRSKAADLVKAWDDVVEGLVYGGLADEMFDHFSKTADLESHSPVIGAAAEYAIIHLAT
FIHHIFVLSPEGQYLLKLLENVHNLIPYKMVKQTLRMGNAASMISAMMRLLLAKLSVTSVTNWIGLTANADDGMNLLQRIISL
VLSWDAGEFRKSADKVERAKDRPSDEMLEAIRQYIAMSRDEHKTVRNASEEHAQSIITAIFNGSNPTLATQLDDQKHAQCLEY
YSALLSVRDRESITTAFCRQPPDLFTATIKDLFAAYEPMIRMVHSQIDLREHVEAGQLFIDEFIKAGKPKKDGSMPTVDDYVG
LFMRNRGLMYRWVHAFAASCPDVWEEMKKWTNDAVLKFRQERKPVQKTSNAEAGEQASNGTVDMSTMDDQLNKLFQSVPEKSQ
KEVLISLDNHAAYLAQVEALSLCRLQRIIDSDDSESGNMSGPGMYLSRWQSLLEDTAITPETPKGPVRHGKDVKNALTMGKIG
VEGTGKAREEALQAAIEEAEEGPEAPDVDVVIKELADGFKKLLQESAGKDISNTG
FUN_006167 CDS scaffold_20 189644 192422 -
hypothetical protein PF14613 ENOG41

MPGAPDVNKPTDIKTKEADVNRKLQVYGIISAFQNGKVPSNDQIDVALSSFLASRALSGRHEKLSAEGQELVKDAANAIKQAK
YLLLSKNEGNLIQDFIWQTTQFDPKSVNTPNAPISKDAAKQDGDQALEGLRTLGQLLITNGQFRKLLSDATVLFRDMAGDAAT
NAAARVRPSQEQLRQIDEPAQDNVWHEAPDFSKDNLKQQAKGFYSGNPKEDAKDVASASINAAAPGQLQQNQQANAQQGTYPQ
VTTGTETGNVPSSSVPASTDAARDAARQTDPQAGKSAAKQAAEAKLDNKVDPETKESILQRNEEYRRKARNYFDKKMPQERKD
QTIWRLKKMILECQQHEDYSQAIQTLLRLAETYSRHGRTLGQGSTDTAKDARGGLAAAERDLRTIIERFANGTSTEDLWEAIG
QIYRDADNDRELKDWFKAMDSYIRRCLLQQGYILEDESTREWDQLYDQGRYLLRNKYRGHTDRVVDEIKFLADQFDQDPQNHA
FAESLQKLFKDLGNDQDGKPVFKPHLLKDLRDVIIPAALENIAYIPIPRIEYTDSQVDAIIENLVLESDNFAPNIFEVSSEHY
FRWGRKKIANKSHQTVEVKVAGIQMDLRDVSFHIKRKQGFPSITDTGVADIILPGDGFSFKMKVSTADKKDRQNYFKVEKVDV
DFNKLQIKVKKSNHKLLFSLFKPFMLKVIRPPLQKAVEKAIKDQCNKFDQLLFQVKQEADRAAAEAKNHPENAPNIYNRYYTA
AQQQFTKGKKKAEAAASDKKANIAMTKEDSIFPNINLPGGISTKATEYKELARKGDKWESPVFSIGSAKKSTDIPSAPKIQRK
AHPVSDLGPKNTSNADYDFYPAGGNTHTGNGALNGGNGLQIGNGAVNGKHPAGFTNGQTGLPTGQAPLTTGQINPV
FUN_006168 CDS scaffold_20 196054 197129 - RRI1 COP9
signalosome catalytic subunit rri1 EOG09263RDD PF01398;PF14464;PF18323
M67A
MEASALKAWELDNNVQLVDPKRDALYNFDADAQKAINNEKAWKQTPDYFKHVRISATALIKMTMHARSGGNLEVMGLMQGYTH
QDTFIVTDAFRLPVEGTETRVNAQGEANEYLVEYLDLCRAQGRQENVVGWYHSHPGYGCWLSGIDVDTEAMQQKWQDPFLAVV
IDPDRTINSGKVDIGAFRTYPEDHQAGNGTATSDGFQAVPLAKAAEFGAHASRYYSLEVSHFKSSLDSHLLELLWHKYWVQTL
SQNPLITNRDYGNKQMLDLSSKIKEATTGITRSRAGQGMMGTSHKSSDKAVDKLAREASLIASKERSGLVANQVKASVFNDLG
SKAEEPTS
FUN_006169 CDS scaffold_20 199266 202437 -
hypothetical protein ENOG41

MAHGGHWDPNSVGNYNQTPPPAVPHQAHGGAYTPGSSVVSYQNQPYTHPYQQAVSPSTSPLSAQSHSYPASPGSLGYQQSIPL
GQTQTHNTMQYQQQSMAYNGQQGVIYQQGSSSGQQDGPMQNKGAIVPVTTTPLTTSTSPNEKEVSEKEFPVKLKGSKHFFDSR
SIYRWRNPVLYQKPGYTYTSSEVKMRHRDYAGRVSFKPRSDRYLHHNDGYLRNMYVACVDAIYEGPGTSTWKRTYVRKVAPMK
VRIATWIIDFSYDPKSWEDWGIMVLRTFPAAIAMALVFWDGKPNVIKRNLAYAPVLYRYHGDAKVWSNLLENRKGLSLMARNN
QIYRMLRPRYLCFLREPFNDENRGVDVRSVVEWENSDGQDINLAYLFVAYSTEHFSHSSEQDMMALHHIAETACRAAKLPAYW
IACSCMRDENELESDVYRISDVLRGSDRMVIAVGRGKGAKAGHSGKANTESLLREWGSRMWTFPEVLLSPGRTISVYTRDGNL
QSPLVVAKNQFAALVWTYMDSDVARHLIDHYLGSISLSRLEQAVLALKCLYSRHTTEYLPGDQAYALMGLLRLRPQVDRTDTA
FQAFSRLSLANDSDRLLERYICTLPRDKDQPWYDMEDAYESSLWDITPYCQVAGIADNDTIIIDGAWGISIRWKTFYPVYWST
GPSWKRYFAALAVEWNGAFFIIAIALIASGASASSSSSSSSSSMYGYSTGASASSGTAMIIPGVIFLLLFVWIWLITPNLVRV

IYGGKFADTQAEMFGFEGHLNAPTIERSIFGGNFGRFSWSTNGSPLSRSIVNDDGERVGVDPYKDPEVRMKVEAAKQARPGDM
RIFTLVDTYNMELTLFEAVRPPVTLMFCASEGGMQRAIGCSYEWETQTMYRETVLRMPTTALNRMGRVPRFRMGIQRPLYPSA
PLNGAV
FUN_006170 CDS scaffold_20 203824 205376 +
hypothetical protein ENOG41

MSWTQRANPYDAFAQEATQTPLAVAFPQWMLNIWEVTHDHPEGRWYLEPNARCLRQRASLRIVFAPLDLPRRDALSQFVELFN
ILNIPSDFTKERLQSVSHSFGRSTDAHGSSSWFHFLCKNIDIKQTPGNAPEIDNRAATMGYHISTLPQADYSWHRAGFFLRVD
NNGCITLVCFGVMQKVRERINEFVAAKAWQHVATDPYILFDLVFEALYFEVDNTVWKMNTVFGPLEHLILEYANSKNIRKMSS
KVPFSAMHNCAKHIIHIAEAIDSCIMLVDATLHNVGNHQHPEQPTLREPIIKQLRETLQYRRSLFMSSQLRLSSLQKRIDNAI
TLSFNLVTQQDSMVMIQDSNSMKVIAAITMIFLPTTGVATVVGSQLFTSEILKDGTTWDVRLTPLFWTMWWISIPLTVFVVLL
AIIWHWWVHTESPTGEVVQVVKRAATFSSSGTRTPPNGNAK
FUN_006171 CDS scaffold_20 205632 206486 -
hypothetical protein PF03443 ENOG41
AA9
MHFLYSLLATAGLAAAHGYVESGTIGGQTYQFYNPYTDPYMNPVPKRISRPIPGNGPVEDVTSIDMQCNGYTAGGIKGSSPAA
LHADAKAGSTVNLKWTLWPDSHVGPVITYMARCPDSGCDTWEPGTQKVWFKIQEAGRDGTSNNWATSPLMKSGNSGVNYTIPE
CIKPGYYLVRHELIALHSASGYPGAQFYPGCHQLKVSGSGSTTPSNLVAFPGAYASTDPGVTYNPYQATTYKIPGPAVFKC
FUN_006172 CDS scaffold_20 207149 208352 +
hypothetical protein ENOG41

MRLPLEVMGLIAEHAAQHIINASPFIQPTNQEEAIKTQEHHRDLLNLVVSCKTVYSHSRHLLYTCLILGNATDIVAALANIVK
HPRVGPSIRHIRCCTELHSETERDKALEYWLLDHSQDAALLQDTLRAEGLLPPWWNETDIPWTCWEGFIYDDCTEVALLALLH
MVAELETLSFQQLDADLITDWEQVRDLRASFAQARSGSQPFLGSLRNLHFGSGTLGGMENFLSNQPHENLEVLVLDGIGIEGF
SRWSRYFEFKGIHLKELYLGTKSTSPQAVIDPLLARREWVNGRWVTPVPKELETQSEVCLEGEEAWRLSVPEARDTMDCLKDF
KNLRVLDVVFDRPPDWPSRPLIALKNIASGSLEVFRVEGYPFRVVPGDFGPCNTEYERAFVPRFDTVE
FUN_006173 CDS scaffold_20 215545 216907 +
hypothetical protein ENOG41

MVKRKDWRETSSLAPSSPPKTTVSLTMRPTTPTSPVKIPTTNNRYSAHPQKRHSSQRKRNSDSRSSDKLSPSLVALLAVTDIP
RHKQLQRRRRRSEKTLTVQEVIDCQHASEKELSWTFGSPLDMLLSPPQEVADDDDSVSDCNIGSSMSTRTYSIDSIPSLDESF
SSECLSSLGSPRTPSPCSRRTNLTPMRKSLEPVISPPGSVDEHPLAGDDLDVDELDFSTLNQPEEAQPKSQFLEPFKPLRSVF
KSNLTASLRALRSAARSFSVLNLPSIPPDDYLTRSILTIDPNVPYADERRPPVSEEMPSAAVRRYLNPTTNARIEPPTKTVFA
GTFAASIQMRTYKIQRSRTAPGSNRNSYPSTSLQSQPAPVPQQPQPQVPTQSVPGMRQREMRENSDFIRIAVMEMAMRKRGKL
DDQRPGRARFALPPRQTSTKPYEMGANGVPARWIPITV
FUN_006174 CDS scaffold_20 220951 222921 -
hypothetical protein PF00083;PF07690

MKLRWWNSTPSTRTNSKMGPSSEAIPIPTDARPIPLPELPEFATGSPRSSEPELESLPNDLTNSLWLPHSHASDSTTRRVYRG
FAEEKEATFFSCCRQYPKAVGWSLLLFLTVVMEAYDKSLVTGLLAFPAFREKYGEPILPKDTPAEDQMYEISPSWQMGLQNAA
IACEIIGLLAHGYITDLIGYRKMMLASLFWMCIAVFPAFFAGSIGVLLTSQALSGLSWGVIEILAATYSAEVVPNALRPFVLS
SINMCWLVGQVIGTGILRSLVHETSQWSYRLPFALQWAWAIPLLFGVWFAPDSPWWLIRHERTDEARKSLTRLTNKNGTDIED
TISLMKHTNKMEKDSNYGGASYRDLFKGVNRRRTEISCIAWVCQALSGWSLTSYAPYFFEQAGFSASISFNLSTACYGVGIIG
GIIAWILLPFVGRRKLYIYGLLAPICLLTAGGVVWVVLSDRTGANWTLGGLIIAMTFIYDMTIGPVCYIVVAEIPSTRLRVKT
VALARVTYNLCLIFNNIIMPKMLNPSAWNIGGASCFLYAGTSFFCLIWCYIRLPETRNLTYLELDILFERKAPTPKFKELQDR
LEDSAYLSMTRAERLTNWHGWLAYS
FUN_006175 CDS scaffold_20 225168 225573 -
hypothetical protein

MRPLAPEIQNCHSKENPHGEMRVVSWYVQLACVADPQSVHHNCQRWCSCVLKWNVLCLGQIDPANFVQGDLELFQDGKVAGSR

VCNANHISSFPPLNESAPQVLYRYGLCVTETETFHSGHAVLDGKLISSNEI
FUN_006176 CDS scaffold_20 227065 227381 +
hypothetical protein ENOG41

MAAIARPALAKPAAYPKALPVVVAVGSFALVASYVTSQLATTSAKFDRSFAKYNTPESEANRARTFDGAIENPRTSLFNILGR
RQ
FUN_006177 CDS scaffold_20 227947 237073 -
hypothetical protein PF10293;PF10305;PF10344;PF10347;PF10351;PF10359
ENOG41
MALLNPTFVFGCIVLLYLSSFVLLAVFRIITGISIQRIGYFSLRRLAYTPRDGCRIEIRGLGLHVHRPTFAQPTWLSIVLSEL
VVTLDINELEGNKQVDVDPEDSDAETSKDKKPDAKTTARQQPQLPRRATSTVVRSETWKKLTNIKEKIKRLHRNISWLKLVDV
VATNSAVNIVDVGNVQVGSFTLAVDTRRKMVDRVRFFARLADRHGKDQQAEWTVSLRSVLFTADGNESMEVLDQLFINVHGHL
YKSMDGLREAAVAIKMGRLHIPFDDLQTCMSRLKQRRAEVKTPGAEEEPVDIFLGKVAQEIDDPGTTNEDLMRTVSDSKEFFS
SILRGIKEIQFAVSFLGMSKKVQSIKPGSTPIQLNAAMKEVGIDLHRLDSKSPAHRMYFPAEAIAHEALAAALSISVGLDDGH
GQPERLLYVPMATTTIKTTLLSKTVEVLRASTVDERNANIMFANSVVTSPSVDLDPHHLPLLIALFKSKPRAPRAQNENHHLI
SRLLPKANYKFSMHEPVVRIKLQPLQKAEDPDDFDLIISSISSISLDVESSHSLMADMHYSLDGSFRLQSHDLYYQTNNGERF
NLITTESLDMKLHLGARPDVYVDVSGNLQSASISMVRPEIIDGLRQIVQELRIDVEPEKGTISRPRGGQNFLRSMPNWLLRAQ
FDASDFNVEIAGIDEEISDDTRGVFLHWDTVSTEYRAHRLDGLQRRASRRRANSRSISQSDSEWTPPSSSPRPKKKPQNIGDG
RKLTLFASGIEAQIMDAVDTLEPEPFLSVPRLELSLSAASDASGPTFNIQVGVRSIRAKYSLYRHYAIGVAVMTLRKAFMQSG
RDIPRTKQHGSYVDIIPTANRLAPPGTPDFDPGSSTITPELVTLDFKAALVQLKADMPHDPAMMLHMYGLELGRQRWSTPYIA
AKLVRLYTEPPRMRNVWARLVSIKSGRLDFRESRRKTAYGGVVDEKMVDIVTEAVRIAVPHEVIVSRVSDNFINVIKAVQQLH
HRFKTGTNEYILEKKPEGPKSVPKVSVRTRNFLFELEDGGFEWKLGMIYRTGLIEQTQRLARENAFKVKLKRVREEEIRRGSS
KFRSRSTFPRGRPEMGDRPSSRSRSADAFEGTDARPSSQYGRIPRYNPNIESLRVNGVSKVTEHYARGHLDMFNAQSWKKRID
RAYQMSQDSMKESRGQFWGANQIPEDMEDSENLLEVPPRPALMAAVISDFHFMIDKPTFPMKDLPDFLHKIGKGMPKDMQYSL
LVPMHLSIAMGEAKVTLRDYPLPLVHIPPMKYGQSAKIQAVSLKANFVVAEEFRGVESTRRVRVNVVPPRSTLPGSSNEGSFA
IEVRRTIGPVKSYSDVYMDVNSALPTRITWGPCYQPAIQDMMMVIESFTKPQFDPSERVGFWDKLRLNFHSRIHMSWKGDGDV
HLALKGTRDPYAMTGNGAGFLMCWRNDIRWNINADDDPKGFISVESGEYILAVPDYSHEVRDQARRLYDAEGHLPEDNYKSEA
SFKKVVMKLSGKVKVMAGLIFERAIEDGQRSFEFRPHYDIVLKNPHFAKPDANGLPYDAMRGFRSRHIHLSIAIHAPADRDWL
SDSPEPSRSYNTIHLTPRFFTHFFNWWGLFGQPLSLPIRQGSLFPGREKNSKKFGRHLATVKYNILLAPLFLSHIYKHKDVED
HSENAVSATGLKVRFDSFSLDLHQRREEFNTLGKGKHGDDTSKTSGIKIHAAQLDLVSADVRAVSASLKGTTTEAIKKGSIAT
LMSESDEKPDLSRFTIPDNDFSWIDMDDFVELDWITPTEPHPDTKILPLAYAPRLTYFRQTDIGGVIAGDPTRTSPFGSEPTH
FCVMRHDDDPRRIQGQLIRDRLDQLGRQMESHDREMGEAELRVIKCDAKDRVAVEEFKAFQKHTDVLQEKKEFLESMLNQAKI
RAPSSTNRSSNDGRKQSGESSIPFQDETMEIPATSEFESEFNNRFVIHNLQLKWNNTLRNIVLRYLHQNSQRRGFTYYLSRPA
IKFILDIVEEQNKAKSNNKPGKAKSTHSRQGGRRSSRASSIHDDETPLDIEERIKRILNDGRKFTSATWGTNAEGFVEGSIED
LVTGIAEEFTPQSSYHIRIIAPQIQLQSDKNKKNVVLATAKGMELKVMEVMEKERISDNVSGLVQRRFLVNMDSVQFFVTHRK
WFQTSLVSMYVGSKYGAPSGSSWPPWVPIEVVFDFQADPFGFKRVVQKTSAMVRYDKFNTLRLKYNDEVNTDGNENASQVNME
NRMDNLWVEFPTARALCNSSQYYALYVIVLDLLMYNEPMEKTRSERLEKIILASDFSDLSGAPAMVQKLQQRIRQMEEIKDHF
QIYAKHLDKKGWQDRLHLEKDLAACEDELFFMMKAITSSQRKYDVNSDDSTGLLKWNITIRDIVWHLMQDSNEPLVELQLKDL
EYDRTDNSDGSHVNLIRVGKVLGLNLLPDATYPEMIAPYIDPEKPHTQEGEHKEMIRVYWYMLEAIAGIPVMDRFEVNLFPIK
VQLEREVGKRLFEYIFPGINGEKSAGTKNDSPFMLKQVTADGEEDEDYEDGNGLTPSYTRQSSSSSFSAGSGSLELRLRPTLT
SNTKTEAPPGKALSVNTGNEGHSFRLFRTPGTSKTMLKKASHESLRSNMGPRVGLTRASTGLSSREGRESRDGDKKSRFGLGK
KHHNEKQQSDDLTKMMTRASQYMTFSSIKMPSVVLCLSYRGKDNRNIEDVHDFVFRMPSIEWENKTWSNLDLALALKKAVVKA
LISHTGALIGNKFSKHRPNAEQRDKLRVLANSSVLIAPSSSGGSQHYPGSLNDSDDSSLYGSSPVDFSRSPPRSIRAGSTHSS
VRPGSAMRRRSRSSSVKSRRSHRNGGTGHGLQAPHGPNVPAFLMMTPPTPIDDHHRNDRHHHGVELLRPATASPQSSIHSMNN
SMYSQQGQTYGITPSNSHASSVNSASTHSSIPVPVAQQHRPGFRRIPTGIERPASSMSNHDSGVRRKNGASNLKDKLSALTSR
IGNRESSSPSQQEDDSEAVDDQLTPAGPISRSRRSSSHTGSRLSWTPSRMKTG
FUN_006178 CDS scaffold_20 239239 240034 -
hypothetical protein ENOG41

MANDRNQEPQMVSVRQRQSSRGLFSPEVVDLVVPPLKFGGYAGTAGILAGVGGSIFQEANPIVWGAFSGFQWFTLGTSFWFTR

SIVVKAWGGEERLKNGDKTIASAIAGTAAGSVGGLIRGPKNILPAMLVFTTVGAGGQMIANRMATRKPKVHDENESFWTRWSP
LKKLTDQEYRDMMSEKMLRIDADIALIDDRIAELKKQAEDEELKGSST
FUN_006179 CDS scaffold_20 241122 243162 +
hypothetical protein PF00076

MAPKKKEQQKMSLGDFLADSFGGGSSWADEVEETSYVTGTQALPPSDRPRYNNNAASSWQDRGFSVREAAPQTLPDKPPYTAH
LGNLSYDVTNDAVGEYLADCGVVNVRLIEDRELQRPKGFGYVEFETLDGLKKALTFDGENFNGRMIKIKVADPPRGGDPGRGD
SIREMGNWDRKGPLPDAPSRGGARDFGDRERRPRDPAFESRPQREFTWERHGPLAPLSPQEGSGSRDGSRPRASPDVLGDRSE
SYRGNRRDSPAWGEAREGGSRPRPERPERPERTATAAEKDMQWRERMRPDAAKAASPDGSAPPSPALSNAAPAQGGRPRLNLQ
KRTVSEAPDASSASQGSDAKASPFGAARPIDTAAREKEIEVKRLQAIQEKKEADEKAKEERKLAKEAAAKAEAEAAEAAEAAE
AAEAAEAKQAAEAAEAKETPEASQETTEPKAEVAEGANGEQKVPIRNREPREGAPKSRATEASSWRSASNDQRGPRGGAGPRG
GRGGRGGIREARAGPLRSNGSAAQQSASTDAEPATPTADDDGWTTVPNKKGRQGRAMAP
FUN_006180 CDS scaffold_20 244176 244797 -
hypothetical protein PF00293 ENOG41

MPTSPSPPLSYTQPASLEAFASTPDVFRAAHPPIEHLIAGALVTNPQGQVLLLRRAAHDSWPLLWEVPGGCVDDSDHGLVAAA
VRELWEEAGLRAKAVKAVVGIAPITEPMPDDPLEKDLEVLYDMLVFRADDGVWGKLTVWVEVESCDEVKIDENEHVEFAWVTE
EEALQNKFKDGKKLEFVSEGVKRNVLEGFRLWKEESEASR
FUN_006181 CDS scaffold_20 245500 247141 -
hypothetical protein PF12430;PF12537

MWPFTRCGEDACEIQPRPVTLASSIAALPFLLICISTSILVVRHVFPRLSSDADSRDGEDHVLPTHAPACLRQVHAEHGAKSW
RRRGAAWTFGLTVGLAATLGALIMDEIIEVVNADSRNLALRITVPSLLFLLVVLVPWLECRSVVSSAGWSFQRTAKGKLPRVA
WGIQTALFMGWLFAFWSVGQAVPEGAMRRTSNWSGSLSEMLTRGCLERIGVVGISLMALLSGFAAVSSPWQAFMDLTTRRKRP
VTDADVARKQAGLDTANEMLLTKRHRLQYLERKTSGPQNGASAKGSGLVGKVMGSLRGPTGDEAEMRSLRLEIAGLETMEANL
ASGVSLLKSHRAATVRASTSIGRILLIPSQLFSLYCVYRIGATTITTIRRAYSPTSSFANTDPINRFLSILARHWDPKLDQLA
WARLISFALSGVILLASANSVVQTFHLFAKWTPGLLRQAQANLALVVGQIAATYVISASLLLRSQLPSALGSAVGSVLRGALS
PAFVDGWFEGWFLVGSLITAIGIWIGRKLGGDDEWDEYGMEEMGAKMS
FUN_006182 CDS scaffold_20 248073 248769 -
hypothetical protein

MKGKQKLGTDNNKHKNSTGDEIAESNDKSSQENNQLPQVTQLDSAAKGGLSSLEEEGDTTELQHLDDLQQPPQEHGASMHSRA
ENKQPKVPSNSKSEDKEGPSSPPFEAPDAASKATQIPSGRPIPTSPTGDQAPSTQDKGSSIGLENLKSPPNTRFPSGDLPRGD
SEEEVQDNDQSGSGDGDGDGDSNNNNNEENDNEPPNVAEARTRPSR
FUN_006183 CDS scaffold_20 248772 251166 -
hypothetical protein PF00582 ENOG41

MIQPNPPKRSDSDVSPRTVNPPRNSASEDAKVDYFPDSNTVDGSNTSVRASDGDDSSARKSSISFAPDPRRSSSCESNQNSAP
GQDSTIQRKSSTGSVSFRRMTNPQLPQGNPQQMNNSRIRASSPNHSRFKKHVAFDNVTTGEPTKNNAISFTLNVRHKGYQARR
RSRCFMVGVDEHTYSDYALQWLLDELVDDGDEVVCVRVVEKELRLTDKQYRDDADSVMKGIVARNGNNRAININIVLEYAVGKLH
TTFQVLIQMYQPAMLIVGTRGRTLGGLQGLVNTRNSFSKYCLQYSPVPVVVVRPTEKRIKKKAKRANDSARQTYVSMLAANAG
KHEADSEASSIYELEVHNSPDEEAHQVARALGLPASFDPTIKPLNHSQIPNRRSPDPTATADPVNETSENQRVVKDDVSVAAE
SDDDDDDDDDSGEFEVMSGQQALDRQKMDQLHKMEVGEAAAFKMKVDEELDEEDDTPSAPKATS
FUN_006184 CDS scaffold_20 255811 257350 -
hypothetical protein PF10255

MATYQNGVAPARAIDDDSDVEEEALVAEYKEHVQYEDGDDLSRTTSLNLTAQTDDIQARLVQAAQPLDFQAPLEAKFQSYDAY
CSLFHFILNSEGPVDLEPPSYYWAWDVIDEFIYQFNSFSSYRMRIARQTTNEEERQALRENPNTWGCYSVLNVLYSLIQRSQI
TEQLAAMKRNEDPLAVAGEYGSKNLYKMLGYFSIIGLLRVHCLLGDFSLALKTLDDIELNKKAMFARVMAAHFTTYYYVGFSY
MMQHRYADAIRMFSHILIYVSRTKNFQKSAQYDSITKKNEQMYALIAICVAFQPTRLDDTIHSALREKYGDQLLKLQRGGPES

LPIFEELFRAACPKFISPVPPDFENPEANIDPVEHHLAVFMDEVKTNMWSPTIKSYLRLYTTMDLNKLAGFLEVKPEELRSWL
LVTKQRTKQLRWQDQGLLEGELVNVSDLDYALQGDLIHISEAKVGRKLVDWYLRNLSRTYN
FUN_006185 CDS scaffold_20 257944 261176 +
hypothetical protein EOG09260BSW PF00009;PF00679;PF03144;PF14492

MPVVIPDKLASLQRQSSDIRNICILAHVDHGKTSLTDALLATNGIISPKLAGKIRYLDSRPDEQIRGITMESSAISLYFAMRR
KAAPDADPEDKEYLVNLIDSPGHIDFSSEVSTASRLCDGAVVLVDAVEGVCSQTVTVLRQTWTEKLKPLLVINKIDRLVTELK
MTPGEAYIHLNKLLEQVNAVLGSFFQGERMEEDLNWRERMEERVNAATAAKESAIADQVSDTGEVQFEERDDEDIYFAPEKNN
VIFSSAIDGWAFTCRQFAAMYEKKLGIKRGIMEKVLWGNFYLDPKTKKILGPKHLKGRNLKPLFVQLVLEPVWTVYQATVGGD
NGQGDRDLLEKVTKSLGIKITPHMLKSRDQKLLMTTIFANWLPLSTALLVSVIESLPSPPAAQAARLPELIEESPGSEHIDQT
IKDAMISFKHEKSDPVVAYVSKMVSIPESELPENKRRAGTQMSGEEARELARKKRAEAARAQAAAGENGVDSMATSMDAINLD
DYAPELEEKKVDPEHLIGFARIYSGTLSVGDKLYVIPPKWSPAEPSADPEPQEVTVTALYMLMGRNLEALDSVPAGCVFGIGG
LEGKILKSGTLCSRRDGAVNLAGVTMLGKPIVRVALEPVNPADLDKMIHGLKLLVQSDPCAEYELLGSGEHVLLTAGELHLER
CLTDLKERFALCDIQPGAPIVPYRETIVRAEEMRPPANKELGRGAVVATTSSKQVTLSLRVQPIPENVTDFLVKNGDAVKRVY
DRGAKKDDESEEIVAEADVAAGNTLSVEDFKKQLKEKLEGGKGRDIWKDSIDKIVAFGPRRTGPNLLIDATADGIFSKAFATE
KTVETAPRADESLHPSHLTDKISYAFQLATAQGPLCNEPLQGVAVFIEDVTLNLAEDDSSARDKLGRLTGEIIRTFQSSLRAG
FMDWSPRLMLAMYSVEIQASTEVLGRVYDVLTRRRGRVNAELMKEGTPFFTIQALVPVAESFGFADEMRKRTSGAAQPQLIFA
GFEILDEDPFWVPFTEDDLEDLGELADKENVAKRYMDGVRRKKGLLVEGRNVATDAEKQKTLKR
FUN_006186 CDS scaffold_20 262613 264098 +
hypothetical protein ENOG41

MDPQTSIEESAAAITEVNLKAFNIEAFTLLGIALLVTALRSCVRIRTVGCRNLWADDYLVILATGIYVIETGLAYSVGNIAQG
LANNSMTDEQRASLQPEDHEYQLRIIGSKIQIALWATYSSLLWILKAAMCTFYYRLTKDLQGHRIRVIIGFGLIISSFVVVQM
NLLLSCRPFDHWWQIFPDPGAFCHAAISPALIWTCLAFNLATDFYLIMIPMPMLWKAAMPWPQKVGLIALFSCGLFVTMAAIL
RVVLLVSDPINGPQLAASWAVRETFVAIMTTNIPMLFPTFKKWAVPIVERAGSSLSVYRTPPNTIVDSRFSGALSLGSLRRKS
RGSSRITCISPMSPTASETEAHMNSIGLIYTIPDSSIKRPKASKTTHKIAAIRLPRIDTSAPADSEHGYGRLSTRSDSSVVSW
SETLVNSPQSEGTESLLSARVLHDGYSKHV
FUN_006187 CDS scaffold_20 264784 266267 +
hypothetical protein ENOG41

MTTTPRTRSKTKETRTVSEASLITPARSTTPESPIAAPIKAKPKSSPVFLPTPPETKRKTTCRDSPVPLKLRKTTSTSRAVTR
PGTLNWLADIEDEDYVPPQSPTPAGSGAIEEDETSENEEEKVSKLGPKSFQCESFTKSWEEIGQQRDFEFFQAYPHDEFSTLC
VEHFMQLAESWCSRRKSMVNHQVQKIVRAGLTHKPDFSKLALPVCDETWTVKSLQDFKVELHKGISDASDRVKLNHIQTLEAT
GERPICELGNQDRPLKCSLCTNYELPCKGKYIEVDKAHCRMDIPPYVLNPFYTTLEKASRPVPDCCHPKALTILMGDKGKDYM
ECEKCCYSQALPQHPLQRPKEYAIKVAGIRIPVKVRPERLCLNSKLGHVDVGGIHIIVDHCVGGPMIKFRSTHFSKAHEMHAL
DFHLEGEINIAEFELPEEHTYDHHESCEVEAEFNELYWQEGALNAITHCSNLFDNCSCH
FUN_006188 CDS scaffold_20 267140 268675 + DOT1 Nucleosomal
histone H3-Lys79 methylase PF08123

MRLFGGKNNKIKVDPPKIRIEKVVVDRPAQKPKPKSTSALSGSASRSSSSHRPSPKPLARQASHSPYPSSSDEKRLERKRKAP
SVTRRSPASDRIEFDKDSDGEDDGWMTLDTKRQRKGTEDGSFVDPNRKLRSVRAFEDRMDSRSFIHAVDVASLEHKCVPVMGA
QKEDVAIRLQYPSLQPREKYELVWGKDKIDAVEASIKVVRHVAETYLTEEEAEPFTNPNGGIIRRLEKASNRNIQDLMGFKAA
LREYNEKLRALVEDGVIAKNLDKMHELPQHLVAFILDQIYDRTVALKVELLSKYENGTDYVYGELLHPFISKILVEQTRMTSG
QVFVDLGSGVGNVVLQAALEIGCESWGCEMMENACNLAEEQKKEFDARCMLWGVRPGKVHLERGDFRKNLPIHEALKRADVVL
VNNKAFTSQLNDDLVRMFLDLKSGCKIVSLKSFVAEKSNNHNINDVGSTILEVEECTYPEGYVSWTNAGGSYFISTRK
FUN_006189 CDS scaffold_20 268876 269623 - ATG16 autophagy
protein 16, interacts with Atg12p-Atg5p PF08614

MPNWRDQYLSSIKDAELSNPVNMELVQACSQMADRISALEAEKNGLEALVANSGTPTAQPTEPSTNDPGVAQLKQDLAEALRS
KGVTEKRLRAGEEELMQLRAKHKADTKSIRDLTADRNSLTTRLKDREYELREKRKLIEQVQDEMIALNLQMSMAEKERDKVKK

ENKELVDRWMKRMAQEADAMNLANEPIFEKGR
FUN_006190	CDS	scaffold_20	270022	272836	+
hypothetical protein	EOG09260FL2	PF04840;PF04841

MDTLHARADWESVGDRWFRKTQQYTAVFDQDLDLDNYIVAGAPYAGALALWRDDTKLLAYQPGRSAKPAIDIYSLAGKKLRSI
PWDNGTIKGLGWSEDETLLVVTTDGTVRCYDLQGDFTQFSLGHGADNYSVESCRFYDHGMVALLGNNSLVTVSSYTEPRPKAL
AKIPESEIHAWSIISPNHTLSRSVEVLLSVDKTVYVVDATDCEDRFLDIGPFSHISVSPDGRYVNLYAVNGKAHVISSDFQER
LFEHDSDSQTPPLYVEWCGSDALIAWEDEVHIIGPGDSSSSYIYDSTRVHVISEHDGARLITNDFCEFLERVPRDTLEVFGQS
SDSSPASILLDAVGQLELESPKADDYIQLIRPNLTGAVDTCVNAAGREFDTYWQKRLLKAASFGKSVLDIYNSDDFVDMCETL
RVLNAVRYYEVGMPLSFEQYHRLTPESLIRRLLNRHEYLLALKIAGYLKLPTDRIYVHWASSKVRVGGEDDDTICRLIVERLS
GKPGISFEEIARAAYQEGRGRLATELLNHEPRGGRQVPLLLSMEEDELALDKAIESGDTDLILSVLLQLKKKLPLAAFFRVIN
ARPTATALVEALAMEEGDNTLLKDLYYQDDRREDGANVFIRESLKQPDARTASDKLALAAKLLADSKESTFEVHALKEATTLL
RMQEAFDRDLTDTFTGLSVNETMFKLIRLGYHGRAKKIQSEFKVPEKVAWWIRLRALVAKRDWNEIEEISKTRRSPIGWEPFF
NLTLQAGNPRLASVFVPKCTSVEAGETITMYEKCGMRVKAAQEAVRLKDSESWERLLEAAGKGSQEGREIERLGQSVFKK
FUN_006191	CDS	scaffold_20	275880	277773	+
hypothetical protein		PF00498;PF13639;PF17123

MFTQPVVSTSTGPPQSSSTSTSRPSRLRGLSYLRNYTQNHLLSRDNHHNSSSTGSAGGTGTASARSTSPNRHGSSLTRSLSHS
PTTSGPSTGAQNTNHLTLVSSTPDPVRNLTSATTITSIPALPPTSVPASVPAAASAATNSTESPRTSSQHSADINDPATAATE
LSMTADTQASAAAGTVDSNQDQPAMTRSRSATTGEGGSSTPETAPSIRFSAYYDPRSTRPSLTFPPISRTLPTGSEKIRVGRY
SERDSHSMSNMPGNQPSAAPVGFKSKVVSRRHCEFWYEDGKWYIKDVKSSSGTFLNHIRLSPPSQESKAFPVNDGDIVQLGID
FKGGEEMIFRCVKMRLELNRGWQNKLNTFNMAAHKRLQTMASSGAAGTNSQDCSICLSSIAPCQSLFVAPCSHTWHFKCVRSL
LNSPQYPIFICPNCRAGADLEADVDELAEEWQQLKNEAEAEASTPAQADTEPETDPVPAAANSGTSTPERSHTADIDHMDVTV
NITPETPQRNEVNHAISEPLPIRNAASAPRRVGQLGDSRTPSPPGGTEGPITPRNNVGPWVFDGSAGRTVSATEGEMTSIDAA
AQPDTSENDIAVR
FUN_006192	CDS	scaffold_20	278741	280316	+
hypothetical protein		PF00675;PF05193
M16B
MKQDPTELDRVTTLPNGLRVASEALPGSFAGVGVYIEGGSRFENDSLRGVSHIMDRLAFKSTSKRSADDMLEQVEALGGNIQC
ASSRESMMYQAATFNNAVPPTIELLAETIRDPQITEEEVAEQIETARYEIREIWSKPELILPELVHTAAFKDNTLGNPLLCPE
ERLGVIDRNTVLAYRDLFYRPERMVVAYAGVEHGEAVRLTEKFFGDMKKGSEQVQEITGSETSESELSDSEASSSSASSSPQT
SSGLLSRFFKHTPSASQNLQNLPSQNDITKPSKYTGGFLSLPAQPPNLSGLPTFTHIHLAFEGLPVASDDIYALATLQTLLGG
GGSFSAGGPGKGMYSRLYTNVLNQHGWVESCMAFNHSYTDSGLFGISASCLPGRTANMLDVMCQELRALTLTTGFSRLQETEV
ARAKNQLRSSLLMNLESRMVELEDLGRSIQVHGRKIPVKDMCRRIENLTVDDLRRVAKMVVGGKVKNPGGGSGAPTVVLQEAQ
AYGVSSHSMSWDQIQDRIDSWKLGST
FUN_006193	CDS	scaffold_20	280771	283528	-
hypothetical protein			ENOG41

MLPRKKARPNPAISAANNDSQTLVSNSTTEAPTSQSQASKASPAVEADHNNNAKAPSTQQIMGDPGSTAQHKSKDVRKTKSWY
GSWQRPSKASASTSVAKENIFGGSVKLNKTSDLSRYEASKGDDNASIRSTAGTIKTMPKVSETKSDVTMTEESAQAESQTQKK
SDPEPSRESSKVMETRESLKDAVIAEPTPPKAPESKTDDNQGQPDTCTEAPVSQTDPQRPAASSGWLGWWSKTPFTETQAAPA
PDVNGTATAEAEATKEVDEPRPVTPPAQRTSTDQVTSPKSIKAPQPTSWFSYWYPAAESVKASEDKQDTQPQPSTDAPSEDVT
MTETPPPHGHEPPPKAGSTWVFWSKESPKSKDSTPMPESGEVAIMGEGSEAHPRPMAECDVSPTKDRVKADVKPKEIQATVKS
GWMKKNKRGRPQSMDLDPPSPSVSGTSTPTSTTPATQDIDKAAVKAASSSPSIAESEPSSKTSTNLLLPSFSSTYHMKENPSI
LKQLTQFLLRTQQAPPNHVFRVDNPPKIRKAIAIGVHGLFPATYLRPMIGQPTGTSLRFAALGAEAIRRWTESHGCDDCEIEK
VALEGEGKIRDRVDNLWNLMLNWIDHIRSADFILIACHSQGVPVSIMLLEKLIDLGIITNAKVGVCAMAGVALGPFPDYKSGL
LMGSAAELWEFGNPESDNSKRFEKALKRVVDYGTRITFIGSIDDQVVPMESAIYSPANHPYIYRAVFIDGRVHAPDFIAHLVG
FALKLRNLGVSDHGLIRELSVPLAGSLYSGAGHSRLYYDDAVYDLAIAHALETAPAGPSPPPCTISPRGGPLTSQNPYVLPWI
MRGLLEENFVRTELSNEADELLRQFDDWKPTNKALKDVKYRLEAVRSKL
FUN_006194	CDS	scaffold_20	283849	289342	+

hypothetical protein   EOG092603YJ   PF00575

MSNLKRKDAPGGNPPAKSAKNTKEARPTKKENVGKDAKPAPKKSAETAEKAPVVSLLKEDEPMFPRGGASVLTPLEQKKIQLE
AKADAMRDEEFNTGNKVQKKKKRKTALKGDKKSDKKTGEEEQAVRIESLSFKNLVKGSLVLAQVTRINNLDLEVALPNNLTGH
ISIVAMSQQLTDRLENETAEKDDEEDDEAEDDEGIDLKSIFAVGQYLRAYVASTAEESTAGKSKRRIELSLRPNEANTGLAKD
DVVPNTTVMASIVSVEDRGFVMDTGIENLGAFLAKSEVDKNIDEQRLQPGAVFLCQVTSKGANGKIAQLSLQQKKIGNPKSVP
ADATTINTFLPGTMADVLISNTDRRGLSGKIMGHLDVTADIIQSGAGPAGVSLDTAYKIGSRVKARVICNFPGAREPKLGISL
LPHITALEKKRPTKSTDSKKNPTQVLPISSLVETCTVRHVEPDIGLFVDIGIPGLSGFVHISRVKDGKVEALYEASGPYKVDS
EHKGRVIGYNEMDGLFQISFEKSVLEQQYLRLEDVPIGAVVTCNIEKVLIDEKGVSGLVVKVAEGITGFVPEQHFSDVKLQHP
EKKFRQGMKVKARVLSTNLSKKQMRLTLKKTLVNSEAPVIKAYEEASVGMQIPGTIIKIQSSGAIIQFFGAVQGFLPISEMSE
AYIKDPKEHFRTGQVVSVHVLDVEPESKRLVVSCKDPSAFGLDKQNALKKLQLGDIVSAKVTQKTEDQVFVELADAQLKAILP
VGHLTDKSTSKNQYALKRIAVGQTLSDLVIIEKNENRRAVILSHKPSLVSAGKDKTLLTSFEDAKEGKIVAGFVRNITVTAVF
VQFGGAVNALLPKARLAADIQDLPDFGMHKHQSIEVKIVSVIPDHKRIVVAPADFDESAELDKKAKASDKPAASDDIEFGTVA
KAKITSIKDTQLNVQLVDSKVQGRIDVSQIFDKWEDIPDPKDPLDKYHKKQTVSVRVMGVHSAKDHRFLPFSHRSLHSVLELT
AKPSDLKAKTLEPLSLENLKLGDTYVAFVNNSSPQHLWVNLSPSVRGRISAMEASDDLSQLNDLEANFPVGSALKVRVTGVDA
RNKRLDLSARSSSSSETVTWSALKQNMVLPGRVTKVNERQVLVKLSELVSGPVHLPDMADDFDTVNTLKQKKGEIVRVSIVEL
DASNKRLRLSTRPSRIMSSTLPVKDKEISDVSQLDAGDIVRGFVKNVSDKGLFVLLGGQVTALVKISNLSDRYLKEWKDHFQI
DQLVKGRVIAVDKQTRHVELSLKSSVVDEDYTPPITYNDIKEGQVVTGKVRKVEEFGAFILIDNSDNVSGLCHRSQMAENPVK
DATKLYKEGDAVKARVLEVDINKRRVTFGLKPSLFEDADTDMDDSDAGVELDDDDSDDDVDMDGAGALLKILGTDNAGDSDED
EDDEDDEDEDDEDEDDDDEEEEEDDDEDVEMEDQPTKKKSKGLGAGKKSEWSADPFDEPESESEEQSKDNEKTKKKRRNRDEI
QVDRTAELDANGPQTSSDYERLLLGQPDSSELWIAYMAFQMQVSELSKAREVAERAIKSINIREETEKLNVWVAYLNLEVAYG
TKQTVEEVFKRACQYNDAQEVHERLASIYIQSEKLKDADALFETMVKKFGAKSPNVWLNYAHFLHATRNDPDRARALLPRATQ
QLGDRHSQTLVSRFAALEFRSPNGEPERGRTMFATLLAAYPKKGDLWSQLLDLEIGLSDADPTAIRDVFDRRTRVKGLKPKMA
EKWFRRWGDWEEKLDPKGKDKVMAKAQEWAVAFKAKKEADAAAAAEDEEMEE
FUN_006195   CDS   scaffold_20   290473 290963 +
hypothetical protein   ENOG41

MRFSIAAASAALLALAEARITGIKVPKEIAVGEPFEAIIVRENYIQSVFDSSIVFGYAPESQYPGTIGQVANSFALGKKESNK
VEPLKKKLTIPEGATKGKGLVTAALLSIYGASGSPTISEYNVTVTFGDKTSKEYASSS
FUN_006196   CDS   scaffold_20   291332 293195 -
hypothetical protein   PF12763   ENOG41

MTPVAAHTTGSSDAAAALRGATLAFNKTKPAPPPPQKDNGALTAATSAGRPRSPTKNLGAQSTGGSTIDNEQYGQNGGSIAAR
LRQLGHSPGQLHPSAARGGTDPKSASFIAATLAASRSASPSPKARTPRRRTSVEGLSSSGDIVDSGPIAPTGSLISMFEQSRA
GDPVKRSSPKRIPAKVPKEPVDGISEEGITQQQERPSLETKPKPKPKPKPKPLTPEPRTPIKPAPEVLSPKPALPPKPVLSPN
PALSPNPALSPKSFMRPKTPPQIITRHATNPMPTPSPDPQKRNPKPREQPKQRPPTPPQPRGISKPKAESKSVLHGKSPTITQ
KRSAPPLEPRLSRPRTTPPTPTEARPTSALTVSSDDTFVSASSVQSPVRESPPVMPVKRHFTGSVPPSPTRDFRRSQFQSSSA
TSLPLESLTSAIVAGSLASARLTPHNTGGSLAPPLPRRQKSPRMMQTLRQPPKPLEEDTDRHKKKGLKLHKGKHAHHEGSRKK
WREEVTPRERKRYEAVWASNRGLFLDLPNAPLDAMNGDPSEYVLNTVVREIWKRSRLPADELAEVWDLVDRKGRGVLGRAEFV
VGMWLIDQRLRGRKIPAKVSDSVWGSANGIQTKKPGHKH
FUN_006197   CDS   scaffold_20   294371 295471 -
hypothetical protein   ENOG41

MGSSMRWLAVAALVSFSLDKTSALEFDLPPSAPEPTSPARLRAIRHVYARADATTETLSITVAPDNTCGFYTPNTSFFFTCTG
GVKCMYENDKYNVAFCGERDFKTACMNSVDALNPDKCDVDCRRNTNIQKCTADTKTECYTIYFPNNIEKYPCHTQSGFSSMNF
PDGVSDFEQSTLGVDVFPALATTEASSTEESTTEASSKSTTAASTTTVTGAPQNDDGEENKDNGGSSTPVGAIAGGVVGGVAI
LVAVGLIIFFIRRRDNKKKAAAQPLMSDQAHTQPQMPYGMAPQQQHMHPYQEWHTGSPPPQGWQHSPSPPIPGAPVLVEAPDS
NTAQIHEMGDGTVKYK
FUN_006198   CDS   scaffold_20   297127 299278 -
hypothetical protein   EOG09260WYQ   PF00749;PF03950

MSDPVAEVTEGTVKLVLDEVTGEQVTRNELKKRTQKRAKKAAAQASREEKAKNQEANPKPAAPKPKVQEPTQLDPDAMFKQGF
LADVYKERPVKPVVTRFPPEPNGYLHLGHAKAIAIDFGFARYHGGKTILRFDDTNPDEEEEVYFEWILKIIRWLGFEPSAITH
SSDNFQKLFDLAKELIKKEKAYVCHCNEAEIKLQRGGKEGKEGPRYRCKHAEQDVETNLQKFQDMHDGKYEPQTAFLRMKQDI
TSGNPQMWDLAAYRIPKKQKPHHRAPEWKIFPTYDFTHCLCDSFEGITHSLCTTEFILSRESYEWLNKSLEVYEPMQRDGTIM
SKRALKKLVEGGIVRGWDDPRLYTLIAIKRRGIPPGALLAFINELGVTTAKTIIQIARFEQTVRRYLENTVPRLMLVLDPVPI
VIEDLEETQELEVPYSPKDPKFGTHKVRLTKTVYIDRSDFREEDIKGYFRLAPGKTVGLLNAPHPIKATSFEKDESGKVTSIK
AVFDKESKPKTYIQWVPEGSIKLEARVHSALFKSDDPTAAEGGFLNDVNPNSEVIYPEALVEEGFKEVRERAPWPVTAGETSE
ASGPESVRFQAMRVAYFAMDSDSTDDKIVLNRIVSLKEDREKN
FUN_006199 CDS scaffold_20 301305 304198 +
hypothetical protein PF00172;PF04082 ENOG41

MSRAGELPAEGKSTATTMEDLNLVLGHKTSRSLADSESLASANRSSSMGCRVCRARKVKCDGRPNGCRNCERLQLECVDDDGS
KSGSRRGSVPVSLRKIRTYRSCTSCRVSKTKCDGDRPRCSRCSARNLECQYDGGSAPRWARNLSKAPTSGSTEEDLESSLDAS
SIAGESLRSHSLIQSRENDVATSHRSSTRDTSSINPPIDFSDDTELSIHSWLISPDLPSGNNIRTVVDHYFANIHPLRCFAFV
HRPSFARLLDKGFESDDEKALLHIICAQGAKFYALSINLEDQDAKASLIRAAGNSWAQKAEMLVLTNFGKISVQRLMTCILLY
DFHFRLGEYTQALMLSGLAVRMAHALQLNVEFSPDILCAEANESSPPAVEKESRRRLMWACYILDAWTGSGVDQLTLLREADI
KIQLPCNERNFRLRIPCVTETLGVGHVLQFLPPAIVPRRPASNMGIMAYYIRIVTLWKRVVRYVKHLNTSPPPWLPESDFAAL
DADLRSWGRHLPEFISYSTDTIYARLESDQLGSLVLLHCTYHHNLLDLYRISMPDLFKLIKPFYFPPNQQEFLQSLQADCFYH
AKQISTILAEAVQHGARYLADSLLPCFAYDSSRVMLYYIARLLDLSRPDAETIVVDAINAVESNSKILRTMAALFPLADSLAT
TTERWLAKIRKTFAQDEHMSDRPLHGDEERSPILTGSPVTSSPDEAMGALGLARLAQGQSSDMKPIDRDAWNKMESGSPTASK
RGAGSVSGASSQPEITPARSKLQHEQPVSQRTPPGATTSHMDHQAVDLSDLQNYLSWDMYGIMEMNDNGLKAGIEDNGMPSWS
AI
FUN_006200 CDS scaffold_20 306887 309304 +
hypothetical protein EOG092628LW PF03999

MVDTSYLAQQVNNSIAQLHGLFDEIGVPDHEREARESELFSALSEALNSQVRLVNSEKKEMVDEAKKIITMVHQMEASLDDSK
RRRDYEDDDDLAITYPLTQCLQQLKEKHIQISRLHKERFEQVKKLVVALESYSSHLEPTFVQIPLPPTGPNQSIPPNFDLSST
YVDKLDNEFTRVYEEYSRRIATVQALADQTIGLWAELGIPQAQQDGAIVKYYRDAPEQLGLHEEDINRLRSKRDRLSDEKKNR
EKKLKELRSSVEALWIKLGVDESETKPFLNANRGCGVRQINEFEDELARLNELKRQNLHLFVEDSRIKLQELWDALYFCEDEM
LEFTPAFSDVYSDALLEAHEREIARLEALKEQRAPTLALIDRHKSLIQERDDLAASSQDASRLMARGQKGEKRDPGKLLREEK
MRKRIAKELPKITADLRKILSKWEDEYGRPFLVYGESYLDEIEASDGPKKGALPPRSKTPAGPPPSTIKPPKSVPNRAATKSA
PPRSMTKTPTAGMPPKRPGHQATGSISSIPGSPARSPSRIPARVPLSNMKHGNNSPERPRAESRADTFRPGPPLMRAPPPKMR
DLMARGDLEAPMNPYKSMGLNSSIVRHVEPEDVYDDRPRTARSNSNNSQYSSHPSNNSQFSSHPSNNSQYYSEESFHDPYSTI
RSQGYRPMAPPRQISGNSMASTNISGSENWETYDDNSEPELDVSDAYYAKLRATRNKRFSPEPGRPTSSQSKRIRGIPPTASY
NGPVMIDQDGNRIISGSEWTDEDAY
FUN_006201 CDS scaffold_20 312521 314107 +
hypothetical protein PF00141
AA2
MKFTVLIAAALAAAPIPEAAAHPGMGETIKEIERIAARSWGSGWGGNKWPGQGNGGGSSSWPGQNNGGSSSSWPGQSNGNGDG
SSSWPGQTNSGGSSSWPGQSSGGGGSGSGNGNGNGWSGDSFDAHSLIGDLVTISDKSLTSVGRDIKKILQDNGNPTSRERYFG
CPSMNSQRCKRDTCCVWQYISNELEDKFRGEAGRCTRWARYAVRIGFHDAGTWSLKTASQGGGADGSIILSNELTRGENMGLQ
QIGEYYQTIYNKYHTQYGFTQVTMADLIQMGSNIAAVTCPLGPRVRSFVGRKDSTKANLGGLLPRVDSDALTLINLFKDKTIG
PDDLVALIGAHTTSQQHHTNIDRDGDPQDSTPGVWDILFYQQTIDQNAPKRVYKFPSDVALANHPLTQPAFEAFASGSTGQAA
WNVDYARAYVRLSLLGVNNINDLTECTKVLPQPVESYRHKDKGRFNKWLQTDDNSWTSKSVSKDVDDGYEISVPENKIPSRRG
PWKTWTSTFKWW
FUN_006202 CDS scaffold_20 316021 317046 -
hypothetical protein PF00300 ENOG41

MSSKWTFKAQPGIFVELADIAHEYPGEKVTTQPNLGLIPDQKYPSDDPDAPDQRDWARLAAYVRWLNENSSDNVSYKVLYLTR

HGLGVHNKMHAQVGSEAWNTRVSFQNGDDKETWFDAFLTEVGEKQAQDLNSFWTDLIEKQGAPQPRIFYTSPLARCLQTTDIV
FSPLMASQSPPQQPIVKELLRERITRHTCDYRRDRTWIAENYPGYKIEDGFEEEDQFTNRVEPETDEEHIVRKQRALEDIFNE
TTKDDDFISLTVHSYAIRAIQAAVGSGVCRTREGTSIAILVRGEKDGEVDGPELDMNDYVSADPEGVSSLRRFSM
FUN_006203 CDS scaffold_20 317559 318641 -
hypothetical protein ENOG41

MGILDDLIPRPTAPPATRTLKRRAVTSSQTTTEEITITIAPDNTCGYISERLGASRACPLNDWCYFFPPVPAQSFTNGGILCC
GETSCQYHATCVNSEEYFVSSKCDGGCEVDNYTLKCTDSASPYCNTVSWEGSTFDYWCNDLDISTAQSAALTYKGQTSREFGT
INERDHSSLLSQMTEAKTEGSVNAEATGTATSQSAATETNKDSGSSGTPVGAIAGGTVGGVAALAIIGVAVFFFLRRKKKSKA
SSSSKYQQAPGGDKPGWNQQQQQGGYYYDPNSPSTGYNGSPNGQYGYQQAGGYPAGHQQPAIHEAGGEAVGSIPQELPDSGCG
KKKPVELA
FUN_006204 CDS scaffold_20 320210 322072 -
hypothetical protein PF14033 ENOG41

MPTPQEIKLPGFGLPISYAGAHFPVVLGMEEGEGEGDWRASTLTIREVCMIKVIEDLTNKPEWWIKVNDDEITAKWKKEAMEM
PWGEYRVYGDFTHAMADACIKELRKKADIYQKTGLIPVMDYASTAIKSDNLVPKDLRDALVTAVSPLENVPEEHKDWHPGSDG
KVLDIVHPSLWPLVYGRSLILPDKRINLEEALSHCGKGVVVPVDNSDDTMWPSSAFSKRFQWLPCDVDLTGVHPRIDSYINNV
HPVKHAELYPVIEKFIEKSLPAWDVIYRWHDDFEVQRVFTKNVRPDCKVPEICGDDWCSAQNRPLDDDEAPRREDEDYEEDYE
ESDRNKRDEEWFRETHVPELPDPKTELEELVKINPSDVKTSGFFGNASRVQVIVKLANIHLTPEKPTYDGGSWHVEGQLNEHI
CATALYYYDCDNITDSRLDFRTAANREDQTVELNYEQGDFDSIERVFAIDPGADLLQNIGSVLTRQDRMLFFPNVYQHHVSPF
ELVDKSRPGHRKILALFLVDPEVPIISTANVPPQQRDWWAEGLLKNDRFNNLPPELTRMVVDNLDFPIDLEDAKKIREELMAE
RTNLQDTLNSDLKNLEWNFCEH
FUN_006205 CDS scaffold_20 323330 324353 +
hypothetical protein ENOG41

MTETEIAVVESPAEAIVIPSAEPVVADAPPAEPIEKSLNSNSNSNINTTSNNKNKNVAQNSSPLARLLKSPSAIDDFVEHLNR
VIQTRRGTDTVLLFTTYAARLIGAILNILGRTTLRHSARKVVEMAFKLPPSTSVVLSTATAPPLATLALNLSKYIQGFTNMLG
EWRTMNRMWGIIGTYLEAKDLILRLRGQKLDANGEKVPAPNRVNTAFMTVQIICNLVYNVGEGACWLTCKGATNLSPKTSAKL
GIMAARSWCAWVALELVRLLVERARRTTSGDVATEEEWKNNWKAEVLGTLPWMPLSAHWSTEEGLMPELMVAAIATWPATNMM
KSVWRQTA
FUN_006206 CDS scaffold_20 325497 327380 +
hypothetical protein EOG0926251E PF03062

MLYFLHRPFVALADTVGAAPDDLKLVTSFLISFPLAALLKRIPDSRPDLKNLFAISVSIFYLVGLFDLWDGVRTLFISAGGTY
CIAKFLRTSPYMPWIGFAFVMGHMSLSHIARQAADDPTKADVTGAQMVLLMKLSAFCWNVADGQLPEEYLSDFQKRNMLKELP
PILDYAGWVLFFPALFAGPSFDFTDYRKWLDTTMFDAPNVDPAKKPPVRKKRKIPRSGGPAAWKAASGLFLIAMFMGLGGSYY
PGLLTSDIYMKYGFLRRVWIMHMVSFTARLKYYGVWYLTEGSCILAGMGYNGVDPVTGKVFWNRLQNIDPWAVETAQNPRGYL
GGWNINTSNWLRNYVYLRVTPRGKKPGFRASLMTFGTSALWHGFYPGYYLSFVLASFIQTAAKHYRRHVRPFFLEPITGNPSP
KKKYYDFVSYLATQLTFTFATAPFLVLTLQGSLLAWSRVYFYAVIWTFLSLVFFSSPGKAALRKQLEKRQGRASAKLVRSIST
DSLTGKEPILGISKDIEGDFNEAVEEIKAEMEMRQRKVKAEIEAHKAKAKTG
FUN_006207 CDS scaffold_20 328169 330827 -
hypothetical protein ENOG41

MSVSPSSSLPRKALHERSNSQNNSQNNRLGIRVVPYSPPRVASDGTVTSKTVTYADASTETDSELAQTDGSPTHSSDSQFETP
SPSLQRARTRSIVLSPSPLKRPTRYPSSEFDQNPAPWLPHYDTDADDHTSYASRPPSREPSTSPRPPSRKRVINVHADKTFSI
LPQDASMSSRVESLWSLPPSFTTGNTSYGRGSMGTYSDDRQSRPASPMTPLTERNASSPVISPRPRDPSTPDNSNSSPWNYRL
FGGLRKVPKTPDVKQKQPQSRDAPPELPLPTLPEVDLPVHEAGPSSQWLTQKTSFQSSLSEQTRSTFSERTNVKTYGFSSPMN
VADLNSLPPSSIHSNIELLGDPSSAEASLHISTRPQTEDSNVNVVTYGGHSASSSVVAVRNRVRPEFSQESLIVPPLQPRKRR
SSDSFALAKSRSRETLRSRSASITSLSTIFTQEATRALFVGPPTILQHAGPSWPDLQNRPQARPHQWSGQLSTVMSESEGGSE
PASRALSLSSVPGRRGSGVGSSHSKHVLSMASSLFGLEEHVETPSHSRSNSLESPAATYTRSMPRDPATGTIRLVRDHDEDGD

GLADLEVYHHPSSRSRMGRFLSSYSSDRSLRSTASFNAAVPAWARVYYGSGERKWLAAQPSMESMYSEYSQSQPPDGQLSRSP
SQDANATNIRNPRRRPREALPHQHSNTGSMDISAAPAHPALAVVRNIKKQTSSIWSPHLARDRRPVSQSIWQPPASQWEARSE
LTGRRNAQVTMFVVGFVFPLAWMFAALIPIQRNNETEDIERNNSTSGLGKRNETSARASGEEDAVFTSAIWWRKVNRGMSLIG
LLILGAIIALIVVGVKQRWGH
FUN_006208 CDS scaffold_20 334434 335577 +
hypothetical protein EOG09263IMF PF02517
M79
MSVLSSAEFAPPQAVALLVVYCLVYVIPLYFSAATRPSPTRSRDAPEAIRARIFVVSLSTAVCSLVTLYLLSSHGAIAVEKPL
HFMGYWPLGLIDAARALWLTALLFAGPLYESLVIDGAAHQWLRLQPLRDLWTDWPTWRNMVAGPITEECLFRSAGVPLLLRSG
ASLTGTIFMSPLIFGLAHLHHFYEFRITHPRTPLPIAIARSLLQLSYTSLFGAYATFLFLRTGSLLAVVLVHTFCNCMGLPRL
WGQLDPYWLPEGDPSVASSRKMWTGVYYVLLVGGLVAWWQNLYSLTETPMALAKF
FUN_006209 CDS scaffold_20 335876 337198 - MMM1 ERMES
complex subunit mmm1 EOG09262X74 PF10296

MEADTCPRLIEPTLSFTQGLIVGQLSVVLVLAAFIKFFIFGDPPSADVTASLRATERRSRTLAHKRSLLSIRSPANRGDRSQD
RSLSRKKSTVLRNPPTLTIGSILSKAYYNVDSHQPESLDWFNVLIAQTIAQFRSDAQHDDAILDSLTKALNGDSRPDFIDEIR
VTELSLGEDFPILSNCRIIPVDEDGVSLAPGKKFDPATAARDGLRLQARMDVDLSDMLTLAVRTKLILNYPKKLSAVLPVELA
VSVIRFSGTLSISFIPSNPSQSTPTRMIFNFLDDYRLDFEIRSLLGSRSKLHNVPKIAQLVEARLHRWFDERAVEPRFQEIAL
PSLWPRKKNTRGPEDASTERSESIGRSRGNDAGRDHREEIRGRGGDAEIRDRGAGGTVRHRRGTRSNDSDDFSMPGSLPDFQV
AAS
FUN_006210 CDS scaffold_20 337633 341853 +
hypothetical protein PF00995;PF01134;PF13932

MDVAQAVSGYINKVVTTSDTSSAKMKILLLDRETVSIVSTAVTQSSLLNHEVYLIDRLDNAAREKMRHLRCLSFVRPSPESIQ
LLIDELRDPKYGEYHLYFTNVVKKSSLERLAEADDHEVVKLVQEHFADYNVINPDLFSFSFTLPQQRIWAGSPDTWNPDSLQR
CSEGLVAVLLSLKKKPLIRYQKTSPLAKKLASEVRYLMTQEDSLFDFRKVDTPPILLVLDRREDPVTPLLTQWTYQAMVHHLL
GIQNGRVDLSDVPDISPEQKEIVLSQDQDPFFKKNMFLNFGDLGGNIKEYVGQFQSKTKNNENIESISDMKRFIEEYPEFRKL
SGNVSKHVTLVSELSRRVAADNLLEVSELEQSLACNDNHGTDVKNIQRLIQSPNVTPESKVGLVALYALRYHKHPSNTLPMLQ
DLLVAAGKVSPREADLVSKVTAYHNSLQASQSQGGISEIFESAGIFSGTGSRFKGLKGVENVYTQHSPLLETTLQNLIKGRLK
DQQYPFVEGGGTTKDKPQDIIVFIVGGATYEEAKMIAELNASSPGVRVVLGGTTIHNSATFLEEVDDAVSSWPESRAILRHKW
TITRRRYLATVSGDLQPYDVVVIGGGHAGAEACAAAARSGARTALVTPKIDNLGTCSCNPSFGGIGKGTIIREIDALDGLAGR
IIDKSGVQFHTLNRRKGAAVWGPRAQIDRELYKKHTKDELSSYPNLSIVLDSVSDIVTEPQELFEGASSCIAGVRLESGQILP
TKKVVITTGTFLGGEIHIGLTAYPAGRLGEAATFGLSKSLRDAGFELGRLKTGTPPRIDAASINYDVLEKQYGDDPPTPFSYL
NETVAIQEQMTCSVTYTTEDTHRIVRENLDKTIHIRETVKGPRYCPSLESKVIRFADKERHIVWLEPEGLDSPVIYPNGLSMT
IPAEAQEEVLRTIPGLENSKMLQPGYGVEYDYIDPRGLKSTLETKAISGLYLAGQINGTTGYEEAAGQGVLAGINAGRSSQGL
PQVFLSRGDGYIGIMVDDLITKGVTEPYRMFTSRSEFRMSARADNADVRLTSKGYGWGVISEQRWSRYRDERQQVDDLTKLLQ
SVAMSPLQWIEKGYHMKRNTQRRDGIDVLRLSNPETRVNIDQLAPVIPSVMDFPPRVRRRVEIESFYAPYIKIAESERKQLTN
DERVKIPLDLNYDNIPGLAMSEKEALRAAKPENLAQARHVEGVTPSGSLRLLAHVRRRPEAYLLEKDPSRGRRTQRTNKN
FUN_006211 CDS scaffold_20 342296 343321 -
hypothetical protein PF01066

MSLSARLAWRATTSCTARSIPALVRSAPRPTIASTPRCLFFTKAQSWLDHKPQQKGLEEKPVGSRPVIQHDNDTNPPRPAAVL
HEDIYTLPNILTFTRLVAAPVIGYLVLHDQHAWAVGLFAYAGVTDLLDGWIARRWDSKTVVGTVIDPMADKTLMTILTICLAA
KGALPIWCAGIILGRDVGLAISAIYYRWISLPPPKTFARYWDFSLPSAEVRPTTISKYNTFLQLALVGVTTAAPLLSTDLSSA
LTVMQYVVAATTIWSGASYIYTKDAVKILNKQNSKKP
FUN_006212 CDS scaffold_20 344772 346176 +
hypothetical protein PF02809;PF06087

MAGSREDPVELGSDMDEDEALRYAIALSLQEQEEQGDQSSQIPSASTSTSHRKGTGSGGASLGLLSLDRKKMEEERLQRLAKR
RRSPEDERSVDEVPPAKRMTPSEPSRTVTATATAHVLPSFVPYPKGAIKRTWAKGYPRTSDDIKIEEVFQKDKLELALLSSYQ

WDDEWLMSKIDPRKTKLLLLAFADSEAQKSEMRSNAPPGIKFVFPAMNGPGAMHSKLQLLKYPDYLRVVVPTANLVPYDWGET
GVMENMVFLIDLPRLKDPATYRQTAFSTELGRFLSATGVGEGMVSSLANYDFSQTKHLGFVYTIPGGHQGDSLKRIGYSGLGT
TVAALGLATDEPIEVDFVVGTIKVAYQSKEH
FUN_006213 CDS scaffold_20 347206 347620 +
hypothetical protein

MSCRNWESGVVVRISNPKNSSTVSASFSTSASASAGASSSTKVKAAEGQARSSVRAGKISGEPDKGCDTSQHNHHHYQMQKQN
QKQKQEEGLTDTDAHTDSDMMSGTVFEGTVPIPMRRPGRRYREGEEPWFYSVQG
FUN_006214 CDS scaffold_20 352121 353114 + SGE1 multidrug
transporter PF09729

MSGTMPLQPTFYGFIGDTTDALIIFEACLSGKLFHVPRRPHDRERQDLIKSGNIFVYEEHASGIKRWTDSISWSPSRILGNYL
LYRELEKPFPPGEKKRARGRNGKSTTQSGGITKSRPRNSVPFQTGLEHGNEYTTVPSDDERHLVGSLVDSYDFKEQGLVKKTI
SITYQGVPHHLVSYYHVEDVKAGLLPSPVDDPRLRGVVPRTELLTGQNFRAPIEEAQHGTYLSGAYMPNMDHQYAPIGFPTHT
QQPALQQQPQQQPQPQHQPQLQYQPQPHQHQPQLQYQPQQQHQPQQQYRPQPQHQPQLQYQPQALHPTAHGYPQSYGQTWW
FUN_006215 CDS scaffold_20 353252 354588 - VRG4_2 GDP-mannose
transporter into the lumen of the Golgi

MTDKKNEDYVVQMGNDNYDKNEKPSFQARSPPPAAYGSSPVGGLEQLEKSPPLSILAYCLSSISMTVVNKYVVSGSFWNLNFF
YLTVQAVVCIGAITLCKQLGLIKVLAPFDSDRARKWFPISLLLVGMIYTSTKSLQFLSVPVYTIFKNLTIIVIAYGEVLWFGG
SVTPLALLSFGLMVLSSIVAAWADIQSAINGDFGTTDSAAAVSTLNAGYAWMGMNVFCSAAYVLGMRKVIKKMNFKDWDTMYY
NNLLTIPVLVVCSLLTEDWSAYNFSRNFPDDTRNKIIIGMIYSGLAAIFISYCSAWCIRVTSSTTYSMVGALNKLPIAISGLV
FFSAPVTFGSVSAIFLGFISGLVYAWSRVRQSMSSGGSLPTVRPTMSASAKSNRDANS
FUN_006216 CDS scaffold_20 355204 356317 -
hypothetical protein PF00106;PF01370;PF08659;PF13561 ENOG41

MDTRTEVSDVTEASSRPRIAQLPASCDPPQPNTPVKQLVWVIFGGTGQMGHSLVKCALSHGDLVTSVGRVFETSLKDMENIHE
NCLGALCDVRARESVNRVIERTLQRFGRIDIVVNCSGYGVIGACEDQDEHDIRNQFETNFMGTLHIIHATLPYFRRQNAGRYL
IFSSTSGALGVPGLGPYCATKYAVEGLIEAMLYETDAFNIKATLIEPGHVRPEEPEMQDTSLPTSWGHFLVKPPSETYGHPTS
PALHARRIVQWLGDKYRPTSAIKCAELVWQLGHCSYPPLRLLLGSYAIESIRDRMRSVTEELEDWKHLNFPAGPDDDREVGEE
AAAAVAPETNEAAAS
FUN_006217 CDS scaffold_20 357440 358078 - ADI1
1,2-dihydroxy-3-keto-5-methylthiopentene dioxygenase PF03079;PF07883

MRAYFYDGLPGDQRLPHNSGMPVSVDDLMNIGVYYYHLPELESVDNLAKERGYKNRDEITVSPQAMGDIYETKVKSFFAEHLH
EDEEIRYIRGGRGYFDVRSKDDDWVRVLLEKDDLLILPPGIYHRFTTDESNYVHAMRLFKEDPKWTPLNRGPDVDKNEHRQEY
VKQFLGEPNQ
FUN_006218 CDS scaffold_20 358765 359576 -
hypothetical protein PF04511

MDGGRLPLEQWFWEMPTCTRWWTTATILTSALVQCQMVTPFQLFYSFRAVFVKSQYWRLLTTFLYFGPFSLDLLFHVYFLQRY
ARLLEESSGRSPAHFSWLLLYSMASLIALSPLVSMPFLGHPLSSTLVYIWSRRNPETRLSFLGLLVFTAPYLPWVLMGFSLVL
HGTIPKDEIMGVVIGHVWYFFSDVYPPLHNGSRPLDPPNWWRRLFEARPRVDADDSTNDIMVAGAREAAPRDL
FUN_006219 CDS scaffold_20 360277 360797 + HH3V Histone
H3-like centromeric protein hH3v PF00125

MPPRKSKASGAGKSRPSDVQAGDPVPVRAKRRYRPGTVALREIRHYQSGTKLLLRKLPFARLVREIALTMRPRDEGLRWQSQA
IMALQEAAEAYMVHLFEDTNLCAIHAKRVTIMQKDIQLARRIRGIWGGLG
FUN_006220 CDS scaffold_20 361307 361850 +
hypothetical protein ENOG41

MHRAFNSRVAFRAMSRGIAPATCVGAFIATRPVIRCDAPTLATGPPPRRRPLEVSPDTVRQLSSGSVAGFGIGVVVALFSRTL
ALLTGLIAFSIHVASRWGLDIPRILGIEKLLKNSSVWNKSKERPLFTASFLVTFILATFVRL
FUN_006221 CDS scaffold_20 361875 364029 -
hypothetical protein PF00557;PF01321;PF16188;PF16189
M24B
MLFQTATSLLRSAPRRLAAASRLSSRRWISSETMTKLDTTSRLTRLRGLMKERNVHIYIVPSEDSHSSEYIADCDARRAYISG
FTGSAGCAVVTLESAALATDGRYFNQATSQLDSNWTLLKQGLQDVPTWQDWSAEQSSGGKNVGVDPTLISGSTAKNLAEKIRK
NGGAELVPVDGNLVDLVWGDERPSRPSEQVIIQPDELAGESVLNKLTKVRQELEKKHSPGFLVSMLDEIAWLFNLRGNDIPYN
PVFFAYATVTLDAAKLYIDEAKLDDKCRSHLTSNKVDIKPYEAIFDDAQALHAAHAEKSKSGDKVPTGNFLISNKGSWALKRA
LGGDSSVDEIRSLIGDAKAIKTEAELKGMRDCHVRDGAALIQYFAWLEDQLVNKKATLDEVQAADKLEEHRKEKKDFVGLSFP
TISSTGANAAIIHYGPERGNCATIDPEAIYLCDSGAQYRDGTTDTTRTLHFGKPTDAEREAYTLVLKGHISLDQAIFPKGTTG
FALDSLARQHLWKNGLDYRHGTGHGVGSFLNVHEGPIGIGTRVQYAEVALAPGNVLSNEPGYYEDGKYGIRIENMVLVKEVKA
KHSFGDKPFLGFEYVTLVPYCRNLIDTTLLTSEEKEWLNTYNAKVLEKTQEYFEGDNVTLAWLKRETQHIE
FUN_006222 CDS scaffold_20 365419 366589 -
hypothetical protein PF15365 ENOG41

MSETPSQPTTTPARRRNGRGNGRPAAQKAYASENDVASIDPARYDRAPRTPQKGAGTDSPHAQLSHTNSKQRPRNANNNNNNN
NKHRGKNGPHSPESARPGRHTPPHQSSSMKSVSAFAGATFHASPAPSALPLPSFVSRSVTESPSVSKTSRDILQEPSPPTDTE
APTPLRQSASGVQESPLDFMFRAHRQEKERQRSESTSSFRPSGLVTESPSAQSPFGPGSIPKPATLPQTARTQTRFQSGGIDS
VELDGTPGRPMGPAFSTPYQERIKAARSNSARSPADLSATQPKPNAVPEDPTEALKKFLFIGNGTNASQSAPTGFAPAPAPAP
APAPPAQHYNYARSGPSVPAPCDGRSNNLQAMENDLRRILKLDLSTGSPGADQRLFS
FUN_006223 CDS scaffold_20 367876 368390 + RBX1 RING-box
protein 1 EOG09265AT5 PF12678;PF12861

MADVEMSDAPVAKKGEGSGSKSVEGKKKFEVKKWNAVALWAWDIVVDNCAICRNHIMDLCIECQANQASATSEECTVAWGICN
HAFHFHCISRWLKARSVCPLDNRDWEFQKYGR
FUN_006224 CDS scaffold_20 368539 368960 -
hypothetical protein PF06645

MAEQALDQIRDVFDGQIDFEGQKLAELLATVLLVISGLISFVVGYILQDIKLAVYLGLGGTALTFLVVVPAWPFYNKHPVKWL
PAGYAYDLASKGNQ
FUN_006455 CDS scaffold_21 3119 4087 +
hypothetical protein PF01116

MPEPIENLRNTNRTLRILEDAVKGNYGVLAAICYNIEHLTAIVKAAEAKRSPLVLLLFPSTLRQLPTLAWAASAAIKSATVPL
SLHLDHAQDVSQIEEVVKTLPFDSIMVDMSHYDHEENLAKTTALTKLCHKRGIAVEAESGRINGGEDGIADTDDLEALFTSPE
EAENFLKAGIDILAPSVGNIHGDYSPKGPDLDLARLSSIDKQIRGHAFMALHGTNDFTAQIMQDCIKAGAVKLNVNKLLLEVW
HVHLKKNAHKPFMQRVDEGIEILQAEVERWIDICGSTGKA
FUN_006456 CDS scaffold_21 4749 6347 -
hypothetical protein PF00023;PF12796;PF13606;PF13637;PF13857
ENOG41
MNHGRIYFRAEAVSRGEIIKCIIDKILDENSLHKEESWEWDYWSEPEPEHNPLQVVYVDVLNLAATCKDIRRPVTKQIYIRDAR
DNHSAALLVSAKRNDIAGVIRALHIGANIHSDDATETVTYYTTGPNAIKTGYRVPLQLENQATAIHWAAFNGHKEIVSLLLNH
GASINHRVSIGTREWFIRQKLPAVQLYKNCTVLDEYPISSTRRAVADNAFLGLRCLGSLQAVDNQYRFMKLEQGANTLYFAVK
GGNIDIAESLIKAGAELMTHTGTGLTALHQAVSNGDISMAKDLLMEGIDPNNADPFGKTPLHFIKCSEEEMAHPAVKMIKTLV
EHGADINRCDLLGNTPLLTHLQDFERNDSVIAEFIRQGAHIYEGFYDDLEAPLNELNQECHGIYRICWPRLRTGSSAA
FUN_006457 CDS scaffold_21 7678 8634 +
hypothetical protein PF00043;PF02798;PF13409;PF13410;PF13417;PF14497

MAQKTKTDITLYTTNTPNGIKPSILLEELNLEYKVYPIKMTENEQKEEWFLKINPNGRIPALTDTLDGKQIRVFESGAMLQYL
VDRYDKDHKLSFPHGSAEHWEMTSWLMWQMGGLGPMQGQANHFKRYAPEKIEYGINRYTNETRRLYRTLDTHLAQTSSGYIVG
DKVTIADISIWGWVSSAKWAGVDLSEFPHLEKWLYKLLERPGFEAGRHVPTRHTAFELAKLSEEELEAKASASKAWVQSGMKE
DAKK
FUN_006458 CDS scaffold_21 8702 10240 -
hypothetical protein PF02666;PF12588

MVHQHGDHHKIPAKHRIHKPGAWLPADHRVHREYMRRVTKYADENPKELVPVLQEFKDFIEKTPRVYMYFIQMFEEIPQKHPY
QSDPTGTPQLRDYEHMLQVLNHIFSTAPEWTDAAESVGMVGVPLCAIFDYPMGTPSGYAAFLDPEVNRMLKKVLNHWGKFLMT
PKSAEVLGDHKQGWFGPVGLNDLVEVANKPNKTNFKFEEVFECDPSAKYYGFKSWDDFFTRKVRDSARPVASPDDDLVIANCC
ESRVYNIERDVKLRDRFFAKGQPYSILDMLAHDPLAEKFAGGTIYQAFLSALSYHRWHTPVSGKVVRAFVQDGTYFSEPLFEG
VGEPGSTEISSAGLSQSQGYLSALATRAIILIEADEPAIGLLAFVGIGMDEVSTCEITVKEGQHVKKGQETGMFHFGGSTHCV
IFRKGVKVEGFPEVGREENVPVRSQLAVVKK
FUN_006459 CDS scaffold_21 11550 13244 -
hypothetical protein ENOG41

MATMAPAPTSTENITPPRSSHGHSDSWGYNMPQKENFDNDMNAGTNHFTTQNGNSNSYHSGMDRVGRKVNSHDNLSSAKLHNS
VDGAGARYISESKTSPALNGNSWGQTFGPQTEDEYSRNAVPIDQEVVVVDEGSKWIHRDKLARIESEELQAAGIFVPRTRNHS
KQRRDRSRSAMRRGTDASENGPRSRKNSTAVEGRSPEIAGESWDLRTPEEIAEEESNAYFTSNSHKGGSRIPVAKVSPAPISV
DRFERASRKTSESPDDERTLTNEKTRPGSAHMMKGFDAMSANSNHNNSLPAPKRTATDTSPKKGATGPRKTSGPSKTGTAATG
RPKTRSGSIGNSISTRPSTRSGELSPGNKAPEGDPPWMINSYKPDPRLPPDQQLLPTVARRLQQEKWEKEGKFGDVYDKDFRP
LNDNELGKPDQLDQADKTEETDEKQRETSPEGEWPLKLEPTKSPTQRAGGYSTMPKISDKPQNSPLPSPRTPLSPNAPNAPQA
PAQEQSKPEPSTERPVQQHPQQPQPAEDDSKGGCGCCVVM
FUN_006460 CDS scaffold_21 15518 16774 +
hypothetical protein PF00149 ENOG41

MAAVMRRRTRIVCISDTHNCQVKLPKGDVLIHAGDLTNQGSHAELAKTVAWLEKQDFEAKIVIAGNHDLTLDPKFYAEHGLYF
HNKKPQSHDECLRLFTSSPSITYLSHESATIRLRSHSGPRTQFKVFGSPYSPRFGLWAFYYDAPQNPSNWSDLTSIWESIPLD
ADIVVTHTPPRTHCDETDERRATGCEALRQALWRVRPQLAVCGHIHDGRGAKRVMWDLSCQNVAYQEESVVRWQDPGEGNNKT
CLIDLTGKKAPSLANDGSHPGRYGSSTREPDVTDVPHFIDSAESSLYTFGSGPADIHASHGTTGLGGDPASPRSDQTALAGRM
GRRETCVVNAAIMKSRYPHIGGKQFNKPIVVDLDLPIWEDEGHIEAP
FUN_006461 CDS scaffold_21 23306 25028 +
hypothetical protein PF00226 ENOG41

MTTSTPDPYEILGVAKDAQLPEIRSAHRKLVLKCHPDKVQDPTLKAQKQDEFQRVQTAYELLADEKARKRYDDQARLAELREQ
MRAKAAASASSRPSASATASPSSSRPTPTSTTYKYEIRTNDRTYKTDRQPSPVRGYSSYSRSYEDEHPRAADIFEGVRTVRRE
KSYQDKTTKREEREREKELERAREKEYARELREKERERERRRADEAIRRAEKENKEAEKEARRAEKKSRDKSRKRDTEEKKR
HTKPYIETYDGEPISSKSEKKKSSKKHDERRERDRSSHREEAPPTTSSMHPPPIPVEYQDKTNAALNYIQASRSKGSYASRHM
VQPPAPTPPPVGSPFAAPADDDDARRSSAKPRRGSTGEKAYKPEVVDDPIEATPPTARPHMHKSATATGAPTTSPPRRTNTMP
NEGYSRHVPGISRAQTFSGAYPEPDPRGRGRTRMQAQVPESDDEDDYERYRPERERKSHRSSKKHRSPESRGEHVMQYRVNDG
RTTLQNSYSRATPTEEAYSYYANQPNVRIVERPSMPSRDAAYSASYGNYPGKVKTSSYGDVQFSEYPAYREAYAA
FUN_006462 CDS scaffold_21 29098 29323 +
hypothetical protein

MPSSIAICKETITAEKKDISAASCLHSASLHMNTVEQQRIEQADDKAVDMLYQAYAQIRAPPTVAPQALVAHPQ
FUN_006463 CDS scaffold_21 29576 29813 +
hypothetical protein

MINPEVAVQLHFSGRRISAVAVSNPAKTLEDHLKSDRRCDDRYVIHAVHHDKASENPSVAYVYADSPKREHDEFQFIL
FUN_006464 tRNA scaffold_21 35929 36022 - tRNA-Glu

FUN_006465 CDS scaffold_21 36935 38822 +
hypothetical protein

MVGEAFETNALFREHVRNSSGHKYWLADDTADDNDSVFTPSLNSSHQTAEDDVFGPFASDGNICNEPCCRHYGTDYKCKSEFN
RHADTGAHRTAANLSKVLLSRIPTASALQAEQEGLRSFRCTSRSCHRHGEVFSTAKAFFNHLKDDEHRDGWYVKFDDDDLDYS
SDRDVLPGIQFYAGGKKGRCVNEKCPRFRFQFDSYGAMKQHSRSLGHVRAEEDLGLTDADESGEEVWKKSAVHGMEVAEDGLL
WKCTKQGCKKFNVVIKGLGNAKSHYNSDTHLMAAEEIATSSDESVEHIEGMEPLKDGDGWRCLKRGCRKFNKLFLYLGNAKKH
ASGDSHALAEEPDSANENLDGLDYSMEEGVWTCVKPGCKRMNAIYQHLASARKHANANFHALAEEPAEEILEDIEGMVVLDGE
SAYMCIKFGCKGSGKTYSNVRSAKLHANARPHMKAGDISAFMPNQFNMDLFATPTRTPRNLLLTPMETEESTIVVTPGSLSAG
RGTNPAGHPAGARNGTSARTTKMIRLGRSSVTTAGSSKHEVLERKNRELESRVAKLEMQMGQVLGLQSPQIQNNAHYQNSQVH
QSAQLFQQIPEVPQAPETTCARVEGLSKFVRAAYRPKMSMPVNEDEL
FUN_006466 CDS scaffold_21 39184 40195 -
hypothetical protein ENOG41

MSTSSPSTFLQNAAQQILNTVLLNFMTADFKNHVFYNSNHRLEEAKQKGVTLSDISIGDDCLPTWTGRTWDPDAESLHDALQG
DALNLLEAQEAGIMVRRDIIWKSNRKLDEARAEGGAAAEEAIPFLLEHDEGNDDHSSPKGSIHEDTDTSEGTLVNVNTEDSDE
DTLMVIDDAETTDDGSDSDMEFVVIGNDDELPDNEIETSNIFDPEPSYDDSDDDKPNIFHGAQVMGVLAQRSIVDLEVYIAED
DEEIQDLPRMELAIDQESYERYFGTDGAIELEIVGGRLICTGELFEEDQEMTLDDVADDLEILSLTASDEE
FUN_006467 CDS scaffold_21 40856 41230 -
hypothetical protein ENOG41

MPSVGNPNGPSKNRLAARASSARKERRKRSQAPKDKVAKADTTRGARPGLLPTSGPRAKLSAKKARKMEKRLAHAMKRKMEAE
GEAEMKDAPEVEGEKAEEVEMGDIQ
FUN_006468 CDS scaffold_21 41615 43206 -
hypothetical protein PF03188;PF10348;PF10355 ENOG41

MVSDYRLKAIAAPSLSKVATIALLVTAVQAHDHDASHIPEGETVSLEPLDTVLWIHIFIQMLAYGVIFPVGMVLGMTKNRWHV
PTQAVGTILAILGFFLGHAHKGREFIPNHVHGKFAHILQLLLAAQVVLGLYLKGHWEKGINGRIRKLIRPCHSFIGKLMPVLS
WTQMIFGGITALGFCQGDHTGQCAAHFIMGGAFIAYGILLTIILVVGQLWIRRHGRSQEFYDSVAIAAWGCVNTFTEHRWGTD
WVKNDWQHTTMGIIWWCAGLAGIWLSKDRDGNPKRNFIPGFVIFITGWAMSAHPQELMVSAATHTMFGYTLMVVGITRIIEIA
FVLRDAHSISEDGRTANSFQFVPVFFMYAAGFLFMGATEEQMNLVAGSAMDHVSYILITYSLASIMFLFVNMLIGLWDRLAHP
VLDTKELGNNHYANGLAVEDGQLRDAQEFELDGLMSDDDDDPSRRMLGPNSEDSAATVGRNQQTRA
FUN_006469 CDS scaffold_21 44701 45250 -
hypothetical protein PF10280 ENOG41

MASPNDIEMAGAESSRNEPFTIEENIRQLNAIDQQIIQLMKHTATALNALTIPKSTETDQNMDPSAEPTKPSLDPGTQKEAFA
SATDQFFRALHTVDVKIKRQVMALEEANIISLAPPPRLNKTGPIIPQPTPNGVGAVGNIGAGWLNSRGTRVERDMEAELWGKA
KEILKEKEAKLESGSR
FUN_006470 CDS scaffold_21 46193 48893 +
hypothetical protein PF00400;PF04494;PF08513

MSGPAQTPGAPQAPGYQGPAPTPPAPTHTTTTAATGQSGSSGGPMSNQNLNQIVTDYLLKRGFNRTEEVFRQESKHLGPDGKP
IYQLANLGPKKYQRAFGLLREWVENNLDIYKFELSKLLWPVFVYSFLELVQHAYTEDAKAFLRDIGPNFQPVHADDLKTLGTI
TLPQHINENPMTKLYRENKYRIPLNQHASGDLFNFLERESDQGGSVIRQVLATYCQIDSTARGPITPFSFEAVYRRSRNMEIE
SVDTNEGIPGVNIGLSNKDVLDPTAPLKLGPLPMDADLRDDVRAELEEEERRIPPPPGKTSLVEEFDQKIKREESADAPSRAE
LPLPPSRPRDIMLEMQKLRENRDRFKIDGATGGVGVPISACMFTFHNTLGSVACMDFSNDGQLVAAGTTESYIRVWSLDGKAL
PTMNAHEKEAKFNSRKLIGHSGPVYDVSFSDAVSGPPQKLFGDEGKSNPAMDTRPKLLLSASADGQIRLWSLESWSCLCLYKS
HDGPAFRTLWGPHGHYFISGGYDKAVRLWMQDHASPQRLLVGHDTAISALAWHPNGLYVFSASDETDKSIRMWSVVTGSCVRV

FTGHNEYITAMECAPNGKILASADCAGNIFFWDLEKGTRIKRSRGHGKGGIWSLSFSVESNVLASGGQDGTVRLWDVELPSDP
QKASQQQTGLDATATGADGTSAGGTVGEASRVTAGTSGPAGTSGTTGTHKKKNKEVMITPDQISAFPTKKTPVMKVKFTRMNL
VIVGGCYDPER
FUN_006471   CDS   scaffold_21   49526   51309   +
hypothetical protein   PF00787;PF09325

MDVEESPWADSSQAASQQASQPEAAASQSSNPAPSASSSAPRPSRGPRRLVAQPTRLEAVDDPLGPLGAATEDNGPAQDAPPV
PPQKEQMVIRTTMPQTQQQRRPADPHHIDDDEDDGPKAPRAPPPVEAAQPSAVRSSNQPSVSVEQAAKPTFQITVGDPHKVGD
LTSSHIVYSVRTKTTSKGYKQPEFEVKRRYRDFLWLYTTLHGNNPGIVVPPPPEKQAVGRFDSNFVESRRAALEKMLNKTAIH
PTLQHDPDLKLFLESETFNVDVKHKERREPIPTESKGVLGSLGINVGGGNKFVEQDDWFHDRKVYLDALENQLKGLLKAMETM
VGQRKMMAEAAGDFSASLHALSTVELSPSLSGPLDALSELQLTIRDVYDRQAQQDVLTFGIILEEYIRLIGSVKQAFGQRQKA
FYSWHAAESELQKKKATQDKLLRQGKSQQDRLNQMSAEVGESERKVHQARLLFEDMGRLMRSELDRFEKEKVEDFKSGVETFL
ESAVEAQKELIEKWETFLMQLDAEDDESVFYRPPVYQQQQQQKGGDTAVDRARARIDEDSD
FUN_006472   CDS   scaffold_21   51655   52135   -
hypothetical protein   ENOG41

MAKFSVSPATLSPLRTTLSTPCSPTRLSLGQVRHATFIPRPRRPYTFTQLVTLSDGSSYTMRTTSPLPIYRATKDTRNTLLWQ
PSEKSLKNVELDEAGKLAAFRERYGRAYDASHAEEGEGDAEAADEGYADLITGYAPSQDANAMRDSGPKKQAKKRK
FUN_006473   CDS   scaffold_21   52833   53343   +
hypothetical protein   PF01613   ENOG41

MRLLPHSVVVCTSNQPGDGPRAMTMSSFTSLTLAPTPLVTFNIATPSRTLDAIRASGGFNVHVLSGDERGANVASHFTKGNVG
GNAFKGLAYSQEDDEAPVLREDGVMFALRCRVLKDAPEGGLLRVRDHVIVVGEVVEMVRVGEEEDEEFGLVYADRRYREVGVV
LEM
FUN_006474   CDS   scaffold_21   55332   57489   +
hypothetical protein   EOG09261G1Y   PF00183;PF02518;PF13589

MSETFEFQAEISQLLSLIINTVYSNKEIFLRELVSNASDALDKIRYKALSDPSQLDSGKDLRIDIIPDKANKTLTIRDTGIGM
TKADLVNNLGTIARSGTKQFMEALTAGADVSMIGQFGVGFYSAYLVADQVRVISKNNDDEQYVWESSAGGTFSITEDTEGEPL
GRGTAIILHLKDEQTEYLNESKIKEVIKKHSEFISYPIYLHVEKETEKEVPDEEAEEVTEEGDDKKPKIEEVDDDEEEKKPKT
KKIKETKIEEEELNKQKPIWTRNPQDISQEEYASFYKSLSNDWEDHLAVKHFSVEGQLEFRAILFVPKRAPFDLFETKKTKNN
IKLYVRRVFITDDATDLIPEWLGFVKGVVDSEDLPLNLSRETLQQNKIMKVIKKNIVKKSLELFQEIAEDKEQFDKFYSAFSK
NLKLGIHEDSQNRSILAKLLRFNSTKSGDELTSLSDYVTRMPEHQNNMYYITGESIKAVSKSPFLDALREKGFEVLFLVDPID
EYAMTQLKEFEGKKLVDITKDFELEETEDEKKAREAEEKEYESLAKALKNVLGDKVEKVVVSHKLGTSPCAIRTGQFGWSANM
ERIMKAQALRDTSMSSYMSSKKTFEISPKSPIVQELKKKVETDGENDRTVKSIVQLLFETSLLVSGFTIDEPAGFADRIHKLV
QLGLNIEEDDSAPAEADAATDAPAAATGDSAMEEVD
FUN_006475   CDS   scaffold_21   58128   60050   -
hypothetical protein   PF03062

MSSSIDPLPNGNGHAGDHDHVIRPRPRKPKHSQVSEVLRMSTAEDSEGHLMPDNMSTGTASGLTSGRSTPVPENAPPSVRVLS
TVRKQTRAEQRRRIFPTIEFASRVSHFDPESDYRDFHGFFNLFWIGLAIMGITSMLRNLKDTGFPLRVQIWGLFTVKLWHLAI
ADFLMVATTAVSLPLNRAARSAKPGSFLSWDKGGIAIQSVYQVAWLSLWIVVPFHFEWTWTSQVFFLLHTMVLLMKMHSYAFY
NGHLSQTEKRLHALDNPFSPHDRRPAYQYPSTDSLANGEATSSGSKKRRRNRSKKRQQAKRSNSDGNKSNEEVDAVNNNLLVP
SPVASGNNSADEVDELREDLARELTSPIGNVTYPNNLTWSNYMDYLLCPTLCYEIEYPRTSHINWISLISKIIATFGCIFLLT
IISEDFILPVLIDASERLDAVPAVSSLSEKSLILAETISWLLFPFMLTFLLVFLVIFEYVLGAFAEITCFADRHFYSDWWNST
DWMEFSREWNIPVYSFLRRHVYSASRPRIGRGAATALTFLISAIGHEIVMACITKKLRGYGFVCQMLQLPIVMLQRTRWVRGK
KTLNNVCFWASMVLGLSMICALYVLV
FUN_006476   CDS   scaffold_21   61532   63114   +
hypothetical protein   ENOG41

MANQTTQQHRMRLRSSGCLRWSYLPQEIRRNILNTIVDQRNPRWSALASVSKEWQSVIGTRNMAKLNLRPSCLEDFEQAVVRK
RDLVRHIYLNVELTEYKCTLCKNKFAVTIAEKNNRLMSKAVTKLLTILSTWNTTGPLTLELNASSASDSKHWFKNFRFNDEHD
APYSRDSVIPTEGMHSDWHDPKHGWENGVRVKPPPISANKRLFGLINLNGPPYSILNVNAVTRFMIRRQFRYRLGPREFASLL
SKFSRLTDLVYEPWPQKYTDSYRYPWDPALIGMHLPRTLKRVILFQDFKQYLTENIRQDLDHSYYAILPIHWSKFNLPGNAKS
IAQTSLRLNELAVSFFIDALNFFKACEEEWTWDYLQSLSLTSSLLLQNIKEQKGRIDSLLITAAKFLLRMPNLTTMALWSGGT
GKACAFIYTRTKHYAHVTWRGTWDLEISRQVLEAWEGVAKLHSVELRVRHEHLQEIIRSHGDAIFYLNLPCQVIEPTSLWQIR
MEDA
FUN_006477 CDS scaffold_21 63595 64507 -
hypothetical protein

MSSKAIDLTGASPPIASIPSHSVKREHSLPIATVPREQVPEQNSYFLYKKECRLAWIKYLGLLGKVKLREGPDRLPFFIGMPP
EHLTFQVDAERLAVIQQTAMSDPRNSLITLGTVLKTLRDRPRHTHIAWDAVQQKLANSHRDQIKHWESASSRNPVILLQSTQA
SSGQTPVHKVIQSAMNRLVGIRTQEDDPPSCPGYVLPMASFERAEMNAEKAKDEIQRRQWADMSLRSLRNFMDAQKNEAVIDK
MVEFQKHYEALAAVGSK
FUN_006478 tRNA scaffold_21 65669 65760 + tRNA-Glu

FUN_006479 tRNA scaffold_21 66226 66324 + tRNA-Glu

FUN_006480 tRNA scaffold_21 67258 67352 - tRNA-Glu

FUN_006481 tRNA scaffold_21 68679 68773 - tRNA-Glu

FUN_006482 CDS scaffold_21 69127 75302 - INO80 Putative
DNA helicase ino80 PF00176;PF00271;PF04851;PF13892

MDQNGYNSSVLQRPPHREDGGDDDRDSRPHHHHHHHHHHNNRRDGDSAGPGPIVGETATASSNAGGANVHQHSTFSLRSP
KSEYRPPPFTSPNGHSHSHSHSHQNTPSAGHSLQSPPRPALPNPYMSSSTGAPGGPVAPSLPPPVGLNSSSPGSSAAGLHQQQ
HHRQPPAPPPAGPPPVSPLHPPVAYYPPGSNHDIYIPRETKPASRGFYDPTTDTTKESRVSDAATPGASWHNANANANAPPVG
TPKTRDPYPYSQSAEQSTPSYYNGSYTSPRDPSYSRPRSPVSHSHQNPPAGSLSPPGRQPLLASPSARHGATANMTSSTNGAS
ALTPYKSDLAAPSPPKPASASTSRANPMSFDSILSSSEPARKPKEQSPLISREVEPEIKQERDSRRDREPKHDSRDSKQTKRS
LEPEADHDTEVEKDVETEPEPFPGRDKEREPPVKKRGGRKSTKGRASDIRDAATPKNGRRLSIKKESPTPRLPAKRQANGLPK
PKTWSAEMEKKIQNAETQIDNRASNLEPDEFDQQQYKERAQKRRRVMDQLDLEHGLSRRAAFATSTGKKLVLHAELGKRRYDD
VFYDEALHEVREQEVYAEKERKKDMQRKRRREKSMAVTMEQKEAALARAEAAEDETERQKHLRDAERASKKAQQTKLILQKGI
KGPARNLELNLEGGTMSSFQASDVESGEAGTPSGKRKGKGRSGPRLKKSKEQKQAEKDSAEAAQAALDAGEELPTKEENRVRI
KIKKSKKDPSAETEKDKDDGDKTEDEPVEKKSKKSKDKDKEKVDDIPDNEKRFMSKGYNQIYDQIWRDMARKDVNKTFKLAVD
SYATKASNLKKTAILASKEAKRWQLRTNKGTKDLQARAKRVMRDMMGFWKRNEREERDLRKAAEKQEIENARKEEADREAARQ
KRKLNFLISQTELYSHFIGKKIKTDEVERSTDNPDVAKDAHQIDQQKLDIDEPTGPVTGKVTNFENLDFEEGSEEALRAAAMA
NAQNAIAEAQKKARDFNNQGLDMDEEGEMNFQNPTGLGDVEIEQPKLINAQLKEYQLKGLNWLVNLYEQGINGILADEMGLGK
TVQSISVMAYLAEKHDIWGPFLVVAPASTLHNWQQEIAKFVPEFKILPYWGGANDRKVLRKFWDRKHTTYRKDAPFHVCVTSY
QLVVSDVAYFQKMRWQYMILDEAQAIKSSQSSRWKALLNFHCRNRLLLTGTPIQNNMQELWALLHFIMPSLFDSHDEFSEWFS
KDIESHAQSNTKLNEDQLKRLHMILKPFMLRRVKKHVQKELGDKIEMDIFCDLTYRQRAYYSNLRNQINIMDLVEKATMGDDQ
DSGTLMNLVMQFRKVCNHPDLFERAEVNSPFACAYFAETASFVREGSEVAVGYSSRNLIEYELPRLIWRKGGRINKAGPDSQV
AGWKNQALNHMMNVWSPDNIRESCDGSKAFSWLRFADTSPYEAYEATHQSLIVRAAKELQKRDRLGYMNVAYSDTEDQNYTPA
HALFQIRARQNRKPLADITSEGILTQLMNVAQSDYNESGLGRLEPAGRPRASAPPIQVSCRSWGSEAERSEALFNAPIRKILY
GPTVFEEKALVEKKLPMELWPTREMLPKPDHEKKGFTNISVPSMRRFVTDSGKLAKLDELLFKLKSEGHRVLLYFQMTRMIDM
MEEYLTYRNYKYCRLDGSTKLEDRRDTVHDFQTRPEIFIFLLSTRAGGLGINLTSADTVIFYDSDWNPTIDSQAMDRAHRLGQ

TKQVTVYRLITRGTIEERIRKRAMQKEEVQRVVIQGGGASVDFSGRRAPENRNRDIAMWLADDEQAEMIERREKELLESGELE
KQQKKKGGKRRKAENTASLDEMYHEGEGNFDDGSKGLSGTATPATAATPGDSDSKGKKGRKGTKRAKTAKQRLAIADGMME
FUN_006483 CDS scaffold_21 77808 79761 - ARB1 ABC transporter ATP-binding protein arb1 EOG092614E6 PF00005;PF12848

MAPSSSKEKRLAKKAAEGKLKGKKGKKTEEPELDAHGNPIKDDDAPATSGAKLEEVKRLTEQMDKHGISDRVTTGVLASTQTS
KDVKITSCSLVFHGRVLITDSTLELTYGRRYGLLGENGCGKSTFLKAIAAREYPIPEHLDLYLLNEGAPPSDLGALEWVVREA
ELELERLDHQAEKLLEDEGPESPVLLDLYEHMDKLDPSTFATRASLILTGLGFNKKTIHKKTKDMSGGWRMRVALAKALFVKP
SVLLLDDPTAHLDLEACVWLEEYLKKWERTLVLVSHSMDFLNGVCSNMIDMRGKQLIYYGGNYDSYSKTRTENETNQMKAYQK
QQDEIVHIKKFIASAGTYANLVRQAKSRQKILDKMEADGFIQPVEQDRVFTFRFADVDKLPPPVLSFDNVTFSYSGKPEDDLY
RNLDLGFDMDSRTALVGPNGVGKSTLLRLMTGKLSPTGGVVTRHTHLKLGLYSQHSSEQLDLTKSALDFVRDKYSEKSQDYQY
WRQQLGKYGLSGDSQTALMGTLSEGQKSRIVFALLAIESPNMLLLDEPTNGLDIPTIDSLADAINAFSGGVIVVSHDFRLLDK
IAKQILVCENQTIRTWDGSISEYKNYLRKKMISAGAV
FUN_006484 CDS scaffold_21 80968 81570 -
hypothetical protein ENOG41

MSPTAPSHGQKVDLGQNAPVKQEGAGTIPNESLAAESLKEGGEFTSNEGIHGENQPTSRSENTSAGRNTNISSAPSSGSASKS
SDSAPKSLAGTAPSYVENQYIKAPGGPHGKNLKEGIDDSNTKDGLKKALGSEPGSQDDPSRLAEQQFQQNQTAAGRDAGPKQS
ELEGKTAFDALNNETSS
FUN_006485 CDS scaffold_21 86070 87742 +
hypothetical protein PF02836;PF03198;PF07983 ENOG41 GH72;CBM43
MKFSAAIVAAAATAASAKLEPITMKGSKLFYSNGTQFFMKGVAYQQDTAAAGETNDKTTKYIDPLADEEACKRDIPLLKQLGT
NIIRTYAINPKADHKACMKLLDDAGIYVISDLSEPSVSINRDDPKWDVELYERYIGVVDELGQYDNVVGFFAGNEVSNNVSNT
QASAFVKAAVRDTKKHIKSKFSRWLGVGYASNDDVDIREQIADYFNCGDDDSRIDYWGYNIYSWCGKSSMQDSGYSDQAKFFE
DYSVPVFFAEYGCNEPDGAAGRIFDETTALYEEKVMTDVFSGGIVYMYFQEANDYGLVKISKNGDAVKQKDFAQLQKKANAAK
PSGVEEDSYKPTGKAATCPEQSKNWKANSVLPPVPDSDLCDCMVKSRSCVPADNLKAKDFNDIFGYICGQDKKICTAINANAT
AGIYGAYSMCSNEAKLAYILDAYYTSQKSAADACDFKGKATTQKAESQDSCKSALASASKINEEVATATHAVASSSTGGSNSS
SEDDENFGLQAASIARVFSLGDFAVGAYMAVAGVVGAGMVLL
FUN_006486 CDS scaffold_21 91987 94247 +
hypothetical protein PF03572 ENOG41

MIPSILLALAGVSFAALEPRQVMHQARNQPSFISSRDLSEAESEPCKFISQAYVEAEPEPGKPVVLAIPPSVGIACLKSVPLD
KKRDLELLDYLEPLIGFQSTLEILADPPKEYLFPGVDVLGGFDKIRSKLENNKYETQYEVMTDLRSLFAAANDGHFDYPPALL
NAFYYVRRGVEFESVSANGIRLPYIFHVIDTSRGNQGLLDYTPSAVKSIDGTPIAEWLEEDASFATSNSQDPDAQYNGLFASI
PRGAVGSSSSTLFTQFEIPDTYTIEFHNGSKLEIVNQLVIPATVDLSGIESGEDFHYYVEITPTNTTTEEPSANERRSTQETD
DDLPGYPKPLVKQSSNAVSAYLLNGEDYKDTAVLSILSFLPIGIDISNPPADFSMTKFILEGQAVILQLIKAAKATGRDKLII
DMSANGGGSVVLAHSIYRLLFPEGEFTGWDRYRANPALEAAAEVDYDNLVKSLITRSEYYPVAPGNKYLETGKEFYGPYTVKG
QNVTAAFQTDKTVPWDESIPAYINGFDPERKPLVAEAPWKPENIIIVTDGICASACGILTGLLTRNHGIRTLALGGRPLNQAM
QAMGGVKGTLLNFNADITSAIANVRANAKTDKETASILSDASSSFPSSQNPPLLPLPAGGVGKVNSLNGYTEDNLEGYPVHFR
YEAANCRLFYTQRMMASPIESWRRARGVAWNNEPCVSGSTTNSDGTIGDKALKYDSRVRSRASGIKGPGELKK
FUN_006487 CDS scaffold_21 94618 95771 +
hypothetical protein PF01569

MPLLPRVNSAVANRSQSKKQKFKTFMRQWFLVNWRDLLAMAVVGAVAFGIYHSPVIITRTFPVTFDATSGDIVYPQWAYPDRG
WILPSWLSGLISIAIPIITYIVAQFRIKSAWDASNAIIGTNWSVILASLFQVTLKQLVGGFRPYFLDVCMPDISLAKTHNKTG
LNGVGFYQIMYTTEICTQPDKSRIQNAITSFPSGHTTAAFAGFGFLFLWLNAKLKVWADHKPAFWKLFLTFLPLLGAVLIAGS
LTIDAAHNWYDILGGGFIGTIMAFASYRSTYASVWDWRFNHLPLQETEAFRYGFDGDLDYAAQTLSMAAGWGGKKVRLPESAS
APAISSESTLRSGAEEIENLDANTRRKRRGPVGDEAV
FUN_006488 CDS scaffold_21 96205 97428 -

hypothetical protein		PF04366		ENOG41

MTLYSTSNKSIENVDPLKQAGQPPSYSIQPDHTEYQQPQPQDRNASSTARPTAGERFHKISSKAGSPLNKVANLFGAEGWWPS
TMDKECSKAARILHSFTSLNSSTSPTEKGPLHPTGLTRKSMVKIPPSVLQSAAGLAIFNVIRAGACHNSLSGGSGIVIARRSD
GTWSPPSSFVVSSLGAGFVFGLDVYDCVCVLNTQEQVAAFTKPRVSFGAEGSVALGPIGTGGSVEAALSKTARPVWSYMKSRG
LWMGVQIDGTIVVTRGDANATFYNERGITAKKILCGDVAWPMAAKPLFEVLQALEGRTDYDRTVVQAVGQVPPPGDSILSEKQ
ERYTDEPQVQETREQTPPEVASRDVKEELPDYEIVKKDEEEKIVDDEKARLAASGY
FUN_006489	tRNA	scaffold_21	97834	97942	+		tRNA-Met

FUN_006490	CDS	scaffold_21	99068	101235	+
hypothetical protein		PF00069;PF07714

MSGRNELKLGLTLWPLMGHLEPRPYRFADGIDGTFQRLDDSLRFAHWIRDWSESHLRQGSAVAGLIDVRSRTLETDLDALRYE
LHEMGSSIVNGVPTYIGELLERIKRCVAELTELVDFYGQNREQGRSDALDALFASQFILDIDERTGYPRSLPNQQGLVQMAIS
IAETLEKTSLNGASLVPQMEAVQSFEEHRPDLERCLFQFARMLEPRYVHTYNKTEDVPYEQKAFEPIAKGSFGAVVRVEHKKT
SEELAMKTFFCNDRSKILQEIGVLEVCNHPNIVKLVEAFTVANDERYEEDNGIIHLILRPWAPYTLEDFLKSTDGKRKDRCRW
FSPGSPQSDLCIYRIMKKLAQAVAYMHGRSIKHKDIKPDNILLHEPNSPNIRPLLTDVGVSKVFIRGGSTNFDDCSYIFLAPE
QVARLESNLRADIWQLGCCFALILGVAKRGRAGRDELWDSFCRTKDRRSCQIASEHEHFMKSLKVLCDESLSRREALSSQLAY
ELVSGMLDKNHETRLEIHQVLSRLDQLVAQMSTVSVGDVEMSYL
FUN_006491	CDS	scaffold_21	101658	102155	+
hypothetical protein		PF16015		ENOG41

MAPAATNSHSETARDNASGFIAYARSSVDRVVPPNSRQKAYDNTASFASSRPILFSFVVSQLLLSFLPLLIFLTFSLSTILFA
LGAAIVFALFWIGVALLVLVPALLVTSSIAVLVWGWAIGSFIIARWLYNHSPVGVQNNEVVKKENGN
FUN_006492	CDS	scaffold_21	102536	104050	-	RPT2	ATPase of
26S proteasome regulatory subunit 4		PF00004;PF07724;PF16450;PF17862

MKDKPKYEPPPRPTTRVGRKKRKAGGTSAAQKLPAVYPTSRCKLRLLRMQRIHDHLLLEEEYVENQERLRKAKAAKEGQSVGT
DADVDRLADERGRVDDMRGSPMGVGTLEELIDDDHAIVSSTTGPEYYVSIMSFVDKDLLEPGASVLLHHKSVSIVGVLTDDAD
PIVSVMKLDKAPTESYADIGGLEQQIQEVRESVELPLLHPELYEEMGIKPPKGVILYGAPGTGKTLLAKAVANQTSATFLRIV
GSELIQKYLGDGPRLVRQLFQVAGENAPSIVFIDEIDAIGTKRYDSTSGGEREVQRTMLELLNQLDGFDDRGDVKVIMATNKI
DTLDPALIRPGRIDRKILFENPDQNTKRKIFTLHTSKMNLNEDVDLEEFISQKDDLSGADIKAICSEAGLLALRERRMRVQMA
DFRSARERVLRTKQEGEPEGLYL
FUN_006493	CDS	scaffold_21	105372	106248	+
hypothetical protein		PF00179;PF09288;PF14461

MSRDRRVAKELKDIEADHDDSGVNASPVNNDLKHLKGTFPAPPDTPYAGGTFTVDIQIPDQYPFKAPAIRFDTKIWHPNISSQ
TGAICLDTLSSNWSPVQTIKTALLSLRMLLECPNPKDPQDAEVAKMMIEDPKRFALMAHEWAVKYAGAPRQDVDLTKYQNDVA
AAPVKDDNARLAGYNRNLVERFVNMGFPLEDVVEAFIHFGIDRNGGRDYELEEAYMGDVVARLLHEN
FUN_006494	CDS	scaffold_21	106853	108081	-	TCA8
Succinate--CoA ligase [ADP-forming] subunit alpha, mitochondrial		EOG092638CT
PF00549;PF02629;PF13607

MQSFTRATRASALQNVGRRAYSSSQYAKTINNLRINADTKVIYQGFTGKQGTFHAQQAIEYGTKVVGGTNPKKAGQTHLGLPV
FKNVGEAVKETGATATGLFVPPPLAAGIEEALEAEIPLVVCITEGIPQHDMVRITDMLKTQSKTRLVGPNCPGIIAPEQCKI
GIMPGFIHKRGRIGIVSRSGTLTYEAVNQTTQAGLGQSLVVGIGGDPFSGTNFIDCLKVFTEDPDTDGIIMIGEIGGSAEEDA
AEFLKQANTVNGGKPVVSFIAGISAPPGRRMGHAGAIVSGGKGGADTKIKALEDAGVIVERSPAGLGKALYNEFARRDLL
FUN_006495	CDS	scaffold_21	108971	110378	-
hypothetical protein		PF07690		ENOG41

MTYNEHNAEDSCLKNQHEKQAQPKTNPEVPRGDAAPAPDDAGLETGGVAHPSEASADDSEANLEFPEGGLEAWLVVFGSFCGM
FSIYGLINTSAVFESYFSENQLRDYSSSQIGWIFSLYLFLVFLVGIQVGPVFDKYGPRLIVASGGILIAASLFILSVCEEFYQ
ILLSYSVVGGLGGALLNSPCYASIAHWFDQKRGLATGVAMTAGSIGGIIFPIFLQKLLPSVGFAWTTRILGFIILGLTVPATL
CMRSRLPRSNKVSSVWPDLSIFKDLKFTFSACGIFFMEWGLFVPLTYIVSYAGRHSGDANSSYTLISILNAGSLLGRFLPGLL
ADKIGRFNVILLTLGLCIISAFALWLPAGDSQAMIIAFAVVFGFASGSNLGLTPVCVGQLCDPRDYGRFISTAFMVASFGTLS
SLPIAGAILEAGDKGDSGWTALIVFDGLSYVLALACFLAARVMAVGWDVKSVY
FUN_006496 CDS scaffold_21 111792 113416 -
hypothetical protein PF01490 ENOG41

MDKTSTLEDSELQTSRTPSRRPWLASAFGGPNVTIGPRIEPLAEHLAHVSESDSTAAEDLVDKQVASEAGNAIQYRTCSWQKT
AGLLFSEYIVLSIMSFPWSYSLLGLVPGLILTAVIAGLVLYTSLVLWEFCLRHPEVRDVCDIGQMIWWNKRWAWWWTAVMFVL
NNTFIQALHVLVGAIYLNTMTEADSIAGCRTVAFAAVVTVISWIGSLPRTFSMLSKLGFASTFFTFISVILATAFAGAQGKPA
GYPEMGEPVISIWTPKTTTLVTGMSAFMNMSYTFIGQTTIPSFIAEMRDPRDFPKALWACTMAEIVTFSVVGSVIYVYTGNQY
MVTPAFGVLTDTYKKVSYSFMVPTIIFVGCLYASVTGRFVFFRMFKDSEHLTSHTFKGWFWWGLVLLISWAASFFIAEIIPFF
SSLLSVMSSLFNCWFGFIFWALAYFRMRNADRKVGRKRNPILDIFSVALNVVIMLTGALYLTLGTYVSVQGIIDQFRAGQVSS
VFSCKSNGL
FUN_006497 CDS scaffold_21 114570 116073 -
hypothetical protein PF01565 ENOG41
AA7
MTTLTPQPSIPQAESLIAAGLEARVLTPTDAEFSARQESYWSNSAKIKPACIVQPRTPEEVASVVKALVSAGQKFAVRSGGHT
NWPGSNNIEGGVTIDLVHLNHVSFDATTETADIGPGCRWREVYAELSKHGRAVAGGREGNVGVAGLLLGGGNAFFTARQGFGC
DNVVSYQVVLANGDIITADEKNNTDLYRVLKGGSSNFGIVTKFTMKAIKSDKVWGGMTFFPKQVIPGAIEALSAFTDNVPNDI
HSNLVTIFTHMPDFKDIVVATLYANMEGIEKPPAYEKWLALPEIMNTVKNTTLTEMAFEYNIPANFYDTWFTCCFKNDTRIIT
KASELHDKLVEELKAFIPDGNFITQCLFQPLPTLFGQRGIEAGGNIMGMDRHKTNGILFLAVVMAQTPEQEAFARPKVQAWIK
DVQEFAATIEGGNLEWTYLNYADKSQDPLGSYGAENIKKMKDAAAKYDPEQVFQKLVPGGFKLSDVKDE
FUN_006498 CDS scaffold_21 117427 118667 +
hypothetical protein ENOG41

MVFSVVLQTLILRDFIRGIMGTHGWEMPITKFALFARALYLLPILYNPVQCGAKMALLLVYRRLAPQKWFHFTIWATSFVVIG
SSIAITFTTIFPCKPVRAGWDITITDKKCIDRPAVYQATAIMGAITDAMVLAVPIPVVIPLQISRRQKIGLICFFGVGGVTVF
TSVMRLIALIRSMGNVDQSWGGGPVLLWIFAEANLSIICATLTTVKPFIKHISPRILGSSYGASKAGMSNPSAPPTIGAISTG
GMPRHDHYERFDDGPMYPLQTVTKAEASKSHENKNGGSRSVTRPMSGDSQGDSDSEKAILQTRTTTVTYS
FUN_006499 CDS scaffold_21 119407 120227 +
hypothetical protein PF12796

MDLLLALGEQKYAEEHPLHYAATKDDATLFRRLLQKPGLRADIDGDPGSGHDCLQTAIFNGNKEIAEALVTLPLSEFDRTRHP
MAAHGAISYLASAATLGRLDILKIMTDTGNVDIFEGDSLGKTQLHHVSDVPIGQTPFHPIDDEGAVKIARFLLEQGAIQVDCE
DNKGQTPLSLAVQHGKRGIVQVLFEVSRDPDSKDQDGNSPFYYAVRNGHGPIAELLANTGKVDCGVRARAESDRRSDQERGPT
FTWHVL
FUN_006500 CDS scaffold_21 120512 121676 -
hypothetical protein PF04616 ENOG41
GH43_26;GH43_27;GH43;GH43_31;GH43_30
MLVLSVITLVLGLCSSFVLGFTNPIRTGSDPHIVYQDGMYYLTSTTWTNVSITKAPTIEGLKTAESQLIWSDRSNATRACNFW
APEMHKVGDTWYVYFSASVCDEGNWDVMLPTLRVYVLKGGKENPLSADYEMMDAIIPPNFNAGMLDATIFKIEGTEYFLFSSV
EGPKSPDGASLWIAKLLSPTTCGNATMIAHPEFEWERSDSPVLEGPYGIISPAGTTYVVVYAADSCNTPAYKLGAMIFTKGSDP
LSPDSWTKLPDPIFETKNGLYGPAHNAFFKSPDGTEDWNVFHANLNAGDRCGPSRKTFIQPLSWKDDMIDLGEPRPVGTEIMP
PSGE
FUN_006501 tRNA scaffold_21 122146 122239 + tRNA-Glu

FUN_006502	CDS	scaffold_21	122573	124059	-	ADH1_2	Alcohol dehydrogenase		PF00107;PF08240

MLSSSNVMRTGILRTGRIATRNTARLARSATAQIQTTRRNFSSSQRTLCASCATSGGQSCVRHNPNSRASAGNITTSFSANSS
LGKTAAYATAASPIIPKEQWAQVLEERGGAVQYKKIPVPTPGSDEVLINVKYSGVCHTDLHAVNGDWPLESKMPLVGGHEGAG
IVVARGELVKDVEIGDHVGLKWLHGSCMSCEQCRQSNESLCSEASLSGYTVDGSFQQYAVGKAAHVARIPKDCDLAGVAPILC
AGLTVYKALKSSGVRPGQSVVIAGAGGGLGVFAIQYAKAMGLRVTALDGGDEKRKVCTELGADTFVDFKTSTDIVGEIKNATG
GLGPDAVLLLAASEKPFQQASQYVKSKGTIVCVGLPANAFVKAPVFDTVVREINIKGSYVGNRQDTAEAIEFYRQGLIKAPYK
IVGLSELQKVYDMMITGNVAGRYVVDTSR
FUN_006503	CDS	scaffold_21	126130	129429	+
hypothetical protein		PF05729		ENOG41

MNDTTGITTAAARLVGITDEAVRFFQQLDHTTNSVAKRVQQLEAFSRLAKEIELKPSANDHDDEEQLGLTKQILLYCYKLISR
ILSQLEWVDNGGRHASTQQGLLGVYAELNNWEVKDPFEDLHREQLCLVAFRKSRFRPLSWASESDSHPLLYSHTFKARELDFL
SLLFVTDPREDHASLESSRGAIVKRTCTWITETSEYRSWLSGSGDRRGLLIQGGEGHGKTMLTMYLTKQLEQLAACMSTDTVL
YFFCNHGNINANSATTVLRGLIWQLCRLRPDHLHHGLKRAVSVDEGRIALATNSVETLWQIFSAMIQDPTTGTVTCAIDGLDE
CDESSIKTLAERFSELLSSTNQDRRNFRILLTSRHPCQQDNLSTEFSVICLDSDAHKHNVQDVQRLINSHVSNIAHEHGWSRG
FQEQMNKILTKRAGGSFLWASLAVSGLKGQDEGSAAKYLNDLAGDNNVIYERSLLAIPLECRQKVRSLLTWAALAYYPLSLGE
LSTLTSDEFSTPEAAFINLKACLGYCRTLLTIKTEIRRFGSGYETIETIQFSHRSVKDHLLQPYVLRTATDTDADLDFFQIIP
DNGHEILASRCLEIMEKGLEVEAERESPVEYAHVLPYASRFWFRHLRECPEKLCNETLEKRALNFLTRNHENRRLWFSYLSNL
RHGREDIPSTASEHHDTIGGLEEINGSDVIFFEHPKDTTSATQLQALQLACLLGITAIVRKILASTSLFTCIRQANIRPLHSY
SRTRPRSEDRLMKVQRGGILQLSTAEIVAMTPIELAVLGGHHKVASLLLDRYPRRSIWQDSSFALATAVSRCDENMVRLLVEG
GAPRIRASRQLEGPICTAVVNRRLDVVKFLCFSEAEIWAQPNSKLGEVTQALLCLSSDANSSSLDGTVFIQYAKVLLCGGASP
DGFNLYGENRGLRYWKRAVMEYLHRHGITLQNLGPYPNRETPLMVFVSSLGISSSLSEPVEAVQFLLGSGASINQTDLKGWSA
LHHVANQIALGRAKKSYEATDDAEEYQLYKIANLLIAAGIDQEMEDREKRRAADILRVVGAPIWNRNISEYESFLRREPLSRM
SLT
FUN_006504	CDS	scaffold_21	130161	130542	-
hypothetical protein

MLSSNVLRALALLVATVNAGPCRPTTTLAGNTIEATSTATVIESTADLTSTIVESAAETETTDIPTTTTAIVEESTTTDAAST
TTSAAVDNTSCSISDDCVANTEFCLVNGLNMCVCLDAVCVTVD
FUN_006505	tRNA	scaffold_21	130688	130778	-		tRNA-Glu

FUN_006506	CDS	scaffold_21	131152	132106	-
hypothetical protein		PF05368;PF13460		ENOG41

MPKYVLTGADGNLGRIAASFATEIAKPGDELVFTTYKDEAIPADLRKSWTDKGAKVVTATYDDIESLKAAFQGAEAVSLISTW
LFGEGRRRQAQTVVDAAKACGVKRVCYTSFCGAGIEAENEEDIPFLPRDHHFIEKIIYASGLEYNIQRNYLYADNIPTLFAPS
WKFCGDRWLVNTHGKAGAYVAREDCGNVLAALLLGRGEPNKVYEITGPKAVTNEEVFKWMCEQTGYSGEIVDLPDEELKRWWL
DHGLPTDFFGDFSKLPMKLCIGDLLCCGEMVAREFMAETNDNVEKLTGRKPIPYQEALLQYKDIFPKP
FUN_006507	CDS	scaffold_21	132728	135075	-
hypothetical protein		PF06985		ENOG41

MASLNLKRTPYPTGDTEDDIRKDPILCRTCWNLYGGSTATAVPLAGIAMHENSGSINANRAATAGNLDEAPAWAGRGRIDYAS
WQISVYLPDVPGTVARGCPSCFLLQEILTKLTKGTLDCNDPELWLDIVFCKGNVLRMNLIRGSPPDDFDMFSSGGGSTDGDLL
ESFEIYTMPGSPCPWPTIGSSMNVERPDWSLPAEIGGHASHIEPDPTSKSSFDRVKSWIKTCKDTHTICTEGEASSNPQLPKR
VIDIGPDTNDGIHIFVHDDATQQITEPYIALSHCWGKTQHLISTRATLDQWKQNIPFNRFAKTFQDAVVISRELGIRYVWIDS
LCIVQDDKQDWEIEAAKMASIYNGAELVLAATGSADGSGGCLFQREPFVTVSGTFPEGKPFEIYGRKVAKHAVFGWNTDPNGS

KGSSNPIVGTTVSEVYDYPLMTRAWCFQERLLATRILHYTKSEMIFDCLSSMECECGALEKHEDDPLVPARRIIKTGHKFITG
TKSYRGSSTNPAAPLKGEIKEFVEHHELWRDLIVQYSQKNITKRTDGLPAVAGLATEWFNKLTGRYLAGLWEKDILNDLRWMP
DEKDSGEEPEYISPSWSWLSVHRGVTWGVESFEFDKFFVTVDFDRTSCPLSGHNQFGAVDSGYIFLTGRIMPTTFSIDGNMVW
LEKPGNNTRKPFDRPDSLWRLRNLKNNELFCLRLSTRMSTRNAWDDDAALVLVKADKEILKKQPEEVQKFENVYQRVGFITNY
MTQGWNHERDSKEMGLYII
FUN_006508 CDS scaffold_21 135624 137521 -
hypothetical protein ENOG41

MARWTQPLLLTASVLLSLSSTGTAHPANNHAIHPKHAVIPPVLPPNAFKRQDAPATEITPVPEVKQAEDVSQPTATVPFEAPI
PAEDTNDWRSVNCEADGVKDHTLYGPDRWKNVDAGDAWNAVIEGWKMNITGGREQKHFSNNVSNFFHGPQDMFCETFSDEGSG
CEDLNLQCHSTSVPAGYFILHSFENIFAMHSNIWNAVGDAVRDNDIGTIASEFGKVPPKEGGLATSILIDIALMAWGMVMGPA
WNKLIGPKFSDSTNAGTLKDTTNDLVKNSLTLTKDILNGRAQDQLGVQNGLTAQMQGLTEGWKDGVLATQKWLFGGSEDGYNQ
LGAMISDASMFGDNWLLDRGDHAKQVKRAINAFLIPMAWQYSPDVIIPFIAESDVACDQDPGWNKEATFTTMYQHWIDDGALK
DGRFCFNGKSYWLLGGRDRNTNCDSKSGDSDCGGFALPPGWQSLKDKKFTDISLDNVIIGSLNSKAANGGKNGFKLDLGDEPS
REVLDSLAGSGIEAPGVFNIPICSMDEIIENYYLWYGNSRDDMTNFPCNV
FUN_006509 CDS scaffold_21 141250 142969 + ELP3 Elongator
subunit EOG09261VI3 PF00583;PF04055;PF16199

MATATMTVTAAPIKKKPDMAPESERFLRCCADVANALIEDHEASKAGRATRDINLNSLRNKLAKKHKLQNIPPLTAIIASIPE
HYKKYILPKLIAKPIRTSSGIAVVAVMCKPHRCPHIAYTGNICVYCPGGPDSDFEYSTQSYTGYEPTSMRAIRARYDPFEQAR
GRVDQLKSLGHSVDKVEYIIMGGTFMSLSESYREEFIAQLHNALSGYQTTNVDEAVEAGEMSNIKCVGITIETRPDYCLQPHL
SDMLRYGCTRLEVGVQSLYEDVARDSNRGHTVAAVAETFCLAKDAGYKVVSHMMPDLPNVGMERDIDQFREYFENPAFRTDGL
KIYPTLVIRGTGLYELWRTGRYQNYTPNGLIDLIARIMALIPPWTRIYRVQRDIPMPLVTSGVENGNLRELALARMKDFGTTC
RDVRTREVGVNEIKNKIRPNQIELVRRDYVANGGWETFLAYEDPKQDILVALLRLRKCTEKYTYREELTGQPTSMVRELHVYG
TAVPVHARDPRKFQHQGFGTLLMEEAERIAIEEHGSDKISVISGVGVRSYYKKLGFWLDGPYMSKWLDGREQPE
FUN_006510 CDS scaffold_21 143483 144827 +
hypothetical protein PF00153 ENOG41

MNNRRNDVPDYGTGILPALPPDVVLPDRDPRSNAATAASAAGVRALSTQLIAFYFRAPAKAFFRTRVDYLAYARTIHQAQTEL
MMKAAANDPSASRLNVFMRQSWLLLRSTTPGVLTSAIRHQGWGIIPNQILPPLIANVGVGAVLYTSYLQILGRLHEESGQARK
RVYPPPHPMHTFTAGLLAGGLQSVVAAPLDAIQARYDHRDLMPNDGSGKPRSMWAFSAEKLREIGLRGIFAGWGLSLAKDSLG
SAIFFSTFEYVKAQGYYRFVTWYYGGLAEDIVDVLAMKRPTHEHRQEEGMRLIRPHYALEPMFLLLGGISASFTQQILLHPLT
HFQVKHWDHLEDLDEKAARLRASRVANPEKPRRRWRMLRAYYHAYQESLAVCRGEAAAEGLTLTKWLYRGFWWNAIRQVPSTS
AGLIIFELIRRKYGLGQEEVRITKDGYDILLH
FUN_006511 CDS scaffold_21 145243 145857 -
hypothetical protein ENOG41

MLGRRILQVVIQNPPAFWLHEFTLRRRIHEVGWSTDQIQIPGDENPAHILQGFLQRPGDVEIGRFLGLVQPQRFVGTILDHGI
EDRKRLGFMYNRLLRWATLVPLGIQGTTFCLPSSDERYSMQLLFQIAAVNHQSRLFWRRYDFESPFRHDGWFKKYPVSGSIEH
FSLGIDYSRLEKEQDRQRD
FUN_006512 CDS scaffold_21 147704 149847 - CCR4
Glucose-repressible alcohol dehydrogenase transcriptional effector EOG09260WGT
PF13855
MYPQHSGQGQHARLNGAGRGIPNLLYNYQHTQHQHPSQTQHHQGLQHDHGMPNANGLGHHSTFTPGVLSNSSPFNPNALQNGH
SAGNRGQPPPNEMWQEQLRMQKDAERAHSAMTDQQQPHYYARLRASENRGIGPPPSASLRAHPDDEDDGVDRRRPLAIEKEAT
RQDWHNLDMSGQGLRNLAPELFKYKFLNELFIASNKLTRLPNAIGELRALRHLDASFNQIIEIPPEIGMCTYLKNLLLFNNNI
QTLPNELGSLHLLEMLGIEGNPLDADVKHKIMEEGTKSLIHTLKENTPMPIPPTPRRPIVIQEDVSPSLERIKVFSWNILCDK
YATPQTYGYTPTRALDWEYRKGCILDELRIRDADFLALQEVSTDAFKEDLSPELAQMDYKGVHWPKSRAKTMSEKDAQSVDGC
AVFYKQSKFILLDKQLIEFATIAINRPDMKNQHDVFNRVMPKDNIAVICFFESRLTGARVILVNAHLTWDSALADVKVIQTGI
LMEHVTKLAEKYARWPAVKDKKMIVLPMGDDEVPVPQAEPGPSQEYRTNTEIPLLVCGDFNSTEDSSVYELMSMGRVPPDHLE

LSSFQYGSFTRDGIEHPFSLRDAYAHIKNTADEMPFTNYTPGFADVIDYIWYSTNTLEVVDLLGPPDPEYLKRIPAFPYWHFP
ADHIQIMSEFVIKGRKEKKHLPDREPDFGPGSRG
FUN_006513 CDS scaffold_21 153349 155218 +
hypothetical protein PF08241;PF13489;PF13649 ENOG41

MSEWSGFYYASANPLPTFARRAKAAEKERQRPETPPRSNSDSHDIAPNSSRARRPFRHTRTSSGSQGSRSSHDSSLVHSTTSP
NTKNGPALAEDDSHYSHSKLLSSDSRQQSSKPVTTTTTTKTASISNKHLPSKSLTGGGASPLHSPVVFNRPVYVDKTRQRDPR
RQSSAATPTASSVDLKNVTMYSQLDAGNKLMSGTTKQVNLDFPRTDSVSSTSGNTTVSTQTMTSTDHSNSDPATKPFIVKNGR
TYYNDPTSAYPLPTDLAELHRQSLRTLLMIQVYGAPVYTPFIRNNPPQRVLEVGCGTGFWSMMCHRYYKERGHNGIQFTGIDI
APLAPGSPGSPVELCKPDKDMKWRFVQHDLRHQPWPFADEEFDLIMVKDTCLMVPSHVYQLFVEEYLRMLKPGGVMEIWDSDP
TFRMLRPHVPSAMPGSEEAEEHEAALDLGAYLMTSKTPLSAPLNSFLVEYNTWLSRTLEGRQLSPVPCSLIGAFLHAEAEVLM
DIKTKRLAIPLSEVRWEREGVGGVVTKDGKSYVEMKAKSTPHQKVEKKTLTPGQSALRKTALLTVAQQVSALEPIIREASGKS
QDEWDAWVAKMMADLMSESGTSWGECLELGVWSSRKRPRKE
FUN_006514 CDS scaffold_21 155751 155970 - ETFRF1 Electron
transfer flavoprotein regulatory factor 1 ENOG41

MTELLYLGREYPLGFDYFRPRLHKAFISKAGERDEDKIRQGIAQAEYVKKEIEAL
FUN_006515 CDS scaffold_21 156334 157671 +
hypothetical protein ENOG41

MLVRGHNALRSSRALFRLPSLVRYRCEIKEGPEPEQKQKQKPKERRTTRPWPSGLQELGAESARPIPADPSSTFTMVNIPNAS
TAYERWPTLKNFKPALQPRTCGTIRPGAGPGGGATVDAYMELSCLPYLYNDLGVMIFEGPHCVQVKIMSNILRSVECEECRLQ
DRDWTTTKAYYEVNYSKWNVYRIKLDPSKHVETSSQNGRRKSSITANGLYDLTNIALEKWKADGTGGPVPVETLPGSLKMYIL
NHTGGPGLANDTINGSYLPQLHVYVATGIRGNTIYMCIEFHTVGTRRLVGVTYLRLRPTFDLSPAKYMAEEPPQRVDGNTDFD
QKFTTIEEKIKAGNAYSEKQAFQPPPPYARSIESSLEFYEKLVPSNTRRNSDIFKAAMFKLYKLEMRLWSQRKRAMKK
FUN_006516 CDS scaffold_21 162274 165124 +
hypothetical protein ENOG41

MPRAPGSSKRQQGAASHRDNRHENGLVGSAKRSSDKKGSGLLDNSLRRSEQGTAGAAGQSTPVNGHANSPYKQCVYDASNDSR
TDDLRRSSLDAVSEMSSDSSNANGLSGVEAGHRQIDVNAMKNADVHRDSGPFDLATTVLKSLPMQDTLAILIILMHVPSLSLT
VIYTIFTFLTFVPPVATSSGMSINLAEIFDGNSTMPSLVTVLCVDFFFLLVWLFLWGPIQDAILDFAKPVIAITLGGGTSARD
GTSRGLTTCFMWVIGHHLLRGTRAHWGRVARHIPEHWRLPQVFNNPVDSASSAYDKMSAYGWIRSVLAIHILTQGIVRYIREW
YLRREKANASVGASDPEAGKPPSVISDTANEGGFATPDTEAGLNSTSTVPTSKKRRKQSTQVRLQQPLWAALASTKIVVVKEY
ELSHAASESAGTNATDIHNLGNAPFNNQPNQIWISYIGSDEVCFNTSHFPEIDDDEPRSVESSGEDSRPANIDTSKPFYVRVN
NAVWQPTRMFPIEESPGESHEGVRWTGDIYGLRPASKYVCEFVDSQTDEVLFSTSIRTVQATQREVDGVPPPVANGQRSLHPD
SPALTLRTSIAAVEAKLSEERSRLKTLRKEYKNRANALKKDNELTDNQLSSAGNHDEKYRQKIRQQETQKAQAERDTQQLADQ
LKNFDTAPELGERKKKVEKQYSSEKKVFEAAQKAFKEYKSGLEKEIKAKEVEKSNLNTRRNKVATRIAKVENELANITDANNR
GLDEAERRNQERSIWQGHVAGIEANYNERLAHVRANNANKSEEIRHLQMQLQSFHEYINSNGMSYDMMPPLDAGHSHLGPPFQ
PTTSSTWNPDPTAPPHYPTGLWSASDNVLPSASAPTLPSMSPWQPPPTAPPFEPRMTRSRGRSSSMLSNVSGFTQSSGEEYAS
PPMDSHRVKHIWASRTRASGGGSSGSGSNGDPTSPR
FUN_006517 CDS scaffold_21 165979 167034 -
hypothetical protein ENOG41

MMALGVGSRAVDLGVHVSISKLSLDCSLGRFQVFKVKRQGIQLSLGTPLTLGIAASYQYTSGVQSTLPLSCVLGISCGQETIE
ECNVPLEIAAIKTLLGQQQDSIEENGELITIAGAKIFELEPVINKIEEEFPIVKNKLRTYCKQTLDSFSDELDDMARADRDKW
DCVHESGKMSELIDKVSEENATLKEKIKTQKSALIKQKIALKEEKDANKQLKSDVASMMRRLVAVEEVVHPAMCEAAEQKLGE
RGV
FUN_006518 CDS scaffold_21 167829 168472 +
hypothetical protein ENOG41

MSRPRPGSRRRRKLRREREEPGEWVPKQEKEPAQAAADPSEASTIESTTEATTSASAPDSKDTTGETKDRHYYYRQEKFERRF
VALDRCIKERVDANIVKGRAAIDADTKKLRRQHKELLTKVKSEAEQIRRAYQVDIRDTKKEINRLERKVDEIQEENETLRDEI
ARERRRMMYERYDGPSHDQ
FUN_006519 CDS scaffold_21 169298 172683 +
hypothetical protein PF10257 ENOG41

MDFWSRLLSPLSSGGTRKDLGHDPAKRLHRFEKQYSLLLSAWRASSNLSRDSDAAETLEIRLQELTNILSDESRRPLPHPCIQ
YCSLKQIYVPIGKIATTSYNEWIIKEAVLFFATLIESEEEAFVENQTFSASLTNLLVRITGINSVRLGLDTESRVVELAFNIT
TKIRLDPSILPAWFKTHRGVALQNREKDENPRDAFVGRTQKADFPLFYILMDYIHHEGKVGDFARTGLLYIIEAASSSGPLEQ
WIVESDLSTLMATGLGALYSQLSRKLVVDHLPHNLPPILAFSDYEHPTSNYEVISSCSPEFQSHLETFLSHLLFWQDVLNHCR
SVEVKSTLLEHFQVIFLQQLLYPSLLESSDIDGGSSVAVLTYLRRILESLDHPDMINLILHYLLALPDNIASARPGSSSSASK
ARKRKSMDLATMLAERAEPAATPLLFNLVDLVLACLRSRNQQTIHVTLQLVSAILKRHHRYAIVTLLRTDNVPIQNATRTVGA
QEQEVEFIMSLAGTIGGEDDFDEVYQLVLRDTMSRLEAHPCSLKLVAPRASTSNQRLPDSLPGAPKEVADHTLRPDDPLLNSL
LDLLETFFVNQVETNLSVTETLVDLAVCGFMKIEGWLTRSPSSYIYNEVEKQRRKSTEDEDVESEDDDDFEDLPSPTPEQKQL
QAMEQCRQRPQWSQASLPRVLNVLRRLEEQVAAYRRSIPRFDELVQQRRDAFRTADAMLHHAGPTPRPTPISEDPPDRPSFEE
VSRNASPSRPSALEGLAHRLLSELGTPSRASSPRGRKELGRSSGSGNATPGKSVLSSKEASHSRGRGTPGRSHSPPRTLDTID
SAQKAREEALARQMAEFAAMDQTILSKKVGLPDVKVEPIPLKFDKRPMPEPMETEVTEEEHAEQAPSEADEDQFEDTVTDLTE
QLHVEVKDEDDAKTEDTETDLSDAETAPEIAEPMEPVELEAEDSANVDAEEGEPAKEVEVNEIEAKDLKVDEPEPSESEYHDT
ETKEDEPEEHKIATPQRETRNLDTPEPETPQPREARENTTTVSVSHVVTNVIILQSFLFELAALIQVRAGLFDEVRFV
FUN_006520 CDS scaffold_21 172923 174135 -
hypothetical protein PF10544;PF13455 ENOG41

MPFVANTPESYLNRSDSKATNGTCRGLTSNGRPCRRAVQQTAPKTDKRGRPVTPDPRDESLYCWQHREQANMSAHSSPGPRAT
STPILEGRTSLDTLADRLGLVDLQEKKKYRRDTDRRKQTAPRPKPQPKQSLMCCCFSVPLQDVQESRPSRPQPRPVQKTSASV
PPKRASKQHLTASQNTSSAKTSRRSRKSTGSQTAQIKDFIPDSVHAQTASALMSELARPFGASEEPGFIYMFWLTPTSQSSSA
PVDAARSLLSPPSPNRPPRSRSASNAVSSFAAADPSTSKNTMLLKIGRAANVQRRLNQWQRQCGHEVEILRYYPYLPGSQESS
GVVPHMTQHVHRVERLVHIELAGMGLKKDAEKCDACGREHREWFEVEATREGIKAVDGVIRRWIEWDETMT
FUN_006521 CDS scaffold_21 176318 177062 + SOD2_1 Superoxide
dismutase [Mn], mitochondrial PF00081;PF02777

MSIGTYSLPALPYAYDALEPSISAQIMELHHSKHHQAYVTNLNAALKNYATATSTNDIAGQIALQSAIKFNGGGHINHSLFWE
NLSPSSSADAKPESAPTLSAEIPKTWGSIQAFQEAFKKTLLGLQGSGWGWLVKDTHGLRIVTTKDQDPVVGGEVPIFGVDMWE
HAYYLQYLNGKAAYVDNVWNVINWKTAEARFTGTREDAFKVLRASI
FUN_006522 CDS scaffold_21 178219 179898 +
hypothetical protein ENOG41

MSFFARLTLVVLWALAVCAQDTVTVTVTDTITNCPSAGLRFYPRAEATVTECALSCYTTVSQCTSTQYIGTSSGIELPTCSSV
ILSESETFTNVISLPTVSIPSTSIFANTSLSYTSISETSSVVSQPAVSIGSSALAGETSSELESTVYGYSTSTATLEVTSEIV
TNLETMPTFAPSFDSSALAPGTTQFPQGAESTTLSEGSTEQPSESLSLGTESVPTKGLSTETYVSRQGSSTLSEDTAASTVPE
RVSTTTLKSVITSQVILTERISTIYTASAEASTFDENTEVVETSSAEGASTETGQGPASAADASSEAVTIPTAESTAAGASTL
TTEAEISTTTPCTSTRHTHIYDNTTIVHSTADQSNDSSALAGSFTSASSLASEYTSTTSEVIEQTTAIGAETSEGQAGVSTSE
YLEGTTETVVGVQTTLPTEFIPIESTATQTDPEIVTSSQPIETPVPSAESTGLQGYDFGPSSNDGWPTASFASTADAATGFTT
[truncated: 530,612 more chars]
